# Forecasting life expectancy, years of life lost, and all-cause and cause-specific mortality for 250 causes of death: reference and alternative scenarios for 2016–40 for 195 countries and territories

**DOI:** 10.1016/S0140-6736(18)31694-5

**Published:** 2018-11-10

**Authors:** Kyle J Foreman, Neal Marquez, Andrew Dolgert, Kai Fukutaki, Nancy Fullman, Madeline McGaughey, Martin A Pletcher, Amanda E Smith, Kendrick Tang, Chun-Wei Yuan, Jonathan C Brown, Joseph Friedman, Jiawei He, Kyle R Heuton, Mollie Holmberg, Disha J Patel, Patrick Reidy, Austin Carter, Kelly Cercy, Abigail Chapin, Dirk Douwes-Schultz, Tahvi Frank, Falko Goettsch, Patrick Y Liu, Vishnu Nandakumar, Marissa B Reitsma, Vince Reuter, Nafis Sadat, Reed J D Sorensen, Vinay Srinivasan, Rachel L Updike, Hunter York, Alan D Lopez, Rafael Lozano, Stephen S Lim, Ali H Mokdad, Stein Emil Vollset, Christopher J L Murray

**Affiliations:** a Institute for Health Metrics and Evaluation, University of Washington, Seattle, WA, USA; b Department of Sociology, University of Washington, Seattle, WA, USA; c School of Public Health, University of California Los Angeles, Los Angeles, CA, USA; d School of Medicine, University of California Los Angeles, Los Angeles, CA, USA; e Baidu, Beijing, China; f OM1, Boston, MA, USA; g Department of Geography, University of British Columbia, Vancouver, BC, Canada; h Wellframe, Boston, MA, USA; i Memorial Sloan Kettering Cancer Center, New York, NY, USA; j School of Population and Global Health, University of Melbourne, Melbourne, VIC, Australia; k National Institute of Public Health, Cuernavaca, Mexico

## Abstract

**Background:**

Understanding potential trajectories in health and drivers of health is crucial to guiding long-term investments and policy implementation. Past work on forecasting has provided an incomplete landscape of future health scenarios, highlighting a need for a more robust modelling platform from which policy options and potential health trajectories can be assessed. This study provides a novel approach to modelling life expectancy, all-cause mortality and cause of death forecasts —and alternative future scenarios—for 250 causes of death from 2016 to 2040 in 195 countries and territories.

**Methods:**

We modelled 250 causes and cause groups organised by the Global Burden of Diseases, Injuries, and Risk Factors Study (GBD) hierarchical cause structure, using GBD 2016 estimates from 1990–2016, to generate predictions for 2017–40. Our modelling framework used data from the GBD 2016 study to systematically account for the relationships between risk factors and health outcomes for 79 independent drivers of health. We developed a three-component model of cause-specific mortality: a component due to changes in risk factors and select interventions; the underlying mortality rate for each cause that is a function of income per capita, educational attainment, and total fertility rate under 25 years and time; and an autoregressive integrated moving average model for unexplained changes correlated with time. We assessed the performance by fitting models with data from 1990–2006 and using these to forecast for 2007–16. Our final model used for generating forecasts and alternative scenarios was fitted to data from 1990–2016. We used this model for 195 countries and territories to generate a reference scenario or forecast through 2040 for each measure by location. Additionally, we generated better health and worse health scenarios based on the 85th and 15th percentiles, respectively, of annualised rates of change across location-years for all the GBD risk factors, income per person, educational attainment, select intervention coverage, and total fertility rate under 25 years in the past. We used the model to generate all-cause age-sex specific mortality, life expectancy, and years of life lost (YLLs) for 250 causes. Scenarios for fertility were also generated and used in a cohort component model to generate population scenarios. For each reference forecast, better health, and worse health scenarios, we generated estimates of mortality and YLLs attributable to each risk factor in the future.

**Findings:**

Globally, most independent drivers of health were forecast to improve by 2040, but 36 were forecast to worsen. As shown by the better health scenarios, greater progress might be possible, yet for some drivers such as high body-mass index (BMI), their toll will rise in the absence of intervention. We forecasted global life expectancy to increase by 4·4 years (95% UI 2·2 to 6·4) for men and 4·4 years (2·1 to 6·4) for women by 2040, but based on better and worse health scenarios, trajectories could range from a gain of 7·8 years (5·9 to 9·8) to a non-significant loss of 0·4 years (–2·8 to 2·2) for men, and an increase of 7·2 years (5·3 to 9·1) to essentially no change (0·1 years [–2·7 to 2·5]) for women. In 2040, Japan, Singapore, Spain, and Switzerland had a forecasted life expectancy exceeding 85 years for both sexes, and 59 countries including China were projected to surpass a life expectancy of 80 years by 2040. At the same time, Central African Republic, Lesotho, Somalia, and Zimbabwe had projected life expectancies below 65 years in 2040, indicating global disparities in survival are likely to persist if current trends hold. Forecasted YLLs showed a rising toll from several non-communicable diseases (NCDs), partly driven by population growth and ageing. Differences between the reference forecast and alternative scenarios were most striking for HIV/AIDS, for which a potential increase of 120·2% (95% UI 67·2–190·3) in YLLs (nearly 118 million) was projected globally from 2016–40 under the worse health scenario. Compared with 2016, NCDs were forecast to account for a greater proportion of YLLs in all GBD regions by 2040 (67·3% of YLLs [95% UI 61·9–72·3] globally); nonetheless, in many lower-income countries, communicable, maternal, neonatal, and nutritional (CMNN) diseases still accounted for a large share of YLLs in 2040 (eg, 53·5% of YLLs [95% UI 48·3–58·5] in Sub-Saharan Africa). There were large gaps for many health risks between the reference forecast and better health scenario for attributable YLLs. In most countries, metabolic risks amenable to health care (eg, high blood pressure and high plasma fasting glucose) and risks best targeted by population-level or intersectoral interventions (eg, tobacco, high BMI, and ambient particulate matter pollution) had some of the largest differences between reference and better health scenarios. The main exception was sub-Saharan Africa, where many risks associated with poverty and lower levels of development (eg, unsafe water and sanitation, household air pollution, and child malnutrition) were projected to still account for substantive disparities between reference and better health scenarios in 2040.

**Interpretation:**

With the present study, we provide a robust, flexible forecasting platform from which reference forecasts and alternative health scenarios can be explored in relation to a wide range of independent drivers of health. Our reference forecast points to overall improvements through 2040 in most countries, yet the range found across better and worse health scenarios renders a precarious vision of the future—a world with accelerating progress from technical innovation but with the potential for worsening health outcomes in the absence of deliberate policy action. For some causes of YLLs, large differences between the reference forecast and alternative scenarios reflect the opportunity to accelerate gains if countries move their trajectories toward better health scenarios—or alarming challenges if countries fall behind their reference forecasts. Generally, decision makers should plan for the likely continued shift toward NCDs and target resources toward the modifiable risks that drive substantial premature mortality. If such modifiable risks are prioritised today, there is opportunity to reduce avoidable mortality in the future. However, CMNN causes and related risks will remain the predominant health priority among lower-income countries. Based on our 2040 worse health scenario, there is a real risk of HIV mortality rebounding if countries lose momentum against the HIV epidemic, jeopardising decades of progress against the disease. Continued technical innovation and increased health spending, including development assistance for health targeted to the world's poorest people, are likely to remain vital components to charting a future where all populations can live full, healthy lives.

**Funding:**

Bill & Melinda Gates Foundation.

## Introduction

Health and social-services planning and investments require consideration of possible future trends in health and corresponding drivers. Many choices have long lag periods between initial investments and their effects, which can unfold over several decades; examples include training in different medical specialties, research and development for new drugs and vaccines, health system infrastructure construction, fiscal solvency of social security or health insurance, and policy implementation. Forecasts and alternative scenarios can help to frame these choices and to identify areas of more or less uncertainty. For instance, determining whether a country is likely to see more deaths from diabetes amid decreases in deaths from tuberculosis can serve as a crucial input to inform national policy dialogues and resource allocation. Quantitative forecasts of mortality and causes of death might be useful in this respect, particularly if they are linked to forecast and posited changes or alternative scenarios derived from the main independent drivers of population health. Such drivers include risk factors (eg, tobacco use, hypertension, air pollution, diet, and sanitation), interventions (eg, HIV treatment and vaccinations), and broader sociodemographic and health system factors.

Health forecasts, sometimes known as reference scenarios, aim to delineate the most likely future health trends. Forecast performance or accuracy can be empirically assessed by withholding data from recent periods and comparing forecasts generated without these data with what actually happened. Nonetheless, even forecasts based on models with a good out-of-time performance cannot anticipate all future drivers of health change. For example, no model from the 1970s would have forecast the HIV epidemic or, more recently, a cure for hepatitis C. Health forecasts are fundamentally imperfect, grounded only in what we know of the past and present; such forecasts can and should be supplemented with alternative scenarios that examine the universe of plausible futures. Alternative scenarios are particularly useful when they are linked to actionable choices that can affect different health drivers. A comprehensive forecast and scenarios framework for mortality and causes of death should possess two key attributes: good out-of-time forecast performance; and preservation of the causal effects for independent drivers and health outcomes that are consistent with known evidence from randomised controlled trials, and cohort or other observational studies.

Many disease-specific forecasts and some alternative scenarios have been published for particular countries or regions.^1^, ^2^, ^3^, ^4^, ^5^, ^6^, ^7^, ^8^ Likewise, demographers in governments and intergovernmental agencies routinely produce forecasts of all-cause mortality using models based on recent time trends.^3^, ^9^ Comprehensive forecasts of all-cause mortality and causes of death have occurred less often: two studies forecast the entire Global Burden of Diseases, Injuries and Risk Factors Study (GBD) cause list while another looked at large cause categories over time.^10^, ^11^, ^12^ Such work forecasted an anticipated continuation of the epidemiologic transition, with a global shift from communicable to non-communicable diseases (NCDs) as the predominant cause of burden. While comprehensive, these studies did not report on forecast performance and did not systematically account for causal pathways of health changes consistent with randomised controlled trials and cohort studies. Some country-specific forecasts show the alarming potential of rising obesity and subsequent declines in life expectancy, particularly driven by increased diabetes-related morbidity and mortality.^13^ As health and social service agencies face an increasing set of complex challenges (eg, population ageing, escalating expenses, and shortages in health-care providers), the value of robust forecasts and alternative scenarios that can chart probable health futures and options for modifying these trajectories rises in tandem.

**Research in context**

**Evidence before this study**

Health forecasts and alternative future scenarios can serve as vital inputs into long-term planning and investments in health, particularly in terms of framing different choices, their potential effects, and the relative certainty associated with each option. Past work to generate health-focused forecasts includes that from the UN Population Division, which routinely produces all-cause mortality forecasts through year 2100, and the Austrian Wittgenstein Center, which produces life expectancy forecasts with different scenarios to the end of the 21st century. Longer-range forecasts have also been developed to assess the potential effects of climate change on mortality. Furthermore, various national agencies produce country-level mortality forecasts, and forecasts for individual causes of death have been produced periodically as well. Comprehensive forecasts of cause-specific and all-cause mortality were developed as part of the Global Burden of Disease Study 1990 (GBD 1990); those methods were then applied for the period from 2002–30. The primary purpose of these past modelling efforts was to generate reference or baseline forecasts of what was likely to occur on the basis of past trends; however, few—if any—offered insights into a range of future scenarios while accounting for independent drivers of potential health changes.

**Added value of this study**

With this study, we provided a completely novel approach to forecasting all-cause and cause-specific mortality and scenario construction. Our modelling framework was designed to leverage the data on risk outcome relationships in the Global Burden of Diseases, Injuries, and Risk Factors Study 2016 such that the relationship between risk factors (eg, smoking) and specific disease outcomes (eg, lung cancer) were consistent with relevant cohort studies and randomised controlled trials. Because this forecasting framework is grounded in 79 independent drivers of health change, we could leverage these models to generate a full suite of alternative scenarios beyond reference forecasts. The overall model has three components: a model for risk-attributable deaths, a model for mortality not explained by risk factors that is a function of the Socio-demographic Index (SDI) and other covariates depending on the cause and time, and an autoregressive integrated moving average process for the unexplained latent trends for each location-age-sex-cause. To ensure the robustness of this framework, we assessed the overall model performance by fitting it to data from 1990–2006 and evaluating its forecasts for 2007–2016. Our model performed better than other widely used methods such as Lee-Carter. Based on our model, we generated reference scenarios for all-cause mortality, life expectancy, and 250 causes of death for 195 countries and territories from 2016–40. Additionally, we assessed better health and worse health scenarios for each metric by country, reflecting the 85th and 15th percentiles, respectively, of the annualised rates of change observed for the drivers across locations in the past.

**Implications of all the available evidence**

Our reference forecast predicted continued declines in global mortality and improvements in life expectancy, though at a slower rate than achieved in the past. Because of faster progress among many lower-SDI countries, absolute disparities between countries are currently projected to narrow by 2040. Nonetheless, the differences between better health and worse health scenarios for 2040 remain substantial, emphasising that the reference forecast is not inevitable and thus policy choices made today can profoundly affect each country's future health trajectories. For most countries, prioritising non-communicable diseases (NCDs) and NCD-related risks in health planning and investment decisions has the potential to markedly reduce premature mortality by 2040. Although NCDs were projected to rise in many low-SDI countries, communicable, maternal, neonatal, and nutritional diseases are likely to remain among the leading causes of early death. Furthermore, based on our 2040 worse health scenario, rebounds in HIV mortality and thus reversals in life expectancy could occur if countries cannot maintain the gains achieved in the past. Continued technical innovation and increased health spending, both domestic and international funding targeted to countries with the most need, are crucial for a future where all people have the opportunity to live full, healthy lives.

The GBD provides a unique resource from which a new generation of health forecasts with alternative scenarios can be developed.^14^, ^15^ GBD has refined the collection and standardisation of detailed health and risk data over several recent publication cycles and now covers, from 1990–2016, 195 countries and territories with data on cause-specific and all-cause mortality, risk factors, and selected interventions.^16^, ^17^ The GBD comparative risk assessment, with its meta-analyses of published studies (ie, randomised trials and cohort and other observational data) on risk-outcome causal relationships, enables the modelling of future disease burden while accounting for drivers of health change.^16^ Additionally, an overall measure of development—the Socio-demographic Index (SDI)—was developed as part of GBD, providing a mechanism for assessing the effects of improved development on health. Drawing from the broader GBD study, we have developed a novel forecasting model, producing reference forecast, better health scenario, and worse health scenario for life expectancy, all-cause mortality, and cause-specific mortality from 250 causes from 2017–40 in 195 countries and territories. By leveraging the relationships between independent drivers of health captured within GBD, we provide a robust platform from which alternative scenarios can be assessed, which could be vital inputs for strategies and investments to improve population health.

## Methods

### Overall forecasting model structure

We modelled 250 causes and cause groups organised by the GBD hierarchical cause structure, using GBD data from 1990–2016 to generate predictions from 2017–2040. Predictions were made for 195 countries and territories modelled in GBD 2016, referred to as locations in this study. For 246 of these causes, we developed a three-component model of cause-specific mortality: a component explained by changes in behavioural, metabolic, and environmental risks, and select interventions quantified in GBD; a component explained by income per person, educational attainment, and total fertility rate under 25 years, which was combined into the SDI metric, and time; and an autoregressive integrated moving average (ARIMA) model to capture the unexplained component correlated over time.^18^ We summarise the overall model and individual components below; further detail and model formulae are in appendix 1 (pp 7–31).

The model's main component captured the prevalence for 65 risk factors reported in GBD 2016 and the relative risks (RRs) between levels of risk exposure and each GBD outcome. GBD 2016 reported RRs for each risk-outcome pair based on meta-analyses of randomised trials and cohort studies.^16^ Details, including how risks operate through each other such as body-mass index (BMI) through systolic blood pressure and cholesterol, are provided elsewhere (appendix 1, pp 12–21). Interventions quantified in GBD currently include antiretroviral therapy (ART) for people living with HIV, prevention of mother-to-child transmission of HIV, met need for family planning with modern contraception methods, and vaccination coverage of diphtheria, tetanus, pertussis, (three doses) and measles; pneumococcal conjugate vaccine; and vaccination coverage of rotavirus; and *Haemophilus influenzae* type B. We refer to these risks, interventions, and measures of development as independent drivers, a term that originates in regression terminology and does not imply that the drivers are independent of each other. For instance, we used SDI rather than its individual components because of their strong correlations with each other.

Our model's second component captured relationships between variations in each cause of death that were not explained by risks and interventions (ie, underlying mortality rate) to three variables reflective of overall development: income per person, educational attainment among populations aged 15 years and older, and total fertility rate under 25 years.^18^ Because of high co-linearity between these three factors, we used SDI, which combines all three into a single measure.^17^ For some causes, other independent variables with strong known relationships for which data were available (ie, age-specific fertility for maternal causes, HIV mortality for maternal HIV, and vehicles per person for road injuries) or risks which could not be quantified in terms of RR because they are part of the disease definition (eg, systolic blood pressure for hypertensive heart disease, fasting plasma glucose [FPG] for diabetes, and alcohol consumption for alcohol-related cirrhosis; other risks are in appendix 1 [p 14]) were added as additional model covariates. For these two model components, we specified the following relationship between the logarithm of cause-specific mortality and drivers of mortality:

$$\begin{array}{c} \log\left(m_{T}\right)∼N\left(\hat{y},\sigma \right)\\ \hat{y}=\alpha_{1\text{a}}+\beta_{0}{\text{SDI}}_{<0.8}+\beta_{1}{\text{SDI}}_{\geq \text{0}\text{.8}}+\theta_{a}t+\log\left(§\right) \end{array}$$

where α_la_ is a location-age-specific intercept, β_0_ is a global effect on an SDI of less than 0·8, β_1_ is a global effect on an SDI of more than 0·8, θ_a_ is an age-specific secular trend, and § is the scalar capturing the effects of all relevant risks or interventions on each cause, with separate models fitted for each cause-sex pair. Thus, on the log scale we modelled the total cause-specific mortality rate (*m*_T_) as the sum of the underlying mortality captured by SDI, the secular trend, and a scalar component that captures the effect of relevant risks or interventions for that cause. We used a broken stick (linear piecewise) spline on SDI (one piece for SDI between 0 and 0·8, another piece for 0·8 to 1·0) to account for rates of change in underlying mortality among high-SDI countries. For several NCDs (eg, diabetes and stroke), for which mortality has substantially decreased in many high-SDI countries, we also included an SDI*time interaction effect; more detail is in appendix 1 (pp 9–12).

Our model's third component captured variation over time that was not explained by independent drivers included in the first two components. This was achieved by fitting ARIMA models to the residual trends that remained from the first two components. Appendix 1 details the ARIMA specifications used and outlines how the ARIMA component was organised hierarchically (ie, a more robust residual trend on all-cause mortality was used to constrain the more sensitive cause-specific residual trends; appendix 1, pp 10–12).

We developed separate models for some causes of death because of their unique nature, largely following GBD methods.^17^ For deaths from stochastic events such as conflict and natural disasters, we randomly sampled past death rates from 1950–2016 for location, age group, and sex 1000 times for each year in the future and then applied an SDI-adjustment factor derived from by how much increases in SDI were associated with reducing mortality from such events in the past. For HIV mortality, we sought to account for its high sensitivity to changes in intervention coverage by projecting incidence hazard, ART, prevention of mother-to-child transmission of HIV, and co-trimoxazole coverage. These forecasts were informed by a frontier analysis to predict ART prices^19^ and translating predicted funding into expected treatment coverage. Incidence hazards were generated for each scenario using a rate of change approach across high-prevalence and low-prevalence countries with additional discounting from forecasted ART coverage (appendix 1, pp 32–40). We then used these inputs and scenarios in Spectrum,^20^, ^21^, ^22^ a cohort component model used by GBD and UNAIDS that applies disease progression parameters to an age-specific and sex-specific population over time; these results provided a full time series of HIV mortality by location through 2040.

We calculated the years of life lost (YLLs)—a measure of premature mortality—by summing up the remaining life expectancy for people dying in each age group. For GBD 2016, the reference life expectancy at birth was 86·6 years, derived from the lowest observed risk of death for each 5-year age group; to avoid problems associated with small numbers, we restricted this calculation to all populations greater than 5 million individuals in 2016. Age-standardised mortality rates and YLL rates were computed using the world standard population developed for the GBD study, which is a time-invariant standard.^17^

### Reference forecasts, and better health and worse health scenarios

For each independent driver—65 risk factors, select interventions, income per person, educational attainment, and total fertility rate under 25 years—we developed reference forecasts through 2040 and two alternative scenarios: better health and worse health. These scenarios corresponded with the relative effect of these drivers on health outcomes; they did not necessarily reflect societal valuations of other non-health consequences. Below, we summarise the models used to forecast independent drivers of health; more details are in appendix 1 (pp 15–31).

We produced better health and worse health scenarios by taking the 85th and 15th percentiles, respectively, of annualised rates of change (ARC) observed across all locations and years in the past and constructed hypothetical future scenarios to show what would happen if each place had that level of change in the future. In cases where the reference scenario was higher than the 85th percentile or lower than the 15th percentile, the reference was used as the better health or worse health scenario, respectively. Further computational details regarding reference forecasts and scenarios are in appendix 1 (p 20).

### Modeling the independent drivers of health

GBD 2015 introduced SEVs, a univariate measure of RR-weighted prevalence of risks that allowed all risk exposures, whether dichotomous, polytomous, or continuous, to be reported on a 0–1 scale.^24^ For SEVs, 0 indicates that no one in the population is at increased risk and 1 indicates that the whole population has levels of exposure associated with the highest risk. We forecast SEVs for each risk by location, age, and sex using the weighted mean of its past ARC, and projecting these rates into the future. These weights allow for more recent trends to be more heavily weighted in forecasts; weight selection was based on out-of-sample predictive validity (appendix 1, pp 16–20). For each cause of death, we combined relevant risks to calculate the population-level RR or scalar, then multiplied this value by the underlying mortality rate in the future (appendix 1, pp 13–15).

We produced reference forecasts for income per person by testing an ensemble pool of 11 520 individual models from 1970–2017, each of which captured relationships between the annual log growth rate in income and a different combination of demographic indicators, time-series components, and weighting functions.^23^ This ensemble pool and its corresponding model selection are described in more detail in appendix 1 (pp 26–28). We generated better health and worse health scenarios by scaling the growth rate to the 85th and 15th percentiles of the residuals from a model of log growth rate against income level, which captured plausible variation in income growth.

Data for mean years of education and the proportion of women of reproductive age who had their need for family planning satisfied with modern methods (ie, contraceptive met need) were from GBD 2017 and were forecasted from the weighted mean of their past ARC in a similar framework as the SEVs for risk factors (appendix 1, pp 28–30).

For age-specific fertility, we first modelled fertility in women aged 20–24 years based on met need for contraception and women's educational attainment in that age group, plus an ARIMA to capture recent latent trends in each location. We modelled fertility in other age groups (10–14 and 15–19 years, and 5-year bins from 25–49 years) based on the age-specific fertility of the 20–24 year-olds, accounting for possible non-linear relationships and age-specific contraceptive met need where applicable. We combined estimates of age-specific fertility to calculate the total fertility rate under 25 years and produced better and worse health scenarios of each fertility metric based on corresponding better and worse health scenarios of estimated female education and met need for family planning (appendix pp 30–32). We forecasted population by using our all-cause mortality and age-specific fertility forecasts as inputs to a cohort component model. Initial population in 2016 and annual net migration projections were derived from UN Population Division estimates.^9^

### Vaccine coverage

We forecasted coverage for the diphtheria, tetanus, and pertussis vaccine, and the measles-containing vaccine, 1 dose using linear models with SDI as an independent variable. *H influenzae* type B vaccine, pneumococcal conjugate vaccine, and rotavirus vaccines, which have not yet been introduced in all countries, were modelled relative to third-dose diphtheria, tetanus, and pertussis vaccine, and were assumed to scale up to this ratio with this vaccination coverage for each country over time. For countries with known introduction dates, we ran spatiotemporal Gaussian process regression on the ratio of vaccine coverage to third-dose diphtheria, tetanus, and pertussis vaccine coverage, mirroring GBD methods.^16^, ^17^, ^25^ For the other countries, we simulated introduction dates based on a Weibull distribution and generated theoretical scale-up curves for each year (appendix 1 pp 22–24).

### Model validation and forecasting analysis

We assessed the overall performance of the forecasting and scenarios framework by fitting models using only data from 1990–2006 and then forecasting from 2007–16. Out-of-sample validation forecasts for 2014–16 were then compared with observed data for 2014–16. We assessed model performance using two metrics: mean error (a measure of bias) and root-mean-squared error (a measure of accuracy). Appendix 1 provides these metrics for life expectancy, all-cause mortality, and cause-specific mortality (pp 43–59). Our model was better at forecasting all-cause mortality and life expectancy than the most-widely used demographic approach, the Lee-Carter method.^26^ To generate forecasts, we re-fit the model for the entire period of 1990–2016.

### Uncertainty analysis

We sought to propagate uncertainty from both model parameters and inputs throughout our estimation process. We sampled correlated draws of each parameter from the variance-covariance matrix of each fitted model when generating predictions. We incorporated uncertainty from the GBD by predicting each independent driver and cause-specific mortality rate in 2016, finding the difference between predicted draws and GBD 2016 draws in log space, then adding that correction factor to 2017–40 forecasts at the draw level. Point estimates were computed as the mean of 1000 draws from the final draw distribution and 95% uncertainty intervals (UIs) from the 2·5 and 97·5 percentiles.

### Role of the funding source

The funder of the study had no role in study design, data collection, data analysis, data interpretation, or writing of the report. The corresponding author had full access to all the data in the study and had final responsibility for the decision to submit for publication.

## Results

### Global trends and patterns through 2040

**Figure 1:**
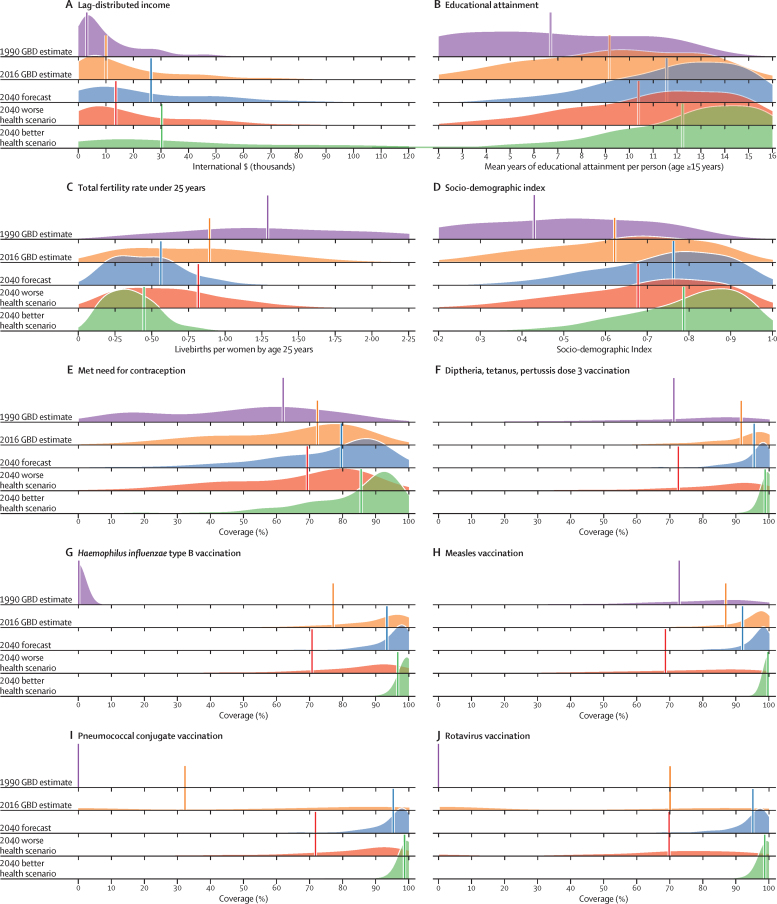

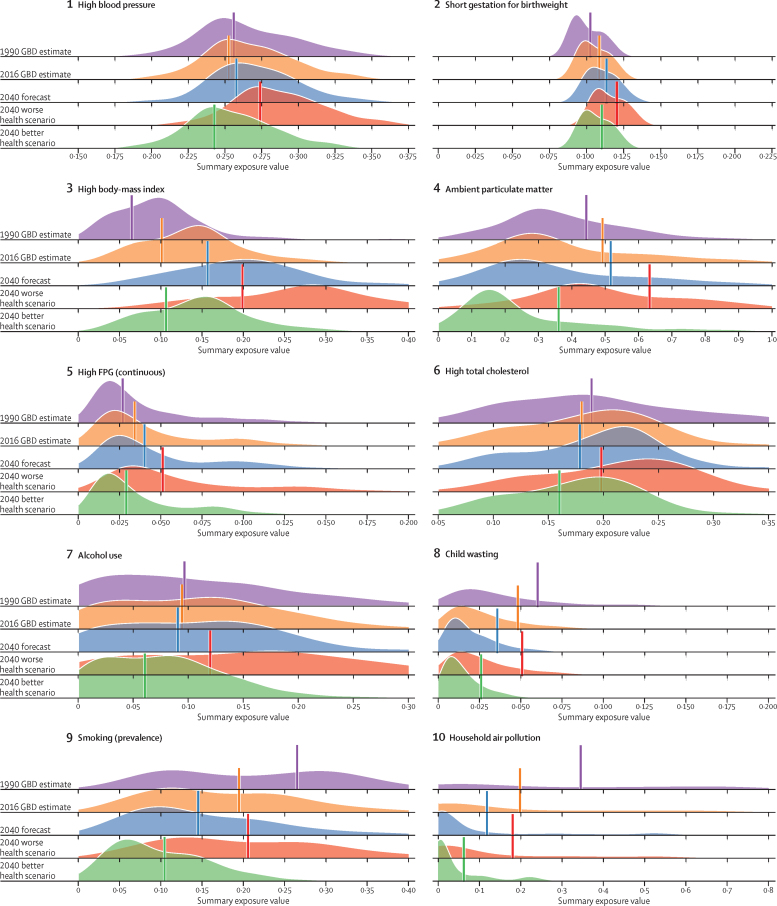

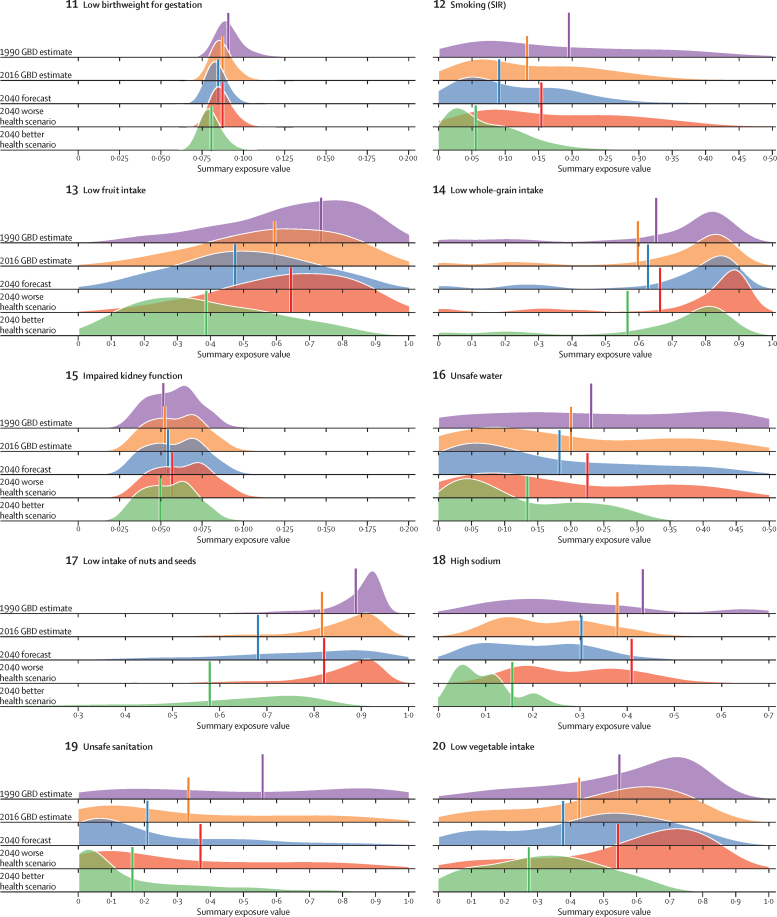
Global distribution across countries of the most influential drivers of health Figure shows: (A) lag-distributed income, (B) educational attainment, (C) total fertility rate under 25 years, (D) Socio-demographic Index, (E) met need for contraception, (F) diphtheria, tetanus, pertussis dose 3 vaccination, (G) *Haemophilus influenzae* type B vaccination, (H) measles vaccination, (I) pneumococcal conjugate vaccination, and (J) rotavirus vaccination. Colours show distinction between year estimates in the past and scenarios in the future. Data are the 1990 Global Burden of Disease Study (GBD) estimate, the 2016 GBD estimate, the 2040 forecast, the 2040 better health scenario, and the 2040 worse health scenario. Shown are the top 20 risks ranked from 1–20 by the number of risk-attributable years of life lost in 2016, and ordered horizontally, across rows. The estimate for each country is the age-standardised mean value across both sexes, and a Gaussian kernel density estimator produced a distribution from the estimates for all countries. Vertical lines represent the global population-weighted average, whereas the density distribution gives each of the 195 countries equal weight. FPG=fasting plasma glucose. SIR=smoking impact ratio.

The course of future all-cause and cause-specific mortality largely will be determined by how trends in key drivers unfold (figure 1). Most of these drivers were projected to improve, whereas 36 were forecast to worsen by 2040. Uneven distributions of progress for drivers such as income per person and education suggested that many locations are unlikely to see conditions improve based on current forecasts. For instance, by 2040, 31 countries were forecast to be still classified as low-income (ie, income per person less than US$1000, per the World Bank), while 36 countries averaged less than 10 years of education among populations aged 25 years and older. The better health scenario highlighted the potential for accelerated progress; nonetheless, eight countries would remain low-income and 31 would average less 10 years of education under this scenario.

With increasing SDI, many countries were projected to see rising metabolic and behavioural risks, including some dietary risks, alcohol, and smoking. Because high BMI has increased almost everywhere since 1990, its continued rise was forecasted through 2040—even in the better health scenario. Similarly, high FPG and systolic blood pressure were projected to increase through 2040; however, because of advances in treatment and expansion of NCD risk management in some countries, the better health scenario reflected the future potential for reducing these metabolic risks. By contrast, continued decreases in risks that generally improve alongside gains in development (eg, low birthweight, child malnutrition, household air pollution, unsafe water and sanitation) were forecasted for the reference and better health scenarios; at the global level, the 2040 worse health scenario ranged from similar levels in 2016 to moderately higher levels of risk exposure. Country-level smoking trends were markedly variable in the past, corresponding with where countries are on the tobacco-epidemic curve and in the scale-up of tobacco control;^27^ subsequently, our better and worse health scenarios showed comparably varied future trajectories. In 2016, the age-standardised prevalence of smoking over age 30 years was 19·4% (95% UI 19·2–19·6) globally, with a reference forecast of 14·5% (14·3–14·7) in 2040, and the better health scenario showed the potential to reduce prevalence to 10·4% (10·3–10·6). Conversely, the worse health scenario portended global stagnation—or potential reversal—for progress against smoking, with a projected age-standardised prevalence of 20·6% (20·3–20·8) in 2040. Globally, ambient particulate matter increased from 1990–2016, but country-level trends showed widening inequalities in levels of exposure, with some countries achieving substantial declines.^28^, ^29^ As a result, our reference forecast showed similar levels of exposure in 2040 as in 2016, whereas our better health scenario showed the potential for substantial reductions.

**Figure 2:**
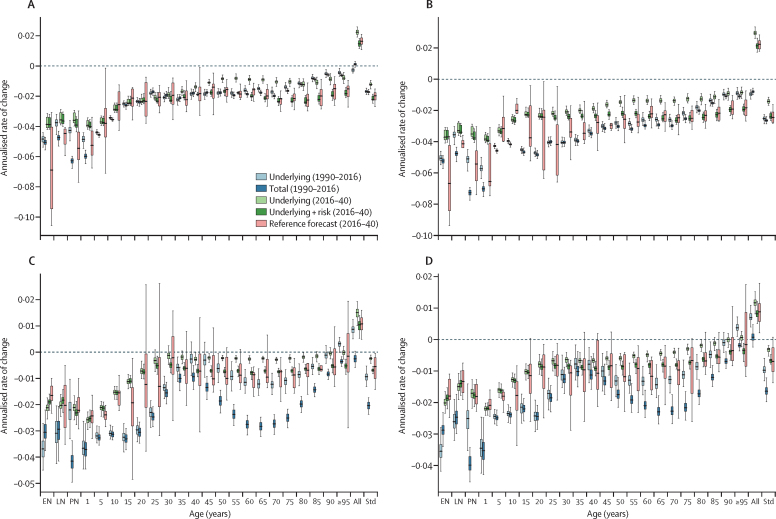
The three model components for China and Australia by age and sex: (A) China, male, all causes; (B) China, female, all causes; (C) Australia, male, all causes; (D) Australia, female, all causes. Figure shows the annualised rates of change (ARCs; errors bars represent 95% UI) for deaths from 1990 to 2016 broken down into the ARC for the underlying death rate and total death rate, and the ARC for the reference scenario for 2016 to 2040 by underlying rate, the underlying rate plus risk attributable mortality, and the underlying rate plus risk attributable mortality plus the autoregressive integrated moving average (ARIMA) component (reference scenario). The ARC is measured in terms of all-cause mortality by location, and rates of change are shown by age group on the x-axis. Rates of change are calculated from 2016 to 2040 in the forecasts and from 1990 to 2016 on past GBD estimates. GBD=Global Burden of Disease Study. EN=early neonatal. LN=late neonatal. PN=post-neonatal. Std=age standardised.

Figure 2 shows examples of all-cause mortality modelling for men and women in China and Australia. For each country and age group, we compared ARCs for underlying and total death rates observed 1990–2016, and then ARCs for predicted death rates from 2017–40 based on the three model components. In China, past ARCs for underlying mortality nearly equalled ARCs for total mortality in most age groups, suggesting that the net effects of risk factors and select interventions were not a large contributor to past progress in China. Conversely, underlying plus risk factor mortality ARC was faster in the future than the ARCs in underlying alone, particularly among adult age groups; this indicates risk factors could have a larger effect on future changes in China's total mortality. Compared with China, different patterns emerged for Australia; for instance, among populations older than 50 years, past ARCs were fuelled partly by favourable risk factor trends, as shown by faster declines in total versus underlying mortality. From 2016–40, Australia's underlying mortality rate was projected to decline less rapidly because of slowed increases in SDI and relative stagnation of risk trends with respect to the all-cause death rate. As with China, the ARIMA component contributed to widening UIs substantially for each age group.

**Figure 3:**
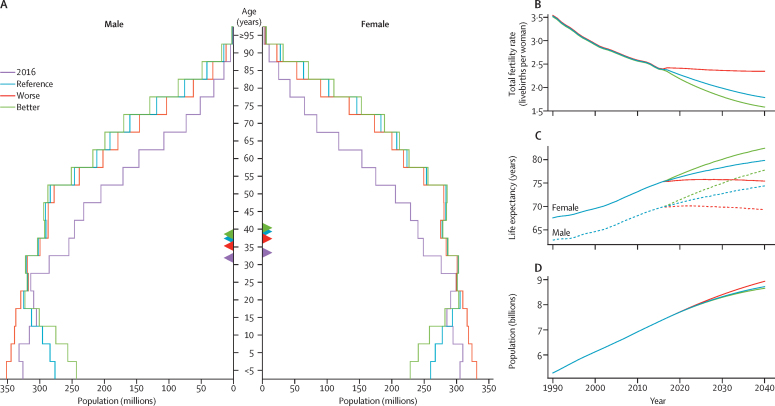
Global distribution of population in 2016 and 2040 reference forecasts, 2040 better health scenario, and 2040 worse health scenario Data are shown (A) by age and sex and by (B) total fertility rate, (C) life expectancy, and (D) population. Triangles within the population pyramid represent the mean age globally for males and females for corresponding years and forecasts. Inlays show total population forecasts, and associated inputs into the population forecast: fertility and life expectancy.

Based on reference forecasts and scenarios from 2016–40 (figure 3), a range of global demographic trends have the potential to unfold. Although total population was projected to increase in each trajectory, estimates spanned from 8·7 billion (95% UI 8·5–8·9) in 2040 under the better health scenario (a 17·2% increase since 2016) to 9·0 billion (8·7–9·2) under the worse health scenario (a 21·0% rise since 2016). This range was primarily driven by differences in forecasted total fertility rate, which would essentially stagnate from 2016–40 under the worse health scenario, and subsequent effects in population age structure. Compared with 2016, all three scenarios point to a much larger proportion of the overall population older than 10 years; it is projected to increase from 82·8% in 2016 to 84·8% (95% UI 84·0–85·5) under the worse health scenario, 87·5% (86·8–88·0) in the reference scenario, and 88·7% (88·2–89·2) in the better health scenario. However, under the age of 25, vastly different population patterns could emerge by 2040. The reference and better health scenarios each showed declines in total population under age 10, from 1·3 billion in 2016 to 1·1 billion (1·0–1·2) and 1·0 billion (0·9–1·1) in 2040, respectively. Conversely, the population under age 10 years in the worse health scenario was expected to increase to 1·4 billion (1·3–1·5) by 2040. This range reflects the effect of forecasted fertility and the potential vast differences in population structure under different scenarios.

Global life expectancy was projected to increase by 4·4 years (95% UI 2·2–6·4) for men and 4·4 years (2·1–6·4) for women by 2040, to 74·3 years (72·1–76·4) and 79·7 years (77·4–81·8), respectively. The better health scenario showed a potential increase of 7·8 years (5·9–9·8) and 7·2 years (5·3–9·1) for male and female life expectancy, rising to 77·8 years (75·7–79·7) for men and 82·5 years (80·5–84·5) for women in 2040. The worse health scenario had both male and female life expectancy plateauing through 2040 (ie, a non-significant loss of 0·4 years [–2·8 to 2·2] for men and a non-significant decrease of 0·1 years [–2·7 to 2·5] for women, to 69·5 years [67·1–72·1] and 75·2 [72·6–78·0], respectively). Compared with the past, global progress in extending life expectancy was forecasted to be slower from 2016–40. This trend resulted from forecasts of slowed advances on key drivers such as SDI; worsening of several risks, particularly high BMI; and stagnated gains on cardiovascular diseases, which was a major factor in historical improvements in life expectancy.

Reference forecasts showed a 37·6% increase (95% UI 22·7–54·0) in total deaths by 2040 (table), rising from 54·7 million in 2016 to 75·3 million (67·3–83·9) in 2040. By contrast, projected total YLLs had a non-significant drop, reflecting the effects of aging and projected life expectancy gains. By 2040, the reference forecast showed that 12·0% (9·3–16·3) of global deaths were due to communicable, maternal, neonatal, and nutritional (CMNN) diseases; 81·0% (75·5–84·4) to NCDs; and 7·0% (5·9–8·0) to injuries. Better and worse health scenarios both showed a continued global transition from CMNN to NCDs, yet how this shift could occur varied considerably across scenarios. For instance, in the better health scenario, 11·3% (9·0–15·7) of deaths and 18·9% (15·4–23·0) of YLLs were from CMNN causes. The worse health scenario predicted 14·9% (11·6–20·0) of deaths and 27·6% (22·9–33·2) of YLLs from CMNN causes in 2040.

**Table:** Number of global deaths and years of life lost (YLLs) in GBD 2016, and in the 2040 forecast, better health, and worse health scenarios for all causes

			**YLLs (thousands)**				**Deaths (thousands)**			
			2016	2040 forecast	2040 better health scenario	2040 worse health scenario	2016	2040 forecast	2040 better health scenario	2040 worse health scenario
**All causes**			**1 585 864·98 (1 559 572·96–1 613 799·53)**	**1 529 212·73 (1 315 165·66–1 744 642·71)**	**1 188 899·29 (1 021 216·03–1 357 596·47)**	**2 170 421·82 (1 858 433·76–2 480 811·12)**	**54 698·58 (54 028·68–55 514·89)**	**75 263·26 (67 310·21–83 866·49)**	**62 570·88 (55 675·51–70 026·74)**	**96 717·00 (86 181·44–107 125·85)**
**Communicable, maternal, neonatal, and nutritional diseases**			**566 351·51 (544 844·20–589 177·03)**	**327 915·23 (259 161·31–411 573·26)**	**224 435·34 (178 473·79–284 556·33)**	**598 656·74 (477 682·48–738 597·66)**	**10 557·99 (10 097·72–11 143·43)**	**9045·20 (7140·61–12 255·51)**	**7062·37 (5620·93–9774·50)**	**14 412·69 (11 287·00–19 346·12)**
	**HIV/AIDS and tuberculosis**		**94 262·23 (91 006·54–97 422·77)**	**61 740·92 (52 484·54–72 255·86)**	**40 791·02 (32 831·31–49 852·17)**	**159 169·97 (127 603·05–198 280·29)**	**2246·81 (2172·76–2314·46)**	**1615·03 (1382·99–1895·90)**	**1097·66 (921·12–1295·95)**	**3693·73 (2974·48–4535·38)**
	Tuberculosis		40 718·82 (38 983·48–42 538·24)	24 447·59 (19 148·30–33 027·79)	15 420·20 (12 129·08–20 306·06)	41 289·76 (31 424·17–57 573·96)	1213·06 (1161·55–1265·42)	873·38 (682·40–1152·23)	592·71 (476·39–758·25)	1390·68 (1053·96–1839·04)
	HIV/AIDS		53 543·41 (50 984·67–56 292·03)	37 293·33 (30 627·08–43 943·07)	25 370·81 (18 112·95–33 054·78)	117 880·21 (88 380·31–154 595·33)	1033·75 (987·35–1081·57)	741·65 (622·58–861·74)	504·95 (391·05–622·73)	2303·05 (1693·81–3021·48)
	**Diarrhoea, lower respiratory, and other common infectious diseases**		**209 304·89 (195 330·83–228 343·14)**	**138 761·43 (93 594·79–213 267·31)**	**93 032·67 (63 217·08–145 739·80)**	**238 083·51 (156 241·30–360 986·16)**	**4805·16 (4381·23–5480·60)**	**5298·39 (3703·14–8351·15)**	**4259·99 (3011·67–6855·53)**	**7704·67 (5126·24–12563·43)**
Diarrhoeal diseases			66 908·74 (56 202·72–85 858·53)	40 524·03 (14 762·73–101 647·27)	28 021·71 (9794·70–76 730·50)	71 076·51 (26 699·92–179 056·22)	1655·94 (1244·07–2366·55)	1611·38 (482·52–4664·63)	1339·44 (429·02–3975·25)	2694·30 (755·15–7522·05)
Intestinal infectious diseases			10 476·49 (5 926·65–17 188·46)	5701·35 (3107·03–9562·97)	5206·44 (2904·21–8598·60)	7 481·47 (4 010·80–12 801·64)	155·45 (87·59–255·41)	95·71 (52·70–159·94)	89·75 (50·46–148·62)	119·73 (64·59–205·69)
	Typhoid fever		8729·56 (4775·28–14 334·44)	4569·82 (2478·06–7700·70)	4207·58 (2336·90–6992·10)	5975·76 (3159·86–10 219·72)	128·17 (70·08–210·19)	75·35 (41·37–126·60)	71·21 (39·51–117·74)	93·95 (50·61–158·55)
	Paratyphoid fever		1596·58 (750·49–3096·66)	1076·47 (490·50–2096·87)	964·15 (444·02–1850·37)	1412·76 (646·14–2803·89)	25·19 (11·75–49·24)	19·36 (8·89–37·78)	17·81 (8·23–34·18)	24·30 (11·23–47·93)
	Other intestinal infectious diseases		150·35 (40·76–410·64)	55·05 (14·42–158·56)	34·71 (9·70–98·90)	92·94 (23·58–268·24)	2·09 (0·63–5·46)	0·99 (0·32–2·53)	0·73 (0·24–1·76)	1·49 (0·45–3·89)
Lower respiratory infections			91 363·09 (84 223·22–97 870·27)	68 700·69 (47 070·49–95 459·61)	43 223·40 (31 094·43–57 744·00)	120 751·92 (78 575·87–169 498·36)	2377·70 (2145·58–2512·81)	3120·44 (2448·88–3873·80)	2442·72 (1975·59–2949·52)	4244·98 (3153·62–5294·84)
Upper respiratory infections			126·68 (105·23–154·86)	80·17 (61·87–105·86)	51·80 (39·82–65·67)	136·50 (104·57–178·61)	2·31 (2·04–2·65)	1·97 (1·66–2·36)	1·43 (1·17–1·74)	2·98 (2·46–3·62)
Otitis media			50·36 (37·49–72·37)	13·59 (8·94–20·10)	13·61 (8·94–20·27)	14·05 (9·34–20·76)	1·08 (0·84–1·47)	0·50 (0·34–0·70)	0·54 (0·36–0·75)	0·46 (0·31–0·65)
Meningitis			20 383·03 (16 781·47–26 724·06)	12 372·35 (8642·93–18 279·73)	9346·04 (6723·01–12 956·00)	20 279·99 (12 993·78–31 788·31)	318·40 (265·22–408·71)	238·61 (177·78–340·30)	203·01 (154·23–286·29)	330·65 (234·15–489·14)
	Pneumococcal meningitis		1268·37 (996·16–1721·54)	739·19 (516·71–1074·29)	627·21 (449·45–885·63)	1036·47 (694·55–1534·35)	23·14 (18·70–30·93)	18·10 (13·43–26·28)	17·05 (12·75–24·90)	21·72 (15·81–32·20)
	*Haemophilus influenzae* type B meningitis		2177·50 (1723·91–2955·18)	2973·90 (979·79–6864·46)	1418·74 (633·61–2857·77)	8137·38 (2178·25–18 545·53)	31·41 (25·37–41·43)	41·39 (17·21–87·12)	23·63 (13·54–41·45)	101·17 (31·39–222·98)
	Meningococcal meningitis		8159·59 (6630·42–10 743·55)	4046·14 (2959·09–5655·49)	3430·39 (2520·21–4817·57)	5143·10 (3722·39–7248·47)	127·42 (105·43–164·01)	84·70 (64·28–122·35)	77·20 (58·18–112·99)	97·61 (73·46–138·20)
	Other meningitis		8777·57 (7123·54–11 853·65)	4613·11 (3358·62–6281·61)	3869·69 (2808·40–5253·24)	5963·03 (4319·87–8332·46)	136·42 (112·68–178·02)	94·43 (71·84–128·98)	85·12 (64·95–118·61)	110·13 (83·98–149·09)
Encephalitis			5053·33 (4020·10–6845·05)	3952·14 (2945·46–5493·69)	3666·49 (2662·00–5292·85)	4877·10 (3706·02–6756·93)	102·87 (83·93–138·39)	129·64 (98·07–184·26)	129·98 (97·00–187·56)	138·68 (108·44–194·11)
Diphtheria			86·90 (62·45–123·42)	37·30 (18·76–68·20)	14·11 (9·10–22·06)	60·55 (32·52–104·75)	1·11 (0·82–1·54)	0·49 (0·27–0·85)	0·21 (0·15–0·31)	0·76 (0·42–1·29)
Whooping cough			6 170·82 (3287·59–10 666·24)	4 754·38 (2208·32–8636·49)	2080·42 (931·29–3736·91)	8243·37 (3998·94–14 647·80)	73·01 (38·90–126·14)	56·42 (26·33–102·48)	24·93 (11·21–44·69)	97·52 (47·47–173·05)
Tetanus			2362·79 (1440·70–3057·88)	465·28 (268·52–739·51)	318·63 (178·66–524·95)	815·99 (456·69–1266·35)	36·69 (22·20–47·21)	7·68 (4·56–11·64)	6·10 (3·42–9·67)	11·59 (6·86–17·24)
Measles			5702·64 (2133·93–12 239·05)	1879·97 (514·03–5457·86)	884·76 (334·63–2351·01)	3889·69 (880·91–10 481·31)	68·12 (25·50–146·06)	26·02 (8·35–68·90)	13·39 (5·65–30·74)	51·03 (14·05–129·25)
Varicella and herpes zoster			620·02 (557·00–693·29)	280·20 (219·32–345·09)	205·28 (160·73–255·27)	456·40 (350·67–558·36)	12·47 (11·38–13·95)	9·53 (7·94–11·31)	8·50 (7·14–10·13)	11·99 (9·92–13·99)
Neglected tropical diseases and malaria			**61 330·01 (50 832·05–73 173·51)**	**29 561·40 (22 780·21–36 729·59)**	**24 157·32 (18 726·60–29 742·99)**	**37 825·85 (29 076·49–47 538·20)**	**843·59 (708·02–988·97)**	**574·29 (447·63–696·86)**	**516·05 (400·92–622·13)**	**667·01 (519·06–806·19)**
Malaria			54 460·50 (44 151·02–66 240·10)	19 913·82 (14 344·62–25 844·54)	15 475·13 (11 573·05–19 663·19)	26 062·22 (18 939·87–34 396·98)	719·55 (594·61–863·03)	332·65 (252·96–419·06)	281·88 (219·50–354·17)	402·52 (306·81–508·98)
Chagas disease			156·13 (146·16–168·66)	96·37 (82·15–116·78)	118·95 (103·62–142·79)	84·88 (72·45–104·49)	7·14 (6·74–7·77)	5·54 (4·75–6·69)	7·02 (6·13–8·29)	4·75 (4·02–5·81)
Leishmaniasis			705·85 (398·32–1 204·23)	260·41 (150·91–442·31)	215·54 (123·60–371·18)	473·69 (285·65–780·94)	13·67 (7·66–23·00)	5·28 (3·08–9·41)	4·51 (2·59–8·22)	8·73 (5·28–15·02)
African trypanosomiasis			126·51 (63·55–212·07)	16·00 (6·76–32·43)	15·65 (6·65–31·17)	17·11 (7·18–34·40)	2·29 (1·16–3·83)	0·30 (0·13–0·61)	0·30 (0·13–0·59)	0·32 (0·14–0·63)
Schistosomiasis			367·36 (333·89–401·84)	287·47 (239·53–349·08)	245·88 (203·86–301·41)	333·76 (280·73–405·68)	10·09 (9·26–10·97)	8·93 (7·73–10·47)	7·97 (6·84–9·36)	9·90 (8·62–11·55)
Cysticercosis			47·24 (39·82–56·34)	21·08 (16·21–27·34)	19·26 (14·60–25·04)	22·91 (17·64–29·57)	1·00 (0·86–1·17)	0·51 (0·40–0·64)	0·48 (0·38–0·61)	0·53 (0·42–0·67)
Cystic echinococcosis			46·04 (36·82–57·76)	19·52 (14·31–26·15)	13·59 (9·96–18·51)	34·91 (25·33–46·70)	1·01 (0·84–1·22)	0·57 (0·44–0·75)	0·43 (0·33–0·59)	0·91 (0·70–1·20)
Dengue			1975·12 (619·25–2751·84)	6658·50 (2782·71–8825·22)	6459·35 (2656·44–8603·02)	7149·65 (2850·34–9664·67)	37·78 (10·91–52·73)	182·47 (72·22–242·60)	184·75 (72·17–246·92)	182·19 (69·91–243·44)
Yellow fever			373·91 (80·80–1 074·88)	151·00 (28·19–486·14)	112·32 (20·43–363·13)	239·63 (46·28–735·94)	5·80 (1·24–16·73)	2·47 (0·46–7·89)	1·87 (0·34–5·98)	3·81 (0·73–11·83)
Rabies			744·18 (383·72–1106·23)	300·82 (154·43–508·23)	187·53 (104·04–310·85)	635·28 (330·52–1034·84)	13·29 (7·16–19·06)	6·13 (3·44–10·11)	4·27 (2·52–7·48)	12·19 (6·97–19·00)
Intestinal nematode infections			385·30 (309·19–484·21)	433·48 (312·40–573·45)	346·78 (252·10–447·89)	558·46 (413·72–720·82)	4·88 (3·98–6·06)	5·88 (4·36–7·65)	4·89 (3·71–6·21)	7·30 (5·53–9·27)
Other neglected tropical diseases			1940·72 (1328·90–2505·52)	1402·93 (958·86–1858·61)	947·33 (662·50–1216·47)	2213·36 (1526·52–2853·69)	27·06 (19·16–33·98)	23·56 (17·54–29·97)	17·68 (13·69–22·23)	33·86 (24·83–42·94)
**Maternal disorders**			**12 817·77 (11 808·37–14 106·37)**	**7 798·31 (6 182·46–10 649·41)**	**5309·20 (4176·87–7291·45)**	**12 187·02 (9652·10–16 660·62)**	**230·61 (212·53–253·39)**	**144·68 (115·16–196·43)**	**98·31 (77·65–134·04)**	**225·12 (178·49–307·18)**
Maternal haemorrhage			4018·54 (3248·03–4975·76)	2257·76 (1643·05–3099·19)	1588·79 (1144·08–2172·40)	3595·49 (2643·75–4943·59)	72·40 (58·51–89·12)	41·78 (30·54–57·34)	29·31 (21·20–40·28)	66·23 (49·16–90·46)
Maternal sepsis and other maternal infections			1093·59 (789·31–1478·23)	568·50 (376·83–841·55)	389·11 (258·50–575·18)	891·03 (583·79–1295·11)	19·53 (14·29–26·19)	10·43 (6·96–15·15)	7·13 (4·75–10·30)	16·26 (10·81–23·56)
Maternal hypertensive disorders			1780·77 (1360·65–2265·89)	1250·12 (896·33–1796·74)	792·36 (569·70–1148·96)	1907·78 (1367·86–2750·31)	31·60 (24·46–39·84)	22·59 (16·28–32·67)	14·32 (10·29–20·90)	34·32 (24·75–49·71)
Maternal obstructed labour and uterine rupture			553·65 (369·57–798·39)	379·32 (236·18–586·43)	242·75 (152·46–374·34)	625·08 (391·74–963·69)	10·25 (6·84–14·57)	7·14 (4·44–10·99)	4·58 (2·86–7·17)	11·73 (7·36–17·88)
Maternal abortion, miscarriage, and ectopic pregnancy			1 081·93 (796·17–1466·60)	669·82 (421·69–1 034·83)	480·38 (305·32–736·48)	1078·37 (678·31–1661·59)	19·70 (14·56–26·12)	12·42 (7·89–19·21)	8·93 (5·71–13·62)	19·91 (12·65–30·74)
Indirect maternal deaths			1987·87 (1463·84–2619·81)	1196·12 (805·84–1823·06)	791·55 (533·07–1211·43)	1774·10 (1200·07–2639·78)	35·74 (26·39–46·78)	22·18 (15·00–33·40)	14·65 (9·89–22·15)	32·73 (22·39–48·55)
Late maternal deaths			228·46 (134·55–370·93)	105·43 (58·28–177·79)	71·14 (39·77–120·02)	159·24 (87·59–269·43)	4·11 (2·45–6·55)	1·96 (1·08–3·30)	1·32 (0·74–2·20)	2·95 (1·59–5·04)
Maternal deaths aggravated by HIV/AIDS			105·42 (66·70–142·91)	430·32 (219·73–765·51)	253·70 (108·50–475·83)	763·37 (401·65–1499·63)	2·02 (1·28–2·73)	8·53 (4·34–14·58)	4·98 (2·30–9·27)	15·03 (8·24–28·61)
Other maternal disorders			1967·54 (1475·03–2540·02)	940·92 (640·79–1337·36)	699·42 (483·11–998·06)	1392·56 (943·04–1990·31)	35·26 (26·84–45·20)	17·64 (12·04–25·17)	13·09 (9·19–18·56)	25·96 (17·77–36·89)
**Neonatal disorders**			**149 832·24 (142 306·49–157 779·95)**	**71 532·70 (50 184·26–101 689·62)**	**46 226·36 (32 810·65–65 649·90)**	**125 242·45 (89 349·23–176 163·80)**	**1731·04 (1644·11–1822·86)**	**826·50 (580·03–1174·69)**	**534·13 (379·23–758·40)**	**1447·02 (1032·61–2035·11)**
Neonatal preterm birth complications			53 703·14 (49 224·85–58 402·33)	23 071·13 (17 788·51–28 231·40)	13 767·25 (10 898·61–16 562·02)	41 394·88 (32 259·50–50 431·42)	620·40 (568·68–674·66)	266·53 (205·53–326·14)	159·06 (125·91–191·37)	478·20 (372·69–582·54)
Neonatal encephalopathy due to birth asphyxia and trauma			45 435·32 (40 396·97–49 877·36)	20 742·99 (11 639·24–35 181·47)	13 856·95 (8078·40–22 995·26)	34 654·55 (20 226·60–57 283·90)	524·89 (466·66–576·25)	239·66 (134·53–406·40)	160·10 (93·35–265·66)	400·38 (233·70–661·74)
Neonatal sepsis and other neonatal infections			21 029·06 (17 740·28–27 500·03)	15 385·72 (10 734·18–22 527·89)	9832·61 (6980·69–14 274·10)	25 900·96 (18 157·23–38 004·22)	242·99 (205·03–317·71)	177·79 (124·06–260·29)	113·63 (80·69–164·92)	299·30 (209·84–439·07)
Haemolytic disease and other neonatal jaundice			4258·58 (3689·17–4937·32)	1167·54 (753·90–1833·25)	783·30 (517·92–1228·71)	2248·73 (1444·02–3482·05)	49·21 (42·63–57·04)	13·49 (8·71–21·18)	9·05 (5·99–14·20)	25·98 (16·69–40·23)
Other neonatal disorders			25 406·13 (22 984·87–27 937·49)	11 165·32 (7419·11–17 176·36)	7986·25 (5468·16–12 253·82)	21 043·32 (13 945·66–31 869·47)	293·56 (265·56–322·79)	129·03 (85·77–198·47)	92·29 (63·21–141·56)	243·16 (161·18–368·19)
**Nutritional deficiencies**			**19 504·73 (17 125·04–22 894·16)**	**8986·65 (7 277·02–11 129·94)**	**6937·91 (5770·91–8577·33)**	**12 969·33 (10 436·93–16 068·38)**	**368·11 (334·00–422·69)**	**327·61 (288·44–384·44)**	**318·31 (288·04–364·30)**	**364·60 (313·47–433·86)**
Protein-energy malnutrition			17 513·99 (15 224·67–20 732·25)	7108·68 (5653·91–8771·70)	5303·04 (4356·63–6438·46)	10 617·12 (8432·41–13 107·99)	308·39 (276·86–355·83)	247·26 (216·27–284·24)	241·13 (217·81–270·52)	277·70 (238·53–327·29)
Iodine deficiency			102·59 (66·06–168·78)	246·57 (145·69–434·01)	185·86 (116·03–285·81)	367·03 (196·01–662·26)	2·23 (1·62–3·14)	9·83 (5·88–15·74)	8·92 (5·12–15·00)	11·62 (7·14–17·83)
Dietary iron deficiency			114·38 (101·14–134·79)	114·98 (96·87–153·19)	107·09 (90·30–147·39)	129·20 (108·78–168·09)	2·97 (2·52–3·75)	4·14 (3·42–6·38)	4·21 (3·44–6·62)	4·17 (3·46–6·34)
Other nutritional deficiencies			1773·77 (1481·20–2040·79)	1516·43 (1186·34–2061·48)	1341·93 (1064·60–1792·05)	1855·99 (1463·36–2463·38)	54·51 (46·04–64·97)	66·37 (54·44–88·52)	64·05 (52·88–83·78)	71·11 (57·82–94·48)
**Other communicable, maternal, neonatal, and nutritional diseases**			**19 299·64 (14 992·73–24 689·33)**	**9533·83 (7866·60–11 573·30)**	**7980·87 (6693·68–9500·80)**	**13 178·60 (10 557·77–16 503·73)**	**332·68 (281·00–395·76)**	**258·70 (230·29–291·30)**	**237·92 (212·52–265·93)**	**310·54 (271·66–355·70)**
Sexually transmitted diseases excluding HIV			9470·11 (5539·12–14 702·01)	2353·54 (1286·32–3755·26)	1816·26 (1018·33–2852·33)	3880·23 (2016·63–6290·97)	115·76 (69·90–176·96)	32·36 (19·79–48·92)	25·93 (16·32–38·06)	50·48 (28·85–78·95)
	Syphilis		9228·17 (5288·03–14 456·12)	2149·74 (1091·03–3541·37)	1626·93 (849·93–2636·56)	3649·30 (1799·73–6059·79)	109·57 (63·52–170·78)	26·51 (14·05–42·90)	20·32 (11·08–32·27)	44·15 (22·43–72·29)
	Chlamydial infection		46·85 (39·26–53·45)	39·71 (31·09–51·74)	36·81 (28·66–48·13)	45·11 (35·47–57·83)	1·19 (0·98–1·33)	1·14 (0·93–1·40)	1·10 (0·89–1·35)	1·23 (1·01–1·50)
	Gonococcal infection		127·41 (105·62–144·48)	108·22 (83·96–141·93)	100·90 (78·10–133·09)	121·99 (94·78–159·15)	3·37 (2·77–3·80)	3·24 (2·62–4·04)	3·13 (2·52–3·92)	3·48 (2·80–4·33)
	Other sexually transmitted infections		67·69 (56·52–76·94)	55·87 (43·17–74·01)	51·61 (39·68–68·71)	63·83 (49·15–83·57)	1·63 (1·35–1·83)	1·46 (1·17–1·86)	1·39 (1·11–1·76)	1·62 (1·30–2·04)
Acute hepatitis			5497·93 (5228·66–5778·42)	3651·32 (3157·93–4232·51)	3315·09 (2847·40–3878·33)	4489·15 (3904·65–5174·41)	134·04 (127·82–139·97)	120·68 (107·87–134·84)	113·68 (100·93–129·30)	139·04 (125·51–154·03)
	Acute hepatitis A		378·87 (302·70–458·94)	97·01 (69·37–127·62)	82·06 (58·49–108·20)	143·00 (96·92–193·09)	5·25 (4·34–6·23)	2·24 (1·72–2·86)	2·03 (1·56–2·65)	2·85 (2·14–3·63)
	Acute hepatitis B		3658·41 (3417·23–3917·82)	2763·19 (2368·78–3243·28)	2498·99 (2121·17–2968·49)	3371·88 (2924·42–3945·13)	100·28 (94·01–106·30)	92·87 (80·77–107·08)	86·46 (74·58–100·76)	108·68 (95·61–123·05)
	Acute hepatitis C		77·22 (60·92–97·72)	87·58 (64·60–116·78)	82·14 (60·30–110·08)	95·12 (70·54–126·20)	2·46 (1·87–3·24)	3·43 (2·54–4·60)	3·32 (2·43–4·50)	3·53 (2·60–4·75)
	Acute hepatitis E		1383·43 (1195·82–1570·28)	703·54 (552·00–884·92)	651·91 (508·79–829·67)	879·16 (692·70–1101·35)	26·05 (22·11–30·43)	22·14 (17·29–28·13)	21·86 (16·77–28·03)	23·98 (18·90–29·98)
Other unspecified infectious diseases			4331·60 (2650·24–5920·94)	3528·96 (2635·59–4463·68)	2849·52 (2175·02–3500·35)	4809·21 (3470·38–6222·55)	82·88 (56·22–103·72)	105·66 (84·27–122·39)	98·30 (79·12–113·04)	121·02 (93·48–142·40)
**Non-communicable diseases**			**819 437·12 (804 360·07–836 584·77)**	**1 029 256·35 (877 741·47–1195 845·93)**	**819 015·96 (700 609·53–945 763·35)**	**1 356 608·44 (1 140 440·87–1 568 374·81)**	**39 529·59 (38 805·36–40 253·20)**	**60 993·19 (51 380·73–70 111·98)**	**50 805·98 (43 602·80–57 387·60)**	**76 275·86 (64 410·32–86 415·63)**
	**Neoplasms**		**208 041·17 (203 600·04–212 089·56)**	**295 591·07 (259 388·84–336 707·00)**	**258 069·82 (228 893·99–292 485·35)**	**350 744·38 (298 153·22–407 340·25)**	**8927·40 (8754·97–9089·24)**	**14 880·85 (13 225·95–16 619·79)**	**13 311·52 (11 909·83–14 907·64)**	**17 115·94 (14 775·33–19 646·90)**
	Lip and oral cavity cancer		4492·64 (4287·46–4677·97)	7162·57 (4962·19–9195·49)	5315·46 (3882·43–6578·28)	9476·18 (6340·46–12 321·76)	176·49 (169·18–183·02)	308·69 (212·18–394·58)	239·79 (173·39–299·24)	393·08 (264·26–510·65)
	Nasopharynx cancer		1866·39 (1770·55–1967·17)	2644·37 (1942·11–3472·28)	1819·45 (1374·36–2322·14)	3951·95 (2714·65–5623·44)	63·75 (60·63–67·02)	102·71 (75·07–135·47)	71·93 (53·88–91·14)	150·02 (102·78–217·92)
	Other pharynx cancer		3151·70 (2895·96–3333·59)	5051·60 (3607·69–6837·82)	4362·45 (3202·40–5683·25)	5323·45 (3755·52–7308·34)	118·63 (109·33–125·14)	216·12 (152·75–286·31)	192·11 (140·71–248·37)	218·15 (153·05–293·43)
	Oesophageal cancer		9164·59 (8913·46–9444·10)	14 987·57 (9099·89–24 302·18)	10 440·66 (6949·61–16 150·58)	22 132·59 (12 409·43–38 688·61)	414·89 (404·39–427·18)	766·50 (488·72–1285·91)	550·23 (381·71–856·95)	1090·35 (630·98–1917·59)
	Stomach cancer		18 045·32 (17 580·05–18 535·04)	18 058·20 (15 116·92–22 424·00)	15 954·31 (13 600·28–19 618·04)	21 012·28 (17 403·68–25 661·16)	834·17 (813·54–855·46)	990·23 (830·57–1230·00)	891·17 (761·40–1101·76)	1112·24 (924·89–1391·95)
	Colon and rectum cancer		16 597·93 (15 919·45–17 213·66)	26 400·77 (19 633·12–37 145·60)	22 132·48 (16 739·89–29 961·84)	31 331·86 (23 624·71–44 545·96)	829·56 (797·30–860·44)	1547·75 (1164·32–2191·05)	1329·82 (1015·91–1803·64)	1774·75 (1348·27–2565·66)
	Liver cancer		20 915·71 (20 029·12–21 730·96)	35 487·07 (27 243·22–49 706·06)	32 930·35 (26 712·73–43 698·83)	37 729·38 (28 311·22–55 428·12)	828·94 (796·16–857·96)	1679·63 (1311·17–2297·91)	1602·50 (1328·19–2098·20)	1720·16 (1315·30–2493·46)
		Liver cancer due to hepatitis B	9704·02 (8495·14–10 846·75)	15 984·92 (11 742·51–22 690·54)	15 042·06 (11 701·98–20 344·07)	16 334·40 (11 643·45–24 443·45)	349·53 (301·96–391·78)	702·26 (515·29–983·42)	678·35 (529·85–899·84)	688·77 (498·29–1007·57)
		Liver cancer due to hepatitis C	3267·80 (2889·48–3621·48)	5875·06 (4443·66–8277·99)	5607·86 (4371·30–7512·71)	6268·45 (4531·63–9172·86)	159·67 (143·42–176·07)	326·72 (251·67–446·02)	321·12 (256·04–419·66)	335·94 (246·15–474·96)
		Liver cancer due to alcohol use	2892·13 (2438·18–3361·38)	5059·21 (3590·52–7105·27)	4239·98 (3157·41–5676·54)	6191·39 (4305·54–9114·66)	129·23 (109·78–150·48)	261·81 (192·09–355·30)	227·63 (173·25–300·21)	305·77 (215·03–436·52)
		Liver cancer due to other causes	5051·76 (4479·84–5703·84)	8567·87 (6168·28–12 352·39)	8040·45 (6069·37–10 948·83)	8935·14 (6118·74–13 697·04)	190·51 (169·75–214·63)	388·84 (283·31–553·06)	375·40 (287·52–502·37)	389·68 (267·60–589·17)
	Gallbladder and biliary tract cancer		3269·80 (2965·89–3487·77)	3731·09 (2854·97–4996·54)	3483·65 (2712·79–4526·05)	3811·83 (2888·07–5246·92)	161·56 (148·71–170·97)	209·08 (164·45–272·63)	201·50 (161·99–255·17)	206·45 (161·22–274·44)
	Pancreatic cancer		8145·03 (7933·68–8359·17)	13 476·41 (11 270·99–16 843·51)	12 987·49 (11 090·65–15 794·67)	14 294·73 (11 576·66–18 465·71)	405·50 (394·38–416·00)	750·64 (636·08–936·87)	740·47 (638·40–902·30)	773·18 (637·08–989·42)
	Larynx cancer		2674·72 (2586·78–2767·61)	3567·34 (2289·39–5400·05)	2380·00 (1668·99–3320·91)	5584·05 (3314·36–8626·26)	111·04 (107·60–114·65)	159·21 (103·66–243·22)	110·20 (78·23–151·96)	242·21 (142·28–377·11)
	Tracheal, bronchus, and lung cancer		35 966·80 (34 937·63–36 978·97)	43 405·12 (32 788·21–57 654·61)	30 316·89 (23 677·54–38 223·24)	70 199·37 (49 909·54–98 211·46)	1706·88 (1659·40–1753·40)	2358·90 (1782·36–3150·61)	1696·70 (1327·29–2181·04)	3687·93 (2631·69–5099·19)
	Malignant skin melanoma		1460·67 (1301·99–1614·12)	2147·97 (1908·51–2427·66)	2440·76 (2180·20–2775·96)	1927·94 (1721·16–2167·34)	61·68 (54·39–66·60)	108·63 (97·40–118·50)	127·26 (113·24–140·58)	93·73 (83·17–101·66)
	Non-melanoma skin cancer		991·73 (953·50–1031·27)	1597·39 (1486·91–1706·77)	1490·00 (1382·03–1610·55)	1649·27 (1559·36–1748·79)	53·06 (51·14–55·18)	103·93 (97·12–110·59)	100·60 (94·52–107·21)	101·89 (93·59–108·96)
	Breast cancer		14 368·87 (13 568·90–15 369·65)	21 012·59 (16 070·25–26 964·36)	18 431·79 (14 335·52–23 244·57)	23 368·20 (17 530·73–30 912·46)	545·59 (516·55–581·67)	911·29 (706·11–1158·90)	809·24 (641·24–1010·41)	993·36 (756·29–1299·51)
	Cervical cancer		7204·07 (5855·64–7673·39)	8272·64 (6620·70–9635·85)	7543·40 (6040·92–8897·39)	9129·20 (7137·97–10 691·02)	247·16 (204·12–263·48)	316·85 (256·86–362·10)	293·02 (241·21–337·30)	341·19 (273·64–391·55)
	Uterine cancer		1973·17 (1875·54–2070·08)	3101·30 (1996·72–4858·57)	2374·50 (1603·40–3611·99)	3907·59 (2479·13–6130·01)	87·53 (83·14–91·97)	158·41 (105·67–242·48)	125·86 (86·78–187·27)	191·49 (125·75–292·72)
	Ovarian cancer		4141·91 (3927·54–4340·62)	6212·55 (4822·50–7996·13)	6040·61 (4750·06–7667·37)	6052·57 (4660·32–7966·76)	165·04 (156·70–172·73)	275·23 (218·67–356·82)	268·72 (215·86–342·99)	265·09 (207·49–349·88)
	Prostate cancer		5540·60 (4536·21–5992·09)	12299·01 (9735·70–13 524·76)	12627·11 (9989·87–13 981·14)	11371·20 (8940·84–12 527·91)	380·92 (320·81–412·87)	927·78 (747·88–1027·50)	978·74 (803·17–1079·98)	826·23 (662·01–926·17)
	Testicular cancer		368·08 (350·82–386·93)	374·95 (336·18–421·94)	348·52 (311·77–394·56)	394·86 (354·79–444·74)	8·65 (8·29–9·03)	10·72 (9·91–11·61)	10·33 (9·49–11·30)	10·79 (9·99–11·69)
	Kidney cancer		2910·03 (2799·88–3016·36)	4617·06 (3532·76–6091·09)	4296·38 (3389·27–5575·18)	5103·22 (3751·68–6883·80)	131·80 (127·34–136·18)	241·41 (187·34–311·99)	232·08 (184·91–297·82)	257·30 (194·84–344·78)
	Bladder cancer		3150·24 (3043·85–3241·97)	4852·95 (3953·78–6358·38)	4545·54 (3818·35–5789·27)	5288·92 (4204·92–7189·43)	186·20 (180·45–191·69)	323·45 (264·72–427·66)	311·75 (262·53–402·52)	339·12 (265·98–470·63)
	Brain and nervous system cancer		7554·07 (6820·68–8181·18)	10 583·46 (9469·21–11 808·96)	10 446·03 (9307·90–11 666·58)	10 514·70 (9387·52–11 631·54)	227·04 (204·78–241·28)	397·20 (353·10–430·25)	401·68 (357·28–438·32)	378·98 (338·06–407·52)
	Thyroid cancer		1043·17 (998·65–1088·04)	1539·73 (1225·48–1998·17)	1420·82 (1157·90–1786·48)	1654·29 (1302·88–2166·24)	42·86 (41·19–44·73)	73·49 (59·55–91·38)	69·58 (57·55–84·89)	75·53 (60·06–94·11)
	Mesothelioma		649·65 (610·14–687·99)	934·98 (852·21–1034·21)	967·52 (875·60–1079·71)	893·53 (818·92–982·37)	30·21 (28·30–31·88)	50·59 (46·70–54·51)	53·81 (49·37–58·28)	46·54 (43·21–49·93)
	Hodgkin's lymphoma		1097·68 (916·41–1300·78)	1007·46 (824·44–1189·40)	870·15 (707·80–1029·18)	1190·30 (961·57–1399·15)	28·74 (24·61–33·79)	31·96 (27·07–37·45)	28·39 (23·84–33·35)	35·94 (29·91–42·08)
	Non-Hodgkin lymphoma		6635·98 (6029·97–6928·74)	9683·47 (8323·51–11 551·64)	9401·25 (8137·88–11 168·66)	9681·24 (8284·78–11 642·21)	239·58 (221·18–247·87)	416·59 (366·81–493·16)	413·54 (365·95–488·33)	398·39 (349·60–474·88)
	Multiple myeloma		2044·37 (1839·22–2262·64)	3628·90 (3014·63–4400·33)	3597·16 (3002·17–4325·75)	3497·68 (2893·87–4289·98)	98·44 (87·38–109·81)	191·08 (162·41–228·03)	193·11 (164·00–229·90)	179·25 (152·20–215·92)
	Leukaemia		9990·00 (9167·13–10 596·07)	11 451·15 (9608·19–13 282·31)	10 613·01 (8963·63–12 239·56)	12 397·52 (10 216·13–14 618·94)	310·17 (286·15–324·38)	473·78 (396·84–552·65)	451·55 (382·29–519·15)	493·24 (407·94–587·57)
		Acute lymphoid leukaemia	2391·03 (2182·08–2644·47)	2195·74 (1851·45–2580·26)	2025·75 (1708·11–2369·05)	2358·88 (1970·57–2792·19)	50·94 (46·16–55·60)	61·78 (52·19–72·75)	58·22 (49·30–67·64)	64·07 (53·51–75·59)
		Chronic lymphoid leukaemia	645·93 (602·60–738·73)	851·73 (675·66–1039·42)	817·32 (656·49–990·52)	890·72 (690·24–1116·05)	35·39 (33·12–40·15)	54·06 (43·84–65·08)	53·38 (43·65–63·76)	54·55 (43·01–67·87)
		Acute myeloid leukaemia	2622·62 (2419·48–2809·79)	3886·68 (3231·10–4538·42)	3765·82 (3143·83–4397·85)	3987·65 (3283·54–4744·19)	85·33 (78·40–89·70)	154·32 (128·94–181·59)	153·02 (129·41–178·12)	154·23 (126·52–183·76)
		Chronic myeloid leukaemia	597·98 (538·80–661·31)	587·66 (473·58–720·41)	524·27 (425·06–634·46)	631·60 (501·24–780·22)	21·94 (20·21–23·76)	23·86 (19·47–28·72)	21·47 (17·71–25·55)	24·95 (20·13–30·45)
		Other leukaemia	3732·44 (3252·39–3950·12)	3929·34 (3272·08–4604·97)	3479·86 (2883·62–4036·85)	4528·66 (3704·88–5387·17)	116·56 (103·31–123·02)	179·76 (150·31–210·19)	165·46 (138·92–191·00)	195·44 (160·26–233·79)
	Other neoplasms		12 626·26 (11 487·26–13 043·60)	18 301·42 (16 278·65–20 373·56)	18 492·06 (16 391·65–20 760·12)	17 874·50 (16 004·21–19 746·99)	431·34 (392·75–443·83)	779·02 (702·08–832·60)	815·84 (734·36–881·38)	719·37 (651·79–761·26)
**Cardiovascular diseases**			**319 638·66 (312 436·72–327 187·01)**	**326 257·96 (234 399·69–423 736·41)**	**220 070·39 (161 641·21–292 676·91)**	**496 321·31 (375 174·81–629 672·75)**	**17 646·59 (17 281·71–18 071·08)**	**21 888·53 (15 543·92–28 468·09)**	**15 888·53 (11 706·11–21 545·02)**	**31 210·25 (22 879·87–40 778·05)**
Rheumatic heart disease			8347·62 (7957·19–8806·00)	5651·84 (4299·48–8799·24)	5 175·09 (3970·48–7913·68)	6844·20 (5188·38–11 167·80)	314·58 (302·34–328·71)	303·21 (222·03–597·74)	290·06 (212·65–552·00)	338·12 (249·04–619·98)
Ischaemic heart disease			167 695·16 (163 400·63–172 479·67)	161 610·26 (94 677·32–237 291·03)	104 342·24 (58 047·10–154 373·64)	247 683·10 (152 717·04–349 779·28)	9480·54 (9230·53–9757·70)	10 872·35 (5646·40–16 897·12)	7442·13 (3798·26–11 930·22)	16 034·02 (8686·24–24 368·53)
Stroke			101 992·79 (99 104·55–105 018·72)	91 064·29 (61 314·72–134 761·46)	58 155·44 (39 025·40–86 818·38)	153 332·71 (102 916·43–234 211·51)	5528·23 (5334·61–5734·68)	5973·78 (3978·57–9391·60)	4231·39 (2826·29–7050·62)	9151·31 (6109·85–14 387·85)
	Ischaemic stroke		40 095·14 (38 501·65–41 842·07)	37 575·58 (22 612·73–66 879·26)	26 057·01 (15 733·98–48 118·92)	56 877·92 (34 814·92–100 588·50)	2690·17 (2571·77–2817·62)	2948·05 (1749·03–5869·85)	2221·88 (1336·04–4661·31)	4055·62 (2312·36–7919·19)
	Intracerebral haemorrhage		61 897·65 (60 240·15–63 722·74)	53 488·71 (35 930·13–79 150·90)	32 098·43 (21 458·26–47 853·48)	96 454·79 (62 636·80–136 941·77)	2838·06 (2748·57–2934·06)	3025·72 (2063·62–4704·00)	2009·51 (1320·53–3092·59)	5095·69 (3308·84–8261·56)
Hypertensive heart disease			14 955·00 (12 105·83–16 330·59)	28 401·65 (15 523·83–68 261·60)	17 688·25 (10 641·16–38 483·51)	44 607·61 (23 102·18–110 344·80)	893·73 (698·62–982·93)	2073·74 (1104·34–4950·72)	1425·58 (808·83–3000·09)	2956·96 (1506·48–7141·03)
Cardiomyopathy and myocarditis			8159·05 (7052·67–9049·67)	8614·12 (6850·32–10 800·69)	7310·79 (5917·79–9097·46)	9442·84 (7566·48–11 901·06)	339·55 (282·60–371·08)	452·78 (352·30–577·61)	404·25 (315·08–517·36)	462·43 (358·49–594·66)
	Myocarditis		1234·37 (992·16–1358·33)	1241·71 (1008·54–1434·20)	1236·63 (994·83–1431·20)	1255·94 (1018·42–1451·88)	46·54 (35·84–51·06)	73·06 (56·59–83·12)	78·48 (61·04–89·37)	66·25 (51·67–76·34)
	Alcoholic cardiomyopathy		2494·33 (1967·43–3151·60)	2202·89 (1597·63–3202·28)	1780·37 (1293·12–2561·52)	2678·03 (1931·36–3885·10)	83·34 (67·20–102·92)	84·18 (65·30–111·05)	72·09 (55·34–94·26)	98·06 (75·50–129·91)
	Other cardiomyopathy		4430·35 (3771·63–4730·05)	5169·52 (3845·95–6907·49)	4293·79 (3269·70–5686·91)	5508·87 (4068·99–7592·64)	209·67 (170·27–224·67)	295·54 (212·19–416·30)	253·67 (184·66–365·95)	298·12 (214·33–429·93)
Atrial fibrillation and flutter			2336·89 (1890·44–2827·80)	4956·87 (3608·07–6594·66)	4662·91 (3404·45–6170·78)	5075·29 (3730·04–6687·38)	239·23 (188·69–293·59)	542·14 (390·42–725·27)	530·02 (381·69–706·25)	526·74 (381·17–709·83)
Aortic aneurysm			2881·76 (2800·85–2975·54)	3800·68 (2921·69–4850·64)	3627·32 (2763·99–4659·23)	3926·29 (2981·58–5043·96)	166·57 (162·05–171·56)	256·06 (190·18–331·65)	257·61 (189·72–333·44)	246·83 (185·38–323·21)
Peripheral artery disease			715·52 (544·36–1007·02)	1611·91 (1148·64–2428·73)	1618·04 (1147·83–2471·70)	1630·97 (1147·44–2510·47)	60·70 (45·37–89·48)	155·90 (105·29–254·65)	165·48 (110·97–263·68)	147·97 (100·12–244·75)
Endocarditis			2329·06 (2067·07–2756·07)	3633·34 (2855·29–4676·19)	3451·77 (2717·91–4385·23)	3777·87 (2954·52–4894·36)	95·97 (82·15–112·83)	189·64 (143·97–258·83)	190·00 (146·75–261·54)	182·88 (138·11–247·39)
Other cardiovascular and circulatory diseases			10 225·83 (9436·43–12 584·08)	16 913·00 (13 669·80–22 174·90)	14 038·53 (11 315·88–18 481·55)	20 000·42 (15 920·54–26 315·57)	527·48 (493·07–627·50)	1068·93 (848·16–1388·61)	952·02 (753·79–1226·29)	1162·99 (911·78–1523·15)
**Chronic respiratory diseases**			**61 574·64 (59 099·41–65 209·15)**	**77 269·92 (55 458·89–109 434·21)**	**59 493·91 (44 169·75–80 335·34)**	**113 915·65 (76 580·63–174 658·72)**	**3542·29 (3403·60–3739·61)**	**5206·46 (3728·95–7593·43)**	**4196·66 (3135·38–5700·53)**	**7266·43 (4938·98–11 328·65)**
Chronic obstructive pulmonary disease			47 146·20 (44 992·80–50 032·34)	62 276·02 (40 821·37–94 079·98)	45 326·76 (30 730·78–64 371·81)	97 677·17 (60 875·40–158 519·41)	2934·33 (2817·24–3120·35)	4410·06 (2958·18–6769·66)	3397·30 (2371·11–4891·82)	6469·10 (4129·74–10 458·76)
Pneumoconiosis			414·94 (391·48– 450·21)	338·53 (293·04– 401·80)	324·33 (280·26–376·64)	375·43 (334·06–423·21)	21·49 (20·45–23·05)	22·21 (19·62–25·48)	22·20 (19·58–25·28)	22·98 (20·92–25·75)
	Silicosis		210·16 (194·31–230·82)	99·85 (74·75–140·02)	85·97 (65·01–111·39)	134·27 (107·93–161·44)	10·40 (9·57–11·68)	5·93 (4·51–8·02)	5·24 (3·99–6·76)	7·46 (6·14–9·03)
	Asbestosis		60·96 (46·29–71·87)	101·51 (81·69–121·61)	113·27 (92·28–135·58)	90·59 (72·16–107·24)	3·50 (2·43–4·06)	7·04 (5·60–8·29)	8·14 (6·64–9·59)	5·99 (4·64–7·00)
	Coal workers' pneumoconiosis		46·60 (30·38–54·24)	36·53 (25·86–47·59)	30·78 (21·20–39·66)	47·29 (32·79–59·58)	2·68 (1·79–3·07)	2·62 (1·93–3·41)	2·30 (1·64–2·97)	3·15 (2·28–3·92)
	Other pneumoconiosis		97·22 (81·95–127·89)	100·64 (79·37–132·32)	94·31 (72·67–125·15)	103·27 (83·58–133·79)	4·91 (4·17–6·57)	6·63 (5·37–8·68)	6·51 (5·21–8·57)	6·37 (5·25–8·27)
Asthma			10 499·32 (8643·21–12 621·19)	7978·51 (5430·34–11 457·49)	6466·43 (4496·48–9044·98)	9829·21 (6670·20–14 144·67)	420·02 (338·81–517·69)	393·43 (275·35–548·69)	333·80 (236·22–454·72)	451·88 (315·32–637·62)
Interstitial lung disease and pulmonary sarcoidosis			2305·43 (1695·61–2717·00)	4983·86 (3623·74–6161·02)	5765·80 (4253·26–7037·41)	4211·19 (3004·43–5145·31)	127·47 (90·82–147·69)	311·25 (224·70–373·04)	372·93 (275·84–442·95)	253·24 (179·66–301·52)
Other chronic respiratory diseases			1208·75 (847·40–1438·67)	1693·01 (1182·15–2087·79)	1610·58 (1141·58–1982·74)	1822·66 (1244·05–2260·81)	38·98 (27·29–45·65)	69·51 (47·95–84·09)	70·43 (48·99–85·25)	69·23 (47·58–84·08)
**Cirrhosis and other chronic liver diseases**			**37 283·07 (35 413·31–41 442·98)**	**48 324·15 (43 655·69–54 757·85)**	**44 158·29 (39 536·81–50 452·38)**	**53 144·00 (48 394·75–60 100·36)**	**1 256·85 (1 197·09–1 376·86)**	**1903·64 (1760·57–2097·77)**	**1787·20 (1637·35–1979·96)**	**2014·49 (1881·85–2228·68)**
Cirrhosis and other chronic liver diseases due to hepatitis B			10 846·50 (9787·89–12 777·41)	14 880·49 (12 899·14–17 732·38)	14 308·72 (12 368·22–16 964·03)	15 697·63 (13 716·23–18 707·04)	365·57 (330·81–422·57)	582·17 (514·27–683·44)	572·65 (502·98–670·06)	593·64 (530·13–690·12)
Cirrhosis and other chronic liver diseases due to hepatitis C			9455·51 (8516·28–10 668·97)	13 019·27 (11 343·29–15 066·04)	12 246·93 (10 638·70–14 290·60)	13 577·82 (11 819·56–15 676·92)	326·75 (295·07–364·97)	509·03 (451·45–576·73)	489·43 (430·09–562·00)	513·82 (455·72–580·25)
Cirrhosis and other chronic liver diseases due to alcohol use			9440·30 (8601·03–10 523·64)	11 865·99 (10 478·07–13 782·63)	9 622·07 (8 405·28–11 257·27)	14 624·74 (13 037·75–16 860·82)	334·89 (306·46–371·88)	477·23 (429·65–541·53)	401·16 (355·94–457·45)	564·56 (512·48–638·30)
Cirrhosis and other chronic liver diseases due to other causes			7540·75 (6769·21–8562·42)	8558·40 (7470·52–9881·86)	7980·57 (6959·23–9248·83)	9243·82 (8098·78–10 702·46)	229·64 (206·21–258·15)	335·21 (296·04–381·00)	323·96 (285·34–370·05)	342·46 (303·34–389·80)
**Digestive diseases**			**27 082·09 (25 735·97–29 026·39)**	**32 690·58 (28 907·29–37 113·89)**	**29 897·69 (26 279·42–33 903·59)**	**36 401·28 (31 908·83–41 640·92)**	**1092·33 (1042·78–1177·81)**	**1729·74 (1570·00–1943·26)**	**1663·32 (1507·42–1872·67)**	**1784·75 (1610·69–2026·13)**
Peptic ulcer disease			5742·34 (5308·86–6470·00)	4833·97 (3951·60–6115·02)	4103·13 (3341·54–5179·42)	6057·19 (4913·18–7741·88)	246·72 (230·08–272·70)	265·44 (222·70–325·05)	234·64 (196·86–288·79)	311·59 (260·83–383·63)
Gastritis and duodenitis			1017·42 (930·59–1148·36)	1557·50 (1342·73–1828·51)	1473·11 (1261·60–1736·97)	1695·38 (1476·53–1980·21)	42·99 (39·34–47·75)	83·70 (73·76–96·59)	82·66 (72·25–95·77)	85·74 (75·37–97·94)
Appendicitis			1886·62 (1684·29–2200·23)	1718·04 (1448·39–2104·71)	1528·72 (1270·13–1907·15)	2065·74 (1744·32–2496·12)	50·19 (44·99–57·43)	63·09 (55·25–76·05)	58·74 (50·52–71·41)	70·14 (61·89–83·92)
Paralytic ileus and intestinal obstruction			7572·53 (6329·25–8263·90)	9730·92 (8188·10–11 223·00)	9618·87 (8084·78–11 195·22)	10 156·62 (8513·88–11 592·13)	254·61 (213·31–280·85)	461·91 (395·14–522·54)	483·94 (413·18–546·22)	437·59 (372·25–495·13)
Inguinal, femoral, and abdominal hernia			954·58 (739·84–1139·71)	1032·91 (779·14–1236·49)	990·40 (744·72–1191·70)	1093·43 (812·53–1305·62)	43·73 (35·57–52·14)	58·61 (45·48–70·29)	58·92 (45·44–71·95)	56·78 (43·53–67·25)
Inflammatory bowel disease			981·64 (819·41–1144·74)	1362·40 (1164·24–1592·17)	1380·50 (1172·15–1617·74)	1324·61 (1120·29–1557·37)	41·61 (34·54–45·10)	74·52 (64·31–83·09)	78·95 (68·17–88·01)	67·52 (58·13–74·87)
Vascular intestinal disorders			1671·00 (1536·99–1908·24)	2663·15 (2380·93–3092·93)	2633·01 (2335·43–3083·41)	2605·71 (2356·03–3009·89)	100·91 (92·87–113·67)	189·61 (172·47–217·30)	194·02 (175·99–223·05)	176·08 (159·46–203·29)
Gallbladder and biliary diseases			1866·75 (1758·57–2184·15)	2746·42 (1825·84–4401·30)	2149·61 (1499·63–3388·95)	3362·05 (2212·26–5489·47)	101·83 (96·11–118·08)	185·22 (126·84–294·36)	153·34 (108·60–241·00)	211·69 (144·05–344·52)
Pancreatitis			3274·24 (2832·81–3650·57)	4186·34 (3122·88–5783·48)	3346·45 (2592·70–4423·28)	5001·22 (3687·57–7144·00)	112·05 (97·41–124·60)	173·19 (135·22–230·31)	144·27 (115·71–183·01)	197·20 (149·84–273·04)
Other digestive diseases			2114·97 (1937·50–2386·83)	2858·93 (2539·42–3237·82)	2673·88 (2367·18–3048·49)	3039·34 (2718·42–3436·44)	97·69 (88·88–108·10)	174·45 (158·26–193·47)	173·84 (156·77–193·47)	170·41 (154·27–189·98)
**Neurological disorders**			**34 154·45 (30 976·21–38 350·74)**	**68 735·01 (56 418·37–85 594·30)**	**69 096·55 (57 167·21–84 437·23)**	**66 280·32 (53 588·93–84 323·67)**	**2825·82 (2497·00–3217·57)**	**6700·00 (5371·09–8457·63)**	**7028·33 (5770·39–8611·84)**	**6065·76 (4737·03–7895·84)**
Alzheimer's disease and other dementias			22 348·78 (19 381·82–26 349·20)	51 671·92 (39 669·40–67 824·33)	52 240·08 (40 873·50–66 836·46)	48 821·61 (36 399·57–66 567·64)	2382·13 (2060·41–2777·61)	5799·48 (4501·54–7517·70)	6061·58 (4818·44–7635·42)	5255·59 (3939·26–7016·09)
Parkinson's disease			2528·14 (1992·32–3147·38)	6183·54 (4879·29–7762·12)	6899·94 (5430·51–8660·22)	5148·20 (4020·79–6444·72)	211·30 (167·77–265·16)	556·39 (434·37–700·10)	635·63 (504·85–800·38)	448·04 (348·35–566·86)
Epilepsy			5945·43 (5555·08–6409·61)	6485·40 (5297·65–8012·26)	5485·80 (4500·93–6728·04)	8031·19 (6557·79–9917·97)	126·05 (118·63–135·52)	173·82 (142·29–207·87)	153·18 (126·19–183·03)	203·13 (168·25–244·83)
Multiple sclerosis			567·35 (517·29–646·89)	697·25 (573·73–836·26)	708·26 (584·01–847·78)	690·80 (564·23–833·25)	18·93 (16·58–21·03)	25·68 (21·46–29·73)	26·57 (22·24–30·75)	24·88 (20·64–28·99)
Motor neuron disease			855·92 (819·37–883·29)	1251·64 (1138·92–1371·90)	1273·70 (1150·47–1408·77)	1197·81 (1100·77–1307·33)	34·33 (33·05–35·36)	57·81 (53·42–61·99)	59·68 (54·64–64·87)	54·11 (50·71–57·80)
Other neurological disorders			1908·83 (1775·71–2020·32)	2445·26 (2193·37–2717·74)	2488·76 (2226·16–2777·72)	2390·72 (2145·26–2642·88)	53·08 (50·93–55·37)	86·82 (80·33–93·07)	91·69 (84·07–99·15)	80·03 (74·93–85·44)
**Mental and substance use disorders**			**12 033·73 (10 748·31–13 076·40)**	**18 167·84 (15 641·39–20 933·89)**	**19 534·61 (16 734·02–22 703·24)**	**17 593·95 (15 172·11–20 256·13)**	**318·28 (283·23–343·69)**	**558·24 (484·16–628·50)**	**614·77 (532·65–696·87)**	**518·51 (457·41–582·18)**
Alcohol use disorders			6213·98 (5164·12–6877·78)	6599·22 (5424·07–7869·17)	6556·62 (5368·97–7877·25)	6845·26 (5642·19–8103·96)	173·89 (145·53–190·90)	209·16 (171·47–246·13)	212·23 (174·20–251·02)	211·37 (175·46–247·60)
Drug use disorders			5787·35 (5264·51–6426·74)	11 533·11 (9885·26–13 320·20)	12 941·35 (11 109·60–15 113·55)	10 713·54 (9246·05–12 353·23)	143·77 (130·28–158·78)	348·40 (302·88–393·12)	401·83 (348·54–458·57)	306·46 (268·71–345·20)
	Opioid use disorders		3656·92 (3098·22–4048·42)	5683·35 (4438·29–6659·70)	5921·06 (4684·93–6998·92)	5504·28 (4289·26–6404·01)	86·22 (72·67–94·68)	157·36 (123·55–181·52)	167·83 (132·77–194·58)	146·72 (116·21–167·61)
	Cocaine use disorders		356·97 (288·96–463·85)	953·71 (720·69–1215·78)	1091·78 (824·50–1385·89)	890·12 (663·37–1127·87)	8·80 (7·06–11·27)	25·84 (19·49–32·37)	29·83 (22·47–37·40)	23·54 (17·66–29·29)
	Amphetamine use disorders		224·23 (185·18–300·34)	855·00 (648·42–1090·67)	1052·37 (789·42–1325·00)	773·21 (569·90–987·33)	5·22 (4·30–6·85)	21·39 (16·58–27·16)	26·51 (20·41–33·16)	18·92 (14·28–23·57)
	Other drug use disorders		1549·23 (1395·62–1961·50)	4041·05 (3369·16–4829·62)	4876·15 (4096·69–5867·03)	3545·94 (3026·08–4235·72)	43·53 (39·37–52·89)	143·81 (121·40–167·46)	177·66 (153·46–208·10)	117·29 (102·55–133·97)
Eating disorders			32·40 (28·77–36·08)	35·52 (29·42–42·49)	36·64 (30·35–44·14)	35·14 (29·23–41·83)	0·61 (0·54–0·68)	0·68 (0·57–0·81)	0·71 (0·59–0·85)	0·67 (0·56–0·80)
**Diabetes, urogenital, blood, and endocrine diseases**			**71 460·46 (69 628·99–73 928·81)**	**125 981·86 (84 913·74–194 272·53)**	**89 084·12 (63 040·21–133 824·31)**	**174 390·14 (116 474·41–274 353·73)**	**3191·13 (3112·85–3271·85)**	**7211·23 (5017·51–10906·78)**	**5469·17 (3964·00–8218·74)**	**9279·91 (6459·64–14223·71)**
Diabetes mellitus			28 650·00 (27 998·10–29 279·38)	50 614·49 (31 560·43–94 467·30)	31 971·13 (20 869·92–57 343·38)	77 138·84 (47 788·43–142 699·07)	1437·71 (1402·66–1471·02)	2971·28 (1888·03–5073·60)	2080·67 (1431·20–3320·51)	4137·34 (2580·99–7112·27)
Acute glomerulonephritis			320·39 (305·40–337·79)	388·09 (336·31–439·55)	364·92 (320·81–418·14)	440·13 (384·97–497·41)	11·02 (10·54–11·52)	18·20 (15·67–21·02)	17·88 (15·65–20·48)	19·33 (16·76–22·29)
Chronic kidney disease			26 260·54 (25 370·98–27 674·31)	52 597·49 (28 451·46–104 731·93)	35 567·28 (19 594·69–71 079·85)	73 173·78 (39 848·19–146 920·38)	1186·56 (1150·74–1236·56)	3087·91 (1525·45–5957·18)	2228·97 (1123·50–4332·23)	4045·89 (1959·75–7733·47)
	Chronic kidney disease due to diabetes mellitus		10 965·18 (9948·04–11 927·79)	24 917·34 (11 799·44–48 620·72)	16 709·19 (8038·31–32 885·79)	34 533·86 (16 526·18–68 545·28)	500·76 (452·44–543·96)	1380·79 (639·60–2950·79)	988·38 (469·96–2168·93)	1822·18 (846·77–3821·40)
	Chronic kidney disease due to hypertension		4927·08 (4406·71–5548·10)	11 581·49 (5442·77–23 307·25)	7927·64 (3937·83–16 374·03)	15 979·13 (7308·61–31 613·59)	299·69 (268·21–335·50)	881·05 (389·42–1805·31)	651·09 (305·37–1436·38)	1133·53 (494·50–2192·35)
	Chronic kidney disease due to glomerulonephritis		4453·76 (3958·42–5035·20)	6424·18 (3363·38–13 140·59)	4283·78 (2396·00–8308·05)	9039·62 (4680·69–18 355·20)	150·13 (133·20–168·89)	297·47 (138·24–582·85)	206·01 (98·19–397·97)	395·29 (187·68–772·38)
	Chronic kidney disease due to other and unspecified causes		5914·52 (5263·11–6715·11)	9 674·47 (4510·04–19 424·46)	6646·67 (3303·54–12 497·23)	13 621·17 (6283·42–27 021·38)	235·98 (206·97–266·43)	528·60 (215·11–1022·62)	383·49 (162·50–743·55)	694·89 (280·07–1357·59)
Urinary diseases and male infertility			5825·72 (5620·25–6028·46)	10 426·34 (9402·77–11 382·93)	10 095·27 (8957·65–11 229·62)	10 399·37 (9547·44–11 184·80)	275·21 (267·02–284·13)	669·43 (617·85–710·49)	675·32 (613·87–724·71)	615·15 (570·59–656·79)
	Urinary tract infections		4040·91 (3794·05–4296·03)	8146·94 (7318·69–8878·02)	7944·33 (7044·95–8800·16)	7911·05 (7243·12–8480·18)	203·55 (193·71–213·94)	547·69 (504·11–586·18)	555·37 (506·70–600·12)	493·84 (454·14–530·40)
	Urolithiasis		415·06 (351·37–568·25)	811·84 (646·14–1124·64)	790·08 (621·17–1105·64)	818·57 (666·73–1135·25)	18·71 (15·94–25·76)	44·93 (36·53–61·39)	44·98 (36·60–61·70)	42·32 (35·15–57·36)
	Other urinary diseases		1369·76 (1187·74–1559·01)	1467·56 (1201·55–1761·18)	1360·85 (1098·17–1660·59)	1669·75 (1377·57–2003·92)	52·94 (45·28–59·29)	76·81 (63·74–89·97)	74·97 (62·38–88·48)	78·98 (65·12–92·63)
Gynaecological diseases			265·48 (239·09–289·12)	279·12 (230·81–331·93)	241·06 (197·72–290·31)	334·72 (279·66–393·50)	8·34 (7·45–9·05)	10·40 (8·85–12·00)	9·54 (8·01–11·12)	11·56 (9·98–13·15)
	Uterine fibroids		87·93 (59·02–109·92)	74·58 (42·95–102·54)	65·28 (36·53–92·17)	92·50 (54·16–127·57)	2·91 (1·97–3·64)	2·83 (1·62–3·87)	2·59 (1·41–3·65)	3·34 (1·99–4·48)
	Polycystic ovarian syndrome		18·70 (7·00–35·78)	13·36 (3·70–28·97)	10·40 (2·76–23·20)	19·06 (5·30–41·01)	0·42 (0·16–0·79)	0·31 (0·09–0·66)	0·24 (0·06–0·53)	0·44 (0·12–0·92)
	Endometriosis		3·05 (1·19–4·51)	3·27 (1·63–5·38)	2·58 (1·27–4·38)	3·88 (1·86–6·40)	0·07 (0·03–0·10)	0·07 (0·04–0·12)	0·06 (0·03–0·10)	0·09 (0·04–0·14)
	Genital prolapse		14·40 (7·54–20·69)	15·46 (7·51–24·26)	13·97 (6·48–22·57)	18·34 (9·01–28·39)	0·90 (0·45–1·31)	1·03 (0·47–1·64)	0·99 (0·42–1·61)	1·12 (0·52–1·78)
	Other gynaecological diseases		141·39 (101·36–171·88)	172·45 (125·49–218·70)	148·84 (104·43–194·13)	200·95 (144·22–255·59)	4·04 (2·89–5·01)	6·16 (4·44–7·72)	5·67 (3·99–7·26)	6·58 (4·74–8·32)
Haemoglobinopathies and haemolytic anaemias			5749·22 (5096·81–6685·28)	5779·97 (4823·26–6949·86)	5004·71 (4189·87–6082·30)	6876·12 (5737·17–8265·52)	127·99 (113·11–149·07)	180·40 (157·09–214·26)	168·20 (145·21–201·63)	194·08 (169·72–231·51)
	Thalassaemias		493·34 (422·79–608·87)	185·08 (141·61–241·57)	183·01 (138·72–240·97)	213·10 (156·39–290·44)	6·26 (5·43–7·66)	2·50 (1·97–3·23)	2·48 (1·93–3·22)	2·82 (2·14–3·78)
	Sickle cell disorders		3800·55 (3296·48–4494·70)	3670·21 (2937·01–4540·83)	3065·67 (2453·93–3794·43)	4500·56 (3614·07–5564·63)	55·30 (48·14–65·79)	62·36 (50·24–77·18)	53·61 (43·24–66·72)	74·28 (60·14–91·35)
	G6PD deficiency		711·78 (610·56–850·22)	1022·63 (848·34–1292·34)	892·99 (735·34–1136·16)	1229·30 (1027·20–1539·85)	17·86 (15·35–21·64)	35·32 (29·59–43·86)	31·86 (26·54–40·36)	40·40 (34·27–50·55)
	Other haemoglobinopathies and haemolytic anaemias		743·55 (651·51–875·17)	902·05 (779·71–1080·70)	863·04 (732·23–1036·06)	933·16 (804·81–1123·60)	48·58 (42·73–56·86)	80·23 (68·35–94·83)	80·25 (68·58–95·62)	76·58 (63·51–91·93)
Endocrine, metabolic, blood, and immune disorders			4389·11 (3902·73–4910·93)	5896·37 (5053·29–6903·26)	5839·74 (4967·77–6882·23)	6027·18 (5178·03–6990·48)	144·30 (122·64–153·63)	273·62 (230·68–300·51)	288·59 (243·68–319·95)	256·55 (217·00–282·38)
**Musculoskeletal disorders**			**2198·24 (1965·60–2494·14)**	**3530·99 (3061·48–4042·74)**	**3408·06 (2966·03–3917·00)**	**3601·89 (3142·03–4088·87)**	**89·23 (78·89–98·13)**	**187·74 (163·59–208·10)**	**185·07 (160·86–204·38)**	**185·36 (160·87–205·67)**
Rheumatoid arthritis			574·17 (487·59–668·99)	1069·42 (867·59–1312·76)	936·38 (760·24–1127·82)	1242·46 (1030·94–1471·88)	31·00 (26·47–35·76)	71·92 (59·20–86·61)	64·97 (52·70–76·69)	79·85 (66·62–93·00)
Other musculoskeletal disorders			1624·07 (1432·71–1864·54)	2461·56 (2126·77–2827·39)	2471·68 (2124·41–2861·18)	2359·43 (2049·62–2718·35)	58·23 (51·06–64·61)	115·82 (98·63–128·38)	120·10 (102·88–134·24)	105·51 (89·55–117·51)
**Other non-communicable diseases**			**45 970·60 (40 880·95–50 868·23)**	**32 706·96 (26 818·36–39 441·15)**	**26 202·54 (21 720·48–30 811·16)**	**44 215·51 (35 429·20–53 711·68)**	**639·68 (576·40–703·63)**	**726·74 (585·19–863·19)**	**661·41 (515·67–787·88)**	**834·47 (683·19–989·65)**
Congenital birth defects			40 707·17 (35 761·86–45 627·57)	24 074·01 (18 539·18–30 075·57)	18 002·10 (14 297·67–21 909·94)	34 762·99 (26 442·21–43 727·31)	498·93 (440·16–556·55)	314·56 (249·48–385·20)	240·53 (195·24–289·37)	442·92 (343·85–548·45)
	Neural-tube defects		3407·47 (2362·11–5150·35)	1061·15 (624·88–1861·94)	657·99 (390·69–1118·30)	1781·49 (1060·23–3037·03)	40·07 (27·87–60·47)	12·82 (7·66–22·25)	8·03 (4·88–13·63)	21·32 (12·86–36·16)
	Congenital heart anomalies		17 809·17 (15 807·17–20 444·43)	9582·98 (7610·01–12 056·60)	7261·75 (5737·97–9101·39)	13 714·66 (10 706·45–17 536·79)	221·28 (197·71–253·82)	127·29 (102·76–158·05)	98·09 (78·47–121·70)	177·95 (141·68–224·40)
	Orofacial clefts		192·20 (107·02–316·42)	92·07 (38·93–179·68)	55·87 (23·43–107·87)	160·23 (65·07–318·93)	2·23 (1·24–3·67)	1·07 (0·45–2·08)	0·65 (0·27–1·25)	1·86 (0·76–3·70)
	Down's syndrome		981·25 (850·19–1274·58)	905·44 (740·93–1209·68)	803·19 (668·14–1024·98)	1122·56 (897·15–1619·83)	14·84 (12·82–18·42)	16·46 (13·77–20·42)	15·30 (13·02–18·44)	19·20 (15·91–25·33)
	Other chromosomal abnormalities		1457·92 (1090·95–2066·93)	1572·56 (1062·32–2306·94)	1159·56 (820·03–1636·12)	2129·18 (1388·96–3207·18)	17·49 (13·20–24·55)	19·37 (13·33–27·96)	14·46 (10·31–20·13)	25·90 (17·10–38·36)
	Congenital musculoskeletal and limb anomalies		722·00 (519·66–1318·57)	464·76 (307·64–918·58)	368·00 (235·38–747·07)	671·80 (421·81–1392·47)	8·75 (6·35–15·70)	5·90 (3·97–11·32)	4·70 (3·08–9·26)	8·35 (5·35–16·88)
	Urogenital congenital anomalies		896·51 (698·38–1105·03)	595·48 (447·91–756·97)	483·71 (358·35–614·73)	819·17 (589·84–1058·92)	12·05 (9·56–14·75)	9·75 (7·62–12·22)	8·27 (6·36–10·39)	12·51 (9·47–15·61)
	Digestive congenital anomalies		2915·74 (2252·55–4540·70)	1647·12 (1145·54–2631·98)	1178·35 (811·76–1918·20)	2451·30 (1682·28–4057·15)	34·29 (26·49–53·18)	19·79 (13·83–31·39)	14·24 (9·87–22·88)	29·20 (20·18–47·96)
	Other congenital birth defects		12 324·90 (8589·29–17 181·30)	8152·45 (4878·04–12 787·23)	6033·69 (3792·40–9057·01)	11 912·59 (6943·72–18 797·61)	147·93 (104·12–204·96)	102·11 (63·06–157·32)	76·79 (49·97–112·97)	146·63 (87·76–228·24)
Skin and subcutaneous diseases			2759·24 (1738·63–3611·70)	7355·32 (4732·72–9613·55)	7203·46 (4600·65–9363·05)	7546·98 (4863·56–9910·67)	111·69 (71·83–144·41)	397·36 (258·31–507·56)	409·31 (263·56–525·96)	369·44 (241·80–474·25)
	Cellulitis		437·34 (235·60–565·93)	1443·55 (794·66–1843·83)	1492·39 (821·12–1925·33)	1333·98 (734·32–1710·25)	18·92 (10·44–25·50)	76·51 (41·72–98·15)	82·21 (45·30–104·36)	66·33 (36·60–86·90)
	Pyoderma		1827·47 (1139·86–2485·19)	4818·78 (3123·23–6557·09)	4641·40 (2960·92–6219·43)	5145·51 (3331·93–6981·72)	62·02 (38·96–83·22)	237·18 (155·02–317·07)	242·04 (155·95–320·59)	226·46 (145·99–305·96)
	Decubitus ulcer		380·63 (245·03–516·80)	880·85 (567·08–1172·62)	861·72 (554·45–1152·73)	853·56 (547·76–1148·56)	26·44 (16·90–35·99)	73·88 (46·79–98·32)	74·98 (47·77–100·00)	67·68 (42·88–91·36)
	Other skin and subcutaneous diseases		113·80 (77·82–160·51)	212·14 (144·62–301·59)	207·94 (142·28–292·44)	213·94 (144·26–305·15)	4·31 (3·04–6·30)	9·80 (6·93–14·21)	10·09 (7·06–14·58)	8·98 (6·34–12·91)
Sudden infant death syndrome			2504·19 (2015·23–3003·09)	1277·63 (893·51–1703·86)	996·98 (692·00–1345·10)	1905·55 (1279·76–2579·13)	29·06 (23·39–34·85)	14·82 (10·36–19·77)	11·57 (8·03–15·61)	22·11 (14·85–29·92)
**Injuries**			**200 076·35 (191 347·73–207 066·54)**	**172 041·16 (149 434·07–202 184·44)**	**145 447·98 (126 628·42–168 892·35)**	**215 156·64 (185 789·15–253 836·82)**	**4610·99 (4364·81–4768·86)**	**5224·86 (4646·81–5933·12)**	**4702·53 (4203·74–5283·76)**	**6028·45 (5314·34–6887·16)**
	**Transport injuries**		**65 706·86 (63 870·89–68 591·16)**	**55 363·63 (45 921·31–74 345·12)**	**41 190·60 (34 986·46–53 622·33)**	**84 357·62 (69 341·11–112 401·83)**	**1437·29 (1400·00–1492·45)**	**1407·24 (1178·42–1823·59)**	**1092·32 (939·13–1379·13)**	**2062·94 (1711·24–2674·50)**
	Road injuries		61 412·07 (59 638·88–64 244·07)	50 143·95 (41 534·85–67 394·49)	36 745·08 (31 189·62–47 577·65)	78 200·45 (64 175·36–104 302·36)	1342·28 (1307·57–1393·72)	1267·79 (1061·78–1646·84)	970·37 (832·19–1226·79)	1904·67 (1580·70–2472·57)
		Pedestrian road injuries	21 740·97 (20 466·35–23 243·54)	14 641·13 (12 077·54–18 853·88)	10 428·09 (8725·63–13 212·33)	24 532·64 (20 113·74–32 068·06)	514·33 (485·76–546·68)	427·09 (358·39–542·63)	318·17 (272·73–393·38)	691·18 (574·20–890·17)
		Cyclist road injuries	3095·55 (2811·61–3487·67)	2619·38 (2050·34–3592·08)	2082·28 (1660·42–2754·55)	3976·06 (3118·63–5499·38)	74·75 (68·50–83·50)	77·86 (61·50–106·74)	65·31 (52·54–85·99)	108·33 (84·87–148·09)
		Motorcyclist road injuries	12 601·43 (11 425·94–13 642·74)	10 798·59 (8472·97–15 147·80)	8968·98 (7194·41–12 180·29)	15 356·65 (11 927·50–21 427·53)	251·26 (227·03–269·91)	240·60 (190·32–333·86)	205·37 (167·23–276·61)	326·89 (255·62–455·63)
		Motor vehicle road injuries	23 391·19 (21 813·46–26 453·91)	21 571·93 (17 414·29–29 312·78)	14 886·66 (12 229·66–19 217·44)	33 511·39 (26 815·96–45 300·46)	488·71 (454·63–549·39)	507·47 (415·00–676·83)	369·59 (305·97–466·16)	756·49 (612·72–1007·09)
		Other road injuries	582·93 (535·13–725·78)	512·91 (402·35–700·93)	379·07 (305·29–506·08)	823·71 (641·88–1143·58)	13·23 (12·19–16·38)	14·78 (11·86–19·93)	11·92 (9·76–15·85)	21·78 (17·16–29·97)
Other transport injuries			4294·79 (3991·27–4796·07)	5219·68 (4163·90–7034·05)	4445·53 (3632·09–5901·24)	6157·18 (4902·16–8421·01)	95·01 (88·78–106·62)	139·45 (112·94–181·35)	121·95 (101·38–158·92)	158·27 (126·41–209·19)
**Unintentional injuries**			**69 727·11 (62 737·61–73 048·22)**	**58 163·98 (50 912·78–65 392·90)**	**52 780·22 (45 978·25–59 385·05)**	**66 745·65 (57 994·95–74 882·82)**	**1803·86 (1587·98–1889·28)**	**2341·06 (2008·65–2571·77)**	**2 290·08 (1 959·00–2 503·37)**	**2 387·32 (2 046·80–2 600·93)**
Falls			16 827·42 (14 324·96–17 828·35)	20 886·11 (17 449·72–22 846·15)	20 493·11 (17 182·95–22 697·00)	21 544·24 (17 931·81–23 369·17)	678·46 (559·21–719·32)	1251·79 (1011·31–1387·09)	1287·01 (1038·06–1422·97)	1175·69 (936·15–1302·65)
Drowning			16 575·72 (15 016·39–17 803·41)	9540·30 (7961·64–11 517·74)	8197·61 (6876·39–9774·78)	11 628·32 (9529·95–13 961·00)	302·93 (272·75–322·39)	237·03 (202·09–281·10)	213·21 (181·29–248·72)	269·91 (225·17–317·83)
Fire, heat, and hot substances			5696·05 (4651·71–6188·51)	3883·45 (3084·15–4685·24)	3223·27 (2531·66–3863·78)	4792·54 (3770·80–5808·24)	132·08 (110·13–141·63)	125·56 (103·54–148·38)	110·45 (91·12–128·06)	141·28 (114·58–167·81)
Poisonings			2851·04 (2118·58–3240·46)	1296·74 (959·52–1620·92)	983·17 (727·91–1188·11)	1792·95 (1276·11–2222·46)	57·08 (42·42–63·58)	32·68 (24·53–40·83)	26·23 (19·55–30·96)	42·21 (30·47–50·81)
Exposure to mechanical forces			7509·63 (6132·22–8051·89)	5290·23 (4319·19–6044·82)	4498·37 (3618·64–5133·86)	6471·13 (5207·72–7369·41)	154·84 (123·96–165·14)	139·52 (112·67–155·15)	125·45 (100·76–140·08)	159·20 (126·92–175·93)
	Unintentional firearm injuries		1123·71 (881·70–1233·13)	846·50 (646·14–1001·76)	696·33 (534·86–825·31)	1006·04 (755·92–1184·61)	22·95 (18·24–24·76)	21·19 (16·67–24·00)	18·54 (14·73–20·97)	23·61 (18·34–26·70)
	Unintentional suffocation		1474·69 (1151·64–1717·10)	814·68 (636·24–1018·58)	641·16 (502·76–781·24)	1074·87 (836·49–1351·88)	22·63 (17·38–26·00)	16·37 (12·54–19·40)	14·09 (10·53–16·57)	19·40 (14·90–23·15)
	Other exposure to mechanical forces		4911·23 (3856·75–5218·17)	3629·05 (2894·26–4145·74)	3160·89 (2479·92–3612·37)	4390·21 (3457·01–4999·36)	109·26 (84·20–115·90)	101·96 (80·43–113·90)	92·82 (72·82–104·27)	116·19 (90·15–128·57)
Adverse effects of medical treatment			4601·97 (3861·10–5157·12)	4614·89 (3822·40–5301·78)	4228·26 (3506·22–4839·86)	5268·64 (4314·89–6074·53)	126·73 (109·32–140·49)	177·37 (152·27–197·86)	173·99 (149·25–194·59)	182·22 (156·41–203·24)
Animal contact			4268·95 (3176·54–4791·49)	3868·17 (2924·74–4701·03)	3481·14 (2581·51–4244·26)	5061·15 (3746·62–6145·44)	91·59 (68·84–102·20)	109·83 (82·41–130·84)	103·55 (77·42–124·56)	132·25 (97·55–156·60)
	Venomous animal contact		3662·04 (2606·92–4190·13)	3159·01 (2276·37–3908·85)	2884·63 (2046·92–3609·19)	4166·84 (2929·13–5179·51)	78·81 (56·81–89·39)	90·76 (65·91–111·03)	86·45 (62·09–106·23)	110·12 (77·74–133·59)
	Non-venomous animal contact		606·90 (479·70–841·74)	709·15 (533·11–986·40)	596·51 (448·58–831·88)	894·31 (666·54–1252·16)	12·78 (10·32–17·37)	19·07 (14·95–25·83)	17·09 (13·24–23·55)	22·13 (17·29–30·26)
Foreign body			4703·00 (4114·40–5317·87)	4174·11 (3501·47–4912·46)	3811·67 (3223·49–4415·19)	4567·32 (3771·24–5483·96)	106·27 (92·49–114·91)	134·38 (112·31–149·53)	134·00 (110·79–149·04)	130·68 (108·40–145·76)
	Pulmonary aspiration and foreign body in airway		4203·16 (3638·86–4809·69)	3706·49 (3094·59–4409·35)	3369·33 (2832·09–3920·11)	4045·38 (3323·02–4900·60)	95·92 (82·50–104·51)	120·87 (99·54–134·29)	120·22 (98·94–133·91)	117·12 (96·82–130·45)
	Foreign body in other body part		499·84 (372·99–586·34)	467·62 (321·20–592·42)	442·34 (310·26–559·55)	521·94 (349·92–664·03)	10·34 (7·86–12·04)	13·51 (9·82–16·18)	13·79 (10·21–16·37)	13·56 (9·66–16·29)
Environmental heat and cold exposure			1920·96 (1216·20–2408·60)	1728·59 (1113·49–2228·05)	1400·07 (871·24–1775·05)	2095·87 (1321·65–2627·11)	55·60 (36·43–71·46)	62·92 (41·44–79·06)	54·70 (34·98–67·01)	71·23 (45·59–87·55)
Other unintentional injuries			4772·37 (4186·14–5000·09)	2881·41 (2502·77–3292·95)	2463·55 (2108·62–2852·29)	3523·50 (3043·77–4015·07)	98·29 (84·22–102·79)	69·99 (60·80–78·49)	61·51 (52·07–69·39)	82·64 (71·27–91·63)
**Self-harm and interpersonal violence**			**54 833·93 (50 105·60–58 459·47)**	**54 502·13 (44 319·70–69 140·81)**	**47 539·51 (39 342·84–59 955·82)**	**59 901·26 (47 778·17–77 176·33)**	**1207·94 (1108·85–1290·98)**	**1393·66 (1126·40–1749·81)**	**1236·80 (1023·74–1523·52)**	**1494·83 (1186·81–1911·21)**
Self-harm			34 621·42 (32 412·04–37 408·58)	37 320·20 (28 815·99–49 427·27)	32 600·62 (26 039·66–41 920·43)	41 608·85 (31 743·20–55 659·49)	817·15 (762·05–883·74)	1031·34 (801·35–1342·58)	917·97 (736·10–1152·60)	1115·17 (850·45–1478·85)
	Self-harm by firearm		2840·07 (2373·75–3578·95)	2486·62 (1649·94–3699·28)	2049·23 (1398·06–2944·41)	2847·55 (1849·47–4298·88)	67·52 (55·39–84·13)	66·42 (45·43–97·31)	55·67 (39·55–79·18)	73·87 (48·79–110·93)
	Self-harm by other specified means		31 781·35 (29 699·54–34 445·40)	34 833·59 (26 961·13–45 891·30)	30 551·39 (24 446·50–39 200·07)	38 761·30 (29 680·83–51 796·56)	749·63 (700·93–812·55)	964·92 (750·23–1 250·29)	862·30 (690·38–1083·26)	1041·30 (794·94–1377·72)
Interpersonal violence			20 212·52 (16 632·13–23 093·86)	17 181·92 (13 451·18–21 891·19)	14 938·89 (11 670·64–18 893·94)	18 292·41 (14 228·41–22 914·68)	390·79 (320·78–453·71)	362·31 (286·16–452·80)	318·83 (254·60–397·78)	379·66 (298·02–472·54)
	Physical violence by firearm		8615·86 (5744·46–9727·92)	6973·53 (4648·78–8926·42)	5820·85 (3953·91–7402·88)	7424·86 (4855·17–9440·71)	160·98 (107·16–182·48)	138·98 (93·71–174·07)	117·43 (80·51–146·03)	146·43 (96·48–182·33)
	Physical violence by sharp object		4876·53 (3900·91–6470·22)	4467·67 (3255·02–6114·85)	3992·53 (2915·38–5434·44)	4536·38 (3302·32–6189·05)	97·39 (78·14–128·54)	96·80 (71·79–129·75)	87·37 (63·93–116·33)	97·10 (72·28–129·61)
	Physical violence by other means		6720·13 (5734·01–8489·35)	5740·72 (4418·98–7707·19)	5125·50 (3984·58–6895·60)	6331·17 (4851·24–8556·87)	132·43 (111·32–168·39)	126·53 (98·32–170·19)	114·03 (89·49–151·37)	136·13 (105·19–184·51)
**Forces of nature, conflict and terrorism, and executions and police conflict**			**9808·44 (6797·54–13 037·71)**	**4011·42 (708·58–18 585·61)**	**3937·66 (690·63–18 633·79)**	**4152·10 (749·22–18 680·14)**	**161·89 (112·58–215·06)**	**82·90 (13·20–397·46)**	**83·32 (13·14–412·86)**	**83·36 (13·53–390·38)**
Exposure to forces of nature			357·59 (217·91–507·75)	1386·15 (117·93–9787·90)	1357·70 (117·14–9343·07)	1440·21 (120·24–10 113·39)	7·06 (4·22–10·13)	31·67 (2·86–199·16)	31·91 (2·87–200·20)	31·65 (2·94–198·20)
Conflict and terrorism			9226·02 (6241·21–12 407·43)	1756·66 (207·67–12 381·20)	1722·46 (204·80–12 441·55)	1835·04 (210·54–12 401·08)	150·46 (101·46–202·67)	33·50 (4·12–267·40)	33·70 (4·14–278·34)	34·03 (4·10–262·88)
Executions and police conflict			224·83 (119·67–261·63)	868·60 (0·00–5251·96)	857·49 (0·00–5157·42)	876·84 (0·00–5 336·91)	4·38 (2·28–5·04)	17·72 (0·00–103·92)	17·71 (0·00–103·30)	17·68 (0·00–104·24)

Because of differences in population and Socio-demographic Index across the scenarios, forecasts of numbers might result in reference values outside the better and worse estimates. Values are reported in thousands (95% UI). Rows in bold type indicate Level 1 and Level 2 causes from the Global Burden of Disease Study (GBD) cause hierarchy. G6PH=glucose-6-phosphate dehydrogenase.

Assessing differences and ranges for reference forecasts compared with better and worse health scenarios identified important opportunities for accelerating progress—or threats of reversing health gains. For instance, the global reference forecast for HIV/AIDS YLLs showed a 30·6% decline (19·1–40·4) from 2016–40. The better health scenario had HIV/AIDS YLLs decreasing more than 50%. Yet if the worse health scenario prevails, a 120·2% increase (67·2–190·3) in YLLs—or nearly 118 million YLLs—from HIV/AIDs could occur by 2040. For most of the leading NCDs, reference forecasts showed rising YLLs by 2040; however, for some causes such as ischaemic heart disease, projections under the better health scenario showed possible declines by 2040. Reference forecasts for chronic obstructive pulmonary disease (COPD) and tracheal, bronchus, and lung cancer had moderate YLL increases from 2016–40, while the better health scenario had small, non-significant decreases for each cause. For many NCDs, including COPD and lung cancer, the worse health scenario pointed to YLL increases exceeding 70% by 2040. Projections across all scenarios showed rising YLLs due to several cancers, including cervical, breast, colon and rectum, and liver cancers, reflecting the effects of both population growth and ageing. Such demographic factors were also pronounced for Alzheimer's disease and other dementias, for which deaths and YLLs more than doubled by 2040 in each scenario; and diabetes, for which YLLs were forecast to rise in all scenarios for 2040 (ie, a 76·7% [10·3–228·8] increase for the reference; 11·6% [–27·3 to 100·5] for better and 169·3% [66·7 to 397·1] for worse. Injury YLLs generally showed potential for sizeable decreases in the future, as exemplified by the reference and better health scenarios for 2040. However, for some injuries, including road injuries and self-harm, 2040 worse health scenarios showed rising YLLs and deaths.

Deaths from lower respiratory infections (LRIs) were projected to increase between 2016 (2·4 million [2·1–2·5]) and 2040 (3·1 million [2·4–3·9]). YLLs for LRIs, however, were forecasted to decrease by 24·8% (–3·4 to 47·9), surpassing the more moderate reduction in projected all-cause YLLs in 2040. These results represent how LRIs have their greatest toll among both children younger than 5 years and elderly people, and thus population ageing may differentially affect overall measures of mortality from LRIs. Relative to the reference forecast, the range between better and worse health scenarios provides a signal on the scope for policy change. Among the leading causes of death and YLLs, the largest range-to-reference ratios included HIV/AIDS, neonatal disorders, road injuries, diarrhoeal diseases, tuberculosis, lung cancer, and stroke. This metric was particularly pronounced for HIV/AIDS, with the ratio exceeding 2 for deaths and YLLs.

**Figure 4:**
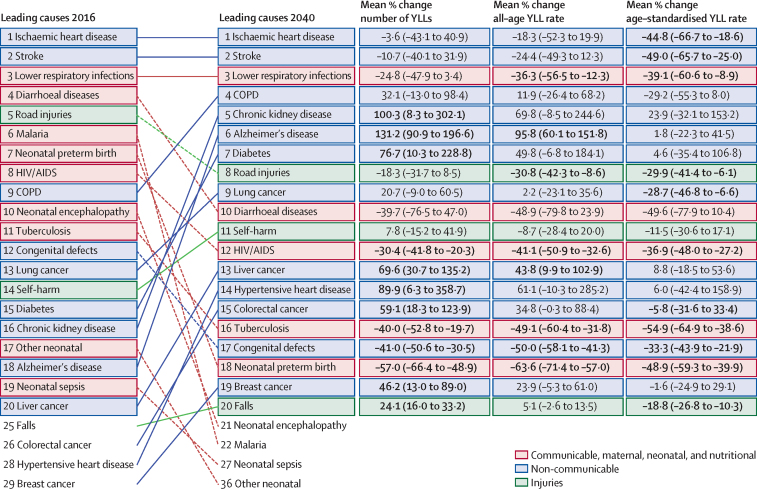
Leading 20 Level 3 causes of YLLs globally in 2016 and 2040 by rank order Figure shows percentage changes in the number of years of life lost (YLLs), all-age, and age-standardised rates. Rectangles are colour-coded based on Global Burden of Disease (GBD) Level 1 cause hierarchy: red=communicable, maternal, neonatal, and nutritional diseases; blue=non-communicable causes; green=injuries. Causes are connected by lines between time periods, with solid lines representing increasing relative rank and dashed lines representing decreasing rank. From 2016 to 2040, three measures of change are shown: percentage change in total number of YLLs, percentage change in the all-age YLL rate, and percentage change in the age-standardised YLL rate. Statistically significant changes are in bold. COPD=chronic obstructive pulmonary disease. Neonatal preterm birth=neonatal disorders due to preterm birth complications.

From 2016–40, the reference forecast showed the potential for major shifts in the leading causes of YLLs (figure 4). While the leading three causes of YLLs in 2016 remained the same in 2040 (ie, ischaemic heart disease, stroke, and LRIs), most of the top ten causes fell in rank by 2040. The primary exception was COPD, which was forecasted to rise from 9th to 4th between 2016 and 2040. Several other NCDs were projected to rise in ranking by 2040, particularly diabetes (from 15th to 7th), chronic kidney disease (from 16th to 5th), and Alzheimer's disease (from 18th to 6th). By comparing forecasted changes in terms of total YLLs, all-age YLL rates, and age-standardised YLL rates, the amount by which shifts in projected population growth and age structure account for changing patterns, as compared with cause-specific mortality rates, can be parsed out. For many NCDs, population growth and ageing fuelled their upward trajectories, with significant increases projected from 2016–40. By contrast, several CMNN causes, including HIV/AIDS, neonatal disorders, and malaria, recorded significant reductions across all three measures as well as relative rank; these trends underscore the potential for continued progress against these causes in parallel with changing demographic patterns. Two causes of injuries—road injuries and self-harm—ranked among the leading 20 causes of death in 2016, but showed divergent trends by 2040. Road injuries not only fell in relative rank (from 5th to 8th), but also saw significant decreases in all-age YLL rates and age-standardised YLL rates by 2040. Conversely, self-harm somewhat rose in relative rank (from 14th to 11th), though this was mainly driven by faster projected reductions for several CMNN causes ranked above self-harm in 2016.

**Figure 5:**
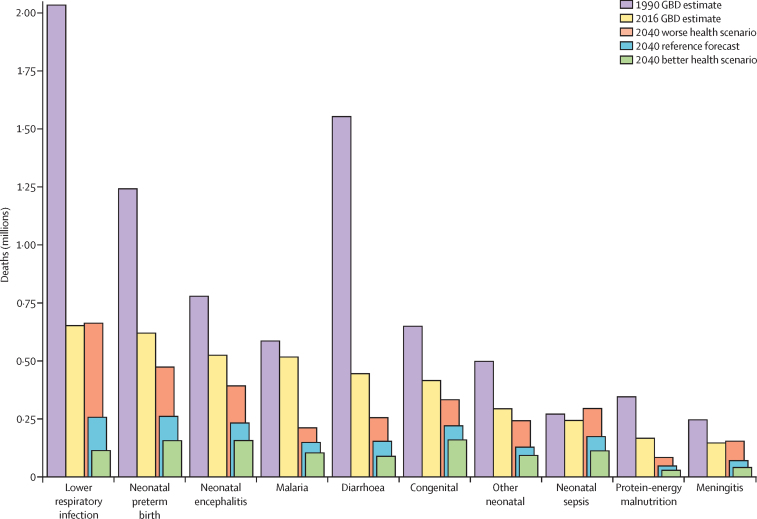
Evolution of leading causes of global under-5 deaths from 1990 to 2016 and in the 2040 reference forecast, 2040 better health scenario, and 2040 worse health scenario Estimates are reported in millions, with 1990 and 2016 estimates based on Global Burden of Disease Study (GBD) 2016 results. Neonatal preterm birth=neonatal disorders due to preterm birth complications. Congenital=congenital defects. Other neonatal=other neonatal disorders.

Despite marked reductions in the leading causes of under-5 deaths from 1990–2016, our 2040 worse health scenarios suggest that such gains could be undone in the future (figure 5). Based on the reference forecast, 2·1 million (95% UI 1·6–2·7) under-5 deaths were projected to occur in 2040, a 57·2% (44·8–67·1) decrease from 2016 (ie, 5·0 million [4·7–5·2]). The most pronounced decreases in under-5 deaths were forecasted for LRIs, malaria, and neonatal disorders due to preterm birth complications. Conversely, far less progress was projected for neonatal sepsis and meningitis under the reference forecast. In considering 2040 scenarios, three causes—LRIs, neonatal sepsis, and meningitis—showed the potential for equalling or exceeding their toll in 2016 under the 2040 worse health scenario. In absolute terms, this was particularly evident for LRIs, with the worse health scenario resulting in 661 000 (233 000–1 173 000) under-5 LRI deaths in 2040. Yet, if all countries could meet the pace of progress established by the better health scenario, under-5 deaths from LRIs could decrease to 115 000 (43 100–264 000) in 2040.

**Figure 6:**
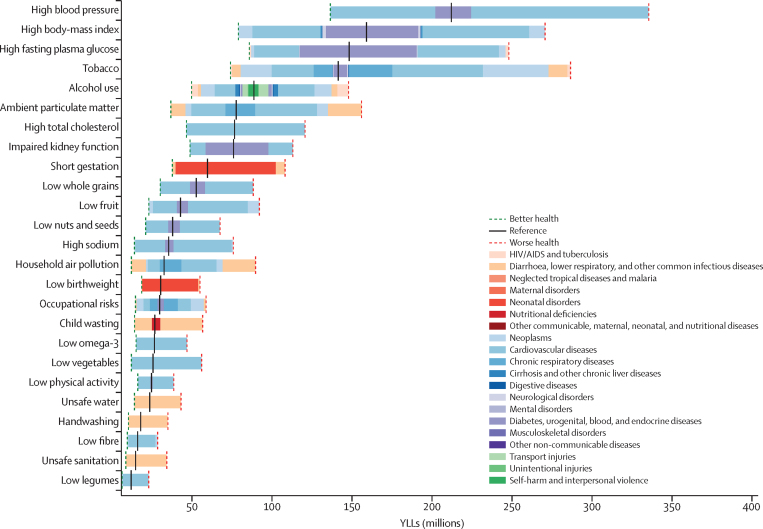
Leading 20 risk factors contributing to the global difference in risk-attributable YLLs between the 2040 reference forecast, 2040 better health scenario, and 2040 worse health scenario The differences between reference and better and worse health scenarios are grouped by Global Burden of Disease Study (GBD) Level 2 causes attributable to risks, which are colour coded to correspond with the causes contributing to the change in years of life lost (YLLs) between scenarios for each risk factor. Black solid vertical lines represent all-cause attributable YLLs in the 2040 reference forecast, red dashed vertical lines represent all-cause attributable YLLs in the 2040 worse health scenario, and green dashed vertical lines all-cause attributable YLLs in the 2040 better health scenario.

Charting how changes in underlying risks could contribute to higher or lower rates of premature mortality across health scenarios showed the large potential for improving future health outcomes by intervening on modifiable risk factors today (figure 6). In 2040, our reference forecasts pointed to three metabolic risks—high blood pressure, high BMI, and high FPG—as among the five leading global risk factors for YLLs. Differences between risk-attributable YLLs in the better and worse health scenarios were at least 2·6-times for these leading metabolic factors, a trend driven by massive variation in YLLs from cardiovascular diseases. Tobacco, the fourth-leading risk factor in 2040, showed a similarly large range across scenarios, reflecting the equally variable past trends in smoking across countries. In fact, based on the 2040 worse health scenarios, tobacco could eclipse high BMI and high FPG to become the 2nd leading risk factor behind high blood pressure. Other risks for which the range in risk-attributable YLLs spanned more than 50 million across better and worse health scenarios included high total cholesterol, ambient particulate matter pollution, household air pollution, alcohol use, and several dietary risks. Short gestational age, ranked as the 9th leading risk factor for attributable YLLs in 2040, was the only risk among the leading ten that mainly affected CMNN diseases. Note that differences in risk-attributable YLLs between reference and scenarios for each risk must be interpreted with the shift of all risk factors and drivers of mortality set to better or worse health, not just the risk in question.

### Super-region and regional findings

**Figure 7:**
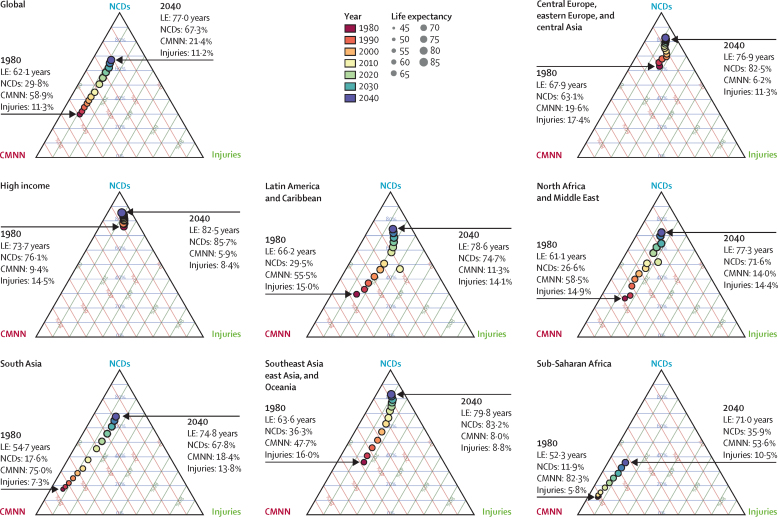
Global and GBD super-region life expectancy and relative contribution of Level 1 GBD cause groups to total YLLs, 1980–2040, for the reference forecast scenario Each ternary plot represents the relative contribution of years of life lost (YLLs) by Level 1 Global Burden of Disease Study (GBD) cause group in a given year and changes in life expectancy over time as depicted by colour-coded circles sized relative to life expectancy. The closer each circle is to a given corner of the ternary plot—communicable, maternal, neonatal, and nutritional (CMNN), non-communicable diseases (NCDs), and injuries—the greater is the proportion of YLLs due to that Level 1 GBD cause. If CMNN, NCDs, and injuries contributed equally to YLLs (ie, each a third), the circle would be positioned in the middle of the ternary plot. LE=life expectancy.

Figure 7 shows the evolution of YLLs, as categorised by CMNN, NCDs, and injuries, in the past and across 2040 projections globally and regionally. While the shift from CMNN to NCD YLLs was evident across GBD super-regions, the magnitude by which such epidemiological transitions varied. The most pronounced changes were forecasted in south Asia, as well in much of Latin America and southeast Asia, east Asia and Oceania, and North Africa and the Middle East. By contrast, high-income countries had comparatively smaller shifts, because such transitions occurred before these periods. By 2040, despite some forecasted changes in burden composition, sub-Saharan Africa still had a greater share of YLLs from CMNN causes than NCDs.

**Figure 8:**
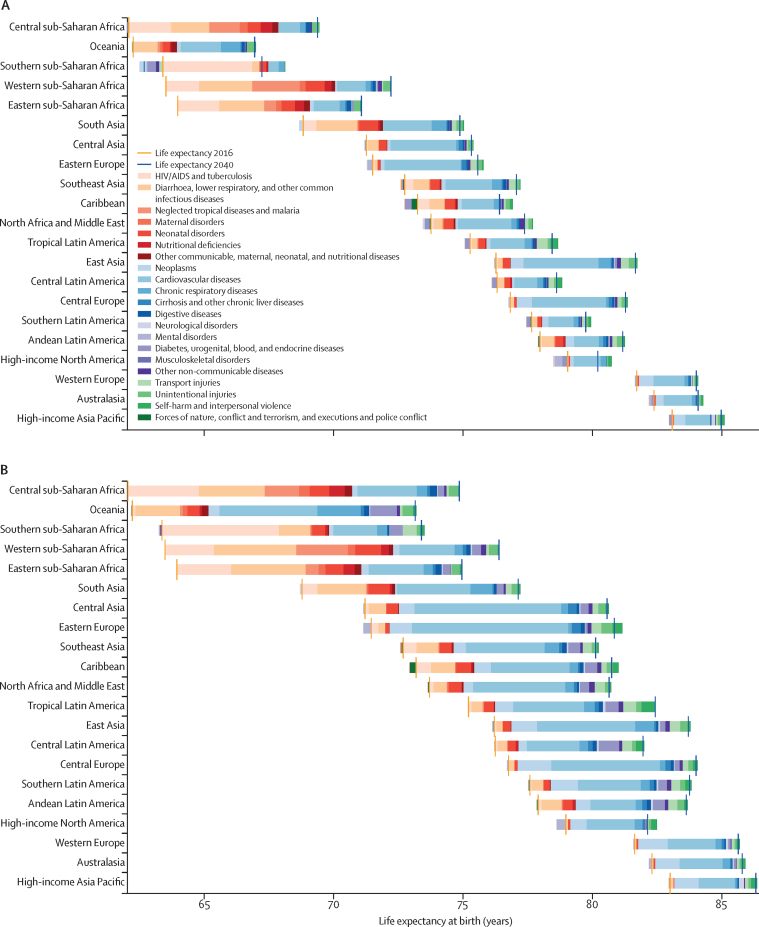

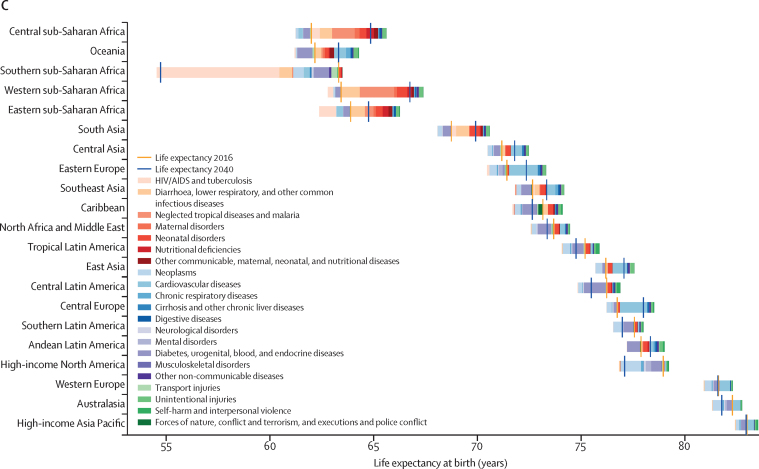
Life expectancy changes from 2016 to 2040, by the 21 GBD Level 2 causes of death and for each GBD region, for the (A) reference forecast, (B) better health scenario, and (C) worse health scenario Regions are based on Global Burden of Disease Study (GBD) 2016 location hierarchy. Blue vertical lines represent the estimated life expectancy in 2040 for both sexes, and orange vertical lines represent GBD 2016 estimated life expectancy in 2016 for both sexes. Horizontal rectangles are colour-coded by GBD Level 2 causes contributing to the difference in life expectancy between 2016 and 2040, with causes to the left of the orange line contributing to a reduction in life expectancy and causes to the right of the orange line contributing to an increase in life expectancy.

By GBD region, how forecasted changes in cause-specific mortality affected life expectancy varied by the reference (figure 8A), better health scenario (8B), and worse health scenario (8C). For the reference, western sub-Saharan Africa, central sub-Saharan Africa, and eastern sub-Saharan Africa recorded the largest projected gains by 2040, whereas as higher-income regions—which already achieved relatively high life expectancies in 2016—were forecast to have the smallest improvements. Although some regional convergence occurred, by 2040, an 18-year gap in life expectancy remained between Oceania and high-income Asia Pacific. Under the better health scenario, a greater chance for global convergence on life expectancy emerged, with the gap between the highest and lowest regional life expectancies decreasing to 13·2 years (95% UI 10·8–15·5). Larger declines in cardiovascular diseases across regions, faster reductions in many CMNN causes, and minimal increases in causes like diabetes underpinned the regional differences in forecasted life expectancies in the reference and better health scenario. Conversely, the combination of worsening risk trends and slowed gains in economic growth, education, and reductions in total fertility rate under 25 years could culminate in reversals in life expectancy improvements and widen regional gaps to 28·2 years (22·5–36·3). This potential was particularly striking in southern sub-Saharan Africa, where the rebound of HIV under the worse health scenario would lead to sizeable declines in life expectancy. Elsewhere, minimal progress against cardiovascular diseases contributed to slowed or negligible life expectancy gains.

### Country-level findings

**Figure 9:**
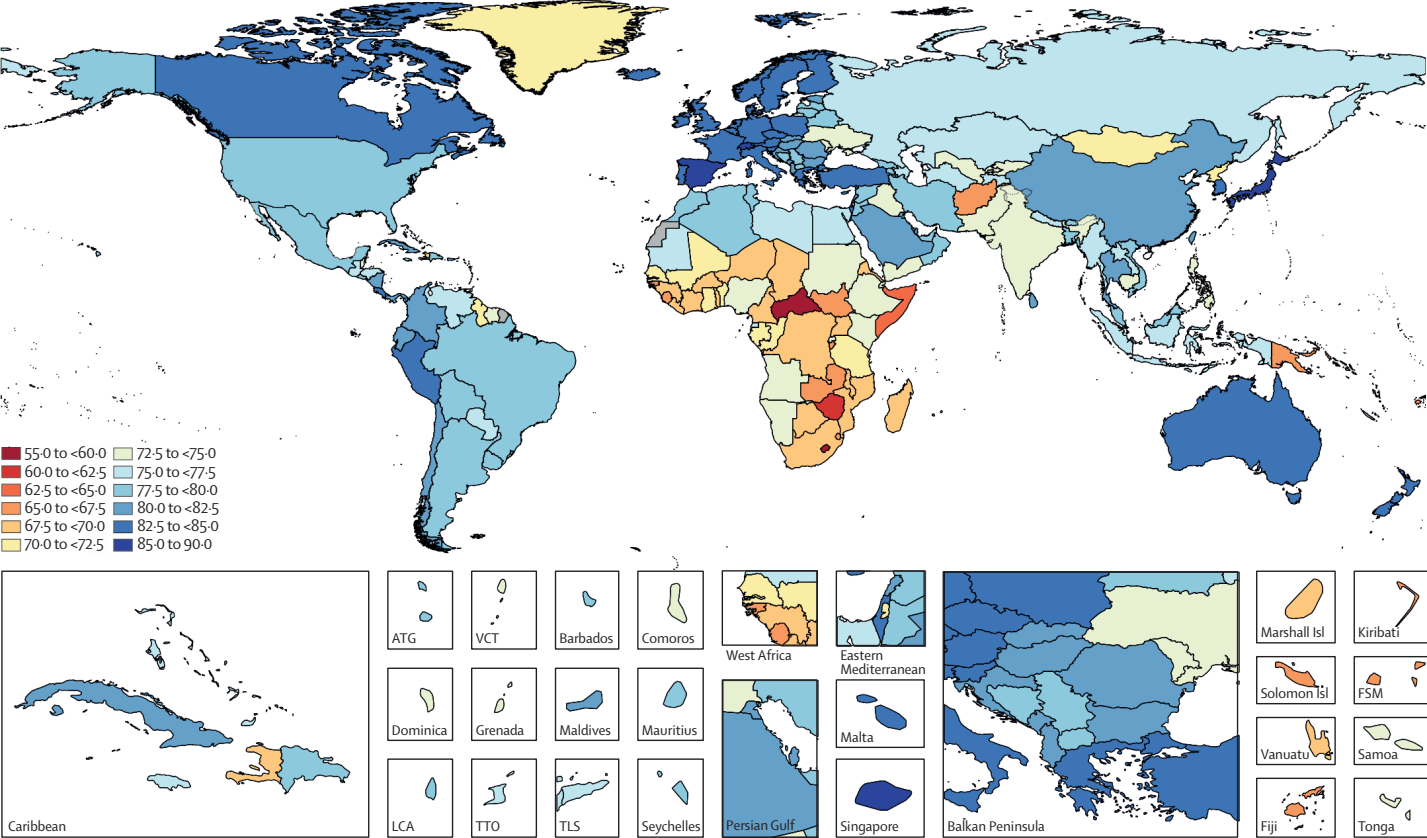
Map of life expectancy for both sexes in 2040 based on the reference forecast Key shown in years. ATG=Antigua and Barbuda. FSM=Federated States of Micronesia. LCA=Saint Lucia. TLS=Timor-Leste. TTO=Trinidad and Tobago. VCT=Saint Vincent and the Grenadines.

Based on the 2040 reference forecast, life expectancy for both sexes combined ranged from 57·3 years (95% UI 48·7–65·3) in Lesotho to 85·8 years (83·6–87·4) in Spain (figure 9). By 2040, 59 countries were projected to meet or exceed a life expectancy of 80 years. Beyond most high-income countries, such locations included those in Latin America (eg, Cuba, Peru, Colombia, and Chile), southeast Asia (eg, Thailand and Sri Lanka), and China. China surpassed a life expectancy of 80 years by 2040 (81·9 years [78·6–84·2]), and also recorded higher levels than the USA (79·8 years [76·3–82·9]). Russia, Tajikistan, Kazakhstan, and other central Asian countries all had forecasted life expectancy between 75 and 80 years by 2040 while India and Pakistan were just below 75 years. Further, reference forecasts put several countries in sub-Saharan Africa on the trajectory to reach similar levels of life expectancy, including Rwanda (74·8 years [66·2–81·1]), Nigeria (74·8 years [71·5–78·3]), and Kenya (73·9 years [67·2–78·1]). In 2040, four countries in sub-Saharan Africa were projected to have life expectancies less than 65 years (Central African Republic, Lesotho, Somalia, and Zimbabwe).

**Figure 10:**
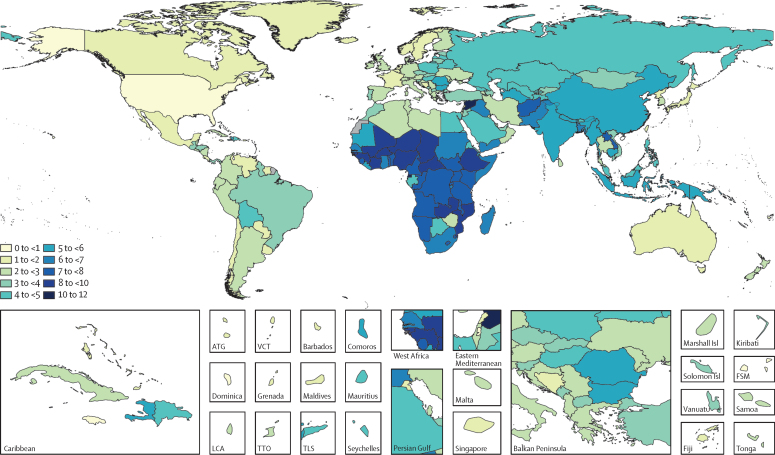
Map of the differences in life expectancy for both sexes from 2016 to 2040 based on the reference forecast Key shown in years. Legend shown in years. ATG=Antigua and Barbuda. FSM=Federated States of Micronesia. LCA=Saint Lucia. TLS=Timor-Leste. TTO=Trinidad and Tobago. VCT=Saint Vincent and the Grenadines.

In terms of projected changes in life expectancy, reference forecasts unveiled striking geographic heterogeneities in much of the world by 2040 (figure 10). The largest absolute gains were primarily found in sub-Saharan Africa, with several countries recording projected gains of 9 years or higher (eg, Equatorial Guinea, Nigeria, Mali, and Mozambique). Some countries in south, southeast, and east Asia also saw substantive gains forecasted (eg, China, Indonesia, Laos), nearing or exceeding a gain of 5 years in 2040. Some countries in North Africa and the Middle East saw potential gains in forecasted lifespans; however, for a subset of them, such as Syria, this was likely a reflection of marked life expectancy declines in the recent past due to its civil war and difficulties with accurately forecasting the effects of war in the future. Bolivia, Dominican Republic, Brazil, and Panama had among the highest forecasted life expectancy gains in Latin America and the Caribbean, each increasing average lifespans by at least 3 years by 2040. Among high-income countries, most saw forecasted increases of 1 to 3 years in life expectancy by 2040; an exception was Portugal, which had a projected gain of 3·5 years (0·5–6·0) in the reference scenario. A map showing differences in country-level life expectancy across 2040 scenarios is in appendix 2 (p 81).

**Figure 11:**
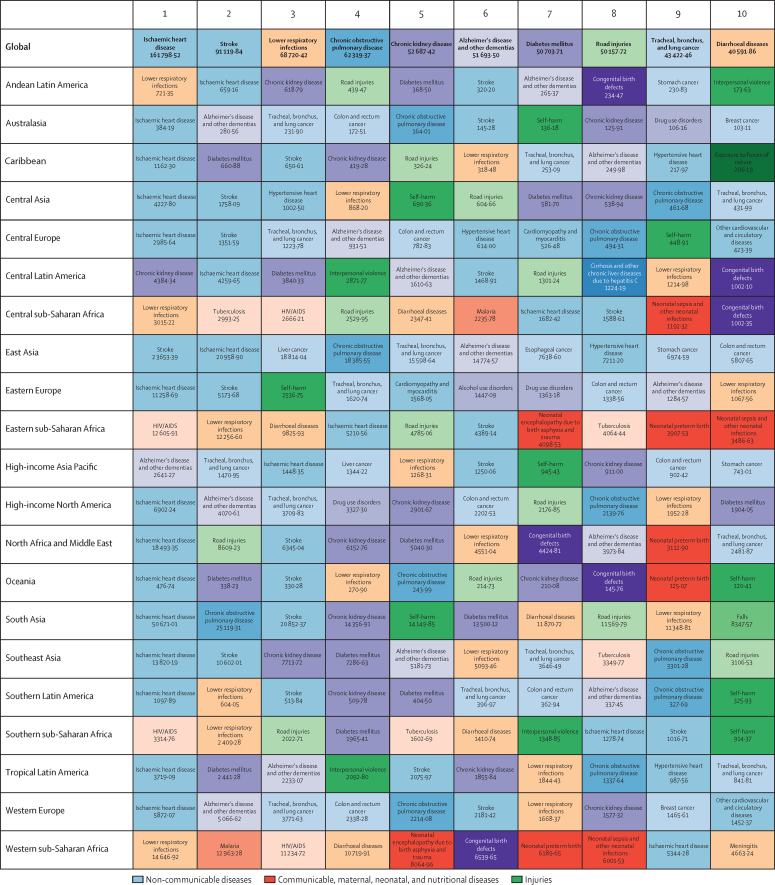
Ten leading causes of YLLs in 2040, globally and by GBD region, based on the reference forecast Values are reported in thousands. Causes are listed at the Global Burden of Disease Study (GBD) Level 3 cause hierarchy from GBD 2016, and are colour coded in accordance with their Level 2 categorisation (shading represents GBD Level 2 cause hierarchy). YLLs=years of life lost.

Reference forecasts showed distinct geographic patterns for the leading causes of YLLs in 2040, stressing the importance of charting country-level trajectories. Figure 11 presents global and regional findings, while country-level results can be found in appendix 2 (pp 82–90). In 2040, 105 of 195 locations had ischaemic heart disease as the leading cause of YLLs, while stroke (in six countries) and diabetes (in 20 countries) were also among the leading NCDs for YLLs. Sub-Saharan Africa was the primary exception, where HIV/AIDS, diarrhoeal diseases, and LRIs were projected to remain the main leading causes of YLLs in 2040. Nonetheless, ischaemic heart disease, stroke, and diabetes emerged as some of the leading ten causes of YLLs in western, southern, and eastern sub-Saharan Africa by 2040, portending a rise in the double burden of disease. Notably, Alzheimer's disease also was among the leading three causes of YLLs for 39 locations in 2040, reflecting the effects of population ageing.

In southeast Asia, east Asia, and Oceania, the projected toll of CMNN diseases generally receded as NCDs were forecasted to become the leading cause of YLLs by 2040. COPD surfaced as a leading cause of YLLs in most south Asian countries, generally out-ranking diabetes and chronic kidney disease by 2040. Beyond the burden of ischaemic heart disease (IHD) and stroke projected for central Europe, eastern Europe, and central Asia, lung and colon cancers frequently ranked among the leading five causes of YLLs in 2040. For high-income countries, Alzheimer's disease was the first-leading or second-leading cause of YLLs in 24 locations by 2040. Within Latin America and the Caribbean, diabetes and chronic kidney disease were forecasted to rank alongside IHD as the leading causes of YLLs in 2040, followed by interpersonal violence as the 4th-leading cause of YLLs. In North Africa and the Middle East, road injuries were forecasted to remain—or rise—as a leading cause of YLLs in many countries by 2040, ranking at least third in 11 of 21 countries. Reference forecasts suggest that conflict and terrorism were likely to still incur high levels of YLLs in North Africa and the Middle East in 2040. Although CMNN diseases were forecasted to remain leading causes of YLLs in most sub-Saharan African countries, all 45 countries included IHD as among the ten causes with the greatest toll in 2040. The reference forecast showed that diabetes could rank between the 2nd and 4th leading cause of YLLs in four countries in this region, although LRIs, HIV/AIDS, and diarrhoeal diseases were projected to remain as the leading cause of premature mortality throughout southern sub-Saharan Africa in 2040.

**Figure 12:**
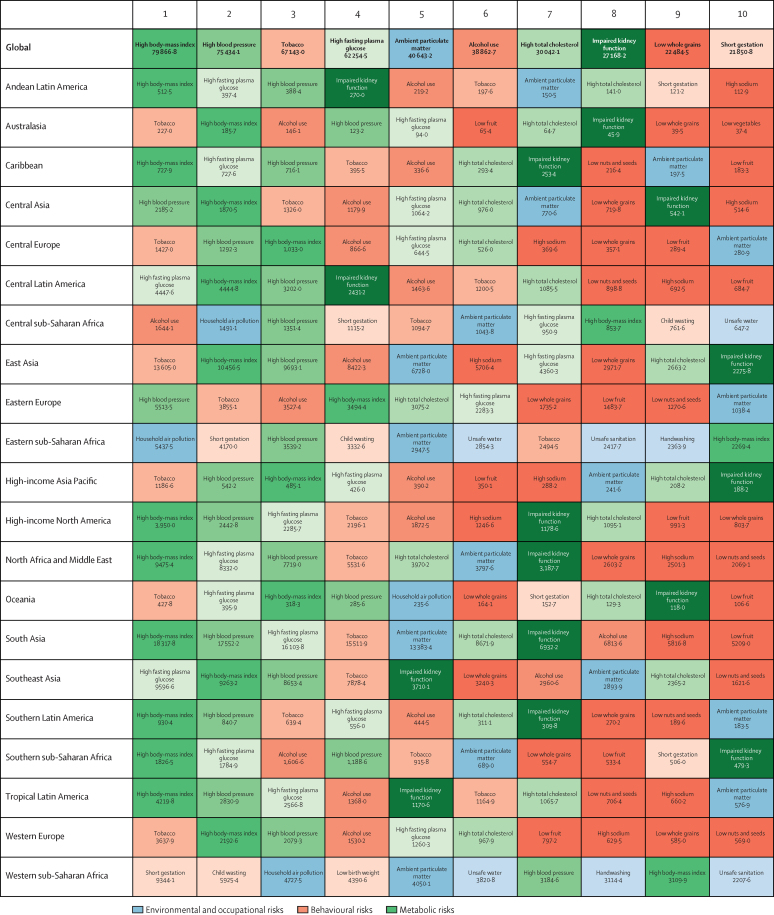
Ten leading risk factors contributing to the largest differences in risk-attributable YLLs between the 2040 reference forecast and 2040 better health scenario, globally and by GBD region Values are reported in thousands. Risks are listed at the Global Burden of Disease Study (GBD) Level 3 risk hierarchy from GBD 2016, and are colour coded in accordance with their Level 1 categorisation (shading represents GBD Level 2 cause hierarchy). YLLs=years of life lost.

By measuring gaps between reference forecasts and 2040 better health scenarios for risk-attributable YLLs (figure 12), we could identify which modifiable risk factors offer the greatest opportunity for averting premature mortality in the future—if each country can move their health trajectories toward the better health scenario. Global and regional results are presented in figure 12, and country-level results are in appendix 2 (pp 58–68). Risk factors are ranked in accordance with the largest differences in attributable YLLs between the reference and better health scenarios, signalling which risks may be the best targets for investment today to have the largest impact on avoidable YLLs in the future. Out of 195 countries and territories, 76 had high BMI as the leading risk for potentially avoidable YLLs by 2040, followed by 48 for tobacco, and 25 for high systolic blood pressure. For most locations, at least one of these risks ranked among the leading three priority targets; sub-Saharan Africa was the main exception, where short gestational age, household air pollution, and child wasting were forecasted to have the among largest gaps between the reference and better health scenarios by 2040.

Overall, a combination of metabolic risk factors amenable to health care (ie, high blood pressure, high FPG, and high total cholesterol) and risks better addressed by public health policies and intersectoral action (ie, tobacco and high BMI) comprised the risk factors with the largest disparities in the reference and better health scenarios by 2040. Divergent patterns emerged for some regions, demonstrating the need to account for country-level risk profiles when planning for long-term intervention and policy. For instance, in southeast Asia, east Asia, and Oceania, ambient particulate matter pollution also had large gaps between reference forecast and 2040 better health scenarios, highlighting the importance of environmental initiatives alongside mitigating leading metabolic and behavioural risks. Alcohol use was consistently among the leading risks on this measure in central Europe, eastern Europe, and central Asia, as well as many high-income countries. Metabolic risk factors, particularly high FPG, dominated Latin America and the Caribbean, while ambient particulate matter pollution and tobacco ranked among the leading risks for redress in North Africa and the Middle East. Among south Asian countries, ambient particulate matter pollution and tobacco also were among the leading ten risks for the gap between reference and better health scenarios. The risk profile for avoidable YLLs differed substantially for most sub-Saharan African countries, with many countries showing large potential for closing reference and better health scenario forecasts for unsafe water, sanitation, and hygiene; child wasting; and household air pollution. Metabolic risks like high FPG or high BMI did not rank among the five leading risks in many sub-Saharan African countries, underscoring an unfinished agenda for risk factors associated with poverty and lower levels of SDI. In appendix 2 (pp 58–79), we report the country-level results for this analysis for both the gaps between the reference and better health scenario, and the equivalent gaps between the reference and worse health scenarios for risk-attributable YLLs, highlighting some of the largest threats for countries if they are unable to maintain their current trajectories. Country-level forecasts by cause, as well as better and worse health scenarios through 2040, are in appendix 2 (pp 91–2554).

## Discussion

This was the first study to forecast a comprehensive set of cause-specific and all-cause mortality and associated indicators using a framework that allows for exploring different scenarios for many risk factors and other independent drivers. In our reference scenario, life expectancy was forecasted to continue increasing globally, and 116 of 195 countries and territories were projected to have significant advances in life expectancy by 2040. Gains were projected to be faster among many low-to-middle SDI countries, indicating that inequalities in life expectancy could narrow by 2040; nonetheless, reference forecasts still had life expectancy ranging from more than 85 years in four countries (Japan, Singapore, Spain, and Switzerland) to less than 60 years in the Central African Republic and Lesotho. Global and regional shifts in the proportion of YLLs caused by CMNN diseases toward NCDs were projected to continue into the future, the main exception being sub-Saharan Africa. For many countries, the gaps between better and worse health scenarios for projected cause-specific mortality and risk-attributable YLLs were massive, representing an equally wide range of future trajectories by 2040 with the potential for tremendous progress or alarming regression in health outcomes.

In the reference scenario, the global shift towards NCDs masks heterogeneous trends for different diseases. Globally, six of the leading ten causes of YLLs were CMNN diseases in 2016; four of these—HIV/AIDS, neonatal disorders due to preterm birth complications, malaria, and neonatal encephalopathy—were projected to fall below the leading 10 causes by 2040. Total LRI YLLs conceal the marked shift projected to occur across age groups, particularly among older populations. Our forecasts of the rapid rise for several NCDs, including COPD, diabetes, chronic kidney disease, Alzheimer's disease, and lung cancer, portend serious health system implications. To the extent that increasing YLLs signal rising disease occurrence and increased demand for health care, these shifts could have significant ramifications on the volume and type of health expenditure in many health systems. The changing nature of premature mortality also has implications for the curricula and specialties for health-care professionals. These shifts evident at the global level are even more striking in some regions and countries such as India or Indonesia.

Our reference forecast was driven to an important extent by trends in the risk factors currently included in our model. We showed how selected risk factor forecasts and scenario selection play out in a single country in appendix 1 (p 22) and point to how the position of the forecast relative to scenarios varies among the risks. Globally, 43 drivers were forecasted to improve under the reference scenario and 36 were projected to worsen. Future trajectories were driven by the balance of these negative and positive forces. In nearly all locations BMI is worsening, and other drivers such as ambient air pollution, FPG, cholesterol, and some components of diet also were forecast to worsen in many places. Each location's trends hinge upon whether progress in reducing risk for drivers such as tobacco or some dietary risks can counteract projected adverse trends, particularly for high BMI. When these trends were translated into attributable mortality in 2040, we found that four risks accounted for more than 100 million YLLs: three metabolic risk factors (high blood pressure, high BMI, and high FPG) and tobacco. In addition to these drivers' effects in the reference scenario, a very wide range of attributable YLLs emerged between their better and worse health scenarios. Both the size of each risk's attributable YLLs and range suggest that these factors should be the focus of policy attention and health-care prioritisation. For instance, there is an important role for expanding access to primary care and risk management for high blood pressure, FPG, and cholesterol; subsequently, efforts to scale up universal health coverage may be one avenue for reducing the contribution of these risks in the future. High BMI, the second-leading future risk in the reference scenario, poses a more complex challenge; while policy options are available, their effect was less clear given that even the better health scenario showed rising burden attributable to high BMI. As demonstrated by the wide range in attributable YLLs due to tobacco and ambient particulate matter air pollution across 2040 scenarios, it is crucial to intensify and strengthen initiatives targeting tobacco and air pollution.

Based on our reference forecast, a subset of countries were projected to remain as low-income in 2040, and still experience relatively low educational attainment, life expectancy below 65 years, and YLLs dominated by CMNN diseases. Many different thresholds can be used to identify this set of countries which will continue not to enjoy the substantive gains in health outcomes observed in many other parts of the world. The exact thresholds are less important than the recognition that, based on past trends of independent drivers and continued relationships between these drivers and health outcomes, a set of countries, including the Central African Republic, Zimbabwe, and Somalia, will continue to be markedly disadvantaged. This phenomenon of the long tail of the distribution was evident in the better health scenario as well (eg, Lesotho was still projected to have a life expectancy less than 65 years under the better health scenario). Further, under the 2040 worse health scenario, global inequalities would substantially widen, with most of these most disadvantaged countries remaining in central and western sub-Saharan Africa and some in Oceania. This long tail has implications for development policy and the need to focus scarce resources toward these countries in an effort to accelerate progress.

Our forecasts suggest continued progress in improving life expectancy. These forecasts, however, do not account for the potential effects of climate change on life expectancy except as mediated through ambient air pollution. Predicted impacts in other studies on extreme weather-related deaths and heat wave deaths are not large enough to have much impact on global life expectancy. WHO estimated that climate change would cause 250 000 additional deaths each year by 2040.^30^, ^31^ Climate change, however, might have a much greater effect on survival on populations in some fragile environments mediated through reduced agricultural output and increased food insecurity. The pathway to large health effects could also be through conflicts and migration that might stem from severe food insecurity. These effects are extremely hard to model using statistical models fit to past data and will require other approaches to quantify their potential effect. Another risk for future health gains not incorporated in these forecasts is the potential rise of antimicrobial resistance, with some claims suggesting millions of deaths due to antimicrobial resistance.^32^, ^33^ The validity of these claims, however, is not well established.^34^, ^35^ We also did not forecast the potential effect of a major global influenza pandemic of the magnitude of the one occurring in 1918; such a pandemic would substantially increase mortality in any given year.^35^ Although our forecasts currently do not reflect these potential negative risks, they also do not account for the potential for accelerated technical innovation, which could lead to striking breakthroughs in prevention or medical treatment (eg, HIV vaccine, cure for cancer). For each cause of death, our models capture the global secular trend in the past 27 years and assume this secular trend continues in the future; in other words, we already assumed the disease-specific pace of innovation will continue. Yet breakthroughs for diseases for which minimal innovation has occurred during the past-quarter century are not captured in these forecasts.

Taken together, our forecasts point to a world where most populations are living longer and many health improvements are likely to occur if current trajectories hold; at the same time, such gains are not without potential important social consequences, particularly if long-term planning and policy design are not fully considered today.^36^ Social security, pensions, and programmes specifically targeting the support of older populations have generally been most solvent when a greater proportion of people are contributing to these initiatives or are active in the workforce than the proportion of people who are immediately benefitting from them. Based our modelling framework, the demographic dynamics projected to unfold involve a decrease in total fertility rate and steady shifts in population toward older ages, which also portends a larger overall volume of disease burden and expansion of morbidity in many places.^6^, ^37^ Countries such as Japan have already experienced the economic toll and stress on social programmes that can occur when demography's balancing act tilts toward older populations and younger cohorts struggle to fill the growing economical and financial gaps left behind as their elders retire. Similar concerns have been recently mounting in China,^38^, ^39^ and in the absence of deliberate action concerning programmes such as social security and pensions, it is likely that more countries will face these very complex demographic challenges in future.

An important finding is that in the reference scenario, we forecasted slower progress in 2040 than that achieved in the past; however, in the better health scenario, global life expectancy improvements exceeded gains that occurred from 1990–2016. This forecasted slowdown in the reference scenario is rooted in a combination of several factors. First, some risks were projected to worsen in the future, most notably high BMI. Second, past progress on other leading risk factors for premature mortality, namely tobacco and ambient particulate matter air pollution, was highly variable^40^ and thus such heterogeneity was projected through 2040. Third, several countries that have already achieved higher levels of life expectancy have also had stagnated gains.^41^, ^42^ Fourth, the relatively new occurrence observed in a number of high-income countries of increases in mortality in younger adults, particularly younger than 50 years, was captured and propagated in our forecasts.^43^ Last, such slowdowns might be related to how we capture the effect of innovation, or the inclusion of global secular trends by age that are supposed to capture changes in health not accounted for by measured risk factors, interventions, or SDI. An important part of this temporal trend might be due to innovations in medical and public health technologies and programmes; however, there might be important interactions between technological innovation and health system capacity to deliver such innovations that are not currently captured in our model.

Gaps between the better and worse health scenarios provide some quantification of the scope for policy impact on future health trajectories. To the extent that we believe the 85th and 15th percentile rates of change for each independent driver could occur in each country, even if we do not yet know the policy mix used to achieve this rate of change, then the wide range of outcomes between better and worse means that policy today can have a huge effect on the future. The future is not inevitable; funding of public health and medical care, social policy and multisectoral action for health can have profound effects on both short-term and long-term outcomes. We hope that the quantification of these scenarios will bring more attention to how different countries have achieved faster progress, and why some countries experience slower gains, for each of the independent drivers. Most importantly, our study shows that the future is highly malleable, but also requires concerted attention and continued priorisation of the key drivers of health.

Our choice of defining the better and worse health scenarios based on the 85th and 15th percentiles of rates of change is arbitrary. We could have selected more ambitious rates of change (ie, the 95th and fifth percentiles), as they also have been used in the past.^44^ We selected the 85th and 15th percentiles to balance the considerations of what is possible and replicable across many contexts, as well as the fact that achieving these percentiles for all drivers at once and consistently for 24 years is quite unlikely by the laws of probability. Furthermore, the fact that annual rates of change for a risk factor or an intervention have been achieved in 15% of countries does not mean that success can be replicated everywhere else. However, rates achieved in 15% of countries are clearly not impossible and can serve as a guide for what is achievable.

These scenarios are meant to define the scope of what is generally possible. However, for specific drivers such as tobacco, there might also be value in analysing the effect of much faster rates of progress (ie, the 99th percentile). The case of tobacco is illustrative, since only 20 countries had rates of change in the tobacco SEV between 1990 and 2016 faster than 2% (eg, the USA and Canada), so these tobacco policy success stories do not define the more conservative 85th percentile. We purposefully developed our forecasting models to support more detailed alternative scenarios for specific risks, with the ultimate goal of enabling any user to explore the effect of change on any independent driver at any given rate of change. Computational speed currently precludes such endless possibilities, but our modelling platform supports this future endeavour.

Before this study, most forecasting models of all-cause or cause-specific mortality used only one covariate, namely time. These trend-based models have the advantage of not needing to forecast time into the future and, depending on the formulation, they can produce reasonable out-of-sample predictive validity. The other tradition has been disease-specific or intervention-specific modelling exercises to assess the effect of changing a singular independent driver in some alternative scenario. Such models have focused on capturing the causal connections between the driver and the outcome of concern, such as tobacco and lung cancer, rather than achieving good out-of-sample predictive validity. In this analysis, we captured the causal connections between 79 drivers and outcomes in addition to correlations between SDI and time with outcomes, and also achieved better out-of-sample predictive validity than previous efforts. We believe achieving reasonable out-of-sample predictive validity and building a modelling framework that allows exploration of myriad combinations of independent drivers is highly useful for policy exploration and dialogue. Balancing these two objectives and producing coherent forecasts for all-cause and cause-specific mortality for a large number of causes has required constructing a complicated and interlocking modelling framework; however, such complexity is necessary to achieve the effective balance provided with this method.

For two causes of death, natural disasters and conflict, developing models with good predictive validity has been challenging. We explored published modelling strategies but they did not provide good out-of-sample predictive validity with our data.^45^ In the absence of more robust modelling strategies, we opted to use an overly simplified approach for the current analysis: sampling for each year in the future the location-specific distribution of the event rate from 1950–2016. In settings of extreme events, such as the Haiti earthquake of 2010 that effectively means the average for the future in those countries is 1/67th of the extreme event occurring in each year in the future. The simple nature of our models for these two causes must be considered when interpreting results for locations which have experienced major events from 1950–2016. To date, however, we have not found a model with more predictive power than this relatively simplistic approach. Another important issue to note in our forecasts related to conflict is that migration is not endogenous to the model. We do not, for example, model increased migration in a draw of the future reference scenario that is higher when there is a conflict. Our migration data is based on the UN Population Division forecasts, which are not linked to any particular model of conflict in our simulations. This important limitation can only be addressed by building a model that predicts migration as a function of conflict, economic factors, and other contextual determinants; additionally, modelling migration is known to be particularly challenging.^46^, ^47^

In the reference scenario, our forecasts include underlying mortality rates and global effects of time interacted with each 5-year age-group. This temporal trend component of the underlying model was meant to capture trends across countries for a given cause that were not captured by risk factors or SDI. Technical innovation is an important component of this temporal trend as are other social policy innovations and trends in risks not currently included in GBD. Although the average pace of technical innovation from 1990–2016 was captured, our model did not explicitly map out the probability of particular technical breakthroughs. For example, our road-injury estimates account for the trends related to SDI, but do not capture the potentially large effects of self-driving or driver-assisted car technology.^48^, ^49^ Any future technical innovations that could contribute to accelerated or abrupt declines in mortality, such as the advent of ART for HIV in 1996, were not captured in the reference scenario. Simulations of the potential effect of major innovations, whether drugs, vaccines, or broader technical innovation, can use the reference scenario as the comparator against which the effect of these developments can be assessed.

A key aspect of our forecasting model was to capture causal relations between the independent drivers and mortality so that we could explore alternative scenarios from different settings of drivers. The causal relations between risk factors and cause-specific mortality builds on the comparative risk factor assessment in GBD 2016. This evidence synthesis also accounts for mediation of the effect of risks through others (ie, the effect of BMI on mortality from ischaemic heart disease through high systolic blood pressure, cholesterol, and FPG).^16^ For the components of SDI (income per person, educational attainment, and total fertility rate under 25 years), we clearly cannot, with the modelling framework used here, claim that this study has demonstrated causality for these three variables. However, findings from some published studies support causal connections between all three and many health outcomes.^50^, ^51^, ^52^, ^53^ Given that the total fertility rate under 25 years in SDI is used a proxy measure of women's empowerment, the question is whether the associations for causes other than maternal and child health outcomes is causal. We clearly cannot prove that they are but believe they are credibly causal given the mechanisms through which women's empowerment might influence many causes.

In this analysis, we developed a modelling framework that produced estimates for all-cause and cause-specific mortality, used 79 covariates as independent drivers, and for risk factors and interventions produced results consistent with evidence on causality from cohort studies and randomised trials. In addition to preserving the causal relationships between independent drivers and their potential effects on future health, our overall results achieved good out-of-sample predictive validity compared with other simpler modelling strategies. There are many opportunities to further refine the modelling strategy. As more time series for intervention coverage are made available, we also can add more interventions as part of the risk factor and intervention-attributable component of the model. In the better and worse health scenarios, we did not explicitly preserve the observed covariance between different independent drivers; however, this kind of relationship could be built into our modelling strategy. The costs of moving from reference forecasts to better health scenarios for the independent drivers were not included in the current analysis. Combining forecasts of health expenditures, health system efficiency, and estimates of costs could produce better forecasts of the independent drivers of health and support analyses on the optimum allocation of resources on a dollar per disability-adjusted life-year basis in the future. Based on the current model, by optimising the computational engine, we could allow users to explore an essentially limitless combination of different trajectories for the risk factors, income per person, educational attainment, and met need for family planning. Such user-driven scenario construction should prove a useful input for policy dialogues in many settings, allowing rapid generation of the effect of changing individual risk factors or combinations of risks with different socioeconomic contexts. In our forecasts we noted larger increases in number of deaths compared to YLLs. This was partly due to how we computed YLLs with the GBD 2016 reference life table, kept constant into the future. In coming forecasting iterations we will consider revision of our current YLL standard to reflect expected progress in the best observed age-specific death rates.

### Limitations

Any forecasting study is subject to several limitations. Many factors, which were not included in our models, could change the nature of future health; consequently, any forecasting effort must acknowledge that this task is extremely challenging. Although our model performed well out-of-sample for 2007–16 fit to data for the period 1990–2006, similar model performance is not guaranteed in the future. Second, our models for forecasting independent drivers of health were relatively simple extrapolations of past trends weighted, to some degree, for recency. Our forecasts were limited by the validity of these simple extrapolations, even though they also had reasonable out-of-sample predictive performance. Third, our model deviated from demographic tradition, wherein only past trends were used to extrapolate to the future (ie, time is the sole independent driver). Proponents of this approach and its many variants argue they have better out-of-sample predictive validity than models that incorporate causal connections. With the present study, we achieved comparatively good, out-of-sample predictive performance while incorporating causal relationships where they have been established (eg, for tobacco and ischaemic heart disease). Fourth, our analysis of the components for each cause of death currently unexplained by risk factors was complicated by high co-linearity between the secular trend, income per person, educational attainment, and total fertility rate under 25 years. To address this co-linearity, the regressions used SDI, a reduced form of the three variables. Fifth, our better and worse health scenarios had comparatively small variations in total fertility rate under 25 years for many locations, which resulted from having the effect of better and worse health scenarios operate through changes in female education and met need for family planning. Other unexplained drivers of fertility change were not reflected in these scenarios. Sixth, we did not model migration related to conflict, natural disasters, or other catastrophic events. More generally, improved modelling of migration and refugees would be an important area of improvement for our population forecasting. Seventh, we modelled each sex separately, a decision informed by issues that arise in many extrapolation models where estimates are modelled for single sex and then the sex ratios for mortality.^54^ We did not impose any relationship regarding male-female differences in age-specific mortality or life expectancy; notably, we did not forecast any reversals where male life expectancy exceeded that of females in 2040. Finally, to capture the causal relations between risks and cause-specific mortality it is important that patterns of mediation of risks through other risks are identified and correctly quantified. GBD offers comprehensive and systematic efforts to use mediation adjustments in its risk factor analysis. Since many mediation pathways remain unknown and others poorly quantified, our mediation matrix might not be fully accurate. However, despite limitations with the mediation matrix, our model's performance still exceeds that of methods like Lee-Carter,^26^ which do not incorporate risk factors at all.

### Conclusion

With this study, we offer a robust yet flexible forecasting platform from which future health scenarios can be examined across countries and over time; a crucial resource for long-term health planning and investments. Based on our reference forecast, health outcomes were generally predicted to improve through 2040; however, as shown by the 2040 better and worse health scenarios, ample room exists for both substantial progress and the reversal of health gains. The intersection of deliberate policy action, technological innovation, and careful attention to rising environmental, social, and geopolitical risks will likely shape the range of possible health trajectories in the future. The considerable range between better and worse health scenarios, even when accounting for 79 health drivers, shows the important role of policy change in improved health for the next generation. Decision makers in most countries ought to plan for a continued shift towards NCDs and target resources to the leading risk factors sensitive to policy change or health care, such as high blood pressure, high FPG, high BMI, tobacco, ambient air pollution, and diet. In the absence of major medical advances, CMNN causes are likely to remain dominant health challenges among the poorest countries, and there is a real risk that the HIV epidemic could rebound in many countries if progress is not maintained. Continued technical innovation and heightened spending on health, inclusive of development assistance for health, will be necessary to prevent millions of people from living in substantially worse conditions than the rest of the world.
