# Supplementary material for: Forecasting life expectancy, years of life lost, and all-cause and cause-specific mortality for 250 causes of death: reference and alternative scenarios for 2016–40 for 195 countries and territories
Source: Lancet. 2018 Nov 10;392(10159):2052–90. doi: 10.1016/S0140-6736(18)31694-5 (PMC6227505; doi:10.1016/S0140-6736(18)31694-5)
Supplement: Supplementary appendix 2 [file mmc2.pdf]

THE LANCET

Supplementary appendix 1

This appendix formed part of the original submission and has been peer reviewed. We post it as supplied by the authors.

Supplement to: Foreman K J, Marquez N, Dolgert A, et al. Forecasting life expectancy, years of life lost, and all-cause and cause-specific mortality for 250 causes of death: reference and alternative scenarios for 2016–40 for 195 countries and territories. *Lancet* 2018; published online October 16. http://dx.doi.org/10.1016/S0140-6736(18)31694-5.

Supplementary Results Appendix

Forecasting life expectancy, years of life lost, all-cause and cause-specific mortality for 250 causes of death: reference and alternative scenarios 2016–2040 for 195 countries and territories

List of Figures and Tables

Figure 1. Swarm plots showing the annualized effect of each covariate on the predicted change in mortality from 2017 to 2040. Where possible, models were fit with a linear spline on SDI with a knot at 0.8 - this is reflected by the two separate SDI columns in many cause-sex pairs. The coefficients for each SDI category only impact mortality for location-years where the SDI falls within that category. Thus, countries with SDI below 0.8 through the entire forecast time series will have non-zero swarm plot values only in the 'SDI < 0.8' column. Likewise, locations with SDI above 0.8 through the time series will only have non-zero swarm plot values in the 'SDI >= 0.8' column. Locations with forecast SDI values in both categories will have non-zero swarm plot values in both columns. Secular trend effect is shown as the average effect taken over ages. Each data point represents a location and is color coded by GBD 2016 super region. Blue = Southeast asia, East Asia, Oceania. Orange = Central Europe, Eastern Europe, Central Asia. Green = High Income. Red = Latin America and Caribbean. Purple = North Africa and Middle East. Brown = South Asia. Pink = Sub-Saharan Africa. SDI = Sociodemographic Index. (Page 3)

Figure 2. Illustration of the components of the mortality modeling process for all-cause mortality for females in Australia. The panels on the left show cause-specific death rates by age, with small multiples showing the time trend from 1990 to 2040 for each age group. The bottom left panel is the underlying mortality component captured by SDI and global secular trends; above that is risk-attributable mortality, captured by the risk factor scalar; second from the top is the sum of the underlying and risk-attributable mortality; and the top left panel shows the final mortality predictions after the ARIMA process, which accounts for latent trends. The panels on the right show the cause- and risk-specific population attributable fractions (PAFs) that go into the risk attributable estimation; the all-risk PAF at the top is used to create the risk factor scalar, and the specific risks are then sorted in descending order of attributable deaths in 2016. (Page 57)

Figure 3. Ten leading risk factors contributing to the difference in YLLs between the forecast and worse health scenario in 2040 by location, representing potential burden with regression to the worse scenario for each risk factor. Values are reported in thousands, with 95% uncertainty intervals in parentheses. YLLs = years of life lost. (Page 58)

Figure 4. Global life expectancy from 1990-2040 in the reference scenario (forecast), better health, and worse health scenarios. Dashed lines represent global male life expectancy, solid lines represent global female life expectancy. Black lines = past data from GBD 2016. Blue = reference scenario. Green = better scenario. Red = worse scenario. Shaded areas represent 95% uncertainty intervals. (Page 68)

Figure 5. Difference between the better and worse life expectancy scenarios in 2040 for both sexes combined. Legend measured in years. *Summary*: Based on the potential trajectories of many independent drivers (ie, risk factors, SDI, and coverage of interventions like ART and vaccination) and their substantive effects on life expectancy, we forecasted gains in survival that could span from nearly 3

years in Japan to more than 20 years in South Africa. In higher-income countries, this range was generally bounded to a 3–4 year gain, even for the better scenario. Conversely, modifying trajectories of independent drivers could result in life expectancy improvements of at least four years in much of sub-Saharan Africa; among locations with large HIV epidemics, such advances could easily exceed 10 years by 2040. ATG=Antigua and Barbuda. BRB=Barbados. COM=Comoros. DMA=Dominica. E Med=Eastern Mediterranean. FJI=Fiji. FSM=Federated States of Micronesia. GRD=Grenada. KIR=Kiribati. LCA=Saint Lucia. MDV=Maldives. MHL=Marshall Islands. MLT=Malta. MUS=Mauritius. SGP=Singapore. SLB=Solomon Islands. SYC=Seychelles. TLS=Timor-Leste. TON=Tonga. TTO=Trinidad and Tobago. VCT=Saint Vincent and the Grenadines. VUT=Vanuatu. W Africa=West Africa. WSM=Samoa. (Page 69)

Table 1. Number of Years of life lost (YLLs) and deaths in GBD 2016, the 2040 forecast (reference scenario), better, and worse scenarios for all causes of death and all locations. Values are reported as counts, or in thousands, as described in the location specific heading, and 95% uncertainty intervals are in parentheses. Due to differences in populations and SDI across the scenarios, forecasts of numbers (and not rates) may result in reference values outside of the better and worse estimates. Highlighted rows indicate level 1 and 2 causes from the GBD cause hierarchy. GBD = Global burden of disease. (Page 70)

Table 2. Correlation between SEV_{rc} and SEV_r in the period 1990-2016. Values were computed as Pearson correlation coefficients. SEV = summary exposure variable. (Page 3,649)

Table 3a. Pearson correlation coefficients among the components of SDI, top 20 risks, and vaccines in GBD 2016 for 1990-2016. Blank cells occur where risk pairs are not estimated on overlapping age groups. (Page 3,659)

Table 3b. Pearson correlation coefficients among the components of SDI, top 20 risks, and vaccines in reference scenario forecasts 2017-2040. Blank cells occur where risk pairs are not estimated on overlapping age groups. (Page 3,660)

Table 3c. Ratio of correlation coefficients among the components of SDI, top 20 risks, and vaccines in reference scenario forecasts (2017-2040) divided by correlations in the past (1990-2016). (Page 3,661)

Supplemental Results Appendix Figure 1. Swarm plots showing the annualized effect of each covariate on the predicted change in mortality from 2017 to 2040. Where possible, models were fit with a linear spline on SDI with a knot at 0.8 - this is reflected by the two separate SDI columns in many cause-sex pairs. The coefficients for each SDI category only impact mortality for location-years where the SDI falls within that category. Thus, countries with SDI below 0.8 through the entire forecast time series will have non-zero swarm plot values only in the 'SDI < 0.8' column. Likewise, locations with SDI above 0.8 through the time series will only have non-zero swarm plot values in the 'SDI >= 0.8' column. A subset of causes also include coefficients for the SDI*Time interaction term. See the methods appendix (pp 9) for more details. Locations with forecasted SDI values in both categories will have non-zero swarm plot values in both columns. Secular trend effect is shown as the average effect taken over ages. Each data point represents a location and is color coded by GBD 2016 super region. Blue = Southeast asia, East Asia, Oceania. Orange = Central Europe, Eastern Europe, Central Asia. Green = High Income. Red = Latin America and Caribbean. Purple = North Africa and Middle East. Brown = South Asia. Pink = Sub-Saharan Africa. SDI = Sociodemographic Index.

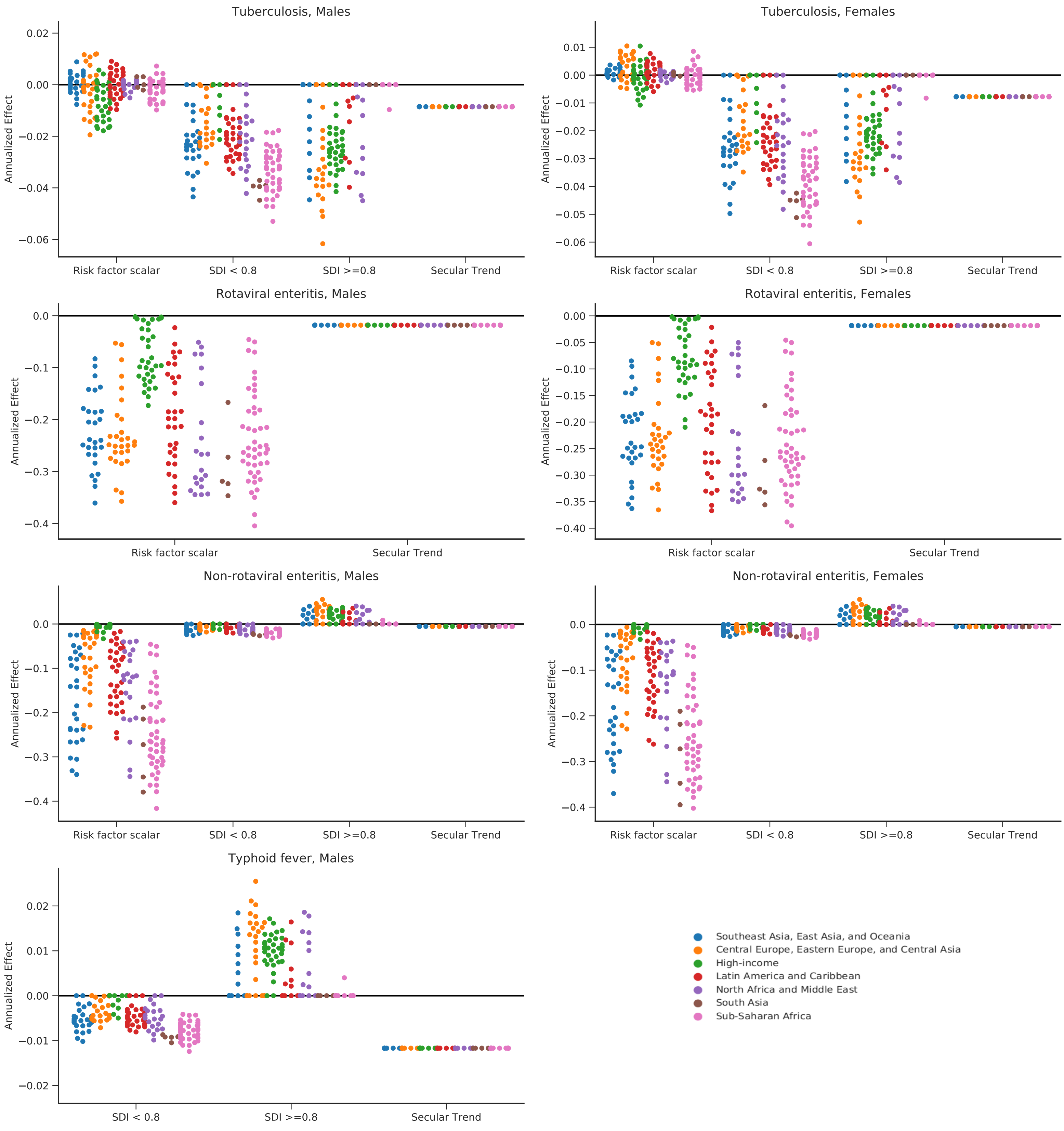

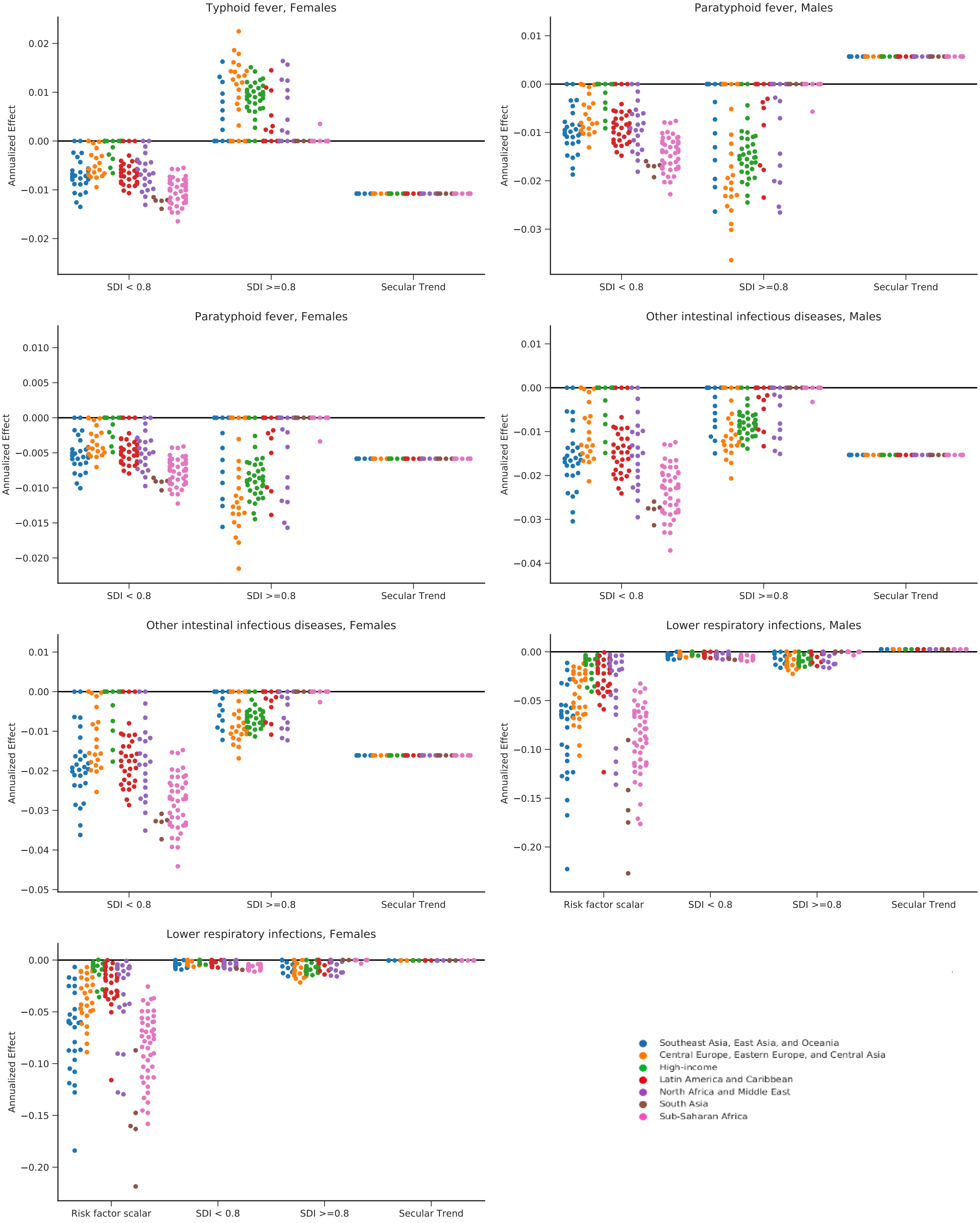

Typhoid fever, Females
Paratyphoid fever, Males
Paratyphoid fever, Females
Other intestinal infectious diseases, Males
Other intestinal infectious diseases, Females
Lower respiratory infections, Males
Lower respiratory infections, Females
Annualized Effect
Risk factor scalar
SDI < 0.8
SDI >=0.8
Secular Trend
Southeast Asia, East Asia, and Oceania
Central Europe, Eastern Europe, and Central Asia
High-income
Latin America and Caribbean
North Africa and Middle East
South Asia
Sub-Saharan Africa

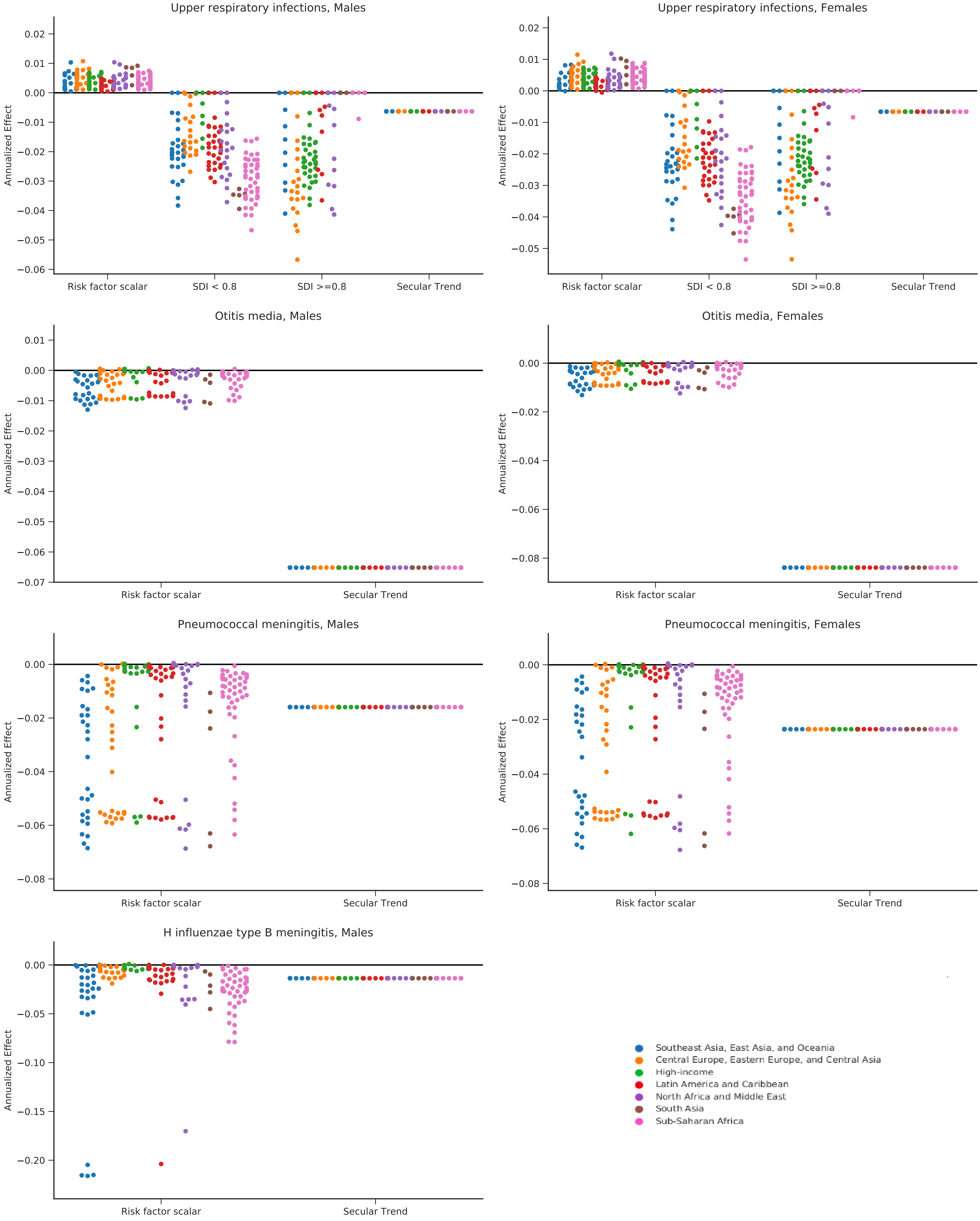

Upper respiratory infections, Males
Annualized Effect
0.02
0.01
0.00
−0.01
−0.02
−0.03
−0.04
−0.05
−0.06
Risk factor scalar
SDI < 0.8
SDI >=0.8
Secular Trend
Upper respiratory infections, Females
Annualized Effect
0.02
0.01
0.00
−0.01
−0.02
−0.03
−0.04
−0.05
Risk factor scalar
SDI < 0.8
SDI >=0.8
Secular Trend
Otitis media, Males
Annualized Effect
0.01
0.00
−0.01
−0.02
−0.03
−0.04
−0.05
−0.06
−0.07
Risk factor scalar
Secular Trend
Otitis media, Females
Annualized Effect
0.00
−0.02
−0.04
−0.06
−0.08
Risk factor scalar
Secular Trend
Pneumococcal meningitis, Males
Annualized Effect
0.00
−0.02
−0.04
−0.06
−0.08
Risk factor scalar
Secular Trend
Pneumococcal meningitis, Females
Annualized Effect
0.00
−0.02
−0.04
−0.06
−0.08
Risk factor scalar
Secular Trend
H influenzae type B meningitis, Males
Annualized Effect
0.00
−0.05
−0.10
−0.15
−0.20
Risk factor scalar
Secular Trend
Southeast Asia, East Asia, and Oceania
Central Europe, Eastern Europe, and Central Asia
High-income
Latin America and Caribbean
North Africa and Middle East
South Asia
Sub-Saharan Africa

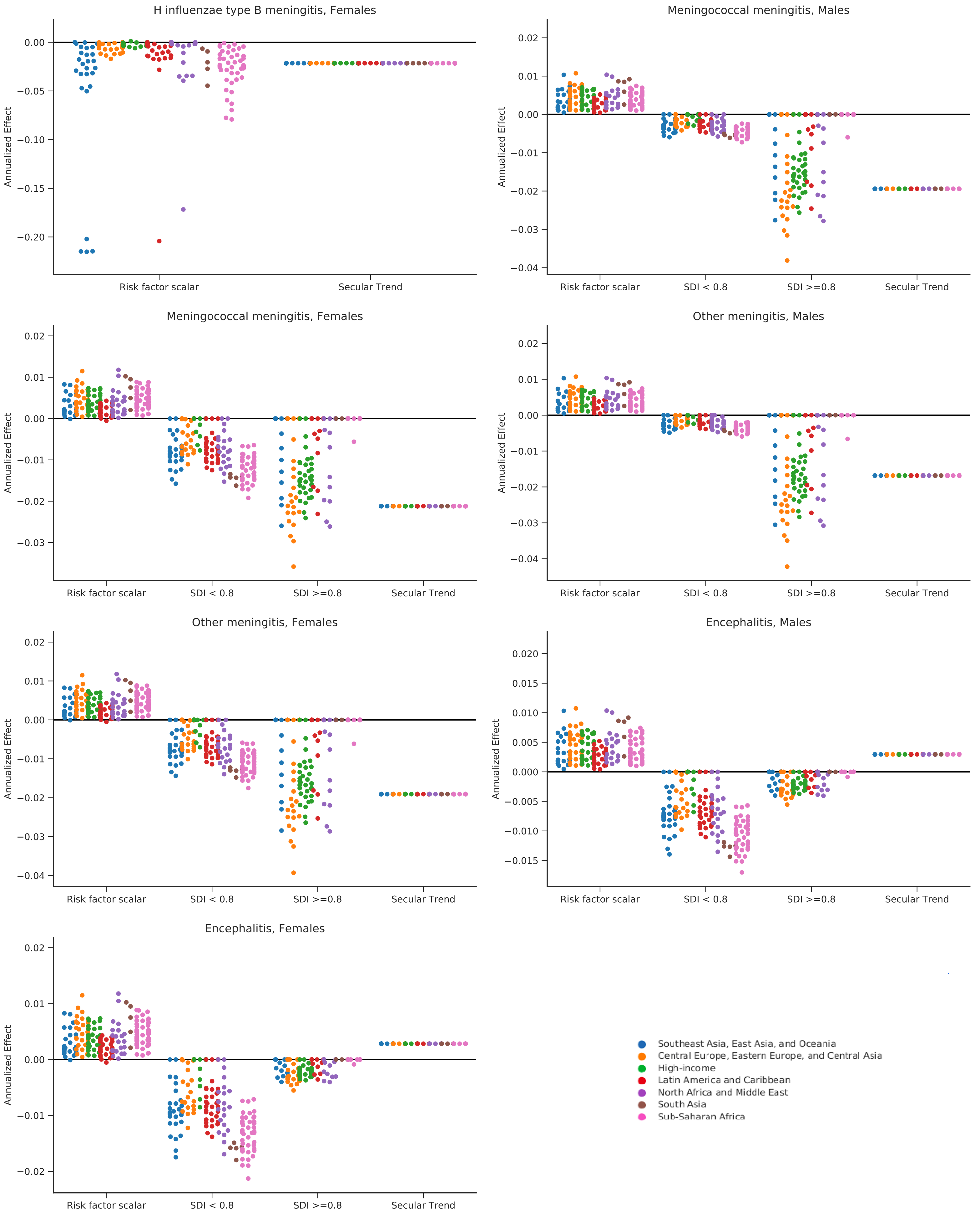

H influenzae type B meningitis, Females
Annualized Effect
0.00
−0.05
−0.10
−0.15
−0.20
Risk factor scalar
Secular Trend
Meningococcal meningitis, Males
0.02
0.01
0.00
−0.01
−0.02
−0.03
−0.04
Risk factor scalar
SDI < 0.8
SDI >=0.8
Secular Trend
Meningococcal meningitis, Females
Other meningitis, Males
Other meningitis, Females
Encephalitis, Males
0.020
0.015
0.010
0.005
0.000
−0.005
−0.010
−0.015
Encephalitis, Females
Southeast Asia, East Asia, and Oceania
Central Europe, Eastern Europe, and Central Asia
High-income
Latin America and Caribbean
North Africa and Middle East
South Asia
Sub-Saharan Africa

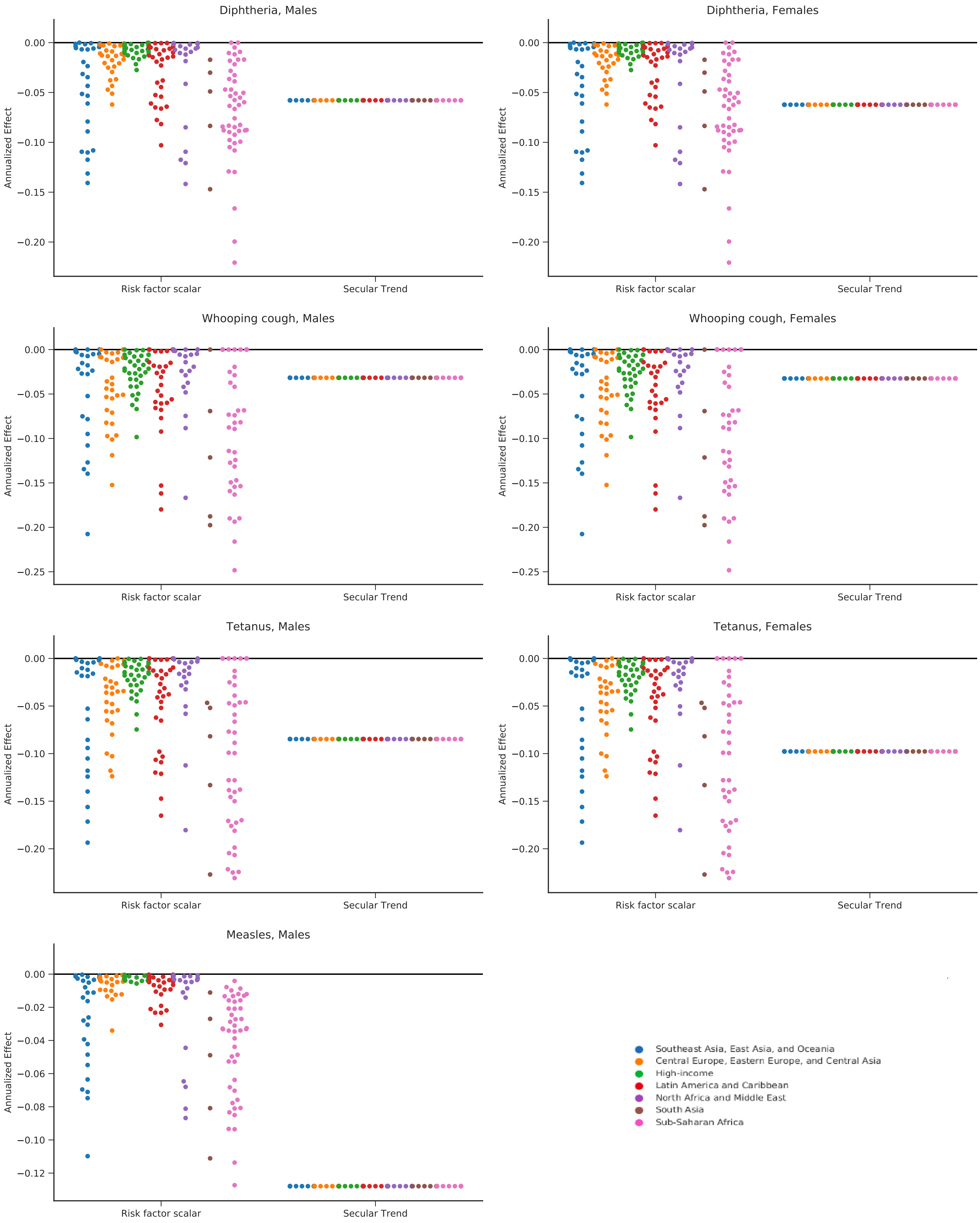

Diphtheria, Males
Diphtheria, Females
Whooping cough, Males
Whooping cough, Females
Tetanus, Males
Tetanus, Females
Measles, Males
Annualized Effect
0.00
−0.02
−0.04
−0.05
−0.06
−0.08
−0.10
−0.12
−0.15
−0.20
−0.25
Risk factor scalar
Secular Trend
Southeast Asia, East Asia, and Oceania
Central Europe, Eastern Europe, and Central Asia
High-income
Latin America and Caribbean
North Africa and Middle East
South Asia
Sub-Saharan Africa

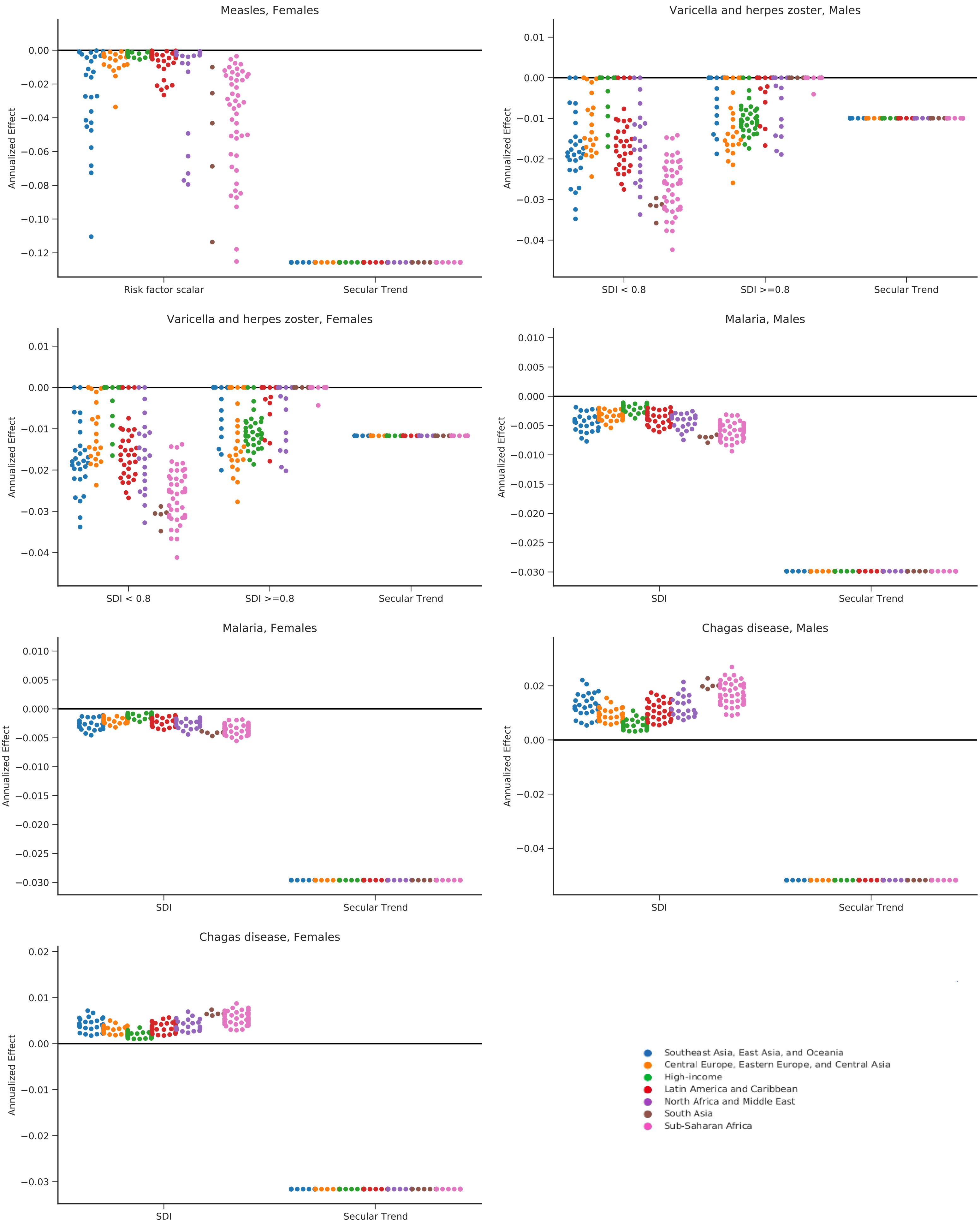
Measles, Females
Varicella and herpes zoster, Males
Varicella and herpes zoster, Females
Malaria, Males
Malaria, Females
Chagas disease, Males
Chagas disease, Females
Annualized Effect
Risk factor scalar
Secular Trend
SDI < 0.8
SDI >=0.8
SDI
0.00
−0.02
−0.04
−0.06
−0.08
−0.10
−0.12
0.01
−0.01
−0.03
0.010
0.005
0.000
−0.005
−0.010
−0.015
−0.020
−0.025
−0.030
0.02
Southeast Asia, East Asia, and Oceania
Central Europe, Eastern Europe, and Central Asia
High-income
Latin America and Caribbean
North Africa and Middle East
South Asia
Sub-Saharan Africa

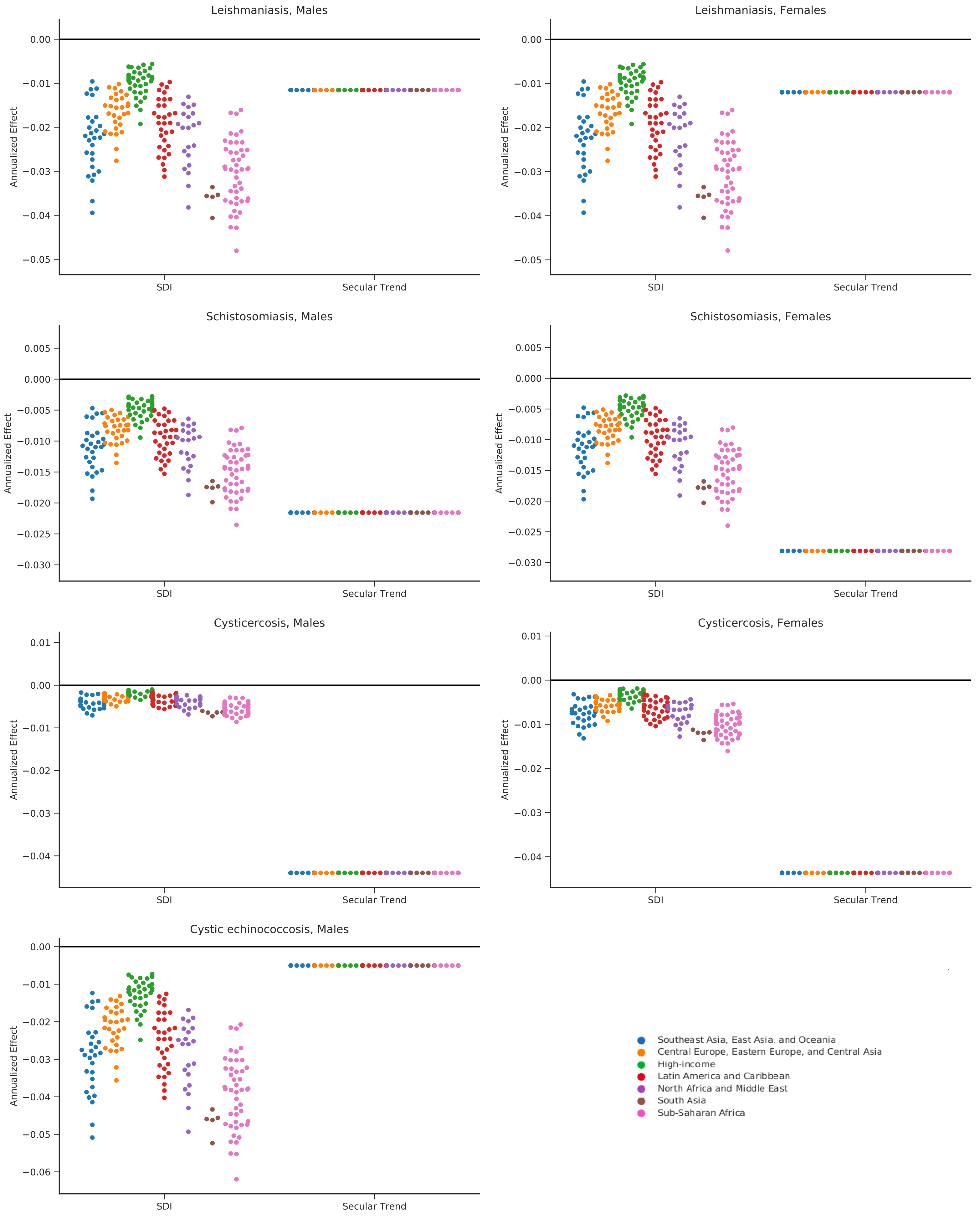
Leishmaniasis, Males
Leishmaniasis, Females
Schistosomiasis, Males
Schistosomiasis, Females
Cysticercosis, Males
Cysticercosis, Females
Cystic echinococcosis, Males
Annualized Effect
SDI
Secular Trend
0.00
−0.01
−0.02
−0.03
−0.04
−0.05
−0.06
0.01
0.005
0.000
−0.005
−0.010
−0.015
−0.020
−0.025
−0.030
Southeast Asia, East Asia, and Oceania
Central Europe, Eastern Europe, and Central Asia
High-income
Latin America and Caribbean
North Africa and Middle East
South Asia
Sub-Saharan Africa

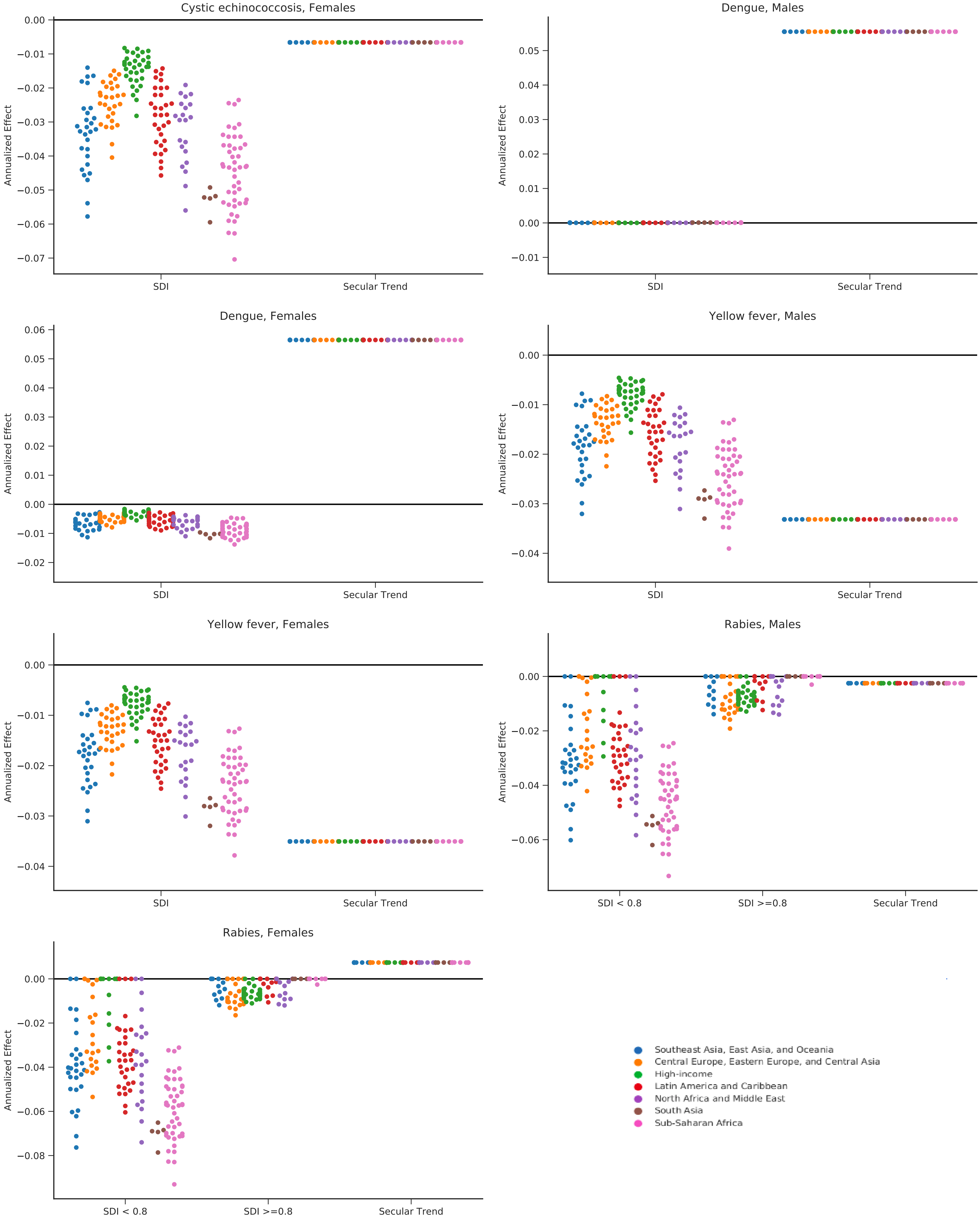

Cystic echinococcosis, Females
Dengue, Males
Dengue, Females
Yellow fever, Males
Yellow fever, Females
Rabies, Males
Rabies, Females
Annualized Effect
SDI
Secular Trend
SDI < 0.8
SDI >=0.8
0.00
−0.01
−0.02
−0.03
−0.04
−0.05
−0.06
−0.07
−0.08
0.01
0.02
0.03
0.04
0.05
0.06
Southeast Asia, East Asia, and Oceania
Central Europe, Eastern Europe, and Central Asia
High-income
Latin America and Caribbean
North Africa and Middle East
South Asia
Sub-Saharan Africa

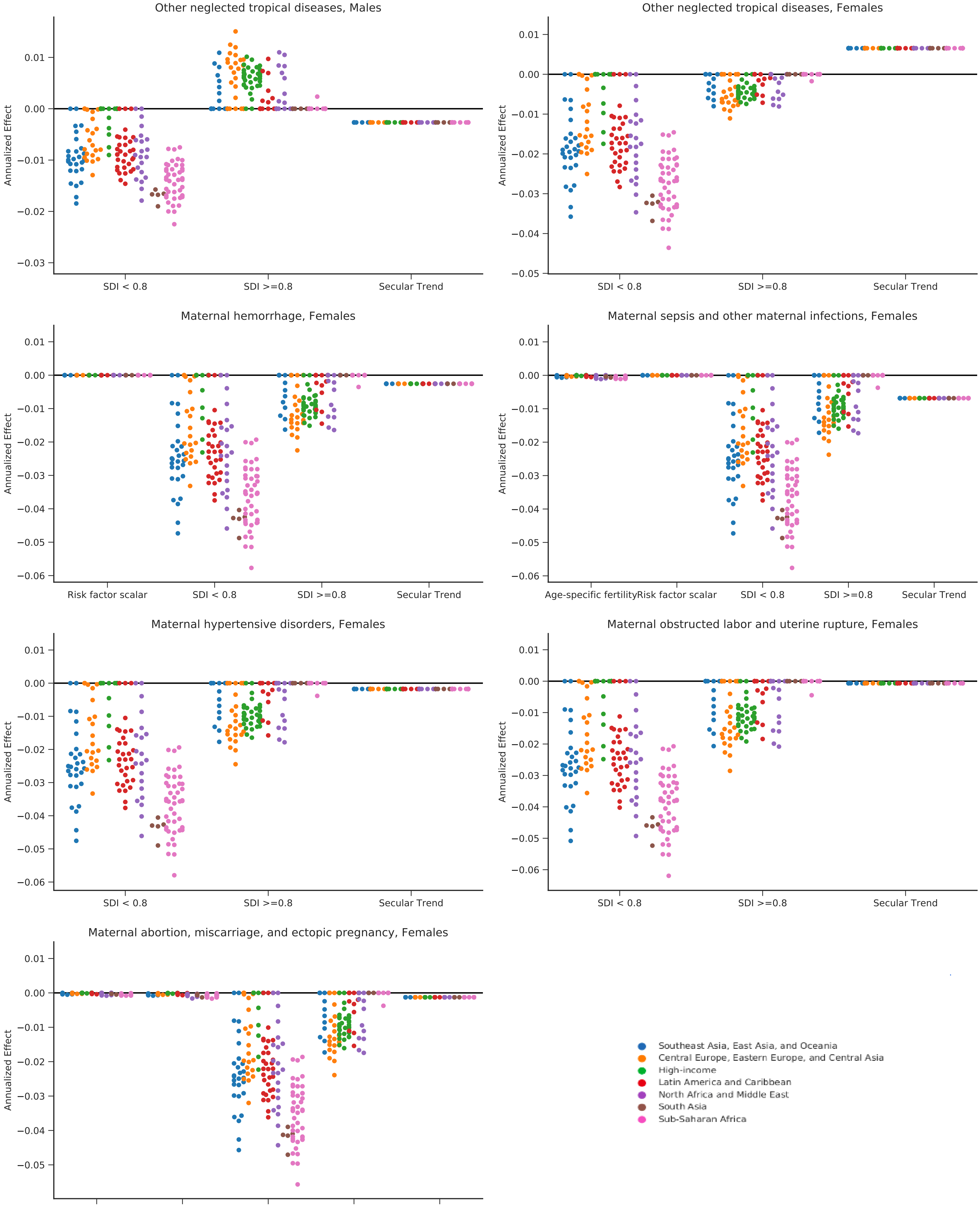

Other neglected tropical diseases, Males
Annualized Effect
0.01
0.00
−0.01
−0.02
−0.03
SDI < 0.8
SDI >=0.8
Secular Trend
Other neglected tropical diseases, Females
Annualized Effect
0.01
0.00
−0.01
−0.02
−0.03
−0.04
−0.05
SDI < 0.8
SDI >=0.8
Secular Trend
Maternal hemorrhage, Females
Annualized Effect
0.01
0.00
−0.01
−0.02
−0.03
−0.04
−0.05
−0.06
Risk factor scalar
SDI < 0.8
SDI >=0.8
Secular Trend
Maternal sepsis and other maternal infections, Females
Annualized Effect
0.01
0.00
−0.01
−0.02
−0.03
−0.04
−0.05
−0.06
Age-specific fertility
Risk factor scalar
SDI < 0.8
SDI >=0.8
Secular Trend
Maternal hypertensive disorders, Females
Annualized Effect
0.01
0.00
−0.01
−0.02
−0.03
−0.04
−0.05
−0.06
SDI < 0.8
SDI >=0.8
Secular Trend
Maternal obstructed labor and uterine rupture, Females
Annualized Effect
0.01
0.00
−0.01
−0.02
−0.03
−0.04
−0.05
−0.06
SDI < 0.8
SDI >=0.8
Secular Trend
Maternal abortion, miscarriage, and ectopic pregnancy, Females
Annualized Effect
0.01
0.00
−0.01
−0.02
−0.03
−0.04
−0.05
Age-specific fertility
Risk factor scalar
SDI < 0.8
SDI >=0.8
Secular Trend
Southeast Asia, East Asia, and Oceania
Central Europe, Eastern Europe, and Central Asia
High-income
Latin America and Caribbean
North Africa and Middle East
South Asia
Sub-Saharan Africa

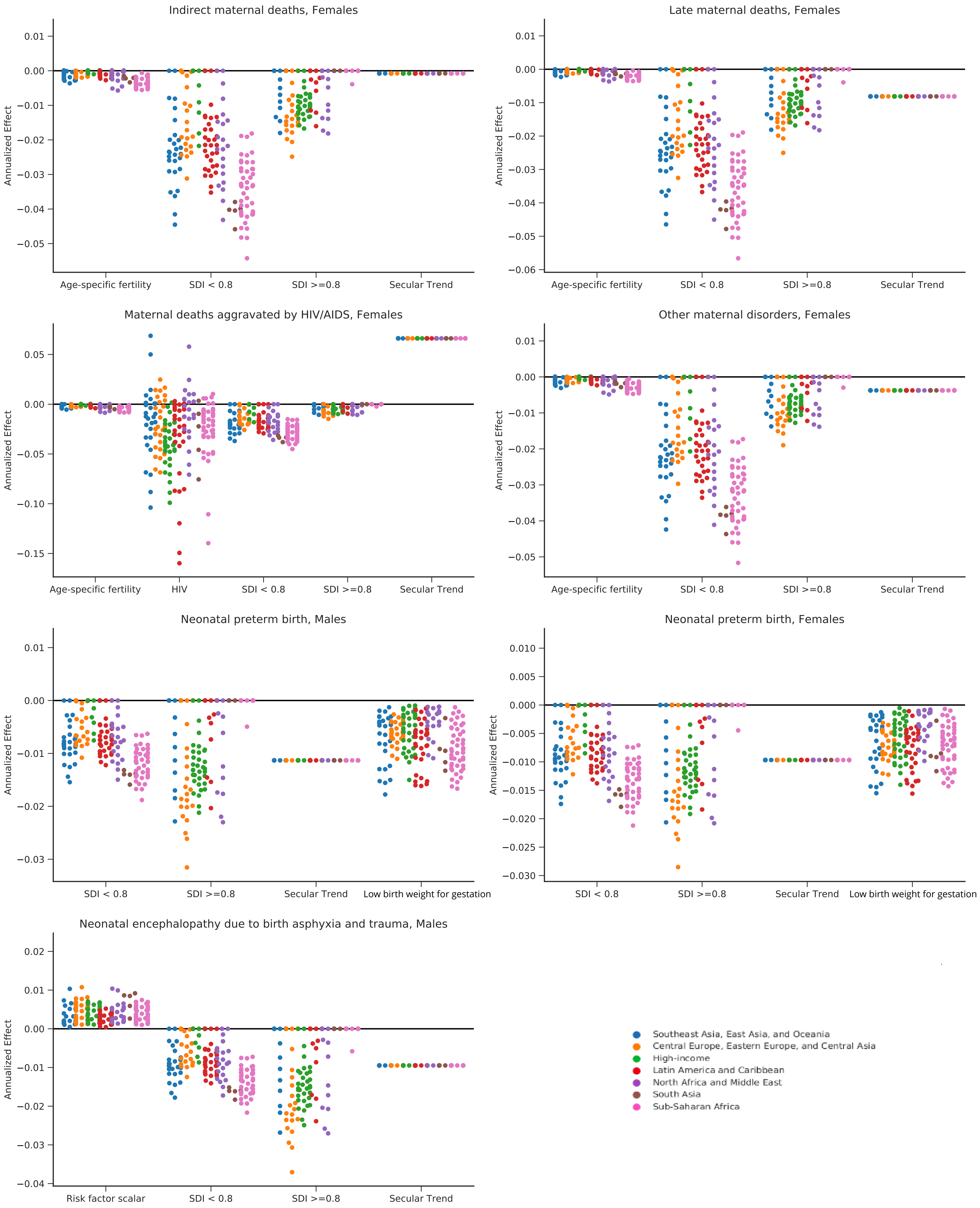

Indirect maternal deaths, Females
Annualized Effect
0.01
0.00
−0.01
−0.02
−0.03
−0.04
−0.05
Age-specific fertility
SDI < 0.8
SDI >=0.8
Secular Trend
Late maternal deaths, Females
Annualized Effect
0.01
0.00
−0.01
−0.02
−0.03
−0.04
−0.05
−0.06
Age-specific fertility
SDI < 0.8
SDI >=0.8
Secular Trend
Maternal deaths aggravated by HIV/AIDS, Females
Annualized Effect
0.05
0.00
−0.05
−0.10
−0.15
Age-specific fertility
HIV
SDI < 0.8
SDI >=0.8
Secular Trend
Other maternal disorders, Females
Annualized Effect
0.01
0.00
−0.01
−0.02
−0.03
−0.04
−0.05
Age-specific fertility
SDI < 0.8
SDI >=0.8
Secular Trend
Neonatal preterm birth, Males
Annualized Effect
0.01
0.00
−0.01
−0.02
−0.03
SDI < 0.8
SDI >=0.8
Secular Trend
Low birth weight for gestation
Neonatal preterm birth, Females
Annualized Effect
0.010
0.005
0.000
−0.005
−0.010
−0.015
−0.020
−0.025
−0.030
SDI < 0.8
SDI >=0.8
Secular Trend
Low birth weight for gestation
Neonatal encephalopathy due to birth asphyxia and trauma, Males
Annualized Effect
0.02
0.01
0.00
−0.01
−0.02
−0.03
−0.04
Risk factor scalar
SDI < 0.8
SDI >=0.8
Secular Trend
Southeast Asia, East Asia, and Oceania
Central Europe, Eastern Europe, and Central Asia
High-income
Latin America and Caribbean
North Africa and Middle East
South Asia
Sub-Saharan Africa

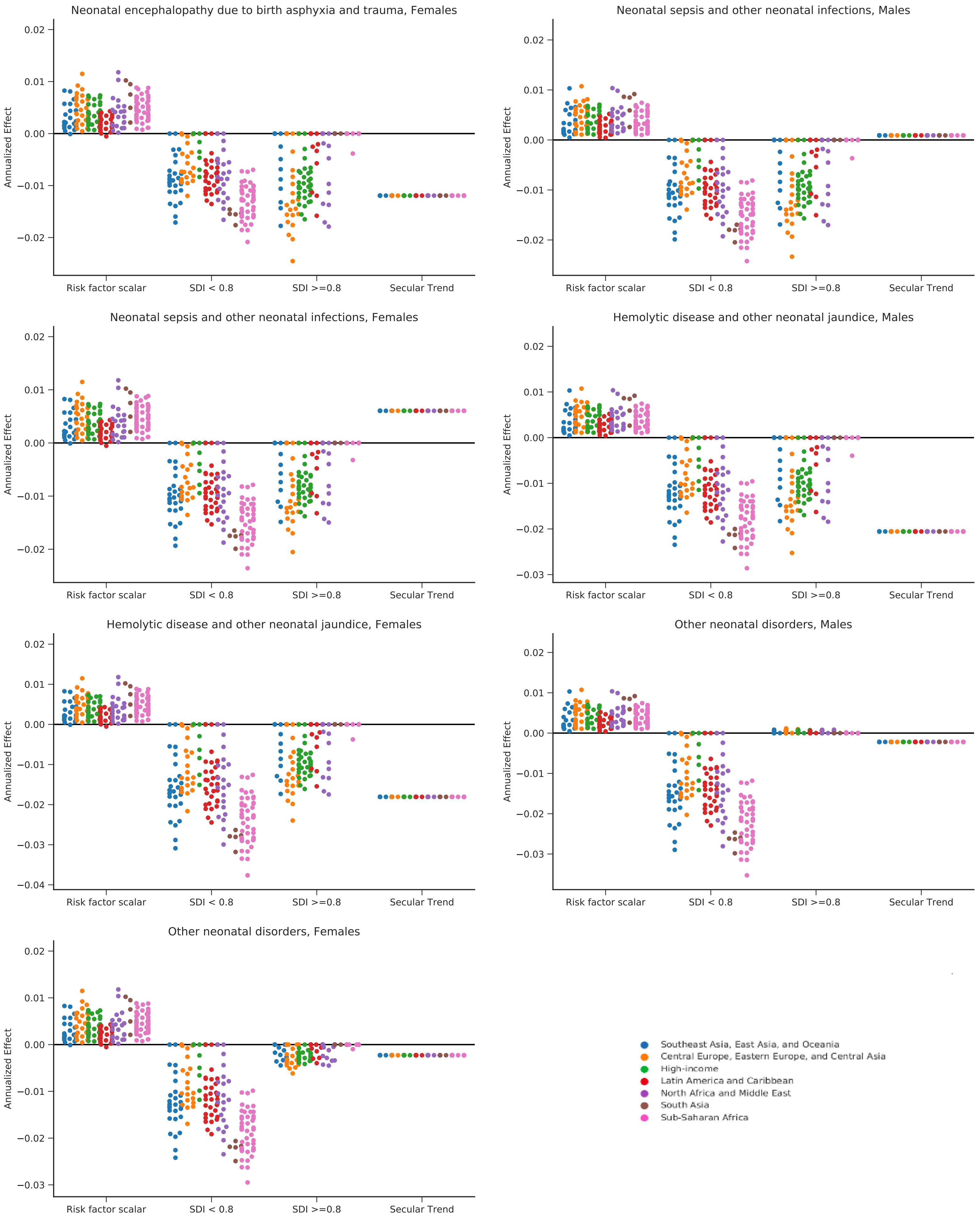
Neonatal encephalopathy due to birth asphyxia and trauma, Females
Neonatal sepsis and other neonatal infections, Males
Neonatal sepsis and other neonatal infections, Females
Hemolytic disease and other neonatal jaundice, Males
Hemolytic disease and other neonatal jaundice, Females
Other neonatal disorders, Males
Other neonatal disorders, Females
Annualized Effect
0.02
0.01
0.00
−0.01
−0.02
−0.03
−0.04
Risk factor scalar
SDI < 0.8
SDI >=0.8
Secular Trend
Southeast Asia, East Asia, and Oceania
Central Europe, Eastern Europe, and Central Asia
High-income
Latin America and Caribbean
North Africa and Middle East
South Asia
Sub-Saharan Africa

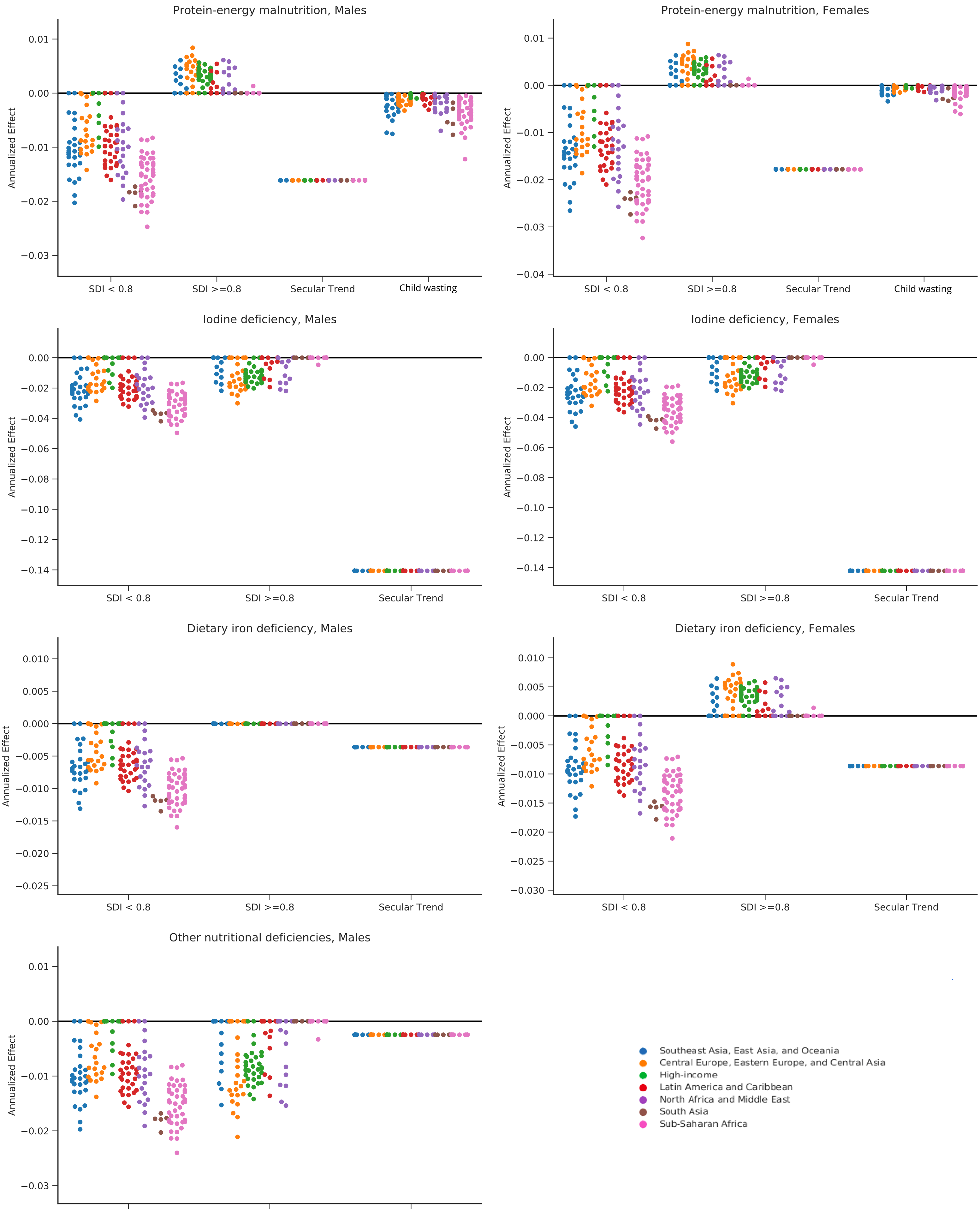
Protein-energy malnutrition, Males
Annualized Effect
0.01
0.00
−0.01
−0.02
−0.03
SDI < 0.8
SDI >=0.8
Secular Trend
Child wasting
Protein-energy malnutrition, Females
Annualized Effect
0.01
0.00
−0.01
−0.02
−0.03
−0.04
SDI < 0.8
SDI >=0.8
Secular Trend
Child wasting
Iodine deficiency, Males
Annualized Effect
0.00
−0.02
−0.04
−0.06
−0.08
−0.10
−0.12
−0.14
SDI < 0.8
SDI >=0.8
Secular Trend
Iodine deficiency, Females
Annualized Effect
0.00
−0.02
−0.04
−0.06
−0.08
−0.10
−0.12
−0.14
SDI < 0.8
SDI >=0.8
Secular Trend
Dietary iron deficiency, Males
Annualized Effect
0.010
0.005
0.000
−0.005
−0.010
−0.015
−0.020
−0.025
SDI < 0.8
SDI >=0.8
Secular Trend
Dietary iron deficiency, Females
Annualized Effect
0.010
0.005
0.000
−0.005
−0.010
−0.015
−0.020
−0.025
−0.030
SDI < 0.8
SDI >=0.8
Secular Trend
Other nutritional deficiencies, Males
Annualized Effect
0.01
0.00
−0.01
−0.02
−0.03
SDI < 0.8
SDI >=0.8
Secular Trend
Southeast Asia, East Asia, and Oceania
Central Europe, Eastern Europe, and Central Asia
High-income
Latin America and Caribbean
North Africa and Middle East
South Asia
Sub-Saharan Africa

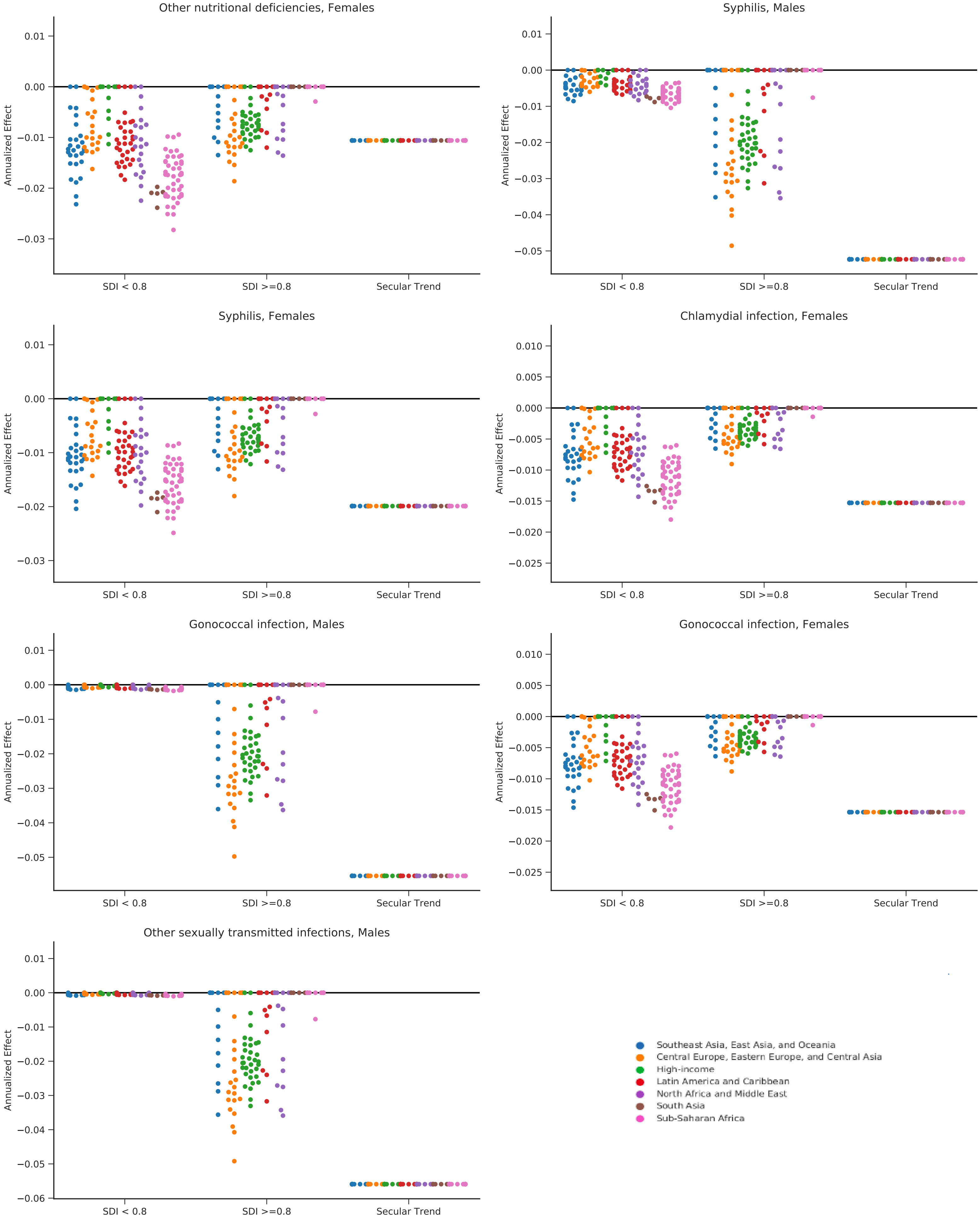

Other nutritional deficiencies, Females
Syphilis, Males
Syphilis, Females
Chlamydial infection, Females
Gonococcal infection, Males
Gonococcal infection, Females
Other sexually transmitted infections, Males
Annualized Effect
SDI < 0.8
SDI >=0.8
Secular Trend
0.01
0.00
−0.01
−0.02
−0.03
−0.04
−0.05
−0.06
0.010
0.005
0.000
−0.005
−0.010
−0.015
−0.020
−0.025
Southeast Asia, East Asia, and Oceania
Central Europe, Eastern Europe, and Central Asia
High-income
Latin America and Caribbean
North Africa and Middle East
South Asia
Sub-Saharan Africa

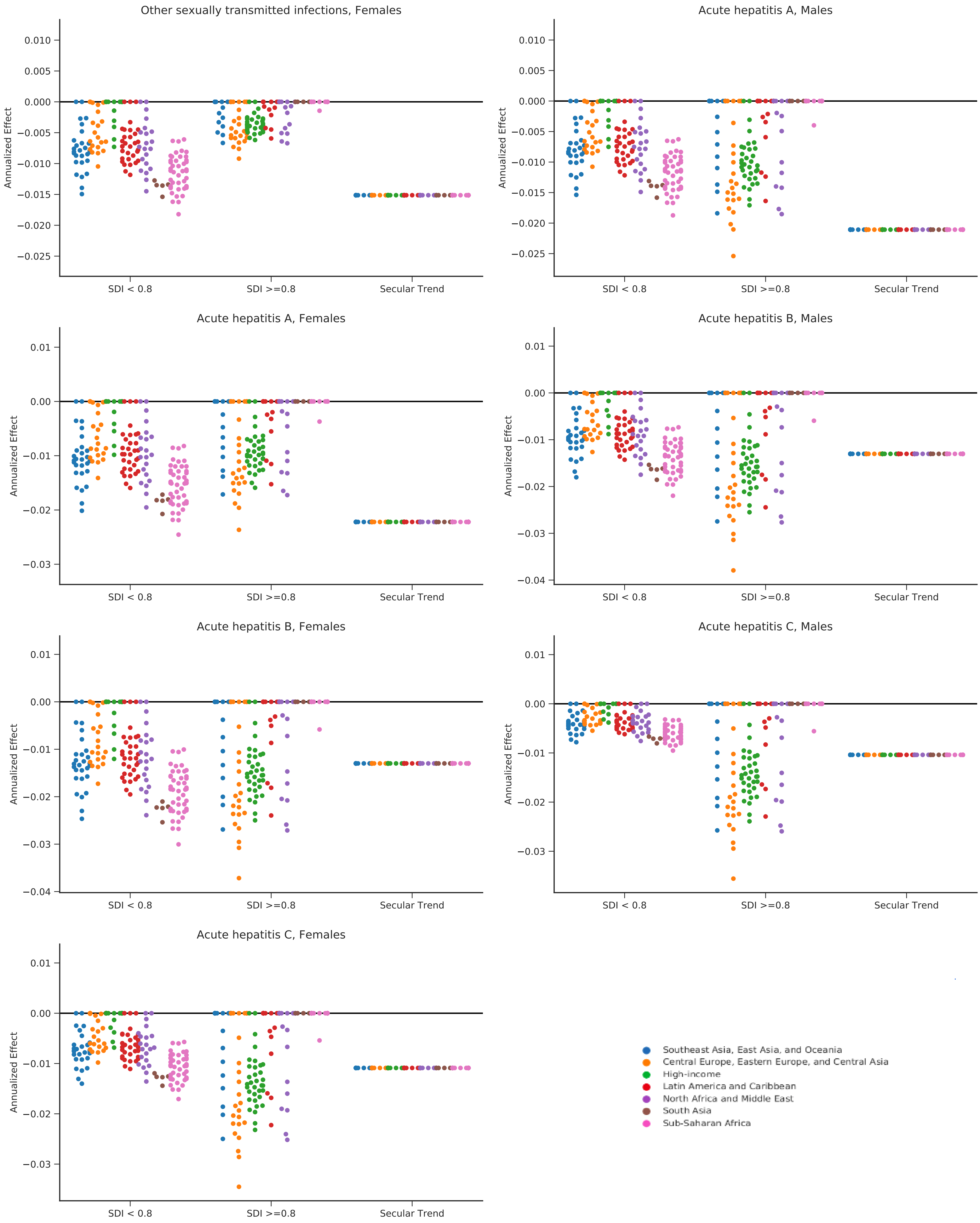
Other sexually transmitted infections, Females
Acute hepatitis A, Males
Acute hepatitis A, Females
Acute hepatitis B, Males
Acute hepatitis B, Females
Acute hepatitis C, Males
Acute hepatitis C, Females
Annualized Effect
SDI < 0.8
SDI >=0.8
Secular Trend
0.010
0.005
0.000
−0.005
−0.010
−0.015
−0.020
−0.025
0.01
0.00
−0.01
−0.02
−0.03
−0.04
Southeast Asia, East Asia, and Oceania
Central Europe, Eastern Europe, and Central Asia
High-income
Latin America and Caribbean
North Africa and Middle East
South Asia
Sub-Saharan Africa

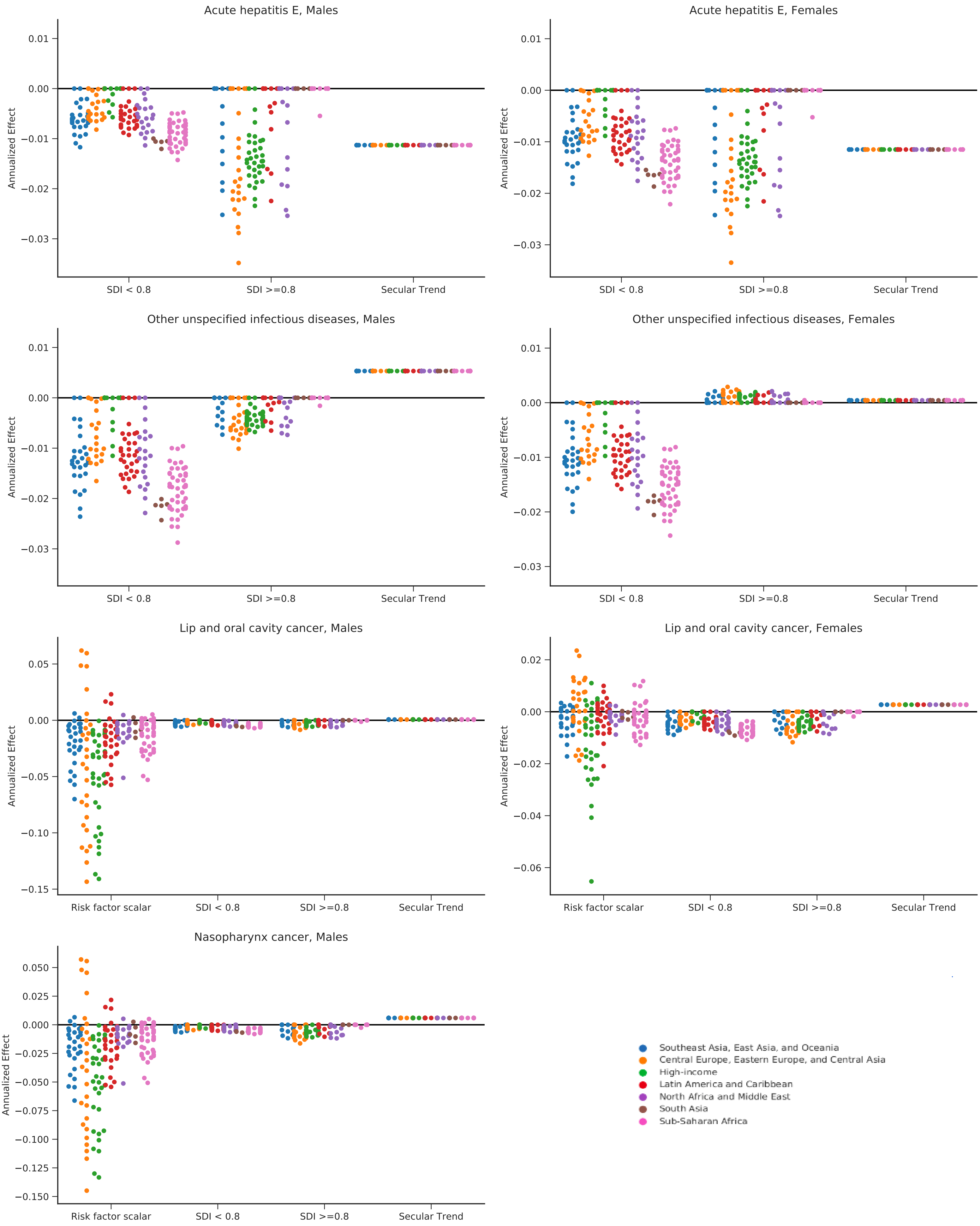
Acute hepatitis E, Males
Acute hepatitis E, Females
Other unspecified infectious diseases, Males
Other unspecified infectious diseases, Females
Lip and oral cavity cancer, Males
Lip and oral cavity cancer, Females
Nasopharynx cancer, Males
Annualized Effect
SDI < 0.8
SDI >=0.8
Secular Trend
Risk factor scalar
0.01
0.00
−0.01
−0.02
−0.03
0.05
−0.05
−0.10
−0.15
0.02
−0.02
−0.04
−0.06
0.050
0.025
0.000
−0.025
−0.050
−0.075
−0.100
−0.125
−0.150
Southeast Asia, East Asia, and Oceania
Central Europe, Eastern Europe, and Central Asia
High-income
Latin America and Caribbean
North Africa and Middle East
South Asia
Sub-Saharan Africa

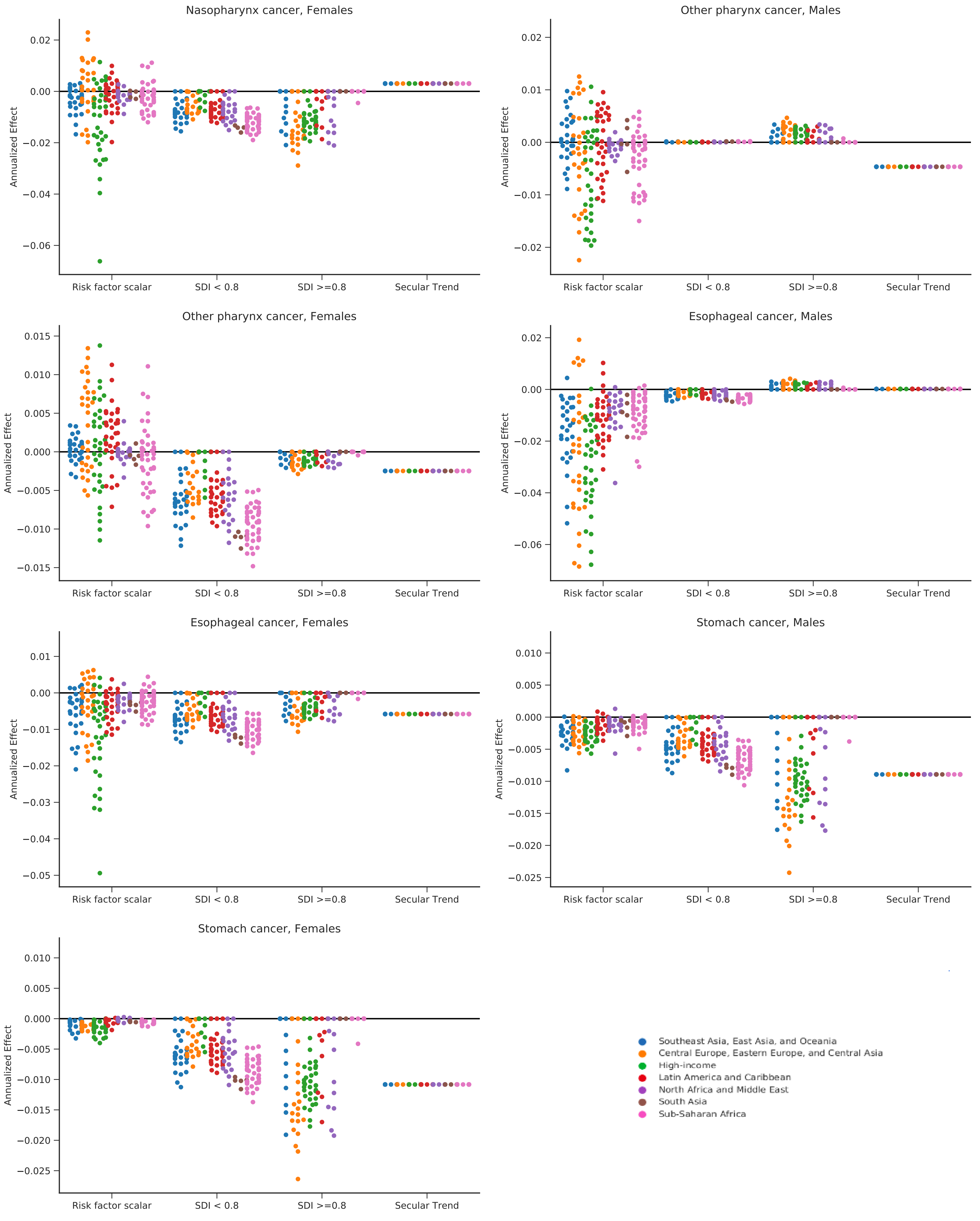
Nasopharynx cancer, Females
Other pharynx cancer, Males
Other pharynx cancer, Females
Esophageal cancer, Males
Esophageal cancer, Females
Stomach cancer, Males
Stomach cancer, Females
Annualized Effect
Risk factor scalar
SDI < 0.8
SDI >=0.8
Secular Trend
Southeast Asia, East Asia, and Oceania
Central Europe, Eastern Europe, and Central Asia
High-income
Latin America and Caribbean
North Africa and Middle East
South Asia
Sub-Saharan Africa

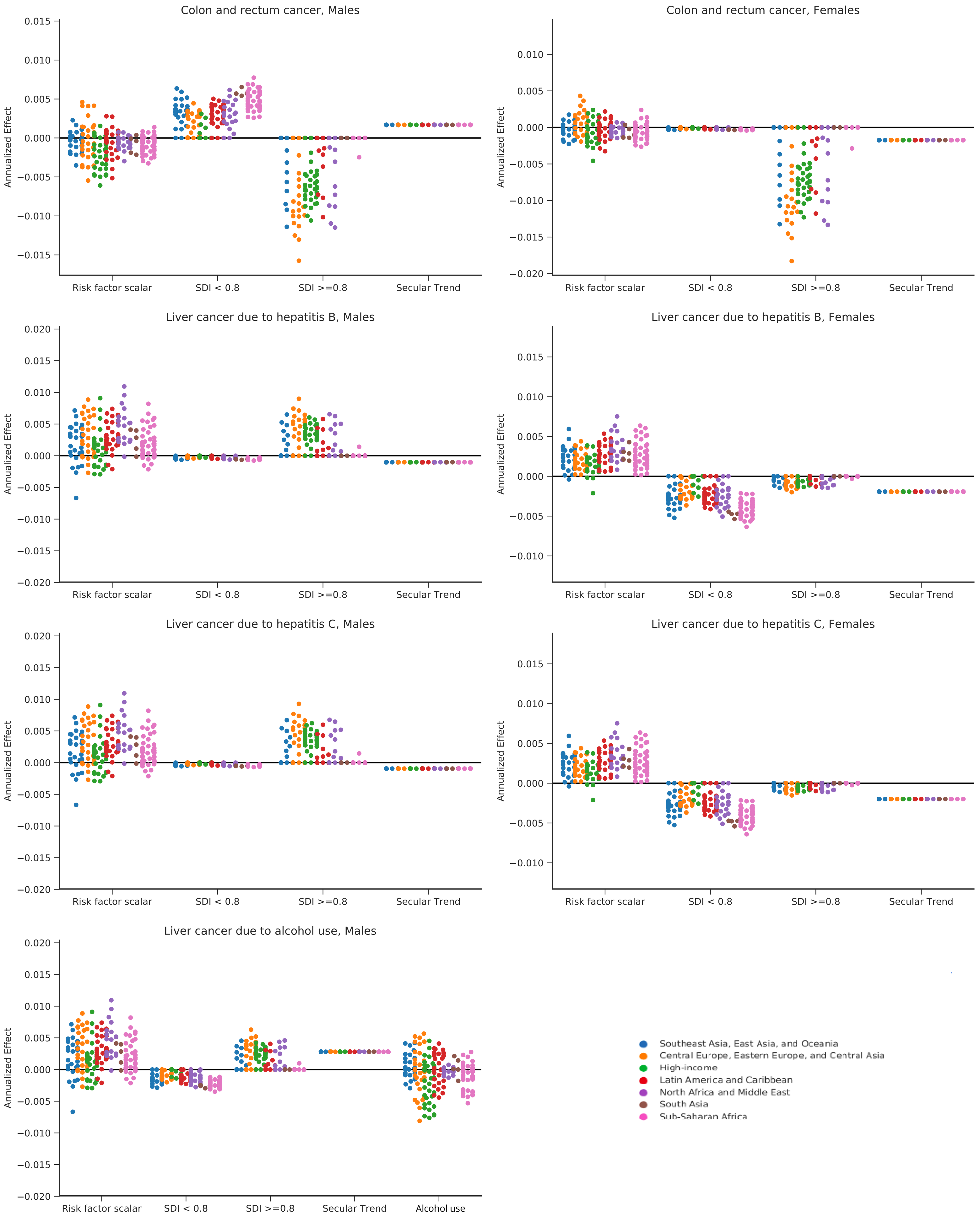

Colon and rectum cancer, Males
Colon and rectum cancer, Females
Liver cancer due to hepatitis B, Males
Liver cancer due to hepatitis B, Females
Liver cancer due to hepatitis C, Males
Liver cancer due to hepatitis C, Females
Liver cancer due to alcohol use, Males
Annualized Effect
Risk factor scalar
SDI < 0.8
SDI >=0.8
Secular Trend
Alcohol use
Southeast Asia, East Asia, and Oceania
Central Europe, Eastern Europe, and Central Asia
High-income
Latin America and Caribbean
North Africa and Middle East
South Asia
Sub-Saharan Africa

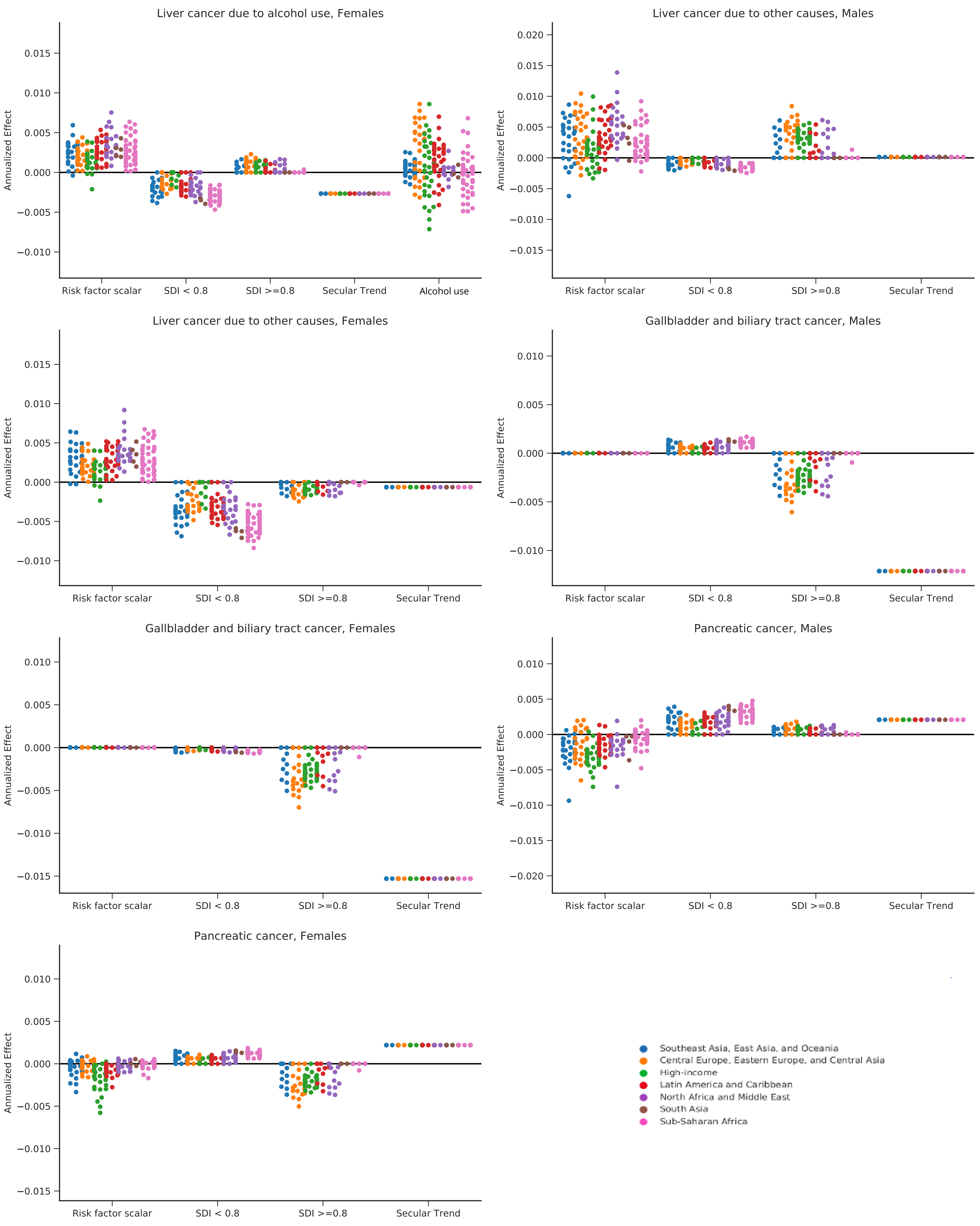
Liver cancer due to alcohol use, Females
Annualized Effect
0.015
0.010
0.005
0.000
−0.005
−0.010
Risk factor scalar
SDI < 0.8
SDI >=0.8
Secular Trend
Alcohol use
Liver cancer due to other causes, Males
Annualized Effect
0.020
0.015
0.010
0.005
0.000
−0.005
−0.010
−0.015
Risk factor scalar
SDI < 0.8
SDI >=0.8
Secular Trend
Liver cancer due to other causes, Females
Annualized Effect
0.015
0.010
0.005
0.000
−0.005
−0.010
Risk factor scalar
SDI < 0.8
SDI >=0.8
Secular Trend
Gallbladder and biliary tract cancer, Males
Annualized Effect
0.010
0.005
0.000
−0.005
−0.010
Risk factor scalar
SDI < 0.8
SDI >=0.8
Secular Trend
Gallbladder and biliary tract cancer, Females
Annualized Effect
0.010
0.005
0.000
−0.005
−0.010
−0.015
Risk factor scalar
SDI < 0.8
SDI >=0.8
Secular Trend
Pancreatic cancer, Males
Annualized Effect
0.010
0.005
0.000
−0.005
−0.010
−0.015
−0.020
Risk factor scalar
SDI < 0.8
SDI >=0.8
Secular Trend
Pancreatic cancer, Females
Annualized Effect
0.010
0.005
0.000
−0.005
−0.010
−0.015
Risk factor scalar
SDI < 0.8
SDI >=0.8
Secular Trend
Southeast Asia, East Asia, and Oceania
Central Europe, Eastern Europe, and Central Asia
High-income
Latin America and Caribbean
North Africa and Middle East
South Asia
Sub-Saharan Africa

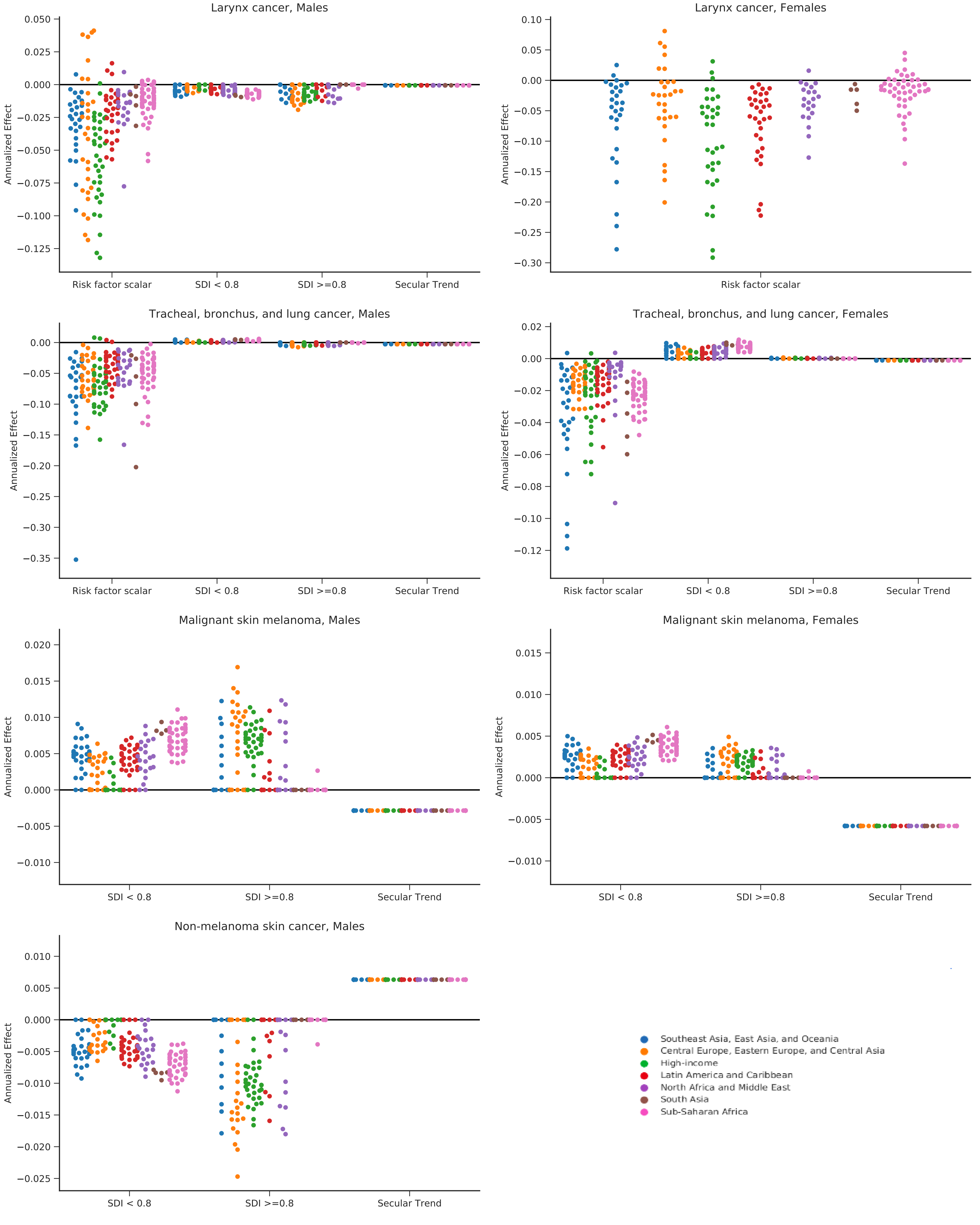

Larynx cancer, Males
Annualized Effect
Risk factor scalar
SDI < 0.8
SDI >=0.8
Secular Trend
Larynx cancer, Females
Annualized Effect
Risk factor scalar
Tracheal, bronchus, and lung cancer, Males
Annualized Effect
Risk factor scalar
SDI < 0.8
SDI >=0.8
Secular Trend
Tracheal, bronchus, and lung cancer, Females
Annualized Effect
Risk factor scalar
SDI < 0.8
SDI >=0.8
Secular Trend
Malignant skin melanoma, Males
Annualized Effect
SDI < 0.8
SDI >=0.8
Secular Trend
Malignant skin melanoma, Females
Annualized Effect
SDI < 0.8
SDI >=0.8
Secular Trend
Non-melanoma skin cancer, Males
Annualized Effect
SDI < 0.8
SDI >=0.8
Secular Trend
Southeast Asia, East Asia, and Oceania
Central Europe, Eastern Europe, and Central Asia
High-income
Latin America and Caribbean
North Africa and Middle East
South Asia
Sub-Saharan Africa

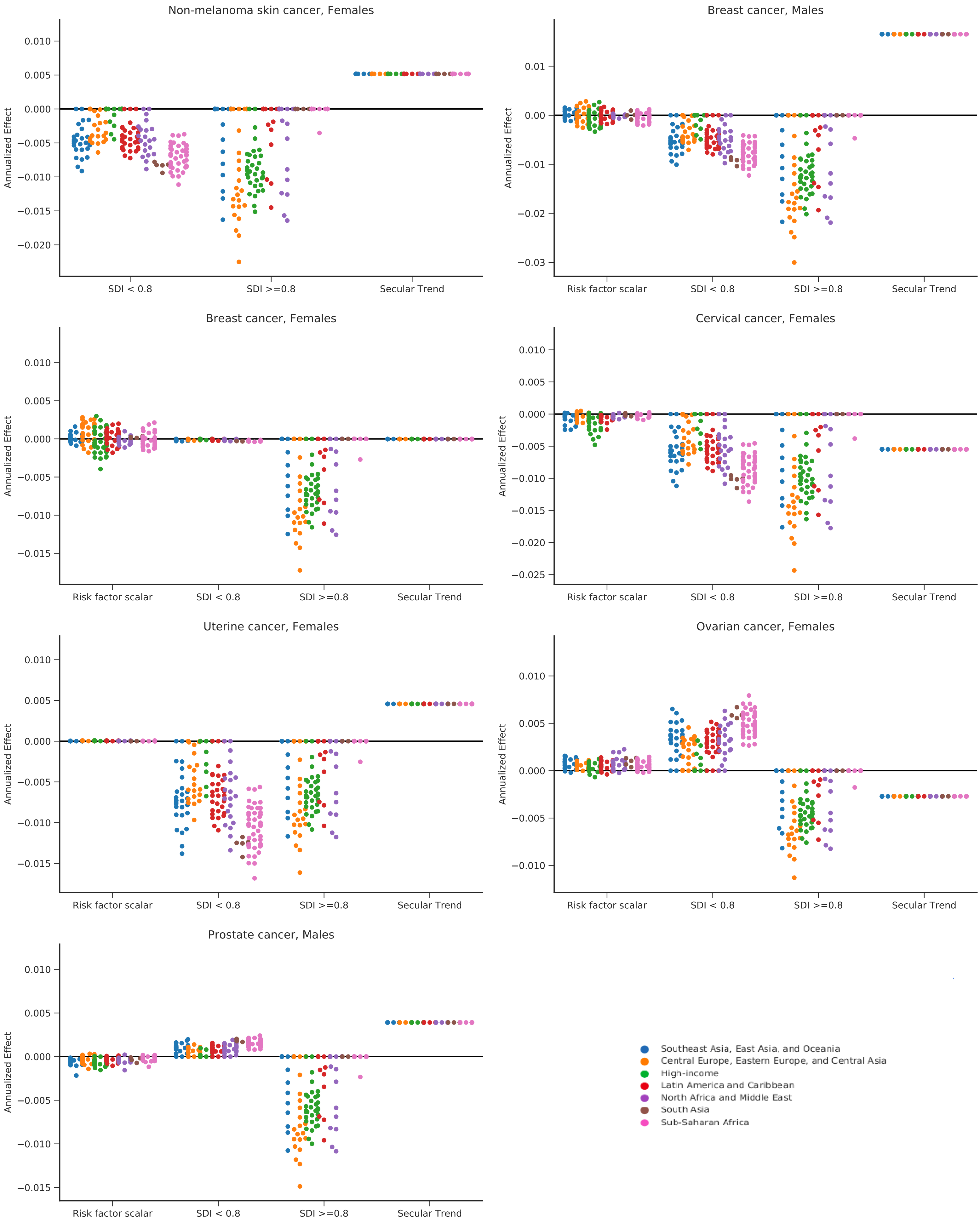
Non-melanoma skin cancer, Females
Annualized Effect
0.010
0.005
0.000
−0.005
−0.010
−0.015
−0.020
SDI < 0.8
SDI >=0.8
Secular Trend
Breast cancer, Males
Annualized Effect
0.01
0.00
−0.01
−0.02
−0.03
Risk factor scalar
SDI < 0.8
SDI >=0.8
Secular Trend
Breast cancer, Females
Annualized Effect
0.010
0.005
0.000
−0.005
−0.010
−0.015
Risk factor scalar
SDI < 0.8
SDI >=0.8
Secular Trend
Cervical cancer, Females
Annualized Effect
0.010
0.005
0.000
−0.005
−0.010
−0.015
−0.020
−0.025
Risk factor scalar
SDI < 0.8
SDI >=0.8
Secular Trend
Uterine cancer, Females
Annualized Effect
0.010
0.005
0.000
−0.005
−0.010
−0.015
Risk factor scalar
SDI < 0.8
SDI >=0.8
Secular Trend
Ovarian cancer, Females
Annualized Effect
0.010
0.005
0.000
−0.005
−0.010
Risk factor scalar
SDI < 0.8
SDI >=0.8
Secular Trend
Prostate cancer, Males
Annualized Effect
0.010
0.005
0.000
−0.005
−0.010
−0.015
Risk factor scalar
SDI < 0.8
SDI >=0.8
Secular Trend
Southeast Asia, East Asia, and Oceania
Central Europe, Eastern Europe, and Central Asia
High-income
Latin America and Caribbean
North Africa and Middle East
South Asia
Sub-Saharan Africa

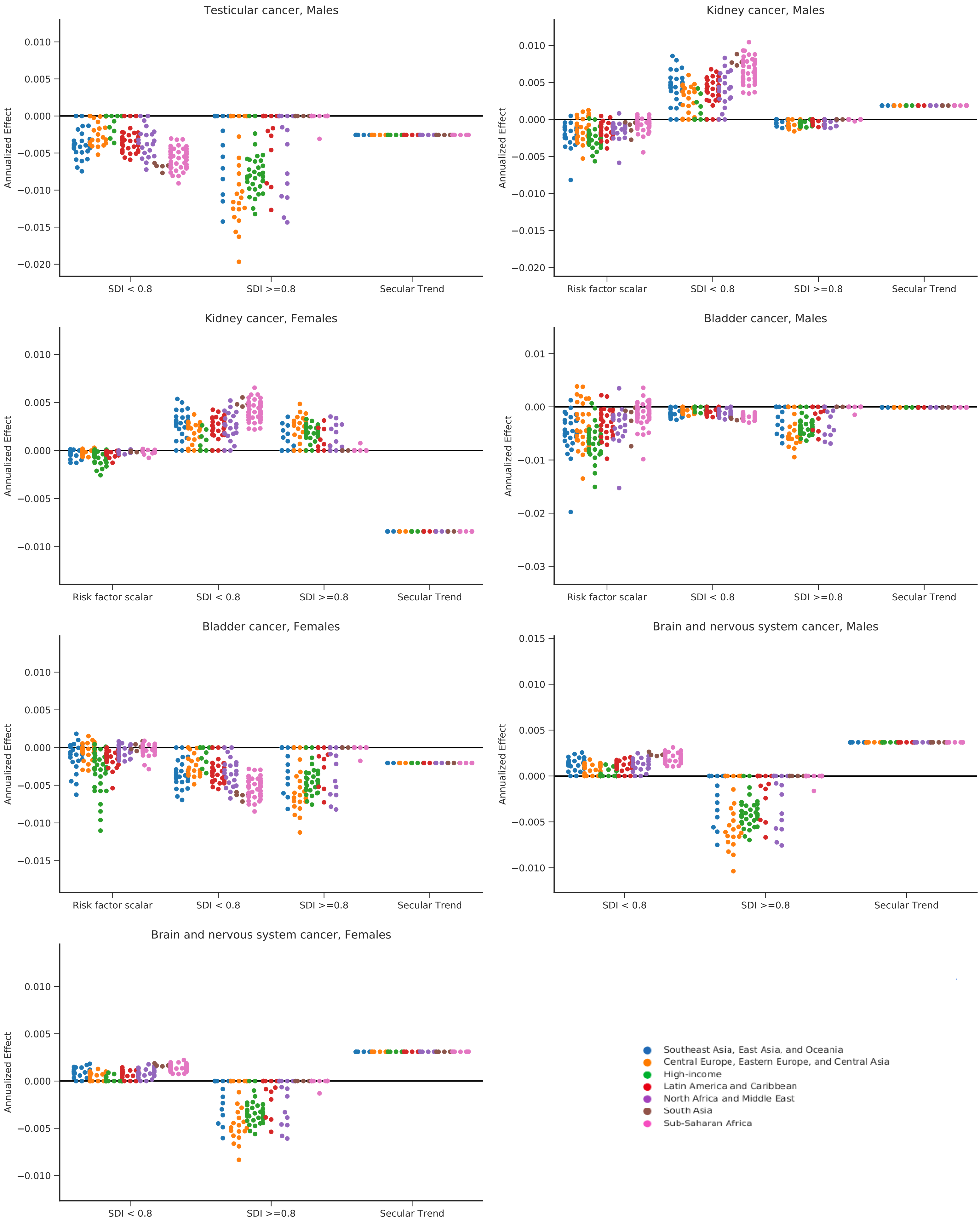
Testicular cancer, Males
Annualized Effect
0.010
0.005
0.000
−0.005
−0.010
−0.015
−0.020
SDI < 0.8
SDI >=0.8
Secular Trend
Kidney cancer, Males
Annualized Effect
0.010
0.005
0.000
−0.005
−0.010
−0.015
−0.020
Risk factor scalar
SDI < 0.8
SDI >=0.8
Secular Trend
Kidney cancer, Females
Annualized Effect
0.010
0.005
0.000
−0.005
−0.010
Risk factor scalar
SDI < 0.8
SDI >=0.8
Secular Trend
Bladder cancer, Males
Annualized Effect
0.01
0.00
−0.01
−0.02
−0.03
Risk factor scalar
SDI < 0.8
SDI >=0.8
Secular Trend
Bladder cancer, Females
Annualized Effect
0.010
0.005
0.000
−0.005
−0.010
−0.015
Risk factor scalar
SDI < 0.8
SDI >=0.8
Secular Trend
Brain and nervous system cancer, Males
Annualized Effect
0.015
0.010
0.005
0.000
−0.005
−0.010
SDI < 0.8
SDI >=0.8
Secular Trend
Brain and nervous system cancer, Females
Annualized Effect
0.010
0.005
0.000
−0.005
−0.010
SDI < 0.8
SDI >=0.8
Secular Trend
Southeast Asia, East Asia, and Oceania
Central Europe, Eastern Europe, and Central Asia
High-income
Latin America and Caribbean
North Africa and Middle East
South Asia
Sub-Saharan Africa

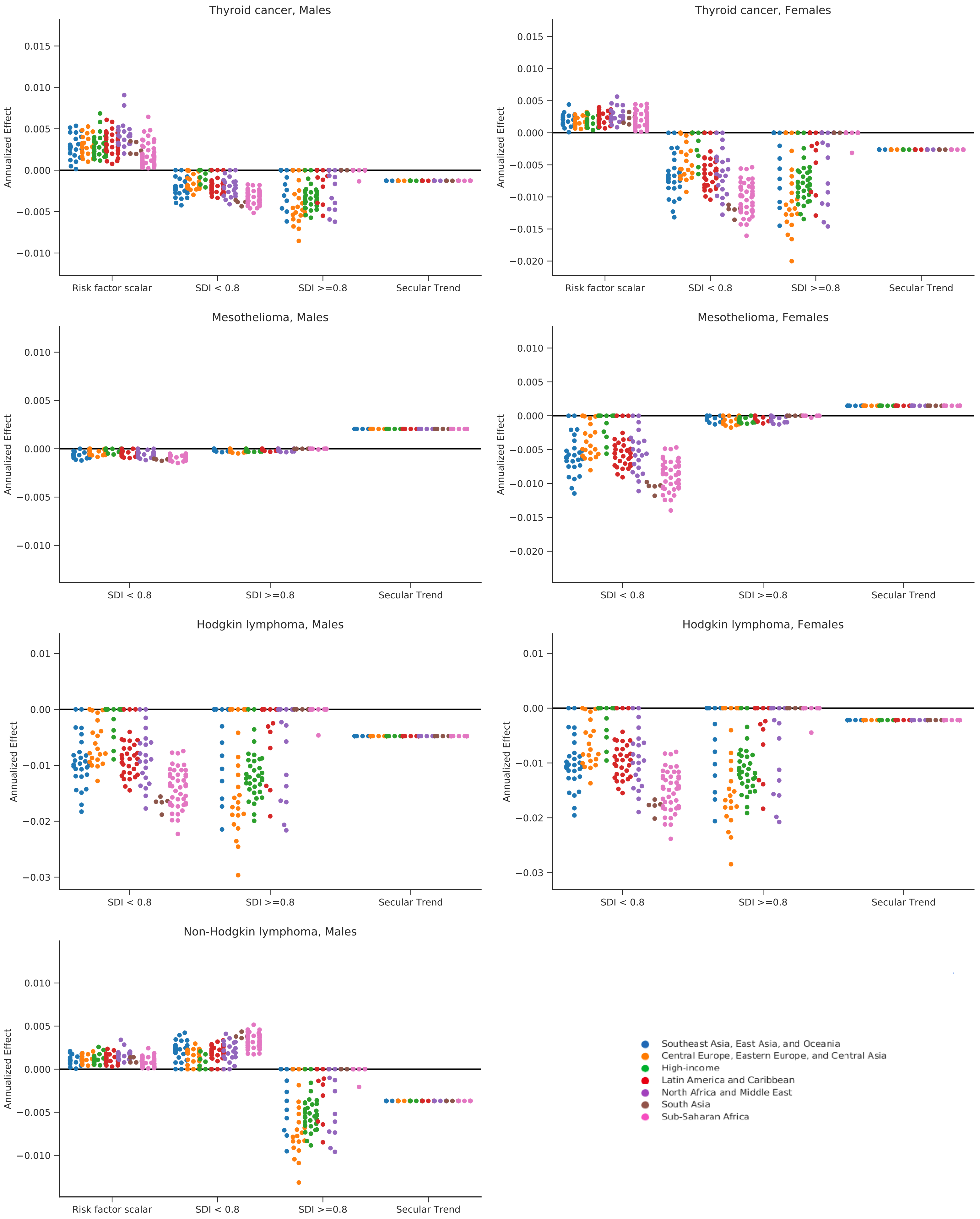

Thyroid cancer, Males
Thyroid cancer, Females
Mesothelioma, Males
Mesothelioma, Females
Hodgkin lymphoma, Males
Hodgkin lymphoma, Females
Non-Hodgkin lymphoma, Males
Annualized Effect
Risk factor scalar
SDI < 0.8
SDI >=0.8
Secular Trend
0.015
0.010
0.005
0.000
−0.005
−0.010
−0.015
−0.020
0.01
0.00
−0.01
−0.02
−0.03
Southeast Asia, East Asia, and Oceania
Central Europe, Eastern Europe, and Central Asia
High-income
Latin America and Caribbean
North Africa and Middle East
South Asia
Sub-Saharan Africa

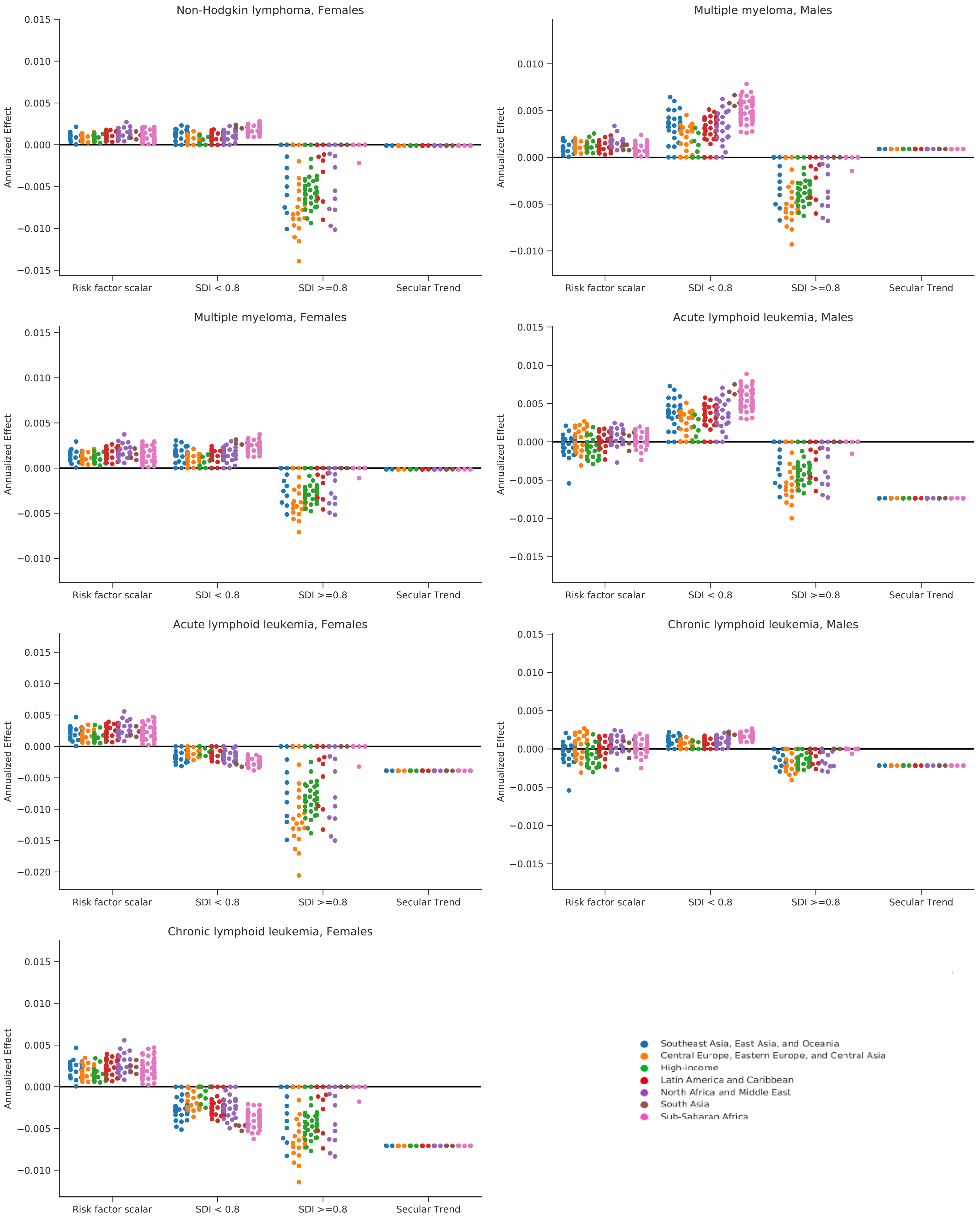

Non-Hodgkin lymphoma, Females
Multiple myeloma, Males
Multiple myeloma, Females
Acute lymphoid leukemia, Males
Acute lymphoid leukemia, Females
Chronic lymphoid leukemia, Males
Chronic lymphoid leukemia, Females
Annualized Effect
0.015
0.010
0.005
0.000
−0.005
−0.010
−0.015
−0.020
Risk factor scalar
SDI < 0.8
SDI >=0.8
Secular Trend
Southeast Asia, East Asia, and Oceania
Central Europe, Eastern Europe, and Central Asia
High-income
Latin America and Caribbean
North Africa and Middle East
South Asia
Sub-Saharan Africa

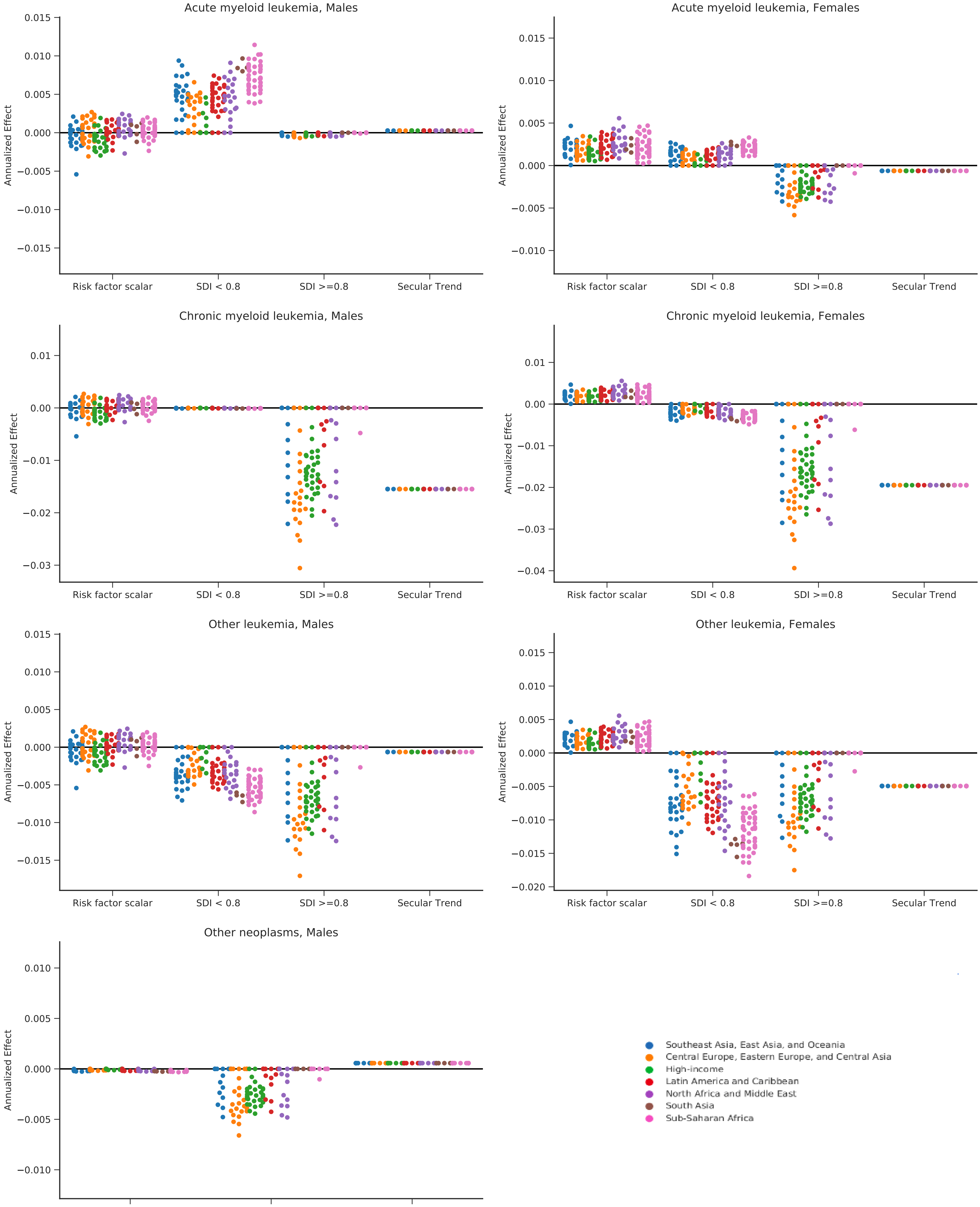

Acute myeloid leukemia, Males
Acute myeloid leukemia, Females
Chronic myeloid leukemia, Males
Chronic myeloid leukemia, Females
Other leukemia, Males
Other leukemia, Females
Other neoplasms, Males
Annualized Effect
Risk factor scalar
SDI < 0.8
SDI >=0.8
Secular Trend
0.015
0.010
0.005
0.000
−0.005
−0.010
−0.015
−0.020
0.01
0.00
−0.01
−0.02
−0.03
−0.04
Southeast Asia, East Asia, and Oceania
Central Europe, Eastern Europe, and Central Asia
High-income
Latin America and Caribbean
North Africa and Middle East
South Asia
Sub-Saharan Africa

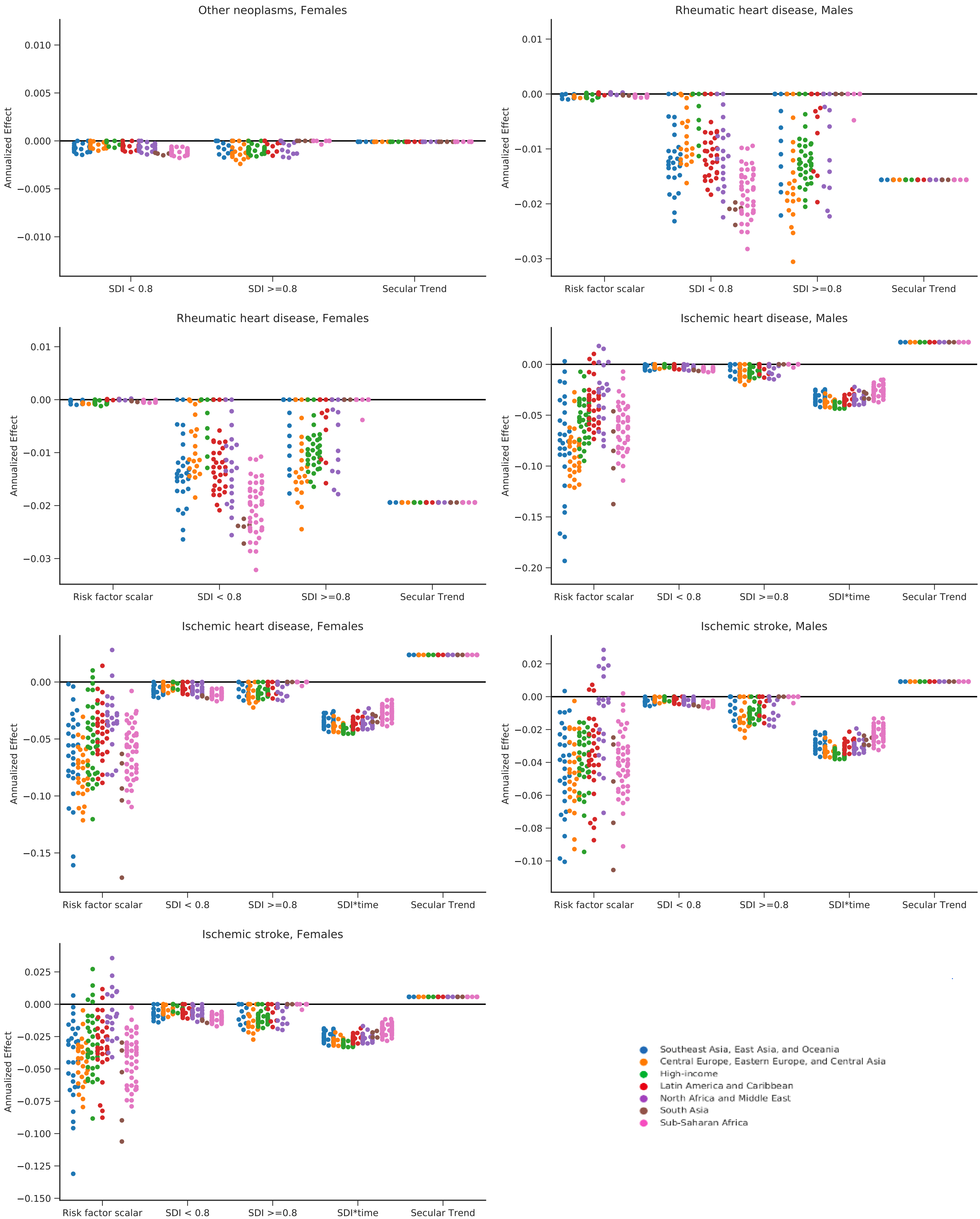

Other neoplasms, Females
Rheumatic heart disease, Males
Rheumatic heart disease, Females
Ischemic heart disease, Males
Ischemic heart disease, Females
Ischemic stroke, Males
Ischemic stroke, Females
Annualized Effect
Risk factor scalar
SDI < 0.8
SDI >=0.8
SDI*time
Secular Trend
Southeast Asia, East Asia, and Oceania
Central Europe, Eastern Europe, and Central Asia
High-income
Latin America and Caribbean
North Africa and Middle East
South Asia
Sub-Saharan Africa

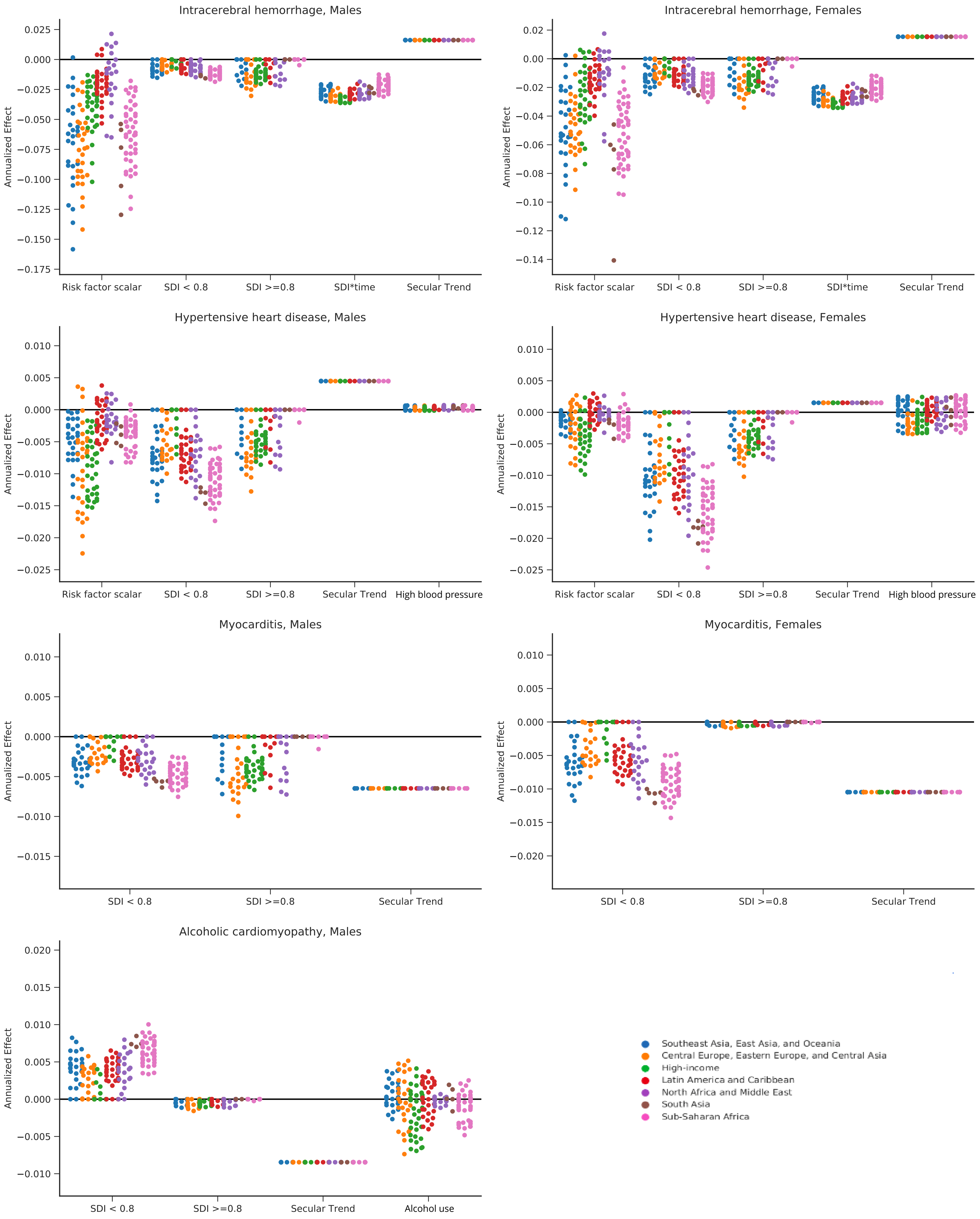
Intracerebral hemorrhage, Males
Annualized Effect
0.025
0.000
−0.025
−0.050
−0.075
−0.100
−0.125
−0.150
−0.175
Risk factor scalar
SDI < 0.8
SDI >=0.8
SDI*time
Secular Trend
Intracerebral hemorrhage, Females
Annualized Effect
0.02
0.00
−0.02
−0.04
−0.06
−0.08
−0.10
−0.12
−0.14
Risk factor scalar
SDI < 0.8
SDI >=0.8
SDI*time
Secular Trend
Hypertensive heart disease, Males
Annualized Effect
0.010
0.005
0.000
−0.005
−0.010
−0.015
−0.020
−0.025
Risk factor scalar
SDI < 0.8
SDI >=0.8
Secular Trend
High blood pressure
Hypertensive heart disease, Females
Annualized Effect
0.010
0.005
0.000
−0.005
−0.010
−0.015
−0.020
−0.025
Risk factor scalar
SDI < 0.8
SDI >=0.8
Secular Trend
High blood pressure
Myocarditis, Males
Annualized Effect
0.010
0.005
0.000
−0.005
−0.010
−0.015
SDI < 0.8
SDI >=0.8
Secular Trend
Myocarditis, Females
Annualized Effect
0.010
0.005
0.000
−0.005
−0.010
−0.015
−0.020
SDI < 0.8
SDI >=0.8
Secular Trend
Alcoholic cardiomyopathy, Males
Annualized Effect
0.020
0.015
0.010
0.005
0.000
−0.005
−0.010
SDI < 0.8
SDI >=0.8
Secular Trend
Alcohol use
Southeast Asia, East Asia, and Oceania
Central Europe, Eastern Europe, and Central Asia
High-income
Latin America and Caribbean
North Africa and Middle East
South Asia
Sub-Saharan Africa

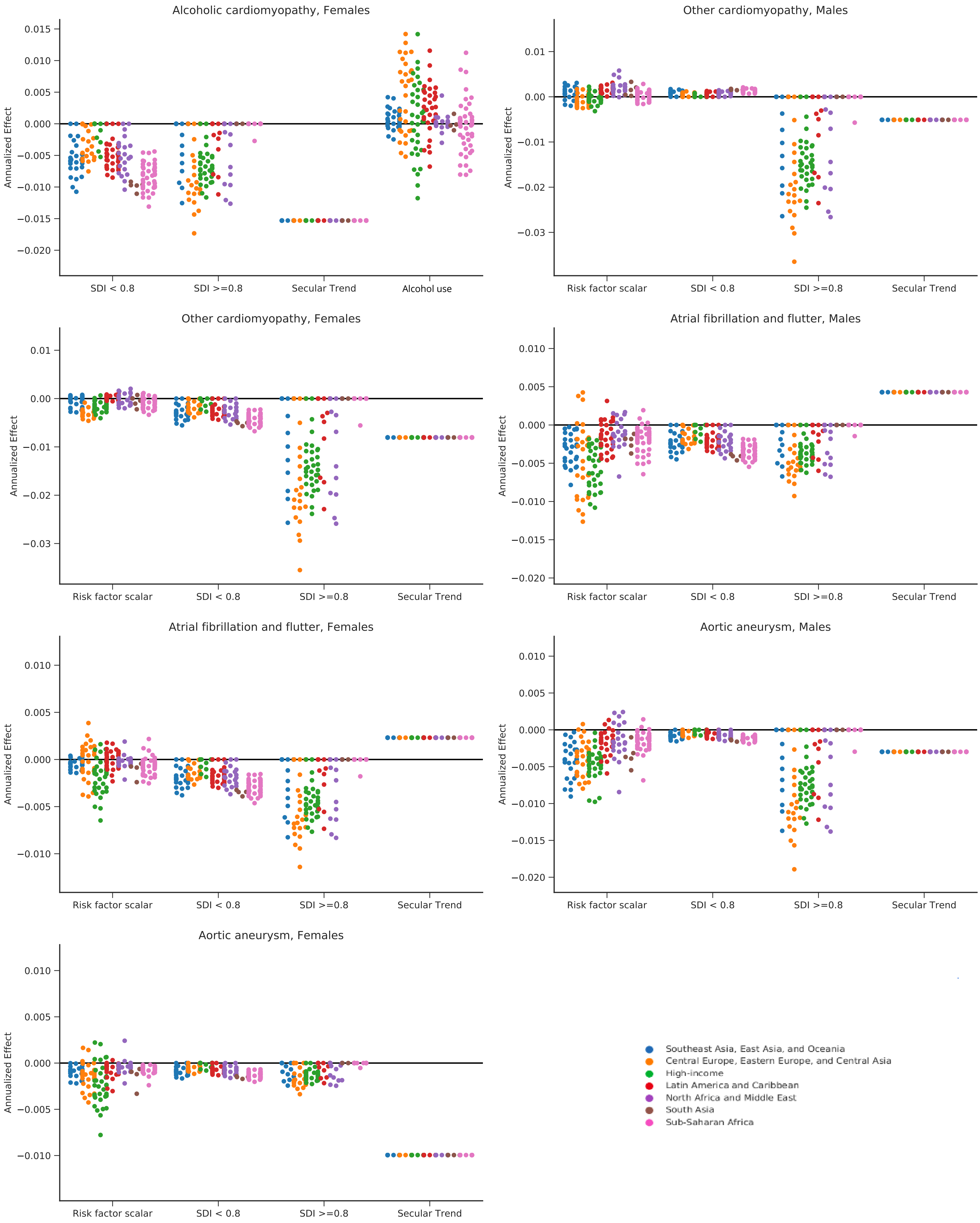

Alcoholic cardiomyopathy, Females
Other cardiomyopathy, Males
Other cardiomyopathy, Females
Atrial fibrillation and flutter, Males
Atrial fibrillation and flutter, Females
Aortic aneurysm, Males
Aortic aneurysm, Females
Annualized Effect
Risk factor scalar
SDI < 0.8
SDI >=0.8
Secular Trend
Alcohol use
Southeast Asia, East Asia, and Oceania
Central Europe, Eastern Europe, and Central Asia
High-income
Latin America and Caribbean
North Africa and Middle East
South Asia
Sub-Saharan Africa

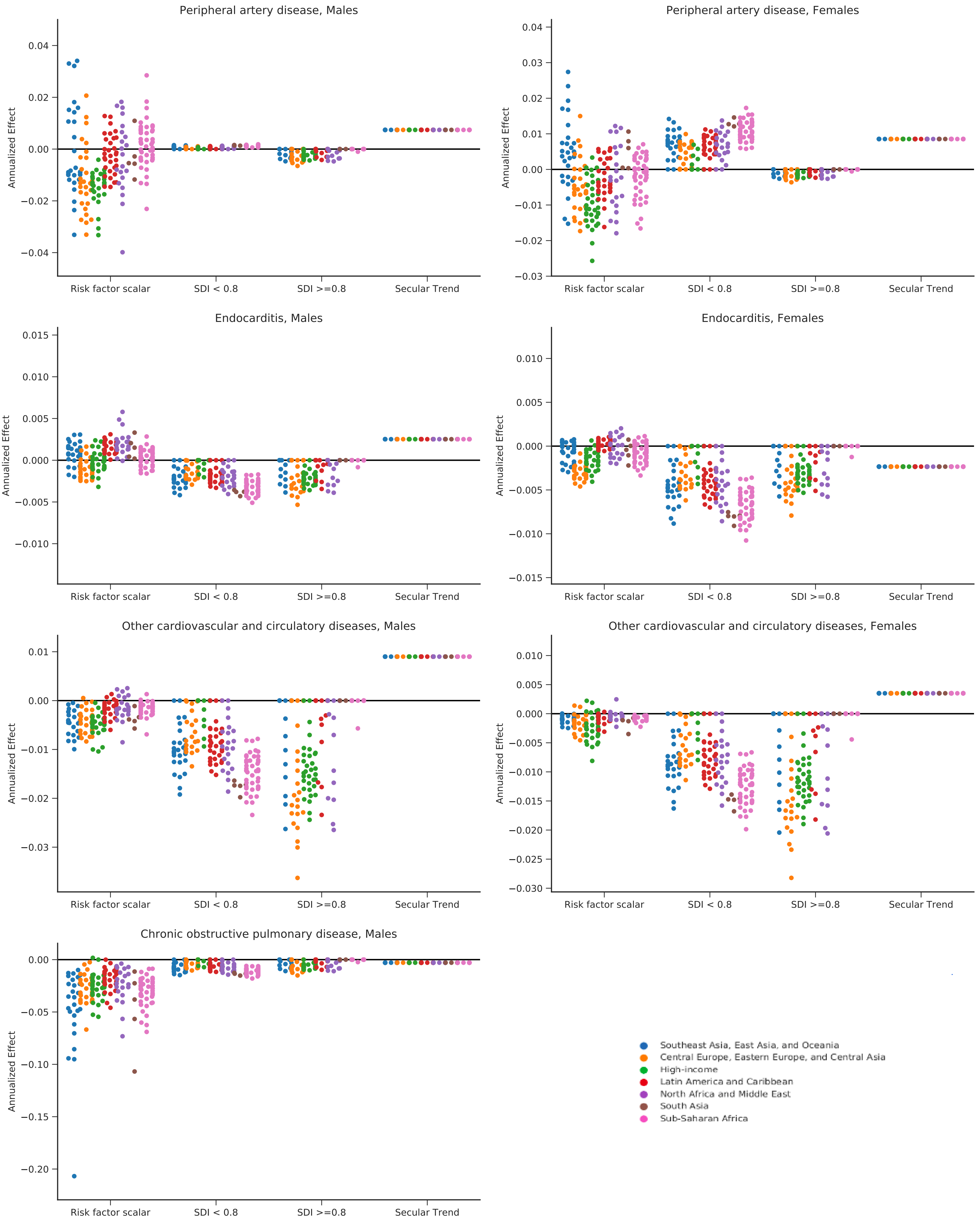

Peripheral artery disease, Males
Peripheral artery disease, Females
Endocarditis, Males
Endocarditis, Females
Other cardiovascular and circulatory diseases, Males
Other cardiovascular and circulatory diseases, Females
Chronic obstructive pulmonary disease, Males
Annualized Effect
Risk factor scalar
SDI < 0.8
SDI >=0.8
Secular Trend
Southeast Asia, East Asia, and Oceania
Central Europe, Eastern Europe, and Central Asia
High-income
Latin America and Caribbean
North Africa and Middle East
South Asia
Sub-Saharan Africa

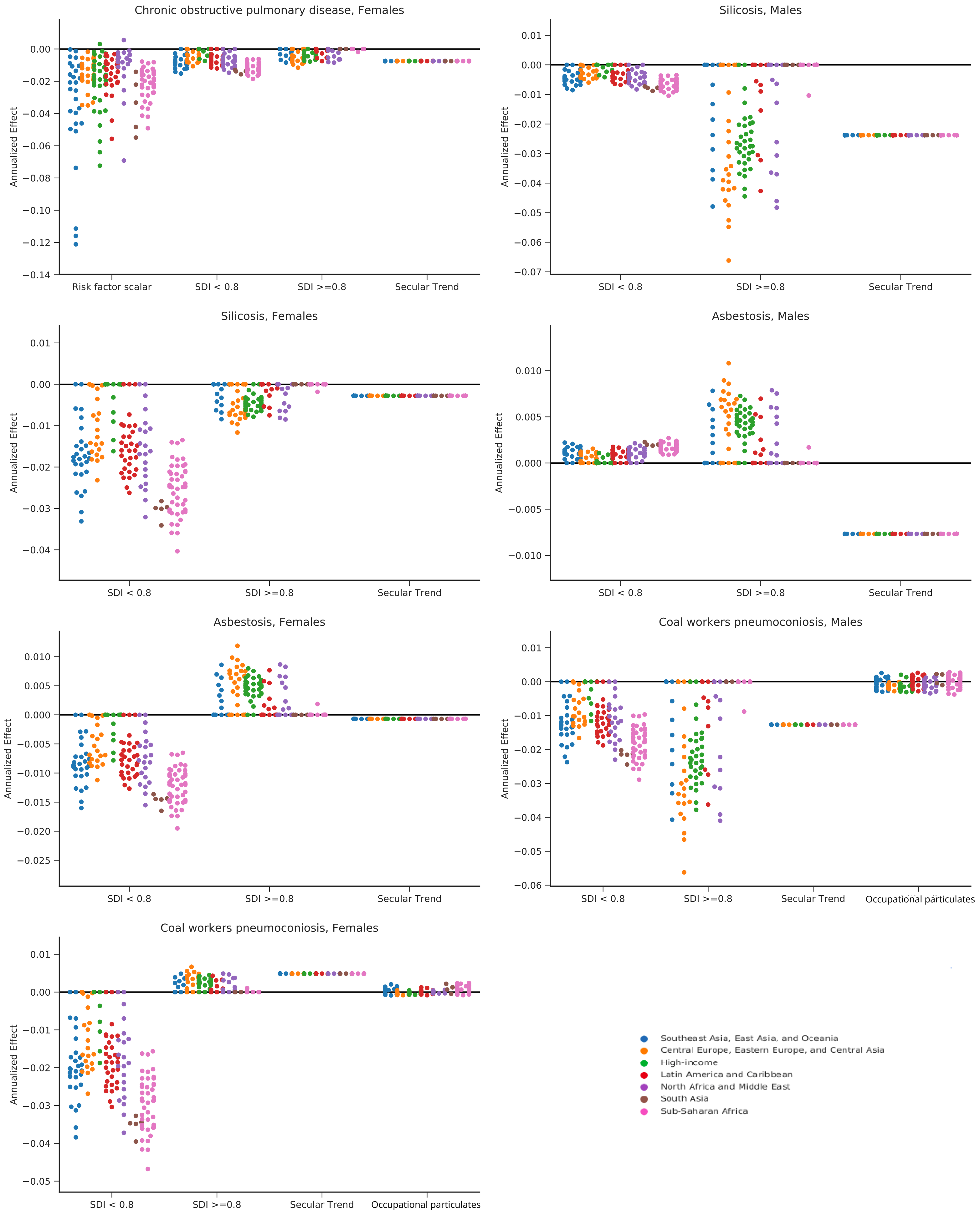
Chronic obstructive pulmonary disease, Females
Silicosis, Males
Silicosis, Females
Asbestosis, Males
Asbestosis, Females
Coal workers pneumoconiosis, Males
Coal workers pneumoconiosis, Females
Annualized Effect
Risk factor scalar
SDI < 0.8
SDI >=0.8
Secular Trend
Occupational particulates
Southeast Asia, East Asia, and Oceania
Central Europe, Eastern Europe, and Central Asia
High-income
Latin America and Caribbean
North Africa and Middle East
South Asia
Sub-Saharan Africa

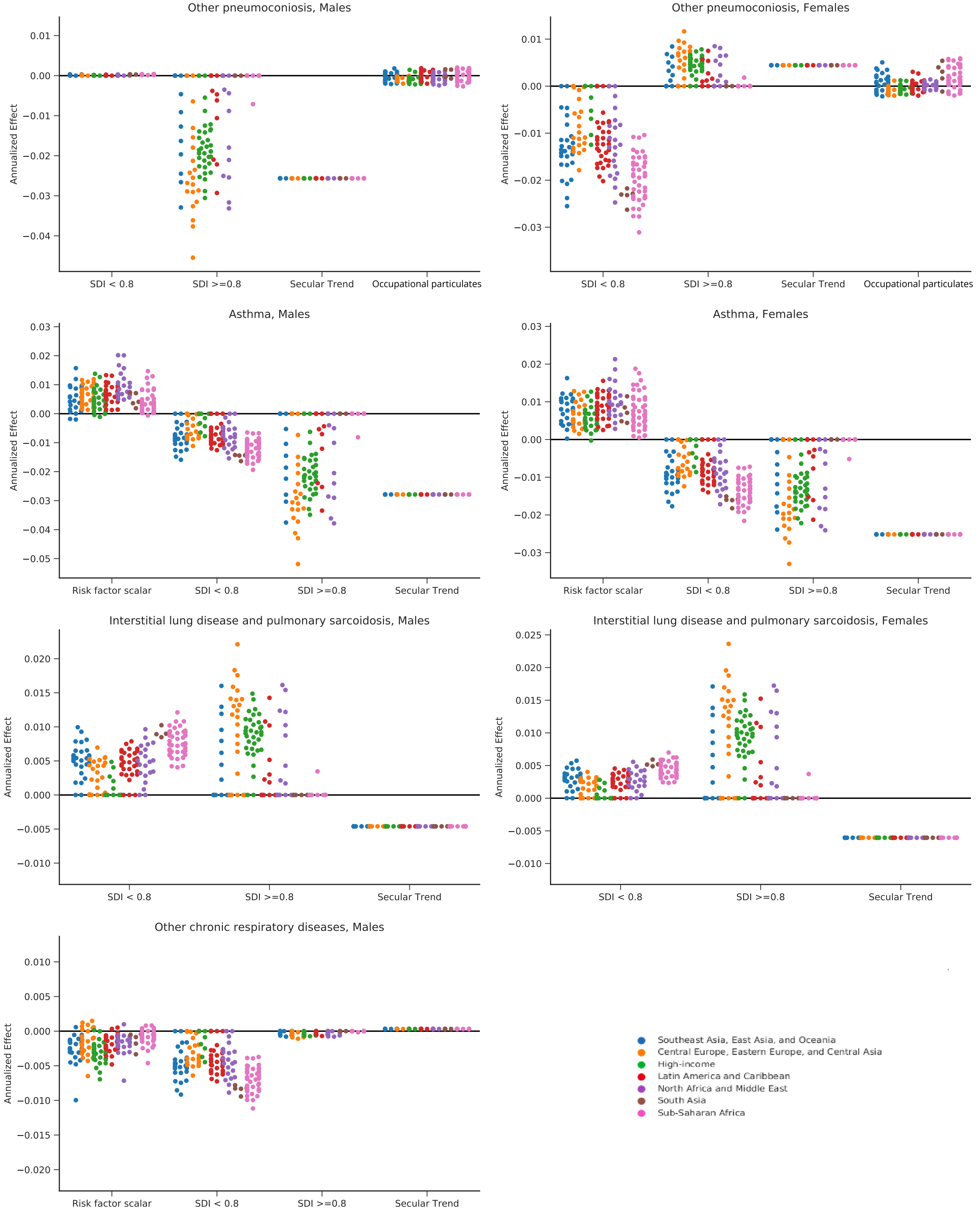

Other pneumoconiosis, Males
Annualized Effect
SDI < 0.8
SDI >=0.8
Secular Trend
Occupational particulates
Other pneumoconiosis, Females
Annualized Effect
SDI < 0.8
SDI >=0.8
Secular Trend
Occupational particulates
Asthma, Males
Annualized Effect
Risk factor scalar
SDI < 0.8
SDI >=0.8
Secular Trend
Asthma, Females
Annualized Effect
Risk factor scalar
SDI < 0.8
SDI >=0.8
Secular Trend
Interstitial lung disease and pulmonary sarcoidosis, Males
Annualized Effect
SDI < 0.8
SDI >=0.8
Secular Trend
Interstitial lung disease and pulmonary sarcoidosis, Females
Annualized Effect
SDI < 0.8
SDI >=0.8
Secular Trend
Other chronic respiratory diseases, Males
Annualized Effect
Risk factor scalar
SDI < 0.8
SDI >=0.8
Secular Trend
Southeast Asia, East Asia, and Oceania
Central Europe, Eastern Europe, and Central Asia
High-income
Latin America and Caribbean
North Africa and Middle East
South Asia
Sub-Saharan Africa

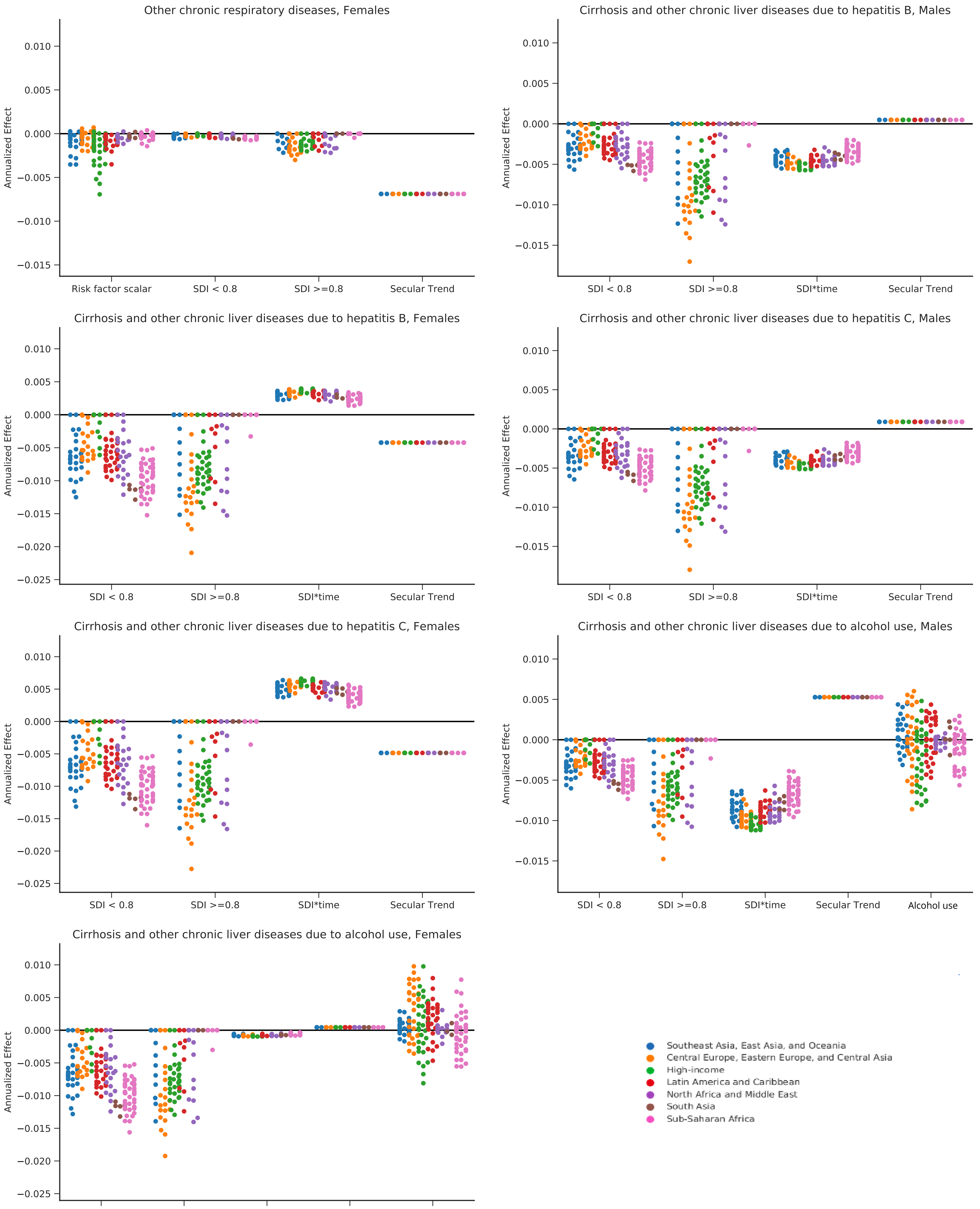
Other chronic respiratory diseases, Females
Annualized Effect
0.010
0.005
0.000
−0.005
−0.010
−0.015
Risk factor scalar
SDI < 0.8
SDI >=0.8
Secular Trend
Cirrhosis and other chronic liver diseases due to hepatitis B, Males
Annualized Effect
0.010
0.005
0.000
−0.005
−0.010
−0.015
SDI < 0.8
SDI >=0.8
SDI*time
Secular Trend
Cirrhosis and other chronic liver diseases due to hepatitis B, Females
Annualized Effect
0.010
0.005
0.000
−0.005
−0.010
−0.015
−0.020
−0.025
SDI < 0.8
SDI >=0.8
SDI*time
Secular Trend
Cirrhosis and other chronic liver diseases due to hepatitis C, Males
Annualized Effect
0.010
0.005
0.000
−0.005
−0.010
−0.015
SDI < 0.8
SDI >=0.8
SDI*time
Secular Trend
Cirrhosis and other chronic liver diseases due to hepatitis C, Females
Annualized Effect
0.010
0.005
0.000
−0.005
−0.010
−0.015
−0.020
−0.025
SDI < 0.8
SDI >=0.8
SDI*time
Secular Trend
Cirrhosis and other chronic liver diseases due to alcohol use, Males
Annualized Effect
0.010
0.005
0.000
−0.005
−0.010
−0.015
SDI < 0.8
SDI >=0.8
SDI*time
Secular Trend
Alcohol use
Cirrhosis and other chronic liver diseases due to alcohol use, Females
Annualized Effect
0.010
0.005
0.000
−0.005
−0.010
−0.015
−0.020
−0.025
SDI < 0.8
SDI >=0.8
SDI*time
Secular Trend
Alcohol use
Southeast Asia, East Asia, and Oceania
Central Europe, Eastern Europe, and Central Asia
High-income
Latin America and Caribbean
North Africa and Middle East
South Asia
Sub-Saharan Africa

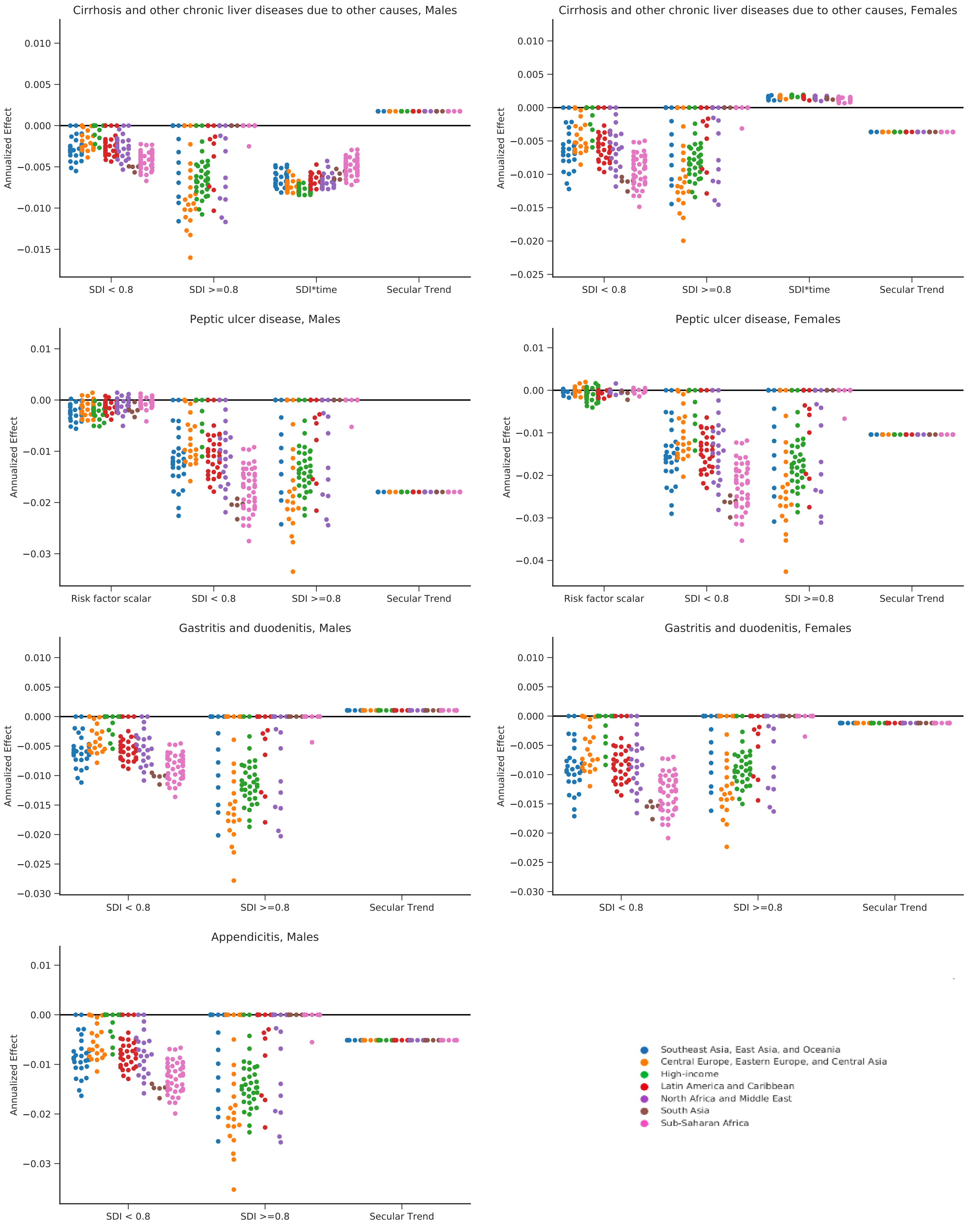
Cirrhosis and other chronic liver diseases due to other causes, Males
Cirrhosis and other chronic liver diseases due to other causes, Females
Peptic ulcer disease, Males
Peptic ulcer disease, Females
Gastritis and duodenitis, Males
Gastritis and duodenitis, Females
Appendicitis, Males
Annualized Effect
Risk factor scalar
SDI < 0.8
SDI >=0.8
SDI*time
Secular Trend
Southeast Asia, East Asia, and Oceania
Central Europe, Eastern Europe, and Central Asia
High-income
Latin America and Caribbean
North Africa and Middle East
South Asia
Sub-Saharan Africa

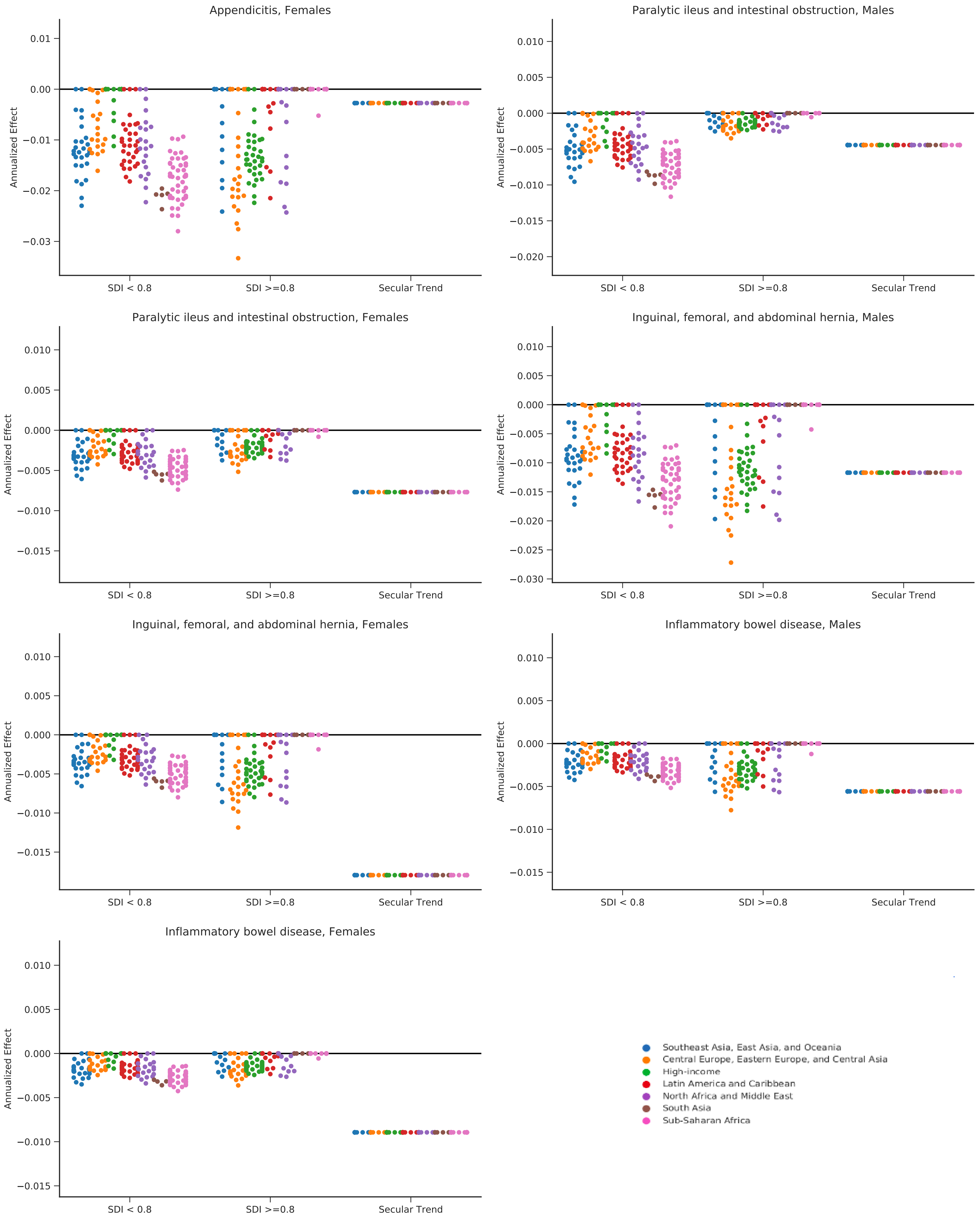

Appendicitis, Females
Paralytic ileus and intestinal obstruction, Males
Paralytic ileus and intestinal obstruction, Females
Inguinal, femoral, and abdominal hernia, Males
Inguinal, femoral, and abdominal hernia, Females
Inflammatory bowel disease, Males
Inflammatory bowel disease, Females
Annualized Effect
SDI < 0.8
SDI >=0.8
Secular Trend
0.01
0.00
−0.01
−0.02
−0.03
0.010
0.005
0.000
−0.005
−0.010
−0.015
−0.020
−0.025
−0.030
Southeast Asia, East Asia, and Oceania
Central Europe, Eastern Europe, and Central Asia
High-income
Latin America and Caribbean
North Africa and Middle East
South Asia
Sub-Saharan Africa

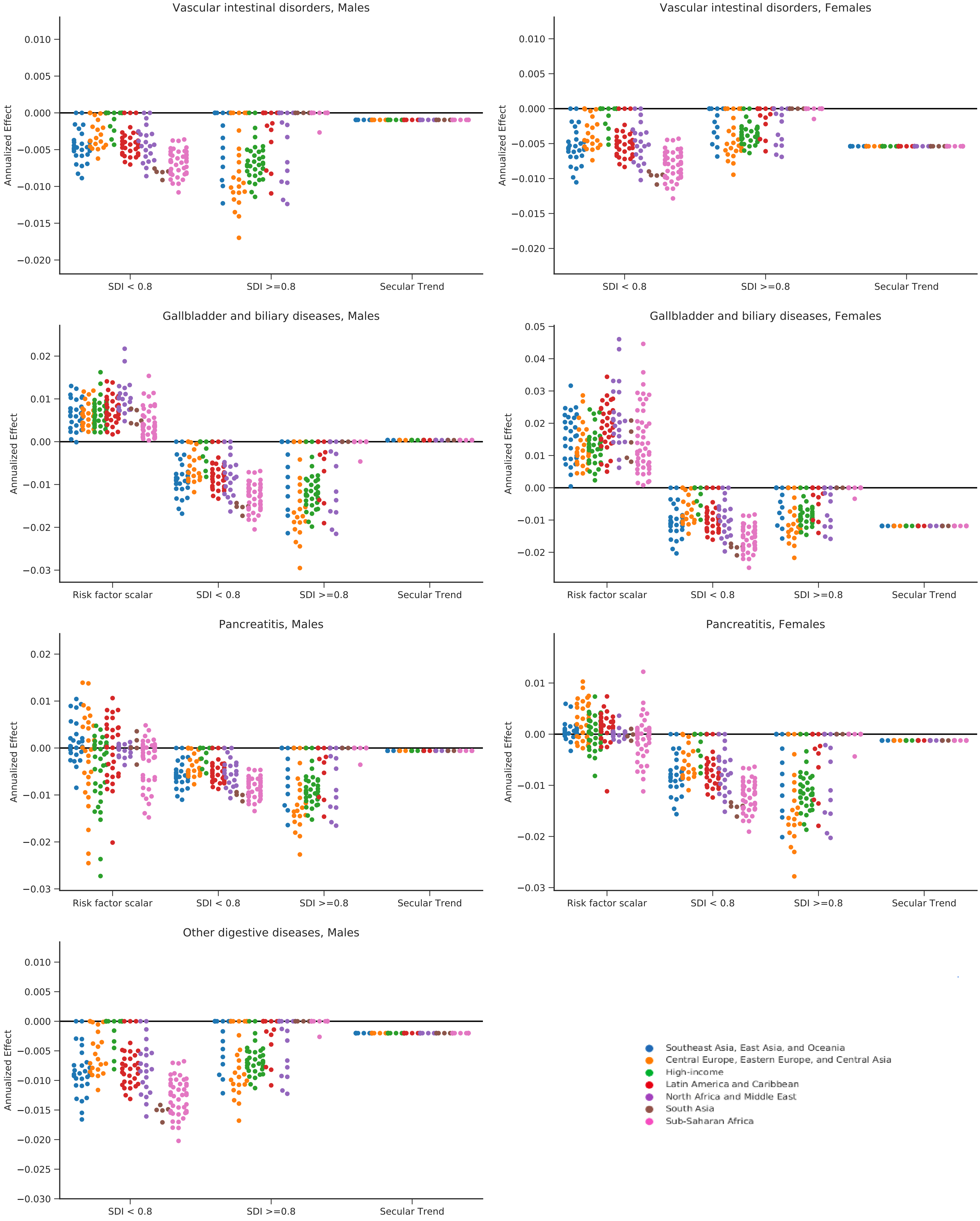

Vascular intestinal disorders, Males
Vascular intestinal disorders, Females
Gallbladder and biliary diseases, Males
Gallbladder and biliary diseases, Females
Pancreatitis, Males
Pancreatitis, Females
Other digestive diseases, Males
Annualized Effect
Risk factor scalar
SDI < 0.8
SDI >=0.8
Secular Trend
Southeast Asia, East Asia, and Oceania
Central Europe, Eastern Europe, and Central Asia
High-income
Latin America and Caribbean
North Africa and Middle East
South Asia
Sub-Saharan Africa

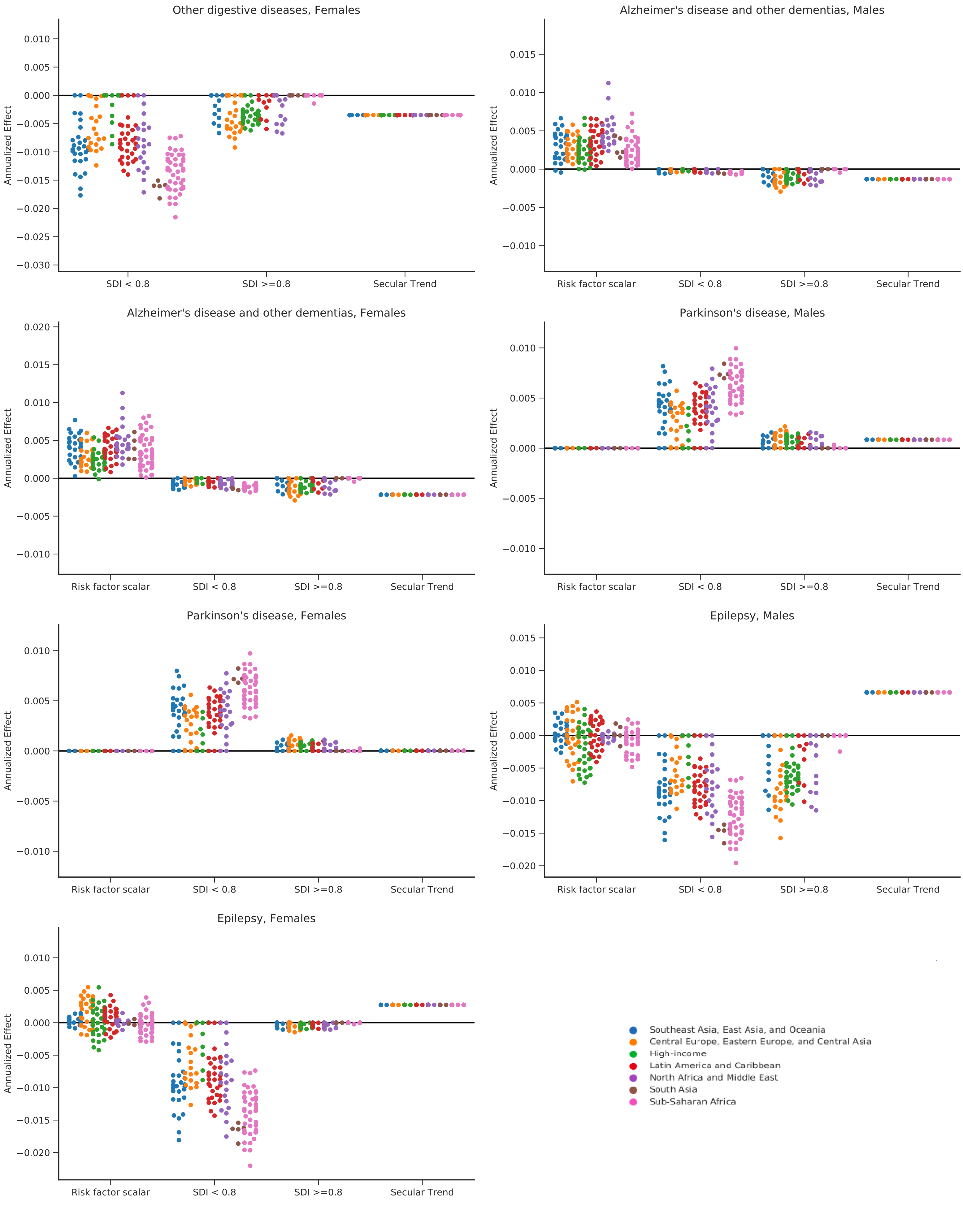

Other digestive diseases, Females
Annualized Effect
0.010
0.005
0.000
−0.005
−0.010
−0.015
−0.020
−0.025
−0.030
SDI < 0.8
SDI >=0.8
Secular Trend
Alzheimer's disease and other dementias, Males
Annualized Effect
0.015
0.010
0.005
0.000
−0.005
−0.010
Risk factor scalar
SDI < 0.8
SDI >=0.8
Secular Trend
Alzheimer's disease and other dementias, Females
Annualized Effect
0.020
0.015
0.010
0.005
0.000
−0.005
−0.010
Risk factor scalar
SDI < 0.8
SDI >=0.8
Secular Trend
Parkinson's disease, Males
Annualized Effect
0.010
0.005
0.000
−0.005
−0.010
Risk factor scalar
SDI < 0.8
SDI >=0.8
Secular Trend
Parkinson's disease, Females
Annualized Effect
0.010
0.005
0.000
−0.005
−0.010
Risk factor scalar
SDI < 0.8
SDI >=0.8
Secular Trend
Epilepsy, Males
Annualized Effect
0.015
0.010
0.005
0.000
−0.005
−0.010
−0.015
−0.020
Risk factor scalar
SDI < 0.8
SDI >=0.8
Secular Trend
Epilepsy, Females
Annualized Effect
0.010
0.005
0.000
−0.005
−0.010
−0.015
−0.020
Risk factor scalar
SDI < 0.8
SDI >=0.8
Secular Trend
Southeast Asia, East Asia, and Oceania
Central Europe, Eastern Europe, and Central Asia
High-income
Latin America and Caribbean
North Africa and Middle East
South Asia
Sub-Saharan Africa

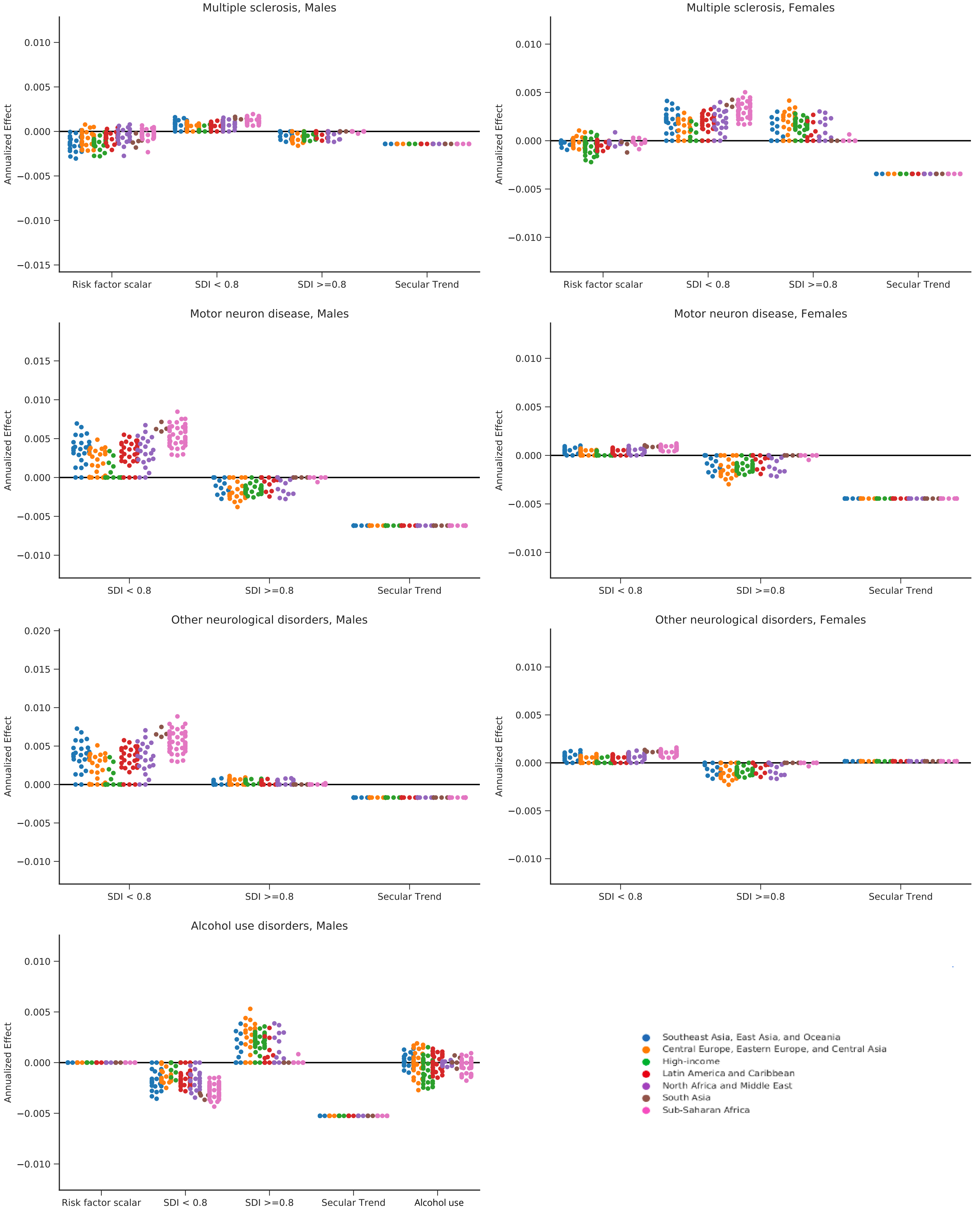
Multiple sclerosis, Males
Multiple sclerosis, Females
Motor neuron disease, Males
Motor neuron disease, Females
Other neurological disorders, Males
Other neurological disorders, Females
Alcohol use disorders, Males
Annualized Effect
Risk factor scalar
SDI < 0.8
SDI >=0.8
Secular Trend
Alcohol use
Southeast Asia, East Asia, and Oceania
Central Europe, Eastern Europe, and Central Asia
High-income
Latin America and Caribbean
North Africa and Middle East
South Asia
Sub-Saharan Africa

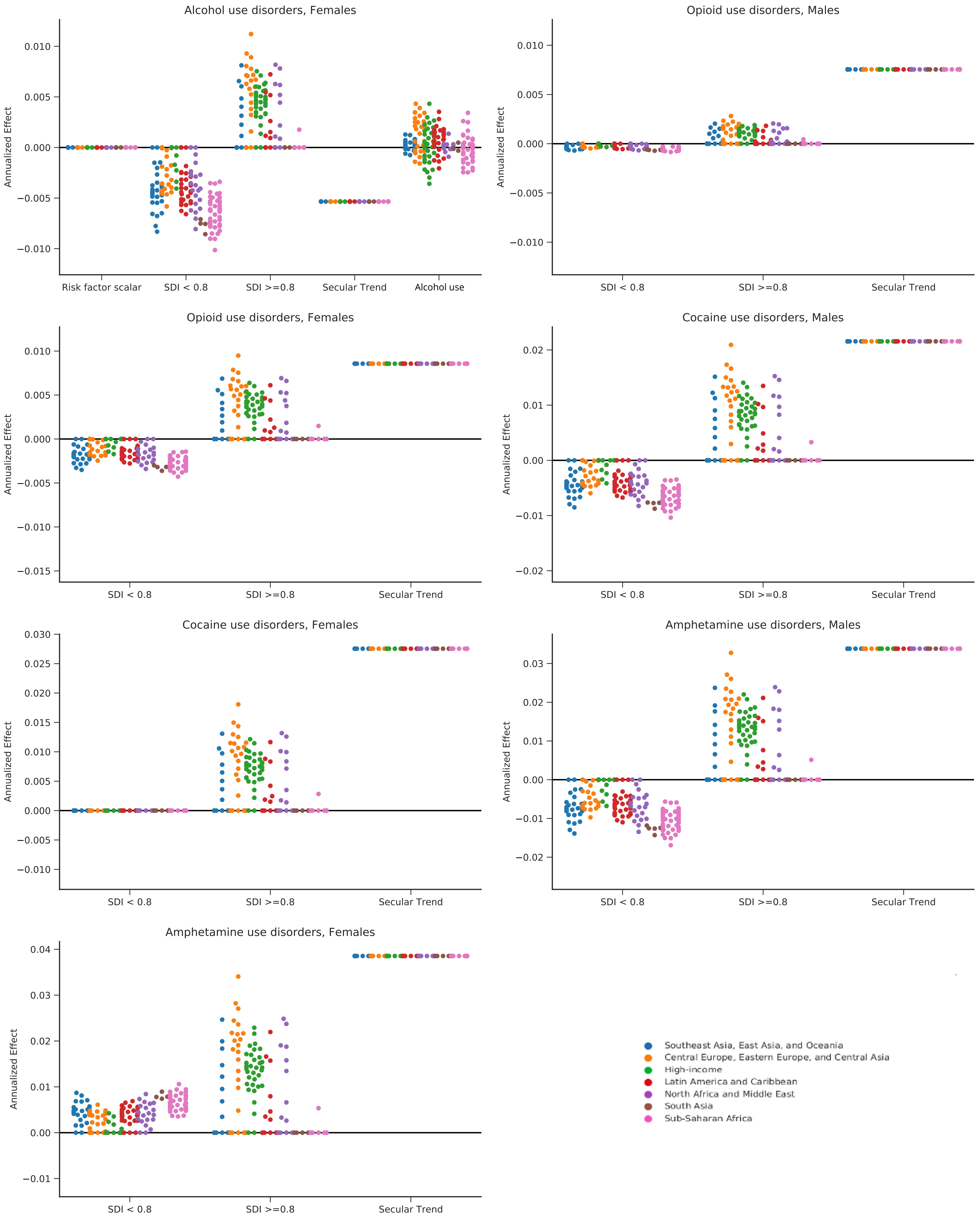
Alcohol use disorders, Females
Annualized Effect
0.010
0.005
0.000
−0.005
−0.010
Risk factor scalar
SDI < 0.8
SDI >=0.8
Secular Trend
Alcohol use
Opioid use disorders, Males
Annualized Effect
0.010
0.005
0.000
−0.005
−0.010
SDI < 0.8
SDI >=0.8
Secular Trend
Opioid use disorders, Females
Annualized Effect
0.010
0.005
0.000
−0.005
−0.010
−0.015
SDI < 0.8
SDI >=0.8
Secular Trend
Cocaine use disorders, Males
Annualized Effect
0.02
0.01
0.00
−0.01
−0.02
SDI < 0.8
SDI >=0.8
Secular Trend
Cocaine use disorders, Females
Annualized Effect
0.030
0.025
0.020
0.015
0.010
0.005
0.000
−0.005
−0.010
SDI < 0.8
SDI >=0.8
Secular Trend
Amphetamine use disorders, Males
Annualized Effect
0.03
0.02
0.01
0.00
−0.01
−0.02
SDI < 0.8
SDI >=0.8
Secular Trend
Amphetamine use disorders, Females
Annualized Effect
0.04
0.03
0.02
0.01
0.00
−0.01
SDI < 0.8
SDI >=0.8
Secular Trend
Southeast Asia, East Asia, and Oceania
Central Europe, Eastern Europe, and Central Asia
High-income
Latin America and Caribbean
North Africa and Middle East
South Asia
Sub-Saharan Africa

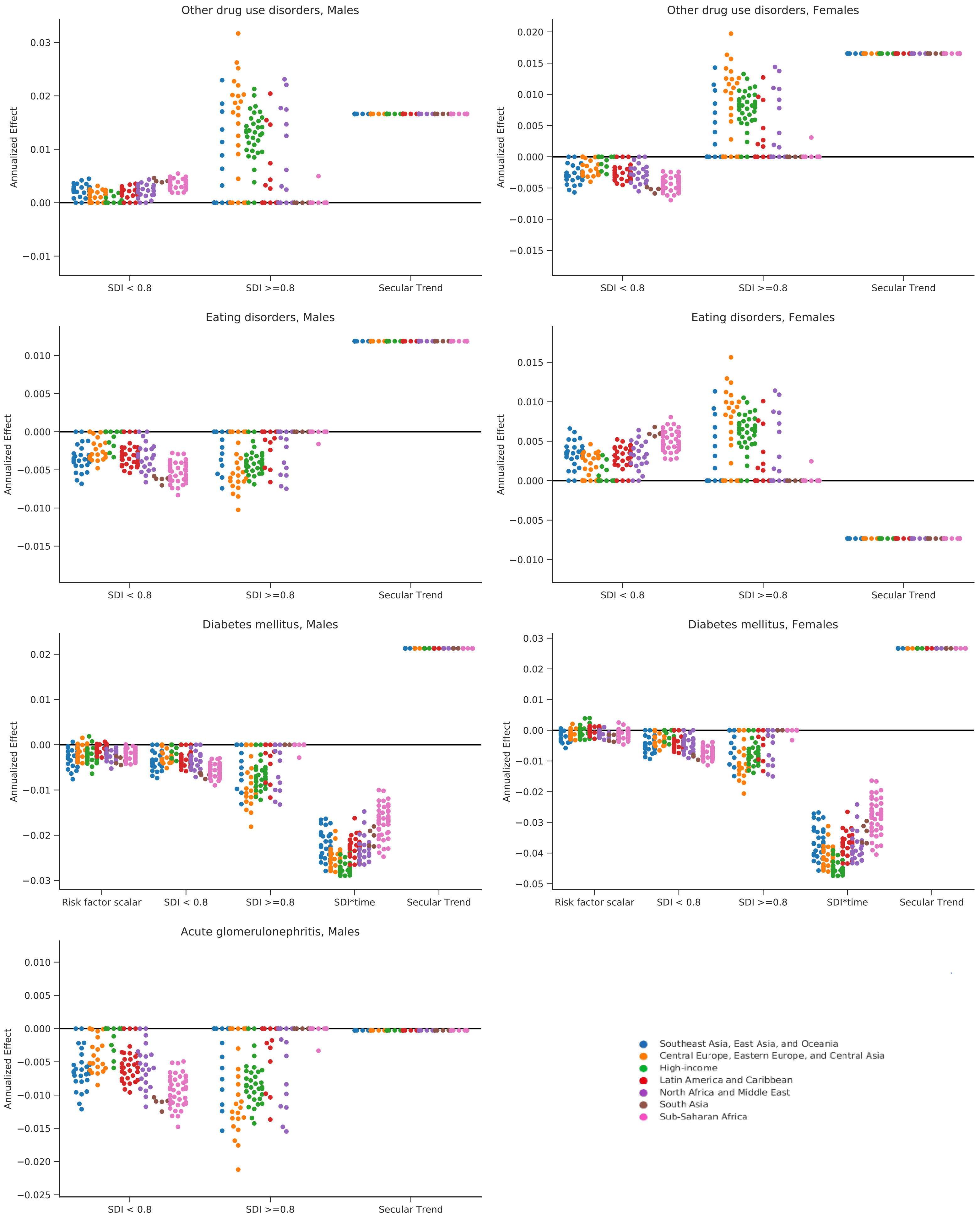
Other drug use disorders, Males
Other drug use disorders, Females
Eating disorders, Males
Eating disorders, Females
Diabetes mellitus, Males
Diabetes mellitus, Females
Acute glomerulonephritis, Males
Annualized Effect
Risk factor scalar
SDI < 0.8
SDI >=0.8
SDI*time
Secular Trend
Southeast Asia, East Asia, and Oceania
Central Europe, Eastern Europe, and Central Asia
High-income
Latin America and Caribbean
North Africa and Middle East
South Asia
Sub-Saharan Africa

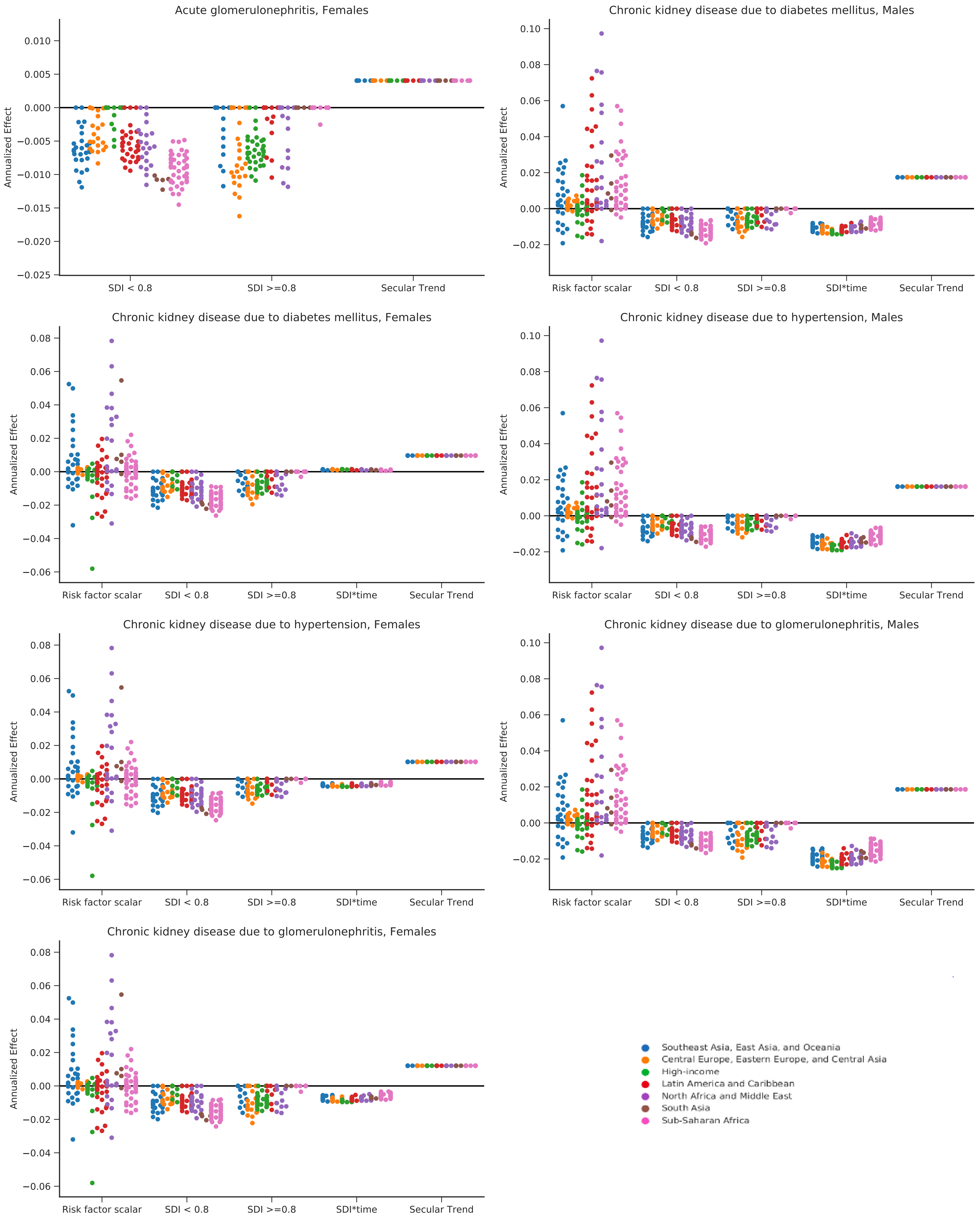

Acute glomerulonephritis, Females
Annualized Effect
SDI < 0.8
SDI >=0.8
Secular Trend
Chronic kidney disease due to diabetes mellitus, Males
Chronic kidney disease due to diabetes mellitus, Females
Chronic kidney disease due to hypertension, Males
Chronic kidney disease due to hypertension, Females
Chronic kidney disease due to glomerulonephritis, Males
Chronic kidney disease due to glomerulonephritis, Females
Risk factor scalar
SDI*time
Southeast Asia, East Asia, and Oceania
Central Europe, Eastern Europe, and Central Asia
High-income
Latin America and Caribbean
North Africa and Middle East
South Asia
Sub-Saharan Africa

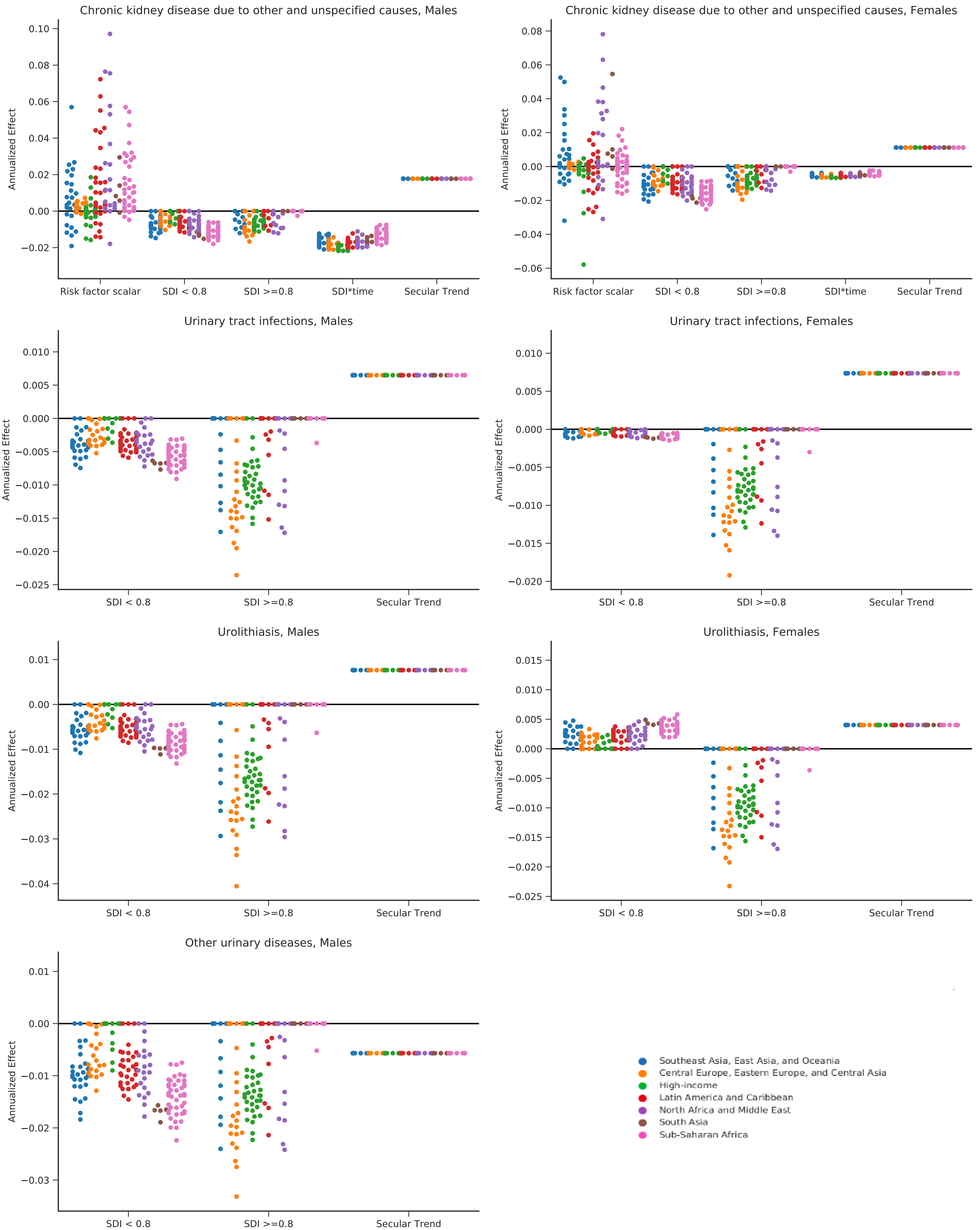
Chronic kidney disease due to other and unspecified causes, Males
Annualized Effect
0.10
0.08
0.06
0.04
0.02
0.00
−0.02
Risk factor scalar
SDI < 0.8
SDI >=0.8
SDI*time
Secular Trend
Chronic kidney disease due to other and unspecified causes, Females
Annualized Effect
0.08
0.06
0.04
0.02
0.00
−0.02
−0.04
−0.06
Risk factor scalar
SDI < 0.8
SDI >=0.8
SDI*time
Secular Trend
Urinary tract infections, Males
Annualized Effect
0.010
0.005
0.000
−0.005
−0.010
−0.015
−0.020
−0.025
SDI < 0.8
SDI >=0.8
Secular Trend
Urinary tract infections, Females
Annualized Effect
0.010
0.005
0.000
−0.005
−0.010
−0.015
−0.020
SDI < 0.8
SDI >=0.8
Secular Trend
Urolithiasis, Males
Annualized Effect
0.01
0.00
−0.01
−0.02
−0.03
−0.04
SDI < 0.8
SDI >=0.8
Secular Trend
Urolithiasis, Females
Annualized Effect
0.015
0.010
0.005
0.000
−0.005
−0.010
−0.015
−0.020
−0.025
SDI < 0.8
SDI >=0.8
Secular Trend
Other urinary diseases, Males
Annualized Effect
0.01
0.00
−0.01
−0.02
−0.03
SDI < 0.8
SDI >=0.8
Secular Trend
Southeast Asia, East Asia, and Oceania
Central Europe, Eastern Europe, and Central Asia
High-income
Latin America and Caribbean
North Africa and Middle East
South Asia
Sub-Saharan Africa

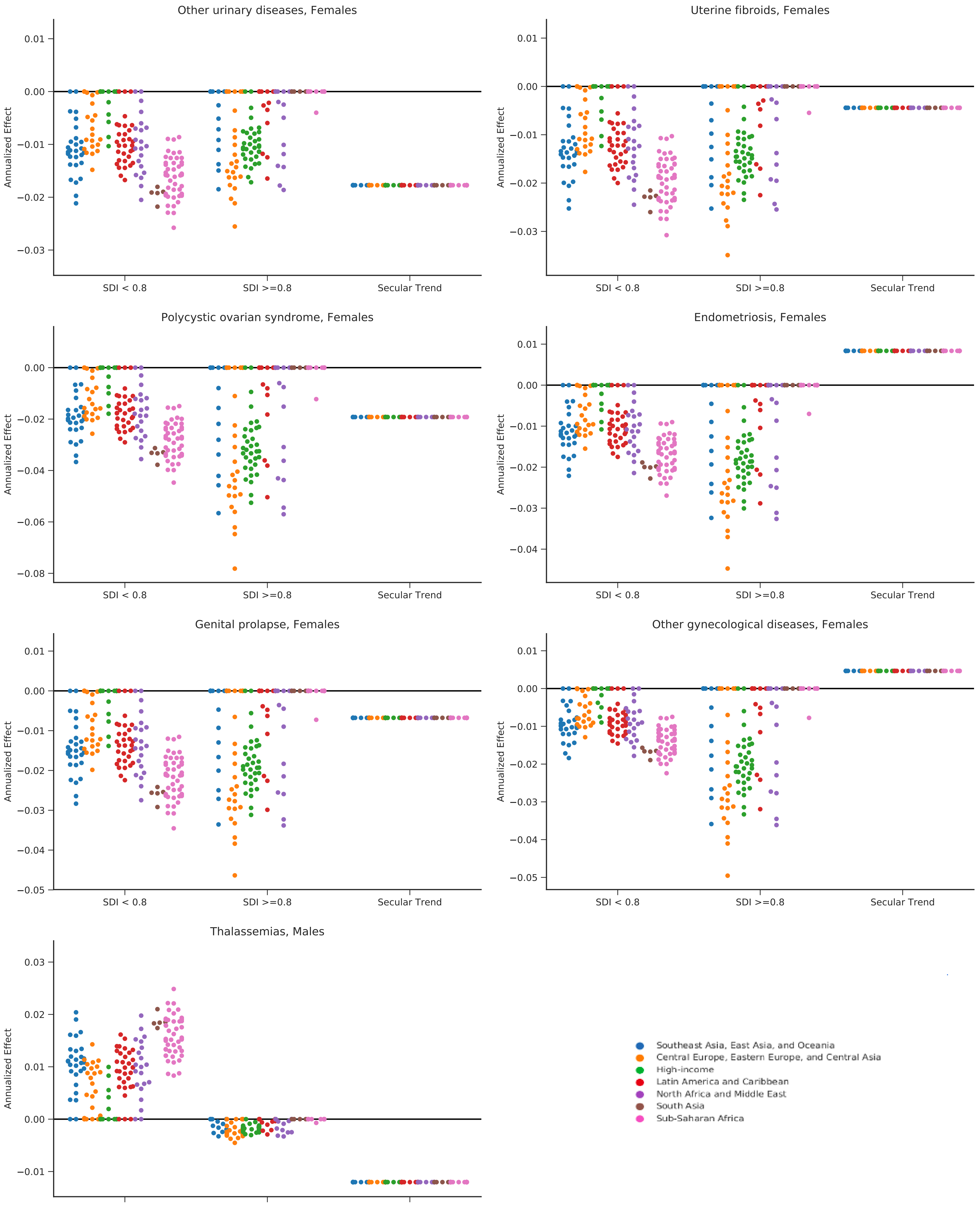

Other urinary diseases, Females
Uterine fibroids, Females
Polycystic ovarian syndrome, Females
Endometriosis, Females
Genital prolapse, Females
Other gynecological diseases, Females
Thalassemias, Males
Annualized Effect
SDI < 0.8
SDI >=0.8
Secular Trend
0.03
0.02
0.01
0.00
−0.01
−0.02
−0.03
−0.04
−0.05
−0.06
−0.08
Southeast Asia, East Asia, and Oceania
Central Europe, Eastern Europe, and Central Asia
High-income
Latin America and Caribbean
North Africa and Middle East
South Asia
Sub-Saharan Africa

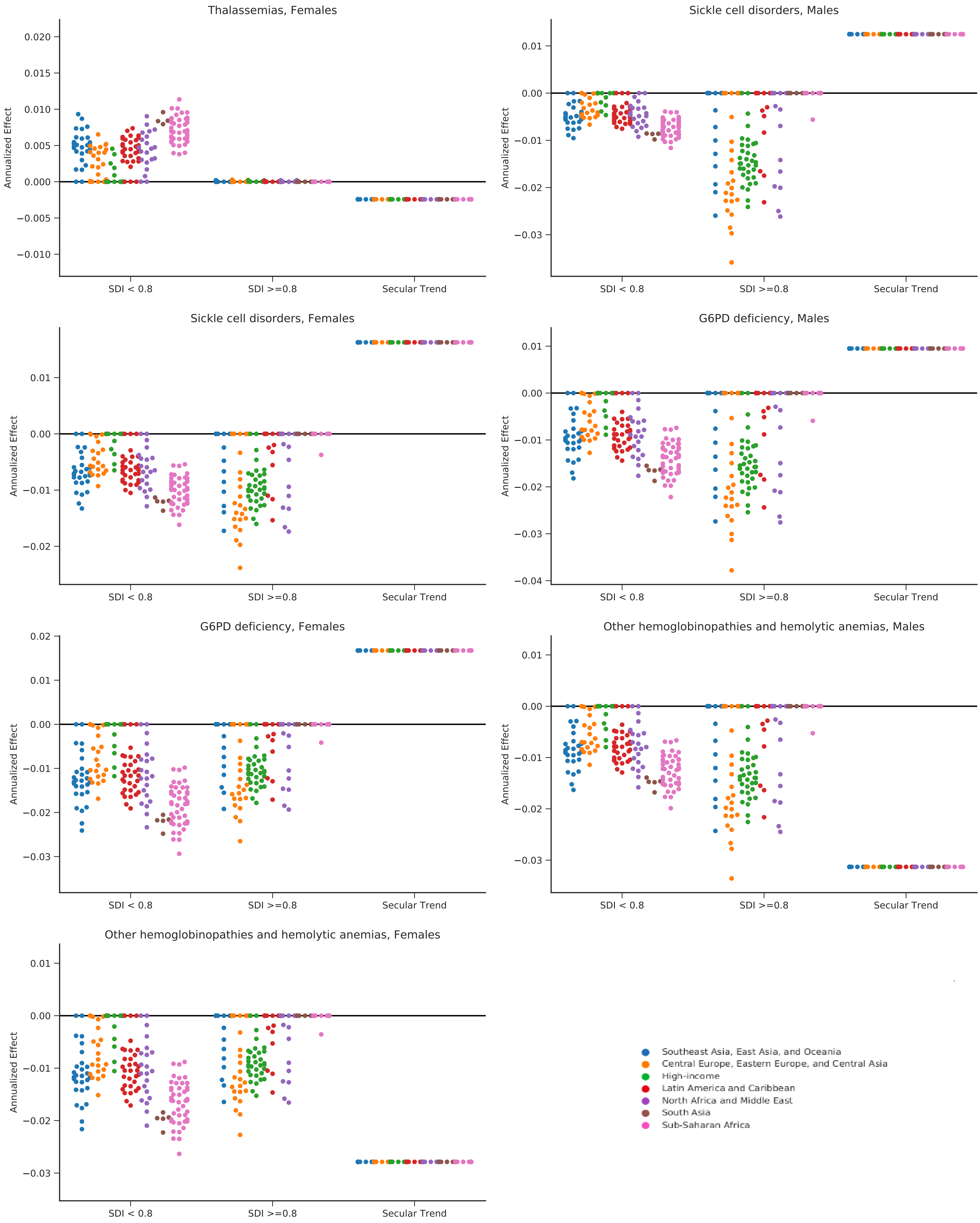

Thalassemias, Females
Sickle cell disorders, Males
Sickle cell disorders, Females
G6PD deficiency, Males
G6PD deficiency, Females
Other hemoglobinopathies and hemolytic anemias, Males
Other hemoglobinopathies and hemolytic anemias, Females
Annualized Effect
SDI < 0.8
SDI >=0.8
Secular Trend
Southeast Asia, East Asia, and Oceania
Central Europe, Eastern Europe, and Central Asia
High-income
Latin America and Caribbean
North Africa and Middle East
South Asia
Sub-Saharan Africa

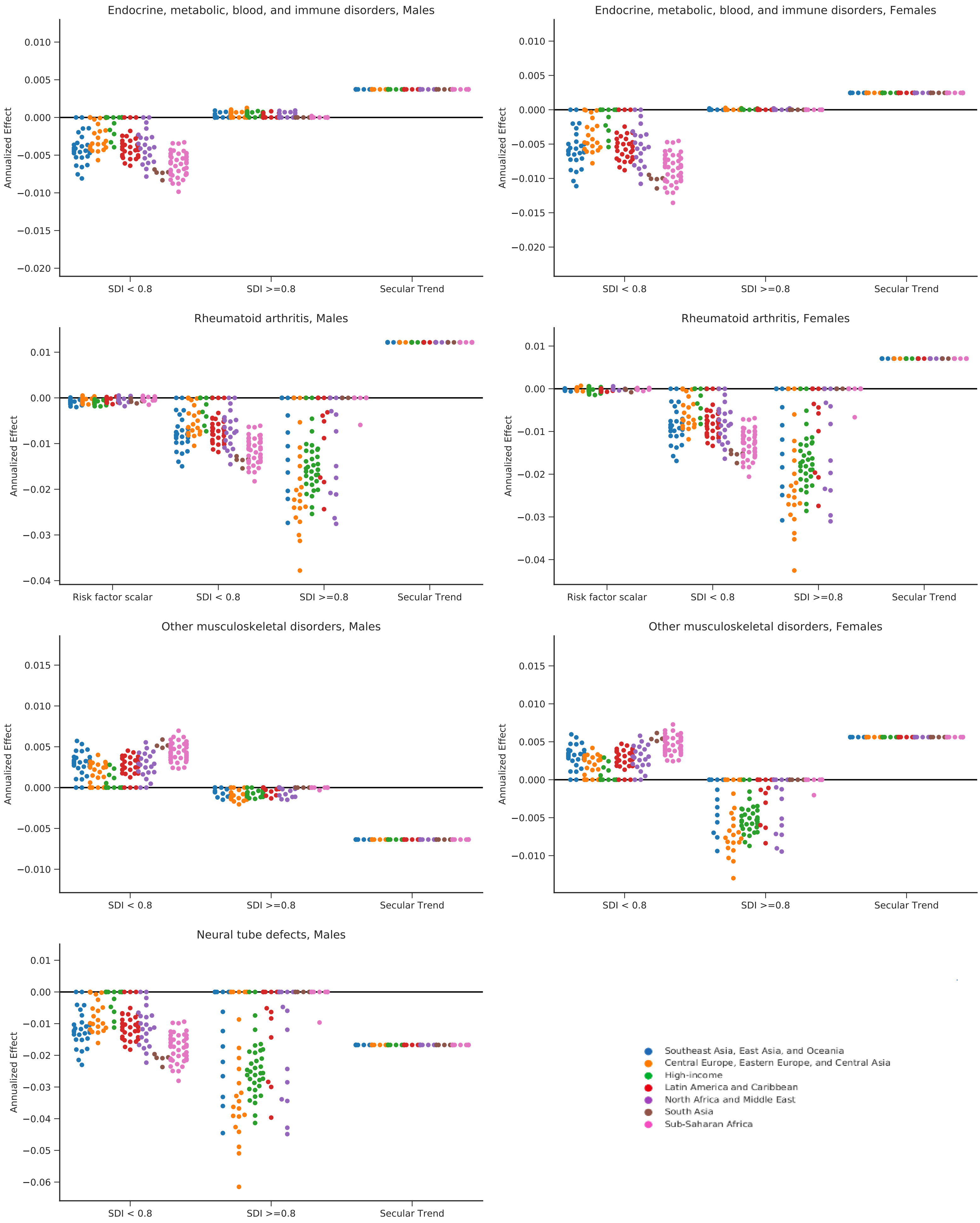
Endocrine, metabolic, blood, and immune disorders, Males
Endocrine, metabolic, blood, and immune disorders, Females
Rheumatoid arthritis, Males
Rheumatoid arthritis, Females
Other musculoskeletal disorders, Males
Other musculoskeletal disorders, Females
Neural tube defects, Males
Annualized Effect
Risk factor scalar
SDI < 0.8
SDI >=0.8
Secular Trend
Southeast Asia, East Asia, and Oceania
Central Europe, Eastern Europe, and Central Asia
High-income
Latin America and Caribbean
North Africa and Middle East
South Asia
Sub-Saharan Africa

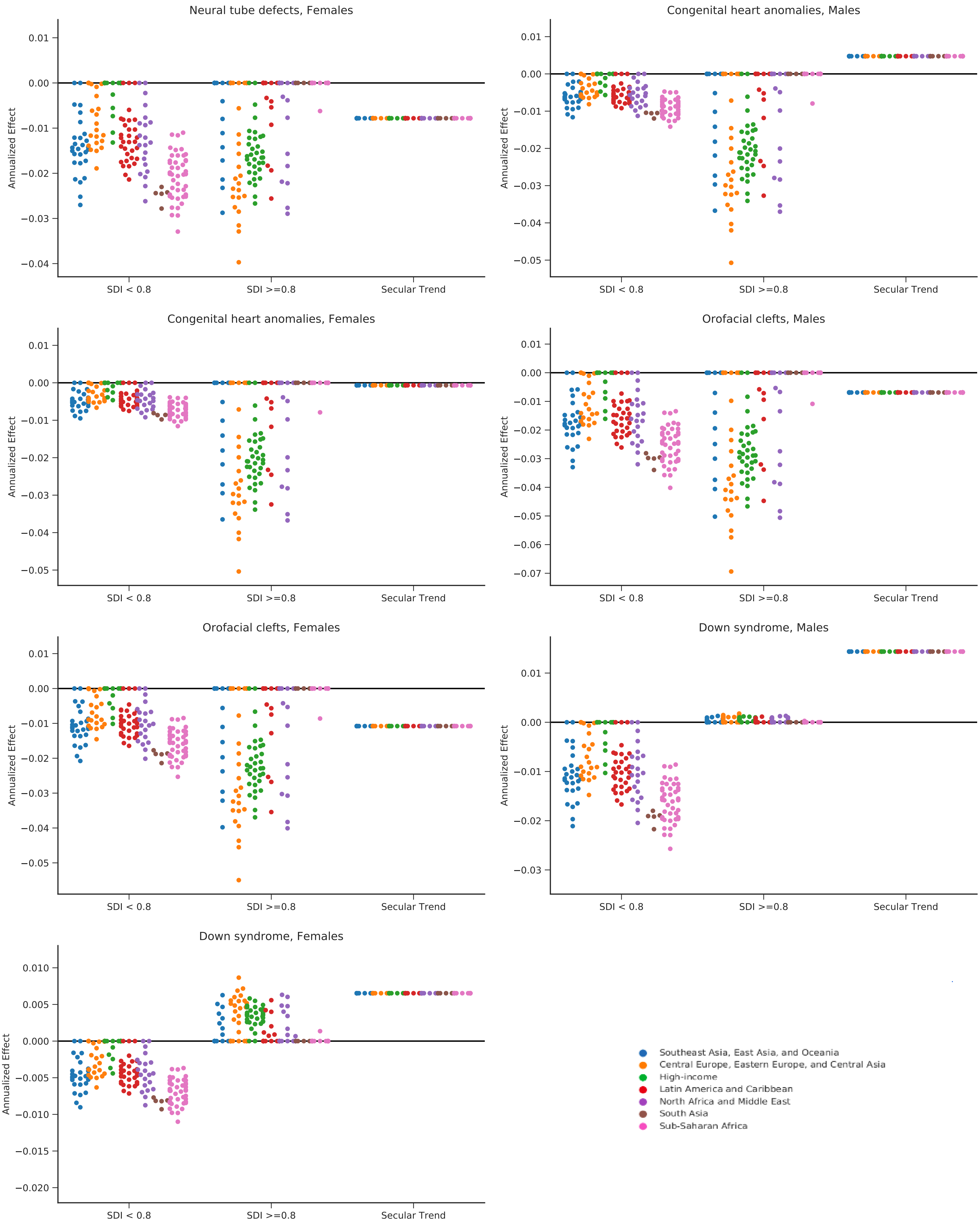
Neural tube defects, Females
Congenital heart anomalies, Males
Congenital heart anomalies, Females
Orofacial clefts, Males
Orofacial clefts, Females
Down syndrome, Males
Down syndrome, Females
Annualized Effect
SDI < 0.8
SDI >=0.8
Secular Trend
0.01
0.00
−0.01
−0.02
−0.03
−0.04
−0.05
−0.06
−0.07
0.010
0.005
0.000
−0.005
−0.010
−0.015
−0.020
Southeast Asia, East Asia, and Oceania
Central Europe, Eastern Europe, and Central Asia
High-income
Latin America and Caribbean
North Africa and Middle East
South Asia
Sub-Saharan Africa

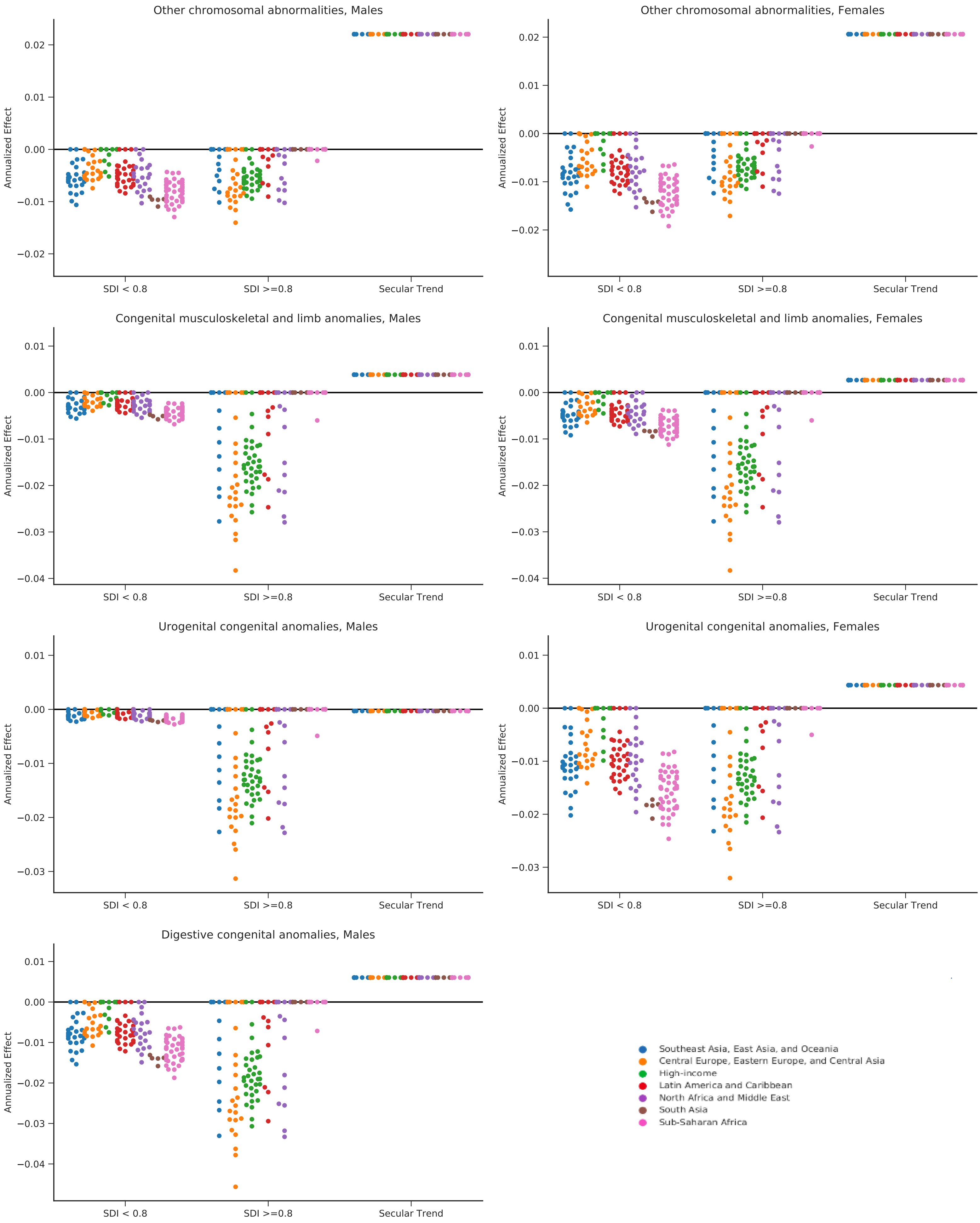

Other chromosomal abnormalities, Males
Other chromosomal abnormalities, Females
Congenital musculoskeletal and limb anomalies, Males
Congenital musculoskeletal and limb anomalies, Females
Urogenital congenital anomalies, Males
Urogenital congenital anomalies, Females
Digestive congenital anomalies, Males
Annualized Effect
SDI < 0.8
SDI >=0.8
Secular Trend
Southeast Asia, East Asia, and Oceania
Central Europe, Eastern Europe, and Central Asia
High-income
Latin America and Caribbean
North Africa and Middle East
South Asia
Sub-Saharan Africa

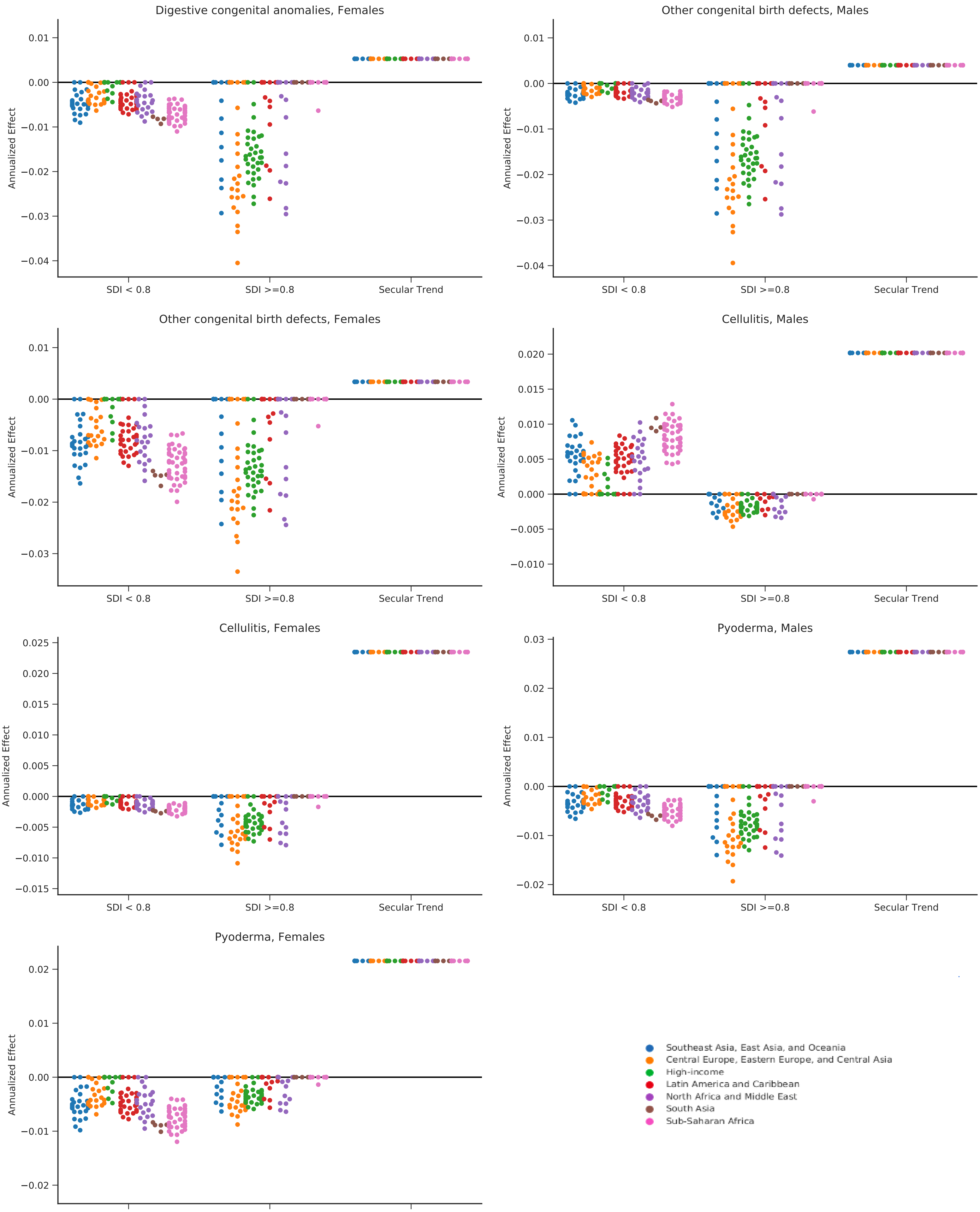

Digestive congenital anomalies, Females
Other congenital birth defects, Males
Other congenital birth defects, Females
Cellulitis, Males
Cellulitis, Females
Pyoderma, Males
Pyoderma, Females
Annualized Effect
SDI < 0.8
SDI >=0.8
Secular Trend
Southeast Asia, East Asia, and Oceania
Central Europe, Eastern Europe, and Central Asia
High-income
Latin America and Caribbean
North Africa and Middle East
South Asia
Sub-Saharan Africa

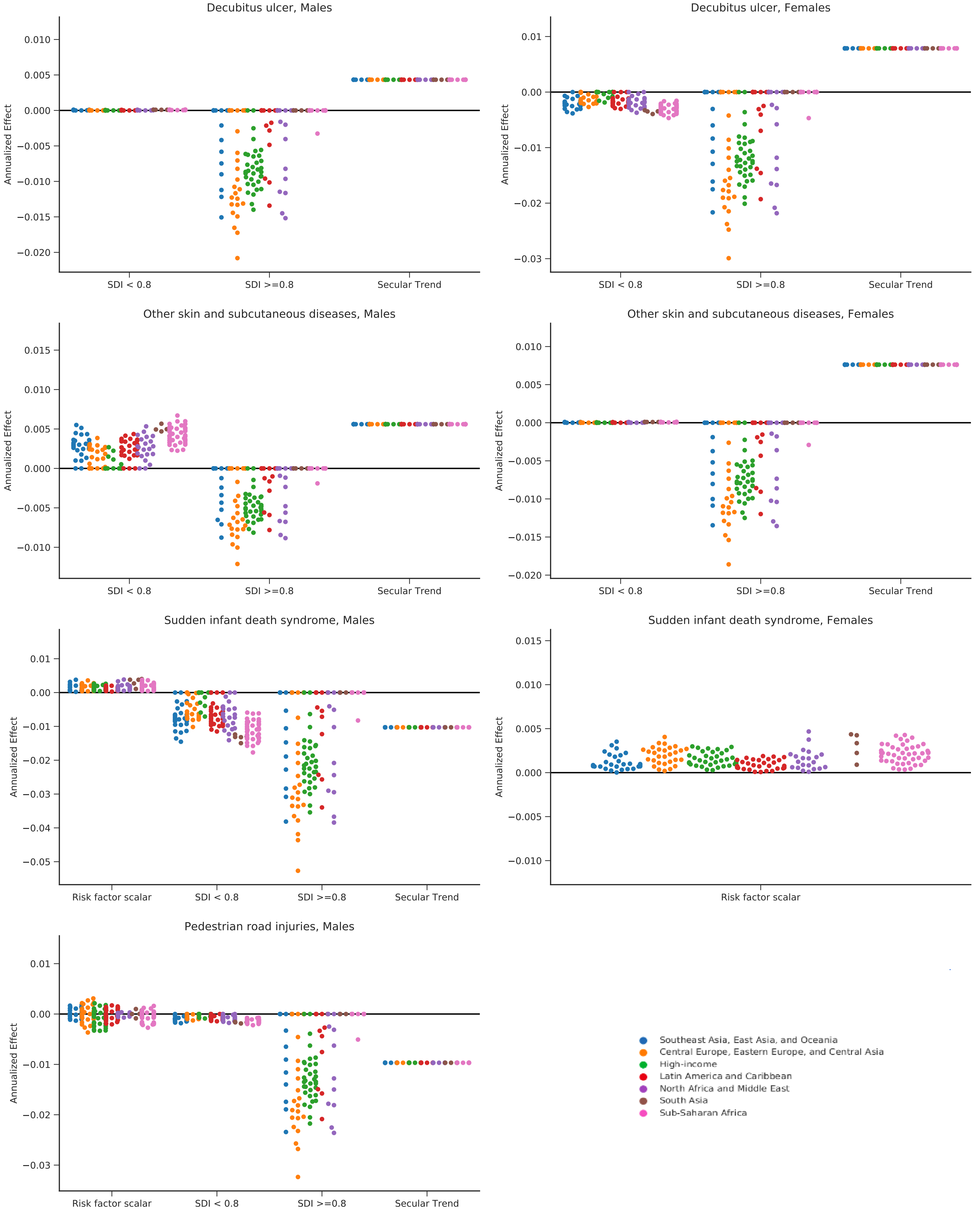
Decubitus ulcer, Males
Annualized Effect
0.010
0.005
0.000
−0.005
−0.010
−0.015
−0.020
SDI < 0.8
SDI >=0.8
Secular Trend
Decubitus ulcer, Females
Annualized Effect
0.01
0.00
−0.01
−0.02
−0.03
SDI < 0.8
SDI >=0.8
Secular Trend
Other skin and subcutaneous diseases, Males
Annualized Effect
0.015
0.010
0.005
0.000
−0.005
−0.010
SDI < 0.8
SDI >=0.8
Secular Trend
Other skin and subcutaneous diseases, Females
Annualized Effect
0.010
0.005
0.000
−0.005
−0.010
−0.015
−0.020
SDI < 0.8
SDI >=0.8
Secular Trend
Sudden infant death syndrome, Males
Annualized Effect
0.01
0.00
−0.01
−0.02
−0.03
−0.04
−0.05
Risk factor scalar
SDI < 0.8
SDI >=0.8
Secular Trend
Sudden infant death syndrome, Females
Annualized Effect
0.015
0.010
0.005
0.000
−0.005
−0.010
Risk factor scalar
Pedestrian road injuries, Males
Annualized Effect
0.01
0.00
−0.01
−0.02
−0.03
Risk factor scalar
SDI < 0.8
SDI >=0.8
Secular Trend
Southeast Asia, East Asia, and Oceania
Central Europe, Eastern Europe, and Central Asia
High-income
Latin America and Caribbean
North Africa and Middle East
South Asia
Sub-Saharan Africa

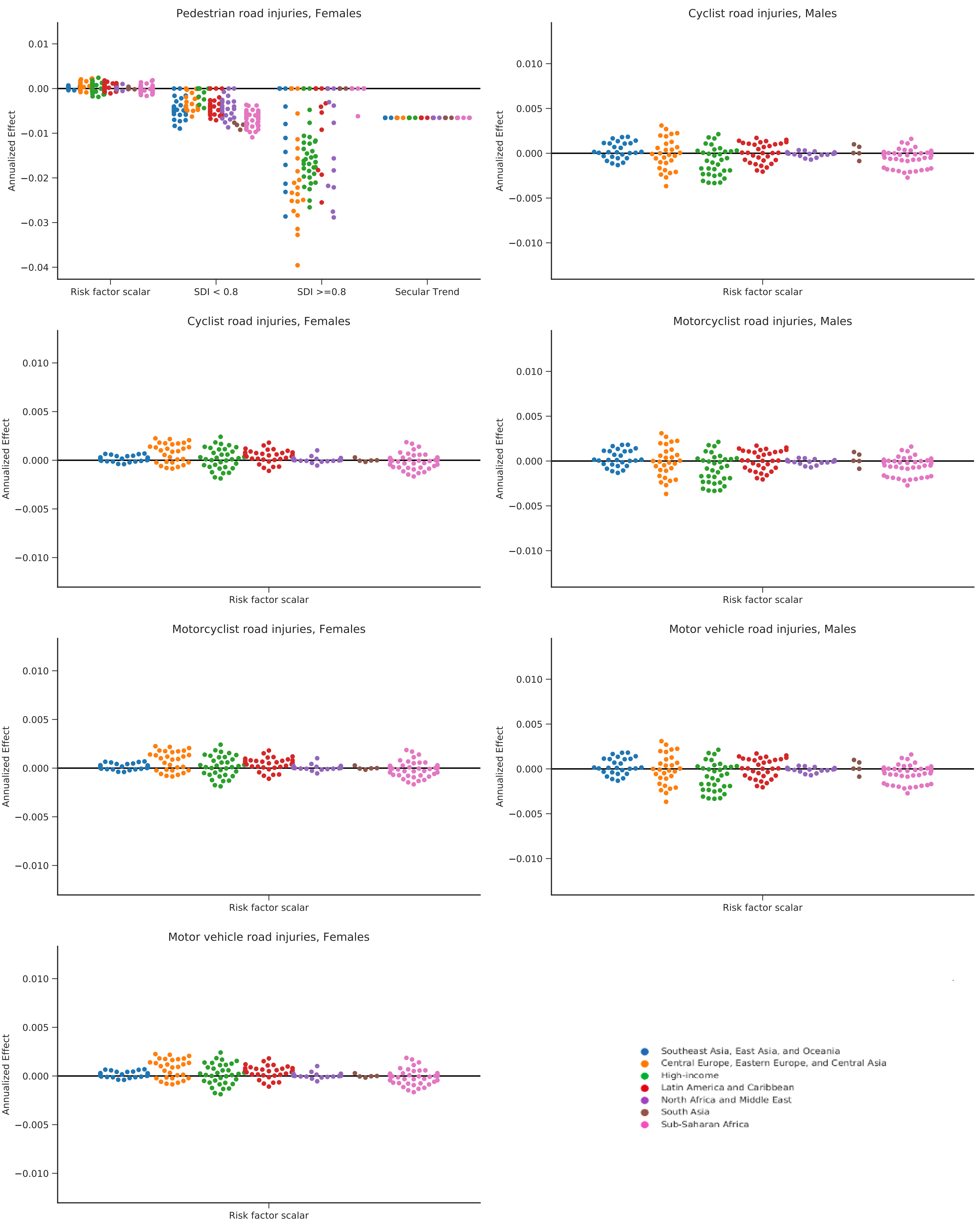

Pedestrian road injuries, Females
Cyclist road injuries, Males
Cyclist road injuries, Females
Motorcyclist road injuries, Males
Motorcyclist road injuries, Females
Motor vehicle road injuries, Males
Motor vehicle road injuries, Females
Annualized Effect
0.01
0.00
−0.01
−0.02
−0.03
−0.04
0.010
0.005
0.000
−0.005
−0.010
Risk factor scalar
SDI < 0.8
SDI >=0.8
Secular Trend
Southeast Asia, East Asia, and Oceania
Central Europe, Eastern Europe, and Central Asia
High-income
Latin America and Caribbean
North Africa and Middle East
South Asia
Sub-Saharan Africa

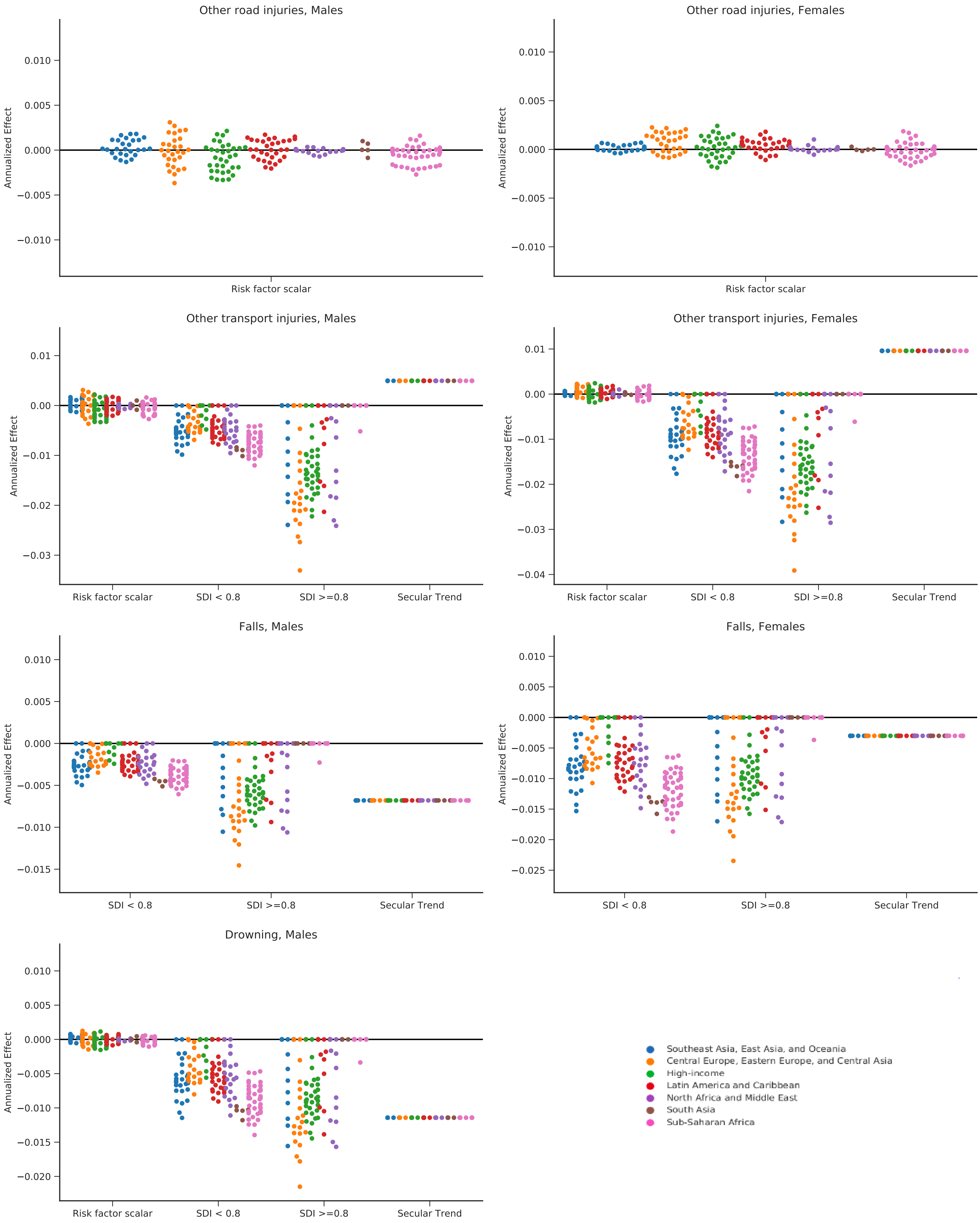

Other road injuries, Males
Other road injuries, Females
Other transport injuries, Males
Other transport injuries, Females
Falls, Males
Falls, Females
Drowning, Males
Annualized Effect
Risk factor scalar
SDI < 0.8
SDI >=0.8
Secular Trend
0.010
0.005
0.000
−0.005
−0.010
−0.015
−0.020
−0.025
0.01
0.00
−0.01
−0.02
−0.03
−0.04
Southeast Asia, East Asia, and Oceania
Central Europe, Eastern Europe, and Central Asia
High-income
Latin America and Caribbean
North Africa and Middle East
South Asia
Sub-Saharan Africa

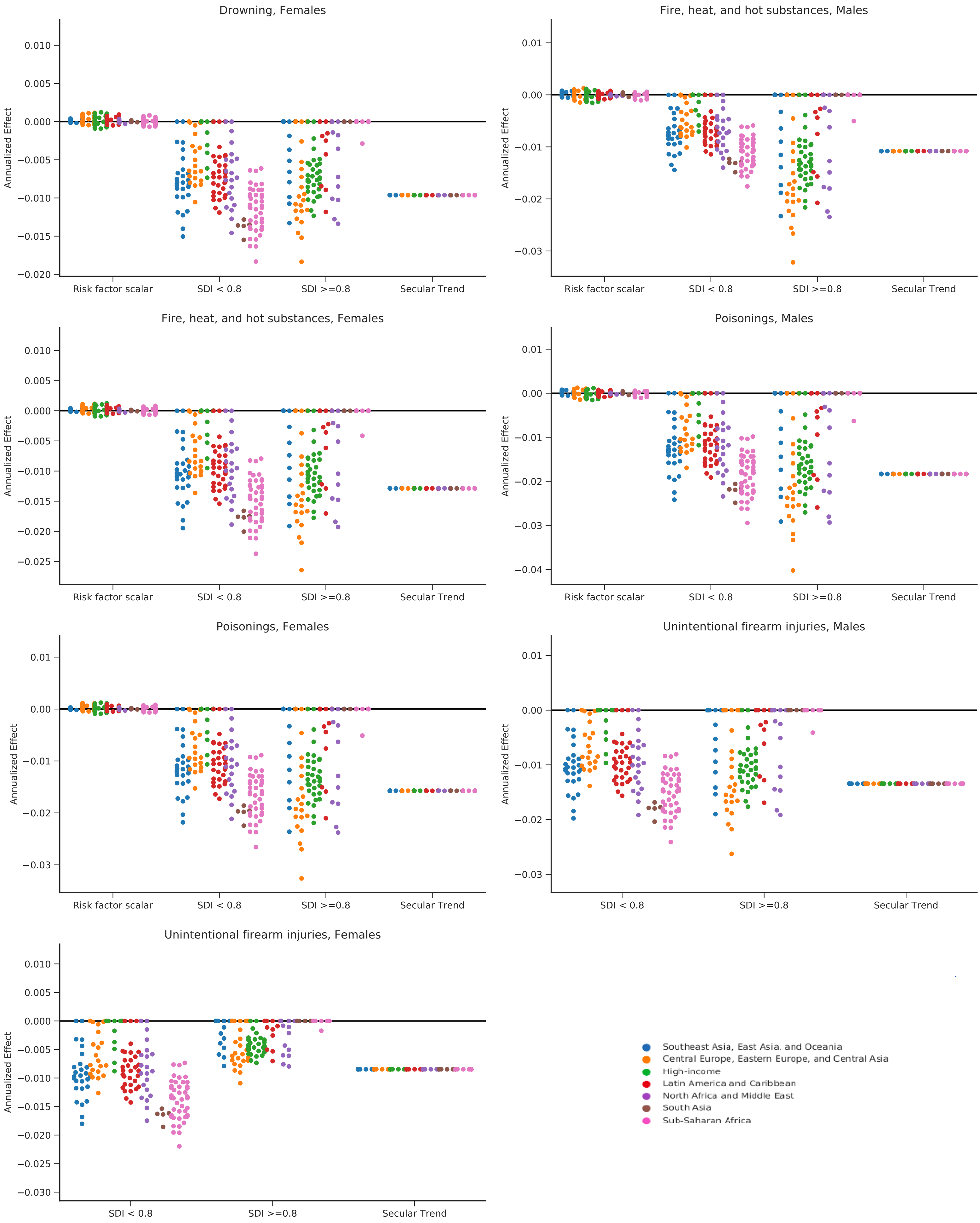
Drowning, Females
Fire, heat, and hot substances, Males
Fire, heat, and hot substances, Females
Poisonings, Males
Poisonings, Females
Unintentional firearm injuries, Males
Unintentional firearm injuries, Females
Annualized Effect
Risk factor scalar
SDI < 0.8
SDI >=0.8
Secular Trend
0.010
0.005
0.000
−0.005
−0.010
−0.015
−0.020
−0.025
−0.030
0.01
0.00
−0.01
−0.02
−0.03
−0.04
Southeast Asia, East Asia, and Oceania
Central Europe, Eastern Europe, and Central Asia
High-income
Latin America and Caribbean
North Africa and Middle East
South Asia
Sub-Saharan Africa

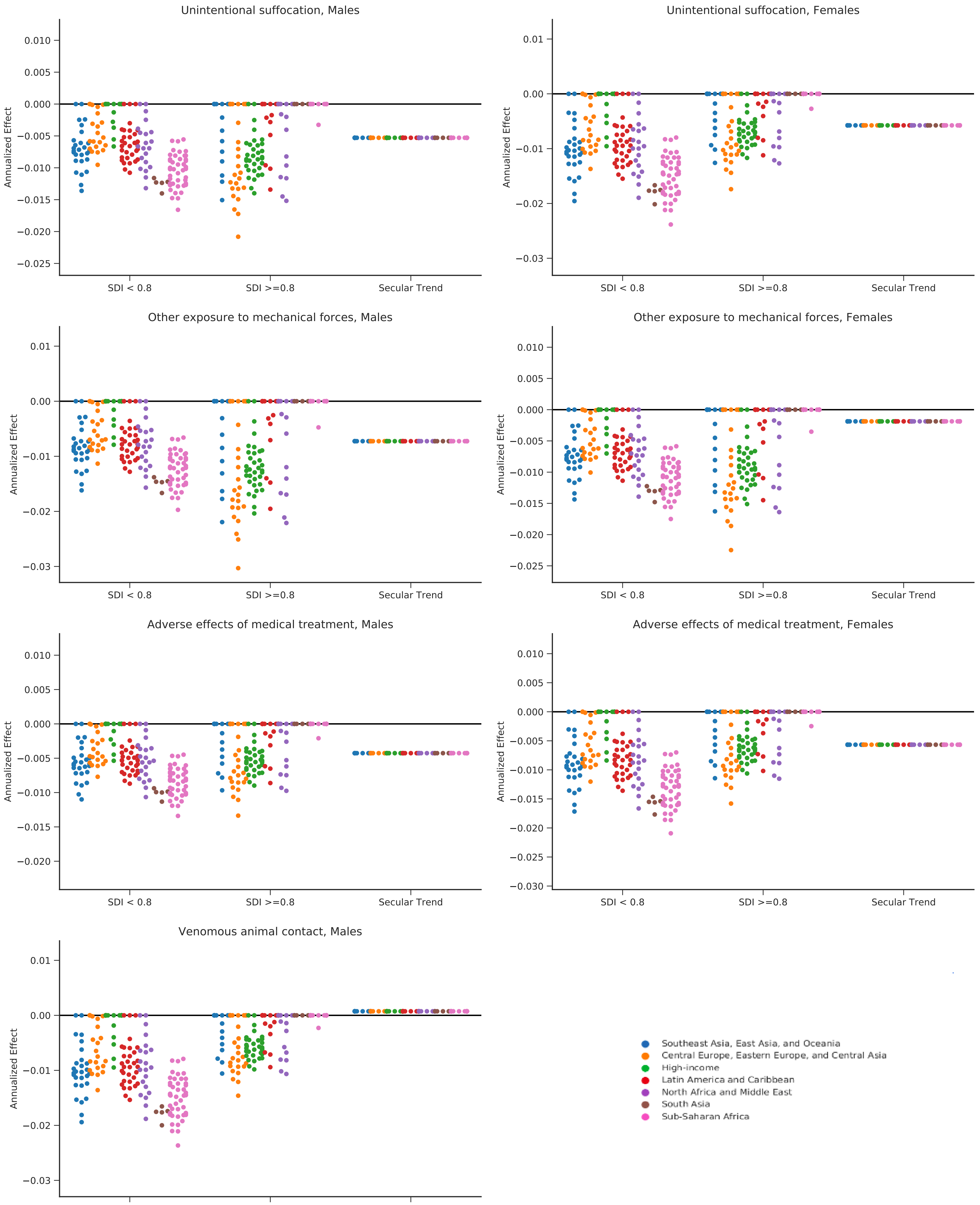
Unintentional suffocation, Males
Unintentional suffocation, Females
Other exposure to mechanical forces, Males
Other exposure to mechanical forces, Females
Adverse effects of medical treatment, Males
Adverse effects of medical treatment, Females
Venomous animal contact, Males
Annualized Effect
SDI < 0.8
SDI >=0.8
Secular Trend
0.010
0.005
0.000
−0.005
−0.010
−0.015
−0.020
−0.025
−0.030
0.01
0.00
−0.01
−0.02
−0.03
Southeast Asia, East Asia, and Oceania
Central Europe, Eastern Europe, and Central Asia
High-income
Latin America and Caribbean
North Africa and Middle East
South Asia
Sub-Saharan Africa

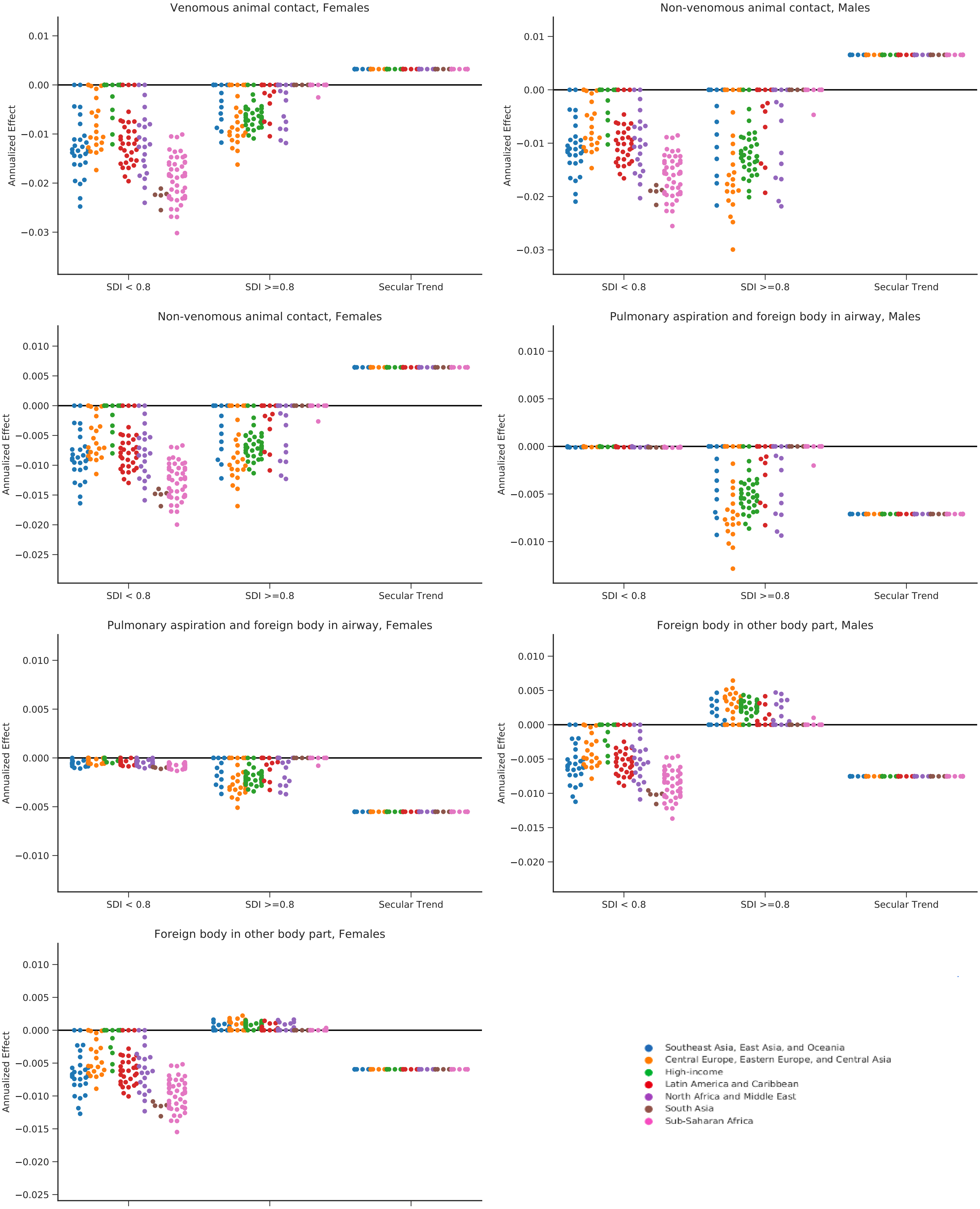

Venomous animal contact, Females
Non-venomous animal contact, Males
Non-venomous animal contact, Females
Pulmonary aspiration and foreign body in airway, Males
Pulmonary aspiration and foreign body in airway, Females
Foreign body in other body part, Males
Foreign body in other body part, Females
Annualized Effect
SDI < 0.8
SDI >=0.8
Secular Trend
0.01
0.00
−0.01
−0.02
−0.03
0.010
0.005
0.000
−0.005
−0.010
−0.015
−0.020
−0.025
Southeast Asia, East Asia, and Oceania
Central Europe, Eastern Europe, and Central Asia
High-income
Latin America and Caribbean
North Africa and Middle East
South Asia
Sub-Saharan Africa

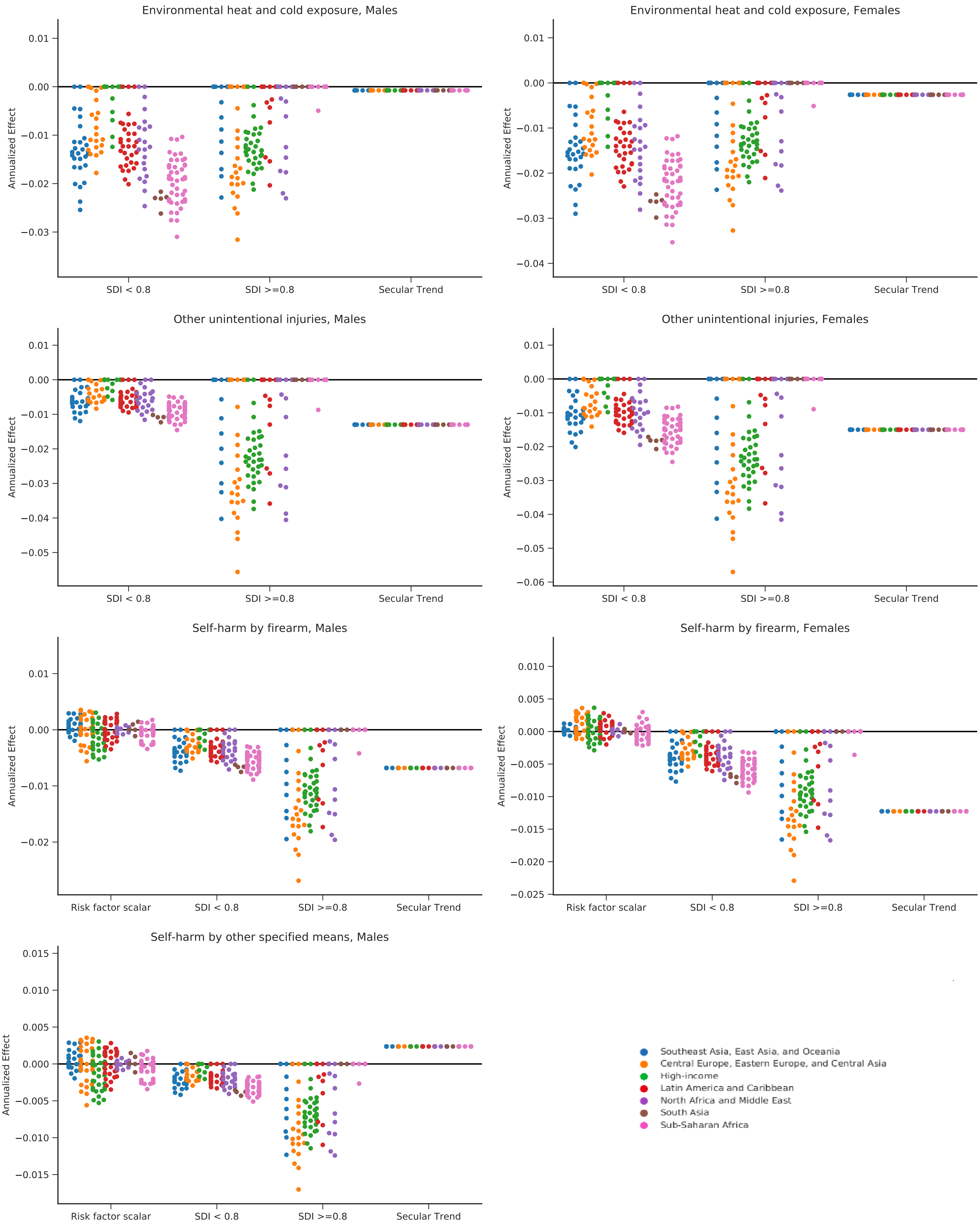

Environmental heat and cold exposure, Males
Environmental heat and cold exposure, Females
Other unintentional injuries, Males
Other unintentional injuries, Females
Self-harm by firearm, Males
Self-harm by firearm, Females
Self-harm by other specified means, Males
Annualized Effect
Risk factor scalar
SDI < 0.8
SDI >=0.8
Secular Trend
Southeast Asia, East Asia, and Oceania
Central Europe, Eastern Europe, and Central Asia
High-income
Latin America and Caribbean
North Africa and Middle East
South Asia
Sub-Saharan Africa

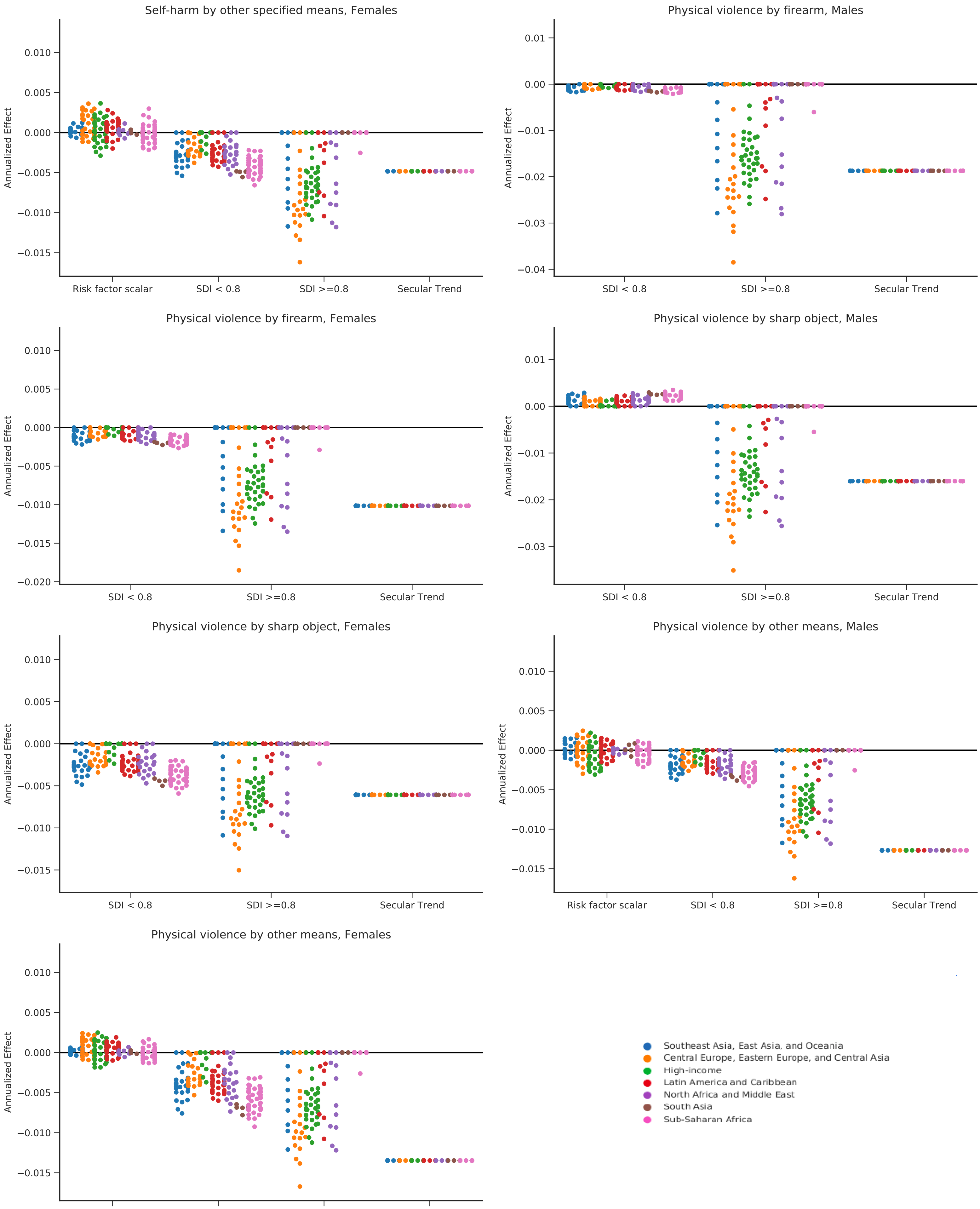
Self-harm by other specified means, Females
Physical violence by firearm, Males
Physical violence by firearm, Females
Physical violence by sharp object, Males
Physical violence by sharp object, Females
Physical violence by other means, Males
Physical violence by other means, Females
Annualized Effect
Risk factor scalar
SDI < 0.8
SDI >=0.8
Secular Trend
Southeast Asia, East Asia, and Oceania
Central Europe, Eastern Europe, and Central Asia
High-income
Latin America and Caribbean
North Africa and Middle East
South Asia
Sub-Saharan Africa

Figure 2. Illustration of the components of the mortality modeling process for all-cause mortality for females in Australia. The panels on the left show cause-specific death rates by age, with small multiples showing the time trend from 1990 to 2040 for each age group. The bottom left panel is the underlying mortality component captured by SDI and global secular trends; above that is risk-attributable mortality, captured by the risk factor scalar; second from the top is the sum of the underlying and risk-attributable mortality; and the top left panel shows the final mortality predictions after the ARIMA process, which accounts for latent trends. The panels on the right show the cause- and risk-specific population attributable fractions (PAFs) that go into the risk attributable estimation; the all-risk PAF at the top is used to create the risk factor scalar, and the specific risks are then sorted in descending order of attributable deaths in 2016.

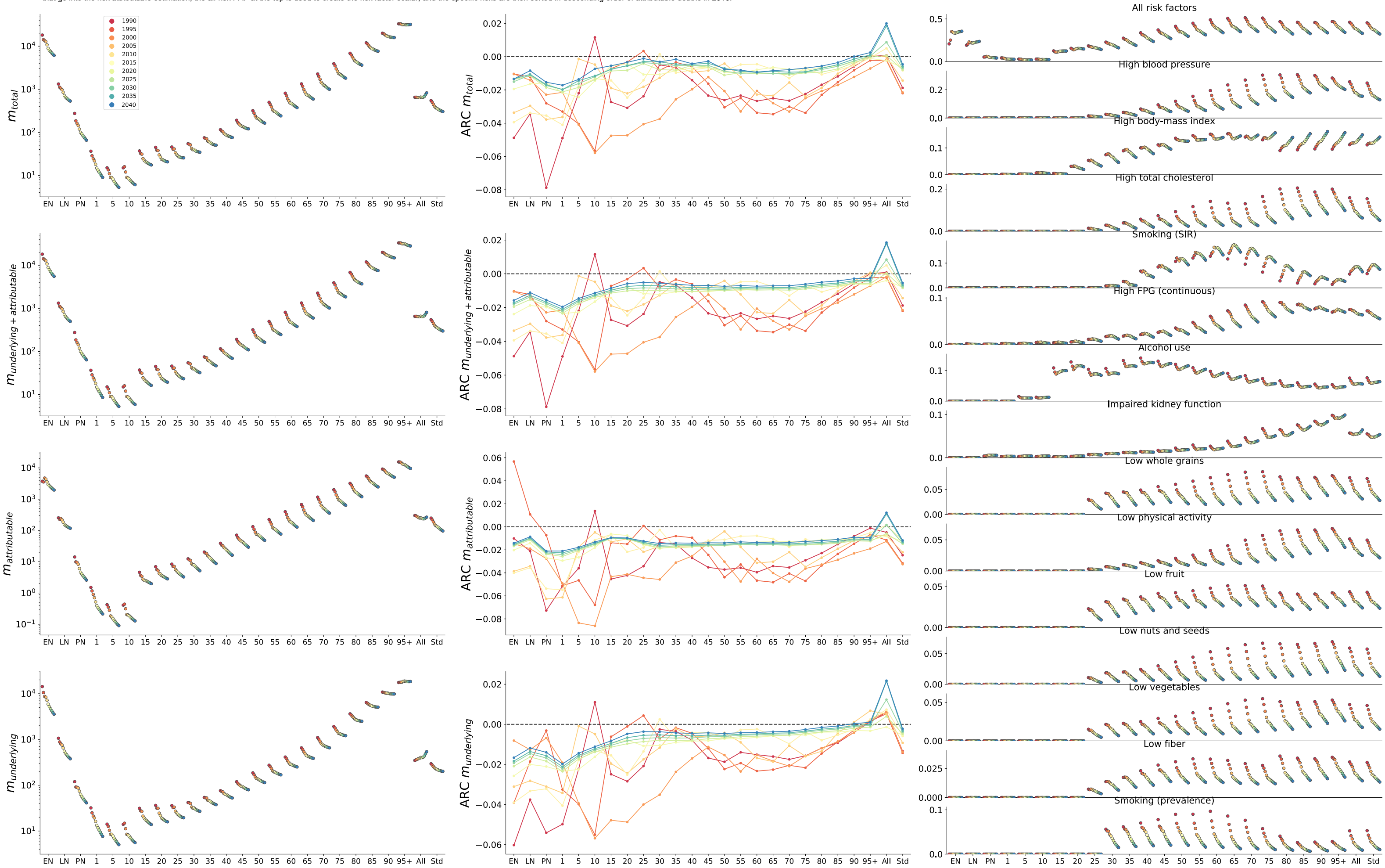

Supplemental Appendix Figure 3. Ten leading risk factors contributing to the greatest difference in risk-attributable years of life lost (YLLs) between the forecast and better health scenario in 2040 by location. Values are reported in thousands, with 95% uncertainty intervals in parentheses.

Location	Risk #1	Risk #2	Risk #3	Risk #4	Risk #5	Risk #6	Risk #7	Risk #8	Risk #9	Risk #10
Global	Tobacco 162,104·0 (104,421·9 to 240,944·4)	High blood pressure 142,907·9 (91,000·2 to 208,810·7)	High body-mass index 115,871·6 (75,375·4 to 180,141·6)	Ambient particulate matter 95,201·9 (70,345·0 to 124,534·8)	High fasting plasma glucose 92,960·5 (55,023·4 to 150,881·2)	High total cholesterol 71,564·6 (43,565·4 to 107,116·1)	Household air pollution 64,509·1 (46,583·5 to 84,250·1)	Alcohol use 59,698·3 (43,368·7 to 82,832·7)	Low fruit 59,033·5 (32,536·0 to 99,619·0)	Short gestation 49,918·8 (34,782·7 to 73,108·4)
Southeast Asia, East Asia, and Oceania	Tobacco 62,345·7 (33,274·6 to 104,797·5)	High blood pressure 48,100·6 (28,685·0 to 70,259·2)	High fasting plasma glucose 29,661·4 (17,247·3 to 45,518·7)	High body-mass index 28,965·3 (15,988·0 to 44,332·8)	Ambient particulate matter 25,549·0 (16,273·3 to 35,360·2)	Low fruit 21,698·1 (12,508·3 to 32,678·3)	High sodium 21,249·5 (9,276·6 to 42,180·0)	Alcohol use 18,651·2 (12,165·3 to 26,465·6)	High total cholesterol 18,208·9 (11,402·3 to 27,175·0)	Low whole grains 17,261·5 (10,160·9 to 28,544·8)
East Asia	Tobacco 46,489·6 (21,004·7 to 86,109·1)	High blood pressure 34,321·3 (19,014·0 to 50,852·2)	High fasting plasma glucose 20,178·9 (10,699·5 to 33,450·3)	High body-mass index 19,441·0 (9,949·2 to 30,960·0)	High sodium 18,726·1 (9,102·4 to 35,415·5)	Ambient particulate matter 18,317·2 (10,509·1 to 26,990·5)	Low fruit 15,479·1 (8,760·5 to 23,064·5)	Alcohol use 13,309·1 (7,844·0 to 20,154·2)	High total cholesterol 12,416·9 (7,038·6 to 19,528·1)	Household air pollution 11,747·3 (6,933·9 to 18,846·4)
China	Tobacco 45,348·8 (20,070·3 to 84,904·1)	High blood pressure 33,588·2 (18,397·3 to 49,931·8)	High fasting plasma glucose 19,624·9 (10,338·3 to 32,694·8)	High body-mass index 18,841·6 (9,632·8 to 29,953·1)	High sodium 18,305·8 (8,996·8 to 34,647·0)	Ambient particulate matter 17,888·7 (10,150·5 to 26,568·6)	Low fruit 15,183·8 (8,586·7 to 22,710·2)	Alcohol use 12,950·6 (7,598·6 to 19,515·3)	High total cholesterol 12,144·3 (6,824·6 to 19,177·9)	Household air pollution 11,446·6 (6,650·2 to 18,414·0)
North Korea	Tobacco 721·2 (460·6 to 1,072·0)	High blood pressure 499·3 (258·2 to 837·8)	Ambient particulate matter 341·1 (215·2 to 460·1)	High body-mass index 311·8 (85·9 to 689·0)	Household air pollution 311·0 (191·0 to 443·2)	High sodium 294·1 (43·9 to 636·9)	High fasting plasma glucose 269·8 (144·9 to 465·5)	Alcohol use 237·2 (112·4 to 534·9)	Low fruit 227·1 (118·2 to 401·2)	Low whole grains 224·6 (116·9 to 382·8)
Taiwan (Province of China)	Tobacco 500·3 (348·0 to 672·1)	High fasting plasma glucose 286·5 (150·8 to 474·6)	High body-mass index 267·9 (144·9 to 422·4)	High blood pressure 231·6 (128·0 to 366·0)	Alcohol use 123·9 (57·9 to 209·9)	Impaired kidney function 102·4 (58·8 to 155·4)	High sodium 99·4 (12·8 to 229·1)	Ambient particulate matter 90·9 (61·1 to 128·9)	High total cholesterol 88·6 (44·4 to 148·2)	Low fruit 68·7 (27·7 to 116·7)
Southeast Asia	Tobacco 15,896·8 (11,402·8 to 20,829·3)	High blood pressure 13,539·1 (8,835·7 to 19,083·9)	High fasting plasma glucose 9,290·5 (5,842·9 to 13,418·1)	High body-mass index 9,065·5 (5,624·5 to 14,361·2)	Ambient particulate matter 7,009·4 (4,996·1 to 9,073·0)	Low fruit 6,054·0 (3,435·8 to 9,292·3)	Low whole grains 5,760·5 (3,595·1 to 8,897·6)	High total cholesterol 5,638·1 (3,509·3 to 7,779·8)	Alcohol use 5,123·1 (3,886·0 to 6,623·4)	Household air pollution 4,628·9 (3,343·7 to 6,435·7)
Cambodia	Tobacco 471·1 (313·7 to 656·4)	Alcohol use 310·0 (215·1 to 429·8)	Household air pollution 306·1 (218·4 to 449·3)	High blood pressure 264·7 (159·8 to 388·5)	Ambient particulate matter 243·1 (174·7 to 354·3)	High body-mass index 215·2 (115·2 to 344·0)	High fasting plasma glucose 198·0 (113·8 to 296·4)	Low fruit 180·7 (114·2 to 292·7)	Low whole grains 155·8 (89·2 to 241·0)	High total cholesterol 133·4 (80·5 to 193·2)
Indonesia	High blood pressure 6,225·2 (3,591·9 to 9,076·5)	Tobacco 4,704·9 (2,790·6 to 6,831·3)	High fasting plasma glucose 3,920·3 (2,215·9 to 6,028·3)	High body-mass index 3,759·6 (2,104·3 to 6,427·2)	Low fruit 2,832·5 (1,490·0 to 4,688·4)	Low whole grains 2,626·8 (1,466·4 to 4,278·9)	Ambient particulate matter 2,584·5 (1,547·0 to 3,569·1)	High total cholesterol 2,415·4 (1,364·7 to 3,446·3)	Low vegetables 2,280·9 (1,182·3 to 3,634·1)	Low nuts and seeds 1,556·8 (894·3 to 2,500·8)
Laos	Tobacco 237·2 (152·7 to 349·7)	High blood pressure 180·7 (118·8 to 258·3)	Household air pollution 169·5 (103·5 to 254·7)	Ambient particulate matter 146·4 (95·8 to 219·2)	High body-mass index 134·3 (76·6 to 207·6)	High fasting plasma glucose 128·7 (73·0 to 203·7)	Child wasting 122·5 (32·4 to 292·0)	Short gestation 118·9 (52·5 to 231·2)	High total cholesterol 93·2 (57·2 to 125·9)	Alcohol use 92·4 (42·7 to 157·5)
Malaysia	Tobacco 789·1 (531·8 to 1,120·3)	High blood pressure 751·1 (438·8 to 1,123·9)	High body-mass index 591·1 (353·7 to 948·6)	High fasting plasma glucose 511·5 (262·9 to 931·7)	High total cholesterol 467·2 (258·6 to 699·1)	Ambient particulate matter 332·0 (237·8 to 448·3)	Low whole grains 298·6 (167·1 to 503·4)	Low vegetables 248·4 (131·3 to 438·1)	Low fruit 216·3 (102·1 to 371·6)	Low nuts and seeds 212·1 (108·0 to 363·9)
Maldives	High blood pressure 6·7 (3·3 to 13·2)	Tobacco 5·4 (2·3 to 10·9)	High body-mass index 5·3 (2·4 to 10·6)	High fasting plasma glucose 4·7 (2·1 to 9·2)	High total cholesterol 4·2 (1·7 to 8·1)	Ambient particulate matter 3·2 (1·5 to 5·8)	Low whole grains 2·6 (1·0 to 5·2)	Impaired kidney function 2·3 (1·2 to 4·1)	Low vegetables 2·1 (0·8 to 4·0)	Low fruit 1·9 (0·7 to 4·2)
Mauritius	High fasting plasma glucose 45·0 (26·9 to 63·4)	High body-mass index 38·7 (23·0 to 56·3)	High blood pressure 35·3 (21·3 to 50·5)	Tobacco 25·9 (15·2 to 38·9)	High total cholesterol 17·4 (9·1 to 27·6)	Impaired kidney function 15·1 (9·2 to 22·6)	Low fruit 13·2 (7·7 to 20·6)	Alcohol use 13·0 (7·8 to 21·2)	Low whole grains 12·2 (6·8 to 19·4)	Ambient particulate matter 10·9 (6·2 to 16·6)
Myanmar	Tobacco 1,924·1 (1,404·3 to 2,569·4)	High blood pressure 967·9 (623·7 to 1,487·7)	Household air pollution 786·4 (492·3 to 1,127·3)	Ambient particulate matter 703·3 (538·5 to 979·4)	High fasting plasma glucose 591·5 (338·3 to 951·3)	High body-mass index 556·5 (320·6 to 938·3)	Low fruit 537·1 (357·4 to 813·0)	Low whole grains 403·3 (256·4 to 643·7)	High total cholesterol 294·1 (178·8 to 457·1)	Low vegetables 279·4 (138·1 to 540·5)
Philippines	Tobacco 3,118·0 (2,188·2 to 4,272·3)	High blood pressure 2,327·2 (1,519·0 to 3,225·2)	High body-mass index 2,124·1 (1,299·4 to 3,423·6)	High fasting plasma glucose 2,013·1 (1,270·3 to 3,023·0)	Alcohol use 1,438·0 (860·7 to 2,061·7)	Ambient particulate matter 1,386·4 (989·2 to 1,900·3)	High total cholesterol 1,107·4 (733·0 to 1,591·7)	Low whole grains 1,061·5 (678·3 to 1,565·3)	Household air pollution 952·1 (610·5 to 1,485·4)	Low fruit 885·8 (403·4 to 1,414·9)
Sri Lanka	High blood pressure 316·2 (171·7 to 520·9)	High fasting plasma glucose 304·9 (157·1 to 509·6)	Tobacco 267·3 (135·5 to 448·2)	High body-mass index 258·0 (116·8 to 478·3)	High total cholesterol 196·4 (105·7 to 330·4)	Ambient particulate matter 150·8 (81·5 to 243·0)	Household air pollution 133·7 (67·1 to 239·5)	Low fruit 130·3 (63·0 to 221·4)	Low whole grains 112·7 (57·0 to 193·0)	Low nuts and seeds 103·8 (50·6 to 177·4)
Seychelles	High blood pressure 2·3 (1·2 to 3·6)	High body-mass index 2·3 (1·3 to 3·6)	Tobacco 2·1 (1·2 to 3·3)	Alcohol use 1·4 (0·8 to 2·4)	High fasting plasma glucose 1·3 (0·6 to 2·2)	High total cholesterol 0·9 (0·4 to 1·5)	Ambient particulate matter 0·8 (0·4 to 1·3)	Low whole grains 0·6 (0·3 to 1·1)	Impaired kidney function 0·6 (0·3 to 0·9)	Low fruit 0·6 (0·3 to 1·0)
Thailand	Tobacco 1,699·4 (1,194·3 to 2,480·6)	Alcohol use 820·5 (495·9 to 1,284·5)	High body-mass index 608·6 (348·7 to 950·2)	High blood pressure 605·1 (366·0 to 964·9)	High fasting plasma glucose 571·2 (311·3 to 961·6)	Ambient particulate matter 484·6 (357·5 to 673·3)	High total cholesterol 302·0 (177·9 to 457·7)	Impaired kidney function 254·7 (138·2 to 414·2)	Low whole grains 244·2 (135·0 to 393·3)	Low fruit 234·9 (104·6 to 421·8)
Timor-Leste	Tobacco 18·0 (10·1 to 31·1)	Short gestation 14·9 (6·2 to 30·5)	High blood pressure 13·9 (7·6 to 23·5)	Household air pollution 13·8 (8·2 to 22·9)	Child wasting 13·1 (3·4 to 30·7)	Low birth weight 10·0 (4·7 to 18·3)	Ambient particulate matter 9·8 (5·8 to 16·9)	High fasting plasma glucose 7·0 (3·5 to 13·4)	High body-mass index 6·8 (2·4 to 12·8)	Low whole grains 5·8 (3·1 to 10·0)
Vietnam	Tobacco 2,671·6 (1,854·9 to 3,725·6)	High blood pressure 1,830·3 (1,137·5 to 2,750·8)	Alcohol use 1,520·5 (954·7 to 2,169·3)	High fasting plasma glucose 1,065·4 (634·6 to 1,635·9)	Ambient particulate matter 946·6 (693·1 to 1,276·9)	Low fruit 926·0 (568·7 to 1,342·2)	High body-mass index 742·5 (335·0 to 1,302·9)	Low whole grains 733·2 (441·6 to 1,183·1)	Household air pollution 674·0 (413·4 to 1,091·1)	High total cholesterol 584·5 (346·7 to 858·9)
Oceania	Tobacco 433·3 (295·7 to 600·3)	High body-mass index 400·6 (284·0 to 524·3)	High fasting plasma glucose 295·5 (190·1 to 382·3)	High blood pressure 265·6 (181·6 to 344·4)	Household air pollution 252·3 (185·7 to 354·8)	Ambient particulate matter 209·8 (151·4 to 286·6)	High total cholesterol 177·0 (111·1 to 237·5)	Alcohol use 162·0 (84·6 to 273·3)	Low fruit 157·6 (106·4 to 226·7)	Low whole grains 138·3 (92·4 to 195·0)
American Samoa	High body-mass index 2·4 (1·7 to 3·3)	High fasting plasma glucose 2·0 (1·2 to 2·9)	Tobacco 1·6 (1·0 to 2·4)	High blood pressure 1·5 (0·9 to 2·2)	Impaired kidney function 0·7 (0·5 to 0·9)	High total cholesterol 0·6 (0·3 to 0·9)	Low whole grains 0·6 (0·4 to 0·9)	Low vegetables 0·4 (0·2 to 0·7)	Low nuts and seeds 0·4 (0·2 to 0·6)	Alcohol use 0·3 (0·1 to 0·7)
Federated States of Micronesia	High body-mass index 4·4 (2·7 to 6·0)	Tobacco 3·5 (1·9 to 5·5)	High fasting plasma glucose 3·3 (1·7 to 5·1)	High blood pressure 2·5 (1·4 to 3·8)	High total cholesterol 1·8 (1·0 to 2·8)	Low whole grains 1·4 (0·8 to 2·0)	Ambient particulate matter 1·2 (0·5 to 2·1)	Low vegetables 1·1 (0·6 to 1·8)	Low fruit 1·1 (0·6 to 1·7)	Impaired kidney function 1·0 (0·5 to 1·7)
Fiji	High body-mass index 35·9 (23·2 to 52·0)	High fasting plasma glucose 28·0 (16·3 to 42·2)	Tobacco 21·0 (11·9 to 34·6)	High blood pressure 20·4 (11·0 to 31·9)	High total cholesterol 15·1 (7·8 to 24·3)	Low whole grains 10·8 (5·8 to 17·5)	Low fruit 10·0 (5·8 to 17·1)	Low vegetables 8·2 (4·4 to 15·2)	Low nuts and seeds 8·0 (3·8 to 14·5)	Impaired kidney function 7·7 (5·0 to 11·1)

Location	Risk #1	Risk #2	Risk #3	Risk #4	Risk #5	Risk #6	Risk #7	Risk #8	Risk #9	Risk #10
Guam	High body-mass index 4·4 (2·7 to 6·2)	Tobacco 3·6 (2·1 to 5·5)	High fasting plasma glucose 3·5 (1·3 to 6·1)	High blood pressure 3·1 (1·5 to 5·1)	High total cholesterol 2·1 (0·9 to 3·6)	Low whole grains 1·3 (0·6 to 2·5)	Ambient particulate matter 1·2 (0·3 to 2·0)	Low vegetables 1·1 (0·5 to 1·9)	Low nuts and seeds 1·0 (0·4 to 1·8)	Impaired kidney function 0·9 (0·2 to 1·4)
Kiribati	High body-mass index 4·5 (2·8 to 6·7)	Tobacco 3·2 (2·0 to 4·8)	High fasting plasma glucose 3·1 (1·3 to 5·1)	High blood pressure 2·1 (1·2 to 3·4)	Household air pollution 1·6 (1·0 to 2·1)	Low whole grains 1·3 (0·6 to 2·2)	High total cholesterol 1·1 (0·6 to 1·9)	Low fruit 1·1 (0·6 to 1·8)	Short gestation 1·0 (0·5 to 2·2)	Low vegetables 1·0 (0·5 to 1·7)
Marshall Islands	High body-mass index 3·2 (1·9 to 4·6)	High fasting plasma glucose 2·2 (1·0 to 3·8)	Tobacco 1·8 (0·9 to 2·7)	High blood pressure 1·2 (0·6 to 1·8)	Low whole grains 0·9 (0·4 to 1·5)	High total cholesterol 0·8 (0·4 to 1·2)	Low fruit 0·7 (0·3 to 1·2)	Ambient particulate matter 0·6 (0·0 to 1·2)	Low nuts and seeds 0·5 (0·2 to 1·0)	Low vegetables 0·5 (0·3 to 0·8)
Northern Mariana Islands	High body-mass index 3·8 (2·4 to 5·6)	Tobacco 3·5 (2·1 to 5·2)	High fasting plasma glucose 3·1 (1·5 to 5·0)	High blood pressure 2·4 (1·2 to 3·6)	High total cholesterol 1·2 (0·6 to 1·9)	Ambient particulate matter 1·1 (0·6 to 1·6)	Low whole grains 1·1 (0·5 to 1·7)	Impaired kidney function 0·8 (0·4 to 1·4)	Low vegetables 0·7 (0·3 to 1·2)	Low fruit 0·6 (0·3 to 1·1)
Papua New Guinea	Tobacco 331·6 (220·3 to 464·2)	High body-mass index 269·6 (185·1 to 380·3)	Household air pollution 210·3 (152·9 to 308·1)	High fasting plasma glucose 199·8 (123·3 to 270·8)	High blood pressure 185·1 (126·3 to 240·9)	Ambient particulate matter 172·1 (124·3 to 230·1)	Alcohol use 139·7 (70·8 to 235·8)	High total cholesterol 125·4 (72·9 to 172·3)	Low fruit 118·9 (76·9 to 166·8)	Child wasting 102·2 (32·4 to 201·2)
Samoa	High body-mass index 3·9 (2·4 to 5·3)	High fasting plasma glucose 2·8 (1·4 to 4·4)	Tobacco 2·7 (1·7 to 3·8)	High blood pressure 2·3 (1·3 to 3·5)	Household air pollution 1·0 (0·6 to 1·5)	Low whole grains 1·0 (0·5 to 1·7)	High total cholesterol 0·9 (0·5 to 1·5)	Impaired kidney function 0·9 (0·3 to 1·4)	Low vegetables 0·8 (0·5 to 1·3)	Alcohol use 0·8 (0·5 to 1·3)
Solomon Islands	High body-mass index 22·6 (16·2 to 29·9)	Tobacco 18·2 (12·7 to 25·7)	High fasting plasma glucose 15·5 (9·1 to 22·8)	High blood pressure 12·3 (7·6 to 18·8)	Household air pollution 10·2 (7·4 to 13·7)	High total cholesterol 7·3 (4·0 to 10·5)	Low fruit 7·3 (4·7 to 10·6)	Low whole grains 7·1 (4·3 to 10·6)	Ambient particulate matter 6·3 (4·4 to 8·8)	Low vegetables 5·3 (3·1 to 8·1)
Tonga	High body-mass index 3·0 (2·2 to 3·9)	Tobacco 2·2 (1·5 to 2·9)	High fasting plasma glucose 2·1 (1·4 to 3·0)	High blood pressure 1·6 (1·0 to 2·2)	High total cholesterol 0·9 (0·5 to 1·3)	Low whole grains 0·8 (0·5 to 1·2)	Impaired kidney function 0·7 (0·4 to 1·0)	Low fruit 0·6 (0·4 to 0·9)	Household air pollution 0·6 (0·3 to 0·8)	Low vegetables 0·5 (0·3 to 0·8)
Vanuatu	High body-mass index 11·9 (7·6 to 16·4)	High blood pressure 9·5 (5·7 to 14·1)	High fasting plasma glucose 8·4 (4·8 to 13·2)	Tobacco 8·2 (4·5 to 12·5)	High total cholesterol 5·8 (3·3 to 8·6)	Household air pollution 5·4 (3·1 to 7·9)	Low whole grains 4·4 (2·5 to 6·9)	Ambient particulate matter 3·8 (2·0 to 6·3)	Low vegetables 3·7 (1·8 to 5·9)	Low fruit 3·5 (2·0 to 5·8)
Central Europe, Eastern Europe, and Central Asia	High body-mass index 10,025·6 (6,283·9 to 18,262·6)	Tobacco 9,200·9 (5,605·6 to 15,532·8)	High blood pressure 9,137·5 (5,003·9 to 15,588·5)	Alcohol use 6,099·7 (4,006·1 to 10,212·1)	High total cholesterol 5,926·5 (3,010·3 to 10,369·6)	Ambient particulate matter 4,758·1 (2,986·8 to 7,614·4)	High fasting plasma glucose 4,164·5 (1,860·9 to 8,186·3)	Low fruit 3,422·0 (1,975·3 to 6,446·4)	Low whole grains 3,401·9 (1,686·8 to 6,402·1)	High sodium 3,278·4 (743·4 to 8,005·2)
Central Asia	High body-mass index 2,410·1 (1,457·3 to 3,892·8)	High blood pressure 2,284·9 (1,395·6 to 3,420·5)	Tobacco 1,784·2 (1,077·9 to 2,706·0)	High total cholesterol 1,328·7 (756·8 to 1,942·1)	Ambient particulate matter 1,272·3 (800·6 to 1,731·8)	Alcohol use 1,143·5 (753·7 to 1,642·4)	High sodium 990·7 (353·2 to 2,004·9)	Low fruit 981·1 (536·1 to 1,639·6)	Low whole grains 911·0 (482·8 to 1,484·0)	High fasting plasma glucose 800·1 (382·4 to 1,431·2)
Armenia	Tobacco 58·6 (35·5 to 89·0)	High body-mass index 51·6 (32·1 to 78·3)	High blood pressure 48·5 (22·8 to 88·0)	High total cholesterol 27·3 (12·6 to 49·6)	High fasting plasma glucose 25·0 (10·2 to 44·8)	High sodium 22·9 (5·7 to 50·8)	Ambient particulate matter 22·3 (13·0 to 32·9)	Low nuts and seeds 17·8 (8·4 to 31·9)	Low whole grains 17·3 (8·1 to 34·7)	Low fruit 16·9 (9·3 to 27·3)
Azerbaijan	High blood pressure 227·5 (122·9 to 381·2)	High body-mass index 217·2 (124·0 to 377·8)	Tobacco 203·0 (107·4 to 333·3)	Ambient particulate matter 144·3 (89·5 to 210·9)	High total cholesterol 129·9 (65·0 to 210·9)	Alcohol use 118·6 (64·6 to 178·9)	High sodium 110·7 (38·6 to 204·0)	Low fruit 100·1 (50·5 to 173·4)	High fasting plasma glucose 92·7 (41·1 to 172·6)	Low nuts and seeds 91·2 (46·4 to 143·5)
Georgia	High body-mass index 80·2 (43·7 to 133·2)	High blood pressure 74·1 (24·6 to 137·4)	Tobacco 53·5 (25·8 to 97·1)	High sodium 37·3 (12·5 to 78·3)	Ambient particulate matter 33·6 (20·0 to 56·7)	Low fruit 31·6 (15·0 to 58·8)	High total cholesterol 31·6 (11·3 to 61·7)	Alcohol use 27·7 (12·6 to 53·6)	Low whole grains 26·3 (8·3 to 59·1)	Low nuts and seeds 24·6 (9·1 to 41·9)
Kazakhstan	High body-mass index 561·3 (282·5 to 1,029·2)	High blood pressure 559·6 (276·6 to 982·4)	Tobacco 556·4 (286·0 to 929·3)	Alcohol use 419·5 (230·1 to 693·1)	High total cholesterol 337·5 (151·8 to 574·4)	Ambient particulate matter 251·1 (138·8 to 408·3)	High sodium 234·7 (72·5 to 561·7)	Low fruit 232·4 (99·6 to 423·8)	Low whole grains 221·6 (93·8 to 415·1)	High fasting plasma glucose 184·4 (78·1 to 396·2)
Kyrgyzstan	High body-mass index 160·1 (78·5 to 275·1)	Tobacco 118·7 (56·0 to 194·3)	High blood pressure 118·6 (49·4 to 201·9)	Alcohol use 102·2 (51·1 to 188·0)	Ambient particulate matter 74·5 (41·7 to 114·7)	High sodium 67·2 (19·8 to 127·7)	High total cholesterol 67·1 (26·5 to 109·1)	Low fruit 57·2 (22·2 to 105·9)	Low whole grains 55·1 (21·9 to 108·9)	High fasting plasma glucose 45·2 (18·1 to 93·9)
Mongolia	High body-mass index 100·3 (55·1 to 169·3)	High blood pressure 81·7 (38·8 to 143·4)	Tobacco 71·8 (37·4 to 122·6)	Low fruit 43·9 (21·5 to 88·1)	Alcohol use 41·5 (21·5 to 72·5)	Ambient particulate matter 38·3 (21·5 to 63·2)	High sodium 36·9 (13·2 to 85·6)	Low vegetables 36·0 (15·7 to 66·1)	Low whole grains 33·0 (14·9 to 63·2)	High total cholesterol 30·7 (14·9 to 49·9)
Tajikistan	High body-mass index 169·2 (101·4 to 286·9)	High blood pressure 141·5 (71·0 to 246·6)	Ambient particulate matter 110·5 (68·7 to 177·4)	Tobacco 92·3 (45·6 to 175·7)	High total cholesterol 79·8 (38·5 to 140·7)	High sodium 72·4 (19·7 to 152·0)	Low fruit 70·3 (35·2 to 137·9)	Low whole grains 60·5 (27·7 to 105·0)	High fasting plasma glucose 57·4 (25·4 to 113·9)	Alcohol use 52·8 (26·0 to 90·5)
Turkmenistan	High blood pressure 203·7 (105·0 to 324·4)	High body-mass index 181·2 (92·7 to 296·8)	Tobacco 112·6 (59·1 to 179·3)	High total cholesterol 103·8 (49·2 to 168·7)	Ambient particulate matter 102·8 (57·5 to 151·1)	Alcohol use 102·2 (54·1 to 165·0)	Low fruit 86·8 (42·3 to 152·6)	High sodium 83·9 (24·5 to 171·5)	Low whole grains 81·9 (36·9 to 147·2)	High fasting plasma glucose 65·3 (22·6 to 123·2)
Uzbekistan	High body-mass index 889·0 (541·1 to 1,433·9)	High blood pressure 826·4 (443·6 to 1,308·1)	Tobacco 543·3 (294·0 to 895·2)	High total cholesterol 515·0 (254·2 to 784·3)	Ambient particulate matter 494·5 (296·5 to 780·8)	High sodium 404·6 (123·5 to 861·3)	Low fruit 343·7 (170·6 to 561·7)	Low whole grains 323·6 (164·8 to 553·8)	Low nuts and seeds 301·6 (129·2 to 538·6)	Alcohol use 291·4 (139·6 to 501·6)
Central Europe	Tobacco 2,427·1 (1,820·1 to 3,184·7)	High body-mass index 2,030·2 (1,428·9 to 2,842·5)	High blood pressure 1,947·8 (1,306·7 to 2,867·4)	Alcohol use 1,413·7 (957·4 to 1,970·7)	High total cholesterol 1,093·5 (683·5 to 1,590·0)	High fasting plasma glucose 923·6 (479·2 to 1,609·2)	Ambient particulate matter 907·5 (658·3 to 1,182·9)	High sodium 710·2 (247·2 to 1,405·8)	Low fruit 697·0 (448·3 to 1,009·0)	Low whole grains 618·3 (377·9 to 989·8)
Albania	High blood pressure 50·7 (27·9 to 80·6)	High body-mass index 48·4 (29·1 to 76·8)	Tobacco 42·4 (24·9 to 65·8)	High total cholesterol 27·4 (15·3 to 45·7)	High sodium 22·0 (6·5 to 43·4)	Ambient particulate matter 21·8 (13·9 to 34·2)	Alcohol use 20·8 (12·2 to 35·4)	Low whole grains 17·7 (9·4 to 31·6)	High fasting plasma glucose 16·6 (7·7 to 32·5)	Low fruit 15·3 (7·7 to 27·2)
Bosnia and Herzegovina	High body-mass index 54·5 (33·1 to 84·0)	High blood pressure 50·6 (23·9 to 79·7)	Tobacco 48·7 (27·3 to 73·7)	Alcohol use 34·0 (20·3 to 53·7)	High total cholesterol 29·1 (14·7 to 48·8)	Ambient particulate matter 28·5 (17·8 to 41·1)	High fasting plasma glucose 24·8 (8·6 to 46·5)	High sodium 24·2 (7·2 to 51·0)	Low fruit 21·1 (11·1 to 34·1)	Low whole grains 18·5 (8·0 to 32·5)
Bulgaria	High body-mass index 168·7 (104·4 to 251·4)	High blood pressure 163·0 (69·5 to 294·2)	Tobacco 140·5 (76·9 to 223·7)	High total cholesterol 91·8 (40·6 to 157·1)	Alcohol use 72·9 (32·3 to 137·0)	High fasting plasma glucose 72·6 (30·7 to 123·9)	Ambient particulate matter 70·9 (45·5 to 109·4)	High sodium 68·3 (17·7 to 159·5)	Low fruit 55·8 (29·9 to 100·1)	Low whole grains 50·9 (21·1 to 92·4)

Location	Risk #1	Risk #2	Risk #3	Risk #4	Risk #5	Risk #6	Risk #7	Risk #8	Risk #9	Risk #10
Croatia	Tobacco<br>83·0<br>(55·4 to 115·8)	High body-mass index<br>59·5<br>(38·2 to 90·6)	Alcohol use<br>55·1<br>(34·7 to 81·2)	High blood pressure<br>52·8<br>(25·1 to 86·1)	High fasting plasma glucose<br>30·8<br>(14·0 to 58·3)	High total cholesterol<br>27·2<br>(12·9 to 45·2)	Ambient particulate matter<br>25·4<br>(16·8 to 36·0)	High sodium<br>23·0<br>(8·2 to 47·4)	Low fruit<br>19·9<br>(12·1 to 30·5)	Low whole grains<br>17·0<br>(8·7 to 30·5)
Czech Republic	Tobacco<br>219·9<br>(162·1 to 299·0)	High body-mass index<br>180·5<br>(127·0 to 252·4)	High blood pressure<br>153·4<br>(86·9 to 238·8)	Alcohol use<br>133·4<br>(92·5 to 187·0)	High total cholesterol<br>100·4<br>(57·1 to 158·8)	High fasting plasma glucose<br>96·7<br>(49·1 to 173·8)	Ambient particulate matter<br>72·7<br>(50·9 to 103·8)	High sodium<br>61·8<br>(16·3 to 118·4)	Low whole grains<br>50·3<br>(31·3 to 85·2)	Low fruit<br>48·1<br>(31·2 to 71·6)
Hungary	Tobacco<br>205·9<br>(145·1 to 285·3)	High body-mass index<br>192·1<br>(134·2 to 267·2)	Alcohol use<br>169·4<br>(102·7 to 281·0)	High blood pressure<br>159·8<br>(90·5 to 266·4)	High total cholesterol<br>92·2<br>(49·3 to 153·2)	High fasting plasma glucose<br>86·1<br>(38·3 to 165·4)	Ambient particulate matter<br>79·2<br>(56·3 to 112·4)	High sodium<br>62·9<br>(21·6 to 119·0)	Low fruit<br>52·5<br>(32·8 to 80·1)	Low whole grains<br>46·2<br>(22·6 to 81·6)
Macedonia	High body-mass index<br>45·2<br>(25·0 to 69·0)	High blood pressure<br>40·9<br>(12·0 to 71·8)	Tobacco<br>39·7<br>(22·9 to 63·5)	High fasting plasma glucose<br>23·6<br>(9·6 to 38·4)	Ambient particulate matter<br>21·5<br>(14·4 to 31·7)	High total cholesterol<br>20·1<br>(8·5 to 33·8)	High sodium<br>17·3<br>(5·1 to 36·2)	Low whole grains<br>14·6<br>(4·8 to 26·2)	Alcohol use<br>13·2<br>(6·2 to 24·7)	Low fruit<br>13·0<br>(6·4 to 22·3)
Montenegro	High body-mass index<br>11·5<br>(6·8 to 16·8)	High blood pressure<br>10·5<br>(4·2 to 19·7)	Tobacco<br>9·1<br>(4·5 to 15·3)	Alcohol use<br>6·0<br>(2·7 to 11·0)	High fasting plasma glucose<br>5·8<br>(2·4 to 10·9)	High total cholesterol<br>4·8<br>(2·3 to 7·9)	Ambient particulate matter<br>4·7<br>(2·8 to 6·8)	High sodium<br>4·5<br>(1·2 to 9·2)	Low fruit<br>4·0<br>(1·8 to 7·7)	Low whole grains<br>3·7<br>(1·5 to 7·0)
Poland	Tobacco<br>975·7<br>(708·5 to 1,296·0)	High body-mass index<br>594·1<br>(410·3 to 843·3)	High blood pressure<br>561·6<br>(303·9 to 876·9)	Alcohol use<br>396·6<br>(268·7 to 586·4)	High total cholesterol<br>344·2<br>(182·9 to 547·1)	Ambient particulate matter<br>291·7<br>(207·1 to 382·6)	High fasting plasma glucose<br>285·8<br>(135·1 to 520·6)	Low fruit<br>240·9<br>(157·2 to 362·3)	High sodium<br>199·3<br>(63·7 to 459·1)	Low whole grains<br>185·3<br>(92·5 to 324·3)
Romania	High blood pressure<br>443·7<br>(193·5 to 774·6)	Tobacco<br>371·1<br>(227·7 to 593·9)	High body-mass index<br>366·0<br>(203·7 to 599·9)	Alcohol use<br>358·6<br>(158·9 to 673·6)	High total cholesterol<br>202·0<br>(98·9 to 389·1)	Ambient particulate matter<br>171·3<br>(109·9 to 281·4)	High sodium<br>162·3<br>(48·8 to 365·1)	High fasting plasma glucose<br>144·5<br>(60·1 to 297·1)	Low fruit<br>129·8<br>(62·1 to 214·1)	Low whole grains<br>128·9<br>(48·2 to 260·7)
Serbia	High body-mass index<br>170·6<br>(99·2 to 258·9)	High blood pressure<br>133·2<br>(48·0 to 237·8)	Tobacco<br>122·2<br>(62·5 to 190·1)	High fasting plasma glucose<br>76·9<br>(29·9 to 150·8)	High total cholesterol<br>74·5<br>(33·3 to 132·9)	Alcohol use<br>69·5<br>(23·6 to 144·7)	Ambient particulate matter<br>63·1<br>(37·7 to 96·4)	High sodium<br>58·5<br>(18·6 to 112·0)	Low fruit<br>53·8<br>(29·2 to 89·9)	Low whole grains<br>44·5<br>(17·0 to 82·2)
Slovakia	Tobacco<br>138·1<br>(93·1 to 203·9)	High body-mass index<br>115·9<br>(73·2 to 177·1)	High blood pressure<br>106·6<br>(50·7 to 172·9)	Alcohol use<br>84·2<br>(51·6 to 125·9)	High total cholesterol<br>68·4<br>(34·4 to 109·5)	High fasting plasma glucose<br>49·9<br>(21·1 to 108·7)	Ambient particulate matter<br>49·0<br>(33·2 to 73·5)	High sodium<br>41·2<br>(9·7 to 82·5)	Low fruit<br>39·0<br>(22·9 to 64·9)	Low whole grains<br>34·6<br>(16·7 to 63·7)
Slovenia	Tobacco<br>35·4<br>(24·1 to 50·0)	Alcohol use<br>22·8<br>(14·2 to 32·5)	High body-mass index<br>22·4<br>(15·3 to 31·3)	High blood pressure<br>20·9<br>(10·8 to 32·2)	High fasting plasma glucose<br>12·3<br>(5·3 to 21·5)	High total cholesterol<br>10·4<br>(5·1 to 16·0)	Ambient particulate matter<br>8·7<br>(5·7 to 11·6)	High sodium<br>8·6<br>(2·1 to 16·6)	Low whole grains<br>5·8<br>(2·7 to 9·6)	Low fruit<br>5·1<br>(2·6 to 8·6)
Eastern Europe	High body-mass index<br>5,585·1<br>(3,061·0 to 11,145·3)	Tobacco<br>4,990·0<br>(2,503·9 to 9,629·8)	High blood pressure<br>4,896·3<br>(2,039·4 to 9,279·8)	Alcohol use<br>3,569·1<br>(1,922·3 to 6,381·1)	High total cholesterol<br>3,511·1<br>(1,436·0 to 7,065·4)	Ambient particulate matter<br>2,573·3<br>(1,457·3 to 4,688·5)	High fasting plasma glucose<br>2,458·5<br>(861·4 to 5,380·0)	Low whole grains<br>1,869·6<br>(719·2 to 4,025·7)	Low fruit<br>1,734·4<br>(760·7 to 3,510·5)	High sodium<br>1,592·1<br>(128·3 to 4,408·2)
Belarus	High body-mass index<br>255·6<br>(132·8 to 433·6)	High blood pressure<br>253·7<br>(106·4 to 410·9)	Tobacco<br>209·7<br>(102·5 to 348·9)	High total cholesterol<br>187·0<br>(74·6 to 309·2)	Alcohol use<br>147·6<br>(76·8 to 235·2)	Ambient particulate matter<br>123·7<br>(59·8 to 197·7)	Low whole grains<br>95·9<br>(40·0 to 173·2)	High fasting plasma glucose<br>95·0<br>(34·2 to 186·5)	Low nuts and seeds<br>94·2<br>(37·8 to 161·1)	Low fruit<br>92·4<br>(41·9 to 162·5)
Estonia	High blood pressure<br>21·0<br>(9·3 to 38·9)	Tobacco<br>20·3<br>(11·5 to 33·8)	High body-mass index<br>18·8<br>(10·9 to 32·2)	Alcohol use<br>13·6<br>(7·2 to 23·0)	High total cholesterol<br>11·1<br>(4·2 to 18·7)	High fasting plasma glucose<br>9·1<br>(3·4 to 18·5)	High sodium<br>7·4<br>(0·6 to 19·5)	Low fruit<br>5·4<br>(2·9 to 9·5)	Low whole grains<br>5·3<br>(2·1 to 9·7)	Low nuts and seeds<br>5·3<br>(1·4 to 9·8)
Latvia	High body-mass index<br>31·2<br>(16·7 to 53·9)	High blood pressure<br>28·3<br>(9·8 to 55·3)	Tobacco<br>25·0<br>(11·9 to 48·9)	Alcohol use<br>20·1<br>(10·4 to 40·4)	High total cholesterol<br>19·3<br>(7·4 to 34·9)	Ambient particulate matter<br>13·5<br>(6·9 to 24·6)	Low fruit<br>11·5<br>(4·4 to 21·8)	High fasting plasma glucose<br>11·4<br>(3·1 to 23·7)	High sodium<br>10·4<br>(0·8 to 28·1)	Low whole grains<br>9·7<br>(3·0 to 20·3)
Lithuania	Tobacco<br>52·3<br>(30·4 to 94·5)	High blood pressure<br>46·5<br>(15·4 to 103·9)	High body-mass index<br>44·5<br>(20·7 to 93·6)	High total cholesterol<br>32·8<br>(10·2 to 68·4)	Alcohol use<br>31·9<br>(17·1 to 66·0)	Ambient particulate matter<br>22·5<br>(12·1 to 42·8)	High fasting plasma glucose<br>19·4<br>(7·1 to 47·1)	Low fruit<br>18·6<br>(8·8 to 39·9)	High sodium<br>16·3<br>(1·5 to 52·0)	Low whole grains<br>15·6<br>(5·0 to 32·2)
Moldova	High body-mass index<br>113·3<br>(59·4 to 225·3)	High blood pressure<br>106·9<br>(50·1 to 183·6)	Tobacco<br>104·3<br>(56·3 to 190·7)	Alcohol use<br>86·3<br>(44·6 to 163·3)	High total cholesterol<br>63·8<br>(29·3 to 116·1)	Ambient particulate matter<br>60·6<br>(31·1 to 106·9)	High fasting plasma glucose<br>45·6<br>(17·1 to 93·8)	Low fruit<br>40·5<br>(17·9 to 85·1)	Low whole grains<br>40·0<br>(16·8 to 81·9)	High sodium<br>37·5<br>(3·2 to 100·5)
Russia	High body-mass index<br>3,744·3<br>(1,921·5 to 8,024·5)	Tobacco<br>3,357·3<br>(1,515·8 to 7,042·0)	High blood pressure<br>3,144·1<br>(1,035·7 to 6,562·4)	Alcohol use<br>2,465·3<br>(1,174·1 to 4,662·0)	High total cholesterol<br>2,254·9<br>(822·6 to 5,084·8)	High fasting plasma glucose<br>1,744·9<br>(595·8 to 4,082·5)	Ambient particulate matter<br>1,635·6<br>(840·3 to 3,188·2)	Low whole grains<br>1,220·6<br>(385·7 to 2,904·2)	Low fruit<br>1,153·4<br>(465·3 to 2,552·2)	Low nuts and seeds<br>1,056·4<br>(427·5 to 2,238·5)
Ukraine	High body-mass index<br>1,373·2<br>(739·6 to 2,419·2)	High blood pressure<br>1,283·8<br>(549·4 to 2,169·5)	Tobacco<br>1,231·5<br>(595·9 to 2,098·5)	High total cholesterol<br>931·9<br>(395·9 to 1,582·4)	Alcohol use<br>837·1<br>(448·7 to 1,433·2)	Ambient particulate matter<br>708·1<br>(360·8 to 1,179·9)	High fasting plasma glucose<br>554·4<br>(196·8 to 1,026·8)	Low whole grains<br>477·6<br>(202·8 to 868·6)	High sodium<br>457·0<br>(36·7 to 1,100·3)	Low fruit<br>409·7<br>(175·0 to 736·0)
High-income	Tobacco<br>20,021·3<br>(15,278·0 to 27,635·0)	High body-mass index<br>12,308·1<br>(8,759·8 to 18,512·6)	High blood pressure<br>8,407·5<br>(5,059·5 to 14,788·0)	Alcohol use<br>8,178·6<br>(5,652·7 to 12,055·9)	High fasting plasma glucose<br>7,995·8<br>(4,067·5 to 14,850·4)	High total cholesterol<br>5,188·1<br>(2,594·9 to 8,854·4)	Ambient particulate matter<br>4,307·5<br>(2,969·3 to 6,338·7)	Low whole grains<br>3,145·8<br>(1,755·1 to 5,733·8)	High sodium<br>2,927·0<br>(587·9 to 6,903·6)	Low fruit<br>2,898·9<br>(1,663·9 to 4,766·6)
High-income Asia Pacific	Tobacco<br>2,193·7<br>(1,537·3 to 3,203·6)	High body-mass index<br>1,108·3<br>(527·9 to 1,911·3)	High blood pressure<br>1,060·5<br>(570·7 to 1,678·8)	High fasting plasma glucose<br>1,052·7<br>(522·9 to 1,895·7)	Alcohol use<br>811·8<br>(481·3 to 1,279·3)	High sodium<br>606·5<br>(282·3 to 1,075·6)	Ambient particulate matter<br>478·2<br>(313·8 to 736·2)	High total cholesterol<br>440·5<br>(215·8 to 789·1)	Impaired kidney function<br>348·8<br>(203·2 to 527·3)	Low whole grains<br>334·8<br>(168·9 to 597·8)
Brunei	Tobacco<br>11·2<br>(6·8 to 19·4)	High fasting plasma glucose<br>9·0<br>(3·0 to 17·9)	High body-mass index<br>6·7<br>(2·9 to 13·6)	High blood pressure<br>6·4<br>(2·6 to 12·2)	High total cholesterol<br>3·7<br>(1·3 to 7·2)	High sodium<br>3·2<br>(1·1 to 7·4)	Low whole grains<br>2·8<br>(1·0 to 5·9)	Impaired kidney function<br>2·3<br>(0·4 to 4·5)	Ambient particulate matter<br>2·2<br>(1·1 to 4·0)	Low fruit<br>2·0<br>(0·8 to 4·2)
Japan	Tobacco<br>1,201·0<br>(869·6 to 1,719·0)	High blood pressure<br>659·3<br>(287·5 to 1,163·2)	High body-mass index<br>639·5<br>(286·9 to 1,072·5)	High fasting plasma glucose<br>587·7<br>(240·5 to 1,120·9)	High sodium<br>419·7<br>(216·1 to 742·2)	Alcohol use<br>410·6<br>(220·6 to 668·6)	High total cholesterol<br>270·0<br>(117·3 to 526·4)	Ambient particulate matter<br>241·2<br>(160·5 to 370·4)	Impaired kidney function<br>229·2<br>(108·4 to 389·1)	Low whole grains<br>182·6<br>(71·6 to 369·3)
South Korea	Tobacco<br>942·7<br>(477·5 to 1,599·2)	High fasting plasma glucose<br>436·9<br>(209·9 to 832·3)	High body-mass index<br>426·8<br>(205·2 to 753·4)	Alcohol use<br>396·7<br>(231·3 to 663·9)	High blood pressure<br>342·0<br>(192·6 to 544·5)	Ambient particulate matter<br>213·9<br>(106·7 to 344·5)	High sodium<br>154·8<br>(34·6 to 318·9)	High total cholesterol<br>141·3<br>(72·7 to 249·4)	Low whole grains<br>138·6<br>(73·4 to 248·6)	Low fruit<br>137·5<br>(67·4 to 241·4)
Singapore	Tobacco<br>65·1<br>(36·8 to 105·9)	High blood pressure<br>43·7<br>(24·9 to 85·7)	High body-mass index<br>34·3<br>(19·4 to 63·2)	High fasting plasma glucose<br>29·0<br>(13·0 to 62·0)	Ambient particulate matter<br>24·9<br>(14·8 to 41·1)	High total cholesterol<br>23·4<br>(11·8 to 46·1)	High sodium<br>20·7<br>(7·6 to 43·6)	Occupational SHS<br>12·9<br>(8·0 to 21·3)	Low whole grains<br>12·3<br>(6·3 to 26·8)	Low nuts and seeds<br>12·3<br>(6·2 to 25·6)

Location	Risk #1	Risk #2	Risk #3	Risk #4	Risk #5	Risk #6	Risk #7	Risk #8	Risk #9	Risk #10
Australasia	Tobacco 393·3 (297·9 to 533·2)	High body-mass index 279·8 (201·4 to 420·2)	High blood pressure 200·0 (120·4 to 343·1)	Alcohol use 193·9 (127·8 to 299·3)	High fasting plasma glucose 143·8 (68·6 to 270·7)	High total cholesterol 127·3 (69·3 to 214·2)	Low whole grains 68·4 (39·7 to 133·0)	Low fruit 62·4 (36·5 to 103·8)	Low nuts and seeds 58·0 (30·1 to 107·0)	Ambient particulate matter 55·9 (34·2 to 89·2)
Australia	Tobacco 335·5 (252·2 to 464·4)	High body-mass index 232·8 (167·2 to 349·2)	High blood pressure 166·3 (97·7 to 285·3)	Alcohol use 164·6 (104·3 to 255·5)	High fasting plasma glucose 124·7 (59·9 to 234·7)	High total cholesterol 108·0 (58·7 to 182·0)	Low whole grains 60·5 (33·7 to 118·0)	Low fruit 52·0 (30·1 to 87·1)	Ambient particulate matter 47·5 (29·3 to 77·0)	Low nuts and seeds 47·2 (24·1 to 89·9)
New Zealand	Tobacco 58·5 (42·4 to 85·3)	High body-mass index 46·9 (34·0 to 68·8)	High blood pressure 33·6 (20·6 to 56·2)	Alcohol use 29·8 (18·4 to 46·2)	High total cholesterol 19·4 (11·0 to 31·3)	High fasting plasma glucose 19·4 (9·9 to 36·9)	Low nuts and seeds 10·8 (5·3 to 18·4)	Low fruit 10·5 (6·4 to 17·8)	High sodium 9·7 (0·0 to 29·0)	Ambient particulate matter 8·5 (5·1 to 13·4)
Western Europe	Tobacco 6,585·9 (5,169·1 to 8,626·2)	High body-mass index 4,349·6 (3,220·7 to 5,917·1)	Alcohol use 3,842·6 (2,881·5 to 5,199·4)	High blood pressure 3,005·1 (1,911·3 to 4,749·2)	High fasting plasma glucose 2,334·2 (1,214·9 to 4,105·0)	High total cholesterol 1,684·5 (940·7 to 2,784·0)	Ambient particulate matter 1,457·9 (1,082·9 to 1,994·3)	Low fruit 940·6 (576·3 to 1,414·5)	Low whole grains 930·8 (550·3 to 1,609·9)	Occupational asbestos 761·3 (507·7 to 1,106·2)
Andorra	Tobacco 1·2 (0·7 to 1·8)	Alcohol use 0·8 (0·5 to 1·2)	High body-mass index 0·7 (0·5 to 1·1)	High blood pressure 0·5 (0·2 to 0·9)	High fasting plasma glucose 0·4 (0·2 to 0·7)	High total cholesterol 0·3 (0·1 to 0·5)	Ambient particulate matter 0·3 (0·1 to 0·4)	Low whole grains 0·2 (0·1 to 0·3)	High sodium 0·2 (0·0 to 0·4)	Occupational asbestos 0·1 (0·1 to 0·2)
Austria	Tobacco 103·8 (73·0 to 141·1)	High body-mass index 102·2 (74·5 to 141·5)	Alcohol use 86·2 (62·0 to 128·8)	High blood pressure 71·0 (41·4 to 116·3)	High fasting plasma glucose 42·5 (20·3 to 74·3)	High total cholesterol 42·3 (21·7 to 66·6)	Ambient particulate matter 32·9 (21·6 to 47·2)	High sodium 22·3 (3·0 to 53·9)	Low whole grains 21·2 (11·9 to 36·9)	Low vegetables 19·6 (9·5 to 33·8)
Belgium	Tobacco 217·8 (160·0 to 295·7)	High body-mass index 118·7 (82·7 to 166·1)	Alcohol use 91·2 (60·5 to 123·7)	High blood pressure 72·5 (33·1 to 129·9)	High fasting plasma glucose 57·5 (23·5 to 106·7)	Ambient particulate matter 53·2 (36·0 to 71·5)	High total cholesterol 42·3 (21·0 to 71·5)	Low fruit 33·4 (21·5 to 49·8)	Occupational asbestos 28·4 (17·3 to 41·1)	Low whole grains 24·1 (12·1 to 45·8)
Cyprus	High body-mass index 11·9 (8·0 to 17·8)	Tobacco 11·9 (7·9 to 16·7)	High fasting plasma glucose 10·6 (5·8 to 17·7)	High blood pressure 9·2 (4·5 to 14·4)	Alcohol use 6·8 (4·2 to 10·3)	High total cholesterol 5·1 (2·3 to 8·3)	Ambient particulate matter 3·8 (2·5 to 5·3)	Low whole grains 3·1 (1·7 to 5·0)	Impaired kidney function 3·1 (1·8 to 4·8)	Low fruit 2·8 (1·7 to 4·2)
Denmark	Tobacco 132·4 (97·2 to 177·2)	Alcohol use 63·9 (43·9 to 87·2)	High body-mass index 51·3 (36·4 to 68·4)	High blood pressure 41·4 (23·8 to 67·3)	High fasting plasma glucose 32·2 (16·7 to 56·9)	Ambient particulate matter 21·5 (14·1 to 30·7)	High total cholesterol 19·5 (10·1 to 35·6)	Occupational asbestos 13·1 (8·2 to 18·8)	Low whole grains 11·0 (6·5 to 20·9)	Low vegetables 10·6 (5·6 to 19·1)
Finland	High body-mass index 57·7 (36·3 to 83·3)	Tobacco 56·5 (39·5 to 78·8)	Alcohol use 46·0 (29·8 to 63·9)	High blood pressure 45·7 (21·9 to 73·0)	High fasting plasma glucose 34·5 (14·2 to 65·0)	High total cholesterol 24·8 (11·2 to 38·6)	Low whole grains 13·6 (6·6 to 24·1)	Low vegetables 13·5 (6·5 to 25·1)	Low fruit 12·4 (6·5 to 20·1)	Ambient particulate matter 11·2 (6·9 to 17·9)
France	Tobacco 878·2 (660·8 to 1,152·6)	Alcohol use 689·8 (475·2 to 940·5)	High body-mass index 521·9 (368·1 to 718·1)	High blood pressure 310·1 (137·5 to 510·0)	High fasting plasma glucose 237·0 (99·5 to 449·4)	High total cholesterol 168·8 (75·3 to 295·3)	Ambient particulate matter 158·2 (106·1 to 216·9)	Low fruit 128·1 (79·1 to 182·1)	Occupational asbestos 122·7 (76·8 to 185·0)	Low whole grains 101·3 (46·1 to 163·5)
Germany	Tobacco 1,289·5 (939·6 to 1,812·5)	High body-mass index 976·6 (726·7 to 1,457·9)	Alcohol use 942·9 (695·7 to 1,292·4)	High blood pressure 746·6 (432·9 to 1,219·1)	High fasting plasma glucose 632·0 (324·6 to 1,133·5)	High total cholesterol 416·1 (209·7 to 756·0)	Ambient particulate matter 343·5 (234·1 to 486·1)	Low fruit 253·8 (162·1 to 377·9)	Low whole grains 223·0 (121·9 to 398·3)	Low nuts and seeds 209·4 (91·5 to 372·3)
Greece	Tobacco 202·6 (141·7 to 286·2)	High body-mass index 127·8 (84·0 to 199·4)	Alcohol use 105·7 (72·8 to 168·5)	High blood pressure 96·3 (47·1 to 168·8)	High fasting plasma glucose 78·3 (36·3 to 156·4)	High total cholesterol 61·8 (33·0 to 102·5)	Ambient particulate matter 50·0 (32·3 to 73·3)	Low whole grains 37·5 (19·3 to 67·9)	High sodium 34·2 (3·4 to 84·7)	Low nuts and seeds 23·6 (10·5 to 45·2)
Iceland	Tobacco 7·7 (5·9 to 10·4)	High body-mass index 3·3 (2·3 to 4·7)	High blood pressure 2·0 (0·8 to 4·0)	High fasting plasma glucose 1·5 (0·6 to 3·1)	High total cholesterol 1·5 (0·6 to 2·9)	Alcohol use 1·4 (0·8 to 2·4)	Ambient particulate matter 0·9 (0·6 to 1·4)	Low fruit 0·8 (0·4 to 1·4)	Low vegetables 0·8 (0·4 to 1·4)	Low whole grains 0·7 (0·3 to 1·4)
Ireland	Tobacco 92·5 (65·2 to 126·5)	High body-mass index 51·8 (32·1 to 76·0)	Alcohol use 50·1 (34·5 to 70·9)	High blood pressure 38·9 (20·7 to 64·1)	High fasting plasma glucose 31·5 (14·5 to 59·3)	High total cholesterol 22·6 (10·8 to 38·2)	Ambient particulate matter 17·2 (11·0 to 25·5)	Low fruit 16·9 (10·0 to 27·4)	Low whole grains 12·3 (6·3 to 21·6)	High sodium 11·2 (1·0 to 28·2)
Israel	Tobacco 103·2 (58·3 to 178·9)	High body-mass index 87·3 (45·5 to 147·3)	High fasting plasma glucose 74·2 (27·5 to 158·7)	High blood pressure 65·7 (28·0 to 123·1)	High total cholesterol 32·2 (12·3 to 62·1)	Impaired kidney function 30·0 (11·9 to 62·6)	Ambient particulate matter 27·7 (14·9 to 49·1)	Low whole grains 20·2 (8·6 to 40·2)	High sodium 18·6 (2·2 to 49·6)	Alcohol use 15·6 (6·9 to 28·9)
Italy	Tobacco 941·1 (672·4 to 1,262·2)	High body-mass index 636·6 (457·2 to 892·9)	Alcohol use 525·5 (367·4 to 755·8)	High blood pressure 401·3 (242·4 to 626·4)	High fasting plasma glucose 257·0 (116·6 to 482·9)	Ambient particulate matter 193·1 (138·8 to 265·4)	High total cholesterol 190·4 (105·9 to 303·1)	Occupational asbestos 141·0 (83·3 to 218·3)	High sodium 127·3 (11·2 to 316·2)	Low whole grains 116·2 (64·0 to 203·0)
Luxembourg	Tobacco 9·8 (6·4 to 14·0)	Alcohol use 6·2 (4·0 to 9·2)	High body-mass index 5·6 (3·4 to 8·2)	High blood pressure 4·9 (2·6 to 8·2)	High fasting plasma glucose 3·4 (1·5 to 6·8)	Ambient particulate matter 2·5 (1·6 to 3·7)	High total cholesterol 2·3 (1·2 to 3·7)	High sodium 1·4 (0·1 to 3·5)	Low whole grains 1·4 (0·7 to 2·3)	Low vegetables 1·2 (0·6 to 2·2)
Malta	Tobacco 5·6 (3·2 to 10·2)	High body-mass index 5·4 (3·2 to 9·2)	High blood pressure 5·0 (2·5 to 9·1)	High fasting plasma glucose 3·5 (1·6 to 6·7)	High total cholesterol 3·0 (1·5 to 5·7)	Ambient particulate matter 1·8 (0·8 to 3·2)	Alcohol use 1·7 (0·9 to 3·5)	Low nuts and seeds 1·5 (0·7 to 3·0)	Low fruit 1·5 (0·8 to 2·9)	Low whole grains 1·4 (0·7 to 3·1)
Netherlands	Tobacco 361·2 (278·4 to 470·3)	High body-mass index 154·5 (107·1 to 226·6)	Alcohol use 115·6 (77·5 to 182·9)	High fasting plasma glucose 111·0 (51·3 to 221·1)	High blood pressure 107·5 (62·2 to 185·5)	Ambient particulate matter 71·6 (50·2 to 98·9)	High total cholesterol 54·9 (27·3 to 94·5)	Occupational asbestos 43·5 (28·1 to 60·7)	Low fruit 38·9 (21·6 to 59·7)	Low whole grains 35·8 (18·9 to 67·5)
Norway	Tobacco 96·6 (72·3 to 132·3)	High body-mass index 42·3 (27·9 to 62·0)	High blood pressure 35·8 (19·2 to 62·6)	High fasting plasma glucose 31·8 (13·9 to 59·5)	Alcohol use 30·5 (19·5 to 45·4)	High total cholesterol 23·2 (11·3 to 41·9)	Ambient particulate matter 15·9 (9·8 to 24·1)	Low fruit 11·1 (5·7 to 19·7)	Low vegetables 11·0 (5·5 to 19·1)	Low nuts and seeds 10·5 (4·6 to 18·2)
Portugal	Alcohol use 121·3 (77·7 to 194·1)	Tobacco 106·9 (73·0 to 163·3)	High body-mass index 106·6 (66·6 to 164·9)	High blood pressure 74·5 (39·8 to 133·4)	High fasting plasma glucose 67·5 (32·7 to 126·2)	High total cholesterol 35·4 (18·4 to 64·2)	Ambient particulate matter 27·4 (15·7 to 53·2)	Low whole grains 25·2 (12·4 to 49·6)	Low fruit 22·1 (10·7 to 41·0)	High sodium 19·6 (2·1 to 60·7)
Spain	Tobacco 538·8 (396·9 to 765·3)	High body-mass index 427·3 (290·9 to 623·3)	Alcohol use 396·8 (262·2 to 553·4)	High blood pressure 266·4 (133·6 to 441·3)	High fasting plasma glucose 235·5 (111·7 to 451·1)	High total cholesterol 140·2 (68·1 to 240·6)	Ambient particulate matter 109·2 (71·7 to 162·7)	Impaired kidney function 73·2 (38·1 to 112·6)	Low whole grains 67·1 (34·8 to 119·2)	Low nuts and seeds 57·2 (28·4 to 108·9)

Location	Risk #1	Risk #2	Risk #3	Risk #4	Risk #5	Risk #6	Risk #7	Risk #8	Risk #9	Risk #10
Sweden	Tobacco 149·2 (112·1 to 199·1)	High body-mass index 89·1 (59·5 to 127·3)	High blood pressure 70·5 (35·8 to 124·6)	Alcohol use 66·1 (39·4 to 99·0)	High fasting plasma glucose 53·4 (21·0 to 118·2)	High total cholesterol 42·5 (21·2 to 72·0)	Low fruit 23·9 (12·5 to 39·7)	Low vegetables 22·2 (11·5 to 38·2)	Low nuts and seeds 21·9 (10·2 to 38·0)	High sodium 21·5 (3·3 to 50·9)
Switzerland	Tobacco 109·0 (68·4 to 167·5)	High body-mass index 74·8 (45·1 to 113·8)	Alcohol use 65·6 (41·4 to 98·0)	High blood pressure 49·7 (29·3 to 85·1)	High fasting plasma glucose 42·2 (18·5 to 78·1)	High total cholesterol 31·6 (14·7 to 55·3)	Ambient particulate matter 23·1 (13·8 to 34·7)	Low fruit 17·9 (10·4 to 30·0)	High sodium 16·9 (1·9 to 43·0)	Low whole grains 15·7 (7·8 to 30·2)
United Kingdom	Tobacco 1,205·6 (917·7 to 1,596·7)	High body-mass index 687·6 (491·2 to 977·0)	High blood pressure 486·4 (278·7 to 819·3)	Alcohol use 433·6 (279·2 to 632·1)	High total cholesterol 324·7 (173·9 to 534·8)	High fasting plasma glucose 313·6 (142·2 to 606·1)	Ambient particulate matter 279·5 (201·7 to 409·1)	Low fruit 199·0 (121·8 to 307·2)	Low whole grains 169·8 (92·6 to 311·7)	Occupational asbestos 140·4 (94·1 to 192·7)
Southern Latin America	Tobacco 1,249·1 (924·5 to 1,753·8)	High body-mass index 907·3 (651·4 to 1,340·0)	High blood pressure 723·9 (412·2 to 1,210·4)	Alcohol use 588·1 (353·7 to 981·3)	High fasting plasma glucose 465·8 (224·6 to 829·4)	Ambient particulate matter 393·2 (278·6 to 533·8)	High total cholesterol 371·4 (206·3 to 578·0)	Low whole grains 274·6 (153·2 to 462·1)	High sodium 250·1 (10·5 to 646·8)	Low fruit 208·8 (95·5 to 354·3)
Argentina	Tobacco 963·6 (715·5 to 1,293·9)	High body-mass index 686·5 (476·3 to 1,000·6)	High blood pressure 542·1 (305·8 to 911·6)	Alcohol use 443·8 (247·0 to 773·8)	High fasting plasma glucose 350·9 (164·6 to 602·3)	Ambient particulate matter 299·7 (208·3 to 414·5)	High total cholesterol 284·5 (147·7 to 452·2)	Low whole grains 204·6 (108·9 to 339·8)	High sodium 176·7 (5·7 to 460·9)	Low vegetables 156·0 (81·0 to 296·4)
Chile	Tobacco 225·5 (134·1 to 379·6)	High body-mass index 181·1 (113·6 to 287·0)	High blood pressure 148·9 (72·0 to 272·3)	Alcohol use 117·6 (65·8 to 203·1)	High fasting plasma glucose 98·8 (44·2 to 190·4)	Ambient particulate matter 76·2 (50·8 to 120·7)	High total cholesterol 69·3 (33·3 to 118·8)	High sodium 61·1 (3·7 to 161·2)	Low whole grains 56·5 (27·9 to 106·1)	Low fruit 51·5 (26·7 to 101·3)
Uruguay	Tobacco 65·8 (50·7 to 87·7)	High body-mass index 40·9 (28·1 to 56·5)	High blood pressure 34·2 (20·7 to 50·7)	Alcohol use 29·5 (16·9 to 46·1)	Ambient particulate matter 18·0 (13·4 to 24·1)	High total cholesterol 17·9 (10·8 to 25·5)	High fasting plasma glucose 17·3 (8·3 to 30·9)	Low fruit 17·2 (10·3 to 26·0)	Low whole grains 13·3 (8·0 to 22·0)	High sodium 12·5 (0·7 to 28·8)
High-income North America	Tobacco 9,799·2 (6,995·0 to 14,358·5)	High body-mass index 5,684·0 (3,603·1 to 9,186·8)	High fasting plasma glucose 4,102·5 (1,860·3 to 8,331·8)	High blood pressure 3,390·1 (1,743·9 to 6,284·1)	Alcohol use 2,673·9 (1,544·6 to 4,130·0)	High total cholesterol 2,594·3 (1,101·7 to 4,925·4)	Ambient particulate matter 1,916·0 (1,102·5 to 3,090·5)	Low whole grains 1,545·6 (743·7 to 3,053·0)	Low fruit 1,383·0 (763·5 to 2,560·5)	High sodium 1,289·6 (205·5 to 3,181·1)
Canada	Tobacco 685·7 (509·5 to 893·9)	High body-mass index 411·7 (285·4 to 577·7)	High fasting plasma glucose 289·0 (127·8 to 546·4)	High blood pressure 259·0 (132·2 to 402·6)	Alcohol use 211·6 (148·7 to 292·5)	High total cholesterol 174·9 (87·5 to 279·1)	Ambient particulate matter 109·3 (75·0 to 154·1)	Low whole grains 103·0 (51·1 to 184·4)	High sodium 100·4 (15·5 to 223·1)	Low nuts and seeds 88·9 (38·0 to 153·1)
Greenland	Tobacco 1·1 (0·6 to 2·1)	High body-mass index 1·0 (0·6 to 1·8)	High fasting plasma glucose 0·6 (0·3 to 1·3)	High blood pressure 0·6 (0·3 to 1·2)	Alcohol use 0·5 (0·2 to 1·1)	High total cholesterol 0·4 (0·2 to 0·8)	Low whole grains 0·3 (0·1 to 0·6)	High sodium 0·3 (0·1 to 0·8)	Low fruit 0·3 (0·1 to 0·6)	Low vegetables 0·2 (0·1 to 0·4)
United States	Tobacco 9,133·0 (6,371·0 to 13,544·5)	High body-mass index 5,271·1 (3,254·0 to 8,623·9)	High fasting plasma glucose 3,825·8 (1,717·7 to 7,903·3)	High blood pressure 3,128·3 (1,569·6 to 5,857·7)	Alcohol use 2,463·9 (1,355·4 to 3,867·8)	High total cholesterol 2,417·5 (1,010·8 to 4,626·6)	Ambient particulate matter 1,810·3 (1,014·6 to 2,936·9)	Low whole grains 1,441·7 (686·2 to 2,855·7)	Low fruit 1,307·1 (706·5 to 2,446·2)	High sodium 1,188·1 (191·8 to 2,980·3)
Latin America and Caribbean	High body-mass index 9,088·1 (6,700·3 to 13,729·2)	Tobacco 8,829·7 (6,571·4 to 12,284·7)	High blood pressure 6,808·3 (4,581·6 to 10,660·0)	High fasting plasma glucose 5,580·3 (3,584·1 to 9,055·4)	Alcohol use 5,264·2 (3,721·0 to 7,178·1)	Ambient particulate matter 3,826·8 (2,703·5 to 5,385·8)	High total cholesterol 3,759·4 (2,269·7 to 6,084·2)	Low vegetables 2,388·3 (1,345·1 to 4,102·7)	Impaired kidney function 2,212·1 (1,500·8 to 3,487·8)	Low nuts and seeds 1,981·9 (1,092·4 to 3,168·0)
Caribbean	High body-mass index 743·2 (516·1 to 1,136·3)	Tobacco 737·6 (516·5 to 1,048·2)	High blood pressure 638·7 (408·8 to 907·9)	High fasting plasma glucose 520·9 (297·2 to 834·4)	Ambient particulate matter 393·3 (280·2 to 534·6)	High total cholesterol 344·0 (195·1 to 498·9)	Alcohol use 289·6 (187·2 to 445·1)	Low vegetables 235·8 (130·6 to 420·1)	Household air pollution 201·1 (131·4 to 290·2)	Low fruit 189·1 (95·2 to 335·9)
Antigua and Barbuda	High body-mass index 1·7 (1·1 to 2·8)	High fasting plasma glucose 1·4 (0·8 to 2·4)	High blood pressure 1·2 (0·7 to 2·0)	High total cholesterol 0·5 (0·3 to 0·9)	Impaired kidney function 0·5 (0·3 to 0·8)	Ambient particulate matter 0·5 (0·3 to 0·7)	Tobacco 0·4 (0·3 to 0·7)	Low vegetables 0·4 (0·2 to 0·6)	Low fruit 0·4 (0·2 to 0·6)	Low whole grains 0·3 (0·2 to 0·7)
The Bahamas	High body-mass index 9·3 (5·7 to 16·1)	High blood pressure 5·6 (2·7 to 10·9)	High fasting plasma glucose 5·5 (2·6 to 10·7)	Alcohol use 5·2 (2·4 to 9·7)	Tobacco 4·9 (2·9 to 8·5)	High total cholesterol 2·5 (1·3 to 4·8)	Ambient particulate matter 2·5 (1·5 to 4·1)	Impaired kidney function 1·8 (0·7 to 3·6)	Low whole grains 1·6 (0·7 to 3·3)	Occupational SHS 1·2 (0·7 to 2·3)
Barbados	High body-mass index 5·5 (3·6 to 7·8)	High fasting plasma glucose 3·4 (1·3 to 5·7)	High blood pressure 2·7 (1·0 to 4·5)	Alcohol use 1·6 (0·9 to 2·8)	Tobacco 1·4 (0·9 to 2·0)	Ambient particulate matter 1·3 (0·7 to 1·9)	High total cholesterol 1·1 (0·5 to 2·0)	Impaired kidney function 0·9 (0·4 to 1·6)	Low whole grains 0·9 (0·4 to 1·6)	Low vegetables 0·9 (0·4 to 1·4)
Belize	High body-mass index 9·4 (5·7 to 16·1)	High fasting plasma glucose 7·5 (4·0 to 14·2)	Tobacco 6·4 (3·8 to 10·7)	High blood pressure 6·3 (3·3 to 11·4)	Alcohol use 4·9 (2·6 to 9·1)	Ambient particulate matter 4·0 (2·5 to 6·4)	High total cholesterol 3·3 (1·6 to 5·6)	Impaired kidney function 2·4 (1·3 to 4·2)	Low vegetables 2·3 (1·1 to 4·7)	Low fruit 2·2 (1·0 to 4·2)
Bermuda	High body-mass index 0·9 (0·5 to 1·6)	Tobacco 0·8 (0·5 to 1·3)	Alcohol use 0·6 (0·3 to 1·2)	High blood pressure 0·6 (0·3 to 1·1)	High fasting plasma glucose 0·6 (0·3 to 1·1)	High total cholesterol 0·3 (0·2 to 0·7)	Ambient particulate matter 0·2 (0·1 to 0·4)	Low vegetables 0·2 (0·1 to 0·3)	Low nuts and seeds 0·2 (0·1 to 0·3)	Occupational SHS 0·2 (0·1 to 0·3)
Cuba	Tobacco 304·3 (225·0 to 404·9)	High body-mass index 150·0 (100·1 to 217·8)	High blood pressure 113·3 (66·3 to 166·9)	High fasting plasma glucose 85·2 (40·6 to 152·9)	Ambient particulate matter 84·8 (58·7 to 108·5)	High total cholesterol 59·1 (32·0 to 92·5)	Alcohol use 43·9 (25·6 to 73·9)	Low vegetables 40·1 (18·0 to 77·8)	Low omega-3 33·7 (9·1 to 66·3)	Low nuts and seeds 32·8 (15·0 to 53·6)
Dominica	High body-mass index 1·3 (0·8 to 2·2)	High fasting plasma glucose 1·1 (0·6 to 2·0)	High blood pressure 1·0 (0·5 to 1·7)	Tobacco 0·8 (0·5 to 1·3)	Alcohol use 0·7 (0·4 to 1·2)	Ambient particulate matter 0·4 (0·2 to 0·7)	Impaired kidney function 0·4 (0·2 to 0·6)	High total cholesterol 0·4 (0·2 to 0·6)	Low fruit 0·3 (0·1 to 0·5)	Low vegetables 0·3 (0·1 to 0·5)
Dominican Republic	High blood pressure 140·1 (78·7 to 241·4)	Tobacco 131·0 (78·3 to 213·8)	High body-mass index 127·9 (70·0 to 249·2)	High fasting plasma glucose 85·9 (43·6 to 155·8)	High total cholesterol 75·7 (41·4 to 139·9)	Ambient particulate matter 64·9 (40·9 to 105·0)	Alcohol use 64·1 (35·5 to 110·2)	Low vegetables 53·3 (27·4 to 101·5)	Short gestation 42·2 (20·4 to 82·7)	Low nuts and seeds 41·3 (19·8 to 80·8)
Grenada	High body-mass index 2·5 (1·7 to 3·8)	High fasting plasma glucose 2·3 (1·4 to 3·6)	High blood pressure 2·0 (1·3 to 3·0)	Tobacco 1·5 (1·0 to 2·2)	Alcohol use 1·3 (0·8 to 2·2)	Ambient particulate matter 1·0 (0·6 to 1·5)	High total cholesterol 0·9 (0·5 to 1·5)	Low fruit 0·8 (0·4 to 1·3)	Impaired kidney function 0·7 (0·4 to 1·1)	Low vegetables 0·7 (0·4 to 1·1)
Guyana	High body-mass index 20·0 (11·9 to 29·2)	High fasting plasma glucose 18·1 (9·2 to 27·3)	High blood pressure 16·7 (8·9 to 24·6)	Tobacco 11·2 (6·0 to 18·2)	Alcohol use 10·2 (5·8 to 17·1)	High total cholesterol 8·9 (4·2 to 14·3)	Ambient particulate matter 8·7 (5·1 to 13·8)	Low fruit 7·4 (3·5 to 13·5)	Low vegetables 6·9 (3·3 to 11·5)	Low nuts and seeds 4·7 (2·2 to 7·3)

Location	Risk #1	Risk #2	Risk #3	Risk #4	Risk #5	Risk #6	Risk #7	Risk #8	Risk #9	Risk #10
Haiti	High body-mass index<br>278·9<br>(168·1 to 425·8)	High blood pressure<br>253·5<br>(146·2 to 375·3)	High fasting plasma glucose<br>212·9<br>(109·6 to 354·3)	Tobacco<br>198·5<br>(119·8 to 311·8)	Household air pollution<br>177·8<br>(109·4 to 270·9)	Ambient particulate matter<br>176·4<br>(117·0 to 248·0)	High total cholesterol<br>141·3<br>(74·2 to 210·4)	Alcohol use<br>117·2<br>(67·3 to 190·8)	Low vegetables<br>95·7<br>(47·6 to 165·0)	Low fruit<br>94·4<br>(45·8 to 175·0)
Jamaica	High body-mass index<br>44·5<br>(29·7 to 63·3)	Tobacco<br>30·5<br>(19·2 to 45·2)	High fasting plasma glucose<br>28·5<br>(11·5 to 47·9)	High blood pressure<br>26·2<br>(13·8 to 42·1)	Ambient particulate matter<br>15·1<br>(9·8 to 22·0)	Alcohol use<br>12·6<br>(6·4 to 22·6)	High total cholesterol<br>10·7<br>(5·8 to 16·4)	Low vegetables<br>9·6<br>(4·9 to 16·9)	Low whole grains<br>8·5<br>(3·1 to 16·8)	Occupational SHS<br>7·9<br>(5·0 to 12·0)
Puerto Rico	High body-mass index<br>42·6<br>(27·5 to 64·4)	High fasting plasma glucose<br>34·5<br>(15·2 to 65·6)	Tobacco<br>28·6<br>(19·2 to 42·5)	High blood pressure<br>25·3<br>(13·3 to 44·9)	Alcohol use<br>15·8<br>(8·1 to 27·2)	High total cholesterol<br>13·5<br>(6·5 to 24·2)	Ambient particulate matter<br>12·5<br>(6·8 to 22·1)	Low fruit<br>11·8<br>(6·1 to 18·9)	Impaired kidney function<br>9·9<br>(4·3 to 18·8)	Low vegetables<br>8·5<br>(4·3 to 14·9)
Saint Lucia	High body-mass index<br>4·1<br>(2·7 to 6·2)	High fasting plasma glucose<br>3·1<br>(1·6 to 5·2)	High blood pressure<br>2·7<br>(1·5 to 4·4)	Alcohol use<br>2·1<br>(1·2 to 3·1)	Tobacco<br>1·8<br>(1·1 to 2·7)	Ambient particulate matter<br>1·1<br>(0·7 to 1·6)	Impaired kidney function<br>1·0<br>(0·5 to 1·7)	High total cholesterol<br>0·9<br>(0·5 to 1·4)	Low fruit<br>0·8<br>(0·4 to 1·4)	Low vegetables<br>0·7<br>(0·4 to 1·2)
Saint Vincent and the Grenadines	High body-mass index<br>2·6<br>(1·9 to 3·7)	High fasting plasma glucose<br>2·3<br>(1·4 to 3·4)	High blood pressure<br>1·9<br>(1·2 to 2·8)	Tobacco<br>1·4<br>(0·9 to 2·1)	High total cholesterol<br>0·9<br>(0·5 to 1·4)	Ambient particulate matter<br>0·9<br>(0·6 to 1·3)	Alcohol use<br>0·8<br>(0·5 to 1·3)	Low vegetables<br>0·7<br>(0·4 to 1·2)	Low fruit<br>0·6<br>(0·3 to 1·1)	Impaired kidney function<br>0·6<br>(0·3 to 0·8)
Suriname	High body-mass index<br>10·5<br>(6·4 to 14·2)	High blood pressure<br>8·0<br>(4·9 to 11·9)	Tobacco<br>8·0<br>(5·0 to 10·9)	High fasting plasma glucose<br>6·8<br>(2·9 to 11·4)	Alcohol use<br>5·6<br>(3·5 to 8·6)	Ambient particulate matter<br>5·3<br>(3·5 to 7·2)	Low fruit<br>4·4<br>(2·2 to 7·2)	High total cholesterol<br>4·0<br>(2·0 to 6·0)	Low vegetables<br>3·2<br>(1·6 to 5·0)	Short gestation<br>2·7<br>(1·2 to 4·9)
Trinidad and Tobago	High body-mass index<br>32·4<br>(21·3 to 47·7)	High fasting plasma glucose<br>31·2<br>(16·0 to 53·7)	High blood pressure<br>25·1<br>(13·2 to 40·2)	Tobacco<br>19·7<br>(11·7 to 30·5)	High total cholesterol<br>14·3<br>(7·4 to 24·4)	Ambient particulate matter<br>11·3<br>(7·1 to 17·0)	Low fruit<br>10·2<br>(5·3 to 16·9)	Low nuts and seeds<br>8·8<br>(4·1 to 14·8)	Low vegetables<br>8·8<br>(4·3 to 15·3)	Impaired kidney function<br>7·3<br>(4·2 to 10·5)
Virgin Islands, U.S.	High body-mass index<br>1·5<br>(1·0 to 2·1)	Tobacco<br>1·0<br>(0·7 to 1·4)	High blood pressure<br>0·9<br>(0·5 to 1·5)	High fasting plasma glucose<br>0·9<br>(0·4 to 1·6)	Alcohol use<br>0·6<br>(0·3 to 0·9)	High total cholesterol<br>0·6<br>(0·3 to 0·9)	Ambient particulate matter<br>0·5<br>(0·3 to 0·7)	Low vegetables<br>0·3<br>(0·2 to 0·5)	Low whole grains<br>0·3<br>(0·2 to 0·6)	Low nuts and seeds<br>0·3<br>(0·1 to 0·5)
Andean Latin America	High body-mass index<br>692·4<br>(461·7 to 1,066·3)	Alcohol use<br>420·9<br>(258·0 to 589·7)	Tobacco<br>399·4<br>(257·5 to 603·7)	High blood pressure<br>355·5<br>(197·8 to 571·4)	Ambient particulate matter<br>323·9<br>(199·8 to 450·4)	High fasting plasma glucose<br>298·5<br>(160·1 to 488·2)	High total cholesterol<br>237·6<br>(127·8 to 391·0)	Short gestation<br>163·5<br>(110·3 to 235·7)	Household air pollution<br>155·8<br>(94·6 to 223·5)	Low vegetables<br>155·4<br>(72·6 to 290·3)
Bolivia	High body-mass index<br>185·0<br>(122·6 to 298·6)	Tobacco<br>122·9<br>(72·7 to 196·1)	Ambient particulate matter<br>103·5<br>(65·7 to 152·4)	High blood pressure<br>98·0<br>(60·7 to 157·2)	High fasting plasma glucose<br>96·3<br>(51·8 to 209·8)	Short gestation<br>85·2<br>(51·7 to 124·7)	High total cholesterol<br>69·5<br>(37·3 to 115·4)	Low birth weight<br>60·2<br>(37·4 to 87·5)	Alcohol use<br>55·9<br>(26·2 to 113·4)	Low fruit<br>55·5<br>(30·6 to 115·4)
Ecuador	High body-mass index<br>228·6<br>(158·3 to 329·4)	Alcohol use<br>127·7<br>(87·3 to 191·9)	High blood pressure<br>108·0<br>(60·1 to 172·1)	Tobacco<br>98·4<br>(63·6 to 146·7)	High fasting plasma glucose<br>95·6<br>(52·9 to 164·2)	Ambient particulate matter<br>68·0<br>(45·4 to 109·6)	High total cholesterol<br>64·9<br>(35·0 to 107·9)	Impaired kidney function<br>45·2<br>(25·3 to 74·0)	Low vegetables<br>42·4<br>(21·4 to 77·2)	Occupational SHS<br>35·0<br>(22·5 to 52·5)
Peru	High body-mass index<br>278·2<br>(158·2 to 438·3)	Alcohol use<br>241·9<br>(122·6 to 363·5)	Tobacco<br>183·9<br>(94·0 to 307·5)	Ambient particulate matter<br>153·7<br>(77·3 to 214·8)	High blood pressure<br>149·8<br>(62·2 to 267·9)	High fasting plasma glucose<br>109·9<br>(45·0 to 200·2)	High total cholesterol<br>103·2<br>(41·0 to 181·8)	Household air pollution<br>100·2<br>(52·5 to 162·4)	Low fruit<br>69·9<br>(30·8 to 130·9)	Low vegetables<br>69·1<br>(25·7 to 139·9)
Central Latin America	High body-mass index<br>4,482·0<br>(3,149·0 to 6,441·7)	High fasting plasma glucose<br>3,017·3<br>(1,764·6 to 5,057·9)	Tobacco<br>2,808·5<br>(1,977·7 to 4,049·2)	High blood pressure<br>2,761·7<br>(1,780·1 to 4,428·4)	Alcohol use<br>2,170·1<br>(1,535·9 to 3,262·7)	Ambient particulate matter<br>1,545·1<br>(1,092·1 to 2,161·2)	High total cholesterol<br>1,537·6<br>(935·0 to 2,485·4)	Impaired kidney function<br>1,365·5<br>(841·4 to 2,152·2)	Low vegetables<br>933·5<br>(522·5 to 1,528·0)	Low nuts and seeds<br>852·9<br>(479·3 to 1,380·1)
Colombia	Tobacco<br>624·4<br>(405·9 to 958·1)	High body-mass index<br>553·6<br>(344·4 to 918·0)	High blood pressure<br>467·3<br>(249·4 to 839·7)	Alcohol use<br>298·2<br>(163·7 to 551·0)	Ambient particulate matter<br>252·1<br>(166·2 to 388·8)	High fasting plasma glucose<br>251·1<br>(115·2 to 513·4)	High total cholesterol<br>234·2<br>(108·6 to 418·2)	Low vegetables<br>159·6<br>(84·4 to 268·9)	Low fruit<br>139·7<br>(64·5 to 265·6)	Low nuts and seeds<br>136·5<br>(64·9 to 255·2)
Costa Rica	High body-mass index<br>51·1<br>(31·0 to 79·7)	High blood pressure<br>44·1<br>(22·8 to 78·3)	High fasting plasma glucose<br>31·2<br>(14·0 to 62·1)	Alcohol use<br>28·9<br>(18·4 to 45·2)	Tobacco<br>28·6<br>(17·2 to 46·8)	High total cholesterol<br>23·0<br>(11·6 to 42·0)	Impaired kidney function<br>17·8<br>(8·5 to 33·4)	Ambient particulate matter<br>16·8<br>(10·2 to 26·5)	Low vegetables<br>13·7<br>(7·4 to 23·2)	Low nuts and seeds<br>10·9<br>(4·6 to 19·8)
El Salvador	High body-mass index<br>78·1<br>(50·2 to 119·3)	High blood pressure<br>54·6<br>(32·4 to 83·6)	High fasting plasma glucose<br>52·3<br>(27·3 to 94·7)	Tobacco<br>37·3<br>(21·3 to 60·8)	Alcohol use<br>34·4<br>(21·4 to 55·3)	High total cholesterol<br>33·5<br>(17·3 to 54·9)	Ambient particulate matter<br>31·5<br>(20·6 to 47·4)	Impaired kidney function<br>31·4<br>(16·9 to 56·6)	Low vegetables<br>20·8<br>(11·3 to 33·9)	Low fruit<br>19·4<br>(10·5 to 34·2)
Guatemala	High body-mass index<br>268·2<br>(157·4 to 427·6)	High fasting plasma glucose<br>220·3<br>(110·6 to 376·1)	Alcohol use<br>173·8<br>(105·6 to 255·0)	Ambient particulate matter<br>166·1<br>(100·1 to 271·0)	High blood pressure<br>156·6<br>(93·3 to 243·5)	Household air pollution<br>143·0<br>(58·4 to 277·2)	Tobacco<br>122·6<br>(68·7 to 202·5)	High total cholesterol<br>102·8<br>(52·9 to 152·0)	Impaired kidney function<br>92·6<br>(51·1 to 154·8)	Low fruit<br>73·9<br>(35·4 to 127·9)
Honduras	High body-mass index<br>174·0<br>(118·8 to 286·9)	High blood pressure<br>147·4<br>(87·7 to 234·3)	High fasting plasma glucose<br>114·8<br>(59·6 to 224·4)	Tobacco<br>110·2<br>(58·2 to 183·7)	High total cholesterol<br>107·1<br>(59·0 to 166·4)	Ambient particulate matter<br>82·1<br>(52·5 to 117·9)	Impaired kidney function<br>65·4<br>(39·6 to 125·5)	Low vegetables<br>58·6<br>(26·5 to 97·5)	Alcohol use<br>53·0<br>(33·1 to 88·5)	Household air pollution<br>52·6<br>(32·1 to 85·3)
Mexico	High body-mass index<br>2,509·1<br>(1,708·6 to 3,541·3)	High fasting plasma glucose<br>1,874·6<br>(1,008·0 to 3,054·3)	High blood pressure<br>1,258·3<br>(717·4 to 2,070·8)	Tobacco<br>1,209·3<br>(831·0 to 1,770·7)	Alcohol use<br>1,096·1<br>(739·7 to 1,583·5)	Impaired kidney function<br>828·1<br>(409·5 to 1,387·4)	High total cholesterol<br>707·7<br>(385·4 to 1,158·8)	Ambient particulate matter<br>657·6<br>(486·6 to 925·7)	Low nuts and seeds<br>388·3<br>(192·4 to 605·2)	Low vegetables<br>382·0<br>(209·2 to 637·9)
Nicaragua	High body-mass index<br>100·3<br>(59·0 to 159·9)	High blood pressure<br>70·2<br>(38·0 to 113·9)	High fasting plasma glucose<br>64·7<br>(31·2 to 116·3)	Tobacco<br>41·6<br>(22·4 to 73·8)	Impaired kidney function<br>39·2<br>(19·3 to 72·7)	Alcohol use<br>38·0<br>(24·6 to 58·6)	Ambient particulate matter<br>30·2<br>(18·1 to 48·3)	High total cholesterol<br>29·3<br>(14·7 to 48·1)	Low fruit<br>25·8<br>(14·1 to 46·2)	Household air pollution<br>23·6<br>(8·9 to 39·6)
Panama	High blood pressure<br>44·6<br>(25·1 to 64·3)	High body-mass index<br>43·3<br>(25·5 to 67·2)	High fasting plasma glucose<br>38·6<br>(20·2 to 68·9)	Tobacco<br>31·6<br>(20·9 to 48·1)	Alcohol use<br>28·9<br>(16·7 to 43·7)	High total cholesterol<br>21·2<br>(12·4 to 32·3)	Ambient particulate matter<br>19·2<br>(12·7 to 26·7)	Impaired kidney function<br>17·9<br>(9·5 to 30·7)	Low fruit<br>16·7<br>(8·1 to 28·4)	Low vegetables<br>13·7<br>(7·2 to 23·7)
Venezuela	High body-mass index<br>693·5<br>(392·1 to 1,216·7)	Tobacco<br>614·4<br>(379·4 to 1,014·8)	High blood pressure<br>521·7<br>(259·5 to 951·0)	Alcohol use<br>435·8<br>(242·5 to 758·1)	High fasting plasma glucose<br>389·7<br>(139·2 to 753·2)	Ambient particulate matter<br>296·0<br>(180·2 to 489·5)	High total cholesterol<br>274·3<br>(129·5 to 497·4)	Low vegetables<br>201·5<br>(104·0 to 353·4)	Low nuts and seeds<br>159·5<br>(72·4 to 294·6)	Impaired kidney function<br>151·5<br>(54·0 to 279·1)

Location	Risk #1	Risk #2	Risk #3	Risk #4	Risk #5	Risk #6	Risk #7	Risk #8	Risk #9	Risk #10
Tropical Latin America	Tobacco 4,937·2 (3,689·4 to 6,690·4)	High body-mass index 3,160·2 (1,929·8 to 5,098·7)	High blood pressure 3,054·3 (1,785·3 to 4,955·6)	Alcohol use 2,410·0 (1,483·7 to 3,653·0)	High fasting plasma glucose 1,737·9 (887·8 to 3,166·5)	High total cholesterol 1,632·9 (959·4 to 2,661·7)	Ambient particulate matter 1,558·8 (1,101·5 to 2,221·8)	Low vegetables 1,063·1 (621·2 to 1,948·3)	Low fruit 875·8 (370·9 to 1,573·4)	Low nuts and seeds 816·2 (406·3 to 1,380·4)
Brazil	Tobacco 4,797·7 (3,564·2 to 6,474·6)	High body-mass index 3,028·6 (1,804·1 to 4,896·9)	High blood pressure 2,940·4 (1,698·0 to 4,824·0)	Alcohol use 2,330·9 (1,438·8 to 3,529·7)	High fasting plasma glucose 1,660·1 (831·1 to 3,044·1)	High total cholesterol 1,573·6 (923·7 to 2,573·5)	Ambient particulate matter 1,499·6 (1,057·4 to 2,145·2)	Low vegetables 1,025·1 (604·5 to 1,873·1)	Low fruit 827·6 (347·7 to 1,489·9)	Low nuts and seeds 780·6 (384·6 to 1,329·9)
Paraguay	Tobacco 148·0 (88·7 to 225·2)	High body-mass index 132·5 (75·7 to 203·0)	High blood pressure 113·2 (63·5 to 176·0)	Alcohol use 83·8 (41·2 to 160·7)	High fasting plasma glucose 80·7 (44·2 to 141·7)	Ambient particulate matter 63·7 (38·3 to 90·2)	High total cholesterol 58·6 (29·6 to 96·1)	Low fruit 51·3 (23·2 to 99·1)	Low vegetables 37·7 (15·5 to 84·0)	Household air pollution 37·4 (20·7 to 59·2)
North Africa and Middle East	High body-mass index 15,106·0 (10,156·5 to 22,423·8)	High blood pressure 12,378·6 (7,757·3 to 17,946·5)	Tobacco 10,925·8 (6,857·6 to 15,646·4)	High fasting plasma glucose 9,961·5 (5,770·4 to 15,302·7)	High total cholesterol 8,291·0 (4,715·4 to 11,475·0)	Ambient particulate matter 8,264·7 (5,648·5 to 11,053·8)	Short gestation 5,312·5 (3,318·4 to 8,335·4)	Low whole grains 4,995·3 (3,042·2 to 8,041·8)	Low nuts and seeds 4,272·3 (2,158·3 to 6,720·9)	Low fruit 3,857·4 (2,012·0 to 6,283·3)
North Africa and Middle East	High body-mass index 15,106·0 (10,156·5 to 22,423·8)	High blood pressure 12,378·6 (7,757·3 to 17,946·5)	Tobacco 10,925·8 (6,857·6 to 15,646·4)	High fasting plasma glucose 9,961·5 (5,770·4 to 15,302·7)	High total cholesterol 8,291·0 (4,715·4 to 11,475·0)	Ambient particulate matter 8,264·7 (5,648·5 to 11,053·8)	Short gestation 5,312·5 (3,318·4 to 8,335·4)	Low whole grains 4,995·3 (3,042·2 to 8,041·8)	Low nuts and seeds 4,272·3 (2,158·3 to 6,720·9)	Low fruit 3,857·4 (2,012·0 to 6,283·3)
Afghanistan	Short gestation 2,176·2 (1,272·3 to 3,416·2)	Household air pollution 2,091·0 (1,214·0 to 2,981·0)	Ambient particulate matter 1,926·2 (1,287·1 to 2,647·3)	High body-mass index 1,780·1 (1,003·9 to 2,620·8)	Child wasting 1,524·3 (529·2 to 2,609·5)	High blood pressure 1,391·2 (741·9 to 2,220·3)	Hib vaccination 1,336·3 (392·2 to 2,882·0)	Tobacco 1,221·8 (633·7 to 1,999·7)	High fasting plasma glucose 1,219·1 (566·6 to 2,079·4)	Low birth weight 1,154·2 (676·4 to 1,700·3)
Algeria	High body-mass index 612·4 (339·3 to 975·6)	High blood pressure 527·0 (278·5 to 852·6)	Tobacco 497·2 (278·0 to 826·5)	High fasting plasma glucose 450·5 (202·7 to 847·0)	High total cholesterol 338·9 (173·4 to 522·9)	Ambient particulate matter 329·8 (212·6 to 482·2)	Low whole grains 247·4 (126·5 to 444·4)	Low fruit 212·1 (98·1 to 360·1)	Low nuts and seeds 208·0 (100·3 to 340·0)	Impaired kidney function 163·2 (86·3 to 241·4)
Bahrain	High body-mass index 37·1 (19·3 to 70·3)	High fasting plasma glucose 31·3 (15·2 to 59·6)	High blood pressure 22·2 (10·6 to 44·8)	Tobacco 20·8 (9·9 to 42·5)	High total cholesterol 17·1 (6·5 to 35·5)	Ambient particulate matter 12·6 (5·9 to 25·2)	Low whole grains 11·0 (4·7 to 25·9)	Impaired kidney function 7·5 (4·1 to 12·3)	Low nuts and seeds 7·3 (2·7 to 18·5)	Low omega-3 5·8 (1·0 to 15·5)
Egypt	High body-mass index 2,979·7 (1,870·2 to 4,598·2)	High blood pressure 2,044·3 (1,132·1 to 3,105·9)	High fasting plasma glucose 1,817·9 (795·1 to 2,844·5)	Tobacco 1,682·1 (876·7 to 2,622·8)	High total cholesterol 1,384·9 (709·9 to 2,086·1)	Ambient particulate matter 1,144·8 (649·7 to 1,703·1)	Low whole grains 792·2 (433·0 to 1,368·7)	Low nuts and seeds 750·7 (373·5 to 1,256·5)	Low omega-3 609·3 (153·8 to 1,137·6)	Impaired kidney function 606·3 (362·6 to 911·0)
Iran	High body-mass index 1,608·6 (876·8 to 2,880·3)	High blood pressure 1,172·9 (497·3 to 2,095·7)	Tobacco 1,142·0 (593·6 to 1,835·0)	High total cholesterol 1,079·7 (491·5 to 1,787·3)	High fasting plasma glucose 988·1 (333·9 to 1,984·8)	Ambient particulate matter 755·3 (403·0 to 1,135·3)	Low whole grains 568·5 (259·7 to 1,038·5)	Low nuts and seeds 432·7 (175·1 to 851·1)	Low omega-3 390·5 (87·4 to 881·4)	High sodium 325·2 (15·7 to 964·1)
Iraq	High body-mass index 1,635·6 (1,097·0 to 2,515·0)	High blood pressure 1,346·5 (850·7 to 2,002·3)	High fasting plasma glucose 1,187·4 (721·5 to 1,920·9)	Tobacco 1,081·6 (635·1 to 1,680·0)	High total cholesterol 850·5 (494·8 to 1,271·7)	Ambient particulate matter 676·4 (458·8 to 978·8)	Low whole grains 537·2 (283·2 to 965·7)	Low fruit 528·7 (333·7 to 884·3)	Short gestation 439·6 (139·5 to 805·5)	Low nuts and seeds 436·3 (205·3 to 710·7)
Jordan	High body-mass index 145·6 (79·9 to 240·0)	High fasting plasma glucose 112·8 (56·5 to 211·2)	High blood pressure 101·5 (53·1 to 180·3)	Tobacco 85·4 (41·6 to 164·5)	High total cholesterol 66·4 (34·4 to 117·6)	Ambient particulate matter 49·2 (29·0 to 85·7)	Impaired kidney function 41·6 (20·2 to 76·0)	Low whole grains 37·3 (18·6 to 68·0)	Low fruit 32·1 (14·6 to 60·7)	Low nuts and seeds 31·4 (14·1 to 62·0)
Kuwait	High body-mass index 93·2 (41·7 to 212·5)	High fasting plasma glucose 70·6 (24·6 to 165·2)	High blood pressure 69·5 (28·2 to 154·7)	High total cholesterol 50·5 (18·9 to 123·6)	Tobacco 48·8 (19·0 to 118·2)	Ambient particulate matter 33·5 (13·9 to 71·9)	Low whole grains 27·7 (10·5 to 70·5)	Low nuts and seeds 21·9 (7·0 to 60·5)	Impaired kidney function 21·5 (9·1 to 45·6)	Low fruit 16·7 (5·0 to 45·7)
Lebanon	High body-mass index 73·8 (41·9 to 142·2)	Tobacco 68·9 (35·3 to 128·3)	High blood pressure 55·0 (24·2 to 110·7)	High fasting plasma glucose 54·2 (23·1 to 112·7)	High total cholesterol 45·4 (17·7 to 94·4)	Ambient particulate matter 27·6 (14·4 to 54·3)	Low whole grains 23·6 (10·3 to 53·2)	Low omega-3 14·8 (2·6 to 45·3)	Impaired kidney function 14·5 (7·7 to 24·6)	High sodium 13·3 (0·7 to 40·8)
Libya	High body-mass index 154·8 (94·2 to 221·9)	High fasting plasma glucose 115·9 (57·8 to 198·1)	High blood pressure 115·9 (64·3 to 170·1)	Tobacco 86·0 (47·6 to 127·9)	High total cholesterol 63·2 (32·2 to 95·0)	Ambient particulate matter 60·8 (37·3 to 83·7)	Impaired kidney function 45·7 (22·7 to 82·8)	Low whole grains 44·2 (21·0 to 68·7)	Low nuts and seeds 31·7 (15·7 to 52·6)	Low omega-3 26·3 (6·1 to 49·8)
Morocco	High blood pressure 779·8 (488·8 to 1,142·8)	High body-mass index 691·8 (461·6 to 1,055·0)	High fasting plasma glucose 673·4 (410·6 to 1,077·7)	Tobacco 532·4 (325·4 to 794·6)	High total cholesterol 458·4 (251·7 to 720·0)	Ambient particulate matter 338·1 (222·4 to 500·9)	Low nuts and seeds 268·5 (135·0 to 443·0)	Low whole grains 256·7 (151·0 to 433·3)	Low omega-3 212·8 (53·8 to 436·1)	Impaired kidney function 200·8 (138·5 to 297·8)
Palestine	High body-mass index 151·0 (74·8 to 266·2)	Tobacco 107·7 (53·3 to 186·8)	High blood pressure 95·0 (42·8 to 159·2)	High fasting plasma glucose 90·9 (35·8 to 168·6)	High total cholesterol 83·8 (33·8 to 144·4)	Ambient particulate matter 64·8 (32·4 to 109·4)	Low whole grains 47·8 (19·3 to 88·9)	Low fruit 43·5 (18·8 to 77·5)	Low vegetables 40·8 (17·2 to 75·8)	Low nuts and seeds 39·7 (14·3 to 77·5)
Oman	High fasting plasma glucose 131·2 (64·7 to 244·0)	High blood pressure 130·6 (62·5 to 229·2)	High body-mass index 129·6 (69·7 to 213·7)	High total cholesterol 83·1 (37·6 to 150·7)	Tobacco 68·9 (32·8 to 114·7)	Ambient particulate matter 61·8 (32·2 to 100·6)	Low whole grains 53·4 (26·6 to 102·8)	Low nuts and seeds 44·6 (20·7 to 80·3)	Low omega-3 29·9 (5·9 to 68·9)	Impaired kidney function 29·0 (16·5 to 43·8)
Qatar	High body-mass index 40·1 (19·5 to 80·7)	High fasting plasma glucose 31·0 (11·8 to 82·1)	High blood pressure 24·5 (9·5 to 60·4)	Tobacco 21·5 (7·9 to 48·8)	High total cholesterol 17·3 (5·6 to 45·1)	Ambient particulate matter 14·0 (5·1 to 30·8)	Low whole grains 11·5 (4·0 to 29·1)	Impaired kidney function 7·8 (3·2 to 18·6)	Alcohol use 6·9 (2·8 to 16·3)	High sodium 5·6 (0·2 to 18·4)
Saudi Arabia	High body-mass index 545·1 (240·5 to 946·3)	High blood pressure 487·4 (210·0 to 880·3)	High fasting plasma glucose 458·7 (176·9 to 845·7)	Tobacco 263·6 (120·6 to 486·1)	High total cholesterol 262·0 (105·2 to 482·1)	Ambient particulate matter 245·9 (108·9 to 458·9)	Impaired kidney function 211·0 (99·4 to 355·1)	Low nuts and seeds 165·1 (65·7 to 309·4)	Low whole grains 159·3 (65·6 to 335·5)	Low fruit 129·8 (50·4 to 262·8)
Sudan	Short gestation 1,262·5 (603·8 to 2,711·9)	High blood pressure 1,189·4 (736·4 to 1,668·5)	High body-mass index 1,125·9 (674·5 to 1,643·8)	Low birth weight 953·1 (446·6 to 2,031·4)	Ambient particulate matter 862·5 (504·0 to 1,318·6)	High fasting plasma glucose 718·7 (359·5 to 1,094·0)	Household air pollution 705·3 (411·2 to 1,122·8)	Child wasting 663·9 (128·8 to 1,637·5)	High total cholesterol 637·0 (347·3 to 993·8)	Tobacco 634·7 (337·8 to 1,046·5)
Syria	High body-mass index 515·2 (285·3 to 888·0)	High blood pressure 431·2 (222·8 to 745·5)	Tobacco 379·3 (184·5 to 676·4)	High total cholesterol 326·2 (150·2 to 573·6)	High fasting plasma glucose 257·6 (114·7 to 501·3)	Ambient particulate matter 235·4 (124·6 to 393·0)	Low whole grains 198·7 (92·9 to 348·6)	Low vegetables 166·0 (73·3 to 291·2)	Low nuts and seeds 160·6 (60·9 to 314·8)	Low fruit 139·9 (56·7 to 289·8)
Tunisia	Tobacco 200·0 (119·6 to 312·0)	High body-mass index 163·6 (106·7 to 269·2)	High blood pressure 144·4 (84·8 to 214·3)	High fasting plasma glucose 135·4 (75·3 to 232·9)	High total cholesterol 98·0 (48·8 to 154·9)	Ambient particulate matter 92·2 (57·9 to 127·5)	Low whole grains 59·8 (31·9 to 101·3)	Impaired kidney function 51·6 (27·6 to 76·8)	Low fruit 49·8 (26·3 to 74·7)	Low nuts and seeds 48·7 (22·7 to 82·9)

Location	Risk #1	Risk #2	Risk #3	Risk #4	Risk #5	Risk #6	Risk #7	Risk #8	Risk #9	Risk #10
Turkey	Tobacco 1,973·4 (1,216·1 to 3,053·8)	High body-mass index 1,453·3 (947·7 to 2,303·9)	High blood pressure 1,250·5 (710·3 to 2,057·5)	High fasting plasma glucose 984·5 (505·0 to 1,713·1)	High total cholesterol 689·4 (337·7 to 1,193·1)	Ambient particulate matter 682·4 (449·5 to 1,078·3)	Low whole grains 430·2 (225·5 to 739·4)	Impaired kidney function 418·7 (257·0 to 683·8)	Low nuts and seeds 316·5 (152·9 to 739·4)	Low omega-3 294·2 (76·6 to 731·2)
United Arab Emirates	High body-mass index 206·5 (122·8 to 312·6)	High fasting plasma glucose 165·1 (89·2 to 286·6)	High blood pressure 148·7 (85·6 to 232·4)	Tobacco 114·3 (62·0 to 185·2)	High total cholesterol 104·0 (52·4 to 162·3)	Ambient particulate matter 85·4 (51·9 to 128·4)	Low whole grains 71·3 (35·0 to 113·3)	Alcohol use 70·5 (43·2 to 126·2)	Impaired kidney function 55·0 (33·9 to 84·7)	Low vegetables 41·1 (19·2 to 84·3)
Yemen	High body-mass index 926·6 (548·7 to 1,426·7)	Tobacco 835·3 (458·0 to 1,253·2)	High blood pressure 815·0 (502·6 to 1,248·1)	Short gestation 653·5 (198·4 to 1,357·9)	High total cholesterol 641·1 (371·0 to 959·7)	Ambient particulate matter 589·0 (363·3 to 831·1)	Low birth weight 511·0 (161·7 to 1,072·2)	High fasting plasma glucose 451·4 (242·4 to 763·7)	Low whole grains 429·0 (234·9 to 723·6)	Low fruit 360·8 (189·0 to 612·1)
South Asia	High blood pressure 44,674·2 (24,369·6 to 68,424·6)	Tobacco 39,681·6 (22,418·4 to 64,380·3)	Ambient particulate matter 30,776·2 (17,965·5 to 45,300·2)	High fasting plasma glucose 28,444·0 (15,239·6 to 50,946·9)	High body-mass index 27,798·4 (15,324·6 to 49,985·1)	Household air pollution 25,745·8 (15,984·8 to 37,850·9)	High total cholesterol 25,488·6 (13,489·6 to 41,297·1)	Low fruit 19,684·4 (9,769·1 to 34,391·7)	Low vegetables 17,762·8 (8,625·6 to 29,536·1)	Low nuts and seeds 15,303·1 (7,915·6 to 27,059·9)
South Asia	High blood pressure 44,674·2 (24,369·6 to 68,424·6)	Tobacco 39,681·6 (22,418·4 to 64,380·3)	Ambient particulate matter 30,776·2 (17,965·5 to 45,300·2)	High fasting plasma glucose 28,444·0 (15,239·6 to 50,946·9)	High body-mass index 27,798·4 (15,324·6 to 49,985·1)	Household air pollution 25,745·8 (15,984·8 to 37,850·9)	High total cholesterol 25,488·6 (13,489·6 to 41,297·1)	Low fruit 19,684·4 (9,769·1 to 34,391·7)	Low vegetables 17,762·8 (8,625·6 to 29,536·1)	Low nuts and seeds 15,303·1 (7,915·6 to 27,059·9)
Bangladesh	Tobacco 3,872·3 (2,454·0 to 5,862·1)	High blood pressure 2,738·1 (1,604·8 to 4,347·6)	Household air pollution 2,249·0 (1,440·1 to 3,328·6)	Ambient particulate matter 2,148·9 (1,488·1 to 3,216·4)	High fasting plasma glucose 1,964·8 (1,103·9 to 3,187·6)	Low fruit 1,573·6 (941·1 to 2,529·4)	High body-mass index 1,557·1 (734·3 to 2,585·4)	Low whole grains 1,314·0 (783·6 to 2,246·2)	High total cholesterol 1,236·7 (769·2 to 1,904·7)	Low vegetables 1,216·8 (617·2 to 2,060·7)
Bhutan	High blood pressure 13·4 (7·7 to 20·4)	High body-mass index 10·2 (5·9 to 17·0)	High total cholesterol 9·0 (4·9 to 13·9)	High fasting plasma glucose 8·4 (4·4 to 13·8)	Ambient particulate matter 8·2 (5·2 to 12·0)	Tobacco 7·9 (4·1 to 13·8)	Low whole grains 6·1 (3·4 to 11·0)	Low fruit 5·9 (3·3 to 9·8)	Low vegetables 5·4 (2·9 to 9·6)	Low nuts and seeds 5·1 (2·5 to 8·2)
India	High blood pressure 36,549·1 (19,086·8 to 57,054·8)	Tobacco 29,654·0 (15,885·7 to 51,226·0)	Ambient particulate matter 24,933·3 (13,375·9 to 37,935·1)	High fasting plasma glucose 22,972·6 (11,604·5 to 43,124·1)	High total cholesterol 21,284·8 (10,114·6 to 36,442·4)	High body-mass index 21,203·3 (10,886·7 to 40,421·5)	Household air pollution 20,160·8 (11,675·8 to 31,355·6)	Low fruit 15,605·1 (7,340·7 to 27,989·5)	Low vegetables 14,500·8 (6,607·1 to 24,563·9)	Low nuts and seeds 12,854·9 (6,254·0 to 24,383·2)
Nepal	Tobacco 741·7 (434·9 to 1,142·0)	High blood pressure 611·6 (376·5 to 875·1)	Household air pollution 477·2 (312·2 to 700·6)	Ambient particulate matter 413·7 (283·3 to 584·6)	High body-mass index 356·3 (197·1 to 597·2)	High fasting plasma glucose 320·5 (173·1 to 512·6)	High total cholesterol 316·7 (182·6 to 461·1)	Low whole grains 264·4 (159·1 to 442·8)	Low fruit 257·9 (146·1 to 404·1)	Low nuts and seeds 214·3 (117·3 to 334·9)
Pakistan	Tobacco 5,243·6 (3,038·1 to 7,865·8)	High blood pressure 4,822·3 (3,142·2 to 6,605·2)	High body-mass index 4,651·1 (2,840·5 to 6,846·1)	Short gestation 3,634·7 (1,595·4 to 6,659·7)	High fasting plasma glucose 3,404·5 (2,171·3 to 5,081·6)	Ambient particulate matter 3,284·9 (2,314·1 to 4,368·3)	High total cholesterol 2,782·7 (1,617·5 to 3,916·5)	Household air pollution 2,779·0 (1,809·6 to 3,872·7)	Low whole grains 2,283·4 (1,365·7 to 3,263·5)	Low fruit 2,211·1 (1,265·3 to 3,589·1)
Sub-Saharan Africa	Short gestation 28,334·8 (19,057·1 to 42,880·9)	Child wasting 21,494·9 (10,638·5 to 32,598·2)	Household air pollution 18,796·7 (12,425·5 to 27,163·8)	Ambient particulate matter 17,663·6 (11,995·8 to 23,848·2)	High blood pressure 13,326·1 (8,129·3 to 20,918·7)	Low birth weight 13,035·9 (9,688·3 to 17,492·8)	Alcohol use 12,794·1 (8,893·9 to 18,978·4)	High body-mass index 12,212·6 (7,191·5 to 20,520·3)	Tobacco 10,789·1 (7,192·5 to 16,197·0)	Handwashing 9,853·9 (5,220·8 to 15,170·3)
Central Sub-Saharan Africa	Short gestation 4,114·3 (2,378·5 to 6,525·8)	Child wasting 3,353·6 (1,409·5 to 6,102·8)	Household air pollution 2,520·4 (1,405·8 to 3,803·0)	Low birth weight 2,442·6 (1,465·5 to 3,400·4)	Ambient particulate matter 2,385·5 (1,568·9 to 3,466·2)	High body-mass index 1,614·8 (859·9 to 2,769·1)	High blood pressure 1,607·2 (808·6 to 2,692·5)	Alcohol use 1,412·7 (679·5 to 2,638·3)	Tobacco 1,306·2 (753·4 to 2,194·0)	Handwashing 1,246·0 (588·5 to 2,126·9)
Angola	Short gestation 988·3 (606·6 to 1,775·8)	Child wasting 780·5 (272·6 to 1,645·8)	Ambient particulate matter 530·5 (322·5 to 888·2)	Low birth weight 502·4 (300·5 to 806·9)	Household air pollution 475·0 (141·0 to 1,086·1)	High blood pressure 429·3 (235·6 to 767·0)	High body-mass index 413·2 (222·0 to 720·4)	Alcohol use 389·0 (166·8 to 757·3)	Handwashing 358·6 (166·5 to 651·1)	Unsafe water 354·4 (135·9 to 732·8)
Central African Republic	Alcohol use 217·3 (68·0 to 450·2)	Child wasting 216·1 (62·2 to 410·3)	Short gestation 177·8 (85·1 to 301·7)	Household air pollution 155·0 (77·8 to 245·6)	Ambient particulate matter 146·2 (83·8 to 223·2)	Low birth weight 109·0 (56·3 to 176·1)	High body-mass index 105·0 (47·1 to 171·1)	High blood pressure 99·3 (36·7 to 181·2)	Tobacco 87·2 (37·5 to 159·1)	High fasting plasma glucose 77·1 (30·0 to 146·7)
Congo	High body-mass index 78·2 (38·2 to 142·7)	High blood pressure 71·4 (32·2 to 133·2)	Household air pollution 64·8 (30·1 to 116·7)	Short gestation 63·8 (33·3 to 117·8)	Ambient particulate matter 63·2 (36·1 to 109·1)	Alcohol use 58·7 (22·2 to 117·9)	Tobacco 52·3 (25·9 to 96·7)	High fasting plasma glucose 46·9 (21·8 to 92·5)	Child wasting 42·7 (10·6 to 115·6)	Low birth weight 39·6 (22·0 to 69·6)
Democratic Republic of the Congo	Short gestation 2,844·6 (1,417·5 to 4,767·0)	Child wasting 2,331·5 (935·3 to 4,240·1)	Household air pollution 1,869·2 (952·8 to 2,954·4)	Low birth weight 1,768·4 (933·4 to 2,651·8)	Ambient particulate matter 1,637·5 (963·8 to 2,391·6)	High blood pressure 975·7 (384·0 to 1,792·3)	High body-mass index 968·2 (456·8 to 1,631·9)	Tobacco 813·4 (389·0 to 1,507·4)	Child underweight 807·0 (377·0 to 1,540·6)	Handwashing 788·3 (380·4 to 1,409·3)
Equatorial Guinea	Short gestation 29·9 (12·1 to 69·6)	Low birth weight 16·9 (7·6 to 34·6)	Alcohol use 13·4 (6·1 to 26·8)	High body-mass index 10·6 (6·1 to 17·3)	Ambient particulate matter 10·3 (5·7 to 17·6)	Household air pollution 8·9 (1·1 to 19·9)	High blood pressure 8·8 (4·4 to 14·1)	High fasting plasma glucose 7·6 (3·9 to 12·8)	Child wasting 6·9 (2·0 to 16·9)	Tobacco 6·1 (2·6 to 12·2)
Gabon	High body-mass index 25·5 (13·5 to 45·1)	Alcohol use 25·5 (14·4 to 43·8)	High blood pressure 20·9 (10·3 to 41·3)	Ambient particulate matter 17·3 (11·0 to 26·9)	Tobacco 16·3 (8·3 to 30·4)	High fasting plasma glucose 16·2 (7·9 to 29·8)	Short gestation 14·7 (6·7 to 26·8)	Low birth weight 9·2 (4·6 to 16·4)	Low whole grains 8·6 (3·9 to 17·8)	High total cholesterol 8·3 (4·0 to 15·3)
Eastern Sub-Saharan Africa	Short gestation 11,590·6 (8,147·2 to 17,403·7)	Household air pollution 7,999·9 (5,173·0 to 11,174·8)	Child wasting 7,662·2 (4,091·9 to 12,157·8)	Ambient particulate matter 6,577·5 (4,383·1 to 9,157·6)	High blood pressure 6,005·3 (3,596·2 to 9,182·7)	Alcohol use 5,348·0 (3,555·1 to 7,492·8)	Low birth weight 4,670·5 (3,577·0 to 6,455·9)	High body-mass index 4,594·5 (2,472·7 to 7,920·0)	Tobacco 4,221·5 (2,592·4 to 6,519·5)	Handwashing 3,925·5 (2,072·0 to 6,256·3)
Burundi	Alcohol use 428·2 (240·0 to 863·8)	Household air pollution 265·1 (139·2 to 392·3)	Child wasting 241·0 (117·3 to 424·1)	Ambient particulate matter 236·2 (146·0 to 337·3)	Short gestation 214·6 (108·3 to 420·2)	High blood pressure 189·3 (85·9 to 347·4)	High body-mass index 137·8 (56·1 to 267·1)	Tobacco 131·2 (68·9 to 221·8)	Handwashing 109·8 (51·5 to 197·6)	Child stunting 101·0 (38·3 to 246·0)
Comoros	High blood pressure 10·8 (5·9 to 16·9)	Short gestation 10·6 (5·7 to 19·4)	Household air pollution 8·2 (4·3 to 14·8)	Tobacco 8·2 (4·9 to 14·0)	Ambient particulate matter 8·1 (4·9 to 13·2)	Unsafe water 7·4 (1·9 to 18·2)	High body-mass index 7·3 (3·8 to 13·2)	Unsafe sanitation 6·9 (2·8 to 14·3)	Child wasting 6·7 (2·9 to 12·0)	Handwashing 6·1 (2·4 to 11·8)
Djibouti	Tobacco 15·5 (8·1 to 24·5)	Ambient particulate matter 13·8 (8·6 to 21·4)	High blood pressure 13·3 (7·4 to 21·9)	Child wasting 13·1 (5·6 to 23·5)	Short gestation 11·6 (4·4 to 22·7)	High body-mass index 10·7 (5·7 to 17·1)	High fasting plasma glucose 6·5 (3·5 to 10·6)	High total cholesterol 6·1 (3·3 to 10·6)	Household air pollution 6·0 (0·3 to 20·2)	Low fruit 5·9 (3·0 to 11·3)
Eritrea	Child wasting 110·1 (47·2 to 196·8)	Household air pollution 104·9 (17·0 to 214·1)	Ambient particulate matter 103·3 (61·7 to 163·3)	Short gestation 95·1 (45·1 to 156·5)	High blood pressure 70·3 (34·6 to 127·4)	Tobacco 64·3 (35·5 to 109·4)	Unsafe water 62·3 (19·5 to 126·1)	Handwashing 60·8 (26·2 to 108·8)	Unsafe sanitation 58·9 (25·1 to 108·4)	High body-mass index 57·8 (27·5 to 93·5)

Location	Risk #1	Risk #2	Risk #3	Risk #4	Risk #5	Risk #6	Risk #7	Risk #8	Risk #9	Risk #10
Ethiopia	Short gestation 3,411·0 (2,396·6 to 4,938·3)	Household air pollution 2,277·8 (1,446·0 to 3,703·5)	Ambient particulate matter 1,830·6 (1,226·5 to 2,639·2)	High blood pressure 1,743·7 (1,036·1 to 3,035·4)	Child wasting 1,702·1 (550·9 to 3,223·9)	Alcohol use 1,195·0 (519·6 to 2,194·7)	Low birth weight 1,158·9 (830·9 to 1,658·2)	High body-mass index 1,046·0 (489·6 to 2,029·4)	Handwashing 1,027·3 (482·1 to 1,663·9)	High fasting plasma glucose 1,015·4 (597·3 to 1,960·0)
Kenya	Short gestation 777·7 (477·1 to 1,389·1)	Unsafe water 752·0 (155·7 to 1,809·6)	Unsafe sanitation 722·3 (298·0 to 1,649·9)	Handwashing 639·0 (271·2 to 1,220·4)	Child wasting 635·7 (242·7 to 1,395·3)	Household air pollution 550·8 (297·3 to 935·8)	Ambient particulate matter 444·5 (242·9 to 829·4)	High blood pressure 367·0 (204·0 to 601·1)	Tobacco 353·6 (200·1 to 607·0)	High body-mass index 304·2 (161·7 to 518·1)
Madagascar	Short gestation 659·4 (374·3 to 1,020·5)	Child wasting 652·1 (415·2 to 943·1)	High blood pressure 515·6 (272·3 to 846·0)	Household air pollution 504·1 (287·7 to 760·8)	Ambient particulate matter 455·4 (327·1 to 584·1)	Tobacco 393·3 (222·3 to 637·0)	High body-mass index 360·8 (166·1 to 645·7)	Child underweight 323·9 (227·3 to 477·9)	Low birth weight 315·0 (178·7 to 469·3)	Low whole grains 231·4 (120·3 to 377·1)
Malawi	Short gestation 543·1 (355·8 to 810·2)	Child wasting 357·8 (150·8 to 718·9)	Household air pollution 321·8 (163·6 to 533·1)	Ambient particulate matter 273·3 (151·9 to 452·2)	Low birth weight 272·8 (198·5 to 391·1)	High blood pressure 221·6 (93·5 to 472·0)	High body-mass index 193·5 (89·2 to 420·6)	Tobacco 191·9 (97·9 to 405·1)	Alcohol use 156·2 (56·1 to 385·8)	Handwashing 151·9 (73·1 to 296·9)
Mozambique	Short gestation 1,079·7 (601·7 to 1,648·8)	Household air pollution 672·0 (419·5 to 1,112·0)	High blood pressure 565·3 (314·7 to 969·9)	Child wasting 514·4 (258·6 to 943·2)	Low birth weight 467·7 (304·2 to 665·3)	Ambient particulate matter 429·1 (274·9 to 739·1)	Tobacco 386·0 (206·4 to 639·1)	High body-mass index 375·6 (199·4 to 623·8)	Alcohol use 320·2 (116·9 to 653·9)	Handwashing 251·8 (131·9 to 432·6)
Rwanda	Alcohol use 294·8 (97·9 to 775·4)	Short gestation 253·6 (155·5 to 363·4)	Household air pollution 216·5 (111·5 to 365·0)	Ambient particulate matter 189·0 (101·1 to 311·6)	Child wasting 187·6 (84·5 to 382·8)	High blood pressure 154·2 (60·2 to 336·7)	High body-mass index 118·3 (40·5 to 262·4)	Low birth weight 113·4 (79·1 to 162·2)	High fasting plasma glucose 102·1 (44·9 to 217·6)	Tobacco 95·0 (42·4 to 195·5)
Somalia	Short gestation 577·2 (360·3 to 910·3)	Child wasting 523·9 (281·2 to 799·4)	Household air pollution 461·3 (282·8 to 665·4)	Ambient particulate matter 342·3 (202·0 to 475·4)	High body-mass index 270·8 (146·9 to 434·4)	High blood pressure 256·2 (148·2 to 420·6)	PCV vaccination 238·1 (130·3 to 338·4)	Low birth weight 233·4 (145·2 to 362·3)	Child underweight 226·4 (124·5 to 365·9)	Tobacco 219·0 (123·4 to 355·4)
South Sudan	Child wasting 921·0 (598·5 to 1,375·1)	Short gestation 902·9 (567·1 to 1,406·0)	Household air pollution 636·7 (440·1 to 888·4)	Ambient particulate matter 495·9 (355·0 to 699·6)	Child underweight 373·8 (240·0 to 607·3)	PCV vaccination 357·9 (230·0 to 546·4)	Low birth weight 341·0 (191·6 to 571·0)	Hib vaccination 328·3 (108·5 to 665·7)	High body-mass index 293·4 (194·3 to 487·6)	Handwashing 249·5 (140·9 to 353·6)
Tanzania	Short gestation 1,195·5 (771·2 to 1,895·7)	Alcohol use 1,014·4 (435·7 to 1,893·9)	Household air pollution 970·9 (596·6 to 1,546·7)	High blood pressure 838·7 (457·3 to 1,363·5)	Child wasting 823·7 (334·7 to 1,625·6)	High body-mass index 757·8 (445·7 to 1,252·2)	Ambient particulate matter 743·8 (493·9 to 1,217·4)	Tobacco 713·7 (398·0 to 1,185·1)	Low birth weight 482·3 (343·7 to 724·8)	Unsafe sanitation 463·1 (225·7 to 923·4)
Uganda	Short gestation 1,488·6 (689·6 to 2,569·6)	Alcohol use 1,278·4 (519·1 to 2,529·6)	Child wasting 885·0 (387·6 to 1,636·4)	Household air pollution 826·8 (469·9 to 1,245·5)	Ambient particulate matter 786·5 (506·1 to 1,106·2)	Low birth weight 689·0 (332·4 to 1,123·0)	High blood pressure 645·9 (386·2 to 1,104·2)	High body-mass index 542·5 (275·7 to 938·8)	Tobacco 538·4 (291·9 to 892·9)	High fasting plasma glucose 406·3 (240·2 to 715·0)
Zambia	Short gestation 409·1 (235·7 to 744·2)	Child wasting 274·8 (135·3 to 547·2)	Ambient particulate matter 274·4 (150·1 to 432·3)	Alcohol use 265·9 (79·0 to 696·4)	Household air pollution 265·7 (124·1 to 433·5)	Tobacco 225·9 (86·0 to 456·0)	High blood pressure 210·2 (89·4 to 355·3)	Low birth weight 177·5 (112·1 to 293·1)	High body-mass index 162·9 (53·5 to 350·7)	Handwashing 146·0 (66·8 to 264·5)
Southern Sub-Saharan Africa	Tobacco 2,010·5 (1,178·6 to 3,628·6)	Alcohol use 1,469·9 (746·9 to 3,243·6)	High body-mass index 1,353·3 (759·0 to 2,525·3)	Ambient particulate matter 1,048·7 (529·3 to 2,349·6)	High blood pressure 924·8 (394·2 to 1,953·5)	High fasting plasma glucose 713·9 (310·1 to 1,524·5)	Child wasting 604·8 (73·0 to 2,502·6)	Household air pollution 530·8 (238·7 to 1,144·4)	Low fruit 428·8 (204·2 to 883·7)	Low whole grains 413·9 (179·3 to 883·2)
Botswana	Alcohol use 69·0 (22·3 to 154·0)	High body-mass index 60·2 (20·4 to 161·7)	Tobacco 57·2 (17·1 to 133·6)	High blood pressure 52·1 (17·4 to 142·2)	High fasting plasma glucose 48·4 (18·8 to 102·7)	Ambient particulate matter 30·0 (11·5 to 72·6)	Low fruit 23·6 (7·4 to 56·4)	Low whole grains 23·2 (7·1 to 61·7)	High total cholesterol 19·5 (5·9 to 47·1)	Household air pollution 18·1 (5·5 to 47·7)
Lesotho	Alcohol use 65·9 (29·2 to 124·6)	High body-mass index 35·9 (18·6 to 67·2)	Ambient particulate matter 26·3 (14·4 to 45·6)	Tobacco 25·9 (5·2 to 50·5)	Household air pollution 23·3 (11·6 to 43·0)	High blood pressure 21·9 (10·1 to 43·0)	High fasting plasma glucose 21·2 (9·4 to 43·0)	Child wasting 21·0 (4·1 to 55·4)	Unsafe water 16·1 (3·8 to 35·3)	Short gestation 14·9 (7·0 to 27·7)
Namibia	Alcohol use 37·3 (14·1 to 69·8)	High blood pressure 29·3 (13·2 to 57·8)	Tobacco 28·8 (14·9 to 54·9)	High body-mass index 27·5 (12·1 to 51·8)	High fasting plasma glucose 23·4 (10·3 to 47·8)	Ambient particulate matter 22·8 (11·8 to 43·9)	Child wasting 20·5 (3·8 to 75·6)	Household air pollution 18·3 (9·2 to 36·5)	Short gestation 16·1 (6·3 to 27·5)	Unsafe water 14·0 (3·8 to 36·7)
South Africa	Tobacco 1,494·6 (860·1 to 2,629·2)	Alcohol use 932·2 (398·7 to 2,491·9)	High body-mass index 880·2 (474·7 to 1,708·1)	Ambient particulate matter 610·1 (347·5 to 1,381·9)	High blood pressure 598·7 (261·8 to 1,276·7)	High fasting plasma glucose 483·7 (203·4 to 1,084·2)	Low fruit 271·6 (128·1 to 553·1)	Low whole grains 261·0 (116·2 to 586·1)	High total cholesterol 252·8 (98·8 to 582·9)	Household air pollution 194·4 (98·6 to 407·5)
Swaziland	Alcohol use 30·0 (9·9 to 63·9)	High body-mass index 26·4 (9·5 to 56·9)	Tobacco 23·6 (8·9 to 53·9)	Ambient particulate matter 19·4 (8·6 to 39·2)	Child wasting 19·0 (2·7 to 60·6)	High fasting plasma glucose 16·7 (5·3 to 36·6)	Household air pollution 16·6 (6·2 to 38·4)	Unsafe water 15·0 (2·1 to 40·1)	High blood pressure 14·6 (5·5 to 30·2)	Unsafe sanitation 14·1 (3·6 to 36·3)
Zimbabwe	Child wasting 477·5 (35·2 to 2,117·7)	Tobacco 350·9 (115·2 to 1,014·1)	Alcohol use 350·2 (67·1 to 1,167·1)	Ambient particulate matter 328·3 (112·2 to 1,068·9)	High body-mass index 316·4 (119·9 to 745·5)	Household air pollution 296·3 (89·2 to 833·0)	High blood pressure 205·5 (56·3 to 580·2)	Short gestation 161·3 (69·9 to 287·8)	Handwashing 153·7 (24·2 to 637·8)	Unsafe sanitation 144·8 (17·6 to 673·1)
Western Sub-Saharan Africa	Short gestation 12,308·2 (7,874·4 to 19,034·4)	Child wasting 10,034·4 (4,997·2 to 14,598·7)	Ambient particulate matter 7,689·3 (5,130·8 to 9,703·6)	Household air pollution 7,594·6 (4,932·8 to 10,290·4)	Low birth weight 5,683·2 (3,904·5 to 8,233·9)	Unsafe sanitation 5,036·0 (2,523·6 to 7,596·7)	High blood pressure 4,794·0 (3,137·7 to 7,371·8)	High body-mass index 4,658·3 (3,001·2 to 7,263·9)	Alcohol use 4,636·7 (3,351·1 to 6,477·1)	Unsafe water 4,598·3 (1,873·9 to 7,860·6)
Benin	Short gestation 384·5 (153·5 to 619·3)	Child wasting 340·1 (169·9 to 606·6)	Ambient particulate matter 246·0 (175·7 to 363·1)	Household air pollution 243·5 (155·3 to 371·3)	High body-mass index 216·4 (147·2 to 331·8)	Low birth weight 193·9 (81·4 to 308·4)	High blood pressure 191·0 (118·0 to 294·4)	Unsafe water 182·3 (74·6 to 335·8)	Unsafe sanitation 168·7 (81·3 to 279·3)	Handwashing 161·3 (81·2 to 271·6)
Burkina Faso	Short gestation 1,091·8 (571·8 to 1,706·9)	Child wasting 1,011·8 (355·0 to 1,722·5)	Household air pollution 790·2 (410·6 to 1,109·5)	Ambient particulate matter 705·5 (399·3 to 991·3)	Low birth weight 524·5 (306·9 to 760·5)	Alcohol use 421·0 (215·1 to 656·5)	Handwashing 347·2 (178·6 to 540·7)	High blood pressure 328·6 (226·9 to 488·7)	Child underweight 311·5 (137·6 to 614·1)	Tobacco 311·3 (182·5 to 494·8)
Cameroon	Short gestation 731·0 (397·7 to 1,185·9)	Child wasting 562·1 (138·0 to 1,564·3)	Ambient particulate matter 560·0 (297·2 to 1,113·7)	Household air pollution 463·7 (207·1 to 960·1)	High body-mass index 441·4 (262·2 to 673·9)	Alcohol use 397·8 (242·4 to 606·4)	Low birth weight 394·8 (229·0 to 618·4)	High blood pressure 317·3 (182·5 to 493·3)	Tobacco 276·0 (144·2 to 520·3)	Hib vaccination 220·0 (65·6 to 607·2)
Cape Verde	High blood pressure 9·2 (4·9 to 16·0)	Tobacco 7·9 (4·3 to 12·8)	High body-mass index 7·1 (4·2 to 10·8)	Alcohol use 7·0 (4·0 to 10·0)	Ambient particulate matter 5·5 (3·1 to 8·5)	High fasting plasma glucose 4·6 (2·4 to 7·9)	Low whole grains 3·5 (1·8 to 5·9)	High total cholesterol 3·4 (1·8 to 5·7)	Low fruit 3·2 (1·7 to 5·5)	Low vegetables 3·0 (1·6 to 5·3)

Location	Risk #1	Risk #2	Risk #3	Risk #4	Risk #5	Risk #6	Risk #7	Risk #8	Risk #9	Risk #10
Chad	Child wasting 1,437·9 (787·1 to 2,122·0)	Short gestation 1,312·8 (712·9 to 2,070·9)	Household air pollution 692·1 (409·0 to 1,103·3)	Ambient particulate matter 653·8 (429·2 to 912·6)	Low birth weight 652·8 (355·1 to 1,031·4)	Unsafe water 550·2 (199·6 to 929·7)	Handwashing 494·6 (297·2 to 760·4)	Child underweight 488·9 (301·7 to 714·8)	Unsafe sanitation 482·8 (279·6 to 730·1)	Hib vaccination 452·0 (155·3 to 842·6)
Cote d'Ivoire	Short gestation 766·2 (460·3 to 1,285·3)	High blood pressure 596·6 (342·5 to 998·7)	Ambient particulate matter 556·3 (357·5 to 816·9)	Household air pollution 541·5 (289·3 to 905·5)	Child wasting 518·2 (195·6 to 1,108·6)	High body-mass index 515·4 (290·5 to 879·7)	Alcohol use 426·1 (198·8 to 750·0)	Tobacco 413·7 (232·8 to 683·5)	Low birth weight 410·5 (259·1 to 621·4)	High fasting plasma glucose 289·6 (147·7 to 508·5)
The Gambia	Short gestation 45·4 (27·6 to 72·5)	High body-mass index 31·6 (21·4 to 45·3)	High blood pressure 26·4 (17·7 to 41·7)	Tobacco 24·2 (15·2 to 38·2)	Ambient particulate matter 24·2 (16·8 to 31·3)	Low birth weight 23·9 (13·9 to 34·4)	Household air pollution 23·5 (16·3 to 31·4)	Child wasting 22·2 (11·8 to 35·3)	Alcohol use 17·6 (8·5 to 32·2)	Unsafe water 11·9 (4·2 to 22·3)
Ghana	Short gestation 464·8 (246·8 to 812·2)	Alcohol use 464·2 (233·7 to 717·6)	High blood pressure 436·3 (274·0 to 648·3)	High body-mass index 404·5 (253·4 to 592·9)	Ambient particulate matter 403·4 (283·5 to 525·4)	Household air pollution 401·0 (257·9 to 547·3)	High fasting plasma glucose 204·1 (119·5 to 340·7)	Tobacco 202·7 (128·0 to 319·2)	Low birth weight 180·7 (102·2 to 294·2)	Low whole grains 179·0 (108·6 to 298·2)
Guinea	Short gestation 654·1 (422·7 to 966·5)	Child wasting 420·1 (192·5 to 767·5)	Household air pollution 400·4 (246·5 to 639·7)	Ambient particulate matter 334·5 (222·1 to 495·1)	Low birth weight 315·4 (214·2 to 422·9)	High blood pressure 240·3 (139·9 to 405·8)	High body-mass index 217·9 (126·2 to 351·3)	Tobacco 184·1 (96·1 to 317·6)	Handwashing 175·2 (92·3 to 266·6)	Unsafe water 147·9 (51·8 to 265·1)
Guinea-Bissau	Short gestation 69·1 (48·2 to 98·9)	High body-mass index 52·6 (33·7 to 76·4)	High blood pressure 46·8 (30·4 to 66·5)	Household air pollution 44·6 (28·3 to 66·4)	Ambient particulate matter 42·4 (31·9 to 59·1)	Child wasting 38·4 (17·2 to 73·2)	Alcohol use 37·6 (20·2 to 62·5)	Low birth weight 35·8 (24·0 to 48·1)	Tobacco 31·1 (18·7 to 48·7)	High fasting plasma glucose 24·4 (14·5 to 40·0)
Liberia	Short gestation 116·6 (78·4 to 165·5)	High body-mass index 102·4 (50·3 to 162·7)	High blood pressure 86·9 (28·3 to 156·0)	Child wasting 84·1 (31·0 to 174·8)	Household air pollution 81·9 (51·2 to 131·4)	Alcohol use 75·3 (35·7 to 134·0)	Unsafe water 64·8 (18·5 to 133·3)	Unsafe sanitation 62·0 (24·1 to 109·5)	Ambient particulate matter 60·0 (38·6 to 96·0)	Low birth weight 59·2 (41·1 to 80·5)
Mali	Short gestation 1,629·0 (922·7 to 2,528·3)	Child wasting 1,138·4 (574·0 to 1,889·7)	Low birth weight 835·8 (521·7 to 1,214·0)	Unsafe water 584·0 (188·0 to 1,045·8)	Unsafe sanitation 521·1 (243·1 to 883·2)	Household air pollution 508·1 (320·6 to 757·2)	Ambient particulate matter 446·3 (296·6 to 686·2)	Handwashing 445·1 (213·1 to 769·0)	Child underweight 380·8 (200·7 to 600·9)	Hib vaccination 342·0 (132·5 to 619·1)
Mauritania	Short gestation 85·5 (55·1 to 120·3)	High body-mass index 53·0 (32·3 to 80·3)	Ambient particulate matter 52·8 (31·5 to 81·9)	High blood pressure 47·2 (26·3 to 78·2)	Household air pollution 39·4 (14·7 to 66·2)	Low birth weight 38·8 (26·6 to 53·2)	Child wasting 38·6 (16·4 to 63·8)	Tobacco 38·5 (18·1 to 70·1)	Unsafe water 29·2 (8·6 to 61·0)	Unsafe sanitation 27·2 (12·9 to 53·5)
Niger	Short gestation 1,550·3 (1,138·6 to 2,152·0)	Child wasting 1,495·0 (977·6 to 2,023·2)	Household air pollution 857·6 (497·7 to 1,170·8)	Ambient particulate matter 802·4 (555·8 to 1,071·1)	Unsafe water 789·9 (381·4 to 1,320·4)	Hib vaccination 752·0 (340·7 to 1,347·1)	Unsafe sanitation 681·0 (404·6 to 1,098·2)	Handwashing 651·0 (347·2 to 938·2)	Low birth weight 637·7 (454·8 to 872·2)	Child underweight 609·4 (401·9 to 885·5)
Nigeria	Short gestation 2,565·2 (940·9 to 5,243·0)	Child wasting 2,306·6 (553·4 to 4,057·1)	Ambient particulate matter 2,255·7 (1,386·1 to 3,367·2)	Alcohol use 2,103·7 (1,201·1 to 3,432·0)	Household air pollution 2,038·9 (1,059·1 to 3,397·8)	Unsafe sanitation 1,979·0 (844·5 to 3,521·0)	Handwashing 1,242·4 (563·3 to 2,054·2)	Unsafe water 1,234·7 (479·2 to 2,612·4)	PCV vaccination 1,189·8 (597·5 to 1,781·0)	Hib vaccination 1,167·4 (260·4 to 2,546·3)
Sao Tome and Principe	High blood pressure 3·3 (1·8 to 6·3)	Household air pollution 2·9 (0·7 to 5·4)	High body-mass index 2·9 (1·6 to 5·0)	Alcohol use 2·7 (1·6 to 4·5)	Tobacco 2·4 (1·2 to 4·4)	Ambient particulate matter 2·0 (1·0 to 3·5)	Short gestation 1·8 (1·1 to 2·8)	Low fruit 1·3 (0·5 to 2·6)	High fasting plasma glucose 1·2 (0·6 to 2·4)	Low whole grains 1·2 (0·6 to 2·4)
Senegal	Short gestation 385·4 (200·3 to 580·4)	High blood pressure 288·4 (172·6 to 443·8)	High body-mass index 284·2 (174·9 to 442·6)	Ambient particulate matter 244·6 (170·1 to 326·3)	Tobacco 214·6 (130·2 to 345·6)	Child wasting 207·9 (87·9 to 410·8)	Household air pollution 193·0 (95·4 to 310·0)	Low birth weight 183·7 (95·9 to 278·2)	High fasting plasma glucose 159·1 (97·6 to 244·2)	Low fruit 127·2 (71·3 to 212·1)
Sierra Leone	Short gestation 338·5 (220·3 to 499·0)	Child wasting 303·9 (139·6 to 557·5)	Household air pollution 257·7 (163·4 to 410·8)	Ambient particulate matter 211·7 (138·0 to 310·6)	Alcohol use 183·9 (103·5 to 295·9)	High blood pressure 171·3 (99·0 to 260·6)	Tobacco 168·3 (96·1 to 271·8)	Low birth weight 159·2 (107·5 to 220·2)	Hib vaccination 137·3 (47·4 to 308·2)	High body-mass index 132·8 (76·3 to 212·3)
Togo	Short gestation 158·1 (95·7 to 239·0)	Household air pollution 154·3 (90·7 to 227·7)	Ambient particulate matter 149·3 (87·5 to 220·8)	High blood pressure 144·2 (69·9 to 267·0)	Tobacco 112·1 (64·5 to 192·6)	High body-mass index 103·0 (41·0 to 201·6)	Child wasting 95·8 (32·1 to 229·9)	Alcohol use 87·3 (42·5 to 164·3)	Low birth weight 83·3 (56·3 to 116·6)	Low fruit 67·1 (31·9 to 129·5)

Appendix Figure 4. Global life expectancy from 1990-2040 in the reference scenario (forecast), better health, and worse health scenarios. Dashed lines represent global male life expectancy, solid lines represent global female life expectancy. Black lines = past data from GBD 2016. Blue = reference scenario. Green = better health scenario. Red = worse health scenario. Shaded areas represent 95% uncertainty intervals.

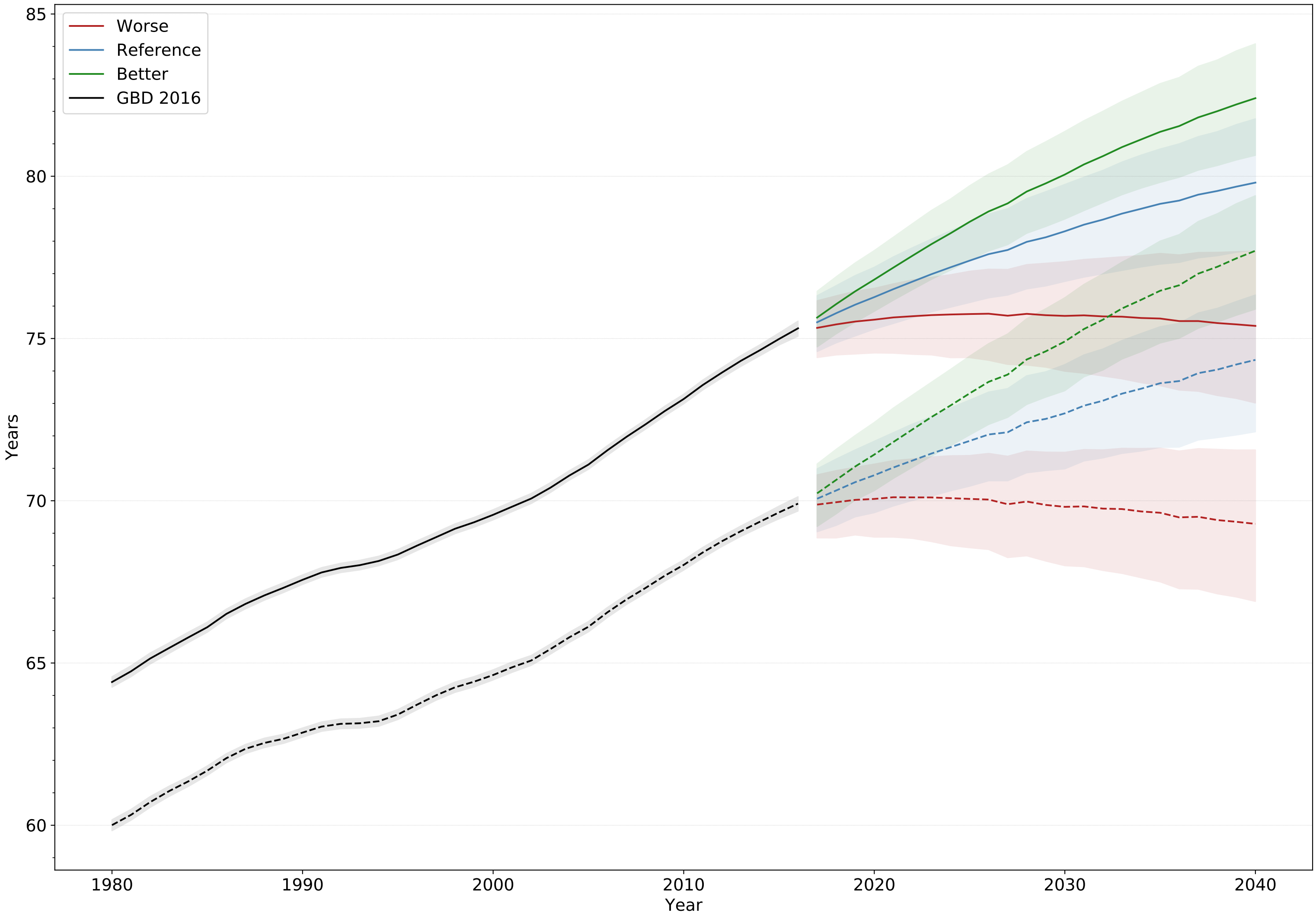

Appendix Figure 5. Difference between the better and worse life expectancy scenarios in 2040 for both sexes combined. Legend measured in years. ATG=Antigua and Barbuda. BRB=Barbados. COM=Comoros. DMA=Dominica. E Med=Eastern Mediterranean. FJI=Fiji. FSM=Federated States of Micronesia. GRD=Grenada. KIR=Kiribati. LCA=Saint Lucia. MDV=Maldives. MHL=Marshall Islands. MLT=Malta. MUS=Mauritius. SGP=Singapore. SLB=Solomon Islands. SYC=Seychelles. TLS=Timor-Leste. TON=Tonga. TTO=Trinidad and Tobago. VCT=Saint Vincent and the Grenadines. VUT=Vanuatu. W Africa=West Africa. WSM=Samoa.

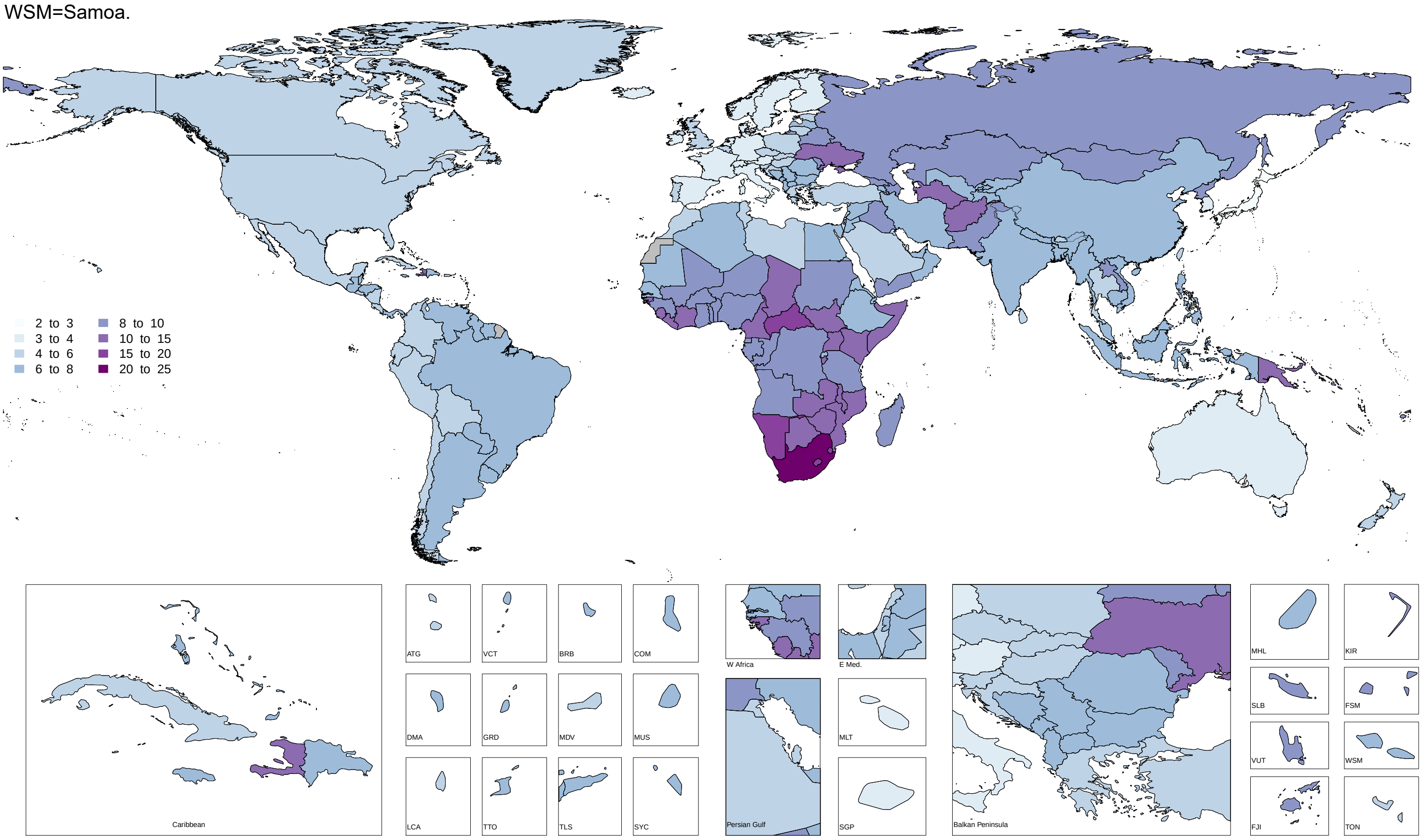

Table 1. Number of Years of life lost (YLLs) and deaths in GBD 2016, the 2040 forecast (reference scenario), better, and worse scenarios for all causes of death and all locations. Values are reported as counts, or in thousands, as described in the location specific heading, and 95% uncertainty intervals are in parentheses. Due to differences in populations and SDI across the scenarios, forecasts of numbers may result in reference values outside of the better and worse estimates. Highlighted rows indicate level 1 and 2 causes from the GBD cause hierarchy. GBD = Global burden of disease.

Global								
Cause	YLLs (in 1000s)				Deaths (in 1000s)			
	2016	2040 Forecast	2040 Better Scenario	2040 Worse Scenario	2016	2040 Forecast	2040 Better Scenario	2040 Worse Scenario
**All causes**	**1,585,864.98 (1,559,572.96 to 1,613,799.53)**	**1,522,929.99 (1,310,033.32 to 1,775,013.45)**	**1,193,905.99 (1,033,011.11 to 1,385,361.63)**	**2,177,435.98 (1,858,580.54 to 2,522,615.79)**	**54,698.58 (54,028.68 to 55,514.89)**	**74,983.79 (66,337.22 to 85,381.93)**	**62,943.02 (55,680.24 to 70,996.21)**	**96,329.12 (84,258.24 to 107,989.38)**
**Communicable, maternal, neonatal, and nutritional diseases**	**566,351.51 (544,844.20 to 589,177.03)**	**326,085.66 (256,204.27 to 416,351.81)**	**223,159.21 (178,311.61 to 284,636.18)**	**596,227.75 (473,441.15 to 745,019.40)**	**10,557.99 (10,097.72 to 11,143.43)**	**9,012.74 (6,969.25 to 12,099.73)**	**7,000.50 (5,462.69 to 9,493.78)**	**14,423.84 (11,187.34 to 19,113.00)**
**HIV/AIDS and tuberculosis**	**94,262.23 (91,006.54 to 97,422.77)**	**61,697.37 (52,307.01 to 73,881.48)**	**40,732.09 (32,189.41 to 49,383.31)**	**158,910.20 (126,153.39 to 197,320.41)**	**2,246.81 (2,172.76 to 2,314.46)**	**1,611.50 (1,371.77 to 1,940.47)**	**1,092.93 (914.99 to 1,309.47)**	**3,687.50 (2,932.53 to 4,490.51)**
Tuberculosis	40,718.82 (38,983.48 to 42,538.24)	24,420.28 (18,423.72 to 33,956.86)	15,369.17 (11,817.48 to 20,793.65)	41,159.88 (30,728.59 to 57,599.09)	1,213.06 (1,161.55 to 1,265.42)	870.10 (668.48 to 1,154.09)	588.14 (456.75 to 756.09)	1,386.82 (1,042.25 to 1,849.57)
HIV/AIDS	53,543.41 (50,984.67 to 56,292.03)	37,277.09 (30,834.47 to 43,840.23)	25,362.91 (18,173.12 to 33,023.26)	117,750.32 (88,538.93 to 154,571.28)	1,033.75 (987.35 to 1,081.57)	741.41 (624.14 to 863.09)	504.78 (389.10 to 619.10)	2,300.68 (1,694.61 to 3,024.39)
**Diarrhea, lower respiratory, and other common infectious diseases**	**209,304.89 (195,330.83 to 228,343.14)**	**138,104.82 (89,879.14 to 210,847.98)**	**92,268.80 (60,763.21 to 143,633.01)**	**236,500.14 (152,036.89 to 359,032.23)**	**4,805.16 (4,381.23 to 5,480.60)**	**5,280.21 (3,533.42 to 8,186.27)**	**4,210.74 (2,884.82 to 6,601.87)**	**7,720.03 (4,938.54 to 12,199.11)**
Diarrheal diseases	66,908.74 (56,202.72 to 85,858.53)	40,222.53 (14,747.38 to 95,244.05)	27,516.48 (9,284.80 to 70,165.36)	70,657.51 (26,572.16 to 165,528.84)	1,655.94 (1,244.07 to 2,366.55)	1,598.66 (422.80 to 4,203.65)	1,310.93 (386.90 to 3,447.50)	2,691.88 (677.37 to 6,926.59)
Intestinal infectious diseases	10,476.49 (5,926.65 to 17,188.46)	5,697.97 (2,996.07 to 9,529.61)	5,197.83 (2,806.16 to 8,652.75)	7,467.83 (3,966.52 to 12,584.03)	155.45 (87.59 to 255.41)	95.66 (51.31 to 159.22)	89.58 (48.83 to 147.93)	119.54 (63.52 to 202.29)
Typhoid fever	8,729.56 (4,775.28 to 14,334.44)	4,566.22 (2,403.51 to 7,789.95)	4,199.70 (2,266.70 to 6,948.52)	5,964.70 (3,085.78 to 10,222.49)	128.17 (70.08 to 210.19)	75.29 (40.33 to 127.66)	71.04 (38.41 to 119.04)	93.78 (48.74 to 160.88)
Paratyphoid fever	1,596.58 (750.49 to 3,096.66)	1,076.66 (504.52 to 2,060.82)	963.33 (445.22 to 1,794.98)	1,409.49 (654.76 to 2,721.05)	25.19 (11.75 to 49.24)	19.37 (8.90 to 36.89)	17.80 (8.14 to 33.68)	24.25 (11.28 to 46.54)
Other intestinal infectious diseases	150.35 (40.76 to 410.64)	55.09 (15.56 to 157.51)	34.81 (10.28 to 96.24)	93.64 (25.33 to 266.36)	2.09 (0.63 to 5.46)	1.00 (0.33 to 2.54)	0.73 (0.24 to 1.81)	1.50 (0.48 to 3.93)
Lower respiratory infections	91,363.09 (84,223.22 to 97,870.27)	68,309.71 (45,673.67 to 99,818.11)	42,962.16 (31,363.08 to 60,256.27)	119,611.44 (74,820.38 to 178,367.34)	2,377.70 (2,145.58 to 2,512.81)	3,114.69 (2,410.77 to 3,963.72)	2,423.08 (1,916.83 to 2,983.71)	4,262.23 (3,130.39 to 5,652.28)
Upper respiratory infections	126.68 (105.23 to 154.86)	79.94 (61.28 to 104.29)	51.73 (40.03 to 64.73)	135.83 (103.28 to 172.32)	2.31 (2.04 to 2.65)	1.97 (1.64 to 2.38)	1.42 (1.18 to 1.71)	2.97 (2.47 to 3.58)
Otitis media	50.36 (37.49 to 72.37)	13.55 (8.98 to 20.06)	13.56 (9.02 to 20.08)	14.00 (9.37 to 20.86)	1.08 (0.84 to 1.47)	0.50 (0.33 to 0.69)	0.53 (0.35 to 0.74)	0.46 (0.30 to 0.63)
Meningitis	20,383.03 (16,781.47 to 26,724.06)	12,356.42 (8,683.57 to 17,687.07)	9,338.58 (6,864.48 to 13,293.94)	20,293.47 (12,922.01 to 31,384.33)	318.40 (265.22 to 408.71)	238.65 (177.63 to 337.91)	202.78 (153.00 to 291.85)	331.33 (232.49 to 484.26)
Pneumococcal meningitis	1,268.37 (996.16 to 1,721.54)	739.53 (520.77 to 1,088.44)	628.57 (452.57 to 912.45)	1,034.23 (704.74 to 1,607.46)	23.14 (18.70 to 30.93)	18.16 (13.38 to 27.09)	17.08 (12.71 to 25.41)	21.78 (15.68 to 32.19)
H influenzae type B meningitis	2,177.50 (1,723.91 to 2,955.18)	2,971.42 (910.55 to 7,133.43)	1,414.02 (624.05 to 3,057.97)	8,177.47 (2,099.59 to 19,717.53)	31.41 (25.37 to 41.43)	41.38 (16.55 to 90.66)	23.57 (13.45 to 42.89)	101.70 (29.58 to 236.28)
Meningococcal meningitis	8,159.59 (6,630.42 to 10,743.55)	4,037.61 (2,903.62 to 5,703.62)	3,426.96 (2,469.64 to 4,886.67)	5,129.39 (3,626.37 to 7,305.05)	127.42 (105.43 to 164.01)	84.65 (62.94 to 123.69)	77.07 (57.20 to 115.20)	97.62 (72.06 to 138.75)
[illegible]	8,777.57 (7,123.54 to [illegible]	4,607.86 (3,365.47 to [illegible]	3,869.04 (2,851.79 to [illegible]	5,952.38 (4,263.80 to [illegible]	136.42 (112.68 to [illegible]	94.47 (71.15 to [illegible]	85.06 (63.90 to [illegible]	110.23 (83.53 to [illegible]

Other meningitis	[illegible] 11,853.65)	[illegible] 6,452.47)	[illegible] 5,418.82)	[illegible] 8,367.38)	[illegible] 178.02)	[illegible] 132.32)	[illegible] 120.86)	[illegible] 150.41)
Encephalitis	5,053.33 (4,020.10 to 6,845.05)	3,945.66 (2,902.24 to 5,401.18)	3,653.69 (2,618.86 to 5,109.70)	4,869.64 (3,669.82 to 6,667.29)	102.87 (83.93 to 138.39)	129.16 (95.02 to 178.48)	128.94 (93.05 to 180.12)	138.74 (106.26 to 190.89)
Diphtheria	86.90 (62.45 to 123.42)	37.76 (19.33 to 70.74)	14.18 (9.40 to 21.83)	61.13 (32.26 to 102.91)	1.11 (0.82 to 1.54)	0.49 (0.27 to 0.88)	0.21 (0.15 to 0.31)	0.77 (0.42 to 1.27)
Whooping cough	6,170.82 (3,287.59 to 10,666.24)	4,811.98 (2,229.65 to 8,486.00)	2,103.48 (970.90 to 3,648.57)	8,308.08 (4,013.36 to 14,473.64)	73.01 (38.90 to 126.14)	57.12 (26.61 to 100.78)	25.21 (11.68 to 43.58)	98.30 (47.56 to 171.23)
Tetanus	2,362.79 (1,440.70 to 3,057.88)	464.53 (262.78 to 725.13)	318.39 (177.14 to 504.89)	823.26 (464.51 to 1,258.58)	36.69 (22.20 to 47.21)	7.67 (4.45 to 11.51)	6.09 (3.37 to 9.15)	11.67 (6.84 to 17.47)
Measles	5,702.64 (2,133.93 to 12,239.05)	1,884.57 (503.32 to 5,723.56)	893.75 (334.97 to 2,554.95)	3,801.20 (805.28 to 11,309.58)	68.12 (25.50 to 146.06)	26.13 (8.50 to 72.17)	13.53 (5.69 to 33.17)	50.07 (13.23 to 137.33)

Global								
Cause	YLLs (in 1000s)				Deaths (in 1000s)			
	2016	2040 Forecast	2040 Better Scenario	2040 Worse Scenario	2016	2040 Forecast	2040 Better Scenario	2040 Worse Scenario
Varicella and herpes zoster	620.02 (557.00 to 693.29)	280.19 (219.26 to 343.62)	204.95 (160.89 to 250.85)	456.75 (357.43 to 556.83)	12.47 (11.38 to 13.95)	9.53 (7.90 to 11.12)	8.43 (7.05 to 9.83)	12.07 (10.11 to 14.14)
**Neglected tropical diseases and malaria**	**61,330.01 (50,832.05 to 73,173.51)**	**29,655.40 (22,532.05 to 37,012.64)**	**24,234.72 (18,735.26 to 29,741.62)**	**37,890.90 (28,987.41 to 47,666.62)**	**843.59 (708.02 to 988.97)**	**575.78 (448.79 to 698.97)**	**516.57 (401.04 to 623.01)**	**668.29 (524.86 to 808.09)**
Malaria	54,460.50 (44,151.02 to 66,240.10)	19,935.15 (14,728.36 to 26,209.68)	15,485.75 (11,797.90 to 19,855.20)	26,072.79 (19,323.25 to 34,516.09)	719.55 (594.61 to 863.03)	332.98 (258.49 to 424.73)	281.72 (223.81 to 356.71)	402.98 (311.91 to 513.28)
Chagas disease	156.13 (146.16 to 168.66)	97.53 (83.94 to 118.72)	119.40 (102.76 to 142.79)	86.15 (73.77 to 106.02)	7.14 (6.74 to 7.77)	5.59 (4.86 to 6.74)	7.00 (6.09 to 8.29)	4.84 (4.18 to 5.89)
Leishmaniasis	705.85 (398.32 to 1,204.23)	261.41 (150.70 to 466.60)	216.61 (121.44 to 392.64)	479.18 (285.91 to 815.58)	13.67 (7.66 to 23.00)	5.28 (3.03 to 10.08)	4.52 (2.55 to 8.58)	8.80 (5.28 to 15.81)
African trypanosomiasis	126.51 (63.55 to 212.07)	15.84 (6.73 to 33.30)	15.50 (6.69 to 31.59)	16.95 (7.32 to 35.11)	2.29 (1.16 to 3.83)	0.30 (0.13 to 0.62)	0.30 (0.13 to 0.61)	0.31 (0.13 to 0.66)
Schistosomiasis	367.36 (333.89 to 401.84)	287.63 (237.74 to 353.77)	246.00 (202.81 to 302.93)	333.78 (279.64 to 408.43)	10.09 (9.26 to 10.97)	8.94 (7.75 to 10.48)	7.96 (6.83 to 9.41)	9.92 (8.62 to 11.59)
Cysticercosis	47.24 (39.82 to 56.34)	21.17 (16.13 to 29.05)	19.36 (14.78 to 26.63)	22.98 (17.60 to 31.19)	1.00 (0.86 to 1.17)	0.51 (0.40 to 0.68)	0.48 (0.38 to 0.64)	0.53 (0.42 to 0.70)
Cystic echinococcosis	46.04 (36.82 to 57.76)	19.61 (14.64 to 26.57)	13.61 (10.05 to 18.50)	35.11 (25.91 to 47.07)	1.01 (0.84 to 1.22)	0.57 (0.44 to 0.75)	0.43 (0.33 to 0.59)	0.92 (0.70 to 1.19)
Dengue	1,975.12 (619.25 to 2,751.84)	6,715.71 (2,724.90 to 8,798.56)	6,513.22 (2,569.42 to 8,534.37)	7,179.34 (2,775.20 to 9,552.14)	37.78 (10.91 to 52.73)	183.41 (71.27 to 240.46)	185.34 (71.17 to 243.61)	182.59 (68.22 to 239.58)
Yellow fever	373.91 (80.80 to 1,074.88)	151.77 (28.54 to 466.60)	113.00 (20.80 to 356.91)	241.11 (47.55 to 733.72)	5.80 (1.24 to 16.73)	2.48 (0.46 to 7.81)	1.88 (0.34 to 6.09)	3.83 (0.76 to 11.93)
Rabies	744.18 (383.72 to 1,106.23)	302.22 (158.43 to 501.90)	188.19 (99.69 to 311.57)	639.35 (338.51 to 1,036.62)	13.29 (7.16 to 19.06)	6.14 (3.47 to 9.98)	4.27 (2.36 to 7.11)	12.22 (6.93 to 19.75)
Intestinal nematode infections	385.30 (309.19 to 484.21)	440.72 (314.83 to 578.85)	352.27 (256.28 to 451.04)	567.00 (419.56 to 732.24)	4.88 (3.98 to 6.06)	5.97 (4.43 to 7.72)	4.96 (3.76 to 6.30)	7.41 (5.63 to 9.41)
Other neglected tropical diseases	1,940.72 (1,328.90 to 2,505.52)	1,406.65 (968.43 to 1,889.21)	951.82 (671.18 to 1,228.97)	2,217.16 (1,520.43 to 2,906.65)	27.06 (19.16 to 33.98)	23.61 (17.82 to 30.55)	17.72 (13.80 to 22.10)	33.94 (25.01 to 43.01)
**Maternal disorders**	**12,817.77 (11,808.37 to 14,106.37)**	**8,181.86 (6,512.04 to 11,242.69)**	**5,486.50 (4,357.58 to 7,465.97)**	**13,140.04 (10,574.85 to 17,585.16)**	**230.61 (212.53 to 253.39)**	**151.90 (120.87 to 206.46)**	**101.74 (80.88 to 137.01)**	**242.65 (195.17 to 323.91)**
Maternal hemorrhage	4,018.54 (3,248.03 to 4,975.76)	2,193.50 (1,592.88 to 3,058.91)	1,540.48 (1,115.58 to 2,147.77)	3,486.55 (2,522.18 to 4,802.31)	72.40 (58.51 to 89.12)	40.81 (29.75 to 56.68)	28.56 (20.70 to 39.75)	64.56 (46.54 to 88.59)
Maternal sepsis and other maternal infections	1,093.59 (789.31 to 1,478.23)	542.90 (354.74 to 812.87)	372.36 (247.53 to 558.14)	853.99 (564.47 to 1,258.95)	19.53 (14.29 to 26.19)	10.02 (6.61 to 14.80)	6.87 (4.60 to 10.20)	15.68 (10.44 to 22.83)
Maternal hypertensive disorders	1,780.77 (1,360.65 to 2,265.89)	1,224.14 (869.31 to 1,732.10)	776.94 (551.14 to 1,111.18)	1,864.05 (1,332.28 to 2,622.50)	31.60 (24.46 to 39.84)	22.21 (15.72 to 31.23)	14.10 (10.06 to 20.09)	33.67 (24.20 to 47.01)
Maternal obstructed labor and uterine rupture	553.65 (369.57 to 798.39)	375.56 (225.76 to 587.43)	240.72 (147.77 to 369.14)	618.69 (379.41 to 951.42)	10.25 (6.84 to 14.57)	7.11 (4.27 to 11.09)	4.56 (2.79 to 7.01)	11.67 (7.04 to 18.00)
Maternal abortion, miscarriage, and ectopic pregnancy	1,081.93 (796.17 to 1,466.60)	654.04 (395.10 to 1,120.68)	467.84 (283.11 to 789.01)	1,063.78 (634.35 to 1,858.59)	19.70 (14.56 to 26.12)	12.23 (7.44 to 20.22)	8.77 (5.35 to 14.69)	19.78 (11.94 to 33.16)

Indirect maternal deaths	1,987.87 (1,463.84 to 2,619.81)	1,441.42 (961.07 to 2,205.51)	923.25 (615.96 to 1,407.32)	2,299.79 (1,536.27 to 3,392.42)	35.74 (26.39 to 46.78)	26.42 (17.73 to 40.04)	16.93 (11.37 to 25.62)	41.86 (28.60 to 61.27)
Late maternal deaths	228.46 (134.55 to 370.93)	113.28 (62.48 to 195.51)	75.58 (42.75 to 131.39)	177.22 (97.54 to 312.23)	4.11 (2.45 to 6.55)	2.10 (1.12 to 3.58)	1.40 (0.77 to 2.35)	3.26 (1.74 to 5.63)
Maternal deaths aggravated by HIV/AIDS	105.42 (66.70 to 142.91)	560.49 (331.36 to 963.13)	308.44 (145.36 to 566.44)	1,104.08 (619.68 to 1,935.98)	2.02 (1.28 to 2.73)	11.03 (6.60 to 18.18)	6.07 (3.04 to 10.56)	21.39 (12.43 to 36.31)
Other maternal disorders	1,967.54 (1,475.03 to 2,540.02)	1,076.52 (738.18 to 1,615.13)	780.88 (541.56 to 1,147.85)	1,671.89 (1,147.61 to 2,421.27)	35.26 (26.84 to 45.20)	19.97 (13.77 to 29.79)	14.48 (10.10 to 21.36)	30.77 (21.11 to 44.46)

Global								
Cause	YLLs (in 1000s)				Deaths (in 1000s)			
	2016	2040 Forecast	2040 Better Scenario	2040 Worse Scenario	2016	2040 Forecast	2040 Better Scenario	2040 Worse Scenario
**Neonatal disorders**	**149,832.24 (142,306.49 to 157,779.95)**	**69,930.49 (47,252.84 to 96,272.91)**	**45,552.86 (31,835.16 to 61,843.31)**	**123,596.22 (85,104.71 to 167,869.80)**	**1,731.04 (1,644.11 to 1,822.86)**	**808.10 (546.12 to 1,112.30)**	**526.42 (368.00 to 714.67)**	**1,428.19 (983.60 to 1,939.63)**
Neonatal preterm birth	53,703.14 (49,224.85 to 58,402.33)	22,605.27 (17,113.81 to 27,929.77)	13,550.82 (10,485.59 to 16,426.41)	41,000.02 (31,135.92 to 50,038.09)	620.40 (568.68 to 674.66)	261.19 (197.78 to 322.71)	156.58 (121.20 to 189.80)	473.70 (359.82 to 578.13)
Neonatal encephalopathy due to birth asphyxia and trauma	45,435.32 (40,396.97 to 49,877.36)	20,129.27 (11,122.89 to 32,453.45)	13,583.56 (7,877.62 to 21,300.38)	33,983.44 (19,302.40 to 52,988.66)	524.89 (466.66 to 576.25)	232.60 (128.60 to 374.92)	156.97 (91.04 to 246.09)	392.68 (223.09 to 612.19)
Neonatal sepsis and other neonatal infections	21,029.06 (17,740.28 to 27,500.03)	15,007.01 (10,102.58 to 21,580.83)	9,688.07 (6,663.61 to 13,633.54)	25,487.17 (17,462.01 to 36,188.97)	242.99 (205.03 to 317.71)	173.44 (116.81 to 249.38)	111.97 (77.04 to 157.55)	294.56 (201.88 to 418.16)
Hemolytic disease and other neonatal jaundice	4,258.58 (3,689.17 to 4,937.32)	1,141.12 (722.81 to 1,725.53)	774.12 (500.71 to 1,152.44)	2,215.00 (1,430.50 to 3,359.89)	49.21 (42.63 to 57.04)	13.19 (8.36 to 19.94)	8.95 (5.79 to 13.32)	25.60 (16.54 to 38.82)
Other neonatal disorders	25,406.13 (22,984.87 to 27,937.49)	11,047.81 (7,297.14 to 16,152.26)	7,956.30 (5,335.44 to 11,371.57)	20,910.59 (13,964.40 to 30,051.45)	293.56 (265.56 to 322.79)	127.69 (84.37 to 186.65)	91.95 (61.68 to 131.40)	241.66 (161.45 to 347.28)
**Nutritional deficiencies**	**19,504.73 (17,125.04 to 22,894.16)**	**8,993.80 (7,420.53 to 11,156.35)**	**6,916.49 (5,817.63 to 8,387.63)**	**13,012.47 (10,674.55 to 15,924.56)**	**368.11 (334.00 to 422.69)**	**327.19 (293.14 to 384.19)**	**315.46 (286.79 to 361.39)**	**366.75 (323.68 to 434.47)**
Protein-energy malnutrition	17,513.99 (15,224.67 to 20,732.25)	7,118.59 (5,790.22 to 8,788.02)	5,290.30 (4,413.97 to 6,431.62)	10,657.06 (8,595.19 to 13,084.27)	308.39 (276.86 to 355.83)	247.19 (219.88 to 286.15)	239.09 (216.83 to 268.95)	279.68 (244.67 to 327.43)
Iodine deficiency	102.59 (66.06 to 168.78)	249.20 (139.65 to 491.27)	186.00 (111.05 to 309.12)	374.16 (192.74 to 732.60)	2.23 (1.62 to 3.14)	9.69 (5.74 to 15.45)	8.69 (4.93 to 14.42)	11.60 (7.07 to 18.46)
Dietary iron deficiency	114.38 (101.14 to 134.79)	114.35 (95.58 to 152.63)	106.45 (89.01 to 144.00)	128.42 (107.59 to 169.05)	2.97 (2.52 to 3.75)	4.21 (3.44 to 6.32)	4.27 (3.45 to 6.55)	4.24 (3.47 to 6.15)
Other nutritional deficiencies	1,773.77 (1,481.20 to 2,040.79)	1,511.66 (1,172.55 to 2,062.48)	1,333.73 (1,041.33 to 1,788.87)	1,852.84 (1,418.86 to 2,489.70)	54.51 (46.04 to 64.97)	66.10 (52.24 to 88.27)	63.41 (51.02 to 83.16)	71.23 (56.97 to 94.79)
**Other communicable, maternal, neonatal, and nutritional diseases**	**19,299.64 (14,992.73 to 24,689.33)**	**9,521.93 (7,846.48 to 11,505.21)**	**7,967.75 (6,684.42 to 9,545.37)**	**13,177.78 (10,549.48 to 16,324.20)**	**332.68 (281.00 to 395.76)**	**258.05 (229.19 to 292.35)**	**236.64 (210.03 to 267.35)**	**310.43 (272.02 to 354.04)**
Sexually transmitted infections excluding HIV	9,470.11 (5,539.12 to 14,702.01)	2,353.20 (1,291.78 to 3,857.42)	1,818.53 (1,038.48 to 2,952.50)	3,892.90 (2,063.83 to 6,584.46)	115.76 (69.90 to 176.96)	32.34 (19.62 to 49.86)	25.92 (16.34 to 39.39)	50.63 (29.00 to 81.98)
Syphilis	9,228.17 (5,288.03 to 14,456.12)	2,149.59 (1,100.32 to 3,655.36)	1,629.77 (854.03 to 2,773.29)	3,662.18 (1,851.97 to 6,298.74)	109.57 (63.52 to 170.78)	26.51 (14.19 to 44.15)	20.34 (11.29 to 33.58)	44.31 (23.04 to 75.28)
Chlamydial infection	46.85 (39.26 to 53.45)	39.72 (31.19 to 53.21)	36.74 (28.61 to 49.04)	45.12 (35.39 to 59.66)	1.19 (0.98 to 1.33)	1.14 (0.92 to 1.44)	1.09 (0.88 to 1.36)	1.23 (0.99 to 1.53)
Gonococcal infection	127.41 (105.62 to 144.48)	108.02 (85.09 to 143.35)	100.51 (78.76 to 133.53)	121.77 (95.74 to 158.88)	3.37 (2.77 to 3.80)	3.23 (2.63 to 4.05)	3.11 (2.50 to 3.87)	3.48 (2.80 to 4.33)
Other sexually transmitted infections	67.69 (56.52 to 76.94)	55.87 (43.66 to 74.06)	51.51 (39.83 to 68.38)	63.82 (50.23 to 83.95)	1.63 (1.35 to 1.83)	1.46 (1.18 to 1.88)	1.38 (1.10 to 1.78)	1.62 (1.29 to 2.07)
Acute hepatitis	5,497.93 (5,228.66 to 5,778.42)	3,644.25 (3,170.61 to 4,242.95)	3,304.68 (2,848.27 to 3,876.01)	4,472.44 (3,922.50 to 5,124.31)	134.04 (127.82 to 139.97)	120.28 (106.41 to 136.72)	112.99 (97.80 to 130.05)	138.57 (124.64 to 153.29)
Acute hepatitis A	378.87 (302.70 to 458.94)	96.18 (68.88 to 126.89)	81.37 (57.77 to 105.91)	141.26 (97.85 to 187.50)	5.25 (4.34 to 6.23)	2.23 (1.70 to 2.86)	2.02 (1.54 to 2.64)	2.83 (2.13 to 3.63)
Acute hepatitis B	3,658.41 (3,417.23 to 3,917.82)	2,758.88 (2,358.95 to 3,253.81)	2,492.70 (2,107.40 to 2,953.49)	3,359.08 (2,898.43 to 3,890.38)	100.28 (94.01 to 106.30)	92.58 (79.56 to 107.29)	85.98 (72.90 to 100.93)	108.25 (94.82 to 122.93)

Acute hepatitis C	77.22 (60.92 to 97.72)	86.78 (64.71 to 115.41)	81.26 (60.15 to 108.58)	94.18 (70.47 to 124.12)	2.46 (1.87 to 3.24)	3.41 (2.51 to 4.52)	3.30 (2.41 to 4.39)	3.51 (2.57 to 4.68)
Acute hepatitis E	1,383.43 (1,195.82 to 1,570.28)	702.41 (538.26 to 877.44)	649.35 (495.18 to 809.59)	877.92 (684.86 to 1,071.57)	26.05 (22.11 to 30.43)	22.06 (16.79 to 28.04)	21.69 (16.32 to 27.79)	23.99 (18.46 to 30.18)
Other unspecified infectious diseases	4,331.60 (2,650.24 to 5,920.94)	3,524.48 (2,629.91 to 4,428.07)	2,844.53 (2,152.54 to 3,528.95)	4,812.45 (3,473.07 to 6,247.23)	82.88 (56.22 to 103.72)	105.43 (83.79 to 122.31)	97.73 (78.55 to 112.38)	121.22 (93.23 to 142.27)
**Non-communicable diseases**	**819,437.12 (804,360.07 to 836,584.77)**	**1,025,432.56 (852,319.43 to 1,231,749.50)**	**825,501.15 (695,151.45 to 972,037.68)**	**1,368,538.62 (1,114,825.74 to 1,655,765.09)**	**39,529.59 (38,805.36 to 40,253.20)**	**60,761.19 (52,144.71 to 70,359.68)**	**51,258.02 (44,270.10 to 58,471.08)**	**75,921.84 (64,644.62 to 87,861.03)**

Global								
Cause	YLLs (in 1000s)				Deaths (in 1000s)			
	2016	2040 Forecast	2040 Better Scenario	2040 Worse Scenario	2016	2040 Forecast	2040 Better Scenario	2040 Worse Scenario
**Neoplasms**	**208,041.17 (203,600.04 to 212,089.56)**	**294,981.85 (255,713.08 to 339,042.71)**	**262,223.01 (227,227.64 to 300,051.84)**	**341,487.93 (291,237.48 to 403,821.99)**	**8,927.40 (8,754.97 to 9,089.24)**	**14,847.21 (13,125.82 to 16,886.59)**	**13,495.92 (12,012.43 to 15,195.22)**	**16,725.84 (14,420.60 to 19,635.10)**
Lip and oral cavity cancer	4,492.64 (4,287.46 to 4,677.97)	7,151.55 (5,213.08 to 9,599.93)	5,310.62 (4,034.69 to 6,935.07)	9,354.59 (6,601.09 to 12,671.37)	176.49 (169.18 to 183.02)	307.93 (227.12 to 406.97)	238.85 (184.05 to 307.54)	389.66 (280.14 to 530.11)
Nasopharynx cancer	1,866.39 (1,770.55 to 1,967.17)	2,654.88 (1,974.93 to 3,602.55)	1,826.85 (1,406.37 to 2,346.40)	3,926.98 (2,782.06 to 5,832.46)	63.75 (60.63 to 67.02)	102.81 (75.99 to 139.70)	71.91 (55.18 to 92.70)	148.94 (104.32 to 221.47)
Other pharynx cancer	3,151.70 (2,895.96 to 3,333.59)	5,014.49 (3,629.42 to 6,781.78)	4,327.70 (3,214.24 to 5,722.80)	5,233.22 (3,753.08 to 7,098.64)	118.63 (109.33 to 125.14)	214.18 (156.01 to 287.14)	189.94 (143.50 to 247.33)	215.19 (154.68 to 291.70)
Esophageal cancer	9,164.59 (8,913.46 to 9,444.10)	14,449.25 (9,456.11 to 21,885.88)	11,380.45 (7,850.48 to 16,348.30)	19,036.49 (11,402.03 to 31,446.01)	414.89 (404.39 to 427.18)	740.35 (492.94 to 1,112.81)	595.60 (419.45 to 847.18)	946.51 (576.08 to 1,528.55)
Stomach cancer	18,045.32 (17,580.05 to 18,535.04)	18,091.70 (14,605.03 to 22,708.19)	15,933.12 (13,163.23 to 19,689.92)	20,916.43 (17,037.78 to 25,731.19)	834.17 (813.54 to 855.46)	989.37 (811.62 to 1,239.90)	886.40 (742.45 to 1,080.84)	1,108.81 (917.93 to 1,349.69)
Colon and rectum cancer	16,597.93 (15,919.45 to 17,213.66)	26,384.70 (19,283.50 to 36,435.60)	23,381.81 (17,278.16 to 32,103.48)	28,885.07 (20,776.56 to 39,531.95)	829.56 (797.30 to 860.44)	1,548.94 (1,145.23 to 2,130.23)	1,399.86 (1,052.15 to 1,912.52)	1,653.17 (1,195.44 to 2,280.94)
Liver cancer	20,915.71 (20,029.12 to 21,730.96)	35,702.03 (26,894.95 to 48,162.19)	33,107.01 (26,056.47 to 42,080.38)	37,658.20 (27,410.97 to 52,817.54)	828.94 (796.16 to 857.96)	1,686.11 (1,295.49 to 2,215.46)	1,604.09 (1,284.18 to 1,997.17)	1,721.54 (1,284.46 to 2,366.28)
Liver cancer due to hepatitis B	9,704.02 (8,495.14 to 10,846.75)	16,111.86 (11,816.45 to 22,737.09)	15,166.34 (11,657.02 to 20,049.62)	16,287.69 (11,529.61 to 23,738.07)	349.53 (301.96 to 391.78)	705.23 (527.29 to 970.49)	680.09 (528.90 to 881.00)	687.15 (498.04 to 977.15)
Liver cancer due to hepatitis C	3,267.80 (2,889.48 to 3,621.48)	5,902.95 (4,412.47 to 8,065.89)	5,623.13 (4,315.99 to 7,396.56)	6,280.23 (4,527.39 to 8,891.89)	159.67 (143.42 to 176.07)	327.99 (249.17 to 435.00)	320.98 (250.88 to 412.47)	337.93 (247.07 to 467.60)
Liver cancer due to alcohol use	2,892.13 (2,438.18 to 3,361.38)	5,072.51 (3,681.31 to 6,917.49)	4,235.69 (3,157.33 to 5,586.01)	6,192.50 (4,308.84 to 8,818.66)	129.23 (109.78 to 150.48)	262.81 (191.26 to 357.78)	227.38 (171.47 to 298.64)	307.39 (215.01 to 433.30)
Liver cancer due to other causes	5,051.76 (4,479.84 to 5,703.84)	8,614.71 (6,091.85 to 12,233.58)	8,081.84 (5,936.45 to 10,960.14)	8,897.78 (6,001.37 to 13,254.43)	190.51 (169.75 to 214.63)	390.08 (276.21 to 548.90)	375.65 (281.01 to 501.66)	389.07 (263.61 to 572.48)
Gallbladder and biliary tract cancer	3,269.80 (2,965.89 to 3,487.77)	3,611.20 (3,145.17 to 4,022.31)	3,688.27 (3,162.69 to 4,164.63)	3,400.82 (2,988.92 to 3,714.28)	161.56 (148.71 to 170.97)	203.26 (182.19 to 221.04)	211.76 (188.43 to 231.61)	186.25 (168.98 to 200.20)
Pancreatic cancer	8,145.03 (7,933.68 to 8,359.17)	13,336.04 (11,162.00 to 16,248.17)	13,261.66 (11,082.71 to 16,045.54)	13,631.93 (11,254.79 to 16,819.87)	405.50 (394.38 to 416.00)	743.82 (630.99 to 898.57)	753.77 (640.84 to 908.45)	743.14 (618.84 to 918.82)
Larynx cancer	2,674.72 (2,586.78 to 2,767.61)	3,612.62 (2,294.72 to 5,452.02)	2,470.61 (1,702.79 to 3,533.37)	5,370.16 (3,194.70 to 8,480.95)	111.04 (107.60 to 114.65)	162.23 (102.91 to 244.76)	115.04 (79.61 to 164.14)	234.92 (140.39 to 372.76)
Tracheal, bronchus, and lung cancer	35,966.80 (34,937.63 to 36,978.97)	44,160.67 (33,168.70 to 60,264.97)	30,833.78 (23,605.89 to 40,601.28)	71,120.77 (49,953.42 to 104,269.67)	1,706.88 (1,659.40 to 1,753.40)	2,394.91 (1,797.79 to 3,274.40)	1,719.52 (1,344.31 to 2,260.73)	3,741.72 (2,626.85 to 5,335.29)
Malignant skin melanoma	1,460.67 (1,301.99 to 1,614.12)	2,156.46 (1,895.50 to 2,469.02)	2,451.85 (2,127.00 to 2,861.31)	1,929.32 (1,712.64 to 2,192.94)	61.68 (54.39 to 66.60)	109.11 (96.47 to 119.44)	127.60 (112.13 to 143.42)	94.40 (83.34 to 102.99)
Non-melanoma skin cancer	991.73 (953.50 to 1,031.27)	1,598.31 (1,463.76 to 1,730.79)	1,488.79 (1,350.60 to 1,625.59)	1,647.05 (1,539.39 to 1,751.23)	53.06 (51.14 to 55.18)	104.01 (97.36 to 110.91)	100.27 (93.46 to 107.10)	102.39 (94.79 to 109.45)
Breast cancer	14,368.87 (13,568.90 to 15,369.65)	20,651.60 (16,232.22 to 26,350.16)	18,801.36 (14,871.73 to 23,767.86)	22,198.76 (17,310.73 to 28,544.28)	545.59 (516.55 to 581.67)	896.25 (715.69 to 1,139.96)	826.49 (665.18 to 1,045.86)	945.49 (745.50 to 1,213.25)
Cervical cancer	7,204.07 (5,855.64 to 7,673.39)	8,339.31 (6,634.14 to 10,021.97)	7,579.00 (6,039.65 to 9,172.39)	9,191.61 (7,300.87 to 10,973.25)	247.16 (204.12 to 263.48)	319.33 (259.55 to 373.38)	293.83 (237.26 to 345.24)	344.63 (277.08 to 402.84)

Uterine cancer	1,973.17 (1,875.54 to 2,070.08)	2,853.28 (2,530.06 to 3,181.37)	2,656.32 (2,318.38 to 2,998.94)	3,030.65 (2,719.16 to 3,326.42)	87.53 (83.14 to 91.97)	146.63 (134.44 to 159.38)	139.16 (125.43 to 153.58)	150.71 (139.50 to 161.75)
Ovarian cancer	4,141.91 (3,927.54 to 4,340.62)	6,223.76 (4,887.67 to 8,092.70)	6,031.34 (4,766.68 to 7,701.00)	6,063.54 (4,702.78 to 7,963.61)	165.04 (156.70 to 172.73)	275.93 (219.30 to 359.91)	268.15 (214.59 to 345.35)	266.86 (208.37 to 355.02)
Prostate cancer	5,540.60 (4,536.21 to 5,992.09)	12,249.22 (9,695.72 to 13,504.91)	12,551.56 (9,860.14 to 13,954.96)	11,345.01 (8,945.48 to 12,564.25)	380.92 (320.81 to 412.87)	924.26 (760.24 to 1,018.33)	971.05 (796.59 to 1,067.71)	828.44 (675.44 to 927.14)
Testicular cancer	368.08 (350.82 to 386.93)	374.58 (330.64 to 422.79)	349.16 (305.28 to 395.72)	392.25 (349.93 to 441.91)	8.65 (8.29 to 9.03)	10.69 (9.75 to 11.64)	10.31 (9.30 to 11.33)	10.72 (9.89 to 11.62)

Global								
Cause	YLLs (in 1000s)				Deaths (in 1000s)			
	2016	2040 Forecast	2040 Better Scenario	2040 Worse Scenario	2016	2040 Forecast	2040 Better Scenario	2040 Worse Scenario
Kidney cancer	2,910.03 (2,799.88 to 3,016.36)	4,442.03 (3,791.65 to 5,165.38)	4,513.28 (3,873.01 to 5,182.87)	4,430.44 (3,706.09 to 5,330.54)	131.80 (127.34 to 136.18)	233.14 (202.88 to 267.82)	242.86 (212.75 to 276.28)	225.72 (190.21 to 268.43)
Bladder cancer	3,150.24 (3,043.85 to 3,241.97)	4,830.92 (3,930.58 to 6,026.27)	4,514.76 (3,758.31 to 5,537.77)	5,272.52 (4,168.55 to 6,791.95)	186.20 (180.45 to 191.69)	322.25 (261.11 to 401.64)	309.20 (257.40 to 377.62)	340.37 (263.84 to 440.45)
Brain and nervous system cancer	7,554.07 (6,820.68 to 8,181.18)	10,590.67 (9,228.00 to 11,866.78)	10,460.97 (9,058.28 to 11,842.27)	10,464.03 (9,185.05 to 11,620.88)	227.04 (204.78 to 241.28)	397.08 (349.20 to 434.07)	401.20 (348.64 to 442.11)	377.86 (333.24 to 408.35)
Thyroid cancer	1,043.17 (998.65 to 1,088.04)	1,537.40 (1,227.77 to 1,955.54)	1,414.36 (1,151.77 to 1,749.19)	1,646.51 (1,310.97 to 2,137.13)	42.86 (41.19 to 44.73)	73.45 (59.65 to 92.63)	69.18 (57.76 to 85.40)	75.60 (60.89 to 97.09)
Mesothelioma	649.65 (610.14 to 687.99)	933.19 (825.68 to 1,050.08)	966.17 (843.79 to 1,105.82)	888.79 (800.63 to 981.13)	30.21 (28.30 to 31.88)	50.49 (45.97 to 55.10)	53.62 (48.10 to 59.77)	46.53 (42.89 to 49.82)
Hodgkin lymphoma	1,097.68 (916.41 to 1,300.78)	1,005.93 (819.45 to 1,212.41)	869.86 (700.20 to 1,049.21)	1,182.18 (955.62 to 1,411.70)	28.74 (24.61 to 33.79)	31.89 (26.58 to 37.86)	28.32 (23.40 to 33.76)	35.73 (29.66 to 42.31)
Non-Hodgkin lymphoma	6,635.98 (6,029.97 to 6,928.74)	9,672.61 (8,254.59 to 11,357.29)	9,387.96 (8,023.17 to 10,891.46)	9,643.14 (8,205.54 to 11,333.58)	239.58 (221.18 to 247.87)	416.38 (362.39 to 480.75)	412.39 (362.11 to 471.54)	398.67 (346.35 to 465.24)
Multiple myeloma	2,044.37 (1,839.22 to 2,262.64)	3,616.57 (3,038.77 to 4,362.64)	3,583.36 (3,010.79 to 4,293.44)	3,477.05 (2,902.49 to 4,257.30)	98.44 (87.38 to 109.81)	190.65 (162.63 to 226.72)	192.24 (164.80 to 227.39)	179.27 (151.68 to 218.80)
Leukemia	9,990.00 (9,167.13 to 10,596.07)	11,444.15 (9,602.38 to 13,611.53)	10,598.74 (8,943.45 to 12,401.56)	12,352.18 (10,246.57 to 14,885.93)	310.17 (286.15 to 324.38)	473.68 (398.28 to 566.11)	450.17 (382.11 to 528.44)	493.85 (405.16 to 604.45)
Acute lymphoid leukemia	2,391.03 (2,182.08 to 2,644.47)	2,197.34 (1,850.10 to 2,588.66)	2,026.63 (1,713.90 to 2,395.85)	2,352.91 (1,973.13 to 2,802.08)	50.94 (46.16 to 55.60)	61.87 (52.46 to 73.45)	58.19 (49.75 to 68.07)	64.02 (53.84 to 76.95)
Chronic lymphoid leukemia	645.93 (602.60 to 738.73)	849.38 (673.93 to 1,048.19)	813.48 (656.34 to 993.87)	888.69 (684.04 to 1,128.29)	35.39 (33.12 to 40.15)	54.07 (43.63 to 67.15)	53.17 (43.69 to 64.71)	54.93 (42.68 to 70.12)
Acute myeloid leukemia	2,622.62 (2,419.48 to 2,809.79)	3,887.26 (3,252.80 to 4,654.11)	3,765.31 (3,178.64 to 4,422.36)	3,977.57 (3,305.01 to 4,844.86)	85.33 (78.40 to 89.70)	154.38 (129.27 to 184.05)	152.76 (129.52 to 179.85)	154.53 (127.01 to 188.59)
Chronic myeloid leukemia	597.98 (538.80 to 661.31)	584.46 (459.75 to 724.74)	521.02 (415.69 to 639.28)	626.45 (490.94 to 792.18)	21.94 (20.21 to 23.76)	23.75 (19.15 to 29.31)	21.31 (17.32 to 25.78)	24.90 (19.63 to 31.28)
Other leukemia	3,732.44 (3,252.39 to 3,950.12)	3,925.71 (3,231.90 to 4,701.87)	3,472.30 (2,875.75 to 4,109.69)	4,506.55 (3,692.16 to 5,503.89)	116.56 (103.31 to 123.02)	179.61 (149.06 to 216.53)	164.73 (137.50 to 195.72)	195.47 (160.18 to 242.92)
Other neoplasms	12,626.26 (11,487.26 to 13,043.60)	18,292.71 (16,087.53 to 20,418.76)	18,482.30 (16,131.17 to 20,877.35)	17,798.24 (15,795.63 to 19,655.43)	431.34 (392.75 to 443.83)	778.07 (699.40 to 833.90)	813.15 (726.88 to 879.26)	718.74 (649.04 to 762.34)
**Cardiovascular diseases**	**319,638.66 (312,436.72 to 327,187.01)**	**330,270.82 (223,007.85 to 484,160.96)**	**214,898.28 (150,792.91 to 305,298.44)**	**547,359.19 (364,191.44 to 782,303.91)**	**17,646.59 (17,281.71 to 18,071.08)**	**21,961.64 (15,848.49 to 30,477.72)**	**15,771.64 (11,679.44 to 21,554.60)**	**32,449.52 (23,117.49 to 44,488.49)**
Rheumatic heart disease	8,347.62 (7,957.19 to 8,806.00)	5,527.34 (4,513.33 to 6,982.17)	4,982.73 (4,092.31 to 6,190.92)	6,697.09 (5,554.20 to 8,331.56)	314.58 (302.34 to 328.71)	295.77 (248.53 to 370.28)	277.23 (235.89 to 340.82)	332.71 (285.73 to 411.38)
Ischemic heart disease	167,695.16 (163,400.63 to 172,479.67)	166,263.04 (95,488.90 to 281,374.22)	99,265.49 (59,905.99 to 165,277.29)	292,876.28 (167,470.36 to 481,197.21)	9,480.54 (9,230.53 to 9,757.70)	10,915.70 (6,720.23 to 17,327.79)	7,275.74 (4,549.47 to 11,115.37)	17,099.72 (10,510.06 to 26,741.87)
Stroke	101,992.79 (99,104.55 to 105,018.72)	90,246.11 (57,622.24 to 141,748.85)	54,283.44 (36,410.30 to 83,743.37)	166,519.06 (106,391.86 to 259,930.08)	5,528.23 (5,334.61 to 5,734.68)	5,916.98 (4,000.37 to 9,122.34)	3,993.99 (2,798.67 to 6,138.62)	9,641.33 (6,451.30 to 14,623.44)
Ischemic stroke	40,095.14 (38,501.65 to 41,842.07)	37,640.13 (22,629.86 to 67,060.62)	24,810.98 (15,483.30 to 43,285.93)	60,559.59 (35,559.35 to 106,756.57)	2,690.17 (2,571.77 to 2,817.62)	2,958.06 (1,816.07 to 5,390.43)	2,135.00 (1,351.22 to 3,813.85)	4,258.82 (2,600.59 to 7,687.31)

Intracerebral hemorrhage	61,897.65 (60,240.15 to 63,722.74)	52,605.98 (31,632.02 to 87,554.53)	29,472.46 (18,785.86 to 46,454.31)	105,959.47 (62,081.29 to 175,634.92)	2,838.06 (2,748.57 to 2,934.06)	2,958.92 (1,921.13 to 4,526.88)	1,858.99 (1,262.85 to 2,715.27)	5,382.50 (3,349.48 to 8,493.69)
Hypertensive heart disease	14,955.00 (12,105.83 to 16,330.59)	28,661.45 (16,859.91 to 57,024.80)	21,881.71 (13,887.97 to 40,909.26)	37,324.70 (21,247.83 to 78,328.22)	893.73 (698.62 to 982.93)	2,144.30 (1,246.74 to 4,660.87)	1,729.62 (1,074.80 to 3,495.96)	2,609.45 (1,462.23 to 5,716.43)
Cardiomyopathy and myocarditis	8,159.05 (7,052.67 to 9,049.67)	8,654.67 (6,462.56 to 11,241.49)	7,362.49 (5,551.26 to 9,471.13)	9,460.78 (7,091.81 to 12,178.70)	339.55 (282.60 to 371.08)	460.77 (341.69 to 611.93)	410.25 (306.88 to 536.78)	473.18 (354.02 to 622.39)
Myocarditis	1,234.37 (992.16 to 1,358.33)	1,256.31 (998.86 to 1,458.17)	1,253.20 (988.34 to 1,456.23)	1,263.46 (1,015.80 to 1,466.97)	46.54 (35.84 to 51.06)	74.69 (57.91 to 84.87)	80.05 (62.47 to 90.66)	67.94 (51.89 to 77.86)

Global								
Cause	YLLs (in 1000s)				Deaths (in 1000s)			
	2016	2040 Forecast	2040 Better Scenario	2040 Worse Scenario	2016	2040 Forecast	2040 Better Scenario	2040 Worse Scenario
Alcoholic cardiomyopathy	2,494.33 (1,967.43 to 3,151.60)	2,170.98 (1,418.52 to 3,213.18)	1,779.23 (1,158.73 to 2,679.31)	2,613.62 (1,723.26 to 3,826.12)	83.34 (67.20 to 102.92)	83.68 (61.50 to 112.28)	72.36 (51.86 to 96.91)	96.72 (71.47 to 128.53)
Other cardiomyopathy	4,430.35 (3,771.63 to 4,730.05)	5,227.37 (3,724.93 to 7,274.23)	4,330.05 (3,106.45 to 6,012.41)	5,583.71 (3,936.22 to 7,870.80)	209.67 (170.27 to 224.67)	302.40 (204.47 to 434.37)	257.83 (176.20 to 366.20)	308.52 (207.91 to 447.90)
Atrial fibrillation and flutter	2,336.89 (1,890.44 to 2,827.80)	4,932.52 (3,721.42 to 6,433.66)	4,753.94 (3,613.08 to 6,200.75)	4,880.80 (3,698.34 to 6,363.65)	239.23 (188.69 to 293.59)	542.61 (410.58 to 697.18)	541.65 (414.36 to 694.07)	511.47 (384.67 to 659.21)
Aortic aneurysm	2,881.76 (2,800.85 to 2,975.54)	3,801.03 (2,993.22 to 4,866.06)	3,532.74 (2,837.93 to 4,483.12)	3,951.56 (3,073.59 to 5,110.70)	166.57 (162.05 to 171.56)	257.01 (214.27 to 320.20)	251.24 (210.69 to 309.07)	250.82 (206.79 to 311.94)
Peripheral artery disease	715.52 (544.36 to 1,007.02)	1,627.71 (1,130.09 to 2,407.72)	1,592.46 (1,112.06 to 2,349.64)	1,655.46 (1,137.68 to 2,447.52)	60.70 (45.37 to 89.48)	158.36 (111.74 to 233.92)	163.63 (114.91 to 244.82)	152.25 (106.60 to 224.92)
Endocarditis	2,329.06 (2,067.07 to 2,756.07)	3,662.17 (2,730.31 to 4,954.04)	3,473.53 (2,587.62 to 4,617.13)	3,796.47 (2,842.21 to 5,134.61)	95.97 (82.15 to 112.83)	193.07 (142.65 to 262.38)	192.28 (143.32 to 259.97)	186.90 (138.04 to 255.70)
Other cardiovascular and circulatory diseases	10,225.83 (9,436.43 to 12,584.08)	16,894.78 (13,308.59 to 22,323.43)	13,769.74 (10,994.84 to 17,999.54)	20,196.99 (15,641.16 to 26,760.19)	527.48 (493.07 to 627.50)	1,077.07 (879.01 to 1,395.65)	936.01 (769.80 to 1,229.05)	1,191.68 (957.54 to 1,571.67)
**Chronic respiratory diseases**	**61,574.64 (59,099.41 to 65,209.15)**	**76,985.71 (55,617.08 to 109,526.60)**	**59,291.19 (44,201.32 to 81,192.42)**	**112,639.19 (76,361.06 to 168,484.72)**	**3,542.29 (3,403.60 to 3,739.61)**	**5,198.60 (3,739.31 to 7,392.03)**	**4,180.87 (3,127.43 to 5,695.41)**	**7,239.62 (4,894.90 to 10,776.19)**
Chronic obstructive pulmonary disease	47,146.20 (44,992.80 to 50,032.34)	62,113.16 (41,814.16 to 94,017.44)	45,257.61 (31,473.99 to 66,186.93)	96,610.46 (62,138.89 to 152,974.16)	2,934.33 (2,817.24 to 3,120.35)	4,407.12 (3,022.34 to 6,552.79)	3,389.85 (2,401.30 to 4,851.60)	6,445.79 (4,158.69 to 10,002.40)
Pneumoconiosis	414.94 (391.48 to 450.21)	330.09 (286.39 to 387.59)	316.41 (272.95 to 370.75)	361.61 (323.19 to 407.45)	21.49 (20.45 to 23.05)	22.20 (19.63 to 25.44)	22.09 (19.51 to 24.97)	22.92 (20.32 to 25.76)
Silicosis	210.16 (194.31 to 230.82)	92.84 (69.58 to 128.33)	80.21 (59.94 to 106.19)	121.97 (99.04 to 147.08)	10.40 (9.57 to 11.68)	6.03 (4.55 to 8.16)	5.35 (4.04 to 7.03)	7.41 (5.99 to 8.90)
Asbestosis	60.96 (46.29 to 71.87)	100.90 (79.54 to 120.14)	112.34 (89.80 to 134.12)	90.09 (69.88 to 106.59)	3.50 (2.43 to 4.06)	6.98 (5.44 to 8.20)	8.04 (6.49 to 9.37)	5.99 (4.61 to 7.05)
Coal workers pneumoconiosis	46.60 (30.38 to 54.24)	36.46 (24.94 to 47.40)	30.61 (20.40 to 39.34)	46.97 (31.77 to 58.25)	2.68 (1.79 to 3.07)	2.61 (1.87 to 3.41)	2.28 (1.62 to 2.99)	3.14 (2.25 to 3.96)
Other pneumoconiosis	97.22 (81.95 to 127.89)	99.89 (78.34 to 130.02)	93.25 (71.06 to 122.60)	102.58 (82.72 to 132.94)	4.91 (4.17 to 6.57)	6.58 (5.30 to 8.48)	6.43 (5.10 to 8.23)	6.37 (5.18 to 8.34)
Asthma	10,499.32 (8,643.21 to 12,621.19)	7,910.13 (5,598.28 to 11,111.76)	6,402.62 (4,603.33 to 8,709.47)	9,682.32 (6,886.61 to 13,820.84)	420.02 (338.81 to 517.69)	390.65 (283.68 to 550.09)	329.79 (242.56 to 445.80)	448.77 (318.04 to 637.23)
Interstitial lung disease and pulmonary sarcoidosis	2,305.43 (1,695.61 to 2,717.00)	4,948.78 (3,555.04 to 6,095.82)	5,713.59 (4,243.76 to 7,027.83)	4,178.20 (2,996.88 to 5,109.79)	127.47 (90.82 to 147.69)	309.56 (224.83 to 370.18)	369.39 (274.56 to 441.77)	253.20 (181.20 to 300.32)
Other chronic respiratory diseases	1,208.75 (847.40 to 1,438.67)	1,683.54 (1,185.90 to 2,049.61)	1,600.96 (1,130.28 to 1,950.65)	1,806.61 (1,260.04 to 2,209.97)	38.98 (27.29 to 45.65)	69.07 (48.38 to 82.90)	69.76 (49.21 to 83.32)	68.93 (47.87 to 83.76)
**Cirrhosis and other chronic liver diseases**	**37,283.07 (35,413.31 to 41,442.98)**	**48,251.38 (43,070.43 to 55,078.20)**	**44,180.66 (38,946.40 to 50,876.11)**	**52,661.56 (47,852.35 to 59,040.38)**	**1,256.85 (1,197.09 to 1,376.86)**	**1,898.70 (1,742.98 to 2,081.23)**	**1,782.33 (1,616.19 to 1,980.76)**	**2,002.12 (1,867.95 to 2,167.41)**
Cirrhosis and other chronic liver diseases due to hepatitis B	10,846.50 (9,787.89 to 12,777.41)	14,891.11 (12,958.76 to 17,554.97)	14,349.35 (12,357.69 to 16,913.95)	15,562.53 (13,709.65 to 17,859.65)	365.57 (330.81 to 422.57)	582.19 (512.95 to 658.05)	572.68 (501.65 to 659.16)	590.11 (525.54 to 673.39)
Cirrhosis and other chronic liver diseases due to hepatitis C	9,455.51 (8,516.28 to 10,668.97)	12,924.91 (11,278.83 to 14,950.89)	12,194.33 (10,525.96 to 14,302.85)	13,391.43 (11,700.38 to 15,293.48)	326.75 (295.07 to 364.97)	504.65 (447.72 to 569.32)	485.93 (425.86 to 552.61)	508.16 (453.32 to 570.77)

Cirrhosis and other chronic liver diseases due to alcohol use	9,440.30 (8,601.03 to 10,523.64)	11,848.21 (10,219.38 to 13,803.08)	9,622.60 (8,177.28 to 11,375.40)	14,480.70 (12,728.71 to 16,496.69)	334.89 (306.46 to 371.88)	476.27 (422.92 to 537.12)	399.85 (350.41 to 455.61)	561.40 (507.09 to 624.65)
Cirrhosis and other chronic liver diseases due to other causes	7,540.75 (6,769.21 to 8,562.42)	8,587.15 (7,486.43 to 9,927.50)	8,014.38 (6,960.50 to 9,339.26)	9,226.90 (8,114.37 to 10,637.26)	229.64 (206.21 to 258.15)	335.59 (298.68 to 383.21)	323.87 (285.85 to 373.96)	342.45 (305.48 to 391.12)
**Digestive diseases**	**27,082.09 (25,735.97 to 29,026.39)**	**32,599.21 (28,374.61 to 37,280.38)**	**29,770.73 (25,823.79 to 34,419.07)**	**36,196.27 (31,824.71 to 41,428.67)**	**1,092.33 (1,042.78 to 1,177.81)**	**1,727.53 (1,540.36 to 1,945.84)**	**1,653.75 (1,468.53 to 1,860.29)**	**1,788.09 (1,589.57 to 2,030.92)**
Peptic ulcer disease	5,742.34 (5,308.86 to 6,470.00)	4,812.98 (3,969.55 to 5,922.66)	4,080.24 (3,327.87 to 5,005.83)	6,000.96 (4,908.67 to 7,394.13)	246.72 (230.08 to 272.70)	264.57 (221.04 to 315.59)	232.87 (195.77 to 277.95)	310.76 (257.89 to 374.28)

Global								
**Cause**	**YLLs (in 1000s)**				**Deaths (in 1000s)**			
	**2016**	**2040 Forecast**	**2040 Better Scenario**	**2040 Worse Scenario**	**2016**	**2040 Forecast**	**2040 Better Scenario**	**2040 Worse Scenario**
Gastritis and duodenitis	1,017.42 (930.59 to 1,148.36)	1,556.96 (1,349.77 to 1,823.14)	1,469.33 (1,251.22 to 1,720.58)	1,686.65 (1,473.21 to 1,937.54)	42.99 (39.34 to 47.75)	83.57 (73.35 to 95.87)	82.10 (71.87 to 94.11)	85.60 (74.66 to 97.08)
Appendicitis	1,886.62 (1,684.29 to 2,200.23)	1,712.92 (1,459.48 to 2,073.38)	1,522.08 (1,266.11 to 1,858.54)	2,052.23 (1,754.72 to 2,467.49)	50.19 (44.99 to 57.43)	62.87 (54.72 to 74.81)	58.31 (49.63 to 70.60)	69.94 (61.32 to 83.00)
Paralytic ileus and intestinal obstruction	7,572.53 (6,329.25 to 8,263.90)	9,676.75 (7,977.25 to 11,342.69)	9,555.16 (7,857.71 to 11,275.76)	10,067.19 (8,299.14 to 11,610.77)	254.61 (213.31 to 280.85)	459.69 (386.68 to 524.61)	479.76 (402.38 to 547.14)	436.41 (365.84 to 496.91)
Inguinal, femoral, and abdominal hernia	954.58 (739.84 to 1,139.71)	1,029.26 (772.20 to 1,242.93)	984.89 (732.02 to 1,198.35)	1,088.37 (812.53 to 1,294.61)	43.73 (35.57 to 52.14)	58.49 (45.47 to 70.50)	58.53 (44.92 to 70.47)	56.96 (43.98 to 68.09)
Inflammatory bowel disease	981.64 (819.41 to 1,144.74)	1,360.14 (1,135.08 to 1,589.42)	1,376.60 (1,141.39 to 1,607.28)	1,320.46 (1,100.04 to 1,532.17)	41.61 (34.54 to 45.10)	74.40 (62.72 to 82.55)	78.55 (65.48 to 87.92)	67.71 (57.23 to 74.85)
Vascular intestinal disorders	1,671.00 (1,536.99 to 1,908.24)	2,650.02 (2,362.55 to 3,103.86)	2,614.25 (2,287.54 to 3,060.76)	2,595.86 (2,352.98 to 3,027.55)	100.91 (92.87 to 113.67)	189.28 (172.08 to 217.71)	192.80 (173.57 to 221.20)	177.14 (160.13 to 203.59)
Gallbladder and biliary diseases	1,866.75 (1,758.57 to 2,184.15)	2,746.41 (1,798.62 to 4,287.99)	2,139.49 (1,477.76 to 3,243.76)	3,365.20 (2,146.96 to 5,344.12)	101.83 (96.11 to 118.08)	186.33 (121.96 to 294.05)	153.05 (105.72 to 233.82)	214.64 (136.93 to 343.92)
Pancreatitis	3,274.24 (2,832.81 to 3,650.57)	4,187.05 (3,108.14 to 5,901.12)	3,351.95 (2,543.76 to 4,488.36)	4,970.58 (3,667.46 to 7,117.28)	112.05 (97.41 to 124.60)	173.22 (133.07 to 233.49)	144.12 (113.81 to 187.88)	196.84 (149.06 to 273.19)
Other digestive diseases	2,114.97 (1,937.50 to 2,386.83)	2,866.74 (2,572.65 to 3,226.40)	2,676.73 (2,371.21 to 3,041.37)	3,048.77 (2,743.93 to 3,421.34)	97.69 (88.88 to 108.10)	175.10 (157.59 to 195.42)	173.67 (155.72 to 195.13)	172.09 (154.78 to 192.16)
**Neurological disorders**	**34,154.45 (30,976.21 to 38,350.74)**	**68,949.07 (56,228.79 to 88,519.59)**	**68,906.82 (57,075.01 to 85,966.09)**	**66,948.50 (53,640.20 to 88,556.24)**	**2,825.82 (2,497.00 to 3,217.57)**	**6,742.06 (5,392.94 to 8,859.90)**	**7,016.38 (5,700.98 to 8,866.01)**	**6,182.30 (4,727.39 to 8,454.55)**
Alzheimer's disease and other dementias	22,348.78 (19,381.82 to 26,349.20)	51,864.33 (39,536.97 to 71,088.18)	52,038.05 (40,594.20 to 68,221.11)	49,508.88 (36,333.84 to 70,793.09)	2,382.13 (2,060.41 to 2,777.61)	5,840.49 (4,445.93 to 7,968.13)	6,051.25 (4,739.88 to 7,903.50)	5,368.20 (3,943.69 to 7,588.65)
Parkinson's disease	2,528.14 (1,992.32 to 3,147.38)	6,185.14 (4,832.59 to 7,782.07)	6,882.42 (5,371.70 to 8,676.76)	5,175.16 (4,040.09 to 6,497.18)	211.30 (167.77 to 265.16)	557.23 (439.44 to 696.73)	634.04 (501.27 to 800.83)	452.69 (352.53 to 574.38)
Epilepsy	5,945.43 (5,555.08 to 6,409.61)	6,505.50 (5,279.96 to 8,225.13)	5,512.54 (4,507.06 to 6,882.85)	8,003.17 (6,511.97 to 10,093.65)	126.05 (118.63 to 135.52)	174.06 (142.59 to 213.64)	153.37 (127.07 to 185.46)	202.48 (165.44 to 249.83)
Multiple sclerosis	567.35 (517.29 to 646.89)	696.15 (573.14 to 848.49)	708.21 (577.50 to 867.00)	685.01 (564.76 to 837.49)	18.93 (16.58 to 21.03)	25.64 (21.15 to 30.29)	26.52 (21.82 to 31.58)	24.75 (20.42 to 29.31)
Motor neuron disease	855.92 (819.37 to 883.29)	1,252.30 (1,122.50 to 1,392.23)	1,274.70 (1,130.42 to 1,441.17)	1,193.96 (1,085.68 to 1,304.16)	34.33 (33.05 to 35.36)	57.84 (52.81 to 63.01)	59.64 (53.74 to 65.97)	54.15 (50.09 to 58.07)
Other neurological disorders	1,908.83 (1,775.71 to 2,020.32)	2,445.65 (2,186.47 to 2,729.75)	2,490.90 (2,209.59 to 2,801.26)	2,382.32 (2,146.23 to 2,656.15)	53.08 (50.93 to 55.37)	86.80 (79.30 to 93.79)	91.56 (82.43 to 100.09)	80.02 (74.20 to 85.88)
**Mental disorders**	**12,033.73 (10,748.31 to 13,076.40)**	**18,220.17 (15,050.12 to 21,722.47)**	**19,666.72 (16,000.10 to 24,036.91)**	**17,488.79 (14,604.30 to 20,671.15)**	**318.28 (283.23 to 343.69)**	**559.02 (476.49 to 641.00)**	**617.27 (517.56 to 725.96)**	**515.62 (443.27 to 588.90)**
Alcohol use disorders	6,213.98 (5,164.12 to 6,877.78)	6,612.03 (5,178.79 to 8,222.62)	6,590.80 (5,106.05 to 8,369.00)	6,792.02 (5,405.84 to 8,330.28)	173.89 (145.53 to 190.90)	209.27 (167.73 to 252.06)	212.70 (168.35 to 260.73)	209.81 (169.75 to 249.82)
Drug use disorders	5,787.35 (5,264.51 to 6,426.74)	11,572.28 (9,526.41 to 13,804.65)	13,038.84 (10,593.17 to 15,878.42)	10,661.48 (8,827.62 to 12,555.62)	143.77 (130.28 to 158.78)	349.07 (294.88 to 401.04)	403.85 (340.62 to 470.06)	305.14 (265.48 to 345.68)
Opioid use disorders	3,656.92 (3,098.22 to 4,048.42)	5,702.43 (4,460.17 to 6,925.60)	5,963.73 (4,599.39 to 7,359.69)	5,477.93 (4,325.13 to 6,559.71)	86.22 (72.67 to 94.68)	157.57 (123.13 to 184.81)	168.54 (131.93 to 200.94)	145.85 (116.10 to 168.80)

Cocaine use disorders	356.97 (288.96 to 463.85)	953.07 (685.45 to 1,225.97)	1,097.38 (786.53 to 1,430.09)	878.96 (626.56 to 1,141.70)	8.80 (7.06 to 11.27)	25.81 (18.68 to 32.30)	29.96 (21.43 to 38.39)	23.23 (16.73 to 29.43)
Amphetamine use disorders	224.23 (185.18 to 300.34)	858.48 (639.80 to 1,123.90)	1,062.23 (782.54 to 1,400.76)	768.41 (571.03 to 993.04)	5.22 (4.30 to 6.85)	21.46 (16.27 to 27.63)	26.74 (20.03 to 34.56)	18.78 (14.36 to 23.79)
Other drug use disorders	1,549.23 (1,395.62 to 1,961.50)	4,058.29 (3,294.08 to 4,860.42)	4,915.51 (3,972.34 to 6,020.99)	3,536.18 (2,922.40 to 4,258.20)	43.53 (39.37 to 52.89)	144.23 (120.76 to 167.36)	178.60 (150.94 to 207.75)	117.27 (102.06 to 133.84)
Eating disorders	32.40 (28.77 to 36.08)	35.87 (29.67 to 44.08)	37.08 (30.42 to 45.90)	35.29 (29.41 to 43.12)	0.61 (0.54 to 0.68)	0.69 (0.57 to 0.85)	0.72 (0.59 to 0.89)	0.68 (0.56 to 0.82)

Global								
Cause	YLLs (in 1000s)				Deaths (in 1000s)			
	2016	2040 Forecast	2040 Better Scenario	2040 Worse Scenario	2016	2040 Forecast	2040 Better Scenario	2040 Worse Scenario
**Diabetes, urogenital, blood, and endocrine diseases**	**71,460.46 (69,628.99 to 73,928.81)**	**119,131.34 (89,406.19 to 164,635.93)**	**97,067.77 (73,407.22 to 132,658.46)**	**146,185.41 (109,119.47 to 197,727.42)**	**3,191.13 (3,112.85 to 3,271.85)**	**6,914.32 (5,166.40 to 9,628.62)**	**5,897.82 (4,470.86 to 8,170.00)**	**7,998.80 (5,939.79 to 11,068.02)**
Diabetes mellitus	28,650.00 (27,998.10 to 29,279.38)	45,425.52 (32,589.44 to 63,374.26)	36,837.12 (26,956.50 to 50,911.65)	57,531.52 (40,787.06 to 81,635.12)	1,437.71 (1,402.66 to 1,471.02)	2,746.60 (2,089.94 to 3,596.09)	2,350.27 (1,808.28 to 3,055.05)	3,253.49 (2,426.94 to 4,349.52)
Acute glomerulonephritis	320.39 (305.40 to 337.79)	389.21 (333.56 to 454.19)	365.29 (308.08 to 431.01)	437.27 (379.21 to 502.30)	11.02 (10.54 to 11.52)	18.13 (15.32 to 21.34)	17.77 (14.95 to 20.79)	19.17 (16.23 to 22.30)
Chronic kidney disease	26,260.54 (25,370.98 to 27,674.31)	50,779.56 (25,297.41 to 96,249.90)	38,578.51 (20,249.76 to 73,279.94)	64,464.09 (31,861.45 to 117,883.54)	1,186.56 (1,150.74 to 1,236.56)	3,008.53 (1,442.28 to 5,800.88)	2,386.76 (1,203.99 to 4,639.85)	3,636.82 (1,709.30 to 6,838.00)
Chronic kidney disease due to diabetes mellitus	10,965.18 (9,948.04 to 11,927.79)	23,916.85 (11,260.66 to 46,446.96)	18,035.82 (9,076.24 to 36,159.73)	30,420.37 (14,141.80 to 57,177.84)	500.76 (452.44 to 543.96)	1,338.16 (610.79 to 2,620.80)	1,049.70 (508.09 to 2,104.82)	1,641.74 (740.92 to 3,089.45)
Chronic kidney disease due to hypertension	4,927.08 (4,406.71 to 5,548.10)	11,243.62 (5,263.48 to 21,923.46)	8,873.22 (4,397.29 to 17,464.59)	13,958.58 (6,482.44 to 26,381.74)	299.69 (268.21 to 335.50)	861.47 (402.13 to 1,658.12)	713.56 (352.75 to 1,443.07)	1,012.06 (467.20 to 1,910.97)
Chronic kidney disease due to glomerulonephritis	4,453.76 (3,958.42 to 5,035.20)	6,123.91 (3,509.30 to 10,815.67)	4,500.87 (2,704.90 to 7,992.55)	7,878.15 (4,515.27 to 13,496.69)	150.13 (133.20 to 168.89)	288.60 (144.50 to 547.60)	216.36 (114.55 to 428.01)	353.05 (175.65 to 644.11)
Chronic kidney disease due to other and unspecified causes	5,914.52 (5,263.11 to 6,715.11)	9,495.17 (5,013.85 to 17,402.99)	7,168.60 (4,011.99 to 13,089.54)	12,206.99 (6,521.83 to 21,388.73)	235.98 (206.97 to 266.43)	520.30 (250.38 to 1,012.77)	407.13 (206.35 to 812.42)	629.97 (306.35 to 1,170.65)
Urinary diseases and male infertility	5,825.72 (5,620.25 to 6,028.46)	10,473.40 (9,404.22 to 11,515.63)	10,099.31 (8,879.84 to 11,289.62)	10,442.37 (9,608.46 to 11,258.00)	275.21 (267.02 to 284.13)	673.38 (620.79 to 723.68)	674.80 (608.73 to 735.92)	623.08 (584.82 to 662.33)
Urinary tract infections	4,040.91 (3,794.05 to 4,296.03)	8,187.29 (7,261.74 to 9,025.34)	7,948.73 (6,918.39 to 8,954.36)	7,956.90 (7,193.46 to 8,663.53)	203.55 (193.71 to 213.94)	551.28 (504.79 to 596.65)	555.11 (499.10 to 609.02)	501.23 (464.85 to 539.76)
Urolithiasis	415.06 (351.37 to 568.25)	813.54 (651.49 to 1,121.28)	787.99 (619.68 to 1,108.63)	816.48 (674.69 to 1,119.00)	18.71 (15.94 to 25.76)	45.04 (37.00 to 61.23)	44.78 (36.34 to 62.01)	42.49 (35.55 to 57.00)
Other urinary diseases	1,369.76 (1,187.74 to 1,559.01)	1,472.57 (1,198.79 to 1,753.96)	1,362.59 (1,093.81 to 1,654.70)	1,668.98 (1,378.79 to 1,961.91)	52.94 (45.28 to 59.29)	77.07 (63.86 to 89.42)	74.91 (61.52 to 88.70)	79.36 (66.28 to 92.17)
Gynecological diseases	265.48 (239.09 to 289.12)	284.37 (230.20 to 349.97)	244.55 (194.19 to 301.96)	339.31 (279.15 to 410.81)	8.34 (7.45 to 9.05)	10.50 (8.84 to 12.39)	9.56 (7.82 to 11.54)	11.70 (10.06 to 13.50)
Uterine fibroids	87.93 (59.02 to 109.92)	74.99 (42.46 to 105.95)	65.36 (35.29 to 93.87)	92.23 (53.35 to 128.68)	2.91 (1.97 to 3.64)	2.83 (1.58 to 3.94)	2.57 (1.35 to 3.66)	3.33 (1.91 to 4.53)
Polycystic ovarian syndrome	18.70 (7.00 to 35.78)	13.66 (3.89 to 29.70)	10.59 (2.92 to 23.31)	19.32 (5.47 to 40.97)	0.42 (0.16 to 0.79)	0.31 (0.09 to 0.67)	0.24 (0.07 to 0.53)	0.44 (0.13 to 0.93)
Endometriosis	3.05 (1.19 to 4.51)	3.34 (1.59 to 5.88)	2.62 (1.23 to 4.65)	3.92 (1.85 to 6.86)	0.07 (0.03 to 0.10)	0.07 (0.03 to 0.13)	0.06 (0.03 to 0.10)	0.09 (0.04 to 0.15)
Genital prolapse	14.40 (7.54 to 20.69)	15.50 (7.69 to 24.14)	13.90 (6.32 to 22.28)	18.41 (9.07 to 28.51)	0.90 (0.45 to 1.31)	1.03 (0.46 to 1.68)	0.98 (0.42 to 1.64)	1.12 (0.51 to 1.80)
Other gynecological diseases	141.39 (101.36 to 171.88)	176.88 (126.87 to 225.07)	152.08 (106.15 to 197.57)	205.42 (146.26 to 259.53)	4.04 (2.89 to 5.01)	6.26 (4.55 to 7.93)	5.72 (4.08 to 7.37)	6.71 (4.78 to 8.43)
Hemoglobinopathies and hemolytic anemias	5,749.22 (5,096.81 to 6,685.28)	5,821.36 (4,886.22 to 7,073.23)	5,032.04 (4,205.76 to 6,187.05)	6,911.70 (5,814.22 to 8,406.36)	127.99 (113.11 to 149.07)	181.21 (159.13 to 216.61)	168.10 (145.00 to 201.59)	195.46 (171.85 to 231.18)
Thalassemias	493.34 (422.79 to 608.87)	184.67 (142.54 to 241.87)	182.72 (140.34 to 237.25)	212.75 (159.42 to 288.85)	6.26 (5.43 to 7.66)	2.50 (1.98 to 3.21)	2.48 (1.95 to 3.17)	2.82 (2.16 to 3.77)

Sickle cell disorders	3,800.55 (3,296.48 to 4,494.70)	3,706.36 (2,989.69 to 4,597.67)	3,095.12 (2,511.01 to 3,853.18)	4,530.22 (3,657.00 to 5,615.16)	55.30 (48.14 to 65.79)	63.04 (51.29 to 78.98)	54.15 (43.97 to 67.90)	74.76 (60.61 to 93.02)
G6PD deficiency	711.78 (610.56 to 850.22)	1,028.18 (845.98 to 1,300.35)	896.58 (730.08 to 1,134.38)	1,230.67 (1,010.25 to 1,531.68)	17.86 (15.35 to 21.64)	35.39 (29.60 to 45.06)	31.84 (26.20 to 41.14)	40.40 (34.04 to 51.04)
Other hemoglobinopathies and hemolytic anemias	743.55 (651.51 to 875.17)	902.14 (780.68 to 1,084.40)	857.62 (729.74 to 1,027.49)	938.05 (802.30 to 1,122.18)	48.58 (42.73 to 56.86)	80.28 (68.59 to 93.82)	79.62 (68.41 to 93.64)	77.48 (63.86 to 92.19)
Endocrine, metabolic, blood, and immune disorders	4,389.11 (3,902.73 to 4,910.93)	5,957.92 (4,939.59 to 7,257.04)	5,910.95 (4,866.98 to 7,266.20)	6,059.16 (5,099.05 to 7,223.37)	144.30 (122.64 to 153.63)	275.97 (225.91 to 307.99)	290.56 (235.23 to 327.44)	259.08 (217.74 to 287.95)

Global								
Cause	YLLs (in 1000s)				Deaths (in 1000s)			
	2016	2040 Forecast	2040 Better Scenario	2040 Worse Scenario	2016	2040 Forecast	2040 Better Scenario	2040 Worse Scenario
**Musculoskeletal disorders**	**2,198.24 (1,965.60 to 2,494.14)**	**3,544.34 (3,028.26 to 4,084.00)**	**3,412.47 (2,914.47 to 3,972.27)**	**3,616.19 (3,093.73 to 4,086.64)**	**89.23 (78.89 to 98.13)**	**188.29 (161.74 to 209.22)**	**184.63 (158.24 to 204.20)**	**186.95 (161.38 to 208.75)**
Rheumatoid arthritis	574.17 (487.59 to 668.99)	1,069.58 (874.32 to 1,284.35)	931.47 (748.40 to 1,122.69)	1,243.81 (1,021.09 to 1,468.71)	31.00 (26.47 to 35.76)	71.89 (58.56 to 85.88)	64.46 (52.01 to 76.38)	80.25 (65.63 to 95.56)
Other musculoskeletal disorders	1,624.07 (1,432.71 to 1,864.54)	2,474.76 (2,081.88 to 2,870.45)	2,481.00 (2,079.01 to 2,888.95)	2,372.38 (2,010.69 to 2,722.34)	58.23 (51.06 to 64.61)	116.40 (98.61 to 128.73)	120.18 (102.11 to 133.71)	106.70 (91.00 to 117.93)
**Other non-communicable diseases**	**45,970.60 (40,880.95 to 50,868.23)**	**32,498.67 (26,323.87 to 39,193.51)**	**26,083.49 (21,424.77 to 30,943.41)**	**43,955.59 (34,887.61 to 53,265.93)**	**639.68 (576.40 to 703.63)**	**723.81 (584.78 to 862.05)**	**657.40 (517.42 to 778.74)**	**832.98 (689.93 to 988.29)**
Congenital birth defects	40,707.17 (35,761.86 to 45,627.57)	23,891.63 (18,373.48 to 29,737.60)	17,921.75 (14,145.59 to 21,991.54)	34,534.25 (26,046.99 to 43,354.80)	498.93 (440.16 to 556.55)	312.59 (246.52 to 380.94)	239.75 (193.49 to 289.11)	440.16 (340.21 to 544.68)
Neural tube defects	3,407.47 (2,362.11 to 5,150.35)	1,042.20 (603.90 to 1,752.97)	652.52 (384.98 to 1,111.26)	1,759.74 (1,058.91 to 2,946.79)	40.07 (27.87 to 60.47)	12.61 (7.42 to 20.90)	7.97 (4.78 to 13.43)	21.07 (12.80 to 35.00)
Congenital heart anomalies	17,809.17 (15,807.17 to 20,444.43)	9,579.33 (7,582.35 to 11,947.30)	7,255.60 (5,769.92 to 9,128.12)	13,693.98 (10,668.49 to 17,478.75)	221.28 (197.71 to 253.82)	127.35 (102.29 to 156.13)	98.12 (79.81 to 120.96)	177.69 (141.58 to 222.47)
Orofacial clefts	192.20 (107.02 to 316.42)	90.98 (38.00 to 181.19)	55.38 (23.34 to 106.52)	159.26 (65.14 to 320.07)	2.23 (1.24 to 3.67)	1.05 (0.44 to 2.10)	0.64 (0.27 to 1.23)	1.85 (0.75 to 3.71)
Down syndrome	981.25 (850.19 to 1,274.58)	906.05 (741.43 to 1,220.88)	805.56 (668.53 to 1,019.28)	1,117.77 (883.88 to 1,613.47)	14.84 (12.82 to 18.42)	16.49 (13.80 to 20.55)	15.36 (12.89 to 18.48)	19.11 (15.91 to 25.18)
Other chromosomal abnormalities	1,457.92 (1,090.95 to 2,066.93)	1,552.71 (1,073.91 to 2,233.07)	1,151.81 (825.40 to 1,588.11)	2,105.68 (1,396.48 to 3,092.50)	17.49 (13.20 to 24.55)	19.15 (13.38 to 27.23)	14.38 (10.46 to 19.67)	25.63 (17.28 to 37.16)
Congenital musculoskeletal and limb anomalies	722.00 (519.66 to 1,318.57)	462.18 (296.11 to 900.62)	366.79 (232.37 to 738.21)	669.17 (409.27 to 1,361.19)	8.75 (6.35 to 15.70)	5.87 (3.88 to 11.19)	4.69 (3.06 to 9.13)	8.32 (5.26 to 16.54)
Urogenital congenital anomalies	896.51 (698.38 to 1,105.03)	595.82 (446.69 to 755.78)	484.30 (361.64 to 607.89)	817.88 (583.79 to 1,049.63)	12.05 (9.56 to 14.75)	9.76 (7.66 to 12.03)	8.28 (6.37 to 10.20)	12.48 (9.37 to 15.46)
Digestive congenital anomalies	2,915.74 (2,252.55 to 4,540.70)	1,641.38 (1,115.98 to 2,545.94)	1,173.89 (797.37 to 1,854.03)	2,447.01 (1,642.12 to 3,919.40)	34.29 (26.49 to 53.18)	19.73 (13.47 to 30.26)	14.19 (9.65 to 22.15)	29.15 (19.65 to 46.33)
Other congenital birth defects	12,324.90 (8,589.29 to 17,181.30)	8,020.98 (4,745.37 to 12,490.21)	5,975.90 (3,706.90 to 8,896.47)	11,763.75 (6,779.80 to 18,205.04)	147.93 (104.12 to 204.96)	100.59 (61.97 to 153.37)	76.12 (48.66 to 111.12)	144.86 (85.12 to 220.79)
Skin and subcutaneous diseases	2,759.24 (1,738.63 to 3,611.70)	7,329.44 (4,706.43 to 9,525.26)	7,164.78 (4,602.76 to 9,252.40)	7,517.44 (4,847.05 to 9,914.42)	111.69 (71.83 to 144.41)	396.40 (257.11 to 511.79)	406.09 (264.55 to 517.10)	370.73 (239.34 to 483.60)
Cellulitis	437.34 (235.60 to 565.93)	1,440.79 (793.09 to 1,849.23)	1,487.57 (815.41 to 1,926.52)	1,330.59 (737.47 to 1,698.73)	18.92 (10.44 to 25.50)	76.44 (42.65 to 97.55)	81.76 (45.22 to 103.09)	66.66 (37.16 to 86.07)
Pyoderma	1,827.47 (1,139.86 to 2,485.19)	4,797.13 (3,110.58 to 6,478.61)	4,613.05 (2,969.57 to 6,185.23)	5,116.54 (3,291.66 to 6,950.87)	62.02 (38.96 to 83.22)	236.38 (154.41 to 317.56)	240.01 (156.10 to 319.71)	226.71 (146.56 to 306.05)
Decubitus ulcer	380.63 (245.03 to 516.80)	879.61 (559.36 to 1,175.21)	856.71 (543.62 to 1,148.37)	856.44 (545.46 to 1,149.14)	26.44 (16.90 to 35.99)	73.79 (46.58 to 98.43)	74.29 (47.41 to 98.90)	68.33 (43.34 to 92.12)
Other skin and subcutaneous diseases	113.80 (77.82 to 160.51)	211.91 (146.21 to 303.33)	207.46 (141.39 to 295.20)	213.87 (145.69 to 304.19)	4.31 (3.04 to 6.30)	9.78 (6.92 to 14.04)	10.03 (7.09 to 14.28)	9.03 (6.28 to 13.05)
Sudden infant death syndrome	2,504.19 (2,015.23 to 3,003.09)	1,277.60 (867.68 to 1,723.21)	996.96 (674.24 to 1,352.29)	1,903.90 (1,269.93 to 2,615.15)	29.06 (23.39 to 34.85)	14.82 (10.07 to 20.00)	11.57 (7.82 to 15.69)	22.09 (14.74 to 30.34)

**Injuries**	**200,076.35 (191,347.73 to 207,066.54)**	**171,411.76 (147,221.44 to 201,646.36)**	**145,245.64 (125,660.86 to 169,190.33)**	**212,669.60 (182,791.13 to 252,362.83)**	**4,610.99 (4,364.81 to 4,768.86)**	**5,209.86 (4,568.75 to 5,950.88)**	**4,684.49 (4,148.96 to 5,287.11)**	**5,983.45 (5,255.04 to 6,852.72)**
**Transport injuries**	**65,706.86 (63,870.89 to 68,591.16)**	**54,924.92 (44,368.75 to 69,968.56)**	**41,113.87 (34,103.85 to 50,773.35)**	**82,310.65 (66,220.67 to 105,581.13)**	**1,437.29 (1,400.00 to 1,492.45)**	**1,398.53 (1,150.12 to 1,745.57)**	**1,088.56 (916.87 to 1,326.40)**	**2,017.82 (1,639.44 to 2,568.95)**
Road injuries	61,412.07 (59,638.88 to 64,244.07)	49,718.06 (40,359.63 to 63,227.72)	36,676.91 (30,362.46 to 45,130.16)	76,184.97 (61,435.51 to 97,702.33)	1,342.28 (1,307.57 to 1,393.72)	1,259.64 (1,035.55 to 1,572.62)	967.22 (814.30 to 1,176.58)	1,860.39 (1,509.07 to 2,367.88)
Pedestrian road injuries	21,740.97 (20,466.35 to 23,243.54)	14,191.12 (11,492.15 to 18,072.23)	10,310.14 (8,555.22 to 12,779.30)	22,613.51 (18,114.64 to 28,802.24)	514.33 (485.76 to 546.68)	419.39 (346.57 to 523.45)	315.07 (265.02 to 383.06)	649.54 (532.26 to 821.22)

Global								
Cause	YLLs (in 1000s)				Deaths (in 1000s)			
	2016	2040 Forecast	2040 Better Scenario	2040 Worse Scenario	2016	2040 Forecast	2040 Better Scenario	2040 Worse Scenario
Cyclist road injuries	3,095.55 (2,811.61 to 3,487.67)	2,619.62 (2,052.39 to 3,502.60)	2,079.93 (1,649.80 to 2,795.53)	3,985.58 (3,103.28 to 5,312.80)	74.75 (68.50 to 83.50)	77.70 (61.07 to 102.46)	65.04 (52.22 to 83.92)	108.46 (84.83 to 143.43)
Motorcyclist road injuries	12,601.43 (11,425.94 to 13,642.74)	10,841.91 (8,362.63 to 14,255.41)	9,022.79 (7,142.92 to 11,502.85)	15,354.66 (11,750.17 to 20,512.54)	251.26 (227.03 to 269.91)	241.44 (187.91 to 316.28)	206.41 (164.05 to 261.30)	326.73 (252.73 to 434.08)
Motor vehicle road injuries	23,391.19 (21,813.46 to 26,453.91)	21,553.36 (17,141.92 to 27,808.12)	14,885.57 (12,034.32 to 18,631.40)	33,410.29 (26,327.08 to 43,129.29)	488.71 (454.63 to 549.39)	506.39 (409.56 to 636.07)	368.85 (303.73 to 456.37)	753.96 (603.18 to 964.84)
Other road injuries	582.93 (535.13 to 725.78)	512.05 (405.99 to 693.66)	378.48 (303.43 to 503.13)	820.92 (641.65 to 1,130.29)	13.23 (12.19 to 16.38)	14.72 (11.73 to 19.94)	11.85 (9.66 to 15.84)	21.70 (17.11 to 30.07)
Other transport injuries	4,294.79 (3,991.27 to 4,796.07)	5,206.86 (4,071.37 to 6,856.82)	4,436.97 (3,574.73 to 5,702.85)	6,125.68 (4,787.08 to 8,054.59)	95.01 (88.78 to 106.62)	138.89 (110.24 to 178.04)	121.35 (98.92 to 153.17)	157.43 (125.36 to 202.14)
**Unintentional injuries**	**69,727.11 (62,737.61 to 73,048.22)**	**58,035.20 (50,169.88 to 65,332.70)**	**52,599.50 (44,898.56 to 59,707.40)**	**66,537.97 (56,941.19 to 74,887.67)**	**1,803.86 (1,587.98 to 1,889.28)**	**2,336.80 (1,979.16 to 2,560.75)**	**2,276.38 (1,917.68 to 2,501.99)**	**2,392.22 (2,018.78 to 2,612.87)**
Falls	16,827.42 (14,324.96 to 17,828.35)	20,843.05 (17,342.11 to 23,095.90)	20,391.98 (16,791.25 to 22,636.74)	21,531.45 (17,847.57 to 23,603.46)	678.46 (559.21 to 719.32)	1,249.81 (1,004.57 to 1,378.99)	1,277.71 (1,032.27 to 1,410.74)	1,182.16 (942.55 to 1,307.96)
Drowning	16,575.72 (15,016.39 to 17,803.41)	9,515.91 (7,898.89 to 11,597.41)	8,174.01 (6,844.77 to 9,793.31)	11,576.48 (9,551.98 to 14,084.69)	302.93 (272.75 to 322.39)	236.61 (196.01 to 282.91)	212.44 (178.23 to 249.07)	269.36 (222.52 to 321.97)
Fire, heat, and hot substances	5,696.05 (4,651.71 to 6,188.51)	3,872.99 (3,080.63 to 4,784.86)	3,215.98 (2,564.56 to 3,918.26)	4,770.36 (3,791.51 to 5,853.21)	132.08 (110.13 to 141.63)	125.38 (102.91 to 150.12)	110.03 (91.18 to 129.99)	141.38 (115.02 to 169.78)
Poisonings	2,851.04 (2,118.58 to 3,240.46)	1,295.50 (949.41 to 1,616.91)	982.82 (709.36 to 1,214.35)	1,787.45 (1,267.17 to 2,247.11)	57.08 (42.42 to 63.58)	32.71 (24.15 to 40.02)	26.22 (19.19 to 31.87)	42.27 (30.66 to 51.64)
Exposure to mechanical forces	7,509.63 (6,132.22 to 8,051.89)	5,291.63 (4,249.96 to 6,156.02)	4,496.95 (3,577.64 to 5,227.79)	6,460.05 (5,169.79 to 7,446.92)	154.84 (123.96 to 165.14)	139.59 (110.61 to 158.03)	125.22 (98.67 to 141.53)	159.31 (125.44 to 178.21)
Unintentional firearm injuries	1,123.71 (881.70 to 1,233.13)	846.76 (620.39 to 1,001.00)	696.08 (513.53 to 824.74)	1,005.56 (738.69 to 1,186.41)	22.95 (18.24 to 24.76)	21.20 (16.26 to 24.02)	18.50 (14.22 to 20.97)	23.65 (18.08 to 26.91)
Unintentional suffocation	1,474.69 (1,151.64 to 1,717.10)	816.50 (633.32 to 1,028.23)	642.35 (497.92 to 795.03)	1,074.29 (829.09 to 1,335.94)	22.63 (17.38 to 26.00)	16.45 (12.31 to 19.92)	14.13 (10.52 to 16.91)	19.49 (14.84 to 23.50)
Other exposure to mechanical forces	4,911.23 (3,856.75 to 5,218.17)	3,628.37 (2,795.58 to 4,199.36)	3,158.52 (2,427.20 to 3,655.87)	4,380.19 (3,354.63 to 5,016.79)	109.26 (84.20 to 115.90)	101.94 (78.94 to 115.50)	92.59 (70.89 to 105.07)	116.16 (89.26 to 130.06)
Adverse effects of medical treatment	4,601.97 (3,861.10 to 5,157.12)	4,591.94 (3,785.40 to 5,318.64)	4,203.13 (3,463.06 to 4,861.45)	5,237.89 (4,301.18 to 6,056.59)	126.73 (109.32 to 140.49)	176.46 (151.58 to 199.37)	172.47 (146.78 to 194.81)	181.90 (156.20 to 203.86)
Animal contact	4,268.95 (3,176.54 to 4,791.49)	3,863.23 (2,882.55 to 4,676.66)	3,476.77 (2,555.46 to 4,202.68)	5,038.65 (3,643.82 to 6,056.01)	91.59 (68.84 to 102.20)	109.59 (81.36 to 130.45)	103.12 (76.21 to 124.02)	131.89 (96.33 to 155.99)
Venomous animal contact	3,662.04 (2,606.92 to 4,190.13)	3,155.89 (2,224.65 to 3,863.75)	2,882.39 (2,012.86 to 3,545.85)	4,147.59 (2,861.95 to 5,075.09)	78.81 (56.81 to 89.39)	90.57 (64.50 to 109.81)	86.11 (61.40 to 104.92)	109.77 (76.76 to 132.75)
Non-venomous animal contact	606.90 (479.70 to 841.74)	707.34 (531.99 to 976.39)	594.38 (445.12 to 810.49)	891.06 (656.39 to 1,252.01)	12.78 (10.32 to 17.37)	19.03 (14.78 to 25.58)	17.01 (13.20 to 23.04)	22.11 (17.06 to 29.85)
Foreign body	4,703.00 (4,114.40 to 5,317.87)	4,158.61 (3,433.49 to 4,910.99)	3,797.98 (3,154.03 to 4,411.37)	4,544.95 (3,710.12 to 5,469.50)	106.27 (92.49 to 114.91)	134.02 (110.12 to 149.25)	133.28 (109.52 to 148.90)	130.69 (108.11 to 145.76)
Pulmonary aspiration and foreign body in airway	4,203.16 (3,638.86 to 4,809.69)	3,691.10 (3,040.60 to 4,424.71)	3,355.72 (2,795.26 to 3,939.14)	4,023.99 (3,261.98 to 4,907.38)	95.92 (82.50 to 104.51)	120.50 (98.40 to 134.58)	119.52 (96.54 to 133.61)	117.10 (95.28 to 131.07)

Foreign body in other body part	499.84 (372.99 to 586.34)	467.51 (320.49 to 592.65)	442.26 (309.15 to 559.28)	520.96 (349.29 to 660.42)	10.34 (7.86 to 12.04)	13.52 (9.89 to 16.36)	13.76 (10.19 to 16.57)	13.59 (9.69 to 16.57)
Environmental heat and cold exposure	1,920.96 (1,216.20 to 2,408.60)	1,720.16 (1,065.90 to 2,230.22)	1,396.72 (836.96 to 1,805.61)	2,074.83 (1,265.09 to 2,598.66)	55.60 (36.43 to 71.46)	62.65 (40.72 to 79.14)	54.48 (34.26 to 68.02)	70.75 (44.91 to 86.51)
Other unintentional injuries	4,772.37 (4,186.14 to 5,000.09)	2,882.20 (2,463.58 to 3,291.20)	2,463.16 (2,101.07 to 2,823.15)	3,515.86 (3,024.45 to 3,970.19)	98.29 (84.22 to 102.79)	69.98 (59.52 to 78.56)	61.40 (51.44 to 69.58)	82.52 (69.70 to 91.31)
**Self-harm and interpersonal violence**	**54,833.93 (50,105.60 to 58,459.47)**	**54,442.32 (42,904.37 to 69,895.61)**	**47,595.98 (38,413.42 to 59,974.15)**	**59,673.40 (46,283.80 to 77,972.05)**	**1,207.94 (1,108.85 to 1,290.98)**	**1,391.68 (1,099.94 to 1,778.88)**	**1,236.30 (1,003.51 to 1,536.72)**	**1,490.14 (1,157.08 to 1,944.05)**

Global								
Cause	YLLs (in 1000s)				Deaths (in 1000s)			
	2016	2040 Forecast	2040 Better Scenario	2040 Worse Scenario	2016	2040 Forecast	2040 Better Scenario	2040 Worse Scenario
Self-harm	34,621.42 (32,412.04 to 37,408.58)	37,326.03 (28,206.16 to 49,963.66)	32,614.98 (25,598.35 to 41,702.33)	41,538.23 (30,701.94 to 56,783.20)	817.15 (762.05 to 883.74)	1,031.09 (784.85 to 1,351.64)	917.14 (725.86 to 1,156.50)	1,113.96 (825.22 to 1,504.06)
Self-harm by firearm	2,840.07 (2,373.75 to 3,578.95)	2,507.22 (1,618.53 to 3,726.99)	2,064.63 (1,373.43 to 2,973.93)	2,869.66 (1,804.74 to 4,438.30)	67.52 (55.39 to 84.13)	66.91 (44.86 to 96.43)	55.99 (38.62 to 77.74)	74.51 (48.10 to 111.72)
Self-harm by other specified means	31,781.35 (29,699.54 to 34,445.40)	34,818.82 (26,435.47 to 46,321.75)	30,550.35 (24,019.59 to 38,980.59)	38,668.58 (28,670.50 to 52,510.59)	749.63 (700.93 to 812.55)	964.18 (736.67 to 1,261.75)	861.15 (681.38 to 1,087.17)	1,039.45 (773.92 to 1,393.11)
Interpersonal violence	20,212.52 (16,632.13 to 23,093.86)	17,116.29 (12,987.52 to 21,492.53)	14,981.00 (11,524.97 to 18,786.98)	18,135.16 (13,792.69 to 22,733.29)	390.79 (320.78 to 453.71)	360.59 (281.23 to 447.02)	319.16 (247.11 to 397.81)	376.18 (293.10 to 468.13)
Physical violence by firearm	8,615.86 (5,744.46 to 9,727.92)	6,933.53 (4,597.26 to 8,866.50)	5,869.14 (3,956.32 to 7,536.96)	7,305.21 (4,739.26 to 9,327.73)	160.98 (107.16 to 182.48)	137.99 (92.61 to 173.13)	118.16 (80.88 to 147.57)	143.86 (94.26 to 179.51)
Physical violence by sharp object	4,876.53 (3,900.91 to 6,470.22)	4,396.69 (3,169.75 to 5,967.74)	3,949.36 (2,839.32 to 5,346.07)	4,446.66 (3,216.35 to 6,069.05)	97.39 (78.14 to 128.54)	95.18 (69.37 to 127.34)	86.28 (62.50 to 114.32)	95.14 (69.57 to 128.43)
Physical violence by other means	6,720.13 (5,734.01 to 8,489.35)	5,786.07 (4,242.44 to 7,838.06)	5,162.51 (3,805.43 to 6,957.57)	6,383.29 (4,673.84 to 8,650.12)	132.43 (111.32 to 168.39)	127.41 (94.48 to 173.55)	114.71 (86.32 to 155.06)	137.18 (101.91 to 185.62)
**Forces of nature, conflict and terrorism, and executions and police conflict**	**9,808.44 (6,797.54 to 13,037.71)**	**4,009.32 (708.41 to 18,516.93)**	**3,936.29 (690.83 to 18,617.09)**	**4,147.59 (748.67 to 18,601.11)**	**161.89 (112.58 to 215.06)**	**82.85 (13.20 to 398.28)**	**83.26 (13.16 to 412.92)**	**83.27 (13.57 to 391.45)**
Exposure to forces of nature	357.59 (217.91 to 507.75)	1,385.45 (117.06 to 9,800.63)	1,357.06 (116.38 to 9,373.33)	1,438.92 (119.16 to 10,127.35)	7.06 (4.22 to 10.13)	31.65 (2.86 to 196.72)	31.88 (2.87 to 196.37)	31.63 (2.93 to 195.78)
Conflict and terrorism	9,226.02 (6,241.21 to 12,407.43)	1,755.63 (207.68 to 12,383.02)	1,721.89 (204.66 to 12,433.62)	1,833.00 (210.50 to 12,389.01)	150.46 (101.46 to 202.67)	33.49 (4.10 to 265.45)	33.68 (4.13 to 277.92)	33.99 (4.08 to 260.94)
Executions and police conflict	224.83 (119.67 to 261.63)	868.25 (0.00 to 5,258.86)	857.34 (0.00 to 5,167.22)	875.67 (0.00 to 5,343.87)	4.38 (2.28 to 5.04)	17.71 (0.00 to 104.08)	17.70 (0.00 to 103.50)	17.65 (0.00 to 104.10)

Southeast Asia, East Asia, and Oceania								
**Cause**	**YLLs (in 1000s)**				**Deaths (in 1000s)**			
	**2016**	**2040 Forecast**	**2040 Better Scenario**	**2040 Worse Scenario**	**2016**	**2040 Forecast**	**2040 Better Scenario**	**2040 Worse Scenario**
**All causes**	**340,730.94 (334,746.11 to 346,653.02)**	**351,963.37 (299,634.52 to 417,811.07)**	**286,541.66 (246,928.57 to 332,002.68)**	**491,368.42 (407,951.53 to 588,073.99)**	**14,263.65 (14,037.66 to 14,492.09)**	**21,660.09 (18,846.39 to 25,076.10)**	**18,477.07 (16,281.33 to 20,863.78)**	**28,533.07 (24,610.42 to 32,887.62)**
**Communicable, maternal, neonatal, and nutritional diseases**	**47,609.16 (45,330.95 to 49,760.23)**	**28,280.71 (23,472.04 to 34,770.50)**	**21,013.30 (17,445.23 to 25,882.54)**	**41,153.29 (33,611.84 to 51,472.87)**	**1,112.78 (1,045.93 to 1,166.86)**	**1,227.95 (992.18 to 1,548.17)**	**1,023.35 (827.16 to 1,281.69)**	**1,641.43 (1,292.01 to 2,102.28)**
**HIV/AIDS and tuberculosis**	**9,125.43 (8,644.28 to 9,679.03)**	**5,339.57 (4,216.10 to 6,841.51)**	**3,418.00 (2,678.33 to 4,322.47)**	**8,901.26 (7,080.14 to 11,300.77)**	**277.74 (264.00 to 292.79)**	**221.35 (170.74 to 286.17)**	**149.40 (117.55 to 191.36)**	**341.97 (262.27 to 447.62)**
Tuberculosis	5,966.85 (5,673.72 to 6,282.79)	3,835.01 (2,781.92 to 5,227.11)	2,466.86 (1,843.62 to 3,379.70)	6,051.00 (4,325.23 to 8,441.69)	208.16 (198.14 to 219.94)	184.06 (135.60 to 248.97)	123.67 (92.63 to 166.15)	277.43 (200.22 to 382.79)
HIV/AIDS	3,158.58 (2,772.28 to 3,632.34)	1,504.56 (1,184.39 to 1,965.33)	951.14 (671.02 to 1,353.56)	2,850.26 (2,320.82 to 3,543.39)	69.58 (60.90 to 80.24)	37.29 (29.44 to 48.93)	25.73 (18.96 to 36.15)	64.54 (52.44 to 80.63)
**Diarrhea, lower respiratory, and other common infectious diseases**	**18,477.07 (17,100.57 to 20,015.23)**	**13,184.89 (9,306.02 to 18,906.38)**	**9,953.89 (7,145.09 to 14,237.71)**	**19,628.10 (13,412.79 to 29,002.31)**	**534.84 (469.21 to 584.75)**	**773.69 (554.86 to 1,082.26)**	**658.08 (476.74 to 913.70)**	**1,036.11 (712.27 to 1,468.69)**
Diarrheal diseases	2,888.10 (2,270.83 to 3,736.04)	1,997.50 (387.94 to 5,629.81)	1,753.20 (403.44 to 5,002.77)	3,010.08 (561.68 to 8,592.92)	82.39 (52.85 to 114.89)	122.23 (21.72 to 360.37)	119.72 (26.83 to 340.28)	170.19 (29.25 to 513.88)
Intestinal infectious diseases	1,610.67 (904.13 to 2,645.30)	845.60 (441.93 to 1,424.15)	807.93 (438.05 to 1,339.95)	950.11 (484.26 to 1,622.60)	24.47 (13.62 to 39.99)	14.77 (7.96 to 24.61)	14.61 (8.05 to 23.75)	16.14 (8.48 to 27.23)
Typhoid fever	1,393.57 (757.93 to 2,284.44)	719.71 (369.74 to 1,222.09)	706.49 (373.15 to 1,166.62)	797.94 (400.71 to 1,378.00)	21.06 (11.53 to 34.63)	12.49 (6.59 to 20.82)	12.71 (6.93 to 20.81)	13.44 (6.85 to 22.86)
Paratyphoid fever	212.90 (100.57 to 409.57)	124.60 (55.42 to 238.26)	100.36 (44.78 to 191.36)	150.52 (66.19 to 291.35)	3.33 (1.55 to 6.40)	2.22 (0.99 to 4.31)	1.85 (0.83 to 3.55)	2.63 (1.18 to 5.12)
Other intestinal infectious diseases	4.20 (1.66 to 9.04)	1.28 (0.48 to 2.81)	1.08 (0.40 to 2.31)	1.64 (0.62 to 3.70)	0.08 (0.03 to 0.17)	0.05 (0.02 to 0.11)	0.05 (0.02 to 0.10)	0.06 (0.02 to 0.13)
Lower respiratory infections	11,116.01 (10,043.76 to 12,011.27)	8,570.74 (6,279.91 to 11,987.39)	6,160.23 (4,659.79 to 8,115.81)	13,099.69 (9,079.02 to 19,521.96)	380.06 (328.34 to 412.92)	598.51 (454.85 to 775.38)	492.52 (375.95 to 621.11)	800.97 (583.82 to 1,077.09)
Upper respiratory infections	14.14 (12.58 to 16.08)	7.60 (5.81 to 9.90)	5.76 (4.29 to 7.64)	11.44 (8.60 to 15.08)	0.38 (0.34 to 0.42)	0.33 (0.25 to 0.44)	0.27 (0.20 to 0.36)	0.48 (0.35 to 0.64)
Otitis media	8.20 (3.79 to 13.48)	3.06 (1.26 to 5.03)	3.13 (1.28 to 5.16)	2.95 (1.21 to 4.96)	0.23 (0.12 to 0.35)	0.15 (0.06 to 0.25)	0.16 (0.07 to 0.26)	0.14 (0.06 to 0.23)
Meningitis	1,179.69 (888.06 to 1,392.26)	472.36 (316.25 to 653.61)	379.13 (265.30 to 502.79)	583.85 (381.81 to 826.66)	20.86 (15.57 to 24.88)	12.96 (9.44 to 17.23)	11.42 (8.27 to 15.56)	14.90 (10.75 to 19.78)
Pneumococcal meningitis	33.52 (24.36 to 42.10)	11.66 (8.41 to 17.45)	11.74 (8.46 to 17.59)	11.80 (8.50 to 17.02)	0.75 (0.56 to 1.01)	0.58 (0.42 to 0.91)	0.62 (0.45 to 0.97)	0.52 (0.39 to 0.82)
H influenzae type B meningitis	94.04 (72.00 to 115.28)	65.90 (25.88 to 155.52)	41.03 (22.70 to 77.21)	106.63 (29.93 to 282.74)	1.57 (1.19 to 1.88)	1.30 (0.76 to 2.42)	1.05 (0.71 to 1.60)	1.72 (0.78 to 3.84)
Meningococcal meningitis	432.73 (321.51 to 520.69)	158.01 (111.27 to 214.04)	133.42 (94.04 to 181.11)	191.13 (136.44 to 255.43)	8.28 (6.14 to 10.40)	5.21 (3.73 to 7.25)	4.70 (3.37 to 6.51)	6.08 (4.39 to 8.37)
Other meningitis	619.41 (464.72 to 739.96)	236.78 (162.44 to 313.27)	192.93 (135.87 to 255.71)	274.30 (185.40 to 359.12)	10.26 (7.61 to 12.24)	5.87 (4.25 to 7.60)	5.06 (3.64 to 6.74)	6.58 (4.74 to 8.57)
Encephalitis	644.08 (488.68 to 739.96)	396.58 (280.95 to 519.67)	357.57 (260.26 to 472.51)	448.83 (310.67 to 589.33)	11.43 (8.97 to 12.92)	11.16 (8.52 to 13.51)	10.79 (8.32 to 12.94)	11.83 (9.08 to 14.36)

Diphtheria	4.24 (3.68 to 4.87)	0.77 (0.46 to 1.26)	0.54 (0.40 to 0.71)	1.23 (0.54 to 2.34)	0.07 (0.06 to 0.07)	0.01 (0.01 to 0.02)	0.01 (0.01 to 0.01)	0.02 (0.01 to 0.03)
Whooping cough	377.17 (145.28 to 774.61)	160.11 (16.63 to 419.73)	39.06 (8.72 to 97.47)	241.21 (23.52 to 618.51)	4.48 (1.73 to 9.20)	1.91 (0.21 to 4.98)	0.49 (0.11 to 1.20)	2.87 (0.30 to 7.32)
Tetanus	188.26 (82.50 to 233.79)	29.96 (11.74 to 42.57)	24.16 (9.15 to 33.99)	35.55 (14.76 to 50.32)	4.38 (1.78 to 5.51)	0.87 (0.32 to 1.21)	0.83 (0.30 to 1.16)	0.89 (0.34 to 1.25)
Measles	387.07 (149.13 to 807.94)	675.38 (140.27 to 2,462.43)	402.37 (112.79 to 1,321.21)	1,210.46 (185.51 to 4,604.13)	4.64 (1.79 to 9.68)	9.39 (2.30 to 30.68)	5.94 (1.88 to 16.99)	16.09 (3.02 to 56.92)

Southeast Asia, East Asia, and Oceania								
**Cause**	**YLLs (in 1000s)**				**Deaths (in 1000s)**			
	**2016**	**2040 Forecast**	**2040 Better Scenario**	**2040 Worse Scenario**	**2016**	**2040 Forecast**	**2040 Better Scenario**	**2040 Worse Scenario**
Varicella and herpes zoster	59.45 (52.94 to 68.10)	25.24 (18.02 to 32.80)	20.83 (15.64 to 27.22)	32.70 (22.33 to 43.28)	1.45 (1.34 to 1.58)	1.41 (1.14 to 1.73)	1.34 (1.09 to 1.64)	1.61 (1.27 to 2.01)
**Neglected tropical diseases and malaria**	**1,640.34 (1,064.77 to 1,995.00)**	**2,402.83 (1,655.56 to 2,917.66)**	**2,165.83 (1,500.10 to 2,601.42)**	**2,593.47 (1,753.60 to 3,166.40)**	**30.58 (20.83 to 36.64)**	**51.92 (37.25 to 61.48)**	**49.38 (35.67 to 58.17)**	**54.04 (38.15 to 64.18)**
Malaria	804.35 (514.13 to 1,079.93)	458.07 (316.64 to 813.22)	439.23 (308.20 to 782.93)	493.35 (336.79 to 887.87)	16.51 (11.22 to 23.22)	12.74 (8.89 to 23.28)	12.69 (8.90 to 22.68)	13.04 (8.98 to 24.04)
Chagas disease	0.00 (0.00 to 0.00)	0.00 (0.00 to 0.00)	0.00 (0.00 to 0.00)	0.00 (0.00 to 0.00)	0.00 (0.00 to 0.00)	0.00 (0.00 to 0.00)	0.00 (0.00 to 0.00)	0.00 (0.00 to 0.00)
Leishmaniasis	2.43 (1.55 to 3.79)	0.84 (0.51 to 1.43)	0.79 (0.49 to 1.32)	1.12 (0.69 to 1.92)	0.07 (0.05 to 0.12)	0.04 (0.02 to 0.06)	0.03 (0.02 to 0.06)	0.05 (0.03 to 0.08)
African trypanosomiasis	0.00 (0.00 to 0.00)	0.00 (0.00 to 0.00)	0.00 (0.00 to 0.00)	0.00 (0.00 to 0.00)	0.00 (0.00 to 0.00)	0.00 (0.00 to 0.00)	0.00 (0.00 to 0.00)	0.00 (0.00 to 0.00)
Schistosomiasis	16.19 (13.94 to 18.70)	10.01 (8.09 to 12.27)	9.67 (7.76 to 11.86)	10.87 (8.86 to 13.32)	0.61 (0.52 to 0.70)	0.50 (0.41 to 0.62)	0.50 (0.40 to 0.62)	0.52 (0.42 to 0.64)
Cysticercosis	2.61 (2.33 to 2.91)	0.88 (0.76 to 1.05)	0.86 (0.73 to 1.02)	0.93 (0.80 to 1.10)	0.07 (0.06 to 0.08)	0.03 (0.03 to 0.04)	0.03 (0.03 to 0.03)	0.03 (0.03 to 0.04)
Cystic echinococcosis	1.88 (1.60 to 2.19)	0.81 (0.62 to 1.06)	0.71 (0.56 to 0.90)	1.17 (0.89 to 1.55)	0.06 (0.05 to 0.07)	0.04 (0.03 to 0.05)	0.04 (0.03 to 0.05)	0.06 (0.04 to 0.07)
Dengue	670.36 (291.48 to 893.13)	1,852.35 (1,055.93 to 2,353.75)	1,643.58 (931.41 to 2,073.00)	1,989.30 (1,122.19 to 2,549.19)	10.21 (4.48 to 13.58)	36.27 (21.39 to 45.44)	33.88 (20.02 to 42.19)	37.73 (21.83 to 47.57)
Yellow fever	0.00 (0.00 to 0.00)	0.00 (0.00 to 0.00)	0.00 (0.00 to 0.00)	0.00 (0.00 to 0.00)	0.00 (0.00 to 0.00)	0.00 (0.00 to 0.00)	0.00 (0.00 to 0.00)	0.00 (0.00 to 0.00)
Rabies	72.97 (26.79 to 88.35)	24.01 (10.32 to 31.31)	19.03 (7.41 to 24.36)	36.86 (16.62 to 49.14)	1.63 (0.61 to 1.93)	0.66 (0.29 to 0.83)	0.56 (0.22 to 0.69)	0.98 (0.44 to 1.26)
Intestinal nematode infections	27.93 (23.48 to 33.28)	25.01 (18.92 to 35.69)	21.84 (16.50 to 31.93)	27.13 (20.47 to 38.44)	0.41 (0.36 to 0.48)	0.48 (0.39 to 0.66)	0.46 (0.37 to 0.63)	0.49 (0.39 to 0.66)
Other neglected tropical diseases	41.63 (37.38 to 55.56)	30.86 (26.08 to 42.45)	30.11 (25.62 to 39.86)	32.75 (27.21 to 48.17)	1.01 (0.89 to 1.30)	1.16 (1.00 to 1.55)	1.19 (1.02 to 1.56)	1.15 (0.99 to 1.64)
**Maternal disorders**	**1,210.64 (1,122.01 to 1,300.40)**	**550.75 (452.68 to 667.23)**	**433.14 (349.61 to 534.48)**	**785.08 (635.69 to 959.69)**	**22.27 (20.61 to 23.94)**	**10.31 (8.46 to 12.55)**	**8.15 (6.60 to 10.10)**	**14.70 (11.94 to 18.03)**
Maternal hemorrhage	398.29 (318.61 to 488.83)	174.13 (128.01 to 234.29)	137.14 (97.86 to 182.54)	247.86 (176.70 to 332.65)	7.29 (5.85 to 8.92)	3.24 (2.37 to 4.37)	2.56 (1.83 to 3.44)	4.61 (3.28 to 6.22)
Maternal sepsis and other maternal infections	68.50 (45.10 to 97.25)	29.81 (18.24 to 45.11)	22.39 (13.50 to 34.35)	41.62 (25.17 to 63.43)	1.30 (0.86 to 1.85)	0.57 (0.35 to 0.86)	0.43 (0.26 to 0.66)	0.80 (0.49 to 1.20)
Maternal hypertensive disorders	139.98 (103.71 to 183.32)	66.01 (45.02 to 93.30)	50.79 (34.27 to 72.06)	93.72 (63.89 to 133.05)	2.56 (1.88 to 3.36)	1.23 (0.84 to 1.74)	0.95 (0.64 to 1.36)	1.75 (1.19 to 2.50)
Maternal obstructed labor and uterine rupture	39.25 (22.53 to 61.23)	18.76 (10.04 to 31.76)	13.68 (7.16 to 23.43)	26.74 (14.27 to 44.97)	0.73 (0.42 to 1.15)	0.35 (0.19 to 0.62)	0.26 (0.14 to 0.45)	0.51 (0.27 to 0.86)
Maternal abortion, miscarriage, and ectopic pregnancy	129.89 (97.19 to 168.27)	59.51 (39.36 to 90.52)	46.72 (30.93 to 71.44)	88.55 (59.99 to 132.47)	2.41 (1.82 to 3.09)	1.13 (0.76 to 1.70)	0.89 (0.60 to 1.34)	1.69 (1.16 to 2.50)

Indirect maternal deaths	145.05 (101.92 to 198.11)	72.45 (46.68 to 105.06)	56.78 (36.32 to 83.44)	103.91 (67.44 to 148.28)	2.62 (1.85 to 3.53)	1.32 (0.86 to 1.90)	1.04 (0.66 to 1.52)	1.90 (1.22 to 2.70)
Late maternal deaths	26.33 (12.99 to 44.78)	10.84 (4.95 to 19.89)	8.23 (3.75 to 15.05)	15.32 (7.07 to 28.20)	0.49 (0.24 to 0.83)	0.20 (0.09 to 0.37)	0.16 (0.07 to 0.29)	0.29 (0.13 to 0.53)
Maternal deaths aggravated by HIV/AIDS	1.01 (0.54 to 1.53)	2.07 (0.96 to 3.56)	1.20 (0.53 to 2.14)	3.18 (1.60 to 5.24)	0.02 (0.01 to 0.03)	0.04 (0.02 to 0.07)	0.02 (0.01 to 0.04)	0.06 (0.03 to 0.10)
Other maternal disorders	262.33 (198.24 to 331.19)	117.17 (80.90 to 165.05)	96.21 (65.98 to 138.03)	164.18 (113.77 to 230.81)	4.85 (3.72 to 6.13)	2.22 (1.53 to 3.11)	1.83 (1.23 to 2.60)	3.10 (2.13 to 4.36)

Cause	YLLs (in 1000s)				Deaths (in 1000s)			
	2016	2040 Forecast	2040 Better Scenario	2040 Worse Scenario	2016	2040 Forecast	2040 Better Scenario	2040 Worse Scenario
**Neonatal disorders**	**14,807.95 (13,781.10 to 15,854.07)**	**5,029.74 (3,619.20 to 6,721.54)**	**3,359.57 (2,362.14 to 4,489.64)**	**7,259.32 (5,117.14 to 9,740.34)**	**171.11 (159.25 to 183.20)**	**58.13 (41.82 to 77.69)**	**38.83 (27.31 to 51.90)**	**83.89 (59.14 to 112.54)**
Neonatal preterm birth	6,918.17 (6,360.64 to 7,560.06)	2,038.75 (1,554.65 to 2,553.05)	1,230.79 (924.77 to 1,561.77)	3,085.58 (2,334.26 to 3,858.41)	79.93 (73.48 to 87.34)	23.56 (17.96 to 29.50)	14.22 (10.69 to 18.04)	35.65 (26.97 to 44.58)
Neonatal encephalopathy due to birth asphyxia and trauma	4,596.70 (4,138.97 to 5,109.37)	1,433.47 (846.14 to 2,218.24)	1,038.49 (597.52 to 1,626.38)	2,036.43 (1,163.16 to 3,199.80)	53.12 (47.83 to 59.04)	16.57 (9.78 to 25.63)	12.00 (6.91 to 18.79)	23.53 (13.44 to 36.97)
Neonatal sepsis and other neonatal infections	1,673.42 (1,388.87 to 2,187.89)	958.06 (669.96 to 1,348.86)	641.81 (446.10 to 905.69)	1,302.88 (904.25 to 1,861.04)	19.34 (16.05 to 25.28)	11.07 (7.74 to 15.59)	7.42 (5.16 to 10.47)	15.06 (10.45 to 21.51)
Hemolytic disease and other neonatal jaundice	146.17 (128.55 to 167.05)	31.00 (19.49 to 49.25)	21.88 (13.52 to 34.78)	45.40 (28.17 to 73.54)	1.69 (1.49 to 1.93)	0.36 (0.23 to 0.57)	0.25 (0.16 to 0.40)	0.52 (0.33 to 0.85)
Other neonatal disorders	1,473.48 (1,313.92 to 1,665.09)	568.46 (374.49 to 826.76)	426.60 (276.18 to 627.83)	789.03 (515.54 to 1,155.01)	17.04 (15.19 to 19.26)	6.57 (4.33 to 9.56)	4.93 (3.19 to 7.26)	9.12 (5.96 to 13.35)
**Nutritional deficiencies**	**788.54 (738.24 to 850.19)**	**795.88 (710.69 to 891.89)**	**827.64 (747.62 to 921.62)**	**804.61 (705.65 to 909.03)**	**39.38 (37.63 to 41.25)**	**74.17 (61.78 to 86.55)**	**83.71 (72.81 to 95.68)**	**67.01 (54.62 to 80.14)**
Protein-energy malnutrition	653.67 (606.77 to 716.51)	640.54 (556.24 to 734.48)	679.63 (602.35 to 772.10)	636.03 (547.16 to 733.21)	33.54 (31.86 to 35.78)	62.41 (51.40 to 74.29)	71.69 (60.72 to 83.15)	55.26 (43.87 to 66.98)
Iodine deficiency	7.99 (5.54 to 12.21)	24.98 (7.72 to 63.10)	23.98 (6.74 to 64.19)	28.22 (9.11 to 64.01)	0.26 (0.18 to 0.36)	2.14 (0.44 to 6.74)	2.20 (0.41 to 7.16)	2.24 (0.51 to 6.28)
Dietary iron deficiency	34.07 (21.23 to 40.68)	34.19 (24.85 to 43.12)	32.09 (24.32 to 40.81)	36.28 (25.54 to 45.75)	0.86 (0.61 to 1.03)	1.40 (1.00 to 1.95)	1.43 (1.01 to 2.06)	1.35 (0.97 to 1.87)
Other nutritional deficiencies	92.81 (80.16 to 104.98)	96.16 (79.19 to 112.42)	91.95 (74.14 to 108.14)	104.09 (85.84 to 122.96)	4.72 (3.83 to 5.27)	8.23 (6.42 to 9.93)	8.40 (6.60 to 10.02)	8.16 (6.20 to 9.90)
**Other communicable, maternal, neonatal, and nutritional diseases**	**1,559.19 (1,286.28 to 1,849.64)**	**977.05 (862.44 to 1,111.82)**	**855.23 (755.41 to 970.72)**	**1,181.45 (1,040.43 to 1,331.65)**	**36.86 (33.45 to 40.45)**	**38.37 (34.92 to 42.53)**	**35.80 (32.57 to 39.54)**	**43.72 (39.54 to 48.05)**
Sexually transmitted infections excluding HIV	405.97 (230.08 to 667.13)	93.23 (56.58 to 156.42)	68.49 (44.28 to 111.41)	112.89 (67.68 to 192.09)	5.58 (3.53 to 8.65)	1.89 (1.43 to 2.63)	1.59 (1.26 to 2.10)	2.15 (1.60 to 3.10)
Syphilis	377.12 (201.55 to 638.06)	74.42 (38.06 to 135.59)	50.71 (26.76 to 94.91)	92.07 (47.23 to 170.86)	4.74 (2.68 to 7.79)	1.11 (0.68 to 1.87)	0.82 (0.54 to 1.33)	1.34 (0.81 to 2.28)
Chlamydial infection	5.36 (4.75 to 6.41)	3.60 (3.00 to 4.26)	3.41 (2.80 to 4.03)	3.98 (3.40 to 4.78)	0.16 (0.13 to 0.18)	0.15 (0.11 to 0.17)	0.15 (0.11 to 0.17)	0.16 (0.12 to 0.18)
Gonococcal infection	15.50 (13.13 to 17.59)	10.34 (8.43 to 12.09)	9.81 (8.01 to 11.37)	11.39 (9.47 to 13.42)	0.47 (0.38 to 0.52)	0.45 (0.33 to 0.51)	0.44 (0.32 to 0.51)	0.47 (0.35 to 0.53)
Other sexually transmitted infections	7.99 (6.88 to 9.26)	4.87 (4.10 to 5.79)	4.56 (3.83 to 5.44)	5.46 (4.63 to 6.55)	0.22 (0.18 to 0.24)	0.17 (0.14 to 0.20)	0.17 (0.13 to 0.20)	0.19 (0.15 to 0.21)
Acute hepatitis	657.92 (627.13 to 695.11)	484.64 (417.25 to 568.19)	417.02 (354.15 to 487.55)	605.73 (530.85 to 689.66)	20.19 (19.32 to 21.20)	19.91 (17.24 to 23.21)	17.63 (15.11 to 20.62)	24.22 (21.29 to 27.25)
Acute hepatitis A	29.14 (25.73 to 33.71)	10.37 (7.85 to 13.57)	8.85 (6.60 to 11.95)	12.83 (9.85 to 16.79)	0.51 (0.44 to 0.60)	0.32 (0.25 to 0.41)	0.29 (0.22 to 0.38)	0.37 (0.29 to 0.48)
Acute hepatitis B	563.28 (532.42 to 595.53)	422.18 (359.51 to 503.98)	362.63 (301.65 to 432.17)	532.10 (456.87 to 612.52)	17.80 (16.83 to 18.80)	16.95 (14.41 to 20.06)	14.89 (12.48 to 17.67)	20.95 (18.18 to 23.95)

Acute hepatitis C	10.35 (7.76 to 14.04)	11.00 (8.01 to 15.12)	9.72 (7.04 to 13.25)	12.65 (9.21 to 17.40)	0.35 (0.26 to 0.49)	0.49 (0.35 to 0.66)	0.44 (0.31 to 0.60)	0.54 (0.39 to 0.75)
Acute hepatitis E	55.15 (46.28 to 65.23)	41.09 (30.92 to 53.86)	35.83 (26.83 to 47.58)	48.14 (36.22 to 62.54)	1.53 (1.19 to 1.92)	2.15 (1.52 to 3.03)	2.00 (1.41 to 2.80)	2.36 (1.66 to 3.24)
Other unspecified infectious diseases	495.30 (285.53 to 637.57)	399.17 (314.11 to 471.72)	369.72 (296.14 to 432.36)	462.83 (347.16 to 562.26)	11.09 (8.14 to 12.81)	16.58 (14.31 to 18.40)	16.59 (14.48 to 18.44)	17.35 (14.77 to 19.51)
**Non-communicable diseases**	**246,583.92 (242,023.90 to 251,401.79)**	**292,437.22 (240,728.21 to 356,004.99)**	**238,808.66 (200,950.18 to 282,467.31)**	**408,234.93 (323,366.77 to 501,514.09)**	**11,998.23 (11,799.19 to 12,215.92)**	**19,255.88 (16,440.50 to 22,707.48)**	**16,391.21 (14,260.54 to 18,826.44)**	**25,443.96 (21,481.30 to 29,836.27)**

Southeast Asia, East Asia, and Oceania								
Cause	YLLs (in 1000s)				Deaths (in 1000s)			
	2016	2040 Forecast	2040 Better Scenario	2040 Worse Scenario	2016	2040 Forecast	2040 Better Scenario	2040 Worse Scenario
**Neoplasms**	**75,254.30 (73,477.23 to 77,033.64)**	**101,002.54 (82,092.42 to 131,090.52)**	**88,381.87 (73,636.20 to 108,836.81)**	**121,572.00 (93,046.67 to 173,364.33)**	**3,062.16 (2,984.27 to 3,135.91)**	**5,309.03 (4,362.01 to 6,769.31)**	**4,733.73 (4,037.00 to 5,789.54)**	**6,200.99 (4,749.09 to 8,532.46)**
Lip and oral cavity cancer	862.64 (822.83 to 898.91)	1,075.94 (761.46 to 1,576.89)	761.30 (573.74 to 1,042.58)	1,551.37 (1,026.86 to 2,438.74)	35.97 (34.33 to 37.37)	56.26 (39.42 to 80.63)	40.94 (31.12 to 55.85)	78.02 (51.62 to 118.64)
Nasopharynx cancer	1,129.59 (1,056.15 to 1,199.72)	1,409.28 (1,000.53 to 2,171.01)	935.24 (689.86 to 1,341.04)	2,195.93 (1,413.65 to 3,877.00)	38.47 (36.06 to 40.78)	58.14 (40.73 to 87.87)	39.28 (28.75 to 55.77)	87.98 (55.92 to 150.28)
Other pharynx cancer	404.86 (348.25 to 454.10)	607.66 (413.37 to 904.62)	506.02 (358.11 to 726.73)	644.30 (424.61 to 971.66)	15.07 (13.10 to 16.73)	27.53 (18.95 to 40.25)	23.43 (16.95 to 33.03)	28.19 (19.10 to 41.66)
Esophageal cancer	4,808.69 (4,611.29 to 5,015.47)	7,907.27 (4,887.60 to 12,913.10)	6,217.72 (4,102.92 to 9,481.69)	10,203.65 (5,439.42 to 18,960.08)	224.17 (215.49 to 233.15)	438.79 (276.19 to 714.81)	350.30 (235.63 to 534.83)	550.11 (300.89 to 991.30)
Stomach cancer	8,587.82 (8,307.91 to 8,866.09)	8,181.80 (6,273.81 to 10,687.79)	7,308.05 (5,728.16 to 9,289.23)	10,165.87 (8,009.81 to 12,711.11)	385.52 (373.02 to 397.54)	467.26 (364.03 to 605.06)	424.24 (336.11 to 527.60)	561.50 (456.10 to 688.27)
Colon and rectum cancer	5,131.45 (4,894.17 to 5,357.75)	8,596.21 (6,344.28 to 11,748.03)	7,573.68 (5,700.69 to 10,352.94)	9,597.28 (7,009.59 to 13,061.87)	229.72 (219.07 to 239.20)	513.89 (379.57 to 697.92)	464.63 (351.00 to 629.61)	551.10 (401.18 to 746.67)
Liver cancer	13,521.84 (12,958.96 to 14,226.59)	21,810.30 (16,130.53 to 29,954.56)	20,347.43 (15,876.86 to 26,289.94)	21,612.00 (15,284.28 to 31,706.94)	507.80 (487.43 to 533.00)	1,040.02 (787.07 to 1,394.90)	990.77 (787.24 to 1,252.06)	996.23 (732.33 to 1,410.25)
Liver cancer due to hepatitis B	7,175.49 (6,310.54 to 7,990.77)	11,106.83 (8,121.86 to 15,895.16)	10,516.95 (8,034.38 to 14,049.67)	10,814.29 (7,513.12 to 16,379.17)	257.96 (224.51 to 287.41)	508.49 (375.17 to 700.67)	491.29 (379.93 to 642.53)	478.43 (339.90 to 699.04)
Liver cancer due to hepatitis C	1,209.80 (1,056.37 to 1,379.97)	2,220.33 (1,646.75 to 3,031.73)	2,119.34 (1,634.76 to 2,709.56)	2,170.82 (1,561.16 to 3,065.22)	52.28 (46.29 to 59.11)	122.11 (92.39 to 163.11)	119.17 (93.05 to 150.86)	115.64 (84.37 to 158.22)
Liver cancer due to alcohol use	1,579.44 (1,277.42 to 1,924.93)	2,821.45 (1,993.96 to 3,916.91)	2,349.33 (1,704.77 to 3,165.79)	3,030.05 (2,097.09 to 4,306.61)	68.01 (55.57 to 81.70)	151.65 (110.05 to 210.64)	130.83 (95.60 to 174.32)	156.47 (109.59 to 223.43)
Liver cancer due to other causes	3,557.12 (3,106.94 to 4,078.63)	5,661.68 (3,906.05 to 8,204.00)	5,361.81 (3,897.43 to 7,478.44)	5,596.84 (3,621.98 to 8,616.19)	129.55 (114.18 to 148.04)	257.76 (179.47 to 365.38)	249.48 (182.09 to 338.32)	245.71 (162.36 to 374.12)
Gallbladder and biliary tract cancer	788.73 (641.11 to 866.65)	868.35 (694.48 to 969.66)	886.85 (708.19 to 997.36)	822.03 (669.34 to 920.87)	37.02 (30.19 to 40.58)	52.89 (43.10 to 58.97)	55.12 (44.80 to 61.42)	48.20 (39.34 to 53.75)
Pancreatic cancer	2,183.40 (2,121.72 to 2,248.85)	3,741.66 (3,096.16 to 4,573.28)	3,746.27 (3,134.35 to 4,511.54)	3,718.61 (2,977.58 to 4,771.76)	95.96 (93.36 to 98.66)	206.94 (172.79 to 252.47)	210.35 (177.57 to 251.09)	199.38 (160.37 to 254.90)
Larynx cancer	579.92 (556.22 to 611.01)	708.10 (384.26 to 1,359.01)	470.58 (283.76 to 845.28)	1,153.54 (539.67 to 2,390.10)	25.06 (24.06 to 26.26)	37.49 (19.75 to 74.30)	25.34 (14.95 to 46.89)	58.55 (27.35 to 118.95)
Tracheal, bronchus, and lung cancer	16,017.49 (15,357.61 to 16,646.70)	19,676.08 (12,585.75 to 34,208.49)	14,076.79 (9,648.09 to 22,372.99)	32,555.88 (18,056.64 to 62,345.17)	726.57 (697.92 to 753.54)	1,099.82 (692.90 to 1,846.61)	805.55 (551.22 to 1,272.80)	1,743.70 (962.48 to 3,093.38)
Malignant skin melanoma	199.51 (169.43 to 221.88)	279.38 (239.62 to 323.81)	306.53 (262.84 to 357.40)	230.04 (200.78 to 262.93)	7.18 (6.19 to 8.08)	14.59 (12.52 to 17.06)	16.40 (14.31 to 19.14)	11.43 (10.02 to 13.23)
Non-melanoma skin cancer	397.27 (376.10 to 419.12)	591.25 (521.88 to 675.42)	565.75 (496.26 to 643.75)	648.83 (587.97 to 710.60)	18.47 (17.54 to 19.43)	37.63 (33.24 to 42.97)	37.06 (32.82 to 42.30)	39.37 (34.16 to 43.85)
Breast cancer	3,554.92 (3,223.18 to 3,871.44)	4,341.05 (3,362.94 to 5,543.76)	3,952.01 (3,116.56 to 4,957.33)	4,835.14 (3,710.95 to 6,241.51)	119.98 (108.73 to 130.83)	190.58 (148.86 to 244.41)	176.42 (140.36 to 221.01)	207.36 (159.93 to 264.59)
Cervical cancer	1,958.31 (1,357.44 to 2,102.86)	1,650.28 (1,156.45 to 2,036.95)	1,499.35 (1,055.41 to 1,828.51)	1,952.55 (1,351.61 to 2,431.23)	66.57 (46.65 to 71.37)	72.15 (51.07 to 88.29)	66.50 (47.50 to 80.09)	83.59 (57.90 to 103.04)

Uterine cancer	557.04 (530.52 to 586.53)	720.19 (626.55 to 813.42)	684.55 (581.52 to 777.77)	799.86 (693.47 to 899.17)	21.24 (20.22 to 22.38)	36.03 (31.98 to 39.82)	34.86 (30.55 to 38.88)	38.88 (34.67 to 42.86)
Ovarian cancer	961.69 (909.90 to 1,018.10)	1,327.99 (1,072.50 to 1,666.67)	1,275.64 (1,028.15 to 1,580.90)	1,334.31 (1,064.58 to 1,705.19)	33.64 (31.86 to 35.49)	59.02 (48.23 to 73.45)	57.28 (47.23 to 70.25)	58.50 (47.17 to 74.31)
Prostate cancer	869.30 (663.27 to 1,034.72)	2,207.49 (1,681.36 to 2,598.81)	2,276.50 (1,725.67 to 2,628.20)	2,062.74 (1,560.66 to 2,431.36)	55.06 (41.69 to 65.18)	168.35 (126.83 to 198.38)	179.08 (135.46 to 207.62)	149.53 (109.23 to 182.12)
Testicular cancer	50.73 (47.68 to 54.71)	50.74 (45.38 to 56.70)	48.66 (43.60 to 54.46)	53.68 (48.39 to 59.57)	1.37 (1.30 to 1.46)	1.96 (1.75 to 2.19)	1.95 (1.75 to 2.20)	1.97 (1.72 to 2.20)

Southeast Asia, East Asia, and Oceania								
**Cause**	**YLLs (in 1000s)**				**Deaths (in 1000s)**			
	**2016**	**2040 Forecast**	**2040 Better Scenario**	**2040 Worse Scenario**	**2016**	**2040 Forecast**	**2040 Better Scenario**	**2040 Worse Scenario**
Kidney cancer	587.56 (556.98 to 613.60)	901.45 (767.58 to 1,078.19)	920.70 (785.21 to 1,088.79)	846.45 (705.60 to 1,044.09)	23.02 (21.84 to 24.00)	47.79 (40.76 to 57.00)	49.93 (43.06 to 58.76)	42.93 (35.46 to 53.01)
Bladder cancer	664.10 (626.57 to 689.37)	1,130.75 (885.16 to 1,427.26)	1,084.90 (874.86 to 1,364.16)	1,231.74 (916.92 to 1,643.87)	36.14 (34.17 to 37.48)	79.15 (62.09 to 100.51)	77.61 (62.66 to 95.92)	82.58 (61.31 to 109.26)
Brain and nervous system cancer	2,484.81 (2,208.10 to 2,763.57)	3,040.84 (2,606.68 to 3,461.35)	3,021.53 (2,585.02 to 3,406.99)	3,028.89 (2,619.48 to 3,381.77)	74.97 (65.80 to 83.57)	130.11 (110.21 to 145.75)	132.28 (112.16 to 147.47)	124.59 (105.88 to 137.76)
Thyroid cancer	275.46 (260.89 to 290.07)	389.50 (324.06 to 493.55)	361.11 (306.35 to 444.82)	409.23 (339.00 to 527.22)	11.53 (10.96 to 12.09)	21.05 (17.69 to 26.24)	20.08 (17.33 to 24.38)	21.28 (17.66 to 26.69)
Mesothelioma	118.33 (109.67 to 124.45)	141.60 (128.56 to 155.23)	145.88 (130.93 to 161.50)	135.10 (123.87 to 147.67)	4.22 (3.91 to 4.46)	6.82 (6.25 to 7.44)	7.19 (6.58 to 7.92)	6.25 (5.69 to 6.83)
Hodgkin lymphoma	172.81 (146.51 to 227.59)	142.38 (118.35 to 194.78)	128.31 (105.46 to 170.71)	170.72 (143.28 to 227.01)	5.28 (4.48 to 6.87)	5.79 (4.78 to 7.84)	5.35 (4.42 to 7.03)	6.76 (5.60 to 8.76)
Non-Hodgkin lymphoma	1,679.90 (1,516.03 to 1,769.63)	2,073.23 (1,813.08 to 2,405.36)	2,003.70 (1,763.61 to 2,288.46)	2,073.81 (1,818.23 to 2,394.61)	56.67 (50.94 to 59.54)	96.61 (85.65 to 110.70)	95.68 (85.28 to 108.25)	93.24 (81.17 to 107.27)
Multiple myeloma	374.85 (333.31 to 428.40)	607.67 (518.47 to 735.15)	597.73 (511.73 to 706.53)	585.77 (502.57 to 711.92)	14.90 (13.37 to 17.17)	31.03 (27.08 to 37.53)	31.16 (27.19 to 37.01)	28.94 (25.02 to 35.12)
Leukemia	3,241.20 (2,836.70 to 3,468.92)	2,706.60 (2,228.33 to 3,346.51)	2,489.04 (2,077.01 to 2,986.84)	3,003.36 (2,443.69 to 3,744.87)	84.50 (72.95 to 89.92)	108.71 (88.56 to 133.64)	102.70 (85.36 to 123.65)	115.94 (94.42 to 145.19)
Acute lymphoid leukemia	742.44 (633.34 to 828.49)	543.50 (457.60 to 653.16)	497.78 (419.01 to 583.60)	584.17 (485.11 to 710.62)	17.20 (14.72 to 19.17)	19.03 (15.85 to 23.00)	17.85 (15.04 to 20.82)	19.77 (16.36 to 24.14)
Chronic lymphoid leukemia	126.90 (111.50 to 144.62)	112.55 (91.44 to 140.18)	108.08 (88.02 to 132.06)	116.16 (92.88 to 148.45)	4.20 (3.70 to 4.79)	5.28 (4.37 to 6.51)	5.20 (4.33 to 6.27)	5.22 (4.21 to 6.63)
Acute myeloid leukemia	451.26 (405.02 to 532.68)	531.21 (445.40 to 648.62)	512.09 (432.52 to 615.02)	526.88 (434.79 to 655.83)	11.47 (10.27 to 13.39)	19.64 (16.47 to 24.06)	19.46 (16.40 to 23.11)	18.72 (15.39 to 23.21)
Chronic myeloid leukemia	80.79 (73.54 to 95.37)	61.43 (49.22 to 78.53)	53.63 (44.00 to 67.17)	71.20 (56.79 to 92.99)	2.39 (2.18 to 2.85)	2.36 (1.89 to 3.03)	2.11 (1.74 to 2.67)	2.67 (2.15 to 3.46)
Other leukemia	1,839.81 (1,507.62 to 1,987.63)	1,457.90 (1,136.00 to 1,866.91)	1,317.46 (1,029.43 to 1,630.87)	1,704.94 (1,344.82 to 2,190.85)	49.24 (39.66 to 53.13)	62.40 (48.95 to 79.71)	58.08 (45.56 to 72.10)	69.57 (54.48 to 89.03)
Other neoplasms	3,090.07 (2,831.53 to 3,187.93)	4,117.50 (3,704.70 to 4,387.59)	4,190.04 (3,753.42 to 4,491.82)	3,949.34 (3,576.16 to 4,186.68)	106.11 (95.99 to 109.51)	202.63 (180.34 to 215.15)	212.24 (189.06 to 225.26)	184.87 (163.71 to 197.37)
**Cardiovascular diseases**	**100,094.59 (97,879.17 to 102,325.05)**	**90,753.25 (62,875.01 to 132,385.88)**	**63,167.81 (45,146.27 to 89,741.03)**	**163,680.32 (111,665.63 to 231,646.27)**	**5,445.59 (5,330.22 to 5,559.99)**	**6,711.14 (4,830.74 to 9,634.18)**	**5,102.74 (3,750.39 to 7,130.16)**	**10,916.34 (7,877.86 to 15,039.49)**
Rheumatic heart disease	2,192.35 (2,101.89 to 2,290.80)	1,723.35 (1,374.82 to 2,206.48)	1,551.74 (1,269.27 to 1,955.22)	2,120.04 (1,751.73 to 2,663.42)	93.41 (89.52 to 97.85)	111.37 (90.14 to 145.23)	104.52 (85.43 to 132.49)	130.05 (107.88 to 167.50)
Ischemic heart disease	42,694.35 (41,522.83 to 43,892.74)	35,969.77 (21,157.24 to 60,347.52)	23,790.15 (14,261.78 to 38,349.98)	68,299.16 (40,049.77 to 109,508.14)	2,409.87 (2,347.62 to 2,482.02)	2,661.78 (1,654.97 to 4,263.41)	1,957.24 (1,232.50 to 2,996.79)	4,613.67 (2,833.96 to 7,199.54)
Stroke	44,845.48 (43,626.95 to 46,208.81)	33,301.42 (21,132.12 to 50,669.52)	21,404.22 (14,404.20 to 32,562.63)	68,899.82 (43,709.32 to 106,620.14)	2,363.25 (2,282.94 to 2,444.45)	2,367.84 (1,554.93 to 3,548.62)	1,681.50 (1,167.24 to 2,485.83)	4,356.74 (2,885.06 to 6,608.64)
Ischemic stroke	14,510.05 (13,994.63 to 15,060.02)	11,717.02 (7,004.19 to 20,663.26)	8,253.94 (5,177.92 to 14,094.76)	21,621.60 (12,823.76 to 36,225.47)	925.31 (886.95 to 963.11)	960.37 (581.17 to 1,710.26)	732.79 (467.07 to 1,263.01)	1,592.03 (968.16 to 2,801.49)

Intracerebral hemorrhage	30,335.43 (29,434.41 to 31,313.55)	21,584.40 (12,932.00 to 34,865.34)	13,150.28 (8,224.88 to 20,721.36)	47,278.23 (27,875.34 to 77,258.73)	1,437.94 (1,389.79 to 1,487.91)	1,407.47 (879.37 to 2,277.14)	948.71 (624.38 to 1,400.06)	2,764.72 (1,718.43 to 4,274.81)
Hypertensive heart disease	5,213.26 (3,976.33 to 5,791.42)	11,200.41 (6,104.32 to 24,837.25)	8,670.97 (5,087.63 to 18,323.47)	14,602.97 (7,641.31 to 34,521.28)	325.55 (241.26 to 365.35)	932.08 (485.58 to 2,222.80)	752.99 (423.31 to 1,748.22)	1,138.58 (562.10 to 2,811.74)
Cardiomyopathy and myocarditis	1,396.17 (1,140.07 to 1,495.33)	1,575.77 (1,153.64 to 1,991.25)	1,459.05 (1,070.03 to 1,844.83)	1,657.45 (1,212.43 to 2,100.47)	52.16 (41.01 to 56.11)	91.21 (63.50 to 117.84)	89.34 (62.76 to 115.88)	89.33 (62.81 to 115.96)
Myocarditis	477.58 (360.72 to 532.19)	471.02 (343.09 to 555.09)	481.72 (349.51 to 571.20)	455.89 (330.20 to 537.45)	17.59 (12.49 to 20.18)	30.99 (21.01 to 38.47)	33.32 (22.61 to 40.98)	27.12 (17.76 to 34.37)

Southeast Asia, East Asia, and Oceania								
**Cause**	**YLLs (in 1000s)**				**Deaths (in 1000s)**			
	**2016**	**2040 Forecast**	**2040 Better Scenario**	**2040 Worse Scenario**	**2016**	**2040 Forecast**	**2040 Better Scenario**	**2040 Worse Scenario**
Alcoholic cardiomyopathy	172.68 (113.20 to 198.80)	195.69 (124.63 to 237.61)	171.82 (109.19 to 210.98)	197.12 (129.56 to 233.49)	5.78 (3.87 to 6.63)	8.32 (5.46 to 9.82)	7.55 (4.86 to 8.97)	8.03 (5.43 to 9.34)
Other cardiomyopathy	745.91 (626.69 to 846.30)	909.06 (618.50 to 1,248.47)	805.50 (567.97 to 1,116.47)	1,004.44 (708.46 to 1,388.76)	28.79 (23.56 to 33.31)	51.90 (33.87 to 75.05)	48.46 (32.01 to 69.41)	54.18 (36.37 to 77.43)
Atrial fibrillation and flutter	584.48 (465.82 to 712.29)	1,529.41 (1,140.74 to 1,988.27)	1,450.68 (1,092.18 to 1,882.59)	1,527.61 (1,135.09 to 1,999.54)	53.96 (42.83 to 66.51)	167.39 (124.91 to 217.85)	164.07 (123.56 to 211.87)	157.41 (114.26 to 209.40)
Aortic aneurysm	495.62 (465.28 to 530.30)	735.27 (591.30 to 921.83)	692.67 (563.32 to 858.01)	780.66 (617.59 to 978.57)	24.94 (23.35 to 26.59)	50.89 (42.69 to 61.84)	50.26 (42.50 to 60.28)	50.11 (41.28 to 60.46)
Peripheral artery disease	41.27 (34.65 to 50.16)	135.04 (100.62 to 179.78)	131.99 (99.89 to 174.92)	132.08 (97.97 to 174.02)	2.92 (2.49 to 3.63)	12.62 (9.58 to 16.95)	12.92 (9.84 to 17.25)	11.39 (8.42 to 15.34)
Endocarditis	567.76 (507.57 to 714.58)	846.50 (641.23 to 1,137.00)	810.17 (616.94 to 1,078.49)	861.14 (651.17 to 1,158.72)	20.21 (18.00 to 24.98)	45.58 (33.70 to 61.84)	45.57 (34.17 to 61.22)	43.65 (32.15 to 59.04)
Other cardiovascular and circulatory diseases	2,063.87 (1,846.00 to 3,112.32)	3,736.31 (2,679.10 to 5,882.62)	3,206.16 (2,316.89 to 5,058.76)	4,799.38 (3,451.35 to 7,381.20)	99.32 (88.55 to 151.65)	270.38 (193.79 to 429.36)	244.33 (176.30 to 392.71)	325.41 (235.88 to 505.80)
**Chronic respiratory diseases**	**18,708.71 (18,080.41 to 19,906.43)**	**24,495.73 (13,480.41 to 45,232.31)**	**17,651.59 (10,447.34 to 29,939.55)**	**38,730.63 (18,805.66 to 76,220.88)**	**1,204.56 (1,163.56 to 1,295.31)**	**1,972.66 (1,039.56 to 3,730.98)**	**1,478.29 (849.30 to 2,660.62)**	**2,966.21 (1,387.07 to 5,858.69)**
Chronic obstructive pulmonary disease	15,700.90 (15,062.47 to 17,070.50)	22,034.96 (11,262.26 to 42,431.12)	15,576.27 (8,622.65 to 27,861.47)	35,955.43 (16,099.77 to 73,130.27)	1,073.46 (1,030.97 to 1,172.89)	1,828.63 (900.24 to 3,578.71)	1,350.45 (738.17 to 2,535.96)	2,811.37 (1,235.17 to 5,717.94)
Pneumoconiosis	206.64 (190.92 to 223.37)	94.01 (68.47 to 136.93)	81.51 (61.28 to 110.63)	128.20 (102.69 to 157.39)	9.07 (8.27 to 9.75)	6.05 (4.45 to 8.49)	5.45 (4.17 to 7.18)	7.66 (6.05 to 9.34)
Silicosis	149.34 (135.92 to 162.35)	50.54 (31.77 to 84.65)	42.71 (28.08 to 65.91)	78.29 (59.02 to 98.76)	6.49 (5.87 to 7.03)	3.11 (1.99 to 5.06)	2.72 (1.77 to 4.12)	4.53 (3.36 to 5.71)
Asbestosis	9.71 (7.50 to 12.55)	12.70 (8.95 to 17.04)	13.88 (9.77 to 18.77)	10.46 (7.57 to 14.12)	0.39 (0.28 to 0.52)	0.83 (0.57 to 1.14)	0.94 (0.67 to 1.29)	0.64 (0.44 to 0.90)
Coal workers pneumoconiosis	18.81 (10.35 to 21.78)	11.38 (6.84 to 17.58)	8.67 (5.09 to 12.94)	17.11 (10.02 to 23.76)	0.97 (0.53 to 1.13)	0.79 (0.46 to 1.22)	0.63 (0.37 to 0.93)	1.11 (0.65 to 1.55)
Other pneumoconiosis	28.78 (25.69 to 36.10)	19.38 (15.28 to 25.48)	16.25 (12.85 to 21.05)	22.34 (18.07 to 28.49)	1.22 (1.09 to 1.48)	1.31 (1.06 to 1.68)	1.15 (0.92 to 1.49)	1.38 (1.12 to 1.76)
Asthma	2,356.60 (1,936.77 to 2,743.64)	1,705.82 (1,203.06 to 2,391.59)	1,271.04 (922.15 to 1,721.01)	2,086.00 (1,443.80 to 2,982.58)	103.43 (81.29 to 121.95)	99.38 (69.74 to 137.01)	78.39 (56.48 to 105.46)	116.78 (79.69 to 165.47)
Interstitial lung disease and pulmonary sarcoidosis	247.21 (205.94 to 309.00)	452.26 (353.75 to 580.45)	521.74 (416.33 to 670.90)	347.66 (280.69 to 442.91)	11.60 (9.50 to 14.55)	27.74 (21.05 to 35.25)	32.87 (25.76 to 41.94)	20.16 (15.82 to 25.80)
Other chronic respiratory diseases	197.36 (119.98 to 239.96)	208.68 (128.97 to 272.40)	201.02 (124.83 to 263.08)	213.34 (132.20 to 277.51)	7.02 (3.93 to 8.85)	10.86 (6.07 to 15.09)	11.13 (6.22 to 15.53)	10.24 (5.80 to 14.11)
**Cirrhosis and other chronic liver diseases**	**9,138.71 (8,588.76 to 10,197.97)**	**10,598.37 (9,543.33 to 11,960.17)**	**9,833.77 (8,796.60 to 11,199.85)**	**11,589.26 (10,580.42 to 13,085.24)**	**313.73 (295.49 to 351.05)**	**466.03 (425.07 to 527.66)**	**444.09 (403.12 to 497.93)**	**491.91 (450.05 to 553.18)**
Cirrhosis and other chronic liver diseases due to hepatitis B	3,716.07 (3,367.26 to 4,407.82)	4,277.50 (3,713.95 to 5,008.27)	4,120.19 (3,585.68 to 4,900.45)	4,602.14 (4,078.78 to 5,393.19)	131.82 (119.99 to 155.44)	193.11 (169.39 to 227.71)	190.14 (167.06 to 225.03)	201.14 (178.60 to 234.69)
Cirrhosis and other chronic liver diseases due to hepatitis C	1,519.11 (1,313.60 to 1,772.15)	1,799.37 (1,534.54 to 2,117.18)	1,715.46 (1,477.07 to 2,008.51)	1,913.76 (1,649.71 to 2,235.65)	49.98 (43.88 to 57.55)	73.01 (63.83 to 84.25)	71.04 (61.90 to 82.37)	75.39 (66.06 to 86.99)

Cirrhosis and other chronic liver diseases due to alcohol use	1,671.12 (1,464.64 to 1,941.90)	2,025.71 (1,752.75 to 2,393.58)	1,626.08 (1,397.88 to 1,901.19)	2,384.26 (2,072.15 to 2,807.00)	58.01 (51.56 to 67.72)	87.81 (76.90 to 102.28)	73.22 (63.48 to 84.89)	99.17 (86.35 to 114.88)
Cirrhosis and other chronic liver diseases due to other causes	2,232.41 (1,982.05 to 2,531.79)	2,495.81 (2,169.48 to 2,894.89)	2,372.04 (2,066.39 to 2,784.74)	2,689.09 (2,350.09 to 3,111.06)	73.92 (65.44 to 83.84)	112.10 (97.03 to 130.04)	109.68 (95.33 to 128.72)	116.20 (99.61 to 136.36)
**Digestive diseases**	**5,795.37 (5,469.33 to 6,221.97)**	**6,728.40 (5,888.31 to 7,782.68)**	**6,132.11 (5,386.38 to 6,982.61)**	**7,579.28 (6,609.75 to 8,740.40)**	**247.53 (234.02 to 265.15)**	**427.11 (368.83 to 488.69)**	**408.09 (359.30 to 462.64)**	**453.98 (387.49 to 528.10)**
Peptic ulcer disease	1,797.50 (1,660.36 to 1,929.34)	1,507.52 (1,205.41 to 1,860.95)	1,270.08 (1,014.57 to 1,575.19)	1,931.12 (1,539.85 to 2,395.27)	83.46 (75.91 to 88.92)	95.18 (75.66 to 117.05)	83.55 (66.85 to 102.72)	117.23 (92.58 to 145.32)

Southeast Asia, East Asia, and Oceania								
Cause	YLLs (in 1000s)				Deaths (in 1000s)			
	2016	2040 Forecast	2040 Better Scenario	2040 Worse Scenario	2016	2040 Forecast	2040 Better Scenario	2040 Worse Scenario
Gastritis and duodenitis	353.98 (324.70 to 382.91)	488.07 (410.03 to 581.97)	467.76 (386.96 to 558.89)	552.78 (464.29 to 644.52)	18.03 (16.51 to 19.42)	34.69 (28.66 to 41.98)	34.39 (28.23 to 41.81)	37.11 (29.59 to 44.48)
Appendicitis	257.81 (233.45 to 329.88)	235.59 (205.31 to 295.83)	204.87 (177.37 to 254.61)	275.59 (242.57 to 344.15)	7.78 (7.07 to 9.78)	11.09 (9.71 to 13.42)	10.19 (8.82 to 12.28)	12.40 (10.86 to 15.05)
Paralytic ileus and intestinal obstruction	1,173.25 (953.31 to 1,321.17)	1,374.98 (1,147.59 to 1,621.01)	1,394.00 (1,163.89 to 1,639.44)	1,355.01 (1,123.69 to 1,598.93)	40.55 (33.85 to 48.48)	85.66 (70.59 to 102.92)	91.31 (76.11 to 109.46)	77.95 (64.02 to 94.95)
Inguinal, femoral, and abdominal hernia	136.09 (85.37 to 162.08)	165.04 (94.03 to 206.71)	158.18 (89.41 to 200.42)	170.70 (99.64 to 215.07)	6.71 (4.00 to 8.17)	11.76 (6.35 to 15.17)	11.84 (6.34 to 15.35)	11.41 (6.29 to 14.92)
Inflammatory bowel disease	176.54 (160.94 to 200.18)	260.14 (232.33 to 308.36)	268.65 (238.67 to 318.86)	245.47 (218.36 to 292.75)	7.73 (7.02 to 9.05)	18.18 (15.73 to 21.81)	19.47 (17.01 to 23.51)	16.02 (13.28 to 19.41)
Vascular intestinal disorders	226.88 (210.75 to 280.03)	398.57 (357.65 to 487.74)	400.40 (356.49 to 487.76)	399.11 (357.82 to 487.50)	13.02 (12.11 to 15.75)	30.64 (27.22 to 37.19)	31.71 (28.24 to 38.05)	29.17 (24.85 to 35.76)
Gallbladder and biliary diseases	440.40 (409.07 to 537.28)	705.64 (481.03 to 1,099.48)	566.83 (409.67 to 829.99)	849.58 (562.60 to 1,329.65)	24.04 (22.32 to 28.58)	53.47 (36.18 to 82.43)	45.39 (32.50 to 65.55)	60.80 (39.64 to 93.88)
Pancreatitis	689.88 (591.30 to 797.96)	872.27 (641.89 to 1,236.12)	699.55 (529.83 to 939.41)	1,037.73 (752.98 to 1,527.72)	23.13 (19.63 to 26.16)	39.86 (29.50 to 55.75)	32.98 (25.09 to 43.69)	45.56 (32.77 to 64.97)
Other digestive diseases	543.05 (498.33 to 620.36)	720.57 (643.27 to 832.79)	701.79 (622.06 to 818.28)	762.20 (680.38 to 886.35)	23.07 (21.40 to 25.74)	46.59 (41.53 to 53.04)	47.26 (41.98 to 54.02)	46.33 (40.46 to 53.53)
**Neurological disorders**	**9,479.77 (8,433.07 to 10,794.61)**	**23,676.66 (19,039.60 to 30,206.14)**	**23,653.32 (19,656.88 to 29,206.24)**	**21,950.44 (17,051.72 to 28,998.84)**	**756.67 (659.26 to 876.33)**	**2,410.78 (1,886.55 to 3,121.03)**	**2,485.24 (2,016.42 to 3,081.22)**	**2,108.82 (1,549.11 to 2,857.72)**
Alzheimer's disease and other dementias	7,143.60 (6,117.14 to 8,458.33)	20,023.91 (15,464.44 to 26,461.03)	19,915.50 (15,904.97 to 25,134.44)	18,575.89 (13,762.69 to 25,721.89)	665.08 (571.57 to 783.78)	2,172.54 (1,660.76 to 2,885.81)	2,227.33 (1,760.94 to 2,831.09)	1,910.54 (1,354.72 to 2,667.70)
Parkinson's disease	727.35 (564.27 to 915.33)	2,137.12 (1,634.73 to 2,722.99)	2,336.43 (1,784.18 to 2,972.23)	1,743.22 (1,315.18 to 2,250.45)	53.58 (41.75 to 67.91)	186.48 (141.99 to 240.80)	208.23 (159.42 to 267.60)	145.67 (108.30 to 191.21)
Epilepsy	1,061.19 (1,006.83 to 1,136.64)	871.75 (708.43 to 1,073.58)	746.03 (616.25 to 899.47)	1,023.20 (830.14 to 1,289.37)	23.08 (21.89 to 24.66)	26.80 (21.99 to 32.79)	23.64 (19.82 to 28.22)	29.98 (24.27 to 36.96)
Multiple sclerosis	62.61 (58.39 to 76.57)	71.11 (60.52 to 88.68)	71.87 (61.01 to 90.56)	68.59 (58.27 to 85.99)	1.87 (1.73 to 2.28)	2.73 (2.37 to 3.37)	2.81 (2.43 to 3.44)	2.55 (2.20 to 3.17)
Motor neuron disease	130.06 (122.11 to 135.29)	181.33 (163.62 to 196.47)	184.69 (165.36 to 201.57)	171.80 (155.98 to 185.04)	4.01 (3.77 to 4.15)	7.33 (6.66 to 7.83)	7.56 (6.86 to 8.17)	6.77 (6.17 to 7.28)
Other neurological disorders	354.97 (337.96 to 373.50)	391.43 (361.42 to 424.35)	398.80 (366.77 to 433.47)	367.74 (341.63 to 396.94)	9.05 (8.73 to 9.40)	14.90 (14.04 to 15.80)	15.67 (14.72 to 16.64)	13.31 (12.50 to 14.12)
**Mental disorders**	**2,437.85 (2,056.33 to 2,802.44)**	**3,559.40 (2,893.71 to 4,218.16)**	**3,794.46 (3,105.92 to 4,414.09)**	**2,998.96 (2,536.12 to 3,393.12)**	**67.82 (55.87 to 75.83)**	**143.18 (114.23 to 168.86)**	**157.83 (127.90 to 185.44)**	**112.62 (93.00 to 125.38)**
Alcohol use disorders	1,108.86 (706.36 to 1,230.46)	915.96 (576.09 to 1,093.53)	882.10 (545.96 to 1,056.74)	872.69 (560.71 to 1,024.57)	30.05 (19.34 to 33.30)	31.99 (21.09 to 37.43)	31.63 (20.81 to 37.25)	29.13 (19.13 to 33.55)
Drug use disorders	1,321.52 (1,181.29 to 1,759.52)	2,637.14 (2,097.24 to 3,255.47)	2,905.93 (2,356.25 to 3,520.54)	2,120.12 (1,816.27 to 2,526.11)	37.63 (33.00 to 47.02)	111.07 (86.86 to 134.98)	126.07 (100.94 to 151.81)	83.37 (70.13 to 95.90)
Opioid use disorders	825.55 (684.18 to 1,146.98)	1,220.67 (937.17 to 1,522.70)	1,282.48 (969.54 to 1,613.83)	1,095.80 (850.83 to 1,384.09)	22.55 (18.04 to 29.34)	47.10 (34.45 to 57.06)	50.84 (36.20 to 61.01)	39.90 (29.31 to 48.00)

Cocaine use disorders	86.71 (67.37 to 132.43)	214.08 (144.42 to 320.18)	237.16 (163.17 to 343.72)	169.02 (122.80 to 241.89)	2.28 (1.77 to 3.43)	7.27 (4.86 to 10.72)	8.15 (5.49 to 11.83)	5.58 (4.01 to 7.88)
Amphetamine use disorders	53.55 (40.63 to 85.71)	181.02 (116.73 to 271.97)	207.91 (140.61 to 309.98)	130.24 (97.43 to 192.60)	1.30 (1.00 to 2.01)	5.48 (3.53 to 8.04)	6.38 (4.28 to 9.37)	3.82 (2.88 to 5.45)
Other drug use disorders	355.70 (286.31 to 512.52)	1,021.37 (732.89 to 1,350.78)	1,178.38 (878.83 to 1,551.68)	725.06 (578.38 to 926.89)	11.49 (9.40 to 15.43)	51.22 (35.63 to 67.33)	60.70 (44.69 to 79.55)	34.07 (27.11 to 42.09)
Eating disorders	7.47 (6.45 to 9.33)	6.30 (5.10 to 8.04)	6.43 (5.13 to 8.22)	6.14 (4.99 to 7.85)	0.14 (0.12 to 0.18)	0.12 (0.10 to 0.16)	0.13 (0.10 to 0.16)	0.12 (0.10 to 0.15)

Southeast Asia, East Asia, and Oceania								
Cause	YLLs (in 1000s)				Deaths (in 1000s)			
	2016	2040 Forecast	2040 Better Scenario	2040 Worse Scenario	2016	2040 Forecast	2040 Better Scenario	2040 Worse Scenario
**Diabetes, urogenital, blood, and endocrine diseases**	**17,374.11 (16,885.59 to 17,982.98)**	**26,634.35 (19,987.47 to 37,147.97)**	**21,966.23 (16,802.94 to 30,122.83)**	**34,094.90 (25,276.84 to 46,850.34)**	**765.48 (743.21 to 788.90)**	**1,645.36 (1,246.53 to 2,290.37)**	**1,418.47 (1,087.78 to 1,947.45)**	**2,009.92 (1,517.85 to 2,767.18)**
Diabetes mellitus	7,697.50 (7,470.37 to 7,957.24)	10,415.01 (7,407.45 to 14,687.07)	8,518.00 (6,094.65 to 11,749.96)	13,930.07 (9,707.13 to 19,877.46)	358.68 (347.88 to 371.33)	638.77 (471.41 to 858.63)	546.17 (407.46 to 723.24)	809.19 (580.66 to 1,114.55)
Acute glomerulonephritis	215.96 (204.71 to 228.00)	260.55 (212.83 to 317.56)	249.93 (200.91 to 305.82)	296.55 (245.64 to 351.28)	7.98 (7.58 to 8.41)	13.48 (10.82 to 16.50)	13.31 (10.71 to 16.25)	14.54 (11.73 to 17.56)
Chronic kidney disease	7,086.08 (6,700.21 to 7,394.10)	12,602.27 (6,617.93 to 23,211.69)	9,972.79 (5,544.61 to 18,202.40)	16,395.54 (8,376.01 to 29,503.96)	308.48 (289.63 to 321.10)	781.30 (400.26 to 1,447.36)	646.49 (349.22 to 1,194.62)	980.63 (489.20 to 1,776.35)
Chronic kidney disease due to diabetes mellitus	3,894.16 (3,540.70 to 4,207.95)	7,204.45 (3,671.79 to 13,494.73)	5,618.99 (3,051.98 to 10,621.91)	9,406.67 (4,742.36 to 17,253.83)	160.01 (143.70 to 174.31)	400.73 (198.03 to 760.42)	324.68 (168.88 to 613.79)	509.46 (247.15 to 942.44)
Chronic kidney disease due to hypertension	1,934.04 (1,706.89 to 2,181.48)	4,027.01 (2,058.61 to 7,592.60)	3,269.16 (1,744.76 to 6,134.93)	5,143.14 (2,519.15 to 9,498.49)	108.01 (95.76 to 120.25)	306.81 (158.49 to 567.01)	261.01 (140.94 to 489.04)	374.35 (184.24 to 677.91)
Chronic kidney disease due to glomerulonephritis	606.97 (517.20 to 709.20)	582.59 (362.32 to 969.03)	456.09 (292.76 to 763.31)	787.90 (475.55 to 1,301.62)	17.24 (14.54 to 20.35)	26.96 (14.73 to 50.77)	21.75 (12.33 to 40.94)	36.07 (19.35 to 66.80)
Chronic kidney disease due to other and unspecified causes	650.90 (551.33 to 762.67)	788.22 (448.94 to 1,387.49)	628.55 (373.93 to 1,091.94)	1,057.83 (599.51 to 1,806.30)	23.21 (19.52 to 27.39)	46.79 (24.22 to 85.35)	39.05 (20.88 to 71.79)	60.75 (31.42 to 110.04)
Urinary diseases and male infertility	947.59 (908.97 to 1,001.21)	1,817.86 (1,667.80 to 1,980.59)	1,735.93 (1,577.84 to 1,898.81)	1,864.67 (1,742.66 to 1,996.45)	45.97 (44.07 to 48.55)	131.88 (120.19 to 145.45)	130.99 (119.34 to 144.00)	127.96 (115.42 to 140.30)
Urinary tract infections	643.14 (595.56 to 700.29)	1,343.06 (1,219.57 to 1,480.73)	1,298.13 (1,171.03 to 1,427.07)	1,341.75 (1,232.60 to 1,467.35)	29.64 (27.55 to 33.37)	96.57 (87.04 to 109.90)	97.12 (87.27 to 110.72)	91.40 (81.22 to 104.24)
Urolithiasis	106.21 (95.12 to 132.44)	221.86 (184.05 to 286.95)	206.51 (169.49 to 261.65)	246.02 (211.67 to 307.54)	5.48 (4.91 to 6.73)	15.57 (12.52 to 19.96)	14.98 (12.13 to 19.29)	16.27 (13.49 to 20.80)
Other urinary diseases	198.24 (169.41 to 234.94)	252.94 (206.32 to 308.69)	231.28 (184.89 to 282.69)	276.90 (228.43 to 337.34)	10.85 (8.72 to 12.22)	19.75 (15.34 to 23.96)	18.90 (14.54 to 23.04)	20.29 (15.68 to 24.69)
Gynecological diseases	89.67 (56.46 to 102.22)	66.19 (43.05 to 82.60)	52.50 (35.38 to 67.70)	80.44 (54.01 to 100.39)	2.66 (1.72 to 3.00)	2.31 (1.53 to 2.81)	1.90 (1.32 to 2.33)	2.77 (1.93 to 3.30)
Uterine fibroids	33.68 (19.16 to 40.08)	18.16 (11.41 to 24.20)	15.45 (9.86 to 20.69)	23.26 (14.60 to 30.69)	1.15 (0.64 to 1.35)	0.74 (0.48 to 0.97)	0.64 (0.40 to 0.85)	0.94 (0.56 to 1.21)
Polycystic ovarian syndrome	2.47 (0.99 to 4.35)	1.18 (0.42 to 2.24)	0.79 (0.27 to 1.53)	1.59 (0.57 to 2.98)	0.06 (0.02 to 0.10)	0.03 (0.01 to 0.05)	0.02 (0.01 to 0.04)	0.04 (0.01 to 0.07)
Endometriosis	1.27 (0.44 to 2.01)	1.00 (0.46 to 1.88)	0.79 (0.37 to 1.48)	1.22 (0.57 to 2.24)	0.03 (0.01 to 0.05)	0.02 (0.01 to 0.04)	0.02 (0.01 to 0.03)	0.03 (0.01 to 0.05)
Genital prolapse	2.75 (1.59 to 4.10)	3.03 (1.75 to 4.79)	2.44 (1.47 to 3.90)	3.63 (2.12 to 5.74)	0.15 (0.08 to 0.21)	0.17 (0.09 to 0.27)	0.14 (0.08 to 0.22)	0.20 (0.11 to 0.31)
Other gynecological diseases	49.51 (33.09 to 58.25)	42.83 (28.88 to 55.66)	33.02 (21.97 to 44.38)	50.74 (34.79 to 65.79)	1.28 (0.89 to 1.49)	1.35 (0.95 to 1.69)	1.07 (0.74 to 1.39)	1.56 (1.11 to 1.94)
Hemoglobinopathies and hemolytic anemias	627.48 (515.31 to 696.84)	543.86 (430.24 to 632.11)	494.72 (387.00 to 578.02)	627.19 (496.83 to 717.28)	18.61 (14.85 to 20.65)	26.33 (20.27 to 30.44)	24.72 (18.91 to 28.97)	29.25 (22.65 to 33.37)
Thalassemias	183.85 (156.15 to 211.55)	59.97 (47.31 to 76.44)	57.10 (44.88 to 72.45)	60.00 (46.75 to 77.09)	2.39 (2.06 to 2.74)	0.85 (0.67 to 1.06)	0.81 (0.65 to 1.01)	0.84 (0.66 to 1.06)

Sickle cell disorders	1.15 (0.94 to 1.35)	0.71 (0.56 to 0.88)	0.61 (0.47 to 0.75)	0.83 (0.65 to 1.01)	0.02 (0.02 to 0.02)	0.02 (0.01 to 0.02)	0.01 (0.01 to 0.02)	0.02 (0.01 to 0.02)
G6PD deficiency	226.27 (180.61 to 260.78)	253.51 (199.07 to 301.23)	222.18 (172.01 to 264.00)	306.87 (239.48 to 364.30)	5.66 (4.48 to 6.53)	8.97 (6.96 to 10.56)	8.01 (6.14 to 9.49)	10.61 (8.25 to 12.37)
Other hemoglobinopathies and hemolytic anemias	216.21 (165.19 to 240.85)	229.66 (173.54 to 271.21)	214.84 (162.10 to 256.16)	259.49 (198.88 to 301.85)	10.53 (8.02 to 11.71)	16.50 (12.33 to 19.39)	15.89 (11.97 to 18.84)	17.78 (13.24 to 20.77)
Endocrine, metabolic, blood, and immune disorders	709.83 (622.88 to 834.84)	928.62 (805.67 to 1,096.48)	942.36 (818.16 to 1,110.98)	900.43 (787.00 to 1,067.38)	23.10 (20.32 to 27.41)	51.29 (44.68 to 60.77)	54.89 (48.02 to 65.03)	45.58 (39.28 to 53.92)

Southeast Asia, East Asia, and Oceania								
Cause	YLLs (in 1000s)				Deaths (in 1000s)			
	2016	2040 Forecast	2040 Better Scenario	2040 Worse Scenario	2016	2040 Forecast	2040 Better Scenario	2040 Worse Scenario
**Musculoskeletal disorders**	**714.60 (608.99 to 889.51)**	**953.05 (788.07 to 1,207.23)**	**909.29 (757.66 to 1,126.56)**	**1,071.27 (909.16 to 1,320.25)**	**24.92 (21.31 to 30.75)**	**52.54 (43.34 to 68.31)**	**51.06 (42.62 to 64.08)**	**58.19 (47.97 to 71.47)**
Rheumatoid arthritis	205.73 (155.90 to 242.59)	362.70 (256.96 to 501.78)	321.86 (234.61 to 432.38)	489.05 (369.49 to 607.31)	10.55 (8.07 to 12.19)	25.87 (18.21 to 35.71)	23.64 (17.49 to 31.77)	33.30 (25.01 to 41.99)
Other musculoskeletal disorders	508.87 (442.31 to 656.56)	590.35 (501.48 to 772.79)	587.43 (498.51 to 764.30)	582.21 (497.75 to 765.93)	14.37 (12.75 to 19.15)	26.67 (22.82 to 37.04)	27.42 (23.63 to 38.21)	24.89 (21.02 to 34.48)
**Other non-communicable diseases**	**7,585.90 (6,933.98 to 8,306.41)**	**4,035.47 (3,369.04 to 4,886.19)**	**3,318.19 (2,749.91 to 3,961.80)**	**4,967.88 (4,150.31 to 6,086.80)**	**109.76 (97.86 to 120.11)**	**118.05 (89.26 to 147.60)**	**111.68 (81.73 to 142.42)**	**124.98 (96.43 to 155.17)**
Congenital birth defects	6,933.23 (6,294.75 to 7,647.88)	2,787.79 (2,240.02 to 3,564.07)	2,107.90 (1,696.53 to 2,671.17)	3,712.87 (2,945.43 to 4,783.81)	86.67 (78.75 to 95.39)	37.90 (30.98 to 47.29)	29.31 (24.02 to 36.49)	49.96 (40.47 to 62.76)
Neural tube defects	281.48 (211.31 to 365.14)	63.12 (41.79 to 91.11)	42.33 (27.94 to 61.34)	90.73 (60.44 to 129.79)	3.33 (2.53 to 4.29)	0.78 (0.53 to 1.09)	0.53 (0.36 to 0.75)	1.12 (0.77 to 1.57)
Congenital heart anomalies	4,405.19 (3,934.01 to 4,906.76)	1,642.64 (1,255.97 to 2,173.83)	1,231.70 (951.54 to 1,601.10)	2,241.12 (1,720.60 to 2,934.57)	55.39 (49.37 to 61.42)	22.18 (17.31 to 28.76)	16.91 (13.22 to 21.74)	30.12 (23.73 to 38.68)
Orofacial clefts	35.63 (26.53 to 52.79)	8.53 (4.99 to 14.26)	5.49 (3.14 to 9.04)	13.83 (8.27 to 22.81)	0.41 (0.31 to 0.61)	0.10 (0.06 to 0.17)	0.06 (0.04 to 0.10)	0.16 (0.10 to 0.26)
Down syndrome	121.34 (101.68 to 159.87)	87.53 (69.76 to 125.72)	77.55 (62.72 to 103.58)	98.16 (78.01 to 143.97)	1.80 (1.55 to 2.32)	1.57 (1.29 to 2.10)	1.45 (1.20 to 1.93)	1.70 (1.40 to 2.33)
Other chromosomal abnormalities	135.04 (102.23 to 258.70)	109.99 (71.22 to 245.41)	86.75 (58.17 to 177.40)	136.68 (87.54 to 303.83)	1.63 (1.25 to 3.08)	1.41 (0.97 to 2.97)	1.13 (0.81 to 2.16)	1.73 (1.18 to 3.63)
Congenital musculoskeletal and limb anomalies	106.25 (90.85 to 127.84)	52.68 (37.84 to 71.98)	40.42 (29.03 to 54.01)	67.63 (48.80 to 90.82)	1.30 (1.11 to 1.56)	0.67 (0.49 to 0.90)	0.53 (0.39 to 0.69)	0.87 (0.64 to 1.15)
Urogenital congenital anomalies	89.02 (75.32 to 115.19)	64.49 (52.62 to 80.34)	53.70 (43.31 to 66.93)	75.54 (62.23 to 96.46)	1.52 (1.28 to 1.91)	1.47 (1.15 to 1.77)	1.28 (0.99 to 1.52)	1.67 (1.34 to 2.03)
Digestive congenital anomalies	522.40 (448.61 to 648.78)	199.33 (143.00 to 286.03)	144.20 (104.34 to 207.55)	271.05 (194.35 to 386.76)	6.14 (5.29 to 7.62)	2.40 (1.73 to 3.43)	1.75 (1.28 to 2.49)	3.26 (2.37 to 4.61)
Other congenital birth defects	1,236.89 (1,025.77 to 1,384.75)	559.49 (400.02 to 714.10)	425.74 (305.39 to 534.76)	718.13 (519.36 to 913.39)	15.14 (12.73 to 16.94)	7.32 (5.37 to 9.19)	5.67 (4.16 to 7.00)	9.34 (7.00 to 11.68)
Skin and subcutaneous diseases	441.31 (285.17 to 611.65)	1,169.75 (774.36 to 1,584.61)	1,150.73 (759.86 to 1,548.63)	1,156.08 (760.13 to 1,588.59)	20.64 (13.00 to 28.39)	79.25 (50.57 to 111.25)	81.68 (52.93 to 112.71)	73.87 (46.47 to 104.22)
Cellulitis	58.92 (39.66 to 94.59)	201.46 (134.86 to 306.60)	208.72 (141.74 to 315.39)	187.17 (125.53 to 294.21)	2.19 (1.42 to 3.41)	11.00 (6.80 to 16.32)	11.94 (7.53 to 17.44)	9.65 (5.91 to 14.62)
Pyoderma	226.90 (145.73 to 324.87)	603.19 (391.04 to 852.12)	579.33 (377.28 to 816.56)	611.34 (394.66 to 863.66)	8.72 (5.54 to 12.72)	36.50 (24.04 to 54.30)	36.98 (24.10 to 54.38)	34.59 (22.55 to 51.49)
Decubitus ulcer	134.59 (79.33 to 182.35)	329.82 (194.03 to 434.38)	327.66 (195.24 to 427.35)	322.62 (188.16 to 426.10)	8.95 (5.10 to 11.89)	29.69 (16.71 to 39.64)	30.60 (17.42 to 40.49)	27.72 (15.11 to 37.11)
Other skin and subcutaneous diseases	20.90 (14.13 to 31.37)	35.28 (24.33 to 51.34)	35.01 (24.11 to 51.08)	34.95 (23.98 to 51.13)	0.78 (0.53 to 1.15)	2.07 (1.41 to 3.08)	2.16 (1.48 to 3.22)	1.92 (1.28 to 2.93)
Sudden infant death syndrome	211.36 (170.36 to 267.32)	77.93 (53.32 to 113.60)	59.57 (40.82 to 88.43)	98.94 (67.40 to 144.91)	2.45 (1.98 to 3.10)	0.90 (0.62 to 1.32)	0.69 (0.47 to 1.03)	1.15 (0.78 to 1.68)

**Injuries**	**46,537.86 (44,215.50 to 47,866.03)**	**31,245.45 (25,865.30 to 41,065.73)**	**26,719.71 (22,362.66 to 35,889.99)**	**41,980.20 (34,747.78 to 54,320.70)**	**1,152.64 (1,067.33 to 1,188.50)**	**1,176.26 (992.68 to 1,440.28)**	**1,062.51 (902.71 to 1,266.05)**	**1,447.68 (1,234.93 to 1,753.20)**
**Transport injuries**	**20,122.41 (19,444.68 to 20,926.09)**	**10,081.09 (7,724.76 to 13,620.59)**	**7,541.46 (5,977.55 to 9,673.86)**	**18,232.84 (14,190.18 to 23,990.84)**	**470.82 (455.11 to 487.14)**	**319.27 (250.19 to 422.91)**	**248.48 (201.99 to 308.98)**	**540.96 (428.49 to 695.45)**
Road injuries	19,141.21 (18,487.90 to 19,875.42)	9,091.86 (6,970.96 to 12,303.36)	6,730.23 (5,335.11 to 8,606.09)	17,007.27 (13,215.99 to 22,390.47)	447.83 (432.41 to 462.85)	288.46 (226.31 to 381.58)	222.44 (181.36 to 275.47)	504.05 (399.80 to 647.09)
Pedestrian road injuries	8,676.21 (8,198.58 to 9,121.23)	3,250.42 (2,389.21 to 4,586.73)	2,256.86 (1,735.55 to 2,992.78)	6,763.29 (5,210.97 to 8,893.65)	217.59 (206.35 to 227.93)	123.38 (94.10 to 169.16)	90.11 (71.54 to 116.71)	236.59 (188.25 to 304.50)

Southeast Asia, East Asia, and Oceania								
Cause	YLLs (in 1000s)				Deaths (in 1000s)			
	2016	2040 Forecast	2040 Better Scenario	2040 Worse Scenario	2016	2040 Forecast	2040 Better Scenario	2040 Worse Scenario
Cyclist road injuries	1,220.20 (1,119.13 to 1,305.52)	757.17 (569.69 to 1,045.32)	588.88 (459.00 to 785.13)	1,238.28 (927.69 to 1,700.19)	31.63 (29.26 to 33.86)	27.34 (20.50 to 37.19)	22.37 (17.52 to 29.28)	39.90 (29.98 to 53.93)
Motorcyclist road injuries	4,166.13 (3,806.24 to 4,625.50)	2,635.34 (1,858.04 to 3,737.79)	2,145.30 (1,577.65 to 2,925.36)	3,953.80 (2,841.48 to 5,459.83)	86.91 (79.20 to 95.48)	65.80 (46.84 to 91.92)	55.09 (41.06 to 73.14)	92.46 (67.03 to 127.63)
Motor vehicle road injuries	4,892.78 (4,375.87 to 5,745.74)	2,341.15 (1,818.88 to 3,073.42)	1,659.92 (1,333.88 to 2,114.30)	4,844.66 (3,758.67 to 6,328.78)	107.36 (95.57 to 126.53)	68.29 (54.28 to 87.21)	51.92 (42.76 to 65.19)	128.96 (101.18 to 165.47)
Other road injuries	185.89 (167.94 to 232.42)	107.78 (82.83 to 149.48)	79.28 (63.26 to 107.30)	207.23 (157.70 to 285.02)	4.33 (3.91 to 5.47)	3.65 (2.81 to 5.15)	2.95 (2.34 to 4.07)	6.15 (4.74 to 8.55)
Other transport injuries	981.20 (896.97 to 1,178.44)	989.23 (748.49 to 1,359.37)	811.23 (634.28 to 1,067.38)	1,225.58 (942.83 to 1,635.21)	22.99 (21.02 to 27.75)	30.81 (23.46 to 41.91)	26.04 (20.48 to 34.34)	36.91 (28.21 to 49.32)
**Unintentional injuries**	**17,078.18 (14,399.82 to 17,930.45)**	**11,325.16 (9,110.64 to 13,143.26)**	**10,464.71 (8,418.24 to 12,050.69)**	**12,904.22 (10,468.48 to 14,735.62)**	**445.15 (356.76 to 470.10)**	**538.51 (409.23 to 620.22)**	**528.08 (400.16 to 601.25)**	**565.06 (426.66 to 642.63)**
Falls	4,710.19 (3,564.64 to 5,099.57)	4,947.88 (3,679.62 to 5,667.55)	4,820.25 (3,541.25 to 5,496.21)	5,221.69 (3,859.40 to 5,865.37)	177.84 (126.16 to 193.81)	319.91 (219.90 to 372.50)	324.34 (226.98 to 374.24)	316.97 (218.07 to 370.01)
Drowning	5,210.58 (4,586.05 to 5,495.28)	2,434.81 (1,989.23 to 2,998.18)	2,141.18 (1,750.57 to 2,626.97)	2,899.96 (2,352.41 to 3,611.12)	99.82 (86.15 to 104.90)	74.17 (59.52 to 89.12)	68.05 (54.97 to 80.81)	83.72 (66.67 to 101.94)
Fire, heat, and hot substances	631.02 (513.38 to 680.48)	431.75 (335.46 to 542.40)	373.96 (292.38 to 458.60)	526.01 (410.12 to 649.95)	17.62 (14.00 to 18.99)	20.71 (15.83 to 26.16)	19.05 (14.44 to 23.52)	23.66 (17.89 to 29.49)
Poisonings	814.56 (465.53 to 936.79)	277.30 (153.64 to 390.61)	231.17 (127.02 to 317.81)	384.06 (211.05 to 516.66)	19.94 (11.25 to 23.04)	10.23 (5.80 to 14.28)	8.85 (4.89 to 12.20)	13.65 (7.42 to 18.24)
Exposure to mechanical forces	2,301.18 (1,521.45 to 2,508.96)	1,070.82 (697.98 to 1,340.73)	952.69 (620.23 to 1,184.33)	1,343.40 (853.86 to 1,619.62)	50.60 (32.59 to 55.14)	34.16 (22.12 to 42.33)	31.66 (20.43 to 38.89)	40.78 (25.49 to 48.07)
Unintentional firearm injuries	84.89 (74.87 to 109.00)	58.36 (50.14 to 73.42)	52.13 (44.74 to 64.80)	66.13 (56.41 to 84.47)	2.18 (1.96 to 2.68)	2.67 (2.28 to 3.14)	2.60 (2.22 to 3.05)	2.74 (2.30 to 3.29)
Unintentional suffocation	478.34 (284.77 to 565.36)	162.77 (95.55 to 226.58)	142.52 (81.14 to 201.66)	201.74 (116.68 to 286.30)	7.62 (4.32 to 8.99)	4.30 (2.22 to 5.64)	4.04 (2.06 to 5.33)	4.93 (2.54 to 6.46)
Other exposure to mechanical forces	1,737.95 (1,086.49 to 1,889.41)	849.70 (524.06 to 1,083.28)	758.03 (466.44 to 944.48)	1,075.52 (651.88 to 1,294.06)	40.79 (24.99 to 44.42)	27.19 (16.81 to 34.24)	25.01 (15.43 to 31.02)	33.10 (19.76 to 39.22)
Adverse effects of medical treatment	594.68 (519.77 to 672.27)	520.45 (449.45 to 606.98)	486.88 (419.15 to 570.97)	566.96 (490.65 to 654.57)	15.22 (13.57 to 17.50)	20.69 (18.44 to 24.13)	20.39 (18.11 to 24.22)	21.24 (18.86 to 24.75)
Animal contact	264.11 (239.45 to 298.43)	272.87 (238.16 to 316.03)	244.22 (210.83 to 280.90)	319.67 (274.73 to 369.25)	6.94 (6.11 to 7.61)	10.06 (8.67 to 11.43)	9.50 (8.04 to 10.83)	11.23 (9.42 to 12.77)
Venomous animal contact	160.61 (133.35 to 178.14)	163.62 (129.96 to 188.72)	147.17 (117.20 to 170.43)	188.08 (148.27 to 219.04)	4.29 (3.51 to 4.73)	6.02 (4.65 to 6.89)	5.73 (4.39 to 6.58)	6.61 (4.91 to 7.64)
Non-venomous animal contact	103.50 (89.93 to 144.13)	109.25 (89.74 to 149.79)	97.05 (80.03 to 134.54)	131.60 (108.66 to 175.06)	2.64 (2.27 to 3.65)	4.04 (3.29 to 5.39)	3.77 (3.02 to 5.09)	4.63 (3.77 to 6.02)
Foreign body	859.49 (736.04 to 1,004.02)	630.58 (514.51 to 735.89)	602.24 (490.73 to 693.54)	647.47 (525.16 to 767.60)	18.45 (15.37 to 20.27)	25.45 (19.51 to 28.78)	26.19 (20.11 to 29.41)	23.60 (17.93 to 27.00)
Pulmonary aspiration and foreign body in airway	759.60 (628.26 to 864.42)	537.24 (416.57 to 632.36)	508.57 (394.17 to 594.46)	554.38 (427.61 to 662.69)	15.97 (12.58 to 17.59)	21.10 (15.12 to 24.30)	21.55 (15.38 to 24.81)	19.72 (14.21 to 22.94)

Foreign body in other body part	99.89 (83.86 to 164.33)	93.33 (77.34 to 142.09)	93.66 (77.83 to 140.48)	93.08 (77.18 to 142.36)	2.48 (2.17 to 3.87)	4.34 (3.64 to 6.15)	4.64 (3.94 to 6.59)	3.88 (3.26 to 5.50)
Environmental heat and cold exposure	153.41 (114.42 to 194.80)	132.79 (95.40 to 172.51)	117.52 (82.12 to 152.10)	172.20 (123.45 to 223.53)	5.09 (3.70 to 6.33)	7.01 (4.85 to 9.18)	6.61 (4.45 to 8.58)	8.49 (5.92 to 11.08)
Other unintentional injuries	1,538.97 (1,096.58 to 1,629.50)	605.93 (442.88 to 789.48)	494.61 (367.62 to 624.61)	822.79 (581.23 to 960.92)	33.64 (23.61 to 35.63)	16.12 (11.77 to 21.06)	13.45 (9.76 to 16.91)	21.72 (15.20 to 24.73)
**Self-harm and interpersonal violence**	**9,062.39 (8,582.78 to 10,185.23)**	**8,407.82 (6,387.06 to 11,133.44)**	**7,295.54 (5,778.69 to 9,334.04)**	**9,397.01 (7,067.93 to 12,708.95)**	**231.34 (218.60 to 255.77)**	**287.90 (217.86 to 380.80)**	**254.92 (201.05 to 324.68)**	**311.45 (233.95 to 425.09)**

Southeast Asia, East Asia, and Oceania								
Cause	YLLs (in 1000s)				Deaths (in 1000s)			
	2016	2040 Forecast	2040 Better Scenario	2040 Worse Scenario	2016	2040 Forecast	2040 Better Scenario	2040 Worse Scenario
Self-harm	6,609.26 (6,279.10 to 7,600.12)	6,652.95 (4,763.25 to 9,199.99)	5,688.83 (4,255.98 to 7,530.96)	7,548.09 (5,348.46 to 10,673.41)	181.74 (171.93 to 203.80)	247.28 (179.35 to 338.45)	217.29 (165.78 to 284.99)	268.97 (192.80 to 375.82)
Self-harm by firearm	139.32 (98.25 to 200.18)	114.80 (70.84 to 181.04)	94.30 (60.31 to 146.08)	133.22 (79.52 to 214.04)	3.31 (2.38 to 4.70)	3.36 (2.18 to 5.12)	2.85 (1.93 to 4.15)	3.77 (2.34 to 5.86)
Self-harm by other specified means	6,469.94 (6,153.96 to 7,401.39)	6,538.15 (4,675.15 to 9,052.84)	5,594.53 (4,187.68 to 7,406.68)	7,414.87 (5,256.61 to 10,510.65)	178.43 (168.62 to 200.36)	243.92 (176.73 to 334.52)	214.44 (163.79 to 282.11)	265.20 (189.91 to 371.98)
Interpersonal violence	2,453.13 (2,068.26 to 2,917.71)	1,754.87 (1,299.16 to 2,163.06)	1,606.71 (1,192.09 to 1,965.41)	1,848.92 (1,359.28 to 2,287.93)	49.60 (41.72 to 58.86)	40.62 (30.38 to 49.21)	37.63 (28.33 to 45.47)	42.48 (31.43 to 52.20)
Physical violence by firearm	638.15 (392.22 to 793.77)	550.00 (281.63 to 749.74)	504.73 (254.28 to 685.12)	560.39 (287.74 to 768.14)	12.91 (7.87 to 16.00)	11.96 (6.23 to 15.89)	11.10 (5.85 to 14.92)	12.11 (6.36 to 16.28)
Physical violence by sharp object	997.94 (826.77 to 1,266.84)	748.91 (530.97 to 947.18)	699.56 (506.07 to 880.54)	780.85 (569.28 to 996.13)	19.87 (16.35 to 25.30)	16.62 (11.92 to 20.82)	15.73 (11.35 to 19.71)	17.23 (12.56 to 21.58)
Physical violence by other means	817.04 (706.44 to 1,108.10)	455.96 (351.76 to 635.94)	402.42 (311.17 to 559.01)	507.68 (387.09 to 708.11)	16.82 (14.48 to 22.68)	12.03 (9.30 to 16.58)	10.81 (8.38 to 14.50)	13.14 (9.96 to 18.23)
**Forces of nature, conflict and terrorism, and executions and police conflict**	**274.88 (183.26 to 373.40)**	**1,431.37 (34.12 to 12,196.53)**	**1,418.00 (33.07 to 12,157.18)**	**1,446.12 (34.43 to 12,218.61)**	**5.33 (3.51 to 7.31)**	**30.58 (0.79 to 256.43)**	**31.02 (0.79 to 268.05)**	**30.22 (0.77 to 251.24)**
Exposure to forces of nature	93.48 (26.71 to 165.45)	239.12 (1.26 to 2,785.01)	234.41 (1.21 to 2,775.15)	239.72 (1.26 to 2,780.61)	2.05 (0.56 to 3.66)	5.96 (0.03 to 67.84)	6.01 (0.03 to 68.70)	5.79 (0.03 to 65.95)
Conflict and terrorism	123.23 (60.21 to 187.67)	774.32 (9.57 to 11,252.61)	773.09 (9.15 to 11,296.45)	787.29 (9.68 to 11,263.34)	2.10 (1.02 to 3.19)	15.98 (0.19 to 237.80)	16.41 (0.19 to 249.99)	15.86 (0.19 to 233.15)
Executions and police conflict	58.17 (47.55 to 65.40)	417.93 (0.00 to 3,034.58)	410.51 (0.00 to 2,995.94)	419.11 (0.00 to 3,047.14)	1.18 (0.93 to 1.33)	8.64 (0.00 to 64.56)	8.60 (0.00 to 64.49)	8.57 (0.00 to 64.20)

East Asia								
**Cause**	**YLLs (in 1000s)**				**Deaths (in 1000s)**			
	**2016**	**2040 Forecast**	**2040 Better Scenario**	**2040 Worse Scenario**	**2016**	**2040 Forecast**	**2040 Better Scenario**	**2040 Worse Scenario**
**All causes**	**217,960.83 (213,386.33 to 222,754.59)**	**217,048.81 (172,074.92 to 272,928.59)**	**180,317.62 (148,463.47 to 221,148.47)**	**313,346.93 (243,951.32 to 405,311.47)**	**10,073.64 (9,874.16 to 10,270.89)**	**14,972.91 (12,450.19 to 17,989.92)**	**12,911.12 (11,023.75 to 15,111.00)**	**20,274.69 (16,817.79 to 24,140.72)**
**Communicable, maternal, neonatal, and nutritional diseases**	**13,650.27 (12,800.25 to 14,587.22)**	**6,022.31 (4,643.77 to 7,992.83)**	**4,878.02 (3,796.12 to 6,251.68)**	**9,217.17 (6,947.54 to 12,398.24)**	**364.21 (340.00 to 396.07)**	**423.35 (326.36 to 545.37)**	**376.87 (302.89 to 466.60)**	**568.40 (417.88 to 746.85)**
**HIV/AIDS and tuberculosis**	**2,435.67 (2,314.58 to 2,609.80)**	**848.31 (587.72 to 1,263.24)**	**603.37 (435.21 to 839.45)**	**1,664.23 (1,156.73 to 2,356.43)**	**75.32 (72.07 to 80.50)**	**41.48 (28.54 to 61.92)**	**30.82 (22.84 to 41.73)**	**72.59 (49.95 to 103.49)**
Tuberculosis	1,123.41 (1,064.11 to 1,254.67)	474.75 (270.81 to 833.40)	296.43 (179.70 to 483.38)	962.87 (564.96 to 1,583.02)	43.52 (41.28 to 48.12)	27.12 (15.78 to 47.81)	17.93 (11.12 to 28.15)	51.65 (30.89 to 83.08)
HIV/AIDS	1,312.26 (1,212.17 to 1,460.70)	373.57 (245.97 to 601.72)	306.94 (202.81 to 494.48)	701.37 (473.28 to 1,100.99)	31.79 (29.51 to 35.09)	14.36 (10.32 to 20.57)	12.89 (9.40 to 18.49)	20.94 (14.71 to 31.36)
**Diarrhea, lower respiratory, and other common infectious diseases**	**4,964.58 (4,572.46 to 5,535.92)**	**3,684.00 (2,672.12 to 5,215.03)**	**3,000.93 (2,226.09 to 4,095.88)**	**5,582.09 (3,884.03 to 8,083.13)**	**193.34 (170.07 to 225.13)**	**321.70 (234.50 to 437.91)**	**284.32 (216.29 to 369.77)**	**434.81 (303.41 to 592.56)**
Diarrheal diseases	278.37 (221.06 to 380.50)	132.55 (35.03 to 356.87)	138.89 (39.65 to 363.68)	153.61 (36.46 to 448.51)	6.44 (4.67 to 10.22)	9.08 (2.54 to 24.52)	10.45 (3.07 to 26.74)	8.97 (2.25 to 27.41)
Intestinal infectious diseases	176.06 (89.80 to 303.94)	63.23 (29.14 to 118.33)	65.91 (30.00 to 123.93)	59.36 (26.23 to 113.49)	2.87 (1.47 to 4.97)	1.41 (0.69 to 2.52)	1.51 (0.74 to 2.64)	1.25 (0.61 to 2.27)
Typhoid fever	147.32 (73.40 to 255.90)	55.07 (24.44 to 104.34)	58.77 (26.02 to 111.73)	47.57 (20.77 to 92.73)	2.38 (1.19 to 4.15)	1.22 (0.58 to 2.17)	1.34 (0.64 to 2.39)	1.00 (0.47 to 1.86)
Paratyphoid fever	28.23 (12.26 to 55.17)	7.96 (3.09 to 16.19)	6.95 (2.77 to 14.38)	11.54 (4.64 to 24.24)	0.47 (0.20 to 0.91)	0.18 (0.07 to 0.36)	0.16 (0.06 to 0.32)	0.24 (0.10 to 0.51)
Other intestinal infectious diseases	0.51 (0.20 to 1.10)	0.20 (0.08 to 0.45)	0.19 (0.07 to 0.44)	0.25 (0.09 to 0.57)	0.01 (0.01 to 0.03)	0.01 (0.01 to 0.03)	0.01 (0.01 to 0.03)	0.02 (0.01 to 0.04)
Lower respiratory infections	3,880.94 (3,512.14 to 4,528.26)	3,219.88 (2,298.20 to 4,655.07)	2,554.16 (1,863.35 to 3,533.38)	4,969.42 (3,390.75 to 7,368.47)	171.30 (148.36 to 203.06)	301.99 (217.18 to 415.54)	263.53 (197.40 to 350.95)	413.08 (283.91 to 566.37)
Upper respiratory infections	7.28 (6.33 to 8.40)	3.23 (2.09 to 4.99)	2.53 (1.54 to 4.02)	5.46 (3.46 to 8.25)	0.23 (0.20 to 0.26)	0.17 (0.11 to 0.27)	0.14 (0.09 to 0.23)	0.28 (0.18 to 0.42)
Otitis media	0.69 (0.59 to 0.80)	0.19 (0.12 to 0.31)	0.20 (0.12 to 0.33)	0.18 (0.11 to 0.29)	0.02 (0.02 to 0.03)	0.02 (0.01 to 0.03)	0.02 (0.01 to 0.04)	0.01 (0.01 to 0.03)
Meningitis	335.59 (245.78 to 445.09)	102.01 (67.95 to 160.44)	92.61 (61.94 to 145.56)	137.61 (93.52 to 209.60)	7.29 (5.35 to 10.25)	4.55 (3.22 to 7.05)	4.32 (3.00 to 6.70)	5.56 (3.95 to 8.64)
Pneumococcal meningitis	26.72 (19.42 to 34.78)	9.66 (6.83 to 15.10)	9.97 (7.01 to 15.45)	9.42 (6.66 to 14.61)	0.63 (0.47 to 0.89)	0.52 (0.38 to 0.84)	0.56 (0.41 to 0.90)	0.46 (0.33 to 0.73)
H influenzae type B meningitis	24.89 (18.02 to 31.83)	11.19 (6.45 to 21.35)	11.20 (6.55 to 20.87)	12.75 (6.63 to 26.86)	0.52 (0.38 to 0.70)	0.40 (0.28 to 0.60)	0.42 (0.29 to 0.63)	0.38 (0.26 to 0.60)
Meningococcal meningitis	156.53 (112.50 to 207.96)	50.29 (32.68 to 81.21)	44.74 (29.28 to 71.95)	70.25 (47.96 to 106.51)	3.74 (2.72 to 5.19)	2.47 (1.70 to 3.92)	2.30 (1.58 to 3.66)	3.17 (2.25 to 4.87)
Other meningitis	127.45 (93.54 to 169.26)	30.87 (19.93 to 50.26)	26.71 (17.09 to 44.03)	45.19 (29.74 to 70.16)	2.40 (1.78 to 3.35)	1.15 (0.78 to 1.91)	1.04 (0.70 to 1.66)	1.55 (1.09 to 2.41)
Encephalitis	217.04 (177.47 to 287.92)	96.66 (63.20 to 142.93)	93.39 (61.55 to 139.40)	109.03 (68.17 to 165.62)	3.80 (3.11 to 4.86)	3.03 (2.28 to 3.86)	3.06 (2.30 to 3.93)	3.14 (2.32 to 4.15)

Diphtheria	0.72 (0.63 to 0.84)	0.08 (0.05 to 0.12)	0.08 (0.05 to 0.12)	0.17 (0.07 to 0.33)	0.01 (0.01 to 0.01)	0.00 (0.00 to 0.00)	0.00 (0.00 to 0.00)	0.00 (0.00 to 0.00)
Whooping cough	23.42 (4.96 to 66.50)	5.38 (0.43 to 19.01)	4.79 (0.39 to 16.17)	39.74 (1.37 to 134.53)	0.28 (0.06 to 0.79)	0.06 (0.01 to 0.23)	0.06 (0.01 to 0.19)	0.47 (0.02 to 1.59)
Tetanus	25.18 (16.72 to 31.62)	2.38 (1.46 to 3.49)	2.41 (1.47 to 3.56)	4.99 (2.52 to 9.31)	0.57 (0.36 to 0.78)	0.09 (0.05 to 0.14)	0.09 (0.05 to 0.14)	0.11 (0.06 to 0.17)
Measles	4.78 (1.37 to 12.20)	51.96 (2.58 to 250.16)	40.01 (2.10 to 171.06)	94.27 (3.31 to 534.05)	0.06 (0.02 to 0.15)	0.76 (0.04 to 3.27)	0.60 (0.03 to 2.38)	1.31 (0.05 to 6.70)

East Asia								
**Cause**	**YLLs (in 1000s)**				**Deaths (in 1000s)**			
	**2016**	**2040 Forecast**	**2040 Better Scenario**	**2040 Worse Scenario**	**2016**	**2040 Forecast**	**2040 Better Scenario**	**2040 Worse Scenario**
Varicella and herpes zoster	14.49 (12.88 to 16.59)	6.44 (4.74 to 8.62)	5.94 (4.36 to 8.18)	8.26 (5.85 to 11.65)	0.47 (0.43 to 0.51)	0.55 (0.41 to 0.72)	0.54 (0.40 to 0.74)	0.62 (0.43 to 0.86)
**Neglected tropical diseases and malaria**	**94.64 (60.43 to 104.50)**	**44.48 (28.65 to 52.82)**	**44.10 (28.61 to 52.18)**	**49.97 (30.74 to 63.59)**	**2.53 (1.69 to 2.77)**	**1.85 (1.25 to 2.15)**	**1.89 (1.29 to 2.18)**	**1.96 (1.28 to 2.40)**
Malaria	7.50 (5.13 to 10.65)	3.04 (2.00 to 4.28)	3.06 (1.99 to 4.21)	3.10 (2.02 to 4.44)	0.19 (0.13 to 0.27)	0.11 (0.08 to 0.16)	0.12 (0.08 to 0.16)	0.11 (0.07 to 0.16)
Chagas disease	0.00 (0.00 to 0.00)	0.00 (0.00 to 0.00)	0.00 (0.00 to 0.00)	0.00 (0.00 to 0.00)	0.00 (0.00 to 0.00)	0.00 (0.00 to 0.00)	0.00 (0.00 to 0.00)	0.00 (0.00 to 0.00)
Leishmaniasis	2.42 (1.54 to 3.78)	0.83 (0.50 to 1.42)	0.78 (0.49 to 1.31)	1.11 (0.68 to 1.91)	0.07 (0.05 to 0.12)	0.04 (0.02 to 0.06)	0.03 (0.02 to 0.06)	0.05 (0.03 to 0.08)
African trypanosomiasis	0.00 (0.00 to 0.00)	0.00 (0.00 to 0.00)	0.00 (0.00 to 0.00)	0.00 (0.00 to 0.00)	0.00 (0.00 to 0.00)	0.00 (0.00 to 0.00)	0.00 (0.00 to 0.00)	0.00 (0.00 to 0.00)
Schistosomiasis	11.97 (10.12 to 14.13)	6.92 (5.48 to 8.63)	6.92 (5.47 to 8.65)	7.50 (5.93 to 9.43)	0.47 (0.40 to 0.56)	0.38 (0.30 to 0.48)	0.39 (0.31 to 0.48)	0.40 (0.31 to 0.49)
Cysticercosis	1.52 (1.38 to 1.69)	0.45 (0.37 to 0.53)	0.45 (0.37 to 0.53)	0.46 (0.39 to 0.54)	0.04 (0.04 to 0.05)	0.02 (0.02 to 0.02)	0.02 (0.02 to 0.02)	0.02 (0.02 to 0.02)
Cystic echinococcosis	1.55 (1.32 to 1.82)	0.64 (0.47 to 0.87)	0.58 (0.44 to 0.76)	0.93 (0.68 to 1.24)	0.05 (0.04 to 0.06)	0.03 (0.03 to 0.05)	0.03 (0.03 to 0.04)	0.05 (0.03 to 0.06)
Dengue	0.60 (0.37 to 0.93)	2.17 (0.83 to 3.40)	2.22 (0.84 to 3.46)	2.10 (0.82 to 3.28)	0.02 (0.01 to 0.04)	0.16 (0.04 to 0.27)	0.17 (0.05 to 0.28)	0.15 (0.04 to 0.25)
Yellow fever	0.00 (0.00 to 0.00)	0.00 (0.00 to 0.00)	0.00 (0.00 to 0.00)	0.00 (0.00 to 0.00)	0.00 (0.00 to 0.00)	0.00 (0.00 to 0.00)	0.00 (0.00 to 0.00)	0.00 (0.00 to 0.00)
Rabies	41.75 (10.88 to 50.08)	11.49 (2.41 to 15.68)	10.44 (2.36 to 13.94)	16.41 (3.55 to 24.83)	0.97 (0.25 to 1.15)	0.36 (0.08 to 0.48)	0.33 (0.08 to 0.43)	0.49 (0.11 to 0.74)
Intestinal nematode infections	1.99 (1.59 to 2.53)	1.18 (0.95 to 1.52)	1.18 (0.95 to 1.52)	1.16 (0.92 to 1.51)	0.04 (0.03 to 0.05)	0.05 (0.04 to 0.05)	0.05 (0.04 to 0.06)	0.04 (0.03 to 0.05)
Other neglected tropical diseases	25.34 (17.53 to 28.10)	17.75 (11.52 to 21.12)	18.47 (12.13 to 22.04)	17.21 (11.08 to 21.04)	0.66 (0.45 to 0.74)	0.70 (0.45 to 0.82)	0.75 (0.48 to 0.88)	0.65 (0.42 to 0.78)
**Maternal disorders**	**159.18 (144.96 to 175.27)**	**46.16 (31.39 to 68.76)**	**32.40 (19.83 to 52.98)**	**60.70 (37.54 to 96.14)**	**2.86 (2.60 to 3.14)**	**0.85 (0.58 to 1.25)**	**0.60 (0.37 to 0.96)**	**1.12 (0.70 to 1.75)**
Maternal hemorrhage	49.22 (41.89 to 57.86)	17.45 (10.79 to 26.63)	11.98 (6.91 to 19.61)	22.59 (13.20 to 36.55)	0.88 (0.75 to 1.03)	0.32 (0.20 to 0.48)	0.22 (0.13 to 0.35)	0.41 (0.24 to 0.66)
Maternal sepsis and other maternal infections	8.53 (6.74 to 10.69)	2.50 (1.52 to 3.82)	1.84 (1.06 to 3.01)	3.37 (1.97 to 5.62)	0.17 (0.14 to 0.21)	0.05 (0.03 to 0.08)	0.04 (0.02 to 0.06)	0.07 (0.04 to 0.12)
Maternal hypertensive disorders	17.91 (14.31 to 22.10)	4.79 (2.81 to 7.72)	3.32 (1.83 to 5.74)	6.35 (3.52 to 11.15)	0.31 (0.25 to 0.39)	0.09 (0.05 to 0.14)	0.06 (0.03 to 0.10)	0.11 (0.06 to 0.20)
Maternal obstructed labor and uterine rupture	6.90 (5.31 to 8.88)	1.99 (1.07 to 3.36)	1.38 (0.71 to 2.43)	2.75 (1.40 to 4.87)	0.12 (0.10 to 0.16)	0.04 (0.02 to 0.06)	0.03 (0.01 to 0.04)	0.05 (0.03 to 0.09)
Maternal abortion, miscarriage, and ectopic pregnancy	18.66 (15.12 to 22.84)	5.27 (3.04 to 8.75)	3.77 (2.11 to 6.48)	7.12 (3.86 to 12.29)	0.34 (0.28 to 0.42)	0.10 (0.06 to 0.17)	0.07 (0.04 to 0.12)	0.14 (0.08 to 0.23)

Indirect maternal deaths	16.17 (11.62 to 22.79)	5.58 (3.11 to 9.28)	3.78 (1.97 to 6.66)	7.35 (3.78 to 12.74)	0.28 (0.20 to 0.39)	0.10 (0.06 to 0.16)	0.07 (0.04 to 0.12)	0.13 (0.07 to 0.23)
Late maternal deaths	1.27 (0.75 to 1.97)	0.39 (0.18 to 0.74)	0.24 (0.11 to 0.46)	0.50 (0.22 to 0.93)	0.02 (0.01 to 0.03)	0.01 (0.00 to 0.01)	0.00 (0.00 to 0.01)	0.01 (0.00 to 0.02)
Maternal deaths aggravated by HIV/AIDS	0.03 (0.02 to 0.06)	0.07 (0.00 to 0.43)	0.04 (0.00 to 0.27)	0.13 (0.02 to 0.56)	0.00 (0.00 to 0.00)	0.00 (0.00 to 0.01)	0.00 (0.00 to 0.00)	0.00 (0.00 to 0.01)
Other maternal disorders	40.49 (34.68 to 47.07)	8.12 (4.84 to 13.22)	6.06 (3.47 to 10.69)	10.55 (5.99 to 18.40)	0.72 (0.62 to 0.84)	0.15 (0.09 to 0.24)	0.11 (0.06 to 0.19)	0.19 (0.11 to 0.33)

East Asia								
Cause	YLLs (in 1000s)				Deaths (in 1000s)			
	2016	2040 Forecast	2040 Better Scenario	2040 Worse Scenario	2016	2040 Forecast	2040 Better Scenario	2040 Worse Scenario
**Neonatal disorders**	**5,327.54 (4,806.34 to 5,887.27)**	**862.79 (412.01 to 1,500.52)**	**654.58 (299.08 to 1,156.89)**	**1,283.74 (587.46 to 2,248.84)**	**61.58 (55.55 to 68.06)**	**9.98 (4.77 to 17.35)**	**7.57 (3.46 to 13.38)**	**14.84 (6.80 to 25.99)**
Neonatal preterm birth	2,705.87 (2,317.75 to 3,065.45)	397.83 (189.02 to 684.45)	269.49 (124.42 to 463.96)	621.22 (280.23 to 1,062.21)	31.27 (26.78 to 35.42)	4.60 (2.19 to 7.91)	3.12 (1.44 to 5.36)	7.18 (3.24 to 12.28)
Neonatal encephalopathy due to birth asphyxia and trauma	1,741.94 (1,540.02 to 2,001.37)	274.81 (106.50 to 540.31)	219.96 (81.86 to 446.51)	418.83 (152.48 to 816.16)	20.14 (17.80 to 23.13)	3.18 (1.23 to 6.25)	2.54 (0.95 to 5.16)	4.84 (1.77 to 9.43)
Neonatal sepsis and other neonatal infections	194.15 (144.72 to 262.41)	44.87 (25.51 to 77.68)	37.77 (21.46 to 64.00)	63.89 (36.17 to 106.48)	2.24 (1.67 to 3.03)	0.52 (0.30 to 0.90)	0.44 (0.25 to 0.74)	0.74 (0.42 to 1.23)
Hemolytic disease and other neonatal jaundice	92.14 (79.59 to 108.45)	12.15 (6.29 to 22.22)	9.80 (4.98 to 17.59)	17.81 (9.22 to 32.69)	1.07 (0.92 to 1.25)	0.14 (0.07 to 0.26)	0.11 (0.06 to 0.20)	0.21 (0.11 to 0.38)
Other neonatal disorders	593.45 (511.60 to 676.94)	133.13 (67.85 to 229.41)	117.56 (57.60 to 205.32)	162.00 (82.07 to 287.29)	6.87 (5.92 to 7.84)	1.54 (0.79 to 2.66)	1.36 (0.67 to 2.38)	1.88 (0.95 to 3.33)
**Nutritional deficiencies**	**232.76 (221.20 to 247.27)**	**288.08 (234.77 to 349.61)**	**314.79 (262.97 to 376.76)**	**250.29 (195.71 to 311.05)**	**14.80 (14.18 to 15.46)**	**34.64 (25.51 to 44.30)**	**39.40 (30.29 to 49.50)**	**27.44 (18.87 to 36.62)**
Protein-energy malnutrition	167.32 (156.65 to 193.78)	227.34 (174.26 to 287.15)	254.12 (202.74 to 310.65)	184.93 (139.46 to 237.32)	11.69 (11.08 to 12.80)	28.91 (19.74 to 38.15)	33.44 (24.66 to 43.12)	21.75 (14.28 to 30.07)
Iodine deficiency	3.45 (2.27 to 5.25)	10.68 (1.24 to 36.72)	10.79 (1.14 to 39.16)	11.35 (1.48 to 35.95)	0.13 (0.08 to 0.19)	1.11 (0.08 to 4.72)	1.16 (0.08 to 5.12)	1.10 (0.10 to 4.47)
Dietary iron deficiency	8.47 (7.51 to 9.21)	7.41 (4.68 to 10.67)	7.74 (4.81 to 11.29)	6.89 (4.52 to 9.73)	0.25 (0.18 to 0.27)	0.44 (0.20 to 0.74)	0.48 (0.22 to 0.79)	0.37 (0.18 to 0.61)
Other nutritional deficiencies	53.52 (34.61 to 58.90)	42.65 (25.98 to 52.75)	42.14 (25.64 to 52.88)	47.12 (29.04 to 59.74)	2.74 (1.70 to 3.01)	4.18 (2.38 to 5.38)	4.31 (2.60 to 5.59)	4.21 (2.49 to 5.61)
**Other communicable, maternal, neonatal, and nutritional diseases**	**435.88 (413.71 to 461.44)**	**248.49 (199.46 to 316.49)**	**227.85 (181.02 to 284.53)**	**326.14 (266.44 to 397.21)**	**13.79 (13.18 to 14.36)**	**12.85 (10.62 to 15.65)**	**12.27 (10.06 to 15.02)**	**15.64 (13.02 to 18.93)**
Sexually transmitted infections excluding HIV	25.47 (20.03 to 34.82)	6.91 (5.16 to 8.94)	6.39 (4.77 to 8.43)	8.27 (6.16 to 10.63)	0.63 (0.50 to 0.75)	0.32 (0.23 to 0.39)	0.32 (0.22 to 0.38)	0.36 (0.26 to 0.43)
Syphilis	16.29 (11.04 to 25.25)	2.72 (1.86 to 4.15)	2.34 (1.64 to 3.55)	3.60 (2.58 to 5.41)	0.33 (0.26 to 0.44)	0.10 (0.08 to 0.14)	0.10 (0.07 to 0.12)	0.13 (0.10 to 0.16)
Chlamydial infection	1.67 (1.24 to 1.84)	0.78 (0.56 to 0.97)	0.76 (0.55 to 0.95)	0.86 (0.62 to 1.09)	0.05 (0.04 to 0.06)	0.04 (0.03 to 0.05)	0.04 (0.03 to 0.05)	0.04 (0.03 to 0.05)
Gonococcal infection	5.02 (3.77 to 5.53)	2.39 (1.71 to 2.98)	2.32 (1.65 to 2.90)	2.65 (1.89 to 3.32)	0.17 (0.13 to 0.19)	0.13 (0.09 to 0.16)	0.13 (0.09 to 0.16)	0.14 (0.09 to 0.17)
Other sexually transmitted infections	2.48 (1.87 to 2.73)	1.01 (0.73 to 1.27)	0.97 (0.70 to 1.23)	1.15 (0.82 to 1.44)	0.08 (0.06 to 0.08)	0.05 (0.03 to 0.06)	0.05 (0.03 to 0.06)	0.05 (0.04 to 0.06)
Acute hepatitis	306.44 (292.54 to 322.86)	165.37 (123.59 to 225.67)	145.72 (107.78 to 194.97)	236.69 (188.43 to 294.31)	10.07 (9.65 to 10.53)	7.95 (6.01 to 10.60)	7.20 (5.40 to 9.55)	10.85 (8.78 to 13.30)
Acute hepatitis A	12.72 (10.99 to 14.98)	3.10 (1.91 to 5.04)	2.75 (1.72 to 4.41)	4.11 (2.59 to 6.65)	0.21 (0.18 to 0.25)	0.11 (0.07 to 0.15)	0.10 (0.07 to 0.15)	0.13 (0.09 to 0.18)
Acute hepatitis B	269.17 (255.77 to 283.11)	148.25 (109.45 to 205.62)	130.27 (93.84 to 176.43)	213.80 (167.13 to 267.91)	9.13 (8.66 to 9.61)	6.95 (5.17 to 9.39)	6.25 (4.61 to 8.37)	9.62 (7.67 to 12.00)

Acute hepatitis C	3.79 (2.76 to 5.25)	2.91 (1.90 to 4.55)	2.61 (1.69 to 3.96)	3.94 (2.65 to 5.87)	0.14 (0.10 to 0.19)	0.15 (0.10 to 0.22)	0.14 (0.09 to 0.20)	0.19 (0.13 to 0.28)
Acute hepatitis E	20.76 (17.53 to 24.64)	11.11 (7.35 to 16.25)	10.09 (6.78 to 14.52)	14.83 (10.25 to 21.12)	0.59 (0.46 to 0.75)	0.75 (0.46 to 1.14)	0.71 (0.46 to 1.10)	0.90 (0.59 to 1.34)
Other unspecified infectious diseases	103.98 (86.83 to 117.98)	76.22 (62.54 to 90.39)	75.74 (61.63 to 90.43)	81.19 (65.18 to 99.64)	3.08 (2.60 to 3.35)	4.57 (3.72 to 5.36)	4.76 (3.90 to 5.58)	4.43 (3.50 to 5.36)
**Non-communicable diseases**	**173,026.33 (169,149.66 to 177,195.21)**	**193,151.62 (149,960.79 to 246,842.57)**	**160,266.01 (129,075.55 to 198,758.69)**	**279,245.65 (210,262.33 to 369,484.14)**	**8,903.33 (8,717.55 to 9,085.42)**	**13,782.08 (11,290.11 to 16,786.20)**	**11,843.14 (9,997.32 to 13,936.70)**	**18,748.83 (15,212.09 to 22,764.69)**

East Asia								
**Cause**	**YLLs (in 1000s)**				**Deaths (in 1000s)**			
	**2016**	**2040 Forecast**	**2040 Better Scenario**	**2040 Worse Scenario**	**2016**	**2040 Forecast**	**2040 Better Scenario**	**2040 Worse Scenario**
**Neoplasms**	**59,793.44 (58,089.53 to 61,531.52)**	**76,328.24 (59,227.84 to 105,900.20)**	**66,540.03 (53,077.85 to 87,157.02)**	**93,249.58 (65,877.63 to 145,081.75)**	**2,477.76 (2,407.18 to 2,549.64)**	**4,143.31 (3,266.05 to 5,567.97)**	**3,672.49 (2,999.59 to 4,691.39)**	**4,905.55 (3,476.31 to 7,164.58)**
Lip and oral cavity cancer	533.98 (506.30 to 559.58)	550.03 (330.12 to 933.84)	373.88 (246.83 to 596.57)	770.50 (417.22 to 1,492.66)	21.75 (20.65 to 22.72)	28.77 (17.46 to 46.95)	19.96 (13.11 to 30.91)	38.78 (20.59 to 71.41)
Nasopharynx cancer	856.25 (787.68 to 920.70)	990.50 (630.83 to 1,712.59)	641.25 (435.43 to 1,009.55)	1,558.60 (868.57 to 3,167.61)	29.46 (27.19 to 31.61)	41.90 (26.36 to 70.49)	27.54 (18.51 to 42.05)	64.05 (35.83 to 123.76)
Other pharynx cancer	139.61 (124.43 to 150.23)	174.68 (116.44 to 259.75)	137.39 (95.21 to 194.90)	169.49 (110.43 to 257.52)	5.43 (4.85 to 5.81)	8.96 (5.96 to 13.14)	7.26 (5.07 to 10.11)	8.39 (5.50 to 12.41)
Esophageal cancer	4,516.66 (4,325.44 to 4,714.13)	7,508.20 (4,598.58 to 12,479.35)	5,908.82 (3,881.01 to 9,082.27)	9,614.28 (4,997.16 to 18,226.62)	212.00 (203.37 to 220.67)	419.24 (260.25 to 689.93)	334.68 (223.50 to 518.97)	522.34 (279.10 to 951.68)
Stomach cancer	7,618.46 (7,354.73 to 7,884.59)	7,010.92 (5,224.69 to 9,414.01)	6,243.79 (4,725.34 to 8,105.08)	8,869.38 (6,879.22 to 11,245.42)	346.23 (334.62 to 357.91)	408.93 (310.36 to 540.70)	369.98 (286.46 to 467.97)	499.18 (400.84 to 621.43)
Colon and rectum cancer	3,887.93 (3,689.91 to 4,070.67)	6,075.40 (4,367.89 to 8,374.21)	5,250.37 (3,867.90 to 7,183.35)	7,023.32 (5,137.59 to 9,590.25)	175.94 (167.56 to 183.69)	377.00 (274.73 to 513.25)	334.92 (250.60 to 448.77)	416.95 (304.65 to 564.76)
Liver cancer	11,803.58 (11,277.51 to 12,399.29)	18,977.42 (13,936.89 to 26,663.43)	17,888.86 (13,694.34 to 23,510.61)	18,400.39 (12,915.21 to 27,755.02)	442.80 (423.24 to 467.36)	910.04 (684.05 to 1,244.60)	873.85 (687.31 to 1,119.49)	853.89 (617.08 to 1,240.25)
Liver cancer due to hepatitis B	6,448.57 (5,683.76 to 7,145.92)	10,015.38 (7,224.63 to 14,356.54)	9,549.52 (7,200.52 to 12,912.42)	9,609.56 (6,576.58 to 14,880.19)	232.35 (202.90 to 257.09)	461.64 (339.40 to 642.85)	448.43 (343.55 to 589.50)	428.35 (300.23 to 636.50)
Liver cancer due to hepatitis C	926.58 (812.75 to 1,062.18)	1,678.55 (1,224.88 to 2,383.35)	1,614.83 (1,230.88 to 2,147.52)	1,601.39 (1,112.00 to 2,358.07)	39.73 (35.31 to 44.89)	93.26 (69.16 to 128.11)	91.40 (69.69 to 119.13)	86.34 (61.19 to 120.95)
Liver cancer due to alcohol use	1,251.31 (1,008.07 to 1,540.88)	2,264.09 (1,561.19 to 3,202.34)	1,928.95 (1,367.45 to 2,650.65)	2,294.01 (1,542.83 to 3,374.99)	54.78 (44.64 to 65.89)	125.67 (87.65 to 175.93)	110.31 (78.17 to 149.59)	123.38 (83.70 to 177.46)
Liver cancer due to other causes	3,177.12 (2,780.34 to 3,659.15)	5,019.41 (3,379.20 to 7,387.25)	4,795.56 (3,432.32 to 6,688.46)	4,895.42 (3,087.79 to 7,716.22)	115.94 (101.96 to 132.92)	229.47 (158.04 to 331.00)	223.72 (161.68 to 307.83)	215.82 (138.93 to 331.97)
Gallbladder and biliary tract cancer	549.02 (454.44 to 625.88)	560.34 (456.19 to 652.80)	574.02 (467.19 to 664.79)	533.76 (435.26 to 621.49)	26.10 (21.76 to 29.69)	35.61 (29.63 to 41.23)	37.21 (30.74 to 42.80)	32.50 (26.59 to 37.69)
Pancreatic cancer	1,691.69 (1,624.11 to 1,757.19)	2,699.53 (2,172.49 to 3,439.59)	2,727.00 (2,194.20 to 3,388.72)	2,671.27 (2,047.16 to 3,557.71)	74.90 (72.00 to 77.88)	153.76 (125.01 to 190.71)	157.14 (128.65 to 192.17)	147.69 (115.04 to 191.75)
Larynx cancer	414.45 (396.25 to 438.35)	478.41 (219.14 to 1,069.25)	309.63 (162.72 to 636.32)	803.16 (301.02 to 1,940.56)	18.20 (17.41 to 19.19)	26.24 (11.54 to 58.75)	17.24 (8.64 to 36.93)	41.97 (15.73 to 99.09)
Tracheal, bronchus, and lung cancer	13,537.41 (12,974.95 to 14,101.33)	15,837.59 (9,188.62 to 30,329.46)	11,408.14 (7,261.70 to 19,698.01)	26,447.03 (12,695.37 to 55,485.09)	616.83 (592.16 to 641.48)	902.10 (512.68 to 1,628.01)	662.25 (419.93 to 1,112.34)	1,442.42 (698.94 to 2,809.13)
Malignant skin melanoma	146.11 (115.44 to 158.65)	189.26 (150.32 to 220.81)	206.57 (163.54 to 241.28)	150.34 (120.32 to 170.55)	5.31 (4.25 to 5.74)	10.44 (8.38 to 12.18)	11.65 (9.48 to 13.48)	7.92 (6.27 to 9.08)
Non-melanoma skin cancer	297.41 (280.74 to 315.44)	397.37 (337.36 to 475.55)	382.73 (323.71 to 455.13)	454.14 (402.58 to 504.79)	14.22 (13.50 to 15.09)	27.07 (23.14 to 32.55)	26.79 (22.99 to 31.70)	29.23 (24.51 to 33.29)
Breast cancer	2,122.33 (1,787.09 to 2,381.83)	2,142.21 (1,534.68 to 2,877.02)	1,954.34 (1,430.40 to 2,565.86)	2,536.14 (1,853.94 to 3,393.86)	73.93 (62.05 to 82.91)	104.39 (76.90 to 137.60)	96.67 (72.45 to 125.34)	119.60 (88.07 to 157.19)
Cervical cancer	1,182.42 (724.50 to 1,354.12)	783.17 (463.67 to 1,092.72)	716.67 (429.83 to 966.40)	988.14 (598.51 to 1,361.97)	40.30 (25.52 to 46.12)	36.99 (22.65 to 50.15)	34.22 (21.52 to 45.17)	45.46 (28.52 to 61.76)

Uterine cancer	366.62 (341.80 to 403.92)	413.69 (333.95 to 498.69)	395.79 (311.33 to 483.98)	472.95 (381.75 to 567.82)	13.96 (13.03 to 15.35)	21.98 (18.42 to 25.81)	21.36 (17.49 to 25.04)	24.28 (20.66 to 28.47)
Ovarian cancer	543.60 (490.98 to 593.22)	625.24 (476.35 to 805.12)	600.14 (451.49 to 768.32)	673.02 (509.21 to 873.37)	19.47 (17.63 to 21.06)	30.36 (23.83 to 38.33)	29.41 (23.11 to 36.72)	31.97 (25.05 to 40.85)
Prostate cancer	555.92 (432.27 to 694.39)	1,247.22 (963.48 to 1,575.47)	1,280.63 (1,001.05 to 1,607.21)	1,205.48 (916.63 to 1,529.97)	35.75 (27.22 to 44.15)	99.97 (75.42 to 127.02)	105.97 (80.79 to 130.26)	90.87 (65.51 to 116.89)
Testicular cancer	22.15 (20.78 to 23.59)	19.51 (16.61 to 23.31)	19.11 (16.33 to 22.82)	21.38 (18.49 to 24.61)	0.68 (0.64 to 0.73)	1.00 (0.82 to 1.20)	1.02 (0.85 to 1.24)	1.02 (0.82 to 1.22)

East Asia								
Cause	YLLs (in 1000s)				Deaths (in 1000s)			
	2016	2040 Forecast	2040 Better Scenario	2040 Worse Scenario	2016	2040 Forecast	2040 Better Scenario	2040 Worse Scenario
Kidney cancer	419.62 (393.21 to 441.85)	582.61 (473.71 to 740.46)	596.49 (486.83 to 744.01)	540.35 (425.26 to 717.68)	16.96 (15.94 to 17.77)	32.97 (26.71 to 40.55)	34.38 (28.55 to 41.69)	29.25 (23.38 to 37.54)
Bladder cancer	493.97 (462.19 to 519.63)	802.53 (602.07 to 1,064.57)	780.72 (610.21 to 1,010.19)	879.14 (621.50 to 1,255.62)	27.60 (25.92 to 28.93)	58.95 (44.87 to 76.84)	58.33 (45.99 to 73.43)	61.74 (43.77 to 84.37)
Brain and nervous system cancer	1,951.46 (1,757.50 to 2,199.63)	2,207.04 (1,912.20 to 2,546.49)	2,203.68 (1,880.84 to 2,547.17)	2,224.22 (1,941.82 to 2,530.36)	60.64 (54.29 to 68.08)	100.61 (86.22 to 113.90)	102.41 (87.36 to 115.84)	97.19 (84.17 to 109.05)
Thyroid cancer	141.99 (125.23 to 151.45)	166.94 (130.54 to 219.44)	157.87 (124.58 to 202.21)	178.13 (138.82 to 239.24)	6.21 (5.34 to 6.63)	10.21 (7.94 to 13.45)	9.94 (7.84 to 12.61)	10.37 (7.96 to 13.77)
Mesothelioma	76.78 (66.07 to 81.69)	81.43 (68.83 to 91.15)	84.34 (70.96 to 94.88)	76.26 (65.61 to 85.07)	2.85 (2.48 to 3.05)	4.30 (3.72 to 4.80)	4.54 (3.92 to 5.06)	3.86 (3.34 to 4.33)
Hodgkin lymphoma	91.77 (75.79 to 119.14)	64.12 (47.94 to 90.64)	59.02 (44.20 to 79.68)	81.89 (64.64 to 107.08)	3.04 (2.46 to 3.92)	3.03 (2.31 to 4.20)	2.85 (2.17 to 3.79)	3.72 (2.91 to 4.81)
Non-Hodgkin lymphoma	1,119.91 (937.11 to 1,195.89)	1,162.77 (931.36 to 1,407.47)	1,123.26 (900.61 to 1,330.63)	1,209.52 (968.47 to 1,438.61)	39.41 (32.84 to 42.07)	59.21 (48.42 to 70.11)	58.40 (47.66 to 67.99)	59.09 (47.64 to 69.70)
Multiple myeloma	269.12 (230.21 to 298.90)	390.30 (322.08 to 469.92)	383.20 (317.05 to 452.37)	384.44 (317.50 to 468.78)	10.83 (9.34 to 12.24)	21.02 (17.81 to 25.08)	21.06 (17.89 to 24.71)	19.92 (16.58 to 24.16)
Leukemia	2,415.31 (2,050.84 to 2,605.25)	1,704.97 (1,342.27 to 2,220.42)	1,585.17 (1,260.99 to 1,976.27)	1,945.59 (1,520.98 to 2,545.79)	62.65 (53.41 to 67.52)	71.71 (57.23 to 92.68)	68.13 (55.28 to 85.30)	78.29 (61.91 to 101.37)
Acute lymphoid leukemia	514.15 (408.47 to 560.61)	257.10 (198.54 to 324.26)	238.72 (183.61 to 294.55)	290.20 (221.49 to 369.01)	11.36 (9.16 to 12.38)	8.81 (6.83 to 11.02)	8.29 (6.47 to 10.18)	9.64 (7.45 to 12.21)
Chronic lymphoid leukemia	113.48 (97.57 to 128.47)	93.64 (74.93 to 119.58)	90.23 (72.61 to 111.26)	96.76 (76.46 to 125.76)	3.77 (3.25 to 4.20)	4.49 (3.66 to 5.59)	4.43 (3.64 to 5.39)	4.44 (3.51 to 5.70)
Acute myeloid leukemia	289.51 (253.27 to 338.09)	280.55 (223.45 to 351.04)	273.18 (219.41 to 334.80)	276.82 (219.73 to 356.94)	7.62 (6.62 to 8.92)	11.79 (9.49 to 14.65)	11.71 (9.44 to 14.34)	11.15 (8.80 to 14.33)
Chronic myeloid leukemia	37.88 (34.23 to 46.77)	20.59 (14.92 to 30.50)	17.94 (13.24 to 24.73)	27.33 (20.92 to 38.71)	1.17 (1.06 to 1.43)	0.93 (0.68 to 1.36)	0.83 (0.62 to 1.13)	1.19 (0.92 to 1.63)
Other leukemia	1,460.28 (1,186.96 to 1,595.71)	1,053.10 (796.69 to 1,402.26)	965.10 (732.87 to 1,238.05)	1,254.48 (952.07 to 1,669.89)	38.74 (31.70 to 42.13)	45.69 (34.88 to 60.05)	42.88 (33.33 to 54.70)	51.87 (40.18 to 68.31)
Other neoplasms	2,027.89 (1,798.66 to 2,119.33)	2,484.83 (2,126.81 to 2,718.63)	2,547.15 (2,172.04 to 2,807.51)	2,367.26 (2,042.81 to 2,569.04)	74.31 (64.32 to 77.63)	136.55 (116.02 to 148.23)	143.34 (122.84 to 155.47)	123.60 (104.88 to 135.60)
**Cardiovascular diseases**	**71,276.84 (69,398.04 to 73,078.07)**	**58,009.35 (37,641.98 to 90,795.71)**	**41,944.91 (27,924.45 to 62,914.87)**	**111,473.85 (73,464.15 to 162,995.41)**	**4,105.07 (4,004.93 to 4,203.12)**	**4,774.93 (3,266.45 to 7,276.58)**	**3,734.46 (2,637.80 to 5,465.14)**	**8,098.96 (5,728.13 to 11,410.00)**
Rheumatic heart disease	1,522.60 (1,437.45 to 1,607.40)	1,281.89 (963.67 to 1,724.58)	1,173.67 (911.43 to 1,520.80)	1,601.79 (1,274.54 to 2,092.38)	74.48 (70.26 to 78.72)	93.82 (73.55 to 125.86)	88.58 (69.93 to 115.03)	110.37 (88.27 to 146.83)
Ischemic heart disease	29,375.42 (28,459.34 to 30,346.98)	20,930.56 (12,348.17 to 34,817.57)	14,951.45 (8,889.40 to 24,667.55)	43,090.65 (25,240.97 to 67,890.38)	1,774.98 (1,722.03 to 1,832.87)	1,777.88 (1,096.98 to 2,846.12)	1,382.09 (867.23 to 2,124.94)	3,256.01 (2,016.14 to 4,988.77)
Stroke	33,843.27 (32,785.23 to 34,970.45)	22,726.82 (13,456.82 to 36,569.36)	15,081.94 (9,297.16 to 23,861.91)	50,190.18 (30,689.96 to 77,985.86)	1,848.93 (1,782.31 to 1,917.49)	1,730.96 (1,078.33 to 2,642.41)	1,258.69 (837.72 to 1,915.66)	3,353.59 (2,158.59 to 5,046.55)
Ischemic stroke	11,715.49 (11,302.51 to 12,166.18)	8,416.87 (4,860.09 to 14,265.60)	6,062.49 (3,653.62 to 10,586.19)	16,627.05 (9,700.38 to 28,168.36)	749.12 (717.14 to 780.96)	710.76 (425.73 to 1,241.60)	552.81 (342.17 to 947.78)	1,247.73 (760.54 to 2,102.13)

Intracerebral hemorrhage	22,127.77 (21,406.00 to 22,908.60)	14,309.96 (7,952.03 to 24,492.59)	9,019.45 (5,278.48 to 14,838.59)	33,563.14 (19,051.87 to 55,404.46)	1,099.81 (1,060.91 to 1,141.08)	1,020.20 (595.13 to 1,653.47)	705.88 (445.07 to 1,074.31)	2,105.85 (1,286.12 to 3,299.54)
Hypertensive heart disease	3,661.51 (2,589.66 to 4,209.66)	8,274.06 (3,794.07 to 20,613.89)	6,361.46 (3,130.14 to 14,987.36)	10,864.08 (4,900.74 to 28,519.53)	248.49 (173.39 to 285.45)	751.97 (343.38 to 1,963.03)	604.74 (303.33 to 1,542.85)	920.71 (396.15 to 2,476.44)
Cardiomyopathy and myocarditis	807.29 (652.43 to 874.05)	742.78 (534.54 to 967.31)	700.97 (502.80 to 896.05)	822.30 (597.02 to 1,048.87)	31.10 (24.29 to 33.85)	49.97 (34.73 to 65.82)	49.73 (35.26 to 63.90)	50.46 (35.02 to 66.28)
Myocarditis	364.86 (258.73 to 418.71)	357.11 (239.66 to 438.25)	370.93 (249.46 to 458.07)	338.81 (225.47 to 419.36)	14.55 (9.76 to 17.12)	26.54 (17.12 to 33.88)	28.73 (18.77 to 36.30)	22.83 (14.15 to 30.06)

Cause	YLLs (in 1000s)				Deaths (in 1000s)			
	**East Asia**							
	2016	2040 Forecast	2040 Better Scenario	2040 Worse Scenario	2016	2040 Forecast	2040 Better Scenario	2040 Worse Scenario
Alcoholic cardiomyopathy	84.61 (52.09 to 107.26)	75.99 (46.41 to 101.63)	59.82 (36.51 to 81.38)	75.33 (44.97 to 97.58)	2.81 (1.79 to 3.61)	3.36 (2.20 to 4.53)	2.77 (1.81 to 3.83)	3.19 (2.05 to 4.20)
Other cardiomyopathy	357.81 (313.39 to 423.78)	309.68 (182.45 to 492.52)	270.23 (171.56 to 417.98)	408.16 (273.42 to 584.06)	13.74 (11.61 to 17.03)	20.06 (11.44 to 32.18)	18.23 (11.15 to 29.30)	24.44 (15.76 to 37.40)
Atrial fibrillation and flutter	430.96 (345.28 to 524.63)	1,108.28 (820.23 to 1,451.96)	1,040.74 (777.26 to 1,365.66)	1,113.61 (830.14 to 1,487.88)	39.99 (31.78 to 49.14)	126.20 (93.16 to 165.95)	122.59 (90.77 to 160.31)	118.76 (83.65 to 160.56)
Aortic aneurysm	287.10 (268.59 to 306.29)	374.43 (285.26 to 495.73)	351.23 (271.79 to 464.96)	418.41 (322.62 to 538.01)	14.26 (13.39 to 15.06)	27.76 (22.33 to 34.79)	27.34 (22.21 to 34.32)	28.12 (22.22 to 35.18)
Peripheral artery disease	31.73 (25.82 to 39.51)	100.41 (72.50 to 136.70)	99.18 (71.71 to 136.97)	100.55 (71.41 to 137.20)	2.26 (1.84 to 2.84)	9.78 (7.13 to 13.49)	10.06 (7.43 to 13.95)	8.97 (6.39 to 12.37)
Endocarditis	145.76 (124.73 to 184.54)	245.18 (176.20 to 342.32)	241.56 (173.87 to 332.50)	244.38 (175.63 to 336.16)	6.99 (5.94 to 8.43)	19.41 (13.70 to 28.07)	19.81 (14.23 to 27.93)	18.15 (12.46 to 26.18)
Other cardiovascular and circulatory diseases	1,171.21 (1,024.50 to 1,797.09)	2,224.94 (1,422.73 to 3,775.96)	1,942.71 (1,253.33 to 3,250.04)	3,027.90 (2,049.83 to 4,919.66)	63.59 (55.48 to 98.97)	187.19 (124.02 to 311.91)	170.83 (114.27 to 283.90)	233.82 (160.03 to 379.36)
**Chronic respiratory diseases**	**13,682.27 (13,144.49 to 14,847.99)**	**19,085.65 (8,552.56 to 39,368.40)**	**13,695.68 (6,918.56 to 26,311.48)**	**30,912.17 (12,059.46 to 69,109.42)**	**955.46 (917.78 to 1,045.94)**	**1,639.70 (734.13 to 3,398.97)**	**1,220.71 (610.26 to 2,401.27)**	**2,506.02 (977.40 to 5,420.85)**
Chronic obstructive pulmonary disease	12,844.32 (12,317.81 to 14,043.12)	18,452.52 (8,024.28 to 38,625.10)	13,082.71 (6,419.95 to 25,519.11)	30,208.63 (11,417.42 to 68,229.50)	913.22 (876.76 to 1,005.67)	1,593.68 (695.57 to 3,346.24)	1,175.25 (575.10 to 2,347.93)	2,456.99 (935.44 to 5,371.89)
Pneumoconiosis	201.83 (186.03 to 218.29)	87.99 (62.65 to 130.71)	75.87 (55.54 to 105.11)	122.28 (96.70 to 151.71)	8.83 (8.04 to 9.51)	5.66 (4.08 to 8.08)	5.07 (3.79 to 6.81)	7.30 (5.71 to 8.95)
Silicosis	148.36 (135.18 to 161.51)	49.68 (30.83 to 83.70)	41.94 (27.25 to 65.06)	77.42 (58.14 to 98.04)	6.44 (5.82 to 6.98)	3.06 (1.92 to 5.02)	2.67 (1.72 to 4.09)	4.48 (3.31 to 5.67)
Asbestosis	8.59 (6.41 to 11.33)	11.00 (7.33 to 14.98)	12.04 (8.04 to 16.55)	8.87 (5.90 to 12.27)	0.34 (0.24 to 0.47)	0.74 (0.49 to 1.02)	0.84 (0.56 to 1.16)	0.56 (0.37 to 0.80)
Coal workers pneumoconiosis	18.55 (10.06 to 21.52)	11.09 (6.57 to 17.30)	8.44 (4.93 to 12.65)	16.76 (9.60 to 23.44)	0.96 (0.52 to 1.12)	0.77 (0.44 to 1.20)	0.61 (0.35 to 0.91)	1.09 (0.61 to 1.52)
Other pneumoconiosis	26.33 (23.12 to 33.41)	16.23 (12.18 to 22.17)	13.46 (10.06 to 18.38)	19.22 (14.96 to 25.35)	1.08 (0.95 to 1.34)	1.09 (0.84 to 1.46)	0.94 (0.71 to 1.28)	1.17 (0.91 to 1.55)
Asthma	406.13 (342.75 to 453.40)	227.39 (139.96 to 376.21)	181.62 (116.27 to 275.65)	341.47 (222.41 to 517.32)	22.89 (18.42 to 25.57)	19.07 (11.96 to 30.87)	15.92 (10.62 to 24.13)	26.57 (17.31 to 39.56)
Interstitial lung disease and pulmonary sarcoidosis	143.57 (113.90 to 185.62)	251.83 (168.82 to 339.40)	288.60 (202.36 to 395.73)	175.55 (131.05 to 237.27)	7.05 (5.41 to 8.81)	16.80 (11.03 to 22.45)	19.75 (13.57 to 26.65)	11.13 (7.92 to 14.42)
Other chronic respiratory diseases	86.42 (42.25 to 114.20)	65.91 (28.78 to 111.89)	66.88 (29.00 to 112.81)	64.23 (28.11 to 111.58)	3.47 (1.60 to 4.67)	4.49 (1.88 to 7.72)	4.73 (1.97 to 8.20)	4.03 (1.71 to 6.92)
**Cirrhosis and other chronic liver diseases**	**4,651.90 (4,353.68 to 5,595.28)**	**4,605.34 (3,915.37 to 5,638.37)**	**4,275.70 (3,617.62 to 5,187.58)**	**5,231.79 (4,570.84 to 6,399.70)**	**170.38 (159.84 to 202.33)**	**227.01 (196.95 to 274.73)**	**216.43 (186.85 to 259.37)**	**247.31 (217.91 to 296.95)**
Cirrhosis and other chronic liver diseases due to hepatitis B	2,290.01 (2,102.30 to 2,822.83)	2,318.77 (1,945.83 to 2,906.84)	2,244.78 (1,885.04 to 2,827.36)	2,591.32 (2,231.07 to 3,188.30)	85.46 (78.34 to 103.43)	115.42 (98.14 to 144.07)	114.00 (97.77 to 140.31)	124.01 (106.77 to 149.32)
Cirrhosis and other chronic liver diseases due to hepatitis C	640.75 (562.35 to 795.84)	571.81 (466.52 to 729.30)	551.38 (451.77 to 705.97)	642.86 (538.98 to 807.02)	22.08 (19.50 to 27.39)	25.59 (21.39 to 32.59)	25.17 (21.13 to 30.99)	27.74 (23.49 to 34.62)

Cirrhosis and other chronic liver diseases due to alcohol use	716.05 (639.37 to 876.79)	740.97 (618.09 to 945.61)	539.98 (451.71 to 673.94)	901.07 (759.22 to 1,113.50)	26.16 (23.59 to 31.43)	35.46 (30.11 to 43.64)	27.40 (23.21 to 33.49)	41.12 (34.88 to 50.30)
Cirrhosis and other chronic liver diseases due to other causes	1,005.09 (862.95 to 1,198.69)	973.79 (796.53 to 1,228.39)	939.57 (765.98 to 1,192.09)	1,096.54 (909.37 to 1,347.71)	36.67 (31.52 to 43.59)	50.53 (40.78 to 62.26)	49.85 (41.13 to 62.49)	54.44 (44.30 to 68.40)
**Digestive diseases**	**2,483.74 (2,349.93 to 2,802.40)**	**2,801.76 (2,327.42 to 3,474.05)**	**2,620.91 (2,205.42 to 3,215.94)**	**3,260.44 (2,717.22 to 4,034.57)**	**121.83 (114.54 to 139.62)**	**214.49 (176.71 to 265.33)**	**208.55 (174.90 to 254.76)**	**232.86 (184.27 to 290.37)**
Peptic ulcer disease	797.53 (732.10 to 882.57)	557.21 (409.17 to 758.35)	491.74 (364.09 to 665.35)	796.99 (608.36 to 1,024.69)	39.78 (36.65 to 43.87)	40.37 (30.03 to 54.29)	36.84 (27.67 to 49.36)	54.59 (41.05 to 70.12)

East Asia								
Cause	YLLs (in 1000s)				Deaths (in 1000s)			
	2016	2040 Forecast	2040 Better Scenario	2040 Worse Scenario	2016	2040 Forecast	2040 Better Scenario	2040 Worse Scenario
Gastritis and duodenitis	290.63 (261.76 to 314.43)	382.62 (308.53 to 472.44)	369.47 (291.69 to 460.44)	443.03 (359.07 to 532.66)	15.34 (13.93 to 16.55)	28.67 (22.80 to 36.00)	28.54 (22.40 to 35.77)	31.13 (23.80 to 38.34)
Appendicitis	49.98 (44.75 to 62.22)	52.23 (41.31 to 68.52)	48.37 (38.43 to 63.53)	66.86 (55.66 to 84.81)	2.14 (1.92 to 2.68)	3.77 (2.96 to 4.98)	3.61 (2.90 to 4.67)	4.51 (3.64 to 5.68)
Paralytic ileus and intestinal obstruction	373.78 (319.04 to 461.35)	443.62 (373.89 to 601.46)	465.32 (391.54 to 628.58)	410.75 (335.77 to 569.78)	15.65 (13.61 to 20.49)	35.69 (28.79 to 47.71)	38.62 (31.62 to 51.32)	30.87 (23.96 to 42.09)
Inguinal, femoral, and abdominal hernia	27.94 (23.05 to 39.54)	29.09 (22.36 to 42.70)	28.97 (22.25 to 42.50)	31.21 (23.48 to 46.17)	1.61 (1.29 to 2.35)	2.64 (1.98 to 3.97)	2.73 (2.04 to 3.99)	2.63 (1.84 to 3.88)
Inflammatory bowel disease	117.36 (99.10 to 132.13)	169.70 (144.55 to 202.30)	176.98 (150.98 to 212.22)	157.91 (132.78 to 190.64)	5.56 (4.89 to 6.66)	13.53 (11.26 to 16.76)	14.55 (12.25 to 18.11)	11.74 (9.17 to 14.68)
Vascular intestinal disorders	125.96 (116.11 to 161.47)	212.89 (185.19 to 265.75)	215.94 (186.30 to 273.57)	215.14 (185.23 to 269.55)	7.58 (6.99 to 9.45)	17.82 (15.19 to 22.44)	18.55 (15.84 to 23.25)	17.01 (13.93 to 21.81)
Gallbladder and biliary diseases	242.84 (219.37 to 341.12)	404.72 (252.75 to 676.97)	341.02 (228.48 to 544.83)	506.51 (311.02 to 867.40)	14.85 (13.50 to 19.96)	35.41 (22.46 to 56.52)	31.06 (20.97 to 47.87)	41.29 (25.09 to 67.18)
Pancreatitis	271.66 (235.75 to 331.83)	294.47 (194.39 to 439.86)	226.66 (159.49 to 323.51)	364.59 (247.42 to 553.42)	10.57 (9.16 to 12.96)	17.14 (11.77 to 25.01)	13.88 (9.94 to 19.65)	20.05 (13.87 to 29.61)
Other digestive diseases	186.06 (168.82 to 209.65)	255.20 (222.10 to 299.66)	256.44 (220.25 to 311.04)	267.45 (228.75 to 321.81)	8.75 (8.04 to 9.95)	19.45 (16.63 to 23.25)	20.17 (17.22 to 24.30)	19.03 (15.36 to 23.62)
**Neurological disorders**	**6,868.10 (6,078.34 to 7,832.91)**	**17,187.76 (13,689.96 to 21,893.03)**	**17,287.62 (14,221.84 to 21,400.98)**	**15,908.42 (12,122.03 to 21,541.42)**	**561.18 (488.36 to 648.77)**	**1,830.60 (1,397.14 to 2,382.34)**	**1,893.73 (1,499.50 to 2,345.63)**	**1,597.76 (1,118.33 to 2,190.32)**
Alzheimer's disease and other dementias	5,304.09 (4,546.76 to 6,262.52)	14,733.60 (11,290.10 to 19,462.95)	14,750.04 (11,774.97 to 18,804.74)	13,717.40 (9,946.48 to 19,349.40)	495.13 (423.61 to 582.79)	1,654.69 (1,243.59 to 2,209.24)	1,703.21 (1,325.42 to 2,169.80)	1,454.70 (993.20 to 2,049.42)
Parkinson's disease	561.22 (435.55 to 707.52)	1,602.53 (1,216.76 to 2,061.21)	1,740.41 (1,332.36 to 2,221.94)	1,297.11 (962.95 to 1,708.33)	41.58 (32.29 to 53.09)	143.68 (107.93 to 186.22)	159.35 (120.82 to 205.05)	111.14 (79.61 to 148.07)
Epilepsy	616.04 (578.24 to 692.07)	436.29 (332.90 to 560.64)	372.95 (289.10 to 475.13)	503.72 (379.91 to 672.59)	13.61 (12.80 to 15.21)	14.92 (11.84 to 18.92)	13.14 (10.63 to 16.31)	16.29 (12.64 to 21.29)
Multiple sclerosis	42.10 (37.05 to 48.78)	43.15 (35.45 to 52.00)	43.69 (35.91 to 52.75)	41.34 (34.13 to 50.48)	1.29 (1.13 to 1.49)	1.79 (1.52 to 2.14)	1.84 (1.55 to 2.18)	1.65 (1.39 to 1.98)
Motor neuron disease	99.83 (95.08 to 104.69)	128.25 (114.37 to 141.94)	130.32 (115.24 to 145.08)	122.09 (109.59 to 134.62)	3.10 (2.96 to 3.23)	5.35 (4.83 to 5.86)	5.51 (4.93 to 6.04)	4.96 (4.47 to 5.41)
Other neurological disorders	244.83 (234.54 to 255.95)	243.94 (221.22 to 268.09)	250.21 (225.79 to 275.41)	226.76 (206.35 to 247.50)	6.47 (6.25 to 6.74)	10.17 (9.44 to 10.90)	10.67 (9.93 to 11.45)	9.01 (8.29 to 9.71)
**Mental disorders**	**1,944.44 (1,616.78 to 2,270.88)**	**2,897.33 (2,260.81 to 3,530.97)**	**3,120.09 (2,479.83 to 3,687.85)**	**2,338.55 (1,915.20 to 2,698.66)**	**55.93 (45.29 to 63.07)**	**124.27 (96.18 to 149.04)**	**138.03 (109.96 to 163.88)**	**94.35 (75.97 to 106.68)**
Alcohol use disorders	816.12 (473.99 to 920.83)	608.48 (318.24 to 762.61)	581.28 (300.28 to 727.71)	553.28 (291.53 to 682.78)	22.57 (13.20 to 25.45)	22.67 (13.24 to 27.55)	22.30 (12.81 to 26.94)	19.73 (11.36 to 23.57)
Drug use disorders	1,124.81 (996.81 to 1,553.17)	2,286.27 (1,769.80 to 2,888.96)	2,536.20 (2,018.00 to 3,122.50)	1,782.74 (1,502.08 to 2,171.98)	33.30 (29.31 to 42.42)	101.55 (77.97 to 124.82)	115.68 (91.13 to 140.90)	74.57 (61.90 to 86.46)
Opioid use disorders	685.87 (563.38 to 999.86)	1,010.56 (750.37 to 1,285.21)	1,068.70 (788.25 to 1,374.83)	888.42 (675.84 to 1,163.60)	19.60 (15.45 to 26.15)	41.71 (29.47 to 51.29)	45.18 (31.05 to 54.87)	34.77 (24.68 to 42.39)

Cocaine use disorders	81.37 (63.65 to 125.50)	200.98 (133.04 to 301.39)	223.37 (152.45 to 323.67)	156.01 (112.31 to 223.33)	2.16 (1.67 to 3.23)	6.91 (4.55 to 10.25)	7.76 (5.17 to 11.31)	5.23 (3.70 to 7.44)
Amphetamine use disorders	48.33 (36.96 to 79.34)	163.96 (100.35 to 252.24)	189.59 (125.61 to 287.86)	113.01 (81.68 to 168.71)	1.19 (0.91 to 1.88)	5.08 (3.19 to 7.62)	5.93 (3.87 to 8.78)	3.42 (2.52 to 4.91)
Other drug use disorders	309.24 (251.56 to 459.60)	910.77 (630.16 to 1,238.88)	1,054.54 (772.34 to 1,408.05)	625.30 (494.04 to 822.64)	10.34 (8.51 to 14.19)	47.85 (32.82 to 63.70)	56.81 (41.18 to 75.31)	31.15 (24.37 to 39.26)
Eating disorders	3.52 (2.65 to 3.91)	2.57 (1.80 to 3.28)	2.61 (1.81 to 3.37)	2.53 (1.82 to 3.22)	0.07 (0.05 to 0.07)	0.05 (0.04 to 0.07)	0.05 (0.04 to 0.07)	0.05 (0.04 to 0.06)

East Asia								
Cause	YLLs (in 1000s)				Deaths (in 1000s)			
	2016	2040 Forecast	2040 Better Scenario	2040 Worse Scenario	2016	2040 Forecast	2040 Better Scenario	2040 Worse Scenario
**Diabetes, urogenital, blood, and endocrine diseases**	**8,106.97 (7,829.73 to 8,487.74)**	**10,491.97 (7,920.09 to 13,802.96)**	**9,222.63 (7,134.23 to 12,002.08)**	**14,516.16 (11,036.31 to 19,016.69)**	**383.59 (370.85 to 398.62)**	**747.93 (582.19 to 974.39)**	**681.56 (532.63 to 869.62)**	**974.10 (752.19 to 1,272.64)**
Diabetes mellitus	3,062.45 (2,955.41 to 3,167.57)	3,850.89 (2,640.21 to 5,658.10)	3,217.77 (2,275.90 to 4,555.65)	5,515.58 (3,762.05 to 7,983.24)	155.91 (150.48 to 161.27)	270.96 (191.99 to 375.34)	236.68 (172.63 to 324.67)	362.33 (253.15 to 503.56)
Acute glomerulonephritis	204.32 (193.36 to 216.49)	246.42 (199.39 to 302.20)	237.37 (188.89 to 292.56)	281.44 (230.44 to 337.28)	7.68 (7.30 to 8.10)	13.03 (10.40 to 16.01)	12.87 (10.20 to 15.77)	14.08 (11.30 to 17.11)
Chronic kidney disease	3,766.13 (3,583.29 to 4,055.26)	5,066.92 (3,086.99 to 8,172.51)	4,445.15 (2,753.86 to 6,988.64)	7,317.74 (4,276.98 to 11,864.99)	177.04 (167.44 to 188.11)	362.45 (216.08 to 581.27)	327.59 (202.75 to 520.26)	497.91 (288.20 to 803.37)
Chronic kidney disease due to diabetes mellitus	1,963.02 (1,764.64 to 2,168.61)	2,651.81 (1,575.12 to 4,387.06)	2,308.98 (1,392.82 to 3,759.91)	3,878.16 (2,184.83 to 6,597.64)	87.18 (77.42 to 96.59)	173.03 (100.07 to 291.36)	154.65 (92.66 to 253.55)	242.46 (136.13 to 408.49)
Chronic kidney disease due to hypertension	1,123.62 (984.97 to 1,276.16)	1,806.71 (1,080.13 to 2,930.34)	1,611.17 (984.62 to 2,548.62)	2,529.06 (1,443.58 to 4,140.42)	64.69 (57.53 to 72.78)	149.78 (88.63 to 250.05)	137.74 (84.09 to 225.49)	198.61 (110.69 to 329.15)
Chronic kidney disease due to glomerulonephritis	308.57 (257.18 to 370.12)	234.19 (140.15 to 384.78)	199.80 (122.19 to 321.89)	357.86 (208.72 to 592.18)	10.19 (8.36 to 12.43)	13.53 (7.67 to 23.55)	11.82 (6.80 to 20.29)	20.08 (11.18 to 34.41)
Chronic kidney disease due to other and unspecified causes	370.91 (307.70 to 445.27)	374.21 (218.87 to 632.79)	325.20 (196.75 to 542.08)	552.65 (316.60 to 930.06)	14.98 (12.27 to 18.14)	26.11 (14.61 to 45.56)	23.38 (13.51 to 39.25)	36.77 (20.01 to 63.12)
Urinary diseases and male infertility	320.04 (306.31 to 339.12)	610.22 (531.17 to 714.60)	594.54 (516.09 to 691.64)	675.90 (607.05 to 749.49)	18.22 (17.41 to 19.24)	57.03 (49.01 to 67.05)	57.51 (49.85 to 67.22)	58.34 (48.54 to 68.07)
Urinary tract infections	196.28 (178.59 to 215.80)	402.38 (349.13 to 464.67)	396.69 (343.58 to 458.94)	428.67 (377.02 to 484.63)	10.80 (9.82 to 11.94)	39.33 (33.49 to 46.32)	40.00 (34.05 to 46.87)	38.94 (32.09 to 46.35)
Urolithiasis	68.12 (59.51 to 82.38)	130.56 (99.81 to 178.22)	122.38 (95.23 to 162.71)	157.73 (130.93 to 197.44)	3.69 (3.20 to 4.47)	10.08 (7.63 to 13.86)	9.78 (7.51 to 13.14)	11.24 (8.76 to 14.60)
Other urinary diseases	55.64 (47.80 to 68.28)	77.28 (56.91 to 105.55)	75.47 (55.73 to 101.56)	89.49 (65.19 to 117.76)	3.73 (3.18 to 4.51)	7.62 (5.41 to 10.61)	7.73 (5.59 to 10.58)	8.15 (5.62 to 11.45)
Gynecological diseases	30.91 (18.29 to 34.74)	10.74 (5.97 to 15.53)	9.32 (5.35 to 13.10)	15.75 (9.35 to 20.64)	1.09 (0.64 to 1.22)	0.53 (0.30 to 0.75)	0.47 (0.27 to 0.66)	0.76 (0.44 to 0.98)
Uterine fibroids	22.40 (10.80 to 26.87)	7.51 (3.92 to 11.04)	6.60 (3.32 to 9.36)	10.70 (5.81 to 14.57)	0.79 (0.38 to 0.95)	0.36 (0.18 to 0.52)	0.32 (0.18 to 0.46)	0.50 (0.27 to 0.69)
Polycystic ovarian syndrome	0.33 (0.26 to 0.43)	0.04 (0.02 to 0.08)	0.03 (0.02 to 0.05)	0.09 (0.05 to 0.13)	0.01 (0.01 to 0.01)	0.00 (0.00 to 0.00)	0.00 (0.00 to 0.00)	0.00 (0.00 to 0.00)
Endometriosis	0.48 (0.08 to 0.67)	0.08 (0.02 to 0.15)	0.07 (0.01 to 0.12)	0.12 (0.02 to 0.22)	0.01 (0.00 to 0.01)	0.00 (0.00 to 0.00)	0.00 (0.00 to 0.00)	0.00 (0.00 to 0.01)
Genital prolapse	0.48 (0.18 to 0.59)	0.25 (0.09 to 0.39)	0.21 (0.07 to 0.32)	0.37 (0.13 to 0.54)	0.03 (0.01 to 0.04)	0.02 (0.01 to 0.03)	0.02 (0.01 to 0.03)	0.03 (0.01 to 0.04)
Other gynecological diseases	7.22 (4.00 to 8.69)	2.86 (1.43 to 4.46)	2.40 (1.23 to 3.69)	4.46 (2.39 to 6.07)	0.25 (0.14 to 0.29)	0.15 (0.08 to 0.23)	0.13 (0.07 to 0.20)	0.23 (0.13 to 0.30)
Hemoglobinopathies and hemolytic anemias	305.20 (250.84 to 345.36)	183.47 (135.93 to 236.84)	170.79 (126.18 to 215.30)	228.00 (174.49 to 276.77)	8.87 (7.05 to 10.13)	9.61 (7.09 to 12.26)	9.17 (6.72 to 11.74)	11.46 (8.63 to 13.78)
Thalassemias	96.56 (83.62 to 115.06)	22.58 (15.77 to 32.13)	22.26 (15.62 to 31.47)	22.54 (15.60 to 31.87)	1.28 (1.11 to 1.52)	0.35 (0.25 to 0.47)	0.34 (0.25 to 0.47)	0.34 (0.25 to 0.46)

Sickle cell disorders	0.49 (0.38 to 0.61)	0.17 (0.11 to 0.24)	0.15 (0.10 to 0.21)	0.22 (0.15 to 0.31)	0.01 (0.01 to 0.01)	0.00 (0.00 to 0.01)	0.00 (0.00 to 0.00)	0.00 (0.00 to 0.01)
G6PD deficiency	94.95 (72.97 to 111.20)	64.59 (45.11 to 88.49)	57.70 (39.35 to 77.66)	88.43 (62.70 to 113.21)	2.36 (1.79 to 2.77)	2.36 (1.64 to 3.23)	2.14 (1.49 to 2.87)	3.17 (2.27 to 3.98)
Other hemoglobinopathies and hemolytic anemias	113.20 (86.58 to 129.83)	96.13 (70.17 to 124.06)	90.68 (65.86 to 116.10)	116.81 (87.62 to 140.31)	5.23 (4.00 to 5.94)	6.90 (4.96 to 8.74)	6.68 (4.86 to 8.53)	7.95 (5.89 to 9.61)
Endocrine, metabolic, blood, and immune disorders	417.92 (350.56 to 489.67)	523.31 (427.29 to 603.80)	547.70 (443.62 to 630.78)	481.74 (394.13 to 558.72)	14.79 (11.97 to 16.84)	34.32 (27.38 to 39.30)	37.26 (30.00 to 42.00)	29.22 (22.50 to 33.93)

Cause	YLLs (in 1000s)				Deaths (in 1000s)			
	**2016**	**2040 Forecast**	**2040 Better Scenario**	**2040 Worse Scenario**	**2016**	**2040 Forecast**	**2040 Better Scenario**	**2040 Worse Scenario**
**Musculoskeletal disorders**	**507.58 (422.56 to 634.82)**	**622.91 (483.13 to 811.35)**	**585.68 (451.24 to 734.48)**	**753.11 (588.58 to 932.69)**	**19.07 (15.65 to 23.03)**	**38.98 (29.65 to 50.55)**	**37.34 (29.06 to 46.72)**	**45.65 (35.23 to 54.98)**
Rheumatoid arthritis	184.91 (138.49 to 215.05)	307.61 (206.55 to 442.94)	273.14 (191.90 to 375.83)	430.78 (313.51 to 537.11)	9.55 (7.14 to 10.94)	22.46 (15.37 to 31.95)	20.49 (14.50 to 28.15)	29.87 (21.61 to 37.36)
Other musculoskeletal disorders	322.67 (270.90 to 427.23)	315.30 (252.55 to 423.36)	312.53 (248.89 to 417.07)	322.33 (256.57 to 446.81)	9.52 (8.10 to 12.57)	16.52 (13.23 to 21.74)	16.86 (13.47 to 22.13)	15.78 (12.34 to 21.28)
**Other non-communicable diseases**	**3,711.04 (3,309.94 to 4,453.76)**	**1,121.32 (806.31 to 1,641.15)**	**972.76 (708.80 to 1,389.44)**	**1,601.58 (1,179.85 to 2,249.92)**	**53.07 (47.42 to 62.60)**	**40.86 (28.41 to 55.87)**	**39.84 (27.31 to 54.50)**	**46.27 (33.78 to 61.21)**
Congenital birth defects	3,524.81 (3,118.23 to 4,259.81)	805.64 (531.51 to 1,292.95)	657.31 (430.75 to 1,064.47)	1,273.30 (881.84 to 1,873.40)	44.88 (39.96 to 54.02)	11.77 (8.20 to 17.95)	9.75 (6.68 to 14.84)	18.25 (13.45 to 25.48)
Neural tube defects	41.74 (33.16 to 55.42)	6.43 (4.06 to 10.27)	4.93 (3.03 to 8.07)	10.74 (6.89 to 16.77)	0.53 (0.43 to 0.70)	0.10 (0.07 to 0.15)	0.08 (0.05 to 0.12)	0.17 (0.12 to 0.25)
Congenital heart anomalies	2,528.52 (2,244.06 to 3,025.96)	538.73 (340.04 to 888.11)	430.38 (274.38 to 702.99)	889.27 (616.79 to 1,326.56)	32.21 (28.83 to 38.30)	7.67 (5.02 to 12.09)	6.18 (4.08 to 9.62)	12.55 (8.97 to 17.83)
Orofacial clefts	27.47 (20.27 to 43.39)	4.15 (2.03 to 7.80)	2.89 (1.34 to 5.52)	7.68 (3.80 to 14.01)	0.32 (0.23 to 0.50)	0.05 (0.02 to 0.09)	0.03 (0.02 to 0.06)	0.09 (0.04 to 0.16)
Down syndrome	47.44 (38.88 to 60.94)	27.67 (21.38 to 38.25)	27.88 (21.76 to 38.12)	27.31 (20.99 to 38.40)	0.79 (0.66 to 1.03)	0.61 (0.47 to 0.84)	0.62 (0.49 to 0.84)	0.59 (0.46 to 0.81)
Other chromosomal abnormalities	37.01 (29.96 to 54.80)	15.87 (10.43 to 24.15)	14.36 (9.40 to 22.30)	19.48 (12.58 to 30.42)	0.47 (0.38 to 0.67)	0.22 (0.15 to 0.33)	0.20 (0.14 to 0.30)	0.27 (0.18 to 0.41)
Congenital musculoskeletal and limb anomalies	43.79 (37.02 to 61.69)	12.23 (7.88 to 19.11)	10.31 (6.73 to 15.60)	17.93 (11.78 to 26.97)	0.56 (0.48 to 0.79)	0.18 (0.13 to 0.28)	0.16 (0.11 to 0.23)	0.27 (0.19 to 0.38)
Urogenital congenital anomalies	33.15 (27.72 to 45.76)	13.27 (9.53 to 18.63)	11.75 (8.20 to 16.04)	17.84 (12.93 to 24.92)	0.57 (0.47 to 0.78)	0.33 (0.24 to 0.45)	0.29 (0.20 to 0.39)	0.43 (0.32 to 0.57)
Digestive congenital anomalies	299.01 (251.06 to 367.80)	61.80 (36.32 to 108.57)	49.63 (28.54 to 84.90)	98.28 (58.57 to 160.03)	3.53 (2.96 to 4.33)	0.76 (0.46 to 1.31)	0.62 (0.37 to 1.04)	1.21 (0.74 to 1.93)
Other congenital birth defects	466.69 (385.11 to 658.77)	125.50 (80.26 to 197.75)	105.17 (66.03 to 168.97)	184.76 (120.76 to 289.21)	5.91 (4.90 to 8.25)	1.85 (1.25 to 2.75)	1.57 (1.05 to 2.38)	2.68 (1.87 to 4.03)
Skin and subcutaneous diseases	127.61 (83.25 to 196.19)	302.36 (191.61 to 455.24)	303.94 (187.60 to 444.66)	309.80 (195.65 to 475.20)	7.51 (4.59 to 11.19)	28.93 (17.25 to 42.18)	29.95 (17.60 to 44.14)	27.80 (16.20 to 41.99)
Cellulitis	14.53 (8.70 to 26.02)	33.65 (16.83 to 53.02)	34.68 (17.17 to 55.05)	31.75 (15.90 to 51.54)	0.57 (0.28 to 0.90)	2.64 (1.06 to 3.83)	2.81 (1.12 to 4.08)	2.36 (0.93 to 3.40)
Pyoderma	51.24 (34.66 to 90.07)	134.96 (87.65 to 221.35)	136.22 (89.14 to 221.48)	137.65 (89.74 to 229.13)	2.48 (1.63 to 4.31)	11.58 (7.35 to 19.17)	12.13 (7.87 to 19.95)	10.89 (6.92 to 18.87)
Decubitus ulcer	49.29 (27.17 to 69.96)	116.07 (58.99 to 164.08)	115.22 (57.63 to 164.36)	122.62 (61.97 to 175.19)	3.93 (2.11 to 5.60)	13.35 (6.64 to 19.50)	13.60 (6.54 to 19.73)	13.30 (6.50 to 19.43)
Other skin and subcutaneous diseases	12.55 (8.41 to 20.78)	17.69 (11.86 to 27.99)	17.82 (11.50 to 28.44)	17.78 (11.55 to 29.17)	0.52 (0.35 to 0.85)	1.35 (0.86 to 2.16)	1.42 (0.91 to 2.22)	1.25 (0.79 to 2.05)
Sudden infant death syndrome	58.61 (49.96 to 71.38)	13.31 (6.89 to 25.15)	11.51 (6.04 to 21.74)	18.49 (9.88 to 32.56)	0.68 (0.58 to 0.83)	0.15 (0.08 to 0.29)	0.13 (0.07 to 0.25)	0.21 (0.11 to 0.38)

**Injuries**	**31,284.24 (29,365.98 to 32,339.28)**	**17,874.89 (13,871.07 to 26,827.69)**	**15,173.60 (11,855.58 to 24,055.07)**	**24,884.11 (19,722.12 to 34,514.34)**	**806.10 (735.68 to 836.50)**	**767.48 (622.74 to 998.73)**	**691.12 (568.88 to 876.35)**	**957.47 (792.01 to 1,222.28)**
**Transport injuries**	**14,066.14 (13,563.90 to 14,621.59)**	**6,127.83 (4,415.02 to 8,747.55)**	**4,518.53 (3,365.87 to 6,035.45)**	**11,180.63 (8,271.29 to 15,326.64)**	**336.96 (324.64 to 349.57)**	**203.46 (150.99 to 281.60)**	**155.28 (119.76 to 204.55)**	**348.45 (265.94 to 465.72)**
Road injuries	13,496.73 (13,028.65 to 13,979.53)	5,692.15 (4,108.91 to 8,108.72)	4,167.01 (3,112.29 to 5,574.33)	10,576.35 (7,854.32 to 14,506.64)	323.21 (311.75 to 333.88)	188.89 (141.37 to 260.01)	143.24 (111.51 to 188.20)	329.02 (251.98 to 439.20)
Pedestrian road injuries	7,469.49 (7,050.72 to 7,879.06)	2,466.37 (1,727.99 to 3,682.68)	1,682.75 (1,221.51 to 2,355.92)	5,382.31 (4,026.06 to 7,309.50)	186.17 (175.83 to 195.51)	93.38 (66.45 to 135.40)	66.85 (49.80 to 91.41)	187.11 (144.93 to 246.60)

East Asia								
Cause	YLLs (in 1000s)				Deaths (in 1000s)			
	2016	2040 Forecast	2040 Better Scenario	2040 Worse Scenario	2016	2040 Forecast	2040 Better Scenario	2040 Worse Scenario
Cyclist road injuries	989.89 (874.21 to 1,056.62)	591.76 (421.95 to 815.01)	461.38 (345.98 to 614.15)	942.91 (674.09 to 1,297.61)	25.87 (22.95 to 27.60)	21.69 (15.66 to 29.73)	17.68 (13.28 to 23.51)	30.81 (22.02 to 41.94)
Motorcyclist road injuries	2,531.59 (2,218.63 to 2,736.05)	1,628.06 (1,085.96 to 2,438.64)	1,311.76 (906.82 to 1,881.55)	2,253.37 (1,509.63 to 3,373.47)	54.94 (47.74 to 59.15)	42.41 (28.97 to 62.13)	35.00 (24.87 to 48.77)	55.42 (37.91 to 80.74)
Motor vehicle road injuries	2,424.57 (2,104.44 to 3,007.71)	967.14 (697.28 to 1,350.29)	682.68 (508.91 to 933.31)	1,926.61 (1,375.68 to 2,662.78)	54.28 (46.92 to 67.40)	30.00 (22.57 to 41.05)	22.59 (17.71 to 29.94)	53.43 (39.66 to 72.17)
Other road injuries	81.19 (66.20 to 90.46)	38.82 (27.13 to 54.23)	28.43 (20.79 to 38.23)	71.16 (48.84 to 100.41)	1.94 (1.59 to 2.17)	1.42 (1.05 to 1.96)	1.12 (0.86 to 1.48)	2.26 (1.62 to 3.17)
Other transport injuries	569.40 (515.56 to 762.12)	435.68 (280.08 to 686.41)	351.52 (235.67 to 530.89)	604.28 (417.51 to 894.16)	13.75 (12.48 to 18.41)	14.57 (9.56 to 22.62)	12.04 (8.30 to 17.97)	19.43 (13.83 to 28.69)
**Unintentional injuries**	**11,488.71 (9,117.19 to 12,201.88)**	**6,515.75 (4,674.52 to 8,074.54)**	**6,108.19 (4,454.72 to 7,440.01)**	**7,725.99 (5,671.24 to 9,220.71)**	**308.86 (235.58 to 329.96)**	**355.54 (251.09 to 428.27)**	**350.77 (249.73 to 417.60)**	**381.49 (271.00 to 451.39)**
Falls	3,355.02 (2,380.39 to 3,677.79)	3,093.87 (2,095.29 to 3,732.14)	3,027.33 (2,041.68 to 3,613.21)	3,354.62 (2,285.26 to 3,897.58)	125.85 (86.33 to 138.02)	217.54 (143.56 to 262.64)	220.87 (147.44 to 264.17)	219.37 (143.71 to 265.45)
Drowning	3,348.89 (2,851.41 to 3,543.09)	1,356.76 (991.79 to 1,771.10)	1,218.07 (891.26 to 1,592.48)	1,668.32 (1,215.68 to 2,193.11)	66.49 (53.43 to 70.21)	48.86 (34.26 to 60.85)	45.45 (31.68 to 56.37)	55.93 (38.88 to 70.07)
Fire, heat, and hot substances	342.63 (271.43 to 371.69)	226.51 (164.52 to 310.40)	200.24 (144.76 to 265.87)	289.35 (208.65 to 373.89)	11.12 (8.50 to 12.08)	14.06 (10.19 to 18.86)	13.07 (9.20 to 16.94)	16.45 (11.65 to 21.16)
Poisonings	668.96 (369.66 to 773.05)	200.58 (106.30 to 296.83)	168.99 (87.56 to 246.42)	289.82 (150.54 to 404.66)	16.78 (9.33 to 19.43)	8.15 (4.40 to 11.75)	7.09 (3.86 to 10.15)	11.19 (5.89 to 15.31)
Exposure to mechanical forces	1,916.83 (1,160.40 to 2,124.57)	767.16 (436.93 to 1,024.57)	694.19 (393.95 to 911.15)	998.84 (551.14 to 1,243.52)	42.84 (25.35 to 47.32)	26.43 (15.45 to 34.29)	24.77 (14.38 to 31.85)	32.31 (18.49 to 39.12)
Unintentional firearm injuries	29.07 (25.36 to 41.65)	19.93 (16.48 to 25.89)	19.28 (15.74 to 24.50)	22.35 (18.04 to 30.44)	0.96 (0.86 to 1.24)	1.61 (1.25 to 1.95)	1.65 (1.29 to 2.01)	1.58 (1.18 to 2.01)
Unintentional suffocation	404.13 (207.84 to 495.78)	116.51 (50.74 to 175.57)	106.15 (46.81 to 165.64)	146.42 (63.78 to 223.51)	6.54 (3.28 to 7.91)	3.50 (1.54 to 4.75)	3.37 (1.50 to 4.60)	4.00 (1.74 to 5.49)
Other exposure to mechanical forces	1,483.63 (872.32 to 1,626.76)	630.73 (350.30 to 844.54)	568.76 (316.92 to 751.57)	830.07 (450.62 to 1,025.71)	35.34 (20.40 to 38.84)	21.32 (11.99 to 28.15)	19.75 (11.12 to 25.55)	26.72 (14.77 to 32.40)
Adverse effects of medical treatment	211.65 (190.71 to 275.61)	160.34 (135.73 to 209.64)	155.30 (129.35 to 207.05)	177.01 (149.65 to 229.90)	5.89 (5.38 to 7.64)	8.27 (7.11 to 10.63)	8.33 (7.06 to 10.78)	8.49 (7.21 to 10.89)
Animal contact	100.24 (90.14 to 117.19)	91.85 (75.47 to 114.24)	86.11 (68.04 to 108.27)	111.91 (89.64 to 140.00)	2.93 (2.60 to 3.40)	4.14 (3.42 to 5.14)	4.01 (3.23 to 4.97)	4.74 (3.82 to 5.89)
Venomous animal contact	53.25 (43.22 to 61.83)	46.42 (34.27 to 57.46)	44.43 (33.26 to 56.09)	54.01 (40.10 to 67.75)	1.62 (1.29 to 1.87)	2.15 (1.57 to 2.66)	2.12 (1.55 to 2.61)	2.36 (1.74 to 2.94)
Non-venomous animal contact	46.99 (41.20 to 63.47)	45.44 (35.33 to 62.10)	41.68 (31.26 to 57.77)	57.91 (44.87 to 79.83)	1.31 (1.14 to 1.80)	1.99 (1.55 to 2.74)	1.89 (1.43 to 2.64)	2.38 (1.86 to 3.26)
Foreign body	343.29 (288.99 to 401.65)	236.61 (188.67 to 285.84)	237.00 (187.93 to 281.79)	236.91 (186.49 to 287.62)	8.48 (6.91 to 9.26)	13.80 (10.57 to 15.92)	14.55 (11.21 to 16.63)	12.39 (9.30 to 14.69)
Pulmonary aspiration and foreign body in airway	310.03 (247.19 to 363.25)	201.56 (146.48 to 246.11)	200.26 (146.75 to 242.18)	205.17 (152.60 to 251.36)	7.54 (5.86 to 8.29)	11.38 (8.01 to 13.32)	11.89 (8.44 to 13.89)	10.41 (7.38 to 12.49)

Foreign body in other body part	33.25 (27.79 to 56.87)	35.05 (27.41 to 54.03)	36.74 (29.09 to 56.25)	31.74 (24.69 to 50.01)	0.93 (0.80 to 1.50)	2.42 (1.82 to 3.56)	2.66 (2.10 to 3.89)	1.97 (1.46 to 2.85)
Environmental heat and cold exposure	126.03 (96.52 to 166.64)	105.86 (74.53 to 142.56)	96.27 (66.93 to 128.81)	140.85 (100.46 to 187.24)	4.43 (3.22 to 5.60)	6.19 (4.19 to 8.28)	5.91 (3.90 to 7.85)	7.57 (5.19 to 10.04)
Other unintentional injuries	1,075.17 (682.63 to 1,150.70)	276.21 (166.77 to 445.95)	224.70 (130.48 to 340.24)	458.36 (281.54 to 562.38)	24.05 (15.23 to 25.71)	8.10 (5.06 to 12.93)	6.72 (3.86 to 9.94)	13.05 (8.15 to 15.51)
**Self-harm and interpersonal violence**	**5,605.68 (5,307.46 to 6,512.43)**	**4,688.40 (3,278.97 to 6,537.13)**	**4,001.66 (2,945.94 to 5,413.95)**	**5,435.08 (3,813.28 to 7,645.45)**	**157.63 (149.20 to 178.57)**	**196.23 (140.81 to 271.36)**	**172.38 (129.14 to 230.61)**	**215.58 (153.99 to 303.87)**

East Asia								
Cause	YLLs (in 1000s)				Deaths (in 1000s)			
	2016	2040 Forecast	2040 Better Scenario	2040 Worse Scenario	2016	2040 Forecast	2040 Better Scenario	2040 Worse Scenario
Self-harm	4,664.99 (4,418.41 to 5,330.07)	4,304.76 (2,942.78 to 6,094.56)	3,658.05 (2,646.00 to 4,993.06)	4,973.81 (3,397.91 to 7,158.52)	137.85 (129.29 to 153.80)	185.28 (131.78 to 260.00)	162.46 (120.36 to 218.85)	202.81 (142.03 to 288.53)
Self-harm by firearm	50.72 (37.86 to 85.95)	27.55 (15.48 to 49.54)	21.86 (13.33 to 38.47)	35.58 (20.23 to 64.20)	1.33 (1.02 to 2.19)	1.08 (0.67 to 1.80)	0.91 (0.61 to 1.50)	1.29 (0.79 to 2.18)
Self-harm by other specified means	4,614.26 (4,371.87 to 5,258.46)	4,277.21 (2,926.50 to 6,056.75)	3,636.19 (2,632.82 to 4,958.74)	4,938.23 (3,366.33 to 7,106.17)	136.51 (127.81 to 151.73)	184.20 (131.06 to 258.55)	161.54 (119.72 to 217.45)	201.51 (141.10 to 286.80)
Interpersonal violence	940.70 (826.78 to 1,383.21)	383.63 (284.82 to 595.73)	343.61 (256.43 to 519.26)	461.27 (353.02 to 716.29)	19.79 (17.45 to 29.02)	10.95 (8.28 to 16.33)	9.92 (7.60 to 14.67)	12.77 (9.94 to 19.05)
Physical violence by firearm	34.94 (23.32 to 67.20)	15.09 (10.25 to 29.24)	13.81 (9.47 to 25.68)	18.86 (13.26 to 36.44)	0.83 (0.56 to 1.60)	0.52 (0.36 to 0.96)	0.49 (0.34 to 0.88)	0.63 (0.45 to 1.15)
Physical violence by sharp object	403.62 (349.79 to 641.45)	159.15 (116.11 to 257.61)	145.45 (107.11 to 231.94)	198.66 (154.83 to 325.89)	8.29 (7.18 to 13.00)	4.17 (3.12 to 6.47)	3.86 (2.92 to 5.88)	5.06 (4.05 to 7.88)
Physical violence by other means	502.13 (441.25 to 730.51)	209.40 (151.14 to 328.32)	184.35 (134.52 to 285.07)	243.75 (176.44 to 379.43)	10.66 (9.34 to 15.20)	6.26 (4.55 to 9.70)	5.58 (4.14 to 8.42)	7.08 (5.19 to 10.98)
**Forces of nature, conflict and terrorism, and executions and police conflict**	**123.71 (58.70 to 195.77)**	**542.92 (0.21 to 10,803.23)**	**545.22 (0.21 to 10,857.03)**	**542.42 (0.20 to 10,805.38)**	**2.65 (1.20 to 4.28)**	**12.24 (0.01 to 230.10)**	**12.69 (0.01 to 242.51)**	**11.95 (0.01 to 225.29)**
Exposure to forces of nature	78.51 (12.46 to 150.48)	87.76 (0.01 to 568.11)	88.30 (0.01 to 570.56)	86.53 (0.01 to 560.49)	1.76 (0.28 to 3.37)	2.60 (0.00 to 16.85)	2.65 (0.00 to 17.20)	2.48 (0.00 to 16.09)
Conflict and terrorism	0.00 (0.00 to 0.00)	435.05 (0.00 to 10,718.77)	436.76 (0.00 to 10,771.51)	435.93 (0.00 to 10,722.31)	0.00 (0.00 to 0.00)	9.16 (0.00 to 227.39)	9.55 (0.00 to 239.59)	9.00 (0.00 to 222.79)
Executions and police conflict	45.20 (39.00 to 52.16)	20.10 (0.00 to 0.00)	20.16 (0.00 to 0.00)	19.96 (0.00 to 0.00)	0.89 (0.77 to 1.02)	0.48 (0.00 to 0.00)	0.49 (0.00 to 0.00)	0.47 (0.00 to 0.00)

China								
**Cause**	**YLLs (in 1000s)**				**Deaths (in 1000s)**			
	**2016**	**2040 Forecast**	**2040 Better Scenario**	**2040 Worse Scenario**	**2016**	**2040 Forecast**	**2040 Better Scenario**	**2040 Worse Scenario**
**All causes**	**208,211.11 (203,732.37 to 212,783.98)**	**205,805.78 (161,956.42 to 260,208.98)**	**171,770.68 (140,312.63 to 211,401.78)**	**299,978.34 (229,103.62 to 389,729.07)**	**9,666.24 (9,471.55 to 9,856.85)**	**14,170.52 (11,663.26 to 17,128.11)**	**12,239.82 (10,419.04 to 14,358.81)**	**19,349.03 (15,960.69 to 23,171.62)**
**Communicable, maternal, neonatal, and nutritional diseases**	**12,347.10 (11,624.18 to 13,202.23)**	**5,294.29 (4,017.38 to 7,150.27)**	**4,368.95 (3,355.55 to 5,646.95)**	**8,321.00 (6,173.39 to 11,280.47)**	**334.18 (313.15 to 365.07)**	**368.77 (285.49 to 481.51)**	**330.47 (262.53 to 413.36)**	**505.83 (367.11 to 669.97)**
**HIV/AIDS and tuberculosis**	**2,309.34 (2,204.74 to 2,433.45)**	**741.11 (506.99 to 1,136.67)**	**543.00 (391.35 to 759.46)**	**1,531.58 (1,062.27 to 2,205.42)**	**71.03 (68.10 to 75.45)**	**36.19 (24.77 to 55.89)**	**27.56 (20.37 to 37.84)**	**66.21 (44.94 to 96.19)**
Tuberculosis	1,036.57 (980.89 to 1,154.17)	397.49 (208.52 to 730.65)	259.27 (148.78 to 440.96)	868.23 (481.68 to 1,461.79)	40.12 (37.99 to 44.44)	22.56 (12.19 to 42.08)	15.26 (8.97 to 24.82)	46.17 (26.70 to 75.72)
HIV/AIDS	1,272.78 (1,189.39 to 1,348.35)	343.62 (227.23 to 550.44)	283.74 (196.06 to 413.64)	663.35 (458.35 to 1,025.22)	30.91 (28.94 to 32.71)	13.63 (9.96 to 19.27)	12.30 (9.27 to 16.57)	20.04 (14.25 to 29.72)
**Diarrhea, lower respiratory, and other common infectious diseases**	**4,474.53 (4,111.93 to 5,008.77)**	**3,275.10 (2,340.14 to 4,751.93)**	**2,686.13 (1,952.55 to 3,682.05)**	**5,055.61 (3,453.26 to 7,417.89)**	**176.78 (157.45 to 208.64)**	**277.52 (204.44 to 385.66)**	**245.30 (187.17 to 326.86)**	**383.81 (262.67 to 536.53)**
Diarrheal diseases	216.95 (173.61 to 306.90)	111.10 (29.35 to 307.47)	127.15 (34.56 to 341.91)	130.47 (29.82 to 403.60)	5.54 (3.95 to 9.21)	7.63 (2.08 to 20.60)	9.33 (2.58 to 24.42)	7.68 (1.77 to 23.34)
Intestinal infectious diseases	172.07 (87.76 to 297.17)	61.19 (27.83 to 115.52)	64.16 (29.05 to 121.07)	57.31 (25.15 to 110.46)	2.80 (1.43 to 4.85)	1.36 (0.66 to 2.45)	1.47 (0.72 to 2.58)	1.21 (0.59 to 2.20)
Typhoid fever	143.93 (71.62 to 250.62)	53.36 (23.42 to 101.99)	57.27 (25.20 to 109.58)	45.87 (19.80 to 89.90)	2.33 (1.16 to 4.07)	1.18 (0.56 to 2.12)	1.30 (0.62 to 2.32)	0.96 (0.45 to 1.80)
Paratyphoid fever	27.63 (11.95 to 54.18)	7.63 (2.94 to 15.74)	6.70 (2.66 to 14.03)	11.20 (4.48 to 23.59)	0.46 (0.20 to 0.89)	0.17 (0.07 to 0.35)	0.15 (0.06 to 0.31)	0.24 (0.10 to 0.50)
Other intestinal infectious diseases	0.50 (0.20 to 1.08)	0.20 (0.07 to 0.44)	0.19 (0.07 to 0.43)	0.24 (0.09 to 0.56)	0.01 (0.01 to 0.03)	0.01 (0.01 to 0.03)	0.01 (0.00 to 0.03)	0.02 (0.01 to 0.04)
Lower respiratory infections	3,510.50 (3,181.07 to 4,125.98)	2,861.66 (2,004.11 to 4,233.72)	2,273.35 (1,629.05 to 3,200.17)	4,516.44 (3,027.60 to 6,772.51)	156.62 (138.09 to 189.57)	260.08 (187.51 to 366.77)	226.36 (166.46 to 305.94)	364.42 (245.24 to 510.65)
Upper respiratory infections	6.45 (5.65 to 7.34)	2.60 (1.54 to 4.26)	2.20 (1.22 to 3.72)	4.79 (2.82 to 7.64)	0.21 (0.18 to 0.23)	0.15 (0.09 to 0.24)	0.13 (0.07 to 0.22)	0.25 (0.15 to 0.40)
Otitis media	0.65 (0.56 to 0.76)	0.14 (0.09 to 0.21)	0.14 (0.09 to 0.22)	0.13 (0.08 to 0.21)	0.02 (0.02 to 0.02)	0.01 (0.01 to 0.01)	0.01 (0.01 to 0.01)	0.01 (0.00 to 0.01)
Meningitis	316.18 (226.12 to 423.74)	95.65 (62.29 to 152.34)	86.89 (57.33 to 139.80)	130.59 (87.38 to 201.05)	6.91 (5.00 to 9.71)	4.29 (3.00 to 6.74)	4.06 (2.79 to 6.39)	5.29 (3.73 to 8.31)
Pneumococcal meningitis	25.62 (18.33 to 33.57)	9.33 (6.60 to 14.70)	9.63 (6.78 to 15.03)	9.05 (6.37 to 14.13)	0.61 (0.45 to 0.86)	0.50 (0.36 to 0.81)	0.54 (0.39 to 0.88)	0.44 (0.32 to 0.71)
H influenzae type B meningitis	23.69 (16.64 to 30.63)	10.78 (6.18 to 20.71)	10.79 (6.28 to 20.15)	11.89 (6.19 to 25.36)	0.49 (0.35 to 0.67)	0.39 (0.27 to 0.58)	0.41 (0.28 to 0.61)	0.36 (0.24 to 0.57)
Meningococcal meningitis	149.65 (106.22 to 201.22)	47.84 (30.62 to 77.61)	42.56 (27.37 to 69.68)	67.76 (45.68 to 103.69)	3.60 (2.61 to 5.04)	2.36 (1.59 to 3.79)	2.19 (1.50 to 3.50)	3.06 (2.15 to 4.73)
Other meningitis	117.22 (83.44 to 157.53)	27.69 (17.28 to 45.61)	23.91 (14.83 to 40.69)	41.89 (26.95 to 66.54)	2.21 (1.60 to 3.10)	1.03 (0.69 to 1.73)	0.93 (0.61 to 1.52)	1.43 (1.00 to 2.28)
Encephalitis	199.38 (158.89 to 268.35)	88.19 (57.11 to 133.87)	86.42 (55.25 to 130.92)	100.26 (62.08 to 154.32)	3.52 (2.82 to 4.55)	2.76 (2.05 to 3.59)	2.81 (2.10 to 3.67)	2.88 (2.09 to 3.87)

Diphtheria	0.66 (0.58 to 0.77)	0.08 (0.05 to 0.11)	0.08 (0.05 to 0.11)	0.12 (0.06 to 0.22)	0.01 (0.01 to 0.01)	0.00 (0.00 to 0.00)	0.00 (0.00 to 0.00)	0.00 (0.00 to 0.00)
Whooping cough	11.72 (1.06 to 39.02)	2.48 (0.09 to 10.34)	2.39 (0.09 to 10.12)	23.78 (0.45 to 94.68)	0.14 (0.01 to 0.47)	0.03 (0.00 to 0.13)	0.03 (0.00 to 0.12)	0.28 (0.01 to 1.12)
Tetanus	22.99 (15.66 to 29.47)	2.21 (1.33 to 3.34)	2.25 (1.36 to 3.40)	4.44 (2.29 to 8.38)	0.53 (0.34 to 0.75)	0.08 (0.05 to 0.13)	0.09 (0.05 to 0.14)	0.10 (0.06 to 0.16)
Measles	4.05 (1.12 to 10.21)	44.25 (0.85 to 214.52)	35.80 (0.83 to 159.79)	79.95 (1.32 to 448.28)	0.05 (0.01 to 0.12)	0.65 (0.02 to 2.86)	0.54 (0.02 to 2.16)	1.12 (0.02 to 5.99)

Cause	YLLs (in 1000s)				Deaths (in 1000s)			
	**2016**	**2040 Forecast**	**2040 Better Scenario**	**2040 Worse Scenario**	**2016**	**2040 Forecast**	**2040 Better Scenario**	**2040 Worse Scenario**
Varicella and herpes zoster	12.93 (11.53 to 14.84)	5.56 (4.00 to 7.63)	5.31 (3.78 to 7.47)	7.34 (5.11 to 10.67)	0.43 (0.40 to 0.47)	0.48 (0.34 to 0.64)	0.48 (0.34 to 0.67)	0.55 (0.37 to 0.79)
**Neglected tropical diseases and malaria**	**91.88 (59.45 to 101.64)**	**41.32 (27.65 to 49.10)**	**41.58 (28.02 to 49.22)**	**46.78 (29.91 to 60.39)**	**2.46 (1.66 to 2.69)**	**1.67 (1.21 to 1.93)**	**1.72 (1.26 to 1.98)**	**1.78 (1.25 to 2.22)**
Malaria	7.41 (5.03 to 10.56)	2.98 (1.95 to 4.23)	3.01 (1.95 to 4.17)	3.04 (1.96 to 4.39)	0.19 (0.13 to 0.27)	0.11 (0.07 to 0.15)	0.12 (0.07 to 0.16)	0.11 (0.07 to 0.16)
Chagas disease	0.00 (0.00 to 0.00)	0.00 (0.00 to 0.00)	0.00 (0.00 to 0.00)	0.00 (0.00 to 0.00)	0.00 (0.00 to 0.00)	0.00 (0.00 to 0.00)	0.00 (0.00 to 0.00)	0.00 (0.00 to 0.00)
Leishmaniasis	2.42 (1.54 to 3.78)	0.83 (0.50 to 1.42)	0.78 (0.49 to 1.31)	1.11 (0.68 to 1.91)	0.07 (0.05 to 0.12)	0.04 (0.02 to 0.06)	0.03 (0.02 to 0.06)	0.05 (0.03 to 0.08)
African trypanosomiasis	0.00 (0.00 to 0.00)	0.00 (0.00 to 0.00)	0.00 (0.00 to 0.00)	0.00 (0.00 to 0.00)	0.00 (0.00 to 0.00)	0.00 (0.00 to 0.00)	0.00 (0.00 to 0.00)	0.00 (0.00 to 0.00)
Schistosomiasis	11.97 (10.12 to 14.13)	6.92 (5.48 to 8.63)	6.92 (5.47 to 8.65)	7.50 (5.93 to 9.43)	0.47 (0.40 to 0.56)	0.38 (0.30 to 0.48)	0.39 (0.31 to 0.48)	0.40 (0.31 to 0.49)
Cysticercosis	1.45 (1.31 to 1.61)	0.42 (0.35 to 0.50)	0.42 (0.35 to 0.50)	0.43 (0.37 to 0.51)	0.04 (0.04 to 0.05)	0.02 (0.01 to 0.02)	0.02 (0.01 to 0.02)	0.02 (0.01 to 0.02)
Cystic echinococcosis	1.51 (1.27 to 1.77)	0.61 (0.44 to 0.84)	0.57 (0.43 to 0.74)	0.89 (0.65 to 1.21)	0.05 (0.04 to 0.06)	0.03 (0.02 to 0.04)	0.03 (0.02 to 0.04)	0.05 (0.03 to 0.06)
Dengue	0.28 (0.15 to 0.84)	0.54 (0.30 to 1.57)	0.55 (0.30 to 1.61)	0.53 (0.29 to 1.58)	0.01 (0.00 to 0.02)	0.02 (0.01 to 0.08)	0.03 (0.01 to 0.08)	0.02 (0.01 to 0.07)
Yellow fever	0.00 (0.00 to 0.00)	0.00 (0.00 to 0.00)	0.00 (0.00 to 0.00)	0.00 (0.00 to 0.00)	0.00 (0.00 to 0.00)	0.00 (0.00 to 0.00)	0.00 (0.00 to 0.00)	0.00 (0.00 to 0.00)
Rabies	40.66 (10.80 to 48.96)	10.84 (2.38 to 14.90)	10.22 (2.35 to 13.76)	15.68 (3.49 to 24.12)	0.95 (0.25 to 1.13)	0.34 (0.08 to 0.46)	0.33 (0.08 to 0.43)	0.48 (0.11 to 0.72)
Intestinal nematode infections	1.79 (1.44 to 2.24)	1.07 (0.85 to 1.39)	1.08 (0.86 to 1.39)	1.05 (0.82 to 1.38)	0.04 (0.03 to 0.04)	0.04 (0.04 to 0.05)	0.04 (0.04 to 0.05)	0.04 (0.03 to 0.04)
Other neglected tropical diseases	24.39 (16.96 to 27.14)	17.10 (11.12 to 20.45)	18.04 (11.88 to 21.59)	16.54 (10.68 to 20.34)	0.64 (0.44 to 0.72)	0.68 (0.44 to 0.80)	0.74 (0.47 to 0.87)	0.63 (0.41 to 0.76)
**Maternal disorders**	**130.45 (117.26 to 145.10)**	**25.09 (13.41 to 44.16)**	**23.09 (11.58 to 42.68)**	**37.83 (17.70 to 71.83)**	**2.34 (2.11 to 2.60)**	**0.47 (0.26 to 0.81)**	**0.43 (0.22 to 0.78)**	**0.70 (0.34 to 1.32)**
Maternal hemorrhage	37.01 (31.82 to 42.79)	8.93 (4.49 to 16.46)	8.27 (3.84 to 16.04)	13.37 (5.81 to 26.35)	0.66 (0.57 to 0.76)	0.16 (0.08 to 0.30)	0.15 (0.07 to 0.29)	0.25 (0.11 to 0.48)
Maternal sepsis and other maternal infections	7.06 (5.75 to 8.58)	1.56 (0.87 to 2.57)	1.43 (0.75 to 2.46)	2.35 (1.21 to 4.38)	0.14 (0.12 to 0.17)	0.03 (0.02 to 0.05)	0.03 (0.02 to 0.05)	0.05 (0.03 to 0.09)
Maternal hypertensive disorders	14.87 (12.21 to 18.12)	2.56 (1.24 to 4.89)	2.35 (1.09 to 4.64)	3.93 (1.70 to 8.13)	0.26 (0.21 to 0.32)	0.05 (0.02 to 0.09)	0.04 (0.02 to 0.08)	0.07 (0.03 to 0.15)
Maternal obstructed labor and uterine rupture	5.77 (4.56 to 7.14)	1.14 (0.56 to 2.15)	1.03 (0.45 to 2.04)	1.83 (0.77 to 3.83)	0.10 (0.08 to 0.13)	0.02 (0.01 to 0.04)	0.02 (0.01 to 0.04)	0.03 (0.02 to 0.07)
Maternal abortion, miscarriage, and ectopic pregnancy	15.88 (13.18 to 18.89)	3.08 (1.67 to 5.89)	2.82 (1.43 to 5.01)	4.73 (2.32 to 8.90)	0.29 (0.24 to 0.35)	0.06 (0.03 to 0.11)	0.05 (0.03 to 0.10)	0.09 (0.05 to 0.17)

Table title: **China**

Indirect maternal deaths	12.83 (9.31 to 18.63)	2.76 (1.28 to 5.67)	2.50 (1.05 to 5.27)	4.27 (1.74 to 8.96)	0.22 (0.16 to 0.32)	0.05 (0.02 to 0.10)	0.04 (0.02 to 0.09)	0.08 (0.03 to 0.16)
Late maternal deaths	0.88 (0.53 to 1.34)	0.15 (0.06 to 0.32)	0.14 (0.05 to 0.29)	0.23 (0.08 to 0.54)	0.02 (0.01 to 0.02)	0.00 (0.00 to 0.01)	0.00 (0.00 to 0.01)	0.00 (0.00 to 0.01)
Maternal deaths aggravated by HIV/AIDS	0.03 (0.01 to 0.05)	0.02 (0.00 to 0.19)	0.02 (0.00 to 0.18)	0.06 (0.01 to 0.38)	0.00 (0.00 to 0.00)	0.00 (0.00 to 0.00)	0.00 (0.00 to 0.00)	0.00 (0.00 to 0.01)
Other maternal disorders	36.12 (31.14 to 41.61)	4.89 (2.53 to 8.99)	4.53 (2.26 to 8.92)	7.05 (3.29 to 13.49)	0.64 (0.55 to 0.74)	0.09 (0.05 to 0.16)	0.08 (0.04 to 0.16)	0.13 (0.06 to 0.24)

China								
Cause	YLLs (in 1000s)				Deaths (in 1000s)			
	2016	2040 Forecast	2040 Better Scenario	2040 Worse Scenario	2016	2040 Forecast	2040 Better Scenario	2040 Worse Scenario
**Neonatal disorders**	**4,722.14 (4,277.91 to 5,224.10)**	**713.47 (289.32 to 1,322.12)**	**565.81 (224.11 to 1,035.08)**	**1,110.54 (439.38 to 2,035.60)**	**54.59 (49.45 to 60.39)**	**8.25 (3.35 to 15.28)**	**6.54 (2.59 to 11.97)**	**12.84 (5.09 to 23.53)**
Neonatal preterm birth	2,411.04 (2,069.95 to 2,697.09)	326.74 (126.64 to 597.34)	230.68 (90.38 to 423.37)	537.29 (205.50 to 976.83)	27.86 (23.92 to 31.17)	3.78 (1.46 to 6.90)	2.67 (1.05 to 4.89)	6.21 (2.38 to 11.29)
Neonatal encephalopathy due to birth asphyxia and trauma	1,524.96 (1,353.06 to 1,778.55)	223.79 (70.77 to 469.94)	186.53 (55.52 to 396.28)	360.53 (112.93 to 743.60)	17.63 (15.64 to 20.55)	2.59 (0.82 to 5.43)	2.16 (0.64 to 4.58)	4.17 (1.31 to 8.59)
Neonatal sepsis and other neonatal infections	178.97 (138.32 to 251.25)	39.69 (21.74 to 71.57)	34.37 (18.62 to 60.19)	58.01 (31.94 to 98.84)	2.07 (1.60 to 2.90)	0.46 (0.25 to 0.83)	0.40 (0.22 to 0.70)	0.67 (0.37 to 1.14)
Hemolytic disease and other neonatal jaundice	79.97 (68.15 to 93.84)	10.00 (4.72 to 18.82)	8.59 (4.11 to 16.37)	15.34 (7.52 to 29.45)	0.93 (0.79 to 1.09)	0.12 (0.05 to 0.22)	0.10 (0.05 to 0.19)	0.18 (0.09 to 0.34)
Other neonatal disorders	527.21 (448.47 to 602.02)	113.26 (51.58 to 204.20)	105.64 (48.39 to 189.40)	139.38 (63.11 to 253.48)	6.10 (5.19 to 6.97)	1.31 (0.60 to 2.37)	1.22 (0.56 to 2.19)	1.61 (0.73 to 2.93)
**Nutritional deficiencies**	**215.58 (205.49 to 227.42)**	**274.27 (220.74 to 336.00)**	**302.26 (251.44 to 363.46)**	**237.33 (182.57 to 297.18)**	**14.17 (13.55 to 14.79)**	**33.18 (24.05 to 42.88)**	**37.91 (29.03 to 48.01)**	**26.16 (17.57 to 35.29)**
Protein-energy malnutrition	153.92 (144.90 to 178.19)	217.51 (164.11 to 277.61)	245.08 (194.49 to 301.03)	175.84 (130.13 to 227.78)	11.20 (10.61 to 12.27)	27.83 (18.93 to 36.92)	32.32 (23.44 to 41.95)	20.81 (13.36 to 29.16)
Iodine deficiency	3.10 (2.07 to 4.64)	9.99 (0.61 to 36.52)	10.23 (0.57 to 38.84)	10.67 (0.82 to 35.55)	0.12 (0.07 to 0.17)	1.05 (0.04 to 4.65)	1.11 (0.04 to 5.10)	1.05 (0.05 to 4.45)
Dietary iron deficiency	8.04 (7.09 to 8.78)	7.01 (4.31 to 10.24)	7.38 (4.47 to 10.80)	6.49 (4.12 to 9.34)	0.24 (0.17 to 0.26)	0.42 (0.18 to 0.72)	0.46 (0.21 to 0.77)	0.36 (0.16 to 0.60)
Other nutritional deficiencies	50.52 (32.48 to 55.44)	39.76 (24.04 to 50.21)	39.57 (23.71 to 50.37)	44.33 (27.20 to 56.84)	2.62 (1.61 to 2.88)	3.88 (2.20 to 5.05)	4.02 (2.40 to 5.25)	3.94 (2.31 to 5.30)
**Other communicable, maternal, neonatal, and nutritional diseases**	**403.17 (382.77 to 424.44)**	**223.92 (173.64 to 290.85)**	**207.08 (160.71 to 263.86)**	**301.32 (242.61 to 372.34)**	**12.81 (12.22 to 13.35)**	**11.49 (9.30 to 14.29)**	**11.00 (8.85 to 13.63)**	**14.32 (11.74 to 17.51)**
Sexually transmitted infections excluding HIV	20.47 (16.49 to 26.42)	5.83 (4.28 to 7.47)	5.52 (4.00 to 7.12)	7.15 (5.21 to 9.22)	0.55 (0.43 to 0.63)	0.28 (0.19 to 0.34)	0.27 (0.19 to 0.33)	0.31 (0.23 to 0.38)
Syphilis	11.88 (8.62 to 17.74)	2.08 (1.44 to 3.15)	1.86 (1.29 to 2.79)	2.92 (2.08 to 4.30)	0.27 (0.21 to 0.35)	0.09 (0.06 to 0.12)	0.08 (0.06 to 0.11)	0.11 (0.08 to 0.14)
Chlamydial infection	1.56 (1.13 to 1.74)	0.70 (0.49 to 0.87)	0.68 (0.48 to 0.87)	0.78 (0.54 to 1.00)	0.05 (0.04 to 0.06)	0.04 (0.03 to 0.04)	0.04 (0.03 to 0.05)	0.04 (0.03 to 0.05)
Gonococcal infection	4.70 (3.45 to 5.21)	2.14 (1.47 to 2.69)	2.09 (1.45 to 2.67)	2.41 (1.68 to 3.05)	0.16 (0.11 to 0.18)	0.11 (0.08 to 0.14)	0.11 (0.08 to 0.14)	0.12 (0.08 to 0.15)
Other sexually transmitted infections	2.33 (1.69 to 2.58)	0.91 (0.63 to 1.16)	0.89 (0.62 to 1.13)	1.05 (0.72 to 1.34)	0.07 (0.05 to 0.08)	0.04 (0.03 to 0.05)	0.04 (0.03 to 0.05)	0.04 (0.03 to 0.05)
Acute hepatitis	287.34 (273.57 to 303.69)	151.37 (108.39 to 212.26)	134.39 (96.87 to 183.19)	222.27 (175.01 to 282.16)	9.45 (9.04 to 9.89)	7.31 (5.38 to 9.96)	6.63 (4.85 to 8.98)	10.19 (8.14 to 12.66)
Acute hepatitis A	11.29 (9.81 to 13.62)	2.74 (1.65 to 4.66)	2.47 (1.51 to 4.13)	3.73 (2.27 to 6.25)	0.19 (0.16 to 0.22)	0.09 (0.06 to 0.14)	0.09 (0.06 to 0.13)	0.12 (0.08 to 0.17)
Acute hepatitis B	252.98 (240.11 to 266.98)	135.87 (96.50 to 192.73)	120.36 (84.60 to 166.24)	201.02 (154.38 to 256.67)	8.59 (8.15 to 9.04)	6.41 (4.64 to 8.89)	5.79 (4.16 to 7.87)	9.07 (7.08 to 11.48)

Acute hepatitis C	3.48 (2.53 to 4.82)	2.64 (1.67 to 4.13)	2.36 (1.49 to 3.66)	3.67 (2.45 to 5.47)	0.13 (0.09 to 0.18)	0.13 (0.09 to 0.21)	0.12 (0.08 to 0.19)	0.18 (0.12 to 0.26)
Acute hepatitis E	19.60 (16.50 to 23.24)	10.13 (6.64 to 15.02)	9.20 (6.08 to 13.49)	13.85 (9.57 to 20.03)	0.55 (0.43 to 0.70)	0.67 (0.41 to 1.02)	0.64 (0.40 to 1.00)	0.83 (0.53 to 1.26)
Other unspecified infectious diseases	95.36 (78.09 to 107.59)	66.71 (53.47 to 80.80)	67.17 (53.91 to 81.49)	71.89 (56.33 to 90.19)	2.80 (2.35 to 3.05)	3.90 (3.15 to 4.64)	4.10 (3.33 to 4.88)	3.82 (2.93 to 4.74)
**Non-communicable diseases**	**165,968.00 (162,300.56 to 169,991.72)**	**184,016.09 (141,760.04 to 236,119.62)**	**153,406.61 (122,629.15 to 191,933.36)**	**268,229.44 (199,382.44 to 358,037.95)**	**8,558.79 (8,378.23 to 8,733.13)**	**13,078.35 (10,619.30 to 16,029.41)**	**11,258.60 (9,452.91 to 13,364.12)**	**17,930.65 (14,474.68 to 21,948.70)**

China								
Cause	YLLs (in 1000s)				Deaths (in 1000s)			
	2016	2040 Forecast	2040 Better Scenario	2040 Worse Scenario	2016	2040 Forecast	2040 Better Scenario	2040 Worse Scenario
**Neoplasms**	**57,405.56 (55,724.40 to 59,141.06)**	**73,481.70 (56,482.02 to 102,953.74)**	**64,111.55 (50,798.86 to 84,844.40)**	**89,971.01 (62,757.93 to 141,389.79)**	**2,378.51 (2,310.36 to 2,448.01)**	**3,976.62 (3,107.04 to 5,391.09)**	**3,525.08 (2,849.09 to 4,528.93)**	**4,714.12 (3,306.56 to 6,970.56)**
Lip and oral cavity cancer	471.60 (448.17 to 492.97)	506.35 (296.56 to 882.49)	346.95 (225.63 to 563.27)	707.28 (366.69 to 1,420.68)	19.59 (18.67 to 20.41)	26.61 (15.54 to 44.76)	18.58 (12.09 to 29.49)	35.70 (18.63 to 67.48)
Nasopharynx cancer	814.88 (744.84 to 882.42)	947.64 (592.34 to 1,661.47)	614.13 (408.73 to 980.36)	1,498.17 (813.46 to 3,109.35)	28.11 (25.79 to 30.33)	40.20 (25.03 to 68.92)	26.45 (17.69 to 41.09)	61.64 (33.59 to 121.12)
Other pharynx cancer	116.02 (103.19 to 125.15)	154.21 (101.86 to 228.59)	121.56 (84.11 to 173.10)	148.60 (96.48 to 225.77)	4.61 (4.11 to 4.94)	7.88 (5.25 to 11.58)	6.39 (4.45 to 8.96)	7.31 (4.73 to 11.02)
Esophageal cancer	4,378.09 (4,186.88 to 4,575.94)	7,339.89 (4,490.96 to 12,268.07)	5,792.78 (3,796.65 to 8,970.11)	9,400.67 (4,849.41 to 17,930.30)	206.21 (197.72 to 214.83)	410.30 (253.16 to 678.56)	328.33 (218.61 to 509.85)	511.00 (268.54 to 933.30)
Stomach cancer	7,355.90 (7,094.72 to 7,624.13)	6,777.56 (4,984.37 to 9,196.54)	6,036.47 (4,535.76 to 7,856.90)	8,617.93 (6,662.30 to 10,920.55)	334.78 (323.44 to 346.91)	395.57 (298.02 to 525.38)	357.53 (274.56 to 453.74)	484.83 (390.14 to 605.61)
Colon and rectum cancer	3,704.45 (3,517.06 to 3,881.33)	5,841.82 (4,190.94 to 8,054.13)	5,025.22 (3,698.41 to 6,891.67)	6,769.18 (4,950.79 to 9,278.51)	167.09 (159.11 to 174.55)	359.34 (261.25 to 489.36)	317.74 (236.61 to 428.40)	397.90 (290.21 to 536.72)
Liver cancer	11,411.23 (10,876.68 to 12,016.77)	18,416.16 (13,474.85 to 25,915.37)	17,372.37 (13,238.49 to 22,883.95)	17,778.93 (12,411.93 to 26,786.93)	426.90 (408.02 to 451.24)	878.56 (658.35 to 1,208.67)	844.28 (662.62 to 1,088.91)	819.65 (588.96 to 1,202.70)
Liver cancer due to hepatitis B	6,237.32 (5,489.41 to 6,916.12)	9,738.64 (6,988.78 to 13,947.41)	9,291.83 (6,959.97 to 12,630.70)	9,306.05 (6,341.89 to 14,455.83)	224.53 (195.82 to 248.91)	447.83 (328.56 to 623.25)	435.32 (331.56 to 575.41)	413.47 (288.41 to 616.25)
Liver cancer due to hepatitis C	849.73 (745.21 to 976.08)	1,546.95 (1,121.29 to 2,203.32)	1,492.11 (1,128.87 to 1,985.52)	1,457.54 (996.36 to 2,177.07)	35.95 (31.78 to 40.76)	84.05 (61.41 to 116.63)	82.64 (62.31 to 108.76)	76.45 (53.51 to 108.92)
Liver cancer due to alcohol use	1,220.95 (981.87 to 1,499.37)	2,218.88 (1,524.57 to 3,148.96)	1,891.33 (1,335.64 to 2,613.02)	2,240.09 (1,499.79 to 3,312.25)	53.37 (43.34 to 64.50)	123.05 (85.37 to 172.11)	108.06 (76.51 to 147.05)	120.33 (81.45 to 173.78)
Liver cancer due to other causes	3,103.22 (2,709.19 to 3,578.61)	4,911.69 (3,297.48 to 7,237.59)	4,697.10 (3,351.76 to 6,536.26)	4,775.24 (2,981.98 to 7,562.33)	113.05 (99.25 to 129.71)	223.62 (153.62 to 322.64)	218.26 (157.41 to 301.32)	209.40 (134.11 to 323.48)
Gallbladder and biliary tract cancer	524.55 (434.40 to 599.66)	536.83 (437.65 to 629.24)	548.58 (447.88 to 639.86)	511.57 (416.74 to 598.34)	24.90 (20.73 to 28.46)	33.92 (28.13 to 39.56)	35.35 (29.07 to 40.84)	30.94 (25.31 to 35.96)
Pancreatic cancer	1,623.54 (1,556.23 to 1,688.22)	2,604.05 (2,075.78 to 3,333.03)	2,631.86 (2,105.61 to 3,289.14)	2,569.62 (1,961.52 to 3,437.86)	71.72 (68.87 to 74.56)	147.46 (119.24 to 183.69)	150.80 (123.02 to 185.51)	141.01 (109.15 to 183.46)
Larynx cancer	397.30 (378.33 to 422.06)	459.22 (204.69 to 1,055.11)	296.18 (151.88 to 621.28)	778.41 (285.15 to 1,920.46)	17.48 (16.67 to 18.50)	25.21 (10.68 to 57.49)	16.51 (8.15 to 36.06)	40.60 (14.75 to 97.37)
Tracheal, bronchus, and lung cancer	12,940.05 (12,373.08 to 13,527.21)	15,058.26 (8,510.82 to 29,607.19)	10,876.07 (6,826.42 to 19,171.18)	25,438.54 (11,833.66 to 54,532.04)	590.55 (565.38 to 615.88)	859.07 (469.38 to 1,587.68)	632.25 (390.94 to 1,084.30)	1,384.23 (643.43 to 2,770.36)
Malignant skin melanoma	140.31 (111.20 to 152.76)	181.71 (144.40 to 213.01)	198.07 (157.04 to 232.19)	143.53 (114.70 to 163.51)	5.07 (4.06 to 5.50)	9.90 (7.89 to 11.66)	11.03 (8.97 to 12.84)	7.45 (5.93 to 8.58)
Non-melanoma skin cancer	289.35 (272.42 to 307.89)	383.79 (323.89 to 462.12)	369.33 (309.62 to 442.33)	441.06 (389.35 to 492.06)	13.82 (13.09 to 14.72)	25.99 (22.01 to 31.32)	25.66 (21.84 to 30.49)	28.20 (23.52 to 32.20)
Breast cancer	2,021.11 (1,708.00 to 2,283.17)	2,058.92 (1,465.14 to 2,779.57)	1,874.99 (1,353.14 to 2,488.03)	2,444.50 (1,776.21 to 3,274.50)	70.45 (58.97 to 79.29)	100.33 (73.38 to 132.75)	92.71 (69.24 to 121.13)	115.20 (84.26 to 151.83)
Cervical cancer	1,129.49 (665.18 to 1,307.05)	738.44 (423.52 to 1,047.58)	678.61 (391.72 to 936.74)	939.68 (549.36 to 1,312.03)	38.36 (23.44 to 44.28)	34.67 (20.59 to 47.86)	32.15 (19.71 to 42.96)	42.96 (26.55 to 58.31)

Uterine cancer	347.97 (322.65 to 385.01)	392.06 (314.34 to 477.40)	376.70 (294.47 to 464.60)	451.23 (360.93 to 547.50)	13.24 (12.32 to 14.60)	20.87 (17.42 to 24.68)	20.32 (16.55 to 24.03)	23.19 (19.53 to 27.32)
Ovarian cancer	519.21 (466.69 to 570.63)	603.97 (456.08 to 781.39)	577.25 (432.64 to 737.16)	651.21 (488.56 to 850.10)	18.59 (16.73 to 20.21)	29.31 (22.88 to 37.15)	28.28 (22.20 to 35.35)	30.91 (24.06 to 39.57)
Prostate cancer	532.73 (419.16 to 671.78)	1,191.62 (929.68 to 1,523.40)	1,219.17 (960.79 to 1,546.31)	1,152.92 (880.44 to 1,479.38)	34.08 (26.18 to 42.65)	94.20 (71.77 to 121.89)	99.55 (76.47 to 124.56)	85.49 (61.68 to 112.28)
Testicular cancer	20.67 (19.26 to 22.09)	18.35 (15.52 to 22.06)	18.01 (15.30 to 21.79)	20.24 (17.49 to 23.40)	0.65 (0.60 to 0.69)	0.96 (0.78 to 1.16)	0.97 (0.81 to 1.20)	0.98 (0.78 to 1.18)

China								
**Cause**	**YLLs (in 1000s)**				**Deaths (in 1000s)**			
	**2016**	**2040 Forecast**	**2040 Better Scenario**	**2040 Worse Scenario**	**2016**	**2040 Forecast**	**2040 Better Scenario**	**2040 Worse Scenario**
Kidney cancer	397.99 (375.46 to 419.61)	553.09 (447.64 to 702.51)	565.61 (462.27 to 705.28)	510.40 (399.35 to 684.43)	15.95 (15.07 to 16.75)	30.77 (24.91 to 38.00)	32.09 (26.46 to 39.28)	27.05 (21.38 to 35.09)
Bladder cancer	468.04 (437.07 to 493.43)	759.66 (569.20 to 1,011.44)	740.88 (574.42 to 969.07)	831.25 (579.39 to 1,205.67)	26.05 (24.44 to 27.32)	55.12 (41.39 to 72.04)	54.69 (42.57 to 69.07)	57.44 (40.07 to 78.99)
Brain and nervous system cancer	1,898.64 (1,720.58 to 2,155.71)	2,149.86 (1,866.40 to 2,493.93)	2,141.61 (1,832.55 to 2,490.87)	2,168.75 (1,900.09 to 2,477.95)	59.12 (53.26 to 66.81)	98.24 (84.60 to 111.78)	99.75 (85.51 to 113.27)	94.96 (82.45 to 107.05)
Thyroid cancer	135.81 (119.25 to 145.42)	159.16 (123.85 to 209.97)	150.37 (117.98 to 192.44)	170.21 (131.77 to 229.58)	5.92 (5.08 to 6.34)	9.68 (7.47 to 12.79)	9.41 (7.34 to 12.01)	9.84 (7.51 to 13.11)
Mesothelioma	74.17 (63.37 to 79.19)	78.02 (65.51 to 87.80)	80.86 (68.20 to 91.25)	73.06 (62.18 to 81.98)	2.75 (2.38 to 2.94)	4.06 (3.50 to 4.56)	4.29 (3.70 to 4.82)	3.65 (3.15 to 4.12)
Hodgkin lymphoma	87.75 (73.50 to 115.04)	60.54 (45.50 to 86.54)	56.17 (42.02 to 76.81)	78.30 (62.27 to 103.42)	2.92 (2.40 to 3.79)	2.89 (2.21 to 4.06)	2.73 (2.08 to 3.67)	3.59 (2.81 to 4.66)
Non-Hodgkin lymphoma	1,074.94 (895.46 to 1,150.27)	1,113.37 (892.97 to 1,354.39)	1,070.37 (856.07 to 1,276.73)	1,160.68 (927.03 to 1,384.84)	37.64 (31.36 to 40.23)	56.17 (46.00 to 66.60)	55.11 (45.17 to 64.44)	56.16 (45.39 to 66.39)
Multiple myeloma	258.68 (223.45 to 290.60)	376.51 (312.35 to 455.35)	368.24 (304.83 to 437.46)	370.96 (307.93 to 452.20)	10.36 (9.08 to 11.90)	20.13 (17.09 to 24.19)	20.08 (17.19 to 23.76)	19.06 (15.95 to 23.26)
Leukemia	2,320.77 (1,979.51 to 2,513.02)	1,627.69 (1,274.48 to 2,131.79)	1,513.11 (1,201.68 to 1,902.06)	1,864.36 (1,446.77 to 2,450.55)	60.20 (51.61 to 65.08)	68.44 (54.46 to 88.63)	64.97 (52.41 to 81.92)	74.90 (59.48 to 97.49)
Acute lymphoid leukemia	492.42 (387.77 to 536.86)	243.40 (186.84 to 309.84)	224.94 (171.25 to 279.48)	275.73 (210.13 to 352.37)	10.88 (8.64 to 11.87)	8.38 (6.45 to 10.54)	7.85 (6.10 to 9.74)	9.20 (7.06 to 11.67)
Chronic lymphoid leukemia	108.47 (93.55 to 123.01)	88.53 (70.59 to 113.69)	85.17 (68.37 to 105.10)	91.43 (71.84 to 119.65)	3.59 (3.13 to 4.04)	4.24 (3.45 to 5.29)	4.17 (3.42 to 5.11)	4.19 (3.30 to 5.38)
Acute myeloid leukemia	275.29 (240.81 to 322.43)	266.41 (211.90 to 335.09)	258.50 (206.38 to 318.60)	262.12 (206.86 to 341.76)	7.23 (6.28 to 8.46)	11.18 (8.99 to 13.99)	11.07 (8.93 to 13.65)	10.52 (8.27 to 13.51)
Chronic myeloid leukemia	35.49 (31.92 to 44.02)	19.06 (13.59 to 28.81)	16.46 (11.97 to 23.03)	25.71 (19.62 to 36.70)	1.10 (0.98 to 1.35)	0.87 (0.63 to 1.30)	0.77 (0.57 to 1.07)	1.12 (0.87 to 1.55)
Other leukemia	1,409.11 (1,162.84 to 1,544.36)	1,010.29 (767.85 to 1,360.32)	928.04 (703.68 to 1,199.07)	1,209.37 (916.17 to 1,615.70)	37.39 (30.98 to 40.81)	43.77 (33.79 to 58.09)	41.11 (31.97 to 53.36)	49.87 (38.72 to 66.45)
Other neoplasms	1,950.32 (1,730.28 to 2,037.97)	2,392.97 (2,049.84 to 2,628.25)	2,450.01 (2,087.19 to 2,707.14)	2,279.78 (1,968.84 to 2,482.25)	71.41 (61.82 to 74.65)	130.78 (111.75 to 142.24)	137.06 (117.43 to 149.03)	118.30 (100.79 to 130.05)
**Cardiovascular diseases**	**68,855.42 (66,991.62 to 70,620.65)**	**55,000.42 (35,211.47 to 86,756.83)**	**40,218.38 (26,752.20 to 60,876.57)**	**107,432.53 (70,306.38 to 158,159.75)**	**3,974.88 (3,877.57 to 4,075.74)**	**4,540.44 (3,064.97 to 6,994.40)**	**3,570.28 (2,505.94 to 5,214.86)**	**7,804.28 (5,485.19 to 11,041.59)**
Rheumatic heart disease	1,448.11 (1,364.94 to 1,533.90)	1,224.75 (910.40 to 1,660.76)	1,130.07 (867.42 to 1,479.57)	1,544.07 (1,221.72 to 2,034.68)	71.29 (67.19 to 75.60)	90.10 (70.33 to 121.86)	85.41 (66.98 to 111.72)	106.75 (85.26 to 142.34)
Ischemic heart disease	28,455.70 (27,500.49 to 29,360.31)	19,799.33 (11,611.30 to 33,137.10)	14,332.90 (8,487.17 to 23,697.43)	41,533.52 (24,338.01 to 65,326.70)	1,722.57 (1,670.39 to 1,777.85)	1,686.66 (1,033.56 to 2,718.17)	1,321.26 (824.25 to 2,031.65)	3,137.10 (1,937.40 to 4,807.66)
Stroke	32,685.80 (31,644.62 to 33,888.79)	21,387.24 (12,371.34 to 34,892.11)	14,450.98 (8,839.81 to 23,005.71)	48,317.20 (29,355.88 to 75,424.69)	1,790.03 (1,725.73 to 1,857.80)	1,642.21 (1,008.34 to 2,521.81)	1,207.05 (795.76 to 1,852.55)	3,238.20 (2,076.92 to 4,884.22)
Ischemic stroke	11,384.99 (10,979.20 to 11,823.26)	8,021.04 (4,606.41 to 13,719.72)	5,829.06 (3,500.21 to 10,151.06)	16,103.48 (9,395.61 to 27,392.25)	728.57 (697.51 to 759.23)	679.31 (404.45 to 1,192.04)	530.79 (326.79 to 910.88)	1,208.47 (734.51 to 2,039.98)

Intracerebral hemorrhage	21,300.81 (20,580.48 to 22,064.97)	13,366.20 (7,342.41 to 23,119.25)	8,621.93 (4,990.70 to 14,167.73)	32,213.71 (18,207.79 to 53,274.62)	1,061.47 (1,023.17 to 1,101.97)	962.90 (554.23 to 1,571.49)	676.27 (422.18 to 1,038.61)	2,029.74 (1,232.72 to 3,176.29)
Hypertensive heart disease	3,547.91 (2,491.71 to 4,102.04)	8,041.07 (3,651.61 to 20,197.89)	6,167.94 (2,996.66 to 14,649.34)	10,567.73 (4,720.54 to 27,925.77)	241.08 (167.70 to 278.28)	726.66 (327.11 to 1,919.92)	582.19 (287.26 to 1,500.76)	889.39 (375.14 to 2,406.76)
Cardiomyopathy and myocarditis	771.13 (619.97 to 835.66)	717.11 (510.29 to 938.52)	675.27 (479.12 to 867.73)	795.93 (571.69 to 1,015.75)	29.98 (23.08 to 32.74)	48.28 (32.67 to 63.65)	47.95 (33.14 to 61.75)	48.80 (33.29 to 64.30)
Myocarditis	353.49 (250.37 to 405.63)	348.33 (232.25 to 428.77)	362.32 (241.55 to 447.62)	330.37 (217.89 to 410.33)	14.23 (9.49 to 16.81)	25.97 (16.52 to 33.42)	28.11 (18.05 to 35.79)	22.31 (13.49 to 29.60)

China								
Cause	YLLs (in 1000s)				Deaths (in 1000s)			
	2016	2040 Forecast	2040 Better Scenario	2040 Worse Scenario	2016	2040 Forecast	2040 Better Scenario	2040 Worse Scenario
Alcoholic cardiomyopathy	81.97 (49.83 to 103.83)	73.64 (44.36 to 99.08)	57.60 (34.66 to 78.90)	72.90 (43.19 to 95.00)	2.72 (1.70 to 3.49)	3.22 (2.04 to 4.38)	2.63 (1.69 to 3.67)	3.05 (1.91 to 4.07)
Other cardiomyopathy	335.68 (291.97 to 398.81)	295.14 (170.84 to 472.96)	255.35 (159.77 to 401.48)	392.66 (260.94 to 563.88)	13.03 (10.94 to 16.30)	19.10 (10.71 to 31.09)	17.20 (10.39 to 28.23)	23.44 (15.05 to 36.32)
Atrial fibrillation and flutter	413.09 (332.06 to 502.32)	1,057.60 (781.57 to 1,383.95)	988.47 (737.16 to 1,298.55)	1,062.89 (789.95 to 1,420.39)	38.23 (30.51 to 46.98)	119.27 (87.87 to 156.74)	115.35 (85.09 to 151.13)	112.00 (78.85 to 152.09)
Aortic aneurysm	264.24 (245.87 to 284.58)	347.56 (261.83 to 462.26)	323.54 (246.42 to 431.56)	390.41 (297.81 to 504.25)	13.04 (12.19 to 13.89)	25.18 (19.99 to 31.75)	24.62 (19.84 to 31.00)	25.57 (20.05 to 32.11)
Peripheral artery disease	31.02 (25.18 to 38.85)	97.94 (70.37 to 133.65)	96.44 (69.89 to 134.29)	98.02 (69.87 to 134.36)	2.20 (1.79 to 2.79)	9.45 (6.87 to 13.09)	9.69 (7.10 to 13.57)	8.65 (6.10 to 12.00)
Endocarditis	127.30 (110.18 to 165.22)	212.43 (152.32 to 298.21)	207.69 (148.97 to 294.00)	211.74 (151.68 to 291.42)	5.92 (5.08 to 7.42)	15.67 (10.99 to 22.81)	15.86 (11.39 to 22.48)	14.53 (9.94 to 20.94)
Other cardiovascular and circulatory diseases	1,111.12 (966.67 to 1,712.59)	2,115.39 (1,334.34 to 3,629.65)	1,845.07 (1,173.93 to 3,123.74)	2,911.02 (1,969.15 to 4,740.44)	60.53 (52.58 to 95.06)	176.96 (115.57 to 300.55)	160.89 (106.35 to 273.22)	223.29 (152.53 to 363.84)
**Chronic respiratory diseases**	**13,077.50 (12,537.87 to 14,263.13)**	**18,153.13 (7,871.53 to 38,373.22)**	**13,193.38 (6,567.12 to 25,826.67)**	**29,704.73 (11,266.44 to 68,329.65)**	**916.11 (878.54 to 1,007.50)**	**1,564.05 (672.27 to 3,312.43)**	**1,174.37 (565.59 to 2,352.48)**	**2,407.97 (893.14 to 5,335.49)**
Chronic obstructive pulmonary disease	12,286.52 (11,764.69 to 13,513.27)	17,563.71 (7,371.23 to 37,672.46)	12,623.55 (6,069.73 to 25,076.47)	29,044.61 (10,671.46 to 67,489.12)	876.27 (839.48 to 969.50)	1,521.64 (638.64 to 3,263.44)	1,132.61 (534.34 to 2,303.20)	2,362.45 (858.43 to 5,289.62)
Pneumoconiosis	191.19 (174.13 to 207.13)	80.74 (55.66 to 121.07)	68.96 (49.25 to 97.49)	115.19 (89.37 to 143.92)	8.34 (7.53 to 9.03)	5.16 (3.61 to 7.55)	4.57 (3.32 to 6.31)	6.82 (5.24 to 8.44)
Silicosis	140.97 (127.41 to 153.75)	45.58 (26.95 to 78.62)	37.98 (23.65 to 61.34)	73.49 (54.28 to 94.01)	6.13 (5.50 to 6.67)	2.82 (1.69 to 4.77)	2.43 (1.53 to 3.83)	4.25 (3.07 to 5.45)
Asbestosis	8.15 (6.10 to 10.73)	10.12 (6.72 to 14.06)	11.10 (7.42 to 15.59)	8.09 (5.42 to 11.39)	0.32 (0.23 to 0.44)	0.66 (0.44 to 0.94)	0.75 (0.50 to 1.07)	0.49 (0.33 to 0.72)
Coal workers pneumoconiosis	17.59 (9.32 to 20.69)	10.20 (5.71 to 16.43)	7.73 (4.18 to 11.93)	15.84 (8.61 to 22.39)	0.90 (0.46 to 1.06)	0.70 (0.39 to 1.12)	0.55 (0.29 to 0.85)	1.02 (0.55 to 1.44)
Other pneumoconiosis	24.48 (21.38 to 30.38)	14.85 (10.94 to 20.51)	12.15 (8.90 to 16.44)	17.77 (13.64 to 23.84)	0.99 (0.86 to 1.23)	0.97 (0.73 to 1.32)	0.83 (0.61 to 1.13)	1.05 (0.81 to 1.40)
Asthma	380.66 (304.40 to 428.17)	210.39 (124.51 to 356.64)	167.06 (104.35 to 261.06)	322.30 (205.84 to 493.52)	21.51 (16.71 to 24.20)	17.58 (10.54 to 29.26)	14.55 (9.34 to 22.41)	24.91 (15.90 to 37.29)
Interstitial lung disease and pulmonary sarcoidosis	135.30 (107.34 to 176.78)	234.93 (155.96 to 321.98)	269.32 (184.50 to 373.62)	161.01 (117.06 to 221.26)	6.61 (5.05 to 8.31)	15.35 (9.98 to 20.86)	18.09 (12.17 to 25.07)	9.93 (6.96 to 13.21)
Other chronic respiratory diseases	83.83 (40.73 to 111.51)	63.36 (26.88 to 109.37)	64.49 (27.15 to 110.62)	61.61 (26.31 to 108.64)	3.38 (1.54 to 4.57)	4.32 (1.75 to 7.52)	4.55 (1.86 to 7.98)	3.85 (1.57 to 6.70)
**Cirrhosis and other chronic liver diseases**	**4,369.89 (4,050.48 to 5,268.66)**	**4,347.61 (3,649.58 to 5,379.37)**	**4,033.74 (3,387.38 to 4,936.54)**	**4,972.31 (4,315.17 to 6,107.23)**	**160.30 (149.23 to 191.35)**	**213.02 (182.93 to 259.58)**	**202.71 (173.59 to 243.84)**	**233.57 (204.20 to 280.97)**
Cirrhosis and other chronic liver diseases due to hepatitis B	2,172.20 (1,976.23 to 2,665.26)	2,207.48 (1,839.54 to 2,789.59)	2,137.23 (1,775.25 to 2,710.30)	2,481.72 (2,118.48 to 3,060.43)	81.23 (74.07 to 98.15)	109.50 (92.28 to 137.31)	108.04 (91.79 to 133.99)	118.31 (101.57 to 143.21)
Cirrhosis and other chronic liver diseases due to hepatitis C	577.53 (505.95 to 736.86)	520.55 (421.09 to 673.74)	501.84 (406.69 to 651.24)	591.83 (492.18 to 752.72)	19.95 (17.56 to 24.96)	23.03 (19.05 to 29.79)	22.61 (18.78 to 28.48)	25.24 (21.23 to 32.01)

Cirrhosis and other chronic liver diseases due to alcohol use	673.95 (599.60 to 833.66)	702.75 (577.16 to 895.84)	509.34 (424.11 to 637.76)	858.82 (718.50 to 1,067.65)	24.65 (22.11 to 29.93)	33.38 (28.13 to 41.38)	25.62 (21.47 to 31.60)	38.90 (32.73 to 47.75)
Cirrhosis and other chronic liver diseases due to other causes	946.21 (805.28 to 1,131.54)	916.83 (739.69 to 1,166.47)	885.32 (715.54 to 1,129.99)	1,039.94 (854.40 to 1,289.80)	34.48 (29.47 to 41.30)	47.11 (37.60 to 58.63)	46.43 (37.92 to 58.68)	51.12 (41.18 to 64.80)
**Digestive diseases**	**2,338.77 (2,207.50 to 2,621.27)**	**2,603.14 (2,143.37 to 3,266.79)**	**2,438.25 (2,029.67 to 3,016.76)**	**3,060.87 (2,520.38 to 3,804.35)**	**114.37 (107.17 to 131.45)**	**195.59 (159.05 to 246.24)**	**189.97 (156.93 to 236.58)**	**214.40 (167.15 to 270.89)**
Peptic ulcer disease	753.87 (688.40 to 832.29)	515.17 (371.35 to 710.47)	458.41 (332.04 to 625.51)	753.36 (572.38 to 976.67)	37.58 (34.56 to 41.25)	37.04 (26.83 to 50.98)	33.89 (24.95 to 46.15)	51.19 (38.19 to 66.64)

China								
**Cause**	**YLLs (in 1000s)**				**Deaths (in 1000s)**			
	**2016**	**2040 Forecast**	**2040 Better Scenario**	**2040 Worse Scenario**	**2016**	**2040 Forecast**	**2040 Better Scenario**	**2040 Worse Scenario**
Gastritis and duodenitis	284.18 (254.48 to 308.45)	372.17 (297.84 to 462.90)	359.90 (282.33 to 451.01)	433.18 (349.21 to 522.82)	15.02 (13.61 to 16.29)	28.01 (22.15 to 35.37)	27.89 (21.85 to 35.07)	30.54 (23.29 to 37.68)
Appendicitis	45.61 (40.46 to 56.60)	46.33 (35.52 to 62.41)	42.93 (32.93 to 58.39)	60.92 (49.97 to 79.28)	1.92 (1.69 to 2.45)	3.20 (2.43 to 4.42)	3.05 (2.34 to 4.16)	3.95 (3.13 to 5.23)
Paralytic ileus and intestinal obstruction	351.99 (302.18 to 437.74)	413.55 (346.80 to 573.74)	434.30 (364.81 to 601.90)	382.92 (310.99 to 543.93)	14.71 (12.88 to 19.64)	32.48 (25.99 to 45.41)	35.16 (28.91 to 49.08)	28.01 (21.57 to 40.24)
Inguinal, femoral, and abdominal hernia	25.73 (21.05 to 35.97)	26.25 (19.83 to 38.15)	26.12 (19.47 to 37.70)	28.50 (20.99 to 42.24)	1.47 (1.17 to 2.15)	2.33 (1.71 to 3.55)	2.40 (1.75 to 3.54)	2.34 (1.59 to 3.48)
Inflammatory bowel disease	110.76 (93.15 to 125.55)	161.09 (135.45 to 194.03)	167.84 (141.32 to 203.36)	149.92 (124.77 to 182.49)	5.23 (4.59 to 6.33)	12.64 (10.38 to 15.85)	13.58 (11.31 to 17.18)	10.95 (8.53 to 13.95)
Vascular intestinal disorders	113.86 (104.07 to 142.37)	187.75 (160.53 to 231.65)	190.06 (161.76 to 240.32)	191.30 (162.43 to 238.83)	6.77 (6.20 to 8.36)	15.01 (12.56 to 18.91)	15.60 (13.05 to 19.44)	14.41 (11.44 to 18.47)
Gallbladder and biliary diseases	225.73 (201.05 to 322.56)	372.46 (225.94 to 638.73)	312.18 (205.83 to 516.21)	470.77 (285.08 to 825.10)	13.69 (12.26 to 18.85)	31.68 (19.52 to 53.04)	27.59 (18.22 to 43.98)	37.24 (22.18 to 62.77)
Pancreatitis	255.09 (219.28 to 309.09)	274.54 (179.89 to 409.42)	210.71 (145.75 to 306.34)	343.00 (229.60 to 521.95)	9.93 (8.52 to 12.11)	15.82 (10.66 to 23.32)	12.76 (9.06 to 18.21)	18.67 (12.79 to 27.53)
Other digestive diseases	171.93 (155.33 to 194.49)	233.81 (200.76 to 276.08)	235.79 (200.92 to 288.32)	247.00 (209.19 to 300.28)	8.05 (7.30 to 9.22)	17.37 (14.55 to 21.10)	18.05 (15.16 to 22.11)	17.11 (13.54 to 21.57)
**Neurological disorders**	**6,617.03 (5,852.04 to 7,559.00)**	**16,539.13 (13,135.52 to 21,026.34)**	**16,603.72 (13,666.84 to 20,597.11)**	**15,272.45 (11,584.30 to 20,675.38)**	**540.03 (470.60 to 624.51)**	**1,744.79 (1,326.00 to 2,278.28)**	**1,801.99 (1,427.56 to 2,237.08)**	**1,515.25 (1,042.00 to 2,100.23)**
Alzheimer's disease and other dementias	5,124.79 (4,392.46 to 6,066.37)	14,192.22 (10,865.75 to 18,748.84)	14,176.60 (11,282.66 to 18,087.91)	13,183.86 (9,544.89 to 18,646.44)	476.90 (407.70 to 560.63)	1,577.40 (1,165.98 to 2,111.01)	1,620.84 (1,251.70 to 2,068.24)	1,379.88 (929.93 to 1,945.67)
Parkinson's disease	542.29 (421.01 to 683.39)	1,544.46 (1,172.45 to 1,992.28)	1,673.40 (1,274.02 to 2,143.69)	1,245.52 (920.42 to 1,640.76)	40.01 (31.13 to 51.07)	137.29 (102.63 to 178.42)	151.99 (114.91 to 196.23)	105.56 (74.75 to 141.63)
Epilepsy	580.81 (542.10 to 651.07)	403.44 (304.49 to 524.60)	347.11 (267.34 to 449.39)	468.67 (351.25 to 631.23)	12.78 (11.97 to 14.36)	13.50 (10.57 to 17.29)	11.91 (9.57 to 14.96)	14.86 (11.42 to 19.66)
Multiple sclerosis	40.24 (35.26 to 46.59)	41.49 (33.86 to 50.16)	41.92 (34.25 to 50.66)	39.70 (32.62 to 48.18)	1.23 (1.08 to 1.42)	1.72 (1.46 to 2.06)	1.76 (1.48 to 2.09)	1.59 (1.32 to 1.91)
Motor neuron disease	96.17 (90.97 to 101.13)	124.36 (109.92 to 137.90)	126.05 (111.04 to 140.48)	118.33 (105.49 to 130.78)	2.99 (2.84 to 3.12)	5.19 (4.66 to 5.68)	5.33 (4.75 to 5.86)	4.81 (4.31 to 5.26)
Other neurological disorders	232.73 (222.79 to 243.58)	233.16 (210.35 to 257.07)	238.63 (214.24 to 263.42)	216.37 (196.45 to 236.47)	6.12 (5.90 to 6.37)	9.68 (8.96 to 10.40)	10.14 (9.42 to 10.92)	8.56 (7.87 to 9.26)
**Mental disorders**	**1,879.29 (1,568.00 to 2,221.29)**	**2,818.42 (2,192.57 to 3,448.06)**	**3,039.36 (2,419.49 to 3,604.67)**	**2,263.26 (1,852.16 to 2,619.20)**	**54.19 (44.06 to 61.79)**	**121.19 (93.32 to 145.89)**	**134.70 (107.32 to 160.21)**	**91.55 (73.77 to 104.01)**
Alcohol use disorders	790.12 (459.94 to 895.55)	592.37 (311.59 to 748.67)	565.97 (291.62 to 712.14)	537.19 (284.76 to 662.14)	21.90 (12.80 to 24.76)	22.12 (12.81 to 27.03)	21.76 (12.46 to 26.28)	19.20 (11.04 to 23.04)
Drug use disorders	1,085.79 (949.07 to 1,525.29)	2,223.57 (1,708.49 to 2,848.68)	2,470.88 (1,964.73 to 3,069.66)	1,723.65 (1,442.38 to 2,122.85)	32.24 (28.25 to 41.53)	99.01 (75.68 to 122.35)	112.89 (88.52 to 137.78)	72.30 (59.83 to 84.56)
Opioid use disorders	663.65 (542.30 to 986.76)	983.56 (728.88 to 1,259.11)	1,040.93 (765.19 to 1,344.47)	862.37 (654.53 to 1,146.65)	19.03 (14.80 to 25.76)	40.76 (28.66 to 50.40)	44.15 (30.29 to 53.84)	33.89 (24.00 to 41.44)

Cocaine use disorders	78.35 (61.87 to 122.31)	195.00 (128.63 to 294.95)	217.51 (148.15 to 317.13)	150.29 (107.75 to 216.81)	2.08 (1.62 to 3.16)	6.72 (4.41 to 10.06)	7.58 (5.04 to 11.08)	5.06 (3.59 to 7.24)
Amphetamine use disorders	46.29 (35.81 to 77.10)	158.50 (95.79 to 247.00)	184.21 (120.90 to 282.96)	107.88 (78.14 to 162.40)	1.15 (0.88 to 1.82)	4.93 (3.03 to 7.47)	5.78 (3.78 to 8.61)	3.28 (2.41 to 4.77)
Other drug use disorders	297.49 (242.65 to 449.44)	886.51 (607.17 to 1,217.67)	1,028.22 (749.04 to 1,382.21)	603.12 (474.09 to 799.50)	9.98 (8.19 to 13.90)	46.60 (31.76 to 62.45)	55.39 (39.70 to 73.82)	30.07 (23.39 to 38.25)
Eating disorders	3.38 (2.52 to 3.76)	2.47 (1.70 to 3.18)	2.51 (1.71 to 3.26)	2.43 (1.73 to 3.08)	0.06 (0.05 to 0.07)	0.05 (0.03 to 0.06)	0.05 (0.03 to 0.07)	0.05 (0.03 to 0.06)

China								
Cause	YLLs (in 1000s)				Deaths (in 1000s)			
	2016	2040 Forecast	2040 Better Scenario	2040 Worse Scenario	2016	2040 Forecast	2040 Better Scenario	2040 Worse Scenario
**Diabetes, urogenital, blood, and endocrine diseases**	**7,569.95 (7,292.39 to 7,930.97)**	**9,509.30 (7,144.42 to 12,520.37)**	**8,367.09 (6,416.11 to 10,854.67)**	**13,384.78 (10,086.64 to 17,514.12)**	**354.02 (341.11 to 367.91)**	**651.03 (503.89 to 858.46)**	**590.53 (459.20 to 753.05)**	**865.53 (667.91 to 1,131.59)**
Diabetes mellitus	2,797.16 (2,700.24 to 2,893.94)	3,460.57 (2,347.22 to 5,124.61)	2,894.56 (2,020.08 to 4,101.62)	5,081.22 (3,444.95 to 7,396.54)	140.84 (135.94 to 145.57)	235.22 (163.83 to 331.89)	204.79 (147.56 to 282.13)	323.81 (223.96 to 450.20)
Acute glomerulonephritis	200.10 (189.38 to 212.34)	240.53 (193.71 to 297.41)	232.19 (183.65 to 287.60)	275.69 (225.23 to 331.23)	7.53 (7.16 to 7.96)	12.78 (10.19 to 15.73)	12.63 (10.02 to 15.52)	13.85 (11.05 to 16.85)
Chronic kidney disease	3,585.70 (3,408.10 to 3,867.63)	4,647.44 (2,866.37 to 7,416.70)	4,085.50 (2,564.73 to 6,363.34)	6,789.39 (3,996.62 to 11,037.92)	167.50 (158.36 to 178.80)	323.01 (196.35 to 521.54)	290.81 (184.15 to 452.53)	448.58 (266.47 to 722.60)
Chronic kidney disease due to diabetes mellitus	1,868.73 (1,681.93 to 2,068.27)	2,442.85 (1,460.21 to 4,031.01)	2,133.03 (1,294.36 to 3,412.65)	3,613.37 (2,071.78 to 6,080.52)	82.61 (73.57 to 91.63)	155.83 (92.46 to 261.73)	138.89 (84.88 to 224.37)	220.80 (127.07 to 365.85)
Chronic kidney disease due to hypertension	1,070.21 (937.57 to 1,222.88)	1,647.27 (1,004.79 to 2,604.61)	1,468.78 (915.76 to 2,289.83)	2,328.83 (1,358.80 to 3,727.80)	61.05 (54.21 to 68.77)	131.75 (80.14 to 211.16)	120.55 (75.33 to 188.06)	176.23 (101.40 to 283.86)
Chronic kidney disease due to glomerulonephritis	293.80 (244.54 to 353.96)	214.70 (128.66 to 356.92)	184.69 (112.52 to 299.10)	333.93 (194.48 to 553.18)	9.67 (7.93 to 11.82)	12.18 (6.97 to 20.87)	10.63 (6.23 to 17.61)	18.38 (10.48 to 31.65)
Chronic kidney disease due to other and unspecified causes	352.96 (291.57 to 425.44)	342.63 (202.06 to 575.15)	298.99 (182.08 to 499.23)	513.25 (297.62 to 858.59)	14.18 (11.57 to 17.24)	23.24 (13.17 to 39.37)	20.74 (12.23 to 34.44)	33.17 (18.47 to 56.35)
Urinary diseases and male infertility	277.21 (265.86 to 293.34)	487.34 (412.33 to 589.20)	469.56 (397.21 to 562.49)	555.30 (491.27 to 618.18)	14.76 (14.22 to 15.47)	38.86 (31.76 to 47.89)	38.79 (32.37 to 47.20)	40.88 (32.25 to 48.05)
Urinary tract infections	165.48 (148.77 to 181.61)	304.63 (254.49 to 366.51)	297.00 (251.77 to 353.08)	333.05 (283.89 to 380.01)	8.31 (7.39 to 9.18)	24.52 (19.91 to 30.11)	24.73 (20.37 to 30.03)	24.76 (19.42 to 29.81)
Urolithiasis	61.27 (53.64 to 77.46)	116.58 (85.20 to 165.07)	108.31 (81.23 to 150.70)	143.68 (117.16 to 187.95)	3.16 (2.75 to 4.00)	8.27 (6.04 to 11.78)	7.94 (5.96 to 11.08)	9.46 (7.33 to 12.65)
Other urinary diseases	50.46 (42.96 to 61.16)	66.12 (46.26 to 92.07)	64.25 (45.69 to 87.26)	78.58 (55.90 to 103.14)	3.30 (2.77 to 3.88)	6.07 (4.16 to 8.72)	6.13 (4.25 to 8.46)	6.66 (4.28 to 9.26)
Gynecological diseases	29.96 (17.33 to 33.89)	10.11 (5.28 to 14.79)	8.86 (4.92 to 12.64)	15.10 (8.74 to 19.94)	1.05 (0.61 to 1.19)	0.51 (0.28 to 0.72)	0.45 (0.25 to 0.64)	0.73 (0.42 to 0.95)
Uterine fibroids	21.79 (10.37 to 26.17)	7.13 (3.67 to 10.63)	6.34 (3.11 to 9.16)	10.31 (5.45 to 14.17)	0.77 (0.37 to 0.92)	0.34 (0.17 to 0.50)	0.31 (0.16 to 0.45)	0.48 (0.25 to 0.68)
Polycystic ovarian syndrome	0.31 (0.22 to 0.41)	0.03 (0.01 to 0.07)	0.02 (0.01 to 0.05)	0.07 (0.04 to 0.12)	0.01 (0.01 to 0.01)	0.00 (0.00 to 0.00)	0.00 (0.00 to 0.00)	0.00 (0.00 to 0.00)
Endometriosis	0.48 (0.08 to 0.66)	0.07 (0.01 to 0.14)	0.06 (0.01 to 0.11)	0.12 (0.02 to 0.21)	0.01 (0.00 to 0.01)	0.00 (0.00 to 0.00)	0.00 (0.00 to 0.00)	0.00 (0.00 to 0.00)
Genital prolapse	0.46 (0.16 to 0.57)	0.23 (0.07 to 0.37)	0.20 (0.06 to 0.31)	0.35 (0.11 to 0.52)	0.03 (0.01 to 0.04)	0.02 (0.00 to 0.03)	0.02 (0.00 to 0.03)	0.03 (0.01 to 0.04)
Other gynecological diseases	6.93 (3.76 to 8.41)	2.65 (1.26 to 4.26)	2.23 (1.09 to 3.51)	4.24 (2.21 to 5.86)	0.24 (0.13 to 0.29)	0.14 (0.07 to 0.22)	0.12 (0.06 to 0.19)	0.22 (0.12 to 0.29)
Hemoglobinopathies and hemolytic anemias	285.49 (233.22 to 320.46)	170.68 (124.77 to 222.89)	159.41 (116.84 to 203.48)	215.16 (163.32 to 261.63)	8.34 (6.60 to 9.51)	8.83 (6.50 to 11.39)	8.43 (6.23 to 10.85)	10.70 (8.10 to 12.99)
Thalassemias	88.35 (75.93 to 103.67)	21.05 (14.42 to 30.56)	20.48 (14.07 to 29.69)	20.98 (14.15 to 30.28)	1.18 (1.02 to 1.37)	0.33 (0.24 to 0.45)	0.32 (0.23 to 0.44)	0.32 (0.23 to 0.44)

Sickle cell disorders	0.46 (0.35 to 0.58)	0.15 (0.10 to 0.22)	0.14 (0.09 to 0.20)	0.20 (0.13 to 0.28)	0.01 (0.01 to 0.01)	0.00 (0.00 to 0.00)	0.00 (0.00 to 0.00)	0.00 (0.00 to 0.01)
G6PD deficiency	90.03 (68.60 to 104.28)	60.17 (41.52 to 84.37)	54.27 (36.48 to 73.88)	83.88 (58.97 to 107.91)	2.24 (1.69 to 2.63)	2.23 (1.54 to 3.08)	2.03 (1.40 to 2.73)	3.03 (2.14 to 3.82)
Other hemoglobinopathies and hemolytic anemias	106.66 (80.80 to 122.27)	89.31 (64.71 to 116.22)	84.51 (61.17 to 109.17)	110.09 (82.32 to 133.33)	4.91 (3.76 to 5.60)	6.28 (4.60 to 8.07)	6.08 (4.45 to 7.86)	7.35 (5.51 to 8.94)
Endocrine, metabolic, blood, and immune disorders	394.32 (331.04 to 462.47)	492.64 (397.00 to 564.27)	517.02 (417.14 to 594.87)	452.91 (367.30 to 521.39)	13.99 (11.25 to 15.84)	31.82 (24.94 to 36.54)	34.62 (27.41 to 39.32)	26.99 (20.27 to 31.32)

China								
**Cause**	**YLLs (in 1000s)**				**Deaths (in 1000s)**			
	**2016**	**2040 Forecast**	**2040 Better Scenario**	**2040 Worse Scenario**	**2016**	**2040 Forecast**	**2040 Better Scenario**	**2040 Worse Scenario**
**Musculoskeletal disorders**	**481.95 (401.01 to 601.61)**	**588.92 (451.70 to 775.31)**	**551.83 (423.38 to 693.99)**	**719.53 (559.38 to 884.09)**	**18.10 (14.82 to 21.86)**	**36.72 (27.89 to 48.05)**	**35.04 (27.28 to 44.19)**	**43.47 (33.33 to 52.75)**
Rheumatoid arthritis	177.59 (130.42 to 205.27)	293.71 (194.57 to 427.72)	260.86 (179.04 to 363.30)	416.83 (303.08 to 521.67)	9.19 (6.79 to 10.37)	21.52 (14.37 to 30.98)	19.60 (13.64 to 27.17)	28.94 (20.68 to 36.41)
Other musculoskeletal disorders	304.36 (255.80 to 407.45)	295.22 (233.63 to 398.18)	290.97 (230.94 to 392.04)	302.70 (239.90 to 423.15)	8.92 (7.63 to 11.86)	15.20 (12.09 to 20.23)	15.44 (12.39 to 20.52)	14.54 (11.27 to 19.92)
**Other non-communicable diseases**	**3,372.63 (3,008.50 to 4,093.15)**	**974.33 (682.75 to 1,456.52)**	**849.31 (600.75 to 1,237.74)**	**1,447.98 (1,053.89 to 2,052.03)**	**48.26 (43.27 to 57.70)**	**34.91 (24.47 to 47.98)**	**33.94 (23.22 to 46.43)**	**40.50 (29.29 to 54.39)**
Congenital birth defects	3,207.68 (2,848.47 to 3,927.89)	697.18 (437.76 to 1,148.33)	573.46 (360.65 to 952.26)	1,156.91 (787.11 to 1,719.32)	41.04 (36.54 to 49.99)	10.35 (7.00 to 16.07)	8.64 (5.75 to 13.37)	16.74 (12.12 to 23.63)
Neural tube defects	33.72 (26.96 to 45.32)	5.07 (3.05 to 8.60)	4.12 (2.40 to 7.18)	9.25 (5.66 to 15.05)	0.44 (0.36 to 0.59)	0.08 (0.05 to 0.14)	0.07 (0.04 to 0.11)	0.15 (0.10 to 0.22)
Congenital heart anomalies	2,317.45 (2,065.57 to 2,838.27)	467.50 (282.89 to 793.52)	375.26 (225.18 to 640.60)	812.86 (551.12 to 1,218.25)	29.65 (26.53 to 36.19)	6.74 (4.27 to 11.05)	5.45 (3.47 to 8.80)	11.55 (8.19 to 16.54)
Orofacial clefts	24.08 (17.95 to 39.99)	3.00 (1.30 to 6.29)	2.26 (0.93 to 4.67)	6.41 (2.90 to 12.09)	0.28 (0.21 to 0.46)	0.03 (0.01 to 0.07)	0.03 (0.01 to 0.05)	0.07 (0.03 to 0.14)
Down syndrome	44.16 (35.69 to 56.31)	25.58 (19.52 to 34.91)	26.16 (20.00 to 35.39)	25.18 (19.05 to 35.27)	0.74 (0.62 to 0.96)	0.57 (0.44 to 0.76)	0.59 (0.46 to 0.78)	0.55 (0.42 to 0.76)
Other chromosomal abnormalities	32.06 (25.17 to 47.53)	13.13 (8.06 to 21.03)	12.07 (7.37 to 19.29)	16.53 (10.10 to 26.79)	0.41 (0.31 to 0.60)	0.19 (0.12 to 0.29)	0.17 (0.11 to 0.26)	0.23 (0.15 to 0.36)
Congenital musculoskeletal and limb anomalies	39.23 (32.22 to 55.94)	10.50 (6.56 to 17.51)	8.88 (5.33 to 14.02)	16.08 (10.08 to 24.99)	0.50 (0.42 to 0.72)	0.16 (0.10 to 0.26)	0.14 (0.09 to 0.21)	0.24 (0.17 to 0.36)
Urogenital congenital anomalies	30.59 (25.05 to 42.14)	12.10 (8.49 to 17.30)	10.78 (7.36 to 15.02)	16.62 (11.98 to 23.52)	0.53 (0.43 to 0.72)	0.30 (0.22 to 0.43)	0.27 (0.19 to 0.37)	0.41 (0.30 to 0.54)
Digestive congenital anomalies	270.64 (218.28 to 335.39)	52.17 (28.36 to 95.05)	42.52 (21.91 to 75.33)	87.87 (48.99 to 147.89)	3.19 (2.58 to 3.95)	0.65 (0.37 to 1.16)	0.53 (0.29 to 0.92)	1.08 (0.63 to 1.79)
Other congenital birth defects	415.75 (335.37 to 603.89)	108.12 (66.38 to 178.21)	91.41 (54.81 to 153.67)	166.12 (104.02 to 270.30)	5.29 (4.31 to 7.56)	1.62 (1.07 to 2.54)	1.38 (0.90 to 2.18)	2.44 (1.65 to 3.76)
Skin and subcutaneous diseases	114.45 (75.67 to 178.85)	265.74 (170.35 to 400.38)	265.91 (165.49 to 388.11)	274.64 (174.18 to 424.55)	6.64 (4.13 to 9.83)	24.43 (14.83 to 35.56)	25.19 (14.86 to 37.46)	23.58 (13.42 to 36.29)
Cellulitis	11.44 (7.40 to 22.52)	23.61 (14.29 to 44.96)	24.14 (14.15 to 45.57)	22.30 (13.33 to 43.10)	0.39 (0.23 to 0.76)	1.48 (0.80 to 2.79)	1.57 (0.84 to 2.91)	1.29 (0.70 to 2.46)
Pyoderma	46.78 (31.67 to 83.16)	121.54 (78.69 to 200.26)	122.51 (79.80 to 201.77)	124.83 (81.86 to 211.96)	2.24 (1.47 to 3.88)	10.03 (6.38 to 17.16)	10.49 (6.69 to 17.69)	9.46 (5.89 to 16.55)
Decubitus ulcer	45.12 (24.64 to 63.25)	105.36 (51.84 to 149.04)	104.03 (50.11 to 149.25)	112.13 (55.33 to 159.80)	3.57 (1.87 to 5.03)	11.82 (5.57 to 17.33)	11.98 (5.34 to 17.59)	11.82 (5.52 to 17.60)
Other skin and subcutaneous diseases	11.11 (7.28 to 18.66)	15.22 (9.74 to 23.63)	15.23 (9.49 to 23.83)	15.39 (9.71 to 24.04)	0.45 (0.29 to 0.72)	1.10 (0.68 to 1.69)	1.15 (0.70 to 1.76)	1.02 (0.62 to 1.64)
Sudden infant death syndrome	50.50 (43.11 to 61.62)	11.41 (5.71 to 21.35)	9.94 (4.97 to 19.39)	16.43 (8.60 to 29.61)	0.59 (0.50 to 0.71)	0.13 (0.07 to 0.25)	0.12 (0.06 to 0.22)	0.19 (0.10 to 0.34)

**Injuries**	**29,896.02 (27,939.92 to 30,919.87)**	**16,495.40 (13,009.70 to 21,163.23)**	**13,995.12 (11,161.49 to 17,527.39)**	**23,427.90 (18,810.84 to 29,376.37)**	**773.27 (704.60 to 803.71)**	**723.40 (585.05 to 894.66)**	**650.75 (536.37 to 778.43)**	**912.56 (756.03 to 1,093.72)**
**Transport injuries**	**13,528.38 (13,023.09 to 14,108.88)**	**5,739.09 (4,078.50 to 8,257.98)**	**4,267.77 (3,151.56 to 5,756.35)**	**10,736.59 (7,879.53 to 14,845.90)**	**324.63 (312.66 to 336.75)**	**191.90 (141.28 to 269.48)**	**146.84 (112.51 to 195.44)**	**335.74 (255.38 to 450.10)**
Road injuries	12,989.94 (12,521.59 to 13,476.48)	5,336.76 (3,796.52 to 7,663.41)	3,943.38 (2,920.09 to 5,321.30)	10,167.99 (7,494.15 to 14,043.59)	311.61 (299.90 to 322.29)	178.35 (132.41 to 249.71)	135.67 (104.83 to 178.98)	317.38 (241.01 to 425.78)
Pedestrian road injuries	7,225.78 (6,797.06 to 7,629.11)	2,315.06 (1,576.73 to 3,509.56)	1,593.46 (1,134.17 to 2,269.85)	5,206.59 (3,873.63 to 7,111.00)	180.36 (169.64 to 189.35)	88.37 (62.34 to 130.49)	63.36 (46.39 to 87.40)	181.48 (139.78 to 240.04)

China								
Cause	YLLs (in 1000s)				Deaths (in 1000s)			
	2016	2040 Forecast	2040 Better Scenario	2040 Worse Scenario	2016	2040 Forecast	2040 Better Scenario	2040 Worse Scenario
Cyclist road injuries	968.37 (858.79 to 1,031.73)	574.07 (409.95 to 793.86)	449.30 (335.94 to 600.17)	923.15 (661.08 to 1,274.56)	25.27 (22.50 to 26.99)	21.00 (15.07 to 28.97)	17.13 (12.80 to 22.81)	30.10 (21.60 to 41.09)
Motorcyclist road injuries	2,399.00 (2,093.86 to 2,605.97)	1,522.58 (993.64 to 2,314.37)	1,233.13 (836.75 to 1,795.07)	2,136.79 (1,418.74 to 3,229.22)	52.06 (44.73 to 56.14)	39.69 (26.55 to 58.55)	32.81 (23.03 to 46.59)	52.52 (35.29 to 76.59)
Motor vehicle road injuries	2,319.09 (2,001.57 to 2,876.08)	889.24 (636.69 to 1,263.65)	640.76 (475.96 to 886.13)	1,833.83 (1,301.84 to 2,543.51)	52.05 (44.72 to 64.47)	27.96 (20.84 to 38.18)	21.30 (16.61 to 28.33)	51.12 (37.68 to 69.64)
Other road injuries	77.70 (64.58 to 87.57)	35.82 (25.24 to 50.45)	26.72 (19.64 to 36.08)	67.63 (46.29 to 95.43)	1.87 (1.56 to 2.11)	1.33 (0.98 to 1.83)	1.06 (0.81 to 1.41)	2.16 (1.55 to 3.04)
Other transport injuries	538.45 (484.02 to 725.48)	402.32 (251.19 to 648.84)	324.40 (212.52 to 495.50)	568.60 (385.10 to 855.19)	13.02 (11.76 to 17.52)	13.55 (8.77 to 21.32)	11.18 (7.58 to 16.78)	18.36 (12.94 to 27.07)
**Unintentional injuries**	**11,007.13 (8,659.05 to 11,715.72)**	**6,215.34 (4,403.49 to 7,750.09)**	**5,846.18 (4,229.32 to 7,174.79)**	**7,420.88 (5,423.05 to 8,880.47)**	**297.56 (225.52 to 318.39)**	**341.16 (236.93 to 413.79)**	**336.75 (236.34 to 404.13)**	**367.56 (258.26 to 437.33)**
Falls	3,243.92 (2,281.43 to 3,568.97)	2,983.12 (1,994.36 to 3,624.64)	2,919.55 (1,955.24 to 3,502.54)	3,245.70 (2,189.00 to 3,788.35)	121.94 (82.94 to 134.19)	209.82 (135.41 to 255.10)	212.88 (139.32 to 256.06)	212.04 (136.53 to 257.98)
Drowning	3,187.77 (2,704.99 to 3,376.79)	1,291.41 (932.84 to 1,700.63)	1,164.98 (840.69 to 1,530.37)	1,599.49 (1,158.01 to 2,109.66)	63.72 (51.17 to 67.37)	47.14 (32.75 to 58.85)	43.94 (30.34 to 54.79)	54.18 (37.28 to 68.05)
Fire, heat, and hot substances	320.65 (249.04 to 348.65)	212.08 (147.96 to 297.78)	188.23 (131.77 to 253.32)	274.44 (193.75 to 356.45)	10.51 (7.90 to 11.44)	13.22 (9.26 to 17.94)	12.29 (8.48 to 16.16)	15.62 (10.81 to 20.28)
Poisonings	641.53 (349.36 to 746.95)	187.70 (94.32 to 282.53)	160.14 (80.41 to 237.14)	276.22 (138.61 to 391.69)	16.18 (8.82 to 18.87)	7.76 (4.02 to 11.30)	6.79 (3.60 to 9.85)	10.78 (5.56 to 14.80)
Exposure to mechanical forces	1,841.29 (1,103.64 to 2,042.18)	725.20 (404.52 to 979.07)	661.46 (371.00 to 876.54)	955.61 (519.94 to 1,202.98)	41.40 (24.27 to 45.76)	25.27 (14.56 to 33.07)	23.79 (13.65 to 30.81)	31.15 (17.60 to 37.83)
Unintentional firearm injuries	27.09 (23.79 to 38.46)	18.33 (15.02 to 23.59)	17.95 (14.52 to 22.62)	20.73 (16.57 to 28.20)	0.91 (0.81 to 1.15)	1.50 (1.14 to 1.85)	1.55 (1.19 to 1.90)	1.48 (1.08 to 1.90)
Unintentional suffocation	379.75 (199.36 to 463.60)	109.59 (48.05 to 167.77)	101.06 (45.00 to 157.72)	139.04 (61.15 to 214.49)	6.23 (3.18 to 7.50)	3.38 (1.47 to 4.59)	3.27 (1.45 to 4.48)	3.88 (1.67 to 5.32)
Other exposure to mechanical forces	1,434.44 (834.64 to 1,573.97)	597.28 (326.07 to 810.31)	542.45 (295.91 to 720.84)	795.84 (425.22 to 988.66)	34.27 (19.55 to 37.68)	20.39 (11.36 to 26.96)	18.96 (10.57 to 24.71)	25.79 (14.12 to 31.45)
Adverse effects of medical treatment	194.85 (174.79 to 254.24)	147.23 (122.13 to 195.30)	143.38 (117.79 to 195.75)	163.90 (136.28 to 216.24)	5.41 (4.89 to 7.15)	7.39 (6.26 to 9.93)	7.45 (6.23 to 10.07)	7.65 (6.40 to 10.27)
Animal contact	96.41 (87.23 to 113.35)	87.62 (71.58 to 109.57)	82.94 (64.86 to 105.17)	107.62 (85.61 to 135.66)	2.83 (2.51 to 3.30)	3.98 (3.29 to 4.98)	3.88 (3.13 to 4.83)	4.59 (3.66 to 5.74)
Venomous animal contact	51.60 (40.97 to 60.31)	44.79 (32.76 to 55.87)	43.22 (32.32 to 54.75)	52.37 (38.78 to 66.17)	1.57 (1.26 to 1.83)	2.09 (1.52 to 2.60)	2.06 (1.52 to 2.55)	2.30 (1.68 to 2.88)
Non-venomous animal contact	44.80 (39.40 to 61.23)	42.82 (32.87 to 59.17)	39.72 (29.31 to 55.85)	55.25 (42.50 to 77.17)	1.25 (1.09 to 1.74)	1.90 (1.45 to 2.64)	1.81 (1.35 to 2.55)	2.28 (1.76 to 3.17)
Foreign body	320.20 (268.05 to 376.57)	223.94 (176.17 to 271.49)	224.31 (176.63 to 267.74)	224.37 (175.93 to 271.66)	7.97 (6.50 to 8.71)	13.03 (9.91 to 15.10)	13.73 (10.47 to 15.76)	11.67 (8.69 to 13.89)
Pulmonary aspiration and foreign body in airway	289.01 (228.06 to 335.39)	190.28 (136.78 to 234.14)	188.84 (136.62 to 229.50)	193.99 (142.05 to 238.30)	7.08 (5.43 to 7.79)	10.69 (7.43 to 12.64)	11.16 (7.80 to 13.10)	9.77 (6.79 to 11.81)

Foreign body in other body part	31.18 (25.67 to 54.48)	33.66 (25.95 to 52.50)	35.47 (27.88 to 55.03)	30.38 (23.43 to 48.33)	0.89 (0.76 to 1.45)	2.33 (1.73 to 3.43)	2.57 (2.02 to 3.77)	1.89 (1.38 to 2.76)
Environmental heat and cold exposure	119.51 (91.81 to 159.29)	99.64 (69.45 to 135.64)	91.78 (63.46 to 124.54)	134.50 (96.10 to 180.46)	4.24 (3.09 to 5.38)	5.90 (4.00 to 8.00)	5.67 (3.69 to 7.56)	7.30 (4.94 to 9.67)
Other unintentional injuries	1,041.00 (667.30 to 1,113.83)	257.40 (153.09 to 424.64)	209.41 (120.08 to 322.97)	439.03 (270.80 to 540.76)	23.35 (14.91 to 24.98)	7.65 (4.72 to 12.46)	6.33 (3.68 to 9.56)	12.58 (7.84 to 15.01)
**Self-harm and interpersonal violence**	**5,273.75 (4,985.26 to 6,136.79)**	**4,425.34 (3,068.26 to 6,193.87)**	**3,764.97 (2,742.76 to 5,109.93)**	**5,156.16 (3,604.56 to 7,258.98)**	**149.25 (141.07 to 168.07)**	**187.12 (133.29 to 258.71)**	**163.89 (121.89 to 219.93)**	**206.18 (146.45 to 289.97)**

China

Cause	YLLs (in 1000s)				Deaths (in 1000s)			
	2016	2040 Forecast	2040 Better Scenario	2040 Worse Scenario	2016	2040 Forecast	2040 Better Scenario	2040 Worse Scenario
Self-harm	4,387.79 (4,155.67 to 5,038.28)	4,072.96 (2,761.88 to 5,809.12)	3,451.02 (2,478.67 to 4,734.89)	4,727.25 (3,215.27 to 6,767.64)	130.56 (121.94 to 145.40)	176.93 (125.45 to 248.16)	154.70 (114.41 to 208.12)	194.19 (135.94 to 276.75)
Self-harm by firearm	46.95 (34.99 to 78.98)	24.72 (13.55 to 44.59)	19.49 (11.41 to 34.75)	32.48 (17.96 to 58.68)	1.24 (0.95 to 2.04)	0.99 (0.60 to 1.67)	0.84 (0.55 to 1.37)	1.20 (0.72 to 1.98)
Self-harm by other specified means	4,340.84 (4,106.33 to 4,955.86)	4,048.24 (2,749.10 to 5,771.78)	3,431.53 (2,467.96 to 4,704.88)	4,694.77 (3,188.56 to 6,714.65)	129.31 (120.80 to 143.71)	175.94 (124.79 to 246.86)	153.86 (113.86 to 206.82)	192.99 (135.12 to 275.14)
Interpersonal violence	885.96 (773.87 to 1,313.42)	352.38 (259.89 to 558.64)	313.95 (231.45 to 487.43)	428.91 (324.42 to 674.60)	18.69 (16.43 to 27.61)	10.19 (7.58 to 15.32)	9.19 (6.90 to 13.78)	12.00 (9.25 to 18.12)
Physical violence by firearm	31.64 (21.21 to 62.23)	13.44 (9.01 to 25.13)	12.19 (8.12 to 22.63)	17.17 (12.08 to 32.85)	0.76 (0.51 to 1.44)	0.48 (0.32 to 0.87)	0.44 (0.29 to 0.80)	0.59 (0.41 to 1.07)
Physical violence by sharp object	384.29 (328.78 to 612.15)	146.28 (105.20 to 243.75)	132.45 (93.98 to 218.24)	185.66 (142.15 to 307.85)	7.91 (6.79 to 12.54)	3.88 (2.85 to 6.16)	3.56 (2.64 to 5.57)	4.77 (3.77 to 7.50)
Physical violence by other means	470.03 (406.81 to 691.53)	192.66 (136.35 to 304.98)	169.31 (122.45 to 266.71)	226.08 (161.71 to 351.77)	10.02 (8.68 to 14.42)	5.83 (4.15 to 9.06)	5.19 (3.82 to 7.90)	6.64 (4.80 to 10.30)
**Forces of nature, conflict and terrorism, and executions and police conflict**	**86.76 (50.77 to 126.14)**	**115.64 (0.01 to 1,284.71)**	**116.20 (0.01 to 1,289.09)**	**114.28 (0.01 to 1,277.85)**	**1.83 (1.02 to 2.72)**	**3.21 (0.00 to 31.13)**	**3.27 (0.00 to 31.45)**	**3.08 (0.00 to 30.53)**
Exposure to forces of nature	43.00 (6.82 to 82.41)	85.93 (0.00 to 568.00)	86.44 (0.00 to 570.45)	84.71 (0.00 to 560.38)	0.97 (0.15 to 1.86)	2.54 (0.00 to 16.85)	2.60 (0.00 to 17.20)	2.43 (0.00 to 16.09)
Conflict and terrorism	0.00 (0.00 to 0.00)	9.61 (0.00 to 91.82)	9.60 (0.00 to 91.52)	9.61 (0.00 to 92.19)	0.00 (0.00 to 0.00)	0.18 (0.00 to 2.28)	0.18 (0.00 to 2.35)	0.18 (0.00 to 2.17)
Executions and police conflict	43.76 (38.03 to 50.70)	20.10 (0.00 to 0.00)	20.16 (0.00 to 0.00)	19.96 (0.00 to 0.00)	0.87 (0.76 to 0.99)	0.48 (0.00 to 0.00)	0.49 (0.00 to 0.00)	0.47 (0.00 to 0.00)

North Korea								
Cause	YLLs (actual counts)				Deaths (actual counts)			
	2016	2040 Forecast	2040 Better Scenario	2040 Worse Scenario	2016	2040 Forecast	2040 Better Scenario	2040 Worse Scenario
**All causes**	**6,512,459.48 (5,869,571.43 to 7,359,191.01)**	**7,120,788.91 (5,545,284.56 to 17,019,415.11)**	**4,814,053.40 (3,674,150.96 to 14,957,174.52)**	**8,391,252.41 (6,485,946.08 to 18,195,585.58)**	**233,049.96 (217,420.58 to 248,809.64)**	**350,064.17 (300,813.52 to 556,985.74)**	**248,090.84 (211,018.63 to 467,690.11)**	**400,414.36 (340,528.27 to 599,969.08)**
**Communicable, maternal, neonatal, and nutritional diseases**	**1,107,254.90 (798,225.18 to 1,561,650.88)**	**426,016.11 (304,014.06 to 606,258.30)**	**242,619.08 (171,483.07 to 381,562.73)**	**535,090.43 (376,569.12 to 770,731.96)**	**18,553.24 (14,415.41 to 24,566.96)**	**13,499.65 (10,025.27 to 18,393.88)**	**8,689.17 (6,545.97 to 12,291.32)**	**15,657.28 (11,501.63 to 21,504.24)**
**HIV/AIDS and tuberculosis**	**103,246.26 (69,207.40 to 237,446.26)**	**92,255.44 (50,825.35 to 225,077.59)**	**48,591.11 (20,873.39 to 180,199.89)**	**112,102.00 (62,259.06 to 243,179.95)**	**3,139.43 (2,309.45 to 6,074.22)**	**3,463.02 (2,123.77 to 6,766.98)**	**1,802.55 (974.12 to 5,051.96)**	**4,030.44 (2,454.57 to 7,136.51)**
Tuberculosis	73,724.83 (61,695.58 to 89,495.18)	63,390.70 (44,165.97 to 90,455.30)	26,428.74 (19,097.04 to 37,418.84)	76,335.55 (52,072.13 to 111,703.75)	2,490.40 (2,108.64 to 2,969.32)	2,770.11 (1,911.85 to 4,008.30)	1,249.18 (887.98 to 1,757.41)	3,200.15 (2,161.43 to 4,691.69)
HIV/AIDS	29,521.44 (1,572.78 to 160,189.23)	28,864.73 (406.82 to 157,179.65)	22,162.36 (57.00 to 153,358.93)	35,766.45 (2,525.55 to 159,826.59)	649.03 (31.02 to 3,684.55)	692.92 (8.46 to 4,022.76)	553.37 (1.58 to 3,834.10)	830.29 (52.94 to 4,175.39)
**Diarrhea, lower respiratory, and other common infectious diseases**	**362,828.83 (251,004.38 to 533,014.81)**	**144,454.40 (87,568.55 to 237,900.20)**	**82,753.85 (54,639.30 to 124,249.64)**	**208,656.93 (120,367.09 to 362,142.55)**	**7,279.28 (5,649.39 to 9,968.01)**	**6,865.94 (4,255.54 to 10,618.78)**	**4,792.58 (3,184.51 to 7,091.41)**	**8,234.93 (5,108.76 to 12,765.35)**
Diarrheal diseases	58,715.53 (32,271.83 to 103,242.24)	12,288.20 (1,969.99 to 31,695.17)	3,849.77 (738.08 to 11,071.46)	15,360.42 (2,731.43 to 38,668.69)	784.20 (462.11 to 1,301.35)	317.57 (63.18 to 878.83)	120.30 (28.70 to 349.91)	363.01 (76.44 to 1,000.83)
Intestinal infectious diseases	2,833.72 (1,459.48 to 5,000.86)	1,366.53 (665.15 to 2,431.12)	1,058.21 (516.71 to 1,852.79)	1,443.05 (689.04 to 2,550.79)	43.30 (22.73 to 75.11)	24.63 (12.57 to 42.63)	19.82 (10.16 to 33.72)	25.40 (12.64 to 44.16)
Typhoid fever	2,412.15 (1,242.34 to 4,278.36)	1,106.90 (527.07 to 2,054.39)	865.36 (411.92 to 1,544.99)	1,169.77 (548.02 to 2,155.57)	36.59 (19.01 to 64.36)	19.77 (10.01 to 34.88)	16.08 (8.33 to 27.54)	20.38 (10.20 to 36.12)
Paratyphoid fever	416.15 (178.87 to 819.17)	258.06 (108.61 to 512.00)	191.95 (81.88 to 379.81)	271.65 (114.36 to 545.45)	6.61 (2.88 to 13.01)	4.81 (2.09 to 9.39)	3.70 (1.61 to 7.13)	4.96 (2.13 to 9.70)
Other intestinal infectious diseases	5.42 (1.93 to 12.82)	1.57 (0.59 to 3.66)	0.90 (0.33 to 2.05)	1.63 (0.61 to 3.77)	0.10 (0.04 to 0.21)	0.06 (0.02 to 0.12)	0.04 (0.01 to 0.08)	0.06 (0.02 to 0.12)
Lower respiratory infections	255,737.53 (171,771.35 to 380,590.37)	109,264.74 (62,797.23 to 182,296.23)	62,791.28 (39,985.22 to 95,129.52)	150,204.78 (80,537.74 to 268,528.02)	5,769.27 (4,484.17 to 7,911.62)	6,096.94 (3,669.62 to 9,577.84)	4,320.20 (2,796.21 to 6,549.40)	7,191.16 (4,246.21 to 11,406.92)
Upper respiratory infections	784.91 (380.68 to 1,583.96)	595.49 (271.72 to 1,093.17)	301.70 (133.87 to 554.49)	626.07 (287.92 to 1,157.38)	17.27 (9.35 to 29.07)	21.39 (9.65 to 40.41)	11.84 (5.21 to 22.79)	21.45 (9.37 to 39.81)
Otitis media	11.37 (4.90 to 29.45)	2.79 (1.03 to 7.96)	2.95 (1.09 to 8.47)	2.72 (1.01 to 7.75)	0.29 (0.13 to 0.73)	0.11 (0.04 to 0.32)	0.13 (0.05 to 0.37)	0.10 (0.04 to 0.29)
Meningitis	15,521.94 (9,897.91 to 23,473.20)	4,777.06 (3,139.50 to 7,455.17)	4,166.10 (2,757.86 to 6,539.21)	5,309.91 (3,298.25 to 8,450.60)	259.86 (184.84 to 381.19)	133.77 (98.13 to 209.54)	128.34 (94.40 to 199.06)	135.06 (97.20 to 209.98)
Pneumococcal meningitis	909.18 (551.16 to 1,436.89)	178.52 (120.20 to 281.39)	184.74 (125.37 to 291.74)	224.30 (142.94 to 358.98)	15.94 (11.07 to 24.06)	6.07 (4.35 to 9.52)	7.03 (5.11 to 10.89)	6.85 (4.82 to 10.62)
H influenzae type B meningitis	1,016.58 (615.76 to 1,589.54)	283.38 (147.27 to 566.89)	277.42 (149.03 to 544.64)	721.76 (189.25 to 2,066.15)	16.51 (11.16 to 24.59)	6.95 (4.58 to 11.39)	7.57 (5.11 to 12.19)	11.77 (5.07 to 27.59)
Meningococcal meningitis	5,590.16 (3,604.19 to 8,533.27)	1,875.80 (1,253.20 to 2,931.72)	1,619.54 (1,094.93 to 2,536.22)	1,881.17 (1,254.50 to 2,965.16)	100.99 (72.74 to 148.93)	59.47 (42.98 to 92.14)	56.85 (41.28 to 87.95)	56.59 (41.19 to 88.11)
Other meningitis	8,006.02 (4,976.60 to 12,534.82)	2,439.35 (1,573.45 to 3,849.15)	2,084.40 (1,343.48 to 3,276.87)	2,482.67 (1,581.69 to 4,045.70)	126.42 (87.16 to 187.17)	61.28 (43.88 to 97.40)	56.89 (41.32 to 91.27)	59.84 (42.41 to 94.85)
Encephalitis	14,020.80 (8,222.62 to 22,670.55)	5,835.76 (3,112.79 to 9,977.59)	4,277.85 (2,370.03 to 7,248.64)	6,188.98 (3,197.33 to 10,437.31)	198.21 (125.50 to 309.82)	119.67 (79.98 to 180.57)	94.09 (64.08 to 139.40)	121.91 (78.54 to 187.05)

Diphtheria	49.34 (24.99 to 90.78)	6.24 (3.18 to 11.95)	6.03 (3.08 to 11.36)	40.24 (4.97 to 134.61)	0.73 (0.42 to 1.23)	0.12 (0.06 to 0.21)	0.11 (0.06 to 0.20)	0.52 (0.10 to 1.65)
Whooping cough	11,287.47 (615.88 to 42,212.35)	2,800.63 (81.97 to 11,882.97)	2,370.44 (72.80 to 9,292.18)	15,470.58 (313.68 to 51,490.76)	133.55 (7.29 to 499.17)	33.39 (1.03 to 141.49)	28.31 (0.96 to 111.46)	182.63 (3.90 to 606.87)
Tetanus	2,071.04 (809.69 to 4,369.54)	156.38 (53.50 to 336.08)	155.34 (53.46 to 323.90)	534.47 (158.66 to 1,439.20)	38.56 (15.23 to 75.89)	3.92 (1.28 to 7.82)	4.19 (1.35 to 8.42)	8.16 (2.71 to 18.76)
Measles	440.20 (114.64 to 1,172.01)	6,746.94 (99.91 to 47,638.18)	3,419.95 (89.21 to 20,444.97)	12,833.01 (136.28 to 90,692.14)	5.24 (1.37 to 13.92)	87.55 (1.63 to 558.43)	45.92 (1.41 to 242.38)	160.52 (2.02 to 1,065.51)

North Korea								
Cause	YLLs (actual counts)				Deaths (actual counts)			
	2016	2040 Forecast	2040 Better Scenario	2040 Worse Scenario	2016	2040 Forecast	2040 Better Scenario	2040 Worse Scenario
Varicella and herpes zoster	1,354.99 (738.65 to 2,367.22)	613.65 (331.88 to 1,020.99)	354.24 (202.28 to 583.46)	642.70 (335.58 to 1,130.14)	28.80 (19.00 to 41.94)	26.89 (16.04 to 41.12)	19.34 (11.23 to 30.44)	25.03 (14.61 to 38.86)
**Neglected tropical diseases and malaria**	**2,275.63 (905.58 to 3,930.26)**	**1,424.56 (567.55 to 2,357.39)**	**743.12 (301.68 to 1,222.29)**	**1,529.90 (608.04 to 2,576.37)**	**45.53 (18.41 to 74.71)**	**38.46 (16.27 to 60.71)**	**21.85 (9.27 to 34.44)**	**39.83 (16.43 to 63.66)**
Malaria	95.65 (79.15 to 117.45)	57.24 (47.19 to 70.08)	52.38 (39.70 to 66.38)	56.66 (46.62 to 70.09)	2.39 (2.03 to 2.85)	1.81 (1.53 to 2.18)	1.77 (1.39 to 2.23)	1.74 (1.47 to 2.10)
Chagas disease	0.00 (0.00 to 0.00)	0.00 (0.00 to 0.00)	0.00 (0.00 to 0.00)	0.00 (0.00 to 0.00)	0.00 (0.00 to 0.00)	0.00 (0.00 to 0.00)	0.00 (0.00 to 0.00)	0.00 (0.00 to 0.00)
Leishmaniasis	0.00 (0.00 to 0.00)	0.00 (0.00 to 0.00)	0.00 (0.00 to 0.00)	0.00 (0.00 to 0.00)	0.00 (0.00 to 0.00)	0.00 (0.00 to 0.00)	0.00 (0.00 to 0.00)	0.00 (0.00 to 0.00)
African trypanosomiasis	0.00 (0.00 to 0.00)	0.00 (0.00 to 0.00)	0.00 (0.00 to 0.00)	0.00 (0.00 to 0.00)	0.00 (0.00 to 0.00)	0.00 (0.00 to 0.00)	0.00 (0.00 to 0.00)	0.00 (0.00 to 0.00)
Schistosomiasis	0.00 (0.00 to 0.00)	0.00 (0.00 to 0.00)	0.00 (0.00 to 0.00)	0.00 (0.00 to 0.00)	0.00 (0.00 to 0.00)	0.00 (0.00 to 0.00)	0.00 (0.00 to 0.00)	0.00 (0.00 to 0.00)
Cysticercosis	72.93 (62.49 to 84.52)	27.54 (22.82 to 32.95)	23.54 (17.80 to 30.07)	27.55 (22.99 to 32.73)	1.87 (1.62 to 2.13)	0.85 (0.71 to 1.01)	0.76 (0.59 to 0.97)	0.83 (0.70 to 0.99)
Cystic echinococcosis	41.49 (32.07 to 52.51)	29.92 (22.31 to 40.35)	11.98 (8.34 to 16.47)	31.78 (23.56 to 42.08)	1.10 (0.87 to 1.39)	1.08 (0.80 to 1.43)	0.46 (0.34 to 0.63)	1.10 (0.83 to 1.46)
Dengue	0.00 (0.00 to 0.00)	0.00 (0.00 to 0.00)	0.00 (0.00 to 0.00)	0.00 (0.00 to 0.00)	0.00 (0.00 to 0.00)	0.00 (0.00 to 0.00)	0.00 (0.00 to 0.00)	0.00 (0.00 to 0.00)
Yellow fever	0.00 (0.00 to 0.00)	0.00 (0.00 to 0.00)	0.00 (0.00 to 0.00)	0.00 (0.00 to 0.00)	0.00 (0.00 to 0.00)	0.00 (0.00 to 0.00)	0.00 (0.00 to 0.00)	0.00 (0.00 to 0.00)
Rabies	1,084.76 (69.03 to 2,640.63)	646.97 (45.49 to 1,477.65)	211.34 (9.39 to 544.60)	723.29 (47.53 to 1,661.98)	21.01 (1.41 to 49.16)	15.50 (1.12 to 34.45)	5.30 (0.25 to 13.46)	16.88 (1.15 to 37.74)
Intestinal nematode infections	172.85 (44.29 to 396.86)	92.59 (36.87 to 189.73)	86.50 (35.24 to 184.30)	96.69 (36.61 to 199.69)	2.55 (0.97 to 5.22)	1.95 (0.99 to 3.29)	2.01 (0.99 to 3.39)	1.93 (0.96 to 3.25)
Other neglected tropical diseases	807.94 (378.10 to 1,317.88)	570.30 (280.93 to 792.46)	357.38 (145.37 to 503.02)	593.94 (298.88 to 830.29)	16.61 (7.67 to 24.46)	17.27 (7.79 to 23.68)	11.55 (4.35 to 16.16)	17.36 (7.96 to 23.67)
**Maternal disorders**	**27,324.90 (19,772.53 to 35,760.01)**	**20,767.08 (13,929.39 to 29,530.89)**	**9,015.59 (5,870.05 to 13,313.74)**	**22,537.62 (14,839.09 to 32,748.58)**	**488.06 (355.77 to 637.01)**	**376.52 (252.41 to 531.46)**	**163.43 (106.74 to 238.58)**	**408.41 (272.49 to 588.12)**
Maternal hemorrhage	11,670.57 (7,549.31 to 16,492.22)	8,404.38 (4,600.00 to 13,737.63)	3,606.09 (1,928.94 to 5,866.99)	9,104.08 (5,026.80 to 15,005.64)	207.63 (134.63 to 292.64)	151.51 (84.85 to 241.25)	65.00 (35.14 to 105.32)	164.08 (90.96 to 265.41)
Maternal sepsis and other maternal infections	1,396.64 (690.22 to 2,468.50)	924.86 (424.91 to 1,750.21)	395.49 (180.82 to 758.12)	1,001.70 (459.70 to 1,875.47)	27.28 (13.61 to 47.41)	18.37 (8.55 to 34.30)	7.86 (3.62 to 14.66)	19.89 (8.98 to 37.12)
Maternal hypertensive disorders	2,883.74 (1,640.70 to 4,703.66)	2,196.41 (1,117.94 to 3,967.22)	932.52 (465.65 to 1,722.97)	2,382.27 (1,206.89 to 4,315.60)	51.14 (28.93 to 83.23)	39.56 (20.19 to 70.60)	16.80 (8.50 to 30.83)	42.90 (21.54 to 76.97)
Maternal obstructed labor and uterine rupture	1,088.00 (518.30 to 1,931.16)	839.20 (336.68 to 1,713.03)	337.60 (133.49 to 697.36)	914.49 (361.51 to 1,911.57)	19.60 (9.34 to 34.86)	15.35 (6.21 to 31.48)	6.17 (2.46 to 12.89)	16.72 (6.54 to 34.46)
Maternal abortion, miscarriage, and ectopic pregnancy	2,690.68 (1,322.57 to 4,491.85)	2,178.14 (966.32 to 4,101.82)	931.98 (402.93 to 1,787.83)	2,363.76 (1,042.69 to 4,493.28)	48.76 (24.37 to 81.15)	40.28 (18.53 to 73.77)	17.23 (7.74 to 32.29)	43.69 (19.98 to 81.23)

Indirect maternal deaths	3,218.23 (1,756.74 to 5,057.76)	2,778.10 (1,285.44 to 4,865.01)	1,230.79 (559.58 to 2,177.19)	3,030.64 (1,413.25 to 5,329.79)	55.88 (30.80 to 87.61)	49.14 (23.00 to 85.78)	21.77 (9.93 to 37.61)	53.57 (24.97 to 94.08)
Late maternal deaths	363.80 (140.53 to 690.61)	238.61 (87.60 to 489.56)	103.07 (36.32 to 216.12)	259.79 (93.11 to 547.20)	6.47 (2.56 to 12.23)	4.31 (1.58 to 8.94)	1.86 (0.66 to 3.94)	4.69 (1.71 to 10.01)
Maternal deaths aggravated by HIV/AIDS	4.58 (0.20 to 26.60)	54.03 (0.13 to 404.69)	24.17 (0.00 to 193.72)	61.38 (0.57 to 440.81)	0.08 (0.00 to 0.48)	0.96 (0.00 to 6.94)	0.43 (0.00 to 3.31)	1.08 (0.01 to 7.67)
Other maternal disorders	4,008.66 (2,244.46 to 6,084.94)	3,153.35 (1,603.66 to 5,545.09)	1,453.87 (733.70 to 2,577.84)	3,419.50 (1,701.54 to 6,035.04)	71.22 (39.95 to 108.33)	57.04 (28.91 to 99.73)	26.30 (13.54 to 46.47)	61.80 (31.31 to 110.30)

North Korea								
Cause	YLLs (actual counts)				Deaths (actual counts)			
	2016	2040 Forecast	2040 Better Scenario	2040 Worse Scenario	2016	2040 Forecast	2040 Better Scenario	2040 Worse Scenario
**Neonatal disorders**	**576,894.27 (374,929.52 to 863,542.00)**	**145,160.32 (81,589.17 to 234,008.19)**	**85,056.18 (48,122.59 to 137,786.46)**	**168,443.74 (94,941.00 to 271,132.78)**	**6,667.20 (4,332.52 to 9,983.92)**	**1,678.14 (943.11 to 2,703.75)**	**983.38 (556.35 to 1,592.58)**	**1,947.26 (1,097.21 to 3,134.39)**
Neonatal preterm birth	283,479.92 (161,282.68 to 460,106.34)	70,017.13 (38,501.44 to 118,584.62)	37,926.37 (19,960.23 to 64,845.75)	82,497.66 (44,352.95 to 139,773.68)	3,275.60 (1,864.52 to 5,318.02)	809.18 (445.06 to 1,370.20)	438.38 (230.71 to 749.55)	953.38 (512.55 to 1,615.27)
Neonatal encephalopathy due to birth asphyxia and trauma	212,771.84 (116,025.01 to 342,568.89)	50,594.40 (20,836.87 to 93,705.11)	33,048.02 (13,957.20 to 60,456.17)	57,795.12 (23,773.32 to 107,135.88)	2,458.69 (1,340.64 to 3,957.92)	584.87 (241.09 to 1,083.00)	382.04 (161.46 to 698.87)	668.11 (274.93 to 1,238.35)
Neonatal sepsis and other neonatal infections	12,853.11 (4,801.20 to 24,614.53)	4,796.69 (1,795.49 to 9,797.26)	3,047.25 (1,133.90 to 6,319.16)	5,439.60 (2,034.63 to 11,342.77)	148.60 (55.53 to 284.58)	55.47 (20.75 to 113.41)	35.24 (13.11 to 73.04)	62.91 (23.52 to 131.17)
Hemolytic disease and other neonatal jaundice	12,102.99 (5,844.40 to 22,778.62)	2,145.23 (860.06 to 4,603.95)	1,206.35 (484.25 to 2,541.30)	2,467.79 (990.44 to 5,318.00)	139.96 (67.56 to 263.39)	24.81 (9.96 to 53.27)	13.95 (5.60 to 29.38)	28.54 (11.45 to 61.52)
Other neonatal disorders	55,686.40 (29,399.13 to 96,947.94)	17,606.87 (8,105.22 to 33,755.64)	9,828.20 (4,544.18 to 18,749.56)	20,243.57 (9,268.38 to 38,697.72)	644.36 (340.37 to 1,121.05)	203.80 (93.87 to 390.85)	113.76 (52.59 to 216.96)	234.31 (107.31 to 448.00)
**Nutritional deficiencies**	**13,961.46 (9,400.97 to 21,622.73)**	**7,504.55 (5,796.77 to 9,916.92)**	**5,878.26 (4,599.38 to 7,680.00)**	**7,252.98 (5,488.98 to 9,843.44)**	**433.73 (355.68 to 540.36)**	**542.41 (386.69 to 722.51)**	**504.78 (373.25 to 663.32)**	**477.58 (330.50 to 655.18)**
Protein-energy malnutrition	10,899.61 (6,737.90 to 17,566.51)	5,116.28 (3,569.28 to 7,278.05)	4,023.59 (2,888.62 to 5,598.70)	4,930.15 (3,383.91 to 7,138.94)	335.79 (267.06 to 430.83)	397.87 (261.98 to 552.12)	374.33 (249.44 to 515.32)	347.86 (221.77 to 494.36)
Iodine deficiency	299.63 (26.70 to 1,163.20)	439.73 (33.71 to 1,430.16)	298.05 (17.51 to 954.67)	435.47 (35.38 to 1,539.20)	8.46 (0.83 to 27.64)	27.05 (0.93 to 99.87)	21.04 (0.55 to 87.00)	24.85 (0.94 to 90.38)
Dietary iron deficiency	299.96 (214.79 to 397.94)	267.71 (163.11 to 426.88)	220.69 (132.27 to 360.14)	264.42 (165.31 to 412.50)	7.93 (4.94 to 13.17)	11.56 (5.21 to 23.13)	10.68 (4.55 to 22.56)	10.76 (4.96 to 21.33)
Other nutritional deficiencies	2,462.26 (1,468.70 to 4,049.88)	1,680.83 (1,117.57 to 2,374.07)	1,335.93 (888.65 to 1,886.58)	1,622.94 (1,057.74 to 2,300.98)	81.55 (53.01 to 113.48)	105.93 (68.31 to 156.96)	98.73 (63.57 to 145.43)	94.12 (58.88 to 141.18)
**Other communicable, maternal, neonatal, and nutritional diseases**	**20,723.55 (15,549.54 to 29,824.63)**	**14,449.75 (11,495.90 to 18,324.25)**	**10,580.97 (8,376.95 to 13,371.88)**	**14,567.26 (11,465.99 to 18,623.41)**	**500.00 (407.59 to 620.88)**	**535.15 (431.78 to 654.74)**	**420.59 (336.89 to 517.56)**	**518.83 (412.66 to 640.78)**
Sexually transmitted infections excluding HIV	4,486.29 (1,740.92 to 9,994.31)	779.25 (385.82 to 1,589.16)	572.79 (304.79 to 1,111.79)	819.79 (399.95 to 1,659.23)	63.45 (30.89 to 129.06)	17.48 (11.18 to 28.19)	14.05 (9.26 to 21.88)	17.60 (11.08 to 29.11)
Syphilis	4,147.53 (1,449.66 to 9,590.91)	562.78 (196.30 to 1,326.28)	398.65 (145.08 to 915.59)	603.22 (200.08 to 1,437.71)	52.65 (20.88 to 116.86)	9.49 (4.67 to 18.77)	7.21 (3.77 to 13.87)	9.82 (4.73 to 19.40)
Chlamydial infection	62.70 (44.05 to 100.01)	40.18 (27.48 to 61.96)	32.17 (21.86 to 49.26)	40.22 (27.13 to 62.01)	1.99 (1.41 to 3.12)	1.48 (1.04 to 2.25)	1.28 (0.90 to 1.97)	1.44 (1.02 to 2.19)
Gonococcal infection	184.63 (130.55 to 287.70)	120.18 (80.60 to 186.71)	97.38 (65.42 to 147.33)	119.93 (79.91 to 184.55)	6.10 (4.34 to 9.29)	4.61 (3.18 to 6.94)	3.98 (2.75 to 5.98)	4.48 (3.08 to 6.77)
Other sexually transmitted infections	91.43 (64.83 to 143.46)	56.12 (38.08 to 84.86)	44.60 (30.54 to 67.05)	56.41 (38.18 to 85.15)	2.71 (1.93 to 4.14)	1.89 (1.31 to 2.83)	1.58 (1.09 to 2.32)	1.86 (1.28 to 2.76)
Acute hepatitis	11,811.25 (9,531.20 to 14,600.61)	9,803.12 (7,370.54 to 12,605.56)	7,242.86 (5,389.71 to 9,275.34)	9,837.14 (7,358.94 to 12,844.89)	339.32 (282.53 to 408.12)	367.06 (279.22 to 461.95)	286.11 (216.26 to 363.91)	357.80 (270.90 to 456.75)
Acute hepatitis A	1,285.40 (621.06 to 2,292.37)	271.50 (138.80 to 514.95)	191.50 (102.69 to 354.26)	285.33 (142.86 to 546.65)	17.46 (9.45 to 29.11)	6.21 (3.91 to 9.83)	4.83 (3.07 to 7.44)	6.21 (3.87 to 9.85)
Acute hepatitis B	9,579.44 (7,625.04 to 11,959.10)	8,788.09 (6,454.49 to 11,426.35)	6,415.66 (4,691.79 to 8,377.47)	8,824.35 (6,397.26 to 11,713.27)	294.59 (241.49 to 355.43)	325.99 (244.27 to 417.75)	248.33 (183.09 to 323.36)	319.32 (236.44 to 409.72)

Acute hepatitis C	156.18 (105.16 to 225.17)	168.69 (106.29 to 264.58)	149.84 (96.17 to 231.95)	165.10 (103.42 to 261.30)	5.50 (3.73 to 7.91)	7.04 (4.48 to 10.85)	6.55 (4.25 to 9.84)	6.71 (4.22 to 10.39)
Acute hepatitis E	790.23 (556.05 to 1,119.83)	574.83 (343.54 to 934.57)	485.87 (288.80 to 791.50)	562.36 (337.46 to 912.85)	21.78 (13.94 to 33.81)	27.82 (14.92 to 47.72)	26.41 (13.92 to 45.18)	25.55 (13.76 to 43.47)
Other unspecified infectious diseases	4,426.01 (2,173.06 to 11,347.51)	3,867.38 (2,497.73 to 6,022.92)	2,765.31 (1,794.78 to 4,272.62)	3,910.33 (2,506.84 to 6,185.11)	97.23 (55.95 to 183.15)	150.61 (99.16 to 224.21)	120.42 (78.64 to 183.00)	143.43 (92.80 to 216.46)
**Non-communicable diseases**	**4,441,525.35 (4,102,385.26 to 4,800,312.92)**	**5,582,339.56 (4,540,703.62 to 6,574,321.14)**	**3,642,199.43 (2,986,520.52 to 4,241,979.49)**	**6,682,812.33 (5,326,705.96 to 7,932,107.23)**	**194,506.37 (181,766.46 to 206,514.89)**	**308,934.87 (268,718.57 to 347,476.20)**	**214,966.42 (186,873.15 to 241,006.14)**	**356,486.22 (305,584.04 to 399,938.10)**

North Korea								
Cause	YLLs (actual counts)				Deaths (actual counts)			
	2016	2040 Forecast	2040 Better Scenario	2040 Worse Scenario	2016	2040 Forecast	2040 Better Scenario	2040 Worse Scenario
**Neoplasms**	**1,308,770.61 (1,188,358.08 to 1,438,673.68)**	**1,663,721.18 (1,311,444.57 to 2,036,922.73)**	**1,409,987.95 (1,126,445.27 to 1,697,199.04)**	**1,818,894.04 (1,440,429.51 to 2,216,083.53)**	**49,006.04 (44,700.37 to 53,387.23)**	**73,992.49 (59,475.63 to 89,614.32)**	**64,851.13 (53,494.56 to 76,854.48)**	**80,333.93 (64,414.80 to 97,858.22)**
Lip and oral cavity cancer	7,508.66 (5,328.49 to 11,103.06)	10,210.91 (5,576.29 to 18,704.75)	7,139.45 (3,932.00 to 12,692.97)	12,875.80 (6,736.16 to 23,828.68)	279.88 (199.46 to 406.13)	432.83 (238.81 to 781.38)	311.45 (173.86 to 541.36)	542.86 (286.17 to 998.78)
Nasopharynx cancer	16,518.50 (11,138.40 to 21,942.97)	23,291.86 (11,779.36 to 40,499.32)	15,427.33 (7,908.31 to 27,176.78)	29,603.06 (14,987.15 to 52,459.87)	514.15 (350.80 to 678.23)	800.64 (408.10 to 1,369.07)	534.00 (274.44 to 927.05)	1,016.45 (509.23 to 1,765.45)
Other pharynx cancer	1,930.43 (1,492.53 to 2,669.10)	2,290.51 (1,347.52 to 3,844.93)	2,030.31 (1,240.60 to 3,345.35)	2,582.39 (1,461.59 to 4,391.56)	69.81 (54.55 to 94.91)	100.42 (59.62 to 166.70)	93.92 (58.17 to 149.80)	109.70 (62.64 to 188.69)
Esophageal cancer	81,938.39 (71,919.78 to 93,374.89)	118,075.67 (66,008.17 to 196,621.80)	81,188.96 (46,637.42 to 134,583.06)	142,679.72 (78,451.61 to 241,998.28)	3,545.10 (3,127.16 to 4,008.22)	5,689.61 (3,246.87 to 9,303.25)	4,013.04 (2,376.87 to 6,422.79)	6,832.41 (3,818.49 to 11,258.17)
Stomach cancer	194,998.99 (170,011.59 to 228,625.99)	183,357.30 (133,030.25 to 246,614.91)	159,787.80 (118,383.31 to 211,396.27)	193,335.02 (134,932.82 to 265,929.07)	7,852.16 (6,842.80 to 9,162.67)	8,696.39 (6,436.88 to 11,460.05)	7,961.12 (6,015.41 to 10,338.48)	9,016.04 (6,482.29 to 12,194.66)
Colon and rectum cancer	65,635.10 (54,877.17 to 79,705.91)	98,569.86 (69,146.12 to 139,015.82)	104,089.29 (73,800.17 to 145,029.88)	100,304.91 (69,424.68 to 142,135.62)	2,642.18 (2,210.19 to 3,177.61)	4,858.74 (3,442.85 to 6,836.33)	5,466.01 (3,877.35 to 7,616.71)	4,790.72 (3,332.62 to 6,717.85)
Liver cancer	183,217.16 (145,482.43 to 237,392.33)	243,056.97 (156,181.02 to 377,589.16)	232,070.76 (152,415.54 to 351,880.32)	261,224.81 (160,678.64 to 419,238.64)	6,509.57 (5,204.14 to 8,352.59)	10,116.93 (6,775.16 to 15,319.59)	10,022.33 (6,959.48 to 14,732.88)	10,695.34 (6,875.16 to 16,700.42)
Liver cancer due to hepatitis B	109,762.16 (85,271.58 to 145,057.41)	139,675.25 (86,697.23 to 220,348.65)	134,509.73 (86,137.89 to 208,767.46)	149,048.27 (90,270.69 to 240,796.03)	3,676.26 (2,887.42 to 4,818.82)	5,472.90 (3,618.15 to 8,320.88)	5,451.06 (3,709.21 to 8,084.48)	5,751.57 (3,618.61 to 9,096.97)
Liver cancer due to hepatitis C	24,517.22 (17,560.64 to 32,781.59)	36,620.68 (22,875.33 to 56,118.45)	35,741.89 (22,949.50 to 55,140.10)	38,643.21 (23,296.77 to 59,808.92)	1,041.00 (768.91 to 1,398.28)	1,807.22 (1,164.07 to 2,731.34)	1,847.82 (1,242.27 to 2,709.72)	1,868.64 (1,147.99 to 2,821.71)
Liver cancer due to alcohol use	13,707.03 (9,568.44 to 19,004.01)	18,695.90 (11,939.05 to 28,752.25)	16,648.63 (10,845.55 to 24,989.40)	21,869.46 (13,461.16 to 34,003.05)	578.84 (398.48 to 814.90)	873.14 (560.92 to 1,333.12)	813.62 (530.65 to 1,213.89)	997.07 (626.78 to 1,560.11)
Liver cancer due to other causes	35,230.75 (26,720.91 to 47,851.08)	48,065.14 (28,398.70 to 79,706.61)	45,170.51 (27,767.82 to 72,621.52)	51,663.86 (29,362.67 to 88,579.21)	1,213.46 (926.19 to 1,632.05)	1,963.66 (1,216.30 to 3,149.95)	1,909.83 (1,240.83 to 2,927.47)	2,078.06 (1,223.38 to 3,433.85)
Gallbladder and biliary tract cancer	10,947.52 (8,405.02 to 14,055.70)	10,689.70 (8,116.73 to 13,712.24)	12,189.80 (9,221.69 to 15,798.02)	9,987.23 (7,496.68 to 12,939.62)	485.68 (377.57 to 617.49)	560.06 (428.16 to 720.52)	677.25 (525.99 to 878.31)	507.69 (383.47 to 658.22)
Pancreatic cancer	26,407.96 (23,263.74 to 29,951.91)	42,383.22 (32,392.37 to 54,322.15)	44,259.45 (33,911.70 to 55,908.00)	43,053.51 (32,620.55 to 55,650.76)	1,091.64 (959.93 to 1,238.70)	2,006.25 (1,574.97 to 2,542.88)	2,182.85 (1,741.51 to 2,731.61)	2,008.55 (1,557.36 to 2,578.45)
Larynx cancer	10,975.88 (7,478.52 to 13,756.12)	13,736.30 (6,868.89 to 24,177.80)	10,060.25 (5,232.45 to 16,813.72)	16,166.37 (7,953.79 to 28,555.48)	425.08 (302.38 to 523.02)	602.11 (310.48 to 1,031.41)	447.28 (236.25 to 740.67)	708.88 (354.08 to 1,233.27)
Tracheal, bronchus, and lung cancer	405,414.06 (334,575.09 to 477,514.94)	575,955.99 (370,341.65 to 814,280.33)	394,302.53 (253,236.65 to 560,126.98)	665,762.19 (439,885.01 to 929,566.28)	16,142.94 (13,513.79 to 18,904.93)	26,051.29 (16,791.28 to 37,388.29)	17,850.23 (11,519.97 to 25,727.62)	30,508.58 (20,074.43 to 43,260.43)
Malignant skin melanoma	2,259.32 (1,733.63 to 3,050.82)	2,488.66 (1,931.02 to 3,375.28)	3,098.50 (2,376.00 to 4,202.45)	2,350.10 (1,819.64 to 3,189.61)	78.17 (61.82 to 102.52)	106.09 (84.05 to 138.96)	142.17 (112.49 to 185.65)	96.55 (76.03 to 127.50)
Non-melanoma skin cancer	5,174.06 (4,187.68 to 6,118.60)	8,630.77 (6,963.36 to 10,296.12)	8,343.06 (6,713.34 to 9,923.93)	8,163.57 (6,541.82 to 9,764.34)	221.25 (182.81 to 257.22)	459.11 (375.81 to 543.37)	481.10 (399.98 to 561.63)	417.19 (332.15 to 500.54)
Breast cancer	43,489.71 (31,370.68 to 65,753.03)	48,749.98 (29,466.46 to 79,182.13)	47,386.71 (29,740.67 to 75,872.16)	51,635.59 (30,976.93 to 84,193.10)	1,446.96 (1,052.70 to 2,152.81)	1,893.31 (1,199.72 to 2,993.22)	1,929.62 (1,261.64 to 2,966.13)	1,966.38 (1,215.55 to 3,152.55)
Cervical cancer	32,980.96 (20,996.15 to 52,433.92)	29,229.73 (14,731.21 to 47,980.49)	23,413.48 (12,150.22 to 38,512.48)	30,472.17 (15,101.69 to 50,898.21)	1,096.29 (677.20 to 1,782.58)	1,110.38 (572.76 to 1,842.26)	929.19 (492.12 to 1,513.96)	1,138.98 (575.62 to 1,942.41)

Uterine cancer	11,181.01 (8,749.03 to 14,263.56)	14,827.08 (11,348.22 to 19,353.02)	12,349.08 (9,347.54 to 16,262.32)	14,722.09 (11,280.77 to 19,456.93)	412.47 (324.04 to 525.56)	632.28 (491.29 to 834.52)	553.52 (434.45 to 724.99)	614.09 (475.53 to 812.62)
Ovarian cancer	9,413.94 (7,476.47 to 11,993.76)	11,355.51 (8,455.34 to 15,336.26)	13,201.56 (9,703.84 to 17,725.59)	11,160.52 (8,309.07 to 15,111.78)	331.55 (266.40 to 417.21)	458.23 (350.90 to 611.71)	553.89 (424.17 to 732.97)	442.38 (335.16 to 595.09)
Prostate cancer	7,715.43 (5,266.58 to 10,060.32)	19,150.22 (13,321.40 to 25,129.50)	23,680.36 (16,513.85 to 30,689.03)	17,274.13 (11,920.72 to 22,854.10)	426.52 (293.47 to 543.76)	1,276.16 (893.91 to 1,674.31)	1,694.56 (1,197.25 to 2,152.47)	1,109.05 (747.08 to 1,487.64)
Testicular cancer	936.53 (594.34 to 1,413.94)	933.42 (574.32 to 1,414.00)	878.02 (546.26 to 1,336.72)	910.63 (557.79 to 1,387.37)	22.34 (15.00 to 32.73)	29.46 (19.49 to 44.28)	30.16 (20.16 to 44.73)	27.34 (17.91 to 41.15)

North Korea								
Cause	YLLs (actual counts)				Deaths (actual counts)			
	2016	2040 Forecast	2040 Better Scenario	2040 Worse Scenario	2016	2040 Forecast	2040 Better Scenario	2040 Worse Scenario
Kidney cancer	7,056.57 (4,865.18 to 9,550.68)	9,192.83 (5,328.85 to 14,855.41)	10,845.12 (6,355.70 to 17,403.05)	9,063.24 (5,277.64 to 14,781.08)	242.11 (166.17 to 328.58)	405.68 (237.54 to 647.96)	502.77 (298.62 to 779.49)	391.80 (229.92 to 635.99)
Bladder cancer	11,145.41 (8,340.69 to 14,068.82)	17,784.54 (11,277.57 to 27,021.87)	16,615.86 (10,749.40 to 24,819.60)	18,372.02 (11,297.44 to 29,015.74)	530.36 (404.08 to 660.18)	1,009.26 (652.60 to 1,511.87)	997.36 (668.51 to 1,433.06)	1,022.81 (637.21 to 1,565.08)
Brain and nervous system cancer	34,815.70 (21,246.71 to 45,467.67)	42,397.54 (25,438.33 to 56,390.73)	47,044.46 (27,806.75 to 62,697.26)	40,929.27 (24,775.36 to 53,848.24)	937.49 (564.69 to 1,217.28)	1,506.88 (882.51 to 1,997.46)	1,771.41 (1,028.84 to 2,366.45)	1,407.96 (844.20 to 1,871.08)
Thyroid cancer	2,958.02 (2,494.13 to 3,498.19)	3,700.28 (2,983.18 to 4,673.76)	3,510.94 (2,838.06 to 4,363.05)	3,673.04 (2,898.49 to 4,850.36)	117.52 (100.03 to 138.94)	180.58 (147.18 to 225.38)	182.80 (150.29 to 227.64)	173.05 (135.93 to 229.79)
Mesothelioma	1,320.82 (1,070.25 to 1,687.09)	1,510.80 (1,214.60 to 1,920.20)	1,479.37 (1,183.99 to 1,934.68)	1,461.18 (1,169.62 to 1,850.53)	44.58 (36.35 to 56.00)	61.07 (49.76 to 76.22)	62.90 (51.81 to 78.71)	57.42 (46.29 to 71.64)
Hodgkin lymphoma	3,131.26 (1,823.23 to 3,943.79)	3,094.38 (1,841.04 to 3,989.28)	2,369.74 (1,427.37 to 3,045.72)	3,076.59 (1,838.53 to 3,976.63)	88.46 (51.36 to 110.23)	112.34 (66.31 to 141.89)	90.99 (54.60 to 115.74)	108.20 (63.38 to 137.81)
Non-Hodgkin lymphoma	18,904.84 (15,707.82 to 22,469.00)	22,253.88 (18,317.41 to 26,723.42)	25,753.07 (20,741.75 to 31,132.19)	21,506.47 (17,740.04 to 26,196.17)	586.39 (488.51 to 687.76)	907.95 (745.36 to 1,093.03)	1,116.05 (904.51 to 1,334.75)	849.09 (698.47 to 1,033.55)
Multiple myeloma	3,730.37 (2,616.80 to 4,837.45)	5,361.44 (3,646.81 to 7,148.90)	6,476.88 (4,290.60 to 8,677.14)	5,132.88 (3,543.16 to 6,913.45)	139.67 (97.61 to 182.86)	242.06 (168.00 to 324.71)	307.38 (202.98 to 413.07)	225.58 (156.59 to 304.11)
Leukemia	67,725.90 (42,927.39 to 84,835.11)	54,682.60 (34,918.73 to 75,618.47)	50,462.20 (32,226.98 to 69,204.44)	56,758.45 (36,148.13 to 79,481.26)	1,500.37 (943.03 to 1,863.97)	1,701.99 (1,073.43 to 2,397.29)	1,639.53 (1,020.61 to 2,289.17)	1,727.44 (1,100.40 to 2,469.65)
Acute lymphoid leukemia	13,678.13 (9,282.11 to 20,211.90)	9,370.67 (6,249.02 to 13,484.07)	9,685.35 (6,530.18 to 13,185.69)	9,681.17 (6,458.33 to 14,105.70)	259.86 (171.30 to 379.55)	240.51 (155.33 to 337.99)	257.93 (165.46 to 352.24)	244.47 (157.06 to 348.85)
Chronic lymphoid leukemia	2,647.23 (1,873.47 to 4,087.89)	2,830.42 (1,918.20 to 4,175.58)	2,860.45 (1,968.91 to 4,103.40)	2,894.34 (1,932.63 to 4,337.13)	78.96 (53.20 to 118.73)	102.91 (67.48 to 155.52)	109.34 (72.80 to 157.32)	102.74 (66.32 to 158.04)
Acute myeloid leukemia	6,617.05 (4,419.42 to 9,387.38)	6,815.41 (4,553.54 to 9,778.57)	7,640.34 (5,143.33 to 10,710.91)	6,951.54 (4,666.33 to 10,043.37)	148.06 (96.76 to 204.91)	208.16 (141.09 to 303.47)	244.07 (164.35 to 344.47)	208.05 (140.60 to 306.33)
Chronic myeloid leukemia	1,602.52 (1,028.95 to 2,290.32)	1,193.80 (695.51 to 1,793.01)	1,173.98 (687.57 to 1,732.77)	1,228.37 (712.77 to 1,885.05)	43.98 (27.73 to 61.06)	39.80 (23.14 to 59.97)	40.76 (23.96 to 59.97)	40.17 (23.07 to 61.87)
Other leukemia	43,180.98 (22,383.90 to 59,581.74)	34,472.30 (17,672.56 to 52,312.31)	29,102.07 (14,347.96 to 45,251.16)	36,003.02 (18,482.47 to 55,088.43)	969.51 (485.30 to 1,311.88)	1,110.61 (560.87 to 1,705.96)	987.42 (472.47 to 1,527.88)	1,132.01 (570.94 to 1,754.04)
Other neoplasms	39,338.11 (33,331.42 to 45,111.27)	46,759.25 (39,308.22 to 53,922.21)	50,533.60 (42,067.97 to 58,311.22)	44,657.09 (37,509.30 to 51,610.33)	1,225.35 (1,031.50 to 1,395.89)	1,984.41 (1,628.55 to 2,317.68)	2,306.25 (1,899.74 to 2,707.65)	1,821.42 (1,495.56 to 2,150.06)
**Cardiovascular diseases**	**1,782,276.02 (1,633,159.19 to 1,922,511.38)**	**2,274,334.15 (1,601,488.75 to 3,196,351.58)**	**1,089,062.80 (773,676.08 to 1,517,841.22)**	**3,007,995.91 (2,009,908.09 to 4,244,202.83)**	**87,999.81 (81,783.88 to 94,430.05)**	**139,254.40 (104,923.87 to 181,080.82)**	**78,361.66 (60,408.92 to 104,160.70)**	**170,416.37 (124,258.30 to 226,662.22)**
Rheumatic heart disease	67,628.89 (51,200.53 to 88,066.32)	49,771.04 (36,534.33 to 66,167.62)	36,180.82 (25,996.45 to 47,929.64)	50,098.90 (36,191.22 to 67,969.81)	2,763.29 (2,063.40 to 3,704.57)	2,759.96 (1,978.99 to 3,686.62)	2,194.48 (1,577.68 to 2,909.63)	2,657.40 (1,876.82 to 3,553.59)
Ischemic heart disease	619,697.11 (532,637.51 to 701,822.31)	831,165.94 (491,870.73 to 1,323,816.97)	379,477.28 (226,622.54 to 614,998.29)	1,094,935.54 (623,262.28 to 1,721,151.37)	31,791.17 (26,973.68 to 35,882.89)	52,341.74 (33,446.86 to 80,439.54)	28,227.98 (18,349.65 to 43,517.92)	63,695.07 (39,990.57 to 98,307.73)
Stroke	949,374.33 (837,486.09 to 1,055,541.57)	1,170,117.92 (754,190.75 to 1,810,056.32)	486,894.91 (317,571.60 to 733,757.41)	1,607,628.82 (998,778.50 to 2,548,390.48)	46,224.20 (40,907.69 to 51,735.57)	68,861.38 (47,548.26 to 97,877.11)	33,825.24 (23,786.77 to 47,391.59)	87,445.13 (58,126.59 to 126,897.73)
Ischemic stroke	258,411.40 (221,801.80 to 296,427.82)	318,480.51 (193,786.42 to 513,999.21)	164,230.54 (101,631.32 to 268,010.12)	411,007.99 (239,356.31 to 665,286.12)	14,919.66 (12,783.44 to 17,081.98)	21,380.48 (13,546.28 to 34,455.49)	12,686.99 (8,351.00 to 20,509.03)	25,699.99 (15,689.91 to 41,684.46)

Intracerebral hemorrhage	690,962.93 (601,811.21 to 781,395.65)	851,637.42 (519,200.44 to 1,378,278.85)	322,664.37 (202,895.64 to 510,781.25)	1,196,620.83 (709,102.59 to 1,960,384.61)	31,304.54 (27,450.80 to 35,611.53)	47,480.91 (31,293.52 to 70,520.64)	21,138.26 (14,554.42 to 30,268.59)	61,745.15 (38,930.37 to 93,380.67)
Hypertensive heart disease	67,895.52 (51,227.38 to 85,456.55)	116,727.10 (68,845.30 to 223,323.92)	88,280.80 (53,509.33 to 159,438.79)	146,254.66 (78,843.74 to 312,512.33)	3,984.89 (2,936.08 to 5,074.70)	8,496.88 (5,028.16 to 16,704.66)	7,060.87 (4,351.21 to 13,332.60)	10,082.26 (5,382.52 to 22,006.20)
Cardiomyopathy and myocarditis	20,518.28 (15,064.33 to 27,177.90)	15,248.00 (10,740.92 to 21,483.18)	15,454.25 (10,714.36 to 21,959.85)	15,319.77 (10,810.07 to 21,921.92)	548.16 (413.14 to 702.25)	666.27 (461.58 to 927.07)	755.06 (516.95 to 1,076.14)	631.28 (439.18 to 886.26)
Myocarditis	9,103.87 (4,872.30 to 13,220.31)	6,143.99 (3,213.14 to 8,816.97)	5,859.17 (2,923.55 to 8,545.22)	5,966.52 (3,147.05 to 8,579.21)	234.15 (124.02 to 324.53)	272.87 (138.15 to 399.34)	298.01 (142.76 to 436.13)	247.09 (125.35 to 362.29)

North Korea								
Cause	YLLs (actual counts)				Deaths (actual counts)			
	2016	2040 Forecast	2040 Better Scenario	2040 Worse Scenario	2016	2040 Forecast	2040 Better Scenario	2040 Worse Scenario
Alcoholic cardiomyopathy	1,090.18 (569.54 to 1,697.24)	940.73 (464.41 to 1,514.24)	1,014.46 (476.75 to 1,653.69)	1,013.88 (500.00 to 1,610.59)	33.85 (18.90 to 52.97)	35.28 (18.67 to 56.41)	40.12 (20.07 to 65.75)	36.65 (19.41 to 58.37)
Other cardiomyopathy	10,324.23 (7,185.53 to 15,658.34)	8,163.28 (5,357.34 to 13,249.07)	8,580.62 (5,683.94 to 13,806.14)	8,339.38 (5,337.30 to 13,585.88)	280.16 (203.98 to 429.36)	358.11 (225.72 to 591.55)	416.94 (266.00 to 672.97)	347.54 (215.39 to 574.63)
Atrial fibrillation and flutter	7,617.86 (5,701.86 to 9,971.07)	14,854.45 (10,796.30 to 19,876.64)	17,234.83 (12,642.95 to 22,822.45)	14,460.15 (10,318.07 to 19,672.35)	648.18 (478.09 to 855.51)	1,390.94 (966.91 to 1,889.53)	1,767.63 (1,279.49 to 2,348.65)	1,285.35 (876.17 to 1,771.81)
Aortic aneurysm	6,009.38 (4,854.62 to 7,584.85)	6,708.12 (5,002.47 to 9,027.10)	7,097.41 (5,301.53 to 9,623.81)	6,962.36 (5,051.72 to 9,462.59)	260.36 (215.40 to 322.88)	368.22 (285.06 to 477.60)	432.64 (340.51 to 559.25)	359.84 (271.12 to 477.82)
Peripheral artery disease	281.51 (138.95 to 422.08)	760.71 (333.59 to 1,266.87)	967.63 (413.27 to 1,598.48)	752.24 (322.12 to 1,245.78)	17.27 (8.15 to 26.12)	56.95 (23.89 to 91.13)	81.70 (34.42 to 131.24)	53.03 (21.90 to 85.56)
Endocarditis	4,213.94 (2,813.60 to 6,757.49)	5,099.49 (3,268.86 to 8,150.32)	5,160.89 (3,307.39 to 8,374.99)	5,132.41 (3,262.37 to 8,331.43)	140.91 (101.10 to 222.69)	270.85 (169.31 to 448.99)	305.32 (193.66 to 506.59)	257.24 (158.74 to 433.99)
Other cardiovascular and circulatory diseases	39,039.18 (28,971.48 to 54,146.78)	63,881.39 (43,869.14 to 92,858.52)	52,313.97 (35,987.17 to 75,166.43)	66,451.06 (44,614.27 to 98,417.00)	1,621.38 (1,224.16 to 2,290.62)	4,041.20 (2,716.15 to 5,837.88)	3,710.74 (2,543.25 to 5,278.91)	3,949.77 (2,606.09 to 5,719.12)
**Chronic respiratory diseases**	**500,304.74 (413,440.30 to 590,505.34)**	**729,850.67 (390,658.49 to 1,275,287.13)**	**348,262.78 (199,655.80 to 566,312.33)**	**915,379.79 (487,009.15 to 1,540,433.91)**	**30,367.34 (24,820.92 to 36,543.87)**	**48,900.90 (25,991.27 to 81,787.58)**	**25,252.32 (14,199.72 to 40,672.69)**	**60,001.13 (31,761.43 to 98,613.93)**
Chronic obstructive pulmonary disease	472,339.55 (388,263.06 to 563,800.64)	708,593.22 (374,506.71 to 1,243,712.24)	328,006.63 (182,256.92 to 544,943.40)	894,021.69 (466,792.52 to 1,519,210.16)	29,185.01 (23,628.67 to 35,372.47)	47,710.24 (24,879.35 to 80,676.13)	24,032.50 (12,998.44 to 39,581.11)	58,851.79 (30,295.56 to 97,377.93)
Pneumoconiosis	7,941.63 (5,572.70 to 11,289.96)	4,940.95 (3,274.99 to 7,488.61)	4,593.34 (2,989.27 to 6,847.16)	4,733.17 (3,170.40 to 7,225.46)	292.33 (204.75 to 411.99)	248.87 (164.42 to 377.76)	244.56 (158.16 to 368.97)	230.18 (153.29 to 346.31)
Silicosis	6,361.86 (4,277.66 to 9,103.87)	3,523.57 (2,173.75 to 5,483.73)	3,378.91 (2,084.96 to 5,277.78)	3,303.04 (2,076.82 to 5,141.14)	232.41 (157.46 to 333.94)	173.77 (105.66 to 273.21)	176.52 (106.29 to 285.41)	157.07 (94.95 to 249.00)
Asbestosis	210.87 (124.58 to 381.20)	249.61 (136.00 to 465.42)	244.73 (131.96 to 469.56)	238.85 (131.14 to 432.97)	7.57 (3.94 to 14.33)	12.71 (6.23 to 23.68)	13.61 (6.61 to 26.52)	11.63 (5.64 to 21.89)
Coal workers pneumoconiosis	426.90 (155.96 to 1,122.28)	507.38 (189.69 to 1,269.73)	349.77 (122.27 to 963.27)	507.92 (188.78 to 1,302.22)	19.14 (6.86 to 50.87)	29.85 (10.72 to 77.25)	22.05 (7.60 to 62.39)	28.73 (10.30 to 73.02)
Other pneumoconiosis	942.01 (481.75 to 1,912.59)	660.39 (362.82 to 1,178.42)	619.92 (331.05 to 1,119.47)	683.36 (383.51 to 1,208.77)	33.20 (17.76 to 66.16)	32.55 (17.96 to 59.32)	32.38 (17.50 to 59.11)	32.76 (18.05 to 59.02)
Asthma	15,580.39 (10,593.27 to 28,244.18)	10,586.67 (6,546.37 to 19,566.19)	8,799.28 (5,480.15 to 16,119.37)	11,196.39 (6,560.91 to 21,365.59)	727.76 (479.79 to 1,442.99)	656.22 (389.33 to 1,239.35)	601.70 (364.93 to 1,107.69)	659.48 (380.59 to 1,274.13)
Interstitial lung disease and pulmonary sarcoidosis	2,643.31 (1,232.24 to 4,763.72)	4,135.90 (1,893.58 to 7,874.82)	5,402.46 (2,392.89 to 10,904.23)	3,807.27 (1,755.57 to 7,190.95)	110.75 (50.99 to 206.29)	216.33 (97.10 to 420.09)	301.66 (133.00 to 607.16)	192.49 (86.76 to 370.98)
Other chronic respiratory diseases	1,799.87 (987.17 to 2,664.22)	1,593.92 (828.16 to 2,435.83)	1,461.07 (778.59 to 2,215.08)	1,621.27 (849.28 to 2,503.50)	51.50 (28.78 to 73.63)	69.25 (36.16 to 107.44)	71.89 (38.71 to 111.10)	67.18 (34.40 to 104.11)
**Cirrhosis and other chronic liver diseases**	**108,887.17 (87,990.49 to 146,797.86)**	**132,009.12 (106,339.56 to 178,481.23)**	**120,069.59 (96,753.22 to 162,229.96)**	**130,428.15 (104,925.65 to 175,181.40)**	**3,676.60 (3,040.69 to 4,833.53)**	**5,364.76 (4,412.40 to 7,003.93)**	**5,150.17 (4,215.81 to 6,712.47)**	**5,143.52 (4,220.44 to 6,751.49)**
Cirrhosis and other chronic liver diseases due to hepatitis B	48,758.81 (38,138.72 to 67,372.68)	61,568.57 (47,962.14 to 83,888.54)	57,531.44 (44,824.76 to 79,372.91)	59,420.07 (46,240.15 to 79,993.53)	1,676.72 (1,359.34 to 2,223.93)	2,535.34 (2,025.40 to 3,426.22)	2,495.44 (1,998.85 to 3,321.36)	2,377.52 (1,894.55 to 3,216.61)
Cirrhosis and other chronic liver diseases due to hepatitis C	19,518.83 (14,141.52 to 28,590.47)	23,463.57 (17,127.92 to 33,513.08)	21,695.71 (15,645.43 to 30,838.05)	22,748.35 (16,515.54 to 32,618.90)	630.88 (470.96 to 902.43)	896.12 (663.52 to 1,287.65)	866.65 (640.93 to 1,240.91)	847.35 (628.37 to 1,215.59)

Cirrhosis and other chronic liver diseases due to alcohol use	15,395.36 (11,819.84 to 21,709.22)	17,685.09 (13,711.44 to 24,830.99)	14,341.44 (11,160.92 to 20,094.86)	19,690.19 (15,102.44 to 27,920.42)	526.47 (418.16 to 721.07)	724.23 (573.17 to 994.06)	625.31 (497.77 to 842.42)	775.66 (603.88 to 1,062.15)
Cirrhosis and other chronic liver diseases due to other causes	25,214.17 (20,492.30 to 32,882.62)	29,291.88 (23,470.80 to 37,599.92)	26,501.01 (21,131.67 to 34,316.20)	28,569.54 (22,784.95 to 36,877.51)	842.53 (694.10 to 1,050.00)	1,209.05 (979.47 to 1,554.14)	1,162.78 (946.27 to 1,479.14)	1,142.99 (918.62 to 1,474.25)
**Digestive diseases**	**81,425.66 (62,324.61 to 108,449.06)**	**88,383.82 (69,109.84 to 121,212.27)**	**73,913.52 (57,660.41 to 98,424.06)**	**87,652.96 (67,845.27 to 120,836.94)**	**3,271.50 (2,585.31 to 4,285.94)**	**5,002.76 (3,773.04 to 6,782.58)**	**4,604.94 (3,587.99 to 6,057.12)**	**4,716.25 (3,483.57 to 6,489.75)**
Peptic ulcer disease	30,980.12 (23,599.43 to 42,570.71)	28,846.03 (20,947.72 to 40,393.27)	20,292.38 (14,706.64 to 28,016.99)	29,033.27 (20,878.79 to 40,679.83)	1,340.99 (1,031.79 to 1,809.52)	1,632.51 (1,179.37 to 2,280.50)	1,247.49 (903.78 to 1,718.32)	1,571.17 (1,116.50 to 2,215.80)

North Korea								
Cause	YLLs (actual counts)				Deaths (actual counts)			
	2016	2040 Forecast	2040 Better Scenario	2040 Worse Scenario	2016	2040 Forecast	2040 Better Scenario	2040 Worse Scenario
Gastritis and duodenitis	6,204.27 (4,125.49 to 8,204.86)	10,079.96 (6,657.84 to 13,783.12)	9,201.53 (6,017.85 to 12,549.92)	9,487.06 (6,133.10 to 13,060.01)	299.95 (196.72 to 416.94)	607.07 (389.94 to 877.37)	605.18 (391.68 to 860.67)	546.27 (341.50 to 801.71)
Appendicitis	2,164.38 (1,521.45 to 3,839.02)	2,319.21 (1,591.98 to 4,093.36)	1,838.10 (1,246.93 to 3,278.55)	2,256.58 (1,537.36 to 4,049.65)	73.52 (50.92 to 128.21)	114.51 (77.43 to 205.36)	99.52 (67.71 to 176.72)	105.99 (71.51 to 191.29)
Paralytic ileus and intestinal obstruction	13,766.37 (8,514.88 to 25,384.91)	10,352.10 (7,896.86 to 13,863.95)	10,208.94 (7,790.95 to 13,417.30)	9,816.07 (7,405.17 to 13,478.85)	364.91 (276.45 to 520.72)	556.29 (415.46 to 747.10)	621.01 (472.24 to 812.51)	493.37 (359.57 to 673.32)
Inguinal, femoral, and abdominal hernia	1,023.85 (620.98 to 2,149.79)	1,091.25 (647.31 to 2,197.47)	1,039.35 (627.53 to 2,106.15)	1,006.13 (595.10 to 2,063.46)	49.02 (30.59 to 99.22)	73.41 (42.16 to 149.22)	78.07 (46.02 to 155.23)	64.06 (36.09 to 132.92)
Inflammatory bowel disease	3,166.12 (2,070.10 to 5,120.41)	3,261.45 (2,325.39 to 4,675.30)	3,540.35 (2,575.92 to 4,853.40)	3,038.09 (2,157.48 to 4,392.99)	114.66 (82.65 to 162.09)	191.57 (136.62 to 267.70)	233.17 (168.94 to 316.42)	168.36 (116.44 to 239.57)
Vascular intestinal disorders	2,772.54 (1,921.48 to 5,420.38)	4,718.33 (3,196.60 to 9,378.74)	4,711.16 (3,195.76 to 9,271.20)	4,359.16 (2,908.60 to 8,736.36)	145.09 (101.66 to 268.44)	297.71 (196.62 to 588.86)	320.21 (219.25 to 608.72)	264.97 (172.60 to 524.83)
Gallbladder and biliary diseases	7,600.00 (5,523.58 to 11,570.92)	10,321.70 (6,462.23 to 16,256.50)	8,731.22 (5,526.20 to 13,762.39)	10,840.16 (6,447.15 to 18,108.25)	395.00 (285.30 to 604.08)	678.15 (414.14 to 1,074.01)	628.42 (389.91 to 992.17)	678.39 (386.28 to 1,147.91)
Pancreatitis	7,097.53 (4,981.47 to 11,721.76)	9,530.47 (5,687.03 to 18,064.04)	7,686.39 (4,738.98 to 13,789.49)	10,301.70 (5,874.53 to 20,008.04)	243.08 (169.62 to 392.88)	412.29 (252.73 to 727.03)	361.46 (231.77 to 616.27)	424.37 (249.10 to 796.06)
Other digestive diseases	6,650.47 (4,595.58 to 10,315.71)	7,863.33 (5,642.99 to 12,015.90)	6,664.12 (4,767.92 to 10,280.79)	7,514.72 (5,277.92 to 11,466.04)	245.27 (174.53 to 373.96)	439.26 (296.51 to 697.03)	410.41 (285.55 to 642.19)	399.29 (268.98 to 634.00)
**Neurological disorders**	**108,037.18 (93,300.35 to 125,259.41)**	**178,017.22 (140,869.26 to 223,941.66)**	**211,463.33 (173,428.53 to 257,432.07)**	**168,549.95 (129,120.37 to 216,557.21)**	**7,170.01 (6,117.78 to 8,608.59)**	**14,568.17 (10,573.57 to 19,435.24)**	**19,520.52 (15,236.33 to 24,500.55)**	**13,060.72 (8,971.16 to 18,078.04)**
Alzheimer's disease and other dementias	70,466.52 (58,364.96 to 85,749.91)	135,823.49 (98,926.90 to 180,435.58)	167,977.13 (130,506.77 to 213,883.09)	126,409.54 (88,042.16 to 173,009.35)	6,098.29 (5,061.53 to 7,511.28)	12,762.99 (8,869.46 to 17,548.34)	17,213.33 (13,002.69 to 22,145.46)	11,402.26 (7,366.48 to 16,351.98)
Parkinson's disease	5,849.73 (4,366.65 to 7,580.43)	13,246.34 (9,553.31 to 17,528.95)	19,305.70 (14,424.77 to 24,922.58)	11,472.80 (8,061.60 to 15,496.85)	418.12 (310.12 to 545.16)	1,043.81 (718.49 to 1,434.17)	1,620.10 (1,185.93 to 2,130.65)	875.97 (579.86 to 1,228.02)
Epilepsy	23,340.75 (18,718.70 to 29,235.01)	20,755.89 (14,765.21 to 28,527.00)	14,924.17 (10,671.25 to 20,719.12)	22,683.30 (15,940.79 to 31,719.94)	453.23 (370.65 to 554.66)	500.30 (369.27 to 665.59)	376.48 (284.94 to 497.93)	535.33 (388.55 to 728.15)
Multiple sclerosis	1,020.84 (672.11 to 1,588.60)	1,076.87 (693.08 to 1,701.62)	1,171.82 (746.88 to 1,843.44)	1,066.59 (680.66 to 1,679.84)	29.41 (19.26 to 45.08)	35.58 (22.64 to 54.15)	40.41 (25.65 to 61.49)	34.50 (21.71 to 53.43)
Motor neuron disease	2,274.26 (1,623.98 to 3,799.90)	2,451.51 (1,741.82 to 3,871.41)	2,798.63 (1,967.12 to 4,458.29)	2,363.26 (1,692.98 to 3,693.28)	59.70 (43.09 to 96.37)	86.38 (60.53 to 133.05)	102.22 (70.68 to 156.84)	81.57 (57.32 to 126.85)
Other neurological disorders	5,085.08 (4,232.32 to 6,165.36)	4,663.13 (3,926.74 to 5,614.17)	5,285.88 (4,451.02 to 6,347.09)	4,554.47 (3,829.66 to 5,471.28)	111.26 (95.48 to 130.92)	139.12 (119.10 to 165.02)	167.98 (141.35 to 199.46)	131.09 (112.44 to 154.45)
**Mental disorders**	**36,310.92 (20,991.98 to 49,715.45)**	**53,556.78 (31,529.20 to 72,678.71)**	**54,526.36 (32,537.20 to 73,338.75)**	**52,575.64 (31,017.29 to 71,534.74)**	**978.73 (578.14 to 1,291.65)**	**1,739.78 (1,086.76 to 2,234.05)**	**1,895.49 (1,181.14 to 2,403.16)**	**1,649.59 (1,018.98 to 2,140.24)**
Alcohol use disorders	12,920.82 (5,662.75 to 20,562.33)	10,265.50 (4,312.26 to 17,330.99)	9,803.07 (4,055.00 to 16,541.15)	10,489.54 (4,397.38 to 17,734.90)	327.51 (151.02 to 510.78)	305.31 (144.10 to 492.07)	305.42 (145.19 to 487.84)	304.19 (143.23 to 492.63)
Drug use disorders	23,312.81 (12,282.21 to 34,047.17)	43,211.76 (24,310.48 to 60,561.95)	44,645.87 (25,477.72 to 61,399.71)	42,006.51 (23,691.11 to 59,376.44)	649.76 (350.07 to 903.22)	1,432.96 (838.21 to 1,882.87)	1,588.59 (959.03 to 2,049.56)	1,343.88 (776.10 to 1,784.19)
Opioid use disorders	13,208.72 (5,552.22 to 20,620.30)	20,659.26 (9,212.70 to 31,408.52)	21,226.84 (9,747.97 to 32,090.95)	20,131.64 (9,015.08 to 30,893.24)	352.34 (158.87 to 514.83)	652.44 (322.61 to 913.01)	712.56 (353.44 to 983.73)	615.52 (298.51 to 878.15)

Cocaine use disorders	1,918.81 (1,023.73 to 3,002.77)	4,377.85 (2,364.11 to 6,866.09)	4,163.98 (2,222.23 to 6,593.99)	4,309.56 (2,338.88 to 6,763.64)	48.24 (25.89 to 75.80)	126.12 (67.71 to 201.01)	123.69 (66.48 to 201.27)	121.98 (66.05 to 195.71)
Amphetamine use disorders	1,128.29 (604.25 to 1,789.64)	3,473.58 (1,731.05 to 5,703.50)	3,251.84 (1,520.14 to 5,450.68)	3,443.73 (1,717.42 to 5,648.10)	27.25 (13.56 to 44.59)	92.40 (44.23 to 157.70)	90.30 (39.85 to 161.61)	90.12 (42.84 to 152.62)
Other drug use disorders	7,056.99 (3,626.95 to 11,012.88)	14,701.06 (7,947.25 to 22,215.61)	16,003.20 (8,813.26 to 23,884.46)	14,121.58 (7,519.09 to 21,440.04)	221.93 (121.06 to 324.23)	562.00 (333.14 to 790.65)	662.04 (395.70 to 909.20)	516.26 (303.28 to 738.53)
Eating disorders	77.29 (49.06 to 109.41)	79.53 (48.50 to 118.05)	77.43 (47.08 to 116.18)	79.59 (48.95 to 117.83)	1.46 (0.93 to 2.07)	1.52 (0.93 to 2.26)	1.48 (0.89 to 2.22)	1.51 (0.93 to 2.24)

Cause	YLLs (actual counts)				Deaths (actual counts)			
	**North Korea**							
	2016	2040 Forecast	2040 Better Scenario	2040 Worse Scenario	2016	2040 Forecast	2040 Better Scenario	2040 Worse Scenario
**Diabetes, urogenital, blood, and endocrine diseases**	**203,496.70 (179,473.02 to 229,421.72)**	**333,109.51 (244,206.47 to 533,356.69)**	**229,902.29 (170,304.27 to 364,081.04)**	**365,766.80 (263,775.15 to 584,838.76)**	**7,919.95 (7,003.24 to 8,942.83)**	**17,495.67 (12,803.01 to 30,527.46)**	**12,913.72 (9,769.75 to 21,045.73)**	**18,571.34 (13,212.22 to 32,907.59)**
Diabetes mellitus	75,503.09 (64,893.93 to 86,761.72)	130,691.85 (86,147.93 to 191,818.30)	87,001.26 (58,733.49 to 127,419.18)	141,182.89 (90,181.20 to 208,969.33)	3,394.22 (2,896.62 to 3,946.70)	7,204.67 (4,928.96 to 10,109.13)	5,163.63 (3,582.52 to 7,264.26)	7,456.33 (4,917.87 to 10,792.50)
Acute glomerulonephritis	4,073.85 (3,137.61 to 5,312.70)	5,782.31 (4,259.79 to 7,794.44)	5,074.26 (3,680.15 to 6,880.26)	5,642.91 (4,155.41 to 7,609.35)	141.76 (109.40 to 186.78)	241.62 (174.93 to 339.76)	229.90 (164.48 to 321.79)	226.21 (162.08 to 313.70)
Chronic kidney disease	92,565.33 (79,394.31 to 107,145.72)	168,838.07 (98,593.82 to 384,124.55)	112,434.72 (69,206.67 to 241,839.38)	191,931.90 (108,362.11 to 433,716.89)	3,572.89 (3,098.06 to 4,166.83)	8,830.97 (4,823.21 to 22,475.87)	6,284.95 (3,690.92 to 14,746.30)	9,771.64 (5,141.79 to 25,598.26)
Chronic kidney disease due to diabetes mellitus	48,873.77 (40,770.71 to 58,300.27)	92,998.09 (54,315.11 to 200,529.55)	61,950.81 (37,162.88 to 128,217.47)	106,412.18 (59,882.03 to 234,886.47)	1,847.55 (1,552.76 to 2,225.45)	4,558.21 (2,480.85 to 11,348.94)	3,207.42 (1,831.31 to 7,581.76)	5,098.86 (2,665.15 to 12,790.83)
Chronic kidney disease due to hypertension	23,004.43 (18,681.56 to 28,004.92)	50,297.02 (26,965.07 to 134,115.62)	34,144.75 (19,502.89 to 82,357.53)	56,738.42 (29,063.37 to 148,561.58)	1,142.37 (936.71 to 1,376.44)	3,181.58 (1,654.23 to 8,260.88)	2,332.97 (1,319.97 to 5,469.42)	3,465.73 (1,720.02 to 9,360.01)
Chronic kidney disease due to glomerulonephritis	9,929.81 (7,782.82 to 12,456.03)	11,320.50 (7,120.45 to 21,219.81)	7,165.51 (4,553.69 to 13,223.97)	12,723.18 (7,710.04 to 24,526.45)	255.21 (201.64 to 320.96)	428.59 (246.59 to 1,028.30)	285.59 (170.63 to 682.98)	476.46 (264.33 to 1,181.93)
Chronic kidney disease due to other and unspecified causes	10,757.32 (8,511.86 to 13,442.33)	14,222.46 (8,468.10 to 29,204.92)	9,173.65 (5,553.18 to 18,532.14)	16,058.12 (9,159.69 to 33,006.70)	327.76 (255.96 to 411.10)	662.59 (362.04 to 1,609.28)	458.97 (260.87 to 1,060.16)	730.59 (385.06 to 1,799.74)
Urinary diseases and male infertility	5,906.44 (4,958.04 to 7,225.28)	9,200.70 (7,655.07 to 11,191.13)	9,302.45 (7,730.69 to 11,370.15)	8,622.26 (7,194.13 to 10,514.18)	227.99 (195.72 to 271.82)	508.24 (406.22 to 619.23)	571.10 (472.95 to 691.48)	449.42 (349.87 to 557.79)
Urinary tract infections	3,622.78 (2,653.84 to 4,644.34)	5,554.26 (4,185.09 to 7,049.16)	5,840.57 (4,428.38 to 7,543.17)	5,233.26 (3,937.74 to 6,625.63)	130.57 (100.10 to 165.00)	296.15 (218.67 to 387.03)	350.36 (267.43 to 447.16)	263.41 (194.12 to 349.39)
Urolithiasis	1,003.31 (566.63 to 1,373.36)	1,856.32 (1,024.09 to 2,570.65)	1,891.84 (1,038.65 to 2,630.01)	1,740.36 (967.65 to 2,422.41)	41.24 (23.14 to 56.37)	94.19 (52.16 to 134.56)	104.85 (57.39 to 146.86)	84.31 (46.79 to 121.78)
Other urinary diseases	1,280.36 (843.59 to 1,972.30)	1,790.11 (1,039.40 to 3,076.17)	1,570.04 (871.63 to 2,753.68)	1,648.64 (963.15 to 2,847.71)	56.18 (37.58 to 85.00)	117.90 (65.46 to 209.72)	115.89 (63.68 to 209.07)	101.71 (56.71 to 181.80)
Gynecological diseases	779.37 (474.62 to 1,131.34)	564.24 (316.40 to 843.97)	393.59 (223.08 to 594.96)	571.89 (315.04 to 875.96)	25.76 (15.87 to 36.71)	21.35 (12.35 to 31.83)	15.76 (9.04 to 23.10)	21.12 (12.07 to 31.77)
Uterine fibroids	532.28 (273.31 to 812.50)	360.95 (165.71 to 604.63)	243.42 (113.43 to 411.31)	366.92 (164.79 to 624.47)	17.75 (9.34 to 26.95)	13.66 (6.39 to 22.51)	9.65 (4.62 to 16.03)	13.60 (6.45 to 22.56)
Polycystic ovarian syndrome	18.60 (6.03 to 43.17)	11.70 (3.47 to 28.08)	6.16 (1.81 to 15.12)	12.40 (3.74 to 29.73)	0.41 (0.13 to 0.98)	0.26 (0.08 to 0.63)	0.14 (0.04 to 0.33)	0.28 (0.08 to 0.66)
Endometriosis	5.34 (1.52 to 15.17)	5.21 (1.36 to 16.04)	3.54 (0.93 to 10.88)	5.39 (1.43 to 16.63)	0.13 (0.03 to 0.37)	0.12 (0.03 to 0.38)	0.08 (0.02 to 0.26)	0.13 (0.03 to 0.39)
Genital prolapse	11.71 (4.46 to 30.52)	10.59 (3.78 to 27.04)	7.04 (2.48 to 18.42)	10.60 (3.74 to 27.83)	0.59 (0.21 to 1.58)	0.55 (0.19 to 1.48)	0.40 (0.14 to 1.08)	0.53 (0.18 to 1.47)
Other gynecological diseases	211.43 (105.47 to 346.71)	175.79 (83.80 to 289.83)	133.43 (64.18 to 220.95)	176.59 (84.28 to 296.43)	6.89 (3.18 to 11.18)	6.76 (3.14 to 11.27)	5.49 (2.61 to 9.07)	6.59 (3.00 to 11.10)
Hemoglobinopathies and hemolytic anemias	14,942.35 (11,098.93 to 20,664.68)	8,504.66 (6,612.59 to 11,587.42)	7,092.05 (5,464.10 to 9,634.74)	8,447.23 (6,578.02 to 11,504.37)	321.35 (253.48 to 428.97)	308.93 (243.73 to 416.48)	262.38 (209.41 to 353.51)	294.54 (228.37 to 398.80)
Thalassemias	7,617.85 (4,560.92 to 12,322.20)	1,394.73 (810.59 to 2,304.35)	1,644.42 (986.30 to 2,679.03)	1,428.49 (825.18 to 2,379.50)	94.21 (57.71 to 149.93)	18.31 (11.13 to 30.31)	21.68 (13.33 to 35.25)	18.70 (11.29 to 31.12)

Sickle cell disorders	25.40 (13.50 to 47.37)	14.19 (7.98 to 24.57)	11.34 (6.38 to 19.49)	14.62 (8.22 to 25.15)	0.40 (0.22 to 0.68)	0.26 (0.15 to 0.43)	0.21 (0.12 to 0.35)	0.27 (0.16 to 0.44)
G6PD deficiency	3,373.16 (2,583.35 to 4,736.47)	3,576.83 (2,636.75 to 5,188.05)	2,600.22 (1,861.92 to 3,798.98)	3,620.39 (2,635.84 to 5,280.06)	74.92 (58.32 to 103.85)	98.68 (74.65 to 138.25)	74.25 (54.92 to 107.04)	97.91 (73.41 to 138.92)
Other hemoglobinopathies and hemolytic anemias	3,925.95 (3,120.05 to 5,328.26)	3,518.91 (2,678.95 to 4,751.60)	2,836.07 (2,167.09 to 3,812.87)	3,383.73 (2,549.55 to 4,596.88)	151.82 (121.10 to 206.61)	191.67 (145.65 to 264.65)	166.24 (129.46 to 225.89)	177.66 (132.78 to 246.02)
Endocrine, metabolic, blood, and immune disorders	9,726.27 (7,093.76 to 15,425.15)	9,527.68 (7,247.30 to 15,367.75)	8,603.95 (6,540.86 to 13,921.92)	9,367.71 (7,069.64 to 15,349.60)	235.99 (180.31 to 379.74)	379.90 (293.99 to 608.76)	386.00 (299.21 to 613.05)	352.06 (271.00 to 570.23)

Cause	North Korea							
	YLLs (actual counts)				Deaths (actual counts)			
	2016	2040 Forecast	2040 Better Scenario	2040 Worse Scenario	2016	2040 Forecast	2040 Better Scenario	2040 Worse Scenario
**Musculoskeletal disorders**	**12,751.44 (9,449.78 to 18,854.20)**	**18,853.30 (13,791.15 to 28,315.66)**	**18,617.34 (13,585.02 to 27,388.37)**	**18,296.77 (13,377.62 to 27,937.10)**	**418.94 (311.20 to 638.84)**	**856.90 (621.05 to 1,297.47)**	**870.42 (638.83 to 1,284.56)**	**801.69 (573.71 to 1,216.82)**
Rheumatoid arthritis	5,216.72 (3,357.88 to 8,511.06)	10,338.32 (6,822.67 to 16,410.65)	8,807.39 (5,622.90 to 14,169.76)	10,009.72 (6,491.45 to 16,081.30)	233.94 (156.29 to 380.31)	589.31 (392.74 to 929.89)	541.01 (357.91 to 843.81)	549.70 (363.78 to 885.12)
Other musculoskeletal disorders	7,534.72 (5,353.21 to 10,962.33)	8,514.98 (5,766.66 to 13,372.85)	9,809.95 (6,765.40 to 15,235.90)	8,287.05 (5,602.41 to 13,031.75)	185.00 (126.41 to 287.25)	267.58 (174.08 to 442.52)	329.40 (222.25 to 531.84)	251.99 (162.89 to 419.70)
**Other non-communicable diseases**	**299,264.92 (193,070.36 to 459,999.16)**	**110,503.82 (70,613.77 to 176,034.96)**	**86,393.47 (56,489.23 to 134,753.32)**	**117,272.33 (73,953.58 to 186,236.84)**	**3,697.43 (2,459.88 to 5,567.36)**	**1,759.05 (1,218.65 to 2,555.96)**	**1,546.05 (1,092.11 to 2,209.81)**	**1,791.69 (1,224.04 to 2,636.59)**
Congenital birth defects	290,836.37 (186,189.81 to 450,221.18)	102,725.16 (64,022.13 to 166,401.20)	78,428.87 (49,046.14 to 126,546.04)	109,803.30 (67,043.77 to 176,985.90)	3,499.33 (2,265.02 to 5,360.90)	1,331.77 (866.31 to 2,091.53)	1,027.71 (674.65 to 1,606.53)	1,415.49 (907.06 to 2,208.79)
Neural tube defects	7,761.93 (3,582.34 to 15,660.98)	1,322.13 (571.27 to 2,949.93)	784.50 (339.93 to 1,761.55)	1,445.94 (618.47 to 3,147.78)	91.25 (42.66 to 183.32)	16.60 (7.66 to 35.71)	9.93 (4.64 to 21.60)	18.07 (8.25 to 38.68)
Congenital heart anomalies	196,885.57 (120,350.61 to 307,049.07)	68,938.18 (41,914.73 to 116,398.53)	53,020.96 (32,664.59 to 88,855.65)	73,607.44 (44,309.95 to 126,857.83)	2,377.59 (1,488.62 to 3,661.43)	896.60 (562.22 to 1,467.20)	696.24 (440.24 to 1,125.81)	952.23 (588.53 to 1,585.43)
Orofacial clefts	3,335.31 (1,026.11 to 8,053.08)	1,143.20 (316.54 to 3,063.56)	620.03 (180.04 to 1,640.97)	1,267.51 (346.49 to 3,477.40)	38.59 (11.88 to 93.09)	13.23 (3.66 to 35.50)	7.18 (2.08 to 18.99)	14.67 (4.01 to 40.24)
Down syndrome	2,282.25 (1,354.04 to 4,059.98)	1,432.21 (889.71 to 2,312.71)	1,053.50 (656.39 to 1,697.02)	1,499.12 (936.93 to 2,474.61)	32.32 (20.41 to 54.09)	25.67 (16.74 to 40.36)	19.33 (12.55 to 30.37)	26.54 (17.09 to 41.79)
Other chromosomal abnormalities	2,237.02 (743.35 to 5,864.05)	1,507.46 (496.54 to 3,814.70)	1,102.97 (363.08 to 2,921.83)	1,615.87 (539.79 to 4,156.47)	26.57 (8.86 to 70.31)	18.87 (6.34 to 49.00)	13.93 (4.67 to 37.63)	20.15 (6.78 to 51.92)
Congenital musculoskeletal and limb anomalies	3,773.66 (1,900.76 to 7,632.24)	1,562.97 (783.04 to 3,224.96)	1,277.67 (647.09 to 2,627.53)	1,656.26 (830.22 to 3,414.34)	45.37 (23.53 to 91.18)	20.15 (10.56 to 41.31)	16.63 (8.93 to 33.31)	21.24 (11.05 to 43.21)
Urogenital congenital anomalies	2,178.38 (1,275.05 to 3,632.72)	1,030.69 (649.11 to 1,652.91)	841.15 (527.15 to 1,352.51)	1,074.28 (672.57 to 1,717.40)	30.16 (18.80 to 49.09)	19.06 (12.79 to 28.40)	16.01 (10.70 to 23.80)	19.49 (13.11 to 29.01)
Digestive congenital anomalies	27,191.38 (13,305.57 to 53,556.52)	9,384.73 (4,214.20 to 19,270.02)	6,886.13 (3,080.16 to 14,109.66)	10,125.52 (4,469.32 to 20,577.67)	316.70 (155.11 to 623.90)	110.84 (50.55 to 225.61)	81.54 (37.14 to 167.00)	119.43 (53.35 to 242.06)
Other congenital birth defects	45,190.87 (25,115.08 to 75,602.21)	16,403.59 (8,784.55 to 27,999.41)	12,841.97 (7,005.49 to 21,783.01)	17,511.34 (9,298.83 to 30,032.79)	540.78 (304.76 to 899.15)	210.74 (118.92 to 349.49)	166.91 (94.85 to 274.90)	223.66 (125.27 to 372.42)
Skin and subcutaneous diseases	3,133.86 (2,000.68 to 6,195.09)	6,269.16 (4,103.51 to 12,301.43)	6,770.37 (4,505.08 to 12,971.99)	5,844.62 (3,796.63 to 11,505.44)	136.66 (88.61 to 280.34)	409.78 (245.89 to 845.49)	504.49 (316.77 to 1,001.93)	357.35 (209.28 to 739.19)
Cellulitis	364.44 (170.52 to 942.41)	775.11 (348.05 to 2,061.33)	896.10 (402.41 to 2,307.19)	735.56 (332.27 to 1,918.02)	11.83 (4.70 to 35.32)	38.53 (14.05 to 109.20)	49.48 (18.11 to 141.02)	34.58 (12.59 to 99.03)
Pyoderma	1,622.92 (959.29 to 3,219.89)	3,287.34 (2,065.74 to 6,261.67)	3,265.11 (2,038.25 to 6,179.52)	3,105.88 (1,913.96 to 5,903.82)	59.10 (32.53 to 126.57)	198.52 (112.99 to 401.95)	225.57 (127.67 to 453.78)	175.04 (98.00 to 351.58)
Decubitus ulcer	836.98 (443.23 to 1,859.44)	1,779.08 (837.14 to 3,984.41)	2,120.27 (1,016.14 to 4,625.68)	1,596.24 (740.48 to 3,599.35)	55.87 (26.85 to 131.61)	151.92 (64.59 to 364.05)	202.42 (93.29 to 466.56)	129.22 (52.83 to 322.78)
Other skin and subcutaneous diseases	309.52 (181.85 to 738.34)	427.64 (253.18 to 981.70)	488.90 (292.87 to 1,099.91)	406.94 (238.94 to 944.51)	9.87 (5.57 to 25.18)	20.81 (11.24 to 52.07)	27.02 (14.74 to 65.08)	18.52 (9.90 to 47.36)
Sudden infant death syndrome	5,294.68 (2,411.83 to 10,365.44)	1,509.49 (553.59 to 3,655.09)	1,194.22 (430.84 to 2,904.14)	1,624.41 (601.13 to 3,856.39)	61.44 (27.98 to 120.32)	17.51 (6.42 to 42.41)	13.85 (5.00 to 33.70)	18.84 (6.97 to 44.74)

**Injuries**	**963,679.23 (826,284.97 to 1,122,201.66)**	**1,112,433.25 (551,250.71 to 11,307,742.85)**	**929,234.89 (407,817.96 to 11,181,627.12)**	**1,173,349.65 (587,660.33 to 11,386,070.09)**	**19,990.35 (17,476.93 to 22,605.76)**	**27,629.65 (15,889.16 to 247,749.50)**	**24,435.25 (13,000.79 to 253,456.49)**	**28,270.86 (16,353.81 to 244,670.56)**
**Transport injuries**	**387,881.88 (310,372.30 to 467,892.59)**	**293,454.07 (204,990.94 to 395,329.64)**	**162,417.88 (112,769.12 to 219,890.37)**	**341,032.97 (234,744.29 to 468,228.87)**	**7,976.84 (6,464.43 to 9,541.23)**	**7,052.00 (5,118.13 to 9,212.03)**	**4,201.34 (3,067.53 to 5,532.81)**	**7,993.89 (5,727.94 to 10,576.33)**
Road injuries	364,076.17 (289,699.92 to 441,411.50)	265,066.39 (182,949.09 to 361,787.42)	139,724.73 (95,418.13 to 192,337.41)	310,979.53 (212,081.71 to 428,938.81)	7,448.95 (6,039.18 to 8,921.65)	6,294.89 (4,571.49 to 8,337.00)	3,571.99 (2,558.09 to 4,767.78)	7,209.66 (5,136.99 to 9,662.80)
Pedestrian road injuries	196,237.03 (143,196.14 to 242,082.68)	126,577.14 (82,430.37 to 173,542.74)	66,889.78 (43,375.26 to 91,671.61)	146,932.88 (95,582.34 to 204,652.77)	4,121.59 (3,095.47 to 5,030.48)	3,299.60 (2,261.92 to 4,356.38)	1,889.99 (1,275.02 to 2,491.50)	3,723.04 (2,529.49 to 4,984.19)

North Korea								
**Cause**	**YLLs (actual counts)**				**Deaths (actual counts)**			
	**2016**	**2040 Forecast**	**2040 Better Scenario**	**2040 Worse Scenario**	**2016**	**2040 Forecast**	**2040 Better Scenario**	**2040 Worse Scenario**
Cyclist road injuries	12,686.74 (6,627.39 to 20,645.76)	11,101.85 (5,130.99 to 18,485.27)	5,843.02 (2,641.53 to 9,810.18)	13,060.05 (6,023.64 to 22,202.69)	279.18 (147.52 to 443.19)	288.62 (137.84 to 471.92)	173.51 (78.78 to 288.38)	328.37 (157.97 to 540.68)
Motorcyclist road injuries	76,527.87 (54,973.96 to 106,965.26)	64,863.93 (39,861.06 to 99,007.01)	40,578.14 (24,663.67 to 62,690.64)	74,267.50 (45,469.80 to 113,577.86)	1,472.86 (1,054.92 to 2,065.39)	1,318.31 (842.39 to 1,956.61)	864.95 (545.94 to 1,275.52)	1,492.20 (936.26 to 2,229.65)
Motor vehicle road injuries	75,949.43 (51,957.39 to 104,889.08)	60,219.94 (38,550.90 to 87,569.30)	25,370.11 (16,009.98 to 36,930.25)	73,929.03 (46,889.05 to 108,246.92)	1,519.54 (1,048.89 to 2,060.83)	1,332.32 (883.59 to 1,900.56)	614.50 (409.41 to 858.87)	1,600.41 (1,041.97 to 2,333.59)
Other road injuries	2,675.11 (810.14 to 3,909.45)	2,303.54 (653.44 to 3,682.03)	1,043.68 (282.72 to 1,614.65)	2,790.07 (798.78 to 4,541.32)	55.78 (17.29 to 79.47)	56.04 (16.08 to 84.85)	29.05 (8.05 to 43.15)	65.65 (18.72 to 103.01)
Other transport injuries	23,805.71 (14,656.28 to 35,916.29)	28,387.68 (16,499.12 to 43,271.72)	22,693.15 (13,176.07 to 33,956.28)	30,053.44 (17,386.09 to 46,325.21)	527.89 (331.28 to 774.69)	757.11 (456.19 to 1,111.05)	629.35 (382.10 to 901.33)	784.23 (465.96 to 1,172.70)
**Unintentional injuries**	**378,659.23 (310,430.63 to 471,088.50)**	**219,199.58 (177,258.91 to 266,616.02)**	**180,828.56 (142,613.70 to 218,653.85)**	**222,635.67 (177,820.37 to 274,651.99)**	**7,491.63 (6,344.52 to 8,829.53)**	**6,703.52 (5,701.02 to 7,735.28)**	**6,171.39 (5,184.55 to 7,120.32)**	**6,448.51 (5,452.27 to 7,468.12)**
Falls	67,656.41 (51,376.24 to 91,109.97)	63,620.09 (48,825.07 to 84,471.98)	60,006.02 (45,888.62 to 79,367.01)	61,893.34 (47,553.22 to 82,924.27)	1,949.49 (1,542.30 to 2,535.95)	2,735.84 (2,167.97 to 3,488.01)	2,862.80 (2,266.72 to 3,611.76)	2,505.97 (1,978.13 to 3,202.71)
Drowning	140,042.37 (107,033.51 to 186,035.92)	55,095.43 (40,177.84 to 73,987.16)	43,378.94 (31,641.20 to 57,965.84)	57,891.21 (41,468.00 to 78,472.38)	2,205.65 (1,765.36 to 2,807.44)	1,232.11 (954.94 to 1,548.88)	1,034.23 (794.98 to 1,304.90)	1,251.29 (960.40 to 1,601.86)
Fire, heat, and hot substances	16,365.69 (11,112.38 to 27,150.44)	10,523.08 (7,039.45 to 17,885.44)	8,255.90 (5,614.35 to 13,984.80)	10,762.95 (7,037.54 to 18,485.81)	375.32 (268.89 to 603.78)	395.82 (277.35 to 665.15)	349.82 (252.32 to 580.29)	380.05 (262.80 to 643.68)
Poisonings	24,103.30 (15,247.10 to 34,159.21)	11,979.95 (7,449.64 to 17,320.08)	8,021.45 (5,084.14 to 11,376.51)	12,590.72 (7,806.48 to 18,519.42)	512.71 (331.14 to 703.05)	340.12 (214.56 to 486.46)	244.15 (155.11 to 342.02)	344.97 (217.01 to 495.59)
Exposure to mechanical forces	66,654.02 (42,462.45 to 96,546.87)	37,924.25 (25,436.39 to 52,879.43)	28,746.78 (19,089.29 to 40,271.41)	38,936.18 (25,860.44 to 54,761.19)	1,197.17 (802.52 to 1,634.87)	878.25 (608.45 to 1,170.38)	701.05 (488.13 to 921.77)	878.16 (608.56 to 1,180.28)
Unintentional firearm injuries	1,522.04 (967.42 to 2,736.92)	1,095.51 (660.88 to 1,975.49)	813.31 (498.48 to 1,443.06)	1,109.24 (665.27 to 1,993.41)	34.07 (23.44 to 57.48)	35.33 (24.13 to 58.72)	30.28 (21.05 to 48.71)	33.58 (22.23 to 55.76)
Unintentional suffocation	23,023.05 (7,796.02 to 47,578.24)	6,563.36 (2,241.73 to 13,592.74)	4,746.23 (1,624.55 to 9,860.58)	7,003.02 (2,359.73 to 14,646.92)	290.04 (103.21 to 579.12)	106.76 (42.06 to 197.71)	82.56 (32.83 to 150.99)	109.98 (42.18 to 205.13)
Other exposure to mechanical forces	42,108.93 (30,090.95 to 55,115.95)	30,265.39 (20,401.74 to 42,403.90)	23,187.24 (15,509.01 to 32,006.87)	30,823.91 (20,776.13 to 43,418.75)	873.07 (637.62 to 1,145.93)	736.17 (514.45 to 987.30)	588.21 (409.82 to 781.35)	734.59 (507.00 to 989.58)
Adverse effects of medical treatment	10,943.23 (7,735.23 to 16,230.88)	6,950.89 (5,334.44 to 10,176.38)	5,658.72 (4,378.37 to 8,247.87)	7,000.84 (5,332.55 to 10,332.79)	219.61 (168.76 to 315.97)	226.37 (186.06 to 322.41)	202.17 (165.97 to 283.76)	216.82 (175.81 to 309.53)
Animal contact	3,419.92 (2,296.37 to 4,721.66)	3,858.81 (2,556.80 to 5,478.73)	2,798.61 (1,853.03 to 4,055.16)	3,909.99 (2,549.83 to 5,508.68)	82.59 (54.34 to 113.11)	124.93 (82.68 to 176.00)	97.54 (64.62 to 138.53)	121.71 (80.19 to 172.50)
Venomous animal contact	1,399.21 (855.56 to 2,074.80)	1,447.16 (866.12 to 2,277.67)	1,039.11 (620.96 to 1,677.21)	1,464.76 (873.19 to 2,317.05)	35.78 (21.23 to 53.41)	49.84 (29.09 to 77.45)	38.55 (22.39 to 60.09)	48.43 (27.81 to 75.96)
Non-venomous animal contact	2,020.71 (1,368.40 to 2,834.37)	2,411.65 (1,562.04 to 3,525.36)	1,759.50 (1,131.22 to 2,654.91)	2,445.23 (1,578.25 to 3,607.93)	46.81 (31.86 to 66.01)	75.09 (50.05 to 109.15)	58.99 (38.51 to 87.47)	73.29 (48.93 to 105.80)
Foreign body	14,795.00 (9,242.48 to 23,386.35)	6,391.04 (4,456.26 to 9,848.69)	6,281.06 (4,476.56 to 9,315.14)	6,417.46 (4,429.83 to 9,950.74)	238.44 (165.74 to 347.40)	194.55 (151.80 to 263.97)	220.73 (172.18 to 295.93)	180.74 (139.30 to 244.03)
Pulmonary aspiration and foreign body in airway	13,064.32 (7,832.24 to 21,321.58)	5,471.14 (3,650.24 to 8,741.90)	5,505.67 (3,817.73 to 8,413.85)	5,485.74 (3,627.47 to 8,855.96)	207.57 (139.85 to 307.82)	163.27 (121.82 to 236.32)	189.63 (143.10 to 272.86)	151.60 (111.71 to 219.12)

Foreign body in other body part	1,730.69 (920.07 to 2,899.65)	919.90 (519.38 to 1,407.64)	775.40 (425.77 to 1,173.72)	931.72 (520.90 to 1,442.58)	30.87 (17.68 to 45.97)	31.29 (18.42 to 42.18)	31.10 (18.12 to 42.10)	29.14 (17.12 to 39.46)
Environmental heat and cold exposure	5,159.37 (3,843.32 to 7,118.06)	5,171.20 (3,722.56 to 7,505.35)	3,444.57 (2,443.35 to 4,995.27)	5,236.98 (3,704.88 to 7,520.80)	132.22 (98.34 to 177.98)	187.40 (136.05 to 265.36)	138.89 (99.16 to 200.95)	179.50 (130.91 to 258.40)
Other unintentional injuries	29,519.91 (14,602.80 to 38,508.66)	17,684.83 (8,540.61 to 24,268.48)	14,236.51 (6,743.81 to 19,721.03)	17,996.02 (8,674.89 to 24,988.88)	578.44 (279.18 to 750.04)	388.13 (190.40 to 526.29)	320.01 (153.65 to 435.59)	389.30 (190.69 to 529.04)
**Self-harm and interpersonal violence**	**170,336.57 (130,620.14 to 220,987.29)**	**173,258.87 (118,131.27 to 248,666.86)**	**157,733.86 (112,573.82 to 220,120.46)**	**182,291.36 (121,578.32 to 267,223.53)**	**3,951.59 (3,069.60 to 5,248.85)**	**4,864.52 (3,432.83 to 6,913.86)**	**4,665.39 (3,386.18 to 6,471.33)**	**4,981.07 (3,388.17 to 7,298.84)**

North Korea								
Cause	YLLs (actual counts)				Deaths (actual counts)			
	2016	2040 Forecast	2040 Better Scenario	2040 Worse Scenario	2016	2040 Forecast	2040 Better Scenario	2040 Worse Scenario
Self-harm	132,155.50 (97,590.03 to 176,588.82)	147,862.32 (97,583.69 to 218,722.59)	133,517.58 (91,132.06 to 193,120.39)	156,265.84 (98,800.76 to 234,293.23)	3,239.83 (2,435.43 to 4,356.27)	4,308.07 (2,958.17 to 6,273.27)	4,123.49 (2,929.09 to 5,841.27)	4,418.12 (2,907.01 to 6,609.16)
Self-harm by firearm	2,724.93 (1,214.43 to 5,814.45)	2,403.84 (945.97 to 5,519.51)	1,999.17 (822.57 to 4,422.19)	2,620.73 (972.00 to 6,144.06)	60.85 (28.94 to 124.78)	60.17 (25.80 to 132.47)	52.53 (24.11 to 110.73)	64.15 (26.17 to 144.41)
Self-harm by other specified means	129,430.56 (96,250.97 to 170,848.27)	145,458.48 (96,163.46 to 214,141.49)	131,518.41 (90,202.80 to 189,607.31)	153,645.11 (97,392.72 to 230,452.43)	3,178.97 (2,404.18 to 4,238.29)	4,247.90 (2,919.04 to 6,196.50)	4,070.96 (2,907.66 to 5,758.90)	4,353.97 (2,879.22 to 6,409.50)
Interpersonal violence	38,181.07 (28,474.36 to 52,755.63)	25,396.55 (17,515.10 to 37,239.08)	24,216.28 (16,813.39 to 35,140.06)	26,025.52 (17,777.13 to 38,185.06)	711.76 (539.08 to 979.55)	556.45 (398.16 to 787.85)	541.90 (389.93 to 753.05)	562.95 (399.44 to 803.28)
Physical violence by firearm	1,688.25 (832.74 to 4,245.54)	1,242.02 (608.44 to 3,208.17)	1,226.53 (601.28 to 3,203.88)	1,234.28 (600.07 to 3,160.48)	35.53 (18.06 to 86.60)	31.39 (16.53 to 74.61)	32.07 (17.15 to 76.70)	30.55 (15.95 to 72.83)
Physical violence by sharp object	15,292.86 (9,560.33 to 21,728.50)	11,479.41 (7,003.62 to 17,571.96)	11,665.11 (7,159.41 to 17,825.02)	11,476.93 (6,969.18 to 17,545.03)	286.30 (179.70 to 396.42)	237.17 (146.56 to 349.56)	244.63 (150.87 to 356.69)	234.67 (144.55 to 345.01)
Physical violence by other means	21,199.95 (15,373.23 to 30,667.46)	12,675.12 (8,281.10 to 19,557.88)	11,324.65 (7,458.12 to 17,421.54)	13,314.30 (8,529.94 to 20,819.49)	389.94 (288.52 to 559.90)	287.90 (192.98 to 427.60)	265.19 (180.81 to 392.96)	297.72 (195.76 to 447.61)
**Forces of nature, conflict and terrorism, and executions and police conflict**	**26,801.55 (5,569.26 to 50,219.92)**	**426,520.73 (0.00 to 10,627,429.65)**	**428,254.59 (0.00 to 10,683,556.18)**	**427,389.64 (0.00 to 10,630,448.42)**	**570.29 (116.88 to 1,072.73)**	**9,009.60 (0.00 to 225,887.27)**	**9,397.13 (0.00 to 238,138.75)**	**8,847.39 (0.00 to 221,283.83)**
Exposure to forces of nature	25,501.31 (4,046.20 to 48,876.38)	1,126.78 (0.00 to 13,963.41)	1,144.75 (0.00 to 14,157.49)	1,121.88 (0.00 to 13,917.40)	545.41 (86.54 to 1,045.34)	28.79 (0.00 to 361.53)	30.91 (0.00 to 388.92)	27.82 (0.00 to 348.80)
Conflict and terrorism	0.00 (0.00 to 0.00)	425,393.95 (0.00 to 10,627,429.65)	427,109.84 (0.00 to 10,683,556.18)	426,267.76 (0.00 to 10,630,448.42)	0.00 (0.00 to 0.00)	8,980.81 (0.00 to 225,887.27)	9,366.22 (0.00 to 238,138.75)	8,819.57 (0.00 to 221,283.83)
Executions and police conflict	1,300.24 (365.08 to 2,680.91)	0.00 (0.00 to 0.00)	0.00 (0.00 to 0.00)	0.00 (0.00 to 0.00)	24.88 (6.66 to 51.23)	0.00 (0.00 to 0.00)	0.00 (0.00 to 0.00)	0.00 (0.00 to 0.00)

Taiwan (Province of China)								
Cause	YLLs (actual counts)				Deaths (actual counts)			
	2016	2040 Forecast	2040 Better Scenario	2040 Worse Scenario	2016	2040 Forecast	2040 Better Scenario	2040 Worse Scenario
**All causes**	**3,237,260.78 (2,828,916.88 to 3,630,385.33)**	**4,122,244.18 (3,392,744.04 to 4,945,896.39)**	**3,732,885.90 (3,100,501.91 to 4,468,086.65)**	**4,977,342.74 (4,057,971.37 to 5,999,796.01)**	**174,355.77 (154,007.14 to 193,599.71)**	**452,320.87 (391,962.32 to 523,840.24)**	**423,215.84 (369,326.65 to 491,616.16)**	**525,244.95 (449,773.93 to 613,305.13)**
**Communicable, maternal, neonatal, and nutritional diseases**	**195,914.26 (135,279.72 to 233,706.66)**	**302,005.64 (148,615.11 to 466,024.98)**	**266,443.74 (135,508.97 to 396,861.35)**	**361,079.01 (181,856.55 to 571,570.28)**	**11,485.87 (6,870.28 to 14,205.62)**	**41,076.10 (19,689.18 to 64,355.16)**	**37,709.81 (18,565.55 to 57,114.24)**	**46,910.36 (22,225.99 to 75,932.14)**
**HIV/AIDS and tuberculosis**	**23,081.03 (20,397.86 to 25,825.46)**	**14,943.25 (8,298.38 to 26,795.69)**	**11,772.65 (7,045.67 to 19,991.03)**	**20,550.33 (11,477.19 to 38,549.26)**	**1,147.58 (1,016.54 to 1,286.68)**	**1,825.19 (958.50 to 3,362.30)**	**1,458.18 (833.52 to 2,511.36)**	**2,345.02 (1,218.26 to 4,548.92)**
Tuberculosis	13,118.79 (11,137.00 to 15,150.09)	13,862.63 (7,242.51 to 25,602.81)	10,732.40 (6,174.52 to 18,761.84)	18,298.23 (9,419.60 to 36,049.49)	912.75 (787.33 to 1,044.30)	1,790.04 (930.33 to 3,321.77)	1,425.10 (804.22 to 2,478.45)	2,278.74 (1,138.03 to 4,460.53)
HIV/AIDS	9,962.24 (8,253.42 to 11,446.99)	1,080.62 (485.48 to 1,972.64)	1,040.25 (509.37 to 1,862.11)	2,252.09 (1,154.50 to 3,569.87)	234.82 (192.21 to 271.04)	35.15 (14.10 to 67.80)	33.08 (14.14 to 63.46)	66.28 (28.88 to 109.56)
**Diarrhea, lower respiratory, and other common infectious diseases**	**127,222.43 (69,769.19 to 162,051.64)**	**264,444.67 (116,996.10 to 416,625.47)**	**232,046.72 (104,725.17 to 356,889.67)**	**317,816.73 (138,787.06 to 510,371.14)**	**9,277.00 (4,690.18 to 11,852.54)**	**37,315.43 (16,669.03 to 59,317.29)**	**34,227.85 (15,775.73 to 53,033.72)**	**42,769.47 (18,851.39 to 70,926.47)**
Diarrheal diseases	2,707.34 (1,659.65 to 3,769.23)	9,165.97 (2,301.77 to 28,530.29)	7,886.65 (2,349.99 to 22,548.13)	7,780.48 (1,889.81 to 24,812.17)	114.44 (56.85 to 172.72)	1,136.55 (285.69 to 3,500.73)	999.51 (297.50 to 2,782.76)	930.18 (222.32 to 2,995.28)
Intestinal infectious diseases	1,158.88 (560.17 to 2,048.99)	667.79 (303.73 to 1,242.74)	694.51 (320.42 to 1,280.44)	605.14 (270.93 to 1,121.55)	20.13 (10.00 to 35.46)	17.74 (8.80 to 30.27)	18.79 (9.35 to 32.89)	15.55 (7.58 to 27.01)
Typhoid fever	976.27 (461.69 to 1,743.45)	600.42 (272.31 to 1,111.25)	629.04 (286.49 to 1,158.87)	532.47 (234.77 to 992.75)	16.82 (8.03 to 29.64)	15.76 (7.76 to 27.17)	16.82 (8.25 to 29.75)	13.49 (6.38 to 23.87)
Paratyphoid fever	180.86 (77.49 to 359.16)	65.53 (25.15 to 140.20)	63.59 (24.25 to 134.66)	70.89 (27.55 to 148.87)	3.23 (1.38 to 6.47)	1.73 (0.70 to 3.57)	1.71 (0.69 to 3.52)	1.82 (0.74 to 3.73)
Other intestinal infectious diseases	1.75 (0.69 to 3.62)	1.84 (0.66 to 4.14)	1.88 (0.67 to 4.25)	1.78 (0.64 to 3.96)	0.07 (0.03 to 0.15)	0.25 (0.08 to 0.59)	0.26 (0.08 to 0.62)	0.23 (0.07 to 0.55)
Lower respiratory infections	114,704.30 (58,305.42 to 148,274.31)	248,960.24 (106,866.65 to 397,287.75)	218,018.02 (95,222.11 to 333,519.02)	302,778.91 (129,317.84 to 492,779.94)	8,907.83 (4,345.94 to 11,469.56)	35,809.79 (15,620.14 to 57,093.59)	32,853.16 (14,765.69 to 50,734.39)	41,469.59 (17,949.96 to 69,203.03)
Upper respiratory infections	44.04 (23.24 to 77.32)	37.98 (15.23 to 78.06)	36.36 (14.28 to 74.69)	43.83 (17.36 to 91.23)	1.65 (0.85 to 2.89)	3.45 (1.09 to 7.80)	3.36 (1.07 to 7.75)	3.83 (1.23 to 8.81)
Otitis media	32.91 (4.91 to 65.72)	52.25 (0.97 to 147.58)	56.48 (1.02 to 159.21)	45.46 (0.89 to 129.34)	3.44 (0.31 to 7.53)	8.52 (0.12 to 25.10)	9.32 (0.13 to 27.33)	7.27 (0.11 to 21.56)
Meningitis	3,891.79 (2,789.47 to 5,151.56)	1,581.63 (1,053.01 to 2,238.97)	1,560.47 (1,031.46 to 2,220.97)	1,708.32 (1,136.06 to 2,395.03)	122.87 (83.09 to 158.20)	126.91 (87.97 to 174.95)	127.68 (89.94 to 175.51)	129.91 (89.91 to 178.37)
Pneumococcal meningitis	191.82 (133.65 to 247.06)	145.73 (95.98 to 203.13)	151.23 (97.46 to 212.43)	140.80 (93.04 to 194.65)	7.12 (4.82 to 9.18)	13.83 (9.39 to 19.69)	14.58 (9.83 to 21.05)	12.91 (8.73 to 18.38)
H influenzae type B meningitis	187.73 (136.05 to 242.50)	128.23 (80.15 to 200.06)	129.80 (81.11 to 197.09)	144.62 (82.21 to 261.53)	5.94 (3.96 to 7.66)	8.94 (6.14 to 12.30)	9.40 (6.46 to 13.13)	8.33 (5.69 to 11.51)
Meningococcal meningitis	1,289.80 (908.28 to 1,706.54)	567.07 (376.87 to 785.98)	558.10 (367.24 to 780.21)	607.37 (403.34 to 841.45)	45.99 (30.85 to 59.01)	51.11 (35.00 to 70.47)	51.09 (35.13 to 70.76)	52.75 (36.12 to 73.21)
Other meningitis	2,222.44 (1,614.47 to 2,964.85)	740.60 (483.35 to 1,065.54)	721.34 (470.57 to 1,033.82)	815.53 (528.77 to 1,178.33)	63.83 (43.61 to 83.30)	53.02 (35.79 to 74.50)	52.62 (35.97 to 73.41)	55.92 (37.57 to 78.17)
Encephalitis	3,638.95 (2,818.76 to 4,520.31)	2,630.73 (1,642.22 to 3,620.18)	2,683.82 (1,628.59 to 3,731.36)	2,582.74 (1,605.32 to 3,581.29)	84.90 (61.17 to 105.80)	149.43 (85.43 to 209.22)	155.57 (88.80 to 218.79)	139.54 (80.12 to 193.59)

Diphtheria	8.31 (5.23 to 12.27)	1.31 (0.63 to 2.61)	1.26 (0.59 to 2.49)	1.84 (0.71 to 4.17)	0.17 (0.10 to 0.27)	0.03 (0.01 to 0.07)	0.03 (0.01 to 0.07)	0.04 (0.02 to 0.08)
Whooping cough	410.19 (38.12 to 1,264.06)	94.43 (2.12 to 432.64)	34.50 (1.32 to 156.87)	497.18 (2.82 to 1,880.30)	4.91 (0.46 to 15.15)	1.13 (0.03 to 5.10)	0.43 (0.02 to 1.88)	5.87 (0.04 to 22.08)
Tetanus	119.93 (106.77 to 133.98)	9.08 (5.81 to 14.01)	9.00 (5.71 to 14.09)	10.56 (6.56 to 17.69)	2.52 (2.22 to 2.84)	0.24 (0.18 to 0.34)	0.25 (0.18 to 0.35)	0.25 (0.18 to 0.35)
Measles	296.79 (89.94 to 728.21)	969.17 (25.46 to 4,182.64)	787.85 (23.76 to 3,194.18)	1,488.07 (36.94 to 6,202.83)	3.58 (1.08 to 8.76)	17.40 (0.41 to 83.91)	14.34 (0.38 to 66.56)	24.65 (0.60 to 109.86)

Taiwan (Province of China)								
Cause	YLLs (actual counts)				Deaths (actual counts)			
	2016	2040 Forecast	2040 Better Scenario	2040 Worse Scenario	2016	2040 Forecast	2040 Better Scenario	2040 Worse Scenario
Varicella and herpes zoster	208.99 (143.49 to 294.55)	274.08 (145.03 to 463.52)	277.81 (147.40 to 470.24)	274.20 (145.45 to 465.16)	10.56 (7.01 to 15.14)	44.24 (20.28 to 80.17)	45.42 (20.66 to 82.97)	42.79 (19.86 to 78.38)
**Neglected tropical diseases and malaria**	**488.94 (183.11 to 754.56)**	**1,733.52 (223.61 to 3,088.16)**	**1,777.54 (228.24 to 3,167.55)**	**1,659.84 (217.40 to 2,973.92)**	**22.25 (4.34 to 37.80)**	**143.25 (13.73 to 255.13)**	**149.01 (14.41 to 263.92)**	**133.79 (12.82 to 240.14)**
Malaria	0.00 (0.00 to 0.00)	0.00 (0.00 to 0.00)	0.00 (0.00 to 0.00)	0.00 (0.00 to 0.00)	0.00 (0.00 to 0.00)	0.00 (0.00 to 0.00)	0.00 (0.00 to 0.00)	0.00 (0.00 to 0.00)
Chagas disease	0.00 (0.00 to 0.00)	0.00 (0.00 to 0.00)	0.00 (0.00 to 0.00)	0.00 (0.00 to 0.00)	0.00 (0.00 to 0.00)	0.00 (0.00 to 0.00)	0.00 (0.00 to 0.00)	0.00 (0.00 to 0.00)
Leishmaniasis	0.00 (0.00 to 0.00)	0.00 (0.00 to 0.00)	0.00 (0.00 to 0.00)	0.00 (0.00 to 0.00)	0.00 (0.00 to 0.00)	0.00 (0.00 to 0.00)	0.00 (0.00 to 0.00)	0.00 (0.00 to 0.00)
African trypanosomiasis	0.00 (0.00 to 0.00)	0.00 (0.00 to 0.00)	0.00 (0.00 to 0.00)	0.00 (0.00 to 0.00)	0.00 (0.00 to 0.00)	0.00 (0.00 to 0.00)	0.00 (0.00 to 0.00)	0.00 (0.00 to 0.00)
Schistosomiasis	0.00 (0.00 to 0.00)	0.00 (0.00 to 0.00)	0.00 (0.00 to 0.00)	0.00 (0.00 to 0.00)	0.00 (0.00 to 0.00)	0.00 (0.00 to 0.00)	0.00 (0.00 to 0.00)	0.00 (0.00 to 0.00)
Cysticercosis	0.00 (0.00 to 0.00)	0.00 (0.00 to 0.00)	0.00 (0.00 to 0.00)	0.00 (0.00 to 0.00)	0.00 (0.00 to 0.00)	0.00 (0.00 to 0.00)	0.00 (0.00 to 0.00)	0.00 (0.00 to 0.00)
Cystic echinococcosis	1.80 (1.38 to 2.29)	1.28 (0.85 to 2.41)	1.27 (0.84 to 2.40)	1.35 (0.88 to 2.55)	0.08 (0.06 to 0.09)	0.13 (0.08 to 0.22)	0.13 (0.08 to 0.23)	0.13 (0.08 to 0.23)
Dengue	312.42 (27.58 to 560.78)	1,631.44 (88.95 to 2,990.68)	1,674.22 (91.00 to 3,068.86)	1,560.21 (86.81 to 2,884.91)	18.32 (0.75 to 33.31)	139.02 (6.92 to 251.44)	144.62 (7.23 to 260.25)	129.83 (6.45 to 236.60)
Yellow fever	0.00 (0.00 to 0.00)	0.00 (0.00 to 0.00)	0.00 (0.00 to 0.00)	0.00 (0.00 to 0.00)	0.00 (0.00 to 0.00)	0.00 (0.00 to 0.00)	0.00 (0.00 to 0.00)	0.00 (0.00 to 0.00)
Rabies	3.17 (1.28 to 4.84)	1.22 (0.75 to 1.98)	1.20 (0.73 to 1.90)	1.29 (0.78 to 2.05)	0.06 (0.03 to 0.08)	0.05 (0.03 to 0.09)	0.06 (0.03 to 0.09)	0.05 (0.03 to 0.09)
Intestinal nematode infections	30.95 (14.37 to 54.79)	20.36 (8.99 to 43.07)	20.76 (9.20 to 43.57)	19.56 (8.74 to 40.82)	0.85 (0.45 to 1.30)	1.51 (0.53 to 3.46)	1.58 (0.55 to 3.57)	1.38 (0.49 to 3.20)
Other neglected tropical diseases	140.60 (117.31 to 165.62)	79.21 (61.11 to 111.64)	80.09 (61.50 to 113.53)	77.43 (59.99 to 108.58)	2.94 (2.50 to 3.38)	2.54 (1.90 to 4.41)	2.62 (1.96 to 4.59)	2.39 (1.81 to 4.11)
**Maternal disorders**	**1,405.12 (1,067.68 to 1,817.35)**	**305.60 (193.24 to 449.39)**	**294.20 (185.33 to 429.41)**	**335.49 (211.67 to 491.37)**	**27.22 (20.63 to 35.43)**	**5.66 (3.62 to 8.16)**	**5.44 (3.47 to 7.87)**	**6.21 (3.95 to 8.92)**
Maternal hemorrhage	541.91 (369.98 to 746.23)	109.88 (61.29 to 178.66)	105.96 (58.42 to 171.32)	120.36 (66.77 to 196.28)	10.45 (7.20 to 14.53)	2.04 (1.16 to 3.22)	1.97 (1.09 to 3.08)	2.24 (1.25 to 3.56)
Maternal sepsis and other maternal infections	72.21 (42.48 to 115.88)	14.50 (7.21 to 26.06)	13.96 (6.97 to 25.32)	15.96 (7.93 to 28.82)	1.54 (0.90 to 2.51)	0.31 (0.15 to 0.56)	0.30 (0.15 to 0.54)	0.34 (0.17 to 0.61)
Maternal hypertensive disorders	153.56 (94.98 to 231.07)	33.10 (17.64 to 55.41)	31.83 (16.95 to 53.60)	36.53 (19.46 to 61.46)	2.91 (1.76 to 4.36)	0.59 (0.32 to 0.98)	0.57 (0.31 to 0.95)	0.66 (0.35 to 1.09)
Maternal obstructed labor and uterine rupture	37.11 (16.87 to 67.84)	9.44 (3.84 to 18.15)	9.03 (3.73 to 17.33)	10.57 (4.36 to 20.73)	0.73 (0.34 to 1.33)	0.18 (0.08 to 0.34)	0.17 (0.07 to 0.32)	0.20 (0.08 to 0.38)
Maternal abortion, miscarriage, and ectopic pregnancy	89.22 (48.72 to 145.20)	19.03 (9.71 to 35.05)	18.19 (9.22 to 33.21)	21.10 (10.70 to 39.89)	1.84 (1.01 to 3.01)	0.38 (0.19 to 0.70)	0.36 (0.18 to 0.68)	0.42 (0.22 to 0.77)

Indirect maternal deaths	126.84 (77.90 to 196.00)	41.53 (20.72 to 75.04)	39.78 (19.94 to 71.65)	46.08 (22.87 to 83.26)	2.39 (1.47 to 3.66)	0.73 (0.37 to 1.30)	0.69 (0.36 to 1.25)	0.80 (0.41 to 1.43)
Late maternal deaths	22.57 (9.84 to 45.39)	5.26 (2.10 to 11.33)	5.05 (2.02 to 10.78)	5.84 (2.37 to 12.78)	0.44 (0.19 to 0.88)	0.10 (0.04 to 0.20)	0.09 (0.04 to 0.19)	0.11 (0.04 to 0.22)
Maternal deaths aggravated by HIV/AIDS	0.04 (0.01 to 0.10)	0.04 (0.00 to 0.51)	0.05 (0.00 to 0.50)	0.09 (0.00 to 0.86)	0.00 (0.00 to 0.00)	0.00 (0.00 to 0.01)	0.00 (0.00 to 0.01)	0.00 (0.00 to 0.01)
Other maternal disorders	361.64 (246.68 to 521.51)	72.81 (41.63 to 117.32)	70.34 (39.99 to 113.64)	78.96 (44.49 to 128.51)	6.93 (4.70 to 10.01)	1.33 (0.77 to 2.15)	1.29 (0.74 to 2.05)	1.44 (0.83 to 2.36)

Taiwan (Province of China)								
**Cause**	**YLLs (actual counts)**				**Deaths (actual counts)**			
	**2016**	**2040 Forecast**	**2040 Better Scenario**	**2040 Worse Scenario**	**2016**	**2040 Forecast**	**2040 Better Scenario**	**2040 Worse Scenario**
**Neonatal disorders**	**28,503.32 (24,433.05 to 32,850.33)**	**4,155.05 (2,304.95 to 7,571.03)**	**3,717.39 (2,069.27 to 6,869.25)**	**4,756.71 (2,646.72 to 8,811.23)**	**329.39 (282.35 to 379.68)**	**48.03 (26.65 to 87.51)**	**42.98 (23.94 to 79.40)**	**54.98 (30.60 to 101.81)**
Neonatal preterm birth	11,351.95 (8,933.75 to 14,098.63)	1,073.98 (596.76 to 1,829.81)	891.13 (501.34 to 1,549.93)	1,426.79 (815.71 to 2,507.03)	131.18 (103.26 to 162.90)	12.42 (6.90 to 21.18)	10.30 (5.80 to 17.96)	16.49 (9.43 to 28.97)
Neonatal encephalopathy due to birth asphyxia and trauma	4,206.28 (2,977.59 to 6,636.23)	430.36 (210.55 to 914.03)	383.11 (185.94 to 828.03)	504.76 (238.44 to 1,074.32)	48.64 (34.44 to 76.68)	4.98 (2.44 to 10.56)	4.43 (2.15 to 9.57)	5.84 (2.76 to 12.42)
Neonatal sepsis and other neonatal infections	2,327.06 (708.34 to 3,212.90)	386.65 (100.54 to 726.68)	349.70 (91.54 to 658.09)	438.87 (112.02 to 820.82)	26.89 (8.19 to 37.12)	4.47 (1.16 to 8.40)	4.04 (1.06 to 7.60)	5.07 (1.30 to 9.48)
Hemolytic disease and other neonatal jaundice	63.54 (45.85 to 85.32)	5.63 (2.78 to 11.10)	5.09 (2.50 to 10.12)	6.43 (3.16 to 12.82)	0.74 (0.53 to 0.99)	0.07 (0.03 to 0.13)	0.06 (0.03 to 0.12)	0.07 (0.04 to 0.15)
Other neonatal disorders	10,554.50 (8,241.15 to 13,017.94)	2,258.42 (1,102.04 to 4,596.23)	2,088.35 (1,037.80 to 4,216.62)	2,379.86 (1,146.38 to 4,883.17)	121.94 (95.22 to 150.40)	26.10 (12.74 to 53.11)	24.14 (12.00 to 48.73)	27.50 (13.25 to 56.45)
**Nutritional deficiencies**	**3,226.73 (2,725.27 to 3,741.43)**	**6,304.26 (5,007.57 to 7,947.39)**	**6,650.35 (5,252.42 to 8,374.20)**	**5,701.38 (4,557.47 to 7,105.96)**	**202.57 (173.17 to 234.34)**	**914.00 (680.05 to 1,209.85)**	**978.09 (727.89 to 1,291.78)**	**801.69 (592.07 to 1,075.31)**
Protein-energy malnutrition	2,506.68 (2,079.57 to 3,042.86)	4,707.89 (3,547.94 to 6,255.71)	5,012.18 (3,768.35 to 6,577.95)	4,155.56 (3,146.33 to 5,579.64)	153.55 (128.30 to 191.42)	683.63 (479.07 to 960.91)	738.62 (516.11 to 1,034.50)	584.39 (399.27 to 831.18)
Iodine deficiency	57.07 (5.51 to 163.25)	253.33 (5.29 to 1,105.69)	261.74 (5.06 to 1,174.70)	239.52 (5.78 to 1,002.79)	2.59 (0.20 to 8.60)	33.65 (0.17 to 210.74)	35.26 (0.17 to 222.97)	30.87 (0.18 to 188.47)
Dietary iron deficiency	127.95 (105.37 to 152.61)	135.85 (87.67 to 224.84)	138.70 (89.56 to 231.69)	130.21 (84.51 to 212.63)	3.05 (2.48 to 3.79)	6.24 (3.37 to 13.19)	6.49 (3.50 to 13.74)	5.78 (3.18 to 12.02)
Other nutritional deficiencies	535.03 (334.92 to 645.57)	1,207.18 (677.13 to 1,653.51)	1,237.73 (680.94 to 1,702.45)	1,176.08 (665.41 to 1,609.81)	43.37 (24.72 to 54.08)	190.49 (103.27 to 273.29)	197.72 (105.67 to 283.81)	180.65 (98.89 to 261.16)
**Other communicable, maternal, neonatal, and nutritional diseases**	**11,986.69 (10,051.34 to 13,961.86)**	**10,119.30 (7,796.24 to 12,658.51)**	**10,184.89 (7,765.47 to 12,732.64)**	**10,258.53 (7,991.06 to 12,705.78)**	**479.86 (392.01 to 555.95)**	**824.54 (629.51 to 1,018.63)**	**848.27 (638.37 to 1,049.44)**	**799.19 (607.51 to 985.14)**
Sexually transmitted infections excluding HIV	507.39 (359.04 to 712.38)	292.30 (219.36 to 383.04)	292.87 (219.19 to 385.84)	292.27 (220.79 to 377.55)	16.79 (12.94 to 20.61)	30.18 (17.05 to 42.21)	30.80 (17.10 to 43.22)	28.92 (16.44 to 40.34)
Syphilis	265.05 (136.97 to 461.03)	78.69 (56.10 to 112.81)	77.47 (54.88 to 109.80)	82.34 (57.89 to 118.10)	6.41 (4.52 to 8.94)	6.89 (4.00 to 9.61)	6.94 (4.02 to 9.69)	6.85 (3.99 to 9.47)
Chlamydial infection	45.74 (35.19 to 60.80)	44.50 (32.52 to 59.58)	44.96 (32.46 to 60.67)	43.56 (32.36 to 57.20)	2.05 (1.53 to 2.55)	5.12 (2.71 to 7.32)	5.25 (2.77 to 7.57)	4.84 (2.59 to 6.92)
Gonococcal infection	133.78 (104.24 to 178.10)	125.49 (91.13 to 167.96)	126.65 (91.98 to 169.96)	123.08 (90.23 to 162.05)	6.00 (4.53 to 7.43)	14.01 (7.74 to 20.38)	14.36 (7.79 to 20.97)	13.27 (7.38 to 19.12)
Other sexually transmitted infections	62.83 (48.21 to 85.18)	43.62 (33.15 to 58.75)	43.80 (33.21 to 59.15)	43.29 (33.18 to 57.50)	2.33 (1.83 to 2.93)	4.16 (2.44 to 6.02)	4.25 (2.48 to 6.17)	3.96 (2.37 to 5.64)
Acute hepatitis	7,292.38 (5,968.01 to 8,749.62)	4,192.01 (2,836.40 to 5,847.88)	4,087.74 (2,781.04 to 5,692.40)	4,579.89 (3,182.32 to 6,320.04)	280.94 (230.94 to 336.66)	280.91 (196.88 to 386.12)	278.29 (194.60 to 383.36)	297.55 (213.72 to 405.81)
Acute hepatitis A	149.21 (93.35 to 214.45)	86.97 (49.01 to 143.01)	86.43 (48.44 to 140.15)	90.52 (51.05 to 150.25)	4.48 (2.97 to 6.46)	6.04 (3.31 to 9.90)	6.09 (3.35 to 9.95)	6.09 (3.35 to 9.96)
Acute hepatitis B	6,612.23 (5,382.70 to 8,021.30)	3,597.99 (2,374.71 to 5,086.53)	3,498.00 (2,348.16 to 4,932.93)	3,955.64 (2,681.65 to 5,575.35)	248.10 (203.54 to 298.06)	219.62 (149.85 to 310.99)	216.56 (146.43 to 308.48)	235.06 (161.17 to 331.52)

Acute hepatitis C	156.49 (101.16 to 234.59)	100.59 (61.63 to 158.28)	98.48 (60.04 to 157.18)	108.97 (67.02 to 169.08)	6.68 (4.39 to 9.65)	6.92 (4.25 to 10.67)	6.87 (4.18 to 10.68)	7.28 (4.51 to 10.98)
Acute hepatitis E	374.45 (233.85 to 553.68)	406.47 (211.01 to 652.32)	404.83 (212.21 to 664.49)	424.75 (227.56 to 683.09)	21.68 (12.67 to 33.71)	48.33 (23.73 to 79.94)	48.77 (24.23 to 82.74)	49.12 (24.34 to 82.57)
Other unspecified infectious diseases	4,186.92 (2,741.71 to 5,090.68)	5,634.99 (3,526.51 to 7,151.94)	5,804.28 (3,609.82 to 7,426.85)	5,386.37 (3,354.58 to 6,856.76)	182.13 (104.75 to 223.48)	513.45 (311.61 to 659.38)	539.18 (328.90 to 693.20)	472.72 (284.43 to 609.70)
**Non-communicable diseases**	**2,616,808.08 (2,275,575.02 to 2,947,759.15)**	**3,553,189.55 (2,880,226.13 to 4,320,529.85)**	**3,217,197.13 (2,627,314.49 to 3,919,290.34)**	**4,333,407.44 (3,468,437.93 to 5,278,378.25)**	**150,026.76 (131,744.93 to 167,722.38)**	**394,793.86 (335,282.04 to 466,347.93)**	**369,570.17 (316,823.29 to 435,812.00)**	**461,698.16 (388,609.90 to 549,061.40)**

Cause	YLLs (actual counts) 2016	YLLs 2040 Forecast	YLLs 2040 Better Scenario	YLLs 2040 Worse Scenario	Deaths (actual counts) 2016	Deaths 2040 Forecast	Deaths 2040 Better Scenario	Deaths 2040 Worse Scenario
**Taiwan (Province of China)**								
**Neoplasms**	**1,079,109.07 (930,279.51 to 1,224,088.25)**	**1,182,814.44 (916,940.22 to 1,512,406.15)**	**1,018,493.84 (804,156.61 to 1,264,973.62)**	**1,459,676.68 (1,104,837.80 to 1,892,870.76)**	**50,243.39 (43,747.22 to 56,602.37)**	**92,696.99 (76,124.18 to 111,972.25)**	**82,559.34 (68,259.81 to 98,296.47)**	**111,097.68 (89,044.91 to 136,523.41)**
Lip and oral cavity cancer	54,868.26 (44,404.32 to 66,045.41)	33,462.45 (15,600.34 to 61,983.99)	19,794.13 (10,090.00 to 35,346.85)	50,344.41 (22,285.01 to 95,276.13)	1,883.95 (1,543.58 to 2,236.74)	1,725.18 (880.28 to 3,038.22)	1,072.03 (603.66 to 1,821.21)	2,540.94 (1,243.73 to 4,534.52)
Nasopharynx cancer	24,859.96 (20,574.42 to 30,082.92)	19,562.23 (9,738.62 to 34,789.23)	11,693.59 (6,288.91 to 20,196.26)	30,827.35 (14,976.46 to 57,044.14)	832.57 (690.23 to 1,004.42)	903.83 (476.73 to 1,583.29)	552.22 (314.39 to 918.63)	1,395.10 (715.73 to 2,435.86)
Other pharynx cancer	21,658.85 (16,840.72 to 26,568.31)	18,175.38 (8,970.77 to 31,391.99)	13,795.84 (7,171.79 to 23,241.66)	18,309.68 (9,109.17 to 30,803.95)	751.56 (586.86 to 910.45)	981.76 (525.22 to 1,652.34)	768.11 (434.91 to 1,267.80)	963.53 (503.28 to 1,651.52)
Esophageal cancer	56,626.75 (46,358.50 to 68,021.13)	50,234.64 (28,197.36 to 85,209.35)	34,844.19 (21,153.32 to 55,815.89)	70,927.58 (37,572.87 to 127,046.90)	2,252.83 (1,880.42 to 2,670.99)	3,248.56 (1,976.03 to 5,265.19)	2,335.11 (1,516.61 to 3,594.86)	4,505.86 (2,608.34 to 7,664.14)
Stomach cancer	67,563.26 (57,732.19 to 78,214.87)	50,007.02 (38,002.84 to 66,313.77)	47,533.86 (36,796.84 to 62,115.10)	58,120.91 (42,821.14 to 78,278.62)	3,599.25 (3,114.44 to 4,134.68)	4,662.60 (3,598.31 to 6,107.64)	4,485.66 (3,539.17 to 5,833.20)	5,337.00 (3,938.01 to 7,152.00)
Colon and rectum cancer	117,846.26 (100,073.40 to 136,199.12)	135,010.46 (93,334.15 to 191,746.63)	121,064.96 (85,464.15 to 170,442.84)	153,831.78 (104,554.22 to 221,883.33)	6,207.49 (5,340.68 to 7,164.17)	12,799.93 (9,078.76 to 17,951.20)	11,707.83 (8,349.23 to 16,439.21)	14,258.14 (9,943.19 to 20,058.93)
Liver cancer	209,134.84 (174,375.03 to 246,309.87)	318,204.59 (209,977.19 to 467,326.47)	284,416.57 (196,977.85 to 397,416.27)	360,242.65 (221,472.08 to 559,984.76)	9,387.32 (7,889.29 to 10,954.34)	21,360.77 (14,994.82 to 29,759.34)	19,554.68 (14,408.50 to 26,151.99)	23,549.92 (15,440.25 to 34,503.40)
Liver cancer due to hepatitis B	101,486.70 (81,534.98 to 123,940.74)	137,056.40 (85,945.58 to 212,108.02)	123,176.20 (81,646.37 to 180,775.76)	154,462.67 (90,350.27 to 250,778.27)	4,140.02 (3,353.76 to 4,982.03)	8,331.85 (5,575.02 to 12,062.87)	7,662.73 (5,378.39 to 10,628.19)	9,134.20 (5,838.12 to 14,021.61)
Liver cancer due to hepatitis C	52,328.26 (42,392.28 to 62,776.37)	94,981.37 (64,368.96 to 135,415.44)	86,979.64 (61,665.00 to 118,704.31)	105,213.20 (67,194.83 to 156,340.85)	2,743.60 (2,188.44 to 3,296.53)	7,403.59 (5,199.11 to 10,214.71)	6,912.78 (5,047.63 to 9,143.72)	8,020.61 (5,307.78 to 11,499.43)
Liver cancer due to alcohol use	16,645.94 (11,981.51 to 22,348.54)	26,514.17 (16,881.27 to 39,322.29)	20,969.96 (13,367.32 to 30,704.72)	32,050.03 (19,482.32 to 49,056.04)	824.91 (543.01 to 1,122.02)	1,743.16 (1,052.97 to 2,586.68)	1,433.65 (862.09 to 2,094.04)	2,050.01 (1,202.51 to 3,100.26)
Liver cancer due to other causes	38,673.94 (30,959.43 to 47,725.76)	59,652.64 (34,934.32 to 95,287.38)	53,290.77 (33,231.96 to 82,273.84)	68,516.76 (37,354.57 to 118,193.87)	1,678.79 (1,359.33 to 2,053.55)	3,882.16 (2,450.72 to 6,050.35)	3,545.53 (2,330.39 to 5,306.83)	4,345.09 (2,531.64 to 7,270.15)
Gallbladder and biliary tract cancer	13,527.80 (9,355.69 to 16,405.34)	12,820.72 (8,910.53 to 15,380.66)	13,250.15 (9,294.15 to 15,941.07)	12,202.61 (8,439.36 to 14,514.94)	720.07 (488.32 to 863.41)	1,133.49 (783.85 to 1,329.48)	1,186.40 (825.88 to 1,389.74)	1,051.93 (715.06 to 1,234.58)
Pancreatic cancer	41,743.46 (35,599.95 to 48,199.94)	53,095.71 (39,773.80 to 69,639.38)	50,887.63 (38,346.48 to 66,413.11)	58,593.98 (43,068.73 to 78,278.28)	2,089.53 (1,793.02 to 2,394.65)	4,293.38 (3,336.88 to 5,480.82)	4,158.66 (3,257.15 to 5,325.10)	4,667.75 (3,549.96 to 6,096.96)
Larynx cancer	6,175.25 (4,957.14 to 7,402.09)	5,453.99 (2,642.01 to 10,063.48)	3,390.27 (1,816.43 to 6,002.18)	8,582.77 (4,009.95 to 16,314.94)	294.75 (241.48 to 349.56)	429.33 (217.53 to 780.54)	280.03 (150.00 to 493.69)	659.32 (316.42 to 1,229.39)
Tracheal, bronchus, and lung cancer	191,950.34 (163,550.47 to 220,295.80)	203,374.84 (135,596.67 to 289,741.27)	137,764.88 (93,415.25 to 194,234.99)	342,723.97 (221,928.70 to 496,581.45)	10,134.37 (8,748.89 to 11,559.04)	16,984.82 (11,674.00 to 23,789.49)	12,145.80 (8,431.47 to 17,018.98)	27,686.40 (18,474.47 to 38,538.09)
Malignant skin melanoma	3,545.04 (2,496.33 to 4,203.09)	5,063.39 (3,201.41 to 6,149.77)	5,403.91 (3,454.80 to 6,616.68)	4,460.43 (2,819.54 to 5,425.64)	154.60 (101.70 to 184.83)	435.88 (278.45 to 527.30)	473.00 (305.54 to 568.31)	372.39 (236.68 to 453.73)
Non-melanoma skin cancer	2,889.56 (2,417.01 to 3,362.20)	4,947.97 (4,173.42 to 5,774.86)	5,050.15 (4,243.43 to 5,985.59)	4,912.69 (4,147.85 to 5,742.50)	174.67 (149.19 to 201.15)	627.96 (505.16 to 747.37)	648.50 (525.75 to 779.92)	606.93 (484.00 to 737.22)
Breast cancer	57,727.40 (37,480.94 to 71,962.67)	34,543.94 (22,507.80 to 48,226.23)	31,955.35 (21,221.58 to 43,730.25)	40,009.49 (25,317.12 to 57,599.37)	2,038.40 (1,373.37 to 2,526.43)	2,167.30 (1,543.28 to 2,926.52)	2,036.29 (1,456.03 to 2,747.67)	2,438.22 (1,673.16 to 3,422.56)
Cervical cancer	19,945.80 (15,376.15 to 26,948.44)	15,506.17 (10,825.59 to 21,429.35)	14,639.16 (10,373.37 to 19,686.48)	17,991.81 (11,953.71 to 25,873.01)	843.50 (654.05 to 1,050.39)	1,200.56 (726.16 to 1,641.13)	1,143.02 (712.87 to 1,548.23)	1,361.20 (806.41 to 1,935.99)

Uterine cancer	7,468.40 (5,691.93 to 9,334.87)	6,797.68 (5,217.71 to 8,513.04)	6,738.58 (5,125.59 to 8,469.98)	6,998.08 (5,330.49 to 8,774.13)	306.17 (238.00 to 377.84)	482.14 (380.72 to 595.19)	483.61 (374.31 to 600.96)	482.65 (380.48 to 593.62)
Ovarian cancer	14,976.59 (11,379.90 to 18,181.36)	9,917.44 (7,164.47 to 13,521.23)	9,693.99 (7,030.09 to 13,199.15)	10,650.53 (7,553.40 to 15,072.69)	547.83 (422.68 to 660.10)	589.62 (443.34 to 789.12)	580.27 (438.44 to 776.07)	623.28 (458.46 to 857.90)
Prostate cancer	15,479.14 (7,956.42 to 20,444.45)	36,452.78 (19,276.79 to 48,110.32)	37,785.71 (20,194.70 to 50,200.54)	35,289.20 (18,492.72 to 46,374.37)	1,249.50 (620.80 to 1,635.18)	4,493.12 (2,409.08 to 5,878.91)	4,718.42 (2,569.77 to 6,127.80)	4,270.94 (2,234.13 to 5,683.67)
Testicular cancer	540.05 (417.12 to 671.41)	222.98 (165.49 to 300.04)	224.36 (166.63 to 299.56)	227.07 (170.22 to 303.75)	13.97 (11.23 to 17.18)	12.57 (9.69 to 16.01)	13.12 (10.12 to 16.87)	12.06 (9.38 to 15.24)

Taiwan (Province of China)								
Cause	YLLs (actual counts)				Deaths (actual counts)			
	2016	2040 Forecast	2040 Better Scenario	2040 Worse Scenario	2016	2040 Forecast	2040 Better Scenario	2040 Worse Scenario
Kidney cancer	14,573.36 (10,969.89 to 17,138.91)	20,330.02 (14,207.39 to 27,356.68)	20,033.52 (14,190.74 to 26,349.90)	20,879.33 (14,010.92 to 28,786.51)	762.65 (573.36 to 898.17)	1,794.43 (1,242.14 to 2,387.50)	1,791.10 (1,264.93 to 2,341.81)	1,811.44 (1,191.78 to 2,449.86)
Bladder cancer	14,784.72 (12,622.66 to 17,196.86)	25,088.33 (17,575.66 to 35,009.74)	23,214.70 (16,600.88 to 32,078.38)	29,517.65 (19,889.58 to 43,265.06)	1,017.52 (873.81 to 1,176.53)	2,815.80 (2,027.84 to 3,895.85)	2,642.30 (1,933.29 to 3,608.38)	3,280.35 (2,266.43 to 4,691.64)
Brain and nervous system cancer	18,006.01 (12,343.42 to 21,851.62)	14,784.28 (10,451.80 to 18,508.85)	15,029.72 (10,600.12 to 18,997.29)	14,545.77 (10,242.48 to 17,999.05)	582.65 (411.90 to 709.66)	856.66 (618.03 to 1,053.19)	885.78 (636.64 to 1,102.40)	817.89 (585.50 to 990.43)
Thyroid cancer	3,229.31 (2,696.84 to 3,802.96)	4,088.85 (3,139.18 to 5,443.67)	3,983.41 (3,123.94 to 5,185.38)	4,252.38 (3,237.69 to 5,766.68)	169.70 (140.35 to 198.98)	354.64 (270.81 to 459.52)	351.27 (273.65 to 445.29)	358.12 (274.73 to 469.45)
Mesothelioma	1,291.23 (1,070.93 to 1,555.51)	1,897.52 (1,482.15 to 2,247.62)	1,998.39 (1,542.84 to 2,393.60)	1,742.24 (1,392.53 to 2,043.16)	62.11 (51.12 to 72.94)	172.01 (112.76 to 207.39)	184.12 (120.20 to 220.58)	153.19 (101.22 to 183.19)
Hodgkin lymphoma	881.19 (479.35 to 1,135.12)	490.22 (275.93 to 653.41)	486.10 (276.11 to 646.50)	516.89 (289.66 to 687.13)	29.87 (16.92 to 37.89)	29.08 (17.31 to 37.10)	29.35 (17.48 to 37.51)	29.73 (17.60 to 37.70)
Non-Hodgkin lymphoma	26,068.11 (20,752.37 to 30,665.92)	27,145.54 (17,936.75 to 33,971.81)	27,131.17 (17,930.96 to 33,985.35)	27,339.88 (17,669.30 to 34,922.13)	1,180.76 (810.26 to 1,416.03)	2,137.00 (1,169.13 to 2,698.18)	2,172.82 (1,188.98 to 2,735.92)	2,086.96 (1,121.13 to 2,673.39)
Multiple myeloma	6,705.12 (3,530.81 to 8,534.88)	8,433.41 (3,829.10 to 11,536.94)	8,484.39 (3,866.56 to 11,534.25)	8,348.29 (3,787.23 to 11,464.61)	326.97 (152.06 to 422.35)	655.59 (276.32 to 898.06)	670.15 (283.53 to 908.13)	630.86 (261.49 to 872.57)
Leukemia	26,806.43 (20,304.12 to 31,210.00)	22,600.13 (13,361.16 to 30,352.01)	21,595.35 (12,768.36 to 28,447.58)	24,468.49 (14,430.62 to 33,728.60)	955.51 (601.34 to 1,143.86)	1,567.42 (738.16 to 2,148.70)	1,519.43 (715.16 to 2,041.59)	1,665.20 (768.85 to 2,362.18)
Acute lymphoid leukemia	8,060.50 (6,407.57 to 9,828.49)	4,327.99 (3,036.04 to 5,930.50)	4,094.22 (2,905.50 to 5,500.39)	4,786.59 (3,316.63 to 6,636.16)	213.29 (169.35 to 259.44)	185.39 (122.23 to 253.34)	176.80 (118.85 to 237.60)	202.19 (135.51 to 280.44)
Chronic lymphoid leukemia	2,367.27 (1,687.77 to 2,994.31)	2,285.44 (1,254.63 to 3,222.57)	2,195.78 (1,226.40 to 3,034.64)	2,429.42 (1,317.22 to 3,499.63)	93.16 (58.42 to 118.70)	148.68 (72.60 to 209.52)	144.89 (71.53 to 198.71)	154.88 (73.58 to 224.98)
Acute myeloid leukemia	7,603.79 (5,855.31 to 9,485.30)	7,321.52 (5,112.44 to 9,868.31)	7,046.90 (4,955.14 to 9,395.73)	7,749.49 (5,313.51 to 10,651.77)	239.45 (180.37 to 298.44)	406.03 (258.68 to 552.18)	396.27 (255.37 to 534.15)	420.55 (263.45 to 590.05)
Chronic myeloid leukemia	788.24 (561.86 to 1,084.69)	327.23 (203.84 to 501.17)	300.27 (188.74 to 457.03)	390.97 (240.09 to 601.39)	27.73 (19.58 to 38.31)	20.59 (12.61 to 30.44)	19.23 (11.78 to 28.75)	23.96 (14.25 to 36.67)
Other leukemia	7,986.62 (3,286.30 to 10,093.79)	8,337.95 (1,788.24 to 12,305.94)	7,958.18 (1,695.71 to 11,376.24)	9,112.03 (1,954.24 to 13,758.12)	381.87 (104.90 to 488.94)	806.73 (131.84 to 1,187.87)	782.25 (126.04 to 1,121.46)	863.63 (140.34 to 1,300.68)
Other neoplasms	38,236.56 (30,039.77 to 44,339.64)	45,101.77 (33,091.96 to 52,252.03)	46,609.79 (34,519.24 to 54,312.24)	42,818.75 (31,156.38 to 49,317.92)	1,673.32 (1,219.07 to 1,938.27)	3,781.58 (2,481.30 to 4,404.71)	3,970.25 (2,629.58 to 4,634.53)	3,480.35 (2,250.30 to 4,048.32)
**Cardiovascular diseases**	**639,146.40 (550,080.85 to 731,859.68)**	**734,603.05 (506,529.40 to 1,060,688.93)**	**637,467.39 (451,693.90 to 906,487.12)**	**1,033,331.98 (675,872.37 to 1,561,168.28)**	**42,186.36 (36,670.51 to 48,061.45)**	**95,236.20 (67,839.48 to 137,151.36)**	**85,817.89 (62,182.67 to 121,732.34)**	**124,271.37 (83,447.32 to 185,270.86)**
Rheumatic heart disease	6,861.02 (5,804.52 to 8,151.17)	7,375.82 (5,990.51 to 9,558.07)	7,419.47 (6,040.28 to 9,538.85)	7,621.03 (6,124.13 to 9,844.00)	421.88 (361.73 to 498.98)	962.31 (762.71 to 1,260.21)	977.65 (780.06 to 1,256.02)	968.21 (750.11 to 1,288.46)
Ischemic heart disease	300,024.59 (253,631.70 to 353,918.28)	300,065.29 (171,909.36 to 486,914.48)	239,075.78 (142,356.48 to 379,284.95)	462,197.02 (251,399.60 to 797,953.29)	20,619.09 (17,731.32 to 24,400.29)	38,879.03 (22,615.85 to 61,502.88)	32,606.59 (19,226.95 to 51,304.50)	55,207.07 (30,824.74 to 94,380.28)
Stroke	208,091.68 (175,683.54 to 242,294.26)	169,470.70 (105,076.40 to 284,220.88)	144,062.27 (92,531.21 to 232,849.14)	265,359.61 (154,843.77 to 480,384.33)	12,674.91 (10,877.58 to 14,526.19)	19,886.41 (12,645.78 to 32,955.18)	17,807.74 (11,641.88 to 29,567.23)	27,937.12 (17,081.03 to 48,726.09)
Ischemic stroke	72,093.07 (60,984.80 to 84,188.83)	77,349.33 (44,116.93 to 157,885.63)	69,207.07 (40,012.31 to 140,722.49)	112,558.37 (60,058.14 to 250,191.95)	5,638.28 (4,838.24 to 6,509.96)	10,075.95 (5,719.39 to 19,007.13)	9,333.51 (5,344.86 to 17,167.75)	13,566.95 (7,482.11 to 26,929.76)

Intracerebral hemorrhage	135,998.61 (114,569.37 to 159,407.82)	92,121.37 (55,953.54 to 142,208.06)	74,855.21 (47,278.34 to 115,364.12)	152,801.24 (85,521.08 to 256,327.83)	7,036.63 (5,970.65 to 8,097.78)	9,810.46 (6,199.13 to 14,942.36)	8,474.23 (5,557.39 to 12,892.15)	14,370.17 (8,392.60 to 23,294.18)
Hypertensive heart disease	45,711.94 (31,189.56 to 54,771.10)	116,260.98 (67,179.32 to 224,833.00)	105,236.42 (63,992.31 to 188,767.09)	150,089.40 (76,055.59 to 323,461.17)	3,417.99 (2,463.91 to 4,041.54)	16,810.23 (9,562.31 to 34,213.69)	15,485.20 (9,101.56 to 29,379.49)	21,245.01 (10,532.79 to 47,298.22)
Cardiomyopathy and myocarditis	15,636.84 (11,065.29 to 18,827.86)	10,420.70 (7,548.41 to 16,264.54)	10,244.68 (7,343.21 to 16,184.19)	11,049.51 (7,983.87 to 16,841.61)	578.25 (466.52 to 784.10)	1,017.83 (622.80 to 1,977.74)	1,030.38 (620.66 to 2,031.68)	1,030.68 (624.21 to 2,007.19)
Myocarditis	2,272.44 (1,403.93 to 2,960.50)	2,637.44 (1,695.83 to 5,018.07)	2,747.88 (1,740.95 to 5,303.06)	2,474.04 (1,622.44 to 4,561.12)	82.66 (60.79 to 137.54)	298.23 (130.38 to 742.54)	317.85 (138.78 to 794.33)	267.54 (115.81 to 661.42)

Taiwan (Province of China)								
**Cause**	**YLLs (actual counts)**				**Deaths (actual counts)**			
	**2016**	**2040 Forecast**	**2040 Better Scenario**	**2040 Worse Scenario**	**2016**	**2040 Forecast**	**2040 Better Scenario**	**2040 Worse Scenario**
Alcoholic cardiomyopathy	1,553.63 (920.65 to 2,734.32)	1,407.82 (981.20 to 2,256.32)	1,201.93 (836.51 to 1,945.81)	1,417.47 (1,001.47 to 2,268.83)	57.94 (40.64 to 97.72)	110.48 (76.27 to 168.08)	99.05 (67.11 to 152.52)	108.04 (75.05 to 163.76)
Other cardiomyopathy	11,810.77 (8,376.92 to 14,568.95)	6,375.44 (4,344.77 to 9,757.44)	6,294.87 (4,359.04 to 9,771.91)	7,158.00 (4,874.06 to 10,853.62)	437.65 (345.24 to 571.00)	609.12 (346.57 to 1,181.41)	613.48 (351.38 to 1,189.78)	655.10 (370.72 to 1,241.48)
Atrial fibrillation and flutter	10,254.63 (7,772.96 to 13,486.59)	35,820.12 (24,970.39 to 49,467.15)	35,032.72 (24,833.06 to 48,103.27)	36,258.77 (24,922.20 to 50,469.54)	1,117.20 (828.28 to 1,476.27)	5,535.02 (3,823.18 to 7,667.08)	5,472.26 (3,843.91 to 7,583.90)	5,481.14 (3,759.86 to 7,652.05)
Aortic aneurysm	16,842.54 (13,845.07 to 20,119.28)	20,162.37 (15,698.09 to 25,973.41)	20,588.43 (16,108.16 to 26,671.67)	21,045.27 (16,192.53 to 27,420.95)	953.71 (795.64 to 1,140.82)	2,206.71 (1,745.79 to 2,835.08)	2,287.04 (1,807.43 to 2,958.98)	2,191.22 (1,703.36 to 2,821.43)
Peripheral artery disease	423.05 (325.98 to 509.91)	1,718.26 (1,075.87 to 2,374.12)	1,771.79 (1,126.76 to 2,440.87)	1,781.61 (1,101.31 to 2,470.34)	40.59 (28.84 to 48.96)	270.95 (159.16 to 383.49)	282.66 (167.48 to 399.72)	271.15 (159.58 to 390.84)
Endocarditis	14,249.81 (8,840.02 to 18,973.82)	27,646.86 (14,578.03 to 41,794.82)	28,706.08 (15,321.06 to 43,210.42)	27,500.68 (14,233.90 to 41,847.97)	922.61 (509.62 to 1,200.01)	3,473.01 (1,666.70 to 5,486.18)	3,642.22 (1,783.97 to 5,794.86)	3,358.26 (1,604.30 to 5,498.55)
Other cardiovascular and circulatory diseases	21,050.31 (16,523.42 to 28,397.49)	45,661.93 (32,867.88 to 64,687.45)	45,329.75 (32,831.09 to 64,263.54)	50,429.08 (34,928.58 to 72,203.63)	1,440.13 (1,139.61 to 1,917.16)	6,194.70 (4,362.48 to 8,675.46)	6,226.16 (4,390.54 to 8,673.61)	6,581.52 (4,515.76 to 9,473.62)
**Chronic respiratory diseases**	**104,465.12 (89,581.58 to 125,378.54)**	**202,668.51 (121,682.41 to 329,603.13)**	**154,031.42 (94,069.55 to 244,522.77)**	**292,065.78 (174,376.52 to 471,002.94)**	**8,980.80 (7,742.50 to 10,892.33)**	**26,747.49 (16,588.18 to 42,888.29)**	**21,087.17 (13,197.72 to 33,821.95)**	**38,053.30 (23,413.61 to 61,754.32)**
Chronic obstructive pulmonary disease	85,457.26 (72,596.37 to 105,836.29)	180,217.33 (102,176.85 to 303,318.86)	131,147.04 (75,566.51 to 218,239.67)	269,997.95 (156,242.11 to 446,290.69)	7,765.47 (6,644.78 to 9,612.33)	24,323.93 (14,578.85 to 39,917.88)	18,603.65 (11,059.98 to 30,814.10)	35,689.11 (21,357.12 to 58,588.65)
Pneumoconiosis	2,699.20 (2,017.90 to 3,536.18)	2,316.88 (1,639.17 to 3,293.08)	2,312.95 (1,624.57 to 3,300.77)	2,357.77 (1,689.93 to 3,250.71)	194.83 (147.79 to 251.66)	252.13 (180.93 to 351.41)	255.67 (182.30 to 358.05)	250.24 (182.83 to 340.76)
Silicosis	1,031.53 (677.26 to 1,466.10)	579.01 (302.57 to 1,049.99)	574.64 (300.31 to 1,059.15)	633.98 (340.79 to 1,125.52)	80.89 (53.01 to 116.62)	62.91 (33.18 to 113.92)	63.05 (32.42 to 113.86)	67.43 (36.19 to 116.71)
Asbestosis	222.56 (89.24 to 369.50)	632.16 (271.94 to 1,066.05)	687.95 (294.65 to 1,155.54)	541.51 (224.20 to 914.07)	14.05 (5.10 to 23.67)	68.09 (30.63 to 112.97)	75.12 (34.04 to 123.12)	56.86 (23.33 to 95.51)
Coal workers pneumoconiosis	533.17 (147.90 to 816.02)	382.88 (137.64 to 699.43)	353.02 (131.94 to 644.77)	415.18 (145.75 to 733.99)	40.53 (10.37 to 62.64)	40.48 (15.83 to 71.04)	38.11 (15.48 to 67.58)	42.71 (16.55 to 73.26)
Other pneumoconiosis	911.95 (645.87 to 1,308.00)	722.82 (455.24 to 1,189.72)	697.34 (428.86 to 1,184.39)	767.10 (490.97 to 1,243.24)	59.37 (41.93 to 91.18)	80.64 (51.00 to 133.12)	79.39 (49.15 to 132.92)	83.24 (53.97 to 133.19)
Asthma	9,885.22 (7,914.24 to 12,114.29)	6,419.10 (4,208.16 to 9,947.83)	5,764.13 (3,869.88 to 8,717.30)	7,974.10 (5,038.25 to 12,581.01)	654.71 (534.08 to 825.01)	837.67 (545.34 to 1,331.17)	766.76 (509.44 to 1,187.39)	1,000.46 (626.72 to 1,627.79)
Interstitial lung disease and pulmonary sarcoidosis	5,628.58 (4,282.60 to 7,183.00)	12,765.92 (8,972.15 to 16,753.00)	13,878.13 (9,808.61 to 18,284.34)	10,738.70 (7,487.13 to 14,126.94)	326.11 (239.93 to 404.67)	1,231.72 (848.83 to 1,630.53)	1,360.17 (951.57 to 1,810.49)	1,007.60 (686.40 to 1,331.16)
Other chronic respiratory diseases	794.86 (477.11 to 1,031.90)	949.28 (594.35 to 1,398.42)	929.17 (588.76 to 1,362.89)	997.26 (618.48 to 1,473.52)	39.69 (25.32 to 52.54)	102.04 (63.59 to 169.66)	100.91 (63.20 to 167.91)	105.89 (65.34 to 175.34)
**Cirrhosis and other chronic liver diseases**	**173,120.90 (139,009.13 to 206,765.60)**	**125,717.37 (101,691.28 to 152,504.59)**	**121,892.65 (98,819.22 to 149,751.78)**	**129,051.00 (105,804.39 to 156,624.00)**	**6,400.24 (5,333.48 to 7,545.01)**	**8,624.59 (7,329.76 to 10,139.00)**	**8,565.58 (7,251.34 to 10,113.74)**	**8,591.19 (7,271.24 to 10,051.73)**
Cirrhosis and other chronic liver diseases due to hepatitis B	69,046.49 (53,388.20 to 83,654.65)	49,717.41 (39,622.19 to 62,585.48)	50,015.05 (39,161.69 to 63,605.79)	50,176.48 (39,813.72 to 63,013.28)	2,559.63 (2,056.02 to 3,081.83)	3,383.17 (2,734.56 to 4,187.95)	3,457.94 (2,783.60 to 4,301.15)	3,321.49 (2,686.77 to 4,083.54)
Cirrhosis and other chronic liver diseases due to hepatitis C	43,704.11 (32,709.42 to 55,515.27)	27,801.08 (20,882.21 to 35,795.19)	27,841.16 (20,928.83 to 36,189.36)	28,282.12 (21,442.28 to 36,467.39)	1,498.81 (1,167.10 to 1,870.75)	1,663.45 (1,303.54 to 2,094.90)	1,693.95 (1,313.36 to 2,150.55)	1,646.29 (1,294.04 to 2,077.15)

Cirrhosis and other chronic liver diseases due to alcohol use	26,705.61 (20,622.91 to 33,319.80)	20,532.58 (16,280.56 to 25,929.18)	16,297.03 (13,066.95 to 20,485.50)	22,565.69 (17,846.96 to 28,296.55)	985.89 (798.53 to 1,200.55)	1,362.48 (1,122.04 to 1,658.13)	1,159.09 (945.84 to 1,413.16)	1,443.88 (1,185.65 to 1,745.57)
Cirrhosis and other chronic liver diseases due to other causes	33,664.70 (27,100.19 to 40,526.99)	27,666.30 (22,213.41 to 34,360.82)	27,739.40 (22,226.30 to 34,379.64)	28,026.72 (22,603.71 to 34,484.97)	1,355.91 (1,116.55 to 1,604.54)	2,215.50 (1,791.96 to 2,691.00)	2,254.60 (1,819.73 to 2,750.87)	2,179.53 (1,760.26 to 2,657.06)
**Digestive diseases**	**63,546.74 (53,909.72 to 73,637.82)**	**110,236.76 (81,516.02 to 136,041.06)**	**108,746.97 (80,244.09 to 132,510.15)**	**111,919.41 (80,986.20 to 140,361.05)**	**4,179.43 (3,324.43 to 4,857.27)**	**13,899.44 (9,437.26 to 17,319.28)**	**13,976.95 (9,585.28 to 17,259.09)**	**13,744.05 (9,116.85 to 17,513.86)**
Peptic ulcer disease	12,678.60 (10,563.86 to 15,101.96)	13,192.10 (10,480.63 to 16,594.63)	13,043.07 (10,502.35 to 16,221.83)	14,593.04 (11,439.49 to 18,538.60)	858.56 (722.23 to 1,015.03)	1,700.52 (1,323.21 to 2,196.24)	1,699.11 (1,343.41 to 2,187.43)	1,830.53 (1,379.35 to 2,420.48)

Taiwan (Province of China)								
Cause	YLLs (actual counts)				Deaths (actual counts)			
	2016	2040 Forecast	2040 Better Scenario	2040 Worse Scenario	2016	2040 Forecast	2040 Better Scenario	2040 Worse Scenario
Gastritis and duodenitis	245.14 (196.91 to 301.94)	363.64 (275.07 to 477.73)	368.69 (277.59 to 484.13)	364.23 (276.48 to 475.33)	14.41 (11.75 to 17.47)	46.11 (33.09 to 63.92)	47.32 (33.90 to 65.23)	44.94 (32.22 to 63.01)
Appendicitis	2,195.76 (1,634.33 to 2,630.41)	3,577.57 (1,769.77 to 4,691.70)	3,602.65 (1,740.76 to 4,810.57)	3,689.65 (1,812.12 to 4,909.60)	149.49 (78.71 to 187.38)	448.80 (190.28 to 611.26)	457.50 (192.07 to 625.80)	451.53 (188.15 to 610.81)
Paralytic ileus and intestinal obstruction	8,018.40 (4,470.30 to 10,124.39)	19,717.48 (8,483.23 to 26,358.05)	20,809.03 (8,874.41 to 27,877.86)	18,019.58 (7,756.36 to 23,919.54)	576.61 (258.16 to 756.75)	2,653.55 (1,002.48 to 3,718.50)	2,831.03 (1,072.61 to 3,957.99)	2,370.89 (887.74 to 3,355.39)
Inguinal, femoral, and abdominal hernia	1,187.89 (866.88 to 1,492.35)	1,755.84 (1,245.88 to 2,330.27)	1,811.02 (1,298.78 to 2,398.39)	1,708.58 (1,213.99 to 2,244.97)	87.48 (62.28 to 110.81)	242.40 (167.88 to 326.65)	253.20 (176.23 to 342.77)	229.66 (157.38 to 308.67)
Inflammatory bowel disease	3,429.57 (2,387.65 to 4,201.26)	5,347.35 (3,788.82 to 6,621.66)	5,606.97 (3,972.83 to 6,962.05)	4,948.62 (3,480.82 to 6,064.67)	209.89 (145.71 to 257.80)	694.18 (474.89 to 906.66)	737.92 (506.95 to 964.32)	624.22 (425.14 to 813.98)
Vascular intestinal disorders	9,325.52 (7,644.02 to 12,670.25)	20,422.81 (16,933.24 to 26,673.76)	21,161.20 (17,384.22 to 27,564.80)	19,485.02 (16,158.49 to 25,484.00)	664.40 (550.72 to 860.54)	2,513.96 (2,021.87 to 3,291.96)	2,634.96 (2,141.16 to 3,458.05)	2,340.90 (1,871.46 to 3,066.12)
Gallbladder and biliary diseases	9,514.13 (6,099.20 to 11,720.63)	21,938.70 (9,248.98 to 35,515.53)	20,104.55 (8,713.99 to 31,540.38)	24,895.01 (10,209.76 to 41,565.08)	767.45 (415.65 to 981.46)	3,052.32 (1,156.25 to 5,045.52)	2,840.15 (1,117.05 to 4,530.61)	3,372.11 (1,249.50 to 5,735.13)
Pancreatitis	9,473.00 (7,580.84 to 12,735.26)	10,397.23 (4,883.53 to 20,644.64)	8,256.43 (4,511.96 to 15,292.12)	11,279.87 (5,236.98 to 22,728.15)	395.68 (315.26 to 508.01)	911.31 (456.58 to 1,774.75)	761.59 (421.63 to 1,349.92)	956.99 (473.41 to 1,958.80)
Other digestive diseases	7,478.74 (6,227.31 to 9,106.78)	13,524.03 (11,021.32 to 16,757.08)	13,983.35 (11,243.39 to 17,372.27)	12,935.81 (10,623.04 to 15,845.93)	455.46 (380.16 to 543.81)	1,636.29 (1,289.31 to 2,075.34)	1,714.18 (1,357.24 to 2,179.21)	1,522.28 (1,202.00 to 1,932.79)
**Neurological disorders**	**143,037.16 (119,406.71 to 170,650.59)**	**470,619.01 (367,547.57 to 611,304.55)**	**472,437.19 (373,046.48 to 604,896.21)**	**467,420.63 (353,105.90 to 637,827.28)**	**13,982.74 (11,592.99 to 16,743.36)**	**71,245.05 (53,845.50 to 93,399.22)**	**72,225.06 (55,493.47 to 91,964.29)**	**69,447.41 (50,818.71 to 95,253.51)**
Alzheimer's disease and other dementias	108,826.97 (88,686.63 to 134,302.25)	405,555.24 (300,084.66 to 543,322.00)	405,460.75 (303,750.79 to 535,975.44)	407,132.81 (292,949.16 to 582,014.21)	12,131.84 (9,877.99 to 14,775.74)	64,531.68 (47,168.74 to 86,462.23)	65,161.13 (48,462.27 to 84,834.29)	63,425.99 (45,054.19 to 89,478.68)
Parkinson's disease	13,076.97 (9,840.22 to 17,270.05)	44,822.49 (33,762.75 to 58,892.01)	47,702.37 (35,982.89 to 62,181.20)	40,121.37 (30,222.62 to 52,903.51)	1,153.87 (868.09 to 1,540.40)	5,348.37 (3,991.63 to 7,027.09)	5,739.21 (4,334.99 to 7,504.09)	4,707.18 (3,515.36 to 6,196.11)
Epilepsy	11,891.91 (10,054.32 to 14,854.01)	12,096.79 (8,598.36 to 17,288.02)	10,915.26 (8,016.25 to 14,916.75)	12,366.96 (8,758.22 to 17,824.93)	381.19 (328.18 to 439.83)	912.00 (589.70 to 1,281.47)	851.63 (553.93 to 1,171.15)	893.68 (581.42 to 1,259.90)
Multiple sclerosis	838.39 (686.76 to 1,073.58)	577.47 (471.83 to 732.94)	589.31 (479.42 to 749.79)	569.21 (464.50 to 725.38)	26.73 (22.27 to 33.56)	31.65 (26.78 to 39.31)	32.82 (27.76 to 41.01)	30.21 (25.58 to 37.47)
Motor neuron disease	1,383.97 (1,154.28 to 1,627.26)	1,443.56 (1,154.61 to 1,721.23)	1,476.41 (1,167.82 to 1,774.60)	1,392.61 (1,120.57 to 1,652.66)	48.65 (40.17 to 56.83)	75.48 (62.18 to 88.05)	78.15 (63.60 to 91.64)	71.26 (59.01 to 82.74)
Other neurological disorders	7,018.97 (5,980.42 to 8,080.35)	6,123.44 (5,058.25 to 7,303.60)	6,293.10 (5,191.13 to 7,557.68)	5,837.66 (4,851.89 to 6,928.74)	240.46 (206.60 to 279.55)	345.87 (300.70 to 405.45)	362.12 (312.62 to 429.06)	319.09 (278.34 to 370.58)
**Mental disorders**	**28,842.45 (17,118.78 to 36,955.01)**	**25,358.22 (18,945.76 to 32,093.14)**	**26,211.70 (19,908.40 to 33,149.02)**	**22,711.44 (17,145.70 to 28,627.73)**	**754.78 (493.73 to 944.71)**	**1,343.05 (1,106.76 to 1,619.68)**	**1,433.44 (1,170.19 to 1,734.47)**	**1,147.64 (927.59 to 1,380.47)**
Alcohol use disorders	13,070.17 (6,325.19 to 17,281.08)	5,848.81 (3,257.60 to 8,314.10)	5,509.11 (3,088.82 to 7,838.82)	5,604.48 (3,096.57 to 7,962.40)	342.77 (179.50 to 447.16)	241.21 (170.20 to 321.08)	234.08 (167.48 to 313.81)	224.31 (157.25 to 299.12)
Drug use disorders	15,705.26 (9,550.46 to 20,300.92)	19,484.94 (14,979.62 to 24,809.70)	20,678.01 (16,032.61 to 26,445.68)	17,082.77 (13,009.04 to 21,704.56)	410.65 (288.62 to 513.67)	1,101.36 (887.94 to 1,342.58)	1,198.87 (961.13 to 1,478.64)	922.84 (735.17 to 1,134.31)
Opioid use disorders	9,008.35 (4,847.81 to 12,163.98)	6,337.93 (4,300.58 to 8,581.37)	6,545.86 (4,527.89 to 8,890.67)	5,926.08 (4,004.69 to 7,944.36)	221.48 (136.11 to 289.81)	297.70 (220.27 to 407.15)	314.93 (231.36 to 438.69)	266.75 (198.24 to 364.54)

Cocaine use disorders	1,102.52 (581.49 to 1,565.50)	1,603.51 (1,058.62 to 2,394.50)	1,695.08 (1,117.14 to 2,561.16)	1,415.29 (931.45 to 2,060.94)	26.50 (15.25 to 37.18)	56.01 (37.46 to 85.45)	60.18 (39.93 to 92.81)	48.26 (32.77 to 72.75)
Amphetamine use disorders	906.88 (395.38 to 1,349.55)	1,984.60 (1,077.95 to 2,816.25)	2,121.55 (1,172.72 to 3,017.15)	1,684.60 (907.59 to 2,447.62)	20.52 (9.78 to 29.63)	58.52 (36.90 to 80.14)	63.33 (40.03 to 86.43)	48.76 (30.63 to 67.45)
Other drug use disorders	4,687.52 (2,953.18 to 6,077.67)	9,558.90 (7,263.42 to 12,599.23)	10,315.51 (7,823.88 to 13,654.17)	8,056.80 (5,926.73 to 10,482.96)	142.15 (106.02 to 178.52)	689.12 (516.51 to 882.08)	760.44 (572.36 to 975.74)	559.07 (416.70 to 726.19)
Eating disorders	67.03 (49.51 to 85.03)	24.47 (16.97 to 36.17)	24.59 (17.03 to 36.58)	24.19 (16.71 to 35.63)	1.36 (0.99 to 1.73)	0.49 (0.33 to 0.72)	0.49 (0.34 to 0.73)	0.49 (0.33 to 0.70)

Taiwan (Province of China)								
Cause	YLLs (actual counts)				Deaths (actual counts)			
	2016	2040 Forecast	2040 Better Scenario	2040 Worse Scenario	2016	2040 Forecast	2040 Better Scenario	2040 Worse Scenario
**Diabetes, urogenital, blood, and endocrine diseases**	**333,519.93 (289,879.38 to 379,485.85)**	**649,553.84 (466,623.76 to 1,090,737.16)**	**625,638.41 (446,304.05 to 1,091,704.31)**	**765,618.45 (528,641.63 to 1,271,404.88)**	**21,649.30 (19,064.99 to 24,276.38)**	**79,402.89 (58,754.43 to 131,447.87)**	**78,114.26 (57,620.47 to 128,987.76)**	**89,993.00 (64,272.81 to 152,083.75)**
Diabetes mellitus	189,779.46 (162,388.60 to 219,479.80)	259,627.94 (188,297.64 to 349,217.54)	236,218.18 (173,499.78 to 314,408.14)	293,176.17 (208,687.04 to 401,358.39)	11,673.81 (10,084.68 to 13,371.08)	28,530.22 (20,641.55 to 38,185.48)	26,728.87 (19,447.31 to 35,075.33)	31,067.13 (22,160.63 to 42,034.52)
Acute glomerulonephritis	144.40 (125.45 to 164.06)	104.44 (78.86 to 135.77)	104.33 (78.48 to 136.11)	106.31 (80.87 to 136.94)	4.51 (3.76 to 5.35)	8.21 (5.15 to 13.45)	8.41 (5.26 to 13.73)	7.90 (5.04 to 12.65)
Chronic kidney disease	87,861.53 (73,188.68 to 101,614.91)	250,641.04 (87,864.08 to 725,696.86)	247,207.98 (87,452.36 to 716,145.93)	336,422.25 (114,778.44 to 857,473.00)	5,964.12 (4,114.54 to 6,869.08)	30,615.39 (11,593.41 to 85,186.87)	30,495.24 (11,399.19 to 85,533.75)	39,565.31 (14,276.37 to 100,407.21)
Chronic kidney disease due to diabetes mellitus	45,418.45 (37,219.71 to 53,607.98)	115,964.92 (39,734.10 to 330,073.28)	113,995.93 (40,027.83 to 327,443.47)	158,377.58 (52,726.67 to 406,931.61)	2,728.75 (2,015.54 to 3,275.86)	12,640.14 (4,568.70 to 33,178.37)	12,554.48 (4,650.86 to 33,272.87)	16,562.06 (5,753.19 to 41,963.18)
Chronic kidney disease due to hypertension	30,412.00 (22,763.15 to 36,523.52)	109,145.77 (37,224.67 to 320,815.75)	108,240.87 (37,644.76 to 319,519.74)	143,493.78 (47,109.04 to 374,975.62)	2,498.59 (1,613.21 to 3,010.60)	14,848.31 (5,440.13 to 42,715.80)	14,852.21 (5,476.35 to 42,264.79)	18,911.97 (6,738.09 to 51,211.20)
Chronic kidney disease due to glomerulonephritis	4,838.25 (3,777.06 to 6,250.46)	8,176.31 (2,845.86 to 23,591.82)	7,943.31 (2,822.08 to 23,327.26)	11,207.46 (3,729.58 to 29,518.60)	262.69 (189.73 to 342.11)	919.17 (301.13 to 2,625.77)	901.87 (300.89 to 2,578.40)	1,219.62 (394.21 to 3,391.01)
Chronic kidney disease due to other and unspecified causes	7,192.84 (5,594.56 to 9,278.17)	17,354.04 (5,816.77 to 50,417.02)	17,027.86 (5,755.11 to 49,916.09)	23,343.43 (7,570.83 to 61,158.10)	474.09 (328.56 to 615.73)	2,207.78 (738.86 to 6,328.44)	2,186.67 (732.37 to 6,258.04)	2,871.66 (928.79 to 7,480.28)
Urinary diseases and male infertility	36,923.42 (31,637.03 to 43,083.83)	113,684.94 (94,911.89 to 138,478.20)	115,675.88 (97,139.26 to 140,947.74)	111,977.37 (93,645.42 to 139,287.22)	3,225.78 (2,764.40 to 3,760.16)	17,658.32 (14,188.06 to 22,060.01)	18,144.87 (14,686.90 to 22,393.14)	17,005.31 (13,561.91 to 21,459.26)
Urinary tract infections	27,176.45 (21,149.44 to 34,297.16)	92,189.57 (71,529.05 to 117,107.00)	93,850.92 (72,554.74 to 119,679.21)	90,395.46 (69,265.77 to 116,393.54)	2,359.57 (1,768.65 to 2,990.02)	14,510.99 (10,707.75 to 18,724.59)	14,913.26 (11,031.42 to 19,322.24)	13,917.39 (10,104.38 to 18,181.20)
Urolithiasis	5,851.17 (2,563.59 to 10,306.07)	12,120.69 (4,662.13 to 25,603.40)	12,174.07 (4,571.01 to 25,520.98)	12,317.77 (4,770.66 to 25,956.02)	491.17 (204.97 to 931.08)	1,716.17 (606.66 to 3,845.81)	1,742.20 (601.65 to 3,872.36)	1,700.76 (591.60 to 3,805.76)
Other urinary diseases	3,895.81 (2,389.64 to 6,236.21)	9,374.68 (4,982.80 to 15,787.60)	9,650.90 (5,041.92 to 16,161.80)	9,264.14 (4,983.60 to 15,636.66)	375.04 (226.69 to 595.20)	1,431.15 (762.22 to 2,411.06)	1,489.40 (796.30 to 2,513.24)	1,387.16 (731.38 to 2,374.45)
Gynecological diseases	170.13 (125.21 to 222.42)	64.15 (45.49 to 86.74)	61.18 (43.52 to 82.47)	72.96 (52.06 to 98.88)	6.96 (5.35 to 8.73)	5.72 (3.84 to 8.22)	5.52 (3.71 to 7.83)	6.33 (4.30 to 9.13)
Uterine fibroids	69.89 (45.28 to 97.33)	17.88 (11.11 to 27.94)	17.20 (10.59 to 27.11)	19.92 (12.24 to 31.52)	2.13 (1.43 to 2.98)	1.15 (0.65 to 2.14)	1.12 (0.64 to 2.07)	1.23 (0.71 to 2.38)
Polycystic ovarian syndrome	7.38 (2.27 to 14.30)	0.55 (0.14 to 1.26)	0.49 (0.13 to 1.12)	0.74 (0.18 to 1.69)	0.17 (0.05 to 0.33)	0.01 (0.00 to 0.03)	0.01 (0.00 to 0.03)	0.02 (0.00 to 0.04)
Endometriosis	3.55 (1.01 to 7.77)	1.01 (0.25 to 2.42)	0.95 (0.23 to 2.27)	1.20 (0.29 to 2.91)	0.09 (0.02 to 0.20)	0.03 (0.01 to 0.06)	0.02 (0.00 to 0.06)	0.03 (0.01 to 0.07)
Genital prolapse	9.16 (5.05 to 16.86)	7.36 (3.10 to 14.15)	7.08 (2.96 to 13.48)	8.22 (3.46 to 16.16)	0.74 (0.35 to 1.36)	0.90 (0.33 to 1.86)	0.88 (0.32 to 1.75)	0.99 (0.38 to 2.02)
Other gynecological diseases	80.15 (58.04 to 109.26)	37.34 (24.63 to 54.67)	35.47 (23.61 to 51.49)	42.87 (28.30 to 62.59)	3.82 (2.70 to 5.06)	3.63 (2.15 to 5.63)	3.49 (2.09 to 5.32)	4.06 (2.39 to 6.18)
Hemoglobinopathies and hemolytic anemias	4,768.04 (3,932.21 to 6,225.80)	4,288.28 (2,677.32 to 5,529.84)	4,293.27 (2,641.29 to 5,541.09)	4,395.86 (2,738.46 to 5,650.07)	213.05 (150.00 to 258.44)	468.62 (213.87 to 677.35)	477.27 (213.94 to 689.66)	463.79 (215.10 to 676.99)
Thalassemias	599.16 (440.00 to 925.74)	136.46 (68.47 to 279.69)	135.24 (67.54 to 278.06)	137.54 (69.29 to 282.10)	7.86 (5.74 to 12.22)	1.95 (0.97 to 4.01)	1.93 (0.96 to 3.97)	1.96 (0.98 to 4.07)

Sickle cell disorders	6.41 (3.89 to 10.16)	2.61 (1.51 to 4.24)	2.52 (1.44 to 4.12)	2.87 (1.64 to 4.76)	0.14 (0.08 to 0.21)	0.07 (0.04 to 0.11)	0.07 (0.04 to 0.11)	0.08 (0.05 to 0.12)
G6PD deficiency	1,546.52 (1,141.55 to 2,324.69)	847.82 (609.41 to 1,171.44)	824.90 (596.40 to 1,156.94)	921.68 (665.81 to 1,297.79)	41.24 (31.62 to 58.58)	37.70 (26.04 to 49.13)	37.25 (25.66 to 48.20)	39.95 (27.93 to 51.90)
Other hemoglobinopathies and hemolytic anemias	2,615.95 (2,013.53 to 3,149.38)	3,301.39 (1,772.29 to 4,539.91)	3,330.62 (1,761.51 to 4,545.16)	3,333.77 (1,800.95 to 4,566.45)	163.81 (104.36 to 208.83)	428.90 (183.14 to 635.53)	438.02 (182.39 to 642.40)	421.80 (182.94 to 629.09)
Endocrine, metabolic, blood, and immune disorders	13,872.96 (11,003.51 to 16,354.45)	21,143.05 (17,573.71 to 27,921.59)	22,077.59 (18,270.59 to 29,549.58)	19,467.54 (16,322.79 to 25,966.52)	561.07 (464.10 to 692.05)	2,116.41 (1,685.42 to 3,179.74)	2,254.09 (1,786.53 to 3,388.26)	1,877.24 (1,488.48 to 2,835.68)

Cause	YLLs (actual counts)				Deaths (actual counts)			
	2016	2040 Forecast	2040 Better Scenario	2040 Worse Scenario	2016	2040 Forecast	2040 Better Scenario	2040 Worse Scenario
**Musculoskeletal disorders**	**12,876.68 (9,809.72 to 15,730.39)**	**15,133.88 (11,844.82 to 18,082.83)**	**15,229.24 (12,018.62 to 18,230.11)**	**15,285.41 (11,781.91 to 18,323.75)**	**545.22 (420.94 to 661.18)**	**1,407.96 (1,102.24 to 1,734.36)**	**1,439.31 (1,138.79 to 1,772.36)**	**1,377.00 (1,054.83 to 1,705.63)**
Rheumatoid arthritis	2,104.62 (1,598.02 to 3,203.60)	3,566.71 (2,489.84 to 5,751.92)	3,472.68 (2,402.69 to 5,759.07)	3,941.08 (2,750.02 to 6,229.08)	129.54 (96.97 to 203.02)	355.34 (235.37 to 607.62)	349.38 (232.71 to 605.30)	383.81 (256.35 to 640.93)
Other musculoskeletal disorders	10,772.07 (7,947.67 to 13,003.23)	11,567.17 (8,158.54 to 14,043.26)	11,756.56 (8,364.85 to 14,304.87)	11,344.33 (7,799.90 to 13,758.84)	415.68 (297.38 to 499.33)	1,052.62 (711.26 to 1,301.25)	1,089.93 (751.05 to 1,338.69)	993.20 (653.21 to 1,237.96)
**Other non-communicable diseases**	**39,143.60 (31,700.35 to 45,221.81)**	**36,484.48 (21,076.77 to 47,672.96)**	**37,048.32 (21,495.03 to 48,610.48)**	**36,326.66 (21,530.04 to 47,214.25)**	**1,104.51 (724.47 to 1,367.53)**	**4,190.19 (2,347.88 to 5,781.49)**	**4,351.17 (2,430.94 to 5,953.66)**	**3,975.51 (2,194.16 to 5,492.41)**
Congenital birth defects	26,292.38 (22,190.74 to 30,785.35)	5,736.69 (3,891.67 to 8,476.19)	5,418.53 (3,670.12 to 8,059.10)	6,578.32 (4,418.39 to 9,817.31)	346.74 (296.30 to 401.98)	89.88 (64.43 to 125.40)	85.61 (61.12 to 119.44)	101.49 (72.35 to 143.21)
Neural tube defects	258.42 (195.73 to 346.79)	31.79 (20.92 to 46.28)	29.43 (19.67 to 42.84)	38.36 (25.35 to 57.59)	3.81 (3.00 to 4.84)	0.58 (0.41 to 0.82)	0.54 (0.39 to 0.75)	0.69 (0.48 to 0.98)
Congenital heart anomalies	14,179.74 (11,160.79 to 17,245.96)	2,290.30 (1,412.80 to 3,672.58)	2,106.02 (1,293.38 to 3,408.17)	2,803.06 (1,753.88 to 4,555.81)	182.50 (148.45 to 219.06)	32.75 (21.32 to 49.69)	30.21 (19.44 to 46.31)	39.87 (25.98 to 61.86)
Orofacial clefts	52.24 (15.41 to 96.91)	5.61 (1.21 to 13.36)	4.97 (1.07 to 11.71)	7.46 (1.67 to 17.36)	0.61 (0.18 to 1.12)	0.06 (0.01 to 0.16)	0.06 (0.01 to 0.14)	0.09 (0.02 to 0.20)
Down syndrome	1,000.49 (779.66 to 1,239.68)	658.31 (489.03 to 878.63)	665.68 (494.64 to 890.08)	637.36 (472.88 to 851.77)	18.08 (14.37 to 22.88)	14.63 (10.89 to 19.32)	14.87 (11.10 to 19.64)	14.06 (10.53 to 18.44)
Other chromosomal abnormalities	2,714.27 (1,918.44 to 3,628.99)	1,235.98 (733.50 to 2,016.83)	1,193.19 (712.02 to 1,972.55)	1,329.27 (779.35 to 2,191.47)	34.32 (25.25 to 46.07)	16.93 (10.29 to 26.65)	16.39 (9.93 to 25.94)	18.13 (10.97 to 29.02)
Congenital musculoskeletal and limb anomalies	785.52 (527.40 to 1,052.57)	165.19 (95.34 to 270.08)	153.95 (89.49 to 254.42)	194.47 (111.09 to 326.83)	9.93 (6.95 to 13.08)	2.36 (1.45 to 3.73)	2.21 (1.36 to 3.50)	2.76 (1.69 to 4.40)
Urogenital congenital anomalies	380.32 (299.36 to 491.10)	136.31 (93.12 to 184.61)	131.02 (87.57 to 177.62)	151.14 (101.79 to 205.23)	7.59 (5.50 to 9.37)	3.66 (2.34 to 5.00)	3.54 (2.19 to 4.83)	4.01 (2.48 to 5.48)
Digestive congenital anomalies	1,175.62 (761.34 to 3,098.75)	241.08 (140.43 to 471.45)	224.00 (131.03 to 443.29)	286.47 (168.18 to 568.54)	15.56 (10.92 to 36.71)	3.92 (2.62 to 6.32)	3.67 (2.41 to 5.82)	4.62 (3.02 to 7.51)
Other congenital birth defects	5,745.76 (4,389.40 to 7,147.09)	972.11 (614.52 to 1,473.95)	910.28 (579.33 to 1,380.13)	1,130.74 (720.86 to 1,754.43)	74.35 (58.05 to 90.94)	14.99 (10.08 to 21.43)	14.12 (9.62 to 20.19)	17.27 (11.70 to 25.50)
Skin and subcutaneous diseases	10,032.99 (5,042.08 to 13,586.32)	30,354.87 (15,799.73 to 40,387.80)	31,257.16 (16,157.26 to 41,634.46)	29,308.14 (15,047.00 to 39,492.36)	725.07 (367.09 to 984.31)	4,095.75 (2,253.61 to 5,675.61)	4,261.24 (2,334.24 to 5,858.94)	3,868.91 (2,094.76 to 5,357.15)
Cellulitis	2,726.55 (840.26 to 4,153.44)	9,265.36 (1,867.77 to 15,393.25)	9,642.07 (1,968.23 to 16,146.07)	8,718.31 (1,777.46 to 14,494.60)	169.38 (31.66 to 283.98)	1,128.88 (186.54 to 2,000.32)	1,189.76 (201.24 to 2,095.80)	1,035.56 (168.25 to 1,857.65)
Pyoderma	2,840.09 (1,464.88 to 4,266.60)	10,128.92 (5,786.69 to 16,076.19)	10,453.02 (6,008.95 to 16,598.83)	9,718.99 (5,366.56 to 15,466.47)	189.62 (93.49 to 284.91)	1,350.90 (769.93 to 2,280.02)	1,411.90 (807.83 to 2,392.16)	1,261.17 (694.04 to 2,152.52)
Decubitus ulcer	3,334.17 (1,654.43 to 4,777.51)	8,923.54 (5,006.88 to 15,763.01)	9,067.43 (5,106.78 to 16,178.01)	8,893.01 (5,006.22 to 15,473.28)	300.10 (166.64 to 459.35)	1,384.10 (779.12 to 2,687.05)	1,416.79 (820.47 to 2,749.35)	1,354.64 (765.06 to 2,615.40)
Other skin and subcutaneous diseases	1,132.19 (675.84 to 1,893.37)	2,037.05 (1,278.54 to 3,753.67)	2,094.65 (1,309.12 to 3,850.47)	1,977.83 (1,243.56 to 3,607.33)	65.97 (39.88 to 113.23)	231.88 (142.40 to 473.73)	242.79 (149.09 to 492.64)	217.54 (133.37 to 440.18)
Sudden infant death syndrome	2,818.23 (1,939.69 to 3,966.44)	392.92 (163.23 to 915.50)	372.63 (153.20 to 871.94)	440.19 (182.50 to 991.53)	32.70 (22.50 to 46.03)	4.56 (1.89 to 10.62)	4.32 (1.78 to 10.12)	5.11 (2.12 to 11.50)

**Injuries**	**424,538.44 (367,058.65 to 483,208.04)**	**267,048.99 (204,244.68 to 352,703.02)**	**249,245.02 (194,606.58 to 321,997.35)**	**282,856.30 (214,232.34 to 374,757.30)**	**12,843.14 (11,146.04 to 14,550.22)**	**16,450.91 (13,664.22 to 20,053.61)**	**15,935.86 (13,357.75 to 19,175.98)**	**16,636.42 (13,663.15 to 20,540.15)**
**Transport injuries**	**149,872.88 (128,205.11 to 174,776.46)**	**95,285.54 (67,815.48 to 139,991.81)**	**88,334.74 (64,760.73 to 126,413.73)**	**103,009.85 (72,607.62 to 152,103.72)**	**4,355.18 (3,716.18 to 5,053.86)**	**4,506.49 (3,365.55 to 6,212.10)**	**4,238.13 (3,267.40 to 5,610.26)**	**4,719.84 (3,495.73 to 6,628.25)**
Road injuries	142,721.99 (122,324.73 to 166,154.81)	90,321.32 (64,622.12 to 132,935.94)	83,905.38 (61,860.29 to 119,944.43)	97,382.24 (69,038.81 to 143,335.41)	4,155.87 (3,560.04 to 4,813.77)	4,247.93 (3,204.28 to 5,858.42)	4,001.49 (3,101.95 to 5,316.32)	4,438.45 (3,299.59 to 6,145.04)
Pedestrian road injuries	47,478.76 (40,096.12 to 58,368.81)	24,735.93 (17,956.66 to 34,814.70)	22,400.16 (16,901.25 to 31,332.69)	28,785.08 (20,677.81 to 41,701.00)	1,690.13 (1,430.15 to 2,061.52)	1,718.55 (1,288.69 to 2,351.35)	1,592.02 (1,227.90 to 2,114.14)	1,910.66 (1,415.39 to 2,639.13)

Taiwan (Province of China)								
Cause	YLLs (actual counts)				Deaths (actual counts)			
	2016	2040 Forecast	2040 Better Scenario	2040 Worse Scenario	2016	2040 Forecast	2040 Better Scenario	2040 Worse Scenario
Cyclist road injuries	8,837.12 (7,291.35 to 10,675.54)	6,590.91 (4,608.67 to 9,987.27)	6,243.23 (4,453.54 to 9,136.83)	6,697.77 (4,652.29 to 10,073.29)	320.87 (260.70 to 391.05)	393.03 (259.17 to 610.20)	377.95 (257.50 to 561.26)	383.28 (252.70 to 590.35)
Motorcyclist road injuries	56,056.84 (46,235.12 to 65,778.23)	40,616.74 (25,969.81 to 63,662.21)	38,051.27 (25,269.84 to 57,778.87)	42,314.09 (27,010.04 to 66,196.71)	1,413.39 (1,142.58 to 1,664.45)	1,400.40 (866.49 to 2,080.15)	1,325.31 (836.12 to 1,882.23)	1,402.90 (871.87 to 2,122.82)
Motor vehicle road injuries	29,529.72 (23,660.13 to 38,442.56)	17,678.13 (11,653.37 to 26,961.03)	16,550.11 (11,237.99 to 24,587.94)	18,847.85 (12,285.02 to 28,540.92)	710.56 (570.86 to 935.98)	704.21 (474.51 to 1,061.08)	675.54 (469.65 to 1,003.25)	710.20 (482.63 to 1,057.46)
Other road injuries	819.55 (632.99 to 1,024.17)	699.61 (454.68 to 1,094.26)	660.61 (441.61 to 1,004.89)	737.45 (473.73 to 1,158.04)	20.92 (16.89 to 26.27)	31.74 (21.79 to 51.97)	30.67 (21.64 to 48.48)	31.41 (21.26 to 50.47)
Other transport injuries	7,150.89 (5,673.09 to 8,705.80)	4,964.23 (3,207.57 to 8,051.09)	4,429.35 (2,967.96 to 6,932.07)	5,627.61 (3,580.86 to 9,145.04)	199.30 (160.16 to 248.38)	258.56 (167.99 to 423.06)	236.63 (158.53 to 389.22)	281.40 (182.50 to 467.89)
**Unintentional injuries**	**102,919.97 (87,931.05 to 117,680.58)**	**81,210.79 (67,491.45 to 95,090.07)**	**81,185.04 (67,387.02 to 95,065.43)**	**82,469.86 (68,478.26 to 97,025.08)**	**3,807.58 (3,171.18 to 4,369.18)**	**7,671.06 (6,484.33 to 8,944.72)**	**7,841.93 (6,693.00 to 9,083.06)**	**7,479.88 (6,220.16 to 8,788.56)**
Falls	43,443.47 (33,341.97 to 51,605.78)	47,127.49 (38,172.27 to 55,198.77)	47,773.83 (38,553.43 to 56,283.02)	47,025.72 (37,843.77 to 55,602.30)	1,952.69 (1,516.47 to 2,303.32)	4,983.23 (4,084.76 to 5,983.47)	5,130.59 (4,227.50 to 6,161.93)	4,819.95 (3,938.67 to 5,877.66)
Drowning	21,078.10 (17,761.31 to 24,765.32)	10,256.45 (7,631.47 to 13,818.34)	9,711.72 (7,326.70 to 12,859.49)	10,932.62 (8,105.96 to 15,008.81)	564.65 (469.32 to 660.58)	486.56 (384.10 to 624.71)	471.51 (377.21 to 598.66)	499.99 (394.22 to 647.70)
Fire, heat, and hot substances	5,618.29 (4,393.99 to 6,549.28)	3,900.77 (2,835.04 to 4,934.77)	3,751.42 (2,706.17 to 4,646.89)	4,140.82 (3,032.76 to 5,322.68)	232.40 (177.19 to 273.77)	442.82 (311.30 to 569.79)	435.43 (310.11 to 557.04)	452.21 (321.51 to 590.32)
Poisonings	3,325.31 (2,474.33 to 4,027.29)	895.80 (654.97 to 1,255.80)	833.42 (618.77 to 1,145.51)	1,013.10 (729.65 to 1,444.22)	91.41 (71.25 to 112.55)	53.82 (41.19 to 72.78)	51.45 (40.04 to 69.33)	58.15 (43.92 to 80.22)
Exposure to mechanical forces	8,884.11 (7,293.18 to 11,395.09)	4,037.65 (3,218.71 to 4,980.12)	3,976.80 (3,196.15 to 4,892.51)	4,288.92 (3,434.39 to 5,314.98)	244.83 (205.60 to 299.26)	277.97 (219.73 to 329.69)	281.30 (220.96 to 334.42)	279.20 (218.61 to 336.40)
Unintentional firearm injuries	449.17 (369.18 to 615.07)	507.82 (405.48 to 622.63)	514.13 (409.21 to 630.35)	502.41 (400.40 to 616.09)	21.30 (17.84 to 25.68)	69.45 (50.82 to 90.72)	71.48 (52.38 to 93.02)	66.06 (48.28 to 87.16)
Unintentional suffocation	1,356.76 (410.33 to 2,220.22)	351.67 (165.80 to 639.82)	342.55 (163.40 to 634.68)	376.17 (171.85 to 691.79)	20.46 (8.54 to 31.45)	9.94 (6.88 to 14.81)	9.93 (6.84 to 15.06)	10.22 (7.29 to 14.79)
Other exposure to mechanical forces	7,078.19 (5,651.25 to 10,272.39)	3,178.16 (2,456.59 to 4,029.89)	3,120.12 (2,435.43 to 3,931.15)	3,410.34 (2,655.26 to 4,414.55)	203.07 (164.89 to 259.94)	198.57 (146.63 to 244.71)	199.89 (145.14 to 244.40)	202.92 (149.95 to 249.80)
Adverse effects of medical treatment	5,851.26 (4,291.14 to 7,169.41)	6,165.98 (2,977.97 to 8,283.59)	6,259.63 (2,988.09 to 8,444.91)	6,103.44 (2,994.54 to 8,174.40)	260.37 (141.21 to 347.19)	651.81 (256.82 to 933.17)	672.68 (264.25 to 959.43)	623.57 (244.11 to 894.88)
Animal contact	419.14 (341.60 to 510.24)	378.01 (293.03 to 469.50)	379.83 (293.19 to 475.26)	385.93 (299.06 to 484.94)	15.22 (12.48 to 18.45)	33.20 (25.28 to 42.05)	34.04 (25.48 to 43.21)	32.60 (24.32 to 41.86)
Venomous animal contact	249.32 (192.27 to 309.91)	174.99 (130.71 to 227.88)	175.87 (132.17 to 228.51)	177.34 (133.29 to 229.98)	7.76 (6.04 to 9.72)	12.27 (8.38 to 16.70)	12.58 (8.59 to 17.10)	11.94 (8.09 to 16.16)
Non-venomous animal contact	169.82 (139.74 to 214.65)	203.02 (151.85 to 265.78)	203.96 (150.43 to 266.52)	208.59 (154.75 to 275.12)	7.46 (5.97 to 9.50)	20.93 (15.05 to 27.54)	21.46 (15.55 to 28.47)	20.67 (14.50 to 27.75)
Foreign body	8,295.57 (6,813.50 to 9,999.41)	6,278.57 (4,774.55 to 7,492.74)	6,405.35 (4,862.37 to 7,619.64)	6,124.23 (4,608.81 to 7,316.31)	268.58 (204.01 to 316.39)	579.01 (440.02 to 731.94)	603.48 (460.55 to 766.61)	540.49 (410.61 to 681.62)
Pulmonary aspiration and foreign body in airway	7,954.55 (6,439.97 to 9,638.98)	5,806.65 (4,372.50 to 6,994.78)	5,914.47 (4,437.29 to 7,133.20)	5,688.71 (4,271.06 to 6,865.52)	255.04 (191.15 to 302.24)	520.21 (390.89 to 645.97)	541.30 (409.53 to 679.04)	488.19 (365.12 to 606.27)

Foreign body in other body part	341.03 (256.19 to 426.84)	471.92 (339.33 to 665.80)	490.88 (351.11 to 682.33)	435.53 (311.70 to 610.49)	13.54 (9.66 to 17.83)	58.80 (40.86 to 93.39)	62.18 (43.23 to 98.61)	52.30 (36.24 to 84.26)
Environmental heat and cold exposure	1,361.72 (944.32 to 1,729.16)	1,052.90 (768.63 to 1,426.53)	1,043.49 (761.06 to 1,410.40)	1,116.55 (809.38 to 1,501.40)	53.59 (37.82 to 67.82)	96.49 (71.74 to 151.67)	97.65 (72.20 to 153.81)	98.23 (73.05 to 152.81)
Other unintentional injuries	4,642.99 (3,746.16 to 6,403.36)	1,117.17 (829.45 to 1,500.04)	1,049.55 (787.86 to 1,385.43)	1,338.53 (996.82 to 1,827.54)	123.84 (100.67 to 157.76)	66.15 (49.82 to 85.12)	63.80 (48.98 to 81.86)	75.48 (56.64 to 99.00)
**Self-harm and interpersonal violence**	**161,594.85 (132,035.39 to 191,079.06)**	**89,793.75 (56,402.02 to 136,228.26)**	**78,960.56 (52,349.28 to 115,967.07)**	**96,628.99 (59,573.33 to 150,378.68)**	**4,434.29 (3,733.37 to 5,205.31)**	**4,246.75 (2,776.03 to 6,437.55)**	**3,828.55 (2,639.54 to 5,641.10)**	**4,411.24 (2,826.15 to 6,795.63)**

Taiwan (Province of China)								
Cause	YLLs (actual counts)				Deaths (actual counts)			
	2016	2040 Forecast	2040 Better Scenario	2040 Worse Scenario	2016	2040 Forecast	2040 Better Scenario	2040 Worse Scenario
Self-harm	145,039.76 (114,243.40 to 171,290.54)	83,935.44 (51,532.10 to 128,731.14)	73,510.44 (48,090.36 to 108,119.06)	90,293.46 (54,714.99 to 141,605.88)	4,052.40 (3,371.32 to 4,761.34)	4,044.14 (2,614.82 to 6,203.93)	3,636.97 (2,485.72 to 5,404.69)	4,198.11 (2,663.76 to 6,521.05)
Self-harm by firearm	1,043.42 (577.21 to 1,414.25)	425.39 (231.38 to 770.24)	365.74 (211.55 to 627.46)	472.82 (249.06 to 850.14)	27.76 (18.00 to 38.41)	27.50 (16.22 to 46.85)	25.14 (15.39 to 42.47)	28.86 (16.43 to 50.89)
Self-harm by other specified means	143,996.34 (113,472.62 to 169,997.73)	83,510.05 (51,299.62 to 128,111.33)	73,144.69 (47,863.85 to 107,604.97)	89,820.64 (54,428.02 to 140,793.91)	4,024.64 (3,350.91 to 4,729.63)	4,016.64 (2,601.18 to 6,127.02)	3,611.83 (2,465.45 to 5,370.12)	4,169.24 (2,647.27 to 6,479.88)
Interpersonal violence	16,555.09 (13,115.33 to 23,727.16)	5,858.31 (3,908.88 to 8,982.32)	5,450.12 (3,649.71 to 8,258.51)	6,335.53 (4,188.29 to 9,790.99)	381.89 (302.99 to 532.70)	202.62 (142.64 to 287.61)	191.58 (137.02 to 265.95)	213.13 (151.76 to 300.92)
Physical violence by firearm	1,610.97 (680.57 to 2,404.79)	403.79 (195.43 to 654.94)	387.57 (187.10 to 634.02)	454.03 (215.28 to 757.40)	37.48 (16.55 to 57.05)	12.92 (7.28 to 21.14)	12.55 (7.08 to 20.81)	14.19 (7.76 to 23.50)
Physical violence by sharp object	4,040.46 (2,912.84 to 7,779.77)	1,389.08 (937.45 to 2,327.40)	1,343.51 (907.44 to 2,246.85)	1,522.53 (1,013.72 to 2,605.82)	93.34 (69.13 to 165.52)	49.27 (33.63 to 67.46)	48.58 (32.61 to 66.69)	52.07 (35.57 to 71.94)
Physical violence by other means	10,903.66 (8,473.23 to 14,830.72)	4,065.43 (2,580.77 to 6,423.70)	3,719.04 (2,402.80 to 5,823.16)	4,358.97 (2,749.86 to 6,824.61)	251.07 (195.02 to 341.03)	140.43 (93.88 to 214.71)	130.45 (89.71 to 193.80)	146.88 (98.51 to 225.25)
**Forces of nature, conflict and terrorism, and executions and police conflict**	**10,150.74 (1,728.76 to 19,324.36)**	**758.91 (0.00 to 2,957.88)**	**764.69 (0.00 to 2,969.46)**	**747.60 (0.00 to 2,934.19)**	**246.09 (41.56 to 468.81)**	**26.61 (0.00 to 102.62)**	**27.25 (0.00 to 104.25)**	**25.47 (0.00 to 99.33)**
Exposure to forces of nature	10,012.22 (1,588.60 to 19,189.64)	706.52 (0.00 to 2,905.08)	711.95 (0.00 to 2,918.86)	695.88 (0.00 to 2,884.02)	243.10 (38.57 to 465.92)	24.97 (0.00 to 100.49)	25.57 (0.00 to 102.50)	23.92 (0.00 to 97.97)
Conflict and terrorism	0.00 (0.00 to 0.00)	52.39 (0.00 to 126.46)	52.74 (0.00 to 127.35)	51.72 (0.00 to 124.61)	0.00 (0.00 to 0.00)	1.64 (0.00 to 4.24)	1.69 (0.00 to 4.38)	1.55 (0.00 to 3.97)
Executions and police conflict	138.53 (113.78 to 170.24)	0.00 (0.00 to 0.00)	0.00 (0.00 to 0.00)	0.00 (0.00 to 0.00)	2.99 (2.39 to 3.74)	0.00 (0.00 to 0.00)	0.00 (0.00 to 0.00)	0.00 (0.00 to 0.00)

Southeast Asia								
Cause	YLLs (in 1000s)				Deaths (in 1000s)			
	2016	2040 Forecast	2040 Better Scenario	2040 Worse Scenario	2016	2040 Forecast	2040 Better Scenario	2040 Worse Scenario
**All causes**	**118,944.33 (115,176.93 to 122,858.72)**	**130,423.53 (113,556.24 to 149,783.19)**	**103,364.11 (90,058.59 to 117,568.46)**	**172,423.84 (148,612.69 to 200,178.73)**	**4,090.64 (3,977.62 to 4,209.01)**	**6,547.56 (5,832.27 to 7,332.34)**	**5,467.11 (4,874.53 to 6,100.14)**	**8,091.63 (7,187.62 to 9,068.59)**
**Communicable, maternal, neonatal, and nutritional diseases**	**32,654.45 (30,819.13 to 34,643.84)**	**21,188.69 (17,583.86 to 26,719.29)**	**15,603.51 (12,851.05 to 19,666.05)**	**30,477.45 (24,679.55 to 38,695.50)**	**727.10 (677.13 to 771.72)**	**783.82 (612.91 to 1,050.45)**	**634.39 (496.87 to 862.79)**	**1,046.02 (813.63 to 1,416.77)**
**HIV/AIDS and tuberculosis**	**6,625.80 (6,166.97 to 7,143.24)**	**4,411.22 (3,475.13 to 5,662.11)**	**2,764.80 (2,109.76 to 3,550.52)**	**7,124.18 (5,720.05 to 9,082.23)**	**200.82 (188.43 to 214.30)**	**178.07 (135.64 to 233.98)**	**117.44 (89.79 to 152.32)**	**266.95 (206.82 to 349.92)**
Tuberculosis	4,813.88 (4,554.07 to 5,076.82)	3,339.33 (2,462.96 to 4,548.58)	2,160.82 (1,619.37 to 2,909.56)	5,060.16 (3,693.95 to 6,938.98)	163.69 (154.88 to 173.62)	156.26 (116.22 to 210.60)	105.40 (79.39 to 140.79)	224.92 (164.61 to 304.11)
HIV/AIDS	1,811.92 (1,439.55 to 2,269.91)	1,071.89 (801.55 to 1,447.39)	603.97 (368.20 to 939.42)	2,064.02 (1,638.30 to 2,669.03)	37.13 (28.92 to 47.35)	21.82 (15.66 to 30.84)	12.05 (6.49 to 20.51)	42.03 (32.57 to 54.91)
**Diarrhea, lower respiratory, and other common infectious diseases**	**12,884.88 (11,665.77 to 14,195.30)**	**8,985.15 (5,954.47 to 13,757.51)**	**6,713.14 (4,549.85 to 10,298.30)**	**13,267.74 (8,670.72 to 20,655.97)**	**329.92 (282.05 to 370.48)**	**439.86 (285.76 to 684.04)**	**366.78 (238.88 to 582.57)**	**584.65 (365.51 to 933.30)**
Diarrheal diseases	2,518.42 (1,959.84 to 3,270.59)	1,790.02 (331.00 to 5,137.68)	1,577.89 (353.56 to 4,508.45)	2,751.05 (495.50 to 7,822.49)	73.48 (46.20 to 101.16)	110.39 (18.56 to 332.27)	107.65 (22.74 to 305.70)	157.53 (26.40 to 476.17)
Intestinal infectious diseases	1,411.74 (786.06 to 2,296.39)	761.83 (398.97 to 1,275.10)	727.87 (392.89 to 1,203.15)	868.77 (443.95 to 1,490.75)	21.28 (11.92 to 34.77)	13.06 (6.98 to 21.93)	12.89 (7.02 to 21.13)	14.56 (7.62 to 24.69)
Typhoid fever	1,226.69 (668.68 to 2,001.13)	647.57 (330.50 to 1,094.35)	635.89 (338.42 to 1,053.35)	732.13 (363.24 to 1,257.21)	18.40 (10.07 to 30.03)	11.02 (5.84 to 18.54)	11.20 (6.09 to 18.34)	12.18 (6.20 to 20.65)
Paratyphoid fever	181.49 (85.67 to 349.83)	113.24 (50.07 to 216.78)	91.13 (40.25 to 173.24)	135.31 (58.89 to 265.86)	2.81 (1.33 to 5.47)	2.00 (0.89 to 3.87)	1.65 (0.75 to 3.15)	2.33 (1.04 to 4.51)
Other intestinal infectious diseases	3.56 (1.36 to 7.72)	1.01 (0.38 to 2.28)	0.85 (0.31 to 1.84)	1.32 (0.49 to 3.04)	0.07 (0.03 to 0.14)	0.04 (0.01 to 0.08)	0.04 (0.01 to 0.08)	0.05 (0.02 to 0.09)
Lower respiratory infections	6,856.31 (6,133.53 to 7,442.28)	5,088.58 (3,575.32 to 7,229.55)	3,482.41 (2,594.68 to 4,619.55)	7,704.65 (5,071.99 to 11,766.35)	201.79 (170.92 to 218.58)	289.66 (210.86 to 378.40)	224.93 (170.36 to 284.91)	378.26 (267.29 to 514.41)
Upper respiratory infections	6.45 (5.37 to 7.88)	3.98 (2.89 to 5.40)	3.04 (2.17 to 4.12)	5.53 (3.99 to 7.44)	0.15 (0.13 to 0.18)	0.15 (0.11 to 0.19)	0.12 (0.08 to 0.16)	0.19 (0.14 to 0.25)
Otitis media	7.45 (3.09 to 12.79)	2.85 (1.12 to 4.89)	2.92 (1.14 to 4.95)	2.76 (1.08 to 4.78)	0.21 (0.10 to 0.32)	0.13 (0.05 to 0.23)	0.14 (0.06 to 0.25)	0.12 (0.05 to 0.22)
Meningitis	797.07 (594.64 to 945.30)	338.23 (220.74 to 459.47)	266.28 (182.35 to 356.97)	408.35 (247.94 to 586.06)	12.89 (9.54 to 15.06)	7.90 (5.57 to 10.17)	6.73 (4.84 to 8.53)	8.76 (6.10 to 11.64)
Pneumococcal meningitis	6.65 (4.45 to 8.99)	1.94 (1.25 to 2.71)	1.73 (1.16 to 2.39)	2.30 (1.43 to 3.41)	0.11 (0.08 to 0.15)	0.06 (0.04 to 0.08)	0.06 (0.04 to 0.08)	0.06 (0.04 to 0.09)
H influenzae type B meningitis	63.87 (47.96 to 78.16)	46.38 (16.27 to 114.59)	27.17 (13.98 to 52.67)	80.95 (19.81 to 221.47)	0.98 (0.74 to 1.18)	0.79 (0.40 to 1.61)	0.58 (0.37 to 0.91)	1.17 (0.45 to 2.85)
Meningococcal meningitis	266.96 (196.78 to 323.66)	102.72 (67.56 to 137.61)	84.99 (58.07 to 114.07)	115.64 (75.54 to 155.19)	4.40 (3.24 to 5.28)	2.64 (1.87 to 3.39)	2.33 (1.65 to 2.98)	2.82 (1.99 to 3.62)
Other meningitis	459.58 (338.13 to 557.01)	187.18 (126.83 to 246.87)	152.39 (105.30 to 202.86)	209.45 (139.42 to 275.06)	7.39 (5.42 to 8.76)	4.41 (3.13 to 5.58)	3.76 (2.70 to 4.80)	4.71 (3.39 to 5.94)
Encephalitis	411.35 (294.56 to 499.21)	282.67 (198.90 to 367.08)	252.60 (183.87 to 328.58)	321.59 (222.67 to 417.80)	7.38 (5.57 to 8.54)	7.83 (6.00 to 9.43)	7.50 (5.78 to 8.98)	8.37 (6.44 to 10.00)

Diphtheria	3.32 (2.79 to 3.92)	0.61 (0.37 to 1.00)	0.42 (0.30 to 0.57)	0.95 (0.41 to 1.79)	0.05 (0.04 to 0.06)	0.01 (0.01 to 0.02)	0.01 (0.01 to 0.01)	0.01 (0.01 to 0.02)
Whooping cough	322.99 (102.03 to 720.74)	120.63 (10.56 to 363.68)	28.71 (5.53 to 80.46)	164.71 (12.56 to 470.78)	3.84 (1.22 to 8.56)	1.44 (0.14 to 4.34)	0.36 (0.08 to 1.00)	1.96 (0.16 to 5.59)
Tetanus	162.40 (64.49 to 206.00)	27.46 (10.16 to 39.68)	21.70 (7.41 to 31.22)	30.43 (12.00 to 43.72)	3.79 (1.39 to 4.82)	0.78 (0.27 to 1.11)	0.74 (0.24 to 1.06)	0.78 (0.28 to 1.11)
Measles	345.23 (131.84 to 722.74)	551.49 (104.68 to 1,970.63)	335.36 (86.13 to 1,054.22)	986.74 (138.65 to 3,747.07)	4.14 (1.58 to 8.65)	7.69 (1.71 to 25.48)	4.97 (1.48 to 13.98)	13.14 (2.32 to 45.98)

Southeast Asia								
Cause	YLLs (in 1000s)				Deaths (in 1000s)			
	2016	2040 Forecast	2040 Better Scenario	2040 Worse Scenario	2016	2040 Forecast	2040 Better Scenario	2040 Worse Scenario
Varicella and herpes zoster	42.16 (36.67 to 50.19)	16.81 (11.44 to 22.83)	13.95 (9.92 to 18.74)	22.20 (14.47 to 30.66)	0.93 (0.83 to 1.07)	0.82 (0.62 to 1.05)	0.77 (0.59 to 0.97)	0.95 (0.72 to 1.22)
**Neglected tropical diseases and malaria**	**1,487.64 (962.69 to 1,819.38)**	**2,309.02 (1,592.05 to 2,815.21)**	**2,080.06 (1,436.19 to 2,507.62)**	**2,492.57 (1,685.95 to 3,045.34)**	**27.01 (18.51 to 32.67)**	**49.07 (35.22 to 58.33)**	**46.58 (33.72 to 55.22)**	**51.08 (36.01 to 60.92)**
Malaria	744.92 (485.27 to 1,005.87)	414.23 (289.57 to 768.45)	401.19 (283.90 to 749.27)	448.21 (308.57 to 834.42)	15.38 (10.59 to 22.06)	11.82 (8.38 to 22.24)	11.84 (8.39 to 21.63)	12.12 (8.42 to 22.88)
Chagas disease	0.00 (0.00 to 0.00)	0.00 (0.00 to 0.00)	0.00 (0.00 to 0.00)	0.00 (0.00 to 0.00)	0.00 (0.00 to 0.00)	0.00 (0.00 to 0.00)	0.00 (0.00 to 0.00)	0.00 (0.00 to 0.00)
Leishmaniasis	0.01 (0.01 to 0.02)	0.01 (0.00 to 0.01)	0.01 (0.00 to 0.01)	0.01 (0.00 to 0.01)	0.00 (0.00 to 0.00)	0.00 (0.00 to 0.00)	0.00 (0.00 to 0.00)	0.00 (0.00 to 0.00)
African trypanosomiasis	0.00 (0.00 to 0.00)	0.00 (0.00 to 0.00)	0.00 (0.00 to 0.00)	0.00 (0.00 to 0.00)	0.00 (0.00 to 0.00)	0.00 (0.00 to 0.00)	0.00 (0.00 to 0.00)	0.00 (0.00 to 0.00)
Schistosomiasis	4.21 (2.97 to 5.76)	3.09 (1.96 to 4.82)	2.75 (1.75 to 4.28)	3.37 (2.16 to 5.12)	0.13 (0.09 to 0.18)	0.12 (0.07 to 0.18)	0.11 (0.07 to 0.17)	0.12 (0.08 to 0.19)
Cysticercosis	0.97 (0.82 to 1.15)	0.37 (0.30 to 0.51)	0.36 (0.28 to 0.49)	0.40 (0.32 to 0.54)	0.02 (0.02 to 0.03)	0.01 (0.01 to 0.01)	0.01 (0.01 to 0.01)	0.01 (0.01 to 0.02)
Cystic echinococcosis	0.30 (0.25 to 0.37)	0.15 (0.11 to 0.23)	0.12 (0.09 to 0.19)	0.23 (0.17 to 0.34)	0.01 (0.01 to 0.01)	0.01 (0.00 to 0.01)	0.01 (0.00 to 0.01)	0.01 (0.01 to 0.01)
Dengue	668.67 (290.39 to 891.34)	1,848.24 (1,053.04 to 2,348.96)	1,639.48 (927.31 to 2,068.28)	1,985.29 (1,119.40 to 2,544.29)	10.17 (4.44 to 13.53)	36.05 (21.25 to 45.14)	33.64 (19.87 to 41.90)	37.52 (21.75 to 47.32)
Yellow fever	0.00 (0.00 to 0.00)	0.00 (0.00 to 0.00)	0.00 (0.00 to 0.00)	0.00 (0.00 to 0.00)	0.00 (0.00 to 0.00)	0.00 (0.00 to 0.00)	0.00 (0.00 to 0.00)	0.00 (0.00 to 0.00)
Rabies	31.16 (16.59 to 41.87)	12.51 (7.56 to 17.48)	8.59 (5.04 to 11.95)	20.44 (12.51 to 27.88)	0.66 (0.37 to 0.87)	0.30 (0.21 to 0.41)	0.23 (0.15 to 0.31)	0.48 (0.32 to 0.65)
Intestinal nematode infections	22.17 (18.48 to 26.41)	18.72 (13.82 to 28.21)	16.91 (12.41 to 26.19)	20.55 (15.34 to 30.39)	0.32 (0.28 to 0.38)	0.36 (0.28 to 0.53)	0.35 (0.27 to 0.51)	0.37 (0.29 to 0.53)
Other neglected tropical diseases	15.22 (11.54 to 30.21)	11.70 (8.26 to 26.28)	10.65 (7.62 to 22.90)	14.07 (9.88 to 31.40)	0.32 (0.26 to 0.60)	0.40 (0.29 to 0.81)	0.40 (0.29 to 0.80)	0.45 (0.32 to 0.93)
**Maternal disorders**	**994.36 (913.12 to 1,078.44)**	**456.08 (369.42 to 561.66)**	**376.69 (298.40 to 470.01)**	**667.91 (538.01 to 824.49)**	**18.39 (16.86 to 19.96)**	**8.59 (6.94 to 10.63)**	**7.12 (5.65 to 8.91)**	**12.56 (10.05 to 15.52)**
Maternal hemorrhage	333.39 (260.72 to 412.24)	143.89 (104.30 to 196.27)	118.69 (83.57 to 160.26)	210.51 (148.81 to 285.52)	6.13 (4.79 to 7.57)	2.69 (1.93 to 3.70)	2.22 (1.56 to 2.99)	3.93 (2.77 to 5.40)
Maternal sepsis and other maternal infections	54.29 (34.60 to 79.99)	23.04 (13.92 to 35.70)	18.42 (10.68 to 29.20)	33.31 (19.66 to 52.33)	1.02 (0.65 to 1.49)	0.44 (0.27 to 0.68)	0.35 (0.21 to 0.55)	0.64 (0.38 to 0.99)
Maternal hypertensive disorders	114.41 (83.41 to 151.45)	54.53 (36.44 to 78.64)	44.12 (28.98 to 64.02)	79.64 (53.09 to 113.68)	2.11 (1.52 to 2.81)	1.02 (0.69 to 1.48)	0.83 (0.55 to 1.20)	1.49 (1.00 to 2.16)
Maternal obstructed labor and uterine rupture	27.87 (14.34 to 45.59)	12.74 (6.30 to 22.66)	10.37 (4.92 to 18.39)	19.30 (9.47 to 34.48)	0.52 (0.27 to 0.87)	0.24 (0.12 to 0.44)	0.20 (0.09 to 0.35)	0.37 (0.18 to 0.67)
Maternal abortion, miscarriage, and ectopic pregnancy	107.77 (78.87 to 142.09)	51.06 (33.69 to 78.44)	41.34 (26.99 to 63.76)	77.69 (52.75 to 118.07)	2.01 (1.47 to 2.62)	0.97 (0.65 to 1.46)	0.79 (0.53 to 1.22)	1.48 (1.02 to 2.21)

Indirect maternal deaths	121.81 (85.65 to 165.41)	60.24 (38.53 to 88.01)	49.84 (31.20 to 74.43)	88.77 (57.10 to 127.90)	2.21 (1.54 to 2.99)	1.11 (0.71 to 1.61)	0.92 (0.57 to 1.36)	1.63 (1.04 to 2.34)
Late maternal deaths	21.89 (10.57 to 37.62)	8.25 (3.71 to 15.29)	6.93 (3.11 to 12.80)	12.24 (5.60 to 22.88)	0.41 (0.19 to 0.69)	0.16 (0.07 to 0.29)	0.13 (0.06 to 0.24)	0.23 (0.10 to 0.43)
Maternal deaths aggravated by HIV/AIDS	0.78 (0.42 to 1.16)	1.39 (0.65 to 2.42)	0.90 (0.39 to 1.64)	2.29 (1.08 to 3.94)	0.01 (0.01 to 0.02)	0.03 (0.01 to 0.05)	0.02 (0.01 to 0.03)	0.04 (0.02 to 0.08)
Other maternal disorders	212.15 (154.89 to 273.42)	100.93 (69.17 to 144.05)	86.08 (58.29 to 124.23)	144.17 (97.96 to 205.27)	3.96 (2.95 to 5.12)	1.92 (1.31 to 2.74)	1.65 (1.10 to 2.36)	2.74 (1.85 to 3.92)

Southeast Asia								
Cause	YLLs (in 1000s)				Deaths (in 1000s)			
	2016	2040 Forecast	2040 Better Scenario	2040 Worse Scenario	2016	2040 Forecast	2040 Better Scenario	2040 Worse Scenario
**Neonatal disorders**	**9,132.97 (8,252.45 to 10,168.53)**	**3,863.61 (2,840.92 to 4,959.00)**	**2,573.70 (1,874.16 to 3,335.01)**	**5,597.50 (4,105.70 to 7,285.11)**	**105.51 (95.34 to 117.47)**	**44.64 (32.83 to 57.29)**	**29.74 (21.66 to 38.53)**	**64.67 (47.44 to 84.17)**
Neonatal preterm birth	4,058.91 (3,610.47 to 4,681.85)	1,516.42 (1,185.24 to 1,887.25)	910.87 (712.64 to 1,139.98)	2,307.39 (1,800.85 to 2,897.31)	46.89 (41.71 to 54.08)	17.52 (13.70 to 21.80)	10.52 (8.23 to 13.17)	26.66 (20.80 to 33.47)
Neonatal encephalopathy due to birth asphyxia and trauma	2,777.67 (2,381.75 to 3,186.47)	1,096.41 (669.04 to 1,631.67)	788.58 (470.68 to 1,183.04)	1,541.41 (933.91 to 2,329.32)	32.09 (27.51 to 36.81)	12.67 (7.73 to 18.85)	9.11 (5.44 to 13.67)	17.81 (10.79 to 26.91)
Neonatal sepsis and other neonatal infections	1,444.87 (1,165.62 to 1,905.21)	873.96 (606.04 to 1,252.45)	585.57 (408.30 to 834.24)	1,191.00 (823.55 to 1,720.99)	16.69 (13.47 to 22.01)	10.10 (7.00 to 14.47)	6.77 (4.72 to 9.64)	13.76 (9.52 to 19.89)
Hemolytic disease and other neonatal jaundice	46.57 (36.19 to 57.87)	14.59 (8.63 to 23.10)	10.32 (6.04 to 16.37)	22.21 (13.17 to 35.85)	0.54 (0.42 to 0.67)	0.17 (0.10 to 0.27)	0.12 (0.07 to 0.19)	0.26 (0.15 to 0.41)
Other neonatal disorders	804.93 (667.12 to 978.98)	362.22 (234.50 to 516.99)	278.36 (179.29 to 400.63)	535.49 (342.57 to 773.53)	9.30 (7.71 to 11.31)	4.19 (2.71 to 5.97)	3.22 (2.07 to 4.63)	6.19 (3.96 to 8.94)
**Nutritional deficiencies**	**517.91 (477.83 to 563.58)**	**483.21 (423.78 to 546.89)**	**498.05 (438.58 to 572.59)**	**525.46 (457.62 to 600.55)**	**23.90 (22.44 to 25.61)**	**38.93 (32.25 to 45.84)**	**43.82 (37.12 to 51.08)**	**38.95 (30.98 to 46.86)**
Protein-energy malnutrition	453.32 (416.68 to 498.27)	396.05 (342.19 to 458.53)	415.91 (359.60 to 485.85)	430.21 (366.41 to 502.24)	21.30 (19.89 to 22.86)	33.09 (26.76 to 39.62)	37.92 (31.23 to 45.18)	33.09 (25.78 to 40.69)
Iodine deficiency	4.43 (2.47 to 8.34)	13.80 (3.36 to 37.95)	12.91 (2.90 to 37.56)	16.30 (4.06 to 45.03)	0.13 (0.07 to 0.21)	1.02 (0.17 to 3.50)	1.02 (0.16 to 3.81)	1.12 (0.21 to 3.73)
Dietary iron deficiency	21.17 (11.37 to 26.26)	20.14 (15.16 to 26.96)	19.63 (14.86 to 26.89)	22.30 (16.33 to 29.47)	0.51 (0.36 to 0.66)	0.78 (0.52 to 1.30)	0.80 (0.53 to 1.37)	0.81 (0.54 to 1.31)
Other nutritional deficiencies	38.98 (33.09 to 55.52)	53.22 (43.31 to 71.17)	49.59 (40.09 to 66.88)	56.66 (46.33 to 76.80)	1.96 (1.65 to 2.67)	4.04 (3.05 to 5.52)	4.07 (3.13 to 5.53)	3.93 (2.96 to 5.33)
**Other communicable, maternal, neonatal, and nutritional diseases**	**1,010.90 (775.22 to 1,247.70)**	**680.40 (591.71 to 779.31)**	**597.07 (520.14 to 681.67)**	**802.09 (690.31 to 927.67)**	**21.54 (18.60 to 24.47)**	**24.65 (22.40 to 27.20)**	**22.91 (20.66 to 25.47)**	**27.15 (24.59 to 30.03)**
Sexually transmitted infections excluding HIV	290.65 (162.79 to 476.89)	62.55 (37.76 to 106.57)	49.38 (31.41 to 81.66)	77.35 (45.06 to 134.50)	3.88 (2.40 to 6.06)	1.27 (0.95 to 1.78)	1.11 (0.87 to 1.49)	1.45 (1.07 to 2.12)
Syphilis	272.46 (144.07 to 458.59)	49.18 (24.33 to 93.60)	36.69 (18.63 to 69.27)	62.50 (30.21 to 120.90)	3.37 (1.87 to 5.54)	0.74 (0.45 to 1.25)	0.59 (0.37 to 0.96)	0.90 (0.52 to 1.59)
Chlamydial infection	3.40 (3.03 to 4.32)	2.57 (2.17 to 3.10)	2.45 (2.04 to 2.95)	2.86 (2.42 to 3.54)	0.10 (0.08 to 0.11)	0.10 (0.08 to 0.12)	0.10 (0.08 to 0.12)	0.11 (0.08 to 0.12)
Gonococcal infection	9.71 (8.41 to 11.64)	7.31 (6.05 to 8.74)	6.95 (5.76 to 8.29)	8.07 (6.85 to 9.72)	0.28 (0.23 to 0.32)	0.30 (0.23 to 0.35)	0.30 (0.22 to 0.35)	0.31 (0.24 to 0.37)
Other sexually transmitted infections	5.07 (4.48 to 6.24)	3.50 (2.94 to 4.27)	3.29 (2.74 to 4.01)	3.93 (3.33 to 4.89)	0.13 (0.11 to 0.15)	0.12 (0.10 to 0.14)	0.12 (0.09 to 0.14)	0.13 (0.10 to 0.15)
Acute hepatitis	343.48 (321.16 to 369.79)	312.13 (268.89 to 363.13)	265.81 (223.49 to 318.39)	361.58 (314.24 to 418.00)	9.94 (9.29 to 10.69)	11.77 (10.25 to 13.41)	10.28 (8.79 to 12.02)	13.19 (11.55 to 15.11)
Acute hepatitis A	15.95 (13.71 to 18.55)	7.05 (5.42 to 9.26)	5.96 (4.52 to 7.94)	8.47 (6.54 to 11.04)	0.29 (0.25 to 0.34)	0.21 (0.16 to 0.28)	0.19 (0.15 to 0.25)	0.24 (0.18 to 0.31)
Acute hepatitis B	288.05 (266.06 to 311.80)	268.04 (225.82 to 316.58)	227.77 (187.59 to 276.50)	312.18 (264.65 to 366.96)	8.52 (7.89 to 9.24)	9.84 (8.41 to 11.43)	8.52 (7.14 to 10.15)	11.17 (9.57 to 12.94)

Acute hepatitis C	6.42 (4.87 to 8.59)	7.96 (5.68 to 11.00)	6.99 (5.06 to 9.62)	8.57 (6.21 to 11.78)	0.21 (0.16 to 0.29)	0.33 (0.24 to 0.45)	0.30 (0.21 to 0.41)	0.35 (0.25 to 0.48)
Acute hepatitis E	33.06 (26.95 to 39.84)	29.08 (21.66 to 38.24)	25.09 (18.35 to 33.40)	32.36 (24.20 to 42.35)	0.92 (0.71 to 1.17)	1.39 (0.98 to 1.91)	1.28 (0.90 to 1.77)	1.43 (1.02 to 2.00)
Other unspecified infectious diseases	376.77 (183.40 to 516.69)	305.73 (226.66 to 371.53)	281.88 (215.91 to 338.85)	363.15 (256.31 to 452.50)	7.72 (5.15 to 9.43)	11.61 (9.88 to 13.22)	11.51 (9.97 to 13.16)	12.51 (10.43 to 14.41)
**Non-communicable diseases**	**71,506.74 (68,956.76 to 74,054.47)**	**96,458.70 (81,594.37 to 113,524.76)**	**76,698.77 (65,794.75 to 88,804.13)**	**125,501.69 (104,074.86 to 149,908.46)**	**3,025.97 (2,926.49 to 3,128.43)**	**5,367.00 (4,693.61 to 6,128.52)**	**4,471.61 (3,942.99 to 5,047.71)**	**6,568.29 (5,674.31 to 7,578.99)**

Southeast Asia								
**Cause**	**YLLs (in 1000s)**				**Deaths (in 1000s)**			
	**2016**	**2040 Forecast**	**2040 Better Scenario**	**2040 Worse Scenario**	**2016**	**2040 Forecast**	**2040 Better Scenario**	**2040 Worse Scenario**
**Neoplasms**	**15,219.19 (14,532.07 to 15,946.79)**	**24,331.01 (21,586.04 to 27,437.32)**	**21,534.07 (19,268.52 to 24,030.28)**	**27,946.88 (24,602.87 to 31,717.64)**	**576.50 (552.60 to 601.41)**	**1,153.50 (1,031.96 to 1,293.42)**	**1,049.49 (945.82 to 1,162.98)**	**1,282.56 (1,130.20 to 1,453.98)**
Lip and oral cavity cancer	324.48 (305.35 to 344.62)	519.92 (386.93 to 699.00)	383.38 (297.88 to 490.93)	772.54 (543.94 to 1,081.94)	14.07 (13.30 to 14.92)	27.27 (20.20 to 37.34)	20.82 (16.11 to 27.05)	38.95 (27.42 to 55.61)
Nasopharynx cancer	269.81 (250.88 to 289.76)	413.03 (307.56 to 548.06)	290.47 (221.26 to 377.94)	628.89 (450.19 to 856.92)	8.90 (8.30 to 9.52)	16.05 (11.92 to 21.06)	11.63 (8.91 to 14.92)	23.67 (16.70 to 32.31)
Other pharynx cancer	261.77 (216.34 to 305.65)	429.00 (283.56 to 648.78)	365.25 (253.38 to 539.61)	469.91 (304.59 to 721.95)	9.52 (7.97 to 10.98)	18.42 (12.47 to 27.54)	16.04 (11.52 to 23.24)	19.64 (13.07 to 29.56)
Esophageal cancer	288.69 (269.03 to 309.76)	394.60 (259.44 to 594.72)	305.72 (212.84 to 435.43)	583.37 (356.25 to 920.74)	12.04 (11.27 to 12.82)	19.38 (13.21 to 28.26)	15.49 (11.20 to 21.65)	27.55 (17.49 to 42.65)
Stomach cancer	948.87 (889.29 to 1,017.90)	1,147.67 (974.30 to 1,369.44)	1,043.51 (892.95 to 1,226.35)	1,272.76 (1,047.96 to 1,543.68)	38.58 (36.40 to 41.15)	57.44 (49.57 to 67.32)	53.41 (46.44 to 61.63)	61.45 (51.24 to 73.86)
Colon and rectum cancer	1,228.52 (1,150.14 to 1,307.49)	2,496.68 (1,844.24 to 3,499.11)	2,298.36 (1,711.59 to 3,189.28)	2,549.21 (1,860.07 to 3,529.61)	53.20 (49.78 to 56.47)	135.91 (99.66 to 190.17)	128.63 (95.69 to 179.54)	133.19 (96.22 to 183.95)
Liver cancer	1,701.21 (1,542.99 to 1,858.61)	2,807.52 (2,107.29 to 3,758.27)	2,435.60 (1,948.13 to 3,091.23)	3,182.07 (2,256.68 to 4,450.77)	64.40 (59.04 to 69.85)	129.06 (99.71 to 168.00)	116.03 (94.38 to 143.05)	141.32 (103.66 to 189.69)
Liver cancer due to hepatitis B	717.63 (581.25 to 869.91)	1,077.38 (763.96 to 1,514.63)	954.65 (726.11 to 1,288.20)	1,188.44 (800.72 to 1,742.79)	25.31 (20.76 to 30.36)	46.37 (33.34 to 63.36)	42.39 (32.03 to 54.95)	49.54 (34.15 to 70.87)
Liver cancer due to hepatitis C	280.68 (229.14 to 338.30)	537.88 (394.27 to 729.23)	500.74 (383.03 to 644.09)	565.10 (408.24 to 778.79)	12.44 (10.38 to 14.90)	28.68 (21.60 to 37.75)	27.60 (21.51 to 34.90)	29.12 (21.32 to 39.08)
Liver cancer due to alcohol use	326.56 (239.95 to 425.43)	555.56 (358.68 to 826.88)	418.84 (282.56 to 594.96)	733.65 (458.77 to 1,111.85)	13.17 (9.65 to 17.07)	25.91 (16.60 to 37.72)	20.46 (13.47 to 28.90)	33.00 (20.71 to 49.48)
Liver cancer due to other causes	376.34 (294.51 to 465.76)	636.71 (419.40 to 936.95)	561.37 (390.84 to 771.35)	694.89 (437.40 to 1,059.23)	13.49 (10.72 to 16.63)	28.10 (18.69 to 40.64)	25.58 (17.85 to 35.06)	29.66 (19.08 to 44.08)
Gallbladder and biliary tract cancer	237.48 (180.21 to 267.68)	305.87 (236.85 to 343.22)	310.45 (240.29 to 351.13)	286.29 (222.01 to 319.08)	10.83 (8.37 to 12.13)	17.20 (13.39 to 19.16)	17.80 (13.78 to 20.01)	15.62 (12.07 to 17.38)
Pancreatic cancer	486.10 (451.92 to 528.47)	1,032.24 (851.34 to 1,272.05)	1,009.04 (847.72 to 1,225.79)	1,037.59 (849.01 to 1,280.21)	20.83 (19.45 to 22.58)	52.76 (43.76 to 64.83)	52.75 (44.45 to 63.77)	51.30 (42.14 to 63.32)
Larynx cancer	162.69 (151.08 to 175.45)	225.85 (143.03 to 342.26)	158.63 (106.46 to 234.87)	345.25 (208.56 to 535.94)	6.76 (6.30 to 7.25)	11.10 (7.10 to 16.93)	8.01 (5.46 to 11.76)	16.39 (9.91 to 25.74)
Tracheal, bronchus, and lung cancer	2,448.49 (2,273.57 to 2,619.30)	3,792.17 (2,929.74 to 4,791.45)	2,641.03 (2,105.66 to 3,265.92)	6,045.64 (4,585.49 to 7,769.12)	108.53 (101.29 to 115.22)	195.92 (153.86 to 247.54)	142.19 (114.26 to 175.90)	298.90 (226.06 to 384.53)
Malignant skin melanoma	52.28 (47.30 to 65.85)	88.29 (78.14 to 114.01)	97.71 (85.38 to 127.06)	78.03 (69.44 to 99.95)	1.83 (1.65 to 2.37)	4.08 (3.64 to 5.38)	4.66 (4.12 to 6.23)	3.45 (3.08 to 4.58)
Non-melanoma skin cancer	97.90 (89.39 to 107.14)	190.38 (169.64 to 212.75)	179.59 (158.37 to 203.53)	191.37 (172.71 to 213.49)	4.18 (3.84 to 4.54)	10.40 (9.47 to 11.45)	10.11 (9.11 to 11.19)	10.01 (9.05 to 11.06)
Breast cancer	1,404.11 (1,282.79 to 1,545.05)	2,156.31 (1,677.81 to 2,795.99)	1,956.78 (1,555.59 to 2,466.47)	2,254.57 (1,720.57 to 2,928.03)	45.17 (41.29 to 50.09)	84.75 (66.66 to 109.33)	78.28 (63.05 to 98.18)	86.31 (66.89 to 112.03)
Cervical cancer	753.41 (594.81 to 838.20)	839.70 (641.39 to 1,011.41)	759.28 (574.47 to 910.88)	935.83 (707.05 to 1,143.35)	25.61 (20.33 to 28.36)	34.32 (26.47 to 40.63)	31.53 (24.25 to 37.45)	37.26 (28.29 to 45.04)

Uterine cancer	184.31 (162.04 to 201.33)	296.86 (255.59 to 336.17)	280.14 (238.93 to 319.01)	317.38 (276.14 to 359.07)	7.08 (6.29 to 7.67)	13.69 (12.13 to 15.30)	13.17 (11.60 to 14.76)	14.25 (12.65 to 15.83)
Ovarian cancer	414.51 (375.55 to 462.32)	697.60 (545.70 to 906.54)	669.70 (536.33 to 848.88)	656.34 (511.94 to 857.60)	14.05 (12.79 to 15.56)	28.48 (22.61 to 36.97)	27.65 (22.44 to 34.87)	26.35 (20.92 to 34.46)
Prostate cancer	307.77 (221.91 to 347.66)	947.00 (685.42 to 1,075.20)	979.77 (714.77 to 1,113.27)	845.73 (611.96 to 966.63)	18.99 (13.74 to 21.34)	67.57 (49.28 to 77.07)	72.06 (53.18 to 81.89)	57.99 (41.60 to 67.13)
Testicular cancer	27.64 (25.06 to 32.13)	30.07 (26.11 to 35.41)	28.48 (24.73 to 33.43)	31.14 (27.20 to 36.59)	0.67 (0.61 to 0.76)	0.93 (0.83 to 1.06)	0.91 (0.81 to 1.04)	0.92 (0.83 to 1.06)

Southeast Asia								
Cause	YLLs (in 1000s)				Deaths (in 1000s)			
	2016	2040 Forecast	2040 Better Scenario	2040 Worse Scenario	2016	2040 Forecast	2040 Better Scenario	2040 Worse Scenario
Kidney cancer	166.33 (154.62 to 182.11)	316.38 (267.58 to 372.39)	321.57 (274.10 to 375.16)	303.69 (254.10 to 366.57)	6.01 (5.58 to 6.58)	14.74 (12.67 to 17.32)	15.45 (13.30 to 18.07)	13.60 (11.38 to 16.29)
Bladder cancer	167.87 (157.61 to 179.25)	324.95 (255.95 to 418.79)	301.21 (243.39 to 379.37)	349.25 (267.55 to 460.61)	8.44 (7.94 to 9.01)	20.06 (15.80 to 25.98)	19.14 (15.49 to 24.11)	20.69 (15.70 to 27.48)
Brain and nervous system cancer	527.62 (407.45 to 627.02)	825.38 (632.09 to 992.84)	809.58 (619.40 to 974.43)	796.35 (609.18 to 958.41)	14.20 (10.68 to 16.78)	29.29 (21.70 to 34.98)	29.64 (21.92 to 35.54)	27.20 (20.05 to 32.48)
Thyroid cancer	131.93 (121.22 to 153.52)	220.50 (182.63 to 282.65)	201.36 (169.13 to 251.00)	228.94 (188.71 to 295.43)	5.27 (4.86 to 6.29)	10.76 (9.07 to 13.60)	10.06 (8.61 to 12.31)	10.83 (9.05 to 13.80)
Mesothelioma	41.01 (38.14 to 47.42)	59.37 (53.65 to 69.60)	60.73 (54.33 to 71.52)	58.08 (52.86 to 67.32)	1.35 (1.25 to 1.57)	2.50 (2.28 to 3.01)	2.63 (2.38 to 3.17)	2.36 (2.16 to 2.82)
Hodgkin lymphoma	79.24 (68.51 to 108.88)	76.31 (62.58 to 106.70)	67.80 (55.19 to 93.99)	86.81 (71.63 to 119.75)	2.19 (1.90 to 3.01)	2.71 (2.25 to 3.76)	2.46 (2.02 to 3.44)	2.98 (2.48 to 4.09)
Non-Hodgkin lymphoma	552.69 (507.87 to 658.96)	900.40 (789.48 to 1,115.81)	869.91 (762.51 to 1,070.59)	854.27 (748.55 to 1,058.47)	17.07 (15.66 to 20.54)	37.10 (32.68 to 46.04)	36.94 (32.62 to 46.38)	33.86 (29.77 to 41.61)
Multiple myeloma	103.52 (92.08 to 131.47)	213.54 (180.33 to 273.09)	210.15 (177.81 to 264.81)	197.61 (166.56 to 252.30)	3.99 (3.56 to 5.07)	9.86 (8.45 to 12.58)	9.92 (8.50 to 12.56)	8.88 (7.55 to 11.44)
Leukemia	805.27 (745.39 to 900.81)	976.88 (823.85 to 1,156.48)	881.77 (754.20 to 1,019.86)	1,031.61 (858.52 to 1,240.71)	21.41 (18.96 to 23.03)	36.40 (30.52 to 43.27)	33.98 (28.78 to 39.53)	37.05 (30.48 to 45.56)
Acute lymphoid leukemia	224.25 (204.99 to 272.72)	282.01 (240.27 to 337.75)	255.24 (220.07 to 306.49)	289.35 (242.64 to 350.61)	5.77 (5.32 to 6.84)	10.14 (8.53 to 12.22)	9.49 (8.09 to 11.42)	10.04 (8.36 to 12.33)
Chronic lymphoid leukemia	13.14 (11.81 to 15.76)	18.49 (14.92 to 22.72)	17.43 (14.40 to 20.98)	18.98 (15.03 to 23.77)	0.43 (0.38 to 0.51)	0.77 (0.63 to 0.95)	0.75 (0.63 to 0.92)	0.76 (0.60 to 0.95)
Acute myeloid leukemia	155.68 (140.73 to 191.23)	241.67 (202.98 to 293.96)	230.06 (195.89 to 274.48)	240.75 (199.84 to 297.21)	3.73 (3.40 to 4.47)	7.65 (6.43 to 9.29)	7.53 (6.40 to 8.99)	7.36 (6.11 to 9.11)
Chronic myeloid leukemia	40.47 (36.09 to 47.05)	38.28 (30.47 to 48.73)	33.24 (26.66 to 41.54)	41.15 (32.20 to 53.63)	1.15 (1.03 to 1.36)	1.35 (1.09 to 1.74)	1.21 (0.98 to 1.54)	1.41 (1.11 to 1.84)
Other leukemia	371.74 (316.94 to 399.72)	396.42 (322.42 to 476.49)	345.81 (279.74 to 405.60)	441.37 (354.17 to 542.93)	10.33 (8.06 to 11.20)	16.50 (12.36 to 20.10)	15.00 (11.37 to 17.95)	17.48 (13.20 to 22.03)
Other neoplasms	1,043.66 (977.41 to 1,121.20)	1,606.50 (1,472.05 to 1,757.17)	1,617.11 (1,469.45 to 1,781.92)	1,556.39 (1,436.18 to 1,689.78)	31.34 (29.48 to 33.79)	65.36 (60.63 to 70.90)	68.11 (62.87 to 74.32)	60.58 (56.63 to 65.57)
**Cardiovascular diseases**	**27,994.40 (26,867.78 to 29,170.75)**	**31,691.81 (22,580.90 to 44,588.37)**	**20,697.94 (15,294.19 to 28,612.17)**	**50,739.34 (35,387.91 to 71,706.72)**	**1,311.38 (1,261.56 to 1,368.22)**	**1,895.11 (1,409.28 to 2,598.13)**	**1,344.98 (1,030.58 to 1,795.85)**	**2,762.42 (2,038.52 to 3,798.04)**
Rheumatic heart disease	612.50 (563.69 to 667.03)	402.87 (343.22 to 477.37)	350.75 (295.14 to 416.83)	477.31 (406.11 to 577.60)	17.57 (16.04 to 19.22)	16.43 (14.22 to 19.23)	15.06 (12.93 to 17.60)	18.54 (15.99 to 21.86)
Ischemic heart disease	12,943.39 (12,379.66 to 13,523.97)	14,525.99 (8,149.34 to 25,328.09)	8,617.86 (4,964.32 to 14,738.56)	24,462.84 (13,936.51 to 41,542.55)	620.73 (594.93 to 650.60)	863.53 (518.17 to 1,434.37)	565.10 (346.38 to 923.19)	1,329.28 (797.68 to 2,189.06)
Stroke	10,724.23 (10,259.73 to 11,216.88)	10,247.32 (6,706.97 to 16,047.68)	6,182.08 (4,264.53 to 9,299.66)	18,220.21 (11,415.52 to 28,636.71)	504.52 (481.49 to 529.72)	624.05 (438.01 to 959.92)	416.42 (299.79 to 616.54)	984.80 (665.29 to 1,507.04)
Ischemic stroke	2,727.40 (2,526.94 to 2,961.48)	3,219.48 (1,936.09 to 6,314.75)	2,147.13 (1,354.80 to 3,978.80)	4,888.32 (2,845.80 to 9,457.97)	173.12 (160.59 to 187.25)	245.63 (149.57 to 526.35)	177.50 (111.97 to 351.69)	339.27 (201.17 to 702.77)

Intracerebral hemorrhage	7,996.83 (7,610.39 to 8,397.26)	7,027.85 (4,398.81 to 11,072.39)	4,034.95 (2,681.68 to 6,129.37)	13,331.90 (7,950.87 to 21,600.78)	331.40 (314.98 to 347.89)	378.42 (252.85 to 577.02)	238.92 (169.29 to 346.57)	645.53 (410.53 to 996.95)
Hypertensive heart disease	1,515.95 (1,244.34 to 1,683.39)	2,867.94 (2,050.61 to 4,258.87)	2,268.74 (1,713.78 to 3,084.02)	3,667.46 (2,462.29 to 6,239.63)	75.62 (62.80 to 83.41)	177.54 (128.36 to 267.93)	146.27 (112.14 to 200.48)	214.91 (146.06 to 361.13)
Cardiomyopathy and myocarditis	566.12 (464.68 to 619.03)	805.43 (589.65 to 1,018.29)	732.44 (536.01 to 925.34)	807.23 (596.43 to 1,024.39)	20.46 (16.35 to 22.57)	40.38 (28.15 to 53.30)	38.71 (26.46 to 51.05)	38.03 (27.13 to 50.79)
Myocarditis	106.51 (88.92 to 131.78)	106.25 (87.53 to 130.39)	103.80 (84.80 to 127.49)	109.49 (90.19 to 133.85)	2.88 (2.37 to 3.43)	4.20 (3.41 to 4.97)	4.34 (3.51 to 5.09)	4.05 (3.31 to 4.80)

Southeast Asia								
**Cause**	**YLLs (in 1000s)**				**Deaths (in 1000s)**			
	**2016**	**2040 Forecast**	**2040 Better Scenario**	**2040 Worse Scenario**	**2016**	**2040 Forecast**	**2040 Better Scenario**	**2040 Worse Scenario**
Alcoholic cardiomyopathy	86.77 (56.69 to 105.02)	118.38 (72.28 to 148.69)	110.75 (65.74 to 142.08)	120.17 (78.07 to 146.99)	2.93 (1.94 to 3.48)	4.91 (3.06 to 6.03)	4.73 (2.88 to 5.89)	4.78 (3.10 to 5.76)
Other cardiomyopathy	372.83 (296.56 to 412.89)	580.79 (397.44 to 779.81)	517.88 (353.54 to 692.97)	577.56 (392.02 to 786.89)	14.65 (11.30 to 16.22)	31.26 (20.53 to 43.83)	29.64 (19.02 to 41.84)	29.20 (19.65 to 41.64)
Atrial fibrillation and flutter	152.00 (121.15 to 184.79)	418.55 (318.49 to 533.78)	407.04 (312.73 to 513.44)	411.57 (309.02 to 534.32)	13.84 (10.87 to 17.12)	40.99 (30.88 to 52.90)	41.22 (31.19 to 52.42)	38.47 (28.35 to 49.78)
Aortic aneurysm	201.47 (182.64 to 227.14)	350.50 (285.35 to 428.30)	331.43 (272.69 to 400.03)	351.78 (280.93 to 440.85)	10.40 (9.45 to 11.59)	22.69 (19.05 to 27.16)	22.44 (18.93 to 26.42)	21.57 (17.68 to 26.31)
Peripheral artery disease	9.24 (7.73 to 11.13)	33.86 (25.79 to 44.69)	32.04 (24.59 to 42.38)	30.76 (23.07 to 40.51)	0.64 (0.54 to 0.80)	2.80 (2.14 to 3.73)	2.82 (2.18 to 3.79)	2.38 (1.81 to 3.15)
Endocarditis	410.97 (366.46 to 517.33)	584.87 (441.48 to 799.19)	553.49 (421.93 to 740.35)	600.21 (448.28 to 826.05)	12.90 (11.50 to 16.16)	25.59 (19.42 to 34.72)	25.18 (19.35 to 34.06)	24.96 (18.77 to 34.15)
Other cardiovascular and circulatory diseases	858.52 (752.56 to 1,255.22)	1,454.48 (1,101.17 to 2,109.52)	1,222.07 (939.08 to 1,749.74)	1,709.96 (1,263.81 to 2,508.29)	34.70 (30.54 to 50.84)	81.12 (63.10 to 119.26)	71.77 (56.83 to 104.46)	89.49 (67.87 to 131.28)
**Chronic respiratory diseases**	**4,716.74 (4,427.58 to 4,973.12)**	**5,031.06 (4,101.34 to 6,248.34)**	**3,728.98 (3,145.33 to 4,482.77)**	**7,324.75 (5,713.31 to 9,573.92)**	**237.44 (220.86 to 250.68)**	**317.10 (261.23 to 389.21)**	**247.43 (210.00 to 294.51)**	**440.45 (347.43 to 568.08)**
Chronic obstructive pulmonary disease	2,690.18 (2,343.46 to 2,973.09)	3,345.79 (2,466.79 to 4,444.63)	2,381.27 (1,828.69 to 3,035.98)	5,415.03 (3,832.14 to 7,547.81)	153.50 (135.29 to 166.78)	224.51 (169.29 to 291.22)	169.76 (133.97 to 213.99)	340.53 (247.30 to 461.70)
Pneumoconiosis	4.10 (3.55 to 4.89)	5.09 (4.44 to 5.87)	4.77 (4.14 to 5.48)	5.01 (4.41 to 5.76)	0.21 (0.18 to 0.24)	0.34 (0.30 to 0.39)	0.33 (0.29 to 0.37)	0.32 (0.28 to 0.38)
Silicosis	0.84 (0.62 to 1.15)	0.71 (0.50 to 0.97)	0.63 (0.43 to 0.87)	0.71 (0.52 to 0.97)	0.04 (0.03 to 0.05)	0.04 (0.03 to 0.06)	0.04 (0.03 to 0.06)	0.04 (0.03 to 0.06)
Asbestosis	0.96 (0.81 to 1.19)	1.46 (1.23 to 1.84)	1.61 (1.36 to 2.01)	1.36 (1.15 to 1.70)	0.04 (0.03 to 0.05)	0.08 (0.06 to 0.11)	0.09 (0.07 to 0.12)	0.07 (0.06 to 0.09)
Coal workers pneumoconiosis	0.24 (0.19 to 0.40)	0.27 (0.21 to 0.46)	0.21 (0.16 to 0.35)	0.32 (0.24 to 0.51)	0.01 (0.01 to 0.02)	0.02 (0.01 to 0.03)	0.02 (0.01 to 0.03)	0.02 (0.02 to 0.03)
Other pneumoconiosis	2.06 (1.73 to 2.51)	2.65 (2.19 to 3.19)	2.31 (1.90 to 2.80)	2.62 (2.16 to 3.13)	0.12 (0.10 to 0.14)	0.20 (0.16 to 0.24)	0.18 (0.15 to 0.22)	0.19 (0.15 to 0.23)
Asthma	1,839.90 (1,474.68 to 2,181.75)	1,389.78 (963.21 to 1,981.24)	1,023.97 (725.11 to 1,389.10)	1,637.18 (1,101.02 to 2,377.73)	76.57 (58.27 to 93.08)	76.68 (52.96 to 108.13)	59.51 (41.35 to 79.99)	86.06 (56.99 to 125.00)
Interstitial lung disease and pulmonary sarcoidosis	95.11 (77.08 to 122.94)	186.63 (155.38 to 241.29)	217.64 (180.79 to 284.83)	158.99 (133.46 to 205.77)	4.32 (3.59 to 5.58)	10.49 (8.76 to 13.82)	12.56 (10.58 to 16.57)	8.62 (7.23 to 11.21)
Other chronic respiratory diseases	87.46 (59.20 to 108.05)	103.77 (69.44 to 130.07)	101.33 (66.79 to 127.67)	108.54 (73.48 to 135.09)	2.85 (1.78 to 3.64)	5.08 (3.01 to 6.75)	5.27 (3.08 to 7.02)	4.92 (2.95 to 6.51)
**Cirrhosis and other chronic liver diseases**	**4,388.64 (4,019.88 to 4,792.19)**	**5,851.18 (5,261.48 to 6,528.87)**	**5,430.82 (4,831.38 to 6,114.36)**	**6,212.13 (5,638.57 to 6,896.25)**	**140.72 (129.74 to 152.61)**	**234.88 (215.69 to 258.02)**	**223.76 (202.77 to 248.04)**	**240.49 (221.26 to 262.96)**
Cirrhosis and other chronic liver diseases due to hepatitis B	1,388.34 (1,196.62 to 1,613.22)	1,899.80 (1,627.74 to 2,239.96)	1,820.47 (1,560.91 to 2,154.21)	1,952.72 (1,671.04 to 2,284.28)	45.29 (39.11 to 52.53)	75.89 (65.40 to 87.81)	74.38 (63.69 to 87.03)	75.40 (64.61 to 86.92)
Cirrhosis and other chronic liver diseases due to hepatitis C	862.48 (729.00 to 1,026.40)	1,203.56 (1,011.03 to 1,414.66)	1,141.92 (953.11 to 1,350.65)	1,247.15 (1,046.05 to 1,465.15)	27.46 (23.52 to 32.07)	46.70 (39.85 to 54.52)	45.19 (38.66 to 53.03)	46.96 (39.94 to 54.63)

Cirrhosis and other chronic liver diseases due to alcohol use	942.17 (794.15 to 1,115.46)	1,267.53 (1,062.27 to 1,502.21)	1,072.04 (895.51 to 1,284.57)	1,461.98 (1,226.18 to 1,740.03)	31.46 (26.93 to 36.89)	51.79 (43.94 to 60.29)	45.35 (38.28 to 53.68)	57.40 (48.67 to 66.90)
Cirrhosis and other chronic liver diseases due to other causes	1,195.64 (1,063.97 to 1,332.51)	1,480.29 (1,289.76 to 1,706.06)	1,396.40 (1,208.11 to 1,613.70)	1,550.28 (1,364.07 to 1,775.22)	36.51 (32.56 to 40.83)	60.50 (52.89 to 68.67)	58.84 (51.00 to 67.18)	60.72 (53.25 to 68.96)
**Digestive diseases**	**3,243.54 (2,947.05 to 3,423.90)**	**3,852.35 (3,329.76 to 4,396.15)**	**3,453.05 (2,966.45 to 3,966.07)**	**4,239.87 (3,659.87 to 4,905.98)**	**123.69 (110.29 to 130.56)**	**210.08 (174.86 to 238.71)**	**197.33 (164.72 to 225.11)**	**218.56 (182.54 to 251.41)**
Peptic ulcer disease	976.10 (860.09 to 1,061.73)	929.09 (733.28 to 1,157.81)	763.58 (596.89 to 955.66)	1,111.65 (872.02 to 1,396.62)	42.91 (37.02 to 46.56)	54.02 (41.65 to 66.96)	46.11 (35.20 to 56.93)	61.84 (47.33 to 77.32)

Southeast Asia								
Cause	YLLs (in 1000s)				Deaths (in 1000s)			
	2016	2040 Forecast	2040 Better Scenario	2040 Worse Scenario	2016	2040 Forecast	2040 Better Scenario	2040 Worse Scenario
Gastritis and duodenitis	62.76 (54.62 to 74.79)	104.63 (88.84 to 125.56)	97.57 (82.06 to 118.69)	108.94 (91.87 to 130.08)	2.67 (2.33 to 3.06)	5.99 (5.07 to 7.01)	5.82 (4.87 to 6.89)	5.94 (4.99 to 6.95)
Appendicitis	199.63 (176.83 to 256.89)	174.30 (148.58 to 221.63)	149.55 (126.34 to 190.69)	199.35 (170.20 to 256.42)	5.44 (4.78 to 6.89)	7.07 (6.07 to 8.54)	6.36 (5.39 to 7.64)	7.64 (6.50 to 9.36)
Paralytic ileus and intestinal obstruction	788.94 (618.56 to 894.90)	920.73 (739.28 to 1,066.58)	920.04 (732.49 to 1,066.77)	933.33 (746.00 to 1,092.02)	24.66 (19.66 to 28.21)	49.67 (39.61 to 58.13)	52.40 (41.71 to 61.53)	46.80 (37.51 to 55.28)
Inguinal, femoral, and abdominal hernia	106.59 (58.53 to 133.40)	134.26 (67.17 to 176.89)	127.65 (62.53 to 170.34)	137.88 (70.69 to 182.67)	5.04 (2.57 to 6.56)	9.03 (4.03 to 12.49)	9.03 (3.97 to 12.46)	8.71 (3.95 to 12.16)
Inflammatory bowel disease	57.06 (50.87 to 71.08)	87.50 (76.47 to 108.33)	88.78 (77.09 to 109.71)	84.69 (74.36 to 105.56)	2.11 (1.89 to 2.51)	4.56 (3.91 to 5.38)	4.82 (4.11 to 5.70)	4.19 (3.59 to 4.98)
Vascular intestinal disorders	99.14 (89.18 to 123.01)	183.16 (161.73 to 220.70)	182.02 (159.77 to 216.19)	181.57 (158.90 to 219.04)	5.37 (4.80 to 6.41)	12.71 (11.05 to 14.95)	13.04 (11.35 to 15.24)	12.06 (10.29 to 14.21)
Gallbladder and biliary diseases	191.52 (157.43 to 209.22)	293.10 (191.14 to 432.83)	219.91 (150.19 to 310.12)	333.41 (214.86 to 498.16)	8.99 (6.95 to 9.85)	17.75 (11.14 to 26.06)	14.08 (9.17 to 19.50)	19.16 (11.86 to 28.59)
Pancreatitis	414.33 (347.47 to 473.28)	572.63 (406.87 to 832.11)	468.90 (344.83 to 646.05)	666.80 (464.41 to 996.73)	12.45 (9.97 to 14.17)	22.56 (15.39 to 33.18)	18.97 (13.17 to 26.55)	25.33 (17.06 to 38.04)
Other digestive diseases	347.47 (308.95 to 403.46)	452.94 (397.81 to 532.66)	435.05 (379.25 to 514.84)	482.25 (423.80 to 569.63)	14.04 (12.64 to 15.96)	26.71 (23.37 to 30.91)	26.71 (23.18 to 30.99)	26.89 (23.20 to 31.05)
**Neurological disorders**	**2,579.25 (2,291.85 to 2,940.95)**	**6,438.11 (5,234.36 to 8,294.16)**	**6,312.24 (5,269.32 to 7,760.43)**	**5,991.28 (4,819.40 to 7,737.28)**	**193.67 (168.51 to 226.22)**	**577.17 (453.05 to 742.25)**	**587.77 (480.33 to 721.16)**	**508.31 (389.25 to 675.08)**
Alzheimer's disease and other dementias	1,822.48 (1,548.30 to 2,180.90)	5,260.21 (4,078.96 to 7,101.85)	5,130.11 (4,104.79 to 6,502.54)	4,830.10 (3,646.70 to 6,573.44)	168.50 (143.92 to 200.85)	515.40 (393.03 to 680.84)	520.99 (413.48 to 659.52)	453.65 (338.53 to 618.30)
Parkinson's disease	164.83 (131.10 to 206.32)	532.11 (412.68 to 673.29)	592.55 (458.97 to 749.68)	444.05 (343.91 to 559.05)	11.90 (9.28 to 14.97)	42.62 (32.88 to 54.55)	48.61 (37.89 to 62.26)	34.39 (26.40 to 44.51)
Epilepsy	434.69 (401.35 to 464.45)	422.69 (347.09 to 523.60)	364.11 (301.91 to 444.27)	504.50 (411.55 to 626.90)	9.27 (8.36 to 9.98)	11.64 (9.51 to 14.40)	10.33 (8.48 to 12.52)	13.41 (10.81 to 16.75)
Multiple sclerosis	20.22 (17.65 to 26.95)	27.53 (22.71 to 37.77)	27.73 (22.97 to 38.04)	26.82 (22.08 to 36.66)	0.58 (0.51 to 0.77)	0.93 (0.79 to 1.25)	0.96 (0.81 to 1.28)	0.89 (0.75 to 1.19)
Motor neuron disease	29.76 (25.15 to 32.98)	52.27 (43.68 to 59.47)	53.46 (44.42 to 61.28)	48.96 (41.02 to 55.35)	0.90 (0.77 to 1.00)	1.94 (1.63 to 2.19)	2.02 (1.70 to 2.29)	1.78 (1.50 to 2.00)
Other neurological disorders	107.28 (94.59 to 119.02)	143.29 (127.92 to 159.18)	144.28 (128.68 to 160.32)	136.84 (121.22 to 152.08)	2.51 (2.29 to 2.71)	4.63 (4.27 to 5.02)	4.87 (4.49 to 5.31)	4.20 (3.85 to 4.56)
**Mental disorders**	**487.35 (398.47 to 550.59)**	**651.80 (557.02 to 769.20)**	**664.38 (564.52 to 784.60)**	**649.91 (556.96 to 764.00)**	**11.75 (9.87 to 13.35)**	**18.67 (16.16 to 21.55)**	**19.55 (16.80 to 22.59)**	**18.03 (15.64 to 20.87)**
Alcohol use disorders	289.19 (227.55 to 351.38)	302.97 (243.53 to 386.11)	296.60 (237.70 to 380.32)	314.63 (254.67 to 396.89)	7.40 (5.97 to 9.11)	9.21 (7.57 to 11.83)	9.23 (7.54 to 11.84)	9.29 (7.62 to 11.73)
Drug use disorders	194.23 (154.19 to 226.43)	345.13 (287.68 to 413.37)	364.00 (302.11 to 434.84)	331.70 (275.57 to 395.82)	4.28 (3.51 to 4.94)	9.39 (8.03 to 10.85)	10.25 (8.68 to 11.95)	8.67 (7.35 to 10.09)
Opioid use disorders	138.22 (108.43 to 161.47)	207.24 (168.04 to 250.58)	210.96 (169.93 to 256.65)	204.51 (165.85 to 246.54)	2.92 (2.35 to 3.39)	5.34 (4.43 to 6.25)	5.60 (4.61 to 6.56)	5.07 (4.21 to 5.98)

Cocaine use disorders	5.16 (3.85 to 7.90)	12.60 (9.70 to 18.48)	13.32 (10.25 to 19.76)	12.51 (9.68 to 18.39)	0.12 (0.09 to 0.18)	0.35 (0.28 to 0.51)	0.38 (0.30 to 0.55)	0.34 (0.27 to 0.49)
Amphetamine use disorders	5.15 (3.87 to 6.94)	16.79 (12.63 to 22.82)	18.08 (13.83 to 24.81)	16.97 (12.45 to 23.07)	0.11 (0.08 to 0.14)	0.40 (0.31 to 0.54)	0.44 (0.34 to 0.59)	0.39 (0.30 to 0.52)
Other drug use disorders	45.69 (32.24 to 55.73)	108.51 (83.12 to 136.07)	121.64 (93.42 to 152.66)	97.71 (73.83 to 120.98)	1.13 (0.82 to 1.32)	3.31 (2.62 to 3.91)	3.83 (3.00 to 4.56)	2.87 (2.23 to 3.39)
Eating disorders	3.92 (3.24 to 5.45)	3.69 (2.83 to 5.35)	3.78 (2.89 to 5.56)	3.58 (2.77 to 5.12)	0.07 (0.06 to 0.10)	0.07 (0.06 to 0.11)	0.07 (0.06 to 0.11)	0.07 (0.05 to 0.10)

Southeast Asia								
Cause	YLLs (in 1000s)				Deaths (in 1000s)			
	2016	2040 Forecast	2040 Better Scenario	2040 Worse Scenario	2016	2040 Forecast	2040 Better Scenario	2040 Worse Scenario
**Diabetes, urogenital, blood, and endocrine diseases**	**8,958.39 (8,594.98 to 9,340.66)**	**15,550.35 (11,131.04 to 23,240.73)**	**12,326.79 (9,074.00 to 18,328.07)**	**18,915.43 (13,476.92 to 27,687.65)**	**370.51 (355.93 to 386.70)**	**872.79 (620.02 to 1,317.45)**	**718.18 (524.20 to 1,081.73)**	**1,009.40 (714.15 to 1,489.55)**
Diabetes mellitus	4,453.80 (4,267.48 to 4,669.88)	6,259.99 (4,377.36 to 8,822.39)	5,086.98 (3,576.05 to 7,126.44)	8,077.84 (5,504.77 to 11,428.23)	195.54 (187.31 to 205.60)	354.20 (256.47 to 472.60)	299.17 (219.49 to 394.56)	432.51 (308.09 to 583.39)
Acute glomerulonephritis	11.33 (9.42 to 13.93)	13.55 (10.77 to 16.99)	12.07 (9.65 to 15.05)	14.52 (11.66 to 18.20)	0.29 (0.24 to 0.35)	0.44 (0.35 to 0.54)	0.42 (0.34 to 0.53)	0.44 (0.36 to 0.55)
Chronic kidney disease	3,223.84 (2,961.56 to 3,422.18)	7,293.82 (3,299.09 to 15,564.10)	5,365.04 (2,612.40 to 11,565.85)	8,797.70 (3,888.69 to 18,038.29)	128.14 (117.21 to 135.46)	409.24 (171.63 to 889.31)	311.89 (138.18 to 699.31)	472.01 (191.93 to 1,003.59)
Chronic kidney disease due to diabetes mellitus	1,878.82 (1,701.21 to 2,040.26)	4,410.04 (1,991.72 to 9,400.94)	3,213.79 (1,537.00 to 6,965.17)	5,361.95 (2,387.02 to 11,211.78)	71.02 (63.64 to 77.45)	222.21 (89.78 to 491.01)	166.07 (72.53 to 385.11)	260.80 (104.30 to 560.19)
Chronic kidney disease due to hypertension	790.03 (689.87 to 896.15)	2,162.87 (902.14 to 4,682.32)	1,617.86 (715.39 to 3,548.11)	2,548.74 (1,046.18 to 5,406.67)	42.40 (36.85 to 47.56)	154.22 (65.05 to 336.50)	121.11 (53.34 to 277.51)	172.70 (69.39 to 365.37)
Chronic kidney disease due to glomerulonephritis	287.09 (240.59 to 337.03)	329.47 (200.15 to 588.16)	244.41 (154.39 to 427.09)	408.08 (246.75 to 714.58)	6.80 (5.73 to 7.92)	12.89 (6.17 to 27.57)	9.57 (4.90 to 20.75)	15.38 (7.35 to 32.21)
Chronic kidney disease due to other and unspecified causes	267.89 (223.36 to 312.22)	391.43 (203.09 to 765.05)	288.98 (158.82 to 574.72)	478.93 (247.13 to 927.09)	7.92 (6.61 to 9.25)	19.93 (8.61 to 43.83)	15.14 (6.91 to 35.23)	23.14 (9.86 to 49.52)
Urinary diseases and male infertility	616.78 (583.22 to 656.31)	1,191.30 (1,089.28 to 1,301.93)	1,126.52 (1,009.86 to 1,245.98)	1,172.55 (1,084.23 to 1,264.82)	27.44 (25.98 to 29.22)	74.31 (67.45 to 81.34)	72.91 (65.35 to 80.39)	69.12 (63.08 to 75.07)
Urinary tract infections	438.77 (408.54 to 479.55)	927.67 (837.27 to 1,021.63)	889.41 (793.14 to 987.02)	900.18 (819.52 to 983.33)	18.61 (17.21 to 21.38)	56.81 (50.63 to 64.83)	56.66 (50.30 to 65.02)	52.07 (46.52 to 59.31)
Urolithiasis	37.74 (31.59 to 50.77)	90.66 (75.23 to 119.11)	83.46 (67.93 to 109.46)	87.67 (73.22 to 115.31)	1.78 (1.50 to 2.38)	5.46 (4.41 to 6.99)	5.17 (4.13 to 6.67)	5.01 (4.08 to 6.44)
Other urinary diseases	140.27 (118.05 to 166.33)	172.96 (136.36 to 209.42)	153.65 (119.56 to 188.50)	184.69 (148.46 to 222.97)	7.05 (5.38 to 8.09)	12.03 (8.64 to 15.01)	11.08 (8.01 to 13.85)	12.05 (8.64 to 14.81)
Gynecological diseases	58.43 (37.21 to 68.45)	55.16 (36.48 to 70.37)	42.96 (28.87 to 56.51)	64.39 (44.79 to 82.07)	1.57 (1.07 to 1.81)	1.77 (1.26 to 2.20)	1.42 (1.01 to 1.83)	2.00 (1.47 to 2.48)
Uterine fibroids	11.23 (7.19 to 15.08)	10.62 (6.15 to 15.56)	8.82 (5.11 to 13.02)	12.53 (7.34 to 18.43)	0.35 (0.23 to 0.47)	0.38 (0.23 to 0.55)	0.32 (0.19 to 0.46)	0.44 (0.26 to 0.63)
Polycystic ovarian syndrome	2.12 (0.69 to 4.03)	1.13 (0.37 to 2.20)	0.76 (0.24 to 1.49)	1.50 (0.49 to 2.90)	0.05 (0.02 to 0.10)	0.03 (0.01 to 0.05)	0.02 (0.01 to 0.04)	0.04 (0.01 to 0.07)
Endometriosis	0.78 (0.34 to 1.45)	0.92 (0.43 to 1.80)	0.73 (0.34 to 1.40)	1.09 (0.50 to 2.08)	0.02 (0.01 to 0.03)	0.02 (0.01 to 0.04)	0.02 (0.01 to 0.03)	0.03 (0.01 to 0.05)
Genital prolapse	2.26 (1.35 to 3.57)	2.77 (1.60 to 4.53)	2.22 (1.27 to 3.64)	3.25 (1.88 to 5.33)	0.11 (0.07 to 0.18)	0.15 (0.08 to 0.24)	0.12 (0.07 to 0.20)	0.17 (0.10 to 0.28)
Other gynecological diseases	42.03 (26.60 to 50.31)	39.72 (26.30 to 52.39)	30.43 (19.16 to 41.70)	46.02 (30.23 to 61.05)	1.03 (0.70 to 1.23)	1.19 (0.83 to 1.52)	0.94 (0.63 to 1.24)	1.33 (0.93 to 1.71)
Hemoglobinopathies and hemolytic anemias	317.59 (260.94 to 349.93)	355.52 (282.14 to 407.56)	320.04 (251.40 to 371.60)	394.21 (312.62 to 450.66)	9.62 (7.60 to 10.58)	16.58 (12.73 to 19.21)	15.43 (11.79 to 18.18)	17.65 (13.50 to 20.33)
Thalassemias	86.12 (70.86 to 100.25)	36.67 (28.72 to 46.15)	34.18 (27.11 to 43.04)	36.73 (28.22 to 47.00)	1.10 (0.91 to 1.27)	0.49 (0.39 to 0.61)	0.46 (0.37 to 0.57)	0.49 (0.38 to 0.62)

Sickle cell disorders	0.65 (0.54 to 0.76)	0.54 (0.43 to 0.66)	0.45 (0.36 to 0.55)	0.60 (0.48 to 0.73)	0.01 (0.01 to 0.01)	0.01 (0.01 to 0.01)	0.01 (0.01 to 0.01)	0.01 (0.01 to 0.02)
G6PD deficiency	129.03 (105.18 to 145.45)	185.87 (145.56 to 218.81)	162.19 (126.63 to 193.44)	215.27 (171.49 to 251.49)	3.25 (2.62 to 3.66)	6.53 (5.05 to 7.62)	5.80 (4.48 to 6.88)	7.37 (5.76 to 8.56)
Other hemoglobinopathies and hemolytic anemias	101.79 (78.74 to 113.82)	132.44 (98.15 to 157.23)	123.22 (91.67 to 148.77)	141.62 (104.65 to 167.89)	5.26 (4.01 to 5.89)	9.55 (6.98 to 11.36)	9.16 (6.79 to 11.09)	9.78 (7.18 to 11.74)
Endocrine, metabolic, blood, and immune disorders	276.63 (249.94 to 332.85)	381.01 (337.03 to 482.85)	373.19 (329.18 to 471.00)	394.23 (350.81 to 499.44)	7.91 (7.25 to 10.25)	16.24 (14.31 to 22.01)	16.93 (14.86 to 22.63)	15.67 (13.87 to 21.15)

Southeast Asia								
Cause	YLLs (in 1000s)				Deaths (in 1000s)			
	2016	2040 Forecast	2040 Better Scenario	2040 Worse Scenario	2016	2040 Forecast	2040 Better Scenario	2040 Worse Scenario
**Musculoskeletal disorders**	**201.74 (181.34 to 246.81)**	**322.20 (279.71 to 417.98)**	**315.36 (273.03 to 410.81)**	**310.37 (270.72 to 398.57)**	**5.74 (5.20 to 7.58)**	**13.37 (11.35 to 19.38)**	**13.50 (11.44 to 19.48)**	**12.36 (10.50 to 17.87)**
Rheumatoid arthritis	20.37 (17.47 to 27.60)	54.26 (42.10 to 73.98)	48.02 (36.63 to 64.99)	57.45 (43.90 to 78.52)	0.98 (0.85 to 1.39)	3.37 (2.56 to 4.66)	3.12 (2.37 to 4.30)	3.40 (2.55 to 4.74)
Other musculoskeletal disorders	181.38 (162.27 to 218.32)	267.93 (233.68 to 348.65)	267.35 (231.18 to 346.54)	252.92 (221.04 to 328.14)	4.76 (4.29 to 6.25)	10.00 (8.57 to 15.10)	10.38 (8.86 to 15.62)	8.96 (7.71 to 13.36)
**Other non-communicable diseases**	**3,717.49 (3,158.55 to 4,153.36)**	**2,738.84 (2,288.19 to 3,190.61)**	**2,235.14 (1,842.19 to 2,594.17)**	**3,171.72 (2,649.42 to 3,741.11)**	**54.57 (46.30 to 60.95)**	**74.32 (55.55 to 90.59)**	**69.62 (51.44 to 84.48)**	**75.70 (58.19 to 90.49)**
Congenital birth defects	3,266.24 (2,669.19 to 3,693.31)	1,837.29 (1,498.13 to 2,243.55)	1,368.29 (1,118.40 to 1,661.44)	2,275.21 (1,824.29 to 2,795.98)	40.06 (33.01 to 45.11)	24.31 (20.06 to 29.36)	18.49 (15.23 to 22.02)	29.67 (24.15 to 35.93)
Neural tube defects	222.74 (150.33 to 294.59)	49.16 (29.37 to 73.41)	34.09 (20.43 to 52.00)	71.03 (42.15 to 105.60)	2.60 (1.76 to 3.44)	0.59 (0.36 to 0.87)	0.41 (0.25 to 0.62)	0.84 (0.51 to 1.24)
Congenital heart anomalies	1,818.53 (1,472.37 to 2,105.74)	1,045.43 (785.78 to 1,308.87)	766.82 (586.72 to 950.41)	1,286.19 (965.65 to 1,613.53)	22.46 (18.26 to 25.89)	13.76 (10.55 to 16.94)	10.27 (7.93 to 12.57)	16.74 (12.82 to 20.71)
Orofacial clefts	8.01 (4.40 to 13.49)	4.26 (2.03 to 8.17)	2.55 (1.20 to 4.64)	5.99 (2.89 to 11.46)	0.09 (0.05 to 0.16)	0.05 (0.02 to 0.09)	0.03 (0.01 to 0.05)	0.07 (0.03 to 0.13)
Down syndrome	66.65 (54.82 to 86.76)	51.15 (40.66 to 65.76)	44.74 (35.81 to 57.95)	60.91 (48.25 to 80.23)	0.92 (0.77 to 1.18)	0.84 (0.68 to 1.09)	0.76 (0.62 to 1.00)	0.97 (0.79 to 1.26)
Other chromosomal abnormalities	92.76 (63.73 to 199.81)	85.37 (53.29 to 213.63)	67.69 (43.96 to 155.20)	107.18 (66.60 to 265.85)	1.10 (0.77 to 2.35)	1.08 (0.72 to 2.53)	0.87 (0.60 to 1.86)	1.34 (0.87 to 3.15)
Congenital musculoskeletal and limb anomalies	59.07 (41.80 to 74.98)	36.34 (25.11 to 48.63)	27.68 (18.74 to 36.66)	45.08 (30.85 to 60.78)	0.70 (0.49 to 0.88)	0.44 (0.31 to 0.58)	0.34 (0.23 to 0.44)	0.54 (0.37 to 0.72)
Urogenital congenital anomalies	51.99 (43.12 to 67.68)	47.06 (38.43 to 60.43)	39.49 (32.07 to 49.56)	53.02 (43.14 to 68.45)	0.90 (0.75 to 1.11)	1.08 (0.87 to 1.31)	0.95 (0.75 to 1.13)	1.18 (0.95 to 1.42)
Digestive congenital anomalies	216.44 (169.44 to 316.04)	130.73 (94.18 to 194.72)	91.02 (65.75 to 132.68)	164.91 (118.14 to 243.83)	2.54 (1.99 to 3.70)	1.56 (1.14 to 2.31)	1.09 (0.80 to 1.59)	1.96 (1.42 to 2.88)
Other congenital birth defects	730.05 (494.55 to 867.97)	387.79 (246.30 to 487.40)	294.21 (188.67 to 367.06)	480.90 (302.98 to 610.51)	8.75 (6.00 to 10.34)	4.90 (3.22 to 6.11)	3.77 (2.51 to 4.64)	6.01 (3.91 to 7.51)
Skin and subcutaneous diseases	304.24 (194.46 to 404.59)	841.72 (558.15 to 1,106.76)	821.48 (548.71 to 1,071.52)	821.52 (541.60 to 1,086.00)	12.80 (8.11 to 17.04)	49.32 (32.00 to 67.02)	50.61 (33.64 to 66.89)	45.17 (28.87 to 61.10)
Cellulitis	43.39 (29.02 to 69.87)	164.83 (113.73 to 263.13)	170.69 (118.93 to 271.23)	152.65 (105.46 to 242.06)	1.59 (1.06 to 2.51)	8.23 (5.56 to 12.82)	8.98 (6.14 to 14.09)	7.18 (4.85 to 11.40)
Pyoderma	169.01 (105.92 to 231.74)	449.33 (281.31 to 596.37)	425.31 (267.38 to 566.39)	455.19 (285.17 to 609.28)	6.03 (3.64 to 8.37)	24.23 (15.34 to 34.27)	24.13 (15.32 to 33.36)	23.07 (14.39 to 32.32)
Decubitus ulcer	83.59 (47.65 to 107.05)	210.15 (122.55 to 274.61)	208.46 (121.55 to 265.61)	196.69 (114.24 to 261.18)	4.93 (2.80 to 6.35)	16.14 (9.33 to 21.14)	16.75 (9.78 to 21.41)	14.25 (8.07 to 19.00)
Other skin and subcutaneous diseases	8.25 (5.20 to 11.21)	17.41 (11.14 to 24.16)	17.02 (10.96 to 23.76)	16.99 (10.81 to 23.67)	0.26 (0.16 to 0.35)	0.72 (0.45 to 1.01)	0.74 (0.46 to 1.04)	0.66 (0.41 to 0.94)
Sudden infant death syndrome	147.01 (111.26 to 194.25)	59.83 (40.06 to 88.64)	45.36 (30.41 to 68.18)	75.00 (49.81 to 112.28)	1.71 (1.29 to 2.25)	0.69 (0.46 to 1.03)	0.53 (0.35 to 0.79)	0.87 (0.58 to 1.30)

**Injuries**	**14,783.13 (14,044.84 to 15,519.49)**	**12,776.14 (10,738.66 to 16,375.55)**	**11,061.83 (9,274.84 to 14,535.02)**	**16,444.71 (13,854.62 to 20,864.26)**	**337.56 (318.38 to 353.81)**	**396.75 (346.00 to 472.55)**	**361.10 (314.19 to 432.80)**	**477.32 (416.93 to 570.03)**
**Transport injuries**	**5,909.51 (5,547.70 to 6,281.89)**	**3,723.15 (3,051.67 to 4,811.74)**	**2,836.54 (2,375.94 to 3,551.10)**	**6,793.80 (5,615.45 to 8,706.09)**	**130.92 (123.00 to 138.96)**	**111.08 (92.67 to 138.65)**	**89.23 (76.16 to 108.01)**	**187.28 (156.78 to 232.72)**
Road injuries	5,512.41 (5,159.57 to 5,874.07)	3,194.72 (2,602.58 to 4,146.99)	2,396.58 (1,993.06 to 2,989.13)	6,200.44 (5,105.24 to 7,896.14)	121.97 (114.27 to 129.97)	95.35 (79.26 to 119.48)	75.65 (64.51 to 91.36)	170.36 (142.76 to 211.48)
Pedestrian road injuries	1,168.76 (1,025.49 to 1,375.91)	732.87 (606.87 to 920.63)	532.31 (445.98 to 654.32)	1,323.99 (1,081.50 to 1,666.10)	30.63 (27.32 to 35.41)	28.87 (24.11 to 35.35)	22.32 (18.98 to 27.06)	48.25 (39.76 to 59.24)

Southeast Asia								
**Cause**	**YLLs (in 1000s)**				**Deaths (in 1000s)**			
	**2016**	**2040 Forecast**	**2040 Better Scenario**	**2040 Worse Scenario**	**2016**	**2040 Forecast**	**2040 Better Scenario**	**2040 Worse Scenario**
Cyclist road injuries	228.78 (168.90 to 305.81)	163.51 (112.74 to 241.79)	125.99 (88.44 to 182.41)	293.20 (200.09 to 430.85)	5.73 (4.16 to 7.71)	5.61 (3.82 to 8.42)	4.66 (3.21 to 6.88)	9.05 (6.06 to 13.62)
Motorcyclist road injuries	1,608.81 (1,395.36 to 1,915.33)	965.13 (724.90 to 1,334.16)	797.95 (611.77 to 1,065.68)	1,653.45 (1,243.78 to 2,269.64)	31.49 (27.42 to 37.54)	22.59 (17.11 to 30.54)	19.39 (15.14 to 25.10)	36.16 (27.40 to 48.83)
Motor vehicle road injuries	2,404.20 (2,095.69 to 2,726.17)	1,269.17 (1,000.59 to 1,656.05)	893.37 (727.95 to 1,117.65)	2,799.30 (2,213.22 to 3,611.63)	51.80 (45.53 to 58.79)	36.14 (29.41 to 45.95)	27.53 (22.96 to 33.99)	73.12 (59.02 to 92.85)
Other road injuries	101.87 (84.81 to 148.63)	64.05 (48.00 to 98.53)	46.96 (36.03 to 70.04)	130.50 (97.61 to 199.48)	2.34 (1.93 to 3.45)	2.14 (1.57 to 3.39)	1.75 (1.32 to 2.75)	3.79 (2.84 to 5.87)
Other transport injuries	397.10 (323.95 to 465.37)	528.42 (398.15 to 698.94)	439.96 (334.03 to 572.83)	593.36 (442.89 to 796.50)	8.95 (7.28 to 10.41)	15.73 (12.05 to 20.59)	13.59 (10.33 to 17.62)	16.92 (12.60 to 22.55)
**Unintentional injuries**	**5,387.92 (5,041.43 to 5,697.85)**	**4,622.57 (4,120.58 to 5,114.71)**	**4,214.18 (3,764.91 to 4,662.07)**	**4,979.49 (4,447.68 to 5,542.69)**	**132.52 (117.36 to 140.14)**	**179.03 (151.30 to 194.42)**	**174.03 (146.35 to 190.64)**	**179.54 (150.15 to 196.85)**
Falls	1,340.37 (1,176.50 to 1,447.81)	1,839.01 (1,524.72 to 2,031.57)	1,779.42 (1,457.19 to 1,974.70)	1,852.08 (1,522.34 to 2,044.57)	51.58 (39.60 to 56.62)	101.93 (77.61 to 114.83)	103.02 (78.02 to 115.92)	97.19 (72.32 to 110.46)
Drowning	1,785.35 (1,626.52 to 1,928.66)	1,014.61 (864.37 to 1,210.85)	875.06 (751.64 to 1,036.93)	1,162.87 (980.03 to 1,392.42)	32.05 (29.87 to 34.72)	24.16 (20.97 to 28.63)	21.69 (18.84 to 25.52)	26.55 (22.82 to 31.52)
Fire, heat, and hot substances	271.77 (222.65 to 298.52)	192.04 (157.51 to 226.37)	164.31 (134.79 to 193.24)	222.26 (180.42 to 263.86)	6.20 (5.11 to 6.74)	6.38 (5.33 to 7.38)	5.76 (4.84 to 6.63)	6.92 (5.76 to 8.13)
Poisonings	133.54 (83.78 to 159.17)	68.34 (40.51 to 90.74)	56.90 (33.73 to 76.14)	84.84 (50.44 to 114.00)	2.94 (1.74 to 3.57)	1.91 (1.11 to 2.49)	1.65 (0.99 to 2.15)	2.28 (1.32 to 3.04)
Exposure to mechanical forces	359.96 (328.90 to 394.36)	279.68 (235.64 to 319.21)	240.81 (201.86 to 276.33)	319.16 (268.06 to 365.92)	7.32 (6.45 to 7.83)	7.25 (6.14 to 8.07)	6.50 (5.54 to 7.29)	7.97 (6.69 to 8.88)
Unintentional firearm injuries	51.05 (44.62 to 62.34)	33.94 (28.64 to 42.10)	29.68 (25.10 to 37.19)	39.02 (32.86 to 48.89)	1.13 (0.99 to 1.35)	0.97 (0.83 to 1.18)	0.88 (0.76 to 1.08)	1.06 (0.92 to 1.29)
Unintentional suffocation	68.97 (57.84 to 88.08)	40.97 (33.58 to 52.72)	32.66 (26.98 to 40.53)	49.61 (40.12 to 63.54)	1.01 (0.88 to 1.20)	0.72 (0.59 to 0.87)	0.61 (0.49 to 0.73)	0.84 (0.70 to 1.01)
Other exposure to mechanical forces	239.94 (191.73 to 263.05)	204.77 (156.58 to 238.24)	178.47 (135.23 to 209.71)	230.53 (176.36 to 268.27)	5.18 (4.15 to 5.65)	5.56 (4.44 to 6.31)	5.01 (4.04 to 5.70)	6.07 (4.78 to 6.87)
Adverse effects of medical treatment	364.37 (293.29 to 402.09)	339.65 (275.16 to 395.50)	315.69 (258.64 to 367.85)	368.52 (299.92 to 428.23)	8.97 (7.50 to 9.93)	11.95 (10.35 to 13.90)	11.66 (10.05 to 13.49)	12.28 (10.56 to 14.06)
Animal contact	157.66 (140.68 to 176.84)	171.77 (144.11 to 199.43)	151.44 (126.47 to 176.00)	198.10 (166.92 to 230.67)	3.86 (3.37 to 4.20)	5.66 (4.76 to 6.46)	5.28 (4.40 to 6.04)	6.23 (5.23 to 7.15)
Venomous animal contact	102.46 (83.29 to 114.73)	110.01 (84.80 to 129.14)	97.60 (74.68 to 115.20)	126.56 (96.86 to 149.05)	2.55 (2.01 to 2.85)	3.66 (2.74 to 4.24)	3.45 (2.59 to 4.02)	4.04 (3.04 to 4.68)
Non-venomous animal contact	55.20 (45.35 to 78.43)	61.76 (49.80 to 87.36)	53.85 (42.78 to 75.31)	71.54 (57.37 to 100.62)	1.30 (1.08 to 1.77)	2.00 (1.59 to 2.64)	1.83 (1.43 to 2.44)	2.20 (1.74 to 2.91)
Foreign body	503.80 (413.27 to 627.29)	380.94 (312.04 to 456.44)	354.28 (291.16 to 417.94)	397.10 (322.94 to 484.36)	9.76 (7.92 to 11.24)	11.39 (8.66 to 12.93)	11.39 (8.62 to 13.03)	10.96 (8.34 to 12.48)
Pulmonary aspiration and foreign body in airway	438.21 (344.84 to 535.99)	323.64 (250.25 to 387.39)	298.10 (231.31 to 354.60)	336.81 (257.36 to 415.43)	8.23 (6.33 to 9.57)	9.48 (6.74 to 11.02)	9.42 (6.66 to 11.03)	9.07 (6.50 to 10.57)

Foreign body in other body part	65.60 (52.18 to 112.44)	57.30 (45.88 to 90.58)	56.18 (45.05 to 87.99)	60.29 (47.96 to 96.23)	1.53 (1.27 to 2.43)	1.90 (1.60 to 2.77)	1.97 (1.64 to 2.84)	1.89 (1.60 to 2.76)
Environmental heat and cold exposure	19.83 (13.50 to 22.47)	17.27 (12.26 to 20.49)	14.72 (10.55 to 17.58)	20.99 (14.93 to 25.00)	0.50 (0.37 to 0.58)	0.60 (0.47 to 0.73)	0.54 (0.42 to 0.67)	0.69 (0.53 to 0.82)
Other unintentional injuries	451.27 (397.57 to 495.28)	319.27 (263.24 to 369.95)	261.54 (215.78 to 307.89)	353.56 (292.94 to 407.57)	9.34 (8.18 to 10.23)	7.80 (6.49 to 8.87)	6.56 (5.53 to 7.51)	8.45 (7.02 to 9.57)
**Self-harm and interpersonal violence**	**3,334.77 (2,974.44 to 3,658.61)**	**3,558.20 (2,816.68 to 4,544.18)**	**3,153.05 (2,540.17 to 3,951.33)**	**3,784.23 (2,948.43 to 4,905.88)**	**71.44 (64.06 to 78.20)**	**88.59 (71.08 to 110.43)**	**79.80 (65.72 to 98.47)**	**92.53 (73.09 to 118.19)**

Southeast Asia								
Cause	YLLs (in 1000s)				Deaths (in 1000s)			
	2016	2040 Forecast	2040 Better Scenario	2040 Worse Scenario	2016	2040 Forecast	2040 Better Scenario	2040 Worse Scenario
Self-harm	1,863.63 (1,690.39 to 2,231.06)	2,231.14 (1,654.59 to 3,011.43)	1,930.68 (1,467.72 to 2,554.71)	2,442.75 (1,751.77 to 3,386.22)	42.38 (38.64 to 49.79)	59.76 (45.00 to 77.88)	52.87 (41.03 to 67.47)	63.69 (46.78 to 85.56)
Self-harm by firearm	81.15 (56.55 to 114.09)	77.83 (48.69 to 124.13)	64.63 (41.39 to 100.68)	86.85 (52.97 to 140.37)	1.83 (1.28 to 2.49)	2.09 (1.36 to 3.11)	1.78 (1.19 to 2.65)	2.27 (1.44 to 3.48)
Self-harm by other specified means	1,782.48 (1,619.55 to 2,130.00)	2,153.31 (1,593.41 to 2,897.81)	1,866.05 (1,416.41 to 2,466.85)	2,355.90 (1,688.00 to 3,244.34)	40.55 (37.00 to 47.61)	57.67 (43.64 to 75.36)	51.09 (39.69 to 65.17)	61.42 (45.19 to 82.57)
Interpersonal violence	1,471.14 (1,053.64 to 1,738.40)	1,327.05 (884.55 to 1,679.44)	1,222.37 (807.03 to 1,556.03)	1,341.48 (873.34 to 1,710.75)	29.05 (20.83 to 34.50)	28.83 (19.05 to 36.16)	26.93 (17.66 to 33.90)	28.85 (18.74 to 36.39)
Physical violence by firearm	594.64 (343.55 to 749.88)	526.12 (254.41 to 721.97)	482.54 (230.44 to 659.18)	532.67 (254.99 to 736.66)	11.92 (6.85 to 15.03)	11.28 (5.60 to 15.19)	10.45 (5.11 to 14.30)	11.31 (5.49 to 15.30)
Physical violence by sharp object	583.54 (390.16 to 718.62)	577.30 (356.11 to 747.66)	541.58 (333.69 to 711.97)	569.77 (345.71 to 740.76)	11.38 (7.57 to 14.00)	12.22 (7.41 to 15.72)	11.64 (7.08 to 15.22)	11.94 (7.12 to 15.46)
Physical violence by other means	292.96 (230.25 to 375.08)	223.64 (167.97 to 304.40)	198.25 (149.26 to 267.42)	239.04 (178.22 to 323.62)	5.76 (4.54 to 7.33)	5.34 (4.01 to 7.09)	4.84 (3.61 to 6.31)	5.59 (4.15 to 7.52)
**Forces of nature, conflict and terrorism, and executions and police conflict**	**150.93 (84.24 to 219.34)**	**872.22 (25.07 to 3,603.41)**	**858.06 (24.37 to 3,467.25)**	**887.19 (25.22 to 3,711.03)**	**2.68 (1.52 to 3.85)**	**18.04 (0.57 to 68.39)**	**18.03 (0.57 to 67.49)**	**17.97 (0.56 to 68.73)**
Exposure to forces of nature	14.95 (9.50 to 20.13)	135.41 (0.25 to 2,242.74)	131.64 (0.24 to 2,219.47)	136.97 (0.25 to 2,269.42)	0.29 (0.19 to 0.40)	3.07 (0.01 to 51.25)	3.07 (0.01 to 51.83)	3.01 (0.01 to 49.96)
Conflict and terrorism	123.09 (60.14 to 187.45)	338.98 (9.49 to 1,605.89)	336.06 (9.07 to 1,599.86)	351.06 (9.60 to 1,646.92)	2.09 (1.02 to 3.19)	6.81 (0.19 to 33.75)	6.85 (0.19 to 34.06)	6.85 (0.19 to 33.68)
Executions and police conflict	12.89 (7.22 to 16.27)	397.83 (0.00 to 3,034.58)	390.35 (0.00 to 2,995.94)	399.16 (0.00 to 3,047.14)	0.29 (0.14 to 0.36)	8.16 (0.00 to 64.56)	8.11 (0.00 to 64.49)	8.10 (0.00 to 64.20)

Cambodia								
Cause	YLLs (actual counts)				Deaths (actual counts)			
	2016	2040 Forecast	2040 Better Scenario	2040 Worse Scenario	2016	2040 Forecast	2040 Better Scenario	2040 Worse Scenario
**All causes**	**3,626,938.11 (3,328,727.61 to 3,951,169.95)**	**3,363,562.47 (2,910,710.87 to 4,371,076.73)**	**2,836,557.46 (2,472,764.61 to 3,802,809.86)**	**4,901,332.56 (4,196,974.27 to 5,991,975.41)**	**98,454.46 (92,446.85 to 104,628.73)**	**140,604.17 (125,539.11 to 161,215.39)**	**122,209.63 (109,565.47 to 142,105.91)**	**185,134.63 (165,905.88 to 209,078.53)**
**Communicable, maternal, neonatal, and nutritional diseases**	**1,465,248.10 (1,256,444.09 to 1,690,326.86)**	**625,675.72 (477,866.46 to 826,264.25)**	**508,311.05 (394,315.60 to 662,794.31)**	**1,199,656.85 (841,779.85 to 1,732,853.47)**	**26,198.76 (23,471.75 to 29,522.26)**	**19,183.27 (14,723.59 to 24,744.42)**	**15,694.95 (12,460.54 to 19,864.09)**	**31,521.82 (23,590.91 to 42,339.77)**
**HIV/AIDS and tuberculosis**	**138,288.73 (103,439.21 to 175,519.46)**	**60,973.90 (31,647.46 to 121,977.35)**	**45,410.56 (22,861.50 to 93,265.72)**	**124,723.07 (63,174.78 to 240,849.79)**	**3,724.45 (2,921.95 to 4,676.00)**	**2,262.74 (1,240.51 to 4,252.56)**	**1,733.50 (974.55 to 3,140.79)**	**4,200.11 (2,239.35 to 7,703.01)**
Tuberculosis	80,473.09 (67,266.13 to 98,758.82)	48,562.27 (24,831.43 to 95,853.38)	34,355.31 (18,732.07 to 63,331.29)	97,195.85 (46,095.87 to 195,441.31)	2,501.41 (2,102.04 to 3,112.59)	1,971.34 (1,081.85 to 3,748.21)	1,464.06 (840.10 to 2,540.86)	3,649.44 (1,838.03 to 7,129.23)
HIV/AIDS	57,815.64 (26,211.48 to 94,285.13)	12,411.63 (709.45 to 55,787.44)	11,055.25 (516.79 to 52,189.59)	27,527.22 (3,311.11 to 84,049.72)	1,223.04 (556.12 to 2,057.63)	291.40 (26.21 to 1,410.26)	269.44 (20.30 to 1,407.60)	550.66 (66.74 to 1,890.49)
**Diarrhea, lower respiratory, and other common infectious diseases**	**668,336.35 (562,010.42 to 788,573.08)**	**306,697.74 (197,983.71 to 461,803.18)**	**223,783.99 (151,013.49 to 333,264.87)**	**655,296.25 (386,961.91 to 1,115,846.93)**	**12,924.33 (11,308.47 to 14,935.77)**	**11,650.21 (8,054.08 to 16,869.93)**	**8,916.08 (6,255.35 to 12,766.59)**	**19,972.72 (13,056.39 to 29,892.06)**
Diarrheal diseases	54,115.00 (37,379.58 to 77,032.09)	21,581.59 (3,773.81 to 68,194.97)	17,091.53 (3,339.55 to 53,296.35)	50,515.92 (9,939.72 to 160,774.68)	1,349.27 (834.17 to 2,090.02)	1,172.82 (189.69 to 4,038.87)	988.31 (178.73 to 3,211.73)	2,395.79 (392.06 to 8,209.12)
Intestinal infectious diseases	50,648.56 (27,558.92 to 85,602.82)	23,365.31 (11,728.85 to 40,713.79)	22,222.41 (11,430.35 to 38,290.52)	32,303.19 (15,644.35 to 57,371.77)	752.82 (410.85 to 1,254.40)	397.72 (205.59 to 668.87)	381.48 (202.13 to 638.70)	524.35 (266.16 to 921.32)
Typhoid fever	43,946.90 (22,951.47 to 75,318.99)	19,687.21 (9,527.60 to 34,994.12)	18,760.03 (9,344.62 to 32,456.19)	27,118.73 (12,780.66 to 49,771.41)	650.05 (342.56 to 1,100.33)	332.39 (162.75 to 572.92)	319.48 (161.70 to 543.28)	436.66 (208.99 to 770.02)
Paratyphoid fever	6,467.90 (2,893.24 to 12,772.91)	3,623.79 (1,589.14 to 7,196.66)	3,413.78 (1,501.10 to 6,787.75)	5,098.94 (2,195.43 to 10,381.56)	98.90 (44.33 to 192.55)	63.64 (28.39 to 124.22)	60.40 (27.19 to 117.51)	85.38 (37.73 to 168.99)
Other intestinal infectious diseases	233.75 (85.62 to 545.07)	54.31 (17.78 to 124.70)	48.60 (16.13 to 113.36)	85.52 (26.65 to 204.12)	3.87 (1.50 to 8.41)	1.70 (0.59 to 3.75)	1.60 (0.54 to 3.58)	2.31 (0.79 to 5.10)
Lower respiratory infections	502,252.00 (412,937.93 to 596,571.09)	222,457.90 (136,920.03 to 359,170.39)	151,439.81 (98,694.60 to 233,840.84)	482,756.00 (253,811.83 to 877,100.06)	9,857.73 (8,479.99 to 11,429.14)	9,324.50 (6,508.76 to 13,308.79)	6,870.19 (4,965.97 to 9,523.82)	15,672.03 (10,051.58 to 23,606.40)
Upper respiratory infections	355.66 (163.72 to 697.00)	183.83 (79.17 to 360.74)	160.04 (68.56 to 310.25)	306.38 (129.43 to 617.77)	7.05 (3.70 to 11.76)	5.65 (2.53 to 10.94)	5.08 (2.20 to 9.84)	8.58 (3.85 to 16.87)
Otitis media	52.81 (28.83 to 97.22)	37.08 (14.18 to 81.45)	38.68 (14.67 to 83.43)	35.27 (13.94 to 78.53)	1.70 (0.83 to 3.07)	1.77 (0.59 to 3.78)	1.93 (0.64 to 4.11)	1.55 (0.54 to 3.32)
Meningitis	27,950.07 (15,791.41 to 43,087.57)	11,281.97 (6,485.60 to 18,076.24)	10,755.87 (6,280.09 to 17,239.43)	17,342.06 (7,985.03 to 29,950.91)	424.26 (260.25 to 636.03)	244.21 (161.62 to 369.59)	241.04 (159.76 to 362.37)	316.17 (188.87 to 503.47)
Pneumococcal meningitis	276.84 (141.89 to 463.88)	87.88 (47.46 to 154.11)	86.26 (46.98 to 149.46)	132.33 (63.11 to 246.61)	4.26 (2.40 to 6.83)	2.33 (1.41 to 3.85)	2.39 (1.45 to 3.98)	2.88 (1.69 to 4.94)
H influenzae type B meningitis	2,218.26 (1,208.49 to 3,474.92)	1,391.02 (447.79 to 3,477.64)	1,175.18 (436.67 to 2,785.08)	5,032.90 (774.66 to 14,457.54)	32.15 (18.79 to 49.21)	23.20 (10.79 to 48.15)	21.10 (10.90 to 40.57)	65.05 (14.67 to 174.99)
Meningococcal meningitis	9,360.76 (5,101.68 to 14,954.14)	3,469.33 (2,035.83 to 5,679.38)	3,361.13 (1,970.47 to 5,482.54)	4,299.63 (2,394.62 to 7,262.98)	145.38 (87.17 to 222.97)	81.84 (53.68 to 129.91)	81.70 (53.27 to 129.68)	92.04 (58.71 to 148.62)
Other meningitis	16,094.21 (9,017.84 to 25,297.10)	6,333.74 (3,720.06 to 10,052.35)	6,133.30 (3,598.00 to 9,790.00)	7,877.20 (4,348.27 to 12,996.14)	242.47 (146.58 to 365.14)	136.84 (89.19 to 209.40)	135.84 (89.18 to 209.22)	156.20 (98.86 to 242.78)
Encephalitis	10,065.62 (6,000.28 to 17,595.72)	6,097.30 (3,625.24 to 10,472.50)	5,775.86 (3,386.21 to 10,004.74)	8,095.26 (4,680.75 to 14,520.64)	166.47 (106.89 to 279.82)	148.17 (97.90 to 244.68)	144.51 (95.48 to 235.64)	178.38 (114.92 to 295.84)

Diphtheria	44.83 (26.10 to 76.15)	13.70 (7.20 to 22.53)	12.23 (6.65 to 20.04)	42.59 (9.33 to 119.71)	0.66 (0.42 to 1.04)	0.26 (0.14 to 0.43)	0.24 (0.13 to 0.40)	0.60 (0.18 to 1.50)
Whooping cough	11,744.60 (883.11 to 42,904.13)	2,084.64 (68.26 to 8,667.11)	1,039.93 (46.61 to 4,345.59)	6,808.33 (142.39 to 28,083.55)	139.26 (10.47 to 507.53)	25.25 (0.93 to 104.55)	12.97 (0.60 to 53.22)	80.86 (1.74 to 332.84)
Tetanus	8,471.35 (4,965.96 to 13,807.48)	1,601.35 (782.76 to 2,750.61)	1,477.70 (728.96 to 2,460.97)	2,707.13 (1,265.86 to 5,557.23)	182.91 (106.37 to 287.79)	49.07 (25.38 to 84.53)	49.44 (24.38 to 84.52)	59.03 (30.13 to 99.87)
Measles	1,100.72 (339.52 to 2,661.64)	17,469.44 (817.07 to 77,426.09)	13,296.66 (780.21 to 53,302.25)	53,529.63 (1,547.81 to 270,035.99)	13.18 (4.06 to 31.81)	259.18 (14.03 to 974.75)	199.64 (13.10 to 671.37)	707.96 (25.73 to 3,276.19)

Cambodia								
Cause	YLLs (actual counts)				Deaths (actual counts)			
	2016	2040 Forecast	2040 Better Scenario	2040 Worse Scenario	2016	2040 Forecast	2040 Better Scenario	2040 Worse Scenario
Varicella and herpes zoster	1,535.14 (919.78 to 2,393.87)	523.63 (289.79 to 885.73)	473.28 (263.18 to 811.89)	854.50 (437.06 to 1,527.50)	29.01 (20.24 to 40.29)	21.61 (13.09 to 33.07)	21.26 (12.59 to 33.20)	27.43 (16.61 to 42.57)
**Neglected tropical diseases and malaria**	**126,259.17 (71,812.29 to 196,016.32)**	**69,533.16 (42,666.07 to 100,929.66)**	**67,351.70 (41,868.14 to 96,536.68)**	**81,739.47 (47,666.66 to 121,861.13)**	**2,222.97 (1,344.78 to 3,295.20)**	**1,562.78 (1,019.52 to 2,364.96)**	**1,536.84 (1,025.68 to 2,287.04)**	**1,735.46 (1,093.71 to 2,679.12)**
Malaria	121,745.49 (66,071.88 to 193,038.17)	59,705.11 (32,270.69 to 91,775.53)	57,672.87 (31,883.13 to 87,817.69)	70,390.36 (36,347.50 to 111,856.76)	2,143.72 (1,266.13 to 3,223.13)	1,317.11 (780.53 to 2,093.49)	1,290.38 (769.97 to 2,056.58)	1,471.48 (841.42 to 2,368.45)
Chagas disease	0.00 (0.00 to 0.00)	0.00 (0.00 to 0.00)	0.00 (0.00 to 0.00)	0.00 (0.00 to 0.00)	0.00 (0.00 to 0.00)	0.00 (0.00 to 0.00)	0.00 (0.00 to 0.00)	0.00 (0.00 to 0.00)
Leishmaniasis	0.00 (0.00 to 0.00)	0.00 (0.00 to 0.00)	0.00 (0.00 to 0.00)	0.00 (0.00 to 0.00)	0.00 (0.00 to 0.00)	0.00 (0.00 to 0.00)	0.00 (0.00 to 0.00)	0.00 (0.00 to 0.00)
African trypanosomiasis	0.00 (0.00 to 0.00)	0.00 (0.00 to 0.00)	0.00 (0.00 to 0.00)	0.00 (0.00 to 0.00)	0.00 (0.00 to 0.00)	0.00 (0.00 to 0.00)	0.00 (0.00 to 0.00)	0.00 (0.00 to 0.00)
Schistosomiasis	117.00 (54.44 to 202.61)	75.54 (32.15 to 140.12)	71.61 (30.18 to 131.76)	91.57 (38.61 to 169.71)	3.48 (1.58 to 6.01)	2.83 (1.11 to 5.50)	2.75 (1.09 to 5.36)	3.24 (1.28 to 6.18)
Cysticercosis	52.39 (45.11 to 60.71)	20.64 (15.82 to 25.17)	20.00 (15.38 to 24.40)	23.31 (18.11 to 28.32)	1.20 (1.04 to 1.38)	0.57 (0.45 to 0.69)	0.56 (0.44 to 0.68)	0.63 (0.49 to 0.75)
Cystic echinococcosis	22.96 (18.15 to 28.86)	8.78 (5.94 to 11.92)	7.26 (5.04 to 9.75)	16.11 (11.11 to 22.25)	0.58 (0.45 to 0.72)	0.30 (0.21 to 0.40)	0.25 (0.18 to 0.34)	0.52 (0.35 to 0.71)
Dengue	3,624.49 (748.10 to 11,754.98)	9,358.39 (2,795.45 to 19,922.41)	9,233.30 (2,779.42 to 19,710.84)	10,700.35 (3,107.82 to 22,829.34)	61.73 (13.43 to 179.65)	230.97 (69.31 to 485.23)	232.12 (69.85 to 488.94)	245.88 (72.26 to 508.95)
Yellow fever	0.00 (0.00 to 0.00)	0.00 (0.00 to 0.00)	0.00 (0.00 to 0.00)	0.00 (0.00 to 0.00)	0.00 (0.00 to 0.00)	0.00 (0.00 to 0.00)	0.00 (0.00 to 0.00)	0.00 (0.00 to 0.00)
Rabies	51.00 (20.32 to 111.25)	14.58 (5.43 to 32.69)	11.47 (4.51 to 24.52)	32.28 (12.45 to 68.55)	1.05 (0.46 to 2.26)	0.40 (0.17 to 0.87)	0.32 (0.14 to 0.70)	0.81 (0.33 to 1.70)
Intestinal nematode infections	191.49 (71.59 to 365.62)	129.59 (55.73 to 250.75)	130.32 (56.39 to 254.16)	159.23 (62.20 to 309.59)	2.80 (1.31 to 4.90)	2.73 (1.46 to 4.47)	2.81 (1.51 to 4.63)	2.96 (1.48 to 5.11)
Other neglected tropical diseases	454.36 (229.04 to 1,085.02)	220.54 (133.24 to 453.80)	204.85 (122.88 to 412.97)	326.25 (190.83 to 658.99)	8.41 (4.77 to 19.52)	7.88 (4.56 to 17.07)	7.63 (4.42 to 16.64)	9.96 (5.72 to 22.99)
**Maternal disorders**	**27,728.92 (20,337.61 to 36,662.87)**	**12,919.15 (8,164.29 to 20,037.09)**	**10,730.21 (6,687.15 to 16,593.48)**	**22,623.86 (14,169.34 to 35,820.20)**	**526.71 (387.96 to 698.33)**	**255.56 (162.72 to 389.88)**	**212.25 (131.83 to 330.13)**	**446.07 (280.23 to 702.61)**
Maternal hemorrhage	3,125.51 (1,992.08 to 4,726.81)	1,689.48 (961.37 to 2,851.86)	1,410.42 (781.71 to 2,376.76)	2,950.21 (1,618.38 to 4,910.97)	58.33 (37.17 to 88.57)	32.65 (18.58 to 54.86)	27.26 (15.05 to 45.69)	56.90 (30.80 to 94.45)
Maternal sepsis and other maternal infections	544.47 (258.67 to 909.41)	314.25 (139.16 to 580.17)	262.36 (114.00 to 494.98)	550.93 (239.81 to 1,000.83)	10.63 (5.10 to 17.72)	6.45 (2.84 to 11.73)	5.38 (2.32 to 10.13)	11.27 (4.96 to 20.94)
Maternal hypertensive disorders	1,001.76 (626.98 to 1,518.93)	593.23 (330.02 to 950.78)	494.63 (265.75 to 814.30)	1,044.87 (563.50 to 1,696.73)	18.84 (11.84 to 28.45)	11.56 (6.50 to 18.71)	9.64 (5.24 to 15.93)	20.31 (11.23 to 32.38)
Maternal obstructed labor and uterine rupture	132.45 (50.42 to 250.69)	81.85 (29.20 to 167.91)	67.40 (24.16 to 139.41)	148.79 (54.14 to 303.95)	2.54 (0.98 to 4.83)	1.63 (0.56 to 3.37)	1.34 (0.48 to 2.81)	2.95 (1.08 to 6.03)
Maternal abortion, miscarriage, and ectopic pregnancy	20,907.84 (15,155.25 to 27,934.19)	8,836.49 (5,305.96 to 14,224.58)	7,329.99 (4,222.16 to 11,630.31)	15,331.16 (8,921.26 to 24,938.69)	398.55 (289.92 to 530.05)	175.95 (105.96 to 280.77)	145.95 (84.52 to 231.88)	304.27 (177.36 to 488.89)

Indirect maternal deaths	925.97 (565.90 to 1,453.33)	577.82 (295.12 to 993.10)	485.71 (245.52 to 858.96)	1,020.50 (529.98 to 1,781.03)	16.94 (10.19 to 26.26)	10.90 (5.62 to 18.39)	9.17 (4.70 to 15.89)	19.14 (9.92 to 32.97)
Late maternal deaths	81.67 (31.88 to 167.95)	47.77 (16.42 to 103.54)	39.99 (13.45 to 88.62)	84.40 (28.80 to 188.84)	1.54 (0.60 to 3.11)	0.93 (0.31 to 1.99)	0.78 (0.26 to 1.69)	1.64 (0.56 to 3.54)
Maternal deaths aggravated by HIV/AIDS	74.88 (36.23 to 132.00)	122.06 (1.12 to 510.83)	83.17 (0.53 to 413.31)	375.10 (14.90 to 1,069.41)	1.57 (0.76 to 2.75)	2.53 (0.02 to 11.15)	1.74 (0.01 to 9.01)	7.67 (0.27 to 23.46)
Other maternal disorders	934.37 (565.02 to 1,470.35)	656.20 (342.11 to 1,122.85)	556.54 (291.52 to 965.78)	1,117.91 (590.50 to 1,924.24)	17.76 (10.84 to 28.07)	12.95 (6.86 to 22.30)	10.99 (5.85 to 19.52)	21.93 (11.57 to 37.73)

Cambodia								
Cause	YLLs (actual counts)				Deaths (actual counts)			
	2016	2040 Forecast	2040 Better Scenario	2040 Worse Scenario	2016	2040 Forecast	2040 Better Scenario	2040 Worse Scenario
**Neonatal disorders**	**420,170.05 (327,106.37 to 528,461.34)**	**120,449.50 (76,861.14 to 185,838.77)**	**109,109.65 (70,380.12 to 171,883.94)**	**242,101.78 (157,808.55 to 374,509.07)**	**4,854.09 (3,778.78 to 6,105.81)**	**1,391.69 (887.95 to 2,147.10)**	**1,260.67 (813.34 to 1,985.70)**	**2,797.13 (1,823.64 to 4,326.67)**
Neonatal preterm birth	194,079.14 (135,525.92 to 265,107.24)	46,699.56 (28,367.87 to 72,347.32)	42,702.02 (26,217.23 to 66,110.29)	107,689.23 (64,786.02 to 168,310.68)	2,241.90 (1,565.89 to 3,062.23)	539.53 (327.82 to 835.67)	493.35 (302.93 to 763.67)	1,244.06 (748.41 to 1,944.13)
Neonatal encephalopathy due to birth asphyxia and trauma	132,398.65 (85,632.35 to 195,183.42)	39,916.42 (20,280.68 to 73,062.76)	36,253.40 (18,272.56 to 67,402.77)	70,891.62 (35,665.40 to 131,869.31)	1,529.52 (989.43 to 2,254.68)	461.18 (234.38 to 844.09)	418.86 (211.11 to 778.71)	819.05 (412.09 to 1,523.16)
Neonatal sepsis and other neonatal infections	42,218.20 (26,882.83 to 65,177.89)	17,342.24 (9,156.19 to 31,094.23)	15,664.16 (8,193.92 to 28,674.36)	31,253.39 (16,061.36 to 56,222.77)	487.76 (310.71 to 752.85)	200.38 (105.79 to 359.17)	180.99 (94.67 to 331.47)	361.11 (185.59 to 649.47)
Hemolytic disease and other neonatal jaundice	3,137.96 (1,524.65 to 5,493.26)	772.22 (296.76 to 1,758.74)	678.29 (261.50 to 1,532.91)	1,507.47 (586.15 to 3,436.55)	36.26 (17.61 to 63.45)	8.92 (3.43 to 20.32)	7.84 (3.02 to 17.71)	17.42 (6.77 to 39.69)
Other neonatal disorders	48,336.10 (27,766.36 to 76,055.26)	15,719.06 (7,398.67 to 29,339.05)	13,811.78 (6,618.71 to 25,577.49)	30,760.08 (14,731.10 to 56,456.93)	558.65 (320.90 to 878.59)	181.67 (85.57 to 338.97)	159.63 (76.55 to 295.61)	355.50 (170.35 to 652.18)
**Nutritional deficiencies**	**13,895.46 (10,473.85 to 18,308.95)**	**9,016.16 (7,202.41 to 11,028.27)**	**8,815.74 (7,090.29 to 10,873.81)**	**11,123.84 (8,670.61 to 14,130.00)**	**463.98 (387.69 to 548.71)**	**573.64 (432.14 to 732.79)**	**593.05 (450.64 to 753.65)**	**599.48 (439.72 to 781.43)**
Protein-energy malnutrition	11,975.96 (8,958.70 to 16,078.28)	7,260.75 (5,644.68 to 9,000.77)	7,111.15 (5,554.07 to 8,865.91)	8,970.72 (6,826.56 to 11,504.84)	400.42 (331.89 to 473.79)	472.51 (350.96 to 612.55)	489.57 (369.06 to 625.11)	491.91 (356.26 to 646.52)
Iodine deficiency	211.40 (17.83 to 694.28)	346.43 (10.34 to 1,111.02)	326.00 (9.81 to 1,086.86)	468.27 (17.86 to 1,389.51)	5.09 (0.45 to 14.40)	19.24 (0.37 to 83.76)	18.96 (0.33 to 84.14)	22.64 (0.60 to 87.53)
Dietary iron deficiency	208.92 (151.94 to 290.47)	130.66 (85.99 to 249.87)	125.02 (82.33 to 242.98)	163.17 (107.78 to 291.56)	3.96 (2.86 to 6.41)	3.76 (2.23 to 9.33)	3.72 (2.18 to 9.16)	4.26 (2.59 to 10.46)
Other nutritional deficiencies	1,499.18 (997.56 to 2,343.05)	1,278.32 (865.46 to 1,938.00)	1,253.57 (848.72 to 1,917.62)	1,521.68 (1,022.19 to 2,307.70)	54.51 (37.19 to 83.04)	78.13 (48.98 to 124.25)	80.80 (50.94 to 126.91)	80.67 (50.25 to 128.21)
**Other communicable, maternal, neonatal, and nutritional diseases**	**70,569.41 (42,385.14 to 159,832.59)**	**46,086.10 (34,440.69 to 69,830.95)**	**43,109.21 (32,219.89 to 64,851.69)**	**62,048.56 (42,759.87 to 107,662.95)**	**1,482.24 (1,109.38 to 2,459.52)**	**1,486.65 (1,223.26 to 1,874.41)**	**1,442.56 (1,179.34 to 1,794.84)**	**1,770.85 (1,401.66 to 2,403.12)**
Sexually transmitted infections excluding HIV	8,045.78 (6,154.66 to 10,391.05)	6,259.29 (4,761.65 to 8,227.43)	6,028.51 (4,562.46 to 7,904.35)	7,335.04 (5,500.42 to 9,668.18)	211.79 (162.84 to 267.83)	236.66 (161.12 to 308.32)	236.49 (160.33 to 304.65)	253.46 (175.85 to 330.33)
Syphilis	3,623.09 (2,224.90 to 5,136.44)	2,250.40 (1,405.89 to 3,366.92)	2,157.66 (1,337.95 to 3,185.71)	2,725.51 (1,696.20 to 4,085.89)	81.80 (53.31 to 111.48)	74.20 (46.77 to 105.15)	73.53 (46.25 to 103.86)	82.36 (51.60 to 115.59)
Chlamydial infection	831.37 (647.49 to 1,209.65)	762.41 (569.35 to 1,059.17)	735.57 (550.12 to 1,000.92)	880.20 (645.64 to 1,256.22)	24.46 (19.37 to 31.65)	31.70 (21.69 to 42.42)	31.93 (21.67 to 42.49)	33.12 (22.85 to 44.67)
Gonococcal infection	2,384.06 (1,893.54 to 3,232.72)	2,221.72 (1,683.66 to 2,971.54)	2,152.55 (1,631.38 to 2,857.11)	2,528.80 (1,899.49 to 3,447.69)	72.94 (55.50 to 92.63)	94.67 (61.47 to 126.68)	95.29 (61.51 to 126.61)	98.77 (66.97 to 133.46)
Other sexually transmitted infections	1,207.27 (946.33 to 1,702.52)	1,024.75 (779.02 to 1,446.93)	982.73 (737.44 to 1,366.44)	1,200.53 (902.21 to 1,701.24)	32.59 (26.11 to 42.86)	36.09 (26.85 to 46.51)	35.74 (26.26 to 46.01)	39.21 (29.32 to 51.13)
Acute hepatitis	26,627.22 (22,379.16 to 31,385.81)	21,571.83 (16,746.57 to 28,051.82)	20,293.57 (15,771.06 to 26,168.28)	26,557.47 (20,158.65 to 35,083.59)	731.77 (616.38 to 859.32)	744.56 (588.64 to 955.50)	718.03 (568.54 to 913.96)	869.74 (676.13 to 1,107.01)
Acute hepatitis A	1,313.67 (759.70 to 1,976.89)	503.01 (316.62 to 763.00)	475.33 (299.00 to 720.15)	671.60 (416.39 to 1,049.69)	22.09 (14.71 to 30.81)	14.90 (9.33 to 22.38)	14.48 (9.11 to 21.74)	17.75 (11.02 to 26.80)
Acute hepatitis B	22,104.13 (18,210.68 to 26,493.57)	18,420.41 (13,970.08 to 24,399.46)	17,234.00 (12,879.45 to 22,624.25)	22,818.00 (16,870.39 to 30,896.59)	621.45 (517.83 to 742.05)	616.08 (472.65 to 801.45)	587.85 (450.56 to 764.81)	734.21 (555.77 to 960.67)

Acute hepatitis C	591.32 (415.84 to 827.94)	651.17 (414.06 to 1,031.56)	636.27 (406.67 to 993.41)	716.13 (454.85 to 1,136.41)	18.56 (13.01 to 26.65)	24.39 (15.72 to 38.17)	24.34 (15.60 to 37.43)	25.64 (16.24 to 40.26)
Acute hepatitis E	2,618.10 (1,868.01 to 3,581.43)	1,997.24 (1,243.56 to 3,024.63)	1,947.97 (1,203.00 to 2,925.53)	2,351.75 (1,500.12 to 3,583.44)	69.67 (47.94 to 99.56)	89.19 (53.01 to 138.03)	91.36 (54.51 to 141.78)	92.14 (54.29 to 146.75)
Other unspecified infectious diseases	35,896.41 (10,010.76 to 126,136.48)	18,254.97 (10,422.17 to 40,523.63)	16,787.13 (9,731.91 to 36,085.49)	28,156.05 (13,483.59 to 74,397.09)	538.68 (237.17 to 1,575.27)	505.42 (366.97 to 763.65)	488.03 (358.31 to 712.06)	647.65 (433.88 to 1,152.49)
**Non-communicable diseases**	**1,643,246.36 (1,524,018.14 to 1,768,307.54)**	**2,193,347.60 (1,902,347.51 to 2,519,944.75)**	**1,839,684.73 (1,620,530.41 to 2,081,545.61)**	**3,014,491.60 (2,575,181.23 to 3,512,854.10)**	**61,724.20 (57,807.86 to 65,905.57)**	**107,241.00 (94,485.05 to 120,554.43)**	**93,359.20 (83,063.91 to 104,274.74)**	**137,033.73 (119,742.44 to 154,860.31)**

Cambodia								
Cause	YLLs (actual counts)				Deaths (actual counts)			
	2016	2040 Forecast	2040 Better Scenario	2040 Worse Scenario	2016	2040 Forecast	2040 Better Scenario	2040 Worse Scenario
**Neoplasms**	**345,624.40 (311,060.07 to 380,390.02)**	**580,650.77 (479,086.27 to 688,260.52)**	**515,273.05 (434,959.86 to 594,626.93)**	**684,069.53 (552,098.02 to 827,012.51)**	**12,077.25 (10,813.24 to 13,188.68)**	**24,660.78 (20,245.85 to 29,442.38)**	**22,528.32 (18,938.43 to 26,134.30)**	**27,685.53 (22,188.18 to 33,918.06)**
Lip and oral cavity cancer	7,884.93 (6,747.79 to 9,264.46)	13,644.40 (8,097.30 to 22,769.74)	9,138.61 (5,834.71 to 14,432.30)	23,013.46 (13,124.57 to 39,042.92)	295.40 (253.11 to 344.11)	597.63 (357.38 to 974.95)	422.92 (269.90 to 647.44)	958.47 (540.17 to 1,635.55)
Nasopharynx cancer	5,856.73 (4,745.59 to 7,228.07)	10,436.58 (6,435.16 to 16,437.77)	6,678.48 (4,373.00 to 10,078.61)	18,240.01 (10,970.01 to 29,236.26)	185.72 (152.07 to 228.61)	373.05 (232.37 to 595.67)	245.22 (163.84 to 368.86)	636.07 (385.88 to 1,013.50)
Other pharynx cancer	3,950.91 (3,229.07 to 5,126.48)	6,436.60 (3,902.70 to 10,435.86)	5,500.65 (3,515.27 to 8,909.29)	8,021.99 (4,717.90 to 13,347.46)	141.86 (117.13 to 181.75)	259.25 (160.28 to 417.19)	226.86 (147.50 to 357.29)	311.67 (184.05 to 515.89)
Esophageal cancer	6,185.87 (5,420.54 to 7,019.26)	9,886.10 (5,819.90 to 16,764.68)	7,261.89 (4,491.71 to 11,734.60)	15,803.06 (8,615.03 to 27,571.91)	238.90 (210.78 to 270.47)	435.72 (255.43 to 728.74)	335.29 (208.83 to 528.70)	663.64 (361.71 to 1,143.53)
Stomach cancer	26,978.33 (23,744.56 to 30,366.66)	33,580.63 (27,275.96 to 40,906.15)	31,833.98 (26,339.67 to 38,085.28)	37,967.88 (29,757.31 to 47,660.35)	1,049.76 (922.12 to 1,194.21)	1,544.13 (1,257.86 to 1,875.86)	1,512.52 (1,254.96 to 1,814.91)	1,652.38 (1,296.18 to 2,070.42)
Colon and rectum cancer	27,700.44 (23,092.09 to 32,659.03)	62,483.52 (41,221.92 to 90,520.53)	60,241.27 (40,817.05 to 86,494.20)	63,120.15 (41,317.74 to 93,100.76)	1,057.59 (872.46 to 1,247.65)	2,938.84 (1,936.73 to 4,168.02)	2,932.97 (1,994.93 to 4,127.45)	2,791.43 (1,831.31 to 4,064.96)
Liver cancer	18,771.93 (15,173.34 to 23,629.52)	33,971.62 (24,374.58 to 47,161.96)	30,084.71 (22,594.04 to 40,943.48)	39,021.36 (26,394.18 to 56,469.69)	696.84 (569.61 to 872.12)	1,489.56 (1,070.27 to 2,043.93)	1,369.66 (1,022.22 to 1,828.02)	1,621.47 (1,099.14 to 2,318.03)
Liver cancer due to hepatitis B	7,522.47 (5,708.11 to 9,902.92)	12,556.89 (8,523.34 to 18,393.62)	11,267.73 (7,981.59 to 15,884.69)	14,059.56 (9,046.52 to 21,466.26)	252.44 (190.19 to 333.48)	499.37 (337.15 to 731.62)	462.77 (324.38 to 648.94)	534.17 (346.13 to 806.99)
Liver cancer due to hepatitis C	5,500.79 (4,090.04 to 7,525.76)	10,968.03 (7,438.82 to 16,073.80)	10,113.14 (7,147.79 to 14,342.39)	11,841.92 (7,665.47 to 17,836.50)	234.70 (175.73 to 317.58)	548.91 (374.68 to 776.99)	525.59 (373.73 to 737.09)	560.13 (367.39 to 822.86)
Liver cancer due to alcohol use	2,600.47 (1,792.79 to 3,722.17)	4,606.70 (2,812.32 to 7,443.21)	3,574.02 (2,260.25 to 5,618.03)	6,458.79 (3,754.31 to 10,605.57)	100.25 (69.85 to 144.11)	197.23 (123.43 to 315.60)	159.72 (104.41 to 246.51)	262.07 (156.95 to 423.51)
Liver cancer due to other causes	3,148.20 (2,134.12 to 4,506.63)	5,840.01 (3,439.96 to 9,283.87)	5,129.82 (3,194.86 to 7,753.79)	6,661.09 (3,741.85 to 10,984.19)	109.45 (74.60 to 155.98)	244.04 (146.24 to 383.15)	221.57 (139.18 to 338.47)	265.09 (149.16 to 433.09)
Gallbladder and biliary tract cancer	4,452.80 (3,564.16 to 5,519.53)	6,445.11 (5,220.82 to 7,937.26)	6,760.38 (5,448.55 to 8,255.77)	5,880.66 (4,700.51 to 7,411.84)	185.84 (151.08 to 228.61)	314.43 (256.37 to 382.13)	338.77 (276.35 to 408.53)	272.30 (219.29 to 340.73)
Pancreatic cancer	10,062.40 (8,985.78 to 11,277.81)	22,946.42 (18,759.63 to 28,128.42)	21,974.09 (18,218.93 to 26,570.31)	23,137.02 (18,436.05 to 28,937.38)	408.62 (367.66 to 456.12)	1,074.56 (881.24 to 1,319.93)	1,063.87 (887.43 to 1,278.44)	1,027.89 (817.78 to 1,283.50)
Larynx cancer	5,074.29 (4,395.80 to 5,904.49)	7,910.80 (3,865.32 to 14,170.91)	4,940.21 (2,598.08 to 8,800.79)	13,219.28 (6,371.00 to 23,613.95)	197.49 (171.80 to 229.92)	342.54 (166.84 to 611.48)	221.67 (113.73 to 391.58)	558.09 (263.02 to 1,003.46)
Tracheal, bronchus, and lung cancer	54,263.94 (45,701.87 to 63,022.37)	95,398.35 (58,633.28 to 150,693.76)	56,103.17 (35,844.46 to 85,284.73)	152,316.77 (92,294.76 to 236,998.73)	2,194.20 (1,818.82 to 2,609.33)	4,301.59 (2,593.46 to 6,853.10)	2,642.76 (1,654.45 to 4,138.94)	6,620.38 (3,969.49 to 10,565.37)
Malignant skin melanoma	1,136.86 (895.82 to 1,447.58)	2,048.78 (1,626.57 to 2,595.19)	2,183.27 (1,721.87 to 2,769.95)	1,775.14 (1,394.89 to 2,241.18)	36.96 (29.54 to 46.42)	84.58 (67.44 to 105.85)	93.05 (73.83 to 116.74)	68.77 (54.05 to 86.65)
Non-melanoma skin cancer	2,131.12 (1,800.72 to 2,453.16)	4,220.41 (3,529.71 to 4,977.76)	4,297.01 (3,576.76 to 5,079.92)	4,276.91 (3,589.79 to 5,025.44)	82.96 (68.93 to 98.08)	202.09 (165.08 to 245.29)	213.29 (174.24 to 261.32)	191.15 (156.38 to 233.58)
Breast cancer	38,563.29 (24,901.43 to 50,952.19)	66,341.71 (41,255.43 to 100,796.92)	62,892.17 (40,310.73 to 90,107.25)	70,689.84 (42,446.12 to 114,026.79)	1,194.39 (795.27 to 1,562.20)	2,394.02 (1,528.60 to 3,521.51)	2,311.83 (1,517.56 to 3,269.22)	2,457.21 (1,514.49 to 3,870.74)
Cervical cancer	24,407.30 (16,258.18 to 30,735.22)	29,213.76 (19,278.89 to 39,317.92)	27,809.88 (18,716.56 to 36,860.46)	33,971.97 (21,843.01 to 47,569.06)	793.66 (538.64 to 980.15)	1,084.71 (753.25 to 1,413.36)	1,053.79 (734.03 to 1,345.25)	1,210.54 (811.07 to 1,629.25)

Uterine cancer	5,116.34 (3,915.86 to 6,386.19)	8,144.55 (6,204.97 to 10,130.29)	7,952.60 (6,137.45 to 9,894.10)	8,970.85 (6,727.25 to 11,285.29)	192.23 (150.46 to 235.21)	357.35 (278.74 to 436.84)	355.92 (282.81 to 433.55)	377.65 (288.81 to 465.72)
Ovarian cancer	8,347.93 (6,557.20 to 10,170.36)	15,689.65 (11,805.31 to 20,830.91)	15,978.05 (12,025.55 to 20,821.60)	14,161.86 (10,467.59 to 19,215.65)	276.20 (216.99 to 333.57)	597.42 (453.73 to 785.49)	617.06 (474.68 to 800.27)	524.03 (391.54 to 697.34)
Prostate cancer	6,793.12 (4,147.92 to 8,790.54)	20,947.09 (12,955.93 to 26,467.01)	22,764.87 (14,107.81 to 28,275.00)	17,813.16 (10,975.99 to 23,163.29)	400.00 (244.00 to 519.12)	1,387.73 (844.03 to 1,790.34)	1,564.43 (957.52 to 1,996.43)	1,105.89 (663.03 to 1,474.53)
Testicular cancer	1,113.95 (820.36 to 1,552.89)	1,184.17 (861.99 to 1,646.32)	1,172.08 (854.81 to 1,635.46)	1,272.25 (921.39 to 1,770.29)	23.75 (18.16 to 32.81)	30.29 (23.28 to 41.62)	30.71 (23.60 to 42.31)	31.07 (23.59 to 42.69)

Cambodia								
Cause	YLLs (actual counts)				Deaths (actual counts)			
	2016	2040 Forecast	2040 Better Scenario	2040 Worse Scenario	2016	2040 Forecast	2040 Better Scenario	2040 Worse Scenario
Kidney cancer	3,139.25 (2,684.02 to 3,743.03)	6,601.34 (4,812.74 to 9,229.86)	6,553.28 (4,873.78 to 8,978.50)	6,273.49 (4,484.93 to 8,934.80)	107.41 (89.15 to 130.43)	281.51 (202.16 to 396.75)	288.70 (210.04 to 394.89)	252.94 (176.29 to 366.50)
Bladder cancer	4,296.66 (3,618.20 to 5,012.70)	8,334.05 (5,988.64 to 11,516.73)	7,622.93 (5,674.88 to 10,100.27)	9,225.74 (6,446.93 to 13,157.25)	199.34 (166.97 to 232.20)	457.40 (324.71 to 637.68)	437.68 (323.64 to 586.22)	473.69 (323.91 to 688.10)
Brain and nervous system cancer	11,326.69 (8,976.99 to 13,470.25)	17,858.05 (13,806.29 to 21,348.75)	18,428.22 (14,246.06 to 22,126.89)	17,377.11 (13,626.12 to 20,755.03)	276.46 (216.28 to 331.48)	572.89 (443.11 to 697.45)	604.68 (464.06 to 734.72)	522.43 (404.33 to 629.68)
Thyroid cancer	2,767.78 (2,235.39 to 3,378.30)	4,519.78 (3,462.91 to 5,805.89)	4,357.02 (3,412.69 to 5,502.90)	4,824.72 (3,611.93 to 6,317.73)	105.66 (86.18 to 127.55)	203.52 (157.45 to 258.04)	201.07 (158.36 to 249.65)	207.39 (156.73 to 267.53)
Mesothelioma	756.77 (606.63 to 1,029.15)	1,246.05 (984.26 to 1,713.44)	1,266.83 (998.37 to 1,751.56)	1,242.78 (988.74 to 1,698.06)	22.64 (18.27 to 30.14)	45.44 (36.32 to 61.00)	47.45 (37.76 to 63.74)	42.99 (34.15 to 57.03)
Hodgkin lymphoma	2,911.38 (2,391.37 to 4,053.02)	2,781.61 (2,209.81 to 3,896.15)	2,650.97 (2,093.59 to 3,724.57)	3,385.92 (2,665.43 to 4,820.70)	73.92 (61.29 to 102.46)	86.98 (69.39 to 120.19)	84.43 (67.64 to 117.51)	100.95 (80.39 to 142.00)
Non-Hodgkin lymphoma	12,002.75 (10,178.18 to 14,176.83)	20,040.03 (16,759.91 to 24,274.28)	20,632.21 (17,209.74 to 24,912.13)	18,805.21 (15,633.32 to 22,838.42)	344.77 (294.07 to 406.56)	751.51 (626.38 to 911.64)	794.23 (661.48 to 954.07)	661.86 (546.41 to 807.53)
Multiple myeloma	2,284.66 (1,878.19 to 2,796.27)	5,046.04 (4,039.37 to 6,385.25)	5,225.66 (4,167.10 to 6,555.02)	4,527.13 (3,598.54 to 5,736.10)	82.68 (68.06 to 100.42)	210.64 (169.38 to 263.49)	222.85 (179.66 to 279.25)	181.39 (143.04 to 227.62)
Leukemia	24,346.83 (19,538.00 to 32,128.65)	28,193.94 (22,178.24 to 37,443.08)	26,877.11 (21,440.12 to 34,994.83)	31,241.94 (24,123.39 to 41,651.41)	572.39 (472.01 to 754.95)	929.24 (724.85 to 1,212.51)	905.12 (719.35 to 1,157.85)	964.70 (736.29 to 1,291.42)
Acute lymphoid leukemia	5,814.39 (4,590.58 to 9,233.29)	6,967.78 (5,419.22 to 10,180.85)	6,813.21 (5,378.78 to 9,904.90)	7,251.55 (5,577.45 to 10,882.15)	133.81 (107.10 to 207.81)	230.70 (178.16 to 343.71)	230.02 (180.22 to 335.37)	223.66 (170.39 to 337.10)
Chronic lymphoid leukemia	393.71 (294.95 to 597.19)	559.12 (408.76 to 837.16)	537.92 (400.18 to 807.17)	579.26 (416.29 to 888.82)	11.70 (8.84 to 17.66)	20.68 (15.24 to 30.17)	20.52 (15.33 to 29.43)	20.23 (14.60 to 29.88)
Acute myeloid leukemia	3,523.19 (2,800.71 to 4,899.28)	5,370.80 (4,118.77 to 7,326.33)	5,274.15 (4,093.09 to 7,114.76)	5,356.97 (4,035.76 to 7,318.73)	77.98 (62.17 to 103.30)	159.73 (121.62 to 215.93)	159.43 (123.42 to 211.89)	150.83 (112.63 to 208.00)
Chronic myeloid leukemia	1,433.40 (1,037.26 to 2,120.52)	1,580.63 (1,121.25 to 2,352.16)	1,488.30 (1,063.70 to 2,211.77)	1,677.28 (1,160.39 to 2,538.62)	39.29 (29.15 to 57.95)	52.65 (37.42 to 77.61)	50.80 (36.46 to 74.73)	53.59 (37.49 to 80.62)
Other leukemia	13,182.14 (9,595.76 to 16,830.67)	13,715.60 (9,779.42 to 18,620.42)	12,763.54 (9,333.15 to 17,035.47)	16,376.88 (11,502.78 to 22,470.86)	309.60 (235.95 to 390.75)	465.47 (340.85 to 627.23)	444.35 (331.88 to 587.18)	516.40 (370.39 to 713.72)
Other neoplasms	22,999.12 (20,340.06 to 25,948.55)	35,099.63 (30,235.96 to 39,741.20)	36,091.42 (31,105.89 to 40,958.63)	34,491.84 (29,795.48 to 39,364.51)	639.60 (559.99 to 717.01)	1,312.17 (1,107.99 to 1,486.00)	1,389.52 (1,172.90 to 1,576.33)	1,197.08 (1,015.33 to 1,364.34)
**Cardiovascular diseases**	**551,605.46 (507,301.16 to 599,852.94)**	**612,666.54 (442,463.31 to 835,111.22)**	**412,876.00 (307,875.55 to 552,877.42)**	**1,124,554.84 (792,508.38 to 1,538,890.37)**	**24,134.51 (22,484.96 to 25,947.03)**	**34,287.29 (25,775.25 to 45,429.48)**	**25,261.57 (19,514.55 to 32,511.71)**	**55,947.66 (41,327.51 to 73,351.45)**
Rheumatic heart disease	14,410.27 (10,818.44 to 18,048.06)	9,004.37 (6,801.16 to 11,684.11)	8,413.45 (6,346.68 to 10,708.21)	11,541.36 (8,497.58 to 15,111.47)	419.16 (315.28 to 525.92)	362.41 (269.30 to 459.40)	351.41 (260.63 to 439.30)	428.95 (313.48 to 546.30)
Ischemic heart disease	206,423.09 (185,272.93 to 230,515.45)	234,855.76 (136,447.09 to 400,529.53)	146,342.05 (87,228.66 to 243,071.49)	455,545.64 (269,260.94 to 714,785.51)	9,065.53 (8,170.63 to 9,985.60)	13,046.53 (7,855.09 to 21,128.54)	9,046.49 (5,593.53 to 14,181.67)	22,337.51 (13,780.47 to 34,472.60)
Stroke	259,410.33 (234,292.42 to 285,523.20)	259,975.62 (162,753.87 to 398,385.47)	161,198.54 (106,576.08 to 246,625.14)	519,477.04 (322,826.77 to 790,180.60)	11,791.03 (10,749.01 to 12,888.66)	14,853.60 (9,983.42 to 21,696.48)	10,250.99 (7,098.81 to 14,638.08)	26,195.43 (17,207.38 to 38,446.32)
Ischemic stroke	66,322.12 (56,459.51 to 77,265.10)	78,304.20 (46,254.45 to 132,164.83)	54,804.48 (33,241.06 to 91,567.95)	126,131.40 (72,123.11 to 211,017.92)	3,904.12 (3,344.71 to 4,521.24)	5,694.12 (3,414.56 to 10,340.34)	4,324.44 (2,706.17 to 7,763.16)	8,276.64 (4,834.87 to 15,154.43)

Intracerebral hemorrhage	193,088.22 (172,650.54 to 213,987.71)	181,671.42 (105,706.84 to 296,436.44)	106,394.05 (64,376.63 to 174,095.12)	393,345.64 (229,505.76 to 637,085.12)	7,886.92 (7,067.21 to 8,721.34)	9,159.49 (5,788.38 to 14,246.86)	5,926.55 (3,868.84 to 9,214.45)	17,918.79 (11,072.34 to 28,518.22)
Hypertensive heart disease	28,701.92 (21,585.59 to 35,401.71)	47,717.30 (28,320.50 to 79,053.89)	38,455.01 (24,267.76 to 58,191.25)	69,084.22 (38,407.00 to 129,829.63)	1,335.81 (1,007.98 to 1,641.35)	2,821.82 (1,702.13 to 4,634.12)	2,385.21 (1,516.12 to 3,578.89)	3,750.40 (2,129.24 to 7,171.66)
Cardiomyopathy and myocarditis	11,616.88 (8,345.88 to 14,577.52)	14,402.35 (9,952.11 to 19,035.66)	14,190.29 (9,782.40 to 18,600.46)	14,931.63 (10,239.46 to 19,689.47)	354.83 (260.86 to 429.95)	627.38 (428.51 to 844.52)	646.03 (442.66 to 865.98)	592.38 (409.78 to 790.40)
Myocarditis	2,634.70 (1,853.45 to 3,665.16)	2,068.91 (1,493.45 to 2,732.31)	2,054.64 (1,482.86 to 2,704.57)	2,298.09 (1,674.89 to 3,050.71)	59.40 (43.75 to 75.99)	70.73 (48.96 to 94.15)	73.32 (50.28 to 97.96)	70.41 (49.26 to 92.57)

Cambodia								
Cause	YLLs (actual counts)				Deaths (actual counts)			
	2016	2040 Forecast	2040 Better Scenario	2040 Worse Scenario	2016	2040 Forecast	2040 Better Scenario	2040 Worse Scenario
Alcoholic cardiomyopathy	1,699.80 (965.15 to 2,400.06)	2,403.34 (1,330.33 to 3,503.16)	2,151.42 (1,159.70 to 3,146.09)	2,819.18 (1,597.26 to 3,983.12)	56.72 (32.75 to 81.10)	93.79 (52.25 to 135.54)	87.05 (47.83 to 126.65)	105.26 (61.18 to 148.69)
Other cardiomyopathy	7,282.37 (4,939.82 to 9,454.44)	9,930.11 (6,547.98 to 13,974.52)	9,984.22 (6,659.70 to 13,800.73)	9,814.35 (6,378.70 to 13,947.31)	238.71 (171.05 to 297.55)	462.86 (308.96 to 653.16)	485.66 (322.35 to 675.61)	416.71 (276.02 to 593.81)
Atrial fibrillation and flutter	2,042.22 (1,578.64 to 2,580.55)	5,639.69 (4,095.01 to 7,491.90)	5,664.03 (4,181.70 to 7,445.37)	5,486.99 (3,936.70 to 7,460.45)	163.76 (126.24 to 205.72)	546.61 (385.34 to 738.70)	572.83 (408.45 to 766.61)	487.41 (328.52 to 679.42)
Aortic aneurysm	2,991.51 (2,499.71 to 3,658.03)	4,801.50 (3,813.27 to 6,123.00)	4,768.49 (3,834.18 to 6,045.97)	4,761.30 (3,743.81 to 6,184.94)	140.17 (119.11 to 169.85)	279.73 (222.25 to 353.89)	292.00 (234.66 to 368.72)	253.09 (199.51 to 325.39)
Peripheral artery disease	105.83 (73.58 to 164.52)	402.55 (263.04 to 741.25)	403.37 (265.02 to 740.52)	346.50 (222.84 to 618.26)	6.43 (4.50 to 10.33)	31.84 (20.21 to 61.95)	34.06 (21.67 to 67.57)	24.31 (15.51 to 46.61)
Endocarditis	6,258.21 (4,970.17 to 8,090.97)	8,059.34 (6,126.96 to 10,840.10)	7,869.84 (5,952.41 to 10,481.03)	8,585.24 (6,471.52 to 11,723.97)	176.01 (140.91 to 224.08)	314.40 (235.67 to 426.18)	319.57 (240.12 to 433.56)	307.44 (230.59 to 422.88)
Other cardiovascular and circulatory diseases	19,645.21 (15,009.58 to 31,382.75)	27,808.05 (20,086.08 to 42,231.45)	25,570.93 (18,840.75 to 38,430.98)	34,794.93 (24,242.54 to 53,026.87)	681.78 (540.87 to 1,069.08)	1,402.96 (1,028.60 to 2,095.73)	1,362.98 (999.44 to 2,073.75)	1,570.74 (1,132.00 to 2,422.78)
**Chronic respiratory diseases**	**88,227.54 (74,506.17 to 100,306.05)**	**79,311.80 (57,093.87 to 108,770.47)**	**55,587.81 (42,508.54 to 71,275.08)**	**129,459.85 (87,279.86 to 191,662.21)**	**4,065.12 (3,386.74 to 4,628.50)**	**4,495.48 (3,211.33 to 6,184.47)**	**3,277.11 (2,442.93 to 4,244.88)**	**6,933.49 (4,649.18 to 10,250.01)**
Chronic obstructive pulmonary disease	45,337.65 (34,273.38 to 55,729.13)	48,419.28 (30,066.06 to 75,419.44)	28,160.20 (17,881.51 to 42,091.15)	92,076.27 (54,406.52 to 149,825.13)	2,327.87 (1,686.61 to 2,843.08)	2,891.29 (1,753.53 to 4,461.96)	1,800.60 (1,138.98 to 2,637.53)	5,129.70 (3,022.29 to 8,297.08)
Pneumoconiosis	62.81 (43.20 to 93.38)	70.74 (49.36 to 102.36)	69.84 (46.98 to 103.17)	71.16 (51.06 to 99.76)	2.99 (2.03 to 4.60)	4.21 (2.85 to 6.21)	4.29 (2.82 to 6.43)	3.96 (2.71 to 5.79)
Silicosis	15.35 (7.87 to 27.91)	14.79 (7.00 to 28.59)	15.25 (7.05 to 29.67)	14.60 (7.21 to 27.44)	0.75 (0.37 to 1.43)	0.87 (0.41 to 1.74)	0.93 (0.43 to 1.88)	0.81 (0.40 to 1.56)
Asbestosis	12.05 (8.37 to 17.03)	15.24 (10.34 to 21.70)	15.75 (10.29 to 22.99)	14.71 (10.30 to 20.54)	0.41 (0.28 to 0.59)	0.68 (0.43 to 1.05)	0.73 (0.44 to 1.16)	0.61 (0.41 to 0.92)
Coal workers pneumoconiosis	4.32 (2.25 to 8.64)	4.47 (2.32 to 8.47)	3.89 (2.00 to 7.36)	5.75 (3.03 to 10.90)	0.22 (0.12 to 0.44)	0.29 (0.14 to 0.56)	0.26 (0.13 to 0.52)	0.35 (0.18 to 0.64)
Other pneumoconiosis	31.09 (19.68 to 47.07)	36.24 (23.15 to 55.89)	34.95 (21.78 to 55.47)	36.09 (23.20 to 54.20)	1.61 (1.02 to 2.45)	2.37 (1.47 to 3.77)	2.38 (1.42 to 3.86)	2.19 (1.36 to 3.46)
Asthma	40,406.38 (28,919.60 to 57,374.27)	26,987.97 (16,772.51 to 43,544.22)	23,367.71 (15,152.29 to 35,583.90)	33,724.03 (20,203.59 to 57,457.32)	1,647.68 (1,138.91 to 2,555.22)	1,411.80 (829.30 to 2,302.81)	1,268.36 (800.27 to 1,963.56)	1,639.75 (918.89 to 2,856.94)
Interstitial lung disease and pulmonary sarcoidosis	1,098.41 (643.17 to 1,745.43)	2,364.00 (1,403.33 to 3,702.48)	2,554.23 (1,514.68 to 3,995.39)	1,974.20 (1,124.41 to 3,097.56)	47.83 (27.41 to 75.54)	121.69 (71.42 to 192.47)	135.14 (78.97 to 212.84)	96.29 (52.88 to 152.00)
Other chronic respiratory diseases	1,322.29 (692.26 to 2,036.71)	1,469.82 (764.80 to 2,283.68)	1,435.82 (749.30 to 2,264.79)	1,614.19 (816.73 to 2,486.23)	38.75 (20.06 to 61.09)	66.49 (28.89 to 118.77)	68.72 (30.28 to 126.39)	63.79 (28.81 to 109.92)
**Cirrhosis and other chronic liver diseases**	**237,376.80 (208,457.87 to 270,583.55)**	**333,986.34 (294,152.94 to 379,952.86)**	**319,714.57 (279,929.34 to 362,839.00)**	**377,910.34 (329,227.61 to 432,956.69)**	**7,217.34 (6,420.57 to 8,108.84)**	**12,143.76 (10,686.34 to 13,755.18)**	**11,944.42 (10,504.49 to 13,537.52)**	**13,070.29 (11,395.21 to 14,889.19)**
Cirrhosis and other chronic liver diseases due to hepatitis B	69,475.69 (55,975.76 to 85,496.78)	103,196.71 (82,991.99 to 127,327.38)	102,649.94 (82,877.66 to 126,691.69)	108,370.03 (86,695.07 to 136,800.03)	2,144.23 (1,760.52 to 2,594.56)	3,720.26 (3,039.99 to 4,494.37)	3,790.94 (3,085.69 to 4,568.89)	3,736.89 (3,013.99 to 4,572.77)
Cirrhosis and other chronic liver diseases due to hepatitis C	44,690.23 (35,326.96 to 55,797.97)	68,657.73 (54,765.78 to 85,121.74)	68,012.98 (54,292.11 to 83,163.33)	72,722.61 (57,026.21 to 91,362.33)	1,364.58 (1,093.95 to 1,652.78)	2,419.02 (1,965.03 to 2,966.24)	2,447.50 (1,995.28 to 2,975.16)	2,463.07 (1,970.93 to 3,036.49)

Cirrhosis and other chronic liver diseases due to alcohol use	51,575.39 (40,641.39 to 65,128.48)	73,532.17 (57,965.20 to 92,629.86)	61,490.63 (48,370.56 to 76,965.13)	101,141.62 (80,207.30 to 125,623.57)	1,661.10 (1,342.15 to 2,039.96)	2,737.94 (2,203.70 to 3,337.20)	2,383.51 (1,918.97 to 2,916.67)	3,557.30 (2,884.11 to 4,314.62)
Cirrhosis and other chronic liver diseases due to other causes	71,635.48 (61,140.20 to 83,476.73)	88,599.73 (74,961.95 to 105,301.87)	87,561.01 (74,175.83 to 103,718.51)	95,676.08 (80,644.34 to 113,870.11)	2,047.43 (1,762.90 to 2,353.94)	3,266.54 (2,750.08 to 3,833.76)	3,322.47 (2,784.50 to 3,917.59)	3,313.03 (2,761.97 to 3,930.13)
**Digestive diseases**	**145,877.73 (123,894.29 to 172,220.30)**	**142,845.30 (113,236.07 to 191,578.59)**	**132,841.64 (106,708.27 to 171,281.56)**	**174,127.16 (135,062.02 to 238,000.32)**	**4,865.19 (4,179.84 to 5,480.12)**	**6,943.65 (5,512.55 to 8,724.16)**	**6,785.79 (5,429.58 to 8,293.60)**	**7,659.62 (5,882.55 to 10,037.78)**
Peptic ulcer disease	51,778.50 (40,221.76 to 62,575.27)	42,736.89 (30,253.03 to 55,590.30)	39,242.87 (28,167.33 to 50,353.64)	55,066.71 (38,687.48 to 71,210.53)	2,127.33 (1,618.32 to 2,533.79)	2,340.34 (1,640.43 to 3,029.75)	2,232.83 (1,583.37 to 2,848.55)	2,798.52 (1,918.76 to 3,601.29)

Cambodia								
Cause	YLLs (actual counts)				Deaths (actual counts)			
	2016	2040 Forecast	2040 Better Scenario	2040 Worse Scenario	2016	2040 Forecast	2040 Better Scenario	2040 Worse Scenario
Gastritis and duodenitis	2,990.40 (2,109.44 to 4,046.93)	4,812.96 (3,235.65 to 6,676.54)	4,816.27 (3,176.86 to 6,704.49)	5,115.39 (3,418.56 to 7,121.88)	118.31 (81.42 to 154.11)	262.40 (168.93 to 368.82)	272.54 (175.34 to 389.17)	258.05 (166.67 to 360.63)
Appendicitis	10,253.43 (8,051.15 to 13,418.60)	7,662.60 (5,846.15 to 10,266.90)	7,296.33 (5,603.82 to 9,832.92)	9,531.43 (7,208.64 to 13,140.38)	247.05 (199.54 to 316.02)	267.42 (210.25 to 347.24)	263.67 (207.05 to 339.94)	304.56 (236.15 to 396.97)
Paralytic ileus and intestinal obstruction	37,048.08 (25,666.79 to 59,831.25)	30,514.86 (23,565.64 to 39,051.20)	30,908.70 (24,125.24 to 39,382.77)	33,323.79 (24,944.81 to 45,736.19)	901.07 (685.68 to 1,189.36)	1,478.01 (1,121.78 to 1,838.21)	1,572.51 (1,191.74 to 1,952.80)	1,378.81 (1,026.94 to 1,737.48)
Inguinal, femoral, and abdominal hernia	2,518.97 (1,666.11 to 3,190.26)	2,795.55 (1,792.96 to 3,623.77)	2,832.05 (1,785.54 to 3,675.41)	2,936.34 (1,898.94 to 3,863.72)	105.95 (65.52 to 134.31)	162.29 (97.87 to 213.14)	172.26 (102.65 to 225.64)	153.12 (94.20 to 202.83)
Inflammatory bowel disease	2,267.98 (1,760.33 to 2,965.92)	2,951.86 (2,281.68 to 3,787.53)	3,033.13 (2,364.79 to 3,873.58)	2,885.17 (2,210.05 to 3,752.42)	73.07 (57.21 to 90.90)	137.79 (104.79 to 170.00)	147.13 (111.98 to 181.80)	123.80 (92.97 to 155.67)
Vascular intestinal disorders	2,285.31 (1,695.98 to 3,430.26)	3,958.24 (2,898.13 to 6,011.98)	4,065.22 (2,965.82 to 6,137.38)	3,919.17 (2,837.99 to 5,937.71)	110.04 (80.04 to 168.10)	251.75 (181.30 to 384.98)	267.64 (192.74 to 406.02)	231.72 (163.35 to 358.87)
Gallbladder and biliary diseases	6,611.56 (5,258.35 to 8,184.19)	7,753.22 (5,044.69 to 11,466.00)	6,655.17 (4,520.75 to 9,320.83)	9,405.88 (5,983.51 to 14,418.37)	274.78 (216.65 to 329.18)	440.00 (280.86 to 656.49)	394.74 (265.75 to 553.58)	490.77 (302.94 to 752.26)
Pancreatitis	16,497.73 (12,069.87 to 20,947.95)	23,892.74 (9,986.49 to 60,386.58)	18,536.72 (8,362.28 to 41,982.47)	34,049.34 (13,623.98 to 86,260.95)	450.22 (324.51 to 585.25)	811.38 (357.47 to 1,923.88)	651.73 (301.66 to 1,469.83)	1,104.45 (454.38 to 2,664.24)
Other digestive diseases	13,625.76 (10,710.91 to 17,433.81)	15,766.37 (11,975.53 to 20,188.71)	15,455.17 (11,853.92 to 19,732.82)	17,893.93 (13,847.63 to 23,123.16)	457.38 (364.26 to 564.50)	792.26 (613.23 to 1,009.31)	810.75 (624.23 to 1,030.55)	815.83 (630.18 to 1,046.97)
**Neurological disorders**	**50,590.54 (44,848.24 to 58,060.15)**	**118,465.81 (96,939.44 to 147,436.65)**	**120,582.59 (101,417.32 to 145,211.90)**	**108,675.99 (86,754.06 to 137,733.11)**	**2,951.14 (2,578.15 to 3,462.45)**	**9,756.26 (7,584.52 to 12,613.80)**	**10,398.59 (8,345.60 to 12,820.37)**	**7,953.81 (5,913.66 to 10,616.50)**
Alzheimer's disease and other dementias	30,814.06 (25,901.42 to 37,157.94)	91,578.56 (70,644.18 to 119,669.46)	93,967.09 (75,221.41 to 119,128.98)	79,160.60 (58,768.12 to 106,514.86)	2,438.55 (2,073.29 to 2,955.39)	8,609.57 (6,404.53 to 11,461.59)	9,163.24 (7,114.38 to 11,580.46)	6,919.45 (4,948.53 to 9,625.60)
Parkinson's disease	2,620.71 (2,007.91 to 3,286.17)	9,025.06 (6,820.62 to 11,377.91)	10,235.99 (7,826.50 to 12,956.63)	6,844.34 (5,091.69 to 8,675.82)	171.69 (130.37 to 217.28)	689.20 (508.23 to 896.50)	804.69 (604.74 to 1,039.51)	492.16 (349.76 to 652.42)
Epilepsy	13,579.03 (10,802.56 to 16,650.57)	12,629.77 (8,137.11 to 19,468.06)	10,976.16 (7,366.12 to 16,551.67)	17,585.14 (11,182.06 to 27,397.05)	259.95 (211.79 to 314.07)	302.37 (199.18 to 459.02)	266.94 (183.90 to 391.42)	401.15 (259.38 to 630.17)
Multiple sclerosis	477.41 (366.27 to 650.58)	759.89 (561.67 to 1,033.91)	763.88 (570.53 to 1,038.37)	737.17 (544.75 to 1,011.15)	12.91 (9.82 to 17.58)	23.38 (17.26 to 32.09)	23.82 (17.60 to 32.26)	22.08 (16.25 to 30.38)
Motor neuron disease	554.74 (393.36 to 719.74)	1,028.82 (718.75 to 1,309.32)	1,070.88 (748.10 to 1,366.45)	950.34 (665.40 to 1,210.51)	15.33 (10.87 to 19.39)	34.70 (24.71 to 44.33)	36.61 (26.00 to 47.33)	30.98 (21.79 to 39.88)
Other neurological disorders	2,544.59 (2,131.15 to 3,058.63)	3,443.71 (2,988.79 to 3,976.90)	3,568.59 (3,083.54 to 4,096.92)	3,398.39 (2,893.54 to 3,981.11)	52.70 (46.17 to 59.41)	97.04 (83.53 to 111.79)	103.28 (88.75 to 119.59)	87.99 (76.37 to 100.75)
**Mental disorders**	**15,371.88 (9,282.79 to 24,641.28)**	**21,171.71 (13,261.39 to 33,374.46)**	**20,516.26 (12,935.16 to 32,287.38)**	**22,667.29 (14,173.94 to 35,801.62)**	**344.52 (218.06 to 528.40)**	**536.64 (357.55 to 793.72)**	**527.47 (354.54 to 775.52)**	**559.01 (365.81 to 838.73)**
Alcohol use disorders	11,208.32 (6,322.62 to 19,922.69)	12,975.16 (7,093.61 to 23,513.18)	12,254.68 (6,705.44 to 22,232.82)	14,389.15 (7,947.02 to 26,165.02)	260.22 (154.77 to 428.95)	343.40 (200.48 to 574.43)	328.95 (191.31 to 547.67)	371.30 (216.48 to 625.06)
Drug use disorders	4,123.00 (2,399.03 to 7,407.06)	8,145.98 (5,119.93 to 13,712.70)	8,210.38 (5,216.20 to 13,664.27)	8,227.77 (5,110.09 to 14,088.58)	83.53 (51.26 to 144.10)	192.22 (129.42 to 303.70)	197.49 (134.14 to 308.93)	186.71 (123.10 to 299.78)
Opioid use disorders	2,647.62 (1,418.83 to 5,073.59)	4,287.42 (2,397.79 to 7,871.18)	4,306.62 (2,427.87 to 7,904.38)	4,391.05 (2,403.84 to 8,346.67)	52.01 (29.13 to 96.79)	98.21 (59.40 to 167.24)	100.66 (61.65 to 168.32)	96.47 (56.37 to 168.72)

Cocaine use disorders	171.74 (86.56 to 426.31)	421.13 (205.90 to 1,083.69)	414.37 (203.97 to 1,064.33)	455.50 (223.59 to 1,165.65)	3.65 (1.94 to 8.83)	10.04 (5.30 to 23.75)	9.98 (5.27 to 23.40)	10.61 (5.56 to 25.19)
Amphetamine use disorders	145.45 (71.97 to 356.65)	428.96 (208.49 to 986.75)	414.21 (198.40 to 937.93)	492.14 (232.85 to 1,116.04)	2.85 (1.48 to 6.89)	9.16 (4.78 to 19.74)	8.92 (4.64 to 19.13)	10.31 (5.24 to 22.32)
Other drug use disorders	1,158.20 (660.94 to 2,073.33)	3,008.48 (1,761.15 to 5,275.12)	3,075.17 (1,812.46 to 5,316.89)	2,889.08 (1,652.79 to 5,193.04)	25.01 (14.98 to 42.73)	74.82 (47.64 to 121.65)	77.93 (50.23 to 125.87)	69.31 (43.07 to 115.28)
Eating disorders	40.55 (27.28 to 68.29)	50.56 (33.28 to 83.29)	51.21 (33.55 to 83.95)	50.37 (33.49 to 81.87)	0.77 (0.52 to 1.30)	1.01 (0.65 to 1.70)	1.03 (0.66 to 1.73)	1.00 (0.66 to 1.65)

Cambodia								
Cause	YLLs (actual counts)				Deaths (actual counts)			
	2016	2040 Forecast	2040 Better Scenario	2040 Worse Scenario	2016	2040 Forecast	2040 Better Scenario	2040 Worse Scenario
**Diabetes, urogenital, blood, and endocrine diseases**	**144,019.18 (130,306.53 to 160,008.16)**	**266,153.14 (180,536.17 to 476,825.19)**	**224,783.37 (156,136.35 to 379,822.11)**	**343,928.42 (236,023.43 to 588,016.38)**	**5,158.54 (4,702.64 to 5,700.04)**	**13,419.76 (8,702.30 to 27,382.58)**	**11,598.33 (7,749.09 to 22,412.66)**	**16,169.96 (10,502.08 to 31,641.11)**
Diabetes mellitus	59,615.04 (51,810.33 to 69,304.22)	97,625.57 (62,155.21 to 148,011.94)	84,018.85 (54,584.20 to 126,547.51)	137,294.28 (84,902.47 to 210,862.17)	2,396.45 (2,089.64 to 2,774.44)	4,839.59 (3,138.26 to 6,991.82)	4,342.68 (2,909.40 to 6,322.70)	6,348.79 (4,012.84 to 9,328.25)
Acute glomerulonephritis	143.57 (117.17 to 177.10)	178.48 (132.27 to 241.93)	175.25 (128.72 to 234.65)	206.80 (154.04 to 279.84)	3.40 (2.65 to 4.28)	6.04 (4.15 to 8.54)	6.12 (4.21 to 8.68)	6.38 (4.42 to 9.07)
Chronic kidney disease	55,493.24 (48,835.05 to 62,598.59)	131,353.96 (61,007.78 to 347,295.76)	103,765.40 (52,183.82 to 271,532.90)	165,833.06 (77,560.23 to 430,031.46)	1,919.14 (1,715.66 to 2,143.84)	6,998.00 (2,702.85 to 21,442.28)	5,616.26 (2,325.33 to 17,134.89)	8,239.93 (3,199.12 to 24,634.02)
Chronic kidney disease due to diabetes mellitus	30,666.61 (26,226.66 to 35,264.39)	76,877.42 (35,746.82 to 207,411.30)	60,289.69 (30,442.11 to 160,097.89)	97,878.52 (46,050.08 to 259,088.00)	1,039.33 (896.33 to 1,186.70)	3,704.49 (1,442.78 to 11,188.02)	2,931.35 (1,223.66 to 8,728.63)	4,469.77 (1,751.00 to 13,032.27)
Chronic kidney disease due to hypertension	13,460.57 (11,206.24 to 16,080.92)	39,999.03 (15,403.85 to 120,163.18)	31,616.76 (13,098.00 to 93,981.58)	48,680.38 (18,435.23 to 142,263.38)	611.38 (514.13 to 713.19)	2,675.64 (950.70 to 8,381.04)	2,183.81 (831.95 to 6,870.01)	3,016.78 (1,042.76 to 9,341.26)
Chronic kidney disease due to glomerulonephritis	6,148.70 (4,916.22 to 7,722.35)	6,885.38 (4,074.46 to 13,904.75)	5,706.80 (3,495.84 to 10,907.75)	9,309.63 (5,560.39 to 17,935.87)	132.58 (108.76 to 161.90)	252.12 (115.91 to 691.10)	204.24 (98.50 to 548.97)	315.81 (150.14 to 816.71)
Chronic kidney disease due to other and unspecified causes	5,217.36 (4,213.76 to 6,465.76)	7,592.13 (3,955.18 to 17,473.75)	6,152.15 (3,354.96 to 14,040.65)	9,964.52 (5,188.82 to 22,625.77)	135.85 (111.21 to 165.16)	365.75 (148.65 to 1,098.07)	296.86 (128.86 to 886.34)	437.57 (178.13 to 1,251.57)
Urinary diseases and male infertility	10,510.48 (9,193.49 to 12,137.87)	15,776.66 (13,489.77 to 18,648.60)	16,070.47 (13,712.02 to 19,069.36)	16,094.68 (13,784.28 to 18,823.43)	371.19 (327.46 to 428.38)	766.16 (654.37 to 909.03)	817.05 (695.57 to 967.11)	707.47 (603.12 to 842.36)
Urinary tract infections	6,866.45 (5,116.21 to 8,548.29)	11,373.64 (8,753.00 to 14,021.37)	11,720.53 (9,045.02 to 14,399.32)	11,153.88 (8,434.09 to 13,829.44)	243.39 (180.56 to 300.79)	562.72 (433.97 to 696.61)	605.99 (466.68 to 749.71)	500.86 (378.79 to 628.89)
Urolithiasis	423.02 (308.54 to 583.72)	862.18 (617.56 to 1,241.43)	882.66 (633.17 to 1,269.66)	862.23 (614.05 to 1,262.74)	16.97 (12.41 to 23.08)	40.57 (29.04 to 59.65)	43.08 (30.98 to 62.95)	37.56 (26.99 to 54.88)
Other urinary diseases	3,221.01 (2,351.28 to 4,568.06)	3,540.84 (2,399.13 to 5,280.80)	3,467.28 (2,335.39 to 5,297.97)	4,078.57 (2,779.53 to 6,066.23)	110.83 (79.26 to 158.95)	162.87 (108.80 to 244.79)	167.98 (111.55 to 254.06)	169.05 (112.92 to 252.23)
Gynecological diseases	644.57 (315.50 to 851.10)	569.05 (289.07 to 803.06)	534.87 (268.83 to 747.29)	702.67 (361.60 to 993.67)	17.34 (8.56 to 22.64)	17.65 (8.75 to 23.86)	17.00 (8.28 to 23.05)	20.72 (10.49 to 28.31)
Uterine fibroids	132.29 (39.30 to 215.07)	107.75 (33.07 to 197.96)	99.91 (30.78 to 180.68)	138.21 (43.41 to 248.84)	4.23 (1.28 to 6.74)	3.70 (1.22 to 6.43)	3.49 (1.12 to 6.10)	4.60 (1.52 to 7.98)
Polycystic ovarian syndrome	18.71 (6.59 to 44.35)	10.30 (3.26 to 27.76)	9.04 (2.81 to 24.46)	15.39 (4.85 to 41.86)	0.45 (0.15 to 1.08)	0.25 (0.08 to 0.65)	0.22 (0.07 to 0.58)	0.37 (0.11 to 0.95)
Endometriosis	7.66 (2.10 to 21.86)	10.16 (2.16 to 31.70)	9.41 (2.02 to 29.75)	12.95 (2.93 to 41.74)	0.18 (0.05 to 0.55)	0.25 (0.05 to 0.80)	0.23 (0.05 to 0.74)	0.31 (0.07 to 1.02)
Genital prolapse	25.24 (8.48 to 43.55)	23.72 (7.56 to 43.20)	22.22 (7.19 to 39.87)	29.93 (9.88 to 54.28)	1.24 (0.40 to 2.08)	1.30 (0.41 to 2.34)	1.25 (0.41 to 2.25)	1.55 (0.51 to 2.80)
Other gynecological diseases	460.67 (239.32 to 627.47)	417.11 (218.10 to 605.57)	394.29 (213.75 to 573.43)	506.19 (259.83 to 752.65)	11.24 (6.11 to 15.08)	12.15 (6.77 to 17.02)	11.82 (6.46 to 16.62)	13.88 (7.60 to 19.99)
Hemoglobinopathies and hemolytic anemias	11,687.50 (9,296.56 to 15,070.41)	13,259.55 (10,665.14 to 17,069.77)	12,885.51 (10,352.61 to 16,789.72)	15,557.86 (12,359.49 to 20,119.19)	307.18 (251.69 to 386.99)	516.67 (420.52 to 655.93)	515.21 (419.65 to 658.75)	570.08 (458.20 to 738.93)
Thalassemias	3,291.13 (2,028.34 to 4,896.04)	1,519.89 (965.87 to 2,246.78)	1,599.55 (1,010.77 to 2,371.25)	1,636.86 (978.25 to 2,449.23)	42.14 (26.75 to 61.71)	20.59 (13.40 to 29.88)	21.66 (13.99 to 31.53)	21.87 (13.53 to 32.63)

Sickle cell disorders	8.98 (6.40 to 12.28)	9.48 (6.47 to 13.74)	9.20 (6.25 to 13.43)	10.91 (7.52 to 16.05)	0.16 (0.12 to 0.22)	0.21 (0.15 to 0.31)	0.21 (0.14 to 0.30)	0.24 (0.17 to 0.35)
G6PD deficiency	6,156.49 (4,908.98 to 7,841.02)	8,840.03 (6,959.10 to 11,747.05)	8,382.70 (6,549.79 to 11,026.40)	10,784.64 (8,330.89 to 14,592.45)	153.00 (121.79 to 195.39)	294.22 (234.75 to 387.19)	284.31 (224.60 to 372.44)	345.07 (270.10 to 456.84)
Other hemoglobinopathies and hemolytic anemias	2,230.89 (1,778.15 to 2,771.10)	2,890.15 (2,294.72 to 3,652.28)	2,894.06 (2,275.96 to 3,660.30)	3,125.45 (2,432.45 to 4,065.36)	111.87 (90.40 to 138.88)	201.65 (158.35 to 261.44)	209.03 (162.84 to 266.33)	202.90 (156.14 to 268.78)
Endocrine, metabolic, blood, and immune disorders	5,924.78 (4,863.00 to 8,779.98)	7,389.87 (5,876.84 to 10,674.83)	7,333.03 (5,819.68 to 10,675.98)	8,239.07 (6,645.46 to 11,999.47)	143.84 (118.34 to 206.87)	275.65 (214.26 to 397.14)	284.01 (220.37 to 411.71)	276.59 (215.68 to 400.78)

Cambodia								
Cause	YLLs (actual counts)				Deaths (actual counts)			
	2016	2040 Forecast	2040 Better Scenario	2040 Worse Scenario	2016	2040 Forecast	2040 Better Scenario	2040 Worse Scenario
**Musculoskeletal disorders**	**3,380.80 (2,851.04 to 4,682.57)**	**5,829.76 (4,814.74 to 8,374.49)**	**6,019.26 (4,966.96 to 8,560.00)**	**5,570.21 (4,587.31 to 7,865.31)**	**87.64 (73.91 to 125.03)**	**207.13 (164.82 to 317.90)**	**219.65 (175.17 to 338.21)**	**187.35 (149.78 to 284.56)**
Rheumatoid arthritis	459.63 (337.55 to 674.75)	968.55 (641.02 to 1,461.07)	944.23 (632.07 to 1,426.45)	1,083.84 (727.66 to 1,628.56)	18.97 (13.42 to 29.14)	51.14 (33.16 to 82.89)	51.32 (32.81 to 84.37)	53.82 (35.06 to 86.34)
Other musculoskeletal disorders	2,921.17 (2,431.06 to 4,104.30)	4,861.21 (3,953.86 to 7,050.19)	5,075.04 (4,129.56 to 7,298.73)	4,486.37 (3,617.30 to 6,507.28)	68.67 (57.18 to 98.42)	155.99 (123.00 to 247.15)	168.34 (132.44 to 265.68)	133.53 (105.93 to 207.35)
**Other non-communicable diseases**	**61,172.03 (45,020.75 to 84,421.03)**	**32,266.44 (23,920.25 to 42,258.06)**	**31,490.18 (23,597.12 to 41,678.59)**	**43,527.96 (31,370.50 to 58,480.12)**	**822.97 (621.00 to 1,097.77)**	**790.26 (597.98 to 1,128.67)**	**817.95 (613.79 to 1,177.01)**	**867.01 (653.95 to 1,178.74)**
Congenital birth defects	51,601.89 (37,072.74 to 72,245.76)	19,922.96 (13,580.20 to 29,170.95)	18,922.66 (12,839.05 to 27,377.70)	30,273.54 (20,345.73 to 44,186.19)	619.14 (449.43 to 860.82)	257.06 (180.06 to 365.49)	244.70 (171.89 to 344.28)	380.76 (259.81 to 544.90)
Neural tube defects	4,909.94 (2,347.66 to 8,701.50)	915.69 (360.37 to 1,918.72)	826.70 (326.52 to 1,741.99)	1,667.57 (631.79 to 3,552.10)	57.01 (27.43 to 100.84)	10.73 (4.26 to 22.31)	9.69 (3.85 to 20.26)	19.47 (7.48 to 41.31)
Congenital heart anomalies	28,495.28 (19,142.94 to 41,958.06)	10,968.36 (7,021.13 to 16,486.91)	10,449.88 (6,716.06 to 15,864.54)	16,322.50 (10,494.84 to 25,011.49)	344.58 (234.72 to 502.00)	142.73 (95.63 to 210.43)	136.19 (91.44 to 202.23)	207.29 (137.02 to 310.74)
Orofacial clefts	115.80 (34.38 to 279.97)	34.28 (8.87 to 86.63)	30.44 (7.84 to 79.41)	65.58 (16.24 to 170.08)	1.34 (0.40 to 3.24)	0.40 (0.10 to 1.00)	0.35 (0.09 to 0.92)	0.76 (0.19 to 1.97)
Down syndrome	492.81 (305.99 to 849.64)	378.37 (232.24 to 631.62)	355.66 (220.34 to 597.06)	553.13 (335.05 to 944.86)	6.63 (4.26 to 11.16)	6.20 (4.10 to 10.00)	5.85 (3.84 to 9.46)	8.55 (5.52 to 13.93)
Other chromosomal abnormalities	696.53 (290.75 to 1,391.21)	562.60 (237.19 to 1,169.54)	529.54 (220.12 to 1,097.72)	900.92 (372.40 to 1,854.27)	8.14 (3.42 to 16.20)	6.77 (2.98 to 13.73)	6.38 (2.79 to 12.93)	10.72 (4.70 to 21.75)
Congenital musculoskeletal and limb anomalies	1,155.05 (518.55 to 2,351.46)	580.22 (230.57 to 1,274.99)	557.72 (221.54 to 1,220.76)	867.52 (348.06 to 1,970.65)	13.49 (6.11 to 27.44)	6.89 (2.81 to 14.97)	6.63 (2.73 to 14.40)	10.24 (4.16 to 23.00)
Urogenital congenital anomalies	742.74 (491.49 to 1,162.58)	463.24 (328.12 to 675.26)	448.29 (318.84 to 648.64)	610.87 (423.12 to 920.77)	11.36 (8.04 to 17.02)	9.85 (7.41 to 13.35)	9.62 (7.22 to 13.00)	11.91 (8.86 to 16.55)
Digestive congenital anomalies	3,024.68 (1,524.10 to 5,868.56)	1,114.87 (515.72 to 2,215.71)	1,052.70 (480.93 to 2,092.77)	1,773.39 (788.95 to 3,569.95)	35.22 (17.78 to 68.24)	13.12 (6.12 to 25.93)	12.39 (5.71 to 24.43)	20.79 (9.31 to 41.65)
Other congenital birth defects	11,969.06 (7,658.45 to 17,881.71)	4,905.34 (3,033.37 to 7,610.21)	4,671.72 (2,907.62 to 7,193.07)	7,512.07 (4,600.97 to 11,633.32)	141.37 (90.91 to 209.81)	60.38 (38.21 to 92.20)	57.59 (36.49 to 87.43)	91.04 (56.61 to 139.12)
Skin and subcutaneous diseases	4,069.68 (2,701.68 to 6,824.68)	10,279.69 (6,975.01 to 16,367.20)	10,590.96 (7,201.00 to 16,802.15)	10,113.57 (6,785.15 to 16,399.57)	139.99 (94.08 to 230.15)	509.26 (341.41 to 828.55)	550.32 (366.97 to 890.60)	449.81 (300.57 to 738.57)
Cellulitis	263.14 (148.05 to 493.50)	1,002.73 (547.98 to 1,777.90)	1,076.78 (593.48 to 1,907.03)	872.13 (481.59 to 1,591.70)	8.96 (5.09 to 16.93)	48.38 (27.81 to 90.76)	54.08 (30.57 to 98.93)	38.72 (22.35 to 73.88)
Pyoderma	2,326.20 (1,505.27 to 4,037.38)	5,695.63 (3,709.17 to 9,573.40)	5,730.22 (3,719.98 to 9,660.19)	5,976.04 (3,893.72 to 10,110.12)	64.35 (41.56 to 110.61)	235.41 (151.56 to 403.19)	247.34 (159.38 to 422.80)	224.02 (144.13 to 392.70)
Decubitus ulcer	1,361.70 (858.54 to 2,310.42)	3,356.32 (2,117.29 to 5,609.29)	3,549.68 (2,249.47 to 5,923.14)	3,050.98 (1,922.61 to 5,255.63)	63.42 (39.64 to 109.35)	216.92 (130.32 to 384.95)	239.67 (145.83 to 415.96)	179.64 (106.57 to 329.53)
Other skin and subcutaneous diseases	118.64 (71.74 to 194.08)	225.01 (134.84 to 352.99)	234.28 (139.32 to 363.35)	214.42 (129.66 to 335.25)	3.26 (1.96 to 5.18)	8.55 (5.07 to 13.65)	9.24 (5.37 to 14.76)	7.43 (4.34 to 12.12)
Sudden infant death syndrome	5,500.47 (2,282.43 to 11,420.10)	2,063.80 (814.07 to 4,453.29)	1,976.57 (773.76 to 4,215.13)	3,140.85 (1,232.69 to 6,541.12)	63.84 (26.48 to 132.55)	23.94 (9.44 to 51.68)	22.93 (8.98 to 48.91)	36.44 (14.30 to 75.91)

**Injuries**	**518,443.65 (460,258.27 to 573,815.97)**	**544,539.15 (376,233.64 to 1,489,978.72)**	**488,561.68 (332,444.52 to 1,433,420.65)**	**687,184.12 (483,659.86 to 1,707,898.17)**	**10,531.50 (9,418.71 to 11,557.02)**	**14,179.90 (10,650.80 to 32,531.47)**	**13,155.48 (9,911.97 to 31,474.60)**	**16,579.07 (12,520.93 to 35,703.98)**
**Transport injuries**	**178,976.60 (150,252.55 to 208,752.75)**	**164,662.25 (117,239.77 to 235,163.03)**	**120,015.20 (87,364.21 to 169,441.05)**	**252,754.95 (175,583.64 to 369,634.20)**	**3,710.70 (3,159.09 to 4,303.34)**	**4,290.04 (3,115.16 to 6,093.74)**	**3,281.46 (2,447.90 to 4,566.66)**	**6,073.67 (4,311.83 to 8,908.96)**
Road injuries	169,904.20 (141,904.79 to 198,136.97)	152,478.92 (108,207.20 to 217,713.66)	108,842.78 (78,634.93 to 152,771.44)	237,740.80 (166,092.85 to 347,208.59)	3,520.10 (2,990.77 to 4,072.55)	3,954.42 (2,887.59 to 5,526.41)	2,964.53 (2,232.59 to 4,083.66)	5,687.71 (4,035.25 to 8,269.33)
Pedestrian road injuries	40,457.47 (33,293.08 to 50,751.17)	34,436.94 (24,555.54 to 49,251.45)	25,989.90 (19,114.87 to 35,638.41)	50,733.11 (34,867.43 to 73,716.48)	968.48 (798.24 to 1,188.29)	1,154.63 (836.18 to 1,636.43)	917.18 (683.26 to 1,242.09)	1,548.33 (1,077.34 to 2,248.81)

Cambodia								
Cause	YLLs (actual counts)				Deaths (actual counts)			
	2016	2040 Forecast	2040 Better Scenario	2040 Worse Scenario	2016	2040 Forecast	2040 Better Scenario	2040 Worse Scenario
Cyclist road injuries	6,500.90 (4,107.18 to 10,769.94)	6,730.30 (3,832.38 to 11,673.72)	4,895.03 (2,821.31 to 8,373.07)	10,087.40 (5,707.59 to 17,649.77)	154.43 (98.11 to 257.49)	201.14 (113.77 to 359.39)	154.92 (87.72 to 270.70)	275.95 (153.33 to 493.30)
Motorcyclist road injuries	44,602.61 (33,324.95 to 59,641.80)	42,092.66 (26,033.04 to 66,053.21)	31,924.50 (20,106.59 to 49,422.74)	61,187.57 (37,772.87 to 96,967.75)	811.94 (614.45 to 1,081.03)	853.60 (534.57 to 1,326.99)	661.29 (424.95 to 1,019.75)	1,193.56 (739.47 to 1,907.49)
Motor vehicle road injuries	74,950.65 (55,414.63 to 91,470.73)	65,779.11 (43,788.11 to 95,122.17)	43,675.54 (29,188.29 to 61,217.00)	110,153.46 (72,737.48 to 160,932.85)	1,513.01 (1,115.73 to 1,840.72)	1,646.31 (1,106.39 to 2,328.85)	1,157.06 (798.53 to 1,603.76)	2,529.29 (1,696.78 to 3,687.00)
Other road injuries	3,392.57 (2,224.11 to 6,517.84)	3,439.91 (1,983.16 to 6,694.52)	2,357.82 (1,403.31 to 4,519.17)	5,579.26 (3,123.97 to 10,910.26)	72.25 (48.51 to 133.23)	98.75 (56.38 to 196.21)	74.08 (43.03 to 147.14)	140.57 (79.14 to 275.72)
Other transport injuries	9,072.40 (5,027.94 to 13,453.95)	12,183.33 (6,298.70 to 20,121.55)	11,172.43 (5,959.81 to 17,837.83)	15,014.14 (7,566.03 to 25,525.88)	190.60 (102.18 to 279.95)	335.62 (166.54 to 554.91)	316.93 (162.15 to 501.26)	385.97 (188.01 to 650.80)
**Unintentional injuries**	**257,883.01 (229,511.60 to 288,277.84)**	**186,936.37 (159,937.22 to 216,071.92)**	**181,816.92 (155,931.49 to 209,241.23)**	**220,767.47 (186,131.44 to 260,253.62)**	**5,232.10 (4,692.97 to 5,779.82)**	**5,940.13 (5,161.94 to 6,708.62)**	**6,032.79 (5,251.91 to 6,770.55)**	**6,247.68 (5,387.43 to 7,242.84)**
Falls	61,948.86 (51,953.18 to 73,107.13)	74,259.98 (60,742.53 to 85,342.16)	74,923.36 (61,026.90 to 85,503.30)	77,210.80 (63,493.04 to 89,447.29)	1,830.20 (1,474.21 to 2,098.97)	3,300.70 (2,577.27 to 3,853.30)	3,473.22 (2,718.65 to 4,029.85)	3,130.30 (2,447.99 to 3,714.86)
Drowning	114,949.73 (94,057.15 to 136,038.84)	55,933.11 (43,092.40 to 72,484.96)	52,568.81 (41,288.64 to 67,276.48)	74,027.78 (56,176.00 to 98,491.29)	1,843.42 (1,585.91 to 2,143.08)	1,164.75 (919.73 to 1,496.25)	1,108.83 (893.24 to 1,407.31)	1,444.16 (1,122.18 to 1,887.76)
Fire, heat, and hot substances	18,196.67 (13,010.44 to 25,569.71)	12,612.07 (8,674.40 to 19,449.17)	11,766.38 (8,183.05 to 17,847.65)	15,983.09 (10,702.11 to 24,461.87)	372.17 (279.36 to 520.29)	358.92 (256.82 to 542.73)	346.43 (251.51 to 509.77)	418.31 (291.51 to 628.75)
Poisonings	5,202.54 (3,590.85 to 7,212.93)	2,575.06 (1,707.28 to 3,848.69)	2,319.84 (1,554.49 to 3,431.56)	3,600.14 (2,302.64 to 5,528.97)	98.62 (69.37 to 136.02)	61.08 (41.62 to 92.29)	56.40 (39.34 to 82.51)	80.11 (53.26 to 123.18)
Exposure to mechanical forces	12,821.76 (8,468.27 to 18,203.43)	8,439.61 (5,797.86 to 12,177.87)	8,001.61 (5,451.84 to 11,702.43)	10,722.78 (7,299.61 to 15,449.66)	233.26 (160.02 to 328.11)	192.34 (139.80 to 273.71)	185.60 (136.02 to 267.17)	230.42 (165.59 to 330.67)
Unintentional firearm injuries	2,116.56 (1,164.01 to 4,564.60)	1,406.51 (777.84 to 3,189.92)	1,325.12 (724.26 to 3,011.37)	1,801.05 (989.76 to 4,125.56)	41.41 (23.94 to 88.61)	34.22 (20.35 to 74.57)	32.99 (19.58 to 72.50)	41.30 (24.20 to 90.42)
Unintentional suffocation	2,780.30 (1,506.15 to 4,616.59)	1,241.36 (758.94 to 1,926.62)	1,165.45 (717.07 to 1,816.56)	1,812.29 (1,069.24 to 2,880.54)	37.36 (21.69 to 59.68)	19.70 (12.96 to 29.65)	18.60 (12.17 to 28.03)	27.39 (17.27 to 41.58)
Other exposure to mechanical forces	7,924.90 (5,270.79 to 10,565.92)	5,791.74 (3,938.79 to 7,817.51)	5,511.04 (3,736.80 to 7,532.43)	7,109.44 (4,836.97 to 9,789.77)	154.49 (106.89 to 203.60)	138.41 (102.67 to 181.75)	134.02 (99.79 to 177.12)	161.73 (118.69 to 216.72)
Adverse effects of medical treatment	11,949.13 (9,224.46 to 14,543.77)	10,255.88 (8,039.88 to 12,191.52)	9,962.60 (7,822.90 to 11,834.44)	12,026.61 (9,308.49 to 14,356.67)	259.64 (210.30 to 300.89)	317.63 (262.62 to 371.26)	318.84 (263.56 to 373.98)	341.72 (276.17 to 398.23)
Animal contact	3,379.79 (2,379.55 to 4,490.27)	3,139.69 (2,126.66 to 4,242.66)	2,960.52 (2,030.24 to 4,026.55)	4,062.83 (2,732.49 to 5,508.97)	72.86 (52.17 to 94.30)	89.46 (62.27 to 118.56)	86.62 (59.90 to 115.17)	107.30 (74.22 to 141.89)
Venomous animal contact	1,796.93 (1,169.12 to 2,582.12)	1,569.38 (1,022.97 to 2,261.01)	1,479.57 (963.54 to 2,135.17)	2,043.54 (1,320.71 to 2,992.50)	39.56 (26.62 to 55.10)	46.62 (31.47 to 66.00)	45.16 (30.47 to 63.56)	56.03 (37.37 to 79.72)
Non-venomous animal contact	1,582.86 (1,012.29 to 2,206.09)	1,570.32 (992.46 to 2,230.42)	1,480.95 (934.43 to 2,098.02)	2,019.29 (1,272.80 to 2,873.44)	33.30 (21.15 to 46.07)	42.84 (27.13 to 61.05)	41.46 (25.79 to 58.88)	51.27 (32.34 to 73.43)
Foreign body	14,758.28 (9,877.31 to 20,791.89)	9,470.51 (6,664.13 to 11,927.48)	9,497.52 (6,696.04 to 11,942.97)	10,798.38 (7,444.48 to 14,330.72)	244.65 (171.73 to 314.41)	230.44 (168.79 to 276.65)	239.10 (175.59 to 286.84)	233.51 (170.60 to 283.89)
Pulmonary aspiration and foreign body in airway	13,378.45 (8,485.81 to 19,413.90)	8,491.32 (5,539.03 to 10,874.48)	8,547.72 (5,631.70 to 10,889.70)	9,630.49 (6,243.59 to 13,050.69)	217.90 (145.04 to 288.36)	204.13 (139.11 to 251.05)	212.88 (144.83 to 261.89)	204.69 (138.85 to 254.56)

Foreign body in other body part	1,379.83 (815.28 to 2,737.64)	979.19 (621.18 to 1,905.47)	949.81 (591.40 to 1,860.27)	1,167.89 (732.69 to 2,249.08)	26.75 (16.86 to 51.49)	26.30 (17.33 to 50.58)	26.23 (17.39 to 51.16)	28.81 (19.06 to 54.86)
Environmental heat and cold exposure	987.44 (678.69 to 1,561.20)	899.88 (616.85 to 1,467.77)	833.20 (563.78 to 1,352.42)	1,211.42 (816.49 to 1,924.00)	22.69 (16.33 to 35.73)	27.31 (19.53 to 44.27)	26.08 (18.66 to 42.50)	34.11 (23.92 to 55.98)
Other unintentional injuries	13,688.82 (10,285.25 to 18,394.63)	9,350.56 (6,885.10 to 12,666.09)	8,983.07 (6,599.54 to 12,175.45)	11,123.63 (8,122.46 to 15,321.34)	254.58 (195.04 to 339.95)	197.50 (149.29 to 264.61)	191.67 (144.57 to 255.40)	227.73 (170.33 to 309.57)
**Self-harm and interpersonal violence**	**81,459.67 (63,819.40 to 98,608.99)**	**89,515.93 (62,544.24 to 124,779.35)**	**82,782.73 (59,260.91 to 112,336.22)**	**102,383.39 (68,824.57 to 147,385.48)**	**1,586.33 (1,248.70 to 1,892.58)**	**1,962.76 (1,385.75 to 2,716.49)**	**1,827.88 (1,326.74 to 2,435.03)**	**2,201.14 (1,501.37 to 3,108.32)**

Cambodia								
Cause	YLLs (actual counts)				Deaths (actual counts)			
	2016	2040 Forecast	2040 Better Scenario	2040 Worse Scenario	2016	2040 Forecast	2040 Better Scenario	2040 Worse Scenario
Self-harm	44,546.23 (35,975.59 to 53,383.87)	57,605.73 (35,965.31 to 87,779.16)	51,409.43 (33,374.11 to 76,617.56)	68,475.43 (41,208.99 to 107,981.57)	922.60 (757.90 to 1,078.83)	1,343.53 (875.16 to 2,024.22)	1,217.67 (819.16 to 1,771.20)	1,551.50 (954.48 to 2,402.87)
Self-harm by firearm	1,683.17 (888.06 to 2,966.55)	1,736.45 (734.59 to 3,495.09)	1,521.36 (676.73 to 3,118.05)	2,133.27 (867.93 to 4,366.74)	34.80 (19.44 to 60.36)	41.30 (18.68 to 81.79)	36.93 (17.35 to 70.52)	48.98 (21.22 to 97.11)
Self-harm by other specified means	42,863.06 (34,615.31 to 51,258.96)	55,869.28 (34,760.22 to 84,767.27)	49,888.07 (32,527.68 to 73,700.89)	66,342.16 (39,767.72 to 104,724.93)	887.80 (730.18 to 1,036.54)	1,302.23 (834.89 to 1,954.08)	1,180.75 (796.39 to 1,704.76)	1,502.52 (926.11 to 2,347.72)
Interpersonal violence	36,913.44 (22,638.12 to 50,395.46)	31,910.21 (18,850.03 to 46,117.82)	31,373.31 (18,587.31 to 44,541.44)	33,907.96 (19,483.01 to 50,095.48)	663.73 (416.37 to 888.40)	619.22 (377.10 to 880.48)	610.21 (377.19 to 846.13)	649.63 (391.82 to 943.87)
Physical violence by firearm	8,586.32 (4,368.98 to 17,412.96)	7,247.56 (3,697.18 to 14,746.36)	7,205.02 (3,701.20 to 14,602.51)	7,619.38 (3,805.38 to 15,575.64)	153.57 (79.55 to 318.21)	137.53 (73.17 to 282.05)	137.01 (72.90 to 280.57)	142.92 (73.92 to 293.15)
Physical violence by sharp object	15,733.52 (9,176.02 to 23,049.07)	14,763.25 (8,117.63 to 22,147.75)	14,904.94 (8,194.16 to 22,198.91)	14,776.60 (8,021.50 to 22,358.63)	282.17 (164.16 to 409.66)	279.48 (159.93 to 411.58)	282.79 (161.77 to 413.93)	276.95 (155.57 to 410.64)
Physical violence by other means	12,593.60 (6,589.17 to 19,411.64)	9,899.40 (4,381.31 to 17,854.62)	9,263.35 (4,254.94 to 16,340.96)	11,511.98 (4,951.20 to 21,353.74)	227.99 (123.71 to 343.29)	202.22 (97.10 to 352.85)	190.41 (95.32 to 324.32)	229.77 (105.98 to 413.25)
**Forces of nature, conflict and terrorism, and executions and police conflict**	**124.36 (103.83 to 166.78)**	**103,424.61 (0.00 to 1,030,424.06)**	**103,946.82 (0.00 to 1,035,989.37)**	**111,278.31 (0.00 to 1,110,930.26)**	**2.37 (1.90 to 3.25)**	**1,986.98 (0.00 to 19,827.88)**	**2,013.34 (0.00 to 20,072.10)**	**2,056.59 (0.00 to 20,574.77)**
Exposure to forces of nature	0.00 (0.00 to 0.00)	715.75 (0.00 to 8,494.32)	721.36 (0.00 to 8,556.96)	778.63 (0.00 to 9,210.69)	0.00 (0.00 to 0.00)	15.58 (0.00 to 185.31)	15.91 (0.00 to 189.33)	15.92 (0.00 to 189.05)
Conflict and terrorism	0.00 (0.00 to 0.00)	100,983.58 (0.00 to 1,030,424.06)	101,491.42 (0.00 to 1,035,989.37)	108,704.84 (0.00 to 1,110,930.26)	0.00 (0.00 to 0.00)	1,937.19 (0.00 to 19,827.88)	1,962.89 (0.00 to 20,072.10)	2,005.88 (0.00 to 20,574.77)
Executions and police conflict	124.36 (103.83 to 166.78)	1,725.28 (0.00 to 43,691.20)	1,734.04 (0.00 to 43,932.19)	1,794.85 (0.00 to 45,455.95)	2.37 (1.90 to 3.25)	34.21 (0.00 to 870.55)	34.54 (0.00 to 879.28)	34.79 (0.00 to 883.77)

Indonesia								
**Cause**	**YLLs (in 1000s)**				**Deaths (in 1000s)**			
	**2016**	**2040 Forecast**	**2040 Better Scenario**	**2040 Worse Scenario**	**2016**	**2040 Forecast**	**2040 Better Scenario**	**2040 Worse Scenario**
**All causes**	**47,754.99 (45,948.40 to 49,440.60)**	**51,505.54 (42,422.39 to 62,455.79)**	**39,996.39 (33,102.58 to 48,390.08)**	**67,113.66 (54,655.13 to 82,061.42)**	**1,537.80 (1,479.67 to 1,598.43)**	**2,533.26 (2,161.73 to 2,922.99)**	**2,066.30 (1,775.46 to 2,385.16)**	**3,106.42 (2,656.26 to 3,633.29)**
**Communicable, maternal, neonatal, and nutritional diseases**	**14,740.03 (13,823.34 to 15,721.37)**	**9,221.28 (7,133.48 to 12,559.02)**	**7,014.73 (5,395.33 to 9,675.24)**	**12,567.97 (9,559.55 to 17,429.16)**	**311.98 (285.96 to 337.15)**	**334.70 (237.59 to 504.52)**	**280.89 (197.03 to 427.91)**	**436.99 (308.12 to 666.82)**
**HIV/AIDS and tuberculosis**	**3,177.45 (3,010.14 to 3,362.69)**	**2,228.38 (1,690.87 to 2,906.12)**	**1,376.62 (996.23 to 1,863.55)**	**3,196.05 (2,460.71 to 4,149.47)**	**97.30 (91.82 to 103.11)**	**93.26 (69.17 to 125.10)**	**60.90 (44.59 to 81.51)**	**129.62 (95.90 to 169.18)**
Tuberculosis	2,797.47 (2,632.55 to 2,972.60)	1,853.43 (1,317.44 to 2,553.75)	1,203.40 (848.41 to 1,699.90)	2,673.80 (1,946.65 to 3,643.13)	89.89 (84.34 to 95.55)	85.73 (61.93 to 116.91)	57.69 (41.56 to 78.47)	118.80 (85.52 to 159.44)
HIV/AIDS	379.97 (329.04 to 454.20)	374.95 (293.67 to 516.82)	173.22 (85.33 to 282.11)	522.25 (405.76 to 741.01)	7.41 (6.39 to 8.87)	7.53 (5.72 to 10.91)	3.21 (1.39 to 5.73)	10.82 (8.16 to 16.27)
**Diarrhea, lower respiratory, and other common infectious diseases**	**5,190.26 (4,519.30 to 5,880.68)**	**3,282.77 (1,693.59 to 6,171.58)**	**2,617.14 (1,365.13 to 5,053.16)**	**4,697.14 (2,416.84 to 9,093.94)**	**115.14 (91.38 to 138.19)**	**152.83 (65.29 to 318.28)**	**135.87 (59.77 to 290.45)**	**205.80 (86.44 to 439.71)**
Diarrheal diseases	1,599.51 (1,159.58 to 2,162.52)	1,267.40 (208.25 to 3,733.74)	1,165.33 (241.15 to 3,408.19)	1,894.09 (320.37 to 5,594.64)	49.68 (28.68 to 70.67)	81.70 (12.25 to 247.00)	81.41 (15.90 to 227.30)	115.43 (16.37 to 338.94)
Intestinal infectious diseases	767.96 (433.88 to 1,226.83)	387.12 (195.02 to 662.05)	392.69 (206.34 to 658.86)	444.03 (219.35 to 777.01)	11.47 (6.49 to 18.39)	6.68 (3.39 to 11.24)	6.98 (3.75 to 11.40)	7.50 (3.84 to 12.81)
Typhoid fever	666.81 (370.57 to 1,059.18)	327.60 (159.51 to 566.77)	345.94 (178.48 to 585.02)	372.57 (178.53 to 649.92)	9.92 (5.51 to 15.71)	5.62 (2.86 to 9.51)	6.12 (3.26 to 10.08)	6.26 (3.20 to 10.78)
Paratyphoid fever	99.62 (47.27 to 185.90)	59.12 (25.59 to 114.61)	46.39 (19.98 to 91.75)	70.93 (30.24 to 139.58)	1.52 (0.72 to 2.86)	1.04 (0.46 to 2.03)	0.84 (0.36 to 1.64)	1.23 (0.54 to 2.41)
Other intestinal infectious diseases	1.53 (0.57 to 3.34)	0.40 (0.14 to 0.92)	0.36 (0.13 to 0.80)	0.53 (0.18 to 1.16)	0.03 (0.01 to 0.06)	0.02 (0.01 to 0.03)	0.01 (0.01 to 0.03)	0.02 (0.01 to 0.04)
Lower respiratory infections	1,735.20 (1,554.54 to 1,944.06)	1,077.16 (710.48 to 1,663.88)	714.92 (498.44 to 1,042.69)	1,604.87 (969.19 to 2,769.71)	38.14 (33.58 to 42.03)	54.80 (38.69 to 75.00)	40.43 (29.91 to 55.59)	70.61 (48.48 to 101.45)
Upper respiratory infections	2.46 (2.03 to 3.04)	1.20 (0.76 to 1.91)	0.88 (0.53 to 1.42)	1.68 (1.04 to 2.65)	0.05 (0.05 to 0.06)	0.04 (0.03 to 0.07)	0.03 (0.02 to 0.05)	0.06 (0.04 to 0.09)
Otitis media	6.18 (1.84 to 11.06)	2.29 (0.61 to 4.29)	2.36 (0.63 to 4.32)	2.21 (0.57 to 4.24)	0.17 (0.07 to 0.28)	0.11 (0.03 to 0.21)	0.12 (0.04 to 0.22)	0.10 (0.03 to 0.19)
Meningitis	287.73 (135.80 to 392.24)	100.90 (46.90 to 167.35)	73.42 (35.60 to 116.36)	120.87 (52.35 to 214.75)	4.31 (2.04 to 5.73)	2.27 (1.12 to 3.33)	1.80 (0.91 to 2.57)	2.51 (1.20 to 3.83)
Pneumococcal meningitis	2.83 (1.28 to 4.39)	0.63 (0.29 to 1.04)	0.53 (0.25 to 0.83)	0.75 (0.32 to 1.32)	0.04 (0.02 to 0.06)	0.02 (0.01 to 0.03)	0.02 (0.01 to 0.02)	0.02 (0.01 to 0.03)
H influenzae type B meningitis	22.69 (10.82 to 32.12)	14.77 (3.19 to 46.89)	7.22 (2.62 to 16.72)	24.75 (3.95 to 83.32)	0.33 (0.16 to 0.45)	0.24 (0.08 to 0.61)	0.15 (0.07 to 0.28)	0.35 (0.09 to 1.04)
Meningococcal meningitis	98.50 (45.79 to 138.48)	31.29 (14.98 to 49.82)	24.46 (11.92 to 38.18)	34.85 (16.20 to 54.93)	1.51 (0.72 to 2.05)	0.78 (0.39 to 1.13)	0.65 (0.33 to 0.93)	0.83 (0.42 to 1.19)
Other meningitis	163.71 (76.04 to 225.56)	54.20 (25.82 to 85.42)	41.21 (20.01 to 63.47)	60.51 (27.62 to 95.67)	2.43 (1.14 to 3.25)	1.23 (0.61 to 1.78)	0.99 (0.49 to 1.41)	1.32 (0.64 to 1.89)
Encephalitis	118.66 (47.49 to 178.90)	66.66 (31.07 to 114.58)	60.60 (29.22 to 101.09)	78.03 (35.00 to 134.30)	1.81 (0.85 to 2.59)	1.55 (0.86 to 2.29)	1.50 (0.87 to 2.15)	1.72 (0.93 to 2.57)

Diphtheria	2.32 (1.93 to 2.81)	0.38 (0.21 to 0.65)	0.26 (0.17 to 0.38)	0.53 (0.23 to 1.05)	0.03 (0.03 to 0.04)	0.01 (0.00 to 0.01)	0.01 (0.00 to 0.01)	0.01 (0.00 to 0.01)
Whooping cough	208.44 (20.68 to 589.91)	82.22 (2.56 to 315.63)	17.22 (1.13 to 65.66)	90.71 (2.71 to 342.93)	2.48 (0.25 to 7.01)	0.98 (0.04 to 3.73)	0.22 (0.02 to 0.80)	1.08 (0.04 to 4.08)
Tetanus	113.23 (23.70 to 151.17)	18.74 (3.49 to 29.71)	14.13 (2.09 to 22.50)	19.55 (3.57 to 31.08)	2.64 (0.48 to 3.49)	0.53 (0.09 to 0.83)	0.50 (0.07 to 0.79)	0.52 (0.09 to 0.82)
Measles	327.47 (121.99 to 690.67)	271.11 (16.43 to 1,004.39)	168.86 (13.58 to 536.31)	430.66 (26.01 to 1,676.54)	3.93 (1.46 to 8.27)	3.76 (0.28 to 12.99)	2.50 (0.24 to 7.16)	5.79 (0.39 to 20.83)

Indonesia								
Cause	YLLs (in 1000s)				Deaths (in 1000s)			
	2016	2040 Forecast	2040 Better Scenario	2040 Worse Scenario	2016	2040 Forecast	2040 Better Scenario	2040 Worse Scenario
Varicella and herpes zoster	21.11 (17.65 to 25.52)	7.59 (4.85 to 11.14)	6.47 (4.36 to 9.34)	9.92 (6.33 to 14.52)	0.44 (0.38 to 0.50)	0.39 (0.27 to 0.55)	0.37 (0.26 to 0.53)	0.46 (0.31 to 0.64)
**Neglected tropical diseases and malaria**	**592.40 (244.17 to 776.82)**	**1,133.61 (547.42 to 1,466.37)**	**1,074.26 (540.66 to 1,377.97)**	**1,202.16 (562.75 to 1,567.15)**	**10.86 (4.83 to 13.77)**	**25.74 (12.93 to 32.23)**	**25.31 (13.10 to 31.45)**	**26.39 (12.97 to 33.02)**
Malaria	209.30 (132.89 to 294.26)	160.96 (105.89 to 381.08)	158.54 (104.81 to 370.51)	166.24 (108.81 to 404.42)	4.93 (3.03 to 6.80)	5.43 (3.59 to 10.47)	5.60 (3.60 to 10.29)	5.34 (3.51 to 10.79)
Chagas disease	0.00 (0.00 to 0.00)	0.00 (0.00 to 0.00)	0.00 (0.00 to 0.00)	0.00 (0.00 to 0.00)	0.00 (0.00 to 0.00)	0.00 (0.00 to 0.00)	0.00 (0.00 to 0.00)	0.00 (0.00 to 0.00)
Leishmaniasis	0.00 (0.00 to 0.00)	0.00 (0.00 to 0.00)	0.00 (0.00 to 0.00)	0.00 (0.00 to 0.00)	0.00 (0.00 to 0.00)	0.00 (0.00 to 0.00)	0.00 (0.00 to 0.00)	0.00 (0.00 to 0.00)
African trypanosomiasis	0.00 (0.00 to 0.00)	0.00 (0.00 to 0.00)	0.00 (0.00 to 0.00)	0.00 (0.00 to 0.00)	0.00 (0.00 to 0.00)	0.00 (0.00 to 0.00)	0.00 (0.00 to 0.00)	0.00 (0.00 to 0.00)
Schistosomiasis	0.05 (0.02 to 0.09)	0.04 (0.01 to 0.09)	0.04 (0.01 to 0.09)	0.04 (0.02 to 0.10)	0.00 (0.00 to 0.00)	0.00 (0.00 to 0.00)	0.00 (0.00 to 0.00)	0.00 (0.00 to 0.00)
Cysticercosis	0.12 (0.05 to 0.23)	0.06 (0.02 to 0.14)	0.05 (0.02 to 0.14)	0.06 (0.02 to 0.14)	0.00 (0.00 to 0.01)	0.00 (0.00 to 0.00)	0.00 (0.00 to 0.00)	0.00 (0.00 to 0.00)
Cystic echinococcosis	0.11 (0.09 to 0.13)	0.06 (0.04 to 0.13)	0.05 (0.04 to 0.11)	0.10 (0.06 to 0.19)	0.00 (0.00 to 0.00)	0.00 (0.00 to 0.00)	0.00 (0.00 to 0.00)	0.00 (0.00 to 0.01)
Dengue	364.62 (52.63 to 547.71)	958.87 (301.99 to 1,318.27)	902.65 (297.59 to 1,230.49)	1,020.22 (309.92 to 1,416.39)	5.62 (0.90 to 8.29)	19.98 (6.87 to 26.78)	19.38 (6.95 to 25.86)	20.69 (6.94 to 27.91)
Yellow fever	0.00 (0.00 to 0.00)	0.00 (0.00 to 0.00)	0.00 (0.00 to 0.00)	0.00 (0.00 to 0.00)	0.00 (0.00 to 0.00)	0.00 (0.00 to 0.00)	0.00 (0.00 to 0.00)	0.00 (0.00 to 0.00)
Rabies	1.84 (0.15 to 2.73)	0.54 (0.11 to 0.99)	0.45 (0.10 to 0.84)	0.93 (0.19 to 1.64)	0.03 (0.00 to 0.05)	0.02 (0.00 to 0.02)	0.01 (0.00 to 0.02)	0.02 (0.00 to 0.04)
Intestinal nematode infections	11.53 (9.38 to 14.05)	9.50 (6.39 to 17.36)	8.86 (5.86 to 16.76)	10.16 (6.86 to 18.05)	0.17 (0.14 to 0.20)	0.19 (0.13 to 0.32)	0.18 (0.13 to 0.32)	0.19 (0.14 to 0.33)
Other neglected tropical diseases	4.82 (3.54 to 9.39)	3.59 (2.29 to 9.08)	3.62 (2.31 to 9.34)	4.41 (2.77 to 11.22)	0.09 (0.07 to 0.18)	0.12 (0.08 to 0.28)	0.13 (0.09 to 0.30)	0.14 (0.09 to 0.33)
**Maternal disorders**	**668.27 (602.07 to 740.67)**	**299.63 (228.93 to 380.98)**	**256.57 (193.82 to 330.66)**	**438.77 (332.31 to 560.72)**	**12.49 (11.21 to 13.84)**	**5.71 (4.34 to 7.37)**	**4.90 (3.70 to 6.37)**	**8.35 (6.33 to 10.70)**
Maternal hemorrhage	222.90 (171.11 to 282.96)	91.98 (60.78 to 132.76)	79.11 (51.23 to 113.61)	135.66 (89.10 to 195.54)	4.14 (3.19 to 5.25)	1.74 (1.15 to 2.53)	1.50 (0.96 to 2.18)	2.57 (1.67 to 3.73)
Maternal sepsis and other maternal infections	29.50 (17.20 to 45.40)	11.70 (5.90 to 19.93)	10.00 (4.96 to 17.39)	17.27 (8.95 to 30.26)	0.57 (0.33 to 0.88)	0.23 (0.12 to 0.39)	0.20 (0.10 to 0.34)	0.34 (0.17 to 0.58)
Maternal hypertensive disorders	77.09 (53.63 to 104.95)	34.39 (21.05 to 52.81)	29.16 (17.78 to 45.28)	51.01 (30.82 to 78.46)	1.44 (0.98 to 1.97)	0.66 (0.39 to 1.03)	0.56 (0.34 to 0.87)	0.97 (0.59 to 1.52)
Maternal obstructed labor and uterine rupture	22.59 (11.25 to 37.70)	10.22 (4.63 to 18.69)	8.50 (3.73 to 15.34)	15.55 (7.03 to 28.77)	0.43 (0.21 to 0.73)	0.20 (0.09 to 0.36)	0.17 (0.07 to 0.30)	0.30 (0.14 to 0.57)
Maternal abortion, miscarriage, and ectopic pregnancy	30.24 (16.67 to 48.19)	15.39 (7.83 to 27.31)	13.07 (6.69 to 22.91)	22.92 (11.76 to 40.73)	0.58 (0.32 to 0.91)	0.30 (0.16 to 0.52)	0.26 (0.13 to 0.45)	0.45 (0.23 to 0.78)

Indirect maternal deaths	90.26 (62.06 to 123.43)	45.25 (27.40 to 67.94)	38.04 (22.49 to 58.33)	66.26 (39.60 to 99.17)	1.65 (1.13 to 2.25)	0.84 (0.49 to 1.25)	0.71 (0.41 to 1.08)	1.23 (0.74 to 1.87)
Late maternal deaths	19.90 (9.62 to 34.04)	7.55 (3.36 to 13.97)	6.36 (2.84 to 11.86)	11.16 (5.04 to 21.04)	0.37 (0.18 to 0.63)	0.14 (0.06 to 0.26)	0.12 (0.05 to 0.23)	0.21 (0.09 to 0.40)
Maternal deaths aggravated by HIV/AIDS	0.37 (0.20 to 0.56)	0.54 (0.22 to 1.06)	0.42 (0.12 to 0.86)	0.77 (0.32 to 1.55)	0.01 (0.00 to 0.01)	0.01 (0.00 to 0.02)	0.01 (0.00 to 0.02)	0.01 (0.01 to 0.03)
Other maternal disorders	175.41 (129.07 to 224.54)	82.61 (54.61 to 119.53)	71.90 (47.76 to 105.78)	118.18 (78.68 to 171.87)	3.30 (2.45 to 4.23)	1.59 (1.04 to 2.31)	1.39 (0.91 to 2.02)	2.27 (1.50 to 3.30)

Indonesia								
Cause	YLLs (in 1000s)				Deaths (in 1000s)			
	2016	2040 Forecast	2040 Better Scenario	2040 Worse Scenario	2016	2040 Forecast	2040 Better Scenario	2040 Worse Scenario
**Neonatal disorders**	**4,247.26 (3,809.86 to 4,858.93)**	**1,630.79 (1,042.08 to 2,177.49)**	**1,060.74 (685.28 to 1,436.58)**	**2,294.89 (1,465.61 to 3,117.51)**	**49.06 (44.01 to 56.13)**	**18.84 (12.04 to 25.15)**	**12.26 (7.92 to 16.60)**	**26.51 (16.93 to 36.01)**
Neonatal preterm birth	1,973.28 (1,732.68 to 2,329.92)	764.68 (515.30 to 1,054.31)	419.29 (278.55 to 573.31)	1,059.63 (707.20 to 1,477.45)	22.79 (20.01 to 26.91)	8.83 (5.95 to 12.18)	4.84 (3.22 to 6.62)	12.24 (8.17 to 17.07)
Neonatal encephalopathy due to birth asphyxia and trauma	1,483.22 (1,274.40 to 1,694.87)	530.68 (277.73 to 825.36)	376.75 (202.01 to 576.59)	748.92 (390.83 to 1,167.83)	17.13 (14.72 to 19.58)	6.13 (3.21 to 9.53)	4.35 (2.33 to 6.66)	8.65 (4.52 to 13.49)
Neonatal sepsis and other neonatal infections	411.63 (304.16 to 623.39)	192.63 (106.60 to 323.47)	144.95 (84.31 to 243.81)	273.41 (150.33 to 469.06)	4.76 (3.51 to 7.20)	2.23 (1.23 to 3.74)	1.67 (0.97 to 2.82)	3.16 (1.74 to 5.42)
Hemolytic disease and other neonatal jaundice	19.12 (14.78 to 24.14)	5.15 (2.54 to 8.98)	3.81 (1.87 to 6.58)	7.72 (3.85 to 13.61)	0.22 (0.17 to 0.28)	0.06 (0.03 to 0.10)	0.04 (0.02 to 0.08)	0.09 (0.04 to 0.16)
Other neonatal disorders	360.00 (262.21 to 516.57)	137.65 (72.16 to 230.64)	115.93 (61.35 to 191.46)	205.21 (105.52 to 345.90)	4.16 (3.03 to 5.97)	1.59 (0.83 to 2.67)	1.34 (0.71 to 2.21)	2.37 (1.22 to 4.00)
**Nutritional deficiencies**	**287.06 (266.00 to 317.47)**	**293.98 (248.05 to 350.32)**	**326.56 (275.52 to 393.25)**	**319.18 (264.59 to 387.62)**	**15.62 (14.48 to 16.99)**	**25.92 (20.34 to 31.76)**	**30.40 (24.43 to 37.05)**	**26.41 (20.02 to 33.42)**
Protein-energy malnutrition	257.76 (238.54 to 287.00)	258.73 (214.91 to 314.39)	292.48 (242.94 to 355.52)	279.19 (226.47 to 343.04)	14.74 (13.64 to 16.04)	23.80 (18.18 to 29.69)	28.26 (22.33 to 34.64)	24.14 (17.90 to 30.87)
Iodine deficiency	2.19 (1.23 to 3.58)	7.64 (0.29 to 31.53)	7.58 (0.26 to 33.15)	9.14 (0.40 to 35.97)	0.06 (0.03 to 0.11)	0.63 (0.02 to 3.08)	0.65 (0.01 to 3.53)	0.71 (0.02 to 3.32)
Dietary iron deficiency	13.07 (5.86 to 16.34)	11.04 (7.48 to 16.22)	10.90 (7.33 to 17.00)	12.42 (8.44 to 17.78)	0.25 (0.17 to 0.32)	0.31 (0.21 to 0.69)	0.33 (0.21 to 0.75)	0.33 (0.22 to 0.70)
Other nutritional deficiencies	14.04 (11.18 to 21.39)	16.57 (12.43 to 25.22)	15.60 (11.58 to 24.10)	18.42 (13.84 to 28.26)	0.58 (0.46 to 0.87)	1.18 (0.85 to 1.77)	1.17 (0.85 to 1.79)	1.22 (0.87 to 1.89)
**Other communicable, maternal, neonatal, and nutritional diseases**	**577.34 (387.34 to 727.47)**	**352.12 (279.25 to 432.93)**	**302.85 (242.59 to 373.62)**	**419.79 (329.35 to 517.60)**	**11.51 (8.93 to 13.41)**	**12.40 (10.39 to 14.49)**	**11.26 (9.40 to 13.39)**	**13.91 (11.58 to 16.19)**
Sexually transmitted infections excluding HIV	170.23 (94.24 to 289.12)	29.40 (14.69 to 55.74)	22.83 (11.77 to 43.86)	36.77 (17.63 to 71.93)	2.18 (1.29 to 3.57)	0.55 (0.37 to 0.86)	0.47 (0.33 to 0.70)	0.64 (0.40 to 1.06)
Syphilis	163.99 (88.03 to 282.62)	24.91 (10.23 to 51.66)	18.60 (7.56 to 39.42)	31.86 (12.71 to 66.49)	2.01 (1.12 to 3.40)	0.36 (0.19 to 0.68)	0.28 (0.15 to 0.52)	0.45 (0.22 to 0.86)
Chlamydial infection	1.09 (0.97 to 1.37)	0.85 (0.69 to 1.03)	0.81 (0.65 to 0.98)	0.93 (0.75 to 1.14)	0.03 (0.03 to 0.04)	0.04 (0.03 to 0.04)	0.04 (0.02 to 0.05)	0.04 (0.03 to 0.04)
Gonococcal infection	3.37 (2.68 to 3.88)	2.46 (1.96 to 2.96)	2.32 (1.84 to 2.85)	2.67 (2.15 to 3.25)	0.09 (0.07 to 0.11)	0.11 (0.07 to 0.13)	0.11 (0.07 to 0.13)	0.11 (0.08 to 0.13)
Other sexually transmitted infections	1.77 (1.45 to 2.08)	1.19 (0.93 to 1.47)	1.10 (0.88 to 1.37)	1.31 (1.04 to 1.61)	0.04 (0.04 to 0.05)	0.04 (0.03 to 0.05)	0.04 (0.03 to 0.05)	0.04 (0.03 to 0.05)
Acute hepatitis	205.25 (188.33 to 225.55)	193.07 (155.79 to 237.39)	160.05 (124.23 to 204.48)	222.93 (182.66 to 272.33)	5.93 (5.43 to 6.52)	7.52 (6.19 to 9.02)	6.40 (5.05 to 7.99)	8.42 (7.03 to 10.08)
Acute hepatitis A	9.79 (8.27 to 11.72)	3.91 (2.79 to 5.86)	3.34 (2.35 to 5.11)	4.63 (3.26 to 6.95)	0.18 (0.15 to 0.21)	0.13 (0.09 to 0.17)	0.11 (0.08 to 0.16)	0.14 (0.10 to 0.19)
Acute hepatitis B	172.90 (156.88 to 191.48)	166.68 (132.56 to 207.67)	137.86 (105.17 to 178.92)	193.62 (155.11 to 241.96)	5.13 (4.65 to 5.71)	6.32 (5.05 to 7.75)	5.33 (4.13 to 6.81)	7.17 (5.81 to 8.80)

Acute hepatitis C	3.39 (2.56 to 4.59)	4.71 (3.26 to 6.73)	3.97 (2.71 to 5.74)	5.02 (3.51 to 7.16)	0.11 (0.08 to 0.16)	0.21 (0.14 to 0.29)	0.18 (0.12 to 0.26)	0.21 (0.15 to 0.31)
Acute hepatitis E	19.17 (15.68 to 23.19)	17.77 (12.82 to 24.12)	14.88 (10.56 to 20.83)	19.66 (14.37 to 26.38)	0.51 (0.40 to 0.64)	0.87 (0.61 to 1.23)	0.78 (0.53 to 1.12)	0.90 (0.63 to 1.28)
Other unspecified infectious diseases	201.85 (43.42 to 301.54)	129.65 (58.29 to 184.45)	119.96 (58.85 to 164.28)	160.08 (66.96 to 231.81)	3.40 (1.13 to 4.72)	4.33 (2.37 to 5.61)	4.40 (2.56 to 5.69)	4.86 (2.61 to 6.43)
**Non-communicable diseases**	**28,564.97 (27,353.32 to 29,797.04)**	**38,659.86 (30,844.93 to 47,745.22)**	**29,715.95 (24,220.80 to 36,331.78)**	**49,377.80 (38,967.13 to 62,618.38)**	**1,127.54 (1,076.12 to 1,180.00)**	**2,080.22 (1,759.23 to 2,464.44)**	**1,673.79 (1,426.29 to 1,963.86)**	**2,512.97 (2,096.41 to 2,996.27)**

Indonesia								
Cause	YLLs (in 1000s)				Deaths (in 1000s)			
	2016	2040 Forecast	2040 Better Scenario	2040 Worse Scenario	2016	2040 Forecast	2040 Better Scenario	2040 Worse Scenario
**Neoplasms**	**4,231.97 (3,995.05 to 4,506.35)**	**7,359.81 (6,333.22 to 8,558.44)**	**6,693.02 (5,704.89 to 7,756.96)**	**7,543.02 (6,510.84 to 8,693.40)**	**147.35 (139.09 to 156.17)**	**334.54 (292.17 to 380.92)**	**313.75 (273.68 to 357.08)**	**330.97 (289.67 to 376.96)**
Lip and oral cavity cancer	74.15 (69.27 to 79.50)	117.65 (93.47 to 148.20)	100.43 (80.93 to 125.39)	137.12 (105.54 to 178.63)	2.61 (2.44 to 2.79)	5.35 (4.29 to 6.71)	4.74 (3.88 to 5.88)	5.94 (4.63 to 7.69)
Nasopharynx cancer	76.96 (57.12 to 98.43)	130.78 (85.21 to 191.20)	105.57 (69.83 to 153.19)	157.23 (101.29 to 233.56)	2.52 (1.85 to 3.21)	5.17 (3.40 to 7.43)	4.29 (2.85 to 6.15)	6.02 (3.87 to 8.79)
Other pharynx cancer	54.13 (48.48 to 59.76)	85.48 (67.27 to 108.35)	86.15 (67.67 to 108.70)	88.67 (67.89 to 114.54)	1.99 (1.78 to 2.20)	3.81 (3.03 to 4.79)	3.93 (3.14 to 4.92)	3.85 (2.99 to 4.94)
Esophageal cancer	46.32 (43.86 to 49.10)	67.72 (49.43 to 95.84)	62.88 (46.42 to 89.19)	82.48 (55.91 to 123.58)	1.69 (1.60 to 1.80)	3.08 (2.26 to 4.29)	2.96 (2.21 to 4.10)	3.60 (2.46 to 5.31)
Stomach cancer	310.69 (282.18 to 342.00)	410.42 (342.07 to 505.71)	363.90 (300.11 to 446.82)	435.92 (362.72 to 542.49)	12.44 (11.33 to 13.60)	20.58 (17.39 to 24.78)	18.75 (15.74 to 22.47)	21.16 (17.75 to 25.73)
Colon and rectum cancer	304.25 (285.39 to 323.19)	660.08 (468.14 to 919.88)	594.89 (421.97 to 830.21)	639.48 (452.27 to 898.87)	11.20 (10.51 to 11.92)	32.63 (22.97 to 45.88)	30.27 (21.78 to 42.64)	30.48 (21.58 to 43.06)
Liver cancer	270.87 (208.99 to 325.20)	545.24 (383.84 to 768.44)	515.87 (381.48 to 682.83)	546.38 (385.83 to 781.45)	10.27 (8.09 to 12.15)	25.29 (18.47 to 34.67)	24.84 (18.85 to 32.26)	24.55 (17.86 to 34.18)
Liver cancer due to hepatitis B	58.52 (40.80 to 80.55)	105.46 (67.58 to 156.30)	99.37 (65.91 to 142.65)	106.40 (68.41 to 159.01)	2.02 (1.38 to 2.78)	4.44 (2.87 to 6.50)	4.34 (2.84 to 6.15)	4.35 (2.80 to 6.41)
Liver cancer due to hepatitis C	95.09 (72.47 to 117.09)	204.53 (146.20 to 283.60)	197.60 (144.73 to 264.05)	202.13 (143.19 to 280.65)	4.06 (3.14 to 4.92)	10.48 (7.70 to 14.14)	10.49 (7.97 to 13.73)	10.04 (7.37 to 13.59)
Liver cancer due to alcohol use	22.31 (16.18 to 28.76)	45.02 (30.13 to 65.02)	41.91 (28.80 to 58.09)	45.82 (30.31 to 66.18)	0.87 (0.65 to 1.10)	2.04 (1.41 to 2.89)	1.95 (1.39 to 2.68)	2.02 (1.37 to 2.88)
Liver cancer due to other causes	94.95 (71.92 to 119.21)	190.23 (121.25 to 295.97)	176.99 (120.30 to 257.00)	192.02 (122.11 to 302.58)	3.33 (2.57 to 4.12)	8.33 (5.51 to 12.75)	8.05 (5.67 to 11.48)	8.14 (5.33 to 12.52)
Gallbladder and biliary tract cancer	66.06 (56.99 to 79.66)	95.39 (79.42 to 114.99)	96.64 (80.28 to 117.85)	89.51 (75.56 to 108.32)	2.78 (2.41 to 3.32)	4.92 (4.19 to 5.91)	5.10 (4.29 to 6.17)	4.48 (3.85 to 5.39)
Pancreatic cancer	177.13 (155.00 to 205.25)	417.08 (320.51 to 550.02)	413.22 (320.20 to 537.77)	393.08 (304.34 to 521.26)	7.17 (6.28 to 8.30)	20.50 (15.95 to 27.18)	20.80 (16.35 to 26.92)	18.73 (14.54 to 24.77)
Larynx cancer	47.96 (39.39 to 61.00)	72.41 (50.55 to 101.24)	56.71 (41.58 to 77.30)	89.12 (60.07 to 128.45)	1.92 (1.60 to 2.42)	3.50 (2.47 to 4.86)	2.83 (2.11 to 3.87)	4.14 (2.81 to 6.01)
Tracheal, bronchus, and lung cancer	345.61 (320.51 to 371.29)	667.12 (498.89 to 871.15)	467.66 (370.09 to 588.73)	877.81 (627.49 to 1,183.03)	13.16 (12.18 to 14.25)	32.04 (24.03 to 41.56)	23.27 (18.51 to 28.79)	40.27 (29.18 to 53.52)
Malignant skin melanoma	19.24 (16.61 to 23.00)	33.48 (27.90 to 42.06)	37.61 (30.95 to 48.21)	29.72 (25.05 to 36.57)	0.65 (0.56 to 0.78)	1.51 (1.29 to 1.88)	1.75 (1.47 to 2.23)	1.29 (1.11 to 1.58)
Non-melanoma skin cancer	34.01 (28.52 to 41.37)	70.96 (57.15 to 89.39)	65.20 (51.01 to 83.19)	72.21 (59.01 to 90.74)	1.36 (1.15 to 1.64)	3.68 (3.05 to 4.54)	3.50 (2.87 to 4.36)	3.61 (3.00 to 4.46)
Breast cancer	549.59 (498.43 to 615.99)	859.92 (637.08 to 1,169.22)	755.27 (566.93 to 1,002.29)	865.20 (644.18 to 1,177.11)	16.46 (14.89 to 19.09)	31.81 (23.73 to 43.27)	28.35 (21.41 to 37.87)	31.26 (23.37 to 42.56)
Cervical cancer	299.66 (213.03 to 348.88)	344.32 (236.85 to 426.10)	305.74 (211.40 to 385.36)	368.74 (259.75 to 455.45)	9.90 (7.14 to 11.48)	13.66 (9.67 to 16.42)	12.35 (8.78 to 15.23)	14.27 (10.17 to 17.04)

Uterine cancer	72.11 (56.73 to 81.81)	122.47 (95.20 to 151.08)	114.88 (88.95 to 142.87)	131.29 (101.95 to 160.39)	2.75 (2.18 to 3.10)	5.66 (4.54 to 6.82)	5.40 (4.26 to 6.61)	5.92 (4.73 to 7.08)
Ovarian cancer	154.17 (129.04 to 194.50)	278.06 (202.23 to 398.53)	254.22 (187.93 to 353.74)	257.50 (187.65 to 369.91)	5.13 (4.30 to 6.45)	11.12 (8.22 to 15.76)	10.29 (7.75 to 14.22)	10.11 (7.42 to 14.47)
Prostate cancer	124.72 (79.54 to 150.93)	434.58 (276.86 to 526.91)	435.83 (281.39 to 530.32)	390.01 (245.59 to 469.16)	7.34 (4.64 to 8.85)	30.36 (19.42 to 36.48)	31.39 (20.32 to 37.65)	26.36 (16.47 to 31.98)
Testicular cancer	10.69 (9.37 to 14.78)	11.72 (9.34 to 16.44)	10.64 (8.49 to 15.23)	12.19 (9.76 to 17.39)	0.25 (0.22 to 0.34)	0.36 (0.29 to 0.49)	0.33 (0.27 to 0.47)	0.36 (0.30 to 0.50)

Indonesia								
Cause	YLLs (in 1000s)				Deaths (in 1000s)			
	2016	2040 Forecast	2040 Better Scenario	2040 Worse Scenario	2016	2040 Forecast	2040 Better Scenario	2040 Worse Scenario
Kidney cancer	60.47 (51.88 to 72.46)	121.82 (98.99 to 153.05)	124.70 (100.64 to 157.02)	111.31 (89.77 to 140.03)	2.11 (1.80 to 2.53)	5.59 (4.54 to 7.00)	5.90 (4.81 to 7.44)	4.93 (3.98 to 6.20)
Bladder cancer	62.07 (51.22 to 71.61)	136.53 (98.78 to 184.45)	128.90 (94.47 to 171.15)	135.04 (98.79 to 183.21)	2.93 (2.43 to 3.41)	8.11 (5.95 to 11.01)	7.92 (5.92 to 10.52)	7.71 (5.62 to 10.61)
Brain and nervous system cancer	212.51 (153.28 to 295.91)	339.39 (241.66 to 467.46)	325.71 (230.90 to 446.89)	327.57 (233.06 to 447.26)	5.40 (3.82 to 7.43)	11.71 (8.20 to 15.77)	11.61 (8.13 to 15.72)	10.91 (7.63 to 14.73)
Thyroid cancer	40.56 (35.42 to 46.86)	72.16 (55.74 to 93.88)	64.10 (50.20 to 81.87)	75.08 (58.51 to 97.85)	1.58 (1.38 to 1.82)	3.42 (2.73 to 4.31)	3.13 (2.50 to 3.91)	3.46 (2.78 to 4.42)
Mesothelioma	14.09 (12.58 to 18.27)	20.98 (17.81 to 27.42)	21.62 (18.10 to 28.09)	20.67 (17.74 to 27.02)	0.44 (0.39 to 0.57)	0.85 (0.73 to 1.09)	0.90 (0.77 to 1.17)	0.81 (0.70 to 1.02)
Hodgkin lymphoma	35.66 (29.13 to 50.78)	35.81 (26.65 to 52.02)	30.74 (22.74 to 44.55)	40.83 (30.59 to 59.68)	0.96 (0.79 to 1.37)	1.26 (0.94 to 1.81)	1.11 (0.83 to 1.59)	1.40 (1.06 to 2.01)
Non-Hodgkin lymphoma	204.99 (183.60 to 251.76)	345.16 (289.63 to 433.82)	319.20 (266.10 to 402.40)	326.06 (275.24 to 403.48)	6.10 (5.46 to 7.44)	13.96 (11.97 to 17.13)	13.35 (11.39 to 16.61)	12.74 (11.01 to 15.68)
Multiple myeloma	40.71 (33.10 to 51.80)	87.75 (66.47 to 114.62)	84.02 (64.11 to 108.87)	80.84 (61.65 to 106.13)	1.50 (1.22 to 1.91)	3.93 (3.06 to 5.10)	3.86 (3.00 to 4.95)	3.53 (2.73 to 4.65)
Leukemia	152.93 (139.22 to 198.65)	217.45 (181.26 to 262.02)	194.68 (162.18 to 234.35)	219.27 (183.13 to 267.27)	4.09 (3.75 to 4.70)	8.33 (6.95 to 9.94)	7.73 (6.43 to 9.09)	8.14 (6.78 to 9.74)
Acute lymphoid leukemia	45.22 (37.76 to 69.51)	63.18 (51.05 to 84.19)	56.23 (45.51 to 74.01)	61.55 (49.34 to 81.69)	1.15 (0.98 to 1.50)	2.25 (1.83 to 2.90)	2.07 (1.70 to 2.66)	2.13 (1.73 to 2.75)
Chronic lymphoid leukemia	3.06 (2.59 to 3.78)	4.66 (3.61 to 6.02)	4.34 (3.41 to 5.52)	4.62 (3.55 to 5.96)	0.10 (0.08 to 0.11)	0.19 (0.15 to 0.24)	0.18 (0.14 to 0.23)	0.18 (0.14 to 0.23)
Acute myeloid leukemia	32.36 (27.83 to 45.56)	53.75 (43.58 to 70.17)	50.47 (41.24 to 65.11)	51.05 (41.46 to 67.22)	0.78 (0.69 to 0.99)	1.72 (1.40 to 2.19)	1.66 (1.36 to 2.10)	1.59 (1.29 to 2.04)
Chronic myeloid leukemia	11.28 (8.70 to 13.98)	11.26 (8.25 to 15.19)	8.90 (6.40 to 12.05)	11.56 (8.38 to 15.50)	0.32 (0.24 to 0.39)	0.39 (0.29 to 0.52)	0.31 (0.23 to 0.42)	0.39 (0.29 to 0.52)
Other leukemia	61.00 (51.91 to 74.73)	84.59 (66.61 to 106.76)	74.73 (58.20 to 93.16)	90.49 (70.66 to 113.25)	1.76 (1.49 to 2.01)	3.79 (2.78 to 4.65)	3.50 (2.57 to 4.28)	3.86 (2.94 to 4.76)
Other neoplasms	369.67 (336.39 to 414.34)	557.88 (488.08 to 632.03)	556.04 (482.10 to 636.63)	542.66 (480.97 to 611.07)	10.66 (9.59 to 11.88)	22.37 (20.00 to 24.89)	23.12 (20.42 to 26.09)	20.91 (18.80 to 23.28)
**Cardiovascular diseases**	**12,844.18 (12,258.74 to 13,530.03)**	**15,387.90 (10,292.50 to 22,760.89)**	**9,617.47 (6,602.93 to 14,032.17)**	**23,763.87 (15,818.81 to 35,287.50)**	**558.74 (531.17 to 589.98)**	**876.53 (627.90 to 1,236.03)**	**589.93 (428.64 to 814.77)**	**1,252.09 (890.73 to 1,766.80)**
Rheumatic heart disease	267.20 (232.94 to 304.41)	190.09 (151.72 to 240.19)	166.50 (131.59 to 211.04)	226.08 (180.37 to 287.72)	7.99 (6.87 to 9.18)	8.07 (6.48 to 9.88)	7.33 (5.85 to 9.06)	9.23 (7.44 to 11.38)
Ischemic heart disease	6,262.91 (5,929.31 to 6,629.48)	7,557.07 (4,008.73 to 13,776.85)	4,262.99 (2,296.87 to 7,741.63)	12,243.91 (6,510.12 to 21,626.96)	273.57 (258.72 to 290.33)	420.86 (236.35 to 728.97)	258.23 (150.20 to 432.98)	633.19 (354.38 to 1,091.91)
Stroke	4,770.94 (4,516.24 to 5,044.50)	4,669.97 (2,980.26 to 7,462.08)	2,721.48 (1,780.31 to 4,174.88)	7,918.39 (4,798.70 to 12,726.16)	212.96 (200.34 to 227.12)	281.19 (190.22 to 446.22)	180.50 (126.92 to 275.16)	427.92 (281.01 to 671.36)
Ischemic stroke	1,171.81 (1,037.73 to 1,322.12)	1,490.34 (831.21 to 3,033.60)	935.72 (558.25 to 1,785.17)	2,202.49 (1,203.78 to 4,363.68)	70.28 (62.58 to 79.27)	111.57 (64.38 to 246.69)	76.19 (45.98 to 157.78)	151.25 (86.02 to 322.59)

Intracerebral hemorrhage	3,599.13 (3,386.46 to 3,836.05)	3,179.63 (1,868.77 to 5,097.36)	1,785.76 (1,099.18 to 2,847.82)	5,715.90 (3,152.61 to 9,651.78)	142.69 (133.37 to 152.97)	169.61 (109.05 to 265.66)	104.31 (70.11 to 156.05)	276.67 (167.91 to 442.56)
Hypertensive heart disease	644.92 (481.25 to 744.77)	1,351.03 (905.12 to 2,073.38)	1,071.84 (741.20 to 1,492.61)	1,658.05 (1,033.92 to 2,840.94)	30.18 (22.66 to 34.51)	80.01 (54.13 to 124.69)	65.83 (46.29 to 92.70)	94.61 (59.24 to 162.34)
Cardiomyopathy and myocarditis	246.53 (180.24 to 281.73)	370.33 (244.30 to 493.06)	317.75 (210.06 to 426.59)	368.41 (245.38 to 492.00)	8.10 (5.98 to 9.25)	16.98 (11.19 to 22.98)	15.06 (9.97 to 20.56)	16.19 (10.81 to 22.06)
Myocarditis	44.54 (34.24 to 56.01)	45.18 (33.71 to 57.66)	44.36 (33.31 to 56.80)	46.89 (35.23 to 59.57)	1.11 (0.85 to 1.37)	1.61 (1.23 to 2.03)	1.66 (1.27 to 2.09)	1.59 (1.21 to 1.97)

Indonesia								
Cause	YLLs (in 1000s)				Deaths (in 1000s)			
	2016	2040 Forecast	2040 Better Scenario	2040 Worse Scenario	2016	2040 Forecast	2040 Better Scenario	2040 Worse Scenario
Alcoholic cardiomyopathy	41.47 (18.39 to 57.80)	55.12 (24.88 to 80.78)	53.54 (23.42 to 79.64)	52.78 (24.35 to 75.81)	1.39 (0.67 to 1.86)	2.30 (1.13 to 3.20)	2.28 (1.08 to 3.24)	2.15 (1.08 to 2.92)
Other cardiomyopathy	160.53 (114.48 to 190.16)	270.03 (166.72 to 378.65)	219.86 (135.65 to 316.71)	268.74 (162.71 to 380.65)	5.61 (4.08 to 6.53)	13.07 (8.14 to 18.76)	11.12 (6.92 to 16.40)	12.44 (7.70 to 18.02)
Atrial fibrillation and flutter	53.68 (42.70 to 66.71)	171.66 (130.29 to 222.67)	165.26 (126.10 to 213.15)	165.23 (123.45 to 218.24)	4.19 (3.33 to 5.20)	15.21 (11.33 to 19.36)	15.19 (11.52 to 19.34)	14.01 (10.31 to 18.16)
Aortic aneurysm	81.17 (68.14 to 104.48)	151.58 (114.30 to 204.00)	136.40 (102.79 to 180.51)	148.56 (110.62 to 200.75)	3.75 (3.12 to 4.81)	8.98 (6.94 to 11.96)	8.44 (6.57 to 11.08)	8.44 (6.48 to 11.28)
Peripheral artery disease	3.11 (2.48 to 4.12)	13.89 (9.85 to 19.94)	12.40 (9.02 to 17.79)	12.19 (8.51 to 17.34)	0.20 (0.16 to 0.26)	1.06 (0.77 to 1.48)	1.00 (0.74 to 1.40)	0.88 (0.64 to 1.20)
Endocarditis	153.46 (131.97 to 201.61)	231.89 (165.33 to 330.02)	219.35 (157.75 to 308.24)	240.26 (170.45 to 347.15)	4.42 (3.79 to 5.74)	9.02 (6.47 to 12.85)	8.88 (6.38 to 12.67)	8.98 (6.37 to 13.00)
Other cardiovascular and circulatory diseases	360.26 (290.75 to 573.91)	680.39 (477.24 to 1,065.95)	543.49 (389.72 to 852.15)	782.79 (540.89 to 1,224.17)	13.38 (10.77 to 21.34)	35.17 (24.96 to 54.21)	29.45 (21.46 to 45.81)	38.63 (27.15 to 60.50)
**Chronic respiratory diseases**	**1,832.92 (1,721.66 to 1,946.62)**	**2,151.10 (1,753.23 to 2,669.20)**	**1,660.62 (1,376.76 to 2,000.11)**	**2,742.05 (2,138.01 to 3,556.85)**	**86.16 (80.36 to 91.82)**	**131.88 (107.33 to 160.23)**	**108.06 (90.85 to 127.28)**	**160.07 (125.03 to 203.92)**
Chronic obstructive pulmonary disease	947.07 (774.53 to 1,097.92)	1,337.19 (941.97 to 1,805.97)	1,051.37 (797.34 to 1,343.88)	1,850.01 (1,274.04 to 2,587.60)	49.93 (41.91 to 57.54)	87.41 (63.70 to 114.04)	73.16 (56.26 to 91.50)	113.32 (79.94 to 155.63)
Pneumoconiosis	1.61 (1.33 to 2.01)	1.97 (1.59 to 2.44)	1.78 (1.44 to 2.21)	1.98 (1.61 to 2.44)	0.08 (0.06 to 0.09)	0.13 (0.10 to 0.16)	0.12 (0.10 to 0.15)	0.13 (0.10 to 0.15)
Silicosis	0.28 (0.16 to 0.44)	0.25 (0.13 to 0.39)	0.19 (0.10 to 0.30)	0.25 (0.14 to 0.39)	0.01 (0.01 to 0.02)	0.02 (0.01 to 0.02)	0.01 (0.01 to 0.02)	0.02 (0.01 to 0.02)
Asbestosis	0.41 (0.31 to 0.52)	0.57 (0.42 to 0.77)	0.64 (0.48 to 0.85)	0.54 (0.40 to 0.72)	0.01 (0.01 to 0.02)	0.03 (0.02 to 0.04)	0.03 (0.02 to 0.05)	0.03 (0.02 to 0.04)
Coal workers pneumoconiosis	0.12 (0.07 to 0.23)	0.13 (0.08 to 0.24)	0.10 (0.06 to 0.18)	0.15 (0.10 to 0.29)	0.01 (0.00 to 0.01)	0.01 (0.01 to 0.02)	0.01 (0.00 to 0.01)	0.01 (0.01 to 0.02)
Other pneumoconiosis	0.81 (0.65 to 1.06)	1.03 (0.78 to 1.36)	0.85 (0.62 to 1.15)	1.03 (0.79 to 1.36)	0.04 (0.03 to 0.05)	0.07 (0.06 to 0.10)	0.06 (0.05 to 0.08)	0.07 (0.05 to 0.10)
Asthma	799.25 (633.30 to 985.54)	673.11 (425.34 to 1,003.96)	449.23 (293.59 to 661.37)	764.01 (483.80 to 1,139.10)	32.92 (25.10 to 41.27)	36.93 (23.73 to 54.14)	25.98 (17.10 to 36.50)	40.24 (25.15 to 59.50)
Interstitial lung disease and pulmonary sarcoidosis	46.89 (35.30 to 63.12)	92.43 (72.16 to 124.34)	111.63 (86.65 to 150.99)	79.73 (62.83 to 106.39)	2.01 (1.55 to 2.70)	5.08 (4.02 to 6.88)	6.31 (4.99 to 8.46)	4.24 (3.37 to 5.61)
Other chronic respiratory diseases	38.10 (22.05 to 51.66)	46.40 (25.97 to 65.44)	46.60 (26.08 to 66.20)	46.32 (26.31 to 64.96)	1.23 (0.68 to 1.75)	2.34 (1.16 to 3.42)	2.49 (1.22 to 3.63)	2.14 (1.08 to 3.15)
**Cirrhosis and other chronic liver diseases**	**1,691.11 (1,540.67 to 1,836.59)**	**2,404.95 (2,073.30 to 2,750.18)**	**2,220.68 (1,886.70 to 2,563.30)**	**2,517.67 (2,213.57 to 2,834.78)**	**53.28 (48.56 to 57.71)**	**98.54 (86.78 to 110.69)**	**93.42 (80.95 to 106.66)**	**100.00 (88.82 to 110.88)**
Cirrhosis and other chronic liver diseases due to hepatitis B	430.49 (378.68 to 492.12)	649.34 (534.97 to 773.89)	604.17 (490.93 to 722.26)	671.14 (557.73 to 789.89)	13.99 (12.30 to 15.90)	26.81 (22.35 to 31.09)	25.57 (20.99 to 29.93)	26.92 (22.63 to 30.94)
Cirrhosis and other chronic liver diseases due to hepatitis C	337.28 (296.90 to 383.87)	514.81 (429.78 to 606.00)	474.55 (390.17 to 567.37)	535.66 (451.99 to 623.94)	10.80 (9.58 to 12.24)	20.62 (17.62 to 23.76)	19.43 (16.19 to 22.84)	20.90 (18.01 to 24.28)

Cirrhosis and other chronic liver diseases due to alcohol use	375.62 (322.68 to 431.23)	527.90 (431.47 to 631.01)	485.24 (396.61 to 594.26)	561.49 (467.39 to 666.94)	12.50 (10.76 to 14.34)	22.16 (18.42 to 25.88)	20.86 (17.17 to 24.86)	22.90 (19.10 to 26.51)
Cirrhosis and other chronic liver diseases due to other causes	547.72 (479.92 to 615.29)	712.90 (600.70 to 845.30)	656.71 (540.90 to 786.21)	749.39 (630.21 to 884.33)	15.99 (14.02 to 18.11)	28.95 (24.52 to 34.14)	27.57 (22.79 to 32.92)	29.29 (24.69 to 34.55)
**Digestive diseases**	**1,797.14 (1,496.31 to 1,933.47)**	**2,163.57 (1,715.38 to 2,532.67)**	**1,945.26 (1,523.63 to 2,296.41)**	**2,353.76 (1,862.98 to 2,760.94)**	**66.39 (54.28 to 71.69)**	**118.36 (90.41 to 137.75)**	**111.05 (85.13 to 130.26)**	**122.69 (94.09 to 143.53)**
Peptic ulcer disease	501.05 (388.65 to 575.98)	498.61 (343.82 to 655.75)	391.33 (261.02 to 516.48)	593.06 (409.30 to 776.59)	21.75 (16.34 to 25.04)	29.58 (19.54 to 38.67)	24.02 (15.39 to 31.67)	33.87 (22.41 to 44.54)

Indonesia								
Cause	YLLs (in 1000s)				Deaths (in 1000s)			
	2016	2040 Forecast	2040 Better Scenario	2040 Worse Scenario	2016	2040 Forecast	2040 Better Scenario	2040 Worse Scenario
Gastritis and duodenitis	31.61 (26.19 to 37.23)	55.84 (43.58 to 69.65)	50.95 (39.64 to 65.19)	58.66 (46.06 to 73.32)	1.33 (1.10 to 1.56)	3.26 (2.50 to 4.05)	3.09 (2.35 to 3.90)	3.28 (2.53 to 4.12)
Appendicitis	110.34 (90.74 to 146.65)	97.06 (75.15 to 127.88)	81.04 (61.66 to 107.30)	111.63 (87.22 to 145.52)	3.02 (2.43 to 3.90)	4.03 (3.10 to 5.06)	3.53 (2.68 to 4.50)	4.41 (3.46 to 5.54)
Paralytic ileus and intestinal obstruction	504.07 (367.75 to 585.63)	586.72 (438.13 to 706.04)	590.49 (440.12 to 712.81)	595.87 (443.52 to 722.17)	15.39 (11.63 to 17.99)	31.59 (24.19 to 37.96)	33.40 (25.47 to 40.28)	30.11 (22.78 to 36.36)
Inguinal, femoral, and abdominal hernia	80.00 (35.89 to 105.83)	103.78 (42.17 to 144.46)	98.30 (38.86 to 138.66)	106.63 (44.18 to 148.89)	3.80 (1.56 to 5.22)	7.15 (2.56 to 10.45)	7.12 (2.52 to 10.41)	6.92 (2.50 to 10.16)
Inflammatory bowel disease	30.71 (25.31 to 39.79)	47.28 (37.10 to 61.14)	47.90 (36.91 to 61.92)	46.00 (36.34 to 59.35)	1.11 (0.89 to 1.38)	2.48 (1.83 to 3.10)	2.61 (1.94 to 3.26)	2.31 (1.72 to 2.89)
Vascular intestinal disorders	54.69 (46.20 to 68.49)	105.04 (88.58 to 126.99)	104.31 (86.06 to 127.22)	104.87 (87.06 to 127.37)	2.88 (2.45 to 3.43)	7.15 (5.85 to 8.60)	7.33 (5.97 to 8.93)	6.85 (5.49 to 8.24)
Gallbladder and biliary diseases	90.94 (69.12 to 103.63)	144.10 (86.35 to 223.39)	105.74 (67.01 to 156.52)	163.19 (96.06 to 255.34)	4.16 (3.01 to 4.76)	8.56 (4.91 to 13.32)	6.72 (4.08 to 9.80)	9.25 (5.30 to 14.43)
Pancreatitis	227.40 (174.05 to 269.31)	301.55 (205.62 to 423.37)	258.39 (175.47 to 354.09)	331.80 (227.38 to 484.58)	6.75 (4.95 to 8.03)	12.07 (7.90 to 16.90)	10.56 (6.81 to 14.51)	12.87 (8.34 to 18.06)
Other digestive diseases	166.34 (137.23 to 207.04)	223.60 (180.43 to 279.75)	216.81 (172.64 to 276.06)	242.03 (193.83 to 302.43)	6.21 (5.18 to 7.54)	12.50 (10.12 to 15.75)	12.67 (10.25 to 15.95)	12.83 (10.26 to 16.11)
**Neurological disorders**	**830.40 (733.13 to 947.96)**	**2,288.16 (1,844.55 to 2,946.60)**	**2,227.69 (1,848.66 to 2,745.38)**	**2,101.76 (1,662.76 to 2,717.80)**	**53.90 (46.32 to 63.57)**	**193.24 (149.03 to 252.73)**	**195.81 (159.93 to 240.92)**	**168.48 (125.63 to 222.25)**
Alzheimer's disease and other dementias	549.10 (460.53 to 662.18)	1,854.15 (1,409.27 to 2,486.21)	1,785.87 (1,419.31 to 2,275.05)	1,681.09 (1,248.12 to 2,293.49)	45.59 (38.31 to 54.90)	171.43 (128.50 to 231.44)	172.02 (136.47 to 218.02)	149.27 (107.31 to 204.28)
Parkinson's disease	52.78 (41.05 to 66.91)	194.39 (148.99 to 249.56)	215.77 (164.00 to 278.09)	162.92 (123.51 to 210.02)	3.49 (2.68 to 4.49)	14.99 (11.26 to 19.54)	17.08 (13.02 to 22.34)	12.16 (8.99 to 15.86)
Epilepsy	162.61 (145.60 to 178.11)	149.26 (122.16 to 178.38)	136.08 (110.88 to 163.65)	171.92 (143.98 to 205.67)	3.26 (2.88 to 3.62)	3.94 (3.25 to 4.65)	3.72 (3.06 to 4.45)	4.39 (3.66 to 5.18)
Multiple sclerosis	6.74 (5.59 to 9.85)	9.65 (7.46 to 13.85)	9.56 (7.38 to 13.47)	9.25 (7.16 to 13.30)	0.19 (0.16 to 0.27)	0.32 (0.25 to 0.46)	0.32 (0.25 to 0.45)	0.30 (0.24 to 0.43)
Motor neuron disease	11.66 (8.02 to 13.90)	20.21 (14.13 to 24.90)	20.37 (14.22 to 25.43)	18.90 (13.25 to 23.16)	0.33 (0.23 to 0.40)	0.73 (0.51 to 0.89)	0.75 (0.53 to 0.93)	0.67 (0.48 to 0.81)
Other neurological disorders	47.51 (38.78 to 57.29)	60.50 (49.29 to 71.56)	60.04 (48.94 to 70.99)	57.69 (46.80 to 68.92)	1.04 (0.88 to 1.18)	1.84 (1.57 to 2.10)	1.91 (1.63 to 2.19)	1.68 (1.44 to 1.91)
**Mental disorders**	**86.78 (62.54 to 97.68)**	**115.22 (83.69 to 139.68)**	**125.02 (90.61 to 152.21)**	**113.92 (82.93 to 136.59)**	**2.02 (1.48 to 2.28)**	**3.40 (2.34 to 4.03)**	**3.80 (2.61 to 4.57)**	**3.27 (2.28 to 3.83)**
Alcohol use disorders	42.66 (28.51 to 52.02)	39.62 (28.34 to 54.86)	41.57 (30.08 to 58.01)	39.76 (28.52 to 54.58)	1.03 (0.74 to 1.35)	1.21 (0.90 to 1.81)	1.31 (0.97 to 1.96)	1.19 (0.88 to 1.76)
Drug use disorders	43.24 (28.80 to 50.00)	74.69 (47.05 to 92.51)	82.50 (51.71 to 102.02)	73.28 (45.79 to 90.00)	0.98 (0.60 to 1.14)	2.17 (1.22 to 2.64)	2.47 (1.40 to 3.02)	2.06 (1.16 to 2.49)
Opioid use disorders	28.23 (18.77 to 32.84)	40.32 (25.14 to 50.96)	41.85 (26.01 to 53.11)	40.10 (25.05 to 50.77)	0.61 (0.38 to 0.71)	1.11 (0.64 to 1.38)	1.19 (0.68 to 1.49)	1.07 (0.61 to 1.32)

Cocaine use disorders	1.30 (0.80 to 2.11)	3.22 (1.89 to 5.19)	3.64 (2.14 to 5.92)	3.25 (1.94 to 5.25)	0.03 (0.02 to 0.05)	0.09 (0.05 to 0.15)	0.10 (0.06 to 0.17)	0.09 (0.05 to 0.14)
Amphetamine use disorders	1.16 (0.70 to 1.51)	3.82 (2.14 to 5.23)	4.62 (2.56 to 6.37)	3.81 (2.23 to 5.12)	0.03 (0.01 to 0.03)	0.09 (0.05 to 0.13)	0.12 (0.06 to 0.16)	0.09 (0.05 to 0.12)
Other drug use disorders	12.56 (6.87 to 14.96)	27.33 (15.88 to 35.53)	32.39 (18.89 to 41.79)	26.11 (15.15 to 33.39)	0.31 (0.16 to 0.37)	0.87 (0.44 to 1.12)	1.06 (0.54 to 1.35)	0.81 (0.40 to 1.02)
Eating disorders	0.88 (0.64 to 1.60)	0.91 (0.61 to 1.78)	0.95 (0.63 to 1.90)	0.88 (0.60 to 1.70)	0.02 (0.01 to 0.03)	0.02 (0.01 to 0.04)	0.02 (0.01 to 0.04)	0.02 (0.01 to 0.03)

Indonesia								
Cause	YLLs (in 1000s)				Deaths (in 1000s)			
	2016	2040 Forecast	2040 Better Scenario	2040 Worse Scenario	2016	2040 Forecast	2040 Better Scenario	2040 Worse Scenario
**Diabetes, urogenital, blood, and endocrine diseases**	**3,830.97 (3,630.65 to 4,018.13)**	**5,850.91 (4,294.95 to 8,065.22)**	**4,496.00 (3,341.44 to 6,145.07)**	**7,151.63 (5,219.91 to 9,884.45)**	**139.84 (132.97 to 147.03)**	**300.68 (218.58 to 421.48)**	**237.47 (176.37 to 327.29)**	**351.54 (255.91 to 487.21)**
Diabetes mellitus	2,300.77 (2,182.52 to 2,428.22)	2,918.61 (1,949.26 to 4,255.27)	2,346.09 (1,570.13 to 3,377.05)	3,799.09 (2,538.36 to 5,517.04)	89.43 (84.57 to 94.52)	150.70 (106.10 to 207.23)	125.47 (89.95 to 169.95)	186.69 (130.68 to 255.74)
Acute glomerulonephritis	1.93 (1.75 to 2.16)	2.29 (1.87 to 2.79)	2.04 (1.64 to 2.51)	2.51 (2.06 to 3.05)	0.05 (0.04 to 0.05)	0.08 (0.06 to 0.10)	0.07 (0.05 to 0.09)	0.08 (0.06 to 0.10)
Chronic kidney disease	1,056.17 (922.85 to 1,120.97)	2,248.26 (1,068.51 to 4,265.26)	1,527.88 (797.21 to 2,790.52)	2,636.08 (1,250.73 to 4,953.58)	35.22 (32.32 to 37.60)	116.28 (49.97 to 233.88)	80.08 (37.76 to 160.74)	131.35 (55.86 to 261.20)
Chronic kidney disease due to diabetes mellitus	585.38 (501.36 to 642.18)	1,324.03 (627.23 to 2,549.93)	890.81 (460.52 to 1,654.94)	1,555.68 (733.05 to 3,012.74)	19.41 (17.19 to 21.48)	62.71 (25.97 to 124.76)	42.37 (19.45 to 84.32)	71.60 (29.78 to 140.51)
Chronic kidney disease due to hypertension	248.82 (213.56 to 291.25)	679.00 (289.37 to 1,329.78)	464.20 (216.64 to 900.22)	783.25 (337.30 to 1,544.22)	10.80 (9.37 to 12.48)	43.52 (17.72 to 88.40)	30.79 (13.51 to 62.80)	48.11 (18.99 to 97.94)
Chronic kidney disease due to glomerulonephritis	116.10 (89.88 to 139.04)	113.90 (69.13 to 184.95)	80.95 (51.13 to 126.04)	139.16 (84.60 to 226.26)	2.43 (1.96 to 2.87)	4.07 (2.04 to 7.52)	2.78 (1.50 to 5.08)	4.79 (2.36 to 8.95)
Chronic kidney disease due to other and unspecified causes	105.87 (85.27 to 125.85)	131.33 (71.32 to 229.82)	91.93 (53.74 to 155.60)	157.98 (85.46 to 276.23)	2.57 (2.12 to 3.05)	5.97 (2.72 to 11.84)	4.13 (2.02 to 8.03)	6.84 (3.08 to 13.30)
Urinary diseases and male infertility	191.26 (179.17 to 205.78)	375.90 (326.44 to 427.26)	344.00 (291.66 to 398.79)	377.41 (334.55 to 424.88)	7.48 (6.98 to 8.05)	20.65 (17.78 to 23.54)	19.69 (16.86 to 22.87)	19.75 (17.15 to 22.36)
Urinary tract infections	137.03 (113.89 to 152.99)	292.82 (242.74 to 341.35)	271.34 (222.81 to 321.75)	288.12 (237.38 to 334.52)	5.29 (4.39 to 5.91)	16.00 (13.25 to 18.80)	15.43 (12.87 to 18.18)	15.00 (12.22 to 17.57)
Urolithiasis	7.83 (5.88 to 11.67)	18.41 (13.04 to 27.93)	15.68 (10.81 to 23.86)	18.66 (13.25 to 28.22)	0.32 (0.24 to 0.48)	0.94 (0.67 to 1.43)	0.83 (0.58 to 1.23)	0.92 (0.65 to 1.40)
Other urinary diseases	46.40 (32.92 to 71.55)	64.67 (43.65 to 102.80)	56.98 (36.99 to 91.19)	70.64 (48.11 to 110.30)	1.87 (1.35 to 2.83)	3.71 (2.52 to 5.71)	3.43 (2.25 to 5.37)	3.84 (2.59 to 5.87)
Gynecological diseases	51.83 (30.65 to 61.54)	47.34 (29.03 to 61.86)	36.43 (22.86 to 49.58)	55.37 (34.77 to 72.59)	1.36 (0.87 to 1.59)	1.49 (0.98 to 1.88)	1.18 (0.79 to 1.55)	1.69 (1.10 to 2.14)
Uterine fibroids	8.78 (4.85 to 12.28)	7.78 (3.61 to 12.54)	6.46 (3.10 to 10.26)	9.27 (4.41 to 14.48)	0.28 (0.16 to 0.39)	0.28 (0.14 to 0.43)	0.24 (0.12 to 0.36)	0.33 (0.16 to 0.50)
Polycystic ovarian syndrome	1.74 (0.35 to 3.74)	0.80 (0.15 to 1.95)	0.52 (0.10 to 1.24)	1.09 (0.21 to 2.56)	0.04 (0.01 to 0.09)	0.02 (0.00 to 0.05)	0.01 (0.00 to 0.03)	0.03 (0.01 to 0.06)
Endometriosis	0.68 (0.23 to 1.35)	0.71 (0.22 to 1.52)	0.55 (0.17 to 1.20)	0.84 (0.28 to 1.77)	0.02 (0.01 to 0.03)	0.02 (0.01 to 0.04)	0.01 (0.00 to 0.03)	0.02 (0.01 to 0.04)
Genital prolapse	1.55 (0.66 to 2.83)	1.76 (0.75 to 3.26)	1.39 (0.58 to 2.64)	2.11 (0.90 to 3.99)	0.08 (0.03 to 0.14)	0.10 (0.04 to 0.19)	0.08 (0.03 to 0.15)	0.11 (0.05 to 0.21)
Other gynecological diseases	39.08 (22.58 to 47.30)	36.29 (22.55 to 49.02)	27.52 (16.00 to 38.63)	42.06 (26.41 to 56.71)	0.95 (0.59 to 1.15)	1.07 (0.71 to 1.40)	0.84 (0.52 to 1.13)	1.20 (0.80 to 1.57)
Hemoglobinopathies and hemolytic anemias	139.08 (95.89 to 159.61)	158.09 (100.91 to 204.38)	139.10 (87.82 to 183.63)	174.17 (109.17 to 223.74)	4.24 (2.70 to 5.00)	7.76 (4.54 to 10.34)	7.07 (4.11 to 9.66)	8.25 (4.81 to 10.88)
Thalassemias	39.63 (28.45 to 47.44)	16.75 (11.10 to 23.19)	15.02 (10.15 to 20.37)	16.65 (10.81 to 23.36)	0.51 (0.37 to 0.60)	0.22 (0.15 to 0.30)	0.20 (0.14 to 0.27)	0.22 (0.15 to 0.30)

Sickle cell disorders	0.23 (0.15 to 0.29)	0.20 (0.12 to 0.27)	0.16 (0.10 to 0.23)	0.22 (0.14 to 0.29)	0.00 (0.00 to 0.01)	0.00 (0.00 to 0.01)	0.00 (0.00 to 0.01)	0.01 (0.00 to 0.01)
G6PD deficiency	49.84 (34.29 to 58.18)	73.89 (45.95 to 98.33)	62.54 (38.77 to 85.36)	85.20 (54.42 to 112.27)	1.27 (0.84 to 1.51)	2.68 (1.58 to 3.65)	2.30 (1.35 to 3.20)	3.02 (1.80 to 4.06)
Other hemoglobinopathies and hemolytic anemias	49.38 (31.06 to 58.53)	67.26 (38.86 to 91.72)	61.38 (35.49 to 85.23)	72.10 (41.98 to 97.74)	2.46 (1.46 to 2.99)	4.85 (2.69 to 6.63)	4.56 (2.53 to 6.40)	5.00 (2.78 to 6.87)
Endocrine, metabolic, blood, and immune disorders	89.92 (62.31 to 99.83)	100.41 (81.90 to 119.84)	100.46 (82.75 to 121.36)	107.00 (86.18 to 126.08)	2.07 (1.67 to 2.34)	3.72 (3.20 to 4.81)	3.92 (3.34 to 5.11)	3.73 (3.20 to 4.73)

Indonesia								
Cause	YLLs (in 1000s)				Deaths (in 1000s)			
	2016	2040 Forecast	2040 Better Scenario	2040 Worse Scenario	2016	2040 Forecast	2040 Better Scenario	2040 Worse Scenario
**Musculoskeletal disorders**	**53.88 (46.57 to 75.88)**	**90.82 (75.23 to 130.46)**	**85.71 (70.57 to 122.33)**	**86.76 (72.76 to 123.42)**	**1.48 (1.27 to 2.16)**	**3.62 (2.99 to 5.45)**	**3.52 (2.88 to 5.34)**	**3.35 (2.77 to 4.97)**
Rheumatoid arthritis	5.32 (4.11 to 7.24)	13.45 (9.01 to 19.83)	10.89 (7.15 to 16.11)	14.71 (10.06 to 21.42)	0.23 (0.18 to 0.33)	0.75 (0.51 to 1.11)	0.62 (0.42 to 0.93)	0.79 (0.55 to 1.15)
Other musculoskeletal disorders	48.57 (41.57 to 69.60)	77.37 (63.61 to 115.13)	74.82 (61.42 to 110.67)	72.05 (59.73 to 105.72)	1.24 (1.06 to 1.84)	2.87 (2.35 to 4.50)	2.90 (2.37 to 4.59)	2.56 (2.08 to 3.98)
**Other non-communicable diseases**	**1,365.61 (1,057.51 to 1,540.80)**	**847.42 (646.32 to 1,044.38)**	**644.49 (481.16 to 788.74)**	**1,003.37 (764.15 to 1,248.23)**	**18.38 (14.50 to 20.63)**	**19.44 (13.81 to 23.60)**	**16.98 (11.41 to 20.78)**	**20.52 (14.78 to 24.75)**
Congenital birth defects	1,221.10 (906.87 to 1,387.14)	609.76 (440.46 to 796.42)	423.56 (308.03 to 544.65)	763.57 (546.25 to 1,011.94)	14.73 (11.03 to 16.69)	7.86 (5.80 to 10.07)	5.54 (4.11 to 7.01)	9.73 (7.18 to 12.71)
Neural tube defects	97.73 (60.52 to 130.92)	20.01 (9.98 to 35.41)	12.94 (6.24 to 22.57)	28.54 (14.34 to 50.50)	1.14 (0.71 to 1.52)	0.24 (0.12 to 0.41)	0.15 (0.08 to 0.26)	0.34 (0.17 to 0.59)
Congenital heart anomalies	644.38 (470.91 to 753.02)	330.22 (232.83 to 443.66)	220.78 (149.85 to 297.21)	411.68 (292.06 to 561.72)	7.85 (5.79 to 9.11)	4.29 (3.10 to 5.63)	2.90 (2.03 to 3.83)	5.28 (3.81 to 7.08)
Orofacial clefts	1.58 (0.43 to 2.91)	0.63 (0.14 to 1.32)	0.38 (0.09 to 0.80)	0.95 (0.21 to 2.01)	0.02 (0.00 to 0.03)	0.01 (0.00 to 0.02)	0.00 (0.00 to 0.01)	0.01 (0.00 to 0.02)
Down syndrome	22.97 (17.34 to 35.77)	14.60 (10.24 to 23.32)	13.78 (9.77 to 21.63)	17.86 (12.32 to 28.68)	0.31 (0.24 to 0.47)	0.24 (0.18 to 0.37)	0.23 (0.17 to 0.35)	0.29 (0.21 to 0.45)
Other chromosomal abnormalities	37.22 (24.04 to 65.36)	26.88 (15.26 to 51.41)	21.38 (12.20 to 40.71)	34.42 (19.04 to 66.17)	0.44 (0.28 to 0.76)	0.32 (0.19 to 0.61)	0.26 (0.15 to 0.48)	0.41 (0.24 to 0.78)
Congenital musculoskeletal and limb anomalies	24.59 (14.89 to 34.12)	14.95 (7.88 to 23.52)	10.53 (5.43 to 16.42)	18.35 (9.63 to 29.05)	0.29 (0.18 to 0.40)	0.18 (0.10 to 0.28)	0.13 (0.07 to 0.19)	0.22 (0.12 to 0.34)
Urogenital congenital anomalies	16.40 (11.48 to 20.33)	12.55 (9.31 to 16.31)	9.90 (7.33 to 12.96)	14.46 (10.85 to 18.95)	0.25 (0.19 to 0.31)	0.27 (0.21 to 0.35)	0.22 (0.17 to 0.29)	0.30 (0.23 to 0.39)
Digestive congenital anomalies	76.78 (55.18 to 107.52)	36.49 (23.42 to 55.86)	24.77 (15.53 to 37.88)	47.32 (29.94 to 74.48)	0.90 (0.65 to 1.25)	0.43 (0.28 to 0.66)	0.29 (0.18 to 0.45)	0.56 (0.36 to 0.87)
Other congenital birth defects	299.45 (187.78 to 377.28)	153.41 (87.77 to 215.22)	109.11 (62.11 to 151.93)	189.98 (109.39 to 269.41)	3.55 (2.25 to 4.46)	1.90 (1.14 to 2.63)	1.36 (0.81 to 1.87)	2.33 (1.39 to 3.26)
Skin and subcutaneous diseases	81.24 (42.31 to 106.81)	214.58 (117.09 to 283.95)	203.73 (110.83 to 267.42)	210.90 (114.84 to 279.88)	2.92 (1.53 to 3.74)	11.30 (6.25 to 14.88)	11.23 (6.22 to 14.69)	10.45 (5.76 to 13.75)
Cellulitis	10.51 (4.72 to 16.76)	35.91 (16.54 to 57.52)	36.22 (16.71 to 57.48)	32.52 (14.72 to 52.78)	0.34 (0.16 to 0.55)	1.73 (0.81 to 2.87)	1.82 (0.87 to 3.00)	1.49 (0.70 to 2.46)
Pyoderma	44.53 (22.48 to 59.47)	114.21 (58.60 to 160.41)	107.71 (55.28 to 149.02)	117.44 (59.88 to 164.75)	1.30 (0.65 to 1.81)	5.19 (2.70 to 7.45)	5.17 (2.72 to 7.35)	5.04 (2.66 to 7.17)
Decubitus ulcer	23.92 (11.35 to 32.07)	60.01 (28.39 to 82.20)	55.63 (26.14 to 77.31)	56.69 (26.93 to 76.98)	1.21 (0.58 to 1.56)	4.21 (1.91 to 5.72)	4.07 (1.80 to 5.59)	3.75 (1.69 to 5.15)
Other skin and subcutaneous diseases	2.27 (1.05 to 2.91)	4.45 (2.02 to 5.98)	4.16 (1.85 to 5.62)	4.25 (1.89 to 5.73)	0.07 (0.03 to 0.09)	0.18 (0.08 to 0.25)	0.18 (0.08 to 0.24)	0.17 (0.07 to 0.23)
Sudden infant death syndrome	63.26 (42.27 to 94.76)	23.09 (12.42 to 43.01)	17.21 (9.25 to 31.75)	28.90 (15.08 to 53.45)	0.73 (0.49 to 1.10)	0.27 (0.14 to 0.50)	0.20 (0.11 to 0.37)	0.34 (0.17 to 0.62)

**Injuries**	**4,449.99 (4,216.09 to 4,703.04)**	**3,624.39 (2,903.27 to 6,406.24)**	**3,265.71 (2,554.42 to 6,095.08)**	**5,167.88 (4,255.53 to 8,089.80)**	**98.28 (93.55 to 103.23)**	**118.33 (99.37 to 178.58)**	**111.63 (92.65 to 173.10)**	**156.46 (134.72 to 218.60)**
**Transport injuries**	**2,056.21 (1,915.59 to 2,274.74)**	**879.02 (748.45 to 1,057.21)**	**803.82 (676.43 to 964.21)**	**2,302.17 (1,976.13 to 2,720.06)**	**44.30 (41.45 to 48.38)**	**29.56 (25.89 to 34.53)**	**27.97 (24.10 to 32.79)**	**67.17 (59.65 to 77.57)**
Road injuries	1,911.14 (1,772.27 to 2,128.95)	666.55 (549.63 to 831.13)	631.08 (520.27 to 782.64)	2,078.83 (1,773.81 to 2,480.93)	41.18 (38.29 to 46.02)	23.19 (19.81 to 28.28)	22.56 (19.23 to 27.52)	60.72 (53.60 to 70.50)
Pedestrian road injuries	421.84 (343.86 to 622.54)	178.96 (143.92 to 259.43)	147.69 (114.33 to 217.23)	471.44 (370.39 to 708.42)	10.58 (8.74 to 15.32)	8.04 (6.49 to 11.01)	6.96 (5.47 to 9.90)	18.36 (14.79 to 25.96)

Indonesia								
Cause	YLLs (in 1000s)				Deaths (in 1000s)			
	2016	2040 Forecast	2040 Better Scenario	2040 Worse Scenario	2016	2040 Forecast	2040 Better Scenario	2040 Worse Scenario
Cyclist road injuries	82.34 (51.39 to 132.53)	41.69 (24.36 to 73.80)	42.27 (25.24 to 75.55)	107.47 (64.69 to 186.08)	2.04 (1.26 to 3.31)	1.71 (1.00 to 3.07)	1.79 (1.06 to 3.22)	3.57 (2.12 to 6.11)
Motorcyclist road injuries	520.22 (412.50 to 689.50)	215.52 (152.26 to 306.40)	211.30 (150.69 to 297.57)	499.93 (364.34 to 701.41)	9.69 (7.57 to 12.90)	5.24 (3.73 to 7.48)	5.25 (3.77 to 7.37)	10.71 (7.86 to 14.89)
Motor vehicle road injuries	851.17 (666.95 to 1,003.12)	219.01 (156.73 to 286.85)	218.32 (158.51 to 284.15)	957.33 (722.97 to 1,205.37)	18.07 (14.02 to 21.27)	7.70 (5.55 to 9.76)	8.02 (5.88 to 10.12)	26.73 (20.33 to 33.00)
Other road injuries	35.56 (26.05 to 56.35)	11.37 (7.51 to 19.83)	11.51 (7.74 to 19.87)	42.66 (29.41 to 70.88)	0.80 (0.58 to 1.29)	0.50 (0.32 to 0.91)	0.53 (0.35 to 0.98)	1.36 (0.94 to 2.30)
Other transport injuries	145.07 (99.69 to 188.69)	212.47 (150.01 to 277.94)	172.74 (118.73 to 230.51)	223.35 (150.72 to 305.60)	3.12 (2.08 to 4.08)	6.37 (4.49 to 8.29)	5.41 (3.67 to 7.16)	6.45 (4.25 to 8.59)
**Unintentional injuries**	**1,735.20 (1,586.25 to 1,855.66)**	**1,697.06 (1,451.70 to 1,948.64)**	**1,508.77 (1,271.45 to 1,744.49)**	**1,807.83 (1,506.42 to 2,099.51)**	**40.53 (35.14 to 43.03)**	**64.89 (52.46 to 73.54)**	**61.53 (49.68 to 70.32)**	**65.43 (51.28 to 74.89)**
Falls	476.05 (424.28 to 509.07)	742.85 (618.65 to 843.14)	695.92 (576.41 to 799.27)	754.78 (619.09 to 863.33)	16.23 (13.03 to 17.58)	39.60 (30.33 to 45.85)	38.83 (29.42 to 45.26)	38.48 (28.14 to 45.45)
Drowning	466.86 (405.39 to 518.90)	281.92 (228.35 to 347.55)	238.40 (191.09 to 295.43)	313.26 (251.43 to 390.47)	7.83 (7.07 to 8.60)	6.20 (5.24 to 7.36)	5.47 (4.58 to 6.48)	6.69 (5.58 to 7.99)
Fire, heat, and hot substances	75.05 (59.14 to 82.28)	57.65 (42.66 to 70.40)	48.11 (35.52 to 59.86)	64.81 (47.32 to 79.31)	1.67 (1.31 to 1.84)	1.85 (1.41 to 2.18)	1.62 (1.22 to 1.96)	1.99 (1.51 to 2.36)
Poisonings	37.90 (20.42 to 45.32)	20.03 (9.58 to 26.23)	15.94 (7.58 to 21.63)	23.90 (11.37 to 31.66)	0.86 (0.41 to 1.04)	0.58 (0.25 to 0.74)	0.48 (0.20 to 0.65)	0.67 (0.28 to 0.87)
Exposure to mechanical forces	125.34 (90.79 to 138.78)	118.95 (79.58 to 146.63)	99.91 (64.33 to 126.01)	133.36 (87.38 to 165.35)	2.57 (1.76 to 2.83)	3.00 (1.95 to 3.63)	2.61 (1.65 to 3.22)	3.29 (2.10 to 3.99)
Unintentional firearm injuries	4.97 (4.47 to 5.93)	3.84 (2.99 to 4.94)	3.36 (2.64 to 4.29)	4.37 (3.37 to 5.65)	0.11 (0.10 to 0.12)	0.11 (0.09 to 0.13)	0.10 (0.08 to 0.12)	0.12 (0.10 to 0.15)
Unintentional suffocation	11.33 (8.87 to 15.97)	6.73 (5.18 to 8.95)	5.65 (4.34 to 7.30)	7.96 (6.01 to 10.98)	0.18 (0.15 to 0.22)	0.16 (0.10 to 0.21)	0.14 (0.08 to 0.20)	0.18 (0.11 to 0.24)
Other exposure to mechanical forces	109.04 (72.31 to 120.85)	108.39 (67.76 to 134.92)	90.89 (55.02 to 115.40)	121.02 (74.25 to 150.73)	2.29 (1.46 to 2.54)	2.74 (1.73 to 3.32)	2.36 (1.45 to 2.94)	3.00 (1.85 to 3.65)
Adverse effects of medical treatment	146.87 (117.85 to 170.00)	139.09 (106.03 to 180.16)	128.22 (97.02 to 167.05)	150.67 (112.80 to 193.52)	3.46 (2.72 to 4.12)	4.71 (3.64 to 5.97)	4.55 (3.52 to 5.82)	4.89 (3.73 to 6.25)
Animal contact	70.00 (63.39 to 77.67)	80.80 (64.26 to 100.60)	71.95 (56.74 to 89.48)	92.46 (73.37 to 114.96)	1.71 (1.49 to 1.89)	2.75 (2.16 to 3.33)	2.59 (2.01 to 3.15)	3.03 (2.36 to 3.71)
Venomous animal contact	42.63 (25.70 to 49.21)	48.51 (27.13 to 62.05)	44.35 (25.07 to 57.41)	55.28 (30.17 to 71.26)	1.08 (0.56 to 1.24)	1.72 (0.89 to 2.16)	1.65 (0.89 to 2.07)	1.89 (0.95 to 2.38)
Non-venomous animal contact	27.37 (22.68 to 40.00)	32.29 (24.11 to 46.25)	27.61 (20.31 to 40.11)	37.19 (27.59 to 53.99)	0.63 (0.50 to 0.90)	1.03 (0.73 to 1.41)	0.93 (0.64 to 1.29)	1.14 (0.81 to 1.57)
Foreign body	193.97 (137.19 to 268.26)	134.69 (98.62 to 181.79)	122.45 (90.79 to 162.72)	141.15 (101.10 to 198.34)	3.30 (2.45 to 4.31)	3.36 (2.57 to 4.19)	3.26 (2.49 to 4.04)	3.29 (2.48 to 4.18)
Pulmonary aspiration and foreign body in airway	176.77 (120.32 to 249.64)	121.34 (84.64 to 166.97)	109.01 (75.91 to 148.31)	126.65 (85.03 to 181.75)	2.94 (2.07 to 3.92)	2.98 (2.18 to 3.75)	2.86 (2.09 to 3.57)	2.90 (2.06 to 3.75)

Foreign body in other body part	17.20 (11.45 to 34.46)	13.36 (8.62 to 26.23)	13.44 (8.69 to 26.14)	14.50 (9.32 to 28.04)	0.35 (0.24 to 0.69)	0.38 (0.26 to 0.74)	0.40 (0.27 to 0.77)	0.39 (0.27 to 0.76)
Environmental heat and cold exposure	4.89 (3.39 to 5.57)	4.38 (3.17 to 5.61)	3.62 (2.56 to 4.71)	5.35 (3.83 to 6.88)	0.11 (0.08 to 0.13)	0.14 (0.11 to 0.19)	0.13 (0.09 to 0.16)	0.17 (0.13 to 0.21)
Other unintentional injuries	138.25 (77.00 to 157.52)	116.71 (64.04 to 150.64)	84.24 (45.74 to 112.30)	128.10 (69.56 to 162.88)	2.79 (1.52 to 3.17)	2.69 (1.54 to 3.41)	1.98 (1.13 to 2.60)	2.94 (1.63 to 3.63)
**Self-harm and interpersonal violence**	**640.85 (558.00 to 705.43)**	**733.98 (597.86 to 900.47)**	**642.87 (517.53 to 792.08)**	**742.08 (600.28 to 910.98)**	**13.06 (11.62 to 14.46)**	**17.16 (14.33 to 20.34)**	**15.39 (12.86 to 18.36)**	**17.18 (14.26 to 20.48)**

Indonesia								
Cause	YLLs (in 1000s)				Deaths (in 1000s)			
	2016	2040 Forecast	2040 Better Scenario	2040 Worse Scenario	2016	2040 Forecast	2040 Better Scenario	2040 Worse Scenario
Self-harm	401.94 (363.20 to 491.83)	520.00 (414.34 to 672.91)	460.70 (366.34 to 587.60)	528.96 (418.77 to 679.93)	8.58 (7.80 to 10.35)	12.57 (10.38 to 15.49)	11.39 (9.43 to 13.93)	12.64 (10.32 to 15.50)
Self-harm by firearm	23.78 (13.10 to 36.53)	25.40 (13.23 to 43.53)	21.51 (11.18 to 36.45)	26.41 (13.81 to 44.99)	0.52 (0.29 to 0.79)	0.65 (0.35 to 1.07)	0.57 (0.31 to 0.92)	0.66 (0.36 to 1.09)
Self-harm by other specified means	378.16 (336.77 to 463.17)	494.60 (394.50 to 635.42)	439.18 (347.75 to 559.82)	502.55 (396.76 to 650.55)	8.06 (7.17 to 9.70)	11.92 (9.87 to 14.74)	10.83 (8.94 to 13.38)	11.97 (9.80 to 14.71)
Interpersonal violence	238.91 (166.49 to 269.99)	213.97 (147.54 to 269.63)	182.18 (125.70 to 229.40)	213.12 (143.98 to 267.49)	4.49 (3.22 to 5.05)	4.59 (3.15 to 5.60)	4.00 (2.76 to 4.88)	4.55 (3.11 to 5.56)
Physical violence by firearm	13.24 (9.69 to 24.16)	11.90 (8.55 to 21.47)	9.92 (7.01 to 17.57)	11.94 (8.45 to 21.58)	0.26 (0.19 to 0.46)	0.27 (0.19 to 0.44)	0.23 (0.15 to 0.37)	0.27 (0.18 to 0.44)
Physical violence by sharp object	101.06 (45.98 to 121.21)	102.37 (46.92 to 137.95)	84.03 (36.97 to 113.74)	98.73 (43.68 to 132.39)	1.88 (0.86 to 2.24)	2.01 (0.93 to 2.63)	1.67 (0.75 to 2.22)	1.94 (0.87 to 2.54)
Physical violence by other means	124.61 (95.07 to 174.17)	99.70 (76.11 to 152.46)	88.22 (66.78 to 133.89)	102.45 (77.14 to 153.37)	2.34 (1.85 to 3.21)	2.31 (1.74 to 3.27)	2.10 (1.57 to 2.90)	2.34 (1.75 to 3.30)
**Forces of nature, conflict and terrorism, and executions and police conflict**	**17.74 (11.40 to 23.53)**	**314.34 (0.00 to 3,030.84)**	**310.24 (0.00 to 2,992.23)**	**315.80 (0.00 to 3,043.39)**	**0.38 (0.23 to 0.50)**	**6.74 (0.00 to 64.48)**	**6.74 (0.00 to 64.40)**	**6.68 (0.00 to 64.12)**
Exposure to forces of nature	11.60 (7.03 to 16.09)	50.00 (0.00 to 87.53)	48.88 (0.00 to 85.59)	50.62 (0.00 to 88.84)	0.22 (0.13 to 0.31)	1.14 (0.00 to 2.00)	1.14 (0.00 to 2.01)	1.12 (0.00 to 1.98)
Conflict and terrorism	0.00 (0.00 to 0.00)	7.44 (0.00 to 40.50)	7.26 (0.00 to 39.34)	7.56 (0.00 to 41.27)	0.00 (0.00 to 0.00)	0.15 (0.00 to 0.80)	0.15 (0.00 to 0.79)	0.15 (0.00 to 0.80)
Executions and police conflict	6.14 (1.59 to 8.61)	256.90 (0.00 to 3,030.84)	254.11 (0.00 to 2,992.23)	257.62 (0.00 to 3,043.39)	0.16 (0.03 to 0.21)	5.44 (0.00 to 64.48)	5.44 (0.00 to 64.40)	5.40 (0.00 to 64.12)

Laos								
Cause	YLLs (actual counts)				Deaths (actual counts)			
	2016	2040 Forecast	2040 Better Scenario	2040 Worse Scenario	2016	2040 Forecast	2040 Better Scenario	2040 Worse Scenario
**All causes**	**2,304,695.31 (1,662,986.58 to 3,221,033.44)**	**1,481,104.20 (1,245,362.69 to 1,775,425.39)**	**1,151,266.87 (983,332.62 to 1,368,970.13)**	**2,457,049.02 (1,959,329.91 to 3,098,415.34)**	**48,468.92 (40,805.46 to 59,296.61)**	**54,241.95 (48,889.09 to 59,993.24)**	**44,056.89 (39,657.71 to 49,154.63)**	**77,419.79 (68,008.08 to 87,911.95)**
**Communicable, maternal, neonatal, and nutritional diseases**	**1,167,983.19 (674,588.27 to 1,915,523.47)**	**354,220.84 (205,599.44 to 612,627.34)**	**258,746.89 (146,372.80 to 485,877.65)**	**792,172.62 (435,856.78 to 1,346,809.99)**	**16,168.12 (10,265.92 to 24,994.52)**	**7,625.46 (4,989.20 to 12,693.28)**	**5,636.64 (3,673.08 to 10,552.24)**	**14,140.55 (8,853.70 to 21,822.22)**
**HIV/AIDS and tuberculosis**	**71,968.11 (47,657.30 to 182,498.87)**	**57,538.51 (19,334.53 to 244,138.42)**	**45,856.58 (12,694.25 to 234,277.12)**	**88,746.26 (36,626.40 to 280,443.74)**	**1,906.83 (1,351.33 to 4,345.87)**	**1,697.77 (668.55 to 6,074.37)**	**1,346.80 (485.30 to 5,846.99)**	**2,624.87 (1,199.79 to 7,196.41)**
Tuberculosis	53,159.69 (41,953.09 to 66,049.82)	29,247.88 (15,424.33 to 51,425.17)	19,329.81 (10,961.71 to 32,582.82)	56,590.20 (28,811.70 to 98,996.47)	1,514.76 (1,244.65 to 1,919.95)	1,057.83 (581.26 to 1,792.81)	737.25 (431.42 to 1,185.85)	1,916.56 (998.94 to 3,275.71)
HIV/AIDS	18,808.42 (258.98 to 135,519.65)	28,290.63 (470.57 to 224,214.33)	26,526.77 (188.07 to 223,786.68)	32,156.06 (1,197.91 to 229,953.49)	392.08 (4.57 to 2,943.41)	639.94 (8.33 to 4,950.95)	609.55 (3.50 to 5,052.46)	708.31 (21.05 to 5,183.78)
**Diarrhea, lower respiratory, and other common infectious diseases**	**557,203.53 (314,585.94 to 915,113.84)**	**160,593.54 (89,384.12 to 268,913.05)**	**102,121.57 (59,737.02 to 166,946.83)**	**419,244.84 (203,276.89 to 784,636.28)**	**7,696.83 (4,815.67 to 11,817.18)**	**4,024.61 (2,515.45 to 6,093.91)**	**2,680.80 (1,750.55 to 3,992.00)**	**7,833.53 (4,473.85 to 12,590.71)**
Diarrheal diseases	81,745.49 (39,358.32 to 147,348.67)	17,506.21 (2,656.90 to 44,117.77)	10,510.27 (1,690.10 to 29,838.39)	33,649.78 (5,810.81 to 82,325.16)	1,175.52 (612.19 to 2,020.20)	511.89 (80.69 to 1,598.48)	336.25 (55.21 to 1,082.55)	881.17 (171.50 to 2,653.92)
Intestinal infectious diseases	29,812.08 (14,951.54 to 53,528.90)	12,010.34 (5,227.52 to 23,261.64)	10,625.66 (4,723.08 to 20,063.53)	16,628.31 (6,793.79 to 33,506.54)	416.13 (212.01 to 734.67)	185.79 (84.04 to 350.26)	166.98 (78.22 to 310.04)	249.16 (106.90 to 488.61)
Typhoid fever	25,898.86 (12,240.98 to 47,302.55)	10,187.70 (4,221.51 to 19,380.14)	9,026.84 (3,910.12 to 16,945.96)	14,026.16 (5,481.14 to 28,094.72)	360.56 (171.88 to 654.56)	156.59 (69.43 to 295.67)	140.94 (63.63 to 263.53)	208.91 (86.88 to 417.96)
Paratyphoid fever	3,614.48 (1,534.89 to 7,490.20)	1,786.25 (731.32 to 3,859.50)	1,567.73 (664.46 to 3,373.34)	2,535.72 (984.96 to 5,582.66)	51.73 (22.21 to 105.82)	28.50 (12.16 to 60.82)	25.42 (11.21 to 53.59)	39.12 (15.96 to 85.62)
Other intestinal infectious diseases	298.74 (82.38 to 795.65)	36.38 (8.14 to 109.60)	31.09 (6.80 to 95.52)	66.44 (12.61 to 201.67)	3.84 (1.14 to 9.75)	0.70 (0.20 to 1.82)	0.63 (0.18 to 1.65)	1.13 (0.31 to 3.10)
Lower respiratory infections	369,093.06 (197,085.13 to 623,692.56)	89,393.43 (41,321.42 to 174,589.76)	50,050.67 (25,833.37 to 94,168.63)	253,552.97 (77,814.75 to 583,210.00)	5,124.87 (3,118.39 to 8,040.68)	2,730.00 (1,680.68 to 4,225.33)	1,712.28 (1,134.02 to 2,552.59)	5,227.53 (2,628.79 to 9,381.25)
Upper respiratory infections	292.12 (82.92 to 876.40)	74.42 (23.86 to 193.68)	61.72 (20.01 to 156.13)	142.51 (42.35 to 404.44)	4.18 (1.60 to 11.53)	1.70 (0.68 to 3.59)	1.48 (0.58 to 3.17)	2.90 (1.09 to 6.66)
Otitis media	97.56 (32.75 to 325.26)	29.34 (9.94 to 67.26)	29.70 (9.96 to 68.73)	30.45 (10.25 to 71.94)	1.96 (0.82 to 4.59)	1.18 (0.37 to 2.78)	1.27 (0.39 to 2.98)	1.08 (0.35 to 2.55)
Meningitis	38,839.98 (16,340.09 to 74,511.71)	9,406.64 (3,396.71 to 18,563.64)	8,654.30 (3,306.25 to 16,675.70)	23,500.90 (5,131.66 to 57,269.15)	491.75 (226.16 to 904.30)	148.79 (74.54 to 263.91)	140.84 (74.08 to 241.12)	314.29 (94.13 to 712.56)
Pneumococcal meningitis	401.66 (132.93 to 890.79)	75.94 (26.35 to 174.32)	72.34 (26.56 to 160.97)	194.14 (47.36 to 530.75)	5.10 (1.92 to 10.74)	1.33 (0.64 to 2.51)	1.33 (0.67 to 2.39)	2.81 (1.00 to 6.74)
H influenzae type B meningitis	3,362.43 (1,307.99 to 6,590.63)	1,983.83 (281.12 to 6,770.05)	1,846.18 (281.81 to 6,230.23)	13,133.62 (932.26 to 43,572.64)	41.67 (17.61 to 79.73)	25.57 (5.43 to 81.40)	24.11 (5.73 to 75.09)	155.28 (13.39 to 509.48)
Meningococcal meningitis	12,914.09 (5,308.90 to 25,512.49)	2,548.99 (1,055.39 to 5,071.38)	2,335.33 (1,022.81 to 4,608.16)	3,541.85 (1,290.64 to 7,932.09)	164.66 (74.58 to 311.63)	43.91 (24.20 to 76.59)	41.76 (23.83 to 71.78)	55.93 (26.81 to 110.72)
Other meningitis	22,161.80 (9,272.86 to 43,085.08)	4,797.88 (1,895.91 to 9,109.92)	4,400.45 (1,812.23 to 8,396.43)	6,631.28 (2,315.34 to 14,144.92)	280.32 (127.89 to 527.69)	77.99 (40.42 to 136.74)	73.65 (40.00 to 123.99)	100.26 (45.77 to 191.29)
Encephalitis	8,968.65 (3,696.30 to 20,327.01)	3,684.26 (1,651.06 to 7,943.28)	3,310.31 (1,525.84 to 7,041.45)	5,367.62 (2,146.50 to 12,722.47)	123.14 (56.84 to 261.75)	68.22 (38.69 to 129.32)	63.64 (37.07 to 117.05)	90.92 (45.94 to 185.34)

Diphtheria	106.75 (44.06 to 251.47)	26.36 (9.43 to 67.79)	25.16 (8.99 to 66.06)	106.34 (12.88 to 351.35)	1.35 (0.60 to 3.05)	0.39 (0.16 to 0.89)	0.38 (0.16 to 0.86)	1.33 (0.22 to 4.22)
Whooping cough	16,109.65 (942.81 to 54,656.85)	4,337.65 (132.15 to 16,451.26)	4,040.15 (144.82 to 14,575.36)	8,140.99 (189.96 to 35,505.98)	190.06 (11.11 to 643.76)	51.49 (1.70 to 192.89)	47.96 (1.82 to 173.65)	96.22 (2.46 to 420.66)
Tetanus	2,980.20 (1,275.13 to 6,020.30)	439.36 (179.52 to 846.79)	424.70 (174.67 to 785.11)	725.55 (295.01 to 1,633.81)	51.80 (23.52 to 91.03)	11.02 (4.08 to 19.10)	11.20 (4.23 to 19.50)	13.86 (6.05 to 26.80)
Measles	7,170.39 (1,935.86 to 19,799.74)	23,221.16 (1,316.49 to 103,975.90)	13,991.59 (1,005.05 to 62,444.33)	76,563.42 (1,881.27 to 365,676.97)	85.40 (23.11 to 235.50)	300.37 (22.69 to 1,256.84)	185.26 (18.20 to 756.57)	935.54 (33.33 to 4,341.44)

Laos								
Cause	YLLs (actual counts)				Deaths (actual counts)			
	2016	2040 Forecast	2040 Better Scenario	2040 Worse Scenario	2016	2040 Forecast	2040 Better Scenario	2040 Worse Scenario
Varicella and herpes zoster	1,987.62 (936.79 to 4,108.52)	464.37 (192.63 to 889.18)	397.35 (176.26 to 775.89)	836.02 (297.16 to 1,721.34)	30.67 (17.84 to 54.60)	13.76 (7.63 to 22.65)	13.26 (7.31 to 21.89)	19.55 (10.07 to 33.54)
**Neglected tropical diseases and malaria**	**7,184.70 (3,653.99 to 13,920.16)**	**5,777.98 (3,266.16 to 10,974.57)**	**5,272.44 (3,036.04 to 9,820.14)**	**7,576.67 (4,169.60 to 14,920.76)**	**103.86 (58.17 to 196.90)**	**105.39 (64.67 to 190.31)**	**100.16 (62.24 to 176.72)**	**127.47 (75.65 to 235.51)**
Malaria	1,214.71 (522.45 to 3,731.90)	669.45 (196.56 to 2,312.99)	641.58 (187.04 to 2,186.92)	766.98 (224.07 to 2,729.99)	21.58 (10.39 to 61.88)	15.48 (4.89 to 51.19)	15.28 (4.77 to 49.19)	16.74 (5.29 to 57.42)
Chagas disease	0.00 (0.00 to 0.00)	0.00 (0.00 to 0.00)	0.00 (0.00 to 0.00)	0.00 (0.00 to 0.00)	0.00 (0.00 to 0.00)	0.00 (0.00 to 0.00)	0.00 (0.00 to 0.00)	0.00 (0.00 to 0.00)
Leishmaniasis	0.00 (0.00 to 0.00)	0.00 (0.00 to 0.00)	0.00 (0.00 to 0.00)	0.00 (0.00 to 0.00)	0.00 (0.00 to 0.00)	0.00 (0.00 to 0.00)	0.00 (0.00 to 0.00)	0.00 (0.00 to 0.00)
African trypanosomiasis	0.00 (0.00 to 0.00)	0.00 (0.00 to 0.00)	0.00 (0.00 to 0.00)	0.00 (0.00 to 0.00)	0.00 (0.00 to 0.00)	0.00 (0.00 to 0.00)	0.00 (0.00 to 0.00)	0.00 (0.00 to 0.00)
Schistosomiasis	173.88 (86.08 to 288.32)	126.24 (41.80 to 265.41)	119.20 (39.04 to 250.39)	152.62 (52.77 to 312.08)	4.74 (2.33 to 7.87)	4.27 (1.42 to 8.69)	4.17 (1.38 to 8.66)	4.89 (1.72 to 9.71)
Cysticercosis	64.58 (49.16 to 81.74)	23.43 (11.61 to 37.87)	22.42 (10.83 to 36.61)	26.85 (13.85 to 42.85)	1.30 (0.98 to 1.62)	0.57 (0.28 to 0.92)	0.56 (0.27 to 0.91)	0.63 (0.32 to 1.00)
Cystic echinococcosis	7.71 (5.77 to 10.21)	2.21 (1.10 to 3.57)	1.77 (0.88 to 2.85)	4.33 (2.22 to 6.78)	0.16 (0.12 to 0.20)	0.06 (0.03 to 0.10)	0.05 (0.03 to 0.09)	0.12 (0.06 to 0.18)
Dengue	2,073.90 (256.21 to 6,856.66)	3,016.30 (817.93 to 7,854.14)	2,767.70 (782.02 to 7,088.13)	3,899.18 (995.61 to 10,487.25)	29.11 (3.90 to 92.52)	55.93 (16.75 to 136.58)	53.42 (16.58 to 127.55)	66.52 (18.48 to 163.50)
Yellow fever	0.00 (0.00 to 0.00)	0.00 (0.00 to 0.00)	0.00 (0.00 to 0.00)	0.00 (0.00 to 0.00)	0.00 (0.00 to 0.00)	0.00 (0.00 to 0.00)	0.00 (0.00 to 0.00)	0.00 (0.00 to 0.00)
Rabies	92.02 (39.27 to 173.92)	22.35 (7.09 to 53.84)	16.64 (5.10 to 42.31)	54.57 (18.39 to 123.61)	1.57 (0.75 to 2.77)	0.48 (0.17 to 1.04)	0.37 (0.13 to 0.83)	1.07 (0.39 to 2.20)
Intestinal nematode infections	3,190.74 (1,630.59 to 5,979.55)	1,795.10 (879.90 to 3,300.30)	1,599.92 (796.13 to 2,958.14)	2,459.27 (1,193.04 to 4,501.88)	40.15 (21.13 to 72.78)	25.82 (13.70 to 43.61)	23.75 (12.52 to 40.54)	33.42 (17.54 to 57.68)
Other neglected tropical diseases	367.14 (143.79 to 893.93)	122.89 (55.22 to 264.47)	103.21 (47.04 to 211.40)	212.88 (87.95 to 478.86)	5.24 (2.49 to 12.17)	2.78 (1.31 to 5.75)	2.55 (1.22 to 5.11)	4.07 (1.91 to 8.77)
**Maternal disorders**	**25,785.18 (18,350.38 to 34,952.02)**	**9,808.06 (5,609.98 to 15,199.87)**	**7,992.50 (4,497.83 to 12,634.33)**	**17,829.61 (10,268.70 to 28,149.72)**	**448.55 (320.09 to 608.09)**	**175.74 (102.92 to 268.91)**	**143.32 (82.19 to 223.75)**	**318.54 (184.97 to 494.66)**
Maternal hemorrhage	9,211.81 (6,080.35 to 13,000.96)	3,459.39 (1,786.49 to 5,550.46)	2,820.92 (1,453.27 to 4,643.27)	6,232.84 (3,225.56 to 10,046.50)	160.24 (104.62 to 223.90)	61.79 (32.20 to 98.39)	50.40 (26.67 to 82.09)	111.11 (58.78 to 176.26)
Maternal sepsis and other maternal infections	632.05 (333.32 to 1,080.76)	222.43 (99.89 to 436.19)	180.91 (80.96 to 359.12)	404.88 (183.38 to 787.71)	11.18 (6.02 to 19.07)	4.09 (1.91 to 7.72)	3.33 (1.54 to 6.31)	7.42 (3.44 to 13.94)
Maternal hypertensive disorders	1,669.54 (1,015.18 to 2,602.36)	646.26 (314.31 to 1,168.34)	525.11 (262.48 to 986.57)	1,173.65 (589.24 to 2,077.97)	28.90 (17.72 to 44.82)	11.53 (5.71 to 20.77)	9.37 (4.76 to 17.24)	20.88 (10.52 to 36.61)
Maternal obstructed labor and uterine rupture	428.51 (208.77 to 752.69)	166.22 (70.04 to 334.20)	133.46 (56.14 to 270.12)	312.97 (127.95 to 634.56)	7.48 (3.76 to 12.90)	3.00 (1.27 to 5.92)	2.41 (1.04 to 4.80)	5.63 (2.40 to 11.27)
Maternal abortion, miscarriage, and ectopic pregnancy	8,184.58 (5,371.41 to 11,687.53)	2,972.76 (1,569.04 to 5,131.09)	2,426.37 (1,249.70 to 4,218.67)	5,426.49 (2,777.54 to 9,142.56)	143.72 (96.24 to 203.74)	54.02 (28.89 to 91.99)	44.12 (23.60 to 75.02)	98.36 (53.05 to 166.78)

Indirect maternal deaths	3,564.37 (2,228.91 to 5,352.34)	1,468.03 (704.05 to 2,583.29)	1,191.45 (553.34 to 2,115.01)	2,718.40 (1,307.90 to 4,785.66)	60.67 (37.94 to 91.22)	25.61 (12.62 to 44.63)	20.81 (10.01 to 36.80)	47.22 (23.58 to 82.64)
Late maternal deaths	160.71 (53.41 to 334.07)	54.82 (15.36 to 117.61)	44.45 (12.84 to 95.87)	101.47 (29.43 to 222.84)	2.79 (0.94 to 5.76)	0.98 (0.28 to 2.08)	0.80 (0.23 to 1.69)	1.81 (0.52 to 3.92)
Maternal deaths aggravated by HIV/AIDS	11.75 (0.96 to 44.48)	28.90 (0.08 to 182.17)	22.57 (0.03 to 154.42)	52.34 (0.36 to 309.92)	0.21 (0.02 to 0.78)	0.53 (0.00 to 3.28)	0.41 (0.00 to 2.79)	0.95 (0.01 to 5.60)
Other maternal disorders	1,921.87 (1,147.18 to 3,043.55)	789.26 (417.17 to 1,379.36)	647.26 (342.55 to 1,165.49)	1,406.58 (723.86 to 2,421.58)	33.37 (19.93 to 52.13)	14.19 (7.58 to 24.58)	11.66 (6.38 to 20.38)	25.16 (13.22 to 42.63)

Laos								
Cause	YLLs (actual counts)				Deaths (actual counts)			
	2016	2040 Forecast	2040 Better Scenario	2040 Worse Scenario	2016	2040 Forecast	2040 Better Scenario	2040 Worse Scenario
**Neonatal disorders**	**474,128.05 (234,901.50 to 834,461.80)**	**109,937.05 (47,936.91 to 207,379.24)**	**88,031.07 (38,777.60 to 166,606.29)**	**243,139.17 (107,299.82 to 453,540.46)**	**5,477.37 (2,713.31 to 9,639.48)**	**1,270.30 (553.94 to 2,396.12)**	**1,017.18 (448.08 to 1,924.91)**	**2,809.24 (1,239.79 to 5,240.30)**
Neonatal preterm birth	230,135.96 (113,111.11 to 419,101.32)	42,256.36 (18,427.37 to 82,636.13)	34,678.02 (15,404.54 to 66,292.31)	110,360.22 (49,265.25 to 211,895.28)	2,658.44 (1,306.60 to 4,841.11)	488.25 (212.91 to 954.82)	400.69 (178.00 to 765.81)	1,275.00 (569.29 to 2,448.08)
Neonatal encephalopathy due to birth asphyxia and trauma	139,076.01 (59,267.79 to 258,715.26)	35,658.80 (13,045.18 to 72,868.29)	28,404.82 (10,412.57 to 57,012.47)	67,895.73 (24,151.22 to 140,844.61)	1,606.59 (684.59 to 2,988.81)	411.98 (150.72 to 841.74)	328.18 (120.32 to 658.80)	784.42 (279.06 to 1,627.00)
Neonatal sepsis and other neonatal infections	45,617.12 (19,040.92 to 91,157.70)	16,599.76 (6,217.28 to 35,750.67)	13,092.71 (4,885.51 to 28,391.10)	32,215.75 (12,158.08 to 69,592.10)	527.02 (219.95 to 1,053.64)	191.81 (71.84 to 413.11)	151.29 (56.45 to 327.94)	372.25 (140.53 to 803.92)
Hemolytic disease and other neonatal jaundice	5,730.65 (2,495.84 to 11,258.22)	1,082.34 (367.36 to 2,485.79)	831.42 (277.28 to 1,892.80)	2,291.68 (783.71 to 5,314.81)	66.21 (28.85 to 130.03)	12.51 (4.25 to 28.72)	9.61 (3.20 to 21.87)	26.48 (9.06 to 61.39)
Other neonatal disorders	53,568.30 (22,804.92 to 100,792.52)	14,339.79 (5,272.34 to 30,844.56)	11,024.10 (4,038.18 to 23,866.64)	30,375.79 (10,965.08 to 65,469.53)	619.10 (263.56 to 1,164.89)	165.74 (60.95 to 356.41)	127.42 (46.66 to 275.76)	351.08 (126.75 to 756.41)
**Nutritional deficiencies**	**12,073.33 (6,707.12 to 21,317.99)**	**3,804.13 (2,651.70 to 5,704.40)**	**3,453.15 (2,458.06 to 4,970.40)**	**5,670.20 (3,544.76 to 9,329.34)**	**242.85 (173.08 to 358.09)**	**175.33 (127.36 to 238.83)**	**180.74 (132.27 to 244.59)**	**200.36 (142.38 to 281.67)**
Protein-energy malnutrition	10,633.87 (5,813.64 to 19,037.59)	3,082.59 (2,100.04 to 4,652.22)	2,794.59 (1,942.02 to 4,071.02)	4,624.12 (2,823.62 to 7,673.77)	212.98 (149.27 to 317.43)	143.92 (104.94 to 197.31)	148.72 (108.76 to 199.39)	164.17 (115.64 to 226.85)
Iodine deficiency	155.30 (3.91 to 823.91)	130.11 (3.35 to 527.97)	113.44 (2.80 to 458.74)	214.74 (6.11 to 1,045.32)	2.53 (0.13 to 10.39)	5.18 (0.12 to 21.23)	5.02 (0.11 to 21.76)	6.77 (0.20 to 25.08)
Dietary iron deficiency	97.46 (64.12 to 148.32)	51.38 (31.34 to 92.69)	47.06 (28.76 to 85.88)	66.70 (40.56 to 116.21)	1.62 (1.09 to 2.56)	1.21 (0.69 to 2.75)	1.16 (0.65 to 2.75)	1.44 (0.83 to 3.08)
Other nutritional deficiencies	1,186.70 (554.51 to 2,351.37)	540.04 (320.77 to 905.63)	498.06 (298.56 to 815.34)	764.64 (443.07 to 1,363.83)	25.73 (15.40 to 44.04)	25.03 (13.67 to 45.17)	25.85 (13.97 to 47.04)	27.98 (15.35 to 49.45)
**Other communicable, maternal, neonatal, and nutritional diseases**	**19,640.30 (8,854.03 to 42,193.35)**	**6,761.57 (4,499.51 to 11,020.08)**	**6,019.58 (4,068.04 to 9,471.31)**	**9,965.87 (6,088.91 to 17,728.29)**	**291.82 (164.48 to 552.88)**	**176.32 (133.78 to 236.50)**	**167.65 (127.41 to 221.93)**	**226.54 (165.23 to 325.84)**
Sexually transmitted infections excluding HIV	10,856.24 (3,018.37 to 28,812.00)	1,435.38 (458.92 to 3,809.13)	1,215.65 (399.49 to 3,195.74)	2,416.11 (673.48 to 6,505.97)	131.01 (39.15 to 340.33)	20.75 (8.86 to 50.06)	18.21 (8.08 to 42.30)	32.54 (11.70 to 81.28)
Syphilis	10,609.35 (2,784.36 to 28,549.86)	1,250.30 (284.02 to 3,605.69)	1,040.82 (243.46 to 3,016.93)	2,195.79 (468.72 to 6,221.96)	125.41 (33.78 to 334.51)	15.73 (4.30 to 43.47)	13.29 (3.84 to 36.55)	26.88 (6.76 to 74.12)
Chlamydial infection	50.81 (29.63 to 78.11)	37.54 (22.05 to 57.12)	35.43 (21.13 to 53.18)	44.82 (25.81 to 69.20)	1.12 (0.66 to 1.67)	1.01 (0.59 to 1.49)	0.99 (0.58 to 1.44)	1.13 (0.66 to 1.68)
Gonococcal infection	126.00 (74.64 to 187.59)	96.61 (58.91 to 144.87)	91.54 (55.95 to 137.54)	114.33 (67.92 to 174.90)	2.96 (1.75 to 4.28)	2.74 (1.68 to 3.96)	2.70 (1.65 to 3.92)	3.06 (1.84 to 4.51)
Other sexually transmitted infections	70.08 (41.26 to 106.61)	50.93 (31.40 to 78.99)	47.87 (29.94 to 73.95)	61.17 (36.65 to 96.08)	1.52 (0.89 to 2.24)	1.27 (0.79 to 1.91)	1.23 (0.76 to 1.80)	1.47 (0.90 to 2.20)
Acute hepatitis	2,950.41 (2,298.71 to 3,837.86)	1,789.10 (1,264.15 to 2,387.77)	1,647.13 (1,152.18 to 2,203.04)	2,319.67 (1,626.74 to 3,084.47)	61.92 (49.63 to 75.25)	49.72 (36.67 to 64.58)	47.13 (34.51 to 61.77)	60.96 (45.17 to 80.32)
Acute hepatitis A	562.08 (201.36 to 1,166.32)	111.61 (42.44 to 274.53)	94.08 (37.58 to 232.56)	194.13 (66.99 to 497.68)	6.98 (2.76 to 14.06)	1.76 (0.89 to 3.76)	1.54 (0.82 to 3.24)	2.78 (1.24 to 6.42)
Acute hepatitis B	1,887.59 (1,454.49 to 2,366.56)	1,433.68 (980.47 to 1,965.92)	1,327.93 (897.39 to 1,819.29)	1,813.58 (1,225.04 to 2,464.46)	45.89 (35.84 to 55.48)	41.02 (29.30 to 54.23)	38.71 (27.08 to 51.32)	50.32 (35.57 to 67.62)

Acute hepatitis C	49.23 (35.19 to 67.23)	44.63 (29.03 to 67.18)	43.03 (27.94 to 64.50)	50.41 (32.90 to 75.52)	1.26 (0.91 to 1.75)	1.43 (0.94 to 2.13)	1.42 (0.94 to 2.12)	1.54 (1.01 to 2.30)
Acute hepatitis E	451.50 (303.83 to 649.59)	199.19 (120.04 to 305.26)	182.09 (110.78 to 274.06)	261.55 (153.79 to 412.77)	7.79 (5.60 to 10.64)	5.52 (3.51 to 8.11)	5.46 (3.44 to 8.06)	6.34 (3.98 to 9.39)
Other unspecified infectious diseases	5,833.65 (2,319.97 to 21,025.48)	3,537.09 (2,288.08 to 6,694.60)	3,156.80 (2,054.73 to 5,728.60)	5,230.09 (3,047.99 to 11,605.18)	98.88 (54.10 to 277.61)	105.86 (75.21 to 148.86)	102.30 (73.22 to 138.45)	133.03 (91.74 to 210.69)
**Non-communicable diseases**	**884,263.83 (735,863.60 to 1,107,348.43)**	**955,846.68 (813,909.21 to 1,116,066.87)**	**745,052.23 (640,822.19 to 862,894.57)**	**1,401,018.30 (1,171,311.12 to 1,653,883.68)**	**27,948.55 (25,473.23 to 30,868.76)**	**42,744.00 (37,755.51 to 48,440.89)**	**34,950.72 (31,339.89 to 39,476.81)**	**57,752.28 (50,494.50 to 65,383.12)**

Laos								
Cause	YLLs (actual counts)				Deaths (actual counts)			
	2016	2040 Forecast	2040 Better Scenario	2040 Worse Scenario	2016	2040 Forecast	2040 Better Scenario	2040 Worse Scenario
**Neoplasms**	**132,640.65 (117,916.10 to 144,849.62)**	**226,876.18 (187,568.37 to 268,682.63)**	**193,876.96 (165,568.00 to 223,011.36)**	**263,008.29 (210,317.34 to 322,392.22)**	**4,304.43 (3,899.92 to 4,692.81)**	**8,983.16 (7,464.14 to 10,573.93)**	**7,958.61 (6,794.24 to 9,130.39)**	**9,894.97 (7,945.52 to 12,042.82)**
Lip and oral cavity cancer	2,540.60 (2,124.98 to 2,966.51)	4,711.17 (2,832.36 to 7,760.12)	3,064.22 (2,014.31 to 4,719.10)	7,335.93 (4,115.65 to 12,324.63)	94.89 (81.92 to 110.31)	197.40 (122.27 to 313.85)	136.64 (92.47 to 202.28)	290.47 (170.90 to 487.08)
Nasopharynx cancer	2,068.88 (1,627.01 to 2,549.13)	3,842.65 (2,314.11 to 6,274.90)	2,405.62 (1,514.04 to 3,761.93)	6,301.76 (3,540.55 to 10,772.29)	63.19 (51.37 to 76.58)	130.07 (80.00 to 209.74)	83.95 (55.08 to 128.70)	207.21 (119.25 to 348.92)
Other pharynx cancer	1,220.54 (904.80 to 1,554.26)	2,150.30 (1,182.86 to 3,641.52)	1,772.30 (1,049.51 to 2,837.05)	2,563.18 (1,397.46 to 4,311.30)	43.60 (32.84 to 54.84)	84.18 (46.80 to 137.63)	71.79 (42.98 to 112.57)	96.71 (52.67 to 159.87)
Esophageal cancer	2,133.79 (1,872.20 to 2,409.17)	3,453.70 (2,121.03 to 5,597.81)	2,413.19 (1,583.37 to 3,674.48)	5,559.10 (3,110.99 to 9,597.30)	81.89 (72.43 to 92.02)	146.24 (92.13 to 228.84)	107.72 (71.88 to 158.58)	223.55 (127.43 to 372.03)
Stomach cancer	8,783.23 (7,484.46 to 10,523.97)	11,383.35 (8,932.13 to 14,287.10)	10,756.19 (8,634.01 to 13,325.66)	13,021.51 (9,858.44 to 16,670.42)	337.83 (293.32 to 406.30)	501.01 (401.83 to 630.61)	493.37 (400.30 to 614.84)	541.49 (423.31 to 695.75)
Colon and rectum cancer	8,774.01 (7,494.23 to 10,452.76)	20,515.20 (13,897.47 to 29,351.96)	19,603.61 (13,621.94 to 27,872.74)	20,496.35 (13,811.02 to 29,302.87)	331.74 (288.46 to 392.97)	922.69 (636.26 to 1,326.04)	922.58 (652.24 to 1,307.89)	866.85 (590.06 to 1,246.78)
Liver cancer	9,177.50 (6,524.29 to 11,283.87)	18,809.21 (12,170.39 to 27,162.04)	15,704.42 (10,673.98 to 21,169.85)	21,230.28 (13,314.56 to 31,946.38)	324.26 (241.66 to 391.47)	751.07 (507.11 to 1,040.72)	658.55 (473.40 to 859.05)	804.55 (527.98 to 1,170.57)
Liver cancer due to hepatitis B	4,403.27 (3,047.26 to 5,709.84)	8,571.02 (5,352.24 to 13,046.47)	7,304.01 (4,806.94 to 10,455.03)	9,509.03 (5,661.09 to 15,156.28)	144.40 (103.00 to 187.38)	318.29 (206.11 to 469.64)	283.12 (190.50 to 392.11)	337.17 (211.87 to 517.74)
Liver cancer due to hepatitis C	1,568.52 (1,012.05 to 2,219.04)	3,543.51 (2,160.61 to 5,328.82)	3,128.70 (1,985.02 to 4,531.99)	3,782.84 (2,237.89 to 5,909.02)	67.57 (45.32 to 93.24)	170.41 (108.24 to 252.58)	159.03 (105.38 to 229.05)	171.29 (104.93 to 259.04)
Liver cancer due to alcohol use	1,343.57 (892.83 to 1,884.96)	3,070.35 (1,828.77 to 4,948.72)	2,250.99 (1,425.40 to 3,419.57)	3,854.91 (2,205.72 to 6,405.38)	51.50 (34.82 to 72.02)	123.88 (77.58 to 195.84)	96.10 (63.13 to 144.95)	147.76 (89.11 to 241.45)
Liver cancer due to other causes	1,862.14 (1,183.17 to 2,560.70)	3,624.34 (2,081.94 to 5,755.97)	3,020.72 (1,825.10 to 4,558.36)	4,083.51 (2,248.86 to 6,706.03)	60.78 (40.04 to 84.82)	138.49 (80.94 to 214.54)	120.30 (73.48 to 179.06)	148.32 (83.34 to 237.94)
Gallbladder and biliary tract cancer	1,535.63 (1,287.66 to 1,819.72)	2,265.92 (1,876.55 to 2,741.24)	2,387.29 (1,984.16 to 2,871.25)	2,085.74 (1,708.56 to 2,531.78)	64.33 (54.45 to 75.53)	106.95 (88.68 to 128.62)	116.64 (96.73 to 137.67)	93.55 (76.02 to 113.53)
Pancreatic cancer	3,601.49 (3,135.28 to 4,039.31)	8,612.16 (6,911.91 to 10,804.99)	8,182.08 (6,746.20 to 9,943.45)	8,635.70 (6,730.63 to 10,968.84)	144.46 (128.31 to 161.01)	384.50 (312.99 to 481.57)	380.22 (319.09 to 461.85)	365.05 (290.20 to 463.95)
Larynx cancer	1,604.49 (1,365.22 to 1,879.75)	2,682.77 (1,345.41 to 4,821.63)	1,645.51 (895.34 to 2,848.12)	4,206.55 (2,011.02 to 7,726.06)	62.32 (53.68 to 72.73)	113.24 (57.14 to 199.35)	71.75 (40.17 to 123.61)	171.82 (85.01 to 308.65)
Tracheal, bronchus, and lung cancer	19,727.30 (16,097.19 to 23,717.09)	39,976.32 (24,231.54 to 60,820.65)	20,624.85 (13,312.29 to 30,474.65)	58,392.85 (34,388.93 to 90,674.76)	774.49 (636.62 to 934.13)	1,702.76 (1,041.21 to 2,591.27)	912.37 (586.71 to 1,341.47)	2,397.94 (1,421.60 to 3,685.45)
Malignant skin melanoma	455.21 (353.40 to 611.37)	877.16 (680.67 to 1,168.88)	937.13 (728.13 to 1,252.40)	762.00 (592.50 to 1,022.91)	14.33 (11.44 to 18.98)	33.95 (26.83 to 45.58)	37.85 (30.19 to 51.05)	27.72 (21.89 to 37.34)
Non-melanoma skin cancer	747.75 (595.82 to 904.05)	1,495.30 (1,162.95 to 1,857.92)	1,516.27 (1,173.44 to 1,902.36)	1,547.04 (1,198.10 to 1,921.52)	28.55 (22.56 to 34.91)	68.53 (52.37 to 86.63)	72.73 (55.98 to 91.22)	66.30 (49.76 to 84.52)
Breast cancer	12,175.52 (7,850.59 to 16,655.50)	22,866.52 (14,707.73 to 32,886.09)	21,011.24 (13,924.86 to 29,617.62)	24,610.76 (15,662.80 to 36,358.55)	367.18 (242.09 to 500.81)	781.83 (511.20 to 1,125.42)	735.95 (489.55 to 1,036.93)	811.05 (519.17 to 1,197.69)
Cervical cancer	8,545.72 (5,920.08 to 10,477.80)	10,987.71 (7,339.50 to 14,730.89)	10,228.72 (6,885.39 to 13,366.69)	12,899.78 (8,352.62 to 18,048.61)	270.98 (189.90 to 331.28)	385.58 (259.79 to 507.01)	369.55 (251.59 to 475.58)	435.17 (289.36 to 592.10)

Uterine cancer	1,703.21 (1,348.68 to 2,055.50)	2,787.26 (2,174.86 to 3,476.42)	2,726.00 (2,128.37 to 3,403.35)	3,126.01 (2,431.54 to 3,921.86)	63.55 (50.38 to 77.35)	116.78 (93.37 to 144.01)	117.38 (93.43 to 145.30)	125.83 (99.48 to 156.90)
Ovarian cancer	2,850.48 (2,324.02 to 3,477.57)	5,877.03 (4,471.14 to 7,859.45)	5,933.75 (4,604.29 to 7,714.07)	5,294.59 (3,990.27 to 7,145.44)	91.39 (74.42 to 111.26)	210.77 (158.67 to 279.85)	217.07 (167.73 to 276.90)	184.93 (138.54 to 248.41)
Prostate cancer	2,368.86 (1,518.59 to 2,984.94)	7,215.93 (4,494.41 to 9,161.63)	7,920.86 (5,113.07 to 9,821.94)	6,159.26 (3,789.87 to 7,959.20)	143.15 (90.77 to 180.28)	497.24 (306.95 to 640.45)	571.03 (359.47 to 722.17)	397.23 (238.45 to 529.01)
Testicular cancer	363.44 (243.75 to 484.35)	442.98 (301.60 to 604.79)	433.76 (293.38 to 592.72)	480.12 (330.09 to 648.34)	7.48 (5.23 to 9.81)	10.65 (7.78 to 13.98)	10.71 (7.92 to 14.07)	11.11 (8.00 to 14.70)

Laos								
**Cause**	**YLLs (actual counts)**				**Deaths (actual counts)**			
	**2016**	**2040 Forecast**	**2040 Better Scenario**	**2040 Worse Scenario**	**2016**	**2040 Forecast**	**2040 Better Scenario**	**2040 Worse Scenario**
Kidney cancer	1,478.43 (1,206.84 to 1,864.71)	2,761.46 (2,050.77 to 3,668.86)	2,739.80 (2,064.80 to 3,521.65)	2,665.46 (1,920.29 to 3,662.37)	43.02 (36.09 to 49.53)	108.35 (77.82 to 146.57)	112.34 (83.16 to 147.87)	97.49 (67.96 to 138.98)
Bladder cancer	1,410.06 (1,150.96 to 1,658.52)	2,865.70 (2,026.12 to 3,968.98)	2,609.66 (1,897.49 to 3,472.67)	3,158.60 (2,129.38 to 4,557.86)	65.74 (53.68 to 77.02)	153.97 (109.22 to 214.49)	148.25 (109.28 to 197.36)	157.86 (107.05 to 228.47)
Brain and nervous system cancer	5,445.25 (4,220.61 to 6,651.98)	7,773.24 (5,932.17 to 9,471.38)	7,814.81 (5,904.57 to 9,507.10)	7,845.23 (6,012.38 to 9,600.35)	112.58 (87.42 to 134.49)	215.28 (161.37 to 258.44)	225.01 (167.70 to 270.90)	202.21 (151.32 to 245.36)
Thyroid cancer	914.71 (707.86 to 1,126.41)	1,604.08 (1,181.67 to 2,151.95)	1,499.32 (1,141.30 to 1,952.19)	1,720.38 (1,239.45 to 2,306.67)	34.43 (27.03 to 42.19)	68.67 (51.03 to 89.92)	66.58 (51.27 to 84.89)	70.42 (50.70 to 92.79)
Mesothelioma	286.35 (223.49 to 372.70)	489.75 (382.29 to 648.44)	495.27 (385.65 to 659.17)	495.81 (386.14 to 657.06)	8.31 (6.65 to 10.57)	16.88 (13.41 to 21.89)	17.69 (14.14 to 22.84)	16.23 (12.92 to 21.05)
Hodgkin lymphoma	1,404.14 (1,069.40 to 1,917.18)	1,253.58 (909.76 to 1,866.82)	1,167.73 (847.47 to 1,730.60)	1,582.83 (1,149.87 to 2,315.19)	31.65 (24.34 to 44.24)	35.13 (25.74 to 53.78)	33.64 (24.68 to 51.01)	42.22 (31.01 to 64.30)
Non-Hodgkin lymphoma	5,531.92 (4,611.09 to 6,677.17)	8,762.99 (7,167.15 to 10,620.30)	8,799.44 (7,284.32 to 10,527.24)	8,365.78 (6,861.52 to 10,103.12)	139.95 (121.49 to 163.70)	294.70 (245.33 to 351.79)	308.03 (256.80 to 364.29)	262.65 (219.06 to 313.24)
Multiple myeloma	793.72 (645.79 to 958.60)	1,897.74 (1,482.67 to 2,361.20)	1,938.19 (1,526.97 to 2,388.62)	1,697.97 (1,335.38 to 2,122.79)	28.36 (23.34 to 33.75)	76.02 (59.63 to 93.88)	79.97 (63.40 to 98.29)	65.37 (50.84 to 82.40)
Leukemia	14,201.06 (10,515.29 to 19,928.49)	13,582.35 (10,565.31 to 17,556.51)	12,485.53 (9,938.61 to 15,998.11)	15,596.18 (11,960.27 to 20,592.13)	272.58 (219.38 to 355.89)	376.85 (301.57 to 472.70)	359.48 (292.45 to 446.15)	401.47 (315.63 to 510.75)
Acute lymphoid leukemia	3,320.56 (2,240.00 to 5,654.53)	3,363.49 (2,510.43 to 5,021.57)	3,164.52 (2,404.22 to 4,651.35)	3,616.97 (2,647.75 to 5,542.92)	62.42 (47.51 to 97.40)	92.74 (72.52 to 133.03)	90.58 (72.11 to 127.41)	91.93 (70.73 to 134.46)
Chronic lymphoid leukemia	162.99 (122.07 to 245.85)	234.80 (168.44 to 340.20)	221.49 (161.33 to 313.97)	244.07 (173.13 to 362.64)	4.52 (3.44 to 6.37)	7.98 (5.87 to 11.05)	7.85 (5.91 to 10.75)	7.84 (5.67 to 11.26)
Acute myeloid leukemia	1,914.50 (1,405.49 to 2,866.76)	2,636.85 (2,014.73 to 3,537.86)	2,510.07 (1,944.79 to 3,346.45)	2,675.74 (2,005.60 to 3,653.80)	36.36 (28.28 to 50.06)	68.16 (53.17 to 89.27)	66.67 (52.85 to 85.95)	65.27 (49.55 to 86.53)
Chronic myeloid leukemia	605.72 (442.98 to 938.13)	675.76 (456.77 to 1,053.14)	624.87 (430.76 to 949.98)	720.97 (478.69 to 1,131.83)	15.34 (11.66 to 23.86)	20.35 (14.21 to 31.34)	19.36 (13.86 to 29.43)	20.87 (14.22 to 32.94)
Other leukemia	8,197.28 (5,648.26 to 11,800.43)	6,671.46 (4,902.16 to 8,961.50)	5,964.58 (4,429.67 to 7,910.99)	8,338.42 (6,016.37 to 11,364.71)	153.94 (116.80 to 203.11)	187.62 (143.47 to 245.03)	175.02 (134.84 to 225.63)	215.55 (160.34 to 284.22)
Other neoplasms	10,797.35 (8,871.39 to 12,962.08)	14,932.67 (12,536.52 to 17,056.02)	15,060.22 (12,637.50 to 17,239.42)	15,171.53 (12,656.60 to 17,386.49)	258.19 (222.14 to 292.00)	491.87 (411.05 to 566.86)	519.79 (433.33 to 595.40)	460.53 (388.58 to 535.92)
**Cardiovascular diseases**	**311,290.28 (277,591.28 to 337,944.31)**	**319,811.95 (235,141.21 to 427,267.17)**	**203,173.50 (152,763.11 to 268,641.52)**	**596,577.80 (435,792.63 to 805,834.92)**	**12,574.23 (11,600.86 to 13,476.81)**	**16,354.11 (12,498.57 to 21,587.56)**	**11,502.24 (9,009.95 to 15,263.92)**	**27,341.07 (20,573.50 to 35,683.58)**
Rheumatic heart disease	12,001.96 (9,618.35 to 14,482.00)	6,260.05 (4,736.57 to 7,857.97)	5,681.09 (4,314.46 to 7,097.43)	8,552.17 (6,431.64 to 10,942.67)	275.43 (218.92 to 333.05)	198.49 (153.66 to 246.66)	189.72 (148.40 to 234.10)	249.99 (190.80 to 315.85)
Ischemic heart disease	139,200.62 (122,505.41 to 154,563.36)	154,703.98 (90,390.05 to 249,815.19)	87,148.32 (51,349.13 to 142,072.37)	298,305.94 (179,435.77 to 453,795.69)	6,017.17 (5,430.31 to 6,699.27)	7,931.85 (4,894.81 to 12,784.14)	5,053.00 (3,143.67 to 8,178.59)	13,823.95 (8,654.05 to 21,431.38)
Stroke	118,036.53 (102,186.57 to 131,435.76)	101,436.26 (66,910.15 to 154,890.77)	60,109.13 (40,632.76 to 88,486.02)	215,476.97 (139,303.51 to 320,570.14)	4,833.98 (4,193.80 to 5,378.12)	5,428.04 (3,690.18 to 8,533.49)	3,656.77 (2,537.51 to 5,786.56)	9,940.70 (6,670.54 to 14,868.60)
Ischemic stroke	29,595.66 (24,977.79 to 34,469.50)	31,763.88 (18,776.81 to 65,135.78)	21,677.97 (12,937.77 to 43,974.44)	53,735.76 (30,452.25 to 106,391.63)	1,709.57 (1,437.42 to 1,991.26)	2,249.46 (1,289.90 to 5,060.63)	1,698.67 (1,002.70 to 3,528.10)	3,374.04 (1,904.57 to 7,300.90)

Intracerebral hemorrhage	88,440.87 (75,211.49 to 101,371.10)	69,672.38 (44,218.27 to 109,316.30)	38,431.16 (25,122.19 to 58,442.89)	161,741.21 (98,869.73 to 255,839.01)	3,124.41 (2,689.99 to 3,491.90)	3,178.57 (2,076.69 to 4,945.96)	1,958.10 (1,365.57 to 2,893.58)	6,566.66 (4,137.60 to 10,375.61)
Hypertensive heart disease	13,874.48 (10,312.92 to 16,649.80)	22,768.49 (14,966.05 to 34,303.82)	17,902.94 (12,484.94 to 24,455.63)	33,740.67 (20,830.90 to 55,877.67)	638.15 (478.36 to 765.73)	1,257.37 (831.62 to 1,915.68)	1,058.28 (730.12 to 1,462.76)	1,728.33 (1,086.15 to 2,928.25)
Cardiomyopathy and myocarditis	9,198.86 (5,561.68 to 14,366.71)	8,912.69 (5,963.34 to 11,935.45)	8,586.12 (5,712.94 to 11,380.27)	9,711.66 (6,351.78 to 13,261.82)	211.78 (140.73 to 282.87)	318.27 (211.26 to 430.65)	326.67 (220.26 to 440.50)	311.71 (204.87 to 420.29)
Myocarditis	2,823.03 (1,528.17 to 5,648.53)	1,534.23 (1,051.71 to 2,248.85)	1,456.62 (1,010.03 to 2,042.56)	1,874.88 (1,273.02 to 3,050.07)	46.83 (30.33 to 79.18)	39.27 (26.94 to 53.47)	39.78 (27.09 to 54.57)	42.49 (29.21 to 58.48)

Laos								
**Cause**	**YLLs (actual counts)**				**Deaths (actual counts)**			
	**2016**	**2040 Forecast**	**2040 Better Scenario**	**2040 Worse Scenario**	**2016**	**2040 Forecast**	**2040 Better Scenario**	**2040 Worse Scenario**
Alcoholic cardiomyopathy	1,022.36 (630.69 to 1,365.78)	1,565.30 (945.70 to 2,154.61)	1,377.16 (824.30 to 1,940.04)	1,707.36 (1,042.97 to 2,298.71)	32.08 (20.03 to 42.87)	55.49 (33.83 to 75.47)	50.76 (31.01 to 69.98)	58.50 (36.08 to 78.57)
Other cardiomyopathy	5,353.47 (2,891.56 to 9,083.92)	5,813.15 (3,537.77 to 8,269.92)	5,752.33 (3,505.65 to 8,019.28)	6,129.42 (3,596.54 to 8,960.68)	132.87 (84.11 to 183.78)	223.51 (143.99 to 320.42)	236.13 (151.78 to 337.45)	210.73 (131.25 to 303.36)
Atrial fibrillation and flutter	952.66 (732.79 to 1,220.63)	2,496.85 (1,809.21 to 3,320.93)	2,541.41 (1,911.76 to 3,341.21)	2,363.21 (1,676.34 to 3,178.43)	78.95 (60.29 to 102.04)	237.16 (163.16 to 322.72)	256.22 (181.80 to 341.07)	206.30 (136.85 to 285.12)
Aortic aneurysm	1,583.87 (1,321.47 to 1,892.82)	2,772.11 (2,163.48 to 3,484.44)	2,692.62 (2,140.47 to 3,338.66)	2,784.93 (2,136.91 to 3,534.16)	70.19 (59.42 to 83.58)	149.65 (117.11 to 186.28)	155.55 (124.53 to 191.36)	137.42 (106.40 to 172.68)
Peripheral artery disease	51.53 (36.91 to 77.58)	212.82 (139.29 to 374.44)	208.92 (137.97 to 375.78)	183.68 (117.33 to 308.51)	3.29 (2.33 to 5.20)	16.42 (10.47 to 30.44)	17.58 (11.39 to 32.78)	12.65 (7.97 to 22.65)
Endocarditis	4,430.12 (3,442.76 to 5,785.91)	5,251.40 (3,747.98 to 7,091.35)	5,054.79 (3,659.69 to 6,807.82)	5,890.63 (4,183.10 to 7,940.41)	103.21 (83.47 to 129.13)	168.72 (124.01 to 229.58)	171.26 (127.89 to 231.20)	174.10 (126.53 to 239.70)
Other cardiovascular and circulatory diseases	11,959.64 (8,127.26 to 19,285.04)	14,997.31 (10,255.34 to 23,407.14)	13,248.17 (9,251.68 to 20,523.67)	19,567.93 (13,203.33 to 30,948.33)	342.08 (248.18 to 520.30)	648.14 (443.47 to 1,020.20)	617.20 (428.96 to 960.63)	755.92 (510.96 to 1,185.96)
**Chronic respiratory diseases**	**59,412.33 (49,062.60 to 70,312.28)**	**55,784.58 (40,727.74 to 76,053.37)**	**35,084.57 (27,511.04 to 44,364.89)**	**83,504.43 (58,397.35 to 118,909.41)**	**2,382.44 (2,016.36 to 2,738.57)**	**2,928.55 (2,162.85 to 4,124.06)**	**1,904.11 (1,483.37 to 2,453.35)**	**4,098.96 (2,865.35 to 6,019.35)**
Chronic obstructive pulmonary disease	25,412.40 (17,905.95 to 31,446.27)	32,508.53 (18,883.63 to 51,792.75)	16,165.07 (9,867.73 to 23,778.06)	54,650.08 (31,435.84 to 89,148.44)	1,260.66 (893.15 to 1,561.59)	1,884.51 (1,141.44 to 3,063.14)	1,004.73 (641.37 to 1,486.68)	2,911.28 (1,694.50 to 4,806.21)
Pneumoconiosis	37.77 (23.59 to 57.74)	44.45 (29.83 to 65.47)	42.48 (27.76 to 63.21)	44.48 (30.66 to 65.00)	1.69 (1.08 to 2.59)	2.53 (1.69 to 3.74)	2.52 (1.65 to 3.77)	2.33 (1.54 to 3.45)
Silicosis	9.16 (3.97 to 17.70)	8.81 (3.58 to 16.59)	9.01 (3.65 to 17.05)	8.94 (3.88 to 16.72)	0.43 (0.19 to 0.80)	0.49 (0.20 to 0.90)	0.52 (0.22 to 0.99)	0.47 (0.20 to 0.86)
Asbestosis	8.17 (5.00 to 11.93)	10.23 (6.30 to 14.77)	10.42 (6.20 to 15.15)	10.24 (6.48 to 14.77)	0.25 (0.15 to 0.35)	0.41 (0.23 to 0.61)	0.43 (0.23 to 0.66)	0.38 (0.23 to 0.55)
Coal workers pneumoconiosis	2.49 (1.15 to 5.31)	2.73 (1.24 to 6.08)	2.24 (1.03 to 5.23)	3.35 (1.59 to 7.08)	0.13 (0.06 to 0.28)	0.17 (0.08 to 0.39)	0.15 (0.07 to 0.33)	0.20 (0.09 to 0.42)
Other pneumoconiosis	17.94 (10.26 to 29.92)	22.68 (13.61 to 36.81)	20.82 (12.22 to 34.51)	21.95 (13.58 to 35.28)	0.88 (0.51 to 1.48)	1.46 (0.87 to 2.48)	1.41 (0.82 to 2.43)	1.29 (0.76 to 2.13)
Asthma	31,972.00 (23,221.49 to 44,917.92)	20,407.45 (12,690.46 to 31,472.77)	16,013.71 (10,757.51 to 23,009.54)	26,006.46 (15,915.41 to 41,074.60)	1,061.86 (758.80 to 1,605.68)	921.58 (574.43 to 1,473.62)	767.06 (517.68 to 1,158.87)	1,080.18 (637.03 to 1,824.25)
Interstitial lung disease and pulmonary sarcoidosis	653.66 (372.36 to 1,051.33)	1,491.96 (856.76 to 2,319.72)	1,610.83 (939.38 to 2,512.17)	1,262.11 (724.56 to 1,967.12)	26.92 (15.45 to 42.75)	72.04 (41.27 to 113.52)	80.83 (46.95 to 127.36)	57.46 (32.66 to 90.99)
Other chronic respiratory diseases	1,336.49 (616.57 to 2,389.53)	1,332.19 (687.13 to 2,102.33)	1,252.47 (651.55 to 1,971.30)	1,541.30 (764.90 to 2,408.20)	31.31 (15.56 to 52.98)	47.89 (23.52 to 88.46)	48.96 (23.87 to 94.31)	47.71 (23.52 to 84.10)
**Cirrhosis and other chronic liver diseases**	**36,048.10 (26,512.63 to 46,539.88)**	**55,619.81 (41,400.99 to 70,885.75)**	**53,078.27 (39,709.68 to 67,490.16)**	**61,922.35 (45,748.52 to 79,587.20)**	**1,007.25 (773.01 to 1,274.33)**	**1,784.28 (1,384.86 to 2,184.00)**	**1,749.65 (1,367.75 to 2,159.93)**	**1,911.19 (1,462.68 to 2,390.35)**
Cirrhosis and other chronic liver diseases due to hepatitis B	13,467.48 (9,424.61 to 17,958.30)	22,701.86 (16,110.93 to 30,529.08)	22,462.48 (16,233.20 to 29,938.43)	24,203.17 (16,836.07 to 32,482.93)	395.66 (290.07 to 513.18)	735.16 (542.78 to 954.53)	744.77 (554.34 to 961.81)	757.59 (555.51 to 979.49)
Cirrhosis and other chronic liver diseases due to hepatitis C	5,822.38 (3,959.96 to 7,905.97)	9,873.52 (6,810.46 to 13,557.62)	9,737.16 (6,717.83 to 13,360.90)	10,613.77 (7,256.52 to 14,678.94)	169.55 (123.01 to 221.87)	314.01 (228.87 to 415.61)	316.11 (232.18 to 418.84)	327.66 (236.69 to 439.35)

Cirrhosis and other chronic liver diseases due to alcohol use	5,981.31 (4,117.59 to 8,025.62)	10,168.05 (7,062.80 to 13,771.81)	8,350.28 (5,841.23 to 11,314.63)	12,723.70 (8,806.06 to 17,331.21)	184.39 (135.03 to 241.20)	340.97 (248.04 to 449.54)	291.39 (211.36 to 383.59)	410.13 (293.40 to 542.82)
Cirrhosis and other chronic liver diseases due to other causes	10,776.94 (8,242.76 to 14,330.71)	12,876.38 (9,681.04 to 16,893.53)	12,528.34 (9,392.28 to 16,311.87)	14,381.71 (10,742.48 to 18,738.84)	257.64 (199.77 to 320.68)	394.14 (305.05 to 492.85)	397.39 (308.50 to 495.50)	415.80 (319.27 to 520.21)
**Digestive diseases**	**45,646.62 (30,880.61 to 69,815.73)**	**35,640.38 (25,636.82 to 46,198.69)**	**32,512.34 (23,266.96 to 41,408.51)**	**44,657.40 (31,423.49 to 58,837.80)**	**1,198.74 (854.46 to 1,525.53)**	**1,427.07 (1,014.04 to 1,935.37)**	**1,390.08 (979.05 to 1,871.23)**	**1,611.35 (1,123.39 to 2,187.22)**
Peptic ulcer disease	12,761.81 (8,910.23 to 16,489.32)	9,801.09 (6,236.73 to 13,649.06)	8,704.63 (5,549.27 to 12,106.98)	13,070.66 (8,276.78 to 18,674.60)	469.30 (325.07 to 603.32)	465.36 (293.17 to 658.80)	434.62 (275.06 to 608.93)	577.11 (358.10 to 832.67)

Laos								
Cause	YLLs (actual counts)				Deaths (actual counts)			
	2016	2040 Forecast	2040 Better Scenario	2040 Worse Scenario	2016	2040 Forecast	2040 Better Scenario	2040 Worse Scenario
Gastritis and duodenitis	710.87 (486.19 to 1,021.55)	1,028.73 (711.83 to 1,425.48)	1,020.17 (704.66 to 1,411.80)	1,137.75 (773.40 to 1,580.85)	24.16 (17.46 to 31.40)	46.57 (32.08 to 64.51)	48.69 (33.59 to 68.32)	47.52 (32.26 to 67.08)
Appendicitis	3,523.24 (2,312.26 to 5,421.43)	2,126.41 (1,467.97 to 3,229.86)	1,957.94 (1,366.56 to 3,057.95)	2,792.01 (1,901.61 to 4,245.05)	68.01 (46.58 to 102.56)	60.09 (41.85 to 99.00)	58.51 (40.93 to 100.48)	72.03 (49.44 to 113.54)
Paralytic ileus and intestinal obstruction	16,466.67 (7,447.69 to 37,744.07)	8,464.61 (5,371.02 to 13,920.80)	8,090.69 (5,145.01 to 12,779.80)	10,628.33 (6,269.40 to 19,300.05)	285.23 (171.93 to 528.45)	297.60 (195.28 to 405.88)	313.55 (206.26 to 423.92)	304.41 (198.00 to 426.71)
Inguinal, femoral, and abdominal hernia	778.89 (425.67 to 1,161.73)	740.78 (393.01 to 1,133.05)	739.15 (387.67 to 1,146.27)	826.00 (435.05 to 1,275.44)	28.27 (15.02 to 43.22)	38.83 (19.35 to 62.30)	41.59 (20.45 to 67.64)	38.44 (19.18 to 62.75)
Inflammatory bowel disease	566.39 (374.38 to 945.47)	657.31 (469.00 to 899.21)	668.30 (476.15 to 909.65)	666.80 (466.56 to 932.34)	15.59 (10.98 to 21.95)	26.17 (19.17 to 34.69)	28.15 (20.43 to 37.18)	24.24 (17.40 to 32.37)
Vascular intestinal disorders	613.85 (443.53 to 878.13)	946.88 (672.14 to 1,384.17)	974.70 (682.58 to 1,433.67)	965.40 (684.83 to 1,412.48)	26.99 (19.35 to 40.08)	54.22 (37.14 to 81.00)	58.86 (40.61 to 88.76)	50.95 (34.20 to 76.05)
Gallbladder and biliary diseases	1,591.91 (1,022.15 to 2,341.72)	1,890.84 (1,026.91 to 3,167.50)	1,487.42 (847.21 to 2,385.19)	2,322.33 (1,245.10 to 3,965.80)	58.63 (38.64 to 86.85)	92.77 (49.03 to 164.00)	78.61 (43.78 to 135.14)	104.43 (54.96 to 180.39)
Pancreatitis	4,351.15 (2,632.44 to 6,548.15)	6,124.62 (3,014.32 to 11,706.39)	5,168.46 (2,655.84 to 9,319.75)	7,569.14 (3,619.30 to 14,859.17)	111.24 (67.50 to 168.31)	184.50 (93.55 to 343.28)	162.11 (85.40 to 286.49)	218.35 (106.05 to 430.28)
Other digestive diseases	4,281.85 (2,634.67 to 7,293.55)	3,859.12 (2,691.72 to 5,187.26)	3,700.88 (2,615.08 to 4,932.57)	4,679.00 (3,232.77 to 6,397.58)	111.31 (79.12 to 151.91)	160.96 (115.43 to 227.23)	165.39 (117.86 to 235.93)	173.88 (123.60 to 243.63)
**Neurological disorders**	**22,274.67 (18,019.24 to 27,290.61)**	**43,484.10 (34,408.37 to 54,357.89)**	**43,238.48 (35,431.96 to 52,213.20)**	**40,948.92 (31,554.29 to 51,789.68)**	**1,156.51 (975.47 to 1,371.58)**	**3,417.58 (2,519.43 to 4,490.93)**	**3,653.41 (2,844.33 to 4,545.89)**	**2,792.90 (1,941.01 to 3,778.28)**
Alzheimer's disease and other dementias	10,932.59 (8,988.96 to 13,298.20)	31,350.27 (22,944.37 to 42,420.40)	31,707.91 (24,584.74 to 40,731.08)	26,935.35 (18,893.88 to 37,061.80)	919.05 (759.70 to 1,126.40)	2,981.36 (2,097.46 to 4,035.51)	3,187.44 (2,358.12 to 4,061.89)	2,382.56 (1,579.33 to 3,376.12)
Parkinson's disease	916.79 (694.32 to 1,154.69)	3,049.45 (2,305.22 to 3,925.56)	3,519.94 (2,712.93 to 4,528.23)	2,324.22 (1,693.72 to 3,061.32)	61.99 (46.84 to 80.63)	234.59 (171.27 to 314.67)	281.74 (208.61 to 370.06)	168.14 (116.40 to 229.32)
Epilepsy	8,111.55 (5,401.25 to 11,694.64)	6,345.32 (4,126.16 to 8,872.62)	5,272.66 (3,441.56 to 7,278.68)	8,892.44 (5,739.27 to 12,607.33)	133.51 (95.76 to 178.14)	132.87 (88.77 to 181.35)	112.75 (76.57 to 150.18)	177.36 (116.62 to 241.99)
Multiple sclerosis	195.09 (144.15 to 265.77)	349.15 (245.42 to 477.75)	346.04 (247.17 to 471.71)	338.98 (238.59 to 465.58)	5.12 (3.90 to 6.95)	10.12 (7.25 to 13.84)	10.19 (7.41 to 14.02)	9.60 (6.90 to 13.23)
Motor neuron disease	359.68 (212.45 to 614.37)	448.46 (356.38 to 570.86)	460.50 (365.65 to 586.47)	435.29 (342.84 to 576.16)	7.24 (5.32 to 10.50)	13.65 (11.14 to 17.36)	14.38 (11.79 to 18.19)	12.48 (10.16 to 15.76)
Other neurological disorders	1,758.97 (1,181.23 to 2,753.18)	1,941.46 (1,559.44 to 2,448.52)	1,931.43 (1,575.95 to 2,366.48)	2,022.65 (1,557.37 to 2,745.59)	29.59 (22.81 to 40.47)	44.98 (38.18 to 51.77)	46.91 (39.91 to 53.51)	42.76 (36.06 to 51.02)
**Mental disorders**	**5,447.64 (3,509.99 to 7,871.68)**	**9,038.21 (5,859.36 to 12,940.67)**	**8,730.84 (5,654.66 to 12,466.52)**	**9,436.05 (6,143.25 to 13,655.83)**	**118.99 (79.65 to 171.37)**	**218.00 (147.47 to 313.22)**	**214.02 (145.25 to 304.67)**	**222.52 (150.00 to 319.46)**
Alcohol use disorders	3,643.29 (2,116.06 to 5,814.86)	5,138.73 (2,799.63 to 8,741.66)	4,827.55 (2,626.64 to 8,110.46)	5,511.09 (3,003.21 to 9,301.91)	83.63 (49.50 to 133.21)	131.77 (73.11 to 218.51)	125.91 (69.87 to 207.98)	138.19 (78.16 to 231.44)
Drug use disorders	1,782.57 (1,076.56 to 2,872.60)	3,868.85 (2,395.29 to 5,872.61)	3,872.51 (2,396.90 to 5,865.34)	3,894.62 (2,408.16 to 5,977.17)	34.97 (21.70 to 56.11)	85.63 (56.58 to 128.31)	87.51 (58.67 to 130.08)	83.75 (55.34 to 127.01)
Opioid use disorders	1,174.74 (673.12 to 1,953.46)	2,090.20 (1,199.24 to 3,410.88)	2,079.54 (1,197.53 to 3,345.51)	2,132.03 (1,223.62 to 3,508.67)	22.36 (13.28 to 36.78)	44.95 (27.92 to 70.52)	45.69 (28.42 to 71.06)	44.48 (27.13 to 70.70)

Cocaine use disorders	63.70 (31.39 to 150.88)	175.29 (89.67 to 386.29)	170.93 (88.10 to 373.95)	190.80 (97.43 to 415.85)	1.31 (0.69 to 3.03)	4.01 (2.18 to 8.54)	3.95 (2.13 to 8.41)	4.28 (2.29 to 9.33)
Amphetamine use disorders	54.75 (27.10 to 117.24)	180.03 (93.48 to 396.97)	172.26 (88.47 to 368.33)	207.33 (107.44 to 449.44)	1.05 (0.55 to 2.24)	3.75 (2.08 to 8.04)	3.62 (1.99 to 7.75)	4.25 (2.32 to 9.08)
Other drug use disorders	489.38 (296.10 to 785.24)	1,423.33 (861.80 to 2,289.67)	1,449.77 (882.45 to 2,327.86)	1,364.46 (812.69 to 2,186.88)	10.24 (6.56 to 16.54)	32.93 (21.18 to 50.62)	34.25 (22.49 to 52.52)	30.74 (19.58 to 47.71)
Eating disorders	21.78 (13.92 to 38.36)	30.63 (19.25 to 50.94)	30.78 (19.79 to 51.39)	30.34 (19.34 to 49.28)	0.40 (0.25 to 0.71)	0.59 (0.38 to 1.00)	0.60 (0.39 to 1.01)	0.58 (0.38 to 0.96)

Laos								
Cause	YLLs (actual counts)				Deaths (actual counts)			
	2016	2040 Forecast	2040 Better Scenario	2040 Worse Scenario	2016	2040 Forecast	2040 Better Scenario	2040 Worse Scenario
**Diabetes, urogenital, blood, and endocrine diseases**	**93,164.37 (82,532.24 to 105,717.48)**	**145,184.47 (99,267.05 to 243,083.30)**	**119,463.24 (84,407.45 to 194,657.32)**	**198,023.71 (134,519.09 to 316,706.29)**	**3,056.48 (2,782.55 to 3,380.18)**	**6,663.62 (4,305.23 to 12,020.28)**	**5,690.28 (3,797.73 to 10,023.64)**	**8,482.77 (5,442.61 to 14,628.34)**
Diabetes mellitus	40,839.02 (36,347.46 to 45,593.23)	56,744.94 (37,575.11 to 80,174.16)	48,431.76 (33,236.31 to 69,036.67)	83,076.25 (53,109.90 to 121,859.40)	1,638.31 (1,452.21 to 1,843.97)	2,762.49 (1,883.59 to 3,730.39)	2,475.12 (1,739.37 to 3,349.01)	3,751.73 (2,452.36 to 5,253.39)
Acute glomerulonephritis	76.05 (60.18 to 100.88)	87.18 (64.91 to 115.94)	81.92 (60.84 to 107.76)	107.37 (80.12 to 145.18)	1.52 (1.21 to 1.94)	2.48 (1.77 to 3.38)	2.46 (1.74 to 3.41)	2.75 (1.99 to 3.68)
Chronic kidney disease	34,357.64 (29,024.47 to 40,334.31)	72,673.96 (30,983.25 to 172,163.76)	55,742.84 (25,191.79 to 131,362.02)	96,635.43 (41,937.56 to 229,660.11)	1,025.69 (898.17 to 1,188.55)	3,340.31 (1,136.52 to 8,571.38)	2,634.54 (970.18 to 6,997.30)	4,149.80 (1,454.71 to 10,539.94)
Chronic kidney disease due to diabetes mellitus	16,779.79 (13,741.79 to 19,932.82)	44,269.14 (18,055.91 to 109,510.18)	33,594.09 (14,550.03 to 80,583.46)	58,711.40 (23,822.68 to 145,205.42)	549.23 (466.96 to 649.69)	1,899.66 (638.35 to 4,997.96)	1,469.91 (530.14 to 3,896.46)	2,404.62 (817.08 to 6,174.52)
Chronic kidney disease due to hypertension	6,412.56 (5,251.77 to 7,681.97)	18,773.48 (6,601.23 to 50,662.15)	14,474.24 (5,444.72 to 39,851.64)	23,996.48 (8,500.52 to 62,278.78)	278.99 (233.46 to 330.64)	1,129.47 (344.89 to 3,102.41)	916.83 (302.02 to 2,767.06)	1,335.63 (400.61 to 3,587.47)
Chronic kidney disease due to glomerulonephritis	5,703.61 (4,249.44 to 7,518.24)	4,728.24 (2,893.36 to 8,478.38)	3,806.38 (2,365.41 to 6,536.17)	6,854.10 (4,237.76 to 11,470.70)	97.62 (76.17 to 123.30)	133.82 (65.65 to 318.67)	106.27 (54.33 to 247.58)	180.07 (93.05 to 396.34)
Chronic kidney disease due to other and unspecified causes	5,461.69 (3,803.36 to 7,888.65)	4,903.10 (2,622.89 to 9,747.72)	3,868.13 (2,164.94 to 7,653.61)	7,073.45 (3,892.68 to 13,378.05)	99.84 (75.72 to 133.58)	177.37 (71.62 to 439.38)	141.54 (60.38 to 374.76)	229.48 (97.51 to 539.95)
Urinary diseases and male infertility	5,354.88 (4,198.54 to 6,846.88)	6,170.86 (5,079.33 to 7,377.60)	6,181.50 (5,134.69 to 7,312.90)	6,652.43 (5,404.51 to 8,043.81)	152.70 (128.83 to 179.31)	271.13 (222.16 to 328.32)	290.74 (238.94 to 350.72)	259.26 (210.11 to 318.19)
Urinary tract infections	3,475.11 (2,385.15 to 4,767.17)	4,450.36 (3,465.40 to 5,392.48)	4,519.67 (3,560.89 to 5,478.05)	4,593.46 (3,475.05 to 5,608.78)	99.29 (74.03 to 121.64)	196.90 (153.39 to 240.91)	213.71 (166.78 to 259.30)	180.56 (138.16 to 224.26)
Urolithiasis	117.81 (85.21 to 162.95)	240.27 (169.40 to 343.84)	243.82 (173.32 to 348.59)	245.86 (172.14 to 356.86)	4.36 (3.19 to 6.18)	10.54 (7.44 to 15.02)	11.22 (7.95 to 15.95)	9.97 (6.96 to 14.60)
Other urinary diseases	1,761.95 (980.35 to 2,867.88)	1,480.23 (871.39 to 2,235.37)	1,418.00 (819.33 to 2,163.77)	1,813.12 (1,064.54 to 2,737.63)	49.05 (30.67 to 71.01)	63.70 (38.12 to 97.01)	65.81 (40.10 to 99.44)	68.72 (41.18 to 104.87)
Gynecological diseases	269.83 (129.60 to 351.17)	241.14 (116.41 to 339.22)	224.09 (107.66 to 319.77)	304.73 (152.90 to 435.10)	6.77 (3.29 to 8.79)	6.65 (3.19 to 9.27)	6.36 (3.10 to 8.98)	8.09 (4.06 to 11.28)
Uterine fibroids	41.31 (16.16 to 72.91)	37.54 (12.92 to 71.29)	34.53 (12.04 to 64.67)	49.10 (17.15 to 91.21)	1.30 (0.52 to 2.38)	1.24 (0.43 to 2.32)	1.16 (0.40 to 2.23)	1.57 (0.56 to 2.93)
Polycystic ovarian syndrome	13.96 (3.67 to 34.82)	7.27 (1.64 to 19.94)	6.31 (1.42 to 18.06)	11.11 (2.54 to 29.31)	0.32 (0.09 to 0.75)	0.17 (0.04 to 0.44)	0.15 (0.03 to 0.38)	0.26 (0.06 to 0.65)
Endometriosis	3.32 (0.88 to 8.57)	5.08 (1.22 to 14.10)	4.66 (1.10 to 13.19)	6.55 (1.54 to 18.15)	0.08 (0.02 to 0.21)	0.12 (0.03 to 0.34)	0.11 (0.02 to 0.32)	0.16 (0.03 to 0.44)
Genital prolapse	13.17 (3.23 to 24.68)	11.42 (2.63 to 22.28)	10.65 (2.45 to 21.07)	14.89 (3.43 to 29.64)	0.65 (0.16 to 1.20)	0.58 (0.13 to 1.10)	0.56 (0.13 to 1.10)	0.71 (0.17 to 1.35)
Other gynecological diseases	198.06 (95.31 to 276.32)	179.83 (80.81 to 264.57)	167.93 (78.74 to 248.53)	223.08 (110.48 to 327.90)	4.42 (2.20 to 6.08)	4.54 (2.10 to 6.59)	4.38 (2.03 to 6.42)	5.39 (2.67 to 7.87)
Hemoglobinopathies and hemolytic anemias	7,312.31 (4,954.96 to 10,467.08)	5,295.63 (4,061.43 to 7,104.24)	5,042.36 (3,855.26 to 6,774.54)	6,314.12 (4,818.80 to 8,491.29)	143.95 (110.86 to 191.37)	163.58 (129.12 to 219.50)	162.30 (128.20 to 218.11)	185.02 (143.15 to 248.12)
Thalassemias	4,197.41 (2,011.32 to 7,121.47)	1,590.10 (873.49 to 2,554.87)	1,520.33 (863.40 to 2,418.41)	1,793.45 (923.88 to 2,915.60)	51.40 (25.68 to 86.44)	20.26 (11.57 to 31.86)	19.43 (11.38 to 30.36)	22.58 (12.08 to 35.96)

Sickle cell disorders	9.64 (6.21 to 13.94)	7.12 (4.66 to 10.40)	6.69 (4.38 to 9.69)	8.65 (5.68 to 12.66)	0.15 (0.10 to 0.21)	0.14 (0.09 to 0.20)	0.13 (0.09 to 0.19)	0.16 (0.11 to 0.23)
G6PD deficiency	2,024.26 (1,527.05 to 2,729.92)	2,620.84 (1,894.93 to 3,663.61)	2,443.08 (1,744.07 to 3,433.03)	3,297.73 (2,384.71 to 4,573.88)	44.91 (34.53 to 60.66)	74.83 (55.95 to 104.69)	71.18 (52.48 to 100.35)	91.04 (66.93 to 126.24)
Other hemoglobinopathies and hemolytic anemias	1,081.00 (862.08 to 1,427.67)	1,077.57 (832.19 to 1,471.57)	1,072.26 (826.38 to 1,445.46)	1,214.29 (924.09 to 1,643.87)	47.50 (38.55 to 63.24)	68.36 (51.85 to 93.79)	71.56 (54.33 to 97.68)	71.24 (52.54 to 98.23)
Endocrine, metabolic, blood, and immune disorders	4,954.64 (2,881.33 to 8,334.48)	3,970.75 (3,042.78 to 5,724.83)	3,758.78 (2,905.22 to 5,314.68)	4,933.38 (3,597.02 to 7,381.48)	87.54 (62.30 to 132.15)	116.98 (92.97 to 164.49)	118.77 (93.79 to 166.51)	126.13 (100.41 to 178.51)

Laos								
Cause	YLLs (actual counts)				Deaths (actual counts)			
	2016	2040 Forecast	2040 Better Scenario	2040 Worse Scenario	2016	2040 Forecast	2040 Better Scenario	2040 Worse Scenario
**Musculoskeletal disorders**	**1,619.49 (1,224.60 to 2,464.81)**	**2,797.15 (2,124.03 to 4,531.34)**	**2,862.74 (2,171.60 to 4,614.29)**	**2,677.53 (2,037.86 to 4,264.77)**	**37.59 (29.19 to 59.49)**	**85.42 (64.49 to 149.24)**	**90.90 (68.34 to 160.49)**	**77.87 (58.90 to 133.89)**
Rheumatoid arthritis	159.00 (114.67 to 252.46)	328.04 (207.32 to 528.95)	315.82 (197.38 to 508.60)	375.49 (236.34 to 611.13)	6.40 (4.43 to 10.58)	16.68 (10.00 to 28.92)	16.75 (9.79 to 28.98)	17.90 (10.90 to 30.63)
Other musculoskeletal disorders	1,460.49 (1,095.41 to 2,237.69)	2,469.11 (1,829.27 to 3,965.22)	2,546.92 (1,907.96 to 4,125.33)	2,302.04 (1,698.12 to 3,647.81)	31.19 (23.94 to 48.74)	68.74 (51.58 to 118.03)	74.15 (55.32 to 128.37)	59.97 (44.94 to 101.52)
**Other non-communicable diseases**	**176,719.68 (71,452.67 to 353,437.19)**	**61,609.83 (29,465.40 to 113,930.16)**	**53,031.29 (26,380.01 to 95,787.60)**	**100,261.83 (44,721.08 to 191,170.25)**	**2,111.91 (889.38 to 4,176.38)**	**882.21 (480.47 to 1,506.78)**	**797.43 (463.10 to 1,310.05)**	**1,318.66 (662.48 to 2,401.70)**
Congenital birth defects	165,769.75 (64,832.31 to 343,041.25)	54,862.26 (23,954.76 to 107,317.60)	46,586.56 (20,973.89 to 88,817.53)	91,700.65 (37,412.04 to 183,358.46)	1,951.40 (769.81 to 4,015.54)	671.98 (303.22 to 1,279.94)	574.04 (267.35 to 1,087.94)	1,105.67 (473.26 to 2,182.17)
Neural tube defects	25,057.39 (7,542.61 to 63,269.04)	3,739.28 (970.79 to 10,471.42)	3,003.97 (770.10 to 8,400.29)	7,427.16 (1,913.96 to 20,278.56)	290.34 (87.68 to 733.51)	43.46 (11.40 to 121.27)	34.93 (9.02 to 97.38)	86.21 (22.37 to 234.91)
Congenital heart anomalies	83,888.63 (32,564.87 to 175,369.56)	28,890.88 (12,303.99 to 56,532.31)	24,739.80 (10,900.24 to 46,648.59)	46,814.17 (19,192.46 to 92,099.69)	994.51 (393.19 to 2,064.98)	359.21 (160.23 to 673.72)	309.61 (140.31 to 574.40)	571.74 (241.58 to 1,107.04)
Orofacial clefts	450.10 (72.99 to 1,297.23)	113.50 (17.89 to 348.18)	89.05 (14.22 to 268.51)	243.58 (36.60 to 778.93)	5.21 (0.85 to 15.01)	1.31 (0.21 to 4.03)	1.03 (0.16 to 3.11)	2.82 (0.42 to 9.01)
Down syndrome	1,512.42 (432.63 to 5,056.60)	880.84 (331.00 to 2,723.33)	744.08 (289.02 to 2,171.82)	1,503.13 (512.94 to 5,117.83)	18.46 (5.78 to 59.63)	12.00 (5.02 to 33.35)	10.31 (4.47 to 26.81)	19.59 (7.47 to 61.91)
Other chromosomal abnormalities	1,793.71 (579.32 to 4,396.86)	1,198.44 (365.80 to 2,865.87)	1,006.28 (317.72 to 2,402.78)	2,084.27 (631.62 to 5,157.93)	20.85 (6.74 to 50.99)	14.11 (4.39 to 33.61)	11.88 (3.83 to 28.02)	24.41 (7.54 to 60.06)
Congenital musculoskeletal and limb anomalies	2,968.26 (742.32 to 8,088.87)	1,339.01 (360.27 to 3,606.03)	1,152.69 (312.40 to 3,031.34)	2,160.34 (567.64 to 6,023.31)	34.55 (8.76 to 93.93)	15.74 (4.28 to 42.01)	13.57 (3.79 to 35.41)	25.29 (6.79 to 70.12)
Urogenital congenital anomalies	1,742.82 (647.90 to 3,913.34)	818.95 (376.71 to 1,615.34)	722.87 (342.54 to 1,415.08)	1,236.63 (534.80 to 2,586.23)	22.53 (9.19 to 48.13)	13.51 (6.94 to 24.79)	12.33 (6.47 to 22.38)	18.74 (9.12 to 36.31)
Digestive congenital anomalies	10,207.40 (3,129.99 to 25,657.84)	3,309.34 (1,049.96 to 8,044.04)	2,774.65 (908.91 to 6,680.17)	5,739.02 (1,813.61 to 14,106.62)	118.72 (36.63 to 297.91)	38.72 (12.40 to 93.64)	32.49 (10.90 to 77.77)	66.99 (21.41 to 164.01)
Other congenital birth defects	38,149.03 (10,761.67 to 99,913.62)	14,572.02 (4,639.15 to 36,400.56)	12,353.19 (4,052.27 to 30,230.24)	24,492.36 (7,292.84 to 63,423.85)	446.22 (128.09 to 1,164.09)	173.91 (57.57 to 429.07)	147.90 (50.15 to 357.71)	289.86 (89.03 to 742.43)
Skin and subcutaneous diseases	1,732.05 (1,121.12 to 2,815.06)	3,951.66 (2,603.43 to 6,226.05)	4,041.60 (2,680.34 to 6,398.10)	4,021.27 (2,643.39 to 6,320.65)	53.53 (35.64 to 87.80)	177.79 (119.13 to 290.74)	195.51 (130.52 to 316.75)	160.32 (106.17 to 264.16)
Cellulitis	143.91 (78.43 to 268.42)	485.35 (272.48 to 886.10)	517.86 (292.49 to 928.94)	433.02 (249.05 to 801.66)	4.32 (2.45 to 8.16)	20.96 (12.10 to 37.72)	23.69 (13.87 to 42.61)	17.06 (9.79 to 32.16)
Pyoderma	1,055.50 (648.59 to 1,769.33)	2,219.14 (1,390.79 to 3,495.66)	2,198.00 (1,358.26 to 3,488.56)	2,430.27 (1,525.74 to 3,824.95)	25.45 (15.94 to 42.64)	82.72 (52.45 to 136.19)	87.54 (55.24 to 144.90)	81.08 (51.75 to 136.66)
Decubitus ulcer	476.33 (292.11 to 820.63)	1,150.08 (708.18 to 1,983.44)	1,226.73 (766.71 to 2,117.51)	1,062.52 (651.59 to 1,821.96)	22.44 (14.03 to 39.15)	70.95 (41.80 to 127.32)	80.86 (48.35 to 142.34)	59.36 (34.32 to 106.36)
Other skin and subcutaneous diseases	56.31 (31.20 to 91.43)	97.09 (56.39 to 151.05)	99.00 (58.27 to 152.68)	95.46 (55.02 to 151.16)	1.33 (0.75 to 2.15)	3.16 (1.84 to 4.94)	3.42 (1.98 to 5.33)	2.82 (1.61 to 4.43)
Sudden infant death syndrome	9,217.88 (3,026.32 to 22,845.49)	2,795.91 (860.25 to 6,926.93)	2,403.13 (739.70 to 5,934.81)	4,539.91 (1,407.99 to 11,254.18)	106.98 (35.10 to 265.20)	32.44 (9.98 to 80.39)	27.88 (8.58 to 68.86)	52.67 (16.33 to 130.61)

**Injuries**	**252,448.28 (203,107.43 to 317,694.29)**	**171,036.68 (133,440.07 to 206,630.59)**	**147,467.76 (115,886.63 to 176,909.36)**	**263,858.10 (206,822.81 to 321,634.58)**	**4,352.25 (3,612.76 to 5,171.74)**	**3,872.50 (3,159.83 to 4,599.75)**	**3,469.53 (2,864.96 to 4,077.38)**	**5,526.96 (4,456.42 to 6,603.69)**
**Transport injuries**	**90,247.59 (71,290.63 to 114,621.80)**	**41,722.73 (29,885.53 to 58,218.38)**	**29,336.40 (21,361.96 to 40,915.19)**	**107,859.69 (78,486.79 to 141,842.63)**	**1,608.12 (1,320.57 to 1,954.06)**	**1,004.56 (732.51 to 1,392.97)**	**748.49 (553.95 to 1,016.81)**	**2,278.53 (1,675.57 to 3,046.01)**
Road injuries	84,763.50 (68,059.67 to 107,724.74)	35,486.81 (25,024.98 to 49,345.29)	23,758.89 (16,829.92 to 32,833.81)	100,152.49 (72,769.79 to 131,583.29)	1,513.26 (1,251.82 to 1,824.68)	858.69 (625.75 to 1,175.35)	613.18 (452.82 to 828.16)	2,110.40 (1,568.89 to 2,782.90)
Pedestrian road injuries	23,940.99 (16,660.90 to 35,519.74)	9,264.43 (6,430.87 to 13,458.32)	6,507.16 (4,599.52 to 9,507.27)	22,274.94 (15,275.86 to 31,452.78)	444.10 (334.39 to 620.90)	267.54 (192.99 to 374.40)	201.66 (148.04 to 279.07)	560.58 (402.92 to 775.00)

Laos								
Cause	YLLs (actual counts)				Deaths (actual counts)			
	2016	2040 Forecast	2040 Better Scenario	2040 Worse Scenario	2016	2040 Forecast	2040 Better Scenario	2040 Worse Scenario
Cyclist road injuries	2,573.73 (1,712.63 to 3,864.38)	1,268.94 (751.19 to 2,044.77)	863.37 (512.85 to 1,380.55)	3,516.55 (2,138.71 to 5,409.56)	52.79 (35.44 to 76.98)	37.50 (22.21 to 60.23)	27.89 (16.73 to 44.89)	85.03 (51.08 to 133.19)
Motorcyclist road injuries	22,837.32 (17,514.36 to 28,783.02)	12,309.47 (7,593.21 to 18,163.86)	8,806.06 (5,489.55 to 13,121.24)	28,761.48 (18,790.53 to 39,896.64)	379.74 (291.80 to 481.36)	243.04 (150.59 to 360.63)	180.12 (112.76 to 263.77)	519.58 (343.99 to 731.51)
Motor vehicle road injuries	33,694.68 (24,203.95 to 45,171.79)	11,938.77 (7,919.88 to 17,720.67)	7,140.75 (4,824.77 to 10,430.48)	43,209.83 (29,655.31 to 60,953.53)	604.97 (445.67 to 775.84)	290.34 (200.29 to 422.30)	188.97 (133.71 to 268.35)	892.07 (630.78 to 1,249.14)
Other road injuries	1,716.78 (1,075.26 to 3,106.58)	705.19 (414.73 to 1,265.92)	441.54 (265.34 to 778.87)	2,389.70 (1,386.71 to 4,411.29)	31.66 (20.40 to 56.62)	20.26 (11.85 to 38.29)	14.54 (8.64 to 27.88)	53.14 (31.48 to 99.67)
Other transport injuries	5,484.09 (2,494.05 to 9,336.69)	6,235.92 (3,055.04 to 9,994.02)	5,577.51 (2,830.19 to 8,739.19)	7,707.20 (3,802.99 to 12,257.08)	94.86 (45.99 to 146.96)	145.88 (70.06 to 227.31)	135.31 (66.78 to 207.48)	168.13 (80.31 to 265.83)
**Unintentional injuries**	**124,862.33 (92,104.77 to 173,296.47)**	**73,375.41 (57,981.55 to 90,336.15)**	**67,755.87 (53,470.34 to 82,995.81)**	**92,793.57 (71,870.69 to 117,822.88)**	**2,040.06 (1,629.28 to 2,627.57)**	**1,705.89 (1,406.71 to 2,014.73)**	**1,659.33 (1,364.33 to 1,947.63)**	**1,964.53 (1,605.51 to 2,392.75)**
Falls	17,191.42 (12,434.55 to 24,858.34)	15,743.50 (12,236.69 to 19,721.39)	15,486.00 (12,061.68 to 19,286.54)	17,358.27 (13,374.43 to 21,992.76)	375.47 (296.65 to 479.00)	523.29 (417.66 to 648.20)	546.92 (441.10 to 680.91)	521.78 (413.47 to 651.82)
Drowning	48,630.94 (35,247.78 to 67,245.94)	23,749.02 (17,650.82 to 32,052.74)	21,189.22 (15,890.84 to 28,290.05)	31,149.63 (22,643.99 to 42,430.34)	720.08 (543.39 to 956.13)	443.33 (334.51 to 584.41)	405.21 (307.99 to 529.08)	552.07 (412.90 to 727.69)
Fire, heat, and hot substances	13,036.28 (8,454.48 to 20,416.10)	7,177.19 (4,856.34 to 11,163.43)	6,435.50 (4,376.28 to 9,890.51)	9,554.54 (6,374.63 to 14,967.84)	217.87 (153.83 to 320.70)	170.74 (119.23 to 266.15)	160.67 (113.55 to 247.01)	208.28 (143.83 to 333.95)
Poisonings	3,516.63 (1,919.07 to 6,315.42)	1,523.85 (878.87 to 2,214.79)	1,319.64 (763.78 to 1,924.06)	2,215.15 (1,219.57 to 3,449.45)	57.90 (33.35 to 94.20)	31.56 (18.92 to 44.55)	28.27 (17.40 to 39.96)	43.06 (24.38 to 62.46)
Exposure to mechanical forces	9,890.35 (6,659.31 to 15,432.71)	5,394.54 (3,631.58 to 7,864.69)	4,901.51 (3,314.56 to 7,219.62)	7,271.65 (4,796.83 to 11,109.27)	154.24 (107.69 to 228.78)	107.14 (72.77 to 156.73)	100.25 (68.77 to 146.11)	135.70 (93.29 to 196.88)
Unintentional firearm injuries	1,466.83 (826.23 to 3,430.31)	902.48 (480.06 to 2,195.26)	823.97 (436.82 to 1,990.42)	1,187.41 (627.13 to 2,912.37)	25.71 (14.92 to 60.95)	19.69 (10.87 to 48.56)	18.54 (10.17 to 46.22)	24.59 (13.36 to 61.26)
Unintentional suffocation	3,297.02 (1,215.99 to 7,269.11)	1,079.96 (542.33 to 2,079.63)	924.08 (470.39 to 1,766.57)	1,753.71 (775.69 to 3,635.02)	40.54 (16.23 to 87.05)	14.80 (8.08 to 26.81)	12.85 (7.28 to 22.71)	23.14 (11.34 to 45.21)
Other exposure to mechanical forces	5,126.50 (3,338.63 to 7,098.35)	3,412.10 (2,143.70 to 4,952.27)	3,153.46 (1,967.58 to 4,571.36)	4,330.53 (2,713.42 to 6,244.73)	87.99 (58.15 to 122.80)	72.65 (47.26 to 105.78)	68.86 (44.62 to 100.54)	87.97 (56.98 to 126.50)
Adverse effects of medical treatment	8,261.63 (5,359.89 to 12,841.99)	5,334.38 (3,822.55 to 6,772.23)	4,998.86 (3,547.70 to 6,372.71)	6,719.67 (4,840.47 to 8,712.94)	142.35 (105.06 to 191.70)	137.96 (99.72 to 178.34)	136.21 (97.04 to 178.19)	157.13 (115.39 to 197.33)
Animal contact	3,245.46 (1,409.02 to 5,836.81)	2,730.80 (1,246.48 to 4,607.05)	2,474.54 (1,134.56 to 4,197.05)	3,700.31 (1,608.55 to 6,504.24)	59.40 (26.94 to 97.92)	66.15 (31.21 to 107.21)	62.55 (29.23 to 99.54)	83.12 (37.88 to 139.29)
Venomous animal contact	1,524.26 (635.60 to 2,983.34)	1,212.61 (523.98 to 2,310.71)	1,096.64 (467.97 to 2,118.47)	1,655.78 (703.58 to 3,247.56)	28.58 (12.45 to 53.11)	30.31 (13.58 to 55.73)	28.70 (12.70 to 52.78)	38.15 (16.75 to 72.15)
Non-venomous animal contact	1,721.20 (663.80 to 3,111.43)	1,518.19 (636.57 to 2,575.63)	1,377.90 (579.71 to 2,359.05)	2,044.53 (820.45 to 3,622.64)	30.81 (12.12 to 50.81)	35.84 (15.03 to 58.01)	33.85 (14.48 to 54.40)	44.96 (18.27 to 74.93)
Foreign body	13,755.73 (6,181.71 to 32,716.07)	6,807.92 (3,968.57 to 12,241.66)	6,359.95 (3,798.04 to 10,829.99)	8,775.96 (4,610.49 to 18,268.22)	187.93 (95.60 to 405.23)	126.33 (85.17 to 185.58)	124.65 (85.54 to 171.08)	145.07 (89.77 to 252.33)
Pulmonary aspiration and foreign body in airway	12,727.04 (5,268.81 to 31,216.32)	6,230.36 (3,333.33 to 11,469.73)	5,826.76 (3,233.51 to 10,214.00)	8,021.46 (3,841.20 to 17,100.85)	171.76 (79.60 to 385.06)	113.64 (71.37 to 172.23)	112.37 (72.65 to 158.76)	130.06 (73.67 to 232.66)

Foreign body in other body part	1,028.69 (489.68 to 2,395.64)	577.56 (326.18 to 1,109.41)	533.19 (308.81 to 1,006.72)	754.49 (400.55 to 1,555.29)	16.17 (8.63 to 34.76)	12.68 (7.76 to 24.11)	12.28 (7.62 to 23.32)	15.01 (8.77 to 28.90)
Environmental heat and cold exposure	597.24 (407.24 to 875.51)	420.77 (278.27 to 594.74)	375.93 (245.11 to 529.43)	607.11 (409.41 to 847.81)	11.08 (8.12 to 15.15)	11.13 (7.65 to 16.54)	10.43 (6.97 to 15.58)	14.71 (10.13 to 21.93)
Other unintentional injuries	6,736.64 (5,021.43 to 9,087.43)	4,493.44 (3,135.58 to 6,481.49)	4,214.73 (2,929.95 to 6,099.66)	5,441.28 (3,864.06 to 7,866.64)	113.74 (84.05 to 154.61)	88.27 (63.30 to 126.03)	84.17 (60.31 to 120.32)	103.61 (74.20 to 150.09)
**Self-harm and interpersonal violence**	**37,269.11 (25,488.04 to 46,994.95)**	**52,728.31 (30,437.97 to 77,665.39)**	**47,228.09 (28,633.11 to 66,919.18)**	**59,798.27 (32,376.28 to 91,099.62)**	**702.84 (490.48 to 878.74)**	**1,100.79 (652.90 to 1,619.73)**	**1,000.51 (620.59 to 1,390.49)**	**1,221.24 (670.60 to 1,848.01)**

Laos								
Cause	YLLs (actual counts)				Deaths (actual counts)			
	2016	2040 Forecast	2040 Better Scenario	2040 Worse Scenario	2016	2040 Forecast	2040 Better Scenario	2040 Worse Scenario
Self-harm	34,729.59 (22,751.73 to 44,505.73)	50,442.19 (28,328.60 to 75,407.10)	45,011.52 (26,307.59 to 64,781.07)	57,402.56 (30,159.64 to 88,605.39)	659.46 (438.98 to 831.32)	1,057.21 (608.86 to 1,580.67)	957.88 (575.10 to 1,350.21)	1,176.37 (627.21 to 1,804.13)
Self-harm by firearm	1,920.13 (450.14 to 4,431.35)	2,243.01 (501.43 to 5,405.17)	1,967.88 (447.43 to 4,631.29)	2,633.92 (585.14 to 6,398.63)	37.48 (9.07 to 86.16)	49.59 (11.31 to 119.58)	44.51 (10.38 to 105.41)	56.53 (12.52 to 139.35)
Self-harm by other specified means	32,809.46 (21,287.65 to 42,052.06)	48,199.17 (26,964.85 to 72,175.56)	43,043.64 (25,254.04 to 62,006.36)	54,768.65 (29,225.58 to 84,679.07)	621.98 (409.66 to 790.67)	1,007.62 (578.86 to 1,488.76)	913.37 (545.96 to 1,294.16)	1,119.84 (609.80 to 1,716.42)
Interpersonal violence	2,539.52 (1,759.55 to 3,387.83)	2,286.12 (1,448.81 to 3,244.06)	2,216.57 (1,394.49 to 3,124.69)	2,395.70 (1,559.60 to 3,327.54)	43.38 (29.82 to 56.90)	43.58 (28.14 to 61.00)	42.63 (27.39 to 59.24)	44.87 (29.60 to 62.29)
Physical violence by firearm	529.87 (293.41 to 1,001.32)	494.18 (259.03 to 975.72)	488.28 (253.29 to 965.42)	507.98 (264.39 to 1,011.87)	9.23 (5.22 to 17.87)	9.26 (4.92 to 18.46)	9.20 (4.94 to 18.23)	9.42 (5.02 to 19.12)
Physical violence by sharp object	952.07 (556.44 to 1,431.53)	998.80 (564.81 to 1,576.95)	1,003.62 (567.97 to 1,582.94)	979.86 (555.07 to 1,543.99)	16.57 (9.82 to 24.98)	18.69 (10.59 to 28.89)	18.91 (10.56 to 28.88)	18.14 (10.46 to 28.28)
Physical violence by other means	1,057.58 (704.51 to 1,515.73)	793.15 (472.21 to 1,235.55)	724.66 (430.97 to 1,122.00)	907.86 (545.88 to 1,395.73)	17.58 (11.84 to 24.72)	15.63 (9.49 to 23.86)	14.51 (8.97 to 21.68)	17.31 (10.63 to 25.99)
**Forces of nature, conflict and terrorism, and executions and police conflict**	**69.26 (51.37 to 92.57)**	**3,210.24 (0.00 to 15,284.61)**	**3,147.40 (0.00 to 14,953.65)**	**3,406.56 (0.00 to 16,247.95)**	**1.23 (0.89 to 1.68)**	**61.26 (0.00 to 287.18)**	**61.20 (0.00 to 286.51)**	**62.66 (0.00 to 295.33)**
Exposure to forces of nature	0.00 (0.00 to 0.00)	291.87 (0.00 to 938.79)	285.70 (0.00 to 914.21)	311.32 (0.00 to 1,010.05)	0.00 (0.00 to 0.00)	6.17 (0.00 to 19.45)	6.21 (0.00 to 19.54)	6.24 (0.00 to 19.80)
Conflict and terrorism	0.00 (0.00 to 0.00)	2,916.93 (0.00 to 15,018.75)	2,860.27 (0.00 to 14,736.35)	3,093.77 (0.00 to 15,936.74)	0.00 (0.00 to 0.00)	55.06 (0.00 to 283.15)	54.96 (0.00 to 282.42)	56.38 (0.00 to 289.95)
Executions and police conflict	69.26 (51.37 to 92.57)	1.44 (0.00 to 23.10)	1.42 (0.00 to 22.89)	1.48 (0.00 to 23.77)	1.23 (0.89 to 1.68)	0.03 (0.00 to 0.44)	0.03 (0.00 to 0.44)	0.03 (0.00 to 0.45)

Malaysia								
Cause	YLLs (in 1000s)				Deaths (in 1000s)			
	2016	2040 Forecast	2040 Better Scenario	2040 Worse Scenario	2016	2040 Forecast	2040 Better Scenario	2040 Worse Scenario
**All causes**	**3,793.39 (3,615.55 to 3,969.12)**	**4,875.64 (4,031.58 to 5,743.91)**	**4,008.98 (3,312.72 to 4,724.29)**	**6,910.45 (5,641.28 to 8,270.76)**	**150.99 (144.50 to 157.75)**	**274.47 (226.49 to 336.25)**	**234.95 (191.59 to 290.26)**	**358.83 (297.28 to 432.45)**
**Communicable, maternal, neonatal, and nutritional diseases**	**634.65 (495.61 to 719.25)**	**821.51 (574.40 to 1,044.72)**	**689.22 (489.29 to 859.78)**	**1,092.69 (758.61 to 1,425.61)**	**21.40 (15.44 to 24.72)**	**41.29 (27.35 to 52.91)**	**36.03 (24.07 to 45.70)**	**51.24 (33.32 to 67.48)**
**HIV/AIDS and tuberculosis**	**84.98 (58.22 to 127.61)**	**41.63 (16.28 to 108.63)**	**31.02 (10.67 to 92.71)**	**81.46 (42.29 to 151.16)**	**2.43 (1.80 to 3.38)**	**1.48 (0.75 to 3.05)**	**1.08 (0.53 to 2.58)**	**2.80 (1.63 to 4.60)**
Tuberculosis	33.14 (29.16 to 37.20)	18.40 (11.44 to 29.32)	11.80 (7.72 to 17.97)	38.81 (23.53 to 65.18)	1.29 (1.14 to 1.46)	0.98 (0.63 to 1.48)	0.66 (0.44 to 0.97)	1.91 (1.18 to 3.14)
HIV/AIDS	51.84 (24.74 to 96.89)	23.24 (2.95 to 87.56)	19.22 (1.84 to 81.58)	42.64 (12.47 to 107.95)	1.14 (0.52 to 2.14)	0.50 (0.05 to 2.04)	0.42 (0.03 to 1.93)	0.88 (0.22 to 2.44)
**Diarrhea, lower respiratory, and other common infectious diseases**	**398.81 (272.45 to 465.56)**	**584.68 (346.68 to 783.01)**	**478.13 (288.94 to 633.24)**	**785.95 (464.52 to 1,100.94)**	**16.35 (10.47 to 19.46)**	**34.70 (20.69 to 46.07)**	**29.95 (17.94 to 39.52)**	**43.05 (25.36 to 59.11)**
Diarrheal diseases	14.68 (9.38 to 20.51)	49.68 (15.36 to 131.63)	55.02 (20.40 to 127.72)	29.08 (7.94 to 79.67)	0.50 (0.28 to 0.76)	3.28 (1.00 to 8.48)	3.85 (1.40 to 9.05)	1.73 (0.47 to 4.95)
Intestinal infectious diseases	22.05 (11.20 to 38.69)	28.02 (13.30 to 52.26)	31.34 (15.21 to 55.78)	22.72 (9.74 to 44.05)	0.34 (0.17 to 0.59)	0.46 (0.22 to 0.84)	0.51 (0.26 to 0.92)	0.37 (0.16 to 0.70)
Typhoid fever	19.20 (9.37 to 34.40)	25.98 (12.17 to 47.92)	29.63 (14.50 to 52.35)	19.88 (8.41 to 38.51)	0.29 (0.15 to 0.52)	0.42 (0.20 to 0.78)	0.49 (0.25 to 0.86)	0.32 (0.14 to 0.61)
Paratyphoid fever	2.84 (1.18 to 5.92)	2.04 (0.77 to 4.59)	1.70 (0.64 to 3.75)	2.83 (1.04 to 6.43)	0.04 (0.02 to 0.09)	0.03 (0.01 to 0.08)	0.03 (0.01 to 0.06)	0.05 (0.02 to 0.10)
Other intestinal infectious diseases	0.01 (0.00 to 0.01)	0.00 (0.00 to 0.01)	0.00 (0.00 to 0.01)	0.00 (0.00 to 0.01)	0.00 (0.00 to 0.00)	0.00 (0.00 to 0.00)	0.00 (0.00 to 0.00)	0.00 (0.00 to 0.00)
Lower respiratory infections	313.89 (193.10 to 373.97)	456.30 (240.95 to 644.63)	345.95 (181.13 to 478.14)	668.28 (355.67 to 986.60)	14.52 (8.75 to 17.54)	29.65 (16.38 to 40.62)	24.35 (13.26 to 33.27)	39.38 (21.99 to 55.22)
Upper respiratory infections	0.08 (0.04 to 0.13)	0.06 (0.02 to 0.12)	0.04 (0.02 to 0.09)	0.10 (0.04 to 0.22)	0.00 (0.00 to 0.00)	0.00 (0.00 to 0.01)	0.00 (0.00 to 0.00)	0.00 (0.00 to 0.01)
Otitis media	0.00 (0.00 to 0.01)	0.00 (0.00 to 0.01)	0.00 (0.00 to 0.01)	0.00 (0.00 to 0.01)	0.00 (0.00 to 0.00)	0.00 (0.00 to 0.00)	0.00 (0.00 to 0.00)	0.00 (0.00 to 0.00)
Meningitis	24.66 (18.35 to 35.67)	11.62 (7.72 to 18.41)	9.62 (6.28 to 15.24)	17.31 (11.39 to 27.51)	0.52 (0.38 to 0.75)	0.34 (0.24 to 0.51)	0.29 (0.20 to 0.44)	0.48 (0.34 to 0.73)
Pneumococcal meningitis	0.22 (0.15 to 0.33)	0.11 (0.07 to 0.17)	0.11 (0.07 to 0.17)	0.11 (0.07 to 0.17)	0.00 (0.00 to 0.01)	0.00 (0.00 to 0.01)	0.00 (0.00 to 0.01)	0.00 (0.00 to 0.01)
H influenzae type B meningitis	1.77 (1.27 to 2.55)	1.76 (0.85 to 3.29)	1.68 (0.87 to 2.98)	2.36 (0.88 to 5.24)	0.04 (0.03 to 0.05)	0.04 (0.03 to 0.06)	0.04 (0.03 to 0.06)	0.05 (0.03 to 0.08)
Meningococcal meningitis	8.56 (6.03 to 12.98)	3.78 (2.47 to 6.33)	3.08 (1.96 to 5.11)	5.58 (3.68 to 8.94)	0.19 (0.13 to 0.28)	0.12 (0.08 to 0.19)	0.10 (0.07 to 0.16)	0.17 (0.12 to 0.26)
Other meningitis	14.10 (10.37 to 20.82)	5.97 (3.91 to 9.74)	4.75 (3.07 to 7.71)	9.25 (6.12 to 15.17)	0.29 (0.21 to 0.42)	0.17 (0.12 to 0.27)	0.14 (0.10 to 0.22)	0.26 (0.18 to 0.40)
Encephalitis	19.24 (14.64 to 24.21)	24.79 (17.28 to 33.49)	24.33 (16.55 to 33.15)	25.64 (17.82 to 34.00)	0.42 (0.32 to 0.50)	0.74 (0.52 to 0.95)	0.74 (0.51 to 0.96)	0.73 (0.52 to 0.93)

Diphtheria	0.01 (0.01 to 0.02)	0.00 (0.00 to 0.01)	0.00 (0.00 to 0.01)	0.01 (0.00 to 0.02)	0.00 (0.00 to 0.00)	0.00 (0.00 to 0.00)	0.00 (0.00 to 0.00)	0.00 (0.00 to 0.00)
Whooping cough	1.26 (0.11 to 4.24)	0.63 (0.01 to 2.67)	0.27 (0.01 to 1.12)	3.28 (0.03 to 13.39)	0.01 (0.00 to 0.05)	0.01 (0.00 to 0.03)	0.00 (0.00 to 0.01)	0.04 (0.00 to 0.16)
Tetanus	0.31 (0.23 to 0.47)	0.06 (0.04 to 0.10)	0.06 (0.04 to 0.10)	0.09 (0.05 to 0.18)	0.01 (0.01 to 0.02)	0.00 (0.00 to 0.00)	0.00 (0.00 to 0.00)	0.00 (0.00 to 0.00)
Measles	1.98 (0.62 to 4.67)	13.12 (0.47 to 44.19)	11.15 (0.37 to 35.24)	18.94 (0.60 to 65.62)	0.02 (0.01 to 0.06)	0.21 (0.01 to 0.79)	0.18 (0.01 to 0.66)	0.29 (0.01 to 1.08)

Malaysia								
Cause	YLLs (in 1000s)				Deaths (in 1000s)			
	2016	2040 Forecast	2040 Better Scenario	2040 Worse Scenario	2016	2040 Forecast	2040 Better Scenario	2040 Worse Scenario
Varicella and herpes zoster	0.65 (0.40 to 1.03)	0.39 (0.19 to 0.73)	0.34 (0.17 to 0.65)	0.50 (0.23 to 0.96)	0.01 (0.01 to 0.02)	0.02 (0.01 to 0.03)	0.02 (0.01 to 0.03)	0.02 (0.01 to 0.03)
**Neglected tropical diseases and malaria**	**23.15 (11.10 to 38.50)**	**100.06 (54.43 to 147.63)**	**97.98 (54.12 to 141.64)**	**103.15 (54.49 to 157.63)**	**0.47 (0.23 to 0.79)**	**2.69 (1.55 to 4.14)**	**2.70 (1.58 to 4.09)**	**2.67 (1.49 to 4.11)**
Malaria	0.76 (0.52 to 1.32)	0.62 (0.39 to 1.26)	0.61 (0.38 to 1.27)	0.62 (0.39 to 1.25)	0.02 (0.01 to 0.03)	0.02 (0.01 to 0.04)	0.02 (0.01 to 0.04)	0.02 (0.01 to 0.04)
Chagas disease	0.00 (0.00 to 0.00)	0.00 (0.00 to 0.00)	0.00 (0.00 to 0.00)	0.00 (0.00 to 0.00)	0.00 (0.00 to 0.00)	0.00 (0.00 to 0.00)	0.00 (0.00 to 0.00)	0.00 (0.00 to 0.00)
Leishmaniasis	0.00 (0.00 to 0.00)	0.00 (0.00 to 0.00)	0.00 (0.00 to 0.00)	0.00 (0.00 to 0.00)	0.00 (0.00 to 0.00)	0.00 (0.00 to 0.00)	0.00 (0.00 to 0.00)	0.00 (0.00 to 0.00)
African trypanosomiasis	0.00 (0.00 to 0.00)	0.00 (0.00 to 0.00)	0.00 (0.00 to 0.00)	0.00 (0.00 to 0.00)	0.00 (0.00 to 0.00)	0.00 (0.00 to 0.00)	0.00 (0.00 to 0.00)	0.00 (0.00 to 0.00)
Schistosomiasis	0.00 (0.00 to 0.00)	0.00 (0.00 to 0.00)	0.00 (0.00 to 0.00)	0.00 (0.00 to 0.00)	0.00 (0.00 to 0.00)	0.00 (0.00 to 0.00)	0.00 (0.00 to 0.00)	0.00 (0.00 to 0.00)
Cysticercosis	0.00 (0.00 to 0.00)	0.00 (0.00 to 0.00)	0.00 (0.00 to 0.00)	0.00 (0.00 to 0.00)	0.00 (0.00 to 0.00)	0.00 (0.00 to 0.00)	0.00 (0.00 to 0.00)	0.00 (0.00 to 0.00)
Cystic echinococcosis	0.00 (0.00 to 0.01)	0.00 (0.00 to 0.01)	0.00 (0.00 to 0.00)	0.01 (0.00 to 0.01)	0.00 (0.00 to 0.00)	0.00 (0.00 to 0.00)	0.00 (0.00 to 0.00)	0.00 (0.00 to 0.00)
Dengue	21.94 (9.76 to 37.41)	98.74 (52.63 to 146.63)	96.65 (51.57 to 140.36)	101.85 (52.90 to 156.48)	0.45 (0.19 to 0.77)	2.66 (1.50 to 4.11)	2.66 (1.54 to 4.07)	2.64 (1.44 to 4.08)
Yellow fever	0.00 (0.00 to 0.00)	0.00 (0.00 to 0.00)	0.00 (0.00 to 0.00)	0.00 (0.00 to 0.00)	0.00 (0.00 to 0.00)	0.00 (0.00 to 0.00)	0.00 (0.00 to 0.00)	0.00 (0.00 to 0.00)
Rabies	0.00 (0.00 to 0.00)	0.00 (0.00 to 0.00)	0.00 (0.00 to 0.00)	0.00 (0.00 to 0.00)	0.00 (0.00 to 0.00)	0.00 (0.00 to 0.00)	0.00 (0.00 to 0.00)	0.00 (0.00 to 0.00)
Intestinal nematode infections	0.25 (0.12 to 0.45)	0.39 (0.20 to 0.67)	0.39 (0.19 to 0.65)	0.39 (0.19 to 0.67)	0.00 (0.00 to 0.01)	0.01 (0.01 to 0.02)	0.01 (0.01 to 0.02)	0.01 (0.00 to 0.01)
Other neglected tropical diseases	0.20 (0.17 to 0.24)	0.31 (0.24 to 0.39)	0.33 (0.25 to 0.42)	0.28 (0.23 to 0.35)	0.00 (0.00 to 0.00)	0.01 (0.01 to 0.01)	0.01 (0.01 to 0.01)	0.01 (0.01 to 0.01)
**Maternal disorders**	**13.24 (10.17 to 16.79)**	**9.43 (5.47 to 14.80)**	**8.28 (4.80 to 13.22)**	**12.11 (7.17 to 19.00)**	**0.25 (0.20 to 0.32)**	**0.18 (0.11 to 0.29)**	**0.16 (0.09 to 0.26)**	**0.24 (0.14 to 0.37)**
Maternal hemorrhage	4.97 (3.47 to 6.81)	3.26 (1.73 to 5.51)	2.88 (1.50 to 4.86)	4.13 (2.21 to 6.93)	0.09 (0.07 to 0.13)	0.06 (0.03 to 0.11)	0.06 (0.03 to 0.09)	0.08 (0.04 to 0.13)
Maternal sepsis and other maternal infections	0.86 (0.47 to 1.41)	0.56 (0.24 to 1.07)	0.49 (0.21 to 0.95)	0.72 (0.32 to 1.36)	0.02 (0.01 to 0.03)	0.01 (0.00 to 0.02)	0.01 (0.00 to 0.02)	0.01 (0.01 to 0.03)
Maternal hypertensive disorders	1.60 (1.06 to 2.26)	1.10 (0.56 to 1.93)	0.96 (0.48 to 1.68)	1.43 (0.74 to 2.48)	0.03 (0.02 to 0.04)	0.02 (0.01 to 0.04)	0.02 (0.01 to 0.03)	0.03 (0.01 to 0.05)
Maternal obstructed labor and uterine rupture	0.19 (0.08 to 0.33)	0.13 (0.05 to 0.30)	0.11 (0.04 to 0.25)	0.18 (0.07 to 0.40)	0.00 (0.00 to 0.01)	0.00 (0.00 to 0.01)	0.00 (0.00 to 0.00)	0.00 (0.00 to 0.01)
Maternal abortion, miscarriage, and ectopic pregnancy	2.55 (1.72 to 3.71)	2.00 (1.03 to 3.50)	1.75 (0.91 to 3.08)	2.63 (1.37 to 4.52)	0.05 (0.03 to 0.07)	0.04 (0.02 to 0.07)	0.04 (0.02 to 0.06)	0.05 (0.03 to 0.09)

Indirect maternal deaths	1.47 (0.97 to 2.12)	1.09 (0.52 to 1.88)	0.94 (0.45 to 1.66)	1.43 (0.69 to 2.54)	0.03 (0.02 to 0.04)	0.02 (0.01 to 0.04)	0.02 (0.01 to 0.03)	0.03 (0.01 to 0.05)
Late maternal deaths	0.11 (0.04 to 0.21)	0.07 (0.02 to 0.15)	0.06 (0.02 to 0.13)	0.09 (0.03 to 0.20)	0.00 (0.00 to 0.00)	0.00 (0.00 to 0.00)	0.00 (0.00 to 0.00)	0.00 (0.00 to 0.00)
Maternal deaths aggravated by HIV/AIDS	0.01 (0.00 to 0.01)	0.03 (0.01 to 0.07)	0.02 (0.00 to 0.06)	0.04 (0.01 to 0.09)	0.00 (0.00 to 0.00)	0.00 (0.00 to 0.00)	0.00 (0.00 to 0.00)	0.00 (0.00 to 0.00)
Other maternal disorders	1.50 (0.95 to 2.15)	1.19 (0.60 to 2.12)	1.07 (0.52 to 1.92)	1.47 (0.75 to 2.59)	0.03 (0.02 to 0.04)	0.02 (0.01 to 0.04)	0.02 (0.01 to 0.04)	0.03 (0.01 to 0.05)

Malaysia								
Cause	YLLs (in 1000s)				Deaths (in 1000s)			
	2016	2040 Forecast	2040 Better Scenario	2040 Worse Scenario	2016	2040 Forecast	2040 Better Scenario	2040 Worse Scenario
**Neonatal disorders**	**86.25 (68.23 to 109.51)**	**49.28 (27.55 to 78.38)**	**38.83 (21.47 to 61.84)**	**69.65 (38.21 to 109.33)**	**1.00 (0.79 to 1.27)**	**0.57 (0.32 to 0.91)**	**0.45 (0.25 to 0.71)**	**0.80 (0.44 to 1.26)**
Neonatal preterm birth	34.56 (24.40 to 46.36)	14.31 (7.79 to 23.12)	9.72 (5.29 to 15.56)	24.38 (13.50 to 38.30)	0.40 (0.28 to 0.54)	0.17 (0.09 to 0.27)	0.11 (0.06 to 0.18)	0.28 (0.16 to 0.44)
Neonatal encephalopathy due to birth asphyxia and trauma	19.97 (13.46 to 28.80)	8.52 (3.99 to 16.03)	6.43 (3.09 to 12.06)	13.08 (5.95 to 25.05)	0.23 (0.16 to 0.33)	0.10 (0.05 to 0.19)	0.07 (0.04 to 0.14)	0.15 (0.07 to 0.29)
Neonatal sepsis and other neonatal infections	14.22 (9.57 to 20.77)	10.79 (5.71 to 18.35)	8.64 (4.61 to 14.84)	14.85 (7.70 to 25.71)	0.16 (0.11 to 0.24)	0.12 (0.07 to 0.21)	0.10 (0.05 to 0.17)	0.17 (0.09 to 0.30)
Hemolytic disease and other neonatal jaundice	0.23 (0.14 to 0.34)	0.10 (0.04 to 0.20)	0.08 (0.03 to 0.16)	0.14 (0.06 to 0.29)	0.00 (0.00 to 0.00)	0.00 (0.00 to 0.00)	0.00 (0.00 to 0.00)	0.00 (0.00 to 0.00)
Other neonatal disorders	17.27 (11.40 to 24.68)	15.56 (7.78 to 27.81)	13.96 (7.06 to 24.58)	17.20 (8.30 to 31.34)	0.20 (0.13 to 0.29)	0.18 (0.09 to 0.32)	0.16 (0.08 to 0.28)	0.20 (0.10 to 0.36)
**Nutritional deficiencies**	**2.05 (1.80 to 2.37)**	**3.38 (2.54 to 4.46)**	**3.58 (2.66 to 4.84)**	**3.09 (2.32 to 4.09)**	**0.10 (0.08 to 0.11)**	**0.27 (0.19 to 0.37)**	**0.30 (0.21 to 0.42)**	**0.22 (0.15 to 0.32)**
Protein-energy malnutrition	1.18 (0.96 to 1.42)	1.98 (1.44 to 2.70)	2.23 (1.61 to 3.07)	1.58 (1.12 to 2.19)	0.06 (0.05 to 0.07)	0.17 (0.11 to 0.25)	0.20 (0.14 to 0.30)	0.12 (0.08 to 0.19)
Iodine deficiency	0.07 (0.01 to 0.19)	0.18 (0.01 to 0.55)	0.18 (0.01 to 0.57)	0.19 (0.01 to 0.51)	0.00 (0.00 to 0.01)	0.01 (0.00 to 0.05)	0.01 (0.00 to 0.06)	0.01 (0.00 to 0.04)
Dietary iron deficiency	0.13 (0.11 to 0.16)	0.16 (0.11 to 0.23)	0.16 (0.11 to 0.24)	0.15 (0.10 to 0.22)	0.00 (0.00 to 0.00)	0.00 (0.00 to 0.01)	0.00 (0.00 to 0.01)	0.00 (0.00 to 0.00)
Other nutritional deficiencies	0.66 (0.55 to 0.90)	1.06 (0.74 to 1.58)	1.01 (0.70 to 1.55)	1.17 (0.83 to 1.72)	0.03 (0.03 to 0.05)	0.08 (0.05 to 0.13)	0.08 (0.05 to 0.13)	0.08 (0.05 to 0.13)
**Other communicable, maternal, neonatal, and nutritional diseases**	**26.16 (19.68 to 35.42)**	**33.06 (23.64 to 45.45)**	**31.41 (22.17 to 43.17)**	**37.28 (26.91 to 52.14)**	**0.80 (0.61 to 0.96)**	**1.39 (1.01 to 1.82)**	**1.37 (1.00 to 1.81)**	**1.46 (1.07 to 1.94)**
Sexually transmitted infections excluding HIV	0.97 (0.38 to 1.28)	0.70 (0.29 to 1.02)	0.66 (0.28 to 0.99)	0.77 (0.32 to 1.09)	0.02 (0.01 to 0.03)	0.02 (0.01 to 0.02)	0.02 (0.01 to 0.02)	0.02 (0.01 to 0.03)
Syphilis	0.26 (0.14 to 0.32)	0.13 (0.07 to 0.20)	0.12 (0.06 to 0.18)	0.17 (0.09 to 0.24)	0.01 (0.00 to 0.01)	0.00 (0.00 to 0.00)	0.00 (0.00 to 0.00)	0.00 (0.00 to 0.01)
Chlamydial infection	0.14 (0.05 to 0.19)	0.11 (0.04 to 0.16)	0.11 (0.04 to 0.16)	0.12 (0.05 to 0.17)	0.00 (0.00 to 0.00)	0.00 (0.00 to 0.00)	0.00 (0.00 to 0.00)	0.00 (0.00 to 0.00)
Gonococcal infection	0.37 (0.12 to 0.50)	0.29 (0.12 to 0.44)	0.28 (0.12 to 0.43)	0.32 (0.12 to 0.45)	0.01 (0.00 to 0.01)	0.01 (0.00 to 0.01)	0.01 (0.00 to 0.01)	0.01 (0.00 to 0.01)
Other sexually transmitted infections	0.20 (0.06 to 0.28)	0.16 (0.06 to 0.23)	0.15 (0.06 to 0.23)	0.17 (0.06 to 0.25)	0.00 (0.00 to 0.01)	0.00 (0.00 to 0.01)	0.00 (0.00 to 0.01)	0.00 (0.00 to 0.01)
Acute hepatitis	11.71 (9.63 to 14.33)	9.46 (6.11 to 14.28)	7.92 (5.08 to 12.26)	13.37 (8.74 to 19.81)	0.38 (0.32 to 0.46)	0.37 (0.25 to 0.53)	0.31 (0.21 to 0.46)	0.50 (0.34 to 0.71)
Acute hepatitis A	0.35 (0.24 to 0.50)	0.29 (0.17 to 0.47)	0.25 (0.15 to 0.42)	0.36 (0.21 to 0.58)	0.01 (0.01 to 0.01)	0.01 (0.01 to 0.02)	0.01 (0.01 to 0.02)	0.01 (0.01 to 0.02)
Acute hepatitis B	9.90 (7.96 to 12.34)	7.84 (4.81 to 12.36)	6.52 (3.99 to 10.49)	11.19 (7.09 to 17.13)	0.32 (0.26 to 0.39)	0.29 (0.19 to 0.43)	0.24 (0.16 to 0.37)	0.40 (0.27 to 0.59)

Acute hepatitis C	0.41 (0.29 to 0.61)	0.37 (0.22 to 0.61)	0.32 (0.19 to 0.52)	0.52 (0.32 to 0.82)	0.01 (0.01 to 0.02)	0.01 (0.01 to 0.02)	0.01 (0.01 to 0.02)	0.02 (0.01 to 0.03)
Acute hepatitis E	1.05 (0.64 to 1.55)	0.97 (0.52 to 1.60)	0.83 (0.45 to 1.39)	1.30 (0.70 to 2.14)	0.04 (0.02 to 0.06)	0.05 (0.03 to 0.08)	0.05 (0.03 to 0.07)	0.06 (0.04 to 0.10)
Other unspecified infectious diseases	13.48 (7.98 to 22.73)	22.90 (14.81 to 33.07)	22.83 (14.96 to 32.71)	23.14 (14.27 to 34.27)	0.40 (0.23 to 0.54)	1.00 (0.64 to 1.37)	1.05 (0.67 to 1.43)	0.94 (0.58 to 1.30)
**Non-communicable diseases**	**2,570.87 (2,424.78 to 2,746.59)**	**3,590.05 (2,863.38 to 4,418.31)**	**2,901.79 (2,340.05 to 3,586.94)**	**5,167.49 (4,050.24 to 6,475.97)**	**115.85 (109.57 to 123.90)**	**218.55 (174.12 to 283.08)**	**185.32 (146.85 to 245.89)**	**288.50 (230.80 to 365.45)**

Malaysia								
Cause	YLLs (in 1000s)				Deaths (in 1000s)			
	2016	2040 Forecast	2040 Better Scenario	2040 Worse Scenario	2016	2040 Forecast	2040 Better Scenario	2040 Worse Scenario
**Neoplasms**	**602.75 (562.17 to 659.59)**	**1,032.93 (867.57 to 1,224.32)**	**863.91 (732.97 to 1,014.69)**	**1,308.96 (1,083.23 to 1,587.84)**	**24.22 (22.56 to 26.59)**	**52.67 (44.73 to 61.89)**	**45.19 (38.56 to 52.84)**	**63.31 (52.71 to 75.84)**
Lip and oral cavity cancer	9.62 (8.57 to 10.67)	15.79 (11.00 to 22.59)	10.87 (8.13 to 14.73)	25.23 (16.18 to 37.21)	0.40 (0.36 to 0.45)	0.81 (0.57 to 1.16)	0.57 (0.43 to 0.78)	1.21 (0.80 to 1.78)
Nasopharynx cancer	25.13 (21.81 to 29.11)	38.14 (24.39 to 56.14)	23.84 (16.08 to 33.59)	71.37 (42.79 to 111.97)	0.86 (0.74 to 1.00)	1.50 (0.96 to 2.17)	0.96 (0.65 to 1.36)	2.64 (1.62 to 4.03)
Other pharynx cancer	4.56 (3.87 to 5.38)	8.91 (6.37 to 12.75)	8.29 (6.12 to 11.60)	9.22 (6.42 to 13.54)	0.20 (0.17 to 0.23)	0.46 (0.33 to 0.66)	0.45 (0.34 to 0.62)	0.45 (0.32 to 0.66)
Esophageal cancer	10.28 (9.19 to 11.46)	18.40 (12.45 to 27.30)	14.40 (10.33 to 20.43)	25.07 (15.97 to 39.52)	0.48 (0.43 to 0.54)	1.02 (0.70 to 1.51)	0.82 (0.59 to 1.14)	1.32 (0.86 to 2.03)
Stomach cancer	26.56 (24.26 to 29.18)	30.14 (24.50 to 36.82)	25.82 (21.09 to 31.08)	39.71 (31.73 to 50.04)	1.27 (1.16 to 1.40)	1.83 (1.50 to 2.23)	1.61 (1.33 to 1.95)	2.29 (1.81 to 2.86)
Colon and rectum cancer	61.89 (54.10 to 69.98)	114.84 (80.23 to 163.56)	100.86 (70.91 to 141.25)	139.64 (97.45 to 199.58)	2.94 (2.56 to 3.31)	7.14 (4.98 to 10.15)	6.48 (4.53 to 9.24)	8.20 (5.70 to 11.64)
Liver cancer	45.15 (39.35 to 51.72)	125.65 (84.90 to 180.44)	108.60 (79.49 to 145.46)	126.93 (82.51 to 188.44)	1.91 (1.68 to 2.19)	6.24 (4.41 to 8.74)	5.57 (4.21 to 7.29)	6.01 (4.08 to 8.59)
Liver cancer due to hepatitis B	28.83 (24.43 to 34.04)	77.42 (51.92 to 114.35)	67.21 (48.59 to 92.17)	77.84 (49.62 to 116.60)	1.18 (0.99 to 1.39)	3.70 (2.56 to 5.30)	3.32 (2.46 to 4.41)	3.55 (2.39 to 5.20)
Liver cancer due to hepatitis C	4.90 (3.71 to 6.46)	15.19 (9.83 to 22.84)	13.50 (9.21 to 19.26)	14.85 (9.31 to 22.30)	0.25 (0.19 to 0.32)	0.91 (0.59 to 1.33)	0.83 (0.57 to 1.17)	0.84 (0.54 to 1.25)
Liver cancer due to alcohol use	3.01 (2.15 to 4.06)	8.79 (5.31 to 14.23)	7.09 (4.59 to 10.70)	9.54 (5.47 to 15.72)	0.13 (0.09 to 0.18)	0.43 (0.26 to 0.66)	0.36 (0.23 to 0.53)	0.44 (0.26 to 0.70)
Liver cancer due to other causes	8.41 (6.19 to 11.26)	24.25 (14.20 to 38.36)	20.81 (13.23 to 30.80)	24.71 (13.79 to 40.37)	0.36 (0.26 to 0.48)	1.21 (0.71 to 1.88)	1.07 (0.68 to 1.57)	1.17 (0.68 to 1.87)
Gallbladder and biliary tract cancer	4.53 (3.95 to 5.91)	6.43 (5.46 to 8.44)	6.49 (5.44 to 8.50)	6.32 (5.45 to 8.31)	0.21 (0.19 to 0.28)	0.38 (0.32 to 0.48)	0.40 (0.33 to 0.50)	0.36 (0.30 to 0.45)
Pancreatic cancer	19.42 (17.75 to 21.41)	43.46 (34.88 to 55.13)	41.46 (33.48 to 51.86)	45.85 (36.28 to 59.23)	0.89 (0.81 to 0.98)	2.39 (1.91 to 3.02)	2.33 (1.87 to 2.90)	2.42 (1.91 to 3.15)
Larynx cancer	4.78 (4.21 to 5.39)	6.38 (3.56 to 10.87)	3.89 (2.33 to 6.38)	11.11 (6.03 to 18.91)	0.23 (0.20 to 0.26)	0.35 (0.20 to 0.61)	0.22 (0.13 to 0.37)	0.57 (0.31 to 0.98)
Tracheal, bronchus, and lung cancer	107.61 (96.37 to 121.10)	194.77 (141.02 to 261.64)	122.94 (92.56 to 161.41)	321.26 (226.37 to 438.62)	4.97 (4.44 to 5.60)	10.66 (7.76 to 14.15)	6.92 (5.20 to 9.05)	16.44 (11.69 to 22.16)
Malignant skin melanoma	2.48 (2.10 to 3.04)	5.19 (4.23 to 6.56)	5.84 (4.72 to 7.40)	4.17 (3.44 to 5.28)	0.09 (0.07 to 0.11)	0.25 (0.20 to 0.31)	0.29 (0.24 to 0.36)	0.19 (0.15 to 0.24)
Non-melanoma skin cancer	3.40 (3.05 to 3.82)	5.75 (4.81 to 6.72)	5.25 (4.39 to 6.25)	6.90 (5.86 to 8.04)	0.16 (0.14 to 0.17)	0.34 (0.29 to 0.40)	0.32 (0.27 to 0.38)	0.38 (0.32 to 0.45)
Breast cancer	64.06 (52.43 to 75.77)	85.64 (62.20 to 114.83)	74.98 (55.79 to 99.22)	107.55 (77.47 to 147.50)	2.14 (1.75 to 2.52)	3.62 (2.58 to 4.89)	3.23 (2.33 to 4.36)	4.37 (3.08 to 6.07)
Cervical cancer	18.52 (15.31 to 23.18)	21.14 (16.13 to 27.92)	18.17 (14.05 to 23.71)	28.05 (20.62 to 37.70)	0.68 (0.57 to 0.84)	0.98 (0.74 to 1.27)	0.86 (0.65 to 1.10)	1.24 (0.92 to 1.67)

Uterine cancer	4.63 (3.83 to 5.52)	7.72 (6.06 to 9.74)	7.20 (5.59 to 9.13)	8.76 (6.85 to 11.07)	0.19 (0.15 to 0.22)	0.40 (0.31 to 0.50)	0.38 (0.29 to 0.48)	0.43 (0.34 to 0.54)
Ovarian cancer	18.19 (15.11 to 21.94)	27.56 (20.60 to 37.61)	25.29 (19.23 to 33.60)	31.00 (22.76 to 43.38)	0.63 (0.53 to 0.75)	1.18 (0.87 to 1.60)	1.10 (0.83 to 1.47)	1.28 (0.95 to 1.78)
Prostate cancer	10.74 (8.48 to 13.61)	25.56 (20.21 to 32.30)	25.05 (19.85 to 31.72)	26.67 (21.07 to 34.08)	0.72 (0.55 to 0.89)	2.01 (1.51 to 2.49)	2.03 (1.53 to 2.50)	1.99 (1.50 to 2.50)
Testicular cancer	1.01 (0.80 to 1.33)	0.85 (0.60 to 1.22)	0.77 (0.54 to 1.11)	1.03 (0.73 to 1.44)	0.02 (0.02 to 0.03)	0.03 (0.02 to 0.03)	0.02 (0.02 to 0.03)	0.03 (0.02 to 0.04)

Malaysia								
Cause	YLLs (in 1000s)				Deaths (in 1000s)			
	2016	2040 Forecast	2040 Better Scenario	2040 Worse Scenario	2016	2040 Forecast	2040 Better Scenario	2040 Worse Scenario
Kidney cancer	7.64 (6.54 to 8.92)	15.84 (12.28 to 20.75)	15.48 (12.29 to 19.97)	16.13 (12.22 to 21.74)	0.30 (0.26 to 0.36)	0.77 (0.60 to 1.02)	0.78 (0.62 to 1.00)	0.75 (0.57 to 1.01)
Bladder cancer	7.17 (6.21 to 8.15)	13.65 (10.14 to 18.87)	12.05 (9.18 to 16.22)	16.02 (11.38 to 23.05)	0.40 (0.35 to 0.46)	0.96 (0.71 to 1.31)	0.87 (0.66 to 1.16)	1.06 (0.76 to 1.52)
Brain and nervous system cancer	16.07 (13.35 to 21.02)	23.94 (19.15 to 33.75)	22.98 (18.42 to 32.46)	25.81 (20.85 to 36.56)	0.43 (0.35 to 0.63)	0.82 (0.65 to 1.29)	0.81 (0.63 to 1.28)	0.85 (0.67 to 1.33)
Thyroid cancer	4.41 (3.82 to 5.23)	8.07 (6.08 to 10.79)	7.02 (5.47 to 9.14)	9.10 (6.82 to 12.18)	0.20 (0.18 to 0.23)	0.47 (0.36 to 0.63)	0.43 (0.33 to 0.56)	0.51 (0.38 to 0.68)
Mesothelioma	1.18 (1.00 to 1.39)	2.05 (1.66 to 2.51)	2.11 (1.69 to 2.60)	1.94 (1.59 to 2.36)	0.04 (0.03 to 0.05)	0.09 (0.07 to 0.11)	0.09 (0.08 to 0.11)	0.08 (0.07 to 0.10)
Hodgkin lymphoma	2.14 (1.66 to 2.68)	1.84 (1.36 to 2.54)	1.57 (1.16 to 2.18)	2.46 (1.82 to 3.41)	0.06 (0.05 to 0.08)	0.06 (0.05 to 0.09)	0.06 (0.04 to 0.08)	0.08 (0.06 to 0.12)
Non-Hodgkin lymphoma	28.03 (24.86 to 33.68)	43.56 (35.73 to 53.95)	39.64 (32.93 to 48.57)	48.53 (39.94 to 60.42)	0.95 (0.84 to 1.13)	1.95 (1.59 to 2.38)	1.83 (1.51 to 2.22)	2.07 (1.70 to 2.53)
Multiple myeloma	5.20 (4.40 to 6.45)	10.68 (8.41 to 13.91)	9.98 (7.94 to 12.76)	11.17 (8.82 to 14.54)	0.21 (0.18 to 0.26)	0.52 (0.41 to 0.65)	0.49 (0.39 to 0.62)	0.52 (0.41 to 0.66)
Leukemia	48.12 (42.47 to 61.05)	64.17 (50.22 to 86.59)	56.22 (44.99 to 74.98)	75.24 (58.00 to 103.03)	1.35 (1.19 to 1.78)	2.50 (1.97 to 3.47)	2.24 (1.78 to 3.04)	2.81 (2.15 to 3.96)
Acute lymphoid leukemia	15.81 (13.32 to 19.68)	18.97 (14.95 to 26.19)	16.42 (13.15 to 22.32)	22.77 (17.95 to 31.59)	0.43 (0.37 to 0.59)	0.70 (0.54 to 1.04)	0.62 (0.48 to 0.90)	0.81 (0.62 to 1.19)
Chronic lymphoid leukemia	1.10 (0.89 to 1.35)	1.62 (1.23 to 2.14)	1.46 (1.13 to 1.92)	1.78 (1.34 to 2.37)	0.04 (0.03 to 0.05)	0.07 (0.06 to 0.10)	0.07 (0.05 to 0.09)	0.08 (0.06 to 0.10)
Acute myeloid leukemia	13.02 (11.15 to 16.62)	21.36 (16.77 to 29.26)	19.54 (15.63 to 26.08)	22.89 (17.74 to 31.99)	0.33 (0.28 to 0.44)	0.72 (0.56 to 1.02)	0.67 (0.53 to 0.95)	0.74 (0.56 to 1.07)
Chronic myeloid leukemia	2.52 (1.97 to 3.29)	1.74 (1.21 to 2.47)	1.32 (0.94 to 1.89)	2.64 (1.83 to 3.83)	0.08 (0.06 to 0.10)	0.07 (0.05 to 0.10)	0.05 (0.04 to 0.07)	0.10 (0.07 to 0.14)
Other leukemia	15.66 (12.94 to 21.66)	20.48 (14.83 to 30.48)	17.48 (12.94 to 25.54)	25.16 (18.16 to 38.23)	0.47 (0.40 to 0.67)	0.95 (0.70 to 1.36)	0.83 (0.63 to 1.17)	1.09 (0.78 to 1.60)
Other neoplasms	40.22 (36.66 to 44.44)	66.83 (58.68 to 76.16)	66.86 (58.30 to 76.80)	66.72 (59.06 to 76.15)	1.27 (1.15 to 1.43)	2.94 (2.55 to 3.44)	3.05 (2.61 to 3.58)	2.76 (2.41 to 3.21)
**Cardiovascular diseases**	**1,166.69 (1,088.69 to 1,250.39)**	**1,131.77 (761.73 to 1,665.52)**	**770.20 (536.49 to 1,098.42)**	**2,137.56 (1,381.42 to 3,174.92)**	**56.45 (52.88 to 60.64)**	**72.29 (48.95 to 107.52)**	**53.48 (36.61 to 79.86)**	**121.56 (81.48 to 177.61)**
Rheumatic heart disease	13.71 (11.85 to 16.00)	9.04 (7.15 to 11.59)	7.98 (6.20 to 10.22)	11.54 (9.02 to 14.62)	0.45 (0.39 to 0.52)	0.47 (0.37 to 0.61)	0.43 (0.34 to 0.56)	0.56 (0.44 to 0.71)
Ischemic heart disease	726.61 (670.95 to 795.57)	678.72 (389.72 to 1,135.29)	423.32 (248.32 to 713.84)	1,363.99 (763.88 to 2,286.97)	36.30 (33.75 to 39.64)	43.80 (25.61 to 72.36)	30.29 (17.78 to 52.52)	77.87 (44.78 to 127.09)
Stroke	292.97 (266.30 to 319.13)	231.61 (150.02 to 356.29)	153.27 (101.00 to 232.15)	500.64 (308.56 to 794.48)	14.30 (13.08 to 15.51)	15.64 (10.21 to 24.08)	11.43 (7.64 to 17.76)	29.07 (18.69 to 45.36)
Ischemic stroke	75.05 (66.62 to 84.81)	69.03 (40.36 to 134.38)	50.17 (29.33 to 98.39)	128.13 (73.26 to 256.77)	4.70 (4.19 to 5.30)	5.83 (3.29 to 11.79)	4.57 (2.54 to 9.17)	9.54 (5.28 to 19.89)

Intracerebral hemorrhage	217.92 (197.93 to 239.29)	162.58 (102.39 to 257.78)	103.11 (67.79 to 155.13)	372.51 (216.57 to 615.58)	9.60 (8.71 to 10.54)	9.81 (6.37 to 15.35)	6.86 (4.65 to 10.40)	19.53 (12.09 to 31.31)
Hypertensive heart disease	12.86 (10.90 to 16.09)	29.66 (20.10 to 47.22)	23.66 (16.82 to 34.88)	36.09 (22.82 to 65.42)	0.63 (0.52 to 0.82)	2.05 (1.36 to 3.46)	1.70 (1.18 to 2.59)	2.28 (1.42 to 4.24)
Cardiomyopathy and myocarditis	22.43 (17.09 to 26.28)	22.23 (15.83 to 30.48)	18.50 (13.34 to 24.94)	30.21 (21.35 to 41.77)	0.70 (0.58 to 0.84)	0.94 (0.67 to 1.39)	0.81 (0.58 to 1.22)	1.21 (0.85 to 1.76)
Myocarditis	2.34 (1.45 to 3.16)	2.57 (1.69 to 3.60)	2.52 (1.69 to 3.53)	2.69 (1.71 to 3.78)	0.05 (0.04 to 0.08)	0.09 (0.06 to 0.15)	0.09 (0.06 to 0.16)	0.09 (0.06 to 0.14)

Malaysia								
Cause	YLLs (in 1000s)				Deaths (in 1000s)			
	2016	2040 Forecast	2040 Better Scenario	2040 Worse Scenario	2016	2040 Forecast	2040 Better Scenario	2040 Worse Scenario
Alcoholic cardiomyopathy	2.00 (1.27 to 2.86)	2.55 (1.59 to 3.66)	2.41 (1.53 to 3.45)	2.73 (1.70 to 3.91)	0.07 (0.05 to 0.10)	0.11 (0.08 to 0.16)	0.11 (0.08 to 0.16)	0.11 (0.08 to 0.16)
Other cardiomyopathy	18.10 (13.75 to 21.12)	17.10 (11.55 to 24.60)	13.57 (9.31 to 19.17)	24.80 (16.52 to 35.53)	0.57 (0.48 to 0.69)	0.74 (0.50 to 1.12)	0.61 (0.41 to 0.95)	1.01 (0.67 to 1.48)
Atrial fibrillation and flutter	4.86 (3.80 to 6.10)	14.21 (10.40 to 19.02)	13.87 (10.16 to 18.44)	14.48 (10.40 to 19.37)	0.41 (0.32 to 0.52)	1.48 (1.05 to 2.02)	1.50 (1.06 to 2.04)	1.41 (0.96 to 1.92)
Aortic aneurysm	14.52 (12.20 to 17.03)	24.79 (19.98 to 31.11)	23.27 (18.97 to 29.07)	27.71 (21.69 to 35.37)	0.80 (0.68 to 0.94)	1.74 (1.41 to 2.17)	1.71 (1.39 to 2.12)	1.80 (1.44 to 2.26)
Peripheral artery disease	0.36 (0.27 to 0.47)	1.24 (0.85 to 1.74)	1.20 (0.83 to 1.69)	1.26 (0.87 to 1.77)	0.02 (0.02 to 0.03)	0.11 (0.07 to 0.16)	0.12 (0.08 to 0.17)	0.11 (0.07 to 0.15)
Endocarditis	30.10 (23.48 to 35.14)	50.93 (34.99 to 76.66)	47.97 (33.26 to 70.57)	52.80 (35.74 to 79.81)	0.88 (0.69 to 1.02)	2.00 (1.39 to 2.80)	1.96 (1.37 to 2.73)	1.95 (1.33 to 2.75)
Other cardiovascular and circulatory diseases	48.27 (34.96 to 57.89)	69.35 (45.54 to 94.78)	57.15 (38.83 to 77.61)	98.84 (63.80 to 138.43)	1.96 (1.37 to 2.34)	4.07 (2.68 to 5.59)	3.53 (2.33 to 4.84)	5.31 (3.35 to 7.28)
**Chronic respiratory diseases**	**129.81 (117.68 to 143.87)**	**175.29 (133.94 to 232.48)**	**119.68 (95.87 to 151.76)**	**270.63 (199.45 to 367.97)**	**6.59 (5.92 to 7.41)**	**11.54 (8.68 to 15.24)**	**8.13 (6.32 to 10.69)**	**16.68 (12.12 to 22.86)**
Chronic obstructive pulmonary disease	86.67 (76.92 to 98.38)	138.17 (99.58 to 193.55)	86.61 (64.92 to 118.36)	224.85 (157.45 to 321.79)	5.05 (4.48 to 5.73)	9.65 (7.02 to 13.30)	6.34 (4.75 to 8.65)	14.56 (10.09 to 20.48)
Pneumoconiosis	0.14 (0.11 to 0.18)	0.14 (0.11 to 0.17)	0.13 (0.10 to 0.17)	0.15 (0.12 to 0.20)	0.01 (0.01 to 0.01)	0.01 (0.01 to 0.01)	0.01 (0.01 to 0.01)	0.01 (0.01 to 0.01)
Silicosis	0.04 (0.02 to 0.05)	0.01 (0.01 to 0.02)	0.01 (0.01 to 0.02)	0.02 (0.01 to 0.04)	0.00 (0.00 to 0.00)	0.00 (0.00 to 0.00)	0.00 (0.00 to 0.00)	0.00 (0.00 to 0.00)
Asbestosis	0.03 (0.02 to 0.05)	0.07 (0.05 to 0.09)	0.07 (0.05 to 0.10)	0.05 (0.04 to 0.07)	0.00 (0.00 to 0.00)	0.00 (0.00 to 0.00)	0.00 (0.00 to 0.01)	0.00 (0.00 to 0.00)
Coal workers pneumoconiosis	0.01 (0.00 to 0.01)	0.01 (0.00 to 0.01)	0.01 (0.00 to 0.01)	0.01 (0.01 to 0.02)	0.00 (0.00 to 0.00)	0.00 (0.00 to 0.00)	0.00 (0.00 to 0.00)	0.00 (0.00 to 0.00)
Other pneumoconiosis	0.06 (0.05 to 0.09)	0.05 (0.04 to 0.07)	0.04 (0.03 to 0.06)	0.06 (0.05 to 0.09)	0.00 (0.00 to 0.00)	0.00 (0.00 to 0.01)	0.00 (0.00 to 0.00)	0.00 (0.00 to 0.01)
Asthma	33.99 (27.92 to 40.49)	17.80 (11.27 to 27.24)	11.34 (7.56 to 16.90)	30.19 (19.04 to 47.51)	1.19 (0.97 to 1.48)	0.84 (0.52 to 1.31)	0.57 (0.37 to 0.86)	1.33 (0.82 to 2.12)
Interstitial lung disease and pulmonary sarcoidosis	5.47 (3.93 to 8.10)	14.09 (9.94 to 21.60)	16.66 (12.10 to 24.97)	10.11 (7.09 to 15.80)	0.23 (0.16 to 0.38)	0.77 (0.52 to 1.30)	0.93 (0.65 to 1.57)	0.52 (0.35 to 0.88)
Other chronic respiratory diseases	3.55 (2.37 to 4.81)	5.10 (3.08 to 7.39)	4.94 (3.00 to 7.02)	5.32 (3.21 to 7.71)	0.12 (0.07 to 0.17)	0.27 (0.14 to 0.46)	0.27 (0.14 to 0.46)	0.27 (0.13 to 0.45)
**Cirrhosis and other chronic liver diseases**	**77.12 (65.88 to 90.29)**	**108.86 (88.33 to 134.13)**	**99.59 (79.82 to 123.87)**	**128.09 (104.31 to 157.73)**	**2.82 (2.43 to 3.29)**	**4.84 (3.95 to 5.90)**	**4.53 (3.67 to 5.58)**	**5.45 (4.46 to 6.64)**
Cirrhosis and other chronic liver diseases due to hepatitis B	34.52 (27.64 to 42.99)	48.94 (36.70 to 62.85)	45.33 (33.87 to 58.42)	56.83 (43.02 to 73.22)	1.27 (1.02 to 1.57)	2.12 (1.62 to 2.69)	2.01 (1.52 to 2.58)	2.37 (1.82 to 2.99)
Cirrhosis and other chronic liver diseases due to hepatitis C	11.18 (8.71 to 14.23)	15.88 (11.81 to 21.09)	14.57 (10.82 to 19.47)	18.77 (13.88 to 24.59)	0.40 (0.31 to 0.51)	0.67 (0.50 to 0.86)	0.62 (0.47 to 0.81)	0.76 (0.57 to 0.98)

Cirrhosis and other chronic liver diseases due to alcohol use	12.61 (9.86 to 16.20)	17.55 (13.42 to 23.35)	15.16 (11.33 to 20.29)	21.79 (16.58 to 28.70)	0.47 (0.37 to 0.58)	0.77 (0.60 to 1.00)	0.69 (0.53 to 0.89)	0.91 (0.71 to 1.19)
Cirrhosis and other chronic liver diseases due to other causes	18.82 (15.55 to 23.31)	26.49 (20.52 to 34.33)	24.53 (18.99 to 31.91)	30.70 (23.91 to 39.68)	0.69 (0.57 to 0.84)	1.28 (1.00 to 1.65)	1.22 (0.95 to 1.58)	1.41 (1.09 to 1.82)
**Digestive diseases**	**89.56 (79.33 to 100.61)**	**130.52 (101.05 to 164.79)**	**117.06 (91.89 to 144.88)**	**154.44 (118.71 to 199.31)**	**4.17 (3.47 to 4.72)**	**8.49 (6.32 to 10.78)**	**8.01 (5.94 to 10.13)**	**9.27 (6.85 to 11.98)**
Peptic ulcer disease	21.93 (18.66 to 25.45)	19.55 (14.94 to 25.47)	16.10 (12.12 to 21.16)	27.89 (21.05 to 36.07)	1.18 (0.99 to 1.41)	1.41 (1.06 to 1.90)	1.21 (0.90 to 1.66)	1.90 (1.40 to 2.53)

Malaysia								
Cause	YLLs (in 1000s)				Deaths (in 1000s)			
	2016	2040 Forecast	2040 Better Scenario	2040 Worse Scenario	2016	2040 Forecast	2040 Better Scenario	2040 Worse Scenario
Gastritis and duodenitis	0.55 (0.45 to 0.66)	0.78 (0.57 to 1.03)	0.70 (0.51 to 0.94)	0.96 (0.71 to 1.26)	0.02 (0.02 to 0.03)	0.04 (0.03 to 0.06)	0.04 (0.03 to 0.05)	0.05 (0.04 to 0.06)
Appendicitis	3.74 (3.08 to 4.76)	3.11 (2.37 to 4.09)	2.62 (1.95 to 3.46)	4.34 (3.27 to 5.78)	0.12 (0.09 to 0.15)	0.14 (0.10 to 0.19)	0.12 (0.09 to 0.17)	0.18 (0.13 to 0.24)
Paralytic ileus and intestinal obstruction	17.31 (12.34 to 21.65)	34.06 (20.17 to 43.69)	35.26 (20.64 to 45.45)	32.03 (19.13 to 40.57)	0.82 (0.46 to 1.03)	2.44 (1.17 to 3.31)	2.63 (1.26 to 3.62)	2.13 (1.02 to 2.93)
Inguinal, femoral, and abdominal hernia	1.92 (1.38 to 2.40)	2.14 (1.43 to 2.81)	2.01 (1.35 to 2.65)	2.45 (1.69 to 3.19)	0.10 (0.07 to 0.12)	0.15 (0.10 to 0.20)	0.15 (0.09 to 0.20)	0.16 (0.10 to 0.21)
Inflammatory bowel disease	1.58 (1.31 to 1.88)	2.64 (2.08 to 3.26)	2.68 (2.09 to 3.33)	2.59 (2.05 to 3.16)	0.07 (0.05 to 0.08)	0.15 (0.11 to 0.19)	0.16 (0.12 to 0.21)	0.14 (0.10 to 0.17)
Vascular intestinal disorders	5.40 (4.58 to 7.48)	10.16 (7.96 to 14.72)	10.00 (7.66 to 14.45)	10.57 (8.35 to 15.17)	0.27 (0.23 to 0.39)	0.72 (0.53 to 1.06)	0.73 (0.53 to 1.11)	0.69 (0.52 to 1.02)
Gallbladder and biliary diseases	8.51 (7.18 to 10.05)	15.18 (9.24 to 24.24)	10.80 (6.84 to 16.95)	19.67 (11.94 to 30.99)	0.43 (0.35 to 0.53)	1.06 (0.61 to 1.81)	0.80 (0.48 to 1.32)	1.27 (0.73 to 2.13)
Pancreatitis	15.21 (12.55 to 18.24)	21.42 (11.69 to 42.35)	15.96 (9.28 to 28.92)	31.21 (15.89 to 63.55)	0.57 (0.41 to 0.68)	1.05 (0.54 to 2.08)	0.82 (0.44 to 1.49)	1.45 (0.72 to 2.89)
Other digestive diseases	13.41 (10.76 to 16.16)	21.47 (16.12 to 28.27)	20.92 (15.69 to 27.89)	22.75 (17.29 to 29.73)	0.60 (0.47 to 0.76)	1.34 (0.98 to 1.87)	1.36 (0.97 to 1.95)	1.31 (0.97 to 1.80)
**Neurological disorders**	**106.47 (93.39 to 122.53)**	**327.67 (249.81 to 442.51)**	**315.90 (245.28 to 411.20)**	**306.72 (228.62 to 427.00)**	**7.56 (6.49 to 8.88)**	**30.61 (22.25 to 43.09)**	**30.62 (22.95 to 41.21)**	**26.78 (19.01 to 38.56)**
Alzheimer's disease and other dementias	78.65 (66.29 to 95.46)	277.56 (200.87 to 393.32)	264.80 (197.13 to 356.97)	258.32 (181.46 to 379.33)	6.58 (5.52 to 7.89)	27.89 (19.76 to 40.21)	27.68 (20.27 to 38.09)	24.42 (16.86 to 36.13)
Parkinson's disease	7.37 (5.60 to 9.48)	23.60 (17.73 to 30.43)	25.85 (19.52 to 33.88)	19.89 (14.90 to 25.54)	0.51 (0.39 to 0.67)	1.93 (1.44 to 2.55)	2.17 (1.63 to 2.86)	1.55 (1.13 to 2.03)
Epilepsy	12.26 (10.89 to 13.96)	13.91 (10.42 to 18.42)	12.58 (9.56 to 16.27)	16.13 (11.91 to 21.94)	0.25 (0.23 to 0.28)	0.37 (0.28 to 0.49)	0.35 (0.27 to 0.45)	0.42 (0.31 to 0.56)
Multiple sclerosis	0.80 (0.68 to 0.95)	1.21 (0.98 to 1.53)	1.22 (0.99 to 1.53)	1.19 (0.97 to 1.50)	0.02 (0.02 to 0.03)	0.04 (0.03 to 0.05)	0.04 (0.03 to 0.05)	0.04 (0.03 to 0.05)
Motor neuron disease	1.37 (1.06 to 1.58)	2.54 (1.95 to 3.03)	2.54 (1.95 to 3.07)	2.53 (1.95 to 2.99)	0.04 (0.03 to 0.05)	0.09 (0.07 to 0.11)	0.09 (0.07 to 0.11)	0.09 (0.07 to 0.10)
Other neurological disorders	6.03 (5.26 to 6.86)	8.86 (7.53 to 10.33)	8.92 (7.57 to 10.46)	8.66 (7.40 to 10.10)	0.14 (0.13 to 0.16)	0.28 (0.24 to 0.32)	0.29 (0.24 to 0.34)	0.26 (0.22 to 0.30)
**Mental disorders**	**35.44 (25.98 to 62.24)**	**77.55 (55.90 to 129.33)**	**85.33 (61.37 to 137.55)**	**65.04 (46.47 to 111.10)**	**0.91 (0.68 to 1.49)**	**2.47 (1.89 to 3.64)**	**2.79 (2.15 to 3.99)**	**1.99 (1.51 to 3.05)**
Alcohol use disorders	17.05 (11.89 to 28.22)	25.86 (16.46 to 46.38)	26.83 (17.00 to 47.35)	23.59 (15.04 to 42.67)	0.47 (0.32 to 0.79)	0.89 (0.59 to 1.53)	0.94 (0.62 to 1.58)	0.77 (0.51 to 1.35)
Drug use disorders	18.20 (12.34 to 35.90)	51.43 (35.99 to 90.92)	58.22 (40.78 to 99.22)	41.23 (28.29 to 75.13)	0.43 (0.31 to 0.77)	1.58 (1.21 to 2.35)	1.85 (1.41 to 2.63)	1.21 (0.91 to 1.87)
Opioid use disorders	13.18 (8.59 to 27.22)	26.07 (16.89 to 49.19)	27.33 (17.87 to 50.79)	23.85 (15.33 to 46.58)	0.31 (0.22 to 0.57)	0.80 (0.57 to 1.26)	0.87 (0.62 to 1.34)	0.69 (0.49 to 1.14)

Cocaine use disorders	0.26 (0.16 to 0.51)	1.41 (0.89 to 2.44)	1.63 (1.03 to 2.80)	1.06 (0.66 to 1.86)	0.01 (0.00 to 0.01)	0.05 (0.03 to 0.07)	0.06 (0.03 to 0.09)	0.03 (0.02 to 0.05)
Amphetamine use disorders	0.15 (0.09 to 0.37)	1.35 (0.77 to 2.76)	1.67 (0.98 to 3.43)	0.89 (0.51 to 1.84)	0.00 (0.00 to 0.01)	0.04 (0.03 to 0.06)	0.05 (0.03 to 0.08)	0.03 (0.02 to 0.04)
Other drug use disorders	4.60 (3.07 to 8.80)	22.60 (14.71 to 40.17)	27.60 (18.13 to 48.70)	15.44 (9.79 to 27.62)	0.12 (0.08 to 0.20)	0.70 (0.50 to 1.08)	0.87 (0.61 to 1.32)	0.46 (0.32 to 0.73)
Eating disorders	0.19 (0.13 to 0.30)	0.26 (0.16 to 0.40)	0.28 (0.17 to 0.43)	0.22 (0.14 to 0.34)	0.00 (0.00 to 0.01)	0.01 (0.00 to 0.01)	0.01 (0.00 to 0.01)	0.00 (0.00 to 0.01)

Malaysia								
Cause	YLLs (in 1000s)				Deaths (in 1000s)			
	2016	2040 Forecast	2040 Better Scenario	2040 Worse Scenario	2016	2040 Forecast	2040 Better Scenario	2040 Worse Scenario
**Diabetes, urogenital, blood, and endocrine diseases**	**239.58 (222.81 to 260.40)**	**460.79 (290.28 to 852.84)**	**398.88 (248.56 to 755.08)**	**617.28 (388.94 to 1,109.90)**	**10.70 (9.91 to 11.61)**	**29.85 (17.43 to 65.30)**	**26.90 (15.26 to 62.05)**	**37.27 (22.27 to 75.11)**
Diabetes mellitus	104.25 (94.39 to 114.95)	140.06 (90.62 to 207.65)	115.07 (75.11 to 173.23)	199.37 (127.87 to 302.84)	4.85 (4.38 to 5.36)	8.69 (5.82 to 12.45)	7.46 (4.96 to 10.76)	11.47 (7.46 to 16.55)
Acute glomerulonephritis	0.16 (0.15 to 0.18)	0.16 (0.12 to 0.21)	0.14 (0.11 to 0.18)	0.20 (0.15 to 0.25)	0.00 (0.00 to 0.00)	0.00 (0.00 to 0.00)	0.00 (0.00 to 0.00)	0.00 (0.00 to 0.01)
Chronic kidney disease	83.57 (74.90 to 93.05)	241.38 (99.86 to 651.23)	208.87 (86.13 to 584.90)	328.30 (132.17 to 852.36)	3.68 (3.30 to 4.08)	16.39 (5.51 to 51.97)	14.77 (5.04 to 50.20)	20.76 (6.98 to 60.64)
Chronic kidney disease due to diabetes mellitus	52.47 (46.17 to 58.92)	145.75 (63.18 to 388.63)	124.05 (52.53 to 332.34)	203.22 (83.07 to 505.88)	2.19 (1.92 to 2.47)	8.75 (3.13 to 26.07)	7.70 (2.77 to 23.84)	11.54 (4.03 to 32.68)
Chronic kidney disease due to hypertension	21.23 (17.66 to 25.09)	77.92 (27.49 to 249.92)	69.79 (24.01 to 232.66)	100.03 (34.96 to 280.96)	1.16 (0.97 to 1.37)	6.53 (2.01 to 23.92)	6.09 (1.87 to 24.52)	7.75 (2.37 to 25.16)
Chronic kidney disease due to glomerulonephritis	4.82 (3.90 to 6.19)	7.09 (3.37 to 17.87)	5.92 (2.83 to 15.41)	10.33 (4.78 to 23.89)	0.14 (0.12 to 0.18)	0.39 (0.14 to 1.26)	0.34 (0.11 to 1.17)	0.54 (0.19 to 1.47)
Chronic kidney disease due to other and unspecified causes	5.06 (4.11 to 6.22)	10.63 (4.51 to 29.90)	9.11 (3.87 to 26.91)	14.72 (6.10 to 38.45)	0.19 (0.15 to 0.23)	0.72 (0.23 to 2.43)	0.65 (0.20 to 2.29)	0.93 (0.30 to 2.92)
Urinary diseases and male infertility	31.00 (27.39 to 34.35)	47.45 (38.96 to 56.33)	43.68 (35.69 to 52.32)	55.74 (46.59 to 65.32)	1.48 (1.30 to 1.64)	3.18 (2.45 to 4.02)	3.05 (2.31 to 3.91)	3.47 (2.72 to 4.28)
Urinary tract infections	19.21 (15.76 to 25.10)	34.91 (27.25 to 45.33)	32.41 (25.17 to 42.60)	40.22 (31.63 to 52.27)	0.83 (0.64 to 1.18)	2.22 (1.60 to 3.15)	2.15 (1.53 to 3.09)	2.37 (1.71 to 3.34)
Urolithiasis	0.56 (0.40 to 0.72)	0.71 (0.43 to 1.00)	0.62 (0.37 to 0.87)	0.93 (0.59 to 1.29)	0.02 (0.02 to 0.03)	0.04 (0.02 to 0.06)	0.04 (0.02 to 0.06)	0.05 (0.03 to 0.08)
Other urinary diseases	11.23 (7.41 to 14.30)	11.84 (6.00 to 18.04)	10.66 (5.36 to 16.45)	14.58 (7.92 to 21.40)	0.62 (0.38 to 0.81)	0.92 (0.42 to 1.49)	0.87 (0.38 to 1.44)	1.04 (0.50 to 1.66)
Gynecological diseases	0.26 (0.19 to 0.35)	0.17 (0.11 to 0.25)	0.13 (0.09 to 0.19)	0.29 (0.19 to 0.44)	0.01 (0.00 to 0.01)	0.01 (0.00 to 0.01)	0.00 (0.00 to 0.01)	0.01 (0.01 to 0.01)
Uterine fibroids	0.01 (0.01 to 0.02)	0.01 (0.01 to 0.02)	0.01 (0.00 to 0.02)	0.02 (0.01 to 0.03)	0.00 (0.00 to 0.00)	0.00 (0.00 to 0.00)	0.00 (0.00 to 0.00)	0.00 (0.00 to 0.00)
Polycystic ovarian syndrome	0.00 (0.00 to 0.01)	0.00 (0.00 to 0.00)	0.00 (0.00 to 0.00)	0.00 (0.00 to 0.01)	0.00 (0.00 to 0.00)	0.00 (0.00 to 0.00)	0.00 (0.00 to 0.00)	0.00 (0.00 to 0.00)
Endometriosis	0.00 (0.00 to 0.00)	0.00 (0.00 to 0.00)	0.00 (0.00 to 0.00)	0.00 (0.00 to 0.00)	0.00 (0.00 to 0.00)	0.00 (0.00 to 0.00)	0.00 (0.00 to 0.00)	0.00 (0.00 to 0.00)
Genital prolapse	0.00 (0.00 to 0.00)	0.00 (0.00 to 0.00)	0.00 (0.00 to 0.00)	0.00 (0.00 to 0.00)	0.00 (0.00 to 0.00)	0.00 (0.00 to 0.00)	0.00 (0.00 to 0.00)	0.00 (0.00 to 0.00)
Other gynecological diseases	0.24 (0.17 to 0.32)	0.16 (0.10 to 0.23)	0.12 (0.08 to 0.17)	0.27 (0.17 to 0.41)	0.01 (0.00 to 0.01)	0.01 (0.00 to 0.01)	0.00 (0.00 to 0.01)	0.01 (0.01 to 0.01)
Hemoglobinopathies and hemolytic anemias	9.77 (7.94 to 11.90)	10.73 (8.10 to 13.94)	9.40 (7.04 to 12.38)	13.94 (10.44 to 18.29)	0.34 (0.28 to 0.43)	0.55 (0.42 to 0.73)	0.50 (0.37 to 0.68)	0.67 (0.50 to 0.89)
Thalassemias	1.44 (1.11 to 1.98)	0.76 (0.52 to 1.11)	0.73 (0.50 to 1.05)	0.81 (0.55 to 1.19)	0.02 (0.01 to 0.03)	0.01 (0.01 to 0.01)	0.01 (0.01 to 0.01)	0.01 (0.01 to 0.02)

Sickle cell disorders	0.03 (0.02 to 0.04)	0.02 (0.01 to 0.03)	0.02 (0.01 to 0.02)	0.03 (0.02 to 0.04)	0.00 (0.00 to 0.00)	0.00 (0.00 to 0.00)	0.00 (0.00 to 0.00)	0.00 (0.00 to 0.00)
G6PD deficiency	4.29 (3.29 to 5.22)	5.53 (3.93 to 7.48)	4.64 (3.31 to 6.28)	7.77 (5.53 to 10.60)	0.11 (0.08 to 0.13)	0.19 (0.14 to 0.27)	0.17 (0.12 to 0.23)	0.27 (0.19 to 0.36)
Other hemoglobinopathies and hemolytic anemias	4.02 (3.34 to 5.15)	4.41 (3.34 to 5.95)	4.01 (3.01 to 5.48)	5.33 (4.01 to 7.27)	0.21 (0.17 to 0.28)	0.34 (0.26 to 0.46)	0.32 (0.24 to 0.44)	0.39 (0.29 to 0.53)
Endocrine, metabolic, blood, and immune disorders	10.56 (9.19 to 12.82)	20.83 (16.99 to 26.27)	21.58 (17.52 to 27.07)	19.43 (15.77 to 24.41)	0.35 (0.30 to 0.43)	1.03 (0.80 to 1.29)	1.11 (0.86 to 1.41)	0.89 (0.71 to 1.12)

Malaysia								
Cause	YLLs (in 1000s)				Deaths (in 1000s)			
	2016	2040 Forecast	2040 Better Scenario	2040 Worse Scenario	2016	2040 Forecast	2040 Better Scenario	2040 Worse Scenario
**Musculoskeletal disorders**	**14.17 (11.19 to 16.74)**	**18.46 (14.61 to 26.89)**	**17.67 (13.94 to 26.03)**	**20.15 (16.07 to 28.84)**	**0.33 (0.27 to 0.51)**	**0.61 (0.43 to 1.34)**	**0.60 (0.42 to 1.36)**	**0.64 (0.45 to 1.35)**
Rheumatoid arthritis	0.35 (0.26 to 0.61)	0.49 (0.31 to 1.03)	0.40 (0.25 to 0.87)	0.75 (0.49 to 1.56)	0.01 (0.01 to 0.03)	0.03 (0.01 to 0.06)	0.02 (0.01 to 0.06)	0.04 (0.02 to 0.10)
Other musculoskeletal disorders	13.82 (10.82 to 16.31)	17.97 (14.19 to 25.98)	17.28 (13.62 to 25.45)	19.40 (15.44 to 27.46)	0.32 (0.26 to 0.49)	0.59 (0.41 to 1.29)	0.58 (0.40 to 1.31)	0.60 (0.43 to 1.26)
**Other non-communicable diseases**	**109.28 (85.60 to 133.79)**	**126.20 (86.16 to 159.93)**	**113.57 (75.96 to 143.49)**	**158.61 (111.38 to 201.30)**	**2.11 (1.53 to 2.53)**	**5.18 (3.06 to 6.81)**	**5.06 (2.92 to 6.65)**	**5.56 (3.38 to 7.24)**
Congenital birth defects	83.01 (61.92 to 107.31)	47.82 (33.02 to 68.76)	37.73 (26.39 to 52.57)	74.49 (51.97 to 104.57)	1.05 (0.80 to 1.34)	0.66 (0.48 to 0.92)	0.53 (0.39 to 0.72)	1.02 (0.74 to 1.38)
Neural tube defects	3.10 (1.51 to 5.47)	0.64 (0.27 to 1.40)	0.45 (0.20 to 0.98)	1.16 (0.47 to 2.54)	0.04 (0.02 to 0.06)	0.01 (0.00 to 0.02)	0.01 (0.00 to 0.01)	0.01 (0.01 to 0.03)
Congenital heart anomalies	44.43 (33.17 to 57.32)	19.62 (12.82 to 28.69)	14.07 (9.08 to 20.54)	35.79 (23.89 to 51.01)	0.57 (0.44 to 0.73)	0.28 (0.19 to 0.39)	0.20 (0.14 to 0.28)	0.50 (0.34 to 0.70)
Orofacial clefts	0.07 (0.02 to 0.15)	0.02 (0.00 to 0.06)	0.01 (0.00 to 0.04)	0.04 (0.01 to 0.12)	0.00 (0.00 to 0.00)	0.00 (0.00 to 0.00)	0.00 (0.00 to 0.00)	0.00 (0.00 to 0.00)
Down syndrome	2.32 (1.49 to 3.28)	3.48 (2.30 to 5.03)	3.51 (2.29 to 5.06)	3.35 (2.16 to 4.87)	0.03 (0.02 to 0.05)	0.06 (0.04 to 0.08)	0.06 (0.04 to 0.08)	0.06 (0.04 to 0.08)
Other chromosomal abnormalities	11.71 (7.03 to 17.91)	12.64 (7.41 to 19.78)	10.93 (6.38 to 16.79)	15.95 (8.97 to 25.18)	0.14 (0.08 to 0.21)	0.17 (0.10 to 0.25)	0.14 (0.08 to 0.21)	0.21 (0.12 to 0.32)
Congenital musculoskeletal and limb anomalies	3.14 (1.07 to 5.17)	1.61 (0.53 to 2.99)	1.22 (0.40 to 2.25)	2.61 (0.88 to 4.84)	0.04 (0.01 to 0.06)	0.02 (0.01 to 0.04)	0.01 (0.01 to 0.03)	0.03 (0.01 to 0.06)
Urogenital congenital anomalies	0.99 (0.60 to 1.43)	0.80 (0.54 to 1.16)	0.66 (0.45 to 0.94)	1.14 (0.79 to 1.64)	0.02 (0.01 to 0.02)	0.02 (0.01 to 0.03)	0.02 (0.01 to 0.02)	0.03 (0.02 to 0.04)
Digestive congenital anomalies	2.37 (1.08 to 3.61)	1.17 (0.55 to 2.02)	0.86 (0.41 to 1.51)	1.99 (0.93 to 3.44)	0.03 (0.01 to 0.04)	0.01 (0.01 to 0.02)	0.01 (0.01 to 0.02)	0.02 (0.01 to 0.04)
Other congenital birth defects	14.89 (7.17 to 23.74)	7.84 (3.96 to 13.70)	6.01 (2.97 to 10.47)	12.45 (6.14 to 20.85)	0.18 (0.09 to 0.28)	0.10 (0.05 to 0.17)	0.08 (0.04 to 0.13)	0.16 (0.08 to 0.26)
Skin and subcutaneous diseases	24.93 (14.42 to 34.73)	77.77 (42.86 to 103.09)	75.32 (41.80 to 99.94)	83.27 (45.63 to 112.91)	1.05 (0.56 to 1.38)	4.51 (2.40 to 6.10)	4.52 (2.43 to 6.12)	4.53 (2.39 to 6.17)
Cellulitis	7.02 (3.54 to 10.96)	28.44 (13.32 to 40.16)	28.59 (13.33 to 40.27)	28.31 (13.33 to 40.76)	0.29 (0.13 to 0.42)	1.56 (0.67 to 2.24)	1.62 (0.69 to 2.34)	1.46 (0.61 to 2.13)
Pyoderma	11.74 (6.43 to 17.28)	35.29 (18.10 to 48.36)	33.73 (17.62 to 46.11)	38.63 (19.91 to 53.09)	0.43 (0.22 to 0.59)	1.90 (0.94 to 2.68)	1.90 (0.93 to 2.71)	1.92 (0.95 to 2.73)
Decubitus ulcer	5.05 (2.55 to 7.09)	11.72 (6.07 to 17.07)	10.81 (5.46 to 15.87)	13.71 (6.94 to 20.01)	0.29 (0.15 to 0.41)	0.94 (0.47 to 1.44)	0.90 (0.45 to 1.39)	1.03 (0.52 to 1.58)
Other skin and subcutaneous diseases	1.11 (0.64 to 1.64)	2.33 (1.34 to 3.43)	2.19 (1.26 to 3.25)	2.61 (1.51 to 3.82)	0.04 (0.02 to 0.06)	0.11 (0.06 to 0.17)	0.11 (0.06 to 0.17)	0.12 (0.06 to 0.18)
Sudden infant death syndrome	1.35 (0.83 to 2.17)	0.61 (0.32 to 1.06)	0.52 (0.27 to 0.92)	0.85 (0.45 to 1.50)	0.02 (0.01 to 0.03)	0.01 (0.00 to 0.01)	0.01 (0.00 to 0.01)	0.01 (0.01 to 0.02)

**Injuries**	**587.88 (539.95 to 636.95)**	**464.08 (366.48 to 594.56)**	**417.97 (334.55 to 524.04)**	**650.27 (512.10 to 846.09)**	**13.75 (12.66 to 14.91)**	**14.64 (12.28 to 17.55)**	**13.60 (11.52 to 16.11)**	**19.09 (15.79 to 23.51)**
**Transport injuries**	**310.77 (275.85 to 344.21)**	**201.95 (148.04 to 281.01)**	**185.56 (138.35 to 253.14)**	**328.88 (240.18 to 464.38)**	**7.06 (6.24 to 7.87)**	**5.79 (4.43 to 7.75)**	**5.41 (4.22 to 7.04)**	**8.93 (6.74 to 12.16)**
Road injuries	276.03 (242.80 to 309.51)	166.03 (120.34 to 231.60)	156.92 (116.11 to 213.71)	276.09 (200.42 to 393.50)	6.23 (5.50 to 6.98)	4.72 (3.63 to 6.27)	4.53 (3.54 to 5.86)	7.42 (5.67 to 9.93)
Pedestrian road injuries	52.65 (43.83 to 62.45)	23.72 (17.32 to 31.04)	19.18 (14.22 to 24.89)	49.25 (35.86 to 66.39)	1.53 (1.26 to 1.82)	0.98 (0.72 to 1.27)	0.82 (0.61 to 1.06)	1.85 (1.38 to 2.40)

Malaysia								
Cause	YLLs (in 1000s)				Deaths (in 1000s)			
	2016	2040 Forecast	2040 Better Scenario	2040 Worse Scenario	2016	2040 Forecast	2040 Better Scenario	2040 Worse Scenario
Cyclist road injuries	11.42 (9.08 to 14.03)	9.64 (6.62 to 13.38)	9.41 (6.55 to 12.89)	13.82 (9.47 to 19.37)	0.29 (0.23 to 0.37)	0.32 (0.22 to 0.45)	0.32 (0.22 to 0.44)	0.42 (0.29 to 0.60)
Motorcyclist road injuries	68.13 (45.74 to 86.70)	52.06 (31.65 to 80.12)	49.93 (30.79 to 75.36)	72.21 (42.94 to 113.25)	1.25 (0.87 to 1.59)	1.09 (0.68 to 1.61)	1.05 (0.68 to 1.52)	1.45 (0.90 to 2.18)
Motor vehicle road injuries	137.72 (112.80 to 161.20)	76.39 (53.22 to 108.09)	74.24 (52.84 to 102.60)	133.84 (92.47 to 195.50)	3.01 (2.45 to 3.53)	2.18 (1.59 to 2.95)	2.18 (1.62 to 2.86)	3.49 (2.50 to 4.81)
Other road injuries	6.11 (4.24 to 7.84)	4.22 (2.69 to 6.13)	4.15 (2.70 to 5.90)	6.98 (4.36 to 10.23)	0.15 (0.10 to 0.19)	0.15 (0.10 to 0.21)	0.15 (0.10 to 0.21)	0.21 (0.14 to 0.31)
Other transport injuries	34.74 (27.96 to 43.99)	35.91 (24.43 to 53.27)	28.64 (20.13 to 43.33)	52.79 (35.33 to 80.99)	0.82 (0.66 to 1.05)	1.07 (0.76 to 1.55)	0.88 (0.64 to 1.27)	1.51 (1.06 to 2.26)
**Unintentional injuries**	**152.38 (140.05 to 165.97)**	**133.79 (110.16 to 164.91)**	**121.79 (100.36 to 149.49)**	**159.94 (129.74 to 197.55)**	**3.78 (3.49 to 4.09)**	**5.12 (4.21 to 6.15)**	**4.90 (4.04 to 5.86)**	**5.66 (4.66 to 6.78)**
Falls	20.54 (17.51 to 23.60)	29.24 (23.29 to 35.19)	27.65 (22.10 to 33.32)	32.52 (26.34 to 38.86)	0.77 (0.64 to 0.88)	1.62 (1.28 to 1.95)	1.59 (1.25 to 1.96)	1.69 (1.33 to 2.05)
Drowning	39.94 (33.79 to 46.13)	26.87 (20.15 to 35.79)	23.49 (17.80 to 31.02)	33.76 (25.21 to 45.39)	0.81 (0.69 to 0.91)	0.73 (0.57 to 0.92)	0.65 (0.52 to 0.81)	0.87 (0.68 to 1.12)
Fire, heat, and hot substances	12.12 (10.52 to 13.87)	8.86 (6.44 to 11.56)	7.52 (5.41 to 9.63)	11.93 (8.68 to 15.65)	0.34 (0.27 to 0.38)	0.39 (0.25 to 0.51)	0.34 (0.22 to 0.44)	0.48 (0.31 to 0.63)
Poisonings	8.25 (6.16 to 11.02)	3.75 (2.48 to 5.42)	2.99 (2.03 to 4.30)	5.68 (3.82 to 8.28)	0.19 (0.14 to 0.24)	0.11 (0.08 to 0.16)	0.09 (0.07 to 0.13)	0.16 (0.11 to 0.23)
Exposure to mechanical forces	12.27 (10.42 to 14.32)	9.22 (6.92 to 12.46)	8.02 (6.09 to 10.68)	11.86 (8.87 to 15.77)	0.26 (0.23 to 0.30)	0.26 (0.20 to 0.33)	0.23 (0.18 to 0.29)	0.32 (0.24 to 0.40)
Unintentional firearm injuries	3.54 (2.71 to 4.86)	2.63 (1.73 to 3.84)	2.31 (1.53 to 3.36)	3.35 (2.21 to 4.94)	0.09 (0.07 to 0.12)	0.09 (0.06 to 0.13)	0.08 (0.06 to 0.12)	0.11 (0.07 to 0.15)
Unintentional suffocation	4.79 (3.67 to 6.40)	3.53 (2.37 to 5.25)	3.09 (2.11 to 4.48)	4.42 (3.00 to 6.48)	0.08 (0.06 to 0.10)	0.07 (0.04 to 0.10)	0.06 (0.04 to 0.08)	0.08 (0.06 to 0.12)
Other exposure to mechanical forces	3.94 (3.25 to 4.66)	3.06 (2.30 to 3.96)	2.61 (1.99 to 3.38)	4.09 (3.09 to 5.30)	0.09 (0.08 to 0.11)	0.10 (0.08 to 0.12)	0.09 (0.07 to 0.11)	0.13 (0.10 to 0.16)
Adverse effects of medical treatment	18.36 (13.85 to 21.65)	20.19 (14.80 to 26.32)	18.89 (13.70 to 24.20)	22.71 (16.67 to 29.01)	0.48 (0.38 to 0.56)	0.73 (0.56 to 0.95)	0.71 (0.54 to 0.93)	0.78 (0.59 to 0.98)
Animal contact	2.47 (2.10 to 2.98)	2.96 (2.12 to 4.25)	2.70 (1.94 to 3.83)	3.50 (2.48 to 5.01)	0.06 (0.06 to 0.08)	0.11 (0.08 to 0.15)	0.10 (0.07 to 0.14)	0.12 (0.09 to 0.16)
Venomous animal contact	1.71 (1.42 to 2.08)	2.09 (1.48 to 2.99)	1.94 (1.36 to 2.78)	2.38 (1.68 to 3.42)	0.05 (0.04 to 0.06)	0.08 (0.06 to 0.11)	0.07 (0.05 to 0.10)	0.08 (0.06 to 0.11)
Non-venomous animal contact	0.77 (0.59 to 1.01)	0.87 (0.57 to 1.34)	0.76 (0.50 to 1.17)	1.12 (0.73 to 1.72)	0.02 (0.01 to 0.02)	0.03 (0.02 to 0.04)	0.03 (0.02 to 0.04)	0.04 (0.02 to 0.05)
Foreign body	27.07 (22.16 to 35.52)	27.44 (22.70 to 33.50)	26.65 (22.17 to 32.35)	28.68 (23.43 to 35.47)	0.60 (0.51 to 0.70)	1.01 (0.70 to 1.24)	1.03 (0.70 to 1.30)	0.96 (0.68 to 1.18)
Pulmonary aspiration and foreign body in airway	22.21 (17.78 to 29.81)	20.91 (16.94 to 26.45)	19.86 (16.12 to 24.89)	22.67 (18.22 to 29.12)	0.48 (0.40 to 0.58)	0.78 (0.51 to 0.98)	0.79 (0.51 to 1.02)	0.77 (0.53 to 0.96)

Foreign body in other body part	4.86 (3.85 to 6.62)	6.53 (4.78 to 8.83)	6.79 (4.98 to 9.16)	6.00 (4.37 to 8.20)	0.12 (0.09 to 0.15)	0.22 (0.15 to 0.30)	0.24 (0.16 to 0.32)	0.19 (0.13 to 0.26)
Environmental heat and cold exposure	0.75 (0.55 to 0.94)	0.62 (0.42 to 0.90)	0.53 (0.36 to 0.75)	0.86 (0.59 to 1.22)	0.02 (0.02 to 0.03)	0.02 (0.02 to 0.04)	0.02 (0.01 to 0.03)	0.03 (0.02 to 0.05)
Other unintentional injuries	10.60 (8.63 to 14.01)	4.63 (3.26 to 6.67)	3.37 (2.38 to 4.75)	8.43 (5.91 to 12.00)	0.25 (0.21 to 0.32)	0.15 (0.11 to 0.20)	0.11 (0.08 to 0.15)	0.26 (0.18 to 0.35)
**Self-harm and interpersonal violence**	**124.51 (105.08 to 167.02)**	**125.01 (90.92 to 175.45)**	**107.30 (78.92 to 149.51)**	**158.10 (112.38 to 226.98)**	**2.90 (2.47 to 3.70)**	**3.66 (2.78 to 4.84)**	**3.23 (2.48 to 4.21)**	**4.43 (3.26 to 5.97)**

Malaysia								
Cause	YLLs (in 1000s)				Deaths (in 1000s)			
	2016	2040 Forecast	2040 Better Scenario	2040 Worse Scenario	2016	2040 Forecast	2040 Better Scenario	2040 Worse Scenario
Self-harm	92.75 (78.51 to 124.25)	105.25 (74.73 to 150.78)	90.37 (65.59 to 126.53)	132.05 (90.62 to 194.20)	2.28 (1.93 to 2.88)	3.21 (2.39 to 4.28)	2.84 (2.16 to 3.72)	3.85 (2.79 to 5.25)
Self-harm by firearm	3.72 (2.67 to 7.03)	2.92 (1.73 to 5.34)	2.35 (1.42 to 4.15)	4.11 (2.40 to 7.78)	0.09 (0.06 to 0.15)	0.08 (0.05 to 0.14)	0.07 (0.04 to 0.11)	0.11 (0.07 to 0.20)
Self-harm by other specified means	89.03 (75.32 to 119.73)	102.33 (72.62 to 145.87)	88.01 (63.70 to 122.76)	127.95 (88.11 to 188.10)	2.19 (1.86 to 2.76)	3.12 (2.33 to 4.19)	2.77 (2.11 to 3.62)	3.74 (2.70 to 5.08)
Interpersonal violence	31.76 (23.02 to 49.99)	19.76 (12.87 to 33.88)	16.93 (11.13 to 29.04)	26.05 (16.91 to 44.79)	0.62 (0.45 to 1.02)	0.45 (0.29 to 0.80)	0.39 (0.26 to 0.71)	0.58 (0.38 to 1.05)
Physical violence by firearm	7.51 (4.44 to 12.99)	3.68 (2.09 to 6.91)	2.98 (1.72 to 5.49)	5.47 (3.12 to 10.11)	0.14 (0.08 to 0.25)	0.08 (0.04 to 0.15)	0.06 (0.04 to 0.12)	0.11 (0.07 to 0.21)
Physical violence by sharp object	9.21 (6.00 to 17.93)	5.19 (3.14 to 10.17)	4.33 (2.65 to 8.55)	7.32 (4.42 to 14.53)	0.18 (0.12 to 0.35)	0.11 (0.07 to 0.22)	0.10 (0.06 to 0.19)	0.16 (0.10 to 0.31)
Physical violence by other means	15.04 (10.58 to 23.48)	10.89 (6.85 to 19.30)	9.63 (6.13 to 17.08)	13.27 (8.24 to 24.21)	0.30 (0.21 to 0.49)	0.26 (0.16 to 0.48)	0.23 (0.15 to 0.43)	0.31 (0.19 to 0.57)
**Forces of nature, conflict and terrorism, and executions and police conflict**	**0.21 (0.16 to 0.28)**	**3.34 (0.00 to 32.57)**	**3.32 (0.00 to 32.41)**	**3.34 (0.00 to 32.57)**	**0.00 (0.00 to 0.01)**	**0.07 (0.00 to 0.67)**	**0.07 (0.00 to 0.67)**	**0.07 (0.00 to 0.65)**
Exposure to forces of nature	0.00 (0.00 to 0.00)	0.30 (0.00 to 2.35)	0.30 (0.00 to 2.35)	0.30 (0.00 to 2.35)	0.00 (0.00 to 0.00)	0.01 (0.00 to 0.05)	0.01 (0.00 to 0.05)	0.01 (0.00 to 0.05)
Conflict and terrorism	0.00 (0.00 to 0.00)	3.04 (0.00 to 32.57)	3.02 (0.00 to 32.41)	3.04 (0.00 to 32.57)	0.00 (0.00 to 0.00)	0.06 (0.00 to 0.67)	0.06 (0.00 to 0.67)	0.06 (0.00 to 0.65)
Executions and police conflict	0.21 (0.16 to 0.28)	0.00 (0.00 to 0.00)	0.00 (0.00 to 0.00)	0.00 (0.00 to 0.00)	0.00 (0.00 to 0.01)	0.00 (0.00 to 0.00)	0.00 (0.00 to 0.00)	0.00 (0.00 to 0.00)

Cause	YLLs (actual counts)				Deaths (actual counts)			
Maldives								
	2016	2040 Forecast	2040 Better Scenario	2040 Worse Scenario	2016	2040 Forecast	2040 Better Scenario	2040 Worse Scenario
**All causes**	**26,809.07 (22,697.02 to 31,546.49)**	**46,609.36 (30,754.88 to 68,895.29)**	**39,588.52 (26,528.99 to 57,326.18)**	**61,217.53 (39,594.66 to 90,084.56)**	**1,128.66 (948.98 to 1,335.99)**	**2,493.38 (1,815.08 to 3,332.05)**	**2,170.76 (1,577.35 to 2,895.89)**	**3,105.16 (2,222.85 to 4,164.18)**
**Communicable, maternal, neonatal, and nutritional diseases**	**3,823.24 (3,268.81 to 4,477.91)**	**3,898.30 (2,428.83 to 5,803.23)**	**3,433.34 (2,130.00 to 5,139.80)**	**4,851.44 (2,945.30 to 7,330.79)**	**78.02 (67.15 to 90.70)**	**127.88 (91.38 to 174.44)**	**113.51 (80.25 to 154.31)**	**150.84 (106.82 to 212.79)**
**HIV/AIDS and tuberculosis**	**238.75 (196.09 to 290.17)**	**323.09 (172.21 to 591.86)**	**254.24 (138.37 to 458.10)**	**474.02 (241.37 to 926.08)**	**9.06 (7.30 to 11.06)**	**14.32 (8.39 to 24.33)**	**11.48 (6.85 to 18.95)**	**20.16 (11.24 to 37.12)**
Tuberculosis	191.54 (149.22 to 243.45)	305.97 (153.37 to 574.07)	240.11 (122.76 to 445.83)	449.11 (214.58 to 901.21)	8.16 (6.43 to 10.16)	14.02 (8.07 to 24.01)	11.22 (6.57 to 18.65)	19.73 (10.92 to 36.78)
HIV/AIDS	47.21 (39.52 to 56.39)	17.12 (11.23 to 25.57)	14.12 (8.63 to 22.10)	24.91 (17.50 to 34.90)	0.90 (0.75 to 1.07)	0.30 (0.19 to 0.47)	0.26 (0.15 to 0.42)	0.42 (0.28 to 0.63)
**Diarrhea, lower respiratory, and other common infectious diseases**	**939.84 (783.17 to 1,162.52)**	**1,268.88 (758.56 to 2,032.70)**	**1,019.95 (627.56 to 1,595.05)**	**1,675.00 (971.61 to 2,841.75)**	**29.93 (24.18 to 36.32)**	**59.94 (39.84 to 86.85)**	**50.07 (33.38 to 71.86)**	**72.26 (47.37 to 106.94)**
Diarrheal diseases	99.10 (69.48 to 142.58)	73.00 (13.89 to 236.35)	58.75 (12.54 to 184.26)	134.14 (24.34 to 426.77)	3.13 (1.82 to 4.78)	3.82 (0.76 to 12.25)	3.23 (0.72 to 10.30)	6.70 (1.15 to 22.22)
Intestinal infectious diseases	77.84 (36.54 to 144.02)	63.42 (20.62 to 153.13)	60.74 (19.99 to 144.59)	71.51 (22.69 to 170.02)	1.16 (0.56 to 2.16)	1.04 (0.37 to 2.40)	1.01 (0.36 to 2.32)	1.16 (0.40 to 2.71)
Typhoid fever	65.89 (30.09 to 124.26)	51.34 (15.32 to 126.50)	49.28 (14.89 to 121.34)	57.71 (17.13 to 143.03)	0.98 (0.46 to 1.82)	0.83 (0.29 to 1.93)	0.81 (0.28 to 1.85)	0.92 (0.31 to 2.14)
Paratyphoid fever	11.80 (4.68 to 24.53)	11.98 (3.36 to 30.59)	11.36 (3.20 to 28.70)	13.68 (3.85 to 35.86)	0.18 (0.07 to 0.37)	0.21 (0.06 to 0.50)	0.20 (0.06 to 0.48)	0.23 (0.07 to 0.57)
Other intestinal infectious diseases	0.14 (0.05 to 0.30)	0.10 (0.03 to 0.25)	0.09 (0.03 to 0.22)	0.12 (0.04 to 0.30)	0.00 (0.00 to 0.01)	0.00 (0.00 to 0.01)	0.00 (0.00 to 0.01)	0.00 (0.00 to 0.01)
Lower respiratory infections	511.90 (421.89 to 623.98)	842.96 (506.52 to 1,385.24)	646.27 (395.55 to 1,024.05)	1,090.90 (622.78 to 1,930.99)	21.01 (16.31 to 26.21)	47.80 (31.05 to 69.61)	38.99 (25.54 to 55.38)	56.04 (35.79 to 83.73)
Upper respiratory infections	0.76 (0.39 to 1.28)	1.17 (0.41 to 2.66)	1.03 (0.36 to 2.36)	1.48 (0.52 to 3.26)	0.02 (0.01 to 0.04)	0.04 (0.02 to 0.09)	0.04 (0.01 to 0.08)	0.05 (0.02 to 0.12)
Otitis media	0.48 (0.27 to 0.83)	0.28 (0.13 to 0.57)	0.29 (0.13 to 0.60)	0.28 (0.13 to 0.58)	0.01 (0.01 to 0.02)	0.01 (0.01 to 0.02)	0.01 (0.01 to 0.02)	0.01 (0.00 to 0.02)
Meningitis	73.32 (55.58 to 106.02)	65.65 (34.92 to 124.50)	64.37 (33.95 to 120.81)	71.04 (37.37 to 132.09)	1.46 (1.08 to 2.09)	1.81 (1.13 to 2.99)	1.83 (1.12 to 3.02)	1.84 (1.15 to 3.00)
Pneumococcal meningitis	0.68 (0.44 to 1.06)	0.35 (0.19 to 0.62)	0.35 (0.19 to 0.62)	0.37 (0.20 to 0.67)	0.01 (0.01 to 0.02)	0.01 (0.01 to 0.02)	0.01 (0.01 to 0.02)	0.01 (0.01 to 0.02)
H influenzae type B meningitis	5.38 (3.83 to 7.88)	5.73 (2.34 to 15.06)	5.02 (2.22 to 12.13)	8.93 (2.73 to 27.71)	0.10 (0.08 to 0.14)	0.13 (0.07 to 0.25)	0.12 (0.07 to 0.23)	0.16 (0.07 to 0.39)
Meningococcal meningitis	25.16 (17.90 to 38.21)	22.12 (11.79 to 42.99)	21.91 (11.52 to 43.59)	22.88 (12.21 to 44.50)	0.53 (0.39 to 0.78)	0.66 (0.41 to 1.12)	0.67 (0.40 to 1.14)	0.66 (0.41 to 1.11)
Other meningitis	42.09 (31.14 to 62.17)	37.45 (19.57 to 68.31)	37.09 (19.16 to 67.93)	38.85 (20.40 to 70.90)	0.81 (0.61 to 1.17)	1.01 (0.61 to 1.65)	1.02 (0.60 to 1.70)	1.01 (0.62 to 1.67)
Encephalitis	110.82 (77.41 to 168.71)	147.40 (80.29 to 262.63)	141.29 (76.25 to 255.76)	161.18 (86.87 to 289.46)	2.30 (1.77 to 3.06)	4.33 (2.72 to 6.42)	4.23 (2.62 to 6.31)	4.53 (2.90 to 6.64)

Diphtheria	0.16 (0.09 to 0.25)	0.06 (0.02 to 0.14)	0.05 (0.02 to 0.11)	0.12 (0.03 to 0.41)	0.00 (0.00 to 0.00)	0.00 (0.00 to 0.00)	0.00 (0.00 to 0.00)	0.00 (0.00 to 0.01)
Whooping cough	55.29 (5.21 to 173.02)	22.58 (0.48 to 109.41)	6.79 (0.28 to 33.54)	54.19 (0.68 to 249.88)	0.66 (0.06 to 2.06)	0.27 (0.01 to 1.32)	0.09 (0.00 to 0.41)	0.65 (0.01 to 2.96)
Tetanus	2.06 (1.72 to 2.45)	0.36 (0.17 to 0.64)	0.35 (0.17 to 0.63)	0.42 (0.20 to 0.76)	0.04 (0.03 to 0.04)	0.01 (0.00 to 0.01)	0.01 (0.00 to 0.01)	0.01 (0.00 to 0.01)
Measles	4.28 (1.13 to 11.47)	49.14 (0.75 to 250.26)	37.50 (0.71 to 182.35)	86.20 (0.83 to 473.01)	0.05 (0.01 to 0.14)	0.70 (0.01 to 3.27)	0.55 (0.01 to 2.54)	1.15 (0.02 to 6.01)

Maldives								
**Cause**	**YLLs (actual counts)**				**Deaths (actual counts)**			
	**2016**	**2040 Forecast**	**2040 Better Scenario**	**2040 Worse Scenario**	**2016**	**2040 Forecast**	**2040 Better Scenario**	**2040 Worse Scenario**
Varicella and herpes zoster	3.85 (2.35 to 6.08)	2.85 (1.23 to 6.41)	2.53 (1.08 to 5.82)	3.55 (1.46 to 7.91)	0.09 (0.06 to 0.12)	0.11 (0.06 to 0.18)	0.10 (0.05 to 0.17)	0.12 (0.06 to 0.21)
**Neglected tropical diseases and malaria**	**381.34 (143.27 to 621.79)**	**1,271.96 (637.77 to 2,136.62)**	**1,265.87 (657.18 to 2,093.55)**	**1,336.71 (649.40 to 2,290.48)**	**6.18 (2.59 to 9.83)**	**28.40 (15.62 to 46.25)**	**28.70 (16.10 to 45.67)**	**28.76 (15.37 to 46.62)**
Malaria	0.00 (0.00 to 0.00)	0.00 (0.00 to 0.00)	0.00 (0.00 to 0.00)	0.00 (0.00 to 0.00)	0.00 (0.00 to 0.00)	0.00 (0.00 to 0.00)	0.00 (0.00 to 0.00)	0.00 (0.00 to 0.00)
Chagas disease	0.00 (0.00 to 0.00)	0.00 (0.00 to 0.00)	0.00 (0.00 to 0.00)	0.00 (0.00 to 0.00)	0.00 (0.00 to 0.00)	0.00 (0.00 to 0.00)	0.00 (0.00 to 0.00)	0.00 (0.00 to 0.00)
Leishmaniasis	0.00 (0.00 to 0.00)	0.00 (0.00 to 0.00)	0.00 (0.00 to 0.00)	0.00 (0.00 to 0.00)	0.00 (0.00 to 0.00)	0.00 (0.00 to 0.00)	0.00 (0.00 to 0.00)	0.00 (0.00 to 0.00)
African trypanosomiasis	0.00 (0.00 to 0.00)	0.00 (0.00 to 0.00)	0.00 (0.00 to 0.00)	0.00 (0.00 to 0.00)	0.00 (0.00 to 0.00)	0.00 (0.00 to 0.00)	0.00 (0.00 to 0.00)	0.00 (0.00 to 0.00)
Schistosomiasis	0.00 (0.00 to 0.00)	0.00 (0.00 to 0.00)	0.00 (0.00 to 0.00)	0.00 (0.00 to 0.00)	0.00 (0.00 to 0.00)	0.00 (0.00 to 0.00)	0.00 (0.00 to 0.00)	0.00 (0.00 to 0.00)
Cysticercosis	0.00 (0.00 to 0.00)	0.00 (0.00 to 0.00)	0.00 (0.00 to 0.00)	0.00 (0.00 to 0.00)	0.00 (0.00 to 0.00)	0.00 (0.00 to 0.00)	0.00 (0.00 to 0.00)	0.00 (0.00 to 0.00)
Cystic echinococcosis	0.00 (0.00 to 0.00)	0.00 (0.00 to 0.00)	0.00 (0.00 to 0.00)	0.00 (0.00 to 0.00)	0.00 (0.00 to 0.00)	0.00 (0.00 to 0.00)	0.00 (0.00 to 0.00)	0.00 (0.00 to 0.00)
Dengue	372.61 (134.37 to 612.85)	1,263.12 (630.94 to 2,125.94)	1,257.61 (646.81 to 2,084.29)	1,326.40 (639.72 to 2,279.66)	6.00 (2.41 to 9.65)	28.12 (15.24 to 45.95)	28.44 (15.82 to 45.39)	28.45 (15.08 to 46.07)
Yellow fever	0.00 (0.00 to 0.00)	0.00 (0.00 to 0.00)	0.00 (0.00 to 0.00)	0.00 (0.00 to 0.00)	0.00 (0.00 to 0.00)	0.00 (0.00 to 0.00)	0.00 (0.00 to 0.00)	0.00 (0.00 to 0.00)
Rabies	0.01 (0.01 to 0.02)	0.01 (0.01 to 0.03)	0.01 (0.00 to 0.02)	0.02 (0.01 to 0.04)	0.00 (0.00 to 0.00)	0.00 (0.00 to 0.00)	0.00 (0.00 to 0.00)	0.00 (0.00 to 0.00)
Intestinal nematode infections	0.07 (0.02 to 0.16)	0.07 (0.02 to 0.21)	0.07 (0.02 to 0.23)	0.07 (0.02 to 0.22)	0.00 (0.00 to 0.00)	0.00 (0.00 to 0.00)	0.00 (0.00 to 0.00)	0.00 (0.00 to 0.00)
Other neglected tropical diseases	8.64 (5.70 to 12.97)	8.75 (4.90 to 15.55)	8.17 (4.43 to 14.81)	10.22 (5.76 to 17.79)	0.18 (0.13 to 0.25)	0.28 (0.18 to 0.41)	0.26 (0.17 to 0.40)	0.31 (0.20 to 0.45)
**Maternal disorders**	**175.69 (111.91 to 272.74)**	**151.72 (33.53 to 453.87)**	**126.98 (27.89 to 385.87)**	**209.15 (45.65 to 620.75)**	**3.08 (1.96 to 4.76)**	**2.68 (0.59 to 8.10)**	**2.24 (0.49 to 6.98)**	**3.69 (0.83 to 10.97)**
Maternal hemorrhage	78.26 (45.47 to 128.35)	61.78 (12.78 to 188.63)	51.62 (10.78 to 160.76)	85.02 (18.01 to 252.71)	1.38 (0.80 to 2.25)	1.10 (0.22 to 3.40)	0.92 (0.19 to 2.84)	1.51 (0.31 to 4.53)
Maternal sepsis and other maternal infections	5.50 (2.13 to 10.59)	4.08 (0.70 to 12.61)	3.40 (0.58 to 10.74)	5.63 (0.98 to 17.49)	0.10 (0.04 to 0.19)	0.07 (0.01 to 0.22)	0.06 (0.01 to 0.19)	0.10 (0.02 to 0.32)
Maternal hypertensive disorders	23.91 (13.02 to 40.42)	20.52 (3.77 to 65.61)	17.12 (3.29 to 55.02)	28.46 (5.59 to 90.84)	0.42 (0.23 to 0.70)	0.36 (0.07 to 1.15)	0.30 (0.06 to 0.97)	0.50 (0.10 to 1.60)
Maternal obstructed labor and uterine rupture	3.46 (1.17 to 7.30)	3.13 (0.51 to 10.51)	2.58 (0.40 to 8.70)	4.42 (0.71 to 14.47)	0.06 (0.02 to 0.13)	0.06 (0.01 to 0.19)	0.05 (0.01 to 0.16)	0.08 (0.01 to 0.26)
Maternal abortion, miscarriage, and ectopic pregnancy	18.51 (9.44 to 33.15)	18.16 (3.67 to 56.65)	15.24 (2.98 to 47.85)	25.40 (5.29 to 78.68)	0.33 (0.17 to 0.58)	0.32 (0.07 to 1.04)	0.27 (0.05 to 0.87)	0.45 (0.09 to 1.38)

Indirect maternal deaths	22.87 (11.80 to 40.35)	21.84 (4.25 to 65.86)	18.28 (3.55 to 55.80)	30.06 (5.77 to 90.96)	0.40 (0.20 to 0.70)	0.38 (0.07 to 1.17)	0.32 (0.06 to 0.95)	0.52 (0.10 to 1.57)
Late maternal deaths	2.60 (0.85 to 5.73)	2.20 (0.35 to 7.31)	1.83 (0.28 to 6.24)	3.05 (0.47 to 10.12)	0.05 (0.02 to 0.10)	0.04 (0.01 to 0.13)	0.03 (0.00 to 0.11)	0.05 (0.01 to 0.18)
Maternal deaths aggravated by HIV/AIDS	0.01 (0.00 to 0.01)	0.12 (0.00 to 1.19)	0.10 (0.00 to 1.03)	0.19 (0.00 to 1.75)	0.00 (0.00 to 0.00)	0.00 (0.00 to 0.02)	0.00 (0.00 to 0.01)	0.00 (0.00 to 0.02)
Other maternal disorders	20.59 (10.29 to 35.17)	19.89 (3.82 to 65.46)	16.82 (3.24 to 56.17)	26.92 (5.21 to 87.06)	0.36 (0.18 to 0.61)	0.35 (0.07 to 1.16)	0.30 (0.06 to 0.99)	0.47 (0.09 to 1.54)

Maldives								
Cause	YLLs (actual counts)				Deaths (actual counts)			
	2016	2040 Forecast	2040 Better Scenario	2040 Worse Scenario	2016	2040 Forecast	2040 Better Scenario	2040 Worse Scenario
**Neonatal disorders**	**1,909.77 (1,534.46 to 2,359.29)**	**612.64 (224.65 to 1,559.87)**	**508.08 (184.46 to 1,280.29)**	**861.36 (319.53 to 2,184.29)**	**22.06 (17.73 to 27.25)**	**7.08 (2.60 to 18.04)**	**5.87 (2.13 to 14.79)**	**9.95 (3.69 to 25.24)**
Neonatal preterm birth	761.40 (551.35 to 1,010.78)	187.67 (68.87 to 469.86)	149.48 (54.28 to 374.04)	283.76 (104.18 to 700.90)	8.79 (6.37 to 11.67)	2.17 (0.80 to 5.43)	1.73 (0.63 to 4.32)	3.28 (1.20 to 8.10)
Neonatal encephalopathy due to birth asphyxia and trauma	376.96 (244.31 to 544.81)	109.55 (33.42 to 311.78)	94.39 (28.95 to 268.01)	143.79 (43.56 to 410.99)	4.36 (2.82 to 6.29)	1.27 (0.39 to 3.60)	1.09 (0.33 to 3.10)	1.66 (0.50 to 4.75)
Neonatal sepsis and other neonatal infections	244.23 (142.24 to 383.51)	112.29 (36.17 to 277.16)	95.50 (31.19 to 235.56)	150.31 (48.33 to 364.74)	2.82 (1.64 to 4.43)	1.30 (0.42 to 3.20)	1.10 (0.36 to 2.72)	1.74 (0.56 to 4.22)
Hemolytic disease and other neonatal jaundice	11.87 (7.57 to 18.64)	4.09 (0.99 to 13.70)	3.33 (0.82 to 11.07)	5.86 (1.40 to 19.06)	0.14 (0.09 to 0.22)	0.05 (0.01 to 0.16)	0.04 (0.01 to 0.13)	0.07 (0.02 to 0.22)
Other neonatal disorders	515.30 (321.72 to 761.47)	199.04 (55.46 to 560.96)	165.38 (47.70 to 458.39)	277.64 (80.85 to 769.44)	5.95 (3.72 to 8.80)	2.30 (0.64 to 6.48)	1.91 (0.55 to 5.30)	3.21 (0.93 to 8.89)
**Nutritional deficiencies**	**112.72 (92.75 to 136.95)**	**159.97 (113.30 to 222.11)**	**153.13 (106.75 to 216.77)**	**174.12 (121.33 to 239.85)**	**5.62 (4.60 to 6.86)**	**11.05 (8.06 to 14.35)**	**10.88 (7.85 to 14.35)**	**11.33 (8.16 to 14.65)**
Protein-energy malnutrition	77.63 (61.76 to 95.84)	88.31 (59.42 to 128.03)	83.83 (55.01 to 122.73)	97.58 (64.54 to 141.00)	3.54 (2.81 to 4.41)	5.91 (4.10 to 8.06)	5.78 (3.97 to 7.98)	6.12 (4.13 to 8.31)
Iodine deficiency	1.15 (0.07 to 3.88)	5.70 (0.10 to 19.76)	5.27 (0.09 to 18.30)	6.56 (0.13 to 22.18)	0.04 (0.00 to 0.14)	0.35 (0.00 to 1.45)	0.33 (0.00 to 1.36)	0.38 (0.01 to 1.58)
Dietary iron deficiency	4.81 (3.22 to 7.60)	7.87 (3.80 to 15.60)	7.73 (3.75 to 15.14)	8.35 (4.10 to 16.12)	0.13 (0.08 to 0.27)	0.28 (0.13 to 0.63)	0.29 (0.13 to 0.63)	0.29 (0.14 to 0.63)
Other nutritional deficiencies	29.12 (22.33 to 37.24)	58.10 (38.12 to 79.53)	56.30 (36.21 to 78.13)	61.63 (41.05 to 84.84)	1.92 (1.31 to 2.48)	4.51 (2.86 to 6.08)	4.48 (2.82 to 6.12)	4.53 (2.89 to 6.12)
**Other communicable, maternal, neonatal, and nutritional diseases**	**65.13 (52.21 to 81.09)**	**110.04 (68.43 to 176.54)**	**105.09 (63.73 to 170.23)**	**121.09 (74.63 to 190.52)**	**2.08 (1.64 to 2.57)**	**4.41 (2.91 to 6.33)**	**4.27 (2.77 to 6.21)**	**4.70 (3.21 to 6.62)**
Sexually transmitted infections excluding HIV	8.51 (6.24 to 11.39)	7.45 (4.30 to 13.16)	7.27 (4.17 to 12.83)	7.92 (4.62 to 14.06)	0.27 (0.19 to 0.36)	0.27 (0.18 to 0.41)	0.27 (0.18 to 0.42)	0.28 (0.18 to 0.42)
Syphilis	4.66 (3.29 to 6.32)	3.38 (1.98 to 5.70)	3.35 (1.92 to 5.77)	3.54 (2.10 to 5.90)	0.16 (0.11 to 0.23)	0.13 (0.08 to 0.20)	0.13 (0.08 to 0.21)	0.13 (0.08 to 0.20)
Chlamydial infection	0.70 (0.43 to 1.06)	0.75 (0.35 to 1.55)	0.72 (0.34 to 1.50)	0.82 (0.39 to 1.69)	0.02 (0.01 to 0.03)	0.03 (0.01 to 0.04)	0.02 (0.01 to 0.04)	0.03 (0.02 to 0.05)
Gonococcal infection	2.06 (1.41 to 3.05)	2.19 (1.10 to 4.33)	2.12 (1.07 to 4.19)	2.35 (1.20 to 4.60)	0.06 (0.04 to 0.08)	0.08 (0.05 to 0.13)	0.08 (0.05 to 0.13)	0.08 (0.05 to 0.13)
Other sexually transmitted infections	1.10 (0.75 to 1.63)	1.12 (0.54 to 2.26)	1.08 (0.52 to 2.17)	1.21 (0.59 to 2.45)	0.03 (0.02 to 0.04)	0.03 (0.02 to 0.06)	0.03 (0.02 to 0.06)	0.04 (0.02 to 0.06)
Acute hepatitis	37.38 (29.91 to 47.08)	59.36 (35.47 to 98.76)	56.61 (32.99 to 95.02)	65.52 (39.39 to 108.46)	1.12 (0.89 to 1.40)	2.09 (1.37 to 3.19)	2.01 (1.30 to 3.07)	2.27 (1.48 to 3.46)
Acute hepatitis A	2.29 (1.56 to 3.26)	1.44 (0.73 to 2.56)	1.38 (0.68 to 2.52)	1.61 (0.80 to 2.94)	0.04 (0.03 to 0.06)	0.04 (0.02 to 0.07)	0.04 (0.02 to 0.07)	0.05 (0.03 to 0.07)
Acute hepatitis B	29.87 (23.06 to 38.20)	50.80 (29.29 to 84.62)	48.28 (27.38 to 81.00)	56.28 (33.16 to 93.73)	0.92 (0.71 to 1.18)	1.76 (1.12 to 2.75)	1.68 (1.05 to 2.66)	1.93 (1.22 to 2.99)

Acute hepatitis C	0.91 (0.63 to 1.30)	1.75 (0.95 to 3.03)	1.73 (0.93 to 3.00)	1.82 (1.00 to 3.12)	0.03 (0.02 to 0.04)	0.07 (0.04 to 0.11)	0.07 (0.04 to 0.11)	0.07 (0.04 to 0.11)
Acute hepatitis E	4.31 (3.17 to 5.67)	5.38 (2.89 to 9.28)	5.23 (2.78 to 9.02)	5.81 (3.09 to 9.90)	0.12 (0.08 to 0.18)	0.23 (0.13 to 0.38)	0.23 (0.13 to 0.38)	0.23 (0.13 to 0.38)
Other unspecified infectious diseases	19.23 (13.97 to 26.59)	43.23 (26.20 to 67.10)	41.21 (24.26 to 65.06)	47.66 (28.80 to 73.21)	0.70 (0.45 to 0.92)	2.04 (1.17 to 2.88)	1.99 (1.10 to 2.84)	2.16 (1.26 to 3.00)
**Non-communicable diseases**	**19,646.16 (16,357.74 to 23,567.15)**	**39,134.71 (25,318.94 to 57,435.96)**	**32,786.04 (21,600.67 to 47,672.64)**	**51,893.22 (32,627.31 to 77,850.00)**	**968.34 (813.30 to 1,157.03)**	**2,240.86 (1,606.64 to 3,021.12)**	**1,936.48 (1,385.07 to 2,612.97)**	**2,808.61 (1,976.83 to 3,816.79)**

Maldives								
Cause	YLLs (actual counts)				Deaths (actual counts)			
	2016	2040 Forecast	2040 Better Scenario	2040 Worse Scenario	2016	2040 Forecast	2040 Better Scenario	2040 Worse Scenario
**Neoplasms**	**3,628.52 (2,971.88 to 4,444.01)**	**8,045.44 (5,414.99 to 11,877.34)**	**7,544.19 (5,075.04 to 11,061.91)**	**8,722.10 (5,873.41 to 12,811.44)**	**149.26 (123.05 to 181.01)**	**377.50 (277.91 to 506.97)**	**361.03 (264.27 to 484.04)**	**398.39 (292.92 to 537.96)**
Lip and oral cavity cancer	170.99 (133.46 to 218.74)	385.24 (227.16 to 649.88)	353.70 (212.93 to 582.45)	486.38 (268.97 to 880.84)	8.42 (6.72 to 10.67)	20.44 (13.11 to 32.11)	19.22 (12.44 to 29.68)	24.67 (14.76 to 41.59)
Nasopharynx cancer	29.34 (21.91 to 38.35)	68.24 (36.22 to 124.26)	57.79 (31.62 to 98.29)	98.72 (47.38 to 191.88)	1.15 (0.85 to 1.50)	2.81 (1.59 to 4.81)	2.42 (1.43 to 3.90)	3.94 (2.03 to 7.28)
Other pharynx cancer	17.32 (13.57 to 22.25)	34.64 (19.63 to 59.33)	33.27 (18.98 to 55.72)	41.75 (22.18 to 76.23)	0.71 (0.56 to 0.90)	1.56 (0.96 to 2.49)	1.51 (0.94 to 2.34)	1.83 (1.07 to 3.12)
Esophageal cancer	87.48 (69.83 to 109.24)	173.01 (103.06 to 277.62)	154.65 (93.64 to 240.01)	226.89 (125.50 to 395.06)	4.00 (3.22 to 4.98)	8.35 (5.33 to 12.56)	7.61 (4.93 to 11.21)	10.57 (6.30 to 17.20)
Stomach cancer	125.81 (100.98 to 154.40)	233.77 (161.89 to 334.65)	227.18 (156.04 to 321.44)	245.77 (169.71 to 352.96)	6.16 (5.02 to 7.52)	12.44 (9.21 to 16.26)	12.34 (9.14 to 16.15)	12.62 (9.39 to 16.58)
Colon and rectum cancer	244.89 (196.82 to 305.76)	635.07 (392.67 to 1,030.27)	645.19 (395.38 to 1,039.74)	651.97 (404.31 to 1,056.91)	10.92 (8.84 to 13.40)	32.02 (21.20 to 48.36)	33.16 (21.89 to 50.03)	31.68 (20.94 to 47.37)
Liver cancer	286.97 (216.40 to 371.50)	791.34 (473.34 to 1,335.99)	660.31 (419.36 to 1,039.79)	821.50 (488.98 to 1,408.64)	12.25 (9.40 to 15.67)	35.82 (22.98 to 56.73)	30.77 (20.66 to 45.16)	36.16 (23.07 to 57.79)
Liver cancer due to hepatitis B	122.66 (86.77 to 165.73)	327.20 (186.17 to 583.53)	273.35 (161.76 to 453.01)	336.10 (191.14 to 600.00)	4.82 (3.36 to 6.60)	13.80 (8.09 to 23.01)	11.85 (7.39 to 18.41)	13.81 (8.05 to 23.27)
Liver cancer due to hepatitis C	55.98 (36.44 to 79.55)	168.04 (92.75 to 278.66)	143.37 (82.21 to 227.40)	169.07 (91.80 to 280.09)	2.83 (1.85 to 4.08)	8.82 (5.14 to 14.21)	7.76 (4.68 to 11.92)	8.61 (5.03 to 13.91)
Liver cancer due to alcohol use	35.24 (23.64 to 51.73)	99.41 (52.00 to 173.90)	81.77 (45.31 to 138.50)	110.56 (57.62 to 193.68)	1.56 (1.03 to 2.21)	4.46 (2.44 to 7.42)	3.76 (2.16 to 6.02)	4.82 (2.59 to 8.03)
Liver cancer due to other causes	73.09 (48.21 to 105.54)	196.69 (103.04 to 367.41)	161.82 (89.05 to 284.57)	205.77 (108.28 to 381.20)	3.04 (1.98 to 4.50)	8.75 (4.81 to 15.30)	7.40 (4.31 to 12.37)	8.91 (4.85 to 15.76)
Gallbladder and biliary tract cancer	43.03 (30.31 to 54.95)	75.11 (50.76 to 101.97)	78.19 (52.44 to 107.75)	70.24 (48.80 to 94.34)	2.15 (1.47 to 2.77)	4.03 (2.78 to 5.17)	4.26 (2.89 to 5.55)	3.66 (2.57 to 4.58)
Pancreatic cancer	135.79 (110.16 to 167.93)	351.10 (233.84 to 510.59)	354.11 (235.75 to 510.17)	342.56 (228.33 to 495.66)	6.32 (5.15 to 7.80)	17.61 (12.60 to 23.91)	18.04 (12.89 to 24.43)	16.68 (11.88 to 22.64)
Larynx cancer	46.68 (34.29 to 61.76)	89.89 (46.10 to 162.16)	70.08 (39.13 to 118.62)	133.63 (61.26 to 249.99)	2.22 (1.66 to 2.86)	4.33 (2.33 to 7.52)	3.43 (2.01 to 5.65)	6.28 (3.03 to 11.45)
Tracheal, bronchus, and lung cancer	438.34 (336.55 to 561.91)	923.98 (516.29 to 1,493.53)	676.73 (407.32 to 1,046.58)	1,369.66 (709.82 to 2,341.59)	21.07 (16.10 to 26.82)	45.99 (26.99 to 72.66)	34.48 (21.42 to 51.48)	66.75 (36.66 to 112.03)
Malignant skin melanoma	24.55 (18.43 to 32.53)	52.93 (35.28 to 76.14)	56.69 (37.54 to 81.53)	47.48 (32.61 to 67.08)	0.97 (0.74 to 1.26)	2.40 (1.72 to 3.13)	2.62 (1.87 to 3.49)	2.08 (1.54 to 2.65)
Non-melanoma skin cancer	22.73 (17.62 to 29.01)	58.95 (43.41 to 76.76)	59.29 (42.90 to 78.40)	58.62 (44.01 to 74.83)	1.15 (0.90 to 1.44)	3.23 (2.56 to 3.89)	3.32 (2.56 to 4.07)	3.09 (2.52 to 3.67)
Breast cancer	361.61 (248.97 to 508.06)	872.75 (429.30 to 1,722.75)	852.73 (422.51 to 1,670.05)	905.94 (440.08 to 1,770.58)	12.52 (8.82 to 17.22)	35.48 (19.85 to 63.02)	35.04 (19.76 to 62.27)	36.02 (20.00 to 63.31)
Cervical cancer	138.62 (85.47 to 198.19)	271.88 (128.80 to 519.19)	262.20 (124.33 to 507.80)	288.40 (138.66 to 547.97)	4.61 (3.00 to 6.58)	10.01 (5.22 to 17.15)	9.72 (5.03 to 16.81)	10.47 (5.51 to 17.98)

Uterine cancer	23.60 (16.41 to 33.85)	57.35 (31.54 to 100.49)	55.16 (30.20 to 98.24)	60.78 (33.78 to 106.34)	0.98 (0.68 to 1.39)	2.74 (1.71 to 4.34)	2.67 (1.65 to 4.28)	2.83 (1.80 to 4.39)
Ovarian cancer	116.06 (77.01 to 169.20)	281.52 (129.46 to 560.11)	278.94 (130.07 to 543.42)	267.92 (124.50 to 528.29)	4.07 (2.66 to 5.86)	11.14 (5.69 to 20.08)	11.13 (5.73 to 19.92)	10.53 (5.45 to 18.95)
Prostate cancer	81.78 (57.14 to 109.12)	243.28 (172.79 to 313.58)	261.00 (185.50 to 341.27)	217.76 (157.74 to 280.09)	6.09 (4.22 to 8.06)	18.19 (13.06 to 23.29)	20.02 (14.21 to 25.66)	15.65 (11.21 to 20.08)
Testicular cancer	3.88 (2.59 to 5.44)	4.99 (2.50 to 9.68)	4.92 (2.43 to 9.65)	5.17 (2.64 to 9.92)	0.08 (0.05 to 0.11)	0.12 (0.07 to 0.22)	0.12 (0.07 to 0.22)	0.12 (0.07 to 0.22)

Maldives								
Cause	YLLs (actual counts)				Deaths (actual counts)			
	2016	2040 Forecast	2040 Better Scenario	2040 Worse Scenario	2016	2040 Forecast	2040 Better Scenario	2040 Worse Scenario
Kidney cancer	56.07 (44.10 to 70.32)	135.46 (87.80 to 190.29)	140.82 (91.25 to 198.14)	127.51 (83.46 to 177.66)	2.26 (1.74 to 2.86)	6.19 (4.24 to 8.24)	6.56 (4.51 to 8.79)	5.65 (3.96 to 7.54)
Bladder cancer	97.53 (75.94 to 125.28)	224.32 (150.57 to 320.23)	218.69 (146.79 to 308.86)	231.55 (155.83 to 334.73)	5.81 (4.59 to 7.38)	13.90 (9.88 to 19.16)	13.89 (9.90 to 18.89)	13.78 (9.78 to 19.29)
Brain and nervous system cancer	141.68 (112.09 to 180.60)	252.89 (162.76 to 373.51)	260.92 (166.47 to 387.67)	242.43 (158.21 to 356.61)	3.96 (3.10 to 5.02)	9.36 (6.29 to 12.85)	9.82 (6.52 to 13.65)	8.67 (5.97 to 11.81)
Thyroid cancer	55.04 (43.13 to 69.34)	145.78 (91.86 to 230.63)	129.01 (85.21 to 196.53)	145.42 (93.41 to 226.41)	2.54 (1.94 to 3.23)	7.22 (4.77 to 10.69)	6.55 (4.47 to 9.45)	7.01 (4.70 to 10.26)
Mesothelioma	33.36 (24.48 to 44.19)	69.35 (45.59 to 98.53)	70.77 (46.22 to 101.98)	67.51 (45.23 to 94.96)	1.19 (0.87 to 1.56)	2.79 (2.01 to 3.68)	2.89 (2.06 to 3.87)	2.63 (1.93 to 3.41)
Hodgkin lymphoma	11.54 (8.72 to 16.37)	15.66 (9.61 to 25.18)	14.85 (9.05 to 24.37)	17.25 (10.87 to 28.02)	0.33 (0.25 to 0.49)	0.57 (0.37 to 0.87)	0.54 (0.36 to 0.85)	0.61 (0.41 to 0.93)
Non-Hodgkin lymphoma	171.06 (135.43 to 219.18)	356.47 (246.00 to 510.86)	351.65 (240.90 to 505.72)	334.11 (234.08 to 476.06)	5.87 (4.63 to 7.30)	15.52 (11.36 to 20.53)	15.64 (11.48 to 20.54)	14.07 (10.51 to 18.35)
Multiple myeloma	50.32 (38.34 to 65.60)	146.00 (96.57 to 213.07)	143.78 (96.26 to 206.45)	134.54 (90.10 to 195.27)	2.11 (1.62 to 2.80)	6.66 (4.68 to 9.24)	6.68 (4.69 to 9.22)	5.99 (4.31 to 8.18)
Leukemia	342.41 (278.04 to 425.21)	531.57 (345.04 to 829.94)	493.13 (326.92 to 758.27)	539.48 (355.56 to 849.84)	9.77 (7.93 to 12.20)	21.64 (14.93 to 30.83)	20.48 (14.37 to 28.83)	21.18 (14.73 to 30.17)
Acute lymphoid leukemia	125.23 (99.52 to 155.39)	186.47 (126.87 to 270.76)	177.87 (121.75 to 252.81)	182.14 (124.62 to 267.28)	3.60 (2.89 to 4.54)	7.67 (5.35 to 10.79)	7.46 (5.32 to 10.48)	7.22 (5.10 to 10.00)
Chronic lymphoid leukemia	6.67 (3.22 to 10.13)	10.62 (5.02 to 19.54)	9.83 (4.71 to 17.75)	10.63 (4.99 to 19.94)	0.21 (0.12 to 0.30)	0.43 (0.24 to 0.71)	0.41 (0.23 to 0.65)	0.41 (0.23 to 0.69)
Acute myeloid leukemia	56.96 (44.29 to 75.17)	96.82 (60.73 to 150.98)	93.04 (57.62 to 142.67)	93.55 (58.89 to 151.38)	1.42 (1.10 to 1.82)	3.31 (2.17 to 4.76)	3.24 (2.12 to 4.66)	3.09 (2.03 to 4.50)
Chronic myeloid leukemia	23.66 (16.03 to 34.47)	30.53 (16.01 to 60.47)	27.39 (14.71 to 51.89)	30.86 (16.28 to 61.30)	0.73 (0.50 to 1.02)	1.22 (0.69 to 2.09)	1.12 (0.64 to 1.82)	1.19 (0.69 to 1.98)
Other leukemia	129.87 (100.81 to 173.56)	207.14 (128.13 to 344.81)	185.00 (116.14 to 301.77)	222.30 (138.77 to 371.11)	3.80 (3.01 to 4.86)	9.02 (6.00 to 13.30)	8.26 (5.52 to 11.97)	9.26 (6.17 to 13.54)
Other neoplasms	270.04 (220.17 to 333.98)	562.91 (371.97 to 785.78)	578.43 (377.70 to 817.95)	541.17 (364.67 to 747.60)	9.59 (7.15 to 12.02)	24.93 (16.74 to 32.85)	26.09 (17.35 to 34.74)	23.16 (15.62 to 29.81)
**Cardiovascular diseases**	**8,132.97 (6,651.64 to 9,985.22)**	**13,136.62 (7,506.56 to 21,919.73)**	**9,227.60 (5,523.24 to 14,782.68)**	**22,079.58 (11,888.84 to 37,627.81)**	**444.48 (369.68 to 536.22)**	**794.88 (489.19 to 1,215.17)**	**603.42 (382.91 to 890.43)**	**1,217.18 (733.94 to 1,882.81)**
Rheumatic heart disease	193.49 (155.77 to 240.27)	192.86 (124.20 to 302.01)	177.73 (114.27 to 277.74)	223.04 (144.87 to 347.23)	6.34 (5.17 to 7.80)	9.10 (6.65 to 12.62)	8.59 (6.31 to 11.76)	10.02 (7.39 to 13.84)
Ischemic heart disease	5,430.29 (4,407.21 to 6,731.93)	8,385.39 (4,090.74 to 15,150.22)	5,501.59 (2,825.91 to 9,613.05)	15,122.72 (7,131.13 to 27,763.88)	304.28 (251.69 to 371.48)	504.86 (270.03 to 843.30)	360.49 (198.13 to 580.90)	830.13 (435.29 to 1,416.77)
Stroke	1,580.84 (1,259.83 to 1,974.56)	2,394.13 (1,377.48 to 3,960.41)	1,609.24 (977.94 to 2,592.51)	4,206.16 (2,298.42 to 7,405.72)	82.90 (66.88 to 101.75)	140.67 (90.35 to 217.29)	103.41 (68.07 to 156.83)	221.83 (134.66 to 360.48)
Ischemic stroke	419.06 (332.57 to 520.93)	662.43 (383.07 to 1,169.55)	492.78 (290.53 to 857.80)	1,007.63 (551.35 to 1,817.48)	29.13 (23.52 to 36.18)	50.18 (29.78 to 88.36)	39.85 (23.71 to 68.76)	70.12 (39.87 to 126.40)

Intracerebral hemorrhage	1,161.78 (925.31 to 1,450.60)	1,731.71 (918.06 to 3,049.31)	1,116.46 (643.82 to 1,897.29)	3,198.53 (1,616.39 to 5,977.84)	53.77 (43.06 to 66.91)	90.49 (55.34 to 142.99)	63.56 (40.91 to 96.39)	151.71 (87.56 to 253.99)
Hypertensive heart disease	316.22 (244.33 to 410.45)	766.46 (471.85 to 1,314.86)	641.56 (411.18 to 1,015.79)	1,049.54 (593.04 to 1,996.54)	18.47 (14.21 to 23.99)	49.80 (31.76 to 82.02)	43.29 (28.16 to 65.89)	65.38 (38.62 to 119.96)
Cardiomyopathy and myocarditis	92.51 (73.84 to 116.29)	171.82 (112.84 to 253.30)	173.40 (113.37 to 257.03)	174.17 (115.43 to 256.48)	4.29 (3.14 to 5.41)	10.40 (6.40 to 14.99)	10.75 (6.64 to 15.68)	10.01 (6.33 to 14.56)
Myocarditis	28.01 (21.64 to 37.27)	40.99 (25.51 to 59.79)	41.12 (25.27 to 60.71)	41.19 (26.51 to 59.17)	1.03 (0.72 to 1.38)	1.97 (1.17 to 2.64)	2.03 (1.18 to 2.77)	1.87 (1.16 to 2.46)

Maldives								
Cause	YLLs (actual counts)				Deaths (actual counts)			
	2016	2040 Forecast	2040 Better Scenario	2040 Worse Scenario	2016	2040 Forecast	2040 Better Scenario	2040 Worse Scenario
Alcoholic cardiomyopathy	6.45 (4.40 to 8.71)	11.12 (6.75 to 16.44)	11.28 (6.75 to 16.90)	11.71 (7.22 to 17.15)	0.28 (0.19 to 0.38)	0.54 (0.35 to 0.74)	0.55 (0.36 to 0.77)	0.56 (0.37 to 0.77)
Other cardiomyopathy	58.05 (45.48 to 72.75)	119.71 (74.40 to 182.90)	120.99 (75.04 to 184.49)	121.27 (76.13 to 186.90)	2.98 (2.11 to 3.85)	7.89 (4.48 to 12.07)	8.17 (4.67 to 12.47)	7.59 (4.35 to 11.75)
Atrial fibrillation and flutter	60.19 (44.50 to 79.39)	164.08 (115.41 to 226.76)	160.17 (111.33 to 219.99)	159.99 (112.24 to 220.08)	5.79 (4.28 to 7.53)	16.08 (11.26 to 21.80)	16.23 (11.35 to 22.08)	14.93 (10.24 to 20.54)
Aortic aneurysm	95.75 (75.15 to 118.71)	213.85 (151.75 to 286.01)	199.92 (141.82 to 265.73)	209.89 (149.23 to 282.75)	5.95 (4.71 to 7.31)	14.19 (10.59 to 18.04)	13.82 (10.32 to 17.51)	13.31 (10.12 to 16.82)
Peripheral artery disease	17.91 (11.42 to 27.42)	78.26 (45.38 to 128.58)	79.21 (46.64 to 129.74)	72.69 (41.77 to 119.36)	1.38 (0.89 to 2.24)	6.69 (3.90 to 11.38)	7.02 (4.11 to 11.90)	5.83 (3.41 to 9.63)
Endocarditis	80.89 (59.43 to 112.90)	138.07 (84.05 to 232.33)	136.16 (82.50 to 225.87)	146.64 (89.37 to 246.37)	2.68 (2.06 to 3.49)	6.09 (3.90 to 8.59)	6.15 (3.93 to 8.72)	6.18 (4.02 to 8.71)
Other cardiovascular and circulatory diseases	264.88 (196.90 to 349.36)	631.70 (377.00 to 955.40)	548.63 (330.30 to 818.51)	714.74 (420.23 to 1,093.72)	12.41 (8.99 to 16.28)	37.00 (23.55 to 52.22)	33.65 (21.54 to 46.73)	39.56 (25.35 to 56.14)
**Chronic respiratory diseases**	**1,880.84 (1,535.08 to 2,312.04)**	**4,086.25 (2,672.04 to 6,196.31)**	**3,367.77 (2,273.37 to 4,873.34)**	**5,004.85 (3,177.41 to 7,775.22)**	**105.49 (86.46 to 128.56)**	**243.69 (174.43 to 331.78)**	**206.99 (149.93 to 276.93)**	**284.48 (196.00 to 397.35)**
Chronic obstructive pulmonary disease	1,329.99 (1,051.44 to 1,644.26)	2,888.35 (1,729.47 to 4,596.16)	2,171.14 (1,341.20 to 3,302.12)	3,870.87 (2,246.52 to 6,412.78)	81.84 (66.12 to 100.21)	182.86 (122.00 to 259.25)	144.92 (98.25 to 202.35)	228.96 (148.93 to 335.35)
Pneumoconiosis	3.35 (2.48 to 4.36)	6.05 (4.04 to 8.48)	5.77 (3.69 to 8.17)	6.38 (4.40 to 8.86)	0.18 (0.13 to 0.23)	0.36 (0.26 to 0.49)	0.35 (0.24 to 0.48)	0.37 (0.26 to 0.49)
Silicosis	0.65 (0.44 to 0.92)	0.95 (0.56 to 1.41)	0.92 (0.53 to 1.39)	1.01 (0.62 to 1.49)	0.03 (0.02 to 0.04)	0.05 (0.03 to 0.07)	0.05 (0.03 to 0.07)	0.05 (0.03 to 0.07)
Asbestosis	0.75 (0.51 to 1.05)	1.70 (1.02 to 2.63)	1.72 (1.01 to 2.69)	1.68 (1.03 to 2.56)	0.03 (0.02 to 0.05)	0.09 (0.06 to 0.14)	0.10 (0.06 to 0.15)	0.09 (0.05 to 0.13)
Coal workers pneumoconiosis	0.41 (0.25 to 0.61)	0.81 (0.45 to 1.35)	0.70 (0.38 to 1.17)	0.98 (0.55 to 1.59)	0.02 (0.01 to 0.03)	0.05 (0.03 to 0.08)	0.04 (0.02 to 0.07)	0.06 (0.03 to 0.09)
Other pneumoconiosis	1.54 (1.11 to 2.08)	2.59 (1.66 to 3.70)	2.43 (1.51 to 3.52)	2.71 (1.78 to 3.85)	0.09 (0.06 to 0.12)	0.17 (0.11 to 0.24)	0.16 (0.10 to 0.23)	0.17 (0.11 to 0.24)
Asthma	140.31 (104.80 to 184.90)	202.16 (103.58 to 387.86)	143.57 (76.78 to 265.89)	227.40 (117.56 to 435.62)	5.46 (4.04 to 7.37)	10.46 (5.79 to 18.45)	7.71 (4.46 to 13.03)	11.28 (6.35 to 19.71)
Interstitial lung disease and pulmonary sarcoidosis	382.79 (291.74 to 511.44)	961.93 (672.65 to 1,355.44)	1,020.57 (704.61 to 1,441.75)	869.98 (610.85 to 1,225.05)	17.48 (13.04 to 25.08)	49.07 (35.66 to 67.03)	53.06 (38.12 to 71.57)	42.91 (31.48 to 60.14)
Other chronic respiratory diseases	24.40 (16.15 to 38.73)	27.76 (15.45 to 47.33)	26.72 (14.85 to 46.02)	30.21 (17.02 to 53.57)	0.53 (0.37 to 0.75)	0.95 (0.55 to 1.44)	0.95 (0.54 to 1.42)	0.96 (0.57 to 1.48)
**Cirrhosis and other chronic liver diseases**	**586.15 (455.18 to 752.00)**	**1,258.18 (825.71 to 1,824.77)**	**1,233.79 (805.45 to 1,805.64)**	**1,317.21 (884.57 to 1,908.86)**	**21.53 (16.68 to 27.32)**	**51.58 (37.50 to 69.46)**	**51.24 (36.47 to 69.56)**	**52.67 (38.77 to 69.85)**
Cirrhosis and other chronic liver diseases due to hepatitis B	203.05 (147.74 to 269.56)	453.82 (293.04 to 682.06)	448.19 (285.72 to 678.32)	464.13 (305.19 to 693.35)	7.61 (5.62 to 9.92)	18.42 (12.89 to 25.54)	18.43 (12.65 to 25.83)	18.41 (13.07 to 25.18)
Cirrhosis and other chronic liver diseases due to hepatitis C	121.22 (88.70 to 164.64)	273.98 (171.66 to 428.98)	269.54 (167.88 to 422.73)	281.85 (179.12 to 435.01)	4.37 (3.21 to 5.83)	10.79 (7.28 to 15.72)	10.73 (7.17 to 15.72)	10.87 (7.43 to 15.67)

Cirrhosis and other chronic liver diseases due to alcohol use	108.86 (77.75 to 148.31)	227.37 (145.07 to 348.46)	219.29 (139.25 to 337.36)	257.21 (166.19 to 387.56)	4.18 (3.07 to 5.53)	9.42 (6.39 to 13.07)	9.22 (6.25 to 12.85)	10.35 (7.23 to 14.14)
Cirrhosis and other chronic liver diseases due to other causes	153.01 (121.04 to 196.87)	303.00 (197.99 to 458.55)	296.76 (192.99 to 451.55)	314.01 (205.90 to 471.47)	5.37 (4.20 to 6.88)	12.95 (9.09 to 18.17)	12.86 (8.94 to 18.33)	13.04 (9.28 to 18.02)
**Digestive diseases**	**188.84 (150.67 to 235.30)**	**374.44 (262.84 to 514.85)**	**357.54 (249.28 to 491.90)**	**396.32 (279.51 to 546.78)**	**9.04 (7.16 to 11.14)**	**21.83 (16.69 to 27.39)**	**21.34 (15.91 to 26.96)**	**22.02 (16.95 to 27.98)**
Peptic ulcer disease	36.45 (27.28 to 46.74)	65.59 (43.60 to 96.10)	56.79 (37.33 to 82.67)	75.93 (50.50 to 108.74)	2.00 (1.50 to 2.53)	4.14 (2.89 to 5.65)	3.67 (2.56 to 5.03)	4.61 (3.27 to 6.25)

Maldives								
Cause	YLLs (actual counts)				Deaths (actual counts)			
	2016	2040 Forecast	2040 Better Scenario	2040 Worse Scenario	2016	2040 Forecast	2040 Better Scenario	2040 Worse Scenario
Gastritis and duodenitis	0.68 (0.50 to 0.89)	1.48 (0.93 to 2.17)	1.46 (0.91 to 2.18)	1.53 (0.98 to 2.20)	0.03 (0.02 to 0.04)	0.08 (0.06 to 0.11)	0.08 (0.06 to 0.11)	0.08 (0.06 to 0.11)
Appendicitis	12.41 (9.17 to 16.13)	22.18 (14.17 to 31.47)	21.28 (13.29 to 30.44)	23.94 (15.51 to 33.82)	0.51 (0.35 to 0.66)	1.16 (0.75 to 1.55)	1.14 (0.72 to 1.55)	1.19 (0.79 to 1.59)
Paralytic ileus and intestinal obstruction	67.05 (48.14 to 88.85)	121.59 (77.71 to 173.15)	121.77 (77.28 to 174.34)	121.49 (77.97 to 170.53)	2.97 (2.13 to 3.91)	7.02 (4.81 to 9.23)	7.22 (4.89 to 9.64)	6.67 (4.61 to 8.70)
Inguinal, femoral, and abdominal hernia	2.33 (1.43 to 3.14)	3.49 (1.65 to 5.15)	3.48 (1.65 to 5.21)	3.52 (1.73 to 5.10)	0.13 (0.06 to 0.18)	0.26 (0.11 to 0.38)	0.27 (0.11 to 0.40)	0.24 (0.10 to 0.36)
Inflammatory bowel disease	12.40 (8.83 to 16.87)	25.12 (16.61 to 35.54)	25.80 (16.66 to 37.02)	24.15 (16.40 to 33.93)	0.62 (0.43 to 0.82)	1.43 (0.98 to 1.88)	1.51 (1.01 to 2.05)	1.31 (0.92 to 1.69)
Vascular intestinal disorders	12.75 (9.50 to 16.41)	36.09 (25.04 to 48.55)	36.14 (24.68 to 48.91)	35.89 (25.42 to 47.51)	0.77 (0.58 to 0.99)	2.44 (1.77 to 3.20)	2.50 (1.78 to 3.27)	2.33 (1.71 to 3.00)
Gallbladder and biliary diseases	5.31 (3.26 to 7.03)	17.55 (7.87 to 30.99)	12.69 (5.98 to 21.55)	18.73 (8.70 to 33.17)	0.34 (0.18 to 0.46)	1.24 (0.52 to 2.09)	0.94 (0.40 to 1.55)	1.25 (0.53 to 2.13)
Pancreatitis	21.59 (15.36 to 30.19)	44.08 (24.43 to 85.58)	42.11 (22.98 to 78.47)	51.72 (26.28 to 117.12)	0.80 (0.57 to 1.07)	1.88 (1.13 to 3.30)	1.83 (1.09 to 3.18)	2.11 (1.19 to 4.46)
Other digestive diseases	17.87 (13.10 to 23.28)	37.26 (25.23 to 52.62)	36.02 (23.69 to 51.52)	39.43 (27.17 to 55.47)	0.88 (0.65 to 1.15)	2.19 (1.56 to 2.91)	2.17 (1.52 to 2.92)	2.22 (1.62 to 2.93)
**Neurological disorders**	**1,093.75 (876.21 to 1,334.50)**	**2,923.90 (2,059.55 to 4,078.25)**	**2,694.73 (1,946.69 to 3,630.41)**	**2,771.17 (1,989.38 to 3,876.47)**	**82.02 (66.01 to 101.16)**	**248.40 (174.60 to 346.84)**	**234.36 (170.64 to 316.86)**	**222.21 (155.38 to 319.16)**
Alzheimer's disease and other dementias	738.64 (574.79 to 925.87)	2,297.82 (1,511.22 to 3,425.52)	2,060.69 (1,432.26 to 2,899.22)	2,132.26 (1,421.91 to 3,164.99)	69.61 (55.46 to 87.30)	217.92 (145.54 to 317.88)	202.00 (139.46 to 283.88)	193.93 (129.87 to 289.27)
Parkinson's disease	73.78 (52.95 to 96.45)	218.28 (159.01 to 289.05)	238.85 (173.53 to 317.59)	186.89 (138.72 to 244.50)	5.65 (3.99 to 7.55)	17.05 (12.50 to 22.87)	19.01 (13.84 to 25.51)	14.13 (10.38 to 18.72)
Epilepsy	200.84 (159.10 to 253.30)	264.01 (151.70 to 447.99)	246.66 (140.44 to 415.44)	315.20 (178.57 to 550.41)	4.47 (3.53 to 5.61)	7.86 (5.04 to 11.97)	7.48 (4.81 to 11.39)	9.05 (5.66 to 14.27)
Multiple sclerosis	5.79 (2.94 to 9.25)	10.54 (4.56 to 21.24)	10.33 (4.50 to 20.75)	10.30 (4.51 to 20.61)	0.16 (0.09 to 0.25)	0.34 (0.17 to 0.62)	0.33 (0.17 to 0.62)	0.32 (0.16 to 0.59)
Motor neuron disease	11.57 (8.98 to 14.44)	31.59 (19.40 to 50.16)	32.74 (19.70 to 52.00)	29.88 (18.60 to 46.61)	0.36 (0.28 to 0.44)	1.08 (0.71 to 1.57)	1.12 (0.73 to 1.65)	1.00 (0.67 to 1.46)
Other neurological disorders	63.13 (52.01 to 76.74)	101.66 (69.75 to 144.48)	105.46 (71.71 to 150.39)	96.65 (67.58 to 137.01)	1.78 (1.47 to 2.15)	4.16 (3.13 to 5.41)	4.41 (3.27 to 5.75)	3.77 (2.88 to 4.80)
**Mental disorders**	**126.92 (86.20 to 214.53)**	**273.29 (151.18 to 490.03)**	**275.71 (151.93 to 490.55)**	**273.14 (153.42 to 494.44)**	**3.05 (2.21 to 4.62)**	**8.07 (5.28 to 12.60)**	**8.26 (5.35 to 12.97)**	**7.83 (5.21 to 12.21)**
Alcohol use disorders	30.45 (19.35 to 44.56)	49.26 (25.86 to 85.89)	49.05 (25.55 to 86.24)	50.90 (27.12 to 87.42)	0.84 (0.55 to 1.20)	1.58 (0.91 to 2.54)	1.58 (0.91 to 2.59)	1.60 (0.94 to 2.53)
Drug use disorders	93.78 (61.04 to 175.34)	220.92 (120.72 to 408.78)	223.46 (121.09 to 409.81)	219.20 (120.52 to 411.65)	2.16 (1.52 to 3.57)	6.43 (4.20 to 9.93)	6.61 (4.32 to 10.40)	6.18 (4.07 to 9.69)
Opioid use disorders	43.33 (22.77 to 78.50)	65.57 (29.88 to 135.98)	65.93 (29.86 to 137.17)	65.97 (29.83 to 133.66)	0.87 (0.50 to 1.47)	1.62 (0.89 to 3.09)	1.66 (0.90 to 3.16)	1.59 (0.86 to 3.01)

Cocaine use disorders	7.30 (4.18 to 14.73)	26.15 (14.07 to 50.56)	25.94 (13.91 to 50.18)	26.68 (14.67 to 50.56)	0.19 (0.12 to 0.32)	0.83 (0.48 to 1.34)	0.84 (0.48 to 1.34)	0.83 (0.49 to 1.34)
Amphetamine use disorders	5.82 (3.49 to 9.17)	21.56 (10.44 to 42.27)	21.24 (10.27 to 42.06)	22.43 (10.99 to 44.08)	0.12 (0.08 to 0.18)	0.52 (0.27 to 0.95)	0.52 (0.27 to 0.96)	0.53 (0.28 to 0.96)
Other drug use disorders	37.33 (21.74 to 83.44)	107.64 (55.18 to 216.07)	110.35 (56.08 to 218.89)	104.12 (53.62 to 212.47)	0.98 (0.65 to 1.75)	3.45 (2.16 to 5.46)	3.60 (2.21 to 5.66)	3.23 (2.04 to 5.27)
Eating disorders	2.70 (1.59 to 4.47)	3.11 (1.21 to 7.13)	3.19 (1.23 to 7.30)	3.04 (1.19 to 6.90)	0.05 (0.03 to 0.08)	0.06 (0.02 to 0.15)	0.06 (0.02 to 0.15)	0.06 (0.02 to 0.14)

Maldives								
Cause	YLLs (actual counts)				Deaths (actual counts)			
	2016	2040 Forecast	2040 Better Scenario	2040 Worse Scenario	2016	2040 Forecast	2040 Better Scenario	2040 Worse Scenario
**Diabetes, urogenital, blood, and endocrine diseases**	**2,859.67 (2,360.22 to 3,469.09)**	**7,739.56 (4,402.69 to 15,595.91)**	**6,827.91 (3,889.03 to 13,982.09)**	**9,929.02 (5,448.83 to 20,327.08)**	**132.66 (110.09 to 159.97)**	**448.28 (260.50 to 982.11)**	**402.35 (232.91 to 905.61)**	**558.23 (317.24 to 1,240.71)**
Diabetes mellitus	785.29 (634.93 to 970.56)	2,053.70 (1,233.29 to 3,306.84)	1,725.97 (1,046.30 to 2,776.93)	2,587.05 (1,532.87 to 4,185.87)	41.12 (33.68 to 50.15)	120.61 (78.40 to 178.73)	104.18 (68.29 to 155.37)	144.47 (91.43 to 219.34)
Acute glomerulonephritis	2.21 (1.82 to 2.67)	2.54 (1.48 to 4.30)	2.45 (1.41 to 4.16)	2.74 (1.62 to 4.55)	0.05 (0.04 to 0.06)	0.09 (0.06 to 0.15)	0.09 (0.05 to 0.14)	0.09 (0.06 to 0.15)
Chronic kidney disease	1,498.95 (1,216.24 to 1,854.32)	4,618.70 (2,013.63 to 12,313.41)	4,049.20 (1,811.66 to 10,787.13)	6,246.33 (2,651.94 to 16,947.66)	70.75 (57.78 to 86.32)	273.53 (116.66 to 823.34)	243.41 (106.11 to 755.08)	360.80 (151.39 to 1,064.30)
Chronic kidney disease due to diabetes mellitus	852.87 (674.90 to 1,074.49)	2,768.67 (1,197.53 to 7,162.19)	2,418.94 (1,058.53 to 6,323.43)	3,754.81 (1,544.39 to 9,905.42)	37.67 (29.84 to 46.96)	148.59 (61.01 to 442.78)	131.39 (55.39 to 394.77)	197.70 (79.17 to 581.85)
Chronic kidney disease due to hypertension	414.62 (323.89 to 531.69)	1,439.05 (609.58 to 4,354.63)	1,270.12 (541.43 to 3,871.07)	1,934.92 (795.36 to 5,755.41)	25.65 (20.09 to 32.58)	103.44 (44.31 to 322.73)	92.94 (40.41 to 289.54)	134.47 (55.64 to 411.52)
Chronic kidney disease due to glomerulonephritis	117.85 (90.32 to 154.21)	181.42 (83.79 to 440.66)	159.09 (72.81 to 386.82)	245.39 (109.04 to 591.26)	3.28 (2.48 to 4.31)	8.42 (3.61 to 24.50)	7.45 (3.22 to 21.36)	11.30 (4.69 to 32.57)
Chronic kidney disease due to other and unspecified causes	113.60 (87.76 to 145.69)	229.56 (101.88 to 590.24)	201.04 (89.61 to 532.14)	311.22 (133.36 to 790.36)	4.14 (3.08 to 5.42)	13.08 (5.64 to 37.00)	11.63 (4.90 to 34.24)	17.33 (7.05 to 49.15)
Urinary diseases and male infertility	97.00 (78.73 to 119.78)	282.92 (198.72 to 394.54)	287.18 (200.55 to 403.56)	275.07 (197.40 to 375.25)	4.91 (4.00 to 5.94)	16.96 (12.71 to 21.43)	17.63 (12.99 to 22.64)	15.77 (12.17 to 19.56)
Urinary tract infections	82.62 (65.97 to 102.75)	250.44 (172.06 to 352.52)	255.00 (173.00 to 362.78)	241.95 (169.93 to 333.45)	4.06 (3.27 to 5.00)	14.86 (10.95 to 19.10)	15.49 (11.23 to 20.22)	13.72 (10.41 to 17.29)
Urolithiasis	4.32 (2.68 to 6.58)	11.55 (6.57 to 19.28)	11.73 (6.63 to 19.87)	11.28 (6.41 to 18.62)	0.21 (0.13 to 0.32)	0.65 (0.39 to 1.00)	0.67 (0.40 to 1.05)	0.60 (0.36 to 0.93)
Other urinary diseases	10.06 (6.72 to 14.05)	20.93 (12.07 to 32.10)	20.46 (11.39 to 31.72)	21.85 (12.99 to 32.35)	0.64 (0.38 to 0.93)	1.46 (0.81 to 2.24)	1.47 (0.80 to 2.30)	1.44 (0.81 to 2.20)
Gynecological diseases	0.26 (0.15 to 0.40)	0.49 (0.19 to 1.05)	0.45 (0.18 to 0.98)	0.55 (0.21 to 1.20)	0.01 (0.00 to 0.01)	0.02 (0.01 to 0.03)	0.02 (0.01 to 0.03)	0.02 (0.01 to 0.04)
Uterine fibroids	0.03 (0.01 to 0.05)	0.05 (0.02 to 0.12)	0.05 (0.02 to 0.11)	0.06 (0.02 to 0.13)	0.00 (0.00 to 0.00)	0.00 (0.00 to 0.00)	0.00 (0.00 to 0.00)	0.00 (0.00 to 0.01)
Polycystic ovarian syndrome	0.04 (0.01 to 0.09)	0.04 (0.01 to 0.12)	0.04 (0.01 to 0.11)	0.05 (0.01 to 0.15)	0.00 (0.00 to 0.00)	0.00 (0.00 to 0.00)	0.00 (0.00 to 0.00)	0.00 (0.00 to 0.00)
Endometriosis	0.01 (0.01 to 0.03)	0.03 (0.01 to 0.09)	0.03 (0.01 to 0.09)	0.04 (0.01 to 0.11)	0.00 (0.00 to 0.00)	0.00 (0.00 to 0.00)	0.00 (0.00 to 0.00)	0.00 (0.00 to 0.00)
Genital prolapse	0.02 (0.01 to 0.03)	0.03 (0.01 to 0.06)	0.03 (0.01 to 0.06)	0.04 (0.02 to 0.07)	0.00 (0.00 to 0.00)	0.00 (0.00 to 0.00)	0.00 (0.00 to 0.00)	0.00 (0.00 to 0.00)
Other gynecological diseases	0.16 (0.09 to 0.26)	0.34 (0.12 to 0.75)	0.32 (0.11 to 0.71)	0.37 (0.13 to 0.83)	0.00 (0.00 to 0.01)	0.01 (0.01 to 0.02)	0.01 (0.01 to 0.02)	0.01 (0.01 to 0.02)
Hemoglobinopathies and hemolytic anemias	145.49 (109.86 to 185.87)	258.02 (167.98 to 382.98)	248.33 (159.43 to 371.65)	276.71 (183.69 to 410.76)	5.41 (4.03 to 7.46)	12.53 (8.72 to 17.83)	12.29 (8.47 to 17.54)	12.99 (9.28 to 18.42)
Thalassemias	33.75 (26.52 to 41.93)	15.71 (8.46 to 27.00)	16.63 (8.83 to 28.65)	14.81 (7.88 to 25.23)	0.48 (0.37 to 0.60)	0.28 (0.15 to 0.47)	0.29 (0.15 to 0.50)	0.26 (0.14 to 0.44)

Sickle cell disorders	0.14 (0.09 to 0.20)	0.32 (0.17 to 0.52)	0.31 (0.17 to 0.51)	0.33 (0.18 to 0.54)	0.00 (0.00 to 0.00)	0.01 (0.01 to 0.02)	0.01 (0.01 to 0.02)	0.01 (0.01 to 0.02)
G6PD deficiency	63.99 (43.89 to 87.21)	158.09 (94.45 to 243.46)	149.28 (87.67 to 233.21)	174.68 (105.53 to 267.89)	1.85 (1.28 to 2.58)	6.15 (3.91 to 9.07)	5.87 (3.69 to 8.81)	6.64 (4.31 to 9.68)
Other hemoglobinopathies and hemolytic anemias	47.60 (35.05 to 66.69)	83.90 (57.14 to 120.44)	82.11 (55.55 to 119.27)	86.89 (60.24 to 124.67)	3.07 (2.23 to 4.49)	6.10 (4.27 to 9.03)	6.11 (4.23 to 9.09)	6.08 (4.37 to 8.82)
Endocrine, metabolic, blood, and immune disorders	330.48 (269.27 to 437.67)	523.18 (344.69 to 810.77)	514.32 (336.11 to 803.77)	540.57 (359.83 to 826.90)	10.41 (8.28 to 14.82)	24.53 (17.71 to 35.73)	24.74 (17.77 to 36.49)	24.09 (17.60 to 35.00)

Maldives								
Cause	YLLs (actual counts)				Deaths (actual counts)			
	2016	2040 Forecast	2040 Better Scenario	2040 Worse Scenario	2016	2040 Forecast	2040 Better Scenario	2040 Worse Scenario
**Musculoskeletal disorders**	**56.04 (39.89 to 80.72)**	**121.84 (69.16 to 212.48)**	**125.80 (70.79 to 219.70)**	**115.70 (66.23 to 197.76)**	**1.71 (1.26 to 2.45)**	**5.29 (3.34 to 8.14)**	**5.55 (3.47 to 8.65)**	**4.84 (3.13 to 7.27)**
Rheumatoid arthritis	3.89 (2.67 to 5.96)	13.04 (8.03 to 21.36)	12.27 (7.48 to 20.65)	13.92 (8.54 to 22.80)	0.18 (0.13 to 0.29)	0.76 (0.48 to 1.24)	0.73 (0.45 to 1.19)	0.79 (0.50 to 1.24)
Other musculoskeletal disorders	52.15 (36.68 to 75.48)	108.81 (59.00 to 193.17)	113.53 (62.08 to 201.96)	101.78 (56.21 to 180.92)	1.53 (1.11 to 2.16)	4.53 (2.81 to 7.12)	4.82 (2.98 to 7.58)	4.05 (2.54 to 6.26)
**Other non-communicable diseases**	**1,092.47 (863.74 to 1,455.34)**	**1,175.20 (726.92 to 1,791.00)**	**1,131.02 (702.78 to 1,707.00)**	**1,284.12 (775.55 to 2,018.84)**	**19.10 (14.98 to 24.24)**	**41.34 (25.05 to 56.42)**	**41.93 (24.96 to 58.09)**	**40.76 (25.31 to 55.91)**
Congenital birth defects	893.58 (689.42 to 1,220.51)	627.91 (322.53 to 1,113.38)	577.22 (299.53 to 1,021.19)	746.33 (372.14 to 1,326.54)	11.44 (8.99 to 15.19)	10.14 (5.79 to 16.20)	9.44 (5.51 to 15.12)	11.73 (6.71 to 19.07)
Neural tube defects	12.65 (6.37 to 25.48)	3.41 (1.40 to 8.58)	2.97 (1.21 to 7.36)	4.40 (1.72 to 11.11)	0.16 (0.09 to 0.31)	0.05 (0.02 to 0.12)	0.05 (0.02 to 0.10)	0.07 (0.03 to 0.15)
Congenital heart anomalies	307.24 (222.40 to 446.22)	193.85 (99.47 to 349.22)	180.34 (93.18 to 320.05)	226.27 (113.85 to 412.30)	4.00 (2.95 to 5.66)	3.07 (1.73 to 5.16)	2.88 (1.61 to 4.85)	3.51 (1.92 to 6.05)
Orofacial clefts	0.20 (0.12 to 0.33)	0.06 (0.02 to 0.15)	0.05 (0.02 to 0.13)	0.08 (0.02 to 0.20)	0.00 (0.00 to 0.00)	0.00 (0.00 to 0.00)	0.00 (0.00 to 0.00)	0.00 (0.00 to 0.00)
Down syndrome	72.31 (45.40 to 105.78)	76.03 (38.16 to 133.54)	69.55 (35.43 to 122.61)	90.75 (45.84 to 161.18)	1.03 (0.68 to 1.46)	1.43 (0.76 to 2.40)	1.32 (0.70 to 2.24)	1.66 (0.89 to 2.84)
Other chromosomal abnormalities	228.04 (142.75 to 384.98)	180.58 (70.55 to 386.23)	163.95 (65.20 to 346.95)	218.91 (85.03 to 477.94)	2.73 (1.74 to 4.54)	2.52 (1.13 to 4.89)	2.31 (1.06 to 4.46)	2.99 (1.34 to 6.00)
Congenital musculoskeletal and limb anomalies	18.77 (10.47 to 32.68)	8.32 (3.36 to 19.03)	7.69 (3.13 to 17.36)	9.80 (3.85 to 22.62)	0.23 (0.13 to 0.39)	0.12 (0.06 to 0.25)	0.11 (0.05 to 0.23)	0.14 (0.06 to 0.29)
Urogenital congenital anomalies	19.32 (14.36 to 27.07)	29.20 (14.00 to 47.80)	27.80 (13.16 to 45.52)	32.15 (15.64 to 52.45)	0.40 (0.27 to 0.52)	0.92 (0.40 to 1.50)	0.89 (0.38 to 1.44)	1.00 (0.44 to 1.59)
Digestive congenital anomalies	40.20 (23.17 to 86.39)	20.89 (8.19 to 45.55)	18.96 (7.52 to 40.19)	25.39 (9.85 to 55.71)	0.48 (0.28 to 1.02)	0.29 (0.13 to 0.58)	0.26 (0.12 to 0.52)	0.34 (0.15 to 0.70)
Other congenital birth defects	194.85 (130.83 to 280.68)	115.57 (51.35 to 227.07)	105.91 (47.60 to 205.46)	138.59 (59.65 to 276.36)	2.41 (1.64 to 3.44)	1.74 (0.92 to 3.05)	1.62 (0.86 to 2.85)	2.03 (1.03 to 3.71)
Skin and subcutaneous diseases	165.09 (103.18 to 261.96)	534.47 (295.86 to 825.25)	542.16 (297.40 to 850.13)	522.30 (293.43 to 818.90)	7.26 (4.08 to 10.67)	31.06 (16.95 to 43.02)	32.36 (17.47 to 45.21)	28.85 (15.92 to 40.56)
Cellulitis	25.14 (12.70 to 54.79)	100.04 (47.52 to 208.24)	102.19 (48.51 to 210.04)	96.13 (44.92 to 203.25)	1.04 (0.48 to 2.13)	5.81 (2.59 to 11.11)	6.06 (2.73 to 11.54)	5.33 (2.41 to 10.30)
Pyoderma	85.23 (48.29 to 135.46)	286.66 (143.75 to 449.37)	286.88 (141.87 to 450.24)	287.19 (147.01 to 453.47)	3.31 (1.73 to 4.89)	15.45 (7.73 to 22.03)	15.88 (7.79 to 22.77)	14.74 (7.41 to 21.15)
Decubitus ulcer	41.75 (23.09 to 68.09)	128.86 (67.17 to 204.89)	133.75 (69.54 to 215.28)	120.50 (63.53 to 193.08)	2.68 (1.38 to 4.05)	9.41 (4.72 to 14.27)	10.00 (4.98 to 15.12)	8.40 (4.23 to 12.81)
Other skin and subcutaneous diseases	12.98 (6.52 to 29.57)	18.92 (7.18 to 44.97)	19.34 (7.31 to 46.33)	18.49 (6.99 to 44.55)	0.24 (0.13 to 0.53)	0.40 (0.18 to 0.91)	0.41 (0.18 to 0.94)	0.38 (0.17 to 0.86)
Sudden infant death syndrome	33.80 (12.74 to 70.64)	12.82 (1.79 to 41.33)	11.64 (1.64 to 37.93)	15.50 (2.16 to 48.98)	0.39 (0.15 to 0.82)	0.15 (0.02 to 0.48)	0.14 (0.02 to 0.44)	0.18 (0.02 to 0.57)

**Injuries**	**3,339.67 (2,732.23 to 4,066.75)**	**3,576.35 (2,291.78 to 5,562.46)**	**3,369.14 (2,139.01 to 5,280.61)**	**4,472.87 (2,824.77 to 6,909.27)**	**82.31 (67.20 to 100.06)**	**124.64 (89.20 to 175.65)**	**120.78 (86.51 to 168.95)**	**145.71 (102.91 to 210.45)**
**Transport injuries**	**928.77 (724.21 to 1,222.18)**	**808.82 (502.97 to 1,292.79)**	**678.80 (426.12 to 1,068.27)**	**1,500.83 (911.84 to 2,420.49)**	**24.45 (19.20 to 31.53)**	**31.26 (21.74 to 45.18)**	**27.83 (19.35 to 40.03)**	**50.12 (34.18 to 74.34)**
Road injuries	868.91 (682.78 to 1,126.93)	728.12 (457.75 to 1,125.86)	602.06 (382.81 to 924.89)	1,408.48 (858.88 to 2,271.12)	23.22 (18.36 to 29.86)	29.14 (20.24 to 41.53)	25.78 (17.80 to 36.33)	47.79 (32.54 to 70.64)
Pedestrian road injuries	150.25 (91.62 to 225.99)	126.99 (68.77 to 222.61)	101.97 (54.54 to 178.37)	239.59 (129.50 to 420.59)	4.54 (2.83 to 6.80)	5.78 (3.35 to 9.63)	4.84 (2.77 to 8.13)	9.92 (5.83 to 16.19)

Maldives								
Cause	YLLs (actual counts)				Deaths (actual counts)			
	2016	2040 Forecast	2040 Better Scenario	2040 Worse Scenario	2016	2040 Forecast	2040 Better Scenario	2040 Worse Scenario
Cyclist road injuries	89.90 (55.20 to 133.13)	88.48 (50.58 to 150.12)	75.27 (43.06 to 132.15)	159.36 (89.35 to 266.87)	3.02 (1.94 to 4.51)	4.26 (2.61 to 7.88)	3.93 (2.41 to 7.52)	6.29 (3.84 to 10.52)
Motorcyclist road injuries	261.86 (162.52 to 380.36)	318.94 (189.54 to 514.32)	284.68 (168.75 to 454.64)	496.85 (284.15 to 829.32)	7.48 (5.23 to 10.24)	12.75 (7.29 to 19.16)	12.11 (6.56 to 18.12)	17.12 (10.53 to 26.92)
Motor vehicle road injuries	351.17 (232.64 to 538.05)	183.68 (94.73 to 340.10)	132.49 (68.39 to 245.94)	489.14 (253.28 to 922.68)	7.81 (5.38 to 11.86)	5.96 (3.49 to 10.21)	4.58 (2.69 to 7.77)	13.72 (7.77 to 23.64)
Other road injuries	15.72 (7.37 to 31.27)	10.03 (4.30 to 23.98)	7.65 (3.27 to 18.48)	23.54 (9.91 to 55.17)	0.38 (0.19 to 0.78)	0.39 (0.17 to 0.95)	0.32 (0.14 to 0.79)	0.73 (0.35 to 1.68)
Other transport injuries	59.86 (32.91 to 119.02)	80.70 (39.36 to 168.73)	76.74 (36.98 to 160.28)	92.35 (42.75 to 205.56)	1.22 (0.75 to 2.36)	2.12 (1.14 to 4.09)	2.06 (1.09 to 3.98)	2.33 (1.23 to 4.73)
**Unintentional injuries**	**1,679.49 (1,380.47 to 2,036.50)**	**1,808.24 (1,218.99 to 2,604.77)**	**1,751.29 (1,177.97 to 2,532.49)**	**1,942.99 (1,304.87 to 2,800.46)**	**41.84 (34.24 to 50.69)**	**68.02 (50.70 to 90.09)**	**67.76 (49.93 to 90.01)**	**69.08 (51.27 to 90.82)**
Falls	345.23 (266.60 to 439.21)	599.98 (423.06 to 825.17)	598.45 (418.24 to 830.67)	603.35 (425.57 to 829.94)	13.11 (10.32 to 16.45)	30.03 (22.88 to 37.94)	30.71 (23.18 to 39.08)	28.85 (21.69 to 36.34)
Drowning	663.95 (534.29 to 820.73)	557.22 (348.54 to 841.09)	530.62 (333.33 to 800.10)	626.11 (388.59 to 936.85)	13.53 (10.95 to 16.72)	16.13 (11.11 to 22.98)	15.65 (10.70 to 22.26)	17.43 (11.75 to 24.90)
Fire, heat, and hot substances	146.81 (115.68 to 197.90)	147.02 (93.73 to 235.40)	139.25 (89.26 to 220.96)	165.16 (103.96 to 263.49)	3.88 (3.07 to 5.12)	5.67 (4.07 to 8.00)	5.53 (3.91 to 7.84)	6.02 (4.29 to 8.59)
Poisonings	9.62 (7.22 to 13.07)	6.73 (3.91 to 11.61)	6.15 (3.49 to 10.59)	8.08 (4.49 to 14.01)	0.20 (0.16 to 0.27)	0.20 (0.13 to 0.31)	0.19 (0.12 to 0.29)	0.23 (0.15 to 0.36)
Exposure to mechanical forces	142.53 (112.48 to 181.60)	130.49 (80.94 to 203.37)	122.82 (76.08 to 192.67)	146.29 (91.09 to 226.58)	2.87 (2.32 to 3.56)	3.80 (2.58 to 5.52)	3.66 (2.47 to 5.36)	4.07 (2.78 to 5.92)
Unintentional firearm injuries	14.74 (11.37 to 19.47)	12.28 (7.19 to 20.11)	11.47 (6.70 to 19.07)	13.86 (8.11 to 22.74)	0.34 (0.26 to 0.44)	0.38 (0.25 to 0.57)	0.36 (0.23 to 0.54)	0.41 (0.27 to 0.62)
Unintentional suffocation	49.79 (33.86 to 74.95)	29.83 (16.53 to 52.30)	27.68 (15.26 to 48.02)	34.96 (19.02 to 62.92)	0.72 (0.51 to 1.04)	0.54 (0.32 to 0.88)	0.50 (0.30 to 0.83)	0.61 (0.36 to 1.02)
Other exposure to mechanical forces	78.00 (61.43 to 100.96)	88.38 (55.01 to 140.81)	83.67 (51.45 to 134.06)	97.47 (61.09 to 156.52)	1.82 (1.45 to 2.31)	2.89 (1.97 to 4.20)	2.79 (1.91 to 4.11)	3.05 (2.10 to 4.40)
Adverse effects of medical treatment	85.61 (67.55 to 111.33)	107.06 (69.99 to 158.57)	103.02 (67.59 to 154.41)	114.43 (75.04 to 170.85)	2.44 (1.95 to 3.01)	4.38 (3.11 to 5.93)	4.32 (3.02 to 5.86)	4.46 (3.20 to 6.05)
Animal contact	42.04 (32.79 to 52.90)	52.08 (31.63 to 83.16)	48.54 (29.73 to 77.65)	59.21 (35.67 to 93.73)	0.98 (0.78 to 1.24)	1.73 (1.18 to 2.55)	1.65 (1.12 to 2.46)	1.88 (1.27 to 2.76)
Venomous animal contact	7.39 (5.10 to 10.32)	7.09 (3.83 to 12.78)	6.52 (3.57 to 11.94)	8.26 (4.52 to 15.02)	0.15 (0.11 to 0.22)	0.20 (0.12 to 0.35)	0.19 (0.11 to 0.34)	0.23 (0.13 to 0.40)
Non-venomous animal contact	34.65 (27.03 to 43.64)	45.00 (27.79 to 71.50)	42.02 (25.74 to 67.44)	50.95 (30.79 to 81.56)	0.83 (0.65 to 1.05)	1.53 (1.05 to 2.26)	1.46 (0.99 to 2.17)	1.65 (1.12 to 2.42)
Foreign body	165.63 (80.27 to 252.07)	127.12 (52.21 to 218.32)	126.11 (52.15 to 215.45)	131.61 (52.04 to 229.42)	2.99 (1.52 to 4.41)	3.53 (1.74 to 5.59)	3.60 (1.78 to 5.68)	3.44 (1.66 to 5.48)
Pulmonary aspiration and foreign body in airway	158.03 (72.10 to 245.06)	119.12 (45.94 to 206.92)	118.39 (45.83 to 205.51)	123.11 (45.51 to 220.16)	2.80 (1.33 to 4.20)	3.24 (1.51 to 5.22)	3.31 (1.57 to 5.30)	3.15 (1.40 to 5.11)

Foreign body in other body part	7.60 (5.76 to 12.10)	7.99 (4.75 to 13.25)	7.72 (4.57 to 12.83)	8.50 (5.12 to 14.30)	0.19 (0.14 to 0.26)	0.29 (0.18 to 0.44)	0.29 (0.18 to 0.43)	0.29 (0.18 to 0.44)
Environmental heat and cold exposure	5.49 (4.03 to 7.20)	7.10 (4.14 to 11.18)	6.47 (3.71 to 10.32)	8.33 (4.87 to 13.33)	0.16 (0.12 to 0.21)	0.27 (0.18 to 0.40)	0.25 (0.16 to 0.38)	0.30 (0.20 to 0.46)
Other unintentional injuries	72.58 (56.78 to 92.90)	73.43 (46.17 to 119.70)	69.86 (43.61 to 113.43)	80.43 (50.61 to 130.28)	1.69 (1.33 to 2.14)	2.28 (1.56 to 3.37)	2.21 (1.48 to 3.26)	2.41 (1.65 to 3.51)
**Self-harm and interpersonal violence**	**729.08 (502.58 to 982.48)**	**920.81 (486.89 to 1,648.16)**	**900.71 (482.57 to 1,613.93)**	**989.57 (513.41 to 1,823.21)**	**15.97 (11.19 to 21.47)**	**24.49 (14.21 to 40.12)**	**24.31 (14.25 to 39.45)**	**25.64 (14.58 to 43.87)**

Maldives								
**Cause**	**YLLs (actual counts)**				**Deaths (actual counts)**			
	**2016**	**2040 Forecast**	**2040 Better Scenario**	**2040 Worse Scenario**	**2016**	**2040 Forecast**	**2040 Better Scenario**	**2040 Worse Scenario**
Self-harm	533.45 (364.41 to 732.75)	747.09 (396.82 to 1,320.14)	727.23 (389.48 to 1,281.64)	811.74 (419.46 to 1,489.03)	12.08 (8.43 to 16.16)	20.41 (12.20 to 33.35)	20.21 (12.09 to 32.38)	21.50 (12.46 to 36.49)
Self-harm by firearm	21.16 (10.75 to 33.06)	24.01 (9.34 to 49.69)	23.11 (9.09 to 47.90)	26.48 (9.76 to 55.39)	0.47 (0.25 to 0.73)	0.64 (0.28 to 1.21)	0.62 (0.28 to 1.17)	0.69 (0.29 to 1.32)
Self-harm by other specified means	512.29 (350.31 to 704.13)	723.09 (383.97 to 1,282.52)	704.13 (376.10 to 1,243.04)	785.26 (406.83 to 1,436.32)	11.61 (8.14 to 15.47)	19.77 (11.82 to 32.18)	19.58 (11.77 to 31.32)	20.81 (12.12 to 35.15)
Interpersonal violence	195.63 (87.30 to 284.11)	173.72 (64.77 to 317.59)	173.48 (64.90 to 317.77)	177.82 (65.08 to 322.16)	3.89 (1.73 to 5.55)	4.09 (1.58 to 7.15)	4.10 (1.60 to 7.20)	4.14 (1.57 to 7.21)
Physical violence by firearm	22.39 (8.88 to 40.95)	19.92 (6.67 to 41.12)	19.87 (6.73 to 41.15)	20.08 (6.63 to 40.89)	0.45 (0.17 to 0.79)	0.46 (0.16 to 0.91)	0.46 (0.16 to 0.91)	0.46 (0.16 to 0.89)
Physical violence by sharp object	97.96 (37.19 to 151.90)	90.58 (30.28 to 170.02)	91.78 (30.96 to 172.56)	89.36 (29.54 to 166.48)	1.90 (0.73 to 2.90)	2.00 (0.68 to 3.63)	2.03 (0.69 to 3.69)	1.95 (0.66 to 3.52)
Physical violence by other means	75.27 (31.92 to 109.47)	63.22 (22.81 to 116.45)	61.83 (22.50 to 114.25)	68.38 (24.10 to 127.27)	1.54 (0.67 to 2.19)	1.63 (0.64 to 2.77)	1.61 (0.62 to 2.74)	1.73 (0.67 to 3.06)
**Forces of nature, conflict and terrorism, and executions and police conflict**	**2.33 (1.88 to 3.00)**	**38.47 (0.00 to 0.00)**	**38.34 (0.00 to 0.00)**	**39.48 (0.00 to 0.00)**	**0.05 (0.04 to 0.06)**	**0.87 (0.00 to 0.00)**	**0.87 (0.00 to 0.00)**	**0.87 (0.00 to 0.00)**
Exposure to forces of nature	0.00 (0.00 to 0.00)	38.47 (0.00 to 0.00)	38.34 (0.00 to 0.00)	39.48 (0.00 to 0.00)	0.00 (0.00 to 0.00)	0.87 (0.00 to 0.00)	0.87 (0.00 to 0.00)	0.87 (0.00 to 0.00)
Conflict and terrorism	0.00 (0.00 to 0.00)	0.00 (0.00 to 0.00)	0.00 (0.00 to 0.00)	0.00 (0.00 to 0.00)	0.00 (0.00 to 0.00)	0.00 (0.00 to 0.00)	0.00 (0.00 to 0.00)	0.00 (0.00 to 0.00)
Executions and police conflict	2.33 (1.88 to 3.00)	0.00 (0.00 to 0.00)	0.00 (0.00 to 0.00)	0.00 (0.00 to 0.00)	0.05 (0.04 to 0.06)	0.00 (0.00 to 0.00)	0.00 (0.00 to 0.00)	0.00 (0.00 to 0.00)

Mauritius								
Cause	YLLs (actual counts)				Deaths (actual counts)			
	2016	2040 Forecast	2040 Better Scenario	2040 Worse Scenario	2016	2040 Forecast	2040 Better Scenario	2040 Worse Scenario
**All causes**	**230,124.00 (204,094.62 to 258,820.18)**	**228,739.88 (177,909.23 to 296,602.88)**	**179,805.70 (139,656.74 to 239,124.51)**	**317,711.95 (245,877.93 to 403,201.49)**	**10,115.32 (9,041.98 to 11,293.91)**	**14,304.04 (11,867.09 to 17,685.94)**	**11,873.79 (9,781.44 to 14,892.93)**	**18,320.34 (15,243.71 to 22,001.06)**
**Communicable, maternal, neonatal, and nutritional diseases**	**20,975.02 (18,617.57 to 23,673.82)**	**15,701.42 (12,632.63 to 20,106.72)**	**12,724.20 (10,431.25 to 16,027.92)**	**20,280.84 (16,040.11 to 26,464.83)**	**534.84 (481.63 to 587.79)**	**754.17 (554.68 to 995.34)**	**665.92 (492.79 to 876.77)**	**882.53 (637.83 to 1,164.58)**
**HIV/AIDS and tuberculosis**	**3,754.86 (3,344.79 to 4,219.48)**	**3,156.06 (2,554.70 to 3,694.96)**	**2,894.47 (2,299.22 to 3,438.23)**	**3,546.46 (2,997.32 to 4,081.10)**	**88.71 (79.18 to 99.08)**	**77.08 (61.12 to 89.84)**	**69.97 (54.46 to 82.38)**	**87.76 (72.17 to 102.51)**
Tuberculosis	365.41 (301.83 to 443.52)	150.58 (84.81 to 258.42)	74.81 (44.93 to 125.08)	290.92 (154.38 to 513.04)	13.24 (11.17 to 15.73)	7.38 (4.35 to 12.25)	3.94 (2.46 to 6.14)	13.20 (7.53 to 22.54)
HIV/AIDS	3,389.45 (2,964.90 to 3,850.12)	3,005.48 (2,421.29 to 3,549.14)	2,819.66 (2,230.10 to 3,359.61)	3,255.54 (2,733.78 to 3,770.06)	75.47 (65.79 to 85.76)	69.70 (54.36 to 82.31)	66.03 (50.76 to 79.01)	74.57 (60.93 to 86.75)
**Diarrhea, lower respiratory, and other common infectious diseases**	**7,584.50 (6,661.25 to 8,608.52)**	**8,575.27 (6,095.75 to 11,992.40)**	**6,842.63 (4,979.26 to 9,616.87)**	**11,178.20 (7,680.88 to 16,178.20)**	**305.99 (266.15 to 346.72)**	**598.28 (403.83 to 834.29)**	**523.14 (359.62 to 730.49)**	**702.19 (472.65 to 980.31)**
Diarrheal diseases	815.01 (694.33 to 971.14)	1,100.85 (299.99 to 3,185.16)	1,276.95 (460.17 to 3,369.06)	812.36 (205.46 to 2,409.44)	28.61 (25.03 to 32.74)	76.98 (21.45 to 233.72)	98.51 (35.73 to 259.09)	51.21 (13.62 to 156.48)
Intestinal infectious diseases	116.47 (54.91 to 218.64)	74.40 (33.20 to 142.81)	85.15 (39.50 to 159.15)	68.86 (30.70 to 134.04)	1.86 (0.88 to 3.42)	1.31 (0.61 to 2.45)	1.53 (0.73 to 2.84)	1.19 (0.54 to 2.28)
Typhoid fever	97.17 (44.10 to 181.30)	64.36 (28.03 to 126.93)	77.59 (35.38 to 146.28)	55.86 (22.96 to 112.07)	1.54 (0.71 to 2.86)	1.13 (0.52 to 2.15)	1.38 (0.65 to 2.61)	0.95 (0.42 to 1.85)
Paratyphoid fever	19.07 (7.73 to 41.36)	9.93 (3.85 to 23.43)	7.45 (2.84 to 16.92)	12.86 (5.07 to 29.43)	0.31 (0.13 to 0.66)	0.18 (0.07 to 0.41)	0.14 (0.05 to 0.30)	0.23 (0.09 to 0.51)
Other intestinal infectious diseases	0.23 (0.09 to 0.48)	0.12 (0.04 to 0.26)	0.10 (0.04 to 0.23)	0.13 (0.05 to 0.29)	0.01 (0.00 to 0.01)	0.01 (0.00 to 0.01)	0.01 (0.00 to 0.01)	0.01 (0.00 to 0.01)
Lower respiratory infections	5,739.42 (4,983.53 to 6,534.65)	6,780.05 (4,871.99 to 9,444.11)	4,968.89 (3,657.57 to 6,636.09)	9,495.88 (6,507.70 to 13,894.98)	256.73 (221.45 to 294.19)	503.34 (344.03 to 676.10)	408.71 (288.68 to 540.96)	630.15 (417.01 to 882.35)
Upper respiratory infections	5.17 (2.74 to 8.75)	3.36 (1.46 to 6.83)	2.05 (0.91 to 4.16)	5.12 (2.29 to 10.21)	0.16 (0.08 to 0.27)	0.15 (0.06 to 0.33)	0.10 (0.04 to 0.21)	0.22 (0.09 to 0.47)
Otitis media	0.09 (0.06 to 0.13)	0.03 (0.02 to 0.05)	0.03 (0.02 to 0.05)	0.03 (0.02 to 0.04)	0.00 (0.00 to 0.01)	0.00 (0.00 to 0.00)	0.00 (0.00 to 0.01)	0.00 (0.00 to 0.00)
Meningitis	495.93 (424.46 to 595.38)	211.46 (157.00 to 287.86)	151.71 (112.13 to 205.21)	298.16 (211.68 to 411.03)	9.97 (8.71 to 11.58)	6.14 (4.97 to 7.55)	4.57 (3.75 to 5.64)	7.99 (6.49 to 9.89)
Pneumococcal meningitis	7.42 (5.71 to 9.61)	2.84 (2.01 to 4.09)	2.80 (1.99 to 3.99)	3.06 (2.05 to 4.54)	0.16 (0.13 to 0.20)	0.11 (0.08 to 0.14)	0.11 (0.08 to 0.14)	0.11 (0.08 to 0.14)
H influenzae type B meningitis	35.53 (28.62 to 44.10)	29.62 (15.04 to 55.96)	26.88 (14.37 to 46.90)	48.67 (17.90 to 105.75)	0.68 (0.57 to 0.80)	0.62 (0.43 to 0.94)	0.60 (0.43 to 0.86)	0.82 (0.44 to 1.50)
Meningococcal meningitis	129.39 (106.92 to 158.39)	51.90 (38.61 to 69.98)	36.36 (27.46 to 48.88)	69.64 (51.73 to 93.26)	2.70 (2.30 to 3.17)	1.69 (1.35 to 2.11)	1.24 (1.00 to 1.58)	2.14 (1.71 to 2.64)
Other meningitis	323.58 (275.38 to 385.13)	127.10 (93.38 to 169.71)	85.67 (64.00 to 115.35)	176.79 (129.45 to 237.89)	6.42 (5.57 to 7.46)	3.73 (2.97 to 4.57)	2.62 (2.14 to 3.23)	4.93 (4.02 to 6.01)
Encephalitis	399.46 (267.49 to 500.09)	295.21 (174.85 to 407.15)	277.19 (164.77 to 382.77)	312.18 (182.46 to 436.01)	8.40 (5.29 to 10.81)	8.51 (4.84 to 11.31)	8.31 (4.71 to 11.15)	8.58 (4.84 to 11.39)

Diphtheria	0.72 (0.46 to 1.07)	0.16 (0.09 to 0.28)	0.14 (0.08 to 0.25)	0.35 (0.12 to 0.91)	0.01 (0.01 to 0.02)	0.00 (0.00 to 0.01)	0.00 (0.00 to 0.01)	0.01 (0.00 to 0.01)
Whooping cough	3.59 (1.39 to 11.39)	1.74 (0.45 to 5.33)	1.05 (0.34 to 3.15)	9.79 (0.83 to 34.50)	0.06 (0.02 to 0.18)	0.03 (0.01 to 0.08)	0.02 (0.01 to 0.06)	0.12 (0.01 to 0.41)
Tetanus	1.24 (0.76 to 1.60)	0.16 (0.09 to 0.25)	0.15 (0.08 to 0.22)	0.35 (0.16 to 0.83)	0.03 (0.02 to 0.04)	0.01 (0.00 to 0.01)	0.01 (0.00 to 0.01)	0.01 (0.00 to 0.01)
Measles	2.12 (1.49 to 3.15)	105.56 (0.59 to 634.93)	77.52 (0.54 to 415.01)	172.33 (0.73 to 1,049.65)	0.03 (0.02 to 0.05)	1.70 (0.01 to 8.76)	1.29 (0.01 to 6.22)	2.60 (0.01 to 14.08)

Mauritius								
**Cause**	**YLLs (actual counts)**				**Deaths (actual counts)**			
	**2016**	**2040 Forecast**	**2040 Better Scenario**	**2040 Worse Scenario**	**2016**	**2040 Forecast**	**2040 Better Scenario**	**2040 Worse Scenario**
Varicella and herpes zoster	5.31 (1.91 to 7.61)	2.28 (0.81 to 3.85)	1.80 (0.67 to 3.08)	2.82 (0.99 to 4.93)	0.13 (0.05 to 0.17)	0.10 (0.04 to 0.17)	0.09 (0.04 to 0.15)	0.11 (0.05 to 0.19)
**Neglected tropical diseases and malaria**	**79.33 (40.21 to 135.68)**	**49.20 (25.62 to 84.35)**	**47.27 (24.28 to 80.12)**	**50.41 (25.92 to 85.62)**	**2.77 (1.33 to 4.84)**	**2.35 (1.07 to 4.06)**	**2.35 (1.06 to 4.16)**	**2.29 (1.04 to 4.05)**
Malaria	6.24 (1.95 to 8.82)	2.85 (0.83 to 4.15)	2.74 (0.80 to 4.01)	2.93 (0.85 to 4.24)	0.16 (0.05 to 0.23)	0.09 (0.03 to 0.13)	0.09 (0.03 to 0.13)	0.09 (0.03 to 0.13)
Chagas disease	0.00 (0.00 to 0.00)	0.00 (0.00 to 0.00)	0.00 (0.00 to 0.00)	0.00 (0.00 to 0.00)	0.00 (0.00 to 0.00)	0.00 (0.00 to 0.00)	0.00 (0.00 to 0.00)	0.00 (0.00 to 0.00)
Leishmaniasis	0.00 (0.00 to 0.00)	0.00 (0.00 to 0.00)	0.00 (0.00 to 0.00)	0.00 (0.00 to 0.00)	0.00 (0.00 to 0.00)	0.00 (0.00 to 0.00)	0.00 (0.00 to 0.00)	0.00 (0.00 to 0.00)
African trypanosomiasis	0.00 (0.00 to 0.00)	0.00 (0.00 to 0.00)	0.00 (0.00 to 0.00)	0.00 (0.00 to 0.00)	0.00 (0.00 to 0.00)	0.00 (0.00 to 0.00)	0.00 (0.00 to 0.00)	0.00 (0.00 to 0.00)
Schistosomiasis	64.73 (25.48 to 121.27)	39.67 (16.13 to 74.44)	37.77 (15.20 to 70.02)	40.78 (16.52 to 77.37)	2.41 (0.98 to 4.45)	1.98 (0.75 to 3.72)	1.97 (0.74 to 3.77)	1.95 (0.74 to 3.66)
Cysticercosis	0.00 (0.00 to 0.00)	0.00 (0.00 to 0.00)	0.00 (0.00 to 0.00)	0.00 (0.00 to 0.00)	0.00 (0.00 to 0.00)	0.00 (0.00 to 0.00)	0.00 (0.00 to 0.00)	0.00 (0.00 to 0.00)
Cystic echinococcosis	0.31 (0.24 to 0.38)	0.14 (0.10 to 0.18)	0.11 (0.08 to 0.13)	0.17 (0.13 to 0.22)	0.01 (0.01 to 0.01)	0.01 (0.01 to 0.01)	0.01 (0.00 to 0.01)	0.01 (0.01 to 0.01)
Dengue	0.15 (0.07 to 0.24)	0.87 (0.41 to 1.54)	0.92 (0.45 to 1.63)	0.81 (0.37 to 1.42)	0.01 (0.00 to 0.02)	0.08 (0.03 to 0.17)	0.09 (0.04 to 0.18)	0.07 (0.03 to 0.15)
Yellow fever	0.00 (0.00 to 0.00)	0.00 (0.00 to 0.00)	0.00 (0.00 to 0.00)	0.00 (0.00 to 0.00)	0.00 (0.00 to 0.00)	0.00 (0.00 to 0.00)	0.00 (0.00 to 0.00)	0.00 (0.00 to 0.00)
Rabies	0.04 (0.03 to 0.05)	0.02 (0.02 to 0.03)	0.02 (0.01 to 0.02)	0.02 (0.02 to 0.03)	0.00 (0.00 to 0.00)	0.00 (0.00 to 0.00)	0.00 (0.00 to 0.00)	0.00 (0.00 to 0.00)
Intestinal nematode infections	0.66 (0.24 to 1.30)	0.68 (0.23 to 1.48)	0.66 (0.23 to 1.42)	0.70 (0.23 to 1.56)	0.01 (0.01 to 0.02)	0.02 (0.01 to 0.03)	0.02 (0.01 to 0.03)	0.02 (0.01 to 0.03)
Other neglected tropical diseases	7.20 (6.20 to 9.45)	4.97 (3.90 to 6.87)	5.06 (3.94 to 7.16)	5.00 (3.90 to 6.92)	0.16 (0.13 to 0.23)	0.16 (0.12 to 0.26)	0.18 (0.13 to 0.29)	0.15 (0.11 to 0.23)
**Maternal disorders**	**384.92 (296.54 to 497.83)**	**140.05 (94.90 to 201.93)**	**112.04 (74.06 to 165.00)**	**172.96 (114.61 to 255.04)**	**7.21 (5.55 to 9.35)**	**2.73 (1.85 to 3.92)**	**2.19 (1.46 to 3.20)**	**3.38 (2.20 to 4.91)**
Maternal hemorrhage	67.93 (45.94 to 94.40)	23.37 (13.95 to 36.76)	18.96 (11.19 to 30.01)	28.49 (16.68 to 45.60)	1.26 (0.85 to 1.74)	0.45 (0.27 to 0.72)	0.37 (0.22 to 0.58)	0.55 (0.33 to 0.89)
Maternal sepsis and other maternal infections	65.81 (45.28 to 93.26)	20.58 (11.92 to 32.60)	16.50 (9.35 to 26.54)	25.37 (14.47 to 41.15)	1.26 (0.88 to 1.79)	0.41 (0.24 to 0.65)	0.33 (0.19 to 0.54)	0.51 (0.29 to 0.83)
Maternal hypertensive disorders	67.32 (46.87 to 93.09)	22.98 (13.05 to 36.63)	18.28 (10.34 to 29.85)	28.54 (16.06 to 46.60)	1.25 (0.87 to 1.73)	0.45 (0.25 to 0.71)	0.35 (0.20 to 0.58)	0.55 (0.31 to 0.89)
Maternal obstructed labor and uterine rupture	9.04 (4.80 to 14.73)	3.24 (1.52 to 6.16)	2.49 (1.15 to 4.78)	4.13 (1.94 to 7.89)	0.17 (0.09 to 0.28)	0.06 (0.03 to 0.12)	0.05 (0.02 to 0.09)	0.08 (0.04 to 0.16)
Maternal abortion, miscarriage, and ectopic pregnancy	70.96 (50.03 to 100.34)	29.93 (17.05 to 49.44)	23.71 (13.61 to 39.87)	37.29 (21.11 to 63.54)	1.35 (0.95 to 1.90)	0.60 (0.34 to 1.00)	0.48 (0.27 to 0.79)	0.75 (0.42 to 1.27)

Indirect maternal deaths	70.78 (48.69 to 98.76)	25.68 (15.28 to 41.96)	20.25 (11.94 to 33.22)	32.11 (18.58 to 53.23)	1.29 (0.88 to 1.80)	0.48 (0.29 to 0.78)	0.38 (0.22 to 0.62)	0.60 (0.35 to 0.98)
Late maternal deaths	2.26 (0.97 to 4.24)	0.75 (0.29 to 1.52)	0.59 (0.23 to 1.19)	0.93 (0.38 to 1.89)	0.04 (0.02 to 0.08)	0.01 (0.01 to 0.03)	0.01 (0.00 to 0.02)	0.02 (0.01 to 0.04)
Maternal deaths aggravated by HIV/AIDS	0.21 (0.10 to 0.40)	0.30 (0.12 to 0.62)	0.25 (0.10 to 0.55)	0.34 (0.14 to 0.74)	0.00 (0.00 to 0.01)	0.01 (0.00 to 0.01)	0.01 (0.00 to 0.01)	0.01 (0.00 to 0.01)
Other maternal disorders	30.61 (19.05 to 44.80)	13.23 (7.39 to 22.15)	11.02 (6.15 to 18.78)	15.77 (8.78 to 26.23)	0.58 (0.36 to 0.85)	0.26 (0.15 to 0.44)	0.22 (0.12 to 0.36)	0.31 (0.17 to 0.52)

Mauritius								
Cause	YLLs (actual counts)				Deaths (actual counts)			
	2016	2040 Forecast	2040 Better Scenario	2040 Worse Scenario	2016	2040 Forecast	2040 Better Scenario	2040 Worse Scenario
**Neonatal disorders**	**8,195.49 (6,478.49 to 10,148.94)**	**3,182.13 (2,098.05 to 4,916.99)**	**2,210.89 (1,450.24 to 3,480.80)**	**4,741.90 (3,130.33 to 7,403.35)**	**94.69 (74.86 to 117.26)**	**36.78 (24.25 to 56.82)**	**25.56 (16.76 to 40.23)**	**54.80 (36.17 to 85.56)**
Neonatal preterm birth	4,471.74 (3,513.20 to 5,645.02)	1,518.02 (1,023.93 to 2,300.11)	937.96 (612.31 to 1,413.47)	2,522.23 (1,674.04 to 3,790.61)	51.66 (40.58 to 65.21)	17.54 (11.83 to 26.58)	10.84 (7.08 to 16.33)	29.14 (19.34 to 43.80)
Neonatal encephalopathy due to birth asphyxia and trauma	1,410.83 (1,056.97 to 1,874.16)	514.01 (302.06 to 816.57)	348.11 (206.73 to 561.75)	754.11 (436.27 to 1,239.47)	16.30 (12.21 to 21.65)	5.94 (3.49 to 9.44)	4.02 (2.39 to 6.49)	8.72 (5.05 to 14.33)
Neonatal sepsis and other neonatal infections	1,022.70 (675.99 to 1,490.76)	629.85 (316.15 to 1,164.37)	468.07 (239.78 to 852.34)	851.50 (426.23 to 1,570.12)	11.82 (7.81 to 17.23)	7.28 (3.66 to 13.45)	5.41 (2.77 to 9.85)	9.84 (4.93 to 18.15)
Hemolytic disease and other neonatal jaundice	119.13 (73.39 to 188.64)	31.92 (14.21 to 71.72)	23.14 (10.36 to 52.02)	44.14 (19.40 to 101.88)	1.38 (0.85 to 2.18)	0.37 (0.16 to 0.83)	0.27 (0.12 to 0.60)	0.51 (0.22 to 1.18)
Other neonatal disorders	1,171.09 (845.69 to 1,552.39)	488.33 (278.20 to 820.69)	433.61 (252.84 to 735.79)	569.92 (325.55 to 980.58)	13.54 (9.78 to 17.94)	5.65 (3.22 to 9.49)	5.01 (2.92 to 8.51)	6.59 (3.77 to 11.34)
**Nutritional deficiencies**	**390.21 (335.90 to 447.37)**	**313.85 (250.76 to 380.27)**	**362.60 (291.61 to 436.53)**	**271.85 (215.95 to 329.13)**	**20.61 (17.74 to 23.73)**	**26.88 (18.95 to 34.74)**	**33.03 (23.84 to 42.17)**	**21.66 (14.68 to 28.34)**
Protein-energy malnutrition	357.92 (303.60 to 412.87)	283.60 (218.09 to 351.06)	333.28 (259.09 to 406.74)	241.00 (183.77 to 298.11)	19.60 (16.71 to 22.64)	25.03 (17.07 to 32.93)	31.12 (22.04 to 40.14)	19.91 (13.20 to 26.67)
Iodine deficiency	6.57 (0.37 to 19.27)	13.38 (0.40 to 46.14)	13.72 (0.32 to 50.67)	12.90 (0.45 to 40.06)	0.25 (0.01 to 0.87)	0.98 (0.01 to 4.32)	1.07 (0.01 to 5.18)	0.89 (0.02 to 3.63)
Dietary iron deficiency	7.12 (3.95 to 12.81)	4.77 (2.74 to 8.41)	4.95 (2.83 to 9.05)	4.66 (2.71 to 7.97)	0.15 (0.08 to 0.31)	0.13 (0.07 to 0.27)	0.14 (0.07 to 0.29)	0.12 (0.07 to 0.24)
Other nutritional deficiencies	18.60 (11.50 to 34.50)	12.09 (6.86 to 24.73)	10.65 (5.87 to 22.35)	13.28 (7.74 to 25.93)	0.61 (0.34 to 1.42)	0.75 (0.36 to 1.81)	0.71 (0.34 to 1.72)	0.75 (0.37 to 1.80)
**Other communicable, maternal, neonatal, and nutritional diseases**	**585.70 (483.50 to 691.29)**	**284.86 (221.08 to 354.95)**	**254.28 (196.06 to 321.65)**	**319.06 (245.52 to 406.94)**	**14.86 (12.46 to 17.36)**	**10.07 (8.09 to 12.00)**	**9.68 (7.68 to 11.52)**	**10.45 (8.31 to 12.62)**
Sexually transmitted infections excluding HIV	54.90 (26.26 to 103.29)	17.30 (9.36 to 31.50)	14.22 (8.15 to 25.60)	21.34 (10.54 to 40.12)	0.96 (0.61 to 1.54)	0.45 (0.32 to 0.66)	0.41 (0.30 to 0.58)	0.50 (0.34 to 0.74)
Syphilis	39.44 (10.75 to 85.57)	10.19 (2.77 to 24.02)	7.52 (2.11 to 18.40)	13.84 (3.59 to 31.98)	0.53 (0.20 to 1.07)	0.17 (0.08 to 0.34)	0.14 (0.07 to 0.26)	0.22 (0.10 to 0.44)
Chlamydial infection	3.10 (2.37 to 3.98)	1.42 (1.04 to 1.88)	1.34 (0.97 to 1.81)	1.49 (1.10 to 1.97)	0.09 (0.07 to 0.11)	0.06 (0.04 to 0.07)	0.06 (0.04 to 0.08)	0.06 (0.04 to 0.07)
Gonococcal infection	8.07 (6.17 to 10.28)	3.81 (2.76 to 5.05)	3.59 (2.59 to 4.83)	4.00 (2.89 to 5.29)	0.23 (0.18 to 0.29)	0.16 (0.12 to 0.21)	0.16 (0.12 to 0.21)	0.16 (0.12 to 0.21)
Other sexually transmitted infections	4.30 (3.28 to 5.52)	1.89 (1.36 to 2.61)	1.76 (1.25 to 2.46)	2.01 (1.45 to 2.75)	0.11 (0.08 to 0.14)	0.06 (0.05 to 0.08)	0.06 (0.05 to 0.08)	0.06 (0.05 to 0.08)
Acute hepatitis	248.79 (202.23 to 300.60)	89.77 (63.45 to 119.89)	66.60 (47.18 to 90.26)	115.26 (82.88 to 151.00)	6.37 (5.24 to 7.60)	2.83 (2.13 to 3.61)	2.15 (1.64 to 2.77)	3.51 (2.72 to 4.47)
Acute hepatitis A	8.75 (5.75 to 12.36)	2.02 (1.20 to 3.26)	1.61 (0.96 to 2.55)	2.49 (1.45 to 4.03)	0.16 (0.11 to 0.22)	0.05 (0.03 to 0.08)	0.04 (0.03 to 0.07)	0.06 (0.04 to 0.09)
Acute hepatitis B	205.43 (163.99 to 250.14)	75.36 (51.69 to 103.14)	55.62 (38.41 to 77.17)	97.07 (68.80 to 130.32)	5.43 (4.44 to 6.56)	2.35 (1.71 to 3.09)	1.77 (1.30 to 2.36)	2.95 (2.22 to 3.86)

Acute hepatitis C	5.70 (4.11 to 7.87)	2.32 (1.54 to 3.44)	1.74 (1.13 to 2.61)	2.92 (1.96 to 4.31)	0.16 (0.11 to 0.22)	0.08 (0.05 to 0.11)	0.06 (0.04 to 0.09)	0.10 (0.07 to 0.14)
Acute hepatitis E	28.91 (20.92 to 38.46)	10.07 (6.08 to 16.73)	7.63 (4.60 to 12.77)	12.78 (7.66 to 21.57)	0.62 (0.44 to 0.84)	0.35 (0.21 to 0.52)	0.28 (0.17 to 0.42)	0.41 (0.25 to 0.61)
Other unspecified infectious diseases	282.01 (215.63 to 343.25)	177.79 (129.94 to 227.40)	173.47 (127.98 to 220.48)	182.46 (131.21 to 235.79)	7.53 (5.93 to 8.97)	6.79 (5.10 to 8.28)	7.12 (5.36 to 8.67)	6.44 (4.77 to 7.92)
**Non-communicable diseases**	**188,031.64 (166,514.59 to 212,591.43)**	**200,619.77 (152,047.42 to 268,049.96)**	**157,024.33 (119,141.00 to 215,519.25)**	**279,183.79 (210,543.21 to 364,163.82)**	**9,069.90 (8,099.10 to 10,148.19)**	**13,147.46 (10,656.30 to 16,581.71)**	**10,866.35 (8,780.52 to 13,962.07)**	**16,890.38 (13,818.07 to 20,692.27)**

Mauritius								
Cause	YLLs (actual counts)				Deaths (actual counts)			
	2016	2040 Forecast	2040 Better Scenario	2040 Worse Scenario	2016	2040 Forecast	2040 Better Scenario	2040 Worse Scenario
**Neoplasms**	**29,867.50 (26,104.88 to 34,080.20)**	**34,813.35 (27,393.18 to 43,820.72)**	**29,558.83 (23,565.27 to 36,544.14)**	**41,327.39 (32,012.78 to 52,163.14)**	**1,248.33 (1,101.81 to 1,413.64)**	**1,923.32 (1,527.78 to 2,382.95)**	**1,694.39 (1,364.69 to 2,046.95)**	**2,173.15 (1,694.06 to 2,732.49)**
Lip and oral cavity cancer	940.19 (787.70 to 1,107.46)	887.30 (529.23 to 1,418.29)	657.95 (421.27 to 1,001.66)	1,404.18 (767.22 to 2,349.73)	38.52 (32.85 to 44.85)	44.82 (28.22 to 67.80)	34.70 (23.41 to 51.32)	66.57 (39.05 to 107.08)
Nasopharynx cancer	330.67 (276.84 to 392.11)	331.39 (202.61 to 531.65)	219.92 (139.60 to 338.22)	582.54 (330.95 to 980.19)	12.38 (10.45 to 14.56)	15.20 (9.59 to 23.81)	10.39 (6.92 to 15.64)	25.44 (14.80 to 41.05)
Other pharynx cancer	285.00 (240.70 to 337.11)	233.53 (144.91 to 361.24)	220.57 (141.66 to 336.69)	276.13 (162.94 to 445.40)	10.89 (9.31 to 12.65)	11.23 (7.16 to 16.69)	11.04 (7.29 to 16.05)	12.68 (7.72 to 19.95)
Esophageal cancer	842.03 (716.86 to 994.01)	844.33 (537.49 to 1,304.94)	713.35 (466.25 to 1,068.66)	1,175.21 (708.74 to 1,916.64)	37.47 (32.18 to 43.44)	46.30 (30.55 to 68.76)	40.45 (27.73 to 58.90)	61.17 (37.65 to 96.23)
Stomach cancer	2,191.77 (1,910.62 to 2,544.35)	1,899.19 (1,510.29 to 2,363.74)	1,540.35 (1,243.06 to 1,888.33)	2,268.54 (1,788.01 to 2,883.83)	101.82 (89.28 to 116.55)	113.81 (90.25 to 139.44)	95.45 (77.15 to 115.89)	130.29 (102.67 to 162.93)
Colon and rectum cancer	3,167.40 (2,695.88 to 3,676.12)	4,084.90 (2,664.81 to 6,268.49)	3,348.63 (2,266.65 to 4,990.07)	4,859.63 (3,166.02 to 7,483.48)	147.20 (127.22 to 169.54)	251.56 (167.81 to 376.87)	214.38 (146.94 to 318.16)	284.27 (187.25 to 430.89)
Liver cancer	1,232.56 (1,053.30 to 1,436.95)	2,085.36 (1,493.18 to 2,915.23)	1,942.21 (1,461.03 to 2,581.18)	2,134.45 (1,483.76 to 3,067.08)	56.04 (48.14 to 64.51)	121.50 (87.74 to 166.69)	117.97 (89.32 to 154.30)	118.52 (83.00 to 169.12)
Liver cancer due to hepatitis B	520.04 (403.37 to 652.59)	804.92 (534.13 to 1,197.49)	756.56 (525.58 to 1,069.01)	813.33 (532.23 to 1,232.15)	21.84 (16.40 to 27.55)	43.50 (28.63 to 63.45)	42.58 (29.10 to 60.26)	42.00 (27.02 to 62.31)
Liver cancer due to hepatitis C	278.54 (185.27 to 386.09)	528.19 (332.79 to 795.19)	508.65 (330.27 to 743.23)	520.03 (319.46 to 805.57)	14.68 (10.04 to 20.18)	35.22 (22.12 to 52.42)	35.28 (22.64 to 51.15)	33.24 (20.49 to 49.93)
Liver cancer due to alcohol use	190.46 (135.24 to 263.23)	307.81 (193.44 to 487.70)	272.42 (175.13 to 411.36)	361.64 (221.45 to 584.69)	8.69 (6.14 to 12.06)	17.14 (10.71 to 26.92)	15.82 (10.15 to 23.50)	19.06 (11.87 to 30.54)
Liver cancer due to other causes	243.52 (171.59 to 331.59)	444.44 (242.90 to 774.73)	404.57 (230.89 to 657.89)	439.45 (234.69 to 756.06)	10.83 (7.59 to 14.69)	25.65 (13.65 to 43.46)	24.30 (13.58 to 39.70)	24.22 (12.69 to 41.21)
Gallbladder and biliary tract cancer	358.32 (306.62 to 419.47)	336.59 (284.07 to 392.58)	332.76 (278.61 to 391.99)	331.71 (282.22 to 386.74)	16.93 (14.55 to 19.65)	20.23 (16.86 to 23.33)	20.66 (17.29 to 23.85)	19.16 (15.77 to 22.09)
Pancreatic cancer	1,501.30 (1,288.18 to 1,734.13)	2,251.27 (1,595.02 to 3,190.71)	2,157.53 (1,568.85 to 2,979.00)	2,255.48 (1,593.79 to 3,208.90)	66.69 (57.63 to 76.67)	124.21 (89.06 to 175.83)	122.46 (90.85 to 168.86)	119.62 (84.97 to 169.62)
Larynx cancer	360.22 (299.87 to 427.05)	358.71 (192.53 to 625.18)	223.10 (130.75 to 368.93)	644.84 (318.89 to 1,178.63)	15.77 (13.21 to 18.62)	19.27 (10.78 to 33.27)	12.38 (7.57 to 20.18)	32.79 (17.01 to 58.22)
Tracheal, bronchus, and lung cancer	3,640.39 (3,151.99 to 4,185.53)	4,688.46 (3,098.17 to 7,058.51)	3,210.60 (2,221.71 to 4,556.23)	7,171.42 (4,558.98 to 10,980.70)	167.64 (145.82 to 191.20)	266.73 (177.99 to 398.46)	189.56 (133.64 to 266.36)	386.18 (249.04 to 584.77)
Malignant skin melanoma	84.07 (68.45 to 108.71)	104.97 (85.14 to 139.21)	122.89 (97.98 to 162.08)	90.02 (73.15 to 119.31)	3.28 (2.72 to 4.34)	5.71 (4.50 to 7.52)	6.96 (5.51 to 9.21)	4.66 (3.69 to 6.19)
Non-melanoma skin cancer	111.34 (94.28 to 131.15)	153.81 (126.38 to 181.89)	131.30 (107.83 to 155.94)	169.87 (141.82 to 200.32)	5.37 (4.63 to 6.20)	9.94 (7.81 to 11.82)	8.85 (7.06 to 10.58)	10.44 (8.05 to 12.47)
Breast cancer	3,973.64 (3,142.48 to 4,917.23)	3,906.80 (2,573.65 to 5,923.16)	3,162.91 (2,132.44 to 4,636.52)	4,608.32 (3,035.40 to 7,133.04)	144.69 (116.26 to 176.61)	185.09 (124.10 to 277.71)	154.49 (107.18 to 222.25)	210.40 (140.66 to 316.26)
Cervical cancer	1,092.03 (853.01 to 1,373.16)	1,003.20 (783.33 to 1,302.81)	815.38 (628.49 to 1,059.59)	1,206.82 (924.78 to 1,604.16)	43.11 (34.53 to 53.54)	48.51 (38.05 to 61.18)	40.52 (31.92 to 51.02)	56.31 (44.74 to 71.94)

Uterine cancer	522.88 (418.77 to 650.92)	663.22 (535.47 to 815.59)	593.25 (475.34 to 731.11)	722.74 (583.23 to 884.78)	24.56 (19.91 to 30.17)	39.54 (31.13 to 47.37)	36.46 (29.01 to 43.86)	41.58 (32.93 to 49.84)
Ovarian cancer	962.48 (773.29 to 1,193.84)	1,066.63 (699.62 to 1,628.81)	921.59 (618.99 to 1,338.00)	1,152.34 (759.09 to 1,766.83)	35.03 (28.36 to 43.11)	47.89 (31.85 to 72.21)	42.17 (29.03 to 61.13)	50.45 (33.78 to 77.85)
Prostate cancer	1,166.72 (797.51 to 1,398.48)	2,280.30 (1,556.58 to 2,760.15)	2,176.97 (1,517.35 to 2,641.57)	2,249.34 (1,507.43 to 2,743.58)	77.20 (50.94 to 91.53)	184.60 (113.83 to 232.81)	182.31 (117.92 to 226.67)	174.82 (102.35 to 226.01)
Testicular cancer	39.91 (31.31 to 49.88)	27.55 (20.19 to 37.88)	23.65 (17.29 to 32.14)	30.87 (22.84 to 41.82)	1.03 (0.83 to 1.26)	0.98 (0.76 to 1.24)	0.87 (0.67 to 1.13)	1.04 (0.82 to 1.32)

Mauritius								
Cause	YLLs (actual counts)				Deaths (actual counts)			
	2016	2040 Forecast	2040 Better Scenario	2040 Worse Scenario	2016	2040 Forecast	2040 Better Scenario	2040 Worse Scenario
Kidney cancer	410.88 (351.70 to 476.79)	571.35 (453.94 to 708.43)	578.00 (459.82 to 711.95)	565.05 (442.48 to 713.15)	16.35 (14.14 to 18.78)	29.87 (23.77 to 36.50)	31.35 (25.28 to 37.75)	28.16 (21.93 to 35.37)
Bladder cancer	499.88 (426.27 to 581.73)	737.02 (499.21 to 1,099.13)	646.54 (453.68 to 927.73)	796.34 (532.53 to 1,210.06)	25.84 (22.39 to 29.71)	48.83 (32.90 to 73.93)	44.32 (31.27 to 64.90)	50.45 (33.16 to 77.72)
Brain and nervous system cancer	733.61 (597.06 to 977.21)	830.90 (656.74 to 1,073.70)	776.93 (614.35 to 1,003.64)	868.93 (699.02 to 1,127.29)	22.05 (18.12 to 28.67)	32.63 (26.92 to 40.98)	31.59 (26.06 to 39.26)	32.67 (27.04 to 40.93)
Thyroid cancer	106.04 (89.43 to 124.89)	128.06 (94.92 to 174.33)	106.96 (82.28 to 140.93)	142.14 (105.00 to 194.34)	4.61 (3.93 to 5.40)	6.97 (5.24 to 9.28)	6.00 (4.60 to 7.69)	7.49 (5.57 to 10.09)
Mesothelioma	49.44 (41.39 to 59.05)	56.59 (46.44 to 67.49)	58.84 (47.84 to 70.64)	53.44 (43.99 to 62.92)	1.89 (1.58 to 2.21)	2.90 (2.31 to 3.40)	3.13 (2.50 to 3.69)	2.62 (2.07 to 3.08)
Hodgkin lymphoma	129.71 (108.43 to 150.74)	76.95 (60.84 to 94.36)	59.97 (47.36 to 76.14)	94.42 (75.17 to 116.74)	3.74 (3.20 to 4.36)	2.88 (2.36 to 3.48)	2.31 (1.87 to 2.87)	3.41 (2.80 to 4.09)
Non-Hodgkin lymphoma	721.06 (616.78 to 848.44)	763.77 (616.49 to 950.68)	670.18 (544.92 to 825.23)	824.16 (667.34 to 1,023.28)	24.43 (21.01 to 28.63)	35.63 (28.98 to 44.04)	32.50 (26.67 to 39.02)	36.69 (29.71 to 45.42)
Multiple myeloma	311.96 (215.83 to 380.18)	394.61 (262.64 to 508.31)	362.45 (245.78 to 457.84)	407.67 (272.74 to 527.42)	12.86 (8.90 to 15.46)	20.01 (13.43 to 25.30)	18.96 (12.89 to 23.55)	19.97 (13.62 to 25.38)
Leukemia	1,626.02 (1,434.30 to 1,859.37)	1,521.49 (1,200.45 to 1,909.12)	1,289.99 (1,037.35 to 1,597.81)	1,727.79 (1,347.42 to 2,181.39)	48.69 (42.67 to 55.97)	67.61 (52.95 to 83.67)	59.51 (47.40 to 72.64)	73.13 (55.85 to 92.09)
Acute lymphoid leukemia	359.44 (311.41 to 423.31)	350.10 (279.31 to 440.67)	296.69 (239.70 to 369.53)	396.69 (318.19 to 500.36)	10.81 (9.34 to 12.45)	15.33 (12.12 to 18.77)	13.45 (10.86 to 16.27)	16.55 (12.93 to 20.55)
Chronic lymphoid leukemia	27.54 (18.34 to 34.73)	27.99 (18.03 to 37.61)	25.55 (16.54 to 34.22)	29.45 (19.66 to 40.16)	1.17 (0.73 to 1.50)	1.54 (0.97 to 2.09)	1.47 (0.91 to 1.99)	1.53 (0.97 to 2.13)
Acute myeloid leukemia	284.56 (241.10 to 333.40)	321.69 (249.39 to 413.41)	296.66 (234.28 to 375.66)	339.81 (260.85 to 443.31)	7.65 (6.48 to 9.02)	12.29 (9.53 to 15.69)	11.76 (9.27 to 14.85)	12.40 (9.54 to 15.96)
Chronic myeloid leukemia	57.66 (47.72 to 70.71)	34.62 (25.18 to 46.58)	24.16 (18.41 to 31.94)	46.10 (33.88 to 62.19)	2.04 (1.70 to 2.48)	1.66 (1.23 to 2.25)	1.20 (0.91 to 1.57)	2.11 (1.56 to 2.78)
Other leukemia	896.82 (768.60 to 1,043.03)	787.10 (610.70 to 993.73)	646.94 (513.98 to 813.83)	915.75 (699.78 to 1,173.44)	27.03 (23.39 to 31.35)	36.80 (27.98 to 46.16)	31.63 (24.64 to 39.20)	40.54 (30.36 to 52.29)
Other neoplasms	2,475.98 (2,116.26 to 2,841.42)	2,525.11 (2,144.28 to 2,913.91)	2,494.06 (2,107.34 to 2,898.86)	2,512.98 (2,144.54 to 2,886.23)	82.25 (70.23 to 94.60)	118.89 (99.18 to 136.09)	122.64 (102.71 to 140.50)	112.16 (93.67 to 128.39)
**Cardiovascular diseases**	**61,282.79 (53,895.35 to 69,521.81)**	**55,042.92 (37,219.16 to 82,423.34)**	**35,473.45 (24,144.61 to 53,882.81)**	**98,540.07 (63,193.29 to 148,471.06)**	**3,304.11 (2,951.50 to 3,690.72)**	**4,021.17 (2,700.82 to 6,361.90)**	**2,856.73 (1,928.81 to 4,821.19)**	**6,317.33 (4,250.08 to 9,917.63)**
Rheumatic heart disease	648.80 (554.73 to 756.36)	358.22 (286.84 to 438.77)	287.48 (233.65 to 349.49)	428.86 (349.71 to 526.22)	24.66 (21.29 to 28.45)	21.54 (16.72 to 25.87)	18.23 (14.28 to 21.79)	24.25 (18.69 to 29.51)
Ischemic heart disease	35,832.81 (31,433.20 to 40,803.13)	30,249.98 (16,969.71 to 57,117.24)	18,028.93 (9,950.51 to 33,218.19)	57,332.63 (30,882.69 to 100,545.64)	1,971.20 (1,757.85 to 2,216.14)	2,216.54 (1,238.23 to 4,258.87)	1,471.84 (795.90 to 3,106.08)	3,659.92 (2,046.21 to 6,690.09)
Stroke	15,533.05 (13,361.76 to 17,864.39)	12,814.44 (7,844.41 to 22,620.33)	7,491.09 (4,689.59 to 15,003.19)	25,893.82 (15,592.44 to 43,061.55)	819.66 (714.72 to 930.31)	921.67 (565.65 to 1,851.51)	602.83 (359.49 to 1,334.69)	1,605.47 (1,013.68 to 2,933.58)
Ischemic stroke	3,917.88 (3,346.03 to 4,515.53)	3,894.58 (2,000.13 to 10,600.31)	2,581.79 (1,334.17 to 7,443.35)	6,644.92 (3,428.28 to 16,213.19)	270.44 (235.83 to 309.69)	351.42 (168.87 to 1,016.27)	252.09 (119.14 to 764.69)	536.56 (259.31 to 1,585.98)

Intracerebral hemorrhage	11,615.17 (10,030.19 to 13,377.94)	8,919.85 (5,234.24 to 14,832.11)	4,909.30 (2,998.90 to 7,881.82)	19,248.90 (10,683.16 to 32,207.49)	549.22 (475.65 to 624.73)	570.24 (347.24 to 964.44)	350.74 (221.30 to 628.44)	1,068.92 (625.13 to 1,794.21)
Hypertensive heart disease	5,046.13 (3,472.47 to 6,482.85)	6,663.29 (4,260.91 to 10,085.31)	5,605.14 (3,750.67 to 7,803.29)	8,860.08 (5,362.55 to 14,759.59)	295.42 (201.14 to 372.80)	523.55 (328.08 to 813.82)	466.25 (302.74 to 667.55)	651.66 (380.50 to 1,111.14)
Cardiomyopathy and myocarditis	1,317.63 (872.05 to 1,638.60)	983.10 (646.75 to 1,343.17)	763.24 (516.34 to 1,022.59)	1,261.07 (814.38 to 1,754.37)	46.75 (35.44 to 55.55)	50.02 (35.94 to 67.06)	40.55 (29.36 to 53.56)	60.28 (42.72 to 81.74)
Myocarditis	112.59 (69.25 to 146.37)	88.75 (58.65 to 115.95)	85.86 (58.42 to 113.04)	90.58 (57.79 to 119.23)	3.18 (2.27 to 4.04)	4.00 (2.98 to 5.43)	4.10 (3.09 to 5.64)	3.80 (2.81 to 5.06)

Mauritius								
**Cause**	**YLLs (actual counts)**				**Deaths (actual counts)**			
	**2016**	**2040 Forecast**	**2040 Better Scenario**	**2040 Worse Scenario**	**2016**	**2040 Forecast**	**2040 Better Scenario**	**2040 Worse Scenario**
Alcoholic cardiomyopathy	189.87 (107.54 to 251.93)	156.66 (93.31 to 212.67)	141.19 (85.30 to 192.96)	192.65 (109.40 to 262.46)	5.83 (3.85 to 7.57)	6.19 (4.50 to 8.51)	5.85 (4.26 to 8.13)	7.11 (4.96 to 9.44)
Other cardiomyopathy	1,015.17 (631.85 to 1,276.65)	737.69 (459.01 to 1,038.74)	536.20 (335.74 to 753.00)	977.84 (597.46 to 1,418.53)	37.74 (27.77 to 45.44)	39.83 (26.71 to 56.28)	30.60 (20.85 to 42.91)	49.37 (33.34 to 70.01)
Atrial fibrillation and flutter	384.57 (291.10 to 494.68)	793.95 (556.76 to 1,058.36)	764.59 (539.66 to 1,007.30)	818.76 (566.22 to 1,093.03)	37.19 (28.20 to 48.43)	87.03 (55.77 to 120.45)	87.12 (56.93 to 119.27)	85.45 (53.42 to 119.95)
Aortic aneurysm	335.72 (287.70 to 392.00)	422.36 (336.85 to 517.59)	375.13 (303.15 to 453.76)	470.21 (371.65 to 588.48)	17.42 (15.13 to 20.09)	29.82 (22.68 to 36.81)	28.13 (21.60 to 34.14)	30.80 (22.77 to 38.72)
Peripheral artery disease	47.51 (32.45 to 66.95)	143.99 (86.94 to 210.44)	128.26 (78.09 to 190.43)	140.57 (84.06 to 205.02)	3.71 (2.38 to 5.27)	14.09 (7.69 to 22.13)	13.47 (7.24 to 21.67)	12.91 (6.98 to 20.09)
Endocarditis	521.80 (358.07 to 653.90)	528.15 (344.67 to 747.54)	497.35 (327.14 to 699.65)	567.59 (370.06 to 811.28)	16.92 (11.41 to 21.18)	24.37 (15.74 to 34.58)	24.14 (15.84 to 34.32)	24.64 (15.92 to 35.00)
Other cardiovascular and circulatory diseases	1,614.77 (1,377.08 to 1,894.37)	2,085.45 (1,565.87 to 2,741.90)	1,532.23 (1,181.94 to 1,975.53)	2,766.49 (2,049.22 to 3,638.11)	71.18 (61.41 to 81.97)	132.55 (99.39 to 169.77)	104.18 (78.80 to 132.33)	161.95 (120.21 to 211.65)
**Chronic respiratory diseases**	**8,550.09 (7,475.59 to 9,718.26)**	**9,091.73 (6,905.95 to 11,401.39)**	**7,348.15 (5,757.99 to 8,917.33)**	**11,920.49 (8,727.76 to 15,679.51)**	**492.93 (437.72 to 552.08)**	**697.13 (499.93 to 892.55)**	**585.99 (437.24 to 713.62)**	**865.30 (598.78 to 1,147.05)**
Chronic obstructive pulmonary disease	4,293.61 (3,592.89 to 4,979.73)	5,285.35 (3,671.25 to 7,262.76)	3,782.13 (2,744.71 to 5,078.19)	7,679.70 (5,115.70 to 10,876.54)	275.71 (237.50 to 315.30)	434.52 (292.88 to 596.50)	333.46 (231.40 to 447.52)	584.62 (374.19 to 836.78)
Pneumoconiosis	7.65 (6.26 to 9.25)	5.71 (4.55 to 6.91)	5.30 (4.23 to 6.64)	6.30 (4.99 to 7.64)	0.36 (0.30 to 0.43)	0.41 (0.30 to 0.53)	0.39 (0.28 to 0.50)	0.45 (0.31 to 0.58)
Silicosis	0.70 (0.44 to 0.95)	0.36 (0.23 to 0.52)	0.24 (0.16 to 0.36)	0.50 (0.32 to 0.71)	0.04 (0.03 to 0.05)	0.03 (0.02 to 0.04)	0.02 (0.01 to 0.03)	0.04 (0.02 to 0.05)
Asbestosis	2.93 (2.39 to 3.82)	2.12 (1.65 to 2.71)	2.42 (1.86 to 3.14)	1.87 (1.47 to 2.33)	0.08 (0.07 to 0.10)	0.10 (0.07 to 0.13)	0.11 (0.09 to 0.16)	0.08 (0.06 to 0.11)
Coal workers pneumoconiosis	0.25 (0.11 to 0.36)	0.16 (0.08 to 0.27)	0.15 (0.08 to 0.27)	0.19 (0.09 to 0.30)	0.01 (0.01 to 0.02)	0.02 (0.01 to 0.03)	0.02 (0.01 to 0.03)	0.02 (0.01 to 0.03)
Other pneumoconiosis	3.77 (2.93 to 4.59)	3.07 (2.30 to 3.95)	2.48 (1.85 to 3.27)	3.74 (2.76 to 4.85)	0.23 (0.19 to 0.27)	0.27 (0.18 to 0.37)	0.24 (0.16 to 0.32)	0.31 (0.20 to 0.43)
Asthma	2,891.08 (2,449.05 to 3,534.63)	1,774.61 (1,135.21 to 2,694.07)	1,073.26 (721.34 to 1,559.90)	2,553.81 (1,635.13 to 3,907.34)	151.93 (130.21 to 183.69)	131.65 (81.68 to 207.98)	84.81 (54.64 to 127.44)	177.71 (109.90 to 280.81)
Interstitial lung disease and pulmonary sarcoidosis	970.61 (550.75 to 1,234.72)	1,599.95 (908.38 to 2,054.72)	2,065.09 (1,258.42 to 2,585.49)	1,245.52 (685.05 to 1,590.98)	50.79 (27.60 to 64.75)	106.39 (57.05 to 138.35)	141.96 (81.00 to 180.61)	79.57 (40.70 to 103.63)
Other chronic respiratory diseases	387.14 (265.58 to 671.80)	426.11 (289.26 to 810.81)	422.37 (287.74 to 816.82)	435.17 (291.26 to 822.01)	14.15 (9.60 to 28.41)	24.16 (14.17 to 52.23)	25.38 (14.68 to 55.24)	22.95 (13.43 to 49.69)
**Cirrhosis and other chronic liver diseases**	**8,717.97 (7,216.12 to 10,578.58)**	**8,410.79 (6,696.01 to 10,395.34)**	**7,155.65 (5,643.83 to 8,923.95)**	**9,833.41 (7,857.60 to 12,074.72)**	**286.01 (238.87 to 344.96)**	**335.98 (275.84 to 400.53)**	**294.50 (244.78 to 352.64)**	**377.79 (315.13 to 449.05)**
Cirrhosis and other chronic liver diseases due to hepatitis B	3,202.55 (2,544.91 to 4,016.34)	3,259.93 (2,449.35 to 4,295.14)	2,822.05 (2,116.25 to 3,733.53)	3,632.91 (2,747.05 to 4,740.80)	105.55 (84.12 to 132.39)	128.23 (100.40 to 161.95)	113.92 (89.33 to 144.57)	138.29 (110.02 to 173.57)
Cirrhosis and other chronic liver diseases due to hepatitis C	1,851.69 (1,416.44 to 2,386.00)	1,816.51 (1,323.77 to 2,343.71)	1,553.73 (1,148.16 to 2,030.21)	2,048.72 (1,510.76 to 2,647.10)	59.66 (46.29 to 75.85)	69.22 (52.49 to 87.33)	60.60 (46.10 to 77.46)	75.81 (58.73 to 94.84)

Cirrhosis and other chronic liver diseases due to alcohol use	1,741.34 (1,272.84 to 2,267.62)	1,500.28 (1,102.21 to 1,988.55)	1,189.74 (881.37 to 1,580.54)	2,103.72 (1,572.96 to 2,816.04)	58.59 (44.22 to 74.78)	61.43 (47.02 to 77.24)	50.79 (39.06 to 64.35)	81.06 (63.06 to 102.49)
Cirrhosis and other chronic liver diseases due to other causes	1,922.39 (1,560.86 to 2,353.39)	1,834.06 (1,454.49 to 2,322.58)	1,590.14 (1,258.41 to 2,011.74)	2,048.05 (1,643.22 to 2,570.05)	62.21 (51.38 to 75.55)	77.10 (63.15 to 93.90)	69.19 (56.32 to 84.59)	82.62 (67.78 to 100.90)
**Digestive diseases**	**3,190.12 (2,646.95 to 3,957.39)**	**2,730.22 (2,031.85 to 3,969.05)**	**2,259.14 (1,714.40 to 3,144.92)**	**3,338.37 (2,411.03 to 5,042.55)**	**124.83 (105.58 to 154.57)**	**150.54 (116.34 to 200.47)**	**132.44 (103.03 to 173.68)**	**169.23 (127.48 to 232.16)**
Peptic ulcer disease	736.90 (609.99 to 940.12)	445.98 (340.25 to 583.95)	323.88 (252.44 to 427.93)	587.11 (452.90 to 762.70)	33.11 (27.88 to 42.70)	29.17 (21.87 to 37.69)	22.13 (16.75 to 28.75)	36.34 (26.40 to 46.88)

Mauritius								
Cause	YLLs (actual counts)				Deaths (actual counts)			
	2016	2040 Forecast	2040 Better Scenario	2040 Worse Scenario	2016	2040 Forecast	2040 Better Scenario	2040 Worse Scenario
Gastritis and duodenitis	95.65 (74.23 to 126.56)	99.07 (73.51 to 132.60)	82.00 (60.84 to 111.29)	114.01 (84.33 to 149.58)	4.28 (3.44 to 5.52)	6.29 (4.45 to 8.52)	5.53 (3.88 to 7.52)	6.77 (4.71 to 9.03)
Appendicitis	88.12 (64.86 to 109.27)	54.52 (38.84 to 69.95)	40.91 (28.83 to 53.22)	68.96 (48.18 to 87.57)	2.92 (2.07 to 3.58)	2.66 (1.83 to 3.34)	2.10 (1.44 to 2.67)	3.17 (2.16 to 3.97)
Paralytic ileus and intestinal obstruction	448.92 (354.37 to 580.78)	488.19 (368.55 to 632.66)	491.71 (375.69 to 637.88)	476.28 (360.71 to 621.19)	18.73 (14.27 to 24.27)	30.24 (22.21 to 39.18)	31.90 (23.71 to 41.71)	27.86 (20.25 to 35.83)
Inguinal, femoral, and abdominal hernia	42.71 (32.59 to 69.19)	34.53 (24.95 to 53.03)	30.23 (21.69 to 46.80)	37.77 (27.59 to 60.75)	2.22 (1.66 to 3.41)	2.48 (1.66 to 3.70)	2.29 (1.54 to 3.39)	2.55 (1.71 to 3.95)
Inflammatory bowel disease	78.44 (49.07 to 101.10)	69.66 (50.92 to 88.30)	68.54 (50.51 to 87.31)	69.38 (50.32 to 87.27)	2.83 (2.07 to 3.49)	3.63 (2.79 to 4.47)	3.70 (2.85 to 4.62)	3.46 (2.65 to 4.24)
Vascular intestinal disorders	134.27 (109.61 to 165.30)	181.65 (144.41 to 223.42)	172.30 (136.93 to 212.55)	184.77 (147.69 to 229.51)	7.58 (6.27 to 9.32)	13.84 (10.47 to 17.41)	13.69 (10.36 to 17.12)	13.42 (10.04 to 17.20)
Gallbladder and biliary diseases	175.18 (148.65 to 216.42)	182.78 (113.74 to 284.73)	123.94 (80.79 to 184.99)	230.19 (142.40 to 362.86)	9.18 (7.87 to 11.10)	13.42 (7.91 to 21.74)	9.74 (6.03 to 15.26)	15.87 (9.09 to 26.48)
Pancreatitis	1,063.39 (818.55 to 1,400.93)	875.83 (433.16 to 1,828.14)	650.06 (341.99 to 1,303.11)	1,255.89 (574.29 to 2,757.32)	30.46 (23.72 to 41.27)	30.63 (17.09 to 62.84)	23.58 (13.67 to 45.82)	41.85 (21.11 to 87.21)
Other digestive diseases	326.55 (234.48 to 408.19)	298.02 (215.28 to 369.79)	275.57 (200.10 to 345.60)	314.01 (225.42 to 390.69)	13.52 (10.01 to 16.82)	18.18 (13.19 to 22.92)	17.79 (13.01 to 22.24)	17.95 (12.86 to 23.09)
**Neurological disorders**	**6,956.52 (5,978.45 to 8,116.85)**	**14,120.33 (9,899.30 to 19,892.13)**	**13,512.70 (9,840.84 to 18,192.85)**	**13,539.56 (9,344.94 to 19,390.66)**	**537.48 (454.07 to 637.42)**	**1,396.52 (907.36 to 2,015.20)**	**1,391.32 (946.41 to 1,904.48)**	**1,271.38 (794.16 to 1,912.87)**
Alzheimer's disease and other dementias	4,878.47 (4,015.16 to 5,863.70)	11,682.89 (7,546.05 to 17,634.22)	11,056.48 (7,587.56 to 15,677.21)	11,074.26 (6,905.20 to 16,805.86)	462.46 (382.14 to 559.13)	1,255.30 (781.55 to 1,875.38)	1,237.80 (815.95 to 1,748.26)	1,142.28 (673.66 to 1,768.04)
Parkinson's disease	463.20 (350.69 to 597.41)	1,089.73 (797.58 to 1,417.38)	1,215.35 (898.98 to 1,571.03)	945.45 (677.79 to 1,225.53)	34.29 (25.93 to 44.88)	93.91 (65.23 to 125.11)	107.78 (76.15 to 141.00)	78.77 (52.66 to 106.67)
Epilepsy	1,157.96 (985.66 to 1,366.63)	859.96 (610.50 to 1,175.63)	749.39 (540.01 to 1,013.41)	1,040.05 (708.10 to 1,477.24)	27.72 (23.59 to 32.64)	28.08 (20.66 to 36.58)	25.60 (19.33 to 32.85)	32.26 (23.01 to 43.34)
Multiple sclerosis	44.32 (33.24 to 55.04)	44.13 (32.84 to 56.50)	44.61 (33.01 to 57.14)	43.87 (32.39 to 56.11)	1.35 (1.02 to 1.67)	1.57 (1.21 to 1.99)	1.62 (1.24 to 2.07)	1.52 (1.17 to 1.93)
Motor neuron disease	94.72 (80.37 to 111.13)	125.01 (102.08 to 149.20)	123.98 (100.67 to 148.77)	123.86 (102.45 to 146.50)	3.24 (2.75 to 3.76)	5.12 (4.23 to 6.06)	5.17 (4.27 to 6.14)	4.95 (4.11 to 5.78)
Other neurological disorders	317.84 (270.02 to 369.71)	318.62 (267.21 to 382.04)	322.89 (268.01 to 387.02)	312.07 (262.06 to 374.76)	8.42 (7.33 to 9.62)	12.55 (10.40 to 14.38)	13.34 (11.12 to 15.32)	11.60 (9.60 to 13.29)
**Mental disorders**	**2,379.22 (1,932.05 to 2,897.66)**	**2,091.25 (1,577.40 to 2,713.49)**	**2,231.93 (1,669.05 to 2,895.71)**	**2,067.55 (1,570.86 to 2,649.13)**	**64.45 (52.41 to 78.21)**	**64.29 (50.12 to 80.28)**	**69.53 (54.08 to 86.91)**	**62.34 (49.22 to 76.84)**
Alcohol use disorders	1,914.68 (1,534.18 to 2,363.04)	1,671.19 (1,226.39 to 2,187.95)	1,714.00 (1,249.92 to 2,233.94)	1,713.45 (1,270.71 to 2,217.46)	53.77 (43.21 to 65.86)	52.48 (40.06 to 65.73)	54.68 (41.36 to 69.20)	52.67 (40.99 to 65.58)
Drug use disorders	463.16 (366.27 to 577.09)	418.72 (304.80 to 566.91)	516.54 (375.60 to 696.56)	352.77 (259.81 to 470.76)	10.65 (8.41 to 13.26)	11.77 (9.03 to 15.18)	14.82 (11.42 to 18.85)	9.64 (7.38 to 12.20)
Opioid use disorders	105.85 (77.22 to 144.89)	99.24 (69.18 to 141.44)	103.88 (72.73 to 148.53)	95.30 (66.19 to 134.78)	2.69 (1.99 to 3.54)	3.36 (2.41 to 4.41)	3.74 (2.70 to 4.89)	3.03 (2.19 to 3.96)

Cocaine use disorders	12.56 (4.98 to 17.45)	16.34 (7.47 to 24.87)	19.35 (9.37 to 29.70)	14.20 (6.49 to 21.65)	0.27 (0.11 to 0.37)	0.39 (0.19 to 0.58)	0.47 (0.24 to 0.69)	0.34 (0.17 to 0.49)
Amphetamine use disorders	8.54 (5.77 to 11.84)	16.13 (10.01 to 24.73)	21.55 (13.50 to 32.96)	12.66 (7.93 to 19.45)	0.19 (0.13 to 0.26)	0.41 (0.27 to 0.60)	0.56 (0.37 to 0.80)	0.31 (0.20 to 0.45)
Other drug use disorders	336.22 (245.37 to 431.59)	287.00 (194.96 to 402.99)	371.76 (250.40 to 514.72)	230.61 (155.20 to 320.52)	7.50 (5.50 to 9.53)	7.61 (5.39 to 10.10)	10.05 (7.10 to 13.19)	5.96 (4.18 to 7.93)
Eating disorders	1.38 (1.12 to 1.73)	1.34 (0.95 to 1.82)	1.39 (0.98 to 1.89)	1.33 (0.92 to 1.81)	0.03 (0.02 to 0.04)	0.03 (0.02 to 0.04)	0.03 (0.02 to 0.04)	0.03 (0.02 to 0.04)

Mauritius								
Cause	YLLs (actual counts)				Deaths (actual counts)			
	2016	2040 Forecast	2040 Better Scenario	2040 Worse Scenario	2016	2040 Forecast	2040 Better Scenario	2040 Worse Scenario
**Diabetes, urogenital, blood, and endocrine diseases**	**61,388.22 (53,656.84 to 69,351.16)**	**70,944.05 (39,569.66 to 137,082.82)**	**56,916.09 (31,064.55 to 116,109.92)**	**94,187.76 (54,209.62 to 166,512.41)**	**2,910.96 (2,566.89 to 3,272.50)**	**4,444.23 (2,603.31 to 8,051.70)**	**3,740.60 (2,081.22 to 7,085.92)**	**5,526.16 (3,318.68 to 9,072.92)**
Diabetes mellitus	39,610.60 (34,313.63 to 44,925.50)	28,381.45 (19,525.03 to 39,303.83)	20,969.06 (14,601.81 to 29,015.91)	36,908.42 (25,201.11 to 51,840.72)	1,950.95 (1,703.04 to 2,201.45)	1,811.15 (1,304.81 to 2,395.43)	1,409.21 (1,031.62 to 1,872.55)	2,233.27 (1,577.89 to 2,999.94)
Acute glomerulonephritis	6.94 (6.09 to 7.90)	5.39 (4.13 to 7.03)	4.54 (3.48 to 5.93)	6.31 (4.82 to 8.18)	0.14 (0.12 to 0.16)	0.13 (0.10 to 0.17)	0.11 (0.09 to 0.15)	0.15 (0.12 to 0.19)
Chronic kidney disease	20,008.94 (17,245.78 to 22,986.56)	40,611.75 (11,395.77 to 110,710.09)	34,107.97 (9,731.38 to 91,896.36)	55,223.51 (16,346.09 to 130,258.73)	889.72 (775.73 to 1,013.75)	2,515.24 (673.54 to 6,445.37)	2,214.59 (585.26 to 5,734.98)	3,176.01 (909.59 to 7,129.52)
Chronic kidney disease due to diabetes mellitus	12,744.21 (10,843.53 to 14,836.95)	25,794.90 (7,189.19 to 71,555.90)	21,307.83 (6,088.99 to 60,744.96)	35,881.29 (10,433.53 to 89,247.93)	530.89 (452.35 to 619.65)	1,417.91 (385.69 to 3,682.18)	1,217.77 (323.60 to 3,184.53)	1,852.59 (539.21 to 4,195.64)
Chronic kidney disease due to hypertension	5,163.02 (4,218.50 to 6,168.96)	11,961.67 (3,240.91 to 30,989.04)	10,498.76 (2,746.56 to 28,320.44)	15,356.80 (4,455.21 to 35,397.94)	282.53 (232.46 to 331.99)	927.57 (232.12 to 2,368.91)	852.97 (210.74 to 2,259.29)	1,102.97 (312.12 to 2,465.74)
Chronic kidney disease due to glomerulonephritis	985.69 (786.24 to 1,215.83)	1,139.92 (396.34 to 2,927.01)	894.36 (313.24 to 2,351.67)	1,628.12 (577.07 to 3,669.51)	32.15 (25.41 to 40.42)	60.51 (17.33 to 156.08)	49.58 (14.23 to 131.38)	81.43 (24.49 to 186.65)
Chronic kidney disease due to other and unspecified causes	1,116.02 (893.83 to 1,397.43)	1,715.26 (539.19 to 4,412.24)	1,407.01 (442.02 to 3,747.43)	2,357.31 (747.84 to 5,495.15)	44.15 (35.14 to 55.28)	109.25 (28.22 to 278.12)	94.28 (23.71 to 251.83)	139.03 (38.94 to 325.51)
Urinary diseases and male infertility	559.81 (488.42 to 637.21)	638.39 (546.00 to 742.97)	557.25 (478.14 to 649.61)	705.96 (605.18 to 812.24)	25.29 (22.35 to 28.65)	44.72 (35.24 to 54.10)	41.34 (32.98 to 50.10)	46.26 (36.49 to 56.94)
Urinary tract infections	462.62 (396.09 to 534.30)	565.60 (479.27 to 662.33)	498.71 (423.17 to 583.42)	619.70 (528.57 to 719.06)	21.06 (18.20 to 24.12)	39.70 (31.44 to 48.41)	37.01 (29.49 to 45.05)	40.80 (31.90 to 50.18)
Urolithiasis	12.96 (10.07 to 18.57)	12.85 (9.19 to 19.83)	10.04 (7.16 to 15.91)	15.71 (11.36 to 23.64)	0.48 (0.37 to 0.72)	0.66 (0.46 to 1.13)	0.54 (0.38 to 0.93)	0.76 (0.54 to 1.25)
Other urinary diseases	84.23 (61.96 to 113.10)	59.94 (40.32 to 85.61)	48.50 (33.05 to 70.42)	70.55 (47.45 to 100.36)	3.74 (2.67 to 5.07)	4.35 (2.59 to 7.11)	3.79 (2.26 to 6.24)	4.69 (2.67 to 7.66)
Gynecological diseases	13.44 (5.48 to 18.46)	6.94 (2.98 to 10.31)	4.65 (1.96 to 7.05)	9.88 (4.32 to 14.58)	0.42 (0.19 to 0.57)	0.30 (0.14 to 0.43)	0.21 (0.10 to 0.30)	0.41 (0.19 to 0.57)
Uterine fibroids	4.28 (1.95 to 6.02)	2.00 (0.95 to 3.16)	1.48 (0.66 to 2.33)	2.62 (1.18 to 4.12)	0.13 (0.06 to 0.18)	0.07 (0.03 to 0.11)	0.05 (0.03 to 0.08)	0.09 (0.04 to 0.14)
Polycystic ovarian syndrome	1.81 (0.60 to 3.01)	0.50 (0.14 to 0.97)	0.25 (0.07 to 0.51)	0.92 (0.27 to 1.75)	0.04 (0.02 to 0.07)	0.01 (0.00 to 0.02)	0.01 (0.00 to 0.01)	0.02 (0.01 to 0.04)
Endometriosis	0.04 (0.01 to 0.07)	0.03 (0.01 to 0.06)	0.02 (0.01 to 0.04)	0.04 (0.01 to 0.08)	0.00 (0.00 to 0.00)	0.00 (0.00 to 0.00)	0.00 (0.00 to 0.00)	0.00 (0.00 to 0.00)
Genital prolapse	0.15 (0.07 to 0.25)	0.11 (0.05 to 0.18)	0.07 (0.04 to 0.13)	0.15 (0.07 to 0.25)	0.01 (0.00 to 0.01)	0.01 (0.00 to 0.01)	0.00 (0.00 to 0.01)	0.01 (0.00 to 0.02)
Other gynecological diseases	7.15 (2.80 to 10.38)	4.31 (1.80 to 6.73)	2.84 (1.14 to 4.49)	6.16 (2.56 to 9.44)	0.24 (0.11 to 0.34)	0.21 (0.09 to 0.32)	0.15 (0.06 to 0.22)	0.29 (0.13 to 0.41)
Hemoglobinopathies and hemolytic anemias	541.67 (457.69 to 682.81)	415.56 (330.06 to 534.92)	336.52 (267.07 to 443.94)	495.89 (394.59 to 635.00)	18.96 (16.09 to 23.96)	21.46 (17.17 to 27.60)	18.05 (14.41 to 23.23)	24.38 (19.49 to 31.64)
Thalassemias	98.56 (75.52 to 127.01)	38.28 (24.38 to 58.72)	35.91 (23.30 to 54.76)	40.71 (25.53 to 63.17)	1.34 (1.00 to 1.75)	0.53 (0.33 to 0.81)	0.50 (0.32 to 0.76)	0.56 (0.35 to 0.87)

Sickle cell disorders	1.01 (0.76 to 1.32)	0.74 (0.51 to 1.03)	0.55 (0.38 to 0.78)	0.95 (0.66 to 1.31)	0.02 (0.02 to 0.03)	0.02 (0.01 to 0.03)	0.02 (0.01 to 0.02)	0.03 (0.02 to 0.04)
G6PD deficiency	226.30 (180.64 to 287.95)	197.90 (146.92 to 266.39)	150.00 (111.58 to 205.03)	251.04 (188.62 to 333.27)	6.11 (4.96 to 7.78)	7.17 (5.46 to 9.55)	5.54 (4.24 to 7.53)	8.86 (6.81 to 11.52)
Other hemoglobinopathies and hemolytic anemias	215.81 (179.04 to 280.99)	178.64 (139.68 to 232.19)	150.07 (116.36 to 197.48)	203.19 (157.92 to 266.58)	11.49 (9.61 to 14.77)	13.74 (10.28 to 17.97)	12.00 (9.04 to 15.75)	14.93 (10.88 to 19.82)
Endocrine, metabolic, blood, and immune disorders	646.82 (529.78 to 1,052.97)	884.57 (711.54 to 1,443.44)	936.10 (749.20 to 1,526.84)	837.79 (678.81 to 1,371.77)	25.47 (20.88 to 41.82)	51.23 (40.35 to 87.20)	57.08 (45.08 to 96.99)	45.69 (36.00 to 78.65)

Mauritius								
Cause	YLLs (actual counts)				Deaths (actual counts)			
	2016	2040 Forecast	2040 Better Scenario	2040 Worse Scenario	2016	2040 Forecast	2040 Better Scenario	2040 Worse Scenario
**Musculoskeletal disorders**	**552.80 (283.14 to 678.03)**	**504.26 (295.43 to 615.92)**	**452.72 (265.77 to 557.16)**	**550.53 (311.92 to 669.71)**	**16.96 (10.18 to 20.46)**	**25.03 (16.46 to 30.13)**	**23.21 (15.35 to 28.19)**	**26.30 (17.08 to 32.00)**
Rheumatoid arthritis	73.74 (48.90 to 108.23)	89.19 (59.78 to 130.87)	64.99 (42.03 to 96.05)	114.80 (76.48 to 166.14)	3.96 (2.65 to 5.62)	6.45 (4.23 to 9.60)	4.87 (3.10 to 7.39)	7.97 (5.12 to 11.58)
Other musculoskeletal disorders	479.05 (234.86 to 594.54)	415.07 (231.06 to 516.90)	387.73 (217.16 to 479.28)	435.73 (233.43 to 542.49)	13.00 (7.30 to 15.93)	18.58 (11.71 to 22.57)	18.34 (11.77 to 22.40)	18.33 (11.06 to 22.53)
**Other non-communicable diseases**	**5,146.40 (3,111.51 to 6,534.52)**	**2,870.88 (1,607.47 to 3,851.66)**	**2,115.67 (1,141.21 to 2,833.48)**	**3,878.66 (2,195.63 to 5,238.64)**	**83.84 (48.47 to 105.68)**	**89.25 (41.55 to 119.74)**	**77.65 (34.78 to 104.52)**	**101.39 (50.15 to 135.07)**
Congenital birth defects	4,270.43 (2,489.47 to 5,510.65)	1,793.26 (1,003.41 to 2,512.05)	1,146.29 (633.49 to 1,625.82)	2,709.64 (1,504.63 to 3,748.98)	53.05 (31.50 to 67.69)	23.15 (13.57 to 31.73)	14.93 (8.81 to 20.71)	34.69 (20.46 to 47.19)
Neural tube defects	88.78 (58.57 to 132.12)	15.14 (8.30 to 25.50)	9.09 (4.95 to 15.84)	24.34 (13.42 to 41.06)	1.05 (0.70 to 1.56)	0.19 (0.11 to 0.31)	0.11 (0.06 to 0.20)	0.30 (0.17 to 0.49)
Congenital heart anomalies	1,999.66 (1,300.04 to 2,552.96)	895.07 (538.81 to 1,265.90)	531.69 (322.33 to 759.88)	1,424.67 (857.25 to 2,005.64)	25.46 (16.52 to 32.13)	11.77 (7.21 to 16.29)	7.03 (4.37 to 9.83)	18.60 (11.31 to 25.85)
Orofacial clefts	0.72 (0.35 to 1.30)	0.31 (0.14 to 0.64)	0.17 (0.07 to 0.33)	0.55 (0.24 to 1.10)	0.01 (0.00 to 0.02)	0.00 (0.00 to 0.01)	0.00 (0.00 to 0.00)	0.01 (0.00 to 0.01)
Down syndrome	42.24 (30.12 to 65.77)	39.37 (26.67 to 59.90)	39.75 (26.97 to 60.48)	39.82 (26.85 to 60.78)	0.69 (0.51 to 0.98)	0.70 (0.48 to 0.97)	0.72 (0.50 to 1.00)	0.70 (0.49 to 0.97)
Other chromosomal abnormalities	47.14 (10.02 to 76.97)	45.67 (10.58 to 78.66)	36.70 (8.23 to 63.21)	56.79 (13.36 to 98.14)	0.55 (0.12 to 0.90)	0.53 (0.13 to 0.92)	0.43 (0.10 to 0.74)	0.66 (0.16 to 1.15)
Congenital musculoskeletal and limb anomalies	48.65 (26.96 to 68.04)	26.06 (13.81 to 38.81)	17.14 (9.12 to 25.73)	38.21 (20.23 to 58.22)	0.58 (0.32 to 0.81)	0.32 (0.17 to 0.47)	0.21 (0.12 to 0.31)	0.46 (0.25 to 0.69)
Urogenital congenital anomalies	55.57 (27.20 to 78.10)	32.60 (16.65 to 49.85)	23.38 (12.31 to 35.25)	44.04 (21.81 to 67.34)	0.84 (0.50 to 1.13)	0.56 (0.34 to 0.81)	0.41 (0.26 to 0.60)	0.73 (0.45 to 1.05)
Digestive congenital anomalies	344.60 (151.01 to 512.26)	130.32 (54.74 to 208.45)	81.90 (33.39 to 134.02)	199.22 (86.16 to 319.22)	4.06 (1.79 to 6.02)	1.55 (0.67 to 2.47)	0.98 (0.41 to 1.58)	2.37 (1.05 to 3.77)
Other congenital birth defects	1,643.08 (782.14 to 2,240.35)	608.71 (282.98 to 890.65)	406.48 (185.28 to 612.38)	882.00 (403.54 to 1,307.39)	19.80 (9.49 to 26.71)	7.53 (3.70 to 10.84)	5.05 (2.42 to 7.43)	10.86 (5.22 to 15.88)
Skin and subcutaneous diseases	567.70 (181.62 to 797.07)	980.13 (338.16 to 1,346.36)	895.96 (306.34 to 1,229.90)	1,035.62 (347.32 to 1,423.94)	27.22 (8.81 to 37.83)	64.97 (21.92 to 92.02)	61.87 (21.71 to 86.21)	65.15 (21.63 to 93.18)
Cellulitis	112.68 (18.36 to 179.43)	245.32 (43.16 to 379.47)	242.00 (43.56 to 375.41)	241.31 (41.43 to 373.79)	4.21 (0.69 to 6.72)	12.95 (2.35 to 20.08)	13.39 (2.47 to 20.64)	12.02 (2.15 to 18.69)
Pyoderma	210.25 (69.09 to 293.35)	403.66 (137.70 to 559.18)	369.40 (125.57 to 514.19)	428.94 (141.20 to 597.73)	8.23 (2.59 to 11.72)	24.29 (8.09 to 35.03)	23.71 (7.84 to 34.03)	23.95 (7.92 to 34.68)
Decubitus ulcer	223.31 (71.85 to 307.87)	307.09 (106.17 to 441.20)	263.01 (92.34 to 377.42)	339.39 (114.31 to 489.02)	13.91 (4.65 to 19.10)	26.40 (8.89 to 39.67)	23.53 (8.06 to 35.12)	27.83 (9.00 to 42.72)
Other skin and subcutaneous diseases	21.46 (6.57 to 30.89)	24.07 (7.13 to 35.00)	21.55 (6.27 to 31.39)	25.98 (7.80 to 37.72)	0.86 (0.24 to 1.27)	1.32 (0.35 to 2.00)	1.24 (0.33 to 1.86)	1.35 (0.36 to 2.02)
Sudden infant death syndrome	308.26 (212.33 to 432.08)	97.48 (53.56 to 159.92)	73.42 (39.47 to 124.15)	133.41 (72.94 to 227.87)	3.58 (2.46 to 5.01)	1.13 (0.62 to 1.85)	0.85 (0.46 to 1.44)	1.55 (0.85 to 2.64)

**Injuries**	**21,117.35 (17,982.13 to 24,721.38)**	**12,418.69 (9,421.82 to 16,664.26)**	**10,057.17 (7,709.01 to 13,213.06)**	**18,247.32 (13,496.82 to 25,294.25)**	**510.58 (435.81 to 597.05)**	**402.40 (326.88 to 503.24)**	**341.52 (281.52 to 417.63)**	**547.42 (437.42 to 703.50)**
**Transport injuries**	**6,734.16 (5,677.68 to 7,856.52)**	**2,445.93 (1,708.53 to 3,481.73)**	**1,740.01 (1,229.16 to 2,437.30)**	**6,332.02 (4,353.99 to 9,257.60)**	**161.40 (137.55 to 187.68)**	**81.04 (60.80 to 110.08)**	**60.53 (45.93 to 81.12)**	**184.85 (134.23 to 255.66)**
Road injuries	6,250.93 (5,256.49 to 7,301.04)	2,007.48 (1,406.07 to 2,863.24)	1,422.18 (1,017.49 to 1,978.26)	5,744.21 (3,971.92 to 8,385.61)	148.58 (126.61 to 173.01)	65.20 (48.62 to 88.11)	48.46 (36.92 to 64.42)	164.84 (119.81 to 226.84)
Pedestrian road injuries	1,307.73 (1,005.54 to 1,559.99)	397.87 (281.22 to 545.42)	224.64 (161.99 to 300.26)	1,213.61 (824.68 to 1,725.20)	36.63 (28.58 to 43.44)	16.25 (11.99 to 21.83)	9.79 (7.27 to 12.71)	43.64 (31.40 to 59.77)

Mauritius								
Cause	YLLs (actual counts)				Deaths (actual counts)			
	2016	2040 Forecast	2040 Better Scenario	2040 Worse Scenario	2016	2040 Forecast	2040 Better Scenario	2040 Worse Scenario
Cyclist road injuries	252.51 (189.32 to 306.58)	106.22 (70.06 to 148.42)	83.40 (55.97 to 115.44)	249.80 (165.01 to 358.64)	6.76 (5.08 to 8.21)	3.91 (2.66 to 5.46)	3.29 (2.25 to 4.53)	7.78 (5.20 to 10.96)
Motorcyclist road injuries	1,605.08 (1,209.46 to 1,971.48)	650.62 (404.48 to 1,006.33)	531.75 (337.87 to 809.89)	1,252.50 (782.67 to 2,003.57)	32.22 (24.26 to 39.33)	15.82 (10.12 to 23.49)	13.44 (8.79 to 19.70)	27.76 (17.71 to 42.65)
Motor vehicle road injuries	2,998.02 (2,454.62 to 3,764.03)	824.68 (585.26 to 1,181.62)	562.14 (406.62 to 792.82)	2,939.17 (2,027.36 to 4,303.71)	70.69 (58.26 to 87.68)	28.04 (20.86 to 37.90)	20.97 (15.93 to 27.89)	82.81 (59.96 to 116.05)
Other road injuries	87.59 (61.58 to 111.74)	28.09 (18.04 to 41.12)	20.24 (13.04 to 28.83)	89.13 (55.46 to 136.63)	2.28 (1.55 to 2.89)	1.18 (0.74 to 1.67)	0.97 (0.61 to 1.36)	2.86 (1.78 to 4.19)
Other transport injuries	483.23 (393.57 to 595.60)	438.45 (292.98 to 666.61)	317.83 (218.05 to 471.78)	587.80 (391.86 to 904.11)	12.82 (10.61 to 15.64)	15.84 (11.08 to 22.95)	12.07 (8.60 to 17.17)	20.01 (13.86 to 29.27)
**Unintentional injuries**	**7,437.53 (6,261.99 to 8,667.96)**	**4,877.50 (3,907.23 to 5,935.17)**	**4,177.86 (3,397.69 to 5,063.03)**	**5,566.77 (4,462.37 to 6,906.33)**	**199.39 (168.84 to 230.61)**	**194.09 (161.09 to 226.45)**	**175.28 (146.39 to 202.34)**	**207.79 (169.54 to 245.56)**
Falls	1,706.55 (1,291.67 to 2,061.48)	1,405.75 (1,102.31 to 1,701.16)	1,264.81 (1,011.73 to 1,538.27)	1,503.32 (1,175.75 to 1,803.10)	56.70 (45.52 to 67.25)	69.52 (56.24 to 82.22)	65.33 (53.23 to 77.51)	70.50 (56.00 to 83.40)
Drowning	1,974.50 (1,649.68 to 2,362.65)	1,068.46 (762.20 to 1,457.48)	873.49 (625.30 to 1,186.66)	1,289.02 (912.32 to 1,816.88)	44.95 (37.83 to 53.46)	31.38 (23.97 to 41.56)	26.44 (20.38 to 34.19)	36.49 (27.59 to 49.96)
Fire, heat, and hot substances	1,151.93 (970.34 to 1,437.15)	677.35 (507.14 to 914.63)	523.73 (399.46 to 719.40)	849.47 (637.45 to 1,146.70)	30.55 (25.63 to 40.73)	27.29 (20.66 to 40.33)	22.34 (17.12 to 33.32)	32.09 (23.95 to 46.86)
Poisonings	52.84 (40.77 to 101.06)	24.56 (17.34 to 47.30)	18.00 (12.81 to 34.37)	32.30 (22.69 to 62.66)	1.48 (1.19 to 2.60)	1.33 (0.85 to 2.04)	1.07 (0.70 to 1.63)	1.56 (0.99 to 2.50)
Exposure to mechanical forces	233.57 (189.27 to 280.50)	129.74 (103.18 to 161.72)	104.25 (82.46 to 130.90)	155.81 (122.69 to 197.43)	5.37 (4.33 to 6.41)	4.62 (3.58 to 5.52)	3.96 (3.03 to 4.69)	5.15 (3.93 to 6.25)
Unintentional firearm injuries	21.69 (17.38 to 39.51)	13.03 (9.93 to 22.41)	11.24 (8.57 to 19.22)	14.68 (11.20 to 25.31)	0.59 (0.48 to 1.03)	0.56 (0.43 to 0.88)	0.52 (0.39 to 0.79)	0.58 (0.44 to 0.92)
Unintentional suffocation	66.34 (36.39 to 90.70)	25.16 (13.63 to 36.18)	20.54 (11.26 to 29.64)	30.34 (15.91 to 44.49)	1.05 (0.56 to 1.39)	0.44 (0.24 to 0.61)	0.37 (0.21 to 0.51)	0.52 (0.28 to 0.73)
Other exposure to mechanical forces	145.54 (120.07 to 188.83)	91.55 (71.85 to 121.15)	72.47 (57.18 to 95.33)	110.79 (87.30 to 148.40)	3.73 (3.00 to 4.59)	3.62 (2.71 to 4.38)	3.08 (2.28 to 3.70)	4.05 (3.01 to 4.97)
Adverse effects of medical treatment	1,192.51 (682.07 to 1,567.12)	885.04 (509.86 to 1,155.69)	803.86 (461.19 to 1,055.63)	945.61 (536.97 to 1,251.43)	38.28 (22.29 to 49.94)	41.53 (24.60 to 52.64)	39.59 (23.90 to 50.30)	41.87 (24.35 to 53.80)
Animal contact	39.46 (24.41 to 51.22)	27.96 (17.03 to 37.66)	23.28 (14.43 to 31.18)	32.59 (19.42 to 44.41)	1.04 (0.63 to 1.32)	1.07 (0.66 to 1.38)	0.96 (0.58 to 1.24)	1.15 (0.69 to 1.53)
Venomous animal contact	11.32 (7.81 to 14.65)	7.72 (4.89 to 10.42)	6.85 (4.35 to 9.31)	8.52 (5.41 to 11.85)	0.28 (0.18 to 0.36)	0.30 (0.17 to 0.42)	0.29 (0.16 to 0.40)	0.31 (0.18 to 0.43)
Non-venomous animal contact	28.15 (16.11 to 36.88)	20.24 (11.48 to 27.92)	16.42 (9.51 to 22.56)	24.07 (13.21 to 33.66)	0.75 (0.43 to 0.98)	0.76 (0.44 to 1.00)	0.66 (0.39 to 0.87)	0.85 (0.48 to 1.13)
Foreign body	871.86 (644.49 to 1,080.47)	554.62 (412.86 to 698.65)	499.99 (376.73 to 624.21)	607.67 (438.54 to 771.83)	16.02 (12.44 to 19.04)	13.85 (11.46 to 16.74)	13.15 (10.99 to 16.07)	14.26 (11.50 to 17.02)
Pulmonary aspiration and foreign body in airway	847.97 (624.58 to 1,052.93)	538.17 (397.77 to 680.19)	482.96 (361.42 to 604.57)	591.63 (425.84 to 752.73)	15.53 (11.99 to 18.47)	13.40 (11.03 to 16.34)	12.67 (10.55 to 15.48)	13.85 (11.14 to 16.60)

Foreign body in other body part	23.90 (13.81 to 30.38)	16.45 (10.30 to 21.93)	17.03 (10.89 to 22.57)	16.04 (9.83 to 21.56)	0.49 (0.31 to 0.61)	0.44 (0.30 to 0.57)	0.48 (0.32 to 0.62)	0.41 (0.27 to 0.53)
Environmental heat and cold exposure	22.78 (16.44 to 39.41)	19.67 (13.31 to 34.17)	15.46 (10.47 to 26.28)	23.71 (15.93 to 41.54)	0.84 (0.57 to 1.39)	1.12 (0.73 to 1.86)	0.94 (0.61 to 1.56)	1.26 (0.82 to 2.09)
Other unintentional injuries	191.52 (154.08 to 233.37)	84.35 (61.01 to 116.61)	51.00 (37.67 to 70.56)	127.25 (94.99 to 170.19)	4.16 (3.40 to 5.02)	2.38 (1.81 to 3.10)	1.50 (1.19 to 1.93)	3.44 (2.68 to 4.36)
**Self-harm and interpersonal violence**	**6,938.96 (5,492.50 to 8,507.86)**	**5,074.99 (3,347.88 to 7,863.90)**	**4,118.95 (2,795.06 to 6,248.97)**	**6,328.43 (4,001.27 to 10,251.03)**	**149.66 (120.10 to 183.39)**	**126.73 (86.54 to 189.89)**	**105.16 (73.23 to 153.29)**	**154.26 (100.27 to 241.10)**

Mauritius								
Cause	YLLs (actual counts)				Deaths (actual counts)			
	2016	2040 Forecast	2040 Better Scenario	2040 Worse Scenario	2016	2040 Forecast	2040 Better Scenario	2040 Worse Scenario
Self-harm	5,363.37 (4,143.99 to 6,610.60)	4,222.57 (2,662.57 to 6,810.34)	3,436.58 (2,223.80 to 5,409.43)	5,284.77 (3,131.66 to 8,890.66)	115.38 (91.14 to 142.91)	105.37 (69.10 to 166.04)	87.69 (59.10 to 132.82)	128.66 (79.18 to 208.80)
Self-harm by firearm	70.29 (41.88 to 110.12)	41.08 (21.05 to 77.52)	31.00 (16.27 to 55.91)	54.99 (25.81 to 110.44)	1.79 (1.12 to 2.68)	1.42 (0.79 to 2.44)	1.14 (0.64 to 1.93)	1.76 (0.92 to 3.28)
Self-harm by other specified means	5,293.09 (4,089.02 to 6,539.14)	4,181.48 (2,635.74 to 6,753.55)	3,405.57 (2,207.18 to 5,358.93)	5,229.78 (3,104.92 to 8,778.04)	113.60 (90.01 to 140.53)	103.95 (68.28 to 163.57)	86.55 (58.27 to 130.91)	126.90 (78.01 to 207.07)
Interpersonal violence	1,575.58 (1,001.74 to 2,280.60)	852.43 (508.12 to 1,338.32)	682.37 (416.61 to 1,053.66)	1,043.67 (610.31 to 1,641.89)	34.28 (22.03 to 48.64)	21.36 (12.82 to 32.23)	17.47 (10.73 to 26.07)	25.60 (15.12 to 39.17)
Physical violence by firearm	146.28 (74.88 to 331.76)	75.01 (38.50 to 161.92)	56.16 (29.23 to 120.31)	95.00 (47.77 to 208.58)	3.13 (1.60 to 7.13)	1.76 (0.91 to 3.69)	1.33 (0.70 to 2.68)	2.20 (1.13 to 4.66)
Physical violence by sharp object	646.86 (250.58 to 949.23)	335.65 (131.26 to 530.65)	255.09 (101.94 to 397.33)	420.85 (161.69 to 671.81)	13.46 (5.25 to 19.72)	7.64 (3.07 to 11.81)	5.90 (2.41 to 8.96)	9.44 (3.68 to 14.98)
Physical violence by other means	782.44 (518.24 to 1,315.67)	441.77 (260.08 to 783.45)	371.12 (221.21 to 642.24)	527.82 (302.54 to 942.73)	17.68 (11.66 to 29.37)	11.97 (7.25 to 19.58)	10.25 (6.27 to 16.50)	13.96 (8.20 to 23.58)
**Forces of nature, conflict and terrorism, and executions and police conflict**	**6.70 (5.94 to 7.56)**	**20.27 (0.00 to 192.10)**	**20.36 (0.00 to 192.46)**	**20.11 (0.00 to 191.33)**	**0.13 (0.12 to 0.15)**	**0.54 (0.00 to 4.41)**	**0.55 (0.00 to 4.49)**	**0.52 (0.00 to 4.31)**
Exposure to forces of nature	0.00 (0.00 to 0.00)	20.27 (0.00 to 192.10)	20.36 (0.00 to 192.46)	20.11 (0.00 to 191.33)	0.00 (0.00 to 0.00)	0.54 (0.00 to 4.41)	0.55 (0.00 to 4.49)	0.52 (0.00 to 4.31)
Conflict and terrorism	0.00 (0.00 to 0.00)	0.00 (0.00 to 0.00)	0.00 (0.00 to 0.00)	0.00 (0.00 to 0.00)	0.00 (0.00 to 0.00)	0.00 (0.00 to 0.00)	0.00 (0.00 to 0.00)	0.00 (0.00 to 0.00)
Executions and police conflict	6.70 (5.94 to 7.56)	0.00 (0.00 to 0.00)	0.00 (0.00 to 0.00)	0.00 (0.00 to 0.00)	0.13 (0.12 to 0.15)	0.00 (0.00 to 0.00)	0.00 (0.00 to 0.00)	0.00 (0.00 to 0.00)

Myanmar								
Cause	YLLs (in 1000s)				Deaths (in 1000s)			
	2016	2040 Forecast	2040 Better Scenario	2040 Worse Scenario	2016	2040 Forecast	2040 Better Scenario	2040 Worse Scenario
**All causes**	**11,665.33 (10,455.12 to 13,504.74)**	**11,460.37 (9,703.03 to 13,460.27)**	**9,983.87 (8,507.09 to 11,647.24)**	**15,592.47 (13,045.55 to 18,558.25)**	**374.00 (347.26 to 416.07)**	**560.83 (498.94 to 619.78)**	**505.84 (451.82 to 555.81)**	**701.30 (622.58 to 777.52)**
**Communicable, maternal, neonatal, and nutritional diseases**	**3,630.09 (2,862.92 to 4,915.68)**	**1,650.35 (1,155.58 to 2,347.34)**	**1,293.81 (916.64 to 1,843.50)**	**2,956.98 (2,029.24 to 4,318.45)**	**71.74 (61.38 to 87.01)**	**50.35 (37.36 to 69.21)**	**40.93 (31.02 to 55.34)**	**81.97 (58.60 to 114.76)**
**HIV/AIDS and tuberculosis**	**734.25 (624.20 to 887.56)**	**357.29 (219.03 to 570.64)**	**236.28 (150.14 to 368.72)**	**860.66 (527.42 to 1,246.02)**	**20.44 (17.78 to 23.94)**	**14.01 (9.20 to 21.15)**	**9.95 (6.80 to 14.27)**	**26.63 (17.56 to 37.61)**
Tuberculosis	458.92 (391.24 to 575.19)	298.10 (176.74 to 491.40)	202.41 (132.13 to 317.48)	463.91 (277.34 to 785.00)	14.40 (12.37 to 17.58)	12.67 (8.13 to 19.31)	9.17 (6.31 to 13.10)	18.40 (11.54 to 28.07)
HIV/AIDS	275.33 (201.06 to 410.94)	59.19 (15.95 to 164.53)	33.87 (7.48 to 113.77)	396.75 (174.77 to 695.00)	6.04 (4.37 to 8.80)	1.34 (0.28 to 3.90)	0.78 (0.13 to 2.75)	8.23 (3.28 to 14.74)
**Diarrhea, lower respiratory, and other common infectious diseases**	**1,315.26 (1,000.95 to 1,807.41)**	**619.43 (353.76 to 1,068.50)**	**464.83 (273.70 to 763.51)**	**1,169.95 (618.29 to 2,170.72)**	**27.35 (22.56 to 34.13)**	**22.75 (12.87 to 38.89)**	**18.34 (10.79 to 30.99)**	**38.71 (20.61 to 68.82)**
Diarrheal diseases	244.96 (160.16 to 356.91)	119.21 (18.33 to 352.33)	87.72 (15.33 to 258.87)	245.53 (39.23 to 709.41)	7.23 (4.26 to 10.95)	6.91 (1.08 to 21.85)	5.47 (0.93 to 16.56)	13.30 (2.02 to 41.05)
Intestinal infectious diseases	128.49 (67.69 to 215.41)	55.05 (25.73 to 101.53)	50.61 (23.53 to 91.40)	66.12 (29.59 to 123.98)	1.96 (1.04 to 3.23)	0.97 (0.48 to 1.71)	0.91 (0.45 to 1.59)	1.13 (0.54 to 2.04)
Typhoid fever	111.59 (56.45 to 190.32)	46.52 (21.10 to 87.32)	42.78 (19.62 to 78.50)	55.72 (24.66 to 107.60)	1.70 (0.89 to 2.84)	0.81 (0.39 to 1.46)	0.76 (0.37 to 1.35)	0.95 (0.44 to 1.74)
Paratyphoid fever	16.66 (7.15 to 33.02)	8.45 (3.49 to 17.59)	7.75 (3.19 to 16.23)	10.30 (4.14 to 21.88)	0.26 (0.11 to 0.51)	0.15 (0.07 to 0.30)	0.14 (0.06 to 0.29)	0.18 (0.08 to 0.38)
Other intestinal infectious diseases	0.25 (0.09 to 0.52)	0.08 (0.03 to 0.18)	0.07 (0.02 to 0.16)	0.11 (0.04 to 0.24)	0.01 (0.00 to 0.01)	0.00 (0.00 to 0.01)	0.00 (0.00 to 0.01)	0.00 (0.00 to 0.01)
Lower respiratory infections	760.14 (544.83 to 1,106.21)	317.14 (180.19 to 605.78)	220.77 (134.95 to 392.06)	619.74 (309.24 to 1,314.17)	15.06 (11.97 to 19.39)	12.18 (8.15 to 18.71)	9.55 (6.62 to 14.21)	20.21 (12.71 to 32.21)
Upper respiratory infections	0.98 (0.50 to 1.70)	0.61 (0.27 to 1.21)	0.53 (0.22 to 1.05)	0.91 (0.39 to 1.78)	0.02 (0.01 to 0.04)	0.02 (0.01 to 0.05)	0.02 (0.01 to 0.04)	0.03 (0.01 to 0.06)
Otitis media	0.34 (0.14 to 0.71)	0.18 (0.06 to 0.42)	0.19 (0.07 to 0.43)	0.17 (0.06 to 0.40)	0.01 (0.01 to 0.03)	0.01 (0.00 to 0.02)	0.01 (0.00 to 0.02)	0.01 (0.00 to 0.02)
Meningitis	96.84 (63.69 to 145.92)	39.08 (22.60 to 63.32)	36.41 (21.21 to 59.18)	48.11 (25.52 to 83.61)	1.61 (1.10 to 2.34)	0.92 (0.60 to 1.34)	0.89 (0.59 to 1.30)	1.02 (0.65 to 1.56)
Pneumococcal meningitis	0.84 (0.51 to 1.44)	0.25 (0.15 to 0.41)	0.24 (0.15 to 0.39)	0.30 (0.17 to 0.53)	0.01 (0.01 to 0.02)	0.01 (0.00 to 0.01)	0.01 (0.00 to 0.01)	0.01 (0.01 to 0.01)
H influenzae type B meningitis	7.31 (4.48 to 11.81)	3.83 (1.55 to 9.16)	3.29 (1.43 to 7.36)	8.83 (1.89 to 26.08)	0.12 (0.08 to 0.18)	0.07 (0.04 to 0.14)	0.07 (0.04 to 0.12)	0.13 (0.05 to 0.33)
Meningococcal meningitis	33.04 (20.82 to 52.26)	12.54 (7.34 to 20.27)	11.79 (7.02 to 19.21)	13.97 (7.76 to 22.82)	0.56 (0.37 to 0.83)	0.32 (0.21 to 0.47)	0.31 (0.21 to 0.46)	0.33 (0.21 to 0.50)
Other meningitis	55.64 (35.78 to 84.77)	22.45 (13.07 to 36.72)	21.08 (12.11 to 33.83)	25.00 (13.91 to 41.12)	0.91 (0.61 to 1.32)	0.52 (0.33 to 0.76)	0.50 (0.33 to 0.75)	0.55 (0.35 to 0.81)
Encephalitis	47.72 (30.58 to 74.15)	27.85 (16.57 to 43.45)	25.62 (15.32 to 40.33)	33.13 (19.31 to 52.24)	0.83 (0.57 to 1.16)	0.75 (0.50 to 1.03)	0.72 (0.49 to 1.00)	0.82 (0.55 to 1.17)

Diphtheria	0.11 (0.07 to 0.17)	0.02 (0.01 to 0.04)	0.02 (0.01 to 0.03)	0.06 (0.01 to 0.18)	0.00 (0.00 to 0.00)	0.00 (0.00 to 0.00)	0.00 (0.00 to 0.00)	0.00 (0.00 to 0.00)
Whooping cough	19.44 (1.52 to 66.52)	4.57 (0.17 to 20.00)	2.08 (0.10 to 8.61)	20.78 (0.27 to 85.89)	0.23 (0.02 to 0.79)	0.06 (0.00 to 0.24)	0.03 (0.00 to 0.11)	0.25 (0.00 to 1.02)
Tetanus	6.48 (2.14 to 11.82)	1.10 (0.28 to 2.08)	1.06 (0.25 to 2.00)	1.56 (0.51 to 3.20)	0.18 (0.05 to 0.32)	0.04 (0.01 to 0.07)	0.04 (0.01 to 0.07)	0.04 (0.01 to 0.08)
Measles	2.14 (0.66 to 5.56)	51.48 (3.30 to 203.98)	37.07 (2.92 to 127.95)	129.56 (4.87 to 546.95)	0.03 (0.01 to 0.07)	0.73 (0.06 to 2.66)	0.54 (0.06 to 1.78)	1.70 (0.09 to 6.84)

Myanmar								
Cause	YLLs (in 1000s)				Deaths (in 1000s)			
	2016	2040 Forecast	2040 Better Scenario	2040 Worse Scenario	2016	2040 Forecast	2040 Better Scenario	2040 Worse Scenario
Varicella and herpes zoster	7.62 (4.59 to 12.51)	3.13 (1.73 to 5.41)	2.77 (1.54 to 4.81)	4.27 (2.24 to 7.84)	0.19 (0.13 to 0.27)	0.17 (0.09 to 0.29)	0.16 (0.09 to 0.28)	0.20 (0.11 to 0.33)
**Neglected tropical diseases and malaria**	**371.20 (241.09 to 518.41)**	**176.50 (119.40 to 249.24)**	**167.84 (115.40 to 234.51)**	**196.80 (131.75 to 282.92)**	**7.35 (5.19 to 10.43)**	**4.55 (3.31 to 7.10)**	**4.42 (3.24 to 6.88)**	**4.86 (3.47 to 7.69)**
Malaria	349.67 (219.81 to 495.49)	156.51 (102.87 to 227.56)	149.39 (99.45 to 218.23)	171.96 (110.61 to 257.10)	6.90 (4.76 to 9.97)	3.99 (2.71 to 6.61)	3.89 (2.67 to 6.41)	4.21 (2.82 to 7.09)
Chagas disease	0.00 (0.00 to 0.00)	0.00 (0.00 to 0.00)	0.00 (0.00 to 0.00)	0.00 (0.00 to 0.00)	0.00 (0.00 to 0.00)	0.00 (0.00 to 0.00)	0.00 (0.00 to 0.00)	0.00 (0.00 to 0.00)
Leishmaniasis	0.00 (0.00 to 0.00)	0.00 (0.00 to 0.00)	0.00 (0.00 to 0.00)	0.00 (0.00 to 0.00)	0.00 (0.00 to 0.00)	0.00 (0.00 to 0.00)	0.00 (0.00 to 0.00)	0.00 (0.00 to 0.00)
African trypanosomiasis	0.00 (0.00 to 0.00)	0.00 (0.00 to 0.00)	0.00 (0.00 to 0.00)	0.00 (0.00 to 0.00)	0.00 (0.00 to 0.00)	0.00 (0.00 to 0.00)	0.00 (0.00 to 0.00)	0.00 (0.00 to 0.00)
Schistosomiasis	0.00 (0.00 to 0.00)	0.00 (0.00 to 0.00)	0.00 (0.00 to 0.00)	0.00 (0.00 to 0.00)	0.00 (0.00 to 0.00)	0.00 (0.00 to 0.00)	0.00 (0.00 to 0.00)	0.00 (0.00 to 0.00)
Cysticercosis	0.25 (0.21 to 0.31)	0.08 (0.06 to 0.10)	0.08 (0.06 to 0.10)	0.09 (0.07 to 0.11)	0.01 (0.01 to 0.01)	0.00 (0.00 to 0.00)	0.00 (0.00 to 0.00)	0.00 (0.00 to 0.00)
Cystic echinococcosis	0.06 (0.05 to 0.07)	0.02 (0.02 to 0.03)	0.02 (0.01 to 0.03)	0.04 (0.03 to 0.05)	0.00 (0.00 to 0.00)	0.00 (0.00 to 0.00)	0.00 (0.00 to 0.00)	0.00 (0.00 to 0.00)
Dengue	5.33 (1.02 to 17.26)	13.56 (3.31 to 37.09)	13.06 (3.25 to 35.02)	14.60 (3.54 to 39.96)	0.10 (0.02 to 0.33)	0.38 (0.10 to 1.03)	0.38 (0.10 to 1.00)	0.39 (0.10 to 1.07)
Yellow fever	0.00 (0.00 to 0.00)	0.00 (0.00 to 0.00)	0.00 (0.00 to 0.00)	0.00 (0.00 to 0.00)	0.00 (0.00 to 0.00)	0.00 (0.00 to 0.00)	0.00 (0.00 to 0.00)	0.00 (0.00 to 0.00)
Rabies	12.80 (4.47 to 21.40)	4.27 (1.36 to 7.19)	3.38 (0.98 to 5.91)	7.69 (2.44 to 13.30)	0.28 (0.11 to 0.45)	0.11 (0.05 to 0.18)	0.09 (0.04 to 0.15)	0.19 (0.08 to 0.32)
Intestinal nematode infections	1.95 (1.18 to 3.08)	1.46 (0.85 to 2.42)	1.39 (0.81 to 2.26)	1.60 (0.90 to 2.69)	0.03 (0.02 to 0.05)	0.03 (0.02 to 0.05)	0.03 (0.02 to 0.05)	0.03 (0.02 to 0.05)
Other neglected tropical diseases	1.12 (0.67 to 2.21)	0.59 (0.39 to 1.10)	0.52 (0.34 to 0.97)	0.82 (0.53 to 1.56)	0.02 (0.01 to 0.04)	0.02 (0.01 to 0.05)	0.02 (0.01 to 0.04)	0.03 (0.02 to 0.06)
**Maternal disorders**	**120.39 (94.48 to 147.67)**	**38.06 (23.49 to 55.07)**	**32.11 (18.89 to 48.18)**	**59.71 (36.62 to 87.20)**	**2.18 (1.72 to 2.68)**	**0.70 (0.44 to 1.01)**	**0.59 (0.35 to 0.88)**	**1.09 (0.67 to 1.59)**
Maternal hemorrhage	43.17 (30.08 to 58.08)	12.75 (7.14 to 21.07)	10.80 (5.75 to 18.01)	20.02 (11.12 to 32.36)	0.78 (0.54 to 1.05)	0.23 (0.13 to 0.38)	0.20 (0.11 to 0.33)	0.37 (0.20 to 0.59)
Maternal sepsis and other maternal infections	8.87 (4.68 to 14.07)	2.40 (1.04 to 4.19)	2.02 (0.87 to 3.69)	3.78 (1.60 to 6.79)	0.16 (0.09 to 0.26)	0.04 (0.02 to 0.08)	0.04 (0.02 to 0.07)	0.07 (0.03 to 0.12)
Maternal hypertensive disorders	13.93 (9.19 to 19.73)	4.26 (2.33 to 7.20)	3.59 (1.89 to 6.26)	6.73 (3.61 to 11.56)	0.25 (0.17 to 0.35)	0.08 (0.04 to 0.13)	0.07 (0.03 to 0.11)	0.12 (0.07 to 0.21)
Maternal obstructed labor and uterine rupture	2.04 (0.86 to 3.70)	0.64 (0.23 to 1.32)	0.53 (0.19 to 1.17)	1.04 (0.38 to 2.20)	0.04 (0.02 to 0.07)	0.01 (0.00 to 0.02)	0.01 (0.00 to 0.02)	0.02 (0.01 to 0.04)
Maternal abortion, miscarriage, and ectopic pregnancy	18.30 (11.35 to 26.46)	6.21 (3.18 to 10.91)	5.26 (2.64 to 9.33)	9.82 (5.04 to 17.31)	0.34 (0.21 to 0.48)	0.12 (0.06 to 0.20)	0.10 (0.05 to 0.17)	0.18 (0.09 to 0.32)

Indirect maternal deaths	16.53 (10.70 to 23.50)	5.85 (3.02 to 9.77)	4.93 (2.49 to 8.67)	9.12 (4.74 to 15.70)	0.29 (0.19 to 0.41)	0.10 (0.05 to 0.18)	0.09 (0.04 to 0.15)	0.16 (0.08 to 0.28)
Late maternal deaths	1.37 (0.52 to 2.73)	0.38 (0.13 to 0.79)	0.32 (0.11 to 0.66)	0.59 (0.21 to 1.27)	0.02 (0.01 to 0.05)	0.01 (0.00 to 0.01)	0.01 (0.00 to 0.01)	0.01 (0.00 to 0.02)
Maternal deaths aggravated by HIV/AIDS	0.19 (0.09 to 0.30)	0.26 (0.04 to 0.67)	0.15 (0.01 to 0.51)	0.50 (0.16 to 1.06)	0.00 (0.00 to 0.01)	0.00 (0.00 to 0.01)	0.00 (0.00 to 0.01)	0.01 (0.00 to 0.02)
Other maternal disorders	15.99 (10.27 to 22.98)	5.31 (2.75 to 8.95)	4.52 (2.32 to 7.83)	8.10 (4.26 to 14.02)	0.29 (0.19 to 0.42)	0.10 (0.05 to 0.16)	0.08 (0.04 to 0.14)	0.15 (0.08 to 0.26)

Myanmar								
Cause	YLLs (in 1000s)				Deaths (in 1000s)			
	2016	2040 Forecast	2040 Better Scenario	2040 Worse Scenario	2016	2040 Forecast	2040 Better Scenario	2040 Worse Scenario
**Neonatal disorders**	**999.93 (582.43 to 1,691.07)**	**400.19 (162.82 to 793.42)**	**336.29 (136.76 to 669.01)**	**604.32 (249.63 to 1,164.36)**	**11.55 (6.73 to 19.54)**	**4.62 (1.88 to 9.17)**	**3.89 (1.58 to 7.73)**	**6.98 (2.89 to 13.45)**
Neonatal preterm birth	363.55 (186.41 to 655.32)	112.33 (44.07 to 228.13)	96.97 (37.96 to 193.91)	204.18 (82.88 to 404.21)	4.20 (2.15 to 7.57)	1.30 (0.51 to 2.64)	1.12 (0.44 to 2.24)	2.36 (0.96 to 4.67)
Neonatal encephalopathy due to birth asphyxia and trauma	441.67 (234.22 to 778.44)	189.43 (62.00 to 404.67)	158.37 (52.19 to 342.00)	259.06 (80.69 to 556.16)	5.10 (2.71 to 8.99)	2.19 (0.72 to 4.67)	1.83 (0.60 to 3.95)	2.99 (0.93 to 6.43)
Neonatal sepsis and other neonatal infections	90.73 (42.94 to 167.80)	52.25 (19.26 to 111.94)	43.50 (15.89 to 92.33)	72.39 (26.85 to 152.85)	1.05 (0.50 to 1.94)	0.60 (0.22 to 1.29)	0.50 (0.18 to 1.07)	0.84 (0.31 to 1.77)
Hemolytic disease and other neonatal jaundice	5.54 (2.27 to 11.58)	1.97 (0.57 to 5.14)	1.60 (0.46 to 4.00)	2.92 (0.84 to 7.70)	0.06 (0.03 to 0.13)	0.02 (0.01 to 0.06)	0.02 (0.01 to 0.05)	0.03 (0.01 to 0.09)
Other neonatal disorders	98.43 (48.54 to 179.43)	44.21 (14.93 to 97.59)	35.85 (12.11 to 77.01)	65.76 (22.49 to 139.97)	1.14 (0.56 to 2.07)	0.51 (0.17 to 1.13)	0.41 (0.14 to 0.89)	0.76 (0.26 to 1.62)
**Nutritional deficiencies**	**34.20 (26.93 to 47.45)**	**35.29 (28.87 to 43.79)**	**34.66 (28.27 to 42.97)**	**37.29 (29.66 to 47.70)**	**1.80 (1.56 to 2.13)**	**2.97 (2.33 to 3.75)**	**3.05 (2.40 to 3.83)**	**2.85 (2.13 to 3.74)**
Protein-energy malnutrition	25.05 (19.05 to 35.98)	23.25 (17.14 to 30.23)	22.94 (17.23 to 30.08)	24.32 (17.70 to 32.67)	1.41 (1.20 to 1.70)	2.15 (1.58 to 2.87)	2.22 (1.61 to 2.92)	2.04 (1.42 to 2.83)
Iodine deficiency	0.31 (0.02 to 1.27)	1.19 (0.02 to 4.77)	1.12 (0.02 to 4.85)	1.38 (0.03 to 5.16)	0.01 (0.00 to 0.05)	0.09 (0.00 to 0.41)	0.09 (0.00 to 0.40)	0.10 (0.00 to 0.41)
Dietary iron deficiency	6.12 (2.79 to 9.66)	7.12 (3.92 to 11.32)	6.89 (3.78 to 11.05)	7.71 (4.18 to 12.27)	0.21 (0.12 to 0.31)	0.39 (0.19 to 0.70)	0.40 (0.20 to 0.71)	0.39 (0.19 to 0.69)
Other nutritional deficiencies	2.72 (1.84 to 4.29)	3.74 (2.49 to 5.91)	3.70 (2.47 to 5.65)	3.88 (2.52 to 6.16)	0.17 (0.12 to 0.26)	0.33 (0.22 to 0.54)	0.34 (0.23 to 0.54)	0.32 (0.20 to 0.52)
**Other communicable, maternal, neonatal, and nutritional diseases**	**54.87 (35.50 to 90.46)**	**23.59 (18.06 to 30.81)**	**21.81 (16.37 to 28.55)**	**28.25 (21.00 to 38.32)**	**1.06 (0.80 to 1.50)**	**0.75 (0.60 to 0.95)**	**0.71 (0.57 to 0.90)**	**0.84 (0.67 to 1.09)**
Sexually transmitted infections excluding HIV	30.42 (12.42 to 64.57)	6.01 (3.06 to 11.54)	5.41 (2.80 to 10.37)	7.53 (3.62 to 15.01)	0.41 (0.20 to 0.82)	0.12 (0.08 to 0.20)	0.11 (0.07 to 0.18)	0.14 (0.09 to 0.24)
Syphilis	27.70 (9.79 to 61.70)	4.40 (1.58 to 9.88)	3.87 (1.37 to 8.77)	5.71 (2.03 to 12.87)	0.34 (0.13 to 0.74)	0.07 (0.03 to 0.13)	0.06 (0.03 to 0.12)	0.08 (0.04 to 0.17)
Chlamydial infection	0.54 (0.40 to 0.84)	0.31 (0.22 to 0.47)	0.30 (0.20 to 0.44)	0.36 (0.25 to 0.54)	0.01 (0.01 to 0.02)	0.01 (0.01 to 0.02)	0.01 (0.01 to 0.02)	0.01 (0.01 to 0.02)
Gonococcal infection	1.42 (1.05 to 2.23)	0.87 (0.58 to 1.27)	0.83 (0.54 to 1.22)	0.98 (0.67 to 1.46)	0.04 (0.03 to 0.06)	0.03 (0.02 to 0.05)	0.03 (0.02 to 0.05)	0.03 (0.02 to 0.05)
Other sexually transmitted infections	0.76 (0.55 to 1.18)	0.43 (0.29 to 0.64)	0.41 (0.27 to 0.62)	0.49 (0.34 to 0.75)	0.02 (0.01 to 0.03)	0.01 (0.01 to 0.02)	0.01 (0.01 to 0.02)	0.01 (0.01 to 0.02)
Acute hepatitis	22.08 (18.13 to 27.51)	14.85 (11.12 to 19.18)	13.85 (10.27 to 18.10)	17.48 (13.06 to 22.86)	0.57 (0.48 to 0.71)	0.49 (0.38 to 0.62)	0.46 (0.36 to 0.59)	0.55 (0.43 to 0.70)
Acute hepatitis A	0.87 (0.52 to 1.38)	0.36 (0.22 to 0.57)	0.33 (0.20 to 0.51)	0.43 (0.27 to 0.70)	0.02 (0.01 to 0.02)	0.01 (0.01 to 0.01)	0.01 (0.01 to 0.01)	0.01 (0.01 to 0.02)
Acute hepatitis B	18.27 (14.88 to 22.84)	12.58 (9.19 to 16.67)	11.70 (8.46 to 15.73)	14.91 (10.86 to 19.87)	0.49 (0.40 to 0.59)	0.41 (0.31 to 0.53)	0.38 (0.29 to 0.51)	0.47 (0.35 to 0.60)

Acute hepatitis C	0.48 (0.34 to 0.67)	0.42 (0.28 to 0.62)	0.41 (0.27 to 0.59)	0.45 (0.30 to 0.66)	0.01 (0.01 to 0.02)	0.02 (0.01 to 0.02)	0.02 (0.01 to 0.02)	0.02 (0.01 to 0.02)
Acute hepatitis E	2.46 (1.74 to 3.39)	1.49 (0.97 to 2.26)	1.42 (0.90 to 2.13)	1.68 (1.07 to 2.54)	0.06 (0.04 to 0.08)	0.06 (0.03 to 0.09)	0.06 (0.03 to 0.09)	0.06 (0.04 to 0.09)
Other unspecified infectious diseases	2.37 (1.33 to 4.90)	2.73 (1.64 to 5.45)	2.55 (1.54 to 5.08)	3.24 (1.91 to 6.56)	0.07 (0.04 to 0.15)	0.14 (0.08 to 0.28)	0.13 (0.08 to 0.27)	0.15 (0.09 to 0.31)
**Non-communicable diseases**	**6,682.44 (6,134.58 to 7,492.25)**	**8,563.29 (7,277.84 to 9,887.09)**	**7,548.68 (6,443.41 to 8,668.12)**	**11,194.76 (9,371.59 to 13,146.17)**	**270.81 (251.21 to 297.63)**	**469.15 (417.49 to 517.55)**	**425.34 (379.38 to 468.00)**	**575.36 (508.03 to 640.78)**

Myanmar								
Cause	YLLs (in 1000s)				Deaths (in 1000s)			
	2016	2040 Forecast	2040 Better Scenario	2040 Worse Scenario	2016	2040 Forecast	2040 Better Scenario	2040 Worse Scenario
**Neoplasms**	**1,812.87 (1,648.79 to 2,028.33)**	**2,553.12 (2,107.40 to 3,046.44)**	**2,403.85 (2,004.52 to 2,850.26)**	**3,077.89 (2,477.61 to 3,765.14)**	**66.43 (60.92 to 73.50)**	**120.52 (102.70 to 139.50)**	**116.34 (99.94 to 132.98)**	**139.89 (116.07 to 165.57)**
Lip and oral cavity cancer	39.18 (33.19 to 47.11)	57.14 (36.54 to 85.17)	45.53 (31.21 to 63.53)	89.36 (53.68 to 138.70)	1.57 (1.35 to 1.87)	2.81 (1.91 to 4.01)	2.34 (1.68 to 3.19)	4.20 (2.68 to 6.23)
Nasopharynx cancer	28.16 (23.16 to 34.56)	38.75 (23.06 to 63.20)	28.80 (18.47 to 44.45)	64.37 (36.88 to 107.26)	0.93 (0.77 to 1.12)	1.50 (0.93 to 2.40)	1.15 (0.75 to 1.78)	2.44 (1.46 to 4.05)
Other pharynx cancer	17.65 (14.55 to 21.08)	26.84 (17.93 to 39.87)	22.79 (16.30 to 32.15)	27.52 (18.36 to 40.75)	0.66 (0.55 to 0.78)	1.19 (0.82 to 1.73)	1.04 (0.76 to 1.44)	1.18 (0.81 to 1.73)
Esophageal cancer	27.22 (23.38 to 32.61)	35.37 (19.48 to 62.98)	29.14 (16.60 to 50.02)	59.25 (30.45 to 110.09)	1.04 (0.91 to 1.22)	1.59 (0.92 to 2.72)	1.36 (0.82 to 2.29)	2.56 (1.37 to 4.57)
Stomach cancer	120.70 (104.84 to 142.91)	140.63 (112.05 to 174.12)	137.46 (110.13 to 169.14)	162.36 (125.33 to 207.60)	4.91 (4.29 to 5.80)	7.23 (5.89 to 8.88)	7.22 (5.87 to 8.77)	7.95 (6.26 to 9.99)
Colon and rectum cancer	159.58 (136.78 to 186.65)	302.43 (212.76 to 438.61)	295.15 (210.71 to 427.92)	301.49 (209.85 to 436.52)	6.46 (5.57 to 7.48)	15.95 (11.40 to 22.77)	15.96 (11.59 to 22.46)	15.15 (10.69 to 21.68)
Liver cancer	96.93 (78.81 to 116.93)	160.35 (113.58 to 223.56)	146.25 (107.67 to 196.34)	181.42 (124.25 to 260.37)	3.78 (3.13 to 4.49)	7.85 (5.81 to 10.50)	7.36 (5.61 to 9.47)	8.48 (6.00 to 11.65)
Liver cancer due to hepatitis B	45.66 (35.22 to 57.48)	69.11 (46.98 to 100.29)	63.38 (44.59 to 86.84)	78.45 (51.79 to 116.29)	1.65 (1.27 to 2.06)	3.16 (2.15 to 4.39)	2.98 (2.07 to 3.99)	3.43 (2.30 to 4.88)
Liver cancer due to hepatitis C	26.82 (20.08 to 34.71)	50.52 (34.99 to 71.09)	46.98 (33.73 to 65.30)	55.51 (37.46 to 80.87)	1.20 (0.92 to 1.52)	2.78 (1.98 to 3.86)	2.66 (1.93 to 3.59)	2.92 (2.00 to 4.15)
Liver cancer due to alcohol use	6.89 (4.70 to 9.97)	12.43 (7.68 to 19.68)	10.43 (6.61 to 16.26)	14.40 (8.63 to 23.93)	0.28 (0.19 to 0.39)	0.59 (0.37 to 0.92)	0.51 (0.33 to 0.77)	0.65 (0.40 to 1.04)
Liver cancer due to other causes	17.57 (12.28 to 24.56)	28.28 (17.33 to 44.93)	25.46 (16.05 to 39.73)	33.07 (19.41 to 55.67)	0.65 (0.45 to 0.89)	1.32 (0.81 to 2.04)	1.22 (0.78 to 1.85)	1.47 (0.88 to 2.35)
Gallbladder and biliary tract cancer	24.38 (19.28 to 30.55)	32.03 (25.81 to 39.86)	33.21 (26.76 to 41.33)	29.52 (23.77 to 36.61)	1.05 (0.85 to 1.30)	1.72 (1.42 to 2.09)	1.81 (1.51 to 2.20)	1.52 (1.25 to 1.86)
Pancreatic cancer	64.88 (57.11 to 73.86)	122.34 (95.90 to 154.92)	121.49 (96.91 to 150.35)	129.89 (100.44 to 167.17)	2.72 (2.42 to 3.06)	6.34 (5.11 to 7.95)	6.40 (5.24 to 7.84)	6.43 (5.13 to 8.13)
Larynx cancer	24.79 (19.56 to 29.68)	30.63 (14.83 to 57.32)	24.12 (12.10 to 44.77)	56.78 (26.81 to 98.34)	0.99 (0.80 to 1.17)	1.43 (0.70 to 2.74)	1.15 (0.58 to 2.17)	2.58 (1.23 to 4.40)
Tracheal, bronchus, and lung cancer	314.59 (271.72 to 371.49)	358.13 (233.30 to 542.20)	279.61 (185.20 to 418.41)	716.91 (462.80 to 1,091.70)	12.57 (11.00 to 14.62)	17.10 (11.29 to 25.08)	13.70 (9.21 to 19.58)	33.67 (21.74 to 50.30)
Malignant skin melanoma	6.52 (5.39 to 8.60)	10.09 (8.18 to 13.46)	10.67 (8.62 to 14.38)	8.81 (7.22 to 11.75)	0.23 (0.19 to 0.30)	0.49 (0.41 to 0.66)	0.53 (0.44 to 0.72)	0.40 (0.34 to 0.55)
Non-melanoma skin cancer	9.46 (7.99 to 10.89)	17.89 (14.94 to 21.12)	18.08 (15.00 to 21.36)	17.70 (14.80 to 20.64)	0.39 (0.33 to 0.44)	0.96 (0.81 to 1.10)	0.99 (0.84 to 1.15)	0.89 (0.75 to 1.03)
Breast cancer	147.11 (119.86 to 189.11)	204.98 (146.08 to 293.87)	198.96 (144.30 to 281.77)	208.60 (147.22 to 302.99)	4.74 (3.89 to 6.04)	8.32 (6.08 to 11.75)	8.18 (6.09 to 11.33)	8.20 (5.91 to 11.56)
Cervical cancer	150.36 (107.16 to 187.86)	155.11 (103.38 to 212.97)	149.25 (99.51 to 201.50)	178.39 (116.22 to 253.41)	5.12 (3.75 to 6.48)	6.50 (4.57 to 8.80)	6.35 (4.43 to 8.56)	7.24 (5.02 to 10.14)

Uterine cancer	34.71 (27.16 to 42.24)	49.12 (37.50 to 61.68)	47.92 (36.39 to 60.61)	53.03 (40.21 to 66.17)	1.36 (1.08 to 1.64)	2.40 (1.91 to 2.94)	2.37 (1.86 to 2.91)	2.51 (1.96 to 3.08)
Ovarian cancer	66.47 (54.95 to 79.10)	102.86 (74.42 to 141.56)	102.10 (76.84 to 136.47)	96.00 (69.26 to 132.39)	2.31 (1.92 to 2.73)	4.38 (3.22 to 6.00)	4.39 (3.32 to 5.80)	4.07 (2.98 to 5.63)
Prostate cancer	29.48 (18.09 to 36.71)	91.22 (57.71 to 114.00)	98.75 (63.04 to 123.34)	78.69 (49.53 to 99.11)	1.76 (1.08 to 2.18)	6.37 (4.11 to 7.92)	7.08 (4.65 to 8.77)	5.13 (3.19 to 6.49)
Testicular cancer	3.18 (2.44 to 4.29)	3.29 (2.41 to 4.60)	3.25 (2.36 to 4.57)	3.40 (2.53 to 4.72)	0.07 (0.06 to 0.09)	0.10 (0.07 to 0.13)	0.10 (0.07 to 0.13)	0.09 (0.07 to 0.13)

Myanmar								
Cause	YLLs (in 1000s)				Deaths (in 1000s)			
	2016	2040 Forecast	2040 Better Scenario	2040 Worse Scenario	2016	2040 Forecast	2040 Better Scenario	2040 Worse Scenario
Kidney cancer	20.90 (17.30 to 24.80)	34.12 (25.63 to 46.10)	35.57 (26.67 to 48.15)	34.57 (25.15 to 48.53)	0.76 (0.63 to 0.90)	1.59 (1.24 to 2.08)	1.70 (1.31 to 2.21)	1.54 (1.14 to 2.13)
Bladder cancer	24.08 (19.98 to 29.34)	40.47 (29.81 to 54.79)	39.51 (29.48 to 52.58)	48.44 (34.58 to 68.31)	1.14 (0.96 to 1.37)	2.42 (1.86 to 3.19)	2.42 (1.87 to 3.13)	2.73 (2.01 to 3.76)
Brain and nervous system cancer	59.10 (46.47 to 70.07)	82.75 (63.47 to 101.70)	84.54 (64.77 to 103.89)	78.40 (60.46 to 95.25)	1.58 (1.22 to 1.86)	3.02 (2.30 to 3.61)	3.16 (2.40 to 3.78)	2.73 (2.08 to 3.25)
Thyroid cancer	16.10 (12.84 to 19.73)	25.42 (19.57 to 33.41)	23.60 (18.43 to 30.37)	26.56 (20.26 to 35.26)	0.65 (0.53 to 0.78)	1.28 (1.01 to 1.64)	1.21 (0.98 to 1.53)	1.29 (1.00 to 1.67)
Mesothelioma	4.97 (4.19 to 6.10)	7.77 (6.35 to 9.61)	7.85 (6.40 to 9.69)	7.70 (6.32 to 9.56)	0.19 (0.16 to 0.23)	0.42 (0.34 to 0.53)	0.43 (0.34 to 0.55)	0.40 (0.31 to 0.50)
Hodgkin lymphoma	13.04 (10.33 to 17.93)	11.39 (8.48 to 16.42)	10.84 (7.90 to 15.75)	13.07 (9.77 to 18.82)	0.36 (0.28 to 0.49)	0.41 (0.31 to 0.59)	0.40 (0.30 to 0.56)	0.45 (0.34 to 0.64)
Non-Hodgkin lymphoma	61.23 (53.36 to 71.10)	93.67 (78.44 to 111.06)	94.30 (79.21 to 110.78)	86.21 (72.89 to 102.23)	1.92 (1.69 to 2.21)	4.04 (3.45 to 4.73)	4.16 (3.55 to 4.83)	3.55 (3.02 to 4.17)
Multiple myeloma	14.25 (11.86 to 16.97)	27.15 (21.82 to 33.94)	27.33 (22.04 to 34.01)	24.46 (19.72 to 30.76)	0.54 (0.46 to 0.65)	1.27 (1.04 to 1.57)	1.30 (1.06 to 1.59)	1.10 (0.90 to 1.38)
Leukemia	116.31 (97.20 to 149.96)	128.66 (99.39 to 171.08)	122.19 (95.48 to 158.91)	139.19 (105.90 to 185.75)	3.05 (2.60 to 3.85)	4.86 (3.86 to 6.29)	4.73 (3.82 to 6.03)	5.04 (3.93 to 6.72)
Acute lymphoid leukemia	28.69 (22.65 to 43.81)	33.45 (25.51 to 49.47)	32.47 (25.05 to 47.00)	34.36 (25.48 to 51.44)	0.74 (0.61 to 1.10)	1.25 (0.97 to 1.88)	1.25 (0.96 to 1.83)	1.24 (0.93 to 1.83)
Chronic lymphoid leukemia	2.31 (1.81 to 3.31)	2.80 (2.05 to 4.00)	2.68 (1.99 to 3.76)	2.91 (2.10 to 4.20)	0.07 (0.06 to 0.11)	0.12 (0.09 to 0.17)	0.12 (0.09 to 0.17)	0.12 (0.09 to 0.17)
Acute myeloid leukemia	19.50 (15.59 to 26.43)	26.23 (19.77 to 35.58)	25.57 (19.59 to 33.90)	26.16 (19.47 to 35.89)	0.48 (0.39 to 0.62)	0.88 (0.67 to 1.18)	0.88 (0.67 to 1.14)	0.85 (0.63 to 1.16)
Chronic myeloid leukemia	6.72 (5.25 to 9.52)	6.15 (4.33 to 8.88)	5.86 (4.20 to 8.27)	6.61 (4.56 to 9.67)	0.20 (0.16 to 0.28)	0.23 (0.17 to 0.34)	0.23 (0.17 to 0.33)	0.24 (0.17 to 0.36)
Other leukemia	59.09 (46.02 to 73.54)	60.03 (43.82 to 80.17)	55.61 (41.18 to 73.50)	69.14 (50.04 to 94.89)	1.56 (1.26 to 1.93)	2.37 (1.80 to 3.12)	2.26 (1.72 to 2.92)	2.60 (1.92 to 3.52)
Other neoplasms	117.51 (100.62 to 135.32)	162.51 (136.78 to 189.37)	165.61 (139.28 to 193.89)	155.81 (131.44 to 180.66)	3.59 (3.12 to 4.10)	7.00 (6.05 to 7.92)	7.31 (6.33 to 8.32)	6.36 (5.54 to 7.21)
**Cardiovascular diseases**	**1,865.61 (1,697.33 to 2,096.67)**	**2,025.74 (1,522.25 to 2,713.30)**	**1,520.88 (1,177.87 to 1,980.57)**	**3,401.82 (2,423.58 to 4,715.63)**	**87.47 (80.33 to 96.16)**	**128.16 (100.57 to 162.81)**	**101.84 (81.66 to 126.27)**	**190.96 (146.55 to 250.01)**
Rheumatic heart disease	65.43 (53.13 to 78.78)	42.31 (33.36 to 53.95)	39.70 (31.32 to 49.89)	50.56 (39.43 to 63.93)	2.10 (1.72 to 2.58)	2.06 (1.65 to 2.57)	1.99 (1.60 to 2.47)	2.30 (1.83 to 2.94)
Ischemic heart disease	535.34 (475.02 to 614.77)	510.41 (324.12 to 800.86)	356.71 (233.09 to 547.38)	998.65 (608.40 to 1,592.72)	26.01 (23.47 to 29.28)	33.19 (21.85 to 51.27)	24.79 (16.62 to 37.51)	56.61 (36.74 to 89.81)
Stroke	881.11 (788.92 to 1,000.42)	847.59 (535.51 to 1,321.67)	573.45 (376.07 to 873.32)	1,607.37 (1,006.13 to 2,517.03)	41.37 (37.28 to 46.28)	51.96 (35.24 to 76.37)	37.94 (26.85 to 54.86)	86.73 (58.12 to 127.83)
Ischemic stroke	251.71 (217.15 to 295.42)	292.19 (182.29 to 480.10)	213.59 (137.68 to 345.49)	473.53 (283.62 to 786.13)	15.12 (13.22 to 17.49)	21.55 (13.55 to 36.42)	16.73 (11.06 to 27.83)	31.41 (19.39 to 53.30)

Intracerebral hemorrhage	629.40 (552.38 to 726.99)	555.40 (322.42 to 942.76)	359.86 (218.65 to 587.00)	1,133.85 (640.36 to 1,934.93)	26.26 (23.24 to 29.81)	30.41 (19.24 to 47.33)	21.21 (13.92 to 31.83)	55.32 (33.66 to 88.02)
Hypertensive heart disease	173.65 (131.28 to 207.41)	305.18 (209.29 to 471.28)	240.52 (171.69 to 335.39)	397.01 (251.42 to 666.42)	9.02 (6.79 to 10.70)	20.84 (14.57 to 32.61)	16.85 (12.19 to 23.77)	25.24 (16.63 to 43.39)
Cardiomyopathy and myocarditis	55.72 (39.22 to 70.64)	74.62 (48.85 to 105.69)	72.92 (47.48 to 102.15)	71.57 (47.03 to 101.11)	2.01 (1.48 to 2.48)	3.90 (2.57 to 5.62)	3.96 (2.60 to 5.70)	3.49 (2.33 to 5.00)
Myocarditis	10.40 (7.16 to 14.55)	8.93 (6.49 to 11.76)	8.83 (6.38 to 11.66)	9.25 (6.74 to 12.34)	0.28 (0.21 to 0.35)	0.37 (0.27 to 0.47)	0.38 (0.28 to 0.49)	0.35 (0.26 to 0.45)

Myanmar								
Cause	YLLs (in 1000s)				Deaths (in 1000s)			
	2016	2040 Forecast	2040 Better Scenario	2040 Worse Scenario	2016	2040 Forecast	2040 Better Scenario	2040 Worse Scenario
Alcoholic cardiomyopathy	8.52 (4.21 to 11.85)	11.16 (5.47 to 15.88)	10.35 (5.06 to 14.85)	10.32 (5.11 to 14.53)	0.31 (0.17 to 0.43)	0.51 (0.27 to 0.72)	0.49 (0.26 to 0.70)	0.45 (0.24 to 0.64)
Other cardiomyopathy	36.81 (25.13 to 47.27)	54.53 (34.70 to 79.68)	53.75 (33.96 to 77.34)	52.01 (32.88 to 76.86)	1.43 (1.00 to 1.77)	3.02 (1.90 to 4.55)	3.09 (1.94 to 4.62)	2.69 (1.72 to 4.08)
Atrial fibrillation and flutter	14.50 (11.19 to 18.44)	40.33 (30.45 to 52.72)	41.04 (31.27 to 52.90)	38.60 (28.62 to 51.11)	1.27 (0.97 to 1.60)	4.06 (3.02 to 5.29)	4.26 (3.23 to 5.51)	3.63 (2.67 to 4.85)
Aortic aneurysm	17.06 (13.81 to 21.09)	26.33 (20.71 to 33.29)	26.87 (21.23 to 34.18)	26.95 (20.67 to 34.73)	0.86 (0.71 to 1.04)	1.73 (1.39 to 2.15)	1.82 (1.45 to 2.27)	1.63 (1.29 to 2.05)
Peripheral artery disease	0.77 (0.60 to 1.03)	2.85 (2.02 to 4.06)	2.79 (2.00 to 3.95)	2.50 (1.77 to 3.56)	0.05 (0.04 to 0.07)	0.25 (0.17 to 0.37)	0.26 (0.18 to 0.38)	0.20 (0.14 to 0.29)
Endocarditis	30.40 (25.10 to 38.91)	38.87 (28.72 to 53.25)	37.26 (27.81 to 50.70)	39.66 (29.11 to 54.54)	0.99 (0.81 to 1.24)	1.84 (1.34 to 2.55)	1.82 (1.33 to 2.51)	1.75 (1.27 to 2.40)
Other cardiovascular and circulatory diseases	91.63 (68.15 to 150.32)	137.25 (96.12 to 215.15)	129.62 (92.69 to 203.25)	168.94 (114.37 to 271.33)	3.78 (2.90 to 6.28)	8.33 (5.94 to 13.51)	8.16 (5.88 to 13.05)	9.37 (6.54 to 15.31)
**Chronic respiratory diseases**	**612.49 (526.98 to 712.27)**	**538.77 (384.49 to 745.97)**	**431.65 (318.73 to 579.94)**	**979.07 (660.35 to 1,437.87)**	**30.35 (26.13 to 34.73)**	**32.81 (24.26 to 43.33)**	**27.29 (20.55 to 35.50)**	**57.33 (40.47 to 80.58)**
Chronic obstructive pulmonary disease	346.68 (262.80 to 422.38)	329.41 (195.72 to 518.70)	246.80 (147.51 to 386.02)	730.44 (423.33 to 1,183.13)	18.98 (14.58 to 22.78)	21.12 (12.99 to 32.20)	16.59 (10.16 to 24.94)	44.23 (27.11 to 68.31)
Pneumoconiosis	0.55 (0.38 to 0.78)	0.67 (0.46 to 0.97)	0.66 (0.44 to 0.96)	0.62 (0.44 to 0.88)	0.03 (0.02 to 0.04)	0.04 (0.03 to 0.06)	0.04 (0.03 to 0.06)	0.04 (0.03 to 0.05)
Silicosis	0.15 (0.08 to 0.27)	0.14 (0.08 to 0.25)	0.15 (0.08 to 0.25)	0.14 (0.07 to 0.23)	0.01 (0.00 to 0.01)	0.01 (0.00 to 0.01)	0.01 (0.00 to 0.02)	0.01 (0.00 to 0.01)
Asbestosis	0.08 (0.05 to 0.11)	0.10 (0.06 to 0.15)	0.11 (0.07 to 0.16)	0.09 (0.06 to 0.14)	0.00 (0.00 to 0.00)	0.01 (0.00 to 0.01)	0.01 (0.00 to 0.01)	0.00 (0.00 to 0.01)
Coal workers pneumoconiosis	0.03 (0.01 to 0.06)	0.03 (0.01 to 0.06)	0.03 (0.01 to 0.05)	0.04 (0.02 to 0.07)	0.00 (0.00 to 0.00)	0.00 (0.00 to 0.00)	0.00 (0.00 to 0.00)	0.00 (0.00 to 0.00)
Other pneumoconiosis	0.30 (0.19 to 0.44)	0.40 (0.25 to 0.59)	0.38 (0.24 to 0.57)	0.35 (0.22 to 0.53)	0.02 (0.01 to 0.02)	0.03 (0.02 to 0.04)	0.03 (0.02 to 0.04)	0.02 (0.01 to 0.03)
Asthma	247.39 (172.97 to 350.47)	181.17 (110.62 to 286.41)	155.41 (98.68 to 234.75)	222.39 (129.40 to 373.66)	10.67 (7.12 to 15.33)	10.19 (6.14 to 16.15)	9.09 (5.66 to 13.65)	11.82 (6.66 to 19.96)
Interstitial lung disease and pulmonary sarcoidosis	9.42 (6.40 to 13.12)	18.91 (12.85 to 26.07)	20.36 (13.66 to 28.21)	15.87 (10.75 to 21.83)	0.43 (0.29 to 0.59)	1.05 (0.72 to 1.46)	1.16 (0.79 to 1.59)	0.84 (0.57 to 1.17)
Other chronic respiratory diseases	8.45 (4.02 to 13.36)	8.60 (4.42 to 14.05)	8.42 (4.32 to 13.71)	9.75 (4.96 to 15.82)	0.25 (0.12 to 0.39)	0.39 (0.18 to 0.70)	0.40 (0.19 to 0.72)	0.41 (0.19 to 0.73)
**Cirrhosis and other chronic liver diseases**	**807.97 (678.78 to 1,012.25)**	**1,006.39 (829.67 to 1,243.87)**	**964.70 (791.69 to 1,196.86)**	**1,044.56 (855.72 to 1,299.84)**	**24.37 (20.72 to 30.14)**	**38.12 (32.38 to 45.07)**	**37.49 (31.63 to 44.40)**	**37.77 (31.87 to 45.24)**
Cirrhosis and other chronic liver diseases due to hepatitis B	274.86 (217.22 to 358.04)	347.50 (274.17 to 446.75)	345.35 (269.73 to 441.69)	355.39 (277.32 to 463.75)	8.42 (6.74 to 10.88)	13.19 (10.62 to 16.47)	13.38 (10.71 to 16.77)	12.88 (10.32 to 16.15)
Cirrhosis and other chronic liver diseases due to hepatitis C	189.15 (145.67 to 248.29)	240.75 (184.17 to 316.02)	238.32 (182.26 to 312.74)	248.79 (190.63 to 326.61)	5.70 (4.48 to 7.32)	8.86 (7.01 to 11.22)	8.93 (7.05 to 11.43)	8.80 (6.93 to 11.24)

Cirrhosis and other chronic liver diseases due to alcohol use	145.97 (111.61 to 193.45)	198.22 (148.81 to 263.96)	164.30 (123.32 to 220.09)	210.31 (157.93 to 286.49)	4.63 (3.59 to 5.98)	7.60 (5.95 to 9.90)	6.61 (5.14 to 8.61)	7.70 (5.98 to 10.04)
Cirrhosis and other chronic liver diseases due to other causes	197.99 (161.77 to 242.01)	219.92 (177.26 to 268.90)	216.73 (173.57 to 267.99)	230.07 (185.52 to 283.20)	5.62 (4.70 to 6.74)	8.47 (7.03 to 10.09)	8.57 (7.10 to 10.30)	8.39 (7.01 to 10.05)
**Digestive diseases**	**220.01 (186.63 to 269.37)**	**234.76 (183.45 to 302.66)**	**216.79 (170.92 to 270.85)**	**260.54 (202.21 to 337.11)**	**7.79 (6.69 to 9.13)**	**11.94 (9.58 to 14.34)**	**11.58 (9.40 to 13.82)**	**12.39 (9.96 to 14.97)**
Peptic ulcer disease	72.61 (56.67 to 92.08)	58.74 (42.56 to 76.86)	55.30 (39.70 to 72.99)	75.01 (53.12 to 100.60)	3.03 (2.38 to 3.73)	3.36 (2.42 to 4.39)	3.25 (2.29 to 4.26)	4.04 (2.90 to 5.35)

Myanmar								
Cause	YLLs (in 1000s)				Deaths (in 1000s)			
	2016	2040 Forecast	2040 Better Scenario	2040 Worse Scenario	2016	2040 Forecast	2040 Better Scenario	2040 Worse Scenario
Gastritis and duodenitis	4.41 (2.98 to 7.05)	6.98 (4.56 to 11.08)	6.97 (4.50 to 11.12)	7.15 (4.66 to 11.43)	0.18 (0.12 to 0.26)	0.39 (0.25 to 0.57)	0.40 (0.26 to 0.59)	0.37 (0.24 to 0.56)
Appendicitis	15.63 (12.33 to 21.84)	12.03 (8.93 to 16.65)	11.46 (8.42 to 16.05)	13.77 (10.25 to 19.37)	0.40 (0.31 to 0.56)	0.46 (0.34 to 0.64)	0.46 (0.33 to 0.62)	0.49 (0.36 to 0.68)
Paralytic ileus and intestinal obstruction	48.11 (33.50 to 74.72)	51.24 (38.48 to 67.82)	51.17 (38.41 to 66.64)	51.91 (39.03 to 70.34)	1.44 (1.11 to 1.86)	2.69 (2.05 to 3.33)	2.81 (2.15 to 3.44)	2.46 (1.88 to 3.09)
Inguinal, femoral, and abdominal hernia	5.11 (3.32 to 6.62)	5.68 (3.40 to 7.27)	5.68 (3.36 to 7.40)	5.79 (3.53 to 7.56)	0.23 (0.14 to 0.29)	0.35 (0.20 to 0.46)	0.36 (0.21 to 0.48)	0.32 (0.19 to 0.43)
Inflammatory bowel disease	3.70 (2.87 to 5.07)	4.86 (3.64 to 6.37)	4.98 (3.71 to 6.46)	4.64 (3.50 to 6.05)	0.13 (0.10 to 0.17)	0.25 (0.19 to 0.32)	0.26 (0.20 to 0.34)	0.22 (0.17 to 0.28)
Vascular intestinal disorders	5.46 (4.26 to 7.58)	9.72 (7.55 to 13.06)	9.91 (7.68 to 13.13)	9.45 (7.31 to 12.79)	0.27 (0.21 to 0.37)	0.64 (0.49 to 0.84)	0.67 (0.52 to 0.87)	0.59 (0.44 to 0.77)
Gallbladder and biliary diseases	10.34 (8.40 to 12.57)	14.71 (9.62 to 22.02)	12.02 (8.34 to 16.77)	16.51 (10.66 to 24.64)	0.46 (0.37 to 0.55)	0.88 (0.58 to 1.30)	0.75 (0.52 to 1.03)	0.92 (0.59 to 1.39)
Pancreatitis	34.48 (25.86 to 44.99)	47.86 (23.78 to 101.50)	36.85 (20.84 to 67.36)	51.63 (25.62 to 112.69)	0.96 (0.72 to 1.22)	1.69 (0.93 to 3.25)	1.38 (0.83 to 2.30)	1.74 (0.94 to 3.38)
Other digestive diseases	20.16 (14.25 to 28.98)	22.94 (16.87 to 31.56)	22.45 (16.49 to 30.55)	24.69 (17.87 to 34.09)	0.70 (0.52 to 0.94)	1.23 (0.91 to 1.64)	1.25 (0.92 to 1.65)	1.22 (0.89 to 1.63)
**Neurological disorders**	**289.90 (252.27 to 335.19)**	**744.53 (602.24 to 955.88)**	**721.63 (599.98 to 887.29)**	**679.19 (540.59 to 869.76)**	**20.44 (17.61 to 23.88)**	**66.28 (52.94 to 84.84)**	**66.52 (55.10 to 81.56)**	**56.62 (43.65 to 73.55)**
Alzheimer's disease and other dementias	208.15 (174.18 to 248.04)	618.33 (473.36 to 823.44)	596.73 (481.80 to 754.44)	558.42 (422.97 to 752.24)	17.86 (15.10 to 21.13)	59.82 (46.56 to 79.04)	59.64 (48.08 to 74.78)	51.18 (38.42 to 67.58)
Parkinson's disease	16.40 (12.52 to 20.86)	54.88 (41.93 to 71.36)	60.47 (46.06 to 78.08)	43.45 (32.72 to 56.75)	1.13 (0.86 to 1.47)	4.40 (3.28 to 5.83)	4.95 (3.76 to 6.52)	3.32 (2.44 to 4.43)
Epilepsy	45.77 (38.16 to 57.11)	45.79 (31.58 to 64.71)	38.37 (27.54 to 52.81)	53.11 (36.98 to 74.87)	0.95 (0.81 to 1.18)	1.18 (0.85 to 1.62)	1.01 (0.75 to 1.33)	1.32 (0.95 to 1.79)
Multiple sclerosis	3.30 (2.69 to 4.47)	3.95 (3.07 to 5.38)	4.02 (3.11 to 5.44)	3.89 (3.01 to 5.36)	0.09 (0.08 to 0.13)	0.13 (0.11 to 0.18)	0.14 (0.11 to 0.18)	0.13 (0.10 to 0.18)
Motor neuron disease	3.61 (3.03 to 4.33)	5.94 (4.81 to 7.25)	6.12 (4.94 to 7.53)	5.51 (4.52 to 6.65)	0.11 (0.09 to 0.13)	0.22 (0.18 to 0.27)	0.23 (0.19 to 0.28)	0.20 (0.17 to 0.24)
Other neurological disorders	12.68 (10.26 to 15.70)	15.63 (12.94 to 18.69)	15.93 (13.15 to 19.06)	14.80 (12.21 to 17.82)	0.30 (0.26 to 0.35)	0.52 (0.46 to 0.60)	0.55 (0.48 to 0.63)	0.47 (0.41 to 0.53)
**Mental disorders**	**53.05 (37.22 to 76.47)**	**64.63 (45.31 to 91.45)**	**62.69 (43.91 to 88.52)**	**64.60 (45.41 to 91.75)**	**1.26 (0.91 to 1.76)**	**1.86 (1.37 to 2.53)**	**1.83 (1.36 to 2.50)**	**1.79 (1.32 to 2.44)**
Alcohol use disorders	36.70 (22.39 to 55.25)	38.04 (21.87 to 59.47)	35.97 (20.48 to 56.06)	38.32 (22.07 to 59.90)	0.90 (0.57 to 1.32)	1.12 (0.68 to 1.66)	1.08 (0.65 to 1.61)	1.10 (0.67 to 1.62)
Drug use disorders	16.08 (11.10 to 26.93)	26.34 (18.35 to 41.92)	26.47 (18.52 to 41.83)	26.04 (18.08 to 41.96)	0.35 (0.25 to 0.58)	0.73 (0.55 to 1.14)	0.75 (0.56 to 1.15)	0.69 (0.52 to 1.09)
Opioid use disorders	10.42 (6.96 to 17.73)	14.10 (9.38 to 22.82)	14.11 (9.43 to 22.75)	14.05 (9.29 to 22.83)	0.22 (0.15 to 0.37)	0.38 (0.27 to 0.59)	0.38 (0.28 to 0.60)	0.36 (0.25 to 0.56)

Cocaine use disorders	0.59 (0.33 to 1.34)	1.28 (0.71 to 2.62)	1.26 (0.70 to 2.59)	1.33 (0.74 to 2.78)	0.01 (0.01 to 0.03)	0.03 (0.02 to 0.07)	0.03 (0.02 to 0.07)	0.03 (0.02 to 0.07)
Amphetamine use disorders	0.50 (0.28 to 1.13)	1.35 (0.75 to 2.84)	1.31 (0.72 to 2.67)	1.46 (0.80 to 3.09)	0.01 (0.01 to 0.02)	0.03 (0.02 to 0.07)	0.03 (0.02 to 0.07)	0.03 (0.02 to 0.07)
Other drug use disorders	4.57 (3.09 to 7.67)	9.62 (6.32 to 16.20)	9.79 (6.44 to 16.35)	9.19 (6.01 to 15.62)	0.11 (0.08 to 0.18)	0.29 (0.21 to 0.46)	0.30 (0.22 to 0.47)	0.26 (0.19 to 0.43)
Eating disorders	0.27 (0.18 to 0.46)	0.25 (0.15 to 0.42)	0.25 (0.16 to 0.43)	0.24 (0.15 to 0.41)	0.01 (0.00 to 0.01)	0.01 (0.00 to 0.01)	0.01 (0.00 to 0.01)	0.00 (0.00 to 0.01)

Myanmar								
Cause	YLLs (in 1000s)				Deaths (in 1000s)			
	2016	2040 Forecast	2040 Better Scenario	2040 Worse Scenario	2016	2040 Forecast	2040 Better Scenario	2040 Worse Scenario
**Diabetes, urogenital, blood, and endocrine diseases**	**700.43 (630.70 to 794.29)**	**1,141.17 (858.57 to 1,514.73)**	**987.97 (753.67 to 1,285.50)**	**1,395.52 (1,033.04 to 1,860.68)**	**27.83 (25.35 to 31.11)**	**62.71 (48.00 to 83.13)**	**55.64 (43.13 to 72.88)**	**71.84 (54.32 to 95.01)**
Diabetes mellitus	316.32 (282.14 to 362.26)	474.21 (312.87 to 706.47)	433.44 (286.78 to 648.80)	633.95 (409.82 to 943.88)	13.76 (12.35 to 15.52)	26.67 (18.57 to 37.82)	25.11 (17.31 to 35.86)	33.32 (22.43 to 48.37)
Acute glomerulonephritis	0.59 (0.50 to 0.72)	0.68 (0.51 to 0.90)	0.65 (0.49 to 0.87)	0.73 (0.56 to 0.97)	0.01 (0.01 to 0.02)	0.03 (0.02 to 0.04)	0.03 (0.02 to 0.04)	0.03 (0.02 to 0.04)
Chronic kidney disease	264.30 (230.30 to 312.89)	498.20 (300.49 to 822.69)	386.82 (248.97 to 609.31)	588.06 (352.97 to 977.71)	9.84 (8.69 to 11.36)	27.11 (15.77 to 46.87)	21.36 (13.18 to 35.75)	29.98 (17.59 to 51.36)
Chronic kidney disease due to diabetes mellitus	156.32 (134.25 to 185.46)	304.52 (183.40 to 517.64)	234.79 (149.98 to 383.48)	362.23 (211.40 to 609.07)	5.58 (4.82 to 6.55)	14.91 (8.70 to 26.99)	11.61 (7.14 to 19.92)	16.83 (9.64 to 29.94)
Chronic kidney disease due to hypertension	62.39 (51.54 to 77.55)	142.98 (83.44 to 244.34)	111.34 (68.57 to 186.41)	163.42 (94.03 to 295.43)	3.09 (2.58 to 3.67)	9.99 (5.79 to 16.95)	7.99 (4.90 to 13.26)	10.62 (6.04 to 18.81)
Chronic kidney disease due to glomerulonephritis	23.90 (18.66 to 30.51)	23.44 (15.24 to 35.97)	19.04 (12.77 to 28.79)	29.27 (19.22 to 44.82)	0.55 (0.44 to 0.68)	0.87 (0.54 to 1.49)	0.70 (0.44 to 1.12)	1.02 (0.62 to 1.68)
Chronic kidney disease due to other and unspecified causes	21.69 (17.21 to 27.86)	27.26 (16.94 to 43.96)	21.66 (14.30 to 33.46)	33.14 (20.51 to 52.64)	0.62 (0.50 to 0.76)	1.34 (0.77 to 2.31)	1.07 (0.64 to 1.80)	1.50 (0.88 to 2.59)
Urinary diseases and male infertility	61.86 (53.22 to 72.77)	100.60 (84.52 to 120.81)	102.18 (85.38 to 122.87)	97.45 (82.16 to 117.31)	2.48 (2.15 to 2.92)	5.78 (4.89 to 6.94)	6.06 (5.09 to 7.31)	5.21 (4.40 to 6.26)
Urinary tract infections	45.78 (35.31 to 55.73)	81.42 (65.43 to 99.65)	83.31 (66.98 to 102.13)	77.22 (61.79 to 94.90)	1.83 (1.41 to 2.19)	4.69 (3.79 to 5.72)	4.95 (3.99 to 6.08)	4.15 (3.31 to 5.08)
Urolithiasis	1.84 (1.27 to 2.73)	3.81 (2.53 to 5.96)	3.90 (2.58 to 6.17)	3.62 (2.44 to 5.58)	0.08 (0.05 to 0.12)	0.21 (0.14 to 0.32)	0.22 (0.14 to 0.34)	0.19 (0.12 to 0.29)
Other urinary diseases	14.25 (8.56 to 23.57)	15.36 (8.83 to 26.14)	14.98 (8.61 to 25.37)	16.62 (9.51 to 28.59)	0.58 (0.35 to 0.97)	0.88 (0.50 to 1.51)	0.89 (0.50 to 1.52)	0.87 (0.49 to 1.53)
Gynecological diseases	0.37 (0.29 to 0.49)	0.35 (0.24 to 0.50)	0.33 (0.22 to 0.47)	0.41 (0.28 to 0.59)	0.01 (0.01 to 0.01)	0.01 (0.01 to 0.02)	0.01 (0.01 to 0.02)	0.01 (0.01 to 0.02)
Uterine fibroids	0.08 (0.04 to 0.13)	0.07 (0.03 to 0.13)	0.07 (0.03 to 0.12)	0.09 (0.04 to 0.16)	0.00 (0.00 to 0.00)	0.00 (0.00 to 0.01)	0.00 (0.00 to 0.00)	0.00 (0.00 to 0.01)
Polycystic ovarian syndrome	0.01 (0.00 to 0.03)	0.01 (0.00 to 0.02)	0.01 (0.00 to 0.01)	0.01 (0.00 to 0.02)	0.00 (0.00 to 0.00)	0.00 (0.00 to 0.00)	0.00 (0.00 to 0.00)	0.00 (0.00 to 0.00)
Endometriosis	0.01 (0.00 to 0.01)	0.01 (0.00 to 0.02)	0.01 (0.00 to 0.02)	0.01 (0.00 to 0.02)	0.00 (0.00 to 0.00)	0.00 (0.00 to 0.00)	0.00 (0.00 to 0.00)	0.00 (0.00 to 0.00)
Genital prolapse	0.02 (0.01 to 0.04)	0.02 (0.01 to 0.04)	0.02 (0.01 to 0.04)	0.03 (0.01 to 0.05)	0.00 (0.00 to 0.00)	0.00 (0.00 to 0.00)	0.00 (0.00 to 0.00)	0.00 (0.00 to 0.00)
Other gynecological diseases	0.25 (0.18 to 0.35)	0.24 (0.15 to 0.36)	0.23 (0.15 to 0.35)	0.28 (0.17 to 0.43)	0.01 (0.00 to 0.01)	0.01 (0.01 to 0.01)	0.01 (0.01 to 0.01)	0.01 (0.01 to 0.01)
Hemoglobinopathies and hemolytic anemias	47.71 (39.59 to 60.80)	56.97 (44.33 to 74.96)	54.59 (42.04 to 72.79)	64.17 (49.45 to 84.93)	1.47 (1.22 to 1.88)	2.68 (2.10 to 3.52)	2.63 (2.03 to 3.48)	2.87 (2.23 to 3.78)
Thalassemias	8.36 (4.63 to 13.78)	3.84 (1.94 to 6.75)	3.78 (1.91 to 6.60)	3.76 (1.86 to 6.63)	0.11 (0.06 to 0.17)	0.05 (0.03 to 0.08)	0.05 (0.03 to 0.08)	0.05 (0.02 to 0.08)

Sickle cell disorders	0.06 (0.04 to 0.09)	0.05 (0.03 to 0.07)	0.05 (0.03 to 0.07)	0.05 (0.03 to 0.08)	0.00 (0.00 to 0.00)	0.00 (0.00 to 0.00)	0.00 (0.00 to 0.00)	0.00 (0.00 to 0.00)
G6PD deficiency	25.84 (20.87 to 34.00)	34.67 (25.97 to 47.36)	32.71 (24.29 to 44.71)	40.62 (30.27 to 55.27)	0.66 (0.54 to 0.87)	1.28 (0.97 to 1.71)	1.22 (0.92 to 1.64)	1.45 (1.10 to 1.93)
Other hemoglobinopathies and hemolytic anemias	13.44 (10.78 to 17.05)	18.41 (13.99 to 23.87)	18.06 (13.74 to 23.81)	19.74 (15.10 to 25.84)	0.70 (0.57 to 0.91)	1.35 (1.02 to 1.80)	1.36 (1.03 to 1.81)	1.37 (1.04 to 1.83)
Endocrine, metabolic, blood, and immune disorders	9.28 (7.49 to 11.93)	10.17 (8.16 to 13.29)	9.96 (7.89 to 12.95)	10.74 (8.60 to 14.00)	0.26 (0.21 to 0.34)	0.43 (0.35 to 0.60)	0.44 (0.35 to 0.61)	0.42 (0.34 to 0.58)

Myanmar								
Cause	YLLs (in 1000s)				Deaths (in 1000s)			
	2016	2040 Forecast	2040 Better Scenario	2040 Worse Scenario	2016	2040 Forecast	2040 Better Scenario	2040 Worse Scenario
**Musculoskeletal disorders**	**19.11 (15.47 to 29.04)**	**30.31 (23.75 to 47.23)**	**31.11 (24.28 to 48.73)**	**28.70 (22.57 to 44.59)**	**0.55 (0.45 to 0.87)**	**1.31 (1.01 to 2.23)**	**1.37 (1.06 to 2.34)**	**1.19 (0.92 to 2.03)**
Rheumatoid arthritis	2.50 (1.80 to 3.74)	5.49 (3.44 to 9.02)	5.31 (3.32 to 8.70)	6.15 (3.89 to 10.07)	0.11 (0.08 to 0.18)	0.32 (0.20 to 0.56)	0.31 (0.19 to 0.55)	0.34 (0.21 to 0.60)
Other musculoskeletal disorders	16.61 (13.27 to 25.30)	24.82 (19.12 to 40.16)	25.80 (19.88 to 41.20)	22.55 (17.52 to 36.51)	0.44 (0.35 to 0.70)	0.99 (0.76 to 1.76)	1.06 (0.80 to 1.89)	0.85 (0.65 to 1.50)
**Other non-communicable diseases**	**301.01 (176.71 to 492.99)**	**223.87 (143.80 to 351.08)**	**207.41 (134.59 to 320.86)**	**262.86 (164.10 to 423.01)**	**4.32 (2.83 to 6.62)**	**5.45 (3.94 to 7.71)**	**5.43 (3.91 to 7.76)**	**5.59 (3.99 to 8.01)**
Congenital birth defects	265.05 (147.72 to 449.83)	163.34 (86.67 to 287.64)	146.44 (77.60 to 256.97)	202.77 (108.41 to 362.29)	3.28 (1.87 to 5.44)	2.14 (1.18 to 3.76)	1.94 (1.06 to 3.35)	2.62 (1.46 to 4.62)
Neural tube defects	24.58 (9.12 to 50.92)	7.03 (1.88 to 17.99)	5.97 (1.63 to 15.49)	9.95 (2.73 to 25.06)	0.29 (0.11 to 0.59)	0.08 (0.02 to 0.21)	0.07 (0.02 to 0.18)	0.12 (0.03 to 0.29)
Congenital heart anomalies	133.01 (71.41 to 241.07)	82.65 (42.17 to 150.69)	74.50 (38.53 to 137.62)	101.47 (51.74 to 187.16)	1.67 (0.92 to 3.02)	1.09 (0.58 to 2.00)	0.99 (0.53 to 1.82)	1.32 (0.70 to 2.42)
Orofacial clefts	0.45 (0.12 to 1.27)	0.23 (0.05 to 0.74)	0.19 (0.04 to 0.60)	0.34 (0.08 to 1.14)	0.01 (0.00 to 0.01)	0.00 (0.00 to 0.01)	0.00 (0.00 to 0.01)	0.00 (0.00 to 0.01)
Down syndrome	8.59 (4.24 to 18.25)	6.05 (3.00 to 12.79)	5.47 (2.74 to 11.22)	7.54 (3.68 to 16.03)	0.12 (0.06 to 0.25)	0.10 (0.05 to 0.20)	0.09 (0.05 to 0.18)	0.12 (0.06 to 0.25)
Other chromosomal abnormalities	11.27 (3.49 to 28.72)	9.75 (2.94 to 24.70)	8.57 (2.56 to 22.13)	12.55 (3.76 to 32.13)	0.13 (0.04 to 0.33)	0.12 (0.04 to 0.29)	0.10 (0.03 to 0.26)	0.15 (0.05 to 0.38)
Congenital musculoskeletal and limb anomalies	4.63 (2.01 to 9.56)	3.53 (1.30 to 7.59)	3.18 (1.17 to 6.80)	4.23 (1.57 to 9.13)	0.05 (0.02 to 0.11)	0.04 (0.02 to 0.09)	0.04 (0.01 to 0.08)	0.05 (0.02 to 0.11)
Urogenital congenital anomalies	3.97 (2.16 to 7.10)	3.42 (1.85 to 6.35)	3.20 (1.74 to 6.00)	3.95 (2.12 to 7.26)	0.07 (0.04 to 0.13)	0.08 (0.05 to 0.15)	0.08 (0.04 to 0.15)	0.09 (0.05 to 0.17)
Digestive congenital anomalies	14.58 (6.38 to 28.36)	9.25 (3.75 to 19.37)	8.15 (3.32 to 16.91)	11.84 (4.71 to 24.49)	0.17 (0.07 to 0.33)	0.11 (0.04 to 0.23)	0.10 (0.04 to 0.20)	0.14 (0.06 to 0.29)
Other congenital birth defects	63.98 (33.96 to 109.69)	41.44 (20.68 to 76.57)	37.20 (18.57 to 68.00)	50.90 (25.25 to 95.15)	0.77 (0.42 to 1.29)	0.52 (0.27 to 0.93)	0.47 (0.24 to 0.84)	0.63 (0.32 to 1.16)
Skin and subcutaneous diseases	21.47 (14.09 to 34.98)	53.08 (35.20 to 84.61)	54.34 (36.11 to 86.57)	50.93 (33.64 to 83.11)	0.87 (0.57 to 1.47)	3.22 (2.07 to 5.42)	3.42 (2.21 to 5.64)	2.86 (1.85 to 4.79)
Cellulitis	1.99 (1.09 to 4.14)	7.08 (3.84 to 14.11)	7.47 (4.15 to 14.68)	6.26 (3.46 to 13.08)	0.08 (0.05 to 0.19)	0.43 (0.24 to 0.93)	0.47 (0.27 to 1.00)	0.36 (0.20 to 0.82)
Pyoderma	11.80 (7.79 to 20.90)	28.10 (18.07 to 49.26)	28.18 (18.03 to 49.28)	28.29 (18.35 to 49.67)	0.40 (0.25 to 0.75)	1.50 (0.94 to 2.70)	1.56 (0.97 to 2.79)	1.39 (0.87 to 2.52)
Decubitus ulcer	7.06 (3.60 to 12.14)	16.80 (8.01 to 28.13)	17.54 (8.30 to 29.30)	15.35 (7.44 to 25.72)	0.37 (0.19 to 0.64)	1.24 (0.58 to 2.15)	1.33 (0.62 to 2.31)	1.06 (0.50 to 1.86)
Other skin and subcutaneous diseases	0.62 (0.34 to 1.04)	1.11 (0.61 to 1.80)	1.15 (0.63 to 1.85)	1.03 (0.56 to 1.70)	0.02 (0.01 to 0.03)	0.05 (0.03 to 0.09)	0.06 (0.03 to 0.09)	0.05 (0.03 to 0.08)
Sudden infant death syndrome	14.49 (6.32 to 27.49)	7.45 (2.90 to 15.15)	6.63 (2.56 to 13.82)	9.16 (3.59 to 18.17)	0.17 (0.07 to 0.32)	0.09 (0.03 to 0.18)	0.08 (0.03 to 0.16)	0.11 (0.04 to 0.21)

**Injuries**	**1,352.80 (1,191.24 to 1,558.65)**	**1,246.73 (1,012.87 to 1,490.10)**	**1,141.37 (928.84 to 1,356.88)**	**1,440.73 (1,170.96 to 1,710.11)**	**31.45 (28.05 to 35.92)**	**41.33 (35.43 to 47.31)**	**39.57 (33.71 to 44.70)**	**43.97 (37.75 to 50.53)**
**Transport injuries**	**587.31 (504.92 to 701.09)**	**536.42 (427.16 to 683.61)**	**457.79 (369.42 to 569.68)**	**693.09 (552.03 to 881.18)**	**13.50 (11.68 to 16.04)**	**16.70 (13.87 to 21.02)**	**14.86 (12.46 to 18.35)**	**19.92 (16.63 to 24.86)**
Road injuries	554.56 (476.55 to 661.33)	493.84 (392.19 to 634.79)	418.78 (337.28 to 529.56)	646.64 (516.90 to 822.70)	12.75 (11.02 to 15.20)	15.29 (12.66 to 19.27)	13.53 (11.37 to 16.76)	18.49 (15.38 to 23.10)
Pedestrian road injuries	145.29 (113.48 to 194.41)	131.59 (98.99 to 188.60)	115.26 (87.50 to 164.98)	164.33 (123.42 to 233.50)	3.97 (3.18 to 5.38)	5.34 (4.12 to 7.38)	4.86 (3.78 to 6.73)	6.17 (4.77 to 8.44)

Myanmar								
Cause	YLLs (in 1000s)				Deaths (in 1000s)			
	2016	2040 Forecast	2040 Better Scenario	2040 Worse Scenario	2016	2040 Forecast	2040 Better Scenario	2040 Worse Scenario
Cyclist road injuries	23.49 (14.68 to 38.55)	23.16 (13.87 to 37.76)	19.90 (11.90 to 32.95)	29.59 (17.71 to 48.80)	0.60 (0.37 to 1.03)	0.78 (0.45 to 1.37)	0.70 (0.41 to 1.22)	0.92 (0.53 to 1.63)
Motorcyclist road injuries	125.08 (93.62 to 163.18)	109.09 (75.35 to 153.42)	93.27 (65.19 to 129.01)	137.06 (96.12 to 189.43)	2.38 (1.79 to 3.12)	2.35 (1.66 to 3.25)	2.05 (1.46 to 2.80)	2.84 (2.02 to 3.90)
Motor vehicle road injuries	249.09 (191.92 to 317.39)	218.26 (160.26 to 295.70)	180.50 (134.25 to 241.15)	300.14 (221.89 to 403.12)	5.52 (4.34 to 6.91)	6.42 (4.92 to 8.46)	5.56 (4.33 to 7.21)	8.08 (6.26 to 10.60)
Other road injuries	11.61 (7.92 to 20.98)	11.74 (7.52 to 21.36)	9.86 (6.39 to 17.97)	15.52 (10.06 to 28.34)	0.28 (0.19 to 0.50)	0.40 (0.26 to 0.76)	0.36 (0.23 to 0.67)	0.47 (0.30 to 0.87)
Other transport injuries	32.75 (18.28 to 48.87)	42.58 (22.91 to 65.60)	39.01 (21.45 to 59.84)	46.45 (24.60 to 71.69)	0.75 (0.42 to 1.10)	1.40 (0.74 to 2.12)	1.33 (0.71 to 1.96)	1.43 (0.75 to 2.16)
**Unintentional injuries**	**563.40 (488.85 to 660.27)**	**477.44 (401.92 to 564.57)**	**465.16 (390.03 to 552.62)**	**508.03 (426.44 to 607.10)**	**13.75 (12.17 to 15.70)**	**18.99 (15.71 to 21.92)**	**19.34 (15.93 to 22.38)**	**18.40 (15.42 to 21.33)**
Falls	189.60 (155.98 to 232.43)	240.26 (196.93 to 286.45)	242.12 (197.64 to 288.14)	237.89 (195.84 to 281.16)	6.42 (5.38 to 7.60)	12.41 (9.56 to 15.02)	12.94 (9.96 to 15.59)	11.37 (8.81 to 13.67)
Drowning	118.76 (93.78 to 148.69)	62.08 (46.56 to 83.44)	56.96 (43.00 to 75.93)	71.81 (53.58 to 97.92)	1.97 (1.62 to 2.41)	1.30 (1.03 to 1.67)	1.22 (0.97 to 1.54)	1.44 (1.14 to 1.86)
Fire, heat, and hot substances	40.96 (24.70 to 57.11)	26.68 (16.54 to 37.46)	24.64 (15.34 to 34.49)	30.81 (18.77 to 44.19)	0.84 (0.55 to 1.08)	0.79 (0.54 to 1.00)	0.75 (0.53 to 0.96)	0.84 (0.58 to 1.09)
Poisonings	18.79 (12.94 to 25.70)	8.75 (5.83 to 12.40)	7.89 (5.26 to 11.18)	10.89 (7.03 to 15.72)	0.39 (0.28 to 0.51)	0.25 (0.18 to 0.34)	0.23 (0.16 to 0.31)	0.29 (0.20 to 0.40)
Exposure to mechanical forces	41.73 (32.73 to 56.66)	26.01 (19.46 to 36.50)	24.38 (18.18 to 34.40)	30.63 (22.60 to 43.32)	0.82 (0.66 to 1.11)	0.68 (0.53 to 0.94)	0.66 (0.51 to 0.91)	0.76 (0.59 to 1.05)
Unintentional firearm injuries	6.32 (4.10 to 13.44)	3.92 (2.39 to 8.42)	3.67 (2.23 to 7.93)	4.65 (2.80 to 9.98)	0.13 (0.09 to 0.28)	0.11 (0.08 to 0.23)	0.11 (0.07 to 0.22)	0.13 (0.08 to 0.26)
Unintentional suffocation	8.12 (4.95 to 13.37)	4.16 (2.54 to 6.89)	3.74 (2.26 to 6.14)	5.28 (3.16 to 8.66)	0.12 (0.08 to 0.18)	0.07 (0.04 to 0.10)	0.06 (0.04 to 0.09)	0.08 (0.05 to 0.13)
Other exposure to mechanical forces	27.29 (21.51 to 35.70)	17.93 (13.48 to 24.22)	16.97 (12.71 to 22.95)	20.70 (15.43 to 28.23)	0.57 (0.46 to 0.74)	0.50 (0.39 to 0.66)	0.49 (0.38 to 0.64)	0.55 (0.43 to 0.72)
Adverse effects of medical treatment	47.09 (36.48 to 57.05)	40.19 (29.82 to 51.65)	38.76 (28.63 to 49.76)	44.44 (33.03 to 56.75)	1.20 (0.92 to 1.48)	1.55 (1.17 to 2.01)	1.54 (1.17 to 2.01)	1.60 (1.21 to 2.07)
Animal contact	13.89 (7.89 to 20.43)	12.38 (6.95 to 18.51)	11.58 (6.47 to 17.48)	14.80 (8.19 to 22.65)	0.32 (0.19 to 0.46)	0.40 (0.24 to 0.57)	0.39 (0.23 to 0.57)	0.45 (0.27 to 0.66)
Venomous animal contact	6.92 (3.89 to 11.32)	5.88 (3.34 to 9.59)	5.48 (3.07 to 8.93)	7.06 (3.98 to 11.68)	0.17 (0.10 to 0.27)	0.20 (0.12 to 0.32)	0.19 (0.11 to 0.31)	0.23 (0.13 to 0.36)
Non-venomous animal contact	6.97 (3.71 to 10.66)	6.51 (3.42 to 10.48)	6.10 (3.20 to 9.87)	7.74 (4.00 to 12.49)	0.16 (0.09 to 0.23)	0.20 (0.11 to 0.31)	0.19 (0.11 to 0.29)	0.22 (0.12 to 0.34)
Foreign body	47.38 (33.28 to 70.55)	32.66 (23.03 to 46.15)	31.80 (22.67 to 44.52)	34.32 (24.01 to 49.91)	0.88 (0.67 to 1.16)	0.91 (0.70 to 1.10)	0.92 (0.71 to 1.11)	0.87 (0.66 to 1.08)
Pulmonary aspiration and foreign body in airway	42.19 (28.27 to 64.52)	28.99 (19.77 to 41.92)	28.29 (19.42 to 40.45)	30.23 (20.01 to 45.31)	0.76 (0.55 to 1.04)	0.79 (0.57 to 0.99)	0.81 (0.59 to 1.00)	0.75 (0.54 to 0.96)

Foreign body in other body part	5.19 (3.27 to 9.90)	3.67 (2.30 to 6.83)	3.51 (2.19 to 6.50)	4.09 (2.52 to 7.54)	0.11 (0.08 to 0.21)	0.11 (0.08 to 0.20)	0.11 (0.08 to 0.20)	0.12 (0.08 to 0.21)
Environmental heat and cold exposure	2.91 (2.25 to 4.03)	2.54 (1.85 to 3.66)	2.35 (1.65 to 3.46)	3.17 (2.26 to 4.64)	0.08 (0.06 to 0.11)	0.10 (0.07 to 0.14)	0.09 (0.07 to 0.14)	0.11 (0.08 to 0.16)
Other unintentional injuries	42.29 (32.37 to 56.41)	25.89 (18.84 to 35.78)	24.69 (17.86 to 34.42)	29.27 (21.06 to 40.88)	0.84 (0.65 to 1.12)	0.61 (0.46 to 0.82)	0.59 (0.44 to 0.80)	0.67 (0.50 to 0.90)
**Self-harm and interpersonal violence**	**201.52 (165.26 to 244.70)**	**183.89 (132.43 to 249.11)**	**169.91 (126.17 to 223.39)**	**190.08 (136.99 to 259.01)**	**4.19 (3.47 to 5.07)**	**4.58 (3.43 to 6.04)**	**4.30 (3.31 to 5.45)**	**4.61 (3.45 to 6.11)**

Myanmar								
Cause	YLLs (in 1000s)				Deaths (in 1000s)			
	2016	2040 Forecast	2040 Better Scenario	2040 Worse Scenario	2016	2040 Forecast	2040 Better Scenario	2040 Worse Scenario
Self-harm	138.39 (111.51 to 171.91)	139.08 (97.45 to 192.62)	126.32 (91.38 to 168.32)	144.49 (101.20 to 199.28)	3.01 (2.48 to 3.73)	3.66 (2.67 to 4.94)	3.39 (2.57 to 4.36)	3.68 (2.67 to 5.04)
Self-harm by firearm	6.86 (2.94 to 11.40)	6.49 (2.47 to 12.60)	5.70 (2.29 to 10.92)	6.87 (2.65 to 13.53)	0.15 (0.07 to 0.24)	0.18 (0.07 to 0.33)	0.16 (0.07 to 0.29)	0.18 (0.07 to 0.34)
Self-harm by other specified means	131.53 (106.55 to 163.17)	132.59 (93.97 to 181.01)	120.62 (88.13 to 160.34)	137.62 (96.81 to 190.05)	2.86 (2.35 to 3.53)	3.48 (2.57 to 4.67)	3.23 (2.47 to 4.14)	3.50 (2.57 to 4.74)
Interpersonal violence	63.14 (41.73 to 84.92)	44.80 (27.91 to 63.84)	43.59 (27.34 to 61.50)	45.59 (28.28 to 65.72)	1.18 (0.80 to 1.57)	0.93 (0.61 to 1.29)	0.91 (0.60 to 1.25)	0.93 (0.61 to 1.30)
Physical violence by firearm	16.50 (8.83 to 31.37)	11.49 (5.76 to 22.33)	11.35 (5.71 to 21.97)	11.75 (5.84 to 22.93)	0.31 (0.17 to 0.59)	0.23 (0.12 to 0.45)	0.23 (0.12 to 0.43)	0.23 (0.12 to 0.45)
Physical violence by sharp object	23.85 (14.41 to 34.69)	18.00 (10.23 to 26.47)	18.03 (10.23 to 26.55)	17.76 (10.05 to 26.34)	0.44 (0.27 to 0.63)	0.36 (0.22 to 0.52)	0.36 (0.22 to 0.52)	0.35 (0.21 to 0.51)
Physical violence by other means	22.79 (13.04 to 33.31)	15.31 (8.10 to 24.57)	14.20 (7.48 to 22.41)	16.07 (8.49 to 25.68)	0.43 (0.25 to 0.63)	0.34 (0.20 to 0.53)	0.32 (0.19 to 0.49)	0.35 (0.21 to 0.55)
**Forces of nature, conflict and terrorism, and executions and police conflict**	**0.57 (0.45 to 0.74)**	**48.98 (0.58 to 101.50)**	**48.52 (0.57 to 100.76)**	**49.54 (0.59 to 102.85)**	**0.01 (0.01 to 0.02)**	**1.07 (0.01 to 2.11)**	**1.08 (0.01 to 2.13)**	**1.05 (0.01 to 2.07)**
Exposure to forces of nature	0.00 (0.00 to 0.00)	32.15 (0.00 to 32.37)	31.86 (0.00 to 32.24)	32.46 (0.00 to 32.69)	0.00 (0.00 to 0.00)	0.73 (0.00 to 0.74)	0.74 (0.00 to 0.75)	0.72 (0.00 to 0.72)
Conflict and terrorism	0.00 (0.00 to 0.00)	16.78 (0.28 to 68.56)	16.60 (0.27 to 67.80)	17.02 (0.28 to 69.61)	0.00 (0.00 to 0.00)	0.33 (0.01 to 1.37)	0.33 (0.01 to 1.37)	0.33 (0.01 to 1.35)
Executions and police conflict	0.57 (0.45 to 0.74)	0.05 (0.00 to 0.61)	0.05 (0.00 to 0.61)	0.05 (0.00 to 0.62)	0.01 (0.01 to 0.02)	0.00 (0.00 to 0.01)	0.00 (0.00 to 0.01)	0.00 (0.00 to 0.01)

Philippines								
**Cause**	**YLLs (in 1000s)**				**Deaths (in 1000s)**			
	**2016**	**2040 Forecast**	**2040 Better Scenario**	**2040 Worse Scenario**	**2016**	**2040 Forecast**	**2040 Better Scenario**	**2040 Worse Scenario**
**All causes**	**20,663.31 (18,600.34 to 22,926.65)**	**24,958.40 (21,054.78 to 30,158.97)**	**18,596.58 (15,563.75 to 22,368.43)**	**32,644.24 (27,513.69 to 38,727.98)**	**626.89 (560.15 to 696.38)**	**1,043.23 (914.25 to 1,218.20)**	**845.19 (733.20 to 994.06)**	**1,286.69 (1,128.36 to 1,462.20)**
**Communicable, maternal, neonatal, and nutritional diseases**	**6,620.14 (5,867.17 to 7,424.92)**	**5,672.84 (4,689.67 to 6,858.83)**	**3,582.91 (3,011.77 to 4,230.00)**	**7,807.05 (6,385.47 to 9,616.83)**	**144.32 (130.43 to 158.73)**	**182.44 (148.81 to 222.28)**	**133.56 (109.14 to 158.94)**	**232.96 (187.15 to 287.22)**
**HIV/AIDS and tuberculosis**	**1,067.29 (941.93 to 1,206.49)**	**922.00 (659.51 to 1,282.30)**	**475.98 (317.44 to 695.59)**	**1,614.61 (1,112.13 to 2,339.82)**	**31.76 (27.71 to 36.12)**	**31.10 (21.60 to 43.52)**	**18.08 (11.96 to 26.86)**	**50.57 (33.75 to 73.49)**
Tuberculosis	857.72 (732.25 to 997.52)	659.35 (412.17 to 995.83)	408.18 (252.55 to 624.88)	1,060.75 (615.90 to 1,679.65)	28.10 (24.13 to 32.49)	26.14 (16.93 to 38.56)	17.10 (10.79 to 25.68)	39.78 (24.14 to 62.01)
HIV/AIDS	209.57 (190.94 to 229.87)	262.65 (162.48 to 364.25)	67.80 (49.96 to 90.53)	553.86 (370.34 to 955.07)	3.67 (3.34 to 4.00)	4.96 (2.85 to 7.12)	0.98 (0.69 to 1.37)	10.79 (6.91 to 18.79)
**Diarrhea, lower respiratory, and other common infectious diseases**	**2,790.87 (2,478.43 to 3,124.49)**	**2,461.76 (1,797.96 to 3,380.88)**	**1,593.89 (1,230.13 to 2,062.52)**	**3,328.26 (2,344.83 to 4,725.11)**	**75.51 (67.54 to 83.61)**	**114.94 (83.87 to 152.72)**	**88.17 (66.87 to 111.75)**	**139.53 (98.61 to 190.90)**
Diarrheal diseases	378.63 (277.98 to 515.14)	174.81 (38.59 to 487.33)	109.16 (27.39 to 307.29)	300.69 (62.58 to 799.97)	6.85 (5.18 to 8.88)	5.50 (1.34 to 15.64)	4.23 (1.11 to 11.87)	8.80 (1.99 to 25.86)
Intestinal infectious diseases	203.02 (109.81 to 343.40)	158.15 (81.49 to 286.96)	124.65 (63.68 to 221.93)	176.01 (89.16 to 318.75)	2.93 (1.59 to 5.02)	2.43 (1.28 to 4.40)	1.99 (1.05 to 3.55)	2.67 (1.39 to 4.85)
Typhoid fever	177.60 (94.30 to 306.29)	134.88 (67.06 to 244.05)	107.20 (53.96 to 189.07)	149.76 (74.56 to 268.05)	2.55 (1.36 to 4.38)	2.06 (1.05 to 3.67)	1.70 (0.88 to 2.95)	2.26 (1.15 to 4.01)
Paratyphoid fever	24.57 (11.15 to 47.66)	22.99 (9.64 to 47.00)	17.25 (7.23 to 34.91)	25.91 (10.76 to 53.37)	0.36 (0.17 to 0.71)	0.37 (0.16 to 0.74)	0.28 (0.12 to 0.57)	0.41 (0.17 to 0.84)
Other intestinal infectious diseases	0.85 (0.31 to 1.89)	0.28 (0.10 to 0.66)	0.20 (0.07 to 0.46)	0.34 (0.12 to 0.80)	0.01 (0.01 to 0.03)	0.01 (0.00 to 0.02)	0.01 (0.00 to 0.02)	0.01 (0.00 to 0.02)
Lower respiratory infections	1,873.68 (1,649.88 to 2,102.83)	1,815.00 (1,259.96 to 2,569.61)	1,164.57 (874.68 to 1,528.61)	2,444.11 (1,596.77 to 3,655.57)	60.75 (54.00 to 67.85)	101.96 (72.26 to 138.84)	78.39 (57.87 to 102.14)	121.87 (82.42 to 171.86)
Upper respiratory infections	1.16 (0.52 to 2.20)	0.98 (0.45 to 1.85)	0.66 (0.29 to 1.25)	1.24 (0.55 to 2.39)	0.02 (0.01 to 0.04)	0.03 (0.01 to 0.05)	0.02 (0.01 to 0.04)	0.04 (0.02 to 0.06)
Otitis media	0.64 (0.45 to 0.89)	0.21 (0.13 to 0.34)	0.20 (0.12 to 0.32)	0.21 (0.13 to 0.35)	0.01 (0.01 to 0.01)	0.01 (0.00 to 0.01)	0.01 (0.00 to 0.01)	0.00 (0.00 to 0.01)
Meningitis	211.96 (176.28 to 251.32)	109.50 (67.54 to 151.29)	80.37 (55.19 to 107.44)	120.18 (71.80 to 170.37)	3.02 (2.55 to 3.52)	1.94 (1.40 to 2.51)	1.57 (1.20 to 1.99)	2.04 (1.44 to 2.67)
Pneumococcal meningitis	1.04 (0.80 to 1.33)	0.39 (0.24 to 0.56)	0.31 (0.21 to 0.43)	0.41 (0.25 to 0.60)	0.01 (0.01 to 0.02)	0.01 (0.01 to 0.01)	0.01 (0.01 to 0.01)	0.01 (0.01 to 0.01)
H influenzae type B meningitis	18.56 (14.86 to 22.90)	17.57 (5.14 to 46.45)	8.12 (4.16 to 15.95)	20.21 (5.20 to 55.91)	0.26 (0.21 to 0.31)	0.25 (0.10 to 0.58)	0.14 (0.09 to 0.23)	0.28 (0.10 to 0.69)
Meningococcal meningitis	72.46 (60.08 to 86.23)	32.98 (21.90 to 44.06)	25.88 (17.71 to 34.29)	36.00 (23.13 to 48.26)	1.04 (0.89 to 1.23)	0.63 (0.48 to 0.79)	0.54 (0.41 to 0.68)	0.66 (0.49 to 0.84)
Other meningitis	119.91 (99.11 to 142.29)	58.56 (39.07 to 78.34)	46.05 (31.98 to 61.61)	63.56 (41.52 to 85.79)	1.70 (1.43 to 1.99)	1.05 (0.79 to 1.34)	0.89 (0.67 to 1.14)	1.10 (0.81 to 1.41)
Encephalitis	86.46 (44.25 to 124.96)	59.14 (32.17 to 87.58)	45.23 (26.17 to 65.50)	67.12 (34.79 to 100.95)	1.25 (0.69 to 1.72)	1.05 (0.66 to 1.45)	0.87 (0.56 to 1.17)	1.16 (0.72 to 1.61)

Diphtheria	0.47 (0.26 to 0.78)	0.11 (0.04 to 0.23)	0.06 (0.03 to 0.09)	0.12 (0.05 to 0.26)	0.01 (0.00 to 0.01)	0.00 (0.00 to 0.00)	0.00 (0.00 to 0.00)	0.00 (0.00 to 0.00)
Whooping cough	2.69 (1.21 to 7.00)	1.91 (0.37 to 6.12)	0.41 (0.16 to 0.88)	2.12 (0.36 to 6.95)	0.04 (0.02 to 0.09)	0.02 (0.01 to 0.08)	0.01 (0.00 to 0.01)	0.03 (0.01 to 0.08)
Tetanus	22.82 (14.75 to 32.38)	4.26 (2.45 to 6.65)	3.45 (1.82 to 5.46)	4.39 (2.53 to 6.83)	0.49 (0.32 to 0.70)	0.09 (0.06 to 0.14)	0.09 (0.05 to 0.13)	0.09 (0.06 to 0.14)
Measles	3.57 (2.03 to 7.18)	134.81 (2.13 to 577.90)	63.16 (1.51 to 226.52)	208.45 (2.40 to 891.69)	0.04 (0.02 to 0.09)	1.83 (0.03 to 7.08)	0.93 (0.02 to 3.01)	2.75 (0.04 to 10.97)

Philippines								
Cause	YLLs (in 1000s)				Deaths (in 1000s)			
	2016	2040 Forecast	2040 Better Scenario	2040 Worse Scenario	2016	2040 Forecast	2040 Better Scenario	2040 Worse Scenario
Varicella and herpes zoster	5.77 (2.33 to 9.16)	2.89 (1.02 to 5.02)	1.97 (0.67 to 3.31)	3.61 (1.27 to 6.26)	0.11 (0.04 to 0.15)	0.08 (0.02 to 0.12)	0.07 (0.02 to 0.10)	0.09 (0.03 to 0.14)
**Neglected tropical diseases and malaria**	**267.55 (172.90 to 381.26)**	**693.88 (488.84 to 912.70)**	**541.67 (397.05 to 694.45)**	**763.81 (528.22 to 1,024.12)**	**3.83 (2.56 to 5.34)**	**10.53 (7.63 to 13.70)**	**8.64 (6.50 to 11.10)**	**11.37 (8.10 to 14.88)**
Malaria	8.62 (3.76 to 30.83)	6.57 (2.27 to 30.68)	6.12 (2.11 to 28.75)	6.81 (2.37 to 31.48)	0.19 (0.08 to 0.71)	0.17 (0.06 to 0.79)	0.16 (0.06 to 0.77)	0.17 (0.06 to 0.80)
Chagas disease	0.00 (0.00 to 0.00)	0.00 (0.00 to 0.00)	0.00 (0.00 to 0.00)	0.00 (0.00 to 0.00)	0.00 (0.00 to 0.00)	0.00 (0.00 to 0.00)	0.00 (0.00 to 0.00)	0.00 (0.00 to 0.00)
Leishmaniasis	0.00 (0.00 to 0.00)	0.00 (0.00 to 0.00)	0.00 (0.00 to 0.00)	0.00 (0.00 to 0.00)	0.00 (0.00 to 0.00)	0.00 (0.00 to 0.00)	0.00 (0.00 to 0.00)	0.00 (0.00 to 0.00)
African trypanosomiasis	0.00 (0.00 to 0.00)	0.00 (0.00 to 0.00)	0.00 (0.00 to 0.00)	0.00 (0.00 to 0.00)	0.00 (0.00 to 0.00)	0.00 (0.00 to 0.00)	0.00 (0.00 to 0.00)	0.00 (0.00 to 0.00)
Schistosomiasis	3.81 (2.55 to 5.41)	2.80 (1.65 to 4.52)	2.48 (1.46 to 4.01)	3.04 (1.84 to 4.76)	0.12 (0.08 to 0.17)	0.11 (0.06 to 0.17)	0.10 (0.06 to 0.16)	0.11 (0.07 to 0.18)
Cysticercosis	0.24 (0.21 to 0.29)	0.10 (0.08 to 0.14)	0.09 (0.07 to 0.13)	0.11 (0.08 to 0.15)	0.01 (0.00 to 0.01)	0.00 (0.00 to 0.00)	0.00 (0.00 to 0.00)	0.00 (0.00 to 0.00)
Cystic echinococcosis	0.05 (0.04 to 0.06)	0.03 (0.02 to 0.04)	0.02 (0.01 to 0.02)	0.04 (0.03 to 0.05)	0.00 (0.00 to 0.00)	0.00 (0.00 to 0.00)	0.00 (0.00 to 0.00)	0.00 (0.00 to 0.00)
Dengue	234.23 (133.31 to 349.73)	670.62 (460.70 to 891.38)	523.77 (375.73 to 675.56)	735.79 (492.14 to 993.71)	3.16 (1.87 to 4.58)	9.98 (7.10 to 12.92)	8.18 (6.08 to 10.39)	10.75 (7.49 to 14.05)
Yellow fever	0.00 (0.00 to 0.00)	0.00 (0.00 to 0.00)	0.00 (0.00 to 0.00)	0.00 (0.00 to 0.00)	0.00 (0.00 to 0.00)	0.00 (0.00 to 0.00)	0.00 (0.00 to 0.00)	0.00 (0.00 to 0.00)
Rabies	12.74 (8.00 to 19.54)	6.28 (3.48 to 10.53)	3.54 (2.11 to 5.78)	9.53 (5.46 to 15.82)	0.24 (0.16 to 0.34)	0.13 (0.07 to 0.20)	0.08 (0.05 to 0.11)	0.19 (0.11 to 0.29)
Intestinal nematode infections	3.43 (2.25 to 4.88)	3.96 (2.44 to 6.27)	3.10 (1.93 to 5.09)	4.22 (2.65 to 6.68)	0.05 (0.03 to 0.06)	0.06 (0.04 to 0.09)	0.05 (0.03 to 0.08)	0.06 (0.04 to 0.09)
Other neglected tropical diseases	4.42 (2.33 to 13.90)	3.52 (1.80 to 12.28)	2.55 (1.35 to 8.86)	4.27 (2.18 to 14.92)	0.08 (0.04 to 0.24)	0.08 (0.04 to 0.30)	0.07 (0.03 to 0.26)	0.09 (0.05 to 0.32)
**Maternal disorders**	**104.26 (77.78 to 133.99)**	**70.15 (42.52 to 105.73)**	**48.28 (28.55 to 74.28)**	**94.43 (55.80 to 143.26)**	**1.86 (1.39 to 2.39)**	**1.26 (0.76 to 1.89)**	**0.87 (0.51 to 1.32)**	**1.69 (1.00 to 2.56)**
Maternal hemorrhage	40.37 (27.73 to 56.14)	26.54 (14.88 to 42.47)	18.37 (10.15 to 29.60)	35.63 (19.28 to 57.21)	0.72 (0.49 to 0.99)	0.48 (0.27 to 0.76)	0.33 (0.18 to 0.54)	0.64 (0.34 to 1.02)
Maternal sepsis and other maternal infections	11.85 (7.84 to 17.16)	6.90 (3.61 to 11.80)	4.74 (2.44 to 8.32)	9.33 (4.74 to 16.26)	0.21 (0.14 to 0.30)	0.12 (0.07 to 0.21)	0.09 (0.04 to 0.15)	0.17 (0.09 to 0.29)
Maternal hypertensive disorders	16.62 (10.76 to 23.66)	12.23 (6.37 to 19.85)	8.39 (4.22 to 13.94)	16.47 (8.63 to 27.12)	0.29 (0.19 to 0.42)	0.22 (0.11 to 0.35)	0.15 (0.08 to 0.25)	0.29 (0.15 to 0.48)
Maternal obstructed labor and uterine rupture	2.18 (1.08 to 3.56)	1.32 (0.54 to 2.50)	0.88 (0.36 to 1.69)	1.81 (0.74 to 3.61)	0.04 (0.02 to 0.06)	0.02 (0.01 to 0.05)	0.02 (0.01 to 0.03)	0.03 (0.01 to 0.06)
Maternal abortion, miscarriage, and ectopic pregnancy	13.97 (9.11 to 20.50)	9.76 (4.92 to 16.61)	6.71 (3.37 to 11.84)	13.18 (6.54 to 23.51)	0.25 (0.16 to 0.36)	0.18 (0.09 to 0.30)	0.12 (0.06 to 0.21)	0.24 (0.12 to 0.42)

Indirect maternal deaths	5.56 (3.30 to 8.78)	4.28 (2.05 to 7.59)	2.91 (1.36 to 5.26)	5.81 (2.73 to 10.37)	0.10 (0.06 to 0.15)	0.08 (0.04 to 0.13)	0.05 (0.02 to 0.09)	0.10 (0.05 to 0.18)
Late maternal deaths	0.09 (0.04 to 0.15)	0.06 (0.03 to 0.12)	0.04 (0.02 to 0.08)	0.08 (0.04 to 0.16)	0.00 (0.00 to 0.00)	0.00 (0.00 to 0.00)	0.00 (0.00 to 0.00)	0.00 (0.00 to 0.00)
Maternal deaths aggravated by HIV/AIDS	0.06 (0.02 to 0.13)	0.19 (0.05 to 0.49)	0.03 (0.00 to 0.17)	0.26 (0.07 to 0.70)	0.00 (0.00 to 0.00)	0.00 (0.00 to 0.01)	0.00 (0.00 to 0.00)	0.00 (0.00 to 0.01)
Other maternal disorders	13.57 (8.75 to 19.74)	8.87 (4.89 to 15.17)	6.21 (3.30 to 10.60)	11.85 (6.26 to 20.56)	0.24 (0.16 to 0.35)	0.16 (0.09 to 0.27)	0.11 (0.06 to 0.19)	0.21 (0.11 to 0.37)

Philippines								
**Cause**	**YLLs (in 1000s)**				**Deaths (in 1000s)**			
	**2016**	**2040 Forecast**	**2040 Better Scenario**	**2040 Worse Scenario**	**2016**	**2040 Forecast**	**2040 Better Scenario**	**2040 Worse Scenario**
**Neonatal disorders**	**2,105.47 (1,717.19 to 2,540.38)**	**1,301.72 (940.20 to 1,746.00)**	**737.32 (551.14 to 969.18)**	**1,758.26 (1,286.63 to 2,350.88)**	**24.33 (19.84 to 29.35)**	**15.04 (10.87 to 20.18)**	**8.52 (6.37 to 11.20)**	**20.32 (14.87 to 27.17)**
Neonatal preterm birth	830.99 (617.66 to 1,059.19)	429.39 (290.95 to 595.34)	220.85 (147.76 to 307.79)	615.05 (416.85 to 851.80)	9.60 (7.13 to 12.24)	4.96 (3.36 to 6.88)	2.55 (1.71 to 3.56)	7.11 (4.82 to 9.84)
Neonatal encephalopathy due to birth asphyxia and trauma	443.93 (328.46 to 569.75)	248.53 (151.56 to 373.41)	148.21 (90.46 to 219.59)	321.46 (197.11 to 486.55)	5.13 (3.80 to 6.58)	2.87 (1.75 to 4.32)	1.71 (1.05 to 2.54)	3.72 (2.28 to 5.62)
Neonatal sepsis and other neonatal infections	698.56 (517.61 to 983.78)	524.69 (334.19 to 772.63)	311.33 (200.71 to 456.35)	686.33 (438.77 to 1,018.95)	8.07 (5.98 to 11.37)	6.06 (3.86 to 8.93)	3.60 (2.32 to 5.27)	7.93 (5.07 to 11.78)
Hemolytic disease and other neonatal jaundice	9.60 (5.90 to 14.64)	4.64 (2.33 to 8.19)	2.63 (1.34 to 4.63)	6.32 (3.13 to 11.22)	0.11 (0.07 to 0.17)	0.05 (0.03 to 0.09)	0.03 (0.02 to 0.05)	0.07 (0.04 to 0.13)
Other neonatal disorders	122.38 (88.70 to 164.48)	94.47 (56.33 to 146.83)	54.31 (32.99 to 84.06)	129.09 (75.55 to 198.04)	1.41 (1.03 to 1.90)	1.09 (0.65 to 1.70)	0.63 (0.38 to 0.97)	1.49 (0.87 to 2.29)
**Nutritional deficiencies**	**151.49 (124.27 to 186.57)**	**113.32 (90.86 to 143.94)**	**95.55 (78.35 to 117.93)**	**123.94 (98.72 to 159.20)**	**4.46 (3.84 to 5.29)**	**6.44 (4.59 to 8.41)**	**6.51 (4.79 to 8.36)**	**6.13 (4.25 to 8.29)**
Protein-energy malnutrition	135.07 (108.44 to 170.07)	85.07 (64.10 to 113.20)	70.27 (53.77 to 91.08)	94.50 (69.82 to 126.10)	3.69 (3.07 to 4.52)	4.51 (3.04 to 6.31)	4.54 (3.08 to 6.22)	4.33 (2.85 to 6.04)
Iodine deficiency	1.09 (0.05 to 4.71)	3.19 (0.08 to 12.01)	2.49 (0.06 to 9.33)	3.69 (0.10 to 13.47)	0.02 (0.00 to 0.08)	0.16 (0.00 to 0.88)	0.15 (0.00 to 0.88)	0.16 (0.00 to 0.86)
Dietary iron deficiency	0.77 (0.50 to 1.55)	0.81 (0.51 to 1.53)	0.67 (0.42 to 1.22)	0.90 (0.56 to 1.74)	0.01 (0.01 to 0.03)	0.02 (0.01 to 0.03)	0.02 (0.01 to 0.03)	0.02 (0.01 to 0.04)
Other nutritional deficiencies	14.57 (9.07 to 23.21)	24.25 (14.47 to 36.14)	22.11 (12.46 to 33.04)	24.84 (14.99 to 36.96)	0.73 (0.39 to 1.24)	1.75 (0.89 to 2.80)	1.80 (0.91 to 2.85)	1.61 (0.81 to 2.61)
**Other communicable, maternal, neonatal, and nutritional diseases**	**133.21 (95.80 to 185.19)**	**110.01 (83.70 to 141.70)**	**90.21 (69.96 to 115.74)**	**123.74 (93.95 to 160.64)**	**2.57 (2.04 to 3.34)**	**3.13 (2.52 to 3.87)**	**2.78 (2.24 to 3.51)**	**3.34 (2.66 to 4.22)**
Sexually transmitted infections excluding HIV	54.84 (22.64 to 106.04)	14.89 (5.25 to 32.46)	9.75 (3.73 to 20.21)	17.87 (6.19 to 38.78)	0.67 (0.30 to 1.27)	0.20 (0.09 to 0.41)	0.14 (0.07 to 0.27)	0.24 (0.10 to 0.49)
Syphilis	53.45 (21.13 to 104.69)	13.80 (4.30 to 31.38)	8.77 (2.78 to 19.23)	16.70 (5.17 to 37.72)	0.64 (0.26 to 1.24)	0.17 (0.06 to 0.38)	0.11 (0.04 to 0.23)	0.20 (0.07 to 0.46)
Chlamydial infection	0.27 (0.21 to 0.35)	0.22 (0.15 to 0.29)	0.20 (0.14 to 0.26)	0.23 (0.17 to 0.31)	0.01 (0.01 to 0.01)	0.01 (0.01 to 0.01)	0.01 (0.00 to 0.01)	0.01 (0.01 to 0.01)
Gonococcal infection	0.72 (0.55 to 0.91)	0.57 (0.41 to 0.76)	0.52 (0.37 to 0.70)	0.61 (0.44 to 0.82)	0.02 (0.01 to 0.02)	0.02 (0.01 to 0.02)	0.02 (0.01 to 0.02)	0.02 (0.01 to 0.02)
Other sexually transmitted infections	0.39 (0.29 to 0.49)	0.29 (0.21 to 0.40)	0.26 (0.18 to 0.36)	0.32 (0.22 to 0.43)	0.01 (0.01 to 0.01)	0.01 (0.01 to 0.01)	0.01 (0.01 to 0.01)	0.01 (0.01 to 0.01)
Acute hepatitis	37.79 (31.23 to 46.07)	40.56 (29.92 to 52.98)	34.45 (25.01 to 46.62)	44.56 (32.85 to 59.14)	0.94 (0.78 to 1.15)	1.21 (0.92 to 1.56)	1.07 (0.81 to 1.41)	1.29 (0.98 to 1.68)
Acute hepatitis A	2.19 (1.33 to 3.12)	1.37 (0.83 to 2.25)	1.00 (0.62 to 1.54)	1.61 (0.95 to 2.65)	0.03 (0.02 to 0.04)	0.03 (0.02 to 0.04)	0.02 (0.01 to 0.03)	0.03 (0.02 to 0.05)
Acute hepatitis B	30.65 (24.75 to 37.85)	34.64 (24.97 to 46.26)	29.60 (20.80 to 40.86)	38.09 (27.24 to 51.57)	0.80 (0.65 to 0.99)	1.04 (0.78 to 1.37)	0.91 (0.66 to 1.23)	1.12 (0.83 to 1.49)

Acute hepatitis C	0.58 (0.42 to 0.81)	0.78 (0.51 to 1.15)	0.71 (0.47 to 1.08)	0.79 (0.52 to 1.17)	0.02 (0.01 to 0.02)	0.03 (0.02 to 0.04)	0.03 (0.02 to 0.04)	0.03 (0.02 to 0.04)
Acute hepatitis E	4.37 (3.23 to 5.74)	3.77 (2.39 to 5.60)	3.13 (2.01 to 4.69)	4.06 (2.57 to 6.07)	0.09 (0.06 to 0.12)	0.11 (0.07 to 0.17)	0.11 (0.07 to 0.16)	0.11 (0.07 to 0.17)
Other unspecified infectious diseases	40.58 (32.27 to 57.61)	54.56 (41.93 to 75.15)	46.01 (35.38 to 63.11)	61.31 (46.64 to 85.51)	0.96 (0.77 to 1.35)	1.72 (1.36 to 2.35)	1.57 (1.24 to 2.15)	1.81 (1.42 to 2.54)
**Non-communicable diseases**	**11,585.78 (10,231.04 to 13,053.12)**	**16,565.83 (13,221.06 to 21,329.52)**	**12,665.65 (10,187.77 to 16,128.67)**	**21,770.45 (17,233.36 to 27,435.77)**	**434.21 (383.98 to 486.49)**	**797.21 (667.57 to 979.39)**	**653.87 (549.24 to 812.58)**	**984.77 (824.31 to 1,173.81)**

Philippines								
**Cause**	**YLLs (in 1000s)**				**Deaths (in 1000s)**			
	**2016**	**2040 Forecast**	**2040 Better Scenario**	**2040 Worse Scenario**	**2016**	**2040 Forecast**	**2040 Better Scenario**	**2040 Worse Scenario**
**Neoplasms**	**2,145.24 (1,867.85 to 2,441.96)**	**3,737.67 (3,067.09 to 4,475.30)**	**3,385.54 (2,790.96 to 3,990.92)**	**4,283.92 (3,441.08 to 5,220.56)**	**72.69 (63.11 to 82.38)**	**152.47 (128.22 to 178.48)**	**145.10 (123.91 to 168.04)**	**168.30 (138.58 to 201.91)**
Lip and oral cavity cancer	39.37 (34.06 to 45.67)	71.53 (45.10 to 109.77)	53.55 (35.72 to 78.32)	115.74 (68.27 to 188.56)	1.58 (1.37 to 1.81)	3.30 (2.19 to 4.87)	2.63 (1.82 to 3.76)	5.02 (3.04 to 7.81)
Nasopharynx cancer	45.22 (38.22 to 52.80)	71.19 (43.48 to 111.78)	49.07 (31.83 to 73.39)	122.69 (70.55 to 201.50)	1.48 (1.26 to 1.71)	2.60 (1.64 to 3.93)	1.89 (1.26 to 2.72)	4.31 (2.53 to 6.70)
Other pharynx cancer	10.36 (8.81 to 12.14)	17.87 (11.81 to 26.03)	16.73 (11.30 to 23.92)	22.06 (13.55 to 33.76)	0.39 (0.34 to 0.45)	0.78 (0.53 to 1.13)	0.75 (0.53 to 1.05)	0.91 (0.57 to 1.38)
Esophageal cancer	21.58 (18.36 to 25.04)	32.92 (21.57 to 50.32)	25.00 (17.35 to 36.27)	52.53 (31.56 to 86.84)	0.85 (0.73 to 0.98)	1.48 (0.99 to 2.18)	1.19 (0.85 to 1.69)	2.24 (1.38 to 3.53)
Stomach cancer	66.93 (57.98 to 77.47)	96.19 (78.04 to 118.78)	88.62 (72.40 to 108.83)	102.98 (81.56 to 130.12)	2.66 (2.32 to 3.04)	4.52 (3.70 to 5.44)	4.37 (3.66 to 5.25)	4.61 (3.67 to 5.73)
Colon and rectum cancer	190.63 (161.79 to 223.58)	381.78 (270.49 to 536.46)	379.69 (268.09 to 530.21)	394.52 (276.24 to 553.88)	7.43 (6.38 to 8.58)	17.94 (12.78 to 25.05)	18.71 (13.42 to 26.16)	17.57 (12.45 to 24.90)
Liver cancer	218.51 (183.38 to 259.39)	348.47 (254.53 to 487.06)	311.75 (238.93 to 406.76)	404.34 (277.30 to 589.06)	7.64 (6.47 to 8.92)	14.28 (10.69 to 18.89)	13.41 (10.57 to 16.92)	15.77 (11.18 to 21.99)
Liver cancer due to hepatitis B	106.06 (84.00 to 131.32)	161.25 (112.61 to 231.05)	145.35 (105.96 to 199.47)	181.49 (120.35 to 269.61)	3.46 (2.72 to 4.28)	6.13 (4.42 to 8.62)	5.78 (4.28 to 7.74)	6.62 (4.52 to 9.62)
Liver cancer due to hepatitis C	34.14 (23.04 to 46.87)	62.36 (39.90 to 92.78)	58.37 (38.01 to 84.15)	67.64 (41.85 to 103.39)	1.45 (0.99 to 1.98)	3.11 (2.00 to 4.51)	3.07 (2.01 to 4.32)	3.21 (1.98 to 4.77)
Liver cancer due to alcohol use	32.21 (22.51 to 44.14)	52.11 (33.04 to 79.53)	44.12 (29.13 to 64.83)	72.41 (43.63 to 114.67)	1.20 (0.85 to 1.64)	2.18 (1.42 to 3.38)	1.95 (1.29 to 2.94)	2.84 (1.75 to 4.52)
Liver cancer due to other causes	46.09 (31.63 to 63.85)	72.75 (44.29 to 114.06)	63.91 (40.35 to 96.39)	82.80 (47.68 to 134.22)	1.53 (1.06 to 2.12)	2.85 (1.72 to 4.50)	2.62 (1.65 to 3.98)	3.10 (1.80 to 5.00)
Gallbladder and biliary tract cancer	9.55 (7.87 to 14.60)	15.73 (13.22 to 24.50)	16.80 (13.99 to 26.31)	14.49 (12.34 to 22.10)	0.40 (0.33 to 0.62)	0.77 (0.65 to 1.20)	0.85 (0.72 to 1.36)	0.68 (0.57 to 1.05)
Pancreatic cancer	54.81 (47.02 to 63.08)	127.88 (99.47 to 163.19)	127.11 (101.00 to 158.65)	130.62 (100.34 to 169.30)	2.21 (1.90 to 2.52)	5.94 (4.72 to 7.55)	6.16 (5.00 to 7.60)	5.81 (4.51 to 7.47)
Larynx cancer	17.68 (14.76 to 20.83)	26.16 (13.48 to 48.72)	17.11 (9.38 to 31.50)	44.01 (21.64 to 79.74)	0.70 (0.59 to 0.82)	1.16 (0.60 to 2.18)	0.79 (0.45 to 1.52)	1.88 (0.93 to 3.38)
Tracheal, bronchus, and lung cancer	315.75 (267.36 to 367.95)	555.32 (376.57 to 784.50)	351.70 (252.42 to 482.56)	881.43 (574.68 to 1,287.86)	12.63 (10.78 to 14.63)	24.44 (17.07 to 33.88)	16.28 (11.87 to 21.83)	37.67 (25.13 to 53.29)
Malignant skin melanoma	6.76 (5.32 to 10.25)	13.44 (10.57 to 20.36)	15.15 (11.79 to 23.29)	12.05 (9.57 to 18.67)	0.24 (0.19 to 0.36)	0.57 (0.46 to 0.86)	0.68 (0.54 to 1.01)	0.49 (0.39 to 0.75)
Non-melanoma skin cancer	11.12 (9.47 to 12.93)	23.33 (20.12 to 26.95)	22.76 (19.19 to 26.95)	22.86 (19.79 to 26.39)	0.42 (0.37 to 0.49)	1.07 (0.93 to 1.21)	1.10 (0.95 to 1.27)	1.00 (0.85 to 1.14)
Breast cancer	256.16 (201.52 to 319.17)	460.46 (326.98 to 635.64)	436.32 (314.47 to 591.35)	488.66 (343.45 to 689.10)	8.23 (6.56 to 10.19)	17.28 (12.80 to 23.35)	16.87 (12.64 to 22.27)	17.75 (12.98 to 24.29)
Cervical cancer	81.54 (63.45 to 102.22)	100.87 (69.22 to 139.40)	90.57 (62.32 to 125.45)	111.25 (74.30 to 162.90)	2.48 (1.95 to 3.07)	3.46 (2.52 to 4.64)	3.19 (2.32 to 4.24)	3.71 (2.65 to 5.19)

Uterine cancer	30.47 (24.30 to 37.77)	56.47 (43.16 to 72.50)	51.98 (39.51 to 67.79)	59.26 (45.25 to 75.49)	1.05 (0.85 to 1.29)	2.23 (1.76 to 2.73)	2.11 (1.67 to 2.62)	2.28 (1.82 to 2.79)
Ovarian cancer	63.14 (49.98 to 78.86)	120.10 (83.77 to 166.27)	124.24 (89.07 to 167.88)	113.58 (79.90 to 157.47)	1.99 (1.58 to 2.46)	4.40 (3.21 to 5.93)	4.66 (3.44 to 6.12)	4.06 (3.02 to 5.57)
Prostate cancer	58.66 (46.25 to 71.51)	160.29 (127.51 to 192.55)	181.11 (147.90 to 216.65)	138.09 (105.29 to 169.60)	3.53 (2.80 to 4.26)	10.83 (8.14 to 13.21)	12.87 (9.84 to 15.25)	8.86 (6.38 to 11.22)
Testicular cancer	3.13 (2.41 to 4.05)	4.70 (3.56 to 6.08)	4.53 (3.39 to 5.94)	4.71 (3.58 to 6.11)	0.08 (0.06 to 0.10)	0.15 (0.11 to 0.19)	0.15 (0.12 to 0.19)	0.14 (0.11 to 0.18)

Philippines								
Cause	YLLs (in 1000s)				Deaths (in 1000s)			
	2016	2040 Forecast	2040 Better Scenario	2040 Worse Scenario	2016	2040 Forecast	2040 Better Scenario	2040 Worse Scenario
Kidney cancer	27.80 (24.36 to 31.75)	51.21 (40.29 to 64.38)	52.40 (41.89 to 64.52)	50.25 (38.76 to 64.90)	0.84 (0.73 to 0.96)	1.99 (1.60 to 2.51)	2.17 (1.77 to 2.67)	1.87 (1.45 to 2.40)
Bladder cancer	13.22 (11.21 to 15.31)	26.35 (19.53 to 35.04)	24.44 (18.79 to 31.43)	28.65 (20.49 to 39.44)	0.60 (0.51 to 0.68)	1.41 (1.04 to 1.87)	1.39 (1.07 to 1.78)	1.44 (1.03 to 1.97)
Brain and nervous system cancer	81.29 (68.03 to 99.03)	157.01 (129.06 to 190.54)	155.21 (127.34 to 188.64)	151.98 (125.91 to 184.88)	1.97 (1.63 to 2.38)	4.57 (3.84 to 5.40)	4.79 (4.00 to 5.65)	4.24 (3.57 to 5.05)
Thyroid cancer	23.51 (19.41 to 28.82)	43.25 (33.54 to 56.90)	40.16 (31.33 to 51.91)	44.82 (34.08 to 60.12)	0.92 (0.77 to 1.12)	1.96 (1.57 to 2.53)	1.89 (1.52 to 2.36)	1.96 (1.55 to 2.57)
Mesothelioma	4.82 (4.00 to 5.79)	8.47 (7.11 to 10.10)	8.57 (7.14 to 10.29)	8.28 (6.95 to 9.85)	0.14 (0.12 to 0.17)	0.29 (0.25 to 0.34)	0.31 (0.26 to 0.36)	0.27 (0.23 to 0.32)
Hodgkin lymphoma	5.23 (4.20 to 10.04)	6.49 (5.10 to 12.64)	5.55 (4.19 to 10.75)	7.07 (5.50 to 13.87)	0.14 (0.11 to 0.25)	0.20 (0.16 to 0.37)	0.18 (0.14 to 0.33)	0.21 (0.17 to 0.39)
Non-Hodgkin lymphoma	71.52 (61.35 to 87.48)	126.69 (105.15 to 156.77)	127.68 (105.89 to 158.01)	121.06 (100.48 to 148.95)	2.01 (1.72 to 2.47)	4.51 (3.80 to 5.63)	4.82 (4.07 to 5.94)	4.11 (3.47 to 5.02)
Multiple myeloma	10.65 (8.17 to 17.63)	24.95 (18.68 to 41.86)	26.44 (19.93 to 44.65)	23.17 (17.36 to 39.08)	0.40 (0.30 to 0.66)	1.06 (0.81 to 1.79)	1.17 (0.89 to 1.98)	0.96 (0.72 to 1.61)
Leukemia	218.63 (192.11 to 248.10)	262.57 (217.11 to 312.51)	233.47 (193.45 to 277.54)	278.21 (228.15 to 336.66)	4.41 (3.88 to 5.00)	7.08 (5.93 to 8.52)	6.75 (5.71 to 8.02)	7.23 (5.97 to 8.90)
Acute lymphoid leukemia	58.43 (48.26 to 69.63)	63.71 (51.78 to 77.58)	56.12 (46.19 to 67.57)	65.83 (53.37 to 80.93)	1.02 (0.88 to 1.18)	1.52 (1.27 to 1.79)	1.45 (1.23 to 1.72)	1.51 (1.25 to 1.82)
Chronic lymphoid leukemia	2.29 (1.81 to 2.86)	3.89 (2.95 to 5.14)	3.78 (2.87 to 4.88)	4.00 (2.99 to 5.33)	0.07 (0.06 to 0.09)	0.15 (0.12 to 0.20)	0.15 (0.12 to 0.20)	0.15 (0.11 to 0.20)
Acute myeloid leukemia	49.24 (41.34 to 58.59)	79.05 (64.41 to 97.58)	75.64 (61.82 to 92.55)	79.68 (64.10 to 99.77)	1.02 (0.87 to 1.18)	2.11 (1.73 to 2.55)	2.14 (1.78 to 2.57)	2.05 (1.66 to 2.54)
Chronic myeloid leukemia	7.88 (6.59 to 9.34)	8.34 (6.33 to 10.76)	7.68 (5.86 to 9.82)	8.81 (6.58 to 11.66)	0.21 (0.18 to 0.25)	0.27 (0.21 to 0.34)	0.26 (0.20 to 0.32)	0.27 (0.21 to 0.36)
Other leukemia	100.78 (86.70 to 116.82)	107.58 (85.70 to 132.47)	90.25 (71.79 to 111.70)	119.90 (95.10 to 149.75)	2.08 (1.80 to 2.40)	3.04 (2.45 to 3.76)	2.74 (2.19 to 3.35)	3.25 (2.57 to 4.10)
Other neoplasms	187.21 (162.26 to 214.93)	345.98 (294.42 to 397.32)	347.84 (294.27 to 403.00)	334.56 (288.03 to 382.92)	5.28 (4.57 to 6.09)	12.20 (10.50 to 13.87)	12.98 (11.20 to 14.74)	11.26 (9.75 to 12.79)
**Cardiovascular diseases**	**4,870.46 (4,230.51 to 5,552.89)**	**5,725.70 (4,002.48 to 8,306.07)**	**3,533.32 (2,522.42 to 5,164.35)**	**9,178.20 (6,183.37 to 12,871.52)**	**206.19 (181.86 to 232.44)**	**308.92 (225.11 to 451.54)**	**213.94 (157.17 to 324.65)**	**446.92 (323.05 to 621.72)**
Rheumatic heart disease	167.49 (142.77 to 194.90)	97.13 (76.01 to 122.33)	76.97 (59.68 to 98.15)	113.04 (87.32 to 145.90)	3.44 (2.93 to 4.04)	2.53 (2.07 to 3.09)	2.15 (1.73 to 2.62)	2.79 (2.26 to 3.49)
Ischemic heart disease	2,419.12 (2,085.79 to 2,759.77)	2,932.78 (1,622.79 to 5,315.49)	1,666.56 (927.34 to 3,007.64)	4,776.90 (2,699.78 to 8,048.37)	107.02 (93.87 to 120.47)	161.53 (92.48 to 296.43)	103.99 (59.68 to 185.06)	239.44 (141.31 to 413.41)
Stroke	1,615.75 (1,399.99 to 1,847.18)	1,601.95 (973.21 to 2,676.32)	860.83 (546.77 to 1,423.54)	2,952.16 (1,736.79 to 4,737.36)	67.16 (58.68 to 76.41)	83.90 (53.73 to 157.52)	51.90 (33.43 to 95.62)	135.60 (83.82 to 231.79)
Ischemic stroke	312.96 (269.77 to 358.61)	399.44 (209.50 to 1,146.07)	254.27 (135.76 to 661.32)	593.07 (306.73 to 1,580.54)	18.14 (15.75 to 20.64)	28.47 (14.76 to 82.02)	20.37 (10.77 to 54.32)	37.87 (19.23 to 106.71)

Intracerebral hemorrhage	1,302.79 (1,129.20 to 1,493.20)	1,202.52 (697.54 to 1,984.76)	606.55 (370.81 to 1,018.50)	2,359.09 (1,252.14 to 4,016.16)	49.03 (42.64 to 56.04)	55.43 (34.57 to 91.87)	31.53 (20.52 to 50.63)	97.73 (56.96 to 167.73)
Hypertensive heart disease	392.18 (283.46 to 525.74)	654.48 (434.53 to 971.51)	519.39 (364.50 to 713.96)	873.91 (543.22 to 1,439.16)	18.51 (13.48 to 24.83)	38.23 (26.08 to 58.30)	32.40 (23.13 to 45.15)	47.37 (30.51 to 78.51)
Cardiomyopathy and myocarditis	92.77 (78.46 to 111.00)	127.74 (94.62 to 163.19)	128.21 (91.04 to 166.44)	126.32 (96.78 to 160.06)	3.50 (2.34 to 4.29)	7.02 (3.63 to 10.60)	7.78 (3.80 to 11.85)	6.21 (3.46 to 9.21)
Myocarditis	18.86 (14.37 to 27.28)	20.36 (15.52 to 28.70)	19.03 (14.54 to 26.39)	20.89 (15.70 to 29.98)	0.45 (0.36 to 0.57)	0.68 (0.49 to 0.87)	0.71 (0.48 to 0.92)	0.64 (0.47 to 0.80)

Philippines								
Cause	YLLs (in 1000s)				Deaths (in 1000s)			
	2016	2040 Forecast	2040 Better Scenario	2040 Worse Scenario	2016	2040 Forecast	2040 Better Scenario	2040 Worse Scenario
Alcoholic cardiomyopathy	15.61 (11.06 to 23.26)	19.61 (13.61 to 28.20)	20.01 (13.77 to 28.44)	22.71 (16.20 to 33.27)	0.43 (0.31 to 0.60)	0.65 (0.43 to 0.85)	0.71 (0.46 to 0.93)	0.69 (0.48 to 0.92)
Other cardiomyopathy	58.30 (46.20 to 68.65)	87.77 (56.09 to 121.07)	89.17 (55.11 to 124.06)	82.72 (54.91 to 113.82)	2.62 (1.53 to 3.31)	5.69 (2.57 to 9.16)	6.37 (2.71 to 10.37)	4.88 (2.28 to 7.83)
Atrial fibrillation and flutter	13.73 (10.53 to 17.13)	37.49 (27.53 to 48.54)	39.32 (29.05 to 50.56)	36.63 (26.48 to 48.43)	1.13 (0.86 to 1.43)	3.48 (2.30 to 4.73)	3.88 (2.70 to 5.22)	3.18 (2.07 to 4.42)
Aortic aneurysm	27.51 (23.34 to 31.83)	49.42 (39.58 to 60.52)	48.52 (39.74 to 58.81)	49.66 (39.23 to 62.17)	1.23 (1.06 to 1.40)	2.69 (2.17 to 3.21)	2.83 (2.34 to 3.37)	2.52 (1.98 to 3.05)
Peripheral artery disease	1.96 (1.35 to 2.73)	6.03 (4.01 to 9.22)	6.08 (4.12 to 9.70)	5.78 (3.78 to 8.65)	0.11 (0.08 to 0.17)	0.43 (0.27 to 0.77)	0.48 (0.30 to 0.92)	0.37 (0.23 to 0.67)
Endocarditis	52.07 (37.74 to 71.13)	65.43 (47.01 to 95.62)	60.37 (43.31 to 88.86)	68.60 (48.89 to 100.47)	1.23 (0.92 to 1.78)	1.99 (1.44 to 2.92)	1.98 (1.44 to 2.96)	1.96 (1.42 to 2.91)
Other cardiovascular and circulatory diseases	87.88 (76.35 to 100.64)	153.25 (117.85 to 197.88)	127.05 (97.91 to 164.10)	175.21 (132.85 to 232.45)	2.86 (2.50 to 3.27)	7.12 (5.49 to 9.04)	6.54 (4.98 to 8.25)	7.47 (5.52 to 9.74)
**Chronic respiratory diseases**	**803.21 (704.59 to 911.70)**	**757.91 (563.63 to 1,009.66)**	**498.71 (392.88 to 628.03)**	**1,129.20 (805.55 to 1,570.18)**	**33.72 (29.73 to 38.13)**	**40.47 (30.03 to 53.50)**	**28.37 (22.33 to 35.73)**	**57.70 (40.95 to 80.71)**
Chronic obstructive pulmonary disease	422.32 (360.98 to 493.65)	501.44 (338.74 to 740.82)	296.44 (212.56 to 410.56)	828.06 (522.58 to 1,282.65)	21.21 (18.32 to 24.34)	29.11 (19.65 to 42.38)	18.70 (13.47 to 25.63)	45.15 (29.65 to 68.54)
Pneumoconiosis	0.51 (0.43 to 0.61)	0.79 (0.66 to 0.93)	0.80 (0.67 to 0.95)	0.78 (0.65 to 0.92)	0.02 (0.02 to 0.03)	0.05 (0.04 to 0.06)	0.05 (0.04 to 0.06)	0.04 (0.03 to 0.06)
Silicosis	0.07 (0.05 to 0.10)	0.07 (0.04 to 0.10)	0.07 (0.04 to 0.09)	0.07 (0.04 to 0.10)	0.00 (0.00 to 0.00)	0.00 (0.00 to 0.01)	0.00 (0.00 to 0.01)	0.00 (0.00 to 0.01)
Asbestosis	0.19 (0.15 to 0.26)	0.38 (0.29 to 0.51)	0.42 (0.32 to 0.56)	0.35 (0.27 to 0.46)	0.01 (0.01 to 0.01)	0.02 (0.01 to 0.03)	0.02 (0.02 to 0.03)	0.02 (0.01 to 0.02)
Coal workers pneumoconiosis	0.04 (0.02 to 0.06)	0.04 (0.02 to 0.06)	0.03 (0.01 to 0.05)	0.05 (0.02 to 0.07)	0.00 (0.00 to 0.00)	0.00 (0.00 to 0.00)	0.00 (0.00 to 0.00)	0.00 (0.00 to 0.01)
Other pneumoconiosis	0.21 (0.17 to 0.26)	0.30 (0.23 to 0.38)	0.29 (0.21 to 0.36)	0.31 (0.24 to 0.39)	0.01 (0.01 to 0.01)	0.02 (0.01 to 0.03)	0.02 (0.01 to 0.03)	0.02 (0.01 to 0.03)
Asthma	363.44 (308.36 to 421.18)	230.19 (171.57 to 311.99)	176.04 (129.49 to 236.24)	274.74 (197.94 to 386.97)	11.98 (10.09 to 13.95)	10.29 (7.61 to 14.39)	8.50 (6.31 to 11.48)	11.56 (8.21 to 16.62)
Interstitial lung disease and pulmonary sarcoidosis	4.16 (3.17 to 6.31)	7.79 (5.99 to 12.09)	9.03 (6.81 to 14.21)	6.86 (5.37 to 10.57)	0.16 (0.13 to 0.27)	0.37 (0.28 to 0.63)	0.46 (0.34 to 0.78)	0.31 (0.24 to 0.52)
Other chronic respiratory diseases	12.77 (9.01 to 23.04)	17.69 (12.57 to 29.63)	16.39 (11.65 to 26.41)	18.77 (13.21 to 31.63)	0.34 (0.24 to 0.58)	0.65 (0.40 to 1.02)	0.67 (0.41 to 1.05)	0.64 (0.40 to 1.02)
**Cirrhosis and other chronic liver diseases**	**390.42 (322.38 to 465.68)**	**582.69 (488.05 to 690.33)**	**551.40 (453.39 to 662.97)**	**614.77 (514.61 to 731.08)**	**12.62 (10.63 to 14.88)**	**21.33 (18.52 to 24.71)**	**20.90 (17.98 to 24.45)**	**21.64 (18.56 to 25.15)**
Cirrhosis and other chronic liver diseases due to hepatitis B	155.42 (123.27 to 192.41)	243.39 (195.13 to 303.25)	235.51 (185.86 to 294.12)	243.06 (194.46 to 303.45)	5.10 (4.10 to 6.29)	8.88 (7.33 to 10.76)	8.86 (7.23 to 10.77)	8.58 (7.04 to 10.47)
Cirrhosis and other chronic liver diseases due to hepatitis C	66.38 (50.49 to 84.73)	105.25 (79.74 to 136.33)	101.01 (75.84 to 132.09)	105.81 (79.96 to 137.67)	2.14 (1.64 to 2.72)	3.75 (2.95 to 4.67)	3.70 (2.91 to 4.67)	3.66 (2.87 to 4.60)

Cirrhosis and other chronic liver diseases due to alcohol use	74.49 (56.83 to 96.59)	101.56 (77.13 to 131.42)	89.94 (67.75 to 115.57)	131.13 (100.33 to 167.73)	2.51 (1.95 to 3.23)	3.86 (3.02 to 4.88)	3.57 (2.75 to 4.50)	4.69 (3.66 to 5.82)
Cirrhosis and other chronic liver diseases due to other causes	94.12 (77.74 to 113.69)	132.49 (109.05 to 161.64)	124.93 (100.66 to 155.39)	134.77 (110.56 to 164.24)	2.86 (2.36 to 3.44)	4.84 (4.03 to 5.77)	4.78 (3.92 to 5.80)	4.72 (3.91 to 5.63)
**Digestive diseases**	**439.07 (366.60 to 589.02)**	**504.86 (414.37 to 683.09)**	**435.90 (353.05 to 585.41)**	**562.02 (451.72 to 769.56)**	**14.93 (12.72 to 18.77)**	**22.11 (18.27 to 27.22)**	**20.42 (16.89 to 25.12)**	**23.12 (18.68 to 29.08)**
Peptic ulcer disease	207.80 (168.82 to 262.67)	201.58 (156.07 to 264.14)	164.54 (123.99 to 215.54)	230.93 (173.27 to 306.03)	8.28 (6.89 to 10.04)	10.09 (7.73 to 13.04)	8.70 (6.50 to 11.17)	10.99 (8.16 to 14.46)

Philippines								
Cause	YLLs (in 1000s)				Deaths (in 1000s)			
	2016	2040 Forecast	2040 Better Scenario	2040 Worse Scenario	2016	2040 Forecast	2040 Better Scenario	2040 Worse Scenario
Gastritis and duodenitis	9.21 (7.11 to 14.37)	15.20 (11.10 to 24.36)	14.10 (10.18 to 23.41)	15.45 (11.23 to 25.02)	0.31 (0.24 to 0.54)	0.70 (0.51 to 1.31)	0.71 (0.51 to 1.29)	0.67 (0.47 to 1.25)
Appendicitis	28.85 (22.80 to 38.23)	26.99 (20.53 to 36.92)	22.50 (16.72 to 31.14)	29.74 (22.39 to 40.82)	0.64 (0.51 to 0.87)	0.79 (0.62 to 1.07)	0.72 (0.54 to 0.96)	0.82 (0.63 to 1.12)
Paralytic ileus and intestinal obstruction	83.45 (65.24 to 106.74)	99.34 (78.88 to 132.43)	92.30 (73.72 to 124.35)	101.04 (79.26 to 135.08)	2.13 (1.72 to 2.87)	3.92 (3.03 to 5.46)	4.08 (3.16 to 5.62)	3.66 (2.84 to 5.13)
Inguinal, femoral, and abdominal hernia	8.84 (6.81 to 13.81)	10.19 (7.79 to 15.28)	9.52 (7.10 to 14.14)	10.32 (7.88 to 15.86)	0.33 (0.26 to 0.51)	0.49 (0.36 to 0.73)	0.49 (0.37 to 0.72)	0.46 (0.34 to 0.69)
Inflammatory bowel disease	7.54 (5.20 to 14.48)	14.39 (10.10 to 26.85)	14.56 (10.24 to 27.15)	13.82 (9.72 to 26.50)	0.25 (0.18 to 0.44)	0.64 (0.47 to 1.03)	0.70 (0.52 to 1.11)	0.58 (0.42 to 0.96)
Vascular intestinal disorders	5.27 (4.26 to 6.57)	10.79 (8.54 to 13.10)	10.95 (8.61 to 13.36)	10.26 (8.06 to 12.49)	0.27 (0.22 to 0.33)	0.68 (0.52 to 0.83)	0.73 (0.56 to 0.88)	0.61 (0.46 to 0.76)
Gallbladder and biliary diseases	26.89 (21.87 to 33.18)	37.43 (24.81 to 55.73)	29.41 (20.08 to 42.14)	44.27 (28.12 to 68.24)	0.95 (0.78 to 1.15)	1.64 (1.12 to 2.51)	1.37 (0.95 to 1.95)	1.82 (1.19 to 2.82)
Pancreatitis	44.09 (31.06 to 90.63)	67.59 (37.49 to 144.12)	58.66 (33.21 to 121.07)	83.84 (41.83 to 189.78)	1.23 (0.91 to 2.30)	2.21 (1.40 to 4.11)	2.01 (1.28 to 3.56)	2.59 (1.48 to 5.15)
Other digestive diseases	17.13 (12.60 to 22.90)	21.35 (15.51 to 28.22)	19.36 (13.87 to 24.75)	22.35 (15.98 to 29.69)	0.53 (0.40 to 0.68)	0.94 (0.69 to 1.16)	0.92 (0.69 to 1.14)	0.91 (0.66 to 1.14)
**Neurological disorders**	**259.38 (218.08 to 304.78)**	**670.28 (523.85 to 857.68)**	**699.35 (565.25 to 863.07)**	**632.66 (481.37 to 827.33)**	**17.34 (14.41 to 20.79)**	**54.86 (38.53 to 72.78)**	**61.33 (45.92 to 77.50)**	**48.18 (31.98 to 67.31)**
Alzheimer's disease and other dementias	180.40 (145.64 to 222.57)	528.78 (391.25 to 710.54)	556.07 (429.27 to 716.88)	489.85 (341.98 to 687.79)	14.94 (12.21 to 18.28)	48.57 (32.72 to 66.54)	54.07 (39.26 to 70.57)	42.63 (27.14 to 61.35)
Parkinson's disease	16.97 (12.78 to 21.55)	54.69 (41.84 to 70.46)	66.08 (50.46 to 83.19)	44.84 (33.14 to 57.98)	1.13 (0.85 to 1.46)	4.07 (2.93 to 5.39)	5.13 (3.80 to 6.58)	3.19 (2.20 to 4.27)
Epilepsy	43.58 (37.39 to 50.82)	52.26 (38.03 to 69.96)	41.96 (30.42 to 55.71)	64.97 (45.29 to 89.05)	0.82 (0.70 to 0.95)	1.16 (0.85 to 1.54)	0.98 (0.72 to 1.29)	1.40 (0.99 to 1.92)
Multiple sclerosis	2.68 (1.95 to 4.26)	4.56 (3.27 to 7.37)	4.66 (3.33 to 7.47)	4.47 (3.22 to 7.26)	0.08 (0.06 to 0.12)	0.14 (0.11 to 0.22)	0.15 (0.11 to 0.23)	0.14 (0.10 to 0.22)
Motor neuron disease	3.41 (2.63 to 4.17)	7.60 (5.88 to 9.44)	8.07 (6.19 to 10.00)	7.09 (5.50 to 8.68)	0.10 (0.08 to 0.12)	0.26 (0.20 to 0.32)	0.28 (0.22 to 0.34)	0.24 (0.19 to 0.28)
Other neurological disorders	12.34 (10.67 to 14.27)	22.38 (19.08 to 26.24)	22.50 (19.21 to 26.24)	21.42 (18.38 to 25.23)	0.27 (0.24 to 0.31)	0.65 (0.57 to 0.74)	0.72 (0.63 to 0.81)	0.59 (0.51 to 0.66)
**Mental disorders**	**75.50 (60.57 to 92.59)**	**147.50 (116.55 to 186.96)**	**146.33 (115.06 to 184.59)**	**152.93 (121.31 to 193.58)**	**1.81 (1.46 to 2.20)**	**3.83 (3.13 to 4.65)**	**3.93 (3.21 to 4.79)**	**3.85 (3.15 to 4.66)**
Alcohol use disorders	50.12 (39.79 to 62.10)	77.37 (60.24 to 100.29)	75.57 (58.36 to 97.76)	83.41 (64.96 to 107.19)	1.27 (1.02 to 1.56)	2.17 (1.75 to 2.70)	2.19 (1.75 to 2.73)	2.26 (1.83 to 2.82)
Drug use disorders	23.53 (17.49 to 31.46)	68.56 (50.51 to 92.62)	69.16 (51.00 to 92.53)	67.99 (49.69 to 91.97)	0.50 (0.37 to 0.67)	1.63 (1.23 to 2.11)	1.71 (1.29 to 2.21)	1.56 (1.17 to 2.03)
Opioid use disorders	19.60 (14.19 to 26.28)	51.44 (36.08 to 70.40)	51.40 (36.31 to 70.06)	51.10 (35.89 to 69.95)	0.40 (0.30 to 0.54)	1.16 (0.84 to 1.54)	1.20 (0.88 to 1.58)	1.11 (0.81 to 1.49)

Cocaine use disorders	0.51 (0.34 to 0.80)	2.12 (1.39 to 3.34)	2.11 (1.38 to 3.30)	2.14 (1.38 to 3.41)	0.01 (0.01 to 0.02)	0.06 (0.04 to 0.09)	0.06 (0.04 to 0.09)	0.06 (0.04 to 0.09)
Amphetamine use disorders	1.00 (0.52 to 1.70)	4.80 (2.58 to 8.53)	4.70 (2.56 to 8.20)	5.01 (2.65 to 8.82)	0.02 (0.01 to 0.03)	0.10 (0.06 to 0.17)	0.10 (0.06 to 0.17)	0.10 (0.06 to 0.18)
Other drug use disorders	2.42 (1.63 to 3.43)	10.20 (6.57 to 14.95)	10.95 (6.88 to 15.96)	9.75 (6.27 to 14.28)	0.07 (0.05 to 0.09)	0.31 (0.20 to 0.43)	0.35 (0.23 to 0.48)	0.28 (0.18 to 0.39)
Eating disorders	1.85 (1.35 to 2.47)	1.57 (1.01 to 2.27)	1.60 (1.02 to 2.34)	1.52 (0.99 to 2.22)	0.03 (0.02 to 0.04)	0.03 (0.02 to 0.04)	0.03 (0.02 to 0.04)	0.03 (0.02 to 0.04)

Philippines								
**Cause**	**YLLs (in 1000s)**				**Deaths (in 1000s)**			
	**2016**	**2040 Forecast**	**2040 Better Scenario**	**2040 Worse Scenario**	**2016**	**2040 Forecast**	**2040 Better Scenario**	**2040 Worse Scenario**
**Diabetes, urogenital, blood, and endocrine diseases**	**1,631.82 (1,423.14 to 1,856.55)**	**3,479.50 (1,968.78 to 7,435.06)**	**2,666.63 (1,530.84 to 5,975.69)**	**4,161.55 (2,304.75 to 8,702.42)**	**60.61 (53.33 to 68.54)**	**170.31 (90.79 to 382.98)**	**138.05 (76.49 to 321.31)**	**192.58 (101.76 to 406.92)**
Diabetes mellitus	573.55 (493.68 to 657.41)	891.82 (618.79 to 1,268.33)	694.40 (485.08 to 969.47)	1,095.23 (750.25 to 1,555.65)	24.18 (21.05 to 27.74)	46.02 (32.56 to 62.95)	38.09 (27.37 to 51.06)	53.07 (36.80 to 72.50)
Acute glomerulonephritis	7.30 (5.49 to 9.79)	9.10 (6.55 to 12.31)	7.98 (5.75 to 10.84)	9.63 (6.87 to 13.14)	0.20 (0.15 to 0.25)	0.29 (0.20 to 0.39)	0.28 (0.20 to 0.38)	0.28 (0.20 to 0.39)
Chronic kidney disease	804.67 (701.32 to 922.55)	2,157.08 (673.16 to 6,153.26)	1,560.49 (514.68 to 4,840.57)	2,636.65 (803.08 to 7,250.26)	28.37 (24.78 to 32.32)	105.26 (24.74 to 323.14)	79.98 (19.70 to 269.70)	122.01 (28.49 to 344.63)
Chronic kidney disease due to diabetes mellitus	468.02 (398.14 to 549.68)	1,351.48 (402.31 to 3,981.10)	968.59 (309.98 to 3,034.45)	1,670.20 (485.03 to 4,757.30)	16.27 (13.89 to 19.03)	60.16 (14.08 to 186.18)	44.62 (10.86 to 146.91)	71.37 (16.54 to 204.27)
Chronic kidney disease due to hypertension	184.47 (151.84 to 222.92)	573.10 (140.65 to 1,762.65)	424.21 (107.78 to 1,455.89)	681.63 (163.69 to 1,941.00)	8.49 (7.11 to 10.06)	36.23 (7.69 to 119.22)	28.69 (6.32 to 99.53)	40.25 (8.50 to 119.33)
Chronic kidney disease due to glomerulonephritis	80.14 (64.97 to 98.10)	110.37 (59.23 to 242.04)	79.87 (43.03 to 175.18)	135.33 (71.81 to 287.96)	1.73 (1.42 to 2.13)	3.63 (1.38 to 9.77)	2.69 (1.04 to 8.06)	4.32 (1.63 to 11.10)
Chronic kidney disease due to other and unspecified causes	72.04 (58.64 to 87.88)	122.13 (52.62 to 297.18)	87.82 (38.21 to 234.55)	149.48 (63.77 to 357.69)	1.87 (1.53 to 2.28)	5.24 (1.52 to 15.51)	3.98 (1.17 to 12.82)	6.07 (1.77 to 16.93)
Urinary diseases and male infertility	130.16 (113.58 to 148.82)	245.37 (213.01 to 280.44)	244.62 (209.09 to 283.78)	235.67 (204.49 to 270.02)	4.65 (4.05 to 5.32)	11.99 (10.10 to 13.76)	12.94 (10.82 to 14.97)	10.67 (8.97 to 12.37)
Urinary tract infections	107.38 (90.14 to 124.63)	200.60 (168.87 to 231.98)	201.20 (166.05 to 234.73)	191.78 (160.97 to 221.52)	3.79 (3.19 to 4.40)	9.97 (8.17 to 11.66)	10.86 (8.95 to 12.74)	8.81 (7.11 to 10.34)
Urolithiasis	16.14 (11.88 to 24.55)	34.88 (25.13 to 53.33)	34.58 (23.93 to 54.15)	33.53 (24.12 to 51.54)	0.63 (0.48 to 0.97)	1.57 (1.11 to 2.44)	1.65 (1.15 to 2.58)	1.43 (1.01 to 2.20)
Other urinary diseases	6.64 (4.33 to 12.09)	9.89 (6.52 to 16.80)	8.84 (5.71 to 15.08)	10.36 (6.81 to 17.67)	0.22 (0.15 to 0.38)	0.45 (0.29 to 0.76)	0.44 (0.28 to 0.76)	0.43 (0.28 to 0.74)
Gynecological diseases	4.01 (2.88 to 6.54)	5.46 (3.53 to 8.87)	4.50 (2.90 to 7.43)	6.16 (3.99 to 10.13)	0.13 (0.10 to 0.19)	0.19 (0.13 to 0.27)	0.17 (0.11 to 0.24)	0.21 (0.15 to 0.30)
Uterine fibroids	2.09 (1.44 to 3.39)	2.50 (1.53 to 4.03)	2.07 (1.24 to 3.43)	2.84 (1.75 to 4.61)	0.06 (0.04 to 0.09)	0.08 (0.05 to 0.13)	0.07 (0.05 to 0.11)	0.09 (0.06 to 0.14)
Polycystic ovarian syndrome	0.32 (0.08 to 0.68)	0.29 (0.06 to 0.69)	0.21 (0.04 to 0.52)	0.36 (0.08 to 0.84)	0.01 (0.00 to 0.02)	0.01 (0.00 to 0.02)	0.00 (0.00 to 0.01)	0.01 (0.00 to 0.02)
Endometriosis	0.07 (0.03 to 0.16)	0.18 (0.07 to 0.41)	0.15 (0.05 to 0.33)	0.21 (0.08 to 0.47)	0.00 (0.00 to 0.00)	0.00 (0.00 to 0.01)	0.00 (0.00 to 0.01)	0.00 (0.00 to 0.01)
Genital prolapse	0.63 (0.39 to 1.04)	0.92 (0.52 to 1.55)	0.75 (0.42 to 1.28)	1.03 (0.57 to 1.72)	0.03 (0.02 to 0.05)	0.05 (0.03 to 0.08)	0.04 (0.02 to 0.07)	0.05 (0.03 to 0.08)
Other gynecological diseases	0.90 (0.57 to 1.95)	1.56 (0.92 to 3.33)	1.32 (0.77 to 2.83)	1.71 (1.00 to 3.68)	0.03 (0.02 to 0.05)	0.05 (0.03 to 0.08)	0.04 (0.03 to 0.07)	0.05 (0.03 to 0.09)
Hemoglobinopathies and hemolytic anemias	41.64 (34.63 to 49.33)	49.91 (39.71 to 62.15)	42.91 (33.16 to 53.98)	53.80 (42.86 to 67.56)	1.06 (0.88 to 1.32)	1.72 (1.42 to 2.21)	1.58 (1.26 to 2.04)	1.76 (1.45 to 2.26)
Thalassemias	8.26 (6.91 to 9.80)	4.79 (3.32 to 6.77)	4.16 (2.96 to 5.76)	4.69 (3.24 to 6.53)	0.10 (0.09 to 0.12)	0.06 (0.04 to 0.09)	0.05 (0.04 to 0.08)	0.06 (0.04 to 0.08)

Sickle cell disorders	0.24 (0.18 to 0.30)	0.18 (0.13 to 0.25)	0.14 (0.10 to 0.19)	0.20 (0.14 to 0.27)	0.00 (0.00 to 0.00)	0.00 (0.00 to 0.00)	0.00 (0.00 to 0.00)	0.00 (0.00 to 0.00)
G6PD deficiency	21.66 (17.17 to 26.09)	31.78 (23.88 to 40.69)	26.45 (19.51 to 34.50)	35.50 (26.82 to 45.50)	0.47 (0.38 to 0.57)	0.86 (0.67 to 1.09)	0.74 (0.56 to 0.95)	0.93 (0.72 to 1.18)
Other hemoglobinopathies and hemolytic anemias	11.48 (9.25 to 14.78)	13.16 (10.37 to 17.61)	12.17 (9.41 to 16.02)	13.41 (10.61 to 18.03)	0.49 (0.40 to 0.65)	0.80 (0.62 to 1.09)	0.78 (0.61 to 1.07)	0.77 (0.59 to 1.07)
Endocrine, metabolic, blood, and immune disorders	70.49 (59.00 to 99.84)	120.76 (99.63 to 164.73)	111.73 (91.42 to 152.75)	124.41 (102.59 to 171.74)	2.02 (1.69 to 2.69)	4.85 (3.92 to 6.28)	5.01 (4.09 to 6.43)	4.58 (3.69 to 6.08)

Philippines								
Cause	YLLs (in 1000s)				Deaths (in 1000s)			
	2016	2040 Forecast	2040 Better Scenario	2040 Worse Scenario	2016	2040 Forecast	2040 Better Scenario	2040 Worse Scenario
**Musculoskeletal disorders**	**46.25 (36.89 to 55.99)**	**86.99 (68.75 to 105.86)**	**87.82 (69.09 to 106.79)**	**83.20 (65.80 to 100.85)**	**1.25 (1.02 to 1.46)**	**3.46 (2.67 to 4.26)**	**3.68 (2.82 to 4.46)**	**3.15 (2.44 to 3.92)**
Rheumatoid arthritis	4.87 (3.82 to 6.20)	19.64 (14.11 to 26.23)	18.44 (12.90 to 24.98)	19.57 (13.50 to 26.80)	0.29 (0.23 to 0.36)	1.39 (0.94 to 1.93)	1.39 (0.94 to 1.92)	1.31 (0.84 to 1.85)
Other musculoskeletal disorders	41.37 (32.72 to 50.43)	67.35 (51.85 to 84.06)	69.38 (53.70 to 86.82)	63.63 (49.06 to 79.71)	0.96 (0.77 to 1.16)	2.07 (1.60 to 2.52)	2.30 (1.78 to 2.77)	1.84 (1.43 to 2.23)
**Other non-communicable diseases**	**924.44 (751.19 to 1,132.15)**	**872.74 (693.39 to 1,092.53)**	**660.65 (522.10 to 835.97)**	**972.00 (770.90 to 1,224.22)**	**13.06 (10.81 to 15.74)**	**19.45 (14.87 to 26.39)**	**18.14 (13.15 to 25.39)**	**19.33 (15.33 to 26.21)**
Congenital birth defects	815.02 (640.98 to 1,016.27)	635.70 (473.07 to 839.79)	428.43 (308.38 to 558.43)	742.12 (554.24 to 969.84)	9.95 (7.89 to 12.27)	8.20 (6.17 to 10.69)	5.69 (4.10 to 7.36)	9.49 (7.15 to 12.20)
Neural tube defects	42.11 (26.92 to 63.88)	11.77 (5.98 to 21.11)	6.75 (3.39 to 12.31)	15.25 (7.67 to 27.08)	0.49 (0.31 to 0.74)	0.14 (0.07 to 0.25)	0.08 (0.04 to 0.15)	0.18 (0.09 to 0.32)
Congenital heart anomalies	498.01 (368.90 to 618.03)	399.89 (267.42 to 535.09)	271.83 (176.42 to 364.31)	463.74 (312.02 to 617.09)	6.13 (4.55 to 7.53)	5.17 (3.53 to 6.80)	3.61 (2.34 to 4.77)	5.95 (4.03 to 7.78)
Orofacial clefts	4.41 (2.10 to 10.38)	2.93 (1.24 to 7.17)	1.60 (0.66 to 4.02)	3.90 (1.67 to 9.79)	0.05 (0.02 to 0.12)	0.03 (0.01 to 0.08)	0.02 (0.01 to 0.05)	0.05 (0.02 to 0.11)
Down syndrome	19.33 (10.93 to 26.77)	17.12 (9.09 to 24.00)	12.66 (6.84 to 17.47)	20.05 (10.52 to 28.15)	0.25 (0.15 to 0.34)	0.25 (0.14 to 0.35)	0.20 (0.11 to 0.27)	0.29 (0.16 to 0.40)
Other chromosomal abnormalities	14.48 (4.14 to 93.94)	24.51 (8.36 to 137.92)	16.70 (6.17 to 85.87)	28.92 (9.57 to 164.87)	0.18 (0.06 to 1.10)	0.33 (0.14 to 1.63)	0.24 (0.11 to 1.02)	0.39 (0.16 to 1.95)
Congenital musculoskeletal and limb anomalies	7.93 (5.37 to 14.20)	7.67 (4.77 to 13.43)	5.20 (3.30 to 9.07)	8.80 (5.49 to 15.73)	0.10 (0.06 to 0.17)	0.10 (0.06 to 0.16)	0.07 (0.04 to 0.11)	0.11 (0.07 to 0.19)
Urogenital congenital anomalies	11.66 (7.43 to 23.25)	13.53 (9.03 to 24.36)	10.52 (6.86 to 18.55)	14.92 (9.95 to 27.59)	0.18 (0.12 to 0.32)	0.25 (0.16 to 0.40)	0.21 (0.13 to 0.32)	0.27 (0.17 to 0.43)
Digestive congenital anomalies	75.20 (44.92 to 145.52)	64.37 (37.93 to 112.81)	40.65 (24.47 to 69.73)	77.23 (45.24 to 135.18)	0.88 (0.53 to 1.71)	0.77 (0.46 to 1.34)	0.49 (0.30 to 0.83)	0.92 (0.55 to 1.60)
Other congenital birth defects	141.89 (72.50 to 196.00)	93.92 (41.35 to 138.45)	62.53 (28.53 to 91.58)	109.30 (48.17 to 160.20)	1.69 (0.86 to 2.32)	1.16 (0.52 to 1.68)	0.79 (0.37 to 1.14)	1.34 (0.61 to 1.94)
Skin and subcutaneous diseases	72.89 (47.21 to 129.66)	218.55 (146.74 to 387.98)	220.24 (150.49 to 372.58)	208.00 (139.59 to 371.28)	2.68 (1.66 to 4.77)	11.03 (6.74 to 17.98)	12.31 (7.65 to 19.60)	9.59 (5.87 to 15.80)
Cellulitis	17.02 (10.66 to 37.29)	68.14 (44.23 to 147.30)	72.07 (47.29 to 157.57)	62.73 (40.91 to 135.19)	0.54 (0.34 to 1.27)	2.96 (1.84 to 6.32)	3.40 (2.17 to 7.34)	2.53 (1.56 to 5.60)
Pyoderma	31.20 (20.14 to 59.23)	79.25 (49.88 to 145.41)	72.51 (45.18 to 133.17)	80.92 (51.56 to 149.00)	0.78 (0.50 to 1.49)	3.08 (1.85 to 5.63)	3.18 (1.84 to 5.71)	2.87 (1.71 to 5.27)
Decubitus ulcer	22.04 (11.87 to 37.15)	64.48 (33.41 to 101.53)	68.99 (34.64 to 107.74)	57.94 (29.82 to 92.54)	1.29 (0.66 to 2.10)	4.77 (2.24 to 7.36)	5.48 (2.57 to 8.23)	3.98 (1.87 to 6.41)
Other skin and subcutaneous diseases	2.63 (1.56 to 5.33)	6.68 (3.83 to 13.68)	6.66 (3.76 to 14.06)	6.41 (3.72 to 12.99)	0.07 (0.04 to 0.15)	0.22 (0.12 to 0.49)	0.24 (0.13 to 0.54)	0.20 (0.11 to 0.43)
Sudden infant death syndrome	36.53 (25.81 to 51.21)	18.48 (10.67 to 29.38)	11.97 (6.99 to 18.60)	21.88 (12.70 to 34.85)	0.42 (0.30 to 0.59)	0.21 (0.12 to 0.34)	0.14 (0.08 to 0.22)	0.25 (0.15 to 0.40)

**Injuries**	**2,457.39 (2,105.97 to 2,867.68)**	**2,719.73 (2,089.52 to 3,954.43)**	**2,348.01 (1,755.77 to 3,495.84)**	**3,066.75 (2,343.02 to 4,378.09)**	**48.36 (41.10 to 56.33)**	**63.57 (50.18 to 87.62)**	**57.76 (44.82 to 81.33)**	**68.97 (54.11 to 95.38)**
**Transport injuries**	**556.94 (470.29 to 650.27)**	**632.34 (474.88 to 845.59)**	**449.25 (337.26 to 614.52)**	**856.46 (631.85 to 1,165.62)**	**11.14 (9.42 to 12.93)**	**14.82 (11.31 to 19.50)**	**11.26 (8.58 to 14.91)**	**19.12 (14.31 to 25.73)**
Road injuries	503.42 (424.74 to 585.28)	549.90 (412.65 to 738.35)	379.18 (282.82 to 515.08)	761.61 (563.83 to 1,036.93)	10.05 (8.49 to 11.67)	12.84 (9.87 to 16.67)	9.50 (7.28 to 12.40)	16.91 (12.71 to 22.66)
Pedestrian road injuries	114.21 (95.29 to 144.40)	121.96 (92.87 to 162.65)	86.54 (64.95 to 117.57)	158.12 (117.69 to 218.02)	2.56 (2.15 to 3.24)	3.73 (2.88 to 4.80)	2.90 (2.21 to 3.78)	4.54 (3.43 to 6.09)

Cause	YLLs (in 1000s)				Deaths (in 1000s)			
	2016	2040 Forecast	2040 Better Scenario	2040 Worse Scenario	2016	2040 Forecast	2040 Better Scenario	2040 Worse Scenario
**Philippines**								
Cyclist road injuries	20.88 (16.63 to 25.40)	21.82 (15.88 to 30.01)	15.37 (11.19 to 21.22)	29.92 (21.36 to 41.78)	0.46 (0.36 to 0.56)	0.55 (0.41 to 0.76)	0.43 (0.32 to 0.58)	0.71 (0.51 to 0.98)
Motorcyclist road injuries	110.32 (81.86 to 135.72)	112.26 (74.20 to 163.65)	85.08 (56.31 to 124.83)	146.65 (95.09 to 218.22)	1.97 (1.45 to 2.42)	2.10 (1.39 to 3.01)	1.64 (1.10 to 2.34)	2.69 (1.75 to 3.96)
Motor vehicle road injuries	246.00 (198.03 to 300.15)	280.28 (202.92 to 393.59)	183.15 (132.12 to 259.37)	407.52 (292.10 to 575.12)	4.81 (3.86 to 5.88)	6.14 (4.54 to 8.48)	4.29 (3.18 to 5.92)	8.55 (6.19 to 11.94)
Other road injuries	12.00 (9.15 to 16.16)	13.57 (9.35 to 19.89)	9.04 (6.19 to 13.37)	19.39 (13.05 to 29.18)	0.24 (0.18 to 0.32)	0.32 (0.23 to 0.47)	0.24 (0.17 to 0.35)	0.43 (0.29 to 0.64)
Other transport injuries	53.52 (43.63 to 65.66)	82.45 (58.89 to 116.05)	70.07 (50.37 to 99.24)	94.84 (66.85 to 134.77)	1.09 (0.90 to 1.33)	1.98 (1.45 to 2.77)	1.76 (1.29 to 2.44)	2.21 (1.59 to 3.13)
**Unintentional injuries**	**778.54 (689.93 to 874.91)**	**681.31 (577.35 to 802.57)**	**576.95 (482.07 to 694.50)**	**741.57 (625.18 to 882.59)**	**15.01 (13.27 to 16.88)**	**18.39 (15.51 to 21.34)**	**17.33 (14.41 to 20.45)**	**18.61 (15.67 to 21.77)**
Falls	120.69 (95.21 to 141.26)	156.77 (121.62 to 185.66)	149.20 (115.55 to 179.73)	156.54 (122.52 to 183.92)	3.48 (2.59 to 4.10)	6.55 (4.51 to 7.77)	6.80 (4.72 to 8.16)	6.07 (4.24 to 7.27)
Drowning	304.34 (266.45 to 348.31)	218.26 (174.08 to 272.82)	175.01 (137.74 to 222.58)	248.12 (195.03 to 315.06)	5.02 (4.41 to 5.70)	4.49 (3.68 to 5.51)	3.87 (3.13 to 4.89)	4.92 (3.97 to 6.22)
Fire, heat, and hot substances	50.32 (42.66 to 60.28)	39.00 (30.94 to 50.12)	31.62 (24.58 to 41.72)	44.35 (34.76 to 58.34)	1.04 (0.88 to 1.20)	1.17 (0.91 to 1.44)	1.06 (0.81 to 1.35)	1.23 (0.95 to 1.56)
Poisonings	11.42 (8.72 to 14.02)	7.14 (5.14 to 9.45)	5.39 (3.82 to 7.27)	8.61 (6.16 to 11.78)	0.21 (0.16 to 0.25)	0.16 (0.12 to 0.21)	0.13 (0.10 to 0.17)	0.19 (0.14 to 0.25)
Exposure to mechanical forces	55.99 (46.57 to 70.51)	44.17 (35.55 to 55.62)	33.98 (27.21 to 42.98)	50.58 (40.81 to 64.51)	0.91 (0.77 to 1.10)	0.85 (0.71 to 1.03)	0.71 (0.58 to 0.87)	0.94 (0.78 to 1.14)
Unintentional firearm injuries	11.53 (9.38 to 14.78)	9.95 (7.50 to 13.31)	8.22 (6.07 to 11.09)	11.14 (8.37 to 14.71)	0.24 (0.19 to 0.30)	0.24 (0.19 to 0.31)	0.21 (0.16 to 0.28)	0.26 (0.20 to 0.34)
Unintentional suffocation	28.31 (21.30 to 41.43)	18.83 (13.58 to 27.77)	13.18 (9.62 to 19.16)	22.30 (16.05 to 32.77)	0.37 (0.28 to 0.53)	0.27 (0.20 to 0.38)	0.19 (0.14 to 0.27)	0.31 (0.23 to 0.45)
Other exposure to mechanical forces	16.15 (12.51 to 19.35)	15.39 (11.62 to 19.27)	12.58 (9.24 to 16.03)	17.14 (12.79 to 21.47)	0.30 (0.23 to 0.36)	0.34 (0.26 to 0.42)	0.30 (0.23 to 0.37)	0.37 (0.27 to 0.45)
Adverse effects of medical treatment	43.56 (30.97 to 54.92)	46.86 (35.47 to 62.28)	41.00 (31.40 to 55.83)	50.05 (37.35 to 65.22)	0.91 (0.69 to 1.22)	1.38 (1.09 to 1.92)	1.32 (1.04 to 1.89)	1.37 (1.09 to 1.88)
Animal contact	29.45 (21.62 to 39.36)	34.62 (25.00 to 49.17)	27.18 (19.58 to 39.92)	39.85 (28.41 to 56.84)	0.56 (0.43 to 0.79)	0.79 (0.59 to 1.15)	0.67 (0.50 to 1.01)	0.87 (0.64 to 1.27)
Venomous animal contact	24.24 (16.54 to 32.21)	27.63 (18.87 to 38.73)	21.64 (14.79 to 31.03)	31.91 (21.71 to 44.79)	0.46 (0.32 to 0.63)	0.62 (0.45 to 0.89)	0.52 (0.38 to 0.78)	0.68 (0.49 to 0.98)
Non-venomous animal contact	5.21 (3.42 to 9.55)	6.98 (4.43 to 12.97)	5.54 (3.54 to 10.46)	7.94 (4.99 to 15.24)	0.10 (0.07 to 0.19)	0.17 (0.11 to 0.31)	0.14 (0.09 to 0.26)	0.18 (0.12 to 0.34)
Foreign body	87.82 (70.91 to 115.18)	73.80 (59.82 to 93.95)	62.88 (52.41 to 77.73)	77.53 (62.15 to 99.78)	1.47 (1.23 to 1.75)	1.65 (1.26 to 1.94)	1.60 (1.18 to 1.90)	1.61 (1.26 to 1.88)
Pulmonary aspiration and foreign body in airway	75.36 (59.92 to 96.57)	61.57 (49.39 to 78.29)	52.28 (42.04 to 64.57)	64.27 (50.95 to 82.74)	1.22 (0.98 to 1.46)	1.35 (0.98 to 1.60)	1.31 (0.92 to 1.59)	1.30 (0.98 to 1.54)

Foreign body in other body part	12.46 (9.18 to 23.21)	12.23 (9.11 to 20.96)	10.60 (7.93 to 17.46)	13.25 (9.81 to 23.01)	0.24 (0.19 to 0.41)	0.30 (0.23 to 0.44)	0.28 (0.22 to 0.41)	0.31 (0.24 to 0.46)
Environmental heat and cold exposure	4.84 (1.96 to 6.53)	4.48 (1.83 to 6.43)	3.59 (1.45 to 5.25)	5.15 (2.04 to 7.45)	0.12 (0.05 to 0.15)	0.14 (0.07 to 0.20)	0.12 (0.06 to 0.17)	0.15 (0.07 to 0.21)
Other unintentional injuries	70.12 (57.57 to 84.04)	56.21 (44.46 to 71.12)	47.10 (33.84 to 62.33)	60.79 (47.99 to 77.70)	1.31 (1.07 to 1.57)	1.21 (0.99 to 1.49)	1.06 (0.78 to 1.36)	1.27 (1.03 to 1.57)
**Self-harm and interpersonal violence**	**994.08 (711.51 to 1,265.84)**	**1,195.53 (809.87 to 1,666.98)**	**1,120.54 (766.03 to 1,511.07)**	**1,256.70 (831.01 to 1,788.46)**	**20.03 (14.09 to 25.45)**	**26.30 (17.77 to 35.97)**	**25.19 (17.20 to 33.97)**	**27.21 (17.87 to 38.10)**

Philippines								
Cause	YLLs (in 1000s)				Deaths (in 1000s)			
	2016	2040 Forecast	2040 Better Scenario	2040 Worse Scenario	2016	2040 Forecast	2040 Better Scenario	2040 Worse Scenario
Self-harm	268.81 (199.62 to 441.20)	408.24 (239.81 to 705.31)	370.01 (225.54 to 635.95)	467.46 (258.66 to 836.34)	5.60 (4.22 to 9.06)	9.46 (5.83 to 15.84)	8.87 (5.64 to 14.59)	10.51 (6.06 to 18.16)
Self-harm by firearm	8.33 (4.92 to 17.75)	10.81 (5.27 to 24.14)	9.58 (4.72 to 21.22)	12.66 (5.77 to 28.21)	0.18 (0.11 to 0.38)	0.27 (0.14 to 0.59)	0.24 (0.13 to 0.53)	0.30 (0.15 to 0.67)
Self-harm by other specified means	260.48 (193.90 to 425.52)	397.43 (234.11 to 679.67)	360.44 (219.63 to 616.08)	454.80 (250.62 to 803.41)	5.42 (4.08 to 8.73)	9.19 (5.68 to 15.21)	8.62 (5.51 to 14.17)	10.21 (5.91 to 17.61)
Interpersonal violence	725.27 (394.97 to 954.45)	787.29 (396.12 to 1,096.14)	750.53 (380.13 to 1,042.47)	789.23 (380.08 to 1,113.06)	14.42 (7.51 to 19.32)	16.85 (8.17 to 23.27)	16.32 (8.05 to 22.69)	16.69 (7.75 to 23.37)
Physical violence by firearm	379.19 (144.16 to 528.54)	396.19 (137.49 to 572.71)	374.02 (125.48 to 542.31)	399.50 (133.41 to 585.38)	7.60 (2.79 to 10.57)	8.44 (2.85 to 12.11)	8.07 (2.72 to 11.71)	8.42 (2.75 to 12.21)
Physical violence by sharp object	305.06 (157.51 to 439.87)	353.44 (175.76 to 516.06)	343.33 (172.17 to 494.06)	347.85 (165.22 to 513.87)	6.05 (3.04 to 8.70)	7.58 (3.65 to 10.98)	7.49 (3.62 to 10.72)	7.37 (3.36 to 10.68)
Physical violence by other means	41.02 (18.51 to 67.85)	37.66 (16.06 to 61.97)	33.18 (14.15 to 54.62)	41.89 (17.23 to 70.18)	0.77 (0.33 to 1.24)	0.83 (0.34 to 1.35)	0.76 (0.32 to 1.23)	0.90 (0.35 to 1.49)
**Forces of nature, conflict and terrorism, and executions and police conflict**	**127.83 (65.67 to 192.10)**	**210.55 (0.00 to 1,218.84)**	**201.28 (0.00 to 1,166.97)**	**212.02 (0.00 to 1,224.88)**	**2.19 (1.13 to 3.28)**	**4.06 (0.00 to 23.53)**	**3.98 (0.00 to 23.16)**	**4.03 (0.00 to 23.37)**
Exposure to forces of nature	0.00 (0.00 to 0.00)	33.89 (0.00 to 250.03)	31.57 (0.00 to 232.21)	34.31 (0.00 to 253.25)	0.00 (0.00 to 0.00)	0.70 (0.00 to 5.07)	0.68 (0.00 to 4.95)	0.69 (0.00 to 5.02)
Conflict and terrorism	123.09 (60.14 to 187.45)	38.11 (0.00 to 76.37)	35.84 (0.00 to 74.42)	38.61 (0.00 to 76.72)	2.09 (1.02 to 3.19)	0.70 (0.00 to 1.23)	0.67 (0.00 to 1.20)	0.69 (0.00 to 1.23)
Executions and police conflict	4.74 (3.19 to 6.88)	138.55 (0.00 to 932.85)	133.87 (0.00 to 898.97)	139.09 (0.00 to 938.56)	0.09 (0.06 to 0.13)	2.66 (0.00 to 17.91)	2.62 (0.00 to 17.67)	2.65 (0.00 to 17.80)

Sri Lanka								
Cause	YLLs (actual counts)				Deaths (actual counts)			
	2016	2040 Forecast	2040 Better Scenario	2040 Worse Scenario	2016	2040 Forecast	2040 Better Scenario	2040 Worse Scenario
**All causes**	**2,829,454.76 (2,313,557.10 to 3,443,950.82)**	**3,178,854.71 (2,278,734.18 to 4,462,870.88)**	**2,599,621.67 (1,870,535.77 to 3,624,344.47)**	**4,070,778.31 (2,903,773.00 to 5,659,029.06)**	**122,696.52 (100,743.32 to 146,912.22)**	**195,808.06 (153,250.44 to 251,553.80)**	**166,208.97 (131,444.22 to 216,624.75)**	**237,470.05 (186,699.77 to 300,778.71)**
**Communicable, maternal, neonatal, and nutritional diseases**	**301,161.18 (244,383.32 to 370,766.01)**	**243,191.58 (171,711.15 to 337,739.32)**	**210,238.91 (148,511.88 to 291,924.28)**	**335,715.94 (229,997.92 to 475,883.99)**	**8,604.33 (7,036.70 to 10,385.10)**	**10,678.89 (7,804.33 to 14,592.18)**	**9,661.09 (7,104.48 to 13,585.11)**	**14,436.49 (10,178.81 to 20,299.14)**
**HIV/AIDS and tuberculosis**	**19,943.28 (15,407.69 to 25,299.18)**	**13,508.91 (7,663.19 to 24,222.23)**	**7,717.61 (4,532.01 to 12,983.89)**	**23,514.82 (14,839.92 to 39,778.54)**	**731.63 (569.32 to 920.00)**	**646.31 (394.75 to 1,112.69)**	**384.36 (239.32 to 614.79)**	**1,020.07 (655.38 to 1,706.76)**
Tuberculosis	17,228.27 (13,050.39 to 22,260.16)	11,738.92 (6,088.10 to 22,206.49)	6,642.40 (3,726.06 to 12,112.63)	18,021.62 (9,598.27 to 34,098.67)	672.50 (517.53 to 862.76)	606.05 (354.64 to 1,074.28)	359.37 (221.15 to 593.09)	898.59 (522.33 to 1,571.97)
HIV/AIDS	2,715.01 (1,244.52 to 4,792.92)	1,769.99 (435.55 to 3,956.55)	1,075.21 (239.33 to 3,257.57)	5,493.20 (2,832.85 to 9,030.65)	59.13 (25.08 to 108.26)	40.26 (7.01 to 98.03)	24.99 (3.70 to 81.64)	121.48 (58.03 to 208.91)
**Diarrhea, lower respiratory, and other common infectious diseases**	**161,808.26 (129,462.82 to 202,299.99)**	**133,297.92 (87,615.53 to 193,741.82)**	**115,769.05 (76,374.65 to 172,553.70)**	**192,655.75 (120,659.15 to 288,595.05)**	**5,930.53 (4,682.51 to 7,462.54)**	**7,639.42 (5,182.43 to 11,281.47)**	**6,925.40 (4,664.56 to 10,617.98)**	**10,752.61 (6,884.84 to 16,761.44)**
Diarrheal diseases	10,879.03 (6,192.90 to 20,849.48)	8,039.19 (1,711.88 to 24,625.93)	10,281.44 (2,394.71 to 31,124.17)	15,513.26 (2,770.27 to 53,572.18)	540.98 (266.63 to 1,024.62)	670.09 (137.60 to 2,030.34)	896.03 (201.66 to 2,637.00)	1,241.72 (200.18 to 4,184.78)
Intestinal infectious diseases	25,185.63 (13,520.10 to 42,630.93)	13,882.65 (6,026.59 to 28,805.66)	14,810.53 (6,527.75 to 30,035.48)	14,759.82 (6,248.69 to 31,368.25)	396.02 (217.59 to 666.57)	247.17 (118.95 to 477.22)	268.71 (130.36 to 506.43)	256.54 (119.71 to 496.09)
Typhoid fever	21,919.57 (11,812.66 to 37,549.06)	11,999.75 (4,988.54 to 25,433.65)	13,188.29 (5,706.30 to 27,108.43)	12,515.73 (4,999.37 to 26,579.60)	343.01 (186.51 to 578.48)	212.39 (99.17 to 416.57)	238.18 (115.70 to 450.69)	216.03 (98.28 to 421.40)
Paratyphoid fever	3,253.18 (1,460.48 to 6,639.86)	1,876.39 (663.08 to 4,402.45)	1,616.07 (568.72 to 3,801.78)	2,236.10 (806.25 to 5,294.52)	52.63 (23.33 to 107.37)	34.42 (13.11 to 71.71)	30.16 (11.44 to 64.95)	40.08 (15.46 to 87.48)
Other intestinal infectious diseases	12.89 (5.00 to 26.67)	6.51 (2.28 to 14.55)	6.17 (2.15 to 13.72)	7.98 (2.83 to 17.37)	0.38 (0.15 to 0.80)	0.37 (0.13 to 0.85)	0.37 (0.13 to 0.84)	0.42 (0.15 to 0.96)
Lower respiratory infections	96,982.54 (75,698.30 to 121,313.41)	84,412.74 (53,524.74 to 125,548.00)	65,650.45 (41,924.80 to 98,152.00)	131,616.70 (77,446.58 to 203,996.92)	4,155.20 (3,236.47 to 5,570.31)	5,583.20 (3,606.51 to 8,840.84)	4,662.51 (2,998.97 to 7,576.38)	8,049.24 (4,928.17 to 12,736.80)
Upper respiratory infections	89.51 (47.65 to 152.41)	66.85 (25.78 to 142.62)	52.95 (19.17 to 113.29)	93.38 (34.56 to 196.46)	2.81 (1.40 to 4.79)	3.25 (1.17 to 7.00)	2.66 (0.91 to 5.78)	4.35 (1.53 to 9.37)
Otitis media	9.17 (4.31 to 18.96)	4.71 (1.66 to 10.81)	5.00 (1.76 to 11.64)	4.32 (1.56 to 10.14)	0.48 (0.22 to 0.98)	0.34 (0.12 to 0.76)	0.38 (0.13 to 0.84)	0.30 (0.10 to 0.67)
Meningitis	15,717.01 (10,730.46 to 27,077.21)	10,104.15 (6,412.52 to 16,994.52)	8,762.69 (5,512.91 to 14,789.08)	11,122.35 (7,189.38 to 18,723.38)	484.67 (316.42 to 719.51)	508.94 (254.50 to 731.14)	458.45 (231.10 to 648.49)	532.66 (267.00 to 757.80)
Pneumococcal meningitis	128.31 (84.43 to 220.40)	68.23 (39.48 to 110.20)	70.91 (40.22 to 115.11)	65.52 (38.43 to 103.66)	4.59 (2.62 to 7.08)	4.38 (1.69 to 7.61)	4.71 (1.83 to 8.22)	4.01 (1.59 to 7.00)
H influenzae type B meningitis	959.46 (637.52 to 1,683.35)	723.47 (430.77 to 1,274.99)	728.83 (439.53 to 1,288.08)	734.14 (419.86 to 1,354.43)	27.76 (18.46 to 41.20)	29.45 (15.82 to 42.45)	31.22 (16.10 to 45.20)	27.39 (15.28 to 40.42)
Meningococcal meningitis	9,299.06 (6,378.61 to 16,026.92)	5,932.43 (3,722.02 to 9,653.63)	5,118.48 (3,180.94 to 8,527.92)	6,548.82 (4,195.82 to 11,010.18)	299.21 (188.72 to 432.18)	317.22 (155.24 to 463.94)	284.58 (137.04 to 406.29)	333.15 (162.18 to 480.31)
Other meningitis	5,330.18 (3,545.23 to 9,144.53)	3,380.02 (2,123.28 to 5,763.33)	2,844.47 (1,768.91 to 4,884.40)	3,773.87 (2,433.99 to 6,593.32)	153.11 (103.33 to 227.65)	157.90 (81.97 to 229.20)	137.94 (71.28 to 198.20)	168.11 (85.84 to 241.59)
Encephalitis	12,003.76 (6,272.34 to 24,330.72)	12,473.67 (7,620.03 to 22,999.23)	12,387.74 (7,666.72 to 22,129.84)	13,531.61 (8,093.67 to 25,713.49)	328.15 (228.52 to 504.13)	555.02 (375.07 to 726.82)	572.21 (387.36 to 748.75)	572.68 (389.95 to 757.28)

Diphtheria	9.58 (6.02 to 14.13)	2.02 (0.85 to 4.01)	2.01 (0.84 to 4.04)	2.43 (0.96 to 5.33)	0.17 (0.10 to 0.25)	0.04 (0.02 to 0.07)	0.04 (0.02 to 0.07)	0.04 (0.02 to 0.08)
Whooping cough	178.92 (11.90 to 658.47)	92.69 (1.89 to 474.90)	89.24 (1.94 to 454.95)	305.39 (3.68 to 1,481.38)	2.13 (0.14 to 7.82)	1.10 (0.02 to 5.65)	1.06 (0.03 to 5.40)	3.61 (0.05 to 17.52)
Tetanus	306.96 (204.42 to 566.50)	45.47 (26.10 to 96.99)	46.32 (26.37 to 99.98)	49.86 (28.09 to 100.19)	8.82 (5.69 to 20.03)	1.73 (1.02 to 4.36)	1.83 (1.07 to 4.51)	1.67 (1.00 to 4.00)
Measles	164.10 (35.22 to 474.40)	4,011.31 (79.60 to 16,517.59)	3,533.23 (74.01 to 13,868.67)	5,451.54 (96.05 to 23,888.29)	1.97 (0.43 to 5.70)	57.80 (1.32 to 225.46)	51.10 (1.25 to 187.41)	77.50 (1.51 to 330.14)

Sri Lanka								
Cause	YLLs (actual counts)				Deaths (actual counts)			
	2016	2040 Forecast	2040 Better Scenario	2040 Worse Scenario	2016	2040 Forecast	2040 Better Scenario	2040 Worse Scenario
Varicella and herpes zoster	282.03 (176.40 to 445.19)	162.47 (77.44 to 295.17)	147.46 (71.14 to 265.64)	205.11 (92.17 to 380.02)	9.12 (6.18 to 12.66)	10.72 (5.43 to 18.36)	10.42 (5.19 to 18.10)	12.31 (6.22 to 21.36)
**Neglected tropical diseases and malaria**	**15,964.72 (5,139.33 to 27,422.69)**	**33,227.05 (13,756.52 to 54,856.53)**	**32,525.62 (13,808.13 to 52,822.69)**	**35,042.14 (13,705.31 to 59,015.46)**	**335.67 (124.09 to 551.77)**	**969.07 (484.09 to 1,436.90)**	**980.27 (505.09 to 1,449.57)**	**975.81 (462.76 to 1,465.91)**
Malaria	0.00 (0.00 to 0.00)	0.00 (0.00 to 0.00)	0.00 (0.00 to 0.00)	0.00 (0.00 to 0.00)	0.00 (0.00 to 0.00)	0.00 (0.00 to 0.00)	0.00 (0.00 to 0.00)	0.00 (0.00 to 0.00)
Chagas disease	0.00 (0.00 to 0.00)	0.00 (0.00 to 0.00)	0.00 (0.00 to 0.00)	0.00 (0.00 to 0.00)	0.00 (0.00 to 0.00)	0.00 (0.00 to 0.00)	0.00 (0.00 to 0.00)	0.00 (0.00 to 0.00)
Leishmaniasis	2.09 (1.10 to 3.27)	0.86 (0.39 to 1.62)	0.79 (0.36 to 1.51)	1.09 (0.50 to 1.95)	0.07 (0.04 to 0.11)	0.04 (0.01 to 0.07)	0.03 (0.01 to 0.07)	0.04 (0.02 to 0.09)
African trypanosomiasis	0.00 (0.00 to 0.00)	0.00 (0.00 to 0.00)	0.00 (0.00 to 0.00)	0.00 (0.00 to 0.00)	0.00 (0.00 to 0.00)	0.00 (0.00 to 0.00)	0.00 (0.00 to 0.00)	0.00 (0.00 to 0.00)
Schistosomiasis	0.00 (0.00 to 0.00)	0.00 (0.00 to 0.00)	0.00 (0.00 to 0.00)	0.00 (0.00 to 0.00)	0.00 (0.00 to 0.00)	0.00 (0.00 to 0.00)	0.00 (0.00 to 0.00)	0.00 (0.00 to 0.00)
Cysticercosis	0.00 (0.00 to 0.00)	0.00 (0.00 to 0.00)	0.00 (0.00 to 0.00)	0.00 (0.00 to 0.00)	0.00 (0.00 to 0.00)	0.00 (0.00 to 0.00)	0.00 (0.00 to 0.00)	0.00 (0.00 to 0.00)
Cystic echinococcosis	0.00 (0.00 to 0.00)	0.00 (0.00 to 0.00)	0.00 (0.00 to 0.00)	0.00 (0.00 to 0.00)	0.00 (0.00 to 0.00)	0.00 (0.00 to 0.00)	0.00 (0.00 to 0.00)	0.00 (0.00 to 0.00)
Dengue	13,444.80 (3,111.04 to 24,496.83)	31,794.38 (11,816.38 to 53,060.86)	31,105.76 (11,730.04 to 51,153.54)	33,263.24 (11,586.69 to 57,021.01)	273.13 (64.81 to 480.91)	913.93 (406.70 to 1,388.29)	922.87 (425.36 to 1,389.66)	912.35 (380.70 to 1,398.79)
Yellow fever	0.00 (0.00 to 0.00)	0.00 (0.00 to 0.00)	0.00 (0.00 to 0.00)	0.00 (0.00 to 0.00)	0.00 (0.00 to 0.00)	0.00 (0.00 to 0.00)	0.00 (0.00 to 0.00)	0.00 (0.00 to 0.00)
Rabies	1,428.77 (742.84 to 2,534.97)	574.57 (250.57 to 1,486.90)	531.70 (232.22 to 1,410.66)	847.44 (379.14 to 2,135.66)	36.78 (20.79 to 72.31)	20.07 (8.27 to 74.58)	19.20 (7.76 to 71.14)	28.04 (12.10 to 92.61)
Intestinal nematode infections	98.24 (37.02 to 185.24)	96.44 (34.71 to 208.16)	94.60 (34.42 to 200.89)	96.29 (34.42 to 206.72)	1.96 (0.98 to 3.16)	2.80 (1.20 to 5.60)	2.88 (1.23 to 5.88)	2.62 (1.12 to 5.07)
Other neglected tropical diseases	990.82 (633.14 to 1,637.06)	760.79 (439.00 to 1,594.83)	792.77 (456.20 to 1,632.09)	834.08 (500.43 to 1,690.22)	23.74 (17.09 to 36.71)	32.23 (18.88 to 77.80)	35.29 (20.31 to 85.19)	32.76 (19.92 to 76.20)
**Maternal disorders**	**5,476.19 (3,516.24 to 7,819.44)**	**2,042.16 (908.07 to 3,915.36)**	**1,819.11 (770.89 to 3,535.45)**	**2,848.67 (1,278.47 to 5,453.25)**	**95.84 (61.41 to 136.84)**	**36.07 (16.03 to 68.74)**	**32.15 (13.85 to 61.78)**	**50.27 (22.51 to 94.94)**
Maternal hemorrhage	1,695.48 (1,013.91 to 2,631.73)	558.32 (229.81 to 1,106.13)	500.66 (200.38 to 1,002.11)	775.66 (318.34 to 1,490.63)	29.77 (17.80 to 46.54)	9.86 (4.05 to 19.15)	8.85 (3.49 to 17.91)	13.69 (5.63 to 26.15)
Maternal sepsis and other maternal infections	350.33 (193.00 to 562.67)	108.89 (41.78 to 217.45)	96.90 (36.33 to 195.88)	152.10 (60.14 to 298.09)	6.21 (3.46 to 9.95)	1.96 (0.76 to 3.81)	1.74 (0.67 to 3.50)	2.73 (1.10 to 5.27)
Maternal hypertensive disorders	610.56 (357.99 to 969.53)	200.56 (81.94 to 410.71)	177.77 (71.71 to 365.41)	281.64 (114.25 to 560.27)	10.64 (6.23 to 16.74)	3.52 (1.45 to 7.15)	3.13 (1.25 to 6.35)	4.94 (2.01 to 9.80)
Maternal obstructed labor and uterine rupture	37.72 (14.24 to 74.14)	13.79 (3.81 to 35.75)	12.00 (3.33 to 31.66)	19.90 (5.67 to 51.25)	0.66 (0.25 to 1.30)	0.25 (0.07 to 0.64)	0.21 (0.06 to 0.56)	0.35 (0.10 to 0.92)
Maternal abortion, miscarriage, and ectopic pregnancy	1,172.88 (693.38 to 1,833.37)	530.62 (211.95 to 1,078.76)	471.57 (183.46 to 970.10)	751.57 (301.17 to 1,501.15)	20.72 (12.19 to 31.87)	9.49 (3.87 to 19.48)	8.44 (3.32 to 17.53)	13.44 (5.55 to 27.48)

Indirect maternal deaths	838.42 (476.66 to 1,408.55)	321.47 (128.95 to 661.98)	282.01 (109.00 to 598.94)	448.83 (181.13 to 916.86)	14.36 (8.20 to 23.88)	5.54 (2.23 to 11.29)	4.86 (1.90 to 10.15)	7.72 (3.15 to 15.69)
Late maternal deaths	29.45 (11.15 to 60.91)	11.25 (3.34 to 28.16)	9.88 (2.81 to 24.63)	15.80 (4.70 to 38.63)	0.51 (0.20 to 1.06)	0.20 (0.06 to 0.49)	0.17 (0.05 to 0.43)	0.28 (0.09 to 0.67)
Maternal deaths aggravated by HIV/AIDS	0.31 (0.11 to 0.69)	0.97 (0.08 to 5.26)	0.76 (0.03 to 4.66)	1.48 (0.23 to 7.76)	0.01 (0.00 to 0.01)	0.02 (0.00 to 0.08)	0.01 (0.00 to 0.07)	0.03 (0.00 to 0.12)
Other maternal disorders	741.03 (431.93 to 1,143.92)	296.31 (121.82 to 594.11)	267.54 (107.27 to 539.10)	401.68 (164.53 to 785.33)	12.94 (7.54 to 20.05)	5.24 (2.14 to 10.59)	4.74 (1.93 to 9.43)	7.09 (2.94 to 13.79)

Sri Lanka								
Cause	YLLs (actual counts)				Deaths (actual counts)			
	2016	2040 Forecast	2040 Better Scenario	2040 Worse Scenario	2016	2040 Forecast	2040 Better Scenario	2040 Worse Scenario
**Neonatal disorders**	**82,489.65 (52,833.77 to 122,367.84)**	**46,375.60 (17,739.82 to 97,239.82)**	**37,462.25 (14,174.24 to 77,707.18)**	**65,540.55 (24,087.54 to 135,707.18)**	**953.16 (610.50 to 1,413.91)**	**535.95 (205.03 to 1,123.50)**	**432.96 (163.91 to 898.18)**	**757.38 (278.53 to 1,567.50)**
Neonatal preterm birth	40,643.71 (24,576.41 to 62,672.24)	16,966.48 (6,115.28 to 34,202.62)	13,324.13 (4,719.51 to 28,079.95)	27,817.19 (9,975.94 to 57,453.54)	469.55 (283.87 to 723.99)	196.04 (70.69 to 395.09)	153.96 (54.52 to 324.42)	321.38 (115.31 to 663.83)
Neonatal encephalopathy due to birth asphyxia and trauma	18,943.04 (10,953.34 to 30,348.09)	11,339.47 (3,613.33 to 28,002.80)	8,759.33 (2,726.72 to 21,696.02)	14,679.92 (4,550.08 to 37,071.68)	218.91 (126.60 to 350.67)	131.06 (41.79 to 323.55)	101.24 (31.54 to 250.70)	169.66 (52.63 to 428.27)
Neonatal sepsis and other neonatal infections	13,420.83 (7,391.91 to 23,610.19)	9,331.78 (3,379.64 to 20,318.05)	7,581.83 (2,709.34 to 16,938.16)	11,926.42 (4,209.02 to 25,840.30)	155.08 (85.42 to 272.84)	107.84 (39.06 to 234.75)	87.62 (31.32 to 195.68)	137.82 (48.68 to 298.54)
Hemolytic disease and other neonatal jaundice	127.68 (72.89 to 217.49)	48.01 (14.47 to 117.24)	38.48 (11.78 to 93.48)	64.03 (19.41 to 157.66)	1.48 (0.84 to 2.51)	0.56 (0.17 to 1.36)	0.45 (0.14 to 1.08)	0.74 (0.23 to 1.83)
Other neonatal disorders	9,354.40 (5,338.29 to 15,673.91)	8,689.86 (3,032.24 to 18,874.44)	7,758.49 (2,742.66 to 16,839.96)	11,053.00 (3,864.05 to 24,062.81)	108.13 (61.70 to 181.12)	100.46 (35.11 to 218.06)	89.69 (31.78 to 194.66)	127.77 (44.73 to 278.06)
**Nutritional deficiencies**	**3,389.02 (2,749.43 to 4,149.96)**	**3,919.75 (3,021.34 to 5,005.45)**	**4,227.73 (3,252.56 to 5,455.26)**	**4,081.16 (3,169.32 to 5,201.82)**	**172.24 (140.13 to 210.05)**	**323.09 (246.54 to 408.31)**	**361.87 (279.88 to 455.48)**	**320.71 (232.94 to 410.91)**
Protein-energy malnutrition	2,843.81 (2,275.29 to 3,474.61)	3,146.25 (2,375.45 to 4,086.52)	3,460.88 (2,604.89 to 4,480.58)	3,234.24 (2,467.54 to 4,170.76)	150.43 (121.64 to 183.93)	272.95 (201.42 to 359.39)	310.98 (227.75 to 402.24)	267.86 (186.41 to 357.54)
Iodine deficiency	71.40 (7.40 to 205.13)	175.15 (6.69 to 628.36)	179.19 (5.86 to 669.68)	194.85 (8.78 to 665.74)	2.89 (0.21 to 9.89)	13.42 (0.25 to 57.17)	14.16 (0.22 to 61.79)	14.35 (0.35 to 58.23)
Dietary iron deficiency	130.85 (96.71 to 183.58)	218.85 (130.20 to 374.27)	224.82 (133.20 to 393.34)	233.58 (141.74 to 387.20)	2.88 (2.08 to 5.09)	6.53 (3.72 to 13.95)	6.86 (3.84 to 15.31)	6.78 (3.95 to 14.14)
Other nutritional deficiencies	342.95 (259.00 to 448.83)	379.50 (264.34 to 519.85)	362.84 (248.67 to 510.62)	418.50 (287.00 to 575.08)	16.05 (11.88 to 21.86)	30.18 (20.65 to 44.53)	29.88 (20.11 to 44.78)	31.72 (21.52 to 46.53)
**Other communicable, maternal, neonatal, and nutritional diseases**	**12,090.07 (9,324.71 to 15,518.63)**	**10,820.20 (7,734.66 to 15,353.76)**	**10,717.54 (7,516.99 to 15,173.81)**	**12,032.85 (8,517.09 to 17,002.89)**	**385.26 (299.64 to 494.86)**	**528.99 (396.42 to 735.12)**	**544.07 (404.13 to 773.52)**	**559.63 (419.76 to 776.51)**
Sexually transmitted infections excluding HIV	554.93 (369.39 to 768.14)	255.18 (161.98 to 370.94)	243.89 (152.79 to 359.34)	280.06 (175.01 to 401.50)	16.57 (10.99 to 23.16)	12.44 (8.17 to 16.82)	12.34 (8.06 to 16.73)	12.98 (8.52 to 17.64)
Syphilis	187.78 (125.43 to 255.32)	68.88 (45.47 to 96.55)	62.00 (40.00 to 89.47)	79.10 (51.52 to 111.73)	5.51 (3.35 to 7.66)	3.25 (2.15 to 4.39)	3.06 (1.97 to 4.19)	3.54 (2.30 to 4.76)
Chlamydial infection	71.18 (45.34 to 103.79)	37.01 (22.00 to 55.61)	36.23 (21.76 to 54.78)	39.82 (24.10 to 59.08)	2.15 (1.43 to 3.10)	1.91 (1.20 to 2.71)	1.94 (1.23 to 2.76)	1.95 (1.23 to 2.77)
Gonococcal infection	194.96 (125.73 to 281.05)	102.59 (62.03 to 151.32)	100.39 (60.25 to 151.31)	110.25 (66.41 to 161.61)	6.15 (4.06 to 8.82)	5.36 (3.46 to 7.39)	5.43 (3.45 to 7.55)	5.49 (3.53 to 7.52)
Other sexually transmitted infections	101.01 (65.84 to 146.09)	46.71 (26.83 to 71.99)	45.27 (25.87 to 69.94)	50.89 (29.63 to 78.36)	2.76 (1.81 to 3.98)	1.91 (1.24 to 2.63)	1.91 (1.24 to 2.66)	2.00 (1.29 to 2.77)
Acute hepatitis	491.75 (379.11 to 629.34)	357.39 (240.70 to 506.02)	306.16 (204.26 to 446.86)	419.25 (293.51 to 594.70)	15.61 (12.03 to 19.94)	16.34 (11.76 to 22.04)	14.32 (10.27 to 20.03)	18.58 (13.66 to 24.96)
Acute hepatitis A	12.43 (8.34 to 18.92)	5.65 (3.27 to 9.42)	5.01 (2.89 to 8.33)	6.56 (3.68 to 10.92)	0.26 (0.18 to 0.38)	0.20 (0.11 to 0.32)	0.19 (0.10 to 0.30)	0.22 (0.13 to 0.36)
Acute hepatitis B	398.32 (297.84 to 518.03)	293.81 (190.82 to 428.91)	250.83 (158.42 to 372.68)	347.25 (233.43 to 494.37)	13.00 (9.77 to 16.74)	12.84 (9.02 to 17.88)	11.15 (7.67 to 15.98)	14.83 (10.61 to 20.55)

Acute hepatitis C	11.12 (7.75 to 16.16)	10.12 (6.16 to 16.18)	8.75 (5.34 to 14.20)	11.17 (7.07 to 17.80)	0.39 (0.26 to 0.56)	0.49 (0.31 to 0.76)	0.43 (0.27 to 0.68)	0.53 (0.34 to 0.82)
Acute hepatitis E	69.89 (51.56 to 93.34)	47.81 (27.61 to 74.39)	41.58 (23.77 to 66.91)	54.28 (32.06 to 84.65)	1.96 (1.33 to 2.84)	2.81 (1.58 to 4.43)	2.55 (1.44 to 4.09)	3.00 (1.69 to 4.70)
Other unspecified infectious diseases	11,043.38 (8,424.42 to 14,321.00)	10,207.63 (7,245.85 to 14,596.00)	10,167.48 (7,083.39 to 14,514.47)	11,333.54 (7,971.45 to 16,058.64)	353.08 (272.97 to 457.99)	500.22 (370.60 to 705.12)	517.41 (379.51 to 745.64)	528.07 (392.54 to 741.89)
**Non-communicable diseases**	**1,996,816.94 (1,621,581.23 to 2,409,608.51)**	**2,473,039.63 (1,747,891.92 to 3,595,460.49)**	**2,009,400.31 (1,432,241.23 to 2,895,031.52)**	**3,186,809.16 (2,215,948.03 to 4,609,606.24)**	**99,946.68 (81,826.62 to 119,396.04)**	**167,987.73 (128,715.69 to 220,292.49)**	**141,823.34 (109,771.85 to 188,821.77)**	**203,605.69 (157,458.34 to 264,350.58)**

Sri Lanka								
Cause	YLLs (actual counts)				Deaths (actual counts)			
	2016	2040 Forecast	2040 Better Scenario	2040 Worse Scenario	2016	2040 Forecast	2040 Better Scenario	2040 Worse Scenario
**Neoplasms**	**371,476.69 (298,801.99 to 452,698.06)**	**516,547.21 (382,323.32 to 688,495.88)**	**469,084.38 (350,349.25 to 618,678.21)**	**564,113.52 (412,409.76 to 750,617.86)**	**15,358.64 (12,529.41 to 18,552.16)**	**28,179.04 (22,489.87 to 34,563.21)**	**26,275.71 (21,070.30 to 32,416.15)**	**29,308.72 (23,103.52 to 36,913.80)**
Lip and oral cavity cancer	21,447.47 (16,633.23 to 26,976.83)	36,362.32 (20,288.95 to 60,919.24)	26,192.70 (15,921.23 to 40,962.58)	45,484.81 (23,722.27 to 78,414.65)	988.18 (783.56 to 1,221.78)	2,058.74 (1,229.90 to 3,240.85)	1,550.02 (1,003.82 to 2,322.88)	2,397.21 (1,354.90 to 3,899.75)
Nasopharynx cancer	6,987.06 (5,108.78 to 9,148.33)	11,294.19 (6,033.02 to 19,161.00)	7,594.81 (4,289.52 to 12,457.77)	15,213.47 (7,693.81 to 26,789.44)	279.20 (207.08 to 362.78)	546.62 (313.25 to 892.21)	382.43 (230.59 to 598.31)	689.41 (376.37 to 1,168.66)
Other pharynx cancer	8,809.25 (6,473.88 to 11,582.52)	14,804.35 (7,844.86 to 24,327.66)	11,489.18 (6,600.28 to 18,463.79)	14,926.26 (7,996.70 to 24,731.43)	364.75 (272.33 to 473.96)	735.07 (411.00 to 1,182.66)	587.74 (358.96 to 901.25)	722.75 (413.92 to 1,148.20)
Esophageal cancer	22,917.83 (18,208.25 to 28,581.65)	28,558.12 (16,833.52 to 46,847.02)	24,315.29 (14,628.72 to 39,303.22)	35,611.29 (19,876.33 to 59,658.93)	997.32 (801.74 to 1,225.80)	1,495.13 (935.73 to 2,326.25)	1,314.60 (845.46 to 2,024.52)	1,768.43 (1,051.95 to 2,807.30)
Stomach cancer	21,244.73 (16,834.24 to 26,031.98)	21,244.69 (15,892.54 to 27,772.22)	19,389.71 (14,343.73 to 25,564.04)	23,212.55 (17,441.71 to 30,637.59)	996.02 (803.29 to 1,209.72)	1,302.68 (1,044.93 to 1,622.09)	1,214.00 (968.24 to 1,529.80)	1,371.91 (1,104.12 to 1,738.30)
Colon and rectum cancer	21,121.17 (16,573.02 to 26,216.04)	38,001.81 (24,233.01 to 58,232.08)	32,604.34 (21,102.84 to 48,340.91)	38,774.96 (25,032.14 to 58,600.24)	1,002.56 (792.20 to 1,224.57)	2,382.03 (1,606.78 to 3,520.62)	2,102.77 (1,437.99 to 3,028.13)	2,348.48 (1,578.00 to 3,473.74)
Liver cancer	15,941.40 (12,412.53 to 20,069.89)	20,339.75 (13,726.10 to 29,271.62)	19,776.04 (13,646.62 to 28,157.01)	21,166.17 (14,190.84 to 30,808.82)	694.39 (550.22 to 869.45)	1,127.39 (805.13 to 1,518.45)	1,122.95 (821.52 to 1,494.72)	1,127.31 (800.25 to 1,554.80)
Liver cancer due to hepatitis B	6,801.21 (4,859.83 to 9,070.48)	7,835.71 (4,950.24 to 11,636.17)	7,842.77 (5,030.03 to 11,505.15)	8,155.21 (5,189.81 to 12,225.53)	277.43 (197.51 to 370.04)	410.06 (263.44 to 586.63)	419.01 (273.83 to 589.74)	410.33 (264.70 to 598.89)
Liver cancer due to hepatitis C	3,251.66 (2,034.11 to 4,742.39)	4,698.29 (2,795.91 to 7,200.78)	4,742.55 (2,841.40 to 7,261.94)	4,765.27 (2,787.70 to 7,291.22)	162.56 (103.25 to 237.84)	298.40 (184.99 to 450.11)	307.29 (191.32 to 458.32)	291.69 (179.37 to 444.74)
Liver cancer due to alcohol use	2,249.23 (1,501.12 to 3,318.41)	2,928.87 (1,706.66 to 4,787.62)	2,404.21 (1,405.07 to 3,950.67)	3,175.15 (1,828.93 to 5,190.34)	99.86 (67.08 to 150.36)	152.82 (92.80 to 239.26)	130.27 (79.41 to 207.18)	159.97 (96.64 to 253.24)
Liver cancer due to other causes	3,639.30 (2,437.23 to 5,356.30)	4,876.88 (2,778.22 to 8,172.24)	4,786.50 (2,797.15 to 7,823.46)	5,070.54 (2,853.86 to 8,487.76)	154.54 (103.06 to 228.03)	266.11 (155.30 to 428.69)	266.38 (158.38 to 420.85)	265.34 (152.06 to 427.39)
Gallbladder and biliary tract cancer	7,870.97 (5,897.92 to 10,588.69)	11,016.10 (8,249.58 to 14,915.57)	11,137.83 (8,256.23 to 15,240.36)	10,428.35 (7,957.60 to 13,752.43)	380.83 (292.78 to 507.88)	677.40 (537.63 to 884.87)	697.84 (542.74 to 919.79)	623.54 (503.58 to 798.00)
Pancreatic cancer	13,516.69 (10,815.42 to 16,634.06)	25,404.55 (17,732.59 to 35,902.73)	25,235.78 (17,716.37 to 34,849.53)	24,289.02 (17,066.40 to 33,802.69)	637.21 (517.17 to 778.35)	1,492.56 (1,109.75 to 2,035.20)	1,510.04 (1,131.56 to 1,999.16)	1,376.22 (1,026.52 to 1,876.88)
Larynx cancer	3,621.84 (2,767.62 to 4,754.31)	4,156.44 (2,298.66 to 7,040.40)	3,060.78 (1,833.47 to 4,901.66)	6,355.39 (3,087.36 to 12,078.60)	155.49 (119.78 to 201.44)	216.72 (132.77 to 340.44)	167.52 (110.57 to 245.83)	301.90 (165.71 to 536.32)
Tracheal, bronchus, and lung cancer	34,183.15 (26,739.42 to 43,393.44)	36,827.86 (23,573.35 to 56,284.35)	30,739.52 (20,476.86 to 45,832.44)	61,646.51 (35,203.72 to 103,559.45)	1,457.97 (1,155.94 to 1,823.66)	1,981.68 (1,355.55 to 2,930.17)	1,712.08 (1,214.13 to 2,429.96)	3,022.86 (1,855.09 to 4,797.45)
Malignant skin melanoma	1,586.07 (1,154.39 to 2,063.05)	1,997.91 (1,450.41 to 2,673.25)	2,201.52 (1,567.78 to 2,973.01)	1,749.40 (1,275.89 to 2,316.42)	59.95 (45.20 to 77.27)	108.04 (84.54 to 135.45)	121.95 (93.52 to 153.79)	91.18 (72.16 to 112.38)
Non-melanoma skin cancer	5,386.71 (4,212.63 to 6,834.41)	8,673.11 (6,730.94 to 11,095.10)	8,091.93 (6,084.81 to 10,629.78)	8,975.40 (7,130.24 to 11,083.60)	249.76 (198.13 to 312.93)	521.93 (435.05 to 613.79)	498.38 (407.23 to 603.20)	521.63 (440.23 to 602.69)
Breast cancer	30,115.15 (20,386.70 to 42,405.68)	38,884.65 (22,574.77 to 63,742.00)	33,963.62 (20,090.98 to 53,266.11)	40,602.82 (23,297.43 to 66,189.23)	1,133.54 (780.83 to 1,564.19)	1,962.70 (1,227.25 to 3,074.49)	1,743.37 (1,100.65 to 2,640.61)	1,988.92 (1,207.44 to 3,114.66)
Cervical cancer	9,584.59 (6,381.07 to 13,589.73)	8,570.96 (5,200.19 to 12,667.68)	7,765.74 (4,698.45 to 11,757.80)	9,500.22 (5,851.30 to 14,078.27)	370.91 (250.11 to 523.81)	426.34 (283.22 to 597.61)	393.32 (254.76 to 560.71)	459.39 (307.46 to 642.74)

Uterine cancer	3,963.23 (2,735.02 to 5,421.84)	5,131.09 (3,329.86 to 7,263.71)	4,870.28 (3,118.69 to 7,063.16)	5,579.10 (3,616.73 to 7,791.29)	180.04 (123.18 to 241.99)	302.80 (215.01 to 399.67)	292.57 (204.38 to 393.13)	320.69 (227.43 to 423.21)
Ovarian cancer	9,642.28 (6,908.10 to 12,900.38)	12,490.38 (7,624.10 to 19,964.10)	11,398.81 (7,070.49 to 17,828.15)	12,272.88 (7,541.26 to 19,287.70)	356.85 (258.27 to 474.67)	585.70 (374.22 to 914.29)	538.06 (352.73 to 819.12)	567.10 (368.84 to 865.87)
Prostate cancer	8,368.58 (5,897.00 to 12,078.60)	20,723.66 (15,558.72 to 29,830.08)	20,746.12 (15,498.97 to 29,489.41)	18,877.40 (14,655.98 to 26,792.48)	546.70 (392.20 to 798.48)	1,631.25 (1,251.42 to 2,310.16)	1,670.59 (1,310.46 to 2,370.13)	1,432.90 (1,079.08 to 2,096.76)
Testicular cancer	829.96 (589.92 to 1,156.89)	701.50 (445.72 to 1,098.62)	653.27 (407.74 to 1,021.84)	736.73 (471.63 to 1,132.59)	22.21 (16.07 to 30.69)	25.56 (18.42 to 34.50)	24.54 (17.66 to 33.36)	25.61 (18.66 to 34.17)

Sri Lanka								
Cause	YLLs (actual counts)				Deaths (actual counts)			
	2016	2040 Forecast	2040 Better Scenario	2040 Worse Scenario	2016	2040 Forecast	2040 Better Scenario	2040 Worse Scenario
Kidney cancer	11,260.46 (8,845.50 to 14,397.18)	25,411.14 (18,620.86 to 33,915.45)	26,323.24 (19,124.50 to 35,879.84)	23,540.85 (17,098.58 to 31,732.35)	480.63 (379.69 to 608.89)	1,365.93 (1,059.96 to 1,716.09)	1,444.69 (1,114.43 to 1,829.27)	1,218.90 (945.90 to 1,532.00)
Bladder cancer	4,759.80 (3,682.82 to 6,046.24)	7,300.62 (5,014.89 to 10,503.68)	7,045.74 (4,878.65 to 9,987.93)	7,361.91 (5,111.69 to 10,543.65)	254.38 (199.89 to 319.54)	500.54 (363.10 to 689.54)	494.71 (363.58 to 671.78)	480.73 (347.04 to 669.39)
Brain and nervous system cancer	15,606.35 (11,808.43 to 20,534.15)	19,841.22 (13,355.39 to 27,397.09)	19,432.76 (12,891.50 to 27,337.80)	19,216.55 (13,194.67 to 26,278.58)	500.52 (352.24 to 680.54)	906.33 (574.60 to 1,248.40)	910.51 (570.01 to 1,257.16)	845.43 (545.37 to 1,158.54)
Thyroid cancer	2,783.53 (2,149.70 to 3,550.83)	3,854.58 (2,660.14 to 5,288.71)	3,553.83 (2,458.15 to 4,895.95)	4,167.03 (2,890.31 to 5,786.83)	120.15 (93.81 to 150.60)	209.64 (154.69 to 279.23)	196.63 (145.42 to 258.36)	220.98 (164.18 to 296.40)
Mesothelioma	2,180.86 (1,587.17 to 2,840.06)	2,527.44 (1,797.94 to 3,348.32)	2,603.60 (1,829.43 to 3,478.62)	2,450.01 (1,772.26 to 3,190.60)	79.54 (59.22 to 101.36)	119.58 (93.04 to 146.59)	125.70 (96.40 to 156.87)	112.93 (89.18 to 136.07)
Hodgkin lymphoma	2,634.49 (1,904.60 to 3,770.25)	2,156.03 (1,454.60 to 3,157.26)	1,923.62 (1,266.63 to 2,867.43)	2,466.62 (1,687.78 to 3,660.19)	88.29 (63.30 to 122.61)	93.42 (63.44 to 128.69)	84.94 (56.27 to 121.80)	103.88 (72.17 to 143.93)
Non-Hodgkin lymphoma	14,297.50 (11,186.53 to 17,879.41)	17,887.35 (13,076.36 to 23,383.60)	16,958.98 (12,279.97 to 22,610.11)	17,535.89 (13,099.35 to 22,625.53)	527.17 (415.05 to 656.13)	901.12 (702.13 to 1,117.33)	873.53 (671.58 to 1,101.18)	854.66 (668.03 to 1,060.03)
Multiple myeloma	4,378.87 (3,334.28 to 5,713.54)	6,957.23 (4,789.97 to 9,297.48)	6,772.41 (4,659.34 to 9,215.86)	6,631.83 (4,660.72 to 8,707.61)	184.57 (140.32 to 237.92)	368.42 (268.17 to 468.43)	365.33 (264.60 to 471.61)	341.91 (251.36 to 429.73)
Leukemia	36,274.74 (28,406.25 to 45,221.59)	41,406.76 (29,752.41 to 55,137.75)	38,839.00 (28,110.57 to 52,623.25)	42,792.33 (31,132.09 to 57,385.51)	1,151.42 (915.80 to 1,431.26)	1,892.98 (1,444.06 to 2,404.46)	1,818.60 (1,391.11 to 2,319.06)	1,883.49 (1,445.61 to 2,404.60)
Acute lymphoid leukemia	11,794.55 (9,246.86 to 15,310.93)	14,979.65 (10,645.07 to 20,049.60)	13,972.45 (9,853.84 to 18,870.54)	15,167.56 (10,859.91 to 20,374.67)	405.99 (320.99 to 524.61)	714.08 (534.87 to 921.60)	681.95 (507.65 to 884.48)	696.03 (525.13 to 890.31)
Chronic lymphoid leukemia	456.53 (329.37 to 622.47)	501.59 (336.32 to 738.51)	479.20 (322.82 to 701.86)	512.88 (343.53 to 775.41)	15.26 (11.44 to 21.36)	24.42 (17.78 to 35.42)	24.24 (17.70 to 34.67)	23.65 (17.14 to 35.05)
Acute myeloid leukemia	9,584.89 (7,421.06 to 12,540.56)	12,395.49 (8,763.99 to 17,187.05)	12,018.16 (8,442.54 to 16,749.99)	12,216.26 (8,599.42 to 16,890.39)	267.19 (207.29 to 356.36)	478.65 (345.35 to 659.42)	475.31 (341.87 to 653.12)	454.76 (331.40 to 631.94)
Chronic myeloid leukemia	1,554.80 (1,163.14 to 2,174.88)	1,134.88 (724.31 to 1,712.11)	957.94 (606.63 to 1,445.48)	1,262.51 (808.50 to 1,884.96)	46.67 (34.64 to 64.78)	45.15 (30.50 to 68.18)	38.90 (26.38 to 59.86)	48.34 (32.89 to 71.31)
Other leukemia	12,883.96 (8,212.24 to 16,805.24)	12,395.16 (7,678.86 to 17,443.68)	11,411.25 (6,902.34 to 16,179.64)	13,633.12 (8,383.80 to 19,239.84)	416.30 (267.55 to 536.27)	630.68 (390.01 to 844.21)	598.20 (370.08 to 809.32)	660.71 (405.25 to 892.57)
Other neoplasms	30,161.98 (24,401.04 to 36,999.27)	44,021.40 (33,367.63 to 55,908.53)	44,403.92 (33,133.73 to 56,950.34)	42,547.76 (32,802.27 to 53,058.82)	1,098.10 (878.82 to 1,343.02)	2,240.74 (1,814.70 to 2,680.31)	2,316.30 (1,839.39 to 2,852.12)	2,088.36 (1,725.61 to 2,440.46)
**Cardiovascular diseases**	**784,987.03 (633,937.58 to 959,677.67)**	**789,710.93 (473,592.36 to 1,450,390.82)**	**548,911.49 (333,160.86 to 1,039,665.32)**	**1,268,524.76 (740,320.38 to 2,220,067.51)**	**42,376.13 (34,717.17 to 50,916.41)**	**56,891.89 (36,329.46 to 108,734.67)**	**41,962.23 (27,400.60 to 77,661.54)**	**83,317.42 (53,605.15 to 143,011.31)**
Rheumatic heart disease	8,549.27 (6,626.28 to 10,955.38)	5,273.56 (3,859.19 to 7,036.06)	4,799.21 (3,417.08 to 6,506.97)	6,165.10 (4,523.16 to 8,225.76)	363.24 (286.38 to 461.10)	331.36 (250.93 to 423.31)	310.05 (232.29 to 406.02)	372.00 (282.05 to 482.48)
Ischemic heart disease	470,690.81 (377,055.56 to 579,944.92)	418,799.87 (196,277.81 to 979,153.25)	269,110.32 (129,043.63 to 590,337.73)	752,310.67 (354,636.93 to 1,589,373.21)	25,175.45 (20,553.29 to 30,186.18)	29,469.38 (14,521.25 to 65,757.15)	20,280.94 (10,241.03 to 44,041.09)	47,688.29 (24,441.50 to 99,664.83)
Stroke	184,637.21 (148,977.66 to 228,182.42)	183,770.57 (94,578.29 to 478,123.94)	117,767.53 (63,163.16 to 296,566.95)	304,600.71 (160,126.71 to 707,982.51)	11,009.58 (8,982.17 to 13,338.98)	14,843.34 (7,709.81 to 40,032.50)	10,377.38 (5,487.22 to 26,675.93)	22,062.59 (11,928.69 to 56,467.04)
Ischemic stroke	85,292.72 (69,135.63 to 104,304.49)	101,599.11 (45,126.07 to 346,027.94)	70,451.23 (31,841.61 to 231,308.06)	151,601.62 (68,451.38 to 501,796.14)	6,202.21 (5,086.02 to 7,454.65)	9,419.17 (4,260.95 to 31,420.28)	6,954.38 (3,202.32 to 20,702.40)	12,978.98 (5,884.23 to 42,415.56)

Intracerebral hemorrhage	99,344.49 (78,779.53 to 124,294.48)	82,171.46 (41,360.00 to 161,082.27)	47,316.30 (25,112.79 to 87,822.48)	152,999.09 (72,386.57 to 297,284.42)	4,807.37 (3,874.11 to 5,935.30)	5,424.17 (2,966.10 to 10,074.07)	3,423.00 (1,957.03 to 6,158.32)	9,083.61 (4,816.63 to 16,891.43)
Hypertensive heart disease	31,809.50 (17,425.38 to 44,069.21)	44,191.63 (21,905.61 to 74,152.00)	35,281.37 (17,958.34 to 54,893.17)	56,048.58 (26,601.44 to 106,815.07)	1,691.24 (977.35 to 2,295.62)	3,119.88 (1,641.61 to 5,230.67)	2,587.18 (1,387.43 to 3,972.19)	3,791.68 (1,873.59 to 7,408.98)
Cardiomyopathy and myocarditis	36,047.85 (26,371.12 to 50,518.64)	54,272.65 (35,053.83 to 82,133.87)	46,425.07 (30,598.06 to 69,640.13)	56,229.66 (36,922.39 to 83,502.01)	1,453.80 (1,052.22 to 2,127.42)	3,061.34 (2,009.12 to 4,747.16)	2,703.15 (1,822.97 to 4,171.70)	3,060.26 (2,037.03 to 4,683.31)
Myocarditis	6,956.34 (5,122.41 to 9,400.30)	6,792.52 (4,731.48 to 9,363.31)	6,784.43 (4,693.89 to 9,359.88)	6,876.40 (4,860.07 to 9,462.40)	232.13 (169.34 to 315.72)	349.86 (249.76 to 489.18)	362.12 (256.58 to 509.09)	336.50 (241.05 to 472.03)

Sri Lanka								
Cause	YLLs (actual counts)				Deaths (actual counts)			
	2016	2040 Forecast	2040 Better Scenario	2040 Worse Scenario	2016	2040 Forecast	2040 Better Scenario	2040 Worse Scenario
Alcoholic cardiomyopathy	3,862.43 (2,596.57 to 5,607.66)	6,792.49 (4,306.99 to 9,700.92)	5,545.90 (3,465.54 to 7,974.05)	6,443.59 (4,189.44 to 9,064.77)	156.42 (107.94 to 221.02)	340.97 (224.08 to 465.12)	292.48 (189.20 to 410.50)	315.26 (210.35 to 426.00)
Other cardiomyopathy	25,229.09 (17,835.10 to 37,848.28)	40,687.65 (24,782.12 to 66,294.46)	34,094.74 (20,569.26 to 55,444.45)	42,909.67 (26,373.81 to 68,523.61)	1,065.25 (743.17 to 1,675.48)	2,370.51 (1,446.91 to 3,910.60)	2,048.56 (1,250.64 to 3,412.62)	2,408.50 (1,469.14 to 3,931.02)
Atrial fibrillation and flutter	6,305.99 (4,586.72 to 8,161.08)	16,374.86 (11,825.94 to 21,875.52)	15,484.25 (11,353.64 to 20,774.84)	16,228.42 (11,596.59 to 22,017.65)	615.24 (454.36 to 812.37)	1,829.44 (1,260.38 to 2,463.91)	1,773.55 (1,222.77 to 2,379.72)	1,747.11 (1,165.53 to 2,380.53)
Aortic aneurysm	6,408.93 (4,977.93 to 7,965.15)	9,449.91 (7,077.51 to 12,115.92)	9,095.49 (6,703.89 to 11,733.95)	9,674.68 (7,252.76 to 12,517.82)	372.89 (294.68 to 456.82)	713.81 (550.21 to 881.51)	708.96 (545.10 to 875.94)	695.41 (528.07 to 867.28)
Peripheral artery disease	458.98 (297.32 to 643.46)	1,446.61 (898.67 to 2,067.73)	1,325.95 (836.21 to 1,887.14)	1,369.39 (850.38 to 1,962.69)	32.50 (21.95 to 43.93)	132.07 (83.76 to 185.50)	126.56 (80.73 to 178.86)	117.13 (73.59 to 165.33)
Endocarditis	12,016.45 (9,056.18 to 15,700.40)	14,141.48 (9,354.59 to 20,741.64)	13,639.50 (8,982.36 to 19,758.65)	14,432.84 (9,506.53 to 21,264.35)	418.16 (319.48 to 535.46)	718.74 (487.16 to 997.53)	718.45 (483.08 to 994.03)	699.99 (477.74 to 990.72)
Other cardiovascular and circulatory diseases	28,062.03 (19,110.11 to 53,861.31)	41,989.78 (25,941.07 to 80,419.11)	35,982.80 (21,852.44 to 68,526.28)	51,464.71 (31,284.99 to 98,942.42)	1,244.02 (838.79 to 2,489.53)	2,672.54 (1,709.60 to 5,345.18)	2,376.01 (1,514.07 to 4,798.86)	3,082.97 (1,979.93 to 6,261.32)
**Chronic respiratory diseases**	**181,506.94 (147,707.55 to 224,511.00)**	**149,586.75 (109,265.15 to 200,906.03)**	**123,101.41 (90,965.62 to 163,454.94)**	**211,724.07 (147,380.26 to 292,196.90)**	**10,417.60 (8,542.81 to 12,775.50)**	**11,155.95 (8,567.66 to 14,153.20)**	**9,519.79 (7,390.98 to 12,024.82)**	**14,544.40 (10,858.77 to 19,243.30)**
Chronic obstructive pulmonary disease	81,527.61 (64,051.69 to 106,029.05)	74,971.85 (49,422.07 to 109,274.06)	62,899.57 (43,051.64 to 89,860.23)	119,577.96 (72,291.85 to 192,956.97)	4,694.39 (3,725.00 to 6,176.75)	5,478.03 (3,955.88 to 7,580.27)	4,842.74 (3,560.09 to 6,539.26)	7,799.14 (5,168.72 to 11,654.09)
Pneumoconiosis	114.86 (84.84 to 153.60)	134.47 (102.09 to 177.09)	127.37 (94.35 to 169.04)	136.78 (104.91 to 180.14)	6.49 (4.85 to 8.47)	10.73 (8.14 to 14.24)	10.39 (7.76 to 13.84)	10.50 (7.59 to 14.02)
Silicosis	13.39 (8.51 to 21.17)	11.92 (7.25 to 18.93)	10.13 (5.99 to 16.79)	13.39 (8.42 to 21.12)	0.74 (0.48 to 1.17)	0.92 (0.56 to 1.50)	0.81 (0.48 to 1.39)	0.99 (0.61 to 1.58)
Asbestosis	31.90 (21.95 to 48.92)	43.61 (29.27 to 66.80)	47.88 (32.45 to 71.01)	40.23 (27.15 to 61.42)	1.43 (0.92 to 2.37)	2.75 (1.76 to 4.35)	3.10 (2.02 to 4.74)	2.42 (1.56 to 3.82)
Coal workers pneumoconiosis	12.94 (7.14 to 21.25)	10.51 (5.62 to 17.97)	8.74 (4.59 to 15.42)	12.89 (7.19 to 21.57)	0.65 (0.39 to 1.03)	0.78 (0.42 to 1.40)	0.70 (0.35 to 1.35)	0.91 (0.50 to 1.60)
Other pneumoconiosis	56.63 (41.07 to 78.45)	68.42 (46.08 to 95.72)	60.62 (39.47 to 87.56)	70.27 (49.07 to 95.39)	3.66 (2.62 to 4.96)	6.28 (4.01 to 9.08)	5.78 (3.54 to 8.68)	6.18 (3.95 to 9.02)
Asthma	93,727.39 (70,629.48 to 117,164.97)	66,362.71 (41,367.94 to 95,359.45)	51,080.18 (31,642.43 to 73,771.60)	84,663.74 (51,210.58 to 124,938.61)	5,458.49 (3,954.93 to 6,848.18)	5,190.14 (3,079.82 to 7,403.02)	4,122.38 (2,437.86 to 5,908.31)	6,324.63 (3,608.03 to 9,286.98)
Interstitial lung disease and pulmonary sarcoidosis	3,456.81 (2,352.41 to 5,213.47)	5,210.98 (3,451.98 to 8,002.60)	6,065.34 (4,026.98 to 9,181.25)	4,397.49 (2,939.44 to 6,748.01)	157.80 (105.83 to 253.62)	308.52 (204.73 to 492.79)	367.52 (245.04 to 573.29)	251.48 (168.39 to 401.21)
Other chronic respiratory diseases	2,680.27 (1,851.61 to 3,846.86)	2,906.74 (1,903.23 to 4,253.85)	2,928.95 (1,880.52 to 4,278.04)	2,948.10 (1,939.95 to 4,361.79)	100.43 (66.07 to 144.90)	168.53 (105.55 to 254.22)	176.76 (110.43 to 265.34)	158.64 (99.58 to 237.59)
**Cirrhosis and other chronic liver diseases**	**142,310.13 (102,961.93 to 193,374.13)**	**148,643.33 (103,465.94 to 207,697.89)**	**130,594.89 (90,712.60 to 185,236.24)**	**159,093.34 (112,788.36 to 221,866.56)**	**4,708.29 (3,479.77 to 6,362.37)**	**6,078.42 (4,581.93 to 8,080.67)**	**5,488.81 (4,087.96 to 7,296.54)**	**6,336.46 (4,822.98 to 8,222.37)**
Cirrhosis and other chronic liver diseases due to hepatitis B	51,043.44 (34,875.81 to 71,636.06)	52,244.64 (35,145.30 to 76,024.75)	48,909.44 (32,355.56 to 72,239.95)	54,906.66 (36,796.33 to 78,429.12)	1,693.76 (1,190.08 to 2,341.10)	2,119.38 (1,538.39 to 2,963.32)	2,019.81 (1,442.75 to 2,831.72)	2,172.37 (1,588.07 to 2,958.94)
Cirrhosis and other chronic liver diseases due to hepatitis C	27,735.08 (18,558.62 to 39,625.45)	28,933.02 (18,538.65 to 42,960.50)	26,909.01 (17,203.02 to 39,242.41)	30,681.45 (19,885.94 to 44,780.66)	900.52 (620.39 to 1,266.14)	1,137.07 (778.94 to 1,590.17)	1,074.43 (736.06 to 1,517.81)	1,179.25 (822.96 to 1,623.52)

Cirrhosis and other chronic liver diseases due to alcohol use	31,711.74 (21,175.24 to 44,537.48)	36,730.03 (23,815.27 to 52,635.69)	25,995.76 (16,973.31 to 37,384.80)	40,812.54 (26,846.90 to 58,194.68)	1,070.21 (751.35 to 1,481.85)	1,476.42 (1,033.35 to 1,982.12)	1,105.89 (777.97 to 1,499.59)	1,598.25 (1,133.43 to 2,119.25)
Cirrhosis and other chronic liver diseases due to other causes	31,819.87 (23,430.29 to 42,986.01)	30,735.64 (21,697.09 to 42,562.31)	28,780.68 (20,091.86 to 40,357.54)	32,692.68 (23,433.30 to 44,501.42)	1,043.81 (776.75 to 1,383.79)	1,345.54 (1,014.83 to 1,728.21)	1,288.68 (952.83 to 1,702.83)	1,386.60 (1,045.83 to 1,765.96)
**Digestive diseases**	**25,413.46 (19,023.93 to 35,381.94)**	**35,187.84 (24,706.37 to 50,945.71)**	**32,701.95 (23,019.15 to 46,772.20)**	**37,422.28 (26,428.42 to 53,685.61)**	**1,171.71 (867.71 to 1,624.40)**	**2,329.00 (1,722.30 to 3,167.93)**	**2,259.09 (1,693.76 to 3,030.89)**	**2,372.06 (1,735.28 to 3,208.98)**
Peptic ulcer disease	1,990.03 (1,403.21 to 2,750.45)	1,815.50 (1,265.86 to 2,653.36)	1,559.42 (1,054.66 to 2,252.74)	2,242.90 (1,568.52 to 3,273.98)	103.90 (73.28 to 145.10)	136.29 (94.67 to 197.30)	120.01 (83.39 to 171.98)	161.81 (111.86 to 230.71)

Sri Lanka								
Cause	YLLs (actual counts)				Deaths (actual counts)			
	2016	2040 Forecast	2040 Better Scenario	2040 Worse Scenario	2016	2040 Forecast	2040 Better Scenario	2040 Worse Scenario
Gastritis and duodenitis	2,729.10 (1,962.90 to 3,654.07)	3,812.60 (2,596.07 to 5,390.87)	3,529.22 (2,364.90 to 5,061.93)	4,083.81 (2,840.81 to 5,734.39)	130.46 (94.91 to 175.50)	247.19 (175.92 to 338.83)	234.99 (164.52 to 325.51)	256.12 (182.50 to 348.03)
Appendicitis	1,763.14 (1,273.74 to 2,354.25)	1,709.53 (1,163.90 to 2,408.64)	1,497.48 (995.16 to 2,127.62)	1,973.51 (1,364.21 to 2,765.07)	61.18 (43.32 to 81.53)	91.89 (61.83 to 122.21)	83.06 (55.97 to 113.38)	101.47 (68.41 to 136.30)
Paralytic ileus and intestinal obstruction	6,179.04 (4,463.27 to 9,423.49)	9,216.95 (6,335.78 to 14,661.82)	9,445.90 (6,424.70 to 15,039.26)	9,102.78 (6,340.68 to 14,181.20)	279.60 (197.28 to 435.25)	629.96 (443.59 to 959.84)	664.73 (470.28 to 1,011.61)	594.41 (413.77 to 910.49)
Inguinal, femoral, and abdominal hernia	1,118.08 (658.69 to 2,118.29)	1,295.37 (756.68 to 2,512.72)	1,231.78 (709.49 to 2,431.94)	1,356.50 (795.50 to 2,640.91)	59.65 (34.94 to 113.61)	95.60 (55.59 to 185.18)	93.67 (54.33 to 182.23)	95.61 (54.79 to 183.20)
Inflammatory bowel disease	586.84 (409.36 to 831.80)	777.99 (536.60 to 1,181.82)	787.95 (535.47 to 1,191.51)	758.95 (532.12 to 1,133.08)	23.87 (16.99 to 35.25)	46.95 (33.95 to 70.11)	48.93 (34.79 to 73.22)	43.92 (31.75 to 65.54)
Vascular intestinal disorders	1,919.68 (1,428.33 to 2,625.40)	3,498.51 (2,565.68 to 4,950.19)	3,468.02 (2,482.91 to 4,957.19)	3,519.09 (2,623.67 to 4,885.11)	109.88 (81.90 to 151.51)	269.17 (204.50 to 364.09)	273.80 (206.13 to 369.77)	261.21 (195.58 to 353.00)
Gallbladder and biliary diseases	999.95 (723.92 to 1,473.21)	1,686.98 (1,021.25 to 2,693.29)	1,377.03 (859.02 to 2,159.63)	2,052.60 (1,216.64 to 3,353.05)	56.26 (41.54 to 75.55)	131.75 (81.40 to 209.27)	110.66 (70.48 to 172.82)	153.20 (91.78 to 251.30)
Pancreatitis	2,820.82 (1,968.69 to 4,172.00)	4,349.45 (1,693.67 to 9,979.55)	2,895.62 (1,271.05 to 6,220.90)	4,931.63 (1,872.48 to 11,173.81)	95.11 (68.15 to 132.53)	191.60 (88.33 to 406.63)	134.05 (69.07 to 255.85)	211.34 (94.61 to 454.98)
Other digestive diseases	5,306.79 (3,835.34 to 7,474.62)	7,024.96 (5,023.20 to 10,003.64)	6,909.55 (4,826.43 to 9,870.02)	7,400.51 (5,327.04 to 10,599.54)	251.79 (181.22 to 356.39)	488.59 (354.21 to 678.60)	495.18 (356.84 to 680.95)	492.97 (353.68 to 686.61)
**Neurological disorders**	**93,236.86 (74,489.66 to 115,784.05)**	**226,819.95 (173,907.88 to 305,668.63)**	**220,087.20 (171,595.01 to 286,308.10)**	**216,791.02 (162,690.11 to 299,709.37)**	**7,690.21 (6,186.36 to 9,473.88)**	**23,313.77 (17,002.60 to 31,343.59)**	**23,249.43 (17,750.18 to 29,814.88)**	**21,424.15 (14,925.79 to 29,916.25)**
Alzheimer's disease and other dementias	68,621.40 (53,346.16 to 87,009.90)	187,594.15 (136,978.33 to 264,611.57)	181,245.09 (136,814.32 to 247,230.36)	179,392.67 (126,827.01 to 260,159.30)	6,679.87 (5,276.57 to 8,345.53)	20,783.19 (14,672.50 to 28,668.86)	20,583.52 (15,124.78 to 27,265.89)	19,197.84 (12,913.47 to 27,577.55)
Parkinson's disease	7,375.87 (5,308.92 to 9,934.17)	21,071.70 (15,571.97 to 27,541.43)	22,787.74 (16,778.56 to 29,894.58)	17,914.67 (13,371.11 to 23,511.68)	557.55 (401.61 to 757.91)	1,850.29 (1,364.01 to 2,443.12)	2,035.93 (1,510.05 to 2,687.70)	1,532.24 (1,086.52 to 2,051.38)
Epilepsy	12,759.25 (10,071.61 to 16,158.18)	12,287.80 (7,605.63 to 19,268.07)	10,101.74 (6,458.78 to 15,336.88)	13,938.11 (8,556.16 to 21,724.89)	318.31 (253.67 to 401.23)	424.91 (290.57 to 619.74)	362.46 (255.40 to 507.85)	463.41 (317.73 to 677.34)
Multiple sclerosis	620.62 (452.41 to 897.09)	671.12 (459.83 to 975.80)	680.47 (462.39 to 996.22)	655.40 (455.14 to 940.01)	19.17 (14.16 to 28.00)	25.72 (18.66 to 37.09)	26.48 (19.03 to 38.36)	24.56 (18.08 to 35.14)
Motor neuron disease	1,234.02 (964.96 to 1,574.09)	1,674.94 (1,222.59 to 2,220.51)	1,683.04 (1,208.68 to 2,257.70)	1,583.66 (1,175.36 to 2,062.67)	40.17 (31.72 to 50.77)	69.60 (52.97 to 89.36)	71.06 (53.42 to 93.79)	64.36 (49.73 to 81.06)
Other neurological disorders	2,625.70 (2,115.05 to 3,257.48)	3,520.24 (2,717.48 to 4,489.34)	3,589.11 (2,763.89 to 4,598.39)	3,306.51 (2,581.90 to 4,214.70)	75.14 (60.79 to 92.13)	160.06 (131.02 to 188.85)	169.98 (138.80 to 203.49)	141.74 (117.18 to 166.93)
**Mental disorders**	**15,428.16 (10,889.35 to 22,587.51)**	**17,102.10 (10,978.10 to 25,130.12)**	**17,206.38 (10,995.35 to 25,232.13)**	**16,579.95 (10,796.18 to 24,107.32)**	**435.27 (310.30 to 619.89)**	**589.54 (407.16 to 817.19)**	**605.66 (415.42 to 844.25)**	**557.51 (390.79 to 764.02)**
Alcohol use disorders	10,782.71 (6,979.27 to 15,506.33)	11,164.37 (6,414.12 to 16,740.45)	10,671.31 (6,065.08 to 16,217.88)	10,974.11 (6,420.97 to 16,390.92)	318.68 (207.29 to 446.02)	397.64 (237.32 to 571.45)	389.16 (228.81 to 569.38)	382.33 (230.16 to 538.23)
Drug use disorders	4,586.79 (3,011.82 to 8,591.26)	5,884.16 (3,652.48 to 10,422.58)	6,481.75 (4,096.45 to 11,364.35)	5,551.29 (3,527.78 to 9,987.37)	115.47 (79.22 to 201.60)	190.88 (130.91 to 310.15)	215.48 (149.97 to 345.39)	174.15 (121.91 to 284.01)
Opioid use disorders	2,757.39 (1,769.67 to 5,037.26)	2,708.81 (1,565.50 to 4,657.94)	2,796.50 (1,627.53 to 4,710.75)	2,645.14 (1,541.22 to 4,597.26)	65.53 (43.40 to 111.30)	81.82 (51.79 to 138.50)	86.27 (54.70 to 146.03)	77.54 (49.35 to 131.17)

Cocaine use disorders	337.39 (212.78 to 579.70)	546.50 (310.28 to 907.86)	610.21 (347.72 to 1,024.67)	529.22 (305.25 to 886.10)	8.91 (5.68 to 14.43)	16.67 (9.62 to 26.66)	18.84 (10.93 to 30.36)	15.85 (9.29 to 25.06)
Amphetamine use disorders	201.60 (126.50 to 388.37)	457.71 (254.90 to 851.63)	540.16 (309.20 to 976.15)	436.22 (249.92 to 815.58)	4.92 (3.16 to 8.67)	12.52 (7.38 to 21.25)	14.94 (8.88 to 25.27)	11.75 (7.15 to 20.09)
Other drug use disorders	1,290.40 (789.85 to 3,013.37)	2,171.14 (1,267.31 to 4,376.71)	2,534.88 (1,492.14 to 4,995.98)	1,940.70 (1,137.59 to 4,098.12)	36.11 (23.97 to 75.23)	79.87 (54.47 to 137.22)	95.43 (65.55 to 164.40)	69.00 (46.79 to 122.29)
Eating disorders	58.66 (43.77 to 82.11)	53.57 (32.58 to 85.69)	53.32 (32.14 to 84.77)	54.56 (33.01 to 87.43)	1.12 (0.82 to 1.61)	1.02 (0.61 to 1.63)	1.02 (0.62 to 1.63)	1.03 (0.62 to 1.64)

Sri Lanka								
Cause	YLLs (actual counts)				Deaths (actual counts)			
	2016	2040 Forecast	2040 Better Scenario	2040 Worse Scenario	2016	2040 Forecast	2040 Better Scenario	2040 Worse Scenario
**Diabetes, urogenital, blood, and endocrine diseases**	**327,195.45 (265,263.04 to 399,622.81)**	**551,766.23 (286,026.78 to 1,168,629.42)**	**436,677.45 (232,159.33 to 994,939.46)**	**668,024.68 (350,306.97 to 1,352,812.98)**	**16,948.54 (13,859.81 to 20,329.04)**	**38,522.61 (20,877.45 to 79,084.69)**	**31,618.89 (17,537.91 to 70,526.11)**	**44,756.97 (24,009.92 to 87,128.06)**
Diabetes mellitus	191,943.70 (154,569.27 to 233,494.87)	231,172.06 (159,445.68 to 325,970.26)	194,370.86 (133,031.90 to 278,892.81)	294,215.77 (203,144.50 to 410,847.78)	10,663.83 (8,680.99 to 12,743.42)	17,131.56 (12,602.51 to 22,999.68)	14,857.26 (10,806.13 to 19,885.81)	20,895.71 (15,092.97 to 28,190.71)
Acute glomerulonephritis	134.53 (103.65 to 183.78)	110.25 (73.41 to 173.83)	100.15 (65.09 to 157.62)	124.00 (82.14 to 198.87)	2.88 (2.26 to 3.71)	3.28 (2.37 to 4.51)	3.09 (2.17 to 4.30)	3.50 (2.52 to 4.86)
Chronic kidney disease	94,034.89 (75,239.15 to 115,856.35)	268,699.51 (48,075.92 to 861,048.73)	193,588.71 (35,594.73 to 741,385.71)	319,713.61 (56,047.60 to 975,045.96)	4,476.42 (3,611.48 to 5,477.27)	18,122.94 (2,795.02 to 58,872.88)	13,608.72 (2,157.38 to 52,144.26)	20,603.40 (3,177.14 to 62,897.21)
Chronic kidney disease due to diabetes mellitus	57,404.03 (45,294.70 to 71,033.81)	163,530.10 (28,874.66 to 540,254.75)	115,889.81 (21,498.06 to 456,192.18)	196,771.35 (34,423.54 to 612,126.68)	2,528.92 (2,004.81 to 3,128.61)	9,649.62 (1,453.73 to 31,742.74)	7,082.91 (1,108.71 to 28,096.95)	11,168.84 (1,705.09 to 34,270.37)
Chronic kidney disease due to hypertension	25,109.04 (19,194.75 to 31,923.74)	84,864.20 (13,419.65 to 278,023.26)	63,027.68 (9,977.65 to 245,947.53)	98,223.90 (15,661.05 to 304,892.70)	1,537.73 (1,200.71 to 1,913.12)	7,176.77 (1,119.03 to 23,046.62)	5,565.02 (883.39 to 21,535.30)	7,933.20 (1,259.97 to 24,381.01)
Chronic kidney disease due to glomerulonephritis	5,581.17 (4,305.76 to 7,193.60)	8,176.51 (2,340.62 to 24,206.96)	5,880.31 (1,778.74 to 19,283.62)	10,091.01 (2,905.32 to 28,859.77)	173.01 (130.21 to 225.34)	460.76 (90.86 to 1,487.29)	335.08 (67.99 to 1,243.15)	543.67 (110.72 to 1,620.40)
Chronic kidney disease due to other and unspecified causes	5,940.65 (4,479.53 to 7,855.00)	12,128.70 (2,667.40 to 36,682.65)	8,790.91 (2,086.83 to 31,790.00)	14,627.35 (3,209.31 to 42,496.83)	236.76 (176.61 to 308.97)	835.79 (147.49 to 2,773.60)	625.70 (111.55 to 2,424.57)	957.69 (169.78 to 3,022.06)
Urinary diseases and male infertility	27,191.81 (21,754.33 to 33,880.33)	33,814.26 (25,481.85 to 42,839.00)	31,436.56 (23,188.82 to 41,487.51)	34,640.75 (26,479.84 to 43,556.84)	1,265.40 (1,029.27 to 1,552.98)	2,263.15 (1,768.46 to 2,772.32)	2,164.58 (1,679.79 to 2,695.67)	2,218.17 (1,724.09 to 2,699.47)
Urinary tract infections	21,279.65 (16,510.37 to 26,723.05)	27,858.06 (20,833.83 to 36,859.13)	26,071.88 (18,917.82 to 34,698.94)	28,103.92 (21,113.16 to 35,923.97)	969.26 (748.34 to 1,219.50)	1,851.21 (1,407.78 to 2,324.83)	1,782.23 (1,331.62 to 2,273.28)	1,786.56 (1,343.04 to 2,217.26)
Urolithiasis	258.27 (167.18 to 450.78)	341.36 (206.48 to 653.25)	300.55 (179.28 to 567.52)	356.70 (219.60 to 681.84)	10.32 (6.87 to 18.76)	19.35 (12.31 to 33.92)	17.58 (11.15 to 30.85)	19.22 (12.54 to 35.15)
Other urinary diseases	5,653.88 (3,503.87 to 8,788.42)	5,614.84 (3,296.29 to 9,131.32)	5,064.12 (2,959.24 to 8,394.59)	6,180.13 (3,714.18 to 9,939.20)	285.82 (182.03 to 425.32)	392.59 (237.95 to 613.42)	364.77 (217.73 to 584.79)	412.39 (251.79 to 639.89)
Gynecological diseases	186.23 (126.78 to 262.40)	185.90 (114.48 to 283.41)	148.47 (88.67 to 231.67)	228.48 (142.97 to 346.58)	5.89 (4.12 to 8.29)	7.99 (5.15 to 11.89)	6.50 (4.07 to 10.07)	9.51 (6.24 to 14.14)
Uterine fibroids	4.03 (1.67 to 10.48)	4.42 (1.71 to 10.86)	3.78 (1.51 to 9.20)	5.41 (2.13 to 13.53)	0.14 (0.07 to 0.32)	0.20 (0.09 to 0.43)	0.18 (0.08 to 0.37)	0.24 (0.11 to 0.51)
Polycystic ovarian syndrome	2.56 (0.60 to 8.10)	1.21 (0.23 to 4.19)	0.83 (0.16 to 2.86)	1.77 (0.35 to 6.04)	0.06 (0.01 to 0.21)	0.03 (0.01 to 0.10)	0.02 (0.00 to 0.07)	0.04 (0.01 to 0.15)
Endometriosis	1.32 (0.51 to 3.54)	1.24 (0.38 to 3.56)	1.01 (0.30 to 2.87)	1.57 (0.49 to 4.43)	0.03 (0.01 to 0.08)	0.03 (0.01 to 0.08)	0.02 (0.01 to 0.07)	0.03 (0.01 to 0.10)
Genital prolapse	4.95 (2.14 to 9.19)	4.96 (1.53 to 10.62)	4.06 (1.21 to 9.03)	6.28 (1.97 to 13.71)	0.27 (0.09 to 0.52)	0.31 (0.08 to 0.68)	0.26 (0.07 to 0.56)	0.39 (0.10 to 0.86)
Other gynecological diseases	173.36 (116.72 to 246.52)	174.07 (107.00 to 264.89)	138.81 (83.33 to 215.96)	213.45 (132.72 to 326.01)	5.39 (3.73 to 7.57)	7.41 (4.77 to 10.96)	6.03 (3.75 to 9.47)	8.80 (5.73 to 13.12)
Hemoglobinopathies and hemolytic anemias	7,095.27 (5,105.70 to 11,648.98)	9,305.55 (6,258.97 to 14,702.70)	8,293.05 (5,418.12 to 13,292.46)	10,578.30 (7,132.30 to 16,951.99)	279.48 (205.07 to 438.79)	507.82 (359.74 to 769.29)	462.72 (314.45 to 714.39)	559.33 (397.07 to 855.54)
Thalassemias	699.35 (393.09 to 1,457.99)	350.06 (159.90 to 794.86)	332.55 (154.49 to 744.53)	344.41 (151.90 to 801.73)	9.33 (5.14 to 19.98)	4.75 (2.16 to 11.02)	4.53 (2.08 to 10.41)	4.67 (2.02 to 11.08)

Sickle cell disorders	7.18 (4.43 to 12.43)	8.06 (4.82 to 13.57)	6.97 (4.05 to 11.78)	9.09 (5.51 to 15.24)	0.16 (0.10 to 0.26)	0.24 (0.15 to 0.40)	0.21 (0.12 to 0.35)	0.27 (0.17 to 0.44)
G6PD deficiency	3,666.54 (2,577.18 to 5,673.34)	5,632.57 (3,611.58 to 8,959.63)	4,906.00 (3,081.88 to 7,839.89)	6,572.28 (4,205.94 to 10,262.41)	114.53 (82.04 to 170.02)	246.18 (159.92 to 377.79)	216.99 (137.38 to 334.64)	280.78 (186.90 to 429.74)
Other hemoglobinopathies and hemolytic anemias	2,722.20 (1,957.48 to 4,547.82)	3,314.85 (2,241.53 to 5,484.43)	3,047.53 (1,997.69 to 5,030.60)	3,652.52 (2,477.51 to 5,938.87)	155.46 (113.80 to 256.79)	256.64 (181.43 to 404.88)	240.99 (167.53 to 387.22)	273.60 (188.76 to 438.62)
Endocrine, metabolic, blood, and immune disorders	6,609.02 (5,087.63 to 8,447.24)	8,478.70 (6,116.02 to 11,247.82)	8,739.66 (6,268.12 to 11,729.59)	8,523.76 (6,228.38 to 11,197.84)	254.64 (196.24 to 323.49)	485.87 (359.00 to 617.96)	516.01 (377.11 to 665.91)	467.35 (347.02 to 588.78)

Sri Lanka								
Cause	YLLs (actual counts)				Deaths (actual counts)			
	2016	2040 Forecast	2040 Better Scenario	2040 Worse Scenario	2016	2040 Forecast	2040 Better Scenario	2040 Worse Scenario
**Musculoskeletal disorders**	**1,547.43 (1,074.33 to 1,974.49)**	**2,272.32 (1,380.24 to 3,018.87)**	**2,194.75 (1,337.14 to 2,934.54)**	**2,218.81 (1,347.82 to 2,947.47)**	**60.50 (38.95 to 78.31)**	**133.49 (78.80 to 170.30)**	**131.77 (78.56 to 169.73)**	**126.05 (73.13 to 160.15)**
Rheumatoid arthritis	273.02 (151.72 to 374.25)	404.88 (228.61 to 583.18)	346.33 (197.18 to 509.18)	464.68 (265.99 to 661.70)	13.96 (8.23 to 19.25)	27.79 (16.29 to 40.67)	24.32 (14.59 to 35.74)	30.79 (18.30 to 44.11)
Other musculoskeletal disorders	1,274.41 (892.85 to 1,630.74)	1,867.45 (1,096.88 to 2,517.23)	1,848.42 (1,096.08 to 2,484.97)	1,754.13 (1,016.82 to 2,369.39)	46.53 (28.69 to 60.46)	105.70 (57.38 to 138.16)	107.45 (60.13 to 139.93)	95.26 (49.90 to 124.55)
**Other non-communicable diseases**	**53,714.78 (36,734.84 to 77,186.07)**	**35,402.96 (21,658.97 to 55,586.25)**	**28,840.40 (17,540.32 to 45,057.96)**	**42,316.73 (25,120.19 to 67,025.25)**	**779.79 (565.28 to 1,065.52)**	**794.02 (573.48 to 1,081.94)**	**711.96 (512.75 to 962.83)**	**861.95 (609.64 to 1,187.27)**
Congenital birth defects	50,524.14 (34,601.38 to 74,238.00)	29,310.43 (16,552.27 to 49,125.63)	22,898.03 (12,450.31 to 38,382.97)	36,311.29 (20,019.05 to 61,434.54)	678.94 (469.73 to 963.13)	427.75 (253.31 to 670.19)	339.68 (200.63 to 545.36)	521.94 (308.47 to 835.32)
Neural tube defects	735.71 (365.49 to 1,356.32)	184.00 (84.20 to 373.47)	137.82 (60.98 to 268.90)	253.40 (111.75 to 507.12)	9.56 (5.08 to 16.85)	2.71 (1.40 to 5.02)	2.06 (1.06 to 3.73)	3.68 (1.88 to 6.85)
Congenital heart anomalies	33,130.60 (18,602.56 to 49,096.29)	17,724.44 (8,702.50 to 30,233.25)	13,343.53 (6,401.79 to 23,352.82)	22,372.82 (10,748.02 to 38,717.78)	437.37 (243.58 to 628.36)	247.06 (124.10 to 406.21)	187.52 (91.56 to 313.86)	309.03 (153.96 to 509.78)
Orofacial clefts	36.01 (8.13 to 102.26)	14.03 (2.49 to 42.29)	9.59 (1.70 to 30.43)	21.22 (3.50 to 69.68)	0.42 (0.09 to 1.19)	0.16 (0.03 to 0.49)	0.11 (0.02 to 0.35)	0.25 (0.04 to 0.81)
Down syndrome	1,442.89 (950.75 to 2,126.00)	1,240.09 (718.15 to 2,049.35)	1,244.52 (730.71 to 2,041.53)	1,364.68 (788.99 to 2,285.23)	22.80 (15.85 to 32.44)	22.10 (13.49 to 34.27)	22.44 (13.66 to 34.43)	24.03 (14.67 to 37.48)
Other chromosomal abnormalities	574.53 (298.43 to 1,014.51)	548.63 (209.23 to 1,160.40)	477.17 (175.70 to 1,001.06)	660.54 (250.54 to 1,384.15)	6.78 (3.54 to 11.91)	6.55 (2.56 to 13.70)	5.71 (2.17 to 11.84)	7.87 (3.02 to 16.29)
Congenital musculoskeletal and limb anomalies	573.32 (300.24 to 1,089.35)	409.98 (182.61 to 856.29)	321.89 (139.14 to 677.84)	498.49 (223.74 to 1,089.42)	6.98 (3.75 to 12.99)	5.08 (2.37 to 10.37)	4.00 (1.84 to 8.17)	6.16 (2.87 to 13.01)
Urogenital congenital anomalies	1,934.76 (1,353.94 to 2,881.95)	1,676.59 (1,054.32 to 2,553.41)	1,423.49 (883.72 to 2,194.88)	1,929.82 (1,222.33 to 3,011.57)	38.41 (28.11 to 53.85)	40.12 (26.17 to 58.04)	34.71 (22.27 to 51.90)	44.99 (29.73 to 64.93)
Digestive congenital anomalies	3,041.77 (1,219.26 to 5,450.80)	1,722.15 (602.68 to 3,622.35)	1,319.18 (438.07 to 2,822.66)	2,184.76 (740.39 to 4,577.68)	35.90 (14.46 to 63.98)	20.53 (7.35 to 42.88)	15.75 (5.41 to 33.30)	26.01 (8.90 to 53.78)
Other congenital birth defects	9,054.56 (5,224.30 to 15,672.03)	5,790.50 (2,961.34 to 10,635.29)	4,620.85 (2,312.50 to 8,280.61)	7,025.56 (3,523.87 to 12,907.62)	120.73 (74.35 to 195.69)	83.43 (47.10 to 141.99)	67.37 (37.94 to 112.53)	99.91 (55.89 to 168.73)
Skin and subcutaneous diseases	2,005.16 (1,129.84 to 2,923.94)	5,433.45 (3,398.64 to 8,316.47)	5,372.35 (3,344.24 to 8,132.56)	5,250.35 (3,342.63 to 8,070.32)	87.10 (54.86 to 137.99)	358.63 (249.99 to 596.87)	365.66 (254.22 to 602.14)	331.25 (230.22 to 556.61)
Cellulitis	738.83 (360.64 to 1,179.18)	2,551.92 (1,274.45 to 3,831.90)	2,597.63 (1,295.46 to 3,939.32)	2,319.61 (1,176.98 to 3,569.36)	35.13 (17.75 to 56.35)	171.80 (93.80 to 275.62)	179.36 (99.21 to 289.06)	150.57 (81.58 to 248.88)
Pyoderma	1,058.95 (587.16 to 1,587.26)	2,493.75 (1,524.29 to 4,067.20)	2,410.04 (1,473.22 to 3,939.93)	2,547.89 (1,569.80 to 4,081.46)	41.81 (26.18 to 70.91)	156.09 (98.67 to 288.61)	156.56 (99.34 to 293.40)	151.66 (99.30 to 275.85)
Decubitus ulcer	141.05 (74.39 to 223.03)	286.81 (153.26 to 518.37)	268.80 (143.41 to 487.51)	283.00 (151.11 to 513.43)	8.07 (4.39 to 14.98)	25.48 (13.62 to 53.27)	24.53 (12.96 to 51.58)	24.12 (12.27 to 51.54)
Other skin and subcutaneous diseases	66.33 (37.74 to 97.68)	100.96 (60.28 to 171.96)	95.88 (57.74 to 160.05)	99.86 (58.81 to 166.57)	2.09 (1.33 to 3.65)	5.26 (3.31 to 10.21)	5.20 (3.25 to 10.30)	4.90 (3.06 to 9.60)
Sudden infant death syndrome	1,185.48 (491.74 to 2,491.92)	659.09 (171.64 to 1,864.05)	570.02 (146.61 to 1,633.40)	755.09 (198.06 to 2,064.92)	13.76 (5.71 to 28.92)	7.65 (1.99 to 21.64)	6.61 (1.70 to 18.96)	8.76 (2.29 to 23.96)

**Injuries**	**531,476.64 (420,892.04 to 672,785.81)**	**462,623.50 (266,309.76 to 807,538.40)**	**379,982.45 (227,325.20 to 676,379.07)**	**548,253.20 (325,754.50 to 958,058.80)**	**14,145.51 (11,286.68 to 17,684.89)**	**17,141.43 (11,961.19 to 25,176.48)**	**14,724.54 (10,523.85 to 22,011.10)**	**19,427.86 (13,558.44 to 28,763.89)**
**Transport injuries**	**119,982.58 (94,067.35 to 152,124.85)**	**66,593.05 (40,847.81 to 112,146.48)**	**44,394.01 (27,429.62 to 70,025.76)**	**127,015.28 (76,001.83 to 213,698.69)**	**3,458.51 (2,722.35 to 4,358.02)**	**2,807.00 (1,944.93 to 4,163.55)**	**1,977.17 (1,409.28 to 2,767.33)**	**4,792.00 (3,300.13 to 7,162.73)**
Road injuries	102,461.37 (80,491.00 to 130,274.09)	47,710.41 (29,447.12 to 79,273.51)	29,573.42 (18,596.87 to 46,730.49)	105,673.01 (63,894.81 to 177,014.30)	2,954.03 (2,322.76 to 3,733.62)	2,042.31 (1,427.95 to 2,989.75)	1,354.25 (966.25 to 1,910.13)	3,964.00 (2,752.16 to 5,896.42)
Pedestrian road injuries	28,712.15 (21,656.19 to 37,084.75)	15,392.68 (10,310.58 to 22,752.47)	8,868.32 (6,149.07 to 12,510.17)	32,873.29 (22,366.75 to 48,917.30)	1,105.90 (839.98 to 1,406.21)	885.54 (612.70 to 1,199.85)	545.55 (387.45 to 719.85)	1,698.06 (1,216.73 to 2,309.89)

Sri Lanka								
**Cause**	**YLLs (actual counts)**				**Deaths (actual counts)**			
	**2016**	**2040 Forecast**	**2040 Better Scenario**	**2040 Worse Scenario**	**2016**	**2040 Forecast**	**2040 Better Scenario**	**2040 Worse Scenario**
Cyclist road injuries	4,982.27 (3,593.10 to 6,689.96)	2,670.29 (1,560.33 to 4,365.26)	1,814.62 (1,092.26 to 2,894.73)	5,254.83 (3,065.21 to 8,827.17)	148.52 (108.26 to 195.43)	111.64 (72.12 to 172.14)	82.59 (54.40 to 122.61)	188.48 (117.35 to 294.38)
Motorcyclist road injuries	25,431.22 (17,640.84 to 34,945.24)	15,069.97 (7,501.37 to 28,195.96)	10,778.85 (5,606.14 to 19,231.35)	25,332.22 (12,563.06 to 48,243.42)	575.17 (412.85 to 769.21)	445.94 (260.62 to 743.45)	343.59 (209.12 to 555.61)	673.90 (385.85 to 1,176.67)
Motor vehicle road injuries	41,473.46 (31,330.91 to 54,721.93)	13,737.32 (8,231.36 to 23,168.19)	7,600.15 (4,841.50 to 12,162.74)	40,100.43 (22,850.18 to 71,864.48)	1,068.79 (809.94 to 1,389.72)	554.14 (362.85 to 817.44)	350.47 (229.04 to 488.67)	1,316.87 (872.47 to 2,032.88)
Other road injuries	1,862.27 (1,310.70 to 2,520.78)	840.15 (502.14 to 1,417.43)	511.48 (318.66 to 805.63)	2,112.24 (1,191.47 to 3,732.42)	55.64 (38.54 to 74.48)	45.05 (27.20 to 66.90)	32.05 (18.50 to 46.23)	86.69 (51.33 to 135.13)
Other transport injuries	17,521.21 (12,126.45 to 23,285.95)	18,882.63 (10,560.47 to 33,002.65)	14,820.59 (8,415.49 to 24,122.76)	21,342.27 (11,627.23 to 36,556.02)	504.48 (380.56 to 661.07)	764.69 (484.07 to 1,174.14)	622.93 (401.73 to 946.02)	827.99 (529.40 to 1,283.30)
**Unintentional injuries**	**158,202.71 (126,502.24 to 199,132.52)**	**124,288.14 (88,959.57 to 173,126.37)**	**113,693.56 (80,935.84 to 160,121.08)**	**134,035.97 (94,742.43 to 187,840.03)**	**4,636.69 (3,762.92 to 5,721.71)**	**6,009.50 (4,886.16 to 7,300.86)**	**5,742.55 (4,676.88 to 7,093.13)**	**6,132.67 (4,947.12 to 7,529.88)**
Falls	51,596.11 (39,043.90 to 66,696.13)	56,132.53 (41,845.26 to 73,052.24)	53,779.34 (39,233.17 to 71,446.74)	57,169.76 (42,666.12 to 74,669.71)	2,024.64 (1,595.81 to 2,537.48)	3,508.73 (2,817.55 to 4,178.60)	3,455.59 (2,789.76 to 4,146.48)	3,443.82 (2,731.69 to 4,155.80)
Drowning	48,883.82 (38,755.20 to 62,989.05)	28,844.89 (17,862.85 to 46,460.18)	25,183.53 (15,809.90 to 40,422.80)	32,079.85 (19,463.95 to 51,634.32)	1,101.52 (877.03 to 1,410.95)	918.60 (643.07 to 1,330.29)	827.66 (585.39 to 1,182.25)	982.02 (684.83 to 1,423.97)
Fire, heat, and hot substances	15,220.36 (11,656.93 to 19,313.12)	9,684.68 (6,608.61 to 13,843.75)	8,326.16 (5,668.47 to 11,977.49)	11,122.24 (7,537.98 to 15,980.68)	415.02 (325.31 to 516.38)	434.67 (308.91 to 587.77)	388.89 (278.14 to 525.02)	472.59 (340.00 to 631.26)
Poisonings	2,747.46 (2,010.93 to 4,220.07)	1,254.19 (738.37 to 2,067.84)	1,019.05 (595.40 to 1,773.63)	1,548.94 (905.41 to 2,634.56)	63.28 (45.45 to 101.46)	41.77 (26.29 to 70.35)	35.19 (22.30 to 58.76)	49.14 (31.30 to 83.71)
Exposure to mechanical forces	11,234.78 (6,384.55 to 15,177.24)	6,403.47 (3,465.99 to 10,502.78)	5,677.28 (3,072.65 to 9,346.81)	7,393.98 (3,955.78 to 12,180.50)	253.93 (158.37 to 342.96)	197.28 (128.70 to 285.36)	180.52 (118.73 to 260.48)	218.22 (139.51 to 321.75)
Unintentional firearm injuries	3,249.78 (1,581.87 to 4,887.11)	1,736.52 (872.37 to 3,129.31)	1,575.00 (793.75 to 2,817.67)	2,005.27 (969.68 to 3,515.74)	75.34 (42.01 to 108.61)	55.18 (35.57 to 83.61)	51.72 (33.60 to 78.03)	61.11 (37.78 to 93.55)
Unintentional suffocation	629.01 (317.17 to 956.83)	345.42 (156.26 to 616.05)	305.98 (139.47 to 548.95)	413.21 (185.02 to 757.24)	10.14 (5.31 to 14.94)	6.28 (3.05 to 10.60)	5.66 (2.80 to 9.50)	7.35 (3.55 to 12.19)
Other exposure to mechanical forces	7,355.99 (3,853.02 to 10,156.95)	4,321.53 (2,176.47 to 7,143.46)	3,796.30 (1,946.53 to 6,314.78)	4,975.50 (2,536.56 to 8,251.09)	168.45 (93.85 to 231.44)	135.81 (82.15 to 196.39)	123.14 (74.77 to 182.03)	149.75 (89.02 to 219.19)
Adverse effects of medical treatment	8,008.49 (5,995.20 to 10,091.46)	7,041.35 (4,727.74 to 9,650.78)	6,753.68 (4,503.29 to 9,584.03)	7,507.22 (5,039.38 to 10,391.35)	259.14 (190.26 to 333.55)	352.34 (256.99 to 454.94)	348.59 (250.29 to 457.04)	359.46 (263.04 to 462.52)
Animal contact	5,993.80 (4,481.86 to 7,658.98)	4,684.28 (3,033.40 to 6,858.82)	4,307.84 (2,732.41 to 6,427.41)	5,407.33 (3,492.28 to 8,132.48)	156.77 (120.34 to 200.90)	176.25 (127.70 to 236.25)	166.67 (119.06 to 231.04)	194.10 (139.60 to 263.80)
Venomous animal contact	3,811.47 (2,626.59 to 4,959.87)	2,588.27 (1,593.76 to 3,924.50)	2,407.50 (1,487.15 to 3,713.08)	3,016.45 (1,845.04 to 4,649.45)	88.53 (64.87 to 114.62)	78.84 (52.38 to 115.05)	75.06 (49.04 to 111.61)	88.73 (58.26 to 128.97)
Non-venomous animal contact	2,182.33 (1,577.90 to 3,119.53)	2,096.01 (1,369.58 to 3,118.25)	1,900.34 (1,234.68 to 2,939.18)	2,390.88 (1,546.09 to 3,582.95)	68.24 (50.78 to 95.90)	97.41 (70.16 to 134.60)	91.60 (64.42 to 127.92)	105.37 (75.88 to 143.40)
Foreign body	3,153.75 (2,369.37 to 4,195.64)	2,680.22 (1,947.91 to 3,592.18)	2,657.08 (1,910.19 to 3,548.15)	2,702.31 (1,950.08 to 3,654.73)	84.93 (63.18 to 107.02)	127.03 (77.13 to 161.69)	132.59 (79.40 to 169.55)	119.95 (75.09 to 152.86)
Pulmonary aspiration and foreign body in airway	2,033.46 (1,476.28 to 2,800.26)	1,740.70 (1,151.31 to 2,385.99)	1,680.06 (1,086.71 to 2,324.67)	1,742.49 (1,166.53 to 2,411.99)	52.78 (36.82 to 67.92)	75.12 (44.75 to 98.80)	76.05 (45.35 to 100.80)	70.34 (42.25 to 92.18)

Foreign body in other body part	1,120.29 (817.43 to 1,737.20)	939.52 (646.64 to 1,389.61)	977.02 (677.92 to 1,443.06)	959.82 (658.30 to 1,441.70)	32.14 (23.47 to 44.97)	51.92 (28.69 to 72.36)	56.54 (30.70 to 78.11)	49.61 (28.13 to 68.99)
Environmental heat and cold exposure	1,771.19 (1,050.84 to 2,413.73)	1,485.55 (873.64 to 2,246.69)	1,300.92 (746.18 to 2,033.10)	1,800.48 (1,053.11 to 2,755.93)	53.55 (35.47 to 72.81)	60.44 (40.56 to 88.51)	54.33 (35.37 to 80.41)	70.54 (46.59 to 101.69)
Other unintentional injuries	9,592.94 (7,117.65 to 13,510.55)	6,076.97 (3,581.90 to 10,575.77)	4,688.70 (2,707.08 to 8,239.26)	7,303.85 (4,401.23 to 12,423.04)	223.93 (165.75 to 315.70)	192.38 (129.02 to 291.16)	152.52 (99.98 to 236.62)	222.84 (154.84 to 345.61)
**Self-harm and interpersonal violence**	**249,808.75 (189,087.45 to 330,844.30)**	**258,767.06 (127,146.56 to 510,093.95)**	**208,978.24 (109,562.13 to 399,158.98)**	**274,164.31 (133,171.43 to 560,289.09)**	**5,976.25 (4,536.09 to 7,903.80)**	**8,006.80 (4,560.21 to 13,878.90)**	**6,682.27 (4,055.56 to 10,948.64)**	**8,191.94 (4,630.28 to 14,536.56)**

Sri Lanka								
Cause	YLLs (actual counts)				Deaths (actual counts)			
	2016	2040 Forecast	2040 Better Scenario	2040 Worse Scenario	2016	2040 Forecast	2040 Better Scenario	2040 Worse Scenario
Self-harm	202,343.24 (153,230.43 to 274,363.63)	231,358.04 (110,437.43 to 466,503.25)	185,511.73 (95,730.57 to 357,799.61)	245,108.12 (115,527.60 to 511,090.28)	4,976.61 (3,766.57 to 6,707.80)	7,317.12 (4,124.14 to 12,794.50)	6,082.52 (3,666.76 to 10,059.20)	7,474.57 (4,129.72 to 13,538.66)
Self-harm by firearm	1,144.57 (715.45 to 1,847.08)	960.52 (381.44 to 2,107.32)	731.27 (310.59 to 1,585.68)	1,062.03 (410.13 to 2,371.10)	27.76 (17.94 to 43.85)	31.44 (15.46 to 61.04)	25.21 (13.38 to 47.48)	33.23 (16.05 to 67.66)
Self-harm by other specified means	201,198.67 (152,456.12 to 272,802.62)	230,397.53 (109,962.23 to 464,397.55)	184,780.46 (95,441.62 to 356,213.74)	244,046.09 (115,061.86 to 509,019.02)	4,948.85 (3,748.37 to 6,671.87)	7,285.67 (4,107.29 to 12,741.74)	6,057.31 (3,651.37 to 9,999.93)	7,441.34 (4,112.31 to 13,480.46)
Interpersonal violence	47,465.51 (33,058.54 to 65,800.95)	27,409.01 (14,286.92 to 48,134.75)	23,466.50 (12,559.54 to 41,041.28)	29,056.19 (15,172.81 to 50,787.41)	999.64 (703.32 to 1,359.20)	689.68 (393.65 to 1,119.17)	599.76 (351.87 to 967.09)	717.37 (408.87 to 1,173.03)
Physical violence by firearm	14,581.43 (8,548.09 to 23,534.06)	7,267.37 (3,562.40 to 14,272.87)	6,135.93 (3,048.08 to 12,399.47)	7,879.72 (3,883.34 to 16,030.73)	284.76 (170.00 to 471.48)	158.07 (82.79 to 296.64)	134.80 (71.63 to 255.81)	169.32 (89.40 to 324.42)
Physical violence by sharp object	17,565.53 (10,861.63 to 25,948.10)	10,569.36 (5,265.72 to 18,940.60)	9,174.76 (4,607.48 to 16,223.86)	11,073.79 (5,491.58 to 19,828.77)	371.02 (223.77 to 538.74)	261.99 (136.57 to 427.46)	230.75 (120.64 to 372.68)	270.17 (140.36 to 443.35)
Physical violence by other means	15,318.54 (9,974.17 to 22,325.23)	9,572.28 (4,746.10 to 17,623.80)	8,155.82 (4,163.74 to 14,725.59)	10,102.68 (4,996.36 to 18,644.93)	343.86 (226.40 to 496.00)	269.62 (145.49 to 465.56)	234.20 (130.29 to 394.99)	277.88 (150.21 to 483.67)
**Forces of nature, conflict and terrorism, and executions and police conflict**	**3,482.60 (1,838.23 to 5,154.66)**	**12,975.26 (0.00 to 35,699.29)**	**12,916.64 (0.00 to 35,438.05)**	**13,037.65 (0.00 to 35,991.14)**	**74.06 (38.91 to 109.62)**	**318.13 (0.00 to 790.97)**	**322.55 (0.00 to 798.90)**	**311.26 (0.00 to 774.88)**
Exposure to forces of nature	3,351.13 (1,698.44 to 5,040.81)	7,917.38 (0.00 to 10,028.00)	7,884.71 (0.00 to 9,994.58)	7,949.12 (0.00 to 10,047.51)	71.27 (36.12 to 107.20)	203.95 (0.00 to 250.66)	206.92 (0.00 to 254.28)	199.28 (0.00 to 245.15)
Conflict and terrorism	0.00 (0.00 to 0.00)	5,039.13 (0.00 to 28,673.58)	5,013.22 (0.00 to 28,613.51)	5,069.72 (0.00 to 28,863.48)	0.00 (0.00 to 0.00)	113.77 (0.00 to 675.75)	115.22 (0.00 to 682.89)	111.58 (0.00 to 666.22)
Executions and police conflict	131.47 (101.97 to 168.43)	18.75 (0.00 to 534.47)	18.71 (0.00 to 533.22)	18.81 (0.00 to 536.47)	2.79 (2.11 to 3.84)	0.41 (0.00 to 11.63)	0.41 (0.00 to 11.71)	0.40 (0.00 to 11.50)

Seychelles								
Cause	YLLs (actual counts)				Deaths (actual counts)			
	2016	2040 Forecast	2040 Better Scenario	2040 Worse Scenario	2016	2040 Forecast	2040 Better Scenario	2040 Worse Scenario
**All causes**	**17,462.30 (15,695.05 to 19,717.60)**	**18,016.32 (14,672.90 to 22,659.86)**	**14,661.12 (12,133.67 to 18,258.45)**	**24,521.27 (19,683.03 to 30,836.85)**	**729.06 (666.17 to 811.88)**	**1,013.07 (881.46 to 1,180.99)**	**865.67 (759.08 to 994.19)**	**1,287.18 (1,118.71 to 1,491.17)**
**Communicable, maternal, neonatal, and nutritional diseases**	**2,930.19 (2,614.88 to 3,295.11)**	**2,240.28 (1,747.70 to 2,849.46)**	**1,764.74 (1,419.69 to 2,194.21)**	**3,358.25 (2,635.43 to 4,348.47)**	**93.30 (82.32 to 104.67)**	**114.98 (91.18 to 140.13)**	**97.10 (78.46 to 117.32)**	**152.29 (119.28 to 192.59)**
**HIV/AIDS and tuberculosis**	**444.08 (404.34 to 499.87)**	**150.69 (111.89 to 208.99)**	**112.68 (83.27 to 149.28)**	**536.72 (436.79 to 650.24)**	**10.17 (9.41 to 11.32)**	**3.71 (2.83 to 4.95)**	**2.67 (2.04 to 3.44)**	**13.67 (10.87 to 17.01)**
Tuberculosis	39.76 (32.75 to 49.34)	18.62 (10.10 to 33.03)	10.75 (6.40 to 18.07)	38.80 (20.47 to 70.79)	1.49 (1.27 to 1.80)	0.90 (0.52 to 1.48)	0.54 (0.34 to 0.86)	1.78 (0.99 to 3.15)
HIV/AIDS	404.32 (367.36 to 460.18)	132.08 (94.63 to 188.85)	101.93 (73.37 to 137.86)	497.93 (401.28 to 610.19)	8.67 (7.98 to 9.75)	2.81 (2.04 to 3.98)	2.13 (1.55 to 2.83)	11.89 (9.37 to 15.07)
**Diarrhea, lower respiratory, and other common infectious diseases**	**1,517.41 (1,302.48 to 1,760.12)**	**1,522.58 (1,110.47 to 2,081.84)**	**1,178.72 (886.54 to 1,563.10)**	**2,116.07 (1,473.67 to 3,002.91)**	**66.91 (56.01 to 77.56)**	**95.12 (72.18 to 120.75)**	**78.94 (60.98 to 98.94)**	**121.16 (89.52 to 161.06)**
Diarrheal diseases	43.38 (27.82 to 63.94)	49.27 (11.87 to 145.46)	57.69 (18.12 to 153.00)	45.78 (10.86 to 136.57)	1.82 (1.07 to 2.97)	3.29 (0.78 to 9.45)	4.10 (1.28 to 10.91)	2.85 (0.66 to 8.64)
Intestinal infectious diseases	10.64 (5.05 to 19.34)	6.78 (2.80 to 12.98)	7.37 (3.06 to 13.99)	6.72 (2.76 to 13.04)	0.17 (0.08 to 0.30)	0.12 (0.05 to 0.22)	0.13 (0.06 to 0.25)	0.11 (0.05 to 0.21)
Typhoid fever	8.85 (4.00 to 16.55)	5.78 (2.27 to 11.10)	6.60 (2.72 to 12.56)	5.42 (2.17 to 10.65)	0.14 (0.06 to 0.25)	0.10 (0.04 to 0.19)	0.12 (0.05 to 0.22)	0.09 (0.04 to 0.17)
Paratyphoid fever	1.72 (0.69 to 3.54)	0.97 (0.36 to 2.15)	0.74 (0.27 to 1.67)	1.26 (0.46 to 2.77)	0.03 (0.01 to 0.06)	0.02 (0.01 to 0.04)	0.01 (0.01 to 0.03)	0.02 (0.01 to 0.05)
Other intestinal infectious diseases	0.07 (0.03 to 0.14)	0.03 (0.01 to 0.07)	0.03 (0.01 to 0.06)	0.04 (0.01 to 0.08)	0.00 (0.00 to 0.00)	0.00 (0.00 to 0.00)	0.00 (0.00 to 0.00)	0.00 (0.00 to 0.00)
Lower respiratory infections	1,233.00 (1,041.64 to 1,451.39)	1,333.61 (937.22 to 1,873.34)	1,009.05 (733.46 to 1,360.66)	1,896.14 (1,289.72 to 2,744.86)	59.56 (48.78 to 69.38)	87.42 (64.76 to 113.45)	71.13 (53.96 to 90.02)	113.18 (82.32 to 153.99)
Upper respiratory infections	0.34 (0.18 to 0.60)	0.22 (0.09 to 0.46)	0.14 (0.06 to 0.30)	0.33 (0.15 to 0.69)	0.01 (0.01 to 0.02)	0.01 (0.00 to 0.02)	0.01 (0.00 to 0.01)	0.01 (0.01 to 0.03)
Otitis media	0.07 (0.05 to 0.10)	0.02 (0.01 to 0.04)	0.03 (0.01 to 0.04)	0.02 (0.01 to 0.04)	0.00 (0.00 to 0.00)	0.00 (0.00 to 0.00)	0.00 (0.00 to 0.00)	0.00 (0.00 to 0.00)
Meningitis	191.29 (148.27 to 290.43)	86.67 (56.43 to 142.75)	65.32 (42.91 to 106.89)	110.07 (74.97 to 177.75)	4.41 (3.42 to 6.70)	2.98 (2.07 to 4.77)	2.34 (1.63 to 3.66)	3.56 (2.62 to 5.59)
Pneumococcal meningitis	1.78 (1.26 to 2.62)	0.68 (0.46 to 1.05)	0.69 (0.46 to 1.07)	0.68 (0.46 to 1.05)	0.05 (0.03 to 0.07)	0.03 (0.02 to 0.05)	0.03 (0.02 to 0.05)	0.03 (0.02 to 0.04)
H influenzae type B meningitis	13.77 (10.57 to 21.14)	7.38 (4.84 to 12.13)	7.26 (4.73 to 11.96)	7.71 (4.92 to 12.65)	0.31 (0.24 to 0.47)	0.23 (0.16 to 0.36)	0.24 (0.17 to 0.38)	0.22 (0.16 to 0.35)
Meningococcal meningitis	63.60 (48.03 to 98.99)	28.65 (18.00 to 49.91)	21.42 (13.25 to 36.64)	36.33 (24.21 to 60.48)	1.54 (1.16 to 2.41)	1.06 (0.71 to 1.75)	0.83 (0.56 to 1.35)	1.26 (0.87 to 2.06)
Other meningitis	112.14 (87.46 to 166.10)	49.96 (31.88 to 83.59)	35.95 (23.56 to 58.51)	65.36 (44.96 to 103.67)	2.52 (1.96 to 3.80)	1.66 (1.12 to 2.73)	1.25 (0.87 to 1.93)	2.05 (1.51 to 3.15)
Encephalitis	30.41 (23.98 to 42.67)	26.28 (19.51 to 35.37)	25.30 (18.49 to 33.94)	27.96 (20.62 to 38.02)	0.73 (0.57 to 0.89)	0.95 (0.67 to 1.20)	0.96 (0.67 to 1.22)	0.96 (0.68 to 1.20)

Diphtheria	0.06 (0.04 to 0.08)	0.01 (0.01 to 0.02)	0.01 (0.01 to 0.02)	0.01 (0.01 to 0.03)	0.00 (0.00 to 0.00)	0.00 (0.00 to 0.00)	0.00 (0.00 to 0.00)	0.00 (0.00 to 0.00)
Whooping cough	0.60 (0.04 to 2.00)	0.29 (0.01 to 1.23)	0.23 (0.01 to 0.99)	0.79 (0.01 to 3.40)	0.01 (0.00 to 0.02)	0.00 (0.00 to 0.01)	0.00 (0.00 to 0.01)	0.01 (0.00 to 0.04)
Tetanus	4.49 (2.37 to 6.21)	0.59 (0.30 to 0.94)	0.58 (0.29 to 0.92)	0.66 (0.33 to 1.04)	0.12 (0.07 to 0.17)	0.02 (0.01 to 0.03)	0.02 (0.01 to 0.03)	0.02 (0.01 to 0.03)
Measles	0.78 (0.23 to 1.95)	17.75 (0.17 to 83.72)	12.12 (0.15 to 49.49)	26.15 (0.21 to 129.60)	0.01 (0.00 to 0.02)	0.27 (0.00 to 1.30)	0.19 (0.00 to 0.87)	0.38 (0.00 to 2.03)

Seychelles								
Cause	YLLs (actual counts)				Deaths (actual counts)			
	2016	2040 Forecast	2040 Better Scenario	2040 Worse Scenario	2016	2040 Forecast	2040 Better Scenario	2040 Worse Scenario
Varicella and herpes zoster	2.35 (1.49 to 3.65)	1.08 (0.59 to 1.87)	0.88 (0.50 to 1.50)	1.41 (0.74 to 2.44)	0.08 (0.05 to 0.11)	0.06 (0.03 to 0.09)	0.05 (0.03 to 0.08)	0.06 (0.04 to 0.10)
**Neglected tropical diseases and malaria**	**61.59 (19.23 to 132.94)**	**138.94 (52.64 to 265.60)**	**134.49 (53.28 to 248.81)**	**144.83 (51.77 to 286.64)**	**1.20 (0.44 to 2.41)**	**3.45 (1.47 to 6.39)**	**3.50 (1.54 to 6.41)**	**3.40 (1.37 to 6.44)**
Malaria	0.00 (0.00 to 0.00)	0.00 (0.00 to 0.00)	0.00 (0.00 to 0.00)	0.00 (0.00 to 0.00)	0.00 (0.00 to 0.00)	0.00 (0.00 to 0.00)	0.00 (0.00 to 0.00)	0.00 (0.00 to 0.00)
Chagas disease	0.00 (0.00 to 0.00)	0.00 (0.00 to 0.00)	0.00 (0.00 to 0.00)	0.00 (0.00 to 0.00)	0.00 (0.00 to 0.00)	0.00 (0.00 to 0.00)	0.00 (0.00 to 0.00)	0.00 (0.00 to 0.00)
Leishmaniasis	0.00 (0.00 to 0.00)	0.00 (0.00 to 0.00)	0.00 (0.00 to 0.00)	0.00 (0.00 to 0.00)	0.00 (0.00 to 0.00)	0.00 (0.00 to 0.00)	0.00 (0.00 to 0.00)	0.00 (0.00 to 0.00)
African trypanosomiasis	0.00 (0.00 to 0.00)	0.00 (0.00 to 0.00)	0.00 (0.00 to 0.00)	0.00 (0.00 to 0.00)	0.00 (0.00 to 0.00)	0.00 (0.00 to 0.00)	0.00 (0.00 to 0.00)	0.00 (0.00 to 0.00)
Schistosomiasis	0.00 (0.00 to 0.00)	0.00 (0.00 to 0.00)	0.00 (0.00 to 0.00)	0.00 (0.00 to 0.00)	0.00 (0.00 to 0.00)	0.00 (0.00 to 0.00)	0.00 (0.00 to 0.00)	0.00 (0.00 to 0.00)
Cysticercosis	0.00 (0.00 to 0.00)	0.00 (0.00 to 0.00)	0.00 (0.00 to 0.00)	0.00 (0.00 to 0.00)	0.00 (0.00 to 0.00)	0.00 (0.00 to 0.00)	0.00 (0.00 to 0.00)	0.00 (0.00 to 0.00)
Cystic echinococcosis	0.00 (0.00 to 0.00)	0.00 (0.00 to 0.00)	0.00 (0.00 to 0.00)	0.00 (0.00 to 0.00)	0.00 (0.00 to 0.00)	0.00 (0.00 to 0.00)	0.00 (0.00 to 0.00)	0.00 (0.00 to 0.00)
Dengue	47.02 (6.53 to 117.84)	125.18 (36.72 to 254.79)	119.87 (36.23 to 237.68)	131.01 (36.06 to 274.82)	0.85 (0.13 to 2.07)	2.97 (0.88 to 6.04)	2.97 (0.88 to 5.93)	2.96 (0.86 to 6.09)
Yellow fever	0.00 (0.00 to 0.00)	0.00 (0.00 to 0.00)	0.00 (0.00 to 0.00)	0.00 (0.00 to 0.00)	0.00 (0.00 to 0.00)	0.00 (0.00 to 0.00)	0.00 (0.00 to 0.00)	0.00 (0.00 to 0.00)
Rabies	0.02 (0.01 to 0.02)	0.01 (0.00 to 0.01)	0.01 (0.00 to 0.01)	0.01 (0.00 to 0.02)	0.00 (0.00 to 0.00)	0.00 (0.00 to 0.00)	0.00 (0.00 to 0.00)	0.00 (0.00 to 0.00)
Intestinal nematode infections	0.29 (0.09 to 0.61)	0.30 (0.11 to 0.66)	0.29 (0.11 to 0.63)	0.32 (0.11 to 0.73)	0.01 (0.00 to 0.01)	0.01 (0.00 to 0.02)	0.01 (0.00 to 0.02)	0.01 (0.00 to 0.02)
Other neglected tropical diseases	14.26 (9.47 to 21.05)	13.44 (7.16 to 24.72)	14.32 (7.52 to 26.69)	13.49 (7.38 to 24.90)	0.34 (0.25 to 0.48)	0.46 (0.26 to 0.89)	0.51 (0.28 to 0.99)	0.44 (0.25 to 0.83)
**Maternal disorders**	**25.30 (17.65 to 36.27)**	**11.23 (6.44 to 18.51)**	**9.13 (5.32 to 14.82)**	**14.77 (8.30 to 24.11)**	**0.47 (0.33 to 0.68)**	**0.21 (0.12 to 0.34)**	**0.17 (0.10 to 0.27)**	**0.27 (0.16 to 0.45)**
Maternal hemorrhage	6.19 (3.98 to 9.47)	2.52 (1.33 to 4.49)	2.08 (1.11 to 3.77)	3.28 (1.69 to 5.90)	0.11 (0.07 to 0.17)	0.05 (0.02 to 0.08)	0.04 (0.02 to 0.07)	0.06 (0.03 to 0.11)
Maternal sepsis and other maternal infections	1.23 (0.62 to 2.09)	0.46 (0.21 to 0.90)	0.38 (0.17 to 0.74)	0.60 (0.26 to 1.18)	0.02 (0.01 to 0.04)	0.01 (0.00 to 0.02)	0.01 (0.00 to 0.01)	0.01 (0.00 to 0.02)
Maternal hypertensive disorders	2.04 (1.24 to 3.22)	0.86 (0.44 to 1.54)	0.69 (0.36 to 1.26)	1.13 (0.55 to 2.05)	0.04 (0.02 to 0.06)	0.02 (0.01 to 0.03)	0.01 (0.01 to 0.02)	0.02 (0.01 to 0.04)
Maternal obstructed labor and uterine rupture	0.26 (0.11 to 0.49)	0.11 (0.04 to 0.24)	0.09 (0.03 to 0.19)	0.15 (0.06 to 0.33)	0.00 (0.00 to 0.01)	0.00 (0.00 to 0.00)	0.00 (0.00 to 0.00)	0.00 (0.00 to 0.01)
Maternal abortion, miscarriage, and ectopic pregnancy	11.91 (8.02 to 17.48)	5.54 (3.00 to 9.59)	4.45 (2.44 to 7.71)	7.30 (3.91 to 12.99)	0.22 (0.15 to 0.33)	0.10 (0.06 to 0.18)	0.08 (0.05 to 0.14)	0.14 (0.07 to 0.24)

Indirect maternal deaths	1.33 (0.73 to 2.18)	0.66 (0.29 to 1.30)	0.53 (0.24 to 1.01)	0.88 (0.40 to 1.72)	0.02 (0.01 to 0.04)	0.01 (0.01 to 0.02)	0.01 (0.00 to 0.02)	0.02 (0.01 to 0.03)
Late maternal deaths	0.15 (0.04 to 0.31)	0.06 (0.02 to 0.14)	0.05 (0.01 to 0.11)	0.08 (0.02 to 0.18)	0.00 (0.00 to 0.01)	0.00 (0.00 to 0.00)	0.00 (0.00 to 0.00)	0.00 (0.00 to 0.00)
Maternal deaths aggravated by HIV/AIDS	0.02 (0.01 to 0.04)	0.03 (0.01 to 0.08)	0.02 (0.00 to 0.06)	0.06 (0.02 to 0.14)	0.00 (0.00 to 0.00)	0.00 (0.00 to 0.00)	0.00 (0.00 to 0.00)	0.00 (0.00 to 0.00)
Other maternal disorders	2.18 (1.31 to 3.40)	1.00 (0.53 to 1.77)	0.85 (0.45 to 1.48)	1.28 (0.65 to 2.29)	0.04 (0.02 to 0.06)	0.02 (0.01 to 0.03)	0.02 (0.01 to 0.03)	0.02 (0.01 to 0.04)

Seychelles								
Cause	YLLs (actual counts)				Deaths (actual counts)			
	2016	2040 Forecast	2040 Better Scenario	2040 Worse Scenario	2016	2040 Forecast	2040 Better Scenario	2040 Worse Scenario
**Neonatal disorders**	**725.23 (588.93 to 893.72)**	**236.66 (139.91 to 359.99)**	**154.13 (90.88 to 230.27)**	**355.79 (206.42 to 535.05)**	**8.38 (6.80 to 10.32)**	**2.73 (1.62 to 4.16)**	**1.78 (1.05 to 2.66)**	**4.11 (2.38 to 6.18)**
Neonatal preterm birth	357.67 (261.18 to 459.99)	108.65 (61.66 to 163.08)	58.82 (32.96 to 90.16)	167.10 (93.62 to 257.69)	4.13 (3.02 to 5.31)	1.26 (0.71 to 1.88)	0.68 (0.38 to 1.04)	1.93 (1.08 to 2.98)
Neonatal encephalopathy due to birth asphyxia and trauma	187.53 (121.05 to 269.61)	50.87 (22.34 to 95.48)	34.73 (15.26 to 63.89)	79.26 (34.49 to 147.81)	2.17 (1.40 to 3.11)	0.59 (0.26 to 1.10)	0.40 (0.18 to 0.74)	0.92 (0.40 to 1.71)
Neonatal sepsis and other neonatal infections	110.14 (71.70 to 166.54)	49.04 (24.95 to 81.29)	36.31 (19.35 to 60.58)	72.11 (37.00 to 123.53)	1.27 (0.83 to 1.92)	0.57 (0.29 to 0.94)	0.42 (0.22 to 0.70)	0.83 (0.43 to 1.43)
Hemolytic disease and other neonatal jaundice	2.61 (1.47 to 4.18)	0.58 (0.24 to 1.26)	0.42 (0.18 to 0.88)	0.88 (0.35 to 1.87)	0.03 (0.02 to 0.05)	0.01 (0.00 to 0.01)	0.00 (0.00 to 0.01)	0.01 (0.00 to 0.02)
Other neonatal disorders	67.27 (42.61 to 103.41)	27.53 (13.87 to 50.17)	23.84 (12.38 to 42.75)	36.43 (17.51 to 67.14)	0.78 (0.49 to 1.19)	0.32 (0.16 to 0.58)	0.28 (0.14 to 0.49)	0.42 (0.20 to 0.78)
**Nutritional deficiencies**	**30.65 (26.96 to 35.00)**	**32.54 (27.11 to 38.96)**	**34.50 (28.61 to 41.34)**	**31.79 (26.53 to 38.24)**	**1.80 (1.60 to 2.06)**	**2.45 (1.95 to 3.03)**	**2.70 (2.17 to 3.32)**	**2.27 (1.77 to 2.86)**
Protein-energy malnutrition	18.12 (14.99 to 21.38)	18.93 (14.22 to 23.81)	21.74 (16.46 to 26.70)	17.11 (12.81 to 21.47)	0.98 (0.82 to 1.15)	1.39 (1.01 to 1.83)	1.68 (1.26 to 2.14)	1.19 (0.85 to 1.56)
Iodine deficiency	0.86 (0.12 to 2.24)	1.68 (0.08 to 5.64)	1.63 (0.06 to 5.69)	1.79 (0.09 to 5.71)	0.04 (0.00 to 0.13)	0.12 (0.00 to 0.45)	0.12 (0.00 to 0.48)	0.12 (0.00 to 0.43)
Dietary iron deficiency	1.75 (1.36 to 2.24)	1.44 (0.99 to 2.16)	1.50 (1.03 to 2.25)	1.47 (1.01 to 2.20)	0.04 (0.03 to 0.06)	0.04 (0.03 to 0.07)	0.05 (0.03 to 0.08)	0.04 (0.03 to 0.07)
Other nutritional deficiencies	9.92 (8.22 to 12.48)	10.49 (7.60 to 14.03)	9.64 (6.99 to 13.00)	11.43 (8.33 to 14.95)	0.74 (0.60 to 0.93)	0.89 (0.61 to 1.26)	0.86 (0.59 to 1.21)	0.92 (0.62 to 1.29)
**Other communicable, maternal, neonatal, and nutritional diseases**	**125.93 (103.31 to 163.09)**	**147.63 (117.45 to 189.12)**	**141.09 (111.95 to 180.78)**	**158.29 (125.48 to 203.62)**	**4.37 (3.75 to 5.23)**	**7.31 (5.89 to 8.85)**	**7.33 (5.94 to 8.82)**	**7.40 (5.96 to 9.04)**
Sexually transmitted infections excluding HIV	8.21 (4.53 to 10.91)	3.30 (1.70 to 4.76)	3.02 (1.54 to 4.32)	3.67 (1.87 to 5.23)	0.24 (0.14 to 0.31)	0.13 (0.07 to 0.16)	0.12 (0.07 to 0.16)	0.13 (0.08 to 0.17)
Syphilis	3.08 (1.58 to 4.16)	0.93 (0.48 to 1.33)	0.76 (0.39 to 1.08)	1.13 (0.57 to 1.62)	0.09 (0.05 to 0.12)	0.04 (0.02 to 0.05)	0.03 (0.02 to 0.04)	0.04 (0.02 to 0.05)
Chlamydial infection	0.99 (0.58 to 1.37)	0.47 (0.24 to 0.67)	0.44 (0.23 to 0.64)	0.50 (0.25 to 0.72)	0.03 (0.02 to 0.04)	0.02 (0.01 to 0.02)	0.02 (0.01 to 0.02)	0.02 (0.01 to 0.02)
Gonococcal infection	2.71 (1.53 to 3.74)	1.29 (0.66 to 1.86)	1.22 (0.63 to 1.78)	1.37 (0.70 to 2.00)	0.08 (0.05 to 0.11)	0.05 (0.03 to 0.07)	0.05 (0.03 to 0.07)	0.05 (0.03 to 0.07)
Other sexually transmitted infections	1.43 (0.80 to 1.98)	0.63 (0.31 to 0.95)	0.59 (0.29 to 0.90)	0.68 (0.33 to 1.03)	0.04 (0.02 to 0.05)	0.02 (0.01 to 0.03)	0.02 (0.01 to 0.03)	0.02 (0.01 to 0.03)
Acute hepatitis	33.07 (26.60 to 42.23)	19.80 (13.84 to 28.59)	15.18 (10.90 to 21.14)	25.00 (18.05 to 33.49)	0.97 (0.80 to 1.21)	0.75 (0.55 to 1.04)	0.59 (0.44 to 0.78)	0.93 (0.70 to 1.18)
Acute hepatitis A	1.29 (0.95 to 1.71)	0.50 (0.31 to 0.78)	0.41 (0.26 to 0.66)	0.61 (0.39 to 0.99)	0.03 (0.02 to 0.04)	0.02 (0.01 to 0.02)	0.01 (0.01 to 0.02)	0.02 (0.01 to 0.03)
Acute hepatitis B	28.25 (22.31 to 36.56)	16.90 (11.76 to 24.84)	12.89 (9.16 to 18.45)	21.45 (15.34 to 29.46)	0.83 (0.67 to 1.05)	0.63 (0.44 to 0.88)	0.49 (0.35 to 0.65)	0.78 (0.58 to 1.01)

Acute hepatitis C	0.76 (0.53 to 1.10)	0.59 (0.36 to 0.93)	0.46 (0.28 to 0.71)	0.72 (0.46 to 1.09)	0.02 (0.02 to 0.04)	0.02 (0.01 to 0.04)	0.02 (0.01 to 0.03)	0.03 (0.02 to 0.04)
Acute hepatitis E	2.77 (1.97 to 3.89)	1.81 (1.08 to 2.81)	1.42 (0.86 to 2.22)	2.21 (1.37 to 3.39)	0.09 (0.06 to 0.13)	0.09 (0.05 to 0.14)	0.07 (0.04 to 0.11)	0.10 (0.06 to 0.16)
Other unspecified infectious diseases	84.64 (66.86 to 118.48)	124.52 (98.52 to 161.45)	122.90 (97.45 to 159.21)	129.62 (101.56 to 170.53)	3.15 (2.64 to 3.92)	6.43 (5.15 to 7.89)	6.62 (5.31 to 8.06)	6.35 (5.04 to 7.90)
**Non-communicable diseases**	**12,252.18 (10,923.55 to 13,977.31)**	**14,173.88 (11,373.64 to 18,346.82)**	**11,728.42 (9,585.03 to 14,821.18)**	**19,108.36 (15,009.24 to 24,820.00)**	**579.42 (526.52 to 640.81)**	**846.01 (724.87 to 1,012.75)**	**728.48 (629.52 to 850.53)**	**1,071.57 (912.15 to 1,271.31)**

Seychelles								
Cause	YLLs (actual counts)				Deaths (actual counts)			
	2016	2040 Forecast	2040 Better Scenario	2040 Worse Scenario	2016	2040 Forecast	2040 Better Scenario	2040 Worse Scenario
**Neoplasms**	**3,341.52 (2,936.98 to 3,862.16)**	**4,429.59 (3,535.25 to 5,476.59)**	**3,911.23 (3,193.69 to 4,734.81)**	**5,411.45 (4,293.08 to 6,703.74)**	**139.77 (124.77 to 158.55)**	**233.72 (199.05 to 273.00)**	**212.68 (182.80 to 245.27)**	**271.94 (227.74 to 320.03)**
Lip and oral cavity cancer	161.98 (133.35 to 196.57)	160.31 (87.20 to 276.61)	124.63 (71.35 to 201.77)	298.71 (148.59 to 536.33)	6.84 (5.79 to 8.13)	8.31 (4.92 to 13.18)	6.68 (4.18 to 10.31)	14.53 (7.58 to 24.44)
Nasopharynx cancer	52.24 (42.54 to 63.96)	51.43 (28.15 to 88.36)	36.54 (20.71 to 59.71)	105.44 (52.98 to 188.88)	1.81 (1.51 to 2.20)	2.20 (1.25 to 3.56)	1.60 (0.95 to 2.49)	4.27 (2.23 to 7.34)
Other pharynx cancer	110.79 (89.03 to 137.75)	120.36 (68.60 to 201.95)	120.07 (68.65 to 195.15)	151.60 (80.57 to 261.31)	4.23 (3.46 to 5.16)	5.62 (3.35 to 9.14)	5.74 (3.49 to 9.01)	6.83 (3.84 to 11.37)
Esophageal cancer	117.80 (97.75 to 143.57)	128.03 (77.64 to 207.35)	111.96 (70.88 to 170.13)	201.48 (110.14 to 353.28)	4.76 (4.02 to 5.67)	6.22 (3.95 to 9.35)	5.58 (3.70 to 8.10)	9.29 (5.29 to 15.23)
Stomach cancer	123.32 (106.30 to 145.94)	117.09 (89.83 to 150.52)	96.88 (76.36 to 121.48)	139.47 (108.75 to 177.78)	5.31 (4.63 to 6.12)	6.21 (4.97 to 7.68)	5.27 (4.34 to 6.29)	7.14 (5.80 to 8.73)
Colon and rectum cancer	430.77 (375.06 to 494.46)	596.21 (409.61 to 850.79)	516.65 (359.76 to 723.52)	719.80 (483.16 to 1,027.00)	19.54 (17.29 to 22.03)	33.34 (23.46 to 46.11)	29.69 (21.18 to 41.06)	38.70 (26.82 to 54.29)
Liver cancer	136.84 (113.13 to 165.97)	181.50 (120.35 to 266.08)	166.82 (116.59 to 232.18)	203.10 (130.11 to 310.13)	5.33 (4.47 to 6.29)	8.83 (6.15 to 12.30)	8.38 (6.18 to 11.16)	9.47 (6.38 to 13.59)
Liver cancer due to hepatitis B	60.74 (45.54 to 79.01)	74.53 (45.57 to 115.96)	68.70 (44.47 to 102.56)	80.59 (48.09 to 129.96)	2.19 (1.63 to 2.81)	3.39 (2.15 to 5.02)	3.22 (2.11 to 4.58)	3.53 (2.19 to 5.48)
Liver cancer due to hepatitis C	26.17 (17.71 to 36.47)	42.31 (25.59 to 65.20)	39.91 (25.04 to 59.45)	44.46 (26.22 to 70.36)	1.23 (0.86 to 1.67)	2.38 (1.46 to 3.57)	2.31 (1.49 to 3.40)	2.40 (1.45 to 3.72)
Liver cancer due to alcohol use	21.70 (14.93 to 30.80)	28.05 (16.15 to 46.36)	24.79 (15.03 to 39.35)	38.09 (20.76 to 63.81)	0.86 (0.60 to 1.18)	1.34 (0.78 to 2.16)	1.22 (0.74 to 1.92)	1.72 (0.98 to 2.84)
Liver cancer due to other causes	28.22 (19.17 to 40.05)	36.61 (21.02 to 62.59)	33.42 (20.20 to 53.88)	39.97 (22.26 to 70.10)	1.05 (0.73 to 1.47)	1.73 (0.99 to 2.86)	1.63 (0.98 to 2.56)	1.81 (1.03 to 3.10)
Gallbladder and biliary tract cancer	27.20 (21.65 to 32.44)	30.76 (24.45 to 36.64)	30.61 (24.23 to 36.88)	29.84 (23.82 to 35.16)	1.30 (1.06 to 1.52)	1.73 (1.41 to 2.01)	1.76 (1.44 to 2.07)	1.64 (1.33 to 1.89)
Pancreatic cancer	124.13 (107.43 to 144.63)	211.01 (160.59 to 269.34)	208.01 (159.55 to 263.51)	217.99 (163.58 to 281.59)	5.42 (4.80 to 6.17)	11.07 (8.79 to 13.86)	11.15 (8.96 to 13.86)	11.04 (8.65 to 13.96)
Larynx cancer	89.86 (73.91 to 112.13)	80.66 (42.16 to 143.44)	53.96 (30.21 to 90.74)	161.71 (77.37 to 305.33)	3.67 (3.07 to 4.41)	4.02 (2.27 to 6.96)	2.75 (1.67 to 4.50)	7.63 (3.93 to 13.87)
Tracheal, bronchus, and lung cancer	295.18 (250.99 to 347.90)	397.48 (273.86 to 568.06)	289.16 (207.10 to 399.73)	672.34 (436.06 to 1,012.87)	12.70 (10.98 to 14.65)	20.74 (14.70 to 28.57)	15.50 (11.49 to 20.82)	33.26 (22.10 to 48.02)
Malignant skin melanoma	17.39 (14.09 to 24.40)	29.04 (22.73 to 41.69)	33.33 (25.93 to 47.81)	24.91 (19.94 to 35.72)	0.68 (0.56 to 0.96)	1.45 (1.18 to 2.07)	1.70 (1.38 to 2.41)	1.20 (0.99 to 1.72)
Non-melanoma skin cancer	12.84 (10.86 to 15.35)	19.06 (15.34 to 23.50)	16.50 (13.48 to 20.15)	20.94 (17.69 to 24.66)	0.57 (0.50 to 0.66)	1.05 (0.88 to 1.24)	0.94 (0.81 to 1.09)	1.11 (0.97 to 1.26)
Breast cancer	323.85 (251.87 to 414.54)	364.25 (246.33 to 527.88)	311.23 (219.11 to 437.66)	428.95 (286.84 to 636.11)	11.62 (9.27 to 14.30)	16.29 (11.56 to 22.78)	14.19 (10.32 to 19.46)	18.68 (13.13 to 26.94)
Cervical cancer	197.53 (147.06 to 298.11)	178.40 (126.15 to 274.52)	148.89 (108.74 to 216.63)	209.83 (154.77 to 318.30)	7.64 (5.95 to 11.14)	8.40 (6.34 to 12.32)	7.17 (5.56 to 10.08)	9.59 (7.48 to 13.81)

Uterine cancer	16.36 (13.09 to 20.12)	26.51 (20.94 to 33.44)	24.14 (19.09 to 30.55)	28.93 (22.74 to 36.33)	0.84 (0.70 to 1.01)	1.54 (1.24 to 1.91)	1.44 (1.16 to 1.77)	1.64 (1.30 to 2.03)
Ovarian cancer	83.53 (64.70 to 102.98)	128.57 (90.96 to 178.17)	116.00 (83.18 to 157.85)	136.51 (95.56 to 192.29)	3.36 (2.66 to 4.04)	6.22 (4.44 to 8.42)	5.71 (4.17 to 7.65)	6.47 (4.57 to 8.98)
Prostate cancer	274.54 (212.54 to 358.77)	616.05 (486.60 to 786.96)	588.39 (472.51 to 746.80)	607.35 (477.14 to 779.44)	17.86 (14.17 to 22.97)	44.44 (34.81 to 56.54)	43.64 (35.08 to 54.87)	42.32 (32.41 to 54.10)
Testicular cancer	4.19 (3.23 to 5.33)	3.22 (2.33 to 4.37)	2.84 (2.11 to 3.90)	3.52 (2.63 to 4.72)	0.12 (0.09 to 0.14)	0.12 (0.10 to 0.16)	0.11 (0.09 to 0.15)	0.13 (0.10 to 0.16)

Seychelles								
Cause	YLLs (actual counts)				Deaths (actual counts)			
	2016	2040 Forecast	2040 Better Scenario	2040 Worse Scenario	2016	2040 Forecast	2040 Better Scenario	2040 Worse Scenario
Kidney cancer	44.52 (37.46 to 53.02)	70.00 (53.70 to 90.61)	70.37 (54.08 to 90.79)	70.22 (52.44 to 94.70)	1.74 (1.48 to 2.03)	3.41 (2.72 to 4.25)	3.52 (2.84 to 4.30)	3.29 (2.54 to 4.24)
Bladder cancer	68.05 (58.59 to 79.10)	105.22 (79.77 to 139.73)	96.17 (74.67 to 125.14)	118.80 (87.73 to 162.50)	3.68 (3.21 to 4.19)	6.74 (5.22 to 8.84)	6.36 (5.01 to 8.16)	7.26 (5.39 to 9.83)
Brain and nervous system cancer	130.51 (106.76 to 173.66)	173.29 (135.81 to 227.89)	164.05 (128.49 to 214.80)	176.81 (140.19 to 232.44)	4.02 (3.31 to 5.26)	7.05 (5.52 to 9.02)	6.87 (5.35 to 8.79)	6.93 (5.53 to 8.90)
Thyroid cancer	6.17 (5.21 to 7.28)	9.81 (7.06 to 13.78)	8.37 (6.26 to 11.19)	10.92 (7.88 to 15.26)	0.27 (0.23 to 0.31)	0.51 (0.38 to 0.69)	0.45 (0.34 to 0.59)	0.55 (0.41 to 0.75)
Mesothelioma	5.72 (4.17 to 7.83)	6.83 (4.90 to 9.43)	7.04 (5.01 to 9.85)	6.53 (4.71 to 8.97)	0.19 (0.14 to 0.27)	0.30 (0.23 to 0.40)	0.32 (0.24 to 0.43)	0.27 (0.21 to 0.37)
Hodgkin lymphoma	15.56 (11.76 to 23.46)	11.91 (8.46 to 18.21)	9.63 (6.99 to 14.75)	14.40 (10.39 to 22.31)	0.49 (0.38 to 0.72)	0.48 (0.36 to 0.73)	0.40 (0.30 to 0.60)	0.57 (0.43 to 0.86)
Non-Hodgkin lymphoma	132.91 (107.84 to 155.86)	171.84 (130.10 to 219.90)	152.83 (116.64 to 194.14)	181.73 (139.06 to 231.48)	4.66 (3.81 to 5.41)	7.91 (6.16 to 9.83)	7.23 (5.69 to 8.95)	8.09 (6.24 to 10.16)
Multiple myeloma	38.49 (28.74 to 46.56)	58.49 (42.00 to 75.53)	54.11 (38.76 to 69.19)	59.53 (42.85 to 76.26)	1.50 (1.15 to 1.77)	2.77 (2.07 to 3.49)	2.62 (1.96 to 3.27)	2.75 (2.04 to 3.52)
Leukemia	195.10 (163.90 to 229.97)	214.53 (159.66 to 281.76)	185.23 (141.57 to 240.68)	245.25 (182.52 to 325.65)	5.91 (4.98 to 6.92)	8.98 (6.90 to 11.72)	7.97 (6.26 to 10.00)	9.89 (7.58 to 12.98)
Acute lymphoid leukemia	52.03 (43.07 to 64.64)	54.88 (40.95 to 73.09)	46.80 (35.75 to 62.55)	62.49 (47.14 to 84.25)	1.52 (1.28 to 1.95)	2.25 (1.74 to 3.10)	1.98 (1.54 to 2.65)	2.47 (1.89 to 3.39)
Chronic lymphoid leukemia	4.13 (3.20 to 5.17)	5.04 (3.61 to 6.91)	4.60 (3.34 to 6.16)	5.46 (3.90 to 7.54)	0.15 (0.12 to 0.18)	0.24 (0.18 to 0.32)	0.22 (0.17 to 0.29)	0.25 (0.18 to 0.33)
Acute myeloid leukemia	45.51 (36.48 to 56.69)	60.62 (44.29 to 82.19)	55.99 (41.40 to 74.87)	64.72 (46.67 to 88.88)	1.24 (1.01 to 1.53)	2.22 (1.64 to 2.97)	2.11 (1.58 to 2.76)	2.29 (1.67 to 3.15)
Chronic myeloid leukemia	10.61 (8.19 to 13.73)	6.85 (4.46 to 10.09)	4.96 (3.44 to 7.22)	8.97 (6.09 to 13.18)	0.33 (0.26 to 0.42)	0.28 (0.19 to 0.40)	0.21 (0.15 to 0.29)	0.35 (0.25 to 0.50)
Other leukemia	82.82 (64.37 to 99.71)	87.14 (60.80 to 118.33)	72.89 (51.53 to 98.47)	103.60 (73.00 to 144.28)	2.68 (2.08 to 3.19)	3.99 (2.86 to 5.31)	3.45 (2.53 to 4.47)	4.54 (3.27 to 6.14)
Other neoplasms	104.16 (88.87 to 123.39)	167.73 (139.62 to 202.02)	166.83 (137.90 to 202.18)	164.84 (138.78 to 195.91)	3.69 (3.18 to 4.28)	7.76 (6.74 to 8.94)	7.94 (6.86 to 9.13)	7.37 (6.48 to 8.48)
**Cardiovascular diseases**	**4,489.24 (3,973.38 to 5,126.46)**	**4,243.85 (3,026.92 to 6,127.54)**	**3,125.41 (2,356.10 to 4,207.99)**	**7,063.26 (4,795.94 to 10,456.43)**	**238.05 (216.28 to 264.15)**	**275.20 (211.50 to 367.29)**	**215.69 (172.92 to 272.33)**	**416.47 (309.55 to 571.63)**
Rheumatic heart disease	55.94 (46.20 to 66.55)	27.90 (21.15 to 37.46)	22.73 (17.30 to 29.43)	33.97 (26.23 to 43.02)	1.74 (1.50 to 2.01)	1.28 (1.03 to 1.61)	1.09 (0.89 to 1.31)	1.48 (1.22 to 1.79)
Ischemic heart disease	1,909.46 (1,648.51 to 2,219.39)	1,445.75 (798.17 to 2,506.97)	883.35 (513.36 to 1,515.37)	2,847.48 (1,547.35 to 4,970.77)	104.05 (91.56 to 116.69)	94.55 (57.07 to 152.00)	62.56 (38.36 to 98.09)	164.90 (97.13 to 267.11)
Stroke	949.90 (814.19 to 1,115.54)	651.34 (393.17 to 1,075.36)	385.86 (246.71 to 587.99)	1,330.68 (783.93 to 2,115.62)	47.19 (41.14 to 53.85)	40.33 (26.22 to 62.73)	26.12 (17.47 to 38.11)	72.20 (46.11 to 108.88)
Ischemic stroke	252.66 (216.54 to 296.50)	210.46 (124.80 to 374.18)	136.57 (82.77 to 237.16)	362.31 (206.89 to 622.31)	17.27 (14.96 to 19.93)	16.16 (9.67 to 29.22)	11.26 (6.85 to 19.22)	25.08 (14.88 to 44.79)

Intracerebral hemorrhage	697.24 (589.74 to 825.78)	440.88 (252.90 to 752.06)	249.29 (155.39 to 413.79)	968.38 (534.26 to 1,646.59)	29.92 (25.82 to 34.59)	24.18 (15.45 to 38.48)	14.86 (10.04 to 22.26)	47.13 (28.85 to 74.62)
Hypertensive heart disease	1,109.72 (897.08 to 1,383.35)	1,531.87 (1,074.71 to 2,295.16)	1,330.71 (990.64 to 1,811.73)	2,148.69 (1,409.79 to 3,454.72)	63.10 (52.67 to 77.93)	102.80 (75.56 to 150.43)	93.42 (73.22 to 122.57)	137.21 (95.17 to 215.11)
Cardiomyopathy and myocarditis	205.42 (170.36 to 249.78)	219.73 (160.68 to 292.85)	193.48 (142.53 to 254.09)	262.82 (195.92 to 341.65)	8.97 (7.15 to 10.58)	12.49 (8.76 to 17.11)	11.29 (8.04 to 15.03)	14.18 (10.10 to 18.71)
Myocarditis	23.92 (18.58 to 32.55)	23.29 (17.29 to 31.23)	22.81 (16.78 to 30.59)	23.74 (17.82 to 31.54)	0.79 (0.62 to 1.01)	1.13 (0.81 to 1.42)	1.16 (0.83 to 1.49)	1.07 (0.78 to 1.34)

Seychelles								
Cause	YLLs (actual counts)				Deaths (actual counts)			
	2016	2040 Forecast	2040 Better Scenario	2040 Worse Scenario	2016	2040 Forecast	2040 Better Scenario	2040 Worse Scenario
Alcoholic cardiomyopathy	85.63 (67.15 to 112.29)	90.66 (66.72 to 119.06)	87.51 (63.85 to 115.56)	110.24 (82.64 to 142.95)	3.40 (2.70 to 4.27)	4.49 (3.33 to 5.59)	4.46 (3.29 to 5.58)	5.20 (3.93 to 6.43)
Other cardiomyopathy	95.86 (74.18 to 116.76)	105.78 (67.20 to 161.27)	83.16 (52.10 to 122.56)	128.85 (83.72 to 183.63)	4.78 (3.36 to 5.83)	6.88 (4.04 to 11.06)	5.66 (3.26 to 8.87)	7.91 (4.69 to 12.13)
Atrial fibrillation and flutter	24.58 (19.00 to 30.64)	49.99 (35.63 to 65.80)	48.39 (34.63 to 64.02)	53.30 (37.64 to 71.34)	2.61 (1.99 to 3.37)	5.10 (3.53 to 6.76)	5.10 (3.62 to 6.78)	5.20 (3.53 to 7.06)
Aortic aneurysm	32.54 (27.22 to 39.04)	48.33 (38.27 to 60.41)	43.81 (34.74 to 53.86)	52.23 (41.02 to 64.96)	1.82 (1.53 to 2.15)	3.23 (2.61 to 3.95)	3.07 (2.49 to 3.72)	3.29 (2.65 to 4.02)
Peripheral artery disease	3.29 (2.07 to 4.90)	9.86 (5.86 to 15.32)	9.37 (5.69 to 14.59)	9.87 (5.80 to 15.53)	0.28 (0.18 to 0.42)	0.89 (0.52 to 1.40)	0.89 (0.53 to 1.40)	0.83 (0.48 to 1.33)
Endocarditis	45.57 (35.66 to 60.01)	51.14 (35.48 to 72.49)	49.57 (34.30 to 69.78)	54.54 (38.08 to 77.75)	1.52 (1.21 to 1.98)	2.32 (1.65 to 3.20)	2.34 (1.65 to 3.27)	2.35 (1.67 to 3.28)
Other cardiovascular and circulatory diseases	152.83 (107.97 to 265.64)	207.95 (130.88 to 366.36)	158.15 (103.42 to 272.41)	269.68 (175.32 to 478.94)	6.77 (4.69 to 11.67)	12.22 (7.99 to 21.56)	9.81 (6.49 to 16.68)	14.82 (9.92 to 25.84)
**Chronic respiratory diseases**	**483.97 (422.64 to 564.17)**	**496.76 (369.98 to 654.16)**	**376.05 (293.64 to 475.12)**	**745.63 (527.29 to 1,040.74)**	**26.77 (23.61 to 30.98)**	**32.60 (25.36 to 41.46)**	**25.72 (20.98 to 31.76)**	**45.92 (34.01 to 61.00)**
Chronic obstructive pulmonary disease	306.89 (262.60 to 370.49)	357.65 (248.00 to 500.74)	261.62 (193.07 to 345.71)	571.45 (374.61 to 841.31)	18.61 (16.11 to 22.41)	24.54 (18.08 to 32.45)	18.86 (14.63 to 24.25)	36.27 (24.86 to 50.48)
Pneumoconiosis	0.48 (0.37 to 0.63)	0.49 (0.36 to 0.63)	0.45 (0.34 to 0.59)	0.52 (0.39 to 0.67)	0.03 (0.02 to 0.03)	0.03 (0.02 to 0.04)	0.03 (0.02 to 0.04)	0.03 (0.02 to 0.04)
Silicosis	0.05 (0.03 to 0.09)	0.04 (0.02 to 0.06)	0.02 (0.02 to 0.04)	0.05 (0.03 to 0.07)	0.00 (0.00 to 0.00)	0.00 (0.00 to 0.00)	0.00 (0.00 to 0.00)	0.00 (0.00 to 0.00)
Asbestosis	0.14 (0.10 to 0.18)	0.19 (0.13 to 0.26)	0.21 (0.15 to 0.29)	0.16 (0.11 to 0.23)	0.01 (0.00 to 0.01)	0.01 (0.01 to 0.01)	0.01 (0.01 to 0.02)	0.01 (0.01 to 0.01)
Coal workers pneumoconiosis	0.02 (0.01 to 0.04)	0.02 (0.01 to 0.03)	0.02 (0.01 to 0.03)	0.02 (0.01 to 0.04)	0.00 (0.00 to 0.00)	0.00 (0.00 to 0.00)	0.00 (0.00 to 0.00)	0.00 (0.00 to 0.00)
Other pneumoconiosis	0.27 (0.19 to 0.36)	0.24 (0.17 to 0.34)	0.20 (0.14 to 0.28)	0.29 (0.20 to 0.39)	0.02 (0.01 to 0.02)	0.02 (0.01 to 0.03)	0.02 (0.01 to 0.02)	0.02 (0.01 to 0.03)
Asthma	144.12 (107.51 to 174.99)	88.02 (49.96 to 144.43)	54.56 (33.75 to 84.42)	129.94 (78.90 to 206.43)	6.72 (5.09 to 8.13)	5.20 (3.08 to 8.29)	3.40 (2.17 to 5.24)	7.28 (4.55 to 11.39)
Interstitial lung disease and pulmonary sarcoidosis	19.04 (12.90 to 32.35)	36.69 (24.29 to 60.15)	45.73 (31.81 to 70.54)	29.29 (19.95 to 48.72)	0.89 (0.61 to 1.51)	2.08 (1.39 to 3.40)	2.66 (1.86 to 4.23)	1.61 (1.08 to 2.63)
Other chronic respiratory diseases	13.43 (8.04 to 18.93)	13.91 (7.75 to 20.70)	13.68 (7.50 to 20.55)	14.43 (8.01 to 21.59)	0.53 (0.28 to 0.84)	0.75 (0.36 to 1.25)	0.77 (0.38 to 1.30)	0.72 (0.36 to 1.20)
**Cirrhosis and other chronic liver diseases**	**643.75 (515.75 to 811.43)**	**592.50 (436.17 to 775.88)**	**520.06 (387.72 to 677.03)**	**693.16 (521.37 to 876.15)**	**21.41 (17.70 to 26.11)**	**24.44 (19.46 to 30.06)**	**21.89 (17.54 to 26.95)**	**27.76 (22.59 to 33.03)**
Cirrhosis and other chronic liver diseases due to hepatitis B	224.87 (169.90 to 295.95)	215.08 (147.80 to 294.75)	190.80 (132.71 to 259.25)	235.69 (167.80 to 319.00)	7.41 (5.70 to 9.47)	8.73 (6.34 to 11.28)	7.89 (5.81 to 10.15)	9.35 (7.02 to 11.92)
Cirrhosis and other chronic liver diseases due to hepatitis C	114.58 (82.64 to 152.23)	108.13 (74.36 to 149.53)	94.88 (66.14 to 134.61)	119.86 (83.88 to 165.75)	3.71 (2.78 to 4.82)	4.28 (3.09 to 5.68)	3.82 (2.78 to 5.13)	4.64 (3.46 to 6.02)

Cirrhosis and other chronic liver diseases due to alcohol use	152.06 (111.53 to 202.31)	124.39 (85.95 to 176.58)	106.05 (73.81 to 149.74)	177.57 (123.53 to 244.14)	5.13 (3.85 to 6.63)	5.21 (3.84 to 6.92)	4.55 (3.41 to 6.03)	7.10 (5.25 to 9.31)
Cirrhosis and other chronic liver diseases due to other causes	152.25 (120.08 to 187.34)	144.90 (108.94 to 191.51)	128.34 (98.48 to 171.84)	160.04 (124.77 to 206.78)	5.17 (4.21 to 6.17)	6.22 (4.96 to 7.78)	5.63 (4.53 to 7.01)	6.67 (5.40 to 8.08)
**Digestive diseases**	**557.16 (475.85 to 657.03)**	**569.88 (448.14 to 731.53)**	**513.64 (410.84 to 652.48)**	**656.22 (511.85 to 876.23)**	**24.00 (20.83 to 28.07)**	**32.42 (26.67 to 39.40)**	**30.48 (25.48 to 36.19)**	**35.14 (28.53 to 43.35)**
Peptic ulcer disease	153.62 (125.42 to 190.58)	103.17 (75.53 to 144.03)	78.26 (57.49 to 107.02)	134.70 (101.60 to 179.81)	6.76 (5.67 to 8.18)	5.87 (4.43 to 7.92)	4.59 (3.51 to 5.98)	7.37 (5.73 to 9.54)

Seychelles								
**Cause**	**YLLs (actual counts)**				**Deaths (actual counts)**			
	**2016**	**2040 Forecast**	**2040 Better Scenario**	**2040 Worse Scenario**	**2016**	**2040 Forecast**	**2040 Better Scenario**	**2040 Worse Scenario**
Gastritis and duodenitis	6.24 (4.65 to 8.16)	7.24 (5.00 to 10.17)	6.18 (4.27 to 8.55)	8.17 (5.78 to 11.37)	0.29 (0.22 to 0.37)	0.43 (0.30 to 0.59)	0.38 (0.26 to 0.52)	0.46 (0.33 to 0.62)
Appendicitis	61.06 (48.94 to 75.52)	48.93 (36.16 to 66.55)	38.50 (27.50 to 50.83)	60.22 (44.99 to 76.42)	2.30 (1.84 to 2.75)	2.49 (1.82 to 3.18)	2.03 (1.45 to 2.58)	2.93 (2.19 to 3.61)
Paralytic ileus and intestinal obstruction	192.80 (162.25 to 237.61)	251.49 (210.38 to 307.38)	255.32 (211.89 to 310.34)	246.07 (206.15 to 301.49)	8.81 (7.48 to 11.05)	15.08 (12.71 to 18.96)	15.83 (13.36 to 19.56)	14.13 (11.82 to 17.89)
Inguinal, femoral, and abdominal hernia	2.70 (1.86 to 4.32)	2.57 (1.69 to 4.19)	2.31 (1.51 to 3.82)	2.79 (1.86 to 4.57)	0.15 (0.10 to 0.24)	0.18 (0.11 to 0.28)	0.16 (0.11 to 0.27)	0.18 (0.11 to 0.30)
Inflammatory bowel disease	6.70 (5.42 to 8.38)	8.71 (6.70 to 11.04)	8.70 (6.59 to 11.06)	8.55 (6.73 to 10.75)	0.28 (0.23 to 0.34)	0.48 (0.38 to 0.59)	0.49 (0.38 to 0.61)	0.45 (0.36 to 0.55)
Vascular intestinal disorders	15.66 (12.02 to 20.94)	25.25 (19.34 to 34.99)	24.11 (18.27 to 33.29)	25.80 (20.02 to 35.40)	0.93 (0.72 to 1.26)	1.75 (1.33 to 2.41)	1.72 (1.29 to 2.34)	1.72 (1.30 to 2.36)
Gallbladder and biliary diseases	14.60 (10.26 to 19.74)	21.14 (10.76 to 36.94)	14.96 (7.75 to 25.86)	27.82 (14.31 to 48.57)	0.79 (0.54 to 1.15)	1.36 (0.72 to 2.41)	1.01 (0.53 to 1.77)	1.71 (0.88 to 2.96)
Pancreatitis	72.78 (44.27 to 100.61)	66.17 (25.07 to 150.63)	52.06 (20.72 to 114.41)	104.88 (37.31 to 253.29)	2.27 (1.40 to 3.11)	2.66 (1.14 to 5.67)	2.15 (0.94 to 4.31)	4.04 (1.58 to 9.16)
Other digestive diseases	31.00 (23.54 to 40.49)	35.20 (26.53 to 46.06)	33.24 (24.98 to 43.76)	37.21 (28.21 to 47.82)	1.43 (1.10 to 1.81)	2.13 (1.64 to 2.74)	2.09 (1.59 to 2.69)	2.15 (1.63 to 2.73)
**Neurological disorders**	**457.45 (391.89 to 534.24)**	**932.10 (708.85 to 1,278.66)**	**903.62 (703.69 to 1,189.81)**	**930.38 (687.69 to 1,307.28)**	**40.52 (34.29 to 48.08)**	**85.01 (62.12 to 116.93)**	**85.20 (64.71 to 111.98)**	**81.06 (56.78 to 115.03)**
Alzheimer's disease and other dementias	344.03 (285.17 to 417.23)	773.45 (552.90 to 1,113.34)	742.10 (551.20 to 1,022.94)	770.66 (525.15 to 1,150.91)	36.01 (29.91 to 43.40)	75.89 (53.58 to 108.05)	75.35 (55.25 to 102.41)	72.74 (48.56 to 106.57)
Parkinson's disease	30.06 (22.81 to 38.24)	79.60 (58.93 to 102.66)	87.47 (65.73 to 112.55)	69.08 (50.85 to 89.39)	2.39 (1.82 to 3.12)	6.48 (4.70 to 8.64)	7.28 (5.40 to 9.67)	5.47 (3.94 to 7.30)
Epilepsy	49.89 (39.21 to 64.75)	38.51 (24.61 to 60.76)	33.23 (21.67 to 52.35)	51.06 (31.65 to 83.99)	1.14 (0.90 to 1.48)	1.12 (0.75 to 1.68)	1.00 (0.68 to 1.49)	1.43 (0.94 to 2.21)
Multiple sclerosis	8.79 (4.98 to 12.83)	9.42 (5.12 to 14.01)	9.66 (5.19 to 14.38)	9.13 (4.98 to 13.55)	0.26 (0.15 to 0.38)	0.35 (0.19 to 0.50)	0.37 (0.20 to 0.52)	0.33 (0.18 to 0.47)
Motor neuron disease	6.04 (4.81 to 7.30)	8.74 (6.68 to 10.91)	8.70 (6.63 to 10.94)	8.53 (6.52 to 10.58)	0.19 (0.15 to 0.23)	0.35 (0.28 to 0.41)	0.35 (0.28 to 0.42)	0.33 (0.26 to 0.39)
Other neurological disorders	18.64 (15.27 to 23.45)	22.38 (17.62 to 28.65)	22.46 (17.63 to 28.78)	21.92 (17.40 to 28.03)	0.51 (0.42 to 0.64)	0.82 (0.68 to 1.01)	0.86 (0.71 to 1.05)	0.77 (0.64 to 0.94)
**Mental disorders**	**122.80 (87.27 to 196.80)**	**150.41 (103.13 to 228.80)**	**169.36 (115.60 to 258.59)**	**139.87 (96.27 to 218.91)**	**3.17 (2.32 to 4.93)**	**4.88 (3.52 to 7.07)**	**5.63 (4.13 to 8.11)**	**4.39 (3.23 to 6.31)**
Alcohol use disorders	56.92 (37.41 to 82.27)	45.06 (25.51 to 69.25)	47.50 (26.14 to 73.42)	46.12 (26.97 to 70.05)	1.48 (0.99 to 2.13)	1.44 (0.86 to 2.13)	1.55 (0.90 to 2.26)	1.44 (0.88 to 2.07)
Drug use disorders	65.56 (38.11 to 130.10)	105.05 (63.67 to 176.13)	121.54 (74.48 to 201.12)	93.46 (56.91 to 162.28)	1.68 (1.02 to 3.10)	3.43 (2.24 to 5.30)	4.08 (2.72 to 6.20)	2.95 (1.91 to 4.57)
Opioid use disorders	41.58 (23.81 to 85.93)	53.33 (32.55 to 90.30)	56.54 (34.71 to 94.66)	50.74 (30.64 to 87.76)	1.01 (0.61 to 1.93)	1.66 (1.08 to 2.65)	1.82 (1.19 to 2.83)	1.53 (0.98 to 2.45)

Cocaine use disorders	2.31 (1.11 to 5.57)	5.79 (2.86 to 12.55)	6.86 (3.45 to 14.38)	5.07 (2.48 to 11.23)	0.06 (0.03 to 0.14)	0.18 (0.09 to 0.37)	0.22 (0.11 to 0.44)	0.16 (0.08 to 0.33)
Amphetamine use disorders	1.83 (0.92 to 4.31)	6.49 (3.16 to 13.62)	8.50 (4.21 to 17.91)	5.22 (2.54 to 11.54)	0.04 (0.02 to 0.10)	0.18 (0.09 to 0.38)	0.24 (0.12 to 0.51)	0.14 (0.07 to 0.32)
Other drug use disorders	19.84 (11.12 to 39.91)	39.44 (22.55 to 68.09)	49.64 (28.85 to 83.40)	32.42 (18.62 to 55.02)	0.56 (0.34 to 1.02)	1.40 (0.88 to 2.19)	1.80 (1.15 to 2.75)	1.12 (0.69 to 1.73)
Eating disorders	0.33 (0.21 to 0.66)	0.30 (0.17 to 0.64)	0.33 (0.18 to 0.70)	0.29 (0.16 to 0.59)	0.01 (0.00 to 0.01)	0.01 (0.00 to 0.01)	0.01 (0.00 to 0.01)	0.01 (0.00 to 0.01)

Seychelles								
Cause	YLLs (actual counts)				Deaths (actual counts)			
	2016	2040 Forecast	2040 Better Scenario	2040 Worse Scenario	2016	2040 Forecast	2040 Better Scenario	2040 Worse Scenario
**Diabetes, urogenital, blood, and endocrine diseases**	**1,688.38 (1,470.68 to 1,925.81)**	**2,507.13 (1,609.44 to 4,813.55)**	**2,023.84 (1,339.71 to 3,980.26)**	**3,123.52 (2,027.86 to 5,796.60)**	**78.59 (70.40 to 87.36)**	**151.09 (98.35 to 279.21)**	**125.59 (84.20 to 243.30)**	**181.00 (118.93 to 332.98)**
Diabetes mellitus	368.13 (316.90 to 421.16)	397.24 (263.07 to 590.90)	303.68 (204.57 to 446.46)	548.11 (357.81 to 841.84)	18.70 (16.48 to 21.09)	24.55 (16.94 to 34.91)	19.44 (13.56 to 26.70)	32.08 (22.01 to 46.60)
Acute glomerulonephritis	1.16 (0.96 to 1.39)	2.03 (1.53 to 2.70)	1.79 (1.35 to 2.37)	2.27 (1.75 to 2.93)	0.04 (0.04 to 0.05)	0.09 (0.07 to 0.13)	0.09 (0.06 to 0.11)	0.10 (0.07 to 0.13)
Chronic kidney disease	899.48 (746.21 to 1,047.76)	1,546.25 (740.18 to 3,862.10)	1,220.54 (604.27 to 3,149.67)	1,958.37 (913.72 to 4,567.70)	41.17 (35.17 to 46.82)	93.21 (43.46 to 226.00)	75.45 (36.63 to 194.20)	114.17 (51.97 to 273.96)
Chronic kidney disease due to diabetes mellitus	560.03 (456.45 to 659.17)	971.07 (457.91 to 2,521.36)	758.63 (372.07 to 2,066.81)	1,242.14 (573.37 to 3,100.09)	23.39 (19.41 to 27.16)	53.38 (24.37 to 135.74)	42.49 (20.36 to 116.22)	66.45 (29.87 to 163.99)
Chronic kidney disease due to hypertension	227.18 (184.50 to 272.05)	453.29 (211.61 to 1,137.19)	368.32 (177.35 to 1,000.05)	557.42 (254.13 to 1,305.75)	13.92 (11.58 to 16.21)	33.31 (15.70 to 84.31)	27.85 (13.66 to 72.27)	39.49 (18.15 to 95.65)
Chronic kidney disease due to glomerulonephritis	55.77 (43.46 to 69.27)	52.43 (28.61 to 113.09)	39.68 (22.19 to 89.41)	69.25 (37.47 to 148.56)	1.66 (1.29 to 2.05)	2.45 (1.18 to 6.01)	1.88 (0.92 to 4.80)	3.16 (1.53 to 7.68)
Chronic kidney disease due to other and unspecified causes	56.50 (43.79 to 70.27)	69.46 (34.37 to 156.33)	53.92 (27.35 to 125.92)	89.56 (43.36 to 193.94)	2.20 (1.71 to 2.73)	4.06 (1.87 to 10.15)	3.23 (1.50 to 8.40)	5.06 (2.27 to 12.46)
Urinary diseases and male infertility	238.82 (208.40 to 274.53)	358.61 (296.25 to 429.05)	316.39 (266.93 to 374.70)	387.81 (335.27 to 450.84)	11.99 (10.66 to 13.43)	23.00 (19.27 to 26.95)	21.10 (18.00 to 24.39)	23.72 (20.12 to 27.34)
Urinary tract infections	220.16 (190.27 to 255.57)	337.09 (279.96 to 403.49)	299.39 (248.83 to 355.05)	362.15 (307.44 to 421.78)	11.09 (9.77 to 12.53)	21.74 (18.26 to 25.47)	20.06 (16.94 to 23.26)	22.29 (18.65 to 25.88)
Urolithiasis	8.99 (5.32 to 15.86)	12.17 (6.36 to 23.13)	9.31 (5.06 to 17.50)	14.73 (8.16 to 28.27)	0.41 (0.24 to 0.75)	0.65 (0.34 to 1.24)	0.51 (0.28 to 1.00)	0.76 (0.41 to 1.49)
Other urinary diseases	9.67 (6.26 to 16.28)	9.36 (5.73 to 16.54)	7.70 (4.71 to 13.48)	10.94 (6.84 to 18.84)	0.50 (0.32 to 0.85)	0.61 (0.37 to 1.07)	0.52 (0.33 to 0.91)	0.67 (0.42 to 1.15)
Gynecological diseases	1.52 (0.74 to 2.02)	0.73 (0.35 to 1.12)	0.51 (0.22 to 0.74)	0.99 (0.44 to 1.41)	0.06 (0.03 to 0.07)	0.03 (0.02 to 0.05)	0.02 (0.01 to 0.03)	0.04 (0.02 to 0.06)
Uterine fibroids	0.30 (0.09 to 0.48)	0.16 (0.05 to 0.27)	0.12 (0.04 to 0.21)	0.20 (0.06 to 0.36)	0.01 (0.00 to 0.02)	0.01 (0.00 to 0.01)	0.01 (0.00 to 0.01)	0.01 (0.00 to 0.01)
Polycystic ovarian syndrome	0.04 (0.01 to 0.09)	0.01 (0.00 to 0.02)	0.00 (0.00 to 0.01)	0.01 (0.00 to 0.04)	0.00 (0.00 to 0.00)	0.00 (0.00 to 0.00)	0.00 (0.00 to 0.00)	0.00 (0.00 to 0.00)
Endometriosis	0.02 (0.01 to 0.05)	0.01 (0.00 to 0.03)	0.01 (0.00 to 0.02)	0.01 (0.00 to 0.04)	0.00 (0.00 to 0.00)	0.00 (0.00 to 0.00)	0.00 (0.00 to 0.00)	0.00 (0.00 to 0.00)
Genital prolapse	0.07 (0.02 to 0.12)	0.04 (0.01 to 0.07)	0.03 (0.01 to 0.05)	0.05 (0.01 to 0.09)	0.00 (0.00 to 0.01)	0.00 (0.00 to 0.00)	0.00 (0.00 to 0.00)	0.00 (0.00 to 0.01)
Other gynecological diseases	1.09 (0.58 to 1.47)	0.51 (0.27 to 0.81)	0.35 (0.18 to 0.53)	0.70 (0.37 to 1.05)	0.04 (0.02 to 0.05)	0.02 (0.01 to 0.04)	0.02 (0.01 to 0.02)	0.03 (0.02 to 0.04)
Hemoglobinopathies and hemolytic anemias	132.53 (104.64 to 161.47)	133.35 (100.98 to 173.90)	108.99 (83.18 to 139.57)	159.24 (123.53 to 199.38)	4.87 (4.00 to 6.17)	6.60 (5.16 to 8.83)	5.56 (4.39 to 7.27)	7.59 (6.12 to 9.82)
Thalassemias	16.96 (12.39 to 23.05)	8.12 (4.92 to 12.46)	7.40 (4.55 to 11.17)	8.64 (5.20 to 13.58)	0.23 (0.17 to 0.33)	0.12 (0.07 to 0.18)	0.11 (0.06 to 0.16)	0.12 (0.07 to 0.19)

Sickle cell disorders	0.20 (0.13 to 0.28)	0.17 (0.10 to 0.27)	0.13 (0.08 to 0.20)	0.21 (0.13 to 0.32)	0.00 (0.00 to 0.01)	0.00 (0.00 to 0.01)	0.00 (0.00 to 0.01)	0.01 (0.00 to 0.01)
G6PD deficiency	67.59 (49.51 to 85.94)	75.64 (51.72 to 104.94)	59.28 (40.54 to 81.12)	94.08 (65.41 to 124.47)	1.85 (1.36 to 2.37)	2.91 (2.03 to 3.97)	2.31 (1.59 to 3.15)	3.53 (2.54 to 4.67)
Other hemoglobinopathies and hemolytic anemias	47.78 (37.79 to 59.81)	49.41 (37.56 to 67.35)	42.17 (32.28 to 56.40)	56.31 (43.54 to 76.36)	2.79 (2.28 to 3.65)	3.57 (2.72 to 5.04)	3.14 (2.43 to 4.34)	3.93 (3.00 to 5.45)
Endocrine, metabolic, blood, and immune disorders	46.74 (37.69 to 73.19)	68.93 (53.62 to 106.23)	71.93 (55.81 to 110.06)	66.74 (53.03 to 103.25)	1.76 (1.45 to 2.63)	3.60 (2.96 to 5.16)	3.93 (3.20 to 5.58)	3.30 (2.70 to 4.82)

Seychelles								
Cause	YLLs (actual counts)				Deaths (actual counts)			
	2016	2040 Forecast	2040 Better Scenario	2040 Worse Scenario	2016	2040 Forecast	2040 Better Scenario	2040 Worse Scenario
**Musculoskeletal disorders**	**8.81 (6.72 to 10.69)**	**13.70 (8.88 to 16.87)**	**13.23 (8.64 to 16.34)**	**13.62 (8.74 to 16.62)**	**0.35 (0.24 to 0.41)**	**0.72 (0.45 to 0.87)**	**0.72 (0.46 to 0.87)**	**0.68 (0.43 to 0.84)**
Rheumatoid arthritis	0.09 (0.05 to 0.12)	0.11 (0.06 to 0.18)	0.08 (0.04 to 0.13)	0.15 (0.07 to 0.24)	0.00 (0.00 to 0.00)	0.01 (0.00 to 0.01)	0.00 (0.00 to 0.01)	0.01 (0.00 to 0.01)
Other musculoskeletal disorders	8.72 (6.63 to 10.58)	13.58 (8.82 to 16.74)	13.15 (8.59 to 16.26)	13.47 (8.68 to 16.46)	0.34 (0.24 to 0.40)	0.71 (0.45 to 0.87)	0.72 (0.45 to 0.86)	0.68 (0.42 to 0.83)
**Other non-communicable diseases**	**459.09 (382.79 to 555.64)**	**237.96 (175.12 to 316.13)**	**171.98 (128.29 to 221.33)**	**331.25 (249.30 to 426.00)**	**6.79 (5.72 to 7.98)**	**5.93 (4.22 to 7.69)**	**4.89 (3.37 to 6.03)**	**7.19 (5.25 to 8.82)**
Congenital birth defects	429.39 (354.84 to 525.36)	186.15 (130.20 to 258.10)	124.77 (91.88 to 164.78)	275.83 (201.77 to 363.67)	5.75 (4.76 to 6.91)	2.97 (2.04 to 4.05)	2.06 (1.45 to 2.71)	4.23 (3.12 to 5.43)
Neural tube defects	11.57 (6.55 to 19.50)	1.88 (0.88 to 3.52)	1.16 (0.56 to 2.18)	3.12 (1.46 to 6.07)	0.14 (0.08 to 0.23)	0.03 (0.01 to 0.05)	0.02 (0.01 to 0.03)	0.04 (0.02 to 0.08)
Congenital heart anomalies	240.36 (182.93 to 313.56)	100.93 (66.10 to 146.77)	62.89 (44.27 to 87.97)	155.86 (111.39 to 212.33)	3.24 (2.52 to 4.11)	1.55 (1.03 to 2.26)	0.99 (0.67 to 1.36)	2.33 (1.68 to 3.09)
Orofacial clefts	0.08 (0.04 to 0.14)	0.02 (0.01 to 0.05)	0.01 (0.00 to 0.03)	0.04 (0.01 to 0.09)	0.00 (0.00 to 0.00)	0.00 (0.00 to 0.00)	0.00 (0.00 to 0.00)	0.00 (0.00 to 0.00)
Down syndrome	9.46 (6.73 to 13.47)	7.92 (5.18 to 11.61)	8.00 (5.26 to 11.54)	8.31 (5.34 to 12.32)	0.16 (0.11 to 0.23)	0.17 (0.10 to 0.25)	0.17 (0.11 to 0.25)	0.17 (0.11 to 0.26)
Other chromosomal abnormalities	12.65 (7.67 to 20.76)	9.12 (4.92 to 15.98)	7.32 (3.97 to 12.37)	12.01 (6.41 to 20.80)	0.16 (0.10 to 0.25)	0.13 (0.07 to 0.21)	0.10 (0.06 to 0.18)	0.16 (0.09 to 0.28)
Congenital musculoskeletal and limb anomalies	8.42 (5.33 to 12.44)	3.74 (2.05 to 6.11)	2.50 (1.40 to 4.02)	5.60 (3.06 to 8.99)	0.10 (0.06 to 0.15)	0.05 (0.03 to 0.08)	0.03 (0.02 to 0.05)	0.07 (0.04 to 0.11)
Urogenital congenital anomalies	10.10 (6.73 to 13.02)	8.61 (4.58 to 12.30)	6.68 (3.50 to 9.32)	10.72 (5.92 to 14.52)	0.23 (0.14 to 0.30)	0.27 (0.13 to 0.39)	0.21 (0.10 to 0.30)	0.32 (0.16 to 0.44)
Digestive congenital anomalies	37.48 (22.28 to 64.00)	15.13 (8.23 to 26.57)	9.73 (5.48 to 15.85)	23.42 (12.94 to 39.78)	0.46 (0.28 to 0.76)	0.21 (0.12 to 0.34)	0.14 (0.08 to 0.21)	0.32 (0.19 to 0.50)
Other congenital birth defects	99.27 (70.48 to 142.95)	38.80 (24.22 to 61.02)	26.47 (17.05 to 39.50)	56.75 (36.19 to 86.14)	1.25 (0.90 to 1.79)	0.57 (0.36 to 0.86)	0.39 (0.25 to 0.57)	0.80 (0.52 to 1.17)
Skin and subcutaneous diseases	20.58 (11.53 to 30.70)	48.34 (26.09 to 68.01)	44.65 (24.59 to 60.68)	50.38 (27.25 to 70.77)	0.94 (0.53 to 1.33)	2.92 (1.56 to 4.02)	2.80 (1.53 to 3.83)	2.90 (1.55 to 4.03)
Cellulitis	2.04 (1.10 to 4.29)	6.47 (3.18 to 11.20)	6.51 (3.15 to 10.98)	6.12 (3.10 to 11.09)	0.09 (0.05 to 0.17)	0.38 (0.19 to 0.64)	0.40 (0.19 to 0.67)	0.35 (0.17 to 0.59)
Pyoderma	14.80 (7.97 to 22.60)	34.27 (17.66 to 48.49)	31.36 (16.26 to 43.82)	36.27 (18.76 to 50.84)	0.58 (0.32 to 0.85)	1.93 (0.98 to 2.69)	1.85 (0.94 to 2.53)	1.94 (0.99 to 2.69)
Decubitus ulcer	3.49 (1.84 to 4.94)	7.15 (3.61 to 10.37)	6.37 (3.09 to 9.26)	7.52 (3.75 to 10.88)	0.25 (0.13 to 0.35)	0.58 (0.29 to 0.86)	0.53 (0.26 to 0.79)	0.59 (0.29 to 0.86)
Other skin and subcutaneous diseases	0.25 (0.15 to 0.37)	0.45 (0.26 to 0.66)	0.42 (0.24 to 0.61)	0.46 (0.27 to 0.69)	0.01 (0.01 to 0.02)	0.02 (0.01 to 0.04)	0.02 (0.01 to 0.03)	0.02 (0.01 to 0.04)
Sudden infant death syndrome	9.12 (5.29 to 15.09)	3.47 (1.72 to 6.60)	2.56 (1.30 to 4.76)	5.04 (2.44 to 9.40)	0.11 (0.06 to 0.18)	0.04 (0.02 to 0.08)	0.03 (0.02 to 0.06)	0.06 (0.03 to 0.11)

**Injuries**	**2,279.93 (1,924.48 to 2,682.66)**	**1,602.16 (1,205.75 to 2,126.76)**	**1,167.96 (889.80 to 1,533.85)**	**2,054.66 (1,554.67 to 2,775.55)**	**56.34 (48.16 to 65.96)**	**52.08 (41.86 to 64.83)**	**40.09 (32.87 to 49.08)**	**63.33 (51.12 to 79.73)**
**Transport injuries**	**643.85 (539.53 to 766.83)**	**477.72 (345.81 to 679.61)**	**213.12 (156.00 to 297.31)**	**696.56 (497.75 to 1,018.75)**	**16.31 (13.93 to 19.12)**	**15.44 (11.85 to 20.43)**	**7.66 (5.95 to 9.97)**	**21.37 (16.24 to 29.24)**
Road injuries	538.15 (443.16 to 642.32)	383.74 (278.74 to 554.62)	142.48 (102.90 to 201.03)	569.31 (406.58 to 844.39)	13.43 (11.31 to 15.84)	12.06 (9.34 to 16.06)	5.02 (3.84 to 6.53)	17.00 (12.81 to 23.15)
Pedestrian road injuries	111.80 (79.48 to 140.03)	74.47 (49.03 to 106.69)	26.10 (16.95 to 36.64)	121.81 (81.67 to 175.74)	3.52 (2.57 to 4.30)	3.21 (2.16 to 4.41)	1.26 (0.85 to 1.69)	4.94 (3.43 to 6.76)

Seychelles								
Cause	YLLs (actual counts)				Deaths (actual counts)			
	2016	2040 Forecast	2040 Better Scenario	2040 Worse Scenario	2016	2040 Forecast	2040 Better Scenario	2040 Worse Scenario
Cyclist road injuries	18.21 (8.50 to 30.24)	16.08 (7.25 to 29.00)	7.51 (3.20 to 13.16)	21.69 (9.59 to 40.26)	0.50 (0.25 to 0.82)	0.55 (0.26 to 0.97)	0.30 (0.13 to 0.51)	0.70 (0.32 to 1.26)
Motorcyclist road injuries	118.35 (77.75 to 168.97)	88.72 (52.17 to 149.36)	46.33 (27.37 to 76.04)	117.98 (67.68 to 202.45)	2.40 (1.60 to 3.44)	1.98 (1.19 to 3.25)	1.13 (0.69 to 1.80)	2.55 (1.51 to 4.26)
Motor vehicle road injuries	280.48 (220.87 to 350.30)	197.02 (139.01 to 284.96)	59.98 (42.09 to 84.70)	296.92 (204.36 to 448.59)	6.76 (5.38 to 8.35)	6.04 (4.47 to 8.21)	2.21 (1.65 to 2.89)	8.46 (6.07 to 11.94)
Other road injuries	9.31 (2.39 to 13.20)	7.44 (1.69 to 12.25)	2.56 (0.60 to 3.98)	10.90 (2.39 to 18.46)	0.25 (0.07 to 0.35)	0.26 (0.07 to 0.40)	0.12 (0.03 to 0.18)	0.35 (0.09 to 0.56)
Other transport injuries	105.70 (76.98 to 135.60)	93.98 (60.88 to 141.87)	70.64 (48.02 to 104.69)	127.26 (82.80 to 189.66)	2.88 (2.19 to 3.63)	3.38 (2.32 to 4.88)	2.64 (1.88 to 3.74)	4.37 (3.02 to 6.37)
**Unintentional injuries**	**1,002.81 (859.93 to 1,174.47)**	**649.22 (502.84 to 830.08)**	**555.74 (438.88 to 702.91)**	**763.00 (603.38 to 976.10)**	**25.53 (22.13 to 29.73)**	**23.52 (19.36 to 28.10)**	**21.07 (17.74 to 24.99)**	**26.04 (21.88 to 31.25)**
Falls	166.65 (138.22 to 200.00)	167.93 (135.66 to 204.17)	154.44 (125.56 to 190.42)	177.49 (145.91 to 212.87)	5.92 (5.09 to 6.96)	8.18 (6.94 to 9.50)	7.78 (6.65 to 8.99)	8.28 (7.09 to 9.53)
Drowning	490.59 (414.62 to 587.17)	273.87 (200.20 to 371.22)	229.80 (171.57 to 311.89)	333.33 (244.54 to 460.89)	10.73 (9.09 to 12.82)	7.90 (6.05 to 10.23)	6.85 (5.32 to 8.79)	9.23 (7.08 to 12.30)
Fire, heat, and hot substances	134.46 (111.91 to 168.04)	79.48 (57.38 to 109.89)	63.18 (47.12 to 85.93)	100.77 (73.76 to 137.71)	3.83 (3.21 to 4.67)	3.22 (2.43 to 4.21)	2.69 (2.08 to 3.44)	3.85 (2.98 to 5.03)
Poisonings	21.98 (16.62 to 27.92)	8.93 (5.94 to 12.96)	6.62 (4.72 to 9.35)	12.22 (8.41 to 17.72)	0.51 (0.40 to 0.66)	0.27 (0.20 to 0.38)	0.21 (0.16 to 0.28)	0.36 (0.26 to 0.50)
Exposure to mechanical forces	60.58 (41.54 to 80.49)	33.31 (20.74 to 48.65)	26.97 (17.03 to 38.54)	40.71 (25.99 to 57.26)	1.40 (0.95 to 1.86)	0.97 (0.62 to 1.35)	0.81 (0.53 to 1.12)	1.14 (0.74 to 1.56)
Unintentional firearm injuries	23.61 (11.94 to 35.33)	11.74 (5.28 to 18.75)	9.70 (4.46 to 15.26)	14.11 (6.32 to 22.80)	0.57 (0.29 to 0.85)	0.37 (0.18 to 0.55)	0.31 (0.16 to 0.47)	0.42 (0.20 to 0.65)
Unintentional suffocation	5.12 (3.55 to 7.55)	2.49 (1.67 to 3.69)	2.03 (1.35 to 3.05)	3.14 (2.07 to 4.89)	0.08 (0.06 to 0.11)	0.04 (0.03 to 0.06)	0.04 (0.02 to 0.05)	0.05 (0.04 to 0.08)
Other exposure to mechanical forces	31.85 (20.33 to 43.74)	19.09 (11.24 to 28.41)	15.24 (8.78 to 21.96)	23.45 (13.95 to 33.58)	0.75 (0.48 to 1.02)	0.56 (0.34 to 0.80)	0.46 (0.28 to 0.64)	0.66 (0.40 to 0.91)
Adverse effects of medical treatment	26.08 (20.47 to 31.99)	22.53 (17.12 to 28.56)	20.67 (15.81 to 26.36)	24.52 (18.88 to 31.04)	0.79 (0.60 to 0.96)	0.94 (0.72 to 1.14)	0.90 (0.70 to 1.10)	0.97 (0.76 to 1.18)
Animal contact	8.74 (7.25 to 10.76)	7.20 (5.47 to 9.58)	6.17 (4.74 to 8.14)	8.48 (6.45 to 11.28)	0.22 (0.19 to 0.28)	0.24 (0.19 to 0.31)	0.21 (0.17 to 0.28)	0.27 (0.21 to 0.34)
Venomous animal contact	4.83 (3.68 to 6.43)	3.82 (2.71 to 5.27)	3.42 (2.43 to 4.72)	4.36 (3.07 to 6.08)	0.12 (0.10 to 0.17)	0.13 (0.09 to 0.18)	0.12 (0.09 to 0.17)	0.14 (0.10 to 0.20)
Non-venomous animal contact	3.91 (2.76 to 5.19)	3.38 (2.24 to 4.96)	2.76 (1.84 to 4.01)	4.12 (2.75 to 6.04)	0.10 (0.07 to 0.13)	0.11 (0.07 to 0.16)	0.09 (0.06 to 0.13)	0.13 (0.09 to 0.18)
Foreign body	60.10 (42.48 to 77.38)	40.03 (27.96 to 52.09)	36.89 (26.12 to 47.32)	43.56 (29.93 to 57.46)	1.30 (0.94 to 1.58)	1.27 (0.92 to 1.55)	1.24 (0.91 to 1.51)	1.27 (0.91 to 1.55)
Pulmonary aspiration and foreign body in airway	55.12 (34.41 to 73.06)	36.33 (23.07 to 48.40)	33.05 (20.94 to 43.54)	39.85 (25.05 to 53.95)	1.18 (0.77 to 1.47)	1.14 (0.74 to 1.44)	1.10 (0.71 to 1.39)	1.15 (0.75 to 1.44)

Foreign body in other body part	4.98 (3.46 to 9.56)	3.70 (2.46 to 7.28)	3.84 (2.56 to 7.49)	3.70 (2.44 to 7.42)	0.12 (0.09 to 0.25)	0.13 (0.09 to 0.25)	0.14 (0.10 to 0.27)	0.12 (0.08 to 0.24)
Environmental heat and cold exposure	7.70 (3.50 to 10.35)	4.78 (2.17 to 6.98)	3.80 (1.75 to 5.52)	6.00 (2.75 to 8.68)	0.22 (0.11 to 0.28)	0.17 (0.09 to 0.24)	0.14 (0.08 to 0.19)	0.21 (0.11 to 0.28)
Other unintentional injuries	25.92 (20.65 to 32.23)	11.16 (7.49 to 16.43)	7.18 (5.08 to 9.77)	15.93 (11.57 to 21.14)	0.60 (0.49 to 0.74)	0.34 (0.23 to 0.48)	0.23 (0.17 to 0.29)	0.47 (0.35 to 0.57)
**Self-harm and interpersonal violence**	**631.02 (511.71 to 778.73)**	**473.63 (313.50 to 711.05)**	**397.55 (268.21 to 581.15)**	**593.50 (379.76 to 912.45)**	**14.45 (11.76 to 17.71)**	**13.09 (9.02 to 18.70)**	**11.32 (7.97 to 15.72)**	**15.88 (10.60 to 23.34)**

Seychelles								
Cause	YLLs (actual counts)				Deaths (actual counts)			
	2016	2040 Forecast	2040 Better Scenario	2040 Worse Scenario	2016	2040 Forecast	2040 Better Scenario	2040 Worse Scenario
Self-harm	345.02 (273.94 to 467.81)	315.82 (195.74 to 519.89)	268.59 (171.85 to 425.33)	403.19 (238.02 to 684.39)	8.23 (6.71 to 10.95)	9.10 (5.98 to 14.02)	7.98 (5.46 to 11.73)	11.17 (7.01 to 17.62)
Self-harm by firearm	11.72 (5.89 to 21.75)	7.50 (2.95 to 15.43)	5.93 (2.47 to 11.50)	10.21 (3.89 to 20.82)	0.29 (0.15 to 0.52)	0.24 (0.10 to 0.47)	0.20 (0.09 to 0.38)	0.31 (0.13 to 0.61)
Self-harm by other specified means	333.30 (265.37 to 452.30)	308.32 (191.18 to 507.70)	262.66 (168.09 to 414.14)	392.98 (232.53 to 664.30)	7.93 (6.48 to 10.53)	8.86 (5.86 to 13.68)	7.78 (5.38 to 11.50)	10.86 (6.84 to 17.14)
Interpersonal violence	285.99 (193.17 to 358.56)	157.81 (96.29 to 227.33)	128.96 (77.96 to 183.31)	190.31 (114.13 to 275.28)	6.22 (4.09 to 7.80)	3.99 (2.34 to 5.54)	3.33 (1.93 to 4.67)	4.71 (2.70 to 6.66)
Physical violence by firearm	61.45 (33.08 to 106.85)	31.39 (16.63 to 56.21)	24.40 (12.86 to 42.94)	38.41 (20.37 to 68.18)	1.30 (0.71 to 2.26)	0.73 (0.38 to 1.27)	0.58 (0.30 to 1.00)	0.88 (0.47 to 1.54)
Physical violence by sharp object	117.55 (72.62 to 159.83)	63.49 (36.30 to 95.11)	49.99 (28.98 to 74.89)	76.28 (42.74 to 114.28)	2.47 (1.50 to 3.37)	1.47 (0.82 to 2.16)	1.18 (0.67 to 1.71)	1.74 (0.95 to 2.58)
Physical violence by other means	106.99 (62.02 to 145.00)	62.93 (32.72 to 101.02)	54.57 (28.95 to 85.55)	75.63 (38.37 to 124.26)	2.45 (1.37 to 3.32)	1.78 (0.93 to 2.69)	1.57 (0.83 to 2.34)	2.09 (1.03 to 3.30)
**Forces of nature, conflict and terrorism, and executions and police conflict**	**2.25 (1.39 to 3.58)**	**1.58 (0.00 to 30.78)**	**1.56 (0.00 to 30.32)**	**1.60 (0.00 to 31.11)**	**0.05 (0.03 to 0.07)**	**0.04 (0.00 to 0.76)**	**0.04 (0.00 to 0.77)**	**0.04 (0.00 to 0.75)**
Exposure to forces of nature	0.00 (0.00 to 0.00)	1.56 (0.00 to 30.73)	1.54 (0.00 to 30.27)	1.58 (0.00 to 31.06)	0.00 (0.00 to 0.00)	0.04 (0.00 to 0.76)	0.04 (0.00 to 0.77)	0.04 (0.00 to 0.75)
Conflict and terrorism	0.00 (0.00 to 0.00)	0.02 (0.00 to 0.18)	0.02 (0.00 to 0.18)	0.02 (0.00 to 0.18)	0.00 (0.00 to 0.00)	0.00 (0.00 to 0.00)	0.00 (0.00 to 0.00)	0.00 (0.00 to 0.00)
Executions and police conflict	2.25 (1.39 to 3.58)	0.00 (0.00 to 0.00)	0.00 (0.00 to 0.00)	0.00 (0.00 to 0.00)	0.05 (0.03 to 0.07)	0.00 (0.00 to 0.00)	0.00 (0.00 to 0.00)	0.00 (0.00 to 0.00)

Thailand								
Cause	YLLs (in 1000s)				Deaths (in 1000s)			
	2016	2040 Forecast	2040 Better Scenario	2040 Worse Scenario	2016	2040 Forecast	2040 Better Scenario	2040 Worse Scenario
**All causes**	**10,680.28 (9,671.60 to 11,603.28)**	**11,904.62 (9,969.55 to 14,104.93)**	**9,609.29 (8,127.56 to 11,364.69)**	**15,102.23 (12,582.24 to 18,054.27)**	**452.45 (413.60 to 491.80)**	**767.56 (637.20 to 910.50)**	**639.09 (528.96 to 758.73)**	**918.27 (766.55 to 1,074.63)**
**Communicable, maternal, neonatal, and nutritional diseases**	**1,659.28 (1,254.20 to 2,035.20)**	**1,286.03 (759.88 to 1,895.70)**	**1,019.99 (606.49 to 1,525.79)**	**1,737.57 (1,047.28 to 2,567.61)**	**63.00 (44.24 to 75.54)**	**82.91 (45.31 to 128.75)**	**67.54 (36.50 to 105.57)**	**105.13 (57.11 to 164.38)**
**HIV/AIDS and tuberculosis**	**589.61 (325.23 to 939.81)**	**282.02 (133.14 to 554.89)**	**212.05 (80.35 to 464.88)**	**437.25 (232.94 to 740.78)**	**16.91 (10.75 to 25.36)**	**13.00 (7.28 to 22.35)**	**8.68 (4.48 to 16.02)**	**18.64 (10.69 to 31.65)**
Tuberculosis	117.96 (101.43 to 137.97)	122.69 (70.68 to 210.73)	68.32 (39.93 to 119.62)	175.24 (97.42 to 323.26)	6.48 (5.60 to 7.62)	9.49 (5.44 to 16.49)	5.48 (3.15 to 9.60)	13.08 (7.16 to 23.55)
HIV/AIDS	471.65 (202.44 to 819.24)	159.33 (30.84 to 412.77)	143.72 (23.74 to 390.82)	262.00 (91.66 to 531.79)	10.44 (4.23 to 18.87)	3.51 (0.53 to 9.79)	3.19 (0.42 to 9.26)	5.56 (1.70 to 11.99)
**Diarrhea, lower respiratory, and other common infectious diseases**	**862.09 (578.45 to 1,026.78)**	**862.88 (416.84 to 1,396.44)**	**681.39 (334.47 to 1,119.93)**	**1,142.20 (537.92 to 1,933.57)**	**41.79 (24.45 to 51.23)**	**63.81 (28.23 to 106.44)**	**52.87 (23.00 to 90.43)**	**80.22 (35.71 to 133.82)**
Diarrheal diseases	76.18 (45.16 to 125.50)	97.15 (16.39 to 328.76)	97.62 (19.96 to 313.77)	121.66 (19.18 to 414.74)	3.90 (2.17 to 6.84)	8.31 (1.35 to 28.97)	8.62 (1.68 to 28.83)	10.04 (1.54 to 34.67)
Intestinal infectious diseases	70.11 (35.24 to 119.55)	32.86 (13.25 to 71.08)	34.90 (14.39 to 75.20)	35.42 (14.04 to 78.78)	1.20 (0.60 to 2.04)	0.71 (0.32 to 1.42)	0.78 (0.35 to 1.56)	0.75 (0.33 to 1.51)
Typhoid fever	60.58 (30.49 to 104.75)	27.52 (10.90 to 59.92)	30.67 (12.57 to 66.18)	29.63 (11.42 to 65.25)	1.03 (0.51 to 1.80)	0.59 (0.26 to 1.18)	0.68 (0.30 to 1.36)	0.62 (0.27 to 1.25)
Paratyphoid fever	9.37 (4.07 to 19.01)	5.26 (1.97 to 12.17)	4.16 (1.51 to 9.34)	5.70 (2.07 to 13.33)	0.16 (0.07 to 0.33)	0.12 (0.05 to 0.25)	0.09 (0.04 to 0.20)	0.12 (0.05 to 0.27)
Other intestinal infectious diseases	0.17 (0.07 to 0.35)	0.08 (0.03 to 0.17)	0.07 (0.03 to 0.15)	0.09 (0.03 to 0.19)	0.01 (0.00 to 0.01)	0.01 (0.00 to 0.01)	0.00 (0.00 to 0.01)	0.01 (0.00 to 0.01)
Lower respiratory infections	637.95 (365.30 to 777.27)	673.88 (287.70 to 1,057.06)	499.07 (212.10 to 778.43)	921.05 (392.35 to 1,496.78)	34.76 (17.86 to 42.97)	52.67 (21.12 to 83.62)	41.60 (16.36 to 66.01)	67.28 (27.17 to 108.75)
Upper respiratory infections	0.26 (0.14 to 0.45)	0.24 (0.10 to 0.49)	0.16 (0.06 to 0.33)	0.27 (0.11 to 0.56)	0.01 (0.00 to 0.01)	0.01 (0.00 to 0.02)	0.01 (0.00 to 0.02)	0.01 (0.01 to 0.03)
Otitis media	0.08 (0.03 to 0.15)	0.06 (0.02 to 0.14)	0.07 (0.02 to 0.14)	0.06 (0.02 to 0.13)	0.00 (0.00 to 0.01)	0.01 (0.00 to 0.01)	0.01 (0.00 to 0.01)	0.00 (0.00 to 0.01)
Meningitis	41.72 (27.41 to 53.41)	23.34 (14.02 to 34.67)	18.29 (10.58 to 29.57)	23.59 (14.34 to 35.30)	1.07 (0.70 to 1.38)	0.90 (0.57 to 1.30)	0.73 (0.45 to 1.08)	0.88 (0.57 to 1.27)
Pneumococcal meningitis	0.41 (0.28 to 0.59)	0.18 (0.11 to 0.27)	0.17 (0.10 to 0.27)	0.17 (0.11 to 0.27)	0.01 (0.01 to 0.02)	0.01 (0.01 to 0.01)	0.01 (0.01 to 0.01)	0.01 (0.01 to 0.01)
H influenzae type B meningitis	3.09 (1.99 to 4.09)	1.46 (0.86 to 2.25)	1.43 (0.83 to 2.17)	1.47 (0.87 to 2.33)	0.08 (0.05 to 0.10)	0.06 (0.04 to 0.08)	0.06 (0.04 to 0.08)	0.05 (0.03 to 0.08)
Meningococcal meningitis	5.17 (3.34 to 7.00)	2.86 (1.68 to 4.53)	2.25 (1.28 to 3.73)	2.89 (1.70 to 4.55)	0.14 (0.09 to 0.19)	0.12 (0.08 to 0.18)	0.10 (0.06 to 0.15)	0.12 (0.08 to 0.17)
Other meningitis	33.05 (21.55 to 42.52)	18.84 (11.25 to 28.17)	14.43 (8.35 to 23.20)	19.06 (11.48 to 28.52)	0.84 (0.55 to 1.08)	0.71 (0.45 to 1.04)	0.56 (0.35 to 0.85)	0.70 (0.45 to 1.00)
Encephalitis	26.61 (21.79 to 33.63)	23.76 (17.41 to 33.24)	22.69 (16.54 to 31.42)	24.81 (18.21 to 35.28)	0.68 (0.57 to 0.83)	0.96 (0.72 to 1.26)	0.94 (0.71 to 1.25)	0.97 (0.73 to 1.27)

Diphtheria	0.05 (0.03 to 0.07)	0.01 (0.01 to 0.02)	0.01 (0.00 to 0.02)	0.01 (0.01 to 0.02)	0.00 (0.00 to 0.00)	0.00 (0.00 to 0.00)	0.00 (0.00 to 0.00)	0.00 (0.00 to 0.00)
Whooping cough	6.49 (0.62 to 20.28)	1.22 (0.04 to 5.55)	0.27 (0.02 to 1.24)	2.14 (0.06 to 9.00)	0.08 (0.01 to 0.24)	0.02 (0.00 to 0.07)	0.00 (0.00 to 0.02)	0.03 (0.00 to 0.11)
Tetanus	1.27 (0.88 to 1.80)	0.20 (0.12 to 0.31)	0.20 (0.11 to 0.31)	0.20 (0.12 to 0.30)	0.05 (0.03 to 0.07)	0.01 (0.00 to 0.02)	0.01 (0.00 to 0.02)	0.01 (0.00 to 0.01)
Measles	0.52 (0.15 to 1.30)	9.55 (0.24 to 34.82)	7.61 (0.21 to 27.70)	12.32 (0.26 to 47.27)	0.01 (0.00 to 0.02)	0.16 (0.00 to 0.69)	0.13 (0.00 to 0.54)	0.20 (0.00 to 0.90)

Thailand								
Cause	YLLs (in 1000s)				Deaths (in 1000s)			
	2016	2040 Forecast	2040 Better Scenario	2040 Worse Scenario	2016	2040 Forecast	2040 Better Scenario	2040 Worse Scenario
Varicella and herpes zoster	0.84 (0.59 to 1.18)	0.61 (0.35 to 0.98)	0.50 (0.29 to 0.83)	0.67 (0.38 to 1.08)	0.04 (0.02 to 0.05)	0.06 (0.03 to 0.10)	0.05 (0.02 to 0.09)	0.06 (0.03 to 0.10)
**Neglected tropical diseases and malaria**	**16.11 (9.35 to 23.62)**	**32.34 (20.32 to 52.53)**	**31.24 (20.02 to 50.42)**	**33.28 (20.62 to 54.59)**	**0.34 (0.23 to 0.49)**	**0.95 (0.65 to 1.59)**	**0.94 (0.64 to 1.58)**	**0.95 (0.65 to 1.55)**
Malaria	1.76 (1.06 to 4.55)	0.96 (0.48 to 3.17)	0.94 (0.46 to 3.09)	0.96 (0.48 to 3.22)	0.04 (0.03 to 0.12)	0.03 (0.02 to 0.11)	0.03 (0.02 to 0.11)	0.03 (0.02 to 0.11)
Chagas disease	0.00 (0.00 to 0.00)	0.00 (0.00 to 0.00)	0.00 (0.00 to 0.00)	0.00 (0.00 to 0.00)	0.00 (0.00 to 0.00)	0.00 (0.00 to 0.00)	0.00 (0.00 to 0.00)	0.00 (0.00 to 0.00)
Leishmaniasis	0.01 (0.01 to 0.02)	0.01 (0.00 to 0.01)	0.01 (0.00 to 0.01)	0.01 (0.00 to 0.01)	0.00 (0.00 to 0.00)	0.00 (0.00 to 0.00)	0.00 (0.00 to 0.00)	0.00 (0.00 to 0.00)
African trypanosomiasis	0.00 (0.00 to 0.00)	0.00 (0.00 to 0.00)	0.00 (0.00 to 0.00)	0.00 (0.00 to 0.00)	0.00 (0.00 to 0.00)	0.00 (0.00 to 0.00)	0.00 (0.00 to 0.00)	0.00 (0.00 to 0.00)
Schistosomiasis	0.00 (0.00 to 0.00)	0.00 (0.00 to 0.00)	0.00 (0.00 to 0.00)	0.00 (0.00 to 0.00)	0.00 (0.00 to 0.00)	0.00 (0.00 to 0.00)	0.00 (0.00 to 0.00)	0.00 (0.00 to 0.00)
Cysticercosis	0.09 (0.07 to 0.11)	0.03 (0.02 to 0.04)	0.03 (0.02 to 0.04)	0.03 (0.02 to 0.04)	0.00 (0.00 to 0.00)	0.00 (0.00 to 0.00)	0.00 (0.00 to 0.00)	0.00 (0.00 to 0.00)
Cystic echinococcosis	0.01 (0.01 to 0.02)	0.01 (0.01 to 0.01)	0.01 (0.00 to 0.01)	0.01 (0.01 to 0.01)	0.00 (0.00 to 0.00)	0.00 (0.00 to 0.00)	0.00 (0.00 to 0.00)	0.00 (0.00 to 0.00)
Dengue	12.24 (4.65 to 19.71)	29.32 (17.13 to 49.53)	28.23 (16.83 to 47.45)	30.14 (17.53 to 51.43)	0.23 (0.11 to 0.36)	0.82 (0.52 to 1.44)	0.81 (0.52 to 1.44)	0.81 (0.52 to 1.43)
Yellow fever	0.00 (0.00 to 0.00)	0.00 (0.00 to 0.00)	0.00 (0.00 to 0.00)	0.00 (0.00 to 0.00)	0.00 (0.00 to 0.00)	0.00 (0.00 to 0.00)	0.00 (0.00 to 0.00)	0.00 (0.00 to 0.00)
Rabies	0.09 (0.02 to 0.14)	0.04 (0.01 to 0.08)	0.04 (0.01 to 0.06)	0.06 (0.01 to 0.10)	0.00 (0.00 to 0.00)	0.00 (0.00 to 0.00)	0.00 (0.00 to 0.00)	0.00 (0.00 to 0.00)
Intestinal nematode infections	0.36 (0.20 to 0.55)	0.33 (0.18 to 0.55)	0.33 (0.18 to 0.55)	0.32 (0.18 to 0.54)	0.01 (0.01 to 0.01)	0.01 (0.01 to 0.02)	0.01 (0.01 to 0.02)	0.01 (0.01 to 0.02)
Other neglected tropical diseases	1.55 (0.93 to 1.95)	1.64 (0.91 to 2.40)	1.66 (0.92 to 2.41)	1.75 (0.99 to 2.53)	0.05 (0.03 to 0.06)	0.08 (0.05 to 0.12)	0.08 (0.05 to 0.12)	0.09 (0.05 to 0.12)
**Maternal disorders**	**8.51 (5.79 to 11.94)**	**4.65 (2.16 to 9.40)**	**3.73 (1.74 to 7.65)**	**5.77 (2.74 to 11.94)**	**0.16 (0.11 to 0.23)**	**0.09 (0.04 to 0.17)**	**0.07 (0.03 to 0.14)**	**0.11 (0.05 to 0.22)**
Maternal hemorrhage	1.72 (1.08 to 2.56)	0.91 (0.40 to 1.90)	0.74 (0.32 to 1.58)	1.13 (0.50 to 2.38)	0.03 (0.02 to 0.05)	0.02 (0.01 to 0.03)	0.01 (0.01 to 0.03)	0.02 (0.01 to 0.04)
Maternal sepsis and other maternal infections	1.08 (0.68 to 1.59)	0.49 (0.22 to 0.94)	0.39 (0.17 to 0.77)	0.60 (0.26 to 1.19)	0.02 (0.01 to 0.03)	0.01 (0.00 to 0.02)	0.01 (0.00 to 0.01)	0.01 (0.01 to 0.02)
Maternal hypertensive disorders	0.83 (0.52 to 1.23)	0.49 (0.21 to 1.01)	0.39 (0.17 to 0.81)	0.61 (0.26 to 1.31)	0.02 (0.01 to 0.02)	0.01 (0.00 to 0.02)	0.01 (0.00 to 0.02)	0.01 (0.00 to 0.02)
Maternal obstructed labor and uterine rupture	0.11 (0.06 to 0.20)	0.06 (0.02 to 0.13)	0.04 (0.01 to 0.10)	0.07 (0.02 to 0.17)	0.00 (0.00 to 0.00)	0.00 (0.00 to 0.00)	0.00 (0.00 to 0.00)	0.00 (0.00 to 0.00)
Maternal abortion, miscarriage, and ectopic pregnancy	3.16 (2.08 to 4.60)	1.78 (0.85 to 3.91)	1.42 (0.66 to 3.14)	2.23 (1.05 to 4.83)	0.06 (0.04 to 0.09)	0.03 (0.02 to 0.07)	0.03 (0.01 to 0.06)	0.04 (0.02 to 0.09)

Indirect maternal deaths	0.95 (0.58 to 1.47)	0.47 (0.18 to 1.00)	0.37 (0.14 to 0.82)	0.58 (0.22 to 1.27)	0.02 (0.01 to 0.03)	0.01 (0.00 to 0.02)	0.01 (0.00 to 0.01)	0.01 (0.00 to 0.02)
Late maternal deaths	0.03 (0.01 to 0.06)	0.02 (0.00 to 0.04)	0.01 (0.00 to 0.03)	0.02 (0.01 to 0.05)	0.00 (0.00 to 0.00)	0.00 (0.00 to 0.00)	0.00 (0.00 to 0.00)	0.00 (0.00 to 0.00)
Maternal deaths aggravated by HIV/AIDS	0.05 (0.02 to 0.08)	0.15 (0.03 to 0.39)	0.13 (0.02 to 0.33)	0.19 (0.05 to 0.48)	0.00 (0.00 to 0.00)	0.00 (0.00 to 0.01)	0.00 (0.00 to 0.01)	0.00 (0.00 to 0.01)
Other maternal disorders	0.59 (0.33 to 0.91)	0.28 (0.12 to 0.60)	0.24 (0.10 to 0.50)	0.35 (0.14 to 0.74)	0.01 (0.01 to 0.02)	0.01 (0.00 to 0.01)	0.00 (0.00 to 0.01)	0.01 (0.00 to 0.01)

Thailand								
**Cause**	**YLLs (in 1000s)**				**Deaths (in 1000s)**			
	**2016**	**2040 Forecast**	**2040 Better Scenario**	**2040 Worse Scenario**	**2016**	**2040 Forecast**	**2040 Better Scenario**	**2040 Worse Scenario**
**Neonatal disorders**	**121.76 (84.10 to 166.52)**	**35.14 (16.37 to 70.76)**	**25.75 (11.86 to 51.54)**	**46.82 (21.59 to 94.62)**	**1.41 (0.97 to 1.92)**	**0.41 (0.19 to 0.82)**	**0.30 (0.14 to 0.60)**	**0.54 (0.25 to 1.09)**
Neonatal preterm birth	51.33 (34.44 to 71.73)	13.21 (5.97 to 26.75)	9.44 (4.37 to 19.18)	20.15 (9.28 to 40.76)	0.59 (0.40 to 0.83)	0.15 (0.07 to 0.31)	0.11 (0.05 to 0.22)	0.23 (0.11 to 0.47)
Neonatal encephalopathy due to birth asphyxia and trauma	21.94 (13.90 to 32.45)	6.62 (2.47 to 16.05)	4.59 (1.67 to 11.06)	7.95 (2.95 to 18.95)	0.25 (0.16 to 0.37)	0.08 (0.03 to 0.19)	0.05 (0.02 to 0.13)	0.09 (0.03 to 0.22)
Neonatal sepsis and other neonatal infections	31.62 (20.12 to 48.02)	10.78 (4.28 to 21.54)	7.96 (3.22 to 16.41)	13.06 (5.15 to 26.31)	0.37 (0.23 to 0.55)	0.12 (0.05 to 0.25)	0.09 (0.04 to 0.19)	0.15 (0.06 to 0.30)
Hemolytic disease and other neonatal jaundice	0.14 (0.08 to 0.21)	0.03 (0.01 to 0.06)	0.02 (0.01 to 0.04)	0.03 (0.01 to 0.07)	0.00 (0.00 to 0.00)	0.00 (0.00 to 0.00)	0.00 (0.00 to 0.00)	0.00 (0.00 to 0.00)
Other neonatal disorders	16.74 (10.36 to 24.51)	4.50 (1.76 to 9.97)	3.74 (1.47 to 8.23)	5.63 (2.17 to 12.54)	0.19 (0.12 to 0.28)	0.05 (0.02 to 0.12)	0.04 (0.02 to 0.10)	0.07 (0.03 to 0.14)
**Nutritional deficiencies**	**9.03 (7.92 to 10.31)**	**15.46 (12.19 to 19.27)**	**16.20 (12.67 to 20.58)**	**15.58 (12.29 to 19.45)**	**0.69 (0.60 to 0.80)**	**1.79 (1.35 to 2.33)**	**1.93 (1.44 to 2.55)**	**1.74 (1.30 to 2.29)**
Protein-energy malnutrition	6.85 (5.96 to 7.95)	11.49 (8.55 to 14.85)	12.54 (9.29 to 16.46)	11.54 (8.47 to 14.99)	0.55 (0.47 to 0.65)	1.38 (0.97 to 1.86)	1.55 (1.10 to 2.10)	1.34 (0.96 to 1.82)
Iodine deficiency	0.20 (0.02 to 0.54)	0.67 (0.01 to 2.46)	0.65 (0.01 to 2.43)	0.70 (0.02 to 2.48)	0.01 (0.00 to 0.03)	0.07 (0.00 to 0.33)	0.07 (0.00 to 0.34)	0.07 (0.00 to 0.33)
Dietary iron deficiency	0.29 (0.24 to 0.36)	0.25 (0.15 to 0.47)	0.25 (0.16 to 0.48)	0.25 (0.16 to 0.46)	0.01 (0.01 to 0.01)	0.01 (0.01 to 0.04)	0.02 (0.01 to 0.04)	0.01 (0.01 to 0.04)
Other nutritional deficiencies	1.70 (1.36 to 2.04)	3.06 (2.16 to 4.02)	2.76 (1.94 to 3.75)	3.09 (2.17 to 4.11)	0.12 (0.09 to 0.14)	0.33 (0.21 to 0.45)	0.30 (0.20 to 0.43)	0.32 (0.21 to 0.44)
**Other communicable, maternal, neonatal, and nutritional diseases**	**52.15 (44.53 to 62.57)**	**53.54 (42.00 to 68.62)**	**49.64 (38.71 to 63.80)**	**56.67 (44.13 to 73.21)**	**1.70 (1.46 to 2.13)**	**2.87 (2.22 to 3.99)**	**2.76 (2.11 to 3.91)**	**2.92 (2.27 to 4.05)**
Sexually transmitted infections excluding HIV	2.14 (1.46 to 2.80)	0.91 (0.65 to 1.29)	0.83 (0.58 to 1.18)	0.96 (0.69 to 1.37)	0.05 (0.04 to 0.06)	0.04 (0.03 to 0.07)	0.04 (0.03 to 0.07)	0.04 (0.03 to 0.07)
Syphilis	0.79 (0.51 to 1.25)	0.26 (0.18 to 0.39)	0.22 (0.14 to 0.32)	0.28 (0.19 to 0.43)	0.01 (0.01 to 0.02)	0.01 (0.01 to 0.02)	0.01 (0.01 to 0.02)	0.01 (0.01 to 0.02)
Chlamydial infection	0.26 (0.16 to 0.35)	0.13 (0.09 to 0.19)	0.12 (0.08 to 0.18)	0.13 (0.09 to 0.19)	0.01 (0.00 to 0.01)	0.01 (0.00 to 0.01)	0.01 (0.00 to 0.01)	0.01 (0.00 to 0.01)
Gonococcal infection	0.70 (0.44 to 0.92)	0.36 (0.25 to 0.51)	0.33 (0.24 to 0.48)	0.37 (0.26 to 0.54)	0.02 (0.01 to 0.02)	0.02 (0.01 to 0.03)	0.02 (0.01 to 0.03)	0.02 (0.01 to 0.03)
Other sexually transmitted infections	0.38 (0.23 to 0.51)	0.17 (0.11 to 0.24)	0.16 (0.10 to 0.23)	0.18 (0.12 to 0.26)	0.01 (0.01 to 0.01)	0.01 (0.00 to 0.01)	0.01 (0.00 to 0.01)	0.01 (0.00 to 0.01)
Acute hepatitis	11.91 (9.77 to 14.45)	9.14 (6.84 to 12.02)	7.17 (5.16 to 9.85)	9.60 (7.13 to 12.78)	0.41 (0.34 to 0.49)	0.43 (0.34 to 0.56)	0.35 (0.26 to 0.46)	0.45 (0.35 to 0.57)
Acute hepatitis A	0.18 (0.13 to 0.25)	0.10 (0.06 to 0.15)	0.08 (0.05 to 0.13)	0.10 (0.06 to 0.16)	0.01 (0.00 to 0.01)	0.01 (0.00 to 0.01)	0.00 (0.00 to 0.01)	0.01 (0.00 to 0.01)
Acute hepatitis B	10.73 (8.70 to 13.17)	7.96 (5.76 to 10.63)	6.20 (4.36 to 8.63)	8.40 (6.07 to 11.29)	0.36 (0.30 to 0.44)	0.35 (0.27 to 0.46)	0.28 (0.20 to 0.37)	0.37 (0.27 to 0.48)

Acute hepatitis C	0.31 (0.21 to 0.47)	0.29 (0.19 to 0.44)	0.23 (0.15 to 0.36)	0.29 (0.19 to 0.44)	0.01 (0.01 to 0.02)	0.01 (0.01 to 0.02)	0.01 (0.01 to 0.02)	0.01 (0.01 to 0.02)
Acute hepatitis E	0.70 (0.42 to 1.05)	0.80 (0.46 to 1.26)	0.65 (0.38 to 1.04)	0.80 (0.47 to 1.27)	0.03 (0.02 to 0.05)	0.06 (0.04 to 0.10)	0.05 (0.03 to 0.09)	0.06 (0.03 to 0.10)
Other unspecified infectious diseases	38.11 (31.73 to 48.02)	43.49 (33.60 to 57.10)	41.65 (32.17 to 54.38)	46.11 (35.50 to 61.15)	1.24 (1.05 to 1.60)	2.39 (1.81 to 3.50)	2.37 (1.78 to 3.50)	2.44 (1.85 to 3.52)
**Non-communicable diseases**	**7,030.48 (6,304.67 to 7,787.54)**	**9,377.20 (7,768.54 to 11,331.19)**	**7,532.03 (6,302.70 to 9,065.26)**	**11,653.93 (9,583.76 to 14,136.10)**	**341.61 (308.99 to 376.34)**	**643.55 (525.74 to 774.60)**	**535.46 (434.32 to 651.76)**	**760.81 (619.64 to 913.33)**

Thailand								
Cause	YLLs (in 1000s)				Deaths (in 1000s)			
	2016	2040 Forecast	2040 Better Scenario	2040 Worse Scenario	2016	2040 Forecast	2040 Better Scenario	2040 Worse Scenario
**Neoplasms**	**2,435.39 (2,177.84 to 2,714.69)**	**3,236.31 (2,652.34 to 3,996.39)**	**2,702.64 (2,262.95 to 3,290.44)**	**4,004.55 (3,212.57 to 5,106.98)**	**103.73 (93.56 to 114.61)**	**182.45 (151.07 to 221.08)**	**156.53 (132.20 to 186.52)**	**220.07 (178.65 to 272.14)**
Lip and oral cavity cancer	62.50 (54.77 to 71.59)	81.07 (50.43 to 128.55)	54.44 (35.72 to 83.70)	140.21 (83.78 to 230.38)	2.95 (2.61 to 3.32)	5.12 (3.24 to 8.11)	3.58 (2.32 to 5.45)	8.71 (5.20 to 14.61)
Nasopharynx cancer	36.72 (30.84 to 43.81)	38.59 (23.08 to 63.46)	22.94 (14.34 to 36.05)	66.46 (38.06 to 109.78)	1.31 (1.11 to 1.55)	1.73 (1.03 to 2.72)	1.06 (0.65 to 1.61)	2.93 (1.68 to 4.72)
Other pharynx cancer	16.79 (14.01 to 19.60)	18.72 (11.93 to 28.99)	16.11 (10.63 to 24.31)	22.71 (14.09 to 36.33)	0.61 (0.52 to 0.71)	0.86 (0.55 to 1.31)	0.75 (0.50 to 1.12)	1.02 (0.63 to 1.62)
Esophageal cancer	66.58 (57.21 to 78.31)	61.73 (38.52 to 96.42)	46.53 (30.80 to 69.71)	99.69 (57.42 to 162.96)	2.77 (2.41 to 3.22)	3.35 (2.14 to 5.06)	2.59 (1.77 to 3.78)	5.30 (3.16 to 8.41)
Stomach cancer	95.59 (84.08 to 110.43)	95.46 (76.74 to 121.48)	78.84 (62.80 to 100.07)	104.88 (81.83 to 135.51)	4.19 (3.72 to 4.80)	5.68 (4.58 to 7.24)	4.78 (3.81 to 6.08)	6.09 (4.76 to 7.85)
Colon and rectum cancer	176.16 (152.71 to 202.03)	314.21 (224.09 to 446.82)	266.58 (191.41 to 380.72)	333.66 (235.99 to 483.55)	8.37 (7.29 to 9.55)	20.57 (14.46 to 29.62)	17.89 (12.68 to 25.39)	21.21 (14.74 to 30.30)
Liver cancer	557.20 (477.50 to 642.39)	817.35 (567.92 to 1,158.24)	685.52 (518.77 to 909.41)	974.68 (658.48 to 1,436.65)	22.93 (19.89 to 26.49)	41.72 (30.47 to 57.36)	36.34 (28.04 to 46.76)	48.37 (33.51 to 68.13)
Liver cancer due to hepatitis B	231.72 (180.94 to 291.59)	315.35 (209.93 to 474.46)	275.45 (193.68 to 390.54)	353.40 (225.47 to 557.05)	8.87 (6.99 to 11.07)	15.16 (10.34 to 22.18)	13.70 (9.73 to 18.78)	16.58 (10.80 to 25.23)
Liver cancer due to hepatitis C	63.63 (47.95 to 82.77)	105.04 (68.32 to 155.12)	94.33 (64.84 to 131.57)	115.37 (72.48 to 176.78)	3.10 (2.34 to 4.06)	6.41 (4.21 to 9.39)	5.94 (4.06 to 8.41)	6.87 (4.34 to 10.27)
Liver cancer due to alcohol use	173.34 (113.11 to 239.63)	269.52 (155.34 to 414.92)	206.47 (119.44 to 306.24)	361.99 (205.20 to 561.39)	7.43 (4.73 to 10.07)	13.76 (7.56 to 20.92)	11.04 (6.12 to 16.30)	17.88 (10.09 to 27.77)
Liver cancer due to other causes	88.51 (62.99 to 119.69)	127.44 (75.34 to 199.88)	109.27 (69.62 to 162.10)	143.93 (81.58 to 234.00)	3.53 (2.52 to 4.66)	6.39 (3.86 to 9.73)	5.66 (3.65 to 8.44)	7.04 (4.05 to 11.19)
Gallbladder and biliary tract cancer	100.71 (46.04 to 130.87)	110.98 (50.34 to 143.59)	110.31 (50.11 to 144.01)	104.51 (45.01 to 135.53)	4.86 (2.16 to 6.24)	6.91 (3.06 to 8.91)	6.98 (3.15 to 9.10)	6.35 (2.64 to 8.12)
Pancreatic cancer	70.03 (61.53 to 79.98)	113.14 (88.40 to 143.14)	108.13 (85.68 to 136.09)	122.26 (94.37 to 156.36)	3.23 (2.85 to 3.66)	6.70 (5.26 to 8.51)	6.50 (5.19 to 8.21)	7.09 (5.56 to 9.01)
Larynx cancer	30.06 (25.41 to 35.60)	32.16 (16.83 to 57.95)	19.41 (10.92 to 33.22)	55.32 (27.08 to 101.85)	1.30 (1.11 to 1.52)	1.81 (0.95 to 3.16)	1.13 (0.63 to 1.94)	3.04 (1.49 to 5.50)
Tracheal, bronchus, and lung cancer	446.20 (389.03 to 550.09)	546.04 (376.93 to 851.87)	378.63 (269.45 to 619.45)	959.08 (639.13 to 1,517.99)	21.12 (18.55 to 25.86)	32.40 (22.59 to 50.95)	23.25 (16.64 to 37.10)	55.18 (37.90 to 87.23)
Malignant skin melanoma	6.22 (4.88 to 11.04)	8.44 (6.25 to 16.44)	9.59 (6.96 to 18.83)	7.74 (5.78 to 14.92)	0.23 (0.18 to 0.45)	0.45 (0.34 to 0.94)	0.52 (0.38 to 1.10)	0.40 (0.30 to 0.82)
Non-melanoma skin cancer	19.44 (16.86 to 22.47)	34.03 (29.41 to 39.52)	29.88 (25.32 to 35.55)	33.24 (28.93 to 37.97)	0.90 (0.80 to 1.04)	2.21 (1.91 to 2.58)	1.99 (1.66 to 2.35)	2.08 (1.82 to 2.38)
Breast cancer	153.04 (122.31 to 185.40)	165.42 (114.71 to 240.87)	142.17 (101.20 to 198.59)	180.82 (121.32 to 277.83)	5.38 (4.30 to 6.42)	7.89 (5.59 to 11.10)	6.88 (4.95 to 9.31)	8.42 (5.76 to 12.51)
Cervical cancer	92.80 (73.94 to 114.24)	90.84 (66.32 to 126.63)	75.89 (55.47 to 105.50)	100.67 (69.79 to 144.80)	3.47 (2.81 to 4.21)	4.51 (3.32 to 6.03)	3.82 (2.80 to 5.17)	4.89 (3.44 to 6.81)

Uterine cancer	12.25 (10.04 to 15.08)	16.80 (13.37 to 21.18)	15.13 (12.03 to 19.45)	17.14 (13.75 to 21.38)	0.53 (0.44 to 0.64)	0.94 (0.76 to 1.17)	0.86 (0.70 to 1.08)	0.94 (0.77 to 1.15)
Ovarian cancer	46.32 (37.46 to 57.83)	60.26 (45.01 to 80.80)	55.63 (41.75 to 74.09)	57.84 (43.39 to 78.45)	1.71 (1.42 to 2.09)	2.94 (2.23 to 3.88)	2.75 (2.11 to 3.58)	2.78 (2.10 to 3.71)
Prostate cancer	38.22 (25.05 to 48.05)	97.11 (66.15 to 123.42)	92.85 (63.69 to 119.39)	90.22 (60.42 to 114.06)	2.60 (1.70 to 3.24)	8.18 (5.28 to 10.78)	7.99 (5.22 to 10.63)	7.38 (4.71 to 9.63)
Testicular cancer	1.51 (1.18 to 1.89)	1.25 (0.88 to 1.80)	1.11 (0.78 to 1.62)	1.24 (0.89 to 1.79)	0.04 (0.03 to 0.05)	0.05 (0.04 to 0.06)	0.04 (0.03 to 0.06)	0.05 (0.03 to 0.06)

Thailand								
Cause	YLLs (in 1000s)				Deaths (in 1000s)			
	2016	2040 Forecast	2040 Better Scenario	2040 Worse Scenario	2016	2040 Forecast	2040 Better Scenario	2040 Worse Scenario
Kidney cancer	18.31 (15.84 to 20.89)	27.85 (21.47 to 36.30)	27.53 (21.73 to 35.13)	28.59 (21.33 to 38.45)	0.75 (0.65 to 0.86)	1.53 (1.20 to 1.98)	1.55 (1.23 to 1.99)	1.53 (1.15 to 2.01)
Bladder cancer	26.77 (23.06 to 30.94)	44.59 (32.64 to 61.28)	39.05 (29.33 to 52.40)	51.17 (36.12 to 72.40)	1.54 (1.33 to 1.78)	3.36 (2.42 to 4.68)	3.01 (2.22 to 4.12)	3.77 (2.64 to 5.39)
Brain and nervous system cancer	75.05 (47.18 to 92.37)	99.52 (58.03 to 128.20)	96.04 (56.20 to 124.76)	94.76 (55.07 to 122.43)	2.49 (1.49 to 3.11)	4.65 (2.56 to 6.03)	4.57 (2.53 to 5.99)	4.31 (2.40 to 5.61)
Thyroid cancer	9.99 (8.13 to 18.81)	18.86 (13.87 to 32.35)	16.08 (12.15 to 27.33)	18.78 (13.88 to 30.73)	0.52 (0.43 to 0.81)	1.24 (0.94 to 1.82)	1.09 (0.83 to 1.60)	1.21 (0.91 to 1.73)
Mesothelioma	7.94 (6.44 to 9.69)	8.94 (7.18 to 11.43)	9.13 (7.30 to 11.77)	8.65 (6.97 to 10.91)	0.26 (0.21 to 0.31)	0.38 (0.31 to 0.50)	0.39 (0.32 to 0.54)	0.36 (0.30 to 0.45)
Hodgkin lymphoma	5.69 (4.23 to 9.84)	5.01 (3.61 to 8.60)	4.12 (2.89 to 7.17)	5.23 (3.78 to 8.83)	0.18 (0.14 to 0.31)	0.20 (0.15 to 0.33)	0.17 (0.12 to 0.28)	0.21 (0.15 to 0.34)
Non-Hodgkin lymphoma	39.94 (29.33 to 86.65)	65.09 (45.59 to 139.37)	59.09 (41.59 to 126.39)	61.43 (43.28 to 127.54)	1.48 (1.09 to 3.15)	3.27 (2.34 to 6.88)	3.04 (2.17 to 6.43)	3.01 (2.17 to 6.18)
Multiple myeloma	12.70 (9.77 to 20.99)	22.00 (15.72 to 37.98)	20.59 (15.04 to 35.98)	20.62 (14.87 to 34.86)	0.51 (0.39 to 0.86)	1.12 (0.81 to 1.96)	1.07 (0.78 to 1.89)	1.03 (0.75 to 1.75)
Leukemia	128.34 (73.05 to 153.61)	127.86 (64.23 to 175.77)	108.55 (55.03 to 146.20)	134.90 (66.18 to 193.12)	4.47 (2.30 to 5.40)	6.52 (3.04 to 9.00)	5.65 (2.62 to 7.69)	6.72 (3.08 to 9.54)
Acute lymphoid leukemia	28.15 (15.59 to 34.16)	41.13 (20.61 to 55.24)	35.31 (17.94 to 46.76)	42.22 (20.71 to 58.66)	1.03 (0.53 to 1.25)	1.90 (0.94 to 2.57)	1.66 (0.83 to 2.19)	1.91 (0.94 to 2.64)
Chronic lymphoid leukemia	1.32 (0.83 to 1.71)	1.36 (0.83 to 2.00)	1.20 (0.75 to 1.72)	1.42 (0.85 to 2.13)	0.04 (0.02 to 0.05)	0.05 (0.03 to 0.07)	0.05 (0.03 to 0.06)	0.05 (0.03 to 0.07)
Acute myeloid leukemia	7.57 (4.95 to 9.42)	10.95 (6.39 to 15.23)	10.13 (5.94 to 13.87)	11.16 (6.43 to 15.88)	0.22 (0.13 to 0.28)	0.42 (0.23 to 0.57)	0.39 (0.22 to 0.52)	0.42 (0.23 to 0.59)
Chronic myeloid leukemia	3.32 (1.84 to 4.36)	2.50 (1.40 to 3.94)	1.85 (1.02 to 2.84)	2.70 (1.44 to 4.47)	0.09 (0.05 to 0.12)	0.08 (0.05 to 0.12)	0.06 (0.04 to 0.09)	0.09 (0.05 to 0.14)
Other leukemia	87.99 (47.58 to 105.92)	71.91 (32.49 to 102.55)	60.06 (27.45 to 83.79)	77.41 (35.18 to 114.05)	3.10 (1.50 to 3.76)	4.06 (1.72 to 5.70)	3.49 (1.48 to 4.86)	4.24 (1.76 to 6.18)
Other neoplasms	82.29 (70.73 to 92.94)	112.99 (95.86 to 131.42)	112.77 (95.30 to 131.78)	108.03 (90.71 to 125.54)	3.04 (2.61 to 3.43)	6.15 (5.20 to 7.19)	6.27 (5.34 to 7.37)	5.70 (4.68 to 6.60)
**Cardiovascular diseases**	**1,886.49 (1,679.26 to 2,162.32)**	**1,870.33 (1,336.95 to 2,556.43)**	**1,254.09 (916.85 to 1,709.81)**	**2,769.97 (1,912.28 to 3,915.04)**	**98.89 (88.82 to 111.98)**	**142.95 (100.50 to 198.96)**	**102.49 (73.24 to 142.42)**	**192.55 (132.79 to 272.57)**
Rheumatic heart disease	11.35 (9.88 to 12.87)	7.25 (5.93 to 8.86)	6.04 (4.85 to 7.48)	7.75 (6.29 to 9.68)	0.43 (0.38 to 0.50)	0.44 (0.36 to 0.55)	0.38 (0.30 to 0.47)	0.46 (0.37 to 0.58)
Ischemic heart disease	850.13 (751.83 to 988.04)	792.31 (450.96 to 1,288.84)	500.20 (293.37 to 824.80)	1,254.19 (700.42 to 2,082.26)	47.59 (42.21 to 55.33)	63.06 (36.50 to 102.43)	42.74 (24.17 to 70.08)	90.53 (51.71 to 151.40)
Stroke	755.66 (667.16 to 855.26)	629.16 (412.11 to 937.98)	365.94 (250.87 to 537.59)	1,034.73 (649.92 to 1,606.28)	36.82 (33.06 to 41.33)	45.46 (30.27 to 66.44)	29.09 (19.89 to 42.09)	66.61 (44.07 to 100.08)
Ischemic stroke	185.73 (165.08 to 210.10)	187.09 (117.31 to 312.51)	121.49 (76.98 to 197.40)	270.31 (160.75 to 464.02)	12.24 (10.82 to 13.76)	16.49 (10.16 to 28.73)	11.45 (7.02 to 19.14)	21.85 (13.00 to 39.85)

Intracerebral hemorrhage	569.93 (497.95 to 647.24)	442.07 (277.55 to 686.61)	244.45 (162.84 to 367.80)	764.43 (453.96 to 1,258.43)	24.58 (21.91 to 27.60)	28.98 (19.13 to 43.06)	17.65 (11.95 to 25.61)	44.76 (27.92 to 69.79)
Hypertensive heart disease	30.76 (26.01 to 39.10)	52.98 (35.64 to 81.69)	40.76 (28.69 to 58.24)	66.18 (41.61 to 113.44)	1.78 (1.51 to 2.20)	4.43 (2.88 to 7.11)	3.49 (2.37 to 5.13)	5.26 (3.21 to 9.42)
Cardiomyopathy and myocarditis	14.53 (11.68 to 20.79)	20.02 (14.95 to 29.38)	17.43 (13.02 to 25.30)	19.51 (14.66 to 28.42)	0.61 (0.50 to 0.84)	1.27 (0.92 to 1.81)	1.13 (0.82 to 1.62)	1.19 (0.87 to 1.67)
Myocarditis	6.21 (4.83 to 9.47)	7.81 (5.96 to 11.18)	7.68 (5.84 to 10.88)	7.65 (5.83 to 10.88)	0.23 (0.19 to 0.32)	0.45 (0.35 to 0.60)	0.45 (0.35 to 0.61)	0.43 (0.33 to 0.56)

Thailand								
Cause	YLLs (in 1000s)				Deaths (in 1000s)			
	2016	2040 Forecast	2040 Better Scenario	2040 Worse Scenario	2016	2040 Forecast	2040 Better Scenario	2040 Worse Scenario
Alcoholic cardiomyopathy	1.01 (0.76 to 1.44)	0.86 (0.59 to 1.23)	0.76 (0.50 to 1.10)	0.97 (0.69 to 1.37)	0.04 (0.03 to 0.05)	0.04 (0.03 to 0.06)	0.04 (0.03 to 0.05)	0.04 (0.03 to 0.06)
Other cardiomyopathy	7.31 (5.83 to 10.62)	11.34 (7.63 to 17.28)	9.00 (5.93 to 14.06)	10.89 (7.27 to 16.50)	0.34 (0.27 to 0.49)	0.78 (0.50 to 1.21)	0.64 (0.40 to 1.03)	0.72 (0.47 to 1.12)
Atrial fibrillation and flutter	23.49 (18.16 to 30.32)	61.38 (44.03 to 85.30)	56.97 (40.55 to 78.54)	64.07 (44.61 to 89.46)	2.32 (1.76 to 3.03)	6.97 (4.91 to 9.92)	6.62 (4.63 to 9.51)	7.03 (4.85 to 9.97)
Aortic aneurysm	25.26 (21.92 to 29.11)	41.91 (34.07 to 52.78)	39.30 (32.04 to 49.18)	42.08 (33.84 to 53.41)	1.62 (1.41 to 1.88)	3.69 (2.93 to 4.73)	3.57 (2.82 to 4.55)	3.54 (2.80 to 4.53)
Peripheral artery disease	0.99 (0.75 to 1.19)	3.24 (2.08 to 4.43)	3.12 (1.98 to 4.28)	3.11 (1.97 to 4.32)	0.08 (0.06 to 0.10)	0.35 (0.22 to 0.49)	0.35 (0.22 to 0.50)	0.32 (0.19 to 0.46)
Endocarditis	78.42 (64.49 to 104.74)	109.78 (81.42 to 150.92)	103.65 (78.07 to 138.72)	108.90 (80.32 to 150.93)	3.08 (2.55 to 3.98)	6.68 (4.86 to 9.32)	6.45 (4.70 to 9.01)	6.41 (4.66 to 9.09)
Other cardiovascular and circulatory diseases	95.90 (79.45 to 129.98)	152.30 (113.15 to 212.09)	120.69 (89.29 to 163.84)	169.44 (122.52 to 238.32)	4.57 (3.81 to 6.05)	10.59 (7.83 to 14.49)	8.67 (6.30 to 11.90)	11.19 (8.20 to 15.53)
**Chronic respiratory diseases**	**412.03 (361.17 to 479.97)**	**502.05 (362.73 to 727.76)**	**332.36 (246.89 to 465.85)**	**831.87 (573.36 to 1,240.17)**	**23.89 (21.02 to 28.12)**	**37.35 (26.83 to 54.24)**	**25.69 (18.80 to 36.90)**	**60.07 (41.89 to 89.39)**
Chronic obstructive pulmonary disease	339.74 (296.11 to 399.22)	435.05 (299.25 to 651.67)	280.39 (198.42 to 406.84)	759.22 (505.06 to 1,168.24)	20.76 (18.18 to 24.37)	33.30 (23.35 to 49.34)	22.45 (15.86 to 33.13)	55.81 (38.18 to 84.39)
Pneumoconiosis	0.49 (0.38 to 0.61)	0.59 (0.46 to 0.73)	0.48 (0.38 to 0.61)	0.58 (0.46 to 0.72)	0.03 (0.02 to 0.03)	0.04 (0.03 to 0.06)	0.04 (0.03 to 0.05)	0.04 (0.03 to 0.06)
Silicosis	0.12 (0.08 to 0.18)	0.07 (0.05 to 0.10)	0.05 (0.03 to 0.07)	0.07 (0.05 to 0.10)	0.00 (0.00 to 0.01)	0.00 (0.00 to 0.01)	0.00 (0.00 to 0.00)	0.00 (0.00 to 0.01)
Asbestosis	0.11 (0.08 to 0.16)	0.14 (0.10 to 0.19)	0.15 (0.11 to 0.21)	0.13 (0.10 to 0.19)	0.00 (0.00 to 0.01)	0.01 (0.01 to 0.01)	0.01 (0.01 to 0.01)	0.01 (0.01 to 0.01)
Coal workers pneumoconiosis	0.01 (0.01 to 0.02)	0.02 (0.01 to 0.04)	0.01 (0.01 to 0.03)	0.02 (0.01 to 0.04)	0.00 (0.00 to 0.00)	0.00 (0.00 to 0.00)	0.00 (0.00 to 0.00)	0.00 (0.00 to 0.00)
Other pneumoconiosis	0.25 (0.19 to 0.31)	0.35 (0.27 to 0.46)	0.27 (0.19 to 0.35)	0.35 (0.27 to 0.45)	0.02 (0.01 to 0.02)	0.03 (0.02 to 0.04)	0.02 (0.02 to 0.03)	0.03 (0.02 to 0.04)
Asthma	55.06 (45.94 to 65.01)	44.93 (30.20 to 66.30)	27.80 (19.32 to 39.91)	50.70 (33.27 to 75.93)	2.49 (2.07 to 2.97)	2.79 (1.86 to 4.09)	1.80 (1.26 to 2.56)	3.05 (2.00 to 4.48)
Interstitial lung disease and pulmonary sarcoidosis	7.64 (6.00 to 11.25)	12.32 (8.87 to 20.86)	14.96 (10.66 to 25.33)	11.26 (8.15 to 18.91)	0.33 (0.24 to 0.55)	0.75 (0.51 to 1.36)	0.92 (0.62 to 1.66)	0.66 (0.46 to 1.22)
Other chronic respiratory diseases	9.10 (6.71 to 13.18)	9.17 (5.98 to 15.24)	8.72 (5.73 to 14.33)	10.12 (6.49 to 16.98)	0.29 (0.21 to 0.45)	0.48 (0.29 to 0.84)	0.47 (0.29 to 0.82)	0.51 (0.31 to 0.89)
**Cirrhosis and other chronic liver diseases**	**419.70 (350.37 to 498.16)**	**419.95 (344.64 to 509.32)**	**359.04 (290.47 to 440.57)**	**450.69 (368.56 to 546.11)**	**13.43 (11.50 to 15.88)**	**17.74 (15.07 to 21.00)**	**15.44 (12.97 to 18.61)**	**18.60 (15.79 to 22.00)**
Cirrhosis and other chronic liver diseases due to hepatitis B	136.59 (104.40 to 175.50)	137.08 (104.23 to 180.12)	122.46 (92.90 to 161.84)	137.55 (103.46 to 180.83)	4.33 (3.36 to 5.52)	5.67 (4.49 to 7.37)	5.13 (4.01 to 6.68)	5.59 (4.40 to 7.23)
Cirrhosis and other chronic liver diseases due to hepatitis C	78.42 (59.77 to 101.05)	78.75 (60.00 to 100.99)	69.68 (52.90 to 90.92)	79.36 (60.48 to 101.72)	2.45 (1.90 to 3.09)	3.14 (2.47 to 3.97)	2.81 (2.20 to 3.61)	3.12 (2.46 to 3.91)

Cirrhosis and other chronic liver diseases due to alcohol use	117.53 (91.57 to 146.15)	110.92 (86.78 to 139.00)	83.88 (65.36 to 104.85)	139.57 (110.47 to 173.39)	3.78 (3.02 to 4.61)	4.66 (3.74 to 5.71)	3.65 (2.89 to 4.49)	5.68 (4.59 to 6.87)
Cirrhosis and other chronic liver diseases due to other causes	87.16 (71.14 to 105.85)	93.20 (74.02 to 115.34)	83.02 (65.86 to 103.08)	94.22 (75.24 to 115.70)	2.88 (2.40 to 3.47)	4.26 (3.45 to 5.10)	3.85 (3.10 to 4.70)	4.21 (3.44 to 5.04)
**Digestive diseases**	**232.61 (191.37 to 270.16)**	**283.43 (217.53 to 360.58)**	**242.14 (188.51 to 302.93)**	**298.54 (225.81 to 387.17)**	**10.38 (8.53 to 11.90)**	**18.72 (14.61 to 23.27)**	**16.62 (13.11 to 20.48)**	**18.90 (14.63 to 23.53)**
Peptic ulcer disease	28.03 (22.71 to 33.55)	22.92 (18.02 to 28.59)	17.57 (13.55 to 22.65)	25.85 (20.08 to 32.63)	1.31 (1.10 to 1.54)	1.64 (1.28 to 2.04)	1.28 (0.97 to 1.64)	1.78 (1.38 to 2.22)

Thailand								
Cause	YLLs (in 1000s)				Deaths (in 1000s)			
	2016	2040 Forecast	2040 Better Scenario	2040 Worse Scenario	2016	2040 Forecast	2040 Better Scenario	2040 Worse Scenario
Gastritis and duodenitis	3.86 (3.08 to 4.68)	5.10 (3.85 to 6.53)	4.40 (3.25 to 5.74)	5.10 (3.87 to 6.48)	0.17 (0.14 to 0.20)	0.32 (0.25 to 0.41)	0.29 (0.21 to 0.37)	0.31 (0.24 to 0.39)
Appendicitis	10.92 (8.14 to 14.08)	9.41 (7.01 to 12.68)	7.54 (5.58 to 10.44)	9.79 (7.32 to 13.30)	0.36 (0.28 to 0.46)	0.50 (0.37 to 0.69)	0.41 (0.30 to 0.58)	0.50 (0.38 to 0.69)
Paralytic ileus and intestinal obstruction	26.33 (21.06 to 36.01)	36.49 (27.98 to 50.90)	36.69 (27.93 to 50.93)	35.50 (27.63 to 49.54)	1.26 (0.98 to 1.67)	2.59 (1.94 to 3.42)	2.67 (1.98 to 3.53)	2.43 (1.87 to 3.25)
Inguinal, femoral, and abdominal hernia	2.86 (2.04 to 3.68)	3.37 (2.30 to 4.41)	2.98 (1.98 to 3.92)	3.33 (2.28 to 4.39)	0.15 (0.10 to 0.18)	0.26 (0.16 to 0.35)	0.24 (0.14 to 0.32)	0.25 (0.15 to 0.33)
Inflammatory bowel disease	4.02 (3.01 to 4.91)	4.51 (3.51 to 5.65)	4.47 (3.45 to 5.64)	4.35 (3.41 to 5.44)	0.16 (0.13 to 0.19)	0.26 (0.21 to 0.33)	0.27 (0.21 to 0.34)	0.24 (0.20 to 0.30)
Vascular intestinal disorders	13.84 (10.28 to 16.40)	20.72 (16.54 to 24.79)	19.60 (15.41 to 23.71)	20.16 (15.96 to 24.14)	0.73 (0.57 to 0.85)	1.58 (1.27 to 1.92)	1.53 (1.21 to 1.89)	1.49 (1.21 to 1.82)
Gallbladder and biliary diseases	28.93 (19.12 to 35.05)	45.97 (23.49 to 72.08)	31.43 (16.75 to 47.19)	49.14 (25.07 to 77.24)	1.52 (0.96 to 1.82)	3.25 (1.60 to 5.38)	2.33 (1.20 to 3.67)	3.36 (1.65 to 5.50)
Pancreatitis	32.95 (23.33 to 41.26)	33.89 (14.54 to 73.08)	23.43 (10.40 to 49.43)	42.71 (17.46 to 92.97)	1.01 (0.70 to 1.24)	1.45 (0.66 to 3.11)	1.04 (0.49 to 2.13)	1.79 (0.79 to 3.97)
Other digestive diseases	80.87 (63.11 to 96.00)	101.06 (78.63 to 122.12)	94.03 (72.26 to 114.14)	102.61 (78.32 to 122.88)	3.73 (2.92 to 4.36)	6.88 (5.42 to 8.21)	6.58 (5.16 to 7.93)	6.75 (5.29 to 8.10)
**Neurological disorders**	**381.74 (321.08 to 455.58)**	**944.36 (704.87 to 1,279.36)**	**897.82 (690.25 to 1,168.08)**	**903.87 (675.95 to 1,242.15)**	**33.78 (28.27 to 40.80)**	**98.98 (71.90 to 137.19)**	**96.51 (71.80 to 128.68)**	**91.71 (66.24 to 128.01)**
Alzheimer's disease and other dementias	309.13 (253.34 to 378.90)	824.49 (593.84 to 1,145.54)	774.02 (579.89 to 1,032.34)	791.01 (567.05 to 1,123.47)	30.29 (24.88 to 37.13)	90.30 (64.12 to 127.85)	87.17 (63.09 to 119.40)	83.96 (59.26 to 119.31)
Parkinson's disease	31.41 (25.21 to 38.48)	84.51 (66.80 to 104.29)	91.56 (72.12 to 113.69)	74.86 (58.25 to 92.12)	2.40 (1.92 to 2.98)	7.38 (5.79 to 9.23)	8.12 (6.28 to 10.22)	6.39 (5.03 to 7.96)
Epilepsy	28.26 (24.16 to 32.62)	20.06 (13.33 to 30.31)	16.64 (11.39 to 24.74)	23.42 (15.36 to 36.58)	0.69 (0.60 to 0.79)	0.67 (0.47 to 0.96)	0.57 (0.41 to 0.80)	0.76 (0.53 to 1.12)
Multiple sclerosis	2.16 (1.81 to 2.82)	2.14 (1.69 to 2.79)	2.18 (1.73 to 2.85)	2.12 (1.68 to 2.78)	0.07 (0.06 to 0.08)	0.08 (0.07 to 0.10)	0.09 (0.07 to 0.11)	0.08 (0.07 to 0.10)
Motor neuron disease	3.63 (3.12 to 4.21)	5.42 (4.58 to 6.39)	5.47 (4.62 to 6.42)	5.11 (4.33 to 5.96)	0.12 (0.11 to 0.14)	0.23 (0.20 to 0.27)	0.23 (0.20 to 0.27)	0.21 (0.18 to 0.25)
Other neurological disorders	7.13 (6.21 to 8.19)	7.75 (6.53 to 9.34)	7.95 (6.66 to 9.64)	7.35 (6.20 to 8.83)	0.21 (0.18 to 0.24)	0.32 (0.28 to 0.37)	0.33 (0.29 to 0.39)	0.29 (0.26 to 0.34)
**Mental disorders**	**107.47 (78.99 to 130.00)**	**87.76 (62.31 to 121.36)**	**89.72 (63.68 to 124.80)**	**90.91 (64.67 to 125.40)**	**2.70 (2.09 to 3.28)**	**2.74 (2.13 to 3.56)**	**2.86 (2.22 to 3.73)**	**2.78 (2.16 to 3.57)**
Alcohol use disorders	79.45 (55.51 to 98.86)	55.23 (37.65 to 76.40)	54.73 (37.24 to 75.81)	58.82 (40.34 to 80.47)	2.08 (1.54 to 2.59)	1.77 (1.30 to 2.34)	1.79 (1.30 to 2.35)	1.86 (1.36 to 2.44)
Drug use disorders	27.64 (19.02 to 35.42)	32.19 (20.33 to 49.47)	34.66 (22.48 to 52.55)	31.75 (20.05 to 48.76)	0.61 (0.46 to 0.77)	0.96 (0.72 to 1.30)	1.06 (0.79 to 1.43)	0.92 (0.68 to 1.25)
Opioid use disorders	24.17 (15.41 to 31.74)	25.32 (14.97 to 40.03)	26.22 (15.53 to 41.21)	25.05 (14.81 to 39.77)	0.51 (0.37 to 0.65)	0.69 (0.49 to 0.99)	0.73 (0.52 to 1.05)	0.67 (0.47 to 0.95)

Cocaine use disorders	0.35 (0.24 to 0.61)	0.61 (0.41 to 1.03)	0.71 (0.47 to 1.18)	0.62 (0.41 to 1.04)	0.01 (0.01 to 0.02)	0.02 (0.01 to 0.03)	0.02 (0.02 to 0.04)	0.02 (0.01 to 0.03)
Amphetamine use disorders	0.51 (0.34 to 0.77)	1.26 (0.73 to 2.15)	1.56 (0.89 to 2.72)	1.28 (0.74 to 2.17)	0.01 (0.01 to 0.02)	0.03 (0.02 to 0.05)	0.04 (0.02 to 0.06)	0.03 (0.02 to 0.05)
Other drug use disorders	2.62 (1.85 to 4.61)	5.00 (3.33 to 8.28)	6.17 (4.13 to 10.11)	4.81 (3.21 to 7.97)	0.08 (0.06 to 0.12)	0.21 (0.15 to 0.29)	0.27 (0.19 to 0.36)	0.20 (0.14 to 0.27)
Eating disorders	0.38 (0.30 to 0.50)	0.34 (0.22 to 0.51)	0.33 (0.22 to 0.50)	0.34 (0.22 to 0.51)	0.01 (0.01 to 0.01)	0.01 (0.00 to 0.01)	0.01 (0.00 to 0.01)	0.01 (0.00 to 0.01)

Thailand								
Cause	YLLs (in 1000s)				Deaths (in 1000s)			
	2016	2040 Forecast	2040 Better Scenario	2040 Worse Scenario	2016	2040 Forecast	2040 Better Scenario	2040 Worse Scenario
**Diabetes, urogenital, blood, and endocrine diseases**	**959.60 (858.54 to 1,080.86)**	**1,752.91 (1,317.58 to 2,510.08)**	**1,402.78 (1,036.98 to 1,909.53)**	**2,026.50 (1,484.07 to 2,983.84)**	**49.12 (44.29 to 54.98)**	**126.43 (93.97 to 176.67)**	**104.05 (76.88 to 143.49)**	**140.88 (103.28 to 205.87)**
Diabetes mellitus	363.34 (319.79 to 419.40)	676.79 (456.20 to 1,007.84)	508.53 (344.26 to 760.06)	809.16 (515.07 to 1,226.70)	18.92 (16.63 to 21.92)	46.53 (31.51 to 66.35)	36.11 (24.45 to 52.57)	53.51 (35.29 to 79.19)
Acute glomerulonephritis	0.35 (0.30 to 0.39)	0.29 (0.21 to 0.39)	0.25 (0.18 to 0.35)	0.30 (0.22 to 0.41)	0.01 (0.01 to 0.01)	0.01 (0.01 to 0.02)	0.01 (0.01 to 0.01)	0.01 (0.01 to 0.01)
Chronic kidney disease	424.82 (371.98 to 481.13)	724.81 (429.68 to 1,502.02)	576.14 (355.70 to 1,146.15)	878.28 (495.32 to 1,892.24)	21.31 (18.25 to 23.98)	52.89 (29.27 to 106.12)	42.94 (25.09 to 81.50)	62.36 (33.44 to 131.59)
Chronic kidney disease due to diabetes mellitus	278.66 (240.23 to 317.55)	439.78 (260.16 to 910.27)	346.00 (214.88 to 690.93)	536.21 (305.45 to 1,148.52)	12.45 (10.58 to 14.17)	27.61 (15.30 to 59.71)	22.03 (12.87 to 44.08)	32.97 (17.45 to 72.26)
Chronic kidney disease due to hypertension	110.16 (91.76 to 128.12)	234.73 (132.43 to 506.58)	191.08 (111.39 to 388.44)	281.07 (150.57 to 625.66)	7.34 (5.97 to 8.62)	21.79 (11.84 to 42.66)	18.16 (10.22 to 33.36)	25.26 (13.34 to 52.26)
Chronic kidney disease due to glomerulonephritis	17.15 (14.17 to 22.05)	20.72 (12.66 to 39.86)	15.89 (9.70 to 30.02)	25.16 (14.55 to 50.12)	0.63 (0.53 to 0.75)	1.25 (0.69 to 2.73)	0.97 (0.55 to 2.05)	1.49 (0.79 to 3.26)
Chronic kidney disease due to other and unspecified causes	18.84 (15.00 to 23.16)	29.59 (16.45 to 60.41)	23.16 (13.54 to 45.59)	35.84 (19.78 to 75.85)	0.89 (0.70 to 1.10)	2.24 (1.17 to 4.53)	1.78 (0.97 to 3.51)	2.64 (1.33 to 5.64)
Urinary diseases and male infertility	122.59 (107.55 to 141.17)	280.79 (236.15 to 332.15)	249.95 (206.81 to 298.88)	268.06 (226.62 to 313.51)	7.12 (6.25 to 8.17)	23.19 (18.55 to 28.10)	21.22 (16.64 to 26.02)	21.29 (17.33 to 25.49)
Urinary tract infections	66.13 (48.33 to 101.76)	198.26 (152.11 to 270.98)	179.99 (137.45 to 244.12)	187.65 (145.09 to 255.72)	3.47 (2.36 to 5.85)	15.76 (11.48 to 22.50)	14.71 (10.53 to 21.14)	14.34 (10.53 to 20.50)
Urolithiasis	9.28 (7.27 to 12.59)	27.77 (17.84 to 36.14)	23.48 (14.37 to 31.75)	26.07 (17.37 to 33.38)	0.60 (0.46 to 0.86)	2.38 (1.33 to 3.31)	2.09 (1.10 to 2.99)	2.14 (1.24 to 2.90)
Other urinary diseases	47.18 (23.81 to 62.45)	54.75 (22.14 to 79.91)	46.48 (18.17 to 69.62)	54.35 (22.24 to 78.91)	3.05 (1.43 to 4.18)	5.04 (1.91 to 7.81)	4.42 (1.62 to 7.03)	4.81 (1.84 to 7.42)
Gynecological diseases	0.64 (0.48 to 0.83)	0.55 (0.37 to 0.83)	0.40 (0.26 to 0.60)	0.59 (0.39 to 0.87)	0.02 (0.01 to 0.02)	0.03 (0.02 to 0.04)	0.02 (0.01 to 0.03)	0.03 (0.02 to 0.04)
Uterine fibroids	0.05 (0.03 to 0.08)	0.05 (0.03 to 0.08)	0.04 (0.02 to 0.07)	0.05 (0.03 to 0.09)	0.00 (0.00 to 0.00)	0.00 (0.00 to 0.01)	0.00 (0.00 to 0.01)	0.00 (0.00 to 0.01)
Polycystic ovarian syndrome	0.01 (0.00 to 0.02)	0.00 (0.00 to 0.01)	0.00 (0.00 to 0.01)	0.01 (0.00 to 0.01)	0.00 (0.00 to 0.00)	0.00 (0.00 to 0.00)	0.00 (0.00 to 0.00)	0.00 (0.00 to 0.00)
Endometriosis	0.00 (0.00 to 0.01)	0.00 (0.00 to 0.01)	0.00 (0.00 to 0.01)	0.00 (0.00 to 0.01)	0.00 (0.00 to 0.00)	0.00 (0.00 to 0.00)	0.00 (0.00 to 0.00)	0.00 (0.00 to 0.00)
Genital prolapse	0.01 (0.00 to 0.01)	0.01 (0.00 to 0.01)	0.00 (0.00 to 0.01)	0.01 (0.00 to 0.01)	0.00 (0.00 to 0.00)	0.00 (0.00 to 0.00)	0.00 (0.00 to 0.00)	0.00 (0.00 to 0.00)
Other gynecological diseases	0.57 (0.42 to 0.75)	0.49 (0.32 to 0.75)	0.35 (0.22 to 0.54)	0.52 (0.34 to 0.79)	0.02 (0.01 to 0.02)	0.02 (0.01 to 0.03)	0.02 (0.01 to 0.02)	0.02 (0.01 to 0.03)
Hemoglobinopathies and hemolytic anemias	14.64 (11.55 to 21.00)	18.79 (13.14 to 29.85)	15.68 (10.66 to 25.02)	19.31 (13.61 to 30.46)	0.52 (0.40 to 0.81)	1.03 (0.69 to 1.73)	0.87 (0.57 to 1.46)	1.03 (0.70 to 1.72)
Thalassemias	3.30 (2.55 to 4.69)	1.71 (0.98 to 2.89)	1.70 (0.96 to 2.89)	1.63 (0.95 to 2.71)	0.05 (0.04 to 0.07)	0.03 (0.02 to 0.05)	0.03 (0.02 to 0.05)	0.03 (0.02 to 0.04)

Sickle cell disorders	0.02 (0.01 to 0.02)	0.02 (0.01 to 0.03)	0.02 (0.01 to 0.03)	0.02 (0.01 to 0.03)	0.00 (0.00 to 0.00)	0.00 (0.00 to 0.00)	0.00 (0.00 to 0.00)	0.00 (0.00 to 0.00)
G6PD deficiency	5.99 (4.68 to 8.49)	9.60 (6.69 to 14.76)	7.62 (5.05 to 12.14)	10.08 (7.00 to 15.40)	0.17 (0.13 to 0.25)	0.40 (0.27 to 0.65)	0.32 (0.21 to 0.55)	0.41 (0.28 to 0.68)
Other hemoglobinopathies and hemolytic anemias	5.33 (4.02 to 8.21)	7.46 (5.06 to 12.62)	6.34 (4.12 to 10.78)	7.57 (5.15 to 12.71)	0.30 (0.22 to 0.50)	0.60 (0.39 to 1.05)	0.52 (0.33 to 0.92)	0.59 (0.38 to 1.02)
Endocrine, metabolic, blood, and immune disorders	33.23 (27.81 to 46.28)	50.89 (39.56 to 73.33)	51.83 (39.97 to 75.04)	50.80 (39.86 to 72.88)	1.22 (1.02 to 1.77)	2.76 (2.14 to 4.25)	2.88 (2.21 to 4.51)	2.66 (2.08 to 4.04)

Thailand								
Cause	YLLs (in 1000s)				Deaths (in 1000s)			
	2016	2040 Forecast	2040 Better Scenario	2040 Worse Scenario	2016	2040 Forecast	2040 Better Scenario	2040 Worse Scenario
**Musculoskeletal disorders**	**45.32 (33.37 to 53.83)**	**56.71 (44.78 to 74.76)**	**53.09 (41.91 to 69.64)**	**54.58 (43.41 to 71.19)**	**1.37 (1.15 to 1.73)**	**2.61 (2.05 to 4.24)**	**2.48 (1.92 to 4.03)**	**2.44 (1.94 to 3.82)**
Rheumatoid arthritis	4.34 (3.42 to 6.62)	8.64 (6.21 to 14.32)	6.63 (4.53 to 11.10)	9.07 (6.54 to 14.72)	0.19 (0.14 to 0.31)	0.50 (0.35 to 0.92)	0.39 (0.26 to 0.74)	0.51 (0.36 to 0.92)
Other musculoskeletal disorders	40.99 (29.09 to 49.06)	48.08 (37.34 to 62.95)	46.46 (36.26 to 61.01)	45.51 (34.65 to 59.35)	1.18 (0.98 to 1.43)	2.11 (1.67 to 3.33)	2.08 (1.63 to 3.33)	1.93 (1.54 to 2.93)
**Other non-communicable diseases**	**150.12 (109.78 to 186.29)**	**223.38 (138.40 to 292.55)**	**198.34 (119.88 to 261.80)**	**222.45 (139.52 to 286.48)**	**4.31 (2.85 to 5.53)**	**13.60 (8.28 to 18.87)**	**12.80 (7.73 to 18.07)**	**12.81 (7.89 to 17.62)**
Congenital birth defects	82.18 (61.62 to 111.19)	44.17 (29.77 to 64.78)	32.15 (21.69 to 47.22)	48.61 (32.38 to 71.86)	1.14 (0.89 to 1.48)	0.76 (0.56 to 1.04)	0.57 (0.41 to 0.79)	0.82 (0.59 to 1.13)
Neural tube defects	2.51 (1.51 to 4.13)	0.61 (0.33 to 1.15)	0.40 (0.21 to 0.76)	0.73 (0.39 to 1.42)	0.03 (0.02 to 0.05)	0.01 (0.01 to 0.01)	0.01 (0.00 to 0.01)	0.01 (0.01 to 0.02)
Congenital heart anomalies	38.76 (27.58 to 53.30)	19.99 (12.42 to 30.91)	13.54 (8.41 to 20.99)	22.15 (13.81 to 34.31)	0.51 (0.38 to 0.68)	0.30 (0.20 to 0.45)	0.21 (0.13 to 0.33)	0.33 (0.21 to 0.50)
Orofacial clefts	0.04 (0.02 to 0.07)	0.01 (0.00 to 0.03)	0.01 (0.00 to 0.02)	0.01 (0.01 to 0.03)	0.00 (0.00 to 0.00)	0.00 (0.00 to 0.00)	0.00 (0.00 to 0.00)	0.00 (0.00 to 0.00)
Down syndrome	2.03 (1.56 to 2.82)	1.65 (1.14 to 2.45)	1.66 (1.13 to 2.49)	1.79 (1.22 to 2.65)	0.04 (0.03 to 0.05)	0.03 (0.02 to 0.05)	0.04 (0.02 to 0.05)	0.04 (0.03 to 0.05)
Other chromosomal abnormalities	4.11 (1.12 to 6.82)	1.90 (0.49 to 3.84)	1.54 (0.41 to 3.12)	2.21 (0.57 to 4.47)	0.05 (0.01 to 0.08)	0.02 (0.01 to 0.04)	0.02 (0.01 to 0.04)	0.03 (0.01 to 0.05)
Congenital musculoskeletal and limb anomalies	2.66 (1.33 to 4.33)	0.89 (0.45 to 1.66)	0.64 (0.32 to 1.19)	0.99 (0.50 to 1.88)	0.03 (0.02 to 0.05)	0.01 (0.01 to 0.02)	0.01 (0.00 to 0.01)	0.01 (0.01 to 0.02)
Urogenital congenital anomalies	3.93 (2.92 to 4.80)	4.71 (2.76 to 5.86)	3.82 (2.25 to 4.89)	4.83 (2.84 to 6.00)	0.11 (0.07 to 0.13)	0.16 (0.08 to 0.20)	0.13 (0.07 to 0.16)	0.16 (0.08 to 0.20)
Digestive congenital anomalies	2.64 (1.49 to 4.95)	1.73 (0.97 to 3.09)	1.20 (0.67 to 2.11)	1.97 (1.07 to 3.51)	0.03 (0.02 to 0.06)	0.03 (0.02 to 0.04)	0.02 (0.01 to 0.03)	0.03 (0.02 to 0.05)
Other congenital birth defects	25.50 (18.28 to 36.21)	12.68 (8.10 to 19.48)	9.36 (5.86 to 14.97)	13.91 (8.79 to 21.78)	0.34 (0.25 to 0.46)	0.20 (0.14 to 0.29)	0.15 (0.10 to 0.22)	0.21 (0.14 to 0.32)
Skin and subcutaneous diseases	64.38 (33.16 to 87.58)	178.47 (96.48 to 243.53)	165.64 (89.42 to 226.30)	172.98 (92.89 to 233.81)	3.13 (1.72 to 4.35)	12.83 (7.45 to 18.15)	12.23 (7.07 to 17.51)	11.98 (7.02 to 16.88)
Cellulitis	1.64 (0.79 to 2.82)	6.04 (2.76 to 10.56)	6.03 (2.81 to 10.41)	5.61 (2.56 to 9.77)	0.07 (0.03 to 0.14)	0.41 (0.20 to 0.80)	0.42 (0.21 to 0.81)	0.37 (0.18 to 0.72)
Pyoderma	49.01 (24.68 to 66.39)	143.09 (75.42 to 194.94)	133.51 (71.14 to 182.75)	139.86 (72.92 to 190.52)	2.24 (1.19 to 3.02)	9.81 (5.53 to 14.12)	9.43 (5.36 to 13.47)	9.24 (5.15 to 12.88)
Decubitus ulcer	13.25 (6.83 to 20.20)	28.57 (15.46 to 47.03)	25.38 (13.37 to 41.77)	26.80 (14.37 to 44.35)	0.80 (0.44 to 1.31)	2.57 (1.37 to 4.53)	2.33 (1.27 to 4.21)	2.32 (1.24 to 4.17)
Other skin and subcutaneous diseases	0.49 (0.30 to 0.78)	0.76 (0.42 to 1.17)	0.71 (0.40 to 1.09)	0.72 (0.41 to 1.11)	0.02 (0.01 to 0.03)	0.04 (0.03 to 0.07)	0.04 (0.02 to 0.07)	0.04 (0.02 to 0.06)
Sudden infant death syndrome	3.56 (2.11 to 5.82)	0.75 (0.31 to 1.59)	0.56 (0.23 to 1.17)	0.86 (0.36 to 1.80)	0.04 (0.02 to 0.07)	0.01 (0.00 to 0.02)	0.01 (0.00 to 0.01)	0.01 (0.00 to 0.02)

**Injuries**	**1,990.52 (1,749.54 to 2,256.47)**	**1,241.39 (861.76 to 1,851.02)**	**1,057.27 (744.47 to 1,567.15)**	**1,710.73 (1,159.14 to 2,653.95)**	**47.84 (42.32 to 53.86)**	**41.10 (31.24 to 55.04)**	**36.09 (27.86 to 47.81)**	**52.33 (38.65 to 72.43)**
**Transport injuries**	**868.34 (749.31 to 994.78)**	**369.31 (230.42 to 610.92)**	**329.36 (211.52 to 531.09)**	**786.52 (482.39 to 1,303.99)**	**20.12 (17.48 to 23.08)**	**11.59 (7.88 to 18.01)**	**10.45 (7.31 to 15.66)**	**22.19 (14.67 to 35.37)**
Road injuries	828.27 (715.54 to 948.82)	326.61 (205.06 to 534.71)	297.69 (193.24 to 475.87)	739.48 (454.28 to 1,222.55)	19.18 (16.69 to 22.00)	10.32 (7.00 to 15.88)	9.49 (6.67 to 14.36)	20.81 (13.84 to 33.08)
Pedestrian road injuries	117.22 (98.98 to 138.24)	49.50 (33.68 to 74.25)	37.54 (25.73 to 56.07)	115.06 (76.01 to 176.04)	3.27 (2.80 to 3.92)	2.15 (1.51 to 3.36)	1.67 (1.17 to 2.55)	4.37 (3.08 to 6.70)

Thailand								
Cause	YLLs (in 1000s)				Deaths (in 1000s)			
	2016	2040 Forecast	2040 Better Scenario	2040 Worse Scenario	2016	2040 Forecast	2040 Better Scenario	2040 Worse Scenario
Cyclist road injuries	19.58 (16.18 to 23.90)	7.13 (4.61 to 11.08)	6.77 (4.51 to 10.25)	16.68 (10.59 to 26.42)	0.52 (0.43 to 0.64)	0.27 (0.18 to 0.44)	0.26 (0.18 to 0.42)	0.52 (0.35 to 0.81)
Motorcyclist road injuries	414.15 (343.39 to 498.91)	206.54 (121.30 to 360.13)	193.25 (118.09 to 325.01)	389.05 (222.06 to 690.03)	9.04 (7.59 to 10.75)	5.81 (3.78 to 9.23)	5.52 (3.71 to 8.47)	9.83 (6.21 to 16.48)
Motor vehicle road injuries	267.80 (206.29 to 326.56)	60.58 (37.96 to 97.82)	57.42 (37.12 to 90.83)	209.48 (124.09 to 345.91)	6.12 (4.91 to 7.44)	1.99 (1.34 to 3.08)	1.94 (1.34 to 2.92)	5.82 (3.83 to 9.09)
Other road injuries	9.52 (6.98 to 11.97)	2.86 (1.78 to 4.78)	2.71 (1.73 to 4.48)	9.21 (5.41 to 16.13)	0.22 (0.18 to 0.28)	0.10 (0.06 to 0.18)	0.10 (0.06 to 0.17)	0.26 (0.16 to 0.44)
Other transport injuries	40.07 (31.93 to 49.87)	42.69 (24.62 to 73.05)	31.66 (18.87 to 54.25)	47.04 (27.08 to 81.34)	0.95 (0.77 to 1.16)	1.28 (0.79 to 2.09)	0.97 (0.61 to 1.55)	1.38 (0.84 to 2.26)
**Unintentional injuries**	**545.65 (475.91 to 615.75)**	**404.39 (317.41 to 527.78)**	**351.85 (275.31 to 456.65)**	**415.26 (322.62 to 543.90)**	**14.62 (12.84 to 16.31)**	**16.80 (13.87 to 20.38)**	**15.21 (12.50 to 18.62)**	**16.54 (13.82 to 20.08)**
Falls	109.43 (90.18 to 129.09)	127.03 (104.69 to 157.17)	115.75 (94.61 to 144.82)	125.37 (103.72 to 155.01)	3.88 (3.33 to 4.50)	7.05 (5.70 to 9.09)	6.54 (5.22 to 8.50)	6.73 (5.52 to 8.56)
Drowning	210.30 (179.94 to 241.29)	110.48 (76.45 to 162.42)	92.27 (65.14 to 134.26)	118.65 (81.64 to 174.47)	4.78 (4.10 to 5.46)	3.26 (2.43 to 4.52)	2.78 (2.11 to 3.82)	3.42 (2.52 to 4.71)
Fire, heat, and hot substances	26.19 (15.58 to 33.01)	15.52 (9.02 to 23.12)	12.32 (7.19 to 18.33)	16.63 (9.60 to 24.95)	0.76 (0.49 to 0.93)	0.66 (0.43 to 0.91)	0.55 (0.35 to 0.75)	0.69 (0.43 to 0.95)
Poisonings	8.38 (4.90 to 10.55)	3.64 (2.05 to 5.86)	2.70 (1.54 to 4.31)	4.07 (2.24 to 6.52)	0.19 (0.12 to 0.24)	0.11 (0.08 to 0.16)	0.09 (0.06 to 0.13)	0.12 (0.08 to 0.18)
Exposure to mechanical forces	31.47 (25.33 to 37.36)	20.32 (14.65 to 27.92)	16.82 (12.13 to 23.64)	21.41 (15.66 to 29.94)	0.75 (0.62 to 0.89)	0.65 (0.52 to 0.84)	0.55 (0.43 to 0.72)	0.67 (0.54 to 0.86)
Unintentional firearm injuries	11.65 (7.35 to 14.88)	6.22 (3.55 to 9.48)	5.23 (2.98 to 7.85)	6.61 (3.73 to 9.94)	0.29 (0.19 to 0.36)	0.20 (0.13 to 0.28)	0.17 (0.11 to 0.24)	0.21 (0.13 to 0.29)
Unintentional suffocation	4.61 (2.49 to 6.31)	1.85 (0.80 to 3.12)	1.57 (0.68 to 2.63)	1.97 (0.87 to 3.34)	0.08 (0.04 to 0.11)	0.04 (0.02 to 0.06)	0.03 (0.02 to 0.05)	0.04 (0.02 to 0.06)
Other exposure to mechanical forces	15.21 (12.45 to 20.29)	12.25 (9.02 to 17.38)	10.03 (7.33 to 14.33)	12.83 (9.55 to 18.17)	0.38 (0.32 to 0.50)	0.41 (0.32 to 0.56)	0.35 (0.27 to 0.47)	0.42 (0.33 to 0.56)
Adverse effects of medical treatment	36.47 (21.39 to 47.64)	31.53 (20.57 to 44.17)	28.63 (19.00 to 40.04)	32.43 (20.98 to 45.29)	0.95 (0.71 to 1.15)	1.11 (0.84 to 1.52)	1.03 (0.77 to 1.42)	1.10 (0.86 to 1.48)
Animal contact	9.53 (7.90 to 11.70)	8.79 (6.57 to 11.81)	7.64 (5.65 to 10.45)	9.34 (6.96 to 12.50)	0.27 (0.22 to 0.33)	0.35 (0.27 to 0.46)	0.31 (0.24 to 0.41)	0.36 (0.27 to 0.47)
Venomous animal contact	6.58 (5.39 to 8.13)	5.60 (4.08 to 7.62)	4.98 (3.62 to 6.90)	5.97 (4.37 to 8.25)	0.18 (0.15 to 0.23)	0.21 (0.16 to 0.29)	0.19 (0.15 to 0.26)	0.22 (0.17 to 0.30)
Non-venomous animal contact	2.95 (2.28 to 3.98)	3.19 (2.21 to 4.62)	2.66 (1.81 to 3.85)	3.37 (2.32 to 4.79)	0.08 (0.07 to 0.11)	0.13 (0.10 to 0.18)	0.12 (0.08 to 0.16)	0.14 (0.10 to 0.19)
Foreign body	56.96 (47.24 to 79.35)	54.75 (42.94 to 69.87)	53.36 (41.93 to 67.45)	53.66 (42.24 to 68.58)	1.76 (1.46 to 2.05)	2.70 (1.79 to 3.59)	2.73 (1.78 to 3.65)	2.52 (1.69 to 3.28)
Pulmonary aspiration and foreign body in airway	39.38 (32.39 to 55.50)	39.09 (29.37 to 49.28)	37.26 (27.54 to 46.79)	37.73 (28.33 to 47.32)	1.25 (0.94 to 1.46)	2.03 (1.12 to 2.80)	2.02 (1.09 to 2.85)	1.86 (1.05 to 2.50)

Foreign body in other body part	17.58 (13.54 to 28.60)	15.66 (11.29 to 25.16)	16.10 (11.68 to 25.59)	15.93 (11.59 to 25.10)	0.51 (0.41 to 0.79)	0.67 (0.52 to 0.99)	0.71 (0.54 to 1.03)	0.66 (0.51 to 0.96)
Environmental heat and cold exposure	1.31 (0.91 to 1.71)	1.00 (0.70 to 1.41)	0.80 (0.55 to 1.12)	1.09 (0.74 to 1.50)	0.04 (0.03 to 0.05)	0.04 (0.03 to 0.06)	0.04 (0.03 to 0.05)	0.05 (0.03 to 0.06)
Other unintentional injuries	55.60 (44.63 to 67.43)	31.34 (21.74 to 46.55)	21.55 (14.22 to 33.24)	32.60 (22.91 to 47.96)	1.24 (1.01 to 1.50)	0.85 (0.63 to 1.17)	0.59 (0.43 to 0.85)	0.87 (0.65 to 1.19)
**Self-harm and interpersonal violence**	**576.16 (473.10 to 671.30)**	**463.65 (280.66 to 807.11)**	**372.02 (233.13 to 630.47)**	**504.92 (294.09 to 914.81)**	**13.10 (10.93 to 15.15)**	**12.59 (8.31 to 20.17)**	**10.31 (7.08 to 16.09)**	**13.49 (8.69 to 22.18)**

Thailand								
Cause	YLLs (in 1000s)				Deaths (in 1000s)			
	2016	2040 Forecast	2040 Better Scenario	2040 Worse Scenario	2016	2040 Forecast	2040 Better Scenario	2040 Worse Scenario
Self-harm	350.93 (289.22 to 409.03)	331.90 (186.70 to 604.73)	266.09 (158.45 to 471.56)	372.13 (202.40 to 705.08)	8.41 (6.99 to 9.75)	9.48 (6.00 to 15.66)	7.78 (5.12 to 12.75)	10.38 (6.35 to 17.74)
Self-harm by firearm	23.48 (12.16 to 31.05)	17.71 (7.60 to 35.53)	13.18 (5.96 to 26.54)	20.22 (8.33 to 41.96)	0.54 (0.29 to 0.71)	0.49 (0.24 to 0.92)	0.37 (0.19 to 0.69)	0.54 (0.26 to 1.04)
Self-harm by other specified means	327.45 (271.01 to 384.11)	314.19 (176.87 to 574.66)	252.91 (150.79 to 444.82)	351.91 (191.56 to 665.20)	7.87 (6.58 to 9.09)	8.99 (5.72 to 14.88)	7.41 (4.89 to 11.99)	9.83 (6.04 to 16.72)
Interpersonal violence	225.23 (157.17 to 280.94)	131.75 (81.57 to 207.73)	105.93 (66.18 to 168.60)	132.79 (80.59 to 210.66)	4.69 (3.34 to 5.83)	3.11 (2.10 to 4.57)	2.53 (1.74 to 3.77)	3.11 (2.11 to 4.51)
Physical violence by firearm	146.69 (90.51 to 187.65)	82.08 (45.79 to 130.79)	64.77 (36.14 to 107.11)	82.11 (45.53 to 131.60)	3.00 (1.92 to 3.83)	1.84 (1.10 to 2.82)	1.46 (0.87 to 2.25)	1.82 (1.09 to 2.81)
Physical violence by sharp object	44.36 (22.84 to 74.06)	28.18 (13.99 to 50.15)	22.96 (11.57 to 40.75)	27.72 (13.50 to 48.91)	0.92 (0.50 to 1.49)	0.67 (0.37 to 1.11)	0.55 (0.31 to 0.90)	0.65 (0.36 to 1.04)
Physical violence by other means	34.18 (22.40 to 57.82)	21.50 (11.73 to 39.41)	18.19 (10.22 to 33.07)	22.96 (12.07 to 41.81)	0.77 (0.52 to 1.25)	0.60 (0.37 to 1.04)	0.52 (0.32 to 0.88)	0.63 (0.38 to 1.10)
**Forces of nature, conflict and terrorism, and executions and police conflict**	**0.37 (0.31 to 0.47)**	**4.04 (0.00 to 17.89)**	**4.04 (0.00 to 17.91)**	**4.02 (0.00 to 17.80)**	**0.01 (0.01 to 0.01)**	**0.11 (0.00 to 0.53)**	**0.12 (0.00 to 0.54)**	**0.11 (0.00 to 0.51)**
Exposure to forces of nature	0.00 (0.00 to 0.00)	2.98 (0.00 to 17.89)	2.98 (0.00 to 17.91)	2.96 (0.00 to 17.80)	0.00 (0.00 to 0.00)	0.09 (0.00 to 0.53)	0.09 (0.00 to 0.54)	0.09 (0.00 to 0.51)
Conflict and terrorism	0.00 (0.00 to 0.00)	0.97 (0.00 to 4.88)	0.97 (0.00 to 4.88)	0.97 (0.00 to 4.88)	0.00 (0.00 to 0.00)	0.02 (0.00 to 0.12)	0.02 (0.00 to 0.13)	0.02 (0.00 to 0.12)
Executions and police conflict	0.37 (0.31 to 0.47)	0.09 (0.00 to 2.50)	0.09 (0.00 to 2.50)	0.09 (0.00 to 2.48)	0.01 (0.01 to 0.01)	0.00 (0.00 to 0.06)	0.00 (0.00 to 0.06)	0.00 (0.00 to 0.06)

Timor-Leste								
Cause	YLLs (actual counts)				Deaths (actual counts)			
	2016	2040 Forecast	2040 Better Scenario	2040 Worse Scenario	2016	2040 Forecast	2040 Better Scenario	2040 Worse Scenario
**All causes**	**220,092.57 (167,674.95 to 287,912.39)**	**189,086.62 (142,261.37 to 244,442.33)**	**143,747.34 (108,200.86 to 185,478.35)**	**282,357.80 (204,377.22 to 377,409.37)**	**5,535.35 (4,571.74 to 6,575.53)**	**7,482.79 (6,391.21 to 8,656.58)**	**6,093.47 (5,236.44 to 7,017.41)**	**9,585.04 (8,009.45 to 11,350.00)**
**Communicable, maternal, neonatal, and nutritional diseases**	**108,961.04 (70,427.13 to 168,410.74)**	**58,968.41 (33,218.21 to 110,056.62)**	**40,205.31 (21,490.78 to 84,254.55)**	**114,043.15 (62,398.92 to 190,111.68)**	**1,697.51 (1,191.90 to 2,485.57)**	**1,254.31 (783.44 to 2,250.96)**	**918.64 (549.30 to 1,901.66)**	**2,156.30 (1,331.11 to 3,462.12)**
**HIV/AIDS and tuberculosis**	**10,753.15 (4,587.97 to 37,566.43)**	**11,163.41 (2,407.30 to 52,634.72)**	**8,731.68 (1,422.41 to 49,447.76)**	**20,500.79 (6,163.17 to 69,165.54)**	**292.11 (148.21 to 856.55)**	**311.53 (99.24 to 1,245.55)**	**239.86 (63.61 to 1,207.77)**	**521.87 (199.30 to 1,597.52)**
Tuberculosis	4,287.10 (2,805.11 to 5,929.16)	2,931.84 (1,748.07 to 4,380.37)	1,783.28 (1,031.23 to 2,744.04)	4,157.00 (2,366.51 to 6,626.38)	150.70 (106.61 to 210.99)	127.55 (84.66 to 187.75)	81.85 (53.49 to 121.54)	170.40 (109.22 to 263.11)
HIV/AIDS	6,466.05 (434.98 to 34,439.33)	8,231.56 (3.13 to 50,146.13)	6,948.40 (0.00 to 48,371.03)	16,343.79 (2,001.66 to 66,170.47)	141.40 (8.36 to 747.58)	183.98 (0.11 to 1,133.40)	158.01 (0.00 to 1,131.77)	351.47 (42.90 to 1,445.41)
**Diarrhea, lower respiratory, and other common infectious diseases**	**52,294.28 (33,927.19 to 77,790.29)**	**21,426.79 (10,360.84 to 41,682.23)**	**13,501.56 (6,671.74 to 25,330.84)**	**47,724.25 (20,839.44 to 92,475.12)**	**803.80 (562.09 to 1,134.55)**	**552.98 (301.04 to 952.25)**	**399.44 (221.31 to 668.40)**	**1,009.39 (525.09 to 1,744.44)**
Diarrheal diseases	19,257.97 (10,408.12 to 32,372.77)	4,389.88 (739.98 to 11,909.07)	2,802.20 (437.10 to 8,043.08)	9,443.93 (2,309.84 to 23,426.45)	271.48 (140.82 to 443.86)	108.77 (16.17 to 315.00)	80.13 (10.73 to 250.31)	223.60 (44.03 to 633.51)
Intestinal infectious diseases	3,352.10 (1,636.65 to 5,764.63)	1,996.19 (809.82 to 4,052.34)	1,680.30 (689.95 to 3,328.94)	2,609.56 (1,014.20 to 5,442.38)	47.16 (23.39 to 81.78)	30.07 (12.81 to 59.87)	25.62 (11.00 to 50.46)	38.34 (15.37 to 80.41)
Typhoid fever	2,928.06 (1,380.47 to 5,145.18)	1,693.89 (671.45 to 3,455.08)	1,433.06 (579.11 to 2,878.30)	2,209.45 (850.93 to 4,548.83)	41.09 (19.50 to 72.42)	25.38 (10.67 to 51.55)	21.75 (9.52 to 42.63)	32.30 (12.90 to 66.84)
Paratyphoid fever	413.90 (173.47 to 879.58)	299.69 (106.45 to 698.58)	245.48 (86.65 to 568.96)	395.97 (134.62 to 922.17)	5.93 (2.51 to 12.46)	4.62 (1.72 to 10.72)	3.83 (1.43 to 8.73)	5.95 (2.15 to 13.67)
Other intestinal infectious diseases	10.14 (3.32 to 26.32)	2.60 (0.67 to 7.33)	1.76 (0.46 to 4.92)	4.14 (0.99 to 11.66)	0.14 (0.05 to 0.34)	0.06 (0.02 to 0.15)	0.05 (0.01 to 0.11)	0.08 (0.02 to 0.20)
Lower respiratory infections	22,983.04 (14,626.24 to 36,159.96)	8,705.95 (3,792.09 to 18,381.66)	5,335.25 (2,552.76 to 10,035.89)	22,948.70 (7,741.41 to 51,876.23)	395.49 (283.43 to 555.59)	324.64 (183.92 to 519.17)	236.42 (141.00 to 355.44)	581.38 (301.58 to 994.50)
Upper respiratory infections	17.15 (5.91 to 42.19)	8.91 (3.23 to 21.34)	5.70 (2.06 to 12.94)	13.94 (4.59 to 36.62)	0.29 (0.13 to 0.58)	0.23 (0.09 to 0.47)	0.16 (0.06 to 0.33)	0.32 (0.12 to 0.67)
Otitis media	2.92 (1.25 to 7.69)	1.05 (0.41 to 2.55)	1.07 (0.42 to 2.55)	1.09 (0.43 to 2.72)	0.07 (0.03 to 0.15)	0.04 (0.02 to 0.10)	0.05 (0.02 to 0.11)	0.04 (0.02 to 0.09)
Meningitis	2,593.68 (1,415.60 to 4,409.52)	1,057.40 (458.89 to 2,053.58)	808.39 (391.57 to 1,452.64)	1,874.22 (671.81 to 4,223.32)	35.26 (20.92 to 56.57)	17.95 (9.43 to 31.32)	15.01 (8.46 to 25.49)	27.59 (12.58 to 55.66)
Pneumococcal meningitis	25.79 (11.63 to 49.95)	6.06 (2.72 to 12.01)	4.80 (2.33 to 8.99)	11.13 (4.30 to 26.05)	0.36 (0.19 to 0.64)	0.12 (0.07 to 0.21)	0.11 (0.06 to 0.18)	0.19 (0.09 to 0.37)
H influenzae type B meningitis	217.99 (106.01 to 401.70)	259.44 (41.97 to 904.19)	111.59 (29.01 to 355.01)	808.16 (95.26 to 2,757.92)	2.86 (1.54 to 4.95)	3.36 (0.77 to 10.84)	1.66 (0.61 to 4.50)	9.74 (1.41 to 32.55)
Meningococcal meningitis	866.30 (465.56 to 1,533.35)	276.38 (136.42 to 498.76)	240.68 (118.80 to 433.83)	368.37 (172.02 to 701.55)	11.96 (6.72 to 19.86)	5.31 (3.06 to 9.06)	4.88 (2.84 to 8.25)	6.42 (3.49 to 11.31)
Other meningitis	1,483.59 (794.51 to 2,521.83)	515.52 (250.45 to 935.89)	451.32 (221.73 to 800.61)	686.55 (322.12 to 1,280.74)	20.08 (11.60 to 32.29)	9.15 (5.14 to 15.55)	8.36 (4.70 to 14.27)	11.24 (6.02 to 19.51)
Encephalitis	839.85 (466.07 to 1,432.02)	533.68 (262.28 to 970.70)	436.32 (220.85 to 803.43)	713.51 (343.06 to 1,334.44)	12.61 (7.49 to 20.74)	10.62 (5.85 to 18.68)	9.13 (5.11 to 15.89)	13.05 (7.12 to 23.10)

Diphtheria	17.13 (8.35 to 32.47)	7.46 (2.48 to 18.20)	3.95 (1.73 to 8.08)	15.16 (4.04 to 39.12)	0.22 (0.11 to 0.41)	0.10 (0.04 to 0.23)	0.06 (0.03 to 0.12)	0.20 (0.06 to 0.48)
Whooping cough	2,410.94 (175.55 to 7,363.77)	1,116.77 (57.06 to 4,074.84)	456.25 (25.00 to 1,771.96)	1,609.21 (80.69 to 5,794.22)	28.56 (2.09 to 87.08)	13.29 (0.68 to 48.40)	5.51 (0.31 to 21.37)	19.09 (0.99 to 68.45)
Tetanus	244.81 (121.38 to 484.57)	62.69 (25.08 to 137.34)	36.62 (15.13 to 75.71)	81.53 (31.01 to 184.94)	5.05 (2.74 to 9.35)	1.36 (0.62 to 2.68)	1.11 (0.51 to 2.26)	1.54 (0.71 to 3.30)
Measles	438.65 (121.80 to 1,081.08)	3,493.55 (167.39 to 14,638.54)	1,898.75 (128.96 to 7,363.20)	8,332.74 (249.07 to 36,211.59)	5.26 (1.47 to 12.94)	43.95 (2.72 to 179.71)	24.65 (2.06 to 88.40)	101.88 (3.79 to 433.13)

Timor-Leste								
Cause	YLLs (actual counts)				Deaths (actual counts)			
	2016	2040 Forecast	2040 Better Scenario	2040 Worse Scenario	2016	2040 Forecast	2040 Better Scenario	2040 Worse Scenario
Varicella and herpes zoster	136.03 (72.12 to 234.90)	53.27 (24.83 to 100.35)	36.76 (18.35 to 70.68)	80.63 (36.11 to 162.77)	2.34 (1.51 to 3.57)	1.95 (1.05 to 3.30)	1.58 (0.85 to 2.72)	2.36 (1.24 to 4.06)
**Neglected tropical diseases and malaria**	**375.91 (172.02 to 834.57)**	**377.51 (176.67 to 828.86)**	**343.34 (161.58 to 755.24)**	**465.54 (212.53 to 1,042.36)**	**6.13 (3.03 to 13.40)**	**7.84 (4.12 to 16.85)**	**7.44 (3.95 to 16.01)**	**8.88 (4.56 to 19.03)**
Malaria	110.38 (47.58 to 298.49)	80.95 (21.81 to 254.66)	76.70 (20.38 to 240.97)	88.20 (24.21 to 284.49)	2.21 (1.04 to 6.05)	2.02 (0.60 to 6.13)	1.97 (0.57 to 6.22)	2.09 (0.62 to 6.55)
Chagas disease	0.00 (0.00 to 0.00)	0.00 (0.00 to 0.00)	0.00 (0.00 to 0.00)	0.00 (0.00 to 0.00)	0.00 (0.00 to 0.00)	0.00 (0.00 to 0.00)	0.00 (0.00 to 0.00)	0.00 (0.00 to 0.00)
Leishmaniasis	0.00 (0.00 to 0.00)	0.00 (0.00 to 0.00)	0.00 (0.00 to 0.00)	0.00 (0.00 to 0.00)	0.00 (0.00 to 0.00)	0.00 (0.00 to 0.00)	0.00 (0.00 to 0.00)	0.00 (0.00 to 0.00)
African trypanosomiasis	0.00 (0.00 to 0.00)	0.00 (0.00 to 0.00)	0.00 (0.00 to 0.00)	0.00 (0.00 to 0.00)	0.00 (0.00 to 0.00)	0.00 (0.00 to 0.00)	0.00 (0.00 to 0.00)	0.00 (0.00 to 0.00)
Schistosomiasis	0.00 (0.00 to 0.00)	0.00 (0.00 to 0.00)	0.00 (0.00 to 0.00)	0.00 (0.00 to 0.00)	0.00 (0.00 to 0.00)	0.00 (0.00 to 0.00)	0.00 (0.00 to 0.00)	0.00 (0.00 to 0.00)
Cysticercosis	0.00 (0.00 to 0.00)	0.00 (0.00 to 0.00)	0.00 (0.00 to 0.00)	0.00 (0.00 to 0.00)	0.00 (0.00 to 0.00)	0.00 (0.00 to 0.00)	0.00 (0.00 to 0.00)	0.00 (0.00 to 0.00)
Cystic echinococcosis	0.70 (0.48 to 0.94)	0.39 (0.15 to 0.66)	0.23 (0.08 to 0.40)	0.56 (0.23 to 0.95)	0.02 (0.01 to 0.02)	0.01 (0.01 to 0.02)	0.01 (0.00 to 0.01)	0.02 (0.01 to 0.03)
Dengue	109.55 (11.58 to 506.12)	190.87 (43.74 to 650.43)	175.22 (41.30 to 589.56)	232.80 (51.45 to 803.45)	1.68 (0.19 to 7.48)	3.90 (0.97 to 12.64)	3.74 (0.96 to 12.03)	4.37 (1.05 to 14.12)
Yellow fever	0.00 (0.00 to 0.00)	0.00 (0.00 to 0.00)	0.00 (0.00 to 0.00)	0.00 (0.00 to 0.00)	0.00 (0.00 to 0.00)	0.00 (0.00 to 0.00)	0.00 (0.00 to 0.00)	0.00 (0.00 to 0.00)
Rabies	10.63 (4.05 to 23.81)	6.07 (1.44 to 19.36)	3.24 (0.71 to 11.69)	9.93 (2.66 to 29.94)	0.21 (0.09 to 0.43)	0.14 (0.04 to 0.40)	0.08 (0.02 to 0.24)	0.22 (0.07 to 0.58)
Intestinal nematode infections	109.51 (26.77 to 239.35)	77.81 (19.62 to 176.96)	71.58 (19.54 to 160.11)	103.90 (25.49 to 242.42)	1.44 (0.44 to 2.95)	1.21 (0.44 to 2.40)	1.16 (0.45 to 2.27)	1.50 (0.51 to 3.16)
Other neglected tropical diseases	35.15 (16.33 to 78.66)	21.42 (7.51 to 47.73)	16.38 (5.52 to 35.24)	30.15 (10.91 to 69.92)	0.57 (0.31 to 1.25)	0.56 (0.21 to 1.21)	0.47 (0.17 to 0.98)	0.68 (0.26 to 1.50)
**Maternal disorders**	**4,832.34 (2,127.94 to 7,080.86)**	**3,318.44 (1,342.30 to 5,476.50)**	**2,074.06 (825.94 to 3,470.22)**	**4,678.43 (1,958.37 to 8,063.37)**	**93.01 (40.77 to 137.83)**	**65.19 (26.60 to 108.37)**	**40.77 (16.19 to 67.67)**	**91.59 (36.98 to 156.99)**
Maternal hemorrhage	1,568.34 (654.42 to 2,406.11)	1,082.90 (365.11 to 1,903.86)	674.57 (228.60 to 1,224.63)	1,501.43 (537.85 to 2,714.20)	29.75 (12.55 to 45.55)	20.95 (7.49 to 36.80)	13.05 (4.54 to 23.60)	29.00 (10.81 to 53.37)
Maternal sepsis and other maternal infections	416.35 (164.13 to 718.09)	267.68 (92.49 to 513.69)	166.45 (58.23 to 320.19)	374.66 (132.01 to 771.18)	8.43 (3.31 to 14.80)	5.54 (1.95 to 10.84)	3.45 (1.21 to 6.91)	7.73 (2.77 to 16.17)
Maternal hypertensive disorders	535.39 (223.54 to 878.19)	386.11 (147.77 to 692.59)	239.16 (86.75 to 437.60)	538.59 (201.90 to 1,002.65)	10.24 (4.33 to 16.70)	7.54 (2.80 to 13.60)	4.67 (1.65 to 8.54)	10.50 (3.89 to 19.35)
Maternal obstructed labor and uterine rupture	83.48 (30.45 to 166.79)	64.48 (18.28 to 144.74)	38.74 (11.18 to 87.89)	91.64 (26.25 to 211.13)	1.64 (0.59 to 3.33)	1.30 (0.37 to 2.99)	0.78 (0.22 to 1.80)	1.84 (0.52 to 4.25)
Maternal abortion, miscarriage, and ectopic pregnancy	806.41 (312.74 to 1,337.66)	489.64 (169.07 to 919.54)	304.17 (104.22 to 583.21)	696.27 (243.26 to 1,384.47)	15.95 (6.39 to 26.39)	9.89 (3.49 to 18.28)	6.14 (2.02 to 11.92)	14.02 (5.15 to 27.51)

Indirect maternal deaths	651.30 (252.29 to 1,047.54)	459.45 (174.79 to 851.60)	287.74 (102.05 to 529.41)	668.09 (246.85 to 1,227.82)	12.01 (4.62 to 19.58)	8.64 (3.27 to 16.02)	5.42 (1.93 to 9.94)	12.49 (4.60 to 22.99)
Late maternal deaths	55.36 (14.17 to 113.57)	35.48 (8.62 to 78.10)	22.02 (5.00 to 47.89)	50.81 (11.87 to 115.05)	1.07 (0.27 to 2.15)	0.70 (0.16 to 1.55)	0.44 (0.10 to 0.98)	1.00 (0.23 to 2.27)
Maternal deaths aggravated by HIV/AIDS	2.46 (0.19 to 8.94)	30.43 (0.00 to 171.59)	17.89 (0.00 to 117.33)	49.05 (1.27 to 259.87)	0.05 (0.00 to 0.17)	0.61 (0.00 to 3.53)	0.36 (0.00 to 2.47)	0.99 (0.03 to 5.42)
Other maternal disorders	713.25 (314.81 to 1,152.89)	502.25 (184.16 to 904.47)	323.33 (119.76 to 587.12)	707.89 (264.26 to 1,285.07)	13.89 (6.08 to 22.76)	10.01 (3.71 to 17.99)	6.46 (2.41 to 11.66)	14.03 (5.53 to 26.37)

Timor-Leste								
Cause	YLLs (actual counts)				Deaths (actual counts)			
	2016	2040 Forecast	2040 Better Scenario	2040 Worse Scenario	2016	2040 Forecast	2040 Better Scenario	2040 Worse Scenario
**Neonatal disorders**	**36,426.16 (17,759.54 to 62,388.18)**	**20,564.41 (8,843.66 to 40,234.44)**	**13,863.40 (6,148.49 to 27,101.46)**	**37,814.00 (16,733.99 to 71,588.84)**	**420.83 (205.16 to 720.67)**	**237.58 (102.19 to 464.83)**	**160.16 (71.03 to 313.07)**	**436.85 (193.35 to 827.01)**
Neonatal preterm birth	16,891.83 (7,704.17 to 31,352.69)	8,846.67 (3,691.69 to 17,685.43)	5,456.02 (2,265.89 to 10,783.44)	16,843.75 (7,164.46 to 32,889.45)	195.13 (89.00 to 362.25)	102.20 (42.65 to 204.28)	63.03 (26.18 to 124.57)	194.58 (82.78 to 379.87)
Neonatal encephalopathy due to birth asphyxia and trauma	11,554.67 (4,969.17 to 22,274.33)	6,286.26 (2,150.79 to 14,288.69)	4,616.84 (1,614.02 to 10,496.48)	11,080.40 (3,835.96 to 24,697.16)	133.48 (57.40 to 257.28)	72.62 (24.85 to 165.06)	53.34 (18.65 to 121.25)	128.00 (44.32 to 285.28)
Neonatal sepsis and other neonatal infections	3,675.44 (1,509.11 to 7,309.78)	2,885.73 (1,026.44 to 6,369.06)	2,082.35 (750.08 to 4,556.98)	5,134.82 (1,866.57 to 11,292.34)	42.46 (17.43 to 84.48)	33.34 (11.86 to 73.57)	24.06 (8.67 to 52.64)	59.32 (21.57 to 130.45)
Hemolytic disease and other neonatal jaundice	295.29 (120.69 to 591.91)	123.64 (38.64 to 299.77)	83.02 (27.45 to 200.04)	231.24 (73.52 to 561.11)	3.41 (1.39 to 6.84)	1.43 (0.45 to 3.46)	0.96 (0.32 to 2.31)	2.67 (0.85 to 6.48)
Other neonatal disorders	4,008.93 (1,725.26 to 8,157.34)	2,422.11 (862.87 to 5,509.14)	1,625.17 (590.88 to 3,679.95)	4,523.79 (1,625.21 to 10,297.90)	46.34 (19.95 to 94.23)	27.99 (9.98 to 63.65)	18.78 (6.83 to 42.51)	52.28 (18.79 to 118.97)
**Nutritional deficiencies**	**1,254.42 (801.51 to 1,938.61)**	**603.84 (409.94 to 866.49)**	**501.17 (341.03 to 710.54)**	**785.72 (498.06 to 1,195.45)**	**34.16 (24.57 to 44.73)**	**38.63 (25.64 to 53.16)**	**36.37 (24.69 to 49.12)**	**39.61 (26.03 to 56.74)**
Protein-energy malnutrition	1,140.23 (708.68 to 1,799.20)	499.46 (332.51 to 716.68)	414.59 (280.58 to 588.31)	656.56 (413.51 to 1,028.09)	30.64 (22.28 to 40.68)	32.78 (21.74 to 46.32)	30.97 (20.97 to 42.75)	33.55 (21.90 to 48.58)
Iodine deficiency	14.53 (0.62 to 65.55)	22.47 (0.78 to 82.44)	17.13 (0.56 to 60.83)	30.73 (1.05 to 123.65)	0.32 (0.02 to 1.35)	1.20 (0.02 to 5.36)	1.02 (0.01 to 4.78)	1.34 (0.03 to 5.54)
Dietary iron deficiency	16.07 (9.42 to 26.84)	12.29 (6.28 to 24.98)	10.53 (5.32 to 21.76)	14.67 (7.66 to 29.90)	0.30 (0.18 to 0.61)	0.34 (0.16 to 0.92)	0.31 (0.15 to 0.88)	0.37 (0.18 to 0.97)
Other nutritional deficiencies	83.59 (47.42 to 146.90)	69.62 (37.75 to 116.47)	58.91 (31.86 to 99.20)	83.77 (45.00 to 146.97)	2.90 (1.60 to 4.99)	4.31 (2.19 to 7.90)	4.07 (2.06 to 7.62)	4.36 (2.19 to 7.92)
**Other communicable, maternal, neonatal, and nutritional diseases**	**3,024.78 (1,379.84 to 6,168.61)**	**1,514.02 (783.66 to 2,629.36)**	**1,190.11 (614.32 to 1,996.17)**	**2,074.43 (1,016.52 to 3,887.75)**	**47.47 (25.15 to 88.58)**	**40.57 (23.30 to 61.36)**	**34.60 (19.99 to 51.50)**	**48.09 (27.20 to 74.71)**
Sexually transmitted infections excluding HIV	1,276.57 (453.98 to 2,574.62)	263.16 (79.26 to 635.86)	204.98 (63.41 to 482.98)	419.28 (119.13 to 996.92)	15.40 (5.72 to 30.57)	3.52 (1.34 to 7.84)	2.82 (1.16 to 6.08)	5.35 (1.77 to 12.22)
Syphilis	1,256.57 (431.81 to 2,551.78)	244.51 (61.96 to 615.97)	188.61 (49.52 to 462.51)	398.73 (97.81 to 972.33)	14.89 (5.24 to 30.10)	2.99 (0.86 to 7.35)	2.33 (0.71 to 5.54)	4.80 (1.31 to 11.56)
Chlamydial infection	3.98 (2.03 to 6.63)	3.77 (1.85 to 6.46)	3.31 (1.63 to 5.82)	4.16 (2.05 to 7.23)	0.10 (0.06 to 0.16)	0.11 (0.06 to 0.17)	0.10 (0.06 to 0.16)	0.11 (0.06 to 0.18)
Gonococcal infection	10.41 (5.39 to 17.14)	9.69 (4.71 to 16.18)	8.55 (4.15 to 14.47)	10.63 (5.24 to 17.79)	0.28 (0.16 to 0.45)	0.29 (0.18 to 0.45)	0.27 (0.17 to 0.42)	0.30 (0.18 to 0.48)
Other sexually transmitted infections	5.60 (2.85 to 9.33)	5.18 (2.58 to 8.99)	4.52 (2.19 to 7.72)	5.76 (2.85 to 9.96)	0.13 (0.08 to 0.22)	0.13 (0.07 to 0.21)	0.12 (0.07 to 0.19)	0.14 (0.08 to 0.23)
Acute hepatitis	254.97 (167.30 to 333.82)	196.62 (112.01 to 292.71)	162.85 (92.83 to 243.94)	232.27 (136.32 to 342.86)	6.18 (4.28 to 8.05)	5.84 (3.77 to 8.21)	5.01 (3.25 to 7.08)	6.52 (4.15 to 9.24)
Acute hepatitis A	36.59 (19.05 to 65.49)	10.54 (4.48 to 23.33)	8.06 (3.57 to 17.05)	16.36 (6.21 to 37.40)	0.49 (0.28 to 0.83)	0.18 (0.10 to 0.34)	0.15 (0.08 to 0.25)	0.25 (0.13 to 0.49)
Acute hepatitis B	164.64 (90.03 to 226.08)	150.44 (79.13 to 231.40)	124.12 (64.73 to 192.87)	172.34 (92.35 to 267.02)	4.64 (2.98 to 6.29)	4.63 (2.86 to 6.73)	3.91 (2.44 to 5.70)	5.15 (3.20 to 7.52)

Acute hepatitis C	5.21 (3.38 to 7.41)	4.98 (2.86 to 7.88)	4.54 (2.64 to 7.24)	5.47 (3.14 to 8.75)	0.15 (0.10 to 0.21)	0.17 (0.10 to 0.26)	0.16 (0.10 to 0.25)	0.17 (0.11 to 0.27)
Acute hepatitis E	48.52 (32.46 to 70.81)	30.66 (16.86 to 50.44)	26.13 (14.19 to 43.42)	38.11 (20.49 to 65.56)	0.90 (0.61 to 1.26)	0.86 (0.51 to 1.31)	0.80 (0.47 to 1.21)	0.94 (0.55 to 1.44)
Other unspecified infectious diseases	1,493.24 (404.72 to 4,849.08)	1,054.23 (415.80 to 2,125.79)	822.28 (330.51 to 1,589.72)	1,422.88 (543.62 to 3,174.35)	25.89 (8.54 to 67.47)	31.21 (14.72 to 49.65)	26.77 (12.78 to 41.46)	36.22 (16.37 to 61.50)
**Non-communicable diseases**	**89,562.85 (68,844.78 to 109,286.67)**	**107,422.03 (78,326.97 to 142,834.50)**	**83,167.34 (61,582.38 to 107,891.02)**	**141,100.79 (100,277.42 to 190,226.94)**	**3,431.86 (2,765.51 to 4,091.95)**	**5,705.19 (4,718.03 to 6,795.33)**	**4,683.09 (3,950.77 to 5,477.03)**	**6,846.68 (5,579.67 to 8,263.75)**

Timor-Leste								
Cause	YLLs (actual counts)				Deaths (actual counts)			
	2016	2040 Forecast	2040 Better Scenario	2040 Worse Scenario	2016	2040 Forecast	2040 Better Scenario	2040 Worse Scenario
**Neoplasms**	**16,167.81 (11,558.38 to 20,292.05)**	**23,700.97 (16,039.63 to 32,415.70)**	**22,698.16 (15,683.83 to 30,278.92)**	**26,951.80 (17,624.68 to 38,067.42)**	**587.78 (452.47 to 732.19)**	**1,027.01 (783.05 to 1,295.24)**	**1,031.24 (799.32 to 1,273.27)**	**1,105.18 (801.93 to 1,465.47)**
Lip and oral cavity cancer	360.27 (236.28 to 512.48)	538.86 (287.85 to 943.01)	459.51 (255.06 to 784.25)	736.95 (356.06 to 1,403.34)	15.19 (10.73 to 20.47)	25.55 (15.40 to 39.22)	23.12 (14.65 to 34.61)	32.51 (17.87 to 54.58)
Nasopharynx cancer	278.13 (168.16 to 422.14)	452.81 (213.24 to 887.76)	369.53 (183.16 to 695.02)	656.03 (275.89 to 1,410.04)	9.69 (6.35 to 14.77)	16.68 (8.73 to 30.57)	14.14 (7.57 to 25.14)	23.13 (10.83 to 46.10)
Other pharynx cancer	163.55 (111.61 to 231.52)	218.31 (136.77 to 336.88)	209.29 (131.43 to 316.78)	222.17 (137.92 to 337.99)	6.53 (4.69 to 9.07)	9.67 (6.46 to 14.05)	9.61 (6.47 to 13.94)	9.50 (6.33 to 13.92)
Esophageal cancer	252.27 (173.73 to 345.12)	368.90 (180.94 to 702.59)	310.64 (161.37 to 561.79)	497.14 (224.81 to 1,042.45)	10.82 (7.92 to 14.47)	17.32 (9.56 to 28.88)	15.28 (8.71 to 25.81)	21.95 (11.23 to 39.46)
Stomach cancer	950.78 (678.02 to 1,222.54)	1,085.55 (750.47 to 1,454.39)	1,011.59 (711.79 to 1,360.95)	1,173.55 (807.92 to 1,608.46)	41.88 (31.16 to 53.16)	55.53 (42.46 to 69.96)	54.21 (41.25 to 68.07)	57.12 (42.86 to 73.71)
Colon and rectum cancer	1,148.77 (816.16 to 1,561.95)	2,198.14 (1,379.78 to 3,367.51)	2,239.51 (1,423.79 to 3,385.62)	2,136.98 (1,305.45 to 3,293.85)	49.34 (36.31 to 65.10)	113.89 (76.74 to 166.65)	121.69 (82.74 to 177.49)	105.20 (69.74 to 155.60)
Liver cancer	947.04 (568.41 to 1,284.14)	1,475.83 (835.54 to 2,263.03)	1,432.60 (815.08 to 2,151.92)	1,634.19 (899.41 to 2,625.98)	38.52 (24.48 to 51.14)	67.09 (43.37 to 94.25)	67.73 (44.26 to 93.48)	70.52 (43.45 to 103.25)
Liver cancer due to hepatitis B	414.25 (239.92 to 574.89)	633.89 (338.86 to 990.10)	616.22 (331.54 to 958.77)	704.83 (360.56 to 1,169.49)	15.77 (9.33 to 21.84)	26.55 (15.96 to 39.21)	26.79 (16.32 to 38.79)	28.14 (16.24 to 44.27)
Liver cancer due to hepatitis C	202.98 (119.04 to 308.43)	328.10 (184.63 to 523.32)	325.68 (185.45 to 514.95)	350.82 (190.75 to 581.80)	9.61 (5.80 to 14.66)	17.85 (10.84 to 26.95)	18.51 (11.41 to 27.82)	18.09 (10.63 to 28.42)
Liver cancer due to alcohol use	91.83 (51.49 to 140.08)	138.51 (73.97 to 231.08)	132.71 (70.71 to 216.64)	155.84 (79.13 to 276.82)	3.97 (2.32 to 6.17)	6.32 (3.60 to 10.33)	6.25 (3.62 to 10.09)	6.80 (3.73 to 11.23)
Liver cancer due to other causes	237.98 (129.47 to 365.07)	375.34 (183.21 to 648.58)	357.98 (176.71 to 615.83)	422.70 (196.98 to 788.52)	9.17 (5.16 to 14.00)	16.36 (8.88 to 25.96)	16.18 (9.01 to 25.59)	17.50 (9.18 to 29.31)
Gallbladder and biliary tract cancer	234.97 (164.98 to 303.37)	266.73 (184.04 to 349.07)	283.60 (195.08 to 378.20)	250.26 (175.21 to 322.82)	10.67 (7.84 to 13.48)	13.91 (10.52 to 17.48)	15.39 (11.63 to 19.42)	12.54 (9.62 to 15.79)
Pancreatic cancer	563.60 (415.71 to 712.57)	1,025.31 (726.57 to 1,391.05)	1,061.01 (749.25 to 1,435.17)	1,043.74 (711.53 to 1,447.49)	24.51 (18.99 to 30.53)	49.82 (38.10 to 63.63)	53.60 (41.47 to 67.53)	48.33 (36.21 to 62.69)
Larynx cancer	165.72 (115.84 to 235.07)	215.71 (99.24 to 432.38)	163.61 (81.41 to 321.33)	339.87 (143.63 to 691.96)	7.30 (5.26 to 10.17)	10.26 (5.30 to 19.88)	8.10 (4.48 to 14.85)	15.51 (7.23 to 29.72)
Tracheal, bronchus, and lung cancer	1,887.70 (1,327.51 to 2,507.26)	2,584.68 (1,340.06 to 4,450.54)	1,853.31 (1,054.49 to 3,154.87)	4,578.90 (2,149.63 to 8,387.02)	80.15 (59.78 to 103.92)	119.08 (69.04 to 184.95)	89.69 (56.06 to 134.44)	200.28 (106.10 to 341.55)
Malignant skin melanoma	56.07 (35.48 to 77.91)	91.29 (57.23 to 130.49)	102.89 (65.98 to 148.27)	83.33 (52.30 to 118.16)	2.01 (1.40 to 2.74)	3.97 (2.94 to 5.31)	4.71 (3.52 to 6.27)	3.43 (2.52 to 4.51)
Non-melanoma skin cancer	109.56 (68.72 to 167.75)	198.11 (127.56 to 296.90)	190.71 (123.88 to 286.88)	198.30 (127.21 to 295.57)	4.67 (3.14 to 6.75)	10.48 (7.48 to 14.39)	10.67 (7.72 to 14.54)	9.92 (7.05 to 13.69)
Breast cancer	1,322.57 (760.64 to 1,947.28)	2,141.28 (1,197.75 to 3,391.48)	2,095.73 (1,195.37 to 3,278.62)	2,192.10 (1,212.50 to 3,518.38)	45.08 (27.47 to 64.70)	80.01 (47.93 to 120.77)	80.95 (49.74 to 118.84)	79.31 (46.87 to 123.21)
Cervical cancer	1,001.01 (600.11 to 1,486.62)	1,171.37 (630.15 to 1,926.17)	1,047.27 (590.49 to 1,684.32)	1,294.89 (684.62 to 2,186.14)	36.26 (22.56 to 53.14)	44.69 (27.13 to 67.18)	41.44 (25.53 to 60.69)	47.68 (28.23 to 74.54)

Uterine cancer	222.79 (147.03 to 330.45)	315.34 (206.50 to 469.83)	286.26 (186.71 to 417.01)	331.42 (220.33 to 490.23)	9.29 (6.29 to 13.77)	14.58 (10.41 to 20.68)	13.75 (9.75 to 19.26)	14.82 (10.53 to 21.04)
Ovarian cancer	452.27 (291.55 to 641.89)	673.30 (407.95 to 1,008.88)	725.41 (434.30 to 1,083.01)	633.91 (383.32 to 955.18)	16.14 (10.82 to 22.45)	25.79 (17.16 to 37.29)	28.48 (18.97 to 40.71)	23.79 (15.53 to 34.20)
Prostate cancer	504.45 (311.50 to 713.36)	1,213.00 (769.13 to 1,595.05)	1,356.75 (870.59 to 1,778.05)	1,087.53 (673.07 to 1,438.20)	30.13 (18.66 to 41.86)	93.33 (59.89 to 120.97)	109.29 (70.69 to 139.71)	79.66 (49.86 to 104.65)
Testicular cancer	33.47 (14.10 to 63.47)	47.13 (20.55 to 95.98)	44.28 (19.40 to 89.91)	49.25 (20.98 to 102.07)	0.78 (0.37 to 1.45)	1.26 (0.70 to 2.42)	1.23 (0.69 to 2.37)	1.26 (0.69 to 2.42)

Timor-Leste								
Cause	YLLs (actual counts)				Deaths (actual counts)			
	2016	2040 Forecast	2040 Better Scenario	2040 Worse Scenario	2016	2040 Forecast	2040 Better Scenario	2040 Worse Scenario
Kidney cancer	194.39 (130.36 to 274.50)	325.26 (186.40 to 523.60)	358.31 (206.19 to 569.89)	326.51 (186.14 to 533.38)	6.69 (4.43 to 9.66)	14.03 (8.47 to 21.05)	16.20 (9.89 to 24.33)	13.18 (7.87 to 20.66)
Bladder cancer	213.49 (152.19 to 291.15)	344.83 (227.63 to 498.34)	337.75 (227.73 to 476.49)	371.27 (235.28 to 563.19)	10.74 (8.01 to 14.27)	21.42 (15.13 to 29.34)	22.13 (16.21 to 29.41)	21.66 (14.75 to 31.11)
Brain and nervous system cancer	765.56 (507.41 to 1,005.17)	1,124.49 (697.84 to 1,590.56)	1,152.17 (710.54 to 1,637.26)	1,171.56 (739.18 to 1,626.80)	17.61 (11.52 to 23.38)	31.45 (20.22 to 43.24)	33.58 (21.59 to 45.97)	30.51 (19.79 to 40.91)
Thyroid cancer	135.93 (94.34 to 185.61)	191.12 (128.27 to 270.42)	178.93 (120.88 to 254.09)	199.27 (133.91 to 281.49)	5.71 (4.02 to 7.64)	9.00 (6.53 to 12.09)	8.75 (6.39 to 11.71)	9.03 (6.53 to 12.15)
Mesothelioma	37.28 (20.53 to 56.78)	57.31 (31.15 to 88.96)	57.06 (31.55 to 89.47)	56.99 (30.83 to 87.75)	1.23 (0.75 to 1.83)	2.18 (1.40 to 3.14)	2.26 (1.46 to 3.29)	2.07 (1.32 to 2.99)
Hodgkin lymphoma	136.51 (84.42 to 218.78)	137.66 (80.26 to 241.84)	115.03 (65.97 to 204.12)	159.23 (93.10 to 272.42)	3.56 (2.28 to 5.94)	4.11 (2.53 to 6.99)	3.54 (2.18 to 6.04)	4.52 (2.81 to 7.62)
Non-Hodgkin lymphoma	782.85 (517.82 to 1,035.80)	1,138.96 (695.88 to 1,570.64)	1,198.82 (735.35 to 1,654.15)	1,133.77 (712.19 to 1,544.92)	22.60 (15.28 to 29.56)	40.01 (27.00 to 51.87)	44.03 (29.94 to 57.17)	37.36 (25.32 to 48.61)
Multiple myeloma	112.17 (75.00 to 159.62)	200.51 (130.45 to 297.67)	218.31 (142.25 to 324.10)	187.85 (121.82 to 278.09)	4.50 (3.14 to 6.24)	8.88 (6.30 to 12.42)	10.00 (7.07 to 13.98)	8.04 (5.64 to 11.22)
Leukemia	1,638.35 (1,157.09 to 2,147.48)	1,776.36 (1,152.20 to 2,457.67)	1,696.21 (1,107.08 to 2,346.08)	2,017.87 (1,305.49 to 2,805.56)	35.35 (25.65 to 44.72)	50.28 (34.95 to 65.75)	50.27 (34.75 to 65.95)	52.95 (36.55 to 70.51)
Acute lymphoid leukemia	442.42 (302.33 to 640.14)	483.09 (313.10 to 705.88)	490.52 (312.87 to 724.98)	530.65 (342.16 to 782.34)	9.38 (6.51 to 13.13)	13.33 (8.80 to 19.13)	14.16 (9.36 to 20.43)	13.56 (9.01 to 19.17)
Chronic lymphoid leukemia	20.43 (11.98 to 29.45)	28.94 (16.95 to 45.17)	28.88 (17.01 to 45.10)	29.79 (16.86 to 46.59)	0.66 (0.43 to 0.93)	1.07 (0.70 to 1.54)	1.12 (0.74 to 1.61)	1.05 (0.68 to 1.53)
Acute myeloid leukemia	232.90 (155.28 to 328.12)	323.66 (201.12 to 475.30)	338.44 (210.60 to 499.80)	343.37 (213.43 to 502.67)	4.77 (3.22 to 6.62)	8.22 (5.35 to 11.91)	8.92 (5.81 to 12.95)	8.22 (5.29 to 11.96)
Chronic myeloid leukemia	53.56 (27.84 to 83.13)	56.78 (28.30 to 93.46)	55.56 (27.86 to 91.93)	60.08 (29.16 to 101.95)	1.53 (0.88 to 2.35)	1.82 (1.04 to 2.88)	1.84 (1.06 to 2.89)	1.85 (1.02 to 3.02)
Other leukemia	889.05 (615.42 to 1,181.57)	883.89 (563.47 to 1,234.25)	782.80 (494.23 to 1,122.05)	1,053.98 (674.37 to 1,486.30)	19.02 (13.36 to 24.44)	25.84 (16.95 to 35.12)	24.22 (15.83 to 32.86)	28.27 (18.51 to 38.80)
Other neoplasms	1,496.30 (1,002.56 to 1,983.81)	2,122.82 (1,343.70 to 2,903.97)	2,142.05 (1,351.02 to 2,943.63)	2,186.99 (1,389.08 to 2,963.12)	40.83 (27.38 to 54.39)	72.77 (48.17 to 95.15)	77.37 (51.35 to 101.84)	69.41 (46.13 to 89.65)
**Cardiovascular diseases**	**34,519.28 (25,787.26 to 42,816.19)**	**39,882.31 (26,022.83 to 59,979.52)**	**23,527.00 (15,744.41 to 34,740.75)**	**60,063.84 (36,877.56 to 89,550.62)**	**1,615.07 (1,283.72 to 1,961.04)**	**2,477.55 (1,765.64 to 3,372.98)**	**1,633.28 (1,209.17 to 2,204.79)**	**3,344.48 (2,337.04 to 4,560.63)**
Rheumatic heart disease	1,136.89 (724.56 to 1,581.59)	756.68 (452.11 to 1,128.17)	597.88 (353.10 to 873.72)	912.74 (548.15 to 1,362.42)	32.15 (20.50 to 45.27)	28.78 (18.42 to 40.15)	24.37 (15.35 to 33.73)	31.88 (20.38 to 45.62)
Ischemic heart disease	16,504.39 (11,877.92 to 20,848.52)	19,528.79 (10,524.79 to 34,587.08)	10,064.02 (5,400.65 to 17,569.80)	31,288.64 (15,997.02 to 54,865.58)	805.52 (615.22 to 998.23)	1,241.87 (722.50 to 2,055.01)	732.59 (428.90 to 1,196.37)	1,766.77 (1,029.39 to 2,897.60)
Stroke	11,952.81 (9,006.00 to 15,398.27)	12,427.57 (7,061.15 to 21,481.76)	6,752.53 (3,959.08 to 12,312.96)	19,520.65 (10,864.71 to 33,527.53)	570.17 (447.16 to 717.97)	785.60 (492.40 to 1,358.11)	489.42 (314.82 to 853.82)	1,093.80 (674.77 to 1,840.80)
Ischemic stroke	2,858.03 (2,188.88 to 3,624.90)	3,408.49 (1,837.67 to 8,091.94)	2,142.90 (1,207.96 to 4,893.86)	4,638.90 (2,505.19 to 10,584.12)	178.51 (139.71 to 224.70)	288.39 (162.72 to 700.02)	202.25 (117.79 to 450.30)	356.46 (199.10 to 844.72)

Intracerebral hemorrhage	9,094.79 (6,585.48 to 12,011.16)	9,019.08 (4,674.58 to 17,317.11)	4,609.63 (2,489.92 to 8,458.36)	14,881.75 (7,421.65 to 27,579.93)	391.66 (298.90 to 509.14)	497.21 (288.23 to 844.37)	287.17 (172.67 to 460.93)	737.34 (419.15 to 1,268.18)
Hypertensive heart disease	1,802.78 (1,210.52 to 2,491.42)	2,727.71 (1,722.95 to 4,356.62)	2,076.54 (1,386.75 to 3,120.31)	3,424.37 (2,021.96 to 6,007.31)	92.07 (64.77 to 122.15)	182.94 (119.74 to 292.06)	149.12 (102.22 to 218.84)	213.95 (130.64 to 386.11)
Cardiomyopathy and myocarditis	825.10 (519.60 to 1,116.99)	986.29 (536.23 to 1,529.60)	962.40 (534.77 to 1,478.99)	1,035.59 (574.30 to 1,594.04)	25.09 (15.95 to 34.30)	42.87 (24.48 to 63.37)	45.31 (26.54 to 65.87)	40.50 (23.20 to 60.34)
Myocarditis	202.80 (128.98 to 289.81)	169.82 (91.48 to 293.62)	155.34 (82.56 to 273.97)	196.87 (108.80 to 325.46)	4.22 (2.55 to 6.32)	5.24 (2.89 to 9.10)	5.21 (2.88 to 9.06)	5.35 (3.07 to 9.02)

Timor-Leste								
Cause	YLLs (actual counts)				Deaths (actual counts)			
	2016	2040 Forecast	2040 Better Scenario	2040 Worse Scenario	2016	2040 Forecast	2040 Better Scenario	2040 Worse Scenario
Alcoholic cardiomyopathy	77.72 (35.06 to 135.38)	103.73 (43.01 to 196.66)	109.19 (44.44 to 200.02)	99.90 (40.89 to 190.78)	2.89 (1.53 to 4.89)	4.36 (2.15 to 7.44)	4.80 (2.35 to 8.14)	4.00 (2.00 to 6.83)
Other cardiomyopathy	544.59 (329.99 to 739.10)	712.75 (389.31 to 1,123.82)	697.88 (387.78 to 1,085.09)	738.82 (399.20 to 1,160.41)	17.99 (11.40 to 24.10)	33.27 (18.88 to 50.45)	35.29 (20.24 to 52.93)	31.15 (17.56 to 47.90)
Atrial fibrillation and flutter	192.94 (134.83 to 261.82)	432.47 (308.87 to 565.50)	457.91 (331.80 to 594.04)	411.63 (291.25 to 547.14)	15.02 (10.66 to 20.29)	46.43 (30.70 to 63.94)	52.42 (36.02 to 70.61)	41.46 (26.32 to 58.40)
Aortic aneurysm	191.54 (128.89 to 292.37)	269.99 (181.82 to 410.78)	269.00 (184.36 to 394.42)	276.84 (181.39 to 430.71)	9.64 (6.86 to 13.91)	17.39 (12.27 to 24.94)	18.62 (13.53 to 26.35)	16.55 (11.57 to 24.39)
Peripheral artery disease	6.71 (3.89 to 10.69)	21.90 (12.81 to 35.37)	23.81 (14.28 to 37.24)	20.14 (11.67 to 32.05)	0.43 (0.25 to 0.69)	2.05 (1.18 to 3.32)	2.46 (1.44 to 4.03)	1.73 (0.94 to 2.77)
Endocarditis	539.80 (337.61 to 767.67)	783.04 (427.07 to 1,270.36)	719.65 (394.59 to 1,160.40)	839.31 (465.75 to 1,354.78)	15.08 (10.16 to 20.96)	27.61 (18.00 to 41.01)	27.10 (17.82 to 40.65)	27.44 (17.60 to 40.93)
Other cardiovascular and circulatory diseases	1,366.31 (918.99 to 2,274.62)	1,947.87 (1,230.20 to 3,146.65)	1,603.25 (1,018.29 to 2,605.61)	2,333.93 (1,424.17 to 3,811.87)	49.89 (34.15 to 83.78)	102.01 (67.81 to 170.38)	91.86 (61.26 to 154.18)	110.40 (71.88 to 184.01)
**Chronic respiratory diseases**	**6,858.53 (5,129.67 to 8,640.53)**	**5,113.05 (3,414.28 to 7,093.99)**	**3,798.13 (2,670.27 to 5,074.42)**	**7,863.86 (4,914.26 to 11,763.76)**	**322.95 (249.77 to 402.81)**	**317.82 (231.45 to 425.47)**	**250.83 (191.22 to 317.81)**	**458.77 (309.92 to 660.76)**
Chronic obstructive pulmonary disease	3,403.98 (2,239.82 to 4,456.76)	2,953.64 (1,729.22 to 4,605.06)	1,949.46 (1,188.76 to 2,866.97)	5,307.06 (2,875.52 to 8,784.76)	186.26 (129.53 to 239.38)	205.21 (129.94 to 309.21)	147.23 (99.73 to 205.23)	336.29 (200.53 to 530.81)
Pneumoconiosis	4.29 (2.67 to 6.53)	4.46 (2.97 to 6.33)	4.39 (2.88 to 6.28)	4.46 (3.04 to 6.38)	0.22 (0.14 to 0.32)	0.32 (0.21 to 0.45)	0.33 (0.22 to 0.47)	0.30 (0.20 to 0.43)
Silicosis	0.83 (0.39 to 1.51)	0.65 (0.33 to 1.12)	0.63 (0.31 to 1.12)	0.64 (0.33 to 1.09)	0.04 (0.02 to 0.08)	0.05 (0.02 to 0.08)	0.05 (0.02 to 0.08)	0.04 (0.02 to 0.07)
Asbestosis	1.03 (0.58 to 1.62)	1.28 (0.75 to 1.95)	1.32 (0.74 to 2.04)	1.25 (0.75 to 1.89)	0.04 (0.02 to 0.06)	0.07 (0.04 to 0.11)	0.07 (0.04 to 0.12)	0.06 (0.04 to 0.10)
Coal workers pneumoconiosis	0.28 (0.13 to 0.65)	0.27 (0.14 to 0.53)	0.21 (0.11 to 0.43)	0.31 (0.16 to 0.61)	0.02 (0.01 to 0.04)	0.02 (0.01 to 0.04)	0.02 (0.01 to 0.04)	0.02 (0.01 to 0.05)
Other pneumoconiosis	2.15 (1.28 to 3.45)	2.25 (1.43 to 3.45)	2.23 (1.40 to 3.37)	2.26 (1.43 to 3.53)	0.12 (0.07 to 0.18)	0.18 (0.11 to 0.29)	0.20 (0.12 to 0.31)	0.17 (0.10 to 0.27)
Asthma	3,157.96 (2,138.30 to 4,514.92)	1,789.27 (1,107.52 to 2,740.66)	1,461.39 (934.27 to 2,162.12)	2,179.62 (1,321.79 to 3,492.35)	126.31 (86.19 to 193.49)	95.04 (61.27 to 143.26)	83.47 (56.14 to 123.15)	106.41 (66.86 to 171.14)
Interstitial lung disease and pulmonary sarcoidosis	115.47 (62.55 to 193.92)	176.63 (98.98 to 288.92)	202.68 (115.94 to 325.51)	158.92 (87.19 to 258.77)	5.29 (2.92 to 9.07)	9.69 (5.68 to 15.90)	11.66 (6.99 to 18.78)	8.30 (4.63 to 13.64)
Other chronic respiratory diseases	176.82 (96.25 to 294.29)	189.05 (100.70 to 319.45)	180.21 (95.74 to 302.52)	213.80 (111.60 to 365.80)	4.87 (2.47 to 8.56)	7.57 (3.61 to 14.09)	8.14 (3.83 to 15.02)	7.47 (3.67 to 13.55)
**Cirrhosis and other chronic liver diseases**	**3,132.34 (1,931.51 to 4,592.30)**	**4,428.28 (2,631.37 to 6,872.10)**	**4,127.08 (2,417.76 to 6,356.77)**	**4,625.11 (2,747.19 to 7,311.55)**	**102.06 (69.99 to 145.62)**	**163.07 (114.67 to 234.34)**	**157.80 (110.83 to 223.25)**	**163.20 (113.51 to 236.53)**
Cirrhosis and other chronic liver diseases due to hepatitis B	1,004.54 (560.90 to 1,590.96)	1,538.52 (860.14 to 2,494.83)	1,454.64 (798.72 to 2,335.11)	1,575.24 (867.67 to 2,617.34)	34.73 (22.64 to 52.91)	57.79 (37.92 to 86.22)	56.47 (37.48 to 83.73)	57.16 (37.30 to 87.17)
Cirrhosis and other chronic liver diseases due to hepatitis C	619.94 (346.69 to 970.15)	954.92 (532.47 to 1,576.21)	896.51 (498.66 to 1,471.71)	983.32 (543.35 to 1,615.02)	21.03 (13.43 to 31.97)	34.70 (22.30 to 53.36)	33.57 (21.34 to 51.09)	34.66 (21.96 to 53.47)

Cirrhosis and other chronic liver diseases due to alcohol use	516.48 (297.09 to 807.01)	740.23 (404.66 to 1,180.76)	674.19 (378.66 to 1,078.72)	780.03 (430.64 to 1,265.44)	18.50 (11.72 to 27.88)	29.10 (19.34 to 42.43)	27.49 (18.33 to 39.99)	29.56 (19.44 to 44.25)
Cirrhosis and other chronic liver diseases due to other causes	991.38 (671.95 to 1,336.12)	1,194.62 (778.58 to 1,731.67)	1,101.74 (720.43 to 1,580.25)	1,286.53 (839.45 to 1,852.22)	27.80 (19.92 to 37.58)	41.48 (29.69 to 56.74)	40.27 (29.01 to 54.05)	41.82 (29.90 to 56.89)
**Digestive diseases**	**4,567.33 (2,946.18 to 6,371.27)**	**4,502.76 (2,719.98 to 6,751.92)**	**3,941.46 (2,423.40 to 5,922.17)**	**5,106.69 (3,109.03 to 7,670.76)**	**152.56 (101.29 to 217.06)**	**214.10 (143.46 to 312.18)**	**201.40 (136.75 to 287.69)**	**220.32 (146.43 to 321.75)**
Peptic ulcer disease	1,168.77 (748.41 to 1,687.53)	952.18 (600.92 to 1,398.03)	748.04 (484.02 to 1,070.80)	1,129.63 (708.90 to 1,685.32)	50.96 (34.69 to 72.59)	54.64 (36.76 to 77.58)	45.75 (30.46 to 64.95)	60.71 (40.17 to 85.67)

Timor-Leste								
**Cause**	**YLLs (actual counts)**				**Deaths (actual counts)**			
	**2016**	**2040 Forecast**	**2040 Better Scenario**	**2040 Worse Scenario**	**2016**	**2040 Forecast**	**2040 Better Scenario**	**2040 Worse Scenario**
Gastritis and duodenitis	76.41 (38.60 to 139.34)	118.85 (54.93 to 227.83)	109.84 (50.74 to 210.95)	123.29 (57.21 to 234.21)	3.10 (1.61 to 5.62)	6.30 (3.25 to 11.04)	6.17 (3.17 to 10.77)	6.13 (3.13 to 10.89)
Appendicitis	342.51 (219.71 to 477.81)	269.87 (166.26 to 415.80)	223.89 (132.00 to 345.52)	321.86 (190.78 to 492.11)	7.89 (5.32 to 11.33)	8.70 (5.87 to 13.01)	7.74 (5.21 to 11.60)	9.41 (6.30 to 14.14)
Paralytic ileus and intestinal obstruction	1,546.76 (931.57 to 2,714.84)	1,316.42 (836.28 to 1,952.88)	1,230.86 (797.08 to 1,785.43)	1,509.97 (927.07 to 2,448.87)	39.56 (25.88 to 55.69)	58.62 (37.87 to 77.47)	60.39 (39.84 to 78.90)	57.14 (37.16 to 76.31)
Inguinal, femoral, and abdominal hernia	95.60 (45.90 to 147.07)	96.76 (44.68 to 151.35)	89.56 (41.56 to 138.70)	101.09 (46.46 to 158.95)	4.20 (1.96 to 6.54)	6.13 (2.79 to 9.28)	6.16 (2.79 to 9.27)	5.83 (2.63 to 9.02)
Inflammatory bowel disease	85.67 (50.42 to 122.73)	107.59 (63.39 to 156.55)	107.90 (63.45 to 156.28)	107.17 (63.69 to 154.90)	2.94 (1.86 to 4.30)	5.02 (3.19 to 7.14)	5.36 (3.46 to 7.58)	4.64 (3.00 to 6.55)
Vascular intestinal disorders	88.40 (51.04 to 147.35)	128.84 (77.25 to 212.38)	126.45 (76.66 to 206.55)	126.85 (76.34 to 212.01)	4.53 (2.64 to 7.62)	8.86 (5.43 to 14.16)	9.26 (5.78 to 14.70)	8.19 (4.93 to 13.37)
Gallbladder and biliary diseases	195.49 (106.50 to 314.20)	208.02 (103.93 to 378.09)	172.56 (87.53 to 302.42)	240.45 (115.30 to 440.04)	8.72 (4.78 to 13.96)	12.45 (6.27 to 21.77)	11.18 (5.89 to 18.91)	13.25 (6.59 to 24.01)
Pancreatitis	485.04 (180.14 to 1,012.94)	745.03 (244.03 to 1,750.75)	641.99 (220.55 to 1,431.23)	829.57 (261.42 to 2,030.64)	14.65 (6.09 to 29.60)	25.19 (9.76 to 55.50)	22.67 (9.01 to 47.89)	26.82 (9.97 to 59.75)
Other digestive diseases	482.70 (281.75 to 781.10)	559.20 (322.18 to 998.54)	490.39 (285.61 to 871.66)	616.81 (353.16 to 1,084.92)	16.00 (9.50 to 28.12)	28.20 (17.30 to 49.31)	26.74 (16.62 to 46.52)	28.20 (16.98 to 49.31)
**Neurological disorders**	**3,412.39 (2,670.07 to 4,222.60)**	**6,650.47 (5,263.64 to 8,164.86)**	**7,059.10 (5,732.57 to 8,532.58)**	**6,331.63 (4,967.80 to 7,960.53)**	**203.19 (160.94 to 251.99)**	**614.46 (434.60 to 797.26)**	**706.87 (529.84 to 876.60)**	**533.22 (365.30 to 723.99)**
Alzheimer's disease and other dementias	2,214.65 (1,681.41 to 2,851.75)	5,056.31 (3,772.34 to 6,556.17)	5,483.16 (4,246.55 to 6,809.20)	4,616.55 (3,357.81 to 6,160.53)	171.88 (133.78 to 218.90)	548.85 (373.20 to 730.02)	632.11 (454.52 to 800.68)	473.10 (309.15 to 659.73)
Parkinson's disease	196.78 (137.56 to 270.63)	495.83 (365.93 to 659.62)	600.53 (444.99 to 794.28)	416.92 (304.87 to 558.91)	12.98 (9.19 to 17.90)	40.96 (29.01 to 56.11)	51.83 (38.30 to 69.36)	32.98 (22.65 to 45.50)
Epilepsy	763.56 (510.77 to 1,021.27)	778.24 (454.13 to 1,197.93)	641.76 (370.71 to 1,004.56)	960.89 (565.47 to 1,464.00)	13.43 (8.74 to 18.07)	16.47 (9.91 to 25.40)	14.03 (8.40 to 21.74)	19.17 (11.68 to 29.24)
Multiple sclerosis	18.21 (9.71 to 28.21)	28.72 (14.88 to 45.23)	29.48 (15.44 to 46.89)	28.49 (14.92 to 45.05)	0.53 (0.31 to 0.82)	0.88 (0.52 to 1.36)	0.92 (0.54 to 1.42)	0.86 (0.50 to 1.33)
Motor neuron disease	34.05 (21.60 to 49.92)	42.85 (25.80 to 63.57)	45.44 (27.53 to 68.08)	43.12 (26.33 to 62.70)	0.86 (0.57 to 1.19)	1.38 (0.87 to 2.09)	1.51 (0.94 to 2.29)	1.31 (0.84 to 1.95)
Other neurological disorders	185.14 (127.68 to 262.07)	248.50 (157.27 to 376.43)	258.73 (159.54 to 391.92)	265.66 (169.87 to 395.90)	3.52 (2.46 to 4.94)	5.91 (3.82 to 8.59)	6.47 (4.19 to 9.39)	5.80 (3.88 to 8.40)
**Mental disorders**	**564.61 (234.63 to 1,051.70)**	**993.12 (401.63 to 1,803.01)**	**981.85 (397.34 to 1,801.29)**	**1,006.47 (409.59 to 1,818.30)**	**13.24 (5.88 to 23.70)**	**24.32 (11.90 to 42.87)**	**24.52 (12.19 to 42.95)**	**24.06 (11.65 to 42.18)**
Alcohol use disorders	343.74 (132.97 to 717.53)	478.24 (187.45 to 1,058.52)	465.10 (183.09 to 1,038.29)	486.79 (188.13 to 1,065.22)	8.81 (3.86 to 17.60)	13.07 (6.12 to 26.67)	12.96 (6.05 to 26.98)	13.00 (6.08 to 26.27)
Drug use disorders	218.61 (86.87 to 405.30)	511.72 (193.77 to 956.60)	513.54 (193.36 to 942.53)	516.44 (199.01 to 951.41)	4.38 (1.87 to 7.93)	11.19 (5.35 to 19.52)	11.50 (5.63 to 20.11)	11.00 (5.23 to 19.28)
Opioid use disorders	147.97 (62.33 to 281.75)	291.40 (116.12 to 566.05)	289.22 (114.53 to 555.36)	297.12 (120.24 to 570.33)	2.86 (1.26 to 5.34)	6.16 (2.95 to 11.30)	6.27 (3.05 to 11.51)	6.11 (2.92 to 11.22)

Cocaine use disorders	6.41 (2.38 to 13.86)	19.84 (7.45 to 44.31)	18.52 (6.96 to 41.86)	20.89 (7.80 to 47.04)	0.14 (0.06 to 0.30)	0.46 (0.20 to 1.01)	0.44 (0.19 to 0.97)	0.48 (0.20 to 1.03)
Amphetamine use disorders	5.59 (2.03 to 11.90)	23.00 (7.38 to 51.81)	20.57 (6.67 to 45.79)	25.03 (7.99 to 56.27)	0.11 (0.04 to 0.24)	0.48 (0.17 to 1.05)	0.43 (0.16 to 0.94)	0.51 (0.18 to 1.13)
Other drug use disorders	58.63 (20.52 to 110.21)	177.48 (58.93 to 328.80)	185.23 (61.73 to 344.71)	173.39 (57.40 to 324.01)	1.27 (0.52 to 2.34)	4.08 (1.73 to 7.20)	4.36 (1.90 to 7.64)	3.89 (1.62 to 6.90)
Eating disorders	2.26 (1.12 to 3.96)	3.15 (1.47 to 5.69)	3.21 (1.49 to 5.86)	3.23 (1.56 to 5.82)	0.04 (0.02 to 0.07)	0.06 (0.03 to 0.11)	0.06 (0.03 to 0.11)	0.06 (0.03 to 0.11)

Timor-Leste								
Cause	YLLs (actual counts)				Deaths (actual counts)			
	2016	2040 Forecast	2040 Better Scenario	2040 Worse Scenario	2016	2040 Forecast	2040 Better Scenario	2040 Worse Scenario
**Diabetes, urogenital, blood, and endocrine diseases**	**7,088.44 (5,244.12 to 8,957.34)**	**13,140.78 (7,404.32 to 26,532.17)**	**9,582.91 (5,577.46 to 18,376.15)**	**15,546.67 (8,957.05 to 31,264.87)**	**269.02 (205.43 to 336.73)**	**725.83 (397.85 to 1,612.24)**	**550.91 (315.41 to 1,261.01)**	**806.02 (448.16 to 1,752.18)**
Diabetes mellitus	2,902.32 (2,085.31 to 3,835.94)	4,179.75 (2,502.12 to 6,401.12)	3,163.07 (1,900.06 to 4,866.41)	5,109.18 (3,022.70 to 8,037.76)	129.81 (96.38 to 170.45)	233.69 (151.91 to 339.50)	187.32 (122.95 to 268.35)	268.96 (173.27 to 390.13)
Acute glomerulonephritis	9.72 (6.83 to 13.08)	11.56 (7.41 to 16.46)	10.15 (6.43 to 14.30)	13.73 (8.78 to 19.53)	0.20 (0.14 to 0.26)	0.30 (0.21 to 0.42)	0.28 (0.19 to 0.39)	0.32 (0.22 to 0.45)
Chronic kidney disease	2,699.49 (1,856.21 to 3,633.94)	7,266.71 (2,525.80 to 20,774.06)	4,834.98 (1,790.59 to 13,849.39)	8,566.96 (2,949.69 to 24,604.67)	97.97 (71.17 to 130.50)	418.89 (114.11 to 1,331.95)	290.23 (83.51 to 1,013.15)	465.02 (124.11 to 1,420.07)
Chronic kidney disease due to diabetes mellitus	1,377.80 (905.63 to 1,914.76)	4,185.44 (1,371.15 to 12,796.10)	2,747.40 (969.17 to 8,232.10)	4,928.94 (1,577.46 to 14,354.21)	52.99 (36.88 to 71.51)	217.86 (57.97 to 700.44)	147.11 (42.36 to 501.74)	246.49 (65.38 to 750.91)
Chronic kidney disease due to hypertension	608.29 (420.69 to 842.96)	2,137.71 (613.68 to 6,857.17)	1,431.09 (433.78 to 4,738.54)	2,437.48 (681.17 to 7,575.32)	30.32 (22.12 to 41.68)	163.69 (38.33 to 564.76)	117.16 (30.26 to 425.30)	175.43 (41.62 to 585.65)
Chronic kidney disease due to glomerulonephritis	362.84 (240.08 to 517.36)	449.68 (235.30 to 861.13)	316.56 (166.07 to 588.09)	574.81 (307.55 to 1,055.30)	6.99 (4.68 to 9.84)	14.80 (5.75 to 40.03)	10.22 (4.11 to 29.63)	17.49 (7.02 to 44.11)
Chronic kidney disease due to other and unspecified causes	350.56 (236.24 to 487.96)	493.88 (224.49 to 1,129.10)	339.94 (164.78 to 744.96)	625.73 (299.21 to 1,333.66)	7.67 (5.54 to 10.67)	22.53 (7.32 to 66.81)	15.75 (5.29 to 49.04)	25.61 (8.64 to 75.09)
Urinary diseases and male infertility	467.18 (346.15 to 587.17)	669.23 (488.23 to 845.69)	652.02 (476.46 to 837.48)	692.25 (505.89 to 879.87)	17.11 (13.23 to 21.44)	37.02 (28.80 to 44.94)	39.14 (30.95 to 47.78)	34.32 (26.79 to 41.69)
Urinary tract infections	321.38 (217.37 to 424.54)	492.46 (348.03 to 643.88)	493.06 (346.83 to 650.56)	501.82 (352.00 to 656.56)	11.46 (7.93 to 14.88)	26.44 (19.32 to 33.34)	28.80 (21.47 to 36.21)	24.11 (17.57 to 30.44)
Urolithiasis	16.98 (11.07 to 25.67)	30.54 (19.32 to 47.42)	30.25 (18.91 to 47.29)	30.63 (19.21 to 47.20)	0.71 (0.46 to 1.08)	1.58 (1.04 to 2.40)	1.67 (1.11 to 2.52)	1.47 (0.97 to 2.20)
Other urinary diseases	128.82 (82.64 to 213.71)	146.22 (87.15 to 249.65)	128.71 (76.37 to 223.28)	159.80 (97.87 to 274.85)	4.94 (3.30 to 8.07)	9.01 (5.49 to 14.78)	8.67 (5.27 to 14.39)	8.74 (5.33 to 14.49)
Gynecological diseases	23.16 (8.35 to 38.17)	23.57 (8.47 to 41.23)	19.59 (6.88 to 34.36)	26.70 (9.56 to 47.34)	0.69 (0.29 to 1.09)	0.73 (0.31 to 1.18)	0.64 (0.26 to 1.01)	0.79 (0.34 to 1.31)
Uterine fibroids	3.98 (1.23 to 8.54)	3.50 (1.02 to 8.35)	2.80 (0.75 to 6.65)	4.04 (1.17 to 9.55)	0.14 (0.05 to 0.30)	0.13 (0.04 to 0.31)	0.11 (0.03 to 0.25)	0.14 (0.04 to 0.34)
Polycystic ovarian syndrome	1.28 (0.26 to 3.97)	0.69 (0.10 to 2.35)	0.48 (0.07 to 1.63)	0.86 (0.14 to 2.89)	0.03 (0.01 to 0.09)	0.02 (0.00 to 0.05)	0.01 (0.00 to 0.04)	0.02 (0.00 to 0.07)
Endometriosis	0.34 (0.07 to 1.15)	0.47 (0.08 to 1.59)	0.38 (0.06 to 1.20)	0.54 (0.09 to 1.79)	0.01 (0.00 to 0.03)	0.01 (0.00 to 0.04)	0.01 (0.00 to 0.03)	0.01 (0.00 to 0.04)
Genital prolapse	2.18 (0.48 to 4.86)	1.48 (0.32 to 3.58)	1.18 (0.25 to 2.84)	1.68 (0.37 to 4.01)	0.11 (0.03 to 0.24)	0.08 (0.02 to 0.19)	0.07 (0.01 to 0.16)	0.09 (0.02 to 0.20)
Other gynecological diseases	15.38 (5.61 to 24.68)	17.43 (6.02 to 31.14)	14.75 (5.00 to 26.30)	19.57 (6.80 to 35.49)	0.39 (0.16 to 0.64)	0.49 (0.20 to 0.80)	0.44 (0.18 to 0.70)	0.53 (0.21 to 0.86)
Hemoglobinopathies and hemolytic anemias	646.04 (463.80 to 872.07)	603.04 (416.98 to 839.84)	552.65 (381.98 to 769.21)	678.23 (466.47 to 940.35)	15.77 (11.55 to 21.50)	22.30 (16.86 to 30.95)	20.60 (15.24 to 28.22)	23.32 (17.33 to 31.74)
Thalassemias	329.25 (214.33 to 484.80)	174.80 (99.96 to 296.24)	187.62 (106.93 to 315.84)	206.96 (116.82 to 355.56)	4.13 (2.71 to 6.00)	2.29 (1.31 to 3.88)	2.46 (1.41 to 4.09)	2.67 (1.51 to 4.57)

Sickle cell disorders	1.02 (0.47 to 2.11)	0.77 (0.37 to 1.43)	0.67 (0.33 to 1.24)	0.95 (0.46 to 1.83)	0.01 (0.01 to 0.03)	0.01 (0.01 to 0.02)	0.01 (0.01 to 0.02)	0.02 (0.01 to 0.03)
G6PD deficiency	177.99 (101.18 to 267.47)	279.24 (162.24 to 433.89)	231.24 (131.83 to 360.88)	316.57 (183.17 to 487.37)	4.54 (2.81 to 6.74)	8.75 (5.70 to 13.10)	7.39 (4.70 to 11.14)	9.63 (6.28 to 14.54)
Other hemoglobinopathies and hemolytic anemias	137.77 (95.51 to 196.01)	148.24 (104.25 to 211.62)	133.11 (92.06 to 189.86)	153.74 (108.60 to 216.34)	7.08 (5.21 to 9.98)	11.25 (8.07 to 15.90)	10.73 (7.62 to 14.97)	11.00 (7.76 to 15.35)
Endocrine, metabolic, blood, and immune disorders	340.53 (231.13 to 506.88)	386.93 (250.85 to 581.41)	350.45 (225.85 to 533.18)	459.62 (297.32 to 691.45)	7.48 (5.15 to 11.02)	12.89 (8.78 to 19.20)	12.69 (8.64 to 19.10)	13.27 (9.09 to 19.74)

Timor-Leste								
Cause	YLLs (actual counts)				Deaths (actual counts)			
	2016	2040 Forecast	2040 Better Scenario	2040 Worse Scenario	2016	2040 Forecast	2040 Better Scenario	2040 Worse Scenario
**Musculoskeletal disorders**	**170.85 (103.69 to 252.71)**	**285.15 (166.77 to 446.32)**	**299.05 (174.24 to 470.86)**	**283.60 (167.73 to 441.99)**	**4.44 (2.84 to 6.78)**	**9.66 (6.24 to 15.49)**	**10.58 (6.88 to 17.28)**	**9.05 (5.87 to 14.23)**
Rheumatoid arthritis	24.54 (14.19 to 37.72)	44.58 (23.59 to 73.88)	39.63 (20.37 to 65.49)	48.14 (25.99 to 79.23)	1.05 (0.59 to 1.68)	2.43 (1.28 to 3.92)	2.28 (1.16 to 3.78)	2.48 (1.33 to 4.02)
Other musculoskeletal disorders	146.31 (84.82 to 221.86)	240.58 (137.37 to 391.00)	259.42 (148.41 to 421.81)	235.46 (136.12 to 375.03)	3.38 (2.10 to 5.39)	7.23 (4.54 to 12.09)	8.30 (5.25 to 13.93)	6.57 (4.13 to 10.91)
**Other non-communicable diseases**	**13,081.28 (7,133.58 to 22,203.12)**	**8,725.14 (5,015.20 to 15,045.38)**	**7,152.60 (4,202.76 to 12,117.57)**	**13,321.12 (7,257.17 to 23,478.31)**	**161.55 (91.66 to 268.41)**	**131.37 (86.43 to 206.31)**	**115.66 (78.04 to 176.11)**	**182.38 (110.84 to 302.47)**
Congenital birth defects	12,103.27 (6,510.86 to 20,855.46)	7,758.54 (4,163.23 to 13,803.96)	6,241.61 (3,428.16 to 10,985.38)	12,150.13 (6,329.31 to 21,985.15)	143.93 (78.28 to 245.66)	94.73 (52.95 to 164.60)	76.60 (43.57 to 131.41)	146.33 (78.10 to 259.75)
Neural tube defects	1,166.30 (487.35 to 2,293.24)	359.14 (124.01 to 843.62)	252.37 (88.51 to 578.29)	630.13 (222.08 to 1,453.05)	13.54 (5.67 to 26.54)	4.18 (1.46 to 9.78)	2.94 (1.04 to 6.71)	7.32 (2.59 to 16.84)
Congenital heart anomalies	6,686.54 (3,670.85 to 11,114.06)	4,075.16 (2,227.40 to 7,138.91)	3,314.51 (1,797.33 to 5,703.93)	6,253.79 (3,304.85 to 11,231.70)	80.02 (44.86 to 131.25)	50.36 (27.84 to 85.97)	41.18 (22.93 to 69.43)	76.10 (40.88 to 133.47)
Orofacial clefts	18.31 (4.79 to 48.73)	10.82 (2.53 to 32.52)	7.18 (1.70 to 21.19)	19.75 (4.49 to 56.83)	0.21 (0.06 to 0.56)	0.13 (0.03 to 0.38)	0.08 (0.02 to 0.24)	0.23 (0.05 to 0.66)
Down syndrome	235.32 (88.16 to 710.00)	223.99 (89.04 to 628.92)	172.16 (70.79 to 483.74)	351.03 (131.29 to 1,021.76)	2.92 (1.18 to 8.50)	2.97 (1.31 to 7.70)	2.32 (1.05 to 6.13)	4.50 (1.86 to 12.60)
Other chromosomal abnormalities	279.09 (92.10 to 777.98)	358.07 (114.14 to 983.51)	280.06 (89.74 to 752.27)	586.22 (188.36 to 1,581.02)	3.25 (1.08 to 9.04)	4.20 (1.36 to 11.43)	3.29 (1.07 to 8.75)	6.84 (2.22 to 18.39)
Congenital musculoskeletal and limb anomalies	211.42 (81.37 to 447.48)	182.28 (64.57 to 387.18)	152.83 (54.84 to 322.40)	282.96 (99.61 to 604.78)	2.47 (0.96 to 5.21)	2.14 (0.78 to 4.52)	1.80 (0.66 to 3.75)	3.31 (1.18 to 7.03)
Urogenital congenital anomalies	144.15 (74.82 to 251.28)	115.67 (62.76 to 202.06)	97.43 (54.25 to 170.80)	165.86 (84.74 to 304.75)	2.02 (1.17 to 3.35)	1.84 (1.13 to 2.95)	1.59 (0.96 to 2.53)	2.45 (1.43 to 4.19)
Digestive congenital anomalies	666.25 (291.26 to 1,392.33)	436.15 (180.06 to 896.61)	342.98 (145.45 to 701.10)	709.83 (287.07 to 1,473.84)	7.77 (3.42 to 16.22)	5.10 (2.14 to 10.43)	4.02 (1.72 to 8.18)	8.29 (3.38 to 17.15)
Other congenital birth defects	2,695.89 (1,093.63 to 5,595.57)	1,997.27 (836.64 to 4,088.94)	1,622.09 (703.98 to 3,339.60)	3,150.55 (1,299.53 to 6,688.18)	31.73 (13.17 to 65.43)	23.81 (10.41 to 48.09)	19.39 (8.73 to 39.26)	37.29 (15.70 to 78.35)
Skin and subcutaneous diseases	262.27 (159.50 to 446.49)	640.25 (392.28 to 1,048.90)	641.82 (399.47 to 1,035.64)	647.65 (392.06 to 1,040.31)	9.31 (5.88 to 15.83)	32.85 (21.46 to 54.12)	35.93 (23.84 to 58.39)	29.98 (19.27 to 50.67)
Cellulitis	23.01 (10.92 to 45.53)	68.15 (31.48 to 132.25)	75.78 (34.16 to 148.20)	64.23 (29.48 to 125.34)	0.71 (0.35 to 1.41)	3.09 (1.51 to 6.02)	3.67 (1.82 to 7.06)	2.68 (1.29 to 5.33)
Pyoderma	161.46 (91.02 to 279.17)	401.68 (223.63 to 670.88)	383.47 (213.51 to 643.41)	423.75 (236.67 to 703.38)	4.72 (2.78 to 8.14)	17.39 (10.44 to 29.30)	17.94 (10.85 to 30.50)	16.61 (10.09 to 28.15)
Decubitus ulcer	69.50 (38.92 to 126.66)	155.36 (94.86 to 279.67)	166.83 (100.55 to 299.23)	144.45 (88.54 to 258.01)	3.65 (2.19 to 6.59)	11.86 (6.84 to 21.27)	13.75 (7.96 to 24.64)	10.22 (5.82 to 18.69)
Other skin and subcutaneous diseases	8.31 (4.45 to 13.89)	15.05 (7.70 to 24.60)	15.75 (8.22 to 25.43)	15.21 (7.87 to 24.95)	0.22 (0.12 to 0.37)	0.51 (0.30 to 0.83)	0.57 (0.33 to 0.93)	0.47 (0.27 to 0.77)
Sudden infant death syndrome	715.74 (295.49 to 1,511.36)	326.36 (111.29 to 747.19)	269.17 (91.46 to 613.02)	523.35 (179.47 to 1,176.94)	8.31 (3.43 to 17.54)	3.79 (1.29 to 8.67)	3.12 (1.06 to 7.11)	6.07 (2.08 to 13.66)

**Injuries**	**21,568.68 (15,083.56 to 27,765.94)**	**22,696.18 (13,447.71 to 33,683.46)**	**20,374.69 (11,823.89 to 30,982.80)**	**27,213.85 (16,583.78 to 39,331.83)**	**405.98 (285.53 to 512.86)**	**523.28 (348.71 to 725.05)**	**491.74 (328.50 to 690.64)**	**582.07 (392.19 to 798.76)**
**Transport injuries**	**6,072.81 (3,930.77 to 8,170.44)**	**6,714.55 (4,265.13 to 9,531.34)**	**5,745.80 (3,603.25 to 8,181.57)**	**9,033.35 (5,728.58 to 12,714.14)**	**120.28 (82.07 to 158.37)**	**159.32 (107.29 to 215.13)**	**144.37 (97.70 to 193.78)**	**195.09 (131.78 to 263.05)**
Road injuries	5,393.66 (3,490.93 to 7,218.02)	5,800.41 (3,588.65 to 8,142.60)	4,944.02 (3,022.51 to 6,931.73)	7,954.78 (4,987.47 to 11,136.82)	107.29 (73.80 to 142.28)	136.57 (92.30 to 182.41)	123.27 (84.33 to 164.35)	170.53 (115.07 to 229.24)
Pedestrian road injuries	1,427.95 (888.69 to 2,055.48)	1,194.30 (743.83 to 1,770.78)	1,023.43 (643.16 to 1,509.58)	1,581.11 (997.60 to 2,314.77)	31.16 (20.61 to 43.83)	36.56 (25.58 to 52.68)	33.79 (23.28 to 48.36)	42.81 (29.85 to 60.53)

Timor-Leste								
Cause	YLLs (actual counts)				Deaths (actual counts)			
	2016	2040 Forecast	2040 Better Scenario	2040 Worse Scenario	2016	2040 Forecast	2040 Better Scenario	2040 Worse Scenario
Cyclist road injuries	111.24 (42.65 to 188.59)	125.28 (47.45 to 216.12)	108.32 (41.43 to 186.70)	170.42 (63.78 to 303.73)	2.53 (1.02 to 4.19)	3.28 (1.31 to 5.53)	3.04 (1.21 to 5.17)	3.99 (1.58 to 6.74)
Motorcyclist road injuries	1,132.84 (673.45 to 1,747.98)	1,411.54 (739.71 to 2,302.09)	1,234.67 (639.85 to 2,034.19)	1,860.79 (1,017.13 to 2,972.38)	19.55 (11.31 to 29.89)	25.90 (13.49 to 42.65)	23.21 (11.96 to 37.82)	32.57 (17.71 to 52.97)
Motor vehicle road injuries	2,598.17 (1,610.85 to 3,541.75)	2,913.87 (1,685.90 to 4,391.72)	2,445.77 (1,405.69 to 3,676.23)	4,123.61 (2,436.29 to 6,139.16)	51.50 (32.66 to 70.60)	66.72 (41.83 to 94.80)	59.40 (37.56 to 84.15)	86.12 (53.98 to 122.02)
Other road injuries	123.46 (39.47 to 209.16)	155.42 (50.21 to 284.90)	131.83 (42.25 to 245.38)	218.85 (72.58 to 384.54)	2.55 (0.86 to 4.48)	4.12 (1.40 to 7.35)	3.84 (1.34 to 6.74)	5.05 (1.72 to 9.00)
Other transport injuries	679.15 (390.02 to 1,053.51)	914.14 (494.42 to 1,455.72)	801.78 (444.02 to 1,265.62)	1,078.57 (600.83 to 1,727.02)	13.00 (7.75 to 19.37)	22.75 (13.19 to 33.28)	21.10 (12.37 to 30.90)	24.57 (14.28 to 36.17)
**Unintentional injuries**	**10,367.56 (7,593.24 to 13,583.72)**	**7,714.45 (5,027.43 to 10,690.27)**	**6,753.10 (4,356.55 to 9,384.29)**	**9,372.55 (6,174.33 to 13,045.32)**	**186.85 (138.55 to 235.54)**	**196.55 (144.03 to 253.21)**	**185.21 (135.15 to 239.11)**	**213.13 (153.78 to 277.66)**
Falls	1,588.52 (1,093.97 to 2,265.54)	1,670.66 (1,082.69 to 2,488.27)	1,588.25 (1,029.38 to 2,378.56)	1,807.36 (1,192.00 to 2,661.31)	42.47 (30.67 to 58.21)	68.70 (51.72 to 93.50)	70.65 (54.05 to 95.04)	66.25 (49.26 to 90.61)
Drowning	4,083.06 (2,865.58 to 5,585.75)	2,475.83 (1,533.97 to 3,670.05)	2,101.87 (1,281.51 to 3,132.26)	3,128.67 (1,941.53 to 4,681.44)	61.92 (44.28 to 83.84)	44.92 (29.58 to 64.79)	39.28 (25.38 to 56.67)	53.52 (34.74 to 76.88)
Fire, heat, and hot substances	990.66 (654.52 to 1,488.48)	698.69 (413.25 to 1,145.81)	583.22 (344.39 to 958.21)	862.54 (512.16 to 1,419.09)	18.67 (12.79 to 28.02)	18.41 (12.26 to 30.15)	16.49 (10.90 to 26.81)	20.42 (13.53 to 33.10)
Poisonings	264.93 (155.33 to 429.00)	156.55 (84.25 to 270.17)	121.04 (64.33 to 211.93)	201.17 (108.34 to 351.28)	4.74 (2.84 to 7.47)	3.42 (2.02 to 5.60)	2.78 (1.63 to 4.63)	4.09 (2.35 to 6.82)
Exposure to mechanical forces	738.30 (471.97 to 1,116.71)	547.88 (307.44 to 897.26)	450.12 (253.48 to 758.29)	699.55 (408.27 to 1,147.71)	12.12 (7.79 to 18.66)	11.04 (6.37 to 18.44)	9.45 (5.51 to 15.75)	13.08 (7.83 to 21.53)
Unintentional firearm injuries	101.45 (50.47 to 247.76)	81.67 (35.64 to 206.44)	66.39 (27.48 to 169.49)	99.85 (42.37 to 251.60)	1.91 (0.94 to 4.80)	1.90 (0.89 to 4.70)	1.62 (0.77 to 4.13)	2.16 (1.00 to 5.41)
Unintentional suffocation	247.01 (117.71 to 469.40)	130.75 (65.64 to 241.42)	102.52 (51.19 to 182.13)	197.20 (92.09 to 386.96)	3.11 (1.58 to 5.70)	1.78 (0.95 to 3.07)	1.42 (0.75 to 2.38)	2.60 (1.30 to 4.78)
Other exposure to mechanical forces	389.85 (238.21 to 648.21)	335.46 (177.69 to 633.37)	281.21 (141.93 to 537.14)	402.50 (216.67 to 735.98)	7.10 (4.31 to 12.11)	7.36 (4.07 to 13.41)	6.41 (3.55 to 11.69)	8.32 (4.62 to 14.99)
Adverse effects of medical treatment	724.96 (505.25 to 970.25)	630.37 (385.45 to 938.13)	549.14 (333.66 to 838.43)	754.14 (480.46 to 1,103.16)	14.65 (10.34 to 19.58)	17.89 (11.81 to 26.16)	16.67 (11.05 to 24.56)	19.17 (12.86 to 27.55)
Animal contact	268.25 (163.99 to 388.87)	285.32 (160.90 to 436.20)	229.11 (125.45 to 356.40)	356.61 (197.70 to 567.14)	5.35 (3.36 to 7.48)	7.13 (4.40 to 10.31)	6.03 (3.59 to 8.87)	8.20 (4.94 to 12.20)
Venomous animal contact	133.79 (77.85 to 207.73)	132.65 (69.28 to 215.68)	105.98 (53.51 to 168.64)	167.30 (87.36 to 277.91)	2.73 (1.62 to 4.08)	3.41 (1.97 to 5.24)	2.88 (1.62 to 4.44)	3.94 (2.25 to 6.26)
Non-venomous animal contact	134.45 (74.70 to 215.48)	152.68 (75.47 to 258.27)	123.13 (60.19 to 209.74)	189.32 (92.52 to 325.86)	2.61 (1.50 to 4.22)	3.71 (2.05 to 6.15)	3.15 (1.74 to 5.21)	4.26 (2.30 to 7.12)
Foreign body	1,214.17 (685.93 to 2,236.84)	822.54 (495.84 to 1,318.78)	765.39 (473.42 to 1,180.79)	1,060.51 (599.54 to 1,868.40)	17.89 (11.33 to 29.53)	15.98 (11.03 to 22.62)	15.91 (11.09 to 22.22)	18.23 (12.03 to 26.92)
Pulmonary aspiration and foreign body in airway	1,119.41 (588.38 to 2,123.34)	751.38 (437.91 to 1,244.01)	703.74 (415.31 to 1,121.69)	971.86 (520.30 to 1,773.44)	16.25 (9.70 to 27.72)	14.33 (9.47 to 20.57)	14.39 (9.58 to 20.55)	16.39 (10.37 to 25.41)

Foreign body in other body part	94.76 (54.44 to 179.34)	71.15 (38.84 to 133.06)	61.65 (32.74 to 114.47)	88.65 (46.73 to 167.52)	1.64 (1.00 to 2.93)	1.65 (0.96 to 3.01)	1.52 (0.88 to 2.79)	1.85 (1.07 to 3.37)
Environmental heat and cold exposure	50.16 (31.70 to 74.79)	48.31 (27.40 to 78.10)	36.92 (20.69 to 59.43)	62.06 (35.24 to 98.60)	1.09 (0.72 to 1.64)	1.43 (0.95 to 2.23)	1.17 (0.76 to 1.87)	1.66 (1.06 to 2.59)
Other unintentional injuries	444.55 (267.93 to 679.39)	378.30 (197.55 to 628.04)	328.04 (170.04 to 573.03)	439.92 (235.75 to 745.54)	7.94 (4.70 to 12.43)	7.63 (4.19 to 12.84)	6.78 (3.76 to 11.73)	8.51 (4.80 to 14.44)
**Self-harm and interpersonal violence**	**5,119.72 (2,743.92 to 7,021.58)**	**7,000.95 (3,498.86 to 10,399.06)**	**6,631.84 (3,385.23 to 9,911.98)**	**7,426.38 (3,690.14 to 11,099.41)**	**98.70 (54.73 to 134.80)**	**144.35 (79.60 to 209.34)**	**139.10 (76.98 to 201.31)**	**149.59 (81.70 to 218.69)**

Timor-Leste								
Cause	YLLs (actual counts)				Deaths (actual counts)			
	2016	2040 Forecast	2040 Better Scenario	2040 Worse Scenario	2016	2040 Forecast	2040 Better Scenario	2040 Worse Scenario
Self-harm	3,307.20 (1,713.45 to 4,726.79)	5,180.29 (2,576.12 to 8,149.78)	4,855.99 (2,373.94 to 7,521.55)	5,502.76 (2,714.80 to 8,684.77)	67.25 (36.77 to 95.19)	110.91 (59.03 to 164.58)	106.26 (57.93 to 157.02)	114.85 (61.05 to 172.29)
Self-harm by firearm	105.33 (39.90 to 236.38)	134.81 (46.54 to 318.40)	122.96 (42.97 to 290.49)	144.76 (49.50 to 339.87)	2.21 (0.83 to 4.90)	3.15 (1.16 to 7.10)	2.96 (1.09 to 6.74)	3.26 (1.18 to 7.44)
Self-harm by other specified means	3,201.87 (1,664.87 to 4,585.97)	5,045.48 (2,519.52 to 7,800.50)	4,733.03 (2,320.62 to 7,336.20)	5,358.01 (2,640.32 to 8,410.37)	65.05 (35.33 to 91.90)	107.77 (57.99 to 160.80)	103.30 (56.94 to 154.28)	111.59 (60.10 to 167.85)
Interpersonal violence	1,812.52 (927.62 to 2,564.47)	1,820.66 (809.94 to 2,787.47)	1,775.85 (795.60 to 2,707.07)	1,923.62 (872.42 to 2,944.46)	31.45 (16.61 to 44.43)	33.43 (16.05 to 50.67)	32.84 (15.70 to 49.88)	34.74 (16.73 to 52.18)
Physical violence by firearm	419.36 (157.67 to 889.62)	442.76 (144.17 to 971.53)	432.04 (140.18 to 953.20)	461.25 (152.44 to 1,012.37)	7.38 (2.64 to 15.73)	8.04 (2.69 to 17.77)	7.87 (2.63 to 17.49)	8.29 (2.81 to 18.19)
Physical violence by sharp object	631.23 (279.19 to 1,009.22)	712.15 (278.48 to 1,207.68)	721.08 (279.91 to 1,219.21)	724.07 (292.91 to 1,217.05)	11.03 (4.88 to 17.88)	12.93 (5.23 to 22.26)	13.16 (5.28 to 22.56)	13.01 (5.35 to 22.34)
Physical violence by other means	761.93 (385.78 to 1,120.99)	665.75 (289.56 to 1,099.04)	622.73 (269.76 to 1,033.58)	738.30 (327.15 to 1,204.01)	13.04 (6.69 to 19.10)	12.47 (5.94 to 20.33)	11.80 (5.58 to 19.18)	13.45 (6.43 to 21.68)
**Forces of nature, conflict and terrorism, and executions and police conflict**	**8.58 (5.88 to 11.18)**	**1,266.23 (0.00 to 10,013.82)**	**1,243.95 (0.00 to 9,835.61)**	**1,381.57 (0.00 to 10,831.78)**	**0.15 (0.10 to 0.20)**	**23.06 (0.00 to 182.67)**	**23.06 (0.00 to 182.97)**	**24.25 (0.00 to 191.50)**
Exposure to forces of nature	0.00 (0.00 to 0.00)	1.52 (0.00 to 28.21)	1.49 (0.00 to 27.59)	1.70 (0.00 to 31.43)	0.00 (0.00 to 0.00)	0.03 (0.00 to 0.53)	0.03 (0.00 to 0.54)	0.03 (0.00 to 0.56)
Conflict and terrorism	0.00 (0.00 to 0.00)	1,264.70 (0.00 to 10,013.82)	1,242.45 (0.00 to 9,835.61)	1,379.88 (0.00 to 10,831.78)	0.00 (0.00 to 0.00)	23.03 (0.00 to 182.67)	23.03 (0.00 to 182.97)	24.22 (0.00 to 191.50)
Executions and police conflict	8.58 (5.88 to 11.18)	0.00 (0.00 to 0.00)	0.00 (0.00 to 0.00)	0.00 (0.00 to 0.00)	0.15 (0.10 to 0.20)	0.00 (0.00 to 0.00)	0.00 (0.00 to 0.00)	0.00 (0.00 to 0.00)

Vietnam								
Cause	YLLs (in 1000s)				Deaths (in 1000s)			
	2016	2040 Forecast	2040 Better Scenario	2040 Worse Scenario	2016	2040 Forecast	2040 Better Scenario	2040 Worse Scenario
**All causes**	**15,131.45 (13,458.78 to 16,798.15)**	**16,967.59 (14,493.98 to 20,288.41)**	**14,006.10 (12,115.96 to 16,293.18)**	**22,628.12 (19,042.89 to 27,057.72)**	**661.38 (613.80 to 715.77)**	**937.00 (848.66 to 1,037.43)**	**809.19 (742.93 to 883.37)**	**1,169.52 (1,053.25 to 1,302.09)**
**Communicable, maternal, neonatal, and nutritional diseases**	**2,299.19 (1,924.98 to 2,714.61)**	**1,198.96 (919.05 to 1,598.23)**	**941.70 (739.73 to 1,217.75)**	**1,797.67 (1,346.17 to 2,463.60)**	**61.30 (53.72 to 71.06)**	**50.65 (37.84 to 70.62)**	**41.18 (31.46 to 55.11)**	**72.06 (52.83 to 102.04)**
**HIV/AIDS and tuberculosis**	**726.82 (545.88 to 949.37)**	**425.69 (257.66 to 758.12)**	**317.15 (197.44 to 522.92)**	**660.59 (397.58 to 1,183.97)**	**25.21 (20.56 to 31.07)**	**19.84 (11.74 to 36.12)**	**14.72 (9.25 to 24.84)**	**29.67 (17.57 to 54.25)**
Tuberculosis	392.92 (325.53 to 480.30)	288.33 (147.31 to 592.06)	200.29 (107.54 to 384.98)	461.85 (234.35 to 961.01)	18.67 (15.88 to 22.21)	17.12 (9.58 to 33.12)	12.39 (7.26 to 22.36)	25.80 (14.04 to 49.99)
HIV/AIDS	333.91 (158.37 to 555.93)	137.36 (58.00 to 260.28)	116.86 (45.66 to 212.31)	198.74 (98.09 to 361.61)	6.54 (2.98 to 11.30)	2.72 (1.10 to 5.39)	2.32 (0.83 to 4.59)	3.87 (1.83 to 7.42)
**Diarrhea, lower respiratory, and other common infectious diseases**	**877.92 (730.95 to 1,058.16)**	**524.04 (384.16 to 732.26)**	**400.91 (303.03 to 546.52)**	**791.31 (562.34 to 1,158.77)**	**26.02 (22.40 to 31.15)**	**25.14 (18.05 to 35.03)**	**21.09 (15.70 to 28.62)**	**35.50 (24.64 to 51.02)**
Diarrheal diseases	37.51 (21.66 to 59.64)	25.43 (4.61 to 83.43)	17.64 (4.01 to 54.95)	44.52 (8.04 to 143.13)	1.96 (0.87 to 3.71)	1.87 (0.31 to 6.80)	1.37 (0.28 to 4.40)	3.03 (0.47 to 10.54)
Intestinal infectious diseases	110.90 (59.89 to 187.86)	48.29 (25.26 to 83.07)	43.29 (22.91 to 73.23)	56.95 (29.12 to 101.16)	1.76 (0.94 to 3.00)	0.92 (0.49 to 1.55)	0.85 (0.46 to 1.44)	1.05 (0.55 to 1.82)
Typhoid fever	96.05 (49.79 to 162.13)	40.59 (20.47 to 68.55)	36.34 (18.95 to 61.71)	47.68 (23.64 to 82.49)	1.51 (0.79 to 2.57)	0.77 (0.40 to 1.30)	0.71 (0.38 to 1.19)	0.87 (0.45 to 1.51)
Paratyphoid fever	14.65 (6.64 to 28.62)	7.64 (3.29 to 14.89)	6.89 (3.06 to 13.28)	9.19 (3.94 to 18.07)	0.24 (0.11 to 0.47)	0.15 (0.07 to 0.30)	0.14 (0.06 to 0.27)	0.18 (0.08 to 0.35)
Other intestinal infectious diseases	0.19 (0.08 to 0.41)	0.07 (0.02 to 0.15)	0.06 (0.02 to 0.13)	0.08 (0.03 to 0.19)	0.01 (0.00 to 0.01)	0.00 (0.00 to 0.01)	0.00 (0.00 to 0.01)	0.00 (0.00 to 0.01)
Lower respiratory infections	536.65 (443.98 to 650.78)	325.22 (223.43 to 479.21)	250.85 (180.63 to 354.45)	529.06 (346.27 to 827.87)	18.69 (15.93 to 23.27)	19.08 (13.44 to 27.66)	16.03 (11.52 to 22.43)	27.67 (18.64 to 40.78)
Upper respiratory infections	0.75 (0.39 to 1.22)	0.55 (0.23 to 1.05)	0.48 (0.20 to 0.92)	0.75 (0.32 to 1.38)	0.02 (0.01 to 0.04)	0.02 (0.01 to 0.05)	0.02 (0.01 to 0.04)	0.03 (0.01 to 0.06)
Otitis media	0.05 (0.02 to 0.10)	0.03 (0.01 to 0.06)	0.03 (0.01 to 0.06)	0.03 (0.01 to 0.06)	0.00 (0.00 to 0.00)	0.00 (0.00 to 0.00)	0.00 (0.00 to 0.00)	0.00 (0.00 to 0.00)
Meningitis	48.29 (34.68 to 72.60)	21.13 (13.88 to 32.69)	18.55 (12.71 to 27.96)	23.44 (14.88 to 37.29)	0.91 (0.70 to 1.29)	0.60 (0.43 to 0.88)	0.57 (0.42 to 0.86)	0.62 (0.45 to 0.91)
Pneumococcal meningitis	0.46 (0.28 to 0.89)	0.14 (0.09 to 0.22)	0.12 (0.08 to 0.18)	0.14 (0.09 to 0.25)	0.01 (0.01 to 0.02)	0.00 (0.00 to 0.01)	0.00 (0.00 to 0.01)	0.00 (0.00 to 0.01)
H influenzae type B meningitis	3.64 (2.45 to 5.81)	2.53 (0.93 to 6.58)	1.49 (0.82 to 2.96)	3.45 (0.98 to 9.66)	0.06 (0.05 to 0.10)	0.05 (0.03 to 0.10)	0.04 (0.03 to 0.06)	0.06 (0.03 to 0.13)
Meningococcal meningitis	16.56 (11.21 to 24.84)	6.79 (4.49 to 10.51)	6.26 (4.12 to 9.82)	7.30 (4.81 to 11.35)	0.33 (0.24 to 0.46)	0.21 (0.15 to 0.33)	0.21 (0.14 to 0.33)	0.22 (0.15 to 0.34)
Other meningitis	27.63 (19.58 to 42.25)	11.67 (7.85 to 17.41)	10.68 (7.21 to 16.02)	12.55 (8.34 to 19.28)	0.51 (0.38 to 0.73)	0.33 (0.23 to 0.49)	0.32 (0.22 to 0.48)	0.34 (0.24 to 0.50)
Encephalitis	80.24 (57.11 to 113.19)	56.81 (39.67 to 81.45)	51.41 (37.21 to 72.53)	64.20 (43.46 to 94.51)	1.75 (1.34 to 2.34)	1.97 (1.53 to 2.54)	1.91 (1.49 to 2.43)	2.09 (1.61 to 2.80)

Diphtheria	0.18 (0.11 to 0.28)	0.04 (0.02 to 0.07)	0.03 (0.02 to 0.05)	0.05 (0.02 to 0.10)	0.00 (0.00 to 0.00)	0.00 (0.00 to 0.00)	0.00 (0.00 to 0.00)	0.00 (0.00 to 0.00)
Whooping cough	54.17 (4.61 to 168.25)	22.29 (0.54 to 69.88)	2.79 (0.15 to 10.15)	28.56 (0.51 to 89.67)	0.64 (0.05 to 2.01)	0.27 (0.01 to 0.83)	0.04 (0.00 to 0.13)	0.34 (0.01 to 1.06)
Tetanus	6.28 (4.40 to 9.60)	0.91 (0.53 to 1.50)	0.78 (0.42 to 1.36)	1.04 (0.62 to 1.68)	0.19 (0.12 to 0.34)	0.04 (0.02 to 0.06)	0.03 (0.02 to 0.06)	0.03 (0.02 to 0.06)
Measles	0.67 (0.19 to 1.68)	22.40 (0.46 to 97.23)	14.27 (0.43 to 49.40)	41.50 (0.66 to 183.62)	0.01 (0.00 to 0.02)	0.32 (0.01 to 1.21)	0.22 (0.01 to 0.71)	0.57 (0.01 to 2.33)

Vietnam								
Cause	YLLs (in 1000s)				Deaths (in 1000s)			
	2016	2040 Forecast	2040 Better Scenario	2040 Worse Scenario	2016	2040 Forecast	2040 Better Scenario	2040 Worse Scenario
Varicella and herpes zoster	2.22 (1.45 to 3.31)	0.95 (0.55 to 1.54)	0.80 (0.49 to 1.24)	1.21 (0.67 to 2.03)	0.08 (0.05 to 0.12)	0.05 (0.03 to 0.09)	0.05 (0.03 to 0.09)	0.06 (0.04 to 0.10)
**Neglected tropical diseases and malaria**	**66.93 (48.26 to 97.59)**	**59.21 (43.83 to 87.64)**	**57.34 (42.50 to 84.51)**	**63.77 (46.61 to 95.35)**	**1.49 (1.10 to 2.17)**	**1.84 (1.37 to 2.92)**	**1.84 (1.36 to 2.92)**	**1.89 (1.41 to 3.02)**
Malaria	51.73 (35.08 to 77.19)	27.56 (16.46 to 48.48)	26.60 (15.97 to 46.80)	29.72 (17.61 to 53.11)	1.13 (0.80 to 1.81)	0.82 (0.49 to 1.64)	0.81 (0.49 to 1.59)	0.84 (0.51 to 1.72)
Chagas disease	0.00 (0.00 to 0.00)	0.00 (0.00 to 0.00)	0.00 (0.00 to 0.00)	0.00 (0.00 to 0.00)	0.00 (0.00 to 0.00)	0.00 (0.00 to 0.00)	0.00 (0.00 to 0.00)	0.00 (0.00 to 0.00)
Leishmaniasis	0.00 (0.00 to 0.00)	0.00 (0.00 to 0.00)	0.00 (0.00 to 0.00)	0.00 (0.00 to 0.00)	0.00 (0.00 to 0.00)	0.00 (0.00 to 0.00)	0.00 (0.00 to 0.00)	0.00 (0.00 to 0.00)
African trypanosomiasis	0.00 (0.00 to 0.00)	0.00 (0.00 to 0.00)	0.00 (0.00 to 0.00)	0.00 (0.00 to 0.00)	0.00 (0.00 to 0.00)	0.00 (0.00 to 0.00)	0.00 (0.00 to 0.00)	0.00 (0.00 to 0.00)
Schistosomiasis	0.00 (0.00 to 0.00)	0.00 (0.00 to 0.00)	0.00 (0.00 to 0.00)	0.00 (0.00 to 0.00)	0.00 (0.00 to 0.00)	0.00 (0.00 to 0.00)	0.00 (0.00 to 0.00)	0.00 (0.00 to 0.00)
Cysticercosis	0.15 (0.12 to 0.18)	0.06 (0.04 to 0.09)	0.06 (0.04 to 0.08)	0.06 (0.04 to 0.09)	0.00 (0.00 to 0.00)	0.00 (0.00 to 0.00)	0.00 (0.00 to 0.00)	0.00 (0.00 to 0.00)
Cystic echinococcosis	0.03 (0.03 to 0.04)	0.02 (0.01 to 0.03)	0.01 (0.01 to 0.02)	0.02 (0.02 to 0.04)	0.00 (0.00 to 0.00)	0.00 (0.00 to 0.00)	0.00 (0.00 to 0.00)	0.00 (0.00 to 0.00)
Dengue	10.63 (3.62 to 28.15)	28.99 (14.88 to 49.71)	28.32 (14.87 to 47.96)	30.60 (15.59 to 52.94)	0.23 (0.08 to 0.53)	0.93 (0.50 to 1.66)	0.94 (0.51 to 1.67)	0.93 (0.49 to 1.63)
Yellow fever	0.00 (0.00 to 0.00)	0.00 (0.00 to 0.00)	0.00 (0.00 to 0.00)	0.00 (0.00 to 0.00)	0.00 (0.00 to 0.00)	0.00 (0.00 to 0.00)	0.00 (0.00 to 0.00)	0.00 (0.00 to 0.00)
Rabies	2.09 (0.77 to 3.41)	0.75 (0.25 to 1.50)	0.61 (0.20 to 1.20)	1.26 (0.43 to 2.35)	0.07 (0.03 to 0.11)	0.03 (0.01 to 0.05)	0.02 (0.01 to 0.04)	0.04 (0.02 to 0.08)
Intestinal nematode infections	1.06 (0.67 to 1.55)	0.95 (0.52 to 1.58)	0.92 (0.51 to 1.49)	1.01 (0.55 to 1.69)	0.02 (0.01 to 0.03)	0.03 (0.01 to 0.04)	0.03 (0.01 to 0.04)	0.03 (0.01 to 0.04)
Other neglected tropical diseases	1.24 (0.81 to 2.41)	0.89 (0.53 to 1.51)	0.82 (0.48 to 1.39)	1.09 (0.66 to 1.95)	0.03 (0.02 to 0.06)	0.04 (0.02 to 0.07)	0.04 (0.02 to 0.07)	0.04 (0.02 to 0.09)
**Maternal disorders**	**15.28 (10.18 to 23.04)**	**5.23 (3.21 to 8.45)**	**4.41 (2.58 to 7.46)**	**7.94 (4.84 to 13.24)**	**0.28 (0.19 to 0.42)**	**0.10 (0.06 to 0.16)**	**0.08 (0.05 to 0.14)**	**0.15 (0.09 to 0.25)**
Maternal hemorrhage	4.52 (2.72 to 7.13)	1.39 (0.77 to 2.39)	1.18 (0.64 to 2.12)	2.11 (1.18 to 3.64)	0.08 (0.05 to 0.13)	0.03 (0.01 to 0.04)	0.02 (0.01 to 0.04)	0.04 (0.02 to 0.07)
Maternal sepsis and other maternal infections	0.11 (0.05 to 0.21)	0.03 (0.01 to 0.07)	0.03 (0.01 to 0.06)	0.05 (0.02 to 0.10)	0.00 (0.00 to 0.00)	0.00 (0.00 to 0.00)	0.00 (0.00 to 0.00)	0.00 (0.00 to 0.00)
Maternal hypertensive disorders	0.43 (0.23 to 0.71)	0.14 (0.07 to 0.23)	0.11 (0.06 to 0.20)	0.21 (0.10 to 0.36)	0.01 (0.00 to 0.01)	0.00 (0.00 to 0.00)	0.00 (0.00 to 0.00)	0.00 (0.00 to 0.01)
Maternal obstructed labor and uterine rupture	0.07 (0.03 to 0.14)	0.02 (0.01 to 0.05)	0.02 (0.01 to 0.04)	0.04 (0.01 to 0.08)	0.00 (0.00 to 0.00)	0.00 (0.00 to 0.00)	0.00 (0.00 to 0.00)	0.00 (0.00 to 0.00)
Maternal abortion, miscarriage, and ectopic pregnancy	8.38 (5.48 to 12.75)	2.97 (1.70 to 5.16)	2.51 (1.40 to 4.39)	4.54 (2.59 to 7.98)	0.15 (0.10 to 0.23)	0.06 (0.03 to 0.10)	0.05 (0.03 to 0.08)	0.09 (0.05 to 0.15)

Indirect maternal deaths	0.96 (0.54 to 1.62)	0.36 (0.18 to 0.66)	0.30 (0.15 to 0.55)	0.54 (0.28 to 0.95)	0.02 (0.01 to 0.03)	0.01 (0.00 to 0.01)	0.01 (0.00 to 0.01)	0.01 (0.01 to 0.02)
Late maternal deaths	0.06 (0.02 to 0.12)	0.02 (0.01 to 0.04)	0.02 (0.00 to 0.03)	0.03 (0.01 to 0.06)	0.00 (0.00 to 0.00)	0.00 (0.00 to 0.00)	0.00 (0.00 to 0.00)	0.00 (0.00 to 0.00)
Maternal deaths aggravated by HIV/AIDS	0.03 (0.01 to 0.05)	0.03 (0.01 to 0.08)	0.03 (0.01 to 0.06)	0.05 (0.01 to 0.11)	0.00 (0.00 to 0.00)	0.00 (0.00 to 0.00)	0.00 (0.00 to 0.00)	0.00 (0.00 to 0.00)
Other maternal disorders	0.73 (0.40 to 1.22)	0.26 (0.13 to 0.46)	0.22 (0.11 to 0.39)	0.38 (0.20 to 0.66)	0.01 (0.01 to 0.02)	0.00 (0.00 to 0.01)	0.00 (0.00 to 0.01)	0.01 (0.00 to 0.01)

Vietnam								
Cause	YLLs (in 1000s)				Deaths (in 1000s)			
	2016	2040 Forecast	2040 Better Scenario	2040 Worse Scenario	2016	2040 Forecast	2040 Better Scenario	2040 Worse Scenario
**Neonatal disorders**	**548.25 (402.91 to 741.29)**	**140.70 (87.66 to 211.08)**	**120.49 (74.06 to 182.03)**	**222.59 (139.12 to 330.77)**	**6.33 (4.65 to 8.56)**	**1.63 (1.01 to 2.44)**	**1.39 (0.86 to 2.10)**	**2.57 (1.61 to 3.82)**
Neonatal preterm birth	317.85 (223.33 to 437.74)	64.19 (37.59 to 100.83)	56.25 (32.64 to 88.12)	115.67 (68.67 to 177.27)	3.67 (2.58 to 5.06)	0.74 (0.43 to 1.17)	0.65 (0.38 to 1.02)	1.34 (0.79 to 2.05)
Neonatal encephalopathy due to birth asphyxia and trauma	62.99 (35.59 to 115.04)	17.49 (8.40 to 35.53)	14.82 (6.98 to 30.41)	23.65 (11.22 to 48.15)	0.73 (0.41 to 1.33)	0.20 (0.10 to 0.41)	0.17 (0.08 to 0.35)	0.27 (0.13 to 0.56)
Neonatal sepsis and other neonatal infections	91.80 (54.60 to 151.84)	34.89 (18.24 to 62.35)	29.50 (15.48 to 53.52)	47.99 (24.42 to 88.82)	1.06 (0.63 to 1.75)	0.40 (0.21 to 0.72)	0.34 (0.18 to 0.62)	0.55 (0.28 to 1.03)
Hemolytic disease and other neonatal jaundice	2.52 (1.46 to 4.10)	0.62 (0.29 to 1.25)	0.51 (0.24 to 1.03)	0.90 (0.41 to 1.78)	0.03 (0.02 to 0.05)	0.01 (0.00 to 0.01)	0.01 (0.00 to 0.01)	0.01 (0.00 to 0.02)
Other neonatal disorders	73.09 (43.05 to 117.70)	23.52 (12.16 to 41.43)	19.41 (9.87 to 34.00)	34.38 (17.19 to 60.94)	0.84 (0.50 to 1.36)	0.27 (0.14 to 0.48)	0.22 (0.11 to 0.39)	0.40 (0.20 to 0.70)
**Nutritional deficiencies**	**2.92 (2.47 to 3.43)**	**2.84 (2.16 to 3.65)**	**2.77 (2.09 to 3.60)**	**3.10 (2.32 to 4.03)**	**0.29 (0.24 to 0.36)**	**0.29 (0.20 to 0.38)**	**0.29 (0.20 to 0.39)**	**0.30 (0.20 to 0.40)**
Protein-energy malnutrition	0.36 (0.27 to 0.47)	0.25 (0.15 to 0.38)	0.24 (0.15 to 0.38)	0.28 (0.17 to 0.43)	0.03 (0.02 to 0.04)	0.03 (0.01 to 0.04)	0.03 (0.01 to 0.04)	0.03 (0.01 to 0.05)
Iodine deficiency	0.11 (0.01 to 0.40)	0.20 (0.01 to 0.91)	0.19 (0.01 to 0.87)	0.23 (0.01 to 1.04)	0.01 (0.00 to 0.04)	0.02 (0.00 to 0.09)	0.02 (0.00 to 0.09)	0.02 (0.00 to 0.10)
Dietary iron deficiency	0.33 (0.20 to 0.51)	0.31 (0.13 to 0.63)	0.30 (0.13 to 0.63)	0.33 (0.15 to 0.68)	0.03 (0.01 to 0.06)	0.03 (0.01 to 0.07)	0.03 (0.01 to 0.07)	0.03 (0.01 to 0.07)
Other nutritional deficiencies	2.13 (1.70 to 2.59)	2.08 (1.32 to 2.86)	2.04 (1.29 to 2.81)	2.26 (1.41 to 3.20)	0.23 (0.18 to 0.29)	0.21 (0.13 to 0.31)	0.21 (0.13 to 0.31)	0.22 (0.13 to 0.32)
**Other communicable, maternal, neonatal, and nutritional diseases**	**61.06 (41.42 to 113.30)**	**41.26 (32.32 to 54.84)**	**38.63 (29.87 to 51.12)**	**48.37 (37.14 to 66.20)**	**1.68 (1.34 to 2.32)**	**1.81 (1.51 to 2.21)**	**1.77 (1.46 to 2.13)**	**1.99 (1.62 to 2.43)**
Sexually transmitted infections excluding HIV	11.25 (3.91 to 26.95)	2.32 (1.10 to 5.26)	2.12 (1.03 to 4.82)	2.86 (1.27 to 6.78)	0.17 (0.08 to 0.35)	0.06 (0.04 to 0.10)	0.06 (0.04 to 0.09)	0.07 (0.04 to 0.12)
Syphilis	10.55 (3.20 to 26.24)	1.79 (0.58 to 4.72)	1.60 (0.53 to 4.28)	2.28 (0.70 to 6.22)	0.14 (0.05 to 0.32)	0.03 (0.02 to 0.07)	0.03 (0.02 to 0.06)	0.04 (0.02 to 0.08)
Chlamydial infection	0.13 (0.10 to 0.19)	0.10 (0.07 to 0.13)	0.10 (0.07 to 0.13)	0.11 (0.08 to 0.14)	0.01 (0.00 to 0.01)	0.01 (0.00 to 0.01)	0.01 (0.00 to 0.01)	0.01 (0.00 to 0.01)
Gonococcal infection	0.39 (0.31 to 0.53)	0.31 (0.22 to 0.41)	0.30 (0.21 to 0.40)	0.33 (0.24 to 0.45)	0.02 (0.01 to 0.02)	0.02 (0.01 to 0.03)	0.02 (0.01 to 0.02)	0.02 (0.01 to 0.03)
Other sexually transmitted infections	0.18 (0.14 to 0.27)	0.13 (0.09 to 0.17)	0.12 (0.09 to 0.16)	0.14 (0.10 to 0.18)	0.01 (0.01 to 0.01)	0.01 (0.00 to 0.01)	0.01 (0.00 to 0.01)	0.01 (0.00 to 0.01)
Acute hepatitis	24.09 (19.63 to 29.58)	20.43 (15.49 to 26.44)	19.37 (14.72 to 25.34)	23.31 (17.71 to 30.22)	0.88 (0.73 to 1.06)	0.91 (0.71 to 1.14)	0.88 (0.69 to 1.11)	0.99 (0.78 to 1.25)
Acute hepatitis A	0.63 (0.44 to 0.87)	0.38 (0.23 to 0.59)	0.36 (0.22 to 0.57)	0.44 (0.27 to 0.67)	0.02 (0.01 to 0.02)	0.02 (0.01 to 0.03)	0.02 (0.01 to 0.03)	0.02 (0.01 to 0.03)
Acute hepatitis B	20.78 (16.78 to 25.89)	17.45 (12.91 to 23.41)	16.46 (11.96 to 22.12)	20.12 (14.70 to 26.86)	0.73 (0.59 to 0.88)	0.73 (0.55 to 0.93)	0.70 (0.53 to 0.91)	0.82 (0.61 to 1.04)

Acute hepatitis C	0.58 (0.39 to 0.85)	0.65 (0.41 to 1.02)	0.64 (0.40 to 0.98)	0.69 (0.43 to 1.06)	0.02 (0.02 to 0.03)	0.03 (0.02 to 0.04)	0.03 (0.02 to 0.04)	0.03 (0.02 to 0.05)
Acute hepatitis E	2.10 (1.45 to 3.01)	1.94 (1.18 to 3.08)	1.91 (1.15 to 3.02)	2.06 (1.25 to 3.27)	0.11 (0.07 to 0.17)	0.13 (0.07 to 0.21)	0.13 (0.07 to 0.22)	0.13 (0.07 to 0.21)
Other unspecified infectious diseases	25.72 (11.78 to 73.50)	18.51 (12.87 to 31.03)	17.14 (12.23 to 27.71)	22.19 (15.13 to 39.72)	0.63 (0.42 to 1.21)	0.84 (0.65 to 1.08)	0.82 (0.63 to 1.06)	0.92 (0.70 to 1.23)
**Non-communicable diseases**	**10,238.39 (9,190.43 to 11,367.95)**	**13,526.02 (11,526.82 to 16,068.25)**	**11,300.37 (9,741.98 to 13,088.43)**	**17,996.97 (15,016.16 to 21,523.99)**	**532.28 (497.79 to 574.21)**	**805.64 (724.65 to 900.24)**	**698.62 (637.70 to 772.90)**	**1,004.71 (894.39 to 1,133.06)**

Vietnam								
Cause	YLLs (in 1000s)				Deaths (in 1000s)			
	2016	2040 Forecast	2040 Better Scenario	2040 Worse Scenario	2016	2040 Forecast	2040 Better Scenario	2040 Worse Scenario
**Neoplasms**	**3,088.23 (2,703.93 to 3,543.14)**	**4,969.71 (4,014.71 to 6,139.40)**	**4,201.78 (3,447.53 to 5,097.21)**	**6,082.08 (4,782.53 to 7,626.88)**	**128.23 (115.72 to 143.10)**	**242.95 (202.79 to 289.22)**	**210.18 (178.19 to 244.90)**	**286.43 (230.59 to 345.44)**
Lip and oral cavity cancer	66.15 (56.00 to 77.53)	119.02 (67.58 to 195.95)	77.81 (46.16 to 123.30)	184.60 (103.88 to 302.45)	3.51 (3.07 to 3.98)	6.87 (3.79 to 11.22)	4.71 (2.70 to 7.47)	9.99 (5.40 to 16.37)
Nasopharynx cancer	42.02 (29.22 to 55.90)	68.39 (34.33 to 119.78)	42.38 (21.79 to 75.56)	104.48 (52.81 to 173.92)	1.25 (0.90 to 1.63)	2.43 (1.27 to 4.15)	1.52 (0.81 to 2.61)	3.70 (1.92 to 6.05)
Other pharynx cancer	143.73 (99.41 to 184.73)	246.37 (130.10 to 434.63)	195.14 (108.24 to 331.95)	272.65 (145.60 to 476.20)	5.11 (3.63 to 6.40)	10.18 (5.44 to 17.59)	8.17 (4.55 to 14.09)	11.01 (5.95 to 19.01)
Esophageal cancer	84.18 (71.38 to 98.58)	134.27 (70.33 to 246.48)	91.88 (51.27 to 159.66)	204.14 (101.37 to 388.14)	3.83 (3.37 to 4.33)	6.67 (3.73 to 11.48)	4.72 (2.80 to 7.69)	9.71 (5.12 to 17.41)
Stomach cancer	268.00 (226.53 to 316.12)	303.03 (226.69 to 390.77)	281.95 (217.38 to 357.24)	346.40 (249.08 to 461.85)	10.58 (9.14 to 12.20)	13.93 (10.67 to 17.62)	13.18 (10.45 to 16.33)	15.44 (11.31 to 20.21)
Colon and rectum cancer	273.41 (232.77 to 316.82)	589.81 (405.68 to 849.45)	537.36 (381.66 to 755.75)	604.62 (416.32 to 875.81)	14.18 (12.27 to 16.08)	34.69 (24.11 to 48.84)	32.65 (23.54 to 45.43)	33.80 (23.30 to 47.26)
Liver cancer	466.05 (351.16 to 620.48)	727.46 (451.24 to 1,153.62)	593.15 (387.24 to 892.51)	856.12 (501.61 to 1,420.58)	16.04 (12.50 to 20.54)	29.81 (19.47 to 44.77)	24.88 (17.11 to 35.18)	34.06 (20.97 to 53.16)
Liver cancer due to hepatitis B	227.00 (162.06 to 322.94)	316.01 (189.04 to 507.93)	273.98 (174.48 to 425.62)	355.05 (201.76 to 600.28)	7.41 (5.46 to 10.13)	12.36 (7.71 to 19.40)	10.95 (7.24 to 16.26)	13.54 (8.03 to 21.80)
Liver cancer due to hepatitis C	45.22 (30.88 to 63.01)	78.86 (46.75 to 123.42)	69.93 (44.06 to 106.25)	86.99 (48.78 to 139.29)	1.90 (1.32 to 2.63)	3.84 (2.30 to 5.93)	3.49 (2.19 to 5.22)	4.08 (2.35 to 6.33)
Liver cancer due to alcohol use	82.25 (55.21 to 118.02)	155.44 (89.65 to 258.24)	99.25 (60.22 to 157.24)	213.93 (117.44 to 366.94)	2.99 (2.08 to 4.26)	6.36 (3.80 to 10.25)	4.20 (2.62 to 6.59)	8.50 (4.84 to 14.21)
Liver cancer due to other causes	111.58 (70.24 to 161.61)	177.16 (97.28 to 293.11)	149.98 (87.46 to 241.18)	200.16 (104.65 to 340.90)	3.75 (2.50 to 5.25)	7.24 (4.10 to 11.64)	6.25 (3.73 to 9.63)	7.94 (4.31 to 13.26)
Gallbladder and biliary tract cancer	17.73 (13.98 to 21.95)	24.24 (18.98 to 29.28)	25.35 (19.86 to 30.72)	22.27 (17.67 to 27.12)	0.87 (0.72 to 1.05)	1.32 (1.06 to 1.57)	1.41 (1.13 to 1.68)	1.18 (0.95 to 1.40)
Pancreatic cancer	70.32 (60.73 to 81.12)	145.49 (112.63 to 186.98)	136.45 (108.20 to 170.37)	153.93 (116.85 to 202.29)	3.32 (2.94 to 3.76)	7.62 (6.03 to 9.61)	7.29 (5.92 to 8.99)	7.75 (6.02 to 9.97)
Larynx cancer	26.46 (20.56 to 34.40)	42.18 (19.03 to 82.11)	26.92 (13.22 to 52.20)	63.17 (27.95 to 121.83)	1.17 (0.94 to 1.48)	2.12 (1.00 to 4.08)	1.37 (0.69 to 2.54)	3.04 (1.38 to 5.74)
Tracheal, bronchus, and lung cancer	804.28 (678.53 to 923.50)	1,282.39 (862.24 to 1,759.30)	921.64 (626.71 to 1,233.05)	1,991.00 (1,310.02 to 2,748.83)	39.37 (32.78 to 44.40)	70.42 (49.04 to 93.55)	52.86 (36.37 to 69.81)	102.30 (69.32 to 137.99)
Malignant skin melanoma	7.69 (5.66 to 9.91)	12.28 (9.32 to 15.50)	13.04 (9.84 to 16.50)	10.85 (8.24 to 13.71)	0.29 (0.22 to 0.36)	0.56 (0.43 to 0.72)	0.61 (0.47 to 0.78)	0.47 (0.37 to 0.61)
Non-melanoma skin cancer	11.95 (9.93 to 14.18)	23.22 (19.65 to 27.16)	23.75 (20.03 to 27.88)	22.83 (19.53 to 26.77)	0.58 (0.50 to 0.67)	1.29 (1.13 to 1.49)	1.36 (1.18 to 1.56)	1.21 (1.04 to 1.41)
Breast cancer	147.31 (112.49 to 192.19)	240.66 (164.69 to 358.57)	221.29 (156.50 to 313.07)	255.71 (167.69 to 401.66)	5.31 (4.22 to 6.68)	10.21 (7.11 to 14.93)	9.53 (6.94 to 13.27)	10.53 (7.01 to 16.20)
Cervical cancer	65.57 (35.47 to 89.38)	74.49 (35.74 to 114.59)	70.21 (33.72 to 103.75)	87.68 (39.17 to 140.73)	2.43 (1.36 to 3.21)	3.13 (1.52 to 4.68)	2.98 (1.51 to 4.34)	3.60 (1.68 to 5.69)

Uterine cancer	18.57 (13.82 to 25.30)	26.60 (19.94 to 36.12)	25.98 (19.38 to 35.27)	28.48 (21.41 to 38.51)	0.74 (0.57 to 0.98)	1.20 (0.93 to 1.57)	1.19 (0.92 to 1.55)	1.26 (0.97 to 1.66)
Ovarian cancer	43.76 (33.97 to 56.60)	71.28 (52.75 to 97.46)	71.64 (54.01 to 95.34)	65.31 (48.41 to 89.28)	1.50 (1.21 to 1.90)	2.91 (2.24 to 3.91)	2.94 (2.30 to 3.90)	2.62 (2.01 to 3.55)
Prostate cancer	26.40 (17.22 to 32.75)	82.84 (53.92 to 102.90)	88.13 (57.60 to 108.95)	73.13 (47.45 to 92.52)	1.82 (1.22 to 2.20)	5.80 (3.93 to 7.20)	6.35 (4.32 to 7.77)	4.89 (3.25 to 6.18)
Testicular cancer	5.72 (4.07 to 7.58)	5.80 (4.08 to 7.89)	5.80 (4.07 to 7.91)	5.93 (4.21 to 8.05)	0.15 (0.11 to 0.19)	0.19 (0.14 to 0.24)	0.19 (0.14 to 0.25)	0.19 (0.14 to 0.24)

Vietnam								
Cause	YLLs (in 1000s)				Deaths (in 1000s)			
	2016	2040 Forecast	2040 Better Scenario	2040 Worse Scenario	2016	2040 Forecast	2040 Better Scenario	2040 Worse Scenario
Kidney cancer	14.61 (11.78 to 17.56)	29.07 (20.71 to 39.07)	28.52 (20.83 to 37.90)	28.70 (19.68 to 40.00)	0.60 (0.49 to 0.71)	1.41 (1.02 to 1.88)	1.41 (1.05 to 1.84)	1.34 (0.93 to 1.84)
Bladder cancer	23.23 (18.18 to 27.50)	42.75 (28.72 to 62.08)	38.03 (26.46 to 53.07)	47.93 (31.07 to 71.27)	1.26 (1.00 to 1.46)	2.55 (1.70 to 3.65)	2.32 (1.61 to 3.21)	2.72 (1.76 to 4.00)
Brain and nervous system cancer	49.45 (37.17 to 60.40)	73.57 (55.05 to 91.87)	75.72 (56.56 to 94.85)	69.65 (51.99 to 86.25)	1.38 (1.07 to 1.68)	2.69 (2.15 to 3.30)	2.82 (2.26 to 3.46)	2.46 (1.95 to 3.00)
Thyroid cancer	30.59 (24.58 to 37.91)	41.87 (32.63 to 56.04)	40.17 (31.96 to 52.59)	42.95 (33.04 to 57.39)	1.12 (0.91 to 1.47)	1.85 (1.46 to 2.54)	1.81 (1.45 to 2.45)	1.84 (1.43 to 2.58)
Mesothelioma	4.66 (3.66 to 6.27)	6.61 (5.16 to 8.84)	6.78 (5.30 to 9.12)	6.37 (5.06 to 8.44)	0.16 (0.13 to 0.22)	0.29 (0.23 to 0.36)	0.30 (0.25 to 0.39)	0.27 (0.21 to 0.34)
Hodgkin lymphoma	10.24 (7.45 to 13.19)	9.22 (6.56 to 12.53)	8.91 (6.30 to 12.09)	10.29 (7.40 to 13.84)	0.29 (0.22 to 0.39)	0.34 (0.24 to 0.47)	0.34 (0.24 to 0.47)	0.37 (0.26 to 0.51)
Non-Hodgkin lymphoma	113.33 (93.63 to 137.32)	175.51 (143.10 to 217.25)	179.67 (146.66 to 222.69)	162.31 (132.73 to 201.67)	3.54 (2.95 to 4.45)	7.24 (5.95 to 9.22)	7.59 (6.26 to 9.65)	6.44 (5.28 to 8.26)
Multiple myeloma	12.04 (8.81 to 14.97)	25.91 (18.23 to 33.03)	26.68 (18.83 to 33.66)	23.33 (16.53 to 29.44)	0.51 (0.37 to 0.64)	1.25 (0.88 to 1.58)	1.31 (0.92 to 1.64)	1.09 (0.78 to 1.38)
Leukemia	62.32 (50.22 to 91.06)	87.32 (62.80 to 125.22)	83.30 (61.81 to 118.19)	88.94 (62.55 to 130.30)	1.95 (1.63 to 2.72)	3.69 (2.69 to 5.15)	3.59 (2.69 to 4.95)	3.62 (2.54 to 5.19)
Acute lymphoid leukemia	26.03 (20.61 to 40.04)	34.71 (25.02 to 51.10)	33.29 (24.58 to 48.62)	34.94 (24.99 to 51.21)	0.78 (0.63 to 1.15)	1.42 (1.03 to 2.11)	1.39 (1.04 to 2.05)	1.38 (0.97 to 2.06)
Chronic lymphoid leukemia	1.98 (1.56 to 2.61)	2.76 (1.99 to 3.77)	2.63 (1.92 to 3.50)	2.82 (1.98 to 3.91)	0.07 (0.06 to 0.10)	0.13 (0.09 to 0.17)	0.12 (0.09 to 0.16)	0.12 (0.09 to 0.17)
Acute myeloid leukemia	18.34 (14.16 to 27.00)	28.75 (20.19 to 41.75)	27.74 (19.94 to 39.69)	28.35 (19.45 to 41.70)	0.50 (0.40 to 0.71)	1.06 (0.75 to 1.53)	1.04 (0.75 to 1.48)	1.01 (0.70 to 1.50)
Chronic myeloid leukemia	5.00 (3.77 to 7.41)	4.71 (3.05 to 7.35)	4.40 (2.93 to 6.81)	4.95 (3.12 to 7.77)	0.16 (0.12 to 0.23)	0.19 (0.12 to 0.29)	0.18 (0.12 to 0.28)	0.19 (0.12 to 0.30)
Other leukemia	10.96 (8.53 to 18.26)	16.39 (10.99 to 24.89)	15.24 (10.42 to 22.98)	17.88 (11.64 to 28.14)	0.44 (0.36 to 0.60)	0.89 (0.60 to 1.33)	0.86 (0.59 to 1.24)	0.92 (0.60 to 1.42)
Other neoplasms	178.44 (151.30 to 208.34)	258.07 (217.12 to 306.96)	264.17 (221.28 to 315.95)	248.30 (209.26 to 294.34)	5.37 (4.55 to 6.37)	10.30 (8.70 to 12.59)	10.78 (9.08 to 13.16)	9.52 (8.03 to 11.71)
**Cardiovascular diseases**	**3,604.67 (3,243.25 to 4,032.74)**	**3,650.27 (2,712.66 to 4,907.76)**	**2,721.63 (2,113.53 to 3,582.60)**	**6,208.48 (4,404.19 to 8,515.04)**	**218.95 (203.91 to 237.69)**	**246.58 (191.69 to 319.49)**	**195.90 (157.72 to 247.39)**	**374.02 (283.56 to 498.10)**
Rheumatic heart disease	50.33 (39.45 to 64.62)	34.48 (26.86 to 46.02)	32.96 (25.60 to 43.87)	39.68 (30.54 to 53.45)	2.03 (1.65 to 2.56)	1.87 (1.49 to 2.45)	1.85 (1.48 to 2.39)	2.04 (1.58 to 2.67)
Ischemic heart disease	1,273.29 (1,133.94 to 1,435.32)	1,156.67 (729.27 to 1,868.30)	852.56 (558.40 to 1,320.66)	2,163.17 (1,299.63 to 3,523.54)	86.80 (79.28 to 95.08)	84.50 (55.39 to 126.30)	66.62 (45.26 to 97.50)	138.28 (88.86 to 215.17)
Stroke	1,815.70 (1,608.02 to 2,055.90)	1,672.04 (1,069.62 to 2,560.15)	1,138.34 (763.58 to 1,713.51)	3,079.68 (1,884.45 to 4,884.53)	102.75 (92.85 to 113.54)	107.38 (73.22 to 156.88)	79.00 (56.85 to 112.53)	175.39 (117.56 to 259.80)
Ischemic stroke	541.48 (473.65 to 615.78)	554.08 (344.71 to 950.15)	414.48 (266.18 to 681.44)	865.67 (517.19 to 1,432.47)	40.33 (35.79 to 45.09)	43.02 (26.98 to 76.05)	34.23 (22.53 to 58.09)	60.88 (37.63 to 107.47)

Intracerebral hemorrhage	1,274.22 (1,107.04 to 1,475.38)	1,117.96 (656.90 to 1,819.67)	723.85 (463.14 to 1,130.01)	2,214.01 (1,247.11 to 3,698.41)	62.41 (55.23 to 70.14)	64.36 (41.24 to 99.10)	44.76 (30.96 to 65.30)	114.51 (70.06 to 185.19)
Hypertensive heart disease	178.93 (126.82 to 250.76)	342.18 (192.45 to 627.67)	266.41 (162.26 to 433.06)	454.26 (241.62 to 926.63)	11.37 (8.03 to 16.01)	23.49 (13.30 to 42.96)	18.85 (11.40 to 30.90)	29.30 (15.48 to 61.29)
Cardiomyopathy and myocarditis	74.84 (53.60 to 98.65)	109.05 (72.42 to 152.75)	104.94 (69.57 to 146.77)	106.13 (71.16 to 150.65)	3.44 (2.54 to 4.36)	6.05 (4.00 to 8.37)	6.09 (4.01 to 8.43)	5.56 (3.70 to 7.76)
Myocarditis	11.39 (8.15 to 15.24)	10.51 (7.39 to 14.25)	10.62 (7.37 to 14.40)	10.55 (7.39 to 14.22)	0.41 (0.29 to 0.54)	0.51 (0.36 to 0.69)	0.54 (0.38 to 0.73)	0.48 (0.34 to 0.65)

Vietnam								
Cause	YLLs (in 1000s)				Deaths (in 1000s)			
	2016	2040 Forecast	2040 Better Scenario	2040 Worse Scenario	2016	2040 Forecast	2040 Better Scenario	2040 Worse Scenario
Alcoholic cardiomyopathy	11.23 (6.12 to 17.55)	17.74 (9.54 to 27.78)	14.06 (7.46 to 21.91)	19.08 (10.68 to 29.81)	0.44 (0.26 to 0.65)	0.80 (0.46 to 1.20)	0.66 (0.37 to 1.00)	0.83 (0.49 to 1.23)
Other cardiomyopathy	52.22 (37.45 to 67.30)	80.79 (52.09 to 114.83)	80.26 (51.82 to 113.17)	76.50 (49.06 to 110.25)	2.59 (1.92 to 3.28)	4.74 (3.02 to 6.88)	4.90 (3.12 to 7.04)	4.25 (2.71 to 6.23)
Atrial fibrillation and flutter	31.78 (24.71 to 39.87)	66.47 (47.41 to 90.35)	64.40 (46.81 to 84.29)	66.00 (46.84 to 90.59)	3.60 (2.75 to 4.66)	6.91 (4.89 to 9.35)	6.89 (4.95 to 9.11)	6.52 (4.49 to 9.00)
Aortic aneurysm	24.30 (20.15 to 29.67)	37.73 (29.60 to 47.34)	38.91 (30.93 to 48.36)	37.85 (28.98 to 48.43)	1.53 (1.30 to 1.82)	2.61 (2.08 to 3.22)	2.79 (2.25 to 3.43)	2.44 (1.91 to 3.05)
Peripheral artery disease	1.36 (0.73 to 2.04)	4.23 (2.15 to 6.72)	4.20 (2.11 to 6.59)	3.71 (1.90 to 5.96)	0.13 (0.06 to 0.19)	0.39 (0.18 to 0.63)	0.40 (0.19 to 0.65)	0.32 (0.15 to 0.52)
Endocarditis	42.61 (33.25 to 57.77)	58.00 (41.20 to 83.18)	55.96 (40.08 to 79.99)	58.46 (40.79 to 84.28)	1.58 (1.27 to 2.09)	2.74 (1.94 to 3.90)	2.75 (1.98 to 3.88)	2.61 (1.85 to 3.72)
Other cardiovascular and circulatory diseases	111.53 (83.00 to 175.63)	169.42 (119.17 to 266.48)	162.96 (115.25 to 253.05)	199.53 (134.58 to 318.72)	5.74 (4.34 to 8.82)	10.64 (7.58 to 16.81)	10.65 (7.67 to 16.70)	11.57 (8.05 to 18.84)
**Chronic respiratory diseases**	**579.36 (448.50 to 668.10)**	**591.76 (415.66 to 779.75)**	**449.23 (317.30 to 573.35)**	**906.30 (602.28 to 1,250.33)**	**38.91 (29.60 to 44.79)**	**42.41 (29.45 to 56.25)**	**33.51 (23.29 to 43.47)**	**60.31 (40.08 to 81.83)**
Chronic obstructive pulmonary disease	386.10 (301.56 to 466.72)	429.81 (283.05 to 612.37)	298.94 (197.24 to 418.15)	727.07 (464.15 to 1,072.50)	28.73 (22.28 to 34.52)	32.26 (21.52 to 45.26)	23.78 (16.04 to 33.21)	49.63 (32.09 to 71.74)
Pneumoconiosis	0.56 (0.42 to 0.77)	0.65 (0.48 to 0.89)	0.65 (0.47 to 0.89)	0.63 (0.46 to 0.86)	0.04 (0.03 to 0.05)	0.05 (0.03 to 0.07)	0.05 (0.03 to 0.07)	0.04 (0.03 to 0.06)
Silicosis	0.14 (0.07 to 0.24)	0.12 (0.06 to 0.22)	0.13 (0.06 to 0.22)	0.12 (0.06 to 0.21)	0.01 (0.00 to 0.01)	0.01 (0.00 to 0.01)	0.01 (0.00 to 0.02)	0.01 (0.00 to 0.01)
Asbestosis	0.09 (0.07 to 0.13)	0.13 (0.08 to 0.20)	0.13 (0.09 to 0.21)	0.12 (0.08 to 0.18)	0.00 (0.00 to 0.01)	0.01 (0.00 to 0.01)	0.01 (0.01 to 0.01)	0.01 (0.00 to 0.01)
Coal workers pneumoconiosis	0.02 (0.01 to 0.04)	0.02 (0.01 to 0.04)	0.02 (0.01 to 0.03)	0.02 (0.01 to 0.05)	0.00 (0.00 to 0.00)	0.00 (0.00 to 0.00)	0.00 (0.00 to 0.00)	0.00 (0.00 to 0.00)
Other pneumoconiosis	0.32 (0.23 to 0.43)	0.38 (0.26 to 0.54)	0.37 (0.25 to 0.52)	0.36 (0.25 to 0.51)	0.02 (0.02 to 0.03)	0.03 (0.02 to 0.04)	0.03 (0.02 to 0.04)	0.03 (0.02 to 0.04)
Asthma	168.32 (105.56 to 243.01)	122.20 (63.78 to 197.54)	108.85 (59.38 to 167.85)	142.43 (73.36 to 244.86)	8.86 (5.20 to 13.27)	7.70 (3.93 to 12.23)	7.10 (3.83 to 10.74)	8.52 (4.31 to 14.23)
Interstitial lung disease and pulmonary sarcoidosis	14.82 (9.92 to 21.18)	28.85 (19.59 to 40.04)	30.98 (21.19 to 42.63)	24.90 (16.54 to 35.91)	0.85 (0.53 to 1.29)	1.78 (1.17 to 2.59)	1.96 (1.32 to 2.78)	1.48 (0.94 to 2.22)
Other chronic respiratory diseases	9.55 (4.97 to 15.02)	10.25 (4.38 to 17.84)	9.81 (4.20 to 16.83)	11.27 (4.75 to 19.55)	0.44 (0.18 to 0.83)	0.63 (0.20 to 1.25)	0.62 (0.21 to 1.23)	0.64 (0.21 to 1.30)
**Cirrhosis and other chronic liver diseases**	**573.50 (461.29 to 759.38)**	**765.16 (629.29 to 982.35)**	**709.40 (584.45 to 911.07)**	**830.22 (678.20 to 1,053.82)**	**20.84 (17.43 to 26.48)**	**33.27 (28.34 to 40.26)**	**31.80 (27.17 to 38.28)**	**34.60 (29.23 to 42.39)**
Cirrhosis and other chronic liver diseases due to hepatitis B	217.84 (164.75 to 298.11)	286.63 (219.33 to 385.88)	285.50 (218.96 to 382.73)	292.01 (221.47 to 392.47)	7.79 (6.14 to 10.21)	12.27 (9.77 to 15.85)	12.53 (10.02 to 16.10)	12.03 (9.45 to 15.59)
Cirrhosis and other chronic liver diseases due to hepatitis C	99.12 (71.12 to 139.34)	135.40 (100.44 to 183.75)	134.31 (100.07 to 179.74)	139.17 (102.58 to 189.68)	3.45 (2.63 to 4.48)	5.59 (4.36 to 7.20)	5.66 (4.44 to 7.23)	5.55 (4.30 to 7.22)

Cirrhosis and other chronic liver diseases due to alcohol use	124.16 (92.95 to 175.78)	186.09 (141.99 to 253.86)	133.56 (99.73 to 182.85)	237.12 (180.96 to 323.20)	4.58 (3.58 to 6.12)	7.98 (6.31 to 10.16)	6.01 (4.69 to 7.66)	9.71 (7.71 to 12.47)
Cirrhosis and other chronic liver diseases due to other causes	132.38 (104.55 to 174.65)	157.05 (124.95 to 204.11)	156.03 (124.56 to 202.81)	161.92 (128.33 to 209.21)	5.02 (4.13 to 6.24)	7.43 (6.06 to 9.13)	7.60 (6.23 to 9.25)	7.32 (5.92 to 9.05)
**Digestive diseases**	**239.72 (201.46 to 310.01)**	**305.83 (229.08 to 447.67)**	**283.91 (218.22 to 396.34)**	**336.75 (249.49 to 499.73)**	**12.49 (10.79 to 15.02)**	**18.85 (15.00 to 25.02)**	**18.35 (14.85 to 23.66)**	**19.59 (15.08 to 26.89)**
Peptic ulcer disease	76.07 (53.47 to 108.32)	69.86 (47.91 to 98.88)	66.44 (45.42 to 91.84)	84.37 (56.86 to 120.54)	4.56 (3.39 to 5.97)	4.77 (3.36 to 6.35)	4.68 (3.31 to 6.17)	5.46 (3.75 to 7.38)

Vietnam								
Cause	YLLs (in 1000s)				Deaths (in 1000s)			
	2016	2040 Forecast	2040 Better Scenario	2040 Worse Scenario	2016	2040 Forecast	2040 Better Scenario	2040 Worse Scenario
Gastritis and duodenitis	6.51 (4.52 to 9.18)	10.63 (7.12 to 15.15)	10.67 (7.12 to 15.23)	10.81 (7.25 to 15.55)	0.38 (0.27 to 0.54)	0.69 (0.47 to 1.01)	0.71 (0.48 to 1.03)	0.67 (0.45 to 0.99)
Appendicitis	14.12 (9.89 to 21.08)	13.51 (9.25 to 20.17)	13.05 (8.98 to 19.39)	14.97 (10.30 to 22.74)	0.53 (0.38 to 0.75)	0.70 (0.48 to 0.98)	0.70 (0.49 to 0.97)	0.73 (0.50 to 1.04)
Paralytic ileus and intestinal obstruction	47.73 (36.88 to 63.32)	60.72 (47.49 to 81.60)	61.77 (48.38 to 82.09)	59.83 (46.39 to 82.11)	2.10 (1.70 to 2.69)	3.81 (2.95 to 4.97)	4.03 (3.15 to 5.24)	3.51 (2.65 to 4.66)
Inguinal, femoral, and abdominal hernia	3.30 (2.01 to 4.26)	3.86 (2.13 to 5.17)	3.94 (2.14 to 5.27)	3.81 (2.12 to 5.11)	0.24 (0.12 to 0.33)	0.31 (0.15 to 0.44)	0.32 (0.16 to 0.47)	0.28 (0.14 to 0.41)
Inflammatory bowel disease	5.89 (4.64 to 7.71)	9.05 (7.08 to 11.60)	9.32 (7.31 to 11.85)	8.62 (6.77 to 11.06)	0.28 (0.23 to 0.35)	0.54 (0.43 to 0.67)	0.57 (0.46 to 0.71)	0.49 (0.39 to 0.62)
Vascular intestinal disorders	9.41 (7.51 to 13.18)	17.55 (13.68 to 24.40)	17.98 (14.05 to 24.89)	17.09 (13.13 to 24.34)	0.68 (0.56 to 0.91)	1.32 (1.03 to 1.78)	1.39 (1.08 to 1.86)	1.23 (0.94 to 1.69)
Gallbladder and biliary diseases	16.31 (13.25 to 20.40)	23.34 (15.39 to 34.00)	20.21 (13.95 to 27.41)	25.66 (16.65 to 38.01)	1.06 (0.85 to 1.29)	1.64 (1.04 to 2.42)	1.47 (1.00 to 2.03)	1.72 (1.08 to 2.54)
Pancreatitis	34.90 (24.16 to 48.02)	63.27 (21.91 to 168.87)	46.83 (17.13 to 121.46)	75.72 (25.95 to 201.40)	1.23 (0.88 to 1.65)	2.81 (1.01 to 7.09)	2.13 (0.84 to 5.12)	3.24 (1.15 to 8.30)
Other digestive diseases	25.48 (18.29 to 37.06)	34.03 (23.95 to 50.12)	33.69 (23.78 to 48.76)	35.88 (24.87 to 52.58)	1.42 (1.04 to 1.89)	2.28 (1.60 to 3.08)	2.34 (1.67 to 3.14)	2.27 (1.56 to 3.13)
**Neurological disorders**	**533.35 (462.72 to 613.85)**	**1,034.04 (842.42 to 1,294.62)**	**1,026.19 (857.85 to 1,231.39)**	**962.86 (764.57 to 1,219.84)**	**47.98 (41.00 to 56.30)**	**92.74 (72.13 to 119.78)**	**95.59 (76.73 to 118.51)**	**80.88 (60.52 to 106.97)**
Alzheimer's disease and other dementias	378.50 (318.34 to 452.38)	813.14 (622.61 to 1,063.39)	813.08 (645.76 to 1,027.11)	735.18 (546.34 to 972.21)	42.46 (35.60 to 50.80)	81.43 (61.09 to 108.10)	83.81 (65.34 to 106.68)	70.53 (51.11 to 96.74)
Parkinson's disease	28.23 (21.92 to 35.80)	83.75 (63.23 to 106.82)	92.72 (70.35 to 118.45)	68.36 (51.28 to 88.05)	2.39 (1.82 to 3.08)	6.81 (5.04 to 8.82)	7.72 (5.81 to 9.97)	5.36 (3.87 to 7.06)
Epilepsy	105.58 (86.08 to 126.38)	107.60 (70.25 to 168.47)	89.94 (61.04 to 136.79)	131.44 (85.69 to 207.20)	2.54 (2.04 to 3.03)	3.39 (2.23 to 5.10)	2.90 (1.97 to 4.21)	3.99 (2.62 to 6.13)
Multiple sclerosis	3.18 (2.30 to 4.43)	4.10 (2.97 to 5.62)	4.17 (3.03 to 5.74)	4.03 (2.89 to 5.56)	0.09 (0.07 to 0.13)	0.15 (0.11 to 0.20)	0.15 (0.11 to 0.20)	0.14 (0.10 to 0.19)
Motor neuron disease	3.79 (3.04 to 4.75)	7.11 (5.65 to 8.84)	7.37 (5.85 to 9.18)	6.57 (5.26 to 8.20)	0.12 (0.10 to 0.15)	0.28 (0.23 to 0.34)	0.29 (0.24 to 0.35)	0.25 (0.20 to 0.30)
Other neurological disorders	14.07 (10.82 to 17.62)	18.35 (14.03 to 22.88)	18.92 (14.40 to 23.57)	17.28 (13.18 to 21.48)	0.38 (0.29 to 0.47)	0.68 (0.52 to 0.84)	0.73 (0.55 to 0.89)	0.61 (0.47 to 0.75)
**Mental disorders**	**89.66 (49.74 to 124.56)**	**107.40 (63.70 to 158.00)**	**104.24 (61.70 to 153.08)**	**109.44 (64.37 to 161.38)**	**2.07 (1.27 to 2.77)**	**2.90 (2.02 to 3.88)**	**2.86 (2.00 to 3.83)**	**2.89 (1.97 to 3.86)**
Alcohol use disorders	35.23 (21.57 to 49.26)	34.86 (20.73 to 49.96)	31.45 (18.58 to 45.27)	37.10 (22.06 to 52.61)	0.92 (0.57 to 1.26)	1.09 (0.65 to 1.50)	1.00 (0.59 to 1.38)	1.13 (0.68 to 1.56)
Drug use disorders	54.21 (24.28 to 81.93)	72.32 (39.29 to 113.68)	72.56 (40.14 to 113.63)	72.13 (38.40 to 113.82)	1.15 (0.63 to 1.64)	1.81 (1.24 to 2.57)	1.85 (1.28 to 2.61)	1.75 (1.18 to 2.51)
Opioid use disorders	35.70 (15.44 to 54.37)	40.12 (20.82 to 63.61)	40.08 (21.21 to 63.28)	40.43 (20.45 to 64.34)	0.72 (0.36 to 1.04)	0.95 (0.62 to 1.40)	0.97 (0.63 to 1.41)	0.93 (0.59 to 1.38)

Cocaine use disorders	1.56 (0.75 to 2.78)	2.73 (1.61 to 4.83)	2.69 (1.59 to 4.75)	2.85 (1.67 to 5.02)	0.03 (0.02 to 0.06)	0.07 (0.04 to 0.12)	0.07 (0.04 to 0.12)	0.07 (0.04 to 0.12)
Amphetamine use disorders	1.41 (0.54 to 2.46)	3.07 (1.49 to 5.12)	3.00 (1.49 to 4.96)	3.29 (1.51 to 5.69)	0.03 (0.01 to 0.05)	0.07 (0.04 to 0.11)	0.07 (0.04 to 0.11)	0.07 (0.04 to 0.12)
Other drug use disorders	15.54 (6.44 to 24.69)	26.39 (12.60 to 43.90)	26.79 (12.79 to 44.63)	25.55 (11.93 to 42.82)	0.37 (0.20 to 0.53)	0.72 (0.45 to 1.04)	0.75 (0.47 to 1.07)	0.68 (0.42 to 0.98)
Eating disorders	0.22 (0.14 to 0.43)	0.22 (0.14 to 0.43)	0.22 (0.14 to 0.43)	0.22 (0.14 to 0.42)	0.00 (0.00 to 0.01)	0.00 (0.00 to 0.01)	0.00 (0.00 to 0.01)	0.00 (0.00 to 0.01)

Vietnam								
Cause	YLLs (in 1000s)				Deaths (in 1000s)			
	2016	2040 Forecast	2040 Better Scenario	2040 Worse Scenario	2016	2040 Forecast	2040 Better Scenario	2040 Worse Scenario
**Diabetes, urogenital, blood, and endocrine diseases**	**958.59 (849.33 to 1,080.61)**	**1,776.26 (1,216.68 to 3,073.33)**	**1,493.12 (1,061.63 to 2,445.27)**	**2,192.62 (1,504.06 to 3,720.41)**	**53.84 (47.93 to 59.61)**	**116.36 (81.37 to 203.22)**	**100.60 (72.54 to 170.17)**	**136.47 (94.58 to 234.02)**
Diabetes mellitus	459.52 (401.85 to 525.48)	725.10 (473.99 to 1,070.85)	625.89 (423.06 to 906.82)	965.02 (621.79 to 1,460.04)	27.56 (24.23 to 31.42)	47.83 (32.55 to 67.11)	42.81 (29.60 to 59.28)	59.78 (38.45 to 85.65)
Acute glomerulonephritis	0.62 (0.51 to 0.75)	0.62 (0.46 to 0.82)	0.60 (0.44 to 0.80)	0.67 (0.51 to 0.88)	0.02 (0.01 to 0.02)	0.02 (0.02 to 0.03)	0.02 (0.02 to 0.03)	0.02 (0.02 to 0.03)
Chronic kidney disease	381.32 (255.86 to 443.66)	882.66 (411.95 to 2,294.95)	696.67 (350.12 to 1,743.39)	1,058.72 (478.82 to 2,681.05)	21.20 (12.42 to 24.13)	58.58 (25.90 to 154.64)	47.33 (21.96 to 124.64)	67.35 (28.88 to 177.81)
Chronic kidney disease due to diabetes mellitus	217.59 (155.00 to 258.74)	517.52 (244.20 to 1,295.33)	406.28 (203.60 to 992.09)	624.92 (287.22 to 1,533.53)	10.36 (6.62 to 12.21)	30.48 (13.11 to 82.70)	24.34 (11.07 to 66.28)	35.51 (14.97 to 94.83)
Chronic kidney disease due to hypertension	111.57 (69.63 to 133.62)	290.72 (129.07 to 795.36)	230.91 (109.83 to 620.99)	342.58 (145.83 to 910.51)	8.73 (4.57 to 10.38)	23.55 (10.16 to 63.46)	19.32 (8.67 to 54.44)	26.53 (11.26 to 70.67)
Chronic kidney disease due to glomerulonephritis	26.03 (19.09 to 32.71)	31.80 (16.21 to 73.20)	25.54 (13.80 to 57.90)	39.39 (20.24 to 88.20)	0.86 (0.56 to 1.08)	1.71 (0.73 to 4.85)	1.37 (0.62 to 3.89)	2.03 (0.87 to 5.67)
Chronic kidney disease due to other and unspecified causes	26.13 (19.20 to 31.73)	42.63 (20.40 to 104.82)	33.95 (17.40 to 83.57)	51.83 (24.73 to 122.91)	1.24 (0.75 to 1.57)	2.84 (1.25 to 7.53)	2.30 (1.05 to 6.16)	3.28 (1.38 to 8.58)
Urinary diseases and male infertility	35.48 (30.76 to 40.68)	81.02 (69.05 to 94.88)	84.22 (72.06 to 98.04)	76.39 (64.85 to 89.08)	2.38 (2.07 to 2.71)	5.91 (4.79 to 7.07)	6.36 (5.23 to 7.54)	5.26 (4.20 to 6.33)
Urinary tract infections	30.53 (26.33 to 35.17)	72.42 (61.17 to 84.45)	75.45 (63.83 to 87.64)	67.81 (57.01 to 79.11)	2.04 (1.76 to 2.37)	5.31 (4.23 to 6.44)	5.72 (4.64 to 6.88)	4.69 (3.71 to 5.75)
Urolithiasis	1.25 (0.87 to 1.73)	3.39 (2.18 to 4.79)	3.54 (2.28 to 5.00)	3.15 (2.01 to 4.44)	0.09 (0.06 to 0.12)	0.23 (0.15 to 0.34)	0.25 (0.16 to 0.37)	0.20 (0.13 to 0.30)
Other urinary diseases	3.69 (2.03 to 6.27)	5.20 (2.70 to 8.99)	5.23 (2.72 to 9.00)	5.42 (2.81 to 9.28)	0.25 (0.14 to 0.42)	0.37 (0.20 to 0.66)	0.39 (0.20 to 0.70)	0.36 (0.19 to 0.64)
Gynecological diseases	0.17 (0.13 to 0.24)	0.17 (0.13 to 0.24)	0.17 (0.12 to 0.23)	0.20 (0.14 to 0.28)	0.01 (0.01 to 0.01)	0.01 (0.01 to 0.01)	0.01 (0.01 to 0.01)	0.01 (0.01 to 0.01)
Uterine fibroids	0.03 (0.01 to 0.04)	0.03 (0.01 to 0.05)	0.03 (0.01 to 0.04)	0.03 (0.02 to 0.06)	0.00 (0.00 to 0.00)	0.00 (0.00 to 0.00)	0.00 (0.00 to 0.00)	0.00 (0.00 to 0.00)
Polycystic ovarian syndrome	0.00 (0.00 to 0.01)	0.00 (0.00 to 0.01)	0.00 (0.00 to 0.00)	0.00 (0.00 to 0.01)	0.00 (0.00 to 0.00)	0.00 (0.00 to 0.00)	0.00 (0.00 to 0.00)	0.00 (0.00 to 0.00)
Endometriosis	0.00 (0.00 to 0.01)	0.00 (0.00 to 0.01)	0.00 (0.00 to 0.01)	0.00 (0.00 to 0.01)	0.00 (0.00 to 0.00)	0.00 (0.00 to 0.00)	0.00 (0.00 to 0.00)	0.00 (0.00 to 0.00)
Genital prolapse	0.01 (0.00 to 0.01)	0.01 (0.00 to 0.01)	0.01 (0.00 to 0.01)	0.01 (0.00 to 0.02)	0.00 (0.00 to 0.00)	0.00 (0.00 to 0.00)	0.00 (0.00 to 0.00)	0.00 (0.00 to 0.00)
Other gynecological diseases	0.13 (0.10 to 0.19)	0.13 (0.09 to 0.19)	0.13 (0.09 to 0.19)	0.15 (0.11 to 0.22)	0.00 (0.00 to 0.01)	0.01 (0.00 to 0.01)	0.01 (0.00 to 0.01)	0.01 (0.00 to 0.01)
Hemoglobinopathies and hemolytic anemias	37.18 (29.32 to 45.26)	31.11 (24.48 to 39.30)	30.30 (23.93 to 37.96)	34.05 (26.92 to 43.03)	1.21 (0.97 to 1.49)	1.56 (1.23 to 2.04)	1.56 (1.22 to 2.04)	1.65 (1.30 to 2.16)
Thalassemias	16.46 (11.28 to 22.88)	5.08 (3.40 to 7.51)	5.05 (3.44 to 7.42)	5.09 (3.27 to 7.74)	0.21 (0.15 to 0.28)	0.07 (0.05 to 0.10)	0.07 (0.05 to 0.10)	0.07 (0.04 to 0.10)

Sickle cell disorders	0.05 (0.04 to 0.07)	0.05 (0.03 to 0.06)	0.04 (0.03 to 0.06)	0.05 (0.04 to 0.07)	0.00 (0.00 to 0.00)	0.00 (0.00 to 0.00)	0.00 (0.00 to 0.00)	0.00 (0.00 to 0.00)
G6PD deficiency	9.01 (6.98 to 11.75)	12.28 (9.37 to 16.40)	11.64 (8.79 to 15.63)	14.25 (10.78 to 19.03)	0.24 (0.19 to 0.31)	0.46 (0.36 to 0.62)	0.45 (0.34 to 0.59)	0.53 (0.41 to 0.71)
Other hemoglobinopathies and hemolytic anemias	11.65 (9.26 to 14.69)	13.70 (10.56 to 18.20)	13.56 (10.54 to 17.84)	14.66 (11.41 to 19.55)	0.76 (0.61 to 0.97)	1.03 (0.79 to 1.37)	1.04 (0.80 to 1.38)	1.05 (0.79 to 1.44)
Endocrine, metabolic, blood, and immune disorders	44.30 (36.40 to 58.31)	55.58 (44.64 to 79.72)	55.26 (44.20 to 79.19)	57.58 (46.43 to 81.86)	1.47 (1.20 to 2.28)	2.45 (1.94 to 4.05)	2.51 (2.00 to 4.21)	2.40 (1.91 to 3.91)

Vietnam								
Cause	YLLs (in 1000s)				Deaths (in 1000s)			
	2016	2040 Forecast	2040 Better Scenario	2040 Worse Scenario	2016	2040 Forecast	2040 Better Scenario	2040 Worse Scenario
**Musculoskeletal disorders**	**15.67 (10.84 to 26.68)**	**26.54 (18.03 to 45.28)**	**27.45 (18.75 to 47.08)**	**25.03 (17.10 to 42.39)**	**0.55 (0.38 to 0.99)**	**1.26 (0.85 to 2.35)**	**1.34 (0.91 to 2.51)**	**1.14 (0.77 to 2.10)**
Rheumatoid arthritis	1.99 (1.28 to 3.21)	4.59 (2.88 to 7.59)	4.52 (2.86 to 7.40)	4.98 (3.14 to 8.30)	0.10 (0.07 to 0.18)	0.27 (0.17 to 0.48)	0.28 (0.17 to 0.49)	0.29 (0.18 to 0.52)
Other musculoskeletal disorders	13.68 (9.27 to 23.74)	21.94 (14.56 to 39.38)	22.93 (15.22 to 40.70)	20.05 (13.31 to 35.91)	0.45 (0.30 to 0.84)	0.98 (0.64 to 1.89)	1.06 (0.70 to 2.01)	0.85 (0.55 to 1.66)
**Other non-communicable diseases**	**555.64 (398.06 to 745.22)**	**299.04 (223.34 to 382.70)**	**283.42 (209.84 to 359.02)**	**343.19 (250.80 to 447.19)**	**8.40 (6.04 to 10.87)**	**8.33 (5.43 to 10.80)**	**8.49 (5.39 to 11.03)**	**8.38 (5.65 to 10.83)**
Congenital birth defects	514.29 (366.22 to 694.61)	219.89 (158.28 to 293.46)	201.88 (147.35 to 267.02)	266.83 (190.21 to 362.96)	6.45 (4.66 to 8.53)	3.16 (2.36 to 4.07)	2.94 (2.21 to 3.79)	3.75 (2.77 to 4.92)
Neural tube defects	20.73 (8.49 to 41.22)	3.83 (1.55 to 8.48)	3.31 (1.35 to 7.23)	5.31 (2.09 to 11.53)	0.25 (0.10 to 0.48)	0.05 (0.02 to 0.10)	0.04 (0.02 to 0.09)	0.07 (0.03 to 0.14)
Congenital heart anomalies	305.20 (215.07 to 404.86)	129.00 (90.90 to 174.42)	118.59 (84.61 to 159.85)	156.29 (107.74 to 211.63)	3.85 (2.74 to 5.01)	1.81 (1.31 to 2.38)	1.68 (1.23 to 2.19)	2.15 (1.54 to 2.83)
Orofacial clefts	0.84 (0.23 to 1.97)	0.26 (0.07 to 0.66)	0.22 (0.06 to 0.57)	0.38 (0.10 to 0.99)	0.01 (0.00 to 0.02)	0.00 (0.00 to 0.01)	0.00 (0.00 to 0.01)	0.00 (0.00 to 0.01)
Down syndrome	7.61 (5.01 to 11.54)	5.34 (3.62 to 8.38)	4.98 (3.38 to 7.79)	6.34 (4.32 to 9.90)	0.12 (0.08 to 0.17)	0.11 (0.07 to 0.17)	0.10 (0.07 to 0.16)	0.12 (0.08 to 0.19)
Other chromosomal abnormalities	10.35 (5.71 to 17.95)	6.68 (3.53 to 11.61)	6.00 (3.22 to 10.21)	8.47 (4.48 to 14.48)	0.12 (0.07 to 0.21)	0.09 (0.05 to 0.15)	0.08 (0.04 to 0.13)	0.11 (0.06 to 0.18)
Congenital musculoskeletal and limb anomalies	11.13 (6.18 to 17.43)	5.10 (2.68 to 8.45)	4.66 (2.47 to 7.73)	6.16 (3.18 to 10.26)	0.13 (0.07 to 0.20)	0.06 (0.03 to 0.10)	0.06 (0.03 to 0.09)	0.08 (0.04 to 0.12)
Urogenital congenital anomalies	10.40 (7.54 to 13.85)	8.84 (6.54 to 11.78)	8.57 (6.29 to 11.44)	9.62 (7.10 to 12.69)	0.19 (0.15 to 0.24)	0.24 (0.18 to 0.32)	0.24 (0.18 to 0.32)	0.26 (0.19 to 0.34)
Digestive congenital anomalies	27.50 (14.17 to 47.06)	10.81 (5.30 to 18.99)	9.69 (4.72 to 16.98)	13.71 (6.60 to 24.69)	0.32 (0.17 to 0.55)	0.13 (0.07 to 0.23)	0.12 (0.06 to 0.20)	0.17 (0.08 to 0.29)
Other congenital birth defects	120.53 (77.13 to 190.70)	50.03 (31.46 to 80.73)	45.86 (28.92 to 72.47)	60.55 (37.15 to 99.67)	1.46 (0.95 to 2.29)	0.67 (0.44 to 1.04)	0.62 (0.41 to 0.95)	0.80 (0.51 to 1.26)
Skin and subcutaneous diseases	30.51 (14.66 to 41.29)	75.73 (39.35 to 101.71)	78.43 (40.70 to 104.79)	72.20 (37.43 to 96.74)	1.83 (0.80 to 2.61)	5.13 (2.48 to 7.28)	5.52 (2.64 to 7.80)	4.58 (2.21 to 6.58)
Cellulitis	3.90 (1.96 to 6.62)	14.45 (7.75 to 23.85)	15.35 (8.15 to 25.12)	12.90 (6.87 to 21.92)	0.21 (0.10 to 0.37)	0.85 (0.46 to 1.45)	0.94 (0.50 to 1.55)	0.72 (0.39 to 1.30)
Pyoderma	15.81 (7.32 to 22.50)	36.99 (18.14 to 52.31)	37.43 (18.14 to 52.94)	37.06 (18.33 to 52.67)	0.73 (0.31 to 1.09)	2.17 (0.98 to 3.17)	2.29 (1.04 to 3.34)	2.04 (0.95 to 3.00)
Decubitus ulcer	9.95 (3.66 to 14.71)	22.71 (8.86 to 34.74)	24.01 (9.39 to 37.01)	20.76 (8.08 to 32.31)	0.86 (0.28 to 1.37)	2.02 (0.70 to 3.38)	2.21 (0.77 to 3.65)	1.75 (0.59 to 2.96)
Other skin and subcutaneous diseases	0.84 (0.36 to 1.25)	1.58 (0.69 to 2.31)	1.64 (0.72 to 2.39)	1.47 (0.65 to 2.16)	0.04 (0.01 to 0.05)	0.08 (0.03 to 0.13)	0.09 (0.04 to 0.14)	0.07 (0.03 to 0.11)
Sudden infant death syndrome	10.85 (5.99 to 19.10)	3.42 (1.58 to 6.88)	3.12 (1.42 to 6.27)	4.16 (1.90 to 8.24)	0.13 (0.07 to 0.22)	0.04 (0.02 to 0.08)	0.04 (0.02 to 0.07)	0.05 (0.02 to 0.10)

**Injuries**	**2,593.87 (2,143.76 to 3,004.68)**	**2,242.60 (1,587.96 to 3,387.55)**	**1,764.02 (1,250.15 to 2,793.23)**	**2,833.49 (1,998.17 to 4,194.43)**	**67.80 (56.33 to 77.60)**	**80.71 (60.74 to 107.12)**	**69.39 (51.89 to 92.04)**	**92.76 (69.78 to 123.66)**
**Transport injuries**	**1,126.35 (899.70 to 1,354.35)**	**815.23 (498.31 to 1,438.66)**	**404.39 (254.52 to 660.79)**	**1,311.82 (805.95 to 2,284.29)**	**25.70 (21.08 to 30.44)**	**24.03 (15.60 to 39.77)**	**12.86 (8.64 to 20.04)**	**36.03 (23.07 to 59.74)**
Road injuries	1,068.82 (855.07 to 1,282.24)	742.51 (457.64 to 1,292.58)	340.04 (217.00 to 551.63)	1,229.74 (760.67 to 2,137.45)	24.31 (19.91 to 28.82)	21.72 (14.11 to 35.36)	10.77 (7.30 to 16.54)	33.54 (21.53 to 54.69)
Pedestrian road injuries	221.43 (159.03 to 281.58)	165.02 (100.64 to 277.79)	82.43 (53.79 to 128.17)	254.60 (153.57 to 435.60)	6.12 (4.56 to 7.58)	6.21 (4.06 to 9.80)	3.34 (2.27 to 4.89)	8.96 (5.73 to 14.40)

Vietnam								
Cause	YLLs (in 1000s)				Deaths (in 1000s)			
	2016	2040 Forecast	2040 Better Scenario	2040 Worse Scenario	2016	2040 Forecast	2040 Better Scenario	2040 Worse Scenario
Cyclist road injuries	56.54 (35.19 to 93.67)	48.78 (25.32 to 90.39)	24.20 (12.95 to 43.18)	75.80 (39.17 to 143.98)	1.45 (0.90 to 2.42)	1.60 (0.84 to 2.93)	0.87 (0.48 to 1.52)	2.31 (1.20 to 4.32)
Motorcyclist road injuries	274.91 (198.75 to 356.78)	196.45 (103.45 to 380.14)	110.46 (58.25 to 210.70)	287.41 (152.64 to 536.93)	5.32 (3.91 to 6.93)	4.37 (2.34 to 8.35)	2.61 (1.40 to 4.82)	6.11 (3.38 to 11.39)
Motor vehicle road injuries	496.08 (359.17 to 629.93)	317.26 (191.54 to 534.55)	116.80 (73.37 to 185.39)	585.80 (350.95 to 996.23)	10.94 (8.09 to 13.74)	9.04 (5.84 to 14.33)	3.71 (2.50 to 5.49)	15.40 (9.72 to 24.64)
Other road injuries	19.87 (12.20 to 35.20)	14.99 (7.82 to 28.73)	6.15 (3.30 to 11.64)	26.13 (13.51 to 51.73)	0.48 (0.30 to 0.85)	0.49 (0.26 to 0.94)	0.24 (0.13 to 0.45)	0.76 (0.40 to 1.49)
Other transport injuries	57.53 (40.65 to 80.00)	72.72 (40.04 to 143.56)	64.35 (37.09 to 122.19)	82.08 (45.20 to 161.87)	1.40 (1.05 to 1.87)	2.31 (1.31 to 4.29)	2.10 (1.25 to 3.71)	2.49 (1.39 to 4.56)
**Unintentional injuries**	**1,051.32 (853.16 to 1,220.52)**	**821.45 (637.98 to 988.69)**	**806.17 (627.74 to 965.19)**	**873.76 (675.28 to 1,053.30)**	**32.47 (24.38 to 37.44)**	**40.32 (28.25 to 47.28)**	**41.46 (29.41 to 48.44)**	**39.65 (27.96 to 46.95)**
Falls	289.51 (211.07 to 344.80)	389.35 (256.18 to 459.46)	397.52 (264.16 to 470.22)	385.66 (254.94 to 460.01)	16.46 (9.45 to 19.78)	26.94 (15.47 to 32.89)	28.40 (16.68 to 34.36)	25.35 (14.36 to 31.53)
Drowning	425.47 (350.78 to 503.81)	200.73 (150.38 to 276.95)	185.05 (139.20 to 250.21)	233.09 (173.44 to 323.18)	7.85 (6.69 to 9.34)	5.52 (4.23 to 7.55)	5.22 (4.06 to 7.06)	6.07 (4.58 to 8.41)
Fire, heat, and hot substances	18.27 (14.07 to 22.36)	12.95 (9.21 to 18.46)	12.02 (8.70 to 16.74)	14.75 (10.22 to 21.38)	0.49 (0.39 to 0.58)	0.50 (0.37 to 0.68)	0.48 (0.36 to 0.64)	0.53 (0.39 to 0.74)
Poisonings	36.98 (13.18 to 57.76)	19.39 (5.26 to 36.01)	17.11 (4.74 to 31.32)	23.95 (6.45 to 45.26)	0.89 (0.30 to 1.37)	0.56 (0.17 to 0.99)	0.50 (0.15 to 0.86)	0.67 (0.19 to 1.22)
Exposure to mechanical forces	58.03 (44.61 to 72.13)	39.56 (29.36 to 50.68)	38.08 (28.32 to 49.28)	44.46 (32.99 to 56.48)	1.34 (1.05 to 1.63)	1.27 (1.02 to 1.54)	1.25 (1.01 to 1.53)	1.37 (1.09 to 1.65)
Unintentional firearm injuries	6.04 (3.40 to 8.66)	3.18 (1.78 to 4.70)	3.02 (1.71 to 4.39)	3.71 (2.04 to 5.44)	0.13 (0.08 to 0.18)	0.09 (0.06 to 0.13)	0.09 (0.06 to 0.12)	0.10 (0.07 to 0.14)
Unintentional suffocation	4.74 (2.96 to 8.01)	2.97 (2.09 to 4.16)	2.84 (1.98 to 3.98)	3.37 (2.38 to 4.78)	0.09 (0.06 to 0.12)	0.08 (0.05 to 0.13)	0.08 (0.04 to 0.13)	0.09 (0.05 to 0.14)
Other exposure to mechanical forces	47.25 (34.45 to 59.17)	33.41 (24.36 to 43.48)	32.22 (23.61 to 42.21)	37.39 (26.99 to 47.92)	1.12 (0.87 to 1.37)	1.10 (0.88 to 1.33)	1.08 (0.87 to 1.32)	1.18 (0.93 to 1.42)
Adverse effects of medical treatment	41.77 (31.60 to 52.22)	36.98 (28.13 to 46.97)	36.49 (27.63 to 46.68)	39.56 (29.72 to 49.80)	1.25 (0.97 to 1.53)	1.58 (1.22 to 1.94)	1.61 (1.24 to 2.01)	1.59 (1.23 to 1.95)
Animal contact	19.34 (14.31 to 23.75)	21.02 (14.24 to 26.88)	20.10 (13.74 to 25.58)	24.22 (16.47 to 31.29)	0.63 (0.42 to 0.78)	0.91 (0.57 to 1.17)	0.89 (0.57 to 1.14)	1.00 (0.62 to 1.31)
Venomous animal contact	13.09 (8.22 to 16.92)	14.61 (7.84 to 20.06)	13.94 (7.52 to 19.08)	16.86 (8.89 to 22.99)	0.46 (0.26 to 0.60)	0.66 (0.34 to 0.91)	0.65 (0.34 to 0.89)	0.74 (0.37 to 1.02)
Non-venomous animal contact	6.25 (4.37 to 8.87)	6.41 (4.49 to 9.08)	6.15 (4.26 to 8.75)	7.36 (5.15 to 10.47)	0.17 (0.13 to 0.25)	0.24 (0.18 to 0.35)	0.24 (0.17 to 0.35)	0.26 (0.19 to 0.39)
Foreign body	56.62 (40.20 to 75.27)	36.54 (25.63 to 48.99)	36.67 (25.86 to 49.38)	37.09 (25.73 to 49.95)	1.21 (0.87 to 1.57)	1.22 (0.89 to 1.56)	1.28 (0.94 to 1.62)	1.15 (0.83 to 1.46)
Pulmonary aspiration and foreign body in airway	51.98 (34.16 to 70.50)	33.37 (21.60 to 45.57)	33.57 (22.23 to 45.87)	33.66 (21.74 to 46.48)	1.09 (0.73 to 1.45)	1.11 (0.75 to 1.45)	1.17 (0.80 to 1.52)	1.03 (0.68 to 1.36)

Foreign body in other body part	4.64 (3.17 to 8.49)	3.17 (2.15 to 5.79)	3.11 (2.06 to 5.79)	3.43 (2.33 to 6.19)	0.12 (0.08 to 0.21)	0.11 (0.08 to 0.21)	0.11 (0.08 to 0.21)	0.12 (0.08 to 0.21)
Environmental heat and cold exposure	1.67 (0.91 to 2.26)	1.32 (0.73 to 1.83)	1.23 (0.69 to 1.74)	1.61 (0.88 to 2.24)	0.05 (0.03 to 0.06)	0.05 (0.03 to 0.06)	0.05 (0.03 to 0.06)	0.06 (0.04 to 0.07)
Other unintentional injuries	103.66 (74.81 to 134.74)	63.61 (44.11 to 86.11)	61.90 (42.53 to 85.26)	69.37 (49.65 to 93.90)	2.30 (1.71 to 2.95)	1.78 (1.29 to 2.28)	1.77 (1.29 to 2.28)	1.86 (1.37 to 2.39)
**Self-harm and interpersonal violence**	**415.68 (332.08 to 508.53)**	**436.95 (273.79 to 680.25)**	**385.17 (252.51 to 585.35)**	**475.68 (288.89 to 754.93)**	**9.62 (7.89 to 11.51)**	**12.79 (8.34 to 19.34)**	**11.46 (7.84 to 16.50)**	**13.52 (8.61 to 20.59)**

Vietnam								
**Cause**	**YLLs (in 1000s)**				**Deaths (in 1000s)**			
	**2016**	**2040 Forecast**	**2040 Better Scenario**	**2040 Worse Scenario**	**2016**	**2040 Forecast**	**2040 Better Scenario**	**2040 Worse Scenario**
Self-harm	319.64 (256.78 to 389.00)	373.73 (220.89 to 610.51)	323.30 (201.03 to 506.87)	411.34 (234.94 to 683.98)	7.74 (6.40 to 9.20)	11.33 (7.13 to 17.88)	10.03 (6.67 to 14.78)	12.05 (7.37 to 19.16)
Self-harm by firearm	10.03 (6.25 to 16.60)	9.24 (4.17 to 18.04)	7.82 (3.78 to 15.26)	10.39 (4.65 to 20.59)	0.25 (0.15 to 0.40)	0.30 (0.14 to 0.55)	0.26 (0.13 to 0.46)	0.32 (0.15 to 0.59)
Self-harm by other specified means	309.61 (249.55 to 378.11)	364.49 (216.06 to 589.14)	315.48 (196.68 to 492.30)	400.95 (229.99 to 663.49)	7.50 (6.21 to 8.94)	11.03 (6.98 to 17.28)	9.77 (6.53 to 14.37)	11.74 (7.22 to 18.64)
Interpersonal violence	96.04 (64.55 to 130.27)	63.22 (40.42 to 96.48)	61.87 (39.82 to 92.70)	64.35 (41.05 to 97.98)	1.87 (1.29 to 2.51)	1.46 (0.98 to 2.13)	1.43 (0.97 to 2.06)	1.46 (0.97 to 2.15)
Physical violence by firearm	7.16 (3.90 to 13.35)	4.55 (2.49 to 8.36)	4.54 (2.47 to 8.46)	4.64 (2.53 to 8.42)	0.14 (0.08 to 0.26)	0.10 (0.06 to 0.19)	0.10 (0.06 to 0.19)	0.10 (0.06 to 0.18)
Physical violence by sharp object	64.26 (36.56 to 91.39)	41.89 (23.12 to 63.70)	42.03 (23.23 to 64.12)	41.56 (22.53 to 62.94)	1.21 (0.71 to 1.70)	0.89 (0.52 to 1.31)	0.90 (0.53 to 1.32)	0.87 (0.49 to 1.28)
Physical violence by other means	24.62 (17.56 to 40.10)	16.78 (9.84 to 30.80)	15.31 (9.22 to 27.77)	18.15 (10.48 to 33.62)	0.52 (0.38 to 0.82)	0.47 (0.29 to 0.82)	0.43 (0.27 to 0.74)	0.49 (0.29 to 0.87)
**Forces of nature, conflict and terrorism, and executions and police conflict**	**0.52 (0.44 to 0.60)**	**168.96 (0.00 to 1,048.82)**	**168.29 (0.00 to 1,044.77)**	**172.22 (0.00 to 1,070.79)**	**0.01 (0.01 to 0.01)**	**3.58 (0.00 to 22.25)**	**3.61 (0.00 to 22.46)**	**3.56 (0.00 to 22.17)**
Exposure to forces of nature	0.00 (0.00 to 0.00)	6.93 (0.00 to 48.93)	6.92 (0.00 to 48.96)	7.03 (0.00 to 49.42)	0.00 (0.00 to 0.00)	0.17 (0.00 to 1.24)	0.17 (0.00 to 1.26)	0.17 (0.00 to 1.21)
Conflict and terrorism	0.00 (0.00 to 0.00)	162.03 (0.00 to 1,048.82)	161.36 (0.00 to 1,044.77)	165.19 (0.00 to 1,070.79)	0.00 (0.00 to 0.00)	3.41 (0.00 to 22.25)	3.44 (0.00 to 22.46)	3.39 (0.00 to 22.17)
Executions and police conflict	0.52 (0.44 to 0.60)	0.00 (0.00 to 0.00)	0.00 (0.00 to 0.00)	0.00 (0.00 to 0.00)	0.01 (0.01 to 0.01)	0.00 (0.00 to 0.00)	0.00 (0.00 to 0.00)	0.00 (0.00 to 0.00)

Oceania								
**Cause**	**YLLs (actual counts)**				**Deaths (actual counts)**			
	**2016**	**2040 Forecast**	**2040 Better Scenario**	**2040 Worse Scenario**	**2016**	**2040 Forecast**	**2040 Better Scenario**	**2040 Worse Scenario**
**All causes**	**3,635,869.45 (3,060,471.99 to 4,327,879.66)**	**4,491,033.47 (3,748,718.20 to 5,405,571.88)**	**2,859,928.15 (2,425,397.99 to 3,397,381.75)**	**5,597,639.05 (4,690,790.36 to 6,657,416.52)**	**90,985.00 (79,517.12 to 103,386.10)**	**139,616.52 (124,665.33 to 155,478.07)**	**98,838.15 (88,657.26 to 110,656.52)**	**166,747.42 (149,290.98 to 184,519.48)**
**Communicable, maternal, neonatal, and nutritional diseases**	**1,260,905.05 (910,793.54 to 1,783,706.06)**	**1,069,707.62 (728,389.33 to 1,577,356.89)**	**531,773.87 (386,700.51 to 740,906.64)**	**1,458,668.93 (968,191.54 to 2,191,962.39)**	**20,180.61 (15,677.97 to 26,599.88)**	**20,779.97 (14,824.80 to 28,712.35)**	**12,088.96 (9,028.70 to 16,361.21)**	**27,017.99 (18,733.33 to 37,894.46)**
**HIV/AIDS and tuberculosis**	**54,211.17 (42,978.40 to 69,632.91)**	**80,033.83 (53,288.60 to 116,467.88)**	**49,835.50 (30,417.28 to 78,643.28)**	**112,844.98 (78,132.10 to 165,355.30)**	**1,237.80 (1,031.85 to 1,519.02)**	**1,796.48 (1,222.09 to 2,505.44)**	**1,132.56 (733.84 to 1,716.46)**	**2,437.15 (1,749.17 to 3,499.08)**
Tuberculosis	21,966.16 (18,945.41 to 25,460.45)	20,927.59 (14,886.52 to 30,183.93)	9,610.03 (6,868.92 to 13,601.40)	27,970.92 (19,262.87 to 42,302.08)	627.98 (546.68 to 720.87)	678.18 (483.90 to 963.10)	338.65 (248.32 to 468.42)	860.83 (596.06 to 1,271.60)
HIV/AIDS	32,245.01 (21,532.22 to 47,352.77)	59,106.24 (34,095.62 to 94,203.82)	40,225.47 (21,301.83 to 68,008.65)	84,874.06 (52,255.59 to 137,989.25)	609.82 (430.25 to 868.98)	1,118.30 (643.74 to 1,795.23)	793.91 (402.60 to 1,359.59)	1,576.31 (987.09 to 2,554.62)
**Diarrhea, lower respiratory, and other common infectious diseases**	**609,555.39 (433,947.64 to 863,713.80)**	**515,743.44 (298,027.07 to 811,971.36)**	**239,815.36 (153,768.99 to 365,111.13)**	**778,273.41 (431,770.08 to 1,286,244.69)**	**10,930.21 (8,573.39 to 14,243.23)**	**12,126.91 (7,564.85 to 18,851.87)**	**6,978.97 (4,493.21 to 10,786.39)**	**16,650.22 (10,002.62 to 26,138.74)**
Diarrheal diseases	87,605.68 (55,612.69 to 130,231.52)	74,931.07 (13,737.02 to 215,634.89)	36,425.72 (7,437.29 to 107,129.83)	105,419.36 (21,426.42 to 298,357.88)	2,311.55 (1,377.90 to 3,590.62)	2,756.73 (496.58 to 7,866.80)	1,625.13 (310.43 to 4,707.45)	3,691.62 (645.85 to 10,430.45)
Intestinal infectious diseases	21,184.08 (10,539.84 to 36,171.89)	20,540.73 (9,263.41 to 37,337.93)	14,144.56 (6,496.30 to 25,260.70)	21,985.18 (9,937.34 to 39,687.62)	304.72 (152.55 to 520.47)	302.88 (144.03 to 533.37)	216.91 (104.03 to 378.03)	321.54 (149.56 to 575.75)
Typhoid fever	18,088.65 (8,546.01 to 32,011.86)	17,073.10 (7,555.71 to 31,524.75)	11,833.05 (5,371.42 to 21,268.52)	18,242.94 (7,950.98 to 34,208.43)	258.95 (124.36 to 457.05)	249.92 (114.51 to 458.21)	180.09 (84.52 to 319.68)	264.92 (118.72 to 491.04)
Paratyphoid fever	2,962.98 (1,294.35 to 6,021.24)	3,401.96 (1,407.18 to 7,113.08)	2,279.20 (966.77 to 4,551.76)	3,669.82 (1,484.62 to 7,680.76)	43.80 (19.22 to 88.84)	51.78 (22.17 to 106.49)	36.14 (15.70 to 72.42)	55.36 (23.26 to 114.36)
Other intestinal infectious diseases	132.45 (44.25 to 318.03)	65.67 (15.70 to 186.39)	32.31 (8.88 to 84.68)	72.43 (16.40 to 202.13)	1.97 (0.68 to 4.51)	1.18 (0.36 to 2.97)	0.68 (0.22 to 1.61)	1.26 (0.38 to 3.26)
Lower respiratory infections	368,629.87 (253,352.93 to 541,652.30)	262,281.35 (136,484.53 to 479,637.67)	123,662.72 (76,599.67 to 200,664.81)	425,626.56 (192,299.54 to 808,759.51)	6,550.66 (4,940.57 to 8,958.73)	6,854.79 (4,385.00 to 10,853.40)	4,060.24 (2,885.56 to 5,874.51)	9,620.35 (5,528.29 to 15,729.17)
Upper respiratory infections	401.81 (202.65 to 804.09)	389.96 (165.00 to 905.59)	191.65 (90.94 to 398.03)	441.71 (182.84 to 1,021.87)	7.25 (4.23 to 12.32)	8.62 (4.12 to 17.40)	4.80 (2.38 to 9.46)	9.43 (4.40 to 19.48)
Otitis media	42.06 (19.24 to 89.03)	13.36 (5.52 to 31.92)	12.72 (5.34 to 31.59)	13.39 (5.56 to 32.48)	0.80 (0.42 to 1.50)	0.34 (0.15 to 0.85)	0.35 (0.17 to 0.79)	0.32 (0.14 to 0.81)
Meningitis	46,070.55 (30,542.77 to 73,971.52)	32,125.33 (15,516.10 to 60,727.81)	20,232.20 (12,114.12 to 32,950.93)	37,896.05 (16,461.52 to 77,581.99)	667.43 (468.31 to 1,010.44)	514.17 (292.49 to 885.30)	370.25 (239.92 to 559.16)	578.01 (304.83 to 1,067.94)
Pneumococcal meningitis	138.76 (85.21 to 236.48)	65.71 (34.48 to 116.58)	45.79 (27.65 to 72.67)	78.18 (38.54 to 148.37)	2.02 (1.33 to 3.19)	1.17 (0.72 to 1.90)	0.95 (0.64 to 1.41)	1.32 (0.78 to 2.24)
H influenzae type B meningitis	5,198.81 (3,201.30 to 9,052.54)	8,328.77 (1,668.17 to 26,138.47)	2,668.46 (1,136.76 to 6,395.56)	12,925.21 (1,908.50 to 41,463.36)	73.04 (48.23 to 118.79)	108.86 (29.02 to 317.85)	43.67 (23.28 to 89.72)	161.96 (32.63 to 500.94)
Meningococcal meningitis	8,910.56 (5,621.61 to 14,812.14)	5,003.43 (2,859.88 to 8,401.17)	3,695.04 (2,279.40 to 5,831.70)	5,232.21 (2,961.38 to 8,761.11)	134.50 (90.94 to 209.52)	90.71 (58.04 to 139.00)	74.16 (49.44 to 110.29)	92.25 (58.10 to 139.62)
Other meningitis	31,822.42 (20,990.79 to 51,789.94)	18,727.42 (10,143.48 to 30,770.30)	13,822.92 (8,301.10 to 21,774.93)	19,660.44 (10,431.75 to 32,767.59)	457.87 (315.98 to 691.79)	313.42 (192.80 to 495.73)	251.47 (163.54 to 374.90)	322.48 (192.38 to 504.50)
Encephalitis	15,178.39 (10,126.74 to 24,891.03)	17,248.55 (9,571.07 to 28,671.90)	11,582.72 (6,945.80 to 17,409.58)	18,208.87 (9,856.08 to 30,355.97)	235.93 (170.20 to 347.92)	306.64 (195.17 to 445.04)	229.07 (156.57 to 310.33)	315.53 (196.46 to 464.96)

Diphtheria	203.94 (108.69 to 367.65)	78.51 (24.50 to 198.90)	31.31 (18.84 to 49.14)	112.85 (25.34 to 318.91)	2.90 (1.73 to 4.82)	1.15 (0.46 to 2.59)	0.60 (0.37 to 0.92)	1.55 (0.48 to 3.95)
Whooping cough	30,387.31 (4,982.41 to 85,827.59)	34,100.70 (1,399.18 to 109,255.64)	5,552.21 (558.52 to 18,118.65)	36,755.20 (1,596.39 to 125,549.97)	359.54 (59.04 to 1,015.29)	403.82 (18.15 to 1,296.66)	67.43 (7.21 to 218.19)	435.02 (19.59 to 1,479.88)
Tetanus	446.51 (207.59 to 937.81)	118.95 (46.61 to 268.77)	46.33 (18.25 to 128.18)	125.75 (48.44 to 289.96)	8.14 (3.71 to 20.43)	2.00 (0.86 to 4.64)	1.24 (0.39 to 3.80)	2.04 (0.88 to 4.53)
Measles	36,661.97 (11,387.41 to 82,423.56)	71,921.73 (5,509.07 to 308,727.97)	26,991.06 (3,912.69 to 99,437.00)	129,451.48 (7,958.25 to 528,254.22)	438.05 (137.48 to 987.29)	937.77 (93.95 to 3,773.66)	379.60 (64.65 to 1,228.33)	1,635.00 (124.22 to 6,349.02)

Oceania								
Cause	YLLs (actual counts)				Deaths (actual counts)			
	2016	2040 Forecast	2040 Better Scenario	2040 Worse Scenario	2016	2040 Forecast	2040 Better Scenario	2040 Worse Scenario
Varicella and herpes zoster	2,743.21 (1,578.94 to 4,865.94)	1,993.21 (735.44 to 4,136.98)	942.15 (413.26 to 1,760.99)	2,237.02 (812.78 to 4,771.07)	43.25 (28.70 to 68.15)	38.00 (20.02 to 66.37)	23.37 (13.98 to 35.45)	39.80 (19.93 to 71.05)
**Neglected tropical diseases and malaria**	**56,202.08 (30,726.63 to 102,318.73)**	**49,335.14 (25,941.91 to 87,735.81)**	**41,668.65 (22,902.24 to 66,333.93)**	**50,921.29 (26,056.55 to 91,310.11)**	**999.15 (549.52 to 1,680.75)**	**996.96 (575.50 to 1,579.07)**	**902.63 (548.83 to 1,317.70)**	**999.26 (554.51 to 1,632.45)**
Malaria	50,965.67 (25,902.84 to 95,619.82)	40,800.85 (18,614.80 to 75,865.33)	34,985.62 (17,271.62 to 59,910.85)	42,035.79 (18,650.21 to 80,497.12)	912.19 (468.86 to 1,590.31)	805.66 (410.25 to 1,388.11)	727.16 (393.22 to 1,150.10)	813.27 (396.78 to 1,406.59)
Chagas disease	0.00 (0.00 to 0.00)	0.00 (0.00 to 0.00)	0.00 (0.00 to 0.00)	0.00 (0.00 to 0.00)	0.00 (0.00 to 0.00)	0.00 (0.00 to 0.00)	0.00 (0.00 to 0.00)	0.00 (0.00 to 0.00)
Leishmaniasis	0.00 (0.00 to 0.00)	0.00 (0.00 to 0.00)	0.00 (0.00 to 0.00)	0.00 (0.00 to 0.00)	0.00 (0.00 to 0.00)	0.00 (0.00 to 0.00)	0.00 (0.00 to 0.00)	0.00 (0.00 to 0.00)
African trypanosomiasis	0.00 (0.00 to 0.00)	0.00 (0.00 to 0.00)	0.00 (0.00 to 0.00)	0.00 (0.00 to 0.00)	0.00 (0.00 to 0.00)	0.00 (0.00 to 0.00)	0.00 (0.00 to 0.00)	0.00 (0.00 to 0.00)
Schistosomiasis	0.00 (0.00 to 0.00)	0.00 (0.00 to 0.00)	0.00 (0.00 to 0.00)	0.00 (0.00 to 0.00)	0.00 (0.00 to 0.00)	0.00 (0.00 to 0.00)	0.00 (0.00 to 0.00)	0.00 (0.00 to 0.00)
Cysticercosis	118.62 (88.24 to 153.87)	61.60 (45.90 to 79.19)	53.79 (39.68 to 69.48)	62.41 (46.46 to 80.34)	2.60 (1.94 to 3.38)	1.51 (1.14 to 1.93)	1.38 (1.03 to 1.76)	1.50 (1.14 to 1.92)
Cystic echinococcosis	21.80 (15.89 to 29.52)	15.42 (10.82 to 21.73)	7.15 (4.90 to 10.00)	17.69 (12.16 to 24.37)	0.50 (0.36 to 0.67)	0.43 (0.31 to 0.60)	0.22 (0.15 to 0.30)	0.48 (0.34 to 0.65)
Dengue	289.66 (130.50 to 485.53)	1,938.51 (1,023.61 to 3,135.77)	1,874.18 (1,013.06 to 2,978.65)	1,909.43 (981.77 to 3,123.75)	7.25 (3.19 to 13.04)	63.05 (34.78 to 106.42)	67.37 (38.37 to 109.69)	58.25 (31.39 to 99.60)
Yellow fever	0.00 (0.00 to 0.00)	0.00 (0.00 to 0.00)	0.00 (0.00 to 0.00)	0.00 (0.00 to 0.00)	0.00 (0.00 to 0.00)	0.00 (0.00 to 0.00)	0.00 (0.00 to 0.00)	0.00 (0.00 to 0.00)
Rabies	13.62 (4.90 to 26.90)	12.02 (3.85 to 23.44)	5.11 (1.68 to 9.42)	14.11 (4.59 to 27.25)	0.40 (0.15 to 0.79)	0.45 (0.15 to 0.84)	0.23 (0.07 to 0.42)	0.48 (0.15 to 0.92)
Intestinal nematode infections	3,732.88 (2,093.87 to 6,401.18)	5,109.05 (2,641.16 to 10,105.85)	3,756.34 (2,038.00 to 7,180.13)	5,416.07 (2,808.81 to 10,660.21)	49.67 (29.30 to 81.81)	73.21 (41.90 to 134.52)	58.86 (35.68 to 99.27)	75.85 (42.74 to 140.31)
Other neglected tropical diseases	1,059.83 (490.56 to 2,097.01)	1,397.69 (743.18 to 2,676.68)	986.46 (562.82 to 1,941.99)	1,465.77 (761.74 to 2,765.93)	26.55 (13.84 to 50.04)	52.65 (28.11 to 103.07)	47.41 (24.84 to 95.95)	49.42 (26.42 to 92.95)
**Maternal disorders**	**55,931.66 (41,675.43 to 74,711.79)**	**48,512.72 (32,255.73 to 69,550.29)**	**24,056.29 (15,810.86 to 34,604.33)**	**56,468.61 (37,562.58 to 80,848.47)**	**1,003.90 (746.13 to 1,336.99)**	**875.92 (583.77 to 1,248.21)**	**436.16 (286.19 to 624.00)**	**1,018.82 (679.46 to 1,445.80)**
Maternal hemorrhage	15,292.81 (9,312.90 to 23,112.26)	12,786.16 (7,255.91 to 20,097.22)	6,471.21 (3,640.43 to 10,389.64)	14,755.31 (8,355.36 to 23,373.71)	274.18 (164.45 to 410.37)	230.56 (129.64 to 364.07)	117.04 (66.51 to 187.22)	265.92 (150.22 to 419.53)
Maternal sepsis and other maternal infections	5,621.30 (2,947.12 to 9,318.43)	4,268.46 (1,932.31 to 7,705.27)	2,128.39 (977.84 to 3,867.57)	4,946.49 (2,270.19 to 8,816.38)	102.78 (53.48 to 170.11)	78.56 (35.78 to 139.24)	39.32 (18.23 to 70.02)	90.97 (41.40 to 161.07)
Maternal hypertensive disorders	7,524.63 (4,369.29 to 11,851.36)	6,691.41 (3,722.89 to 11,260.47)	3,350.40 (1,842.09 to 5,589.19)	7,731.63 (4,241.99 to 13,217.28)	134.63 (78.41 to 212.86)	120.61 (66.25 to 204.20)	60.61 (32.66 to 101.89)	139.27 (76.14 to 238.76)
Maternal obstructed labor and uterine rupture	4,452.13 (2,074.17 to 8,164.66)	4,032.29 (1,687.66 to 8,016.17)	1,936.23 (820.89 to 3,929.06)	4,691.64 (2,003.24 to 9,187.92)	80.64 (37.12 to 144.96)	73.46 (31.04 to 144.96)	35.39 (15.05 to 71.86)	85.41 (36.12 to 164.03)
Maternal abortion, miscarriage, and ectopic pregnancy	3,328.26 (1,502.10 to 6,247.02)	3,173.13 (1,257.80 to 6,721.35)	1,615.65 (619.18 to 3,356.27)	3,743.65 (1,468.12 to 7,802.08)	61.04 (27.92 to 113.55)	58.45 (23.50 to 123.03)	29.89 (11.98 to 60.77)	68.92 (27.53 to 142.93)

Indirect maternal deaths	6,934.17 (3,576.98 to 11,437.92)	6,630.74 (3,212.67 to 12,209.54)	3,165.87 (1,528.69 to 5,853.79)	7,794.56 (3,777.76 to 14,489.07)	122.53 (63.27 to 204.62)	117.62 (57.32 to 216.38)	56.46 (27.17 to 105.96)	138.12 (67.01 to 256.52)
Late maternal deaths	3,143.58 (1,313.48 to 5,958.81)	2,202.79 (888.35 to 4,522.90)	1,059.70 (423.94 to 2,139.82)	2,583.08 (1,049.11 to 5,175.46)	56.07 (24.16 to 103.63)	39.37 (15.48 to 79.87)	19.03 (7.60 to 38.36)	46.12 (18.82 to 91.35)
Maternal deaths aggravated by HIV/AIDS	198.90 (97.00 to 349.94)	607.48 (169.08 to 1,557.37)	257.26 (27.35 to 704.88)	762.58 (272.47 to 1,868.13)	3.78 (1.86 to 6.64)	11.43 (3.15 to 28.01)	4.88 (0.48 to 13.10)	14.35 (5.23 to 33.11)
Other maternal disorders	9,435.89 (5,244.94 to 15,056.94)	8,120.27 (4,229.46 to 13,996.87)	4,071.56 (2,057.69 to 6,956.93)	9,459.68 (4,857.29 to 16,464.41)	168.25 (94.45 to 272.45)	145.85 (75.23 to 250.54)	73.54 (36.99 to 124.96)	169.73 (87.50 to 294.08)

Oceania								
Cause	YLLs (actual counts)				Deaths (actual counts)			
	2016	2040 Forecast	2040 Better Scenario	2040 Worse Scenario	2016	2040 Forecast	2040 Better Scenario	2040 Worse Scenario
**Neonatal disorders**	**336,964.43 (201,838.98 to 543,278.66)**	**303,341.19 (172,158.16 to 511,630.57)**	**131,282.11 (74,770.72 to 219,031.63)**	**378,074.22 (212,069.33 to 624,531.59)**	**3,894.96 (2,333.69 to 6,279.18)**	**3,507.62 (1,990.86 to 5,917.26)**	**1,518.30 (865.03 to 2,531.49)**	**4,371.48 (2,453.46 to 7,220.00)**
Neonatal preterm birth	148,732.45 (85,157.36 to 251,643.75)	124,501.73 (67,573.98 to 214,410.98)	50,430.72 (27,567.59 to 87,093.19)	156,968.99 (85,648.86 to 272,396.87)	1,718.66 (984.12 to 2,908.44)	1,439.01 (781.00 to 2,477.03)	583.00 (318.84 to 1,006.70)	1,814.16 (990.17 to 3,147.79)
Neonatal encephalopathy due to birth asphyxia and trauma	73,907.65 (40,668.60 to 126,773.94)	62,241.24 (31,232.80 to 115,811.85)	29,945.51 (15,296.76 to 55,271.06)	76,183.11 (38,505.33 to 141,670.93)	854.47 (470.10 to 1,465.25)	720.09 (361.42 to 1,339.60)	346.50 (177.02 to 639.09)	881.30 (445.94 to 1,637.74)
Neonatal sepsis and other neonatal infections	32,740.05 (15,086.04 to 58,961.18)	39,226.00 (17,670.21 to 75,805.92)	18,467.76 (8,452.09 to 35,041.90)	47,997.07 (21,809.25 to 92,555.97)	378.42 (174.34 to 681.31)	453.60 (204.44 to 876.79)	213.58 (97.72 to 405.22)	555.00 (252.31 to 1,069.46)
Hemolytic disease and other neonatal jaundice	7,409.22 (3,520.17 to 15,303.63)	4,261.01 (1,731.23 to 9,381.56)	1,759.22 (712.86 to 3,968.11)	5,378.75 (2,182.55 to 11,818.07)	85.60 (40.67 to 176.81)	49.24 (20.01 to 108.39)	20.33 (8.24 to 45.85)	62.15 (25.23 to 136.56)
Other neonatal disorders	74,175.06 (39,558.53 to 132,666.37)	73,111.22 (36,305.36 to 136,385.89)	30,678.90 (15,369.61 to 56,763.70)	91,546.30 (45,297.37 to 168,066.92)	857.80 (457.33 to 1,533.34)	845.69 (420.00 to 1,576.92)	354.90 (177.88 to 656.38)	1,058.86 (524.00 to 1,942.79)
**Nutritional deficiencies**	**36,978.72 (20,692.32 to 63,397.93)**	**24,587.76 (14,833.55 to 41,009.87)**	**14,808.61 (9,898.19 to 22,958.47)**	**28,868.88 (16,401.38 to 50,398.70)**	**616.91 (411.39 to 933.06)**	**599.60 (425.08 to 835.39)**	**493.95 (369.33 to 643.72)**	**612.38 (413.64 to 893.78)**
Protein-energy malnutrition	32,232.63 (16,676.74 to 58,147.38)	17,156.95 (9,151.50 to 30,945.94)	9,592.27 (5,716.37 to 16,328.83)	20,896.84 (10,479.73 to 39,061.25)	507.60 (319.54 to 810.23)	406.00 (265.85 to 598.87)	328.18 (231.91 to 442.97)	421.36 (259.02 to 666.05)
Iodine deficiency	94.16 (19.28 to 351.13)	497.24 (52.38 to 2,714.27)	283.02 (35.83 to 1,238.42)	573.22 (54.33 to 3,550.92)	1.84 (0.51 to 5.39)	13.46 (1.81 to 47.27)	10.08 (1.38 to 37.96)	13.73 (1.87 to 50.95)
Dietary iron deficiency	4,409.25 (2,411.43 to 8,253.63)	6,639.57 (3,818.61 to 11,958.53)	4,716.65 (2,953.72 to 7,705.56)	7,092.00 (3,829.29 to 13,217.63)	100.91 (64.46 to 155.58)	169.76 (106.58 to 254.46)	146.28 (92.38 to 221.18)	167.49 (105.73 to 260.99)
Other nutritional deficiencies	242.68 (156.85 to 461.80)	294.00 (179.49 to 528.06)	216.67 (137.44 to 383.22)	306.83 (187.21 to 558.42)	6.56 (4.57 to 10.86)	10.38 (6.45 to 17.46)	9.40 (5.73 to 16.20)	9.79 (6.09 to 16.51)
**Other communicable, maternal, neonatal, and nutritional diseases**	**111,061.60 (54,193.06 to 210,856.22)**	**48,153.54 (29,874.53 to 77,248.48)**	**30,307.35 (20,531.25 to 45,399.38)**	**53,217.53 (32,558.07 to 86,929.48)**	**1,497.69 (824.57 to 2,680.24)**	**876.48 (626.47 to 1,235.91)**	**626.39 (474.46 to 820.55)**	**928.69 (659.87 to 1,349.96)**
Sexually transmitted infections excluding HIV	89,513.57 (34,557.49 to 185,574.23)	23,773.68 (8,742.41 to 48,621.88)	12,713.14 (5,068.73 to 25,634.72)	27,269.85 (10,041.15 to 56,605.30)	1,064.40 (422.04 to 2,177.77)	294.72 (117.86 to 584.14)	162.94 (73.03 to 313.31)	335.86 (133.68 to 676.96)
Syphilis	88,052.95 (32,954.90 to 183,627.62)	22,514.97 (7,638.42 to 47,207.68)	11,676.16 (4,063.22 to 24,630.09)	25,966.66 (8,845.33 to 55,408.46)	1,032.71 (388.24 to 2,151.35)	266.90 (91.90 to 555.66)	139.67 (49.71 to 291.15)	307.25 (106.22 to 650.95)
Chlamydial infection	285.12 (146.87 to 411.01)	247.13 (127.76 to 357.94)	202.80 (106.71 to 301.18)	256.18 (131.87 to 371.25)	6.13 (3.12 to 8.86)	5.41 (2.79 to 7.87)	4.51 (2.36 to 6.63)	5.57 (2.87 to 8.06)
Gonococcal infection	752.08 (383.49 to 1,091.83)	646.78 (329.33 to 946.06)	534.87 (275.19 to 803.04)	668.91 (345.79 to 979.90)	16.43 (8.25 to 23.71)	14.42 (7.45 to 20.98)	12.12 (6.33 to 17.85)	14.80 (7.77 to 21.46)
Other sexually transmitted infections	423.43 (220.22 to 615.49)	364.80 (185.29 to 547.58)	299.31 (155.19 to 448.03)	378.10 (193.23 to 565.02)	9.13 (4.66 to 13.19)	7.99 (3.97 to 11.97)	6.64 (3.42 to 9.88)	8.23 (4.19 to 12.23)
Acute hepatitis	7,484.09 (5,838.23 to 9,336.86)	7,148.86 (5,223.37 to 9,703.34)	5,496.04 (3,984.39 to 7,383.92)	7,456.17 (5,406.24 to 10,044.77)	164.52 (129.33 to 204.43)	178.28 (131.12 to 235.94)	142.58 (104.41 to 189.12)	182.22 (134.08 to 240.70)
Acute hepatitis A	450.86 (270.98 to 749.60)	224.61 (125.68 to 389.29)	139.15 (83.64 to 223.40)	248.88 (136.38 to 432.15)	6.43 (4.17 to 9.95)	3.86 (2.44 to 5.94)	2.70 (1.78 to 3.95)	4.12 (2.56 to 6.44)
Acute hepatitis B	5,624.73 (4,334.02 to 7,172.27)	5,888.29 (4,212.76 to 8,125.02)	4,583.53 (3,252.88 to 6,263.28)	6,113.97 (4,306.16 to 8,366.27)	134.49 (105.30 to 170.44)	152.88 (111.39 to 203.98)	122.09 (88.18 to 164.68)	156.23 (112.86 to 211.11)

Acute hepatitis C	129.49 (87.03 to 187.43)	141.34 (89.45 to 212.38)	124.09 (78.56 to 188.83)	142.15 (88.61 to 215.63)	3.05 (2.03 to 4.33)	3.90 (2.49 to 5.96)	3.59 (2.28 to 5.51)	3.83 (2.44 to 5.91)
Acute hepatitis E	1,279.01 (839.16 to 1,882.17)	894.61 (529.35 to 1,425.75)	649.26 (394.89 to 987.27)	951.18 (564.37 to 1,531.85)	20.55 (13.90 to 29.54)	17.64 (10.80 to 26.19)	14.20 (8.77 to 20.93)	18.04 (10.97 to 26.81)
Other unspecified infectious diseases	14,063.94 (9,250.65 to 27,084.64)	17,231.00 (12,035.59 to 25,770.00)	12,098.18 (8,744.14 to 16,935.60)	18,491.51 (12,803.28 to 28,623.10)	268.77 (196.91 to 428.40)	403.48 (306.98 to 544.60)	320.87 (247.99 to 413.85)	410.61 (311.68 to 558.92)
**Non-communicable diseases**	**1,923,696.60 (1,658,075.45 to 2,218,282.77)**	**2,826,905.34 (2,356,994.82 to 3,340,874.56)**	**1,843,873.71 (1,554,195.02 to 2,180,307.62)**	**3,487,585.48 (2,911,324.13 to 4,098,275.80)**	**62,360.57 (55,208.82 to 70,612.91)**	**106,806.06 (94,550.13 to 120,595.27)**	**76,459.78 (67,847.88 to 86,024.76)**	**126,834.76 (111,815.09 to 141,057.43)**

Oceania								
Cause	YLLs (actual counts)				Deaths (actual counts)			
	2016	2040 Forecast	2040 Better Scenario	2040 Worse Scenario	2016	2040 Forecast	2040 Better Scenario	2040 Worse Scenario
**Neoplasms**	**215,239.78 (178,864.53 to 256,652.69)**	**343,290.38 (273,260.97 to 431,746.64)**	**307,768.17 (252,873.76 to 373,956.27)**	**375,535.17 (293,510.75 to 478,232.39)**	**6,744.95 (5,731.64 to 7,927.60)**	**12,222.44 (9,957.95 to 15,092.88)**	**11,757.00 (9,920.94 to 13,970.23)**	**12,872.85 (10,277.53 to 16,196.10)**
Lip and oral cavity cancer	3,587.86 (2,993.38 to 4,384.81)	5,985.65 (3,767.56 to 9,819.49)	4,036.98 (2,719.41 to 6,229.89)	8,334.39 (4,883.43 to 14,463.71)	122.43 (104.83 to 146.59)	220.39 (144.28 to 348.53)	162.35 (114.18 to 243.47)	290.03 (174.29 to 479.65)
Nasopharynx cancer	3,080.92 (2,246.88 to 4,086.87)	5,754.66 (2,941.02 to 10,859.42)	3,512.12 (1,943.74 to 6,336.82)	8,436.65 (4,083.23 to 16,264.54)	92.86 (68.67 to 121.38)	184.67 (98.15 to 339.32)	117.87 (66.47 to 205.85)	263.27 (127.58 to 502.10)
Other pharynx cancer	3,013.09 (2,142.66 to 4,197.60)	3,982.95 (2,418.86 to 6,425.84)	3,390.27 (2,170.55 to 5,299.25)	4,898.88 (2,715.65 to 8,317.15)	98.22 (71.80 to 134.30)	142.53 (89.22 to 221.87)	128.61 (84.92 to 197.17)	168.37 (96.99 to 275.74)
Esophageal cancer	2,806.05 (2,391.92 to 3,313.60)	4,475.10 (2,726.17 to 7,156.18)	3,185.00 (2,063.13 to 4,865.07)	6,000.76 (3,315.99 to 10,302.43)	101.62 (87.77 to 117.93)	175.15 (108.16 to 272.15)	134.13 (89.82 to 198.44)	223.76 (127.73 to 368.40)
Stomach cancer	18,779.83 (15,553.03 to 22,297.58)	23,213.81 (18,316.27 to 29,419.48)	20,751.69 (16,619.00 to 25,627.90)	23,728.23 (18,346.32 to 30,696.05)	636.57 (537.01 to 743.22)	884.60 (719.10 to 1,079.08)	849.97 (701.78 to 1,011.53)	866.10 (688.00 to 1,077.87)
Colon and rectum cancer	12,766.97 (9,844.40 to 17,143.29)	24,134.83 (15,401.55 to 37,735.82)	24,952.84 (16,574.95 to 38,120.18)	24,754.34 (15,463.26 to 39,423.52)	460.76 (369.62 to 602.73)	979.80 (636.66 to 1,528.39)	1,086.12 (734.19 to 1,653.08)	956.68 (609.86 to 1,515.47)
Liver cancer	14,084.94 (11,400.71 to 18,454.82)	25,356.17 (17,184.15 to 37,199.06)	22,970.73 (16,298.92 to 32,370.49)	29,532.86 (18,812.77 to 44,836.08)	466.04 (382.79 to 602.67)	920.64 (641.18 to 1,327.97)	890.77 (645.61 to 1,235.12)	1,024.71 (671.61 to 1,518.95)
Liver cancer due to hepatitis B	8,084.34 (6,411.20 to 10,707.63)	14,068.39 (9,541.68 to 21,016.98)	12,783.83 (9,090.97 to 18,350.53)	16,283.62 (10,289.51 to 24,922.88)	252.99 (203.67 to 331.57)	481.00 (333.91 to 691.45)	463.90 (331.86 to 653.40)	534.81 (344.33 to 799.95)
Liver cancer due to hepatitis C	2,007.68 (1,531.84 to 2,829.02)	3,910.59 (2,729.62 to 5,910.59)	3,764.00 (2,718.83 to 5,509.08)	4,330.62 (2,837.59 to 6,683.79)	80.59 (62.68 to 112.48)	171.83 (122.42 to 254.91)	178.12 (130.19 to 256.77)	180.56 (121.71 to 269.28)
Liver cancer due to alcohol use	971.27 (730.46 to 1,349.09)	1,806.86 (1,177.14 to 2,754.08)	1,538.22 (1,050.37 to 2,323.53)	2,393.85 (1,465.19 to 3,786.98)	35.15 (27.08 to 47.85)	69.29 (46.11 to 105.04)	63.16 (44.55 to 92.76)	86.79 (55.07 to 137.10)
Liver cancer due to other causes	3,021.65 (2,379.97 to 3,868.87)	5,570.32 (3,520.36 to 8,717.95)	4,884.67 (3,207.20 to 7,334.60)	6,524.77 (3,842.37 to 10,719.83)	97.31 (77.78 to 124.54)	198.52 (121.49 to 315.55)	185.59 (120.76 to 280.72)	222.54 (128.41 to 364.27)
Gallbladder and biliary tract cancer	1,777.09 (1,306.98 to 2,472.84)	2,129.60 (1,597.44 to 2,866.20)	2,376.11 (1,802.08 to 3,215.07)	1,975.35 (1,467.44 to 2,688.09)	67.04 (51.02 to 90.39)	88.84 (67.37 to 116.84)	105.11 (80.85 to 137.45)	79.46 (58.99 to 105.77)
Pancreatic cancer	4,713.38 (4,039.51 to 5,537.72)	9,891.60 (7,104.09 to 13,575.93)	10,222.11 (7,630.43 to 13,645.68)	9,752.98 (6,988.09 to 13,582.69)	184.18 (160.56 to 212.56)	416.20 (304.61 to 574.33)	456.36 (345.61 to 604.85)	394.69 (284.03 to 550.97)
Larynx cancer	2,483.95 (2,052.47 to 2,961.78)	3,839.62 (2,058.40 to 6,536.62)	2,327.60 (1,358.84 to 3,974.15)	5,135.68 (2,636.55 to 8,674.09)	85.06 (71.82 to 99.76)	145.33 (78.69 to 243.68)	92.11 (54.81 to 154.07)	187.90 (98.24 to 317.43)
Tracheal, bronchus, and lung cancer	26,994.64 (21,870.65 to 34,952.79)	46,318.63 (26,683.77 to 79,695.00)	27,627.89 (17,465.04 to 46,005.71)	63,215.98 (35,880.55 to 105,732.83)	970.35 (801.39 to 1,227.15)	1,794.87 (1,064.45 to 3,063.73)	1,114.95 (724.98 to 1,822.16)	2,381.40 (1,396.43 to 4,038.35)
Malignant skin melanoma	1,027.07 (749.38 to 1,566.70)	1,826.22 (1,274.26 to 2,899.12)	2,255.64 (1,565.67 to 3,734.65)	1,665.57 (1,171.09 to 2,614.25)	35.22 (26.35 to 52.69)	70.69 (51.00 to 109.48)	93.60 (66.90 to 148.61)	61.77 (44.36 to 94.30)
Non-melanoma skin cancer	1,780.86 (1,339.82 to 2,320.80)	3,498.88 (2,680.24 to 4,439.56)	3,433.64 (2,620.89 to 4,387.14)	3,330.24 (2,545.45 to 4,220.11)	66.55 (52.51 to 82.73)	151.80 (120.20 to 186.89)	164.95 (131.36 to 202.50)	136.25 (106.73 to 169.47)
Breast cancer	26,153.35 (16,143.86 to 38,364.86)	42,526.28 (23,212.16 to 68,839.89)	40,889.32 (23,322.80 to 65,020.63)	44,422.13 (23,750.67 to 74,421.49)	795.94 (512.41 to 1,138.14)	1,441.62 (825.78 to 2,307.47)	1,459.38 (873.51 to 2,261.21)	1,457.00 (812.55 to 2,375.99)
Cervical cancer	21,200.48 (12,859.85 to 29,032.93)	27,409.19 (16,495.24 to 39,490.30)	23,400.87 (14,461.11 to 33,291.52)	28,579.74 (16,518.79 to 42,200.89)	613.44 (381.16 to 825.73)	848.09 (529.43 to 1,196.49)	750.96 (482.77 to 1,039.50)	864.48 (524.92 to 1,242.43)

Uterine cancer	5,775.62 (4,148.59 to 7,453.74)	9,647.35 (7,053.54 to 12,429.58)	8,626.69 (6,424.81 to 11,010.37)	9,529.55 (6,825.92 to 12,307.43)	197.16 (146.96 to 248.81)	358.67 (269.23 to 448.56)	334.46 (256.49 to 418.86)	345.79 (251.16 to 437.36)
Ovarian cancer	2,864.41 (2,180.13 to 3,933.42)	5,142.44 (3,357.22 to 8,034.45)	5,791.29 (3,897.19 to 8,937.22)	4,944.47 (3,203.70 to 7,796.46)	96.44 (74.91 to 130.40)	187.12 (123.25 to 291.54)	218.40 (148.46 to 332.43)	175.89 (114.29 to 274.07)
Prostate cancer	4,933.72 (3,518.76 to 6,820.59)	13,265.36 (9,736.40 to 18,704.54)	16,093.22 (12,056.64 to 22,152.38)	11,531.66 (8,236.17 to 16,733.59)	272.11 (196.52 to 360.17)	808.57 (591.25 to 1,135.32)	1,047.32 (789.02 to 1,417.57)	673.26 (477.35 to 975.27)
Testicular cancer	913.04 (720.19 to 1,132.86)	1,161.05 (882.67 to 1,477.06)	1,065.30 (812.24 to 1,358.45)	1,162.07 (888.71 to 1,479.82)	19.84 (15.98 to 24.22)	27.48 (21.82 to 33.52)	26.34 (20.92 to 32.54)	26.70 (21.14 to 32.65)

Oceania								
Cause	YLLs (actual counts)				Deaths (actual counts)			
	2016	2040 Forecast	2040 Better Scenario	2040 Worse Scenario	2016	2040 Forecast	2040 Better Scenario	2040 Worse Scenario
Kidney cancer	1,321.98 (1,111.05 to 1,628.66)	2,453.87 (1,874.97 to 3,279.09)	2,639.18 (2,034.15 to 3,542.64)	2,413.35 (1,823.87 to 3,307.03)	39.57 (33.75 to 48.49)	85.67 (65.38 to 113.50)	100.71 (76.81 to 129.72)	80.35 (60.68 to 108.27)
Bladder cancer	1,924.08 (1,547.83 to 2,573.64)	3,270.39 (2,090.10 to 5,252.66)	2,976.63 (2,000.43 to 4,672.94)	3,351.92 (2,091.24 to 5,413.87)	78.24 (64.40 to 102.67)	145.63 (96.75 to 233.52)	143.11 (99.20 to 219.71)	142.23 (91.90 to 226.81)
Brain and nervous system cancer	4,952.84 (3,860.14 to 6,022.50)	8,412.67 (6,250.75 to 10,352.98)	8,266.52 (6,111.60 to 10,204.87)	8,311.34 (6,195.02 to 10,233.11)	108.35 (83.33 to 130.61)	209.73 (153.65 to 255.32)	225.95 (164.14 to 276.66)	198.79 (146.29 to 240.91)
Thyroid cancer	1,304.40 (1,010.29 to 1,675.23)	2,052.82 (1,503.94 to 2,794.96)	1,884.97 (1,404.34 to 2,537.61)	2,151.91 (1,527.79 to 3,030.30)	47.85 (37.55 to 60.53)	83.31 (62.40 to 112.19)	81.00 (61.50 to 106.84)	84.17 (60.87 to 116.39)
Mesothelioma	485.35 (354.76 to 715.73)	796.59 (582.61 to 1,210.36)	807.89 (585.57 to 1,256.22)	762.75 (554.20 to 1,156.88)	15.45 (11.54 to 22.32)	27.53 (20.50 to 40.60)	29.25 (21.56 to 44.30)	25.58 (18.82 to 37.41)
Hodgkin lymphoma	1,679.49 (1,224.22 to 2,137.22)	1,942.72 (1,409.28 to 2,567.56)	1,488.49 (1,069.08 to 1,969.46)	2,021.52 (1,441.83 to 2,653.98)	37.72 (27.90 to 47.77)	48.57 (36.37 to 62.97)	39.76 (29.57 to 51.38)	48.87 (36.28 to 63.53)
Non-Hodgkin lymphoma	6,438.39 (5,294.38 to 8,081.37)	10,058.39 (8,108.13 to 12,965.81)	10,534.85 (8,593.01 to 13,605.67)	10,014.89 (7,989.45 to 13,060.31)	166.10 (140.01 to 206.83)	302.61 (248.03 to 381.70)	348.15 (286.39 to 441.45)	287.36 (232.51 to 371.12)
Multiple myeloma	2,027.50 (1,444.10 to 2,543.89)	3,826.75 (2,717.13 to 5,063.62)	4,377.43 (3,144.75 to 5,732.00)	3,717.45 (2,618.11 to 4,990.76)	72.14 (52.75 to 88.93)	150.12 (108.55 to 197.14)	181.01 (133.69 to 236.89)	140.85 (101.85 to 185.00)
Leukemia	19,455.58 (13,965.31 to 26,048.90)	24,745.80 (17,272.93 to 33,896.59)	22,104.11 (15,839.44 to 29,429.87)	26,155.57 (18,119.22 to 36,590.59)	394.12 (288.24 to 508.57)	595.31 (432.42 to 803.46)	585.00 (434.51 to 764.33)	607.50 (429.75 to 835.62)
Acute lymphoid leukemia	3,713.51 (2,448.28 to 6,113.96)	4,391.76 (2,830.87 to 7,300.25)	3,822.43 (2,490.43 to 5,984.48)	4,628.08 (2,942.27 to 7,751.28)	60.30 (41.64 to 95.34)	79.97 (53.49 to 124.45)	75.10 (51.12 to 111.86)	83.03 (55.04 to 130.74)
Chronic lymphoid leukemia	258.68 (183.93 to 354.50)	415.72 (275.47 to 603.52)	428.01 (284.37 to 619.59)	420.40 (272.76 to 620.92)	9.55 (6.80 to 13.06)	17.25 (11.40 to 24.82)	19.13 (12.78 to 27.68)	16.65 (10.77 to 24.54)
Acute myeloid leukemia	5,854.77 (4,130.61 to 7,765.15)	8,987.84 (6,213.64 to 12,148.63)	8,851.29 (6,253.87 to 11,586.07)	9,306.94 (6,439.40 to 12,699.13)	115.36 (81.47 to 147.42)	208.42 (149.29 to 267.97)	224.77 (164.13 to 281.25)	208.79 (147.48 to 273.81)
Chronic myeloid leukemia	2,383.23 (1,517.90 to 3,552.34)	2,566.18 (1,531.94 to 4,187.21)	2,458.23 (1,486.29 to 3,934.93)	2,715.44 (1,592.47 to 4,448.50)	64.30 (42.54 to 95.55)	76.67 (47.78 to 122.22)	76.90 (48.59 to 121.67)	78.78 (47.93 to 129.29)
Other leukemia	7,245.39 (4,295.01 to 11,100.52)	8,384.30 (4,743.77 to 13,491.93)	6,544.15 (3,778.04 to 10,286.05)	9,084.70 (5,035.85 to 14,787.08)	144.61 (91.41 to 212.45)	213.00 (128.70 to 323.82)	189.09 (116.84 to 284.29)	220.25 (129.59 to 339.43)
Other neoplasms	16,912.92 (13,532.87 to 19,803.24)	26,170.99 (21,202.04 to 30,616.68)	25,778.80 (20,618.23 to 30,111.71)	25,702.97 (20,732.40 to 30,163.89)	403.61 (322.42 to 466.38)	726.89 (590.74 to 836.39)	789.30 (634.98 to 913.00)	679.65 (548.93 to 785.86)
**Cardiovascular diseases**	**771,046.03 (668,412.66 to 897,990.75)**	**1,052,081.26 (752,825.71 to 1,416,253.62)**	**524,965.11 (389,829.48 to 726,950.72)**	**1,467,119.10 (1,078,164.76 to 1,900,838.04)**	**26,207.28 (22,979.80 to 29,963.13)**	**41,094.06 (30,968.33 to 54,396.30)**	**23,299.91 (18,058.05 to 32,705.21)**	**54,951.90 (41,190.41 to 70,219.40)**
Rheumatic heart disease	56,346.95 (42,754.75 to 71,771.54)	38,586.19 (27,688.41 to 51,424.85)	27,326.40 (19,859.33 to 35,807.81)	40,944.55 (29,214.02 to 53,767.51)	1,331.70 (986.46 to 1,703.21)	1,124.37 (821.76 to 1,446.88)	880.20 (659.60 to 1,117.05)	1,135.31 (810.38 to 1,472.68)
Ischemic heart disease	351,076.73 (300,417.26 to 417,582.51)	513,215.89 (278,546.88 to 849,699.67)	220,831.18 (119,905.67 to 399,752.50)	745,666.56 (435,078.23 to 1,130,446.09)	12,764.30 (11,099.77 to 14,850.75)	20,361.40 (11,785.81 to 33,814.18)	10,051.46 (5,825.64 to 17,866.05)	28,384.81 (17,237.55 to 44,885.87)
Stroke	257,759.27 (216,803.67 to 303,915.22)	327,274.45 (199,003.59 to 496,628.41)	140,202.43 (86,930.60 to 231,030.12)	489,427.08 (302,936.22 to 704,516.95)	8,675.12 (7,362.17 to 10,037.92)	12,831.13 (8,151.77 to 20,922.16)	6,392.84 (4,151.26 to 11,430.22)	18,357.02 (11,684.44 to 27,342.50)
Ischemic stroke	61,272.08 (51,687.15 to 71,135.32)	80,679.27 (45,831.50 to 178,662.03)	44,317.25 (24,154.78 to 101,652.96)	106,231.89 (60,287.78 to 216,814.83)	2,636.10 (2,243.95 to 3,035.51)	3,977.10 (2,119.33 to 10,251.60)	2,483.19 (1,311.31 to 6,527.52)	5,020.87 (2,649.92 to 11,568.66)

Intracerebral hemorrhage	196,487.19 (163,800.23 to 234,660.84)	246,595.18 (136,317.79 to 411,290.32)	95,885.18 (54,830.87 to 169,734.82)	383,195.19 (222,681.62 to 584,727.64)	6,039.02 (5,070.89 to 7,075.54)	8,854.03 (5,133.82 to 15,307.64)	3,909.65 (2,431.66 to 6,813.00)	13,336.15 (7,712.40 to 21,686.84)
Hypertensive heart disease	32,851.46 (21,476.29 to 42,301.77)	58,411.18 (36,860.22 to 86,056.67)	40,780.06 (26,905.76 to 56,032.34)	71,429.93 (41,795.63 to 116,075.83)	1,269.14 (860.35 to 1,603.31)	2,571.47 (1,660.04 to 3,710.42)	1,983.39 (1,348.10 to 2,681.44)	2,953.64 (1,792.62 to 4,770.35)
Cardiomyopathy and myocarditis	21,853.33 (13,339.30 to 30,545.46)	27,563.01 (16,666.00 to 38,411.62)	25,637.24 (15,943.09 to 35,018.89)	27,923.06 (16,727.13 to 39,437.12)	558.97 (362.58 to 739.60)	871.24 (557.07 to 1,191.38)	898.09 (583.10 to 1,226.74)	836.47 (527.48 to 1,161.35)
Myocarditis	6,059.09 (4,048.63 to 8,807.76)	7,659.18 (5,245.65 to 10,879.67)	6,993.80 (4,806.40 to 9,867.61)	7,584.61 (5,148.47 to 10,782.97)	164.55 (112.65 to 238.23)	257.36 (175.46 to 373.61)	259.19 (176.07 to 377.73)	241.55 (164.01 to 353.26)

Oceania								
Cause	YLLs (actual counts)				Deaths (actual counts)			
	2016	2040 Forecast	2040 Better Scenario	2040 Worse Scenario	2016	2040 Forecast	2040 Better Scenario	2040 Worse Scenario
Alcoholic cardiomyopathy	1,155.87 (581.84 to 2,023.75)	1,318.18 (663.18 to 2,192.47)	1,251.66 (626.93 to 2,001.96)	1,624.44 (803.84 to 2,790.26)	35.39 (18.71 to 58.94)	45.26 (23.50 to 71.39)	46.35 (23.72 to 71.29)	52.22 (26.75 to 86.35)
Other cardiomyopathy	14,638.37 (8,255.36 to 20,660.41)	18,585.65 (10,297.93 to 27,528.81)	17,391.78 (9,921.97 to 25,605.71)	18,714.01 (10,144.55 to 28,163.39)	359.03 (219.95 to 486.75)	568.62 (332.02 to 861.14)	592.54 (355.03 to 891.14)	542.71 (310.75 to 826.48)
Atrial fibrillation and flutter	1,132.33 (875.96 to 1,440.83)	2,579.14 (1,866.97 to 3,399.84)	2,902.46 (2,138.02 to 3,765.66)	2,435.30 (1,715.29 to 3,276.10)	85.14 (65.74 to 109.00)	205.88 (138.60 to 278.87)	257.67 (182.35 to 341.12)	181.63 (120.23 to 252.64)
Aortic aneurysm	6,663.96 (4,652.10 to 9,652.95)	10,343.35 (6,955.12 to 15,283.85)	10,016.08 (6,882.78 to 14,700.11)	10,461.93 (6,801.64 to 15,733.24)	254.19 (187.37 to 357.57)	442.15 (321.13 to 624.84)	477.65 (355.01 to 666.78)	420.67 (295.12 to 603.87)
Peripheral artery disease	280.32 (148.46 to 520.79)	765.31 (365.25 to 1,701.97)	765.17 (387.97 to 1,638.61)	761.01 (350.95 to 1,742.26)	13.73 (7.64 to 23.05)	40.37 (20.57 to 84.43)	46.63 (25.35 to 96.20)	37.35 (18.61 to 78.38)
Endocarditis	10,415.75 (8,218.39 to 13,243.97)	16,451.58 (12,185.26 to 21,454.37)	15,123.99 (11,272.24 to 19,949.14)	16,554.32 (12,260.18 to 22,019.08)	301.94 (243.08 to 381.77)	572.10 (424.06 to 779.59)	587.70 (438.40 to 799.72)	542.67 (403.43 to 734.87)
Other cardiovascular and circulatory diseases	32,665.94 (21,609.95 to 42,239.78)	56,891.17 (37,525.63 to 76,074.89)	41,380.10 (27,319.22 to 54,457.23)	61,515.36 (39,566.52 to 83,638.73)	953.05 (639.85 to 1,208.68)	2,073.96 (1,369.81 to 2,710.52)	1,724.29 (1,173.72 to 2,219.18)	2,102.34 (1,366.49 to 2,795.83)
**Chronic respiratory diseases**	**300,590.44 (249,064.55 to 353,244.89)**	**379,023.00 (280,928.78 to 524,652.53)**	**226,931.54 (181,397.26 to 287,699.64)**	**493,710.12 (349,268.34 to 722,492.73)**	**11,114.96 (9,133.00 to 13,077.65)**	**15,862.04 (11,700.13 to 21,663.61)**	**10,144.40 (8,095.80 to 12,451.74)**	**19,742.79 (13,917.91 to 27,978.48)**
Chronic obstructive pulmonary disease	160,889.66 (122,179.09 to 198,223.81)	236,641.86 (148,565.55 to 375,652.29)	112,286.72 (78,176.15 to 162,889.93)	331,767.90 (201,730.77 to 549,304.79)	6,375.96 (4,781.12 to 7,838.13)	10,447.32 (6,858.01 to 16,168.11)	5,436.29 (3,853.11 to 7,621.77)	13,846.15 (8,706.73 to 21,616.76)
Pneumoconiosis	701.40 (466.30 to 993.35)	923.75 (604.35 to 1,340.82)	874.21 (560.30 to 1,259.40)	907.03 (596.61 to 1,322.39)	30.02 (20.29 to 41.96)	47.68 (29.98 to 70.93)	51.72 (32.00 to 76.80)	43.80 (27.50 to 65.25)
Silicosis	138.88 (68.94 to 226.53)	157.64 (75.84 to 268.20)	143.74 (67.14 to 245.99)	149.92 (71.19 to 258.80)	6.59 (3.34 to 10.61)	8.98 (4.04 to 15.93)	9.36 (4.00 to 16.88)	7.99 (3.56 to 13.97)
Asbestosis	155.76 (98.44 to 269.33)	239.70 (148.00 to 402.99)	237.24 (144.74 to 380.18)	230.25 (141.90 to 392.99)	5.32 (3.25 to 8.95)	10.06 (5.84 to 17.19)	11.20 (6.46 to 19.05)	9.10 (5.24 to 15.78)
Coal workers pneumoconiosis	18.34 (7.27 to 34.98)	28.27 (10.02 to 56.53)	20.76 (7.16 to 41.23)	29.74 (10.42 to 60.76)	0.89 (0.35 to 1.67)	1.63 (0.54 to 3.28)	1.38 (0.44 to 2.76)	1.61 (0.53 to 3.22)
Other pneumoconiosis	388.42 (243.62 to 584.09)	498.14 (299.79 to 758.86)	472.47 (271.09 to 728.74)	497.12 (301.17 to 757.82)	17.21 (10.81 to 25.55)	27.01 (15.64 to 43.34)	29.78 (16.45 to 48.58)	25.11 (14.48 to 40.93)
Asthma	107,307.31 (78,572.11 to 144,369.93)	88,647.02 (57,655.78 to 137,262.22)	65,456.13 (43,140.24 to 98,823.39)	107,347.40 (66,065.87 to 174,372.05)	3,801.30 (2,765.06 to 5,172.41)	3,627.56 (2,309.14 to 5,820.11)	2,955.80 (1,934.85 to 4,574.79)	4,144.00 (2,518.62 to 6,776.44)
Interstitial lung disease and pulmonary sarcoidosis	8,352.48 (3,519.79 to 17,903.42)	13,804.42 (5,892.54 to 28,311.60)	15,502.33 (6,911.58 to 30,814.66)	13,117.96 (5,553.83 to 27,143.19)	219.83 (96.11 to 467.84)	450.65 (179.68 to 930.92)	561.58 (227.15 to 1,114.24)	406.13 (163.66 to 846.82)
Other chronic respiratory diseases	23,339.60 (13,258.05 to 34,370.64)	39,005.95 (22,642.91 to 59,085.22)	32,812.16 (20,207.07 to 47,341.15)	40,569.83 (22,602.48 to 63,094.82)	687.85 (395.93 to 1,006.34)	1,288.82 (763.94 to 1,956.08)	1,139.01 (711.14 to 1,649.45)	1,302.72 (748.17 to 2,020.50)
**Cirrhosis and other chronic liver diseases**	**91,267.10 (74,017.17 to 113,428.49)**	**141,857.87 (115,620.23 to 174,729.94)**	**127,253.74 (103,492.20 to 156,241.08)**	**145,341.14 (118,811.62 to 180,307.39)**	**2,389.46 (1,954.03 to 2,945.91)**	**4,133.10 (3,460.41 to 4,960.30)**	**3,906.30 (3,254.19 to 4,688.49)**	**4,111.10 (3,435.13 to 4,992.99)**
Cirrhosis and other chronic liver diseases due to hepatitis B	35,519.07 (28,175.86 to 44,939.85)	58,929.34 (46,576.65 to 73,153.72)	54,951.05 (43,428.47 to 68,437.76)	58,101.13 (45,975.92 to 73,136.09)	992.70 (797.68 to 1,234.99)	1,803.86 (1,472.00 to 2,207.45)	1,758.07 (1,436.59 to 2,136.13)	1,729.41 (1,412.30 to 2,117.03)
Cirrhosis and other chronic liver diseases due to hepatitis C	14,520.00 (11,485.05 to 18,665.40)	23,997.64 (19,140.66 to 30,585.91)	22,164.16 (17,721.62 to 28,346.41)	23,755.72 (18,861.22 to 30,408.65)	398.45 (320.07 to 503.33)	713.79 (583.42 to 881.97)	684.20 (559.11 to 846.55)	690.15 (563.03 to 857.26)

Cirrhosis and other chronic liver diseases due to alcohol use	11,366.00 (8,830.16 to 14,608.03)	17,204.28 (13,589.34 to 22,024.95)	14,061.88 (11,175.20 to 17,961.83)	21,208.70 (16,553.38 to 27,395.53)	330.95 (262.44 to 418.89)	548.98 (447.65 to 687.88)	473.78 (384.54 to 588.85)	647.82 (524.02 to 816.83)
Cirrhosis and other chronic liver diseases due to other causes	29,862.03 (24,331.09 to 37,152.35)	41,726.60 (33,468.95 to 51,774.78)	36,076.64 (29,112.73 to 44,812.76)	42,275.58 (33,793.90 to 53,076.57)	667.35 (549.84 to 819.30)	1,066.46 (886.80 to 1,294.14)	990.25 (824.36 to 1,191.31)	1,043.72 (866.99 to 1,277.40)
**Digestive diseases**	**62,683.55 (49,401.20 to 87,969.70)**	**74,290.93 (59,234.79 to 104,455.10)**	**58,147.26 (46,375.71 to 81,216.19)**	**78,960.95 (62,396.04 to 111,307.11)**	**1,761.11 (1,446.93 to 2,441.80)**	**2,540.22 (2,000.62 to 3,487.02)**	**2,207.96 (1,736.12 to 3,007.52)**	**2,555.54 (1,995.37 to 3,543.87)**
Peptic ulcer disease	22,110.76 (16,630.75 to 32,407.69)	21,218.16 (15,252.37 to 31,256.85)	14,758.32 (10,498.98 to 21,500.55)	22,484.77 (15,908.38 to 33,639.91)	683.17 (521.51 to 985.02)	790.90 (561.06 to 1,158.51)	601.18 (428.25 to 858.84)	799.53 (550.29 to 1,179.79)

Oceania								
**Cause**	**YLLs (actual counts)**				**Deaths (actual counts)**			
	**2016**	**2040 Forecast**	**2040 Better Scenario**	**2040 Worse Scenario**	**2016**	**2040 Forecast**	**2040 Better Scenario**	**2040 Worse Scenario**
Gastritis and duodenitis	482.67 (313.94 to 860.98)	821.32 (531.57 to 1,497.87)	724.14 (470.61 to 1,303.22)	812.23 (518.86 to 1,506.05)	16.07 (10.57 to 27.74)	31.54 (20.11 to 54.61)	30.44 (19.54 to 51.84)	29.63 (18.67 to 52.74)
Appendicitis	7,916.85 (5,555.50 to 11,668.62)	9,059.93 (6,478.91 to 13,614.51)	6,945.70 (5,027.38 to 10,445.67)	9,377.34 (6,613.84 to 14,251.29)	185.58 (137.32 to 272.60)	256.08 (195.93 to 384.72)	215.81 (165.30 to 322.40)	252.50 (191.63 to 380.19)
Paralytic ileus and intestinal obstruction	9,314.01 (5,622.73 to 16,107.04)	10,627.66 (7,084.30 to 17,116.88)	8,643.43 (6,085.62 to 13,669.51)	10,924.53 (7,117.39 to 17,987.23)	189.27 (133.01 to 294.89)	299.51 (217.27 to 458.68)	297.18 (218.16 to 446.73)	281.84 (201.02 to 436.49)
Inguinal, femoral, and abdominal hernia	1,356.38 (1,015.06 to 2,173.39)	1,678.71 (1,235.18 to 2,664.70)	1,555.40 (1,147.01 to 2,435.69)	1,601.90 (1,174.31 to 2,561.34)	52.74 (39.09 to 79.81)	78.20 (54.66 to 116.61)	80.37 (56.42 to 119.30)	70.11 (48.33 to 105.90)
Inflammatory bowel disease	2,026.28 (1,330.73 to 3,603.70)	2,939.33 (1,999.84 to 4,994.52)	2,884.95 (2,014.01 to 4,683.67)	2,868.16 (1,927.96 to 4,929.07)	52.80 (37.56 to 84.51)	91.31 (66.27 to 136.88)	98.56 (73.12 to 142.80)	84.49 (60.28 to 131.51)
Vascular intestinal disorders	1,578.12 (1,026.35 to 2,133.44)	2,519.61 (1,712.24 to 3,254.73)	2,440.16 (1,721.88 to 3,144.23)	2,402.42 (1,585.20 to 3,106.81)	57.52 (41.38 to 74.25)	111.05 (79.81 to 144.75)	118.17 (88.12 to 153.88)	100.33 (70.01 to 131.42)
Gallbladder and biliary diseases	5,683.97 (4,329.27 to 8,162.47)	7,817.54 (4,760.47 to 13,302.69)	5,906.74 (3,613.52 to 9,856.83)	9,662.95 (5,549.56 to 16,833.95)	186.25 (140.72 to 262.76)	302.67 (177.54 to 519.64)	251.34 (149.91 to 417.02)	352.94 (196.45 to 625.86)
Pancreatitis	3,277.70 (2,301.60 to 4,806.71)	5,169.05 (2,607.63 to 10,555.89)	3,988.57 (2,098.09 to 7,916.59)	6,334.32 (2,905.92 to 13,544.17)	86.22 (60.15 to 124.92)	153.91 (80.84 to 293.74)	128.97 (70.37 to 239.10)	178.65 (85.37 to 370.13)
Other digestive diseases	8,936.82 (6,537.75 to 13,215.66)	12,439.63 (9,125.46 to 18,326.13)	10,299.84 (7,492.96 to 15,257.06)	12,492.34 (9,138.63 to 18,881.60)	251.48 (190.85 to 363.22)	425.05 (312.01 to 605.51)	385.94 (279.00 to 545.58)	405.53 (297.37 to 581.62)
**Neurological disorders**	**26,692.77 (22,581.20 to 32,195.04)**	**50,789.65 (39,355.11 to 66,931.64)**	**53,467.16 (42,325.72 to 69,134.37)**	**50,748.43 (38,418.92 to 67,914.37)**	**1,286.79 (1,092.34 to 1,521.81)**	**3,013.77 (2,068.48 to 4,256.50)**	**3,740.63 (2,707.59 to 5,076.26)**	**2,750.52 (1,842.12 to 4,092.21)**
Alzheimer's disease and other dementias	12,471.69 (10,179.84 to 15,289.01)	30,098.65 (19,772.39 to 45,461.14)	35,350.40 (24,909.04 to 51,015.10)	28,387.79 (17,488.62 to 44,986.43)	968.49 (793.76 to 1,187.03)	2,447.18 (1,529.31 to 3,713.31)	3,132.82 (2,133.83 to 4,462.15)	2,189.55 (1,298.45 to 3,449.43)
Parkinson's disease	928.14 (709.64 to 1,177.50)	2,477.10 (1,806.03 to 3,264.32)	3,476.48 (2,603.02 to 4,494.94)	2,057.80 (1,472.28 to 2,733.52)	62.37 (47.54 to 80.28)	176.73 (123.86 to 239.27)	264.11 (193.67 to 351.71)	141.21 (96.11 to 194.58)
Epilepsy	9,898.27 (7,088.06 to 13,919.71)	12,762.28 (8,410.98 to 19,322.55)	8,966.21 (5,852.12 to 13,632.90)	14,982.01 (9,697.62 to 23,089.75)	177.25 (128.54 to 246.53)	244.33 (164.52 to 360.88)	178.55 (120.24 to 265.68)	283.43 (184.95 to 435.60)
Multiple sclerosis	265.65 (144.43 to 517.36)	436.85 (233.56 to 851.82)	451.44 (243.98 to 886.02)	432.40 (230.94 to 844.24)	7.13 (3.96 to 13.73)	12.44 (6.94 to 23.90)	13.22 (7.53 to 25.65)	12.10 (6.67 to 23.28)
Motor neuron disease	422.15 (338.10 to 572.38)	809.77 (624.18 to 1,174.27)	914.95 (701.15 to 1,333.91)	760.58 (590.97 to 1,102.66)	12.89 (10.49 to 17.82)	27.97 (21.56 to 40.82)	33.08 (25.06 to 48.53)	25.57 (19.74 to 37.72)
Other neurological disorders	2,706.88 (2,260.16 to 3,227.93)	4,205.00 (3,388.15 to 5,086.03)	4,307.68 (3,469.56 to 5,268.76)	4,127.84 (3,344.82 to 4,964.61)	58.66 (49.48 to 70.08)	105.12 (84.91 to 127.64)	118.85 (94.59 to 144.93)	98.67 (80.05 to 119.63)
**Mental disorders**	**5,431.57 (3,075.79 to 12,057.82)**	**10,271.32 (5,596.70 to 24,044.46)**	**9,983.49 (5,467.25 to 23,105.33)**	**10,496.60 (5,662.76 to 24,603.77)**	**120.52 (70.12 to 264.82)**	**239.40 (135.98 to 538.24)**	**243.73 (140.24 to 533.53)**	**238.63 (133.79 to 541.08)**
Alcohol use disorders	3,161.15 (1,575.62 to 9,881.70)	4,505.65 (1,842.00 to 16,540.90)	4,224.40 (1,701.32 to 15,559.86)	4,776.63 (1,961.98 to 17,431.08)	72.70 (37.73 to 218.25)	107.57 (47.12 to 374.13)	103.84 (46.08 to 358.45)	111.90 (48.89 to 389.48)
Drug use disorders	2,249.03 (1,277.88 to 4,077.90)	5,730.27 (3,166.85 to 10,191.63)	5,724.48 (3,195.42 to 10,140.26)	5,684.58 (3,111.57 to 10,206.06)	47.40 (27.44 to 82.89)	131.14 (73.91 to 224.85)	139.21 (79.62 to 235.01)	126.04 (70.03 to 218.34)
Opioid use disorders	1,304.60 (668.66 to 2,629.48)	2,866.02 (1,287.52 to 5,822.86)	2,811.78 (1,285.45 to 5,709.03)	2,865.08 (1,276.75 to 5,876.14)	25.49 (13.16 to 50.82)	59.44 (26.71 to 118.96)	60.43 (27.82 to 118.62)	58.28 (25.84 to 118.55)

Cocaine use disorders	170.16 (81.01 to 272.68)	498.56 (229.28 to 848.58)	467.09 (211.17 to 807.74)	499.61 (232.03 to 843.62)	3.53 (1.70 to 5.51)	11.04 (5.28 to 18.41)	10.76 (5.07 to 18.31)	10.82 (5.23 to 17.90)
Amphetamine use disorders	67.54 (36.12 to 105.69)	268.90 (139.05 to 437.04)	245.70 (124.81 to 402.30)	271.61 (141.14 to 435.71)	1.47 (0.81 to 2.24)	6.07 (3.20 to 9.51)	5.69 (2.97 to 8.81)	6.03 (3.20 to 9.42)
Other drug use disorders	706.73 (367.74 to 1,256.10)	2,096.79 (1,038.26 to 3,813.64)	2,199.92 (1,111.91 to 3,998.91)	2,048.27 (1,001.03 to 3,767.63)	16.90 (9.26 to 28.71)	54.60 (28.09 to 96.71)	62.34 (32.85 to 107.08)	50.92 (25.77 to 91.64)
Eating disorders	21.38 (14.98 to 32.58)	35.40 (23.01 to 54.86)	34.61 (22.80 to 55.10)	35.39 (23.06 to 53.80)	0.42 (0.29 to 0.65)	0.69 (0.45 to 1.07)	0.68 (0.45 to 1.10)	0.69 (0.45 to 1.07)

Cause	YLLs (actual counts)				Deaths (actual counts)			
	2016	2040 Forecast	2040 Better Scenario	2040 Worse Scenario	2016	2040 Forecast	2040 Better Scenario	2040 Worse Scenario
**Oceania**								
**Diabetes, urogenital, blood, and endocrine diseases**	**292,849.16 (255,490.77 to 336,784.95)**	**592,034.57 (421,378.06 to 897,325.50)**	**416,803.32 (294,574.94 to 674,081.22)**	**663,305.94 (461,454.95 to 981,566.70)**	**10,590.97 (9,313.34 to 11,978.18)**	**24,640.07 (17,582.71 to 37,219.78)**	**18,735.69 (13,454.38 to 29,866.91)**	**26,420.41 (18,482.97 to 38,348.17)**
Diabetes mellitus	172,970.23 (150,915.76 to 198,399.83)	304,130.97 (205,715.52 to 426,753.36)	213,253.56 (144,566.30 to 300,370.51)	336,650.43 (219,421.91 to 477,574.65)	6,813.79 (6,011.54 to 7,734.49)	13,609.57 (9,513.64 to 18,153.55)	10,313.91 (7,280.16 to 13,838.35)	14,357.39 (9,775.86 to 19,593.29)
Acute glomerulonephritis	296.36 (177.45 to 467.20)	578.22 (303.48 to 1,045.22)	495.48 (252.47 to 902.80)	580.90 (304.49 to 1,046.88)	8.27 (4.77 to 13.26)	17.13 (8.49 to 31.52)	15.33 (7.49 to 28.30)	16.85 (8.32 to 30.94)
Chronic kidney disease	90,590.27 (71,370.62 to 115,998.65)	241,530.47 (73,901.16 to 609,556.36)	162,608.82 (50,219.87 to 449,597.45)	280,106.96 (86,317.13 to 649,942.81)	3,027.50 (2,421.88 to 3,813.12)	9,599.48 (2,563.65 to 24,619.81)	7,009.74 (1,799.20 to 20,060.94)	10,703.69 (2,922.85 to 24,776.37)
Chronic kidney disease due to diabetes mellitus	49,120.84 (38,308.59 to 64,052.91)	142,593.20 (39,876.88 to 373,447.41)	96,220.84 (27,514.60 to 273,006.32)	166,559.35 (47,129.84 to 405,296.48)	1,668.00 (1,311.63 to 2,136.12)	5,493.04 (1,402.73 to 14,312.42)	3,959.14 (1,001.24 to 11,402.85)	6,198.94 (1,602.43 to 14,741.84)
Chronic kidney disease due to hypertension	18,844.62 (14,443.11 to 25,021.30)	57,426.78 (14,677.40 to 152,198.80)	40,131.77 (10,464.83 to 116,258.47)	65,346.06 (16,736.05 to 153,520.14)	818.33 (646.71 to 1,061.02)	2,808.60 (681.76 to 7,400.15)	2,158.20 (526.51 to 6,277.56)	3,041.47 (771.65 to 7,203.49)
Chronic kidney disease due to glomerulonephritis	10,920.21 (8,131.27 to 14,614.39)	18,926.62 (8,642.80 to 41,451.41)	11,882.04 (5,439.24 to 27,472.77)	21,951.16 (9,784.75 to 45,643.99)	244.12 (183.26 to 324.22)	542.76 (195.06 to 1,322.92)	364.42 (130.47 to 959.06)	615.55 (224.08 to 1,414.33)
Chronic kidney disease due to other and unspecified causes	11,704.60 (8,631.60 to 15,727.14)	22,583.87 (8,959.38 to 50,974.70)	14,374.16 (5,618.20 to 34,527.44)	26,250.39 (10,223.31 to 53,810.63)	297.05 (219.79 to 389.38)	755.08 (233.70 to 1,830.05)	527.98 (158.48 to 1,415.69)	847.72 (268.20 to 1,864.60)
Urinary diseases and male infertility	9,661.07 (7,686.26 to 12,795.78)	16,335.48 (13,018.29 to 22,147.51)	14,872.18 (11,947.11 to 20,135.22)	16,220.50 (12,933.98 to 21,971.64)	257.17 (217.39 to 328.86)	544.59 (452.47 to 705.77)	572.89 (472.67 to 748.90)	503.07 (414.45 to 660.45)
Urinary tract infections	7,315.28 (5,600.81 to 9,944.31)	13,005.03 (10,078.62 to 17,948.56)	12,035.90 (9,420.75 to 16,594.96)	12,897.19 (9,985.82 to 18,009.21)	188.07 (152.37 to 241.36)	426.21 (348.32 to 561.11)	462.04 (376.22 to 604.94)	391.34 (316.24 to 519.11)
Urolithiasis	278.96 (199.03 to 447.54)	637.95 (434.56 to 1,021.18)	677.05 (459.52 to 1,100.02)	610.00 (417.65 to 972.51)	8.68 (6.16 to 13.85)	21.85 (14.76 to 35.71)	24.78 (16.60 to 40.12)	20.06 (13.45 to 32.58)
Other urinary diseases	2,066.82 (1,291.51 to 2,863.47)	2,692.50 (1,481.66 to 3,761.26)	2,159.22 (1,165.21 to 3,015.75)	2,713.31 (1,463.05 to 3,782.79)	60.42 (38.70 to 81.15)	96.53 (52.54 to 135.03)	86.08 (46.12 to 122.32)	91.68 (49.74 to 129.39)
Gynecological diseases	250.22 (143.73 to 351.00)	295.27 (162.84 to 444.52)	222.73 (122.56 to 335.65)	308.24 (173.12 to 459.39)	6.01 (3.54 to 8.36)	7.33 (4.28 to 10.79)	5.65 (3.26 to 8.29)	7.56 (4.39 to 11.09)
Uterine fibroids	29.65 (5.89 to 59.28)	32.20 (5.76 to 70.72)	23.52 (4.31 to 53.67)	33.76 (6.20 to 74.92)	0.82 (0.18 to 1.62)	0.92 (0.18 to 1.97)	0.69 (0.14 to 1.51)	0.95 (0.19 to 2.04)
Polycystic ovarian syndrome	6.02 (2.22 to 14.03)	4.75 (1.59 to 12.24)	2.87 (0.96 to 7.26)	5.22 (1.82 to 12.69)	0.15 (0.05 to 0.34)	0.11 (0.04 to 0.29)	0.07 (0.02 to 0.17)	0.13 (0.04 to 0.31)
Endometriosis	1.70 (0.75 to 3.69)	2.35 (0.77 to 6.41)	1.73 (0.56 to 4.96)	2.48 (0.82 to 6.80)	0.04 (0.02 to 0.08)	0.05 (0.02 to 0.16)	0.04 (0.01 to 0.12)	0.06 (0.02 to 0.17)
Genital prolapse	10.46 (2.36 to 24.16)	12.69 (2.48 to 30.39)	9.06 (1.76 to 21.65)	13.28 (2.64 to 32.22)	0.33 (0.08 to 0.66)	0.39 (0.08 to 0.81)	0.30 (0.06 to 0.63)	0.39 (0.08 to 0.82)
Other gynecological diseases	202.39 (110.17 to 289.70)	243.28 (135.19 to 369.90)	185.55 (101.24 to 283.18)	253.51 (138.60 to 387.21)	4.67 (2.61 to 6.61)	5.85 (3.27 to 8.76)	4.55 (2.56 to 6.79)	6.03 (3.37 to 9.15)
Hemoglobinopathies and hemolytic anemias	4,209.95 (3,163.04 to 5,341.08)	4,863.15 (3,672.86 to 6,297.00)	3,889.64 (2,931.00 to 5,051.84)	4,979.23 (3,757.95 to 6,547.20)	97.89 (75.78 to 125.09)	139.26 (107.51 to 184.98)	117.64 (90.40 to 154.53)	137.14 (106.64 to 183.36)
Thalassemias	1,070.40 (627.52 to 1,558.82)	720.83 (427.83 to 1,080.42)	663.15 (419.11 to 976.52)	732.95 (429.37 to 1,087.93)	13.58 (8.14 to 19.39)	9.36 (5.71 to 13.98)	8.72 (5.58 to 12.56)	9.49 (5.70 to 13.97)

Sickle cell disorders	4.45 (2.96 to 6.11)	4.90 (3.13 to 7.20)	3.83 (2.40 to 5.65)	5.14 (3.26 to 7.55)	0.07 (0.05 to 0.10)	0.08 (0.05 to 0.12)	0.06 (0.04 to 0.09)	0.09 (0.05 to 0.12)
G6PD deficiency	2,108.53 (1,602.36 to 2,756.77)	3,045.37 (2,245.54 to 4,068.13)	2,288.20 (1,675.72 to 3,071.57)	3,181.09 (2,351.69 to 4,291.33)	46.15 (35.05 to 60.26)	77.27 (58.19 to 104.39)	60.22 (44.90 to 80.65)	78.99 (59.74 to 106.11)
Other hemoglobinopathies and hemolytic anemias	1,026.57 (769.98 to 1,362.65)	1,092.05 (819.81 to 1,507.59)	934.46 (703.10 to 1,273.37)	1,060.05 (796.47 to 1,464.21)	38.09 (29.77 to 50.76)	52.56 (40.25 to 72.13)	48.63 (37.47 to 67.21)	48.58 (36.58 to 67.49)
Endocrine, metabolic, blood, and immune disorders	14,871.06 (11,656.79 to 19,388.62)	24,301.01 (18,554.22 to 32,603.57)	21,460.92 (16,276.74 to 28,717.13)	24,459.68 (18,698.87 to 32,561.46)	380.34 (302.05 to 490.35)	722.72 (554.28 to 963.83)	700.53 (534.37 to 941.41)	694.72 (537.31 to 919.95)

Cause	YLLs (actual counts)				Deaths (actual counts)			
	**Oceania**							
	2016	2040 Forecast	2040 Better Scenario	2040 Worse Scenario	2016	2040 Forecast	2040 Better Scenario	2040 Worse Scenario
**Musculoskeletal disorders**	**4,986.63 (3,077.89 to 6,871.83)**	**7,948.01 (4,693.74 to 11,317.57)**	**8,253.05 (4,938.87 to 11,799.33)**	**7,790.68 (4,629.97 to 11,133.93)**	**103.13 (66.23 to 140.14)**	**188.67 (118.21 to 260.83)**	**208.76 (131.27 to 285.70)**	**178.58 (111.82 to 247.87)**
Rheumatoid arthritis	412.93 (253.85 to 573.84)	829.31 (494.08 to 1,201.78)	700.40 (408.44 to 1,022.41)	828.24 (494.59 to 1,200.37)	13.38 (8.06 to 18.54)	33.30 (18.80 to 49.25)	30.80 (16.88 to 45.27)	31.69 (18.33 to 46.72)
Other musculoskeletal disorders	4,573.70 (2,800.63 to 6,371.36)	7,118.70 (4,136.26 to 10,192.56)	7,552.65 (4,425.21 to 10,842.28)	6,962.45 (4,046.34 to 9,961.20)	89.76 (56.66 to 122.42)	155.37 (94.33 to 216.36)	177.97 (111.24 to 244.09)	146.89 (88.16 to 205.14)
**Other non-communicable diseases**	**152,909.56 (73,485.14 to 268,591.79)**	**175,318.35 (95,206.92 to 298,995.33)**	**110,300.88 (68,821.79 to 174,862.55)**	**194,577.34 (102,810.42 to 338,137.37)**	**2,041.39 (1,106.79 to 3,399.51)**	**2,872.28 (1,910.15 to 4,395.09)**	**2,215.41 (1,600.51 to 3,159.60)**	**3,012.43 (1,911.41 to 4,762.20)**
Congenital birth defects	138,406.76 (59,043.50 to 251,002.95)	144,865.84 (65,374.36 to 261,628.77)	82,300.40 (39,874.30 to 144,203.92)	164,359.01 (71,084.55 to 301,309.50)	1,677.74 (736.39 to 2,993.73)	1,815.41 (853.96 to 3,224.16)	1,067.79 (544.43 to 1,811.03)	2,045.02 (930.40 to 3,667.21)
Neural tube defects	16,745.97 (5,733.24 to 38,881.78)	7,537.44 (2,284.28 to 19,349.46)	3,315.69 (1,000.37 to 8,698.14)	8,966.98 (2,665.89 to 23,307.30)	194.38 (66.82 to 450.20)	87.82 (26.89 to 224.51)	38.78 (11.86 to 101.10)	104.40 (31.42 to 270.11)
Congenital heart anomalies	56,042.56 (30,318.88 to 97,674.91)	58,483.58 (31,744.07 to 98,316.22)	34,504.55 (19,720.15 to 55,981.60)	65,655.84 (35,116.37 to 112,220.62)	689.54 (379.34 to 1,183.12)	746.21 (420.36 to 1,240.10)	456.67 (267.57 to 723.61)	831.39 (460.60 to 1,385.57)
Orofacial clefts	149.04 (58.66 to 315.62)	128.57 (45.28 to 303.26)	54.48 (20.78 to 122.84)	158.67 (55.77 to 385.83)	1.73 (0.68 to 3.66)	1.49 (0.52 to 3.52)	0.63 (0.24 to 1.42)	1.84 (0.65 to 4.47)
Down syndrome	7,166.04 (1,783.34 to 27,788.78)	8,712.54 (2,308.01 to 32,468.20)	4,926.77 (1,476.65 to 17,354.00)	9,930.72 (2,580.47 to 37,010.94)	90.73 (24.61 to 340.97)	117.81 (34.44 to 413.20)	70.93 (23.35 to 227.35)	132.41 (37.08 to 467.13)
Other chromosomal abnormalities	5,149.43 (1,519.99 to 13,424.71)	8,747.86 (2,375.24 to 21,752.85)	4,699.99 (1,366.98 to 11,333.20)	10,026.38 (2,755.75 to 25,093.49)	60.68 (18.04 to 157.35)	104.25 (28.56 to 256.68)	56.84 (16.65 to 136.02)	119.14 (33.10 to 296.89)
Congenital musculoskeletal and limb anomalies	3,319.90 (1,029.83 to 8,532.21)	4,104.83 (1,345.75 to 10,622.29)	2,424.78 (841.06 to 6,156.69)	4,617.57 (1,479.61 to 12,006.86)	39.62 (12.43 to 102.07)	49.67 (16.48 to 128.68)	29.92 (10.62 to 75.37)	55.67 (18.09 to 143.64)
Urogenital congenital anomalies	3,820.36 (1,434.59 to 7,224.40)	4,151.75 (1,684.30 to 7,644.38)	2,463.50 (1,076.73 to 4,298.08)	4,673.40 (1,878.42 to 8,669.04)	49.69 (19.63 to 90.98)	58.25 (25.80 to 103.06)	37.54 (17.82 to 61.41)	64.26 (27.91 to 113.99)
Digestive congenital anomalies	6,702.30 (2,412.61 to 14,420.23)	6,798.86 (2,452.90 to 15,031.07)	3,549.82 (1,346.39 to 7,624.64)	7,856.43 (2,787.63 to 17,445.08)	78.44 (28.38 to 168.26)	80.28 (29.22 to 176.43)	42.35 (16.36 to 90.67)	92.60 (33.22 to 203.84)
Other congenital birth defects	39,311.16 (10,118.15 to 93,542.29)	46,200.42 (13,080.32 to 110,368.38)	26,360.82 (8,286.96 to 59,074.47)	52,473.03 (14,708.32 to 125,168.89)	472.93 (125.19 to 1,115.95)	569.63 (167.98 to 1,335.32)	334.12 (111.03 to 734.07)	643.30 (187.38 to 1,496.73)
Skin and subcutaneous diseases	8,934.89 (5,480.18 to 13,772.16)	25,666.59 (16,463.08 to 39,137.81)	25,300.59 (16,350.88 to 38,210.75)	24,769.19 (15,824.88 to 37,534.26)	299.08 (183.98 to 467.46)	1,001.38 (643.37 to 1,515.89)	1,116.31 (721.67 to 1,703.82)	904.23 (580.38 to 1,395.17)
Cellulitis	929.07 (361.75 to 1,428.58)	2,980.66 (1,094.43 to 4,704.13)	3,353.36 (1,223.76 to 5,359.06)	2,776.28 (1,015.72 to 4,395.51)	32.24 (12.38 to 48.75)	124.19 (47.61 to 196.28)	153.68 (57.85 to 243.71)	109.01 (41.35 to 171.65)
Pyoderma	6,374.28 (3,874.90 to 9,906.17)	18,907.41 (11,925.38 to 29,613.40)	17,792.98 (11,195.63 to 27,764.41)	18,499.85 (11,673.98 to 29,083.66)	192.54 (117.80 to 303.04)	680.93 (448.76 to 1,076.43)	716.44 (466.08 to 1,134.84)	626.48 (411.66 to 980.01)
Decubitus ulcer	1,547.01 (838.57 to 3,027.23)	3,600.98 (1,804.48 to 7,450.31)	3,979.90 (2,069.14 to 8,051.14)	3,316.90 (1,611.26 to 7,009.19)	72.54 (41.21 to 141.48)	192.11 (97.59 to 392.00)	241.55 (127.15 to 478.56)	164.86 (81.49 to 334.77)
Other skin and subcutaneous diseases	84.54 (49.35 to 134.02)	177.53 (104.34 to 277.14)	174.35 (102.61 to 275.89)	176.16 (103.96 to 280.22)	1.75 (1.05 to 2.81)	4.15 (2.52 to 6.55)	4.64 (2.78 to 7.28)	3.87 (2.36 to 6.17)
Sudden infant death syndrome	5,567.90 (2,744.07 to 10,765.85)	4,785.92 (2,147.98 to 9,631.39)	2,699.90 (1,227.42 to 5,231.54)	5,449.14 (2,468.31 to 10,841.74)	64.58 (31.83 to 124.88)	55.49 (24.90 to 111.74)	31.31 (14.24 to 60.69)	63.18 (28.62 to 125.73)

**Injuries**	**451,267.80 (381,662.95 to 530,079.84)**	**594,420.50 (471,510.60 to 751,670.48)**	**484,280.57 (385,861.96 to 611,457.32)**	**651,384.64 (510,345.88 to 830,810.22)**	**8,443.83 (7,106.27 to 9,943.33)**	**12,030.49 (9,745.06 to 15,076.63)**	**10,289.41 (8,393.05 to 12,753.09)**	**12,894.67 (10,332.53 to 16,189.25)**
**Transport injuries**	**139,103.92 (115,509.14 to 166,276.15)**	**230,119.96 (175,707.07 to 302,472.49)**	**186,385.62 (142,918.77 to 241,818.77)**	**258,412.47 (192,598.58 to 352,395.48)**	**2,738.66 (2,290.47 to 3,278.39)**	**4,726.82 (3,647.52 to 6,181.36)**	**3,971.62 (3,096.58 to 5,149.62)**	**5,225.11 (3,910.30 to 7,078.78)**
Road injuries	124,921.27 (103,500.80 to 149,892.53)	204,982.39 (156,444.24 to 273,501.54)	166,639.49 (127,442.63 to 219,282.17)	230,472.51 (170,408.29 to 314,266.80)	2,466.27 (2,053.57 to 2,963.39)	4,215.51 (3,209.35 to 5,541.90)	3,554.63 (2,732.73 to 4,619.64)	4,665.32 (3,486.24 to 6,286.53)
Pedestrian road injuries	36,298.85 (28,800.38 to 44,634.23)	51,173.55 (37,157.23 to 69,482.80)	41,793.57 (30,865.10 to 56,356.70)	56,990.56 (40,375.75 to 80,011.79)	740.75 (596.53 to 919.39)	1,122.15 (826.18 to 1,523.97)	949.61 (705.72 to 1,267.97)	1,228.86 (888.42 to 1,711.71)

Oceania								
Cause	YLLs (actual counts)				Deaths (actual counts)			
	2016	2040 Forecast	2040 Better Scenario	2040 Worse Scenario	2016	2040 Forecast	2040 Better Scenario	2040 Worse Scenario
Cyclist road injuries	1,206.21 (996.49 to 1,455.68)	1,904.96 (1,423.69 to 2,484.96)	1,500.90 (1,114.50 to 1,972.08)	2,170.54 (1,594.70 to 2,894.31)	24.54 (20.44 to 29.70)	40.51 (30.77 to 52.79)	33.72 (25.67 to 44.13)	45.03 (33.30 to 59.94)
Motorcyclist road injuries	23,770.09 (17,017.76 to 31,255.95)	42,154.26 (27,161.38 to 62,303.13)	35,591.00 (22,888.04 to 52,311.90)	46,982.26 (29,342.23 to 71,717.72)	443.95 (315.15 to 582.74)	798.21 (511.00 to 1,173.59)	690.10 (442.42 to 1,012.90)	882.16 (554.69 to 1,357.50)
Motor vehicle road injuries	60,950.88 (49,135.56 to 75,345.76)	104,836.32 (77,691.16 to 137,962.63)	83,863.49 (62,697.55 to 109,822.03)	118,761.79 (86,005.45 to 159,248.94)	1,207.34 (968.23 to 1,486.72)	2,160.97 (1,618.47 to 2,819.95)	1,803.56 (1,363.27 to 2,335.26)	2,404.80 (1,765.84 to 3,198.80)
Other road injuries	2,695.23 (1,324.83 to 3,668.28)	4,913.31 (2,247.07 to 7,180.87)	3,890.52 (1,747.73 to 5,686.12)	5,567.38 (2,571.52 to 8,369.64)	49.69 (24.52 to 67.07)	93.67 (42.36 to 135.54)	77.65 (34.97 to 112.05)	104.46 (47.32 to 154.86)
Other transport injuries	14,182.65 (8,168.72 to 22,199.61)	25,137.57 (13,972.36 to 39,855.65)	19,746.13 (10,982.66 to 31,442.91)	27,939.95 (14,850.62 to 44,916.21)	272.39 (161.62 to 424.20)	511.31 (291.61 to 816.99)	416.99 (238.76 to 670.11)	559.78 (311.47 to 912.78)
**Unintentional injuries**	**194,281.27 (160,913.52 to 233,716.08)**	**186,835.93 (151,158.14 to 230,341.11)**	**142,340.17 (115,000.93 to 171,864.90)**	**198,751.90 (159,499.30 to 247,971.18)**	**3,533.13 (2,977.60 to 4,137.10)**	**3,936.29 (3,290.56 to 4,633.42)**	**3,281.34 (2,754.81 to 3,831.89)**	**4,031.12 (3,349.92 to 4,797.17)**
Falls	12,639.32 (8,910.59 to 18,444.54)	14,997.49 (10,989.07 to 21,200.17)	13,501.44 (10,087.97 to 18,750.43)	14,996.78 (10,902.51 to 21,549.43)	296.37 (223.27 to 406.49)	439.28 (333.28 to 593.94)	447.24 (345.87 to 597.27)	411.72 (309.13 to 560.06)
Drowning	74,138.12 (58,766.87 to 93,177.55)	63,439.04 (46,448.77 to 83,668.33)	48,055.10 (35,828.01 to 62,201.03)	68,776.03 (49,636.49 to 92,214.19)	1,233.80 (980.16 to 1,518.60)	1,153.15 (876.26 to 1,486.08)	913.17 (702.02 to 1,156.33)	1,232.00 (916.85 to 1,621.36)
Fire, heat, and hot substances	16,258.41 (12,170.50 to 21,641.61)	13,206.97 (9,507.87 to 18,124.42)	9,406.32 (6,944.06 to 12,877.92)	14,408.99 (10,222.21 to 19,781.30)	288.51 (223.73 to 374.58)	272.21 (211.80 to 365.31)	214.05 (167.55 to 284.64)	285.87 (218.91 to 385.62)
Poisonings	11,887.43 (5,713.74 to 19,491.32)	8,375.57 (4,062.54 to 13,886.12)	5,271.25 (2,576.52 to 8,500.68)	9,398.03 (4,500.26 to 15,625.49)	207.17 (102.35 to 309.63)	164.87 (82.00 to 259.49)	115.35 (58.63 to 172.09)	178.23 (87.68 to 282.58)
Exposure to mechanical forces	23,943.53 (19,040.91 to 29,580.44)	23,976.93 (18,306.65 to 30,822.82)	17,685.54 (13,473.56 to 22,734.40)	25,401.08 (19,370.62 to 32,770.03)	429.31 (345.19 to 539.27)	483.56 (377.14 to 621.29)	383.67 (298.87 to 508.32)	496.62 (385.41 to 641.53)
Unintentional firearm injuries	4,716.78 (3,137.45 to 6,742.91)	4,484.72 (2,777.68 to 6,762.73)	3,172.41 (1,974.28 to 4,701.18)	4,769.77 (2,983.23 to 7,200.01)	86.97 (59.06 to 120.01)	95.51 (61.29 to 137.57)	73.68 (47.59 to 106.20)	97.78 (62.59 to 141.71)
Unintentional suffocation	5,149.65 (3,245.84 to 7,478.82)	5,293.28 (3,308.20 to 7,855.42)	3,705.49 (2,349.35 to 5,518.75)	5,719.00 (3,553.56 to 8,664.54)	77.42 (50.18 to 109.34)	83.60 (53.68 to 123.96)	60.84 (39.23 to 90.31)	89.30 (56.87 to 132.14)
Other exposure to mechanical forces	14,077.10 (10,826.42 to 18,784.11)	14,198.93 (10,480.90 to 19,527.42)	10,807.64 (7,989.91 to 15,251.21)	14,912.30 (11,146.32 to 20,381.29)	264.91 (202.51 to 363.17)	304.45 (225.73 to 434.76)	249.15 (184.57 to 361.86)	309.54 (230.37 to 440.98)
Adverse effects of medical treatment	18,163.96 (13,475.87 to 23,779.47)	20,454.15 (15,626.40 to 26,460.91)	15,892.19 (12,410.47 to 20,030.12)	21,427.71 (16,072.44 to 28,255.34)	354.14 (278.22 to 439.79)	465.71 (379.58 to 561.15)	402.54 (330.02 to 478.32)	465.90 (377.56 to 566.81)
Animal contact	5,992.63 (3,986.70 to 8,473.22)	9,244.10 (6,477.10 to 13,187.24)	6,666.90 (4,902.20 to 9,310.15)	9,657.75 (6,676.90 to 14,020.11)	144.40 (104.28 to 188.22)	263.64 (198.27 to 354.02)	215.01 (163.66 to 288.41)	260.37 (196.06 to 356.68)
Venomous animal contact	4,759.24 (3,033.15 to 7,233.69)	7,192.98 (4,786.32 to 10,759.62)	5,145.18 (3,608.65 to 7,452.61)	7,509.78 (4,942.78 to 11,480.39)	115.92 (79.96 to 156.67)	208.52 (148.23 to 292.44)	169.03 (121.84 to 235.16)	205.66 (145.93 to 295.30)
Non-venomous animal contact	1,233.39 (740.55 to 2,255.30)	2,051.12 (1,191.08 to 3,609.28)	1,521.72 (929.75 to 2,553.26)	2,147.96 (1,243.65 to 3,742.84)	28.47 (17.91 to 49.66)	55.12 (34.00 to 91.02)	45.98 (29.29 to 74.09)	54.70 (33.00 to 91.93)
Foreign body	11,761.58 (7,351.23 to 18,698.48)	13,027.46 (8,369.27 to 19,383.49)	10,958.84 (7,344.62 to 15,210.99)	13,454.52 (8,493.58 to 20,640.65)	199.68 (135.71 to 282.37)	260.94 (184.29 to 339.24)	253.16 (181.26 to 314.87)	255.15 (178.58 to 338.76)
Pulmonary aspiration and foreign body in airway	10,807.35 (6,496.20 to 17,530.75)	12,046.61 (7,474.27 to 18,209.85)	10,216.69 (6,745.42 to 14,220.39)	12,410.78 (7,578.16 to 19,079.84)	183.52 (120.04 to 263.40)	243.08 (166.21 to 314.81)	238.70 (167.02 to 297.62)	236.56 (160.60 to 314.42)

Foreign body in other body part	954.23 (532.00 to 1,609.49)	980.85 (535.26 to 1,686.45)	742.15 (422.08 to 1,230.60)	1,043.74 (575.54 to 1,802.16)	16.16 (9.47 to 26.51)	17.86 (10.37 to 29.25)	14.46 (8.62 to 23.19)	18.59 (10.70 to 30.65)
Environmental heat and cold exposure	7,523.04 (4,266.35 to 10,825.41)	9,656.17 (5,227.32 to 15,112.16)	6,527.71 (3,551.92 to 9,921.36)	10,364.39 (5,672.61 to 15,982.85)	150.83 (87.42 to 208.18)	217.10 (122.65 to 317.92)	157.36 (87.15 to 231.34)	225.23 (126.01 to 332.35)
Other unintentional injuries	11,973.24 (8,448.81 to 15,457.63)	10,458.05 (7,246.67 to 14,035.76)	8,374.86 (5,684.96 to 11,261.38)	10,866.63 (7,500.99 to 14,592.68)	228.93 (160.44 to 296.38)	215.83 (149.59 to 286.94)	179.78 (123.93 to 242.59)	220.03 (151.82 to 290.78)
**Self-harm and interpersonal violence**	**117,817.57 (87,745.22 to 144,359.76)**	**161,227.23 (111,352.59 to 215,844.79)**	**140,830.05 (99,442.97 to 186,304.12)**	**177,705.82 (118,163.06 to 251,618.74)**	**2,170.94 (1,617.94 to 2,643.91)**	**3,067.07 (2,147.78 to 4,102.61)**	**2,745.72 (1,973.81 to 3,608.98)**	**3,340.10 (2,241.33 to 4,672.82)**

Oceania								
Cause	YLLs (actual counts)				Deaths (actual counts)			
	2016	2040 Forecast	2040 Better Scenario	2040 Worse Scenario	2016	2040 Forecast	2040 Better Scenario	2040 Worse Scenario
Self-harm	78,267.01 (53,610.58 to 98,559.99)	117,045.22 (70,020.11 to 170,168.87)	100,098.37 (61,175.39 to 143,000.97)	131,537.90 (77,079.24 to 201,233.14)	1,447.61 (1,005.93 to 1,810.57)	2,233.21 (1,380.67 to 3,229.41)	1,963.39 (1,240.20 to 2,774.29)	2,475.08 (1,491.22 to 3,742.95)
Self-harm by firearm	7,339.04 (1,807.47 to 15,410.47)	9,418.08 (2,227.89 to 21,043.02)	7,814.90 (1,852.15 to 17,179.45)	10,799.34 (2,487.00 to 24,620.30)	139.63 (35.98 to 291.67)	183.97 (43.72 to 400.84)	155.34 (37.09 to 333.19)	208.93 (47.86 to 465.23)
Self-harm by other specified means	70,927.96 (46,945.01 to 89,189.17)	107,627.15 (63,001.24 to 154,923.48)	92,283.47 (55,468.97 to 131,135.38)	120,738.55 (69,015.23 to 182,564.44)	1,307.99 (871.88 to 1,647.52)	2,049.25 (1,227.21 to 2,921.50)	1,808.05 (1,121.82 to 2,534.08)	2,266.15 (1,328.47 to 3,391.54)
Interpersonal violence	39,550.56 (24,754.71 to 50,693.09)	44,182.01 (25,858.88 to 60,232.28)	40,731.68 (23,873.21 to 55,257.36)	46,167.92 (26,766.15 to 64,205.92)	723.33 (450.09 to 923.19)	833.86 (492.71 to 1,123.90)	782.33 (468.02 to 1,048.00)	865.02 (500.65 to 1,192.60)
Physical violence by firearm	7,869.91 (3,797.24 to 13,209.87)	8,791.09 (4,157.08 to 15,007.15)	8,384.04 (3,962.72 to 14,316.92)	8,862.87 (4,198.87 to 15,103.83)	143.35 (69.78 to 240.63)	162.08 (78.25 to 278.37)	156.33 (75.45 to 266.95)	162.62 (77.84 to 281.80)
Physical violence by sharp object	10,092.78 (6,147.60 to 13,863.09)	12,467.09 (7,474.27 to 17,665.22)	12,533.67 (7,506.51 to 17,700.33)	12,418.44 (7,418.19 to 17,785.07)	186.53 (113.33 to 255.88)	235.26 (141.41 to 333.25)	238.97 (144.06 to 336.54)	233.20 (139.34 to 332.79)
Physical violence by other means	21,587.87 (12,830.85 to 28,891.80)	22,923.82 (11,896.55 to 34,893.02)	19,813.97 (10,206.79 to 30,146.29)	24,886.62 (12,823.92 to 38,696.06)	393.45 (230.01 to 522.07)	436.53 (228.06 to 654.64)	387.02 (204.10 to 579.50)	469.20 (241.45 to 727.91)
**Forces of nature, conflict and terrorism, and executions and police conflict**	**65.03 (55.80 to 75.17)**	**16,237.38 (0.00 to 170,272.96)**	**14,724.73 (0.00 to 154,718.56)**	**16,514.46 (0.00 to 172,944.69)**	**1.09 (0.95 to 1.25)**	**300.30 (0.00 to 3,137.51)**	**290.74 (0.00 to 3,061.58)**	**298.35 (0.00 to 3,096.88)**
Exposure to forces of nature	0.00 (0.00 to 0.00)	15,950.31 (0.00 to 170,256.85)	14,461.63 (0.00 to 154,703.41)	16,222.88 (0.00 to 172,927.99)	0.00 (0.00 to 0.00)	295.39 (0.00 to 3,137.23)	286.04 (0.00 to 3,061.31)	293.43 (0.00 to 3,096.59)
Conflict and terrorism	0.00 (0.00 to 0.00)	287.08 (0.00 to 3,068.10)	263.10 (0.00 to 2,793.43)	291.57 (0.00 to 3,109.13)	0.00 (0.00 to 0.00)	4.91 (0.00 to 52.30)	4.70 (0.00 to 50.00)	4.92 (0.00 to 52.25)
Executions and police conflict	65.03 (55.80 to 75.17)	0.00 (0.00 to 0.00)	0.00 (0.00 to 0.00)	0.00 (0.00 to 0.00)	1.09 (0.95 to 1.25)	0.00 (0.00 to 0.00)	0.00 (0.00 to 0.00)	0.00 (0.00 to 0.00)

American Samoa								
Cause	YLLs (actual counts)				Deaths (actual counts)			
	2016	2040 Forecast	2040 Better Scenario	2040 Worse Scenario	2016	2040 Forecast	2040 Better Scenario	2040 Worse Scenario
**All causes**	**11,198.21 (9,569.29 to 12,985.06)**	**19,509.42 (15,556.70 to 24,492.43)**	**12,686.54 (10,141.66 to 15,880.83)**	**23,073.78 (18,329.94 to 29,462.02)**	**345.83 (295.12 to 401.89)**	**839.26 (710.38 to 978.09)**	**583.07 (492.31 to 691.39)**	**963.72 (815.21 to 1,139.26)**
**Communicable, maternal, neonatal, and nutritional diseases**	**1,479.15 (1,199.59 to 2,057.79)**	**1,641.47 (1,223.76 to 2,448.66)**	**1,067.47 (783.59 to 1,836.91)**	**1,912.79 (1,432.00 to 2,777.69)**	**29.82 (24.27 to 42.09)**	**53.20 (41.43 to 74.02)**	**39.67 (30.62 to 60.16)**	**58.97 (45.41 to 80.92)**
**HIV/AIDS and tuberculosis**	**144.33 (24.68 to 737.96)**	**208.41 (27.78 to 1,060.48)**	**113.99 (5.93 to 892.73)**	**285.58 (56.31 to 1,139.60)**	**3.30 (0.68 to 16.03)**	**5.32 (0.99 to 26.71)**	**2.97 (0.25 to 23.60)**	**6.98 (1.79 to 27.55)**
Tuberculosis	18.10 (14.37 to 21.84)	25.73 (16.52 to 40.56)	7.53 (4.94 to 11.34)	31.01 (19.39 to 50.21)	0.55 (0.45 to 0.66)	1.03 (0.67 to 1.61)	0.32 (0.21 to 0.48)	1.19 (0.77 to 1.91)
HIV/AIDS	126.22 (6.17 to 719.66)	182.68 (4.01 to 1,034.63)	106.47 (0.00 to 886.83)	254.57 (26.53 to 1,110.05)	2.75 (0.12 to 15.51)	4.29 (0.08 to 25.65)	2.65 (0.00 to 23.36)	5.79 (0.60 to 26.50)
**Diarrhea, lower respiratory, and other common infectious diseases**	**625.49 (514.70 to 756.76)**	**870.02 (662.96 to 1,182.02)**	**585.37 (445.96 to 783.25)**	**999.78 (747.03 to 1,376.44)**	**16.18 (13.32 to 19.71)**	**35.18 (26.87 to 47.22)**	**25.98 (19.82 to 35.86)**	**38.79 (28.98 to 53.09)**
Diarrheal diseases	41.74 (27.16 to 62.12)	55.09 (14.30 to 154.61)	82.19 (27.11 to 205.19)	66.94 (16.03 to 190.15)	1.31 (0.75 to 2.04)	2.98 (0.75 to 8.30)	4.90 (1.55 to 12.06)	3.43 (0.77 to 9.93)
Intestinal infectious diseases	54.00 (26.52 to 94.98)	42.93 (20.53 to 72.60)	49.11 (25.29 to 78.20)	46.09 (21.73 to 79.34)	0.78 (0.39 to 1.37)	0.69 (0.33 to 1.17)	0.81 (0.41 to 1.30)	0.73 (0.35 to 1.25)
Typhoid fever	46.27 (21.96 to 81.90)	35.44 (16.69 to 60.62)	45.02 (22.84 to 72.44)	38.04 (17.85 to 65.97)	0.67 (0.32 to 1.17)	0.56 (0.27 to 0.98)	0.74 (0.37 to 1.21)	0.59 (0.28 to 1.03)
Paratyphoid fever	7.71 (3.18 to 15.81)	7.48 (3.08 to 14.73)	4.08 (1.64 to 7.83)	8.03 (3.30 to 15.78)	0.12 (0.05 to 0.23)	0.12 (0.05 to 0.24)	0.07 (0.03 to 0.13)	0.13 (0.05 to 0.26)
Other intestinal infectious diseases	0.02 (0.01 to 0.04)	0.02 (0.01 to 0.04)	0.01 (0.00 to 0.02)	0.02 (0.01 to 0.04)	0.00 (0.00 to 0.00)	0.00 (0.00 to 0.00)	0.00 (0.00 to 0.00)	0.00 (0.00 to 0.00)
Lower respiratory infections	449.23 (367.77 to 545.95)	704.47 (523.80 to 988.99)	412.54 (297.07 to 575.91)	810.45 (586.85 to 1,171.41)	12.76 (10.40 to 15.83)	30.03 (22.67 to 41.09)	19.28 (14.19 to 26.51)	33.06 (24.47 to 46.83)
Upper respiratory infections	0.33 (0.17 to 0.57)	0.52 (0.19 to 1.12)	0.18 (0.07 to 0.41)	0.56 (0.21 to 1.22)	0.01 (0.00 to 0.01)	0.02 (0.01 to 0.04)	0.01 (0.00 to 0.01)	0.02 (0.01 to 0.04)
Otitis media	0.01 (0.00 to 0.02)	0.00 (0.00 to 0.01)	0.00 (0.00 to 0.01)	0.00 (0.00 to 0.01)	0.00 (0.00 to 0.00)	0.00 (0.00 to 0.00)	0.00 (0.00 to 0.00)	0.00 (0.00 to 0.00)
Meningitis	46.13 (35.56 to 64.75)	32.90 (23.28 to 50.01)	17.50 (12.19 to 27.08)	34.21 (23.90 to 51.75)	0.79 (0.61 to 1.11)	0.75 (0.56 to 1.13)	0.42 (0.31 to 0.66)	0.75 (0.56 to 1.12)
Pneumococcal meningitis	0.13 (0.09 to 0.19)	0.06 (0.04 to 0.09)	0.06 (0.04 to 0.09)	0.06 (0.04 to 0.09)	0.00 (0.00 to 0.00)	0.00 (0.00 to 0.00)	0.00 (0.00 to 0.00)	0.00 (0.00 to 0.00)
H influenzae type B meningitis	4.75 (3.50 to 6.78)	2.82 (1.73 to 4.98)	2.57 (1.65 to 4.21)	3.11 (1.77 to 6.01)	0.08 (0.06 to 0.11)	0.06 (0.04 to 0.10)	0.06 (0.05 to 0.10)	0.06 (0.04 to 0.11)
Meningococcal meningitis	9.35 (6.60 to 13.82)	6.88 (4.72 to 10.87)	3.59 (2.36 to 5.68)	7.08 (4.85 to 11.17)	0.17 (0.12 to 0.25)	0.18 (0.13 to 0.27)	0.10 (0.07 to 0.16)	0.17 (0.12 to 0.26)
Other meningitis	31.90 (24.33 to 45.58)	23.14 (16.19 to 35.13)	11.28 (7.61 to 17.79)	23.96 (16.62 to 36.30)	0.54 (0.41 to 0.77)	0.51 (0.37 to 0.77)	0.26 (0.18 to 0.41)	0.51 (0.37 to 0.77)
Encephalitis	11.91 (9.16 to 15.86)	17.59 (12.53 to 24.10)	14.94 (10.50 to 20.83)	18.30 (13.03 to 25.34)	0.24 (0.18 to 0.31)	0.46 (0.34 to 0.61)	0.41 (0.31 to 0.56)	0.46 (0.35 to 0.62)

Diphtheria	0.06 (0.04 to 0.09)	0.02 (0.01 to 0.03)	0.01 (0.01 to 0.02)	0.02 (0.01 to 0.05)	0.00 (0.00 to 0.00)	0.00 (0.00 to 0.00)	0.00 (0.00 to 0.00)	0.00 (0.00 to 0.00)
Whooping cough	4.31 (0.31 to 13.61)	2.81 (0.11 to 11.14)	0.58 (0.04 to 2.27)	5.98 (0.19 to 22.98)	0.05 (0.00 to 0.16)	0.03 (0.00 to 0.13)	0.01 (0.00 to 0.03)	0.07 (0.00 to 0.27)
Tetanus	0.46 (0.40 to 0.54)	0.07 (0.05 to 0.10)	0.06 (0.04 to 0.09)	0.08 (0.05 to 0.13)	0.01 (0.01 to 0.01)	0.00 (0.00 to 0.00)	0.00 (0.00 to 0.00)	0.00 (0.00 to 0.00)
Measles	14.92 (4.74 to 36.08)	11.76 (0.03 to 66.49)	7.25 (0.02 to 34.43)	15.05 (0.04 to 88.00)	0.18 (0.06 to 0.44)	0.17 (0.00 to 0.90)	0.11 (0.00 to 0.48)	0.21 (0.00 to 1.18)

American Samoa								
Cause	YLLs (actual counts)				Deaths (actual counts)			
	2016	2040 Forecast	2040 Better Scenario	2040 Worse Scenario	2016	2040 Forecast	2040 Better Scenario	2040 Worse Scenario
Varicella and herpes zoster	2.38 (1.44 to 3.76)	1.87 (0.90 to 3.39)	1.00 (0.50 to 1.78)	2.09 (0.97 to 3.81)	0.04 (0.03 to 0.06)	0.06 (0.03 to 0.09)	0.04 (0.02 to 0.06)	0.06 (0.03 to 0.09)
**Neglected tropical diseases and malaria**	**15.38 (5.10 to 24.18)**	**94.21 (34.93 to 146.49)**	**93.78 (36.45 to 141.59)**	**92.59 (33.71 to 143.68)**	**0.42 (0.14 to 0.66)**	**3.85 (1.50 to 6.07)**	**4.00 (1.70 to 6.17)**	**3.66 (1.39 to 5.81)**
Malaria	5.32 (0.34 to 10.54)	9.44 (0.27 to 22.78)	9.05 (0.26 to 21.56)	9.39 (0.27 to 22.67)	0.13 (0.01 to 0.25)	0.26 (0.01 to 0.59)	0.25 (0.01 to 0.58)	0.25 (0.01 to 0.58)
Chagas disease	0.00 (0.00 to 0.00)	0.00 (0.00 to 0.00)	0.00 (0.00 to 0.00)	0.00 (0.00 to 0.00)	0.00 (0.00 to 0.00)	0.00 (0.00 to 0.00)	0.00 (0.00 to 0.00)	0.00 (0.00 to 0.00)
Leishmaniasis	0.00 (0.00 to 0.00)	0.00 (0.00 to 0.00)	0.00 (0.00 to 0.00)	0.00 (0.00 to 0.00)	0.00 (0.00 to 0.00)	0.00 (0.00 to 0.00)	0.00 (0.00 to 0.00)	0.00 (0.00 to 0.00)
African trypanosomiasis	0.00 (0.00 to 0.00)	0.00 (0.00 to 0.00)	0.00 (0.00 to 0.00)	0.00 (0.00 to 0.00)	0.00 (0.00 to 0.00)	0.00 (0.00 to 0.00)	0.00 (0.00 to 0.00)	0.00 (0.00 to 0.00)
Schistosomiasis	0.00 (0.00 to 0.00)	0.00 (0.00 to 0.00)	0.00 (0.00 to 0.00)	0.00 (0.00 to 0.00)	0.00 (0.00 to 0.00)	0.00 (0.00 to 0.00)	0.00 (0.00 to 0.00)	0.00 (0.00 to 0.00)
Cysticercosis	0.12 (0.09 to 0.15)	0.10 (0.06 to 0.14)	0.09 (0.05 to 0.13)	0.10 (0.06 to 0.14)	0.00 (0.00 to 0.00)	0.00 (0.00 to 0.00)	0.00 (0.00 to 0.00)	0.00 (0.00 to 0.00)
Cystic echinococcosis	0.07 (0.06 to 0.10)	0.12 (0.07 to 0.17)	0.06 (0.04 to 0.09)	0.13 (0.07 to 0.19)	0.00 (0.00 to 0.00)	0.00 (0.00 to 0.01)	0.00 (0.00 to 0.00)	0.00 (0.00 to 0.01)
Dengue	8.29 (2.85 to 14.36)	80.24 (30.57 to 129.04)	79.91 (32.39 to 123.99)	78.65 (29.35 to 127.39)	0.24 (0.08 to 0.43)	3.36 (1.29 to 5.53)	3.48 (1.43 to 5.55)	3.19 (1.20 to 5.32)
Yellow fever	0.00 (0.00 to 0.00)	0.00 (0.00 to 0.00)	0.00 (0.00 to 0.00)	0.00 (0.00 to 0.00)	0.00 (0.00 to 0.00)	0.00 (0.00 to 0.00)	0.00 (0.00 to 0.00)	0.00 (0.00 to 0.00)
Rabies	0.02 (0.01 to 0.03)	0.03 (0.01 to 0.06)	0.02 (0.01 to 0.04)	0.04 (0.01 to 0.06)	0.00 (0.00 to 0.00)	0.00 (0.00 to 0.00)	0.00 (0.00 to 0.00)	0.00 (0.00 to 0.00)
Intestinal nematode infections	0.00 (0.00 to 0.00)	0.00 (0.00 to 0.00)	0.00 (0.00 to 0.00)	0.00 (0.00 to 0.00)	0.00 (0.00 to 0.00)	0.00 (0.00 to 0.00)	0.00 (0.00 to 0.00)	0.00 (0.00 to 0.00)
Other neglected tropical diseases	1.56 (1.02 to 2.14)	4.27 (1.94 to 6.08)	4.65 (2.14 to 6.66)	4.29 (1.94 to 6.02)	0.05 (0.03 to 0.07)	0.22 (0.09 to 0.33)	0.26 (0.11 to 0.39)	0.21 (0.09 to 0.32)
**Maternal disorders**	**71.62 (50.15 to 95.54)**	**69.88 (40.86 to 114.26)**	**39.12 (23.07 to 62.84)**	**78.36 (46.40 to 124.80)**	**1.38 (0.96 to 1.85)**	**1.30 (0.76 to 2.13)**	**0.73 (0.42 to 1.17)**	**1.46 (0.87 to 2.32)**
Maternal hemorrhage	23.01 (14.08 to 34.29)	22.44 (12.05 to 39.86)	12.76 (6.63 to 21.85)	24.98 (13.35 to 43.58)	0.44 (0.28 to 0.66)	0.42 (0.22 to 0.74)	0.24 (0.12 to 0.41)	0.46 (0.25 to 0.81)
Maternal sepsis and other maternal infections	7.53 (3.59 to 13.40)	6.22 (2.45 to 13.21)	3.46 (1.35 to 7.26)	6.95 (2.83 to 14.73)	0.15 (0.07 to 0.26)	0.12 (0.05 to 0.25)	0.07 (0.03 to 0.14)	0.13 (0.05 to 0.28)
Maternal hypertensive disorders	10.01 (5.63 to 16.08)	10.03 (4.85 to 18.34)	5.49 (2.61 to 10.21)	11.19 (5.38 to 20.57)	0.19 (0.11 to 0.31)	0.19 (0.09 to 0.35)	0.10 (0.05 to 0.19)	0.21 (0.10 to 0.38)
Maternal obstructed labor and uterine rupture	4.95 (2.21 to 9.50)	5.11 (1.79 to 11.09)	2.61 (0.91 to 5.58)	5.73 (2.03 to 12.26)	0.10 (0.04 to 0.18)	0.10 (0.03 to 0.21)	0.05 (0.02 to 0.11)	0.11 (0.04 to 0.23)
Maternal abortion, miscarriage, and ectopic pregnancy	5.11 (2.43 to 9.01)	5.17 (2.07 to 10.30)	2.85 (1.15 to 5.79)	5.82 (2.35 to 11.45)	0.10 (0.05 to 0.17)	0.10 (0.04 to 0.20)	0.05 (0.02 to 0.11)	0.11 (0.04 to 0.22)

Indirect maternal deaths	7.04 (3.74 to 11.30)	7.68 (3.32 to 14.54)	4.15 (1.82 to 8.00)	8.74 (3.90 to 16.64)	0.13 (0.07 to 0.22)	0.14 (0.06 to 0.27)	0.08 (0.03 to 0.15)	0.16 (0.07 to 0.31)
Late maternal deaths	2.65 (1.11 to 5.12)	2.11 (0.78 to 4.64)	1.14 (0.41 to 2.51)	2.39 (0.87 to 5.22)	0.05 (0.02 to 0.10)	0.04 (0.01 to 0.09)	0.02 (0.01 to 0.05)	0.04 (0.02 to 0.10)
Maternal deaths aggravated by HIV/AIDS	0.01 (0.00 to 0.08)	0.18 (0.00 to 1.27)	0.09 (0.00 to 0.79)	0.22 (0.00 to 1.51)	0.00 (0.00 to 0.00)	0.00 (0.00 to 0.02)	0.00 (0.00 to 0.01)	0.00 (0.00 to 0.03)
Other maternal disorders	11.31 (6.35 to 18.36)	10.94 (5.35 to 20.03)	6.58 (3.20 to 11.98)	12.34 (5.91 to 22.06)	0.22 (0.12 to 0.36)	0.20 (0.10 to 0.37)	0.12 (0.06 to 0.22)	0.23 (0.11 to 0.41)

American Samoa								
Cause	YLLs (actual counts)				Deaths (actual counts)			
	2016	2040 Forecast	2040 Better Scenario	2040 Worse Scenario	2016	2040 Forecast	2040 Better Scenario	2040 Worse Scenario
**Neonatal disorders**	**356.06 (260.61 to 471.24)**	**254.03 (140.25 to 422.31)**	**120.35 (66.27 to 196.44)**	**302.51 (170.08 to 502.39)**	**4.12 (3.01 to 5.45)**	**2.94 (1.62 to 4.88)**	**1.39 (0.77 to 2.27)**	**3.50 (1.97 to 5.81)**
Neonatal preterm birth	157.09 (112.77 to 209.72)	103.13 (56.97 to 164.62)	37.84 (21.05 to 60.76)	123.06 (68.28 to 197.95)	1.82 (1.30 to 2.42)	1.19 (0.66 to 1.90)	0.44 (0.24 to 0.70)	1.42 (0.79 to 2.29)
Neonatal encephalopathy due to birth asphyxia and trauma	94.41 (64.65 to 132.98)	64.22 (31.16 to 118.98)	27.87 (13.72 to 50.55)	75.74 (36.83 to 139.10)	1.09 (0.75 to 1.54)	0.74 (0.36 to 1.38)	0.32 (0.16 to 0.58)	0.88 (0.43 to 1.61)
Neonatal sepsis and other neonatal infections	26.34 (16.03 to 42.13)	25.48 (12.01 to 47.94)	12.74 (6.17 to 23.85)	30.17 (14.21 to 57.03)	0.30 (0.19 to 0.49)	0.29 (0.14 to 0.55)	0.15 (0.07 to 0.28)	0.35 (0.16 to 0.66)
Hemolytic disease and other neonatal jaundice	1.30 (0.74 to 2.16)	0.73 (0.30 to 1.51)	0.35 (0.14 to 0.74)	0.87 (0.35 to 1.85)	0.02 (0.01 to 0.03)	0.01 (0.00 to 0.02)	0.00 (0.00 to 0.01)	0.01 (0.00 to 0.02)
Other neonatal disorders	76.92 (50.07 to 108.00)	60.46 (27.60 to 110.27)	41.54 (19.91 to 74.69)	72.68 (33.38 to 133.57)	0.89 (0.58 to 1.25)	0.70 (0.32 to 1.28)	0.48 (0.23 to 0.86)	0.84 (0.39 to 1.54)
**Nutritional deficiencies**	**32.06 (24.03 to 43.19)**	**33.29 (26.12 to 41.77)**	**35.43 (28.74 to 42.72)**	**33.95 (26.22 to 43.27)**	**0.98 (0.82 to 1.16)**	**1.95 (1.58 to 2.34)**	**2.41 (1.97 to 2.91)**	**1.84 (1.46 to 2.22)**
Protein-energy malnutrition	28.28 (20.50 to 39.41)	27.07 (20.85 to 35.18)	30.58 (24.45 to 37.95)	27.64 (20.83 to 37.11)	0.86 (0.72 to 1.04)	1.64 (1.29 to 2.01)	2.15 (1.70 to 2.65)	1.54 (1.19 to 1.92)
Iodine deficiency	0.31 (0.02 to 1.04)	1.28 (0.04 to 4.27)	1.08 (0.02 to 3.89)	1.33 (0.04 to 4.49)	0.01 (0.00 to 0.02)	0.07 (0.00 to 0.26)	0.06 (0.00 to 0.29)	0.06 (0.00 to 0.24)
Dietary iron deficiency	0.50 (0.36 to 0.80)	0.54 (0.37 to 0.83)	0.53 (0.37 to 0.81)	0.57 (0.39 to 0.89)	0.01 (0.01 to 0.01)	0.01 (0.01 to 0.02)	0.02 (0.01 to 0.02)	0.01 (0.01 to 0.02)
Other nutritional deficiencies	2.97 (2.22 to 3.82)	4.41 (3.28 to 5.65)	3.24 (2.39 to 4.22)	4.42 (3.31 to 5.67)	0.10 (0.07 to 0.12)	0.23 (0.16 to 0.30)	0.18 (0.13 to 0.25)	0.21 (0.15 to 0.29)
**Other communicable, maternal, neonatal, and nutritional diseases**	**234.21 (86.14 to 444.55)**	**111.63 (62.79 to 185.32)**	**79.43 (47.41 to 118.50)**	**120.02 (66.41 to 203.14)**	**3.45 (1.66 to 5.91)**	**2.67 (1.62 to 3.76)**	**2.18 (1.36 to 2.97)**	**2.74 (1.65 to 3.89)**
Sexually transmitted infections excluding HIV	170.09 (20.29 to 385.96)	38.46 (4.83 to 109.74)	18.85 (2.81 to 51.77)	44.17 (5.24 to 126.28)	2.03 (0.27 to 4.55)	0.48 (0.08 to 1.32)	0.25 (0.06 to 0.64)	0.55 (0.09 to 1.51)
Syphilis	167.41 (17.61 to 383.73)	36.55 (2.68 to 107.91)	17.28 (1.31 to 50.26)	42.21 (3.02 to 124.43)	1.97 (0.21 to 4.50)	0.44 (0.04 to 1.27)	0.21 (0.02 to 0.60)	0.50 (0.04 to 1.47)
Chlamydial infection	0.51 (0.34 to 0.72)	0.36 (0.23 to 0.55)	0.30 (0.18 to 0.46)	0.37 (0.23 to 0.56)	0.01 (0.01 to 0.02)	0.01 (0.01 to 0.01)	0.01 (0.00 to 0.01)	0.01 (0.01 to 0.01)
Gonococcal infection	1.39 (0.93 to 1.96)	0.99 (0.65 to 1.49)	0.82 (0.53 to 1.23)	1.02 (0.66 to 1.55)	0.03 (0.02 to 0.04)	0.02 (0.02 to 0.03)	0.02 (0.01 to 0.03)	0.02 (0.02 to 0.03)
Other sexually transmitted infections	0.78 (0.52 to 1.11)	0.56 (0.36 to 0.86)	0.46 (0.29 to 0.70)	0.58 (0.37 to 0.89)	0.02 (0.01 to 0.02)	0.01 (0.01 to 0.02)	0.01 (0.01 to 0.02)	0.01 (0.01 to 0.02)
Acute hepatitis	5.21 (3.92 to 6.83)	5.17 (3.43 to 7.44)	2.75 (1.79 to 4.07)	5.33 (3.50 to 7.65)	0.13 (0.10 to 0.17)	0.17 (0.11 to 0.24)	0.09 (0.06 to 0.14)	0.17 (0.11 to 0.24)
Acute hepatitis A	0.19 (0.13 to 0.27)	0.10 (0.06 to 0.16)	0.06 (0.03 to 0.10)	0.10 (0.06 to 0.17)	0.00 (0.00 to 0.00)	0.00 (0.00 to 0.00)	0.00 (0.00 to 0.00)	0.00 (0.00 to 0.00)
Acute hepatitis B	4.30 (3.16 to 5.74)	4.47 (2.89 to 6.54)	2.35 (1.47 to 3.53)	4.61 (3.00 to 6.70)	0.11 (0.08 to 0.15)	0.15 (0.09 to 0.21)	0.08 (0.05 to 0.12)	0.15 (0.10 to 0.21)

Acute hepatitis C	0.09 (0.06 to 0.13)	0.12 (0.07 to 0.19)	0.07 (0.04 to 0.11)	0.12 (0.07 to 0.19)	0.00 (0.00 to 0.00)	0.00 (0.00 to 0.01)	0.00 (0.00 to 0.00)	0.00 (0.00 to 0.01)
Acute hepatitis E	0.63 (0.44 to 0.87)	0.48 (0.28 to 0.79)	0.26 (0.15 to 0.44)	0.50 (0.29 to 0.82)	0.01 (0.01 to 0.02)	0.02 (0.01 to 0.03)	0.01 (0.01 to 0.02)	0.02 (0.01 to 0.02)
Other unspecified infectious diseases	58.91 (33.95 to 76.11)	68.00 (36.62 to 95.34)	57.83 (32.34 to 79.54)	70.51 (38.43 to 98.05)	1.29 (0.71 to 1.67)	2.02 (1.12 to 2.70)	1.84 (1.06 to 2.46)	2.02 (1.13 to 2.70)
**Non-communicable diseases**	**8,132.49 (6,812.04 to 9,569.66)**	**15,864.36 (12,435.40 to 19,709.72)**	**10,299.57 (8,067.72 to 12,804.70)**	**19,029.11 (14,595.71 to 23,996.58)**	**283.71 (240.75 to 330.61)**	**736.67 (617.43 to 857.42)**	**508.96 (425.70 to 602.90)**	**853.75 (708.06 to 1,001.32)**

Cause	YLLs (actual counts)				Deaths (actual counts)			
	**American Samoa**							
	2016	2040 Forecast	2040 Better Scenario	2040 Worse Scenario	2016	2040 Forecast	2040 Better Scenario	2040 Worse Scenario
**Neoplasms**	**1,738.97 (1,435.48 to 2,063.42)**	**3,584.23 (2,715.82 to 4,683.64)**	**2,780.16 (2,110.28 to 3,606.71)**	**3,987.30 (2,970.00 to 5,283.60)**	**57.81 (48.51 to 67.80)**	**159.45 (126.18 to 202.22)**	**128.36 (102.68 to 161.60)**	**172.58 (133.66 to 223.53)**
Lip and oral cavity cancer	20.53 (15.98 to 25.77)	47.98 (27.54 to 84.21)	27.92 (17.44 to 44.15)	61.71 (33.76 to 113.00)	0.73 (0.58 to 0.90)	2.07 (1.25 to 3.40)	1.25 (0.83 to 1.88)	2.58 (1.46 to 4.49)
Nasopharynx cancer	24.69 (19.94 to 30.21)	47.20 (27.50 to 81.92)	21.39 (13.39 to 34.68)	63.14 (35.17 to 114.28)	0.75 (0.61 to 0.91)	1.74 (1.05 to 2.92)	0.82 (0.54 to 1.26)	2.27 (1.31 to 4.01)
Other pharynx cancer	30.99 (23.71 to 39.97)	51.15 (33.36 to 78.45)	44.39 (30.19 to 64.92)	55.30 (34.95 to 87.70)	1.02 (0.79 to 1.29)	2.12 (1.43 to 3.17)	1.91 (1.35 to 2.71)	2.25 (1.47 to 3.46)
Esophageal cancer	13.06 (10.44 to 15.89)	27.60 (16.27 to 45.02)	18.39 (12.06 to 27.81)	34.48 (19.57 to 59.49)	0.49 (0.40 to 0.58)	1.26 (0.78 to 2.01)	0.87 (0.59 to 1.28)	1.53 (0.90 to 2.49)
Stomach cancer	133.44 (108.76 to 161.23)	202.16 (157.16 to 261.86)	128.63 (98.52 to 164.34)	210.44 (160.69 to 277.39)	4.64 (3.84 to 5.52)	9.14 (7.37 to 11.41)	6.07 (4.87 to 7.52)	9.27 (7.31 to 11.74)
Colon and rectum cancer	133.93 (109.21 to 162.06)	328.66 (210.51 to 512.59)	243.93 (160.26 to 374.83)	344.24 (217.48 to 539.97)	4.84 (4.00 to 5.80)	15.44 (10.07 to 23.88)	12.02 (7.99 to 18.48)	15.72 (10.17 to 24.86)
Liver cancer	149.19 (117.88 to 182.81)	300.84 (183.84 to 479.83)	285.85 (180.94 to 437.82)	377.69 (214.38 to 625.87)	4.84 (3.90 to 5.84)	12.44 (7.92 to 19.39)	12.25 (8.11 to 18.19)	15.09 (8.94 to 24.26)
Liver cancer due to hepatitis B	86.64 (67.40 to 107.95)	161.58 (96.94 to 254.66)	153.85 (95.35 to 235.52)	201.66 (112.48 to 330.41)	2.66 (2.10 to 3.27)	6.31 (3.89 to 9.79)	6.20 (3.96 to 9.26)	7.63 (4.36 to 12.16)
Liver cancer due to hepatitis C	20.00 (15.40 to 25.28)	50.04 (30.52 to 77.79)	48.57 (30.69 to 73.51)	60.44 (34.92 to 97.60)	0.79 (0.61 to 0.98)	2.44 (1.56 to 3.68)	2.47 (1.62 to 3.57)	2.84 (1.73 to 4.42)
Liver cancer due to alcohol use	12.23 (9.03 to 16.01)	27.15 (15.86 to 44.56)	24.43 (14.85 to 38.86)	35.16 (19.37 to 59.50)	0.43 (0.32 to 0.54)	1.16 (0.70 to 1.84)	1.08 (0.68 to 1.70)	1.45 (0.83 to 2.36)
Liver cancer due to other causes	30.32 (23.00 to 38.95)	62.08 (32.59 to 112.54)	59.00 (32.63 to 103.87)	80.43 (39.51 to 154.69)	0.96 (0.74 to 1.21)	2.53 (1.38 to 4.53)	2.50 (1.40 to 4.40)	3.17 (1.60 to 6.02)
Gallbladder and biliary tract cancer	13.81 (10.42 to 17.80)	18.07 (14.24 to 22.46)	17.34 (13.56 to 21.91)	17.33 (13.78 to 21.39)	0.49 (0.38 to 0.63)	0.83 (0.68 to 1.01)	0.83 (0.67 to 1.02)	0.78 (0.65 to 0.95)
Pancreatic cancer	47.69 (39.08 to 57.24)	128.81 (87.03 to 190.12)	121.42 (83.60 to 177.58)	136.00 (90.33 to 204.94)	1.80 (1.49 to 2.13)	5.96 (4.06 to 8.77)	5.79 (4.02 to 8.43)	6.17 (4.13 to 9.31)
Larynx cancer	12.51 (10.00 to 15.53)	25.20 (13.00 to 45.19)	11.96 (6.56 to 21.75)	33.09 (16.44 to 60.35)	0.44 (0.36 to 0.54)	1.17 (0.62 to 2.14)	0.57 (0.32 to 1.01)	1.49 (0.78 to 2.69)
Tracheal, bronchus, and lung cancer	247.20 (199.87 to 300.87)	604.72 (371.18 to 950.75)	352.75 (223.16 to 550.07)	837.30 (503.03 to 1,343.26)	9.32 (7.67 to 11.22)	28.45 (17.82 to 44.39)	16.95 (10.93 to 26.03)	38.38 (23.76 to 60.42)
Malignant skin melanoma	12.28 (8.79 to 16.08)	23.14 (16.81 to 29.10)	32.54 (23.29 to 41.54)	21.67 (15.79 to 27.15)	0.43 (0.31 to 0.55)	1.05 (0.76 to 1.28)	1.56 (1.11 to 1.94)	0.96 (0.69 to 1.17)
Non-melanoma skin cancer	14.22 (11.38 to 17.58)	31.32 (25.93 to 37.12)	22.25 (18.10 to 27.02)	30.27 (25.20 to 35.81)	0.55 (0.45 to 0.65)	1.64 (1.39 to 1.85)	1.25 (1.06 to 1.45)	1.52 (1.28 to 1.73)
Breast cancer	212.75 (152.81 to 277.38)	373.00 (227.97 to 597.04)	271.17 (168.68 to 431.40)	391.97 (237.53 to 636.05)	6.46 (4.71 to 8.28)	14.88 (9.33 to 23.50)	11.20 (7.17 to 17.32)	15.32 (9.44 to 24.60)
Cervical cancer	42.83 (27.03 to 58.89)	63.70 (35.13 to 99.29)	40.54 (22.66 to 62.26)	66.79 (36.64 to 105.81)	1.28 (0.78 to 1.75)	2.36 (1.30 to 3.57)	1.52 (0.85 to 2.29)	2.45 (1.34 to 3.75)

Uterine cancer	60.16 (43.65 to 80.15)	129.46 (92.20 to 171.34)	99.63 (70.42 to 133.89)	129.24 (93.11 to 170.16)	2.05 (1.49 to 2.71)	5.50 (4.04 to 7.18)	4.32 (3.15 to 5.74)	5.43 (4.06 to 7.02)
Ovarian cancer	54.04 (39.25 to 70.35)	151.41 (90.03 to 242.69)	129.75 (78.31 to 204.68)	152.67 (90.63 to 246.95)	2.04 (1.51 to 2.63)	7.11 (4.24 to 11.39)	6.20 (3.72 to 9.82)	7.09 (4.20 to 11.53)
Prostate cancer	77.85 (58.70 to 103.47)	307.82 (243.49 to 388.34)	270.63 (213.68 to 339.76)	287.14 (219.75 to 368.20)	4.08 (3.15 to 5.28)	20.19 (15.84 to 25.16)	18.68 (14.85 to 22.93)	18.31 (13.87 to 23.22)
Testicular cancer	1.35 (0.99 to 1.76)	1.65 (1.19 to 2.21)	1.19 (0.85 to 1.60)	1.65 (1.20 to 2.21)	0.03 (0.02 to 0.04)	0.04 (0.03 to 0.06)	0.03 (0.02 to 0.04)	0.04 (0.03 to 0.05)

American Samoa								
Cause	YLLs (actual counts)				Deaths (actual counts)			
	2016	2040 Forecast	2040 Better Scenario	2040 Worse Scenario	2016	2040 Forecast	2040 Better Scenario	2040 Worse Scenario
Kidney cancer	12.23 (9.83 to 15.07)	26.34 (19.06 to 35.83)	26.31 (19.66 to 34.95)	26.92 (19.26 to 37.60)	0.38 (0.31 to 0.46)	1.11 (0.85 to 1.47)	1.16 (0.91 to 1.48)	1.10 (0.82 to 1.51)
Bladder cancer	18.20 (14.87 to 22.01)	37.36 (23.45 to 57.97)	28.56 (18.48 to 43.55)	41.27 (25.10 to 65.70)	0.72 (0.60 to 0.86)	1.86 (1.20 to 2.90)	1.48 (0.99 to 2.25)	2.00 (1.24 to 3.21)
Brain and nervous system cancer	46.07 (36.97 to 57.95)	75.14 (59.82 to 98.29)	65.62 (51.77 to 86.74)	74.11 (59.29 to 96.08)	1.07 (0.85 to 1.40)	2.32 (1.87 to 3.07)	2.13 (1.69 to 2.85)	2.23 (1.82 to 2.96)
Thyroid cancer	8.44 (6.66 to 10.47)	19.70 (13.94 to 27.48)	14.75 (10.37 to 20.57)	21.76 (15.16 to 30.29)	0.33 (0.27 to 0.41)	0.93 (0.67 to 1.25)	0.72 (0.52 to 0.97)	1.00 (0.71 to 1.34)
Mesothelioma	5.25 (4.11 to 6.57)	9.45 (7.31 to 11.66)	9.57 (7.36 to 11.98)	9.23 (7.21 to 11.33)	0.17 (0.14 to 0.21)	0.38 (0.30 to 0.46)	0.39 (0.31 to 0.49)	0.36 (0.29 to 0.44)
Hodgkin lymphoma	4.95 (3.69 to 6.58)	7.04 (4.76 to 10.29)	4.19 (2.81 to 6.07)	7.21 (4.85 to 10.52)	0.13 (0.09 to 0.17)	0.23 (0.16 to 0.32)	0.14 (0.10 to 0.20)	0.23 (0.16 to 0.32)
Non-Hodgkin lymphoma	49.90 (38.52 to 60.74)	87.50 (66.28 to 115.50)	72.14 (54.17 to 94.68)	90.32 (67.63 to 120.58)	1.35 (1.06 to 1.64)	3.26 (2.53 to 4.22)	2.84 (2.16 to 3.65)	3.26 (2.51 to 4.26)
Multiple myeloma	22.50 (15.31 to 30.29)	52.64 (34.28 to 74.71)	48.87 (31.61 to 69.66)	54.52 (34.93 to 77.88)	0.78 (0.54 to 1.03)	2.24 (1.54 to 3.07)	2.15 (1.46 to 2.97)	2.28 (1.54 to 3.16)
Leukemia	65.22 (53.10 to 78.63)	87.32 (65.50 to 117.43)	65.16 (49.02 to 87.13)	96.82 (71.11 to 132.75)	1.52 (1.24 to 1.85)	2.87 (2.15 to 3.84)	2.25 (1.71 to 2.98)	3.10 (2.27 to 4.24)
Acute lymphoid leukemia	17.07 (13.30 to 22.43)	18.92 (13.76 to 26.14)	13.19 (9.58 to 18.36)	20.93 (15.04 to 29.60)	0.32 (0.25 to 0.42)	0.48 (0.35 to 0.67)	0.36 (0.26 to 0.50)	0.52 (0.37 to 0.74)
Chronic lymphoid leukemia	2.01 (1.46 to 2.55)	4.83 (3.29 to 6.68)	4.23 (2.89 to 5.76)	5.26 (3.53 to 7.46)	0.07 (0.05 to 0.09)	0.23 (0.15 to 0.31)	0.21 (0.15 to 0.28)	0.24 (0.16 to 0.34)
Acute myeloid leukemia	16.55 (12.95 to 20.72)	26.35 (19.48 to 35.02)	24.22 (18.09 to 32.09)	28.92 (20.95 to 39.29)	0.36 (0.28 to 0.45)	0.78 (0.58 to 1.04)	0.76 (0.58 to 0.99)	0.84 (0.61 to 1.14)
Chronic myeloid leukemia	13.30 (10.34 to 17.31)	17.27 (11.95 to 25.03)	9.61 (6.48 to 14.14)	19.27 (13.10 to 28.62)	0.39 (0.31 to 0.50)	0.64 (0.45 to 0.91)	0.36 (0.26 to 0.52)	0.70 (0.48 to 1.01)
Other leukemia	16.29 (12.37 to 20.38)	19.96 (14.01 to 27.67)	13.91 (9.77 to 19.29)	22.43 (15.53 to 31.91)	0.38 (0.29 to 0.48)	0.75 (0.53 to 1.04)	0.56 (0.40 to 0.77)	0.81 (0.57 to 1.14)
Other neoplasms	203.67 (165.33 to 242.63)	317.85 (257.87 to 385.00)	303.32 (244.10 to 372.10)	313.02 (256.35 to 377.66)	5.10 (4.22 to 6.12)	10.87 (9.12 to 12.82)	11.00 (9.14 to 13.08)	10.39 (8.82 to 12.19)
**Cardiovascular diseases**	**2,715.85 (2,249.12 to 3,242.12)**	**4,449.25 (3,067.28 to 6,140.78)**	**2,028.43 (1,462.01 to 2,779.30)**	**6,084.32 (4,117.94 to 8,561.64)**	**99.59 (84.02 to 116.74)**	**209.76 (157.28 to 281.10)**	**106.27 (81.81 to 140.88)**	**272.89 (197.61 to 369.15)**
Rheumatic heart disease	113.67 (93.17 to 136.99)	93.59 (72.59 to 118.66)	56.75 (43.32 to 73.97)	96.36 (74.48 to 124.28)	3.08 (2.53 to 3.67)	3.68 (3.04 to 4.40)	2.40 (1.97 to 2.91)	3.64 (3.01 to 4.42)
Ischemic heart disease	1,333.43 (1,092.32 to 1,608.92)	1,999.41 (1,104.20 to 3,423.18)	782.98 (442.33 to 1,382.55)	3,019.32 (1,670.02 to 5,109.24)	50.49 (42.60 to 59.66)	94.43 (56.79 to 153.44)	41.39 (24.80 to 71.20)	135.17 (79.31 to 217.88)
Stroke	727.59 (603.39 to 866.89)	1,168.67 (718.82 to 1,836.93)	410.89 (262.54 to 652.67)	1,717.07 (1,041.37 to 2,700.65)	27.22 (22.72 to 32.18)	55.30 (37.20 to 83.01)	22.19 (15.28 to 33.44)	76.93 (50.27 to 116.22)
Ischemic stroke	176.47 (144.72 to 211.10)	299.01 (171.37 to 554.03)	125.42 (75.71 to 233.21)	412.60 (228.51 to 761.56)	8.18 (6.84 to 9.67)	17.08 (10.17 to 33.08)	8.05 (4.95 to 15.76)	22.36 (12.52 to 46.00)

Intracerebral hemorrhage	551.12 (453.90 to 658.39)	869.67 (499.35 to 1,463.08)	285.47 (171.61 to 469.05)	1,304.47 (722.67 to 2,191.55)	19.03 (15.86 to 22.69)	38.21 (23.92 to 59.68)	14.14 (9.23 to 21.75)	54.56 (32.50 to 88.36)
Hypertensive heart disease	128.55 (99.43 to 170.94)	349.81 (251.79 to 501.91)	236.76 (179.37 to 318.99)	393.38 (273.18 to 577.24)	5.07 (4.03 to 6.58)	17.85 (13.39 to 24.83)	13.04 (10.30 to 17.02)	19.30 (14.00 to 28.14)
Cardiomyopathy and myocarditis	162.46 (124.69 to 199.09)	273.64 (192.99 to 373.66)	165.75 (114.89 to 224.66)	272.95 (190.73 to 376.79)	4.88 (3.80 to 5.95)	11.24 (8.06 to 15.05)	7.24 (5.12 to 9.65)	10.84 (7.58 to 14.42)
Myocarditis	10.70 (7.44 to 14.81)	18.69 (12.80 to 25.72)	17.57 (12.18 to 23.87)	18.34 (12.71 to 25.22)	0.34 (0.23 to 0.47)	0.80 (0.55 to 1.08)	0.79 (0.56 to 1.06)	0.76 (0.52 to 1.04)

American Samoa								
Cause	YLLs (actual counts)				Deaths (actual counts)			
	2016	2040 Forecast	2040 Better Scenario	2040 Worse Scenario	2016	2040 Forecast	2040 Better Scenario	2040 Worse Scenario
Alcoholic cardiomyopathy	7.14 (4.69 to 9.84)	11.07 (7.03 to 15.25)	9.76 (5.92 to 13.61)	11.27 (7.28 to 15.49)	0.23 (0.16 to 0.32)	0.45 (0.29 to 0.62)	0.42 (0.27 to 0.58)	0.45 (0.29 to 0.61)
Other cardiomyopathy	144.61 (110.44 to 177.75)	243.88 (166.39 to 341.05)	138.41 (91.04 to 195.87)	243.35 (164.73 to 343.78)	4.31 (3.34 to 5.28)	9.99 (6.94 to 13.68)	6.02 (4.09 to 8.30)	9.63 (6.65 to 13.10)
Atrial fibrillation and flutter	8.72 (6.78 to 11.09)	32.43 (24.61 to 42.30)	29.56 (22.58 to 37.93)	31.31 (23.51 to 41.55)	0.69 (0.54 to 0.91)	2.65 (1.95 to 3.49)	2.61 (1.97 to 3.37)	2.46 (1.78 to 3.30)
Aortic aneurysm	27.93 (22.70 to 33.89)	56.55 (45.93 to 68.50)	46.21 (37.34 to 55.79)	57.00 (45.54 to 70.08)	1.12 (0.93 to 1.34)	2.88 (2.44 to 3.41)	2.60 (2.18 to 3.02)	2.78 (2.33 to 3.36)
Peripheral artery disease	7.31 (3.31 to 10.47)	17.82 (7.92 to 27.57)	15.62 (7.17 to 23.50)	18.98 (8.31 to 29.85)	0.32 (0.16 to 0.43)	1.00 (0.49 to 1.48)	0.97 (0.50 to 1.39)	1.01 (0.49 to 1.53)
Endocarditis	63.05 (48.33 to 79.19)	111.87 (77.55 to 152.99)	100.10 (69.78 to 134.22)	112.67 (77.63 to 154.21)	1.94 (1.52 to 2.39)	4.72 (3.36 to 6.18)	4.53 (3.27 to 5.84)	4.57 (3.24 to 6.08)
Other cardiovascular and circulatory diseases	143.15 (106.75 to 184.66)	345.45 (241.49 to 467.35)	183.81 (130.79 to 246.16)	365.28 (247.94 to 499.56)	4.78 (3.60 to 6.10)	16.01 (11.38 to 21.00)	9.31 (6.95 to 12.10)	16.19 (11.22 to 21.49)
**Chronic respiratory diseases**	**479.68 (393.76 to 572.56)**	**841.39 (608.82 to 1,145.03)**	**504.43 (375.69 to 669.83)**	**1,111.45 (778.64 to 1,538.97)**	**17.10 (14.25 to 20.23)**	**39.26 (29.48 to 51.56)**	**23.74 (18.36 to 30.17)**	**50.53 (37.08 to 67.18)**
Chronic obstructive pulmonary disease	294.56 (242.85 to 356.24)	601.53 (409.79 to 858.68)	313.89 (220.15 to 435.11)	834.67 (552.35 to 1,229.26)	11.35 (9.50 to 13.52)	29.78 (21.42 to 40.44)	16.18 (12.08 to 21.55)	39.85 (27.38 to 55.08)
Pneumoconiosis	1.69 (1.24 to 2.30)	1.90 (1.40 to 2.52)	1.96 (1.39 to 2.67)	2.11 (1.54 to 2.83)	0.07 (0.05 to 0.08)	0.11 (0.08 to 0.14)	0.12 (0.09 to 0.15)	0.12 (0.09 to 0.15)
Silicosis	0.13 (0.07 to 0.21)	0.15 (0.08 to 0.24)	0.10 (0.05 to 0.17)	0.15 (0.08 to 0.24)	0.01 (0.00 to 0.01)	0.01 (0.01 to 0.02)	0.01 (0.00 to 0.01)	0.01 (0.01 to 0.01)
Asbestosis	0.46 (0.31 to 0.70)	0.51 (0.35 to 0.77)	0.63 (0.43 to 0.93)	0.51 (0.34 to 0.78)	0.01 (0.01 to 0.02)	0.02 (0.01 to 0.04)	0.03 (0.02 to 0.05)	0.02 (0.01 to 0.03)
Coal workers pneumoconiosis	0.01 (0.01 to 0.02)	0.02 (0.01 to 0.04)	0.02 (0.01 to 0.03)	0.02 (0.01 to 0.04)	0.00 (0.00 to 0.00)	0.00 (0.00 to 0.00)	0.00 (0.00 to 0.00)	0.00 (0.00 to 0.00)
Other pneumoconiosis	1.09 (0.79 to 1.50)	1.21 (0.85 to 1.68)	1.21 (0.80 to 1.74)	1.42 (1.00 to 1.97)	0.04 (0.03 to 0.06)	0.07 (0.05 to 0.10)	0.08 (0.05 to 0.11)	0.08 (0.06 to 0.11)
Asthma	103.53 (81.85 to 131.52)	107.78 (61.05 to 178.65)	48.84 (27.64 to 83.29)	139.05 (79.03 to 226.95)	3.47 (2.72 to 4.39)	4.81 (2.86 to 7.68)	2.35 (1.36 to 3.84)	5.90 (3.47 to 9.31)
Interstitial lung disease and pulmonary sarcoidosis	23.94 (17.37 to 31.32)	36.77 (26.99 to 47.31)	56.51 (41.13 to 73.13)	35.49 (25.41 to 45.51)	0.64 (0.46 to 0.83)	1.33 (0.98 to 1.73)	2.16 (1.58 to 2.77)	1.25 (0.91 to 1.61)
Other chronic respiratory diseases	55.96 (37.69 to 76.31)	93.40 (59.01 to 137.75)	83.23 (54.21 to 119.06)	100.14 (61.83 to 152.29)	1.58 (1.04 to 2.19)	3.22 (2.03 to 4.67)	2.94 (1.92 to 4.12)	3.41 (2.08 to 5.09)
**Cirrhosis and other chronic liver diseases**	**207.96 (164.22 to 258.36)**	**310.62 (249.51 to 383.57)**	**226.25 (178.94 to 283.60)**	**311.91 (252.35 to 382.82)**	**5.69 (4.52 to 6.98)**	**10.84 (9.05 to 12.91)**	**8.20 (6.72 to 9.90)**	**10.65 (8.90 to 12.66)**
Cirrhosis and other chronic liver diseases due to hepatitis B	83.10 (64.12 to 106.18)	129.97 (101.67 to 161.58)	96.76 (73.50 to 123.11)	128.79 (101.23 to 160.41)	2.36 (1.84 to 2.98)	4.64 (3.78 to 5.59)	3.58 (2.86 to 4.35)	4.51 (3.69 to 5.43)
Cirrhosis and other chronic liver diseases due to hepatitis C	33.10 (25.21 to 43.60)	51.07 (39.37 to 64.78)	37.21 (28.24 to 48.11)	50.75 (38.98 to 64.23)	0.92 (0.71 to 1.18)	1.75 (1.43 to 2.13)	1.31 (1.03 to 1.62)	1.71 (1.40 to 2.08)

Cirrhosis and other chronic liver diseases due to alcohol use	29.94 (22.50 to 38.52)	44.82 (34.91 to 57.56)	30.72 (23.37 to 39.56)	47.12 (37.07 to 60.31)	0.88 (0.68 to 1.12)	1.65 (1.33 to 2.02)	1.18 (0.95 to 1.46)	1.69 (1.37 to 2.08)
Cirrhosis and other chronic liver diseases due to other causes	61.82 (50.46 to 74.05)	84.76 (67.50 to 105.59)	61.55 (48.36 to 78.04)	85.26 (67.86 to 106.09)	1.52 (1.25 to 1.83)	2.79 (2.30 to 3.35)	2.13 (1.74 to 2.58)	2.74 (2.26 to 3.28)
**Digestive diseases**	**202.99 (165.71 to 242.52)**	**341.91 (270.11 to 429.44)**	**233.86 (183.57 to 291.55)**	**365.96 (286.90 to 463.22)**	**6.41 (5.26 to 7.57)**	**14.91 (12.16 to 18.05)**	**10.81 (8.79 to 13.10)**	**15.45 (12.45 to 18.93)**
Peptic ulcer disease	40.40 (32.56 to 49.48)	54.17 (42.28 to 69.05)	27.01 (20.36 to 35.13)	56.86 (43.79 to 73.48)	1.40 (1.14 to 1.70)	2.54 (2.06 to 3.18)	1.34 (1.06 to 1.69)	2.59 (2.08 to 3.25)

American Samoa								
Cause	YLLs (actual counts)				Deaths (actual counts)			
	2016	2040 Forecast	2040 Better Scenario	2040 Worse Scenario	2016	2040 Forecast	2040 Better Scenario	2040 Worse Scenario
Gastritis and duodenitis	0.97 (0.74 to 1.27)	2.19 (1.56 to 3.12)	1.50 (1.05 to 2.17)	2.16 (1.52 to 3.10)	0.04 (0.03 to 0.05)	0.11 (0.08 to 0.15)	0.08 (0.06 to 0.11)	0.10 (0.07 to 0.14)
Appendicitis	23.70 (18.12 to 29.31)	28.65 (21.42 to 35.70)	16.00 (11.59 to 20.33)	28.94 (21.99 to 36.10)	0.62 (0.48 to 0.76)	1.10 (0.84 to 1.32)	0.65 (0.48 to 0.80)	1.07 (0.83 to 1.28)
Paralytic ileus and intestinal obstruction	13.77 (10.90 to 17.83)	22.63 (17.53 to 28.98)	21.63 (16.69 to 28.02)	22.31 (17.40 to 28.70)	0.38 (0.30 to 0.48)	1.00 (0.76 to 1.27)	1.04 (0.79 to 1.30)	0.95 (0.73 to 1.19)
Inguinal, femoral, and abdominal hernia	5.63 (4.13 to 7.45)	9.83 (7.18 to 13.33)	7.51 (5.44 to 10.31)	9.52 (6.97 to 12.94)	0.23 (0.17 to 0.31)	0.54 (0.40 to 0.73)	0.43 (0.32 to 0.60)	0.50 (0.38 to 0.69)
Inflammatory bowel disease	16.69 (12.67 to 22.18)	32.57 (24.94 to 43.28)	30.36 (23.10 to 39.78)	31.74 (24.19 to 42.31)	0.47 (0.37 to 0.62)	1.25 (1.01 to 1.60)	1.24 (1.00 to 1.58)	1.18 (0.94 to 1.52)
Vascular intestinal disorders	6.15 (4.71 to 7.75)	14.68 (11.86 to 17.54)	12.44 (9.79 to 15.00)	14.15 (11.43 to 16.84)	0.24 (0.20 to 0.30)	0.78 (0.65 to 0.92)	0.70 (0.58 to 0.83)	0.73 (0.61 to 0.87)
Gallbladder and biliary diseases	38.43 (27.98 to 47.87)	74.05 (38.31 to 127.30)	43.84 (22.90 to 75.96)	94.52 (48.50 to 158.98)	1.32 (0.97 to 1.63)	3.48 (1.83 to 5.78)	2.18 (1.13 to 3.64)	4.24 (2.26 to 6.93)
Pancreatitis	25.98 (19.76 to 32.96)	45.95 (29.75 to 79.90)	27.67 (18.80 to 44.06)	48.69 (30.45 to 88.46)	0.71 (0.55 to 0.91)	1.66 (1.16 to 2.66)	1.06 (0.77 to 1.61)	1.71 (1.16 to 2.87)
Other digestive diseases	31.27 (25.38 to 38.78)	57.21 (45.60 to 72.64)	45.89 (36.16 to 58.33)	57.07 (45.61 to 71.98)	0.98 (0.81 to 1.20)	2.45 (2.03 to 3.02)	2.07 (1.70 to 2.53)	2.38 (1.96 to 2.92)
**Neurological disorders**	**214.36 (179.59 to 250.08)**	**556.36 (401.17 to 813.26)**	**548.33 (397.57 to 791.75)**	**585.27 (408.02 to 870.05)**	**10.46 (8.77 to 12.40)**	**36.78 (25.14 to 56.37)**	**39.88 (27.71 to 59.50)**	**37.26 (24.43 to 57.89)**
Alzheimer's disease and other dementias	89.86 (71.91 to 110.46)	372.46 (226.34 to 624.33)	379.21 (233.01 to 623.81)	398.10 (225.26 to 678.24)	7.53 (6.12 to 9.23)	30.38 (18.83 to 49.57)	33.10 (21.05 to 52.59)	31.10 (18.27 to 51.39)
Parkinson's disease	7.77 (5.86 to 9.92)	33.19 (25.00 to 42.60)	40.69 (30.97 to 52.39)	29.97 (22.67 to 38.44)	0.51 (0.39 to 0.67)	2.40 (1.78 to 3.13)	3.07 (2.34 to 4.00)	2.11 (1.56 to 2.75)
Epilepsy	80.44 (62.66 to 96.64)	88.29 (56.19 to 132.75)	63.86 (41.11 to 93.12)	96.34 (60.60 to 147.61)	1.52 (1.17 to 1.84)	1.90 (1.27 to 2.80)	1.42 (0.96 to 2.01)	2.04 (1.34 to 3.04)
Multiple sclerosis	2.30 (1.35 to 4.18)	3.40 (2.03 to 6.10)	3.52 (2.09 to 6.33)	3.39 (2.01 to 6.13)	0.06 (0.04 to 0.11)	0.11 (0.07 to 0.19)	0.12 (0.07 to 0.20)	0.11 (0.07 to 0.19)
Motor neuron disease	4.36 (3.46 to 5.35)	11.49 (9.05 to 14.35)	11.71 (9.08 to 14.70)	11.00 (8.70 to 13.53)	0.14 (0.11 to 0.17)	0.45 (0.36 to 0.55)	0.47 (0.37 to 0.57)	0.43 (0.35 to 0.52)
Other neurological disorders	29.64 (24.72 to 35.13)	47.53 (39.32 to 56.74)	49.34 (40.71 to 59.21)	46.47 (38.65 to 55.53)	0.71 (0.59 to 0.85)	1.55 (1.30 to 1.81)	1.69 (1.42 to 1.98)	1.47 (1.24 to 1.72)
**Mental disorders**	**39.56 (30.10 to 52.42)**	**77.17 (58.49 to 99.78)**	**104.01 (78.14 to 135.55)**	**76.64 (58.12 to 98.98)**	**0.89 (0.69 to 1.18)**	**2.10 (1.66 to 2.64)**	**3.02 (2.40 to 3.87)**	**2.03 (1.61 to 2.57)**
Alcohol use disorders	14.70 (10.17 to 21.03)	17.45 (11.41 to 25.49)	18.67 (12.10 to 27.43)	17.70 (11.52 to 26.00)	0.35 (0.24 to 0.50)	0.47 (0.32 to 0.68)	0.52 (0.36 to 0.77)	0.47 (0.32 to 0.68)
Drug use disorders	24.24 (17.76 to 32.73)	59.03 (43.96 to 77.21)	84.49 (63.10 to 111.05)	58.26 (43.24 to 76.20)	0.53 (0.40 to 0.71)	1.61 (1.26 to 2.05)	2.49 (1.93 to 3.21)	1.55 (1.20 to 1.98)
Opioid use disorders	12.25 (8.49 to 17.37)	23.20 (16.09 to 33.17)	25.43 (17.33 to 37.02)	23.10 (16.02 to 33.04)	0.25 (0.18 to 0.35)	0.54 (0.39 to 0.75)	0.61 (0.44 to 0.85)	0.53 (0.38 to 0.74)

Cocaine use disorders	1.45 (0.93 to 2.18)	4.39 (2.68 to 7.04)	6.10 (3.75 to 9.61)	4.42 (2.70 to 7.13)	0.03 (0.02 to 0.05)	0.11 (0.07 to 0.17)	0.16 (0.10 to 0.24)	0.11 (0.07 to 0.17)
Amphetamine use disorders	0.55 (0.34 to 0.82)	2.40 (1.41 to 3.62)	4.10 (2.38 to 6.24)	2.40 (1.43 to 3.66)	0.01 (0.01 to 0.02)	0.06 (0.04 to 0.09)	0.11 (0.06 to 0.16)	0.06 (0.04 to 0.09)
Other drug use disorders	9.98 (7.09 to 14.25)	29.04 (20.18 to 40.86)	48.85 (34.59 to 69.03)	28.34 (19.74 to 40.34)	0.24 (0.18 to 0.33)	0.90 (0.67 to 1.23)	1.62 (1.20 to 2.16)	0.85 (0.63 to 1.16)
Eating disorders	0.63 (0.42 to 0.95)	0.69 (0.42 to 1.10)	0.85 (0.50 to 1.39)	0.68 (0.42 to 1.09)	0.01 (0.01 to 0.02)	0.01 (0.01 to 0.02)	0.02 (0.01 to 0.03)	0.01 (0.01 to 0.02)

American Samoa								
Cause	YLLs (actual counts)				Deaths (actual counts)			
	2016	2040 Forecast	2040 Better Scenario	2040 Worse Scenario	2016	2040 Forecast	2040 Better Scenario	2040 Worse Scenario
**Diabetes, urogenital, blood, and endocrine diseases**	**2,016.53 (1,674.09 to 2,400.17)**	**4,696.54 (3,412.32 to 6,928.19)**	**3,119.06 (2,215.21 to 4,653.72)**	**5,497.49 (3,968.00 to 7,906.34)**	**74.03 (62.19 to 87.21)**	**225.80 (174.21 to 320.98)**	**156.92 (118.08 to 234.44)**	**256.40 (195.38 to 351.23)**
Diabetes mellitus	1,075.03 (881.37 to 1,290.65)	2,291.92 (1,544.31 to 3,259.54)	1,284.49 (860.28 to 1,892.24)	2,565.95 (1,703.96 to 3,763.34)	42.38 (35.07 to 50.04)	115.65 (80.83 to 158.95)	68.77 (48.75 to 94.72)	124.69 (86.33 to 174.81)
Acute glomerulonephritis	2.72 (1.80 to 3.95)	7.03 (3.98 to 12.65)	5.04 (2.87 to 8.86)	6.98 (3.97 to 12.54)	0.09 (0.06 to 0.13)	0.26 (0.15 to 0.48)	0.19 (0.11 to 0.34)	0.26 (0.14 to 0.47)
Chronic kidney disease	592.57 (488.23 to 711.35)	1,682.68 (694.48 to 3,975.22)	1,170.90 (476.04 to 2,798.55)	2,221.02 (903.77 to 4,763.09)	21.13 (17.66 to 25.00)	80.20 (33.53 to 180.85)	59.42 (24.24 to 134.52)	103.16 (42.89 to 218.37)
Chronic kidney disease due to diabetes mellitus	346.42 (277.30 to 422.91)	1,032.07 (412.30 to 2,481.47)	711.34 (285.10 to 1,739.76)	1,369.54 (544.77 to 3,089.62)	12.00 (9.85 to 14.48)	46.21 (18.42 to 105.73)	33.44 (13.32 to 78.78)	60.09 (24.24 to 128.13)
Chronic kidney disease due to hypertension	128.71 (101.67 to 161.44)	424.52 (174.78 to 1,002.90)	315.84 (128.44 to 759.89)	559.05 (223.40 to 1,232.47)	5.85 (4.73 to 7.20)	24.82 (10.45 to 59.28)	19.75 (8.09 to 48.00)	31.33 (13.06 to 69.32)
Chronic kidney disease due to glomerulonephritis	55.73 (42.58 to 70.62)	98.13 (45.73 to 226.43)	59.87 (27.11 to 136.40)	125.83 (57.03 to 272.62)	1.44 (1.09 to 1.83)	3.58 (1.53 to 8.36)	2.31 (0.97 to 5.50)	4.60 (1.95 to 10.09)
Chronic kidney disease due to other and unspecified causes	61.70 (48.52 to 77.09)	127.96 (55.15 to 273.72)	83.85 (34.72 to 183.97)	166.60 (70.11 to 354.94)	1.85 (1.44 to 2.34)	5.59 (2.34 to 12.50)	3.92 (1.56 to 9.15)	7.14 (2.93 to 14.93)
Urinary diseases and male infertility	83.62 (69.14 to 101.04)	239.13 (197.01 to 287.81)	179.00 (145.06 to 220.42)	231.27 (191.25 to 276.92)	2.77 (2.32 to 3.32)	11.37 (9.74 to 13.13)	9.13 (7.72 to 10.78)	10.60 (9.03 to 12.26)
Urinary tract infections	76.12 (62.12 to 91.64)	220.35 (181.29 to 266.14)	167.13 (135.66 to 206.60)	212.93 (175.74 to 254.47)	2.53 (2.09 to 3.03)	10.51 (8.99 to 12.20)	8.57 (7.23 to 10.14)	9.79 (8.35 to 11.37)
Urolithiasis	2.79 (1.98 to 3.96)	8.93 (6.18 to 12.76)	6.02 (4.04 to 8.87)	8.58 (6.03 to 12.35)	0.09 (0.07 to 0.13)	0.40 (0.28 to 0.57)	0.28 (0.19 to 0.40)	0.37 (0.27 to 0.53)
Other urinary diseases	4.71 (3.30 to 6.50)	9.85 (6.46 to 13.97)	5.84 (3.79 to 8.27)	9.76 (6.41 to 13.75)	0.15 (0.11 to 0.21)	0.46 (0.31 to 0.64)	0.29 (0.19 to 0.41)	0.44 (0.30 to 0.60)
Gynecological diseases	2.34 (1.46 to 3.42)	2.21 (1.33 to 3.35)	0.97 (0.58 to 1.52)	2.27 (1.37 to 3.41)	0.06 (0.04 to 0.09)	0.07 (0.05 to 0.09)	0.03 (0.02 to 0.05)	0.07 (0.05 to 0.09)
Uterine fibroids	0.14 (0.06 to 0.27)	0.13 (0.06 to 0.24)	0.07 (0.03 to 0.13)	0.13 (0.06 to 0.25)	0.00 (0.00 to 0.01)	0.01 (0.00 to 0.01)	0.00 (0.00 to 0.01)	0.01 (0.00 to 0.01)
Polycystic ovarian syndrome	0.01 (0.00 to 0.04)	0.01 (0.00 to 0.03)	0.00 (0.00 to 0.01)	0.01 (0.00 to 0.03)	0.00 (0.00 to 0.00)	0.00 (0.00 to 0.00)	0.00 (0.00 to 0.00)	0.00 (0.00 to 0.00)
Endometriosis	0.30 (0.09 to 0.61)	0.34 (0.09 to 0.69)	0.15 (0.04 to 0.31)	0.36 (0.09 to 0.72)	0.01 (0.00 to 0.01)	0.01 (0.00 to 0.02)	0.00 (0.00 to 0.01)	0.01 (0.00 to 0.02)
Genital prolapse	0.07 (0.04 to 0.12)	0.09 (0.05 to 0.20)	0.04 (0.02 to 0.09)	0.10 (0.05 to 0.20)	0.00 (0.00 to 0.01)	0.00 (0.00 to 0.01)	0.00 (0.00 to 0.00)	0.00 (0.00 to 0.01)
Other gynecological diseases	1.82 (1.11 to 2.74)	1.64 (0.95 to 2.59)	0.71 (0.41 to 1.15)	1.68 (0.97 to 2.64)	0.05 (0.03 to 0.07)	0.05 (0.03 to 0.07)	0.02 (0.01 to 0.03)	0.05 (0.03 to 0.07)
Hemoglobinopathies and hemolytic anemias	6.12 (4.33 to 7.71)	8.53 (5.71 to 11.28)	5.03 (3.30 to 6.75)	8.59 (5.79 to 11.29)	0.16 (0.11 to 0.20)	0.33 (0.22 to 0.42)	0.20 (0.14 to 0.26)	0.32 (0.22 to 0.40)
Thalassemias	0.87 (0.66 to 1.11)	0.40 (0.26 to 0.58)	0.36 (0.24 to 0.51)	0.41 (0.26 to 0.60)	0.01 (0.01 to 0.01)	0.01 (0.00 to 0.01)	0.01 (0.00 to 0.01)	0.01 (0.00 to 0.01)

Sickle cell disorders	0.01 (0.00 to 0.01)	0.01 (0.00 to 0.01)	0.00 (0.00 to 0.01)	0.01 (0.00 to 0.01)	0.00 (0.00 to 0.00)	0.00 (0.00 to 0.00)	0.00 (0.00 to 0.00)	0.00 (0.00 to 0.00)
G6PD deficiency	3.64 (2.47 to 4.78)	5.38 (3.47 to 7.53)	2.92 (1.79 to 4.18)	5.51 (3.53 to 7.62)	0.08 (0.06 to 0.11)	0.16 (0.10 to 0.22)	0.09 (0.06 to 0.13)	0.16 (0.11 to 0.22)
Other hemoglobinopathies and hemolytic anemias	1.60 (1.13 to 2.07)	2.74 (1.88 to 3.60)	1.76 (1.20 to 2.34)	2.67 (1.84 to 3.52)	0.06 (0.04 to 0.08)	0.16 (0.11 to 0.20)	0.11 (0.07 to 0.14)	0.15 (0.11 to 0.19)
Endocrine, metabolic, blood, and immune disorders	254.14 (189.77 to 313.41)	465.04 (349.15 to 576.43)	473.63 (355.77 to 594.32)	461.42 (347.37 to 572.03)	7.44 (5.52 to 9.15)	17.93 (13.66 to 21.68)	19.17 (14.79 to 23.24)	17.32 (13.32 to 20.83)

American Samoa								
Cause	YLLs (actual counts)				Deaths (actual counts)			
	2016	2040 Forecast	2040 Better Scenario	2040 Worse Scenario	2016	2040 Forecast	2040 Better Scenario	2040 Worse Scenario
**Musculoskeletal disorders**	**49.81 (37.67 to 62.57)**	**74.20 (54.55 to 97.12)**	**64.96 (48.14 to 84.90)**	**72.60 (53.42 to 93.77)**	**1.12 (0.85 to 1.40)**	**2.27 (1.71 to 2.82)**	**2.11 (1.59 to 2.62)**	**2.15 (1.61 to 2.67)**
Rheumatoid arthritis	1.49 (0.97 to 2.07)	3.98 (2.45 to 5.75)	2.01 (1.21 to 3.05)	4.02 (2.48 to 5.73)	0.05 (0.03 to 0.07)	0.19 (0.12 to 0.27)	0.10 (0.06 to 0.15)	0.19 (0.12 to 0.26)
Other musculoskeletal disorders	48.32 (36.43 to 60.75)	70.23 (51.37 to 91.74)	62.95 (46.32 to 82.28)	68.58 (50.31 to 89.25)	1.07 (0.81 to 1.33)	2.08 (1.56 to 2.60)	2.01 (1.52 to 2.50)	1.97 (1.48 to 2.46)
**Other non-communicable diseases**	**466.78 (354.49 to 577.20)**	**932.69 (641.79 to 1,225.71)**	**690.09 (452.77 to 937.09)**	**936.15 (643.31 to 1,233.55)**	**10.60 (7.55 to 13.66)**	**35.52 (22.97 to 49.73)**	**29.64 (18.45 to 41.62)**	**33.80 (21.93 to 47.37)**
Congenital birth defects	219.90 (167.73 to 277.62)	188.89 (122.72 to 269.12)	85.80 (54.84 to 121.71)	208.23 (134.33 to 297.69)	2.92 (2.26 to 3.62)	2.83 (1.94 to 3.85)	1.35 (0.91 to 1.86)	3.06 (2.10 to 4.18)
Neural tube defects	4.48 (2.59 to 7.52)	1.53 (0.72 to 2.99)	0.58 (0.27 to 1.14)	1.77 (0.83 to 3.49)	0.05 (0.03 to 0.09)	0.02 (0.01 to 0.04)	0.01 (0.00 to 0.02)	0.02 (0.01 to 0.04)
Congenital heart anomalies	109.46 (81.34 to 141.08)	92.83 (59.68 to 134.61)	34.91 (21.32 to 50.44)	101.97 (64.77 to 149.10)	1.45 (1.10 to 1.83)	1.35 (0.91 to 1.87)	0.52 (0.34 to 0.73)	1.47 (0.98 to 2.05)
Orofacial clefts	0.06 (0.03 to 0.10)	0.05 (0.02 to 0.10)	0.01 (0.01 to 0.03)	0.06 (0.02 to 0.12)	0.00 (0.00 to 0.00)	0.00 (0.00 to 0.00)	0.00 (0.00 to 0.00)	0.00 (0.00 to 0.00)
Down syndrome	10.35 (7.08 to 14.33)	10.82 (6.43 to 16.37)	10.50 (6.49 to 15.53)	11.67 (6.96 to 17.68)	0.16 (0.12 to 0.21)	0.20 (0.13 to 0.30)	0.21 (0.13 to 0.29)	0.21 (0.14 to 0.31)
Other chromosomal abnormalities	7.17 (4.08 to 11.31)	6.73 (3.14 to 12.07)	4.15 (1.93 to 7.36)	7.65 (3.50 to 13.53)	0.09 (0.05 to 0.14)	0.09 (0.04 to 0.15)	0.06 (0.03 to 0.10)	0.10 (0.05 to 0.17)
Congenital musculoskeletal and limb anomalies	6.43 (4.37 to 9.26)	6.29 (3.60 to 10.23)	2.89 (1.64 to 4.55)	6.92 (3.93 to 11.30)	0.08 (0.06 to 0.12)	0.09 (0.05 to 0.14)	0.04 (0.02 to 0.07)	0.09 (0.06 to 0.15)
Urogenital congenital anomalies	11.12 (8.02 to 16.17)	10.84 (7.25 to 16.00)	5.76 (3.82 to 8.47)	11.60 (7.62 to 17.31)	0.17 (0.13 to 0.24)	0.21 (0.14 to 0.29)	0.11 (0.08 to 0.16)	0.21 (0.15 to 0.30)
Digestive congenital anomalies	10.85 (6.42 to 16.39)	7.75 (3.69 to 14.08)	2.96 (1.44 to 5.46)	8.86 (4.22 to 16.44)	0.13 (0.08 to 0.20)	0.10 (0.05 to 0.17)	0.04 (0.02 to 0.07)	0.11 (0.05 to 0.20)
Other congenital birth defects	59.99 (43.11 to 80.24)	52.03 (32.60 to 80.28)	24.05 (14.96 to 36.74)	57.73 (35.74 to 89.03)	0.78 (0.58 to 1.02)	0.77 (0.52 to 1.11)	0.37 (0.24 to 0.54)	0.84 (0.56 to 1.22)
Skin and subcutaneous diseases	214.34 (123.55 to 307.35)	720.66 (440.14 to 1,012.18)	594.04 (364.99 to 827.00)	701.21 (425.98 to 987.31)	7.31 (4.36 to 10.65)	32.43 (19.83 to 46.45)	28.17 (16.96 to 40.06)	30.44 (18.47 to 43.87)
Cellulitis	35.75 (14.47 to 53.59)	155.71 (64.74 to 221.15)	150.83 (64.91 to 211.97)	148.56 (61.70 to 213.31)	1.14 (0.45 to 1.67)	6.65 (2.72 to 9.52)	6.79 (2.86 to 9.58)	6.17 (2.49 to 8.89)
Pyoderma	130.53 (75.56 to 190.95)	432.80 (261.63 to 657.14)	347.98 (217.57 to 527.00)	427.15 (258.60 to 650.94)	3.72 (2.16 to 5.61)	17.14 (10.42 to 26.14)	14.65 (8.92 to 22.19)	16.38 (9.95 to 25.08)
Decubitus ulcer	46.66 (23.97 to 78.93)	129.48 (69.49 to 224.17)	93.16 (51.21 to 160.03)	122.82 (65.97 to 213.53)	2.42 (1.25 to 4.05)	8.57 (4.50 to 14.87)	6.66 (3.60 to 11.70)	7.82 (4.05 to 13.71)
Other skin and subcutaneous diseases	1.40 (0.84 to 2.25)	2.66 (1.69 to 4.28)	2.07 (1.32 to 3.18)	2.67 (1.70 to 4.30)	0.03 (0.02 to 0.04)	0.07 (0.04 to 0.11)	0.06 (0.04 to 0.09)	0.06 (0.04 to 0.11)
Sudden infant death syndrome	32.54 (19.66 to 50.98)	23.15 (7.64 to 45.47)	10.25 (3.15 to 19.35)	26.72 (8.82 to 52.59)	0.38 (0.23 to 0.59)	0.27 (0.09 to 0.53)	0.12 (0.04 to 0.22)	0.31 (0.10 to 0.61)

**Injuries**	**1,586.58 (1,299.87 to 1,878.88)**	**2,003.60 (1,473.06 to 2,584.17)**	**1,319.50 (931.65 to 1,640.03)**	**2,131.88 (1,563.22 to 2,812.48)**	**32.30 (26.55 to 38.49)**	**49.38 (38.41 to 61.05)**	**34.44 (25.93 to 41.18)**	**51.01 (39.37 to 64.52)**
**Transport injuries**	**327.38 (265.44 to 394.96)**	**472.02 (356.07 to 632.29)**	**248.27 (190.67 to 326.36)**	**548.85 (409.24 to 743.84)**	**6.68 (5.45 to 8.03)**	**11.29 (8.78 to 14.69)**	**6.36 (5.04 to 8.09)**	**12.71 (9.87 to 16.68)**
Road injuries	271.03 (221.33 to 324.92)	367.34 (277.36 to 492.99)	193.68 (149.33 to 255.12)	438.94 (327.72 to 597.23)	5.48 (4.46 to 6.57)	8.62 (6.71 to 11.24)	4.90 (3.89 to 6.24)	9.98 (7.74 to 13.16)
Pedestrian road injuries	89.74 (69.91 to 111.19)	100.51 (71.59 to 138.42)	37.81 (27.02 to 50.74)	116.68 (81.64 to 162.54)	1.84 (1.42 to 2.28)	2.50 (1.84 to 3.31)	1.00 (0.73 to 1.30)	2.82 (2.08 to 3.78)

American Samoa								
Cause	YLLs (actual counts)				Deaths (actual counts)			
	2016	2040 Forecast	2040 Better Scenario	2040 Worse Scenario	2016	2040 Forecast	2040 Better Scenario	2040 Worse Scenario
Cyclist road injuries	12.02 (8.98 to 15.26)	13.72 (9.65 to 18.68)	8.40 (5.97 to 11.22)	16.34 (11.39 to 22.67)	0.25 (0.19 to 0.32)	0.35 (0.25 to 0.46)	0.24 (0.18 to 0.32)	0.40 (0.28 to 0.54)
Motorcyclist road injuries	34.58 (22.52 to 44.63)	45.30 (27.10 to 66.78)	31.16 (18.99 to 45.49)	51.88 (30.99 to 77.68)	0.66 (0.43 to 0.85)	0.91 (0.56 to 1.33)	0.65 (0.40 to 0.93)	1.03 (0.62 to 1.51)
Motor vehicle road injuries	128.83 (104.21 to 158.42)	199.66 (148.88 to 270.54)	111.60 (85.83 to 147.91)	244.16 (181.11 to 333.47)	2.61 (2.12 to 3.21)	4.68 (3.66 to 6.14)	2.89 (2.30 to 3.69)	5.52 (4.27 to 7.36)
Other road injuries	5.85 (4.16 to 7.50)	8.14 (5.41 to 11.47)	4.71 (3.17 to 6.58)	9.88 (6.55 to 14.02)	0.12 (0.08 to 0.15)	0.19 (0.13 to 0.26)	0.12 (0.08 to 0.16)	0.22 (0.15 to 0.30)
Other transport injuries	56.35 (43.72 to 70.86)	104.68 (75.48 to 143.74)	54.59 (38.98 to 74.40)	109.91 (78.46 to 154.37)	1.20 (0.94 to 1.50)	2.67 (2.02 to 3.59)	1.45 (1.09 to 1.94)	2.73 (2.05 to 3.74)
**Unintentional injuries**	**812.71 (661.84 to 975.18)**	**855.02 (660.67 to 1,063.16)**	**577.51 (446.39 to 720.39)**	**880.86 (681.37 to 1,096.78)**	**16.90 (13.72 to 20.25)**	**23.54 (19.44 to 27.82)**	**17.10 (14.15 to 20.24)**	**23.43 (19.54 to 27.80)**
Falls	73.72 (56.97 to 92.20)	101.43 (79.38 to 122.46)	79.93 (62.20 to 98.38)	100.11 (78.59 to 119.83)	1.99 (1.57 to 2.46)	3.96 (3.24 to 4.55)	3.32 (2.70 to 3.87)	3.75 (3.06 to 4.34)
Drowning	284.04 (222.68 to 345.04)	246.60 (176.41 to 328.29)	159.13 (113.93 to 212.61)	258.30 (186.59 to 347.35)	5.29 (4.13 to 6.48)	5.24 (3.84 to 6.84)	3.48 (2.55 to 4.53)	5.42 (4.00 to 7.15)
Fire, heat, and hot substances	30.82 (24.01 to 37.79)	31.19 (22.34 to 41.72)	17.59 (12.52 to 23.31)	32.75 (23.04 to 44.11)	0.64 (0.50 to 0.78)	0.85 (0.65 to 1.08)	0.51 (0.39 to 0.65)	0.86 (0.65 to 1.09)
Poisonings	30.85 (20.46 to 39.65)	20.01 (12.96 to 28.53)	9.34 (5.92 to 13.36)	21.39 (13.72 to 30.57)	0.62 (0.41 to 0.79)	0.54 (0.37 to 0.73)	0.27 (0.18 to 0.37)	0.55 (0.37 to 0.75)
Exposure to mechanical forces	88.20 (68.50 to 108.14)	93.38 (67.96 to 119.67)	56.62 (41.05 to 74.21)	96.68 (70.36 to 123.36)	1.78 (1.39 to 2.19)	2.35 (1.83 to 2.87)	1.50 (1.16 to 1.89)	2.37 (1.82 to 2.90)
Unintentional firearm injuries	9.70 (7.26 to 15.30)	9.50 (6.44 to 15.47)	5.97 (4.01 to 9.52)	9.88 (6.76 to 15.93)	0.20 (0.15 to 0.31)	0.26 (0.19 to 0.42)	0.18 (0.12 to 0.28)	0.27 (0.19 to 0.43)
Unintentional suffocation	16.24 (10.96 to 20.55)	16.54 (10.18 to 22.73)	10.79 (6.62 to 15.01)	17.40 (10.58 to 23.87)	0.27 (0.18 to 0.34)	0.30 (0.19 to 0.40)	0.20 (0.13 to 0.27)	0.31 (0.20 to 0.42)
Other exposure to mechanical forces	62.26 (47.40 to 78.36)	67.34 (49.34 to 87.55)	39.86 (28.50 to 52.44)	69.40 (50.08 to 89.55)	1.31 (1.00 to 1.64)	1.79 (1.39 to 2.20)	1.12 (0.85 to 1.41)	1.79 (1.38 to 2.21)
Adverse effects of medical treatment	105.45 (81.32 to 129.45)	123.92 (91.59 to 158.57)	94.90 (69.57 to 121.66)	126.53 (94.00 to 160.84)	2.38 (1.84 to 2.91)	3.74 (2.91 to 4.57)	3.07 (2.37 to 3.74)	3.68 (2.88 to 4.48)
Animal contact	8.58 (6.00 to 11.02)	11.45 (7.02 to 16.53)	7.65 (4.73 to 11.08)	11.85 (7.33 to 17.07)	0.20 (0.14 to 0.25)	0.36 (0.23 to 0.48)	0.26 (0.16 to 0.35)	0.36 (0.23 to 0.48)
Venomous animal contact	2.50 (1.59 to 3.54)	3.37 (1.91 to 5.42)	2.42 (1.38 to 3.89)	3.51 (1.94 to 5.70)	0.06 (0.04 to 0.08)	0.10 (0.06 to 0.14)	0.08 (0.05 to 0.11)	0.10 (0.06 to 0.14)
Non-venomous animal contact	6.08 (4.22 to 7.73)	8.08 (4.94 to 11.64)	5.24 (3.03 to 7.58)	8.34 (5.07 to 11.99)	0.14 (0.10 to 0.18)	0.26 (0.17 to 0.35)	0.18 (0.11 to 0.25)	0.26 (0.17 to 0.35)
Foreign body	101.57 (78.28 to 133.31)	114.48 (86.86 to 151.05)	94.34 (72.76 to 122.10)	115.97 (87.10 to 154.01)	1.97 (1.55 to 2.55)	3.22 (2.64 to 4.14)	2.89 (2.39 to 3.66)	3.11 (2.54 to 3.99)
Pulmonary aspiration and foreign body in airway	96.61 (74.18 to 128.96)	109.93 (83.04 to 144.71)	89.91 (68.65 to 117.74)	111.24 (83.49 to 147.80)	1.88 (1.46 to 2.47)	3.12 (2.55 to 4.04)	2.79 (2.29 to 3.56)	3.01 (2.45 to 3.89)

Foreign body in other body part	4.96 (2.83 to 6.83)	4.55 (2.42 to 7.18)	4.43 (2.41 to 6.80)	4.73 (2.49 to 7.37)	0.09 (0.05 to 0.13)	0.10 (0.05 to 0.15)	0.10 (0.06 to 0.15)	0.10 (0.05 to 0.15)
Environmental heat and cold exposure	66.37 (46.55 to 84.45)	89.73 (59.88 to 126.60)	49.16 (32.32 to 68.91)	93.87 (62.91 to 130.15)	1.57 (1.12 to 2.00)	2.76 (1.97 to 3.75)	1.60 (1.11 to 2.21)	2.81 (2.01 to 3.79)
Other unintentional injuries	23.10 (17.36 to 29.10)	22.84 (16.09 to 29.89)	8.84 (5.89 to 12.33)	23.42 (16.67 to 30.85)	0.47 (0.35 to 0.59)	0.53 (0.39 to 0.68)	0.21 (0.15 to 0.28)	0.53 (0.39 to 0.69)
**Self-harm and interpersonal violence**	**446.05 (355.92 to 557.93)**	**541.85 (392.64 to 744.13)**	**364.88 (270.48 to 496.24)**	**564.88 (404.50 to 790.26)**	**8.72 (6.89 to 11.00)**	**11.72 (8.75 to 15.70)**	**8.15 (6.20 to 10.80)**	**12.04 (8.92 to 16.38)**

American Samoa								
Cause	YLLs (actual counts)				Deaths (actual counts)			
	2016	2040 Forecast	2040 Better Scenario	2040 Worse Scenario	2016	2040 Forecast	2040 Better Scenario	2040 Worse Scenario
Self-harm	232.95 (178.62 to 284.05)	328.06 (219.13 to 474.73)	222.41 (154.26 to 306.22)	345.86 (228.61 to 508.44)	4.57 (3.51 to 5.58)	7.14 (4.98 to 10.01)	5.02 (3.61 to 6.70)	7.41 (5.05 to 10.53)
Self-harm by firearm	22.55 (13.01 to 32.74)	26.78 (14.59 to 47.65)	15.37 (8.63 to 25.60)	28.39 (15.12 to 50.39)	0.46 (0.27 to 0.66)	0.58 (0.32 to 0.99)	0.34 (0.19 to 0.55)	0.61 (0.33 to 1.04)
Self-harm by other specified means	210.40 (162.36 to 256.51)	301.28 (202.54 to 431.88)	207.04 (142.60 to 281.33)	317.47 (211.03 to 465.64)	4.12 (3.16 to 4.99)	6.57 (4.61 to 9.12)	4.68 (3.40 to 6.21)	6.80 (4.68 to 9.65)
Interpersonal violence	213.10 (164.27 to 299.95)	213.78 (152.28 to 314.35)	142.46 (101.63 to 206.12)	219.02 (156.87 to 325.63)	4.14 (3.18 to 5.84)	4.58 (3.33 to 6.64)	3.13 (2.27 to 4.41)	4.64 (3.36 to 6.76)
Physical violence by firearm	34.16 (20.38 to 71.76)	31.95 (17.89 to 69.18)	17.53 (9.73 to 37.73)	32.15 (17.97 to 69.84)	0.66 (0.39 to 1.37)	0.65 (0.38 to 1.36)	0.36 (0.20 to 0.76)	0.64 (0.38 to 1.37)
Physical violence by sharp object	45.62 (32.49 to 67.51)	49.22 (34.90 to 73.78)	30.74 (21.33 to 46.68)	49.15 (34.61 to 74.33)	0.89 (0.63 to 1.31)	1.00 (0.71 to 1.48)	0.63 (0.44 to 0.94)	0.99 (0.71 to 1.47)
Physical violence by other means	133.32 (97.66 to 178.37)	132.62 (88.94 to 198.07)	94.20 (64.67 to 139.17)	137.72 (91.02 to 208.73)	2.60 (1.89 to 3.49)	2.94 (2.00 to 4.26)	2.14 (1.52 to 3.07)	3.00 (2.03 to 4.41)
**Forces of nature, conflict and terrorism, and executions and police conflict**	**0.44 (0.37 to 0.51)**	**134.71 (0.00 to 290.06)**	**128.84 (0.00 to 276.12)**	**137.29 (0.00 to 296.11)**	**0.01 (0.01 to 0.01)**	**2.83 (0.00 to 6.00)**	**2.84 (0.00 to 5.96)**	**2.82 (0.00 to 5.98)**
Exposure to forces of nature	0.00 (0.00 to 0.00)	128.98 (0.00 to 257.65)	123.36 (0.00 to 245.25)	131.44 (0.00 to 263.02)	0.00 (0.00 to 0.00)	2.73 (0.00 to 5.40)	2.73 (0.00 to 5.37)	2.71 (0.00 to 5.37)
Conflict and terrorism	0.00 (0.00 to 0.00)	5.73 (0.00 to 66.00)	5.48 (0.00 to 62.87)	5.85 (0.00 to 67.34)	0.00 (0.00 to 0.00)	0.11 (0.00 to 1.24)	0.11 (0.00 to 1.22)	0.11 (0.00 to 1.25)
Executions and police conflict	0.44 (0.37 to 0.51)	0.00 (0.00 to 0.00)	0.00 (0.00 to 0.00)	0.00 (0.00 to 0.00)	0.01 (0.01 to 0.01)	0.00 (0.00 to 0.00)	0.00 (0.00 to 0.00)	0.00 (0.00 to 0.00)

Federated States of Micronesia								
Cause	YLLs (actual counts)				Deaths (actual counts)			
	2016	2040 Forecast	2040 Better Scenario	2040 Worse Scenario	2016	2040 Forecast	2040 Better Scenario	2040 Worse Scenario
**All causes**	**25,655.19 (21,136.79 to 31,233.88)**	**38,372.22 (26,954.38 to 54,579.52)**	**25,991.80 (19,234.55 to 35,223.34)**	**48,835.08 (33,195.11 to 74,205.69)**	**833.21 (693.26 to 1,003.22)**	**1,269.58 (1,033.11 to 1,580.24)**	**960.07 (803.22 to 1,170.38)**	**1,513.59 (1,203.27 to 1,955.20)**
**Communicable, maternal, neonatal, and nutritional diseases**	**4,564.32 (3,225.53 to 6,938.19)**	**7,806.03 (3,221.78 to 21,591.85)**	**3,406.14 (1,902.79 to 6,762.14)**	**12,126.75 (4,345.12 to 33,893.38)**	**96.30 (69.45 to 147.17)**	**170.57 (84.86 to 394.28)**	**91.99 (57.71 to 162.43)**	**247.10 (104.65 to 627.26)**
**HIV/AIDS and tuberculosis**	**918.04 (241.02 to 3,259.51)**	**4,714.95 (541.83 to 18,143.34)**	**1,373.24 (213.54 to 4,901.18)**	**8,358.12 (922.98 to 30,384.24)**	**22.08 (7.11 to 73.28)**	**90.08 (13.59 to 317.72)**	**30.16 (6.21 to 102.53)**	**153.91 (20.85 to 550.17)**
Tuberculosis	205.80 (153.88 to 268.31)	209.08 (132.86 to 323.02)	102.76 (66.18 to 154.81)	255.77 (156.79 to 400.75)	6.49 (4.96 to 8.36)	6.82 (4.56 to 9.95)	3.57 (2.42 to 5.07)	7.91 (5.21 to 11.64)
HIV/AIDS	712.24 (34.12 to 3,065.98)	4,505.88 (330.93 to 17,973.20)	1,270.48 (102.81 to 4,823.07)	8,102.36 (637.46 to 30,140.11)	15.59 (0.69 to 67.34)	83.26 (6.66 to 311.58)	26.60 (2.45 to 99.34)	146.00 (12.06 to 541.05)
**Diarrhea, lower respiratory, and other common infectious diseases**	**1,841.99 (1,360.50 to 2,464.34)**	**1,871.49 (1,201.39 to 2,835.88)**	**1,272.74 (838.48 to 1,869.08)**	**2,343.31 (1,465.07 to 3,659.22)**	**47.95 (35.16 to 64.01)**	**58.84 (38.36 to 83.99)**	**46.46 (31.40 to 65.16)**	**69.57 (44.37 to 104.47)**
Diarrheal diseases	106.48 (67.21 to 180.09)	86.86 (18.82 to 272.75)	48.26 (11.77 to 144.36)	118.63 (23.03 to 370.52)	3.93 (2.32 to 7.45)	4.24 (0.88 to 13.16)	2.74 (0.63 to 8.41)	5.35 (0.99 to 17.35)
Intestinal infectious diseases	132.11 (68.21 to 237.40)	97.13 (46.15 to 176.16)	72.68 (35.66 to 131.07)	103.80 (48.82 to 191.08)	1.94 (1.01 to 3.44)	1.53 (0.75 to 2.77)	1.18 (0.58 to 2.13)	1.61 (0.78 to 2.95)
Typhoid fever	113.24 (57.87 to 205.49)	79.95 (37.34 to 149.83)	60.08 (29.42 to 111.42)	85.42 (39.44 to 161.25)	1.66 (0.85 to 3.01)	1.25 (0.60 to 2.34)	0.97 (0.48 to 1.78)	1.31 (0.63 to 2.50)
Paratyphoid fever	18.80 (8.07 to 38.03)	17.12 (6.83 to 35.69)	12.57 (5.01 to 26.19)	18.33 (7.18 to 38.31)	0.28 (0.12 to 0.57)	0.28 (0.11 to 0.58)	0.21 (0.09 to 0.45)	0.30 (0.12 to 0.63)
Other intestinal infectious diseases	0.07 (0.03 to 0.15)	0.05 (0.02 to 0.12)	0.03 (0.01 to 0.07)	0.05 (0.02 to 0.13)	0.00 (0.00 to 0.00)	0.00 (0.00 to 0.00)	0.00 (0.00 to 0.00)	0.00 (0.00 to 0.00)
Lower respiratory infections	1,302.21 (950.30 to 1,756.90)	1,339.08 (810.10 to 2,158.18)	962.65 (608.43 to 1,451.40)	1,704.84 (1,001.83 to 2,845.28)	37.50 (26.41 to 51.40)	47.32 (30.49 to 69.82)	38.84 (26.08 to 54.65)	56.06 (34.88 to 85.09)
Upper respiratory infections	2.34 (1.18 to 3.92)	2.87 (0.90 to 6.91)	1.66 (0.51 to 4.01)	3.10 (0.94 to 7.48)	0.06 (0.03 to 0.10)	0.08 (0.03 to 0.18)	0.05 (0.02 to 0.11)	0.08 (0.03 to 0.20)
Otitis media	0.05 (0.02 to 0.11)	0.02 (0.01 to 0.06)	0.02 (0.01 to 0.06)	0.02 (0.01 to 0.05)	0.00 (0.00 to 0.00)	0.00 (0.00 to 0.00)	0.00 (0.00 to 0.00)	0.00 (0.00 to 0.00)
Meningitis	149.40 (95.13 to 244.77)	99.73 (53.94 to 179.41)	80.93 (45.39 to 146.12)	107.28 (56.61 to 197.50)	2.48 (1.58 to 4.04)	1.97 (1.12 to 3.56)	1.74 (1.01 to 3.15)	2.03 (1.15 to 3.68)
Pneumococcal meningitis	0.42 (0.25 to 0.70)	0.23 (0.13 to 0.44)	0.21 (0.11 to 0.39)	0.25 (0.14 to 0.47)	0.01 (0.00 to 0.01)	0.01 (0.00 to 0.01)	0.01 (0.00 to 0.01)	0.01 (0.00 to 0.01)
H influenzae type B meningitis	15.36 (9.35 to 25.45)	15.53 (4.90 to 40.39)	9.14 (4.28 to 18.91)	20.71 (5.38 to 58.05)	0.25 (0.16 to 0.41)	0.25 (0.10 to 0.56)	0.18 (0.09 to 0.35)	0.30 (0.11 to 0.75)
Meningococcal meningitis	30.05 (18.02 to 50.89)	18.50 (9.94 to 34.40)	15.67 (8.45 to 28.88)	18.97 (10.22 to 34.39)	0.53 (0.32 to 0.89)	0.41 (0.23 to 0.75)	0.37 (0.21 to 0.67)	0.40 (0.22 to 0.73)
Other meningitis	103.56 (65.74 to 168.58)	65.47 (36.42 to 118.06)	55.90 (31.52 to 102.64)	67.35 (37.65 to 122.11)	1.69 (1.07 to 2.77)	1.31 (0.74 to 2.38)	1.18 (0.68 to 2.18)	1.31 (0.75 to 2.40)
Encephalitis	24.69 (17.16 to 34.36)	27.42 (16.37 to 44.57)	21.56 (12.83 to 34.84)	28.36 (16.81 to 46.20)	0.48 (0.33 to 0.66)	0.62 (0.39 to 0.96)	0.52 (0.33 to 0.80)	0.62 (0.39 to 0.96)

Diphtheria	0.39 (0.24 to 0.61)	0.12 (0.06 to 0.21)	0.10 (0.05 to 0.17)	0.14 (0.07 to 0.29)	0.01 (0.00 to 0.01)	0.00 (0.00 to 0.00)	0.00 (0.00 to 0.00)	0.00 (0.00 to 0.00)
Whooping cough	107.58 (8.59 to 346.37)	70.77 (1.62 to 239.27)	9.86 (0.58 to 36.93)	78.00 (1.45 to 268.40)	1.28 (0.10 to 4.12)	0.85 (0.02 to 2.85)	0.13 (0.01 to 0.48)	0.93 (0.02 to 3.20)
Tetanus	0.88 (0.68 to 1.15)	0.14 (0.09 to 0.21)	0.12 (0.07 to 0.17)	0.15 (0.10 to 0.23)	0.02 (0.01 to 0.02)	0.00 (0.00 to 0.00)	0.00 (0.00 to 0.00)	0.00 (0.00 to 0.00)
Measles	7.46 (2.21 to 18.11)	142.02 (6.86 to 509.71)	71.88 (3.89 to 211.80)	193.14 (8.28 to 692.11)	0.09 (0.03 to 0.22)	2.08 (0.11 to 6.83)	1.17 (0.07 to 3.50)	2.74 (0.13 to 8.99)

Federated States of Micronesia								
Cause	YLLs (actual counts)				Deaths (actual counts)			
	2016	2040 Forecast	2040 Better Scenario	2040 Worse Scenario	2016	2040 Forecast	2040 Better Scenario	2040 Worse Scenario
Varicella and herpes zoster	8.39 (4.73 to 14.03)	5.32 (2.29 to 10.08)	3.03 (1.44 to 5.38)	5.85 (2.39 to 11.63)	0.17 (0.11 to 0.25)	0.14 (0.08 to 0.24)	0.10 (0.05 to 0.16)	0.14 (0.07 to 0.23)
**Neglected tropical diseases and malaria**	**11.64 (7.60 to 17.51)**	**35.75 (25.21 to 50.06)**	**34.35 (23.87 to 48.12)**	**35.15 (24.39 to 49.42)**	**0.34 (0.24 to 0.47)**	**1.31 (0.91 to 1.84)**	**1.39 (0.97 to 1.95)**	**1.20 (0.82 to 1.68)**
Malaria	0.00 (0.00 to 0.00)	0.00 (0.00 to 0.00)	0.00 (0.00 to 0.00)	0.00 (0.00 to 0.00)	0.00 (0.00 to 0.00)	0.00 (0.00 to 0.00)	0.00 (0.00 to 0.00)	0.00 (0.00 to 0.00)
Chagas disease	0.00 (0.00 to 0.00)	0.00 (0.00 to 0.00)	0.00 (0.00 to 0.00)	0.00 (0.00 to 0.00)	0.00 (0.00 to 0.00)	0.00 (0.00 to 0.00)	0.00 (0.00 to 0.00)	0.00 (0.00 to 0.00)
Leishmaniasis	0.00 (0.00 to 0.00)	0.00 (0.00 to 0.00)	0.00 (0.00 to 0.00)	0.00 (0.00 to 0.00)	0.00 (0.00 to 0.00)	0.00 (0.00 to 0.00)	0.00 (0.00 to 0.00)	0.00 (0.00 to 0.00)
African trypanosomiasis	0.00 (0.00 to 0.00)	0.00 (0.00 to 0.00)	0.00 (0.00 to 0.00)	0.00 (0.00 to 0.00)	0.00 (0.00 to 0.00)	0.00 (0.00 to 0.00)	0.00 (0.00 to 0.00)	0.00 (0.00 to 0.00)
Schistosomiasis	0.00 (0.00 to 0.00)	0.00 (0.00 to 0.00)	0.00 (0.00 to 0.00)	0.00 (0.00 to 0.00)	0.00 (0.00 to 0.00)	0.00 (0.00 to 0.00)	0.00 (0.00 to 0.00)	0.00 (0.00 to 0.00)
Cysticercosis	0.00 (0.00 to 0.00)	0.00 (0.00 to 0.00)	0.00 (0.00 to 0.00)	0.00 (0.00 to 0.00)	0.00 (0.00 to 0.00)	0.00 (0.00 to 0.00)	0.00 (0.00 to 0.00)	0.00 (0.00 to 0.00)
Cystic echinococcosis	0.12 (0.09 to 0.17)	0.16 (0.10 to 0.25)	0.08 (0.05 to 0.13)	0.18 (0.11 to 0.28)	0.00 (0.00 to 0.00)	0.00 (0.00 to 0.01)	0.00 (0.00 to 0.00)	0.01 (0.00 to 0.01)
Dengue	3.11 (1.38 to 6.09)	20.16 (11.29 to 34.10)	20.76 (12.08 to 34.78)	19.45 (10.77 to 32.91)	0.09 (0.04 to 0.17)	0.73 (0.42 to 1.25)	0.83 (0.49 to 1.35)	0.66 (0.37 to 1.14)
Yellow fever	0.00 (0.00 to 0.00)	0.00 (0.00 to 0.00)	0.00 (0.00 to 0.00)	0.00 (0.00 to 0.00)	0.00 (0.00 to 0.00)	0.00 (0.00 to 0.00)	0.00 (0.00 to 0.00)	0.00 (0.00 to 0.00)
Rabies	0.05 (0.02 to 0.09)	0.05 (0.02 to 0.10)	0.02 (0.01 to 0.05)	0.06 (0.03 to 0.12)	0.00 (0.00 to 0.00)	0.00 (0.00 to 0.00)	0.00 (0.00 to 0.00)	0.00 (0.00 to 0.00)
Intestinal nematode infections	3.38 (1.03 to 7.37)	5.68 (2.26 to 11.04)	5.34 (2.26 to 9.91)	5.90 (2.22 to 11.74)	0.06 (0.02 to 0.11)	0.11 (0.05 to 0.19)	0.12 (0.06 to 0.19)	0.11 (0.05 to 0.19)
Other neglected tropical diseases	4.98 (3.16 to 7.65)	9.70 (5.58 to 14.57)	8.14 (4.29 to 12.52)	9.57 (5.48 to 14.42)	0.19 (0.12 to 0.29)	0.46 (0.23 to 0.76)	0.44 (0.21 to 0.74)	0.42 (0.21 to 0.71)
**Maternal disorders**	**140.98 (78.77 to 219.39)**	**158.86 (63.16 to 354.17)**	**83.62 (32.43 to 192.38)**	**178.79 (70.79 to 398.12)**	**2.53 (1.43 to 3.91)**	**3.05 (1.17 to 6.63)**	**1.61 (0.60 to 3.70)**	**3.43 (1.32 to 7.66)**
Maternal hemorrhage	43.91 (21.99 to 71.04)	46.85 (16.52 to 107.79)	24.49 (8.24 to 56.38)	52.34 (18.14 to 120.15)	0.79 (0.38 to 1.27)	0.89 (0.31 to 2.08)	0.47 (0.15 to 1.10)	1.00 (0.34 to 2.27)
Maternal sepsis and other maternal infections	14.76 (5.52 to 29.53)	16.13 (4.37 to 41.59)	8.43 (2.21 to 22.27)	18.09 (4.87 to 47.41)	0.27 (0.11 to 0.54)	0.32 (0.08 to 0.83)	0.17 (0.04 to 0.44)	0.36 (0.09 to 0.93)
Maternal hypertensive disorders	19.04 (9.39 to 32.72)	22.04 (7.35 to 53.67)	11.42 (3.79 to 28.77)	24.66 (8.38 to 60.56)	0.34 (0.17 to 0.59)	0.42 (0.14 to 1.03)	0.22 (0.07 to 0.55)	0.47 (0.16 to 1.14)
Maternal obstructed labor and uterine rupture	9.97 (3.79 to 19.03)	11.95 (3.38 to 31.50)	5.94 (1.63 to 16.15)	13.48 (3.87 to 35.10)	0.18 (0.07 to 0.34)	0.23 (0.07 to 0.61)	0.12 (0.03 to 0.31)	0.26 (0.07 to 0.68)
Maternal abortion, miscarriage, and ectopic pregnancy	6.42 (2.25 to 13.24)	8.49 (2.46 to 21.62)	4.49 (1.30 to 11.49)	9.66 (2.84 to 24.63)	0.12 (0.04 to 0.24)	0.17 (0.05 to 0.42)	0.09 (0.03 to 0.23)	0.19 (0.06 to 0.48)

Indirect maternal deaths	16.03 (7.02 to 28.42)	18.70 (6.28 to 44.12)	10.01 (3.38 to 24.00)	21.28 (7.38 to 51.88)	0.28 (0.12 to 0.51)	0.35 (0.12 to 0.84)	0.19 (0.06 to 0.47)	0.40 (0.14 to 1.01)
Late maternal deaths	6.47 (2.57 to 12.75)	5.98 (1.73 to 16.28)	3.14 (0.92 to 8.51)	6.77 (2.00 to 18.25)	0.12 (0.05 to 0.23)	0.11 (0.03 to 0.31)	0.06 (0.02 to 0.17)	0.13 (0.04 to 0.35)
Maternal deaths aggravated by HIV/AIDS	0.20 (0.02 to 0.73)	1.21 (0.02 to 8.08)	0.52 (0.00 to 3.83)	1.55 (0.03 to 9.34)	0.00 (0.00 to 0.01)	0.02 (0.00 to 0.15)	0.01 (0.00 to 0.07)	0.03 (0.00 to 0.17)
Other maternal disorders	24.20 (11.37 to 41.86)	27.49 (9.48 to 64.77)	15.19 (5.20 to 36.99)	30.96 (10.65 to 72.81)	0.43 (0.21 to 0.75)	0.53 (0.18 to 1.26)	0.29 (0.10 to 0.72)	0.59 (0.21 to 1.43)

Federated States of Micronesia								
Cause	YLLs (actual counts)				Deaths (actual counts)			
	2016	2040 Forecast	2040 Better Scenario	2040 Worse Scenario	2016	2040 Forecast	2040 Better Scenario	2040 Worse Scenario
**Neonatal disorders**	**1,202.17 (756.07 to 1,833.89)**	**727.14 (392.22 to 1,272.10)**	**420.26 (225.18 to 709.65)**	**896.92 (485.30 to 1,561.49)**	**13.89 (8.74 to 21.19)**	**8.41 (4.54 to 14.70)**	**4.86 (2.60 to 8.20)**	**10.37 (5.61 to 18.06)**
Neonatal preterm birth	527.65 (302.11 to 832.88)	304.26 (150.89 to 527.27)	160.10 (81.62 to 282.43)	372.05 (187.67 to 649.99)	6.10 (3.49 to 9.62)	3.52 (1.74 to 6.09)	1.85 (0.94 to 3.26)	4.30 (2.17 to 7.51)
Neonatal encephalopathy due to birth asphyxia and trauma	291.87 (160.42 to 489.27)	155.12 (70.25 to 297.99)	100.82 (46.67 to 185.67)	190.88 (84.87 to 368.66)	3.37 (1.85 to 5.66)	1.79 (0.81 to 3.44)	1.17 (0.54 to 2.15)	2.21 (0.98 to 4.26)
Neonatal sepsis and other neonatal infections	135.01 (68.35 to 239.89)	105.70 (47.46 to 194.07)	66.80 (31.71 to 122.63)	130.57 (59.07 to 239.75)	1.56 (0.79 to 2.77)	1.22 (0.55 to 2.25)	0.77 (0.37 to 1.42)	1.51 (0.68 to 2.77)
Hemolytic disease and other neonatal jaundice	11.64 (5.57 to 22.83)	4.83 (1.78 to 11.31)	2.80 (1.06 to 6.30)	6.08 (2.20 to 14.07)	0.13 (0.06 to 0.26)	0.06 (0.02 to 0.13)	0.03 (0.01 to 0.07)	0.07 (0.03 to 0.16)
Other neonatal disorders	235.99 (125.59 to 400.83)	157.23 (71.22 to 307.26)	89.73 (41.73 to 171.88)	197.35 (90.17 to 389.15)	2.73 (1.45 to 4.64)	1.82 (0.82 to 3.55)	1.04 (0.48 to 1.99)	2.28 (1.04 to 4.50)
**Nutritional deficiencies**	**121.61 (82.95 to 181.33)**	**78.29 (57.94 to 105.73)**	**59.37 (45.88 to 76.38)**	**80.58 (58.31 to 112.81)**	**4.03 (3.21 to 5.00)**	**3.76 (2.49 to 5.02)**	**3.44 (2.31 to 4.59)**	**3.43 (2.27 to 4.69)**
Protein-energy malnutrition	116.57 (78.56 to 176.44)	71.32 (52.61 to 97.23)	54.11 (41.17 to 70.51)	73.43 (52.38 to 104.00)	3.87 (3.07 to 4.80)	3.49 (2.25 to 4.72)	3.21 (2.13 to 4.31)	3.17 (2.05 to 4.44)
Iodine deficiency	1.12 (0.07 to 3.40)	2.85 (0.07 to 9.68)	1.98 (0.04 to 7.05)	2.97 (0.08 to 10.52)	0.03 (0.00 to 0.11)	0.12 (0.00 to 0.55)	0.10 (0.00 to 0.51)	0.12 (0.00 to 0.49)
Dietary iron deficiency	1.01 (0.70 to 1.47)	1.01 (0.63 to 1.71)	0.81 (0.49 to 1.41)	1.06 (0.66 to 1.76)	0.02 (0.01 to 0.04)	0.02 (0.01 to 0.05)	0.02 (0.01 to 0.04)	0.02 (0.01 to 0.05)
Other nutritional deficiencies	2.92 (1.91 to 4.24)	3.11 (1.89 to 4.85)	2.47 (1.50 to 3.86)	3.12 (1.91 to 4.94)	0.11 (0.07 to 0.16)	0.12 (0.08 to 0.20)	0.11 (0.07 to 0.17)	0.12 (0.07 to 0.18)
**Other communicable, maternal, neonatal, and nutritional diseases**	**327.89 (142.03 to 619.02)**	**219.55 (131.36 to 345.33)**	**162.55 (98.69 to 251.55)**	**233.87 (135.84 to 373.63)**	**5.47 (3.17 to 9.03)**	**5.12 (3.48 to 7.38)**	**4.07 (2.79 to 5.80)**	**5.20 (3.46 to 7.47)**
Sexually transmitted infections excluding HIV	193.48 (18.78 to 485.53)	57.86 (12.77 to 149.88)	41.08 (10.08 to 98.82)	65.59 (13.06 to 174.88)	2.39 (0.34 to 5.83)	0.83 (0.25 to 1.95)	0.61 (0.21 to 1.35)	0.92 (0.26 to 2.24)
Syphilis	184.22 (9.76 to 477.44)	46.22 (3.53 to 137.36)	31.52 (2.65 to 88.93)	53.69 (3.84 to 161.77)	2.19 (0.15 to 5.61)	0.57 (0.06 to 1.64)	0.39 (0.05 to 1.06)	0.66 (0.07 to 1.94)
Chlamydial infection	1.82 (0.98 to 2.87)	2.24 (1.00 to 4.46)	1.84 (0.82 to 3.69)	2.30 (1.04 to 4.58)	0.04 (0.02 to 0.06)	0.05 (0.02 to 0.10)	0.04 (0.02 to 0.08)	0.05 (0.02 to 0.10)
Gonococcal infection	4.76 (2.61 to 7.50)	6.01 (2.55 to 12.05)	4.95 (2.12 to 9.99)	6.15 (2.63 to 12.05)	0.11 (0.06 to 0.17)	0.14 (0.06 to 0.26)	0.11 (0.05 to 0.22)	0.14 (0.06 to 0.27)
Other sexually transmitted infections	2.68 (1.46 to 4.22)	3.38 (1.43 to 6.73)	2.77 (1.19 to 5.51)	3.46 (1.50 to 6.85)	0.06 (0.03 to 0.09)	0.07 (0.03 to 0.15)	0.06 (0.03 to 0.12)	0.08 (0.03 to 0.15)
Acute hepatitis	32.12 (23.08 to 43.20)	41.16 (25.30 to 65.66)	32.27 (19.88 to 51.61)	41.95 (25.61 to 67.01)	0.90 (0.66 to 1.19)	1.17 (0.75 to 1.79)	0.94 (0.61 to 1.44)	1.17 (0.76 to 1.80)
Acute hepatitis A	1.00 (0.57 to 1.68)	0.77 (0.41 to 1.34)	0.60 (0.32 to 1.04)	0.81 (0.43 to 1.40)	0.02 (0.01 to 0.03)	0.02 (0.01 to 0.03)	0.02 (0.01 to 0.03)	0.02 (0.01 to 0.03)
Acute hepatitis B	27.22 (19.09 to 37.49)	36.74 (21.95 to 59.45)	28.61 (17.10 to 46.44)	37.45 (22.50 to 61.36)	0.78 (0.57 to 1.05)	1.04 (0.67 to 1.61)	0.83 (0.53 to 1.29)	1.04 (0.67 to 1.63)

Acute hepatitis C	0.63 (0.40 to 0.96)	0.82 (0.47 to 1.42)	0.74 (0.41 to 1.28)	0.81 (0.46 to 1.42)	0.02 (0.01 to 0.03)	0.03 (0.01 to 0.04)	0.02 (0.01 to 0.04)	0.02 (0.01 to 0.04)
Acute hepatitis E	3.26 (2.20 to 4.83)	2.83 (1.51 to 4.79)	2.31 (1.24 to 3.89)	2.89 (1.54 to 4.90)	0.08 (0.05 to 0.12)	0.08 (0.05 to 0.14)	0.07 (0.04 to 0.12)	0.08 (0.04 to 0.13)
Other unspecified infectious diseases	102.29 (66.00 to 190.57)	120.53 (75.72 to 189.41)	89.20 (56.08 to 139.09)	126.33 (78.58 to 198.70)	2.18 (1.55 to 3.38)	3.13 (2.15 to 4.61)	2.52 (1.72 to 3.68)	3.11 (2.11 to 4.57)
**Non-communicable diseases**	**17,266.65 (13,575.70 to 21,551.00)**	**26,019.13 (18,508.35 to 37,192.13)**	**18,794.24 (13,553.67 to 26,758.84)**	**31,877.56 (22,270.83 to 44,970.78)**	**662.60 (538.35 to 808.48)**	**1,001.36 (817.36 to 1,260.92)**	**783.40 (645.96 to 975.94)**	**1,165.15 (940.38 to 1,455.78)**

Federated States of Micronesia								
Cause	YLLs (actual counts)				Deaths (actual counts)			
	2016	2040 Forecast	2040 Better Scenario	2040 Worse Scenario	2016	2040 Forecast	2040 Better Scenario	2040 Worse Scenario
**Neoplasms**	**2,449.12 (1,802.82 to 3,228.60)**	**4,111.53 (2,631.01 to 6,410.34)**	**3,763.76 (2,502.64 to 5,633.87)**	**4,563.65 (2,844.05 to 7,321.26)**	**86.94 (65.63 to 112.24)**	**144.54 (102.21 to 200.91)**	**140.18 (104.06 to 188.81)**	**154.32 (106.10 to 220.46)**
Lip and oral cavity cancer	40.82 (29.25 to 56.62)	71.84 (36.04 to 143.74)	51.34 (27.81 to 95.49)	97.68 (44.97 to 212.64)	1.54 (1.16 to 2.07)	2.50 (1.44 to 4.47)	1.91 (1.20 to 3.12)	3.20 (1.71 to 6.30)
Nasopharynx cancer	30.96 (18.79 to 47.82)	65.80 (24.39 to 144.94)	43.94 (17.67 to 91.96)	94.21 (32.01 to 219.00)	1.03 (0.64 to 1.56)	2.00 (0.81 to 4.21)	1.38 (0.61 to 2.74)	2.78 (1.04 to 6.04)
Other pharynx cancer	31.05 (19.74 to 46.21)	44.99 (25.82 to 78.49)	40.11 (23.34 to 68.99)	48.31 (26.54 to 86.16)	1.13 (0.76 to 1.64)	1.58 (1.02 to 2.52)	1.48 (0.97 to 2.31)	1.63 (1.00 to 2.65)
Esophageal cancer	33.08 (24.94 to 43.23)	52.44 (25.92 to 99.48)	36.13 (19.74 to 64.52)	68.42 (31.27 to 137.43)	1.31 (1.02 to 1.68)	1.92 (1.06 to 3.42)	1.40 (0.84 to 2.31)	2.39 (1.20 to 4.59)
Stomach cancer	183.22 (138.98 to 236.72)	214.74 (145.20 to 314.13)	190.05 (131.00 to 274.13)	223.51 (148.88 to 335.11)	7.17 (5.65 to 9.05)	8.06 (6.11 to 10.69)	7.61 (5.89 to 9.93)	8.04 (5.92 to 10.95)
Colon and rectum cancer	180.94 (125.12 to 251.13)	327.56 (188.58 to 563.17)	338.24 (200.09 to 560.50)	330.61 (185.88 to 563.93)	7.18 (5.24 to 9.71)	12.92 (7.74 to 20.62)	14.20 (8.86 to 21.95)	12.42 (7.38 to 20.03)
Liver cancer	159.56 (115.17 to 216.84)	329.27 (185.44 to 558.07)	282.10 (167.94 to 451.32)	401.79 (209.90 to 710.14)	5.82 (4.32 to 7.79)	11.32 (6.89 to 17.51)	10.29 (6.65 to 14.97)	13.20 (7.47 to 21.63)
Liver cancer due to hepatitis B	89.58 (63.41 to 125.93)	186.15 (101.16 to 324.06)	160.14 (93.38 to 267.92)	226.77 (115.82 to 412.96)	3.09 (2.25 to 4.23)	6.04 (3.56 to 9.55)	5.47 (3.46 to 8.36)	7.07 (3.87 to 11.78)
Liver cancer due to hepatitis C	23.47 (16.99 to 32.55)	44.90 (27.02 to 70.28)	40.50 (25.10 to 62.70)	52.45 (29.32 to 86.93)	1.04 (0.77 to 1.41)	1.91 (1.21 to 2.87)	1.86 (1.24 to 2.69)	2.10 (1.27 to 3.28)
Liver cancer due to alcohol use	10.63 (7.27 to 15.30)	23.15 (12.66 to 39.16)	19.74 (11.60 to 32.25)	29.78 (15.06 to 53.72)	0.42 (0.30 to 0.60)	0.83 (0.49 to 1.31)	0.75 (0.47 to 1.14)	1.02 (0.55 to 1.68)
Liver cancer due to other causes	35.88 (25.52 to 49.86)	75.07 (38.10 to 135.11)	61.73 (33.39 to 105.76)	92.80 (43.01 to 179.35)	1.27 (0.93 to 1.73)	2.54 (1.35 to 4.45)	2.21 (1.25 to 3.62)	3.01 (1.48 to 5.46)
Gallbladder and biliary tract cancer	18.93 (12.37 to 28.34)	21.52 (14.04 to 33.46)	23.68 (15.36 to 36.65)	20.09 (13.18 to 31.32)	0.80 (0.54 to 1.17)	0.88 (0.62 to 1.25)	1.02 (0.72 to 1.46)	0.79 (0.55 to 1.13)
Pancreatic cancer	68.45 (49.55 to 94.21)	133.47 (80.69 to 217.63)	133.99 (84.57 to 211.85)	136.54 (81.04 to 224.84)	2.91 (2.18 to 3.89)	5.39 (3.41 to 8.18)	5.72 (3.79 to 8.44)	5.30 (3.25 to 8.22)
Larynx cancer	29.61 (22.28 to 38.36)	41.59 (18.56 to 80.54)	26.63 (12.53 to 51.20)	55.58 (24.62 to 109.76)	1.14 (0.88 to 1.45)	1.53 (0.74 to 2.87)	1.01 (0.51 to 1.89)	1.98 (0.94 to 3.73)
Tracheal, bronchus, and lung cancer	346.23 (237.93 to 487.58)	615.10 (250.69 to 1,244.36)	422.69 (188.70 to 821.08)	864.75 (350.57 to 1,735.93)	13.87 (9.97 to 19.19)	22.84 (10.76 to 42.49)	16.17 (8.25 to 29.14)	31.25 (14.13 to 58.33)
Malignant skin melanoma	13.87 (9.15 to 21.69)	23.49 (15.74 to 37.44)	28.61 (18.97 to 46.25)	21.55 (14.57 to 33.72)	0.53 (0.36 to 0.80)	0.87 (0.62 to 1.30)	1.12 (0.79 to 1.68)	0.76 (0.54 to 1.12)
Non-melanoma skin cancer	21.12 (13.65 to 30.29)	40.59 (26.15 to 60.53)	39.59 (25.56 to 58.89)	38.40 (24.61 to 56.88)	0.95 (0.68 to 1.28)	1.77 (1.19 to 2.34)	1.88 (1.30 to 2.46)	1.58 (1.05 to 2.11)
Breast cancer	312.22 (150.89 to 510.36)	592.55 (242.16 to 1,144.02)	581.72 (248.39 to 1,116.37)	614.67 (241.43 to 1,219.77)	10.53 (5.24 to 16.89)	19.38 (8.77 to 35.70)	19.93 (9.46 to 35.35)	19.49 (8.55 to 35.57)
Cervical cancer	147.67 (76.30 to 224.89)	246.37 (109.57 to 495.71)	209.89 (97.80 to 407.29)	261.22 (112.76 to 567.24)	4.76 (2.57 to 7.01)	7.34 (3.76 to 13.06)	6.46 (3.41 to 11.18)	7.62 (3.69 to 14.62)

Uterine cancer	69.99 (45.41 to 99.08)	123.92 (73.97 to 190.16)	109.39 (66.05 to 170.33)	122.37 (72.42 to 185.91)	2.59 (1.70 to 3.59)	4.40 (2.83 to 6.27)	4.03 (2.63 to 5.84)	4.25 (2.67 to 6.09)
Ovarian cancer	36.35 (22.97 to 61.28)	69.10 (35.82 to 134.69)	76.35 (39.99 to 147.46)	67.21 (34.84 to 127.74)	1.33 (0.86 to 2.17)	2.45 (1.35 to 4.52)	2.80 (1.57 to 5.02)	2.33 (1.27 to 4.27)
Prostate cancer	79.39 (57.72 to 107.32)	133.87 (97.60 to 180.22)	160.65 (121.22 to 213.08)	117.05 (83.06 to 162.48)	4.77 (3.59 to 6.33)	8.82 (5.77 to 11.94)	11.25 (7.71 to 14.74)	7.34 (4.67 to 10.33)
Testicular cancer	8.21 (5.54 to 11.51)	9.61 (5.99 to 13.99)	8.97 (5.58 to 13.25)	9.51 (6.02 to 13.80)	0.18 (0.12 to 0.25)	0.23 (0.15 to 0.32)	0.22 (0.15 to 0.31)	0.22 (0.14 to 0.31)

Federated States of Micronesia								
Cause	YLLs (actual counts)				Deaths (actual counts)			
	2016	2040 Forecast	2040 Better Scenario	2040 Worse Scenario	2016	2040 Forecast	2040 Better Scenario	2040 Worse Scenario
Kidney cancer	17.68 (13.18 to 23.03)	37.63 (22.93 to 63.18)	40.75 (25.79 to 66.19)	38.27 (22.84 to 66.09)	0.61 (0.47 to 0.80)	1.28 (0.84 to 1.97)	1.47 (1.01 to 2.19)	1.25 (0.79 to 1.97)
Bladder cancer	24.23 (16.86 to 34.07)	37.20 (19.65 to 64.68)	32.85 (18.40 to 54.83)	39.87 (20.31 to 71.65)	1.10 (0.81 to 1.51)	1.63 (0.96 to 2.61)	1.54 (0.96 to 2.40)	1.66 (0.95 to 2.74)
Brain and nervous system cancer	62.78 (45.79 to 91.15)	108.58 (74.22 to 168.00)	114.37 (77.07 to 180.40)	105.81 (72.55 to 163.11)	1.55 (1.13 to 2.27)	2.89 (2.06 to 4.40)	3.20 (2.29 to 4.92)	2.72 (1.97 to 4.14)
Thyroid cancer	13.69 (9.54 to 19.19)	20.53 (13.24 to 31.86)	18.23 (11.96 to 27.69)	21.86 (13.71 to 34.98)	0.55 (0.39 to 0.75)	0.81 (0.54 to 1.14)	0.76 (0.52 to 1.06)	0.83 (0.54 to 1.20)
Mesothelioma	5.57 (3.79 to 7.94)	8.52 (5.57 to 12.70)	8.56 (5.51 to 13.07)	8.17 (5.35 to 12.11)	0.19 (0.14 to 0.28)	0.28 (0.19 to 0.39)	0.29 (0.20 to 0.42)	0.26 (0.18 to 0.36)
Hodgkin lymphoma	13.31 (8.56 to 18.44)	16.02 (9.09 to 26.29)	12.71 (7.17 to 20.79)	16.34 (9.34 to 26.51)	0.32 (0.21 to 0.44)	0.41 (0.25 to 0.63)	0.34 (0.21 to 0.52)	0.41 (0.25 to 0.61)
Non-Hodgkin lymphoma	75.77 (54.14 to 103.29)	120.74 (82.26 to 181.06)	128.95 (87.35 to 193.12)	121.19 (82.18 to 181.87)	2.16 (1.58 to 2.92)	3.73 (2.71 to 5.15)	4.25 (3.13 to 5.86)	3.58 (2.56 to 4.97)
Multiple myeloma	30.26 (19.58 to 42.37)	56.37 (33.79 to 84.56)	62.60 (38.10 to 93.70)	55.96 (33.80 to 85.14)	1.17 (0.78 to 1.60)	2.10 (1.35 to 2.97)	2.45 (1.59 to 3.40)	2.01 (1.30 to 2.89)
Leukemia	197.67 (145.58 to 263.41)	257.14 (165.72 to 397.04)	243.13 (159.19 to 372.76)	279.44 (177.09 to 444.29)	4.57 (3.30 to 6.15)	6.81 (4.47 to 10.13)	6.81 (4.60 to 9.83)	7.17 (4.54 to 11.05)
Acute lymphoid leukemia	37.65 (27.52 to 52.24)	40.39 (26.76 to 63.32)	38.66 (25.84 to 59.14)	43.70 (28.89 to 69.59)	0.69 (0.50 to 0.96)	0.85 (0.56 to 1.33)	0.84 (0.56 to 1.28)	0.91 (0.59 to 1.45)
Chronic lymphoid leukemia	4.02 (2.64 to 5.97)	5.57 (3.36 to 8.92)	5.55 (3.45 to 8.74)	5.83 (3.39 to 9.49)	0.16 (0.11 to 0.23)	0.23 (0.14 to 0.35)	0.25 (0.16 to 0.37)	0.23 (0.14 to 0.36)
Acute myeloid leukemia	71.57 (50.00 to 94.69)	113.14 (72.33 to 168.75)	115.76 (74.64 to 170.61)	121.43 (76.35 to 186.46)	1.55 (1.07 to 2.03)	2.84 (1.88 to 4.16)	3.07 (2.09 to 4.33)	2.96 (1.90 to 4.46)
Chronic myeloid leukemia	22.76 (12.08 to 34.91)	24.91 (11.22 to 46.36)	23.33 (10.86 to 41.48)	27.33 (12.06 to 52.56)	0.68 (0.38 to 1.03)	0.75 (0.38 to 1.33)	0.74 (0.38 to 1.25)	0.80 (0.39 to 1.45)
Other leukemia	61.67 (40.07 to 89.61)	73.13 (39.62 to 130.14)	59.83 (32.49 to 103.57)	81.15 (42.75 to 148.01)	1.50 (0.99 to 2.16)	2.14 (1.21 to 3.49)	1.91 (1.11 to 3.05)	2.26 (1.24 to 3.78)
Other neoplasms	196.49 (146.87 to 260.30)	290.98 (205.21 to 425.31)	297.55 (207.95 to 442.74)	283.24 (200.36 to 411.12)	5.21 (3.92 to 6.75)	8.43 (6.36 to 11.31)	9.21 (6.88 to 12.23)	7.86 (5.90 to 10.46)
**Cardiovascular diseases**	**7,465.57 (5,813.17 to 9,446.40)**	**9,573.65 (5,940.19 to 14,742.94)**	**5,404.89 (3,551.98 to 8,234.98)**	**13,356.47 (8,176.75 to 19,593.16)**	**302.76 (244.41 to 373.33)**	**378.78 (276.28 to 523.33)**	**241.43 (182.41 to 328.97)**	**495.19 (355.22 to 669.80)**
Rheumatic heart disease	375.09 (282.73 to 495.77)	254.34 (169.64 to 383.11)	189.19 (126.88 to 287.07)	262.44 (176.05 to 401.57)	10.49 (8.09 to 13.69)	8.04 (5.98 to 10.82)	6.48 (4.90 to 8.58)	7.89 (5.83 to 10.79)
Ischemic heart disease	3,513.52 (2,675.24 to 4,572.63)	4,690.37 (2,162.24 to 8,777.98)	2,337.29 (1,121.17 to 4,771.72)	7,054.66 (3,582.38 to 11,483.56)	147.19 (117.08 to 185.42)	182.05 (99.33 to 304.87)	102.88 (57.30 to 187.40)	257.73 (146.07 to 399.35)
Stroke	2,426.13 (1,878.74 to 3,103.44)	2,741.00 (1,517.51 to 4,749.05)	1,320.97 (762.40 to 2,332.78)	4,029.61 (2,179.09 to 6,810.57)	99.72 (78.67 to 125.04)	111.62 (71.80 to 176.88)	62.58 (40.81 to 100.38)	152.34 (92.64 to 238.62)
Ischemic stroke	681.64 (522.77 to 869.85)	672.12 (371.61 to 1,198.67)	401.26 (221.86 to 780.98)	872.04 (483.60 to 1,448.74)	34.34 (27.44 to 42.50)	34.86 (19.62 to 70.09)	23.72 (13.19 to 50.17)	42.64 (24.02 to 81.95)

Intracerebral hemorrhage	1,744.48 (1,334.78 to 2,254.40)	2,068.88 (1,024.68 to 4,017.98)	919.71 (473.37 to 1,863.38)	3,157.57 (1,510.87 to 5,780.60)	65.38 (50.75 to 83.66)	76.76 (44.13 to 133.13)	38.86 (23.57 to 69.25)	109.69 (58.76 to 190.03)
Hypertensive heart disease	390.75 (253.50 to 534.70)	660.45 (415.78 to 1,003.32)	471.72 (310.39 to 682.95)	756.04 (450.07 to 1,255.27)	17.18 (11.49 to 22.79)	29.35 (19.61 to 42.41)	22.81 (15.90 to 31.70)	31.44 (19.74 to 51.38)
Cardiomyopathy and myocarditis	230.58 (140.06 to 315.61)	327.10 (186.93 to 500.76)	325.36 (188.55 to 497.11)	319.45 (179.08 to 497.31)	7.47 (4.54 to 10.26)	11.07 (6.54 to 15.68)	11.77 (7.02 to 17.06)	10.32 (5.98 to 15.03)
Myocarditis	58.34 (37.72 to 88.25)	76.73 (47.46 to 118.85)	72.84 (44.51 to 111.36)	74.49 (45.81 to 115.20)	1.91 (1.27 to 2.79)	2.64 (1.75 to 3.68)	2.68 (1.77 to 3.69)	2.46 (1.60 to 3.44)

Federated States of Micronesia								
Cause	YLLs (actual counts)				Deaths (actual counts)			
	2016	2040 Forecast	2040 Better Scenario	2040 Worse Scenario	2016	2040 Forecast	2040 Better Scenario	2040 Worse Scenario
Alcoholic cardiomyopathy	12.95 (5.84 to 20.55)	15.48 (6.24 to 25.19)	16.77 (6.70 to 27.40)	15.66 (6.27 to 25.66)	0.46 (0.21 to 0.70)	0.54 (0.25 to 0.83)	0.61 (0.27 to 0.94)	0.52 (0.23 to 0.81)
Other cardiomyopathy	159.29 (91.64 to 220.83)	234.89 (121.41 to 380.60)	235.75 (125.62 to 378.01)	229.31 (117.88 to 378.74)	5.10 (2.91 to 7.02)	7.88 (4.34 to 11.84)	8.47 (4.77 to 12.79)	7.35 (3.98 to 11.32)
Atrial fibrillation and flutter	21.07 (15.53 to 27.62)	35.55 (24.16 to 48.30)	40.55 (27.90 to 54.05)	32.46 (22.05 to 44.61)	1.80 (1.35 to 2.34)	3.03 (1.72 to 4.41)	3.76 (2.25 to 5.30)	2.61 (1.47 to 3.94)
Aortic aneurysm	85.88 (55.18 to 123.55)	120.32 (74.73 to 178.92)	117.63 (76.83 to 172.19)	121.61 (73.48 to 183.21)	3.78 (2.63 to 5.27)	5.18 (3.49 to 7.23)	5.65 (3.96 to 7.71)	4.90 (3.18 to 6.92)
Peripheral artery disease	4.59 (2.75 to 7.92)	11.64 (6.12 to 22.24)	12.01 (6.54 to 20.81)	11.48 (5.79 to 22.55)	0.26 (0.16 to 0.42)	0.64 (0.36 to 1.12)	0.77 (0.43 to 1.30)	0.58 (0.32 to 1.04)
Endocarditis	104.54 (73.02 to 144.06)	173.66 (110.64 to 266.64)	163.00 (105.06 to 250.64)	170.89 (107.84 to 264.31)	3.54 (2.54 to 4.74)	6.21 (4.27 to 8.84)	6.35 (4.40 to 9.00)	5.79 (3.97 to 8.30)
Other cardiovascular and circulatory diseases	313.42 (192.96 to 419.30)	559.21 (328.24 to 860.60)	427.16 (253.56 to 631.67)	597.83 (342.14 to 958.48)	11.32 (6.90 to 14.84)	21.58 (13.11 to 30.23)	18.39 (11.42 to 25.06)	21.59 (12.91 to 31.30)
**Chronic respiratory diseases**	**1,444.82 (1,107.26 to 1,837.41)**	**1,804.61 (1,105.61 to 2,993.76)**	**1,217.97 (790.30 to 1,898.37)**	**2,436.84 (1,405.82 to 4,227.25)**	**55.50 (44.12 to 69.42)**	**65.60 (43.72 to 98.05)**	**46.30 (33.00 to 64.75)**	**85.55 (53.67 to 134.52)**
Chronic obstructive pulmonary disease	848.40 (647.14 to 1,082.73)	1,108.02 (588.95 to 1,958.48)	645.21 (369.92 to 1,090.15)	1,641.52 (813.53 to 3,103.65)	35.19 (27.92 to 43.89)	42.94 (25.63 to 68.36)	26.78 (17.27 to 39.69)	60.56 (33.91 to 101.67)
Pneumoconiosis	3.73 (2.53 to 5.45)	4.20 (2.72 to 6.63)	3.89 (2.57 to 5.93)	4.16 (2.65 to 6.59)	0.18 (0.13 to 0.26)	0.21 (0.14 to 0.32)	0.22 (0.14 to 0.33)	0.19 (0.13 to 0.30)
Silicosis	0.48 (0.25 to 0.85)	0.43 (0.21 to 0.79)	0.38 (0.19 to 0.69)	0.41 (0.19 to 0.78)	0.03 (0.01 to 0.05)	0.03 (0.01 to 0.05)	0.03 (0.01 to 0.05)	0.02 (0.01 to 0.04)
Asbestosis	0.96 (0.60 to 1.75)	1.42 (0.84 to 2.69)	1.38 (0.83 to 2.49)	1.37 (0.79 to 2.56)	0.04 (0.02 to 0.07)	0.06 (0.03 to 0.11)	0.06 (0.04 to 0.11)	0.05 (0.03 to 0.10)
Coal workers pneumoconiosis	0.07 (0.03 to 0.14)	0.08 (0.03 to 0.19)	0.06 (0.02 to 0.13)	0.08 (0.03 to 0.19)	0.00 (0.00 to 0.01)	0.00 (0.00 to 0.01)	0.00 (0.00 to 0.01)	0.00 (0.00 to 0.01)
Other pneumoconiosis	2.21 (1.47 to 3.30)	2.26 (1.43 to 3.66)	2.07 (1.31 to 3.13)	2.30 (1.44 to 3.79)	0.11 (0.08 to 0.16)	0.12 (0.07 to 0.19)	0.13 (0.08 to 0.20)	0.12 (0.07 to 0.18)
Asthma	370.43 (236.14 to 523.77)	331.28 (177.50 to 586.06)	231.83 (129.98 to 402.76)	414.80 (214.52 to 740.41)	13.63 (9.26 to 19.16)	12.11 (6.89 to 20.57)	9.26 (5.50 to 15.33)	14.29 (7.93 to 24.66)
Interstitial lung disease and pulmonary sarcoidosis	68.37 (42.86 to 107.58)	113.11 (65.70 to 186.58)	131.22 (77.07 to 211.43)	107.93 (63.01 to 179.82)	1.88 (1.19 to 3.02)	3.28 (2.04 to 5.18)	4.02 (2.57 to 6.26)	3.01 (1.86 to 4.83)
Other chronic respiratory diseases	153.89 (92.89 to 232.03)	247.99 (127.53 to 431.50)	205.82 (110.88 to 345.71)	268.43 (134.38 to 490.60)	4.63 (2.73 to 6.98)	7.05 (3.82 to 12.19)	6.02 (3.39 to 9.79)	7.49 (3.95 to 13.40)
**Cirrhosis and other chronic liver diseases**	**588.50 (415.61 to 830.82)**	**1,002.94 (654.86 to 1,471.87)**	**918.27 (596.79 to 1,361.70)**	**1,000.74 (651.59 to 1,463.66)**	**17.01 (12.28 to 23.57)**	**28.99 (20.66 to 40.00)**	**27.53 (19.62 to 37.67)**	**28.20 (19.84 to 38.99)**
Cirrhosis and other chronic liver diseases due to hepatitis B	223.56 (152.12 to 325.42)	420.87 (270.00 to 623.13)	393.17 (251.83 to 583.48)	412.30 (263.77 to 609.52)	6.92 (4.96 to 9.80)	12.47 (8.70 to 17.59)	12.03 (8.48 to 16.91)	11.93 (8.32 to 16.86)
Cirrhosis and other chronic liver diseases due to hepatitis C	89.69 (60.26 to 129.47)	176.37 (109.89 to 271.09)	163.59 (102.85 to 250.87)	173.33 (108.63 to 266.56)	2.70 (1.87 to 3.85)	5.07 (3.46 to 7.42)	4.83 (3.28 to 7.06)	4.89 (3.31 to 7.18)

Cirrhosis and other chronic liver diseases due to alcohol use	73.52 (49.85 to 107.12)	134.04 (86.28 to 202.02)	119.07 (76.90 to 178.62)	143.33 (90.66 to 218.57)	2.39 (1.68 to 3.37)	4.10 (2.87 to 5.88)	3.77 (2.64 to 5.34)	4.25 (2.95 to 6.12)
Cirrhosis and other chronic liver diseases due to other causes	201.73 (146.31 to 269.97)	271.66 (181.11 to 386.25)	242.44 (161.25 to 345.76)	271.78 (182.22 to 386.18)	5.00 (3.71 to 6.53)	7.34 (5.42 to 9.86)	6.89 (5.11 to 9.27)	7.13 (5.20 to 9.56)
**Digestive diseases**	**478.17 (316.82 to 755.46)**	**612.77 (389.66 to 1,005.84)**	**502.25 (323.47 to 818.52)**	**641.49 (406.20 to 1,068.30)**	**16.28 (11.34 to 25.21)**	**22.05 (15.41 to 33.96)**	**19.44 (14.03 to 29.62)**	**21.97 (15.20 to 34.16)**
Peptic ulcer disease	130.39 (85.58 to 209.84)	120.20 (77.42 to 198.28)	85.36 (55.31 to 143.10)	125.63 (78.98 to 212.02)	4.81 (3.31 to 7.54)	4.66 (3.20 to 7.20)	3.57 (2.49 to 5.54)	4.65 (3.15 to 7.16)

Federated States of Micronesia								
Cause	YLLs (actual counts)				Deaths (actual counts)			
	2016	2040 Forecast	2040 Better Scenario	2040 Worse Scenario	2016	2040 Forecast	2040 Better Scenario	2040 Worse Scenario
Gastritis and duodenitis	3.10 (1.56 to 5.70)	5.21 (2.44 to 9.82)	4.62 (2.22 to 8.63)	5.08 (2.38 to 9.65)	0.12 (0.06 to 0.22)	0.21 (0.11 to 0.36)	0.20 (0.10 to 0.34)	0.19 (0.10 to 0.35)
Appendicitis	60.40 (37.42 to 98.51)	79.36 (45.75 to 137.51)	63.23 (36.89 to 109.15)	80.44 (46.07 to 138.74)	1.66 (1.07 to 2.68)	2.38 (1.54 to 3.86)	2.02 (1.34 to 3.27)	2.31 (1.47 to 3.79)
Paralytic ileus and intestinal obstruction	57.25 (38.26 to 90.81)	69.47 (47.31 to 116.79)	65.30 (44.66 to 109.04)	68.01 (45.66 to 112.43)	1.71 (1.22 to 2.79)	2.51 (1.79 to 4.10)	2.61 (1.89 to 4.28)	2.28 (1.61 to 3.78)
Inguinal, femoral, and abdominal hernia	10.73 (6.85 to 19.17)	12.26 (7.92 to 22.52)	11.38 (7.39 to 20.48)	11.61 (7.43 to 21.00)	0.49 (0.34 to 0.84)	0.58 (0.39 to 1.00)	0.59 (0.40 to 0.98)	0.52 (0.34 to 0.90)
Inflammatory bowel disease	20.76 (12.70 to 35.60)	34.13 (19.56 to 59.09)	34.34 (20.16 to 59.25)	32.83 (18.83 to 57.76)	0.63 (0.42 to 1.03)	1.10 (0.74 to 1.77)	1.18 (0.80 to 1.86)	1.01 (0.66 to 1.62)
Vascular intestinal disorders	18.08 (10.97 to 26.88)	28.59 (17.87 to 42.83)	27.91 (17.82 to 41.87)	27.00 (16.55 to 40.97)	0.81 (0.53 to 1.18)	1.30 (0.88 to 1.87)	1.36 (0.94 to 1.96)	1.17 (0.77 to 1.69)
Gallbladder and biliary diseases	63.74 (39.40 to 101.04)	99.79 (46.49 to 204.04)	70.80 (33.95 to 139.32)	123.43 (56.32 to 253.80)	2.42 (1.61 to 3.71)	3.85 (1.93 to 7.26)	2.96 (1.55 to 5.49)	4.49 (2.22 to 8.47)
Pancreatitis	35.61 (21.77 to 60.32)	58.69 (33.26 to 109.46)	50.86 (29.27 to 93.67)	63.55 (34.69 to 124.96)	1.05 (0.66 to 1.78)	1.79 (1.09 to 3.15)	1.63 (1.02 to 2.84)	1.86 (1.10 to 3.39)
Other digestive diseases	78.11 (44.31 to 126.73)	105.06 (58.38 to 177.21)	88.46 (50.42 to 148.35)	103.91 (56.86 to 174.40)	2.57 (1.59 to 4.09)	3.69 (2.34 to 5.79)	3.32 (2.19 to 5.20)	3.49 (2.18 to 5.51)
**Neurological disorders**	**360.86 (290.04 to 445.40)**	**600.56 (433.52 to 848.52)**	**636.26 (464.76 to 878.67)**	**589.34 (417.93 to 849.84)**	**22.11 (17.77 to 27.02)**	**39.63 (25.40 to 61.38)**	**46.86 (30.55 to 68.61)**	**36.26 (22.36 to 57.96)**
Alzheimer's disease and other dementias	207.54 (161.15 to 261.47)	378.39 (224.93 to 623.50)	419.75 (270.30 to 646.07)	361.88 (206.35 to 614.89)	17.99 (14.21 to 22.28)	32.72 (18.43 to 54.41)	39.01 (23.60 to 60.37)	29.72 (16.26 to 50.95)
Parkinson's disease	16.34 (11.97 to 21.76)	31.76 (22.49 to 42.86)	43.61 (31.68 to 57.50)	26.67 (18.74 to 36.28)	1.20 (0.87 to 1.61)	2.38 (1.53 to 3.38)	3.45 (2.35 to 4.75)	1.92 (1.20 to 2.79)
Epilepsy	91.17 (63.97 to 126.62)	113.62 (65.37 to 183.99)	88.61 (50.47 to 144.82)	126.71 (70.95 to 209.60)	1.70 (1.18 to 2.36)	2.35 (1.39 to 3.76)	1.88 (1.10 to 3.03)	2.58 (1.47 to 4.22)
Multiple sclerosis	3.00 (1.47 to 5.82)	6.05 (2.69 to 13.15)	6.28 (2.80 to 13.53)	5.99 (2.63 to 13.29)	0.09 (0.04 to 0.16)	0.17 (0.08 to 0.35)	0.18 (0.09 to 0.37)	0.16 (0.08 to 0.34)
Motor neuron disease	7.88 (5.83 to 10.84)	16.39 (11.96 to 23.36)	18.73 (13.50 to 27.07)	15.38 (11.24 to 21.92)	0.27 (0.21 to 0.37)	0.54 (0.42 to 0.76)	0.64 (0.49 to 0.89)	0.50 (0.38 to 0.69)
Other neurological disorders	34.93 (27.40 to 44.07)	54.36 (39.56 to 73.47)	59.29 (42.98 to 80.81)	52.71 (38.56 to 71.16)	0.87 (0.68 to 1.09)	1.47 (1.13 to 1.87)	1.70 (1.30 to 2.16)	1.38 (1.06 to 1.75)
**Mental disorders**	**68.92 (42.71 to 109.60)**	**137.01 (76.19 to 239.15)**	**136.34 (76.44 to 237.34)**	**136.37 (75.83 to 237.63)**	**1.56 (0.99 to 2.49)**	**3.27 (1.94 to 5.39)**	**3.36 (2.02 to 5.50)**	**3.18 (1.87 to 5.29)**
Alcohol use disorders	34.24 (18.38 to 64.58)	55.21 (24.90 to 118.25)	54.04 (24.07 to 117.12)	55.89 (25.37 to 119.70)	0.82 (0.46 to 1.51)	1.36 (0.65 to 2.78)	1.36 (0.65 to 2.78)	1.36 (0.65 to 2.79)
Drug use disorders	34.36 (22.31 to 52.91)	81.07 (48.49 to 133.25)	81.57 (48.42 to 134.58)	79.75 (47.89 to 130.96)	0.73 (0.49 to 1.11)	1.89 (1.21 to 2.96)	1.99 (1.27 to 3.11)	1.81 (1.16 to 2.85)
Opioid use disorders	20.54 (12.45 to 33.73)	38.70 (21.00 to 68.72)	38.53 (20.85 to 68.15)	38.25 (20.89 to 67.70)	0.40 (0.24 to 0.66)	0.82 (0.46 to 1.44)	0.84 (0.48 to 1.45)	0.80 (0.45 to 1.40)

Cocaine use disorders	2.14 (1.12 to 3.36)	6.74 (3.26 to 11.90)	6.33 (3.03 to 11.13)	6.71 (3.22 to 11.86)	0.04 (0.02 to 0.07)	0.15 (0.08 to 0.25)	0.14 (0.07 to 0.25)	0.15 (0.08 to 0.25)
Amphetamine use disorders	0.95 (0.49 to 1.49)	4.03 (1.93 to 6.98)	3.81 (1.78 to 6.71)	4.00 (1.92 to 6.87)	0.02 (0.01 to 0.03)	0.09 (0.05 to 0.16)	0.09 (0.04 to 0.15)	0.09 (0.05 to 0.15)
Other drug use disorders	10.73 (6.69 to 16.51)	31.60 (18.17 to 54.20)	32.91 (18.99 to 56.39)	30.79 (17.73 to 53.30)	0.27 (0.18 to 0.40)	0.82 (0.51 to 1.32)	0.91 (0.57 to 1.44)	0.77 (0.47 to 1.25)
Eating disorders	0.33 (0.19 to 0.52)	0.74 (0.36 to 1.30)	0.73 (0.35 to 1.34)	0.73 (0.36 to 1.28)	0.01 (0.00 to 0.01)	0.02 (0.01 to 0.03)	0.02 (0.01 to 0.03)	0.02 (0.01 to 0.03)

Federated States of Micronesia								
Cause	YLLs (actual counts)				Deaths (actual counts)			
	2016	2040 Forecast	2040 Better Scenario	2040 Worse Scenario	2016	2040 Forecast	2040 Better Scenario	2040 Worse Scenario
**Diabetes, urogenital, blood, and endocrine diseases**	**3,613.85 (2,768.11 to 4,589.63)**	**7,183.72 (4,388.05 to 12,433.92)**	**5,322.97 (3,190.50 to 9,618.19)**	**8,123.22 (4,906.30 to 13,501.54)**	**146.24 (115.38 to 182.28)**	**293.96 (194.91 to 482.11)**	**233.27 (152.15 to 407.10)**	**316.82 (206.45 to 500.91)**
Diabetes mellitus	2,082.94 (1,590.38 to 2,644.32)	3,133.78 (1,944.88 to 4,777.37)	2,191.01 (1,388.14 to 3,307.32)	3,377.44 (2,073.39 to 5,306.39)	89.23 (69.67 to 110.71)	133.30 (88.68 to 189.85)	100.54 (67.38 to 140.43)	136.74 (87.69 to 200.39)
Acute glomerulonephritis	2.17 (1.44 to 3.18)	3.55 (2.03 to 6.44)	3.11 (1.72 to 5.65)	3.52 (2.04 to 6.41)	0.06 (0.04 to 0.09)	0.10 (0.05 to 0.18)	0.09 (0.05 to 0.17)	0.10 (0.05 to 0.17)
Chronic kidney disease	1,195.86 (875.78 to 1,611.32)	3,428.35 (932.91 to 9,034.24)	2,541.04 (676.84 to 7,032.70)	4,137.99 (1,142.31 to 9,849.60)	46.23 (34.41 to 61.02)	139.91 (37.74 to 350.15)	111.46 (28.43 to 294.77)	160.69 (44.08 to 364.42)
Chronic kidney disease due to diabetes mellitus	665.96 (474.67 to 915.59)	2,121.19 (535.76 to 5,698.27)	1,571.19 (394.00 to 4,565.61)	2,582.92 (669.80 to 6,285.98)	25.22 (18.53 to 33.67)	81.64 (21.18 to 210.58)	64.29 (15.93 to 175.69)	95.22 (25.17 to 221.65)
Chronic kidney disease due to hypertension	281.24 (204.71 to 385.75)	802.83 (204.64 to 2,175.76)	613.46 (151.07 to 1,718.56)	946.48 (244.43 to 2,262.34)	13.85 (10.24 to 18.42)	40.73 (10.48 to 102.21)	33.86 (8.19 to 89.36)	45.13 (12.33 to 101.45)
Chronic kidney disease due to glomerulonephritis	121.03 (83.35 to 170.56)	224.80 (81.34 to 543.52)	156.77 (54.95 to 396.29)	270.96 (96.85 to 634.27)	3.15 (2.20 to 4.36)	7.16 (2.22 to 18.10)	5.31 (1.58 to 13.74)	8.38 (2.65 to 19.59)
Chronic kidney disease due to other and unspecified causes	127.64 (88.61 to 178.51)	279.53 (87.88 to 714.40)	199.63 (60.82 to 536.26)	337.64 (107.24 to 794.17)	4.02 (2.82 to 5.57)	10.37 (2.94 to 26.90)	8.01 (2.16 to 22.12)	11.97 (3.51 to 28.30)
Urinary diseases and male infertility	118.50 (88.43 to 158.56)	232.95 (163.14 to 348.32)	237.36 (164.16 to 360.87)	223.78 (156.78 to 335.03)	4.17 (3.18 to 5.51)	8.69 (6.64 to 11.48)	9.69 (7.40 to 12.90)	7.89 (6.00 to 10.41)
Urinary tract infections	103.82 (74.86 to 140.02)	208.31 (141.99 to 317.41)	214.68 (145.58 to 332.22)	199.92 (136.66 to 305.05)	3.63 (2.72 to 4.83)	7.77 (5.85 to 10.44)	8.79 (6.64 to 11.83)	7.04 (5.26 to 9.44)
Urolithiasis	3.71 (2.10 to 6.10)	8.65 (4.39 to 16.10)	9.32 (4.71 to 17.70)	8.21 (4.21 to 15.25)	0.13 (0.08 to 0.22)	0.31 (0.17 to 0.52)	0.35 (0.19 to 0.60)	0.28 (0.16 to 0.48)
Other urinary diseases	10.98 (6.53 to 16.72)	15.99 (8.84 to 26.28)	13.37 (7.35 to 22.18)	15.65 (8.58 to 25.38)	0.41 (0.25 to 0.61)	0.62 (0.36 to 1.00)	0.55 (0.32 to 0.91)	0.57 (0.33 to 0.93)
Gynecological diseases	1.34 (0.61 to 2.11)	2.33 (0.93 to 4.90)	1.84 (0.73 to 3.84)	2.39 (0.95 to 5.07)	0.03 (0.02 to 0.05)	0.06 (0.03 to 0.12)	0.05 (0.02 to 0.09)	0.06 (0.03 to 0.12)
Uterine fibroids	0.14 (0.04 to 0.28)	0.21 (0.05 to 0.49)	0.15 (0.03 to 0.37)	0.21 (0.05 to 0.50)	0.00 (0.00 to 0.01)	0.01 (0.00 to 0.01)	0.00 (0.00 to 0.01)	0.01 (0.00 to 0.01)
Polycystic ovarian syndrome	0.02 (0.01 to 0.06)	0.03 (0.01 to 0.08)	0.02 (0.00 to 0.05)	0.03 (0.01 to 0.09)	0.00 (0.00 to 0.00)	0.00 (0.00 to 0.00)	0.00 (0.00 to 0.00)	0.00 (0.00 to 0.00)
Endometriosis	0.01 (0.00 to 0.03)	0.02 (0.01 to 0.06)	0.01 (0.00 to 0.04)	0.02 (0.01 to 0.06)	0.00 (0.00 to 0.00)	0.00 (0.00 to 0.00)	0.00 (0.00 to 0.00)	0.00 (0.00 to 0.00)
Genital prolapse	0.04 (0.01 to 0.08)	0.05 (0.01 to 0.12)	0.03 (0.01 to 0.09)	0.05 (0.01 to 0.12)	0.00 (0.00 to 0.00)	0.00 (0.00 to 0.00)	0.00 (0.00 to 0.00)	0.00 (0.00 to 0.00)
Other gynecological diseases	1.12 (0.49 to 1.81)	2.03 (0.77 to 4.40)	1.62 (0.61 to 3.50)	2.08 (0.77 to 4.49)	0.03 (0.01 to 0.04)	0.05 (0.02 to 0.10)	0.04 (0.02 to 0.08)	0.05 (0.02 to 0.10)
Hemoglobinopathies and hemolytic anemias	34.00 (24.15 to 46.53)	40.15 (25.48 to 63.98)	32.53 (20.72 to 50.80)	40.57 (25.69 to 64.28)	0.92 (0.66 to 1.22)	1.21 (0.83 to 1.78)	1.02 (0.71 to 1.49)	1.18 (0.81 to 1.74)
Thalassemias	5.19 (3.60 to 7.12)	2.77 (1.74 to 4.10)	2.99 (1.96 to 4.34)	2.83 (1.79 to 4.18)	0.07 (0.05 to 0.10)	0.04 (0.02 to 0.06)	0.04 (0.03 to 0.06)	0.04 (0.02 to 0.06)

Sickle cell disorders	0.03 (0.02 to 0.05)	0.04 (0.02 to 0.07)	0.03 (0.02 to 0.06)	0.04 (0.02 to 0.08)	0.00 (0.00 to 0.00)	0.00 (0.00 to 0.00)	0.00 (0.00 to 0.00)	0.00 (0.00 to 0.00)
G6PD deficiency	18.77 (12.63 to 26.38)	27.19 (15.55 to 46.05)	20.83 (11.86 to 35.08)	27.92 (16.16 to 46.49)	0.42 (0.29 to 0.59)	0.68 (0.42 to 1.09)	0.53 (0.33 to 0.85)	0.69 (0.42 to 1.11)
Other hemoglobinopathies and hemolytic anemias	10.00 (6.99 to 13.83)	10.15 (7.05 to 14.48)	8.67 (6.05 to 12.38)	9.79 (6.80 to 14.00)	0.42 (0.31 to 0.57)	0.49 (0.35 to 0.67)	0.45 (0.33 to 0.61)	0.45 (0.32 to 0.62)
Endocrine, metabolic, blood, and immune disorders	179.04 (129.60 to 242.90)	342.61 (229.33 to 496.13)	316.08 (214.42 to 456.71)	337.52 (223.72 to 490.55)	5.59 (4.15 to 7.46)	10.70 (7.83 to 14.62)	10.42 (7.66 to 14.12)	10.17 (7.45 to 13.96)

Federated States of Micronesia								
Cause	YLLs (actual counts)				Deaths (actual counts)			
	2016	2040 Forecast	2040 Better Scenario	2040 Worse Scenario	2016	2040 Forecast	2040 Better Scenario	2040 Worse Scenario
**Musculoskeletal disorders**	**71.75 (39.85 to 105.40)**	**113.61 (58.17 to 187.27)**	**122.39 (61.93 to 205.53)**	**110.76 (56.69 to 183.31)**	**1.58 (0.89 to 2.27)**	**2.83 (1.56 to 4.30)**	**3.22 (1.77 to 4.90)**	**2.67 (1.47 to 4.13)**
Rheumatoid arthritis	4.19 (2.21 to 6.36)	7.74 (3.97 to 12.40)	6.65 (3.35 to 10.68)	7.64 (3.91 to 12.30)	0.16 (0.08 to 0.24)	0.34 (0.17 to 0.51)	0.31 (0.16 to 0.48)	0.32 (0.16 to 0.48)
Other musculoskeletal disorders	67.56 (37.07 to 99.10)	105.87 (53.02 to 177.84)	115.75 (57.59 to 196.25)	103.12 (51.05 to 174.21)	1.42 (0.79 to 2.04)	2.50 (1.34 to 3.90)	2.91 (1.58 to 4.48)	2.35 (1.25 to 3.72)
**Other non-communicable diseases**	**725.09 (493.08 to 1,050.85)**	**878.74 (605.88 to 1,233.57)**	**769.13 (533.90 to 1,061.05)**	**918.68 (625.11 to 1,296.24)**	**12.63 (9.15 to 17.01)**	**21.71 (15.23 to 29.11)**	**21.81 (15.31 to 29.15)**	**20.99 (14.71 to 28.30)**
Congenital birth defects	582.31 (368.92 to 883.28)	504.05 (311.62 to 776.75)	389.95 (243.61 to 589.04)	558.30 (341.81 to 866.90)	7.55 (4.96 to 11.10)	7.34 (4.63 to 11.13)	5.84 (3.69 to 8.77)	7.98 (5.01 to 12.09)
Neural tube defects	27.69 (13.22 to 51.59)	9.36 (3.57 to 20.31)	5.77 (2.20 to 12.50)	11.07 (4.16 to 23.96)	0.33 (0.16 to 0.60)	0.12 (0.05 to 0.25)	0.07 (0.03 to 0.15)	0.14 (0.05 to 0.29)
Congenital heart anomalies	283.19 (183.64 to 422.76)	237.48 (148.39 to 367.61)	185.50 (116.19 to 289.11)	260.67 (161.94 to 405.71)	3.73 (2.48 to 5.41)	3.51 (2.20 to 5.42)	2.81 (1.76 to 4.31)	3.78 (2.35 to 5.85)
Orofacial clefts	0.34 (0.13 to 0.90)	0.22 (0.07 to 0.62)	0.13 (0.04 to 0.35)	0.26 (0.09 to 0.75)	0.00 (0.00 to 0.01)	0.00 (0.00 to 0.01)	0.00 (0.00 to 0.00)	0.00 (0.00 to 0.01)
Down syndrome	16.21 (9.42 to 27.01)	22.64 (12.06 to 38.25)	17.39 (9.11 to 29.70)	24.58 (12.98 to 41.77)	0.23 (0.14 to 0.36)	0.40 (0.21 to 0.68)	0.31 (0.17 to 0.55)	0.42 (0.22 to 0.72)
Other chromosomal abnormalities	17.20 (8.20 to 33.94)	23.70 (10.27 to 45.29)	16.91 (7.64 to 32.22)	27.24 (11.57 to 52.57)	0.21 (0.10 to 0.40)	0.30 (0.14 to 0.55)	0.22 (0.10 to 0.40)	0.34 (0.15 to 0.64)
Congenital musculoskeletal and limb anomalies	17.34 (9.35 to 31.10)	15.16 (7.46 to 28.97)	12.20 (5.99 to 23.79)	16.80 (8.09 to 32.13)	0.22 (0.12 to 0.39)	0.20 (0.10 to 0.40)	0.17 (0.08 to 0.33)	0.22 (0.11 to 0.43)
Urogenital congenital anomalies	21.24 (10.97 to 35.23)	20.48 (10.94 to 33.69)	16.55 (8.95 to 26.97)	22.18 (11.57 to 36.82)	0.32 (0.17 to 0.49)	0.37 (0.20 to 0.59)	0.31 (0.17 to 0.49)	0.39 (0.21 to 0.62)
Digestive congenital anomalies	33.71 (16.54 to 62.95)	23.82 (10.49 to 46.94)	17.01 (7.64 to 33.22)	27.43 (11.81 to 54.27)	0.40 (0.20 to 0.74)	0.30 (0.13 to 0.57)	0.21 (0.10 to 0.41)	0.34 (0.15 to 0.66)
Other congenital birth defects	165.39 (96.26 to 270.00)	151.19 (87.34 to 247.80)	118.51 (69.83 to 191.80)	168.05 (95.20 to 277.53)	2.11 (1.26 to 3.37)	2.15 (1.26 to 3.46)	1.73 (1.03 to 2.67)	2.34 (1.36 to 3.81)
Skin and subcutaneous diseases	123.78 (73.41 to 191.23)	363.42 (211.12 to 546.65)	370.59 (219.54 to 552.75)	347.24 (200.97 to 526.66)	4.85 (2.84 to 7.43)	14.24 (8.75 to 20.40)	15.87 (9.84 to 22.70)	12.86 (7.88 to 18.46)
Cellulitis	23.71 (8.50 to 38.90)	87.53 (33.53 to 139.82)	100.48 (39.20 to 159.67)	81.88 (31.29 to 131.37)	0.88 (0.31 to 1.43)	3.23 (1.23 to 5.07)	3.97 (1.56 to 6.19)	2.88 (1.09 to 4.52)
Pyoderma	79.12 (47.99 to 126.59)	230.36 (137.47 to 373.04)	219.98 (128.75 to 355.99)	223.42 (133.51 to 356.05)	2.73 (1.69 to 4.36)	8.36 (5.20 to 13.01)	8.64 (5.38 to 13.36)	7.68 (4.79 to 11.97)
Decubitus ulcer	19.51 (10.21 to 37.22)	42.85 (22.29 to 89.00)	47.37 (24.84 to 93.50)	39.31 (19.54 to 80.68)	1.21 (0.65 to 2.27)	2.59 (1.20 to 5.46)	3.19 (1.54 to 6.46)	2.23 (1.03 to 4.72)
Other skin and subcutaneous diseases	1.44 (0.81 to 2.31)	2.67 (1.52 to 4.35)	2.75 (1.57 to 4.40)	2.64 (1.53 to 4.28)	0.03 (0.02 to 0.05)	0.07 (0.04 to 0.11)	0.08 (0.05 to 0.12)	0.06 (0.04 to 0.10)
Sudden infant death syndrome	19.01 (7.78 to 38.91)	11.26 (4.14 to 25.13)	8.60 (3.31 to 18.25)	13.14 (4.81 to 29.28)	0.22 (0.09 to 0.45)	0.13 (0.05 to 0.29)	0.10 (0.04 to 0.21)	0.15 (0.06 to 0.34)

**Injuries**	**3,824.22 (2,978.11 to 4,841.08)**	**4,547.06 (3,088.96 to 6,338.63)**	**3,791.42 (2,590.35 to 5,310.73)**	**4,830.77 (3,254.19 to 6,819.80)**	**74.30 (57.94 to 94.42)**	**97.65 (69.22 to 134.20)**	**84.68 (60.44 to 115.78)**	**101.34 (70.93 to 141.14)**
**Transport injuries**	**1,037.33 (761.96 to 1,358.88)**	**1,467.25 (993.37 to 2,082.17)**	**1,055.38 (708.02 to 1,510.84)**	**1,592.32 (1,074.47 to 2,277.47)**	**20.18 (14.82 to 26.37)**	**30.95 (21.54 to 42.93)**	**23.44 (16.38 to 32.51)**	**32.89 (22.71 to 46.18)**
Road injuries	924.83 (676.66 to 1,210.07)	1,283.92 (880.90 to 1,819.43)	900.64 (608.28 to 1,299.02)	1,395.52 (947.36 to 2,000.62)	17.97 (13.20 to 23.55)	26.98 (18.87 to 37.49)	20.00 (14.03 to 27.75)	28.71 (19.91 to 40.47)
Pedestrian road injuries	80.76 (55.11 to 110.65)	94.80 (60.02 to 141.69)	69.83 (44.34 to 105.76)	102.27 (63.83 to 154.85)	1.63 (1.13 to 2.22)	2.12 (1.41 to 3.06)	1.64 (1.10 to 2.36)	2.24 (1.45 to 3.28)

Federated States of Micronesia								
Cause	YLLs (actual counts)				Deaths (actual counts)			
	2016	2040 Forecast	2040 Better Scenario	2040 Worse Scenario	2016	2040 Forecast	2040 Better Scenario	2040 Worse Scenario
Cyclist road injuries	15.56 (9.53 to 22.08)	24.19 (13.73 to 37.28)	17.26 (9.74 to 26.69)	26.12 (14.66 to 40.12)	0.32 (0.19 to 0.45)	0.53 (0.31 to 0.80)	0.41 (0.24 to 0.61)	0.56 (0.32 to 0.86)
Motorcyclist road injuries	111.64 (68.98 to 160.44)	154.64 (87.30 to 236.24)	123.95 (70.95 to 189.55)	166.70 (93.08 to 259.16)	2.02 (1.24 to 2.87)	2.99 (1.72 to 4.54)	2.46 (1.42 to 3.74)	3.20 (1.80 to 4.95)
Motor vehicle road injuries	703.82 (519.36 to 940.67)	991.67 (677.00 to 1,438.58)	676.97 (459.57 to 999.01)	1,080.02 (728.78 to 1,577.22)	13.77 (10.24 to 18.22)	20.97 (14.68 to 29.56)	15.22 (10.73 to 21.58)	22.32 (15.51 to 31.74)
Other road injuries	13.05 (7.19 to 19.45)	18.63 (9.62 to 29.92)	12.64 (6.58 to 20.25)	20.41 (10.46 to 33.55)	0.24 (0.13 to 0.35)	0.37 (0.20 to 0.57)	0.26 (0.15 to 0.41)	0.39 (0.21 to 0.62)
Other transport injuries	112.50 (65.36 to 166.94)	183.32 (95.46 to 291.21)	154.74 (82.02 to 246.51)	196.79 (101.59 to 316.50)	2.21 (1.28 to 3.25)	3.96 (2.17 to 6.21)	3.44 (1.90 to 5.37)	4.18 (2.25 to 6.68)
**Unintentional injuries**	**1,369.58 (1,065.88 to 1,716.30)**	**1,325.88 (896.18 to 1,836.32)**	**1,112.73 (757.40 to 1,531.76)**	**1,374.31 (939.38 to 1,892.36)**	**27.81 (21.47 to 35.08)**	**31.31 (22.74 to 41.09)**	**27.81 (20.32 to 36.49)**	**31.34 (22.62 to 41.40)**
Falls	115.28 (79.57 to 169.00)	143.84 (94.04 to 215.74)	136.22 (90.20 to 204.55)	141.22 (92.00 to 212.85)	3.39 (2.39 to 4.87)	4.76 (3.41 to 6.86)	4.88 (3.49 to 6.96)	4.42 (3.12 to 6.40)
Drowning	593.12 (442.54 to 767.09)	491.64 (317.42 to 704.22)	407.34 (259.36 to 581.02)	521.14 (335.64 to 754.57)	10.62 (7.91 to 13.78)	10.03 (6.59 to 14.27)	8.50 (5.55 to 12.03)	10.49 (6.81 to 15.07)
Fire, heat, and hot substances	85.70 (56.46 to 120.19)	72.06 (41.99 to 113.81)	56.67 (32.88 to 88.83)	76.44 (43.42 to 121.38)	1.74 (1.15 to 2.43)	1.69 (1.04 to 2.54)	1.41 (0.87 to 2.12)	1.72 (1.05 to 2.62)
Poisonings	60.41 (36.88 to 85.90)	38.58 (21.54 to 60.75)	27.21 (15.41 to 43.02)	41.89 (23.46 to 66.70)	1.21 (0.77 to 1.68)	0.90 (0.55 to 1.34)	0.68 (0.42 to 1.01)	0.93 (0.56 to 1.42)
Exposure to mechanical forces	160.15 (115.81 to 210.61)	164.38 (104.59 to 245.66)	130.65 (83.05 to 192.30)	169.97 (107.13 to 247.84)	3.17 (2.30 to 4.18)	3.68 (2.42 to 5.30)	3.06 (2.03 to 4.38)	3.70 (2.44 to 5.25)
Unintentional firearm injuries	24.45 (17.74 to 34.42)	19.98 (12.12 to 29.95)	15.18 (9.23 to 22.90)	20.76 (12.76 to 31.03)	0.51 (0.38 to 0.71)	0.48 (0.31 to 0.69)	0.38 (0.25 to 0.56)	0.48 (0.30 to 0.69)
Unintentional suffocation	35.13 (22.72 to 49.73)	33.42 (18.63 to 52.75)	25.79 (14.40 to 40.75)	35.27 (19.70 to 55.36)	0.56 (0.36 to 0.80)	0.59 (0.33 to 0.93)	0.46 (0.26 to 0.74)	0.61 (0.34 to 0.97)
Other exposure to mechanical forces	100.57 (67.74 to 139.22)	110.98 (65.57 to 167.20)	89.68 (53.69 to 135.68)	113.94 (67.17 to 168.71)	2.10 (1.43 to 2.88)	2.62 (1.69 to 3.77)	2.21 (1.43 to 3.16)	2.61 (1.65 to 3.77)
Adverse effects of medical treatment	140.25 (97.04 to 189.92)	182.92 (105.88 to 275.70)	153.73 (89.47 to 234.55)	186.43 (108.62 to 281.76)	3.23 (2.24 to 4.41)	4.70 (3.05 to 6.63)	4.20 (2.74 to 5.93)	4.62 (2.94 to 6.63)
Animal contact	8.99 (5.95 to 13.57)	15.46 (8.48 to 28.70)	11.86 (6.41 to 21.78)	15.95 (8.77 to 28.99)	0.22 (0.15 to 0.33)	0.42 (0.25 to 0.70)	0.34 (0.21 to 0.58)	0.41 (0.25 to 0.70)
Venomous animal contact	2.68 (1.57 to 4.78)	3.90 (1.74 to 8.33)	2.89 (1.32 to 6.15)	4.06 (1.83 to 8.77)	0.07 (0.04 to 0.11)	0.10 (0.05 to 0.20)	0.08 (0.04 to 0.16)	0.10 (0.05 to 0.20)
Non-venomous animal contact	6.31 (4.23 to 9.54)	11.55 (6.36 to 20.45)	8.97 (4.97 to 15.59)	11.90 (6.70 to 20.67)	0.16 (0.11 to 0.23)	0.32 (0.19 to 0.52)	0.26 (0.16 to 0.43)	0.31 (0.19 to 0.52)
Foreign body	88.25 (55.03 to 123.28)	96.11 (58.34 to 133.95)	93.69 (56.76 to 129.30)	96.76 (58.55 to 134.53)	1.77 (1.17 to 2.33)	2.34 (1.49 to 3.07)	2.47 (1.59 to 3.23)	2.24 (1.39 to 2.94)
Pulmonary aspiration and foreign body in airway	82.22 (49.78 to 116.26)	89.34 (53.06 to 123.80)	88.03 (52.63 to 121.14)	89.77 (53.08 to 125.15)	1.66 (1.06 to 2.22)	2.20 (1.38 to 2.88)	2.35 (1.49 to 3.06)	2.09 (1.29 to 2.76)

Foreign body in other body part	6.03 (3.40 to 9.84)	6.77 (3.11 to 13.13)	5.66 (2.58 to 11.01)	6.99 (3.23 to 13.41)	0.11 (0.06 to 0.18)	0.14 (0.07 to 0.27)	0.12 (0.06 to 0.23)	0.14 (0.07 to 0.27)
Environmental heat and cold exposure	37.29 (23.49 to 51.66)	48.42 (27.08 to 73.98)	34.46 (19.33 to 53.90)	50.55 (28.43 to 78.42)	0.85 (0.55 to 1.15)	1.20 (0.70 to 1.77)	0.89 (0.53 to 1.31)	1.22 (0.71 to 1.80)
Other unintentional injuries	80.14 (52.11 to 110.92)	72.48 (40.11 to 112.70)	60.90 (33.58 to 94.37)	73.96 (40.71 to 114.75)	1.60 (1.02 to 2.19)	1.59 (0.92 to 2.42)	1.37 (0.79 to 2.09)	1.59 (0.91 to 2.41)
**Self-harm and interpersonal violence**	**1,416.55 (1,012.05 to 1,855.21)**	**1,725.35 (1,093.30 to 2,557.95)**	**1,595.85 (1,032.88 to 2,364.71)**	**1,835.09 (1,148.33 to 2,809.84)**	**26.30 (18.59 to 34.54)**	**34.83 (22.53 to 51.08)**	**32.87 (21.53 to 48.16)**	**36.55 (22.93 to 55.51)**

Federated States of Micronesia								
Cause	YLLs (actual counts)				Deaths (actual counts)			
	2016	2040 Forecast	2040 Better Scenario	2040 Worse Scenario	2016	2040 Forecast	2040 Better Scenario	2040 Worse Scenario
Self-harm	1,040.08 (715.99 to 1,381.80)	1,336.16 (809.50 to 2,057.86)	1,221.97 (747.39 to 1,857.02)	1,436.47 (841.63 to 2,310.17)	19.33 (13.25 to 25.78)	26.92 (16.86 to 40.79)	25.17 (15.92 to 37.64)	28.52 (17.26 to 45.31)
Self-harm by firearm	66.96 (23.71 to 128.25)	78.19 (23.62 to 178.82)	69.46 (21.50 to 157.85)	85.02 (25.30 to 197.05)	1.28 (0.44 to 2.47)	1.64 (0.50 to 3.72)	1.48 (0.46 to 3.32)	1.77 (0.52 to 4.04)
Self-harm by other specified means	973.12 (681.50 to 1,288.46)	1,257.97 (772.43 to 1,899.29)	1,152.50 (709.67 to 1,729.81)	1,351.44 (807.40 to 2,142.11)	18.05 (12.41 to 23.77)	25.27 (15.82 to 37.39)	23.69 (15.09 to 34.76)	26.76 (16.34 to 41.62)
Interpersonal violence	376.46 (240.22 to 522.00)	389.19 (217.69 to 587.21)	373.89 (209.64 to 567.19)	398.63 (220.76 to 599.26)	6.97 (4.46 to 9.80)	7.91 (4.48 to 11.99)	7.71 (4.36 to 11.57)	8.03 (4.49 to 12.04)
Physical violence by firearm	84.65 (39.26 to 140.39)	88.27 (38.86 to 151.67)	86.04 (37.57 to 147.46)	88.11 (38.88 to 151.12)	1.54 (0.71 to 2.49)	1.76 (0.77 to 3.00)	1.73 (0.77 to 2.96)	1.75 (0.76 to 2.97)
Physical violence by sharp object	100.88 (58.56 to 152.85)	116.24 (60.29 to 188.98)	118.09 (61.16 to 193.90)	115.03 (59.92 to 187.41)	1.87 (1.08 to 2.82)	2.34 (1.20 to 3.78)	2.39 (1.24 to 3.84)	2.30 (1.18 to 3.71)
Physical violence by other means	190.93 (110.77 to 268.25)	184.68 (88.51 to 295.43)	169.77 (81.23 to 270.73)	195.48 (93.74 to 313.71)	3.57 (2.06 to 5.06)	3.81 (1.90 to 6.02)	3.58 (1.78 to 5.65)	3.98 (1.94 to 6.40)
**Forces of nature, conflict and terrorism, and executions and police conflict**	**0.77 (0.60 to 0.96)**	**28.59 (0.00 to 143.99)**	**27.46 (0.00 to 136.13)**	**29.05 (0.00 to 145.95)**	**0.01 (0.01 to 0.02)**	**0.56 (0.00 to 2.78)**	**0.56 (0.00 to 2.74)**	**0.56 (0.00 to 2.77)**
Exposure to forces of nature	0.00 (0.00 to 0.00)	28.59 (0.00 to 143.99)	27.46 (0.00 to 136.13)	29.05 (0.00 to 145.95)	0.00 (0.00 to 0.00)	0.56 (0.00 to 2.78)	0.56 (0.00 to 2.74)	0.56 (0.00 to 2.77)
Conflict and terrorism	0.00 (0.00 to 0.00)	0.00 (0.00 to 0.00)	0.00 (0.00 to 0.00)	0.00 (0.00 to 0.00)	0.00 (0.00 to 0.00)	0.00 (0.00 to 0.00)	0.00 (0.00 to 0.00)	0.00 (0.00 to 0.00)
Executions and police conflict	0.77 (0.60 to 0.96)	0.00 (0.00 to 0.00)	0.00 (0.00 to 0.00)	0.00 (0.00 to 0.00)	0.01 (0.01 to 0.02)	0.00 (0.00 to 0.00)	0.00 (0.00 to 0.00)	0.00 (0.00 to 0.00)

Fiji								
Cause	YLLs (actual counts)				Deaths (actual counts)			
	2016	2040 Forecast	2040 Better Scenario	2040 Worse Scenario	2016	2040 Forecast	2040 Better Scenario	2040 Worse Scenario
**All causes**	**250,826.89 (197,552.17 to 305,128.31)**	**283,134.24 (214,937.71 to 365,353.38)**	**204,615.87 (154,243.95 to 270,105.19)**	**342,300.83 (254,553.98 to 446,915.19)**	**8,320.37 (6,589.74 to 10,086.31)**	**11,030.82 (9,656.70 to 12,624.57)**	**8,788.68 (7,537.59 to 10,317.42)**	**12,639.36 (10,923.36 to 14,580.80)**
**Communicable, maternal, neonatal, and nutritional diseases**	**37,565.59 (27,692.07 to 50,196.36)**	**45,200.14 (25,142.23 to 86,490.36)**	**26,909.15 (16,146.65 to 47,678.30)**	**56,683.12 (30,397.28 to 111,539.12)**	**734.57 (578.28 to 925.49)**	**991.81 (679.01 to 1,582.93)**	**718.02 (526.10 to 1,044.53)**	**1,175.29 (771.61 to 1,942.65)**
**HIV/AIDS and tuberculosis**	**1,732.24 (1,332.98 to 2,195.42)**	**1,714.07 (1,167.11 to 2,457.23)**	**795.46 (489.52 to 1,248.33)**	**2,279.73 (1,564.04 to 3,154.62)**	**49.09 (38.16 to 62.19)**	**48.76 (33.94 to 68.51)**	**24.10 (15.42 to 36.55)**	**61.79 (43.73 to 85.24)**
Tuberculosis	1,210.80 (916.90 to 1,516.83)	869.61 (529.45 to 1,335.57)	424.65 (252.20 to 680.41)	1,041.62 (623.92 to 1,623.09)	38.39 (29.07 to 48.41)	31.57 (20.28 to 47.23)	16.52 (10.31 to 26.17)	36.14 (22.68 to 55.09)
HIV/AIDS	521.44 (264.79 to 890.58)	844.46 (451.38 to 1,552.55)	370.80 (146.47 to 789.24)	1,238.11 (725.24 to 2,029.97)	10.70 (5.23 to 19.12)	17.19 (8.72 to 32.68)	7.59 (2.68 to 17.70)	25.64 (14.39 to 43.05)
**Diarrhea, lower respiratory, and other common infectious diseases**	**19,662.11 (14,700.89 to 25,794.11)**	**23,289.88 (11,122.79 to 51,458.68)**	**14,274.52 (8,204.06 to 26,955.83)**	**31,414.39 (13,915.11 to 72,113.05)**	**455.46 (355.15 to 579.32)**	**641.96 (416.77 to 1,101.08)**	**490.65 (344.38 to 745.16)**	**783.92 (480.34 to 1,403.67)**
Diarrheal diseases	2,615.58 (1,606.75 to 3,843.04)	2,414.79 (462.99 to 9,389.45)	1,613.41 (393.69 to 5,314.07)	3,158.30 (508.78 to 12,825.86)	73.03 (43.18 to 107.19)	95.66 (22.23 to 286.23)	80.77 (21.76 to 229.32)	112.68 (22.95 to 355.62)
Intestinal infectious diseases	648.83 (293.93 to 1,206.69)	607.99 (196.63 to 1,525.43)	527.93 (184.11 to 1,302.17)	634.95 (200.11 to 1,554.74)	9.64 (4.48 to 17.87)	8.98 (3.18 to 21.03)	8.03 (3.07 to 18.50)	9.30 (3.26 to 21.99)
Typhoid fever	555.29 (246.56 to 1,069.89)	509.06 (161.87 to 1,264.94)	458.93 (154.00 to 1,135.97)	531.01 (164.65 to 1,337.25)	8.21 (3.70 to 15.56)	7.47 (2.61 to 17.71)	6.94 (2.55 to 16.00)	7.73 (2.61 to 18.48)
Paratyphoid fever	92.97 (36.89 to 192.77)	98.56 (29.58 to 264.97)	68.75 (20.78 to 179.54)	103.54 (30.26 to 264.31)	1.42 (0.57 to 2.91)	1.51 (0.50 to 3.69)	1.08 (0.35 to 2.65)	1.57 (0.51 to 3.77)
Other intestinal infectious diseases	0.57 (0.20 to 1.26)	0.37 (0.08 to 1.14)	0.24 (0.06 to 0.71)	0.40 (0.08 to 1.23)	0.01 (0.00 to 0.02)	0.01 (0.00 to 0.02)	0.01 (0.00 to 0.01)	0.01 (0.00 to 0.02)
Lower respiratory infections	12,739.10 (9,436.48 to 16,979.20)	14,295.88 (7,446.21 to 33,547.45)	9,242.87 (5,613.28 to 16,731.21)	18,754.07 (9,205.42 to 48,126.01)	319.72 (240.79 to 426.11)	453.10 (318.91 to 703.72)	355.48 (259.04 to 502.69)	543.78 (361.33 to 926.89)
Upper respiratory infections	12.13 (5.95 to 22.27)	12.89 (4.71 to 30.21)	7.14 (2.60 to 16.14)	14.19 (4.97 to 34.16)	0.28 (0.15 to 0.46)	0.33 (0.14 to 0.69)	0.20 (0.08 to 0.41)	0.35 (0.14 to 0.73)
Otitis media	0.41 (0.21 to 0.72)	0.09 (0.04 to 0.22)	0.09 (0.05 to 0.21)	0.09 (0.04 to 0.22)	0.01 (0.00 to 0.01)	0.00 (0.00 to 0.01)	0.00 (0.00 to 0.01)	0.00 (0.00 to 0.00)
Meningitis	1,587.99 (1,084.39 to 2,270.48)	1,231.13 (480.52 to 2,898.05)	803.14 (347.55 to 1,773.15)	1,542.25 (508.09 to 4,001.95)	24.80 (17.77 to 35.57)	20.35 (10.32 to 40.26)	14.65 (8.11 to 26.89)	23.63 (10.59 to 53.76)
Pneumococcal meningitis	4.23 (2.68 to 6.34)	2.78 (1.08 to 6.78)	2.31 (1.03 to 5.03)	3.29 (1.17 to 8.51)	0.07 (0.05 to 0.10)	0.05 (0.03 to 0.10)	0.05 (0.03 to 0.09)	0.06 (0.03 to 0.12)
H influenzae type B meningitis	157.62 (100.74 to 237.52)	311.64 (44.93 to 1,131.87)	157.98 (38.29 to 498.75)	593.76 (59.30 to 2,313.25)	2.39 (1.61 to 3.48)	4.10 (0.93 to 13.76)	2.36 (0.88 to 6.38)	7.36 (1.06 to 27.41)
Meningococcal meningitis	475.37 (328.76 to 703.28)	284.10 (131.32 to 589.91)	201.14 (97.27 to 403.48)	293.08 (131.41 to 624.43)	7.73 (5.50 to 11.12)	5.43 (3.27 to 9.46)	4.21 (2.57 to 7.02)	5.40 (3.17 to 9.67)
Other meningitis	950.77 (645.44 to 1,350.29)	632.61 (278.12 to 1,341.96)	441.72 (199.65 to 905.63)	652.11 (280.05 to 1,422.42)	14.61 (10.26 to 20.95)	10.77 (5.83 to 19.97)	8.04 (4.48 to 14.41)	10.82 (5.67 to 20.26)
Encephalitis	320.25 (238.60 to 424.56)	392.05 (216.38 to 685.34)	323.09 (184.93 to 544.48)	403.33 (222.15 to 717.30)	6.07 (4.56 to 7.81)	8.21 (5.47 to 12.17)	7.30 (5.02 to 10.47)	8.18 (5.47 to 12.19)

Diphtheria	3.09 (1.74 to 5.27)	1.12 (0.32 to 3.44)	0.59 (0.28 to 1.21)	2.26 (0.34 to 9.13)	0.05 (0.03 to 0.08)	0.02 (0.01 to 0.04)	0.01 (0.01 to 0.02)	0.03 (0.01 to 0.11)
Whooping cough	272.62 (19.38 to 974.03)	432.53 (5.93 to 2,206.33)	65.45 (2.53 to 318.97)	643.43 (6.03 to 3,335.29)	3.25 (0.23 to 11.59)	5.13 (0.08 to 26.14)	0.80 (0.03 to 3.84)	7.61 (0.08 to 39.62)
Tetanus	5.80 (4.53 to 7.16)	0.96 (0.48 to 1.89)	0.77 (0.39 to 1.52)	1.14 (0.54 to 2.27)	0.10 (0.08 to 0.12)	0.02 (0.01 to 0.03)	0.01 (0.01 to 0.02)	0.02 (0.01 to 0.03)
Measles	1,349.25 (426.64 to 3,122.35)	3,799.60 (118.43 to 22,754.68)	1,628.84 (82.61 to 9,086.89)	6,151.10 (129.48 to 34,639.72)	16.29 (5.17 to 37.36)	47.79 (1.84 to 275.74)	21.66 (1.33 to 109.72)	75.96 (2.38 to 409.02)

Fiji								
Cause	YLLs (actual counts)				Deaths (actual counts)			
	2016	2040 Forecast	2040 Better Scenario	2040 Worse Scenario	2016	2040 Forecast	2040 Better Scenario	2040 Worse Scenario
Varicella and herpes zoster	107.05 (62.98 to 183.44)	100.86 (30.31 to 305.48)	61.20 (20.77 to 173.27)	109.28 (30.29 to 342.64)	2.22 (1.51 to 3.21)	2.36 (1.18 to 4.99)	1.73 (0.89 to 3.26)	2.37 (1.11 to 5.27)
**Neglected tropical diseases and malaria**	**173.28 (102.65 to 259.54)**	**873.07 (436.42 to 1,706.55)**	**786.88 (413.67 to 1,488.66)**	**875.04 (424.31 to 1,734.14)**	**4.58 (2.98 to 6.40)**	**25.72 (16.27 to 39.75)**	**26.09 (17.06 to 39.18)**	**24.13 (15.01 to 38.10)**
Malaria	0.00 (0.00 to 0.00)	0.00 (0.00 to 0.00)	0.00 (0.00 to 0.00)	0.00 (0.00 to 0.00)	0.00 (0.00 to 0.00)	0.00 (0.00 to 0.00)	0.00 (0.00 to 0.00)	0.00 (0.00 to 0.00)
Chagas disease	0.00 (0.00 to 0.00)	0.00 (0.00 to 0.00)	0.00 (0.00 to 0.00)	0.00 (0.00 to 0.00)	0.00 (0.00 to 0.00)	0.00 (0.00 to 0.00)	0.00 (0.00 to 0.00)	0.00 (0.00 to 0.00)
Leishmaniasis	0.00 (0.00 to 0.00)	0.00 (0.00 to 0.00)	0.00 (0.00 to 0.00)	0.00 (0.00 to 0.00)	0.00 (0.00 to 0.00)	0.00 (0.00 to 0.00)	0.00 (0.00 to 0.00)	0.00 (0.00 to 0.00)
African trypanosomiasis	0.00 (0.00 to 0.00)	0.00 (0.00 to 0.00)	0.00 (0.00 to 0.00)	0.00 (0.00 to 0.00)	0.00 (0.00 to 0.00)	0.00 (0.00 to 0.00)	0.00 (0.00 to 0.00)	0.00 (0.00 to 0.00)
Schistosomiasis	0.00 (0.00 to 0.00)	0.00 (0.00 to 0.00)	0.00 (0.00 to 0.00)	0.00 (0.00 to 0.00)	0.00 (0.00 to 0.00)	0.00 (0.00 to 0.00)	0.00 (0.00 to 0.00)	0.00 (0.00 to 0.00)
Cysticercosis	0.00 (0.00 to 0.00)	0.00 (0.00 to 0.00)	0.00 (0.00 to 0.00)	0.00 (0.00 to 0.00)	0.00 (0.00 to 0.00)	0.00 (0.00 to 0.00)	0.00 (0.00 to 0.00)	0.00 (0.00 to 0.00)
Cystic echinococcosis	0.45 (0.33 to 0.60)	0.43 (0.27 to 0.63)	0.26 (0.17 to 0.39)	0.47 (0.30 to 0.68)	0.01 (0.01 to 0.02)	0.01 (0.01 to 0.02)	0.01 (0.01 to 0.01)	0.02 (0.01 to 0.02)
Dengue	64.22 (25.52 to 127.37)	523.72 (240.13 to 1,021.73)	497.61 (237.55 to 939.07)	514.40 (234.19 to 994.28)	1.64 (0.68 to 3.45)	16.19 (8.93 to 28.39)	17.10 (9.54 to 29.11)	14.94 (8.01 to 25.95)
Yellow fever	0.00 (0.00 to 0.00)	0.00 (0.00 to 0.00)	0.00 (0.00 to 0.00)	0.00 (0.00 to 0.00)	0.00 (0.00 to 0.00)	0.00 (0.00 to 0.00)	0.00 (0.00 to 0.00)	0.00 (0.00 to 0.00)
Rabies	1.05 (0.35 to 1.78)	1.45 (0.33 to 3.16)	0.85 (0.19 to 1.75)	1.66 (0.36 to 3.67)	0.05 (0.01 to 0.10)	0.08 (0.01 to 0.16)	0.05 (0.01 to 0.11)	0.08 (0.01 to 0.17)
Intestinal nematode infections	38.66 (10.07 to 91.16)	173.02 (21.90 to 627.89)	144.73 (21.37 to 524.59)	178.10 (21.63 to 639.95)	0.58 (0.22 to 1.21)	2.41 (0.56 to 7.89)	2.12 (0.56 to 6.70)	2.44 (0.53 to 7.91)
Other neglected tropical diseases	68.90 (40.54 to 108.73)	174.45 (76.14 to 338.09)	143.43 (66.02 to 257.35)	180.42 (79.64 to 359.68)	2.29 (1.39 to 3.25)	7.04 (3.14 to 11.36)	6.82 (3.04 to 11.14)	6.66 (3.03 to 10.66)
**Maternal disorders**	**734.56 (435.11 to 1,181.07)**	**453.03 (228.02 to 853.41)**	**287.40 (138.55 to 552.62)**	**519.58 (251.96 to 1,004.11)**	**13.74 (8.14 to 22.13)**	**8.57 (4.29 to 15.67)**	**5.45 (2.65 to 10.15)**	**9.81 (4.85 to 18.62)**
Maternal hemorrhage	116.09 (57.99 to 212.55)	83.71 (35.00 to 175.75)	53.69 (22.88 to 112.46)	95.66 (40.04 to 203.11)	2.14 (1.08 to 3.93)	1.56 (0.66 to 3.27)	1.00 (0.43 to 2.08)	1.78 (0.76 to 3.70)
Maternal sepsis and other maternal infections	64.40 (34.05 to 114.41)	41.99 (18.32 to 85.80)	26.71 (11.55 to 55.32)	48.11 (20.75 to 100.23)	1.22 (0.65 to 2.18)	0.81 (0.35 to 1.64)	0.51 (0.22 to 1.05)	0.92 (0.41 to 1.90)
Maternal hypertensive disorders	54.45 (28.01 to 95.84)	43.52 (19.28 to 85.64)	27.52 (11.62 to 56.15)	49.85 (21.05 to 102.33)	1.00 (0.52 to 1.75)	0.81 (0.36 to 1.64)	0.51 (0.22 to 1.06)	0.93 (0.40 to 1.90)
Maternal obstructed labor and uterine rupture	31.71 (15.47 to 59.78)	24.35 (9.46 to 51.26)	14.86 (5.54 to 32.40)	28.15 (10.75 to 61.31)	0.59 (0.29 to 1.10)	0.46 (0.18 to 0.97)	0.28 (0.10 to 0.60)	0.53 (0.21 to 1.15)
Maternal abortion, miscarriage, and ectopic pregnancy	358.95 (210.84 to 594.73)	173.13 (83.20 to 336.08)	109.28 (51.64 to 216.51)	198.63 (95.75 to 397.73)	6.79 (3.97 to 11.29)	3.33 (1.64 to 6.43)	2.11 (0.98 to 4.12)	3.82 (1.86 to 7.49)

Indirect maternal deaths	38.60 (20.20 to 70.55)	30.87 (13.81 to 66.45)	19.29 (8.54 to 42.18)	35.63 (15.81 to 77.23)	0.70 (0.37 to 1.31)	0.56 (0.25 to 1.17)	0.35 (0.16 to 0.74)	0.65 (0.30 to 1.37)
Late maternal deaths	7.64 (2.78 to 16.66)	5.03 (1.66 to 11.95)	3.13 (0.98 to 8.02)	5.80 (1.79 to 14.22)	0.14 (0.05 to 0.30)	0.09 (0.03 to 0.22)	0.06 (0.02 to 0.15)	0.11 (0.03 to 0.26)
Maternal deaths aggravated by HIV/AIDS	0.38 (0.14 to 0.82)	1.42 (0.28 to 4.54)	0.71 (0.04 to 2.78)	1.70 (0.37 to 5.37)	0.01 (0.00 to 0.02)	0.03 (0.01 to 0.08)	0.01 (0.00 to 0.05)	0.03 (0.01 to 0.09)
Other maternal disorders	62.34 (32.79 to 112.95)	49.01 (22.02 to 99.69)	32.20 (13.57 to 66.69)	56.05 (23.95 to 114.55)	1.15 (0.60 to 2.09)	0.91 (0.42 to 1.80)	0.60 (0.26 to 1.26)	1.04 (0.45 to 2.12)

Fiji								
Cause	YLLs (actual counts)				Deaths (actual counts)			
	2016	2040 Forecast	2040 Better Scenario	2040 Worse Scenario	2016	2040 Forecast	2040 Better Scenario	2040 Worse Scenario
**Neonatal disorders**	**11,994.64 (7,394.67 to 18,286.63)**	**15,633.28 (6,909.97 to 30,448.27)**	**8,175.19 (3,535.51 to 16,328.85)**	**18,196.04 (8,079.83 to 35,553.15)**	**138.63 (85.45 to 211.45)**	**180.89 (79.95 to 352.59)**	**94.62 (40.92 to 188.70)**	**210.52 (93.62 to 411.62)**
Neonatal preterm birth	6,087.05 (3,557.55 to 9,524.77)	6,789.23 (3,178.00 to 13,388.99)	3,021.68 (1,355.77 to 6,057.42)	7,747.23 (3,597.67 to 15,104.94)	70.33 (41.10 to 110.08)	78.48 (36.75 to 154.86)	34.94 (15.67 to 70.09)	89.55 (41.61 to 174.54)
Neonatal encephalopathy due to birth asphyxia and trauma	3,160.06 (1,897.96 to 5,025.99)	4,004.69 (1,641.23 to 8,736.56)	2,270.44 (906.93 to 4,912.91)	4,707.61 (1,905.86 to 10,263.07)	36.53 (21.94 to 58.10)	46.37 (19.02 to 100.96)	26.29 (10.49 to 56.85)	54.50 (22.12 to 119.22)
Neonatal sepsis and other neonatal infections	1,518.80 (817.36 to 2,626.19)	2,842.32 (1,091.90 to 6,021.06)	1,663.26 (662.97 to 3,511.67)	3,353.95 (1,335.10 to 7,008.25)	17.56 (9.45 to 30.36)	32.91 (12.65 to 69.67)	19.26 (7.68 to 40.65)	38.83 (15.45 to 81.03)
Hemolytic disease and other neonatal jaundice	87.58 (44.38 to 159.95)	58.35 (18.04 to 150.00)	31.76 (9.84 to 82.96)	71.16 (21.73 to 189.39)	1.01 (0.51 to 1.85)	0.67 (0.21 to 1.73)	0.37 (0.11 to 0.96)	0.82 (0.25 to 2.19)
Other neonatal disorders	1,141.16 (634.81 to 1,895.41)	1,938.69 (710.66 to 4,243.76)	1,188.06 (429.10 to 2,628.42)	2,316.09 (838.88 to 5,150.83)	13.20 (7.34 to 21.91)	22.45 (8.25 to 49.13)	13.76 (4.96 to 30.46)	26.82 (9.75 to 59.67)
**Nutritional deficiencies**	**662.86 (413.32 to 1,099.95)**	**559.43 (247.16 to 1,304.49)**	**437.40 (219.97 to 971.46)**	**599.75 (244.92 to 1,440.91)**	**15.35 (11.76 to 20.62)**	**17.81 (12.86 to 27.37)**	**17.53 (13.24 to 24.33)**	**17.07 (11.65 to 27.72)**
Protein-energy malnutrition	628.38 (382.64 to 1,058.68)	509.08 (212.75 to 1,229.72)	399.34 (192.20 to 902.43)	546.47 (213.89 to 1,378.03)	14.42 (11.01 to 19.58)	16.20 (11.31 to 25.53)	16.11 (11.97 to 22.72)	15.50 (10.34 to 25.63)
Iodine deficiency	9.40 (0.61 to 37.64)	24.37 (0.83 to 134.78)	17.34 (0.54 to 87.47)	26.55 (0.84 to 153.30)	0.23 (0.02 to 0.71)	0.74 (0.02 to 2.87)	0.64 (0.01 to 2.58)	0.73 (0.02 to 2.73)
Dietary iron deficiency	5.03 (3.66 to 6.56)	5.69 (2.94 to 11.31)	4.76 (2.54 to 9.15)	5.99 (3.03 to 11.98)	0.09 (0.07 to 0.12)	0.10 (0.06 to 0.17)	0.09 (0.06 to 0.15)	0.10 (0.06 to 0.18)
Other nutritional deficiencies	20.06 (13.79 to 28.67)	20.29 (12.74 to 33.25)	15.97 (10.40 to 24.76)	20.72 (12.88 to 34.12)	0.61 (0.44 to 0.84)	0.77 (0.54 to 1.08)	0.69 (0.48 to 0.96)	0.73 (0.51 to 1.03)
**Other communicable, maternal, neonatal, and nutritional diseases**	**2,605.90 (1,964.51 to 3,387.78)**	**2,677.37 (1,762.02 to 4,045.40)**	**2,152.31 (1,400.16 to 3,183.14)**	**2,798.59 (1,793.23 to 4,216.38)**	**57.74 (43.97 to 72.92)**	**68.11 (51.78 to 89.21)**	**59.58 (46.00 to 77.26)**	**68.05 (51.37 to 89.58)**
Sexually transmitted infections excluding HIV	351.44 (137.68 to 737.29)	233.69 (75.69 to 644.88)	164.77 (59.72 to 426.91)	251.80 (79.28 to 710.53)	5.58 (2.78 to 10.33)	3.77 (1.67 to 8.67)	2.84 (1.37 to 6.07)	3.99 (1.68 to 9.39)
Syphilis	242.90 (38.54 to 631.91)	162.72 (22.22 to 571.69)	103.19 (15.94 to 361.35)	178.46 (23.63 to 632.74)	3.15 (0.74 to 7.67)	2.12 (0.44 to 6.90)	1.39 (0.34 to 4.39)	2.31 (0.45 to 7.63)
Chlamydial infection	21.31 (12.82 to 33.58)	14.00 (7.90 to 24.00)	12.10 (6.68 to 20.61)	14.48 (8.01 to 24.21)	0.47 (0.29 to 0.75)	0.32 (0.19 to 0.53)	0.28 (0.17 to 0.46)	0.33 (0.19 to 0.54)
Gonococcal infection	55.87 (33.52 to 89.60)	36.47 (20.30 to 60.65)	31.76 (17.43 to 52.96)	37.64 (20.68 to 62.91)	1.26 (0.77 to 2.01)	0.86 (0.51 to 1.39)	0.76 (0.45 to 1.22)	0.88 (0.52 to 1.42)
Other sexually transmitted infections	31.37 (18.81 to 50.11)	20.51 (11.55 to 34.73)	17.72 (9.82 to 30.30)	21.22 (11.86 to 36.13)	0.70 (0.42 to 1.11)	0.47 (0.27 to 0.78)	0.41 (0.24 to 0.68)	0.48 (0.28 to 0.79)
Acute hepatitis	340.83 (245.38 to 460.45)	258.55 (167.46 to 382.31)	196.43 (119.75 to 292.83)	263.47 (167.44 to 383.29)	8.43 (6.03 to 11.40)	7.14 (4.83 to 10.05)	5.59 (3.57 to 8.08)	7.11 (4.74 to 9.91)
Acute hepatitis A	15.38 (9.60 to 23.01)	9.14 (3.94 to 19.92)	6.44 (2.85 to 13.64)	9.73 (4.10 to 21.63)	0.26 (0.17 to 0.36)	0.17 (0.09 to 0.31)	0.13 (0.07 to 0.23)	0.18 (0.09 to 0.33)
Acute hepatitis B	271.40 (187.97 to 376.56)	211.80 (135.55 to 313.49)	162.24 (96.27 to 249.57)	215.02 (135.80 to 314.99)	7.13 (4.97 to 9.88)	6.08 (4.00 to 8.66)	4.75 (3.00 to 7.07)	6.06 (3.95 to 8.60)

Acute hepatitis C	6.77 (4.42 to 10.06)	5.88 (3.43 to 9.36)	4.80 (2.68 to 7.79)	5.80 (3.39 to 9.25)	0.18 (0.12 to 0.28)	0.18 (0.11 to 0.28)	0.15 (0.09 to 0.24)	0.17 (0.10 to 0.26)
Acute hepatitis E	47.27 (31.26 to 71.84)	31.73 (15.28 to 62.96)	22.96 (11.35 to 44.42)	32.92 (15.55 to 65.90)	0.87 (0.57 to 1.29)	0.71 (0.40 to 1.23)	0.56 (0.31 to 0.97)	0.71 (0.39 to 1.23)
Other unspecified infectious diseases	1,913.62 (1,407.44 to 2,471.48)	2,185.13 (1,437.10 to 3,332.86)	1,791.10 (1,186.54 to 2,640.39)	2,283.31 (1,493.20 to 3,436.73)	43.73 (32.98 to 55.87)	57.20 (44.00 to 74.85)	51.15 (39.59 to 66.50)	56.95 (43.52 to 74.25)
**Non-communicable diseases**	**187,511.23 (145,269.43 to 230,853.97)**	**214,547.39 (166,933.48 to 271,570.14)**	**161,421.01 (122,538.66 to 210,212.76)**	**258,517.01 (199,865.39 to 329,958.37)**	**7,081.82 (5,601.16 to 8,602.85)**	**9,538.47 (8,319.53 to 10,949.94)**	**7,697.77 (6,590.09 to 9,121.79)**	**10,907.79 (9,434.55 to 12,624.31)**

Fiji								
Cause	YLLs (actual counts)				Deaths (actual counts)			
	2016	2040 Forecast	2040 Better Scenario	2040 Worse Scenario	2016	2040 Forecast	2040 Better Scenario	2040 Worse Scenario
**Neoplasms**	**23,541.26 (17,649.08 to 30,175.03)**	**29,863.46 (22,015.15 to 40,043.55)**	**27,637.91 (20,489.51 to 37,276.29)**	**31,180.99 (22,812.84 to 41,852.04)**	**791.38 (604.84 to 1,003.39)**	**1,163.57 (915.56 to 1,487.90)**	**1,141.10 (899.73 to 1,446.28)**	**1,164.88 (908.66 to 1,496.37)**
Lip and oral cavity cancer	490.37 (370.84 to 627.08)	676.94 (407.06 to 1,042.95)	509.38 (331.33 to 738.08)	819.49 (460.40 to 1,313.01)	18.11 (13.94 to 22.73)	27.95 (18.64 to 41.30)	22.73 (16.11 to 31.64)	31.68 (19.90 to 48.43)
Nasopharynx cancer	123.61 (90.45 to 162.55)	185.85 (107.12 to 295.89)	123.73 (75.13 to 190.97)	237.26 (128.68 to 404.27)	4.00 (2.96 to 5.19)	6.43 (3.90 to 10.04)	4.50 (2.91 to 6.65)	7.84 (4.51 to 13.04)
Other pharynx cancer	248.22 (181.38 to 329.16)	260.53 (172.14 to 385.89)	226.27 (154.65 to 322.43)	269.14 (175.19 to 405.33)	8.76 (6.55 to 11.38)	10.58 (7.41 to 14.99)	9.64 (7.02 to 13.12)	10.60 (7.24 to 15.55)
Esophageal cancer	427.18 (313.06 to 554.17)	502.82 (300.69 to 804.58)	420.02 (258.45 to 661.88)	602.43 (339.16 to 996.38)	16.73 (12.51 to 21.31)	21.75 (13.80 to 33.41)	19.26 (12.58 to 29.04)	24.63 (14.64 to 38.48)
Stomach cancer	1,044.96 (791.04 to 1,337.16)	921.76 (717.14 to 1,142.91)	810.65 (613.86 to 1,027.65)	917.59 (711.19 to 1,155.27)	40.06 (31.18 to 50.01)	42.03 (36.12 to 49.33)	38.82 (32.12 to 46.45)	40.11 (34.02 to 47.62)
Colon and rectum cancer	1,593.07 (1,212.15 to 2,045.77)	2,290.96 (1,421.10 to 3,645.17)	2,141.17 (1,334.41 to 3,471.74)	2,263.84 (1,375.72 to 3,660.33)	62.42 (48.84 to 77.90)	106.15 (67.91 to 167.86)	104.63 (67.82 to 163.66)	100.49 (64.15 to 159.18)
Liver cancer	1,779.59 (1,262.37 to 2,348.29)	2,277.30 (1,454.88 to 3,461.60)	2,195.57 (1,454.91 to 3,214.82)	2,608.10 (1,589.73 to 4,196.87)	63.82 (45.30 to 82.25)	92.51 (62.23 to 134.48)	93.26 (63.69 to 132.41)	100.86 (63.50 to 154.16)
Liver cancer due to hepatitis B	937.88 (647.00 to 1,256.32)	1,147.37 (729.06 to 1,777.89)	1,111.53 (717.21 to 1,662.91)	1,314.31 (785.99 to 2,173.42)	31.81 (22.39 to 42.52)	43.78 (28.93 to 64.30)	44.16 (29.88 to 64.20)	47.92 (29.96 to 74.18)
Liver cancer due to hepatitis C	273.92 (183.00 to 366.27)	375.82 (239.48 to 556.37)	375.87 (248.06 to 539.28)	411.73 (252.09 to 637.91)	11.56 (7.88 to 15.31)	18.34 (12.00 to 26.30)	19.19 (12.76 to 27.10)	19.14 (11.96 to 28.72)
Liver cancer due to alcohol use	143.44 (94.14 to 199.34)	193.56 (115.34 to 307.41)	168.47 (102.93 to 257.66)	229.93 (129.79 to 379.02)	5.49 (3.62 to 7.49)	8.03 (4.91 to 12.43)	7.40 (4.76 to 10.94)	9.07 (5.37 to 14.50)
Liver cancer due to other causes	424.34 (280.63 to 590.80)	560.55 (309.93 to 987.91)	539.70 (301.66 to 926.09)	652.13 (337.19 to 1,207.07)	14.95 (10.22 to 20.48)	22.36 (12.79 to 38.83)	22.52 (13.17 to 38.28)	24.72 (13.42 to 44.47)
Gallbladder and biliary tract cancer	212.88 (152.38 to 279.30)	189.34 (143.92 to 234.07)	198.84 (149.07 to 249.58)	175.44 (135.78 to 215.08)	8.65 (6.26 to 11.09)	8.93 (7.15 to 10.47)	9.77 (7.80 to 11.50)	8.01 (6.37 to 9.33)
Pancreatic cancer	611.08 (463.76 to 780.85)	1,000.59 (661.37 to 1,514.54)	1,073.04 (711.62 to 1,652.65)	970.88 (641.48 to 1,469.90)	25.18 (19.41 to 31.66)	46.27 (31.64 to 68.87)	51.55 (35.71 to 76.38)	43.22 (29.35 to 65.10)
Larynx cancer	216.15 (155.69 to 281.36)	235.38 (130.93 to 427.57)	162.57 (99.50 to 271.77)	323.36 (156.46 to 635.01)	8.46 (6.25 to 10.87)	10.27 (6.22 to 17.77)	7.61 (5.08 to 11.77)	13.22 (6.94 to 25.17)
Tracheal, bronchus, and lung cancer	1,297.11 (974.63 to 1,645.49)	1,697.56 (1,023.27 to 2,784.61)	1,463.20 (907.13 to 2,312.55)	2,402.23 (1,316.92 to 4,047.33)	51.95 (39.65 to 65.08)	76.90 (48.03 to 123.53)	69.61 (44.72 to 106.49)	102.44 (60.25 to 167.10)
Malignant skin melanoma	121.72 (80.43 to 230.98)	165.68 (117.26 to 300.95)	200.87 (140.41 to 366.42)	150.23 (107.91 to 271.91)	4.50 (3.04 to 8.48)	7.30 (5.55 to 13.18)	9.33 (7.04 to 17.06)	6.35 (4.82 to 11.39)
Non-melanoma skin cancer	99.20 (70.87 to 134.10)	152.32 (123.55 to 183.86)	139.61 (109.71 to 171.46)	142.59 (116.42 to 171.66)	4.09 (3.06 to 5.33)	7.89 (6.70 to 9.25)	7.75 (6.43 to 9.24)	7.01 (5.88 to 8.33)
Breast cancer	3,946.18 (2,131.15 to 5,844.02)	4,854.14 (2,383.99 to 8,508.74)	4,487.10 (2,187.77 to 7,862.62)	4,923.85 (2,354.14 to 8,762.61)	130.29 (70.97 to 188.39)	191.42 (94.97 to 325.50)	184.43 (90.97 to 313.75)	188.14 (90.19 to 323.46)
Cervical cancer	2,821.01 (1,595.51 to 4,229.77)	2,599.82 (1,399.20 to 3,971.72)	2,198.41 (1,185.01 to 3,456.50)	2,659.31 (1,405.81 to 4,141.45)	88.28 (50.35 to 129.18)	90.91 (50.73 to 129.33)	79.24 (44.90 to 114.33)	90.81 (51.13 to 131.11)

Uterine cancer	592.82 (393.20 to 840.13)	734.80 (505.46 to 993.84)	651.76 (437.30 to 895.69)	729.51 (504.71 to 983.19)	22.06 (14.75 to 31.56)	31.25 (23.07 to 39.67)	28.52 (20.81 to 37.16)	30.41 (22.74 to 38.10)
Ovarian cancer	318.30 (210.05 to 457.55)	451.44 (251.04 to 775.91)	455.29 (255.05 to 782.68)	449.74 (248.40 to 781.83)	10.37 (6.94 to 15.04)	16.68 (9.90 to 28.16)	17.27 (10.19 to 29.42)	16.29 (9.47 to 27.38)
Prostate cancer	737.65 (279.43 to 1,127.78)	1,424.00 (608.43 to 1,910.94)	1,538.19 (669.67 to 2,063.69)	1,228.85 (510.23 to 1,685.35)	41.46 (16.40 to 62.32)	90.97 (39.38 to 124.22)	102.34 (45.59 to 137.07)	75.77 (31.69 to 107.07)
Testicular cancer	146.52 (92.41 to 216.43)	121.27 (77.43 to 187.37)	108.42 (68.24 to 164.28)	119.25 (76.47 to 185.54)	3.54 (2.29 to 5.07)	3.42 (2.48 to 4.67)	3.20 (2.27 to 4.36)	3.22 (2.35 to 4.40)

Fiji								
Cause	YLLs (actual counts)				Deaths (actual counts)			
	2016	2040 Forecast	2040 Better Scenario	2040 Worse Scenario	2016	2040 Forecast	2040 Better Scenario	2040 Worse Scenario
Kidney cancer	144.61 (109.91 to 183.39)	238.91 (176.16 to 315.40)	257.10 (191.03 to 338.77)	229.58 (169.37 to 305.01)	4.79 (3.66 to 6.03)	9.03 (7.33 to 11.17)	10.43 (8.48 to 12.83)	8.25 (6.70 to 10.39)
Bladder cancer	268.36 (201.57 to 337.89)	326.60 (204.33 to 524.27)	320.08 (202.64 to 504.31)	325.68 (199.72 to 517.49)	11.64 (8.99 to 14.45)	16.33 (10.70 to 25.41)	16.77 (11.11 to 25.80)	15.55 (10.03 to 24.44)
Brain and nervous system cancer	529.71 (364.34 to 708.69)	858.25 (534.92 to 1,353.13)	786.04 (502.43 to 1,205.24)	849.03 (524.80 to 1,359.62)	12.72 (9.17 to 16.83)	21.76 (15.42 to 29.55)	21.74 (15.80 to 28.71)	20.58 (14.53 to 28.28)
Thyroid cancer	231.00 (137.37 to 306.78)	302.23 (193.09 to 412.90)	263.90 (169.99 to 364.71)	321.68 (201.86 to 447.97)	8.96 (5.39 to 11.85)	13.37 (8.62 to 17.84)	12.16 (8.10 to 16.14)	13.76 (8.75 to 18.76)
Mesothelioma	65.06 (44.82 to 88.24)	76.28 (54.93 to 97.55)	79.00 (56.49 to 102.93)	71.85 (52.44 to 90.27)	2.27 (1.57 to 3.06)	2.89 (2.20 to 3.51)	3.10 (2.34 to 3.81)	2.65 (2.03 to 3.18)
Hodgkin lymphoma	146.73 (86.72 to 206.08)	119.25 (69.62 to 174.35)	90.38 (52.03 to 135.39)	123.23 (71.94 to 182.82)	3.42 (2.14 to 4.62)	3.19 (2.12 to 4.22)	2.57 (1.72 to 3.52)	3.16 (2.11 to 4.25)
Non-Hodgkin lymphoma	672.65 (474.29 to 861.77)	871.38 (588.99 to 1,208.95)	820.05 (558.96 to 1,133.67)	870.09 (583.65 to 1,220.56)	18.87 (13.79 to 23.93)	28.12 (21.44 to 35.00)	28.55 (22.07 to 35.31)	26.86 (20.63 to 33.69)
Multiple myeloma	255.48 (169.59 to 344.12)	365.54 (245.17 to 493.16)	377.78 (252.37 to 504.99)	358.30 (234.96 to 493.77)	9.67 (6.48 to 12.75)	15.83 (11.34 to 20.42)	17.03 (12.37 to 21.73)	14.99 (10.43 to 19.54)
Leukemia	2,148.42 (1,629.35 to 2,735.48)	2,966.48 (1,905.50 to 4,491.69)	2,689.28 (1,771.38 to 3,958.20)	3,094.04 (1,984.30 to 4,683.14)	48.39 (37.15 to 61.07)	75.78 (57.07 to 99.93)	74.57 (56.81 to 97.49)	76.00 (56.11 to 101.32)
Acute lymphoid leukemia	329.72 (234.04 to 453.07)	473.63 (240.40 to 915.44)	398.72 (212.82 to 757.74)	492.58 (250.57 to 946.62)	5.93 (4.32 to 7.97)	8.65 (5.38 to 14.77)	7.79 (5.00 to 12.78)	8.86 (5.50 to 15.00)
Chronic lymphoid leukemia	26.41 (18.44 to 37.49)	35.07 (25.17 to 49.71)	35.60 (25.37 to 49.38)	34.94 (24.68 to 49.96)	1.02 (0.73 to 1.44)	1.61 (1.17 to 2.17)	1.74 (1.27 to 2.36)	1.51 (1.10 to 2.08)
Acute myeloid leukemia	1,177.88 (886.04 to 1,494.79)	1,782.32 (1,142.96 to 2,674.03)	1,706.75 (1,104.53 to 2,488.27)	1,845.55 (1,197.92 to 2,775.74)	26.07 (19.75 to 32.91)	44.54 (33.49 to 58.15)	46.32 (35.34 to 59.90)	44.39 (32.73 to 59.30)
Chronic myeloid leukemia	121.18 (86.64 to 161.29)	105.45 (70.49 to 152.89)	88.72 (58.14 to 130.47)	111.37 (73.36 to 161.10)	3.85 (2.80 to 5.06)	3.86 (2.72 to 5.34)	3.36 (2.32 to 4.75)	3.93 (2.75 to 5.57)
Other leukemia	493.23 (357.55 to 646.03)	570.00 (360.47 to 853.87)	459.49 (291.14 to 680.94)	609.59 (379.66 to 921.96)	11.53 (8.68 to 14.76)	17.12 (12.77 to 22.92)	15.36 (11.34 to 20.60)	17.30 (12.49 to 23.20)
Other neoplasms	2,251.62 (1,586.84 to 2,823.62)	2,996.04 (1,969.07 to 4,318.54)	2,850.19 (1,938.78 to 3,976.15)	2,944.43 (1,895.29 to 4,276.69)	57.92 (42.07 to 73.55)	87.64 (66.77 to 108.07)	90.72 (70.31 to 111.08)	81.96 (62.08 to 101.60)
**Cardiovascular diseases**	**71,651.27 (54,394.03 to 89,689.67)**	**67,146.86 (48,227.97 to 94,053.55)**	**43,569.96 (30,897.79 to 60,725.73)**	**95,250.14 (63,685.61 to 137,731.85)**	**2,754.23 (2,150.61 to 3,381.47)**	**3,055.27 (2,447.05 to 3,926.87)**	**2,152.40 (1,700.08 to 2,720.34)**	**4,032.64 (3,042.09 to 5,397.19)**
Rheumatic heart disease	3,558.60 (2,736.46 to 4,464.29)	2,047.62 (1,501.01 to 2,786.65)	1,553.10 (1,099.70 to 2,193.42)	2,110.48 (1,538.16 to 2,947.64)	98.81 (76.67 to 123.55)	74.49 (63.08 to 88.63)	61.32 (50.24 to 74.24)	72.54 (60.91 to 87.83)
Ischemic heart disease	38,797.20 (28,295.04 to 49,982.37)	32,117.96 (17,847.21 to 55,390.56)	18,163.50 (10,284.78 to 31,593.89)	53,194.62 (28,929.80 to 88,795.29)	1,515.00 (1,153.19 to 1,895.93)	1,445.70 (907.14 to 2,275.72)	892.45 (565.14 to 1,428.00)	2,202.36 (1,344.23 to 3,488.81)
Stroke	14,003.41 (10,862.45 to 17,578.47)	11,181.98 (6,718.17 to 17,612.80)	6,202.11 (3,821.12 to 9,895.19)	17,061.07 (10,109.05 to 27,782.76)	568.83 (449.33 to 709.67)	543.55 (366.59 to 813.31)	332.35 (224.05 to 535.85)	773.07 (507.48 to 1,196.88)
Ischemic stroke	4,278.18 (3,307.61 to 5,386.90)	3,372.43 (1,969.15 to 5,955.16)	2,136.87 (1,250.60 to 3,835.25)	4,816.34 (2,717.68 to 8,388.09)	206.86 (163.13 to 258.65)	196.57 (118.72 to 360.65)	134.82 (82.17 to 246.53)	262.22 (151.15 to 499.06)

Intracerebral hemorrhage	9,725.23 (7,522.73 to 12,335.14)	7,809.56 (4,282.39 to 13,545.06)	4,065.24 (2,204.07 to 6,921.24)	12,244.73 (6,679.81 to 21,274.52)	361.96 (283.30 to 455.68)	346.97 (213.31 to 542.39)	197.53 (122.88 to 312.67)	510.86 (301.67 to 831.73)
Hypertensive heart disease	4,790.80 (3,269.84 to 6,315.48)	6,968.82 (4,663.90 to 9,874.19)	5,248.81 (3,575.45 to 7,182.44)	7,735.13 (4,890.27 to 12,318.76)	206.45 (142.16 to 265.71)	362.46 (255.12 to 504.19)	289.77 (209.50 to 380.82)	380.48 (253.96 to 599.16)
Cardiomyopathy and myocarditis	2,482.81 (1,859.81 to 3,240.15)	3,039.46 (2,144.45 to 4,181.56)	2,674.67 (1,875.17 to 3,746.12)	2,953.00 (2,105.35 to 4,132.12)	76.44 (57.98 to 99.28)	111.93 (87.65 to 145.89)	106.84 (82.96 to 138.74)	103.05 (80.80 to 134.33)
Myocarditis	1,066.26 (756.64 to 1,517.69)	1,277.24 (937.15 to 1,828.26)	1,223.48 (890.93 to 1,726.46)	1,231.58 (902.20 to 1,733.02)	34.65 (25.03 to 48.28)	50.28 (39.84 to 67.73)	51.54 (40.75 to 68.85)	46.13 (36.43 to 62.07)

Fiji								
Cause	YLLs (actual counts)				Deaths (actual counts)			
	2016	2040 Forecast	2040 Better Scenario	2040 Worse Scenario	2016	2040 Forecast	2040 Better Scenario	2040 Worse Scenario
Alcoholic cardiomyopathy	83.26 (53.98 to 121.95)	84.93 (54.20 to 123.61)	80.09 (50.19 to 117.75)	82.41 (53.36 to 119.33)	2.85 (1.90 to 4.12)	3.33 (2.27 to 4.63)	3.35 (2.24 to 4.70)	3.09 (2.15 to 4.24)
Other cardiomyopathy	1,333.29 (977.54 to 1,713.57)	1,677.30 (1,089.21 to 2,499.32)	1,371.10 (862.24 to 2,068.44)	1,639.02 (1,061.46 to 2,486.03)	38.94 (29.12 to 50.55)	58.32 (40.72 to 80.40)	51.94 (35.28 to 72.78)	53.83 (37.33 to 74.80)
Atrial fibrillation and flutter	140.96 (101.82 to 187.82)	293.77 (221.64 to 379.10)	311.67 (237.17 to 399.72)	269.01 (196.56 to 350.88)	10.96 (8.11 to 14.56)	26.32 (18.37 to 36.16)	29.59 (21.44 to 39.44)	23.02 (15.30 to 31.93)
Aortic aneurysm	920.39 (694.02 to 1,174.85)	1,095.56 (889.70 to 1,339.16)	1,071.65 (870.24 to 1,315.22)	1,095.31 (879.19 to 1,366.39)	39.03 (30.32 to 48.83)	55.69 (47.81 to 64.45)	58.65 (50.59 to 67.94)	52.29 (43.72 to 62.38)
Peripheral artery disease	21.05 (14.16 to 29.18)	47.19 (30.68 to 69.24)	52.72 (33.90 to 77.11)	45.10 (29.29 to 67.10)	1.18 (0.82 to 1.65)	3.34 (2.05 to 4.83)	4.05 (2.45 to 5.84)	2.99 (1.80 to 4.37)
Endocarditis	1,908.63 (1,405.27 to 2,524.85)	2,738.92 (1,938.22 to 3,824.27)	2,510.76 (1,791.90 to 3,491.95)	2,696.04 (1,892.61 to 3,837.77)	61.60 (46.09 to 83.39)	106.62 (80.17 to 145.29)	106.25 (79.57 to 146.84)	99.30 (74.28 to 135.00)
Other cardiovascular and circulatory diseases	5,027.43 (3,690.97 to 6,828.74)	7,615.59 (5,446.80 to 10,454.75)	5,780.98 (4,056.73 to 8,067.76)	8,090.37 (5,610.52 to 11,418.34)	175.91 (130.53 to 240.19)	325.17 (255.38 to 432.03)	271.13 (206.75 to 358.69)	323.53 (246.53 to 432.27)
**Chronic respiratory diseases**	**10,907.93 (8,198.80 to 13,669.03)**	**9,845.34 (7,148.95 to 13,144.05)**	**7,887.87 (5,770.83 to 10,479.68)**	**11,803.16 (8,236.46 to 16,113.20)**	**411.82 (313.76 to 512.12)**	**423.61 (335.47 to 538.92)**	**353.30 (277.95 to 447.62)**	**486.18 (370.76 to 632.84)**
Chronic obstructive pulmonary disease	3,026.27 (2,236.09 to 3,863.01)	3,097.87 (2,107.43 to 4,417.86)	2,387.37 (1,669.53 to 3,295.12)	4,217.92 (2,614.28 to 6,514.04)	128.51 (96.51 to 161.94)	152.66 (112.04 to 203.71)	126.55 (96.37 to 162.49)	192.18 (131.95 to 276.29)
Pneumoconiosis	6.54 (4.75 to 8.83)	6.35 (4.98 to 8.16)	6.18 (4.75 to 8.03)	6.25 (4.89 to 8.07)	0.31 (0.23 to 0.41)	0.38 (0.29 to 0.49)	0.38 (0.30 to 0.50)	0.35 (0.27 to 0.47)
Silicosis	1.02 (0.64 to 1.52)	0.81 (0.52 to 1.22)	0.67 (0.41 to 1.04)	0.77 (0.50 to 1.21)	0.05 (0.03 to 0.08)	0.05 (0.03 to 0.08)	0.05 (0.03 to 0.07)	0.05 (0.03 to 0.08)
Asbestosis	1.72 (1.14 to 2.46)	2.01 (1.39 to 2.87)	2.23 (1.57 to 3.15)	1.88 (1.32 to 2.66)	0.06 (0.04 to 0.10)	0.09 (0.06 to 0.14)	0.11 (0.07 to 0.17)	0.08 (0.06 to 0.13)
Coal workers pneumoconiosis	0.28 (0.16 to 0.48)	0.28 (0.15 to 0.51)	0.23 (0.13 to 0.43)	0.31 (0.16 to 0.53)	0.01 (0.01 to 0.02)	0.02 (0.01 to 0.03)	0.01 (0.01 to 0.03)	0.02 (0.01 to 0.03)
Other pneumoconiosis	3.53 (2.44 to 4.91)	3.24 (2.25 to 4.39)	3.05 (2.05 to 4.17)	3.29 (2.32 to 4.43)	0.18 (0.13 to 0.25)	0.21 (0.14 to 0.30)	0.21 (0.14 to 0.30)	0.20 (0.14 to 0.29)
Asthma	5,739.12 (4,286.52 to 7,327.05)	3,813.69 (2,427.22 to 5,955.42)	2,595.46 (1,595.98 to 4,151.21)	4,658.80 (2,910.62 to 7,376.25)	216.15 (161.87 to 272.12)	172.76 (115.22 to 258.98)	125.94 (81.44 to 196.77)	199.26 (128.58 to 302.52)
Interstitial lung disease and pulmonary sarcoidosis	325.70 (219.24 to 456.92)	405.03 (259.98 to 613.86)	484.97 (308.88 to 708.92)	380.33 (241.19 to 582.52)	8.93 (6.06 to 12.32)	12.75 (9.29 to 16.64)	16.50 (11.64 to 21.60)	11.34 (8.11 to 14.79)
Other chronic respiratory diseases	1,810.30 (1,123.32 to 2,898.09)	2,522.40 (1,658.03 to 3,851.49)	2,413.89 (1,585.60 to 3,598.24)	2,539.87 (1,652.45 to 3,985.63)	57.92 (35.90 to 92.50)	85.06 (57.69 to 127.56)	83.92 (56.97 to 123.03)	83.05 (56.21 to 128.17)
**Cirrhosis and other chronic liver diseases**	**2,627.80 (1,917.75 to 3,409.55)**	**2,774.51 (2,063.25 to 3,706.56)**	**2,398.29 (1,743.59 to 3,213.89)**	**2,759.35 (2,056.47 to 3,635.09)**	**76.30 (57.29 to 98.63)**	**93.61 (77.11 to 114.40)**	**84.77 (68.40 to 105.66)**	**90.05 (74.40 to 109.92)**
Cirrhosis and other chronic liver diseases due to hepatitis B	878.20 (629.08 to 1,191.15)	970.02 (724.04 to 1,295.64)	875.53 (636.42 to 1,201.21)	944.47 (704.64 to 1,240.65)	27.50 (20.36 to 36.71)	34.68 (28.14 to 43.28)	32.42 (26.11 to 41.11)	32.76 (26.75 to 40.14)
Cirrhosis and other chronic liver diseases due to hepatitis C	421.34 (295.64 to 588.17)	470.96 (340.25 to 643.45)	420.48 (300.60 to 585.33)	460.84 (336.36 to 626.15)	12.79 (9.22 to 17.29)	16.05 (12.65 to 20.55)	14.77 (11.48 to 19.23)	15.31 (12.25 to 19.39)

Cirrhosis and other chronic liver diseases due to alcohol use	377.76 (268.92 to 510.62)	424.68 (313.59 to 568.82)	327.94 (236.79 to 441.00)	441.10 (329.57 to 587.26)	12.22 (8.98 to 16.15)	15.35 (12.43 to 18.95)	12.60 (9.98 to 15.74)	15.36 (12.34 to 19.00)
Cirrhosis and other chronic liver diseases due to other causes	950.50 (723.95 to 1,211.94)	908.84 (650.86 to 1,255.12)	774.34 (543.76 to 1,084.86)	912.94 (650.62 to 1,272.04)	23.80 (18.30 to 30.22)	27.54 (22.13 to 34.37)	24.99 (19.72 to 31.73)	26.62 (21.24 to 33.18)
**Digestive diseases**	**3,462.53 (2,583.17 to 4,378.67)**	**3,438.29 (2,573.34 to 4,418.86)**	**2,824.64 (2,118.47 to 3,662.90)**	**3,534.30 (2,657.15 to 4,663.47)**	**114.78 (87.08 to 143.00)**	**136.96 (114.22 to 160.57)**	**121.54 (101.23 to 145.00)**	**133.74 (110.43 to 158.32)**
Peptic ulcer disease	914.59 (663.50 to 1,169.87)	608.46 (463.39 to 775.18)	445.34 (322.11 to 604.79)	636.94 (489.15 to 829.75)	33.07 (24.47 to 42.03)	27.11 (21.89 to 32.94)	20.99 (16.02 to 27.06)	27.13 (21.76 to 33.75)

Fiji								
Cause	YLLs (actual counts)				Deaths (actual counts)			
	2016	2040 Forecast	2040 Better Scenario	2040 Worse Scenario	2016	2040 Forecast	2040 Better Scenario	2040 Worse Scenario
Gastritis and duodenitis	22.42 (15.75 to 30.69)	32.44 (22.46 to 44.94)	28.00 (19.03 to 40.30)	31.02 (21.86 to 43.19)	0.82 (0.58 to 1.12)	1.44 (1.03 to 2.06)	1.32 (0.93 to 1.91)	1.31 (0.94 to 1.84)
Appendicitis	439.92 (328.76 to 564.07)	399.85 (284.98 to 543.25)	304.83 (209.01 to 427.01)	404.99 (285.31 to 556.12)	12.04 (9.06 to 15.36)	13.88 (10.85 to 17.12)	11.36 (8.62 to 14.66)	13.35 (10.43 to 16.59)
Paralytic ileus and intestinal obstruction	692.07 (497.39 to 938.75)	870.80 (588.24 to 1,361.13)	793.17 (558.79 to 1,183.35)	862.53 (573.74 to 1,399.62)	20.99 (15.64 to 28.66)	31.11 (24.91 to 41.51)	31.83 (25.81 to 42.07)	28.63 (22.69 to 39.36)
Inguinal, femoral, and abdominal hernia	97.91 (57.83 to 136.11)	75.89 (45.94 to 98.88)	67.11 (40.65 to 89.69)	71.58 (43.88 to 94.43)	4.16 (2.49 to 5.63)	3.97 (2.47 to 5.04)	3.75 (2.32 to 4.81)	3.56 (2.19 to 4.54)
Inflammatory bowel disease	69.01 (46.78 to 96.05)	82.06 (56.84 to 119.02)	80.54 (56.43 to 117.36)	78.75 (54.62 to 115.24)	2.12 (1.50 to 2.82)	3.07 (2.41 to 3.90)	3.23 (2.48 to 4.09)	2.81 (2.20 to 3.57)
Vascular intestinal disorders	109.94 (79.51 to 144.76)	147.65 (114.69 to 188.96)	139.13 (106.56 to 179.97)	139.93 (109.83 to 180.27)	4.36 (3.27 to 5.62)	7.36 (6.05 to 8.89)	7.43 (5.97 to 8.93)	6.67 (5.40 to 8.08)
Gallbladder and biliary diseases	356.10 (244.88 to 472.80)	366.60 (199.09 to 623.10)	266.95 (145.47 to 456.87)	444.61 (235.21 to 756.38)	13.08 (9.05 to 17.24)	16.45 (9.13 to 26.81)	12.76 (7.04 to 21.41)	18.91 (10.31 to 31.53)
Pancreatitis	159.99 (107.68 to 228.79)	232.59 (119.30 to 479.32)	159.51 (88.92 to 298.45)	246.91 (122.13 to 516.80)	4.66 (3.30 to 6.57)	7.67 (4.73 to 14.08)	5.81 (3.91 to 9.42)	7.74 (4.56 to 14.56)
Other digestive diseases	600.58 (449.44 to 775.48)	621.95 (463.14 to 816.98)	540.06 (389.79 to 716.77)	617.07 (456.88 to 812.85)	19.48 (14.77 to 25.05)	24.91 (20.21 to 30.18)	23.05 (18.53 to 28.18)	23.63 (19.31 to 28.69)
**Neurological disorders**	**3,207.37 (2,538.82 to 3,947.59)**	**5,579.56 (4,002.07 to 8,046.08)**	**5,582.11 (4,001.24 to 7,987.99)**	**5,586.61 (3,941.77 to 8,184.58)**	**165.92 (131.39 to 206.76)**	**375.84 (251.71 to 584.00)**	**421.85 (283.75 to 635.96)**	**355.62 (229.96 to 563.82)**
Alzheimer's disease and other dementias	1,615.35 (1,232.78 to 2,084.21)	3,520.97 (2,186.37 to 5,924.97)	3,804.76 (2,390.14 to 6,163.92)	3,471.74 (2,037.30 to 5,997.03)	126.73 (98.54 to 163.48)	313.03 (189.38 to 520.72)	356.75 (222.02 to 574.41)	296.33 (172.19 to 506.52)
Parkinson's disease	150.58 (108.79 to 200.37)	338.57 (258.13 to 434.18)	422.19 (326.03 to 536.77)	286.90 (215.83 to 373.43)	10.22 (7.48 to 13.61)	25.84 (18.91 to 34.56)	33.53 (24.83 to 43.93)	21.27 (14.93 to 28.96)
Epilepsy	1,124.78 (839.60 to 1,430.35)	1,271.67 (715.08 to 2,074.51)	908.36 (530.60 to 1,489.23)	1,389.74 (780.20 to 2,297.08)	20.64 (15.22 to 26.36)	24.24 (14.79 to 37.64)	17.94 (11.17 to 27.51)	26.07 (15.78 to 40.84)
Multiple sclerosis	39.13 (17.36 to 85.24)	46.57 (21.13 to 102.20)	48.61 (22.02 to 107.75)	45.96 (20.69 to 100.24)	1.12 (0.51 to 2.42)	1.45 (0.70 to 3.08)	1.55 (0.74 to 3.37)	1.41 (0.67 to 3.00)
Motor neuron disease	47.31 (35.44 to 60.64)	73.65 (55.90 to 93.09)	77.45 (58.19 to 99.47)	69.61 (53.66 to 86.68)	1.54 (1.16 to 1.96)	2.61 (2.07 to 3.17)	2.83 (2.22 to 3.50)	2.41 (1.93 to 2.91)
Other neurological disorders	230.21 (178.95 to 287.21)	328.12 (220.20 to 475.02)	320.74 (221.50 to 448.50)	322.66 (214.87 to 472.88)	5.68 (4.45 to 7.07)	8.67 (6.89 to 10.84)	9.26 (7.42 to 11.42)	8.14 (6.42 to 10.24)
**Mental disorders**	**268.28 (176.82 to 417.40)**	**435.79 (285.12 to 686.03)**	**464.61 (303.82 to 725.68)**	**428.86 (280.62 to 681.52)**	**6.39 (4.37 to 9.30)**	**11.63 (8.52 to 16.34)**	**13.09 (9.61 to 18.07)**	**11.04 (8.13 to 15.72)**
Alcohol use disorders	78.54 (47.74 to 124.72)	75.08 (47.16 to 118.87)	73.00 (45.44 to 115.60)	74.96 (47.24 to 118.01)	2.02 (1.28 to 3.05)	2.16 (1.46 to 3.08)	2.18 (1.46 to 3.12)	2.09 (1.43 to 3.01)
Drug use disorders	186.16 (122.72 to 295.83)	355.33 (229.54 to 590.23)	386.15 (251.40 to 629.92)	348.52 (225.89 to 585.22)	4.30 (2.93 to 6.36)	9.37 (6.75 to 13.49)	10.80 (7.79 to 15.27)	8.85 (6.43 to 12.90)
Opioid use disorders	113.08 (71.48 to 194.01)	177.91 (105.49 to 316.19)	181.51 (107.03 to 318.43)	175.90 (104.13 to 314.28)	2.39 (1.58 to 3.86)	4.14 (2.75 to 6.59)	4.39 (2.96 to 6.86)	3.98 (2.64 to 6.38)

Cocaine use disorders	13.54 (7.60 to 28.13)	35.03 (18.22 to 77.52)	37.36 (19.43 to 82.83)	34.87 (17.88 to 77.41)	0.30 (0.18 to 0.57)	0.84 (0.49 to 1.65)	0.92 (0.54 to 1.79)	0.81 (0.46 to 1.65)
Amphetamine use disorders	7.32 (4.18 to 15.30)	27.59 (14.09 to 60.08)	31.74 (15.84 to 70.88)	27.30 (14.21 to 59.35)	0.17 (0.10 to 0.31)	0.64 (0.38 to 1.24)	0.75 (0.42 to 1.47)	0.63 (0.37 to 1.22)
Other drug use disorders	52.21 (34.56 to 77.63)	114.79 (73.17 to 172.59)	135.53 (85.63 to 202.10)	110.46 (70.26 to 165.66)	1.44 (0.98 to 2.05)	3.75 (2.67 to 5.10)	4.74 (3.37 to 6.53)	3.43 (2.44 to 4.66)
Eating disorders	3.58 (2.29 to 6.20)	5.39 (3.10 to 9.31)	5.46 (3.12 to 9.76)	5.37 (3.11 to 9.25)	0.07 (0.04 to 0.12)	0.10 (0.06 to 0.18)	0.11 (0.06 to 0.20)	0.10 (0.06 to 0.18)

Fiji								
Cause	YLLs (actual counts)				Deaths (actual counts)			
	2016	2040 Forecast	2040 Better Scenario	2040 Worse Scenario	2016	2040 Forecast	2040 Better Scenario	2040 Worse Scenario
**Diabetes, urogenital, blood, and endocrine diseases**	**64,206.56 (49,571.56 to 80,073.09)**	**82,596.63 (61,954.60 to 109,361.11)**	**62,433.25 (44,894.62 to 86,737.69)**	**94,168.37 (70,392.27 to 125,259.55)**	**2,640.18 (2,078.96 to 3,234.63)**	**4,038.09 (3,356.66 to 4,974.00)**	**3,214.53 (2,555.73 to 4,059.40)**	**4,392.47 (3,610.82 to 5,434.84)**
Diabetes mellitus	49,446.78 (38,078.14 to 61,724.75)	59,494.84 (39,029.40 to 85,149.97)	44,498.45 (28,556.66 to 66,580.75)	66,079.95 (42,866.78 to 98,646.36)	2,093.87 (1,643.91 to 2,580.91)	3,033.33 (2,197.87 to 4,017.61)	2,390.51 (1,696.96 to 3,225.60)	3,200.57 (2,225.58 to 4,385.98)
Acute glomerulonephritis	6.83 (5.14 to 9.23)	10.28 (5.68 to 19.11)	7.65 (4.24 to 13.77)	10.87 (5.88 to 20.18)	0.13 (0.10 to 0.16)	0.18 (0.12 to 0.29)	0.14 (0.09 to 0.23)	0.19 (0.12 to 0.31)
Chronic kidney disease	12,306.82 (9,370.21 to 15,573.75)	19,681.28 (9,601.65 to 43,927.01)	14,882.82 (7,398.38 to 33,206.72)	24,713.99 (11,846.55 to 51,731.10)	471.96 (365.11 to 595.33)	877.69 (459.29 to 1,715.22)	699.80 (371.16 to 1,386.21)	1,073.73 (544.83 to 1,981.77)
Chronic kidney disease due to diabetes mellitus	6,815.47 (5,119.88 to 8,848.25)	11,636.07 (5,352.82 to 27,154.23)	8,821.20 (4,060.40 to 20,472.64)	14,733.32 (6,527.38 to 31,852.80)	259.69 (198.10 to 333.06)	494.02 (246.02 to 1,006.41)	390.02 (196.24 to 805.88)	610.93 (293.47 to 1,174.67)
Chronic kidney disease due to hypertension	2,901.86 (2,161.89 to 3,817.30)	4,804.37 (2,471.82 to 10,162.93)	3,769.87 (1,930.31 to 7,785.41)	6,004.34 (2,949.38 to 12,321.55)	139.38 (106.03 to 179.18)	272.81 (143.01 to 525.99)	226.13 (120.86 to 431.32)	327.58 (165.00 to 605.93)
Chronic kidney disease due to glomerulonephritis	1,216.25 (891.09 to 1,583.51)	1,422.01 (740.94 to 2,978.88)	990.12 (502.52 to 2,180.40)	1,736.26 (892.65 to 3,633.85)	31.32 (22.85 to 40.95)	43.97 (22.92 to 89.44)	32.33 (17.04 to 66.75)	53.77 (27.46 to 106.11)
Chronic kidney disease due to other and unspecified causes	1,373.24 (1,003.76 to 1,819.05)	1,818.83 (898.91 to 3,842.41)	1,301.62 (656.35 to 2,757.14)	2,240.08 (1,098.73 to 4,534.42)	41.57 (30.42 to 55.61)	66.90 (34.72 to 136.09)	51.32 (26.73 to 106.07)	81.45 (40.62 to 158.31)
Urinary diseases and male infertility	1,012.96 (785.81 to 1,266.24)	1,682.18 (1,270.08 to 2,240.43)	1,494.91 (1,137.98 to 1,949.65)	1,637.98 (1,231.26 to 2,240.61)	33.33 (26.26 to 41.20)	68.69 (58.73 to 80.13)	68.32 (57.03 to 80.72)	62.46 (52.92 to 73.90)
Urinary tract infections	888.12 (681.80 to 1,122.87)	1,561.73 (1,171.18 to 2,094.99)	1,397.05 (1,053.82 to 1,825.01)	1,520.67 (1,130.28 to 2,095.38)	28.82 (22.47 to 36.23)	63.62 (53.99 to 74.53)	63.89 (53.59 to 75.81)	57.81 (48.80 to 68.79)
Urolithiasis	16.89 (12.16 to 22.29)	23.16 (16.41 to 31.40)	20.44 (13.86 to 28.21)	22.19 (15.83 to 30.18)	0.58 (0.41 to 0.77)	0.89 (0.64 to 1.17)	0.84 (0.58 to 1.13)	0.81 (0.59 to 1.07)
Other urinary diseases	107.95 (71.11 to 153.97)	97.29 (65.81 to 141.42)	77.42 (51.36 to 113.93)	95.12 (62.75 to 138.63)	3.93 (2.64 to 5.48)	4.18 (2.91 to 5.91)	3.59 (2.39 to 5.30)	3.83 (2.65 to 5.37)
Gynecological diseases	66.92 (35.40 to 100.26)	58.37 (29.34 to 94.60)	41.23 (19.74 to 70.49)	60.61 (30.12 to 98.91)	1.67 (0.89 to 2.49)	1.55 (0.84 to 2.45)	1.11 (0.57 to 1.85)	1.60 (0.85 to 2.52)
Uterine fibroids	1.38 (0.59 to 2.50)	1.02 (0.41 to 1.93)	0.74 (0.29 to 1.43)	1.06 (0.43 to 2.05)	0.04 (0.02 to 0.08)	0.03 (0.01 to 0.07)	0.03 (0.01 to 0.05)	0.04 (0.01 to 0.07)
Polycystic ovarian syndrome	0.29 (0.03 to 0.80)	0.17 (0.01 to 0.50)	0.09 (0.01 to 0.28)	0.18 (0.02 to 0.53)	0.01 (0.00 to 0.02)	0.00 (0.00 to 0.01)	0.00 (0.00 to 0.01)	0.00 (0.00 to 0.01)
Endometriosis	0.10 (0.04 to 0.27)	0.10 (0.03 to 0.25)	0.07 (0.02 to 0.17)	0.10 (0.03 to 0.26)	0.00 (0.00 to 0.01)	0.00 (0.00 to 0.01)	0.00 (0.00 to 0.00)	0.00 (0.00 to 0.01)
Genital prolapse	0.29 (0.09 to 0.59)	0.18 (0.06 to 0.39)	0.13 (0.04 to 0.26)	0.19 (0.06 to 0.41)	0.01 (0.00 to 0.02)	0.01 (0.00 to 0.01)	0.00 (0.00 to 0.01)	0.01 (0.00 to 0.01)
Other gynecological diseases	64.85 (34.30 to 97.79)	56.90 (28.78 to 92.28)	40.20 (19.09 to 69.10)	59.06 (29.38 to 97.03)	1.61 (0.87 to 2.41)	1.51 (0.81 to 2.38)	1.08 (0.55 to 1.80)	1.55 (0.81 to 2.47)
Hemoglobinopathies and hemolytic anemias	543.58 (392.32 to 715.55)	495.47 (345.24 to 659.84)	399.81 (271.31 to 551.04)	497.59 (346.89 to 663.01)	14.90 (10.74 to 19.64)	16.89 (13.04 to 21.23)	14.18 (10.59 to 18.23)	16.27 (12.55 to 20.35)
Thalassemias	97.34 (67.15 to 132.18)	78.48 (35.02 to 146.26)	71.94 (32.84 to 134.14)	78.36 (35.00 to 144.58)	1.29 (0.90 to 1.73)	1.03 (0.47 to 1.90)	0.95 (0.45 to 1.77)	1.02 (0.47 to 1.89)

Sickle cell disorders	0.61 (0.37 to 0.91)	0.55 (0.29 to 0.90)	0.42 (0.22 to 0.70)	0.57 (0.30 to 0.93)	0.01 (0.01 to 0.02)	0.01 (0.01 to 0.02)	0.01 (0.00 to 0.01)	0.01 (0.01 to 0.02)
G6PD deficiency	240.49 (164.32 to 325.01)	246.79 (160.45 to 353.94)	184.99 (112.75 to 270.86)	255.74 (164.51 to 359.77)	5.52 (3.79 to 7.46)	6.70 (4.57 to 9.07)	5.15 (3.33 to 7.25)	6.80 (4.64 to 9.19)
Other hemoglobinopathies and hemolytic anemias	205.14 (144.70 to 273.81)	169.64 (131.08 to 216.97)	142.46 (105.85 to 187.87)	162.92 (126.59 to 207.42)	8.08 (5.85 to 10.74)	9.15 (7.19 to 11.80)	8.07 (6.20 to 10.57)	8.44 (6.61 to 10.81)
Endocrine, metabolic, blood, and immune disorders	822.68 (607.39 to 1,050.66)	1,174.23 (845.44 to 1,556.68)	1,108.37 (802.92 to 1,447.33)	1,167.39 (834.33 to 1,542.42)	24.32 (18.10 to 31.12)	39.74 (31.63 to 48.27)	40.47 (32.39 to 49.46)	37.65 (29.62 to 45.59)

Fiji								
Cause	YLLs (actual counts)				Deaths (actual counts)			
	2016	2040 Forecast	2040 Better Scenario	2040 Worse Scenario	2016	2040 Forecast	2040 Better Scenario	2040 Worse Scenario
**Musculoskeletal disorders**	**316.25 (226.37 to 433.00)**	**394.95 (263.88 to 591.17)**	**383.93 (260.57 to 575.31)**	**383.28 (255.07 to 578.07)**	**7.24 (5.26 to 9.68)**	**11.12 (8.53 to 14.58)**	**11.58 (8.95 to 15.03)**	**10.31 (7.91 to 13.82)**
Rheumatoid arthritis	16.67 (11.59 to 26.10)	29.90 (20.79 to 42.40)	23.20 (15.29 to 34.05)	29.70 (20.68 to 41.98)	0.59 (0.41 to 0.87)	1.33 (0.96 to 1.80)	1.10 (0.75 to 1.59)	1.27 (0.91 to 1.71)
Other musculoskeletal disorders	299.59 (211.22 to 410.43)	365.05 (239.06 to 548.35)	360.73 (241.45 to 538.53)	353.58 (230.78 to 537.13)	6.66 (4.81 to 8.96)	9.79 (7.37 to 12.99)	10.48 (8.00 to 13.73)	9.04 (6.79 to 12.25)
**Other non-communicable diseases**	**7,321.99 (4,691.37 to 10,880.04)**	**12,472.00 (6,036.78 to 23,207.71)**	**8,238.44 (4,293.77 to 14,788.50)**	**13,421.95 (6,348.62 to 25,439.03)**	**113.57 (78.07 to 156.86)**	**228.75 (145.79 to 361.64)**	**183.61 (120.27 to 271.97)**	**230.86 (140.80 to 379.65)**
Congenital birth defects	6,320.42 (3,815.29 to 9,757.74)	9,871.01 (3,907.04 to 19,995.22)	5,817.36 (2,293.64 to 11,793.90)	10,901.81 (4,336.36 to 22,061.18)	79.18 (49.34 to 119.13)	121.87 (51.44 to 240.60)	73.10 (31.15 to 143.12)	133.96 (56.41 to 264.40)
Neural tube defects	204.76 (97.36 to 389.58)	126.05 (43.61 to 298.58)	62.42 (20.57 to 155.79)	145.84 (48.90 to 358.79)	2.41 (1.16 to 4.56)	1.49 (0.53 to 3.48)	0.74 (0.25 to 1.83)	1.72 (0.59 to 4.19)
Congenital heart anomalies	3,868.00 (2,295.92 to 6,140.12)	5,895.81 (2,292.66 to 12,329.17)	3,359.53 (1,233.52 to 6,893.70)	6,495.11 (2,499.36 to 13,818.83)	48.49 (29.66 to 74.83)	72.73 (29.99 to 147.43)	42.08 (16.70 to 83.43)	79.79 (32.59 to 163.73)
Orofacial clefts	10.97 (1.64 to 38.20)	10.60 (1.52 to 44.24)	5.37 (0.69 to 22.16)	12.07 (1.77 to 50.71)	0.13 (0.02 to 0.45)	0.12 (0.02 to 0.52)	0.06 (0.01 to 0.26)	0.14 (0.02 to 0.59)
Down syndrome	353.63 (154.42 to 641.26)	511.35 (159.02 to 1,265.06)	374.15 (124.26 to 916.32)	565.01 (169.57 to 1,390.95)	4.65 (2.27 to 8.06)	6.67 (2.45 to 15.56)	5.03 (1.97 to 11.49)	7.31 (2.62 to 17.03)
Other chromosomal abnormalities	323.46 (164.98 to 601.72)	736.78 (262.91 to 1,796.81)	468.30 (165.62 to 1,133.70)	823.72 (294.42 to 1,988.45)	3.87 (2.04 to 7.17)	8.73 (3.14 to 21.07)	5.58 (2.02 to 13.40)	9.74 (3.52 to 23.25)
Congenital musculoskeletal and limb anomalies	70.63 (37.70 to 125.02)	144.54 (51.18 to 311.91)	87.35 (30.70 to 187.48)	158.45 (55.96 to 342.11)	0.86 (0.47 to 1.49)	1.72 (0.62 to 3.67)	1.05 (0.39 to 2.21)	1.88 (0.69 to 4.02)
Urogenital congenital anomalies	259.31 (144.90 to 402.44)	404.38 (186.53 to 784.51)	254.08 (119.46 to 491.87)	444.03 (199.16 to 879.69)	3.69 (2.20 to 5.43)	5.57 (2.91 to 10.07)	3.68 (1.92 to 6.49)	6.02 (3.06 to 11.21)
Digestive congenital anomalies	351.18 (126.69 to 634.78)	574.32 (170.07 to 1,348.29)	318.29 (93.91 to 765.48)	644.29 (193.65 to 1,546.35)	4.13 (1.50 to 7.42)	6.73 (2.01 to 15.70)	3.74 (1.12 to 8.93)	7.54 (2.29 to 18.02)
Other congenital birth defects	878.48 (505.24 to 1,421.27)	1,467.17 (571.26 to 3,190.14)	887.88 (352.40 to 1,921.46)	1,613.29 (625.25 to 3,542.22)	10.96 (6.52 to 17.43)	18.10 (7.41 to 38.34)	11.13 (4.79 to 23.35)	19.81 (8.07 to 42.14)
Skin and subcutaneous diseases	927.23 (512.64 to 1,375.43)	2,443.41 (1,405.08 to 3,500.73)	2,325.01 (1,362.86 to 3,287.82)	2,344.22 (1,335.29 to 3,398.73)	33.53 (19.68 to 50.99)	105.05 (64.90 to 157.35)	109.39 (68.95 to 163.51)	94.86 (58.21 to 142.67)
Cellulitis	280.90 (136.81 to 465.17)	799.40 (407.85 to 1,244.10)	841.23 (433.26 to 1,281.44)	743.23 (385.03 to 1,170.75)	10.06 (5.00 to 16.58)	35.46 (19.13 to 55.40)	40.20 (21.64 to 61.51)	31.20 (16.93 to 49.20)
Pyoderma	567.98 (304.34 to 826.14)	1,492.68 (822.56 to 2,254.22)	1,339.25 (752.47 to 1,981.42)	1,460.94 (785.53 to 2,221.90)	19.41 (11.15 to 29.56)	59.86 (36.61 to 90.75)	59.11 (36.72 to 91.24)	55.13 (33.41 to 85.22)
Decubitus ulcer	71.83 (36.07 to 114.48)	138.00 (69.40 to 228.85)	132.67 (66.78 to 221.03)	126.81 (61.82 to 210.24)	3.91 (1.99 to 6.54)	9.41 (4.64 to 17.92)	9.76 (5.02 to 18.23)	8.23 (4.03 to 15.89)
Other skin and subcutaneous diseases	6.52 (3.24 to 9.54)	13.34 (6.15 to 23.86)	11.85 (5.66 to 20.47)	13.24 (6.08 to 24.13)	0.15 (0.08 to 0.22)	0.33 (0.17 to 0.51)	0.32 (0.17 to 0.50)	0.30 (0.16 to 0.49)
Sudden infant death syndrome	74.34 (35.99 to 135.57)	157.58 (33.69 to 429.68)	96.07 (20.60 to 259.58)	175.93 (39.03 to 477.31)	0.86 (0.42 to 1.57)	1.83 (0.39 to 4.99)	1.11 (0.24 to 3.01)	2.04 (0.45 to 5.54)

**Injuries**	**25,750.07 (19,272.17 to 32,329.10)**	**23,386.70 (15,007.94 to 35,649.51)**	**16,285.72 (10,523.72 to 24,195.83)**	**27,100.69 (17,099.24 to 42,443.96)**	**503.98 (373.60 to 634.75)**	**500.54 (359.54 to 692.46)**	**372.88 (273.38 to 506.39)**	**556.28 (392.06 to 785.49)**
**Transport injuries**	**6,011.21 (4,460.15 to 7,706.37)**	**4,719.01 (2,954.10 to 7,211.28)**	**1,724.75 (1,127.48 to 2,602.32)**	**7,586.31 (4,661.93 to 11,734.12)**	**118.96 (88.11 to 151.24)**	**104.30 (73.00 to 148.02)**	**44.17 (32.19 to 59.75)**	**155.39 (105.79 to 223.46)**
Road injuries	5,418.86 (3,999.04 to 6,947.91)	3,956.84 (2,459.51 to 6,116.93)	1,190.56 (773.86 to 1,807.41)	6,781.79 (4,170.61 to 10,503.50)	106.57 (78.19 to 136.10)	86.43 (59.45 to 124.25)	30.78 (22.60 to 42.09)	137.22 (92.59 to 199.21)
Pedestrian road injuries	1,114.66 (817.52 to 1,456.05)	683.36 (418.19 to 1,054.79)	229.35 (140.17 to 358.94)	1,005.99 (600.43 to 1,578.57)	22.11 (15.98 to 28.86)	15.64 (10.53 to 22.49)	5.80 (3.97 to 8.17)	21.98 (14.44 to 32.62)

Fiji								
Cause	YLLs (actual counts)				Deaths (actual counts)			
	2016	2040 Forecast	2040 Better Scenario	2040 Worse Scenario	2016	2040 Forecast	2040 Better Scenario	2040 Worse Scenario
Cyclist road injuries	148.38 (104.45 to 196.88)	102.80 (64.65 to 155.43)	33.41 (22.51 to 47.33)	175.65 (105.98 to 276.90)	3.09 (2.24 to 4.04)	2.51 (1.75 to 3.48)	1.09 (0.81 to 1.46)	3.79 (2.55 to 5.40)
Motorcyclist road injuries	709.99 (434.74 to 1,017.90)	566.69 (284.80 to 1,029.57)	263.84 (134.82 to 471.12)	809.29 (409.90 to 1,441.98)	13.22 (8.18 to 18.95)	11.08 (5.81 to 19.61)	5.66 (3.13 to 9.50)	15.12 (7.89 to 26.83)
Motor vehicle road injuries	3,322.51 (2,427.08 to 4,350.10)	2,518.02 (1,593.85 to 3,862.28)	641.07 (432.24 to 942.13)	4,634.23 (2,878.47 to 7,207.33)	65.81 (48.34 to 85.44)	55.38 (39.38 to 78.98)	17.59 (13.33 to 23.57)	93.31 (63.81 to 136.19)
Other road injuries	123.31 (82.20 to 169.75)	85.97 (48.97 to 133.78)	22.89 (14.56 to 34.22)	156.62 (86.56 to 249.55)	2.34 (1.57 to 3.19)	1.83 (1.18 to 2.66)	0.64 (0.44 to 0.86)	3.02 (1.86 to 4.59)
Other transport injuries	592.36 (437.39 to 775.12)	762.16 (467.83 to 1,198.46)	534.19 (329.28 to 822.02)	804.52 (488.45 to 1,263.44)	12.39 (9.19 to 15.96)	17.87 (12.43 to 25.27)	13.40 (9.38 to 19.00)	18.18 (12.52 to 25.95)
**Unintentional injuries**	**11,775.01 (8,964.63 to 14,671.56)**	**10,150.41 (6,464.90 to 16,064.98)**	**7,802.41 (5,019.41 to 11,999.63)**	**10,610.56 (6,644.82 to 16,839.37)**	**229.33 (173.80 to 285.02)**	**220.98 (165.25 to 300.35)**	**184.61 (139.98 to 245.30)**	**221.22 (163.87 to 305.54)**
Falls	881.05 (630.11 to 1,171.24)	871.71 (626.98 to 1,202.62)	772.71 (548.44 to 1,040.43)	859.33 (605.35 to 1,187.85)	24.02 (17.92 to 30.91)	31.22 (25.38 to 37.31)	30.25 (24.29 to 36.41)	28.90 (23.25 to 35.05)
Drowning	4,517.60 (3,337.45 to 5,881.79)	3,340.47 (1,961.31 to 5,612.02)	2,498.88 (1,482.89 to 4,123.66)	3,537.66 (2,073.33 to 5,992.36)	79.39 (57.97 to 101.67)	61.86 (40.02 to 94.76)	48.20 (30.83 to 72.63)	64.26 (41.33 to 98.63)
Fire, heat, and hot substances	1,848.57 (1,325.97 to 2,409.63)	1,314.69 (754.88 to 2,217.28)	933.31 (548.27 to 1,538.84)	1,399.86 (787.27 to 2,450.31)	36.40 (26.90 to 47.33)	29.55 (21.06 to 42.07)	22.93 (16.59 to 32.35)	30.08 (21.09 to 44.47)
Poisonings	254.27 (170.07 to 352.49)	158.21 (91.59 to 269.15)	101.28 (57.73 to 172.49)	171.75 (97.64 to 305.45)	4.99 (3.47 to 6.76)	3.50 (2.45 to 5.25)	2.47 (1.71 to 3.62)	3.62 (2.44 to 5.56)
Exposure to mechanical forces	1,292.05 (933.25 to 1,692.60)	1,134.29 (671.65 to 1,802.73)	845.07 (494.87 to 1,324.76)	1,198.57 (703.58 to 1,908.18)	23.46 (17.22 to 30.20)	22.03 (14.79 to 31.79)	17.40 (11.61 to 24.57)	22.56 (14.85 to 32.37)
Unintentional firearm injuries	43.82 (31.60 to 57.81)	36.29 (20.86 to 62.97)	27.01 (15.79 to 45.97)	38.59 (21.55 to 69.37)	0.88 (0.65 to 1.12)	0.81 (0.57 to 1.19)	0.66 (0.47 to 0.96)	0.82 (0.56 to 1.25)
Unintentional suffocation	657.31 (419.22 to 896.01)	593.18 (316.35 to 1,017.51)	438.03 (229.98 to 750.03)	633.80 (322.02 to 1,092.97)	10.36 (6.79 to 13.99)	9.40 (5.31 to 15.31)	7.15 (4.13 to 11.55)	9.92 (5.50 to 16.44)
Other exposure to mechanical forces	590.92 (430.72 to 768.38)	504.81 (324.10 to 748.11)	380.04 (239.91 to 574.55)	526.18 (327.75 to 798.66)	12.23 (8.95 to 15.90)	11.83 (8.63 to 15.84)	9.59 (6.81 to 12.98)	11.81 (8.36 to 16.06)
Adverse effects of medical treatment	787.31 (573.93 to 1,015.53)	815.21 (535.83 to 1,255.10)	663.21 (441.14 to 994.74)	843.12 (551.30 to 1,327.19)	17.60 (13.10 to 22.42)	20.62 (15.75 to 26.71)	18.35 (13.98 to 23.50)	20.26 (15.36 to 26.74)
Animal contact	92.08 (65.69 to 123.03)	120.81 (75.03 to 189.32)	90.43 (56.56 to 144.72)	126.89 (77.09 to 201.32)	2.19 (1.59 to 2.92)	3.24 (2.35 to 4.49)	2.67 (1.94 to 3.70)	3.21 (2.28 to 4.53)
Venomous animal contact	21.71 (13.47 to 34.23)	26.31 (14.04 to 48.36)	19.63 (10.74 to 35.75)	28.17 (14.60 to 52.89)	0.48 (0.31 to 0.73)	0.63 (0.40 to 1.00)	0.52 (0.34 to 0.81)	0.64 (0.39 to 1.05)
Non-venomous animal contact	70.37 (49.80 to 94.48)	94.50 (58.82 to 148.02)	70.80 (44.51 to 110.99)	98.73 (60.37 to 156.41)	1.71 (1.23 to 2.25)	2.61 (1.91 to 3.59)	2.15 (1.56 to 2.97)	2.57 (1.85 to 3.63)
Foreign body	1,354.86 (921.65 to 1,884.78)	1,763.04 (920.80 to 3,386.50)	1,460.69 (817.12 to 2,646.67)	1,815.58 (920.97 to 3,487.61)	25.76 (18.94 to 34.55)	34.45 (23.56 to 53.87)	31.81 (22.94 to 46.02)	33.71 (22.38 to 54.62)
Pulmonary aspiration and foreign body in airway	1,218.36 (816.79 to 1,734.94)	1,629.87 (849.40 to 3,174.36)	1,350.99 (751.71 to 2,499.68)	1,673.86 (834.52 to 3,303.32)	23.36 (17.01 to 31.56)	32.14 (21.96 to 50.22)	29.80 (21.27 to 43.09)	31.30 (20.77 to 51.22)

Foreign body in other body part	136.50 (77.88 to 201.77)	133.17 (61.12 to 268.34)	109.70 (51.60 to 213.50)	141.72 (62.83 to 291.74)	2.40 (1.46 to 3.60)	2.31 (1.21 to 4.12)	2.01 (1.09 to 3.47)	2.41 (1.23 to 4.35)
Environmental heat and cold exposure	176.92 (104.86 to 258.79)	174.16 (99.19 to 276.86)	123.07 (69.19 to 199.28)	184.45 (105.36 to 296.97)	4.00 (2.46 to 5.71)	4.38 (2.81 to 6.27)	3.30 (2.15 to 4.82)	4.43 (2.75 to 6.49)
Other unintentional injuries	570.31 (420.33 to 747.54)	457.82 (298.28 to 671.88)	313.76 (187.80 to 495.49)	473.35 (302.00 to 688.81)	11.51 (8.40 to 14.98)	10.13 (7.13 to 14.00)	7.24 (4.75 to 10.84)	10.20 (7.13 to 14.09)
**Self-harm and interpersonal violence**	**7,957.91 (5,583.52 to 10,413.25)**	**8,397.01 (4,952.06 to 14,164.67)**	**6,643.50 (3,980.32 to 10,715.29)**	**8,783.69 (5,054.70 to 15,083.31)**	**155.57 (109.23 to 204.70)**	**172.65 (108.30 to 275.86)**	**141.48 (91.18 to 214.21)**	**177.12 (109.06 to 286.67)**

Fiji								
Cause	YLLs (actual counts)				Deaths (actual counts)			
	2016	2040 Forecast	2040 Better Scenario	2040 Worse Scenario	2016	2040 Forecast	2040 Better Scenario	2040 Worse Scenario
Self-harm	5,479.65 (3,855.79 to 7,365.91)	6,294.36 (3,490.15 to 11,184.40)	4,938.11 (2,870.93 to 8,373.80)	6,614.69 (3,630.62 to 11,938.61)	108.42 (75.52 to 145.06)	131.31 (79.25 to 218.26)	106.90 (67.68 to 167.97)	135.16 (80.35 to 228.85)
Self-harm by firearm	54.60 (35.77 to 95.78)	46.84 (24.38 to 89.45)	34.75 (18.66 to 63.51)	49.44 (25.28 to 95.70)	1.11 (0.73 to 1.90)	1.01 (0.55 to 1.75)	0.77 (0.43 to 1.34)	1.04 (0.56 to 1.88)
Self-harm by other specified means	5,425.05 (3,821.97 to 7,311.83)	6,247.53 (3,464.99 to 11,105.80)	4,903.36 (2,852.31 to 8,309.05)	6,565.25 (3,597.43 to 11,862.36)	107.31 (74.80 to 143.67)	130.30 (78.55 to 216.46)	106.13 (67.24 to 166.71)	134.12 (79.81 to 227.12)
Interpersonal violence	2,478.26 (1,433.53 to 3,353.79)	2,102.65 (1,020.79 to 3,420.20)	1,705.38 (829.16 to 2,726.43)	2,169.00 (1,049.35 to 3,560.25)	47.15 (27.12 to 64.25)	41.34 (20.59 to 63.84)	34.58 (17.49 to 52.68)	41.96 (20.92 to 65.51)
Physical violence by firearm	161.07 (84.13 to 264.96)	133.53 (63.99 to 227.91)	109.47 (53.45 to 185.79)	134.87 (63.54 to 228.20)	3.04 (1.60 to 5.05)	2.56 (1.30 to 4.18)	2.15 (1.07 to 3.58)	2.55 (1.28 to 4.16)
Physical violence by sharp object	303.60 (167.54 to 429.50)	259.15 (136.94 to 402.81)	221.91 (113.83 to 352.51)	258.91 (135.54 to 401.05)	5.76 (3.22 to 8.13)	5.05 (2.69 to 7.59)	4.41 (2.32 to 6.91)	5.00 (2.64 to 7.55)
Physical violence by other means	2,013.59 (1,135.25 to 2,749.38)	1,709.96 (795.13 to 2,874.56)	1,374.00 (659.06 to 2,226.59)	1,775.22 (820.97 to 2,993.24)	38.35 (21.59 to 52.53)	33.73 (16.25 to 53.16)	28.02 (13.81 to 43.25)	34.42 (16.57 to 54.27)
**Forces of nature, conflict and terrorism, and executions and police conflict**	**5.94 (4.57 to 7.55)**	**120.28 (0.00 to 1,282.10)**	**115.06 (0.00 to 1,231.01)**	**120.13 (0.00 to 1,276.66)**	**0.12 (0.08 to 0.16)**	**2.62 (0.00 to 28.38)**	**2.62 (0.00 to 28.49)**	**2.55 (0.00 to 27.51)**
Exposure to forces of nature	0.00 (0.00 to 0.00)	119.17 (0.00 to 1,282.10)	114.00 (0.00 to 1,231.01)	119.02 (0.00 to 1,276.66)	0.00 (0.00 to 0.00)	2.59 (0.00 to 28.38)	2.60 (0.00 to 28.49)	2.53 (0.00 to 27.51)
Conflict and terrorism	0.00 (0.00 to 0.00)	1.11 (0.00 to 10.11)	1.06 (0.00 to 9.69)	1.11 (0.00 to 10.13)	0.00 (0.00 to 0.00)	0.02 (0.00 to 0.19)	0.02 (0.00 to 0.19)	0.02 (0.00 to 0.19)
Executions and police conflict	5.94 (4.57 to 7.55)	0.00 (0.00 to 0.00)	0.00 (0.00 to 0.00)	0.00 (0.00 to 0.00)	0.12 (0.08 to 0.16)	0.00 (0.00 to 0.00)	0.00 (0.00 to 0.00)	0.00 (0.00 to 0.00)

Guam								
Cause	YLLs (actual counts)				Deaths (actual counts)			
	2016	2040 Forecast	2040 Better Scenario	2040 Worse Scenario	2016	2040 Forecast	2040 Better Scenario	2040 Worse Scenario
**All causes**	**36,001.58 (31,401.26 to 40,685.71)**	**38,504.17 (30,199.84 to 48,047.88)**	**28,473.13 (22,614.27 to 35,371.34)**	**45,799.13 (35,913.40 to 57,248.41)**	**1,459.79 (1,308.88 to 1,631.59)**	**1,980.26 (1,708.35 to 2,265.58)**	**1,624.93 (1,398.11 to 1,889.94)**	**2,235.32 (1,904.18 to 2,555.25)**
**Communicable, maternal, neonatal, and nutritional diseases**	**4,500.79 (3,542.18 to 5,881.02)**	**3,596.54 (2,142.55 to 5,661.06)**	**2,402.92 (1,547.59 to 3,969.69)**	**4,096.90 (2,494.60 to 6,423.34)**	**105.54 (87.12 to 136.92)**	**120.68 (87.72 to 164.28)**	**100.12 (75.01 to 138.24)**	**128.41 (92.27 to 173.93)**
**HIV/AIDS and tuberculosis**	**417.64 (141.91 to 1,700.88)**	**404.63 (72.52 to 1,924.88)**	**219.04 (25.71 to 1,561.53)**	**540.34 (124.20 to 2,053.54)**	**11.97 (5.21 to 45.18)**	**11.59 (3.03 to 49.74)**	**6.63 (1.30 to 42.49)**	**14.75 (4.65 to 52.19)**
Tuberculosis	131.14 (103.88 to 157.58)	68.99 (42.51 to 114.97)	33.71 (22.11 to 51.38)	85.64 (50.76 to 146.26)	5.11 (4.02 to 6.05)	3.18 (2.02 to 5.11)	1.68 (1.16 to 2.52)	3.75 (2.31 to 6.10)
HIV/AIDS	286.49 (13.36 to 1,559.77)	335.64 (10.24 to 1,854.90)	185.33 (0.00 to 1,526.39)	454.70 (47.53 to 1,992.80)	6.86 (0.28 to 40.14)	8.41 (0.22 to 46.48)	4.95 (0.00 to 40.80)	10.99 (1.14 to 48.55)
**Diarrhea, lower respiratory, and other common infectious diseases**	**1,792.96 (1,469.16 to 2,158.76)**	**1,662.91 (1,156.09 to 2,485.95)**	**1,208.36 (894.31 to 1,677.57)**	**1,848.70 (1,259.85 to 2,866.10)**	**60.05 (50.90 to 72.02)**	**79.26 (59.02 to 106.19)**	**67.11 (50.81 to 89.15)**	**83.06 (59.99 to 113.46)**
Diarrheal diseases	39.28 (26.14 to 56.64)	118.11 (31.64 to 351.72)	131.39 (47.17 to 347.32)	101.05 (26.56 to 313.56)	1.76 (1.01 to 2.75)	7.89 (2.02 to 23.25)	10.05 (3.36 to 26.08)	6.41 (1.47 to 19.94)
Intestinal infectious diseases	85.11 (28.54 to 191.20)	140.77 (47.00 to 301.75)	145.65 (57.36 to 294.77)	133.52 (44.69 to 289.25)	1.34 (0.45 to 3.01)	2.18 (0.76 to 4.56)	2.37 (0.95 to 4.72)	2.05 (0.72 to 4.42)
Typhoid fever	72.86 (24.57 to 166.05)	127.46 (41.19 to 273.66)	137.33 (53.45 to 278.05)	119.17 (39.78 to 258.14)	1.14 (0.38 to 2.60)	1.97 (0.69 to 4.05)	2.23 (0.88 to 4.39)	1.82 (0.64 to 3.86)
Paratyphoid fever	12.20 (3.70 to 29.79)	13.28 (3.67 to 35.85)	8.29 (2.52 to 21.55)	14.32 (4.06 to 37.90)	0.20 (0.06 to 0.47)	0.21 (0.06 to 0.55)	0.14 (0.04 to 0.35)	0.23 (0.07 to 0.59)
Other intestinal infectious diseases	0.05 (0.02 to 0.12)	0.03 (0.01 to 0.08)	0.03 (0.01 to 0.06)	0.04 (0.01 to 0.08)	0.00 (0.00 to 0.00)	0.00 (0.00 to 0.00)	0.00 (0.00 to 0.00)	0.00 (0.00 to 0.00)
Lower respiratory infections	1,504.75 (1,228.59 to 1,809.78)	1,300.29 (877.88 to 2,038.93)	866.97 (623.91 to 1,217.07)	1,502.93 (994.19 to 2,364.62)	54.15 (45.26 to 65.68)	67.04 (46.89 to 92.43)	53.08 (38.28 to 70.88)	72.37 (51.19 to 102.36)
Upper respiratory infections	0.68 (0.35 to 1.22)	0.54 (0.21 to 1.12)	0.29 (0.12 to 0.61)	0.61 (0.23 to 1.24)	0.02 (0.01 to 0.03)	0.02 (0.01 to 0.04)	0.01 (0.00 to 0.02)	0.02 (0.01 to 0.04)
Otitis media	0.02 (0.01 to 0.05)	0.01 (0.00 to 0.03)	0.01 (0.00 to 0.03)	0.01 (0.00 to 0.03)	0.00 (0.00 to 0.00)	0.00 (0.00 to 0.00)	0.00 (0.00 to 0.00)	0.00 (0.00 to 0.00)
Meningitis	94.09 (68.27 to 135.21)	38.97 (22.49 to 66.06)	24.29 (14.74 to 39.82)	42.20 (24.27 to 69.45)	1.69 (1.29 to 2.37)	0.90 (0.59 to 1.36)	0.64 (0.43 to 0.99)	0.94 (0.62 to 1.43)
Pneumococcal meningitis	0.30 (0.20 to 0.47)	0.11 (0.07 to 0.19)	0.10 (0.06 to 0.17)	0.11 (0.07 to 0.18)	0.01 (0.00 to 0.01)	0.00 (0.00 to 0.01)	0.00 (0.00 to 0.01)	0.00 (0.00 to 0.00)
H influenzae type B meningitis	10.79 (7.15 to 16.42)	5.64 (2.79 to 12.00)	4.58 (2.50 to 8.44)	5.65 (2.72 to 12.30)	0.19 (0.13 to 0.28)	0.12 (0.08 to 0.20)	0.12 (0.08 to 0.19)	0.12 (0.07 to 0.20)
Meningococcal meningitis	18.51 (12.48 to 27.86)	7.63 (4.32 to 12.81)	4.75 (2.85 to 8.07)	8.28 (4.82 to 14.10)	0.37 (0.27 to 0.53)	0.21 (0.13 to 0.33)	0.15 (0.10 to 0.22)	0.22 (0.14 to 0.34)
Other meningitis	64.49 (46.36 to 92.46)	25.58 (14.47 to 43.01)	14.85 (8.74 to 24.60)	28.15 (15.68 to 46.96)	1.12 (0.85 to 1.61)	0.56 (0.36 to 0.88)	0.37 (0.24 to 0.56)	0.60 (0.39 to 0.95)
Encephalitis	15.93 (12.57 to 21.16)	22.81 (15.54 to 33.87)	20.38 (13.82 to 29.82)	22.74 (15.57 to 33.63)	0.36 (0.29 to 0.48)	0.61 (0.47 to 0.85)	0.61 (0.46 to 0.84)	0.59 (0.45 to 0.81)

Diphtheria	0.16 (0.10 to 0.26)	0.05 (0.02 to 0.12)	0.03 (0.02 to 0.06)	0.05 (0.02 to 0.12)	0.00 (0.00 to 0.00)	0.00 (0.00 to 0.00)	0.00 (0.00 to 0.00)	0.00 (0.00 to 0.00)
Whooping cough	7.84 (0.72 to 27.08)	8.58 (0.30 to 35.63)	1.27 (0.05 to 4.89)	9.60 (0.32 to 39.22)	0.09 (0.01 to 0.32)	0.10 (0.00 to 0.42)	0.02 (0.00 to 0.06)	0.11 (0.00 to 0.47)
Tetanus	1.00 (0.84 to 1.16)	0.19 (0.11 to 0.33)	0.14 (0.08 to 0.24)	0.19 (0.11 to 0.35)	0.02 (0.02 to 0.02)	0.00 (0.00 to 0.01)	0.00 (0.00 to 0.00)	0.00 (0.00 to 0.01)
Measles	37.94 (11.21 to 95.12)	27.45 (0.03 to 216.96)	14.52 (0.02 to 103.57)	30.45 (0.03 to 241.91)	0.45 (0.13 to 1.14)	0.35 (0.00 to 2.68)	0.19 (0.00 to 1.27)	0.38 (0.00 to 2.92)

Guam								
Cause	YLLs (actual counts)				Deaths (actual counts)			
	2016	2040 Forecast	2040 Better Scenario	2040 Worse Scenario	2016	2040 Forecast	2040 Better Scenario	2040 Worse Scenario
Varicella and herpes zoster	6.18 (3.81 to 10.10)	5.14 (2.12 to 10.66)	3.42 (1.54 to 6.64)	5.35 (2.15 to 10.89)	0.17 (0.11 to 0.24)	0.18 (0.10 to 0.30)	0.15 (0.08 to 0.27)	0.18 (0.09 to 0.30)
**Neglected tropical diseases and malaria**	**41.53 (10.26 to 77.62)**	**47.81 (13.29 to 83.04)**	**47.78 (13.60 to 81.11)**	**46.42 (12.43 to 81.68)**	**1.45 (0.40 to 2.52)**	**2.30 (0.72 to 3.59)**	**2.55 (0.86 to 3.87)**	**2.14 (0.64 to 3.41)**
Malaria	28.54 (2.63 to 62.90)	23.11 (1.41 to 52.69)	22.34 (1.31 to 51.19)	23.02 (1.42 to 53.08)	0.75 (0.06 to 1.66)	0.65 (0.03 to 1.47)	0.65 (0.03 to 1.46)	0.63 (0.03 to 1.43)
Chagas disease	0.00 (0.00 to 0.00)	0.00 (0.00 to 0.00)	0.00 (0.00 to 0.00)	0.00 (0.00 to 0.00)	0.00 (0.00 to 0.00)	0.00 (0.00 to 0.00)	0.00 (0.00 to 0.00)	0.00 (0.00 to 0.00)
Leishmaniasis	0.00 (0.00 to 0.00)	0.00 (0.00 to 0.00)	0.00 (0.00 to 0.00)	0.00 (0.00 to 0.00)	0.00 (0.00 to 0.00)	0.00 (0.00 to 0.00)	0.00 (0.00 to 0.00)	0.00 (0.00 to 0.00)
African trypanosomiasis	0.00 (0.00 to 0.00)	0.00 (0.00 to 0.00)	0.00 (0.00 to 0.00)	0.00 (0.00 to 0.00)	0.00 (0.00 to 0.00)	0.00 (0.00 to 0.00)	0.00 (0.00 to 0.00)	0.00 (0.00 to 0.00)
Schistosomiasis	0.00 (0.00 to 0.00)	0.00 (0.00 to 0.00)	0.00 (0.00 to 0.00)	0.00 (0.00 to 0.00)	0.00 (0.00 to 0.00)	0.00 (0.00 to 0.00)	0.00 (0.00 to 0.00)	0.00 (0.00 to 0.00)
Cysticercosis	0.00 (0.00 to 0.00)	0.00 (0.00 to 0.00)	0.00 (0.00 to 0.00)	0.00 (0.00 to 0.00)	0.00 (0.00 to 0.00)	0.00 (0.00 to 0.00)	0.00 (0.00 to 0.00)	0.00 (0.00 to 0.00)
Cystic echinococcosis	0.00 (0.00 to 0.00)	0.00 (0.00 to 0.00)	0.00 (0.00 to 0.00)	0.00 (0.00 to 0.00)	0.00 (0.00 to 0.00)	0.00 (0.00 to 0.00)	0.00 (0.00 to 0.00)	0.00 (0.00 to 0.00)
Dengue	0.14 (0.04 to 0.24)	0.88 (0.20 to 1.46)	0.88 (0.20 to 1.49)	0.85 (0.19 to 1.42)	0.01 (0.00 to 0.01)	0.05 (0.01 to 0.08)	0.05 (0.01 to 0.09)	0.04 (0.01 to 0.08)
Yellow fever	0.00 (0.00 to 0.00)	0.00 (0.00 to 0.00)	0.00 (0.00 to 0.00)	0.00 (0.00 to 0.00)	0.00 (0.00 to 0.00)	0.00 (0.00 to 0.00)	0.00 (0.00 to 0.00)	0.00 (0.00 to 0.00)
Rabies	0.03 (0.02 to 0.04)	0.03 (0.02 to 0.05)	0.03 (0.01 to 0.04)	0.03 (0.02 to 0.05)	0.00 (0.00 to 0.00)	0.00 (0.00 to 0.00)	0.00 (0.00 to 0.00)	0.00 (0.00 to 0.00)
Intestinal nematode infections	1.17 (0.40 to 2.66)	2.06 (0.57 to 5.60)	1.67 (0.55 to 4.25)	2.04 (0.55 to 5.59)	0.02 (0.01 to 0.04)	0.04 (0.02 to 0.08)	0.04 (0.02 to 0.07)	0.04 (0.02 to 0.08)
Other neglected tropical diseases	11.65 (6.00 to 16.45)	21.73 (9.52 to 31.03)	22.87 (10.18 to 31.95)	20.48 (8.69 to 29.69)	0.67 (0.30 to 1.00)	1.57 (0.61 to 2.45)	1.82 (0.76 to 2.74)	1.43 (0.54 to 2.26)
**Maternal disorders**	**64.95 (42.36 to 94.17)**	**49.05 (26.85 to 80.15)**	**36.88 (20.07 to 61.11)**	**52.51 (28.76 to 86.98)**	**1.27 (0.82 to 1.87)**	**0.97 (0.52 to 1.62)**	**0.73 (0.39 to 1.23)**	**1.04 (0.56 to 1.75)**
Maternal hemorrhage	9.00 (4.98 to 14.36)	6.78 (3.27 to 12.61)	5.25 (2.48 to 10.05)	7.22 (3.47 to 13.61)	0.17 (0.10 to 0.28)	0.13 (0.06 to 0.25)	0.10 (0.05 to 0.20)	0.14 (0.06 to 0.27)
Maternal sepsis and other maternal infections	4.00 (1.76 to 7.67)	2.75 (1.09 to 5.83)	2.09 (0.80 to 4.45)	2.94 (1.15 to 6.18)	0.08 (0.03 to 0.16)	0.06 (0.02 to 0.12)	0.04 (0.02 to 0.09)	0.06 (0.02 to 0.12)
Maternal hypertensive disorders	4.06 (2.19 to 6.89)	3.09 (1.40 to 5.75)	2.33 (1.05 to 4.41)	3.31 (1.50 to 6.16)	0.08 (0.04 to 0.14)	0.06 (0.03 to 0.12)	0.05 (0.02 to 0.09)	0.06 (0.03 to 0.12)
Maternal obstructed labor and uterine rupture	2.17 (0.89 to 4.16)	1.72 (0.59 to 3.75)	1.26 (0.44 to 2.79)	1.86 (0.67 to 4.00)	0.04 (0.02 to 0.08)	0.03 (0.01 to 0.07)	0.02 (0.01 to 0.06)	0.04 (0.01 to 0.08)
Maternal abortion, miscarriage, and ectopic pregnancy	34.04 (21.68 to 50.12)	25.73 (13.41 to 44.25)	19.18 (9.83 to 33.46)	27.54 (14.10 to 48.62)	0.68 (0.42 to 1.02)	0.52 (0.26 to 0.91)	0.39 (0.19 to 0.69)	0.55 (0.27 to 0.99)

Indirect maternal deaths	3.99 (2.15 to 6.70)	3.25 (1.48 to 6.14)	2.36 (1.06 to 4.48)	3.51 (1.55 to 6.65)	0.08 (0.04 to 0.13)	0.06 (0.03 to 0.12)	0.05 (0.02 to 0.09)	0.07 (0.03 to 0.13)
Late maternal deaths	1.33 (0.52 to 2.57)	0.81 (0.29 to 1.73)	0.60 (0.21 to 1.27)	0.87 (0.31 to 1.86)	0.03 (0.01 to 0.05)	0.02 (0.01 to 0.03)	0.01 (0.00 to 0.02)	0.02 (0.01 to 0.04)
Maternal deaths aggravated by HIV/AIDS	0.01 (0.00 to 0.06)	0.15 (0.00 to 1.02)	0.09 (0.00 to 0.77)	0.16 (0.00 to 1.09)	0.00 (0.00 to 0.00)	0.00 (0.00 to 0.02)	0.00 (0.00 to 0.01)	0.00 (0.00 to 0.02)
Other maternal disorders	6.32 (3.44 to 10.19)	4.78 (2.18 to 8.61)	3.73 (1.70 to 6.74)	5.08 (2.34 to 9.15)	0.12 (0.07 to 0.20)	0.09 (0.04 to 0.17)	0.07 (0.03 to 0.13)	0.10 (0.04 to 0.18)

Guam								
Cause	YLLs (actual counts)				Deaths (actual counts)			
	2016	2040 Forecast	2040 Better Scenario	2040 Worse Scenario	2016	2040 Forecast	2040 Better Scenario	2040 Worse Scenario
**Neonatal disorders**	**1,680.65 (1,200.12 to 2,250.74)**	**1,162.75 (345.94 to 2,368.44)**	**648.10 (195.61 to 1,286.77)**	**1,342.12 (406.21 to 2,699.56)**	**19.42 (13.87 to 26.02)**	**13.44 (4.00 to 27.37)**	**7.49 (2.26 to 14.87)**	**15.52 (4.69 to 31.22)**
Neonatal preterm birth	718.71 (481.04 to 1,005.74)	329.11 (99.10 to 694.62)	158.35 (48.94 to 326.59)	454.23 (141.00 to 932.06)	8.30 (5.56 to 11.62)	3.80 (1.15 to 8.03)	1.83 (0.57 to 3.77)	5.25 (1.63 to 10.77)
Neonatal encephalopathy due to birth asphyxia and trauma	326.48 (205.31 to 475.66)	206.07 (53.36 to 443.98)	100.19 (26.53 to 214.60)	229.67 (58.50 to 504.98)	3.77 (2.37 to 5.50)	2.38 (0.62 to 5.13)	1.16 (0.31 to 2.48)	2.66 (0.68 to 5.84)
Neonatal sepsis and other neonatal infections	192.60 (113.01 to 305.90)	181.33 (45.59 to 416.26)	96.00 (24.41 to 218.65)	197.57 (50.06 to 450.48)	2.23 (1.31 to 3.53)	2.10 (0.53 to 4.81)	1.11 (0.28 to 2.53)	2.28 (0.58 to 5.21)
Hemolytic disease and other neonatal jaundice	12.39 (6.30 to 21.02)	5.38 (1.21 to 13.23)	2.76 (0.64 to 6.61)	5.90 (1.35 to 14.32)	0.14 (0.07 to 0.24)	0.06 (0.01 to 0.15)	0.03 (0.01 to 0.08)	0.07 (0.02 to 0.17)
Other neonatal disorders	430.48 (278.28 to 627.76)	440.85 (130.68 to 933.35)	290.80 (87.75 to 617.50)	454.75 (134.49 to 972.62)	4.98 (3.22 to 7.26)	5.10 (1.51 to 10.80)	3.36 (1.02 to 7.13)	5.26 (1.56 to 11.23)
**Nutritional deficiencies**	**94.77 (71.84 to 126.57)**	**111.88 (79.89 to 161.46)**	**126.16 (97.01 to 165.33)**	**102.03 (69.79 to 148.58)**	**4.09 (3.50 to 4.73)**	**8.71 (5.75 to 11.78)**	**11.75 (8.18 to 15.34)**	**7.50 (4.73 to 10.37)**
Protein-energy malnutrition	91.72 (69.43 to 123.11)	106.11 (73.89 to 154.54)	120.85 (91.59 to 160.02)	96.41 (65.82 to 141.72)	3.99 (3.42 to 4.63)	8.42 (5.37 to 11.47)	11.43 (7.65 to 14.97)	7.23 (4.43 to 10.18)
Iodine deficiency	0.82 (0.04 to 2.92)	3.28 (0.09 to 12.76)	3.14 (0.07 to 12.84)	3.14 (0.09 to 12.37)	0.03 (0.00 to 0.10)	0.21 (0.00 to 1.06)	0.23 (0.00 to 1.30)	0.19 (0.00 to 0.97)
Dietary iron deficiency	0.86 (0.68 to 1.08)	1.19 (0.75 to 1.82)	1.11 (0.73 to 1.65)	1.17 (0.74 to 1.78)	0.02 (0.01 to 0.02)	0.02 (0.02 to 0.03)	0.02 (0.02 to 0.03)	0.02 (0.02 to 0.03)
Other nutritional deficiencies	1.37 (1.08 to 1.73)	1.30 (0.90 to 1.80)	1.06 (0.76 to 1.44)	1.31 (0.92 to 1.81)	0.05 (0.04 to 0.07)	0.06 (0.04 to 0.09)	0.06 (0.04 to 0.09)	0.06 (0.04 to 0.09)
**Other communicable, maternal, neonatal, and nutritional diseases**	**408.29 (154.88 to 829.00)**	**157.51 (91.24 to 280.27)**	**116.61 (74.01 to 189.14)**	**164.78 (93.31 to 292.77)**	**7.28 (4.16 to 12.24)**	**4.39 (3.01 to 6.15)**	**3.84 (2.72 to 5.12)**	**4.40 (2.98 to 6.26)**
Sexually transmitted infections excluding HIV	264.97 (5.60 to 693.20)	54.32 (4.24 to 168.39)	30.28 (3.55 to 94.44)	58.60 (4.20 to 185.23)	3.17 (0.14 to 8.16)	0.69 (0.10 to 2.02)	0.41 (0.09 to 1.17)	0.74 (0.11 to 2.24)
Syphilis	261.08 (1.44 to 689.20)	51.16 (0.85 to 164.55)	27.39 (0.66 to 91.32)	55.38 (0.87 to 181.49)	3.07 (0.04 to 8.07)	0.61 (0.02 to 1.95)	0.33 (0.02 to 1.09)	0.66 (0.02 to 2.16)
Chlamydial infection	0.75 (0.51 to 1.06)	0.62 (0.36 to 1.00)	0.56 (0.33 to 0.92)	0.63 (0.37 to 1.02)	0.02 (0.01 to 0.03)	0.02 (0.01 to 0.02)	0.02 (0.01 to 0.02)	0.02 (0.01 to 0.02)
Gonococcal infection	2.02 (1.36 to 2.82)	1.64 (0.94 to 2.67)	1.50 (0.85 to 2.49)	1.67 (0.96 to 2.72)	0.05 (0.04 to 0.07)	0.04 (0.03 to 0.07)	0.04 (0.03 to 0.06)	0.04 (0.03 to 0.07)
Other sexually transmitted infections	1.12 (0.75 to 1.57)	0.91 (0.52 to 1.47)	0.83 (0.47 to 1.37)	0.93 (0.53 to 1.50)	0.03 (0.02 to 0.04)	0.02 (0.01 to 0.04)	0.02 (0.01 to 0.03)	0.02 (0.01 to 0.04)
Acute hepatitis	89.38 (68.69 to 114.50)	50.09 (31.90 to 74.46)	34.64 (21.90 to 51.96)	54.00 (34.01 to 80.28)	2.54 (2.00 to 3.19)	1.60 (1.05 to 2.26)	1.16 (0.76 to 1.66)	1.69 (1.12 to 2.39)
Acute hepatitis A	3.97 (2.60 to 5.77)	1.10 (0.62 to 1.88)	0.79 (0.47 to 1.29)	1.15 (0.65 to 2.00)	0.07 (0.05 to 0.10)	0.03 (0.02 to 0.05)	0.02 (0.01 to 0.04)	0.03 (0.02 to 0.05)
Acute hepatitis B	75.00 (56.43 to 96.72)	43.52 (27.44 to 65.88)	30.11 (18.88 to 45.76)	47.02 (29.66 to 71.55)	2.20 (1.70 to 2.81)	1.38 (0.90 to 1.99)	0.99 (0.64 to 1.43)	1.47 (0.96 to 2.12)

Acute hepatitis C	1.74 (1.19 to 2.55)	1.00 (0.58 to 1.62)	0.71 (0.41 to 1.15)	1.07 (0.62 to 1.74)	0.05 (0.04 to 0.08)	0.04 (0.02 to 0.05)	0.03 (0.02 to 0.04)	0.04 (0.02 to 0.06)
Acute hepatitis E	8.67 (5.99 to 12.07)	4.47 (2.37 to 7.32)	3.03 (1.62 to 4.92)	4.76 (2.52 to 7.85)	0.22 (0.15 to 0.31)	0.15 (0.08 to 0.26)	0.12 (0.06 to 0.20)	0.16 (0.09 to 0.26)
Other unspecified infectious diseases	53.94 (28.43 to 68.59)	53.10 (30.08 to 70.12)	51.69 (30.68 to 68.19)	52.17 (29.31 to 68.58)	1.57 (0.85 to 1.94)	2.10 (1.19 to 2.66)	2.28 (1.35 to 2.90)	1.97 (1.09 to 2.51)
**Non-communicable diseases**	**26,996.18 (23,564.41 to 30,865.59)**	**30,660.41 (23,746.88 to 39,085.35)**	**23,197.78 (17,950.76 to 29,544.25)**	**36,988.29 (28,198.02 to 47,196.10)**	**1,251.21 (1,120.49 to 1,399.03)**	**1,748.97 (1,488.08 to 2,034.27)**	**1,442.03 (1,218.52 to 1,718.02)**	**1,989.17 (1,664.90 to 2,308.53)**

Guam								
Cause	YLLs (actual counts)				Deaths (actual counts)			
	2016	2040 Forecast	2040 Better Scenario	2040 Worse Scenario	2016	2040 Forecast	2040 Better Scenario	2040 Worse Scenario
**Neoplasms**	**5,777.64 (5,006.67 to 6,713.27)**	**7,831.74 (5,973.41 to 10,166.62)**	**6,015.40 (4,680.10 to 7,631.16)**	**8,941.26 (6,759.41 to 11,810.47)**	**248.02 (218.95 to 282.27)**	**394.47 (312.08 to 491.90)**	**319.11 (263.44 to 384.84)**	**434.66 (340.76 to 549.43)**
Lip and oral cavity cancer	123.62 (102.50 to 146.70)	162.32 (94.83 to 277.77)	102.61 (64.50 to 164.33)	203.70 (114.25 to 359.79)	5.50 (4.70 to 6.38)	8.16 (5.08 to 12.71)	5.48 (3.68 to 8.20)	9.79 (5.93 to 16.00)
Nasopharynx cancer	180.88 (142.13 to 230.56)	241.47 (123.11 to 449.71)	134.61 (73.68 to 238.70)	320.23 (152.70 to 610.79)	6.61 (5.29 to 8.35)	9.35 (5.08 to 16.18)	5.43 (3.08 to 9.07)	12.06 (6.26 to 21.77)
Other pharynx cancer	51.20 (42.03 to 61.47)	67.50 (46.30 to 102.46)	65.87 (46.50 to 93.99)	69.72 (45.50 to 112.27)	2.15 (1.81 to 2.55)	3.32 (2.42 to 4.77)	3.41 (2.56 to 4.70)	3.33 (2.32 to 4.98)
Esophageal cancer	113.70 (91.91 to 138.38)	162.13 (88.65 to 293.23)	119.28 (71.32 to 203.47)	192.69 (102.47 to 357.69)	5.05 (4.17 to 6.03)	8.01 (4.69 to 13.71)	6.17 (3.90 to 10.15)	9.21 (5.20 to 16.14)
Stomach cancer	201.04 (171.40 to 236.79)	159.51 (119.73 to 207.08)	120.12 (93.11 to 152.60)	172.41 (126.67 to 228.43)	9.29 (8.13 to 10.65)	8.97 (6.93 to 11.34)	7.15 (5.76 to 8.70)	9.39 (6.99 to 12.12)
Colon and rectum cancer	570.96 (481.46 to 671.34)	734.23 (499.09 to 1,077.32)	592.08 (415.68 to 853.59)	794.32 (533.93 to 1,186.09)	26.73 (23.05 to 30.60)	41.86 (28.67 to 61.17)	35.74 (25.29 to 50.46)	43.60 (29.88 to 64.07)
Liver cancer	525.02 (424.02 to 640.93)	803.16 (482.89 to 1,366.45)	731.73 (464.65 to 1,166.54)	958.76 (522.65 to 1,693.87)	20.83 (17.23 to 24.80)	36.39 (23.05 to 57.20)	34.86 (23.81 to 51.83)	41.55 (24.56 to 67.67)
Liver cancer due to hepatitis B	281.77 (222.49 to 349.56)	415.31 (246.37 to 702.54)	379.76 (236.88 to 624.60)	495.38 (267.50 to 890.29)	10.55 (8.51 to 12.82)	17.55 (11.02 to 28.45)	16.82 (11.27 to 25.99)	20.09 (11.67 to 33.49)
Liver cancer due to hepatitis C	77.19 (61.33 to 95.36)	124.54 (78.15 to 195.59)	117.50 (77.61 to 174.44)	143.35 (84.14 to 233.50)	3.68 (3.02 to 4.39)	7.01 (4.57 to 10.29)	6.96 (4.83 to 9.84)	7.70 (4.66 to 11.66)
Liver cancer due to alcohol use	47.89 (35.77 to 63.03)	73.93 (43.52 to 125.72)	64.20 (40.86 to 103.01)	92.23 (49.71 to 166.94)	1.97 (1.53 to 2.51)	3.36 (2.07 to 5.39)	3.08 (2.03 to 4.64)	4.00 (2.31 to 6.74)
Liver cancer due to other causes	118.16 (89.62 to 152.94)	189.38 (99.61 to 341.33)	170.26 (95.83 to 289.31)	227.81 (107.32 to 433.93)	4.62 (3.60 to 5.79)	8.47 (4.71 to 14.23)	8.00 (4.79 to 12.95)	9.76 (5.03 to 17.85)
Gallbladder and biliary tract cancer	34.46 (28.55 to 42.85)	31.59 (25.97 to 38.84)	32.18 (26.24 to 39.94)	30.13 (25.08 to 37.28)	1.65 (1.38 to 2.01)	1.78 (1.47 to 2.14)	1.89 (1.57 to 2.26)	1.65 (1.36 to 2.00)
Pancreatic cancer	224.05 (192.66 to 259.38)	366.61 (257.40 to 535.02)	326.77 (233.66 to 460.07)	380.65 (265.56 to 557.60)	10.50 (9.20 to 11.95)	19.41 (14.09 to 27.71)	17.94 (13.49 to 24.81)	19.68 (14.01 to 28.22)
Larynx cancer	60.73 (50.06 to 73.25)	67.51 (35.02 to 118.80)	39.36 (21.44 to 67.62)	84.66 (42.56 to 153.76)	2.81 (2.36 to 3.30)	3.58 (1.92 to 6.28)	2.17 (1.21 to 3.64)	4.35 (2.25 to 7.83)
Tracheal, bronchus, and lung cancer	1,558.84 (1,341.29 to 1,847.32)	2,309.83 (1,478.92 to 3,410.67)	1,340.58 (889.68 to 1,927.84)	2,937.40 (1,859.71 to 4,344.66)	72.01 (62.63 to 84.16)	120.73 (79.65 to 171.05)	73.03 (50.49 to 102.78)	148.97 (97.86 to 213.39)
Malignant skin melanoma	34.15 (26.29 to 43.60)	51.86 (39.45 to 66.30)	67.25 (50.62 to 85.53)	46.02 (35.35 to 57.44)	1.50 (1.16 to 1.87)	2.77 (2.04 to 3.44)	3.81 (2.84 to 4.71)	2.37 (1.74 to 2.93)
Non-melanoma skin cancer	37.92 (31.10 to 46.19)	45.33 (37.05 to 54.57)	39.38 (32.49 to 46.90)	44.19 (35.88 to 52.75)	1.96 (1.68 to 2.28)	3.03 (2.31 to 3.72)	2.86 (2.30 to 3.47)	2.81 (2.11 to 3.46)
Breast cancer	411.74 (315.63 to 517.59)	485.59 (294.57 to 736.82)	380.07 (242.78 to 555.16)	525.84 (314.73 to 807.89)	16.32 (13.06 to 19.96)	23.59 (15.38 to 34.73)	19.42 (13.29 to 27.32)	24.77 (16.08 to 36.61)
Cervical cancer	103.50 (75.85 to 145.44)	101.96 (58.86 to 163.30)	73.49 (45.49 to 112.11)	112.77 (64.74 to 184.88)	3.63 (2.70 to 5.01)	3.99 (2.55 to 6.06)	2.98 (2.04 to 4.24)	4.32 (2.72 to 6.74)

Uterine cancer	68.37 (52.69 to 83.66)	92.56 (68.35 to 120.52)	82.20 (60.26 to 107.35)	93.77 (69.33 to 122.04)	2.79 (2.21 to 3.36)	4.41 (3.50 to 5.40)	4.06 (3.21 to 4.98)	4.38 (3.48 to 5.37)
Ovarian cancer	90.87 (71.06 to 113.53)	112.62 (73.21 to 175.09)	98.98 (65.05 to 149.50)	115.72 (74.45 to 179.62)	3.69 (2.90 to 4.52)	5.14 (3.39 to 7.96)	4.63 (3.12 to 6.90)	5.20 (3.45 to 8.07)
Prostate cancer	215.72 (161.37 to 267.26)	354.12 (264.58 to 440.65)	341.51 (257.17 to 423.00)	332.72 (239.59 to 413.35)	14.28 (10.71 to 17.58)	27.24 (19.49 to 34.74)	27.59 (20.46 to 34.60)	24.84 (16.81 to 32.09)
Testicular cancer	7.32 (5.38 to 9.41)	6.19 (4.15 to 8.74)	5.22 (3.51 to 7.26)	6.27 (4.21 to 8.85)	0.20 (0.15 to 0.25)	0.20 (0.14 to 0.26)	0.18 (0.13 to 0.23)	0.19 (0.14 to 0.25)

Guam								
Cause	YLLs (actual counts)				Deaths (actual counts)			
	2016	2040 Forecast	2040 Better Scenario	2040 Worse Scenario	2016	2040 Forecast	2040 Better Scenario	2040 Worse Scenario
Kidney cancer	71.48 (59.07 to 87.87)	119.63 (81.36 to 174.17)	111.83 (78.61 to 157.14)	121.65 (80.06 to 181.32)	2.90 (2.46 to 3.48)	5.75 (4.10 to 8.00)	5.72 (4.28 to 7.59)	5.65 (3.89 to 8.10)
Bladder cancer	95.30 (79.61 to 113.33)	111.32 (70.42 to 174.58)	87.60 (58.43 to 131.35)	120.54 (74.57 to 195.35)	4.79 (4.08 to 5.62)	6.56 (4.28 to 10.00)	5.43 (3.71 to 7.86)	6.87 (4.39 to 10.76)
Brain and nervous system cancer	99.85 (80.37 to 122.52)	133.25 (99.07 to 172.09)	118.62 (89.16 to 153.05)	132.37 (98.17 to 171.34)	2.85 (2.30 to 3.50)	4.37 (3.49 to 5.41)	4.23 (3.38 to 5.25)	4.22 (3.37 to 5.25)
Thyroid cancer	26.47 (21.62 to 31.13)	30.95 (22.43 to 42.51)	27.15 (19.90 to 37.32)	33.44 (23.61 to 47.00)	1.23 (1.00 to 1.43)	1.69 (1.24 to 2.27)	1.55 (1.16 to 2.07)	1.76 (1.28 to 2.43)
Mesothelioma	15.33 (10.77 to 19.77)	18.56 (13.11 to 23.85)	19.83 (13.73 to 25.97)	17.40 (12.30 to 22.01)	0.60 (0.43 to 0.76)	0.80 (0.58 to 0.99)	0.90 (0.66 to 1.11)	0.73 (0.54 to 0.90)
Hodgkin lymphoma	14.86 (11.35 to 21.58)	12.31 (8.06 to 17.76)	9.18 (6.05 to 13.66)	12.83 (8.36 to 18.76)	0.42 (0.33 to 0.61)	0.40 (0.29 to 0.56)	0.32 (0.24 to 0.46)	0.41 (0.29 to 0.58)
Non-Hodgkin lymphoma	190.94 (156.56 to 237.76)	238.36 (182.25 to 308.38)	209.18 (160.28 to 273.09)	247.33 (186.74 to 326.30)	6.69 (5.62 to 8.21)	10.19 (8.11 to 12.77)	9.65 (7.84 to 12.01)	10.18 (8.02 to 12.86)
Multiple myeloma	82.47 (66.49 to 100.40)	108.62 (83.82 to 138.22)	104.95 (80.95 to 132.50)	110.18 (83.84 to 142.02)	3.65 (2.94 to 4.39)	5.74 (4.32 to 7.19)	5.88 (4.48 to 7.20)	5.60 (4.14 to 7.19)
Leukemia	288.49 (240.12 to 348.62)	344.86 (244.52 to 477.42)	287.19 (207.99 to 386.41)	375.25 (261.43 to 532.53)	8.50 (7.15 to 10.42)	12.91 (9.34 to 17.76)	11.67 (8.76 to 15.53)	13.55 (9.56 to 19.15)
Acute lymphoid leukemia	48.56 (37.07 to 62.43)	46.57 (29.97 to 70.02)	35.72 (23.27 to 53.43)	50.92 (32.92 to 75.71)	1.04 (0.82 to 1.34)	1.13 (0.77 to 1.63)	0.92 (0.64 to 1.31)	1.23 (0.82 to 1.80)
Chronic lymphoid leukemia	10.63 (8.36 to 13.75)	12.51 (8.68 to 17.83)	11.46 (8.12 to 15.92)	13.25 (8.96 to 19.18)	0.51 (0.41 to 0.66)	0.73 (0.49 to 1.08)	0.70 (0.49 to 1.00)	0.74 (0.48 to 1.09)
Acute myeloid leukemia	119.40 (96.82 to 147.37)	176.76 (126.00 to 243.70)	157.09 (112.57 to 213.19)	189.77 (133.09 to 266.54)	3.25 (2.67 to 4.05)	6.03 (4.42 to 8.12)	5.83 (4.39 to 7.63)	6.25 (4.47 to 8.76)
Chronic myeloid leukemia	28.40 (22.64 to 36.08)	17.77 (11.15 to 27.20)	11.71 (7.92 to 17.10)	21.05 (13.45 to 33.10)	1.03 (0.83 to 1.29)	0.74 (0.49 to 1.10)	0.51 (0.36 to 0.73)	0.85 (0.56 to 1.26)
Other leukemia	81.50 (66.31 to 100.61)	91.25 (60.88 to 135.12)	71.21 (49.07 to 103.67)	100.26 (65.83 to 151.01)	2.67 (2.20 to 3.26)	4.28 (2.89 to 6.27)	3.71 (2.64 to 5.16)	4.48 (2.99 to 6.70)
Other neoplasms	278.37 (235.49 to 329.10)	357.77 (286.57 to 432.48)	346.57 (276.95 to 418.78)	348.30 (280.31 to 422.21)	8.93 (7.65 to 10.34)	14.12 (12.03 to 16.20)	14.97 (12.79 to 17.23)	13.23 (11.16 to 15.26)
**Cardiovascular diseases**	**12,475.72 (10,871.57 to 14,352.14)**	**10,857.31 (7,612.43 to 15,867.25)**	**6,934.46 (5,034.01 to 10,005.30)**	**15,022.17 (9,991.10 to 22,430.89)**	**617.08 (550.92 to 689.38)**	**657.38 (495.37 to 923.15)**	**475.04 (363.55 to 696.23)**	**825.44 (605.72 to 1,164.09)**
Rheumatic heart disease	197.64 (166.82 to 233.10)	108.05 (82.01 to 138.81)	86.21 (66.42 to 109.46)	110.57 (83.94 to 141.65)	6.94 (5.99 to 8.02)	5.41 (4.18 to 6.72)	4.80 (3.76 to 5.85)	5.27 (3.97 to 6.62)
Ischemic heart disease	7,764.84 (6,679.55 to 9,006.75)	6,177.90 (3,559.53 to 10,879.25)	3,487.93 (2,001.48 to 6,308.68)	9,323.83 (5,241.70 to 16,072.21)	391.59 (346.94 to 439.19)	370.00 (221.78 to 647.97)	238.59 (140.39 to 453.61)	502.22 (303.30 to 849.87)
Stroke	2,475.15 (2,140.20 to 2,863.33)	1,773.39 (1,033.05 to 3,100.19)	1,002.08 (618.07 to 1,963.29)	2,587.56 (1,459.70 to 4,455.60)	121.17 (106.56 to 138.11)	107.07 (64.97 to 221.22)	69.98 (43.26 to 158.37)	140.56 (83.34 to 272.22)
Ischemic stroke	943.81 (818.39 to 1,099.38)	693.66 (369.82 to 1,781.89)	438.02 (232.40 to 1,272.75)	935.11 (476.12 to 2,199.93)	55.83 (48.59 to 64.04)	50.30 (25.51 to 142.14)	35.70 (17.89 to 107.28)	62.19 (31.51 to 186.38)

Intracerebral hemorrhage	1,531.34 (1,306.54 to 1,780.05)	1,079.73 (599.01 to 1,920.95)	564.06 (337.41 to 940.94)	1,652.45 (848.93 to 2,996.71)	65.34 (56.58 to 74.73)	56.78 (34.73 to 99.23)	34.28 (21.71 to 60.16)	78.37 (43.67 to 141.34)
Hypertensive heart disease	826.89 (661.16 to 1,117.76)	1,419.13 (1,055.41 to 2,005.85)	1,192.78 (921.51 to 1,620.56)	1,571.24 (1,076.84 to 2,497.17)	42.58 (34.86 to 57.28)	92.62 (67.29 to 128.63)	84.10 (63.21 to 112.75)	96.91 (65.74 to 152.82)
Cardiomyopathy and myocarditis	360.69 (274.09 to 434.27)	305.45 (216.13 to 402.56)	249.88 (175.75 to 322.87)	313.61 (221.74 to 421.64)	13.66 (10.46 to 16.25)	14.95 (10.55 to 20.51)	13.33 (9.49 to 17.96)	14.68 (10.19 to 20.20)
Myocarditis	71.90 (46.66 to 93.57)	82.03 (55.04 to 107.12)	83.70 (57.01 to 108.70)	77.99 (51.87 to 102.36)	2.86 (1.89 to 3.73)	4.17 (2.82 to 5.42)	4.60 (3.18 to 5.94)	3.81 (2.56 to 4.98)

Guam								
**Cause**	**YLLs (actual counts)**				**Deaths (actual counts)**			
	**2016**	**2040 Forecast**	**2040 Better Scenario**	**2040 Worse Scenario**	**2016**	**2040 Forecast**	**2040 Better Scenario**	**2040 Worse Scenario**
Alcoholic cardiomyopathy	30.50 (19.88 to 41.66)	27.56 (17.12 to 37.23)	27.25 (16.92 to 36.92)	27.41 (17.22 to 36.89)	1.25 (0.83 to 1.67)	1.33 (0.85 to 1.80)	1.40 (0.89 to 1.90)	1.27 (0.80 to 1.70)
Other cardiomyopathy	258.29 (196.81 to 313.68)	195.86 (125.84 to 277.89)	138.93 (88.84 to 198.11)	208.21 (135.21 to 297.03)	9.55 (7.31 to 11.52)	9.45 (6.15 to 14.11)	7.33 (4.65 to 10.86)	9.60 (6.26 to 14.41)
Atrial fibrillation and flutter	46.46 (35.99 to 58.27)	89.64 (63.62 to 119.12)	93.62 (68.33 to 121.30)	85.78 (60.31 to 115.91)	4.34 (3.36 to 5.58)	9.35 (6.04 to 12.86)	10.35 (7.12 to 13.96)	8.64 (5.33 to 12.12)
Aortic aneurysm	211.51 (177.72 to 252.50)	223.44 (180.97 to 275.15)	205.19 (168.42 to 247.85)	229.74 (181.69 to 291.97)	10.66 (9.22 to 12.40)	14.26 (10.88 to 17.28)	14.50 (11.49 to 17.21)	13.73 (10.40 to 17.16)
Peripheral artery disease	31.06 (13.10 to 42.23)	44.23 (17.25 to 66.05)	41.50 (16.22 to 61.50)	44.12 (16.98 to 67.03)	1.92 (0.84 to 2.53)	3.53 (1.35 to 5.34)	3.70 (1.43 to 5.53)	3.29 (1.24 to 5.05)
Endocarditis	154.72 (121.83 to 184.93)	221.37 (157.68 to 294.88)	216.81 (154.25 to 290.41)	216.86 (154.23 to 287.93)	6.37 (5.13 to 7.61)	11.65 (8.15 to 16.43)	12.51 (8.79 to 17.54)	10.90 (7.59 to 15.43)
Other cardiovascular and circulatory diseases	406.77 (307.64 to 508.61)	494.71 (345.46 to 654.46)	358.46 (255.70 to 460.42)	538.86 (364.76 to 743.98)	17.85 (13.41 to 22.12)	28.53 (19.09 to 37.15)	23.18 (15.90 to 29.90)	29.25 (19.33 to 39.13)
**Chronic respiratory diseases**	**1,526.29 (1,295.12 to 1,794.95)**	**1,998.40 (1,493.82 to 2,697.11)**	**1,613.03 (1,243.00 to 2,078.23)**	**2,307.11 (1,671.95 to 3,174.80)**	**75.65 (65.60 to 87.03)**	**112.35 (86.33 to 144.13)**	**90.99 (72.70 to 112.42)**	**126.70 (93.84 to 165.56)**
Chronic obstructive pulmonary disease	914.63 (771.55 to 1,076.66)	1,229.39 (815.57 to 1,818.70)	731.69 (499.37 to 1,047.89)	1,555.38 (1,008.30 to 2,310.75)	50.78 (43.98 to 58.88)	77.70 (54.22 to 106.75)	49.19 (35.00 to 66.10)	93.69 (64.22 to 130.05)
Pneumoconiosis	4.77 (3.80 to 5.95)	4.79 (3.69 to 6.29)	5.43 (4.17 to 7.15)	4.67 (3.56 to 6.13)	0.33 (0.26 to 0.41)	0.39 (0.27 to 0.55)	0.47 (0.33 to 0.64)	0.37 (0.24 to 0.53)
Silicosis	0.55 (0.38 to 0.76)	0.30 (0.17 to 0.49)	0.24 (0.13 to 0.38)	0.30 (0.17 to 0.47)	0.04 (0.03 to 0.06)	0.03 (0.01 to 0.05)	0.02 (0.01 to 0.04)	0.03 (0.01 to 0.05)
Asbestosis	1.19 (0.82 to 1.64)	1.92 (1.35 to 2.64)	2.47 (1.77 to 3.33)	1.70 (1.16 to 2.34)	0.06 (0.04 to 0.09)	0.13 (0.09 to 0.20)	0.18 (0.12 to 0.27)	0.11 (0.07 to 0.18)
Coal workers pneumoconiosis	0.09 (0.04 to 0.15)	0.07 (0.03 to 0.13)	0.07 (0.03 to 0.13)	0.08 (0.04 to 0.14)	0.01 (0.00 to 0.01)	0.01 (0.00 to 0.01)	0.01 (0.00 to 0.01)	0.01 (0.00 to 0.01)
Other pneumoconiosis	2.94 (2.29 to 3.68)	2.50 (1.73 to 3.53)	2.66 (1.79 to 3.88)	2.60 (1.79 to 3.67)	0.22 (0.17 to 0.28)	0.23 (0.14 to 0.34)	0.25 (0.16 to 0.39)	0.23 (0.14 to 0.35)
Asthma	171.49 (132.83 to 212.65)	84.87 (50.78 to 136.15)	50.99 (31.32 to 79.41)	109.82 (63.79 to 178.36)	8.28 (6.47 to 10.34)	5.08 (3.06 to 8.19)	3.37 (2.07 to 5.52)	6.17 (3.67 to 10.18)
Interstitial lung disease and pulmonary sarcoidosis	264.59 (207.73 to 335.96)	478.68 (365.00 to 626.73)	640.85 (504.12 to 807.40)	430.35 (326.31 to 560.25)	10.13 (8.12 to 13.02)	21.58 (16.88 to 27.53)	30.77 (24.67 to 37.88)	18.77 (14.46 to 24.19)
Other chronic respiratory diseases	170.82 (91.38 to 243.16)	200.67 (101.54 to 310.64)	184.08 (94.42 to 283.26)	206.88 (104.54 to 323.38)	6.12 (3.25 to 8.86)	7.60 (3.89 to 11.73)	7.20 (3.73 to 11.04)	7.71 (3.92 to 12.00)
**Cirrhosis and other chronic liver diseases**	**1,207.99 (963.42 to 1,493.26)**	**1,181.83 (888.61 to 1,524.74)**	**1,010.30 (759.77 to 1,308.76)**	**1,197.60 (905.93 to 1,541.11)**	**40.03 (33.04 to 48.67)**	**45.44 (37.43 to 54.96)**	**41.08 (33.94 to 50.03)**	**44.64 (36.47 to 53.57)**
Cirrhosis and other chronic liver diseases due to hepatitis B	500.20 (394.37 to 629.89)	500.04 (373.15 to 651.53)	435.90 (323.85 to 572.66)	499.47 (373.17 to 654.10)	17.26 (14.17 to 21.67)	19.80 (16.10 to 24.34)	18.11 (14.70 to 22.43)	19.21 (15.67 to 23.48)
Cirrhosis and other chronic liver diseases due to hepatitis C	196.95 (150.29 to 251.85)	198.31 (139.30 to 265.32)	169.99 (120.62 to 229.30)	199.46 (143.66 to 262.50)	6.47 (5.17 to 8.06)	7.30 (5.75 to 9.16)	6.52 (5.15 to 8.22)	7.17 (5.63 to 8.96)

Cirrhosis and other chronic liver diseases due to alcohol use	182.00 (141.09 to 235.32)	174.42 (130.89 to 233.21)	145.25 (109.20 to 191.93)	185.03 (136.92 to 245.71)	6.38 (5.12 to 7.99)	7.02 (5.69 to 8.71)	6.20 (5.01 to 7.63)	7.17 (5.69 to 8.82)
Cirrhosis and other chronic liver diseases due to other causes	328.84 (271.33 to 394.28)	309.07 (232.98 to 396.97)	259.16 (194.27 to 334.77)	313.64 (235.73 to 400.50)	9.92 (8.34 to 11.74)	11.32 (9.29 to 13.59)	10.26 (8.49 to 12.27)	11.09 (9.03 to 13.17)
**Digestive diseases**	**368.98 (310.13 to 431.09)**	**388.38 (312.85 to 473.79)**	**335.56 (269.68 to 407.96)**	**401.82 (319.16 to 496.73)**	**16.00 (13.72 to 18.38)**	**21.58 (17.31 to 26.06)**	**20.18 (16.55 to 24.14)**	**21.43 (16.87 to 26.45)**
Peptic ulcer disease	68.47 (55.77 to 83.43)	40.92 (30.47 to 54.80)	28.87 (21.79 to 37.90)	44.09 (32.64 to 59.35)	3.16 (2.63 to 3.76)	2.43 (1.84 to 3.19)	1.85 (1.44 to 2.36)	2.51 (1.86 to 3.30)

Guam								
Cause	YLLs (actual counts)				Deaths (actual counts)			
	2016	2040 Forecast	2040 Better Scenario	2040 Worse Scenario	2016	2040 Forecast	2040 Better Scenario	2040 Worse Scenario
Gastritis and duodenitis	1.71 (1.33 to 2.17)	2.14 (1.50 to 2.93)	1.81 (1.28 to 2.47)	2.14 (1.52 to 2.96)	0.09 (0.07 to 0.10)	0.13 (0.09 to 0.19)	0.12 (0.09 to 0.17)	0.13 (0.09 to 0.18)
Appendicitis	51.86 (41.78 to 64.15)	43.66 (31.12 to 58.14)	32.23 (23.44 to 42.67)	45.51 (32.28 to 59.93)	1.92 (1.59 to 2.34)	2.14 (1.58 to 2.76)	1.72 (1.28 to 2.20)	2.14 (1.61 to 2.73)
Paralytic ileus and intestinal obstruction	48.76 (37.45 to 65.50)	63.97 (49.91 to 84.61)	64.83 (50.54 to 84.42)	60.58 (47.08 to 80.69)	1.89 (1.52 to 2.47)	3.48 (2.72 to 4.46)	3.92 (3.09 to 4.99)	3.15 (2.41 to 4.09)
Inguinal, femoral, and abdominal hernia	9.74 (7.34 to 14.77)	8.82 (6.52 to 13.78)	7.97 (5.83 to 12.24)	8.52 (6.28 to 13.21)	0.54 (0.40 to 0.83)	0.60 (0.42 to 0.94)	0.58 (0.41 to 0.90)	0.56 (0.39 to 0.88)
Inflammatory bowel disease	17.64 (13.09 to 22.11)	21.48 (15.89 to 27.45)	21.83 (16.03 to 28.01)	20.53 (15.17 to 26.23)	0.72 (0.54 to 0.88)	1.09 (0.83 to 1.38)	1.18 (0.89 to 1.49)	1.01 (0.76 to 1.27)
Vascular intestinal disorders	32.40 (25.40 to 38.39)	44.08 (34.78 to 52.52)	43.37 (33.88 to 52.34)	42.25 (33.00 to 49.98)	1.75 (1.40 to 2.06)	3.02 (2.32 to 3.65)	3.15 (2.45 to 3.82)	2.82 (2.10 to 3.43)
Gallbladder and biliary diseases	50.03 (39.45 to 60.73)	59.24 (34.33 to 96.96)	42.76 (25.33 to 70.61)	73.16 (40.76 to 121.71)	2.38 (1.89 to 2.86)	3.52 (2.02 to 5.90)	2.73 (1.60 to 4.63)	4.13 (2.27 to 6.90)
Pancreatitis	28.92 (22.08 to 36.83)	33.24 (20.19 to 62.01)	25.35 (16.14 to 42.88)	36.45 (20.64 to 74.89)	1.03 (0.80 to 1.27)	1.43 (0.95 to 2.36)	1.17 (0.81 to 1.79)	1.50 (0.94 to 2.70)
Other digestive diseases	59.45 (47.75 to 72.37)	70.83 (56.04 to 86.92)	66.54 (52.17 to 81.78)	68.59 (54.23 to 83.77)	2.51 (2.05 to 3.03)	3.74 (2.98 to 4.56)	3.76 (2.98 to 4.58)	3.50 (2.76 to 4.26)
**Neurological disorders**	**797.10 (676.34 to 933.27)**	**1,591.29 (1,141.17 to 2,295.71)**	**1,652.53 (1,225.82 to 2,262.66)**	**1,579.39 (1,099.23 to 2,351.68)**	**62.85 (52.78 to 75.33)**	**146.49 (94.96 to 217.61)**	**161.92 (112.23 to 225.63)**	**140.86 (87.50 to 215.09)**
Alzheimer's disease and other dementias	570.25 (464.27 to 695.20)	1,243.78 (809.41 to 1,943.64)	1,293.21 (885.09 to 1,929.88)	1,246.27 (779.88 to 2,014.47)	53.75 (44.10 to 65.87)	128.91 (78.34 to 199.62)	141.67 (93.83 to 206.52)	124.90 (72.21 to 197.43)
Parkinson's disease	50.82 (38.05 to 66.50)	109.42 (81.04 to 142.75)	130.95 (98.29 to 168.05)	97.13 (71.55 to 126.49)	3.98 (2.94 to 5.23)	9.33 (6.66 to 12.60)	11.60 (8.58 to 15.33)	8.11 (5.57 to 11.20)
Epilepsy	57.06 (46.68 to 68.32)	66.08 (39.54 to 109.58)	54.66 (34.26 to 88.87)	70.73 (41.08 to 121.10)	1.21 (1.01 to 1.45)	1.60 (1.06 to 2.51)	1.41 (0.97 to 2.11)	1.66 (1.06 to 2.69)
Multiple sclerosis	6.11 (3.57 to 11.78)	7.71 (4.21 to 14.89)	7.82 (4.32 to 15.06)	7.70 (4.18 to 14.89)	0.19 (0.11 to 0.37)	0.26 (0.15 to 0.49)	0.27 (0.16 to 0.52)	0.26 (0.15 to 0.48)
Motor neuron disease	28.45 (23.25 to 34.00)	49.90 (40.08 to 60.11)	51.96 (41.45 to 63.24)	47.18 (38.13 to 56.22)	1.14 (0.93 to 1.35)	2.29 (1.89 to 2.68)	2.47 (2.01 to 2.92)	2.11 (1.73 to 2.48)
Other neurological disorders	84.41 (70.15 to 99.57)	114.41 (89.39 to 146.32)	113.93 (89.08 to 141.57)	110.39 (86.48 to 139.93)	2.57 (2.19 to 2.99)	4.11 (3.49 to 4.80)	4.50 (3.80 to 5.28)	3.82 (3.24 to 4.51)
**Mental disorders**	**127.20 (97.17 to 163.72)**	**248.06 (180.76 to 330.71)**	**305.23 (225.64 to 404.11)**	**231.30 (168.96 to 309.91)**	**3.48 (2.73 to 4.43)**	**8.06 (6.23 to 10.45)**	**10.81 (8.55 to 13.93)**	**7.20 (5.61 to 9.22)**
Alcohol use disorders	68.38 (47.00 to 98.24)	77.39 (48.02 to 118.80)	83.90 (51.36 to 130.34)	75.32 (46.26 to 114.75)	1.80 (1.26 to 2.58)	2.21 (1.48 to 3.29)	2.50 (1.66 to 3.71)	2.10 (1.40 to 3.11)
Drug use disorders	58.55 (45.49 to 74.50)	170.20 (124.34 to 227.24)	220.89 (164.74 to 292.45)	155.50 (114.65 to 209.73)	1.68 (1.34 to 2.13)	5.84 (4.44 to 7.54)	8.31 (6.44 to 10.66)	5.10 (3.85 to 6.65)
Opioid use disorders	29.64 (21.81 to 39.83)	58.39 (39.78 to 85.18)	62.82 (43.07 to 91.96)	56.19 (38.30 to 81.58)	0.71 (0.54 to 0.95)	1.58 (1.15 to 2.25)	1.81 (1.32 to 2.67)	1.47 (1.08 to 2.09)

Cocaine use disorders	3.42 (1.61 to 5.01)	11.34 (5.69 to 18.05)	14.37 (7.25 to 22.28)	10.43 (5.21 to 16.16)	0.09 (0.04 to 0.12)	0.31 (0.16 to 0.46)	0.41 (0.22 to 0.61)	0.27 (0.15 to 0.41)
Amphetamine use disorders	2.69 (1.09 to 4.07)	12.52 (5.35 to 20.33)	18.01 (7.94 to 28.68)	11.15 (4.78 to 17.95)	0.07 (0.03 to 0.10)	0.32 (0.15 to 0.51)	0.48 (0.23 to 0.72)	0.28 (0.13 to 0.44)
Other drug use disorders	22.80 (17.15 to 29.32)	87.95 (60.56 to 121.44)	125.69 (88.90 to 170.85)	77.73 (54.09 to 108.23)	0.82 (0.63 to 1.07)	3.63 (2.55 to 4.87)	5.61 (4.04 to 7.42)	3.07 (2.16 to 4.22)
Eating disorders	0.27 (0.20 to 0.36)	0.48 (0.28 to 0.75)	0.45 (0.27 to 0.68)	0.48 (0.29 to 0.74)	0.01 (0.00 to 0.01)	0.01 (0.01 to 0.01)	0.01 (0.01 to 0.01)	0.01 (0.01 to 0.01)

Guam								
Cause	YLLs (actual counts)				Deaths (actual counts)			
	2016	2040 Forecast	2040 Better Scenario	2040 Worse Scenario	2016	2040 Forecast	2040 Better Scenario	2040 Worse Scenario
**Diabetes, urogenital, blood, and endocrine diseases**	**3,468.25 (2,973.50 to 3,983.77)**	**5,415.09 (3,142.46 to 10,286.26)**	**4,507.97 (2,526.42 to 8,960.09)**	**6,109.53 (3,534.80 to 10,754.96)**	**165.03 (144.92 to 186.24)**	**326.67 (191.33 to 606.36)**	**289.16 (166.25 to 569.95)**	**352.79 (204.50 to 612.84)**
Diabetes mellitus	1,658.93 (1,423.81 to 1,912.01)	1,546.96 (1,059.92 to 2,138.63)	1,157.93 (791.04 to 1,613.41)	1,718.33 (1,153.96 to 2,461.67)	83.52 (73.08 to 94.61)	97.49 (64.74 to 134.15)	78.41 (53.50 to 105.77)	103.23 (68.49 to 144.32)
Acute glomerulonephritis	4.44 (2.95 to 6.57)	4.21 (2.53 to 7.02)	3.45 (2.02 to 5.78)	4.25 (2.56 to 7.14)	0.14 (0.09 to 0.20)	0.13 (0.08 to 0.22)	0.11 (0.07 to 0.19)	0.13 (0.08 to 0.22)
Chronic kidney disease	1,269.14 (1,071.25 to 1,486.91)	3,082.38 (818.12 to 8,252.17)	2,592.64 (664.88 to 7,237.07)	3,627.42 (994.93 to 8,582.65)	60.17 (51.99 to 69.16)	189.02 (43.08 to 495.19)	169.22 (38.17 to 469.71)	211.92 (51.71 to 491.98)
Chronic kidney disease due to diabetes mellitus	716.31 (590.47 to 856.10)	1,806.39 (463.18 to 4,903.02)	1,497.97 (368.81 to 4,191.40)	2,153.72 (564.07 to 5,164.31)	32.23 (27.06 to 37.54)	101.97 (23.61 to 271.27)	89.21 (19.98 to 248.20)	116.13 (28.19 to 273.93)
Chronic kidney disease due to hypertension	334.39 (272.89 to 407.84)	921.90 (218.85 to 2,444.78)	816.22 (191.27 to 2,263.19)	1,054.37 (266.67 to 2,503.28)	19.89 (16.57 to 23.78)	67.76 (15.31 to 177.35)	63.65 (13.68 to 174.38)	73.87 (17.85 to 176.99)
Chronic kidney disease due to glomerulonephritis	98.19 (75.22 to 123.49)	139.66 (50.99 to 335.26)	105.90 (37.93 to 265.95)	166.60 (60.53 to 373.78)	3.24 (2.49 to 4.12)	6.81 (1.89 to 17.63)	5.54 (1.49 to 15.19)	7.85 (2.24 to 18.59)
Chronic kidney disease due to other and unspecified causes	120.25 (94.59 to 151.34)	214.43 (65.79 to 540.51)	172.55 (51.73 to 457.25)	252.73 (81.07 to 572.71)	4.82 (3.82 to 5.96)	12.49 (3.01 to 33.11)	10.83 (2.51 to 30.38)	14.06 (3.64 to 32.82)
Urinary diseases and male infertility	203.15 (173.17 to 235.94)	326.07 (265.90 to 401.24)	281.03 (233.86 to 339.40)	324.64 (265.91 to 397.19)	9.30 (8.01 to 10.73)	20.16 (15.65 to 24.31)	19.14 (15.12 to 22.77)	19.25 (14.56 to 23.50)
Urinary tract infections	187.36 (159.38 to 218.13)	309.25 (250.61 to 379.59)	267.76 (221.24 to 324.66)	307.57 (252.26 to 379.39)	8.63 (7.38 to 9.95)	19.27 (14.81 to 23.26)	18.38 (14.39 to 21.94)	18.38 (13.87 to 22.43)
Urolithiasis	3.75 (2.62 to 5.86)	5.96 (3.72 to 9.57)	4.73 (2.92 to 7.60)	6.12 (3.83 to 9.80)	0.16 (0.11 to 0.24)	0.29 (0.19 to 0.47)	0.24 (0.16 to 0.39)	0.29 (0.19 to 0.46)
Other urinary diseases	12.05 (8.30 to 17.93)	10.87 (7.15 to 17.09)	8.54 (5.64 to 13.34)	10.95 (7.32 to 16.97)	0.52 (0.36 to 0.76)	0.61 (0.39 to 0.92)	0.52 (0.34 to 0.78)	0.58 (0.39 to 0.88)
Gynecological diseases	0.56 (0.35 to 0.81)	0.38 (0.19 to 0.68)	0.23 (0.12 to 0.39)	0.43 (0.22 to 0.73)	0.01 (0.01 to 0.02)	0.01 (0.01 to 0.02)	0.01 (0.00 to 0.01)	0.01 (0.01 to 0.02)
Uterine fibroids	0.03 (0.02 to 0.05)	0.02 (0.01 to 0.04)	0.02 (0.01 to 0.03)	0.02 (0.01 to 0.04)	0.00 (0.00 to 0.00)	0.00 (0.00 to 0.00)	0.00 (0.00 to 0.00)	0.00 (0.00 to 0.00)
Polycystic ovarian syndrome	0.01 (0.00 to 0.04)	0.01 (0.00 to 0.02)	0.00 (0.00 to 0.01)	0.01 (0.00 to 0.02)	0.00 (0.00 to 0.00)	0.00 (0.00 to 0.00)	0.00 (0.00 to 0.00)	0.00 (0.00 to 0.00)
Endometriosis	0.01 (0.00 to 0.02)	0.01 (0.00 to 0.02)	0.00 (0.00 to 0.01)	0.01 (0.00 to 0.02)	0.00 (0.00 to 0.00)	0.00 (0.00 to 0.00)	0.00 (0.00 to 0.00)	0.00 (0.00 to 0.00)
Genital prolapse	0.02 (0.01 to 0.03)	0.01 (0.01 to 0.02)	0.01 (0.00 to 0.01)	0.01 (0.01 to 0.02)	0.00 (0.00 to 0.00)	0.00 (0.00 to 0.00)	0.00 (0.00 to 0.00)	0.00 (0.00 to 0.00)
Other gynecological diseases	0.49 (0.29 to 0.72)	0.33 (0.16 to 0.60)	0.20 (0.10 to 0.35)	0.37 (0.19 to 0.67)	0.01 (0.01 to 0.02)	0.01 (0.01 to 0.02)	0.01 (0.00 to 0.01)	0.01 (0.01 to 0.02)
Hemoglobinopathies and hemolytic anemias	43.47 (31.70 to 54.32)	36.32 (24.36 to 48.65)	27.86 (19.08 to 36.65)	37.28 (25.46 to 48.64)	1.51 (1.10 to 1.86)	1.61 (1.14 to 2.03)	1.32 (0.93 to 1.66)	1.59 (1.13 to 2.01)
Thalassemias	5.41 (3.99 to 7.09)	2.62 (1.47 to 4.33)	2.08 (1.15 to 3.43)	2.64 (1.49 to 4.36)	0.07 (0.05 to 0.09)	0.04 (0.02 to 0.06)	0.03 (0.02 to 0.05)	0.04 (0.02 to 0.06)

Sickle cell disorders	0.03 (0.02 to 0.05)	0.03 (0.02 to 0.05)	0.02 (0.01 to 0.03)	0.03 (0.02 to 0.05)	0.00 (0.00 to 0.00)	0.00 (0.00 to 0.00)	0.00 (0.00 to 0.00)	0.00 (0.00 to 0.00)
G6PD deficiency	21.97 (14.98 to 29.00)	19.97 (12.26 to 29.18)	14.33 (9.11 to 20.45)	21.11 (13.30 to 29.41)	0.57 (0.39 to 0.74)	0.62 (0.40 to 0.86)	0.46 (0.30 to 0.63)	0.64 (0.42 to 0.87)
Other hemoglobinopathies and hemolytic anemias	16.06 (11.69 to 20.28)	13.70 (9.76 to 17.57)	11.43 (8.25 to 14.77)	13.49 (9.61 to 17.41)	0.86 (0.64 to 1.08)	0.95 (0.68 to 1.25)	0.83 (0.60 to 1.08)	0.91 (0.63 to 1.20)
Endocrine, metabolic, blood, and immune disorders	288.57 (223.13 to 358.79)	418.77 (315.78 to 521.07)	444.82 (337.60 to 557.60)	397.17 (300.33 to 493.61)	10.38 (8.05 to 12.64)	18.23 (13.85 to 21.44)	20.94 (16.30 to 24.64)	16.65 (12.61 to 19.76)

Guam								
Cause	YLLs (actual counts)				Deaths (actual counts)			
	2016	2040 Forecast	2040 Better Scenario	2040 Worse Scenario	2016	2040 Forecast	2040 Better Scenario	2040 Worse Scenario
**Musculoskeletal disorders**	**96.26 (75.75 to 123.66)**	**120.87 (85.67 to 164.51)**	**108.11 (78.31 to 147.69)**	**121.62 (85.98 to 167.13)**	**2.85 (2.27 to 3.81)**	**4.64 (3.47 to 6.35)**	**4.58 (3.49 to 6.18)**	**4.47 (3.33 to 6.14)**
Rheumatoid arthritis	6.02 (4.00 to 8.44)	6.68 (4.11 to 10.30)	4.52 (2.76 to 6.95)	7.19 (4.35 to 11.09)	0.28 (0.18 to 0.39)	0.38 (0.23 to 0.58)	0.27 (0.16 to 0.42)	0.40 (0.24 to 0.61)
Other musculoskeletal disorders	90.24 (71.03 to 116.81)	114.19 (80.54 to 156.42)	103.59 (74.88 to 142.27)	114.42 (80.81 to 158.38)	2.57 (2.05 to 3.41)	4.25 (3.21 to 5.83)	4.30 (3.29 to 5.89)	4.07 (3.08 to 5.60)
**Other non-communicable diseases**	**1,150.75 (858.72 to 1,502.46)**	**1,027.43 (590.46 to 1,621.34)**	**715.19 (447.49 to 1,010.88)**	**1,076.51 (611.48 to 1,705.94)**	**20.21 (15.34 to 25.54)**	**31.89 (20.31 to 44.25)**	**29.15 (18.01 to 39.63)**	**30.97 (19.74 to 43.19)**
Congenital birth defects	903.71 (653.59 to 1,215.86)	571.79 (229.58 to 1,073.51)	293.65 (123.34 to 518.02)	632.26 (252.36 to 1,157.36)	11.29 (8.34 to 14.96)	7.51 (3.32 to 13.49)	4.04 (1.96 to 6.77)	8.27 (3.70 to 14.47)
Neural tube defects	57.02 (31.70 to 95.81)	12.49 (3.35 to 30.64)	5.20 (1.38 to 12.70)	14.42 (3.94 to 36.82)	0.67 (0.37 to 1.12)	0.15 (0.04 to 0.36)	0.06 (0.02 to 0.15)	0.17 (0.05 to 0.43)
Congenital heart anomalies	394.65 (281.31 to 547.08)	208.22 (82.09 to 394.97)	93.39 (40.25 to 166.79)	238.06 (96.04 to 440.33)	5.01 (3.67 to 6.73)	2.79 (1.24 to 5.07)	1.31 (0.63 to 2.21)	3.19 (1.42 to 5.68)
Orofacial clefts	4.48 (1.03 to 11.39)	2.11 (0.26 to 7.48)	0.70 (0.09 to 2.36)	2.55 (0.33 to 9.07)	0.05 (0.01 to 0.13)	0.02 (0.00 to 0.09)	0.01 (0.00 to 0.03)	0.03 (0.00 to 0.11)
Down syndrome	23.37 (15.56 to 33.87)	33.72 (17.65 to 58.57)	29.20 (16.58 to 48.35)	33.08 (17.27 to 57.37)	0.33 (0.24 to 0.46)	0.54 (0.33 to 0.84)	0.50 (0.32 to 0.75)	0.53 (0.32 to 0.83)
Other chromosomal abnormalities	71.82 (35.35 to 109.26)	91.53 (25.11 to 194.19)	54.47 (15.03 to 113.16)	96.11 (26.51 to 199.42)	0.85 (0.42 to 1.29)	1.10 (0.31 to 2.28)	0.66 (0.19 to 1.34)	1.15 (0.33 to 2.34)
Congenital musculoskeletal and limb anomalies	31.93 (20.36 to 47.30)	21.62 (7.52 to 43.53)	10.57 (3.74 to 20.82)	24.04 (8.32 to 48.69)	0.39 (0.25 to 0.57)	0.27 (0.10 to 0.53)	0.13 (0.05 to 0.25)	0.30 (0.11 to 0.58)
Urogenital congenital anomalies	23.10 (10.51 to 35.35)	14.97 (5.15 to 30.60)	8.25 (3.12 to 15.74)	16.29 (5.67 to 33.41)	0.33 (0.16 to 0.47)	0.23 (0.10 to 0.42)	0.14 (0.06 to 0.23)	0.25 (0.10 to 0.46)
Digestive congenital anomalies	54.94 (33.05 to 85.29)	30.75 (9.34 to 66.26)	13.71 (4.33 to 28.75)	34.69 (10.99 to 73.48)	0.65 (0.39 to 1.00)	0.37 (0.12 to 0.78)	0.17 (0.05 to 0.34)	0.42 (0.14 to 0.87)
Other congenital birth defects	242.39 (165.22 to 343.21)	156.38 (62.12 to 298.82)	78.15 (31.50 to 141.40)	173.01 (68.92 to 329.69)	3.02 (2.13 to 4.18)	2.04 (0.90 to 3.74)	1.06 (0.49 to 1.82)	2.25 (1.00 to 4.07)
Skin and subcutaneous diseases	170.24 (96.33 to 249.66)	419.68 (243.13 to 592.17)	402.65 (236.87 to 563.29)	405.16 (235.60 to 565.55)	8.02 (4.53 to 11.55)	23.97 (13.60 to 33.94)	24.89 (14.31 to 35.01)	22.25 (12.57 to 32.05)
Cellulitis	47.09 (19.99 to 78.66)	133.37 (62.84 to 216.70)	138.97 (66.72 to 219.79)	124.42 (57.56 to 201.47)	2.17 (1.01 to 3.59)	7.27 (3.60 to 11.27)	8.08 (4.15 to 12.63)	6.51 (3.20 to 10.25)
Pyoderma	105.18 (57.02 to 158.84)	255.48 (139.02 to 373.10)	237.39 (127.61 to 350.36)	250.13 (135.39 to 361.23)	4.59 (2.35 to 6.75)	14.06 (7.09 to 20.86)	14.39 (7.21 to 21.35)	13.21 (6.72 to 19.77)
Decubitus ulcer	16.38 (7.29 to 26.24)	28.10 (11.84 to 47.60)	24.05 (10.09 to 39.72)	27.84 (11.75 to 46.73)	1.22 (0.53 to 1.93)	2.55 (0.96 to 4.38)	2.34 (0.87 to 4.01)	2.44 (0.89 to 4.27)
Other skin and subcutaneous diseases	1.60 (0.91 to 2.51)	2.73 (1.52 to 4.36)	2.24 (1.28 to 3.48)	2.78 (1.57 to 4.40)	0.05 (0.03 to 0.07)	0.09 (0.05 to 0.14)	0.08 (0.05 to 0.13)	0.09 (0.05 to 0.14)
Sudden infant death syndrome	76.80 (44.22 to 124.96)	35.96 (6.28 to 85.76)	18.89 (3.32 to 43.72)	39.09 (7.12 to 94.83)	0.89 (0.51 to 1.45)	0.42 (0.07 to 1.00)	0.22 (0.04 to 0.51)	0.45 (0.08 to 1.10)

**Injuries**	**4,504.60 (3,735.31 to 5,310.67)**	**4,247.22 (2,890.29 to 6,274.89)**	**2,872.43 (2,005.01 to 4,126.02)**	**4,713.94 (3,159.64 to 7,068.54)**	**103.04 (86.96 to 121.58)**	**110.62 (83.36 to 148.59)**	**82.78 (63.67 to 109.55)**	**117.74 (86.02 to 162.36)**
**Transport injuries**	**1,343.07 (1,102.61 to 1,577.10)**	**1,401.47 (959.71 to 2,092.02)**	**667.44 (470.08 to 977.02)**	**1,656.03 (1,115.54 to 2,531.79)**	**31.23 (26.14 to 36.37)**	**36.40 (26.98 to 50.57)**	**20.32 (15.71 to 27.04)**	**41.08 (29.63 to 58.68)**
Road injuries	1,216.10 (992.13 to 1,430.27)	1,271.14 (877.83 to 1,901.84)	580.81 (411.42 to 850.20)	1,511.03 (1,014.05 to 2,302.63)	28.01 (23.29 to 32.67)	32.43 (24.00 to 45.00)	17.45 (13.41 to 23.28)	36.85 (26.61 to 52.89)
Pedestrian road injuries	388.11 (303.23 to 478.08)	233.68 (145.94 to 351.97)	91.86 (59.85 to 134.98)	288.08 (176.68 to 449.41)	9.33 (7.33 to 11.28)	6.60 (4.43 to 9.40)	2.97 (2.11 to 4.09)	7.79 (5.18 to 11.27)

Guam								
Cause	YLLs (actual counts)				Deaths (actual counts)			
	2016	2040 Forecast	2040 Better Scenario	2040 Worse Scenario	2016	2040 Forecast	2040 Better Scenario	2040 Worse Scenario
Cyclist road injuries	29.08 (22.24 to 36.17)	33.38 (22.32 to 49.51)	17.28 (11.85 to 24.84)	38.48 (25.24 to 58.64)	0.74 (0.57 to 0.90)	0.95 (0.68 to 1.36)	0.63 (0.45 to 0.86)	1.03 (0.71 to 1.50)
Motorcyclist road injuries	176.00 (111.37 to 230.44)	182.22 (95.19 to 316.15)	112.09 (60.51 to 190.64)	205.86 (105.37 to 368.25)	3.46 (2.19 to 4.52)	3.69 (2.01 to 6.11)	2.46 (1.39 to 3.90)	4.08 (2.15 to 7.02)
Motor vehicle road injuries	610.43 (490.09 to 754.77)	805.33 (550.86 to 1,186.84)	352.22 (244.98 to 505.54)	959.02 (643.41 to 1,450.40)	14.19 (11.57 to 17.57)	20.77 (15.30 to 28.82)	11.16 (8.65 to 14.69)	23.49 (16.93 to 33.68)
Other road injuries	12.48 (9.14 to 16.31)	16.53 (10.35 to 26.01)	7.34 (4.81 to 11.23)	19.61 (11.96 to 31.60)	0.28 (0.21 to 0.35)	0.42 (0.28 to 0.61)	0.23 (0.17 to 0.33)	0.46 (0.31 to 0.70)
Other transport injuries	126.97 (95.15 to 160.92)	130.33 (81.15 to 197.43)	86.63 (56.30 to 129.84)	145.00 (90.04 to 235.66)	3.23 (2.50 to 4.00)	3.96 (2.67 to 5.80)	2.87 (2.00 to 4.00)	4.23 (2.81 to 6.31)
**Unintentional injuries**	**1,220.66 (1,016.86 to 1,427.41)**	**928.71 (673.85 to 1,229.04)**	**732.71 (544.52 to 955.68)**	**964.07 (700.74 to 1,286.94)**	**30.97 (26.27 to 35.88)**	**31.03 (24.51 to 37.95)**	**27.48 (22.39 to 32.83)**	**30.66 (24.06 to 37.53)**
Falls	200.86 (164.61 to 241.49)	199.07 (153.53 to 251.90)	176.18 (136.44 to 217.61)	197.41 (152.13 to 247.62)	7.88 (6.59 to 9.26)	10.80 (8.20 to 13.43)	10.41 (8.17 to 12.77)	10.24 (7.56 to 12.86)
Drowning	446.25 (362.49 to 537.89)	298.76 (196.55 to 435.33)	223.08 (149.83 to 313.06)	319.66 (210.63 to 458.24)	8.85 (7.20 to 10.68)	6.53 (4.54 to 9.02)	5.11 (3.57 to 6.86)	6.88 (4.71 to 9.53)
Fire, heat, and hot substances	54.67 (43.97 to 68.99)	35.71 (23.40 to 52.56)	24.93 (16.80 to 35.30)	38.47 (24.51 to 56.94)	1.38 (1.14 to 1.71)	1.16 (0.85 to 1.59)	0.91 (0.69 to 1.19)	1.19 (0.85 to 1.62)
Poisonings	42.93 (28.38 to 56.25)	17.29 (10.42 to 26.29)	10.80 (6.71 to 15.92)	19.08 (11.02 to 29.05)	1.04 (0.69 to 1.32)	0.54 (0.36 to 0.76)	0.39 (0.27 to 0.51)	0.57 (0.37 to 0.78)
Exposure to mechanical forces	131.99 (108.01 to 159.53)	100.89 (68.79 to 138.11)	74.40 (51.79 to 98.86)	105.96 (72.75 to 143.07)	3.08 (2.55 to 3.69)	2.87 (2.14 to 3.68)	2.32 (1.80 to 2.87)	2.90 (2.18 to 3.69)
Unintentional firearm injuries	21.31 (15.52 to 35.52)	16.23 (10.02 to 27.41)	12.28 (7.63 to 20.42)	16.84 (10.23 to 29.03)	0.54 (0.39 to 0.86)	0.49 (0.32 to 0.78)	0.41 (0.28 to 0.64)	0.49 (0.32 to 0.78)
Unintentional suffocation	25.40 (17.35 to 33.63)	18.83 (11.11 to 29.06)	13.83 (8.25 to 21.14)	19.79 (11.62 to 30.92)	0.41 (0.29 to 0.53)	0.33 (0.20 to 0.49)	0.25 (0.16 to 0.37)	0.34 (0.21 to 0.50)
Other exposure to mechanical forces	85.27 (68.06 to 104.98)	65.83 (44.21 to 90.97)	48.29 (33.43 to 65.84)	69.34 (47.54 to 96.02)	2.13 (1.74 to 2.63)	2.06 (1.54 to 2.68)	1.66 (1.30 to 2.08)	2.07 (1.56 to 2.68)
Adverse effects of medical treatment	130.12 (95.19 to 159.09)	122.03 (86.82 to 163.73)	104.92 (74.66 to 140.11)	123.13 (86.41 to 166.32)	3.68 (2.72 to 4.43)	4.48 (3.27 to 5.59)	4.29 (3.16 to 5.32)	4.31 (3.15 to 5.45)
Animal contact	3.29 (2.43 to 4.01)	3.95 (2.51 to 5.44)	3.16 (1.97 to 4.32)	4.06 (2.61 to 5.59)	0.10 (0.07 to 0.12)	0.15 (0.10 to 0.20)	0.14 (0.09 to 0.18)	0.15 (0.10 to 0.20)
Venomous animal contact	0.95 (0.67 to 1.24)	1.11 (0.68 to 1.64)	0.92 (0.56 to 1.34)	1.14 (0.68 to 1.68)	0.02 (0.02 to 0.03)	0.03 (0.02 to 0.05)	0.03 (0.02 to 0.04)	0.03 (0.02 to 0.05)
Non-venomous animal contact	2.34 (1.71 to 2.86)	2.83 (1.82 to 3.87)	2.24 (1.41 to 3.08)	2.92 (1.86 to 4.03)	0.08 (0.06 to 0.10)	0.12 (0.08 to 0.16)	0.11 (0.07 to 0.14)	0.12 (0.08 to 0.16)
Foreign body	116.68 (84.09 to 157.19)	103.90 (67.34 to 156.00)	85.90 (58.20 to 123.01)	104.32 (66.53 to 160.98)	2.72 (2.03 to 3.47)	3.12 (2.28 to 4.05)	2.96 (2.21 to 3.78)	2.97 (2.15 to 3.90)
Pulmonary aspiration and foreign body in airway	113.80 (81.72 to 154.14)	101.39 (65.11 to 152.97)	83.49 (56.44 to 119.85)	101.85 (65.04 to 157.39)	2.66 (1.99 to 3.41)	3.06 (2.23 to 4.00)	2.90 (2.17 to 3.72)	2.92 (2.10 to 3.83)

Foreign body in other body part	2.89 (1.78 to 3.89)	2.51 (1.57 to 3.67)	2.41 (1.54 to 3.49)	2.47 (1.54 to 3.60)	0.06 (0.03 to 0.07)	0.06 (0.04 to 0.08)	0.06 (0.04 to 0.08)	0.06 (0.03 to 0.08)
Environmental heat and cold exposure	24.46 (13.85 to 31.97)	20.06 (10.62 to 28.87)	14.59 (7.96 to 20.72)	21.14 (11.53 to 30.27)	0.68 (0.39 to 0.87)	0.67 (0.37 to 0.92)	0.53 (0.29 to 0.72)	0.67 (0.38 to 0.92)
Other unintentional injuries	69.42 (50.53 to 89.65)	27.05 (15.78 to 41.62)	14.75 (8.94 to 21.91)	30.85 (18.54 to 47.15)	1.56 (1.17 to 1.97)	0.71 (0.44 to 1.04)	0.42 (0.27 to 0.59)	0.78 (0.50 to 1.12)
**Self-harm and interpersonal violence**	**1,939.94 (1,568.57 to 2,343.35)**	**1,872.93 (1,173.36 to 3,049.66)**	**1,430.90 (932.47 to 2,214.74)**	**2,050.08 (1,244.07 to 3,477.62)**	**40.82 (33.16 to 49.57)**	**42.29 (28.14 to 65.05)**	**34.07 (23.69 to 49.46)**	**45.11 (29.18 to 72.04)**

Guam								
**Cause**	**YLLs (actual counts)**				**Deaths (actual counts)**			
	**2016**	**2040 Forecast**	**2040 Better Scenario**	**2040 Worse Scenario**	**2016**	**2040 Forecast**	**2040 Better Scenario**	**2040 Worse Scenario**
Self-harm	1,575.83 (1,240.69 to 1,915.00)	1,623.20 (986.31 to 2,724.30)	1,246.35 (792.85 to 1,972.19)	1,781.36 (1,057.80 to 3,097.07)	33.40 (26.64 to 40.49)	36.83 (23.98 to 57.94)	29.87 (20.33 to 44.21)	39.33 (24.76 to 63.91)
Self-harm by firearm	124.87 (80.29 to 182.58)	98.96 (49.08 to 201.21)	69.35 (35.52 to 134.01)	111.68 (53.29 to 225.70)	2.72 (1.76 to 4.05)	2.22 (1.15 to 4.36)	1.60 (0.85 to 2.93)	2.47 (1.24 to 4.84)
Self-harm by other specified means	1,450.96 (1,140.32 to 1,767.58)	1,524.24 (938.71 to 2,531.24)	1,177.00 (752.08 to 1,867.01)	1,669.68 (994.79 to 2,888.37)	30.68 (24.48 to 37.31)	34.61 (22.59 to 54.09)	28.27 (19.43 to 41.77)	36.86 (23.53 to 59.82)
Interpersonal violence	364.10 (272.54 to 473.92)	249.72 (164.03 to 368.25)	184.55 (121.42 to 272.34)	268.72 (174.66 to 402.82)	7.42 (5.63 to 9.60)	5.45 (3.71 to 7.89)	4.20 (2.92 to 6.03)	5.78 (3.91 to 8.46)
Physical violence by firearm	112.85 (73.77 to 177.01)	66.82 (39.74 to 114.20)	45.56 (27.56 to 76.68)	72.69 (42.99 to 122.19)	2.21 (1.47 to 3.45)	1.35 (0.83 to 2.25)	0.94 (0.58 to 1.57)	1.46 (0.88 to 2.41)
Physical violence by sharp object	107.68 (71.43 to 156.80)	72.36 (43.92 to 112.24)	52.42 (32.16 to 79.65)	77.75 (47.16 to 120.99)	2.16 (1.45 to 3.10)	1.50 (0.94 to 2.31)	1.11 (0.70 to 1.67)	1.60 (1.00 to 2.48)
Physical violence by other means	143.57 (102.01 to 190.60)	110.54 (67.46 to 172.67)	86.58 (54.17 to 133.05)	118.28 (70.32 to 190.39)	3.04 (2.20 to 3.98)	2.60 (1.68 to 3.93)	2.15 (1.42 to 3.20)	2.73 (1.72 to 4.20)
**Forces of nature, conflict and terrorism, and executions and police conflict**	**0.95 (0.79 to 1.13)**	**44.11 (0.00 to 411.76)**	**41.37 (0.00 to 387.72)**	**43.76 (0.00 to 410.11)**	**0.02 (0.01 to 0.02)**	**0.91 (0.00 to 8.86)**	**0.90 (0.00 to 8.82)**	**0.88 (0.00 to 8.62)**
Exposure to forces of nature	0.00 (0.00 to 0.00)	13.69 (0.00 to 379.52)	12.87 (0.00 to 356.91)	13.56 (0.00 to 376.52)	0.00 (0.00 to 0.00)	0.30 (0.00 to 8.56)	0.30 (0.00 to 8.57)	0.29 (0.00 to 8.29)
Conflict and terrorism	0.00 (0.00 to 0.00)	30.42 (0.00 to 401.49)	28.50 (0.00 to 376.98)	30.20 (0.00 to 398.82)	0.00 (0.00 to 0.00)	0.61 (0.00 to 8.11)	0.60 (0.00 to 8.03)	0.59 (0.00 to 7.87)
Executions and police conflict	0.95 (0.79 to 1.13)	0.00 (0.00 to 0.00)	0.00 (0.00 to 0.00)	0.00 (0.00 to 0.00)	0.02 (0.01 to 0.02)	0.00 (0.00 to 0.00)	0.00 (0.00 to 0.00)	0.00 (0.00 to 0.00)

Kiribati								
Cause	YLLs (actual counts)				Deaths (actual counts)			
	2016	2040 Forecast	2040 Better Scenario	2040 Worse Scenario	2016	2040 Forecast	2040 Better Scenario	2040 Worse Scenario
**All causes**	**40,544.58 (33,927.65 to 50,795.86)**	**46,406.26 (38,675.42 to 56,192.07)**	**30,239.97 (25,728.61 to 35,677.70)**	**57,067.03 (47,364.78 to 69,375.90)**	**1,046.51 (927.26 to 1,205.07)**	**1,455.83 (1,295.38 to 1,636.52)**	**1,035.74 (933.45 to 1,146.34)**	**1,698.45 (1,512.85 to 1,909.63)**
**Communicable, maternal, neonatal, and nutritional diseases**	**13,312.63 (9,106.40 to 20,435.63)**	**9,230.16 (6,166.01 to 14,161.69)**	**4,996.40 (3,387.85 to 7,367.15)**	**13,118.97 (8,569.90 to 20,176.89)**	**233.65 (180.53 to 319.80)**	**197.21 (138.51 to 274.97)**	**117.85 (84.77 to 166.62)**	**262.57 (179.75 to 379.78)**
**HIV/AIDS and tuberculosis**	**1,586.22 (1,335.09 to 1,874.97)**	**1,265.88 (839.94 to 1,821.20)**	**560.71 (393.00 to 770.66)**	**1,693.37 (1,102.34 to 2,396.51)**	**44.06 (36.80 to 51.89)**	**38.85 (26.08 to 57.73)**	**18.46 (12.85 to 25.78)**	**49.35 (31.53 to 70.95)**
Tuberculosis	1,566.41 (1,316.02 to 1,857.07)	1,247.66 (825.41 to 1,803.24)	549.85 (380.72 to 763.24)	1,657.27 (1,079.03 to 2,364.63)	43.66 (36.47 to 51.56)	38.51 (25.80 to 57.32)	18.26 (12.67 to 25.62)	48.69 (31.01 to 70.37)
HIV/AIDS	19.82 (14.42 to 32.72)	18.22 (7.53 to 41.99)	10.86 (4.45 to 29.25)	36.10 (17.23 to 65.40)	0.39 (0.28 to 0.67)	0.33 (0.13 to 0.83)	0.20 (0.07 to 0.60)	0.66 (0.29 to 1.26)
**Diarrhea, lower respiratory, and other common infectious diseases**	**5,296.80 (3,715.51 to 7,691.01)**	**3,593.00 (2,085.56 to 6,137.32)**	**1,982.87 (1,240.09 to 3,173.57)**	**5,358.46 (2,996.63 to 9,422.83)**	**103.99 (80.05 to 137.40)**	**94.11 (52.83 to 153.95)**	**59.06 (34.79 to 96.19)**	**129.64 (70.39 to 223.87)**
Diarrheal diseases	1,167.12 (697.20 to 1,802.84)	704.32 (107.46 to 1,930.18)	363.00 (61.27 to 1,085.76)	1,172.89 (199.15 to 3,090.07)	31.68 (17.10 to 50.59)	28.46 (4.15 to 80.16)	17.96 (2.64 to 53.23)	44.14 (6.97 to 126.03)
Intestinal infectious diseases	197.91 (94.16 to 355.65)	159.24 (69.40 to 299.02)	118.00 (52.56 to 218.78)	185.37 (79.62 to 351.36)	2.86 (1.39 to 5.07)	2.38 (1.10 to 4.43)	1.82 (0.87 to 3.35)	2.72 (1.25 to 5.06)
Typhoid fever	169.57 (80.01 to 300.01)	132.27 (55.52 to 250.84)	98.67 (43.26 to 183.27)	153.83 (64.43 to 298.52)	2.44 (1.17 to 4.25)	1.97 (0.90 to 3.70)	1.51 (0.71 to 2.76)	2.25 (1.00 to 4.33)
Paratyphoid fever	27.85 (11.91 to 57.26)	26.74 (10.69 to 55.05)	19.20 (7.60 to 40.27)	31.25 (12.49 to 65.51)	0.41 (0.18 to 0.84)	0.41 (0.17 to 0.83)	0.31 (0.13 to 0.62)	0.47 (0.19 to 0.98)
Other intestinal infectious diseases	0.49 (0.16 to 1.26)	0.23 (0.06 to 0.67)	0.12 (0.04 to 0.35)	0.29 (0.07 to 0.84)	0.01 (0.00 to 0.02)	0.00 (0.00 to 0.01)	0.00 (0.00 to 0.01)	0.01 (0.00 to 0.01)
Lower respiratory infections	2,161.77 (1,480.81 to 3,146.20)	1,534.52 (875.52 to 2,850.00)	820.29 (531.43 to 1,316.14)	2,136.65 (1,088.92 to 4,531.44)	45.34 (34.15 to 59.36)	45.51 (29.74 to 68.81)	27.92 (19.63 to 38.78)	57.02 (34.91 to 92.19)
Upper respiratory infections	7.42 (3.16 to 16.37)	6.03 (2.57 to 13.31)	3.11 (1.33 to 6.79)	7.52 (2.96 to 17.13)	0.14 (0.07 to 0.26)	0.14 (0.07 to 0.28)	0.08 (0.04 to 0.15)	0.17 (0.08 to 0.34)
Otitis media	2.76 (0.72 to 5.60)	0.51 (0.10 to 1.13)	0.53 (0.10 to 1.19)	0.50 (0.10 to 1.11)	0.07 (0.02 to 0.14)	0.01 (0.00 to 0.03)	0.02 (0.00 to 0.04)	0.01 (0.00 to 0.03)
Meningitis	1,036.62 (638.08 to 1,689.63)	508.78 (252.51 to 918.83)	385.58 (212.42 to 627.74)	628.75 (276.75 to 1,222.29)	14.92 (9.53 to 22.96)	8.38 (4.77 to 13.33)	6.88 (4.25 to 10.11)	9.76 (5.13 to 17.07)
Pneumococcal meningitis	3.16 (1.86 to 5.39)	1.24 (0.63 to 2.20)	1.09 (0.60 to 1.81)	1.48 (0.70 to 2.81)	0.05 (0.03 to 0.07)	0.02 (0.01 to 0.04)	0.02 (0.01 to 0.03)	0.03 (0.01 to 0.04)
H influenzae type B meningitis	118.72 (70.10 to 205.05)	88.72 (23.80 to 270.19)	54.11 (21.17 to 130.40)	146.65 (28.36 to 492.86)	1.68 (1.06 to 2.72)	1.25 (0.45 to 3.39)	0.88 (0.43 to 1.80)	1.92 (0.51 to 5.98)
Meningococcal meningitis	86.36 (52.16 to 144.21)	37.60 (20.54 to 61.81)	29.94 (17.22 to 46.67)	42.72 (22.58 to 73.66)	1.30 (0.83 to 2.03)	0.69 (0.42 to 1.04)	0.60 (0.37 to 0.86)	0.74 (0.44 to 1.16)
Other meningitis	828.38 (507.17 to 1,337.07)	381.22 (203.49 to 642.99)	300.45 (171.61 to 482.24)	437.90 (222.79 to 776.34)	11.89 (7.64 to 18.21)	6.42 (3.81 to 9.83)	5.38 (3.30 to 7.81)	7.07 (4.02 to 11.32)
Encephalitis	38.78 (17.67 to 69.95)	35.46 (15.14 to 65.44)	26.11 (11.47 to 45.57)	40.42 (16.37 to 78.00)	0.66 (0.31 to 1.09)	0.71 (0.32 to 1.17)	0.57 (0.25 to 0.90)	0.77 (0.34 to 1.32)

Diphtheria	0.80 (0.40 to 1.56)	0.20 (0.07 to 0.49)	0.11 (0.06 to 0.19)	0.36 (0.07 to 1.12)	0.01 (0.01 to 0.02)	0.00 (0.00 to 0.01)	0.00 (0.00 to 0.00)	0.00 (0.00 to 0.01)
Whooping cough	187.88 (13.64 to 640.07)	102.16 (1.84 to 419.43)	16.02 (0.82 to 63.61)	214.30 (2.46 to 893.27)	2.23 (0.16 to 7.62)	1.22 (0.02 to 4.95)	0.20 (0.01 to 0.80)	2.54 (0.03 to 10.53)
Tetanus	1.06 (0.85 to 1.35)	0.17 (0.10 to 0.27)	0.14 (0.08 to 0.22)	0.21 (0.12 to 0.38)	0.02 (0.01 to 0.02)	0.00 (0.00 to 0.00)	0.00 (0.00 to 0.00)	0.00 (0.00 to 0.01)
Measles	466.43 (155.28 to 1,041.91)	524.92 (30.41 to 2,451.62)	241.08 (22.65 to 957.98)	949.76 (43.77 to 4,211.25)	5.58 (1.86 to 12.41)	6.92 (0.52 to 29.44)	3.37 (0.40 to 11.97)	12.08 (0.67 to 49.36)

Cause	YLLs (actual counts)				Deaths (actual counts)			
	2016	2040 Forecast	2040 Better Scenario	2040 Worse Scenario	2016	2040 Forecast	2040 Better Scenario	2040 Worse Scenario
Varicella and herpes zoster	28.25 (14.68 to 52.36)	16.71 (6.21 to 36.35)	8.90 (3.68 to 18.12)	21.73 (7.13 to 51.08)	0.49 (0.31 to 0.77)	0.36 (0.19 to 0.61)	0.24 (0.13 to 0.38)	0.41 (0.20 to 0.77)
**Neglected tropical diseases and malaria**	**23.60 (12.55 to 41.69)**	**59.33 (32.33 to 123.81)**	**52.94 (28.99 to 107.74)**	**63.66 (34.79 to 131.48)**	**0.61 (0.32 to 1.03)**	**1.96 (1.05 to 4.11)**	**2.02 (1.09 to 4.08)**	**1.88 (0.99 to 3.93)**
Malaria	0.00 (0.00 to 0.00)	0.00 (0.00 to 0.00)	0.00 (0.00 to 0.00)	0.00 (0.00 to 0.00)	0.00 (0.00 to 0.00)	0.00 (0.00 to 0.00)	0.00 (0.00 to 0.00)	0.00 (0.00 to 0.00)
Chagas disease	0.00 (0.00 to 0.00)	0.00 (0.00 to 0.00)	0.00 (0.00 to 0.00)	0.00 (0.00 to 0.00)	0.00 (0.00 to 0.00)	0.00 (0.00 to 0.00)	0.00 (0.00 to 0.00)	0.00 (0.00 to 0.00)
Leishmaniasis	0.00 (0.00 to 0.00)	0.00 (0.00 to 0.00)	0.00 (0.00 to 0.00)	0.00 (0.00 to 0.00)	0.00 (0.00 to 0.00)	0.00 (0.00 to 0.00)	0.00 (0.00 to 0.00)	0.00 (0.00 to 0.00)
African trypanosomiasis	0.00 (0.00 to 0.00)	0.00 (0.00 to 0.00)	0.00 (0.00 to 0.00)	0.00 (0.00 to 0.00)	0.00 (0.00 to 0.00)	0.00 (0.00 to 0.00)	0.00 (0.00 to 0.00)	0.00 (0.00 to 0.00)
Schistosomiasis	0.00 (0.00 to 0.00)	0.00 (0.00 to 0.00)	0.00 (0.00 to 0.00)	0.00 (0.00 to 0.00)	0.00 (0.00 to 0.00)	0.00 (0.00 to 0.00)	0.00 (0.00 to 0.00)	0.00 (0.00 to 0.00)
Cysticercosis	0.00 (0.00 to 0.00)	0.00 (0.00 to 0.00)	0.00 (0.00 to 0.00)	0.00 (0.00 to 0.00)	0.00 (0.00 to 0.00)	0.00 (0.00 to 0.00)	0.00 (0.00 to 0.00)	0.00 (0.00 to 0.00)
Cystic echinococcosis	0.00 (0.00 to 0.00)	0.00 (0.00 to 0.00)	0.00 (0.00 to 0.00)	0.00 (0.00 to 0.00)	0.00 (0.00 to 0.00)	0.00 (0.00 to 0.00)	0.00 (0.00 to 0.00)	0.00 (0.00 to 0.00)
Dengue	3.31 (0.27 to 21.99)	20.37 (4.10 to 105.50)	20.03 (4.88 to 93.16)	21.03 (3.97 to 112.74)	0.09 (0.01 to 0.60)	0.71 (0.13 to 3.47)	0.79 (0.17 to 3.45)	0.66 (0.12 to 3.32)
Yellow fever	0.00 (0.00 to 0.00)	0.00 (0.00 to 0.00)	0.00 (0.00 to 0.00)	0.00 (0.00 to 0.00)	0.00 (0.00 to 0.00)	0.00 (0.00 to 0.00)	0.00 (0.00 to 0.00)	0.00 (0.00 to 0.00)
Rabies	0.27 (0.01 to 0.47)	0.52 (0.01 to 1.16)	0.27 (0.00 to 0.65)	0.60 (0.01 to 1.37)	0.01 (0.00 to 0.02)	0.03 (0.00 to 0.06)	0.02 (0.00 to 0.04)	0.03 (0.00 to 0.07)
Intestinal nematode infections	0.75 (0.13 to 2.13)	1.49 (0.34 to 3.76)	1.41 (0.33 to 3.50)	1.72 (0.35 to 4.44)	0.01 (0.00 to 0.03)	0.02 (0.01 to 0.05)	0.02 (0.01 to 0.05)	0.02 (0.01 to 0.06)
Other neglected tropical diseases	19.27 (7.40 to 32.42)	36.94 (8.86 to 60.59)	31.23 (6.73 to 50.99)	40.31 (9.50 to 69.18)	0.50 (0.19 to 0.73)	1.20 (0.26 to 1.93)	1.20 (0.24 to 1.95)	1.17 (0.27 to 1.87)
**Maternal disorders**	**263.40 (179.72 to 369.08)**	**236.94 (150.04 to 359.46)**	**113.12 (70.21 to 172.51)**	**292.18 (178.05 to 448.99)**	**4.96 (3.37 to 6.95)**	**4.50 (2.87 to 6.90)**	**2.15 (1.34 to 3.25)**	**5.53 (3.38 to 8.52)**
Maternal hemorrhage	87.93 (53.65 to 134.26)	76.87 (42.74 to 128.03)	36.61 (20.48 to 60.95)	93.80 (50.99 to 157.36)	1.65 (1.01 to 2.52)	1.45 (0.80 to 2.41)	0.69 (0.39 to 1.14)	1.77 (0.95 to 2.97)
Maternal sepsis and other maternal infections	16.54 (6.59 to 31.66)	13.28 (5.11 to 26.98)	6.30 (2.43 to 13.27)	16.30 (6.22 to 32.96)	0.32 (0.13 to 0.60)	0.26 (0.10 to 0.53)	0.12 (0.05 to 0.26)	0.32 (0.12 to 0.64)
Maternal hypertensive disorders	35.39 (16.70 to 61.80)	33.40 (14.68 to 62.83)	15.76 (6.98 to 28.19)	40.79 (18.37 to 75.80)	0.67 (0.32 to 1.16)	0.63 (0.28 to 1.19)	0.30 (0.13 to 0.54)	0.77 (0.34 to 1.45)
Maternal obstructed labor and uterine rupture	16.69 (6.72 to 31.17)	15.86 (5.94 to 32.77)	7.17 (2.50 to 15.25)	19.62 (7.13 to 41.04)	0.32 (0.13 to 0.60)	0.31 (0.11 to 0.64)	0.14 (0.05 to 0.29)	0.38 (0.13 to 0.78)
Maternal abortion, miscarriage, and ectopic pregnancy	12.61 (4.74 to 26.98)	13.36 (4.69 to 30.60)	6.32 (2.07 to 14.56)	16.48 (5.42 to 38.21)	0.24 (0.09 to 0.53)	0.26 (0.09 to 0.60)	0.12 (0.04 to 0.28)	0.32 (0.11 to 0.74)

Indirect maternal deaths	29.05 (13.92 to 52.82)	27.41 (12.63 to 50.25)	13.13 (5.96 to 24.17)	34.67 (15.27 to 65.05)	0.54 (0.26 to 0.95)	0.52 (0.24 to 0.94)	0.25 (0.11 to 0.46)	0.65 (0.29 to 1.23)
Late maternal deaths	17.63 (6.90 to 34.35)	13.36 (4.88 to 27.32)	6.30 (2.34 to 12.81)	16.74 (6.07 to 33.69)	0.33 (0.13 to 0.64)	0.25 (0.09 to 0.52)	0.12 (0.04 to 0.24)	0.31 (0.11 to 0.62)
Maternal deaths aggravated by HIV/AIDS	0.01 (0.00 to 0.03)	0.13 (0.02 to 0.63)	0.06 (0.01 to 0.27)	0.17 (0.03 to 0.84)	0.00 (0.00 to 0.00)	0.00 (0.00 to 0.01)	0.00 (0.00 to 0.00)	0.00 (0.00 to 0.01)
Other maternal disorders	47.56 (23.51 to 77.95)	43.27 (20.63 to 76.75)	21.49 (10.16 to 36.76)	53.61 (26.42 to 94.05)	0.90 (0.44 to 1.47)	0.82 (0.39 to 1.44)	0.41 (0.19 to 0.70)	1.01 (0.49 to 1.77)

Kiribati								
Cause	YLLs (actual counts)				Deaths (actual counts)			
	2016	2040 Forecast	2040 Better Scenario	2040 Worse Scenario	2016	2040 Forecast	2040 Better Scenario	2040 Worse Scenario
**Neonatal disorders**	**4,646.47 (2,347.66 to 8,283.25)**	**3,236.06 (1,518.27 to 5,936.57)**	**1,734.42 (822.06 to 3,305.19)**	**4,681.04 (2,246.87 to 8,643.23)**	**53.74 (27.17 to 95.78)**	**37.45 (17.57 to 68.70)**	**20.08 (9.52 to 38.25)**	**54.17 (26.01 to 100.00)**
Neonatal preterm birth	1,723.29 (829.03 to 3,305.33)	1,140.25 (515.69 to 2,291.31)	561.99 (248.43 to 1,097.50)	1,658.34 (740.77 to 3,247.32)	19.92 (9.58 to 38.20)	13.19 (5.96 to 26.49)	6.50 (2.88 to 12.70)	19.18 (8.56 to 37.56)
Neonatal encephalopathy due to birth asphyxia and trauma	1,578.09 (707.26 to 3,126.62)	1,028.06 (430.08 to 2,105.82)	608.19 (252.16 to 1,266.29)	1,460.16 (613.92 to 3,027.40)	18.26 (8.19 to 36.17)	11.91 (4.99 to 24.39)	7.04 (2.92 to 14.68)	16.91 (7.10 to 35.07)
Neonatal sepsis and other neonatal infections	279.17 (108.47 to 706.81)	276.94 (102.11 to 680.54)	158.80 (57.97 to 384.38)	396.56 (149.41 to 978.29)	3.23 (1.25 to 8.17)	3.21 (1.18 to 7.90)	1.84 (0.67 to 4.45)	4.59 (1.73 to 11.32)
Hemolytic disease and other neonatal jaundice	134.79 (56.20 to 291.87)	59.44 (20.20 to 142.67)	30.78 (10.62 to 73.69)	87.87 (30.42 to 210.08)	1.56 (0.65 to 3.37)	0.69 (0.23 to 1.65)	0.36 (0.12 to 0.85)	1.02 (0.35 to 2.43)
Other neonatal disorders	931.13 (404.59 to 1,873.78)	731.37 (292.24 to 1,453.46)	374.65 (151.00 to 777.14)	1,078.12 (424.17 to 2,262.11)	10.77 (4.68 to 21.68)	8.47 (3.39 to 16.81)	4.34 (1.75 to 9.00)	12.48 (4.91 to 26.18)
**Nutritional deficiencies**	**1,013.23 (575.50 to 1,765.02)**	**445.90 (265.88 to 787.82)**	**278.07 (178.57 to 455.47)**	**572.21 (325.16 to 1,042.43)**	**17.79 (12.37 to 26.53)**	**12.06 (8.90 to 16.67)**	**9.82 (7.47 to 12.54)**	**12.89 (9.08 to 19.11)**
Protein-energy malnutrition	987.65 (559.30 to 1,737.17)	413.11 (241.07 to 743.30)	255.91 (160.54 to 421.53)	533.27 (286.38 to 993.06)	17.19 (11.92 to 25.89)	11.11 (8.02 to 15.48)	9.05 (6.86 to 11.72)	11.89 (8.25 to 17.83)
Iodine deficiency	3.28 (0.11 to 15.78)	11.90 (0.20 to 79.72)	6.93 (0.11 to 39.29)	15.21 (0.20 to 110.91)	0.07 (0.00 to 0.24)	0.34 (0.01 to 1.41)	0.25 (0.00 to 1.23)	0.37 (0.01 to 1.65)
Dietary iron deficiency	1.19 (0.80 to 2.10)	1.17 (0.73 to 2.28)	0.91 (0.57 to 1.66)	1.31 (0.83 to 2.51)	0.02 (0.01 to 0.04)	0.03 (0.02 to 0.05)	0.02 (0.01 to 0.05)	0.03 (0.02 to 0.05)
Other nutritional deficiencies	21.11 (11.30 to 34.90)	19.72 (11.03 to 31.49)	14.32 (8.12 to 21.25)	22.41 (11.91 to 37.52)	0.51 (0.31 to 0.74)	0.59 (0.35 to 0.85)	0.50 (0.29 to 0.70)	0.60 (0.35 to 0.88)
**Other communicable, maternal, neonatal, and nutritional diseases**	**482.91 (289.17 to 884.84)**	**393.05 (268.24 to 623.06)**	**274.27 (193.63 to 408.80)**	**458.05 (300.95 to 785.15)**	**8.50 (5.98 to 13.37)**	**8.28 (6.30 to 11.43)**	**6.26 (4.86 to 8.24)**	**9.10 (6.71 to 13.34)**
Sexually transmitted infections excluding HIV	135.82 (38.76 to 362.95)	51.86 (19.27 to 126.58)	34.70 (14.13 to 81.75)	65.28 (22.18 to 169.26)	1.78 (0.63 to 4.42)	0.76 (0.35 to 1.63)	0.53 (0.27 to 1.08)	0.93 (0.39 to 2.15)
Syphilis	120.13 (23.23 to 349.34)	39.31 (7.81 to 114.91)	24.71 (5.48 to 72.07)	51.95 (9.71 to 156.53)	1.44 (0.31 to 4.12)	0.49 (0.12 to 1.38)	0.31 (0.08 to 0.87)	0.64 (0.15 to 1.87)
Chlamydial infection	3.08 (2.04 to 5.14)	2.48 (1.55 to 4.19)	1.97 (1.26 to 3.30)	2.64 (1.66 to 4.47)	0.07 (0.04 to 0.11)	0.05 (0.03 to 0.09)	0.04 (0.03 to 0.07)	0.06 (0.04 to 0.10)
Gonococcal infection	8.06 (5.35 to 13.28)	6.44 (4.06 to 11.15)	5.15 (3.23 to 8.80)	6.83 (4.24 to 11.73)	0.18 (0.12 to 0.29)	0.14 (0.09 to 0.24)	0.11 (0.07 to 0.20)	0.15 (0.09 to 0.26)
Other sexually transmitted infections	4.54 (3.01 to 7.46)	3.62 (2.22 to 6.26)	2.87 (1.80 to 4.80)	3.86 (2.41 to 6.44)	0.10 (0.06 to 0.16)	0.08 (0.05 to 0.13)	0.06 (0.04 to 0.10)	0.08 (0.05 to 0.14)
Acute hepatitis	109.36 (80.13 to 152.00)	100.40 (68.65 to 144.80)	77.67 (53.05 to 110.79)	107.49 (72.50 to 158.24)	2.58 (1.88 to 3.56)	2.55 (1.78 to 3.60)	2.03 (1.43 to 2.83)	2.67 (1.83 to 3.88)
Acute hepatitis A	4.81 (2.68 to 8.69)	2.49 (1.28 to 5.13)	1.73 (0.91 to 3.36)	3.02 (1.46 to 6.37)	0.07 (0.05 to 0.13)	0.05 (0.03 to 0.08)	0.03 (0.02 to 0.06)	0.05 (0.03 to 0.10)
Acute hepatitis B	90.18 (64.28 to 125.74)	87.15 (58.00 to 128.37)	67.30 (44.61 to 96.38)	92.54 (61.22 to 139.21)	2.24 (1.61 to 3.14)	2.26 (1.53 to 3.24)	1.79 (1.24 to 2.51)	2.36 (1.59 to 3.45)

Acute hepatitis C	1.78 (1.10 to 2.83)	1.76 (1.03 to 2.90)	1.60 (0.93 to 2.65)	1.81 (1.04 to 2.97)	0.04 (0.03 to 0.07)	0.05 (0.03 to 0.08)	0.05 (0.03 to 0.08)	0.05 (0.03 to 0.08)
Acute hepatitis E	12.59 (7.33 to 21.33)	9.00 (4.97 to 15.62)	7.04 (4.02 to 11.74)	10.13 (5.42 to 17.88)	0.22 (0.13 to 0.35)	0.19 (0.11 to 0.31)	0.16 (0.10 to 0.26)	0.20 (0.12 to 0.33)
Other unspecified infectious diseases	237.73 (123.46 to 593.01)	240.79 (150.80 to 439.68)	161.91 (106.32 to 273.25)	285.27 (167.86 to 565.27)	4.14 (2.63 to 8.24)	4.97 (3.66 to 7.39)	3.69 (2.77 to 5.23)	5.51 (3.88 to 9.09)
**Non-communicable diseases**	**23,267.69 (20,239.53 to 27,042.20)**	**32,475.63 (27,274.57 to 38,756.17)**	**21,775.75 (18,590.18 to 25,607.76)**	**38,604.70 (32,489.52 to 45,868.72)**	**740.14 (658.56 to 833.78)**	**1,166.45 (1,030.34 to 1,320.88)**	**846.18 (756.79 to 950.99)**	**1,334.48 (1,174.57 to 1,513.94)**

Kiribati								
Cause	YLLs (actual counts)				Deaths (actual counts)			
	2016	2040 Forecast	2040 Better Scenario	2040 Worse Scenario	2016	2040 Forecast	2040 Better Scenario	2040 Worse Scenario
**Neoplasms**	**2,849.18 (2,468.26 to 3,276.19)**	**4,161.49 (3,418.95 to 5,087.43)**	**3,590.39 (2,991.63 to 4,273.10)**	**4,474.59 (3,629.03 to 5,569.77)**	**88.79 (77.97 to 100.78)**	**142.50 (118.79 to 172.59)**	**131.84 (112.59 to 154.98)**	**147.70 (120.40 to 182.12)**
Lip and oral cavity cancer	255.44 (199.27 to 316.67)	420.09 (258.64 to 639.77)	253.98 (174.73 to 357.49)	489.63 (297.23 to 778.64)	8.64 (6.97 to 10.38)	14.64 (9.73 to 21.72)	9.78 (7.12 to 13.35)	16.45 (10.55 to 25.08)
Nasopharynx cancer	26.83 (21.31 to 32.94)	52.62 (29.31 to 91.14)	28.10 (17.39 to 43.87)	62.99 (34.76 to 110.96)	0.81 (0.63 to 1.01)	1.61 (0.92 to 2.74)	0.91 (0.57 to 1.39)	1.89 (1.04 to 3.25)
Other pharynx cancer	32.48 (26.62 to 38.87)	41.27 (29.35 to 58.52)	36.00 (26.42 to 49.88)	43.59 (30.41 to 62.58)	1.07 (0.89 to 1.27)	1.47 (1.08 to 2.02)	1.37 (1.03 to 1.83)	1.50 (1.07 to 2.10)
Esophageal cancer	69.30 (58.49 to 81.90)	102.33 (58.82 to 170.81)	63.11 (40.93 to 97.42)	116.24 (64.59 to 195.19)	2.60 (2.21 to 3.03)	3.99 (2.38 to 6.35)	2.71 (1.79 to 4.09)	4.37 (2.51 to 7.02)
Stomach cancer	215.53 (182.77 to 253.03)	221.42 (172.14 to 288.48)	190.33 (155.40 to 234.58)	225.44 (172.27 to 296.51)	7.34 (6.32 to 8.48)	8.12 (6.57 to 10.13)	7.57 (6.36 to 9.02)	7.98 (6.33 to 10.13)
Colon and rectum cancer	149.62 (122.40 to 178.07)	248.10 (164.30 to 376.83)	266.15 (175.16 to 400.58)	247.48 (160.90 to 380.11)	5.39 (4.43 to 6.39)	9.82 (6.44 to 15.07)	11.39 (7.51 to 17.27)	9.38 (6.01 to 14.49)
Liver cancer	282.25 (223.73 to 338.36)	481.44 (312.10 to 725.54)	404.03 (282.05 to 572.60)	563.23 (347.26 to 893.53)	9.23 (7.42 to 11.00)	16.65 (11.26 to 24.27)	15.11 (10.85 to 21.02)	18.71 (11.96 to 28.46)
Liver cancer due to hepatitis B	164.45 (127.45 to 200.61)	272.82 (178.04 to 418.20)	228.68 (157.28 to 332.88)	317.93 (194.44 to 511.12)	5.08 (3.98 to 6.16)	8.89 (6.01 to 13.27)	7.98 (5.77 to 11.35)	10.01 (6.33 to 15.66)
Liver cancer due to hepatitis C	38.54 (29.78 to 47.94)	67.29 (44.04 to 99.51)	60.56 (41.56 to 86.24)	75.61 (47.63 to 116.82)	1.56 (1.23 to 1.93)	2.92 (2.00 to 4.24)	2.86 (2.03 to 3.99)	3.12 (2.01 to 4.74)
Liver cancer due to alcohol use	19.75 (14.64 to 25.85)	35.81 (22.08 to 57.42)	29.18 (19.14 to 43.89)	43.29 (24.87 to 71.44)	0.71 (0.53 to 0.92)	1.33 (0.87 to 2.02)	1.18 (0.81 to 1.71)	1.53 (0.94 to 2.41)
Liver cancer due to other causes	59.51 (46.78 to 74.46)	105.51 (60.18 to 176.78)	85.61 (52.99 to 136.93)	126.40 (68.23 to 224.28)	1.87 (1.51 to 2.32)	3.52 (2.03 to 5.84)	3.08 (1.91 to 4.92)	4.06 (2.20 to 7.08)
Gallbladder and biliary tract cancer	17.35 (13.83 to 21.19)	18.03 (14.67 to 21.63)	20.41 (16.51 to 24.65)	16.79 (13.73 to 20.20)	0.70 (0.56 to 0.85)	0.79 (0.63 to 0.96)	0.95 (0.77 to 1.15)	0.71 (0.57 to 0.87)
Pancreatic cancer	70.95 (60.39 to 83.02)	130.29 (91.32 to 187.95)	134.80 (96.32 to 192.65)	127.49 (86.97 to 189.33)	2.93 (2.49 to 3.43)	5.65 (3.91 to 8.28)	6.26 (4.45 to 8.91)	5.36 (3.66 to 8.07)
Larynx cancer	26.28 (22.03 to 31.42)	39.88 (21.57 to 69.01)	21.31 (12.61 to 37.67)	45.87 (23.96 to 78.82)	0.94 (0.79 to 1.11)	1.49 (0.80 to 2.64)	0.85 (0.50 to 1.50)	1.68 (0.87 to 2.95)
Tracheal, bronchus, and lung cancer	313.70 (263.18 to 377.51)	498.02 (306.55 to 755.44)	269.92 (176.61 to 401.46)	586.87 (352.55 to 893.23)	11.50 (9.76 to 13.56)	19.10 (11.98 to 29.30)	10.90 (7.26 to 16.31)	22.24 (13.40 to 34.21)
Malignant skin melanoma	12.75 (9.20 to 17.42)	18.33 (13.70 to 24.78)	23.14 (16.95 to 31.44)	16.77 (12.48 to 22.58)	0.44 (0.32 to 0.59)	0.68 (0.52 to 0.90)	0.92 (0.70 to 1.24)	0.60 (0.44 to 0.81)
Non-melanoma skin cancer	16.42 (13.46 to 19.98)	27.10 (22.49 to 32.63)	26.63 (21.97 to 32.00)	26.27 (21.75 to 31.44)	0.64 (0.53 to 0.77)	1.18 (0.95 to 1.43)	1.30 (1.04 to 1.59)	1.08 (0.85 to 1.30)
Breast cancer	300.30 (213.05 to 412.20)	425.92 (267.00 to 641.57)	419.77 (269.00 to 624.40)	431.07 (265.91 to 660.55)	9.41 (6.72 to 12.88)	14.85 (9.43 to 22.78)	15.47 (10.12 to 23.54)	14.56 (8.98 to 22.71)
Cervical cancer	100.72 (60.83 to 270.16)	129.09 (72.56 to 365.07)	107.48 (61.33 to 301.97)	135.19 (74.82 to 379.24)	2.87 (1.69 to 8.28)	3.89 (2.17 to 11.65)	3.35 (1.91 to 10.04)	4.00 (2.22 to 12.21)

Uterine cancer	24.27 (17.97 to 31.89)	38.26 (29.43 to 49.06)	33.52 (25.14 to 43.63)	38.35 (29.28 to 48.86)	0.92 (0.68 to 1.21)	1.58 (1.20 to 2.01)	1.44 (1.08 to 1.86)	1.54 (1.17 to 1.95)
Ovarian cancer	39.73 (30.56 to 50.16)	62.58 (42.15 to 94.31)	70.83 (47.81 to 103.83)	61.66 (40.65 to 94.94)	1.33 (1.02 to 1.69)	2.26 (1.53 to 3.46)	2.65 (1.81 to 3.96)	2.17 (1.43 to 3.36)
Prostate cancer	38.45 (25.77 to 48.01)	75.24 (49.91 to 94.22)	96.03 (67.27 to 119.35)	65.68 (43.84 to 84.33)	2.07 (1.44 to 2.61)	4.42 (2.95 to 5.73)	6.12 (4.29 to 7.77)	3.70 (2.42 to 4.94)
Testicular cancer	42.42 (30.96 to 56.89)	45.91 (33.02 to 61.54)	42.22 (30.40 to 56.59)	46.65 (33.33 to 62.67)	0.92 (0.68 to 1.19)	1.05 (0.77 to 1.36)	1.01 (0.75 to 1.32)	1.04 (0.77 to 1.35)

Kiribati								
Cause	YLLs (actual counts)				Deaths (actual counts)			
	2016	2040 Forecast	2040 Better Scenario	2040 Worse Scenario	2016	2040 Forecast	2040 Better Scenario	2040 Worse Scenario
Kidney cancer	47.05 (27.96 to 61.23)	82.12 (40.74 to 122.80)	87.05 (44.73 to 124.91)	79.34 (40.19 to 119.25)	1.42 (0.83 to 1.81)	2.76 (1.43 to 4.02)	3.22 (1.73 to 4.45)	2.55 (1.34 to 3.76)
Bladder cancer	16.10 (13.76 to 18.87)	24.13 (16.30 to 36.22)	21.73 (15.21 to 31.91)	24.47 (16.23 to 37.61)	0.70 (0.60 to 0.81)	1.12 (0.76 to 1.70)	1.11 (0.77 to 1.65)	1.09 (0.71 to 1.70)
Brain and nervous system cancer	76.90 (56.62 to 107.96)	112.91 (84.00 to 154.04)	116.69 (88.17 to 156.70)	115.43 (84.99 to 158.33)	1.72 (1.30 to 2.33)	2.85 (2.17 to 3.76)	3.17 (2.45 to 4.22)	2.76 (2.07 to 3.65)
Thyroid cancer	5.27 (4.30 to 6.36)	7.64 (5.67 to 10.37)	6.93 (5.28 to 9.29)	8.33 (5.97 to 11.54)	0.20 (0.16 to 0.24)	0.32 (0.24 to 0.43)	0.30 (0.23 to 0.40)	0.33 (0.24 to 0.46)
Mesothelioma	3.62 (2.99 to 4.38)	4.82 (4.02 to 5.78)	4.71 (3.89 to 5.69)	4.75 (3.98 to 5.72)	0.12 (0.10 to 0.14)	0.16 (0.14 to 0.19)	0.17 (0.14 to 0.20)	0.16 (0.13 to 0.18)
Hodgkin lymphoma	19.44 (9.16 to 27.69)	20.04 (9.37 to 29.29)	14.98 (7.15 to 21.41)	21.93 (10.08 to 32.10)	0.42 (0.21 to 0.58)	0.48 (0.24 to 0.67)	0.38 (0.19 to 0.53)	0.50 (0.25 to 0.70)
Non-Hodgkin lymphoma	142.30 (54.93 to 192.92)	200.28 (78.90 to 277.78)	213.55 (85.63 to 295.17)	207.49 (80.06 to 290.24)	3.51 (1.43 to 4.64)	5.69 (2.39 to 7.76)	6.63 (2.80 to 9.00)	5.57 (2.31 to 7.73)
Multiple myeloma	17.59 (14.12 to 22.79)	29.90 (22.71 to 39.98)	34.18 (26.18 to 45.52)	29.74 (22.13 to 40.39)	0.63 (0.51 to 0.81)	1.16 (0.89 to 1.55)	1.40 (1.09 to 1.87)	1.11 (0.84 to 1.50)
Leukemia	190.59 (141.35 to 244.97)	211.31 (152.78 to 280.88)	194.25 (142.91 to 253.84)	232.12 (167.57 to 311.96)	3.77 (2.90 to 4.71)	4.90 (3.63 to 6.49)	4.81 (3.63 to 6.26)	5.16 (3.78 to 6.84)
Acute lymphoid leukemia	38.58 (25.73 to 59.28)	37.99 (25.79 to 57.92)	35.95 (24.75 to 53.33)	42.22 (28.15 to 64.81)	0.61 (0.43 to 0.91)	0.69 (0.49 to 1.02)	0.67 (0.48 to 0.98)	0.74 (0.52 to 1.10)
Chronic lymphoid leukemia	2.64 (1.83 to 3.61)	3.67 (2.42 to 5.34)	3.70 (2.45 to 5.32)	3.77 (2.45 to 5.56)	0.10 (0.07 to 0.13)	0.15 (0.10 to 0.22)	0.16 (0.11 to 0.24)	0.15 (0.09 to 0.22)
Acute myeloid leukemia	55.06 (34.03 to 79.37)	77.12 (47.98 to 112.09)	78.61 (49.95 to 111.72)	82.14 (51.08 to 120.80)	1.06 (0.66 to 1.49)	1.72 (1.07 to 2.47)	1.87 (1.19 to 2.60)	1.77 (1.10 to 2.57)
Chronic myeloid leukemia	25.88 (18.30 to 33.92)	24.72 (16.39 to 36.29)	22.83 (15.43 to 32.98)	26.44 (17.24 to 39.23)	0.70 (0.52 to 0.92)	0.73 (0.50 to 1.05)	0.70 (0.49 to 1.00)	0.76 (0.51 to 1.12)
Other leukemia	68.43 (44.75 to 90.43)	67.81 (44.22 to 93.44)	53.16 (34.84 to 73.14)	77.54 (50.44 to 106.58)	1.30 (0.89 to 1.68)	1.61 (1.10 to 2.29)	1.39 (0.96 to 1.96)	1.75 (1.18 to 2.48)
Other neoplasms	295.52 (197.84 to 381.84)	392.42 (274.51 to 494.92)	388.57 (277.76 to 487.79)	403.74 (272.25 to 516.00)	6.54 (4.84 to 8.07)	9.82 (7.62 to 11.81)	10.59 (8.51 to 12.70)	9.53 (7.22 to 11.54)
**Cardiovascular diseases**	**8,619.48 (7,510.63 to 9,884.77)**	**10,640.17 (7,595.37 to 14,189.88)**	**5,172.32 (3,719.07 to 6,915.89)**	**14,235.85 (10,256.13 to 18,554.20)**	**288.53 (253.56 to 327.51)**	**386.34 (293.25 to 504.36)**	**206.60 (159.72 to 270.80)**	**505.17 (381.64 to 648.93)**
Rheumatic heart disease	561.64 (464.05 to 689.76)	341.03 (270.89 to 428.15)	239.42 (191.08 to 301.52)	376.36 (295.53 to 483.13)	14.45 (12.03 to 17.56)	10.11 (8.26 to 12.31)	7.72 (6.37 to 9.41)	10.61 (8.56 to 13.15)
Ischemic heart disease	3,395.83 (2,939.24 to 3,946.72)	4,450.44 (2,484.43 to 6,727.79)	1,787.00 (942.59 to 3,032.69)	6,235.58 (3,695.60 to 9,055.07)	116.24 (101.81 to 133.14)	158.94 (93.60 to 244.68)	70.11 (39.56 to 116.87)	220.48 (134.27 to 326.43)
Stroke	3,284.19 (2,831.08 to 3,781.45)	3,859.87 (2,360.35 to 5,812.86)	1,602.81 (954.70 to 2,528.86)	5,425.81 (3,428.11 to 7,927.60)	113.43 (98.89 to 128.27)	144.04 (94.34 to 209.66)	66.49 (42.63 to 103.85)	197.38 (127.93 to 284.28)
Ischemic stroke	1,022.20 (890.11 to 1,188.69)	1,118.12 (690.68 to 1,787.02)	565.01 (336.47 to 946.42)	1,445.67 (906.07 to 2,232.36)	42.62 (37.36 to 48.88)	51.12 (31.92 to 88.33)	28.35 (17.47 to 50.54)	64.57 (38.68 to 107.25)

Intracerebral hemorrhage	2,261.99 (1,931.82 to 2,638.21)	2,741.75 (1,525.69 to 4,493.72)	1,037.80 (568.68 to 1,775.00)	3,980.14 (2,213.30 to 6,093.78)	70.81 (60.58 to 82.10)	92.91 (55.68 to 148.57)	38.14 (22.50 to 61.95)	132.82 (77.55 to 203.71)
Hypertensive heart disease	383.23 (251.30 to 486.28)	582.11 (353.88 to 846.67)	382.31 (250.39 to 526.59)	688.22 (398.21 to 1,101.45)	14.74 (9.66 to 18.55)	24.58 (15.12 to 35.50)	17.69 (11.40 to 24.00)	27.60 (16.39 to 43.64)
Cardiomyopathy and myocarditis	296.40 (163.81 to 412.08)	351.28 (187.57 to 498.39)	337.51 (182.72 to 474.84)	358.60 (190.91 to 515.26)	7.97 (4.42 to 10.96)	10.75 (5.82 to 14.93)	11.17 (6.08 to 15.35)	10.42 (5.57 to 14.58)
Myocarditis	148.26 (93.42 to 208.15)	171.28 (106.53 to 238.53)	160.33 (100.73 to 221.38)	172.96 (108.60 to 239.94)	4.20 (2.72 to 5.87)	5.50 (3.55 to 7.64)	5.56 (3.60 to 7.65)	5.28 (3.43 to 7.30)

Kiribati								
Cause	YLLs (actual counts)				Deaths (actual counts)			
	2016	2040 Forecast	2040 Better Scenario	2040 Worse Scenario	2016	2040 Forecast	2040 Better Scenario	2040 Worse Scenario
Alcoholic cardiomyopathy	5.36 (3.26 to 7.78)	4.92 (3.01 to 6.97)	4.68 (2.85 to 6.56)	5.05 (3.06 to 7.09)	0.16 (0.10 to 0.23)	0.16 (0.10 to 0.23)	0.16 (0.10 to 0.23)	0.16 (0.10 to 0.23)
Other cardiomyopathy	142.78 (49.94 to 212.19)	175.08 (58.10 to 285.88)	172.51 (58.41 to 274.87)	180.60 (59.97 to 296.84)	3.61 (1.29 to 5.24)	5.09 (1.72 to 7.89)	5.44 (1.91 to 8.27)	4.98 (1.65 to 7.83)
Atrial fibrillation and flutter	21.70 (16.71 to 28.30)	41.00 (30.02 to 53.93)	44.43 (33.55 to 57.53)	38.45 (27.79 to 51.50)	1.62 (1.24 to 2.13)	3.17 (2.21 to 4.34)	3.82 (2.79 to 5.11)	2.79 (1.87 to 3.89)
Aortic aneurysm	27.46 (22.71 to 33.29)	35.89 (27.39 to 47.49)	33.43 (26.18 to 43.33)	36.21 (27.09 to 48.76)	1.02 (0.85 to 1.22)	1.42 (1.11 to 1.78)	1.48 (1.21 to 1.80)	1.36 (1.06 to 1.74)
Peripheral artery disease	4.45 (2.73 to 8.36)	11.66 (6.46 to 24.95)	12.93 (7.30 to 26.11)	11.61 (6.27 to 24.60)	0.22 (0.13 to 0.44)	0.64 (0.34 to 1.33)	0.83 (0.44 to 1.65)	0.60 (0.32 to 1.25)
Endocarditis	189.13 (138.04 to 236.45)	273.07 (192.32 to 361.50)	247.58 (176.72 to 322.34)	284.44 (199.01 to 380.32)	5.34 (4.10 to 6.56)	8.70 (6.33 to 11.11)	8.63 (6.38 to 10.90)	8.55 (6.25 to 10.94)
Other cardiovascular and circulatory diseases	455.47 (308.90 to 580.89)	693.80 (474.23 to 952.10)	484.90 (344.35 to 639.26)	780.55 (523.42 to 1,066.81)	13.50 (9.67 to 16.60)	23.98 (16.82 to 32.54)	18.67 (13.45 to 24.86)	25.38 (17.15 to 34.72)
**Chronic respiratory diseases**	**2,171.14 (1,769.06 to 2,680.95)**	**2,424.14 (1,788.49 to 3,328.22)**	**1,579.83 (1,220.95 to 2,055.62)**	**2,839.39 (2,070.56 to 4,014.05)**	**72.57 (59.90 to 88.37)**	**86.16 (64.63 to 116.00)**	**58.24 (46.31 to 73.92)**	**98.30 (72.23 to 135.16)**
Chronic obstructive pulmonary disease	727.41 (559.55 to 930.21)	948.07 (583.48 to 1,565.27)	432.48 (287.04 to 667.50)	1,161.20 (707.86 to 1,952.82)	28.17 (22.12 to 35.48)	38.44 (24.22 to 61.76)	19.00 (13.01 to 28.12)	45.71 (28.43 to 75.98)
Pneumoconiosis	9.27 (6.90 to 12.96)	9.78 (7.30 to 13.35)	10.93 (7.96 to 15.00)	9.05 (6.75 to 12.49)	0.40 (0.30 to 0.55)	0.50 (0.35 to 0.70)	0.62 (0.43 to 0.87)	0.43 (0.30 to 0.62)
Silicosis	0.48 (0.25 to 0.83)	0.39 (0.18 to 0.77)	0.45 (0.21 to 0.88)	0.35 (0.16 to 0.68)	0.03 (0.01 to 0.05)	0.03 (0.01 to 0.05)	0.03 (0.01 to 0.07)	0.02 (0.01 to 0.05)
Asbestosis	3.33 (2.36 to 4.58)	4.80 (3.39 to 6.64)	5.23 (3.50 to 7.50)	4.50 (3.24 to 6.10)	0.14 (0.09 to 0.20)	0.23 (0.15 to 0.34)	0.28 (0.17 to 0.42)	0.21 (0.14 to 0.30)
Coal workers pneumoconiosis	0.01 (0.01 to 0.02)	0.01 (0.01 to 0.02)	0.01 (0.00 to 0.01)	0.01 (0.01 to 0.02)	0.00 (0.00 to 0.00)	0.00 (0.00 to 0.00)	0.00 (0.00 to 0.00)	0.00 (0.00 to 0.00)
Other pneumoconiosis	5.44 (3.72 to 8.42)	4.57 (2.84 to 7.49)	5.25 (3.19 to 8.80)	4.19 (2.64 to 6.86)	0.24 (0.16 to 0.37)	0.24 (0.13 to 0.41)	0.31 (0.17 to 0.53)	0.20 (0.11 to 0.35)
Asthma	836.60 (624.43 to 1,137.76)	595.72 (356.89 to 970.83)	407.50 (254.40 to 642.31)	768.07 (453.80 to 1,256.43)	28.00 (21.46 to 37.65)	22.49 (13.66 to 35.98)	16.86 (10.80 to 26.03)	27.31 (16.26 to 43.47)
Interstitial lung disease and pulmonary sarcoidosis	74.94 (46.08 to 125.84)	93.52 (60.95 to 150.70)	108.95 (71.70 to 169.99)	91.64 (58.46 to 148.79)	1.69 (1.11 to 2.62)	2.46 (1.63 to 3.81)	3.12 (2.10 to 4.77)	2.28 (1.50 to 3.58)
Other chronic respiratory diseases	522.91 (366.43 to 758.57)	777.05 (511.91 to 1,154.96)	619.96 (429.36 to 888.07)	809.42 (522.86 to 1,226.28)	14.31 (9.89 to 20.82)	22.27 (14.72 to 33.39)	18.64 (12.88 to 26.71)	22.58 (14.66 to 34.78)
**Cirrhosis and other chronic liver diseases**	**929.96 (597.87 to 1,309.56)**	**1,275.69 (824.29 to 1,784.00)**	**1,139.83 (742.38 to 1,585.51)**	**1,311.90 (845.51 to 1,826.51)**	**24.53 (16.66 to 34.04)**	**36.00 (24.93 to 48.82)**	**33.84 (23.92 to 45.56)**	**35.89 (24.54 to 48.90)**
Cirrhosis and other chronic liver diseases due to hepatitis B	350.71 (216.05 to 506.89)	510.36 (318.86 to 737.12)	472.39 (300.15 to 673.29)	511.99 (318.94 to 737.05)	9.94 (6.55 to 14.13)	15.11 (10.11 to 21.20)	14.63 (9.91 to 20.25)	14.78 (9.75 to 20.64)
Cirrhosis and other chronic liver diseases due to hepatitis C	139.93 (86.92 to 208.46)	204.99 (128.89 to 305.61)	187.82 (119.86 to 274.42)	206.47 (129.20 to 304.56)	3.89 (2.53 to 5.69)	5.90 (3.88 to 8.51)	5.62 (3.81 to 7.95)	5.82 (3.77 to 8.39)

Cirrhosis and other chronic liver diseases due to alcohol use	120.41 (74.33 to 176.78)	170.59 (107.08 to 249.12)	142.40 (90.84 to 204.89)	184.26 (114.68 to 267.00)	3.57 (2.35 to 5.08)	5.26 (3.51 to 7.41)	4.63 (3.18 to 6.42)	5.49 (3.64 to 7.76)
Cirrhosis and other chronic liver diseases due to other causes	318.92 (214.83 to 433.72)	389.74 (267.35 to 528.47)	337.23 (231.24 to 452.27)	409.18 (277.50 to 549.75)	7.13 (5.08 to 9.38)	9.73 (7.08 to 12.70)	8.96 (6.60 to 11.60)	9.80 (7.06 to 12.77)
**Digestive diseases**	**1,306.56 (759.30 to 2,706.30)**	**1,520.17 (853.70 to 3,098.44)**	**1,204.44 (680.37 to 2,475.50)**	**1,661.02 (939.25 to 3,413.90)**	**38.81 (21.93 to 77.51)**	**53.08 (28.77 to 103.67)**	**46.00 (25.15 to 89.04)**	**54.65 (29.74 to 109.26)**
Peptic ulcer disease	371.83 (206.20 to 787.66)	329.28 (164.76 to 693.57)	218.47 (110.78 to 447.18)	361.68 (183.80 to 748.82)	12.41 (6.82 to 25.16)	12.89 (6.29 to 26.20)	9.23 (4.55 to 17.81)	13.50 (6.66 to 27.44)

Kiribati								
Cause	YLLs (actual counts)				Deaths (actual counts)			
	2016	2040 Forecast	2040 Better Scenario	2040 Worse Scenario	2016	2040 Forecast	2040 Better Scenario	2040 Worse Scenario
Gastritis and duodenitis	121.31 (62.82 to 229.08)	176.91 (89.77 to 337.71)	154.45 (79.81 to 288.73)	179.28 (92.17 to 342.40)	4.06 (2.05 to 7.46)	6.61 (3.27 to 12.46)	6.24 (3.16 to 11.76)	6.39 (3.16 to 12.19)
Appendicitis	136.08 (77.17 to 300.70)	148.87 (82.05 to 330.30)	112.44 (61.89 to 247.19)	160.87 (88.75 to 353.30)	3.31 (1.85 to 7.11)	4.32 (2.34 to 9.28)	3.57 (1.94 to 7.46)	4.40 (2.41 to 9.54)
Paralytic ileus and intestinal obstruction	245.08 (112.75 to 543.80)	275.25 (128.55 to 629.09)	241.88 (113.80 to 557.76)	298.83 (137.08 to 675.33)	5.51 (2.67 to 12.94)	7.94 (3.75 to 18.82)	8.11 (3.89 to 19.22)	7.74 (3.63 to 18.19)
Inguinal, femoral, and abdominal hernia	23.79 (12.04 to 57.46)	26.35 (12.94 to 62.58)	24.82 (12.20 to 56.89)	25.81 (12.80 to 62.02)	1.00 (0.48 to 2.21)	1.25 (0.57 to 2.68)	1.31 (0.62 to 2.66)	1.14 (0.53 to 2.57)
Inflammatory bowel disease	34.90 (15.64 to 108.56)	49.12 (22.25 to 142.25)	48.99 (22.69 to 137.47)	48.81 (21.80 to 145.03)	0.99 (0.47 to 2.73)	1.60 (0.76 to 4.08)	1.75 (0.86 to 4.31)	1.49 (0.70 to 3.89)
Vascular intestinal disorders	14.71 (8.07 to 25.56)	23.85 (12.83 to 41.09)	23.58 (12.57 to 40.25)	22.85 (12.38 to 39.32)	0.64 (0.33 to 1.13)	1.21 (0.61 to 2.16)	1.31 (0.66 to 2.32)	1.10 (0.55 to 1.97)
Gallbladder and biliary diseases	117.07 (62.11 to 249.53)	168.87 (62.51 to 401.82)	118.70 (45.91 to 280.57)	220.84 (82.14 to 530.83)	4.14 (2.10 to 8.32)	6.93 (2.56 to 16.06)	5.35 (1.99 to 12.18)	8.51 (3.10 to 19.85)
Pancreatitis	102.80 (55.01 to 234.18)	147.44 (71.25 to 353.08)	118.81 (59.03 to 280.58)	160.69 (75.46 to 392.10)	2.64 (1.36 to 6.19)	4.21 (1.94 to 10.26)	3.63 (1.74 to 8.65)	4.38 (2.00 to 10.85)
Other digestive diseases	138.99 (75.36 to 283.38)	174.25 (90.63 to 346.72)	142.31 (74.43 to 278.27)	181.35 (96.49 to 364.40)	4.12 (2.14 to 7.80)	6.13 (3.05 to 11.49)	5.49 (2.65 to 10.13)	6.00 (3.04 to 11.37)
**Neurological disorders**	**541.35 (450.86 to 642.18)**	**849.80 (652.21 to 1,129.36)**	**850.22 (655.64 to 1,131.94)**	**888.38 (669.92 to 1,214.13)**	**24.61 (20.47 to 29.66)**	**46.51 (31.72 to 68.60)**	**55.72 (38.46 to 79.36)**	**44.35 (29.34 to 66.20)**
Alzheimer's disease and other dementias	227.96 (182.29 to 285.54)	454.33 (280.98 to 724.91)	521.17 (335.07 to 796.55)	449.50 (253.28 to 751.46)	18.16 (14.61 to 22.83)	37.01 (22.40 to 58.81)	46.15 (29.27 to 70.00)	34.63 (20.09 to 56.51)
Parkinson's disease	14.85 (11.01 to 19.62)	31.77 (22.61 to 41.69)	46.48 (34.41 to 60.55)	26.41 (18.82 to 35.33)	1.03 (0.76 to 1.38)	2.28 (1.58 to 3.08)	3.56 (2.59 to 4.76)	1.83 (1.24 to 2.56)
Epilepsy	243.04 (189.90 to 307.98)	290.00 (215.93 to 381.50)	204.42 (154.56 to 265.63)	337.62 (242.83 to 439.96)	4.19 (3.37 to 5.11)	5.32 (4.00 to 6.84)	3.85 (2.92 to 4.89)	6.08 (4.47 to 7.98)
Multiple sclerosis	3.24 (1.79 to 5.97)	4.88 (2.64 to 8.90)	4.97 (2.73 to 8.92)	4.84 (2.60 to 8.88)	0.09 (0.05 to 0.16)	0.14 (0.08 to 0.24)	0.14 (0.08 to 0.25)	0.13 (0.07 to 0.24)
Motor neuron disease	8.81 (6.81 to 11.60)	12.72 (9.96 to 16.16)	14.41 (11.35 to 18.32)	12.24 (9.59 to 15.59)	0.24 (0.19 to 0.30)	0.40 (0.32 to 0.51)	0.48 (0.38 to 0.61)	0.38 (0.30 to 0.47)
Other neurological disorders	43.45 (34.08 to 56.86)	56.10 (45.03 to 70.50)	58.76 (47.91 to 72.16)	57.76 (45.74 to 73.48)	0.90 (0.73 to 1.11)	1.35 (1.12 to 1.63)	1.54 (1.27 to 1.83)	1.31 (1.07 to 1.58)
**Mental disorders**	**130.38 (66.23 to 185.11)**	**176.44 (93.95 to 243.20)**	**174.29 (92.46 to 239.11)**	**177.44 (94.12 to 245.35)**	**2.71 (1.43 to 3.75)**	**3.95 (2.23 to 5.29)**	**4.09 (2.32 to 5.43)**	**3.86 (2.16 to 5.20)**
Alcohol use disorders	22.90 (15.20 to 48.98)	25.72 (16.05 to 57.95)	24.37 (14.91 to 55.71)	26.28 (16.33 to 58.86)	0.55 (0.37 to 1.13)	0.66 (0.43 to 1.41)	0.65 (0.42 to 1.41)	0.65 (0.42 to 1.42)
Drug use disorders	107.17 (38.71 to 161.17)	150.25 (60.50 to 219.95)	149.45 (60.81 to 217.93)	150.67 (60.58 to 222.50)	2.15 (0.79 to 3.18)	3.28 (1.39 to 4.70)	3.43 (1.48 to 4.83)	3.20 (1.35 to 4.57)
Opioid use disorders	99.33 (31.15 to 152.98)	130.88 (42.79 to 199.42)	129.45 (42.78 to 195.33)	131.52 (42.74 to 200.99)	1.96 (0.62 to 2.95)	2.77 (0.95 to 4.11)	2.86 (0.98 to 4.19)	2.72 (0.92 to 4.04)

Cocaine use disorders	1.31 (0.53 to 3.92)	3.40 (1.45 to 9.67)	3.20 (1.37 to 9.32)	3.47 (1.48 to 9.85)	0.03 (0.01 to 0.08)	0.07 (0.03 to 0.20)	0.07 (0.03 to 0.20)	0.07 (0.03 to 0.20)
Amphetamine use disorders	0.67 (0.28 to 2.00)	2.55 (1.19 to 6.31)	2.57 (1.18 to 6.34)	2.55 (1.16 to 6.32)	0.02 (0.01 to 0.04)	0.06 (0.03 to 0.13)	0.07 (0.03 to 0.13)	0.06 (0.02 to 0.13)
Other drug use disorders	5.87 (3.60 to 9.64)	13.42 (8.22 to 21.63)	14.23 (8.55 to 22.96)	13.12 (8.03 to 21.21)	0.15 (0.10 to 0.25)	0.38 (0.25 to 0.60)	0.43 (0.29 to 0.67)	0.35 (0.23 to 0.56)
Eating disorders	0.31 (0.15 to 0.57)	0.48 (0.24 to 0.90)	0.47 (0.24 to 0.93)	0.48 (0.24 to 0.90)	0.01 (0.00 to 0.01)	0.01 (0.00 to 0.02)	0.01 (0.00 to 0.02)	0.01 (0.00 to 0.02)

Kiribati								
Cause	YLLs (actual counts)				Deaths (actual counts)			
	2016	2040 Forecast	2040 Better Scenario	2040 Worse Scenario	2016	2040 Forecast	2040 Better Scenario	2040 Worse Scenario
**Diabetes, urogenital, blood, and endocrine diseases**	**4,759.64 (4,147.46 to 5,502.01)**	**9,175.16 (6,959.42 to 12,253.44)**	**6,310.90 (4,823.08 to 8,428.47)**	**10,295.80 (7,779.38 to 13,606.48)**	**171.55 (149.90 to 196.31)**	**371.47 (289.77 to 478.25)**	**273.52 (217.81 to 355.33)**	**399.88 (314.20 to 506.69)**
Diabetes mellitus	3,350.62 (2,855.95 to 3,921.32)	6,247.28 (4,074.19 to 9,011.79)	4,102.51 (2,732.80 to 5,981.42)	6,830.38 (4,510.49 to 9,900.37)	128.60 (110.05 to 149.58)	262.09 (176.09 to 359.21)	184.68 (129.13 to 257.93)	275.62 (186.59 to 384.42)
Acute glomerulonephritis	1.45 (1.17 to 1.81)	2.08 (1.57 to 2.83)	1.76 (1.31 to 2.40)	2.23 (1.70 to 3.00)	0.05 (0.04 to 0.06)	0.06 (0.05 to 0.09)	0.07 (0.05 to 0.10)	0.06 (0.05 to 0.09)
Chronic kidney disease	821.93 (693.15 to 964.38)	2,073.71 (768.21 to 5,061.37)	1,441.19 (547.27 to 3,618.68)	2,576.62 (956.41 to 5,963.92)	27.69 (23.53 to 32.51)	83.17 (29.46 to 206.66)	62.94 (22.55 to 169.71)	98.73 (36.37 to 226.83)
Chronic kidney disease due to diabetes mellitus	455.37 (380.09 to 544.20)	1,256.41 (427.90 to 3,186.28)	874.05 (309.00 to 2,327.76)	1,567.06 (532.17 to 3,805.13)	15.62 (13.14 to 18.61)	48.54 (16.55 to 123.60)	36.32 (12.78 to 97.68)	58.25 (20.16 to 138.09)
Chronic kidney disease due to hypertension	164.37 (128.76 to 204.03)	468.24 (163.78 to 1,198.50)	339.08 (120.31 to 898.23)	570.67 (203.59 to 1,345.50)	7.25 (5.76 to 8.86)	23.65 (7.97 to 62.39)	18.80 (6.31 to 52.32)	27.19 (9.43 to 66.39)
Chronic kidney disease due to glomerulonephritis	98.27 (75.67 to 124.44)	160.98 (79.12 to 346.24)	104.11 (51.04 to 235.83)	202.24 (99.88 to 411.80)	2.18 (1.70 to 2.68)	4.63 (1.90 to 10.91)	3.22 (1.35 to 8.20)	5.66 (2.41 to 12.45)
Chronic kidney disease due to other and unspecified causes	103.93 (80.40 to 131.18)	188.09 (83.95 to 429.79)	123.95 (54.21 to 295.51)	236.65 (104.74 to 515.95)	2.63 (2.07 to 3.24)	6.34 (2.46 to 15.76)	4.60 (1.71 to 12.26)	7.63 (2.96 to 17.50)
Urinary diseases and male infertility	152.43 (124.12 to 190.52)	237.01 (201.82 to 280.27)	230.72 (196.89 to 268.33)	242.41 (201.50 to 295.81)	4.22 (3.61 to 4.91)	8.05 (6.85 to 9.37)	8.89 (7.63 to 10.33)	7.52 (6.34 to 8.82)
Urinary tract infections	110.48 (85.96 to 143.95)	168.26 (135.61 to 210.35)	162.91 (132.76 to 197.74)	174.49 (137.72 to 227.54)	2.85 (2.34 to 3.40)	5.57 (4.55 to 6.71)	6.26 (5.13 to 7.52)	5.20 (4.20 to 6.26)
Urolithiasis	22.85 (15.33 to 31.40)	46.79 (31.18 to 62.84)	50.15 (33.47 to 67.06)	44.84 (30.22 to 60.31)	0.81 (0.54 to 1.10)	1.73 (1.15 to 2.30)	1.96 (1.33 to 2.62)	1.59 (1.08 to 2.13)
Other urinary diseases	19.10 (13.89 to 27.01)	21.96 (14.82 to 33.24)	17.66 (11.75 to 28.11)	23.09 (15.78 to 35.23)	0.56 (0.42 to 0.82)	0.75 (0.50 to 1.26)	0.67 (0.44 to 1.16)	0.74 (0.49 to 1.22)
Gynecological diseases	2.14 (1.16 to 3.07)	2.48 (1.41 to 3.69)	1.84 (1.00 to 2.75)	2.64 (1.46 to 3.91)	0.07 (0.03 to 0.09)	0.08 (0.04 to 0.11)	0.06 (0.03 to 0.09)	0.08 (0.04 to 0.12)
Uterine fibroids	0.39 (0.13 to 0.69)	0.41 (0.12 to 0.81)	0.30 (0.09 to 0.59)	0.43 (0.13 to 0.82)	0.02 (0.00 to 0.03)	0.02 (0.00 to 0.04)	0.01 (0.00 to 0.03)	0.02 (0.00 to 0.04)
Polycystic ovarian syndrome	0.28 (0.09 to 0.57)	0.21 (0.06 to 0.50)	0.12 (0.03 to 0.29)	0.24 (0.07 to 0.57)	0.01 (0.00 to 0.01)	0.01 (0.00 to 0.01)	0.00 (0.00 to 0.01)	0.01 (0.00 to 0.01)
Endometriosis	0.04 (0.01 to 0.08)	0.05 (0.01 to 0.12)	0.04 (0.01 to 0.08)	0.06 (0.01 to 0.13)	0.00 (0.00 to 0.00)	0.00 (0.00 to 0.00)	0.00 (0.00 to 0.00)	0.00 (0.00 to 0.00)
Genital prolapse	0.19 (0.03 to 0.35)	0.19 (0.03 to 0.36)	0.13 (0.02 to 0.26)	0.21 (0.03 to 0.39)	0.01 (0.00 to 0.01)	0.01 (0.00 to 0.02)	0.01 (0.00 to 0.01)	0.01 (0.00 to 0.02)
Other gynecological diseases	1.24 (0.70 to 1.86)	1.62 (0.89 to 2.52)	1.25 (0.69 to 1.96)	1.71 (0.95 to 2.64)	0.03 (0.02 to 0.05)	0.05 (0.02 to 0.08)	0.04 (0.02 to 0.06)	0.05 (0.02 to 0.08)
Hemoglobinopathies and hemolytic anemias	79.85 (58.29 to 102.14)	87.42 (62.90 to 111.06)	69.09 (48.99 to 89.00)	93.42 (66.78 to 118.94)	1.88 (1.39 to 2.35)	2.49 (1.77 to 3.19)	2.05 (1.45 to 2.63)	2.55 (1.81 to 3.29)
Thalassemias	18.69 (11.95 to 28.15)	10.56 (6.40 to 16.57)	11.12 (7.03 to 17.15)	11.66 (6.98 to 18.34)	0.24 (0.15 to 0.35)	0.14 (0.08 to 0.21)	0.15 (0.09 to 0.22)	0.15 (0.09 to 0.23)

Sickle cell disorders	0.06 (0.04 to 0.09)	0.07 (0.04 to 0.11)	0.05 (0.03 to 0.08)	0.07 (0.04 to 0.12)	0.00 (0.00 to 0.00)	0.00 (0.00 to 0.00)	0.00 (0.00 to 0.00)	0.00 (0.00 to 0.00)
G6PD deficiency	42.45 (28.96 to 55.65)	58.09 (38.82 to 76.98)	42.43 (28.02 to 56.89)	62.89 (42.20 to 83.79)	0.93 (0.63 to 1.21)	1.45 (0.99 to 1.88)	1.10 (0.73 to 1.44)	1.54 (1.05 to 2.02)
Other hemoglobinopathies and hemolytic anemias	18.65 (13.74 to 24.54)	18.71 (13.22 to 25.67)	15.49 (10.87 to 20.92)	18.80 (13.15 to 25.62)	0.72 (0.53 to 0.95)	0.90 (0.62 to 1.29)	0.81 (0.56 to 1.12)	0.86 (0.58 to 1.24)
Endocrine, metabolic, blood, and immune disorders	351.21 (233.21 to 683.73)	525.18 (350.58 to 1,018.93)	463.78 (313.34 to 869.36)	548.09 (363.60 to 1,073.40)	9.05 (6.10 to 17.12)	15.53 (10.48 to 29.63)	14.83 (10.14 to 28.04)	15.32 (10.22 to 29.34)

Kiribati								
Cause	YLLs (actual counts)				Deaths (actual counts)			
	2016	2040 Forecast	2040 Better Scenario	2040 Worse Scenario	2016	2040 Forecast	2040 Better Scenario	2040 Worse Scenario
**Musculoskeletal disorders**	**36.84 (17.21 to 48.93)**	**53.24 (25.17 to 71.08)**	**57.33 (26.60 to 76.05)**	**52.94 (24.97 to 70.77)**	**0.76 (0.35 to 0.99)**	**1.26 (0.59 to 1.61)**	**1.46 (0.66 to 1.84)**	**1.20 (0.56 to 1.55)**
Rheumatoid arthritis	2.62 (1.31 to 5.84)	4.11 (2.03 to 8.57)	3.36 (1.68 to 7.36)	4.32 (2.11 to 8.94)	0.07 (0.04 to 0.14)	0.15 (0.07 to 0.27)	0.13 (0.06 to 0.26)	0.15 (0.07 to 0.27)
Other musculoskeletal disorders	34.22 (15.41 to 45.68)	49.12 (22.50 to 65.98)	53.97 (24.92 to 71.73)	48.62 (22.17 to 65.72)	0.69 (0.31 to 0.90)	1.12 (0.51 to 1.44)	1.32 (0.59 to 1.68)	1.06 (0.47 to 1.37)
**Other non-communicable diseases**	**1,923.16 (772.81 to 3,355.55)**	**2,199.34 (1,096.68 to 3,675.88)**	**1,696.21 (899.85 to 2,740.80)**	**2,667.39 (1,254.36 to 4,632.93)**	**27.28 (13.14 to 44.57)**	**39.19 (23.75 to 58.10)**	**34.89 (22.89 to 50.82)**	**43.45 (24.65 to 67.90)**
Congenital birth defects	1,701.20 (589.29 to 3,079.59)	1,711.33 (652.86 to 3,140.08)	1,226.95 (480.22 to 2,191.65)	2,170.76 (821.06 to 4,004.47)	20.82 (7.44 to 37.16)	21.83 (8.65 to 38.36)	15.94 (6.57 to 27.26)	27.26 (10.64 to 48.99)
Neural tube defects	29.60 (9.83 to 62.32)	12.48 (4.59 to 27.26)	7.07 (2.80 to 14.91)	16.75 (5.84 to 38.05)	0.36 (0.13 to 0.75)	0.16 (0.07 to 0.34)	0.10 (0.04 to 0.19)	0.21 (0.08 to 0.46)
Congenital heart anomalies	597.14 (210.41 to 1,098.98)	583.90 (231.16 to 1,048.59)	410.90 (167.75 to 714.10)	736.87 (275.53 to 1,327.65)	7.40 (2.67 to 13.33)	7.60 (3.12 to 13.14)	5.46 (2.26 to 9.14)	9.43 (3.73 to 16.31)
Orofacial clefts	25.04 (0.62 to 88.99)	15.97 (0.44 to 59.23)	7.58 (0.22 to 30.41)	23.12 (0.61 to 90.18)	0.29 (0.01 to 1.03)	0.19 (0.01 to 0.69)	0.09 (0.00 to 0.35)	0.27 (0.01 to 1.05)
Down syndrome	62.91 (20.19 to 213.26)	83.03 (30.51 to 265.24)	56.81 (21.83 to 179.60)	105.65 (36.87 to 344.87)	0.80 (0.28 to 2.60)	1.13 (0.47 to 3.40)	0.79 (0.35 to 2.37)	1.40 (0.55 to 4.32)
Other chromosomal abnormalities	24.06 (7.43 to 56.29)	41.22 (12.91 to 98.01)	27.77 (9.09 to 63.40)	53.57 (16.12 to 128.68)	0.31 (0.10 to 0.68)	0.55 (0.19 to 1.22)	0.39 (0.14 to 0.83)	0.70 (0.23 to 1.58)
Congenital musculoskeletal and limb anomalies	30.78 (10.75 to 60.17)	31.18 (11.53 to 63.60)	22.68 (8.59 to 45.12)	39.97 (14.51 to 81.46)	0.37 (0.13 to 0.71)	0.38 (0.14 to 0.75)	0.28 (0.11 to 0.53)	0.48 (0.18 to 0.96)
Urogenital congenital anomalies	39.95 (14.85 to 78.41)	36.63 (14.85 to 71.10)	26.77 (10.98 to 50.27)	46.04 (18.18 to 91.86)	0.51 (0.20 to 0.96)	0.51 (0.22 to 0.91)	0.39 (0.17 to 0.66)	0.62 (0.26 to 1.14)
Digestive congenital anomalies	107.72 (33.25 to 220.03)	101.33 (34.91 to 210.34)	65.67 (23.40 to 133.08)	133.51 (44.93 to 281.98)	1.29 (0.41 to 2.61)	1.24 (0.44 to 2.52)	0.82 (0.30 to 1.60)	1.62 (0.55 to 3.36)
Other congenital birth defects	783.99 (262.89 to 1,451.61)	805.58 (292.91 to 1,532.45)	601.69 (223.01 to 1,108.75)	1,015.28 (365.33 to 1,947.01)	9.49 (3.29 to 17.28)	10.07 (3.78 to 18.54)	7.64 (2.94 to 13.74)	12.54 (4.61 to 23.62)
Skin and subcutaneous diseases	177.88 (108.96 to 263.60)	453.48 (286.28 to 670.33)	442.35 (282.50 to 661.55)	452.37 (284.96 to 669.30)	5.96 (3.72 to 9.34)	16.96 (11.14 to 27.13)	18.63 (12.48 to 29.62)	15.68 (10.16 to 25.01)
Cellulitis	7.84 (2.13 to 13.18)	26.06 (6.89 to 44.61)	29.85 (7.99 to 49.01)	24.65 (6.42 to 42.34)	0.28 (0.06 to 0.49)	1.08 (0.25 to 1.88)	1.38 (0.33 to 2.36)	0.95 (0.22 to 1.68)
Pyoderma	137.15 (77.24 to 204.43)	359.92 (216.34 to 527.37)	337.29 (202.30 to 505.31)	364.85 (216.46 to 533.97)	4.08 (2.33 to 6.28)	12.27 (7.61 to 19.04)	12.75 (7.95 to 20.05)	11.60 (7.25 to 17.94)
Decubitus ulcer	31.75 (19.55 to 61.35)	65.23 (40.13 to 134.57)	72.85 (45.82 to 147.97)	60.56 (37.38 to 128.85)	1.56 (0.98 to 3.02)	3.56 (2.02 to 7.43)	4.43 (2.59 to 8.94)	3.08 (1.72 to 6.54)
Other skin and subcutaneous diseases	1.15 (0.71 to 2.15)	2.27 (1.41 to 4.13)	2.35 (1.49 to 4.14)	2.32 (1.45 to 4.25)	0.03 (0.02 to 0.05)	0.06 (0.04 to 0.10)	0.07 (0.04 to 0.12)	0.05 (0.03 to 0.10)
Sudden infant death syndrome	44.08 (17.11 to 97.89)	34.53 (10.86 to 85.59)	26.91 (8.59 to 68.04)	44.26 (13.99 to 109.43)	0.51 (0.20 to 1.14)	0.40 (0.13 to 0.99)	0.31 (0.10 to 0.79)	0.51 (0.16 to 1.27)

**Injuries**	**3,964.26 (3,246.57 to 4,841.99)**	**4,700.47 (3,732.83 to 5,832.08)**	**3,467.82 (2,766.57 to 4,262.13)**	**5,343.36 (4,183.83 to 6,671.95)**	**72.71 (59.60 to 87.88)**	**92.16 (74.18 to 112.51)**	**71.71 (57.78 to 87.22)**	**101.40 (80.64 to 125.73)**
**Transport injuries**	**860.47 (694.30 to 1,073.76)**	**1,075.01 (822.07 to 1,423.36)**	**387.07 (292.72 to 508.75)**	**1,377.25 (1,050.60 to 1,813.06)**	**15.61 (12.83 to 18.97)**	**20.57 (16.13 to 26.42)**	**8.35 (6.51 to 10.61)**	**25.31 (19.77 to 32.87)**
Road injuries	804.87 (645.70 to 1,006.70)	987.82 (763.35 to 1,299.02)	319.17 (239.96 to 420.96)	1,279.24 (981.08 to 1,691.24)	14.65 (12.08 to 17.77)	18.95 (14.97 to 24.35)	7.05 (5.49 to 8.97)	23.54 (18.38 to 30.59)
Pedestrian road injuries	57.96 (39.68 to 79.84)	65.66 (43.87 to 90.11)	23.44 (16.06 to 32.53)	81.90 (53.87 to 114.83)	1.05 (0.75 to 1.39)	1.28 (0.91 to 1.72)	0.49 (0.37 to 0.64)	1.55 (1.08 to 2.11)

Kiribati								
Cause	YLLs (actual counts)				Deaths (actual counts)			
	2016	2040 Forecast	2040 Better Scenario	2040 Worse Scenario	2016	2040 Forecast	2040 Better Scenario	2040 Worse Scenario
Cyclist road injuries	15.13 (10.60 to 21.48)	17.85 (12.00 to 25.03)	4.92 (3.44 to 6.87)	23.86 (15.80 to 34.06)	0.27 (0.20 to 0.37)	0.33 (0.24 to 0.46)	0.11 (0.08 to 0.16)	0.43 (0.30 to 0.59)
Motorcyclist road injuries	320.00 (235.37 to 413.26)	423.97 (309.70 to 565.61)	181.64 (131.79 to 243.83)	523.33 (379.29 to 707.46)	5.90 (4.50 to 7.43)	8.19 (6.14 to 10.80)	4.00 (3.00 to 5.24)	9.70 (7.23 to 12.96)
Motor vehicle road injuries	398.29 (293.88 to 528.67)	463.65 (325.60 to 627.23)	105.40 (70.78 to 146.65)	627.32 (444.56 to 855.43)	7.21 (5.36 to 9.27)	8.86 (6.30 to 11.49)	2.37 (1.67 to 3.07)	11.47 (8.18 to 15.08)
Other road injuries	13.50 (7.90 to 20.49)	16.69 (9.04 to 26.06)	3.77 (2.01 to 6.02)	22.83 (12.36 to 35.48)	0.22 (0.14 to 0.34)	0.29 (0.16 to 0.44)	0.07 (0.04 to 0.11)	0.38 (0.21 to 0.59)
Other transport injuries	55.59 (34.45 to 78.11)	87.19 (54.45 to 125.28)	67.90 (43.02 to 96.14)	98.00 (60.46 to 143.32)	0.97 (0.64 to 1.31)	1.62 (1.06 to 2.25)	1.30 (0.88 to 1.79)	1.77 (1.15 to 2.53)
**Unintentional injuries**	**1,431.51 (992.35 to 1,909.07)**	**1,273.25 (853.46 to 1,649.63)**	**995.77 (666.09 to 1,268.67)**	**1,418.12 (947.53 to 1,870.28)**	**26.56 (18.43 to 34.19)**	**27.51 (18.67 to 34.05)**	**23.37 (15.57 to 28.82)**	**28.99 (19.44 to 36.17)**
Falls	101.55 (48.34 to 145.87)	105.21 (50.90 to 142.31)	95.86 (46.01 to 127.17)	109.51 (52.16 to 151.90)	2.42 (1.19 to 3.29)	3.22 (1.55 to 4.24)	3.27 (1.57 to 4.25)	3.08 (1.47 to 4.10)
Drowning	304.72 (213.89 to 427.16)	227.55 (151.86 to 311.35)	175.20 (114.16 to 236.43)	257.94 (171.07 to 364.69)	4.97 (3.53 to 6.57)	4.11 (2.76 to 5.38)	3.28 (2.18 to 4.28)	4.53 (3.04 to 6.11)
Fire, heat, and hot substances	82.77 (36.60 to 125.42)	60.60 (26.51 to 90.76)	44.16 (18.83 to 66.39)	69.43 (30.06 to 106.71)	1.44 (0.69 to 2.11)	1.21 (0.55 to 1.75)	0.95 (0.42 to 1.36)	1.32 (0.59 to 1.93)
Poisonings	169.86 (50.79 to 304.37)	99.44 (28.98 to 174.16)	64.52 (19.30 to 109.39)	118.84 (35.01 to 215.77)	2.96 (0.94 to 4.87)	1.97 (0.64 to 3.19)	1.40 (0.46 to 2.22)	2.22 (0.70 to 3.74)
Exposure to mechanical forces	128.60 (76.98 to 169.53)	120.11 (67.43 to 160.87)	90.30 (48.81 to 120.41)	134.83 (76.11 to 184.65)	2.29 (1.36 to 2.91)	2.38 (1.35 to 3.09)	1.91 (1.04 to 2.48)	2.56 (1.45 to 3.36)
Unintentional firearm injuries	15.45 (10.31 to 24.52)	11.83 (7.98 to 17.99)	8.39 (5.70 to 12.81)	13.66 (9.05 to 20.58)	0.27 (0.18 to 0.43)	0.24 (0.16 to 0.36)	0.18 (0.13 to 0.28)	0.26 (0.18 to 0.39)
Unintentional suffocation	17.19 (10.35 to 28.60)	17.68 (10.11 to 29.57)	12.30 (7.01 to 20.63)	20.87 (11.87 to 34.72)	0.25 (0.15 to 0.40)	0.27 (0.16 to 0.45)	0.19 (0.11 to 0.34)	0.31 (0.18 to 0.51)
Other exposure to mechanical forces	95.96 (39.41 to 131.77)	90.60 (33.18 to 127.46)	69.61 (25.15 to 96.97)	100.31 (36.75 to 142.69)	1.77 (0.73 to 2.36)	1.87 (0.72 to 2.55)	1.53 (0.58 to 2.07)	1.99 (0.76 to 2.73)
Adverse effects of medical treatment	485.03 (317.84 to 666.88)	502.13 (340.16 to 678.41)	404.63 (272.20 to 539.74)	550.56 (365.87 to 740.99)	9.46 (6.65 to 12.51)	11.24 (8.06 to 14.59)	9.79 (6.95 to 12.51)	11.68 (8.30 to 15.21)
Animal contact	9.37 (5.35 to 13.36)	12.30 (7.20 to 17.67)	8.71 (5.20 to 12.36)	14.02 (8.06 to 20.56)	0.19 (0.12 to 0.25)	0.28 (0.18 to 0.37)	0.22 (0.14 to 0.29)	0.30 (0.19 to 0.41)
Venomous animal contact	4.80 (2.74 to 7.19)	5.74 (3.13 to 8.80)	3.89 (2.13 to 5.95)	6.62 (3.59 to 10.25)	0.09 (0.06 to 0.14)	0.12 (0.07 to 0.19)	0.09 (0.06 to 0.14)	0.14 (0.08 to 0.21)
Non-venomous animal contact	4.56 (2.29 to 6.99)	6.56 (3.49 to 9.95)	4.81 (2.56 to 7.22)	7.40 (3.86 to 11.54)	0.09 (0.05 to 0.13)	0.15 (0.09 to 0.22)	0.12 (0.07 to 0.17)	0.16 (0.09 to 0.23)
Foreign body	42.06 (23.23 to 92.56)	42.62 (24.16 to 84.69)	37.39 (22.13 to 69.34)	48.32 (26.38 to 100.86)	0.70 (0.41 to 1.43)	0.84 (0.52 to 1.55)	0.82 (0.52 to 1.44)	0.88 (0.53 to 1.69)
Pulmonary aspiration and foreign body in airway	34.66 (19.69 to 72.59)	34.92 (20.60 to 66.41)	31.40 (19.40 to 55.00)	39.53 (22.39 to 78.90)	0.58 (0.36 to 1.10)	0.70 (0.45 to 1.21)	0.71 (0.47 to 1.20)	0.73 (0.45 to 1.30)

Foreign body in other body part	7.40 (3.01 to 22.49)	7.71 (3.16 to 23.22)	5.99 (2.48 to 18.35)	8.79 (3.57 to 26.17)	0.12 (0.05 to 0.37)	0.14 (0.06 to 0.42)	0.11 (0.05 to 0.35)	0.15 (0.06 to 0.45)
Environmental heat and cold exposure	48.89 (18.65 to 71.10)	55.33 (20.87 to 81.58)	36.65 (13.62 to 53.62)	62.86 (23.42 to 92.32)	1.03 (0.38 to 1.44)	1.28 (0.47 to 1.88)	0.91 (0.33 to 1.31)	1.40 (0.52 to 2.02)
Other unintentional injuries	58.66 (24.78 to 74.83)	47.95 (19.31 to 63.12)	38.37 (15.02 to 51.43)	51.79 (20.67 to 68.47)	1.11 (0.49 to 1.41)	0.98 (0.40 to 1.27)	0.81 (0.33 to 1.07)	1.03 (0.43 to 1.35)
**Self-harm and interpersonal violence**	**1,671.26 (1,304.37 to 2,102.04)**	**2,342.24 (1,736.29 to 3,145.74)**	**2,075.31 (1,593.12 to 2,671.86)**	**2,537.59 (1,829.56 to 3,471.54)**	**30.52 (24.14 to 38.34)**	**43.90 (32.72 to 58.23)**	**39.80 (30.73 to 50.91)**	**46.91 (34.00 to 64.38)**

Kiribati								
Cause	YLLs (actual counts)				Deaths (actual counts)			
	2016	2040 Forecast	2040 Better Scenario	2040 Worse Scenario	2016	2040 Forecast	2040 Better Scenario	2040 Worse Scenario
Self-harm	1,458.92 (1,113.94 to 1,858.08)	2,121.31 (1,493.93 to 2,908.60)	1,862.15 (1,368.49 to 2,455.75)	2,308.21 (1,594.58 to 3,243.98)	26.71 (20.49 to 34.09)	39.75 (28.53 to 54.00)	35.71 (26.51 to 46.71)	42.67 (29.79 to 60.24)
Self-harm by firearm	12.08 (7.15 to 19.22)	13.86 (7.72 to 22.86)	11.70 (6.52 to 18.89)	15.17 (8.26 to 25.47)	0.23 (0.14 to 0.37)	0.27 (0.15 to 0.46)	0.24 (0.13 to 0.38)	0.29 (0.16 to 0.50)
Self-harm by other specified means	1,446.84 (1,107.15 to 1,844.73)	2,107.45 (1,484.47 to 2,897.33)	1,850.45 (1,362.71 to 2,439.53)	2,293.03 (1,584.42 to 3,218.86)	26.48 (20.33 to 33.79)	39.48 (28.33 to 53.55)	35.47 (26.34 to 46.32)	42.37 (29.59 to 59.74)
Interpersonal violence	212.34 (153.58 to 320.53)	220.93 (154.78 to 341.73)	213.16 (151.05 to 331.32)	229.38 (160.24 to 355.57)	3.81 (2.77 to 5.77)	4.15 (2.93 to 6.43)	4.09 (2.93 to 6.33)	4.24 (2.98 to 6.59)
Physical violence by firearm	42.43 (18.42 to 65.27)	42.83 (18.16 to 68.43)	41.08 (17.27 to 66.16)	44.21 (19.22 to 70.64)	0.75 (0.33 to 1.17)	0.79 (0.34 to 1.27)	0.77 (0.33 to 1.24)	0.80 (0.35 to 1.28)
Physical violence by sharp object	99.43 (59.88 to 208.93)	109.91 (64.32 to 233.00)	111.93 (66.69 to 231.43)	111.00 (65.50 to 238.23)	1.80 (1.07 to 3.80)	2.07 (1.21 to 4.34)	2.13 (1.28 to 4.46)	2.06 (1.21 to 4.40)
Physical violence by other means	70.48 (47.26 to 97.60)	68.19 (43.17 to 98.85)	60.16 (37.61 to 86.87)	74.17 (47.51 to 107.92)	1.25 (0.84 to 1.71)	1.30 (0.83 to 1.87)	1.19 (0.75 to 1.70)	1.38 (0.88 to 2.00)
**Forces of nature, conflict and terrorism, and executions and police conflict**	**1.02 (0.81 to 1.30)**	**9.97 (0.00 to 259.47)**	**9.66 (0.00 to 253.95)**	**10.41 (0.00 to 266.82)**	**0.02 (0.01 to 0.02)**	**0.18 (0.00 to 4.41)**	**0.18 (0.00 to 4.35)**	**0.18 (0.00 to 4.51)**
Exposure to forces of nature	0.00 (0.00 to 0.00)	5.31 (0.00 to 0.00)	5.10 (0.00 to 0.00)	5.62 (0.00 to 0.00)	0.00 (0.00 to 0.00)	0.10 (0.00 to 0.00)	0.10 (0.00 to 0.00)	0.10 (0.00 to 0.00)
Conflict and terrorism	0.00 (0.00 to 0.00)	4.66 (0.00 to 0.00)	4.56 (0.00 to 0.00)	4.79 (0.00 to 0.00)	0.00 (0.00 to 0.00)	0.08 (0.00 to 0.00)	0.08 (0.00 to 0.00)	0.08 (0.00 to 0.00)
Executions and police conflict	1.02 (0.81 to 1.30)	0.00 (0.00 to 0.00)	0.00 (0.00 to 0.00)	0.00 (0.00 to 0.00)	0.02 (0.01 to 0.02)	0.00 (0.00 to 0.00)	0.00 (0.00 to 0.00)	0.00 (0.00 to 0.00)

Marshall Islands								
Cause	YLLs (actual counts)				Deaths (actual counts)			
	2016	2040 Forecast	2040 Better Scenario	2040 Worse Scenario	2016	2040 Forecast	2040 Better Scenario	2040 Worse Scenario
**All causes**	**19,596.28 (16,391.82 to 23,274.90)**	**24,794.41 (19,745.57 to 30,325.05)**	**17,292.71 (13,766.30 to 21,376.53)**	**29,345.06 (23,222.05 to 36,220.52)**	**541.48 (468.10 to 617.93)**	**854.55 (732.31 to 992.62)**	**641.50 (548.77 to 748.05)**	**976.55 (829.55 to 1,135.19)**
**Communicable, maternal, neonatal, and nutritional diseases**	**4,127.56 (2,684.49 to 6,401.42)**	**2,721.96 (1,706.81 to 4,432.39)**	**1,566.78 (1,050.28 to 2,556.76)**	**3,555.17 (2,170.65 to 5,712.29)**	**65.23 (47.64 to 93.59)**	**57.05 (40.58 to 82.73)**	**39.16 (28.82 to 55.22)**	**70.95 (48.94 to 102.08)**
**HIV/AIDS and tuberculosis**	**261.06 (148.37 to 694.95)**	**307.45 (121.23 to 984.20)**	**199.44 (59.59 to 843.47)**	**399.99 (165.66 to 1,191.59)**	**6.46 (3.92 to 16.08)**	**7.80 (3.55 to 22.50)**	**5.08 (1.83 to 19.56)**	**9.72 (4.44 to 25.28)**
Tuberculosis	157.60 (117.72 to 195.20)	143.65 (83.31 to 218.99)	66.76 (41.13 to 99.17)	174.25 (101.19 to 274.19)	4.33 (3.18 to 5.31)	4.45 (2.67 to 6.77)	2.24 (1.40 to 3.33)	5.19 (3.10 to 8.24)
HIV/AIDS	103.46 (5.95 to 535.34)	163.80 (9.99 to 854.52)	132.68 (4.41 to 776.36)	225.74 (23.51 to 999.92)	2.13 (0.11 to 11.41)	3.35 (0.15 to 18.43)	2.84 (0.07 to 17.41)	4.52 (0.43 to 19.98)
**Diarrhea, lower respiratory, and other common infectious diseases**	**1,709.49 (1,157.13 to 2,652.97)**	**1,350.28 (825.18 to 2,326.76)**	**774.12 (523.43 to 1,173.91)**	**1,802.53 (1,037.49 to 3,289.38)**	**30.99 (22.93 to 43.50)**	**33.30 (22.69 to 49.90)**	**24.05 (17.35 to 33.08)**	**41.98 (27.36 to 67.38)**
Diarrheal diseases	86.25 (52.78 to 147.51)	42.15 (9.68 to 115.65)	25.32 (6.12 to 75.07)	73.70 (15.02 to 213.37)	2.11 (1.31 to 3.67)	1.69 (0.37 to 5.10)	1.23 (0.28 to 3.75)	2.77 (0.51 to 8.24)
Intestinal infectious diseases	68.53 (32.46 to 127.00)	45.44 (18.04 to 88.02)	33.28 (13.34 to 62.27)	50.33 (19.70 to 97.62)	0.97 (0.46 to 1.78)	0.68 (0.29 to 1.32)	0.51 (0.22 to 0.95)	0.74 (0.30 to 1.43)
Typhoid fever	58.95 (27.17 to 110.81)	37.78 (14.08 to 76.38)	27.82 (10.76 to 52.87)	41.80 (15.61 to 85.07)	0.83 (0.39 to 1.56)	0.56 (0.22 to 1.09)	0.43 (0.17 to 0.79)	0.61 (0.24 to 1.21)
Paratyphoid fever	9.50 (3.79 to 20.09)	7.63 (2.71 to 16.76)	5.43 (1.92 to 12.03)	8.49 (2.99 to 18.92)	0.14 (0.06 to 0.29)	0.12 (0.04 to 0.26)	0.09 (0.03 to 0.19)	0.13 (0.05 to 0.28)
Other intestinal infectious diseases	0.08 (0.03 to 0.18)	0.03 (0.01 to 0.08)	0.02 (0.01 to 0.05)	0.04 (0.01 to 0.10)	0.00 (0.00 to 0.00)	0.00 (0.00 to 0.00)	0.00 (0.00 to 0.00)	0.00 (0.00 to 0.00)
Lower respiratory infections	1,046.87 (722.40 to 1,572.32)	859.02 (502.88 to 1,470.49)	556.14 (366.79 to 856.28)	1,172.12 (631.11 to 2,176.96)	21.35 (15.69 to 28.98)	25.34 (17.03 to 38.72)	19.68 (14.04 to 27.75)	31.63 (20.13 to 52.70)
Upper respiratory infections	1.61 (0.78 to 3.10)	1.38 (0.49 to 2.92)	0.78 (0.28 to 1.70)	1.58 (0.58 to 3.40)	0.03 (0.02 to 0.05)	0.03 (0.01 to 0.07)	0.02 (0.01 to 0.04)	0.04 (0.01 to 0.08)
Otitis media	0.05 (0.02 to 0.13)	0.01 (0.01 to 0.04)	0.01 (0.01 to 0.04)	0.02 (0.01 to 0.04)	0.00 (0.00 to 0.00)	0.00 (0.00 to 0.00)	0.00 (0.00 to 0.00)	0.00 (0.00 to 0.00)
Meningitis	142.97 (86.96 to 229.60)	79.43 (37.53 to 152.38)	54.69 (30.17 to 95.63)	95.22 (42.92 to 195.01)	2.08 (1.32 to 3.33)	1.33 (0.71 to 2.46)	1.03 (0.61 to 1.82)	1.51 (0.79 to 2.89)
Pneumococcal meningitis	0.42 (0.23 to 0.74)	0.17 (0.09 to 0.31)	0.12 (0.07 to 0.23)	0.20 (0.10 to 0.39)	0.01 (0.00 to 0.01)	0.00 (0.00 to 0.01)	0.00 (0.00 to 0.00)	0.00 (0.00 to 0.01)
H influenzae type B meningitis	15.83 (8.89 to 26.91)	21.26 (4.31 to 70.87)	7.10 (3.00 to 17.30)	32.65 (5.51 to 114.73)	0.22 (0.13 to 0.37)	0.28 (0.08 to 0.89)	0.12 (0.06 to 0.25)	0.41 (0.09 to 1.38)
Meningococcal meningitis	27.68 (15.68 to 47.01)	12.32 (6.41 to 23.03)	10.05 (5.43 to 18.90)	13.20 (6.88 to 24.12)	0.42 (0.25 to 0.69)	0.24 (0.13 to 0.45)	0.21 (0.12 to 0.39)	0.25 (0.14 to 0.45)
Other meningitis	99.04 (60.15 to 160.85)	45.68 (23.48 to 79.65)	37.41 (20.67 to 64.97)	49.16 (25.51 to 86.87)	1.43 (0.89 to 2.30)	0.81 (0.45 to 1.44)	0.70 (0.41 to 1.26)	0.85 (0.47 to 1.49)
Encephalitis	17.24 (11.26 to 25.65)	16.18 (9.40 to 25.05)	12.39 (7.43 to 18.81)	17.41 (10.09 to 26.97)	0.29 (0.20 to 0.42)	0.32 (0.20 to 0.48)	0.27 (0.17 to 0.39)	0.34 (0.21 to 0.50)

Diphtheria	1.55 (0.85 to 2.83)	0.47 (0.20 to 1.09)	0.27 (0.15 to 0.46)	0.59 (0.23 to 1.43)	0.02 (0.01 to 0.04)	0.01 (0.00 to 0.02)	0.01 (0.00 to 0.01)	0.01 (0.00 to 0.02)
Whooping cough	237.24 (21.94 to 731.33)	140.43 (8.05 to 453.17)	32.84 (2.25 to 113.44)	160.80 (9.88 to 542.87)	2.81 (0.26 to 8.65)	1.67 (0.10 to 5.40)	0.40 (0.03 to 1.37)	1.91 (0.12 to 6.45)
Tetanus	0.90 (0.57 to 1.60)	0.14 (0.08 to 0.28)	0.09 (0.05 to 0.15)	0.16 (0.09 to 0.31)	0.02 (0.01 to 0.03)	0.00 (0.00 to 0.00)	0.00 (0.00 to 0.00)	0.00 (0.00 to 0.01)
Measles	96.63 (25.79 to 238.96)	160.88 (3.93 to 638.28)	55.80 (3.58 to 173.81)	224.96 (4.77 to 895.72)	1.16 (0.31 to 2.87)	2.13 (0.07 to 7.66)	0.84 (0.06 to 2.54)	2.93 (0.07 to 10.70)

Marshall Islands								
Cause	YLLs (actual counts)				Deaths (actual counts)			
	2016	2040 Forecast	2040 Better Scenario	2040 Worse Scenario	2016	2040 Forecast	2040 Better Scenario	2040 Worse Scenario
Varicella and herpes zoster	9.66 (4.60 to 18.45)	4.75 (1.93 to 10.31)	2.50 (1.21 to 4.87)	5.65 (2.22 to 12.13)	0.15 (0.09 to 0.25)	0.10 (0.05 to 0.16)	0.06 (0.04 to 0.10)	0.10 (0.06 to 0.19)
**Neglected tropical diseases and malaria**	**7.78 (4.65 to 12.14)**	**25.70 (15.55 to 38.74)**	**24.57 (14.86 to 36.67)**	**26.02 (15.69 to 39.34)**	**0.18 (0.12 to 0.26)**	**0.82 (0.54 to 1.17)**	**0.87 (0.58 to 1.22)**	**0.78 (0.51 to 1.12)**
Malaria	0.00 (0.00 to 0.00)	0.00 (0.00 to 0.00)	0.00 (0.00 to 0.00)	0.00 (0.00 to 0.00)	0.00 (0.00 to 0.00)	0.00 (0.00 to 0.00)	0.00 (0.00 to 0.00)	0.00 (0.00 to 0.00)
Chagas disease	0.00 (0.00 to 0.00)	0.00 (0.00 to 0.00)	0.00 (0.00 to 0.00)	0.00 (0.00 to 0.00)	0.00 (0.00 to 0.00)	0.00 (0.00 to 0.00)	0.00 (0.00 to 0.00)	0.00 (0.00 to 0.00)
Leishmaniasis	0.00 (0.00 to 0.00)	0.00 (0.00 to 0.00)	0.00 (0.00 to 0.00)	0.00 (0.00 to 0.00)	0.00 (0.00 to 0.00)	0.00 (0.00 to 0.00)	0.00 (0.00 to 0.00)	0.00 (0.00 to 0.00)
African trypanosomiasis	0.00 (0.00 to 0.00)	0.00 (0.00 to 0.00)	0.00 (0.00 to 0.00)	0.00 (0.00 to 0.00)	0.00 (0.00 to 0.00)	0.00 (0.00 to 0.00)	0.00 (0.00 to 0.00)	0.00 (0.00 to 0.00)
Schistosomiasis	0.00 (0.00 to 0.00)	0.00 (0.00 to 0.00)	0.00 (0.00 to 0.00)	0.00 (0.00 to 0.00)	0.00 (0.00 to 0.00)	0.00 (0.00 to 0.00)	0.00 (0.00 to 0.00)	0.00 (0.00 to 0.00)
Cysticercosis	0.00 (0.00 to 0.00)	0.00 (0.00 to 0.00)	0.00 (0.00 to 0.00)	0.00 (0.00 to 0.00)	0.00 (0.00 to 0.00)	0.00 (0.00 to 0.00)	0.00 (0.00 to 0.00)	0.00 (0.00 to 0.00)
Cystic echinococcosis	0.00 (0.00 to 0.00)	0.00 (0.00 to 0.00)	0.00 (0.00 to 0.00)	0.00 (0.00 to 0.00)	0.00 (0.00 to 0.00)	0.00 (0.00 to 0.00)	0.00 (0.00 to 0.00)	0.00 (0.00 to 0.00)
Dengue	3.30 (1.09 to 6.18)	18.70 (8.06 to 31.78)	18.79 (8.33 to 31.01)	18.63 (7.97 to 32.03)	0.07 (0.03 to 0.14)	0.58 (0.27 to 0.95)	0.64 (0.32 to 1.03)	0.54 (0.25 to 0.90)
Yellow fever	0.00 (0.00 to 0.00)	0.00 (0.00 to 0.00)	0.00 (0.00 to 0.00)	0.00 (0.00 to 0.00)	0.00 (0.00 to 0.00)	0.00 (0.00 to 0.00)	0.00 (0.00 to 0.00)	0.00 (0.00 to 0.00)
Rabies	0.03 (0.01 to 0.06)	0.02 (0.01 to 0.06)	0.01 (0.00 to 0.03)	0.03 (0.01 to 0.06)	0.00 (0.00 to 0.00)	0.00 (0.00 to 0.00)	0.00 (0.00 to 0.00)	0.00 (0.00 to 0.00)
Intestinal nematode infections	1.10 (0.21 to 3.00)	1.46 (0.38 to 3.37)	1.26 (0.37 to 2.79)	1.60 (0.38 to 3.77)	0.02 (0.00 to 0.04)	0.02 (0.01 to 0.05)	0.02 (0.01 to 0.04)	0.03 (0.01 to 0.05)
Other neglected tropical diseases	3.36 (2.07 to 5.37)	5.51 (2.97 to 8.77)	4.50 (2.22 to 7.17)	5.76 (3.14 to 9.18)	0.09 (0.06 to 0.12)	0.22 (0.11 to 0.35)	0.21 (0.10 to 0.34)	0.21 (0.11 to 0.33)
**Maternal disorders**	**128.42 (84.87 to 192.93)**	**81.98 (31.43 to 147.63)**	**43.01 (16.06 to 80.02)**	**96.64 (37.55 to 179.48)**	**2.36 (1.54 to 3.54)**	**1.54 (0.61 to 2.74)**	**0.81 (0.31 to 1.48)**	**1.82 (0.72 to 3.33)**
Maternal hemorrhage	41.75 (24.20 to 67.66)	25.24 (9.10 to 47.71)	13.22 (4.64 to 25.63)	29.60 (10.56 to 58.07)	0.76 (0.44 to 1.23)	0.47 (0.17 to 0.91)	0.25 (0.09 to 0.48)	0.55 (0.20 to 1.08)
Maternal sepsis and other maternal infections	13.47 (5.95 to 24.64)	7.92 (2.33 to 16.99)	4.12 (1.16 to 9.12)	9.30 (2.71 to 21.10)	0.25 (0.11 to 0.47)	0.15 (0.05 to 0.33)	0.08 (0.02 to 0.18)	0.18 (0.05 to 0.40)
Maternal hypertensive disorders	18.21 (9.83 to 30.76)	11.81 (3.85 to 23.67)	6.14 (1.86 to 12.82)	13.86 (4.39 to 30.05)	0.33 (0.18 to 0.56)	0.22 (0.07 to 0.44)	0.12 (0.04 to 0.24)	0.26 (0.09 to 0.56)
Maternal obstructed labor and uterine rupture	9.21 (3.92 to 18.21)	6.29 (1.81 to 14.98)	3.15 (0.86 to 7.73)	7.45 (1.95 to 18.06)	0.17 (0.07 to 0.33)	0.12 (0.03 to 0.27)	0.06 (0.02 to 0.14)	0.14 (0.04 to 0.34)
Maternal abortion, miscarriage, and ectopic pregnancy	4.98 (1.75 to 10.23)	3.65 (1.05 to 8.53)	1.91 (0.53 to 4.66)	4.33 (1.27 to 10.32)	0.09 (0.03 to 0.19)	0.07 (0.02 to 0.16)	0.04 (0.01 to 0.09)	0.08 (0.02 to 0.20)

Indirect maternal deaths	13.76 (7.05 to 23.84)	9.69 (3.20 to 19.54)	5.09 (1.64 to 10.54)	11.57 (3.67 to 23.61)	0.25 (0.13 to 0.43)	0.18 (0.06 to 0.36)	0.09 (0.03 to 0.20)	0.21 (0.07 to 0.44)
Late maternal deaths	5.52 (2.29 to 11.01)	2.97 (0.85 to 6.77)	1.54 (0.40 to 3.68)	3.53 (0.99 to 8.27)	0.10 (0.04 to 0.20)	0.06 (0.02 to 0.13)	0.03 (0.01 to 0.07)	0.07 (0.02 to 0.16)
Maternal deaths aggravated by HIV/AIDS	0.06 (0.00 to 0.30)	0.47 (0.00 to 3.07)	0.25 (0.00 to 1.76)	0.57 (0.01 to 3.56)	0.00 (0.00 to 0.01)	0.01 (0.00 to 0.06)	0.00 (0.00 to 0.03)	0.01 (0.00 to 0.07)
Other maternal disorders	21.46 (11.03 to 36.91)	13.94 (4.80 to 27.86)	7.59 (2.52 to 15.76)	16.42 (5.70 to 33.54)	0.39 (0.20 to 0.66)	0.26 (0.09 to 0.52)	0.14 (0.05 to 0.29)	0.31 (0.11 to 0.61)

Marshall Islands								
Cause	YLLs (actual counts)				Deaths (actual counts)			
	2016	2040 Forecast	2040 Better Scenario	2040 Worse Scenario	2016	2040 Forecast	2040 Better Scenario	2040 Worse Scenario
**Neonatal disorders**	**1,550.94 (762.20 to 2,668.79)**	**754.74 (334.58 to 1,402.81)**	**384.83 (171.68 to 715.40)**	**1,003.43 (445.57 to 1,906.96)**	**17.92 (8.81 to 30.84)**	**8.73 (3.87 to 16.22)**	**4.45 (1.99 to 8.27)**	**11.60 (5.16 to 22.05)**
Neonatal preterm birth	769.24 (359.04 to 1,404.53)	346.64 (145.11 to 669.44)	164.89 (68.89 to 321.72)	466.18 (196.09 to 893.02)	8.89 (4.15 to 16.23)	4.01 (1.68 to 7.74)	1.91 (0.80 to 3.72)	5.39 (2.27 to 10.32)
Neonatal encephalopathy due to birth asphyxia and trauma	342.11 (148.68 to 657.60)	156.47 (59.36 to 327.37)	88.95 (33.56 to 188.31)	203.63 (76.60 to 429.34)	3.96 (1.72 to 7.60)	1.81 (0.69 to 3.78)	1.03 (0.39 to 2.18)	2.36 (0.89 to 4.97)
Neonatal sepsis and other neonatal infections	158.60 (71.73 to 318.06)	102.02 (41.33 to 215.56)	56.48 (23.02 to 119.89)	133.45 (54.21 to 281.13)	1.83 (0.83 to 3.68)	1.18 (0.48 to 2.49)	0.65 (0.27 to 1.39)	1.54 (0.63 to 3.25)
Hemolytic disease and other neonatal jaundice	14.41 (6.19 to 30.02)	4.86 (1.52 to 12.27)	2.47 (0.76 to 6.06)	6.52 (2.10 to 16.44)	0.17 (0.07 to 0.35)	0.06 (0.02 to 0.14)	0.03 (0.01 to 0.07)	0.08 (0.02 to 0.19)
Other neonatal disorders	266.57 (119.67 to 533.18)	144.75 (57.44 to 321.34)	72.05 (29.15 to 159.80)	193.66 (77.11 to 427.15)	3.08 (1.38 to 6.16)	1.67 (0.66 to 3.72)	0.83 (0.34 to 1.85)	2.24 (0.89 to 4.95)
**Nutritional deficiencies**	**124.67 (68.75 to 238.88)**	**54.36 (34.40 to 94.21)**	**36.20 (25.10 to 56.95)**	**62.06 (37.11 to 109.99)**	**2.35 (1.63 to 3.63)**	**1.82 (1.36 to 2.46)**	**1.57 (1.21 to 2.01)**	**1.81 (1.30 to 2.55)**
Protein-energy malnutrition	121.58 (65.69 to 236.22)	50.63 (31.40 to 88.57)	33.52 (23.07 to 53.27)	57.98 (34.22 to 105.29)	2.27 (1.58 to 3.56)	1.70 (1.23 to 2.33)	1.47 (1.12 to 1.92)	1.69 (1.20 to 2.41)
Iodine deficiency	0.81 (0.05 to 3.50)	1.72 (0.05 to 7.57)	1.10 (0.03 to 4.30)	1.96 (0.06 to 9.41)	0.02 (0.00 to 0.06)	0.06 (0.00 to 0.21)	0.05 (0.00 to 0.18)	0.06 (0.00 to 0.21)
Dietary iron deficiency	0.60 (0.43 to 0.84)	0.53 (0.34 to 0.90)	0.42 (0.27 to 0.73)	0.57 (0.35 to 0.95)	0.01 (0.01 to 0.02)	0.01 (0.01 to 0.03)	0.01 (0.01 to 0.02)	0.01 (0.01 to 0.03)
Other nutritional deficiencies	1.67 (1.18 to 2.40)	1.49 (1.01 to 2.14)	1.16 (0.80 to 1.68)	1.56 (1.07 to 2.26)	0.05 (0.03 to 0.07)	0.05 (0.04 to 0.08)	0.05 (0.03 to 0.07)	0.05 (0.04 to 0.08)
**Other communicable, maternal, neonatal, and nutritional diseases**	**345.21 (165.70 to 643.93)**	**147.42 (97.09 to 229.00)**	**104.61 (70.69 to 156.41)**	**164.50 (103.07 to 260.15)**	**4.97 (2.83 to 8.53)**	**3.04 (2.20 to 4.12)**	**2.33 (1.70 to 3.17)**	**3.25 (2.25 to 4.61)**
Sexually transmitted infections excluding HIV	230.76 (68.76 to 508.63)	41.66 (12.27 to 98.97)	26.55 (9.01 to 60.34)	50.00 (14.07 to 119.91)	2.78 (0.87 to 6.01)	0.56 (0.20 to 1.23)	0.37 (0.15 to 0.79)	0.66 (0.22 to 1.49)
Syphilis	224.18 (62.97 to 504.81)	36.34 (7.58 to 94.98)	22.19 (4.99 to 55.70)	44.44 (9.13 to 114.78)	2.64 (0.76 to 5.90)	0.44 (0.10 to 1.13)	0.27 (0.07 to 0.66)	0.54 (0.12 to 1.36)
Chlamydial infection	1.29 (0.64 to 2.02)	1.03 (0.43 to 1.79)	0.85 (0.36 to 1.48)	1.08 (0.46 to 1.85)	0.03 (0.01 to 0.04)	0.02 (0.01 to 0.04)	0.02 (0.01 to 0.03)	0.02 (0.01 to 0.04)
Gonococcal infection	3.37 (1.68 to 5.33)	2.74 (1.14 to 4.79)	2.26 (0.95 to 3.98)	2.86 (1.22 to 4.98)	0.07 (0.04 to 0.11)	0.06 (0.03 to 0.11)	0.05 (0.02 to 0.09)	0.06 (0.03 to 0.11)
Other sexually transmitted infections	1.91 (0.97 to 3.02)	1.54 (0.67 to 2.67)	1.26 (0.55 to 2.17)	1.62 (0.71 to 2.78)	0.04 (0.02 to 0.06)	0.03 (0.01 to 0.06)	0.03 (0.01 to 0.05)	0.04 (0.02 to 0.06)
Acute hepatitis	39.79 (30.62 to 50.58)	32.01 (22.02 to 45.07)	24.93 (16.76 to 35.68)	33.87 (22.61 to 48.14)	0.83 (0.64 to 1.04)	0.77 (0.54 to 1.06)	0.61 (0.42 to 0.87)	0.80 (0.55 to 1.11)
Acute hepatitis A	1.62 (0.92 to 2.76)	0.73 (0.40 to 1.29)	0.52 (0.29 to 0.87)	0.81 (0.44 to 1.46)	0.02 (0.01 to 0.04)	0.01 (0.01 to 0.02)	0.01 (0.01 to 0.02)	0.02 (0.01 to 0.02)
Acute hepatitis B	30.26 (23.10 to 38.99)	27.11 (18.35 to 38.71)	21.18 (14.05 to 30.88)	28.51 (19.01 to 41.57)	0.68 (0.52 to 0.87)	0.67 (0.46 to 0.94)	0.53 (0.36 to 0.76)	0.69 (0.47 to 1.00)

Acute hepatitis C	0.68 (0.42 to 1.01)	0.58 (0.36 to 0.91)	0.51 (0.32 to 0.80)	0.59 (0.36 to 0.94)	0.01 (0.01 to 0.02)	0.02 (0.01 to 0.02)	0.01 (0.01 to 0.02)	0.02 (0.01 to 0.02)
Acute hepatitis E	7.24 (4.34 to 11.26)	3.60 (1.91 to 6.09)	2.72 (1.49 to 4.53)	3.95 (2.05 to 6.76)	0.11 (0.07 to 0.16)	0.07 (0.04 to 0.11)	0.06 (0.03 to 0.09)	0.07 (0.04 to 0.12)
Other unspecified infectious diseases	74.66 (43.57 to 170.17)	73.76 (47.51 to 117.19)	53.13 (34.83 to 83.48)	80.63 (50.77 to 127.93)	1.37 (0.90 to 2.47)	1.71 (1.20 to 2.54)	1.34 (0.94 to 1.95)	1.79 (1.22 to 2.65)
**Non-communicable diseases**	**12,893.79 (10,950.25 to 14,957.55)**	**19,142.63 (14,849.33 to 24,282.81)**	**13,347.17 (10,443.50 to 17,134.85)**	**22,685.69 (17,615.44 to 28,555.34)**	**429.27 (368.84 to 492.08)**	**738.98 (629.13 to 870.55)**	**553.06 (469.30 to 653.27)**	**844.72 (712.55 to 988.09)**

Cause	YLLs (actual counts)				Deaths (actual counts)			
	**Marshall Islands**							
	2016	2040 Forecast	2040 Better Scenario	2040 Worse Scenario	2016	2040 Forecast	2040 Better Scenario	2040 Worse Scenario
**Neoplasms**	**1,279.04 (1,012.31 to 1,548.85)**	**1,994.97 (1,386.03 to 2,762.21)**	**1,831.12 (1,314.61 to 2,463.62)**	**2,223.17 (1,507.30 to 3,163.78)**	**39.06 (31.03 to 46.87)**	**69.81 (50.30 to 95.93)**	**68.01 (50.78 to 90.12)**	**75.48 (52.39 to 107.38)**
Lip and oral cavity cancer	20.64 (15.64 to 27.36)	37.32 (19.46 to 70.66)	24.59 (14.67 to 41.85)	46.64 (22.61 to 91.74)	0.68 (0.53 to 0.87)	1.29 (0.74 to 2.28)	0.92 (0.60 to 1.46)	1.56 (0.82 to 2.94)
Nasopharynx cancer	18.24 (12.07 to 26.67)	40.34 (16.43 to 89.44)	23.66 (10.94 to 47.61)	52.17 (19.79 to 120.21)	0.54 (0.37 to 0.79)	1.24 (0.54 to 2.66)	0.76 (0.38 to 1.47)	1.58 (0.64 to 3.50)
Other pharynx cancer	15.62 (8.72 to 22.74)	21.28 (10.68 to 37.22)	17.11 (9.23 to 27.82)	22.60 (11.06 to 40.32)	0.50 (0.29 to 0.72)	0.75 (0.40 to 1.27)	0.64 (0.36 to 1.00)	0.78 (0.40 to 1.33)
Esophageal cancer	18.93 (14.24 to 24.78)	33.47 (16.44 to 65.44)	22.27 (12.68 to 40.48)	41.52 (19.01 to 84.52)	0.67 (0.52 to 0.86)	1.23 (0.64 to 2.31)	0.87 (0.53 to 1.50)	1.48 (0.73 to 2.93)
Stomach cancer	97.17 (79.00 to 118.50)	106.75 (81.42 to 137.66)	95.62 (73.72 to 120.38)	111.78 (83.49 to 148.03)	3.26 (2.71 to 3.90)	3.97 (3.17 to 4.95)	3.78 (3.01 to 4.63)	4.02 (3.15 to 5.16)
Colon and rectum cancer	89.60 (64.25 to 119.03)	172.89 (97.28 to 291.82)	179.86 (103.72 to 297.56)	173.66 (97.58 to 296.48)	3.09 (2.27 to 4.09)	6.68 (3.94 to 11.33)	7.41 (4.47 to 12.40)	6.48 (3.82 to 11.14)
Liver cancer	91.17 (68.97 to 119.21)	173.86 (104.70 to 281.16)	153.52 (97.52 to 237.61)	216.10 (119.34 to 374.80)	2.95 (2.29 to 3.82)	6.08 (3.78 to 9.53)	5.69 (3.75 to 8.54)	7.26 (4.21 to 12.06)
Liver cancer due to hepatitis B	52.38 (39.28 to 70.22)	95.92 (57.11 to 154.94)	85.25 (53.11 to 131.02)	119.19 (65.67 to 204.37)	1.61 (1.22 to 2.13)	3.17 (2.00 to 4.90)	2.97 (1.96 to 4.41)	3.81 (2.23 to 6.30)
Liver cancer due to hepatitis C	11.62 (8.68 to 15.45)	23.29 (14.37 to 36.49)	21.79 (14.13 to 33.27)	27.79 (16.33 to 46.37)	0.46 (0.35 to 0.61)	1.00 (0.66 to 1.50)	1.00 (0.68 to 1.45)	1.13 (0.70 to 1.78)
Liver cancer due to alcohol use	6.55 (4.80 to 9.02)	13.68 (7.98 to 23.30)	11.22 (6.99 to 18.58)	17.32 (9.44 to 30.93)	0.23 (0.18 to 0.32)	0.50 (0.31 to 0.82)	0.44 (0.29 to 0.68)	0.61 (0.35 to 1.09)
Liver cancer due to other causes	20.61 (15.30 to 28.26)	40.98 (21.31 to 77.59)	35.27 (19.66 to 64.02)	51.80 (24.40 to 102.80)	0.65 (0.49 to 0.89)	1.40 (0.74 to 2.64)	1.28 (0.71 to 2.32)	1.71 (0.82 to 3.35)
Gallbladder and biliary tract cancer	8.99 (6.37 to 12.89)	10.16 (7.33 to 14.27)	11.18 (8.01 to 15.80)	9.60 (7.01 to 13.42)	0.34 (0.25 to 0.47)	0.42 (0.32 to 0.57)	0.49 (0.36 to 0.66)	0.39 (0.29 to 0.52)
Pancreatic cancer	29.86 (23.47 to 37.05)	62.21 (38.00 to 98.84)	65.51 (41.49 to 102.73)	63.04 (38.29 to 102.25)	1.15 (0.92 to 1.41)	2.55 (1.61 to 4.05)	2.83 (1.83 to 4.44)	2.50 (1.56 to 4.00)
Larynx cancer	15.42 (11.65 to 19.38)	23.23 (11.25 to 45.96)	13.84 (7.56 to 24.81)	31.03 (14.36 to 61.37)	0.52 (0.41 to 0.64)	0.84 (0.43 to 1.65)	0.52 (0.30 to 0.92)	1.10 (0.53 to 2.17)
Tracheal, bronchus, and lung cancer	164.45 (108.07 to 230.48)	302.18 (119.16 to 639.05)	214.71 (96.97 to 421.35)	419.17 (161.05 to 892.65)	5.84 (3.95 to 8.03)	11.41 (4.90 to 23.78)	8.41 (4.06 to 16.36)	15.50 (6.39 to 32.59)
Malignant skin melanoma	6.92 (4.88 to 10.83)	11.92 (8.64 to 18.28)	14.55 (10.37 to 23.19)	11.04 (8.06 to 16.76)	0.23 (0.17 to 0.36)	0.44 (0.33 to 0.66)	0.57 (0.43 to 0.87)	0.40 (0.30 to 0.58)
Non-melanoma skin cancer	10.67 (6.97 to 15.03)	19.46 (12.98 to 26.59)	18.91 (12.52 to 26.04)	18.92 (12.68 to 25.93)	0.39 (0.27 to 0.53)	0.81 (0.57 to 1.04)	0.85 (0.61 to 1.10)	0.75 (0.53 to 0.98)
Breast cancer	147.47 (71.97 to 243.10)	223.30 (93.24 to 427.84)	220.55 (93.87 to 418.54)	233.40 (95.68 to 458.94)	4.41 (2.22 to 7.10)	7.51 (3.38 to 13.84)	7.78 (3.56 to 14.12)	7.62 (3.34 to 14.39)
Cervical cancer	79.48 (42.62 to 118.30)	91.76 (45.72 to 149.65)	80.09 (40.18 to 130.03)	95.36 (46.71 to 157.89)	2.26 (1.25 to 3.29)	2.81 (1.50 to 4.34)	2.52 (1.36 to 3.88)	2.87 (1.48 to 4.54)

Uterine cancer	32.34 (21.57 to 44.16)	48.24 (31.27 to 68.59)	42.29 (26.99 to 60.47)	48.52 (30.98 to 69.04)	1.09 (0.75 to 1.45)	1.79 (1.22 to 2.43)	1.63 (1.10 to 2.23)	1.77 (1.22 to 2.40)
Ovarian cancer	14.69 (9.44 to 24.94)	25.90 (12.72 to 49.83)	29.46 (14.35 to 56.34)	25.80 (12.58 to 50.55)	0.49 (0.32 to 0.80)	0.95 (0.49 to 1.81)	1.12 (0.57 to 2.10)	0.93 (0.48 to 1.78)
Prostate cancer	31.63 (23.75 to 44.14)	78.05 (58.72 to 107.34)	94.06 (72.81 to 129.10)	70.87 (52.68 to 99.58)	1.72 (1.31 to 2.33)	4.59 (3.40 to 6.25)	5.86 (4.50 to 7.92)	4.04 (2.92 to 5.63)
Testicular cancer	5.02 (3.50 to 6.63)	6.11 (4.12 to 8.38)	5.65 (3.80 to 7.73)	6.18 (4.19 to 8.49)	0.10 (0.07 to 0.14)	0.14 (0.10 to 0.18)	0.13 (0.09 to 0.17)	0.14 (0.10 to 0.18)

Marshall Islands								
Cause	YLLs (actual counts)				Deaths (actual counts)			
	2016	2040 Forecast	2040 Better Scenario	2040 Worse Scenario	2016	2040 Forecast	2040 Better Scenario	2040 Worse Scenario
Kidney cancer	11.08 (8.54 to 14.34)	20.95 (14.10 to 30.79)	22.80 (15.83 to 32.30)	21.28 (13.99 to 32.68)	0.32 (0.25 to 0.42)	0.71 (0.52 to 1.01)	0.83 (0.61 to 1.14)	0.70 (0.49 to 1.04)
Bladder cancer	11.28 (7.87 to 16.24)	18.83 (9.58 to 35.71)	17.46 (9.25 to 31.81)	20.08 (9.94 to 39.20)	0.45 (0.33 to 0.64)	0.81 (0.43 to 1.52)	0.81 (0.45 to 1.45)	0.83 (0.43 to 1.63)
Brain and nervous system cancer	38.05 (29.55 to 52.44)	54.85 (42.72 to 75.91)	55.87 (43.53 to 77.78)	54.96 (42.65 to 75.51)	0.80 (0.63 to 1.10)	1.37 (1.11 to 1.88)	1.49 (1.21 to 2.04)	1.33 (1.08 to 1.80)
Thyroid cancer	6.28 (4.52 to 8.31)	9.36 (6.32 to 13.69)	8.48 (5.77 to 12.26)	10.35 (6.81 to 15.44)	0.22 (0.16 to 0.29)	0.37 (0.25 to 0.52)	0.35 (0.25 to 0.49)	0.39 (0.27 to 0.56)
Mesothelioma	2.95 (2.02 to 4.29)	4.58 (3.18 to 6.62)	4.66 (3.24 to 6.94)	4.46 (3.10 to 6.36)	0.09 (0.06 to 0.13)	0.15 (0.11 to 0.21)	0.16 (0.11 to 0.23)	0.15 (0.10 to 0.20)
Hodgkin lymphoma	8.61 (5.73 to 11.16)	8.75 (5.63 to 12.06)	6.85 (4.39 to 9.60)	9.21 (5.84 to 13.14)	0.19 (0.13 to 0.24)	0.22 (0.15 to 0.29)	0.18 (0.12 to 0.24)	0.22 (0.15 to 0.30)
Non-Hodgkin lymphoma	44.73 (34.42 to 59.02)	64.22 (47.39 to 86.22)	67.81 (50.45 to 89.96)	66.12 (48.39 to 89.50)	1.09 (0.86 to 1.41)	1.90 (1.47 to 2.48)	2.15 (1.69 to 2.77)	1.88 (1.44 to 2.49)
Multiple myeloma	13.83 (10.15 to 17.83)	26.20 (18.66 to 35.36)	29.47 (21.24 to 39.29)	26.43 (18.39 to 36.62)	0.48 (0.36 to 0.61)	1.00 (0.74 to 1.31)	1.18 (0.88 to 1.52)	0.98 (0.71 to 1.32)
Leukemia	129.13 (97.16 to 166.75)	143.46 (98.38 to 197.73)	134.95 (94.34 to 182.33)	157.93 (106.49 to 220.56)	2.56 (1.97 to 3.23)	3.52 (2.48 to 4.77)	3.51 (2.54 to 4.61)	3.77 (2.61 to 5.26)
Acute lymphoid leukemia	26.68 (18.25 to 39.50)	24.79 (16.08 to 37.70)	23.37 (15.48 to 35.67)	27.15 (17.42 to 41.69)	0.43 (0.31 to 0.62)	0.48 (0.32 to 0.72)	0.47 (0.33 to 0.70)	0.52 (0.35 to 0.77)
Chronic lymphoid leukemia	1.82 (1.28 to 2.52)	2.75 (1.78 to 4.05)	2.79 (1.83 to 4.03)	2.94 (1.84 to 4.47)	0.06 (0.05 to 0.09)	0.11 (0.07 to 0.16)	0.12 (0.08 to 0.17)	0.11 (0.07 to 0.17)
Acute myeloid leukemia	45.61 (32.70 to 59.42)	61.50 (41.53 to 82.72)	62.62 (43.52 to 83.49)	66.49 (44.44 to 91.16)	0.87 (0.63 to 1.11)	1.43 (1.00 to 1.86)	1.55 (1.12 to 1.99)	1.51 (1.05 to 2.01)
Chronic myeloid leukemia	12.59 (7.22 to 18.29)	12.99 (6.60 to 21.69)	12.31 (6.36 to 20.04)	14.41 (7.15 to 24.81)	0.33 (0.19 to 0.47)	0.38 (0.20 to 0.61)	0.37 (0.20 to 0.58)	0.41 (0.21 to 0.67)
Other leukemia	42.44 (29.50 to 57.91)	41.42 (24.33 to 61.89)	33.86 (20.20 to 49.94)	46.93 (27.15 to 70.99)	0.86 (0.61 to 1.15)	1.12 (0.71 to 1.62)	1.00 (0.64 to 1.42)	1.22 (0.75 to 1.81)
Other neoplasms	114.78 (89.96 to 145.76)	155.31 (114.99 to 193.07)	155.34 (114.81 to 195.49)	154.94 (114.85 to 193.25)	2.60 (2.04 to 3.23)	4.25 (3.35 to 5.08)	4.57 (3.62 to 5.46)	4.08 (3.18 to 4.88)
**Cardiovascular diseases**	**3,927.36 (3,299.19 to 4,577.42)**	**5,176.54 (3,489.61 to 7,245.53)**	**2,771.18 (1,912.91 to 3,919.25)**	**6,938.47 (4,611.08 to 9,670.05)**	**133.79 (114.12 to 153.99)**	**199.04 (143.66 to 266.96)**	**117.62 (88.07 to 160.18)**	**257.05 (182.47 to 349.33)**
Rheumatic heart disease	232.00 (184.83 to 284.24)	128.41 (94.42 to 166.00)	93.47 (68.30 to 124.01)	138.26 (99.59 to 181.52)	5.45 (4.32 to 6.61)	3.81 (3.00 to 4.67)	3.00 (2.38 to 3.70)	3.94 (3.06 to 4.95)
Ischemic heart disease	1,868.06 (1,513.93 to 2,280.83)	2,601.98 (1,392.00 to 4,295.41)	1,220.15 (621.92 to 2,160.30)	3,687.04 (1,966.78 to 5,883.36)	66.96 (55.88 to 80.90)	99.88 (56.81 to 163.61)	51.86 (29.76 to 89.74)	136.79 (77.58 to 219.16)
Stroke	1,227.82 (1,011.78 to 1,457.65)	1,516.96 (868.93 to 2,477.60)	701.64 (408.23 to 1,193.41)	2,113.48 (1,208.77 to 3,394.39)	42.14 (35.19 to 49.64)	59.27 (37.16 to 93.66)	30.87 (20.11 to 50.90)	79.26 (48.80 to 124.44)
Ischemic stroke	299.64 (248.56 to 357.60)	361.72 (206.84 to 660.17)	203.30 (117.96 to 386.76)	467.42 (262.42 to 879.96)	13.06 (11.01 to 15.38)	17.94 (10.04 to 37.70)	11.26 (6.54 to 24.79)	22.18 (12.12 to 45.94)

Intracerebral hemorrhage	928.18 (758.84 to 1,112.87)	1,155.24 (586.11 to 2,072.42)	498.34 (259.91 to 922.92)	1,646.07 (835.76 to 2,810.99)	29.09 (23.93 to 34.61)	41.33 (23.42 to 71.40)	19.60 (11.70 to 33.33)	57.08 (31.10 to 94.39)
Hypertensive heart disease	186.48 (112.25 to 238.04)	327.03 (196.40 to 469.81)	229.06 (142.96 to 308.99)	370.17 (209.33 to 584.88)	7.10 (4.35 to 8.95)	14.13 (8.83 to 20.19)	10.71 (7.00 to 14.44)	15.26 (9.16 to 24.41)
Cardiomyopathy and myocarditis	135.88 (88.13 to 183.52)	168.50 (108.71 to 246.37)	163.08 (106.85 to 237.12)	169.05 (108.27 to 248.31)	3.54 (2.34 to 4.79)	5.39 (3.49 to 7.64)	5.59 (3.68 to 7.90)	5.20 (3.34 to 7.47)
Myocarditis	33.85 (24.26 to 48.03)	38.62 (27.52 to 52.97)	35.88 (25.97 to 48.64)	38.74 (27.36 to 53.31)	0.90 (0.65 to 1.27)	1.27 (0.93 to 1.71)	1.27 (0.93 to 1.69)	1.23 (0.90 to 1.64)

Marshall Islands								
Cause	YLLs (actual counts)				Deaths (actual counts)			
	2016	2040 Forecast	2040 Better Scenario	2040 Worse Scenario	2016	2040 Forecast	2040 Better Scenario	2040 Worse Scenario
Alcoholic cardiomyopathy	7.19 (3.55 to 11.39)	9.07 (4.49 to 14.55)	8.98 (4.49 to 14.43)	9.08 (4.50 to 14.48)	0.22 (0.11 to 0.34)	0.31 (0.16 to 0.46)	0.32 (0.17 to 0.48)	0.30 (0.15 to 0.45)
Other cardiomyopathy	94.84 (57.43 to 130.37)	120.80 (72.01 to 188.76)	118.22 (72.24 to 183.43)	121.23 (71.44 to 188.97)	2.41 (1.48 to 3.33)	3.81 (2.24 to 5.83)	4.00 (2.41 to 6.09)	3.67 (2.13 to 5.75)
Atrial fibrillation and flutter	6.67 (5.13 to 8.56)	14.78 (11.11 to 19.86)	16.49 (12.52 to 21.63)	13.87 (10.08 to 18.93)	0.50 (0.38 to 0.65)	1.16 (0.81 to 1.63)	1.42 (1.03 to 1.93)	1.04 (0.70 to 1.47)
Aortic aneurysm	40.31 (25.39 to 57.75)	60.81 (38.15 to 87.73)	59.95 (39.02 to 85.46)	62.04 (37.93 to 91.30)	1.51 (1.01 to 2.12)	2.53 (1.73 to 3.47)	2.74 (1.96 to 3.69)	2.45 (1.64 to 3.41)
Peripheral artery disease	2.50 (1.54 to 3.80)	6.84 (3.84 to 11.81)	7.17 (4.22 to 11.82)	6.79 (3.78 to 11.96)	0.12 (0.08 to 0.17)	0.35 (0.22 to 0.56)	0.42 (0.26 to 0.67)	0.33 (0.20 to 0.52)
Endocarditis	55.29 (41.29 to 74.85)	80.34 (56.62 to 113.30)	73.60 (51.75 to 103.22)	81.51 (57.59 to 115.74)	1.52 (1.18 to 1.98)	2.70 (1.97 to 3.63)	2.69 (1.97 to 3.60)	2.61 (1.90 to 3.54)
Other cardiovascular and circulatory diseases	172.34 (108.72 to 225.97)	270.88 (167.71 to 372.88)	206.58 (128.93 to 279.94)	296.26 (178.33 to 423.30)	4.95 (3.08 to 6.42)	9.83 (6.13 to 13.22)	8.32 (5.23 to 10.90)	10.18 (6.16 to 14.10)
**Chronic respiratory diseases**	**811.71 (657.53 to 979.80)**	**979.36 (652.96 to 1,457.61)**	**679.46 (488.21 to 943.47)**	**1,308.88 (811.78 to 2,060.06)**	**26.09 (21.20 to 31.44)**	**34.88 (23.81 to 50.82)**	**25.29 (18.83 to 34.11)**	**45.52 (29.26 to 69.73)**
Chronic obstructive pulmonary disease	451.49 (355.48 to 550.16)	605.93 (344.46 to 995.10)	364.38 (230.43 to 551.55)	876.09 (462.93 to 1,502.87)	15.82 (12.76 to 19.16)	23.08 (14.02 to 36.39)	14.85 (10.01 to 21.63)	32.21 (18.29 to 53.02)
Pneumoconiosis	1.84 (1.26 to 2.64)	2.01 (1.37 to 2.96)	1.96 (1.34 to 2.86)	2.05 (1.41 to 2.99)	0.07 (0.05 to 0.11)	0.09 (0.07 to 0.14)	0.10 (0.07 to 0.16)	0.09 (0.06 to 0.14)
Silicosis	0.21 (0.10 to 0.38)	0.20 (0.09 to 0.36)	0.18 (0.08 to 0.33)	0.20 (0.09 to 0.36)	0.01 (0.00 to 0.02)	0.01 (0.01 to 0.02)	0.01 (0.01 to 0.02)	0.01 (0.01 to 0.02)
Asbestosis	0.53 (0.34 to 0.85)	0.70 (0.44 to 1.21)	0.71 (0.44 to 1.16)	0.69 (0.43 to 1.19)	0.02 (0.01 to 0.03)	0.03 (0.02 to 0.05)	0.03 (0.02 to 0.05)	0.03 (0.02 to 0.05)
Coal workers pneumoconiosis	0.03 (0.01 to 0.07)	0.04 (0.01 to 0.08)	0.03 (0.01 to 0.06)	0.04 (0.01 to 0.10)	0.00 (0.00 to 0.00)	0.00 (0.00 to 0.00)	0.00 (0.00 to 0.00)	0.00 (0.00 to 0.00)
Other pneumoconiosis	1.07 (0.72 to 1.56)	1.06 (0.70 to 1.59)	1.04 (0.66 to 1.57)	1.12 (0.74 to 1.69)	0.04 (0.03 to 0.07)	0.05 (0.04 to 0.08)	0.06 (0.04 to 0.09)	0.05 (0.04 to 0.08)
Asthma	213.25 (127.05 to 296.66)	157.37 (81.12 to 259.58)	115.01 (60.49 to 190.69)	205.37 (103.12 to 345.58)	6.54 (3.95 to 9.08)	5.58 (2.92 to 9.12)	4.43 (2.37 to 7.12)	6.92 (3.47 to 11.53)
Interstitial lung disease and pulmonary sarcoidosis	46.92 (25.73 to 81.90)	66.08 (37.58 to 111.95)	74.49 (43.39 to 121.56)	64.35 (35.73 to 111.14)	1.06 (0.60 to 1.85)	1.80 (1.07 to 3.09)	2.16 (1.30 to 3.55)	1.69 (0.99 to 2.90)
Other chronic respiratory diseases	98.20 (57.19 to 152.15)	147.98 (83.98 to 251.84)	123.63 (73.42 to 200.22)	161.03 (87.28 to 284.96)	2.60 (1.51 to 4.03)	4.32 (2.47 to 7.41)	3.74 (2.28 to 6.10)	4.61 (2.53 to 8.16)
**Cirrhosis and other chronic liver diseases**	**403.17 (280.41 to 657.16)**	**572.21 (392.13 to 959.70)**	**513.21 (352.58 to 848.98)**	**579.68 (393.25 to 980.51)**	**10.29 (7.24 to 16.89)**	**16.11 (11.58 to 26.46)**	**15.00 (10.93 to 24.31)**	**15.98 (11.40 to 26.45)**
Cirrhosis and other chronic liver diseases due to hepatitis B	154.27 (101.32 to 264.11)	238.32 (156.88 to 407.20)	221.51 (148.21 to 372.80)	238.31 (156.65 to 412.90)	4.21 (2.85 to 7.12)	6.96 (4.79 to 11.72)	6.68 (4.70 to 11.07)	6.83 (4.70 to 11.66)
Cirrhosis and other chronic liver diseases due to hepatitis C	61.42 (39.91 to 106.71)	95.55 (62.06 to 165.42)	88.07 (57.60 to 149.88)	95.84 (62.00 to 166.09)	1.65 (1.10 to 2.82)	2.72 (1.86 to 4.54)	2.58 (1.79 to 4.27)	2.68 (1.82 to 4.53)

Cirrhosis and other chronic liver diseases due to alcohol use	52.11 (34.06 to 90.48)	81.02 (53.37 to 142.36)	65.28 (42.85 to 114.30)	84.00 (54.83 to 149.14)	1.49 (1.01 to 2.53)	2.44 (1.68 to 4.12)	2.06 (1.44 to 3.46)	2.48 (1.70 to 4.30)
Cirrhosis and other chronic liver diseases due to other causes	135.37 (97.55 to 203.12)	157.31 (107.86 to 243.38)	138.35 (94.83 to 214.10)	161.52 (109.88 to 252.38)	2.95 (2.15 to 4.36)	3.99 (2.94 to 5.99)	3.69 (2.72 to 5.50)	3.99 (2.89 to 6.04)
**Digestive diseases**	**284.91 (197.74 to 423.67)**	**322.64 (223.90 to 500.78)**	**262.01 (181.72 to 410.25)**	**346.56 (236.67 to 546.93)**	**8.02 (5.75 to 11.83)**	**11.05 (8.07 to 16.61)**	**9.67 (7.29 to 14.53)**	**11.41 (8.20 to 17.19)**
Peptic ulcer disease	75.01 (52.01 to 114.62)	62.28 (41.50 to 95.33)	45.36 (30.40 to 70.72)	66.51 (43.95 to 103.35)	2.33 (1.65 to 3.51)	2.35 (1.62 to 3.46)	1.83 (1.29 to 2.75)	2.41 (1.65 to 3.62)

Marshall Islands								
Cause	YLLs (actual counts)				Deaths (actual counts)			
	2016	2040 Forecast	2040 Better Scenario	2040 Worse Scenario	2016	2040 Forecast	2040 Better Scenario	2040 Worse Scenario
Gastritis and duodenitis	1.59 (0.79 to 2.84)	2.39 (1.18 to 4.32)	2.11 (1.08 to 3.84)	2.40 (1.19 to 4.43)	0.05 (0.03 to 0.09)	0.09 (0.05 to 0.16)	0.09 (0.05 to 0.15)	0.09 (0.05 to 0.16)
Appendicitis	38.71 (25.22 to 60.19)	39.34 (25.62 to 61.06)	31.12 (20.49 to 48.38)	41.17 (26.27 to 65.69)	0.90 (0.60 to 1.39)	1.12 (0.75 to 1.69)	0.95 (0.64 to 1.45)	1.13 (0.75 to 1.74)
Paralytic ileus and intestinal obstruction	37.69 (23.15 to 64.66)	38.98 (26.30 to 64.82)	34.88 (23.97 to 57.29)	40.11 (26.66 to 66.92)	0.84 (0.58 to 1.35)	1.24 (0.89 to 2.00)	1.26 (0.91 to 2.03)	1.19 (0.85 to 1.92)
Inguinal, femoral, and abdominal hernia	5.73 (3.72 to 10.44)	6.53 (4.31 to 11.55)	5.96 (3.98 to 10.41)	6.42 (4.22 to 11.58)	0.22 (0.15 to 0.38)	0.30 (0.20 to 0.50)	0.29 (0.20 to 0.49)	0.28 (0.19 to 0.48)
Inflammatory bowel disease	12.19 (6.91 to 20.81)	17.29 (10.18 to 29.16)	17.08 (10.33 to 28.20)	17.06 (9.92 to 28.73)	0.31 (0.19 to 0.51)	0.53 (0.34 to 0.84)	0.55 (0.37 to 0.87)	0.50 (0.32 to 0.80)
Vascular intestinal disorders	10.51 (5.74 to 15.71)	16.48 (9.85 to 23.91)	15.88 (9.64 to 22.89)	16.05 (9.39 to 23.31)	0.38 (0.22 to 0.55)	0.71 (0.45 to 1.00)	0.73 (0.47 to 1.02)	0.66 (0.41 to 0.94)
Gallbladder and biliary diseases	34.71 (22.52 to 53.62)	46.99 (23.74 to 86.24)	34.69 (18.39 to 62.97)	61.52 (30.59 to 117.27)	1.11 (0.76 to 1.67)	1.78 (0.95 to 3.15)	1.41 (0.80 to 2.50)	2.21 (1.14 to 4.00)
Pancreatitis	22.38 (13.39 to 38.78)	34.64 (18.33 to 76.10)	26.80 (15.25 to 53.44)	36.34 (18.75 to 80.93)	0.57 (0.36 to 0.97)	0.99 (0.56 to 1.99)	0.81 (0.50 to 1.50)	1.01 (0.56 to 2.09)
Other digestive diseases	46.39 (26.21 to 74.21)	57.72 (34.30 to 93.13)	48.14 (29.20 to 78.36)	58.98 (34.42 to 95.68)	1.29 (0.78 to 2.02)	1.96 (1.27 to 3.03)	1.74 (1.17 to 2.69)	1.93 (1.23 to 2.97)
**Neurological disorders**	**167.32 (136.69 to 199.58)**	**299.09 (218.47 to 422.66)**	**310.73 (228.53 to 441.59)**	**304.65 (217.58 to 438.63)**	**7.60 (6.36 to 9.08)**	**17.11 (11.42 to 26.27)**	**20.56 (13.89 to 31.33)**	**16.49 (10.52 to 26.30)**
Alzheimer's disease and other dementias	70.66 (56.37 to 89.13)	167.16 (98.03 to 285.35)	192.27 (118.46 to 321.48)	168.43 (95.57 to 294.43)	5.46 (4.40 to 6.78)	13.43 (7.66 to 22.59)	16.65 (10.01 to 27.23)	12.92 (7.05 to 22.57)
Parkinson's disease	5.97 (4.45 to 7.84)	15.54 (11.54 to 19.96)	21.14 (15.99 to 26.95)	13.49 (9.83 to 17.56)	0.39 (0.29 to 0.52)	1.08 (0.76 to 1.42)	1.55 (1.15 to 2.00)	0.91 (0.63 to 1.22)
Epilepsy	63.09 (43.57 to 83.17)	75.45 (43.78 to 118.39)	53.17 (31.39 to 82.70)	82.49 (47.67 to 133.07)	1.13 (0.78 to 1.48)	1.48 (0.88 to 2.31)	1.07 (0.65 to 1.64)	1.59 (0.95 to 2.52)
Multiple sclerosis	1.71 (0.89 to 3.19)	2.71 (1.37 to 5.08)	2.84 (1.43 to 5.41)	2.69 (1.35 to 4.99)	0.04 (0.02 to 0.08)	0.07 (0.04 to 0.13)	0.08 (0.04 to 0.14)	0.07 (0.04 to 0.13)
Motor neuron disease	4.06 (3.09 to 5.59)	8.23 (6.34 to 11.33)	9.39 (7.25 to 12.96)	7.81 (6.00 to 10.60)	0.12 (0.10 to 0.17)	0.28 (0.22 to 0.38)	0.33 (0.26 to 0.45)	0.26 (0.21 to 0.35)
Other neurological disorders	21.84 (17.10 to 27.58)	30.00 (23.51 to 37.44)	31.93 (25.09 to 39.60)	29.74 (23.30 to 37.25)	0.46 (0.37 to 0.56)	0.77 (0.63 to 0.91)	0.87 (0.73 to 1.03)	0.73 (0.61 to 0.87)
**Mental disorders**	**43.84 (28.67 to 64.01)**	**76.76 (49.67 to 114.51)**	**75.04 (48.82 to 111.88)**	**76.68 (49.70 to 114.36)**	**0.96 (0.63 to 1.40)**	**1.80 (1.22 to 2.64)**	**1.83 (1.24 to 2.67)**	**1.77 (1.19 to 2.58)**
Alcohol use disorders	22.72 (13.14 to 39.94)	29.17 (15.46 to 55.98)	27.35 (14.37 to 52.32)	29.32 (15.60 to 56.18)	0.52 (0.30 to 0.92)	0.71 (0.39 to 1.37)	0.68 (0.38 to 1.32)	0.71 (0.38 to 1.35)
Drug use disorders	20.89 (13.93 to 33.55)	47.21 (31.01 to 75.28)	47.32 (31.18 to 75.07)	46.98 (31.00 to 75.10)	0.43 (0.29 to 0.69)	1.09 (0.74 to 1.71)	1.14 (0.79 to 1.77)	1.05 (0.72 to 1.67)
Opioid use disorders	12.19 (7.44 to 21.05)	23.26 (13.20 to 42.08)	23.01 (13.08 to 41.35)	23.27 (13.27 to 42.23)	0.23 (0.14 to 0.41)	0.48 (0.29 to 0.86)	0.49 (0.29 to 0.86)	0.47 (0.28 to 0.85)

Cocaine use disorders	1.39 (0.78 to 2.31)	3.99 (2.09 to 6.85)	3.73 (1.96 to 6.45)	4.03 (2.12 to 6.88)	0.03 (0.02 to 0.05)	0.09 (0.05 to 0.15)	0.09 (0.05 to 0.15)	0.09 (0.05 to 0.15)
Amphetamine use disorders	0.56 (0.25 to 0.91)	2.23 (1.01 to 3.83)	2.09 (0.89 to 3.66)	2.25 (1.00 to 3.92)	0.01 (0.01 to 0.02)	0.05 (0.02 to 0.09)	0.05 (0.02 to 0.09)	0.05 (0.02 to 0.09)
Other drug use disorders	6.75 (4.22 to 11.23)	17.73 (10.83 to 30.31)	18.50 (11.50 to 31.63)	17.43 (10.54 to 29.52)	0.16 (0.10 to 0.26)	0.46 (0.30 to 0.77)	0.51 (0.34 to 0.85)	0.44 (0.29 to 0.74)
Eating disorders	0.23 (0.14 to 0.36)	0.38 (0.21 to 0.58)	0.37 (0.20 to 0.58)	0.39 (0.21 to 0.59)	0.00 (0.00 to 0.01)	0.01 (0.00 to 0.01)	0.01 (0.00 to 0.01)	0.01 (0.00 to 0.01)

Marshall Islands								
Cause	YLLs (actual counts)				Deaths (actual counts)			
	2016	2040 Forecast	2040 Better Scenario	2040 Worse Scenario	2016	2040 Forecast	2040 Better Scenario	2040 Worse Scenario
**Diabetes, urogenital, blood, and endocrine diseases**	**5,329.92 (4,421.60 to 6,299.90)**	**9,134.63 (6,670.30 to 12,783.29)**	**6,421.92 (4,634.29 to 9,302.10)**	**10,263.90 (7,352.85 to 14,242.61)**	**193.91 (163.88 to 226.50)**	**376.54 (296.62 to 483.57)**	**282.96 (222.57 to 374.50)**	**408.16 (319.81 to 518.77)**
Diabetes mellitus	4,297.12 (3,552.14 to 5,120.12)	6,537.02 (4,334.98 to 9,249.83)	4,581.45 (3,103.71 to 6,427.34)	7,309.78 (4,795.25 to 10,402.21)	161.73 (136.12 to 190.69)	279.85 (197.10 to 374.67)	210.78 (151.12 to 279.14)	300.72 (210.55 to 410.09)
Acute glomerulonephritis	1.32 (0.92 to 1.90)	1.99 (1.16 to 3.27)	1.73 (1.00 to 2.87)	2.03 (1.20 to 3.30)	0.03 (0.02 to 0.05)	0.06 (0.03 to 0.09)	0.05 (0.03 to 0.08)	0.06 (0.03 to 0.09)
Chronic kidney disease	798.71 (565.69 to 1,114.87)	2,245.84 (620.01 to 6,376.75)	1,514.63 (418.50 to 4,641.18)	2,599.28 (709.67 to 6,811.63)	26.13 (18.67 to 36.49)	85.37 (23.62 to 229.40)	60.82 (16.73 to 179.73)	96.51 (26.56 to 242.36)
Chronic kidney disease due to diabetes mellitus	457.12 (319.89 to 645.91)	1,418.25 (368.19 to 4,178.11)	957.20 (245.29 to 3,030.78)	1,643.48 (421.04 to 4,417.46)	15.08 (10.70 to 21.33)	51.83 (14.02 to 143.71)	36.61 (9.67 to 109.93)	58.92 (15.63 to 149.87)
Chronic kidney disease due to hypertension	153.97 (105.99 to 220.00)	482.94 (129.07 to 1,309.79)	332.31 (89.87 to 985.77)	554.75 (146.72 to 1,420.92)	6.57 (4.60 to 9.37)	22.77 (6.30 to 58.73)	16.83 (4.59 to 46.30)	25.28 (6.92 to 60.34)
Chronic kidney disease due to glomerulonephritis	92.56 (64.21 to 134.70)	158.42 (53.35 to 437.83)	103.23 (34.14 to 306.25)	184.24 (63.49 to 480.70)	2.06 (1.42 to 2.98)	4.55 (1.37 to 12.86)	3.08 (0.91 to 9.01)	5.23 (1.59 to 13.36)
Chronic kidney disease due to other and unspecified causes	95.06 (64.99 to 138.02)	186.23 (57.54 to 507.42)	121.89 (37.19 to 363.42)	216.81 (68.38 to 555.40)	2.42 (1.67 to 3.52)	6.22 (1.78 to 16.50)	4.30 (1.20 to 12.57)	7.08 (2.09 to 18.00)
Urinary diseases and male infertility	79.04 (63.36 to 102.25)	130.63 (103.48 to 168.59)	127.94 (100.39 to 167.06)	129.75 (103.18 to 166.49)	2.17 (1.78 to 2.70)	4.69 (3.89 to 5.72)	5.07 (4.20 to 6.17)	4.41 (3.63 to 5.41)
Urinary tract infections	67.74 (52.03 to 89.20)	114.05 (87.99 to 149.08)	113.21 (87.03 to 148.29)	113.08 (87.01 to 147.96)	1.82 (1.46 to 2.32)	4.09 (3.32 to 5.06)	4.51 (3.65 to 5.56)	3.83 (3.09 to 4.74)
Urolithiasis	2.17 (1.27 to 3.38)	4.55 (2.59 to 7.54)	4.76 (2.62 to 7.92)	4.42 (2.53 to 7.30)	0.07 (0.04 to 0.10)	0.16 (0.09 to 0.25)	0.17 (0.10 to 0.28)	0.15 (0.09 to 0.24)
Other urinary diseases	9.13 (5.58 to 13.72)	12.02 (6.66 to 19.68)	9.97 (5.50 to 16.26)	12.25 (6.72 to 20.19)	0.28 (0.17 to 0.41)	0.44 (0.25 to 0.71)	0.39 (0.22 to 0.64)	0.43 (0.24 to 0.70)
Gynecological diseases	0.85 (0.41 to 1.30)	0.91 (0.39 to 1.63)	0.71 (0.31 to 1.30)	0.96 (0.41 to 1.78)	0.02 (0.01 to 0.03)	0.02 (0.01 to 0.04)	0.02 (0.01 to 0.03)	0.02 (0.01 to 0.04)
Uterine fibroids	0.07 (0.01 to 0.14)	0.07 (0.02 to 0.15)	0.05 (0.01 to 0.11)	0.07 (0.02 to 0.16)	0.00 (0.00 to 0.00)	0.00 (0.00 to 0.00)	0.00 (0.00 to 0.00)	0.00 (0.00 to 0.00)
Polycystic ovarian syndrome	0.02 (0.00 to 0.07)	0.02 (0.00 to 0.06)	0.01 (0.00 to 0.04)	0.02 (0.00 to 0.06)	0.00 (0.00 to 0.00)	0.00 (0.00 to 0.00)	0.00 (0.00 to 0.00)	0.00 (0.00 to 0.00)
Endometriosis	0.01 (0.00 to 0.01)	0.01 (0.00 to 0.02)	0.01 (0.00 to 0.01)	0.01 (0.00 to 0.02)	0.00 (0.00 to 0.00)	0.00 (0.00 to 0.00)	0.00 (0.00 to 0.00)	0.00 (0.00 to 0.00)
Genital prolapse	0.02 (0.01 to 0.05)	0.02 (0.01 to 0.06)	0.01 (0.00 to 0.04)	0.02 (0.01 to 0.06)	0.00 (0.00 to 0.00)	0.00 (0.00 to 0.00)	0.00 (0.00 to 0.00)	0.00 (0.00 to 0.00)
Other gynecological diseases	0.73 (0.33 to 1.11)	0.80 (0.33 to 1.44)	0.63 (0.26 to 1.17)	0.84 (0.34 to 1.58)	0.02 (0.01 to 0.03)	0.02 (0.01 to 0.03)	0.02 (0.01 to 0.03)	0.02 (0.01 to 0.04)
Hemoglobinopathies and hemolytic anemias	21.58 (16.55 to 27.88)	21.13 (15.33 to 27.92)	17.16 (12.40 to 23.08)	22.01 (15.78 to 29.59)	0.49 (0.38 to 0.64)	0.61 (0.46 to 0.79)	0.52 (0.38 to 0.67)	0.62 (0.46 to 0.81)
Thalassemias	4.62 (3.05 to 6.93)	2.08 (1.22 to 3.23)	2.16 (1.29 to 3.34)	2.16 (1.27 to 3.37)	0.06 (0.04 to 0.09)	0.03 (0.02 to 0.04)	0.03 (0.02 to 0.04)	0.03 (0.02 to 0.04)

Sickle cell disorders	0.02 (0.01 to 0.03)	0.02 (0.01 to 0.03)	0.02 (0.01 to 0.03)	0.02 (0.01 to 0.04)	0.00 (0.00 to 0.00)	0.00 (0.00 to 0.00)	0.00 (0.00 to 0.00)	0.00 (0.00 to 0.00)
G6PD deficiency	12.01 (8.89 to 15.77)	14.40 (10.03 to 19.84)	11.03 (7.54 to 15.10)	15.20 (10.51 to 21.44)	0.26 (0.19 to 0.33)	0.36 (0.26 to 0.48)	0.28 (0.20 to 0.39)	0.37 (0.27 to 0.51)
Other hemoglobinopathies and hemolytic anemias	4.93 (3.60 to 6.68)	4.63 (3.34 to 6.16)	3.95 (2.84 to 5.34)	4.62 (3.29 to 6.20)	0.18 (0.13 to 0.24)	0.22 (0.16 to 0.29)	0.20 (0.15 to 0.27)	0.21 (0.15 to 0.28)
Endocrine, metabolic, blood, and immune disorders	131.31 (93.76 to 171.44)	197.11 (141.58 to 264.68)	178.30 (129.83 to 237.32)	200.08 (142.14 to 269.14)	3.34 (2.47 to 4.28)	5.94 (4.43 to 7.46)	5.71 (4.32 to 7.16)	5.82 (4.31 to 7.40)

Marshall Islands								
Cause	YLLs (actual counts)				Deaths (actual counts)			
	2016	2040 Forecast	2040 Better Scenario	2040 Worse Scenario	2016	2040 Forecast	2040 Better Scenario	2040 Worse Scenario
**Musculoskeletal disorders**	**45.27 (25.39 to 66.86)**	**60.52 (30.51 to 96.23)**	**64.62 (33.14 to 102.30)**	**59.61 (30.03 to 95.86)**	**0.91 (0.50 to 1.32)**	**1.46 (0.77 to 2.17)**	**1.64 (0.89 to 2.41)**	**1.39 (0.74 to 2.10)**
Rheumatoid arthritis	2.01 (1.01 to 3.13)	3.54 (1.70 to 5.66)	3.04 (1.48 to 4.89)	3.58 (1.75 to 5.76)	0.07 (0.03 to 0.10)	0.15 (0.07 to 0.24)	0.14 (0.06 to 0.22)	0.14 (0.07 to 0.23)
Other musculoskeletal disorders	43.26 (24.17 to 64.51)	56.98 (28.48 to 91.43)	61.58 (31.62 to 98.91)	56.03 (28.25 to 90.36)	0.85 (0.47 to 1.25)	1.31 (0.70 to 2.01)	1.50 (0.82 to 2.27)	1.25 (0.66 to 1.92)
**Other non-communicable diseases**	**601.25 (323.02 to 1,025.72)**	**525.91 (321.74 to 819.48)**	**417.87 (265.36 to 619.72)**	**584.08 (348.94 to 939.92)**	**8.63 (5.16 to 13.82)**	**11.18 (7.40 to 15.67)**	**10.48 (7.00 to 14.42)**	**11.48 (7.61 to 16.36)**
Congenital birth defects	518.25 (261.78 to 926.88)	341.69 (167.89 to 609.89)	236.39 (119.77 to 416.82)	400.97 (197.33 to 725.81)	6.41 (3.39 to 11.32)	4.60 (2.37 to 7.92)	3.29 (1.75 to 5.64)	5.30 (2.71 to 9.36)
Neural tube defects	37.00 (11.88 to 85.92)	9.94 (2.73 to 24.88)	5.31 (1.47 to 13.07)	12.60 (3.39 to 32.65)	0.43 (0.14 to 1.00)	0.12 (0.03 to 0.29)	0.06 (0.02 to 0.15)	0.15 (0.04 to 0.38)
Congenital heart anomalies	208.89 (101.74 to 375.30)	141.85 (68.44 to 263.95)	100.66 (49.32 to 180.88)	164.22 (78.06 to 307.03)	2.63 (1.31 to 4.63)	1.96 (0.98 to 3.52)	1.44 (0.73 to 2.51)	2.23 (1.09 to 4.03)
Orofacial clefts	0.31 (0.09 to 0.85)	0.16 (0.04 to 0.48)	0.08 (0.02 to 0.23)	0.20 (0.05 to 0.61)	0.00 (0.00 to 0.01)	0.00 (0.00 to 0.01)	0.00 (0.00 to 0.00)	0.00 (0.00 to 0.01)
Down syndrome	18.04 (7.65 to 36.87)	14.26 (6.38 to 28.53)	9.92 (4.47 to 19.07)	16.42 (7.12 to 33.47)	0.24 (0.11 to 0.48)	0.23 (0.11 to 0.44)	0.17 (0.08 to 0.31)	0.25 (0.12 to 0.51)
Other chromosomal abnormalities	17.18 (5.75 to 39.07)	15.40 (4.86 to 34.62)	9.78 (3.11 to 22.13)	18.68 (5.83 to 41.92)	0.20 (0.07 to 0.46)	0.19 (0.06 to 0.42)	0.12 (0.04 to 0.27)	0.23 (0.07 to 0.50)
Congenital musculoskeletal and limb anomalies	13.59 (5.65 to 28.98)	10.00 (3.81 to 21.40)	7.18 (2.85 to 15.55)	11.69 (4.37 to 25.42)	0.16 (0.07 to 0.35)	0.13 (0.05 to 0.27)	0.09 (0.04 to 0.20)	0.15 (0.06 to 0.32)
Urogenital congenital anomalies	14.73 (5.88 to 27.28)	10.59 (4.38 to 20.12)	7.72 (3.31 to 14.39)	12.19 (5.07 to 23.78)	0.20 (0.08 to 0.35)	0.17 (0.07 to 0.30)	0.13 (0.06 to 0.23)	0.19 (0.08 to 0.34)
Digestive congenital anomalies	49.06 (15.82 to 114.28)	28.43 (8.48 to 69.86)	18.02 (5.54 to 43.60)	34.52 (10.10 to 85.26)	0.58 (0.19 to 1.33)	0.35 (0.11 to 0.85)	0.22 (0.07 to 0.53)	0.42 (0.13 to 1.02)
Other congenital birth defects	159.44 (67.35 to 301.98)	111.05 (44.63 to 210.25)	77.71 (31.91 to 144.22)	130.44 (51.90 to 254.65)	1.95 (0.84 to 3.62)	1.46 (0.61 to 2.70)	1.05 (0.46 to 1.88)	1.69 (0.70 to 3.19)
Skin and subcutaneous diseases	63.13 (38.74 to 95.42)	174.00 (108.00 to 254.10)	174.76 (108.14 to 252.51)	170.59 (105.26 to 250.81)	2.00 (1.22 to 3.02)	6.46 (4.01 to 9.58)	7.11 (4.47 to 10.44)	6.03 (3.75 to 8.87)
Cellulitis	13.03 (5.28 to 20.11)	44.25 (18.36 to 65.71)	50.22 (21.40 to 74.93)	42.00 (17.45 to 62.79)	0.44 (0.17 to 0.67)	1.71 (0.70 to 2.54)	2.10 (0.89 to 3.06)	1.56 (0.63 to 2.32)
Pyoderma	41.99 (26.39 to 66.39)	112.62 (70.86 to 174.42)	106.12 (65.13 to 164.23)	112.33 (70.75 to 175.69)	1.20 (0.78 to 1.96)	3.86 (2.46 to 6.13)	3.94 (2.53 to 6.26)	3.68 (2.37 to 5.84)
Decubitus ulcer	7.26 (3.52 to 14.43)	15.70 (7.32 to 32.03)	16.98 (7.89 to 34.13)	14.82 (6.83 to 30.43)	0.34 (0.16 to 0.67)	0.86 (0.39 to 1.77)	1.03 (0.48 to 2.08)	0.76 (0.34 to 1.57)
Other skin and subcutaneous diseases	0.84 (0.47 to 1.34)	1.43 (0.80 to 2.30)	1.44 (0.82 to 2.33)	1.45 (0.81 to 2.35)	0.02 (0.01 to 0.03)	0.03 (0.02 to 0.05)	0.04 (0.02 to 0.06)	0.03 (0.02 to 0.05)
Sudden infant death syndrome	19.88 (7.98 to 40.92)	10.22 (3.34 to 23.75)	6.72 (2.17 to 14.90)	12.52 (4.13 to 28.74)	0.23 (0.09 to 0.47)	0.12 (0.04 to 0.28)	0.08 (0.03 to 0.17)	0.15 (0.05 to 0.33)

**Injuries**	**2,574.93 (2,125.53 to 3,079.64)**	**2,929.82 (2,174.56 to 3,783.13)**	**2,378.75 (1,803.91 to 3,020.98)**	**3,104.20 (2,312.12 to 4,048.22)**	**46.97 (38.77 to 55.63)**	**58.52 (44.91 to 74.11)**	**49.28 (38.38 to 61.10)**	**60.88 (46.64 to 77.98)**
**Transport injuries**	**698.39 (556.00 to 884.81)**	**943.79 (672.16 to 1,284.96)**	**697.58 (504.33 to 940.25)**	**1,025.17 (728.59 to 1,409.25)**	**12.81 (10.19 to 16.06)**	**18.65 (13.62 to 24.77)**	**14.37 (10.72 to 18.93)**	**19.88 (14.41 to 26.81)**
Road injuries	611.42 (484.42 to 781.64)	820.00 (577.03 to 1,124.94)	599.54 (426.50 to 810.34)	893.79 (627.03 to 1,242.17)	11.21 (8.92 to 14.29)	16.16 (11.65 to 21.59)	12.34 (9.12 to 16.22)	17.28 (12.45 to 23.31)
Pedestrian road injuries	140.13 (95.45 to 186.94)	146.91 (91.74 to 213.61)	109.78 (69.42 to 158.27)	158.71 (97.88 to 233.55)	2.56 (1.79 to 3.37)	2.99 (1.92 to 4.21)	2.32 (1.55 to 3.22)	3.17 (2.04 to 4.59)

Marshall Islands								
Cause	YLLs (actual counts)				Deaths (actual counts)			
	2016	2040 Forecast	2040 Better Scenario	2040 Worse Scenario	2016	2040 Forecast	2040 Better Scenario	2040 Worse Scenario
Cyclist road injuries	8.52 (5.34 to 11.74)	11.85 (7.44 to 17.74)	8.71 (5.51 to 12.77)	12.97 (8.04 to 19.53)	0.16 (0.10 to 0.22)	0.24 (0.15 to 0.35)	0.19 (0.12 to 0.27)	0.26 (0.16 to 0.38)
Motorcyclist road injuries	60.81 (38.07 to 87.18)	90.81 (52.29 to 138.75)	72.65 (42.76 to 109.57)	96.46 (55.55 to 149.48)	1.08 (0.68 to 1.57)	1.67 (0.96 to 2.54)	1.36 (0.80 to 2.04)	1.75 (1.02 to 2.71)
Motor vehicle road injuries	394.54 (308.70 to 517.64)	559.41 (387.47 to 776.57)	400.56 (277.45 to 551.45)	613.54 (425.81 to 858.12)	7.28 (5.76 to 9.44)	11.06 (7.91 to 15.07)	8.32 (6.06 to 11.21)	11.87 (8.42 to 16.26)
Other road injuries	7.42 (4.60 to 10.43)	11.02 (6.32 to 16.41)	7.84 (4.65 to 11.33)	12.13 (6.94 to 18.08)	0.13 (0.08 to 0.18)	0.21 (0.12 to 0.30)	0.15 (0.09 to 0.21)	0.22 (0.13 to 0.32)
Other transport injuries	86.98 (55.76 to 119.42)	123.78 (75.22 to 179.36)	98.04 (59.71 to 141.26)	131.38 (79.28 to 193.56)	1.60 (1.05 to 2.16)	2.50 (1.58 to 3.58)	2.04 (1.32 to 2.89)	2.60 (1.64 to 3.82)
**Unintentional injuries**	**998.36 (794.54 to 1,225.88)**	**817.89 (601.30 to 1,035.06)**	**664.33 (491.11 to 826.35)**	**866.43 (635.19 to 1,100.36)**	**18.17 (14.66 to 21.83)**	**17.53 (13.40 to 21.28)**	**15.09 (11.77 to 18.17)**	**18.02 (13.77 to 21.99)**
Falls	76.66 (52.78 to 111.07)	82.28 (56.97 to 117.68)	76.98 (53.54 to 110.04)	82.98 (57.38 to 120.16)	1.80 (1.27 to 2.52)	2.42 (1.75 to 3.32)	2.46 (1.81 to 3.33)	2.33 (1.68 to 3.22)
Drowning	439.19 (331.48 to 556.74)	319.11 (219.36 to 431.88)	253.00 (176.07 to 343.04)	340.41 (231.30 to 465.73)	7.45 (5.63 to 9.28)	6.05 (4.23 to 8.05)	4.92 (3.45 to 6.53)	6.36 (4.40 to 8.54)
Fire, heat, and hot substances	63.37 (43.58 to 87.51)	42.94 (26.04 to 65.65)	32.30 (19.56 to 49.56)	46.51 (28.18 to 70.86)	1.12 (0.78 to 1.51)	0.91 (0.60 to 1.33)	0.73 (0.49 to 1.06)	0.96 (0.62 to 1.40)
Poisonings	45.78 (25.83 to 69.41)	26.20 (13.88 to 41.71)	17.52 (9.42 to 26.93)	29.20 (15.51 to 46.90)	0.81 (0.47 to 1.18)	0.54 (0.30 to 0.82)	0.39 (0.23 to 0.57)	0.58 (0.33 to 0.88)
Exposure to mechanical forces	110.26 (82.89 to 140.91)	96.31 (65.90 to 130.50)	75.14 (51.71 to 100.91)	102.83 (69.80 to 138.78)	2.00 (1.49 to 2.58)	1.99 (1.42 to 2.62)	1.62 (1.17 to 2.12)	2.07 (1.47 to 2.72)
Unintentional firearm injuries	17.30 (12.20 to 24.23)	13.30 (8.25 to 19.79)	9.93 (6.11 to 14.72)	14.33 (8.80 to 21.20)	0.32 (0.24 to 0.44)	0.29 (0.19 to 0.42)	0.23 (0.15 to 0.33)	0.31 (0.20 to 0.43)
Unintentional suffocation	26.71 (15.99 to 39.21)	22.64 (12.70 to 34.10)	17.25 (9.66 to 26.21)	24.61 (13.50 to 37.92)	0.41 (0.25 to 0.59)	0.38 (0.22 to 0.56)	0.30 (0.17 to 0.44)	0.41 (0.23 to 0.62)
Other exposure to mechanical forces	66.25 (46.23 to 88.07)	60.38 (39.92 to 84.42)	47.96 (32.04 to 67.37)	63.89 (42.11 to 88.88)	1.27 (0.88 to 1.66)	1.31 (0.90 to 1.80)	1.09 (0.75 to 1.48)	1.36 (0.93 to 1.83)
Adverse effects of medical treatment	100.75 (73.10 to 130.53)	100.02 (68.63 to 136.73)	82.45 (57.04 to 113.11)	105.24 (72.32 to 144.22)	1.99 (1.49 to 2.52)	2.35 (1.73 to 3.07)	2.07 (1.53 to 2.69)	2.39 (1.76 to 3.12)
Animal contact	5.31 (3.74 to 7.99)	6.74 (4.23 to 10.95)	5.07 (3.15 to 8.33)	7.22 (4.44 to 11.84)	0.11 (0.08 to 0.17)	0.17 (0.11 to 0.26)	0.14 (0.09 to 0.21)	0.17 (0.11 to 0.27)
Venomous animal contact	1.62 (0.97 to 2.84)	1.76 (0.93 to 3.20)	1.28 (0.67 to 2.30)	1.91 (1.00 to 3.52)	0.03 (0.02 to 0.06)	0.04 (0.02 to 0.07)	0.03 (0.02 to 0.06)	0.04 (0.02 to 0.08)
Non-venomous animal contact	3.69 (2.61 to 5.54)	4.98 (3.02 to 8.11)	3.78 (2.28 to 6.26)	5.32 (3.23 to 8.66)	0.08 (0.06 to 0.12)	0.13 (0.08 to 0.20)	0.10 (0.07 to 0.16)	0.13 (0.08 to 0.20)
Foreign body	69.94 (42.11 to 105.59)	62.72 (38.38 to 88.25)	58.16 (36.76 to 79.34)	65.57 (39.63 to 95.59)	1.19 (0.77 to 1.67)	1.34 (0.88 to 1.73)	1.37 (0.89 to 1.72)	1.34 (0.88 to 1.76)
Pulmonary aspiration and foreign body in airway	65.30 (38.37 to 100.00)	58.88 (35.60 to 84.22)	55.06 (34.10 to 76.18)	61.46 (36.25 to 91.02)	1.11 (0.69 to 1.57)	1.27 (0.80 to 1.64)	1.30 (0.83 to 1.65)	1.26 (0.80 to 1.67)

Foreign body in other body part	4.64 (2.88 to 7.81)	3.84 (2.04 to 6.40)	3.10 (1.65 to 5.24)	4.11 (2.19 to 6.99)	0.08 (0.05 to 0.13)	0.07 (0.04 to 0.12)	0.06 (0.03 to 0.10)	0.08 (0.04 to 0.13)
Environmental heat and cold exposure	29.86 (17.36 to 40.66)	34.40 (19.18 to 50.19)	24.23 (13.46 to 35.90)	37.26 (21.20 to 55.12)	0.61 (0.36 to 0.82)	0.78 (0.45 to 1.10)	0.57 (0.33 to 0.83)	0.83 (0.47 to 1.18)
Other unintentional injuries	57.24 (37.39 to 77.31)	47.18 (29.01 to 68.46)	39.49 (24.23 to 57.22)	49.21 (30.58 to 70.90)	1.09 (0.72 to 1.47)	0.97 (0.60 to 1.35)	0.83 (0.52 to 1.17)	0.99 (0.62 to 1.40)
**Self-harm and interpersonal violence**	**877.67 (659.08 to 1,088.40)**	**1,168.14 (778.94 to 1,681.28)**	**1,016.85 (716.13 to 1,389.68)**	**1,212.60 (797.75 to 1,767.29)**	**15.98 (11.96 to 19.90)**	**22.34 (14.93 to 31.71)**	**19.81 (13.92 to 26.67)**	**22.98 (15.21 to 32.81)**

Marshall Islands								
Cause	YLLs (actual counts)				Deaths (actual counts)			
	2016	2040 Forecast	2040 Better Scenario	2040 Worse Scenario	2016	2040 Forecast	2040 Better Scenario	2040 Worse Scenario
Self-harm	634.10 (435.83 to 798.34)	914.38 (561.21 to 1,387.72)	779.04 (497.80 to 1,109.98)	954.29 (581.02 to 1,469.17)	11.55 (8.03 to 14.51)	17.50 (10.78 to 26.00)	15.23 (9.96 to 21.24)	18.08 (11.00 to 27.46)
Self-harm by firearm	40.89 (13.98 to 88.16)	51.20 (14.99 to 121.73)	41.94 (12.42 to 98.01)	53.66 (15.26 to 128.01)	0.78 (0.27 to 1.68)	1.01 (0.30 to 2.40)	0.84 (0.26 to 1.91)	1.05 (0.31 to 2.49)
Self-harm by other specified means	593.21 (410.49 to 748.39)	863.18 (541.54 to 1,304.77)	737.11 (479.06 to 1,056.71)	900.63 (557.72 to 1,368.78)	10.77 (7.55 to 13.51)	16.49 (10.33 to 24.39)	14.39 (9.60 to 20.05)	17.03 (10.63 to 25.48)
Interpersonal violence	243.58 (151.98 to 322.58)	253.76 (144.48 to 361.76)	237.81 (135.39 to 337.68)	258.31 (146.47 to 367.65)	4.43 (2.75 to 5.93)	4.84 (2.78 to 6.91)	4.58 (2.65 to 6.49)	4.90 (2.79 to 6.96)
Physical violence by firearm	56.90 (25.00 to 90.68)	58.71 (25.20 to 97.01)	56.82 (24.54 to 93.47)	59.23 (25.35 to 98.14)	1.03 (0.45 to 1.63)	1.10 (0.47 to 1.80)	1.07 (0.47 to 1.74)	1.11 (0.47 to 1.80)
Physical violence by sharp object	65.43 (39.33 to 94.65)	74.77 (42.79 to 110.86)	76.25 (43.66 to 112.33)	74.46 (42.57 to 110.29)	1.20 (0.73 to 1.73)	1.42 (0.82 to 2.11)	1.46 (0.85 to 2.15)	1.41 (0.82 to 2.10)
Physical violence by other means	121.24 (69.34 to 171.69)	120.28 (57.43 to 196.35)	104.73 (50.37 to 167.55)	124.63 (59.39 to 202.94)	2.19 (1.25 to 3.07)	2.32 (1.13 to 3.71)	2.05 (1.01 to 3.22)	2.38 (1.16 to 3.81)
**Forces of nature, conflict and terrorism, and executions and police conflict**	**0.50 (0.40 to 0.62)**	**0.00 (0.00 to 0.00)**	**0.00 (0.00 to 0.00)**	**0.00 (0.00 to 0.00)**	**0.01 (0.01 to 0.01)**	**0.00 (0.00 to 0.00)**	**0.00 (0.00 to 0.00)**	**0.00 (0.00 to 0.00)**
Exposure to forces of nature	0.00 (0.00 to 0.00)	0.00 (0.00 to 0.00)	0.00 (0.00 to 0.00)	0.00 (0.00 to 0.00)	0.00 (0.00 to 0.00)	0.00 (0.00 to 0.00)	0.00 (0.00 to 0.00)	0.00 (0.00 to 0.00)
Conflict and terrorism	0.00 (0.00 to 0.00)	0.00 (0.00 to 0.00)	0.00 (0.00 to 0.00)	0.00 (0.00 to 0.00)	0.00 (0.00 to 0.00)	0.00 (0.00 to 0.00)	0.00 (0.00 to 0.00)	0.00 (0.00 to 0.00)
Executions and police conflict	0.50 (0.40 to 0.62)	0.00 (0.00 to 0.00)	0.00 (0.00 to 0.00)	0.00 (0.00 to 0.00)	0.01 (0.01 to 0.01)	0.00 (0.00 to 0.00)	0.00 (0.00 to 0.00)	0.00 (0.00 to 0.00)

Northern Mariana Islands								
Cause	YLLs (actual counts)				Deaths (actual counts)			
	2016	2040 Forecast	2040 Better Scenario	2040 Worse Scenario	2016	2040 Forecast	2040 Better Scenario	2040 Worse Scenario
**All causes**	**11,344.71 (9,312.87 to 13,568.84)**	**25,128.79 (19,160.40 to 32,884.60)**	**15,140.81 (11,714.39 to 20,359.84)**	**31,851.06 (24,287.23 to 41,130.06)**	**305.21 (255.44 to 362.14)**	**1,056.02 (857.11 to 1,321.84)**	**672.69 (544.84 to 872.56)**	**1,303.89 (1,042.68 to 1,620.13)**
**Communicable, maternal, neonatal, and nutritional diseases**	**1,309.99 (888.93 to 2,455.95)**	**1,607.41 (1,134.09 to 2,830.87)**	**1,032.93 (710.25 to 2,161.66)**	**1,992.56 (1,441.97 to 3,239.03)**	**27.33 (19.59 to 51.39)**	**59.71 (44.62 to 92.02)**	**42.44 (31.23 to 72.45)**	**71.21 (53.25 to 104.13)**
**HIV/AIDS and tuberculosis**	**300.72 (59.73 to 1,525.14)**	**345.81 (80.00 to 1,647.54)**	**174.21 (13.80 to 1,370.37)**	**467.96 (144.13 to 1,763.28)**	**6.38 (1.50 to 32.57)**	**9.93 (2.97 to 42.39)**	**4.99 (0.58 to 36.08)**	**13.06 (4.71 to 44.92)**
Tuberculosis	44.91 (34.86 to 55.68)	75.71 (46.58 to 120.61)	18.20 (11.68 to 27.18)	100.58 (59.66 to 164.57)	1.20 (0.94 to 1.47)	3.14 (1.96 to 4.90)	0.79 (0.52 to 1.16)	4.07 (2.45 to 6.68)
HIV/AIDS	255.81 (13.04 to 1,490.15)	270.10 (10.18 to 1,565.34)	156.01 (0.00 to 1,343.46)	367.38 (50.49 to 1,639.70)	5.18 (0.24 to 31.52)	6.80 (0.19 to 39.46)	4.21 (0.00 to 35.28)	8.99 (1.07 to 40.72)
**Diarrhea, lower respiratory, and other common infectious diseases**	**490.38 (372.98 to 628.87)**	**913.08 (667.51 to 1,252.91)**	**606.75 (453.36 to 826.13)**	**1,141.40 (805.09 to 1,672.23)**	**12.33 (9.79 to 15.45)**	**39.28 (29.18 to 53.05)**	**27.66 (20.65 to 38.27)**	**47.43 (34.35 to 66.62)**
Diarrheal diseases	16.91 (10.51 to 26.21)	50.95 (13.10 to 152.91)	106.82 (37.74 to 264.45)	42.91 (10.65 to 136.12)	0.51 (0.29 to 0.81)	2.71 (0.65 to 8.18)	6.07 (2.11 to 15.32)	2.21 (0.52 to 7.12)
Intestinal infectious diseases	46.41 (15.67 to 105.64)	41.37 (15.01 to 91.49)	60.45 (25.97 to 118.24)	40.86 (14.34 to 92.76)	0.72 (0.24 to 1.65)	0.75 (0.28 to 1.68)	1.12 (0.47 to 2.25)	0.73 (0.26 to 1.64)
Typhoid fever	39.41 (13.11 to 89.17)	35.24 (12.91 to 78.74)	57.36 (24.79 to 111.46)	33.86 (11.90 to 75.96)	0.61 (0.20 to 1.40)	0.63 (0.23 to 1.41)	1.06 (0.45 to 2.15)	0.60 (0.21 to 1.35)
Paratyphoid fever	6.99 (2.01 to 17.58)	6.12 (1.94 to 14.53)	3.07 (1.05 to 7.06)	6.99 (2.16 to 17.29)	0.11 (0.03 to 0.29)	0.12 (0.04 to 0.28)	0.06 (0.02 to 0.14)	0.13 (0.04 to 0.32)
Other intestinal infectious diseases	0.01 (0.01 to 0.03)	0.01 (0.00 to 0.03)	0.01 (0.00 to 0.02)	0.01 (0.00 to 0.03)	0.00 (0.00 to 0.00)	0.00 (0.00 to 0.00)	0.00 (0.00 to 0.00)	0.00 (0.00 to 0.00)
Lower respiratory infections	388.69 (295.69 to 496.29)	788.76 (566.29 to 1,100.50)	417.36 (297.53 to 575.07)	1,021.32 (708.54 to 1,515.81)	10.43 (8.22 to 13.13)	34.98 (25.49 to 48.04)	19.87 (13.87 to 27.57)	43.59 (31.17 to 62.87)
Upper respiratory infections	0.36 (0.17 to 0.66)	0.60 (0.21 to 1.41)	0.18 (0.06 to 0.41)	0.72 (0.26 to 1.65)	0.01 (0.00 to 0.02)	0.02 (0.01 to 0.05)	0.01 (0.00 to 0.01)	0.03 (0.01 to 0.06)
Otitis media	0.01 (0.00 to 0.01)	0.00 (0.00 to 0.01)	0.01 (0.00 to 0.01)	0.00 (0.00 to 0.01)	0.00 (0.00 to 0.00)	0.00 (0.00 to 0.00)	0.00 (0.00 to 0.00)	0.00 (0.00 to 0.00)
Meningitis	17.86 (12.10 to 26.87)	11.28 (7.17 to 17.76)	5.25 (3.36 to 8.39)	12.76 (8.26 to 19.57)	0.34 (0.23 to 0.51)	0.31 (0.19 to 0.48)	0.15 (0.09 to 0.23)	0.34 (0.22 to 0.51)
Pneumococcal meningitis	0.05 (0.03 to 0.08)	0.03 (0.02 to 0.05)	0.03 (0.02 to 0.05)	0.03 (0.02 to 0.04)	0.00 (0.00 to 0.00)	0.00 (0.00 to 0.00)	0.00 (0.00 to 0.00)	0.00 (0.00 to 0.00)
H influenzae type B meningitis	1.86 (1.23 to 2.91)	1.07 (0.67 to 1.68)	1.07 (0.65 to 1.68)	1.08 (0.67 to 1.75)	0.04 (0.02 to 0.05)	0.03 (0.02 to 0.05)	0.03 (0.02 to 0.05)	0.03 (0.02 to 0.05)
Meningococcal meningitis	3.62 (2.31 to 5.66)	2.42 (1.53 to 3.85)	1.06 (0.65 to 1.68)	2.73 (1.70 to 4.22)	0.07 (0.05 to 0.11)	0.07 (0.04 to 0.11)	0.03 (0.02 to 0.05)	0.08 (0.05 to 0.12)
Other meningitis	12.32 (8.20 to 18.59)	7.76 (4.83 to 12.13)	3.09 (1.92 to 4.83)	8.92 (5.77 to 13.79)	0.23 (0.15 to 0.36)	0.21 (0.13 to 0.33)	0.09 (0.05 to 0.13)	0.23 (0.15 to 0.36)
Encephalitis	9.66 (6.87 to 13.35)	13.37 (10.06 to 17.96)	12.06 (8.85 to 16.45)	13.64 (10.26 to 18.31)	0.18 (0.13 to 0.24)	0.39 (0.30 to 0.51)	0.36 (0.27 to 0.48)	0.39 (0.30 to 0.51)

Diphtheria	0.04 (0.03 to 0.07)	0.01 (0.01 to 0.03)	0.01 (0.01 to 0.03)	0.02 (0.01 to 0.03)	0.00 (0.00 to 0.00)	0.00 (0.00 to 0.00)	0.00 (0.00 to 0.00)	0.00 (0.00 to 0.00)
Whooping cough	3.20 (0.27 to 10.14)	1.20 (0.06 to 5.18)	0.46 (0.03 to 1.95)	2.64 (0.11 to 11.31)	0.04 (0.00 to 0.12)	0.01 (0.00 to 0.06)	0.01 (0.00 to 0.02)	0.03 (0.00 to 0.13)
Tetanus	0.65 (0.51 to 0.80)	0.08 (0.06 to 0.11)	0.08 (0.05 to 0.11)	0.09 (0.06 to 0.14)	0.01 (0.01 to 0.01)	0.00 (0.00 to 0.00)	0.00 (0.00 to 0.00)	0.00 (0.00 to 0.00)
Measles	4.89 (1.06 to 14.14)	4.42 (0.01 to 25.20)	3.51 (0.01 to 18.50)	5.28 (0.01 to 30.84)	0.06 (0.01 to 0.17)	0.07 (0.00 to 0.36)	0.05 (0.00 to 0.27)	0.08 (0.00 to 0.43)

Northern Mariana Islands								
Cause	YLLs (actual counts)				Deaths (actual counts)			
	2016	2040 Forecast	2040 Better Scenario	2040 Worse Scenario	2016	2040 Forecast	2040 Better Scenario	2040 Worse Scenario
Varicella and herpes zoster	1.68 (0.83 to 3.22)	1.04 (0.55 to 1.94)	0.57 (0.31 to 1.05)	1.16 (0.60 to 2.18)	0.03 (0.02 to 0.05)	0.03 (0.02 to 0.05)	0.02 (0.01 to 0.04)	0.04 (0.02 to 0.06)
**Neglected tropical diseases and malaria**	**13.28 (2.40 to 26.55)**	**14.44 (3.70 to 25.97)**	**15.62 (4.40 to 26.59)**	**14.17 (3.46 to 25.88)**	**0.29 (0.06 to 0.56)**	**0.53 (0.17 to 0.84)**	**0.63 (0.22 to 0.96)**	**0.50 (0.15 to 0.81)**
Malaria	11.81 (1.03 to 24.84)	9.89 (0.54 to 20.92)	9.41 (0.52 to 19.76)	9.94 (0.55 to 20.97)	0.24 (0.02 to 0.51)	0.28 (0.02 to 0.58)	0.27 (0.01 to 0.56)	0.28 (0.01 to 0.58)
Chagas disease	0.00 (0.00 to 0.00)	0.00 (0.00 to 0.00)	0.00 (0.00 to 0.00)	0.00 (0.00 to 0.00)	0.00 (0.00 to 0.00)	0.00 (0.00 to 0.00)	0.00 (0.00 to 0.00)	0.00 (0.00 to 0.00)
Leishmaniasis	0.00 (0.00 to 0.00)	0.00 (0.00 to 0.00)	0.00 (0.00 to 0.00)	0.00 (0.00 to 0.00)	0.00 (0.00 to 0.00)	0.00 (0.00 to 0.00)	0.00 (0.00 to 0.00)	0.00 (0.00 to 0.00)
African trypanosomiasis	0.00 (0.00 to 0.00)	0.00 (0.00 to 0.00)	0.00 (0.00 to 0.00)	0.00 (0.00 to 0.00)	0.00 (0.00 to 0.00)	0.00 (0.00 to 0.00)	0.00 (0.00 to 0.00)	0.00 (0.00 to 0.00)
Schistosomiasis	0.00 (0.00 to 0.00)	0.00 (0.00 to 0.00)	0.00 (0.00 to 0.00)	0.00 (0.00 to 0.00)	0.00 (0.00 to 0.00)	0.00 (0.00 to 0.00)	0.00 (0.00 to 0.00)	0.00 (0.00 to 0.00)
Cysticercosis	0.13 (0.10 to 0.16)	0.08 (0.06 to 0.10)	0.07 (0.05 to 0.10)	0.08 (0.06 to 0.10)	0.00 (0.00 to 0.00)	0.00 (0.00 to 0.00)	0.00 (0.00 to 0.00)	0.00 (0.00 to 0.00)
Cystic echinococcosis	0.00 (0.00 to 0.00)	0.00 (0.00 to 0.00)	0.00 (0.00 to 0.00)	0.00 (0.00 to 0.00)	0.00 (0.00 to 0.00)	0.00 (0.00 to 0.00)	0.00 (0.00 to 0.00)	0.00 (0.00 to 0.00)
Dengue	0.02 (0.01 to 0.04)	0.16 (0.07 to 0.33)	0.17 (0.07 to 0.34)	0.16 (0.07 to 0.31)	0.00 (0.00 to 0.00)	0.01 (0.00 to 0.01)	0.01 (0.00 to 0.01)	0.01 (0.00 to 0.01)
Yellow fever	0.00 (0.00 to 0.00)	0.00 (0.00 to 0.00)	0.00 (0.00 to 0.00)	0.00 (0.00 to 0.00)	0.00 (0.00 to 0.00)	0.00 (0.00 to 0.00)	0.00 (0.00 to 0.00)	0.00 (0.00 to 0.00)
Rabies	0.01 (0.00 to 0.01)	0.01 (0.01 to 0.02)	0.01 (0.00 to 0.01)	0.01 (0.01 to 0.02)	0.00 (0.00 to 0.00)	0.00 (0.00 to 0.00)	0.00 (0.00 to 0.00)	0.00 (0.00 to 0.00)
Intestinal nematode infections	0.00 (0.00 to 0.00)	0.00 (0.00 to 0.00)	0.00 (0.00 to 0.00)	0.00 (0.00 to 0.00)	0.00 (0.00 to 0.00)	0.00 (0.00 to 0.00)	0.00 (0.00 to 0.00)	0.00 (0.00 to 0.00)
Other neglected tropical diseases	1.31 (1.00 to 1.75)	4.30 (2.47 to 5.78)	5.97 (3.22 to 8.09)	3.98 (2.27 to 5.34)	0.04 (0.03 to 0.05)	0.23 (0.13 to 0.32)	0.34 (0.18 to 0.48)	0.21 (0.11 to 0.29)
**Maternal disorders**	**46.02 (28.56 to 69.64)**	**30.12 (18.29 to 45.55)**	**17.68 (10.65 to 26.46)**	**33.14 (19.82 to 50.01)**	**0.85 (0.54 to 1.30)**	**0.59 (0.35 to 0.88)**	**0.34 (0.21 to 0.53)**	**0.64 (0.39 to 0.97)**
Maternal hemorrhage	15.29 (8.53 to 24.84)	9.11 (4.92 to 14.98)	5.45 (2.86 to 8.93)	9.97 (5.39 to 16.53)	0.28 (0.16 to 0.45)	0.18 (0.10 to 0.28)	0.11 (0.06 to 0.17)	0.19 (0.10 to 0.31)
Maternal sepsis and other maternal infections	4.62 (1.87 to 8.81)	2.86 (1.17 to 5.50)	1.67 (0.67 to 3.32)	3.14 (1.27 to 6.06)	0.09 (0.04 to 0.16)	0.06 (0.02 to 0.11)	0.03 (0.01 to 0.07)	0.06 (0.03 to 0.13)
Maternal hypertensive disorders	6.46 (3.41 to 11.62)	4.28 (2.19 to 7.29)	2.43 (1.21 to 4.17)	4.71 (2.41 to 8.34)	0.12 (0.06 to 0.21)	0.08 (0.04 to 0.14)	0.05 (0.02 to 0.08)	0.09 (0.05 to 0.16)
Maternal obstructed labor and uterine rupture	3.24 (1.40 to 6.12)	2.23 (0.92 to 4.32)	1.17 (0.48 to 2.34)	2.49 (1.05 to 4.96)	0.06 (0.03 to 0.11)	0.04 (0.02 to 0.09)	0.02 (0.01 to 0.05)	0.05 (0.02 to 0.10)
Maternal abortion, miscarriage, and ectopic pregnancy	2.57 (1.09 to 4.94)	2.00 (0.84 to 3.84)	1.14 (0.49 to 2.23)	2.19 (0.95 to 4.26)	0.05 (0.02 to 0.09)	0.04 (0.02 to 0.08)	0.02 (0.01 to 0.05)	0.04 (0.02 to 0.09)

Indirect maternal deaths	4.74 (2.28 to 8.50)	3.41 (1.60 to 5.75)	1.91 (0.91 to 3.24)	3.80 (1.76 to 6.52)	0.09 (0.04 to 0.15)	0.06 (0.03 to 0.11)	0.04 (0.02 to 0.06)	0.07 (0.03 to 0.13)
Late maternal deaths	1.75 (0.64 to 3.77)	0.99 (0.33 to 2.10)	0.55 (0.19 to 1.15)	1.10 (0.37 to 2.28)	0.03 (0.01 to 0.07)	0.02 (0.01 to 0.04)	0.01 (0.00 to 0.02)	0.02 (0.01 to 0.04)
Maternal deaths aggravated by HIV/AIDS	0.01 (0.00 to 0.06)	0.09 (0.00 to 0.63)	0.05 (0.00 to 0.41)	0.10 (0.00 to 0.68)	0.00 (0.00 to 0.00)	0.00 (0.00 to 0.01)	0.00 (0.00 to 0.01)	0.00 (0.00 to 0.01)
Other maternal disorders	7.36 (3.89 to 12.39)	5.17 (2.62 to 8.70)	3.31 (1.70 to 5.66)	5.64 (2.85 to 9.68)	0.14 (0.07 to 0.23)	0.10 (0.05 to 0.17)	0.06 (0.03 to 0.11)	0.11 (0.06 to 0.19)

Northern Mariana Islands								
Cause	YLLs (actual counts)				Deaths (actual counts)			
	2016	2040 Forecast	2040 Better Scenario	2040 Worse Scenario	2016	2040 Forecast	2040 Better Scenario	2040 Worse Scenario
**Neonatal disorders**	**185.97 (83.26 to 339.22)**	**87.35 (26.22 to 234.48)**	**43.95 (12.79 to 124.13)**	**108.69 (31.72 to 295.92)**	**2.15 (0.96 to 3.92)**	**1.01 (0.30 to 2.71)**	**0.51 (0.15 to 1.43)**	**1.26 (0.37 to 3.42)**
Neonatal preterm birth	78.62 (34.74 to 150.32)	33.41 (10.01 to 92.94)	11.27 (3.49 to 30.32)	42.26 (12.66 to 115.36)	0.91 (0.40 to 1.74)	0.39 (0.12 to 1.07)	0.13 (0.04 to 0.35)	0.49 (0.15 to 1.33)
Neonatal encephalopathy due to birth asphyxia and trauma	43.83 (18.35 to 86.49)	17.60 (4.64 to 50.86)	7.30 (1.90 to 20.97)	22.82 (5.75 to 69.83)	0.51 (0.21 to 1.00)	0.20 (0.05 to 0.59)	0.08 (0.02 to 0.24)	0.26 (0.07 to 0.81)
Neonatal sepsis and other neonatal infections	20.94 (8.31 to 42.33)	11.28 (3.05 to 33.42)	5.68 (1.54 to 16.79)	14.19 (3.76 to 42.49)	0.24 (0.10 to 0.49)	0.13 (0.04 to 0.39)	0.07 (0.02 to 0.19)	0.16 (0.04 to 0.49)
Hemolytic disease and other neonatal jaundice	2.16 (0.80 to 4.65)	0.73 (0.14 to 2.56)	0.35 (0.07 to 1.20)	0.93 (0.18 to 3.13)	0.03 (0.01 to 0.05)	0.01 (0.00 to 0.03)	0.00 (0.00 to 0.01)	0.01 (0.00 to 0.04)
Other neonatal disorders	40.41 (17.01 to 77.36)	24.32 (6.35 to 77.38)	19.36 (5.24 to 58.96)	28.49 (7.37 to 90.69)	0.47 (0.20 to 0.89)	0.28 (0.07 to 0.89)	0.22 (0.06 to 0.68)	0.33 (0.09 to 1.05)
**Nutritional deficiencies**	**28.84 (20.18 to 41.91)**	**49.92 (41.26 to 59.86)**	**66.05 (54.70 to 77.85)**	**46.59 (37.99 to 56.71)**	**1.01 (0.82 to 1.22)**	**3.25 (2.58 to 3.93)**	**4.75 (3.82 to 5.65)**	**2.90 (2.27 to 3.58)**
Protein-energy malnutrition	27.18 (18.97 to 39.86)	46.19 (37.10 to 56.22)	62.70 (51.44 to 74.49)	42.85 (34.11 to 52.58)	0.97 (0.79 to 1.18)	3.07 (2.36 to 3.79)	4.59 (3.64 to 5.52)	2.73 (2.09 to 3.38)
Iodine deficiency	0.27 (0.01 to 0.91)	1.88 (0.04 to 6.82)	1.83 (0.02 to 7.87)	1.84 (0.04 to 6.56)	0.01 (0.00 to 0.02)	0.10 (0.00 to 0.44)	0.11 (0.00 to 0.54)	0.10 (0.00 to 0.41)
Dietary iron deficiency	0.60 (0.44 to 0.79)	0.63 (0.44 to 0.91)	0.69 (0.49 to 0.98)	0.63 (0.44 to 0.90)	0.01 (0.01 to 0.01)	0.02 (0.01 to 0.02)	0.02 (0.01 to 0.03)	0.02 (0.01 to 0.02)
Other nutritional deficiencies	0.80 (0.58 to 1.05)	1.22 (0.84 to 1.70)	0.83 (0.57 to 1.16)	1.27 (0.89 to 1.78)	0.02 (0.01 to 0.03)	0.06 (0.04 to 0.08)	0.04 (0.03 to 0.06)	0.06 (0.04 to 0.08)
**Other communicable, maternal, neonatal, and nutritional diseases**	**244.79 (132.54 to 422.49)**	**166.69 (121.09 to 224.60)**	**108.67 (81.03 to 141.51)**	**180.61 (130.96 to 247.14)**	**4.33 (2.86 to 6.49)**	**5.12 (3.82 to 6.66)**	**3.55 (2.68 to 4.53)**	**5.42 (4.04 to 7.10)**
Sexually transmitted infections excluding HIV	122.74 (12.45 to 303.32)	23.11 (8.40 to 54.41)	13.63 (6.15 to 28.55)	26.81 (8.92 to 66.08)	1.53 (0.26 to 3.64)	0.38 (0.19 to 0.76)	0.25 (0.14 to 0.45)	0.43 (0.20 to 0.89)
Syphilis	114.08 (2.38 to 294.55)	16.05 (1.78 to 47.24)	7.68 (1.14 to 22.17)	19.52 (1.90 to 58.23)	1.35 (0.05 to 3.45)	0.21 (0.04 to 0.58)	0.10 (0.03 to 0.28)	0.25 (0.05 to 0.71)
Chlamydial infection	1.68 (1.10 to 2.39)	1.39 (0.88 to 2.10)	1.18 (0.74 to 1.80)	1.44 (0.92 to 2.18)	0.04 (0.02 to 0.05)	0.03 (0.02 to 0.05)	0.03 (0.02 to 0.04)	0.03 (0.02 to 0.05)
Gonococcal infection	4.49 (2.96 to 6.42)	3.64 (2.26 to 5.61)	3.08 (1.92 to 4.72)	3.76 (2.33 to 5.80)	0.10 (0.06 to 0.14)	0.09 (0.06 to 0.14)	0.08 (0.05 to 0.12)	0.09 (0.06 to 0.14)
Other sexually transmitted infections	2.49 (1.64 to 3.56)	2.02 (1.28 to 3.12)	1.70 (1.06 to 2.59)	2.09 (1.32 to 3.19)	0.05 (0.03 to 0.08)	0.05 (0.03 to 0.08)	0.04 (0.03 to 0.06)	0.05 (0.03 to 0.08)
Acute hepatitis	78.88 (57.94 to 103.19)	81.67 (55.75 to 115.89)	37.48 (24.39 to 54.18)	91.51 (62.52 to 128.90)	1.87 (1.37 to 2.47)	2.61 (1.80 to 3.66)	1.22 (0.82 to 1.73)	2.89 (2.03 to 4.04)
Acute hepatitis A	1.69 (1.08 to 2.44)	1.26 (0.73 to 2.08)	0.75 (0.43 to 1.25)	1.36 (0.79 to 2.23)	0.03 (0.02 to 0.05)	0.04 (0.02 to 0.06)	0.02 (0.01 to 0.04)	0.04 (0.02 to 0.07)
Acute hepatitis B	70.54 (51.00 to 92.87)	73.25 (48.81 to 104.70)	33.25 (21.43 to 48.62)	82.20 (55.13 to 117.18)	1.68 (1.22 to 2.24)	2.32 (1.57 to 3.31)	1.07 (0.70 to 1.54)	2.59 (1.77 to 3.68)

Acute hepatitis C	1.20 (0.76 to 1.79)	1.86 (1.08 to 2.99)	0.89 (0.51 to 1.48)	2.07 (1.20 to 3.37)	0.03 (0.02 to 0.05)	0.06 (0.04 to 0.10)	0.03 (0.02 to 0.05)	0.07 (0.04 to 0.11)
Acute hepatitis E	5.46 (3.44 to 7.80)	5.30 (2.86 to 9.01)	2.59 (1.36 to 4.50)	5.89 (3.16 to 9.93)	0.12 (0.07 to 0.18)	0.18 (0.09 to 0.31)	0.09 (0.05 to 0.16)	0.19 (0.10 to 0.34)
Other unspecified infectious diseases	43.16 (29.16 to 57.89)	61.91 (42.94 to 79.96)	57.56 (41.87 to 73.74)	62.28 (42.76 to 81.30)	0.93 (0.64 to 1.23)	2.13 (1.45 to 2.69)	2.08 (1.50 to 2.61)	2.10 (1.41 to 2.67)
**Non-communicable diseases**	**7,396.96 (5,987.97 to 8,956.89)**	**20,830.90 (15,462.11 to 28,139.50)**	**12,564.12 (9,499.03 to 17,370.25)**	**26,919.38 (19,898.94 to 35,751.17)**	**224.72 (186.52 to 266.66)**	**922.16 (728.95 to 1,189.45)**	**585.42 (463.88 to 790.62)**	**1,153.40 (905.38 to 1,458.59)**

Northern Mariana Islands								
Cause	YLLs (actual counts)				Deaths (actual counts)			
	2016	2040 Forecast	2040 Better Scenario	2040 Worse Scenario	2016	2040 Forecast	2040 Better Scenario	2040 Worse Scenario
**Neoplasms**	**1,827.58 (1,486.94 to 2,207.04)**	**5,592.19 (4,128.53 to 7,452.97)**	**3,870.50 (2,925.76 to 5,119.77)**	**6,953.11 (4,995.96 to 9,562.74)**	**53.63 (44.35 to 64.20)**	**237.66 (179.89 to 311.17)**	**168.53 (131.28 to 218.29)**	**289.67 (213.44 to 391.22)**
Lip and oral cavity cancer	68.36 (52.25 to 87.09)	209.24 (115.63 to 355.63)	113.48 (67.13 to 180.18)	279.68 (148.24 to 489.96)	2.02 (1.59 to 2.53)	8.79 (5.13 to 14.56)	4.95 (3.11 to 7.46)	11.41 (6.32 to 19.25)
Nasopharynx cancer	27.49 (20.15 to 35.54)	96.66 (48.73 to 183.87)	41.00 (21.79 to 74.78)	137.22 (66.74 to 269.95)	0.77 (0.57 to 0.98)	3.55 (1.82 to 6.54)	1.55 (0.85 to 2.67)	4.93 (2.43 to 9.23)
Other pharynx cancer	18.66 (14.45 to 24.14)	57.79 (36.47 to 92.19)	50.85 (34.63 to 74.16)	60.01 (36.70 to 99.75)	0.54 (0.42 to 0.69)	2.25 (1.46 to 3.55)	2.05 (1.42 to 2.99)	2.30 (1.45 to 3.78)
Esophageal cancer	24.06 (18.05 to 30.72)	94.35 (46.85 to 172.34)	67.97 (36.91 to 118.13)	117.73 (55.58 to 219.87)	0.78 (0.61 to 0.98)	4.10 (2.14 to 7.17)	3.04 (1.73 to 5.08)	5.00 (2.51 to 9.12)
Stomach cancer	105.16 (82.94 to 130.26)	206.79 (151.66 to 275.38)	115.47 (85.20 to 152.88)	238.09 (169.32 to 329.58)	3.16 (2.56 to 3.84)	9.15 (6.91 to 11.92)	5.27 (3.99 to 6.76)	10.34 (7.51 to 13.84)
Colon and rectum cancer	108.09 (82.66 to 137.46)	380.90 (249.61 to 588.49)	248.39 (164.58 to 371.28)	425.16 (270.85 to 667.11)	3.35 (2.65 to 4.16)	17.43 (11.53 to 26.66)	11.83 (8.03 to 17.49)	19.02 (12.29 to 29.07)
Liver cancer	127.61 (97.03 to 164.97)	356.27 (223.20 to 556.42)	365.02 (232.32 to 556.59)	454.17 (261.10 to 759.42)	3.63 (2.83 to 4.62)	14.39 (9.19 to 21.88)	15.16 (10.06 to 22.25)	17.87 (10.41 to 28.98)
Liver cancer due to hepatitis B	75.74 (57.76 to 99.71)	187.05 (118.64 to 305.35)	192.60 (123.88 to 302.57)	237.19 (136.03 to 406.61)	2.07 (1.59 to 2.70)	7.23 (4.64 to 11.30)	7.63 (5.07 to 11.58)	8.95 (5.24 to 15.06)
Liver cancer due to hepatitis C	14.24 (10.60 to 18.89)	56.38 (35.25 to 87.57)	58.71 (37.83 to 89.64)	69.90 (40.57 to 114.39)	0.48 (0.37 to 0.63)	2.55 (1.67 to 3.89)	2.74 (1.85 to 4.08)	3.07 (1.86 to 4.90)
Liver cancer due to alcohol use	9.20 (6.56 to 12.60)	33.63 (20.30 to 54.64)	31.65 (19.55 to 50.16)	43.74 (24.19 to 75.29)	0.28 (0.20 to 0.38)	1.36 (0.85 to 2.18)	1.32 (0.85 to 2.04)	1.73 (0.99 to 2.89)
Liver cancer due to other causes	28.44 (20.19 to 38.29)	79.21 (42.65 to 140.84)	82.06 (46.48 to 141.67)	103.33 (50.95 to 197.22)	0.80 (0.58 to 1.06)	3.25 (1.83 to 5.76)	3.46 (2.03 to 5.91)	4.13 (2.11 to 7.87)
Gallbladder and biliary tract cancer	7.48 (5.81 to 9.51)	19.23 (15.25 to 24.03)	17.76 (13.82 to 22.12)	18.86 (15.01 to 23.34)	0.25 (0.19 to 0.31)	0.85 (0.68 to 1.06)	0.81 (0.64 to 1.01)	0.82 (0.66 to 1.02)
Pancreatic cancer	62.39 (49.97 to 76.16)	271.06 (182.70 to 389.97)	245.73 (169.16 to 352.13)	300.93 (198.69 to 442.61)	2.03 (1.67 to 2.45)	11.78 (8.09 to 16.95)	10.94 (7.66 to 15.39)	12.86 (8.58 to 18.95)
Larynx cancer	19.59 (14.87 to 24.66)	66.86 (32.61 to 120.08)	32.27 (16.10 to 55.58)	91.77 (43.00 to 168.72)	0.63 (0.49 to 0.77)	2.98 (1.51 to 5.28)	1.46 (0.78 to 2.45)	4.01 (1.98 to 7.26)
Tracheal, bronchus, and lung cancer	418.90 (330.11 to 521.02)	1,754.08 (1,056.54 to 2,725.02)	1,008.29 (619.23 to 1,539.12)	2,571.94 (1,530.60 to 4,070.05)	14.12 (11.41 to 17.29)	79.24 (47.55 to 120.19)	46.44 (28.25 to 70.56)	112.93 (66.97 to 174.21)
Malignant skin melanoma	9.65 (6.72 to 13.36)	27.25 (19.24 to 36.97)	41.90 (29.52 to 57.12)	24.61 (17.76 to 33.33)	0.29 (0.20 to 0.40)	1.16 (0.83 to 1.54)	1.85 (1.33 to 2.47)	1.03 (0.75 to 1.34)
Non-melanoma skin cancer	18.08 (13.66 to 23.30)	57.15 (44.22 to 72.80)	35.69 (27.14 to 45.76)	59.02 (46.12 to 73.64)	0.61 (0.49 to 0.76)	2.71 (2.21 to 3.26)	1.79 (1.47 to 2.17)	2.72 (2.25 to 3.23)
Breast cancer	186.25 (132.45 to 250.56)	446.57 (278.01 to 669.50)	282.62 (179.40 to 424.15)	514.89 (310.46 to 811.62)	5.01 (3.60 to 6.68)	15.96 (10.20 to 23.89)	10.34 (6.71 to 15.51)	18.14 (11.26 to 28.16)
Cervical cancer	90.16 (55.31 to 127.22)	189.29 (105.14 to 296.25)	104.34 (59.74 to 159.94)	218.26 (119.42 to 352.85)	2.35 (1.44 to 3.29)	6.37 (3.53 to 9.92)	3.54 (2.01 to 5.38)	7.28 (3.98 to 11.71)

Uterine cancer	38.01 (26.48 to 52.56)	137.43 (92.12 to 186.89)	98.65 (66.81 to 134.29)	143.09 (96.87 to 195.95)	1.14 (0.81 to 1.55)	5.04 (3.46 to 6.80)	3.68 (2.56 to 4.97)	5.22 (3.63 to 6.97)
Ovarian cancer	23.02 (16.54 to 30.71)	80.73 (51.84 to 122.73)	62.38 (40.48 to 93.30)	87.11 (55.08 to 134.28)	0.66 (0.48 to 0.88)	2.86 (1.86 to 4.35)	2.24 (1.48 to 3.34)	3.07 (1.96 to 4.75)
Prostate cancer	27.87 (20.16 to 36.41)	229.19 (165.23 to 293.83)	179.56 (129.10 to 233.00)	229.02 (163.93 to 291.34)	1.39 (1.00 to 1.73)	13.98 (10.09 to 17.41)	11.38 (8.17 to 14.07)	13.68 (9.89 to 17.06)
Testicular cancer	3.15 (2.00 to 4.38)	3.18 (2.19 to 4.38)	2.12 (1.46 to 2.94)	3.31 (2.27 to 4.48)	0.07 (0.04 to 0.09)	0.10 (0.07 to 0.13)	0.07 (0.05 to 0.09)	0.10 (0.07 to 0.13)

Northern Mariana Islands								
Cause	YLLs (actual counts)				Deaths (actual counts)			
	2016	2040 Forecast	2040 Better Scenario	2040 Worse Scenario	2016	2040 Forecast	2040 Better Scenario	2040 Worse Scenario
Kidney cancer	26.65 (19.46 to 35.28)	83.57 (53.31 to 127.77)	78.10 (51.98 to 115.31)	90.06 (55.65 to 143.08)	0.75 (0.55 to 0.98)	3.51 (2.32 to 5.22)	3.36 (2.33 to 4.81)	3.71 (2.38 to 5.70)
Bladder cancer	14.83 (11.90 to 18.32)	52.79 (33.39 to 82.53)	37.41 (24.45 to 56.94)	62.27 (37.61 to 100.32)	0.53 (0.44 to 0.65)	2.46 (1.61 to 3.82)	1.80 (1.22 to 2.68)	2.84 (1.77 to 4.49)
Brain and nervous system cancer	52.54 (40.73 to 67.52)	101.89 (78.60 to 139.54)	85.13 (64.96 to 117.44)	102.95 (79.74 to 141.64)	1.19 (0.91 to 1.58)	3.44 (2.69 to 4.79)	2.96 (2.29 to 4.15)	3.42 (2.68 to 4.76)
Thyroid cancer	4.22 (3.36 to 5.27)	15.17 (11.00 to 20.79)	11.05 (8.17 to 15.15)	17.40 (12.24 to 24.60)	0.14 (0.11 to 0.17)	0.66 (0.49 to 0.91)	0.50 (0.37 to 0.68)	0.74 (0.53 to 1.05)
Mesothelioma	2.44 (1.81 to 3.15)	6.67 (5.05 to 8.40)	6.95 (5.18 to 8.86)	6.43 (4.91 to 8.02)	0.07 (0.05 to 0.09)	0.26 (0.20 to 0.32)	0.28 (0.21 to 0.35)	0.25 (0.19 to 0.31)
Hodgkin lymphoma	5.40 (3.78 to 7.19)	6.48 (4.29 to 8.83)	3.48 (2.25 to 4.70)	6.98 (4.81 to 9.44)	0.12 (0.08 to 0.16)	0.22 (0.14 to 0.29)	0.12 (0.08 to 0.16)	0.24 (0.16 to 0.31)
Non-Hodgkin lymphoma	65.14 (49.47 to 82.88)	111.74 (84.01 to 144.56)	85.00 (64.73 to 109.43)	120.47 (91.45 to 157.58)	1.54 (1.18 to 1.94)	4.29 (3.34 to 5.41)	3.39 (2.64 to 4.26)	4.53 (3.49 to 5.76)
Multiple myeloma	32.85 (25.25 to 41.90)	113.96 (86.81 to 148.38)	98.74 (75.01 to 127.94)	120.93 (89.83 to 161.65)	1.02 (0.80 to 1.27)	4.82 (3.76 to 6.07)	4.31 (3.38 to 5.40)	5.00 (3.86 to 6.44)
Leukemia	133.76 (100.85 to 172.09)	219.56 (153.08 to 312.08)	166.38 (116.34 to 237.32)	256.69 (173.72 to 377.72)	3.01 (2.30 to 3.86)	8.02 (5.67 to 11.34)	6.31 (4.49 to 8.83)	9.18 (6.27 to 13.48)
Acute lymphoid leukemia	29.95 (21.82 to 39.77)	36.38 (25.98 to 50.65)	24.52 (17.62 to 34.20)	42.98 (30.24 to 61.04)	0.57 (0.43 to 0.74)	0.98 (0.71 to 1.36)	0.69 (0.49 to 0.95)	1.16 (0.81 to 1.65)
Chronic lymphoid leukemia	1.91 (1.09 to 2.63)	7.05 (3.48 to 11.32)	6.07 (2.99 to 9.62)	8.02 (3.92 to 13.18)	0.06 (0.04 to 0.09)	0.30 (0.16 to 0.46)	0.27 (0.14 to 0.40)	0.33 (0.17 to 0.53)
Acute myeloid leukemia	50.81 (37.16 to 71.76)	94.30 (63.60 to 142.28)	87.79 (59.92 to 131.02)	106.33 (69.08 to 167.69)	1.11 (0.81 to 1.59)	3.38 (2.34 to 5.09)	3.27 (2.32 to 4.81)	3.73 (2.47 to 5.82)
Chronic myeloid leukemia	22.27 (14.70 to 31.52)	26.18 (16.02 to 41.11)	11.04 (6.83 to 16.88)	33.51 (20.11 to 53.29)	0.55 (0.37 to 0.77)	0.92 (0.58 to 1.39)	0.40 (0.25 to 0.60)	1.15 (0.72 to 1.78)
Other leukemia	28.81 (20.69 to 38.59)	55.65 (34.26 to 84.34)	36.95 (22.95 to 55.06)	65.84 (39.19 to 103.22)	0.71 (0.51 to 0.94)	2.44 (1.54 to 3.69)	1.69 (1.08 to 2.51)	2.80 (1.68 to 4.41)
Other neoplasms	105.79 (82.88 to 130.28)	196.33 (158.91 to 238.00)	184.77 (147.10 to 224.56)	194.06 (157.78 to 234.06)	2.47 (1.94 to 3.06)	7.28 (5.96 to 8.70)	7.12 (5.77 to 8.45)	7.05 (5.79 to 8.36)
**Cardiovascular diseases**	**2,426.51 (1,922.08 to 2,950.75)**	**5,484.96 (3,667.74 to 8,186.41)**	**2,157.81 (1,534.10 to 3,094.58)**	**8,404.98 (5,454.33 to 12,663.02)**	**76.64 (62.93 to 91.60)**	**243.83 (174.84 to 345.48)**	**105.48 (79.68 to 143.51)**	**353.62 (246.12 to 501.97)**
Rheumatic heart disease	92.02 (70.64 to 116.36)	81.40 (63.23 to 106.81)	46.50 (35.67 to 60.63)	87.80 (66.97 to 113.77)	2.26 (1.76 to 2.83)	3.17 (2.55 to 4.01)	1.90 (1.53 to 2.40)	3.34 (2.66 to 4.21)
Ischemic heart disease	1,102.90 (861.38 to 1,375.74)	2,324.16 (1,230.07 to 4,123.20)	793.24 (428.81 to 1,494.31)	3,922.50 (2,020.91 to 6,892.34)	35.69 (28.90 to 43.23)	101.30 (57.14 to 174.76)	38.01 (21.95 to 67.11)	161.27 (89.36 to 271.43)
Stroke	794.49 (627.94 to 981.12)	1,848.22 (1,005.62 to 3,207.09)	543.65 (327.14 to 915.79)	3,000.75 (1,630.84 to 5,178.09)	25.25 (20.46 to 30.54)	82.78 (49.69 to 139.67)	27.48 (17.47 to 47.39)	127.02 (74.58 to 210.66)
Ischemic stroke	145.24 (114.96 to 180.98)	393.78 (208.00 to 833.15)	141.18 (78.62 to 307.69)	599.30 (308.34 to 1,170.72)	5.78 (4.77 to 6.89)	21.07 (11.47 to 46.68)	8.44 (4.72 to 19.09)	30.32 (15.79 to 63.91)

Intracerebral hemorrhage	649.25 (506.06 to 806.24)	1,454.44 (737.62 to 2,704.64)	402.47 (226.06 to 704.57)	2,401.45 (1,220.05 to 4,387.70)	19.47 (15.63 to 23.79)	61.70 (34.94 to 110.94)	19.04 (11.56 to 31.60)	96.69 (53.49 to 174.25)
Hypertensive heart disease	72.67 (53.92 to 102.98)	291.03 (205.03 to 429.74)	199.45 (146.53 to 284.86)	341.76 (223.73 to 560.99)	2.49 (1.90 to 3.49)	13.68 (9.93 to 20.18)	9.91 (7.51 to 14.08)	15.54 (10.55 to 25.20)
Cardiomyopathy and myocarditis	134.59 (94.77 to 173.71)	248.72 (164.96 to 344.45)	146.93 (99.75 to 197.32)	269.72 (178.83 to 383.39)	3.61 (2.53 to 4.62)	10.19 (6.79 to 13.90)	6.26 (4.19 to 8.29)	10.81 (7.19 to 15.04)
Myocarditis	22.59 (14.14 to 31.73)	51.47 (31.08 to 70.71)	47.83 (30.72 to 64.36)	50.68 (30.26 to 69.99)	0.62 (0.37 to 0.85)	2.11 (1.27 to 2.84)	2.03 (1.30 to 2.68)	2.04 (1.22 to 2.77)

Northern Mariana Islands								
Cause	YLLs (actual counts)				Deaths (actual counts)			
	2016	2040 Forecast	2040 Better Scenario	2040 Worse Scenario	2016	2040 Forecast	2040 Better Scenario	2040 Worse Scenario
Alcoholic cardiomyopathy	10.41 (5.94 to 14.66)	20.34 (11.11 to 28.91)	18.28 (9.66 to 26.06)	20.31 (11.06 to 28.98)	0.29 (0.16 to 0.40)	0.82 (0.44 to 1.17)	0.77 (0.40 to 1.08)	0.80 (0.43 to 1.15)
Other cardiomyopathy	101.59 (71.24 to 131.85)	176.90 (110.85 to 261.46)	80.83 (48.61 to 119.61)	198.72 (121.22 to 300.94)	2.70 (1.90 to 3.49)	7.26 (4.55 to 10.49)	3.47 (2.11 to 5.12)	7.97 (5.00 to 11.60)
Atrial fibrillation and flutter	6.85 (5.31 to 8.73)	42.90 (31.98 to 56.40)	36.99 (27.96 to 47.73)	44.37 (32.18 to 59.32)	0.53 (0.41 to 0.68)	3.12 (2.31 to 4.07)	2.90 (2.18 to 3.72)	3.10 (2.30 to 4.08)
Aortic aneurysm	34.94 (27.65 to 43.58)	105.25 (83.26 to 133.12)	85.54 (68.29 to 106.88)	113.74 (89.12 to 147.03)	1.26 (1.03 to 1.52)	5.03 (4.08 to 6.13)	4.36 (3.53 to 5.24)	5.25 (4.21 to 6.53)
Peripheral artery disease	7.64 (4.23 to 10.49)	38.38 (20.09 to 56.94)	32.54 (17.05 to 47.24)	42.49 (21.92 to 64.77)	0.39 (0.22 to 0.51)	2.13 (1.11 to 3.03)	1.99 (1.05 to 2.78)	2.26 (1.18 to 3.27)
Endocarditis	37.08 (26.95 to 47.98)	82.39 (56.69 to 112.02)	70.77 (49.09 to 95.72)	84.40 (57.68 to 117.52)	1.00 (0.73 to 1.28)	3.34 (2.29 to 4.48)	3.03 (2.11 to 4.05)	3.35 (2.29 to 4.52)
Other cardiovascular and circulatory diseases	143.33 (101.92 to 183.70)	422.52 (286.27 to 566.24)	202.20 (139.11 to 263.26)	497.46 (327.45 to 686.70)	4.18 (2.98 to 5.26)	19.07 (13.14 to 24.97)	9.64 (6.60 to 12.37)	21.69 (14.60 to 29.33)
**Chronic respiratory diseases**	**474.30 (369.19 to 588.16)**	**1,238.50 (837.99 to 1,754.13)**	**829.38 (600.78 to 1,112.89)**	**1,750.07 (1,140.86 to 2,518.48)**	**14.70 (11.93 to 17.79)**	**56.94 (40.10 to 78.66)**	**37.63 (28.10 to 49.46)**	**78.47 (53.26 to 110.45)**
Chronic obstructive pulmonary disease	286.51 (220.85 to 356.71)	910.88 (561.46 to 1,372.70)	453.26 (286.42 to 672.80)	1,391.57 (829.94 to 2,111.44)	9.60 (7.81 to 11.70)	43.98 (28.66 to 63.88)	22.57 (14.96 to 32.60)	64.49 (40.81 to 94.75)
Pneumoconiosis	2.79 (2.10 to 3.67)	6.44 (4.83 to 8.36)	6.50 (4.84 to 8.66)	6.47 (4.85 to 8.53)	0.12 (0.09 to 0.15)	0.38 (0.28 to 0.51)	0.38 (0.29 to 0.50)	0.37 (0.27 to 0.50)
Silicosis	0.28 (0.17 to 0.43)	0.46 (0.26 to 0.73)	0.18 (0.10 to 0.30)	0.51 (0.30 to 0.79)	0.01 (0.01 to 0.02)	0.03 (0.02 to 0.05)	0.01 (0.01 to 0.02)	0.03 (0.02 to 0.05)
Asbestosis	0.84 (0.58 to 1.18)	2.96 (1.80 to 4.35)	4.25 (2.95 to 5.88)	2.69 (1.60 to 4.04)	0.03 (0.02 to 0.04)	0.16 (0.09 to 0.24)	0.24 (0.16 to 0.34)	0.14 (0.08 to 0.22)
Coal workers pneumoconiosis	0.01 (0.01 to 0.02)	0.04 (0.02 to 0.06)	0.03 (0.01 to 0.05)	0.04 (0.02 to 0.07)	0.00 (0.00 to 0.00)	0.00 (0.00 to 0.00)	0.00 (0.00 to 0.00)	0.00 (0.00 to 0.00)
Other pneumoconiosis	1.66 (1.20 to 2.23)	2.98 (1.97 to 4.41)	2.05 (1.30 to 3.04)	3.23 (2.18 to 4.70)	0.07 (0.06 to 0.10)	0.18 (0.12 to 0.28)	0.12 (0.08 to 0.20)	0.19 (0.13 to 0.29)
Asthma	67.99 (50.82 to 90.40)	76.57 (46.16 to 124.47)	30.15 (17.71 to 50.17)	112.40 (65.04 to 185.02)	2.00 (1.51 to 2.58)	3.25 (2.02 to 5.17)	1.33 (0.82 to 2.10)	4.59 (2.77 to 7.39)
Interstitial lung disease and pulmonary sarcoidosis	76.75 (56.35 to 100.26)	142.45 (106.16 to 184.55)	245.60 (186.55 to 311.61)	129.01 (96.57 to 169.18)	1.90 (1.43 to 2.45)	5.45 (4.06 to 7.13)	9.72 (7.61 to 12.12)	4.85 (3.66 to 6.23)
Other chronic respiratory diseases	40.25 (22.70 to 59.14)	102.16 (52.93 to 167.10)	93.86 (49.25 to 151.36)	110.61 (56.36 to 183.95)	1.09 (0.62 to 1.59)	3.89 (2.00 to 6.32)	3.62 (1.89 to 5.67)	4.16 (2.12 to 6.87)
**Cirrhosis and other chronic liver diseases**	**544.41 (407.45 to 712.36)**	**968.07 (741.44 to 1,219.17)**	**655.42 (503.00 to 841.40)**	**1,007.96 (794.63 to 1,268.45)**	**13.75 (10.44 to 17.85)**	**34.40 (27.60 to 42.16)**	**23.97 (19.05 to 29.52)**	**35.29 (28.63 to 43.10)**
Cirrhosis and other chronic liver diseases due to hepatitis B	225.38 (161.98 to 301.60)	410.79 (308.33 to 525.91)	283.52 (210.74 to 371.82)	423.79 (322.81 to 542.35)	5.82 (4.27 to 7.70)	14.98 (11.66 to 18.75)	10.61 (8.24 to 13.50)	15.24 (12.01 to 19.08)
Cirrhosis and other chronic liver diseases due to hepatitis C	91.20 (66.29 to 121.55)	163.39 (121.94 to 215.39)	109.78 (81.31 to 144.95)	169.59 (129.55 to 220.06)	2.33 (1.72 to 3.08)	5.70 (4.43 to 7.27)	3.92 (3.03 to 5.03)	5.85 (4.61 to 7.31)

Cirrhosis and other chronic liver diseases due to alcohol use	80.32 (58.08 to 108.08)	156.39 (116.75 to 201.65)	100.71 (75.27 to 131.79)	166.58 (127.12 to 214.02)	2.15 (1.58 to 2.86)	5.66 (4.41 to 7.13)	3.77 (2.93 to 4.77)	5.94 (4.70 to 7.48)
Cirrhosis and other chronic liver diseases due to other causes	147.51 (112.59 to 189.62)	237.51 (182.53 to 300.91)	161.41 (124.70 to 207.33)	248.00 (194.21 to 312.44)	3.44 (2.66 to 4.37)	8.05 (6.40 to 9.89)	5.67 (4.47 to 6.98)	8.27 (6.65 to 10.12)
**Digestive diseases**	**179.73 (138.02 to 225.93)**	**404.48 (317.45 to 512.23)**	**278.53 (221.34 to 350.76)**	**448.65 (347.58 to 574.32)**	**5.05 (3.98 to 6.23)**	**17.73 (14.26 to 22.01)**	**12.82 (10.45 to 15.70)**	**19.18 (15.21 to 24.10)**
Peptic ulcer disease	30.46 (23.69 to 38.52)	53.99 (40.48 to 69.65)	24.02 (18.15 to 30.61)	62.06 (47.08 to 79.75)	0.94 (0.76 to 1.16)	2.43 (1.91 to 3.08)	1.14 (0.90 to 1.42)	2.73 (2.12 to 3.42)

Northern Mariana Islands								
Cause	YLLs (actual counts)				Deaths (actual counts)			
	2016	2040 Forecast	2040 Better Scenario	2040 Worse Scenario	2016	2040 Forecast	2040 Better Scenario	2040 Worse Scenario
Gastritis and duodenitis	0.56 (0.40 to 0.75)	1.70 (1.18 to 2.31)	1.02 (0.71 to 1.38)	1.76 (1.23 to 2.40)	0.02 (0.01 to 0.02)	0.08 (0.06 to 0.11)	0.05 (0.04 to 0.07)	0.08 (0.06 to 0.11)
Appendicitis	13.39 (9.70 to 17.38)	19.34 (13.89 to 25.34)	9.49 (6.99 to 12.56)	21.03 (15.80 to 27.56)	0.33 (0.25 to 0.42)	0.75 (0.56 to 0.97)	0.39 (0.29 to 0.50)	0.80 (0.62 to 1.02)
Paralytic ileus and intestinal obstruction	16.24 (12.06 to 22.80)	47.46 (36.90 to 63.81)	47.26 (36.47 to 63.28)	45.79 (35.78 to 61.71)	0.45 (0.35 to 0.62)	2.19 (1.69 to 2.91)	2.30 (1.77 to 3.01)	2.05 (1.58 to 2.73)
Inguinal, femoral, and abdominal hernia	1.62 (1.20 to 2.19)	3.67 (2.67 to 5.18)	2.45 (1.77 to 3.37)	3.74 (2.68 to 5.22)	0.05 (0.04 to 0.07)	0.19 (0.13 to 0.26)	0.13 (0.09 to 0.18)	0.19 (0.13 to 0.26)
Inflammatory bowel disease	16.88 (11.18 to 24.05)	32.64 (23.52 to 44.75)	29.94 (21.61 to 41.06)	31.90 (23.09 to 44.06)	0.42 (0.29 to 0.58)	1.32 (1.00 to 1.74)	1.26 (0.95 to 1.66)	1.26 (0.97 to 1.66)
Vascular intestinal disorders	10.43 (7.70 to 13.46)	36.32 (28.31 to 45.38)	28.94 (22.58 to 36.49)	36.09 (28.33 to 45.32)	0.35 (0.27 to 0.44)	1.78 (1.45 to 2.20)	1.48 (1.19 to 1.83)	1.73 (1.40 to 2.15)
Gallbladder and biliary diseases	35.30 (26.62 to 45.32)	86.49 (48.65 to 144.14)	48.18 (27.71 to 80.35)	119.54 (65.85 to 199.08)	1.04 (0.80 to 1.30)	3.95 (2.29 to 6.47)	2.30 (1.37 to 3.69)	5.26 (2.98 to 8.51)
Pancreatitis	20.33 (14.22 to 27.85)	40.71 (24.98 to 72.69)	22.40 (14.36 to 36.06)	44.28 (26.73 to 85.51)	0.51 (0.36 to 0.69)	1.49 (0.96 to 2.57)	0.87 (0.60 to 1.38)	1.59 (1.03 to 2.86)
Other digestive diseases	34.53 (26.69 to 44.23)	82.16 (64.16 to 105.61)	64.83 (50.41 to 83.36)	82.46 (64.33 to 107.20)	0.95 (0.73 to 1.21)	3.55 (2.78 to 4.55)	2.90 (2.28 to 3.69)	3.50 (2.72 to 4.53)
**Neurological disorders**	**151.35 (124.03 to 181.13)**	**696.63 (515.03 to 984.47)**	**705.16 (528.79 to 975.03)**	**743.04 (523.76 to 1,099.11)**	**8.07 (6.77 to 9.61)**	**45.58 (33.16 to 65.10)**	**49.28 (37.13 to 67.85)**	**47.34 (32.82 to 70.33)**
Alzheimer's disease and other dementias	67.43 (54.74 to 83.39)	502.62 (337.29 to 775.64)	508.26 (348.39 to 764.33)	553.76 (347.84 to 899.61)	5.94 (4.88 to 7.26)	37.27 (25.35 to 56.57)	40.12 (28.35 to 58.66)	39.51 (25.21 to 62.34)
Parkinson's disease	6.71 (5.07 to 8.73)	56.19 (41.36 to 72.82)	66.89 (49.55 to 86.84)	51.55 (38.23 to 66.92)	0.41 (0.32 to 0.53)	3.72 (2.73 to 4.81)	4.61 (3.44 to 5.93)	3.35 (2.42 to 4.36)
Epilepsy	35.69 (26.57 to 45.25)	42.38 (27.37 to 63.93)	31.42 (21.26 to 45.66)	44.96 (28.44 to 69.05)	0.69 (0.52 to 0.87)	1.10 (0.75 to 1.63)	0.84 (0.60 to 1.18)	1.15 (0.77 to 1.72)
Multiple sclerosis	3.22 (2.07 to 5.66)	6.01 (3.79 to 10.70)	6.30 (3.97 to 11.13)	6.04 (3.82 to 10.68)	0.08 (0.05 to 0.14)	0.19 (0.13 to 0.33)	0.21 (0.13 to 0.36)	0.19 (0.12 to 0.34)
Motor neuron disease	7.64 (5.56 to 9.98)	24.94 (18.82 to 31.30)	24.54 (18.30 to 31.09)	24.15 (18.47 to 30.28)	0.22 (0.17 to 0.29)	1.00 (0.78 to 1.24)	1.00 (0.77 to 1.25)	0.95 (0.75 to 1.18)
Other neurological disorders	30.65 (24.03 to 38.26)	64.50 (51.31 to 78.43)	67.75 (53.27 to 83.28)	62.59 (50.04 to 75.31)	0.73 (0.57 to 0.91)	2.29 (1.86 to 2.77)	2.49 (2.00 to 3.05)	2.17 (1.78 to 2.61)
**Mental disorders**	**114.19 (71.21 to 158.23)**	**146.00 (98.91 to 202.94)**	**194.47 (135.80 to 264.29)**	**138.51 (94.88 to 191.87)**	**2.57 (1.64 to 3.59)**	**4.44 (3.15 to 6.00)**	**6.27 (4.53 to 8.28)**	**4.13 (2.94 to 5.54)**
Alcohol use disorders	91.57 (53.85 to 133.08)	94.28 (56.83 to 145.84)	107.73 (65.16 to 166.89)	91.18 (56.25 to 140.01)	2.10 (1.27 to 3.05)	2.78 (1.75 to 4.28)	3.26 (2.08 to 5.15)	2.65 (1.69 to 4.06)
Drug use disorders	22.32 (15.49 to 29.73)	51.45 (38.08 to 66.81)	86.49 (64.73 to 111.58)	47.06 (35.24 to 60.80)	0.47 (0.34 to 0.62)	1.65 (1.25 to 2.14)	3.01 (2.27 to 3.85)	1.47 (1.12 to 1.90)
Opioid use disorders	11.68 (7.69 to 16.46)	16.42 (10.85 to 23.14)	18.89 (12.23 to 27.05)	15.87 (10.57 to 22.27)	0.23 (0.16 to 0.32)	0.45 (0.30 to 0.61)	0.53 (0.35 to 0.74)	0.42 (0.29 to 0.58)

Cocaine use disorders	2.30 (1.43 to 3.55)	6.68 (4.31 to 10.06)	10.86 (7.14 to 16.34)	6.13 (3.91 to 9.24)	0.05 (0.03 to 0.07)	0.20 (0.13 to 0.30)	0.33 (0.22 to 0.50)	0.18 (0.11 to 0.27)
Amphetamine use disorders	0.98 (0.56 to 1.45)	5.09 (2.85 to 7.73)	11.07 (6.53 to 16.47)	4.48 (2.51 to 6.69)	0.02 (0.01 to 0.03)	0.15 (0.08 to 0.22)	0.32 (0.19 to 0.47)	0.13 (0.07 to 0.19)
Other drug use disorders	7.36 (5.14 to 10.04)	23.25 (16.32 to 31.76)	45.68 (32.83 to 61.82)	20.57 (14.57 to 27.81)	0.17 (0.12 to 0.23)	0.87 (0.61 to 1.25)	1.82 (1.30 to 2.58)	0.74 (0.51 to 1.05)
Eating disorders	0.30 (0.20 to 0.42)	0.27 (0.17 to 0.40)	0.26 (0.17 to 0.38)	0.28 (0.18 to 0.41)	0.01 (0.00 to 0.01)	0.01 (0.00 to 0.01)	0.01 (0.00 to 0.01)	0.01 (0.00 to 0.01)

Northern Mariana Islands								
Cause	YLLs (actual counts)				Deaths (actual counts)			
	2016	2040 Forecast	2040 Better Scenario	2040 Worse Scenario	2016	2040 Forecast	2040 Better Scenario	2040 Worse Scenario
**Diabetes, urogenital, blood, and endocrine diseases**	**1,358.72 (1,106.41 to 1,639.30)**	**5,760.30 (3,429.25 to 11,281.76)**	**3,470.17 (2,013.76 to 7,194.58)**	**6,909.10 (4,119.55 to 12,521.51)**	**44.36 (37.09 to 52.57)**	**261.38 (161.74 to 492.72)**	**164.62 (97.00 to 331.73)**	**305.53 (186.43 to 535.56)**
Diabetes mellitus	693.68 (561.36 to 843.30)	2,185.08 (1,434.18 to 3,186.45)	1,141.52 (742.70 to 1,666.72)	2,622.19 (1,681.45 to 3,968.71)	24.32 (20.19 to 29.09)	102.96 (69.65 to 146.76)	56.47 (37.82 to 80.48)	119.40 (77.96 to 174.07)
Acute glomerulonephritis	1.14 (0.81 to 1.68)	2.06 (1.29 to 3.27)	1.35 (0.84 to 2.18)	2.15 (1.36 to 3.43)	0.03 (0.02 to 0.04)	0.07 (0.04 to 0.11)	0.04 (0.03 to 0.08)	0.07 (0.04 to 0.12)
Chronic kidney disease	500.04 (399.46 to 616.73)	3,111.87 (826.35 to 8,925.34)	1,913.63 (511.61 to 5,828.19)	3,825.39 (1,032.15 to 9,689.00)	15.61 (12.72 to 19.00)	139.46 (35.91 to 387.63)	90.83 (23.35 to 274.59)	167.57 (45.07 to 425.36)
Chronic kidney disease due to diabetes mellitus	299.08 (231.75 to 375.20)	1,940.53 (497.22 to 5,680.54)	1,174.01 (302.85 to 3,513.32)	2,402.64 (629.58 to 6,239.11)	9.04 (7.13 to 11.17)	82.81 (21.01 to 237.90)	52.47 (13.45 to 162.99)	100.64 (26.29 to 255.54)
Chronic kidney disease due to hypertension	96.15 (74.46 to 124.83)	807.70 (205.71 to 2,340.55)	537.87 (140.54 to 1,662.34)	973.84 (251.57 to 2,521.92)	3.91 (3.14 to 4.93)	41.60 (10.66 to 116.62)	29.55 (7.50 to 87.06)	48.70 (13.01 to 123.20)
Chronic kidney disease due to glomerulonephritis	52.25 (38.53 to 69.69)	148.28 (47.65 to 416.11)	77.88 (25.79 to 232.33)	183.31 (59.44 to 471.82)	1.23 (0.92 to 1.62)	5.75 (1.64 to 16.49)	3.16 (0.89 to 9.46)	7.02 (2.03 to 18.01)
Chronic kidney disease due to other and unspecified causes	52.55 (38.68 to 69.46)	215.36 (60.19 to 615.54)	123.87 (35.11 to 370.48)	265.61 (75.31 to 688.39)	1.44 (1.09 to 1.86)	9.29 (2.45 to 25.23)	5.65 (1.47 to 16.45)	11.20 (3.06 to 27.72)
Urinary diseases and male infertility	43.78 (34.77 to 53.85)	173.85 (141.70 to 209.29)	114.29 (93.76 to 138.79)	179.13 (146.68 to 214.38)	1.30 (1.07 to 1.57)	7.98 (6.69 to 9.39)	5.49 (4.64 to 6.51)	8.03 (6.71 to 9.50)
Urinary tract infections	40.26 (32.09 to 49.62)	163.75 (133.39 to 197.50)	108.59 (88.76 to 131.54)	168.40 (138.42 to 202.31)	1.20 (0.99 to 1.44)	7.55 (6.33 to 8.93)	5.25 (4.42 to 6.20)	7.58 (6.35 to 9.02)
Urolithiasis	1.68 (1.15 to 2.54)	5.73 (3.78 to 8.92)	3.39 (2.19 to 5.42)	6.09 (4.02 to 9.52)	0.05 (0.03 to 0.07)	0.23 (0.15 to 0.35)	0.14 (0.09 to 0.22)	0.24 (0.16 to 0.38)
Other urinary diseases	1.83 (1.27 to 2.55)	4.37 (2.65 to 6.38)	2.31 (1.40 to 3.39)	4.64 (2.87 to 6.80)	0.05 (0.03 to 0.07)	0.20 (0.12 to 0.30)	0.11 (0.07 to 0.17)	0.20 (0.13 to 0.31)
Gynecological diseases	0.12 (0.08 to 0.18)	0.18 (0.11 to 0.28)	0.06 (0.04 to 0.10)	0.21 (0.13 to 0.32)	0.00 (0.00 to 0.00)	0.01 (0.00 to 0.01)	0.00 (0.00 to 0.00)	0.01 (0.00 to 0.01)
Uterine fibroids	0.01 (0.01 to 0.02)	0.03 (0.01 to 0.05)	0.01 (0.01 to 0.02)	0.03 (0.01 to 0.06)	0.00 (0.00 to 0.00)	0.00 (0.00 to 0.00)	0.00 (0.00 to 0.00)	0.00 (0.00 to 0.00)
Polycystic ovarian syndrome	0.01 (0.00 to 0.03)	0.01 (0.00 to 0.04)	0.00 (0.00 to 0.01)	0.02 (0.00 to 0.05)	0.00 (0.00 to 0.00)	0.00 (0.00 to 0.00)	0.00 (0.00 to 0.00)	0.00 (0.00 to 0.00)
Endometriosis	0.01 (0.00 to 0.01)	0.01 (0.00 to 0.03)	0.00 (0.00 to 0.01)	0.01 (0.01 to 0.03)	0.00 (0.00 to 0.00)	0.00 (0.00 to 0.00)	0.00 (0.00 to 0.00)	0.00 (0.00 to 0.00)
Genital prolapse	0.01 (0.00 to 0.02)	0.01 (0.01 to 0.02)	0.00 (0.00 to 0.01)	0.01 (0.01 to 0.02)	0.00 (0.00 to 0.00)	0.00 (0.00 to 0.00)	0.00 (0.00 to 0.00)	0.00 (0.00 to 0.00)
Other gynecological diseases	0.08 (0.05 to 0.13)	0.11 (0.06 to 0.19)	0.04 (0.02 to 0.06)	0.13 (0.07 to 0.22)	0.00 (0.00 to 0.00)	0.00 (0.00 to 0.01)	0.00 (0.00 to 0.00)	0.00 (0.00 to 0.01)
Hemoglobinopathies and hemolytic anemias	11.23 (8.17 to 14.76)	18.22 (13.12 to 24.36)	10.54 (7.58 to 14.45)	19.53 (13.99 to 25.83)	0.26 (0.19 to 0.34)	0.67 (0.49 to 0.89)	0.40 (0.29 to 0.53)	0.71 (0.52 to 0.93)
Thalassemias	1.14 (0.80 to 1.58)	0.52 (0.34 to 0.78)	0.47 (0.31 to 0.69)	0.55 (0.35 to 0.82)	0.02 (0.01 to 0.02)	0.01 (0.00 to 0.01)	0.01 (0.00 to 0.01)	0.01 (0.01 to 0.01)

Sickle cell disorders	0.02 (0.01 to 0.03)	0.03 (0.01 to 0.04)	0.01 (0.01 to 0.02)	0.03 (0.02 to 0.04)	0.00 (0.00 to 0.00)	0.00 (0.00 to 0.00)	0.00 (0.00 to 0.00)	0.00 (0.00 to 0.00)
G6PD deficiency	7.01 (4.94 to 9.38)	11.17 (7.77 to 15.43)	6.06 (4.14 to 8.46)	12.13 (8.35 to 16.43)	0.15 (0.11 to 0.20)	0.34 (0.24 to 0.47)	0.19 (0.13 to 0.26)	0.37 (0.26 to 0.50)
Other hemoglobinopathies and hemolytic anemias	3.07 (2.16 to 4.21)	6.50 (4.66 to 8.83)	4.00 (2.88 to 5.47)	6.83 (4.90 to 9.20)	0.10 (0.07 to 0.13)	0.32 (0.23 to 0.42)	0.20 (0.15 to 0.27)	0.33 (0.24 to 0.44)
Endocrine, metabolic, blood, and immune disorders	108.72 (82.47 to 140.76)	269.05 (208.06 to 344.89)	288.78 (224.76 to 365.81)	260.50 (201.62 to 333.75)	2.85 (2.20 to 3.65)	10.24 (8.04 to 12.91)	11.38 (9.02 to 14.31)	9.75 (7.69 to 12.15)

Northern Mariana Islands								
Cause	YLLs (actual counts)				Deaths (actual counts)			
	2016	2040 Forecast	2040 Better Scenario	2040 Worse Scenario	2016	2040 Forecast	2040 Better Scenario	2040 Worse Scenario
**Musculoskeletal disorders**	**32.79 (24.51 to 46.00)**	**48.75 (37.93 to 67.18)**	**35.16 (27.58 to 48.72)**	**51.05 (39.90 to 70.77)**	**0.70 (0.53 to 0.98)**	**1.60 (1.31 to 2.16)**	**1.17 (0.95 to 1.59)**	**1.65 (1.34 to 2.22)**
Rheumatoid arthritis	3.09 (2.26 to 4.24)	11.51 (8.39 to 15.67)	5.11 (3.58 to 7.05)	12.84 (9.32 to 17.47)	0.08 (0.06 to 0.11)	0.53 (0.39 to 0.70)	0.24 (0.18 to 0.33)	0.57 (0.43 to 0.76)
Other musculoskeletal disorders	29.70 (21.96 to 42.89)	37.24 (28.13 to 54.43)	30.05 (23.12 to 43.15)	38.21 (28.96 to 55.93)	0.61 (0.46 to 0.89)	1.08 (0.85 to 1.54)	0.92 (0.74 to 1.32)	1.08 (0.85 to 1.57)
**Other non-communicable diseases**	**287.37 (190.30 to 418.80)**	**491.02 (312.66 to 660.38)**	**367.52 (228.29 to 496.24)**	**512.92 (334.13 to 691.46)**	**5.24 (3.69 to 7.10)**	**18.61 (11.01 to 24.50)**	**15.66 (9.15 to 20.92)**	**18.52 (11.01 to 24.47)**
Congenital birth defects	169.79 (104.86 to 268.44)	112.02 (72.33 to 178.42)	49.11 (32.19 to 75.11)	132.56 (85.99 to 215.86)	2.39 (1.59 to 3.54)	2.05 (1.42 to 2.95)	0.96 (0.67 to 1.34)	2.36 (1.64 to 3.46)
Neural tube defects	8.77 (2.95 to 19.44)	1.92 (0.73 to 4.90)	0.52 (0.23 to 1.24)	2.48 (0.91 to 6.59)	0.11 (0.04 to 0.23)	0.03 (0.01 to 0.07)	0.01 (0.00 to 0.02)	0.04 (0.02 to 0.08)
Congenital heart anomalies	79.04 (50.63 to 121.70)	49.96 (32.53 to 76.95)	15.74 (10.15 to 23.42)	60.82 (40.62 to 94.51)	1.17 (0.80 to 1.68)	0.91 (0.62 to 1.30)	0.30 (0.20 to 0.42)	1.09 (0.76 to 1.58)
Orofacial clefts	0.10 (0.04 to 0.22)	0.04 (0.01 to 0.10)	0.01 (0.00 to 0.02)	0.05 (0.01 to 0.14)	0.00 (0.00 to 0.00)	0.00 (0.00 to 0.00)	0.00 (0.00 to 0.00)	0.00 (0.00 to 0.00)
Down syndrome	5.99 (3.60 to 9.02)	7.93 (5.15 to 11.88)	8.80 (5.85 to 13.21)	7.89 (5.06 to 11.74)	0.10 (0.06 to 0.14)	0.18 (0.12 to 0.28)	0.21 (0.14 to 0.30)	0.18 (0.12 to 0.27)
Other chromosomal abnormalities	12.05 (5.16 to 21.62)	11.57 (5.49 to 22.29)	7.38 (3.64 to 13.76)	13.04 (6.06 to 25.74)	0.15 (0.07 to 0.27)	0.19 (0.09 to 0.33)	0.13 (0.07 to 0.21)	0.20 (0.10 to 0.36)
Congenital musculoskeletal and limb anomalies	5.53 (2.43 to 10.67)	2.64 (1.23 to 5.54)	1.02 (0.50 to 2.10)	3.21 (1.47 to 7.07)	0.07 (0.03 to 0.13)	0.04 (0.02 to 0.07)	0.01 (0.01 to 0.03)	0.04 (0.02 to 0.09)
Urogenital congenital anomalies	5.34 (3.22 to 8.76)	5.79 (3.83 to 8.30)	2.91 (1.92 to 4.20)	6.46 (4.30 to 9.38)	0.09 (0.06 to 0.13)	0.15 (0.10 to 0.21)	0.08 (0.05 to 0.11)	0.17 (0.11 to 0.23)
Digestive congenital anomalies	10.87 (4.27 to 22.11)	4.99 (1.96 to 11.54)	1.64 (0.66 to 3.70)	6.29 (2.41 to 14.36)	0.13 (0.06 to 0.26)	0.07 (0.03 to 0.14)	0.02 (0.01 to 0.05)	0.08 (0.03 to 0.18)
Other congenital birth defects	42.11 (23.73 to 71.55)	27.18 (16.28 to 45.83)	11.09 (6.82 to 18.31)	32.32 (18.89 to 56.07)	0.57 (0.35 to 0.92)	0.48 (0.32 to 0.73)	0.20 (0.14 to 0.31)	0.56 (0.36 to 0.87)
Skin and subcutaneous diseases	83.41 (45.78 to 123.83)	367.94 (201.80 to 517.77)	313.81 (176.04 to 439.74)	366.14 (202.60 to 505.41)	2.45 (1.32 to 3.58)	16.43 (8.98 to 22.16)	14.65 (8.02 to 19.83)	15.99 (8.79 to 21.97)
Cellulitis	29.38 (12.43 to 45.45)	161.67 (72.37 to 230.70)	152.11 (70.96 to 215.06)	157.31 (69.86 to 226.12)	0.86 (0.36 to 1.29)	7.22 (3.22 to 10.14)	7.10 (3.18 to 9.94)	6.86 (3.07 to 9.62)
Pyoderma	49.42 (27.37 to 77.72)	191.54 (106.33 to 289.25)	152.78 (85.62 to 232.44)	193.33 (107.87 to 296.16)	1.39 (0.74 to 2.15)	8.36 (4.64 to 12.60)	7.00 (3.83 to 10.62)	8.26 (4.49 to 12.45)
Decubitus ulcer	3.66 (1.80 to 7.19)	13.11 (6.23 to 24.30)	7.71 (3.68 to 13.61)	13.83 (6.53 to 26.37)	0.18 (0.08 to 0.35)	0.80 (0.37 to 1.49)	0.51 (0.22 to 0.93)	0.82 (0.37 to 1.49)
Other skin and subcutaneous diseases	0.96 (0.55 to 1.59)	1.62 (0.93 to 2.50)	1.21 (0.67 to 1.80)	1.68 (0.95 to 2.65)	0.02 (0.01 to 0.03)	0.05 (0.03 to 0.08)	0.04 (0.02 to 0.07)	0.05 (0.03 to 0.08)
Sudden infant death syndrome	34.17 (14.10 to 66.30)	11.06 (2.98 to 27.32)	4.60 (1.33 to 11.81)	14.23 (3.79 to 34.25)	0.40 (0.16 to 0.77)	0.13 (0.03 to 0.32)	0.05 (0.02 to 0.14)	0.16 (0.04 to 0.40)

**Injuries**	**2,637.76 (1,964.10 to 3,318.53)**	**2,690.48 (1,976.02 to 3,588.31)**	**1,543.76 (1,152.91 to 1,998.32)**	**2,939.12 (2,140.68 to 3,904.92)**	**53.16 (39.67 to 66.55)**	**74.15 (58.11 to 94.64)**	**44.83 (35.39 to 55.91)**	**79.28 (61.47 to 101.28)**
**Transport injuries**	**728.62 (539.01 to 933.02)**	**892.05 (633.50 to 1,242.14)**	**427.00 (307.32 to 578.03)**	**1,001.49 (703.00 to 1,420.49)**	**14.69 (10.88 to 18.80)**	**24.24 (17.96 to 33.26)**	**12.15 (9.21 to 16.06)**	**26.74 (19.59 to 37.15)**
Road injuries	645.56 (479.08 to 828.30)	772.59 (544.96 to 1,063.54)	374.63 (271.18 to 505.26)	866.31 (608.98 to 1,199.99)	12.93 (9.57 to 16.70)	20.65 (15.24 to 27.90)	10.52 (7.97 to 13.85)	22.76 (16.70 to 31.32)
Pedestrian road injuries	278.08 (200.04 to 362.52)	278.52 (188.37 to 399.45)	85.79 (57.55 to 121.66)	330.61 (223.59 to 478.16)	5.72 (4.12 to 7.38)	8.00 (5.65 to 11.11)	2.60 (1.79 to 3.54)	9.32 (6.46 to 13.11)

Northern Mariana Islands								
Cause	YLLs (actual counts)				Deaths (actual counts)			
	2016	2040 Forecast	2040 Better Scenario	2040 Worse Scenario	2016	2040 Forecast	2040 Better Scenario	2040 Worse Scenario
Cyclist road injuries	13.82 (9.83 to 18.13)	22.80 (15.83 to 31.83)	14.16 (9.94 to 19.39)	24.50 (16.98 to 34.03)	0.29 (0.21 to 0.38)	0.66 (0.47 to 0.92)	0.45 (0.32 to 0.60)	0.69 (0.49 to 0.97)
Motorcyclist road injuries	69.52 (41.00 to 99.60)	77.05 (45.53 to 118.96)	52.49 (30.62 to 81.14)	82.11 (48.30 to 127.92)	1.34 (0.79 to 1.91)	1.70 (1.04 to 2.58)	1.21 (0.73 to 1.83)	1.79 (1.10 to 2.73)
Motor vehicle road injuries	278.19 (201.56 to 368.37)	386.66 (273.42 to 547.87)	217.79 (157.51 to 295.56)	420.90 (295.69 to 600.41)	5.47 (3.97 to 7.25)	10.09 (7.39 to 13.94)	6.14 (4.66 to 8.15)	10.75 (7.81 to 14.97)
Other road injuries	5.96 (3.74 to 8.67)	7.56 (4.60 to 11.82)	4.39 (2.75 to 6.48)	8.20 (4.97 to 12.91)	0.12 (0.07 to 0.17)	0.20 (0.12 to 0.30)	0.13 (0.08 to 0.18)	0.21 (0.13 to 0.31)
Other transport injuries	83.06 (58.99 to 110.93)	119.45 (79.83 to 177.34)	52.37 (35.24 to 74.33)	135.18 (90.16 to 199.19)	1.76 (1.25 to 2.33)	3.59 (2.43 to 5.21)	1.63 (1.13 to 2.30)	3.99 (2.69 to 5.78)
**Unintentional injuries**	**901.36 (688.66 to 1,112.92)**	**842.30 (659.36 to 1,056.34)**	**531.51 (417.34 to 659.46)**	**900.49 (693.97 to 1,146.71)**	**18.68 (14.25 to 23.02)**	**26.50 (21.71 to 31.63)**	**17.83 (14.53 to 21.28)**	**27.57 (22.30 to 33.16)**
Falls	89.15 (64.81 to 116.64)	143.92 (111.24 to 180.59)	104.13 (79.64 to 129.14)	147.44 (113.37 to 183.91)	2.41 (1.81 to 3.07)	6.43 (5.09 to 7.91)	4.90 (3.88 to 5.96)	6.41 (5.00 to 7.87)
Drowning	425.13 (313.33 to 540.00)	336.99 (240.16 to 458.64)	206.01 (149.88 to 275.90)	363.62 (261.45 to 492.50)	8.29 (6.05 to 10.59)	8.58 (6.30 to 11.31)	5.38 (3.98 to 7.03)	9.13 (6.78 to 12.04)
Fire, heat, and hot substances	38.96 (27.70 to 51.73)	31.01 (21.67 to 42.18)	15.88 (11.10 to 21.27)	34.23 (23.72 to 46.53)	0.77 (0.56 to 1.02)	0.96 (0.70 to 1.27)	0.52 (0.38 to 0.68)	1.03 (0.76 to 1.37)
Poisonings	17.74 (11.27 to 23.93)	12.14 (7.65 to 17.60)	5.11 (3.32 to 7.09)	13.80 (8.63 to 19.60)	0.37 (0.23 to 0.50)	0.39 (0.24 to 0.54)	0.17 (0.11 to 0.23)	0.43 (0.27 to 0.59)
Exposure to mechanical forces	82.78 (62.13 to 104.53)	81.03 (62.23 to 103.95)	48.19 (36.65 to 61.52)	87.02 (66.03 to 112.20)	1.69 (1.28 to 2.14)	2.47 (1.97 to 3.08)	1.53 (1.21 to 1.90)	2.60 (2.05 to 3.25)
Unintentional firearm injuries	22.85 (16.66 to 31.68)	25.54 (18.62 to 34.97)	17.24 (12.51 to 23.29)	26.89 (19.50 to 36.68)	0.47 (0.35 to 0.63)	0.84 (0.62 to 1.13)	0.59 (0.44 to 0.76)	0.86 (0.64 to 1.15)
Unintentional suffocation	9.51 (6.46 to 13.00)	7.94 (5.32 to 11.42)	4.93 (3.29 to 7.08)	8.58 (5.72 to 12.36)	0.17 (0.12 to 0.23)	0.16 (0.11 to 0.23)	0.10 (0.07 to 0.14)	0.17 (0.12 to 0.24)
Other exposure to mechanical forces	50.42 (35.40 to 66.35)	47.56 (34.64 to 63.47)	26.03 (19.13 to 34.15)	51.55 (37.36 to 66.58)	1.05 (0.74 to 1.39)	1.48 (1.12 to 1.87)	0.85 (0.65 to 1.06)	1.56 (1.18 to 1.97)
Adverse effects of medical treatment	76.38 (56.46 to 98.89)	85.81 (60.97 to 113.55)	62.67 (44.56 to 81.76)	89.32 (63.87 to 117.74)	1.65 (1.20 to 2.14)	2.87 (2.01 to 3.67)	2.21 (1.55 to 2.80)	2.92 (2.05 to 3.71)
Animal contact	4.55 (3.19 to 6.09)	6.23 (4.22 to 8.51)	3.98 (2.72 to 5.41)	6.61 (4.61 to 8.96)	0.10 (0.07 to 0.14)	0.21 (0.15 to 0.28)	0.14 (0.10 to 0.19)	0.22 (0.15 to 0.29)
Venomous animal contact	1.48 (0.93 to 2.17)	1.75 (1.16 to 2.48)	1.28 (0.87 to 1.78)	1.81 (1.20 to 2.59)	0.03 (0.02 to 0.05)	0.06 (0.04 to 0.09)	0.05 (0.03 to 0.07)	0.06 (0.04 to 0.08)
Non-venomous animal contact	3.06 (2.12 to 4.19)	4.48 (2.94 to 6.24)	2.70 (1.77 to 3.76)	4.80 (3.16 to 6.71)	0.07 (0.05 to 0.10)	0.15 (0.10 to 0.21)	0.10 (0.06 to 0.13)	0.16 (0.11 to 0.22)
Foreign body	64.48 (46.37 to 88.32)	74.27 (51.97 to 93.37)	61.45 (42.55 to 77.50)	75.59 (53.12 to 95.19)	1.29 (0.92 to 1.67)	2.57 (1.72 to 3.19)	2.23 (1.49 to 2.78)	2.53 (1.69 to 3.12)
Pulmonary aspiration and foreign body in airway	58.28 (39.82 to 80.38)	67.68 (46.60 to 85.84)	54.46 (37.15 to 70.03)	69.05 (47.64 to 88.27)	1.18 (0.82 to 1.54)	2.40 (1.58 to 3.00)	2.05 (1.36 to 2.56)	2.37 (1.56 to 2.94)

Foreign body in other body part	6.20 (3.83 to 9.35)	6.59 (3.87 to 9.96)	6.99 (4.18 to 10.28)	6.55 (3.87 to 9.96)	0.12 (0.07 to 0.17)	0.16 (0.09 to 0.25)	0.18 (0.10 to 0.26)	0.16 (0.09 to 0.25)
Environmental heat and cold exposure	13.04 (8.07 to 18.18)	17.04 (10.58 to 24.48)	8.59 (5.28 to 12.33)	18.62 (11.63 to 26.77)	0.29 (0.18 to 0.40)	0.57 (0.36 to 0.81)	0.30 (0.19 to 0.42)	0.61 (0.39 to 0.85)
Other unintentional injuries	89.15 (59.19 to 121.69)	53.85 (35.50 to 76.31)	15.50 (10.21 to 21.59)	64.24 (42.91 to 88.87)	1.81 (1.21 to 2.46)	1.45 (1.00 to 1.98)	0.43 (0.30 to 0.58)	1.70 (1.20 to 2.28)
**Self-harm and interpersonal violence**	**1,007.17 (724.49 to 1,312.48)**	**949.43 (638.51 to 1,369.61)**	**578.68 (399.31 to 790.49)**	**1,030.36 (681.32 to 1,505.59)**	**19.78 (14.11 to 25.71)**	**23.26 (16.37 to 32.60)**	**14.71 (10.62 to 19.59)**	**24.82 (17.19 to 34.65)**

Northern Mariana Islands								
Cause	YLLs (actual counts)				Deaths (actual counts)			
	2016	2040 Forecast	2040 Better Scenario	2040 Worse Scenario	2016	2040 Forecast	2040 Better Scenario	2040 Worse Scenario
Self-harm	658.69 (438.12 to 874.59)	705.89 (439.43 to 1,087.39)	439.33 (286.94 to 628.45)	765.06 (469.08 to 1,197.11)	12.97 (8.63 to 17.15)	17.22 (11.41 to 25.31)	11.12 (7.76 to 15.41)	18.34 (11.98 to 27.07)
Self-harm by firearm	39.33 (19.91 to 60.77)	37.66 (18.23 to 69.44)	18.94 (9.27 to 33.52)	42.02 (19.76 to 77.54)	0.81 (0.42 to 1.25)	0.94 (0.46 to 1.69)	0.48 (0.24 to 0.85)	1.04 (0.51 to 1.90)
Self-harm by other specified means	619.35 (413.49 to 818.72)	668.23 (416.31 to 1,025.20)	420.39 (273.96 to 599.14)	723.04 (439.86 to 1,119.52)	12.16 (8.10 to 16.14)	16.28 (10.75 to 23.80)	10.63 (7.47 to 14.51)	17.30 (11.34 to 25.44)
Interpersonal violence	348.48 (254.70 to 475.98)	243.53 (167.54 to 333.83)	139.35 (97.93 to 189.51)	265.29 (182.14 to 364.58)	6.81 (4.99 to 9.30)	6.04 (4.26 to 8.00)	3.59 (2.56 to 4.75)	6.49 (4.55 to 8.73)
Physical violence by firearm	95.18 (57.01 to 167.83)	55.56 (33.09 to 97.99)	24.91 (14.66 to 44.50)	62.47 (35.77 to 109.59)	1.87 (1.12 to 3.30)	1.24 (0.75 to 2.17)	0.56 (0.33 to 0.99)	1.39 (0.82 to 2.40)
Physical violence by sharp object	108.35 (69.43 to 156.40)	71.24 (45.81 to 104.81)	37.13 (25.11 to 52.04)	78.30 (51.42 to 113.28)	2.11 (1.37 to 3.06)	1.63 (1.08 to 2.35)	0.86 (0.58 to 1.20)	1.79 (1.19 to 2.52)
Physical violence by other means	144.95 (99.44 to 199.73)	116.74 (73.91 to 168.61)	77.31 (49.81 to 108.01)	124.53 (77.95 to 181.32)	2.83 (1.86 to 3.89)	3.16 (1.97 to 4.43)	2.17 (1.37 to 2.94)	3.31 (2.08 to 4.65)
**Forces of nature, conflict and terrorism, and executions and police conflict**	**0.62 (0.49 to 0.75)**	**6.71 (0.00 to 89.68)**	**6.57 (0.00 to 87.42)**	**6.78 (0.00 to 90.88)**	**0.01 (0.01 to 0.01)**	**0.14 (0.00 to 1.74)**	**0.14 (0.00 to 1.73)**	**0.14 (0.00 to 1.74)**
Exposure to forces of nature	0.00 (0.00 to 0.00)	3.75 (0.00 to 0.00)	3.68 (0.00 to 0.00)	3.79 (0.00 to 0.00)	0.00 (0.00 to 0.00)	0.08 (0.00 to 0.00)	0.08 (0.00 to 0.00)	0.08 (0.00 to 0.00)
Conflict and terrorism	0.00 (0.00 to 0.00)	2.96 (0.00 to 42.83)	2.89 (0.00 to 41.49)	2.99 (0.00 to 43.42)	0.00 (0.00 to 0.00)	0.06 (0.00 to 0.83)	0.06 (0.00 to 0.82)	0.06 (0.00 to 0.83)
Executions and police conflict	0.62 (0.49 to 0.75)	0.00 (0.00 to 0.00)	0.00 (0.00 to 0.00)	0.00 (0.00 to 0.00)	0.01 (0.01 to 0.01)	0.00 (0.00 to 0.00)	0.00 (0.00 to 0.00)	0.00 (0.00 to 0.00)

Papua New Guinea								
Cause	YLLs (actual counts)				Deaths (actual counts)			
	2016	2040 Forecast	2040 Better Scenario	2040 Worse Scenario	2016	2040 Forecast	2040 Better Scenario	2040 Worse Scenario
**All causes**	**2,930,710.52 (2,344,333.03 to 3,632,438.48)**	**3,353,096.36 (2,721,090.66 to 4,100,799.25)**	**2,078,518.28 (1,710,672.71 to 2,522,082.46)**	**4,208,660.66 (3,436,439.21 to 5,101,487.62)**	**69,162.32 (57,603.28 to 80,915.45)**	**96,853.03 (84,535.90 to 110,290.90)**	**66,326.97 (58,375.64 to 75,845.59)**	**117,165.40 (102,022.27 to 132,136.01)**
**Communicable, maternal, neonatal, and nutritional diseases**	**1,114,318.76 (764,328.38 to 1,636,184.87)**	**882,448.27 (572,026.33 to 1,331,897.35)**	**426,461.39 (302,905.80 to 603,273.78)**	**1,212,317.73 (775,628.81 to 1,845,875.84)**	**17,461.69 (13,065.99 to 23,758.94)**	**16,273.92 (11,257.52 to 23,245.88)**	**9,073.45 (6,515.22 to 12,536.61)**	**21,442.94 (14,461.40 to 31,061.68)**
**HIV/AIDS and tuberculosis**	**43,270.03 (32,871.11 to 58,793.36)**	**60,187.05 (37,510.03 to 93,142.63)**	**39,608.42 (22,408.86 to 66,150.48)**	**85,286.94 (57,481.85 to 125,379.18)**	**942.87 (756.99 to 1,228.60)**	**1,292.15 (852.79 to 1,917.16)**	**855.31 (493.32 to 1,367.62)**	**1,777.76 (1,239.55 to 2,533.04)**
Tuberculosis	14,611.76 (11,683.86 to 18,072.74)	12,948.32 (8,303.44 to 20,557.36)	5,786.45 (3,806.10 to 9,065.90)	18,056.96 (11,142.91 to 30,533.29)	409.04 (330.73 to 501.22)	406.65 (271.48 to 625.96)	197.89 (133.83 to 294.41)	538.53 (345.24 to 865.39)
HIV/AIDS	28,658.27 (18,779.03 to 43,881.83)	47,238.73 (25,663.66 to 78,883.43)	33,821.96 (16,674.55 to 59,817.36)	67,229.98 (41,525.20 to 104,496.15)	533.83 (376.09 to 798.62)	885.50 (489.81 to 1,502.63)	657.42 (307.01 to 1,154.81)	1,239.23 (777.42 to 1,927.99)
**Diarrhea, lower respiratory, and other common infectious diseases**	**543,650.64 (368,776.67 to 789,205.01)**	**424,521.62 (242,324.36 to 681,228.63)**	**188,351.72 (116,679.55 to 289,956.25)**	**651,658.20 (349,983.00 to 1,087,478.34)**	**9,472.74 (7,131.26 to 12,695.95)**	**9,352.04 (5,655.16 to 14,893.02)**	**5,071.20 (3,184.50 to 8,093.75)**	**13,130.20 (7,782.65 to 20,809.85)**
Diarrheal diseases	78,711.89 (48,375.41 to 119,546.92)	62,602.70 (11,720.96 to 179,790.32)	29,528.91 (5,854.68 to 86,914.86)	87,555.45 (18,004.58 to 249,265.21)	2,075.11 (1,214.14 to 3,261.80)	2,186.14 (378.39 to 6,368.50)	1,240.44 (212.15 to 3,680.50)	2,929.53 (518.01 to 8,299.96)
Intestinal infectious diseases	18,337.64 (8,978.41 to 31,716.27)	17,288.94 (7,581.71 to 31,758.88)	11,679.99 (5,329.36 to 21,277.63)	18,459.44 (8,010.16 to 33,526.55)	263.11 (130.84 to 455.64)	252.85 (115.55 to 457.16)	177.42 (83.03 to 316.95)	268.11 (120.00 to 490.87)
Typhoid fever	15,649.22 (7,225.69 to 27,842.69)	14,371.62 (6,052.60 to 26,979.96)	9,759.23 (4,323.16 to 17,593.47)	15,317.56 (6,345.10 to 28,831.94)	223.46 (105.40 to 398.85)	208.68 (91.61 to 384.36)	147.13 (67.00 to 264.05)	220.93 (97.15 to 411.29)
Paratyphoid fever	2,559.56 (1,104.17 to 5,257.19)	2,856.89 (1,144.28 to 6,061.80)	1,891.46 (773.60 to 3,872.44)	3,075.26 (1,212.41 to 6,538.87)	37.75 (16.43 to 76.92)	43.11 (18.07 to 90.23)	29.70 (12.64 to 59.45)	46.05 (18.77 to 96.38)
Other intestinal infectious diseases	128.86 (42.90 to 310.49)	60.42 (14.11 to 174.49)	29.31 (7.89 to 78.21)	66.61 (14.53 to 189.12)	1.90 (0.64 to 4.40)	1.05 (0.31 to 2.70)	0.59 (0.19 to 1.42)	1.13 (0.33 to 2.93)
Lower respiratory infections	327,927.86 (214,611.11 to 504,875.90)	209,672.18 (100,956.30 to 392,368.72)	92,436.27 (53,225.70 to 158,662.73)	352,696.89 (148,121.08 to 695,046.31)	5,559.89 (3,988.33 to 7,838.99)	5,045.49 (3,009.04 to 8,446.15)	2,772.00 (1,864.40 to 4,211.38)	7,370.21 (3,898.42 to 12,397.15)
Upper respiratory infections	342.94 (144.96 to 736.18)	308.54 (102.56 to 792.13)	146.11 (52.87 to 338.89)	348.91 (111.86 to 905.41)	5.99 (3.00 to 11.03)	6.46 (2.35 to 14.44)	3.46 (1.32 to 7.64)	7.08 (2.57 to 16.00)
Otitis media	36.91 (14.40 to 82.81)	11.13 (3.97 to 28.40)	10.41 (3.80 to 27.44)	11.20 (3.97 to 28.71)	0.69 (0.31 to 1.38)	0.27 (0.11 to 0.70)	0.28 (0.11 to 0.68)	0.26 (0.10 to 0.69)
Meningitis	40,254.94 (25,242.60 to 68,280.85)	26,892.76 (12,013.46 to 53,150.22)	16,439.60 (9,549.69 to 27,640.17)	31,703.30 (13,036.38 to 68,767.98)	578.29 (386.78 to 924.77)	419.99 (225.14 to 749.61)	293.16 (181.39 to 455.82)	474.26 (233.83 to 903.48)
Pneumococcal meningitis	122.06 (69.45 to 218.21)	54.41 (27.14 to 103.65)	36.47 (21.17 to 59.04)	65.01 (30.94 to 129.07)	1.76 (1.11 to 2.91)	0.94 (0.55 to 1.60)	0.73 (0.47 to 1.11)	1.07 (0.59 to 1.87)
H influenzae type B meningitis	4,576.09 (2,620.91 to 8,401.54)	7,224.14 (1,300.29 to 23,344.30)	2,134.05 (883.35 to 5,259.06)	11,093.19 (1,534.36 to 35,812.21)	63.77 (40.21 to 109.28)	93.21 (23.06 to 284.12)	34.29 (17.58 to 74.39)	138.03 (25.74 to 428.86)
Meningococcal meningitis	7,720.80 (4,589.82 to 13,422.73)	4,078.13 (2,213.45 to 7,195.30)	2,968.30 (1,737.83 to 4,930.33)	4,265.39 (2,300.47 to 7,612.39)	115.07 (74.02 to 188.82)	71.41 (43.08 to 113.06)	57.33 (36.66 to 86.36)	72.98 (43.02 to 116.65)
Other meningitis	27,835.99 (17,279.82 to 47,612.36)	15,536.07 (7,951.02 to 26,768.39)	11,300.79 (6,444.43 to 18,260.05)	16,279.71 (8,149.78 to 28,883.27)	397.69 (266.20 to 630.02)	254.44 (148.46 to 411.97)	200.81 (125.62 to 310.68)	262.18 (149.74 to 424.66)
Encephalitis	14,440.20 (9,400.67 to 24,230.80)	15,516.18 (8,257.04 to 26,404.59)	10,249.32 (5,915.16 to 15,921.84)	16,401.44 (8,546.00 to 28,319.71)	222.26 (157.11 to 335.98)	268.12 (165.47 to 400.52)	196.12 (128.94 to 272.41)	277.02 (167.56 to 420.47)

Diphtheria	182.58 (88.03 to 345.99)	67.79 (18.78 to 181.19)	25.40 (14.12 to 42.14)	98.33 (19.18 to 292.45)	2.57 (1.41 to 4.53)	0.98 (0.35 to 2.34)	0.48 (0.27 to 0.78)	1.33 (0.36 to 3.62)
Whooping cough	27,062.28 (2,245.43 to 83,162.46)	29,867.84 (829.01 to 99,889.62)	4,699.04 (245.70 to 16,394.67)	31,588.77 (789.61 to 114,769.38)	320.09 (26.69 to 984.20)	353.56 (10.87 to 1,185.48)	57.01 (3.08 to 198.52)	373.77 (9.73 to 1,356.21)
Tetanus	416.00 (183.71 to 903.80)	107.62 (39.55 to 252.36)	38.87 (14.14 to 109.58)	113.25 (40.90 to 270.43)	7.54 (3.28 to 19.54)	1.76 (0.72 to 4.07)	1.03 (0.27 to 3.20)	1.79 (0.73 to 4.03)
Measles	33,570.19 (10,054.61 to 75,616.94)	60,549.13 (3,273.37 to 262,085.90)	22,355.34 (2,334.80 to 82,193.51)	110,849.68 (4,699.15 to 473,885.00)	400.89 (120.58 to 902.08)	787.40 (62.07 to 3,264.51)	312.95 (42.68 to 1,030.93)	1,396.22 (75.33 to 5,664.04)

Papua New Guinea								
Cause	YLLs (actual counts)				Deaths (actual counts)			
	2016	2040 Forecast	2040 Better Scenario	2040 Worse Scenario	2016	2040 Forecast	2040 Better Scenario	2040 Worse Scenario
Varicella and herpes zoster	2,367.22 (1,248.30 to 4,455.89)	1,636.80 (562.06 to 3,676.83)	742.46 (301.81 to 1,487.41)	1,831.53 (619.43 to 4,235.86)	36.32 (22.06 to 60.33)	29.02 (13.75 to 54.72)	16.85 (9.11 to 27.77)	30.62 (13.94 to 59.81)
**Neglected tropical diseases and malaria**	**54,865.95 (30,138.67 to 100,447.37)**	**43,801.04 (22,715.45 to 80,100.33)**	**36,573.63 (19,664.56 to 59,499.70)**	**45,365.19 (22,842.85 to 83,236.41)**	**962.90 (540.72 to 1,626.95)**	**841.63 (469.10 to 1,380.52)**	**747.06 (442.26 to 1,127.43)**	**850.87 (462.23 to 1,432.04)**
Malaria	50,134.59 (25,728.78 to 94,288.40)	37,629.93 (17,400.82 to 70,309.50)	32,065.35 (16,045.21 to 55,396.91)	38,849.43 (17,427.75 to 74,809.99)	889.31 (468.15 to 1,547.71)	726.94 (377.28 to 1,265.84)	650.21 (358.99 to 1,035.55)	736.44 (365.73 to 1,276.30)
Chagas disease	0.00 (0.00 to 0.00)	0.00 (0.00 to 0.00)	0.00 (0.00 to 0.00)	0.00 (0.00 to 0.00)	0.00 (0.00 to 0.00)	0.00 (0.00 to 0.00)	0.00 (0.00 to 0.00)	0.00 (0.00 to 0.00)
Leishmaniasis	0.00 (0.00 to 0.00)	0.00 (0.00 to 0.00)	0.00 (0.00 to 0.00)	0.00 (0.00 to 0.00)	0.00 (0.00 to 0.00)	0.00 (0.00 to 0.00)	0.00 (0.00 to 0.00)	0.00 (0.00 to 0.00)
African trypanosomiasis	0.00 (0.00 to 0.00)	0.00 (0.00 to 0.00)	0.00 (0.00 to 0.00)	0.00 (0.00 to 0.00)	0.00 (0.00 to 0.00)	0.00 (0.00 to 0.00)	0.00 (0.00 to 0.00)	0.00 (0.00 to 0.00)
Schistosomiasis	0.00 (0.00 to 0.00)	0.00 (0.00 to 0.00)	0.00 (0.00 to 0.00)	0.00 (0.00 to 0.00)	0.00 (0.00 to 0.00)	0.00 (0.00 to 0.00)	0.00 (0.00 to 0.00)	0.00 (0.00 to 0.00)
Cysticercosis	118.38 (87.99 to 153.59)	57.07 (42.47 to 73.45)	49.71 (36.63 to 64.25)	57.90 (43.09 to 74.59)	2.59 (1.94 to 3.37)	1.38 (1.04 to 1.77)	1.25 (0.94 to 1.61)	1.37 (1.04 to 1.76)
Cystic echinococcosis	21.15 (15.26 to 28.68)	13.60 (9.45 to 19.41)	6.20 (4.17 to 8.79)	15.67 (10.62 to 21.75)	0.48 (0.34 to 0.65)	0.37 (0.26 to 0.52)	0.18 (0.13 to 0.25)	0.41 (0.29 to 0.57)
Dengue	98.23 (33.95 to 263.11)	503.55 (187.22 to 1,361.73)	461.38 (169.42 to 1,274.82)	500.93 (181.38 to 1,350.78)	2.06 (0.73 to 5.66)	14.39 (5.17 to 37.46)	15.46 (5.67 to 39.25)	13.24 (4.77 to 34.76)
Yellow fever	0.00 (0.00 to 0.00)	0.00 (0.00 to 0.00)	0.00 (0.00 to 0.00)	0.00 (0.00 to 0.00)	0.00 (0.00 to 0.00)	0.00 (0.00 to 0.00)	0.00 (0.00 to 0.00)	0.00 (0.00 to 0.00)
Rabies	11.42 (3.83 to 24.18)	8.40 (2.74 to 18.11)	3.22 (1.05 to 6.81)	9.98 (3.09 to 21.39)	0.31 (0.11 to 0.66)	0.27 (0.09 to 0.58)	0.12 (0.04 to 0.27)	0.29 (0.09 to 0.63)
Intestinal nematode infections	3,607.72 (1,977.95 to 6,235.85)	4,620.86 (2,312.63 to 9,261.81)	3,356.26 (1,737.69 to 6,524.83)	4,905.23 (2,470.67 to 9,886.55)	47.82 (27.50 to 79.34)	65.27 (35.95 to 123.47)	51.56 (29.91 to 88.64)	67.87 (36.66 to 129.74)
Other neglected tropical diseases	874.47 (329.13 to 1,898.66)	967.62 (418.37 to 2,149.15)	631.52 (295.36 to 1,475.13)	1,026.06 (436.42 to 2,187.48)	20.33 (8.83 to 43.17)	33.01 (15.03 to 75.46)	28.28 (12.53 to 69.04)	31.24 (14.39 to 69.07)
**Maternal disorders**	**51,684.48 (37,184.94 to 70,062.44)**	**42,624.43 (27,350.70 to 62,216.28)**	**20,923.39 (13,246.62 to 30,677.72)**	**49,555.73 (31,937.17 to 72,092.53)**	**925.17 (666.74 to 1,259.87)**	**766.84 (491.10 to 1,121.58)**	**377.85 (238.47 to 554.12)**	**890.95 (572.71 to 1,300.55)**
Maternal hemorrhage	14,082.96 (8,372.37 to 21,736.89)	11,162.63 (6,082.18 to 17,921.16)	5,598.40 (3,008.37 to 9,208.97)	12,861.78 (7,016.98 to 20,812.20)	251.92 (147.32 to 385.65)	200.62 (108.90 to 322.34)	100.89 (54.41 to 164.70)	231.03 (125.26 to 370.91)
Maternal sepsis and other maternal infections	5,197.42 (2,711.16 to 8,752.19)	3,732.70 (1,665.84 to 6,836.98)	1,841.88 (815.77 to 3,422.12)	4,320.46 (1,947.16 to 7,790.02)	94.74 (48.47 to 158.66)	68.40 (30.68 to 122.83)	33.87 (14.88 to 61.53)	79.11 (35.50 to 142.60)
Maternal hypertensive disorders	6,993.47 (3,985.53 to 11,193.28)	5,892.67 (3,114.79 to 10,099.51)	2,925.77 (1,546.66 to 5,011.51)	6,799.39 (3,635.73 to 11,851.66)	124.83 (71.53 to 200.89)	105.86 (55.23 to 182.52)	52.74 (27.54 to 90.57)	122.07 (64.26 to 214.22)
Maternal obstructed labor and uterine rupture	4,173.24 (1,900.78 to 7,746.24)	3,580.17 (1,480.36 to 7,194.02)	1,706.00 (701.24 to 3,531.18)	4,160.14 (1,709.40 to 8,230.47)	75.43 (34.05 to 138.56)	65.02 (26.41 to 128.90)	31.07 (12.89 to 64.20)	75.50 (31.19 to 148.58)
Maternal abortion, miscarriage, and ectopic pregnancy	2,747.42 (1,056.81 to 5,551.49)	2,652.77 (926.82 to 5,958.04)	1,323.39 (457.10 to 2,876.82)	3,133.45 (1,092.19 to 6,947.60)	50.04 (20.03 to 99.74)	48.55 (17.01 to 107.22)	24.30 (8.75 to 51.06)	57.33 (20.75 to 125.81)

Indirect maternal deaths	6,522.66 (3,299.76 to 10,873.17)	5,918.48 (2,852.04 to 11,020.47)	2,800.51 (1,308.98 to 5,254.25)	6,946.97 (3,297.63 to 13,016.06)	115.03 (58.60 to 193.77)	104.69 (50.21 to 195.41)	49.78 (23.43 to 95.03)	122.76 (58.61 to 231.25)
Late maternal deaths	2,976.64 (1,226.98 to 5,638.13)	1,970.69 (765.45 to 4,095.28)	941.38 (364.60 to 1,926.90)	2,307.61 (900.51 to 4,695.79)	53.00 (22.39 to 98.33)	35.12 (13.68 to 72.36)	16.85 (6.51 to 33.72)	41.09 (16.08 to 82.92)
Maternal deaths aggravated by HIV/AIDS	196.91 (95.91 to 348.08)	556.81 (150.74 to 1,442.66)	233.78 (17.23 to 646.14)	699.77 (243.92 to 1,721.46)	3.74 (1.83 to 6.60)	10.47 (2.81 to 26.09)	4.43 (0.30 to 12.05)	13.16 (4.55 to 30.36)
Other maternal disorders	8,793.76 (4,693.82 to 14,275.35)	7,157.50 (3,642.80 to 12,712.00)	3,552.28 (1,719.54 to 6,233.68)	8,326.16 (4,210.25 to 14,863.96)	156.44 (85.19 to 255.42)	128.11 (64.57 to 225.29)	63.91 (30.70 to 111.55)	148.89 (74.53 to 261.08)

Papua New Guinea								
Cause	YLLs (actual counts)				Deaths (actual counts)			
	2016	2040 Forecast	2040 Better Scenario	2040 Worse Scenario	2016	2040 Forecast	2040 Better Scenario	2040 Worse Scenario
**Neonatal disorders**	**289,451.32 (156,785.28 to 492,821.25)**	**252,801.37 (131,751.93 to 453,153.89)**	**106,270.59 (55,493.67 to 192,076.21)**	**314,066.70 (162,925.89 to 556,329.43)**	**3,345.77 (1,812.20 to 5,695.94)**	**2,923.08 (1,524.14 to 5,241.24)**	**1,228.96 (642.04 to 2,220.59)**	**3,631.24 (1,883.73 to 6,433.80)**
Neonatal preterm birth	127,147.10 (64,956.43 to 230,536.12)	103,477.07 (50,939.79 to 187,901.71)	41,061.15 (20,140.53 to 76,229.76)	130,423.08 (63,409.89 to 238,398.81)	1,469.26 (750.71 to 2,663.55)	1,196.00 (589.08 to 2,171.75)	474.68 (232.78 to 880.90)	1,507.36 (732.91 to 2,753.99)
Neonatal encephalopathy due to birth asphyxia and trauma	61,796.82 (28,781.27 to 116,120.73)	50,277.26 (21,511.27 to 101,029.56)	23,438.15 (10,352.08 to 46,877.34)	61,132.34 (26,411.38 to 121,979.97)	714.44 (332.88 to 1,341.92)	581.63 (248.96 to 1,167.42)	271.18 (119.84 to 542.84)	707.15 (305.31 to 1,410.62)
Neonatal sepsis and other neonatal infections	27,909.61 (11,337.66 to 53,626.08)	32,062.78 (12,375.68 to 66,290.80)	14,511.66 (5,593.64 to 29,812.02)	39,095.63 (15,268.97 to 79,957.99)	322.58 (131.03 to 619.65)	370.71 (142.99 to 766.26)	167.79 (64.63 to 344.78)	452.01 (176.45 to 923.96)
Hemolytic disease and other neonatal jaundice	6,822.12 (3,042.82 to 14,289.60)	3,826.82 (1,462.81 to 8,636.38)	1,552.99 (596.08 to 3,533.73)	4,804.28 (1,821.65 to 10,967.98)	78.82 (35.16 to 165.09)	44.22 (16.90 to 99.76)	17.94 (6.89 to 40.83)	55.51 (21.05 to 126.69)
Other neonatal disorders	65,775.66 (31,552.57 to 123,077.19)	63,157.44 (28,694.48 to 123,209.64)	25,706.64 (11,525.93 to 49,854.44)	78,611.37 (35,262.98 to 152,216.39)	760.66 (364.83 to 1,423.38)	730.51 (331.90 to 1,424.31)	297.36 (133.37 to 576.48)	909.21 (408.11 to 1,760.82)
**Nutritional deficiencies**	**32,674.41 (16,730.56 to 58,496.14)**	**20,651.58 (11,437.80 to 36,282.54)**	**11,949.74 (7,346.42 to 19,383.76)**	**24,480.42 (12,659.46 to 45,077.94)**	**519.30 (316.52 to 826.11)**	**454.22 (303.21 to 667.76)**	**356.96 (255.79 to 489.50)**	**473.80 (300.33 to 733.33)**
Protein-energy malnutrition	28,173.52 (12,707.04 to 53,647.39)	14,076.27 (6,507.85 to 27,160.57)	7,430.04 (3,852.52 to 13,618.29)	17,398.12 (7,494.38 to 34,954.97)	416.34 (235.06 to 717.23)	290.81 (168.58 to 464.73)	219.76 (140.00 to 318.39)	311.35 (167.02 to 536.61)
Iodine deficiency	62.94 (1.58 to 313.12)	381.00 (2.17 to 2,498.60)	204.72 (1.26 to 1,157.49)	443.09 (2.11 to 3,331.11)	1.10 (0.04 to 4.44)	9.27 (0.08 to 39.10)	6.61 (0.05 to 30.53)	9.59 (0.07 to 44.42)
Dietary iron deficiency	4,281.25 (2,353.68 to 8,076.97)	6,004.73 (3,346.55 to 11,053.83)	4,181.80 (2,558.15 to 6,933.38)	6,440.69 (3,410.41 to 12,503.01)	97.88 (62.11 to 152.98)	147.91 (91.15 to 226.40)	125.04 (76.81 to 189.82)	146.99 (91.08 to 232.12)
Other nutritional deficiencies	156.71 (77.07 to 361.19)	189.58 (92.06 to 404.98)	133.18 (68.27 to 281.80)	198.52 (95.09 to 433.47)	3.99 (2.16 to 7.73)	6.23 (2.96 to 12.12)	5.56 (2.55 to 10.80)	5.88 (2.80 to 11.20)
**Other communicable, maternal, neonatal, and nutritional diseases**	**98,721.94 (43,810.12 to 196,458.54)**	**37,861.18 (21,115.71 to 64,876.82)**	**22,783.92 (13,770.41 to 36,791.77)**	**41,904.55 (22,780.16 to 73,682.50)**	**1,292.92 (633.83 to 2,445.04)**	**643.96 (415.92 to 980.77)**	**436.11 (298.73 to 616.11)**	**688.12 (439.71 to 1,077.60)**
Sexually transmitted infections excluding HIV	82,424.28 (29,244.09 to 171,592.66)	20,619.26 (6,638.69 to 44,125.61)	10,806.38 (3,820.62 to 22,938.03)	23,504.66 (7,287.92 to 51,027.90)	977.58 (355.37 to 2,012.64)	253.42 (89.39 to 528.63)	136.75 (52.82 to 279.52)	287.37 (99.36 to 607.00)
Syphilis	81,260.07 (28,246.52 to 170,708.62)	19,668.28 (5,884.50 to 43,231.06)	10,033.00 (3,124.75 to 22,075.88)	22,518.82 (6,455.90 to 50,362.88)	952.39 (333.14 to 1,992.14)	232.61 (71.28 to 507.95)	119.58 (38.56 to 260.53)	265.92 (77.98 to 590.52)
Chlamydial infection	227.18 (100.45 to 339.98)	186.91 (83.09 to 284.88)	151.39 (67.73 to 234.77)	193.99 (87.51 to 292.46)	4.87 (2.14 to 7.31)	4.05 (1.84 to 6.22)	3.33 (1.51 to 5.16)	4.18 (1.92 to 6.37)
Gonococcal infection	599.49 (267.52 to 908.76)	488.34 (217.12 to 747.38)	398.65 (177.75 to 623.48)	505.73 (225.85 to 779.53)	13.05 (5.82 to 19.58)	10.77 (4.88 to 16.43)	8.92 (4.02 to 13.77)	11.08 (5.03 to 17.09)
Other sexually transmitted infections	337.54 (151.17 to 511.06)	275.73 (121.84 to 429.47)	223.34 (99.50 to 348.56)	286.11 (129.23 to 441.53)	7.27 (3.25 to 10.91)	5.99 (2.60 to 9.39)	4.91 (2.17 to 7.79)	6.18 (2.78 to 9.66)
Acute hepatitis	5,972.38 (4,302.61 to 7,852.32)	5,319.91 (3,478.75 to 7,625.84)	4,012.08 (2,590.97 to 5,755.60)	5,560.63 (3,569.84 to 7,952.58)	127.36 (91.84 to 168.13)	128.05 (84.22 to 180.04)	100.42 (65.32 to 143.27)	131.38 (85.60 to 185.55)
Acute hepatitis A	386.59 (207.57 to 681.26)	179.08 (86.28 to 332.27)	105.71 (55.85 to 184.62)	198.97 (95.97 to 373.93)	5.36 (3.16 to 8.76)	2.92 (1.61 to 4.87)	1.94 (1.15 to 3.04)	3.14 (1.71 to 5.33)
Acute hepatitis B	4,389.27 (3,117.90 to 5,913.52)	4,322.83 (2,750.53 to 6,330.53)	3,310.04 (2,092.39 to 4,790.14)	4,496.24 (2,855.93 to 6,523.20)	102.66 (72.37 to 138.35)	109.12 (70.34 to 155.78)	85.63 (54.56 to 123.67)	111.85 (71.91 to 162.14)

Acute hepatitis C	104.89 (65.38 to 160.62)	105.93 (61.46 to 166.39)	91.82 (53.61 to 144.21)	106.98 (62.66 to 170.74)	2.37 (1.46 to 3.49)	2.81 (1.65 to 4.44)	2.56 (1.49 to 4.04)	2.77 (1.63 to 4.38)
Acute hepatitis E	1,091.63 (668.24 to 1,683.84)	712.07 (383.25 to 1,194.33)	504.51 (276.30 to 822.73)	758.44 (411.86 to 1,288.63)	16.96 (10.64 to 25.06)	13.20 (7.43 to 20.73)	10.29 (5.91 to 15.89)	13.61 (7.69 to 21.74)
Other unspecified infectious diseases	10,325.28 (5,668.42 to 23,335.36)	11,922.01 (7,283.62 to 19,839.23)	7,965.45 (5,168.07 to 12,297.27)	12,839.26 (7,968.93 to 21,779.98)	187.98 (118.11 to 340.40)	262.49 (179.39 to 386.42)	198.94 (138.86 to 276.05)	269.38 (183.10 to 402.53)
**Non-communicable diseases**	**1,449,482.06 (1,192,023.16 to 1,734,187.77)**	**1,996,390.84 (1,619,516.15 to 2,404,831.11)**	**1,263,927.41 (1,035,604.46 to 1,533,106.62)**	**2,475,828.96 (2,023,513.83 to 2,975,526.06)**	**44,899.71 (37,775.27 to 52,659.95)**	**71,162.31 (61,372.30 to 81,672.46)**	**49,186.93 (42,702.08 to 56,401.97)**	**85,581.20 (73,659.91 to 97,217.31)**

Papua New Guinea								
Cause	YLLs (actual counts)				Deaths (actual counts)			
	2016	2040 Forecast	2040 Better Scenario	2040 Worse Scenario	2016	2040 Forecast	2040 Better Scenario	2040 Worse Scenario
**Neoplasms**	**149,171.00 (116,518.02 to 188,674.90)**	**230,928.12 (175,859.72 to 300,323.24)**	**205,036.17 (161,044.39 to 256,853.73)**	**254,623.92 (190,780.27 to 336,237.71)**	**4,478.66 (3,561.63 to 5,577.55)**	**7,844.97 (6,161.95 to 9,949.98)**	**7,468.31 (6,151.17 to 9,093.09)**	**8,359.59 (6,415.84 to 10,846.42)**
Lip and oral cavity cancer	2,190.12 (1,674.06 to 2,855.88)	3,582.35 (1,895.12 to 6,633.42)	2,363.72 (1,364.76 to 4,114.64)	5,315.60 (2,610.42 to 10,213.16)	71.46 (55.95 to 92.62)	125.88 (70.06 to 223.36)	90.29 (55.28 to 152.06)	177.50 (90.14 to 325.31)
Nasopharynx cancer	2,293.88 (1,486.87 to 3,207.02)	4,214.74 (1,968.90 to 8,295.84)	2,543.90 (1,271.68 to 4,870.47)	6,366.50 (2,779.05 to 13,049.48)	67.33 (44.01 to 92.89)	132.49 (64.33 to 258.01)	83.33 (41.85 to 153.14)	195.22 (86.33 to 394.01)
Other pharynx cancer	2,257.32 (1,447.21 to 3,338.58)	2,812.35 (1,548.83 to 4,755.90)	2,381.67 (1,370.30 to 3,893.96)	3,612.43 (1,823.40 to 6,382.92)	71.81 (47.03 to 103.74)	97.27 (55.75 to 160.99)	87.27 (52.72 to 141.38)	120.31 (61.84 to 207.78)
Esophageal cancer	1,766.77 (1,378.83 to 2,260.04)	2,795.01 (1,572.11 to 4,837.64)	1,938.86 (1,173.53 to 3,162.38)	3,915.66 (1,994.54 to 7,085.91)	60.92 (48.52 to 76.36)	104.65 (60.52 to 172.67)	77.86 (48.84 to 120.52)	140.40 (74.69 to 243.11)
Stomach cancer	14,479.29 (11,411.38 to 17,928.78)	17,012.51 (12,950.12 to 22,109.39)	15,142.57 (11,828.96 to 19,265.76)	17,409.84 (13,035.93 to 23,089.89)	475.80 (379.07 to 581.30)	622.01 (490.84 to 773.76)	595.16 (477.43 to 727.10)	610.43 (473.62 to 778.46)
Colon and rectum cancer	8,388.13 (5,877.90 to 12,149.93)	15,816.20 (9,922.52 to 25,619.70)	16,509.43 (10,796.93 to 25,685.59)	16,504.26 (10,045.39 to 26,792.63)	288.40 (210.07 to 411.05)	609.93 (392.10 to 984.65)	685.06 (457.91 to 1,070.61)	606.02 (378.53 to 996.61)
Liver cancer	8,351.41 (5,928.21 to 12,351.55)	14,985.87 (9,567.58 to 23,852.10)	13,647.98 (9,165.18 to 21,187.89)	17,299.93 (10,490.33 to 28,600.79)	267.11 (192.27 to 395.47)	527.88 (352.75 to 835.07)	514.23 (356.43 to 791.56)	583.28 (365.85 to 946.79)
Liver cancer due to hepatitis B	4,838.78 (3,396.55 to 7,380.95)	8,379.66 (5,361.80 to 13,593.87)	7,656.80 (5,024.59 to 11,921.58)	9,571.33 (5,843.23 to 16,116.26)	146.53 (103.37 to 222.05)	278.24 (183.94 to 436.95)	270.04 (184.82 to 409.57)	305.87 (191.47 to 501.28)
Liver cancer due to hepatitis C	1,249.40 (873.44 to 1,996.47)	2,420.11 (1,589.47 to 4,025.04)	2,339.94 (1,604.79 to 3,830.34)	2,654.43 (1,631.15 to 4,511.56)	48.38 (34.56 to 77.24)	102.42 (68.17 to 167.97)	107.06 (74.28 to 169.96)	106.61 (67.91 to 178.50)
Liver cancer due to alcohol use	567.77 (377.63 to 902.63)	1,066.18 (657.01 to 1,805.74)	911.17 (595.65 to 1,508.25)	1,474.29 (854.33 to 2,570.08)	19.90 (13.64 to 31.34)	39.89 (25.24 to 66.05)	36.54 (24.26 to 59.05)	52.05 (31.31 to 87.75)
Liver cancer due to other causes	1,695.45 (1,177.04 to 2,493.89)	3,119.93 (1,891.44 to 5,298.26)	2,740.07 (1,735.42 to 4,468.83)	3,599.87 (2,040.40 to 6,344.51)	52.29 (37.09 to 76.33)	107.33 (64.30 to 181.81)	100.58 (63.48 to 164.17)	118.75 (66.62 to 212.53)
Gallbladder and biliary tract cancer	1,262.13 (845.11 to 1,876.06)	1,448.42 (1,016.10 to 2,050.34)	1,623.27 (1,154.26 to 2,280.95)	1,345.57 (937.19 to 1,906.42)	45.98 (32.23 to 66.41)	57.74 (40.93 to 79.92)	68.81 (49.91 to 93.69)	51.71 (36.22 to 72.07)
Pancreatic cancer	2,929.26 (2,323.33 to 3,638.59)	6,238.85 (4,396.62 to 8,656.76)	6,394.83 (4,714.37 to 8,617.10)	6,157.91 (4,250.67 to 8,623.18)	109.58 (89.31 to 134.36)	251.03 (181.64 to 346.50)	273.79 (205.37 to 365.27)	238.26 (170.64 to 333.16)
Larynx cancer	1,828.66 (1,438.26 to 2,258.75)	2,720.90 (1,348.60 to 4,777.19)	1,619.61 (862.92 to 2,926.31)	3,671.51 (1,776.76 to 6,474.94)	59.89 (47.45 to 73.51)	98.87 (50.05 to 173.89)	60.94 (33.92 to 108.94)	129.48 (63.02 to 226.91)
Tracheal, bronchus, and lung cancer	19,404.72 (14,735.28 to 26,543.48)	33,169.59 (18,144.70 to 58,929.85)	19,198.11 (11,485.63 to 33,636.58)	45,347.52 (24,450.74 to 79,124.90)	667.27 (514.57 to 898.38)	1,245.02 (700.54 to 2,216.18)	745.01 (458.98 to 1,283.49)	1,659.16 (916.92 to 2,895.89)
Malignant skin melanoma	645.94 (391.06 to 1,131.08)	1,163.63 (710.68 to 2,051.34)	1,437.57 (868.43 to 2,629.62)	1,063.77 (654.25 to 1,819.46)	20.99 (13.10 to 36.39)	42.25 (26.34 to 74.02)	56.10 (35.03 to 101.44)	37.02 (23.21 to 62.92)
Non-melanoma skin cancer	1,316.50 (921.93 to 1,808.05)	2,476.19 (1,803.81 to 3,248.52)	2,422.09 (1,771.51 to 3,185.66)	2,363.62 (1,718.87 to 3,073.49)	46.34 (33.95 to 60.93)	100.71 (77.15 to 127.72)	109.18 (83.35 to 137.58)	90.79 (68.53 to 117.41)
Breast cancer	17,275.08 (8,687.20 to 28,590.48)	27,499.01 (13,112.60 to 47,913.14)	26,516.46 (13,463.91 to 43,802.57)	29,069.11 (13,675.43 to 51,433.96)	506.59 (268.99 to 821.22)	883.45 (438.31 to 1,518.75)	898.48 (475.18 to 1,475.81)	905.18 (441.44 to 1,584.18)
Cervical cancer	15,712.56 (8,259.55 to 22,841.69)	19,503.58 (10,286.21 to 30,410.34)	16,610.09 (9,230.75 to 25,273.66)	20,357.46 (10,370.93 to 31,643.28)	444.28 (240.41 to 643.54)	582.84 (317.87 to 880.40)	514.74 (292.27 to 762.89)	595.52 (311.62 to 918.58)

Uterine cancer	4,183.62 (2,793.60 to 5,729.76)	6,691.82 (4,628.88 to 8,993.89)	5,981.95 (4,202.66 to 8,038.09)	6,600.86 (4,423.86 to 8,912.93)	139.74 (96.70 to 186.74)	240.97 (168.02 to 318.45)	225.07 (162.42 to 294.62)	231.83 (157.36 to 308.69)
Ovarian cancer	1,879.13 (1,286.54 to 2,806.93)	3,364.55 (2,124.23 to 5,493.41)	3,847.11 (2,477.12 to 6,166.13)	3,215.00 (2,020.93 to 5,266.80)	62.61 (43.66 to 92.45)	120.22 (75.91 to 194.06)	142.69 (93.35 to 225.18)	112.26 (70.40 to 183.71)
Prostate cancer	2,924.14 (2,115.28 to 4,558.16)	8,001.40 (5,713.52 to 12,214.15)	9,853.71 (7,438.30 to 14,606.26)	6,955.52 (4,812.64 to 10,886.20)	152.69 (112.87 to 229.25)	465.12 (321.28 to 705.08)	615.61 (459.81 to 892.74)	386.27 (252.90 to 610.42)
Testicular cancer	596.86 (426.57 to 797.74)	776.50 (538.14 to 1,064.92)	711.98 (493.95 to 981.36)	779.44 (546.40 to 1,066.65)	12.39 (9.09 to 16.30)	17.21 (12.57 to 22.64)	16.40 (11.92 to 21.51)	16.87 (12.30 to 22.36)

Papua New Guinea								
Cause	YLLs (actual counts)				Deaths (actual counts)			
	2016	2040 Forecast	2040 Better Scenario	2040 Worse Scenario	2016	2040 Forecast	2040 Better Scenario	2040 Worse Scenario
Kidney cancer	817.28 (616.28 to 1,086.10)	1,542.11 (1,081.96 to 2,216.20)	1,642.28 (1,169.85 to 2,322.90)	1,523.68 (1,066.41 to 2,200.67)	22.64 (17.23 to 30.23)	50.86 (35.67 to 71.95)	59.53 (41.66 to 82.39)	48.00 (33.42 to 69.24)
Bladder cancer	1,236.31 (900.69 to 1,807.54)	2,131.45 (1,312.47 to 3,615.41)	1,907.30 (1,235.29 to 3,116.55)	2,190.69 (1,320.17 to 3,764.10)	47.75 (35.51 to 68.56)	90.14 (57.22 to 149.98)	87.30 (58.99 to 141.59)	88.33 (54.90 to 147.88)
Brain and nervous system cancer	3,393.24 (2,322.99 to 4,434.61)	5,676.49 (3,727.43 to 7,332.69)	5,550.08 (3,605.74 to 7,176.39)	5,612.89 (3,678.02 to 7,241.77)	71.57 (46.71 to 93.58)	136.01 (85.15 to 174.39)	146.47 (90.98 to 189.71)	129.28 (81.81 to 165.33)
Thyroid cancer	844.42 (582.16 to 1,171.68)	1,271.15 (870.82 to 1,818.50)	1,180.45 (826.22 to 1,654.42)	1,323.60 (876.35 to 1,955.53)	29.85 (20.70 to 41.33)	49.06 (34.16 to 69.25)	48.48 (34.39 to 66.91)	49.20 (32.86 to 71.52)
Mesothelioma	333.47 (218.10 to 541.52)	548.36 (368.50 to 885.30)	552.63 (370.61 to 919.67)	526.63 (355.75 to 846.49)	10.23 (6.73 to 16.32)	18.24 (12.48 to 28.59)	19.25 (13.06 to 31.08)	17.02 (11.73 to 26.50)
Hodgkin lymphoma	1,296.98 (941.20 to 1,687.86)	1,461.61 (1,020.15 to 2,005.67)	1,113.09 (775.66 to 1,523.32)	1,520.69 (1,059.72 to 2,078.97)	28.70 (21.17 to 37.30)	35.38 (25.90 to 47.52)	28.74 (20.87 to 38.70)	35.68 (25.87 to 48.06)
Non-Hodgkin lymphoma	4,312.14 (3,360.12 to 5,784.20)	6,682.09 (5,175.76 to 8,955.99)	7,028.97 (5,498.45 to 9,553.32)	6,641.27 (5,071.76 to 9,015.97)	105.64 (83.46 to 142.58)	189.56 (149.30 to 249.47)	220.62 (174.00 to 291.07)	179.82 (139.61 to 241.99)
Multiple myeloma	1,308.91 (878.50 to 1,762.56)	2,482.10 (1,689.89 to 3,436.93)	2,881.96 (1,983.96 to 3,948.41)	2,404.41 (1,629.00 to 3,324.61)	44.73 (31.01 to 59.65)	93.34 (65.38 to 129.51)	114.47 (81.30 to 157.09)	87.40 (60.32 to 121.34)
Leukemia	14,260.74 (9,191.15 to 20,530.93)	17,067.96 (11,088.47 to 24,680.54)	15,003.27 (10,056.58 to 21,043.06)	18,033.18 (11,547.82 to 26,516.73)	277.47 (183.25 to 383.92)	387.58 (261.97 to 541.05)	375.15 (261.62 to 510.84)	397.00 (262.98 to 569.08)
Acute lymphoid leukemia	2,783.97 (1,611.38 to 5,109.05)	3,143.41 (1,800.74 to 5,632.47)	2,704.71 (1,592.94 to 4,595.30)	3,306.26 (1,895.36 to 5,962.53)	43.90 (26.73 to 76.15)	55.34 (34.19 to 93.98)	51.46 (32.05 to 83.11)	57.45 (35.02 to 98.69)
Chronic lymphoid leukemia	173.88 (108.66 to 256.46)	273.30 (166.62 to 417.91)	281.48 (172.99 to 426.40)	276.04 (164.55 to 430.97)	6.16 (3.80 to 9.08)	10.80 (6.58 to 16.44)	11.99 (7.42 to 18.18)	10.45 (6.20 to 16.31)
Acute myeloid leukemia	3,689.49 (2,346.13 to 5,453.14)	5,443.64 (3,495.19 to 7,632.01)	5,341.66 (3,514.31 to 7,226.85)	5,619.21 (3,620.31 to 8,010.09)	68.02 (43.05 to 97.30)	116.81 (76.26 to 161.14)	125.59 (84.58 to 166.93)	117.33 (76.56 to 162.87)
Chronic myeloid leukemia	1,887.52 (1,131.05 to 2,956.08)	1,929.80 (1,104.73 to 3,231.81)	1,859.50 (1,073.48 to 3,058.46)	2,038.22 (1,132.93 to 3,457.80)	49.73 (30.23 to 77.62)	55.65 (33.35 to 91.87)	56.16 (34.15 to 91.10)	57.17 (32.98 to 95.86)
Other leukemia	5,725.88 (3,056.84 to 9,391.08)	6,277.81 (3,232.94 to 10,793.74)	4,815.92 (2,529.98 to 8,189.74)	6,793.46 (3,462.41 to 11,964.36)	109.66 (61.89 to 172.15)	148.99 (83.06 to 240.51)	129.95 (73.50 to 205.28)	154.61 (83.55 to 251.43)
Other neoplasms	11,682.00 (8,978.58 to 14,404.41)	17,791.31 (14,006.26 to 21,448.92)	17,431.25 (13,814.56 to 20,980.19)	17,495.40 (13,822.14 to 21,188.91)	268.91 (207.82 to 330.29)	469.27 (377.28 to 559.09)	508.28 (408.58 to 603.89)	440.35 (350.72 to 526.46)
**Cardiovascular diseases**	**578,900.52 (478,359.21 to 698,622.91)**	**750,821.91 (529,042.55 to 1,026,458.65)**	**360,429.94 (259,403.78 to 505,098.62)**	**1,048,497.63 (756,624.25 to 1,358,476.32)**	**18,809.31 (15,656.19 to 22,305.02)**	**27,930.50 (20,504.26 to 37,575.21)**	**15,129.07 (11,414.42 to 21,610.39)**	**37,741.24 (27,800.17 to 49,041.53)**
Rheumatic heart disease	45,941.03 (32,784.79 to 61,134.66)	29,653.73 (20,002.62 to 41,365.28)	20,703.59 (14,168.49 to 28,199.64)	31,543.10 (21,276.90 to 42,891.14)	1,054.56 (731.13 to 1,421.01)	816.43 (561.51 to 1,102.07)	628.13 (443.29 to 832.90)	829.78 (560.08 to 1,114.21)
Ischemic heart disease	254,670.74 (206,650.02 to 317,547.91)	364,873.57 (192,314.89 to 612,304.09)	150,899.27 (79,579.03 to 278,287.76)	524,438.26 (305,652.87 to 802,297.36)	8,916.89 (7,328.77 to 10,899.96)	13,922.02 (7,847.47 to 24,178.16)	6,568.16 (3,686.05 to 12,190.29)	19,351.14 (11,679.44 to 31,109.77)
Stroke	205,014.73 (163,969.89 to 251,962.21)	243,252.59 (145,157.75 to 374,024.88)	101,284.44 (61,880.92 to 170,004.07)	365,675.16 (224,535.32 to 528,417.06)	6,619.78 (5,320.25 to 7,979.71)	9,105.51 (5,571.33 to 15,036.80)	4,378.54 (2,776.55 to 7,958.65)	13,202.54 (8,323.64 to 19,896.38)
Ischemic stroke	46,365.44 (37,010.69 to 56,689.40)	57,458.68 (31,616.07 to 130,904.37)	31,055.55 (16,506.18 to 72,079.04)	75,432.08 (42,660.91 to 152,513.85)	1,915.17 (1,546.20 to 2,294.09)	2,710.48 (1,390.29 to 7,226.51)	1,659.81 (847.03 to 4,391.81)	3,433.32 (1,766.32 to 7,956.51)

Intracerebral hemorrhage	158,649.29 (126,859.39 to 195,566.31)	185,793.92 (99,367.43 to 314,511.03)	70,228.89 (39,353.24 to 125,921.75)	290,243.08 (166,259.78 to 443,819.08)	4,704.62 (3,748.43 to 5,731.18)	6,395.03 (3,582.32 to 11,227.61)	2,718.72 (1,619.74 to 4,860.50)	9,769.22 (5,571.89 to 15,796.85)
Hypertensive heart disease	22,247.42 (12,685.31 to 30,880.31)	37,577.40 (20,901.98 to 59,223.88)	25,534.88 (15,418.96 to 37,590.46)	47,413.35 (25,219.58 to 80,195.47)	815.01 (477.67 to 1,120.81)	1,545.65 (895.51 to 2,339.89)	1,165.87 (713.32 to 1,662.21)	1,839.61 (1,005.00 to 3,077.95)
Cardiomyopathy and myocarditis	15,847.00 (8,275.01 to 23,900.52)	18,694.10 (10,279.80 to 27,492.10)	17,255.48 (9,773.84 to 24,725.71)	19,170.28 (10,299.77 to 28,535.29)	377.20 (215.41 to 540.90)	546.40 (313.80 to 788.28)	562.68 (334.13 to 796.82)	533.08 (299.36 to 778.29)
Myocarditis	4,074.07 (2,291.92 to 6,476.00)	4,753.36 (2,793.39 to 7,266.54)	4,227.32 (2,494.19 to 6,409.80)	4,753.81 (2,758.46 to 7,305.21)	101.84 (57.17 to 160.54)	145.30 (82.85 to 231.14)	143.64 (80.75 to 229.55)	137.97 (78.57 to 218.65)

Papua New Guinea								
Cause	YLLs (actual counts)				Deaths (actual counts)			
	2016	2040 Forecast	2040 Better Scenario	2040 Worse Scenario	2016	2040 Forecast	2040 Better Scenario	2040 Worse Scenario
Alcoholic cardiomyopathy	874.25 (385.69 to 1,682.35)	941.88 (434.64 to 1,680.30)	882.58 (411.55 to 1,499.30)	1,228.03 (557.00 to 2,262.12)	25.87 (11.80 to 46.90)	30.99 (14.62 to 52.55)	31.29 (14.90 to 51.28)	37.90 (18.03 to 66.67)
Other cardiomyopathy	10,898.68 (5,383.26 to 16,693.32)	12,998.87 (6,755.48 to 19,832.26)	12,145.57 (6,573.12 to 18,550.81)	13,188.43 (6,692.06 to 20,555.61)	249.49 (135.73 to 362.01)	370.11 (200.04 to 572.84)	387.75 (217.95 to 597.20)	357.21 (191.90 to 561.47)
Atrial fibrillation and flutter	685.36 (507.32 to 906.77)	1,485.05 (1,037.75 to 2,008.72)	1,678.78 (1,234.43 to 2,230.16)	1,423.62 (972.69 to 1,976.28)	48.18 (35.99 to 63.24)	110.97 (71.79 to 155.35)	142.45 (96.90 to 193.37)	98.53 (61.19 to 142.98)
Aortic aneurysm	4,598.35 (2,801.77 to 7,358.56)	6,969.35 (4,276.60 to 11,140.90)	6,661.52 (4,226.27 to 10,416.51)	7,088.11 (4,238.60 to 11,350.64)	165.78 (106.78 to 260.39)	278.24 (181.86 to 423.93)	297.96 (202.86 to 446.66)	266.84 (170.48 to 408.86)
Peripheral artery disease	155.04 (62.19 to 376.14)	486.17 (184.95 to 1,239.50)	473.13 (192.77 to 1,159.32)	488.37 (182.39 to 1,265.27)	6.67 (2.79 to 14.97)	23.38 (9.20 to 57.39)	26.42 (11.28 to 63.67)	21.85 (8.38 to 54.22)
Endocarditis	6,842.54 (4,941.69 to 9,268.11)	10,209.16 (7,386.77 to 13,850.99)	9,297.43 (6,650.66 to 12,670.47)	10,352.97 (7,448.42 to 14,122.92)	187.06 (135.83 to 253.47)	326.88 (231.94 to 450.45)	335.09 (238.05 to 467.76)	313.37 (223.84 to 437.44)
Other cardiovascular and circulatory diseases	22,898.30 (13,849.82 to 32,050.11)	37,620.77 (22,613.16 to 52,746.84)	26,641.42 (16,419.53 to 36,699.15)	40,904.42 (24,404.55 to 58,666.73)	618.18 (370.36 to 848.64)	1,255.01 (756.42 to 1,726.48)	1,023.77 (637.07 to 1,398.83)	1,284.48 (750.05 to 1,794.53)
**Chronic respiratory diseases**	**265,309.15 (213,103.93 to 319,415.77)**	**303,894.72 (218,526.82 to 431,863.78)**	**178,483.74 (138,862.27 to 232,012.56)**	**397,503.48 (270,598.61 to 590,169.87)**	**9,804.70 (7,780.74 to 11,775.55)**	**12,480.57 (9,039.66 to 17,403.30)**	**7,820.44 (6,102.09 to 9,852.41)**	**15,624.15 (10,757.24 to 22,529.47)**
[truncated: 393,988 more chars]
