# Supplementary material for: Forecasting life expectancy, years of life lost, and all-cause and cause-specific mortality for 250 causes of death: reference and alternative scenarios for 2016–40 for 195 countries and territories
Source: Lancet. 2018 Nov 10;392(10159):2052–90. doi: 10.1016/S0140-6736(18)31694-5 (PMC6227505; doi:10.1016/S0140-6736(18)31694-5)
Supplement: Supplementary appendix 1 [file mmc1.pdf]

THE LANCET

Supplementary appendix 1

This appendix formed part of the original submission and has been peer reviewed. We post it as supplied by the authors.

Supplement to: Foreman K J, Marquez N, Dolgert A, et al. Forecasting life expectancy, years of life lost, and all-cause and cause-specific mortality for 250 causes of death: reference and alternative scenarios for 2016–40 for 195 countries and territories. *Lancet* 2018; published online October 16. http://dx.doi.org/10.1016/S0140-6736(18)31694-5.

Methods Appendix: Forecasting life expectancy, years of life lost, all-cause, and cause-specific mortality for 250 causes of death: reference and alternative scenarios 2016–2040 for 195 countries and territories

Preamble

This appendix provides further methodological detail and more detailed results for "Forecasting life expectancy, years of life lost, all-cause, and cause-specific mortality for 250 causes of death: reference and alternative scenarios 2016–2040 for 195 countries and territories." It includes detailed tables and information on data source in an effort to maximize transparency in our estimation processes and provide a comprehensive description of analytical steps.

Table of Contents

List of Abbreviations 3

1. List of Figures and Tables 4

2. Introduction to independent-driver mortality forecasting 5

3. Overview of the forecasting model and scenarios 7

4. The Forecasting Model 9

Cause-Specific Mortality Modeling 9

Accounting for risk factors 9

Basic model 9

Smoothing priors 10

Modeling Latent Trends 10

Cascading Mortality Models 10

5. Modelling relationships between drivers and mortality 12

Modeling overview by cause and sex 12

Mediation, Scalars, and Population Attributable Fractions (PAFs) 13

6. Forecasting independent drivers of health 15

Risk Factors – Summary Exposure Values 15

Reference scenario or forecast 16

Better and worse scenarios for the risk factors 20

Smoking impact ratio (SIR) 21

Vaccines 22

Reference, better, and worse scenarios for DTP3 and measles 22

Reference, better, and worse scenarios for the ratio vaccines (Rotavirus, PCV, Hib) 24

Vehicles per Capita 25

Better and worse scenarios 25

Sociodemographic index 25

Income per Capita 26

Education 28

Fertility 30

Youngest and Oldest Age Groups 31

Better and Worse Scenarios 31

7. Causes of Death Forecasted Outside the Main Framework 32

HIV/AIDS 32
Anti-retroviral therapy for HIV/AIDS coverage 32
Cross-walking Cross-Sectional and Spectrum CD4 Definitions 32
Modeling ART Coverage Frontier as a Function of Income and CD4 Count 33
ART Price Forecasts 34
Forecasting ART Prices 34
Forecasting Scenarios of Spectrum Inputs 36
Forecasting ART Coverage 37
HIV Incidence Hazard 38
War, legal interventions and disasters 39
8. Population forecasts 40
Key Features of the Approach 42
Migration 42
Interpolation and Disaggregation 42
Oldest Ages 42
Internal Verification of Population Model 43
Better and Worse Scenarios 43
9. Model Performance – Out of time predictive validity 43
10. References 60

List of Abbreviations

Abbreviation	Description
ARC	Annualized rate of change
ARIMA	Auto-regressive integrated moving average
ART	Anti-retroviral therapy
ASFR	Age-specific fertility rate
CCMP	Cohort component method of projection
CF	Correction factor
COPD	Chronic obstructive pulmonary disease
CPS	Cigarettes per smoker
CV	Coefficient of variation
DAH	Development assistance for health
DTP-3	Diphtheria-Tetanus-Pertussis dose 3

FPG	Fasting plasma glucose
GBD	Global burden of disease
GDP	Gross domestic product
GHES	Government health expenditure per capita
GPRM	Global price reporting mechanism
HIB	Haemophilus influenzae type B
HIV	Human immunodeficiency virus
IPV	Intimate partner violence
IQR	Interquartile range
LDI	Lag-distributed income
MCV	Meningococcal conjugate vaccine
MEDU	Maternal education
MF	Mediation factor
OOS	Out-of-sample
PAF	Population attributable fraction
PAH	polycyclic aromatic hydrocarbons
PCV	Pneumococcal conjugate vaccine
PMTCT	Prevention of mother-to-child-transmission
PUFA	Polyunsaturated fatty acids
RMSE	Root mean square error
RR	Relative risk
SDI	Sociodemographic index
SEV	Summary exposure value
SHS	Secondhand smoking
SIR	Smoking impact ratio
ST-GPR	Spatio-temporal Gaussian process regression
TFR	Total fertility rate
VPC	Vehicles per capita
WPP	World population prospects

1. List of Figures and Tables

Title	Page
Appendix Figure 1. Flow diagram outlining the mortality forecasting model	8
Appendix Table 1. Omega weights selected for forecasting risk factors, education, fertility, and vaccines	17
Appendix Figure 2. Summary Exposure Value (SEV) forecasts for a sample country and subset of risks	22

Appendix Figure 3. Vaccine coverage for forecast, better, and worse scenarios for a sample country	24
Appendix Table 2. Sociodemographic Index (SDI) indicator scales	26
Appendix Figure 4. GDP per capita forecast, better, and worse scenarios for four sample countries	27
Appendix Figure 5. Education forecast, better, and worse scenarios for four sample countries	29
Appendix Figure 6. Categorical backcast of CD4 survey microdata using modelled progression curves	33
Appendix Figure 7. Predicted probabilities of ART coverage for each individual shown as points	34
Appendix Figure 8. Median and IQR of ART price over time globally	35
Appendix Figure 9. Scenarios of exogenous HIV input projections for sample country (Zambia)	37
Appendix Figure 10. Population forecast by GBD super region	41
Appendix Figure 11. Global Population forecast (reference scenario) by age group	41
Appendix Table 3. Predictive validity for life expectancy forecasts and comparison to Lee Carter.	44
Appendix Figure 12. Out of sample life expectancy predictions for six sample countries.	44
Appendix Table 4. Predictive validity for all-cause mortality forecasts and comparison to Lee Carter.	45
Appendix Figure 13. Out of sample predictions for all- cause mortality for six sample countries.	46
Appendix Table 5. Predictive validity for cause-specific mortality forecasts	48
Appendix Figure 14. Out of sample predictions for two sample causes and six sample countries.	58

2. Introduction to independent-driver mortality forecasting

Models of future mortality used for forecasts (or reference scenarios) or alternative scenarios can be characterized by three attributes. First, whether they are developed to forecast all-cause mortality or all-cause mortality and a set of component causes that aggregate to all-cause mortality. Second, what variables are used to predict the outcomes: time alone or multiple covariates that usually includes time. Third, whether the modeled relationships are developed solely to have good predictive power out of sample or whether the relationships are meant to capture accurately causal connections between covariates on the outcome. Many demographic studies predict life expectancy as a single indicator and use time as the sole covariate.[1–6] A more limited set of studies have predicted age-specific mortality and an even more limited set predict age-specific all-cause mortality and cause-specific all-cause mortality at the same time.[7,8]

Our model belongs to the category of health forecast models where we use many independent variables in addition to time. The advantage of this approach is that we can use the estimated models not only for making a forecast or reference scenario but for other independent scenarios as well. We have designed our model structure, which is outlined below, to capture a range of causal connections included in the GBD risk factor assessment, select interventions with a critical impact on disease-specific outcomes and other broader socio-economic drivers. Each independent driver used in the analysis must also be forecasted in the reference scenario. These drivers include gross domestic product (GDP), education, fertility, individual risk factors (smoking, sanitation, blood pressure, diet, air pollution and many more), interventions (vaccines, other large scale treatment or prevention programs, e.g. antiretroviral treatment [ART] for HIV, prevention of mother-to-child-transmission [PMTCT] of HIV, or malaria prevention programs), and other health system factors.

There is a long and rich tradition of forecasting life expectancy or age-specific mortality using only time as an independent variable, but less work has been done on models attempting to forecast mortality based on causal connections to independent drivers, several of them within the global burden of disease framework.[7,8] There are many reasons for this. First, using covariates in addition to time requires a knowledge-synthesis of causal links between independent drivers and mortality; second, it requires standardized data and methods for forecasting these independent drivers. Put simply, time is easy to forecast as an independent driver whereas smoking prevalence or GDP per capita is more complicated. Depending on the detail of the independent drivers, models with more covariates may also need to deal with how covariates such as different risk factors influence each other such as how obesity influences systolic blood pressure. The great advantage of models with more covariates, especially ones where the causal connections between the independent drivers and the outcomes are supported by trial or cohort data as well as statistical cross-sectional time series associations, is the use of these models for developing alternative scenarios.

The Global Burden of Disease Study (GBD) has been refined through recent cycles and now holds the key components needed for global (195 countries, GBD regions) independent driver forecasting of mortality. In GBD 2013 and GBD 2015, three important innovations were introduced. First the Sociodemographic Index (SDI) which is a composite measure of income per capita, mean years of education, and total fertility under 25 years (TFU25), was estimated for all years from 1990 till present. Details on how the SDI is forecasted are given in Section 6. Second, the Summary Exposure Value (SEV) summarizes population exposure for a risk factor into a single measure ranging from 0 to 1 (no exposure to all population members fully exposed), for any risk factor, whether its exposure distribution is represented by a binary, multi-category, or continuous variable (or combinations of these).[9] Finally, mediation of some risks through other risks (for example high body-mass index through high systolic blood pressure and high cholesterol) were accounted for, starting in GBD 2013, in estimating disease burden for clusters of risks and all risks. In this paper we make combined use of the time series of cause-specific mortality for 195 countries and territories, by age and sex, for 205 causes from 1990 to 2016 with the comparative risk assessment including 65 risk factors, five vaccines, several other interventions, and the Sociodemographic index (SDI).[10–12]

As noted, models that use independent drivers in addition to time can be used specify scenarios based on posited future settings of the independent drivers or subsets of them. Below we provide a detailed overview of the independent drivers forecasting model. We start with giving an overview of the three component forecasting model.

3. Overview of the forecasting model and scenarios

The forecasting model is cause-specific and separate for males and females. It also generates estimates of all-cause mortality that are the sum of cause-specific estimates. The logarithm of the cause-specific mortality is modeled as the sum of three components: 1) underlying mortality; 2) a scalar that captures the combined effects of risk factors on the specific cause, accounting for mediation; and 3) and ARIMA model for the unexplained residual. The underlying mortality is modeled with terms for development (SDI), calendar time, and cause-specific covariates if appropriate. The scalar is a function of all the GBD risk factors relevant to each cause and select interventions tracked in the GBD. The third component is ARIMA forecasting of the residuals of the first two components model (underlying mortality plus combined effect of risk factors). Details are given in Section 4 on the forecasting model. Needed input for this modelling approach are forecasts from 2017 to 2040 for SDI, all individuals risk factors, vaccine interventions, ART coverage, and PMTCT coverage. Later in Section 6, we give the details of how we forecasted the independent drivers.

We develop a forecast or reference scenario which is meant to represent the most likely future trajectory of health given past trends of the independent drivers and the observed past relationships between independent drivers and each cause of death. The forecast is not what will happen only what would most likely happen if past trends and relationships continue into the future. To provide users of the forecasts the credible range of outcomes that might be observed, we also generate a better and worse scenario. In the better scenario, we set the pace of change for each of the independent drivers to the 85th percentile of rates of change observed in the past across locations and years. The better scenario is constructed from the 85th percentile rate of change for all the independent drivers; by chance this is very unlikely to occur in any location. However, it provides a useful upper bound to what might be achieved through policy or other actions to increase the pace of improvement for each of the independent drivers. Faster progress is possible; by definition for any given independent driver 15% of countries experienced faster rates of improvement. We construct a worse scenario using the 15th percentile of rate of change for each of the independent drivers in a similar fashion. Appendix Figure 1 provides an overview of the inputs and outputs of our forecasting model.

Appendix Figure 1. Flow diagram outlining the three component forecasting model. Data inputs are sourced from the Global Burden of Disease Study.

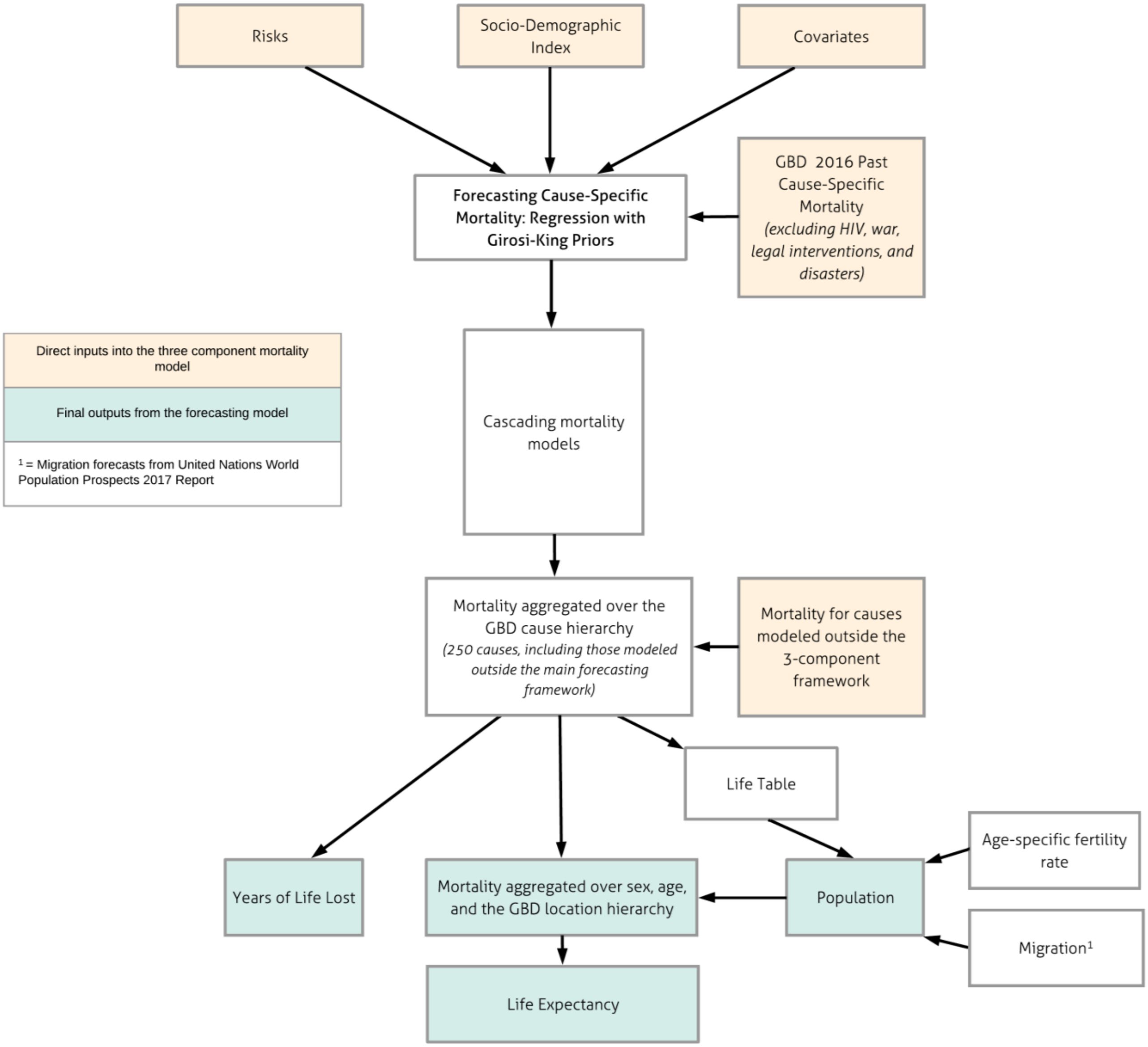

4. The Forecasting Model

Cause-Specific Mortality Modeling

Accounting for risk factors

We took advantage of the relationships between drivers and mortality that we will describe in detail in Section 5 to come up with an additive relationship (in log space) between underlying mortality rate m_U, the risk factor scalar $\mathbb{S}$, and total cause-specific mortality m_T:

$$m_T = m_U \times \mathbb{S}$$

Taking the logarithm gives

$$\ln(m_T) = \ln(m_U) + \ln(\mathbb{S}).$$

We thus accounted for risk factors by including $\ln(\mathbb{S})$ as an offset when modeling total cause-specific mortality.

Basic model

We assumed that underlying mortality m_U could be estimated as a function of SDI, location, age, and time. We used a model with location-age-specific intercepts α, a global effect on SDI β, and age-specific effects on the secular trend θ_a.

$$\ln(m_T) \sim \mathcal{N}(\mathfrak{y}, \sigma)$$

$$\mathfrak{y} = \alpha_{la} + \beta_0 SDI_{<0.8} + \beta_1 SDI_{\geq 0.8} + \theta_a t + \ln(\mathbb{S})$$

For some causes, other independent variables with strong known relationships for which data are available (ie, age-specific fertility for maternal causes, HIV mortality for maternal HIV, vehicles per capita for road injuries) or risk factors which cannot be quantified in terms of RR because they are part of the disease definition (eg, systolic blood pressure for hypertensive heart disease, fasting plasma glucose [FPG] for diabetes, alcohol consumption for alcohol-related cirrhosis; others are listed later in this appendix [pp 14]) were added as additional covariates to the model.

In addition, for a few non-communicable diseases with strong evidence of recent accelerated progress beyond what would be expected by SDI alone, we include an SDI*time interaction effect to capture this. These causes include ischaemic heart disease, diabetes, and all of the child causes of cirrhosis, stroke, and chronic kidney disease. Due to the collinearity of SDI, time, and other covariates in some causes of death, several models with the above formulation did not converge. To address this, all models with one or more coefficients whose standard deviation was more than 1000 times the absolute value of the median coefficient value were rerun without SDI. If the new formulation also did not converge, all covariates besides time were dropped from the underlying mortality formulation and the model was run once more.

Additionally, because SDI is included as an input to the vaccine forecasts, it was excluded as a covariate for all vaccine-dependent causes of death except lower respiratory infections.

Supplementary Results Appendix Figure 1 provides plots of the contributions to future rates of change in cause-specific mortality for each of the independent drivers in the model. These are generated by multiplying the annualized rates of change from 2016 to 2040 in each independent driver by that driver's coefficient, thus simulating the relative contribution of each independent driver to the forecast. These values are summarized across ages by country, with one point shown for each country and colored by GBD super region to show geographic trends.

Smoothing priors

By including the scalar $\mathbb{S}$ as an offset in the model, we were able to place Girosi-King (2008) type priors on the total cause-specific mortality while modeling underlying mortality.[13] These biased the model towards parameterizations that produce consistent age patterns over time by adding a penalty function based on the dot product of the first derivatives of adjacent age groups over time.

Modeling Latent Trends

The residuals ϵ from the basic model represent latent trends in total cause-specific mortality not captured by risk factors, SDI, and global secular trends.

$$\epsilon = \ln(m_T) - \hat{y}$$

We forecasted the latent trends by using an $ARIMA_{1,0,0}$ model, which combines an autoregressive model to capture overall trends and a differencing step to ensure stationarity and reflect expanding uncertainty in the future. However, running independent ARIMA models on the residuals of every cause, location, and age is not very robust and can lead to extreme forecasts. Therefore, we used a pooled model, which enabled us to share ARIMA parameters within geographic super-regions (s).

$$\epsilon_{lat} \sim \mathcal{N}(\hat{\epsilon}_{lat}, \sigma)$$

$$\hat{\epsilon}_{lat} = \psi_{sa}\hat{\epsilon}_{lat-1} + \delta_{sat}$$

$$\delta_{sat} \sim \mathcal{N}(0, \tau_{sa})$$

Cascading Mortality Models

In addition to using the above framework to model cause-specific latent trends, we also modeled the residuals at higher levels of the cause hierarchy in order to prevent a few unusual cause-specific trends from dominating our all-cause forecasts.

First, we generated all-cause total mortality predictions and residuals by summing up cause-specific forecasts based on risk factors, SDI and global secular trends.

$$\hat{Y} = \ln(\sum_{c}^{C} e^{\hat{y}_c})$$

$$E = \ln(M) - \hat{Y}$$

We then used an equally-weighted blend of ARIMA models to forecast these latent trends in the same way as the cause-specific forecasts. The latent trends in the summed all-cause are more robust than those from the cause-specific models alone, so modeling them at the location-age-sex specific level is more feasible. For the blend, we took an equal number of draws from each of four ARIMA specifications: ARIMA(1, 0, 0), ARIMA(1, 1, 0), ARIMA(1, 0, 0) + constant, and ARIMA(1, 1, 0) + constant. These correspond to

$$E_{lat} \sim \mathcal{N}(\hat{E}_{lat}, \sigma)$$

with ARIMA(1, 0, 0):

$$\hat{E}_{lat} = \psi_{la}\hat{E}_{lat-1} + \delta_{lat}$$
$$\delta_{lat} \sim \mathcal{N}(0, \tau_{la}),$$

ARIMA(1, 1, 0):

$$\hat{E}_{lat} = \hat{E}_{lat-1} + \psi_{la}(\hat{E}_{lat-1} - \hat{E}_{lat-2}) + \delta_{lat}$$
$$\delta_{lat} \sim \mathcal{N}(0, \tau_{la}),$$

ARIMA(1, 0, 0) + constant:

$$\hat{E}_{lat} = \psi_{la}\hat{E}_{lat-1} + \delta_{lat}$$
$$\delta_{lat} \sim \mathcal{N}(\delta_{la}, \tau_{la}),$$

ARIMA(1, 1, 0) + constant:

$$\hat{E}_{lat} = \hat{E}_{lat-1} + \psi_{la}\left(\hat{E}_{lat-1} - \hat{E}_{lat-2}\right) + \delta_{lat}$$
$$\delta_{lat} \sim \mathcal{N}(\delta_{la}, \tau_{la}),$$

By adding our estimated all-cause latent trends to the sum of our cause-specific forecasts (*Note*: not including cause-specific latent trends), we then generated robust all-cause mortality forecasts.

$$\hat{M} = e^{\hat{Y} + \hat{E}}$$

We then repeated this method, though without the blend, for successive levels of the cause hierarchy. For instance, we calculated group I, II, and III mortality by finding residuals at each level and then forecasting them using the pooled AR1 [ARIMA (1,0,0)] method described for the cause-specific models.

Then, we generated robust forecasts of cause-specific mortality at each level by generating a forecasted cause fraction and multiplying it by the forecasted parent cause mortality.

$$\hat{m}_c = e^{\hat{y}_c + \hat{\epsilon}_c}$$

$$CS\hat{M}F_c = \frac{\hat{m}_c}{\sum_i^C \hat{m}_i}$$

$$m_c^* = \hat{M} \times CS\hat{M}F_c$$

We did this successively down the cause hierarchy, so that child causes were constrained to the parent, but the parent causes still reflected the risk factors and underlying trends driving each child cause. Latent trends for group I causes were pooled at the region-age-sex level, and latent trends for groups II, III, and IV were pooled at the super-region-age-sex level.

Figure 2 in the main paper shows the annualized rate of change by age for all-cause mortality in China and Australia. It provides a comparison of the ARC from 1990 to 2016 to the ARC implied all three components of the model. We show the ARC for the underlying mortality model, the ARC for the underlying model and attributable mortality and the ARC for the underlying mortality, attributable mortality from risk factors and the ARIMA model.

5. Modelling relationships between drivers and mortality

Modeling overview by cause and sex

We forecasted cause-specific mortality rates m by cause of death c, location l, age group a, and year t. This calculation uses population at mid-year to calculate mortality rate.

$$m_{clat} = \frac{\text{deaths}_{clat}}{\text{pop}_{lat}}$$

The all-cause mortality rate M is the sum of cause-specific mortality rate m_c by location, age group, and year.

$$M_{lat} = \sum_c^C m_{clat}$$

Mediation, Scalars, and Population Attributable Fractions (PAFs)

We generated an estimated risk-specific (r) $P\hat{A}F$ in the future by converting the forecasted $S\hat{E}V$ values to $P\hat{A}F$ as follows (see pages 15-22 for an explanation of $S\hat{E}V$ forecasts):

$$P\hat{A}F_{rclat} = 1 - \frac{1}{S\hat{E}V_{rlat} \times (RR_{rc}^{max} - 1) + 1}$$

$P\hat{A}F$ estimates depend on $S\hat{E}V$, which is not cause-specific; therefore we expect a bias in logit-transformed space, which is the space where exposures are modelled. We try to correct for this bias by forcing our estimated values to agree with the GBD in the year 2016. This is done by first taking a reference PAF directly computed from exposure and cause-specific relative risks available in the GBD:

$$PAF_{rclat} = \frac{\sum_x^X p_{xrlat} \times RR_{xrc} - 1}{\sum_x^X p_{xrlat} \times RR_{xrc}}$$

where x corresponds to the different exposure levels of the risk factor. This is followed by calculating the correction factor CF via comparing (in logit space) the GBD PAF to the $S\hat{E}V$-derived estimated $P\hat{A}F$ in the reference year 2016:

$$CF_{rcla} = \text{logit}(PAF_{rcla2016}) - \text{logit}(P\hat{A}F_{rcla2016})$$

This correction factor is necessary because the $S\hat{E}V$ is summarized across all of the causes of death related to that risk factor. If there are different patterns of relative risk by exposure level for different causes of death for the same risk factor, there is some information loss attributable to this dimensionality reduction. Since that correction factor is relatively stable over time, we can simply add it to each year in the forecast to approximate the cause-risk-specific $P\hat{A}F$ accounting for these different relative risk curves.

We applied the correction factor to the estimated $P\hat{A}F$ to come up with an adjusted estimated PAF^*:

$$PAF_{rclat}^* = \text{expit}(\text{logit}(P\hat{A}F_{rclat}) + CF_{rcla})$$

To properly estimate the joint PAF of all risks, one must take into account of how one risk factor is mediated through other risk factors. The fraction of one risk that is mediated through another is called Mediation Factor (MF).[11] Using risk mediation factors provided in the GBD 2016, we computed the joint (adjusted) PAF of all risks for a cause:

$$PAF_{clat} = 1 - \prod_r^R (1 - PAF_{rclat}^* \times \prod_s^S (1 - MF_{rsc}))$$

Where $s \in S$ denotes the risks that impact the cause *c* via *r*, and $r \in R$ are all the risks associated with cause *c*. Since PAF is the ratio of risk-attributable cause-specific deaths to total cause-specific deaths, we can relate total cause-specific mortality m_T to underlying cause-specific mortality m_U.

$$PAF = \frac{m_A}{m_T}$$
$$\&$$
$$m_T = m_A + m_U$$
$$\therefore$$
$$m_T = m_U \times \frac{1}{1 - PAF}$$

Finally, we generated a risk factor scalar $\mathbb{S}$, corresponding to the ratio of total cause-specific mortality to underlying cause-specific mortality.

$$\mathbb{S}_{clat} = \frac{1}{1 - PAF_{clat}}$$

For some cause-risk factor pairs, the PAF is 1 because exposure to the risk factor is part of the disease definition itself. These are systolic blood pressure for hypertensive heart disease and hypertensive chronic kidney disease, fasting plasma glucose for diabetes mellitus, alcohol consumption for alcohol-related cirrhosis of the liver, underweight for protein energy malnutrition deaths, iron deficiency for aenemia deaths, alcohol consumption for alcohol-related cardiomyopathy, impaired kidney function for chronic kidney disease, low birth-weight for preterm birth complication deaths, and occupational exposure to particulates and silica for certain subtypes of pneumonia deaths. In these cases, the risk factors are excluded from the risk-factor scalar and instead their SEVs are included as additional covariates in the mortality model. In addition, we model rotavirus mortality separate from other diarrhoeal disease mortality since we include rotavirus vaccination coverage forecasts as an independent driver. We then sum up other diarrhoeal and rotavirus diarrhoeal deaths.

Supplementary Results Appendix Figure 2 illustrates the components of the mortality modeling process for female all -cause mortality in Australia. The panels on the left show cause-specific death rates by age, with small multiples showing the time trend from 1990 to 2040 for each age group. The bottom left panel is the underlying mortality component captured by SDI and global secular trends; above that is risk-attributable mortality, captured by the risk factor scalar; second from the top is the sum of the underlying and risk-attributable mortality; and the top left panel shows the final mortality predictions after the ARIMA process, which accounts for latent trends. The panels on the right show the cause- and risk-specific population attributable fractions (PAFs) that go into the risk attributable estimation; the all-risk PAF at the top is used

to create the risk factor scalar, and the specific risks are then sorted in descending order of attributable deaths in 2016.

6. Forecasting independent drivers of health

Risk Factors – Summary Exposure Values

In GBD 2016 the attributable burden of 67 individual risk factors was estimated.[11] These risk factors, which have been assessed through GBD's comparative risk assessment framework, serve as drivers in the cause-specific mortality forecasting.

The GBD 2015 introduced Summary Exposure Values (SEVs), a univariate measure of risk-weighted exposure.[9] The SEV is the relative risk-weighted prevalence of exposure, where 0 is no risk in population and 1 is the entire population at maximum risk.

$$SEV_{rc} = \frac{\int_l^L p_l \, RR_l dl - RR_{min}}{(RR_{max} - 1)RR_{min}}$$

where l denotes a category of exposure, as in low, medium, or high. If we set $RR_{min} = 1$

$$SEV_{rc} = \frac{\int_l^L p_l \, RR_l dl - 1}{RR_{max} - 1}$$

Where RR_{max} is the relative risk at the highest level of exposure in theory or observed globally.

Stated in terms of a population attributable fraction (PAF), the SEV is

$$SEV_{rc} = \frac{PAF_{rc}}{(1 - PAF_{rc})(RR_{max} - 1)}$$

In order to only have one SEV per risk factor, we averaged it across the causes which are affected by each risk factor:

$$SEV_r = \frac{1}{N(c)} \sum_c SEV_{rc}$$

For our forecasting work we computed a SEV for each risk factor, because SEVs have the advantage of being a univariate variable that is simple to forecast while still capturing the complex relationships between risk factors and causes of death. These SEVs are then used to generate population attributable fractions (PAFs) and cause-specific scalars, using the methods detailed in Section 5, pages 9-11.

Reference scenario or forecast

For each risk factor (r), we calculated the annual change in the logit of the SEV for every location (l), age (a), sex (s), and past year (t = 1991, … ,2016). In order to dampen the effect of noisy data we replaced annual changes (first differences) outside the 15th and 85th percentiles with those percentile-values, respectively.

$$d_{l,a,s,t,r} = \mathrm{logit}\left(SEV_{l,a,s,t,r}\right) - \mathrm{logit}\left(SEV_{l,a,s,t-1,r}\right)$$

We then computed the annualized rate of change for each country, age, and sex by calculating the weighted mean of the first difference over time, where the weights w_t are determined by a recency-weighting parameter ω, and scaled to sum to 1.

$$\delta_{l,a,s,r} = \mathrm{mean}\left(d_{l,a,s,r,T}, w_{r,t}\right)$$

$$w_{r,t} = (t - t_{initial} + 1)^{\omega_r}$$

In order to select weighting parameters ω, we used data from just 1990 to 2006 to project each risk factor to 2016 using values of ω ranging, in increments of 0.25, from 0 to 10. We then calculated the root-mean-square error ($RMSE$) out-of-sample. We ignored all ω values larger than the ω value with the minimum RMSE. We also ignored lower ω values that had an RMSE value 5% and beyond greater than the minimum RMSE. We took the reciprocal of the RMSE values for the remaining ω values, and defined the resulting values as the probabilities of selecting their corresponding ω values. So the probability of selecting ω_i would be

$$P(\omega_i) = \frac{\frac{1}{RMSE_{\omega_i}}}{\sum_{\omega}^{\vec{\omega}} \frac{1}{RMSE_{\omega}}} ,$$

Where $\vec{\omega}$ is the list of all tested ω values. We used these probabilities to produce a multinomial distribution of ω values. We forecasted SEVs for each risk factor using a random sample of values from this distribution. This approach helped us avoid choosing values for ω that were based on inconsequential differences in the RMSE, placing more weight on the earlier part of the time series by biasing the distribution of ω values toward zero, and add uncertainty to the SEV forecasts.

Appendix Table 1. Omega weights selected for forecasting independent drivers. For education, fertility, vehicles per capita, and vaccine coverage, values are the omega weights selected. For risk factors, values are the mean, 2.5th percentile, and 97.5th percentile of the distribution of omega values.	
**Risk Factor**	**Weight Distribution Summary**
Unsafe sanitation	5.659<br>(1.750 to 9.500)
No access to handwashing facility	6.997<br>(4.250 to 9.750)
Ambient particulate matter pollution	0.000<br>(0.000 to 0.000)
Household air pollution from solid fuels	6.222<br>(2.750 to 9.750)
Ambient ozone pollution	0.620<br>(0.000 to 1.250)
Residential radon	0.000<br>(0.000 to 0.000)
Lead exposure in bone	0.000<br>(0.000 to 0.000)
Occupational exposure to asbestos	0.126<br>(0.000 to 0.250)
Occupational exposure to arsenic	2.883<br>(0.000 to 5.750)
Occupational exposure to benzene	4.774<br>(0.250 to 9.500)
Occupational exposure to beryllium	0.000<br>(0.000 to 0.000)
Occupational exposure to cadmium	3.572<br>(0.000 to 7.000)
Occupational exposure to chromium	4.724<br>(0.000 to 9.500)
Occupational exposure to diesel engine exhaust	4.746<br>(0.000 to 9.500)
Occupational exposure to secondhand smoke	4.830<br>(0.250 to 9.500)
Occupational exposure to formaldehyde	3.731<br>(0.000 to 7.256)
Occupational exposure to nickel	4.043<br>(0.000 to 8.006)
Occupational exposure to polycyclic aromatic hydrocarbons	1.584<br>(0.000 to 3.250)
Occupational exposure to silica	4.439<br>(0.000 to 9.000)

Risk Factor	Weight Distribution Summary
Occupational exposure to sulfuric acid	4.521<br>(0.000 to 8.750)
Occupational exposure to trichloroethylene	0.742<br>(0.000 to 1.500)
Occupational asthmagens	5.549<br>(1.500 to 9.500)
Occupational particulate matter, gases, and fumes	0.000<br>(0.000 to 0.000)
Non-exclusive breastfeeding	8.650<br>(7.500 to 9.750)
Discontinued breastfeeding	5.390<br>(1.250 to 9.500)
Child underweight	5.717<br>(0.250 to 9.750)
Child wasting	6.802<br>(3.750 to 9.750)
Child stunting	7.653<br>(5.500 to 9.750)
Short gestation for birth weight	5.288<br>(0.750 to 9.506)
Low birth weight for gestation	4.823<br>(0.244 to 9.500)
Iron deficiency	4.912<br>(0.000 to 9.250)
Vitamin A deficiency	7.423<br>(5.000 to 9.750)
Zinc deficiency	6.600<br>(3.250 to 9.750)
Smoking (SIR approach)	4.287<br>(0.500 to 7.750)
Smoking (prevalence approach)	0.124<br>(0.000 to 0.250)
Smokeless tobacco	0.000<br>(0.000 to 0.000)
Secondhand smoke	0.000<br>(0.000 to 0.000)
Alcohol use	4.416<br>(0.000 to 8.500)
Suicide due to drug use disorders	5.085<br>(0.250 to 9.500)
Diet low in fruits	6.646<br>(3.500 to 9.750)
Diet low in vegetables	0.133<br>(0.000 to 0.250)
Diet low in legumes	5.767<br>(1.750 to 9.750)
Diet low in whole grains	7.359<br>(5.000 to 9.750)

Risk Factor	Weight Distribution Summary
Diet low in nuts and seeds	3.315<br>(0.000 to 6.500)
Diet low in milk	7.075<br>(4.500 to 9.750)
Diet high in red meat	6.356<br>(4.250 to 8.500)
Diet high in processed meat	2.730<br>(0.250 to 5.250)
Diet high in sugar-sweetened beverages	0.750<br>(0.500 to 1.000)
Diet low in fiber	4.682<br>(0.250 to 9.000)
Diet low in calcium	5.222<br>(0.750 to 9.750)
Diet low in seafood omega-3 fatty acids	3.425<br>(0.500 to 6.250)
Diet low in polyunsaturated fatty acids	6.819<br>(3.750 to 9.750)
Diet high in trans fatty acids	5.993<br>(2.750 to 9.250)
Diet high in sodium	0.124<br>(0.000 to 0.250)
Childhood sexual abuse against females	0.000<br>(0.000 to 0.000)
Childhood sexual abuse against males	0.000<br>(0.000 to 0.000)
Intimate partner violence (exposure approach)	5.481<br>(1.500 to 9.750)
Low physical activity	0.000<br>(0.000 to 0.000)
High fasting plasma glucose (continuous)	5.040<br>(0.000 to 9.750)
High fasting plasma glucose (categorical)	7.383<br>(4.750 to 9.750)
High total cholesterol	5.699<br>(1.750 to 9.500)
High systolic blood pressure	4.766<br>(0.000 to 9.750)
High body-mass index	0.128<br>(0.000 to 0.250)
Impaired kidney function	7.301<br>(4.750 to 9.750)
Education	4.750
Met need for contraception	0.000
Vehicles per capita	0.000

Risk Factor	Weight Distribution Summary
Diphtheria-Tetanus-Pertussis vaccine (DTP) dose 3	1.000
Measles containing vaccine (MCV1)	1.000
Haemophilus influenzae type B vaccine (Hib)	1.000
Pneumococcal conjugate vaccine (PCV3)	1.000
Rotavirus vaccine	1.000

Finally, to make our reference scenario for each SEV, we simply applied the weighted mean first differences into the future, starting from 2016.

$$SEV_{l,a,s,t,r} = \text{expit}\left(\text{logit}(SEV_{l,a,s,2016,r}) + \delta_{l,a,s,r} \times (t - 2016)\right)$$

Better and worse scenarios for the risk factors

To generate better and worse scenarios for SEVs, we chose 85th and 15th percentiles of weighted annualized rates of change across the 195 countries in the past. As we did for the reference, we replaced annual changes outside the 15th and 85th percentiles with those percentile-values, respectively. We used the temporal weights from the reference scenario as described above,

$$\delta_{a,s,r}{}^{[better]} = \text{percentile}_{85th}(d_{L,a,s,r,T}, w_{r,T})$$

$$\delta_{a,s,r}{}^{[worse]} = \text{percentile}_{15th}(d_{L,a,s,r,T}, w_{r,T})$$

We then applied those common rates of change into the future to produce better and worse scenarios for each country.

$$SEV^{[better]}{}_{l,a,s,t,r} = \text{expit}\left(\text{logit}(SEV_{l,a,s,2016,r}) + \delta^{[better]}{}_{a,s,r} \times (t - 2016)\right)$$

$$SEV^{[worse]}{}_{l,a,s,t,r} = \text{expit}\left(\text{logit}(SEV_{l,a,s,2016,r}) + \delta^{[worse]}{}_{a,s,r} \times (t - 2016)\right)$$

In cases where the reference scenario falls outside the better or worse scenarios, we shifted the appropriate scenario to the reference. This ensures that countries with greater than 85th percentile rates of change will not regress in the better scenario, and countries with less than 15th percentile rates of change will not improve to 15th in the worse scenario. Appendix Figure 2 also illustrates the better and worse scenarios for some examples of SEVs.

Smoking impact ratio (SIR)

We used a different approach for forecasting the smoking impact ratio (SIR), a population smoking exposure measure based on the ratio of observed lung cancer mortality compared to non-smoker lung cancer rates which has been used in GBD to estimate smoking attributable burden of cancers and Chronic Obstructive Pulmonary Disease (COPD).[14,15] Because the SIR is derived from one of the outputs of our forecasting model, namely lung cancer mortality, we cannot base the SIR forecasts on that. To keep smoking prevalence and SIR correlated we fit a simple model for the SIR forecasts with 5-year lagged past and forecasted smoking prevalence and cigarettes per smoker,[13]

$$logit(SEV_{SIR,a,t,s,l}) \sim \alpha + \beta_{SEV_{SDP},a-1,t-5,s,l} SEV_{SDP,a-1,t-5,s,l} + \beta_{CPS,a-1,t-1,s,l} CPS_{a-1,t-5,s,l} + \theta t + \varphi_r + \sigma_{sr}$$

where $SEV_{SIR,a,t,s,l}$ is the age-year-sex-location-specific SEV for SIR, $\beta_{SEV_{SDP},a-1,t-5,s,l}$ is the effect of lagged-by-age-and-year age-year-sex-location-specific SEV for smoking prevalence (SDP), $\beta_{CPS,a-1,t-1,s,l}$is the effect of lagged-by-age-and-year age-year-sex-country-specific rate of cigarettes per smoker (CPS), θt is a time variable, φ_r is the region-specific intercept, σ_{sr} is super region-specific intercept, and α is the global intercept. Since there is no age group prior to the first age group, the values for the first age group cannot be lagged by age, so all occurrences of $a - 1$ were replaced with a for the first age group.

Cigarettes per smoker were calculated by dividing past cigarettes per capita by past smoking prevalence. Log (cigarettes per smoker) were forecasted using the weighted ARC method as previously explained, but with a weight-parameter of zero, weighting all past years equally.

Better and worse scenarios

The reference forecast and better and worse scenarios of the SIR SEV are each forecasted separately using the respective scenarios of the covariates in the model. For example, the better scenario of SIR uses the better scenarios of cigarettes per smoker and the Smoking Direct Prevalence SEV as covariates.

Appendix Figure 2 illustrates the results of SEV forecasting process for six risk factors in the United States.

Appendix Figure 2. Summary Exposure Variable (SEV) forecasts (reference), better, and worse scenarios for five example risks (prevalence of smoking, ambient particular matter, high blood pressure, high body mass index, high total cholesterol, and alcohol use in the United States. SEV values are age-standardized. Light blue line represents estimates from GBD 2016.

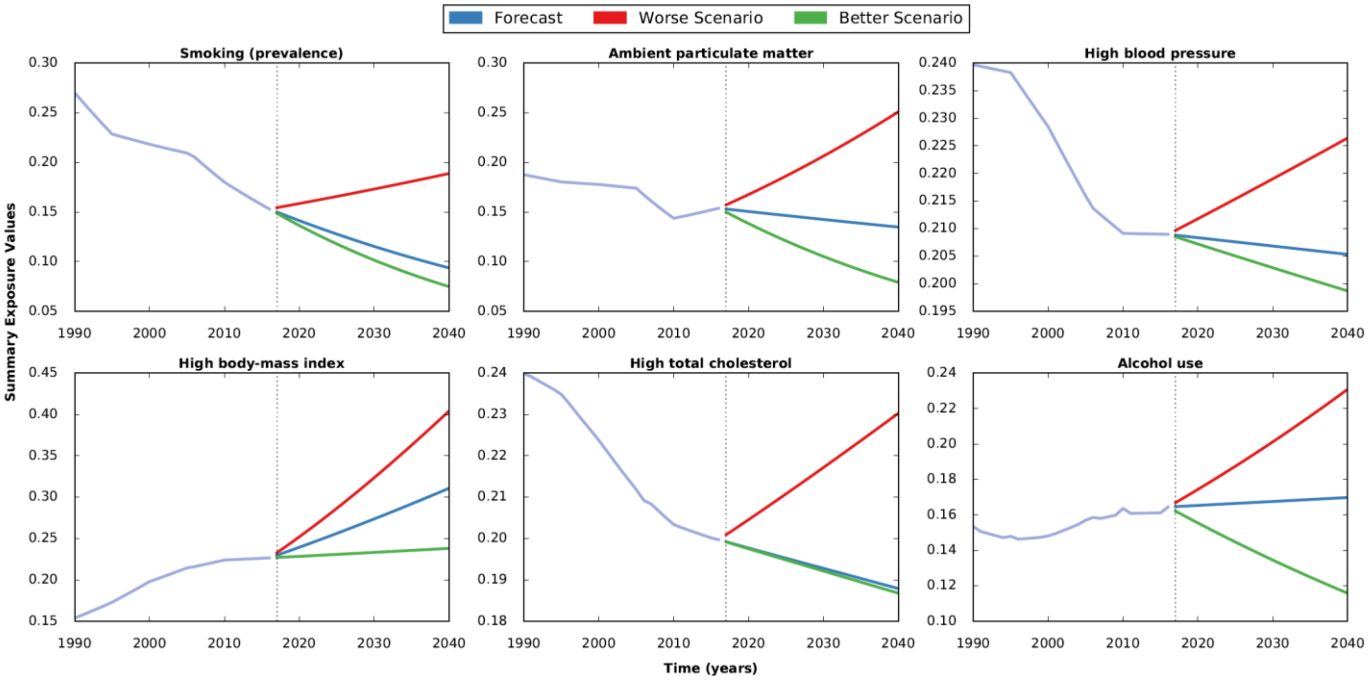

Vaccines

We forecasted coverage for the following five vaccines: diphtheria-tetanus-pertussis (DTP) dose 3, measles (MCV1), rotavirus, pneumococcal (PCV3), and Haemophilus influenzae type B (HIB3). These were divided into two types: simple vaccines and ratio vaccines, based on how they are modeled in the GBD.[16] The simple vaccines, DTP3 and measles, have been introduced in every GBD country, while the ratio vaccines were first introduced more recently and have not yet been added to the routine schedule in all countries. These newer generation vaccines therefore require the additional step of forecasting introduction dates. Due to the typical scheduling of rotavirus, PCV, and Hib vaccine administration programs, coverage for these vaccines was assumed to converge to DTP3 coverage over time (and thus cannot exceed DTP3 coverage).

Reference, better, and worse scenarios for DTP3 and measles

For DTP3 and measles, the models are set up simply for each country as:

$$\text{logit}(coverage) = \alpha + \beta SDI$$

In other words, we predict future coverage using SDI and make sure that it is correctly shifted to match up with the past coverage.

We then used the annualized rate-of-change (AROC) method to forecast better and worse scenarios. This method forecasts rates by taking a weighted quantile of annualized rates-of-change from past data, then projecting that weighted quantile out into future years. A reference scenario is constructed using the weighted median of past annualized rate-of-change across all past years. Better and worse scenarios are constructed using weighted 15th and 85th quantiles of past annualized rates-of-change across all locations and all past years. The 15th and 85th quantiles are then adjusted using the difference between the simple model output and the reference scenario from the AROC method; the difference is added to each scenario so that they align with the simple model output.

Appendix Figure 3 illustrates the results of the vaccine coverage forecast, better and worse scenarios for a sample country (Benin).

Appendix Figure 3. Vaccine coverage forecasts for diphtheria-tetanus-pertussis (DTP) dose 3, measles-containing vaccine (MCV1), rotavirus, pneumococcal conjugate vaccine (PCV3), and haemophilus influenza type B (Hib3) in Benin. Past data use estimates from GBD 2016.

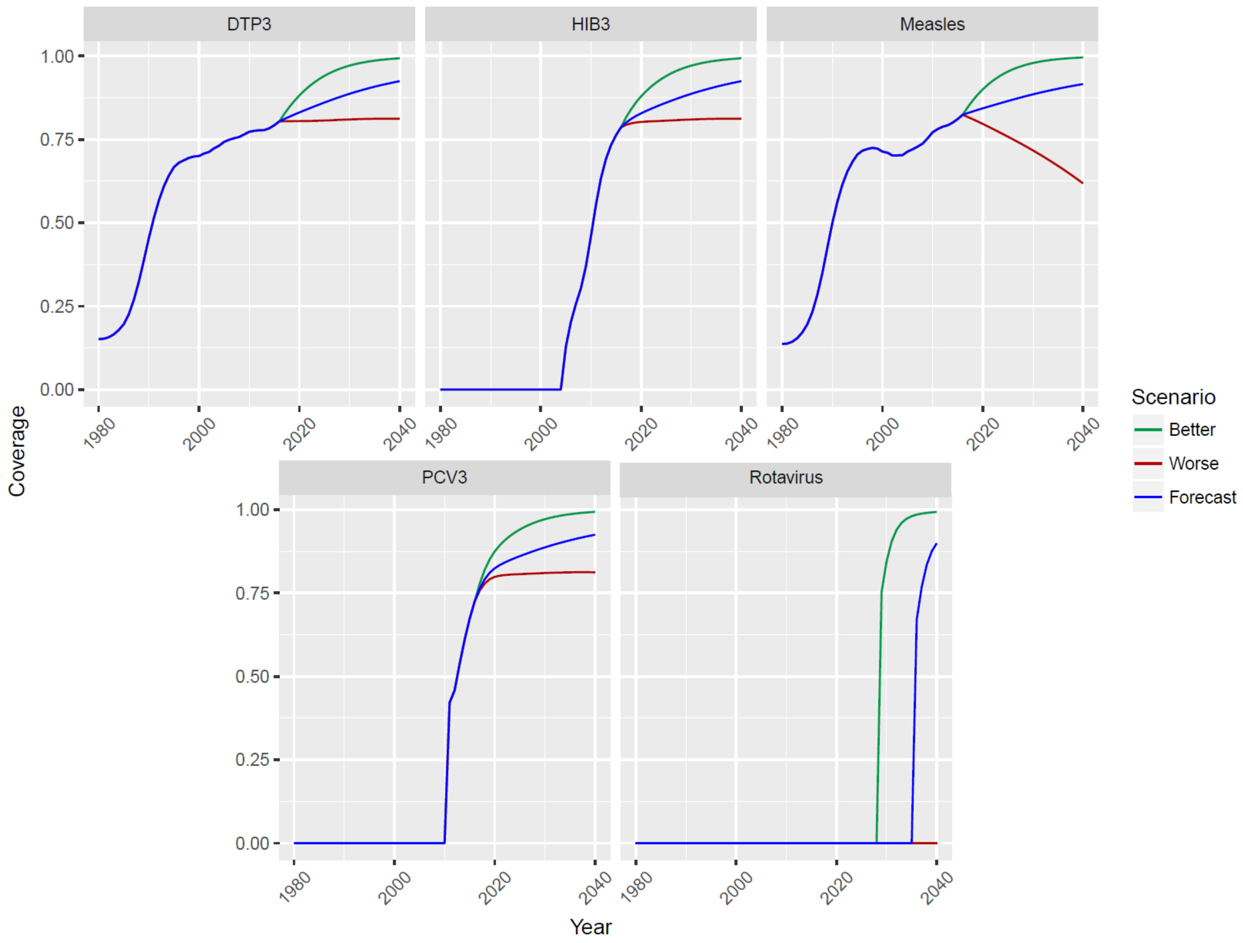

Reference, better, and worse scenarios for the ratio vaccines (Rotavirus, PCV, Hib)

For the ratio vaccines (rotavirus, PCV, Hib), we implement different methods for countries with known vaccine introduction years and countries that have yet to introduce the vaccine. For those that already have introduction years, spatio-temporal Gaussian process regression (ST-GPR) models of coverage ratios (relative to DTP3) are extrapolated through the year 2040 using LDI (lag distributed income) as a covariate.

For countries without introduction years, survival analysis using a Weibull distribution parameterized with SDI; GAVI eligibility status;[17] DTP3 coverage; and Hib introduction date information, as Hib was the first of the three newer generation vaccines to be introduced. The Weibull distribution is used since the "hazard" of a vaccine being introduced is assumed to increase over time, but never to go to infinity. The introduction dates are then simulated using the parameterized Weibull distribution, where the reference scenario is the median of the intro dates, and the better and worse scenarios are the 15th and 85th percentiles, respectively, as later introduction is worse. Scale-up curves for the ratio vaccines relative to DTP3 are generated

for each potential introduction year and matched to the corresponding introduction years. Finally, these ratios are multiplied by forecasted DTP3 coverage to obtain the final coverage forecasts.

Vehicles per Capita

The number of motor vehicles per capita (VPC) was used as a covariate in the cause-specific model forecasting road traffic accidents. To obtain the future time series, VPC were forecasted using a similar weighted ARC approach as we used for SEVs (see pages 15-22). Differences in the approach were that we forecasted in log space, and rather than sampling from a distribution of omega values we just used the omega closest in value to the omega with an RMSE within 5% of the smallest RMSE, and that was also smaller than the omega with smallest RMSE.. The ω value used for VPC is provided in Appendix Table 1.

Better and worse scenarios

To construct better and worse scenarios of VPC, we again used the weighted 85th (better) and 15th (worse) percentiles across the 195 countries in the past. In cases where the reference scenario falls outside the better or worse scenarios, we shifted the appropriate scenario to the reference.

Sociodemographic index

The sociodemographic index (SDI) is a key covariate in a series of GBD analyses. It was introduced in its current form in GBD 2015 and places each country (and sub-national locations) on the global development continuum, scaled from zero to one (highest development) based on income per capita, mean years of education, and total fertility rate. In GBD 2016 the SDI was estimated for each GBD location from 1970 to 2016. In the forecasting model we use the SDI as a driver for future mortality and need to project the SDI for the forecasting years 2017 to 2040. Each of the component drivers are forecasted separately and combined to the future SDI values in a final step presented here. In this paper we use the GBD2017 formulation of SDI where total fertility rate under 25 years (TFU25) is used instead of TFR.[12]

Each underlying indicator Z_i is scaled 0 to 1 by taking the raw values X_i and scaling from the worst to best possible values, with the best and worst values for each indicator listed in the Table below.

$$Z_i = \frac{X_i - worst_i}{best_i - worst_i}$$

Appendix Table 2 presents the scaling values used for the inputs into the sociodemographic index.[10]

Appendix Table 2. Sociodemographic Index (SDI) Scaling Values

Indicator	Worst	Best
**Lag-distributed income (in $ per capita, log space)**	$250	$60,000
**Education (mean years per capita)**	0	17
**Total fertility rate under 25 years (TFU25)**	3.0	0.0

Then, a composite SDI ranging from zero to one was computed by the geometric mean of the three underlying indicators.

$$SDI = \left(\prod_{i}^{I} Z_i\right)^{\frac{1}{I}}$$

Income per Capita

As a component of the SDI and a key covariate, GBD maintains a time series of gross domestic product (GDP) per capita.

Reference scenario

We estimated reference scenarios for gross domestic product (GDP) per capita (in 2015 US dollars) by testing an ensemble of 11,520 individual models on data from 1970 to 2017 for 195 countries. We now describe the model ensemble.

We used the first difference of log GDP per capita (*i.e.* the annual log growth rate) as the dependent variable for all models. Each individual model included a different combination of independent variables (demographic indicators, from the UN World Population Prospects 2017 report, including proportion of population under 20 years of age, proportion of population 65 years or older, and total fertility rate), time series methods (autoregressive terms, auto-correlated residuals, and a convergence term reflecting slow-down in growth rates at higher income levels), and weighting functions to better capture recent trends (using four possible temporal decay functions).

$$y_{l,t} = \ln(GDPpc_{l,t}) - \ln(GDPpc_{l,t-1})$$

We fit all of these models on data from 1970 to 2007 and make out-of-sample (OOS) predictions for 2008 to 2017 and then evaluate their predictive validity. This gave us essentially two trajectories between 2008 through 2017: the observed values and the out-of-sample predictions. For each year, we computed the squared error (the difference) between these two lines, and average these errors for each of the neighboring years. So for example, we had squared errors for 10 data points between 2007 and 2017, and the first mean squared error sum is just the squared error at 2007 and 2008; the second is the mean of the sum of squared errors for 2007, 2008 and 2009; the third is the mean of the sum of squared errors for 2008,

2009 and 2010, and so on, until the last year which uses the sum of squared errors for 2016 and 2017. We then took the square root of this new series to get the running root mean squared errors (RMSE) for a given country.

We assessed a country's 2007 RMSE values for each sub-model, and identified the best 10% of the sub-models (that is, the lowest 10% RMSE values). We repeated this for every country-year out-of-sample. For a specific country, we may potentially have a completely different set of sub-models for each of the OOS years. Then, for the 10% of the sub-models selected in the first year OOS, we only used those models to predict the first year of forecast for each country. The set of 10% of the sub-models selected in the second year OOS were used to predict the second year of forecast for each country, and so on, until the last year OOS model selections were used to compute the forecast the remaining years (2028-2040). This allowed us to narrow down every country's trajectory with the best performing sub-models for each year.

Among the selected sets of sub-models, we computed a total of 1,000 posterior parameter draws to be evenly distributed across each sub-model, country and year, and generated 1,000 reference scenario forecasts of GDP per capita for each country and year between 2018 and 2040. Appendix Figure 4 illustrates the results of GDP per capita forecasts, better, and worse scenarios for four sample countries.

Appendix Figure 4. Gross domestic product (GDP) per capita forecasts (reference), better, and worse scenarios for four example countries: Brazil, India, Germany, and South Africa. Light blue line represents estimates from GBD 2016.

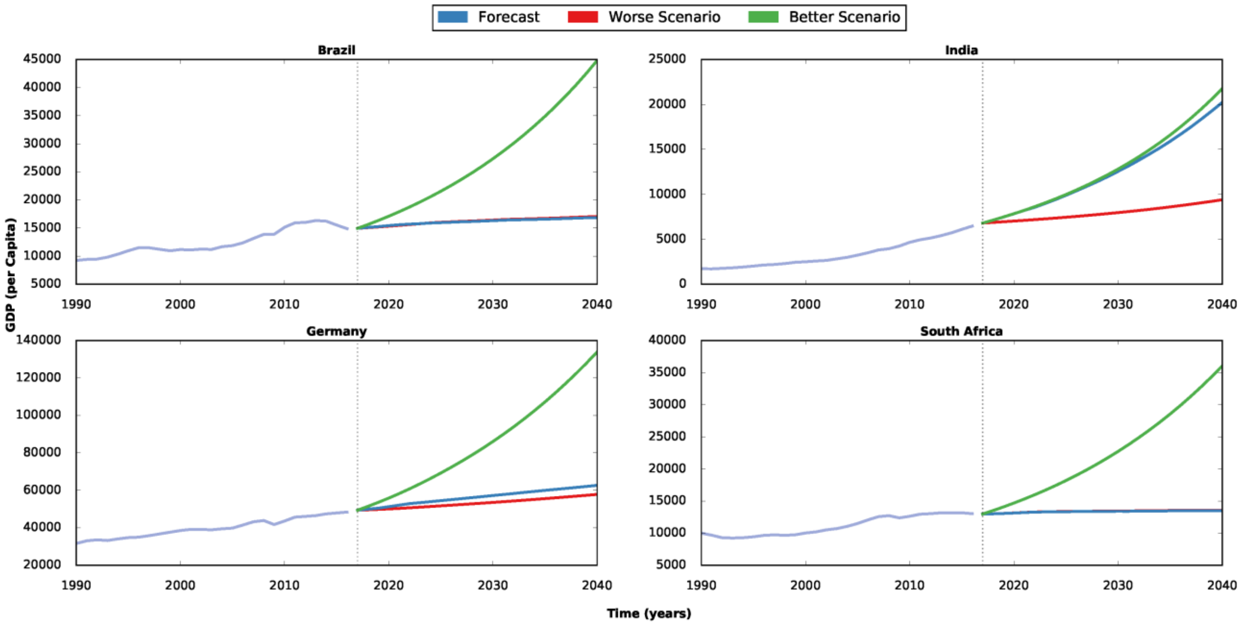

Better and worse scenarios

In order to determine what the possible better and worse growth rates for each country would be, we ran a simple 25-year long-term growth regression of the difference of GDP

per capita (between 1994 and 2017) against the level value of 1994 GDP per capita (all in log spaces). The specification was as follows:

$$\ln GDPpc_{\{l,2017\}} - \ln GDPpc_{\{l,1994\}} = \alpha + \beta \ln GDPpc_{\{l,1994\}} + \varepsilon_i$$

The following steps were used to estimate the future (2018-2040) better and worse trajectory for each country's GDP per capita, following the regression above:

1. We computed the 85th and 15th percentiles of the empirical residuals ε_i across countries, as $Q_{0.85}(\varepsilon)$ and $Q_{0.15}(\varepsilon)$ respectively, where $Q_p(.)$ is a quantile function for a percentile *p*.
2. We computed the starting annualized growth rate from the fitted scenario regression, such that, for country *i*:

$$Better\ Growth\ Rate = \exp(\hat{\alpha}) \times \exp Q_{0.85}(\varepsilon) \times \left(Y_{\{i,2015\}}^{\{\hat{\beta}\}}\right)^{\left\{\frac{1}{2017-1994}\right\}}$$

$$Worse\ Growth\ Rate = \exp(\hat{\alpha}) \times \exp Q_{0.15}(\varepsilon) \times \left(Y_{\{i,2015\}}^{\{\hat{\beta}\}}\right)^{\left\{\frac{1}{2017-1994}\right\}}$$

3. We recursively created better and worse trajectories, conditional on the updated growth rates every year, such that:

$$\mathrm{GDP}_{\{\mathrm{i,t+1}\}} = \mathrm{GDP}_{\{\mathrm{i,t}\}} \times \exp(\hat{\alpha}) \times \exp Q_{0.15}(\varepsilon) \times \left(Y_{\{i,t\}}^{\{\hat{\beta}\}}\right)$$

where $\exp(\hat{\alpha}) \times \exp Q_{0.15}(\varepsilon) \times \left(Y_{\{i,t\}}^{\{\hat{\beta}\}}\right)$ is the conditional growth rate for a single year.

Education

The second component of the SDI is education. In GBD, education is measured as average years of completed education and a complete time series is maintained for all GBD locations and years. We use updated education data from GBD 2017.

Reference scenario

A time series of age-sex-location-specific years of education was forecasted using a similar weighted ARC approach as we used for SEVs (see pages 15-16). We divided all mean years of education values by 18 years of education (the set maximum years of education attainable by an individual) to normalized values between 0 and 1 for the transformation to logit space. Further, rather than sampling from a distribution of omega values we used the lower bound of

the range of omega values with RMSE values within 5% of the minimum RMSE. The ω value used for education is provided in Appendix Table 1.

Appendix Figure 5 illustrates the education forecasts, better, and worse scenarios for four sample countries.

Appendix Figure 5. Years of education per capita for the forecast (reference scenario), better health, and worse health scenarios for four example countries: Germany, Brazil, India, South Africa. Black line represents GBD 2017 estimates.

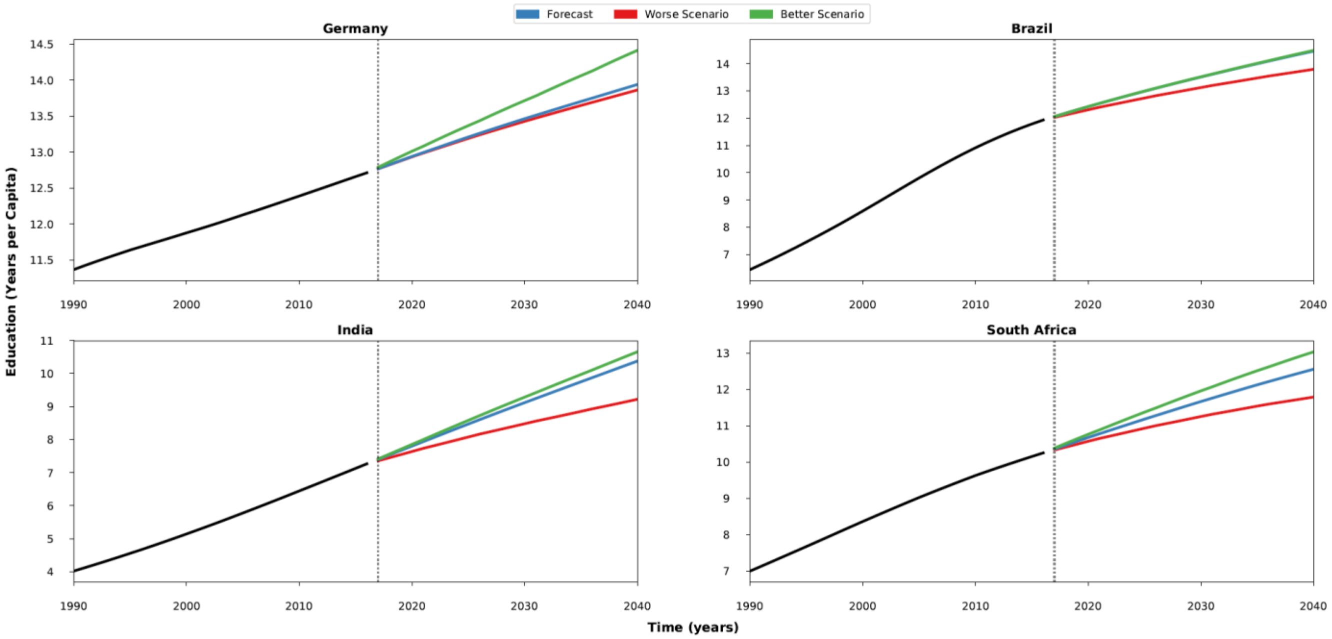

Better and worse scenarios

Scenarios of education (EDU) were based on the annual rate of change of education in logit space for each location, age, sex, and past year (1990 to 2016):

$$d_{l,a,s,t} = Logit(EDU_{l,a,s,t}) - Logit(EDU_{l,a,s,t-1})$$

Using time specific weights $w_t = (t - 1990 + 1)^{1.3}$, the 85th and 15th weighted percentiles of $d_{l,a,s,t}$ were calculated for each age and sex across all locations and years, where the 85th and 15th percentiles correspond to the better and worse scenarios, respectively. The percentiles $\delta_{a,s}^{[scenario]}$ were then applied to the last year of education or the reference scenario of education to generate better and worse scenarios of education:

$$\text{Logit}(\text{EDU}_{l,a,s,t}^{[\text{scenario}]}) = \text{Logit}(\text{EDU}_{l,a,s,\tau_a}) \cdot \delta_{a,s}^{[\text{scenario}]} \cdot (t - \tau_a)$$

where $\tau_a = \max(2016, 2016 + a - 25)$. τ_a was used to introduce an age specific lag effect, since the model assumes that education does not change after age 25 years. This means that scenarios for 35 year olds will not take effect until 10 years after the start of the forecast, while scenarios for ages under 25 will take effect immediately.

Fertility

Total fertility rate for women under the age of 25 (TFU25) is the third component of SDI. TFU25 is computed from age specific fertility rate (ASFR) forecasts. Age-specific maternal education as well as availability of contraception (met need for contraception, family planning) were used as covariates in the estimation ASFR.

Contraceptive met need

We consider the proportion of women of reproductive age (15-49 years) who have their need for family planning satisfied with modern methods.[16]

Reference scenario

The time series of age-sex-location-specific contraceptive met need data from 1990 to 2016 was forecasted through 2040 using a similar weighted ARC approach as we used for SEVs (see pages 15-16). The only difference in the approach was that rather than sampling from a distribution of ω values we used the smallest ω with an RMSE within 5% of the minimum RMSE. The ω value used for contraceptive met need is provided in Appendix Table 1.

Better and worse scenarios

To generate better and worse scenarios of met need for contraception, we used a similar method to the reference scenario, except we looked at the rates of change globally to find what are conceivable better and worse case scenarios based on empirical evidence from the past (85th and 15th percentiles of weighted ARC across countries in the past, utilizing the same ω as the reference scenario).

In cases where the better scenario was above the reference scenario, or the worse scenario was below the reference scenario, the reference scenario was substituted in to reflect the fact that that country's recent trajectory has over- or under-performed the better or worse scenarios.

Fertility Scenarios

Fertility scenarios were modeled using age specific fertility rates (ASFR) and then aggregated to total fertility rate (TFR) and total fertility for women under the age of 25 (TFU25) scenarios:

$$\text{TFR} = \sum^{a} n_a \cdot \text{ASFR}_a$$

Reference Scenario

There are four separate models for ASFR, corresponding to four sets of age groups: 15 to 19 year-olds; 20 to 24 year-olds; 25 to 49 year-olds split into five-year age groups; and the terminal age groups 10 to 14 year-olds and 50 to 54 year-olds.

We first modeled the fixed effect for logit ASFR for 20 to 24 year-olds using maternal education (MEDU) and met need for contraception as independent variables:

$$\text{Logit}(\text{ASFR}_{a,l,t}) \sim \alpha_0 + \beta_a \text{MEDU}_{a,l,t} + \gamma_a \text{met\_need}_{a,l,t}$$

Then we used an autoregressive model to estimate the region-specific errors of ASFR for 20 to 24 year olds:

$$\hat{E}_{rat} = \psi_{ra} \hat{E}_{rat-1} + \delta_{rat}$$

where $\delta_{rat} \sim \mathcal{N}(0, \tau_{ra})$ and r represents a region.

The adjusted estimate for logit ASFR of 20 to 24 year-olds was then:

$$\text{Logit}(\text{ASFR}^*_{a,l,t}) = \text{Logit}(\text{ASFR}_{a,l,t}) + \hat{E}_{rat}$$

Next, we used $\text{Logit}(ASFR^*_{a_{20-24},l,t})$ as a covariate for modeling the 15 to 19 year-olds:

$$\text{Logit}(\text{ASFR}_{a,l,t}) \sim \alpha_0 + \beta_a \text{Logit}(\text{ASFR}^*_{a_{20-24},l,t})$$

Next, we used $\text{Logit}(\text{ASFR}_{a_{20-24},l,t})$ as a covariate for modeling each 5-year age group (a) between 25 to 49 years of age, with an age-specific piecewise linear spline with one knot, and met need as an additional covariate:

$$\begin{aligned}\text{Logit}(\text{ASFR}_{a,l,t}) \sim \quad & \alpha_0 + \beta_a \text{met\_need}_{a,l,t} \\ & + \gamma_a \min(\text{Logit}(\text{ASFR}^*_{a_{20-24},l,t}, \kappa_a) \\ & + \lambda_a \max(\text{Logit}(0, \text{ASFR}^*_{a,l,t} - \kappa_a)\end{aligned}$$

The knots were picked by fitting a LOWESS regression on $\text{Logit}(\text{ASFR}_a)$ and $\text{Logit}(\text{ASFR}_{a_{20-24}})$ and choosing the value corresponding to the largest second derivative in order to identify the inflection point. Final results were intercept-shifted to the GBD 2017 value in 2016 in order to remove discontinuities.

Youngest and Oldest Age Groups

ASFR for the youngest and oldest age groups were estimated by holding the location-specific ratio of the adjacent ASFR in 2016 constant throughout the forecast.

$$\begin{aligned} ASFR_{a_{10-14},t,l} &= ASFR_{a_{15-19},t,l} * \frac{ASFR_{a_{10-14},2016,l}}{ASFR_{a_{15-19},2016,l}} \\ ASFR_{a_{50-54},t,l} &= ASFR_{a_{45-49},t,l} * \frac{ASFR_{a_{50-54},2016,l}}{ASFR_{a_{45-49},2016,l}} \end{aligned}$$

Better and Worse Scenarios

To forecast better and worse scenarios of fertility, the model fits from the reference scenario were used with new inputs corresponding to the scenario being forecasted. Better and worse scenarios of maternal education and met need were used to generate scenarios of ASFR for 20

to 24 year-olds. These in turn were used to generate better and worse scenarios for the other age groups.

7. Causes of Death Forecasted Outside the Main Framework

We follow the practice of the GBD cause-of-death estimation to model HIV/AIDS and war, terrorism, and legal interventions outside of the main cause-of-death modeling framework. HIV/AIDS forecasting make use of Spectrum software developed by UNAIDS and modified in GBD.[10]

HIV/AIDS

Anti-retroviral therapy for HIV/AIDS coverage

In recent years, we have seen a massive scale up of ART treatment among low-income nations, who through large internal investments and substantial development assistance have been able to scale up ART access considerably. For that reason, if the past trends in ART coverage for each country are simply scaled up in projections using a logistic curve, all countries would be projected to achieve 100% coverage by 2040. Given limitations on coverage by health system capacity, and due to the cost of treatment, we bound ART projections with a frontier by income level to reflect resource availability.

Cross-walking Cross-Sectional and Spectrum CD4 Definitions

In order to model the relationship between income and ART coverage, we must also consider CD4 count as a major stratifying variable, since individuals who are sicker (with lower CD4 counts) are more likely to have received a diagnosis and treatment. Survey data provides cross-sectional CD4 count information; however, the Spectrum modeling framework tracks individuals by categorical CD4 count at the initiation of treatment. Therefore, in order to model the relationship between CD4-specific ART coverage and income usable in Spectrum, we cross-walked cross-sectional CD4 values to CD4 at treatment initiation.

We extracted information on the average CD4 progression over time after the initiation of ART treatment from a number of cohort studies.[18–25] We used a natural spline model to parameterize CD4 count response to treatment over time. Our outcome variable $Y_{i,ts}$was the difference in the average CD4 count for a cohort i at time t from the value at the beginning of treatment, time t0:

$$Y_{i,t} = CD4_{i,t} - CD4_{i,t0}$$

We model this change over time using the following model:

$$Y_{i,t,s} = S_1 t * CD4_{i,t0} + S_2 t$$

Where $S_2 t$ is a natural spline on the number of months since treatment initiation, and $(S_1 t * CD4_{i,t0})$ is a natural spline on the number of months interacted with the starting average CD4 count of the cohort. Both spline bases use knots at 3, 12, 24, and 36 months. The model was

fit, for each of the CD4 cut-points used to define compartmental categories in the Spectrum modeling framework (0-49,50-99,100-199,200-249,250-349, 350-500, and 500+).

We then use the progression curves from this model to categorically backcast each individual observed in our cross-sectional survey data sources to one of the aforementioned categories (Appendix Figure 5).

Appendix Figure 6. Categorical backcast of survey microdata using modelled progression curves.

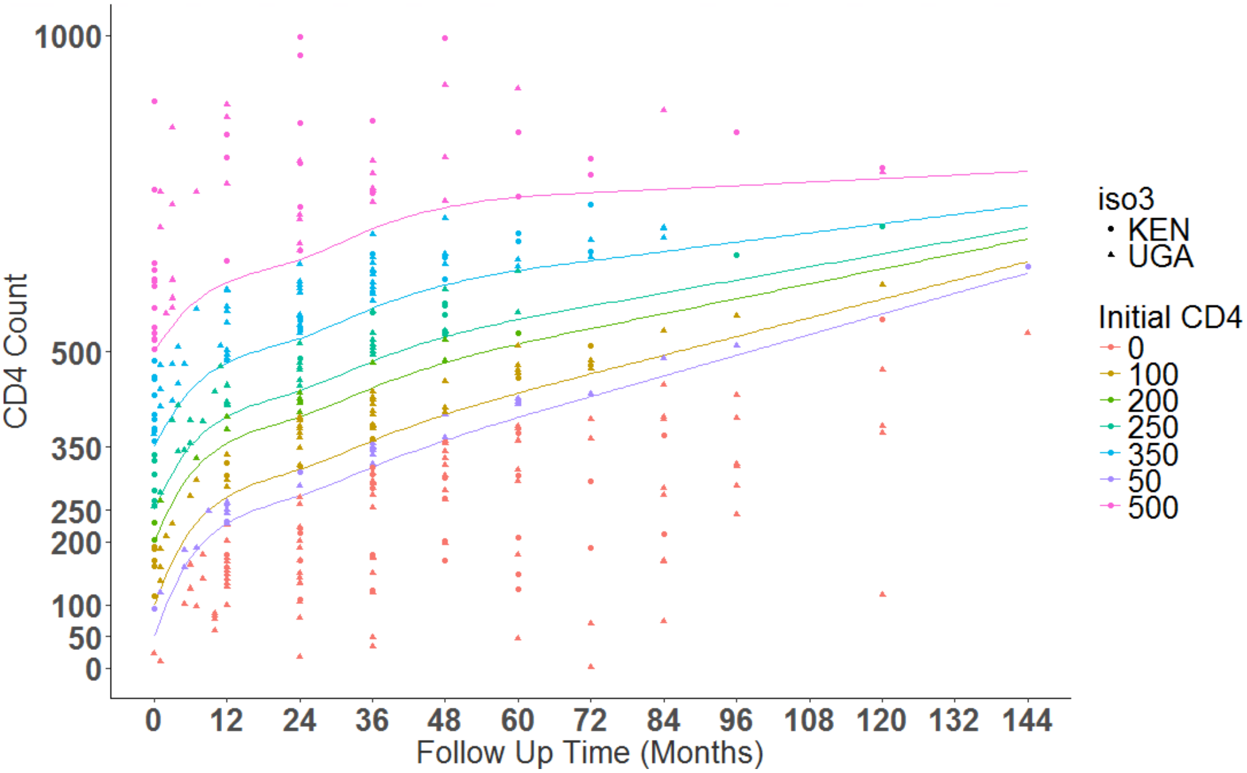

Modeling ART Coverage Frontier as a Function of Income and CD4 Count

To obtain realistic forecasts of ART coverage it is important to place bounds on the coverage relative to resources that are expected to be available. We identified two publically available survey datasets, the 2011 Uganda and 2012 Kenya AIDS Indicator Survey, that provide person-level information regarding the distribution of ART coverage by CD4 count. CD4 information for each participant was obtained from laboratory test values, and cross-walked to the Spectrum definition as described in the previous section. As a proxy for income, we used a household asset index based on assets present in the respondent's home, converted to international dollars.[26] A logistic curve describing the relationship between ART coverage and income is then fit, controlling for CD4 count, age and sex, using a logistic regression:

$$logit(P_{coverage}) = \beta_0 + \beta_1 Income + \beta_2 CD4 + \beta_3 Age + \beta_4 Female$$

We used the predicted probabilities from this model to fit a stochastic frontier analysis, which estimates the maximum possible coverage for a given degree of income and CD4 count (Appendix Figure 6). Formally, we estimate:

$$\log(logit(P_{coverage}) + offset) = \beta_0 + \beta_1 Income + \beta_2 CD4$$

Appendix Figure 7. Predicted probabilities of coverage for each individual shown as points. Frontier of coverage as a function of income is shown with lines. Color indicates categorical CD4 count.

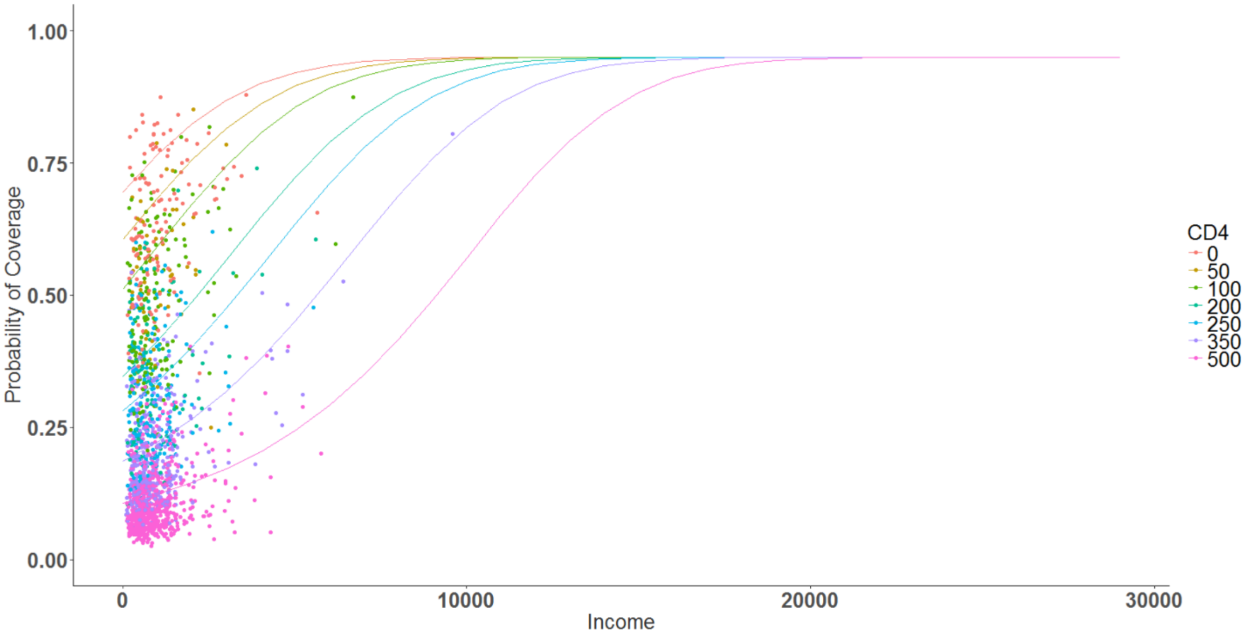

ART Price Forecasts

Forecasting ART Prices

In order to forecast ART coverage, an understanding of the cost of ART treatment over time is necessary. We created estimates and projections of the average cost of ART treatment using data from the Global Price Reporting Mechanism (GPRM).[27] From the GPRM we obtained 1,175 country-years of data representing the average cost of ART in dollars per person per year, covering 130 countries and spanning 2004-2016. We used a stochastic frontier analysis and Gaussian process regression modelling framework to complete the time series and project the estimates through 2040.

Stochastic Frontier Analysis

In order to bound the future minimum cost plausibly, we use a stochastic frontier analysis to model the minimum ART price possible over time.[28] First we create the outcome variable by transforming cost, by rescaling to an inverse zero to one scale, where 0 is the lowest observed cost and 1 is the highest. This is necessary as the stochastic frontier analysis function is used to

find a maximum value. Therefore, the outcome must be rescaled to find a minimum cost frontier. We then take the logit of this transformed cost variable, which creates our outcome variable:

$$Y_{c,t} = logit\left(\frac{Cost_{i,t} - \min(Cost_{i,t})}{range(Cost_{i,t})} + offset\right)$$

We then fit a stochastic frontier analysis, with time as the independent variable, assuming a truncated normal distribution for the extent to which countries fall short of obtaining the minimum achievable ART price.

Gaussian Process Regression

We used Gaussian process regression (GPR) to complete the time series and make projections through the year 2040. GPR has been used extensively in the Global Burden of Disease estimation framework as a data synthesis tool.[29,30] The mean function is a linear model which models the log of the difference between the cost frontier and the current cost, as a function of lag-distributed GDP per capita (LDI) and super-region secular trends:[31]

$$\log\left(Cost_{c,t} - frontier_t\right) = \beta_0 + \beta_1 LDI + \beta_{2,s} t$$

Consistent with prior implementations of GPR, a Matérn covariance function was used to smooth the residuals from the first stage mean function, and produce complete time series with uncertainty.[30] Appendix Figure 7 shows the median and IQR of ART cost globally, as well as the cost frontier.

Appendix Figure 8. Median and IQR of ART price over time globally, alongside the cost frontier as a dashed line. All series are shown in USD.

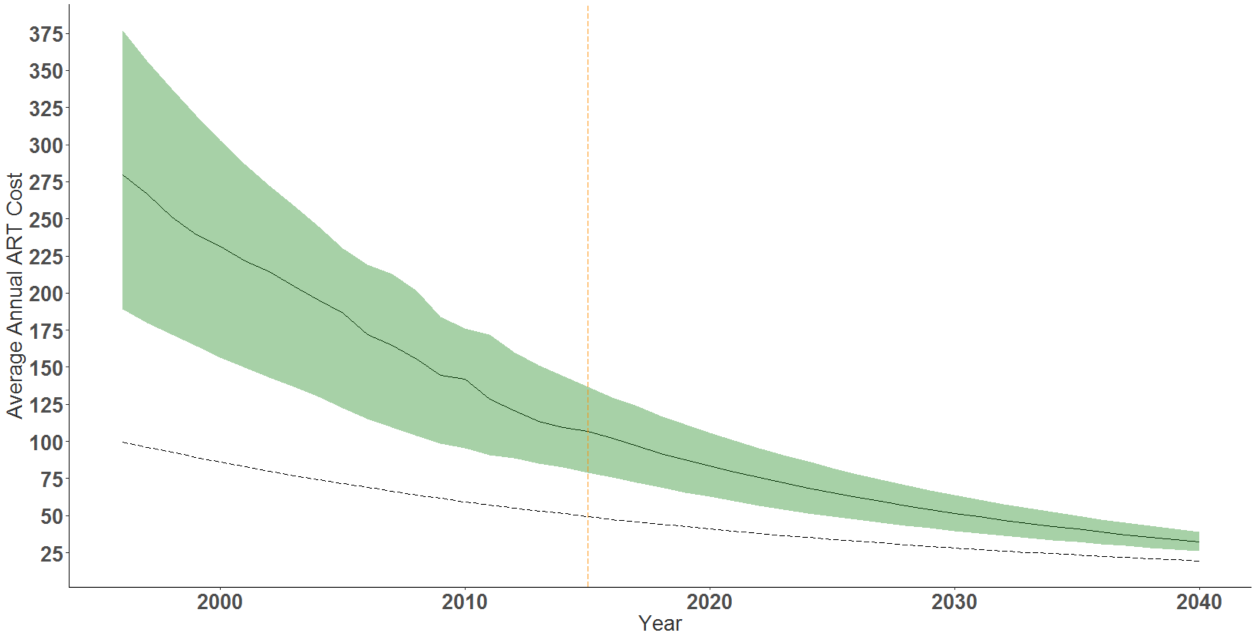

Forecasting Scenarios of Spectrum Inputs

A number of inputs to the ART forecasting, incidence hazard forecasting, and Spectrum HIV modeling systems are treated as exogenous inputs. Projection scenarios for these inputs were created using a rate of change approach, consistent with that used across the forecasting platform. These inputs include:

- ART Price
- Lag Distributed GDP per capita
- HIV-specific development assistance for health
- Government Health Expenditure per capita
- Child ART coverage
- Cotrimoxazole coverage among children
- Coverage of medication used to prevent mother-to-child transmission of HIV (PMTCT) prenatally and postnatally

For each indicator, the distribution of the rate of change across countries was calculated. The time series in each indicator was projected for three scenarios. The ‘reference’ scenario assumed each country grows in the future at the 50th percentile of the past rate of change across countries, the ‘worse’ scenario assumes growth at the 15th percentile, and the ‘better’ scenario assumed growth at the 85th percentile. For ART price, the ‘better’ and ‘worse’ scenarios are flipped, since decreases in price should be considered ‘better’ for health outcomes.

Appendix Figure 9. Scenarios of exogenous input projections for sample country (Zambia)

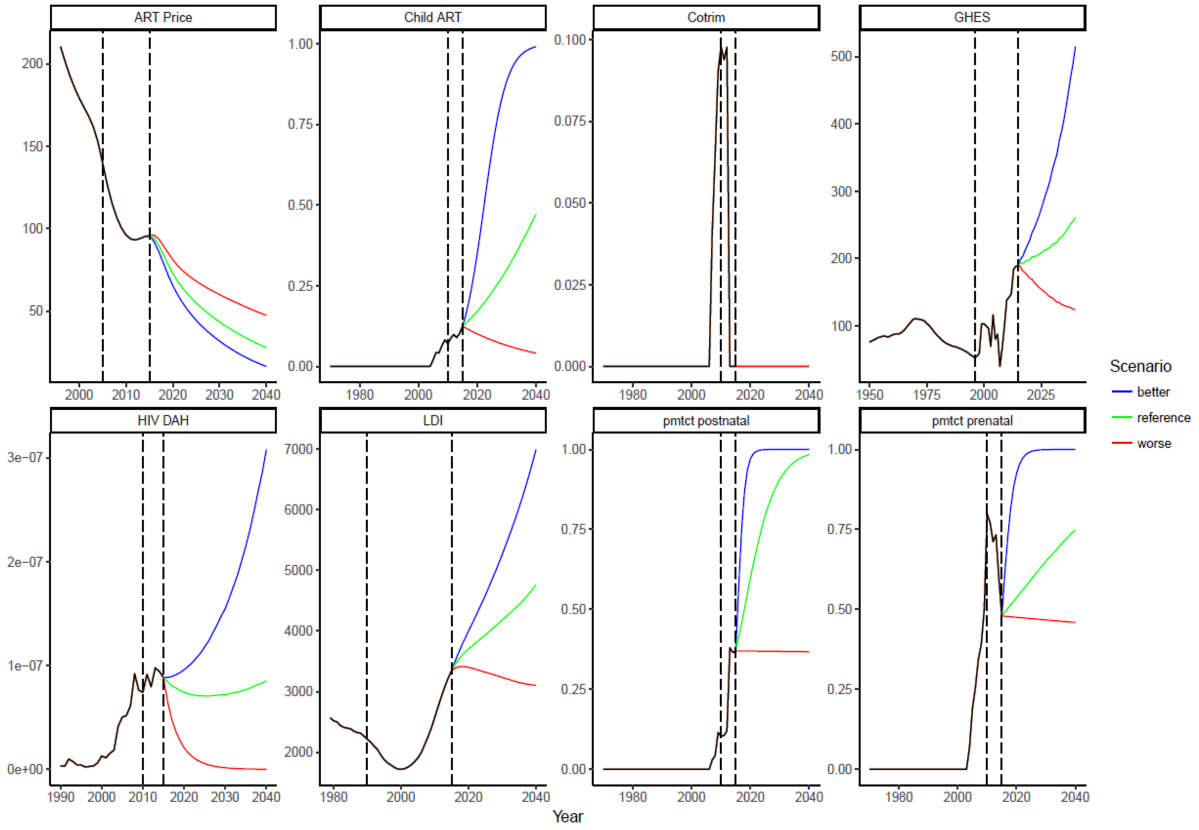

Some of the series had previously existing 'reference' projections, such as ART price (described above), or LDI (published separately). For these indicators, the projections for each scenario were scaled so that the original 'reference' scenario projections were used, and the 'better' and 'worse' projections fall on either side of the 'reference.' Inputs that represent a coverage indicator, including PMTCT, cotrimoxazole, and ART, were forecasted in logit space, while the remaining indicators were modeled in log space. Figure 7 shows an example of forecasted exogenous inputs for Zambia.

Forecasting ART Coverage

ART coverage is projected using the ART bounds described above in the ART coverage frontiers section, as well as the HIV-specific development assistance for health (DAH) and Government Health Expenditure per capita (GHES) exogenous inputs, which are projected as described in the previous section. In order to account for the changing costs of ART over time, the DAH and GHES covariates are rescaled to "dose equivalents," by dividing by ART cost. The relationship between country-year specific ART coverage is then modelled with a slope on dose-equivalents of GHES, a slope on dose-equivalents of DAH (using an indicator variable to remove the countries that are never recipients of DAH), and fixed intercepts for each CD4 group.

$$logit(ART_{c,t}) = \beta_1 GHESdoses + (\beta_2 DAHdoses * I_{DAHrecipient}) + (\beta_3 CD4_{0-49} * I_{0-49}) \ldots (\beta_9 CD4_{500+} * I_{500+})$$

Projected ART values were bounded using the frontiers estimated as described above, or the largest value observed in the past for the time series in question, whichever is larger. Scenarios of ART coverage are created by using scenario specific ART bounds, as well as scenario specific DAH, GHES, and ART price series. We then forecast ART coverage at the granularity it is used in Spectrum, specific to single-year age and sex groups, as well as draws used in Spectrum to propagate uncertainty:

$$logit(ART_{c,,a,s,t,d} = \beta_1 GHESdoses + (\beta_2 DAHdoses * I_{DAHrecipient}) + (\beta_3 CD4_{0-49} * I_{0-49}) \ldots (\beta_9 CD4_{500+} * I_{500+}) + \emptyset_{c,a,s,t,d}$$

where $\emptyset_{c,a,s,t,d}$ is a country-year-age-sex-draw specific intercept shift term, used to ensure no disjunctions in the first year of the forecasts by removing the difference from year 2016 to year 2017 from all forecasted estimates for each time series.

HIV Incidence Hazard

Incidence hazard, the rate of new infections among the susceptible population, is a key input to the Spectrum modeling process. We forecast incidence hazard using ART projections as well as a rate of change approach similar to those described above with respect to the trend in the counterfactual incidence hazard, the expected hazard if ART coverage were zero. A time series of incidence hazard from 1970 through 2016 for each location is taken from GBD 2016 final estimates, then counterfactual incidence hazard is calculated as:

$$Hazard\ Counterfactual_{c,a,s,t,i} = \frac{Hazard_{c,a,s,t,i}}{1-(ART_{c,a,s,t,i} * Viral\ Suppression_{c,a,s,t,i})}$$

$$Viral\ Suppression \sim U(.6,.8)$$

Where ART is the proportion of HIV+ individuals receiving ART, hazard is the number of new HIV infections over population at risk, and viral suppression is the proportion of individuals taking ART who achieve viral suppression. We assumed that a mean of 70% of the on-ART population reached viral suppression and created uncertainty by taking draws from a uniform distribution ranging from 60% - 80%, aligning with assumptions in the EPP model developed by UNAIDs.

Consistent with the approach taken to forecast the independent drivers, projections scenarios for the secular trend in the counterfactual hazard are created by calculating the rate of change across countries over the previous five years, and applying the 15th, 50th, and 85th percentile to each country to create the 'worse,' 'reference,' and 'better' scenarios respectively. The final projected hazard rates therefore decreases in response to improvements in ART coverage, as well as change due to the underlying secular trend in the counterfactual hazard.

Projections of HIV incidence, prevalence, and mortality

In order to produce age- and sex-specific estimates of HIV incidence, prevalence, and mortality, we input projected incidence hazard, ART, PMTCT, and Cotrimoxazole coverage, as well as a number of other predicted demographic inputs, into the Spectrum model. Spectrum is a cohort component model originally developed by UNAIDs that we have modified to incorporate CD4-

specific probability of treatment in addition to a number of other methods developments made for GBD.[31,32] Spectrum ages a population over time using demographic parameters while applying HIV incidence, disease progression, treatment coverage, and mortality. Our final results are age-,sex-, location-specific Spectrum outputs through 2040.

War, legal interventions and disasters

Deaths due to stochastic events including wars, terrorism, legal interventions, and natural disasters were forecasted for each year in the future by randomly sampling from past death rates from 1950 to 2016,

$$\hat{m}_{s,a,f,l} = m_{s,a,r,l},$$

$$\forall a \in A, \forall s \in S, \forall l \in L$$

where $\hat{m}_{s,a,f,l}$ is the age-sex-location-specific mortality rate for the future year $f \in [2017, 2040]$, and $m_{s,a,r,l}$ is the age-sex-location-specific mortality rate of random past year $r \sim U(1950{,}2016)$, A is all of the ages, S is both of the sexes, and L is all of the countries. To maintain correlation among ages, sexes, and countries, a single past year r was randomly selected for a given future year f. For example, if we randomly selected the year 1997 for the year 2030, then the mortality rate for Canadian 20-24 year-old females in 2030 would be inferred from the mortality Canadian 20-24 year-old females in 1997, while the mortality rate for Japanese 40-44 year-old males in 2030 would be inferred from the mortality Japanese 40-44 year-old males in 1997.

In the next step, we applied an SDI-adjustment factor derived from the degree to which increases in SDI have reduced mortality from those events in the past. To do this, we fit a local regression (LOESS) on past mortality rates, $m_{s,a,t_p,l}$, where $t_p \in [1950, 2016]$, and past and forecasted SDI, $SDI_{t,l}$, where $t \in [1950, 2040]$. We extended the traditional LOESS regression to allow us to extrapolate to SDI forecasts. This regression was performed for each sex and age groups separately, but across all years and countries together. The model is

$$\ln\left(m^*_{s,a,t,l}\right) \sim \beta_{s,a} SDI_{t,l}$$

where $m^*_{s,a,t,l}$ is the LOESS-predicted sex-age-year-location-specific mortality rate, $SDI_{t,l}$ is the year-location-specific SDI, and $\beta_{s,a}$ is the sex-age-specific effect of SDI on the mortality rate. Here $t \in [2017, 2040]$, so $m^*_{s,a,t,l}$ has values for both past and future years.

We used these LOESS-predictions to generate an adjustment factor for the randomly selected past mortality rate. The adjustment factor is the ratio of the LOESS-predicted mortality for the future year divided by the LOESS-predicted mortality of the year that was randomly selected to represent that future year's mortality rate. In log space, this is calculated as

$$\phi_{s,a,f,l} = \ln\left(m^*_{s,a,f,l}\right) - \ln\left(m^*_{s,a,r,l}\right)$$

where $m^*_{s,a,f,l}$ is the LOESS-predicted mortality rate of the future year f and $m^*_{s,a,r,l}$ is the LOESS-predicted mortality rate of the randomly selected past year r.

The final estimate of the future year's mortality rate was the observed value of the randomly selected past year multiplied by the correction factor.

$$\widehat{m}^*{}_{s,a,f,l} = \exp(\phi_{s,a,f,l} + \ln(\widehat{m}_{s,a,f,l}))$$

8. Population forecasts

There are four inputs to population forecasts. GBD 2016's population estimate comes from United Nations World Population Prospects (WPP) numbers, as does future migration estimates.[10] Our own estimates of mortality rates and our own estimates of age-specific fertility rates, as described above, were used. Population forecasting uses the cohort-component method of projection (CCMP).[33] This method calculates future populations, N_{t+1}, from current populations, N_t, using a linear model,

$$N_{t+1} = L_t N_t + M_t$$

where L_t is the Leslie matrix and M_t is migration. The time step of the CCMP is usually chosen as five-year or one-year intervals, to match the granularity of ages in population data. In order to capture behavior for the youngest ages, this forecast used a one-week time step which required interpolation and disaggregation to one-week age intervals. The inputs were age-specific fertility forecasts, mortality rate forecasts, and migration.

Appendix Figures 10 and 11 illustrate results of population forecasts by GBD super region and age group, respectively.

Appendix Figure 10. Global population from 1990 to 2040 in the forecast (reference scenario) by Global Burden of Disease (GBD) super region. Values are in billions.

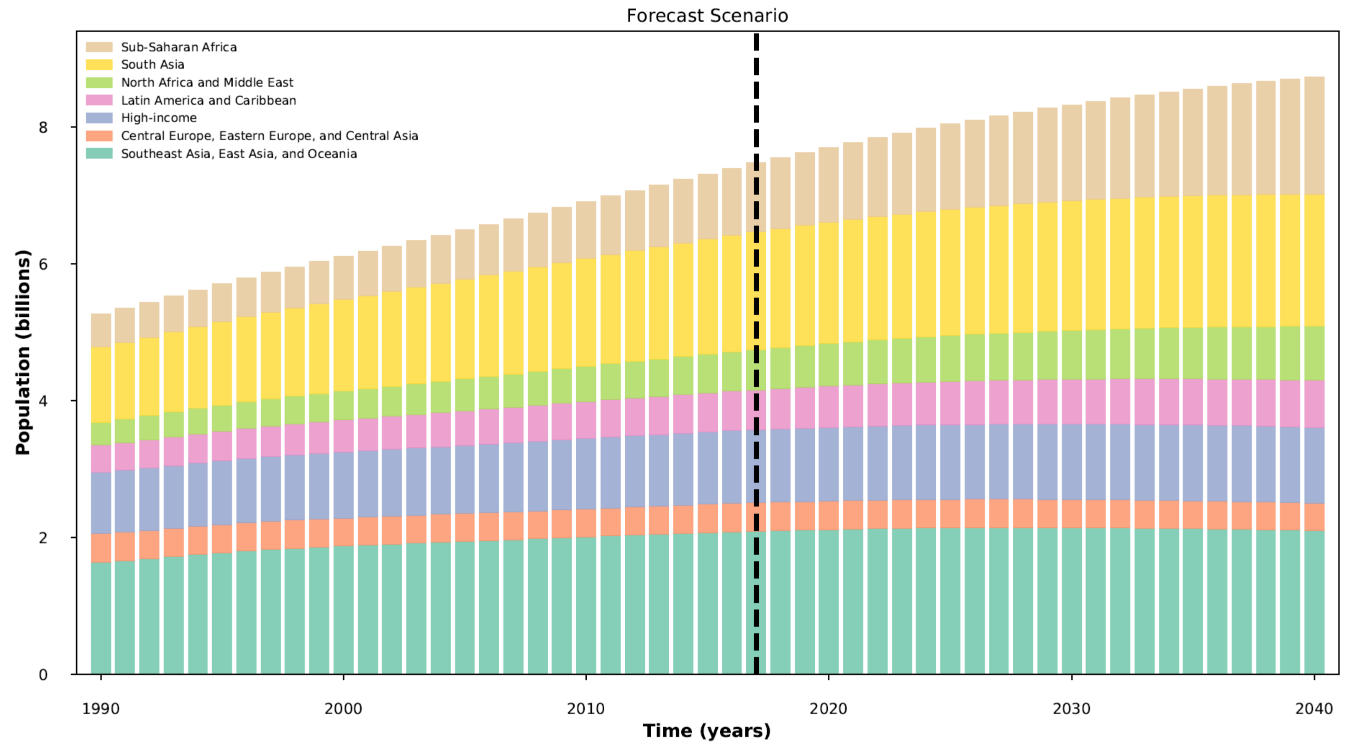

Appendix Figure 11. Global Population from 1990 to 2040 in the forecast (reference scenario) by age group. Values are in billions of persons. Age is measured in years.

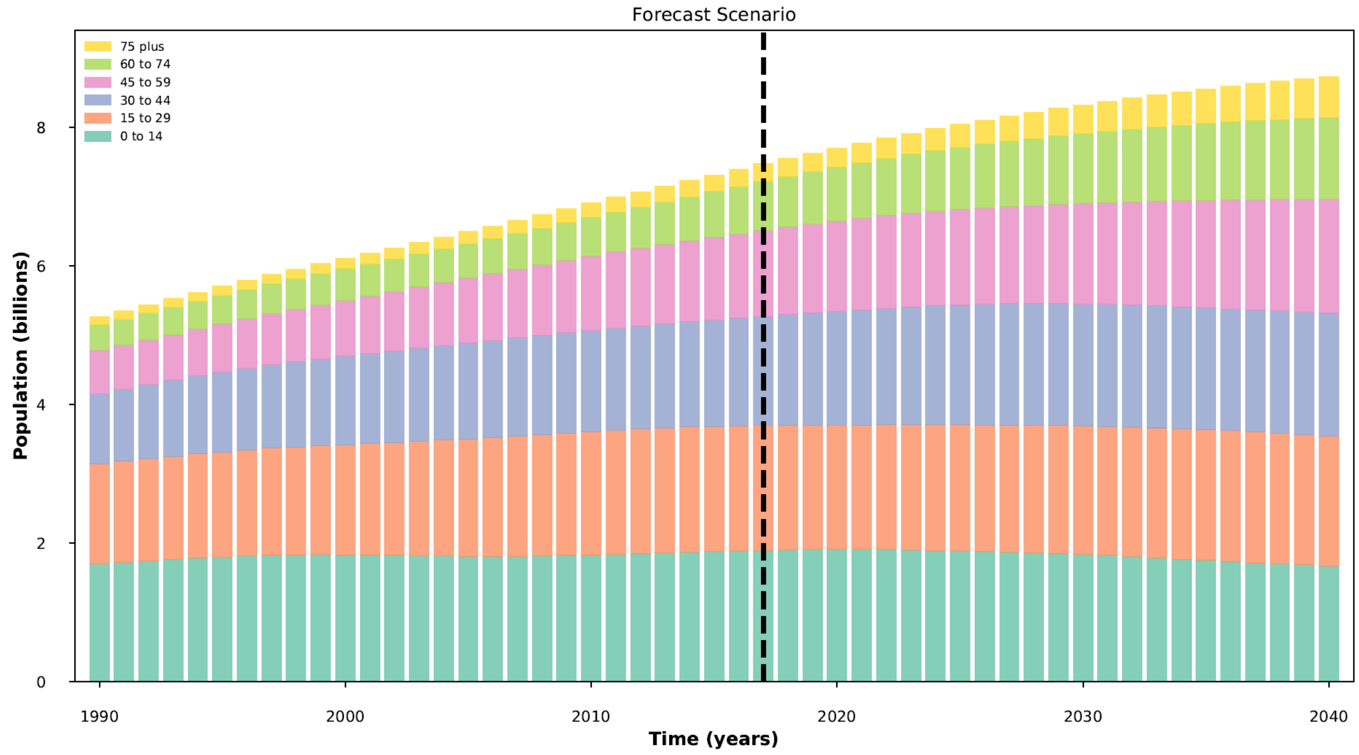

Key Features of the Approach

Migration

We interpolated single year age estimates of WPP 2017 deaths using their reported 5-year-age deaths, lifetables and single year age populations. Using these, we were able to obtain single year estimates of migration in single year age groups by subtracting WPP population between each age year and adding deaths. Because population is reported as a mid-year estimate, migration for a year will consist of migration in the first half of the year plus migration in the second half of the year. Migration in the second half of the year is the change in population not due to deaths,

$$migration_{a,t,2} = \frac{\left(population_{a+1,t+1} - population_{a,t}\right) + \frac{deaths_{a,t} + deaths_{a+1,t+1}}{2}}{2}$$

Migration in the first half of the year is calculated from the previous year's population and deaths.

$$migration_{a,t,1} = migration_{a-1,t-1,2}$$

There isn't a separate account for immigration versus emigration, and the net migration is applied at each weekly time step.

Interpolation and Disaggregation

The input past population was disaggregated using a penalized-component linear model.[34] As described in Preston et al, mortality rates are converted to survivorship ratios in the Leslie matrix, and these are disaggregated using Hyman splines.[33] The age-specific fertility rates were considered constant across each knot, where knots are described in Section 6. Similarly, migration is considered constant across each age group given in the WPP 2017 inputs.[33]

Oldest Ages

The oldest, half-open age interval poses a particular challenge because cubic spline interpolation fails to capture the asymptotic trend of mortality rate. For that age group, the Human Mortality Database used a technique from Thatcher to estimate population for the half-open interval from previous populations in the next-oldest intervals, which they call the survivor ratio method.[35] This algorithm applies the survivor ratio method as a correction after the CCMP is used. The survivor method relies on past data, in this case data for years 2015 and 2016 because data back to 1990 were available as estimates, and there is a 25-year lag of past data required. Using notation where $N_{95+,t}$ is the number of people older than 95 in year, t, the version of the survivor ratio method used relies on a model where five-year survivorship, s, for each five-year period after 95 is the same.

$$N_{95+,t} = \sum_{i=1}^{5} s^i N_{90-94,t-5i}$$

We looked at past data to fit s and applied the equation above to future years. This method is more stable than a simple ratio of N_{95+}/N_{90-94} when birth rates change rapidly.

Internal Verification of Population Model

We checked our implementation by constructing initial population, future fertility rate, and future mortality rate for a stable population model. We then compared the theoretical predicted future population with calculations in order to verify precision within 0.3 percent for each age group by 2040. This means that the chosen interpolations and approximations will correctly reflect input data to the CCMP.

Better and Worse Scenarios

The populations code makes better and worse scenarios that correspond to better ASFR and mortality rates and worse ASFR and mortality rates. Input past population has draws to express uncertainty but not better and worse scenarios. Input migrations have neither scenarios nor draws.

9. Model Performance – Out of time predictive validity

The overall performance of the forecasting framework was evaluated by fitting models using only data from 1990 to 2006 and forecasting for the period 2007 to 2016. War, disaster, terrorism, and HIV were excluded from the out-of-sample analysis. Forecasts for the period 2014 to 2016 were compared to the observed data for 2014 to 2016. Performance was evaluated using two metrics: mean coefficient of variation and median coefficient of variation. The tables below present the results of these analyses for life expectancy, all-cause mortality and cause-specific mortality.

We compare our model with the Lee-Carter model for all-cause mortality and the corresponding life expectancy.[4] Cause-specific trends were not stable enough to produce reliable Lee-Carter estimates, so we omit comparisons at this level.

Appendix Table 3 shows predictive validity metrics for sex-specific life expectancy for our model (IHME) and the Lee-Carter model (LC). These reflect the coefficient of variation (CV), computed as the mean and median scaled root mean squared error (RMSE) divided by the observed values, of the predictions over evaluation years (2014-2016), and countries.

Appendix Table 3. **Predictive validity metrics for life expectancy, with the mean and median computed across locations, with comparison to Lee Carter model. Accuracy is measured with coefficient of variation (CV).**				
**Sex**	**IHME Median CV**	**LC Median CV**	**IHME Mean CV**	**LC Mean CV**
Female	0.007	0.012	0.011	0.022
Male	0.008	0.013	0.012	0.026

Appendix Figure 12 shows life expectancy out of sample predictions for six sample countries: Nigeria, Germany, United States, China, India, and Brazil.

Appendix Figure 12. Comparison of out-of-sample predictions and past data for life expectancy for six sample countries. GBD =Global Burden of Disease.

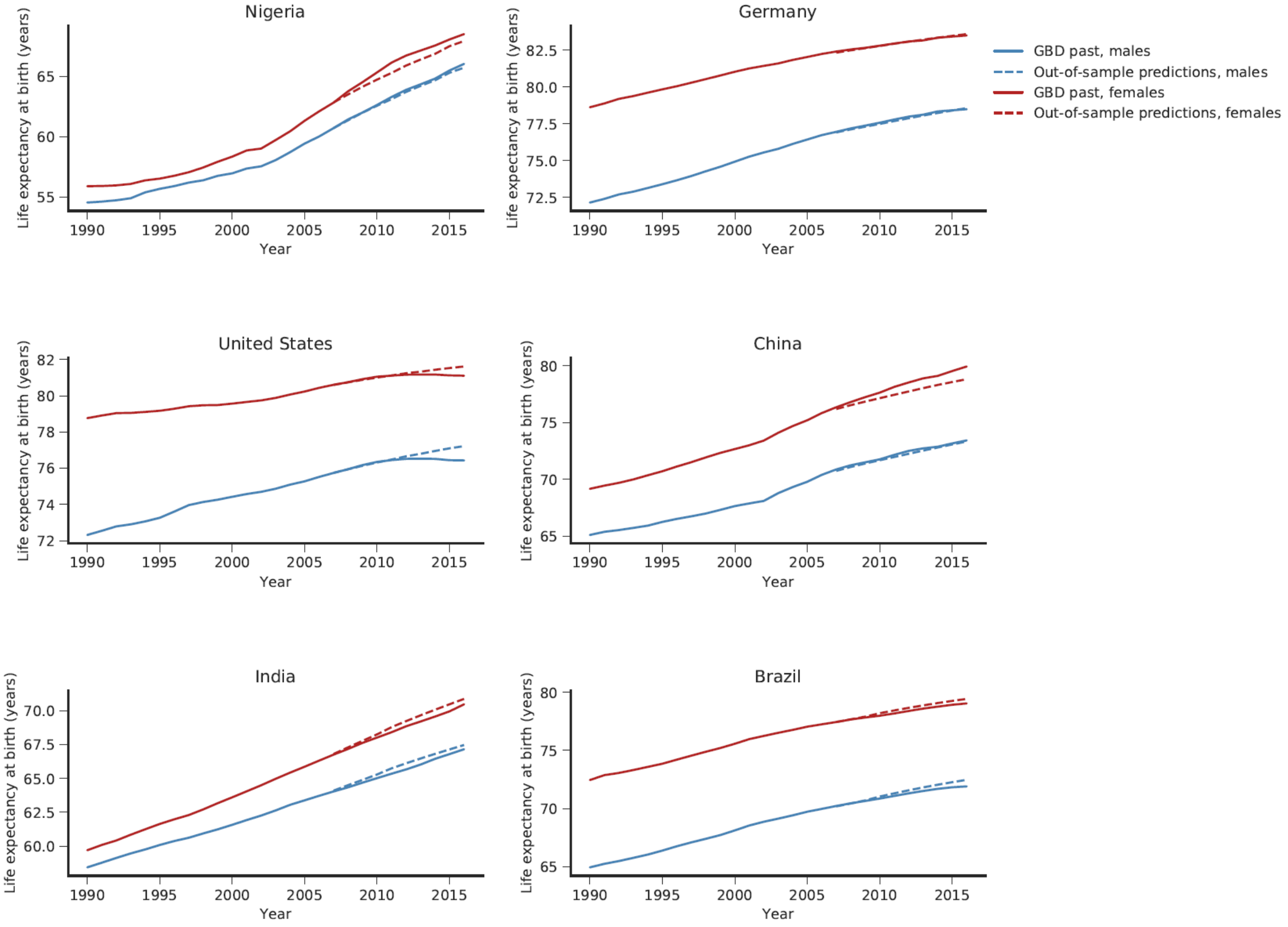

Appendix Table 4 shows the coefficient of variation (RMSE divided by observed values) for age-sex-specific all-cause mortality. Mean and median values are taken across locations in these calculations as well.

Appendix Table 4. **Predictive validity for HIV- and shock-subtracted all-cause mortality forecasts and comparison to Lee Carter. Mean and median values computed across locations. CV = Coefficient of variation. IHME = Institute for Health Metrics and Evaluation (the present model). LC = Lee Carter Model.**					
**Age**	**Sex**	**IHME Median CV**	**LC Median CV**	**IHME Mean CV**	**LC Mean CV**
Early Neonatal	Male	0.075	0.181	0.133	0.232
	Female	0.073	0.177	0.130	0.215
Late Neonatal	Male	0.079	0.159	0.117	0.198
	Female	0.082	0.156	0.119	0.193
Post Neonatal	Male	0.099	0.165	0.151	0.221
	Female	0.098	0.183	0.207	0.230
1 to 4	Male	0.088	0.170	0.135	0.237
	Female	0.099	0.181	0.155	0.246
5 to 9	Male	0.093	0.120	0.153	0.215
	Female	0.084	0.166	0.155	0.244
10 to 14	Male	0.066	0.112	0.110	0.163
	Female	0.088	0.130	0.128	0.194
15 to 19	Male	0.079	0.123	0.127	0.198
	Female	0.073	0.136	0.126	0.211
20 to 24	Male	0.077	0.145	0.138	0.216
	Female	0.072	0.146	0.129	0.232
25 to 29	Male	0.076	0.151	0.125	0.211
	Female	0.079	0.136	0.123	0.234
30 to 34	Male	0.079	0.130	0.114	0.195
	Female	0.075	0.125	0.119	0.211
35 to 39	Male	0.079	0.120	0.110	0.189
	Female	0.085	0.122	0.109	0.182
40 to 44	Male	0.074	0.123	0.112	0.192
	Female	0.068	0.101	0.100	0.161
45 to 49	Male	0.073	0.129	0.114	0.194
	Female	0.058	0.104	0.091	0.158
50 to 54	Male	0.068	0.114	0.102	0.179
	Female	0.058	0.115	0.091	0.173
55 to 59	Male	0.064	0.109	0.094	0.165
	Female	0.061	0.099	0.096	0.164
60 to 64	Male	0.056	0.104	0.075	0.157
	Female	0.067	0.106	0.089	0.165
65 to 69	Male	0.051	0.102	0.075	0.148
	Female	0.066	0.091	0.093	0.163
70 to 74	Male	0.046	0.090	0.067	0.133
	Female	0.060	0.083	0.091	0.156
75 to 79	Male	0.050	0.084	0.070	0.121
	Female	0.050	0.086	0.085	0.145
80 to 84	Male	0.047	0.071	0.061	0.103
	Female	0.053	0.080	0.083	0.123
85 to 89	Male	0.043	0.064	0.066	0.095
	Female	0.046	0.064	0.073	0.109

Age	Sex	IHME Median CV	LC Median CV	IHME Mean CV	LC Mean CV
90 to 94	Male	0.042	0.046	0.070	0.091
	Female	0.046	0.057	0.071	0.096
95 plus	Male	0.039	0.048	0.070	0.089
	Female	0.040	0.054	0.072	0.091

Appendix Figures 13a and 13b show all-cause out of sample predictions for six sample countries: Nigeria, Germany, United States, China, India, and Brazil.

Appendix Figure 13a. Comparison of out-of-sample predictions and past data for all-cause mortality for females, by age group.

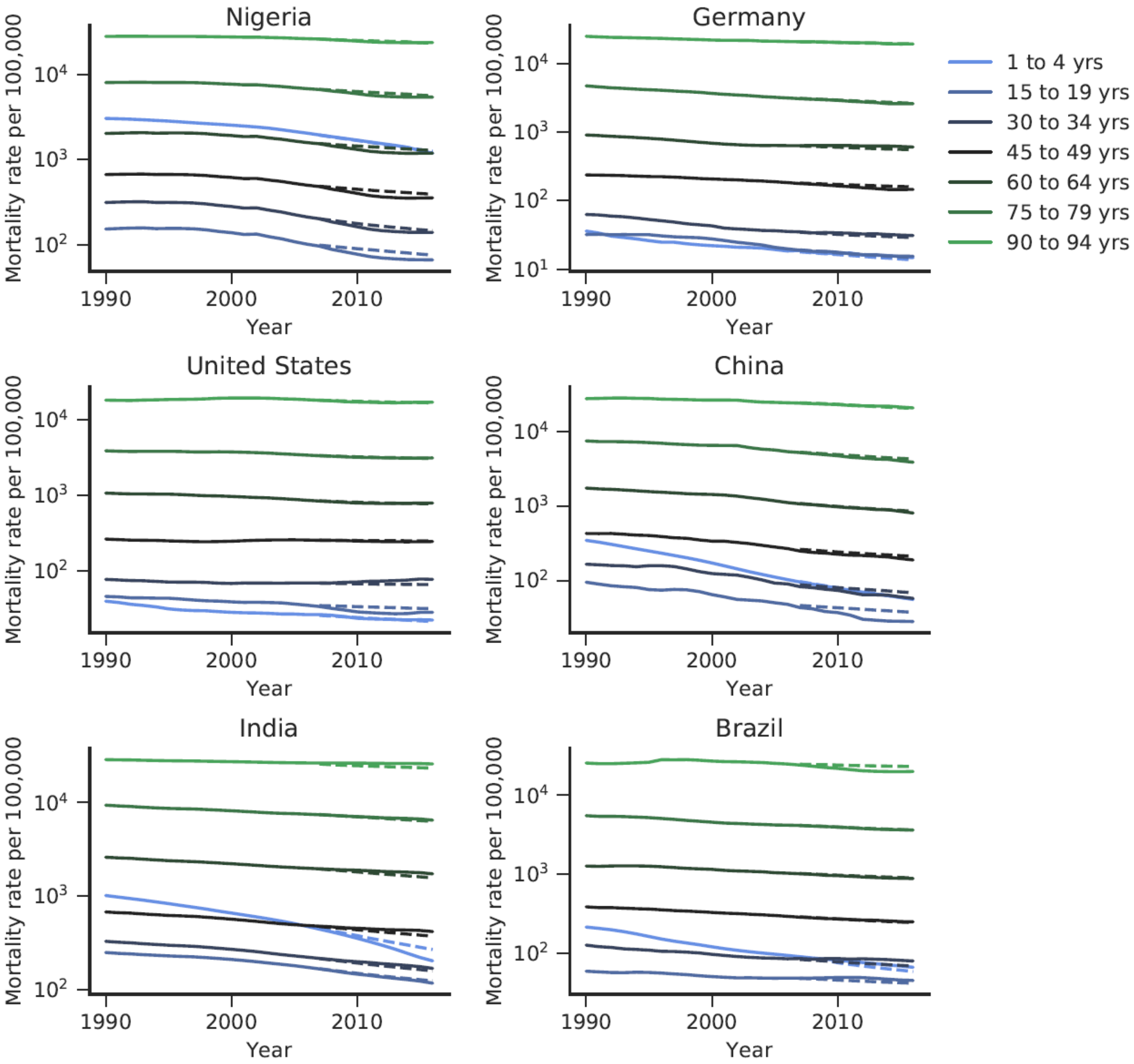

Appendix Figure 13b. Comparison of out-of-sample predictions and past data for all-cause mortality for males, by age group.

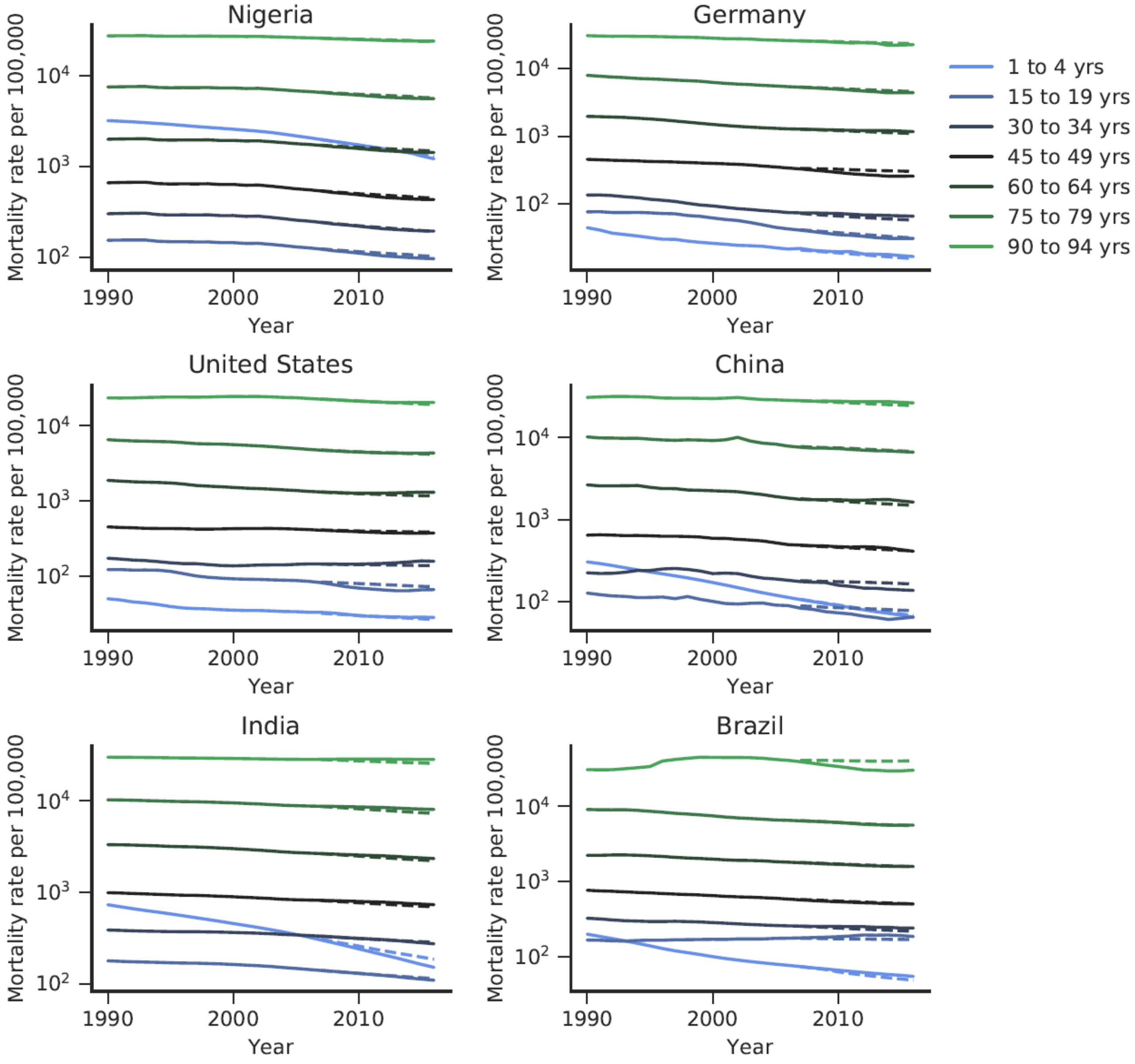

Appendix Table 5 shows the coefficient of variation (CV, root mean squared error [RMSE] divided by the observed values) for cause-sex-specific age-standardized mortality. Both the mean and median are taken across locations and presented. Cause-specific Lee-Carter model predictions are not included, and HIV and stochastic events are subtracted from aggregate cause comparisons.

Appendix Table 5. Predictive validity metrics for cause-sex-specific age-standardized mortality. Cause-specific Lee-Carter model predictions are not included, and HIV, war, disaster, and terrorism are subtracted from aggregate cause comparison. CV = coefficient of variation. For some neglected tropical diseases, many locations have a mortality rate of (or very close to) zero. In such cases, the denominator for the coefficient of variation is so small that even for a very small RMSE, the CV grows extremely large. In cases where the CV is above 10^10, and both the actual and predicted mortality are essentially zero in the locations where the CV is large, we have replaced the CV with a hypen ("-") in the table. Blank cells indicate causes not modeled for that particular sex. Highlighted rows indicate level 1 and 2 causes from the GBD cause hierarchy.

Cause	Median CV Male	Median CV Female	Mean CV Male	Mean CV Female
**All causes**	**0.044**	**0.047**	**0.069**	**0.079**
**Communicable, maternal, neonatal, and nutritional diseases**	**0.071**	**0.067**	**0.101**	**0.104**
**HIV/AIDS and tuberculosis**	**0.166**	**0.166**	**0.242**	**0.226**
Tuberculosis	0.166	0.166	0.242	0.226
**Diarrhea, lower respiratory, and other common infectious diseases**	**0.081**	**0.104**	**0.109**	**0.129**
Diarrheal diseases	0.16	0.212	0.237	0.282
Rotaviral enteritis	0.275	0.317	0.355	0.471
Non-rotaviral enteritis	0.17	0.217	0.24	0.283
Intestinal infectious diseases	0.128	0.145	0.151	0.18
Typhoid fever	0.151	0.188	0.197	0.217
Paratyphoid fever	0.152	0.157	0.212	0.187
Other intestinal infectious diseases	0.178	0.193	0.24	0.256
Lower respiratory infections	0.106	0.131	0.13	0.15
Upper respiratory infections	0.145	0.149	0.204	0.201
Otitis media	0.416	0.478	0.484	0.57
Meningitis	0.187	0.166	0.196	0.178
Pneumococcal meningitis	0.309	0.299	0.309	0.288
H influenzae type B meningitis	0.226	0.269	0.286	0.307
Meningococcal meningitis	0.166	0.155	0.204	0.219
Other meningitis	0.168	0.16	0.218	0.187
Encephalitis	0.121	0.108	0.133	0.137

Cause	Median CV Male	Median CV Female	Mean CV Male	Mean CV Female
Diphtheria	0.277	0.306	0.415	0.442
Whooping cough	0.396	0.39	0.496	0.524
Tetanus	0.262	0.379	0.348	0.471
Measles	25.752	21.519	214528.795	185749.774
Varicella and herpes zoster	0.142	0.158	0.172	0.197
**Neglected tropical diseases and malaria**	**0.202**	**0.201**	**0.341**	**0.345**
Malaria	1	0.992	-	-
Chagas disease	-	-	0.914	0.909
Leishmaniasis	-	-	0.888	0.86
African trypanosomiasis	-	-	inf	inf
Schistosomiasis	-	-	0.735	0.742
Cysticercosis	0.253	0.241	0.433	0.419
Cystic echinococcosis	0.496	0.476	0.554	0.56
Dengue	0.964	0.898	0.868	0.842
Yellow fever	1	1	0.807	0.808
Rabies	0.275	0.248	0.385	0.382
Intestinal nematode infections	1	1	0.941	1.028
Other neglected tropical diseases	0.192	0.168	0.226	0.21
**Maternal disorders**		**0.154**		**0.231**
Maternal hemorrhage		0.181		0.255
Maternal sepsis and other maternal infections		0.179		0.253
Maternal hypertensive disorders		0.173		0.25
Maternal obstructed labor and uterine rupture		0.169		0.264
Maternal abortion, miscarriage, and ectopic pregnancy		0.26		0.403
Indirect maternal deaths		0.203		0.284
Late maternal deaths		0.177		0.257

Cause	Median CV Male	Median CV Female	Mean CV Male	Mean CV Female
Maternal deaths aggravated by HIV/AIDS		1.468		13.073
Other maternal disorders		0.175		0.242
**Neonatal disorders**	**0.081**	**0.081**	**0.132**	**0.13**
Neonatal preterm birth	0.097	0.103	0.139	0.147
Neonatal encephalopathy due to birth asphyxia and trauma	0.096	0.08	0.162	0.149
Neonatal sepsis and other neonatal infections	0.15	0.142	0.22	0.206
Hemolytic disease and other neonatal jaundice	0.267	0.183	0.412	0.296
Other neonatal disorders	0.177	0.164	0.296	0.29
**Nutritional deficiencies**	**0.131**	**0.143**	**0.199**	**0.223**
Protein-energy malnutrition	0.156	0.156	0.229	0.252
Iodine deficiency	0.865	0.8	1.28	1.053
Dietary iron deficiency	0.142	0.214	0.23	0.257
Other nutritional deficiencies	0.153	0.185	0.22	0.274
**Other communicable, maternal, neonatal, and nutritional diseases**	**0.152**	**0.135**	**0.181**	**0.171**
Sexually transmitted diseases excluding HIV	0.262	0.219	0.473	0.298
Syphilis	0.263	0.263	0.475	0.387
Chlamydial infection	-	0.109	-	0.176
Gonococcal infection	0.356	0.114	0.512	0.179
Other sexually transmitted diseases	0.346	0.116	0.514	0.177
Acute hepatitis	0.136	0.157	0.287	0.324
Acute hepatitis A	0.207	0.199	0.308	0.322
Acute hepatitis B	0.14	0.161	0.293	0.334
Acute hepatitis C	0.159	0.171	0.294	0.344
Acute hepatitis E	0.147	0.157	0.287	0.322
Other unspecified infectious diseases	0.12	0.121	0.159	0.159
**Non-communicable diseases**	**0.056**	**0.056**	**0.071**	**0.082**

Cause	Median CV Male	Median CV Female	Mean CV Male	Mean CV Female
**Neoplasms**	**0.051**	**0.054**	**0.08**	**0.08**
Lip and oral cavity cancer	0.094	0.097	0.125	0.127
Nasopharynx cancer	0.124	0.12	0.188	0.191
Other pharynx cancer	0.091	0.089	0.107	0.124
Esophageal cancer	0.102	0.109	0.141	0.157
Stomach cancer	0.083	0.09	0.102	0.121
Colon and rectum cancer	0.07	0.064	0.103	0.102
Liver cancer	0.086	0.073	0.114	0.107
Liver cancer due to hepatitis B	0.086	0.075	0.112	0.104
Liver cancer due to hepatitis C	0.089	0.081	0.115	0.109
Liver cancer due to alcohol use	0.107	0.098	0.132	0.134
Liver cancer due to other causes	0.091	0.074	0.113	0.107
Gallbladder and biliary tract cancer	0.1	0.085	0.114	0.11
Pancreatic cancer	0.059	0.067	0.079	0.086
Larynx cancer	0.096	0.241	0.148	0.367
Tracheal, bronchus, and lung cancer	0.091	0.093	0.135	0.122
Malignant skin melanoma	0.079	0.069	0.102	0.093
Non-melanoma skin cancer	0.067	0.091	0.098	0.143
Breast cancer	0.172	0.073	0.216	0.106
Cervical cancer		0.078		0.1
Uterine cancer		0.086		0.123
Ovarian cancer		0.068		0.094
Prostate cancer	0.064		0.087	
Testicular cancer	0.099		0.155	
Kidney cancer	0.064	0.104	0.093	0.119
Bladder cancer	0.075	0.07	0.095	0.103

Cause	Median CV Male	Median CV Female	Mean CV Male	Mean CV Female
Brain and nervous system cancer	0.055	0.06	0.074	0.084
Thyroid cancer	0.067	0.085	0.103	0.125
Mesothelioma	0.099	0.091	0.122	0.141
Hodgkin lymphoma	0.08	0.084	0.111	0.128
Non-Hodgkin lymphoma	0.053	0.064	0.075	0.088
Multiple myeloma	0.062	0.06	0.09	0.087
Leukemia	0.059	0.073	0.081	0.097
Acute lymphoid leukemia	0.08	0.072	0.104	0.108
Chronic lymphoid leukemia	0.094	0.083	0.133	0.134
Acute myeloid leukemia	0.071	0.082	0.094	0.12
Chronic myeloid leukemia	0.108	0.112	0.159	0.158
Other leukemia	0.089	0.092	0.128	0.134
Other neoplasms	0.064	0.073	0.087	0.097
**Cardiovascular diseases**	**0.065**	**0.067**	**0.085**	**0.101**
Rheumatic heart disease	0.12	0.124	0.153	0.157
Ischemic heart disease	0.082	0.087	0.102	0.116
Stroke	0.078	0.097	0.111	0.141
Ischemic stroke	0.083	0.101	0.116	0.145
Intracerebral hemorrhage	0.079	0.098	0.13	0.164
Hypertensive heart disease	0.082	0.097	0.123	0.148
Cardiomyopathy and myocarditis	0.09	0.1	0.117	0.128
Myocarditis	0.115	0.107	0.144	0.167
Alcoholic cardiomyopathy	0.123	0.15	0.172	0.228
Other cardiomyopathy	0.103	0.116	0.139	0.145
Atrial fibrillation and flutter	0.053	0.061	0.069	0.074
Aortic aneurysm	0.078	0.1	0.102	0.118

Cause	Median CV Male	Median CV Female	Mean CV Male	Mean CV Female
Peripheral artery disease	0.11	0.136	0.153	0.174
Endocarditis	0.074	0.072	0.09	0.091
Other cardiovascular and circulatory diseases	0.106	0.075	0.155	0.126
**Chronic respiratory diseases**	**0.095**	**0.11**	**0.157**	**0.164**
Chronic obstructive pulmonary disease	0.096	0.115	0.172	0.177
Pneumoconiosis	0.178	0.122	0.244	0.269
Silicosis	0.26	0.184	0.386	0.414
Asbestosis	0.155	0.136	0.232	0.222
Coal workers pneumoconiosis	0.191	0.164	0.301	0.352
Other pneumoconiosis	0.17	0.147	0.256	0.391
Asthma	0.158	0.167	0.192	0.207
Interstitial lung disease and pulmonary sarcoidosis	0.127	0.14	0.164	0.172
Other chronic respiratory diseases	0.095	0.142	0.134	0.169
**Cirrhosis and other chronic liver diseases**	**0.082**	**0.088**	**0.126**	**0.13**
Cirrhosis and other chronic liver diseases due to hepatitis B	0.083	0.087	0.128	0.128
Cirrhosis and other chronic liver diseases due to hepatitis C	0.095	0.107	0.141	0.154
Cirrhosis and other chronic liver diseases due to alcohol use	0.082	0.092	0.131	0.138
Cirrhosis and other chronic liver diseases due to other causes	0.093	0.089	0.131	0.132
**Digestive diseases**	**0.072**	**0.07**	**0.089**	**0.095**
Peptic ulcer disease	0.105	0.087	0.125	0.141
Gastritis and duodenitis	0.111	0.151	0.172	0.202
Appendicitis	0.098	0.1	0.124	0.131
Paralytic ileus and intestinal obstruction	0.063	0.071	0.087	0.084
Inguinal, femoral, and abdominal hernia	0.105	0.115	0.133	0.129
Inflammatory bowel disease	0.117	0.108	0.153	0.163
Vascular intestinal disorders	0.069	0.1	0.101	0.131

Cause	Median CV Male	Median CV Female	Mean CV Male	Mean CV Female
Gallbladder and biliary diseases	0.121	0.125	0.134	0.148
Pancreatitis	0.097	0.09	0.142	0.146
Other digestive diseases	0.079	0.095	0.111	0.137
**Neurological disorders**	**0.041**	**0.047**	**0.067**	**0.069**
Alzheimer disease and other dementias	0.044	0.053	0.062	0.072
Parkinson disease	0.052	0.057	0.073	0.077
Epilepsy	0.095	0.079	0.167	0.142
Multiple sclerosis	0.074	0.072	0.1	0.11
Motor neuron disease	0.077	0.083	0.116	0.13
Other neurological disorders	0.095	0.122	0.113	0.173
**Mental disorders**	**0.092**	**0.148**	**0.152**	**0.219**
Alcohol use disorders	0.103	0.144	0.148	0.214
Drug use disorders	0.169	0.153	0.244	0.271
Opioid use disorders	0.127	0.126	0.209	0.218
Cocaine use disorders	0.294	0.239	0.502	0.475
Amphetamine use disorders	0.404	0.279	0.624	0.543
Other drug use disorders	0.238	0.21	0.34	0.346
Eating disorders	0.138	0.142	0.227	0.239
**Diabetes, urogenital, blood, and endocrine diseases**	**0.137**	**0.146**	**0.172**	**0.187**
Diabetes mellitus	0.13	0.135	0.193	0.203
Acute glomerulonephritis	0.127	0.113	0.274	0.288
Chronic kidney disease	0.32	0.297	0.346	0.393
Chronic kidney disease due to diabetes mellitus	0.331	0.3	0.379	0.414
Chronic kidney disease due to hypertension	0.365	0.339	0.384	0.434
Chronic kidney disease due to glomerulonephritis	0.244	0.28	0.302	0.351
Chronic kidney disease due to other causes	0.264	0.274	0.301	0.35

Cause	Median CV Male	Median CV Female	Mean CV Male	Mean CV Female
Urinary diseases and male infertility	0.132	0.14	0.174	0.196
Urinary tract infections	0.152	0.156	0.201	0.21
Urolithiasis	0.148	0.147	0.212	0.206
Other urinary diseases	0.123	0.169	0.176	0.242
Gynecological diseases		0.157		0.216
Uterine fibroids		0.178		0.354
Polycystic ovarian syndrome		0.153		0.236
Endometriosis		0.181		0.325
Genital prolapse		0.159		0.31
Other gynecological diseases		0.195		0.278
Hemoglobinopathies and hemolytic anemias	0.092	0.1	0.12	0.136
Thalassemias	0.165	0.159	0.21	0.202
Sickle cell disorders	0.109	0.125	0.147	0.163
G6PD deficiency	0.132	0.133	0.175	0.179
Other hemoglobinopathies and hemolytic anemias	0.109	0.108	0.132	0.152
Endocrine, metabolic, blood, and immune disorders	0.134	0.131	0.162	0.153
**Musculoskeletal disorders**	**0.084**	**0.129**	**0.137**	**0.16**
Rheumatoid arthritis	0.254	0.162	0.32	0.212
Other musculoskeletal disorders	0.08	0.123	0.122	0.177
**Other non-communicable diseases**	**0.094**	**0.096**	**0.124**	**0.122**
Congenital birth defects	0.109	0.099	0.137	0.135
Neural tube defects	0.208	0.202	0.288	0.284
Congenital heart anomalies	0.099	0.106	0.138	0.154
Orofacial clefts	0.21	0.187	0.347	0.296
Down syndrome	0.202	0.138	0.311	0.185
Other chromosomal abnormalities	0.222	0.224	0.319	0.309

Cause	Median CV Male	Median CV Female	Mean CV Male	Mean CV Female
Congenital musculoskeletal and limb anomalies	0.166	0.154	0.211	0.206
Urogenital congenital anomalies	0.11	0.107	0.149	0.158
Digestive congenital anomalies	0.16	0.165	0.225	0.254
Other congenital birth defects	0.131	0.12	0.16	0.155
Skin and subcutaneous diseases	0.172	0.19	0.21	0.204
Cellulitis	0.113	0.142	0.17	0.156
Pyoderma	0.232	0.205	0.28	0.226
Decubitus ulcer	0.153	0.222	0.278	0.317
Other skin and subcutaneous diseases	0.115	0.178	0.18	0.231
Sudden infant death syndrome	0.179	0.192	0.227	0.254
**Injuries**	**0.056**	**0.069**	**0.1**	**0.105**
**Transport injuries**	**0.114**	**0.115**	**0.164**	**0.163**
Road injuries	0.113	0.116	0.167	0.163
Pedestrian road injuries	0.091	0.116	0.151	0.158
Cyclist road injuries	0.142	0.152	0.223	0.201
Motorcyclist road injuries	0.176	0.242	0.251	0.303
Motor vehicle road injuries	0.124	0.111	0.174	0.168
Other road injuries	0.172	0.136	0.244	0.178
Other transport injuries	0.143	0.153	0.205	0.235
**Unintentional injuries**	**0.065**	**0.066**	**0.094**	**0.096**
Falls	0.075	0.083	0.106	0.119
Drowning	0.086	0.096	0.13	0.144
Fire, heat, and hot substances	0.081	0.08	0.121	0.115
Poisonings	0.158	0.129	0.182	0.15
Exposure to mechanical forces	0.079	0.077	0.105	0.105
Unintentional firearm injuries	0.119	0.098	0.162	0.155

Cause	Median CV Male	Median CV Female	Mean CV Male	Mean CV Female
Unintentional suffocation	0.106	0.127	0.152	0.161
Other exposure to mechanical forces	0.075	0.07	0.109	0.107
Adverse effects of medical treatment	0.076	0.068	0.097	0.104
Animal contact	0.113	0.126	0.161	0.181
Venomous animal contact	0.112	0.129	0.159	0.202
Non-venomous animal contact	0.126	0.128	0.186	0.19
Foreign body	0.077	0.078	0.101	0.11
Pulmonary aspiration and foreign body in airway	0.084	0.089	0.106	0.115
Foreign body in other body part	0.098	0.087	0.185	0.138
Environmental heat and cold exposure	0.109	0.109	0.159	0.16
Other unintentional injuries	0.122	0.158	0.179	0.217
**Self-harm and interpersonal violence**	**0.084**	**0.09**	**0.121**	**0.132**
Self-harm	0.079	0.108	0.132	0.158
Self-harm by firearm	0.105	0.16	0.166	0.216
Self-harm by other specified means	0.078	0.109	0.135	0.159
Interpersonal violence	0.129	0.098	0.152	0.127
Physical violence by firearm	0.164	0.119	0.195	0.145
Physical violence by sharp object	0.113	0.123	0.157	0.147
Physical violence by other means	0.137	0.103	0.148	0.132

Appendix Figures 14a and 14b show out-of-sample predictions for two sample causes (tuberculosis and ischaemic heart disease) for six sample countries: Nigeria, Germany, United States, China, India, and Brazil.

Appendix Figure 14a. Comparison of out-of-sample predictions and past data for ischaemic heart disease, males

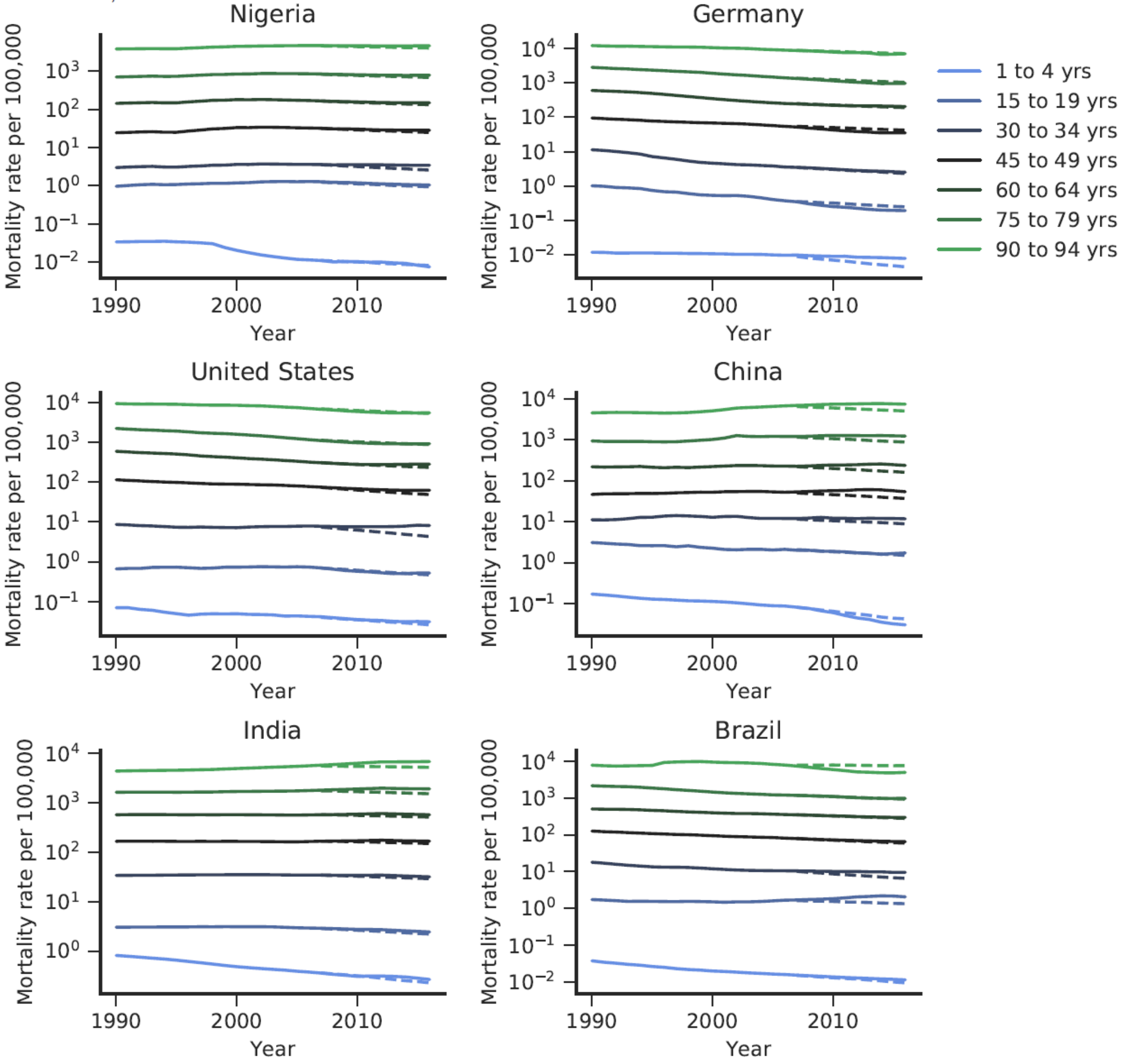

Appendix Figure 14b. Comparison of out-of-sample predictions and past data for tuberculosis, females by age group

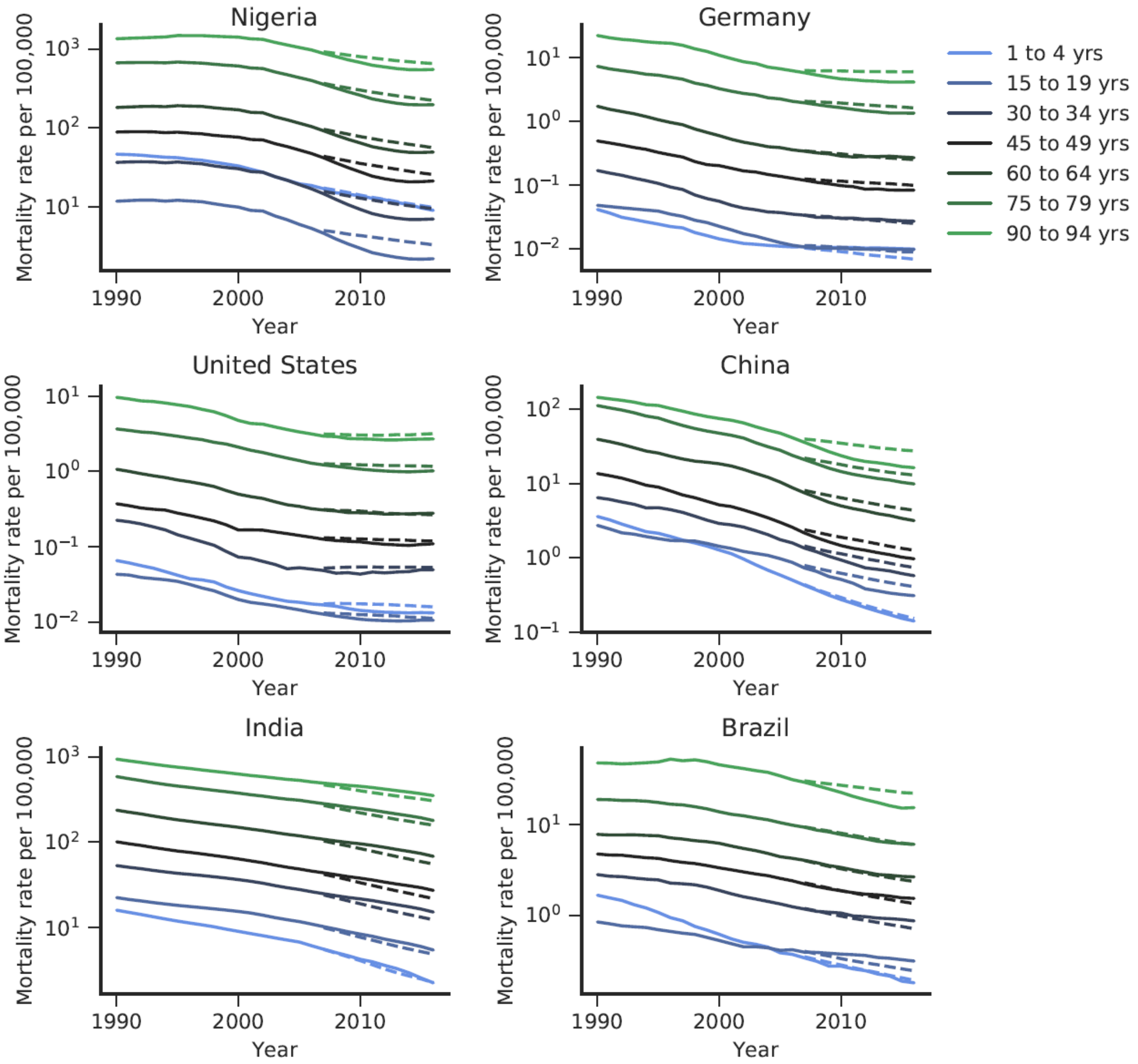

10. References

1 World Population Prospects: The 2017 Revision, Key Findings and Advance Tables. United Nations, Department of Economic and Social Affairs, Population Division, 2017.

2 World Population Prospects: The 2017 Revision, Methodology of the United Nations Population Estimates and Projections. United Nations, Department of Economic and Social Affairs, Population Division, 2017.

3 Kontis V, Bennett JE, Mathers CD, Li G, Foreman K, Ezzati M. Future life expectancy in 35 industrialised countries: projections with a Bayesian model ensemble. *The Lancet* 2017; **389**: 1323–35.

4 Lee RD, Carter LR. Modeling and Forecasting U. S. Mortality. *J Am Stat Assoc* 1992; **87**: 659–71.

5 Girosi F, King G. Understanding the Lee-Carter Mortality Forecasting Method. ; : 35.

6 Soneji S, King G. Statistical Security for Social Security. *Demography* 2012; **49**: 1037–60.

7 Mathers CD, Loncar D. Projections of Global Mortality and Burden of Disease from 2002 to 2030. *PLOS Med* 2006; **3**: e442.

8 Murray CJ, Lopez AD. Alternative projections of mortality and disability by cause 1990–2020: Global Burden of Disease Study. *The Lancet* 1997; **349**: 1498–504.

9 Forouzanfar MH, Afshin A, Alexander LT, *et al.* Global, regional, and national comparative risk assessment of 79 behavioural, environmental and occupational, and metabolic risks or clusters of risks, 1990–2015: a systematic analysis for the Global Burden of Disease Study 2015. *The Lancet* 2016; **388**: 1659–724.

10 Naghavi M, Abajobir AA, Abbafati C, *et al.* Global, regional, and national age-sex specific mortality for 264 causes of death, 1980–2016: a systematic analysis for the Global Burden of Disease Study 2016. *The Lancet* 2017; **390**: 1151–210.

11 Gakidou E, Afshin A, Abajobir AA, *et al.* Global, regional, and national comparative risk assessment of 84 behavioural, environmental and occupational, and metabolic risks or clusters of risks, 1990–2016: a systematic analysis for the Global Burden of Disease Study 2016. *The Lancet* 2017; **390**: 1345–422.

12 GBD Collaborator Network. Population and fertility by age and sex for 195 countries and territories 1950–2017: a systematic analysis for the Global Burden of Disease 2017. *The Lancet* 2018.

13 Girosi F, King G. Demographic Forecasting. Princeton: Princeton University Press, 2008.

14 Peto R, Lopez AD, Boreham J, Thun M, Heath C. Mortality from tobacco in developed countries: indirect estimation from national vital statistics. *Lancet Lond Engl* 1992; **339**: 1268–78.

15 Reitsma MB, Fullman N, Ng M, *et al.* Smoking prevalence and attributable disease burden in 195 countries and territories, 1990–2015: a systematic analysis from the Global Burden of Disease Study 2015. *The Lancet* 2017; **389**: 1885–906.

16 Fullman N, Barber RM, Abajobir AA, *et al.* Measuring progress and projecting attainment on the basis of past trends of the health-related Sustainable Development Goals in 188 countries: an analysis from the Global Burden of Disease Study 2016. *The Lancet* 2017; **390**: 1423–59.

17 Countries eligible for support. Gavi Vaccine Alliance. https://www.gavi.org/support/sustainability/countries-eligible-for-support/ (accessed April 10, 2018).

18 Egger S, Petoumenos K, Kamarulzaman A, *et al.* Long-term patterns in CD4 response is determined by an interaction between baseline CD4 cell count, viral load and time: the Asia Pacific HIV Observational Database (APHOD). *J Acquir Immune Defic Syndr 1999* 2009; **50**: 513–20.

19 Li T, Tubiana R, Katlama C, Calvez V, Mohand HA, Autran B. Long-lasting recovery in CD4 T-cell function and viral-load reduction after highly active antiretroviral therapy in advanced HIV-1 disease. *The Lancet* 1998; **351**: 1682–6.

20 Mocroft A, Phillips A, Gatell J, *et al.* Normalisation of CD4 counts in patients with HIV-1 infection and maximum virological suppression who are taking combination antiretroviral therapy: an observational cohort study. *The Lancet* 2007; **370**: 407–13.

21 Montarroyos UR, Miranda-Filho DB, César CC, *et al.* Factors Related to Changes in CD4+ T-Cell Counts over Time in Patients Living with HIV/AIDS: A Multilevel Analysis. *PLOS ONE* 2014; **9**: e84276.

22 Smith CJ, Sabin CA, Youle MS, *et al.* Factors Influencing Increases in CD4 Cell Counts of HIV-Positive Persons Receiving Long-Term Highly Active Antiretroviral Therapy. *J Infect Dis* 2004; **190**: 1860–8.

23 Tarwater PM, Margolick JB, Jin J, *et al.* Increase and plateau of CD4 T-cell counts in the 3(1/2) years after initiation of potent antiretroviral therapy. *J Acquir Immune Defic Syndr 1999* 2001; **27**: 168–75.

24 Zhou J, Kumarasamy N, Ditangco R, *et al.* The TREAT Asia HIV Observational Database. *J Acquir Immune Defic Syndr 1999* 2005; **38**: 174–9.

25 The potential for CD4 cell increases in HIV-positive individ... : AIDS. https://journals.lww.com/aidsonline/Fulltext/2003/05020/The_potential_for_CD4_cell_increases_in.4.aspx (accessed April 17, 2018).

26 Ferguson BD, Tandon A, Gakidou E, Murray CJL. Estimating Permanent Income Using Indicator Variables. ; : 24.

27 WHO. Global Price Reporting Mechanism for HIV, tuberculosis and malaria. World Health Organization http://www.who.int/hiv/amds/gprm/en/.

28 Bogetoft P, Otto L. Benchmarking with DEA, SFA, and R. New York: Springer-Verlag, 2011 //www.springer.com/us/book/9781441979605 (accessed April 20, 2018).

29 Ng M, Fleming T, Robinson M, *et al.* Global, regional, and national prevalence of overweight and obesity in children and adults during 1980-2013: a systematic analysis for the Global Burden of Disease Study 2013. *Lancet Lond Engl* 2014; **384**: 766–81.

30 Ng M, Freeman MK, Fleming TD, *et al.* Smoking prevalence and cigarette consumption in 187 countries, 1980-2012. *JAMA* 2014; **311**: 183–92.

31 Stover J. Updates to the Spectrum Model to Estimate Key HIV Indicators for Adults and Children. *AIDS* 2014; : S427–S434.

32 Vos T, Allen C, Arora M, *et al.* Global, regional, and national incidence, prevalence, and years lived with disability for 310 diseases and injuries, 1990–2015: a systematic analysis for the Global Burden of Disease Study 2015. *The Lancet* 2016; **388**: 1545–602.

33 Preston SH, Vierboom YC, Stokes A. The role of obesity in exceptionally slow US mortality improvement. *Proc Natl Acad Sci U S A* 2018; **115**: 957–61.

34 Rizzi S, Gampe J, Eilers PHC. Efficient Estimation of Smooth Distributions From Coarsely Grouped Data. *Am J Epidemiol* 2015; **182**: 138–47.

35 Thatcher R, Kannisto V, Andreev KF. The Survivor Ratio Method for Estimating Numbers at High Ages. *Demogr Res* 2002; **6**: 1–18.
